# Supplementary material for: Identification of Novel lncRNA and Differentially Expressed Genes (DEGs) of Testicular Tissues among Cattle, Yak, and Cattle-Yak Associated with Male Infertility
Source: Animals (Basel). 2021 Aug 17;11(8):2420. doi: 10.3390/ani11082420 (PMC8388754; doi:10.3390/ani11082420)
Supplement: Supplementary file 1 [file animals-11-02420-s001.zip › Table S1_cnci_result.pdf]

Transcript	index	score	start	end	length
MSTRG.12.1	noncoding	-0.0274432	180	375	2283
MSTRG.12.2	noncoding	-0.1024	87	165	676
MSTRG.12.3	noncoding	-0.0274432	180	375	1262
MSTRG.32.2	coding	0.388	0	399	4334
MSTRG.33.1	noncoding	-0.065536	12	78	420
MSTRG.34.2	coding	0.131	204	777	1149
MSTRG.35.1	noncoding	-0.0729088	69	282	867
MSTRG.39.3	noncoding	-0.045056	486	558	1065
MSTRG.39.6	coding	0.35	0	1419	10401
MSTRG.59.3	noncoding	-0.0897024	942	1347	1764
MSTRG.60.1	noncoding	-0.012288	1746	1884	2252
MSTRG.61.1	noncoding	-0.016384	516	603	1132
MSTRG.60.2	noncoding	-0.045056	357	474	4486
MSTRG.63.2	coding	0.117	1434	3267	4052
MSTRG.63.4	coding	0.111	1431	3204	3991
MSTRG.63.6	noncoding	-0.0954368	1851	2610	6056
MSTRG.63.7	noncoding	-0.069632	1434	2496	3748
MSTRG.63.5	coding	0.08	1563	3210	3494
MSTRG.63.8	coding	0.111	1851	3624	3902
MSTRG.63.3	coding	0.117	1851	3684	4087
MSTRG.65.10	coding	0.127	0	6756	8160
MSTRG.65.11	coding	0.127	0	6795	8199
MSTRG.65.4	coding	0.126	0	6675	8008
MSTRG.65.5	coding	0.127	0	6807	8193
MSTRG.65.6	coding	0.125	0	6696	8029
MSTRG.65.8	coding	0.127	0	6807	8140
MSTRG.65.9	coding	0.127	0	6786	8190
MSTRG.65.14	coding	0.148	0	5412	7836
MSTRG.65.15	coding	0.148	0	5382	7806
MSTRG.65.16	coding	0.148	0	5412	7815
MSTRG.66.1	coding	0.131	402	3387	4138
MSTRG.66.2	coding	0.133	348	3354	4105
MSTRG.66.3	coding	0.134	282	3291	4040
MSTRG.66.4	coding	0.132	246	3246	3995
MSTRG.68.2	coding	0.061	594	1407	3679
MSTRG.42.1	noncoding	-0.0438272	1638	2070	5454
MSTRG.56.2	coding	0.258	96	1785	4773
MSTRG.56.4	coding	0.26	60	1758	4711
MSTRG.56.6	coding	0.326	36	1437	7508
MSTRG.56.7	coding	0.259	6	1698	2812
MSTRG.56.8	coding	0.258	6	1692	2870
MSTRG.52.1	coding	0.096	285	4803	7093
MSTRG.52.2	coding	0.103	0	4695	6984
MSTRG.53.1	coding	0.229	0	1209	1555
MSTRG.53.2	coding	0.288	954	1389	1632
MSTRG.70.1	coding	0.278	96	1002	3716
MSTRG.73.2	coding	0.127	0	3639	3795
MSTRG.78.1	noncoding	-0.0053248	7014	7161	7956
MSTRG.79.2	noncoding	-0.0749568	0	198	2529
MSTRG.78.2	coding	0.105	1482	1743	8273
MSTRG.80.1	noncoding	-0.008192	69	174	328
MSTRG.81.1	coding	0.048	606	687	1462
MSTRG.79.3	noncoding	-0.0253952	987	1155	9939

MSTRG.82.1	noncoding	-0.057344	141	156	446
MSTRG.79.4	noncoding	-0.0442368	414	588	3123
MSTRG.79.5	noncoding	-0.0503808	5457	5613	6284
MSTRG.79.6	noncoding	-0.036864	2301	2403	3198
MSTRG.83.1	coding	0.096	870	1233	3477
MSTRG.79.7	noncoding	-0.014336	228	432	9751
MSTRG.85.1	noncoding	-0.028672	441	531	1564
MSTRG.86.1	noncoding	-0.057344	2067	2172	6606
MSTRG.96.9	coding	0.136	909	2250	3025
MSTRG.96.10	coding	0.136	1071	2412	3187
MSTRG.96.6	coding	0.136	2091	3432	4207
MSTRG.96.8	coding	0.136	1305	2646	3423
MSTRG.96.3	coding	0.136	1428	2769	3545
MSTRG.97.1	noncoding	-0.0339968	477	663	2424
MSTRG.97.2	coding	0.082	381	1155	2332
MSTRG.91.1	noncoding	-0.0004096	609	747	1940
MSTRG.103.6	coding	0.052	183	1533	3212
MSTRG.103.2	coding	0.068	0	1587	7991
MSTRG.103.5	coding	0.055	183	1608	7127
MSTRG.103.4	coding	0.329	5031	5376	5797
MSTRG.104.1	coding	0.211	0	351	1278
MSTRG.104.3	coding	0.211	0	351	822
MSTRG.111.1	noncoding	-0.2048	267	306	462
MSTRG.117.6	coding	0.086	0	1530	1795
MSTRG.118.5	coding	0.079	24	243	509
MSTRG.123.1	noncoding	-0.0335872	0	45	386
MSTRG.127.1	noncoding	-0.0004096	453	453	672
MSTRG.131.1	noncoding	-0.008192	0	51	226
MSTRG.132.1	noncoding	-0.073728	0	57	2022
MSTRG.134.1	noncoding	-0.04096	114	141	408
MSTRG.151.1	noncoding	-0.057344	2358	2391	2839
MSTRG.188.1	coding	0.069	14730	15276	18614
MSTRG.189.1	noncoding	-0.028672	1506	1608	3356
MSTRG.193.1	noncoding	-0.06144	312	405	669
MSTRG.196.1	coding	0.083	636	864	1087
MSTRG.223.1	noncoding	-0.0118784	0	96	670
MSTRG.241.1	coding	0.125	1092	1389	1743
MSTRG.158.1	coding	0.178	0	1368	9706
MSTRG.158.6	coding	0.065	42	1095	3741
MSTRG.158.5	coding	0.08	0	1065	3712
MSTRG.158.4	coding	0.164	0	1452	3958
MSTRG.158.8	coding	0.037	120	3204	3405
MSTRG.158.7	coding	0.199	0	1281	3554
MSTRG.158.9	coding	0.126	87	351	4805
MSTRG.160.1	noncoding	-0.141312	0	54	1441
MSTRG.160.3	coding	0.113	996	4215	7489
MSTRG.160.4	coding	0.113	924	4143	7684
MSTRG.160.5	coding	0.113	936	4155	6419
MSTRG.160.6	coding	0.113	1659	4878	7735
MSTRG.161.1	noncoding	-0.0126976	309	318	806
MSTRG.162.1	noncoding	-0.014336	6939	7068	11244
MSTRG.162.2	noncoding	-0.014336	924	1053	4935
MSTRG.160.7	noncoding	-0.090112	144	216	1232
MSTRG.163.1	coding	0.102	417	1638	2378

MSTRG.163.3	coding	0.112	411	1500	4264
MSTRG.164.1	coding	0.096	135	741	948
MSTRG.166.2	coding	0.01	387	627	1809
MSTRG.168.2	coding	0.22	0	243	518
MSTRG.169.1	noncoding	-0.024576	27	156	333
MSTRG.175.3	coding	0.125	684	4800	5067
MSTRG.175.4	noncoding	-0.1339392	2361	2376	3094
MSTRG.175.5	coding	0.111	132	3807	3963
MSTRG.175.6	coding	0.1	210	3024	3343
MSTRG.265.8	noncoding	-0.0167936	4050	4377	4630
MSTRG.265.9	coding	0.144	15942	16527	16818
MSTRG.266.1	noncoding	-0.032768	1335	1395	1593
MSTRG.267.1	coding	0.051	4101	5100	7874
MSTRG.268.1	noncoding	-0.02048	1059	1140	5779
MSTRG.269.1	noncoding	-0.43008	1149	1167	1804
MSTRG.268.2	noncoding	-0.02048	1059	1140	2022
MSTRG.270.1	noncoding	-0.024576	435	531	5102
MSTRG.271.1	noncoding	-0.032768	3642	3651	4432
MSTRG.272.1	noncoding	-0.126976	1494	1557	10685
MSTRG.273.1	noncoding	-0.094208	1518	1590	3854
MSTRG.274.1	noncoding	-0.196608	231	237	4757
MSTRG.275.1	noncoding	-0.049152	831	930	2568
MSTRG.276.1	noncoding	-0.008192	243	321	2355
MSTRG.277.1	noncoding	-0.032768	3078	3204	6352
MSTRG.277.2	noncoding	-0.0008192	1017	1164	4614
MSTRG.278.1	coding	0.146	2712	2988	15264
MSTRG.265.10	noncoding	-0.0065536	3369	3483	5365
MSTRG.265.11	coding	0.092	6969	7191	15534
MSTRG.279.1	coding	0.124	13302	13479	13704
MSTRG.265.12	noncoding	-0.028672	5052	5154	10450
MSTRG.280.1	noncoding	-0.0004096	1044	1152	5462
MSTRG.281.1	noncoding	-0.06144	1539	1647	14521
MSTRG.282.1	noncoding	-0.0016384	3396	3519	7437
MSTRG.283.1	noncoding	-0.032768	4446	4467	5415
MSTRG.284.1	noncoding	-0.045056	717	807	6419
MSTRG.284.2	noncoding	-0.045056	642	732	4997
MSTRG.265.14	noncoding	-0.036864	2187	2325	31168
MSTRG.285.1	noncoding	-0.0004096	726	738	6274
MSTRG.250.1	noncoding	-0.0139264	762	975	3949
MSTRG.255.1	coding	0.074	1404	1632	3932
MSTRG.258.1	noncoding	-0.024576	1815	1923	7201
MSTRG.261.1	noncoding	-0.0147456	180	414	1785
MSTRG.262.1	noncoding	-0.012288	2616	2715	4299
MSTRG.263.1	noncoding	-0.1245184	1764	1770	2450
MSTRG.297.1	noncoding	-0.002048	615	729	987
MSTRG.307.1	coding	0.24	0	1239	11283
MSTRG.308.1	noncoding	-0.036864	144	201	3366
MSTRG.312.1	noncoding	-0.053248	417	498	900
MSTRG.314.1	noncoding	-0.0495616	1137	2031	2186
MSTRG.314.3	coding	0.168	1419	1800	5604
MSTRG.325.3	coding	0.127	27	2019	2349
MSTRG.325.4	coding	0.128	27	2022	2546
MSTRG.326.1	noncoding	-0.053248	3762	3840	4429
MSTRG.327.1	coding	0.058	330	534	5165

MSTRG.324.1	noncoding	-0.0167936	87	132	377
MSTRG.328.1	noncoding	-0.032768	375	399	1297
MSTRG.328.2	noncoding	-0.049152	3363	3477	4067
MSTRG.332.1	coding	0.156	276	555	5526
MSTRG.348.1	noncoding	-0.0004096	123	123	277
MSTRG.351.1	coding	0.132	150	1530	2447
MSTRG.352.1	noncoding	-0.0004096	4071	4161	6301
MSTRG.353.1	noncoding	-0.057344	75	141	1004
MSTRG.356.1	noncoding	-0.0094208	3	336	1752
MSTRG.361.1	noncoding	-0.0004096	0	0	224
MSTRG.375.2	coding	0.072	1230	9657	11388
MSTRG.375.5	coding	0.064	897	8874	10605
MSTRG.377.1	noncoding	-0.08192	165	228	1084
MSTRG.377.2	noncoding	-0.02048	402	510	1407
MSTRG.379.3	coding	0.246	87	1434	1947
MSTRG.379.5	coding	0.25	33	1347	1795
MSTRG.369.1	noncoding	-0.016384	399	408	921
MSTRG.370.1	noncoding	-0.118784	57	60	213
MSTRG.371.1	coding	0.05	42	813	1977
MSTRG.381.3	coding	0.127	0	2463	5793
MSTRG.396.1	coding	0.157	4092	6756	7683
MSTRG.397.1	noncoding	-0.0077824	5460	5577	11618
MSTRG.396.3	coding	0.157	864	3558	4020
MSTRG.398.1	noncoding	-0.036864	4266	4347	7300
MSTRG.399.1	noncoding	-0.028672	2232	2337	4279
MSTRG.400.1	noncoding	-0.0094208	3039	3144	5086
MSTRG.401.1	noncoding	-0.0004096	786	852	3548
MSTRG.404.3	coding	0.138	858	1827	3800
MSTRG.404.4	coding	0.119	606	1254	2391
MSTRG.404.6	coding	0.15	372	1245	3203
MSTRG.404.5	coding	0.15	900	1509	3481
MSTRG.404.7	coding	0.164	360	1188	3160
MSTRG.404.8	noncoding	-0.0004096	609	750	3547
MSTRG.391.3	coding	0.124	0	1929	2350
MSTRG.392.2	noncoding	-0.049152	177	339	2025
MSTRG.393.2	noncoding	-0.053248	336	339	822
MSTRG.405.1	coding	0.116	162	1269	1840
MSTRG.405.7	coding	0.104	18	1746	2317
MSTRG.405.3	coding	0.142	0	1356	1940
MSTRG.405.4	coding	0.104	18	1749	2331
MSTRG.405.5	coding	0.142	0	1353	1937
MSTRG.405.6	coding	0.143	0	1365	1949
MSTRG.405.8	coding	0.112	138	1338	1920
MSTRG.405.9	noncoding	-0.12288	615	705	1719
MSTRG.407.1	coding	0.071	81	1242	1465
MSTRG.416.1	noncoding	-0.0180224	207	366	916
MSTRG.423.2	coding	0.097	0	309	2210
MSTRG.424.1	coding	0.182	84	2028	7023
MSTRG.424.2	coding	0.182	471	2415	6720
MSTRG.424.3	coding	0.119	1266	2310	6242
MSTRG.426.1	coding	0.093	735	1254	5075
MSTRG.427.1	noncoding	-0.0688128	4587	5211	5367
MSTRG.429.1	coding	0.077	135	2538	3857
MSTRG.429.2	coding	0.075	0	2583	3432

MSTRG.449.1	noncoding	-0.0004096	0	0	301
MSTRG.451.1	coding	0.054	7182	8157	16139
MSTRG.451.2	coding	0.11	2217	2442	4912
MSTRG.452.1	noncoding	-0.0053248	630	906	1063
MSTRG.457.1	noncoding	-0.1024	12123	12222	13686
MSTRG.435.3	noncoding	-0.0159744	972	1266	1434
MSTRG.439.1	noncoding	-0.0454656	1224	1302	1614
MSTRG.438.2	noncoding	-0.0049152	1341	1446	3811
MSTRG.440.1	noncoding	-0.006144	1725	1815	8222
MSTRG.441.1	coding	0.147	2880	3120	5551
MSTRG.467.6	noncoding	-0.014336	7716	7809	8476
MSTRG.467.7	noncoding	-0.02048	579	639	795
MSTRG.468.1	noncoding	-0.0790528	3918	4107	28602
MSTRG.468.2	noncoding	-0.0774144	12954	13305	21333
MSTRG.468.3	noncoding	-0.032768	357	432	4826
MSTRG.468.4	noncoding	-0.065536	1665	1734	2649
MSTRG.468.5	coding	0.039	6279	6945	9859
MSTRG.470.1	noncoding	-0.0004096	0	0	1555
MSTRG.467.10	coding	0.151	3192	5808	8230
MSTRG.467.9	coding	0.151	3069	5778	8202
MSTRG.467.11	coding	0.153	1068	3192	8946
MSTRG.471.1	noncoding	-0.028672	1890	1983	2752
MSTRG.472.1	noncoding	-0.167936	5799	5853	6260
MSTRG.463.1	coding	0.174	0	522	4928
MSTRG.463.4	coding	0.109	0	423	2748
MSTRG.464.1	noncoding	-0.1024	648	723	2608
MSTRG.474.1	noncoding	-0.004096	1188	1386	1811
MSTRG.479.1	coding	0.419	0	1071	1304
MSTRG.481.2	noncoding	-0.0004096	0	3	533
MSTRG.481.1	noncoding	-0.0724992	282	348	513
MSTRG.487.2	coding	0.079	1968	2802	3659
MSTRG.488.3	coding	0.042	246	999	2220
MSTRG.484.1	noncoding	-0.0004096	0	0	310
MSTRG.493.1	noncoding	-0.012288	297	330	884
MSTRG.497.1	noncoding	-0.028672	54	129	311
MSTRG.506.1	noncoding	-0.0012288	267	399	872
MSTRG.508.3	noncoding	-0.0966656	0	714	3919
MSTRG.508.4	noncoding	-0.0966656	0	714	4202
MSTRG.508.2	noncoding	-0.1466368	0	339	2701
MSTRG.508.5	noncoding	-0.1384448	0	324	10720
MSTRG.509.1	noncoding	-0.0008192	726	936	1125
MSTRG.512.2	noncoding	-0.135168	399	444	761
MSTRG.513.4	coding	0.205	0	3207	6094
MSTRG.514.4	noncoding	-0.0106496	2913	3084	3432
MSTRG.514.3	noncoding	-0.0106496	3420	3591	3938
MSTRG.514.5	noncoding	-0.0106496	3246	3417	3707
MSTRG.515.1	noncoding	-0.0151552	858	1026	4307
MSTRG.521.1	coding	0.079	879	1404	2518
MSTRG.521.3	noncoding	-0.0360448	1716	2049	2203
MSTRG.521.4	noncoding	-0.03072	1833	2139	2292
MSTRG.521.6	noncoding	-0.159744	654	705	1818
MSTRG.521.7	coding	0.079	789	1314	2428
MSTRG.526.3	coding	0.183	0	600	2484
MSTRG.526.1	coding	0.183	0	600	5409

MSTRG.526.2	noncoding	-0.1232896	0	327	10942
MSTRG.527.2	noncoding	-0.0294912	1950	2394	2549
MSTRG.533.2	noncoding	-0.0659456	285	306	620
MSTRG.533.3	coding	0.121	39	2448	2975
MSTRG.535.1	coding	0.151	216	630	1805
MSTRG.535.4	coding	0.149	228	654	2959
MSTRG.538.1	coding	0.16	330	2115	4464
MSTRG.538.2	coding	0.162	720	2508	4857
MSTRG.538.3	coding	0.16	285	2070	4420
MSTRG.538.4	coding	0.162	558	2346	4694
MSTRG.539.1	noncoding	-0.045056	2289	2394	2739
MSTRG.570.1	coding	0.482	1524	3669	4124
MSTRG.570.15	coding	0.482	18141	20286	28296
MSTRG.570.14	coding	0.489	579	2718	10728
MSTRG.570.16	coding	0.489	14736	16875	24886
MSTRG.570.18	coding	0.489	1056	3195	11197
MSTRG.570.17	coding	0.489	1203	3342	11343
MSTRG.570.19	coding	0.489	1545	3684	11680
MSTRG.570.20	coding	0.482	2010	4155	12149
MSTRG.570.22	coding	0.489	18240	20379	28372
MSTRG.570.21	coding	0.482	1428	3573	11567
MSTRG.570.23	coding	0.482	1332	3477	11470
MSTRG.571.1	coding	0.086	1119	1713	1869
MSTRG.572.1	noncoding	-0.0385024	741	1065	1690
MSTRG.570.24	noncoding	-0.0544768	4245	4377	12937
MSTRG.573.1	noncoding	-0.0004096	0	0	884
MSTRG.574.1	noncoding	-0.0016384	1554	1692	1848
MSTRG.575.1	noncoding	-0.0004096	0	0	265
MSTRG.576.1	noncoding	-0.090112	996	1086	1934
MSTRG.577.1	noncoding	-0.032768	3114	3177	3572
MSTRG.578.1	noncoding	-0.0167936	1425	1530	12679
MSTRG.570.25	noncoding	-0.0442368	1221	1254	2096
MSTRG.579.1	coding	0.108	3276	3342	6774
MSTRG.580.1	coding	0.087	3051	3486	3642
MSTRG.581.1	noncoding	-0.049152	1716	1791	9689
MSTRG.582.1	noncoding	-0.0196608	6420	6582	7226
MSTRG.583.1	noncoding	-0.0991232	414	444	792
MSTRG.584.1	noncoding	-0.06144	1179	1221	1597
MSTRG.546.1	coding	0.387	114	813	1478
MSTRG.546.2	coding	0.334	0	849	1548
MSTRG.547.1	coding	0.281	264	1257	1414
MSTRG.560.4	coding	0.147	693	1893	3605
MSTRG.560.1	coding	0.23	0	837	3265
MSTRG.560.6	coding	0.147	474	1674	1888
MSTRG.561.1	noncoding	-0.08192	1482	1509	2009
MSTRG.562.1	noncoding	-0.073728	489	519	2221
MSTRG.564.1	noncoding	-0.0045056	375	432	827
MSTRG.601.2	coding	0.123	678	1482	1650
MSTRG.615.6	coding	0.13	282	1161	1420
MSTRG.615.4	coding	0.147	243	1284	1543
MSTRG.615.5	coding	0.153	153	1284	1544
MSTRG.616.1	noncoding	-0.04096	204	210	1868
MSTRG.617.1	noncoding	-0.016384	0	36	2030
MSTRG.622.2	coding	0.036	513	930	6793

MSTRG.622.3	coding	0.036	513	930	5259
MSTRG.622.4	coding	0.037	603	966	6825
MSTRG.623.1	noncoding	-0.1077248	201	222	1389
MSTRG.624.2	coding	0.272	174	999	3651
MSTRG.624.3	coding	0.28	0	864	3503
MSTRG.625.1	noncoding	-0.0077824	561	825	1091
MSTRG.627.1	coding	0.084	657	930	2349
MSTRG.628.1	coding	0.229	132	1371	2622
MSTRG.628.2	coding	0.231	78	1296	2721
MSTRG.628.3	coding	0.229	162	1401	1946
MSTRG.628.6	coding	0.231	798	2016	3441
MSTRG.628.5	coding	0.231	30	1248	1994
MSTRG.639.1	noncoding	-0.0339968	1755	1947	4455
MSTRG.639.2	noncoding	-0.0159744	1953	2103	12639
MSTRG.645.1	noncoding	-0.0643072	936	981	2423
MSTRG.646.1	coding	0.041	5811	7812	9004
MSTRG.646.3	coding	0.03	3	915	3301
MSTRG.646.4	noncoding	-0.0667648	0	507	4504
MSTRG.633.4	coding	0.14	1575	1854	2317
MSTRG.633.5	coding	0.115	1386	1749	2214
MSTRG.633.6	coding	0.115	1416	1779	2171
MSTRG.633.7	coding	0.14	1515	1794	2186
MSTRG.635.1	noncoding	-0.057344	471	537	810
MSTRG.635.2	noncoding	-0.073728	81	177	475
MSTRG.650.1	coding	0.112	1884	3003	8225
MSTRG.665.2	noncoding	-0.1851392	0	81	1579
MSTRG.665.3	noncoding	-0.008192	969	972	1257
MSTRG.671.6	coding	0.464	0	3564	5177
MSTRG.671.9	coding	0.446	633	2148	2301
MSTRG.671.10	coding	0.454	0	3795	3950
MSTRG.671.12	coding	0.454	0	3795	3950
MSTRG.671.15	coding	0.455	0	3759	3914
MSTRG.671.16	coding	0.455	0	3759	3914
MSTRG.671.18	coding	0.168	264	822	1357
MSTRG.672.1	coding	0.076	2952	4074	6181
MSTRG.667.3	coding	0.046	588	1284	2241
MSTRG.691.1	coding	0.422	4992	12093	13843
MSTRG.691.2	coding	0.42	4992	12120	13870
MSTRG.691.5	coding	0.442	0	4878	11375
MSTRG.691.6	coding	0.445	0	4851	12421
MSTRG.691.3	coding	0.404	0	8817	14952
MSTRG.691.7	noncoding	-0.008192	39	45	341
MSTRG.691.8	coding	0.419	1368	8661	9053
MSTRG.691.9	noncoding	-0.0782336	22089	22398	23036
MSTRG.691.10	coding	0.423	690	7698	9449
MSTRG.691.11	coding	0.419	588	7884	13172
MSTRG.691.12	noncoding	-0.0385024	1866	1896	2639
MSTRG.693.1	noncoding	-0.0401408	1065	1563	10826
MSTRG.694.1	noncoding	-0.016384	1527	1650	2144
MSTRG.691.13	coding	0.42	2391	9591	9745
MSTRG.691.14	coding	0.42	6702	13575	15325
MSTRG.691.15	coding	0.421	807	7713	9401
MSTRG.691.17	coding	0.391	23253	28689	30379
MSTRG.691.18	coding	0.097	1158	1728	15278

MSTRG.695.1	noncoding	-0.0540672	4383	4692	5304
MSTRG.691.21	noncoding	-0.1056768	1488	1749	14645
MSTRG.691.20	coding	0.368	1239	3429	5117
MSTRG.691.22	coding	0.364	1239	3663	4118
MSTRG.696.1	noncoding	-0.008192	2796	2931	6548
MSTRG.675.2	coding	0.131	930	5190	10001
MSTRG.681.5	coding	0.083	333	2637	2790
MSTRG.681.4	coding	0.078	867	3135	3288
MSTRG.684.1	coding	0.076	102	1197	1615
MSTRG.684.4	coding	0.096	0	1176	1406
MSTRG.699.1	coding	0.02	45	1704	2576
MSTRG.699.3	coding	0.022	45	1701	4028
MSTRG.699.4	coding	0.041	678	1707	2441
MSTRG.702.1	coding	0.074	24	243	3322
MSTRG.704.4	coding	0.033	2847	3306	4609
MSTRG.704.6	noncoding	-0.012288	1278	1326	2959
MSTRG.704.7	coding	0.259	0	228	13606
MSTRG.685.2	noncoding	-0.02048	6366	6459	6877
MSTRG.685.1	noncoding	-0.02048	4560	4653	5119
MSTRG.685.3	coding	0.011	708	984	4736
MSTRG.687.5	coding	0.335	399	2865	5563
MSTRG.687.6	coding	0.18	237	1248	1552
MSTRG.705.6	coding	0.392	4395	6228	7076
MSTRG.705.3	coding	0.471	0	3051	3897
MSTRG.705.4	coding	0.431	5661	9156	10002
MSTRG.705.5	coding	0.438	1299	4464	5311
MSTRG.705.1	coding	0.466	1332	4419	5266
MSTRG.705.7	coding	0.463	1260	4392	5212
MSTRG.705.8	coding	0.466	1197	4284	5104
MSTRG.706.3	coding	0.075	0	2346	5291
MSTRG.706.4	noncoding	-0.0372736	1335	1614	5449
MSTRG.707.1	noncoding	-0.02048	0	117	653
MSTRG.708.1	coding	0.262	159	1119	5127
MSTRG.708.5	coding	0.26	2037	2856	3198
MSTRG.708.4	coding	0.251	273	1350	1556
MSTRG.708.3	coding	0.26	159	978	1320
MSTRG.710.3	coding	0.184	0	636	6744
MSTRG.713.3	coding	0.053	558	1386	1801
MSTRG.714.1	coding	0.136	81	528	1915
MSTRG.714.2	coding	0.248	0	801	2194
MSTRG.715.1	coding	0.073	99	630	3601
MSTRG.717.1	noncoding	-0.0004096	0	0	516
MSTRG.718.1	noncoding	-0.086016	1113	1188	1535
MSTRG.730.7	coding	0.059	327	1395	2727
MSTRG.731.1	noncoding	-0.0311296	249	294	719
MSTRG.732.1	noncoding	-0.016384	2136	2232	6110
MSTRG.733.1	noncoding	-0.02048	891	996	1395
MSTRG.727.2	coding	0.434	207	1230	1672
MSTRG.727.3	coding	0.429	189	1200	1640
MSTRG.727.4	coding	0.434	363	1386	1826
MSTRG.728.1	noncoding	-0.0872448	0	699	2248
MSTRG.728.2	noncoding	-0.0876544	6	690	1835
MSTRG.728.3	noncoding	-0.1048576	0	555	1645
MSTRG.735.1	noncoding	-0.0106496	606	627	1746

MSTRG.739.1	coding	0.095	3549	3798	4526
MSTRG.740.1	noncoding	-0.0106496	3186	3348	3842
MSTRG.741.1	noncoding	-0.0057344	741	939	3971
MSTRG.743.2	coding	0.165	228	882	1318
MSTRG.752.5	coding	0.082	249	561	972
MSTRG.752.6	coding	0.082	312	624	918
MSTRG.755.1	noncoding	-0.024576	0	66	518
MSTRG.756.1	noncoding	-0.028672	2784	2910	3083
MSTRG.758.1	noncoding	-0.0372736	4614	4764	5125
MSTRG.774.1	coding	0.082	3072	3285	7359
MSTRG.776.1	noncoding	-0.036864	5757	5859	6943
MSTRG.777.1	noncoding	-0.116736	783	810	2218
MSTRG.770.1	coding	0.127	147	399	552
MSTRG.771.1	noncoding	-0.2777088	192	198	437
MSTRG.771.2	noncoding	-0.069632	33	39	228
MSTRG.778.1	noncoding	-0.02048	75	204	363
MSTRG.782.2	coding	0.183	636	1965	18584
MSTRG.782.3	coding	0.187	237	1980	17253
MSTRG.782.4	coding	0.183	627	1956	2281
MSTRG.782.5	coding	0.187	207	1950	3474
MSTRG.784.1	coding	0.102	0	1350	1690
MSTRG.784.2	coding	0.158	0	756	2254
MSTRG.786.1	coding	0.26	39	726	2289
MSTRG.794.2	noncoding	-0.0176128	165	276	535
MSTRG.803.3	coding	0.249	405	1458	1988
MSTRG.804.1	noncoding	-0.0913408	525	534	1257
MSTRG.804.2	noncoding	-0.0004096	0	0	404
MSTRG.805.1	coding	0.17	0	120	2354
MSTRG.806.1	coding	0.297	2619	3126	3284
MSTRG.806.4	coding	0.297	1386	1893	2048
MSTRG.801.1	noncoding	-0.0299008	291	420	800
MSTRG.813.2	coding	0.036	0	1824	6871
MSTRG.817.1	coding	0.248	321	2007	5449
MSTRG.831.2	coding	0.081	1053	5328	5847
MSTRG.831.4	coding	0.092	258	3627	3823
MSTRG.833.1	noncoding	-0.057344	15	24	236
MSTRG.835.1	coding	0.285	0	597	4143
MSTRG.836.2	coding	0.372	1386	2886	3156
MSTRG.837.4	coding	0.345	633	4845	5318
MSTRG.839.2	coding	0.221	228	882	1037
MSTRG.840.3	coding	0.267	0	1209	1451
MSTRG.840.2	coding	0.349	0	906	2211
MSTRG.841.3	coding	0.302	54	3999	4153
MSTRG.841.2	coding	0.302	381	4326	4479
MSTRG.854.3	coding	0.288	417	2724	4121
MSTRG.855.1	noncoding	-0.118784	4395	4911	5141
MSTRG.855.2	noncoding	-0.012288	5130	5271	5732
MSTRG.848.3	coding	0.039	369	1368	6790
MSTRG.848.5	coding	0.039	2610	3609	4597
MSTRG.848.6	coding	0.039	459	1458	2444
MSTRG.848.8	coding	0.039	489	1488	2476
MSTRG.859.4	coding	0.197	0	468	845
MSTRG.859.6	noncoding	-0.016384	732	822	1525
MSTRG.862.2	coding	0.054	1440	2433	6923

MSTRG.862.5	noncoding	-0.1867776	333	363	1214
MSTRG.862.7	noncoding	-0.176128	354	399	1333
MSTRG.862.6	coding	0.054	1350	2343	4094
MSTRG.862.3	coding	0.054	1440	2433	4270
MSTRG.862.8	noncoding	-0.0462848	6	198	1114
MSTRG.874.1	noncoding	-0.0667648	774	831	2596
MSTRG.892.3	coding	0.261	621	2586	7443
MSTRG.892.2	coding	0.261	432	2397	7254
MSTRG.892.1	coding	0.259	237	2865	7720
MSTRG.897.2	noncoding	-0.0024576	954	1056	5388
MSTRG.914.4	coding	0.372	591	1812	2234
MSTRG.914.3	coding	0.45	0	1626	6733
MSTRG.914.5	coding	0.39	609	1641	2062
MSTRG.915.2	coding	0.449	0	2394	2892
MSTRG.915.3	coding	0.457	135	2412	2908
MSTRG.908.1	noncoding	-0.0004096	0	0	232
MSTRG.920.1	noncoding	-0.0008192	5247	5364	6148
MSTRG.921.1	noncoding	-0.0847872	0	75	591
MSTRG.945.1	coding	0.455	0	357	629
MSTRG.934.4	coding	0.156	216	1026	1661
MSTRG.951.3	coding	0.048	1371	2379	4108
MSTRG.952.1	noncoding	-0.0004096	0	0	342
MSTRG.956.4	coding	0.173	0	1245	2429
MSTRG.957.1	noncoding	-0.0643072	15681	15876	16602
MSTRG.959.1	noncoding	-0.016384	162	249	834
MSTRG.965.1	noncoding	-0.012288	537	678	926
MSTRG.965.2	noncoding	-0.167936	546	567	3161
MSTRG.965.3	noncoding	-0.0151552	336	384	538
MSTRG.979.3	coding	0.115	147	3285	11959
MSTRG.979.7	coding	0.122	351	786	2961
MSTRG.980.3	noncoding	-0.057344	0	249	4964
MSTRG.981.1	noncoding	-0.032768	7713	7815	9865
MSTRG.980.5	noncoding	-0.02048	19965	20079	25559
MSTRG.980.6	noncoding	-0.02048	13440	13554	19375
MSTRG.980.7	coding	0.277	3411	7236	9626
MSTRG.985.1	noncoding	-0.0053248	462	672	1485
MSTRG.967.2	coding	0.148	267	660	3871
MSTRG.972.2	noncoding	-0.0106496	180	366	1216
MSTRG.974.1	coding	0.389	105	1818	2327
MSTRG.975.1	noncoding	-0.0311296	2355	2571	2726
MSTRG.991.1	noncoding	-0.057344	0	90	873
MSTRG.991.2	noncoding	-0.057344	0	90	923
MSTRG.991.3	noncoding	-0.057344	0	90	656
MSTRG.994.1	coding	0.269	270	1743	1949
MSTRG.994.2	coding	0.269	774	2247	2431
MSTRG.994.3	coding	0.269	288	1761	1925
MSTRG.997.1	coding	0.027	45	1776	6877
MSTRG.1000.1	coding	0.121	324	3417	3712
MSTRG.1000.2	coding	0.115	582	3630	3927
MSTRG.1005.5	noncoding	-0.2732032	0	150	3684
MSTRG.1005.6	coding	0.379	843	2559	4311
MSTRG.1002.3	noncoding	-0.0073728	432	591	1227
MSTRG.1002.4	noncoding	-0.0073728	393	552	1188
MSTRG.1008.4	coding	0.158	456	897	1081

MSTRG.1013.4	coding	0.03	528	2376	2651
MSTRG.1013.7	coding	0.03	585	2436	2691
MSTRG.1013.8	coding	0.03	585	2433	2687
MSTRG.1013.9	coding	0.063	1089	1740	2613
MSTRG.1012.2	noncoding	-0.114688	1830	1878	4015
MSTRG.1012.1	noncoding	-0.114688	1830	1878	2210
MSTRG.1029.1	coding	0.036	924	2088	2592
MSTRG.1029.3	coding	0.048	42	1239	2629
MSTRG.1031.1	coding	0.338	72	1038	7550
MSTRG.1031.2	coding	0.338	777	1743	7277
MSTRG.1031.3	coding	0.343	777	1242	2575
MSTRG.1041.2	noncoding	-0.0651264	3357	3699	4311
MSTRG.1044.3	coding	0.006	1836	2844	3261
MSTRG.1036.3	coding	0.213	0	1677	3272
MSTRG.1037.1	coding	0.159	918	1443	1931
MSTRG.1060.2	coding	0.099	273	792	1073
MSTRG.1063.1	coding	0.207	186	783	1393
MSTRG.1063.2	coding	0.2	0	591	1201
MSTRG.1066.1	coding	0.143	441	987	1454
MSTRG.1066.2	coding	0.143	441	987	1498
MSTRG.1066.3	coding	0.122	75	456	962
MSTRG.1065.1	noncoding	-0.1732608	0	15	227
MSTRG.1070.1	noncoding	-0.02048	279	318	473
MSTRG.1085.2	coding	0.273	0	453	4713
MSTRG.1086.2	coding	0.121	282	1194	2682
MSTRG.1096.1	coding	0.069	147	2286	3021
MSTRG.1097.1	noncoding	-0.049152	543	585	2344
MSTRG.1096.2	coding	0.057	0	1962	8562
MSTRG.1108.1	noncoding	-0.0380928	0	45	275
MSTRG.1119.1	noncoding	-0.0004096	4017	4179	7339
MSTRG.1132.1	coding	0.155	5760	6033	13698
MSTRG.1125.1	coding	0.097	1605	2283	4084
MSTRG.1141.1	noncoding	-0.02048	549	666	1539
MSTRG.1149.1	noncoding	-0.0442368	294	342	2740
MSTRG.1150.1	noncoding	-0.008192	54	177	457
MSTRG.1157.6	coding	0.139	1569	2340	5509
MSTRG.1157.7	coding	0.121	19953	20805	22857
MSTRG.1157.8	coding	0.108	1368	2166	3188
MSTRG.1157.10	coding	0.103	1089	1971	3019
MSTRG.1157.9	coding	0.125	1089	1980	3003
MSTRG.1158.1	noncoding	-0.06144	1503	1536	2676
MSTRG.1158.2	noncoding	-0.06144	1491	1524	3976
MSTRG.1159.1	noncoding	-0.014336	6735	6849	8625
MSTRG.1173.1	noncoding	-0.02048	1716	1839	3213
MSTRG.1174.1	noncoding	-0.0004096	2358	2499	3382
MSTRG.1175.1	noncoding	-0.0483328	9735	9948	21537
MSTRG.1161.2	coding	0.319	0	1071	2093
MSTRG.1161.3	noncoding	-0.0569344	879	1188	2927
MSTRG.1164.2	noncoding	-0.16384	681	693	1275
MSTRG.1167.1	noncoding	-0.114688	72	129	285
MSTRG.1184.1	noncoding	-0.012288	573	663	1977
MSTRG.1189.2	noncoding	-0.1335296	21	633	815
MSTRG.1190.1	noncoding	-0.0110592	330	366	677
MSTRG.1194.2	coding	0.087	696	801	1625

MSTRG.1196.2	coding	0.06	480	1380	2186
MSTRG.1207.1	noncoding	-0.831488	144	147	304
MSTRG.1209.1	noncoding	-0.1007616	0	3	201
MSTRG.1214.2	noncoding	-0.008192	786	855	1041
MSTRG.1215.1	noncoding	-0.0012288	393	564	1241
MSTRG.1211.1	noncoding	-0.0479232	57	204	360
MSTRG.1236.4	coding	0.191	2655	3309	5657
MSTRG.1245.2	coding	0.026	444	2082	2239
MSTRG.1250.2	noncoding	-0.0335872	1524	1707	2835
MSTRG.1251.1	noncoding	-0.0344064	369	1002	1162
MSTRG.1252.4	noncoding	-0.008192	312	456	938
MSTRG.1253.1	noncoding	-0.077824	84	147	411
MSTRG.1259.2	coding	0.366	0	189	1089
MSTRG.1260.1	noncoding	-0.045056	1200	1293	1804
MSTRG.1261.2	noncoding	-0.012288	696	831	1242
MSTRG.1261.5	coding	0.176	384	1296	1450
MSTRG.1261.6	coding	0.1	855	1386	1541
MSTRG.1254.1	noncoding	-0.073728	405	438	726
MSTRG.1263.1	coding	0.059	0	2424	5495
MSTRG.1267.4	coding	0.324	1923	3279	4855
MSTRG.1269.1	noncoding	-0.0282624	30	321	478
MSTRG.1272.1	coding	0.598	0	123	5637
MSTRG.1272.4	coding	0.598	0	123	5009
MSTRG.1272.6	coding	0.598	0	123	3588
MSTRG.1272.7	coding	0.598	0	123	5724
MSTRG.1278.2	coding	0.522	0	381	2526
MSTRG.1278.3	coding	0.453	0	198	5293
MSTRG.1279.2	coding	0.17	2733	3984	4337
MSTRG.1288.3	coding	0.062	363	3435	3937
MSTRG.1288.4	coding	0.153	2364	3021	3521
MSTRG.1288.5	coding	0.135	1716	2307	2809
MSTRG.1285.1	noncoding	-0.016384	201	396	720
MSTRG.1309.1	noncoding	-0.18432	774	840	2948
MSTRG.1310.2	noncoding	-0.02048	783	891	1897
MSTRG.1310.1	noncoding	-0.135168	249	291	879
MSTRG.1310.3	noncoding	-0.08192	117	132	286
MSTRG.1311.1	coding	0.109	99	852	10779
MSTRG.1311.7	coding	0.11	1719	2472	3115
MSTRG.1311.5	coding	0.114	1521	2283	2926
MSTRG.1311.6	coding	0.104	1665	2412	3055
MSTRG.1311.8	coding	0.11	5247	5961	6596
MSTRG.1311.9	coding	0.103	1788	2475	3104
MSTRG.1311.10	coding	0.102	195	1245	1873
MSTRG.1309.2	noncoding	-0.18432	1011	1077	2817
MSTRG.1312.1	noncoding	-0.192512	1539	1623	1779
MSTRG.1314.2	coding	0.022	126	882	1478
MSTRG.1317.1	noncoding	-0.049152	1569	1653	1912
MSTRG.1318.2	coding	0.058	1137	2523	2861
MSTRG.1318.3	coding	0.059	354	2451	3458
MSTRG.1319.1	noncoding	-0.0004096	1101	1128	2063
MSTRG.1320.1	noncoding	-0.004096	1845	1965	3196
MSTRG.1321.1	noncoding	-0.0208896	654	798	1464
MSTRG.1323.1	noncoding	-0.0090112	3039	3192	10102
MSTRG.1318.4	noncoding	-0.0008192	4455	4602	4913

MSTRG.1325.1	coding	0.329	0	1554	5485
MSTRG.1332.2	noncoding	-0.0843776	111	831	10108
MSTRG.1338.1	coding	0.146	0	2343	3433
MSTRG.1338.2	coding	0.143	0	2187	3005
MSTRG.1338.4	coding	0.14	369	2424	3063
MSTRG.1338.3	coding	0.151	0	2298	2937
MSTRG.1338.7	coding	0.151	213	1233	2533
MSTRG.1338.8	coding	0.181	603	1326	2338
MSTRG.1339.1	noncoding	-0.0651264	873	1140	1295
MSTRG.1344.2	coding	0.423	138	714	3722
MSTRG.1346.1	coding	0.236	0	780	4771
MSTRG.1351.5	coding	0.08	885	4152	5113
MSTRG.1351.6	coding	0.08	1056	4323	5392
MSTRG.1352.6	coding	0.259	18	762	2937
MSTRG.1352.2	noncoding	-0.024576	1932	2127	2768
MSTRG.1352.4	noncoding	-0.024576	1983	2178	2552
MSTRG.1352.9	noncoding	-0.0700416	2346	2544	2699
MSTRG.1352.11	noncoding	-0.0294912	477	795	1166
MSTRG.1352.12	noncoding	-0.0700416	837	1035	1188
MSTRG.1352.10	noncoding	-0.024576	426	621	939
MSTRG.1352.13	noncoding	-0.0700416	870	1068	1222
MSTRG.1352.14	noncoding	-0.0258048	405	891	1044
MSTRG.1352.15	coding	0.308	0	636	1326
MSTRG.1355.4	coding	0.224	342	2031	2229
MSTRG.1363.1	noncoding	-0.0544768	0	309	1382
MSTRG.1367.2	noncoding	-0.016384	5745	5847	6001
MSTRG.1367.3	noncoding	-0.03072	3690	3852	4006
MSTRG.1380.9	coding	0.086	1290	1464	2339
MSTRG.1380.7	coding	0.064	0	2391	3406
MSTRG.1380.5	coding	0.069	0	2838	3330
MSTRG.1380.8	coding	0.065	654	2352	3368
MSTRG.1380.10	coding	0.015	162	447	2202
MSTRG.1380.11	noncoding	-0.1019904	0	528	1353
MSTRG.1382.1	noncoding	-0.06144	285	366	1423
MSTRG.1384.4	coding	0.09	2061	3963	5851
MSTRG.1384.3	coding	0.053	2859	8340	10228
MSTRG.1384.5	noncoding	-0.014336	1872	1914	2261
MSTRG.1387.1	coding	0.207	0	1152	1400
MSTRG.1391.1	coding	0.233	666	1374	2349
MSTRG.1395.10	coding	0.113	792	2661	2814
MSTRG.1395.11	coding	0.113	384	2253	2407
MSTRG.1395.12	coding	0.113	321	2190	2343
MSTRG.1395.13	coding	0.111	477	2493	2648
MSTRG.1395.14	coding	0.113	225	2094	2249
MSTRG.1395.15	coding	0.113	477	2346	2501
MSTRG.1395.17	coding	0.132	321	1458	2380
MSTRG.1395.18	coding	0.111	315	2331	2484
MSTRG.1395.19	coding	0.114	285	2298	2452
MSTRG.1395.20	coding	0.132	477	1614	2538
MSTRG.1395.21	coding	0.118	372	2220	2375
MSTRG.1395.22	coding	0.118	303	2151	2306
MSTRG.1395.23	coding	0.114	291	2304	2458
MSTRG.1395.24	coding	0.116	303	2298	2453
MSTRG.1396.1	noncoding	-0.0319488	72	273	3276

MSTRG.1396.2	noncoding	-0.0319488	12	213	2871
MSTRG.1394.1	noncoding	-0.17408	54	60	290
MSTRG.1398.1	coding	0.313	0	1530	2247
MSTRG.1398.2	coding	0.231	417	1335	1881
MSTRG.1407.1	noncoding	-0.0004096	78	204	581
MSTRG.1408.1	coding	0.498	78	1110	1263
MSTRG.1414.3	coding	0.317	0	282	1118
MSTRG.1415.1	coding	0.268	480	2274	2427
MSTRG.1415.3	coding	0.313	432	1965	2959
MSTRG.1416.2	coding	0.417	459	1596	1785
MSTRG.1419.4	noncoding	-0.0749568	1860	2109	2481
MSTRG.1419.3	coding	0.174	0	1242	1761
MSTRG.1420.1	noncoding	-0.0454656	57	243	1157
MSTRG.1421.1	coding	0.2	240	1434	2498
MSTRG.1426.2	coding	0.457	0	2409	2591
MSTRG.1432.3	noncoding	-0.098304	39	303	766
MSTRG.1432.2	coding	0.268	231	540	1002
MSTRG.1433.1	coding	0.347	0	426	2566
MSTRG.1433.5	coding	0.283	2313	4281	6677
MSTRG.1429.2	coding	0.43	243	1077	6349
MSTRG.1429.3	coding	0.443	699	1557	6732
MSTRG.1428.1	coding	0.505	0	309	573
MSTRG.1436.1	coding	0.415	0	1260	8252
MSTRG.1436.4	noncoding	-0.1212416	0	963	5668
MSTRG.1436.2	coding	0.415	0	1260	8206
MSTRG.1436.3	coding	0.391	3678	4917	11908
MSTRG.1437.1	noncoding	-0.0413696	1059	1428	2673
MSTRG.1438.1	noncoding	-0.0446464	1449	2739	8281
MSTRG.1436.7	coding	0.351	354	1782	5744
MSTRG.1436.6	coding	0.351	1209	2637	8608
MSTRG.1439.2	coding	0.421	9	3150	4070
MSTRG.1440.1	noncoding	-0.0610304	0	411	894
MSTRG.1441.1	coding	0.353	102	912	1392
MSTRG.1447.1	noncoding	-0.0282624	1125	1674	2899
MSTRG.1449.1	coding	0.435	216	1923	2207
MSTRG.1450.1	coding	0.389	0	1122	2879
MSTRG.1451.2	noncoding	-0.0565248	0	753	906
MSTRG.1451.3	noncoding	-0.073728	0	534	953
MSTRG.1454.5	coding	0.371	972	1455	2016
MSTRG.1455.2	noncoding	-0.0610304	33	726	1196
MSTRG.1455.1	noncoding	-0.0843776	132	684	992
MSTRG.1460.1	noncoding	-0.0237568	348	744	1037
MSTRG.1463.2	coding	0.371	93	1347	1501
MSTRG.1464.1	noncoding	-0.106496	1824	2370	2525
MSTRG.1465.2	coding	0.424	465	1188	2144
MSTRG.1467.2	coding	0.372	0	3993	4285
MSTRG.1469.1	coding	0.475	51	1788	2465
MSTRG.1470.1	noncoding	-0.0933888	0	345	1880
MSTRG.1475.3	noncoding	-0.0475136	0	690	4992
MSTRG.1475.2	coding	0.383	195	1413	1949
MSTRG.1475.4	coding	0.383	231	1449	1986
MSTRG.1475.5	coding	0.383	312	1530	2068
MSTRG.1475.6	coding	0.393	231	1374	1911
MSTRG.1477.1	noncoding	-0.0299008	7812	7989	10235

MSTRG.1473.1	coding	0.515	15	3165	4010
MSTRG.1473.2	coding	0.522	0	3093	3939
MSTRG.1494.2	coding	0.539	99	783	940
MSTRG.1495.1	noncoding	-0.0323584	0	771	927
MSTRG.1496.1	coding	0.279	849	6138	6383
MSTRG.1497.1	coding	0.351	0	2112	4239
MSTRG.1497.2	coding	0.349	0	2109	2352
MSTRG.1499.1	coding	0.302	9	909	4282
MSTRG.1500.3	coding	0.276	363	1380	1648
MSTRG.1501.5	coding	0.429	102	9360	9914
MSTRG.1501.1	coding	0.424	102	9414	9970
MSTRG.1501.2	coding	0.429	102	9252	9811
MSTRG.1501.3	coding	0.428	102	9234	9793
MSTRG.1501.4	coding	0.425	102	9432	9991
MSTRG.1501.6	coding	0.425	102	9522	10081
MSTRG.1501.9	coding	0.429	102	9255	9808
MSTRG.1501.10	coding	0.425	87	9420	9972
MSTRG.1501.11	coding	0.424	87	9396	9948
MSTRG.1501.14	coding	0.454	3423	5778	6479
MSTRG.1481.1	noncoding	-0.036864	0	60	667
MSTRG.1483.2	coding	0.295	0	780	2973
MSTRG.1484.1	noncoding	-0.0794624	66	381	1225
MSTRG.1512.6	coding	0.121	402	1605	1759
MSTRG.1512.5	noncoding	-0.0700416	984	1329	1484
MSTRG.1512.7	noncoding	-0.0507904	34794	35139	35438
MSTRG.1512.10	coding	0.117	6135	6537	12550
MSTRG.1512.8	noncoding	-0.1093632	29484	29832	49985
MSTRG.1512.9	noncoding	-0.0401408	12297	12663	12905
MSTRG.1512.11	noncoding	-0.055296	12315	12525	33095
MSTRG.1512.12	noncoding	-0.1093632	3096	3444	16815
MSTRG.1512.13	noncoding	-0.0323584	1308	1836	20386
MSTRG.1512.14	noncoding	-0.1093632	29484	29832	48143
MSTRG.1512.15	noncoding	-0.1093632	17298	17646	31165
MSTRG.1512.16	noncoding	-0.0417792	7680	7908	9064
MSTRG.1512.17	noncoding	-0.114688	1905	1980	3323
MSTRG.1512.18	noncoding	-0.1093632	1599	1947	6011
MSTRG.1512.19	coding	0.07	2772	2892	9369
MSTRG.1514.1	coding	0.296	858	1287	1464
MSTRG.1509.3	coding	0.366	315	7053	7455
MSTRG.1511.2	coding	0.283	0	1500	3101
MSTRG.1511.3	coding	0.265	0	1614	6025
MSTRG.1511.5	coding	0.271	810	2220	15714
MSTRG.1516.1	noncoding	-0.0028672	78	405	675
MSTRG.1518.1	noncoding	-0.002048	600	702	1247
MSTRG.1519.1	noncoding	-0.1097728	414	636	790
MSTRG.1520.2	coding	0.219	195	1158	4414
MSTRG.1535.1	noncoding	-0.0483328	3027	3225	3533
MSTRG.1536.1	noncoding	-0.057344	93	165	469
MSTRG.1537.4	coding	0.02	285	411	626
MSTRG.1538.1	noncoding	-0.1753088	0	78	4020
MSTRG.1538.7	coding	0.097	4203	8319	8474
MSTRG.1538.8	coding	0.032	366	900	1819
MSTRG.1538.9	coding	0.131	2595	4089	5089
MSTRG.1538.10	coding	0.104	453	3489	4773

MSTRG.1538.12	coding	0.111	4626	5763	6762
MSTRG.1538.13	coding	0.111	4743	5982	6981
MSTRG.1526.2	coding	0.401	0	951	2749
MSTRG.1531.6	noncoding	-0.045056	324	426	2134
MSTRG.1531.5	noncoding	-0.0188416	8391	8568	9026
MSTRG.1531.4	coding	0.133	0	2205	5035
MSTRG.1531.7	coding	0.119	12624	14808	17349
MSTRG.1532.1	noncoding	-0.004096	960	1149	1785
MSTRG.1533.1	noncoding	-0.06144	492	591	2264
MSTRG.1531.8	coding	0.122	282	2397	5227
MSTRG.1531.9	coding	0.124	282	2424	5254
MSTRG.1534.1	coding	0.069	2610	3027	4151
MSTRG.1531.10	coding	0.125	2331	4473	7302
MSTRG.1528.1	noncoding	-0.0397312	1098	1989	2444
MSTRG.1528.3	noncoding	-0.0290816	2034	2913	3067
MSTRG.1530.1	noncoding	-0.0466944	156	966	6984
MSTRG.1544.1	coding	0.336	231	1629	3160
MSTRG.1544.2	coding	0.297	0	1719	2859
MSTRG.1545.1	noncoding	-0.0004096	0	0	224
MSTRG.1549.1	noncoding	-0.0016384	285	417	778
MSTRG.1550.5	coding	0.165	0	2778	2949
MSTRG.1550.6	coding	0.165	0	3576	4408
MSTRG.1550.7	coding	0.233	1500	1989	2473
MSTRG.1554.2	coding	0.653	0	1269	2473
MSTRG.1555.1	noncoding	-0.024576	1065	1170	1969
MSTRG.1556.1	noncoding	-0.0401408	1152	1527	5429
MSTRG.1562.3	coding	0.068	567	933	1100
MSTRG.1562.4	coding	0.248	1707	2034	2307
MSTRG.1562.5	coding	0.13	600	891	1068
MSTRG.1563.1	coding	0.368	156	789	1491
MSTRG.1563.3	coding	0.36	138	714	4021
MSTRG.1558.2	noncoding	-0.0503808	231	1413	1568
MSTRG.1560.1	noncoding	-0.036864	1626	2025	2208
MSTRG.1576.2	coding	0.269	114	1653	2053
MSTRG.1578.4	coding	0.124	0	2133	4764
MSTRG.1578.7	coding	0.129	468	3555	5253
MSTRG.1578.9	noncoding	-0.0172032	807	924	2431
MSTRG.1578.11	noncoding	-0.04096	7854	8106	9741
MSTRG.1578.10	noncoding	-0.04096	3930	4182	5818
MSTRG.1578.12	coding	0.065	3705	4773	11419
MSTRG.1578.13	coding	0.01	10881	11028	15302
MSTRG.1578.14	noncoding	-0.0602112	5856	6156	6310
MSTRG.1567.3	coding	0.157	792	1545	2045
MSTRG.1567.4	coding	0.157	717	1470	1952
MSTRG.1567.5	coding	0.124	408	1140	1662
MSTRG.1567.7	noncoding	-0.028672	3	114	2342
MSTRG.1568.2	coding	0.33	123	1335	2314
MSTRG.1568.4	coding	0.335	66	1308	2289
MSTRG.1568.6	coding	0.4	0	1638	2618
MSTRG.1568.5	coding	0.391	0	1740	2719
MSTRG.1573.1	noncoding	-0.053248	3780	4023	4177
MSTRG.1574.2	coding	0.085	0	372	1227
MSTRG.1574.3	coding	0.085	0	372	2708
MSTRG.1589.1	noncoding	-0.0782336	0	480	693

MSTRG.1590.1	noncoding	-0.0319488	72	711	1556
MSTRG.1592.2	coding	0.33	549	1626	5648
MSTRG.1592.1	coding	0.298	3015	4431	8452
MSTRG.1592.4	coding	0.095	552	825	2086
MSTRG.1592.5	coding	0.129	702	1338	2898
MSTRG.1592.6	coding	0.129	378	1014	1956
MSTRG.1593.1	noncoding	-0.012288	0	210	698
MSTRG.1592.7	noncoding	-0.065536	111	294	2751
MSTRG.1592.8	noncoding	-0.0294912	60	192	1994
MSTRG.1594.1	coding	0.067	585	1365	1661
MSTRG.1595.1	noncoding	-0.0237568	1815	1920	2133
MSTRG.1596.1	noncoding	-0.0004096	210	450	3306
MSTRG.1597.1	noncoding	-0.159744	726	765	942
MSTRG.1598.1	noncoding	-0.0462848	0	72	3230
MSTRG.1596.2	coding	0.089	7650	7857	8377
MSTRG.1583.1	noncoding	-0.0004096	0	0	265
MSTRG.1583.2	noncoding	-0.2031616	0	27	360
MSTRG.1585.1	noncoding	-0.0315392	0	165	515
MSTRG.1600.1	noncoding	-0.1155072	0	69	365
MSTRG.1603.1	coding	0.32	60	1056	1210
MSTRG.1604.1	noncoding	-0.0004096	0	0	345
MSTRG.1606.1	noncoding	-0.0380928	171	570	1398
MSTRG.1610.1	coding	0.13	180	609	990
MSTRG.1610.3	coding	0.133	1458	2304	2587
MSTRG.1610.4	coding	0.191	159	1047	1328
MSTRG.1613.3	noncoding	-0.0212992	3177	3333	6703
MSTRG.1614.1	noncoding	-0.03072	198	540	1502
MSTRG.1614.2	noncoding	-0.03072	198	540	1548
MSTRG.1614.3	coding	0.16	0	909	1194
MSTRG.1614.4	coding	0.082	870	2670	2953
MSTRG.1618.1	coding	0.314	2118	4974	8654
MSTRG.1618.2	noncoding	-0.032768	57	258	808
MSTRG.1618.4	coding	0.376	0	1527	3394
MSTRG.1618.3	coding	0.355	0	1923	3874
MSTRG.1619.1	noncoding	-0.0647168	834	1371	2077
MSTRG.1619.2	noncoding	-0.0397312	834	1407	2461
MSTRG.1623.2	coding	0.24	663	1710	2107
MSTRG.1637.4	noncoding	-0.012288	705	825	5416
MSTRG.1637.5	coding	0.262	402	1677	6652
MSTRG.1632.1	coding	0.03	1998	2550	6583
MSTRG.1632.2	coding	0.03	1101	1653	5764
MSTRG.1644.3	coding	0.201	5961	14190	14784
MSTRG.1642.3	coding	0.046	786	1395	2996
MSTRG.1640.1	noncoding	-0.036864	726	834	2030
MSTRG.1647.3	coding	0.323	0	669	1893
MSTRG.1650.1	noncoding	-0.0425984	9198	9996	10151
MSTRG.1650.2	noncoding	-0.0610304	9177	9720	10044
MSTRG.1650.3	noncoding	-0.0638976	9144	9627	9992
MSTRG.1653.1	coding	0.222	0	813	3005
MSTRG.1654.1	coding	0.216	48	720	3414
MSTRG.1654.2	coding	0.214	48	717	3411
MSTRG.1654.3	coding	0.215	243	966	3722
MSTRG.1657.1	noncoding	-0.0032768	441	567	873
MSTRG.1659.1	noncoding	-0.0770048	0	189	496

MSTRG.1662.1	coding	0.175	0	3207	13359
MSTRG.1662.4	coding	0.183	3	3090	3972
MSTRG.1662.7	coding	0.178	93	3129	4011
MSTRG.1662.9	coding	0.182	0	3063	3889
MSTRG.1662.10	coding	0.177	690	3777	4658
MSTRG.1662.12	coding	0.175	45	2967	3849
MSTRG.1662.13	coding	0.185	111	2310	3192
MSTRG.1663.1	coding	0.149	0	1224	5349
MSTRG.1663.5	coding	0.278	0	360	516
MSTRG.1663.4	noncoding	-0.090112	1341	1410	2251
MSTRG.1663.3	coding	0.201	0	147	1583
MSTRG.1665.1	noncoding	-0.02048	0	66	4198
MSTRG.1667.1	noncoding	-0.032768	75	87	3095
MSTRG.1671.1	coding	0.501	63	1128	2883
MSTRG.1673.5	coding	0.331	180	1542	5206
MSTRG.1673.4	coding	0.289	0	2676	4595
MSTRG.1673.3	coding	0.3	0	2448	6046
MSTRG.1675.1	coding	0.154	6000	6258	8938
MSTRG.1676.1	noncoding	-0.0004096	0	0	244
MSTRG.1677.1	noncoding	-0.1703936	213	222	974
MSTRG.1678.1	noncoding	-0.0028672	0	96	288
MSTRG.1681.1	coding	0.115	687	1374	1599
MSTRG.1685.2	coding	0.179	336	1047	3059
MSTRG.1685.3	coding	0.191	120	753	1709
MSTRG.1689.1	noncoding	-0.016384	0	27	322
MSTRG.1691.1	noncoding	-0.032768	1428	1458	2246
MSTRG.1692.1	noncoding	-0.0004096	339	498	696
MSTRG.1697.2	noncoding	-0.0466944	141	342	2098
MSTRG.1697.1	noncoding	-0.06144	141	234	812
MSTRG.1697.3	coding	0.154	5334	5577	6450
MSTRG.1697.4	noncoding	-0.0761856	0	162	1056
MSTRG.1709.2	coding	0.045	2400	3165	4127
MSTRG.1709.4	coding	0.045	2574	3339	4327
MSTRG.1709.6	coding	0.045	2580	3345	3986
MSTRG.1709.8	coding	0.071	1269	1548	3246
MSTRG.1716.2	coding	0.078	807	1095	1647
MSTRG.1716.1	coding	0.041	453	924	1475
MSTRG.1716.3	coding	0.041	324	795	1110
MSTRG.1717.1	coding	0.097	372	618	1816
MSTRG.1725.1	coding	0.079	498	699	4991
MSTRG.1726.1	noncoding	-0.0446464	4641	5136	5290
MSTRG.1721.1	noncoding	-0.0004096	0	0	321
MSTRG.1721.2	noncoding	-0.0954368	0	3	395
MSTRG.1729.2	noncoding	-0.0004096	255	393	1897
MSTRG.1730.1	coding	0.176	4056	4440	6301
MSTRG.1731.1	noncoding	-0.086016	1431	1509	1819
MSTRG.1734.1	noncoding	-0.0258048	2997	3084	5731
MSTRG.1734.2	noncoding	-0.0139264	105	234	1688
MSTRG.1727.1	noncoding	-0.0512	1575	1728	10124
MSTRG.1727.2	noncoding	-0.053248	567	651	894
MSTRG.1740.3	coding	0.119	297	1389	2155
MSTRG.1740.2	coding	0.127	588	1212	1977
MSTRG.1745.1	coding	0.083	16800	17361	20894
MSTRG.1745.3	coding	0.064	0	1263	20967

MSTRG.1753.1	noncoding	-0.0708608	108	120	391
MSTRG.1753.2	noncoding	-0.1306624	120	144	458
MSTRG.1768.1	noncoding	-0.028672	117	234	1135
MSTRG.1768.2	noncoding	-0.028672	552	669	1541
MSTRG.1768.3	noncoding	-0.1429504	0	60	1370
MSTRG.1770.2	coding	0.386	30	342	624
MSTRG.1772.1	noncoding	-0.0483328	3366	3786	4283
MSTRG.1772.3	noncoding	-0.038912	3225	3627	3975
MSTRG.1772.4	coding	0.072	714	936	1532
MSTRG.1773.1	noncoding	-0.059392	117	318	474
MSTRG.1774.1	noncoding	-0.0602112	30	45	227
MSTRG.1777.2	coding	0.109	120	1497	1734
MSTRG.1777.3	coding	0.09	114	1449	2956
MSTRG.1782.1	coding	0.12	468	4152	5481
MSTRG.1782.5	coding	0.095	627	3780	4910
MSTRG.1782.4	coding	0.125	0	2640	6376
MSTRG.1783.1	noncoding	-0.0475136	1686	1767	6921
MSTRG.1784.1	noncoding	-0.0483328	5226	5472	11439
MSTRG.1787.1	noncoding	-0.0004096	0	0	506
MSTRG.1787.2	noncoding	-0.0004096	0	0	499
MSTRG.1779.1	noncoding	-0.0004096	150	150	401
MSTRG.1780.1	noncoding	-0.0221184	1431	1749	1963
MSTRG.1791.2	coding	0.041	384	924	2074
MSTRG.1791.3	coding	0.544	0	264	1980
MSTRG.1798.1	noncoding	-0.012288	1275	1377	4198
MSTRG.1799.1	coding	0.051	108	615	5681
MSTRG.1799.2	noncoding	-0.0765952	3285	3720	3875
MSTRG.1799.3	coding	0.067	0	519	2052
MSTRG.1799.4	coding	0.066	219	723	1095
MSTRG.1799.7	noncoding	-0.0098304	2040	2193	2938
MSTRG.1799.6	coding	0.124	0	678	1423
MSTRG.1798.2	noncoding	-0.012288	2043	2145	4969
MSTRG.1798.3	noncoding	-0.045056	2523	2607	10417
MSTRG.1799.8	noncoding	-0.088064	0	339	4560
MSTRG.1805.1	coding	0.197	573	1182	3623
MSTRG.1805.3	coding	0.11	306	1362	2674
MSTRG.1801.1	noncoding	-0.0237568	402	459	616
MSTRG.1801.2	noncoding	-0.0237568	366	423	580
MSTRG.1801.3	noncoding	-0.372736	207	213	1731
MSTRG.1804.1	coding	0.18	165	1683	1837
MSTRG.1819.1	noncoding	-0.0741376	0	67068	68416
MSTRG.1811.3	coding	0.001	522	711	1003
MSTRG.1814.1	coding	0.144	297	2949	7597
MSTRG.1814.3	coding	0.144	375	3027	4405
MSTRG.1814.4	coding	0.144	471	3123	4501
MSTRG.1814.2	coding	0.144	555	3207	4586
MSTRG.1814.7	coding	0.144	1611	4263	5234
MSTRG.1814.8	noncoding	-3.80928	1161	1350	1507
MSTRG.1818.1	coding	0.262	0	897	4596
MSTRG.1818.3	coding	0.261	0	900	4599
MSTRG.1832.1	coding	0.039	333	546	7304
MSTRG.1832.3	coding	0.013	408	939	2076
MSTRG.1833.2	noncoding	-0.1032192	0	147	2442
MSTRG.1833.1	noncoding	-0.045056	888	990	1146

MSTRG.1833.3	noncoding	-0.0188416	1350	1503	1660
MSTRG.1834.1	noncoding	-0.014336	513	588	3092
MSTRG.1840.1	noncoding	-0.1155072	0	288	1429
MSTRG.1829.1	noncoding	-0.032768	3054	3156	3310
MSTRG.1841.5	coding	0.043	342	786	940
MSTRG.1847.1	coding	0.147	1233	1467	2215
MSTRG.1847.2	noncoding	-0.0303104	2553	2754	3565
MSTRG.1869.1	noncoding	-0.012288	3297	3411	3871
MSTRG.1868.11	noncoding	-0.028672	29379	29547	35981
MSTRG.1868.12	noncoding	-0.008192	177	309	7977
MSTRG.1868.13	noncoding	-0.0049152	2058	2127	2349
MSTRG.1873.1	noncoding	-0.0692224	918	921	1407
MSTRG.1874.1	noncoding	-0.0036864	7773	7920	34390
MSTRG.1874.2	noncoding	-0.0155648	639	882	16937
MSTRG.1849.4	coding	0.025	459	1665	2149
MSTRG.1850.1	noncoding	-0.004096	1854	2040	2928
MSTRG.1856.4	coding	0.081	1656	2106	3473
MSTRG.1857.1	coding	0.034	480	1602	6096
MSTRG.1860.1	coding	0.057	84	1071	1638
MSTRG.1860.5	noncoding	-0.0167936	6711	6864	7782
MSTRG.1860.6	noncoding	-0.0278528	0	162	3087
MSTRG.1860.3	coding	0.057	657	1644	2209
MSTRG.1860.4	coding	0.044	0	1446	2013
MSTRG.1862.1	noncoding	-0.057344	336	417	583
MSTRG.1860.7	coding	0.045	5070	5757	6323
MSTRG.1864.1	noncoding	-0.0004096	1545	1545	2720
MSTRG.1865.1	noncoding	-0.106496	1017	1110	1854
MSTRG.1866.1	noncoding	-0.0417792	339	528	9772
MSTRG.1867.2	noncoding	-0.0569344	2988	3678	3832
MSTRG.1867.3	noncoding	-0.0544768	3111	3732	3887
MSTRG.1891.2	coding	0.113	0	1377	6858
MSTRG.1895.2	coding	0.173	0	1002	5378
MSTRG.1895.3	noncoding	-0.0077824	246	396	551
MSTRG.1895.5	coding	0.138	1041	1299	1454
MSTRG.1895.4	coding	0.16	0	900	1542
MSTRG.1900.3	coding	0.052	0	1449	1825
MSTRG.1900.4	coding	0.031	357	1548	1924
MSTRG.1900.5	coding	0.059	0	1173	1481
MSTRG.1900.6	noncoding	-0.016384	0	75	1189
MSTRG.1902.4	coding	0.081	105	2025	2255
MSTRG.1906.1	noncoding	-0.024576	543	654	1615
MSTRG.1907.2	coding	0.065	465	1365	3629
MSTRG.1907.3	coding	0.083	402	1374	3639
MSTRG.1907.4	coding	0.083	432	1404	3563
MSTRG.1911.1	noncoding	-0.045056	0	180	626
MSTRG.1912.2	noncoding	-0.0438272	3093	3699	3853
MSTRG.1912.4	noncoding	-0.0360448	768	987	1143
MSTRG.1912.7	noncoding	-0.0360448	6672	6891	7046
MSTRG.1914.1	noncoding	-0.0004096	603	747	1316
MSTRG.1912.8	noncoding	-0.02048	477	504	1807
MSTRG.1912.9	coding	0.032	2841	3588	6839
MSTRG.1909.2	coding	0.084	243	1452	2261
MSTRG.1910.1	noncoding	-0.0421888	63	201	625
MSTRG.1922.1	coding	0.225	99	681	934

MSTRG.1922.4	coding	0.192	1104	1479	1635
MSTRG.1922.6	coding	0.192	1107	1482	1639
MSTRG.1922.5	coding	0.129	93	861	1018
MSTRG.1923.1	noncoding	-0.016384	87	138	346
MSTRG.1924.1	noncoding	-0.0004096	39	186	938
MSTRG.1924.2	noncoding	-0.0094208	123	228	796
MSTRG.1928.3	coding	0.144	303	3375	5115
MSTRG.1928.5	coding	0.144	222	3294	5122
MSTRG.1928.7	coding	0.176	1053	3249	4930
MSTRG.1928.8	noncoding	-0.045056	294	345	1421
MSTRG.1928.9	coding	0.21	1569	2574	3890
MSTRG.1928.10	noncoding	-0.01024	4065	4215	7864
MSTRG.1930.1	noncoding	-0.004096	4725	4860	7548
MSTRG.1928.11	noncoding	-0.0004096	255	354	4852
MSTRG.1928.12	noncoding	-0.0622592	11697	11889	17946
MSTRG.1928.13	noncoding	-0.0622592	10119	10311	10750
MSTRG.1920.4	coding	0.497	0	540	7991
MSTRG.1920.5	coding	0.497	0	540	7974
MSTRG.1920.6	noncoding	-0.008192	1746	1875	4192
MSTRG.1920.7	coding	0.497	0	540	6852
MSTRG.1921.1	noncoding	-0.0233472	309	414	3176
MSTRG.1918.3	coding	0.002	792	1326	2545
MSTRG.1940.1	coding	0.053	729	927	10100
MSTRG.1940.2	coding	0.111	1017	1656	9950
MSTRG.1940.5	coding	0.045	4476	4803	6731
MSTRG.1940.4	coding	0.107	4494	4929	6856
MSTRG.1941.1	noncoding	-0.0212992	3876	4071	8733
MSTRG.1943.1	coding	0.113	3291	3525	3704
MSTRG.1943.2	noncoding	-0.0249856	1083	1203	1891
MSTRG.1943.4	noncoding	-0.0544768	0	144	1308
MSTRG.1945.2	coding	0.131	0	585	1047
MSTRG.1948.1	noncoding	-0.0004096	0	0	1342
MSTRG.1949.1	noncoding	-0.04096	960	1035	1361
MSTRG.1951.1	coding	0.144	633	2640	3042
MSTRG.1934.1	coding	0.124	69	13989	15307
MSTRG.1953.1	noncoding	-0.0090112	2073	2292	2578
MSTRG.1954.1	noncoding	-0.0315392	3516	3846	4334
MSTRG.1995.1	noncoding	-0.094208	2457	2586	3928
MSTRG.2012.1	noncoding	-0.016384	1479	1551	3912
MSTRG.2013.1	noncoding	-0.045056	72	144	371
MSTRG.2022.1	noncoding	-0.139264	2979	3021	3387
MSTRG.2023.1	noncoding	-0.0417792	7506	7674	10824
MSTRG.2023.2	noncoding	-0.0417792	4443	4611	5156
MSTRG.2025.1	noncoding	-0.229376	2709	2736	3127
MSTRG.2028.1	noncoding	-0.008192	1602	1722	1877
MSTRG.1959.1	coding	0.145	2160	4050	4793
MSTRG.1959.3	coding	0.085	15	3801	4330
MSTRG.1960.1	noncoding	-0.0954368	2370	2553	2707
MSTRG.1962.1	noncoding	-0.0811008	0	345	921
MSTRG.1962.2	noncoding	-0.0761856	0	339	738
MSTRG.1968.3	coding	0.08	3	3237	5275
MSTRG.1968.8	coding	0.092	0	3804	4030
MSTRG.1972.2	coding	0.081	0	438	2627
MSTRG.1972.6	noncoding	-0.0323584	1572	1761	6478

MSTRG.1972.4	noncoding	-0.008192	2055	2238	2498
MSTRG.1972.3	coding	0.058	201	453	1845
MSTRG.1972.8	noncoding	-0.008192	2001	2184	2408
MSTRG.1972.10	coding	0.058	99	351	1536
MSTRG.1972.9	noncoding	-0.0323584	1572	1761	6594
MSTRG.1972.11	noncoding	-0.008192	1515	1698	1859
MSTRG.1970.1	noncoding	-0.0004096	0	0	222
MSTRG.1989.1	noncoding	-0.065536	63	96	745
MSTRG.1989.2	noncoding	-0.065536	3	36	500
MSTRG.1990.1	noncoding	-0.006144	213	261	558
MSTRG.2059.1	noncoding	-0.08192	93	123	629
MSTRG.2062.3	coding	0.025	921	1257	1854
MSTRG.2062.4	coding	0.025	4182	4518	5070
MSTRG.2062.6	coding	0.099	2901	3198	4410
MSTRG.2062.5	noncoding	-0.04096	51	387	2511
MSTRG.2063.1	noncoding	-0.077824	2046	2121	3876
MSTRG.2060.1	noncoding	-0.016384	0	48	400
MSTRG.2068.4	coding	0.066	153	1203	1525
MSTRG.2068.5	coding	0.068	144	1185	1604
MSTRG.2068.6	coding	0.073	0	1053	1471
MSTRG.2068.7	coding	0.141	0	1344	1762
MSTRG.2068.9	coding	0.144	0	1335	1753
MSTRG.2068.8	coding	0.142	0	1332	4016
MSTRG.2070.1	noncoding	-0.1052672	198	201	368
MSTRG.2071.1	coding	0.086	1059	1272	2108
MSTRG.2067.1	noncoding	-0.135168	0	24	243
MSTRG.2067.2	noncoding	-0.1753088	0	138	325
MSTRG.2066.1	noncoding	-0.0598016	0	177	647
MSTRG.2072.2	coding	0.168	0	1539	2205
MSTRG.2079.1	noncoding	-0.0229376	0	159	3616
MSTRG.2079.2	noncoding	-0.14336	345	363	706
MSTRG.2085.1	coding	0.136	54	315	1067
MSTRG.2085.2	coding	0.136	738	999	2137
MSTRG.2086.1	noncoding	-0.0004096	0	0	311
MSTRG.2088.6	coding	0.056	1803	2106	2335
MSTRG.2088.4	coding	0.056	2490	2793	3021
MSTRG.2088.5	coding	0.056	1881	2184	2412
MSTRG.2088.8	coding	0.103	3117	3363	3585
MSTRG.2083.1	noncoding	-0.0385024	6081	6264	7792
MSTRG.2095.1	coding	0.044	912	1893	5285
MSTRG.2095.2	coding	0.209	0	141	1006
MSTRG.2098.1	noncoding	-0.0004096	390	513	2846
MSTRG.2097.6	coding	0.07	1020	6705	8174
MSTRG.2097.7	coding	0.109	735	4113	5707
MSTRG.2097.8	coding	0.106	735	4188	6896
MSTRG.2110.8	coding	0.199	972	3621	4183
MSTRG.2110.1	coding	0.179	0	3246	3808
MSTRG.2110.5	coding	0.179	0	3243	3805
MSTRG.2111.1	noncoding	-0.004096	990	1131	1872
MSTRG.2110.9	coding	0.187	0	333	1546
MSTRG.2112.1	noncoding	-0.282624	162	189	751
MSTRG.2113.1	noncoding	-0.02048	540	645	1749
MSTRG.2114.1	coding	0.085	954	2124	2395
MSTRG.2116.1	noncoding	-0.024576	684	726	1198

MSTRG.2114.2	coding	0.085	945	2115	2385
MSTRG.2108.1	noncoding	-0.016384	279	414	570
MSTRG.2119.3	coding	0.196	1116	3153	4434
MSTRG.2118.1	coding	0.279	0	87	242
MSTRG.2126.1	coding	0.076	39	252	406
MSTRG.2148.1	coding	0.085	981	1140	1296
MSTRG.2152.1	noncoding	-0.018432	264	342	910
MSTRG.2154.1	noncoding	-0.1384448	0	90	2752
MSTRG.2153.6	noncoding	-0.008192	2745	2889	5155
MSTRG.2153.3	noncoding	-0.0643072	0	75	1857
MSTRG.2153.5	noncoding	-0.0643072	0	78	1836
MSTRG.2153.4	noncoding	-0.0843776	774	1413	3044
MSTRG.2153.7	coding	0.196	438	1185	2620
MSTRG.2155.1	coding	0.064	498	3183	4273
MSTRG.2155.2	coding	0.068	801	3522	4613
MSTRG.2155.3	coding	0.071	267	2607	3697
MSTRG.2155.4	coding	0.065	279	3015	4106
MSTRG.2155.7	noncoding	-0.0147456	285	573	729
MSTRG.2155.8	coding	0.22	549	789	1625
MSTRG.2155.9	coding	0.07	1227	1551	1982
MSTRG.2144.1	noncoding	-0.012288	381	516	958
MSTRG.2145.1	noncoding	-0.0004096	429	543	998
MSTRG.2146.1	noncoding	-0.0270336	9393	9624	11452
MSTRG.2146.2	noncoding	-0.0270336	18321	18552	20377
MSTRG.2175.1	coding	0.324	255	18666	18820
MSTRG.2175.2	coding	0.329	75	4605	4765
MSTRG.2177.2	coding	0.051	3129	6474	8891
MSTRG.2177.1	coding	0.051	3165	6510	9062
MSTRG.2177.6	coding	0.051	3126	6471	11614
MSTRG.2177.4	coding	0.051	3165	6510	10896
MSTRG.2177.7	coding	0.051	3123	6468	15564
MSTRG.2177.8	coding	0.051	3138	6483	8421
MSTRG.2177.10	coding	0.051	3498	6843	11306
MSTRG.2177.12	coding	0.051	1971	5316	9858
MSTRG.2177.11	coding	0.051	3066	6411	10874
MSTRG.2157.1	coding	0.158	210	1200	1520
MSTRG.2166.1	noncoding	-0.016384	432	504	752
MSTRG.2167.1	noncoding	-0.028672	123	141	385
MSTRG.2179.2	coding	0.46	78	2223	6115
MSTRG.2179.3	coding	0.46	54	2199	2781
MSTRG.2179.4	coding	0.514	768	1965	5771
MSTRG.2200.2	coding	0.095	4248	6162	6842
MSTRG.2200.3	coding	0.095	3891	5805	6484
MSTRG.2200.5	coding	0.081	4140	6123	6527
MSTRG.2201.1	noncoding	-0.016384	1722	1848	2333
MSTRG.2202.1	noncoding	-0.077824	657	744	1955
MSTRG.2203.1	coding	0.066	2577	2775	3579
MSTRG.2210.3	noncoding	-0.012288	3747	3831	6201
MSTRG.2211.1	noncoding	-0.0008192	1056	1116	4060
MSTRG.2210.4	noncoding	-0.0212992	3456	3537	8054
MSTRG.2212.1	coding	0.099	5526	6267	6762
MSTRG.2213.3	coding	0.096	2139	2361	3989
MSTRG.2196.1	noncoding	-0.0004096	0	0	467
MSTRG.2219.1	noncoding	-0.0110592	4992	5190	9913

MSTRG.2220.2	coding	0.195	1320	4905	6033
MSTRG.2221.1	noncoding	-0.008192	17460	17592	17829
MSTRG.2221.2	noncoding	-0.090112	339	423	981
MSTRG.2222.1	noncoding	-0.0086016	10608	10728	12317
MSTRG.2222.2	noncoding	-0.0086016	5283	5403	6978
MSTRG.2220.3	coding	0.144	2490	5640	7010
MSTRG.2220.4	coding	0.101	189	2628	4190
MSTRG.2223.3	noncoding	-0.602112	612	630	787
MSTRG.2223.4	coding	0.092	3690	3960	9652
MSTRG.2223.5	noncoding	-0.008192	5829	6000	6259
MSTRG.2223.6	coding	0.092	780	1050	5490
MSTRG.2224.1	noncoding	-0.004096	546	576	2448
MSTRG.2225.1	noncoding	-0.0192512	270	486	1188
MSTRG.2230.1	noncoding	-0.0524288	333	396	774
MSTRG.2240.1	noncoding	-0.0004096	0	0	216
MSTRG.2241.1	noncoding	-0.114688	540	543	884
MSTRG.2248.2	coding	0.061	945	1380	1849
MSTRG.2248.4	coding	0.061	945	1380	3828
MSTRG.2248.6	coding	0.1	1065	1389	1641
MSTRG.2250.1	noncoding	-0.0045056	0	96	885
MSTRG.2252.1	coding	0.038	609	2370	2612
MSTRG.2257.2	coding	0.33	750	1695	2372
MSTRG.2257.3	coding	0.321	270	1380	2058
MSTRG.2265.1	noncoding	-0.0479232	3177	3489	6939
MSTRG.2265.2	coding	0.097	1557	1833	3522
MSTRG.2265.3	noncoding	-0.0479232	6072	6384	9834
MSTRG.2265.4	coding	0.097	636	912	2553
MSTRG.2265.6	noncoding	-0.0479232	2901	3213	3865
MSTRG.2265.5	coding	0.097	3834	4110	5727
MSTRG.2262.2	noncoding	-0.0946176	0	519	2777
MSTRG.2268.4	coding	0.073	423	2967	3906
MSTRG.2268.3	coding	0.067	390	3366	4306
MSTRG.2268.5	coding	0.071	1002	3498	4439
MSTRG.2269.1	noncoding	-0.08192	1701	1806	2404
MSTRG.2273.1	noncoding	-0.0016384	243	462	706
MSTRG.2278.1	noncoding	-0.114688	504	618	1907
MSTRG.2280.1	coding	0.096	0	2796	6410
MSTRG.2281.1	noncoding	-0.008192	93	234	2766
MSTRG.2282.1	noncoding	-0.053248	357	402	558
MSTRG.2283.1	noncoding	-0.036864	1206	1362	2458
MSTRG.2284.1	coding	0.069	1239	1464	3430
MSTRG.2270.4	coding	0.073	1968	2577	5619
MSTRG.2270.5	coding	0.073	2385	2994	6037
MSTRG.2288.1	coding	0.262	0	2109	7913
MSTRG.2288.3	coding	0.224	516	2457	8256
MSTRG.2289.1	noncoding	-0.0069632	3753	3885	7857
MSTRG.2285.1	noncoding	-0.04096	126	198	405
MSTRG.2311.1	noncoding	-0.0004096	0	0	453
MSTRG.2350.1	coding	0.328	81	1053	1445
MSTRG.2352.2	coding	0.157	1257	3372	3569
MSTRG.2352.3	coding	0.241	786	2028	2279
MSTRG.2352.4	coding	0.238	786	1935	2186
MSTRG.2355.1	noncoding	-0.002048	21	246	418
MSTRG.2361.1	coding	0.107	1659	3057	5682

MSTRG.2361.2	coding	0.107	1503	2901	6918
MSTRG.2361.4	coding	0.107	960	2358	7441
MSTRG.2361.5	coding	0.109	1059	2535	10589
MSTRG.2361.3	coding	0.107	960	2358	10368
MSTRG.2362.1	noncoding	-0.065536	408	471	1616
MSTRG.2364.1	coding	0.278	0	480	6197
MSTRG.2364.4	coding	0.113	0	1272	8154
MSTRG.2364.6	coding	0.147	429	1749	8973
MSTRG.2365.1	noncoding	-0.02048	693	801	2277
MSTRG.2364.7	coding	0.116	2754	4323	8748
MSTRG.2364.8	noncoding	-0.0069632	11388	11637	12542
MSTRG.2364.9	coding	0.143	372	801	1331
MSTRG.2366.1	noncoding	-0.004096	936	1134	3360
MSTRG.2364.11	noncoding	-0.016384	5400	5532	7645
MSTRG.2371.1	coding	0.395	0	5016	6802
MSTRG.2381.1	coding	0.261	366	4764	7775
MSTRG.2381.10	coding	0.262	420	4080	7089
MSTRG.2381.7	coding	0.257	111	3813	6821
MSTRG.2381.4	coding	0.258	819	4497	7506
MSTRG.2381.6	coding	0.263	1128	4764	7774
MSTRG.2381.5	coding	0.262	282	4656	7666
MSTRG.2381.2	coding	0.263	507	4878	7886
MSTRG.2381.3	coding	0.264	744	5091	8101
MSTRG.2381.9	coding	0.261	981	5379	8389
MSTRG.2381.13	coding	0.256	51	3807	6806
MSTRG.2381.12	coding	0.262	1062	4776	7775
MSTRG.2381.15	coding	0.256	750	4506	7408
MSTRG.2381.17	coding	0.259	750	4401	7402
MSTRG.2381.16	coding	0.216	312	2919	11949
MSTRG.2381.14	coding	0.264	1071	5418	8418
MSTRG.2381.18	coding	0.264	978	5325	8325
MSTRG.2381.19	coding	0.209	312	2904	3846
MSTRG.2387.1	noncoding	-0.0925696	120	129	464
MSTRG.2385.1	noncoding	-0.0221184	87	216	444
MSTRG.2391.1	noncoding	-0.0004096	0	0	209
MSTRG.2392.1	noncoding	-0.0004096	81	81	275
MSTRG.2398.5	coding	0.168	0	576	823
MSTRG.2412.1	noncoding	-0.0086016	690	843	4321
MSTRG.2412.2	coding	0.062	3777	4023	4315
MSTRG.2412.3	coding	0.038	3396	4119	7458
MSTRG.2415.1	coding	0.047	1329	1803	5963
MSTRG.2412.4	coding	0.021	2751	2904	6076
MSTRG.2417.1	noncoding	-0.0004096	1833	1974	2620
MSTRG.2417.2	noncoding	-0.008192	1770	1905	2528
MSTRG.2418.1	noncoding	-0.004096	4791	4935	10784
MSTRG.2419.2	noncoding	-0.1097728	0	624	1726
MSTRG.2419.5	noncoding	-0.1175552	450	963	1120
MSTRG.2419.6	noncoding	-0.1241088	57	549	1767
MSTRG.2419.4	noncoding	-0.1241088	57	549	2081
MSTRG.2403.1	coding	0.024	234	351	737
MSTRG.2407.1	noncoding	-0.0528384	0	12	737
MSTRG.2426.1	noncoding	-0.049152	504	510	875
MSTRG.2431.1	noncoding	-0.0421888	9	12	215
MSTRG.2433.1	noncoding	-0.008192	0	33	278

MSTRG.2438.1	noncoding	-0.0077824	48	231	871
MSTRG.2439.4	coding	0.145	477	1224	1377
MSTRG.2435.3	coding	0.001	585	1125	1312
MSTRG.2446.3	coding	0.241	975	2292	2700
MSTRG.2447.1	coding	0.066	1935	2028	3737
MSTRG.2475.4	coding	0.174	0	1644	2264
MSTRG.2475.5	coding	0.138	57	1278	1469
MSTRG.2476.1	noncoding	-0.0659456	0	597	873
MSTRG.2476.2	noncoding	-0.0004096	117	249	527
MSTRG.2477.1	coding	0.053	156	291	561
MSTRG.2479.4	coding	0.048	1809	2808	2975
MSTRG.2480.1	noncoding	-0.02048	837	975	7270
MSTRG.2479.5	coding	0.069	0	573	2012
MSTRG.2479.6	coding	0.02	27	519	1967
MSTRG.2479.7	coding	0.02	27	519	1990
MSTRG.2479.8	coding	0.02	27	519	1738
MSTRG.2479.9	noncoding	-0.0483328	1410	1980	2134
MSTRG.2479.10	coding	0.096	96	357	2669
MSTRG.2479.13	coding	0.02	21	513	1516
MSTRG.2479.14	coding	0.02	27	519	2826
MSTRG.2479.15	noncoding	-0.0483328	1812	2382	2538
MSTRG.2479.16	coding	0.02	21	513	1149
MSTRG.2479.17	coding	0.02	27	519	2256
MSTRG.2485.2	coding	0.144	195	3213	4145
MSTRG.2485.3	coding	0.147	162	3246	4180
MSTRG.2460.1	coding	0.231	111	2043	2196
MSTRG.2464.2	coding	0.132	0	390	3126
MSTRG.2468.1	noncoding	-0.006144	1470	1617	4664
MSTRG.2489.3	coding	0.153	579	1278	2551
MSTRG.2492.2	coding	0.186	0	2586	4224
MSTRG.2493.1	coding	0.177	114	1884	3688
MSTRG.2498.2	coding	0.163	192	1758	6338
MSTRG.2509.3	coding	0.039	1056	1296	1601
MSTRG.2509.5	coding	0.079	1041	1392	1585
MSTRG.2509.4	coding	0.093	1008	1299	1993
MSTRG.2510.1	noncoding	-0.0446464	273	561	2183
MSTRG.2510.2	noncoding	-0.0446464	219	507	1539
MSTRG.2513.3	noncoding	-0.0114688	4332	4482	4638
MSTRG.2513.5	noncoding	-0.0405504	6372	6624	6780
MSTRG.2514.3	noncoding	-0.0446464	735	933	1158
MSTRG.2515.1	noncoding	-0.0147456	2097	2214	4414
MSTRG.2499.1	noncoding	-0.0217088	0	3	232
MSTRG.2508.1	noncoding	-0.004096	195	282	1821
MSTRG.2517.2	coding	0.095	0	519	1997
MSTRG.2517.3	coding	0.05	0	1104	1799
MSTRG.2519.3	coding	0.125	2391	4881	6290
MSTRG.2519.4	coding	0.124	2391	4884	6161
MSTRG.2529.1	coding	0.111	0	3063	10322
MSTRG.2529.2	noncoding	-0.083968	1578	1923	2077
MSTRG.2530.1	noncoding	-0.053248	1353	1440	1627
MSTRG.2566.1	noncoding	-0.0856064	0	279	1724
MSTRG.2567.1	noncoding	-0.0196608	174	246	2063
MSTRG.2568.2	coding	0.153	1374	1626	2112
MSTRG.2569.1	coding	0.016	423	1473	3309

MSTRG.2569.2	coding	0.019	2199	2412	3411
MSTRG.2569.4	noncoding	-0.0077824	2406	2619	4865
MSTRG.2569.6	coding	0.01	12	192	1160
MSTRG.2569.5	noncoding	-0.0077824	1908	2121	2831
MSTRG.2572.1	noncoding	-0.0380928	213	300	1443
MSTRG.2569.7	coding	0.047	5748	8730	17256
MSTRG.2569.8	coding	0.047	4578	7560	15390
MSTRG.2569.9	coding	0.047	4077	7059	8461
MSTRG.2575.1	noncoding	-0.02048	1416	1419	1896
MSTRG.2576.4	noncoding	-0.3346432	0	135	292
MSTRG.2576.3	coding	0.244	0	1026	13110
MSTRG.2577.1	coding	0.039	1221	1623	2095
MSTRG.2576.5	coding	0.115	78	1299	5395
MSTRG.2576.6	noncoding	-0.0315392	2205	2427	8095
MSTRG.2576.7	noncoding	-0.0344064	2289	2439	14455
MSTRG.2579.2	coding	0.231	0	3969	7772
MSTRG.2579.3	coding	0.22	0	2019	2385
MSTRG.2541.2	noncoding	-0.2392064	0	129	873
MSTRG.2542.1	noncoding	-0.0421888	189	198	370
MSTRG.2543.1	noncoding	-0.024576	213	240	419
MSTRG.2544.1	noncoding	-0.008192	696	822	1911
MSTRG.2545.1	noncoding	-0.008192	1671	1686	2925
MSTRG.2536.1	noncoding	-0.077824	81	150	447
MSTRG.2540.1	noncoding	-0.018432	393	456	649
MSTRG.2561.4	coding	0.215	60	705	870
MSTRG.2564.3	coding	0.082	1791	2187	2567
MSTRG.2582.2	coding	0.294	135	864	7056
MSTRG.2582.3	coding	0.294	159	888	6873
MSTRG.2588.1	noncoding	-0.0651264	924	1416	1648
MSTRG.2589.2	coding	0.118	150	1095	5827
MSTRG.2589.3	noncoding	-0.1040384	102	474	1849
MSTRG.2589.4	coding	0.147	0	792	1265
MSTRG.2590.2	noncoding	-0.0536576	1581	1815	1972
MSTRG.2590.1	coding	0.342	0	192	1934
MSTRG.2590.3	coding	0.043	144	684	2050
MSTRG.2591.5	noncoding	-0.0045056	120	483	5570
MSTRG.2591.6	noncoding	-0.129024	0	69	462
MSTRG.2594.1	coding	0.22	168	1314	1834
MSTRG.2596.4	coding	0.16	945	6099	9241
MSTRG.2596.3	coding	0.146	0	5979	9312
MSTRG.2597.2	noncoding	-0.034816	63	177	910
MSTRG.2597.1	noncoding	-0.679936	132	138	797
MSTRG.2598.1	noncoding	-0.110592	51	78	233
MSTRG.2602.1	noncoding	-0.0057344	351	390	677
MSTRG.2602.2	coding	0.12	150	219	505
MSTRG.2602.3	coding	0.12	147	216	502
MSTRG.2602.10	noncoding	-0.06144	4689	4800	5075
MSTRG.2602.11	noncoding	-0.053248	405	492	5067
MSTRG.2602.12	noncoding	-0.032768	117	282	1362
MSTRG.2601.1	noncoding	-0.014336	138	207	420
MSTRG.2601.2	noncoding	-0.045056	15	111	324
MSTRG.2605.3	coding	0.153	438	1893	5675
MSTRG.2605.4	coding	0.153	135	1590	5372
MSTRG.2605.5	coding	0.241	1380	10770	11145

MSTRG.2605.6	coding	0.153	330	1785	5565
MSTRG.2605.7	coding	0.239	0	9702	13368
MSTRG.2605.8	coding	0.257	18	4122	4417
MSTRG.2605.10	coding	0.253	0	6399	6774
MSTRG.2605.9	coding	0.269	0	4554	6599
MSTRG.2605.11	coding	0.247	1092	3048	3327
MSTRG.2609.7	coding	0.045	2415	3036	4551
MSTRG.2609.8	coding	0.045	2241	2862	4377
MSTRG.2609.9	coding	0.045	2451	3072	4516
MSTRG.2610.2	noncoding	-0.028672	1143	1209	6722
MSTRG.2611.1	coding	0.088	2715	2955	4492
MSTRG.2607.3	coding	0.252	0	1086	1595
MSTRG.2682.1	noncoding	-0.018432	2139	2256	4907
MSTRG.2614.1	noncoding	-0.0004096	0	0	276
MSTRG.2632.1	noncoding	-0.016384	741	861	1097
MSTRG.2633.1	noncoding	-0.1019904	0	6	513
MSTRG.2634.3	noncoding	-0.012288	549	570	1206
MSTRG.2635.1	noncoding	-0.028672	1587	1641	3104
MSTRG.2636.1	noncoding	-0.053248	312	399	1735
MSTRG.2638.1	noncoding	-0.008192	783	861	1017
MSTRG.2639.1	coding	0.075	444	1857	6591
MSTRG.2640.1	noncoding	-0.02048	381	471	5197
MSTRG.2641.1	noncoding	-0.036864	192	285	1839
MSTRG.2642.1	noncoding	-0.0004096	0	0	508
MSTRG.2645.1	noncoding	-0.0192512	5100	5250	5407
MSTRG.2645.2	noncoding	-0.0004096	4038	4182	8683
MSTRG.2645.3	noncoding	-0.0585728	396	453	868
MSTRG.2647.1	noncoding	-0.196608	324	360	593
MSTRG.2647.2	noncoding	-0.0434176	303	480	879
MSTRG.2649.1	noncoding	-0.0421888	342	360	952
MSTRG.2668.3	coding	0.358	0	5427	10210
MSTRG.2668.4	coding	0.356	0	5406	10189
MSTRG.2668.5	coding	0.36	0	5556	10313
MSTRG.2668.6	coding	0.336	0	4200	8973
MSTRG.2668.7	coding	0.336	279	4380	6121
MSTRG.2706.2	coding	0.422	48	912	1067
MSTRG.2706.3	coding	0.396	15	1236	1391
MSTRG.2711.3	noncoding	-0.008192	273	282	580
MSTRG.2711.4	coding	0.236	627	1038	1481
MSTRG.2714.3	coding	0.28	90	1395	1566
MSTRG.2714.4	coding	0.258	2424	3489	3661
MSTRG.2715.1	coding	0.275	81	3939	4526
MSTRG.2715.4	coding	0.275	99	3954	4320
MSTRG.2715.5	coding	0.261	870	3624	3991
MSTRG.2717.2	coding	0.423	0	1257	1412
MSTRG.2720.1	noncoding	-0.043008	150	333	1287
MSTRG.2721.2	coding	0.558	327	864	1019
MSTRG.2721.3	coding	0.515	84	723	878
MSTRG.2724.3	coding	0.224	465	1461	2268
MSTRG.2740.1	coding	0.259	216	2283	2897
MSTRG.2742.1	coding	0.24	378	1089	1549
MSTRG.2742.4	coding	0.287	147	1047	1515
MSTRG.2742.5	coding	0.287	57	957	1425
MSTRG.2730.2	coding	0.447	171	1884	2039

MSTRG.2732.1	coding	0.379	33	1029	2793
MSTRG.2732.2	coding	0.373	30	909	1898
MSTRG.2732.5	coding	0.362	18	1035	1204
MSTRG.2743.1	noncoding	-0.1241088	306	2652	3290
MSTRG.2744.1	noncoding	-0.0397312	0	420	798
MSTRG.2747.4	coding	0.319	186	1200	1568
MSTRG.2750.1	noncoding	-0.0393216	12	318	589
MSTRG.2750.2	noncoding	-0.0610304	36	387	576
MSTRG.2750.3	noncoding	-0.0589824	141	441	630
MSTRG.2749.5	coding	0.584	0	2982	7260
MSTRG.2757.1	noncoding	-0.012288	69	126	386
MSTRG.2761.1	noncoding	-0.0004096	0	0	256
MSTRG.2762.1	noncoding	-0.057344	0	12	435
MSTRG.2766.2	coding	0.119	162	1767	2032
MSTRG.2770.1	noncoding	-0.049152	60	93	315
MSTRG.2771.3	noncoding	-0.0053248	159	270	819
MSTRG.2771.2	noncoding	-0.004096	36	177	759
MSTRG.2772.1	coding	0.238	0	1341	2986
MSTRG.2774.2	coding	0.373	225	1242	2222
MSTRG.2774.3	coding	0.365	204	1197	2022
MSTRG.2775.1	noncoding	-0.0172032	222	549	2961
MSTRG.2777.3	coding	0.064	489	2592	3112
MSTRG.2778.1	noncoding	-0.1273856	0	78	413
MSTRG.2778.2	noncoding	-0.0004096	0	0	341
MSTRG.2804.3	noncoding	-0.04096	138	237	1492
MSTRG.2806.5	noncoding	-0.0503808	7467	8103	8758
MSTRG.2806.6	noncoding	-0.0503808	4932	5568	6113
MSTRG.2806.7	noncoding	-0.0417792	10752	11241	11784
MSTRG.2806.8	noncoding	-0.270336	78	105	370
MSTRG.2807.1	noncoding	-0.0401408	4494	4641	7118
MSTRG.2808.1	noncoding	-0.049152	6	168	1546
MSTRG.2809.1	coding	0.08	4737	5106	8207
MSTRG.2810.2	noncoding	-0.059392	5034	5631	7254
MSTRG.2811.8	coding	0.085	3159	3981	4207
MSTRG.2811.10	coding	0.055	1425	2088	2314
MSTRG.2788.4	coding	0.131	0	1698	8272
MSTRG.2788.5	coding	0.115	0	1779	9350
MSTRG.2788.6	coding	0.242	591	1074	2494
MSTRG.2788.7	coding	0.242	591	1074	2421
MSTRG.2793.3	coding	0.076	744	1089	1619
MSTRG.2798.2	coding	0.281	60	1938	2979
MSTRG.2822.10	coding	0.079	2346	5406	5894
MSTRG.2822.6	coding	0.091	189	5874	6362
MSTRG.2829.1	coding	0.28	435	2058	3146
MSTRG.2830.1	noncoding	-0.024576	1200	1353	2173
MSTRG.2799.1	coding	0.12	72	1650	2962
MSTRG.2800.2	coding	0.125	168	2322	2803
MSTRG.2817.1	noncoding	-0.0167936	630	783	4693
MSTRG.2819.1	noncoding	-0.0417792	0	198	627
MSTRG.2819.2	noncoding	-0.094208	168	252	450
MSTRG.2819.3	noncoding	-0.0294912	0	375	573
MSTRG.2819.4	noncoding	-0.045056	0	195	1800
MSTRG.2820.2	noncoding	-0.1318912	0	678	3233
MSTRG.2820.3	coding	0.178	480	1587	3960

MSTRG.2816.4	coding	0.24	330	1392	5290
MSTRG.2816.3	coding	0.226	396	1455	5352
MSTRG.2816.1	coding	0.233	249	1353	5252
MSTRG.2816.5	noncoding	-0.036864	3909	4038	4657
MSTRG.2814.1	noncoding	-0.0212992	528	600	754
MSTRG.2821.2	coding	0.174	0	2850	3094
MSTRG.2821.3	coding	0.159	39	2844	3347
MSTRG.2833.3	coding	0.336	1134	3303	4202
MSTRG.2833.4	noncoding	-0.065536	891	1614	2512
MSTRG.2833.5	noncoding	-0.0630784	24	945	1843
MSTRG.2836.2	coding	0.324	93	2166	2637
MSTRG.2837.1	noncoding	-0.0671744	2430	2751	12154
MSTRG.2837.2	noncoding	-0.0671744	666	987	4995
MSTRG.2843.2	noncoding	-0.1626112	63	426	1190
MSTRG.2840.3	coding	0.363	189	1083	2248
MSTRG.2848.2	coding	0.374	165	2838	3914
MSTRG.2848.3	coding	0.373	507	3144	3300
MSTRG.2848.4	coding	0.239	231	1278	1434
MSTRG.2848.6	coding	0.411	1206	3234	4513
MSTRG.2848.7	noncoding	-0.0917504	0	102	257
MSTRG.2845.1	noncoding	-0.0380928	0	666	969
MSTRG.2845.2	noncoding	-0.0339968	0	585	888
MSTRG.2850.5	coding	0.216	876	1506	4449
MSTRG.2850.4	coding	0.207	78	1092	4335
MSTRG.2850.6	noncoding	-0.0667648	0	147	1901
MSTRG.2850.7	coding	0.203	5019	5649	8626
MSTRG.2850.8	coding	0.197	4485	5103	7773
MSTRG.2846.1	noncoding	-0.02048	1254	1359	1666
MSTRG.2857.2	noncoding	-0.1421312	0	1578	3734
MSTRG.2857.4	coding	0.357	189	1110	2339
MSTRG.2852.4	coding	0.403	12351	13572	15093
MSTRG.2852.3	coding	0.403	12384	13605	15127
MSTRG.2858.2	coding	0.368	0	621	3215
MSTRG.2859.1	noncoding	-0.0135168	0	18	352
MSTRG.2865.3	coding	0.295	0	1737	2754
MSTRG.2866.8	coding	0.101	522	1704	1980
MSTRG.2871.1	coding	0.152	0	1248	3691
MSTRG.2873.1	noncoding	-0.038912	4596	5310	5655
MSTRG.2870.1	coding	0.111	249	1485	2085
MSTRG.2879.1	coding	0.462	0	690	1530
MSTRG.2879.3	coding	0.476	0	636	1148
MSTRG.2884.3	coding	0.374	792	2139	2631
MSTRG.2889.3	noncoding	-0.0851968	93	438	1395
MSTRG.2889.4	noncoding	-0.0724992	0	168	1127
MSTRG.2894.2	noncoding	-0.0004096	39	168	322
MSTRG.2897.1	coding	0.383	2049	3411	6919
MSTRG.2898.2	coding	0.25	6	3357	3611
MSTRG.2898.1	coding	0.251	6	3408	3814
MSTRG.2899.1	noncoding	-0.0684032	1215	1479	1920
MSTRG.2899.3	noncoding	-0.0667648	477	732	1161
MSTRG.2899.2	noncoding	-0.0442368	273	945	1098
MSTRG.2898.4	coding	0.201	186	1494	1688
MSTRG.2900.4	coding	0.422	243	1131	1540
MSTRG.2900.5	noncoding	-0.1093632	78	369	778

MSTRG.2906.4	coding	0.224	222	4923	5090
MSTRG.2906.5	coding	0.183	57	3198	3363
MSTRG.2907.3	coding	0.367	300	1860	2392
MSTRG.2909.1	coding	0.301	516	978	1347
MSTRG.2909.2	coding	0.226	0	636	1005
MSTRG.2910.1	coding	0.204	96	798	3603
MSTRG.2910.4	coding	0.194	516	1200	2015
MSTRG.2912.2	coding	0.466	0	1236	2245
MSTRG.2913.1	noncoding	-0.034816	291	366	962
MSTRG.2914.1	coding	0.338	243	1023	2420
MSTRG.2914.2	coding	0.366	42	846	2202
MSTRG.2921.1	noncoding	-0.0090112	525	672	889
MSTRG.2922.1	noncoding	-0.0294912	93	267	793
MSTRG.2922.2	noncoding	-0.0196608	0	168	688
MSTRG.2920.1	noncoding	-0.0004096	0	0	256
MSTRG.2924.2	coding	0.51	0	4539	5218
MSTRG.2924.3	coding	0.473	2172	4626	5295
MSTRG.2927.3	noncoding	-0.2220032	591	1506	13020
MSTRG.2932.2	coding	0.223	0	2454	5404
MSTRG.2932.6	coding	0.319	0	633	2985
MSTRG.2929.1	noncoding	-0.1568768	0	447	781
MSTRG.2935.3	coding	0.235	672	2307	2542
MSTRG.2943.3	coding	0.366	81	780	1010
MSTRG.2943.4	coding	0.388	774	1251	1481
MSTRG.2943.5	coding	0.393	477	957	1189
MSTRG.2943.7	coding	0.349	534	978	1135
MSTRG.2943.6	coding	0.349	81	867	1022
MSTRG.2950.1	coding	0.299	441	1614	2659
MSTRG.2950.2	coding	0.299	243	1416	2281
MSTRG.2950.3	coding	0.293	1185	2424	3184
MSTRG.2951.1	noncoding	-0.0299008	546	747	903
MSTRG.2956.4	coding	0.217	468	1608	3995
MSTRG.2956.5	coding	0.215	321	1452	3839
MSTRG.2956.6	coding	0.162	2190	4236	6402
MSTRG.2956.7	coding	0.162	876	2922	5042
MSTRG.2957.1	noncoding	-0.147456	3498	3564	4411
MSTRG.2948.3	coding	0.155	69	1626	5344
MSTRG.2960.1	noncoding	-0.0602112	306	483	1130
MSTRG.2968.1	coding	0.202	0	801	3035
MSTRG.2968.2	coding	0.155	153	978	3213
MSTRG.2968.3	coding	0.157	75	975	3149
MSTRG.2973.1	coding	0.212	45	513	669
MSTRG.2978.3	coding	0.474	462	2499	2669
MSTRG.2978.1	coding	0.469	1530	3540	3890
MSTRG.2978.2	coding	0.46	168	2259	2610
MSTRG.2979.1	noncoding	-0.0004096	0	0	440
MSTRG.2980.1	noncoding	-0.0851968	1482	1851	2458
MSTRG.2983.1	coding	0.003	1155	1395	2242
MSTRG.2995.1	noncoding	-0.1142784	0	288	1344
MSTRG.2996.3	coding	0.297	21	1002	3132
MSTRG.2996.4	coding	0.29	9	996	3075
MSTRG.3004.4	coding	0.445	348	1257	1414
MSTRG.3004.6	coding	0.483	405	1707	1864
MSTRG.3004.7	coding	0.437	555	1494	1650

MSTRG.3001.1	coding	0.368	681	1326	1820
MSTRG.3012.4	coding	0.408	96	1131	1386
MSTRG.3012.5	coding	0.421	579	1611	1866
MSTRG.3012.6	coding	0.408	192	1167	1421
MSTRG.3017.3	coding	0.242	771	1236	3063
MSTRG.3017.7	coding	0.242	642	1107	2383
MSTRG.3014.2	coding	0.486	9	1656	4018
MSTRG.3021.2	coding	0.25	774	2235	2606
MSTRG.3026.1	noncoding	-0.0872448	135	153	359
MSTRG.3027.2	coding	0.524	39	1515	4023
MSTRG.3027.3	coding	0.558	0	1311	3739
MSTRG.3027.4	noncoding	-0.1376256	81	366	1394
MSTRG.3028.1	coding	0.209	0	1086	4390
MSTRG.3028.2	coding	0.195	0	726	4148
MSTRG.3028.3	coding	0.207	570	936	3934
MSTRG.3028.4	noncoding	-0.0774144	0	909	4711
MSTRG.3028.5	coding	0.201	600	1002	1527
MSTRG.3029.1	noncoding	-0.0442368	927	1266	1423
MSTRG.3031.6	coding	0.037	54	3018	3947
MSTRG.3031.10	coding	0.031	75	2796	3718
MSTRG.3031.7	coding	0.035	54	2973	3895
MSTRG.3031.9	coding	0.036	54	3015	3937
MSTRG.3036.2	coding	0.143	354	858	1293
MSTRG.3037.1	coding	0.327	387	1626	2104
MSTRG.3037.5	coding	0.324	387	1677	2861
MSTRG.3037.6	coding	0.326	387	1419	1928
MSTRG.3039.3	coding	0.134	309	1428	2263
MSTRG.3043.1	coding	0.153	18	447	6325
MSTRG.3043.7	coding	0.401	0	201	4368
MSTRG.3043.4	coding	0.401	0	201	2381
MSTRG.3043.6	coding	0.401	0	201	2371
MSTRG.3043.3	coding	0.401	0	201	6157
MSTRG.3043.10	coding	0.133	3186	3402	7064
MSTRG.3043.11	coding	0.114	3186	3399	7061
MSTRG.3043.12	coding	0.114	3186	3399	4314
MSTRG.3043.9	coding	0.145	1182	1401	2314
MSTRG.3043.13	noncoding	-0.0212992	1185	1350	2190
MSTRG.3042.1	noncoding	-0.0733184	0	741	1012
MSTRG.3050.1	noncoding	-0.1306624	123	156	501
MSTRG.3054.2	coding	0.25	3	630	800
MSTRG.3055.2	coding	0.391	0	372	1939
MSTRG.3044.2	coding	0.248	117	2286	4773
MSTRG.3056.2	coding	0.478	0	444	601
MSTRG.3059.1	coding	0.412	0	2640	4040
MSTRG.3060.1	coding	0.399	0	1035	3533
MSTRG.3061.1	noncoding	-0.059392	1149	1545	1907
MSTRG.3061.2	noncoding	-0.0438272	780	1110	1471
MSTRG.3064.6	coding	0.145	48	2358	3070
MSTRG.3064.5	coding	0.147	66	1593	19187
MSTRG.3066.1	noncoding	-0.0897024	0	633	1184
MSTRG.3068.4	coding	0.164	1008	3000	3438
MSTRG.3068.5	coding	0.164	570	2562	3001
MSTRG.3068.3	coding	0.164	738	2730	3167
MSTRG.3065.1	noncoding	-0.237568	0	114	464

MSTRG.3070.1	noncoding	-0.0106496	1539	1659	2168
MSTRG.3076.1	coding	0.336	204	3921	7673
MSTRG.3085.2	coding	0.477	0	11736	12360
MSTRG.3087.2	noncoding	-0.073728	90	567	724
MSTRG.3087.1	noncoding	-0.0536576	90	519	691
MSTRG.3093.1	noncoding	-0.0380928	12	225	393
MSTRG.3092.4	coding	0.472	0	2427	3274
MSTRG.3092.5	noncoding	-0.1056768	240	861	1709
MSTRG.3088.2	coding	0.475	111	1884	2463
MSTRG.3088.3	coding	0.475	111	1884	2461
MSTRG.3113.1	noncoding	-0.069632	0	525	3625
MSTRG.3114.1	coding	0.185	57	6048	6502
MSTRG.3115.1	noncoding	-0.0135168	6351	6558	12912
MSTRG.3116.1	noncoding	-0.065536	11889	11982	17077
MSTRG.3116.2	noncoding	-0.065536	10965	11058	13185
MSTRG.3114.2	coding	0.19	246	4929	5864
MSTRG.3114.4	coding	0.195	216	4845	5713
MSTRG.3114.3	coding	0.196	240	4878	5813
MSTRG.3114.5	coding	0.195	216	4842	5777
MSTRG.3114.7	coding	0.19	1110	5793	6662
MSTRG.3114.13	coding	0.196	324	4956	5891
MSTRG.3114.8	coding	0.196	204	4839	5774
MSTRG.3114.9	coding	0.222	2808	5937	6872
MSTRG.3117.1	noncoding	-0.057344	1566	1662	3470
MSTRG.3118.1	noncoding	-0.0454656	651	753	3973
MSTRG.3114.14	coding	0.191	1833	6525	7394
MSTRG.3114.15	coding	0.191	1152	5838	6706
MSTRG.3122.1	noncoding	-0.0421888	114	117	546
MSTRG.3124.3	coding	0.17	270	1794	4095
MSTRG.3124.4	coding	0.164	342	1785	3422
MSTRG.3124.5	coding	0.164	327	1770	3408
MSTRG.3124.8	coding	0.193	1416	2079	3718
MSTRG.3100.1	noncoding	-0.0688128	0	591	2860
MSTRG.3101.1	coding	0.549	0	3810	4100
MSTRG.3101.3	coding	0.552	0	3744	4036
MSTRG.3101.2	coding	0.553	0	3741	8631
MSTRG.3101.4	coding	0.561	0	3591	3884
MSTRG.3101.5	coding	0.389	0	1224	1515
MSTRG.3104.1	noncoding	-0.036864	21	117	1983
MSTRG.3101.6	coding	0.47	300	1701	2023
MSTRG.3096.2	coding	0.322	129	1224	2463
MSTRG.3106.3	coding	0.416	60	3738	3892
MSTRG.3106.4	coding	0.425	0	3597	3752
MSTRG.3106.5	coding	0.428	0	3474	3629
MSTRG.3106.6	coding	0.406	333	3576	3730
MSTRG.3106.7	coding	0.392	0	1536	1690
MSTRG.3106.8	noncoding	-0.0135168	453	798	953
MSTRG.3109.1	coding	0.223	48	711	1417
MSTRG.3111.1	noncoding	-0.034816	333	555	757
MSTRG.3125.3	coding	0.393	0	561	1736
MSTRG.3126.2	coding	0.403	0	126	638
MSTRG.3127.3	coding	0.364	0	873	1366
MSTRG.3127.4	coding	0.355	0	912	1404
MSTRG.3130.1	coding	0.59	0	699	1064

MSTRG.3130.2	coding	0.51	135	1167	4167
MSTRG.3130.3	coding	0.51	135	1167	4060
MSTRG.3132.1	coding	0.439	57	999	2769
MSTRG.3132.3	coding	0.292	66	1518	1972
MSTRG.3133.1	coding	0.503	0	3003	5271
MSTRG.3133.3	coding	0.488	0	3129	4412
MSTRG.3133.4	coding	0.488	0	3129	4326
MSTRG.3133.5	coding	0.487	0	3132	4329
MSTRG.3134.1	noncoding	-0.032768	1146	1275	2278
MSTRG.3144.2	coding	0.439	0	15324	15753
MSTRG.3136.1	noncoding	-0.0667648	1065	1368	2374
MSTRG.3137.1	noncoding	-0.022528	222	480	972
MSTRG.3135.1	noncoding	-0.0446464	366	750	988
MSTRG.3135.2	noncoding	-0.0421888	114	453	684
MSTRG.3141.1	coding	0.342	27	417	654
MSTRG.3146.1	noncoding	-0.0208896	126	192	548
MSTRG.3146.2	noncoding	-0.0290816	354	615	993
MSTRG.3153.1	noncoding	-0.002048	54	195	631
MSTRG.3154.1	coding	0.549	15	3945	4439
MSTRG.3154.4	coding	0.546	279	4161	4416
MSTRG.3154.5	coding	0.479	33	1806	1961
MSTRG.3155.1	noncoding	-0.063488	0	1098	1717
MSTRG.3151.1	noncoding	-0.032768	204	318	473
MSTRG.3152.1	coding	0.446	102	591	927
MSTRG.3157.2	coding	0.338	519	1242	1490
MSTRG.3157.3	coding	0.345	444	1113	1360
MSTRG.3157.5	coding	0.347	333	1065	1313
MSTRG.3163.1	noncoding	-0.0221184	0	1332	1505
MSTRG.3166.3	coding	0.305	0	756	7976
MSTRG.3166.6	coding	0.244	24	897	1214
MSTRG.3166.7	coding	0.305	0	753	1071
MSTRG.3159.4	coding	0.234	102	1887	5642
MSTRG.3179.7	coding	0.1	3555	3774	9743
MSTRG.3179.9	noncoding	-0.06144	1401	1425	1972
MSTRG.3179.10	noncoding	-0.004096	6513	6723	10496
MSTRG.3179.11	coding	0.09	2025	2097	2632
MSTRG.3183.1	coding	0.144	606	1572	3120
MSTRG.3173.1	noncoding	-0.065536	312	399	1416
MSTRG.3176.1	noncoding	-0.0147456	6768	7203	7359
MSTRG.3177.1	noncoding	-0.004096	201	264	417
MSTRG.3178.3	noncoding	-0.0827392	0	252	3435
MSTRG.3185.1	coding	0.357	0	1566	4959
MSTRG.3185.2	coding	0.304	210	1875	10303
MSTRG.3186.1	coding	0.034	11370	12138	14103
MSTRG.3185.3	coding	0.216	120	552	4189
MSTRG.3186.6	coding	0.101	8340	9354	9912
MSTRG.3186.4	coding	0.102	1638	2844	3402
MSTRG.3186.9	coding	0.074	1638	2322	3208
MSTRG.3186.10	coding	0.047	933	2175	3156
MSTRG.3186.11	noncoding	-0.045056	3036	3156	3310
MSTRG.3186.12	noncoding	-0.0118784	2064	2229	5425
MSTRG.3187.1	noncoding	-0.008192	2085	2181	5376
MSTRG.3188.1	noncoding	-0.0024576	4572	4716	5010
MSTRG.3189.1	noncoding	-0.0790528	213	678	1178

MSTRG.3190.2	noncoding	-0.0835584	99	333	1471
MSTRG.3190.3	noncoding	-0.079872	99	330	1468
MSTRG.3197.1	coding	0.149	390	1110	1440
MSTRG.3198.1	noncoding	-0.0315392	411	480	1153
MSTRG.3214.1	noncoding	-0.0503808	3774	4482	4637
MSTRG.3221.1	noncoding	-0.04096	549	618	1186
MSTRG.3226.1	noncoding	-0.0577536	291	870	1025
MSTRG.3228.2	coding	0.412	747	2610	4508
MSTRG.3215.1	noncoding	-0.0077824	933	1134	1290
MSTRG.3217.1	coding	0.078	276	465	769
MSTRG.3218.1	noncoding	-0.0200704	3393	3561	4544
MSTRG.3220.1	noncoding	-0.0004096	0	0	358
MSTRG.3234.2	coding	0.41	0	867	1164
MSTRG.3248.1	noncoding	-0.049152	4776	4860	7473
MSTRG.3249.1	noncoding	-0.002048	6453	6615	9651
MSTRG.3243.1	noncoding	-0.012288	177	237	833
MSTRG.3244.1	noncoding	-0.0401408	45	189	430
MSTRG.3245.1	noncoding	-0.0004096	0	0	282
MSTRG.3252.1	noncoding	-0.151552	2421	2484	2916
MSTRG.3252.3	noncoding	-0.151552	2391	2454	2874
MSTRG.3252.4	noncoding	-0.151552	2391	2454	4189
MSTRG.3252.5	noncoding	-0.151552	2373	2436	4043
MSTRG.3254.1	noncoding	-0.0282624	168	315	801
MSTRG.3259.3	coding	0.144	1383	2799	3403
MSTRG.3259.4	coding	0.126	1209	2331	3350
MSTRG.3261.1	coding	0.565	0	444	887
MSTRG.3263.1	coding	0.084	6747	7116	11233
MSTRG.3264.1	noncoding	-0.139264	72	99	615
MSTRG.3267.2	coding	0.378	186	2736	3207
MSTRG.3267.4	coding	0.401	0	2334	2866
MSTRG.3269.1	noncoding	-0.0401408	2025	2163	2428
MSTRG.3270.2	coding	0.091	0	1926	6842
MSTRG.3270.3	coding	0.377	960	1845	6005
MSTRG.3271.1	coding	0.144	2148	2511	4799
MSTRG.3279.1	noncoding	-0.0139264	3957	4161	4980
MSTRG.3282.2	coding	0.057	0	315	1064
MSTRG.3282.3	noncoding	-0.0053248	102	213	962
MSTRG.3282.4	noncoding	-0.0053248	159	270	1019
MSTRG.3282.6	coding	0.018	1968	2304	2608
MSTRG.3282.5	coding	0.018	123	459	763
MSTRG.3282.7	coding	0.001	78	384	688
MSTRG.3294.1	noncoding	-0.0004096	354	480	634
MSTRG.3294.2	noncoding	-0.0802816	0	138	293
MSTRG.3296.4	coding	0.248	51	540	1045
MSTRG.3296.3	coding	0.213	174	606	1112
MSTRG.3297.5	coding	0.388	90	1059	1646
MSTRG.3300.1	coding	0.221	0	1104	5175
MSTRG.3300.2	noncoding	-0.1110016	0	555	1160
MSTRG.3287.1	noncoding	-0.032768	54	132	364
MSTRG.3307.1	coding	0.54	534	2277	5616
MSTRG.3307.4	coding	0.546	1080	2199	6048
MSTRG.3307.2	coding	0.432	2928	5241	8418
MSTRG.3307.3	coding	0.539	534	2280	5456
MSTRG.3307.6	coding	0.535	519	1944	5089

MSTRG.3312.1	coding	0.336	16308	18039	22351
MSTRG.3314.1	noncoding	-0.0090112	6195	6288	6724
MSTRG.3317.2	coding	0.408	1311	3012	3411
MSTRG.3317.3	coding	0.397	678	2553	2952
MSTRG.3317.4	coding	0.407	894	2469	2869
MSTRG.3317.5	coding	0.405	0	2541	2939
MSTRG.3317.6	coding	0.43	678	2580	2979
MSTRG.3320.1	noncoding	-0.0503808	6072	6855	7012
MSTRG.3321.4	coding	0.366	0	855	1447
MSTRG.3321.3	coding	0.427	285	945	1536
MSTRG.3321.6	coding	0.446	174	804	1564
MSTRG.3333.1	noncoding	-0.14336	1293	1317	1564
MSTRG.3339.3	coding	0.519	3084	4998	9998
MSTRG.3339.4	coding	0.511	57	1671	2520
MSTRG.3340.1	noncoding	-0.0098304	2895	3039	4615
MSTRG.3342.2	coding	0.191	99	390	888
MSTRG.3342.1	coding	0.169	333	627	1127
MSTRG.3343.1	noncoding	-0.06144	852	915	1179
MSTRG.3343.2	noncoding	-0.0139264	2334	2445	3455
MSTRG.3346.1	noncoding	-0.0495616	0	60	298
MSTRG.3354.3	coding	0.234	0	120	274
MSTRG.3357.1	coding	0.398	1341	6024	6946
MSTRG.3357.2	coding	0.402	1341	6144	7066
MSTRG.3358.1	noncoding	-1.032192	1794	1929	2728
MSTRG.3357.4	coding	0.405	1365	5172	6096
MSTRG.3359.6	noncoding	-0.0782336	0	297	798
MSTRG.3364.1	coding	0.332	708	2061	2265
MSTRG.3364.3	coding	0.325	666	2100	2303
MSTRG.3364.4	coding	0.35	570	1371	2762
MSTRG.3364.5	coding	0.39	300	945	2340
MSTRG.3364.6	coding	0.365	423	1146	2541
MSTRG.3364.7	noncoding	-0.1306624	378	840	2039
MSTRG.3371.1	coding	0.273	111	1407	1934
MSTRG.3373.2	coding	0.444	693	4017	5028
MSTRG.3374.3	noncoding	-0.0618496	6663	7218	13965
MSTRG.3375.1	noncoding	-0.024576	0	183	682
MSTRG.3381.2	noncoding	-0.1441792	48	558	987
MSTRG.3384.1	coding	0.298	0	2217	3026
MSTRG.3389.3	coding	0.33	0	1059	7560
MSTRG.3389.2	coding	0.376	309	1653	3550
MSTRG.3389.4	coding	0.356	735	2460	4331
MSTRG.3390.2	coding	0.16	252	675	847
MSTRG.3415.2	coding	0.161	75	729	1168
MSTRG.3415.3	coding	0.174	33	993	2842
MSTRG.3418.1	coding	0.12	93	1374	1886
MSTRG.3420.6	coding	0.15	3474	8811	10753
MSTRG.3420.7	coding	0.131	3474	8193	10570
MSTRG.3420.8	coding	0.149	3516	8673	10654
MSTRG.3421.1	noncoding	-0.0004096	357	504	2496
MSTRG.3424.1	coding	0.299	105	2430	2586
MSTRG.3400.2	coding	0.366	0	1659	4137
MSTRG.3400.6	coding	0.31	441	1446	3937
MSTRG.3400.3	coding	0.377	0	1533	4013
MSTRG.3400.4	coding	0.31	441	1446	3928

MSTRG.3401.2	noncoding	-0.0905216	732	957	3141
MSTRG.3402.2	coding	0.348	288	1254	1456
MSTRG.3427.2	coding	0.459	0	1380	1589
MSTRG.3429.1	coding	0.466	0	1731	2351
MSTRG.3434.8	coding	0.34	513	1935	3248
MSTRG.3435.1	coding	0.403	0	2532	3689
MSTRG.3435.3	coding	0.402	693	3000	4157
MSTRG.3438.1	noncoding	-0.0364544	495	777	1707
MSTRG.3444.1	coding	0.288	6528	9990	12624
MSTRG.3444.3	coding	0.292	6495	9885	12487
MSTRG.3444.5	coding	0.288	222	3684	6318
MSTRG.3444.7	coding	0.288	792	4254	6482
MSTRG.3444.12	coding	0.288	2811	6273	8907
MSTRG.3444.13	coding	0.292	45	3174	5807
MSTRG.3441.3	coding	0.343	0	1059	1403
MSTRG.3441.4	coding	0.322	219	1023	1365
MSTRG.3446.1	noncoding	-0.0524288	306	579	734
MSTRG.3470.3	coding	0.181	156	852	1175
MSTRG.3471.3	coding	0.157	465	3615	4444
MSTRG.3471.1	coding	0.157	96	3246	4073
MSTRG.3471.5	coding	0.157	84	3234	3434
MSTRG.3471.4	coding	0.163	87	3321	3661
MSTRG.3471.10	coding	0.146	12	3975	4195
MSTRG.3471.11	coding	0.164	96	3381	3599
MSTRG.3471.12	coding	0.146	0	3900	4110
MSTRG.3471.13	coding	0.163	465	3699	3907
MSTRG.3471.14	coding	0.155	798	3954	4148
MSTRG.3471.16	noncoding	-0.0499712	63	261	2112
MSTRG.3472.3	coding	0.248	3	333	1318
MSTRG.3474.1	noncoding	-0.049152	714	1014	1688
MSTRG.3475.1	coding	0.398	2886	3627	4985
MSTRG.3475.3	coding	0.398	24	765	1867
MSTRG.3477.1	noncoding	-0.022528	3	30	598
MSTRG.3477.2	noncoding	-0.0004096	0	0	454
MSTRG.3478.5	coding	0.104	0	5526	5822
MSTRG.3478.7	coding	0.099	0	4674	6515
MSTRG.3482.1	coding	0.309	0	597	1162
MSTRG.3447.2	noncoding	-0.0200704	675	927	1238
MSTRG.3454.1	noncoding	-0.0290816	120	402	571
MSTRG.3463.2	coding	0.408	288	621	789
MSTRG.3469.2	noncoding	-0.0548864	48	210	723
MSTRG.3493.2	noncoding	-0.0008192	81	228	1320
MSTRG.3497.1	noncoding	-0.0708608	117	219	374
MSTRG.3498.2	coding	0.175	39	1653	2980
MSTRG.3504.1	noncoding	-0.024576	2721	2868	4022
MSTRG.3509.1	noncoding	-0.0618496	327	348	632
MSTRG.3521.1	coding	0.427	5034	7611	8349
MSTRG.3521.2	coding	0.417	303	3009	3747
MSTRG.3521.3	coding	0.414	228	2979	3716
MSTRG.3521.4	coding	0.421	228	2871	3608
MSTRG.3521.5	coding	0.414	228	2979	3716
MSTRG.3521.6	coding	0.421	237	2880	3617
MSTRG.3521.7	coding	0.433	438	2970	3706
MSTRG.3521.11	coding	0.421	744	3066	4437

MSTRG.3530.4	noncoding	-0.0462848	66	390	965
MSTRG.3536.2	noncoding	-0.0311296	498	675	924
MSTRG.3536.3	coding	0.076	408	585	833
MSTRG.3536.4	noncoding	-0.0311296	339	516	765
MSTRG.3536.5	coding	0.144	96	444	689
MSTRG.3536.6	coding	0.076	504	681	869
MSTRG.3543.3	coding	0.231	999	2628	4173
MSTRG.3544.1	coding	0.3	522	1863	2445
MSTRG.3544.2	coding	0.3	774	2115	2698
MSTRG.3544.4	coding	0.3	510	1851	2332
MSTRG.3544.3	coding	0.305	1695	2637	3220
MSTRG.3544.7	coding	0.296	1494	2409	2993
MSTRG.3546.1	coding	0.356	6	1149	2102
MSTRG.3547.5	coding	0.293	48	3828	4341
MSTRG.3547.4	coding	0.293	123	3897	4409
MSTRG.3547.6	coding	0.271	1011	1473	2195
MSTRG.3542.4	coding	0.145	321	2436	4597
MSTRG.3550.3	coding	0.192	621	954	3064
MSTRG.3548.1	noncoding	-0.0106496	675	879	1087
MSTRG.3557.2	coding	0.174	384	4473	8057
MSTRG.3557.3	coding	0.172	384	4551	8135
MSTRG.3558.1	noncoding	-0.126976	1392	1446	2072
MSTRG.3559.1	noncoding	-0.032768	4437	4608	17056
MSTRG.3561.4	coding	0.215	90	1371	1878
MSTRG.3561.5	coding	0.255	384	1851	2637
MSTRG.3561.6	coding	0.259	549	2586	2741
MSTRG.3561.7	coding	0.241	0	2160	2314
MSTRG.3563.3	noncoding	-0.0299008	54	480	1301
MSTRG.3563.4	coding	0.128	72	588	1367
MSTRG.3565.1	coding	0.258	171	594	1991
MSTRG.3562.1	coding	0.292	51	807	3427
MSTRG.3566.3	coding	0.385	0	1167	1354
MSTRG.3566.2	coding	0.368	0	1212	1400
MSTRG.3570.2	coding	0.245	309	2499	5934
MSTRG.3580.1	coding	0.565	0	354	2561
MSTRG.3580.2	coding	0.608	0	348	2174
MSTRG.3588.6	coding	0.256	42	1254	1497
MSTRG.3588.7	coding	0.197	177	1155	1378
MSTRG.3592.6	coding	0.186	144	699	1280
MSTRG.3593.3	noncoding	-0.0303104	249	564	1287
MSTRG.3598.1	coding	0.035	579	777	1311
MSTRG.3602.1	coding	0.124	315	495	1831
MSTRG.3604.2	coding	0.314	0	1353	3733
MSTRG.3604.3	coding	0.314	0	1356	3736
MSTRG.3604.4	coding	0.3	318	1047	3697
MSTRG.3604.5	coding	0.262	360	861	3239
MSTRG.3604.6	coding	0.24	264	750	3123
MSTRG.3606.2	coding	0.161	0	630	1583
MSTRG.3606.3	coding	0.159	0	636	1589
MSTRG.3612.3	noncoding	-0.0004096	2313	2439	2594
MSTRG.3612.2	noncoding	-0.004096	3507	3651	6649
MSTRG.3612.4	coding	0.014	696	822	3023
MSTRG.3614.1	noncoding	-0.024576	153	213	923
MSTRG.3623.1	coding	0.108	111	1425	11680

MSTRG.3623.2	coding	0.112	66	1386	1563
MSTRG.3623.7	coding	0.162	1278	1923	2479
MSTRG.3631.1	noncoding	-0.0004096	0	0	505
MSTRG.3633.4	noncoding	-0.04096	60	297	7667
MSTRG.3642.2	coding	0.272	174	1977	3242
MSTRG.3642.3	coding	0.278	174	1758	3023
MSTRG.3650.1	noncoding	-0.0008192	1263	1326	2020
MSTRG.3651.1	noncoding	-0.0372736	882	1140	3572
MSTRG.3652.1	coding	0.17	393	987	3048
MSTRG.3654.1	noncoding	-0.024576	150	237	2208
MSTRG.3658.1	noncoding	-0.0077824	0	102	355
MSTRG.3659.2	coding	0.311	225	1155	1487
MSTRG.3663.1	noncoding	-0.0319488	0	342	1300
MSTRG.3664.1	noncoding	-0.004096	81	156	384
MSTRG.3675.1	noncoding	-0.0966656	7488	8250	8445
MSTRG.3675.2	noncoding	-0.0966656	6942	7704	7898
MSTRG.3675.3	noncoding	-0.0966656	6363	7125	7318
MSTRG.3675.4	coding	0.051	132	1170	5368
MSTRG.3675.5	coding	0.152	1038	1386	5608
MSTRG.3675.6	coding	0.06	816	1953	6134
MSTRG.3675.7	coding	0.047	816	1866	3627
MSTRG.3675.8	noncoding	-0.1015808	6129	6873	7054
MSTRG.3675.9	noncoding	-0.1015808	5766	6510	6708
MSTRG.3675.10	coding	0.058	804	2559	5238
MSTRG.3675.11	coding	0.048	372	1365	7489
MSTRG.3676.1	noncoding	-0.0249856	687	1023	5543
MSTRG.3674.1	noncoding	-0.0004096	0	0	255
MSTRG.3679.1	coding	0.277	0	2094	2371
MSTRG.3679.2	coding	0.322	639	2442	2718
MSTRG.3679.3	coding	0.369	1032	2238	2515
MSTRG.3685.1	coding	0.54	0	3657	6906
MSTRG.3685.2	coding	0.546	609	4149	7582
MSTRG.3687.2	coding	0.107	0	510	4358
MSTRG.3694.1	noncoding	-0.1875968	0	444	921
MSTRG.3702.2	coding	0.067	4563	5706	6702
MSTRG.3702.3	coding	0.07	2826	4302	5242
MSTRG.3704.1	noncoding	-0.0004096	0	0	593
MSTRG.3707.2	coding	0.112	1026	2970	5165
MSTRG.3707.3	noncoding	-0.0376832	0	123	346
MSTRG.3708.2	noncoding	-0.24576	159	210	982
MSTRG.3710.1	coding	0.046	171	3087	7925
MSTRG.3712.1	noncoding	-0.016384	3237	3279	4536
MSTRG.3710.4	coding	0.035	498	2883	7422
MSTRG.3710.5	coding	0.069	1650	2685	7522
MSTRG.3716.2	coding	0.145	0	3351	7527
MSTRG.3716.3	coding	0.127	0	3477	7653
MSTRG.3716.5	coding	0.137	435	3966	8341
MSTRG.3716.6	coding	0.149	0	3627	8003
MSTRG.3716.7	coding	0.116	99	3501	7676
MSTRG.3716.9	coding	0.115	99	3300	3456
MSTRG.3725.1	noncoding	-0.0479232	2568	3000	8224
MSTRG.3733.3	noncoding	-0.0008192	333	597	919
MSTRG.3737.2	coding	0.072	903	2310	3876
MSTRG.3721.1	coding	0.213	822	1128	4753

MSTRG.3721.2	coding	0.217	822	1125	1845
MSTRG.3722.1	coding	0.335	0	1647	1873
MSTRG.3748.4	coding	0.417	1569	2199	2718
MSTRG.3749.6	coding	0.404	138	3093	3315
MSTRG.3749.7	coding	0.401	0	3024	3246
MSTRG.3749.3	coding	0.405	24	3021	3243
MSTRG.3749.5	coding	0.4	126	3126	3349
MSTRG.3755.1	noncoding	-0.028672	675	741	3044
MSTRG.3764.2	coding	0.111	360	777	4984
MSTRG.3765.1	noncoding	-0.090112	651	654	1041
MSTRG.3765.2	noncoding	-0.012288	1137	1254	2916
MSTRG.3767.1	noncoding	-0.036864	912	984	1140
MSTRG.3768.1	noncoding	-0.0110592	501	585	1081
MSTRG.3769.1	noncoding	-0.036864	594	699	1333
MSTRG.3770.1	noncoding	-0.0004096	0	0	925
MSTRG.3773.2	coding	0.342	336	798	1145
MSTRG.3773.3	coding	0.342	336	798	4940
MSTRG.3773.4	coding	0.349	2016	2505	6649
MSTRG.3777.2	noncoding	-0.024576	33	156	559
MSTRG.3778.1	coding	0.149	273	1854	2126
MSTRG.3756.2	coding	0.032	321	489	968
MSTRG.3780.4	coding	0.094	2325	2796	4142
MSTRG.3780.5	noncoding	-0.0294912	249	342	1362
MSTRG.3784.1	noncoding	-0.049152	456	513	776
MSTRG.3788.1	noncoding	-0.049152	1131	1188	2165
MSTRG.3789.1	coding	0.256	1077	3864	6286
MSTRG.3789.3	coding	0.258	1077	3837	10170
MSTRG.3790.1	noncoding	-0.0991232	1113	1557	2168
MSTRG.3800.1	coding	0.31	108	783	1010
MSTRG.3802.2	coding	0.371	0	690	1194
MSTRG.3802.3	coding	0.382	0	690	1194
MSTRG.3802.4	coding	0.379	0	690	1193
MSTRG.3802.5	coding	0.379	0	690	1193
MSTRG.3804.1	coding	0.556	600	3249	4148
MSTRG.3807.1	noncoding	-0.0585728	0	366	1490
MSTRG.3813.1	noncoding	-0.1265664	0	798	1521
MSTRG.3817.3	coding	0.404	144	5382	6378
MSTRG.3821.2	coding	0.27	54	3153	4799
MSTRG.3821.1	coding	0.268	189	3282	6604
MSTRG.3821.4	coding	0.279	0	3036	3747
MSTRG.3831.5	coding	0.062	87	1680	2136
MSTRG.3832.3	coding	0.071	339	2235	2852
MSTRG.3832.4	coding	0.071	657	2553	3170
MSTRG.3832.5	coding	0.071	1266	3162	3779
MSTRG.3833.1	noncoding	-0.016384	705	828	985
MSTRG.3834.1	coding	0.064	276	2016	2662
MSTRG.3834.2	coding	0.059	234	1941	6434
MSTRG.3839.1	coding	0.124	390	1347	2323
MSTRG.3825.2	coding	0.142	0	1458	3901
MSTRG.3825.1	coding	0.142	0	1464	3823
MSTRG.3828.1	coding	0.111	0	1134	1449
MSTRG.3842.1	noncoding	-0.1335296	0	249	6432
MSTRG.3842.2	noncoding	-0.1335296	0	249	6580
MSTRG.3842.3	coding	0.063	4392	4620	5386

MSTRG.3842.4	noncoding	-0.0004096	183	252	3144
MSTRG.3842.6	coding	0.177	57	1104	1963
MSTRG.3855.1	noncoding	-0.049152	315	429	4547
MSTRG.3854.3	coding	0.129	666	1602	4598
MSTRG.3856.1	noncoding	-0.0004096	627	765	1055
MSTRG.3852.1	coding	0.568	0	1080	1365
MSTRG.3852.2	coding	0.568	0	1080	2109
MSTRG.3868.1	noncoding	-0.0004096	0	0	1703
MSTRG.3969.1	coding	0.016	2184	2334	4659
MSTRG.3970.1	noncoding	-0.024576	360	435	3249
MSTRG.3971.1	noncoding	-0.135168	2034	2112	2968
MSTRG.3975.1	noncoding	-0.0012288	1218	1251	3862
MSTRG.3976.1	coding	0.038	174	1212	2004
MSTRG.3977.1	noncoding	-0.049152	4485	4572	6824
MSTRG.3978.1	noncoding	-0.0319488	1416	1650	5107
MSTRG.3979.1	noncoding	-0.0036864	1392	1506	3486
MSTRG.3980.1	noncoding	-0.12288	69	81	457
MSTRG.3972.5	noncoding	-0.02048	4020	4149	11297
MSTRG.3981.1	noncoding	-0.126976	1023	1089	1480
MSTRG.3982.1	noncoding	-0.008192	1656	1659	1843
MSTRG.3983.1	noncoding	-0.024576	357	447	3232
MSTRG.3983.2	noncoding	-0.0196608	162	273	1166
MSTRG.3984.1	noncoding	-0.0425984	3171	3327	5506
MSTRG.3984.2	noncoding	-0.0028672	1854	1971	3276
MSTRG.3985.1	coding	0.07	1833	2541	6073
MSTRG.3985.2	noncoding	-0.004096	2382	2418	4108
MSTRG.3972.6	noncoding	-0.0270336	2817	2979	6526
MSTRG.3986.1	noncoding	-0.016384	1254	1329	1506
MSTRG.3987.1	noncoding	-0.0212992	0	60	5423
MSTRG.3987.2	noncoding	-0.0086016	180	252	4246
MSTRG.3987.3	noncoding	-0.0086016	243	315	3742
MSTRG.3987.4	noncoding	-0.0004096	0	0	919
MSTRG.3989.1	noncoding	-0.0135168	2397	2601	5822
MSTRG.3972.9	noncoding	-0.02048	3003	3087	4446
MSTRG.3991.1	noncoding	-0.0274432	342	387	1074
MSTRG.3877.1	noncoding	-0.0196608	1140	1176	3647
MSTRG.3883.1	noncoding	-0.0606208	30	51	322
MSTRG.3889.2	coding	0.102	597	1443	3206
MSTRG.3890.1	coding	0.117	153	2088	2641
MSTRG.3893.1	noncoding	-0.1949696	2538	3408	3562
MSTRG.3895.3	coding	0.124	108	1827	2062
MSTRG.3895.4	coding	0.147	690	2130	2362
MSTRG.3895.6	coding	0.12	54	1260	1415
MSTRG.3895.7	coding	0.128	690	1866	2019
MSTRG.3896.1	noncoding	-0.008192	159	174	339
MSTRG.3897.1	noncoding	-0.1052672	0	204	1433
MSTRG.3900.2	coding	0.038	1224	1782	3595
MSTRG.3902.2	coding	0.06	84	1500	3980
MSTRG.3903.2	coding	0.123	492	822	2628
MSTRG.3903.3	coding	0.123	432	762	2503
MSTRG.3903.4	coding	0.123	492	822	2325
MSTRG.3903.5	coding	0.02	219	363	6908
MSTRG.3910.2	coding	0.029	195	2841	4152
MSTRG.3911.3	coding	0.042	660	1278	2492

MSTRG.3913.3	coding	0.238	918	2145	4599
MSTRG.3918.1	noncoding	-0.0004096	0	0	688
MSTRG.3935.6	coding	0.052	0	1038	3045
MSTRG.3935.5	noncoding	-0.0135168	1839	2016	3091
MSTRG.3935.7	noncoding	-0.0135168	1818	1995	2975
MSTRG.3935.8	coding	0.059	219	789	1962
MSTRG.3935.9	coding	0.102	0	558	2073
MSTRG.3935.11	noncoding	-0.004096	213	318	2747
MSTRG.3936.1	noncoding	-0.012288	408	486	816
MSTRG.3937.1	noncoding	-0.024576	2433	2535	3504
MSTRG.3930.1	noncoding	-0.11264	0	9	858
MSTRG.3934.1	noncoding	-0.057344	1068	1140	2109
MSTRG.3939.2	coding	0.229	0	402	1098
MSTRG.3939.4	noncoding	-0.0004096	84	174	660
MSTRG.3940.3	coding	0.1	0	867	5947
MSTRG.3940.6	coding	0.148	462	681	3306
MSTRG.3940.7	coding	0.148	495	714	3309
MSTRG.3941.1	noncoding	-0.0057344	0	3	492
MSTRG.3954.1	noncoding	-0.08192	285	288	448
MSTRG.3959.2	coding	0.081	0	1872	2156
MSTRG.3960.1	noncoding	-0.045056	333	375	827
MSTRG.3963.3	coding	0.045	159	3732	4101
MSTRG.3964.1	noncoding	-0.16384	303	324	836
MSTRG.3963.6	coding	0.042	282	2346	2716
MSTRG.3965.2	coding	0.227	0	735	6797
MSTRG.3993.2	coding	0.112	30	582	5195
MSTRG.3995.1	noncoding	-0.200704	264	315	469
MSTRG.3997.3	noncoding	-0.0577536	0	522	4775
MSTRG.3997.2	coding	0.175	0	570	24021
MSTRG.4000.1	noncoding	-0.0004096	297	435	1893
MSTRG.4003.1	coding	0.134	1587	3030	4423
MSTRG.4003.3	coding	0.133	384	1512	5162
MSTRG.4003.4	coding	0.135	582	2046	5696
MSTRG.4004.1	noncoding	-0.065536	4545	4629	5092
MSTRG.4006.1	coding	0.078	9	465	7922
MSTRG.4006.2	coding	0.077	9	459	2182
MSTRG.4010.2	coding	0.046	1872	5223	6017
MSTRG.4010.1	coding	0.046	1266	4617	5410
MSTRG.4010.3	coding	0.046	8736	12087	16470
MSTRG.4011.1	noncoding	-0.0008192	447	588	1904
MSTRG.4010.6	noncoding	-0.045056	327	387	3121
MSTRG.4014.1	noncoding	-0.0479232	0	492	3180
MSTRG.4017.1	coding	0.056	963	1218	3984
MSTRG.4017.3	coding	0.056	582	837	2570
MSTRG.4008.3	coding	0.152	1179	2445	2598
MSTRG.4020.1	noncoding	-0.012288	3738	3810	6337
MSTRG.4023.2	coding	0.006	1611	1758	3674
MSTRG.4024.2	coding	0.003	222	438	623
MSTRG.4024.4	coding	0.03	222	327	568
MSTRG.4024.5	coding	0.021	216	381	537
MSTRG.4026.1	coding	0.073	1236	3078	3612
MSTRG.4026.2	coding	0.073	1092	2934	3468
MSTRG.4026.3	coding	0.073	1296	3138	3463
MSTRG.4026.4	coding	0.073	2757	4599	4878

MSTRG.4026.5	coding	0.073	1326	3168	3440
MSTRG.4026.6	coding	0.073	1080	2922	3170
MSTRG.4028.5	coding	0.216	408	2655	6035
MSTRG.4028.6	coding	0.207	231	2586	4436
MSTRG.4028.7	coding	0.21	297	2598	4446
MSTRG.4028.8	coding	0.21	201	2496	5876
MSTRG.4028.9	coding	0.207	171	2532	4382
MSTRG.4030.3	noncoding	-0.0086016	1770	1923	3434
MSTRG.4036.1	noncoding	-0.0479232	33	426	6704
MSTRG.4037.1	noncoding	-0.0004096	0	0	314
MSTRG.4047.3	noncoding	-0.0786432	318	648	5667
MSTRG.4047.4	noncoding	-0.0397312	54	300	1669
MSTRG.4047.5	noncoding	-0.0479232	5274	5469	5624
MSTRG.4047.6	noncoding	-0.0487424	4527	4698	4852
MSTRG.4047.7	noncoding	-0.0602112	0	297	4202
MSTRG.4047.8	coding	0.079	6591	6978	8437
MSTRG.4047.9	coding	0.016	2733	3009	3825
MSTRG.4038.1	noncoding	-0.090112	1626	1710	1866
MSTRG.4051.1	coding	0.089	3363	3540	3991
MSTRG.4051.2	noncoding	-0.0454656	7047	7230	11712
MSTRG.4052.4	coding	0.121	396	1350	2075
MSTRG.4053.1	coding	0.152	813	1167	2594
MSTRG.4055.1	noncoding	-0.057344	393	651	806
MSTRG.4058.1	coding	0.258	330	2634	6582
MSTRG.4058.5	coding	0.265	330	2523	5031
MSTRG.4072.1	coding	0.292	36	1797	2120
MSTRG.4072.3	coding	0.238	0	189	344
MSTRG.4074.1	coding	0.166	0	216	369
MSTRG.4079.1	coding	0.236	0	153	2367
MSTRG.4080.1	noncoding	-0.022528	186	270	427
MSTRG.4081.1	coding	0.215	438	1191	3913
MSTRG.4082.1	noncoding	-0.0475136	75	240	611
MSTRG.4083.1	coding	0.242	117	939	1718
MSTRG.4068.2	coding	0.09	204	963	3405
MSTRG.4070.1	noncoding	-0.0004096	0	0	399
MSTRG.4092.3	noncoding	-0.0991232	2304	2853	17295
MSTRG.4092.4	coding	0.12	9069	9315	10095
MSTRG.4093.1	noncoding	-0.004096	2691	2913	3740
MSTRG.4093.2	noncoding	-0.053248	57	96	271
MSTRG.4094.1	noncoding	-0.0032768	1926	1971	4358
MSTRG.4095.1	noncoding	-0.012288	507	648	1479
MSTRG.4096.1	noncoding	-0.0004096	0	0	4472
MSTRG.4092.5	coding	0.021	4569	4866	8876
MSTRG.4092.6	noncoding	-0.0360448	8004	8418	8574
MSTRG.4092.7	noncoding	-0.114688	1170	1221	1377
MSTRG.4092.8	noncoding	-0.1605632	0	60	2232
MSTRG.4099.1	coding	0.358	0	267	6881
MSTRG.4104.1	noncoding	-0.04096	258	321	1411
MSTRG.4108.1	coding	0.061	411	1929	2319
MSTRG.4108.2	coding	0.037	357	1716	2106
MSTRG.4108.3	coding	0.037	2550	3909	4191
MSTRG.4108.6	noncoding	-0.1175552	996	1023	2034
MSTRG.4108.5	coding	0.061	135	1653	2044
MSTRG.4116.1	coding	0.075	1341	1620	4131

MSTRG.4122.3	coding	0.031	4026	4611	4829
MSTRG.4122.2	coding	0.038	750	2403	2987
MSTRG.4122.1	coding	0.071	1320	3864	8733
MSTRG.4125.3	coding	0.274	258	1164	2518
MSTRG.4129.4	coding	0.076	516	1749	2607
MSTRG.4129.7	coding	0.076	552	1785	2643
MSTRG.4129.8	coding	0.076	1281	2514	3375
MSTRG.4129.9	coding	0.074	1281	2511	3369
MSTRG.4130.1	noncoding	-0.02048	5349	5478	9426
MSTRG.4143.2	coding	0.084	552	819	2137
MSTRG.4143.4	noncoding	-0.0004096	0	0	426
MSTRG.4143.3	noncoding	-0.036864	1308	1488	1895
MSTRG.4144.1	noncoding	-0.0004096	0	0	366
MSTRG.4143.6	coding	0.09	2109	2322	4200
MSTRG.4146.2	coding	0.055	387	549	1475
MSTRG.4147.1	noncoding	-0.049152	204	321	496
MSTRG.4152.2	coding	0.097	165	369	1494
MSTRG.4131.1	noncoding	-0.0212992	147	342	496
MSTRG.4132.3	coding	0.067	159	1233	3703
MSTRG.4132.5	coding	0.067	159	1233	3482
MSTRG.4132.6	coding	0.066	159	1230	1571
MSTRG.4133.1	noncoding	-0.0499712	1083	1290	9020
MSTRG.4134.1	noncoding	-0.0069632	213	489	4348
MSTRG.4139.1	noncoding	-0.053248	4371	4449	5900
MSTRG.4163.2	coding	0.249	4560	5226	7000
MSTRG.4163.1	coding	0.057	120	5286	7061
MSTRG.4163.3	coding	0.178	3	969	2609
MSTRG.4163.4	coding	0.07	276	4410	5810
MSTRG.4163.5	coding	0.249	3696	4362	5763
MSTRG.4163.6	coding	0.249	6156	6873	7637
MSTRG.4163.7	coding	0.07	288	4422	5138
MSTRG.4162.1	noncoding	-0.0004096	0	0	216
MSTRG.4178.5	coding	0.047	5292	6090	8196
MSTRG.4178.3	coding	0.047	5301	6099	9969
MSTRG.4178.6	coding	0.089	120	741	1220
MSTRG.4178.4	coding	0.069	360	741	1221
MSTRG.4178.8	coding	0.048	405	804	1283
MSTRG.4178.7	coding	0.089	933	1554	2033
MSTRG.4183.1	noncoding	-0.065536	267	297	2082
MSTRG.4186.1	noncoding	-0.0434176	1965	2031	7118
MSTRG.4186.2	noncoding	-0.0434176	1344	1410	3836
MSTRG.4186.3	noncoding	-0.028672	1233	1317	1528
MSTRG.4187.1	noncoding	-0.0004096	528	573	833
MSTRG.4203.3	noncoding	-0.139264	354	399	1252
MSTRG.4203.5	noncoding	-0.08192	318	321	830
MSTRG.4203.2	noncoding	-0.028672	2457	2511	2675
MSTRG.4203.6	noncoding	-0.0110592	765	792	1142
MSTRG.4203.7	noncoding	-0.045056	279	318	2374
MSTRG.4213.3	coding	0.174	225	537	1406
MSTRG.4214.1	noncoding	-0.014336	1026	1155	2634
MSTRG.4216.1	noncoding	-0.0004096	0	0	592
MSTRG.4217.3	coding	0.085	57	339	894
MSTRG.4226.1	coding	0.094	150	1881	4110
MSTRG.4226.2	coding	0.029	66	1377	2213

MSTRG.4227.1	noncoding	-0.02048	339	381	910
MSTRG.4226.3	coding	0.014	396	1230	2215
MSTRG.4228.7	coding	0.143	2478	2889	8390
MSTRG.4228.3	noncoding	-0.0557056	2478	2868	7748
MSTRG.4229.4	coding	0.165	0	237	4684
MSTRG.4229.2	coding	0.165	0	237	5730
MSTRG.4229.3	noncoding	-0.067584	0	237	3002
MSTRG.4230.1	noncoding	-0.0004096	123	207	936
MSTRG.4231.1	noncoding	-0.0004096	0	0	219
MSTRG.4219.1	noncoding	-0.151552	0	45	289
MSTRG.4246.3	coding	0.01	348	456	1489
MSTRG.4250.5	noncoding	-0.028672	111	228	2986
MSTRG.4256.1	coding	0.236	0	1101	3455
MSTRG.4256.3	coding	0.254	0	1098	7198
MSTRG.4256.2	coding	0.266	0	1080	7201
MSTRG.4256.4	coding	0.239	0	1143	7356
MSTRG.4257.1	noncoding	-0.0606208	0	156	378
MSTRG.4260.3	coding	0.223	303	1110	2205
MSTRG.4260.4	coding	0.13	2106	3555	3833
MSTRG.4261.3	coding	0.058	846	2043	4077
MSTRG.4261.2	coding	0.058	765	1962	4018
MSTRG.4261.4	coding	0.058	681	1878	3911
MSTRG.4262.1	noncoding	-0.118784	6	90	348
MSTRG.4293.3	noncoding	-0.053248	702	786	941
MSTRG.4293.4	noncoding	-0.053248	1083	1167	1323
MSTRG.4293.5	noncoding	-0.053248	867	951	1107
MSTRG.4293.6	noncoding	-0.053248	921	1005	1161
MSTRG.4293.7	noncoding	-0.053248	804	888	1043
MSTRG.4296.1	noncoding	-0.008192	441	522	1111
MSTRG.4301.2	coding	0.367	162	2064	2969
MSTRG.4301.1	coding	0.363	141	1917	2822
MSTRG.4301.3	coding	0.367	135	2037	2924
MSTRG.4301.4	coding	0.365	114	2007	2483
MSTRG.4301.5	coding	0.395	0	1695	2032
MSTRG.4301.6	coding	0.367	120	2022	2860
MSTRG.4302.1	noncoding	-0.0802816	390	1092	2460
MSTRG.4303.2	noncoding	-0.045056	3393	3498	4891
MSTRG.4304.2	noncoding	-0.057344	1158	1212	1468
MSTRG.4297.1	noncoding	-0.004096	474	504	799
MSTRG.4298.1	noncoding	-0.0139264	654	738	894
MSTRG.4312.5	noncoding	-0.1552384	0	186	1450
MSTRG.4312.4	coding	0.077	2742	6291	7020
MSTRG.4312.2	coding	0.062	0	6297	9709
MSTRG.4312.1	coding	0.062	0	6297	15143
MSTRG.4312.3	coding	0.062	0	6297	11509
MSTRG.4310.2	noncoding	-0.028672	48	126	2552
MSTRG.4317.2	coding	0.156	267	1728	8555
MSTRG.4319.1	coding	0.074	8859	9396	23271
MSTRG.4319.2	coding	0.074	46020	46557	60402
MSTRG.4319.3	noncoding	-0.0401408	2139	2295	9501
MSTRG.4319.4	noncoding	-0.034816	780	1041	24222
MSTRG.4319.5	noncoding	-0.032768	0	90	1952
MSTRG.4325.2	coding	0.271	261	1125	3448
MSTRG.4325.3	coding	0.345	0	288	823

MSTRG.4326.2	noncoding	-0.008192	1089	1350	1696
MSTRG.4328.2	coding	0.192	0	1029	8106
MSTRG.4328.6	coding	0.233	0	822	5472
MSTRG.4328.5	coding	0.192	0	1290	1443
MSTRG.4328.7	coding	0.256	0	687	1676
MSTRG.4328.11	coding	0.263	0	681	7757
MSTRG.4329.3	coding	0.163	0	2262	5574
MSTRG.4330.1	noncoding	-0.0004096	0	0	519
MSTRG.4335.2	coding	0.077	0	2352	6217
MSTRG.4341.1	noncoding	-0.0135168	480	642	1028
MSTRG.4341.2	noncoding	-0.028672	51	207	579
MSTRG.4341.3	noncoding	-0.0237568	285	441	1079
MSTRG.4343.1	coding	0.19	0	1776	3457
MSTRG.4344.1	noncoding	-0.065536	558	633	1772
MSTRG.4340.1	noncoding	-0.0004096	264	264	421
MSTRG.4346.2	coding	0.088	90	345	3365
MSTRG.4345.1	coding	0.283	282	1620	3128
MSTRG.4359.2	coding	0.147	0	7875	10549
MSTRG.4357.1	coding	0.474	0	1521	4134
MSTRG.4358.2	coding	0.306	144	1119	1318
MSTRG.4360.2	coding	0.333	54	1491	3906
MSTRG.4361.1	coding	0.156	318	1251	4747
MSTRG.4361.3	coding	0.17	354	999	1797
MSTRG.4363.1	coding	0.473	324	2262	3835
MSTRG.4363.2	coding	0.516	0	1809	8114
MSTRG.4363.3	coding	0.5	690	2490	2786
MSTRG.4367.4	noncoding	-0.0372736	6264	6717	7030
MSTRG.4367.5	noncoding	-0.0380928	2298	2871	40314
MSTRG.4369.1	noncoding	-0.0671744	20808	21129	25459
MSTRG.4365.2	noncoding	-0.0765952	24	237	523
MSTRG.4371.1	noncoding	-0.008192	633	762	1055
MSTRG.4371.2	noncoding	-0.008192	615	744	1035
MSTRG.4375.1	noncoding	-0.0126976	567	726	971
MSTRG.4372.2	coding	0.01	1110	1350	4535
MSTRG.4382.1	noncoding	-0.0004096	777	786	942
MSTRG.4388.1	coding	0.443	0	2238	7138
MSTRG.4388.3	coding	0.476	0	2661	7561
MSTRG.4388.9	coding	0.432	0	2277	7177
MSTRG.4384.2	coding	0.202	18	336	767
MSTRG.4390.1	noncoding	-0.0598016	1131	1416	2047
MSTRG.4390.2	noncoding	-0.0598016	948	1233	1610
MSTRG.4390.3	noncoding	-0.0598016	891	1176	1547
MSTRG.4399.1	noncoding	-0.0004096	0	0	572
MSTRG.4400.2	coding	0.121	0	1233	2298
MSTRG.4401.2	noncoding	-0.0512	8964	9285	10257
MSTRG.4401.3	coding	0.028	1236	2871	10085
MSTRG.4399.3	coding	0.431	0	822	1451
MSTRG.4401.5	noncoding	-0.0512	10449	10770	11743
MSTRG.4401.8	noncoding	-0.0512	9945	10266	11124
MSTRG.4401.9	noncoding	-0.0118784	9663	9831	12847
MSTRG.4401.11	noncoding	-0.0180224	5595	5754	10238
MSTRG.4401.12	noncoding	-0.0180224	5595	5754	10301
MSTRG.4401.10	noncoding	-0.0118784	9651	9819	12938
MSTRG.4401.13	noncoding	-0.0360448	9984	10281	10437

MSTRG.4401.14	noncoding	-0.0360448	9921	10218	10374
MSTRG.4401.16	noncoding	-0.0528384	7122	7365	7520
MSTRG.4403.1	coding	0.103	1011	1530	2843
MSTRG.4403.3	coding	0.042	639	4932	7094
MSTRG.4404.1	noncoding	-0.004096	846	981	2672
MSTRG.4408.1	noncoding	-0.077824	2028	2094	2370
MSTRG.4409.2	coding	0.179	429	2877	3130
MSTRG.4409.3	coding	0.183	132	2418	2671
MSTRG.4411.1	coding	0.399	582	2682	10320
MSTRG.4411.2	coding	0.399	543	2643	8836
MSTRG.4412.1	coding	0.082	0	4599	4766
MSTRG.4412.2	coding	0.086	0	4428	4582
MSTRG.4412.3	coding	0.077	210	4230	4386
MSTRG.4413.1	coding	0.078	111	711	5657
MSTRG.4413.2	coding	0.06	207	621	3466
MSTRG.4413.5	coding	0.062	357	582	1108
MSTRG.4414.2	coding	0.203	255	651	1495
MSTRG.4418.1	coding	0.201	165	3507	4176
MSTRG.4420.9	coding	0.057	3453	4779	5535
MSTRG.4420.10	coding	0.083	8298	10476	11231
MSTRG.4420.11	coding	0.057	3609	4935	5419
MSTRG.4421.1	noncoding	-0.12288	3255	3297	5550
MSTRG.4422.1	noncoding	-0.0352256	27	204	359
MSTRG.4422.2	noncoding	-0.0258048	24	192	346
MSTRG.4423.4	coding	0.135	0	2226	3173
MSTRG.4423.5	coding	0.122	576	3165	3930
MSTRG.4424.1	noncoding	-0.12288	84	99	255
MSTRG.4426.4	coding	0.399	558	1767	2799
MSTRG.4428.2	coding	0.221	0	531	2232
MSTRG.4429.1	noncoding	-0.012288	678	888	1936
MSTRG.4431.1	noncoding	-0.196608	660	681	1648
MSTRG.4432.2	coding	0.177	1026	1503	1968
MSTRG.4434.1	coding	0.063	1011	1128	1995
MSTRG.4446.2	coding	0.2	0	411	989
MSTRG.4446.1	coding	0.288	0	1467	1676
MSTRG.4446.4	coding	0.286	0	1497	1684
MSTRG.4448.1	coding	0.092	12906	13419	28325
MSTRG.4448.2	coding	0.017	3864	4434	6046
MSTRG.4449.1	noncoding	-0.0356352	819	912	6784
MSTRG.4450.1	noncoding	-0.073728	381	438	835
MSTRG.4451.1	noncoding	-0.0094208	1173	1308	2684
MSTRG.4452.1	coding	0.079	654	846	2549
MSTRG.4454.1	noncoding	-0.083968	498	561	938
MSTRG.4456.1	noncoding	-0.090112	57	75	248
MSTRG.4443.3	noncoding	-0.0282624	711	975	1192
MSTRG.4445.1	noncoding	-0.024576	1446	1713	3568
MSTRG.4462.1	noncoding	-0.0569344	774	957	10178
MSTRG.4462.2	noncoding	-0.0745472	3222	3405	12615
MSTRG.4463.1	noncoding	-0.0282624	1290	1683	4867
MSTRG.4464.1	noncoding	-0.016384	93	234	970
MSTRG.4465.1	noncoding	-0.114688	33	39	304
MSTRG.4467.4	coding	0.165	192	1068	4463
MSTRG.4470.3	coding	0.668	0	291	3630
MSTRG.4470.4	coding	0.686	0	282	3761

MSTRG.4470.5	coding	0.686	0	282	2230
MSTRG.4473.3	coding	0.357	468	1419	2841
MSTRG.4471.2	noncoding	-0.0299008	0	414	570
MSTRG.4471.1	noncoding	-0.0610304	330	591	840
MSTRG.4474.5	coding	0.251	1977	4320	4474
MSTRG.4474.4	coding	0.341	1467	3582	3737
MSTRG.4474.7	coding	0.341	12927	15042	15197
MSTRG.4474.8	coding	0.237	0	1029	9348
MSTRG.4474.10	coding	0.193	2460	3399	4745
MSTRG.4474.12	coding	0.234	1518	2235	4098
MSTRG.4474.13	coding	0.234	1518	2235	3517
MSTRG.4474.14	coding	0.234	1518	2235	3899
MSTRG.4474.11	coding	0.234	1518	2235	4795
MSTRG.4479.6	noncoding	-0.0114688	501	696	1018
MSTRG.4479.4	noncoding	-0.0667648	474	1392	1576
MSTRG.4479.7	noncoding	-0.0958464	543	1014	1330
MSTRG.4479.2	noncoding	-0.0606208	0	1557	1879
MSTRG.4479.1	noncoding	-0.0561152	78	300	624
MSTRG.4480.2	coding	0.205	0	339	2453
MSTRG.4481.4	coding	0.269	615	2838	10153
MSTRG.4481.5	coding	0.252	621	2904	12175
MSTRG.4481.1	coding	0.268	624	2793	12065
MSTRG.4481.2	coding	0.268	624	2793	11335
MSTRG.4477.2	coding	0.424	345	2301	2997
MSTRG.4477.3	coding	0.418	627	2493	3189
MSTRG.4477.4	coding	0.413	339	2355	3050
MSTRG.4487.1	coding	0.259	0	1281	4772
MSTRG.4488.1	noncoding	-0.032768	9783	9852	14126
MSTRG.4488.2	noncoding	-0.032768	6111	6180	7700
MSTRG.4488.3	noncoding	-0.032768	477	546	1556
MSTRG.4494.1	coding	0.071	15159	15723	15951
MSTRG.4495.1	coding	0.118	5130	5505	6450
MSTRG.4494.8	coding	0.032	2364	2847	3001
MSTRG.4494.9	coding	0.148	960	1218	2030
MSTRG.4496.1	coding	0.127	0	150	2031
MSTRG.4494.10	coding	0.068	9690	10248	11302
MSTRG.4494.11	noncoding	-0.0004096	99	195	7615
MSTRG.4498.1	noncoding	-0.0045056	2517	2670	8408
MSTRG.4494.12	coding	0.161	543	1797	2845
MSTRG.4494.13	coding	0.026	1044	1254	1827
MSTRG.4499.3	coding	0.191	0	1140	1754
MSTRG.4483.6	coding	0.232	336	2586	3492
MSTRG.4483.4	coding	0.209	27	1464	6414
MSTRG.4483.5	coding	0.236	96	2400	3306
MSTRG.4483.3	coding	0.239	555	2799	3710
MSTRG.4503.1	noncoding	-0.049152	270	393	546
MSTRG.4504.5	coding	0.271	2655	4281	5273
MSTRG.4506.1	noncoding	-0.0118784	135	279	473
MSTRG.4515.1	coding	0.374	0	3831	4499
MSTRG.4516.2	noncoding	-0.0057344	81	150	509
MSTRG.4516.1	noncoding	-0.0057344	81	150	562
MSTRG.4520.2	coding	0.3	2439	4446	5089
MSTRG.4520.4	coding	0.308	2439	4323	4812
MSTRG.4521.1	noncoding	-0.008192	327	465	853

MSTRG.4533.11	coding	0.316	528	1200	2131
MSTRG.4533.7	coding	0.3	492	1089	2021
MSTRG.4533.10	coding	0.444	0	396	1950
MSTRG.4533.3	coding	0.365	66	915	1847
MSTRG.4533.1	coding	0.352	96	1230	2161
MSTRG.4533.2	coding	0.374	96	1152	2083
MSTRG.4533.4	coding	0.315	0	1233	2165
MSTRG.4533.8	coding	0.345	96	1026	1957
MSTRG.4533.6	coding	0.371	171	1023	1956
MSTRG.4533.5	coding	0.426	0	846	1778
MSTRG.4523.3	coding	0.229	507	1185	1706
MSTRG.4523.4	coding	0.229	495	1173	1543
MSTRG.4524.1	noncoding	-0.0540672	864	1245	6065
MSTRG.4525.1	noncoding	-0.0405504	1239	1632	2100
MSTRG.4538.1	noncoding	-0.02048	237	336	492
MSTRG.4540.1	coding	0.379	126	624	781
MSTRG.4541.2	coding	0.263	183	1134	1454
MSTRG.4549.1	noncoding	-0.110592	438	444	686
MSTRG.4552.4	coding	0.346	609	3486	4189
MSTRG.4553.1	noncoding	-0.0126976	774	1023	3148
MSTRG.4543.2	coding	0.608	72	5547	7212
MSTRG.4543.3	coding	0.612	2184	7779	8005
MSTRG.4562.2	coding	0.239	0	702	6993
MSTRG.4562.3	coding	0.222	1350	2100	4153
MSTRG.4564.1	coding	0.312	180	1761	2217
MSTRG.4555.2	coding	0.387	138	3894	4100
MSTRG.4557.1	noncoding	-0.2052096	6	15	348
MSTRG.4559.3	coding	0.237	486	783	1214
MSTRG.4571.1	coding	0.146	306	684	2916
MSTRG.4572.1	noncoding	-0.014336	4020	4224	6422
MSTRG.4574.1	noncoding	-0.0438272	24	234	1177
MSTRG.4578.1	noncoding	-0.0536576	63	252	673
MSTRG.4578.3	noncoding	-0.0905216	0	207	629
MSTRG.4579.2	coding	0.135	0	852	1048
MSTRG.4580.2	coding	0.178	342	864	1306
MSTRG.4592.1	coding	0.308	615	1686	2223
MSTRG.4592.2	coding	0.296	348	1413	1786
MSTRG.4594.1	coding	0.178	2703	4233	4929
MSTRG.4594.2	coding	0.156	0	3828	4526
MSTRG.4594.4	coding	0.156	1194	3321	3902
MSTRG.4594.6	coding	0.188	1281	1917	2323
MSTRG.4594.5	coding	0.148	2256	4668	5073
MSTRG.4594.7	coding	0.184	8442	9423	9802
MSTRG.4595.1	noncoding	-0.073728	1662	1713	6305
MSTRG.4594.9	noncoding	-0.1024	3651	3720	9913
MSTRG.4594.10	coding	0.162	0	2025	2687
MSTRG.4594.11	noncoding	-0.0294912	333	732	2205
MSTRG.4585.3	coding	0.098	168	573	1971
MSTRG.4585.4	noncoding	-0.0610304	216	372	526
MSTRG.4585.5	coding	0.096	966	1890	4827
MSTRG.4583.1	noncoding	-0.0700416	0	288	571
MSTRG.4588.1	noncoding	-0.0004096	462	606	1024
MSTRG.4600.1	noncoding	-0.0299008	9408	9621	9778
MSTRG.4600.2	noncoding	-0.0008192	1068	1215	2909

MSTRG.4600.3	noncoding	-0.0913408	2949	3483	3639
MSTRG.4601.3	coding	0.22	420	2562	3312
MSTRG.4601.4	coding	0.216	531	2706	3455
MSTRG.4602.1	coding	0.056	438	723	4597
MSTRG.4603.1	noncoding	-0.0090112	792	855	1334
MSTRG.4601.8	coding	0.161	1167	1848	2596
MSTRG.4601.9	coding	0.135	345	996	1744
MSTRG.4604.1	coding	0.537	4767	5910	6088
MSTRG.4604.3	coding	0.537	366	1509	1687
MSTRG.4605.7	coding	0.363	93	1197	1428
MSTRG.4605.8	coding	0.355	2967	4089	4319
MSTRG.4605.4	coding	0.35	306	1461	1691
MSTRG.4605.6	coding	0.35	798	1953	2183
MSTRG.4605.9	noncoding	-0.1568768	279	1146	1302
MSTRG.4605.10	noncoding	-0.032768	1653	1767	4583
MSTRG.4608.3	noncoding	-0.147456	141	198	353
MSTRG.4608.6	coding	0.162	72	1233	2166
MSTRG.4609.2	coding	0.233	669	1395	3855
MSTRG.4609.1	coding	0.232	3720	4449	6909
MSTRG.4611.1	noncoding	-0.0004096	0	0	229
MSTRG.4615.2	coding	0.107	45	225	458
MSTRG.4616.2	coding	0.077	4053	4995	6152
MSTRG.4617.1	noncoding	-0.06144	5664	5787	16053
MSTRG.4618.1	coding	0.074	3042	3234	3389
MSTRG.4616.3	noncoding	-0.0360448	1497	1920	2085
MSTRG.4627.9	coding	0.236	417	1794	3008
MSTRG.4627.10	coding	0.241	3	1443	2657
MSTRG.4627.7	coding	0.24	3	1446	2660
MSTRG.4620.2	coding	0.315	0	609	3127
MSTRG.4632.3	noncoding	-0.0028672	459	609	3531
MSTRG.4634.9	coding	0.328	756	2697	3107
MSTRG.4634.10	coding	0.299	3	1569	1724
MSTRG.4634.13	coding	0.322	0	1416	1824
MSTRG.4706.9	coding	0.337	621	12858	13984
MSTRG.4706.6	coding	0.337	0	16314	17442
MSTRG.4706.4	coding	0.331	0	16257	17385
MSTRG.4706.27	coding	0.336	1200	10431	11242
MSTRG.4706.18	coding	0.338	2445	14589	15399
MSTRG.4706.25	coding	0.27	252	19977	20787
MSTRG.4706.19	coding	0.278	0	22353	23164
MSTRG.4706.12	coding	0.331	13260	29991	30801
MSTRG.4706.14	coding	0.33	27600	43602	44413
MSTRG.4706.24	coding	0.331	1725	17898	18709
MSTRG.4706.16	coding	0.272	267	22578	23388
MSTRG.4706.15	coding	0.273	87	22461	23272
MSTRG.4706.30	noncoding	-0.0937984	0	366	13445
MSTRG.4706.31	noncoding	-0.0753664	1152	1503	1658
MSTRG.4706.32	noncoding	-0.086016	6093	6399	6555
MSTRG.4706.33	noncoding	-0.0933888	36123	37311	50619
MSTRG.4648.1	noncoding	-0.0606208	171	552	1878
MSTRG.4649.1	noncoding	-0.0299008	144	414	1117
MSTRG.4649.2	noncoding	-0.0299008	144	414	1026
MSTRG.4651.1	coding	0.349	0	963	1673
MSTRG.4660.3	coding	0.17	585	2898	3118

MSTRG.4654.1	noncoding	-0.124928	132	1131	2845
MSTRG.4654.2	noncoding	-0.1175552	132	1272	2986
MSTRG.4666.1	coding	0.521	0	708	2699
MSTRG.4673.2	coding	0.295	849	1860	2171
MSTRG.4673.3	coding	0.305	189	1281	1590
MSTRG.4675.2	coding	0.457	0	1653	3169
MSTRG.4679.3	coding	0.279	0	2136	2291
MSTRG.4679.2	coding	0.272	378	2154	2309
MSTRG.4679.5	coding	0.275	0	1671	1825
MSTRG.4680.6	coding	0.197	258	2883	4299
MSTRG.4680.7	noncoding	-0.0319488	9045	9201	9427
MSTRG.4680.8	noncoding	-0.0131072	1809	1944	3350
MSTRG.4677.1	coding	0.177	510	972	1722
MSTRG.4677.4	coding	0.178	639	1095	1843
MSTRG.4682.1	coding	0.478	0	1176	1778
MSTRG.4682.2	coding	0.344	0	705	1206
MSTRG.4687.1	coding	0.434	579	2136	2343
MSTRG.4687.5	coding	0.502	591	1875	2080
MSTRG.4687.4	coding	0.434	258	1815	2019
MSTRG.4689.1	coding	0.372	78	2616	8850
MSTRG.4689.2	coding	0.387	0	2700	8186
MSTRG.4691.1	noncoding	-0.1867776	0	117	432
MSTRG.4692.2	coding	0.498	27	2286	3369
MSTRG.4698.1	noncoding	-0.0004096	0	0	1774
MSTRG.4700.1	noncoding	-0.0577536	1917	2145	6070
MSTRG.4701.1	noncoding	-0.053248	2664	2730	4573
MSTRG.4703.2	coding	0.145	147	417	5559
MSTRG.4703.1	coding	0.145	183	453	3961
MSTRG.4703.3	noncoding	-0.0385024	69	252	2030
MSTRG.4704.1	noncoding	-0.0024576	504	657	1738
MSTRG.4703.4	coding	0.145	147	417	5349
MSTRG.4718.3	noncoding	-0.0045056	327	468	1415
MSTRG.4718.6	noncoding	-0.024576	2280	2442	5826
MSTRG.4718.8	noncoding	-0.0016384	351	498	2822
MSTRG.4705.2	noncoding	-0.0589824	294	912	1279
MSTRG.4709.2	noncoding	-0.0495616	516	873	1028
MSTRG.4709.1	noncoding	-0.0495616	288	645	799
MSTRG.4711.4	coding	0.296	63	3183	3339
MSTRG.4711.5	coding	0.297	63	3156	3312
MSTRG.4711.6	coding	0.298	0	3171	3328
MSTRG.4710.1	coding	0.332	0	957	2186
MSTRG.4725.1	coding	0.248	351	4203	5869
MSTRG.4725.2	coding	0.248	267	4119	7017
MSTRG.4725.12	noncoding	-0.14336	300	309	647
MSTRG.4725.10	coding	0.248	129	3963	5628
MSTRG.4725.11	coding	0.246	1818	5661	7325
MSTRG.4725.9	coding	0.246	129	3972	6870
MSTRG.4725.13	coding	0.247	1650	5481	7147
MSTRG.4725.16	coding	0.264	1023	4155	7054
MSTRG.4725.17	coding	0.267	102	3279	4943
MSTRG.4725.18	coding	0.262	189	3321	4985
MSTRG.4725.19	coding	0.264	96	3264	6163
MSTRG.4725.20	coding	0.262	204	3327	6226
MSTRG.4725.21	coding	0.268	183	2685	6053

MSTRG.4725.22	coding	0.263	162	3297	4961
MSTRG.4722.3	coding	0.471	0	255	4744
MSTRG.4729.1	coding	0.124	180	555	854
MSTRG.4734.4	coding	0.374	162	2703	2962
MSTRG.4734.5	coding	0.51	63	2052	3295
MSTRG.4735.1	noncoding	-0.069632	438	669	825
MSTRG.4735.2	coding	0.437	465	1308	2390
MSTRG.4730.1	coding	0.257	12	393	596
MSTRG.4731.1	noncoding	-0.049152	408	513	772
MSTRG.4738.1	noncoding	-0.0233472	408	414	569
MSTRG.4741.2	noncoding	-0.028672	1698	2082	7133
MSTRG.4744.1	noncoding	-0.022528	1389	1677	3928
MSTRG.4746.1	noncoding	-0.0110592	159	351	799
MSTRG.4747.3	coding	0.509	0	3711	11286
MSTRG.4750.1	noncoding	-0.038912	135	222	377
MSTRG.4751.1	noncoding	-0.004096	324	390	668
MSTRG.4754.1	coding	0.263	6	2550	2877
MSTRG.4754.4	coding	0.264	51	2604	2759
MSTRG.4754.5	coding	0.322	0	1185	1659
MSTRG.4754.6	coding	0.305	1233	2187	2990
MSTRG.4754.7	coding	0.302	810	1758	1977
MSTRG.4752.1	coding	0.501	0	1407	1788
MSTRG.4757.1	noncoding	-0.065536	5640	5835	7550
MSTRG.4758.2	coding	0.235	183	543	1622
MSTRG.4758.3	coding	0.11	51	303	1382
MSTRG.4761.1	coding	0.413	78	8298	9094
MSTRG.4761.3	coding	0.413	75	8241	8905
MSTRG.4761.5	coding	0.414	102	8328	8755
MSTRG.4762.1	noncoding	-0.0520192	312	474	765
MSTRG.4763.1	coding	0.462	0	459	10596
MSTRG.4767.2	noncoding	-0.2613248	0	237	5213
MSTRG.4768.1	noncoding	-0.0053248	420	741	1469
MSTRG.4767.6	coding	0.325	18	1299	2023
MSTRG.4769.1	noncoding	-0.0516096	30	270	3712
MSTRG.4767.7	coding	0.3	3813	6258	10996
MSTRG.4772.2	noncoding	-0.0077824	5337	5526	5680
MSTRG.4766.1	noncoding	-0.0704512	174	423	711
MSTRG.4766.2	noncoding	-0.0704512	165	414	816
MSTRG.4766.3	noncoding	-0.032768	54	177	673
MSTRG.4774.3	coding	0.533	0	864	1330
MSTRG.4774.2	coding	0.531	0	867	1491
MSTRG.4781.2	coding	0.383	702	4590	6759
MSTRG.4785.3	coding	0.538	0	1869	2805
MSTRG.4785.6	coding	0.526	0	1101	5226
MSTRG.4782.1	coding	0.54	0	1065	7010
MSTRG.4782.3	coding	0.549	0	1035	6612
MSTRG.4787.1	coding	0.49	0	3750	8756
MSTRG.4787.2	coding	0.474	57	2145	6636
MSTRG.4787.3	coding	0.487	0	3729	4956
MSTRG.4788.1	noncoding	-0.0684032	669	1296	1647
MSTRG.4789.1	noncoding	-0.0258048	246	735	991
MSTRG.4796.1	coding	0.295	1104	2541	4545
MSTRG.4800.2	coding	0.714	0	1356	2094
MSTRG.4811.1	noncoding	-0.0540672	1716	2541	2696

MSTRG.4811.3	noncoding	-0.0544768	1488	2307	2461
MSTRG.4813.1	coding	0.616	180	5316	7790
MSTRG.4814.1	noncoding	-0.0376832	312	7005	7159
MSTRG.4815.1	noncoding	-0.022528	174	360	514
MSTRG.4826.4	coding	0.308	537	1038	1552
MSTRG.4826.5	coding	0.252	273	1164	1679
MSTRG.4826.2	coding	0.251	1269	1620	2136
MSTRG.4826.3	coding	0.426	0	4335	5877
MSTRG.4826.10	coding	0.427	0	4431	4950
MSTRG.4816.1	noncoding	-0.0720896	132	486	1268
MSTRG.4818.1	coding	0.446	123	1116	2993
MSTRG.4818.2	coding	0.446	828	1821	3696
MSTRG.4818.4	coding	0.446	453	1446	3321
MSTRG.4819.1	noncoding	-0.0405504	0	156	434
MSTRG.4830.2	coding	0.345	0	1881	2244
MSTRG.4831.4	coding	0.585	132	1581	1735
MSTRG.4831.5	coding	0.586	66	1518	1671
MSTRG.4836.1	coding	0.482	60	1740	2638
MSTRG.4836.2	coding	0.498	0	1638	2535
MSTRG.4843.1	noncoding	-0.188416	87	132	286
MSTRG.4844.1	noncoding	-0.0770048	0	78	232
MSTRG.4848.1	noncoding	-0.06144	276	336	703
MSTRG.4849.1	coding	0.33	0	798	1968
MSTRG.4860.1	noncoding	-0.047104	1488	1644	1799
MSTRG.4861.1	coding	0.265	138	1164	1499
MSTRG.4861.2	coding	0.248	138	3801	5086
MSTRG.4870.1	noncoding	-0.0720896	18	432	589
MSTRG.4876.1	coding	0.119	0	681	1728
MSTRG.4877.1	noncoding	-0.045056	1686	1809	2621
MSTRG.4882.1	noncoding	-0.0540672	0	342	1272
MSTRG.4882.2	noncoding	-0.0159744	939	1119	1288
MSTRG.4873.1	noncoding	-0.0172032	657	762	917
MSTRG.4886.1	coding	0.266	207	1056	2961
MSTRG.4887.1	coding	0.324	0	3279	4175
MSTRG.4887.2	coding	0.447	0	990	2445
MSTRG.4895.2	coding	0.103	66	711	3962
MSTRG.4903.1	noncoding	-0.0028672	258	318	662
MSTRG.4903.2	coding	0.061	210	630	2669
MSTRG.4921.2	coding	0.132	0	11448	12812
MSTRG.4921.3	coding	0.133	0	11463	11889
MSTRG.4922.2	coding	0.131	0	1203	1419
MSTRG.4924.1	noncoding	-0.0131072	1152	1158	1464
MSTRG.4925.1	noncoding	-0.335872	612	663	818
MSTRG.4928.3	coding	0.094	366	3216	5461
MSTRG.4928.4	coding	0.094	276	3126	3741
MSTRG.4929.1	noncoding	-0.0458752	198	333	4115
MSTRG.4928.6	noncoding	-0.032768	243	351	505
MSTRG.4930.1	noncoding	-0.024576	1614	1704	5370
MSTRG.4928.7	coding	0.135	432	1956	4539
MSTRG.4912.2	coding	0.097	198	492	1343
MSTRG.4913.1	coding	0.116	810	1044	3173
MSTRG.4913.3	coding	0.365	0	360	5062
MSTRG.4913.4	coding	0.152	858	1110	5603
MSTRG.4920.1	noncoding	-0.045056	27	144	466

MSTRG.4942.1	noncoding	-0.0131072	195	204	451
MSTRG.4950.1	noncoding	-0.069632	537	651	1196
MSTRG.4951.2	noncoding	-0.02048	1137	1260	3316
MSTRG.4953.1	noncoding	-0.0917504	0	891	1402
MSTRG.4961.1	coding	0.202	468	4404	10354
MSTRG.4961.7	coding	0.264	0	1869	7744
MSTRG.4961.4	coding	0.202	213	4149	10120
MSTRG.4961.5	coding	0.202	213	4149	10100
MSTRG.4961.6	coding	0.215	0	2484	8455
MSTRG.4961.10	coding	0.205	507	4389	10337
MSTRG.4961.8	coding	0.202	507	4443	10393
MSTRG.4961.9	coding	0.202	507	4443	10391
MSTRG.4961.12	coding	0.177	450	1773	2572
MSTRG.4961.11	coding	0.231	450	3717	9559
MSTRG.4961.13	coding	0.202	417	4353	10323
MSTRG.4961.15	coding	0.202	390	4326	10186
MSTRG.4961.14	coding	0.202	390	4326	10298
MSTRG.4962.1	noncoding	-0.131072	1239	1278	2006
MSTRG.4963.1	noncoding	-0.786432	564	702	2256
MSTRG.4964.1	noncoding	-0.077824	6069	6141	8243
MSTRG.4965.1	noncoding	-0.004096	2550	2640	9619
MSTRG.4965.2	noncoding	-0.0004096	3444	3588	4819
MSTRG.4965.3	noncoding	-0.0016384	27	228	3053
MSTRG.4966.1	noncoding	-0.126976	0	69	225
MSTRG.4967.1	noncoding	-0.0811008	357	396	1793
MSTRG.4961.17	coding	0.122	2781	3096	3919
MSTRG.4955.1	coding	0.049	675	831	1276
MSTRG.4956.1	noncoding	-0.073728	48	114	280
MSTRG.4959.3	coding	0.214	312	2100	3051
MSTRG.4960.1	noncoding	-0.004096	189	216	540
MSTRG.4975.2	noncoding	-0.04096	513	627	958
MSTRG.4980.10	coding	0.138	78	1818	2051
MSTRG.4980.4	coding	0.138	951	2556	2788
MSTRG.4980.9	coding	0.148	66	1701	1935
MSTRG.4980.8	coding	0.139	399	2043	2277
MSTRG.4980.5	coding	0.139	279	1893	2127
MSTRG.4980.2	coding	0.139	429	2073	2306
MSTRG.4981.2	noncoding	-0.1523712	0	252	5990
MSTRG.4985.6	coding	0.108	3024	3720	4422
MSTRG.4985.8	coding	0.095	237	927	1630
MSTRG.4990.3	noncoding	-0.0454656	24	195	16036
MSTRG.4990.4	noncoding	-0.0487424	0	156	1043
MSTRG.4991.1	noncoding	-0.0016384	1179	1338	7490
MSTRG.4998.3	coding	0.177	0	1245	3796
MSTRG.4998.4	coding	0.182	3189	4200	6751
MSTRG.4998.5	coding	0.18	3189	4197	6748
MSTRG.4998.6	coding	0.159	4218	4683	5448
MSTRG.4998.7	coding	0.103	237	426	793
MSTRG.4988.6	coding	0.188	108	1386	1661
MSTRG.5003.1	noncoding	-0.0118784	330	525	680
MSTRG.5020.1	noncoding	-0.0028672	156	231	495
MSTRG.5021.2	noncoding	-0.1605632	0	93	6109
MSTRG.5046.1	noncoding	-0.03072	366	435	637
MSTRG.5052.1	noncoding	-0.0118784	1551	1602	2744

MSTRG.5077.2	coding	0.153	324	1704	2276
MSTRG.5077.3	coding	0.153	258	1638	2210
MSTRG.5078.1	noncoding	-0.1024	114	129	448
MSTRG.5106.1	noncoding	-0.057344	99	102	410
MSTRG.5095.2	noncoding	-0.008192	579	717	931
MSTRG.5137.1	coding	0.103	2637	3621	11272
MSTRG.5138.1	noncoding	-0.0004096	0	0	569
MSTRG.5137.3	noncoding	-0.036864	1272	1347	2845
MSTRG.5137.2	noncoding	-0.0090112	10695	10824	12163
MSTRG.5137.4	coding	0.135	531	2919	3498
MSTRG.5137.5	noncoding	-0.0278528	1470	1563	3280
MSTRG.5137.6	noncoding	-0.049152	417	459	1418
MSTRG.5140.2	noncoding	-0.053248	165	252	762
MSTRG.5137.7	coding	0.135	348	2736	3315
MSTRG.5137.8	coding	0.135	312	2700	3279
MSTRG.5137.10	noncoding	-0.0106496	945	984	1259
MSTRG.5133.1	coding	0.084	609	2499	3560
MSTRG.5133.3	coding	0.084	840	2730	6448
MSTRG.5133.4	coding	0.07	582	864	2151
MSTRG.5134.1	coding	0.069	465	705	2177
MSTRG.5135.1	noncoding	-0.069632	273	327	3290
MSTRG.5160.1	noncoding	-0.036864	537	666	5343
MSTRG.5192.2	coding	0.162	123	2019	2903
MSTRG.5192.1	coding	0.157	123	2781	2992
MSTRG.5192.3	coding	0.153	114	597	807
MSTRG.5192.4	noncoding	-0.0094208	12	159	371
MSTRG.5196.1	noncoding	-0.036864	330	435	590
MSTRG.5201.1	noncoding	-0.049152	0	90	394
MSTRG.5209.1	noncoding	-0.0004096	0	0	392
MSTRG.5236.1	coding	0.189	1878	14502	17583
MSTRG.5236.3	coding	0.201	4128	13854	14323
MSTRG.5237.1	noncoding	-0.0110592	1950	2109	4180
MSTRG.5236.6	coding	0.257	5241	8937	9360
MSTRG.5238.5	coding	0.299	171	1749	1986
MSTRG.5217.3	coding	0.216	255	2637	6802
MSTRG.5217.4	coding	0.222	9	1767	2225
MSTRG.5218.1	noncoding	-0.147456	2769	2820	3968
MSTRG.5226.1	noncoding	-0.01024	2607	2790	4973
MSTRG.5247.1	noncoding	-0.0212992	4020	4173	4346
MSTRG.5249.2	coding	0.123	66	849	4447
MSTRG.5249.3	coding	0.038	480	1194	1590
MSTRG.5249.4	coding	0.068	480	981	4579
MSTRG.5250.1	noncoding	-0.0524288	189	465	620
MSTRG.5250.2	noncoding	-0.0749568	7539	7839	7994
MSTRG.5232.1	coding	0.162	858	2409	3443
MSTRG.5240.3	noncoding	-0.0012288	1044	1191	2764
MSTRG.5252.4	coding	0.185	6282	6477	7232
MSTRG.5252.3	coding	0.185	1026	1221	1975
MSTRG.5252.10	noncoding	-0.0360448	465	756	2147
MSTRG.5256.1	noncoding	-0.0004096	348	501	1053
MSTRG.5257.1	coding	0.015	381	603	7802
MSTRG.5257.3	noncoding	-0.0397312	1254	1389	3649
MSTRG.5257.5	coding	0.021	5472	5670	7925
MSTRG.5257.6	coding	0.021	1983	2181	3927

MSTRG.5258.1	noncoding	-0.0004096	114	114	1786
MSTRG.5260.1	coding	0.111	1542	1773	1940
MSTRG.5261.4	coding	0.082	0	471	2263
MSTRG.5261.5	coding	0.084	0	459	1829
MSTRG.5265.1	noncoding	-0.0331776	1263	1335	1709
MSTRG.5254.1	coding	0.097	0	363	580
MSTRG.5285.2	coding	0.238	216	3474	3649
MSTRG.5285.3	coding	0.2	183	3564	3739
MSTRG.5285.4	coding	0.241	342	3114	3289
MSTRG.5286.1	noncoding	-0.126976	1659	1701	1856
MSTRG.5285.5	coding	0.242	0	2736	2911
MSTRG.5288.2	coding	0.308	4959	5934	6939
MSTRG.5289.1	noncoding	-0.012288	3759	3870	5178
MSTRG.5293.1	coding	0.073	879	2031	2211
MSTRG.5297.5	coding	0.045	567	816	2319
MSTRG.5298.1	noncoding	-0.018432	3939	4317	6027
MSTRG.5297.6	noncoding	-0.0004096	3960	4104	5386
MSTRG.5297.7	noncoding	-0.098304	258	342	499
MSTRG.5297.8	noncoding	-0.004096	351	450	606
MSTRG.5299.3	noncoding	-0.045056	600	648	1136
MSTRG.5300.1	noncoding	-0.233472	309	342	1686
MSTRG.5301.1	coding	0.122	42	2385	3737
MSTRG.5301.2	noncoding	-0.0212992	0	144	1935
MSTRG.5301.4	coding	0.114	48	2109	3762
MSTRG.5301.8	coding	0.114	48	2109	4569
MSTRG.5301.7	coding	0.114	48	2109	3540
MSTRG.5301.3	coding	0.114	48	2109	3648
MSTRG.5301.6	coding	0.114	48	2109	3696
MSTRG.5301.5	coding	0.114	48	2109	4054
MSTRG.5305.1	noncoding	-0.0106496	8799	8952	18955
MSTRG.5305.2	noncoding	-0.086016	1326	1359	2600
MSTRG.5306.1	noncoding	-0.0004096	1827	2052	2876
MSTRG.5307.1	noncoding	-0.004096	597	669	1464
MSTRG.5271.1	noncoding	-0.0004096	0	0	232
MSTRG.5275.1	noncoding	-0.1138688	1071	1554	2697
MSTRG.5275.2	noncoding	-0.1138688	1119	1602	2697
MSTRG.5276.2	coding	0.114	444	2460	4794
MSTRG.5283.2	coding	0.12	0	1797	7622
MSTRG.5283.3	noncoding	-0.0688128	843	1065	1245
MSTRG.5284.1	noncoding	-0.008192	483	624	2514
MSTRG.5310.2	coding	0.091	1695	2784	3703
MSTRG.5310.3	coding	0.097	858	1725	2776
MSTRG.5312.1	noncoding	-0.0495616	168	387	1500
MSTRG.5317.1	noncoding	-0.024576	3288	3423	3712
MSTRG.5324.1	coding	0.211	0	1461	2214
MSTRG.5324.2	coding	0.226	312	3477	3815
MSTRG.5324.3	coding	0.242	246	669	1255
MSTRG.5331.1	coding	0.062	4827	5058	6251
MSTRG.5358.2	noncoding	-0.0024576	1944	1965	2462
MSTRG.5359.2	coding	0.414	219	666	1760
MSTRG.5359.3	coding	0.414	219	666	2634
MSTRG.5363.2	coding	0.169	246	1131	6094
MSTRG.5363.4	coding	0.169	210	1095	6065
MSTRG.5363.5	coding	0.169	45	930	16144

MSTRG.5365.2	coding	0.031	87	1305	3074
MSTRG.5354.2	coding	0.104	165	9891	10254
MSTRG.5354.3	coding	0.12	156	1185	1868
MSTRG.5370.2	noncoding	-0.057344	555	645	1541
MSTRG.5382.1	noncoding	-0.0249856	633	1143	1347
MSTRG.5384.1	noncoding	-0.2121728	4068	4104	4441
MSTRG.5383.7	coding	0.035	2400	2829	4084
MSTRG.5385.2	noncoding	-0.002048	420	711	5402
MSTRG.5386.1	coding	0.187	66	2136	7133
MSTRG.5386.3	coding	0.174	561	2307	5435
MSTRG.5386.4	coding	0.186	141	2130	2285
MSTRG.5386.5	coding	0.19	180	2196	2349
MSTRG.5386.7	coding	0.189	180	2289	2442
MSTRG.5386.9	noncoding	-0.0720896	240	270	652
MSTRG.5387.1	noncoding	-0.008192	90	213	6983
MSTRG.5388.1	noncoding	-0.098304	11085	11160	11313
MSTRG.5389.1	noncoding	-0.04096	3537	3732	6218
MSTRG.5391.1	noncoding	-0.0098304	42	252	3290
MSTRG.5377.3	coding	0.126	285	2202	5132
MSTRG.5377.2	coding	0.126	321	2238	5168
MSTRG.5377.1	coding	0.126	54	2574	5505
MSTRG.5392.1	coding	0.07	195	774	4538
MSTRG.5392.2	coding	0.066	1017	1563	3404
MSTRG.5393.2	noncoding	-0.1437696	318	1470	4599
MSTRG.5401.3	noncoding	-0.1028096	0	546	1064
MSTRG.5401.2	noncoding	-0.1024	0	561	1317
MSTRG.5401.1	noncoding	-0.0638976	0	276	604
MSTRG.5402.3	coding	0.058	0	1860	3087
MSTRG.5402.4	coding	0.136	3312	3627	3959
MSTRG.5403.1	noncoding	-0.1171456	30	66	424
MSTRG.5404.2	coding	0.068	0	2400	4952
MSTRG.5404.1	coding	0.079	99	2838	10759
MSTRG.5404.3	coding	0.063	0	2358	6024
MSTRG.5405.1	noncoding	-0.0004096	387	477	699
MSTRG.5404.4	coding	0.129	0	351	812
MSTRG.5408.4	coding	0.103	423	672	1052
MSTRG.5399.1	coding	0.07	18	954	2055
MSTRG.5399.2	coding	0.071	0	930	2029
MSTRG.5412.1	noncoding	-0.0536576	1293	1536	3753
MSTRG.5413.2	coding	0.24	0	5316	5623
MSTRG.5414.1	noncoding	-0.032768	15	30	218
MSTRG.5416.3	coding	0.065	450	2292	3812
MSTRG.5417.1	coding	0.075	87	480	745
MSTRG.5419.7	coding	0.086	99	834	2465
MSTRG.5419.6	coding	0.057	399	837	7888
MSTRG.5419.8	coding	0.08	786	1317	3049
MSTRG.5440.1	noncoding	-0.016384	213	327	483
MSTRG.5445.2	coding	0.124	0	993	1467
MSTRG.5491.1	noncoding	-0.032768	315	384	2099
MSTRG.5493.1	coding	0.143	156	471	735
MSTRG.5493.2	coding	0.084	756	1443	2658
MSTRG.5470.2	coding	0.387	0	294	3495
MSTRG.5471.1	noncoding	-0.192512	1560	1590	1922
MSTRG.5478.3	coding	0.085	186	1434	1606

MSTRG.5480.1	coding	0.084	0	1221	1546
MSTRG.5514.1	coding	0.192	228	5325	6000
MSTRG.5515.1	noncoding	-0.028672	84	207	362
MSTRG.5558.2	coding	0.066	1989	2289	7634
MSTRG.5560.1	noncoding	-0.0622592	696	735	2993
MSTRG.5561.1	noncoding	-0.098304	615	666	1509
MSTRG.5561.2	noncoding	-0.032768	807	939	1360
MSTRG.5562.1	noncoding	-0.0004096	1500	1644	6840
MSTRG.5563.1	noncoding	-0.0200704	252	333	4051
MSTRG.5564.1	noncoding	-0.0004096	0	0	1691
MSTRG.5565.1	noncoding	-0.016384	390	480	857
MSTRG.5558.3	noncoding	-0.0065536	33	132	699
MSTRG.5532.1	noncoding	-0.0671744	0	159	8488
MSTRG.5533.1	coding	0.084	774	936	1364
MSTRG.5533.2	noncoding	-0.0053248	888	933	2665
MSTRG.5539.1	noncoding	-0.0626688	0	81	834
MSTRG.5540.1	noncoding	-0.0356352	1512	1644	3328
MSTRG.5538.4	noncoding	-0.0667648	507	699	1946
MSTRG.5543.1	coding	0.079	327	1596	9345
MSTRG.5543.4	coding	0.064	288	1305	2693
MSTRG.5543.2	coding	0.103	309	1272	3801
MSTRG.5543.3	coding	0.068	126	855	3384
MSTRG.5543.7	coding	0.059	330	1029	3560
MSTRG.5543.8	coding	0.06	4422	5139	6256
MSTRG.5543.9	noncoding	-0.057344	1092	1383	7960
MSTRG.5549.1	noncoding	-0.0233472	27	156	413
MSTRG.5550.2	coding	0.233	0	531	2442
MSTRG.5552.1	noncoding	-0.053248	525	579	1037
MSTRG.5555.4	noncoding	-0.16384	0	258	1848
MSTRG.5555.2	noncoding	-0.16384	0	258	4540
MSTRG.5555.3	noncoding	-0.16384	0	258	3435
MSTRG.5570.2	coding	0.127	606	1542	2094
MSTRG.5570.4	coding	0.11	126	1689	2150
MSTRG.5572.2	coding	0.193	0	492	998
MSTRG.5574.5	coding	0.364	0	1041	2471
MSTRG.5574.6	coding	0.322	324	1563	3043
MSTRG.5574.7	coding	0.241	0	285	1848
MSTRG.5575.3	noncoding	-0.0937984	0	486	4179
MSTRG.5575.4	coding	0.082	813	1227	3647
MSTRG.5584.1	noncoding	-0.0745472	1437	1752	1956
MSTRG.5591.1	coding	0.3	42	1890	6724
MSTRG.5591.2	coding	0.299	42	1971	6757
MSTRG.5595.3	coding	0.141	0	1020	1306
MSTRG.5594.1	noncoding	-0.012288	168	303	753
MSTRG.5605.2	noncoding	-0.1343488	657	1530	1771
MSTRG.5606.2	noncoding	-0.032768	507	612	865
MSTRG.5608.1	noncoding	-0.0872448	0	171	796
MSTRG.5610.3	coding	0.174	54	1695	6879
MSTRG.5615.1	noncoding	-0.0278528	1935	2031	2636
MSTRG.5618.3	coding	0.242	0	789	943
MSTRG.5612.1	coding	0.198	909	2091	2273
MSTRG.5627.1	coding	0.13	6204	6489	73520
MSTRG.5628.1	noncoding	-0.053248	171	180	334
MSTRG.5629.1	noncoding	-0.0229376	702	864	1526

MSTRG.5629.2	noncoding	-0.0335872	0	132	768
MSTRG.5627.3	coding	0.28	36	675	3079
MSTRG.5627.4	noncoding	-0.0028672	7719	7860	11190
MSTRG.5627.5	coding	0.13	11742	12027	14765
MSTRG.5627.6	noncoding	-0.004096	3753	3891	14449
MSTRG.5627.7	coding	0.275	6672	7164	9507
MSTRG.5627.8	noncoding	-0.004096	5331	5469	85626
MSTRG.5627.9	noncoding	-0.004096	5331	5469	75975
MSTRG.5627.10	noncoding	-0.0561152	10275	10497	18506
MSTRG.5627.11	noncoding	-0.0561152	10281	10503	18512
MSTRG.5627.12	noncoding	-0.0561152	10254	10476	18468
MSTRG.5627.13	coding	0.285	6477	6843	15972
MSTRG.5627.14	noncoding	-0.0438272	27111	27459	36838
MSTRG.5631.1	noncoding	-0.012288	63	156	6298
MSTRG.5632.1	noncoding	-0.0106496	6984	7134	15990
MSTRG.5633.1	noncoding	-0.075776	1929	2109	31966
MSTRG.5633.2	noncoding	-0.0139264	4017	4308	7531
MSTRG.5634.1	coding	0.056	120	354	2177
MSTRG.5632.2	noncoding	-0.0249856	4503	4797	13911
MSTRG.5634.2	noncoding	-0.0729088	0	81	5592
MSTRG.5635.3	coding	0.098	726	2856	3862
MSTRG.5636.1	noncoding	-0.04096	2787	2886	6166
MSTRG.5621.2	coding	0.072	4734	5517	6459
MSTRG.5624.1	coding	0.065	4689	5445	50521
MSTRG.5624.2	noncoding	-0.004096	870	1035	2221
MSTRG.5638.1	noncoding	-0.0606208	129	366	1710
MSTRG.5643.3	coding	0.062	93	504	1729
MSTRG.5642.4	coding	0.395	0	774	23956
MSTRG.5642.5	noncoding	-0.0290816	8613	8769	10560
MSTRG.5661.1	noncoding	-0.067584	0	207	1077
MSTRG.5668.3	noncoding	-0.01024	633	783	1617
MSTRG.5669.3	coding	0.103	0	1794	4153
MSTRG.5669.4	coding	0.096	1560	3147	3403
MSTRG.5669.5	coding	0.129	0	1698	1956
MSTRG.5670.1	noncoding	-0.049152	540	600	1085
MSTRG.5671.1	noncoding	-0.032768	3558	3642	4447
MSTRG.5672.1	noncoding	-0.0110592	87	243	554
MSTRG.5667.1	noncoding	-0.16384	0	21	708
MSTRG.5681.1	coding	0.062	27	2934	14479
MSTRG.5681.5	coding	0.087	453	2427	2802
MSTRG.5681.10	coding	0.028	444	864	1236
MSTRG.5681.7	coding	0.121	0	675	2554
MSTRG.5681.11	coding	0.028	336	753	1974
MSTRG.5722.15	noncoding	-0.0208896	75	483	4345
MSTRG.5726.1	noncoding	-0.004096	1200	1242	1718
MSTRG.5726.2	noncoding	-0.065536	3054	3096	5275
MSTRG.5722.17	noncoding	-0.077824	252	438	2459
MSTRG.5722.18	coding	0.001	414	519	4420
MSTRG.5727.1	coding	0.109	3288	3513	4233
MSTRG.5728.1	noncoding	-0.024576	6648	6735	7866
MSTRG.5729.1	noncoding	-0.499712	1023	1032	1601
MSTRG.5730.4	coding	0.026	426	759	1044
MSTRG.5722.19	noncoding	-0.0520192	114	414	4134
MSTRG.5722.20	coding	0.102	3894	4086	17240

MSTRG.5722.21	coding	0.105	3378	3672	5335
MSTRG.5734.1	noncoding	-0.098304	477	546	1851
MSTRG.5722.22	noncoding	-0.0557056	717	1053	4816
MSTRG.5687.2	noncoding	-0.1794048	0	93	2961
MSTRG.5690.2	coding	0.185	0	591	1083
MSTRG.5690.3	coding	0.171	0	549	1047
MSTRG.5695.3	noncoding	-0.0413696	12156	12810	12963
MSTRG.5696.1	noncoding	-0.0282624	4626	5109	6404
MSTRG.5695.5	coding	0.325	2412	4038	6752
MSTRG.5695.6	coding	0.306	2763	4734	7448
MSTRG.5693.1	noncoding	-0.004096	366	405	963
MSTRG.5699.1	noncoding	-0.0135168	6	228	505
MSTRG.5704.3	coding	0.417	1011	2214	2521
MSTRG.5707.14	coding	0.418	417	1266	1420
MSTRG.5707.17	coding	0.403	1530	2796	2950
MSTRG.5707.15	coding	0.4	546	2010	2164
MSTRG.5707.11	coding	0.402	336	1587	1740
MSTRG.5707.13	coding	0.402	186	1437	1590
MSTRG.5707.16	coding	0.402	273	1524	1678
MSTRG.5707.6	coding	0.391	255	1698	1852
MSTRG.5707.2	coding	0.391	333	1776	1929
MSTRG.5707.5	coding	0.391	240	1683	1836
MSTRG.5707.9	coding	0.391	390	1833	1986
MSTRG.5711.3	coding	0.27	0	267	1124
MSTRG.5713.3	coding	0.354	357	3363	4168
MSTRG.5713.4	coding	0.356	945	3915	4722
MSTRG.5713.6	coding	0.32	5211	8550	9356
MSTRG.5713.5	coding	0.377	63	2547	4064
MSTRG.5717.2	noncoding	-0.0401408	147	1272	1563
MSTRG.5717.1	coding	0.452	336	2463	5213
MSTRG.5717.3	noncoding	-0.04096	291	906	1061
MSTRG.5717.4	coding	0.453	351	3006	4497
MSTRG.5717.5	coding	0.441	351	2994	3255
MSTRG.5715.4	coding	0.459	0	588	3640
MSTRG.5715.3	coding	0.489	282	822	989
MSTRG.5716.1	coding	0.323	0	615	3269
MSTRG.5740.3	coding	0.138	0	2007	2266
MSTRG.5742.1	noncoding	-0.0004096	0	0	469
MSTRG.5745.3	coding	0.196	1488	2022	3582
MSTRG.5745.2	coding	0.217	2010	2796	4354
MSTRG.5746.8	coding	0.278	39	384	798
MSTRG.5746.9	coding	0.276	414	783	1195
MSTRG.5747.1	coding	0.118	1779	2286	5455
MSTRG.5746.10	coding	0.276	1869	2238	2650
MSTRG.5782.1	noncoding	-0.047104	1350	1389	2018
MSTRG.5757.2	noncoding	-0.065536	45	126	771
MSTRG.5757.1	noncoding	-0.065536	45	126	1036
MSTRG.5761.1	coding	0.31	0	4866	8275
MSTRG.5761.2	coding	0.305	189	5274	8710
MSTRG.5763.2	noncoding	-0.0233472	3636	3789	4195
MSTRG.5762.1	noncoding	-0.077824	87	117	416
MSTRG.5835.1	noncoding	-0.004096	165	297	2548
MSTRG.5835.2	noncoding	-0.0159744	1758	2358	9557
MSTRG.5835.3	coding	0.114	87	432	3582

MSTRG.5835.4	coding	0.077	174	642	19096
MSTRG.5835.5	noncoding	-0.0233472	11376	11505	15510
MSTRG.5835.6	coding	0.099	99	537	4542
MSTRG.5835.7	noncoding	-0.0352256	11148	11319	34385
MSTRG.5835.9	coding	0.027	162	441	1857
MSTRG.5836.1	noncoding	-0.008192	1083	1281	3063
MSTRG.5835.11	noncoding	-0.0688128	6618	6915	7172
MSTRG.5837.1	coding	0.05	4497	4671	14643
MSTRG.5838.1	noncoding	-0.012288	1911	2019	3189
MSTRG.5835.13	noncoding	-0.0090112	612	699	3935
MSTRG.5835.12	coding	0.073	174	390	7636
MSTRG.5835.14	coding	0.124	87	273	7511
MSTRG.5835.15	coding	0.033	162	432	7723
MSTRG.5835.16	coding	0.068	99	339	3272
MSTRG.5835.17	coding	0.081	348	570	3450
MSTRG.5835.18	coding	0.033	162	432	3347
MSTRG.5835.20	coding	0.071	2271	2970	16486
MSTRG.5835.22	noncoding	-0.077824	4356	4428	7240
MSTRG.5835.23	coding	0.113	4854	5199	27623
MSTRG.5835.25	noncoding	-0.0688128	23343	23640	23947
MSTRG.5835.26	coding	0.02	633	861	6035
MSTRG.5835.27	noncoding	-0.0688128	18615	18912	19207
MSTRG.5835.28	noncoding	-0.02048	2823	2952	3109
MSTRG.5835.29	coding	0.099	1128	1350	9740
MSTRG.5835.30	coding	0.099	1293	1515	2359
MSTRG.5840.1	coding	0.083	159	387	1854
MSTRG.5797.1	coding	0.225	729	5823	8148
MSTRG.5797.6	coding	0.136	1176	4050	4411
MSTRG.5797.4	coding	0.126	2751	5805	6168
MSTRG.5797.3	coding	0.126	693	3747	4108
MSTRG.5797.5	coding	0.126	327	3381	3742
MSTRG.5797.7	coding	0.126	129	3183	3428
MSTRG.5798.1	coding	0.024	6540	6681	8945
MSTRG.5802.3	noncoding	-0.118784	2016	2061	3805
MSTRG.5803.1	noncoding	-0.04096	0	402	1248
MSTRG.5805.1	coding	0.268	0	348	7041
MSTRG.5799.1	noncoding	-0.0004096	144	207	381
MSTRG.5815.1	noncoding	-0.0700416	0	15	362
MSTRG.5832.1	noncoding	-0.0700416	0	57	395
MSTRG.5834.1	coding	0.109	156	1359	2113
MSTRG.5845.3	coding	0.089	1971	2832	3264
MSTRG.5845.4	coding	0.097	1461	1863	3108
MSTRG.5856.2	coding	0.112	1542	2490	2646
MSTRG.5858.1	noncoding	-0.094208	405	423	820
MSTRG.5860.1	coding	0.04	69	201	1193
MSTRG.5862.1	noncoding	-0.098304	102	606	5740
MSTRG.5864.1	coding	0.065	0	1692	3274
MSTRG.5865.1	noncoding	-0.0004096	1806	2010	5128
MSTRG.5866.1	noncoding	-0.06144	1098	1188	1632
MSTRG.5867.1	noncoding	-0.0741376	0	510	947
MSTRG.5872.5	coding	0.042	510	735	1922
MSTRG.5873.1	noncoding	-0.12288	3234	3243	4213
MSTRG.5874.2	noncoding	-0.008192	1107	1215	1725
MSTRG.5874.3	noncoding	-0.008192	438	546	1054

MSTRG.5874.5	noncoding	-0.008192	1101	1209	1451
MSTRG.5874.4	noncoding	-0.008192	1143	1251	1761
MSTRG.5877.1	coding	0.211	972	2832	3070
MSTRG.5879.1	noncoding	-0.0442368	1242	1332	2903
MSTRG.5885.2	coding	0.187	246	1683	2345
MSTRG.5886.1	noncoding	-0.0167936	60	219	389
MSTRG.5890.2	coding	0.057	435	993	4046
MSTRG.5890.3	coding	0.033	171	1230	2755
MSTRG.5891.1	coding	0.646	9	1314	3537
MSTRG.5891.2	coding	0.646	0	1293	3316
MSTRG.5891.3	coding	0.645	189	1464	3273
MSTRG.5896.2	coding	0.297	0	1221	12861
MSTRG.5897.1	noncoding	-0.0651264	2565	2745	11595
MSTRG.5901.1	noncoding	-0.004096	516	654	2102
MSTRG.5904.1	coding	0.333	2037	2973	4055
MSTRG.5904.2	coding	0.153	54	1050	2038
MSTRG.5904.4	coding	0.329	1548	2421	3504
MSTRG.5907.1	coding	0.578	0	8358	9154
MSTRG.5908.1	noncoding	-0.0434176	162	1008	1826
MSTRG.5908.2	noncoding	-0.04096	21	669	1027
MSTRG.5907.3	coding	0.569	84	6771	7435
MSTRG.5907.4	coding	0.568	63	6831	7489
MSTRG.5909.2	coding	0.108	1350	2580	3119
MSTRG.5909.3	coding	0.108	1626	2856	3395
MSTRG.5909.4	coding	0.054	159	504	2392
MSTRG.5910.1	coding	0.347	429	1356	2320
MSTRG.5915.1	coding	0.235	345	2184	5362
MSTRG.5915.4	coding	0.485	0	333	1214
MSTRG.5915.3	coding	0.196	0	1890	5067
MSTRG.5916.1	coding	0.071	2124	2622	5224
MSTRG.5917.2	coding	0.143	594	849	1127
MSTRG.5918.1	noncoding	-0.0004096	0	0	280
MSTRG.5919.1	coding	0.134	4551	4800	7350
MSTRG.5920.1	coding	0.04	6204	6621	11623
MSTRG.5924.1	noncoding	-0.057344	0	21	2104
MSTRG.5924.6	coding	0.22	0	1587	6413
MSTRG.5924.7	coding	0.192	0	1359	1731
MSTRG.5924.8	coding	0.176	828	2022	2447
MSTRG.5921.1	noncoding	-0.02048	297	420	1084
MSTRG.5922.3	coding	0.039	1215	1365	2427
MSTRG.5922.4	noncoding	-0.0315392	5301	5487	6628
MSTRG.5923.1	noncoding	-0.065536	1911	1992	9673
MSTRG.5925.1	coding	0.17	66	1602	2215
MSTRG.5925.2	coding	0.17	102	1638	2393
MSTRG.5925.4	coding	0.17	66	1602	3197
MSTRG.5925.3	coding	0.161	72	1209	3067
MSTRG.5925.5	coding	0.17	102	1638	3183
MSTRG.5927.1	coding	0.086	2619	4293	6777
MSTRG.5927.2	coding	0.09	3948	4539	7821
MSTRG.5932.1	coding	0.062	45	372	998
MSTRG.5934.1	coding	0.172	1089	2232	2419
MSTRG.5934.3	coding	0.349	693	1932	2157
MSTRG.5934.4	coding	0.349	741	1980	2205
MSTRG.5935.2	coding	0.175	165	1086	2342

MSTRG.5936.1	coding	0.213	0	2532	2978
MSTRG.5939.1	noncoding	-0.0094208	9	42	406
MSTRG.5940.1	noncoding	-0.049152	93	171	326
MSTRG.5940.2	noncoding	-0.0077824	141	708	935
MSTRG.5939.2	noncoding	-0.0024576	123	132	296
MSTRG.5940.4	noncoding	-0.065536	141	183	338
MSTRG.5939.3	noncoding	-0.008192	219	423	709
MSTRG.5939.4	noncoding	-0.0622592	0	216	461
MSTRG.5940.5	noncoding	-0.0131072	63	117	311
MSTRG.5942.1	noncoding	-0.0106496	15	27	223
MSTRG.5944.1	noncoding	-0.0417792	360	657	1044
MSTRG.5945.1	noncoding	-0.036864	78	99	263
MSTRG.5946.1	noncoding	-0.036864	576	687	844
MSTRG.5945.2	noncoding	-0.036864	60	81	247
MSTRG.5949.2	noncoding	-0.02048	351	480	733
MSTRG.5966.1	noncoding	-0.012288	810	897	1444
MSTRG.5965.5	coding	0.106	327	2037	8375
MSTRG.5969.1	noncoding	-0.299008	1065	1086	1458
MSTRG.5965.6	coding	0.087	552	1110	1433
MSTRG.5965.7	noncoding	-0.016384	1032	1140	1645
MSTRG.5965.8	coding	0.074	3666	4362	6508
MSTRG.5970.1	noncoding	-0.0004096	0	0	741
MSTRG.5972.1	noncoding	-0.02048	444	534	3267
MSTRG.5951.1	noncoding	-0.0208896	993	1248	1613
MSTRG.5953.1	noncoding	-0.0311296	621	1080	1234
MSTRG.5955.2	coding	0.249	69	381	1200
MSTRG.5956.3	coding	0.376	0	1128	2600
MSTRG.5956.2	coding	0.371	0	1281	6812
MSTRG.5956.4	noncoding	-0.0110592	1860	1956	2677
MSTRG.5958.1	noncoding	-0.02048	2967	3069	5275
MSTRG.5956.6	coding	0.303	264	1398	2587
MSTRG.5956.7	coding	0.355	246	1644	2984
MSTRG.5959.1	noncoding	-0.0258048	2964	3045	6012
MSTRG.5960.1	noncoding	-0.0036864	2781	2829	3483
MSTRG.5961.1	noncoding	-0.16384	822	882	4294
MSTRG.5962.1	noncoding	-0.065536	1017	1020	2202
MSTRG.5956.8	coding	0.404	210	921	1813
MSTRG.5985.4	noncoding	-0.0249856	12	783	2562
MSTRG.5985.7	coding	0.087	27	924	2673
MSTRG.5985.5	coding	0.055	1185	2235	3406
MSTRG.5985.8	coding	0.107	0	678	3748
MSTRG.5986.1	noncoding	-0.004096	4620	4677	5464
MSTRG.5989.1	coding	0.146	0	393	1382
MSTRG.5990.2	coding	0.335	312	807	1833
MSTRG.5989.4	coding	0.235	114	618	1496
MSTRG.5984.1	noncoding	-0.0610304	360	558	2710
MSTRG.5991.1	coding	0.352	159	1209	1480
MSTRG.5991.3	coding	0.117	27	231	522
MSTRG.5992.1	coding	0.286	192	2043	6469
MSTRG.5994.1	noncoding	-0.012288	441	564	1203
MSTRG.5995.1	noncoding	-0.0454656	384	918	1073
MSTRG.5997.1	noncoding	-0.065536	48	66	287
MSTRG.5999.1	coding	0.164	33	297	503
MSTRG.6004.5	coding	0.048	270	2076	2489

MSTRG.6004.4	coding	0.067	294	2313	2725
MSTRG.6005.1	noncoding	-0.0090112	321	435	5677
MSTRG.6006.1	noncoding	-0.0221184	3393	3414	4577
MSTRG.6004.7	coding	0.045	273	1878	2947
MSTRG.6007.1	noncoding	-0.065536	564	639	1634
MSTRG.6008.1	coding	0.001	5247	5418	6249
MSTRG.6008.2	noncoding	-0.0507904	0	36	639
MSTRG.6009.3	coding	0.165	2607	6246	6538
MSTRG.6001.2	noncoding	-0.0479232	15	258	413
MSTRG.6010.2	coding	0.211	93	324	4061
MSTRG.6020.4	coding	0.101	156	3060	3737
MSTRG.6029.2	noncoding	-0.1650688	207	804	1093
MSTRG.6029.3	coding	0.379	99	789	1052
MSTRG.6029.4	coding	0.109	36	2709	5935
MSTRG.6030.1	noncoding	-0.0212992	0	129	594
MSTRG.6033.2	coding	0.163	0	333	1130
MSTRG.6035.1	noncoding	-0.055296	0	426	4197
MSTRG.6036.2	coding	0.137	0	1392	2796
MSTRG.6036.3	coding	0.11	201	1131	2536
MSTRG.6037.1	noncoding	-0.032768	855	945	5866
MSTRG.6040.1	coding	0.388	0	3624	15070
MSTRG.6040.2	coding	0.379	0	3702	15005
MSTRG.6040.3	coding	0.387	0	3621	14792
MSTRG.6040.5	coding	0.369	351	2640	3077
MSTRG.6040.4	coding	0.389	0	3543	3980
MSTRG.6040.6	coding	0.37	522	2169	2325
MSTRG.6040.7	coding	0.371	282	2187	2343
MSTRG.6040.8	coding	0.369	0	1947	2102
MSTRG.6040.9	coding	0.406	0	1260	1921
MSTRG.6040.10	coding	0.402	0	1197	1536
MSTRG.6040.11	coding	0.407	0	1218	1614
MSTRG.6042.1	coding	0.33	315	1698	1899
MSTRG.6043.3	coding	0.271	0	1467	2827
MSTRG.6045.2	coding	0.066	513	2328	4417
MSTRG.6050.1	coding	0.312	609	2067	4752
MSTRG.6050.3	coding	0.301	57	1323	3263
MSTRG.6050.4	coding	0.312	531	1989	3533
MSTRG.6050.7	coding	0.259	51	816	1320
MSTRG.6056.1	coding	0.353	984	4029	4443
MSTRG.6058.1	noncoding	-0.0434176	2724	3756	3910
MSTRG.6058.2	noncoding	-0.0401408	3579	3945	4101
MSTRG.6060.3	noncoding	-0.1056768	0	249	4233
MSTRG.6060.2	noncoding	-0.0987136	0	258	4638
MSTRG.6061.2	coding	0.101	0	2343	9546
MSTRG.6061.4	coding	0.055	0	1935	7844
MSTRG.6061.5	noncoding	-0.0151552	951	1104	4541
MSTRG.6061.6	coding	0.102	0	2286	10573
MSTRG.6061.7	coding	0.104	0	2223	5589
MSTRG.6061.10	coding	0.046	0	1953	2109
MSTRG.6061.11	coding	0.049	696	1896	2051
MSTRG.6061.12	coding	0.106	0	2046	2490
MSTRG.6061.9	coding	0.125	0	1776	2476
MSTRG.6062.1	noncoding	-0.008192	975	1116	2702
MSTRG.6063.1	noncoding	-0.036864	219	303	1855

MSTRG.6055.2	coding	0.034	315	636	889
MSTRG.6055.3	coding	0.034	141	462	714
MSTRG.6065.2	coding	0.004	450	783	1249
MSTRG.6066.1	coding	0.306	1245	3819	6257
MSTRG.6067.2	coding	0.013	276	666	1119
MSTRG.6068.2	coding	0.088	123	741	1045
MSTRG.6073.1	coding	0.272	0	327	673
MSTRG.6074.1	noncoding	-0.0516096	138	342	500
MSTRG.6075.1	coding	0.326	330	837	1004
MSTRG.6076.1	coding	0.454	0	504	797
MSTRG.6077.1	coding	0.292	0	291	446
MSTRG.6077.2	coding	0.457	0	507	746
MSTRG.6088.1	coding	0.267	0	876	3446
MSTRG.6088.5	coding	0.307	0	573	728
MSTRG.6089.2	coding	0.311	0	1146	3653
MSTRG.6089.3	coding	0.301	0	975	4034
MSTRG.6096.1	noncoding	-0.0004096	0	0	268
MSTRG.6100.1	noncoding	-0.0352256	2883	3333	7369
MSTRG.6104.1	noncoding	-0.0356352	459	849	1311
MSTRG.6105.1	noncoding	-0.0004096	339	483	818
MSTRG.6112.1	coding	0.309	129	837	4678
MSTRG.6113.1	noncoding	-0.0004096	273	489	852
MSTRG.6121.3	coding	0.289	10767	12924	13239
MSTRG.6121.5	coding	0.291	168	2247	2559
MSTRG.6121.7	coding	0.329	10791	12117	12292
MSTRG.6121.9	coding	0.276	2853	5109	5283
MSTRG.6121.10	coding	0.274	3123	5283	5436
MSTRG.6119.1	coding	0.353	297	2067	3877
MSTRG.6119.3	coding	0.353	1323	3093	3594
MSTRG.6124.3	coding	0.44	231	1620	2815
MSTRG.6125.2	coding	0.443	198	2010	4326
MSTRG.6125.3	coding	0.44	0	1911	4227
MSTRG.6125.4	coding	0.472	198	1887	4203
MSTRG.6130.4	coding	0.411	114	2145	3713
MSTRG.6127.1	noncoding	-0.0667648	0	96	378
MSTRG.6133.1	noncoding	-0.0176128	42	198	373
MSTRG.6133.2	noncoding	-0.02048	72	156	311
MSTRG.6134.1	noncoding	-0.1060864	0	324	479
MSTRG.6136.1	noncoding	-0.094208	453	510	663
MSTRG.6136.2	coding	0.514	330	1227	1734
MSTRG.6139.6	coding	0.477	201	2112	3237
MSTRG.6140.2	noncoding	-0.0446464	0	210	661
MSTRG.6141.2	coding	0.405	204	1764	2646
MSTRG.6143.1	coding	0.259	246	1005	2011
MSTRG.6145.3	coding	0.275	423	1239	1752
MSTRG.6149.1	coding	0.235	81	810	1921
MSTRG.6154.3	coding	0.294	0	666	1028
MSTRG.6163.1	coding	0.293	15	825	1491
MSTRG.6163.2	coding	0.298	72	879	1545
MSTRG.6163.4	coding	0.283	429	1551	2217
MSTRG.6163.5	coding	0.277	0	1191	1856
MSTRG.6161.2	coding	0.26	0	2223	2386
MSTRG.6161.3	coding	0.618	879	2610	3165
MSTRG.6167.1	coding	0.411	1029	3087	3616

MSTRG.6167.3	coding	0.411	744	2802	3328
MSTRG.6169.2	coding	0.497	0	1557	1712
MSTRG.6170.1	noncoding	-0.0147456	1707	1809	2052
MSTRG.6171.1	noncoding	-0.0466944	162	519	981
MSTRG.6171.2	noncoding	-0.0495616	162	549	879
MSTRG.6173.2	coding	0.073	6084	6495	6809
MSTRG.6173.3	noncoding	-0.0507904	54	234	2960
MSTRG.6174.7	coding	0.255	0	1098	1912
MSTRG.6174.1	coding	0.24	0	1011	1824
MSTRG.6174.6	coding	0.222	0	1005	1819
MSTRG.6174.9	noncoding	-0.0958464	0	153	316
MSTRG.6174.10	noncoding	-0.008192	45	165	325
MSTRG.6186.2	coding	0.088	306	828	1649
MSTRG.6190.2	coding	0.234	3	15054	16909
MSTRG.6190.1	coding	0.234	3	15093	16949
MSTRG.6190.10	coding	0.247	294	13764	15065
MSTRG.6190.11	coding	0.247	615	14088	15388
MSTRG.6190.15	coding	0.251	273	13482	14784
MSTRG.6190.12	coding	0.247	276	13746	15047
MSTRG.6190.5	coding	0.248	285	13734	15034
MSTRG.6190.14	coding	0.25	906	14151	15452
MSTRG.6190.9	coding	0.236	0	14811	16113
MSTRG.6190.6	coding	0.233	6	15069	16370
MSTRG.6190.4	coding	0.234	3	15069	16371
MSTRG.6190.8	coding	0.234	3	15063	16365
MSTRG.6190.17	coding	0.127	309	1695	3994
MSTRG.6192.1	noncoding	-0.032768	1008	1386	2744
MSTRG.6193.1	noncoding	-0.028672	726	807	3128
MSTRG.6179.1	noncoding	-0.06144	675	771	3253
MSTRG.6179.2	noncoding	-0.1445888	0	63	738
MSTRG.6180.6	coding	0.116	228	609	4482
MSTRG.6180.7	coding	0.304	0	348	6111
MSTRG.6180.5	coding	0.248	0	414	4354
MSTRG.6180.2	coding	0.38	0	1269	2715
MSTRG.6180.4	coding	0.38	0	1269	2705
MSTRG.6180.8	noncoding	-0.0520192	768	1035	5823
MSTRG.6181.1	noncoding	-0.0180224	1296	2328	2674
MSTRG.6181.2	noncoding	-0.036864	570	837	3632
MSTRG.6180.10	noncoding	-0.0835584	456	726	976
MSTRG.6180.11	noncoding	-0.08192	513	774	1024
MSTRG.6180.9	noncoding	-0.08192	570	831	1081
MSTRG.6182.1	noncoding	-0.1073152	0	267	1174
MSTRG.6184.1	noncoding	-0.0475136	1239	1395	2402
MSTRG.6196.1	noncoding	-0.139264	45	93	246
MSTRG.6201.1	coding	0.368	54	1578	2924
MSTRG.6204.2	coding	0.424	525	3039	3491
MSTRG.6204.3	coding	0.347	1002	4479	4807
MSTRG.6206.1	noncoding	-0.0167936	234	414	598
MSTRG.6212.1	coding	0.314	108	4263	10373
MSTRG.6212.2	coding	0.316	0	4122	9838
MSTRG.6212.3	noncoding	-0.176128	840	927	1080
MSTRG.6212.8	coding	0.286	486	2691	4920
MSTRG.6220.1	noncoding	-0.0360448	228	297	450
MSTRG.6222.3	coding	0.323	366	1194	1349

MSTRG.6222.4	coding	0.444	537	1041	1194
MSTRG.6223.1	noncoding	-0.0528384	1209	1497	5618
MSTRG.6231.1	coding	0.445	195	1764	2583
MSTRG.6232.2	coding	0.323	0	3219	4675
MSTRG.6232.4	coding	0.284	0	3306	4763
MSTRG.6233.1	noncoding	-0.083968	477	699	1972
MSTRG.6233.2	noncoding	-0.073728	1425	1647	2418
MSTRG.6235.5	coding	0.336	246	933	1088
MSTRG.6235.4	coding	0.276	27	1077	1876
MSTRG.6235.6	coding	0.274	0	1194	8169
MSTRG.6235.7	noncoding	-0.0303104	1929	2514	7907
MSTRG.6235.8	coding	0.185	4830	6147	6304
MSTRG.6235.9	coding	0.174	4800	6588	7384
MSTRG.6238.2	coding	0.482	0	3219	4774
MSTRG.6240.1	noncoding	-0.0385024	327	1455	3461
MSTRG.6238.3	noncoding	-0.1048576	0	678	834
MSTRG.6242.1	noncoding	-0.04096	621	741	1273
MSTRG.6237.2	noncoding	-0.036864	2337	2838	3586
MSTRG.6237.3	coding	0.292	1113	2328	3004
MSTRG.6237.4	coding	0.313	504	2505	3280
MSTRG.6237.5	coding	0.257	1017	4023	4800
MSTRG.6251.1	coding	0.301	0	3006	5923
MSTRG.6251.2	coding	0.28	0	1626	9067
MSTRG.6251.3	coding	0.266	1821	4359	7187
MSTRG.6251.4	coding	0.291	13683	15921	18747
MSTRG.6251.5	coding	0.318	4230	5598	8424
MSTRG.6251.7	coding	0.318	1563	2931	3287
MSTRG.6251.8	noncoding	-0.0364544	4086	4332	5815
MSTRG.6252.1	noncoding	-0.0630784	111	549	3487
MSTRG.6253.1	coding	0.346	102	3885	5208
MSTRG.6253.2	coding	0.346	162	3942	4854
MSTRG.6253.3	coding	0.365	501	4032	5354
MSTRG.6253.5	coding	0.394	33	3225	4087
MSTRG.6248.1	noncoding	-0.0557056	396	591	773
MSTRG.6267.3	noncoding	-0.004096	111	231	1254
MSTRG.6271.1	coding	0.079	132	4581	7105
MSTRG.6271.3	coding	0.056	1383	4458	6848
MSTRG.6272.1	coding	0.132	2787	3261	3658
MSTRG.6273.2	coding	0.044	444	1515	5670
MSTRG.6273.3	coding	0.029	1560	2136	6257
MSTRG.6274.2	coding	0.064	60	324	744
MSTRG.6285.1	noncoding	-0.0331776	225	507	1856
MSTRG.6288.1	noncoding	-0.0004096	120	120	403
MSTRG.6312.1	coding	0.503	0	861	10245
MSTRG.6312.2	coding	0.327	0	1689	3656
MSTRG.6314.1	noncoding	-0.331776	51	66	2004
MSTRG.6312.8	coding	0.525	234	762	6546
MSTRG.6315.1	noncoding	-0.028672	8199	8268	11184
MSTRG.6316.1	noncoding	-0.0118784	1092	1170	6954
MSTRG.6314.2	noncoding	-0.004096	5760	5838	11130
MSTRG.6314.3	noncoding	-0.004096	1983	2061	6799
MSTRG.6314.4	noncoding	-0.004096	2220	2298	6131
MSTRG.6317.1	noncoding	-0.0188416	3120	3264	4392
MSTRG.6316.2	coding	0.087	11703	12024	15599

MSTRG.6318.1	noncoding	-0.0057344	18	42	3067
MSTRG.6316.3	noncoding	-0.0622592	2187	2220	2505
MSTRG.6316.4	coding	0.05	87	174	1528
MSTRG.6316.5	noncoding	-0.0364544	6177	6387	20842
MSTRG.6316.6	noncoding	-0.0045056	2919	3111	8309
MSTRG.6319.1	noncoding	-0.012288	81	219	467
MSTRG.6319.2	noncoding	-0.08192	3	81	330
MSTRG.6319.3	noncoding	-0.012288	48	186	484
MSTRG.6320.1	noncoding	-0.0004096	0	0	1277
MSTRG.6323.2	noncoding	-0.02048	3693	3861	13730
MSTRG.6308.4	noncoding	-0.0282624	3204	3417	6026
MSTRG.6327.1	coding	0.05	159	2601	3707
MSTRG.6328.1	noncoding	-0.008192	345	417	945
MSTRG.6332.2	coding	0.311	0	228	1230
MSTRG.6332.3	coding	0.311	0	228	4765
MSTRG.6333.1	noncoding	-0.0180224	159	309	566
MSTRG.6334.2	coding	0.158	303	804	1188
MSTRG.6348.1	noncoding	-0.045056	861	879	1033
MSTRG.6348.2	noncoding	-0.0282624	2016	2115	6248
MSTRG.6349.2	coding	0.204	267	4332	6205
MSTRG.6349.1	coding	0.204	267	4356	7983
MSTRG.6349.3	coding	0.203	249	4356	8029
MSTRG.6349.4	coding	0.206	243	4308	7935
MSTRG.6349.5	coding	0.206	243	4308	6119
MSTRG.6349.6	coding	0.201	225	4356	6282
MSTRG.6349.7	coding	0.206	171	4236	6109
MSTRG.6349.8	coding	0.206	171	4236	8513
MSTRG.6350.1	noncoding	-0.0499712	207	534	689
MSTRG.6351.1	noncoding	-0.036864	5508	5532	7826
MSTRG.6351.3	coding	0.322	0	1242	6332
MSTRG.6355.5	noncoding	-0.26624	270	306	2498
MSTRG.6355.4	coding	0.454	0	507	9869
MSTRG.6356.1	noncoding	-0.024576	1071	1140	1294
MSTRG.6357.1	noncoding	-0.110592	321	378	953
MSTRG.6355.6	noncoding	-0.0057344	429	474	1396
MSTRG.6358.1	noncoding	-0.118784	1101	1185	3894
MSTRG.6359.1	noncoding	-0.02048	2568	2670	3620
MSTRG.6359.2	noncoding	-0.012288	3117	3192	5656
MSTRG.6355.7	noncoding	-0.3076096	0	189	1799
MSTRG.6360.1	noncoding	-0.057344	1413	1461	1990
MSTRG.6363.1	coding	0.085	2025	3018	3563
MSTRG.6364.1	coding	0.101	1380	1596	1978
MSTRG.6384.1	noncoding	-0.0479232	822	1008	3449
MSTRG.6385.1	noncoding	-0.028672	1098	1188	2381
MSTRG.6386.1	noncoding	-0.016384	2571	2673	4194
MSTRG.6387.1	noncoding	-0.053248	2676	2724	4306
MSTRG.6388.1	noncoding	-0.028672	2358	2484	5356
MSTRG.6389.1	coding	0.049	162	648	9231
MSTRG.6389.2	coding	0.047	2040	2526	3648
MSTRG.6389.3	coding	0.067	354	933	1769
MSTRG.6383.2	coding	0.099	1686	4332	6647
MSTRG.6383.3	coding	0.077	1659	2679	3488
MSTRG.6383.4	noncoding	-0.0372736	273	678	3037
MSTRG.6379.1	noncoding	-0.0004096	0	0	548

MSTRG.6391.1	noncoding	-0.1404928	2724	3153	5116
MSTRG.6404.2	coding	0.047	687	2820	5754
MSTRG.6404.3	coding	0.047	1020	3147	6302
MSTRG.6433.11	coding	0.015	780	1077	1954
MSTRG.6433.12	coding	0.017	780	1182	1937
MSTRG.6433.15	coding	0.015	780	1077	1820
MSTRG.6433.3	coding	0.015	519	816	1695
MSTRG.6433.4	coding	0.042	648	1128	1872
MSTRG.6433.5	coding	0.039	648	1233	1989
MSTRG.6433.6	coding	0.042	648	1128	2006
MSTRG.6433.8	coding	0.015	879	1176	1920
MSTRG.6433.13	coding	0.015	1071	1368	2245
MSTRG.6433.10	coding	0.042	888	1368	2246
MSTRG.6433.14	coding	0.017	1119	1521	2277
MSTRG.6433.16	coding	0.015	1953	2250	2993
MSTRG.6433.7	coding	0.017	1173	1575	2331
MSTRG.6433.9	coding	0.042	888	1368	2112
MSTRG.6435.2	coding	0.063	201	8361	8595
MSTRG.6436.2	noncoding	-0.0110592	105	249	3048
MSTRG.6430.2	noncoding	-0.1196032	0	105	4769
MSTRG.6442.1	noncoding	-0.032768	2160	2268	3290
MSTRG.6454.2	coding	0.173	48	447	1536
MSTRG.6456.1	noncoding	-0.028672	135	222	606
MSTRG.6456.2	noncoding	-0.012288	54	156	593
MSTRG.6457.4	noncoding	-0.0004096	2340	2466	2765
MSTRG.6457.2	noncoding	-0.0004096	2448	2574	2873
MSTRG.6457.3	noncoding	-0.0004096	2940	3066	3366
MSTRG.6457.5	noncoding	-0.0004096	2442	2568	2866
MSTRG.6464.3	coding	0.112	174	519	1591
MSTRG.6478.1	coding	0.124	456	2067	4657
MSTRG.6497.2	coding	0.043	2058	2364	2846
MSTRG.6497.3	coding	0.11	39	342	2263
MSTRG.6497.4	coding	0.132	0	3972	5380
MSTRG.6497.5	coding	0.132	0	3972	5704
MSTRG.6497.6	coding	0.133	0	3978	5602
MSTRG.6497.7	coding	0.104	0	2880	11885
MSTRG.6497.9	coding	0.129	636	777	2207
MSTRG.6497.10	noncoding	-0.0770048	5496	5565	8200
MSTRG.6497.11	coding	0.103	0	2856	6114
MSTRG.6501.1	coding	0.082	0	2331	4041
MSTRG.6501.2	coding	0.117	915	2415	4126
MSTRG.6501.3	coding	0.117	1275	2775	3130
MSTRG.6501.4	coding	0.117	810	2310	3711
MSTRG.6501.5	coding	0.117	1335	2835	3913
MSTRG.6501.6	noncoding	-0.0004096	447	552	3167
MSTRG.6501.7	coding	0.117	162	1632	1988
MSTRG.6501.8	coding	0.079	735	1614	2637
MSTRG.6502.4	coding	0.079	813	2769	3323
MSTRG.6503.1	coding	0.078	276	1602	2171
MSTRG.6487.2	coding	0.04	2229	2523	4074
MSTRG.6487.1	coding	0.04	1848	2142	3693
MSTRG.6487.3	coding	0.04	1899	2193	3734
MSTRG.6489.1	noncoding	-0.0753664	1179	1533	1688
MSTRG.6494.1	coding	0.117	582	897	1562

MSTRG.6496.2	coding	0.067	147	627	895
MSTRG.6505.2	coding	0.235	0	1467	2484
MSTRG.6507.1	coding	0.316	0	1023	2289
MSTRG.6507.2	coding	0.309	0	972	2204
MSTRG.6508.2	noncoding	-0.1343488	1206	1767	3635
MSTRG.6513.1	coding	0.311	0	807	1720
MSTRG.6513.2	coding	0.299	0	687	1602
MSTRG.6515.1	noncoding	-0.0303104	0	186	423
MSTRG.6520.1	coding	0.196	0	378	1054
MSTRG.6522.3	coding	0.143	441	1563	1994
MSTRG.6522.4	coding	0.143	108	1230	1662
MSTRG.6530.1	coding	0.239	243	1230	3211
MSTRG.6530.2	coding	0.239	276	1263	2704
MSTRG.6530.7	coding	0.235	351	1311	1857
MSTRG.6530.11	coding	0.263	0	888	1273
MSTRG.6530.13	coding	0.235	684	1644	2025
MSTRG.6530.12	coding	0.235	819	1779	2160
MSTRG.6530.14	coding	0.235	0	1203	1683
MSTRG.6530.15	noncoding	-0.0188416	93	276	1019
MSTRG.6524.1	coding	0.295	60	1431	4706
MSTRG.6524.2	coding	0.288	14130	15690	18420
MSTRG.6524.3	coding	0.183	0	2397	17780
MSTRG.6524.6	coding	0.199	0	2478	2893
MSTRG.6524.7	coding	0.181	264	2649	3055
MSTRG.6524.8	coding	0.178	156	2490	2897
MSTRG.6526.1	coding	0.181	933	2046	2200
MSTRG.6527.2	coding	0.273	444	1029	1184
MSTRG.6527.3	coding	0.273	264	849	1004
MSTRG.6538.3	coding	0.4	177	1707	1867
MSTRG.6544.1	coding	0.34	0	4398	4951
MSTRG.6544.3	coding	0.334	0	4440	4992
MSTRG.6540.2	coding	0.232	0	378	2129
MSTRG.6546.1	coding	0.522	51	1272	1802
MSTRG.6550.2	coding	0.558	0	1227	1771
MSTRG.6550.3	coding	0.582	99	1251	1793
MSTRG.6550.5	coding	0.57	0	1194	1707
MSTRG.6550.6	coding	0.582	63	1215	1729
MSTRG.6551.1	coding	0.354	0	375	3271
MSTRG.6551.2	coding	0.548	0	180	2445
MSTRG.6551.3	coding	0.507	0	258	2994
MSTRG.6554.1	coding	0.392	0	2010	2557
MSTRG.6555.1	noncoding	-0.0380928	345	615	772
MSTRG.6562.1	coding	0.374	0	5943	11808
MSTRG.6562.3	noncoding	-0.129024	84	411	7926
MSTRG.6568.1	noncoding	-0.0331776	1014	1233	1989
MSTRG.6567.6	noncoding	-0.034816	126	375	955
MSTRG.6564.2	coding	0.276	0	414	728
MSTRG.6564.3	noncoding	-0.167936	0	375	2083
MSTRG.6577.3	coding	0.391	0	1029	3077
MSTRG.6577.2	coding	0.398	0	1980	4618
MSTRG.6582.3	coding	0.225	0	1617	3012
MSTRG.6582.2	coding	0.225	0	1617	3448
MSTRG.6582.1	coding	0.225	0	1638	2136
MSTRG.6582.4	coding	0.207	1029	2649	3140

MSTRG.6585.1	noncoding	-0.034816	984	1377	1570
MSTRG.6587.1	noncoding	-0.0114688	291	381	2663
MSTRG.6589.2	noncoding	-0.2732032	0	93	2073
MSTRG.6591.2	coding	0.075	975	2562	2889
MSTRG.6591.3	coding	0.063	0	2007	2321
MSTRG.6592.1	coding	0.108	108	7902	8172
MSTRG.6592.2	coding	0.109	81	7875	11450
MSTRG.6594.2	coding	0.163	87	2130	3313
MSTRG.6594.3	coding	0.165	396	2604	3931
MSTRG.6598.1	noncoding	-0.032768	729	885	2967
MSTRG.6601.4	coding	0.18	0	192	498
MSTRG.6605.1	coding	0.473	18	1023	3084
MSTRG.6606.2	coding	0.184	0	378	3036
MSTRG.6614.1	noncoding	-0.018432	510	1110	2293
MSTRG.6614.2	noncoding	-0.0540672	3717	4251	5597
MSTRG.6612.3	coding	0.167	279	2505	2745
MSTRG.6624.2	coding	0.394	27	1377	1566
MSTRG.6624.3	coding	0.39	237	1665	1853
MSTRG.6624.4	coding	0.356	369	1848	2003
MSTRG.6624.5	coding	0.385	27	1284	1440
MSTRG.6624.7	coding	0.348	525	1908	2064
MSTRG.6624.6	coding	0.338	438	1929	2084
MSTRG.6624.8	coding	0.361	27	1092	1398
MSTRG.6624.9	coding	0.345	27	729	1256
MSTRG.6624.10	coding	0.317	108	1644	1800
MSTRG.6624.11	coding	0.373	462	1740	1894
MSTRG.6626.1	noncoding	-0.0004096	0	0	301
MSTRG.6627.1	coding	0.34	81	1617	3234
MSTRG.6627.2	coding	0.373	0	1443	1714
MSTRG.6627.3	coding	0.369	0	1521	1792
MSTRG.6627.4	coding	0.374	0	1383	1633
MSTRG.6627.5	coding	0.381	0	1452	1722
MSTRG.6627.6	coding	0.379	0	1440	1710
MSTRG.6627.7	coding	0.376	0	1356	1626
MSTRG.6622.2	coding	0.369	111	1719	2011
MSTRG.6622.3	noncoding	-0.1056768	0	231	408
MSTRG.6622.4	coding	0.388	0	1527	1823
MSTRG.6633.1	coding	0.294	333	17988	20646
MSTRG.6633.2	coding	0.293	333	17952	20610
MSTRG.6633.7	coding	0.278	105	12081	12236
MSTRG.6633.5	coding	0.297	105	17436	20093
MSTRG.6634.2	coding	0.334	1200	1851	2538
MSTRG.6629.2	coding	0.413	0	582	888
MSTRG.6636.3	coding	0.349	648	1734	2680
MSTRG.6636.4	coding	0.373	12	1839	2784
MSTRG.6636.5	coding	0.349	732	1818	2764
MSTRG.6636.6	coding	0.349	504	1590	2536
MSTRG.6638.2	coding	0.504	117	1200	1730
MSTRG.6639.2	coding	0.095	0	195	480
MSTRG.6639.3	noncoding	-0.036864	57	153	439
MSTRG.6640.3	coding	0.09	333	2136	2291
MSTRG.6640.4	coding	0.091	11187	13032	13186
MSTRG.6644.4	coding	0.158	438	1248	1912
MSTRG.6644.6	coding	0.124	621	1329	2067

MSTRG.6644.7	coding	0.158	567	1377	2114
MSTRG.6644.8	coding	0.149	708	1401	2070
MSTRG.6652.1	noncoding	-0.1040384	0	780	1024
MSTRG.6660.1	coding	0.241	486	1296	2623
MSTRG.6660.2	coding	0.241	282	1092	2348
MSTRG.6660.5	coding	0.242	4050	4860	6047
MSTRG.6661.5	coding	0.385	123	2616	3093
MSTRG.6661.7	coding	0.385	342	2850	3327
MSTRG.6661.3	coding	0.383	24	2580	3058
MSTRG.6662.6	coding	0.1	849	2307	4431
MSTRG.6662.7	coding	0.1	882	2340	4464
MSTRG.6657.1	coding	0.394	1146	3726	3930
MSTRG.6670.1	noncoding	-0.0004096	396	465	2087
MSTRG.6681.1	coding	0.143	12288	12498	12823
MSTRG.6682.1	coding	0.034	5076	6021	8820
MSTRG.6685.1	coding	0.463	951	1923	2726
MSTRG.6678.2	noncoding	-0.0004096	0	0	214
MSTRG.6698.1	coding	0.046	84	201	355
MSTRG.6705.1	coding	0.091	279	927	12135
MSTRG.6708.1	noncoding	-0.0524288	5568	5676	8962
MSTRG.6709.1	noncoding	-0.002048	2028	2163	6153
MSTRG.6710.1	noncoding	-0.0925696	1440	1467	3506
MSTRG.6707.3	coding	0.099	3615	4644	5798
MSTRG.6707.4	coding	0.087	3351	4332	7136
MSTRG.6711.1	noncoding	-0.04096	1554	1701	2089
MSTRG.6713.1	noncoding	-0.0004096	0	0	262
MSTRG.6714.1	noncoding	-0.0004096	648	819	2288
MSTRG.6714.2	coding	0.04	342	456	1951
MSTRG.6714.3	coding	0.04	3720	3834	5183
MSTRG.6714.4	coding	0.04	342	456	3760
MSTRG.6714.5	noncoding	-0.022528	345	372	1478
MSTRG.6715.1	noncoding	-0.045056	3093	3198	4309
MSTRG.6694.1	noncoding	-0.0004096	2052	2238	3255
MSTRG.6694.2	noncoding	-0.0208896	1305	1416	1648
MSTRG.6695.1	noncoding	-0.049152	48	123	1786
MSTRG.6694.3	noncoding	-0.008192	7872	7971	8893
MSTRG.6694.4	noncoding	-0.036864	2118	2154	3408
MSTRG.6726.2	noncoding	-0.0618496	0	459	3320
MSTRG.6739.2	noncoding	-0.012288	5685	5847	7327
MSTRG.6739.4	noncoding	-0.04096	381	489	643
MSTRG.6740.2	noncoding	-0.02048	2061	2169	5005
MSTRG.6741.3	coding	0.075	330	1173	1362
MSTRG.6741.4	coding	0.115	735	1110	1298
MSTRG.6741.5	coding	0.079	0	834	1559
MSTRG.6744.1	noncoding	-0.0385024	123	498	1320
MSTRG.6744.2	noncoding	-0.0565248	651	834	1976
MSTRG.6748.3	noncoding	-0.0770048	0	3039	3796
MSTRG.6750.2	coding	0.042	1002	1206	2407
MSTRG.6756.1	noncoding	-0.114688	2865	2955	5811
MSTRG.6757.1	coding	0.089	1881	2520	2848
MSTRG.6760.1	noncoding	-0.016384	2436	2631	5922
MSTRG.6762.1	noncoding	-0.0004096	861	1074	10197
MSTRG.6763.1	coding	0.137	144	345	1930
MSTRG.6761.4	coding	0.04	1662	2343	3628

MSTRG.6761.5	coding	0.04	1605	2286	3571
MSTRG.6761.6	coding	0.04	1503	2184	3434
MSTRG.6764.1	noncoding	-0.057344	162	192	451
MSTRG.6774.1	noncoding	-0.036864	4740	4836	5154
MSTRG.6775.1	coding	0.073	4689	4851	5694
MSTRG.6776.1	coding	0.156	1791	2061	2775
MSTRG.6777.1	coding	0.101	798	1125	1765
MSTRG.6776.2	noncoding	-0.0421888	330	333	1429
MSTRG.6777.2	noncoding	-0.053248	21	60	546
MSTRG.6778.1	noncoding	-0.069632	2127	2190	3076
MSTRG.6777.3	coding	0.101	2562	2889	3585
MSTRG.6777.5	coding	0.048	5040	5220	5860
MSTRG.6780.1	noncoding	-0.0176128	1608	1773	2408
MSTRG.6781.3	noncoding	-0.0913408	0	66	946
MSTRG.6781.4	noncoding	-0.0360448	18	174	1626
MSTRG.6785.2	noncoding	-0.0376832	1941	2022	2176
MSTRG.6786.1	noncoding	-0.0557056	1572	1773	2240
MSTRG.6786.2	noncoding	-0.135168	90	102	708
MSTRG.6792.5	coding	0.157	837	1479	1710
MSTRG.6797.3	coding	0.063	624	3258	3776
MSTRG.6797.4	coding	0.011	36	540	1669
MSTRG.6798.1	noncoding	-0.053248	1830	1926	2354
MSTRG.6800.1	noncoding	-0.0172032	129	276	5361
MSTRG.6806.2	coding	0.118	1461	2553	3331
MSTRG.6804.1	coding	0.226	42	552	965
MSTRG.6817.3	coding	0.067	3624	4167	5689
MSTRG.6823.1	noncoding	-0.049152	21	240	3707
MSTRG.6811.1	noncoding	-0.08192	48	117	1592
MSTRG.6811.2	noncoding	-0.049152	375	399	1500
MSTRG.6857.1	noncoding	-0.118784	0	837	17223
MSTRG.6857.2	coding	0.235	2562	4587	5758
MSTRG.6857.3	coding	0.248	0	3912	5066
MSTRG.6857.4	coding	0.234	2502	4524	6051
MSTRG.6857.5	noncoding	-0.008192	40386	40659	40847
MSTRG.6857.6	noncoding	-0.0159744	5760	5925	19909
MSTRG.6857.7	coding	0.062	11499	12078	31708
MSTRG.6857.8	noncoding	-0.0049152	25845	25983	80663
MSTRG.6859.1	noncoding	-0.0262144	3621	3789	5162
MSTRG.6859.2	coding	0.151	5655	5937	10485
MSTRG.6859.3	noncoding	-0.0106496	17910	18036	20457
MSTRG.6859.4	coding	0.151	13467	13749	18157
MSTRG.6860.1	noncoding	-0.0258048	4107	4290	9631
MSTRG.6861.1	coding	0.105	14136	14376	15402
MSTRG.6861.2	coding	0.105	14082	14322	16720
MSTRG.6857.9	noncoding	-0.012288	24258	24396	45971
MSTRG.6857.10	noncoding	-0.0217088	11229	11403	23811
MSTRG.6857.11	noncoding	-0.0217088	11718	11892	16785
MSTRG.6857.12	noncoding	-0.0217088	11769	11943	16837
MSTRG.6857.13	noncoding	-0.0159744	516	681	6125
MSTRG.6857.14	noncoding	-0.0372736	192	252	1269
MSTRG.6857.15	noncoding	-0.0139264	10200	10332	41708
MSTRG.6863.1	coding	0.032	5784	6129	17379
MSTRG.6864.1	noncoding	-0.0188416	3534	3687	19751
MSTRG.6864.2	noncoding	-0.0004096	981	1029	2902

MSTRG.6857.16	noncoding	-0.0004096	153	252	3542
MSTRG.6865.1	noncoding	-0.0024576	1302	1398	4823
MSTRG.6857.17	coding	0.23	1584	4851	6504
MSTRG.6857.19	coding	0.203	12	1335	1713
MSTRG.6866.1	coding	0.097	4617	5277	5618
MSTRG.6857.20	coding	0.231	732	2367	3785
MSTRG.6857.21	coding	0.238	666	2577	4197
MSTRG.6857.22	coding	0.231	213	1845	2221
MSTRG.6857.23	coding	0.232	348	1935	3016
MSTRG.6827.2	noncoding	-0.1048576	0	321	2708
MSTRG.6837.1	noncoding	-0.0008192	162	276	2037
MSTRG.6838.1	noncoding	-0.03072	285	351	854
MSTRG.6839.1	coding	0.068	1257	1422	2214
MSTRG.6845.1	noncoding	-0.02048	135	147	305
MSTRG.6851.1	coding	0.1	2298	2502	2707
MSTRG.6851.2	noncoding	-0.0004096	498	540	2155
MSTRG.6852.1	noncoding	-0.0258048	150	267	2171
MSTRG.6869.1	coding	0.128	264	528	2475
MSTRG.6871.2	noncoding	-0.0360448	99	105	820
MSTRG.6873.2	coding	0.546	0	333	3154
MSTRG.6873.3	coding	0.546	0	333	3009
MSTRG.6884.1	coding	0.23	693	3849	8610
MSTRG.6885.1	noncoding	-0.012288	312	426	5434
MSTRG.6886.1	coding	0.06	1890	3246	5426
MSTRG.6887.1	noncoding	-0.008192	795	1029	6580
MSTRG.6888.1	noncoding	-0.002048	7401	7509	12726
MSTRG.6901.1	noncoding	-0.0094208	960	1113	3211
MSTRG.6906.7	coding	0.165	0	621	7343
MSTRG.6906.8	coding	0.189	0	531	1999
MSTRG.6906.9	coding	0.1	4113	4494	5712
MSTRG.6906.10	coding	0.189	0	531	983
MSTRG.6907.1	coding	0.137	1515	1716	3509
MSTRG.6902.1	noncoding	-0.0004096	0	0	239
MSTRG.6919.1	noncoding	-0.016384	2868	2922	3718
MSTRG.6920.1	noncoding	-0.008192	1479	1629	5443
MSTRG.6921.1	noncoding	-0.073728	243	282	3224
MSTRG.6922.4	noncoding	-0.0950272	6	594	4523
MSTRG.6922.3	coding	0.221	6	2883	5210
MSTRG.6922.5	noncoding	-0.024576	1509	1629	15303
MSTRG.6923.1	noncoding	-0.016384	471	474	739
MSTRG.6925.1	noncoding	-0.077824	1149	1218	1374
MSTRG.6925.2	noncoding	-0.032768	144	225	1452
MSTRG.6925.3	noncoding	-0.032768	195	276	910
MSTRG.6926.1	noncoding	-0.110592	135	213	560
MSTRG.6934.1	coding	0.104	402	987	1984
MSTRG.6939.3	coding	0.225	387	705	1378
MSTRG.6941.1	coding	0.338	63	1224	1703
MSTRG.6941.2	coding	0.333	63	1218	1697
MSTRG.6943.1	coding	0.216	1194	1788	2104
MSTRG.6943.2	coding	0.216	1485	2079	2396
MSTRG.6943.4	coding	0.213	720	1269	1586
MSTRG.6945.2	coding	0.31	288	2310	2695
MSTRG.6947.3	coding	0.214	120	3123	3884
MSTRG.6947.4	coding	0.218	120	3027	3788

MSTRG.6947.6	coding	0.222	0	3126	3889
MSTRG.6948.1	noncoding	-0.0073728	144	330	6522
MSTRG.6947.7	coding	0.214	1209	4212	4973
MSTRG.6947.8	coding	0.22	12	1725	2488
MSTRG.6956.3	coding	0.325	426	2499	6712
MSTRG.6962.4	coding	0.161	195	549	808
MSTRG.6962.5	coding	0.191	192	762	1019
MSTRG.6966.4	coding	0.277	0	5541	7943
MSTRG.6967.3	coding	0.234	81	495	6244
MSTRG.6970.3	coding	0.429	21	3561	4230
MSTRG.6973.5	coding	0.306	93	3117	3270
MSTRG.6974.1	coding	0.337	12	660	853
MSTRG.6975.1	coding	0.271	48	1287	2875
MSTRG.6985.1	coding	0.404	0	3240	8955
MSTRG.6986.1	coding	0.247	75	807	1253
MSTRG.6986.4	coding	0.254	36	696	1047
MSTRG.6988.1	noncoding	-0.032768	369	468	1067
MSTRG.6997.1	noncoding	-0.0413696	867	1293	1503
MSTRG.6997.2	noncoding	-0.0413696	660	1086	1296
MSTRG.6995.2	noncoding	-0.053248	0	78	474
MSTRG.6995.4	noncoding	-0.008192	228	318	541
MSTRG.6995.3	noncoding	-0.02048	117	207	557
MSTRG.6995.5	noncoding	-0.008192	240	330	544
MSTRG.6977.1	coding	0.187	156	492	686
MSTRG.6977.2	coding	0.131	165	762	1005
MSTRG.6977.3	coding	0.133	156	756	957
MSTRG.6977.4	coding	0.176	0	504	901
MSTRG.6977.5	noncoding	-0.0790528	294	540	786
MSTRG.6980.3	coding	0.209	294	1893	2490
MSTRG.6980.2	coding	0.206	504	2181	2777
MSTRG.6981.6	coding	0.292	57	612	3244
MSTRG.6981.4	coding	0.271	663	1248	3878
MSTRG.6981.5	coding	0.271	720	1305	3937
MSTRG.7007.1	coding	0.236	2886	3813	5009
MSTRG.7007.2	coding	0.286	0	2214	8271
MSTRG.7007.4	coding	0.181	498	1488	2445
MSTRG.7007.5	coding	0.282	54	2283	7614
MSTRG.7008.2	coding	0.206	198	663	970
MSTRG.7007.11	coding	0.263	603	2982	3410
MSTRG.6982.1	coding	0.315	174	903	1452
MSTRG.6983.1	noncoding	-0.02048	846	858	1210
MSTRG.6982.3	coding	0.328	222	756	1305
MSTRG.7005.3	coding	0.123	93	573	948
MSTRG.7006.1	coding	0.456	0	1170	3179
MSTRG.7012.2	coding	0.28	855	1632	1808
MSTRG.7019.1	noncoding	-0.0024576	1029	1173	3721
MSTRG.7035.2	coding	0.08	420	1974	4148
MSTRG.7041.4	noncoding	-0.118784	315	375	884
MSTRG.7044.1	noncoding	-0.008192	594	675	895
MSTRG.7050.1	noncoding	-0.0004096	0	0	478
MSTRG.7050.3	noncoding	-0.0004096	0	0	623
MSTRG.7050.2	noncoding	-0.008192	342	423	2291
MSTRG.7054.1	noncoding	-0.0925696	0	72	225
MSTRG.7075.4	coding	0.216	0	1122	2073

MSTRG.7075.5	coding	0.04	183	318	1093
MSTRG.7079.1	coding	0.085	0	1599	1826
MSTRG.7083.1	noncoding	-0.0503808	393	423	580
MSTRG.7077.3	coding	0.034	606	1188	1556
MSTRG.7091.3	coding	0.05	1746	2838	3934
MSTRG.7091.5	noncoding	-0.0745472	3723	3996	4363
MSTRG.7091.4	noncoding	-0.08192	3915	4206	4575
MSTRG.7096.1	noncoding	-0.0172032	1323	1566	2799
MSTRG.7102.1	coding	0.102	0	336	3395
MSTRG.7102.2	coding	0.069	153	765	3816
MSTRG.7102.3	noncoding	-0.024576	2877	3006	3245
MSTRG.7103.1	coding	0.037	330	702	1785
MSTRG.7104.1	coding	0.204	159	2928	3162
MSTRG.7110.1	noncoding	-0.032768	111	207	405
MSTRG.7111.3	coding	0.562	0	285	1591
MSTRG.7111.4	coding	0.567	0	237	1557
MSTRG.7111.5	coding	0.561	0	234	1332
MSTRG.7118.2	coding	0.034	27	240	1004
MSTRG.7122.3	coding	0.2	180	567	796
MSTRG.7130.2	coding	0.234	0	5619	6180
MSTRG.7130.3	coding	0.235	144	5604	5984
MSTRG.7130.4	coding	0.279	249	1584	2144
MSTRG.7135.3	noncoding	-0.03072	210	393	838
MSTRG.7135.4	noncoding	-0.0004096	156	297	451
MSTRG.7138.2	coding	0.419	0	306	3340
MSTRG.7141.1	coding	0.207	0	1773	3478
MSTRG.7141.3	coding	0.153	12	1479	3425
MSTRG.7142.1	noncoding	-0.0237568	66	324	4798
MSTRG.7147.3	coding	0.06	264	387	1097
MSTRG.7147.2	coding	0.154	885	1155	2980
MSTRG.7146.1	noncoding	-0.0004096	0	0	255
MSTRG.7154.2	coding	0.215	0	1932	2799
MSTRG.7154.5	coding	0.215	0	1932	2814
MSTRG.7154.6	noncoding	-0.0196608	2823	3003	3158
MSTRG.7156.1	coding	0.415	1311	2640	6242
MSTRG.7161.1	coding	0.224	228	2316	3931
MSTRG.7161.2	coding	0.219	228	2280	12280
MSTRG.7162.1	coding	0.103	498	852	1811
MSTRG.7164.1	coding	0.052	315	774	1256
MSTRG.7165.1	coding	0.069	171	411	1867
MSTRG.7165.2	coding	0.076	1623	2259	3498
MSTRG.7166.1	noncoding	-0.0360448	3192	3561	3716
MSTRG.7169.1	noncoding	-0.0356352	750	840	1097
MSTRG.7177.1	noncoding	-0.016384	2301	2382	3241
MSTRG.7178.1	coding	0.202	0	246	2058
MSTRG.7178.2	coding	0.226	0	264	641
MSTRG.7177.2	noncoding	-0.0188416	1485	1632	3338
MSTRG.7177.3	noncoding	-0.0188416	1485	1632	3196
MSTRG.7177.4	noncoding	-0.0188416	1485	1632	3283
MSTRG.7177.8	noncoding	-0.0188416	1413	1560	3178
MSTRG.7177.7	noncoding	-0.0188416	1449	1596	3476
MSTRG.7177.6	noncoding	-0.0188416	1932	2079	3875
MSTRG.7177.5	noncoding	-0.0188416	1938	2085	3792
MSTRG.7177.9	noncoding	-0.004096	0	24	2502

MSTRG.7177.10	noncoding	-0.0188416	1449	1596	3260
MSTRG.7177.11	noncoding	-0.0188416	1449	1596	2717
MSTRG.7177.12	noncoding	-0.0188416	1449	1596	2893
MSTRG.7177.15	noncoding	-0.0643072	0	87	1682
MSTRG.7177.16	noncoding	-0.024576	0	159	1620
MSTRG.7177.13	noncoding	-0.0188416	1449	1596	3279
MSTRG.7177.14	noncoding	-0.0188416	1449	1596	3365
MSTRG.7177.18	noncoding	-0.08192	11958	12054	13479
MSTRG.7177.19	noncoding	-0.06144	3204	3288	4866
MSTRG.7179.1	noncoding	-0.053248	3051	3123	7196
MSTRG.7172.1	noncoding	-0.0315392	7248	7380	9074
MSTRG.7176.7	coding	0.157	1431	2628	3360
MSTRG.7176.4	coding	0.144	0	2691	3424
MSTRG.7176.5	coding	0.157	1263	2460	3192
MSTRG.7176.6	coding	0.151	1263	2481	3213
MSTRG.7183.1	coding	0.242	0	1515	9818
MSTRG.7183.3	coding	0.242	0	1515	4823
MSTRG.7189.1	noncoding	-0.0733184	9258	9744	9899
MSTRG.7191.3	coding	0.142	327	1260	3166
MSTRG.7194.1	noncoding	-0.032768	873	993	1975
MSTRG.7195.1	coding	0.291	75	939	4695
MSTRG.7195.2	coding	0.293	108	990	2541
MSTRG.7196.1	coding	0.11	8520	8826	13350
MSTRG.7195.3	coding	0.262	252	756	4367
MSTRG.7186.1	noncoding	-0.06144	0	30	481
MSTRG.7197.3	coding	0.248	0	1752	3426
MSTRG.7200.2	coding	0.647	0	2247	2438
MSTRG.7200.3	coding	0.638	0	2349	2541
MSTRG.7202.1	noncoding	-0.006144	2847	3012	3279
MSTRG.7206.4	coding	0.277	0	1308	5888
MSTRG.7206.5	coding	0.217	237	1305	3293
MSTRG.7206.6	coding	0.217	333	1398	1602
MSTRG.7207.1	noncoding	-0.002048	4173	4320	4524
MSTRG.7206.7	noncoding	-0.1753088	0	189	9169
MSTRG.7211.1	noncoding	-0.03072	669	1203	1773
MSTRG.7213.1	noncoding	-0.0884736	0	435	1418
MSTRG.7216.2	coding	0.476	183	1818	2031
MSTRG.7220.2	coding	0.33	168	723	1202
MSTRG.7220.3	coding	0.233	36	360	864
MSTRG.7220.4	noncoding	-0.0741376	246	441	598
MSTRG.7225.1	coding	0.227	0	1839	4424
MSTRG.7227.1	coding	0.411	171	960	2806
MSTRG.7232.2	noncoding	-0.024576	16698	17427	17882
MSTRG.7232.3	noncoding	-0.024576	12051	12780	13235
MSTRG.7232.1	coding	0.565	354	3066	19555
MSTRG.7232.4	coding	0.565	321	3033	17949
MSTRG.7232.5	coding	0.565	4890	7602	22517
MSTRG.7232.6	noncoding	-0.024576	15192	15921	16380
MSTRG.7235.3	coding	0.537	0	3222	4014
MSTRG.7235.4	coding	0.568	0	2511	2668
MSTRG.7245.1	noncoding	-0.09216	336	546	700
MSTRG.7258.7	coding	0.033	1752	3378	4183
MSTRG.7258.6	coding	0.045	102	1956	2760
MSTRG.7258.5	coding	0.042	123	2010	2814

MSTRG.7260.1	noncoding	-0.0516096	1008	1164	3204
MSTRG.7262.3	coding	0.08	360	2526	5589
MSTRG.7262.5	noncoding	-0.049152	651	765	7545
MSTRG.7263.1	coding	0.135	537	762	13470
MSTRG.7263.2	noncoding	-0.004096	4017	4155	8104
MSTRG.7264.1	noncoding	-0.397312	1155	1170	2102
MSTRG.7266.9	coding	0.069	2892	3978	6237
MSTRG.7266.12	noncoding	-0.0946176	0	102	2219
MSTRG.7268.1	noncoding	-0.233472	240	297	452
MSTRG.7269.5	noncoding	-0.004096	498	627	782
MSTRG.7269.7	noncoding	-0.004096	435	564	718
MSTRG.7269.3	noncoding	-0.057344	465	564	1679
MSTRG.7269.4	noncoding	-0.0176128	6	168	769
MSTRG.7269.6	noncoding	-0.028672	4134	4362	9114
MSTRG.7269.8	noncoding	-0.036864	2622	2673	3271
MSTRG.7269.9	noncoding	-0.0249856	2946	3066	3608
MSTRG.7270.1	noncoding	-0.016384	414	534	1678
MSTRG.7271.1	noncoding	-0.049152	309	426	4198
MSTRG.7277.1	noncoding	-0.006144	1218	1368	1535
MSTRG.7278.1	noncoding	-0.02048	1398	1521	6771
MSTRG.7279.1	noncoding	-0.032768	1098	1215	8112
MSTRG.7276.3	coding	0.113	9990	10365	11344
MSTRG.7276.4	coding	0.118	4344	6096	11063
MSTRG.7281.1	noncoding	-0.0692224	0	78	287
MSTRG.7281.2	noncoding	-0.0692224	0	78	540
MSTRG.7286.10	coding	0.126	0	1575	3536
MSTRG.7286.3	coding	0.066	0	2958	3495
MSTRG.7286.5	coding	0.069	0	2898	3435
MSTRG.7286.7	coding	0.093	0	2691	3229
MSTRG.7286.8	coding	0.135	0	1446	3285
MSTRG.7286.9	coding	0.081	0	2775	3312
MSTRG.7286.11	coding	0.064	0	2844	3381
MSTRG.7286.17	coding	0.088	600	1086	1623
MSTRG.7292.1	coding	0.241	0	1368	3316
MSTRG.7292.3	coding	0.283	312	1101	1301
MSTRG.7293.3	coding	0.071	3	948	6223
MSTRG.7296.1	coding	0.362	2580	4836	6432
MSTRG.7297.3	coding	0.201	258	3201	5024
MSTRG.7297.5	coding	0.199	258	3201	5024
MSTRG.7298.1	noncoding	-0.0004096	1095	1197	1752
MSTRG.7300.1	noncoding	-0.028672	3615	3747	6445
MSTRG.7297.6	coding	0.11	0	342	1926
MSTRG.7297.7	noncoding	-0.0180224	1377	1539	6933
MSTRG.7295.2	noncoding	-0.0356352	282	450	2327
MSTRG.7303.1	noncoding	-0.0004096	3027	3159	5645
MSTRG.7306.1	coding	0.248	0	315	2926
MSTRG.7306.2	coding	0.141	411	729	1074
MSTRG.7316.1	coding	0.064	957	1563	4327
MSTRG.7317.1	noncoding	-0.077824	1812	1893	2818
MSTRG.7322.4	coding	0.158	558	1764	2250
MSTRG.7322.5	coding	0.158	252	1458	1946
MSTRG.7323.1	noncoding	-0.0004096	0	0	475
MSTRG.7323.2	noncoding	-0.012288	351	405	880
MSTRG.7325.1	noncoding	-0.0008192	30	177	402

MSTRG.7320.1	noncoding	-0.057344	18	36	436
MSTRG.7320.2	noncoding	-0.053248	84	141	628
MSTRG.7321.1	noncoding	-0.147456	96	141	305
MSTRG.7333.5	coding	0.055	0	1164	2181
MSTRG.7335.2	coding	0.226	417	2511	2956
MSTRG.7335.4	coding	0.226	429	2523	2969
MSTRG.7335.5	coding	0.228	714	2787	3233
MSTRG.7335.8	coding	0.228	735	2829	3276
MSTRG.7335.9	coding	0.218	654	2853	3300
MSTRG.7335.10	coding	0.236	312	1974	2420
MSTRG.7335.11	coding	0.344	417	855	1302
MSTRG.7335.12	coding	0.358	417	876	1323
MSTRG.7335.13	coding	0.359	231	702	1148
MSTRG.7340.2	coding	0.218	0	819	4856
MSTRG.7340.3	coding	0.099	750	984	4604
MSTRG.7340.4	noncoding	-0.016384	3156	3264	4366
MSTRG.7341.1	noncoding	-0.14336	60	108	264
MSTRG.7342.1	noncoding	-0.0483328	3306	3735	3974
MSTRG.7343.1	coding	0.108	3117	3342	3840
MSTRG.7344.4	noncoding	-0.008192	1671	1776	2252
MSTRG.7346.1	noncoding	-0.032768	144	174	550
MSTRG.7348.1	coding	0.228	0	105	258
MSTRG.7350.1	coding	0.125	177	966	1792
MSTRG.7354.2	coding	0.055	1002	1152	3600
MSTRG.7359.1	noncoding	-0.0004096	174	174	383
MSTRG.7370.1	noncoding	-0.0139264	1764	1974	5079
MSTRG.7371.1	noncoding	-0.028672	858	972	7826
MSTRG.7369.3	coding	0.131	90	378	1542
MSTRG.7372.1	noncoding	-0.02048	5157	5265	6608
MSTRG.7373.1	noncoding	-0.0004096	1083	1164	3861
MSTRG.7374.1	noncoding	-0.012288	7695	7770	7923
MSTRG.7366.2	coding	0.152	0	3327	3877
MSTRG.7365.1	noncoding	-0.0004096	0	0	392
MSTRG.7390.1	noncoding	-0.04096	735	792	1094
MSTRG.7392.1	noncoding	-0.114688	849	900	4953
MSTRG.7393.1	coding	0.265	0	84	239
MSTRG.7394.1	noncoding	-0.0483328	0	135	745
MSTRG.7376.3	coding	0.085	795	993	1902
MSTRG.7376.5	coding	0.085	717	915	1825
MSTRG.7382.1	noncoding	-0.036864	1290	1374	3073
MSTRG.7385.1	noncoding	-0.004096	828	849	1196
MSTRG.7387.1	coding	0.136	1500	2331	5962
MSTRG.7415.2	coding	0.159	294	2670	3930
MSTRG.7415.4	noncoding	-0.0053248	7152	7218	9285
MSTRG.7415.3	coding	0.159	7335	9711	10971
MSTRG.7415.5	coding	0.083	7626	7887	15571
MSTRG.7415.6	noncoding	-0.0352256	555	720	11258
MSTRG.7415.7	coding	0.159	2193	4569	5828
MSTRG.7421.3	coding	0.383	0	615	1146
MSTRG.7423.4	coding	0.126	543	1128	1467
MSTRG.7423.2	coding	0.126	777	1362	1701
MSTRG.7423.3	coding	0.126	507	1092	1430
MSTRG.7426.1	coding	0.264	774	3744	4384
MSTRG.7431.1	coding	0.163	666	1275	1589

MSTRG.7431.2	coding	0.228	483	1230	1839
MSTRG.7432.1	coding	0.174	462	1230	2181
MSTRG.7432.9	coding	0.122	1386	1806	6151
MSTRG.7432.5	coding	0.105	0	3372	8305
MSTRG.7432.6	coding	0.11	1755	5421	9765
MSTRG.7432.7	coding	0.109	0	3801	8146
MSTRG.7434.1	noncoding	-0.008192	408	429	582
MSTRG.7429.1	noncoding	-0.0253952	426	591	1623
MSTRG.7429.2	noncoding	-0.028672	336	456	1949
MSTRG.7437.1	noncoding	-0.1236992	0	18	266
MSTRG.7438.1	coding	0.24	222	699	2902
MSTRG.7439.4	coding	0.214	126	1005	1197
MSTRG.7441.1	coding	0.327	21	1947	5113
MSTRG.7448.1	noncoding	-0.0106496	531	660	1726
MSTRG.7448.2	noncoding	-0.006144	462	612	767
MSTRG.7452.1	coding	0.357	1086	2970	6167
MSTRG.7452.3	coding	0.357	738	2622	5766
MSTRG.7454.1	noncoding	-0.036864	1875	1977	3527
MSTRG.7451.2	coding	0.111	492	1407	1980
MSTRG.7455.1	coding	0.171	0	4605	5305
MSTRG.7455.2	coding	0.184	0	4857	10131
MSTRG.7456.6	coding	0.148	237	1266	2138
MSTRG.7456.7	coding	0.148	234	1263	1788
MSTRG.7456.8	coding	0.173	66	1134	1659
MSTRG.7458.1	noncoding	-0.008192	180	267	423
MSTRG.7457.3	noncoding	-0.0016384	4056	4200	7432
MSTRG.7462.1	noncoding	-0.036864	36	51	381
MSTRG.7467.1	coding	0.356	162	1263	1966
MSTRG.7465.1	coding	0.168	204	1485	2955
MSTRG.7470.1	noncoding	-0.0004096	0	0	298
MSTRG.7474.2	coding	0.295	48	540	808
MSTRG.7476.1	noncoding	-0.083968	0	42	457
MSTRG.7487.1	noncoding	-0.385024	435	444	815
MSTRG.7485.2	coding	0.09	93	1353	1625
MSTRG.7499.1	coding	0.324	33	2997	3933
MSTRG.7496.1	coding	0.102	402	618	855
MSTRG.7501.1	noncoding	-0.0393216	540	654	2269
MSTRG.7505.1	coding	0.247	732	2196	4735
MSTRG.7505.2	coding	0.247	888	2352	4892
MSTRG.7518.1	noncoding	-0.118784	174	240	3186
MSTRG.7529.1	noncoding	-0.0131072	360	528	703
MSTRG.7529.2	noncoding	-0.0131072	345	513	925
MSTRG.7531.1	noncoding	-0.0208896	318	483	990
MSTRG.7534.1	noncoding	-0.0229376	0	129	291
MSTRG.7537.4	coding	0.432	177	2154	3230
MSTRG.7538.1	coding	0.314	51	927	1080
MSTRG.7539.1	coding	0.397	30	2178	2384
MSTRG.7541.1	coding	0.249	195	1014	1310
MSTRG.7542.1	noncoding	-0.0139264	870	1035	1271
MSTRG.7539.2	coding	0.405	312	2379	2804
MSTRG.7544.2	coding	0.325	0	456	1015
MSTRG.7551.1	coding	0.279	234	4086	4557
MSTRG.7551.2	coding	0.292	243	3876	4347
MSTRG.7552.1	noncoding	-0.0581632	342	573	729

MSTRG.7552.3	noncoding	-0.0217088	24	513	668
MSTRG.7552.2	noncoding	-0.0065536	39	480	634
MSTRG.7556.2	coding	0.542	666	3168	3799
MSTRG.7557.1	coding	0.228	942	1803	2050
MSTRG.7557.2	coding	0.226	801	1659	1906
MSTRG.7557.3	coding	0.224	546	1386	1632
MSTRG.7557.4	noncoding	-0.073728	630	1161	1397
MSTRG.7557.5	coding	0.227	807	1665	1912
MSTRG.7557.6	coding	0.226	537	1392	1638
MSTRG.7560.3	coding	0.513	18	699	1593
MSTRG.7562.6	coding	0.33	78	1827	2828
MSTRG.7562.7	coding	0.349	537	2337	3336
MSTRG.7562.8	coding	0.361	57	1533	2533
MSTRG.7563.1	coding	0.262	249	1308	2133
MSTRG.7563.2	coding	0.434	0	429	1444
MSTRG.7563.4	coding	0.422	0	573	933
MSTRG.7573.4	coding	0.445	0	5691	6126
MSTRG.7573.5	coding	0.442	0	5676	6111
MSTRG.7585.1	noncoding	-0.0487424	378	636	7673
MSTRG.7572.1	coding	0.291	0	1332	2161
MSTRG.7572.2	coding	0.251	165	1296	2125
MSTRG.7580.2	coding	0.285	0	1665	2347
MSTRG.7588.2	coding	0.271	129	870	1383
MSTRG.7591.2	coding	0.402	261	8637	8870
MSTRG.7591.3	coding	0.402	129	8505	8741
MSTRG.7591.4	coding	0.287	0	942	1175
MSTRG.7592.1	coding	0.371	0	1191	1415
MSTRG.7594.1	noncoding	-0.008192	189	327	614
MSTRG.7597.2	noncoding	-0.069632	81	141	422
MSTRG.7597.1	noncoding	-0.0004096	0	0	221
MSTRG.7600.1	coding	0.301	252	2799	3060
MSTRG.7604.3	coding	0.287	507	1752	8045
MSTRG.7604.4	coding	0.287	447	1692	9449
MSTRG.7604.5	noncoding	-0.0815104	5055	5265	5692
MSTRG.7606.1	coding	0.59	0	813	6388
MSTRG.7608.2	coding	0.24	480	1242	4683
MSTRG.7611.1	coding	0.373	81	900	4131
MSTRG.7617.2	coding	0.387	159	1644	3403
MSTRG.7618.3	noncoding	-0.0376832	78	711	1443
MSTRG.7618.4	noncoding	-0.0376832	264	897	1610
MSTRG.7619.3	coding	0.34	426	2778	3627
MSTRG.7621.1	noncoding	-0.1134592	0	735	1193
MSTRG.7615.2	coding	0.468	756	1971	3262
MSTRG.7615.1	coding	0.457	150	1437	2728
MSTRG.7615.3	coding	0.482	0	1290	2048
MSTRG.7615.4	coding	0.481	237	1185	1906
MSTRG.7615.5	coding	0.476	150	1395	2115
MSTRG.7616.2	noncoding	-0.0454656	12	711	882
MSTRG.7625.1	coding	0.572	36	2589	3886
MSTRG.7625.2	coding	0.561	36	2664	3939
MSTRG.7626.1	coding	0.269	234	951	1809
MSTRG.7626.2	coding	0.269	369	1086	1929
MSTRG.7630.2	coding	0.313	387	1944	6315
MSTRG.7630.3	coding	0.309	1998	5097	9173

MSTRG.7632.3	coding	0.285	21	450	1596
MSTRG.7637.1	noncoding	-0.0278528	714	1413	3105
MSTRG.7637.4	noncoding	-0.0720896	1653	1992	3135
MSTRG.7637.5	noncoding	-0.026624	627	1320	3274
MSTRG.7638.1	coding	0.323	705	3015	3888
MSTRG.7638.10	coding	0.276	0	7131	9552
MSTRG.7638.11	coding	0.284	0	6657	9078
MSTRG.7638.5	coding	0.278	0	7131	9552
MSTRG.7638.6	coding	0.278	0	7134	9555
MSTRG.7638.7	coding	0.275	0	7215	9636
MSTRG.7638.8	coding	0.28	0	7050	9471
MSTRG.7638.9	coding	0.288	0	6378	8799
MSTRG.7639.1	coding	0.055	72	1020	1382
MSTRG.7638.12	coding	0.234	3063	6357	8778
MSTRG.7640.2	coding	0.24	18	1257	1893
MSTRG.7640.3	coding	0.295	942	1500	2097
MSTRG.7642.1	noncoding	-0.0720896	1884	2136	2291
MSTRG.7642.2	noncoding	-0.0913408	27342	27768	27921
MSTRG.7642.3	noncoding	-0.0720896	11706	11958	12111
MSTRG.7642.4	noncoding	-0.0520192	5229	5415	5571
MSTRG.7634.2	coding	0.483	0	1356	1736
MSTRG.7635.1	coding	0.209	270	1467	3925
MSTRG.7635.2	coding	0.209	258	1455	3466
MSTRG.7635.3	coding	0.209	141	1329	1820
MSTRG.7635.5	coding	0.216	5316	6549	9004
MSTRG.7636.1	noncoding	-0.0139264	837	978	1923
MSTRG.7636.2	noncoding	-0.0139264	33	174	1083
MSTRG.7650.1	coding	0.472	2595	7269	8326
MSTRG.7646.1	coding	0.342	522	1263	1947
MSTRG.7647.1	noncoding	-0.045056	120	285	441
MSTRG.7659.8	coding	0.348	132	894	1169
MSTRG.7660.3	coding	0.353	498	1104	1595
MSTRG.7660.5	coding	0.351	714	1317	1797
MSTRG.7666.1	coding	0.485	2703	8556	11014
MSTRG.7666.3	coding	0.498	0	5799	8099
MSTRG.7666.4	coding	0.503	0	5685	7967
MSTRG.7666.5	coding	0.483	90	5970	6124
MSTRG.7666.6	noncoding	-0.0512	2022	2265	5581
MSTRG.7665.1	coding	0.367	264	1386	2049
MSTRG.7668.2	coding	0.505	858	2814	5022
MSTRG.7668.3	coding	0.499	831	2838	3625
MSTRG.7668.4	coding	0.495	9	1404	1559
MSTRG.7673.1	noncoding	-0.0393216	369	1074	1328
MSTRG.7674.2	coding	0.472	0	654	1408
MSTRG.7675.2	coding	0.586	0	1041	1374
MSTRG.7680.1	noncoding	-0.0356352	1059	1098	1583
MSTRG.7686.3	coding	0.586	0	1299	1972
MSTRG.7694.1	noncoding	-0.065536	102	171	602
MSTRG.7705.5	coding	0.452	0	2304	3867
MSTRG.7703.1	coding	0.516	168	2136	6273
MSTRG.7707.1	coding	0.264	0	1107	1499
MSTRG.7707.3	coding	0.262	0	1104	1496
MSTRG.7711.4	coding	0.398	1428	1998	5275
MSTRG.7711.5	noncoding	-0.1216512	0	516	3770

MSTRG.7711.6	noncoding	-0.1409024	0	618	3897
MSTRG.7711.3	coding	0.38	0	852	4130
MSTRG.7711.1	coding	0.247	3270	4242	7521
MSTRG.7717.2	coding	0.306	798	1698	1880
MSTRG.7717.1	coding	0.306	936	1836	2393
MSTRG.7717.3	coding	0.367	294	1278	4535
MSTRG.7717.4	coding	0.433	273	681	7281
MSTRG.7717.5	noncoding	-0.0598016	240	450	630
MSTRG.7717.8	coding	0.611	0	480	1110
MSTRG.7714.2	coding	0.445	930	2751	4349
MSTRG.7718.1	coding	0.623	0	1641	4693
MSTRG.7722.2	coding	0.403	765	1671	4410
MSTRG.7722.3	coding	0.36	765	1779	4145
MSTRG.7723.1	noncoding	-0.0733184	261	1005	8901
MSTRG.7722.5	coding	0.457	0	1251	4648
MSTRG.7722.6	coding	0.403	258	1164	4663
MSTRG.7723.2	noncoding	-0.0733184	888	1632	10290
MSTRG.7730.5	coding	0.129	765	5982	18041
MSTRG.7730.6	coding	0.131	4587	9891	22672
MSTRG.7731.1	noncoding	-0.008192	2826	2946	3676
MSTRG.7733.3	coding	0.481	39	2262	3142
MSTRG.7737.1	noncoding	-0.053248	939	1047	1474
MSTRG.7738.1	coding	0.212	150	1398	4079
MSTRG.7739.4	noncoding	-0.0475136	0	60	314
MSTRG.7740.2	coding	0.16	75	351	804
MSTRG.7742.2	coding	0.341	0	132	1486
MSTRG.7742.3	noncoding	-0.0405504	1029	1362	1515
MSTRG.7735.1	noncoding	-0.0602112	0	123	492
MSTRG.7744.2	coding	0.215	0	7518	8776
MSTRG.7745.1	noncoding	-0.172032	1911	1965	3654
MSTRG.7747.2	coding	0.395	1014	3429	5317
MSTRG.7747.3	coding	0.415	684	2814	4702
MSTRG.7747.4	coding	0.421	864	3048	4936
MSTRG.7747.1	coding	0.422	957	3171	5309
MSTRG.7747.5	coding	0.437	0	1788	4042
MSTRG.7751.3	coding	0.523	258	1995	3407
MSTRG.7751.6	coding	0.571	0	1716	3000
MSTRG.7751.7	coding	0.585	0	1467	1820
MSTRG.7751.8	coding	0.585	0	1467	1864
MSTRG.7751.9	coding	0.581	0	1581	1737
MSTRG.7752.1	noncoding	-0.0442368	87	675	1071
MSTRG.7752.2	noncoding	-0.0503808	84	1101	1973
MSTRG.7755.1	coding	0.583	0	825	3924
MSTRG.7755.3	coding	0.524	240	1032	4132
MSTRG.7743.1	coding	0.352	114	1773	4575
MSTRG.7758.1	noncoding	-0.172032	96	144	300
MSTRG.7762.2	noncoding	-0.088064	0	732	1945
MSTRG.7760.2	coding	0.32	255	576	1659
MSTRG.7767.1	noncoding	-0.0126976	453	579	735
MSTRG.7767.2	noncoding	-0.04096	372	480	634
MSTRG.7769.2	coding	0.405	183	786	3141
MSTRG.7770.1	noncoding	-0.0397312	627	1317	1472
MSTRG.7770.2	noncoding	-0.0749568	0	486	652
MSTRG.7771.4	coding	0.464	1086	1641	2557

MSTRG.7772.3	coding	0.336	282	2619	6010
MSTRG.7773.2	noncoding	-0.0835584	498	771	4879
MSTRG.7780.2	noncoding	-0.0004096	105	267	547
MSTRG.7782.1	coding	0.242	2091	2562	2742
MSTRG.7782.2	coding	0.209	2793	3243	3399
MSTRG.7782.3	coding	0.209	510	960	1117
MSTRG.7782.4	coding	0.209	6933	7383	7538
MSTRG.7782.5	coding	0.187	6384	6852	7009
MSTRG.7782.8	noncoding	-0.0004096	2646	3213	3369
MSTRG.7782.6	coding	0.209	7917	8367	8524
MSTRG.7782.7	coding	0.209	4554	5004	5161
MSTRG.7782.9	coding	0.209	3051	3501	3657
MSTRG.7782.10	coding	0.187	1140	1608	1764
MSTRG.7782.12	coding	0.209	1824	2274	2429
MSTRG.7782.13	coding	0.219	2094	2583	2755
MSTRG.7782.14	coding	0.124	1113	1338	1493
MSTRG.7783.1	noncoding	-0.0356352	0	330	659
MSTRG.7787.1	coding	0.319	123	1119	2490
MSTRG.7790.3	coding	0.315	24	1611	3709
MSTRG.7791.1	noncoding	-0.0299008	567	882	1678
MSTRG.7794.3	noncoding	-0.0499712	201	1137	1291
MSTRG.7795.1	noncoding	-0.0278528	3240	3699	3855
MSTRG.7796.3	coding	0.429	66	1272	1876
MSTRG.7796.5	coding	0.376	9156	10557	13092
MSTRG.7796.6	noncoding	-0.04096	489	1011	8387
MSTRG.7796.7	noncoding	-0.04096	363	885	1809
MSTRG.7797.1	noncoding	-0.024576	1572	1737	2654
MSTRG.7799.2	coding	0.542	3192	4719	6380
MSTRG.7800.1	noncoding	-0.0053248	306	606	760
MSTRG.7801.2	noncoding	-0.0434176	168	468	3619
MSTRG.7801.1	noncoding	-0.0679936	33	207	4185
MSTRG.7803.1	noncoding	-0.1355776	0	366	928
MSTRG.7804.2	coding	0.18	99	486	1022
MSTRG.7805.1	coding	0.469	0	735	3200
MSTRG.7805.3	coding	0.26	0	1278	2034
MSTRG.7805.4	noncoding	-0.0606208	309	1683	1838
MSTRG.7805.5	coding	0.289	0	1188	1940
MSTRG.7809.1	noncoding	-0.0413696	63	1194	4900
MSTRG.7812.1	noncoding	-0.0114688	6261	6411	12473
MSTRG.7815.1	coding	0.418	192	1266	1655
MSTRG.7815.3	coding	0.346	1851	3939	4326
MSTRG.7814.1	coding	0.617	0	741	2395
MSTRG.7818.2	coding	0.268	165	1611	1793
MSTRG.7818.3	noncoding	-0.0339968	39	120	293
MSTRG.7819.1	noncoding	-0.0012288	81	228	523
MSTRG.7821.2	noncoding	-0.1114112	0	264	623
MSTRG.7825.2	coding	0.628	0	1152	1713
MSTRG.7832.1	noncoding	-0.0823296	0	261	6796
MSTRG.7832.2	noncoding	-0.0417792	2541	2730	3354
MSTRG.7840.1	coding	0.051	375	594	2164
MSTRG.7841.3	coding	0.55	270	1173	2493
MSTRG.7842.2	coding	0.118	606	3720	4268
MSTRG.7843.2	coding	0.25	135	1026	1419
MSTRG.7844.1	coding	0.23	36	1281	2562

MSTRG.7844.4	noncoding	-0.0708608	0	333	983
MSTRG.7844.3	coding	0.24	0	1161	2511
MSTRG.7844.5	coding	0.237	2127	3360	4637
MSTRG.7844.6	coding	0.232	240	1455	2733
MSTRG.7848.1	noncoding	-0.0331776	495	1503	2378
MSTRG.7848.2	noncoding	-0.0045056	0	147	301
MSTRG.7854.5	noncoding	-0.057344	312	3468	5738
MSTRG.7859.2	coding	0.157	0	6945	9682
MSTRG.7859.3	coding	0.159	0	6945	10841
MSTRG.7859.1	coding	0.158	0	7011	10908
MSTRG.7859.4	coding	0.167	0	6879	10580
MSTRG.7859.8	coding	0.139	402	5379	7859
MSTRG.7859.9	coding	0.152	402	5394	7877
MSTRG.7859.7	coding	0.137	0	6504	8982
MSTRG.7859.5	coding	0.161	0	6564	12084
MSTRG.7859.6	coding	0.161	0	7011	9490
MSTRG.7859.10	coding	0.158	0	7011	9086
MSTRG.7859.11	coding	0.166	0	6954	9102
MSTRG.7859.12	coding	0.158	0	6945	8374
MSTRG.7860.1	noncoding	-0.049152	519	606	987
MSTRG.7859.14	noncoding	-0.0208896	354	408	630
MSTRG.7859.13	coding	0.165	0	6954	7888
MSTRG.7859.15	coding	0.17	402	3258	4278
MSTRG.7859.16	coding	0.174	0	7908	8371
MSTRG.7863.1	noncoding	-0.0004096	0	0	250
MSTRG.7855.7	coding	0.206	2436	3306	3794
MSTRG.7868.7	noncoding	-0.094208	0	492	1366
MSTRG.7870.1	noncoding	-0.0548864	2091	2682	2837
MSTRG.7866.2	coding	0.432	60	1083	3036
MSTRG.7866.3	coding	0.371	99	1281	3229
MSTRG.7874.1	noncoding	-0.0008192	45	336	508
MSTRG.7873.2	coding	0.423	609	1515	1727
MSTRG.7879.1	noncoding	-0.0991232	2085	2337	5207
MSTRG.7878.1	noncoding	-0.0557056	3201	3450	4913
MSTRG.7881.1	noncoding	-0.0512	0	567	862
MSTRG.7881.2	noncoding	-0.0364544	117	357	512
MSTRG.7884.1	noncoding	-0.0270336	126	339	588
MSTRG.7891.1	noncoding	-0.0098304	831	933	1315
MSTRG.7898.2	coding	0.457	486	1896	2253
MSTRG.7889.1	noncoding	-0.0483328	0	3018	3174
MSTRG.7903.1	coding	0.421	7014	9882	10084
MSTRG.7903.2	coding	0.355	3558	4536	9937
MSTRG.7903.3	coding	0.293	1227	2202	4344
MSTRG.7903.4	coding	0.348	3753	4674	11340
MSTRG.7905.6	coding	0.339	3627	5772	6280
MSTRG.7905.7	coding	0.145	5187	6135	11256
MSTRG.7915.10	coding	0.435	279	1131	1543
MSTRG.7915.11	coding	0.464	417	3165	3380
MSTRG.7915.5	coding	0.465	417	3162	3917
MSTRG.7915.6	coding	0.469	279	2994	3749
MSTRG.7915.7	coding	0.464	417	3153	3913
MSTRG.7915.9	coding	0.468	417	3123	3883
MSTRG.7915.12	coding	0.457	396	1656	4830
MSTRG.7917.3	coding	0.578	0	5670	5894

MSTRG.7917.4	coding	0.58	0	5493	5654
MSTRG.7913.1	noncoding	-0.0876544	297	807	961
MSTRG.7910.3	coding	0.305	111	357	512
MSTRG.7921.1	coding	0.439	2220	4557	5063
MSTRG.7921.2	coding	0.439	1815	4152	4638
MSTRG.7921.4	coding	0.436	132	2493	2971
MSTRG.7923.1	coding	0.559	0	5247	7122
MSTRG.7923.2	coding	0.496	159	2793	4310
MSTRG.7922.2	coding	0.241	0	924	1311
MSTRG.7922.3	coding	0.235	0	651	1281
MSTRG.7926.2	noncoding	-0.0528384	348	441	1027
MSTRG.7930.1	noncoding	-0.012288	198	330	3442
MSTRG.7933.1	noncoding	-0.196608	0	252	620
MSTRG.7933.2	coding	0.473	0	198	548
MSTRG.7932.1	noncoding	-0.0004096	0	0	250
MSTRG.7939.1	coding	0.218	0	522	1196
MSTRG.7939.2	coding	0.236	990	1374	5918
MSTRG.7939.3	coding	0.244	675	1104	5673
MSTRG.7939.4	noncoding	-0.0192512	1101	1254	1923
MSTRG.7935.2	coding	0.196	0	1740	2398
MSTRG.7943.1	noncoding	-0.0004096	0	0	884
MSTRG.7944.3	noncoding	-0.104448	0	519	4570
MSTRG.7944.4	noncoding	-0.104448	0	519	5544
MSTRG.7944.5	noncoding	-0.104448	0	519	3951
MSTRG.7944.6	coding	0.109	2904	3096	3457
MSTRG.7945.1	noncoding	-0.024576	135	147	1819
MSTRG.7944.7	noncoding	-0.0352256	1677	1797	2017
MSTRG.7958.1	noncoding	-0.0004096	0	0	1215
MSTRG.7962.1	coding	0.048	4482	4743	5490
MSTRG.7984.1	noncoding	-0.0106496	771	1026	1306
MSTRG.7985.1	noncoding	-0.016384	189	312	739
MSTRG.7990.1	noncoding	-0.0004096	1254	1332	3483
MSTRG.7995.1	coding	0.071	5154	5991	15776
MSTRG.7996.1	coding	0.069	3888	4581	5064
MSTRG.7996.2	coding	0.102	4032	4308	4573
MSTRG.7997.1	noncoding	-0.090112	1530	1629	4484
MSTRG.7998.1	noncoding	-0.014336	1677	1842	6987
MSTRG.7988.15	coding	0.167	285	900	1070
MSTRG.7988.13	coding	0.193	0	771	2225
MSTRG.7988.12	coding	0.218	0	822	1308
MSTRG.7988.14	coding	0.221	0	852	1197
MSTRG.7988.11	coding	0.164	285	2733	6916
MSTRG.7988.16	coding	0.21	186	873	1366
MSTRG.7988.18	coding	0.218	0	738	1037
MSTRG.7988.20	noncoding	-0.0024576	1563	1707	4479
MSTRG.7988.22	noncoding	-0.0421888	6012	6396	7327
MSTRG.8002.1	noncoding	-0.0262144	2259	2517	2927
MSTRG.8002.2	noncoding	-0.0364544	5922	6429	7786
MSTRG.8016.1	noncoding	-0.0069632	126	330	2624
MSTRG.8019.1	coding	0.02	4077	4254	4512
MSTRG.8020.1	coding	0.026	1029	3810	4920
MSTRG.8022.1	noncoding	-0.118784	321	324	1025
MSTRG.8023.1	noncoding	-0.02048	447	516	5107
MSTRG.8020.4	noncoding	-0.036864	612	702	906

MSTRG.8020.5	noncoding	-0.131072	507	582	785
MSTRG.8025.1	noncoding	-0.0356352	4536	4638	5547
MSTRG.8024.7	noncoding	-0.0393216	2340	2532	2952
MSTRG.8026.1	coding	0.174	3381	6855	10249
MSTRG.8027.1	noncoding	-0.0311296	0	3	623
MSTRG.8029.1	coding	0.226	294	3279	3629
MSTRG.8029.2	coding	0.226	174	3159	3510
MSTRG.8031.1	noncoding	-0.0200704	1764	1923	4857
MSTRG.8032.2	coding	0.111	0	636	2866
MSTRG.8010.1	noncoding	-0.012288	0	60	214
MSTRG.8060.1	noncoding	-0.004096	36	129	1416
MSTRG.8071.1	noncoding	-0.110592	120	180	334
MSTRG.8074.1	coding	0.117	216	366	1275
MSTRG.8096.1	coding	0.207	1404	8904	11359
MSTRG.8096.2	coding	0.278	0	5994	8449
MSTRG.8096.3	coding	0.282	897	6366	8776
MSTRG.8096.4	coding	0.282	1641	5994	8403
MSTRG.8096.11	coding	0.284	1395	5766	8173
MSTRG.8096.12	coding	0.283	9942	15450	17856
MSTRG.8096.6	coding	0.282	96	5580	7988
MSTRG.8096.10	coding	0.282	1071	6507	8915
MSTRG.8096.5	coding	0.284	315	5817	8225
MSTRG.8096.7	coding	0.284	315	5832	8240
MSTRG.8096.8	coding	0.283	2076	7569	9975
MSTRG.8096.9	coding	0.283	471	6075	8481
MSTRG.8096.13	coding	0.276	0	3243	4644
MSTRG.8096.14	coding	0.285	0	5685	7085
MSTRG.8097.1	noncoding	-0.004096	4056	4146	5545
MSTRG.8098.1	noncoding	-0.0487424	3786	3990	20310
MSTRG.8098.2	noncoding	-0.004096	16095	16239	19103
MSTRG.8099.1	noncoding	-0.0053248	8073	8220	14009
MSTRG.8098.3	noncoding	-0.135168	2040	2094	3733
MSTRG.8100.1	noncoding	-0.0004096	3642	3840	24349
MSTRG.8096.15	noncoding	-0.151552	15612	15714	25384
MSTRG.8096.16	noncoding	-0.028672	11745	11883	28985
MSTRG.8096.17	noncoding	-0.028672	11820	11958	26229
MSTRG.8096.18	noncoding	-0.028672	11739	11877	26274
MSTRG.8096.19	noncoding	-0.018432	15912	16071	22992
MSTRG.8096.20	noncoding	-0.018432	12438	12597	19100
MSTRG.8096.21	noncoding	-0.045056	7749	7863	8019
MSTRG.8096.22	noncoding	-0.008192	9219	9450	12057
MSTRG.8083.1	noncoding	-0.0729088	126	402	3601
MSTRG.8086.1	noncoding	-0.02048	189	222	528
MSTRG.8103.1	coding	0.059	417	828	1426
MSTRG.8109.3	coding	0.115	0	1527	1797
MSTRG.8110.2	coding	0.119	423	948	4061
MSTRG.8110.4	coding	0.149	3108	3342	3938
MSTRG.8110.6	coding	0.113	585	1341	4454
MSTRG.8110.5	noncoding	-0.0929792	3108	3837	3992
MSTRG.8113.1	noncoding	-0.1056768	0	30	531
MSTRG.8119.1	noncoding	-0.0385024	1560	2247	2749
MSTRG.8119.2	noncoding	-0.0212992	11907	12111	18777
MSTRG.8130.1	noncoding	-0.0565248	279	1065	2360
MSTRG.8131.3	coding	0.314	0	465	2210

MSTRG.8131.4	coding	0.314	0	456	2491
MSTRG.8131.5	coding	0.067	429	1239	2067
MSTRG.8131.6	coding	0.067	429	1284	2026
MSTRG.8131.7	coding	0.052	87	924	1666
MSTRG.8117.3	coding	0.236	102	1200	1787
MSTRG.8143.1	coding	0.099	111	1122	1396
MSTRG.8145.1	noncoding	-0.0847872	66	87	792
MSTRG.8146.1	noncoding	-0.0090112	183	213	370
MSTRG.8147.2	coding	0.063	540	1158	1725
MSTRG.8160.2	noncoding	-0.0077824	27	105	260
MSTRG.8160.1	noncoding	-0.0483328	2649	2871	13637
MSTRG.8160.3	noncoding	-0.0180224	930	969	2629
MSTRG.8160.4	noncoding	-0.0483328	2505	2727	12349
MSTRG.8165.1	coding	0.066	0	7938	8216
MSTRG.8165.3	coding	0.07	0	7647	7947
MSTRG.8171.1	noncoding	-0.0004096	0	0	352
MSTRG.8171.2	noncoding	-0.1859584	0	42	475
MSTRG.8172.11	noncoding	-0.0172032	2736	2874	4324
MSTRG.8172.7	noncoding	-0.0098304	471	582	1350
MSTRG.8172.6	noncoding	-0.0053248	633	777	2336
MSTRG.8172.9	noncoding	-0.0053248	120	264	1821
MSTRG.8172.10	noncoding	-0.0053248	804	948	2532
MSTRG.8172.5	noncoding	-0.0098304	471	582	2168
MSTRG.8172.8	coding	0.104	9513	9735	15150
MSTRG.8172.4	noncoding	-0.0053248	114	258	1847
MSTRG.8172.3	coding	0.217	0	192	2474
MSTRG.8172.13	noncoding	-0.032768	87	192	2683
MSTRG.8172.12	noncoding	-0.0053248	10398	10542	11308
MSTRG.8172.14	noncoding	-0.012288	2403	2553	4432
MSTRG.8172.15	noncoding	-0.086016	6318	6426	7152
MSTRG.8185.2	noncoding	-0.0004096	2067	2166	2412
MSTRG.8185.1	noncoding	-0.0503808	1113	1431	4507
MSTRG.8185.3	noncoding	-0.02048	468	507	1764
MSTRG.8195.1	coding	0.282	0	3633	4468
MSTRG.8195.2	coding	0.271	177	3966	4620
MSTRG.8190.2	noncoding	-0.0004096	0	0	488
MSTRG.8198.1	noncoding	-0.0004096	0	0	382
MSTRG.8201.1	noncoding	-0.0311296	174	324	3043
MSTRG.8201.2	noncoding	-0.016384	114	216	6363
MSTRG.8202.1	noncoding	-0.045056	1458	1575	5647
MSTRG.8200.2	coding	0.127	1011	2244	4812
MSTRG.8200.3	noncoding	-0.1245184	1716	1734	1942
MSTRG.8203.1	noncoding	-0.106496	96	117	278
MSTRG.8209.1	noncoding	-0.0466944	828	1041	2319
MSTRG.8209.2	noncoding	-0.0434176	186	192	746
MSTRG.8210.1	noncoding	-0.1847296	0	21	324
MSTRG.8218.1	coding	0.162	39	2247	3615
MSTRG.8219.1	noncoding	-0.06144	93	186	341
MSTRG.8224.3	coding	0.086	420	678	1400
MSTRG.8221.1	noncoding	-0.0249856	84	99	372
MSTRG.8221.2	noncoding	-0.0274432	0	117	388
MSTRG.8238.1	coding	0.082	375	624	1860
MSTRG.8238.2	coding	0.082	960	1209	2445
MSTRG.8238.5	coding	0.076	0	471	1707

MSTRG.8238.6	coding	0.082	870	1119	2354
MSTRG.8238.7	coding	0.082	567	816	2052
MSTRG.8238.8	coding	0.082	900	1149	2385
MSTRG.8239.1	noncoding	-0.02048	4131	4242	9995
MSTRG.8241.3	noncoding	-0.1417216	0	261	10966
MSTRG.8241.2	noncoding	-0.0454656	0	792	3177
MSTRG.8241.4	coding	0.148	0	984	1305
MSTRG.8242.1	noncoding	-0.022528	17562	17847	43372
MSTRG.8241.5	coding	0.15	1377	2121	2442
MSTRG.8241.6	coding	0.136	219	1104	1426
MSTRG.8244.1	noncoding	-0.038912	420	540	828
MSTRG.8241.7	coding	0.158	294	984	1306
MSTRG.8241.8	coding	0.186	1806	2160	2481
MSTRG.8246.2	coding	0.105	126	336	933
MSTRG.8246.6	noncoding	-0.065536	711	801	1398
MSTRG.8246.5	coding	0.072	57	1182	1778
MSTRG.8246.4	coding	0.072	138	1266	1863
MSTRG.8248.1	noncoding	-0.057344	1287	1374	3355
MSTRG.8246.7	noncoding	-0.004096	1494	1569	1949
MSTRG.8249.1	noncoding	-0.0008192	93	216	1079
MSTRG.8255.1	noncoding	-0.0106496	0	54	281
MSTRG.8283.3	coding	0.099	429	2019	4924
MSTRG.8283.5	coding	0.099	507	2097	4455
MSTRG.8283.6	coding	0.099	231	1821	4156
MSTRG.8283.7	coding	0.1	3141	4503	4771
MSTRG.8283.8	noncoding	-0.0483328	231	402	662
MSTRG.8283.9	noncoding	-0.0483328	228	399	5221
MSTRG.8283.10	noncoding	-0.0700416	162	465	3112
MSTRG.8283.12	noncoding	-0.028672	9339	9435	17695
MSTRG.8283.13	noncoding	-0.006144	6852	7002	11259
MSTRG.8285.3	coding	0.071	1389	3681	5948
MSTRG.8395.1	noncoding	-0.0004096	0	0	231
MSTRG.8397.1	noncoding	-0.016384	309	387	649
MSTRG.8409.1	noncoding	-0.151552	5241	5331	6064
MSTRG.8410.1	noncoding	-0.1024	846	876	1072
MSTRG.8411.1	coding	0.062	4869	5094	7198
MSTRG.8412.1	noncoding	-0.057344	1146	1191	1995
MSTRG.8417.2	noncoding	-0.069632	2028	2079	3330
MSTRG.8419.1	noncoding	-0.065536	678	702	2285
MSTRG.8394.2	noncoding	-0.0557056	402	585	6244
MSTRG.8394.3	coding	0.136	84	243	644
MSTRG.8420.1	noncoding	-0.0036864	1647	1794	7284
MSTRG.8422.1	noncoding	-0.032768	12	87	2437
MSTRG.8423.1	noncoding	-0.02048	930	936	1181
MSTRG.8424.1	coding	0.147	2574	2847	9786
MSTRG.8425.1	noncoding	-0.016384	1056	1140	2449
MSTRG.8394.4	noncoding	-0.02048	126	186	1662
MSTRG.8394.5	noncoding	-0.0180224	4962	5073	5581
MSTRG.8394.6	noncoding	-0.0049152	11028	11196	17583
MSTRG.8394.7	noncoding	-0.032768	462	489	2977
MSTRG.8394.8	noncoding	-0.0262144	0	90	1181
MSTRG.8394.9	noncoding	-0.0241664	1305	1404	3858
MSTRG.8282.1	noncoding	-0.0004096	0	0	216
MSTRG.8309.1	coding	0.063	1866	2523	3274

MSTRG.8315.1	noncoding	-0.012288	195	321	613
MSTRG.8316.1	noncoding	-0.073728	156	222	1918
MSTRG.8315.2	noncoding	-0.0626688	3414	3936	14760
MSTRG.8324.3	noncoding	-0.0294912	225	423	733
MSTRG.8324.4	noncoding	-0.0253952	84	279	591
MSTRG.8333.2	coding	0.229	180	675	1633
MSTRG.8333.3	coding	0.093	2784	3234	5997
MSTRG.8333.4	coding	0.23	180	654	6342
MSTRG.8333.5	coding	0.108	2499	2937	6861
MSTRG.8337.1	coding	0.1	159	309	462
MSTRG.8338.2	coding	0.455	0	633	1279
MSTRG.8346.2	coding	0.283	366	1518	2267
MSTRG.8349.6	coding	0.062	162	522	6783
MSTRG.8351.1	noncoding	-0.0004096	342	606	848
MSTRG.8351.5	noncoding	-0.06144	2946	3027	6597
MSTRG.8352.3	coding	0.082	0	2736	2983
MSTRG.8352.4	coding	0.086	216	1029	1266
MSTRG.8353.1	coding	0.039	174	585	8096
MSTRG.8353.2	coding	0.069	0	747	9030
MSTRG.8353.4	coding	0.041	5553	5796	9057
MSTRG.8353.6	coding	0.041	5442	5685	8424
MSTRG.8353.5	noncoding	-0.0618496	0	189	4570
MSTRG.8353.7	noncoding	-0.0618496	0	189	2534
MSTRG.8353.8	coding	0.078	0	474	2467
MSTRG.8354.1	noncoding	-0.0090112	1755	1962	3323
MSTRG.8355.1	coding	0.06	108	279	1092
MSTRG.8356.1	noncoding	-0.0413696	1662	1695	2103
MSTRG.8340.1	noncoding	-0.311296	171	207	985
MSTRG.8343.1	noncoding	-0.0851968	36	45	271
MSTRG.8359.5	coding	0.118	540	831	1147
MSTRG.8359.3	coding	0.118	2826	3117	3819
MSTRG.8359.4	coding	0.118	552	843	1708
MSTRG.8360.1	noncoding	-0.12288	123	138	337
MSTRG.8365.1	noncoding	-0.135168	1575	1632	3590
MSTRG.8358.1	noncoding	-0.090112	372	399	638
MSTRG.8386.1	coding	0.156	585	3900	4416
MSTRG.8387.1	noncoding	-0.024576	81	192	455
MSTRG.8392.1	noncoding	-0.0155648	81	144	714
MSTRG.8432.1	noncoding	-0.008192	3492	3633	12798
MSTRG.8430.1	noncoding	-0.151552	162	186	879
MSTRG.8444.1	coding	0.036	0	1269	3691
MSTRG.8444.2	coding	0.02	78	1311	1883
MSTRG.8444.3	coding	0.067	6	543	2364
MSTRG.8444.7	coding	0.361	15	399	2536
MSTRG.8444.5	coding	0.124	15	1437	3291
MSTRG.8444.6	coding	0.124	15	1437	2538
MSTRG.8444.8	coding	0.023	150	1368	2745
MSTRG.8444.9	coding	0.023	186	1404	3256
MSTRG.8444.10	coding	0.023	177	1395	2509
MSTRG.8444.11	coding	0.023	165	1383	2482
MSTRG.8445.1	coding	0.036	288	2961	3167
MSTRG.8445.3	coding	0.122	1389	1884	2091
MSTRG.8437.1	coding	0.337	207	2313	4008
MSTRG.8439.2	coding	0.349	0	183	1096

MSTRG.8446.5	coding	0.084	1389	2469	2670
MSTRG.8446.7	coding	0.084	213	1293	1494
MSTRG.8460.1	noncoding	-0.0004096	6	6	235
MSTRG.8482.1	coding	0.077	1815	2766	2937
MSTRG.8486.1	noncoding	-0.02048	426	549	733
MSTRG.8488.1	noncoding	-0.0323584	264	324	480
MSTRG.8488.2	noncoding	-0.0036864	135	174	615
MSTRG.8488.3	noncoding	-0.0036864	135	174	941
MSTRG.8488.4	noncoding	-0.047104	303	393	548
MSTRG.8495.1	noncoding	-0.0159744	183	330	762
MSTRG.8494.5	coding	0.26	0	2898	3154
MSTRG.8493.1	noncoding	-0.0008192	45	69	225
MSTRG.8499.1	coding	0.245	0	1176	4661
MSTRG.8506.1	coding	0.245	0	1659	11951
MSTRG.8507.1	noncoding	-0.0016384	156	231	1901
MSTRG.8508.1	noncoding	-0.0507904	1215	1476	3036
MSTRG.8509.1	noncoding	-0.0004096	1122	1188	1725
MSTRG.8512.2	noncoding	-0.1101824	0	270	3617
MSTRG.8512.3	noncoding	-0.1101824	0	270	3716
MSTRG.8512.4	coding	0.036	0	1881	3114
MSTRG.8502.3	coding	0.116	4020	7008	8455
MSTRG.8515.3	coding	0.138	426	957	1838
MSTRG.8517.2	coding	0.115	309	921	1264
MSTRG.8520.1	noncoding	-0.0270336	1686	1884	2123
MSTRG.8521.1	noncoding	-0.065536	753	825	1719
MSTRG.8522.1	noncoding	-0.02048	42	99	500
MSTRG.8524.1	noncoding	-0.0151552	2031	2193	3197
MSTRG.8526.2	coding	0.04	2427	5856	8650
MSTRG.8527.1	coding	0.128	2610	2907	5316
MSTRG.8526.3	noncoding	-0.100352	0	504	1140
MSTRG.8528.1	noncoding	-0.086016	117	138	959
MSTRG.8529.1	coding	0.084	4443	5718	7129
MSTRG.8529.2	coding	0.084	4578	5853	7231
MSTRG.8529.3	coding	0.084	4407	5682	7061
MSTRG.8529.4	coding	0.084	4425	5700	11546
MSTRG.8529.5	coding	0.084	4467	5742	6557
MSTRG.8529.7	coding	0.084	4236	5511	6889
MSTRG.8534.9	coding	0.639	438	2049	4551
MSTRG.8534.10	coding	0.574	918	2736	5129
MSTRG.8534.11	coding	0.575	693	2508	2801
MSTRG.8536.1	noncoding	-0.036864	1272	1365	13322
MSTRG.8536.2	noncoding	-0.036864	624	717	4170
MSTRG.8538.5	coding	0.135	0	717	2111
MSTRG.8539.3	coding	0.322	270	2382	5492
MSTRG.8539.4	coding	0.322	348	2460	5570
MSTRG.8539.5	coding	0.313	276	2451	3609
MSTRG.8543.1	noncoding	-0.094208	444	531	705
MSTRG.8544.1	noncoding	-0.0004096	540	627	841
MSTRG.8542.2	noncoding	-0.008192	111	132	3250
MSTRG.8547.1	noncoding	-0.0479232	381	450	1638
MSTRG.8548.3	noncoding	-0.0876544	0	1476	5560
MSTRG.8548.2	coding	0.331	0	1287	5371
MSTRG.8556.1	noncoding	-0.016384	108	180	335
MSTRG.8557.3	coding	0.072	564	2199	6111

MSTRG.8557.2	coding	0.081	6297	8673	12584
MSTRG.8557.5	coding	0.095	6150	7767	8576
MSTRG.8554.1	noncoding	-0.0598016	0	81	633
MSTRG.8555.1	noncoding	-0.0131072	201	243	786
MSTRG.8565.1	noncoding	-0.0167936	306	453	1716
MSTRG.8565.2	noncoding	-0.049152	519	606	1010
MSTRG.8566.1	noncoding	-0.0004096	60	150	464
MSTRG.8566.2	noncoding	-0.045056	60	96	296
MSTRG.8585.1	noncoding	-0.0008192	0	63	300
MSTRG.8587.1	noncoding	-0.0004096	0	0	356
MSTRG.8589.1	noncoding	-0.02048	846	975	2917
MSTRG.8586.1	noncoding	-0.0315392	285	411	567
MSTRG.8591.2	coding	0.01	33	525	2986
MSTRG.8594.2	coding	0.161	465	4302	5679
MSTRG.8595.1	noncoding	-0.0004096	0	0	322
MSTRG.8598.3	coding	0.197	0	411	3546
MSTRG.8598.4	coding	0.196	0	414	3549
MSTRG.8600.3	noncoding	-0.0352256	2931	3510	6484
MSTRG.8601.1	noncoding	-0.073728	171	255	924
MSTRG.8604.1	noncoding	-0.094208	111	555	1159
MSTRG.8604.2	noncoding	-0.1200128	0	246	847
MSTRG.8605.4	noncoding	-0.086016	0	102	1167
MSTRG.8605.8	noncoding	-0.036864	660	807	971
MSTRG.8605.5	noncoding	-0.036864	1173	1320	1483
MSTRG.8605.6	noncoding	-0.0094208	1698	1926	4345
MSTRG.8606.3	coding	0.134	0	468	4280
MSTRG.8606.5	coding	0.009	6	423	4198
MSTRG.8606.4	coding	0.021	6	438	4249
MSTRG.8608.1	noncoding	-0.032768	36	216	7482
MSTRG.8611.3	coding	0.261	0	1419	1859
MSTRG.8613.1	noncoding	-0.14336	378	408	593
MSTRG.8617.1	noncoding	-0.0466944	15	249	3792
MSTRG.8614.2	coding	0.385	633	1143	2232
MSTRG.8614.3	coding	0.382	546	1053	1422
MSTRG.8624.10	coding	0.122	63	1716	4645
MSTRG.8624.11	coding	0.113	63	1698	4728
MSTRG.8624.12	coding	0.113	63	1698	4684
MSTRG.8624.13	coding	0.117	63	1740	4726
MSTRG.8620.1	noncoding	-0.069632	246	249	413
MSTRG.8629.1	noncoding	-0.012288	261	357	902
MSTRG.8630.1	coding	0.084	843	1083	1943
MSTRG.8630.2	noncoding	-0.008192	249	453	3983
MSTRG.8632.1	noncoding	-0.0004096	0	0	420
MSTRG.8645.5	noncoding	-0.0999424	615	900	3214
MSTRG.8645.4	noncoding	-0.0999424	615	900	3497
MSTRG.8645.6	coding	0.244	492	777	3949
MSTRG.8646.1	noncoding	-0.0458752	216	243	1103
MSTRG.8642.2	coding	0.251	0	213	760
MSTRG.8647.1	coding	0.127	168	2664	7171
MSTRG.8647.3	coding	0.127	159	2655	3307
MSTRG.8648.1	coding	0.082	222	1755	5178
MSTRG.8650.2	coding	0.347	429	4251	5011
MSTRG.8650.1	coding	0.321	0	4362	6989
MSTRG.8650.4	noncoding	-0.0159744	378	381	652

MSTRG.8650.3	coding	0.34	105	4134	6532
MSTRG.8656.3	coding	0.055	807	1185	3794
MSTRG.8657.1	coding	0.053	879	2508	5607
MSTRG.8657.2	coding	0.053	456	2085	3486
MSTRG.8658.1	coding	0.066	7272	8244	9642
MSTRG.8658.3	coding	0.066	2304	3276	5900
MSTRG.8657.3	coding	0.053	573	2202	5300
MSTRG.8657.4	coding	0.054	573	2046	5144
MSTRG.8657.6	noncoding	-0.07168	4605	4851	5546
MSTRG.8657.7	noncoding	-0.0651264	1968	2220	2714
MSTRG.8659.2	noncoding	-0.0192512	564	606	1058
MSTRG.8660.2	coding	0.146	627	2433	4024
MSTRG.8660.3	coding	0.152	495	2352	3942
MSTRG.8660.4	coding	0.147	915	2718	4308
MSTRG.8660.10	coding	0.159	405	1449	5145
MSTRG.8660.11	coding	0.151	33	1917	3501
MSTRG.8660.8	coding	0.148	270	2040	3590
MSTRG.8660.9	coding	0.141	405	2124	3672
MSTRG.8652.1	coding	0.14	471	1620	2233
MSTRG.8652.2	coding	0.359	111	540	1883
MSTRG.8674.2	coding	0.192	1311	4266	5024
MSTRG.8674.1	coding	0.187	0	3171	6975
MSTRG.8674.3	coding	0.183	1239	4155	7957
MSTRG.8674.4	coding	0.191	0	3165	6969
MSTRG.8674.8	coding	0.262	108	1488	2152
MSTRG.8674.7	coding	0.183	1716	4632	6522
MSTRG.8677.5	noncoding	-0.0987136	0	111	628
MSTRG.8677.3	coding	0.217	0	123	751
MSTRG.8677.4	noncoding	-0.02048	0	6	457
MSTRG.8677.8	coding	0.21	0	135	809
MSTRG.8677.9	coding	0.208	0	138	812
MSTRG.8677.10	coding	0.213	0	132	653
MSTRG.8678.2	coding	0.4	30	1233	2276
MSTRG.8680.3	coding	0.07	0	1797	3626
MSTRG.8680.2	coding	0.08	0	3348	3654
MSTRG.8686.1	noncoding	-0.0004096	0	0	321
MSTRG.8701.1	coding	0.14	189	3975	10370
MSTRG.8701.2	coding	0.139	159	3984	11309
MSTRG.8716.1	coding	0.135	501	2139	2466
MSTRG.8713.1	noncoding	-0.0110592	0	27	233
MSTRG.8732.1	noncoding	-0.0745472	2235	2295	2657
MSTRG.8733.1	noncoding	-0.0004096	0	0	511
MSTRG.8733.2	coding	0.171	474	831	2303
MSTRG.8733.3	coding	0.075	450	657	2130
MSTRG.8734.1	coding	0.118	2256	2646	3940
MSTRG.8729.1	coding	0.146	0	348	635
MSTRG.8742.1	coding	0.259	0	1059	3023
MSTRG.8742.3	coding	0.135	180	2406	2660
MSTRG.8742.6	coding	0.265	69	1239	4582
MSTRG.8742.7	coding	0.31	69	1053	4528
MSTRG.8742.8	coding	0.243	528	1569	3534
MSTRG.8742.9	coding	0.29	528	1383	3480
MSTRG.8764.1	noncoding	-0.077824	213	273	1789
MSTRG.8765.1	coding	0.128	1275	1608	5157

MSTRG.8765.2	coding	0.128	15243	15576	17517
MSTRG.8766.1	noncoding	-0.004096	3714	3831	6057
MSTRG.8765.4	noncoding	-0.0049152	675	903	1299
MSTRG.8748.4	coding	0.256	612	2331	3499
MSTRG.8748.6	coding	0.25	612	2208	5390
MSTRG.8768.3	coding	0.186	0	417	2314
MSTRG.8769.1	coding	0.147	0	801	6751
MSTRG.8785.1	coding	0.262	0	318	2739
MSTRG.8785.2	coding	0.171	0	930	2575
MSTRG.8785.3	coding	0.309	0	387	2210
MSTRG.8786.7	coding	0.073	1770	6096	9533
MSTRG.8786.13	coding	0.096	3111	8190	11629
MSTRG.8786.8	coding	0.077	6003	10332	13769
MSTRG.8786.12	coding	0.131	0	4500	7937
MSTRG.8786.10	coding	0.124	168	5673	9110
MSTRG.8786.11	coding	0.136	174	4578	9067
MSTRG.8786.9	coding	0.11	78	5328	8765
MSTRG.8786.15	noncoding	-0.086016	3513	3636	6405
MSTRG.8787.1	noncoding	-0.096256	0	561	11769
MSTRG.8789.1	coding	0.102	246	981	1276
MSTRG.8803.1	coding	0.099	678	915	3921
MSTRG.8808.2	coding	0.06	2982	4569	6778
MSTRG.8809.1	coding	0.149	0	1431	1730
MSTRG.8809.4	coding	0.176	423	1551	3609
MSTRG.8818.3	coding	0.139	438	1353	1669
MSTRG.8815.1	noncoding	-0.004096	84	279	494
MSTRG.8815.2	noncoding	-0.008192	258	465	679
MSTRG.8815.3	noncoding	-0.0036864	258	480	694
MSTRG.8815.4	noncoding	-0.002048	84	264	418
MSTRG.8825.6	coding	0.258	474	3015	3552
MSTRG.8825.7	coding	0.307	1245	3066	3256
MSTRG.8825.8	coding	0.104	474	1182	2210
MSTRG.8826.4	coding	0.321	0	5598	5986
MSTRG.8823.1	noncoding	-0.0425984	237	537	1111
MSTRG.8833.1	noncoding	-0.053248	282	507	1772
MSTRG.8833.2	coding	0.089	579	1578	2463
MSTRG.8833.3	noncoding	-0.053248	282	507	2047
MSTRG.8833.4	coding	0.089	579	1578	2389
MSTRG.8833.5	coding	0.089	579	1578	3484
MSTRG.8833.6	coding	0.089	579	1578	4438
MSTRG.8833.7	noncoding	-0.053248	282	507	4886
MSTRG.8833.8	coding	0.089	450	1449	4451
MSTRG.8830.1	noncoding	-0.0610304	123	387	541
MSTRG.8835.1	noncoding	-0.043008	600	717	2581
MSTRG.8836.1	noncoding	-0.0237568	0	183	2358
MSTRG.8838.1	coding	0.042	579	1341	2220
MSTRG.8839.1	coding	0.076	108	834	1116
MSTRG.8842.1	coding	0.408	0	1125	1350
MSTRG.8846.3	coding	0.245	0	3351	4404
MSTRG.8846.4	coding	0.237	0	3147	4204
MSTRG.8846.5	coding	0.237	0	3147	7556
MSTRG.8846.6	coding	0.246	0	3036	7445
MSTRG.8849.1	noncoding	-0.548864	225	231	602
MSTRG.8844.1	coding	0.376	0	606	960

MSTRG.8844.2	coding	0.376	0	606	1384
MSTRG.8853.1	noncoding	-0.012288	318	336	491
MSTRG.8853.2	noncoding	-0.045056	12	24	422
MSTRG.8853.3	noncoding	-0.024576	444	504	660
MSTRG.8853.4	noncoding	-0.0548864	258	267	420
MSTRG.8857.1	noncoding	-0.0004096	0	0	207
MSTRG.8862.1	coding	0.207	150	780	1214
MSTRG.8862.2	noncoding	-0.1028096	0	144	2083
MSTRG.8863.1	coding	0.112	18912	19158	19986
MSTRG.8864.1	coding	0.036	1653	2103	2975
MSTRG.8865.1	noncoding	-0.18432	384	438	2992
MSTRG.8881.2	noncoding	-0.0724992	333	537	1297
MSTRG.8888.1	noncoding	-0.0352256	9855	10104	12999
MSTRG.8893.1	noncoding	-0.0110592	924	1176	4407
MSTRG.8894.1	noncoding	-0.01024	10302	10455	31076
MSTRG.8898.11	coding	0.124	759	3195	4591
MSTRG.8898.12	coding	0.116	21	3588	4108
MSTRG.8898.13	coding	0.126	774	4026	4535
MSTRG.8902.1	coding	0.137	1560	2112	5031
MSTRG.8896.1	noncoding	-0.012288	396	639	981
MSTRG.8912.1	coding	0.28	81	387	4510
MSTRG.8912.2	coding	0.28	87	393	2039
MSTRG.8913.1	noncoding	-0.02048	33	159	674
MSTRG.8913.2	noncoding	-0.02048	33	159	773
MSTRG.8914.1	coding	0.136	876	1113	2223
MSTRG.8914.2	coding	0.18	876	1062	1643
MSTRG.8908.1	noncoding	-0.0630784	960	1626	2673
MSTRG.8916.1	noncoding	-0.034816	0	135	538
MSTRG.8919.5	coding	0.167	330	10848	14067
MSTRG.8919.3	coding	0.174	54	11280	14499
MSTRG.8919.1	coding	0.161	33	11595	14812
MSTRG.8919.2	coding	0.172	222	11532	14750
MSTRG.8919.6	coding	0.172	222	11577	14795
MSTRG.8919.7	coding	0.175	30	11301	14022
MSTRG.8919.8	coding	0.161	123	11439	14162
MSTRG.8919.9	coding	0.172	180	11490	14110
MSTRG.8920.1	coding	0.305	0	222	458
MSTRG.8920.2	coding	0.283	0	312	467
MSTRG.8918.2	coding	0.327	102	2115	2660
MSTRG.8932.2	coding	0.205	285	1584	2445
MSTRG.8941.1	coding	0.091	336	1056	13154
MSTRG.8943.1	coding	0.098	0	6450	10198
MSTRG.8943.2	coding	0.096	0	5883	9631
MSTRG.8943.3	coding	0.107	0	5835	5991
MSTRG.8943.4	coding	0.103	0	5292	5448
MSTRG.8943.5	coding	0.107	0	5841	5997
MSTRG.8943.6	coding	0.145	0	891	3830
MSTRG.8943.7	coding	0.095	0	5781	8069
MSTRG.8943.8	coding	0.085	705	6708	6863
MSTRG.8943.9	coding	0.08	702	6180	6392
MSTRG.8943.10	coding	0.093	699	6144	6301
MSTRG.8943.11	coding	0.084	777	6192	6404
MSTRG.8943.12	coding	0.085	1227	6480	6635
MSTRG.8943.13	coding	0.089	468	5394	5607

MSTRG.8923.1	noncoding	-0.0258048	2412	2547	2703
MSTRG.8923.4	coding	0.154	213	738	1192
MSTRG.8923.3	noncoding	-0.0258048	1341	1476	1633
MSTRG.8924.1	noncoding	-0.0360448	153	606	759
MSTRG.8928.5	coding	0.313	4248	6480	9773
MSTRG.8929.1	noncoding	-0.0339968	9087	9390	9545
MSTRG.8949.1	noncoding	-0.18432	267	321	2269
MSTRG.8949.2	noncoding	-0.0892928	321	327	520
MSTRG.8951.2	coding	0.563	273	3048	3728
MSTRG.8952.1	noncoding	-0.02048	90	111	315
MSTRG.8954.1	noncoding	-0.0524288	2667	2859	5014
MSTRG.8955.1	noncoding	-0.016384	39	177	822
MSTRG.8957.1	noncoding	-0.1441792	0	39	534
MSTRG.8961.2	noncoding	-0.0258048	51	264	578
MSTRG.8962.2	noncoding	-0.0851968	18	243	398
MSTRG.8962.3	coding	0.282	0	483	7436
MSTRG.8966.1	noncoding	-0.04096	471	588	1101
MSTRG.8967.3	coding	0.228	147	1197	1483
MSTRG.8971.2	coding	0.507	0	1866	2799
MSTRG.8972.2	coding	0.15	210	3396	6772
MSTRG.8976.1	noncoding	-0.0004096	0	0	297
MSTRG.8981.1	noncoding	-0.024576	9	102	410
MSTRG.8994.1	coding	0.256	339	3150	7114
MSTRG.8994.6	noncoding	-0.1830912	0	1185	5355
MSTRG.8994.2	coding	0.237	81	2427	6390
MSTRG.8994.4	coding	0.262	258	2886	6849
MSTRG.8994.7	noncoding	-0.200704	777	801	1314
MSTRG.8995.1	noncoding	-0.045056	3828	3939	5347
MSTRG.8996.1	noncoding	-0.032768	447	543	696
MSTRG.8996.2	noncoding	-0.1380352	0	96	759
MSTRG.8996.3	noncoding	-0.159744	531	564	718
MSTRG.9002.2	coding	0.17	939	1170	3060
MSTRG.9000.2	noncoding	-0.0339968	0	210	851
MSTRG.9000.3	coding	0.069	1320	1551	2867
MSTRG.9000.4	coding	0.069	1329	1560	2892
MSTRG.9003.2	coding	0.143	0	687	4262
MSTRG.9010.1	coding	0.152	102	2019	4819
MSTRG.9010.2	coding	0.16	912	3042	5740
MSTRG.9010.3	coding	0.16	891	3021	3811
MSTRG.9010.4	coding	0.16	672	2802	3588
MSTRG.9010.5	coding	0.16	762	2892	3600
MSTRG.9010.6	coding	0.16	1578	3708	4494
MSTRG.9010.7	coding	0.16	507	2637	3418
MSTRG.9010.9	coding	0.087	4992	5628	6494
MSTRG.9010.10	noncoding	-0.0499712	39	918	4923
MSTRG.9010.11	noncoding	-0.0475136	828	1473	1627
MSTRG.9010.12	noncoding	-0.0483328	30	714	1900
MSTRG.9010.13	noncoding	-0.0335872	201	711	866
MSTRG.9005.1	noncoding	-0.049152	138	240	395
MSTRG.9008.1	noncoding	-0.024576	204	213	515
MSTRG.9011.1	coding	0.256	180	603	1085
MSTRG.9025.1	coding	0.345	30	1047	1200
MSTRG.9027.2	noncoding	-0.0057344	570	699	1113
MSTRG.9029.3	noncoding	-0.0806912	15090	15591	15751

MSTRG.9029.4	coding	0.246	2445	6591	8624
MSTRG.9029.5	noncoding	-0.0364544	6576	7296	7846
MSTRG.9034.1	coding	0.675	102	1323	1477
MSTRG.9034.2	coding	0.687	0	1173	3875
MSTRG.9036.2	coding	0.445	534	1035	1900
MSTRG.9036.3	coding	0.447	264	822	1686
MSTRG.9038.1	noncoding	-0.0229376	51	273	998
MSTRG.9040.3	coding	0.462	0	2367	7476
MSTRG.9040.4	coding	0.475	282	2514	2702
MSTRG.9041.1	coding	0.445	0	1698	1854
MSTRG.9041.2	coding	0.453	0	1386	2137
MSTRG.9042.1	noncoding	-0.0208896	147	594	757
MSTRG.9042.2	noncoding	-0.0630784	297	534	693
MSTRG.9043.1	noncoding	-0.1441792	0	108	368
MSTRG.9045.2	noncoding	-0.1736704	0	183	887
MSTRG.9046.2	noncoding	-0.1593344	0	642	1829
MSTRG.9050.1	coding	0.371	0	2064	2242
MSTRG.9051.1	noncoding	-0.0704512	369	2589	2858
MSTRG.9053.3	noncoding	-0.1253376	0	342	498
MSTRG.9053.5	noncoding	-0.1200128	0	339	495
MSTRG.9053.6	noncoding	-0.0761856	0	861	1015
MSTRG.9053.4	coding	0.438	0	4146	4312
MSTRG.9053.7	noncoding	-0.1077248	753	1773	2002
MSTRG.9054.1	noncoding	-0.0598016	417	843	1000
MSTRG.9055.1	coding	0.406	0	9762	9922
MSTRG.9055.4	coding	0.406	159	9621	10147
MSTRG.9058.1	coding	0.322	0	2085	4231
MSTRG.9058.2	coding	0.311	0	1455	2835
MSTRG.9058.3	coding	0.229	0	1683	2966
MSTRG.9058.4	coding	0.218	1911	2823	4313
MSTRG.9058.8	coding	0.218	4512	5424	5725
MSTRG.9058.10	coding	0.25	0	936	1774
MSTRG.9058.11	coding	0.319	0	1593	1764
MSTRG.9058.5	coding	0.266	0	987	1427
MSTRG.9058.6	coding	0.276	0	1296	1748
MSTRG.9058.7	coding	0.252	0	1170	1327
MSTRG.9058.9	coding	0.315	0	1641	1812
MSTRG.9058.12	coding	0.276	0	1296	1998
MSTRG.9058.14	coding	0.319	0	1512	1876
MSTRG.9059.3	noncoding	-0.0954368	0	174	768
MSTRG.9059.2	coding	0.455	0	1827	2931
MSTRG.9062.6	coding	0.287	108	1956	3778
MSTRG.9056.2	noncoding	-0.0356352	2535	3753	3908
MSTRG.9081.6	noncoding	-0.0745472	2550	2886	3138
MSTRG.9081.7	coding	0.416	0	4773	5036
MSTRG.9081.5	coding	0.355	3264	7596	7858
MSTRG.9081.3	coding	0.415	0	4821	5084
MSTRG.9081.1	coding	0.434	0	4536	4800
MSTRG.9081.4	coding	0.425	0	4650	4914
MSTRG.9081.8	coding	0.421	1131	5799	5954
MSTRG.9081.9	coding	0.444	0	4455	4610
MSTRG.9081.10	coding	0.392	4920	7212	7584
MSTRG.9081.11	coding	0.479	2358	3375	3778
MSTRG.9081.12	noncoding	-0.0720896	0	1131	6322

MSTRG.9082.1	coding	0.081	10653	11970	14778
MSTRG.9082.2	coding	0.041	2661	3561	4575
MSTRG.9083.1	noncoding	-0.0995328	0	39	1162
MSTRG.9084.1	noncoding	-0.0761856	0	873	4332
MSTRG.9083.10	coding	0.346	492	957	3135
MSTRG.9083.12	coding	0.346	507	972	3152
MSTRG.9083.13	coding	0.346	510	975	3155
MSTRG.9083.15	coding	0.346	1242	1707	3887
MSTRG.9083.9	noncoding	-0.0557056	0	1350	3530
MSTRG.9083.4	coding	0.346	1257	1722	3902
MSTRG.9083.8	coding	0.346	222	687	2867
MSTRG.9083.7	coding	0.346	1296	1761	3940
MSTRG.9083.16	noncoding	-0.0217088	510	546	1128
MSTRG.9085.2	coding	0.624	0	474	1601
MSTRG.9072.1	noncoding	-0.0770048	0	606	873
MSTRG.9075.2	noncoding	-0.047104	129	939	2387
MSTRG.9089.1	coding	0.297	231	597	753
MSTRG.9090.2	coding	0.485	0	1437	1987
MSTRG.9090.3	coding	0.432	0	1950	2501
MSTRG.9091.4	coding	0.397	177	4077	4230
MSTRG.9091.5	coding	0.333	900	2742	7831
MSTRG.9091.6	coding	0.342	0	1860	6947
MSTRG.9091.8	noncoding	-0.0425984	5211	5712	10980
MSTRG.9092.1	noncoding	-0.0618496	2232	2592	2954
MSTRG.9093.4	coding	0.387	1194	3567	3809
MSTRG.9094.2	coding	0.458	0	1059	2217
MSTRG.9097.1	noncoding	-0.0561152	1263	3159	3495
MSTRG.9097.2	noncoding	-0.0897024	0	777	1076
MSTRG.9103.5	coding	0.395	0	2958	10161
MSTRG.9103.2	coding	0.404	0	2637	10023
MSTRG.9104.3	coding	0.545	1716	2880	3751
MSTRG.9104.4	coding	0.55	75	1242	2113
MSTRG.9104.5	coding	0.547	336	1518	2388
MSTRG.9104.7	coding	0.544	573	1734	2604
MSTRG.9104.6	coding	0.532	324	1443	2315
MSTRG.9104.8	noncoding	-0.059392	405	1548	4429
MSTRG.9100.2	noncoding	-0.0589824	15	237	659
MSTRG.9107.8	coding	0.494	426	2109	2516
MSTRG.9107.6	coding	0.361	0	2493	2901
MSTRG.9108.1	noncoding	-0.1134592	0	420	610
MSTRG.9111.1	noncoding	-0.129024	87	348	504
MSTRG.9112.2	coding	0.171	237	1149	1325
MSTRG.9113.1	noncoding	-0.094208	0	543	877
MSTRG.9110.1	coding	0.293	0	321	713
MSTRG.9116.3	coding	0.359	0	2718	3091
MSTRG.9125.6	coding	0.435	699	1893	2594
MSTRG.9125.2	coding	0.279	0	1920	2621
MSTRG.9125.3	coding	0.47	225	1998	2700
MSTRG.9125.4	coding	0.273	0	1959	2660
MSTRG.9125.5	coding	0.4	840	2103	2804
MSTRG.9126.4	coding	0.479	507	3285	7735
MSTRG.9127.1	noncoding	-0.059392	15072	18660	31197
MSTRG.9120.2	coding	0.278	219	1329	1913
MSTRG.9120.3	coding	0.232	306	894	5300

MSTRG.9123.1	coding	0.184	0	651	1171
MSTRG.9123.2	coding	0.17	0	603	1107
MSTRG.9129.1	coding	0.183	123	534	871
MSTRG.9129.2	coding	0.229	0	624	853
MSTRG.9128.1	noncoding	-0.0602112	0	1545	1701
MSTRG.9133.3	coding	0.222	1362	2685	2840
MSTRG.9136.1	noncoding	-0.0544768	141	384	610
MSTRG.9138.1	coding	0.067	0	1917	3197
MSTRG.9138.3	coding	0.059	12	1932	4835
MSTRG.9139.1	noncoding	-0.036864	0	90	1099
MSTRG.9138.4	noncoding	-0.0790528	1743	2001	2184
MSTRG.9140.1	noncoding	-0.0630784	6429	6792	7114
MSTRG.9140.3	noncoding	-0.0724992	648	1029	1348
MSTRG.9142.1	noncoding	-0.0004096	0	0	393
MSTRG.9145.3	coding	0.305	66	2256	3158
MSTRG.9145.5	coding	0.317	240	2106	3007
MSTRG.9145.2	coding	0.32	456	2403	3315
MSTRG.9145.4	coding	0.32	240	2232	3142
MSTRG.9145.6	coding	0.301	117	1095	2552
MSTRG.9147.1	noncoding	-0.012288	72	147	3501
MSTRG.9146.4	coding	0.114	498	1245	3094
MSTRG.9146.5	coding	0.141	498	990	4194
MSTRG.9144.3	coding	0.289	0	585	1794
MSTRG.9144.2	coding	0.254	0	810	3881
MSTRG.9157.3	coding	0.293	0	1320	2116
MSTRG.9157.4	noncoding	-0.0315392	1626	1953	2159
MSTRG.9153.3	coding	0.228	618	2316	4248
MSTRG.9148.1	noncoding	-0.0626688	2502	3243	3397
MSTRG.9150.1	coding	0.428	0	633	788
MSTRG.9150.2	coding	0.428	0	630	785
MSTRG.9175.2	coding	0.067	501	1143	2479
MSTRG.9175.4	coding	0.083	207	1434	2324
MSTRG.9175.10	coding	0.043	435	1116	1781
MSTRG.9175.9	coding	0.067	60	702	1811
MSTRG.9175.8	coding	0.043	462	1143	2033
MSTRG.9167.2	coding	0.435	0	3435	4115
MSTRG.9168.1	noncoding	-0.0282624	63	246	417
MSTRG.9188.1	noncoding	-0.0073728	87	243	871
MSTRG.9190.1	coding	0.29	669	978	1189
MSTRG.9190.3	coding	0.344	153	462	619
MSTRG.9190.2	coding	0.398	0	285	440
MSTRG.9194.1	noncoding	-0.0561152	2319	2709	4099
MSTRG.9194.2	noncoding	-0.0118784	1020	1932	2088
MSTRG.9193.3	coding	0.477	0	591	10040
MSTRG.9202.1	coding	0.399	0	3219	3374
MSTRG.9203.1	coding	0.436	0	270	423
MSTRG.9205.1	coding	0.566	0	4713	4867
MSTRG.9205.2	coding	0.46	0	1158	1315
MSTRG.9206.1	noncoding	-0.057344	2010	2355	2617
MSTRG.9209.2	coding	0.411	132	864	1956
MSTRG.9209.3	coding	0.378	1881	2700	3649
MSTRG.9211.3	coding	0.283	0	570	780
MSTRG.9211.4	coding	0.247	1056	1509	1720
MSTRG.9217.2	noncoding	-0.0557056	297	525	1068

MSTRG.9230.1	noncoding	-0.0008192	519	561	716
MSTRG.9231.1	coding	0.506	204	2073	4094
MSTRG.9231.3	coding	0.51	1545	3303	3571
MSTRG.9233.1	coding	0.423	0	1323	4013
MSTRG.9233.5	coding	0.439	0	1206	3897
MSTRG.9235.3	coding	0.413	0	585	1753
MSTRG.9235.4	coding	0.355	0	696	2477
MSTRG.9239.6	coding	0.371	576	2625	5819
MSTRG.9239.9	coding	0.374	177	2124	2881
MSTRG.9239.8	coding	0.307	0	2739	3497
MSTRG.9240.1	noncoding	-0.118784	0	414	2203
MSTRG.9249.4	coding	0.557	660	2028	2584
MSTRG.9249.5	coding	0.544	315	1812	2361
MSTRG.9250.6	coding	0.25	276	3582	3909
MSTRG.9250.7	coding	0.247	276	3543	5883
MSTRG.9250.8	coding	0.247	276	3543	4003
MSTRG.9250.10	coding	0.248	114	3387	3714
MSTRG.9250.9	coding	0.241	114	3270	6784
MSTRG.9258.3	coding	0.5	228	2334	2559
MSTRG.9258.5	coding	0.405	0	2607	2827
MSTRG.9259.2	coding	0.45	0	1809	6857
MSTRG.9260.1	noncoding	-0.1093632	0	117	646
MSTRG.9260.2	noncoding	-0.0458752	102	141	485
MSTRG.9256.3	coding	0.332	0	1041	1365
MSTRG.9257.3	coding	0.412	123	567	748
MSTRG.9264.2	coding	0.551	0	366	522
MSTRG.9266.1	coding	0.446	468	1101	2186
MSTRG.9266.2	coding	0.39	294	819	1956
MSTRG.9266.3	coding	0.371	195	480	1550
MSTRG.9267.2	coding	0.539	0	1305	1882
MSTRG.9267.3	coding	0.488	0	1380	1958
MSTRG.9268.2	noncoding	-0.2035712	0	447	928
MSTRG.9269.1	noncoding	-0.0589824	576	1407	1562
MSTRG.9298.1	coding	0.281	54	4833	5327
MSTRG.9298.18	coding	0.213	216	10893	11096
MSTRG.9298.10	coding	0.229	216	6597	9865
MSTRG.9298.17	coding	0.267	216	4359	5064
MSTRG.9298.20	coding	0.224	216	7650	7805
MSTRG.9298.11	coding	0.214	216	11313	11530
MSTRG.9298.12	coding	0.213	216	10983	11187
MSTRG.9298.13	coding	0.302	216	4710	4924
MSTRG.9298.15	coding	0.168	873	10065	10278
MSTRG.9298.16	coding	0.211	216	11118	11332
MSTRG.9298.9	coding	0.294	294	4866	5080
MSTRG.9298.24	coding	0.24	0	3489	3985
MSTRG.9298.26	coding	0.242	0	3927	4080
MSTRG.9298.25	coding	0.318	0	3069	3274
MSTRG.9298.30	coding	0.163	0	975	1130
MSTRG.9298.31	coding	0.163	0	975	1130
MSTRG.9298.29	coding	0.179	0	5532	5736
MSTRG.9298.33	coding	0.294	117	4689	4903
MSTRG.9298.34	coding	0.299	192	4686	5181
MSTRG.9298.35	coding	0.191	54	2202	2407
MSTRG.9298.36	coding	0.192	54	2172	2377

MSTRG.9278.2	coding	0.325	120	786	1007
MSTRG.9281.1	noncoding	-0.0319488	393	774	927
MSTRG.9283.2	coding	0.539	12	807	1100
MSTRG.9284.1	noncoding	-0.0217088	108	333	636
MSTRG.9285.2	coding	0.381	93	642	804
MSTRG.9287.2	noncoding	-0.0561152	276	654	811
MSTRG.9290.1	coding	0.427	0	3666	5238
MSTRG.9291.1	coding	0.494	0	2973	7747
MSTRG.9291.3	coding	0.453	84	927	5338
MSTRG.9295.1	coding	0.569	252	2274	10724
MSTRG.9295.2	coding	0.532	9	2193	10643
MSTRG.9295.4	coding	0.575	339	2346	3550
MSTRG.9296.1	noncoding	-0.098304	0	375	1850
MSTRG.9303.2	coding	0.235	723	1035	1661
MSTRG.9304.1	coding	0.52	0	1209	1715
MSTRG.9305.2	noncoding	-0.0741376	528	1302	1476
MSTRG.9305.3	noncoding	-0.0475136	177	711	866
MSTRG.9304.3	coding	0.42	24	1512	2159
MSTRG.9306.1	noncoding	-0.0688128	30	591	957
MSTRG.9306.2	noncoding	-0.0864256	441	678	1043
MSTRG.9307.2	coding	0.48	0	1098	1626
MSTRG.9308.4	coding	0.233	69	987	1321
MSTRG.9312.11	coding	0.536	0	6456	7435
MSTRG.9312.12	coding	0.531	0	6477	7456
MSTRG.9312.13	coding	0.538	0	6438	7417
MSTRG.9312.14	coding	0.533	0	6450	7429
MSTRG.9312.3	coding	0.541	0	6408	7387
MSTRG.9312.4	coding	0.538	0	6441	7420
MSTRG.9312.9	coding	0.539	0	6432	7411
MSTRG.9312.15	coding	0.521	0	6627	7605
MSTRG.9312.23	coding	0.574	0	1107	3838
MSTRG.9312.24	noncoding	-0.0643072	1110	1500	15200
MSTRG.9312.25	noncoding	-0.1036288	0	189	836
MSTRG.9310.2	coding	0.431	6	3096	4134
MSTRG.9315.1	noncoding	-0.0507904	4626	4995	13155
MSTRG.9316.1	noncoding	-0.1499136	3	9	219
MSTRG.9315.3	noncoding	-0.0507904	4164	4533	14832
MSTRG.9321.3	noncoding	-0.0811008	0	585	14721
MSTRG.9321.4	noncoding	-0.0811008	0	585	19190
MSTRG.9322.1	noncoding	-0.0331776	1596	1812	6551
MSTRG.9325.2	noncoding	-0.049152	0	261	7606
MSTRG.9327.4	coding	0.216	558	2436	3587
MSTRG.9335.3	noncoding	-0.049152	1038	1881	8683
MSTRG.9335.4	noncoding	-0.012288	2487	4203	8252
MSTRG.9333.2	coding	0.543	906	2271	3018
MSTRG.9338.1	noncoding	-0.0004096	285	396	1776
MSTRG.9361.4	coding	0.428	141	3498	4447
MSTRG.9361.6	coding	0.434	0	3216	4165
MSTRG.9361.7	coding	0.44	0	3216	4166
MSTRG.9361.8	coding	0.437	1083	4026	4975
MSTRG.9361.9	coding	0.437	1239	4182	5131
MSTRG.9361.10	coding	0.437	477	3420	4369
MSTRG.9361.11	coding	0.463	0	2913	3862
MSTRG.9361.13	coding	0.443	150	2694	3643

MSTRG.9361.14	coding	0.446	0	2589	3538
MSTRG.9365.1	noncoding	-0.016384	0	153	525
MSTRG.9364.12	noncoding	-0.004096	867	1038	3366
MSTRG.9364.13	coding	0.327	27	1014	3123
MSTRG.9367.2	coding	0.416	318	2406	10602
MSTRG.9367.3	coding	0.405	267	3414	5963
MSTRG.9367.4	coding	0.443	210	3861	5532
MSTRG.9367.6	coding	0.392	6	2778	2933
MSTRG.9367.9	coding	0.41	210	3081	5709
MSTRG.9367.10	coding	0.406	45	2181	7623
MSTRG.9368.1	noncoding	-0.004096	2487	2607	10623
MSTRG.9367.12	coding	0.405	23028	25830	25987
MSTRG.9343.3	noncoding	-0.188416	0	141	528
MSTRG.9349.1	noncoding	-0.0356352	21	192	2549
MSTRG.9353.1	coding	0.163	87	276	501
MSTRG.9358.4	coding	0.348	45	1083	2411
MSTRG.9358.1	coding	0.357	114	1020	2348
MSTRG.9358.3	coding	0.214	216	1233	2563
MSTRG.9358.5	coding	0.208	0	1068	2396
MSTRG.9358.6	coding	0.483	0	750	2078
MSTRG.9360.3	coding	0.368	810	2232	2413
MSTRG.9369.3	coding	0.476	2175	3462	3683
MSTRG.9369.4	coding	0.475	2004	3294	3514
MSTRG.9369.5	coding	0.465	1158	1854	2075
MSTRG.9374.1	coding	0.553	0	4572	5218
MSTRG.9374.2	coding	0.458	0	5583	7393
MSTRG.9374.3	coding	0.55	735	4857	7914
MSTRG.9374.5	coding	0.551	606	4713	5102
MSTRG.9374.4	coding	0.497	711	5055	6853
MSTRG.9374.7	coding	0.529	411	4719	5107
MSTRG.9372.2	coding	0.452	2859	3804	5343
MSTRG.9372.3	coding	0.375	2253	3519	5004
MSTRG.9373.3	noncoding	-0.1343488	228	789	3708
MSTRG.9377.2	noncoding	-0.1306624	42	1170	1330
MSTRG.9386.6	noncoding	-0.159744	258	1086	1242
MSTRG.9386.4	coding	0.507	258	3387	4693
MSTRG.9379.1	noncoding	-0.053248	534	582	738
MSTRG.9382.1	noncoding	-0.1335296	246	609	765
MSTRG.9385.2	coding	0.447	420	1707	1903
MSTRG.9385.3	coding	0.459	15	1320	1516
MSTRG.9396.1	coding	0.577	822	2454	3847
MSTRG.9396.2	coding	0.635	0	1545	3551
MSTRG.9396.3	coding	0.595	0	1719	3707
MSTRG.9398.4	coding	0.418	309	2301	2456
MSTRG.9398.3	coding	0.444	303	2220	2377
MSTRG.9394.1	coding	0.472	0	1338	5941
MSTRG.9394.2	coding	0.474	0	1338	5654
MSTRG.9394.3	coding	0.448	0	1284	5529
MSTRG.9394.4	coding	0.504	0	1197	4045
MSTRG.9393.1	noncoding	-0.002048	984	1128	2154
MSTRG.9405.6	coding	0.419	0	495	797
MSTRG.9407.3	coding	0.398	303	1221	1387
MSTRG.9410.1	coding	0.527	12	2151	2509
MSTRG.9420.2	coding	0.533	0	645	800

MSTRG.9422.1	coding	0.507	126	2862	7773
MSTRG.9422.4	coding	0.507	120	2856	3257
MSTRG.9423.2	coding	0.41	348	1461	1627
MSTRG.9423.3	coding	0.403	174	1101	1267
MSTRG.9423.4	coding	0.394	195	1083	1247
MSTRG.9425.2	coding	0.375	177	927	1139
MSTRG.9411.2	noncoding	-0.049152	93	306	714
MSTRG.9413.2	noncoding	-0.0622592	246	432	586
MSTRG.9428.2	coding	0.09	0	765	3386
MSTRG.9436.1	coding	0.068	558	16767	20970
MSTRG.9436.2	coding	0.077	558	17877	21306
MSTRG.9436.3	coding	0.105	558	19305	20731
MSTRG.9436.4	coding	0.078	558	17541	20970
MSTRG.9436.5	coding	0.08	558	10419	13848
MSTRG.9436.6	coding	0.127	558	12528	13954
MSTRG.9436.10	coding	0.167	735	9075	9779
MSTRG.9436.11	coding	0.162	735	9408	10112
MSTRG.9436.15	coding	0.104	735	7215	9922
MSTRG.9436.12	coding	0.112	480	18093	18799
MSTRG.9436.13	coding	0.078	480	15609	18318
MSTRG.9436.14	coding	0.112	480	16839	17545
MSTRG.9436.9	coding	0.08	480	15984	18693
MSTRG.9436.16	coding	0.068	552	16761	20048
MSTRG.9436.17	coding	0.068	552	16284	19571
MSTRG.9436.18	coding	0.106	552	20067	20770
MSTRG.9436.19	coding	0.107	552	19731	20434
MSTRG.9431.3	coding	0.147	1932	3852	5175
MSTRG.9431.4	coding	0.147	180	2067	4146
MSTRG.9445.1	coding	0.075	7416	8181	8490
MSTRG.9446.2	noncoding	-0.012288	1926	2064	2297
MSTRG.9446.3	noncoding	-0.0512	54	264	709
MSTRG.9446.4	noncoding	-0.0507904	54	255	700
MSTRG.9446.6	noncoding	-0.012288	294	432	601
MSTRG.9449.1	coding	0.108	60	738	3028
MSTRG.9449.2	coding	0.211	30	348	551
MSTRG.9449.4	coding	0.212	105	345	1612
MSTRG.9449.3	coding	0.118	0	606	2779
MSTRG.9449.5	noncoding	-0.073728	312	378	532
MSTRG.9445.3	coding	0.142	7536	7920	18147
MSTRG.9445.4	noncoding	-0.0741376	1143	1500	2291
MSTRG.9445.6	noncoding	-0.0036864	372	666	1269
MSTRG.9453.1	noncoding	-0.0303104	354	534	759
MSTRG.9453.2	noncoding	-0.0303104	348	528	744
MSTRG.9453.3	noncoding	-0.0303104	1725	1905	2119
MSTRG.9434.2	coding	0.285	0	741	1218
MSTRG.9437.2	coding	0.23	0	3333	3489
MSTRG.9441.1	coding	0.348	468	1893	2245
MSTRG.9458.2	coding	0.387	105	5097	5502
MSTRG.9458.4	coding	0.398	1464	6237	6508
MSTRG.9457.1	coding	0.383	0	1185	2487
MSTRG.9464.2	noncoding	-0.0569344	6	243	586
MSTRG.9464.1	noncoding	-0.0483328	84	366	3160
MSTRG.9464.3	noncoding	-0.0483328	69	351	3064
MSTRG.9464.4	noncoding	-0.0466944	72	621	774

MSTRG.9464.5	noncoding	-0.0700416	1101	1458	1718
MSTRG.9460.1	coding	0.616	0	1293	3357
MSTRG.9471.5	coding	0.467	102	1845	3902
MSTRG.9471.6	coding	0.432	123	2085	2525
MSTRG.9471.7	coding	0.462	378	2070	2511
MSTRG.9471.8	coding	0.437	27	1548	1841
MSTRG.9473.1	coding	0.519	0	2004	7883
MSTRG.9473.2	coding	0.528	522	2409	3020
MSTRG.9473.3	coding	0.47	276	2493	8243
MSTRG.9473.4	coding	0.544	27	1854	2542
MSTRG.9473.5	coding	0.521	0	1995	2308
MSTRG.9468.2	coding	0.385	0	1143	1609
MSTRG.9468.3	coding	0.402	546	1206	1674
MSTRG.9481.4	coding	0.338	318	2322	3794
MSTRG.9481.11	noncoding	-0.0589824	1617	1947	2101
MSTRG.9481.17	coding	0.338	537	2541	3993
MSTRG.9481.14	coding	0.338	300	2304	3776
MSTRG.9481.15	coding	0.338	165	2157	3629
MSTRG.9481.16	coding	0.338	300	2292	3764
MSTRG.9481.9	coding	0.338	276	2268	3740
MSTRG.9481.10	coding	0.338	300	2898	6445
MSTRG.9481.18	coding	0.322	1026	2814	6359
MSTRG.9481.7	coding	0.338	276	2874	6421
MSTRG.9477.2	noncoding	-0.067584	183	612	1342
MSTRG.9478.4	coding	0.396	465	1227	1406
MSTRG.9482.7	coding	0.494	2406	3750	4155
MSTRG.9484.1	noncoding	-0.0331776	33	303	749
MSTRG.9486.2	coding	0.275	0	1548	2652
MSTRG.9493.6	coding	0.648	3060	6111	6649
MSTRG.9493.7	coding	0.645	921	4023	4276
MSTRG.9493.8	coding	0.681	0	2019	2271
MSTRG.9496.4	coding	0.53	0	6144	6424
MSTRG.9498.2	coding	0.463	78	927	1662
MSTRG.9498.8	coding	0.294	276	729	914
MSTRG.9499.1	coding	0.236	1551	2946	3160
MSTRG.9499.2	coding	0.393	1455	2136	2350
MSTRG.9499.4	coding	0.393	1740	2421	2634
MSTRG.9499.5	coding	0.393	615	1296	1511
MSTRG.9500.1	coding	0.371	468	1572	2472
MSTRG.9500.3	coding	0.346	138	1323	2698
MSTRG.9500.4	coding	0.394	357	1365	2739
MSTRG.9503.1	coding	0.488	54	1875	2169
MSTRG.9503.2	coding	0.492	108	1911	2204
MSTRG.9503.3	coding	0.488	87	1905	2197
MSTRG.9503.4	coding	0.489	18	1845	2139
MSTRG.9503.6	coding	0.501	174	1926	2219
MSTRG.9505.2	coding	0.468	1134	1893	2076
MSTRG.9505.3	coding	0.508	1728	3108	3292
MSTRG.9505.4	coding	0.525	1329	2967	3149
MSTRG.9505.5	coding	0.26	0	2514	4086
MSTRG.9505.6	coding	0.466	0	1272	1953
MSTRG.9509.1	coding	0.263	0	474	1099
MSTRG.9509.3	noncoding	-0.0856064	0	321	631
MSTRG.9516.4	coding	0.523	1266	3948	5786

MSTRG.9516.6	noncoding	-0.079872	1812	2175	2380
MSTRG.9516.8	noncoding	-0.008192	1317	1449	1603
MSTRG.9516.7	coding	0.511	204	3222	5061
MSTRG.9518.2	coding	0.509	0	4053	4566
MSTRG.9518.3	coding	0.516	0	3990	4298
MSTRG.9522.1	coding	0.665	0	2508	3639
MSTRG.9523.2	coding	0.229	63	285	440
MSTRG.9526.2	coding	0.533	180	1857	2011
MSTRG.9532.1	noncoding	-0.12288	273	303	583
MSTRG.9534.1	coding	0.32	0	1275	5014
MSTRG.9535.1	noncoding	-0.0589824	0	345	6213
MSTRG.9535.2	noncoding	-0.0606208	0	300	1584
MSTRG.9535.3	noncoding	-0.0712704	141	345	6955
MSTRG.9536.1	coding	0.36	0	4920	5671
MSTRG.9543.1	noncoding	-0.0856064	552	912	1065
MSTRG.9543.2	noncoding	-0.08192	372	711	865
MSTRG.9550.1	noncoding	-0.073728	315	1026	1206
MSTRG.9547.1	coding	0.416	0	1683	3993
MSTRG.9554.1	coding	0.17	378	987	1343
MSTRG.9555.1	noncoding	-0.0192512	372	459	1054
MSTRG.9556.1	noncoding	-0.0393216	603	1119	1310
MSTRG.9556.2	noncoding	-0.0393216	954	1470	1628
MSTRG.9556.3	noncoding	-0.0385024	714	1215	1372
MSTRG.9563.1	coding	0.288	69	1800	2165
MSTRG.9563.2	coding	0.297	0	1767	2006
MSTRG.9564.2	coding	0.447	912	2688	2842
MSTRG.9565.4	coding	0.423	0	1638	1795
MSTRG.9565.5	coding	0.425	0	1518	1851
MSTRG.9566.2	coding	0.212	87	1209	1624
MSTRG.9566.3	coding	0.267	0	876	1828
MSTRG.9574.3	coding	0.325	393	3786	5756
MSTRG.9574.4	coding	0.352	396	2706	3679
MSTRG.9574.5	coding	0.31	96	2781	4750
MSTRG.9574.6	coding	0.321	18	2574	4273
MSTRG.9574.7	coding	0.377	0	2124	3335
MSTRG.9576.1	coding	0.565	132	1692	1849
MSTRG.9576.2	coding	0.558	60	1659	1816
MSTRG.9576.3	coding	0.56	162	1740	1895
MSTRG.9567.2	coding	0.481	0	123	451
MSTRG.9567.3	coding	0.565	0	288	625
MSTRG.9567.4	noncoding	-0.0352256	207	501	656
MSTRG.9568.2	coding	0.497	0	555	1116
MSTRG.9571.1	noncoding	-0.0561152	0	297	977
MSTRG.9577.4	coding	0.273	249	1116	1886
MSTRG.9586.3	coding	0.355	870	2781	3949
MSTRG.9587.2	noncoding	-0.1220608	51	516	2032
MSTRG.9589.3	coding	0.485	153	2010	2184
MSTRG.9589.5	coding	0.486	108	1197	1350
MSTRG.9590.2	coding	0.362	2139	3129	3354
MSTRG.9590.3	coding	0.325	2106	2976	5967
MSTRG.9590.5	coding	0.24	585	1353	1510
MSTRG.9590.7	coding	0.171	654	1938	2215
MSTRG.9590.6	coding	0.171	786	2070	2346
MSTRG.9591.1	coding	0.664	63	2640	2807

MSTRG.9591.3	coding	0.588	0	3039	3207
MSTRG.9598.10	noncoding	-0.0872448	1077	1887	2505
MSTRG.9598.3	noncoding	-0.1077248	1077	1503	2211
MSTRG.9598.4	noncoding	-0.1077248	189	615	1324
MSTRG.9598.6	noncoding	-0.0958464	189	597	1287
MSTRG.9598.7	noncoding	-0.0999424	390	1032	1651
MSTRG.9598.8	noncoding	-0.0958464	156	564	1255
MSTRG.9598.9	noncoding	-0.0999424	1659	2301	2921
MSTRG.9599.1	coding	0.29	399	798	981
MSTRG.9599.3	coding	0.29	252	651	836
MSTRG.9599.4	noncoding	-0.0753664	294	522	824
MSTRG.9600.12	coding	0.374	7119	8604	8791
MSTRG.9600.8	coding	0.358	0	2679	2864
MSTRG.9600.13	coding	0.358	726	4320	4505
MSTRG.9600.7	coding	0.347	87	2745	2931
MSTRG.9600.9	coding	0.346	87	2745	2931
MSTRG.9600.16	coding	0.27	0	318	1980
MSTRG.9602.2	coding	0.418	708	2328	2672
MSTRG.9594.1	coding	0.565	0	897	1203
MSTRG.9594.2	coding	0.31	9	495	860
MSTRG.9594.3	coding	0.486	9	876	1241
MSTRG.9594.5	coding	0.484	144	1245	1623
MSTRG.9597.1	coding	0.502	123	3699	5300
MSTRG.9597.2	coding	0.514	123	3597	5198
MSTRG.9597.3	coding	0.502	609	4185	5782
MSTRG.9597.4	coding	0.514	1263	4737	5819
MSTRG.9604.3	coding	0.528	384	2712	4186
MSTRG.9604.4	coding	0.522	0	2376	2587
MSTRG.9605.1	noncoding	-0.004096	222	231	454
MSTRG.9605.2	noncoding	-0.0258048	30	87	313
MSTRG.9606.1	coding	0.348	210	846	999
MSTRG.9610.1	noncoding	-0.0417792	114	351	581
MSTRG.9610.2	noncoding	-0.0827392	72	435	718
MSTRG.9612.2	coding	0.584	501	1056	3155
MSTRG.9612.3	coding	0.584	216	771	2871
MSTRG.9613.1	noncoding	-0.012288	120	249	570
MSTRG.9612.4	noncoding	-0.006144	426	621	849
MSTRG.9603.1	noncoding	-0.045056	150	234	640
MSTRG.9617.3	coding	0.574	207	1425	1787
MSTRG.9619.2	coding	0.431	558	3354	3845
MSTRG.9620.1	coding	0.282	240	792	958
MSTRG.9620.2	coding	0.271	66	756	922
MSTRG.9624.3	coding	0.466	21	960	1492
MSTRG.9625.1	noncoding	-0.067584	882	1308	2048
MSTRG.9626.1	coding	0.443	9	1047	1764
MSTRG.9626.2	coding	0.488	0	951	1646
MSTRG.9630.2	coding	0.409	42	1839	2132
MSTRG.9634.3	noncoding	-0.0106496	1917	2184	2339
MSTRG.9634.4	noncoding	-0.0282624	591	801	1098
MSTRG.9633.3	coding	0.378	0	318	1496
MSTRG.9633.2	coding	0.378	0	318	1785
MSTRG.9638.1	coding	0.456	0	2166	2344
MSTRG.9640.4	coding	0.516	0	1848	3904
MSTRG.9649.4	coding	0.473	156	1695	1926

MSTRG.9643.1	coding	0.591	0	1083	1238
MSTRG.9651.6	coding	0.337	15972	16803	17190
MSTRG.9650.1	noncoding	-0.002048	39	186	340
MSTRG.9653.1	coding	0.662	252	1230	3385
MSTRG.9653.2	coding	0.67	2373	3372	5509
MSTRG.9655.3	coding	0.399	60	2292	2530
MSTRG.9657.1	coding	0.333	0	225	1283
MSTRG.9658.4	coding	0.041	2820	4140	4658
MSTRG.9661.1	noncoding	-0.0425984	177	966	1177
MSTRG.9662.4	coding	0.386	609	1725	2825
MSTRG.9663.3	coding	0.49	306	1320	1636
MSTRG.9663.4	coding	0.49	504	1518	1833
MSTRG.9665.1	noncoding	-0.0679936	966	1776	3453
MSTRG.9665.2	noncoding	-0.0679936	1206	2016	3938
MSTRG.9665.3	noncoding	-0.0679936	963	1773	3451
MSTRG.9665.5	noncoding	-0.0323584	153	1770	3403
MSTRG.9667.2	coding	0.582	861	2148	4386
MSTRG.9667.3	coding	0.616	0	1215	3983
MSTRG.9675.2	coding	0.405	0	513	688
MSTRG.9675.3	coding	0.433	0	570	739
MSTRG.9675.4	coding	0.375	0	330	506
MSTRG.9670.2	coding	0.659	0	1662	3778
MSTRG.9671.2	coding	0.152	258	858	1013
MSTRG.9676.2	coding	0.436	0	5073	7608
MSTRG.9676.3	coding	0.416	447	1059	1782
MSTRG.9685.1	noncoding	-0.0131072	0	114	1507
MSTRG.9689.1	coding	0.44	54	1122	3011
MSTRG.9689.3	coding	0.456	21	1035	2752
MSTRG.9696.2	coding	0.122	57	2094	9125
MSTRG.9696.5	coding	0.116	339	2265	3139
MSTRG.9692.1	coding	0.247	117	1428	3016
MSTRG.9693.2	coding	0.409	0	354	1457
MSTRG.9691.2	coding	0.418	0	744	5984
MSTRG.9706.1	coding	0.368	360	1914	2456
MSTRG.9706.5	coding	0.357	138	1686	2229
MSTRG.9708.1	noncoding	-0.08192	162	726	1310
MSTRG.9719.1	coding	0.204	0	579	789
MSTRG.9728.1	noncoding	-0.028672	1536	1713	4329
MSTRG.9740.1	noncoding	-0.0356352	0	285	590
MSTRG.9742.1	coding	0.305	231	2310	2788
MSTRG.9742.2	coding	0.301	174	2265	2743
MSTRG.9742.3	coding	0.292	261	2334	2812
MSTRG.9742.4	coding	0.301	168	2268	2745
MSTRG.9742.8	coding	0.291	99	2175	3674
MSTRG.9742.9	coding	0.296	66	2121	2600
MSTRG.9742.13	coding	0.289	1443	3468	3947
MSTRG.9742.15	noncoding	-0.07168	594	945	8157
MSTRG.9742.17	coding	0.175	153	504	995
MSTRG.9742.18	coding	0.183	114	591	1669
MSTRG.9743.1	noncoding	-0.065536	450	540	1285
MSTRG.9743.2	coding	0.134	135	384	991
MSTRG.9745.1	coding	0.097	6	852	4452
MSTRG.9745.3	coding	0.061	3714	4377	7179
MSTRG.9745.6	coding	0.036	1845	3870	4236

MSTRG.9732.2	coding	0.142	531	783	3354
MSTRG.9734.2	noncoding	-0.045056	1197	1296	1724
MSTRG.9734.5	noncoding	-0.172032	267	300	462
MSTRG.9734.4	noncoding	-0.0462848	459	480	662
MSTRG.9735.1	coding	0.187	0	261	541
MSTRG.9738.1	coding	0.174	258	1965	3166
MSTRG.9747.1	noncoding	-0.323584	294	306	734
MSTRG.9753.1	noncoding	-0.0413696	189	348	801
MSTRG.9756.1	noncoding	-0.0380928	3	24	240
MSTRG.9760.1	coding	0.154	150	813	1148
MSTRG.9760.2	coding	0.15	150	795	950
MSTRG.9760.3	noncoding	-0.0294912	150	405	596
MSTRG.9773.1	coding	0.187	246	1779	4335
MSTRG.9774.1	coding	0.123	0	1185	3883
MSTRG.9774.2	coding	0.124	0	1182	2871
MSTRG.9774.4	coding	0.126	0	1206	2866
MSTRG.9775.1	noncoding	-0.008192	2604	2733	4464
MSTRG.9780.1	noncoding	-0.024576	213	306	628
MSTRG.9798.1	noncoding	-0.0004096	0	0	247
MSTRG.9815.16	noncoding	-0.077824	882	921	1463
MSTRG.9815.18	noncoding	-0.016384	13611	13731	14104
MSTRG.9815.19	noncoding	-0.0180224	1818	1941	4246
MSTRG.9815.20	coding	0.072	5784	6243	12184
MSTRG.9815.21	coding	0.072	5784	6243	10165
MSTRG.9816.1	noncoding	-0.114688	66	138	291
MSTRG.9815.22	noncoding	-0.0004096	2520	2628	3871
MSTRG.9815.23	noncoding	-0.065536	2616	2703	9434
MSTRG.9815.24	noncoding	-0.0004096	150	294	1198
MSTRG.9815.25	noncoding	-0.0004096	0	129	19800
MSTRG.9815.26	noncoding	-0.028672	921	1071	4764
MSTRG.9815.27	noncoding	-0.0167936	3351	3474	4613
MSTRG.9817.1	noncoding	-0.0589824	31596	31842	36278
MSTRG.9817.2	noncoding	-0.147456	285	294	537
MSTRG.9811.1	noncoding	-0.0004096	0	0	450
MSTRG.9812.2	noncoding	-0.0831488	0	276	4186
MSTRG.9814.2	coding	0.121	0	2976	4649
MSTRG.9827.1	coding	0.073	381	1575	2180
MSTRG.9827.3	noncoding	-0.002048	342	483	648
MSTRG.9849.1	coding	0.307	582	1617	3284
MSTRG.9854.3	coding	0.058	4299	5379	11619
MSTRG.9858.2	noncoding	-0.098304	2154	2445	2600
MSTRG.9859.1	coding	0.114	1770	2058	3894
MSTRG.9859.2	coding	0.114	2985	3273	4656
MSTRG.9862.1	noncoding	-0.217088	279	318	2769
MSTRG.9863.1	noncoding	-0.012288	1326	1497	2198
MSTRG.9878.1	noncoding	-0.012288	183	336	2388
MSTRG.9884.1	noncoding	-0.004096	342	456	1221
MSTRG.9889.1	noncoding	-0.036864	2043	2130	5289
MSTRG.9889.2	noncoding	-0.0237568	990	1119	4037
MSTRG.9890.3	coding	0.243	819	1704	1880
MSTRG.9891.1	coding	0.32	156	1668	3892
MSTRG.9891.2	coding	0.315	0	1314	3269
MSTRG.9891.3	coding	0.315	0	1314	3225
MSTRG.9891.4	coding	0.338	0	1380	3289

MSTRG.9891.6	coding	0.316	0	1317	3272
MSTRG.9891.7	coding	0.316	0	1317	3228
MSTRG.9891.8	coding	0.315	0	1314	3199
MSTRG.9891.9	coding	0.315	0	1314	3102
MSTRG.9891.10	coding	0.338	0	1383	3139
MSTRG.9891.11	coding	0.338	0	1383	3165
MSTRG.9893.1	noncoding	-0.2048	267	315	468
MSTRG.9894.1	coding	0.471	0	2718	2900
MSTRG.9894.2	coding	0.478	0	2760	2943
MSTRG.9894.3	coding	0.49	0	2496	2679
MSTRG.9898.1	noncoding	-0.0262144	18	381	647
MSTRG.9898.2	noncoding	-0.0479232	69	357	636
MSTRG.9902.6	coding	0.106	24	1884	2220
MSTRG.9902.5	coding	0.134	135	2040	2375
MSTRG.9902.9	noncoding	-0.0270336	4251	4665	4998
MSTRG.9902.8	coding	0.138	63	1422	1755
MSTRG.9902.10	coding	0.151	0	1383	1686
MSTRG.9902.11	coding	0.176	0	867	1170
MSTRG.9899.3	coding	0.205	9	636	2158
MSTRG.9899.4	coding	0.155	195	540	2061
MSTRG.9899.5	coding	0.398	0	291	2183
MSTRG.9906.2	coding	0.138	4293	7707	9366
MSTRG.9913.2	coding	0.327	387	2115	2270
MSTRG.9913.6	coding	0.349	564	1257	1594
MSTRG.9915.2	coding	0.453	90	2271	2448
MSTRG.9918.1	noncoding	-0.057344	234	468	625
MSTRG.9919.2	coding	0.387	0	969	1518
MSTRG.9919.3	coding	0.416	33	891	1455
MSTRG.9919.4	coding	0.267	462	1413	1962
MSTRG.9924.3	coding	0.352	51	1083	1698
MSTRG.9929.1	coding	0.053	618	840	3319
MSTRG.9929.6	coding	0.053	699	921	2320
MSTRG.9929.5	coding	0.088	612	744	2142
MSTRG.9934.3	coding	0.43	714	2064	3167
MSTRG.9941.2	coding	0.518	0	1440	1894
MSTRG.9944.1	coding	0.265	363	1596	4973
MSTRG.9944.2	coding	0.257	54	900	3660
MSTRG.9944.3	coding	0.308	0	1122	1937
MSTRG.9948.1	noncoding	-0.0098304	1578	1773	2131
MSTRG.9961.1	noncoding	-0.024576	228	351	508
MSTRG.9962.3	coding	0.487	795	1920	3487
MSTRG.9962.4	coding	0.487	645	1770	3328
MSTRG.9963.2	coding	0.299	1059	2433	4092
MSTRG.9960.1	noncoding	-0.0770048	309	552	1375
MSTRG.9972.3	coding	0.457	108	1134	2182
MSTRG.9972.4	coding	0.475	339	1923	2972
MSTRG.9974.1	noncoding	-0.004096	231	366	522
MSTRG.9977.1	noncoding	-0.0360448	159	660	2871
MSTRG.9977.2	noncoding	-0.024576	780	975	1286
MSTRG.9984.1	coding	0.539	0	3567	5234
MSTRG.9984.2	coding	0.539	0	3567	5678
MSTRG.9985.2	coding	0.41	0	1020	1242
MSTRG.9986.1	coding	0.288	0	900	1908
MSTRG.9986.3	coding	0.41	0	510	2022

MSTRG.9987.3	coding	0.472	633	1896	3387
MSTRG.9987.4	coding	0.502	537	1932	3425
MSTRG.9990.1	noncoding	-0.0086016	147	177	333
MSTRG.9993.2	coding	0.394	48	2361	2518
MSTRG.9994.1	coding	0.327	0	1023	1185
MSTRG.9997.1	coding	0.477	495	1389	1721
MSTRG.9997.2	coding	0.477	237	1131	1502
MSTRG.9997.3	coding	0.524	210	2040	2915
MSTRG.9997.4	coding	0.48	1251	2295	3169
MSTRG.9997.5	coding	0.506	0	1071	1352
MSTRG.10000.1	coding	0.468	0	888	1154
MSTRG.10001.1	coding	0.532	0	1761	2520
MSTRG.10001.4	coding	0.554	0	1587	1968
MSTRG.10002.1	coding	0.309	1407	2283	3107
MSTRG.10005.2	coding	0.441	1668	2607	3161
MSTRG.10005.1	coding	0.467	1497	2352	2905
MSTRG.10008.4	coding	0.535	5313	7335	8009
MSTRG.10009.2	coding	0.343	0	2097	4950
MSTRG.10011.1	coding	0.346	39	489	949
MSTRG.10012.2	coding	0.442	348	1851	3207
MSTRG.10012.3	coding	0.442	345	1848	3717
MSTRG.10012.4	coding	0.426	327	1956	3313
MSTRG.10013.1	noncoding	-0.0417792	186	540	694
MSTRG.10014.1	coding	0.304	72	591	2227
MSTRG.10014.3	coding	0.304	3	522	2614
MSTRG.10016.1	coding	0.475	51	693	2112
MSTRG.10020.4	coding	0.31	0	1074	1327
MSTRG.10026.10	coding	0.457	0	1983	2456
MSTRG.10026.13	coding	0.502	0	1176	1627
MSTRG.10026.11	coding	0.51	0	1812	1966
MSTRG.10026.12	coding	0.508	0	1815	1969
MSTRG.10026.18	coding	0.504	0	1824	1979
MSTRG.10024.2	coding	0.374	243	1092	3349
MSTRG.10024.3	coding	0.394	243	1038	3295
MSTRG.10025.1	noncoding	-0.0004096	0	0	531
MSTRG.10031.1	noncoding	-0.0626688	402	774	1170
MSTRG.10041.2	coding	0.446	138	4464	4753
MSTRG.10041.3	coding	0.474	1125	4395	4683
MSTRG.10041.7	coding	0.454	0	4350	4586
MSTRG.10042.1	noncoding	-0.0344064	618	999	1600
MSTRG.10043.2	coding	0.427	0	3678	4476
MSTRG.10043.5	coding	0.426	759	4113	4269
MSTRG.10043.6	coding	0.427	777	4059	4213
MSTRG.10045.2	noncoding	-0.086016	1890	2502	2657
MSTRG.10037.3	coding	0.493	0	1221	2345
MSTRG.10038.1	noncoding	-0.0516096	93	738	4682
MSTRG.10038.2	noncoding	-0.0495616	93	1392	1904
MSTRG.10049.1	noncoding	-0.0380928	2355	2823	2978
MSTRG.10050.2	coding	0.482	0	96	703
MSTRG.10052.2	coding	0.105	2640	4929	10686
MSTRG.10053.1	coding	0.088	399	870	1559
MSTRG.10051.2	noncoding	-0.0905216	0	156	637
MSTRG.10058.1	noncoding	-0.012288	3312	3411	3609
MSTRG.10060.1	coding	0.405	384	2706	4786

MSTRG.10078.4	coding	0.117	2175	2391	3578
MSTRG.10078.5	coding	0.117	2298	2514	3702
MSTRG.10078.3	coding	0.038	516	1215	4343
MSTRG.10079.1	noncoding	-0.0458752	7920	8277	8499
MSTRG.10079.2	noncoding	-0.028672	87	204	723
MSTRG.10079.3	noncoding	-0.045056	606	666	820
MSTRG.10082.2	coding	0.097	189	384	3852
MSTRG.10087.3	coding	0.1	120	2055	2359
MSTRG.10091.1	coding	0.445	666	1554	1709
MSTRG.10091.2	coding	0.445	621	1509	1663
MSTRG.10091.3	coding	0.446	39	912	1067
MSTRG.10092.1	noncoding	-0.0651264	489	681	1187
MSTRG.10094.8	coding	0.403	141	1323	1816
MSTRG.10094.6	coding	0.401	177	1401	1895
MSTRG.10094.7	coding	0.4	213	1428	1920
MSTRG.10094.9	coding	0.402	141	1320	1807
MSTRG.10094.10	coding	0.399	186	1407	1869
MSTRG.10094.11	coding	0.399	354	1575	2038
MSTRG.10100.1	noncoding	-0.0425984	33	294	964
MSTRG.10100.2	noncoding	-0.0557056	0	96	766
MSTRG.10101.1	noncoding	-0.0720896	117	678	898
MSTRG.10101.3	noncoding	-0.0507904	426	648	909
MSTRG.10104.2	noncoding	-0.0217088	9567	9975	13849
MSTRG.10105.2	noncoding	-0.0319488	0	210	3218
MSTRG.10107.1	coding	0.452	8739	10620	11339
MSTRG.10102.2	noncoding	-0.02048	1962	2151	7489
MSTRG.10103.1	noncoding	-0.0434176	354	501	1101
MSTRG.10121.1	coding	0.343	1287	3147	7841
MSTRG.10121.3	coding	0.342	1620	3357	7955
MSTRG.10121.4	coding	0.109	2637	2925	3190
MSTRG.10122.1	coding	0.072	1704	3600	4192
MSTRG.10122.2	coding	0.072	1620	3516	3985
MSTRG.10122.3	coding	0.074	1326	3225	3703
MSTRG.10122.4	coding	0.052	2028	3318	3797
MSTRG.10123.1	noncoding	-0.0004096	2199	2391	8867
MSTRG.10122.5	coding	0.065	732	2115	2586
MSTRG.10122.6	coding	0.071	312	1446	2417
MSTRG.10122.7	coding	0.054	663	1956	2426
MSTRG.10122.8	coding	0.072	144	1785	2256
MSTRG.10133.9	noncoding	-0.012288	72	180	3905
MSTRG.10133.3	coding	0.067	372	2922	4689
MSTRG.10133.6	coding	0.066	5526	8172	9938
MSTRG.10133.5	coding	0.065	6777	9393	11159
MSTRG.10133.11	coding	0.072	372	3018	3436
MSTRG.10133.12	coding	0.067	6633	9183	9585
MSTRG.10112.2	noncoding	-0.032768	93	183	398
MSTRG.10112.3	coding	0.217	180	3573	4954
MSTRG.10112.4	coding	0.208	180	3828	4541
MSTRG.10112.5	coding	0.203	180	4047	4672
MSTRG.10112.10	coding	0.205	180	3045	3653
MSTRG.10112.11	coding	0.219	423	2970	3578
MSTRG.10112.12	coding	0.212	180	2826	3164
MSTRG.10115.4	coding	0.141	381	1554	2425
MSTRG.10115.3	coding	0.141	645	1818	2690

MSTRG.10115.5	noncoding	-0.0212992	309	534	1224
MSTRG.10116.2	noncoding	-0.0712704	819	1959	4722
MSTRG.10134.1	noncoding	-0.0462848	0	207	1505
MSTRG.10138.3	noncoding	-0.004096	111	159	313
MSTRG.10138.2	coding	0.334	231	2886	3591
MSTRG.10139.1	noncoding	-0.028672	1524	1608	5083
MSTRG.10138.7	coding	0.362	663	2457	3161
MSTRG.10142.1	noncoding	-0.131072	1113	1161	2050
MSTRG.10146.6	coding	0.422	213	1563	1797
MSTRG.10146.5	coding	0.429	429	1761	1994
MSTRG.10147.1	noncoding	-0.02048	963	1047	1969
MSTRG.10148.1	coding	0.087	678	2754	4006
MSTRG.10148.2	coding	0.092	675	2844	4612
MSTRG.10148.3	coding	0.099	663	2883	4135
MSTRG.10148.7	coding	0.099	456	2676	3928
MSTRG.10148.9	coding	0.099	708	2928	4180
MSTRG.10148.8	coding	0.099	477	2697	4967
MSTRG.10148.6	coding	0.087	477	2553	10450
MSTRG.10148.4	coding	0.099	477	2697	7953
MSTRG.10143.2	coding	0.403	0	444	760
MSTRG.10143.3	coding	0.414	0	543	979
MSTRG.10155.1	noncoding	-0.0499712	0	456	611
MSTRG.10159.1	coding	0.451	882	3192	5098
MSTRG.10160.1	coding	0.434	0	1953	3172
MSTRG.10162.1	noncoding	-0.0782336	90	120	744
MSTRG.10172.2	noncoding	-0.034816	243	447	749
MSTRG.10172.5	coding	0.581	0	1266	5665
MSTRG.10172.6	noncoding	-0.0106496	525	708	863
MSTRG.10173.1	noncoding	-0.024576	66	171	2906
MSTRG.10173.2	noncoding	-0.192512	162	189	2083
MSTRG.10172.8	noncoding	-0.0462848	156	483	697
MSTRG.10172.9	noncoding	-0.0487424	306	603	1127
MSTRG.10172.10	noncoding	-0.034816	30	324	580
MSTRG.10172.11	coding	0.2	0	345	870
MSTRG.10175.1	coding	0.145	0	312	4985
MSTRG.10175.2	noncoding	-0.0536576	6201	7014	11334
MSTRG.10175.3	coding	0.212	0	348	1882
MSTRG.10175.4	coding	0.145	0	312	728
MSTRG.10175.5	coding	0.145	0	312	1089
MSTRG.10178.6	coding	0.108	0	4281	7482
MSTRG.10178.15	coding	0.13	0	2229	8245
MSTRG.10178.7	coding	0.072	0	7782	8267
MSTRG.10178.8	coding	0.108	0	4281	7628
MSTRG.10178.12	coding	0.125	0	2787	8197
MSTRG.10178.11	coding	0.132	0	2844	8851
MSTRG.10178.9	coding	0.107	0	5040	8725
MSTRG.10178.13	coding	0.125	0	2781	9402
MSTRG.10178.1	coding	0.107	0	5040	9485
MSTRG.10178.14	coding	0.111	0	5097	9397
MSTRG.10178.16	coding	0.107	0	5040	13864
MSTRG.10178.3	coding	0.111	0	5097	13972
MSTRG.10178.10	coding	0.107	0	5040	12117
MSTRG.10178.5	coding	0.125	0	2787	13917
MSTRG.10178.17	coding	0.083	504	5118	9564

MSTRG.10178.18	coding	0.081	177	4809	9510
MSTRG.10178.19	coding	0.043	1254	6639	7125
MSTRG.10178.20	coding	0.089	1254	3954	7251
MSTRG.10178.21	coding	0.089	1254	3954	12830
MSTRG.10178.22	coding	0.091	801	2898	7529
MSTRG.10178.24	coding	0.086	1236	3936	7285
MSTRG.10178.23	coding	0.087	174	2841	7285
MSTRG.10180.2	coding	0.276	816	1557	3679
MSTRG.10164.2	coding	0.18	594	1464	1923
MSTRG.10164.1	coding	0.264	0	1875	2507
MSTRG.10164.3	coding	0.142	192	1476	1934
MSTRG.10183.4	coding	0.294	81	618	3320
MSTRG.10184.1	noncoding	-0.0405504	2043	2097	3567
MSTRG.10185.1	noncoding	-0.0421888	0	114	684
MSTRG.10195.1	noncoding	-0.0557056	147	354	1377
MSTRG.10198.1	coding	0.257	84	2208	3340
MSTRG.10197.5	noncoding	-0.0004096	678	816	1081
MSTRG.10197.6	noncoding	-0.1859584	0	420	2360
MSTRG.10208.1	noncoding	-0.0180224	10125	10263	13561
MSTRG.10209.1	coding	0.177	0	270	1210
MSTRG.10197.7	noncoding	-0.1695744	3204	4041	4428
MSTRG.10197.8	noncoding	-0.098304	279	330	798
MSTRG.10197.9	noncoding	-0.1712128	627	1440	1596
MSTRG.10213.1	noncoding	-0.1462272	465	1455	4194
MSTRG.10214.1	noncoding	-0.02048	609	717	1122
MSTRG.10215.1	noncoding	-0.186368	0	120	957
MSTRG.10230.1	noncoding	-0.016384	1815	1977	3238
MSTRG.10231.1	coding	0.307	3552	6585	8229
MSTRG.10231.2	coding	0.311	24	3060	4702
MSTRG.10232.1	noncoding	-0.1097728	0	93	249
MSTRG.10234.1	coding	0.055	234	1716	2035
MSTRG.10234.2	noncoding	-0.0782336	0	528	2167
MSTRG.10231.4	coding	0.303	309	3543	5186
MSTRG.10234.3	noncoding	-0.077824	477	552	1025
MSTRG.10235.1	noncoding	-0.008192	1878	1989	2146
MSTRG.10231.9	coding	0.312	99	3072	4716
MSTRG.10236.1	noncoding	-0.1335296	4143	4611	4768
MSTRG.10231.12	coding	0.326	303	3285	4929
MSTRG.10231.16	coding	0.33	4860	7182	7459
MSTRG.10231.15	coding	0.333	4860	7794	9437
MSTRG.10231.18	coding	0.33	3384	5706	5983
MSTRG.10231.19	coding	0.309	3423	4173	4326
MSTRG.10237.1	noncoding	-0.0249856	2220	2367	4934
MSTRG.10238.1	coding	0.166	4539	4725	5164
MSTRG.10239.3	coding	0.294	51	3945	5492
MSTRG.10239.4	coding	0.31	0	4101	5648
MSTRG.10222.1	noncoding	-0.0684032	627	1041	6273
MSTRG.10219.1	noncoding	-0.0217088	0	54	262
MSTRG.10219.2	noncoding	-0.0217088	0	54	374
MSTRG.10219.3	noncoding	-0.0217088	0	54	318
MSTRG.10243.7	coding	0.158	213	1398	6884
MSTRG.10243.8	coding	0.16	2028	3198	8910
MSTRG.10255.1	noncoding	-0.0004096	0	0	463
MSTRG.10256.3	coding	0.324	0	1428	5088

MSTRG.10259.1	noncoding	-0.075776	7845	8178	10772
MSTRG.10260.1	noncoding	-0.0004096	228	387	8119
MSTRG.10261.1	noncoding	-0.0679936	1626	1707	2742
MSTRG.10266.2	coding	0.103	270	1380	2974
MSTRG.10268.1	noncoding	-0.0004096	0	0	548
MSTRG.10270.1	noncoding	-0.0004096	0	0	276
MSTRG.10273.2	coding	0.255	0	1095	1367
MSTRG.10273.4	coding	0.256	162	900	1171
MSTRG.10273.3	coding	0.25	87	822	1424
MSTRG.10273.5	coding	0.256	183	921	1121
MSTRG.10275.1	noncoding	-0.0004096	834	843	997
MSTRG.10278.2	coding	0.076	69	3237	6314
MSTRG.10281.7	coding	0.12	0	3087	3241
MSTRG.10281.9	coding	0.117	0	3216	3735
MSTRG.10281.6	coding	0.112	0	3207	4219
MSTRG.10281.4	coding	0.116	0	3222	4245
MSTRG.10281.5	coding	0.132	0	3126	4149
MSTRG.10281.8	coding	0.116	0	3285	4308
MSTRG.10282.2	coding	0.274	108	936	1528
MSTRG.10282.3	coding	0.278	696	1854	2432
MSTRG.10282.4	coding	0.287	351	1506	2023
MSTRG.10284.1	coding	0.477	0	2013	4977
MSTRG.10284.4	coding	0.441	0	1644	2080
MSTRG.10290.1	noncoding	-0.049152	999	1050	1204
MSTRG.10290.2	noncoding	-0.016384	1125	1221	2820
MSTRG.10302.4	coding	0.199	1593	4035	4528
MSTRG.10305.1	coding	0.268	0	3699	19064
MSTRG.10305.2	noncoding	-0.0004096	2877	2994	5882
MSTRG.10306.1	noncoding	-0.0004096	0	0	425
MSTRG.10319.2	coding	0.088	0	2055	7856
MSTRG.10322.2	coding	0.191	3657	4227	5525
MSTRG.10322.1	coding	0.191	699	1269	2569
MSTRG.10317.1	noncoding	-0.016384	0	27	415
MSTRG.10326.3	coding	0.428	42	756	1518
MSTRG.10330.3	coding	0.224	147	540	703
MSTRG.10331.1	coding	0.523	735	3435	4667
MSTRG.10331.2	coding	0.41	1305	4764	5996
MSTRG.10329.3	coding	0.353	2040	4518	12507
MSTRG.10343.1	coding	0.209	7335	8796	12577
MSTRG.10344.1	noncoding	-0.014336	879	1047	1371
MSTRG.10343.4	coding	0.235	381	1551	3966
MSTRG.10343.5	coding	0.209	10617	12078	12695
MSTRG.10335.1	coding	0.51	546	1965	2554
MSTRG.10335.3	coding	0.51	375	1794	2382
MSTRG.10336.2	coding	0.535	0	2064	2464
MSTRG.10337.1	noncoding	-0.0724992	0	153	331
MSTRG.10341.2	coding	0.207	168	1428	2198
MSTRG.10341.3	coding	0.28	153	852	1059
MSTRG.10342.1	noncoding	-0.0466944	249	345	693
MSTRG.10350.1	coding	0.377	0	1443	5701
MSTRG.10351.1	coding	0.363	9	3918	5292
MSTRG.10351.2	coding	0.342	9	4005	5377
MSTRG.10351.3	coding	0.309	0	8634	9775
MSTRG.10356.3	coding	0.159	498	1521	1872

MSTRG.10356.4	coding	0.218	0	1317	1684
MSTRG.10362.2	coding	0.215	534	792	1382
MSTRG.10366.2	coding	0.143	135	1335	2236
MSTRG.10370.2	coding	0.453	0	1026	1943
MSTRG.10370.3	coding	0.36	1275	1890	5627
MSTRG.10373.3	coding	0.357	1515	4242	7640
MSTRG.10373.2	coding	0.374	0	3483	8269
MSTRG.10373.5	coding	0.135	13692	14367	14684
MSTRG.10374.1	noncoding	-0.0475136	1587	1833	2789
MSTRG.10375.1	coding	0.062	0	987	1156
MSTRG.10377.1	noncoding	-0.0004096	0	0	249
MSTRG.10379.1	noncoding	-0.0487424	0	15	246
MSTRG.10372.1	noncoding	-0.0045056	4359	4668	5914
MSTRG.10372.2	noncoding	-0.0045056	2562	2871	3549
MSTRG.10437.2	coding	0.169	1800	2556	5515
MSTRG.10437.3	coding	0.169	879	1635	10034
MSTRG.10437.4	noncoding	-0.0360448	5271	5595	6047
MSTRG.10437.5	coding	0.146	474	945	7309
MSTRG.10437.7	noncoding	-0.0049152	393	588	1281
MSTRG.10437.6	noncoding	-0.0118784	7164	7248	9005
MSTRG.10437.8	coding	0.169	20154	20910	21390
MSTRG.10437.9	coding	0.169	735	1491	5585
MSTRG.10437.10	noncoding	-0.0118784	4326	4410	6216
MSTRG.10437.11	noncoding	-0.0135168	2277	2316	4189
MSTRG.10437.12	coding	0.053	19767	20880	52618
MSTRG.10437.13	coding	0.053	19488	20601	37827
MSTRG.10437.14	coding	0.169	16104	16860	20955
MSTRG.10437.15	coding	0.15	7503	7968	12874
MSTRG.10437.16	coding	0.065	22773	23067	27092
MSTRG.10437.17	noncoding	-0.08192	2364	2466	9419
MSTRG.10439.1	noncoding	-0.0004096	972	972	2606
MSTRG.10437.18	coding	0.067	12432	13176	14501
MSTRG.10440.1	noncoding	-0.0188416	2919	3087	4713
MSTRG.10437.19	coding	0.076	930	1560	13019
MSTRG.10437.20	noncoding	-0.0135168	4773	4905	6143
MSTRG.10441.1	coding	0.01	6972	7554	9486
MSTRG.10442.1	coding	0.033	7554	8325	12586
MSTRG.10443.1	noncoding	-0.1024	894	939	3694
MSTRG.10445.1	noncoding	-0.004096	678	762	1835
MSTRG.10437.21	coding	0.126	750	978	1153
MSTRG.10447.1	noncoding	-0.049152	2145	2175	2833
MSTRG.10437.22	noncoding	-0.0147456	4155	4248	4884
MSTRG.10381.1	coding	0.001	69	213	715
MSTRG.10381.3	noncoding	-0.0049152	129	258	475
MSTRG.10381.4	coding	0.168	138	315	532
MSTRG.10381.5	coding	0.077	186	351	568
MSTRG.10381.6	coding	0.077	486	651	849
MSTRG.10386.1	noncoding	-0.0344064	528	540	824
MSTRG.10391.1	noncoding	-0.0507904	978	1308	1472
MSTRG.10383.1	noncoding	-0.0872448	1380	1812	2605
MSTRG.10392.1	coding	0.17	0	459	647
MSTRG.10399.2	coding	0.313	225	1221	2509
MSTRG.10399.3	coding	0.105	39	693	1150
MSTRG.10400.1	noncoding	-0.0282624	654	753	3106

MSTRG.10401.3	coding	0.071	348	768	2189
MSTRG.10410.2	noncoding	-0.0708608	144	150	642
MSTRG.10419.2	coding	0.222	0	966	3306
MSTRG.10419.3	coding	0.222	579	1566	3904
MSTRG.10419.4	coding	0.268	405	2112	4450
MSTRG.10420.1	noncoding	-0.012288	13188	13329	27797
MSTRG.10419.6	coding	0.455	0	432	15468
MSTRG.10407.1	coding	0.051	3207	4302	4493
MSTRG.10407.2	coding	0.098	570	1350	1533
MSTRG.10407.3	noncoding	-0.02048	672	810	1371
MSTRG.10409.1	coding	0.102	528	1200	2592
MSTRG.10470.8	coding	0.428	924	1524	1677
MSTRG.10470.10	coding	0.441	378	972	1128
MSTRG.10470.17	coding	0.423	11565	12129	12285
MSTRG.10470.18	noncoding	-0.028672	168	216	1929
MSTRG.10470.20	noncoding	-0.0196608	2088	2241	7485
MSTRG.10470.22	coding	0.058	7554	8817	10289
MSTRG.10470.23	coding	0.058	1761	3024	24071
MSTRG.10470.24	noncoding	-0.03072	5886	5967	13925
MSTRG.10421.2	noncoding	-0.1093632	153	423	1468
MSTRG.10424.2	noncoding	-0.0344064	270	273	430
MSTRG.10424.3	coding	0.496	0	591	6930
MSTRG.10428.1	noncoding	-0.0004096	3564	3678	3964
MSTRG.10427.3	coding	0.329	75	558	5177
MSTRG.10430.2	coding	0.416	27	1008	1188
MSTRG.10455.1	coding	0.165	405	2493	3957
MSTRG.10455.2	coding	0.176	426	2376	3777
MSTRG.10455.4	coding	0.19	861	2550	3217
MSTRG.10456.1	coding	0.123	681	1149	1381
MSTRG.10456.3	coding	0.125	0	507	739
MSTRG.10456.5	coding	0.123	48	516	748
MSTRG.10456.6	coding	0.112	27	480	711
MSTRG.10456.7	coding	0.148	474	804	1037
MSTRG.10456.4	coding	0.123	0	504	5899
MSTRG.10464.1	coding	0.349	0	3867	4095
MSTRG.10466.1	noncoding	-0.0425984	0	294	447
MSTRG.10435.2	coding	0.172	363	900	3127
MSTRG.10488.3	coding	0.17	0	909	1301
MSTRG.10488.5	coding	0.17	0	909	2724
MSTRG.10488.2	coding	0.17	0	909	1396
MSTRG.10488.4	coding	0.17	0	909	1109
MSTRG.10489.1	noncoding	-0.0118784	201	354	891
MSTRG.10481.1	noncoding	-0.032768	0	24	219
MSTRG.10491.1	noncoding	-0.0278528	216	291	519
MSTRG.10507.1	coding	0.426	153	7887	9137
MSTRG.10508.1	noncoding	-0.008192	27	129	507
MSTRG.10507.3	coding	0.431	9	3126	3317
MSTRG.10513.4	noncoding	-0.0626688	1113	1149	1305
MSTRG.10516.1	noncoding	-0.0557056	4245	4701	4929
MSTRG.10511.1	coding	0.421	0	645	1310
MSTRG.10522.3	noncoding	-0.0630784	624	1173	1488
MSTRG.10523.1	noncoding	-0.0073728	708	975	1559
MSTRG.10524.1	noncoding	-0.0004096	0	0	338
MSTRG.10525.1	noncoding	-0.0524288	369	534	1987

MSTRG.10526.3	coding	0.065	225	786	1234
MSTRG.10526.5	coding	0.053	714	1263	1712
MSTRG.10539.3	noncoding	-0.079872	2115	2151	4467
MSTRG.10539.4	noncoding	-0.0131072	1533	1653	5398
MSTRG.10542.1	noncoding	-0.487424	321	351	1048
MSTRG.10542.2	noncoding	-0.0159744	4923	5121	7456
MSTRG.10543.1	noncoding	-0.151552	189	207	443
MSTRG.10573.1	noncoding	-0.0417792	0	135	446
MSTRG.10577.1	noncoding	-0.0004096	60	60	239
MSTRG.10582.1	noncoding	-0.1134592	726	744	2107
MSTRG.10583.1	noncoding	-0.012288	1086	1263	2200
MSTRG.10584.1	noncoding	-0.0004096	0	0	1810
MSTRG.10590.2	coding	0.084	549	840	1011
MSTRG.10590.3	noncoding	-0.004096	2628	2772	3654
MSTRG.10595.3	coding	0.164	267	1584	3494
MSTRG.10595.4	coding	0.187	738	1959	3802
MSTRG.10595.5	coding	0.187	324	1545	3387
MSTRG.10595.6	coding	0.187	864	2085	3085
MSTRG.10595.7	coding	0.164	264	1581	2579
MSTRG.10604.1	noncoding	-0.0004096	0	0	366
MSTRG.10633.2	coding	0.103	51	1329	4261
MSTRG.10634.1	noncoding	-0.0905216	0	6	1088
MSTRG.10635.1	noncoding	-0.0049152	4086	4233	5796
MSTRG.10633.3	noncoding	-0.045056	2823	2934	3597
MSTRG.10633.4	coding	0.086	54	1029	7406
MSTRG.10633.5	coding	0.042	180	459	8181
MSTRG.10636.1	noncoding	-0.036864	249	297	497
MSTRG.10640.1	noncoding	-0.106496	2559	2649	5382
MSTRG.10641.1	noncoding	-0.0004096	366	456	1206
MSTRG.10639.2	coding	0.061	1626	3978	5691
MSTRG.10642.2	coding	0.06	489	768	3018
MSTRG.10643.1	coding	0.01	264	408	817
MSTRG.10642.3	noncoding	-0.01024	279	486	1376
MSTRG.10623.2	coding	0.158	291	969	1270
MSTRG.10623.3	coding	0.158	183	861	1160
MSTRG.10626.2	coding	0.069	483	4314	5246
MSTRG.10629.2	coding	0.029	597	774	1110
MSTRG.10629.3	coding	0.029	555	732	1066
MSTRG.10646.1	coding	0.214	3	354	776
MSTRG.10646.2	coding	0.252	264	1239	1393
MSTRG.10652.1	coding	0.226	120	2505	4685
MSTRG.10653.1	noncoding	-0.0479232	45	504	734
MSTRG.10652.5	coding	0.296	0	1089	3269
MSTRG.10654.1	coding	0.179	87	2715	5179
MSTRG.10659.1	coding	0.071	639	831	2529
MSTRG.10659.2	noncoding	-0.0004096	51	57	765
MSTRG.10665.1	coding	0.359	0	1677	4865
MSTRG.10667.4	coding	0.053	3396	8064	10819
MSTRG.10667.5	coding	0.056	624	5328	6475
MSTRG.10668.1	noncoding	-0.094208	0	300	5796
MSTRG.10668.2	noncoding	-0.018432	4797	5079	5233
MSTRG.10680.1	noncoding	-0.002048	174	453	838
MSTRG.10680.2	noncoding	-0.1048576	642	888	1042
MSTRG.10680.3	coding	0.313	2988	3315	3472

MSTRG.10680.4	noncoding	-0.077824	0	171	8626
MSTRG.10685.1	noncoding	-0.147456	129	141	360
MSTRG.10688.1	coding	0.118	0	618	2099
MSTRG.10700.2	noncoding	-0.0045056	966	1113	1595
MSTRG.10700.3	noncoding	-0.141312	2040	2286	2441
MSTRG.10700.4	noncoding	-0.141312	2154	2400	2556
MSTRG.10701.1	noncoding	-0.04096	2055	2151	5105
MSTRG.10700.5	noncoding	-0.141312	2595	2841	2996
MSTRG.10703.1	coding	0.132	546	3483	3996
MSTRG.10703.13	coding	0.15	0	2436	2949
MSTRG.10713.1	coding	0.134	1704	2457	3076
MSTRG.10713.5	coding	0.134	936	1689	2250
MSTRG.10713.4	coding	0.134	969	1722	2285
MSTRG.10713.6	coding	0.134	1437	2190	2412
MSTRG.10715.1	noncoding	-0.0417792	501	582	1986
MSTRG.10707.1	noncoding	-0.024576	405	450	1025
MSTRG.10712.2	noncoding	-0.0192512	1194	1392	4892
MSTRG.10718.1	coding	0.184	411	777	1151
MSTRG.10725.1	noncoding	-0.0454656	6414	6603	6803
MSTRG.10725.2	noncoding	-0.057344	4077	4185	4339
MSTRG.10761.1	noncoding	-0.0708608	786	804	1986
MSTRG.10764.1	noncoding	-0.1306624	108	147	422
MSTRG.10766.1	coding	0.254	78	681	1627
MSTRG.10766.2	coding	0.254	78	681	1629
MSTRG.10767.1	noncoding	-0.200704	27	60	215
MSTRG.10768.1	noncoding	-0.073728	246	288	507
MSTRG.10769.1	noncoding	-0.045056	2382	2604	8278
MSTRG.10765.1	noncoding	-0.012288	2376	2544	2701
MSTRG.10765.2	noncoding	-0.0380928	219	345	745
MSTRG.10772.1	noncoding	-0.0589824	597	1008	1162
MSTRG.10775.1	noncoding	-0.065536	711	732	1082
MSTRG.10776.1	noncoding	-0.352256	207	222	2408
MSTRG.10776.2	noncoding	-0.0770048	1479	1785	5510
MSTRG.10777.1	noncoding	-0.043008	486	711	2631
MSTRG.10774.2	noncoding	-0.1511424	1089	1131	2463
MSTRG.10778.1	coding	0.11	1650	1965	2662
MSTRG.10774.3	coding	0.124	1902	2073	2335
MSTRG.10774.4	noncoding	-0.141312	0	1317	3602
MSTRG.10774.5	noncoding	-0.1396736	0	1356	2679
MSTRG.10779.1	coding	0.058	4470	4686	6090
MSTRG.10774.6	noncoding	-0.02048	6612	6726	8745
MSTRG.10780.1	noncoding	-0.045056	87	174	1067
MSTRG.10782.1	noncoding	-0.0364544	186	651	808
MSTRG.10786.2	coding	0.158	102	1305	1494
MSTRG.10786.4	coding	0.127	0	1410	5126
MSTRG.10787.1	coding	0.091	11472	12204	12714
MSTRG.10789.1	noncoding	-0.0344064	5715	5910	8220
MSTRG.10790.1	noncoding	-0.014336	126	291	2951
MSTRG.10791.1	noncoding	-0.004096	5253	5469	7222
MSTRG.10792.1	noncoding	-0.024576	468	579	4062
MSTRG.10793.3	coding	0.134	1530	3459	3616
MSTRG.10793.2	coding	0.134	3120	5049	5205
MSTRG.10794.1	noncoding	-0.0290816	132	249	557
MSTRG.10794.2	noncoding	-0.0311296	120	252	409

MSTRG.10795.4	coding	0.084	2643	5811	8523
MSTRG.10795.5	coding	0.085	2289	5526	8238
MSTRG.10796.1	noncoding	-0.0032768	24	180	333
MSTRG.10806.4	coding	0.026	1032	1689	2300
MSTRG.10807.1	noncoding	-0.0155648	729	1077	1644
MSTRG.10808.2	coding	0.423	423	957	4471
MSTRG.10808.5	noncoding	-0.1011712	0	414	2688
MSTRG.10808.6	coding	0.419	1716	2292	5805
MSTRG.10808.7	coding	0.423	918	1452	4966
MSTRG.10809.1	noncoding	-0.1560576	0	81	1525
MSTRG.10811.1	noncoding	-0.0626688	78	291	1117
MSTRG.10812.1	noncoding	-0.0004096	0	0	298
MSTRG.10820.1	coding	0.223	345	1353	4387
MSTRG.10825.1	coding	0.067	171	486	5123
MSTRG.10830.1	noncoding	-0.0004096	591	831	1723
MSTRG.10831.1	noncoding	-0.049152	1458	1497	3551
MSTRG.10829.4	coding	0.035	4785	5250	6549
MSTRG.10829.5	noncoding	-0.0053248	894	1014	2534
MSTRG.10837.1	coding	0.518	0	2160	5018
MSTRG.10837.2	coding	0.525	0	1653	4511
MSTRG.10837.4	coding	0.479	0	900	1716
MSTRG.10838.1	noncoding	-0.0319488	696	831	1738
MSTRG.10840.2	coding	0.361	117	2694	4448
MSTRG.10840.3	noncoding	-0.1097728	6855	7152	7305
MSTRG.10843.5	coding	0.199	6576	7341	9464
MSTRG.10844.2	coding	0.096	2082	3099	4557
MSTRG.10844.1	coding	0.091	2973	3705	5163
MSTRG.10845.1	noncoding	-0.0208896	1329	1482	2507
MSTRG.10847.1	noncoding	-0.12288	90	141	940
MSTRG.10848.2	coding	0.136	1041	2010	2269
MSTRG.10848.3	coding	0.123	1044	2139	2386
MSTRG.10848.4	coding	0.135	36	1257	1504
MSTRG.10851.3	coding	0.564	324	1452	6743
MSTRG.10852.1	noncoding	-0.0589824	0	162	376
MSTRG.10853.1	noncoding	-0.0004096	0	93	283
MSTRG.10857.1	noncoding	-0.0004096	0	0	213
MSTRG.10867.3	coding	0.228	0	1065	4574
MSTRG.10876.7	coding	0.103	366	1278	1985
MSTRG.10876.8	coding	0.103	504	1416	2123
MSTRG.10876.6	coding	0.103	258	1170	2259
MSTRG.10880.3	coding	0.048	111	2073	2507
MSTRG.10882.1	noncoding	-0.024576	432	534	1642
MSTRG.10882.2	noncoding	-0.0483328	840	1062	3206
MSTRG.10882.4	coding	0.159	816	1059	3263
MSTRG.10882.5	coding	0.105	1182	1482	2372
MSTRG.10882.6	noncoding	-0.038912	9	228	2432
MSTRG.10882.7	coding	0.18	429	675	2933
MSTRG.10882.8	noncoding	-0.057344	348	378	823
MSTRG.10888.2	coding	0.261	579	1470	1943
MSTRG.10888.4	coding	0.278	0	1236	1709
MSTRG.10889.1	noncoding	-0.002048	11442	11559	14448
MSTRG.10889.2	coding	0.306	981	1275	1671
MSTRG.10890.1	noncoding	-0.0299008	0	306	769
MSTRG.10889.5	coding	0.317	20649	20991	23935

MSTRG.10889.7	coding	0.353	132	675	18926
MSTRG.10889.8	coding	0.441	1695	2397	7120
MSTRG.10889.9	coding	0.438	765	1452	2982
MSTRG.10899.1	coding	0.422	141	1131	7557
MSTRG.10901.1	noncoding	-0.026624	12102	12882	13566
MSTRG.10902.1	noncoding	-0.0770048	1692	1920	5507
MSTRG.10903.1	noncoding	-0.004096	12468	12609	16157
MSTRG.10896.2	coding	0.189	222	795	980
MSTRG.10896.3	coding	0.152	222	960	1145
MSTRG.10906.1	coding	0.111	0	171	498
MSTRG.10907.2	coding	0.108	0	885	1056
MSTRG.10907.3	noncoding	-0.0376832	345	702	874
MSTRG.10910.1	noncoding	-0.0004096	0	0	365
MSTRG.10922.3	coding	0.339	0	4731	9147
MSTRG.10922.5	coding	0.331	0	3258	9212
MSTRG.10923.1	noncoding	-0.049152	72	78	1318
MSTRG.10925.1	coding	0.182	456	1605	3910
MSTRG.10925.9	coding	0.174	375	1494	4012
MSTRG.10928.1	noncoding	-0.0196608	618	975	4875
MSTRG.10913.1	noncoding	-0.047104	0	54	473
MSTRG.10919.1	coding	0.37	204	1770	5118
MSTRG.10921.1	noncoding	-0.0401408	195	402	648
MSTRG.10935.1	coding	0.239	264	1767	3387
MSTRG.10935.2	coding	0.269	0	1563	2877
MSTRG.10936.2	coding	0.557	174	1641	3643
MSTRG.10941.2	coding	0.091	651	1830	3254
MSTRG.10942.2	noncoding	-0.0180224	531	678	3952
MSTRG.10943.1	noncoding	-0.0323584	1443	1590	2856
MSTRG.10944.1	coding	0.423	0	2022	7261
MSTRG.10950.3	coding	0.055	1089	2688	4509
MSTRG.10950.4	coding	0.062	84	1683	3502
MSTRG.10956.1	noncoding	-0.008192	87	246	548
MSTRG.10958.1	noncoding	-0.008192	1212	1236	1447
MSTRG.10961.1	noncoding	-0.1024	21	39	1055
MSTRG.10971.1	noncoding	-0.0495616	315	381	1101
MSTRG.10976.1	noncoding	-0.06144	120	222	654
MSTRG.10976.2	noncoding	-0.036864	195	243	680
MSTRG.10976.3	noncoding	-0.0004096	183	183	355
MSTRG.10984.1	coding	0.112	465	1503	2347
MSTRG.10984.3	coding	0.01	2922	3114	3757
MSTRG.10990.4	noncoding	-0.0372736	16647	16821	26689
MSTRG.10990.3	noncoding	-0.045056	10155	10263	18650
MSTRG.10990.5	noncoding	-0.0372736	3597	3774	7349
MSTRG.11012.1	coding	0.156	33	1476	4066
MSTRG.11012.2	coding	0.154	9	1455	4046
MSTRG.11039.1	noncoding	-0.0462848	3465	3543	9137
MSTRG.11040.1	noncoding	-0.1212416	0	282	6134
MSTRG.11041.1	coding	0.073	696	909	3811
MSTRG.11042.1	coding	0.042	3315	4068	8032
MSTRG.11043.1	coding	0.108	3192	3669	6260
MSTRG.11044.1	noncoding	-0.088064	486	795	2980
MSTRG.11045.1	noncoding	-0.012288	231	342	1171
MSTRG.11046.1	noncoding	-0.12288	129	150	384
MSTRG.11036.2	coding	0.048	744	1935	3769

MSTRG.11036.1	coding	0.047	366	1989	2571
MSTRG.11037.1	noncoding	-0.028672	9	126	281
MSTRG.11050.1	noncoding	-0.0487424	618	825	1316
MSTRG.11050.2	noncoding	-0.0495616	105	126	287
MSTRG.11055.1	noncoding	-0.049152	42	267	495
MSTRG.11057.1	noncoding	-0.0004096	0	0	293
MSTRG.11060.1	coding	0.14	303	1107	1261
MSTRG.11071.2	coding	0.033	1686	5541	9194
MSTRG.11071.3	coding	0.041	1737	4824	8777
MSTRG.11071.4	coding	0.041	1686	4773	8726
MSTRG.11071.5	coding	0.045	4431	7131	10014
MSTRG.11072.1	coding	0.033	3282	3453	4933
MSTRG.11073.2	noncoding	-0.012288	879	1023	1631
MSTRG.11063.1	coding	0.316	0	1278	1590
MSTRG.11063.3	coding	0.309	207	1461	1749
MSTRG.11076.2	coding	0.23	117	1080	3703
MSTRG.11100.1	noncoding	-0.024576	10134	10251	12271
MSTRG.11101.1	noncoding	-0.024576	498	558	3843
MSTRG.11090.1	noncoding	-0.0671744	366	708	1086
MSTRG.11091.5	coding	0.125	0	2151	3814
MSTRG.11091.6	coding	0.092	468	2184	3849
MSTRG.11096.2	coding	0.298	0	732	1157
MSTRG.11098.1	coding	0.111	3	1854	9705
MSTRG.11098.2	coding	0.146	0	1155	2122
MSTRG.11103.2	noncoding	-0.077824	1140	1236	1393
MSTRG.11103.3	noncoding	-0.012288	1302	1398	1802
MSTRG.11109.1	noncoding	-0.0004096	0	0	207
MSTRG.11154.2	coding	0.168	126	5778	7557
MSTRG.11156.1	noncoding	-0.008192	0	69	336
MSTRG.11158.1	noncoding	-0.043008	579	828	4158
MSTRG.11161.1	noncoding	-0.012288	114	366	1191
MSTRG.11161.4	noncoding	-0.0475136	0	84	552
MSTRG.11170.1	coding	0.069	1707	2154	2986
MSTRG.11171.1	noncoding	-0.004096	219	285	1597
MSTRG.11169.3	coding	0.172	1068	1968	2178
MSTRG.11177.1	coding	0.208	123	2829	3037
MSTRG.11163.2	noncoding	-0.0016384	2094	2139	2438
MSTRG.11185.1	coding	0.116	1494	4707	8646
MSTRG.11186.1	noncoding	-0.036864	648	699	2641
MSTRG.11188.3	coding	0.082	0	2802	5994
MSTRG.11190.3	coding	0.092	138	4323	6759
MSTRG.11191.1	coding	0.26	477	1152	2751
MSTRG.11194.2	noncoding	-0.024576	273	381	702
MSTRG.11199.1	noncoding	-0.0073728	1644	1746	2694
MSTRG.11194.4	noncoding	-0.0180224	0	234	736
MSTRG.11230.1	noncoding	-0.0888832	411	465	702
MSTRG.11235.1	noncoding	-0.0761856	867	1263	1416
MSTRG.11235.2	coding	0.205	0	447	3069
MSTRG.11243.1	coding	0.275	8205	9519	14645
MSTRG.11243.3	coding	0.195	972	3273	7639
MSTRG.11243.6	noncoding	-0.057344	195	204	447
MSTRG.11243.8	coding	0.199	234	2508	2668
MSTRG.11219.1	noncoding	-0.0069632	1077	1134	1629
MSTRG.11226.1	coding	0.231	462	2556	3182

MSTRG.11226.3	coding	0.222	66	2058	2683
MSTRG.11221.1	noncoding	-0.0589824	63	81	697
MSTRG.11221.2	noncoding	-0.008192	474	555	808
MSTRG.11248.3	coding	0.349	0	4824	5795
MSTRG.11248.9	coding	0.371	246	4614	5538
MSTRG.11248.8	coding	0.362	594	5106	6030
MSTRG.11248.6	coding	0.369	1068	5490	6412
MSTRG.11248.7	coding	0.363	342	4875	5799
MSTRG.11249.1	noncoding	-0.0524288	13023	13203	13478
MSTRG.11257.1	noncoding	-0.0004096	0	0	420
MSTRG.11256.1	noncoding	-0.028672	33	150	304
MSTRG.11279.1	noncoding	-0.098304	309	360	649
MSTRG.11283.1	coding	0.049	33	1173	2564
MSTRG.11283.2	coding	0.049	0	1140	1992
MSTRG.11284.1	coding	0.279	0	471	1253
MSTRG.11292.1	coding	0.298	0	3045	3846
MSTRG.11293.1	noncoding	-0.0073728	1677	1833	3027
MSTRG.11294.2	noncoding	-0.3284992	0	1140	6249
MSTRG.11294.3	coding	0.257	354	693	4399
MSTRG.11294.4	coding	0.345	279	1104	5177
MSTRG.11300.3	coding	0.112	1050	1251	1925
MSTRG.11301.2	noncoding	-0.02048	2280	2409	5142
MSTRG.11301.1	noncoding	-0.02048	4107	4236	6384
MSTRG.11301.3	noncoding	-0.135168	2082	2145	2428
MSTRG.11301.4	noncoding	-0.016384	6627	6747	7062
MSTRG.11301.5	noncoding	-0.0200704	960	1074	2483
MSTRG.11302.1	noncoding	-0.028672	357	456	1094
MSTRG.11302.2	noncoding	-0.0008192	2745	2883	9992
MSTRG.11303.1	noncoding	-0.0004096	453	453	607
MSTRG.11304.2	coding	0.047	207	1125	1993
MSTRG.11304.3	noncoding	-0.0188416	276	678	946
MSTRG.11373.3	coding	0.106	0	2271	3657
MSTRG.11373.4	coding	0.072	1989	2229	6336
MSTRG.11373.8	noncoding	-0.0004096	345	495	1824
MSTRG.11377.1	coding	0.29	1263	4944	12541
MSTRG.11377.2	coding	0.278	1290	5046	6866
MSTRG.11378.1	noncoding	-0.0004096	90	231	1192
MSTRG.11377.3	coding	0.26	108	4914	7246
MSTRG.11377.4	coding	0.276	2013	5886	7555
MSTRG.11377.5	coding	0.278	108	3864	10212
MSTRG.11377.7	coding	0.276	513	4590	4744
MSTRG.11377.6	coding	0.262	660	4848	5001
MSTRG.11377.8	coding	0.147	15333	16164	17452
MSTRG.11377.9	coding	0.156	8832	9813	11104
MSTRG.11377.10	noncoding	-0.024576	729	828	1120
MSTRG.11379.1	noncoding	-0.237568	69	102	2736
MSTRG.11377.11	coding	0.117	2592	3048	9210
MSTRG.11381.1	noncoding	-0.090112	1542	1563	3044
MSTRG.11382.1	noncoding	-0.028672	2145	2250	2594
MSTRG.11377.18	coding	0.039	144	924	3619
MSTRG.11377.19	coding	0.039	171	951	26685
MSTRG.11377.20	coding	0.038	384	1170	15415
MSTRG.11377.21	coding	0.039	177	957	5204
MSTRG.11377.22	noncoding	-0.0761856	1371	1380	1536

MSTRG.11377.23	noncoding	-0.016384	120	213	8068
MSTRG.11377.24	coding	0.093	26949	27474	34337
MSTRG.11377.25	coding	0.093	18270	18795	25658
MSTRG.11377.26	coding	0.156	318	402	2255
MSTRG.11377.27	noncoding	-0.045056	477	567	3447
MSTRG.11309.1	noncoding	-0.0823296	1548	2235	2806
MSTRG.11307.2	noncoding	-0.0835584	633	639	1210
MSTRG.11313.1	noncoding	-0.155648	1344	1413	2700
MSTRG.11313.2	noncoding	-0.0782336	0	372	15851
MSTRG.11313.3	noncoding	-0.110592	3336	3822	3975
MSTRG.11313.4	noncoding	-0.108544	4344	4827	4980
MSTRG.11319.1	noncoding	-0.0004096	654	810	964
MSTRG.11319.2	noncoding	-0.0401408	546	708	871
MSTRG.11321.1	coding	0.105	0	2529	4637
MSTRG.11326.1	noncoding	-0.0258048	198	291	466
MSTRG.11328.2	coding	0.3	0	726	1758
MSTRG.11328.4	coding	0.302	0	450	4590
MSTRG.11331.1	coding	0.071	135	576	1046
MSTRG.11335.1	coding	0.203	0	123	335
MSTRG.11336.2	coding	0.282	0	1635	5582
MSTRG.11336.3	coding	0.208	363	1752	4344
MSTRG.11338.2	noncoding	-0.002048	81	228	3267
MSTRG.11338.3	noncoding	-0.1060864	0	522	3980
MSTRG.11350.1	coding	0.127	0	1089	22679
MSTRG.11350.5	coding	0.12	0	1071	5554
MSTRG.11350.6	coding	0.12	0	1071	5728
MSTRG.11350.4	coding	0.12	0	1071	9411
MSTRG.11350.7	coding	0.172	1761	2145	10148
MSTRG.11351.1	coding	0.17	1893	2148	4643
MSTRG.11352.1	noncoding	-0.118784	813	843	1777
MSTRG.11350.8	coding	0.055	1431	1761	5691
MSTRG.11357.1	coding	0.09	1092	1362	1754
MSTRG.11358.1	coding	0.086	873	1155	5726
MSTRG.11359.1	noncoding	-0.0118784	7119	7263	9327
MSTRG.11360.1	noncoding	-0.036864	5778	5889	20756
MSTRG.11360.2	noncoding	-0.049152	3540	3597	4492
MSTRG.11344.2	noncoding	-0.04096	1146	1233	4891
MSTRG.11345.1	noncoding	-0.0139264	4713	4872	8738
MSTRG.11345.2	noncoding	-0.047104	5763	5958	9873
MSTRG.11346.1	noncoding	-0.08192	291	375	661
MSTRG.11347.1	noncoding	-0.0065536	4377	4518	17052
MSTRG.11348.3	noncoding	-0.0352256	1767	1932	2738
MSTRG.11363.1	noncoding	-0.004096	420	558	1839
MSTRG.11365.1	noncoding	-0.08192	1662	1764	3861
MSTRG.11366.1	noncoding	-0.004096	1350	1488	4509
MSTRG.11367.1	noncoding	-0.049152	4137	4338	15468
MSTRG.11367.2	noncoding	-0.049152	5901	6102	14052
MSTRG.11400.1	coding	0.224	186	3900	17998
MSTRG.11400.2	coding	0.23	186	3768	6963
MSTRG.11400.3	coding	0.233	0	3426	4969
MSTRG.11400.4	noncoding	-0.0446464	6198	6357	16586
MSTRG.11400.5	noncoding	-0.0561152	186	2445	18993
MSTRG.11403.1	coding	0.047	255	4242	4479
MSTRG.11404.1	coding	0.064	2724	4353	4604

MSTRG.11404.2	coding	0.064	2880	4509	4765
MSTRG.11408.1	coding	0.126	918	4665	6047
MSTRG.11410.1	coding	0.155	0	1257	1702
MSTRG.11411.1	coding	0.093	807	1077	1278
MSTRG.11411.2	noncoding	-0.0004096	129	147	705
MSTRG.11413.3	coding	0.268	246	1035	5726
MSTRG.11413.4	coding	0.232	1494	2088	6778
MSTRG.11406.3	coding	0.361	0	1362	2623
MSTRG.11414.1	noncoding	-0.073728	1206	1326	1865
MSTRG.11417.1	noncoding	-0.088064	1233	1626	1780
MSTRG.11417.2	noncoding	-0.0606208	918	1164	1994
MSTRG.11417.3	noncoding	-0.0606208	615	861	2681
MSTRG.11417.4	noncoding	-0.0606208	2832	3078	4220
MSTRG.11418.4	coding	0.189	0	3222	3516
MSTRG.11427.1	coding	0.347	11211	15180	16481
MSTRG.11427.3	coding	0.347	11424	15393	16774
MSTRG.11427.4	coding	0.347	11352	15321	16621
MSTRG.11429.1	coding	0.212	144	3531	5799
MSTRG.11430.1	noncoding	-0.0385024	105	417	1710
MSTRG.11431.2	coding	0.316	243	4041	4600
MSTRG.11431.3	coding	0.32	372	4191	4750
MSTRG.11432.1	coding	0.283	249	2205	2411
MSTRG.11433.1	noncoding	-0.0421888	57	246	695
MSTRG.11436.1	coding	0.201	576	1443	1896
MSTRG.11436.2	coding	0.213	576	1416	1807
MSTRG.11437.3	coding	0.253	141	981	1308
MSTRG.11438.1	coding	0.538	0	1050	1206
MSTRG.11439.3	coding	0.492	522	8163	8550
MSTRG.11441.4	coding	0.364	1650	2619	3068
MSTRG.11441.2	coding	0.263	0	1743	3256
MSTRG.11441.3	coding	0.212	177	1956	3469
MSTRG.11442.1	noncoding	-0.0192512	510	723	1666
MSTRG.11443.4	coding	0.215	0	1200	2086
MSTRG.11443.5	coding	0.226	0	1164	2596
MSTRG.11443.7	coding	0.219	0	1182	2050
MSTRG.11443.8	coding	0.186	0	1257	2126
MSTRG.11443.6	coding	0.186	0	1257	2687
MSTRG.11421.3	coding	0.222	0	342	551
MSTRG.11423.1	noncoding	-0.026624	180	666	1646
MSTRG.11424.2	coding	0.334	1809	4128	4732
MSTRG.11449.2	noncoding	-0.008192	1485	1641	1809
MSTRG.11457.1	coding	0.37	153	924	6580
MSTRG.11457.2	coding	0.397	0	699	4549
MSTRG.11457.3	coding	0.368	0	447	1020
MSTRG.11460.1	noncoding	-0.192512	738	768	1554
MSTRG.11462.12	coding	0.149	2364	2586	5737
MSTRG.11462.7	coding	0.149	990	1212	4362
MSTRG.11462.8	noncoding	-0.0700416	4413	4836	5154
MSTRG.11462.9	coding	0.149	1947	2169	5318
MSTRG.11462.13	coding	0.149	1209	1431	4581
MSTRG.11462.14	coding	0.149	2847	3069	3499
MSTRG.11462.15	noncoding	-0.131072	105	204	2030
MSTRG.11463.1	noncoding	-0.0823296	519	1458	1615
MSTRG.11464.1	noncoding	-0.028672	4389	4512	6437

MSTRG.11465.1	noncoding	-0.0004096	0	0	371
MSTRG.11466.1	coding	0.05	708	2523	2676
MSTRG.11466.2	coding	0.05	3858	5673	5828
MSTRG.11472.1	coding	0.068	54	1665	3375
MSTRG.11472.4	coding	0.138	36	708	1060
MSTRG.11472.2	coding	0.068	36	1647	2744
MSTRG.11472.5	coding	0.126	15	681	2638
MSTRG.11472.6	noncoding	-0.045056	5082	5154	6357
MSTRG.11472.7	noncoding	-0.077824	6927	6993	15536
MSTRG.11472.8	coding	0.036	4554	4779	7009
MSTRG.11473.1	noncoding	-0.024576	3042	3066	6908
MSTRG.11473.2	noncoding	-0.0004096	3123	3165	6347
MSTRG.11473.3	noncoding	-0.0004096	1602	1644	5245
MSTRG.11474.1	noncoding	-0.02048	3111	3231	3861
MSTRG.11473.4	noncoding	-0.024576	1053	1077	4920
MSTRG.11472.9	noncoding	-0.0913408	324	360	821
MSTRG.11472.10	coding	0.046	2568	3183	3647
MSTRG.11475.1	noncoding	-0.0131072	831	909	1802
MSTRG.11469.1	coding	0.321	150	1119	2258
MSTRG.11470.5	coding	0.307	1029	3099	3565
MSTRG.11476.3	coding	0.398	246	1689	3562
MSTRG.11476.2	coding	0.339	2835	5850	7352
MSTRG.11476.4	coding	0.399	12	1425	3272
MSTRG.11476.5	coding	0.395	2478	3867	5715
MSTRG.11477.1	coding	0.274	0	4839	5048
MSTRG.11478.1	noncoding	-0.0024576	1215	1347	4861
MSTRG.11483.2	coding	0.246	0	753	1145
MSTRG.11489.1	noncoding	-0.159744	141	147	302
MSTRG.11490.5	coding	0.377	0	4692	4847
MSTRG.11490.6	coding	0.352	1092	4917	5071
MSTRG.11496.2	coding	0.217	843	3015	3430
MSTRG.11499.2	coding	0.168	828	1779	2860
MSTRG.11499.4	noncoding	-0.0344064	828	1158	1457
MSTRG.11492.7	coding	0.064	387	1416	1617
MSTRG.11492.6	coding	0.064	600	1629	1832
MSTRG.11492.9	coding	0.064	591	1620	1822
MSTRG.11492.11	coding	0.064	1068	2097	2299
MSTRG.11492.12	coding	0.064	369	1398	1601
MSTRG.11492.5	coding	0.064	381	1410	1611
MSTRG.11492.8	coding	0.064	501	1530	1733
MSTRG.11492.10	coding	0.064	804	1833	2036
MSTRG.11492.4	coding	0.064	537	1566	1768
MSTRG.11505.1	coding	0.336	297	1290	1767
MSTRG.11505.2	coding	0.267	552	1686	2443
MSTRG.11507.1	coding	0.187	2601	3045	4790
MSTRG.11507.6	coding	0.356	1947	3018	5961
MSTRG.11507.8	coding	0.43	3225	3783	6725
MSTRG.11507.3	coding	0.337	0	1470	4411
MSTRG.11507.7	coding	0.449	1836	2343	5284
MSTRG.11507.2	coding	0.356	2349	3420	6363
MSTRG.11507.4	coding	0.356	732	1803	4746
MSTRG.11507.9	coding	0.449	2910	3417	6251
MSTRG.11508.1	coding	0.333	630	2208	4806
MSTRG.11508.4	coding	0.363	984	1686	5301

MSTRG.11508.5	coding	0.364	0	1545	1765
MSTRG.11510.1	noncoding	-0.02048	4383	4530	4684
MSTRG.11513.1	coding	0.375	192	1530	1766
MSTRG.11514.1	noncoding	-0.0638976	171	324	846
MSTRG.11514.2	noncoding	-0.0589824	204	354	587
MSTRG.11515.1	coding	0.53	0	546	701
MSTRG.11520.1	coding	0.209	123	4233	5259
MSTRG.11534.1	coding	0.027	255	1281	3623
MSTRG.11537.1	noncoding	-0.0303104	441	606	1196
MSTRG.11539.3	coding	0.195	171	1725	2639
MSTRG.11540.4	noncoding	-0.0344064	0	195	1356
MSTRG.11530.2	coding	0.267	0	564	1268
MSTRG.11551.1	noncoding	-0.0704512	2640	2922	5804
MSTRG.11551.2	noncoding	-0.0704512	2571	2853	4985
MSTRG.11553.1	coding	0.206	30	873	1031
MSTRG.11549.5	noncoding	-0.014336	0	123	890
MSTRG.11549.4	noncoding	-0.0421888	10065	10512	12379
MSTRG.11542.1	noncoding	-0.0299008	4407	4848	5586
MSTRG.11545.1	noncoding	-0.036864	402	570	1131
MSTRG.11556.2	noncoding	-0.0724992	534	936	1421
MSTRG.11560.1	noncoding	-0.0004096	27	183	562
MSTRG.11568.1	coding	0.107	540	1239	1973
MSTRG.11570.1	noncoding	-0.0155648	504	540	1108
MSTRG.11571.1	noncoding	-0.018432	126	165	435
MSTRG.11584.1	noncoding	-0.161792	117	345	1007
MSTRG.11586.1	coding	0.213	0	1032	2866
MSTRG.11589.2	coding	0.533	516	2976	3629
MSTRG.11590.3	coding	0.403	120	1116	1836
MSTRG.11591.2	coding	0.146	0	3021	7480
MSTRG.11594.3	coding	0.338	4608	6690	7580
MSTRG.11595.1	noncoding	-0.0360448	0	264	666
MSTRG.11595.2	noncoding	-0.0004096	30	174	521
MSTRG.11594.4	coding	0.338	1638	3720	4472
MSTRG.11606.4	coding	0.38	174	2814	3673
MSTRG.11606.10	coding	0.382	339	3021	3882
MSTRG.11599.1	coding	0.398	162	2718	3759
MSTRG.11599.2	coding	0.297	111	1326	1600
MSTRG.11610.1	coding	0.341	273	1635	1857
MSTRG.11610.2	coding	0.341	195	1557	1778
MSTRG.11611.2	coding	0.278	264	1992	5902
MSTRG.11611.4	coding	0.281	258	1962	5893
MSTRG.11613.1	noncoding	-0.0200704	147	174	328
MSTRG.11615.1	coding	0.41	0	1071	3468
MSTRG.11616.1	noncoding	-0.012288	216	327	484
MSTRG.11618.2	coding	0.406	0	663	3304
MSTRG.11617.1	noncoding	-0.0290816	48	201	531
MSTRG.11622.1	noncoding	-0.0147456	2265	2427	4331
MSTRG.11622.2	noncoding	-0.028672	138	252	1220
MSTRG.11636.2	coding	0.073	720	1809	2736
MSTRG.11643.2	noncoding	-0.0241664	4392	4608	4763
MSTRG.11643.3	coding	0.064	0	1404	2083
MSTRG.11643.4	coding	0.047	435	1569	2247
MSTRG.11645.1	noncoding	-0.0495616	0	90	464
MSTRG.11646.1	coding	0.271	0	570	1373

MSTRG.11649.4	coding	0.47	237	1479	4197
MSTRG.11656.3	coding	0.521	0	1824	2709
MSTRG.11657.2	coding	0.26	0	708	3658
MSTRG.11662.1	noncoding	-0.012288	63	159	901
MSTRG.11663.4	coding	0.229	672	1860	2574
MSTRG.11663.3	coding	0.369	51	768	1516
MSTRG.11664.2	noncoding	-0.073728	279	378	745
MSTRG.11666.1	coding	0.205	1323	1836	5596
MSTRG.11666.5	coding	0.212	2511	2976	3714
MSTRG.11666.3	coding	0.171	492	936	1675
MSTRG.11666.4	coding	0.134	456	1356	1594
MSTRG.11659.1	noncoding	-0.053248	669	963	1118
MSTRG.11659.2	noncoding	-0.0372736	1320	1533	1689
MSTRG.11659.3	noncoding	-0.016384	603	729	936
MSTRG.11659.4	noncoding	-0.0425984	5580	5889	10398
MSTRG.11674.2	coding	0.155	534	906	1870
MSTRG.11675.1	coding	0.34	171	777	1010
MSTRG.11675.3	coding	0.267	75	540	695
MSTRG.11676.3	coding	0.159	2124	3186	3470
MSTRG.11678.1	coding	0.334	0	576	3631
MSTRG.11678.5	coding	0.339	0	672	3486
MSTRG.11678.3	coding	0.339	0	672	4324
MSTRG.11679.1	noncoding	-0.180224	231	267	1559
MSTRG.11680.1	coding	0.186	360	3660	5589
MSTRG.11680.3	coding	0.186	1311	4611	5523
MSTRG.11677.3	coding	0.206	1011	2223	4310
MSTRG.11683.1	coding	0.264	177	1647	2688
MSTRG.11684.1	coding	0.186	1524	4266	6897
MSTRG.11684.4	coding	0.156	3930	8244	8749
MSTRG.11687.2	coding	0.278	102	810	1121
MSTRG.11696.1	noncoding	-0.0069632	444	597	1581
MSTRG.11697.1	noncoding	-0.0765952	393	1182	1473
MSTRG.11697.2	coding	0.304	0	969	1260
MSTRG.11702.1	coding	0.121	663	4350	5242
MSTRG.11702.3	coding	0.118	240	3963	4351
MSTRG.11703.1	noncoding	-0.036864	312	342	495
MSTRG.11707.1	coding	0.24	0	249	2183
MSTRG.11708.1	noncoding	-0.118784	444	543	700
MSTRG.11708.2	noncoding	-0.0290816	1038	1302	1889
MSTRG.11717.1	coding	0.209	129	1092	2199
MSTRG.11724.2	coding	0.129	249	1527	2238
MSTRG.11724.7	coding	0.115	66	1305	1895
MSTRG.11724.6	coding	0.129	789	2067	2658
MSTRG.11727.1	noncoding	-0.0176128	0	75	761
MSTRG.11728.1	coding	0.131	294	2136	7105
MSTRG.11731.1	noncoding	-0.0004096	0	0	210
MSTRG.11738.1	coding	0.092	0	2208	2638
MSTRG.11738.3	coding	0.11	1065	1977	2407
MSTRG.11738.4	noncoding	-0.0700416	0	117	3864
MSTRG.11738.6	noncoding	-0.036864	2862	2988	4305
MSTRG.11738.7	coding	0.099	3840	4803	5231
MSTRG.11738.8	coding	0.099	2298	3261	3691
MSTRG.11738.9	coding	0.096	1383	2235	2797
MSTRG.11734.3	coding	0.444	0	1068	4234

MSTRG.11737.2	noncoding	-0.065536	369	468	657
MSTRG.11739.1	coding	0.108	0	318	1867
MSTRG.11745.7	coding	0.085	528	3555	6734
MSTRG.11745.8	noncoding	-0.0806912	309	384	1098
MSTRG.11746.1	coding	0.044	1182	1539	2153
MSTRG.11747.1	noncoding	-0.008192	186	297	1439
MSTRG.11763.3	noncoding	-0.012288	1503	1641	1873
MSTRG.11763.4	coding	0.066	129	1593	1905
MSTRG.11764.1	noncoding	-0.0438272	81	312	1314
MSTRG.11763.5	coding	0.387	0	546	1799
MSTRG.11765.1	noncoding	-0.0253952	1416	1473	1815
MSTRG.11763.7	noncoding	-0.008192	480	873	1171
MSTRG.11756.2	coding	0.262	0	5013	8007
MSTRG.11756.6	coding	0.26	0	4038	4490
MSTRG.11761.1	noncoding	-0.028672	462	492	863
MSTRG.11782.1	noncoding	-0.036864	7578	7641	10628
MSTRG.11783.1	coding	0.295	420	753	2871
MSTRG.11783.4	noncoding	-0.131072	531	879	1755
MSTRG.11783.3	coding	0.186	0	702	1578
MSTRG.11792.1	coding	0.093	0	1383	7174
MSTRG.11792.3	coding	0.074	828	1581	4177
MSTRG.11792.2	coding	0.1	0	1512	3975
MSTRG.11792.4	coding	0.104	0	1425	1844
MSTRG.11792.13	coding	0.027	3147	3312	8311
MSTRG.11793.1	noncoding	-0.0004096	0	0	618
MSTRG.11794.1	noncoding	-0.0794624	2592	2691	4649
MSTRG.11785.1	coding	0.381	0	237	2947
MSTRG.11786.2	noncoding	-0.026624	60	261	570
MSTRG.11788.1	coding	0.183	180	987	1434
MSTRG.11788.2	coding	0.183	348	1155	1602
MSTRG.11788.4	coding	0.183	120	927	1226
MSTRG.11795.1	noncoding	-0.008192	783	828	1049
MSTRG.11805.1	noncoding	-0.14336	63	87	262
MSTRG.11806.1	coding	0.095	306	3579	4648
MSTRG.11807.1	noncoding	-0.036864	294	339	1580
MSTRG.11810.2	coding	0.023	360	1137	2124
MSTRG.11814.4	coding	0.197	618	1206	1363
MSTRG.11800.1	noncoding	-0.0274432	39	201	777
MSTRG.11802.3	noncoding	-0.12288	0	441	932
MSTRG.11802.4	coding	0.404	0	405	772
MSTRG.11802.5	coding	0.337	2646	3156	3396
MSTRG.11820.3	coding	0.445	114	1797	3766
MSTRG.11819.1	noncoding	-0.0012288	159	288	806
MSTRG.11834.2	coding	0.257	0	1137	1411
MSTRG.11834.3	coding	0.166	0	606	1412
MSTRG.11838.1	noncoding	-0.008192	465	549	1533
MSTRG.11841.4	noncoding	-0.22528	1260	1284	2559
MSTRG.11842.3	coding	0.083	3000	5184	5576
MSTRG.11842.6	coding	0.083	1449	3633	4023
MSTRG.11821.2	noncoding	-0.0589824	690	1194	3285
MSTRG.11824.2	noncoding	-0.0372736	9657	9852	12045
MSTRG.11824.3	noncoding	-0.0512	15378	15615	16861
MSTRG.11824.4	noncoding	-0.0319488	1530	1686	3269
MSTRG.11824.5	noncoding	-0.0372736	9657	9852	22513

MSTRG.11825.3	coding	0.064	294	1347	1512
MSTRG.11825.4	coding	0.075	42	636	1611
MSTRG.11827.1	noncoding	-0.0114688	0	84	296
MSTRG.11827.3	noncoding	-0.016384	435	564	717
MSTRG.11827.2	noncoding	-0.0598016	435	717	918
MSTRG.11828.1	noncoding	-0.0004096	0	0	201
MSTRG.11847.2	coding	0.214	420	1737	2074
MSTRG.11852.1	coding	0.093	0	807	4016
MSTRG.11852.2	coding	0.247	36	300	1320
MSTRG.11852.3	coding	0.136	9	804	2077
MSTRG.11852.4	noncoding	-0.0397312	909	1089	1477
MSTRG.11852.5	noncoding	-0.0483328	729	957	2667
MSTRG.11860.1	noncoding	-0.098304	387	420	679
MSTRG.11861.1	coding	0.36	60	1083	1840
MSTRG.11854.1	noncoding	-0.004096	0	231	386
MSTRG.11867.3	coding	0.188	417	999	4018
MSTRG.11867.4	noncoding	-0.018432	168	321	592
MSTRG.11867.5	noncoding	-0.065536	204	240	507
MSTRG.11872.1	coding	0.149	450	1362	2033
MSTRG.11873.2	coding	0.431	159	777	1594
MSTRG.11873.4	coding	0.441	159	750	1968
MSTRG.11873.3	coding	0.352	159	1611	5734
MSTRG.11873.7	coding	0.32	0	633	5405
MSTRG.11873.8	coding	0.346	0	519	2004
MSTRG.11873.6	coding	0.309	0	936	1716
MSTRG.11875.1	noncoding	-0.0356352	135	297	453
MSTRG.11876.1	coding	0.116	0	1119	3640
MSTRG.11876.3	coding	0.253	1485	2460	4195
MSTRG.11882.3	noncoding	-0.016384	1743	1929	3069
MSTRG.11882.4	coding	0.04	1785	1971	3111
MSTRG.11889.1	coding	0.394	12	2898	4324
MSTRG.11889.2	coding	0.404	0	2823	5020
MSTRG.11890.1	noncoding	-0.004096	267	399	633
MSTRG.11901.1	noncoding	-0.0004096	1158	1281	3458
MSTRG.11901.2	noncoding	-0.0004096	1353	1476	2615
MSTRG.11902.1	noncoding	-0.0004096	192	192	490
MSTRG.11923.1	noncoding	-0.0344064	0	102	332
MSTRG.11924.1	noncoding	-0.0139264	153	270	688
MSTRG.11929.1	noncoding	-0.03072	0	126	447
MSTRG.11950.1	noncoding	-0.0004096	0	0	366
MSTRG.11951.1	noncoding	-0.0425984	150	297	1397
MSTRG.11960.1	noncoding	-0.0155648	297	309	699
MSTRG.11968.1	coding	0.07	8556	8778	10591
MSTRG.11969.1	noncoding	-0.0016384	5091	5241	6336
MSTRG.11970.1	noncoding	-0.0380928	4464	4644	11970
MSTRG.11973.4	coding	0.581	99	1371	1533
MSTRG.11973.3	coding	0.586	132	1392	1554
MSTRG.11973.2	coding	0.273	78	396	558
MSTRG.11973.1	coding	0.569	0	1350	1512
MSTRG.11974.1	coding	0.385	156	1452	1698
MSTRG.11974.2	coding	0.38	0	1329	1560
MSTRG.11974.3	coding	0.399	0	1275	1519
MSTRG.11973.7	noncoding	-0.200704	39	90	244
MSTRG.11973.8	noncoding	-0.094208	0	99	426

MSTRG.11973.9	noncoding	-0.0172032	0	168	2213
MSTRG.11973.10	noncoding	-0.069632	0	108	276
MSTRG.11973.11	noncoding	-0.036864	0	123	364
MSTRG.11973.12	noncoding	-0.151552	0	54	211
MSTRG.11973.13	noncoding	-0.077824	0	108	600
MSTRG.11973.14	noncoding	-0.065536	0	111	608
MSTRG.11973.15	noncoding	-0.0192512	0	174	754
MSTRG.11973.16	noncoding	-0.0004096	123	255	497
MSTRG.11973.17	noncoding	-0.094208	0	99	254
MSTRG.11973.18	noncoding	-0.0499712	0	120	399
MSTRG.11973.19	noncoding	-0.208896	0	36	222
MSTRG.11973.20	noncoding	-0.057344	36	114	355
MSTRG.11973.21	noncoding	-0.110592	0	75	390
MSTRG.11973.22	noncoding	-0.110592	0	72	367
MSTRG.11973.23	noncoding	-0.200704	0	51	338
MSTRG.11973.24	noncoding	-0.098304	0	84	329
MSTRG.11958.1	coding	0.192	579	1929	4650
MSTRG.11958.2	coding	0.195	684	2043	4762
MSTRG.11972.1	noncoding	-0.0663552	4785	5151	5457
MSTRG.11978.2	coding	0.287	0	456	710
MSTRG.11978.1	coding	0.326	0	2880	6706
MSTRG.11979.1	noncoding	-0.0618496	177	261	497
MSTRG.11978.4	coding	0.326	0	2523	7302
MSTRG.11978.5	coding	0.326	0	2514	7481
MSTRG.11985.2	coding	0.112	237	9588	10252
MSTRG.11986.4	coding	0.298	1152	2190	2530
MSTRG.11987.1	noncoding	-0.0118784	378	426	839
MSTRG.11995.1	coding	0.148	225	5607	5816
MSTRG.11996.1	noncoding	-0.0139264	981	1038	2981
MSTRG.11995.6	coding	0.148	147	5529	6942
MSTRG.11995.5	coding	0.127	897	4494	10021
MSTRG.11995.7	coding	0.16	1656	5790	8996
MSTRG.11995.8	coding	0.16	1281	5415	5607
MSTRG.11995.9	coding	0.16	1443	5577	5765
MSTRG.11991.1	noncoding	-0.094208	1389	1470	6406
MSTRG.11994.2	coding	0.161	339	1224	1610
MSTRG.11994.3	coding	0.154	267	1119	1483
MSTRG.12000.1	coding	0.039	2829	3273	4986
MSTRG.12004.5	coding	0.057	3954	5304	10116
MSTRG.12004.6	coding	0.201	0	1140	8900
MSTRG.12004.7	coding	0.05	1731	2112	2631
MSTRG.12004.8	coding	0.037	627	1434	1954
MSTRG.12004.9	coding	0.066	645	1875	2363
MSTRG.12004.10	coding	0.057	396	1746	1978
MSTRG.12005.1	noncoding	-0.016384	1371	1452	2940
MSTRG.12004.11	coding	0.189	78	1083	1238
MSTRG.12006.4	coding	0.15	531	1326	2522
MSTRG.12007.1	noncoding	-0.086016	60	135	674
MSTRG.12008.1	noncoding	-0.0004096	0	0	415
MSTRG.12009.1	noncoding	-0.06144	99	144	299
MSTRG.12019.2	noncoding	-0.006144	294	435	838
MSTRG.12019.3	noncoding	-0.0196608	270	426	791
MSTRG.12027.2	coding	0.241	78	597	3763
MSTRG.12023.1	coding	0.134	0	1038	4083

MSTRG.12030.1	coding	0.362	0	1311	2287
MSTRG.12030.3	coding	0.354	666	1608	2584
MSTRG.12034.4	coding	0.116	279	1398	1737
MSTRG.12035.1	coding	0.397	342	4041	4468
MSTRG.12039.1	noncoding	-0.0004096	0	57	936
MSTRG.12040.1	noncoding	-0.045056	3060	3138	4121
MSTRG.12041.3	coding	0.59	0	2451	3364
MSTRG.12042.1	noncoding	-0.049152	1536	1617	5931
MSTRG.12043.1	noncoding	-0.0200704	1320	1575	5105
MSTRG.12044.1	noncoding	-0.0438272	2730	2940	4867
MSTRG.12045.3	coding	0.281	1254	2460	3373
MSTRG.12045.4	coding	0.305	0	4494	5406
MSTRG.12047.3	coding	0.413	309	1737	1893
MSTRG.12053.2	coding	0.293	372	1011	2200
MSTRG.12053.3	coding	0.293	1221	1860	6639
MSTRG.12054.1	noncoding	-0.0823296	1206	1392	4529
MSTRG.12057.4	noncoding	-0.0417792	72	330	712
MSTRG.12055.5	noncoding	-0.0811008	3285	3525	6420
MSTRG.12055.6	noncoding	-0.034816	3456	3849	9077
MSTRG.12055.7	noncoding	-0.069632	816	909	1064
MSTRG.12055.8	noncoding	-0.0425984	2646	2823	12406
MSTRG.12055.9	noncoding	-0.008192	789	873	4767
MSTRG.12055.10	noncoding	-0.0192512	789	834	1898
MSTRG.12055.11	noncoding	-0.0290816	1515	1830	2869
MSTRG.12055.12	noncoding	-0.155648	1521	1563	2418
MSTRG.12055.13	coding	0.044	2610	2775	9720
MSTRG.12055.14	noncoding	-0.049152	6075	6195	21455
MSTRG.12059.1	noncoding	-0.008192	228	432	7323
MSTRG.12049.1	noncoding	-0.0253952	303	450	696
MSTRG.12050.1	noncoding	-0.045056	435	528	923
MSTRG.12051.1	noncoding	-0.172032	318	345	587
MSTRG.12069.1	noncoding	-0.0073728	1119	1314	2497
MSTRG.12069.2	coding	0.424	765	2076	10380
MSTRG.12069.5	coding	0.358	420	1059	2038
MSTRG.12070.1	noncoding	-0.028672	816	1536	12775
MSTRG.12069.7	noncoding	-0.012288	1872	1956	3219
MSTRG.12072.3	coding	0.547	0	1836	2273
MSTRG.12072.10	coding	0.524	60	1920	2355
MSTRG.12072.9	coding	0.529	1815	3678	4114
MSTRG.12072.12	coding	0.522	8217	10182	11355
MSTRG.12080.1	coding	0.427	66	2307	2763
MSTRG.12079.1	noncoding	-0.02048	252	264	445
MSTRG.12088.1	noncoding	-0.0004096	0	126	647
MSTRG.12093.2	coding	0.214	138	1932	6139
MSTRG.12095.3	coding	0.229	0	1500	2180
MSTRG.12098.1	coding	0.077	1683	2586	5531
MSTRG.12099.2	coding	0.378	0	993	2889
MSTRG.12096.1	noncoding	-0.110592	87	138	292
MSTRG.12100.1	coding	0.148	2637	4446	4972
MSTRG.12100.2	coding	0.119	1332	4491	5002
MSTRG.12100.3	coding	0.202	2736	3942	5160
MSTRG.12102.1	noncoding	-0.032768	243	288	442
MSTRG.12108.4	coding	0.279	288	6972	7468
MSTRG.12108.1	coding	0.282	288	7035	7646

MSTRG.12108.9	coding	0.285	618	7110	7721
MSTRG.12108.8	coding	0.279	288	6972	8044
MSTRG.12108.12	coding	0.111	66	597	753
MSTRG.12104.3	coding	0.488	501	3018	3254
MSTRG.12121.2	coding	0.749	0	264	1452
MSTRG.12121.5	coding	0.203	0	1101	1256
MSTRG.12123.5	coding	0.221	207	1392	1614
MSTRG.12123.2	coding	0.221	465	1650	1873
MSTRG.12123.4	coding	0.225	0	1632	1854
MSTRG.12123.1	coding	0.221	1002	2187	2410
MSTRG.12123.3	coding	0.178	84	1836	2058
MSTRG.12123.6	noncoding	-0.012288	6	144	472
MSTRG.12124.1	noncoding	-0.004096	114	411	567
MSTRG.12124.2	noncoding	-0.034816	93	2019	2175
MSTRG.12125.1	coding	0.522	39	483	638
MSTRG.12114.2	coding	0.573	1377	1791	2331
MSTRG.12130.3	coding	0.401	1452	2910	3468
MSTRG.12131.1	noncoding	-0.0319488	0	72	598
MSTRG.12138.1	noncoding	-0.114688	0	210	1258
MSTRG.12145.1	noncoding	-0.0479232	1191	1542	1795
MSTRG.12146.1	noncoding	-0.0126976	141	318	474
MSTRG.12147.1	noncoding	-0.04096	198	288	443
MSTRG.12152.1	noncoding	-0.0032768	0	120	712
MSTRG.12153.2	noncoding	-0.0618496	0	345	1671
MSTRG.12157.3	coding	0.175	1365	1539	2455
MSTRG.12157.4	coding	0.197	0	909	1823
MSTRG.12157.2	noncoding	-0.1015808	0	1218	2133
MSTRG.12157.5	coding	0.21	0	315	672
MSTRG.12159.2	coding	0.12	123	660	3165
MSTRG.12159.1	coding	0.135	123	960	1877
MSTRG.12159.3	coding	0.13	87	528	1134
MSTRG.12160.1	noncoding	-0.0499712	3474	4020	7134
MSTRG.12161.1	noncoding	-0.0712704	570	723	1487
MSTRG.12168.3	noncoding	-0.0372736	2562	2736	3366
MSTRG.12188.1	noncoding	-0.0380928	42	252	665
MSTRG.12189.1	noncoding	-0.188416	1341	1410	1567
MSTRG.12189.3	noncoding	-0.1277952	0	225	2433
MSTRG.12195.1	noncoding	-0.057344	7680	7776	7929
MSTRG.12196.1	noncoding	-0.016384	552	675	2274
MSTRG.12197.2	noncoding	-0.118784	852	909	1760
MSTRG.12197.3	coding	0.075	0	1308	3135
MSTRG.12197.4	coding	0.083	2403	3858	4138
MSTRG.12197.5	noncoding	-0.083968	2847	3342	4186
MSTRG.12198.1	noncoding	-0.02048	1356	1437	3164
MSTRG.12202.1	noncoding	-0.04096	57	63	217
MSTRG.12233.1	noncoding	-0.0253952	1236	1254	1742
MSTRG.12238.1	noncoding	-0.16384	3837	3873	4955
MSTRG.12246.2	noncoding	-0.0405504	3873	4257	4677
MSTRG.12208.1	noncoding	-0.0598016	0	63	523
MSTRG.12214.5	noncoding	-0.0106496	2889	3015	9228
MSTRG.12214.6	noncoding	-0.004096	1254	1419	1700
MSTRG.12214.8	coding	0.14	2343	2685	4234
MSTRG.12215.1	noncoding	-0.0446464	327	444	1417
MSTRG.12207.1	noncoding	-0.04096	45	255	1664

MSTRG.12218.5	coding	0.267	600	1455	2335
MSTRG.12218.6	coding	0.267	483	1338	2217
MSTRG.12224.1	noncoding	-0.036864	786	849	1025
MSTRG.12228.1	coding	0.204	72	900	1074
MSTRG.12274.1	noncoding	-0.0458752	0	51	414
MSTRG.12277.1	noncoding	-0.0028672	684	918	7243
MSTRG.12278.2	noncoding	-0.0401408	60	234	388
MSTRG.12279.1	noncoding	-0.0647168	543	1332	1774
MSTRG.12280.1	coding	0.066	2358	3336	4965
MSTRG.12280.2	noncoding	-0.0479232	72	609	8008
MSTRG.12280.3	noncoding	-0.0479232	72	609	4293
MSTRG.12281.1	noncoding	-0.0315392	579	1602	2042
MSTRG.12281.2	noncoding	-0.065536	1608	1932	2919
MSTRG.12285.2	coding	0.169	0	1107	2253
MSTRG.12288.2	coding	0.229	45	1368	1581
MSTRG.12288.3	coding	0.217	60	1821	2024
MSTRG.12302.2	coding	0.202	0	984	1647
MSTRG.12303.1	noncoding	-0.04096	144	261	1269
MSTRG.12302.3	coding	0.202	0	984	2913
MSTRG.12302.4	coding	0.052	0	618	1201
MSTRG.12302.5	coding	0.202	0	984	2178
MSTRG.12320.2	coding	0.236	3720	4305	4462
MSTRG.12320.4	coding	0.256	3636	4185	4338
MSTRG.12320.5	coding	0.06	5814	6240	7520
MSTRG.12320.7	coding	0.06	2343	2769	4101
MSTRG.12322.1	coding	0.165	0	4011	8462
MSTRG.12323.1	noncoding	-0.0004096	270	360	930
MSTRG.12322.3	coding	0.043	150	375	719
MSTRG.12347.5	coding	0.029	120	5535	6775
MSTRG.12347.6	coding	0.023	120	5598	10564
MSTRG.12347.7	coding	0.059	6162	6915	10374
MSTRG.12347.8	coding	0.064	5178	5820	6116
MSTRG.12351.1	noncoding	-0.086016	465	522	1882
MSTRG.12352.1	noncoding	-0.004096	330	471	963
MSTRG.12340.2	coding	0.07	669	2292	3009
MSTRG.12340.4	coding	0.07	900	2523	2956
MSTRG.12340.3	coding	0.087	1128	1860	3458
MSTRG.12341.1	noncoding	-0.0098304	2997	3183	4798
MSTRG.12342.1	noncoding	-0.049152	2031	2136	2550
MSTRG.12353.1	noncoding	-0.043008	0	39	408
MSTRG.12356.1	noncoding	-0.0106496	258	378	587
MSTRG.12355.2	coding	0.163	0	1065	2518
MSTRG.12360.3	coding	0.39	0	1341	5210
MSTRG.12359.1	noncoding	-0.0442368	0	102	277
MSTRG.12416.1	noncoding	-0.0004096	528	651	2328
MSTRG.12425.1	coding	0.081	438	711	1161
MSTRG.12426.2	noncoding	-0.016384	13632	13767	14279
MSTRG.12433.1	noncoding	-0.155648	927	933	2024
MSTRG.12434.4	coding	0.097	0	7182	7554
MSTRG.12434.5	coding	0.096	1971	9084	9457
MSTRG.12434.7	coding	0.099	0	7284	7656
MSTRG.12434.8	coding	0.109	1962	7725	8098
MSTRG.12435.2	coding	0.19	108	1686	2197
MSTRG.12439.2	coding	0.135	192	1716	3322

MSTRG.12440.1	noncoding	-0.0110592	2397	2571	6524
MSTRG.12441.1	noncoding	-0.0237568	6402	6486	8970
MSTRG.12442.1	noncoding	-0.016384	2586	2718	5460
MSTRG.12442.2	noncoding	-0.016384	1911	2043	5923
MSTRG.12441.2	noncoding	-0.0237568	4626	4710	7173
MSTRG.12439.3	noncoding	-0.086016	4215	4302	5173
MSTRG.12439.4	coding	0.035	1161	1461	6643
MSTRG.12384.2	coding	0.236	1380	3018	4324
MSTRG.12384.1	coding	0.227	1380	2850	4238
MSTRG.12384.3	coding	0.32	369	1299	2509
MSTRG.12389.1	noncoding	-0.0192512	252	462	1043
MSTRG.12390.3	coding	0.08	369	1110	1349
MSTRG.12391.1	coding	0.137	3516	3849	5150
MSTRG.12391.2	coding	0.116	2712	2961	3116
MSTRG.12392.1	noncoding	-0.004096	51	102	353
MSTRG.12395.1	noncoding	-0.0086016	114	252	427
MSTRG.12407.1	coding	0.109	210	855	2049
MSTRG.12400.2	noncoding	-0.004096	1158	1353	1509
MSTRG.12447.5	coding	0.212	198	2844	4009
MSTRG.12448.1	coding	0.001	1227	1383	2203
MSTRG.12449.1	noncoding	-0.022528	1734	1857	5588
MSTRG.12450.1	noncoding	-0.02048	3129	3303	7779
MSTRG.12444.1	noncoding	-0.02048	441	612	1615
MSTRG.12445.2	noncoding	-0.0520192	216	417	775
MSTRG.12474.3	noncoding	-0.0114688	483	636	1438
MSTRG.12474.5	noncoding	-0.0262144	327	546	1365
MSTRG.12474.2	noncoding	-0.0106496	162	330	1147
MSTRG.12474.4	noncoding	-0.0262144	918	1137	1954
MSTRG.12474.1	noncoding	-0.0057344	264	405	1224
MSTRG.12474.7	noncoding	-0.0262144	345	564	1370
MSTRG.12474.6	noncoding	-0.0151552	168	348	1138
MSTRG.12474.9	noncoding	-0.0262144	660	879	1505
MSTRG.12474.8	noncoding	-0.0057344	342	483	1110
MSTRG.12475.1	coding	0.074	1065	1317	2887
MSTRG.12475.2	coding	0.074	957	1209	2780
MSTRG.12475.3	coding	0.074	960	1212	2784
MSTRG.12475.5	coding	0.074	3354	3606	5176
MSTRG.12476.1	coding	0.103	0	1632	2800
MSTRG.12476.2	coding	0.103	0	1632	2222
MSTRG.12476.3	coding	0.108	0	1695	3191
MSTRG.12476.7	coding	0.126	0	1335	2395
MSTRG.12476.8	coding	0.128	0	1608	2492
MSTRG.12476.6	coding	0.127	0	1170	2231
MSTRG.12476.9	coding	0.126	216	1530	2414
MSTRG.12494.1	noncoding	-0.012288	1311	1410	2751
MSTRG.12495.1	noncoding	-0.004096	624	726	2235
MSTRG.12495.2	noncoding	-0.0364544	0	162	3721
MSTRG.12504.1	noncoding	-0.249856	351	402	557
MSTRG.12504.2	noncoding	-0.290816	660	705	858
MSTRG.12505.1	coding	0.084	1470	1695	14178
MSTRG.12506.1	noncoding	-0.012288	3111	3225	5354
MSTRG.12507.1	noncoding	-0.0004096	9	72	449
MSTRG.12505.2	coding	0.023	2697	2868	4118
MSTRG.12501.1	coding	0.193	0	3678	4254

MSTRG.12501.3	coding	0.11	426	771	1242
MSTRG.12509.1	noncoding	-0.028672	1083	1290	1796
MSTRG.12510.2	coding	0.078	0	2274	2764
MSTRG.12510.3	coding	0.064	672	2685	6952
MSTRG.12510.4	coding	0.064	480	2016	2507
MSTRG.12517.3	coding	0.17	0	1338	1653
MSTRG.12521.1	noncoding	-0.06144	192	303	554
MSTRG.12525.2	coding	0.093	909	2427	3830
MSTRG.12525.1	coding	0.093	1230	2748	4149
MSTRG.12525.3	coding	0.093	1578	3096	4494
MSTRG.12528.3	noncoding	-0.04096	1041	1149	1393
MSTRG.12537.2	coding	0.315	0	1188	3109
MSTRG.12553.1	coding	0.088	1695	2025	2358
MSTRG.12558.1	noncoding	-0.059392	21	36	1334
MSTRG.12558.2	noncoding	-0.0413696	87	96	600
MSTRG.12558.3	noncoding	-0.0290816	1443	1815	2613
MSTRG.12560.1	noncoding	-0.0303104	4341	4626	18790
MSTRG.12550.2	coding	0.069	489	1770	2197
MSTRG.12550.3	coding	0.07	180	1458	1947
MSTRG.12569.1	noncoding	-0.036864	243	276	472
MSTRG.12572.1	noncoding	-0.135168	153	183	339
MSTRG.12573.1	noncoding	-0.0004096	0	0	247
MSTRG.12576.3	noncoding	-0.057344	306	369	628
MSTRG.12576.4	noncoding	-0.057344	327	390	648
MSTRG.12577.7	coding	0.159	0	306	1497
MSTRG.12577.5	coding	0.159	0	306	1641
MSTRG.12577.4	noncoding	-0.0741376	318	414	1492
MSTRG.12578.4	coding	0.301	4077	4770	5308
MSTRG.12588.1	coding	0.069	5913	9543	10685
MSTRG.12588.2	coding	0.069	6042	9672	10813
MSTRG.12588.3	coding	0.069	5913	9543	10652
MSTRG.12588.5	coding	0.069	5607	9237	10379
MSTRG.12589.1	noncoding	-0.069632	498	573	854
MSTRG.12589.2	noncoding	-0.024576	2325	2409	2802
MSTRG.12589.3	noncoding	-0.0004096	0	48	1502
MSTRG.12589.4	noncoding	-0.028672	483	603	820
MSTRG.12591.1	noncoding	-0.1081344	3012	3258	3693
MSTRG.12595.2	coding	0.108	0	465	620
MSTRG.12596.3	noncoding	-0.0950272	1551	1878	2452
MSTRG.12596.4	noncoding	-0.034816	8748	8949	9526
MSTRG.12596.5	noncoding	-0.02048	135	288	3536
MSTRG.12599.1	noncoding	-0.0512	1122	1305	4843
MSTRG.12598.4	coding	0.241	4926	5721	9348
MSTRG.12600.1	noncoding	-0.0004096	1467	1686	4065
MSTRG.12598.5	coding	0.241	4200	4995	6129
MSTRG.12598.6	noncoding	-0.086016	132	165	1516
MSTRG.12598.8	coding	0.241	1410	2205	6392
MSTRG.12598.9	coding	0.241	915	1710	5338
MSTRG.12601.1	noncoding	-0.032768	492	537	894
MSTRG.12602.1	noncoding	-0.04096	1035	1284	1752
MSTRG.12602.3	noncoding	-0.0012288	60	207	896
MSTRG.12602.2	noncoding	-0.008192	60	189	494
MSTRG.12602.4	noncoding	-0.0212992	48	264	761
MSTRG.12603.1	coding	0.241	366	798	1508

MSTRG.12603.2	coding	0.253	0	648	1389
MSTRG.12603.3	noncoding	-0.0987136	618	1050	1896
MSTRG.12603.4	coding	0.242	252	693	1277
MSTRG.12607.2	coding	0.171	1641	2376	3699
MSTRG.12607.3	coding	0.171	558	1293	2563
MSTRG.12611.5	coding	0.094	789	5943	6578
MSTRG.12611.3	coding	0.089	351	5793	6449
MSTRG.12611.7	coding	0.085	183	5928	6585
MSTRG.12612.1	noncoding	-0.036864	42	144	1533
MSTRG.12612.2	noncoding	-0.053248	459	537	2525
MSTRG.12617.2	coding	0.284	0	1407	1565
MSTRG.12617.3	coding	0.279	0	699	1569
MSTRG.12619.1	noncoding	-0.1605632	0	276	1964
MSTRG.12618.2	noncoding	-0.12288	0	72	1129
MSTRG.12618.3	noncoding	-0.0581632	1398	1578	7364
MSTRG.12619.2	noncoding	-0.16384	0	267	1867
MSTRG.12619.3	noncoding	-0.16384	0	267	1903
MSTRG.12642.1	noncoding	-0.004096	1224	1446	3471
MSTRG.12643.1	noncoding	-0.024576	321	420	4675
MSTRG.12646.3	noncoding	-0.012288	777	864	1136
MSTRG.12647.1	noncoding	-0.0098304	3102	3252	3628
MSTRG.12642.2	noncoding	-0.06144	117	180	3100
MSTRG.12648.1	noncoding	-0.0004096	0	0	1757
MSTRG.12646.5	coding	0.076	0	1398	1790
MSTRG.12646.4	coding	0.076	0	1398	2204
MSTRG.12646.6	noncoding	-0.036864	636	720	1279
MSTRG.12652.3	coding	0.162	1095	2328	2483
MSTRG.12652.4	noncoding	-0.0417792	102	177	1353
MSTRG.12623.3	coding	0.415	1347	2661	7618
MSTRG.12623.4	coding	0.422	1077	2379	7329
MSTRG.12623.6	coding	0.413	7599	8982	9662
MSTRG.12623.5	coding	0.429	0	1416	2096
MSTRG.12625.2	coding	0.033	885	1422	1585
MSTRG.12625.3	noncoding	-0.012288	1002	1143	1296
MSTRG.12627.1	coding	0.232	144	1101	1469
MSTRG.12627.2	coding	0.241	195	1047	2362
MSTRG.12633.2	coding	0.394	0	438	3025
MSTRG.12635.1	coding	0.075	69	2376	4392
MSTRG.12655.1	coding	0.275	0	270	468
MSTRG.12660.1	coding	0.052	462	1158	1529
MSTRG.12661.2	coding	0.11	162	876	1142
MSTRG.12664.1	coding	0.208	591	954	2568
MSTRG.12674.3	coding	0.196	351	780	950
MSTRG.12676.1	coding	0.157	417	6609	8622
MSTRG.12677.2	coding	0.081	747	1305	2056
MSTRG.12680.1	noncoding	-0.0208896	3	165	318
MSTRG.12681.2	coding	0.266	0	2370	2630
MSTRG.12682.1	coding	0.079	873	1635	1788
MSTRG.12685.1	noncoding	-0.0401408	4107	4434	6955
MSTRG.12685.2	noncoding	-0.069632	0	441	5199
MSTRG.12686.1	noncoding	-0.0004096	0	0	362
MSTRG.12683.2	coding	0.064	0	1401	2892
MSTRG.12709.4	coding	0.072	2067	2379	2543
MSTRG.12711.1	noncoding	-0.0086016	1053	1350	5705

MSTRG.12711.2	noncoding	-0.0704512	13215	13437	23852
MSTRG.12711.3	coding	0.129	7428	7731	23540
MSTRG.12709.6	noncoding	-0.008192	4017	4083	6235
MSTRG.12713.1	noncoding	-0.0036864	6861	6948	7513
MSTRG.12715.1	coding	0.118	600	891	11289
MSTRG.12701.1	noncoding	-0.0004096	0	0	224
MSTRG.12716.1	coding	0.182	81	2703	4592
MSTRG.12716.2	coding	0.174	81	1791	4483
MSTRG.12716.3	coding	0.205	2790	4485	4862
MSTRG.12717.1	noncoding	-0.0274432	474	564	2546
MSTRG.12716.4	coding	0.075	6492	6747	9199
MSTRG.12718.1	noncoding	-0.032768	1884	2001	3824
MSTRG.12716.5	noncoding	-0.12288	0	42	1502
MSTRG.12705.2	noncoding	-0.1650688	0	60	265
MSTRG.12705.4	noncoding	-0.0372736	264	369	1367
MSTRG.12705.3	coding	0.066	264	7122	7677
MSTRG.12705.5	coding	0.087	186	3204	3704
MSTRG.12730.2	noncoding	-0.0688128	1656	1857	2060
MSTRG.12731.3	noncoding	-0.0118784	2772	2937	3091
MSTRG.12731.2	coding	0.155	1194	2289	5635
MSTRG.12731.4	noncoding	-0.032768	2079	2184	3291
MSTRG.12731.6	coding	0.129	471	1233	6719
MSTRG.12731.7	coding	0.176	738	1176	6661
MSTRG.12731.8	coding	0.129	471	1233	5640
MSTRG.12720.2	noncoding	-0.0487424	612	978	1277
MSTRG.12721.3	coding	0.067	309	1257	1897
MSTRG.12722.1	noncoding	-0.0004096	0	0	330
MSTRG.12725.1	noncoding	-0.0004096	0	0	420
MSTRG.12725.2	noncoding	-0.0004096	0	0	425
MSTRG.12738.3	coding	0.083	210	861	1017
MSTRG.12746.1	coding	0.049	48	705	1569
MSTRG.12759.1	noncoding	-0.026624	6	27	331
MSTRG.12768.1	noncoding	-0.036864	618	624	786
MSTRG.12769.1	noncoding	-0.028672	24	126	1218
MSTRG.12791.1	coding	0.044	1584	5889	7581
MSTRG.12791.2	coding	0.044	1605	5910	6889
MSTRG.12792.1	coding	0.232	120	16674	18091
MSTRG.12792.2	coding	0.232	300	16854	18269
MSTRG.12792.3	coding	0.232	546	17100	18130
MSTRG.12792.6	noncoding	-0.0008192	726	855	1882
MSTRG.12793.2	noncoding	-0.008192	18	156	1978
MSTRG.12794.1	noncoding	-0.0229376	486	864	1021
MSTRG.12794.2	coding	0.206	0	120	648
MSTRG.12777.1	coding	0.247	138	1467	4425
MSTRG.12778.1	noncoding	-0.0909312	0	231	1858
MSTRG.12784.1	noncoding	-0.376832	177	186	596
MSTRG.12785.2	coding	0.192	5523	5730	5971
MSTRG.12785.3	coding	0.192	6804	7011	7237
MSTRG.12785.4	coding	0.192	5127	5334	5574
MSTRG.12789.3	coding	0.047	339	1722	4282
MSTRG.12800.1	coding	0.35	0	327	1198
MSTRG.12800.2	coding	0.351	0	300	1046
MSTRG.12800.3	coding	0.351	0	300	1050
MSTRG.12804.2	noncoding	-0.1007616	1461	2115	2271

MSTRG.12805.3	coding	0.202	1014	1644	1798
MSTRG.12806.2	coding	0.132	297	1578	2497
MSTRG.12806.1	coding	0.151	0	1413	2882
MSTRG.12806.4	coding	0.124	69	1665	2584
MSTRG.12806.5	coding	0.133	417	1803	3273
MSTRG.12808.2	coding	0.024	168	1050	1597
MSTRG.12810.1	noncoding	-0.032768	3960	4080	12164
MSTRG.12813.1	coding	0.062	807	1203	2631
MSTRG.12816.1	noncoding	-0.0004096	327	471	1115
MSTRG.12829.1	coding	0.141	0	1698	7464
MSTRG.12829.6	coding	0.114	237	1845	3597
MSTRG.12829.4	coding	0.125	0	1767	2295
MSTRG.12829.7	coding	0.097	12	1386	3136
MSTRG.12830.4	coding	0.019	150	2547	2860
MSTRG.12831.1	noncoding	-0.0057344	3747	3927	4084
MSTRG.12835.1	noncoding	-0.0004096	0	0	293
MSTRG.12826.1	noncoding	-0.014336	942	1455	1610
MSTRG.12826.2	noncoding	-0.0405504	1266	1605	1844
MSTRG.12827.1	coding	0.149	0	129	329
MSTRG.12839.2	coding	0.23	84	909	2292
MSTRG.12847.3	coding	0.121	45	2139	2294
MSTRG.12847.4	coding	0.12	45	2133	2288
MSTRG.12848.1	noncoding	-0.086016	966	1017	1419
MSTRG.12851.2	coding	0.116	66	1359	2970
MSTRG.12851.3	coding	0.113	174	963	2431
MSTRG.12863.6	coding	0.264	1998	4716	6078
MSTRG.12863.7	coding	0.268	2154	4875	6238
MSTRG.12863.14	coding	0.263	1941	4806	5872
MSTRG.12863.16	coding	0.264	2073	4941	6008
MSTRG.12863.11	coding	0.266	789	3525	4590
MSTRG.12863.13	coding	0.265	1071	3804	4870
MSTRG.12863.15	coding	0.227	1104	3147	4213
MSTRG.12863.12	coding	0.264	72	2979	4045
MSTRG.12863.18	coding	0.264	90	3126	3411
MSTRG.12863.17	coding	0.226	1182	3222	3509
MSTRG.12863.19	noncoding	-0.036864	5916	6042	7169
MSTRG.12864.1	noncoding	-0.008192	4149	4284	6043
MSTRG.12863.20	noncoding	-0.118784	162	165	465
MSTRG.12888.1	noncoding	-0.024576	222	345	3284
MSTRG.12890.2	noncoding	-0.043008	2610	2805	2959
MSTRG.12890.4	noncoding	-0.135168	237	291	2282
MSTRG.12907.3	coding	0.081	33	1236	2814
MSTRG.12907.2	coding	0.073	81	495	6797
MSTRG.12907.4	coding	0.078	1026	1704	2580
MSTRG.12907.5	noncoding	-0.0278528	2331	2454	3373
MSTRG.12907.6	coding	0.089	33	474	5679
MSTRG.12912.3	coding	0.093	831	3930	4084
MSTRG.12912.4	coding	0.176	243	1503	3583
MSTRG.12914.1	noncoding	-0.0495616	0	189	900
MSTRG.12933.4	coding	0.299	1335	2016	3603
MSTRG.12933.6	coding	0.291	2064	3171	4757
MSTRG.12933.5	coding	0.219	570	2022	3609
MSTRG.12933.3	coding	0.215	4341	5973	7558
MSTRG.12933.7	coding	0.215	4464	6099	7684

MSTRG.12933.8	coding	0.263	249	2295	3880
MSTRG.12934.1	noncoding	-0.032768	507	573	2128
MSTRG.12919.1	noncoding	-0.008192	342	420	1708
MSTRG.12920.1	coding	0.091	132	1581	1791
MSTRG.12920.2	coding	0.093	444	1932	2140
MSTRG.12925.1	noncoding	-0.073728	111	183	348
MSTRG.12925.2	noncoding	-0.1093632	0	12	284
MSTRG.12926.1	noncoding	-0.0004096	0	0	302
MSTRG.12938.1	noncoding	-0.0618496	48	330	1043
MSTRG.12947.3	coding	0.233	231	4800	6974
MSTRG.12947.2	coding	0.235	519	4956	7130
MSTRG.12971.1	coding	0.063	8187	9999	12458
MSTRG.12978.1	coding	0.054	69	231	746
MSTRG.12978.2	noncoding	-0.028672	246	354	18716
MSTRG.12978.3	noncoding	-0.024576	7824	7923	12222
MSTRG.12983.5	coding	0.082	0	2247	2596
MSTRG.12984.1	noncoding	-0.3117056	165	177	378
MSTRG.12939.1	coding	0.491	0	1728	5109
MSTRG.12939.2	coding	0.326	105	2160	3184
MSTRG.12939.3	coding	0.461	1059	2394	3418
MSTRG.12940.1	noncoding	-0.131072	930	990	2957
MSTRG.12939.4	coding	0.466	450	1797	3151
MSTRG.12939.5	coding	0.465	3540	4812	5835
MSTRG.12988.1	coding	0.128	4713	5079	5831
MSTRG.13003.1	noncoding	-0.08192	963	1020	2893
MSTRG.13008.1	noncoding	-0.0004096	663	804	2202
MSTRG.13008.2	noncoding	-0.02048	1482	1617	1978
MSTRG.13009.1	noncoding	-0.0004096	558	603	1894
MSTRG.13010.1	noncoding	-0.147456	30	72	228
MSTRG.13012.1	noncoding	-0.0057344	0	93	1092
MSTRG.13013.1	coding	0.013	2058	2148	3299
MSTRG.13013.2	noncoding	-0.0782336	12942	13290	13443
MSTRG.13013.6	coding	0.362	0	90	853
MSTRG.13013.4	coding	0.46	0	105	8489
MSTRG.13013.5	coding	0.252	0	288	7190
MSTRG.13014.1	noncoding	-0.0167936	828	978	1225
MSTRG.13022.1	noncoding	-0.0663552	4689	4902	27763
MSTRG.13022.2	noncoding	-0.0028672	1428	1557	4485
MSTRG.13025.2	noncoding	-0.004096	5121	5256	24787
MSTRG.13025.4	coding	0.278	53049	54117	60730
MSTRG.13025.5	noncoding	-0.06144	8259	8358	12443
MSTRG.13025.7	noncoding	-0.038912	5970	6234	12028
MSTRG.13025.8	noncoding	-0.038912	6693	6957	12361
MSTRG.13025.9	coding	0.205	11175	12909	19710
MSTRG.13025.10	noncoding	-0.006144	837	1137	6661
MSTRG.13025.11	coding	0.278	10278	11346	18149
MSTRG.13025.12	noncoding	-0.0786432	4887	5265	7247
MSTRG.13030.3	coding	0.193	435	3465	3967
MSTRG.13030.1	coding	0.2	435	10677	12150
MSTRG.13034.1	noncoding	-0.0004096	0	0	287
MSTRG.13034.2	noncoding	-0.0004096	132	132	417
MSTRG.13034.3	coding	0.031	72	156	412
MSTRG.13040.4	coding	0.106	633	5634	7808
MSTRG.13040.6	coding	0.108	369	5433	5955

MSTRG.13039.1	noncoding	-0.036864	72	81	238
MSTRG.13048.1	coding	0.074	0	3495	9162
MSTRG.13049.1	noncoding	-0.036864	168	195	892
MSTRG.13055.1	noncoding	-0.0036864	1062	1125	1968
MSTRG.13053.1	noncoding	-0.0004096	0	0	565
MSTRG.13056.2	coding	0.306	0	1152	3764
MSTRG.13061.1	coding	0.057	1410	2436	3057
MSTRG.13061.3	coding	0.057	8760	9786	10573
MSTRG.13062.1	noncoding	-0.0094208	321	456	2397
MSTRG.13061.5	noncoding	-0.0458752	0	102	3296
MSTRG.13060.1	noncoding	-0.0540672	0	114	2265
MSTRG.13063.1	coding	0.563	0	84	1895
MSTRG.13065.2	coding	0.093	177	519	1165
MSTRG.13066.3	coding	0.064	90	987	1696
MSTRG.13068.1	noncoding	-0.0004096	0	0	253
MSTRG.13077.4	coding	0.21	0	690	1830
MSTRG.13078.1	noncoding	-0.016384	558	678	1142
MSTRG.13079.2	coding	0.483	0	933	1146
MSTRG.13082.3	noncoding	-0.0405504	426	534	688
MSTRG.13082.4	noncoding	-0.0253952	36	240	1076
MSTRG.13082.2	coding	0.09	165	810	1313
MSTRG.13087.1	coding	0.359	372	5610	8486
MSTRG.13087.2	coding	0.35	111	5505	8382
MSTRG.13087.4	coding	0.352	2823	7800	10676
MSTRG.13087.6	coding	0.351	591	5484	8355
MSTRG.13091.2	noncoding	-0.0995328	3	1062	9218
MSTRG.13096.2	noncoding	-0.004096	633	765	4805
MSTRG.13106.2	coding	0.258	0	26739	27569
MSTRG.13106.1	coding	0.258	75	26925	27755
MSTRG.13106.3	coding	0.259	75	26649	27747
MSTRG.13107.1	noncoding	-0.0524288	546	846	1003
MSTRG.13100.4	coding	0.12	234	630	1024
MSTRG.13110.4	coding	0.075	0	4218	7654
MSTRG.13110.3	coding	0.074	0	4224	5840
MSTRG.13110.5	coding	0.074	0	4224	4920
MSTRG.13110.6	coding	0.064	330	2430	3600
MSTRG.13110.7	coding	0.037	96	3678	4504
MSTRG.13111.1	noncoding	-0.012288	966	1059	1287
MSTRG.13112.1	noncoding	-0.028672	270	351	2596
MSTRG.13115.1	coding	0.324	705	5715	7305
MSTRG.13117.1	noncoding	-0.006144	54	126	442
MSTRG.13115.2	coding	0.324	186	5196	6807
MSTRG.13118.2	noncoding	-0.0913408	1014	1452	1768
MSTRG.13119.1	noncoding	-0.008192	8403	8490	9309
MSTRG.13120.1	noncoding	-0.0004096	0	0	938
MSTRG.13118.4	coding	0.117	0	312	2585
MSTRG.13118.5	noncoding	-0.0315392	0	204	1808
MSTRG.13143.4	coding	0.269	0	6648	8153
MSTRG.13143.6	coding	0.233	0	2226	4991
MSTRG.13143.5	coding	0.278	0	5427	5581
MSTRG.13143.7	coding	0.284	0	5367	6870
MSTRG.13144.1	noncoding	-0.0131072	363	522	12965
MSTRG.13143.8	coding	0.268	2058	7455	7608
MSTRG.13143.9	coding	0.272	543	5793	5947

MSTRG.13145.1	noncoding	-0.0262144	0	66	972
MSTRG.13146.1	noncoding	-0.0745472	0	93	4938
MSTRG.13143.10	noncoding	-0.022528	291	333	840
MSTRG.13148.1	noncoding	-0.0159744	2160	2307	4702
MSTRG.13149.1	noncoding	-0.0167936	3255	3321	7817
MSTRG.13150.1	noncoding	-0.0139264	375	477	3081
MSTRG.13151.1	noncoding	-0.036864	2637	2700	5709
MSTRG.13152.1	noncoding	-0.0466944	678	876	4471
MSTRG.13152.2	noncoding	-0.0466944	2040	2238	5686
MSTRG.13143.11	noncoding	-0.0331776	2826	2997	4039
MSTRG.13143.12	coding	0.307	1056	3603	3756
MSTRG.13130.1	noncoding	-0.0139264	87	240	416
MSTRG.13134.1	noncoding	-0.0004096	1551	1764	2196
MSTRG.13137.1	noncoding	-0.0466944	0	1218	2571
MSTRG.13138.3	coding	0.349	0	741	2768
MSTRG.13138.5	coding	0.39	0	465	2030
MSTRG.13138.6	coding	0.385	0	375	1079
MSTRG.13139.1	noncoding	-0.0241664	3543	4026	9449
MSTRG.13139.2	noncoding	-0.0397312	1500	1713	5368
MSTRG.13139.3	noncoding	-0.0290816	9324	9516	13889
MSTRG.13139.4	noncoding	-0.0221184	399	549	7270
MSTRG.13139.5	coding	0.122	5382	5673	22132
MSTRG.13128.2	coding	0.311	528	1353	3036
MSTRG.13128.3	coding	0.291	183	1113	2743
MSTRG.13128.4	coding	0.299	393	1200	2829
MSTRG.13154.1	noncoding	-0.0241664	465	588	844
MSTRG.13154.2	noncoding	-0.04096	0	21	388
MSTRG.13154.3	noncoding	-0.014336	135	513	1007
MSTRG.13154.4	noncoding	-0.0196608	60	528	1029
MSTRG.13155.2	coding	0.141	426	2040	2395
MSTRG.13162.1	noncoding	-0.0331776	1638	1875	3269
MSTRG.13163.1	noncoding	-0.03072	813	1104	9280
MSTRG.13164.1	coding	0.093	20598	20847	23923
MSTRG.13161.6	coding	0.48	1257	4326	6216
MSTRG.13161.4	coding	0.397	0	3969	6057
MSTRG.13161.7	coding	0.504	0	2622	6196
MSTRG.13161.5	coding	0.505	0	4203	13916
MSTRG.13165.1	noncoding	-0.0344064	129	285	439
MSTRG.13159.2	coding	0.313	0	1665	4674
MSTRG.13159.3	coding	0.278	99	1623	4633
MSTRG.13159.4	coding	0.284	513	2049	3551
MSTRG.13166.1	coding	0.232	534	2352	2756
MSTRG.13170.3	noncoding	-0.075776	81	402	607
MSTRG.13171.1	coding	0.302	54	669	3596
MSTRG.13177.1	coding	0.147	1092	1716	2146
MSTRG.13177.4	noncoding	-0.0602112	1038	1662	3666
MSTRG.13177.5	noncoding	-0.0524288	132	1041	1471
MSTRG.13174.1	coding	0.437	0	7527	8993
MSTRG.13181.3	coding	0.294	0	3957	5365
MSTRG.13181.4	coding	0.297	0	4992	5282
MSTRG.13181.5	coding	0.289	0	3864	5112
MSTRG.13179.1	noncoding	-0.0425984	0	318	768
MSTRG.13179.3	noncoding	-0.0815104	96	441	595
MSTRG.13179.2	noncoding	-0.0663552	456	777	932

MSTRG.13180.1	noncoding	-0.0557056	1626	2106	2305
MSTRG.13184.1	coding	0.23	153	1518	3860
MSTRG.13184.2	coding	0.23	435	1800	1981
MSTRG.13199.1	coding	0.212	177	1146	1410
MSTRG.13199.2	coding	0.249	168	942	1124
MSTRG.13201.1	noncoding	-0.032768	27	66	894
MSTRG.13183.1	noncoding	-0.0200704	45	252	891
MSTRG.13206.2	noncoding	-0.0475136	0	372	526
MSTRG.13208.1	coding	0.249	0	888	13581
MSTRG.13208.4	coding	0.269	0	786	7705
MSTRG.13209.1	noncoding	-0.028672	3294	3423	12566
MSTRG.13209.2	coding	0.063	1998	2910	15529
MSTRG.13203.1	noncoding	-0.0851968	162	480	785
MSTRG.13217.1	noncoding	-0.0004096	0	0	202
MSTRG.13221.2	coding	0.253	0	870	1312
MSTRG.13222.1	coding	0.275	324	3498	4573
MSTRG.13222.2	coding	0.302	615	3504	9868
MSTRG.13222.3	coding	0.31	663	3450	7950
MSTRG.13222.6	coding	0.21	3090	3783	3946
MSTRG.13223.1	noncoding	-0.057344	1503	1626	3376
MSTRG.13224.1	noncoding	-0.0864256	1149	1377	2284
MSTRG.13222.7	noncoding	-0.014336	1962	2115	3417
MSTRG.13233.1	coding	0.531	0	2247	5182
MSTRG.13237.1	noncoding	-0.0364544	990	1083	1690
MSTRG.13241.3	coding	0.207	252	1074	1880
MSTRG.13241.4	coding	0.131	0	1605	2412
MSTRG.13241.5	coding	0.119	0	1599	2405
MSTRG.13241.6	coding	0.238	1365	1986	2794
MSTRG.13243.1	coding	0.24	6	1266	1565
MSTRG.13243.2	coding	0.317	0	444	741
MSTRG.13243.3	coding	0.316	0	435	734
MSTRG.13244.3	coding	0.39	0	1674	1981
MSTRG.13261.4	coding	0.508	0	5322	7130
MSTRG.13261.5	coding	0.507	0	5325	7133
MSTRG.13261.6	coding	0.508	0	5322	7130
MSTRG.13261.7	coding	0.507	0	5328	7136
MSTRG.13261.1	coding	0.507	0	5331	7531
MSTRG.13261.8	coding	0.489	423	5304	7112
MSTRG.13261.9	coding	0.493	165	4917	6726
MSTRG.13261.10	coding	0.495	1338	5889	7698
MSTRG.13261.11	coding	0.494	276	4827	6637
MSTRG.13261.12	coding	0.493	192	4749	6559
MSTRG.13261.13	coding	0.498	1023	5607	7415
MSTRG.13261.14	coding	0.495	1107	5679	7488
MSTRG.13263.1	noncoding	-0.0643072	972	1263	1416
MSTRG.13266.1	noncoding	-0.0823296	222	774	948
MSTRG.13267.1	noncoding	-0.045056	2823	4830	5126
MSTRG.13267.2	noncoding	-0.0987136	0	549	1251
MSTRG.13267.3	noncoding	-0.1191936	0	495	804
MSTRG.13267.4	noncoding	-0.172032	0	270	940
MSTRG.13254.2	noncoding	-0.0770048	579	759	916
MSTRG.13254.1	noncoding	-0.069632	132	183	466
MSTRG.13253.3	coding	0.351	0	897	2497
MSTRG.13253.4	coding	0.378	111	927	2687

MSTRG.13259.2	coding	0.523	0	2769	3093
MSTRG.13260.1	noncoding	-0.0671744	1821	2439	3742
MSTRG.13268.1	noncoding	-0.0581632	267	639	2438
MSTRG.13268.2	coding	0.236	165	2361	2695
MSTRG.13268.3	noncoding	-0.0835584	429	1008	1339
MSTRG.13268.4	coding	0.086	1209	1431	7702
MSTRG.13268.5	coding	0.214	33	639	964
MSTRG.13269.1	noncoding	-0.0401408	1425	1635	1957
MSTRG.13268.8	noncoding	-0.0425984	1437	1701	3986
MSTRG.13270.1	noncoding	-0.0258048	501	2124	2279
MSTRG.13273.4	coding	0.2	345	1494	1855
MSTRG.13281.1	coding	0.056	807	1695	2797
MSTRG.13281.2	noncoding	-0.0200704	12	18	271
MSTRG.13282.2	noncoding	-0.114688	0	18	231
MSTRG.13284.8	coding	0.241	0	531	4839
MSTRG.13284.6	coding	0.134	549	1842	2024
MSTRG.13284.9	noncoding	-0.032768	1812	1962	4048
MSTRG.13284.10	coding	0.189	897	1713	1895
MSTRG.13277.1	noncoding	-0.0360448	87	336	901
MSTRG.13277.2	noncoding	-0.0897024	0	243	996
MSTRG.13286.3	coding	0.28	2583	3090	3792
MSTRG.13287.1	noncoding	-0.0528384	609	708	1077
MSTRG.13303.1	coding	0.032	48	180	554
MSTRG.13305.1	noncoding	-0.151552	0	12	243
MSTRG.13314.1	coding	0.128	0	1332	15639
MSTRG.13314.3	coding	0.063	1008	1818	7923
MSTRG.13314.2	coding	0.06	0	918	6102
MSTRG.13323.1	noncoding	-0.0049152	1980	2115	2411
MSTRG.13323.2	noncoding	-0.0278528	1650	1746	1901
MSTRG.13320.3	coding	0.069	1392	3192	3889
MSTRG.13320.4	coding	0.096	7932	8436	9217
MSTRG.13320.5	coding	0.084	819	2733	3430
MSTRG.13320.6	noncoding	-0.0241664	14514	14673	14926
MSTRG.13320.7	coding	0.106	750	1176	1748
MSTRG.13320.9	noncoding	-0.0094208	2331	2427	2673
MSTRG.13324.1	noncoding	-0.0274432	4947	5154	14321
MSTRG.13325.1	noncoding	-0.08192	1419	1491	2589
MSTRG.13327.1	noncoding	-0.024576	1002	1140	4484
MSTRG.13329.1	noncoding	-0.03072	1836	1998	2182
MSTRG.13331.1	noncoding	-0.0253952	102	618	965
MSTRG.13332.1	noncoding	-0.192512	567	591	1041
MSTRG.13332.2	noncoding	-0.192512	564	588	977
MSTRG.13332.3	noncoding	-0.004096	180	285	2329
MSTRG.13334.1	noncoding	-0.053248	267	351	848
MSTRG.13334.4	noncoding	-0.0262144	1926	2055	2209
MSTRG.13318.1	coding	0.158	0	2103	2326
MSTRG.13345.1	noncoding	-0.069632	1563	1641	2559
MSTRG.13356.1	coding	0.083	510	2214	3997
MSTRG.13357.1	noncoding	-0.0094208	2280	2430	3200
MSTRG.13356.3	coding	0.075	1551	2781	4237
MSTRG.13356.4	coding	0.083	696	2400	3824
MSTRG.13356.5	coding	0.108	504	912	9374
MSTRG.13364.2	coding	0.168	135	576	4031
MSTRG.13365.1	noncoding	-0.0004096	0	132	310

MSTRG.13366.3	noncoding	-0.0188416	327	504	3279
MSTRG.13367.1	noncoding	-0.02048	0	66	440
MSTRG.13367.2	noncoding	-0.02048	0	66	426
MSTRG.13369.2	coding	0.192	585	969	1561
MSTRG.13369.4	coding	0.192	378	762	1354
MSTRG.13369.3	coding	0.192	381	765	1573
MSTRG.13378.1	noncoding	-0.0335872	597	846	1772
MSTRG.13378.2	noncoding	-0.008192	231	345	1099
MSTRG.13381.2	noncoding	-0.008192	180	318	970
MSTRG.13389.1	coding	0.127	102	2319	5551
MSTRG.13391.6	coding	0.11	555	1656	2218
MSTRG.13391.3	coding	0.089	606	2043	2607
MSTRG.13396.1	noncoding	-0.0032768	2448	2580	3253
MSTRG.13397.1	noncoding	-0.0229376	15882	16047	16732
MSTRG.13398.1	noncoding	-0.012288	4131	4197	6856
MSTRG.13401.1	noncoding	-0.03072	1281	1434	5315
MSTRG.13409.2	coding	0.249	102	4677	5574
MSTRG.13410.1	noncoding	-0.0208896	1071	1383	2171
MSTRG.13410.2	noncoding	-0.0208896	1071	1383	1976
MSTRG.13409.3	coding	0.248	732	5106	6065
MSTRG.13409.4	coding	0.256	222	4020	4917
MSTRG.13409.5	coding	0.258	60	3687	4646
MSTRG.13409.8	coding	0.253	519	3849	4745
MSTRG.13409.6	coding	0.255	609	3918	4876
MSTRG.13409.7	coding	0.256	519	3834	4794
MSTRG.13409.9	coding	0.255	456	3765	4663
MSTRG.13411.1	noncoding	-0.0385024	2514	2757	5880
MSTRG.13411.4	coding	0.181	114	2034	2341
MSTRG.13423.1	noncoding	-0.0028672	0	81	236
MSTRG.13425.1	noncoding	-0.0274432	17091	17391	19056
MSTRG.13427.1	noncoding	-0.0196608	1500	1668	9357
MSTRG.13427.2	coding	0.289	0	198	2109
MSTRG.13427.4	coding	0.289	0	198	2215
MSTRG.13427.3	noncoding	-0.0487424	1644	2034	2190
MSTRG.13427.6	coding	0.071	957	1281	1710
MSTRG.13427.8	coding	0.15	651	1215	2364
MSTRG.13427.9	noncoding	-0.0147456	1071	1257	1410
MSTRG.13427.10	noncoding	-0.0446464	993	1221	1375
MSTRG.13427.11	coding	0.129	0	231	514
MSTRG.13428.1	noncoding	-0.008192	12624	12693	13897
MSTRG.13428.2	noncoding	-0.065536	3237	3315	7330
MSTRG.13429.1	coding	0.096	5151	5358	15878
MSTRG.13430.1	noncoding	-0.0004096	1731	1887	2562
MSTRG.13431.1	noncoding	-0.008192	2727	2814	13439
MSTRG.13431.2	noncoding	-0.0380928	714	735	2707
MSTRG.13432.1	noncoding	-0.0004096	1920	2058	8932
MSTRG.13413.2	coding	0.182	0	516	815
MSTRG.13414.1	coding	0.084	222	462	2412
MSTRG.13418.1	noncoding	-0.0004096	444	522	2165
MSTRG.13420.1	coding	0.142	339	642	3499
MSTRG.13421.2	coding	0.32	117	996	4973
MSTRG.13419.1	noncoding	-0.04096	18	132	456
MSTRG.13419.2	noncoding	-0.065536	141	225	610
MSTRG.13419.3	noncoding	-0.02048	141	246	536

MSTRG.13433.1	noncoding	-0.0274432	270	447	624
MSTRG.13437.2	coding	0.078	663	1146	1836
MSTRG.13440.1	noncoding	-0.0577536	1494	1881	2267
MSTRG.13449.1	noncoding	-0.0987136	903	1344	1500
MSTRG.13450.3	coding	0.329	3	2310	4178
MSTRG.13450.2	coding	0.273	3	3339	8418
MSTRG.13451.1	noncoding	-0.0036864	81	120	4089
MSTRG.13454.2	noncoding	-0.028672	0	186	1270
MSTRG.13456.1	noncoding	-0.0069632	342	489	5479
MSTRG.13456.2	noncoding	-0.024576	6513	6597	7857
MSTRG.13457.1	noncoding	-0.073728	636	765	5339
MSTRG.13459.1	noncoding	-0.0004096	0	0	233
MSTRG.13461.6	noncoding	-0.073728	1095	1185	1342
MSTRG.13461.5	noncoding	-0.073728	2787	2877	3032
MSTRG.13462.1	noncoding	-0.0004096	366	366	593
MSTRG.13481.1	noncoding	-0.0581632	99	159	315
MSTRG.13486.2	coding	0.357	414	1098	2853
MSTRG.13492.1	noncoding	-0.1093632	0	396	1622
MSTRG.13506.1	noncoding	-0.057344	300	360	689
MSTRG.13506.2	noncoding	-0.0708608	426	984	2372
MSTRG.13506.3	noncoding	-0.0659456	84	705	2147
MSTRG.13506.4	noncoding	-0.0708608	96	654	1808
MSTRG.13510.1	coding	0.037	2115	4869	8978
MSTRG.13510.2	coding	0.037	1743	4497	6152
MSTRG.13510.3	coding	0.037	1743	4494	5873
MSTRG.13510.4	coding	0.037	2121	4872	6337
MSTRG.13510.5	coding	0.047	1743	3768	5935
MSTRG.13510.6	coding	0.041	2421	4617	6125
MSTRG.13497.2	coding	0.279	1542	3105	4025
MSTRG.13504.1	noncoding	-0.0004096	18	165	509
MSTRG.13512.4	coding	0.102	390	1044	2063
MSTRG.13512.6	coding	0.098	333	1065	2083
MSTRG.13512.3	coding	0.099	72	1167	2311
MSTRG.13512.7	coding	0.106	333	849	1993
MSTRG.13512.8	coding	0.106	450	966	2111
MSTRG.13512.9	coding	0.106	390	1188	2333
MSTRG.13518.1	coding	0.433	0	1626	6002
MSTRG.13518.3	coding	0.376	48	579	4004
MSTRG.13519.1	coding	0.165	0	840	3161
MSTRG.13520.4	coding	0.134	432	2277	2567
MSTRG.13531.1	noncoding	-0.0004096	1725	1764	2193
MSTRG.13532.1	noncoding	-0.0356352	132	297	2267
MSTRG.13532.3	noncoding	-0.012288	252	357	1171
MSTRG.13532.6	noncoding	-0.0356352	132	297	2342
MSTRG.13538.2	coding	0.363	24	1938	3369
MSTRG.13540.4	coding	0.425	0	924	1091
MSTRG.13543.1	coding	0.15	195	456	1152
MSTRG.13554.1	coding	0.066	10074	10656	11100
MSTRG.13555.1	noncoding	-0.114688	1044	1047	1242
MSTRG.13560.2	noncoding	-0.09216	0	711	1004
MSTRG.13560.3	coding	0.258	36	603	1240
MSTRG.13562.1	noncoding	-0.06144	60	363	534
MSTRG.13564.1	coding	0.347	222	3360	4119
MSTRG.13564.2	coding	0.348	249	3378	5158

MSTRG.13563.1	noncoding	-0.0651264	525	1044	1750
MSTRG.13563.2	noncoding	-0.034816	528	876	1304
MSTRG.13566.3	coding	0.174	459	4614	4799
MSTRG.13568.5	coding	0.132	948	2184	2777
MSTRG.13568.6	coding	0.127	81	1302	1893
MSTRG.13570.1	coding	0.305	60	3378	8162
MSTRG.13578.1	coding	0.185	6183	8256	13202
MSTRG.13578.4	coding	0.206	342	1887	6728
MSTRG.13578.2	coding	0.185	5886	7959	12801
MSTRG.13578.3	coding	0.185	6156	8229	13071
MSTRG.13578.6	coding	0.185	6036	8109	12951
MSTRG.13578.7	coding	0.185	6159	8232	13074
MSTRG.13578.8	coding	0.185	5943	8016	12821
MSTRG.13578.9	coding	0.185	6102	8175	12980
MSTRG.13578.10	coding	0.185	6681	8754	13508
MSTRG.13578.11	coding	0.185	5106	7179	11930
MSTRG.13579.1	noncoding	-0.0417792	1482	1587	2688
MSTRG.13580.1	noncoding	-0.0004096	0	0	577
MSTRG.13580.2	noncoding	-0.0004096	0	0	391
MSTRG.13578.12	coding	0.071	441	2418	3768
MSTRG.13578.13	coding	0.068	2760	4791	6119
MSTRG.13578.14	coding	0.069	6243	8217	9513
MSTRG.13578.15	coding	0.071	486	2463	3758
MSTRG.13578.16	coding	0.067	1794	3354	4320
MSTRG.13581.1	noncoding	-0.073728	93	159	398
MSTRG.13583.1	noncoding	-0.045056	975	1095	4186
MSTRG.13584.2	noncoding	-0.057344	822	936	1192
MSTRG.13572.3	coding	0.457	87	1764	2468
MSTRG.13573.4	coding	0.234	159	1299	2347
MSTRG.13573.5	coding	0.247	159	1239	2287
MSTRG.13573.3	coding	0.252	423	2082	2747
MSTRG.13573.7	coding	0.29	513	1926	2593
MSTRG.13573.1	coding	0.251	498	2154	2820
MSTRG.13575.2	coding	0.425	0	1347	3081
MSTRG.13576.4	coding	0.296	654	1896	2187
MSTRG.13576.3	coding	0.296	423	1665	1957
MSTRG.13576.5	coding	0.273	0	906	1855
MSTRG.13576.6	coding	0.302	1080	1656	1946
MSTRG.13576.7	coding	0.233	423	1818	2110
MSTRG.13576.8	coding	0.273	0	906	1864
MSTRG.13576.9	coding	0.27	0	1002	1711
MSTRG.13577.2	noncoding	-0.0065536	12	153	650
MSTRG.13577.1	noncoding	-0.0065536	12	153	934
MSTRG.13577.5	noncoding	-0.0028672	0	600	900
MSTRG.13577.9	noncoding	-0.02048	111	219	502
MSTRG.13577.10	noncoding	-0.02048	360	468	867
MSTRG.13577.11	noncoding	-0.012288	339	603	904
MSTRG.13577.6	noncoding	-0.012288	150	414	714
MSTRG.13577.7	noncoding	-0.1728512	0	78	729
MSTRG.13577.8	noncoding	-0.012288	222	486	786
MSTRG.13577.12	noncoding	-0.049152	360	435	673
MSTRG.13589.3	coding	0.122	0	1611	4795
MSTRG.13589.4	coding	0.15	0	270	890
MSTRG.13589.7	coding	0.473	0	780	2452

MSTRG.13589.6	coding	0.27	0	1857	10320
MSTRG.13591.1	noncoding	-0.032768	0	15	1840
MSTRG.13593.5	coding	0.201	15	1581	2425
MSTRG.13593.6	coding	0.202	510	2088	2912
MSTRG.13593.7	coding	0.22	297	1671	2516
MSTRG.13594.1	noncoding	-0.065536	2478	2556	3405
MSTRG.13593.8	coding	0.22	2301	3675	4500
MSTRG.13595.2	coding	0.347	0	978	2175
MSTRG.13595.1	coding	0.34	0	966	2231
MSTRG.13596.1	noncoding	-0.004096	678	726	1064
MSTRG.13597.1	noncoding	-0.086016	555	597	1326
MSTRG.13586.1	coding	0.158	99	4737	7327
MSTRG.13586.2	coding	0.161	0	4533	5258
MSTRG.13586.3	coding	0.16	0	4425	7014
MSTRG.13587.1	noncoding	-0.0180224	1332	1506	2988
MSTRG.13598.2	coding	0.081	1440	1632	1872
MSTRG.13599.1	coding	0.23	12	960	3702
MSTRG.13599.2	coding	0.246	0	837	1420
MSTRG.13599.3	coding	0.244	54	894	1693
MSTRG.13599.4	noncoding	-0.1040384	18	477	1778
MSTRG.13602.1	coding	0.496	381	1311	2754
MSTRG.13602.3	coding	0.495	0	1104	2549
MSTRG.13602.5	coding	0.475	0	1107	2552
MSTRG.13600.1	noncoding	-0.0540672	309	1227	2628
MSTRG.13603.1	noncoding	-0.0217088	0	201	414
MSTRG.13603.2	noncoding	-0.004096	471	594	824
MSTRG.13608.3	coding	0.313	324	2337	4939
MSTRG.13608.2	coding	0.346	0	2094	3797
MSTRG.13608.6	coding	0.333	0	2016	2332
MSTRG.13608.5	coding	0.332	0	2028	2405
MSTRG.13605.1	coding	0.348	15	2766	3198
MSTRG.13612.2	coding	0.233	156	459	807
MSTRG.13614.2	coding	0.433	414	1980	2257
MSTRG.13618.3	coding	0.418	450	1056	1428
MSTRG.13618.1	coding	0.401	870	1638	3203
MSTRG.13618.2	coding	0.401	981	1749	3793
MSTRG.13627.2	coding	0.307	744	4359	5816
MSTRG.13627.3	coding	0.307	96	3711	4930
MSTRG.13630.1	coding	0.507	0	1245	1526
MSTRG.13637.2	coding	0.603	0	1041	3378
MSTRG.13657.4	coding	0.298	240	798	3515
MSTRG.13658.8	coding	0.142	0	1701	2719
MSTRG.13666.4	noncoding	-0.0421888	855	1095	1250
MSTRG.13666.3	noncoding	-0.100352	1032	1257	1414
MSTRG.13666.5	noncoding	-0.0344064	852	1029	1186
MSTRG.13666.2	noncoding	-0.0679936	966	1296	1450
MSTRG.13667.1	coding	0.274	15	3885	4591
MSTRG.13667.5	coding	0.394	4428	5964	6670
MSTRG.13667.6	coding	0.286	0	3663	4370
MSTRG.13667.13	coding	0.394	4554	6102	6776
MSTRG.13667.12	coding	0.395	3279	4812	5518
MSTRG.13652.1	coding	0.385	771	1704	2000
MSTRG.13652.2	coding	0.385	528	1461	1752
MSTRG.13652.3	noncoding	-0.0823296	0	84	426

MSTRG.13653.2	coding	0.342	0	927	1263
MSTRG.13653.3	coding	0.327	96	630	967
MSTRG.13669.2	coding	0.32	150	1830	2260
MSTRG.13671.1	noncoding	-0.0311296	138	312	1515
MSTRG.13672.6	coding	0.321	0	1362	1711
MSTRG.13672.5	coding	0.294	0	1413	1771
MSTRG.13672.4	coding	0.328	0	1230	1592
MSTRG.13682.1	noncoding	-0.098304	72	111	932
MSTRG.13684.1	noncoding	-0.004096	153	162	334
MSTRG.13687.2	noncoding	-0.090112	21	78	387
MSTRG.13690.1	coding	0.189	507	990	1717
MSTRG.13690.2	coding	0.222	777	1272	1900
MSTRG.13690.5	coding	0.214	348	777	1380
MSTRG.13690.3	coding	0.189	342	825	1394
MSTRG.13690.4	coding	0.186	270	750	1353
MSTRG.13711.1	coding	0.239	267	8046	13519
MSTRG.13711.2	coding	0.246	120	6996	7460
MSTRG.13711.3	coding	0.151	180	12459	13601
MSTRG.13711.4	coding	0.237	171	7923	8387
MSTRG.13711.5	coding	0.239	237	8016	13491
MSTRG.13711.6	coding	0.239	24	6825	7295
MSTRG.13713.1	coding	0.166	48	1731	1930
MSTRG.13713.3	coding	0.155	48	1761	1960
MSTRG.13713.2	coding	0.156	222	1938	2137
MSTRG.13706.1	coding	0.239	705	1533	1926
MSTRG.13706.18	coding	0.218	381	1251	1643
MSTRG.13706.20	coding	0.237	591	1419	2156
MSTRG.13706.17	coding	0.219	297	1164	1918
MSTRG.13709.1	noncoding	-0.036864	4932	5022	5719
MSTRG.13706.22	coding	0.237	6984	7809	8201
MSTRG.13727.1	coding	0.277	2289	3381	4278
MSTRG.13727.2	coding	0.274	687	1776	2497
MSTRG.13727.3	coding	0.274	120	1209	2105
MSTRG.13727.4	coding	0.275	126	1215	2107
MSTRG.13725.4	coding	0.181	13563	14268	14520
MSTRG.13725.6	coding	0.093	342	528	3769
MSTRG.13725.7	coding	0.109	408	1167	7428
MSTRG.13725.10	coding	0.073	192	1122	2491
MSTRG.13725.12	coding	0.109	441	1200	2084
MSTRG.13725.13	coding	0.109	408	1167	2052
MSTRG.13729.1	noncoding	-0.135168	117	186	579
MSTRG.13714.3	coding	0.31	0	432	822
MSTRG.13717.1	noncoding	-0.016384	252	270	427
MSTRG.13723.1	coding	0.186	750	1473	14612
MSTRG.13738.1	coding	0.123	318	1155	1309
MSTRG.13746.1	noncoding	-0.098304	27	66	680
MSTRG.13746.2	noncoding	-0.110592	54	66	431
MSTRG.13755.2	coding	0.199	0	243	472
MSTRG.13755.1	coding	0.21	4632	8013	10666
MSTRG.13755.3	coding	0.21	1233	4629	7685
MSTRG.13755.5	coding	0.224	0	3351	4895
MSTRG.13755.6	coding	0.223	0	3306	5957
MSTRG.13756.1	noncoding	-0.002048	3801	3888	8521
MSTRG.13758.2	noncoding	-0.069632	3858	3969	4123

MSTRG.13758.3	noncoding	-0.0024576	11778	11931	12094
MSTRG.13758.4	noncoding	-0.0024576	1950	2103	2268
MSTRG.13750.1	noncoding	-0.032768	0	57	349
MSTRG.13760.1	coding	0.093	1590	1878	3236
MSTRG.13760.2	noncoding	-0.098304	1485	1542	2170
MSTRG.13759.2	coding	0.159	75	657	1190
MSTRG.13761.2	coding	0.342	186	6732	8413
MSTRG.13767.1	noncoding	-0.0155648	78	240	2323
MSTRG.13768.4	coding	0.153	240	4395	6001
MSTRG.13768.3	coding	0.153	474	4629	6243
MSTRG.13768.2	coding	0.153	441	4596	6228
MSTRG.13768.5	coding	0.15	441	2163	2957
MSTRG.13769.1	noncoding	-0.02048	588	720	1719
MSTRG.13768.7	coding	0.19	306	1374	3006
MSTRG.13765.1	noncoding	-0.0499712	510	807	3803
MSTRG.13765.2	noncoding	-0.0499712	381	678	3416
MSTRG.13764.1	coding	0.39	0	1125	1380
MSTRG.13780.2	noncoding	-0.0319488	114	300	1546
MSTRG.13782.1	noncoding	-0.0155648	2049	2169	5994
MSTRG.13780.3	noncoding	-0.147456	5016	5037	5589
MSTRG.13783.1	noncoding	-0.0004096	417	528	843
MSTRG.13784.1	noncoding	-0.053248	2223	2253	3620
MSTRG.13808.1	coding	0.171	423	1083	9578
MSTRG.13808.3	coding	0.156	51	846	1873
MSTRG.13808.4	coding	0.156	51	846	1479
MSTRG.13808.2	coding	0.156	51	846	5053
MSTRG.13808.5	noncoding	-0.0270336	11592	11772	30793
MSTRG.13809.1	noncoding	-0.022528	783	924	3023
MSTRG.13811.1	noncoding	-0.004096	840	969	1608
MSTRG.13819.3	coding	0.224	54	612	1350
MSTRG.13820.1	noncoding	-0.294912	339	357	633
MSTRG.13813.1	noncoding	-0.0004096	0	0	437
MSTRG.13822.1	noncoding	-0.0135168	897	993	1996
MSTRG.13829.1	noncoding	-0.04096	66	75	244
MSTRG.13831.2	coding	0.089	1182	1410	1564
MSTRG.13831.3	coding	0.113	2358	3666	3820
MSTRG.13832.1	noncoding	-0.0004096	459	630	1141
MSTRG.13831.4	coding	0.125	438	1377	2074
MSTRG.13836.1	coding	0.307	405	1614	3442
MSTRG.13837.1	noncoding	-0.131072	4707	4767	6296
MSTRG.13838.5	coding	0.28	432	3678	3930
MSTRG.13838.6	coding	0.233	2145	4200	4526
MSTRG.13834.2	coding	0.052	30	3279	3766
MSTRG.13844.2	coding	0.341	13296	14697	17341
MSTRG.13844.4	coding	0.347	0	2244	4332
MSTRG.13845.1	coding	0.121	15	1041	2037
MSTRG.13846.1	coding	0.321	279	1281	1704
MSTRG.13846.2	coding	0.306	300	1260	1684
MSTRG.13854.3	coding	0.357	0	1341	2126
MSTRG.13849.2	coding	0.389	0	819	1795
MSTRG.13858.2	coding	0.198	0	5232	6679
MSTRG.13859.1	noncoding	-0.0004096	0	0	2133
MSTRG.13862.3	noncoding	-0.0339968	3828	4083	7126
MSTRG.13862.2	coding	0.113	405	1533	4567

MSTRG.13862.4	coding	0.113	333	1461	2203
MSTRG.13862.5	noncoding	-0.0868352	90	153	1135
MSTRG.13867.1	coding	0.08	549	1890	2965
MSTRG.13870.4	coding	0.318	0	3102	3872
MSTRG.13871.3	coding	0.393	510	2994	4186
MSTRG.13871.4	coding	0.377	816	3084	3679
MSTRG.13877.3	coding	0.09	486	8868	11597
MSTRG.13877.4	coding	0.09	486	8721	11450
MSTRG.13877.5	coding	0.084	4095	8715	11446
MSTRG.13884.4	coding	0.343	18	4686	6890
MSTRG.13884.6	coding	0.347	465	5031	7299
MSTRG.13884.5	coding	0.347	465	5031	7321
MSTRG.13884.7	coding	0.347	465	5031	7317
MSTRG.13884.8	coding	0.337	33	3714	5865
MSTRG.13885.2	coding	0.439	0	330	1285
MSTRG.13885.3	coding	0.415	0	681	1238
MSTRG.13882.1	coding	0.351	72	1494	4432
MSTRG.13881.1	coding	0.381	72	840	2323
MSTRG.13886.2	coding	0.313	0	2124	4768
MSTRG.13886.5	coding	0.298	51	1509	1860
MSTRG.13888.1	coding	0.155	399	639	827
MSTRG.13900.1	noncoding	-0.0004096	72	192	420
MSTRG.13902.1	coding	0.243	150	558	1100
MSTRG.13903.3	coding	0.096	1659	5427	8137
MSTRG.13904.1	noncoding	-0.110592	156	174	375
MSTRG.13908.1	coding	0.184	0	13383	17807
MSTRG.13908.2	coding	0.167	3684	15108	16660
MSTRG.13910.2	coding	0.255	3513	7074	7793
MSTRG.13910.5	coding	0.131	1797	5217	6489
MSTRG.13910.6	coding	0.133	534	3771	4301
MSTRG.13913.3	coding	0.059	492	3534	5468
MSTRG.13913.4	noncoding	-0.032768	2850	2946	5779
MSTRG.13891.1	coding	0.117	141	372	920
MSTRG.13899.1	noncoding	-0.049152	645	651	1414
MSTRG.13918.2	coding	0.202	768	1059	1296
MSTRG.13918.3	coding	0.239	0	321	558
MSTRG.13919.2	coding	0.356	627	1821	2782
MSTRG.13919.1	coding	0.356	1053	2247	4283
MSTRG.13940.1	noncoding	-0.0598016	2856	3723	4225
MSTRG.13941.2	noncoding	-0.118784	0	618	2032
MSTRG.13941.3	noncoding	-0.131072	0	549	1812
MSTRG.13942.1	noncoding	-0.0405504	576	762	1157
MSTRG.13942.2	noncoding	-0.053248	135	210	1709
MSTRG.13942.3	noncoding	-0.0126976	141	258	727
MSTRG.13942.4	noncoding	-0.0233472	258	516	1017
MSTRG.13942.5	noncoding	-0.0294912	126	282	437
MSTRG.13942.6	noncoding	-0.0262144	0	357	514
MSTRG.13942.7	noncoding	-0.053248	129	204	574
MSTRG.13942.10	noncoding	-0.008192	0	135	290
MSTRG.13942.11	noncoding	-0.0376832	183	321	564
MSTRG.13942.12	noncoding	-0.0131072	213	315	559
MSTRG.13942.8	noncoding	-0.02048	153	255	482
MSTRG.13942.9	noncoding	-0.0004096	213	327	571
MSTRG.13942.13	noncoding	-0.0270336	210	324	574

MSTRG.13942.17	noncoding	-0.0290816	129	321	569
MSTRG.13942.18	noncoding	-0.0004096	66	144	431
MSTRG.13942.19	noncoding	-0.065536	0	66	453
MSTRG.13942.20	noncoding	-0.008192	144	285	505
MSTRG.13942.14	noncoding	-0.0057344	177	324	576
MSTRG.13942.15	noncoding	-0.1220608	0	57	310
MSTRG.13942.16	noncoding	-0.0004096	0	144	476
MSTRG.13942.21	noncoding	-0.0114688	66	288	539
MSTRG.13942.22	noncoding	-0.0303104	129	315	541
MSTRG.13942.25	noncoding	-0.073728	0	66	450
MSTRG.13942.27	noncoding	-0.014336	63	186	431
MSTRG.13942.28	noncoding	-0.0004096	120	219	506
MSTRG.13942.29	noncoding	-0.012288	0	129	407
MSTRG.13942.30	noncoding	-0.0176128	0	150	317
MSTRG.13942.31	noncoding	-0.0036864	168	273	517
MSTRG.13942.23	noncoding	-0.06144	252	336	560
MSTRG.13942.24	noncoding	-0.0274432	198	339	583
MSTRG.13942.26	noncoding	-0.057344	237	315	540
MSTRG.13942.32	noncoding	-0.0004096	210	321	566
MSTRG.13942.33	noncoding	-0.110592	0	72	226
MSTRG.13927.1	coding	0.219	0	585	4329
MSTRG.13928.2	coding	0.039	0	2994	4413
MSTRG.13944.3	coding	0.04	591	1275	3163
MSTRG.13945.1	noncoding	-0.073728	711	756	1034
MSTRG.13946.3	coding	0.123	132	1899	2206
MSTRG.13929.2	noncoding	-0.1007616	0	393	2321
MSTRG.13930.2	noncoding	-0.008192	993	1134	3570
MSTRG.13952.1	coding	0.341	150	1227	3172
MSTRG.13952.2	coding	0.339	150	1173	1351
MSTRG.13952.3	coding	0.299	150	600	1141
MSTRG.13955.1	noncoding	-0.21504	21	27	703
MSTRG.13962.2	noncoding	-0.0458752	69	357	842
MSTRG.13964.1	noncoding	-0.0217088	0	90	515
MSTRG.13965.1	noncoding	-0.024576	186	234	573
MSTRG.13969.2	noncoding	-0.0192512	159	189	941
MSTRG.13971.1	noncoding	-0.004096	0	144	564
MSTRG.13981.1	noncoding	-0.045056	426	549	957
MSTRG.13981.2	noncoding	-0.045056	435	558	1043
MSTRG.13982.1	coding	0.26	189	609	1761
MSTRG.13983.1	noncoding	-0.0405504	1914	2037	2196
MSTRG.13984.1	coding	0.405	2388	4158	8561
MSTRG.13984.3	coding	0.387	4020	5925	9352
MSTRG.13984.4	coding	0.403	2388	4146	5030
MSTRG.13986.1	noncoding	-0.0151552	1392	1422	4276
MSTRG.13980.2	noncoding	-0.0712704	1347	1530	2438
MSTRG.14009.1	coding	0.109	3288	14670	15198
MSTRG.14009.2	coding	0.109	3273	14655	16435
MSTRG.14009.3	coding	0.11	3261	14601	15522
MSTRG.14009.4	coding	0.11	3228	14613	15160
MSTRG.14010.7	coding	0.059	2379	2625	2779
MSTRG.14010.6	coding	0.06	204	1524	1679
MSTRG.14010.8	coding	0.066	960	1182	1337
MSTRG.14010.9	coding	0.067	1560	1986	10060
MSTRG.13999.1	noncoding	-0.03072	252	633	1012

MSTRG.14003.1	noncoding	-0.0421888	0	528	862
MSTRG.14003.4	noncoding	-0.026624	165	249	424
MSTRG.14001.2	coding	0.211	0	876	1870
MSTRG.14014.1	noncoding	-0.0004096	72	72	250
MSTRG.14034.2	noncoding	-0.137216	0	141	3261
MSTRG.14035.1	noncoding	-0.012288	2433	2529	3195
MSTRG.14036.1	noncoding	-0.0339968	819	969	24748
MSTRG.14037.1	coding	0.121	1830	2061	4259
MSTRG.14036.2	noncoding	-0.0249856	12732	12882	13036
MSTRG.14038.1	noncoding	-0.0319488	156	270	2409
MSTRG.14038.2	noncoding	-0.0176128	1047	1206	6039
MSTRG.14039.1	noncoding	-0.0024576	14970	15141	24583
MSTRG.14039.2	coding	0.077	7539	8091	14630
MSTRG.14041.1	noncoding	-0.0585728	18348	18702	29071
MSTRG.14034.3	coding	0.215	1836	2976	5279
MSTRG.14034.5	noncoding	-0.045056	2364	2436	6592
MSTRG.14034.6	coding	0.217	123	1287	3602
MSTRG.14043.1	noncoding	-0.053248	1623	1683	2018
MSTRG.14026.1	coding	0.073	795	999	1305
MSTRG.14029.2	noncoding	-0.0159744	678	816	972
MSTRG.14030.1	noncoding	-0.0516096	39	192	2576
MSTRG.14059.4	coding	0.146	1293	2046	4551
MSTRG.14059.3	coding	0.146	2580	3333	4782
MSTRG.14059.5	coding	0.144	1176	1926	3374
MSTRG.14059.6	coding	0.146	1143	1896	3345
MSTRG.14060.2	noncoding	-0.0004096	0	0	772
MSTRG.14063.1	noncoding	-0.057344	126	240	1197
MSTRG.14066.3	coding	0.356	0	651	3681
MSTRG.14047.1	noncoding	-0.0282624	126	150	954
MSTRG.14050.4	coding	0.12	789	1302	1466
MSTRG.14069.1	coding	0.181	0	1104	5119
MSTRG.14069.2	coding	0.137	1599	2472	2838
MSTRG.14075.1	coding	0.079	1329	2148	3187
MSTRG.14075.6	coding	0.079	1107	1926	3938
MSTRG.14076.1	noncoding	-0.073728	153	258	1690
MSTRG.14077.1	coding	0.102	5433	6588	7032
MSTRG.14077.2	coding	0.111	0	528	880
MSTRG.14079.1	noncoding	-0.0208896	3207	3444	4130
MSTRG.14079.2	noncoding	-0.0004096	315	453	1810
MSTRG.14081.1	noncoding	-0.0110592	162	210	1976
MSTRG.14082.1	noncoding	-0.0385024	390	591	847
MSTRG.14083.1	noncoding	-0.008192	5064	5190	5587
MSTRG.14084.1	coding	0.128	3903	4176	5103
MSTRG.14085.1	coding	0.091	17847	18483	20800
MSTRG.14093.10	noncoding	-0.0868352	4680	5202	5357
MSTRG.14093.11	noncoding	-0.049152	243	345	4620
MSTRG.14093.4	noncoding	-0.049152	447	570	4843
MSTRG.14093.5	noncoding	-0.0692224	5277	5871	6027
MSTRG.14093.6	noncoding	-0.0376832	54	204	4442
MSTRG.14093.7	noncoding	-0.0376832	54	204	4479
MSTRG.14093.9	noncoding	-0.049152	420	522	4760
MSTRG.14093.12	noncoding	-0.049152	4389	4491	8766
MSTRG.14094.1	noncoding	-0.0114688	2517	2709	3934
MSTRG.14095.1	noncoding	-0.0004096	2169	2265	2474

MSTRG.14099.1	coding	0.072	1896	2463	3720
MSTRG.14100.2	coding	0.091	0	663	1616
MSTRG.14089.2	coding	0.074	4260	5259	10189
MSTRG.14109.1	noncoding	-0.0004096	0	0	562
MSTRG.14115.3	coding	0.114	0	1647	5607
MSTRG.14126.1	noncoding	-0.0004096	0	0	241
MSTRG.14127.1	coding	0.046	1164	1431	2463
MSTRG.14130.1	noncoding	-0.0086016	465	585	1699
MSTRG.14141.6	noncoding	-0.0315392	4035	4443	4597
MSTRG.14141.9	noncoding	-0.0712704	3672	3903	4057
MSTRG.14141.7	noncoding	-0.0315392	4449	4857	5012
MSTRG.14141.11	noncoding	-0.0462848	624	765	2697
MSTRG.14142.3	coding	0.264	1233	6726	13047
MSTRG.14142.4	coding	0.266	885	6303	7598
MSTRG.14143.1	noncoding	-0.0294912	6	87	431
MSTRG.14144.2	coding	0.242	591	1230	2289
MSTRG.14147.1	noncoding	-0.0237568	10977	11259	13995
MSTRG.14147.2	noncoding	-0.0253952	2886	3084	4757
MSTRG.14153.2	coding	0.267	117	660	1437
MSTRG.14153.3	coding	0.284	60	639	1417
MSTRG.14157.1	noncoding	-0.0004096	1752	1989	18927
MSTRG.14157.2	noncoding	-0.043008	25308	25503	45615
MSTRG.14157.4	noncoding	-0.043008	22764	22959	43449
MSTRG.14157.3	noncoding	-0.043008	31071	31266	50292
MSTRG.14157.5	noncoding	-0.043008	31326	31521	46747
MSTRG.14167.1	noncoding	-0.028672	117	216	1278
MSTRG.14172.1	noncoding	-0.049152	0	15	286
MSTRG.14177.1	noncoding	-0.0004096	0	0	341
MSTRG.14182.3	coding	0.028	414	567	1018
MSTRG.14183.4	noncoding	-0.032768	1272	1362	2501
MSTRG.14183.6	noncoding	-0.012288	69	183	2367
MSTRG.14183.7	noncoding	-0.012288	579	714	2493
MSTRG.14183.5	noncoding	-0.012288	579	714	1369
MSTRG.14183.1	coding	0.091	1548	1761	2028
MSTRG.14183.8	coding	0.091	1488	1701	1968
MSTRG.14186.2	coding	0.203	534	1302	1794
MSTRG.14190.2	coding	0.101	1500	4053	5941
MSTRG.14191.1	noncoding	-0.045056	81	162	828
MSTRG.14192.1	noncoding	-0.118784	117	183	337
MSTRG.14193.1	coding	0.122	381	1545	3897
MSTRG.14193.2	noncoding	-0.0581632	2784	2994	6207
MSTRG.14193.3	coding	0.155	5274	7389	7556
MSTRG.14194.3	coding	0.344	0	510	1570
MSTRG.14194.2	coding	0.327	0	525	1445
MSTRG.14189.2	coding	0.145	1773	5172	5609
MSTRG.14189.3	coding	0.142	1650	5217	5753
MSTRG.14198.1	noncoding	-0.0241664	6003	6255	12674
MSTRG.14198.2	noncoding	-0.0708608	3555	3843	5207
MSTRG.14198.3	noncoding	-0.0708608	2532	2820	6696
MSTRG.14198.4	noncoding	-0.0708608	402	690	2098
MSTRG.14198.5	noncoding	-0.0118784	702	852	3223
MSTRG.14199.3	coding	0.05	0	273	478
MSTRG.14201.4	coding	0.133	0	894	1359
MSTRG.14201.5	coding	0.404	0	255	1639

MSTRG.14201.3	coding	0.465	0	225	1752
MSTRG.14201.8	coding	0.042	519	1119	1685
MSTRG.14201.7	coding	0.042	519	1119	10677
MSTRG.14213.1	noncoding	-0.110592	96	123	554
MSTRG.14219.1	noncoding	-0.0909312	0	162	3894
MSTRG.14220.2	coding	0.412	0	513	6195
MSTRG.14223.1	coding	0.013	4884	5253	5656
MSTRG.14227.1	noncoding	-0.036864	210	315	470
MSTRG.14221.1	coding	0.293	330	1392	1850
MSTRG.14221.2	coding	0.276	0	1104	1260
MSTRG.14221.3	coding	0.297	579	1461	1615
MSTRG.14233.2	noncoding	-0.0602112	255	435	656
MSTRG.14233.3	noncoding	-0.0155648	246	408	629
MSTRG.14233.1	noncoding	-0.012288	1521	1644	2196
MSTRG.14233.4	noncoding	-0.0684032	258	429	615
MSTRG.14235.1	noncoding	-0.0131072	0	57	517
MSTRG.14251.3	noncoding	-0.0589824	471	672	1364
MSTRG.14251.4	noncoding	-0.0090112	9048	9189	9358
MSTRG.14251.5	noncoding	-0.036864	1128	1131	1834
MSTRG.14257.1	coding	0.157	23169	23517	25364
MSTRG.14249.6	noncoding	-0.110592	471	513	825
MSTRG.14249.7	noncoding	-0.0057344	51	120	841
MSTRG.14249.5	noncoding	-0.02048	717	846	1054
MSTRG.14249.8	noncoding	-0.0057344	87	156	873
MSTRG.14248.2	coding	0.256	36	270	4165
MSTRG.14266.3	coding	0.049	2196	3336	3944
MSTRG.14269.2	noncoding	-0.069632	4332	4908	5223
MSTRG.14269.4	noncoding	-0.073728	627	708	2971
MSTRG.14272.2	coding	0.033	153	1770	2936
MSTRG.14277.1	noncoding	-0.0335872	1878	2454	2608
MSTRG.14278.1	noncoding	-0.004096	1095	1203	3387
MSTRG.14287.6	coding	0.122	150	3933	4502
MSTRG.14287.7	coding	0.126	213	4029	4658
MSTRG.14287.8	coding	0.121	213	3942	4571
MSTRG.14287.10	coding	0.121	321	4050	4680
MSTRG.14287.11	coding	0.121	774	4587	5216
MSTRG.14287.12	coding	0.121	372	4101	4730
MSTRG.14287.9	coding	0.121	546	4275	4904
MSTRG.14287.13	coding	0.121	702	4515	5129
MSTRG.14288.1	noncoding	-0.0155648	1701	1863	2144
MSTRG.14287.14	coding	0.097	78	1734	2364
MSTRG.14280.1	noncoding	-0.0004096	321	465	1774
MSTRG.14281.1	noncoding	-0.004096	546	696	1162
MSTRG.14281.3	coding	0.277	0	495	1064
MSTRG.14281.4	noncoding	-0.004096	546	696	1171
MSTRG.14281.5	noncoding	-0.024576	2214	2316	3737
MSTRG.14282.1	noncoding	-0.016384	879	1038	15650
MSTRG.14282.2	noncoding	-0.016384	879	1038	2515
MSTRG.14283.1	coding	0.053	3396	3789	5451
MSTRG.14284.1	noncoding	-0.0253952	1800	1977	6317
MSTRG.14285.1	noncoding	-0.016384	144	228	1923
MSTRG.14290.1	coding	0.081	165	348	5307
MSTRG.14290.4	noncoding	-0.1253376	0	297	6505
MSTRG.14290.5	coding	0.134	2163	2523	3039

MSTRG.14290.6	noncoding	-0.124928	0	300	2304
MSTRG.14290.3	coding	0.134	2031	2391	2972
MSTRG.14290.7	coding	0.134	2154	2514	3033
MSTRG.14291.1	noncoding	-0.0221184	0	132	364
MSTRG.14304.1	noncoding	-0.008192	48	210	426
MSTRG.14304.2	noncoding	-0.0131072	234	399	615
MSTRG.14308.1	noncoding	-0.055296	570	792	3005
MSTRG.14295.1	noncoding	-0.0217088	2586	2772	4300
MSTRG.14296.2	coding	0.221	384	2310	3109
MSTRG.14297.1	coding	0.244	0	126	280
MSTRG.14298.1	noncoding	-0.14336	51	60	458
MSTRG.14310.2	coding	0.058	153	690	2276
MSTRG.14311.1	noncoding	-0.0630784	3369	3756	4007
MSTRG.14311.2	noncoding	-0.0053248	825	930	1084
MSTRG.14311.3	coding	0.051	0	291	3777
MSTRG.14311.4	noncoding	-0.043008	3303	3444	3601
MSTRG.14313.2	noncoding	-0.0397312	2808	3024	6880
MSTRG.14317.2	coding	0.443	0	219	609
MSTRG.14318.2	noncoding	-0.012288	3666	3792	3950
MSTRG.14319.1	coding	0.05	465	5013	5317
MSTRG.14319.2	coding	0.045	156	5040	5301
MSTRG.14319.4	coding	0.054	0	4428	4797
MSTRG.14319.3	coding	0.044	633	5049	5310
MSTRG.14319.12	coding	0.051	81	4134	4396
MSTRG.14319.13	coding	0.07	495	2607	2845
MSTRG.14319.14	coding	0.088	30	1806	2068
MSTRG.14320.1	noncoding	-0.01024	123	207	1962
MSTRG.14324.1	noncoding	-0.0217088	669	831	14426
MSTRG.14325.1	coding	0.458	1635	3213	4783
MSTRG.14325.2	coding	0.544	0	1113	2438
MSTRG.14326.1	noncoding	-0.0004096	0	0	322
MSTRG.14330.1	noncoding	-0.0303104	0	306	1057
MSTRG.14331.1	coding	0.041	1368	2493	5992
MSTRG.14331.3	coding	0.071	2625	2832	4301
MSTRG.14334.2	noncoding	-0.059392	90	327	2813
MSTRG.14349.2	coding	0.069	456	936	2733
MSTRG.14350.1	noncoding	-0.0282624	0	270	2845
MSTRG.14354.1	noncoding	-0.0319488	564	630	3991
MSTRG.14343.1	noncoding	-0.1585152	0	849	2574
MSTRG.14359.2	coding	0.054	387	2211	3999
MSTRG.14360.2	noncoding	-0.0856064	0	120	1335
MSTRG.14367.4	coding	0.069	6	1692	2031
MSTRG.14367.5	coding	0.068	6	1695	1973
MSTRG.14368.1	noncoding	-0.0004096	0	0	802
MSTRG.14369.1	noncoding	-0.0126976	1569	1638	4955
MSTRG.14401.1	noncoding	-0.0004096	390	390	631
MSTRG.14409.7	coding	0.161	219	20850	21313
MSTRG.14409.8	coding	0.162	207	20760	21225
MSTRG.14409.9	coding	0.161	324	20955	21876
MSTRG.14409.10	noncoding	-0.057344	1326	1410	3372
MSTRG.14409.11	coding	0.17	279	17994	18914
MSTRG.14409.12	coding	0.174	1506	17772	18692
MSTRG.14409.13	coding	0.196	177	13554	14019
MSTRG.14409.14	coding	0.196	174	13548	14466

MSTRG.14411.3	coding	0.086	1584	3255	3485
MSTRG.14411.4	coding	0.085	1578	3267	3497
MSTRG.14411.5	coding	0.086	2145	3816	4047
MSTRG.14411.6	coding	0.085	1584	3273	3502
MSTRG.14412.1	coding	0.287	0	2730	3088
MSTRG.14376.1	coding	0.122	891	1923	2350
MSTRG.14376.2	coding	0.122	960	1992	6733
MSTRG.14379.1	noncoding	-0.1798144	450	471	1308
MSTRG.14376.3	noncoding	-0.0004096	0	0	332
MSTRG.14379.2	noncoding	-0.0626688	0	129	3153
MSTRG.14380.1	coding	0.685	204	1911	2066
MSTRG.14380.2	coding	0.628	105	840	1562
MSTRG.14371.4	coding	0.094	6	1002	1222
MSTRG.14385.1	noncoding	-0.0180224	126	336	841
MSTRG.14386.1	noncoding	-0.0434176	45	54	207
MSTRG.14387.1	noncoding	-0.036864	0	108	490
MSTRG.14395.1	coding	0.057	1107	1320	4927
MSTRG.14398.1	coding	0.073	1044	1461	2245
MSTRG.14399.1	noncoding	-0.012288	2469	2553	3945
MSTRG.14419.1	noncoding	-0.0413696	2031	2070	5369
MSTRG.14421.1	noncoding	-0.0159744	324	504	854
MSTRG.14422.1	coding	0.125	0	165	1271
MSTRG.14424.1	noncoding	-0.028672	204	333	677
MSTRG.14424.2	noncoding	-0.028672	204	333	920
MSTRG.14426.2	noncoding	-0.0892928	0	147	826
MSTRG.14426.3	noncoding	-0.0888832	0	282	1217
MSTRG.14427.3	coding	0.104	780	2589	5468
MSTRG.14427.4	coding	0.119	414	2301	5179
MSTRG.14428.1	coding	0.146	2982	3240	3854
MSTRG.14429.1	noncoding	-0.0004096	159	261	804
MSTRG.14414.1	noncoding	-0.028672	393	513	4151
MSTRG.14434.1	coding	0.397	0	4218	6569
MSTRG.14435.1	coding	0.365	0	519	1126
MSTRG.14436.2	noncoding	-0.04096	237	312	518
MSTRG.14437.1	coding	0.28	0	687	1877
MSTRG.14436.4	coding	0.154	138	1200	1611
MSTRG.14437.5	coding	0.28	0	687	1462
MSTRG.14439.2	coding	0.173	12	933	2736
MSTRG.14439.3	coding	0.173	12	933	2280
MSTRG.14454.3	coding	0.303	165	6336	6492
MSTRG.14455.1	coding	0.067	1644	2106	3540
MSTRG.14461.1	noncoding	-0.0262144	1257	1287	2012
MSTRG.14440.1	coding	0.02	48	198	414
MSTRG.14441.2	coding	0.433	0	1044	2828
MSTRG.14442.1	coding	0.578	33	2844	3001
MSTRG.14442.3	coding	0.603	36	2643	3075
MSTRG.14442.4	coding	0.603	117	2808	3239
MSTRG.14442.5	coding	0.601	93	2715	3148
MSTRG.14442.6	coding	0.604	174	2850	3282
MSTRG.14445.2	noncoding	-0.159744	177	249	402
MSTRG.14445.1	noncoding	-0.049152	312	465	894
MSTRG.14445.3	noncoding	-0.1675264	0	159	477
MSTRG.14446.1	noncoding	-0.0724992	0	132	650
MSTRG.14447.1	coding	0.35	0	2889	4432

MSTRG.14448.1	noncoding	-0.024576	3570	3615	5441
MSTRG.14453.2	coding	0.313	3	2229	3957
MSTRG.14453.4	coding	0.331	228	2292	3943
MSTRG.14453.5	coding	0.322	0	1818	3527
MSTRG.14486.1	coding	0.129	471	1050	4917
MSTRG.14486.3	coding	0.129	429	1008	4875
MSTRG.14486.4	coding	0.129	438	1017	4882
MSTRG.14487.1	coding	0.647	366	2559	8511
MSTRG.14487.2	coding	0.636	87	2424	8350
MSTRG.14488.3	noncoding	-0.0004096	8991	9135	9289
MSTRG.14488.5	coding	0.178	366	1800	5074
MSTRG.14491.3	noncoding	-0.0421888	954	1155	2099
MSTRG.14491.5	noncoding	-0.0421888	504	705	2617
MSTRG.14491.8	noncoding	-0.0421888	504	705	2564
MSTRG.14491.7	noncoding	-0.0733184	2325	2670	2824
MSTRG.14491.10	noncoding	-0.0421888	20016	20217	21114
MSTRG.14491.6	noncoding	-0.0421888	7017	7218	7990
MSTRG.14491.4	noncoding	-0.0421888	1173	1374	2147
MSTRG.14491.11	noncoding	-0.0421888	213	414	1186
MSTRG.14489.1	coding	0.34	48	1236	1641
MSTRG.14489.3	coding	0.35	207	1392	1795
MSTRG.14489.4	coding	0.352	108	1296	3136
MSTRG.14492.2	noncoding	-0.0421888	66	255	4294
MSTRG.14492.3	noncoding	-0.0528384	3462	3639	8824
MSTRG.14492.4	noncoding	-0.032768	2286	2400	2634
MSTRG.14493.1	noncoding	-0.0106496	5838	5985	6843
MSTRG.14492.6	noncoding	-0.0212992	216	339	839
MSTRG.14492.5	noncoding	-0.028672	909	978	9320
MSTRG.14495.1	coding	0.222	129	663	2648
MSTRG.14508.5	noncoding	-0.2400256	0	132	4364
MSTRG.14508.7	coding	0.208	450	2058	3758
MSTRG.14508.8	coding	0.156	0	4224	4866
MSTRG.14508.4	coding	0.233	0	2247	6299
MSTRG.14508.6	coding	0.151	3153	6420	7922
MSTRG.14508.9	coding	0.214	282	1995	3555
MSTRG.14509.1	noncoding	-0.0212992	1710	1863	2599
MSTRG.14508.10	coding	0.216	474	1962	3463
MSTRG.14513.1	noncoding	-0.0118784	4140	4239	4460
MSTRG.14512.3	coding	0.31	2103	7449	7666
MSTRG.14512.4	coding	0.31	429	5775	5992
MSTRG.14515.1	noncoding	-0.0192512	1662	1827	4293
MSTRG.14515.2	noncoding	-0.069632	159	198	603
MSTRG.14515.3	noncoding	-0.032768	156	219	2310
MSTRG.14516.1	noncoding	-0.012288	312	483	778
MSTRG.14528.1	noncoding	-0.0004096	8856	8991	9916
MSTRG.14528.3	noncoding	-0.004096	300	438	3182
MSTRG.14528.5	noncoding	-0.094208	27	111	1511
MSTRG.14529.1	noncoding	-0.0659456	3201	3390	12243
MSTRG.14529.2	noncoding	-0.02048	1422	1518	8271
MSTRG.14530.1	noncoding	-0.0126976	687	840	11594
MSTRG.14529.3	noncoding	-0.0004096	4143	4290	8467
MSTRG.14529.4	noncoding	-0.04096	1365	1467	2712
MSTRG.14529.5	noncoding	-0.098304	1302	1365	1519
MSTRG.14531.1	noncoding	-0.012288	6186	6360	20672

MSTRG.14531.2	noncoding	-0.0069632	132	147	386
MSTRG.14533.1	noncoding	-0.069632	2313	2391	2795
MSTRG.14534.1	noncoding	-0.024576	23433	23598	58941
MSTRG.14534.2	noncoding	-0.004096	6465	6609	11168
MSTRG.14534.3	noncoding	-0.0106496	1374	1527	2065
MSTRG.14534.4	noncoding	-0.032768	4464	4581	5919
MSTRG.14533.2	noncoding	-0.028672	2136	2244	4796
MSTRG.14535.1	noncoding	-0.0008192	114	195	390
MSTRG.14517.3	coding	0.331	426	1473	3035
MSTRG.14517.4	coding	0.286	7257	7566	12112
MSTRG.14518.1	noncoding	-0.034816	0	99	1266
MSTRG.14519.1	noncoding	-0.1028096	0	66	247
MSTRG.14526.3	coding	0.315	627	2943	3609
MSTRG.14536.1	coding	0.145	0	165	1459
MSTRG.14538.2	coding	0.01	5304	5409	5968
MSTRG.14538.3	noncoding	-0.0557056	0	117	1144
MSTRG.14538.4	coding	0.135	4752	5508	7428
MSTRG.14538.5	coding	0.194	711	987	2350
MSTRG.14539.5	coding	0.196	381	1593	2979
MSTRG.14539.7	coding	0.19	231	1461	2210
MSTRG.14540.1	coding	0.235	282	1803	3726
MSTRG.14540.11	coding	0.25	1014	2388	3412
MSTRG.14540.9	coding	0.228	11616	13266	14290
MSTRG.14540.12	coding	0.216	126	1995	3020
MSTRG.14540.13	coding	0.223	189	1935	2960
MSTRG.14540.10	coding	0.223	732	2478	3503
MSTRG.14540.4	coding	0.221	378	2271	3296
MSTRG.14540.3	coding	0.223	234	1980	3005
MSTRG.14540.6	coding	0.223	231	1977	3001
MSTRG.14540.7	coding	0.238	4011	4863	5887
MSTRG.14540.2	coding	0.221	3351	5244	6268
MSTRG.14540.8	coding	0.221	3357	5250	6274
MSTRG.14540.14	noncoding	-0.028672	6141	6258	6450
MSTRG.14541.1	noncoding	-0.045056	900	927	1348
MSTRG.14543.5	coding	0.297	246	2922	3424
MSTRG.14544.1	coding	0.413	0	1893	2498
MSTRG.14553.1	coding	0.177	3	1977	5934
MSTRG.14553.2	coding	0.372	546	4275	6734
MSTRG.14553.3	coding	0.189	0	1896	3669
MSTRG.14558.2	coding	0.075	933	1380	2013
MSTRG.14560.3	coding	0.106	258	1173	2474
MSTRG.14560.7	noncoding	-0.08192	120	210	3460
MSTRG.14563.6	coding	0.217	1197	2445	6278
MSTRG.14563.4	coding	0.217	222	1470	5305
MSTRG.14563.5	coding	0.217	219	1467	5302
MSTRG.14564.6	noncoding	-0.057344	0	279	1803
MSTRG.14564.4	coding	0.122	453	1896	2885
MSTRG.14564.5	coding	0.096	441	1509	2685
MSTRG.14564.2	coding	0.099	441	1632	2745
MSTRG.14565.1	coding	0.215	258	1686	3277
MSTRG.14564.7	noncoding	-0.012288	138	189	346
MSTRG.14564.8	coding	0.095	357	1449	3097
MSTRG.14565.2	coding	0.234	0	1497	3297
MSTRG.14566.4	coding	0.138	2775	2991	5616

MSTRG.14567.1	noncoding	-0.135168	114	204	12577
MSTRG.14568.1	coding	0.079	4041	4503	5680
MSTRG.14569.1	noncoding	-0.0221184	354	405	634
MSTRG.14556.2	noncoding	-0.0991232	0	204	947
MSTRG.14557.1	coding	0.373	42	5817	6683
MSTRG.14574.4	coding	0.256	276	2400	7166
MSTRG.14576.2	coding	0.105	30	447	638
MSTRG.14579.4	coding	0.184	0	6345	7209
MSTRG.14580.1	coding	0.276	42	1254	1937
MSTRG.14580.3	coding	0.3	276	1203	2639
MSTRG.14570.2	coding	0.462	0	756	1404
MSTRG.14573.2	noncoding	-0.0507904	0	300	1102
MSTRG.14583.1	coding	0.426	0	552	1611
MSTRG.14593.1	coding	0.275	117	1017	1682
MSTRG.14593.3	coding	0.306	330	1980	2643
MSTRG.14593.4	coding	0.304	330	1998	3804
MSTRG.14593.5	coding	0.277	327	1299	12577
MSTRG.14593.7	coding	0.292	8994	9681	10330
MSTRG.14600.1	coding	0.228	531	1371	1908
MSTRG.14600.8	coding	0.225	639	1497	2068
MSTRG.14600.11	coding	0.179	639	3948	4683
MSTRG.14600.5	coding	0.167	639	4080	4842
MSTRG.14600.6	coding	0.191	639	3588	4323
MSTRG.14600.7	coding	0.172	639	3987	4761
MSTRG.14600.2	coding	0.167	639	4083	4892
MSTRG.14600.3	coding	0.172	639	3984	4766
MSTRG.14600.9	coding	0.185	639	3684	4493
MSTRG.14600.12	coding	0.115	4299	6120	11975
MSTRG.14601.1	noncoding	-0.045056	141	192	806
MSTRG.14600.13	noncoding	-0.03072	2472	2913	3647
MSTRG.14600.14	noncoding	-0.0274432	1563	2103	2848
MSTRG.14603.2	coding	0.263	930	2160	3303
MSTRG.14605.3	noncoding	-0.0688128	2274	2550	2886
MSTRG.14605.4	noncoding	-0.0315392	885	1047	4031
MSTRG.14605.2	coding	0.272	528	1056	1210
MSTRG.14605.5	coding	0.353	6444	6765	6920
MSTRG.14591.4	coding	0.272	345	954	1483
MSTRG.14591.5	coding	0.266	0	1047	1460
MSTRG.14589.1	noncoding	-0.0098304	234	417	701
MSTRG.14590.1	noncoding	-0.008192	30	150	411
MSTRG.14592.1	coding	0.519	21	1521	2061
MSTRG.14592.2	coding	0.518	192	1713	2253
MSTRG.14608.1	coding	0.286	891	2256	8090
MSTRG.14608.2	coding	0.286	777	2142	7975
MSTRG.14608.5	coding	0.286	789	2154	4089
MSTRG.14608.4	coding	0.286	2115	3480	5415
MSTRG.14610.1	noncoding	-0.0229376	1680	1878	2742
MSTRG.14608.7	noncoding	-0.059392	2085	2481	5154
MSTRG.14612.1	coding	0.138	105	300	1006
MSTRG.14615.1	coding	0.229	3336	4293	7039
MSTRG.14615.2	coding	0.229	3657	4614	7361
MSTRG.14615.3	coding	0.151	0	1536	4282
MSTRG.14615.5	coding	0.229	1974	2931	5678
MSTRG.14619.3	coding	0.334	360	2763	3196

MSTRG.14619.2	coding	0.305	0	2736	3170
MSTRG.14619.4	coding	0.336	366	2769	3202
MSTRG.14620.1	coding	0.383	0	1044	1488
MSTRG.14620.2	coding	0.393	0	990	1433
MSTRG.14620.3	coding	0.295	0	1317	1762
MSTRG.14620.4	coding	0.383	273	1044	1484
MSTRG.14620.5	noncoding	-0.063488	165	672	993
MSTRG.14620.6	coding	0.454	0	624	1055
MSTRG.14616.3	coding	0.416	0	1164	8345
MSTRG.14624.4	coding	0.303	138	1587	2686
MSTRG.14625.3	coding	0.234	189	1230	1536
MSTRG.14629.1	coding	0.24	0	1485	3277
MSTRG.14629.3	coding	0.211	0	1731	2218
MSTRG.14626.2	coding	0.413	156	1575	2039
MSTRG.14630.1	noncoding	-0.0004096	108	195	649
MSTRG.14633.1	noncoding	-0.086016	114	177	975
MSTRG.14638.1	noncoding	-0.0151552	0	45	372
MSTRG.14645.1	coding	0.237	471	3657	5163
MSTRG.14646.1	noncoding	-0.0196608	852	1011	2073
MSTRG.14648.2	noncoding	-0.0544768	9	255	937
MSTRG.14648.3	coding	0.317	597	2364	2527
MSTRG.14651.1	coding	0.137	357	1389	2910
MSTRG.14651.3	coding	0.137	420	1452	4399
MSTRG.14651.4	coding	0.145	438	1374	1695
MSTRG.14647.1	noncoding	-0.053248	120	129	284
MSTRG.14658.1	noncoding	-0.0847872	1161	1203	1916
MSTRG.14656.1	noncoding	-0.14336	9	36	570
MSTRG.14672.1	coding	0.058	3	204	359
MSTRG.14673.1	noncoding	-0.073728	168	210	364
MSTRG.14675.2	coding	0.032	1719	2322	5357
MSTRG.14678.1	noncoding	-0.016384	291	375	849
MSTRG.14686.1	noncoding	-0.06144	339	369	633
MSTRG.14687.1	noncoding	-0.073728	291	333	1037
MSTRG.14687.2	noncoding	-0.028672	240	291	1342
MSTRG.14688.2	coding	0.35	1128	3099	8266
MSTRG.14692.1	noncoding	-0.0303104	183	318	795
MSTRG.14692.2	noncoding	-0.0663552	288	525	1002
MSTRG.14695.1	noncoding	-0.0004096	390	558	758
MSTRG.14696.5	coding	0.506	0	243	6654
MSTRG.14696.6	noncoding	-0.1024	222	279	5272
MSTRG.14697.1	noncoding	-0.012288	1161	1287	1440
MSTRG.14699.2	noncoding	-0.0815104	18894	19530	19685
MSTRG.14699.1	noncoding	-0.0692224	10068	11328	30953
MSTRG.14699.3	noncoding	-0.0692224	11763	13023	27471
MSTRG.14699.4	noncoding	-0.0692224	13845	15105	34020
MSTRG.14699.5	noncoding	-0.0692224	12270	13530	32094
MSTRG.14699.6	noncoding	-0.0868352	18810	19500	19654
MSTRG.14701.8	noncoding	-0.0004096	687	852	1005
MSTRG.14701.4	coding	0.076	0	1644	1911
MSTRG.14701.5	coding	0.077	0	1572	1839
MSTRG.14701.7	coding	0.081	0	1920	2187
MSTRG.14701.9	coding	0.101	408	1227	1494
MSTRG.14702.1	coding	0.39	69	3897	8441
MSTRG.14703.1	noncoding	-0.0114688	1665	1788	5121

MSTRG.14707.5	coding	0.175	0	1719	2155
MSTRG.14707.4	coding	0.173	0	1722	3013
MSTRG.14707.2	coding	0.173	0	1722	3144
MSTRG.14708.2	coding	0.072	0	4635	13168
MSTRG.14708.6	coding	0.086	339	1284	6512
MSTRG.14708.7	coding	0.116	1101	1689	3935
MSTRG.14709.1	coding	0.043	3012	3915	4068
MSTRG.14710.1	noncoding	-0.02048	1284	1398	2482
MSTRG.14708.9	noncoding	-0.135168	0	762	2459
MSTRG.14711.1	noncoding	-0.0032768	2163	2235	4558
MSTRG.14708.10	noncoding	-0.0401408	225	426	2320
MSTRG.14704.2	coding	0.215	0	1446	2109
MSTRG.14704.3	coding	0.179	195	1302	1965
MSTRG.14704.4	coding	0.181	573	1947	2609
MSTRG.14706.1	noncoding	-0.0004096	1095	1230	1678
MSTRG.14715.1	coding	0.415	105	1428	7646
MSTRG.14715.6	coding	0.405	249	1581	3021
MSTRG.14715.7	coding	0.405	294	1626	3067
MSTRG.14715.8	coding	0.405	384	1716	3155
MSTRG.14716.1	noncoding	-0.0466944	438	567	1780
MSTRG.14717.1	noncoding	-0.0647168	7896	8301	14685
MSTRG.14718.1	noncoding	-0.0774144	417	795	1487
MSTRG.14719.1	noncoding	-0.0221184	2094	2499	3967
MSTRG.14720.1	noncoding	-0.0356352	4212	4776	5785
MSTRG.14721.1	noncoding	-0.0401408	12522	12783	14929
MSTRG.14722.1	noncoding	-0.0036864	3078	3225	4408
MSTRG.14723.1	noncoding	-0.0692224	0	237	2156
MSTRG.14727.1	noncoding	-0.0077824	432	606	1874
MSTRG.14727.2	noncoding	-0.0233472	114	252	874
MSTRG.14730.1	noncoding	-0.0114688	1377	1563	3175
MSTRG.14731.2	coding	0.244	96	1917	2296
MSTRG.14734.2	coding	0.479	81	1299	1636
MSTRG.14735.2	coding	0.447	735	3657	4541
MSTRG.14735.3	coding	0.413	168	3597	4480
MSTRG.14736.1	noncoding	-0.0516096	99	744	2373
MSTRG.14745.1	noncoding	-0.0258048	2358	2898	4225
MSTRG.14746.1	noncoding	-0.0483328	1857	2214	2594
MSTRG.14748.1	noncoding	-0.0540672	447	1179	1975
MSTRG.14749.1	noncoding	-0.0405504	1587	2145	2301
MSTRG.14751.1	noncoding	-0.0393216	0	300	3353
MSTRG.14750.3	coding	0.402	351	1530	1920
MSTRG.14750.2	coding	0.375	210	1797	2187
MSTRG.14750.5	coding	0.341	327	1221	1377
MSTRG.14750.4	coding	0.327	84	1386	1541
MSTRG.14750.6	noncoding	-0.0954368	858	1209	1425
MSTRG.14753.3	coding	0.36	0	408	6965
MSTRG.14754.1	noncoding	-0.1552384	348	351	513
MSTRG.14753.4	coding	0.515	0	135	2913
MSTRG.14769.4	coding	0.069	534	5715	7215
MSTRG.14771.1	coding	0.135	330	666	1584
MSTRG.14772.1	coding	0.179	993	1878	2085
MSTRG.14772.2	coding	0.175	699	1968	2176
MSTRG.14772.4	coding	0.179	816	1701	1909
MSTRG.14773.1	noncoding	-0.0258048	924	1038	4384

MSTRG.14773.2	noncoding	-0.0016384	741	882	6897
MSTRG.14775.1	noncoding	-0.090112	492	594	1412
MSTRG.14777.1	noncoding	-0.016384	6162	6222	6492
MSTRG.14778.1	noncoding	-0.008192	1263	1419	1872
MSTRG.14780.2	noncoding	-0.0561152	0	483	4929
MSTRG.14780.3	noncoding	-0.0598016	0	471	4747
MSTRG.14782.6	coding	0.115	441	1071	1653
MSTRG.14785.4	coding	0.155	0	558	874
MSTRG.14785.5	coding	0.104	0	555	873
MSTRG.14787.1	noncoding	-0.028672	147	264	834
MSTRG.14789.4	coding	0.126	0	3069	3393
MSTRG.14790.3	coding	0.152	0	1956	2175
MSTRG.14790.4	coding	0.167	117	1395	2171
MSTRG.14791.2	noncoding	-0.083968	0	126	545
MSTRG.14792.2	coding	0.35	0	288	869
MSTRG.14793.2	coding	0.184	144	1284	1530
MSTRG.14796.1	coding	0.476	0	909	1065
MSTRG.14800.1	coding	0.336	24	1092	1246
MSTRG.14801.2	coding	0.372	369	2796	3025
MSTRG.14808.2	coding	0.355	0	1890	3592
MSTRG.14808.4	coding	0.387	99	1731	3435
MSTRG.14818.2	coding	0.335	240	2814	5405
MSTRG.14818.4	coding	0.332	519	3093	4832
MSTRG.14819.1	coding	0.38	96	6192	6523
MSTRG.14821.2	coding	0.48	294	1305	3639
MSTRG.14821.3	coding	0.48	159	1170	3575
MSTRG.14821.5	coding	0.47	54	942	3293
MSTRG.14814.1	noncoding	-0.053248	117	159	315
MSTRG.14815.3	coding	0.34	189	2001	2448
MSTRG.14827.1	noncoding	-0.0446464	333	669	824
MSTRG.14828.3	coding	0.21	0	3630	4539
MSTRG.14828.4	coding	0.208	309	3693	4132
MSTRG.14828.2	coding	0.233	0	2559	4396
MSTRG.14828.6	coding	0.135	2739	3870	4907
MSTRG.14830.5	coding	0.553	657	2370	4000
MSTRG.14835.1	noncoding	-0.038912	1629	1785	2155
MSTRG.14835.2	noncoding	-0.0405504	0	498	7155
MSTRG.14834.5	noncoding	-0.0397312	978	1140	2827
MSTRG.14840.1	coding	0.119	1221	2178	9392
MSTRG.14840.5	coding	0.098	345	1809	2196
MSTRG.14833.7	coding	0.181	750	2697	3158
MSTRG.14833.3	coding	0.181	534	2481	2941
MSTRG.14841.1	coding	0.147	0	474	825
MSTRG.14842.3	noncoding	-0.0155648	198	372	934
MSTRG.14842.2	noncoding	-0.012288	570	720	903
MSTRG.14842.1	noncoding	-0.0045056	1029	1176	1691
MSTRG.14842.5	noncoding	-0.012288	462	612	876
MSTRG.14844.2	noncoding	-0.0536576	1359	1680	1834
MSTRG.14846.1	coding	0.181	0	3612	4440
MSTRG.14846.3	coding	0.178	0	3777	4223
MSTRG.14849.1	coding	0.367	0	3792	9106
MSTRG.14849.2	coding	0.365	0	3768	5082
MSTRG.14849.3	coding	0.337	72	4233	5544
MSTRG.14852.1	noncoding	-0.0053248	2271	2493	13698

MSTRG.14852.2	noncoding	-0.032768	318	453	5867
MSTRG.14853.1	noncoding	-0.0147456	48	147	312
MSTRG.14852.3	noncoding	-0.012288	351	471	1522
MSTRG.14854.2	noncoding	-0.0110592	189	432	1374
MSTRG.14854.1	noncoding	-0.0110592	189	432	1560
MSTRG.14854.3	noncoding	-0.0339968	186	405	3461
MSTRG.14854.4	noncoding	-0.0339968	186	405	3461
MSTRG.14854.5	noncoding	-0.0339968	186	405	2200
MSTRG.14855.1	coding	0.538	0	477	12426
MSTRG.14856.1	noncoding	-0.0401408	0	42	424
MSTRG.14864.1	noncoding	-0.004096	240	384	541
MSTRG.14866.2	coding	0.17	0	1761	2620
MSTRG.14866.3	coding	0.186	0	1734	2530
MSTRG.14867.5	noncoding	-0.016384	1458	1488	2545
MSTRG.14867.6	coding	0.455	0	495	9554
MSTRG.14865.2	coding	0.202	0	837	1086
MSTRG.14869.2	coding	0.367	0	3921	4760
MSTRG.14869.5	coding	0.391	0	3402	4241
MSTRG.14869.1	coding	0.368	0	3930	7323
MSTRG.14869.3	coding	0.379	0	3738	7131
MSTRG.14868.1	coding	0.217	651	1797	1962
MSTRG.14871.2	noncoding	-0.0221184	975	1149	1666
MSTRG.14871.1	noncoding	-0.0221184	975	1149	1700
MSTRG.14871.3	noncoding	-0.0221184	93	267	992
MSTRG.14872.2	coding	0.394	690	1572	2112
MSTRG.14872.3	coding	0.238	192	1164	3364
MSTRG.14873.2	coding	0.353	420	1521	3023
MSTRG.14873.3	coding	0.356	0	1374	2771
MSTRG.14875.1	noncoding	-0.012288	297	429	583
MSTRG.14883.1	noncoding	-0.135168	816	873	1402
MSTRG.14884.1	coding	0.042	1851	2580	3068
MSTRG.14885.1	noncoding	-0.0036864	195	243	632
MSTRG.14877.1	noncoding	-0.192512	78	132	425
MSTRG.14878.1	noncoding	-0.0172032	837	981	1137
MSTRG.14890.2	coding	0.132	0	651	2428
MSTRG.14892.2	noncoding	-0.086016	45	102	535
MSTRG.14905.1	coding	0.191	0	1275	1752
MSTRG.14907.2	noncoding	-0.0741376	195	666	820
MSTRG.14909.2	coding	0.19	0	903	1252
MSTRG.14909.3	noncoding	-0.0704512	168	909	1256
MSTRG.14909.4	noncoding	-0.049152	1266	1611	1766
MSTRG.14909.5	coding	0.334	24	957	1174
MSTRG.14911.1	coding	0.248	0	1197	1356
MSTRG.14913.2	coding	0.439	78	4056	4447
MSTRG.14913.3	coding	0.438	78	4077	4468
MSTRG.14921.1	noncoding	-0.043008	2367	2550	3288
MSTRG.14920.2	coding	0.39	396	1629	5415
MSTRG.14914.2	noncoding	-0.1019904	0	162	1997
MSTRG.14915.1	coding	0.189	0	153	471
MSTRG.14915.3	noncoding	-0.038912	1860	2079	2396
MSTRG.14915.4	noncoding	-0.0172032	834	1008	1327
MSTRG.14930.4	coding	0.14	51	603	946
MSTRG.14930.5	noncoding	-0.0356352	4077	4371	4714
MSTRG.14930.6	noncoding	-0.0393216	120	426	775

MSTRG.14931.1	noncoding	-0.0073728	2304	2448	3457
MSTRG.14932.1	noncoding	-0.0151552	1164	1347	3170
MSTRG.14933.1	noncoding	-0.0208896	156	657	812
MSTRG.14933.2	noncoding	-0.0438272	156	747	902
MSTRG.14939.1	noncoding	-0.028672	87	345	591
MSTRG.14940.2	coding	0.182	303	2022	2279
MSTRG.14944.1	coding	0.088	306	1134	2145
MSTRG.14945.1	coding	0.051	0	10803	11997
MSTRG.14945.5	coding	0.047	0	11166	11551
MSTRG.14945.6	coding	0.119	6798	9612	9953
MSTRG.14946.1	noncoding	-0.0180224	255	396	1374
MSTRG.14947.1	noncoding	-0.0188416	1671	1773	3467
MSTRG.14945.7	coding	0.039	0	10005	16241
MSTRG.14948.1	noncoding	-0.0004096	0	0	2904
MSTRG.14945.8	coding	0.022	270	4956	7353
MSTRG.14945.9	coding	0.04	0	6450	7532
MSTRG.14942.2	coding	0.338	162	642	1163
MSTRG.14942.3	coding	0.277	21	672	1192
MSTRG.14942.6	coding	0.335	969	1479	1998
MSTRG.14942.5	coding	0.341	0	735	1025
MSTRG.14942.4	coding	0.249	21	759	4599
MSTRG.14958.1	noncoding	-0.077824	594	675	948
MSTRG.14957.2	coding	0.222	87	1053	4368
MSTRG.14959.1	noncoding	-0.0028672	531	612	1848
MSTRG.14962.2	coding	0.583	1917	3084	5774
MSTRG.14960.1	noncoding	-0.0139264	171	180	336
MSTRG.14965.4	coding	0.379	3771	5526	5831
MSTRG.14965.3	coding	0.495	114	3132	5266
MSTRG.14965.1	coding	0.471	0	4611	6724
MSTRG.14965.2	coding	0.47	930	5439	7551
MSTRG.14965.5	coding	0.472	0	4563	6676
MSTRG.14966.1	noncoding	-0.04096	1908	2607	8481
MSTRG.14968.7	coding	0.103	6489	10428	10701
MSTRG.14968.4	coding	0.105	615	4530	4927
MSTRG.14968.5	coding	0.105	627	4542	4937
MSTRG.14968.8	coding	0.105	2268	6183	6580
MSTRG.14969.1	noncoding	-0.004096	1062	1089	1312
MSTRG.14970.1	noncoding	-0.0729088	120	351	506
MSTRG.14975.1	noncoding	-0.0868352	0	60	649
MSTRG.14986.2	noncoding	-0.083968	879	1119	2547
MSTRG.14986.3	coding	0.237	0	726	2155
MSTRG.14987.2	coding	0.333	0	3975	4611
MSTRG.14989.1	coding	0.111	0	960	1896
MSTRG.14989.2	noncoding	-0.0610304	1335	1818	1974
MSTRG.14989.3	noncoding	-0.0581632	1224	1683	1837
MSTRG.14990.1	noncoding	-0.1060864	669	1629	1783
MSTRG.14992.2	noncoding	-0.1163264	57	756	2248
MSTRG.15000.1	noncoding	-0.098304	444	516	674
MSTRG.15001.1	noncoding	-0.0258048	783	918	2500
MSTRG.15007.4	coding	0.024	0	13104	13518
MSTRG.15007.5	coding	0.026	0	13209	13363
MSTRG.15007.7	coding	0.037	1485	1740	3745
MSTRG.15007.8	coding	0.015	3258	9360	9776
MSTRG.14997.1	noncoding	-0.1499136	0	24	226

MSTRG.14998.1	noncoding	-0.0004096	858	858	1079
MSTRG.15017.1	noncoding	-0.016384	87	111	1143
MSTRG.15018.1	noncoding	-0.0282624	552	633	4151
MSTRG.15021.1	coding	0.175	0	2148	13004
MSTRG.15021.4	coding	0.127	1092	3039	6044
MSTRG.15022.1	noncoding	-0.098304	1389	1422	1812
MSTRG.15023.4	coding	0.031	732	1170	2468
MSTRG.15023.5	coding	0.031	720	1158	2154
MSTRG.15033.1	coding	0.061	939	3393	4243
MSTRG.15033.2	coding	0.061	1230	3684	4336
MSTRG.15033.5	coding	0.061	1401	3855	4086
MSTRG.15026.1	noncoding	-0.53294177	207	249	580
MSTRG.15028.1	noncoding	-0.004096	162	435	1938
MSTRG.15038.2	noncoding	-0.1339392	0	225	669
MSTRG.15046.1	coding	0.369	0	717	2180
MSTRG.15046.4	coding	0.369	0	717	2946
MSTRG.15046.5	coding	0.504	0	408	2403
MSTRG.15046.6	coding	0.504	0	408	2551
MSTRG.15046.7	coding	0.098	738	1065	2263
MSTRG.15047.1	noncoding	-0.0557056	0	1122	6794
MSTRG.15047.2	noncoding	-0.0294912	552	861	5812
MSTRG.15046.9	noncoding	-0.0258048	3306	3375	4736
MSTRG.15050.6	noncoding	-0.0352256	234	492	1500
MSTRG.15050.2	noncoding	-0.0331776	234	501	1517
MSTRG.15050.4	noncoding	-0.0282624	234	498	1175
MSTRG.15050.7	noncoding	-0.0479232	234	477	1727
MSTRG.15051.1	coding	0.04	2157	2286	2439
MSTRG.15051.2	coding	0.201	2559	2931	9590
MSTRG.15051.3	coding	0.237	2361	2751	3208
MSTRG.15051.4	coding	0.201	2553	2925	3381
MSTRG.15052.2	noncoding	-0.098304	183	189	448
MSTRG.15054.1	coding	0.231	0	204	687
MSTRG.15043.1	noncoding	-0.04096	348	408	1763
MSTRG.15039.1	noncoding	-0.036864	0	15	274
MSTRG.15055.2	coding	0.083	0	1299	8891
MSTRG.15055.4	coding	0.058	435	906	5129
MSTRG.15055.6	coding	0.049	576	1464	4326
MSTRG.15055.7	coding	0.037	495	1320	3679
MSTRG.15055.8	coding	0.049	630	1518	3060
MSTRG.15057.1	noncoding	-0.0004096	150	258	635
MSTRG.15058.1	noncoding	-0.0172032	390	576	731
MSTRG.15045.1	noncoding	-0.124928	330	333	700
MSTRG.15068.1	noncoding	-0.0712704	48	81	237
MSTRG.15072.1	noncoding	-0.004096	162	249	403
MSTRG.15072.2	noncoding	-0.008192	246	303	468
MSTRG.15080.1	noncoding	-0.0569344	1299	1422	3362
MSTRG.15083.4	coding	0.082	0	3033	3643
MSTRG.15083.3	coding	0.074	0	3174	4264
MSTRG.15083.5	noncoding	-0.1032192	0	429	3241
MSTRG.15084.1	noncoding	-0.008192	735	843	3290
MSTRG.15085.1	noncoding	-0.012288	153	288	878
MSTRG.15090.1	coding	0.453	0	1290	2720
MSTRG.15090.2	coding	0.709	0	675	17513
MSTRG.15091.1	noncoding	-0.0024576	804	942	2250

MSTRG.15092.1	noncoding	-0.008192	669	753	1958
MSTRG.15093.10	coding	0.09	0	1356	4967
MSTRG.15093.9	coding	0.083	0	1632	5353
MSTRG.15093.11	coding	0.059	570	1950	2192
MSTRG.15093.12	coding	0.062	219	1599	1840
MSTRG.15097.3	coding	0.105	2508	3366	5919
MSTRG.15097.4	noncoding	-0.077824	1893	1962	2115
MSTRG.15100.2	coding	0.079	6	2730	6492
MSTRG.15105.1	coding	0.125	450	1863	6746
MSTRG.15105.2	coding	0.117	201	1257	3127
MSTRG.15105.5	coding	0.087	267	1191	4445
MSTRG.15105.3	coding	0.126	186	1875	10841
MSTRG.15105.4	coding	0.119	186	1719	6437
MSTRG.15105.6	coding	0.121	0	1287	2499
MSTRG.15105.8	coding	0.113	5148	6456	6933
MSTRG.15105.9	noncoding	-0.077824	3546	3612	7848
MSTRG.15106.1	noncoding	-0.069632	9378	9510	10859
MSTRG.15106.2	noncoding	-0.0208896	5553	5709	8542
MSTRG.15106.3	noncoding	-0.0200704	1356	1512	3626
MSTRG.15107.1	coding	0.146	417	1788	6926
MSTRG.15108.1	coding	0.065	0	519	1574
MSTRG.15108.2	coding	0.089	0	675	1480
MSTRG.15109.1	noncoding	-0.1396736	0	66	222
MSTRG.15109.2	noncoding	-0.0987136	0	168	322
MSTRG.15105.11	noncoding	-0.008192	1683	1728	2225
MSTRG.15124.5	coding	0.118	141	1770	4675
MSTRG.15126.1	noncoding	-0.0659456	411	510	898
MSTRG.15127.5	noncoding	-0.13312	0	84	803
MSTRG.15127.4	noncoding	-0.13312	0	84	3620
MSTRG.15127.3	noncoding	-0.0045056	4248	4404	13780
MSTRG.15127.1	coding	0.103	22527	22818	23661
MSTRG.15129.1	noncoding	-0.233472	2316	2373	2971
MSTRG.15131.1	noncoding	-0.024576	1251	1299	7208
MSTRG.15132.1	coding	0.007	1185	1314	3050
MSTRG.15118.3	coding	0.057	174	867	1548
MSTRG.15119.1	noncoding	-0.172032	36	60	290
MSTRG.15141.1	noncoding	-0.0364544	375	540	6694
MSTRG.15141.2	coding	0.369	273	3105	5737
MSTRG.15141.4	coding	0.389	783	2760	4945
MSTRG.15138.1	noncoding	-0.0053248	1476	1650	1887
MSTRG.15138.2	noncoding	-0.073728	99	126	1284
MSTRG.15145.1	coding	0.302	0	693	3875
MSTRG.15146.4	noncoding	-0.06144	189	243	5788
MSTRG.15146.5	noncoding	-0.008192	69	201	1914
MSTRG.15146.6	noncoding	-0.008192	69	201	1297
MSTRG.15146.7	noncoding	-0.0114688	69	249	1154
MSTRG.15146.10	noncoding	-0.2179072	126	138	733
MSTRG.15146.13	noncoding	-0.008192	42	174	1645
MSTRG.15146.11	noncoding	-0.045056	6	279	1001
MSTRG.15146.15	noncoding	-0.008192	69	201	1462
MSTRG.15146.8	noncoding	-0.008192	69	201	1678
MSTRG.15146.14	noncoding	-0.2179072	126	138	755
MSTRG.15146.12	noncoding	-0.2179072	126	138	758
MSTRG.15147.2	noncoding	-0.147456	939	951	1292

MSTRG.15149.1	coding	0.103	1317	1935	4899
MSTRG.15151.1	coding	0.098	564	783	1019
MSTRG.15152.1	coding	0.122	927	1278	1501
MSTRG.15143.1	noncoding	-0.045056	336	519	1047
MSTRG.15156.3	coding	0.533	0	1932	2164
MSTRG.15156.4	coding	0.422	468	1164	3230
MSTRG.15153.1	coding	0.178	0	1254	1824
MSTRG.15153.2	coding	0.197	0	1119	1540
MSTRG.15167.2	coding	0.395	0	207	1059
MSTRG.15174.3	noncoding	-0.2248704	3837	4119	4973
MSTRG.15175.1	noncoding	-0.049152	3057	3132	3384
MSTRG.15176.1	noncoding	-0.0069632	111	261	2558
MSTRG.15174.4	noncoding	-0.0004096	12384	12486	14199
MSTRG.15174.5	coding	0.117	1083	1413	13283
MSTRG.15177.1	noncoding	-0.0004096	0	0	2083
MSTRG.15181.1	coding	0.23	0	2358	7800
MSTRG.15181.5	noncoding	-0.0299008	0	231	3333
MSTRG.15183.2	coding	0.171	282	645	886
MSTRG.15184.1	coding	0.197	0	780	1996
MSTRG.15184.2	coding	0.131	480	1713	3413
MSTRG.15185.1	noncoding	-0.0004096	6024	6138	6646
MSTRG.15184.3	coding	0.168	0	1758	2602
MSTRG.15184.5	coding	0.205	0	741	2028
MSTRG.15184.9	coding	0.254	0	276	768
MSTRG.15184.10	noncoding	-0.0385024	10725	11262	11418
MSTRG.15188.1	noncoding	-0.0606208	15	48	1409
MSTRG.15188.2	noncoding	-0.0004096	1251	1404	1557
MSTRG.15190.1	coding	0.257	57	1650	3920
MSTRG.15190.6	coding	0.246	57	396	3421
MSTRG.15191.1	noncoding	-0.02048	4497	4605	7421
MSTRG.15192.1	noncoding	-0.028672	6744	6855	13063
MSTRG.15194.1	noncoding	-0.034816	12909	13203	14302
MSTRG.15219.1	coding	0.297	495	5025	8178
MSTRG.15219.2	coding	0.289	234	1143	4185
MSTRG.15219.3	coding	0.309	0	4410	7295
MSTRG.15220.1	noncoding	-0.012288	0	78	240
MSTRG.15221.1	noncoding	-0.0012288	0	3	202
MSTRG.15227.1	noncoding	-0.04096	690	729	2725
MSTRG.15253.1	noncoding	-0.151552	1434	1443	1599
MSTRG.15197.1	noncoding	-0.045056	30	54	209
MSTRG.15199.1	coding	0.224	0	156	416
MSTRG.15209.3	coding	0.053	0	1170	3046
MSTRG.15210.1	noncoding	-0.0475136	8259	8442	9596
MSTRG.15210.2	noncoding	-0.0004096	930	1071	2612
MSTRG.15209.4	noncoding	-0.0622592	0	102	621
MSTRG.15212.1	noncoding	-0.1417216	0	12	231
MSTRG.15213.2	noncoding	-0.0835584	0	207	417
MSTRG.15268.1	coding	0.058	471	630	922
MSTRG.15268.2	noncoding	-0.045056	471	585	923
MSTRG.15284.1	coding	0.122	1242	1497	1933
MSTRG.15287.5	coding	0.485	0	7962	9132
MSTRG.15287.6	coding	0.522	2994	10155	11326
MSTRG.15291.1	coding	0.314	363	1227	1586
MSTRG.15296.3	noncoding	-0.012288	2322	2424	2772

MSTRG.15302.2	coding	0.073	381	5346	8977
MSTRG.15312.1	noncoding	-0.0487424	2592	2769	8711
MSTRG.15304.1	noncoding	-0.004096	582	651	1395
MSTRG.15328.2	noncoding	-0.012288	363	432	947
MSTRG.15333.1	noncoding	-0.0802816	60	75	658
MSTRG.15338.1	noncoding	-0.077824	219	309	922
MSTRG.15347.1	coding	0.033	453	963	9644
MSTRG.15353.1	coding	0.041	627	1017	2313
MSTRG.15359.2	coding	0.132	1533	2265	4802
MSTRG.15360.1	noncoding	-0.069632	6075	6186	15509
MSTRG.15360.2	coding	0.086	165	342	497
MSTRG.15362.1	noncoding	-0.049152	2733	2826	3175
MSTRG.15357.1	noncoding	-0.0712704	102	381	610
MSTRG.15368.2	coding	0.281	0	1512	1992
MSTRG.15368.3	coding	0.293	0	1401	1815
MSTRG.15375.2	coding	0.137	0	9030	10062
MSTRG.15379.2	coding	0.37	207	2313	4215
MSTRG.15381.1	coding	0.276	51	402	652
MSTRG.15381.2	coding	0.458	0	453	700
MSTRG.15381.3	coding	0.359	0	483	728
MSTRG.15380.1	noncoding	-0.0167936	306	480	990
MSTRG.15380.2	noncoding	-0.032768	543	636	851
MSTRG.15383.1	noncoding	-0.098304	225	276	431
MSTRG.15386.3	coding	0.369	249	1092	1384
MSTRG.15386.5	coding	0.344	6519	7650	7941
MSTRG.15394.2	noncoding	-0.0651264	45	312	599
MSTRG.15394.1	noncoding	-0.073728	45	342	2656
MSTRG.15399.1	noncoding	-0.077824	405	468	706
MSTRG.15405.4	coding	0.332	291	3516	4925
MSTRG.15405.6	coding	0.391	399	2430	4521
MSTRG.15402.1	noncoding	-0.0176128	336	393	647
MSTRG.15416.3	noncoding	-0.069632	12	24	291
MSTRG.15416.4	coding	0.071	885	1302	1929
MSTRG.15421.1	coding	0.079	6	1731	2559
MSTRG.15421.2	coding	0.104	2388	2874	3588
MSTRG.15422.1	noncoding	-0.02048	147	243	654
MSTRG.15423.1	noncoding	-0.024576	156	267	710
MSTRG.15426.4	coding	0.148	0	597	4400
MSTRG.15427.2	noncoding	-0.065536	243	342	909
MSTRG.15429.6	coding	0.297	0	1968	2428
MSTRG.15429.7	coding	0.284	0	2187	2647
MSTRG.15429.8	coding	0.264	0	2208	2668
MSTRG.15431.1	coding	0.377	345	5421	6386
MSTRG.15431.3	coding	0.377	3	5067	6033
MSTRG.15433.3	coding	0.162	0	1686	2884
MSTRG.15433.5	noncoding	-0.0385024	744	960	10434
MSTRG.15436.2	coding	0.304	0	1605	2137
MSTRG.15436.3	coding	0.399	0	1563	2096
MSTRG.15442.1	noncoding	-0.096256	1962	2733	3195
MSTRG.15444.2	noncoding	-0.0311296	108	174	672
MSTRG.15444.3	noncoding	-0.0221184	648	885	3217
MSTRG.15447.5	noncoding	-0.0974848	1632	1908	2745
MSTRG.15447.4	coding	0.292	627	1626	2462
MSTRG.15447.6	coding	0.275	600	1461	2298

MSTRG.15447.8	coding	0.275	609	1470	2307
MSTRG.15447.9	noncoding	-0.0589824	78	264	2434
MSTRG.15450.2	coding	0.311	867	1902	2965
MSTRG.15450.3	coding	0.272	243	1563	2578
MSTRG.15457.1	noncoding	-0.0679936	3276	3768	3925
MSTRG.15467.2	coding	0.147	66	5325	5517
MSTRG.15469.1	noncoding	-0.0380928	123	126	382
MSTRG.15470.1	noncoding	-0.0004096	0	0	201
MSTRG.15471.1	noncoding	-0.0311296	75	90	248
MSTRG.15486.2	coding	0.335	0	1725	2612
MSTRG.15486.4	coding	0.26	0	417	582
MSTRG.15490.1	noncoding	-0.0053248	948	1056	4286
MSTRG.15491.1	noncoding	-0.1114112	249	261	705
MSTRG.15492.1	noncoding	-0.0131072	19707	19857	24385
MSTRG.15492.2	noncoding	-0.016384	2988	3159	17879
MSTRG.15493.1	coding	0.07	7527	7704	9873
MSTRG.15494.1	noncoding	-0.0827392	1671	1710	4026
MSTRG.15486.5	coding	0.349	1194	2226	7387
MSTRG.15494.2	noncoding	-0.016384	1317	1356	3078
MSTRG.15497.1	coding	0.139	15651	15987	17787
MSTRG.15499.1	noncoding	-0.08192	1536	1635	5518
MSTRG.15499.2	noncoding	-0.016384	678	744	965
MSTRG.15500.1	noncoding	-0.0004096	864	1008	5984
MSTRG.15502.1	coding	0.092	2937	3408	6800
MSTRG.15503.1	noncoding	-0.032768	1596	1785	3354
MSTRG.15502.2	coding	0.129	1407	1704	2046
MSTRG.15477.1	noncoding	-0.0004096	15	15	468
MSTRG.15480.2	noncoding	-0.004096	18	123	3289
MSTRG.15532.2	coding	0.037	6564	11406	12713
MSTRG.15532.1	coding	0.037	6747	11589	12896
MSTRG.15532.4	coding	0.043	4404	6756	7549
MSTRG.15532.5	coding	0.037	5928	10770	11562
MSTRG.15532.3	coding	0.037	6105	10947	11739
MSTRG.15532.6	coding	0.039	7185	11898	12618
MSTRG.15533.1	noncoding	-0.077824	2895	2973	3794
MSTRG.15532.8	noncoding	-0.0446464	81	1029	6647
MSTRG.15534.1	noncoding	-0.0417792	477	555	1701
MSTRG.15535.1	coding	0.081	3120	3315	3584
MSTRG.15536.1	noncoding	-0.004096	1590	1701	6905
MSTRG.15535.3	coding	0.081	3033	3228	4187
MSTRG.15535.4	coding	0.081	3117	3312	4271
MSTRG.15538.1	noncoding	-0.116736	3045	3084	6229
MSTRG.15517.1	noncoding	-0.012288	2106	2475	2687
MSTRG.15518.4	coding	0.152	0	1653	2595
MSTRG.15518.5	coding	0.152	0	1653	3270
MSTRG.15518.6	coding	0.078	72	1467	4756
MSTRG.15518.8	coding	0.09	294	1587	2915
MSTRG.15518.7	coding	0.092	459	1620	2648
MSTRG.15519.1	noncoding	-0.0794624	1155	1356	1511
MSTRG.15521.2	coding	0.294	0	339	5932
MSTRG.15521.3	coding	0.097	1098	1275	3976
MSTRG.15528.3	noncoding	-0.024576	138	243	398
MSTRG.15540.2	coding	0.12	375	3792	4033
MSTRG.15540.8	coding	0.11	243	1254	2323

MSTRG.15540.7	coding	0.083	243	3273	3471
MSTRG.15547.1	noncoding	-0.0270336	3204	3366	6125
MSTRG.15547.2	noncoding	-0.0270336	2688	2850	3745
MSTRG.15547.3	noncoding	-0.0270336	3336	3498	4395
MSTRG.15548.1	coding	0.166	3933	4332	5981
MSTRG.15548.2	coding	0.163	795	1182	1336
MSTRG.15548.3	coding	0.163	621	1008	1165
MSTRG.15549.1	coding	0.197	0	177	373
MSTRG.15554.1	noncoding	-0.0311296	531	774	1885
MSTRG.15572.1	noncoding	-0.006144	216	303	1045
MSTRG.15571.3	noncoding	-0.016384	2133	2274	5703
MSTRG.15571.4	noncoding	-0.016384	408	486	1151
MSTRG.15563.1	noncoding	-0.057344	81	195	4687
MSTRG.15574.1	coding	0.099	210	567	972
MSTRG.15575.1	noncoding	-0.028672	1248	1308	1463
MSTRG.15594.4	coding	0.2	342	2166	5511
MSTRG.15595.1	coding	0.078	279	888	1719
MSTRG.15595.2	coding	0.119	324	711	2486
MSTRG.15596.2	coding	0.395	960	1878	4643
MSTRG.15596.3	coding	0.262	222	1962	4602
MSTRG.15600.2	coding	0.139	18	591	2301
MSTRG.15601.1	noncoding	-0.028672	402	594	858
MSTRG.15602.1	noncoding	-0.0004096	342	435	1772
MSTRG.15602.2	noncoding	-0.0176128	252	528	2131
MSTRG.15603.3	coding	0.081	45	1302	2969
MSTRG.15611.2	coding	0.129	162	852	1310
MSTRG.15614.2	coding	0.159	174	588	888
MSTRG.15618.1	coding	0.037	810	1527	1684
MSTRG.15619.2	coding	0.256	213	1587	3075
MSTRG.15619.3	coding	0.195	198	918	1343
MSTRG.15619.4	coding	0.234	150	852	2340
MSTRG.15625.1	coding	0.244	222	3591	12625
MSTRG.15625.2	coding	0.245	222	3594	4594
MSTRG.15628.2	coding	0.14	0	819	3037
MSTRG.15630.3	noncoding	-0.0548864	0	246	547
MSTRG.15630.2	coding	0.264	594	1221	4743
MSTRG.15638.2	coding	0.19	1152	2745	3725
MSTRG.15638.3	coding	0.19	1440	3033	3611
MSTRG.15638.4	coding	0.053	249	1386	1852
MSTRG.15638.8	coding	0.043	756	888	1044
MSTRG.15638.10	noncoding	-0.0188416	135	258	3082
MSTRG.15644.1	coding	0.282	573	2619	2970
MSTRG.15644.4	noncoding	-0.0917504	135	408	1106
MSTRG.15644.7	noncoding	-0.0917504	57	330	2051
MSTRG.15644.5	noncoding	-0.088064	57	324	1023
MSTRG.15644.6	noncoding	-0.0917504	57	330	1205
MSTRG.15650.1	noncoding	-0.028672	159	240	787
MSTRG.15651.1	coding	0.087	438	852	2886
MSTRG.15656.1	noncoding	-0.2048	1491	1566	2368
MSTRG.15671.1	noncoding	-0.0385024	243	516	870
MSTRG.15654.1	coding	0.059	465	2097	2367
MSTRG.15654.2	coding	0.054	474	2091	3056
MSTRG.15677.1	noncoding	-0.0438272	0	393	2193
MSTRG.15678.2	coding	0.12	0	948	11983

MSTRG.15679.1	noncoding	-0.0262144	2010	2040	3739
MSTRG.15678.3	coding	0.194	0	1044	2758
MSTRG.15678.4	coding	0.141	0	966	3569
MSTRG.15672.1	noncoding	-0.04096	6	105	260
MSTRG.15674.1	coding	0.332	87	840	1303
MSTRG.15675.1	noncoding	-0.0454656	276	417	652
MSTRG.15681.2	noncoding	-0.0434176	0	159	543
MSTRG.15681.3	noncoding	-0.0479232	594	720	875
MSTRG.15681.5	coding	0.173	252	1305	1679
MSTRG.15681.4	coding	0.173	492	1545	1921
MSTRG.15682.1	noncoding	-0.057344	69	147	2250
MSTRG.15683.1	noncoding	-0.053248	3162	3237	6218
MSTRG.15681.6	noncoding	-0.06144	363	420	576
MSTRG.15684.1	coding	0.159	78	825	1064
MSTRG.15681.8	noncoding	-0.0212992	486	648	804
MSTRG.15685.2	coding	0.24	234	2328	4566
MSTRG.15692.1	noncoding	-0.0094208	51	57	228
MSTRG.15694.3	noncoding	-0.1150976	18	912	19246
MSTRG.15694.1	noncoding	-0.1138688	18	912	18271
MSTRG.15694.2	noncoding	-0.1114112	18	888	18251
MSTRG.15694.4	noncoding	-0.114688	18	888	18310
MSTRG.15701.4	coding	0.298	249	1965	2350
MSTRG.15701.5	coding	0.297	0	1722	2086
MSTRG.15703.5	coding	0.267	474	1284	1604
MSTRG.15703.4	coding	0.293	474	1347	1842
MSTRG.15703.6	coding	0.292	474	1344	1839
MSTRG.15703.7	coding	0.266	474	1281	1628
MSTRG.15705.3	coding	0.28	0	1482	1646
MSTRG.15707.2	coding	0.185	114	510	873
MSTRG.15707.4	coding	0.185	132	528	851
MSTRG.15714.3	coding	0.215	0	330	1658
MSTRG.15714.4	coding	0.215	0	330	1587
MSTRG.15715.1	coding	0.324	2268	3174	3912
MSTRG.15715.5	coding	0.333	1146	2265	3001
MSTRG.15715.6	coding	0.324	1407	2313	3051
MSTRG.15719.2	coding	0.075	108	354	957
MSTRG.15720.1	coding	0.279	690	2733	3508
MSTRG.15720.2	coding	0.279	891	2934	3709
MSTRG.15720.3	coding	0.279	471	2514	3291
MSTRG.15708.1	coding	0.316	0	306	4083
MSTRG.15709.1	noncoding	-0.0733184	33	885	2210
MSTRG.15711.3	coding	0.32	0	387	575
MSTRG.15722.4	coding	0.222	669	2253	2936
MSTRG.15722.10	coding	0.212	621	2031	2434
MSTRG.15722.9	coding	0.257	0	2211	2613
MSTRG.15724.2	coding	0.308	0	2148	3112
MSTRG.15726.5	coding	0.281	93	1173	1683
MSTRG.15726.6	coding	0.279	93	1128	1594
MSTRG.15723.2	coding	0.385	51	1950	2288
MSTRG.15723.3	coding	0.388	0	1206	1558
MSTRG.15747.3	coding	0.094	60	4542	8162
MSTRG.15750.2	noncoding	-0.1257472	1596	2280	2918
MSTRG.15750.3	coding	0.433	555	2787	5430
MSTRG.15750.4	coding	0.431	0	2481	3437

MSTRG.15751.5	coding	0.448	111	1032	1404
MSTRG.15751.4	coding	0.491	1188	2271	2642
MSTRG.15752.3	coding	0.267	0	297	4788
MSTRG.15752.4	coding	0.107	603	1125	8665
MSTRG.15752.5	coding	0.107	5046	5568	9446
MSTRG.15754.2	coding	0.546	0	2748	3090
MSTRG.15756.4	coding	0.102	306	1065	2477
MSTRG.15766.3	coding	0.092	123	4320	4807
MSTRG.15766.4	coding	0.092	123	4440	4939
MSTRG.15766.10	coding	0.093	144	4371	4530
MSTRG.15766.8	coding	0.091	144	4428	4587
MSTRG.15766.9	coding	0.094	144	4374	4533
MSTRG.15767.1	noncoding	-0.012288	1323	1395	2904
MSTRG.15766.11	noncoding	-0.016384	954	1059	4456
MSTRG.15766.12	noncoding	-0.0004096	0	0	274
MSTRG.15766.13	noncoding	-0.0360448	207	225	1054
MSTRG.15768.1	noncoding	-0.02048	849	882	1452
MSTRG.15769.1	coding	0.146	1395	1695	2978
MSTRG.15773.1	noncoding	-0.077824	0	318	6448
MSTRG.15773.2	noncoding	-0.0954368	0	432	8359
MSTRG.15776.1	noncoding	-0.0499712	5454	5625	5779
MSTRG.15776.2	coding	0.109	609	1275	2522
MSTRG.15778.3	coding	0.123	111	387	2021
MSTRG.15778.2	noncoding	-0.0352256	645	894	2610
MSTRG.15781.1	noncoding	-0.0077824	0	54	1016
MSTRG.15781.5	coding	0.234	459	924	1458
MSTRG.15781.7	coding	0.17	48	441	638
MSTRG.15782.1	coding	0.075	225	1167	4182
MSTRG.15785.1	noncoding	-0.0282624	21	48	334
MSTRG.15786.1	noncoding	-0.0004096	861	1038	1213
MSTRG.15786.2	noncoding	-0.004096	6	48	242
MSTRG.15786.3	noncoding	-0.02048	1035	1143	1632
MSTRG.15788.1	noncoding	-0.036864	675	735	2033
MSTRG.15791.1	noncoding	-0.0380928	360	432	917
MSTRG.15793.1	coding	0.078	909	1254	2573
MSTRG.15795.1	noncoding	-0.0004096	0	0	343
MSTRG.15796.1	noncoding	-0.0630784	6915	7089	7244
MSTRG.15796.2	noncoding	-0.024576	2712	2820	10375
MSTRG.15796.3	noncoding	-0.024576	2616	2724	5598
MSTRG.15797.4	noncoding	-0.0004096	156	300	581
MSTRG.15797.6	noncoding	-0.0004096	156	300	1299
MSTRG.15798.1	noncoding	-0.0004096	0	0	970
MSTRG.15804.2	coding	0.428	42	1236	3188
MSTRG.15804.3	coding	0.428	42	1236	1588
MSTRG.15804.5	coding	0.432	0	1146	3021
MSTRG.15800.1	noncoding	-0.055296	2847	3549	4106
MSTRG.15801.1	coding	0.478	0	1128	2202
MSTRG.15809.10	coding	0.069	21	2325	5328
MSTRG.15810.1	noncoding	-0.0004096	0	0	295
MSTRG.15811.1	coding	0.078	105	1029	3224
MSTRG.15812.1	noncoding	-0.049152	1113	1125	2054
MSTRG.15813.2	coding	0.101	3498	3855	9314
MSTRG.15813.1	coding	0.095	3570	3852	9379
MSTRG.15813.3	coding	0.095	2949	3231	7099

MSTRG.15813.4	coding	0.095	2847	3129	7144
MSTRG.15809.11	coding	0.054	573	975	4260
MSTRG.15806.1	noncoding	-0.02048	1914	1992	2358
MSTRG.15807.1	coding	0.121	216	1005	1665
MSTRG.15816.1	noncoding	-0.096256	0	126	635
MSTRG.15816.2	noncoding	-0.0536576	0	78	547
MSTRG.15814.1	noncoding	-0.0270336	921	1017	1952
MSTRG.15817.1	coding	0.263	0	1302	1937
MSTRG.15821.1	noncoding	-0.0004096	0	0	225
MSTRG.15822.3	coding	0.269	129	1680	1874
MSTRG.15826.1	noncoding	-0.034816	540	699	953
MSTRG.15832.1	noncoding	-0.02048	36	153	351
MSTRG.15834.1	noncoding	-0.012288	225	315	483
MSTRG.15834.2	noncoding	-0.0004096	222	282	565
MSTRG.15835.1	noncoding	-0.0606208	726	816	970
MSTRG.15835.2	noncoding	-0.0352256	126	129	458
MSTRG.15841.1	coding	0.139	108	2211	3093
MSTRG.15841.2	coding	0.242	597	1407	1562
MSTRG.15843.2	coding	0.342	0	255	2749
MSTRG.15846.6	coding	0.264	51	504	1120
MSTRG.15846.5	coding	0.274	198	657	1274
MSTRG.15847.1	noncoding	-0.098304	1248	1257	1425
MSTRG.15848.1	coding	0.367	141	1074	1544
MSTRG.15848.2	coding	0.334	615	1662	2131
MSTRG.15848.3	coding	0.376	0	951	1420
MSTRG.15858.1	coding	0.04	249	363	1678
MSTRG.15858.2	coding	0.127	0	627	2155
MSTRG.15858.3	coding	0.043	189	1122	2179
MSTRG.15868.1	coding	0.333	3417	6210	6431
MSTRG.15868.6	coding	0.33	591	3372	5891
MSTRG.15868.9	coding	0.327	288	3033	5553
MSTRG.15868.7	coding	0.331	846	3675	6195
MSTRG.15868.3	coding	0.333	108	2937	5457
MSTRG.15868.10	coding	0.329	486	3258	3431
MSTRG.15867.3	noncoding	-0.1196032	81	492	1749
MSTRG.15873.2	coding	0.237	909	2160	2979
MSTRG.15874.2	coding	0.309	0	1161	1317
MSTRG.15879.2	coding	0.136	417	2187	2908
MSTRG.15886.2	coding	0.155	276	1815	4308
MSTRG.15890.1	noncoding	-0.114688	96	135	320
MSTRG.15891.2	coding	0.471	0	714	1860
MSTRG.15896.2	coding	0.316	2061	2535	2954
MSTRG.15902.5	coding	0.344	219	2796	3058
MSTRG.15902.3	coding	0.342	312	3123	3386
MSTRG.15902.4	coding	0.386	0	3087	3350
MSTRG.15902.6	coding	0.341	1008	3615	3850
MSTRG.15903.1	noncoding	-0.0520192	0	201	722
MSTRG.15897.1	coding	0.051	132	321	2102
MSTRG.15898.1	noncoding	-0.0495616	2502	2685	2839
MSTRG.15899.3	coding	0.15	0	1350	3138
MSTRG.15899.2	coding	0.128	492	1425	2197
MSTRG.15899.4	coding	0.155	0	1305	2094
MSTRG.15909.4	coding	0.143	2451	7590	9162
MSTRG.15909.1	coding	0.105	201	4188	4613

MSTRG.15909.6	coding	0.143	87	5226	6799
MSTRG.15907.1	noncoding	-0.0004096	0	0	493
MSTRG.15917.2	coding	0.279	648	1629	5884
MSTRG.15917.3	coding	0.256	276	1092	5347
MSTRG.15917.6	coding	0.256	873	1689	1931
MSTRG.15917.7	coding	0.292	363	1227	1470
MSTRG.15917.5	coding	0.245	492	1545	1787
MSTRG.15917.8	coding	0.272	936	1917	2151
MSTRG.15917.9	coding	0.256	687	1503	1705
MSTRG.15917.10	noncoding	-0.038912	2580	2850	3006
MSTRG.15914.2	coding	0.455	0	1365	3332
MSTRG.15913.1	noncoding	-0.0446464	489	522	994
MSTRG.15921.1	noncoding	-0.0004096	0	0	513
MSTRG.15929.1	coding	0.462	45	4659	6697
MSTRG.15929.4	coding	0.491	0	4146	6237
MSTRG.15930.1	noncoding	-0.0692224	534	900	2895
MSTRG.15929.5	coding	0.463	699	4794	6830
MSTRG.15929.7	coding	0.474	4416	8346	9739
MSTRG.15929.9	coding	0.467	4416	8412	9805
MSTRG.15929.10	coding	0.461	4359	8466	10504
MSTRG.15929.11	coding	0.468	588	4629	6720
MSTRG.15932.1	coding	0.405	0	2184	10762
MSTRG.15932.2	coding	0.457	0	774	1034
MSTRG.15931.1	noncoding	-0.0548864	0	204	359
MSTRG.15936.2	coding	0.234	201	678	915
MSTRG.15936.3	coding	0.182	204	531	768
MSTRG.15937.3	coding	0.282	0	378	754
MSTRG.15935.1	coding	0.411	0	816	1645
MSTRG.15940.1	coding	0.247	186	870	1464
MSTRG.15940.4	coding	0.193	87	531	689
MSTRG.15943.1	noncoding	-0.090112	0	102	494
MSTRG.15961.2	coding	0.073	4008	5121	8410
MSTRG.15962.3	coding	0.171	369	3426	4424
MSTRG.15962.2	coding	0.174	270	3408	4160
MSTRG.15962.7	coding	0.166	11112	14145	15142
MSTRG.15963.1	noncoding	-0.0098304	951	1131	5240
MSTRG.15964.1	noncoding	-0.012288	7413	7497	9389
MSTRG.15952.1	coding	0.28	0	3189	3706
MSTRG.15955.1	noncoding	-0.012288	1251	1299	3073
MSTRG.15956.1	coding	0.074	3618	3888	6436
MSTRG.15957.1	noncoding	-0.018432	2685	2850	3004
MSTRG.15958.1	noncoding	-0.028672	1611	1695	3338
MSTRG.15951.1	noncoding	-0.1421312	0	45	426
MSTRG.15969.2	coding	0.243	0	354	1819
MSTRG.15969.3	coding	0.203	9	372	1838
MSTRG.15969.4	coding	0.203	234	597	2062
MSTRG.15973.1	noncoding	-0.090112	0	57	311
MSTRG.15974.1	coding	0.527	252	1827	3377
MSTRG.15974.5	coding	0.526	153	1497	2014
MSTRG.15978.5	coding	0.211	9606	10647	14287
MSTRG.15978.7	coding	0.182	3078	3348	3523
MSTRG.15978.6	coding	0.107	16803	17259	23994
MSTRG.15979.1	noncoding	-0.02048	774	822	2702
MSTRG.15980.1	coding	0.135	30	216	429

MSTRG.15981.1	noncoding	-0.024576	3768	3900	5408
MSTRG.15978.8	noncoding	-0.004096	6681	6786	8066
MSTRG.15982.1	noncoding	-0.008192	93	156	670
MSTRG.15978.9	noncoding	-0.1036288	0	186	4319
MSTRG.15976.2	coding	0.273	0	894	1280
MSTRG.15990.4	coding	0.338	477	2346	3806
MSTRG.15994.1	coding	0.141	240	1365	1538
MSTRG.15996.1	noncoding	-0.0004096	0	0	430
MSTRG.15997.1	noncoding	-0.0630784	207	414	946
MSTRG.15998.4	coding	0.211	348	1938	2225
MSTRG.15998.3	coding	0.229	0	1446	1733
MSTRG.15998.10	coding	0.289	7119	7797	8087
MSTRG.15998.11	coding	0.238	432	1782	2074
MSTRG.15998.9	coding	0.238	459	1809	2099
MSTRG.15999.1	noncoding	-0.0094208	75	276	580
MSTRG.16000.1	noncoding	-0.0917504	252	279	609
MSTRG.16003.1	coding	0.256	0	1014	1701
MSTRG.16004.1	noncoding	-0.006144	732	987	2706
MSTRG.16006.1	noncoding	-0.0770048	0	168	322
MSTRG.16005.2	noncoding	-0.0782336	2625	3402	3557
MSTRG.16012.1	noncoding	-0.0024576	60	216	627
MSTRG.16013.2	coding	0.238	39	3321	3727
MSTRG.16014.1	noncoding	-0.0413696	624	807	1162
MSTRG.16015.2	coding	0.368	0	1335	1837
MSTRG.16019.1	coding	0.108	819	2472	8210
MSTRG.16019.2	coding	0.145	831	1596	3785
MSTRG.16022.2	coding	0.642	0	2337	3750
MSTRG.16027.2	coding	0.392	0	3186	3425
MSTRG.16031.2	coding	0.275	78	2250	2418
MSTRG.16034.2	noncoding	-0.0483328	552	732	1329
MSTRG.16035.1	noncoding	-0.073728	192	270	778
MSTRG.16039.1	coding	0.433	1629	3324	4579
MSTRG.16039.2	coding	0.433	159	1677	2933
MSTRG.16041.1	coding	0.368	0	606	876
MSTRG.16042.2	noncoding	-0.0004096	561	687	841
MSTRG.16042.3	noncoding	-0.045056	144	333	708
MSTRG.16042.4	noncoding	-0.0786432	0	207	805
MSTRG.16044.3	coding	0.484	2265	3675	4911
MSTRG.16049.2	coding	0.453	0	900	4387
MSTRG.16050.3	coding	0.437	0	756	1172
MSTRG.16050.4	coding	0.396	0	435	1783
MSTRG.16052.1	noncoding	-0.04096	6717	7116	7813
MSTRG.16052.2	noncoding	-0.0643072	4857	5325	6044
MSTRG.16052.3	noncoding	-0.0643072	3840	4308	5605
MSTRG.16056.1	noncoding	-0.0598016	4293	4584	4868
MSTRG.16057.12	coding	0.441	192	2148	2360
MSTRG.16057.15	coding	0.441	186	2142	2355
MSTRG.16057.11	coding	0.366	0	3255	4740
MSTRG.16057.14	coding	0.358	0	3312	4797
MSTRG.16057.17	coding	0.451	555	2739	5223
MSTRG.16057.19	coding	0.482	774	3237	4724
MSTRG.16057.20	coding	0.47	156	2088	3575
MSTRG.16067.2	coding	0.438	0	1266	1427
MSTRG.16058.1	coding	0.171	0	1248	1867

MSTRG.16064.2	coding	0.492	0	1197	3723
MSTRG.16064.3	coding	0.455	228	1461	2680
MSTRG.16064.4	coding	0.501	99	1134	1835
MSTRG.16064.7	coding	0.474	0	1206	3732
MSTRG.16069.2	coding	0.352	174	1812	2238
MSTRG.16069.5	coding	0.404	132	1464	1997
MSTRG.16069.6	coding	0.378	996	2466	2997
MSTRG.16069.8	coding	0.398	996	1875	2411
MSTRG.16072.1	noncoding	-0.0344064	48	303	459
MSTRG.16079.2	coding	0.331	0	4938	5458
MSTRG.16079.4	coding	0.332	291	5184	5705
MSTRG.16074.1	coding	0.417	792	3066	4290
MSTRG.16074.3	coding	0.37	54	765	2819
MSTRG.16081.1	coding	0.523	0	1878	4134
MSTRG.16082.2	coding	0.391	432	1479	2817
MSTRG.16082.3	coding	0.385	153	1143	2480
MSTRG.16082.4	noncoding	-0.0339968	774	1953	3498
MSTRG.16076.2	coding	0.508	0	1200	1363
MSTRG.16083.1	noncoding	-0.0434176	3204	3612	4056
MSTRG.16083.2	noncoding	-0.034816	1152	1338	1784
MSTRG.16083.3	noncoding	-0.1024	420	486	887
MSTRG.16088.2	coding	0.477	189	921	1103
MSTRG.16088.4	coding	0.465	462	1056	1238
MSTRG.16085.1	coding	0.549	0	1350	1852
MSTRG.16092.3	coding	0.509	1563	4455	5902
MSTRG.16093.3	coding	0.364	339	768	3717
MSTRG.16104.1	coding	0.369	5220	7527	9365
MSTRG.16104.4	coding	0.369	3324	5631	7471
MSTRG.16104.5	coding	0.408	4548	8013	9852
MSTRG.16102.1	coding	0.428	0	918	4637
MSTRG.16109.4	coding	0.182	0	6135	7098
MSTRG.16110.1	noncoding	-0.0024576	588	669	930
MSTRG.16113.5	coding	0.38	0	5859	11038
MSTRG.16113.6	coding	0.383	126	5835	9130
MSTRG.16113.8	coding	0.383	126	5832	9127
MSTRG.16113.7	coding	0.363	126	6969	10949
MSTRG.16113.9	coding	0.399	3	5301	8596
MSTRG.16113.11	coding	0.406	1734	6720	10106
MSTRG.16113.12	coding	0.379	177	2025	5321
MSTRG.16113.13	coding	0.356	1689	3927	8280
MSTRG.16113.14	coding	0.383	774	2322	7505
MSTRG.16117.2	coding	0.441	0	3396	5646
MSTRG.16117.3	coding	0.445	1449	3285	5436
MSTRG.16118.1	noncoding	-0.0258048	459	750	925
MSTRG.16123.1	noncoding	-0.0741376	1668	2055	2218
MSTRG.16128.1	coding	0.072	579	8484	13631
MSTRG.16128.5	coding	0.071	417	8262	11064
MSTRG.16128.6	coding	0.071	471	8268	11072
MSTRG.16129.1	coding	0.105	1041	1749	7920
MSTRG.16129.2	coding	0.108	1065	1737	7779
MSTRG.16129.3	noncoding	-0.065536	1221	1311	3815
MSTRG.16132.2	coding	0.277	57	2223	2530
MSTRG.16132.3	coding	0.289	441	2472	3012
MSTRG.16133.1	noncoding	-0.0073728	12	210	9437

MSTRG.16133.2	coding	0.271	408	2406	7142
MSTRG.16133.4	coding	0.34	0	2037	13592
MSTRG.16136.1	coding	0.373	0	1770	1927
MSTRG.16136.2	coding	0.419	0	1368	1566
MSTRG.16138.2	coding	0.291	0	3003	7927
MSTRG.16138.5	coding	0.273	0	2565	7774
MSTRG.16138.6	coding	0.291	0	3003	3644
MSTRG.16144.1	noncoding	-0.0290816	435	753	1032
MSTRG.16144.4	coding	0.358	432	1134	1427
MSTRG.16148.2	coding	0.541	0	4665	5028
MSTRG.16148.5	coding	0.535	0	4641	4796
MSTRG.16149.1	noncoding	-0.0712704	0	708	1931
MSTRG.16159.1	coding	0.319	0	3117	3634
MSTRG.16159.2	coding	0.324	0	2805	3012
MSTRG.16159.3	coding	0.322	0	2640	3403
MSTRG.16159.4	coding	0.328	0	2610	3374
MSTRG.16159.5	coding	0.312	600	3615	4061
MSTRG.16162.1	noncoding	-0.0585728	0	1452	1609
MSTRG.16163.5	coding	0.403	390	4599	5256
MSTRG.16167.2	coding	0.297	0	2154	2961
MSTRG.16170.5	coding	0.325	192	7377	8677
MSTRG.16174.2	coding	0.477	78	822	1346
MSTRG.16191.3	coding	0.512	1356	3282	3437
MSTRG.16191.4	coding	0.507	1665	3639	3793
MSTRG.16191.5	coding	0.505	1062	2625	2778
MSTRG.16182.2	coding	0.207	0	1359	2155
MSTRG.16196.2	coding	0.539	0	2514	4848
MSTRG.16198.1	coding	0.541	309	2742	5729
MSTRG.16198.2	coding	0.49	0	2772	5596
MSTRG.16199.1	noncoding	-0.0466944	201	1251	1408
MSTRG.16195.3	coding	0.404	177	1242	1574
MSTRG.16201.2	coding	0.266	570	3885	5415
MSTRG.16201.3	coding	0.265	69	3426	4956
MSTRG.16201.6	coding	0.271	0	3132	4636
MSTRG.16202.1	noncoding	-0.034816	396	1806	3148
MSTRG.16201.7	coding	0.247	258	3459	4988
MSTRG.16203.1	coding	0.465	0	1641	2579
MSTRG.16207.1	noncoding	-0.0303104	99	879	3073
MSTRG.16210.2	coding	0.245	0	675	974
MSTRG.16211.1	coding	0.377	216	1578	2114
MSTRG.16214.2	noncoding	-0.0888832	0	459	889
MSTRG.16214.3	coding	0.39	0	264	694
MSTRG.16215.1	noncoding	-0.0503808	0	60	391
MSTRG.16217.1	coding	0.382	5658	9885	10519
MSTRG.16218.3	coding	0.375	1206	3627	3879
MSTRG.16218.4	coding	0.375	1101	3522	3780
MSTRG.16221.4	coding	0.494	0	2202	2409
MSTRG.16221.3	coding	0.495	693	2166	2373
MSTRG.16222.1	noncoding	-0.1150976	780	2322	2518
MSTRG.16222.3	noncoding	-0.13312	0	957	1112
MSTRG.16219.1	noncoding	-0.0208896	177	390	682
MSTRG.16226.5	noncoding	-0.0851968	216	669	825
MSTRG.16239.3	coding	0.409	0	2109	2680
MSTRG.16239.4	coding	0.437	0	1863	2597

MSTRG.16244.1	coding	0.455	282	1107	1808
MSTRG.16244.3	coding	0.448	324	1803	2193
MSTRG.16244.5	coding	0.42	1992	3687	4077
MSTRG.16242.7	noncoding	-0.0516096	15	246	400
MSTRG.16233.1	coding	0.447	3357	4290	4446
MSTRG.16233.2	coding	0.259	156	621	776
MSTRG.16248.2	coding	0.495	0	1731	2235
MSTRG.16248.3	coding	0.483	0	1782	2286
MSTRG.16237.1	noncoding	-0.0770048	186	606	847
MSTRG.16252.3	coding	0.348	564	1575	1746
MSTRG.16252.4	coding	0.348	855	1866	2039
MSTRG.16252.1	coding	0.308	255	1398	1569
MSTRG.16253.2	noncoding	-0.1142784	786	1623	1779
MSTRG.16253.6	coding	0.266	0	999	1155
MSTRG.16253.7	noncoding	-0.1142784	216	1053	1208
MSTRG.16254.1	coding	0.486	0	2577	3076
MSTRG.16261.2	coding	0.283	57	8868	9022
MSTRG.16261.6	coding	0.51	192	3930	4358
MSTRG.16261.5	coding	0.511	0	3927	4356
MSTRG.16263.5	coding	0.306	0	1455	1641
MSTRG.16263.3	coding	0.529	0	618	1891
MSTRG.16263.4	coding	0.431	0	1389	1577
MSTRG.16263.2	coding	0.267	630	1944	2132
MSTRG.16258.1	noncoding	-0.0663552	255	462	1887
MSTRG.16258.2	noncoding	-0.0294912	354	558	1985
MSTRG.16264.6	coding	0.427	723	3225	8035
MSTRG.16265.1	noncoding	-0.0667648	0	150	1471
MSTRG.16264.10	coding	0.452	0	2421	11663
MSTRG.16264.9	coding	0.479	0	4017	4402
MSTRG.16264.11	noncoding	-0.0229376	159	666	857
MSTRG.16269.1	noncoding	-0.0401408	1536	2457	4053
MSTRG.16269.2	noncoding	-0.0339968	1491	2760	2917
MSTRG.16269.3	noncoding	-0.0421888	1359	2625	3957
MSTRG.16270.1	coding	0.469	723	2295	6811
MSTRG.16270.2	coding	0.469	657	2229	6743
MSTRG.16270.3	coding	0.469	585	2157	7592
MSTRG.16273.1	noncoding	-0.1024	300	351	758
MSTRG.16274.2	coding	0.059	1110	1596	1975
MSTRG.16275.1	noncoding	-0.045056	102	168	416
MSTRG.16276.1	noncoding	-0.0786432	0	474	1766
MSTRG.16278.2	noncoding	-0.02048	9	147	3228
MSTRG.16284.1	noncoding	-0.024576	0	3	220
MSTRG.16288.1	noncoding	-0.0114688	804	984	4296
MSTRG.16291.1	noncoding	-0.028672	1287	1365	2298
MSTRG.16296.1	noncoding	-0.0004096	381	381	894
MSTRG.16299.1	noncoding	-0.008192	0	72	234
MSTRG.16299.2	noncoding	-0.004096	0	84	272
MSTRG.16302.1	noncoding	-0.0106496	138	273	680
MSTRG.16307.1	noncoding	-0.0364544	978	1326	1719
MSTRG.16319.2	coding	0.036	2994	3609	8888
MSTRG.16319.3	coding	0.07	30	990	6248
MSTRG.16319.4	coding	0.07	30	987	6127
MSTRG.16319.5	coding	0.07	30	987	6230
MSTRG.16319.6	coding	0.07	27	984	5999

MSTRG.16320.2	noncoding	-0.008192	1869	1995	4243
MSTRG.16321.1	coding	0.173	1962	3744	5529
MSTRG.16326.1	coding	0.349	132	1920	2321
MSTRG.16327.1	coding	0.165	234	1266	2014
MSTRG.16328.1	noncoding	-0.0237568	891	954	1120
MSTRG.16329.3	coding	0.059	363	2634	4686
MSTRG.16329.4	noncoding	-0.0045056	387	552	4811
MSTRG.16330.2	coding	0.176	0	5586	11258
MSTRG.16332.1	coding	0.149	258	3159	4290
MSTRG.16334.5	coding	0.064	105	5655	8920
MSTRG.16336.1	noncoding	-0.0004096	0	0	212
MSTRG.16337.1	coding	0.108	264	486	1114
MSTRG.16339.2	noncoding	-0.2224128	0	126	2852
MSTRG.16349.1	noncoding	-0.0290816	2787	2934	5659
MSTRG.16345.2	noncoding	-0.1019904	0	252	6023
MSTRG.16345.3	noncoding	-0.0434176	2124	2340	5904
MSTRG.16343.1	noncoding	-0.094208	249	255	645
MSTRG.16340.1	noncoding	-0.069632	81	129	450
MSTRG.16351.1	noncoding	-0.0884736	21	549	1114
MSTRG.16351.2	noncoding	-0.1204224	0	162	916
MSTRG.16352.4	noncoding	-0.1110016	1353	1593	2813
MSTRG.16353.1	noncoding	-0.006144	5994	6054	7663
MSTRG.16354.1	noncoding	-0.016384	2187	2247	2841
MSTRG.16358.2	coding	0.094	0	1227	1869
MSTRG.16358.3	coding	0.107	0	1374	1572
MSTRG.16361.2	noncoding	-0.0802816	33	303	9125
MSTRG.16361.1	coding	0.121	33	861	4406
MSTRG.16361.3	coding	0.096	0	723	1968
MSTRG.16361.5	coding	0.099	0	561	1181
MSTRG.16361.6	noncoding	-0.03072	4155	4458	8011
MSTRG.16361.7	noncoding	-0.008192	408	504	1617
MSTRG.16364.2	coding	0.102	498	2454	4535
MSTRG.16366.1	coding	0.345	141	879	1102
MSTRG.16369.4	coding	0.05	732	1068	3380
MSTRG.16370.1	coding	0.014	756	837	2701
MSTRG.16370.2	noncoding	-0.0319488	903	993	1763
MSTRG.16375.3	coding	0.044	792	1353	1633
MSTRG.16378.1	noncoding	-0.04096	66	189	343
MSTRG.16388.1	noncoding	-0.024576	1422	1503	2009
MSTRG.16389.1	coding	0.002	8706	8811	14558
MSTRG.16389.2	noncoding	-0.0446464	213	423	2125
MSTRG.16388.2	noncoding	-0.159744	204	261	852
MSTRG.16397.3	coding	0.013	1104	1773	3164
MSTRG.16399.2	coding	0.298	0	276	2423
MSTRG.16410.1	noncoding	-0.0004096	0	0	278
MSTRG.16418.1	noncoding	-0.0815104	1305	1722	2009
MSTRG.16419.6	coding	0.074	57	264	1730
MSTRG.16419.5	coding	0.093	174	711	1839
MSTRG.16419.7	coding	0.023	1338	4722	7045
MSTRG.16419.8	coding	0.158	4119	4473	6795
MSTRG.16419.10	coding	0.021	90	3342	5663
MSTRG.16422.1	coding	0.259	345	1395	2017
MSTRG.16422.2	coding	0.214	345	1725	2060
MSTRG.16422.3	coding	0.259	345	1395	1702

MSTRG.16422.6	coding	0.259	216	1266	1998
MSTRG.16423.4	noncoding	-0.028672	1302	1545	4109
MSTRG.16423.2	coding	0.048	2124	2871	3173
MSTRG.16423.3	coding	0.048	1656	2403	2705
MSTRG.16423.5	noncoding	-0.0835584	3060	3459	7208
MSTRG.16423.7	coding	0.025	795	2697	2998
MSTRG.16423.8	coding	0.048	3381	4128	4429
MSTRG.16423.10	noncoding	-0.0835584	90	489	4919
MSTRG.16423.12	coding	0.025	2124	3033	3748
MSTRG.16425.2	noncoding	-0.045056	306	396	5988
MSTRG.16425.1	coding	0.263	129	1572	6193
MSTRG.16425.3	coding	0.278	129	1638	11317
MSTRG.16425.9	coding	0.278	129	1641	2839
MSTRG.16425.10	coding	0.278	249	1761	2054
MSTRG.16427.1	coding	0.1	291	519	741
MSTRG.16417.1	noncoding	-0.0974848	0	282	497
MSTRG.16429.2	noncoding	-0.0536576	0	48	507
MSTRG.16431.3	coding	0.225	348	1257	2022
MSTRG.16431.4	coding	0.225	279	1188	1844
MSTRG.16470.1	coding	0.105	4476	5490	7901
MSTRG.16472.1	noncoding	-0.0053248	2505	2610	3431
MSTRG.16473.2	noncoding	-0.0167936	204	369	893
MSTRG.16446.1	noncoding	-0.0155648	114	279	433
MSTRG.16448.5	coding	0.483	0	261	2791
MSTRG.16448.6	coding	0.376	0	198	1617
MSTRG.16448.2	coding	0.294	0	1206	2030
MSTRG.16448.4	coding	0.294	0	1206	2037
MSTRG.16448.8	coding	0.229	741	1053	11671
MSTRG.16448.9	coding	0.274	1224	1746	8833
MSTRG.16452.3	coding	0.434	369	4758	6509
MSTRG.16452.5	coding	0.425	12	4539	6194
MSTRG.16452.6	coding	0.424	777	5301	6928
MSTRG.16453.2	coding	0.1	360	1446	4004
MSTRG.16457.1	coding	0.273	0	1065	4206
MSTRG.16461.2	coding	0.133	210	906	1098
MSTRG.16463.2	coding	0.376	222	2115	2908
MSTRG.16463.3	coding	0.307	0	2121	5542
MSTRG.16463.4	coding	0.271	0	2118	5541
MSTRG.16464.1	noncoding	-0.0114688	7854	8007	31071
MSTRG.16465.1	noncoding	-0.008192	36	165	1125
MSTRG.16482.1	noncoding	-0.0004096	0	3	520
MSTRG.16484.1	coding	0.084	0	1869	2703
MSTRG.16486.2	coding	0.045	243	1038	1861
MSTRG.16486.3	coding	0.133	441	966	2912
MSTRG.16491.1	noncoding	-0.0098304	486	639	1209
MSTRG.16492.1	noncoding	-0.07168	0	36	566
MSTRG.16492.2	coding	0.176	324	1134	1406
MSTRG.16498.7	noncoding	-0.0626688	156	723	2732
MSTRG.16498.8	noncoding	-0.0626688	156	723	2756
MSTRG.16498.13	noncoding	-0.0630784	12	621	2775
MSTRG.16498.14	noncoding	-0.0626688	156	723	2879
MSTRG.16498.15	noncoding	-0.059392	156	759	2734
MSTRG.16498.16	coding	0.001	48	225	624
MSTRG.16502.2	noncoding	-0.0749568	0	90	634

MSTRG.16507.1	noncoding	-0.0004096	0	0	303
MSTRG.16511.1	coding	0.163	0	1482	2990
MSTRG.16511.2	coding	0.169	0	1479	2853
MSTRG.16511.7	coding	0.156	441	1794	2158
MSTRG.16514.1	coding	0.09	162	2136	2858
MSTRG.16514.2	coding	0.084	42	2091	2815
MSTRG.16515.3	coding	0.093	153	1266	2247
MSTRG.16539.4	coding	0.166	1830	3630	5561
MSTRG.16539.5	coding	0.144	312	2604	4535
MSTRG.16539.2	coding	0.107	1176	7224	9155
MSTRG.16539.6	coding	0.103	2742	8934	10865
MSTRG.16539.7	coding	0.07	1176	4902	5150
MSTRG.16542.1	noncoding	-0.110592	231	243	652
MSTRG.16539.8	coding	0.084	1176	3876	4445
MSTRG.16565.1	noncoding	-0.004096	90	180	1594
MSTRG.16584.1	noncoding	-0.004096	1056	1140	1663
MSTRG.16584.2	noncoding	-0.004096	2571	2655	3099
MSTRG.16516.1	coding	0.154	0	360	1339
MSTRG.16520.1	noncoding	-0.08192	801	882	3030
MSTRG.16535.6	coding	0.149	0	576	1453
MSTRG.16535.7	coding	0.025	1863	2013	3516
MSTRG.16537.1	noncoding	-0.0073728	3747	3900	4932
MSTRG.16590.2	coding	0.086	3804	4224	5784
MSTRG.16590.4	coding	0.055	231	699	4056
MSTRG.16590.3	coding	0.086	3789	4209	5732
MSTRG.16590.6	noncoding	-0.028672	4146	4263	4419
MSTRG.16597.2	coding	0.264	246	9237	10377
MSTRG.16597.3	coding	0.24	60	933	1332
MSTRG.16597.4	noncoding	-0.22528	714	771	927
MSTRG.16597.5	noncoding	-0.1462272	0	144	585
MSTRG.16597.6	noncoding	-0.1232896	0	93	914
MSTRG.16613.1	noncoding	-0.016384	348	423	1035
MSTRG.16616.1	coding	0.109	5058	5307	9321
MSTRG.16616.2	coding	0.109	10524	10773	14762
MSTRG.16616.3	coding	0.082	2121	2898	12960
MSTRG.16619.1	noncoding	-0.0004096	5955	6036	6746
MSTRG.16620.1	noncoding	-0.0290816	12621	12831	16497
MSTRG.16616.5	coding	0.066	807	1251	1407
MSTRG.16616.6	coding	0.072	2238	2688	2842
MSTRG.16616.7	coding	0.06	1752	2187	2342
MSTRG.16616.8	coding	0.06	4356	4791	4944
MSTRG.16620.2	noncoding	-0.0290816	12003	12213	27710
MSTRG.16620.3	noncoding	-0.0135168	30609	31155	41475
MSTRG.16620.4	noncoding	-0.0135168	22722	23268	32042
MSTRG.16620.5	noncoding	-0.0135168	48099	48645	57416
MSTRG.16620.6	noncoding	-0.0135168	45279	45825	54571
MSTRG.16622.1	noncoding	-0.067584	30	258	3789
MSTRG.16587.1	noncoding	-0.0364544	0	102	352
MSTRG.16601.1	noncoding	-0.0180224	2268	2763	3069
MSTRG.16602.1	noncoding	-0.02048	4065	4185	5489
MSTRG.16603.3	coding	0.16	1578	1842	2137
MSTRG.16603.4	coding	0.102	480	885	1181
MSTRG.16604.2	coding	0.438	0	498	2095
MSTRG.16629.2	coding	0.253	438	1029	2626

MSTRG.16629.3	coding	0.253	759	1350	3670
MSTRG.16630.1	noncoding	-0.016384	3987	4125	8817
MSTRG.16631.3	noncoding	-0.0217088	6432	6849	11110
MSTRG.16629.4	coding	0.253	303	894	2488
MSTRG.16629.5	coding	0.253	1374	1965	4245
MSTRG.16632.1	noncoding	-0.1593344	0	9	219
MSTRG.16642.1	coding	0.343	306	2763	7191
MSTRG.16642.2	coding	0.284	480	2160	6532
MSTRG.16644.1	coding	0.444	12	1080	3753
MSTRG.16645.2	coding	0.05	603	909	1179
MSTRG.16653.5	coding	0.104	0	2295	2448
MSTRG.16653.6	coding	0.111	0	2238	2391
MSTRG.16653.7	coding	0.11	0	2481	2781
MSTRG.16653.8	coding	0.114	0	2319	2619
MSTRG.16653.9	coding	0.392	0	621	2777
MSTRG.16653.10	coding	0.016	2904	3462	4163
MSTRG.16654.1	noncoding	-0.008192	3351	3489	4363
MSTRG.16652.1	coding	0.288	363	2322	4496
MSTRG.16660.1	noncoding	-0.0712704	1563	2427	5024
MSTRG.16660.3	coding	0.261	1866	2334	4138
MSTRG.16660.4	noncoding	-0.0638976	45	1203	2913
MSTRG.16660.5	coding	0.206	0	1332	1499
MSTRG.16665.3	coding	0.125	1476	2508	5710
MSTRG.16665.4	coding	0.125	2037	3069	6272
MSTRG.16671.5	noncoding	-0.016384	516	567	855
MSTRG.16672.2	coding	0.239	0	2247	2436
MSTRG.16672.5	coding	0.257	435	2376	3562
MSTRG.16672.7	coding	0.256	198	2133	2322
MSTRG.16669.1	noncoding	-0.0495616	0	225	3778
MSTRG.16669.2	noncoding	-0.077824	2595	2703	3032
MSTRG.16670.1	coding	0.113	279	2907	3962
MSTRG.16674.1	noncoding	-0.0724992	5364	5673	6253
MSTRG.16676.1	coding	0.187	594	1443	2031
MSTRG.16676.3	coding	0.174	0	1431	3759
MSTRG.16677.4	coding	0.21	0	1164	2188
MSTRG.16677.3	coding	0.172	0	1677	4097
MSTRG.16678.1	noncoding	-0.012288	27	129	518
MSTRG.16679.2	noncoding	-0.0708608	7242	7440	8231
MSTRG.16679.1	noncoding	-0.0249856	0	1134	1926
MSTRG.16679.3	noncoding	-0.0315392	7668	8007	8541
MSTRG.16679.4	noncoding	-0.012288	753	894	1429
MSTRG.16679.5	noncoding	-0.053248	717	918	1138
MSTRG.16685.2	coding	0.532	459	2202	5776
MSTRG.16687.1	noncoding	-0.06144	117	351	1419
MSTRG.16684.1	coding	0.052	384	765	960
MSTRG.16691.1	noncoding	-0.0434176	405	1005	2010
MSTRG.16694.5	noncoding	-0.1114112	0	270	3508
MSTRG.16692.1	noncoding	-0.0770048	0	228	384
MSTRG.16693.1	noncoding	-0.0643072	0	309	555
MSTRG.16702.1	noncoding	-0.0188416	7008	7164	10870
MSTRG.16702.4	coding	0.307	0	444	2394
MSTRG.16702.5	noncoding	-0.0004096	432	543	832
MSTRG.16704.3	noncoding	-0.004096	114	288	547
MSTRG.16702.10	coding	0.132	1128	2682	3850

MSTRG.16702.12	coding	0.237	0	381	4665
MSTRG.16702.13	noncoding	-0.0098304	3306	3453	3731
MSTRG.16709.4	coding	0.397	306	3195	7931
MSTRG.16709.7	noncoding	-0.0806912	0	162	3733
MSTRG.16696.4	coding	0.332	7017	7665	9618
MSTRG.16712.1	coding	0.127	261	4356	10394
MSTRG.16712.2	coding	0.126	147	3981	10017
MSTRG.16712.3	coding	0.127	264	4359	8223
MSTRG.16712.5	coding	0.128	3204	7314	9483
MSTRG.16712.4	coding	0.127	306	4401	6568
MSTRG.16712.6	coding	0.126	234	4068	5869
MSTRG.16712.7	coding	0.125	0	4308	6109
MSTRG.16712.8	coding	0.128	348	4065	4884
MSTRG.16712.9	coding	0.129	0	4344	4579
MSTRG.16712.10	coding	0.129	0	4461	4694
MSTRG.16712.11	coding	0.055	1038	2652	3670
MSTRG.16713.1	coding	0.037	1197	1713	2620
MSTRG.16713.2	coding	0.219	1671	2445	3174
MSTRG.16713.4	noncoding	-0.094208	0	42	2642
MSTRG.16713.11	coding	0.368	30	1287	13666
MSTRG.16719.2	coding	0.189	0	4242	6215
MSTRG.16722.1	coding	0.075	2187	3075	5600
MSTRG.16722.2	coding	0.071	2253	3135	5255
MSTRG.16722.3	coding	0.075	2190	3078	5138
MSTRG.16722.5	coding	0.071	3894	4776	6550
MSTRG.16722.6	coding	0.074	783	1260	6850
MSTRG.16726.1	noncoding	-0.1024	828	912	1249
MSTRG.16733.1	noncoding	-0.008192	351	468	1337
MSTRG.16733.3	coding	0.03	168	582	1023
MSTRG.16745.1	noncoding	-0.0331776	108	162	561
MSTRG.16747.1	noncoding	-0.02048	63	81	497
MSTRG.16754.2	noncoding	-0.04096	1701	1764	2039
MSTRG.16757.3	coding	0.138	411	1686	2735
MSTRG.16757.4	coding	0.138	288	1563	3088
MSTRG.16748.2	coding	0.181	42	1125	2837
MSTRG.16749.1	noncoding	-0.0380928	0	117	274
MSTRG.16749.2	noncoding	-0.0520192	0	84	335
MSTRG.16750.1	noncoding	-0.086016	252	354	691
MSTRG.16761.3	noncoding	-0.0380928	282	348	1280
MSTRG.16764.2	coding	0.153	1086	3021	4190
MSTRG.16765.1	coding	0.02	1638	1743	2168
MSTRG.16766.1	noncoding	-0.0176128	1308	1380	1793
MSTRG.16767.1	noncoding	-0.036864	2202	2277	2482
MSTRG.16769.1	noncoding	-0.0651264	1101	1107	2486
MSTRG.16772.2	coding	0.155	0	3807	4586
MSTRG.16772.3	coding	0.142	84	3798	4650
MSTRG.16772.4	coding	0.14	81	3759	4583
MSTRG.16772.5	coding	0.142	147	3840	4617
MSTRG.16776.1	noncoding	-0.0004096	57	177	530
MSTRG.16799.10	coding	0.129	2766	5601	6535
MSTRG.16799.7	coding	0.128	2790	5634	6569
MSTRG.16799.8	coding	0.129	3078	5913	6848
MSTRG.16799.9	coding	0.129	3126	5961	6895
MSTRG.16799.3	coding	0.128	3312	6156	7089

MSTRG.16799.2	coding	0.085	708	11157	14952
MSTRG.16799.11	coding	0.095	0	10857	14652
MSTRG.16799.4	coding	0.095	72	10980	14783
MSTRG.16799.5	coding	0.094	72	11088	14882
MSTRG.16799.1	coding	0.095	0	11040	14845
MSTRG.16801.1	noncoding	-0.0004096	0	0	2241
MSTRG.16799.12	coding	0.206	3348	3798	4549
MSTRG.16799.13	coding	0.059	1203	4929	5332
MSTRG.16802.1	noncoding	-0.098304	912	996	1751
MSTRG.16803.1	noncoding	-0.0339968	174	507	1257
MSTRG.16780.1	noncoding	-0.0004096	0	0	235
MSTRG.16784.2	coding	0.173	327	1674	2759
MSTRG.16785.1	noncoding	-0.0004096	0	0	230
MSTRG.16787.1	noncoding	-0.0004096	0	0	214
MSTRG.16789.1	noncoding	-0.0004096	0	0	278
MSTRG.16823.10	coding	0.032	99	2625	6335
MSTRG.16823.8	coding	0.032	114	2640	6346
MSTRG.16823.1	coding	0.039	75	3291	7000
MSTRG.16823.5	coding	0.041	75	3252	6961
MSTRG.16823.6	coding	0.041	75	3261	6967
MSTRG.16823.7	coding	0.037	75	3363	7069
MSTRG.16823.9	coding	0.034	75	3276	5832
MSTRG.16823.2	coding	0.041	75	3261	6971
MSTRG.16823.3	coding	0.038	78	3336	7044
MSTRG.16823.11	coding	0.037	75	3363	6265
MSTRG.16823.12	noncoding	-0.075776	1467	1662	3149
MSTRG.16824.1	noncoding	-0.063488	0	483	873
MSTRG.16824.2	noncoding	-0.0745472	0	546	1614
MSTRG.16824.3	noncoding	-0.0733184	0	537	1082
MSTRG.16816.2	coding	0.133	0	630	3154
MSTRG.16816.3	coding	0.14	147	633	3156
MSTRG.16817.1	noncoding	-0.0012288	1623	1785	2135
MSTRG.16818.1	noncoding	-0.0004096	1506	1692	2815
MSTRG.16818.2	noncoding	-0.155648	57	78	830
MSTRG.16820.1	noncoding	-0.057344	417	516	741
MSTRG.16835.1	noncoding	-0.0004096	0	0	235
MSTRG.16835.2	noncoding	-0.0004096	0	0	275
MSTRG.16840.1	noncoding	-0.0929792	3891	4314	6151
MSTRG.16847.1	noncoding	-0.0294912	612	846	1001
MSTRG.16847.2	noncoding	-0.0212992	147	330	484
MSTRG.16855.1	noncoding	-0.16384	282	309	641
MSTRG.16878.1	noncoding	-0.028672	72	87	337
MSTRG.16879.1	noncoding	-0.1835008	0	36	237
MSTRG.16884.1	noncoding	-0.022528	21	180	1462
MSTRG.17027.1	coding	0.154	204	1869	8577
MSTRG.17027.4	coding	0.161	198	1512	12607
MSTRG.17027.5	coding	0.153	198	1635	8911
MSTRG.17027.3	coding	0.144	198	1800	3694
MSTRG.17027.6	coding	0.149	192	1821	10959
MSTRG.17028.1	coding	0.086	1008	1191	3864
MSTRG.17027.8	coding	0.133	25944	27093	29632
MSTRG.17027.12	coding	0.115	1656	2811	5170
MSTRG.17027.13	coding	0.149	624	1866	4339
MSTRG.17027.20	coding	0.163	2247	3099	4431

MSTRG.17027.16	coding	0.137	315	1158	21252
MSTRG.17027.17	coding	0.148	9810	10677	16054
MSTRG.17027.21	coding	0.146	3933	4875	8258
MSTRG.17027.22	coding	0.178	32298	32976	34059
MSTRG.17027.23	noncoding	-0.08192	2487	2529	4062
MSTRG.17031.1	noncoding	-0.0262144	3195	3279	14391
MSTRG.17027.24	coding	0.165	3150	3846	6689
MSTRG.17027.25	coding	0.12	129	1272	3631
MSTRG.17027.26	coding	0.063	7515	8109	19184
MSTRG.17027.27	coding	0.063	4920	5514	14006
MSTRG.17027.28	noncoding	-0.008192	2109	2202	2641
MSTRG.17033.1	noncoding	-0.0004096	0	0	1679
MSTRG.17027.29	noncoding	-0.090112	1545	1620	9257
MSTRG.17027.30	noncoding	-0.036864	1233	1338	7910
MSTRG.17034.1	noncoding	-0.053248	333	405	11327
MSTRG.17035.1	noncoding	-0.036864	2874	2961	3445
MSTRG.17036.1	noncoding	-0.0249856	6750	6825	12077
MSTRG.17037.1	noncoding	-0.032768	2034	2139	2973
MSTRG.17038.1	noncoding	-0.008192	129	234	4062
MSTRG.17039.1	coding	0.02	1389	1482	7025
MSTRG.17040.1	noncoding	-0.049152	132	231	2726
MSTRG.17041.1	coding	0.023	5307	5487	9233
MSTRG.17042.1	noncoding	-0.049152	2013	2100	2598
MSTRG.17042.2	coding	0.078	1398	1770	5725
MSTRG.17027.31	noncoding	-0.053248	3816	3840	4277
MSTRG.17027.32	noncoding	-0.0651264	6141	6483	25720
MSTRG.17027.34	coding	0.04	21348	22314	30300
MSTRG.17027.33	noncoding	-0.077824	17493	17583	23637
MSTRG.17027.35	noncoding	-0.0004096	0	0	489
MSTRG.17027.37	coding	0.085	10497	10761	25677
MSTRG.17027.36	coding	0.086	5433	5574	9163
MSTRG.17027.38	coding	0.04	6867	7833	12520
MSTRG.17027.40	coding	0.04	6552	8079	9537
MSTRG.17027.39	noncoding	-0.053248	9249	9339	9760
MSTRG.17027.41	coding	0.085	4200	4464	13188
MSTRG.17027.42	coding	0.085	4161	4425	7632
MSTRG.17027.43	noncoding	-0.012288	6045	6180	9410
MSTRG.17027.44	noncoding	-0.131072	3381	3450	4906
MSTRG.17044.1	noncoding	-0.557056	3285	3291	4513
MSTRG.17027.45	noncoding	-0.026624	4593	4698	9382
MSTRG.17027.46	coding	0.086	4734	4875	6387
MSTRG.17045.1	noncoding	-0.090112	3135	3177	5245
MSTRG.17046.1	noncoding	-0.0200704	1557	1596	3780
MSTRG.17047.1	noncoding	-0.0323584	5793	5988	12441
MSTRG.17048.1	noncoding	-0.0319488	1848	1884	4629
MSTRG.17049.1	noncoding	-0.065536	300	312	891
MSTRG.17050.1	noncoding	-0.008192	612	672	3230
MSTRG.17051.1	noncoding	-0.1024	2232	2262	4105
MSTRG.16890.1	coding	0.357	519	2184	3377
MSTRG.16890.2	coding	0.284	72	741	896
MSTRG.16899.2	coding	0.134	1275	4269	5677
MSTRG.16906.1	coding	0.075	0	3366	3590
MSTRG.16906.3	coding	0.037	57	567	844
MSTRG.16907.3	coding	0.168	474	735	2346

MSTRG.16907.1	coding	0.142	474	903	2065
MSTRG.16907.2	noncoding	-0.034816	1104	2076	7217
MSTRG.16907.4	noncoding	-0.034816	1101	2073	7214
MSTRG.16907.5	noncoding	-0.0434176	3108	3291	5076
MSTRG.16907.6	coding	0.143	294	594	5504
MSTRG.16907.8	coding	0.142	819	1248	2481
MSTRG.16907.7	noncoding	-0.034816	2691	3663	8708
MSTRG.16907.9	noncoding	-0.024576	1860	1935	3211
MSTRG.16911.1	noncoding	-0.0126976	357	393	1209
MSTRG.16912.1	coding	0.151	2139	2385	4070
MSTRG.16915.1	coding	0.09	75	2913	3860
MSTRG.16918.2	coding	0.01	309	450	1088
MSTRG.16918.1	coding	0.01	309	450	1677
MSTRG.16912.2	coding	0.061	240	1827	2157
MSTRG.16912.3	coding	0.049	48	1587	1915
MSTRG.16902.3	coding	0.178	0	735	2260
MSTRG.16902.4	coding	0.171	0	735	2260
MSTRG.16919.1	noncoding	-0.0004096	0	0	330
MSTRG.16920.1	noncoding	-0.008192	867	1002	2288
MSTRG.16922.1	noncoding	-0.151552	216	258	530
MSTRG.16930.6	coding	0.263	456	3036	3376
MSTRG.16927.1	coding	0.248	117	1086	1518
MSTRG.16928.1	noncoding	-0.0446464	3	39	291
MSTRG.16935.7	coding	0.186	1386	1965	2495
MSTRG.16936.1	noncoding	-0.03072	6447	6747	15963
MSTRG.16935.8	coding	0.186	1371	1953	2574
MSTRG.16938.1	noncoding	-0.0221184	3363	3531	4193
MSTRG.16940.1	coding	0.563	0	480	2199
MSTRG.16940.2	coding	0.594	0	438	2155
MSTRG.16940.3	noncoding	-0.3407872	0	279	3249
MSTRG.16940.4	noncoding	-0.3616768	0	219	5668
MSTRG.16945.1	coding	0.162	438	3726	8493
MSTRG.16948.1	noncoding	-0.004096	4521	4626	5733
MSTRG.16949.2	coding	0.378	288	1194	5161
MSTRG.16953.4	coding	0.308	0	6144	7369
MSTRG.16954.1	noncoding	-0.0065536	1917	2076	2783
MSTRG.16959.1	coding	0.151	0	1383	2474
MSTRG.16959.2	coding	0.139	105	1230	2321
MSTRG.16961.1	noncoding	-0.0417792	480	828	6532
MSTRG.16962.1	noncoding	-0.077824	78	126	827
MSTRG.16965.1	coding	0.196	405	1266	2682
MSTRG.16965.2	coding	0.218	84	987	2402
MSTRG.16965.4	noncoding	-0.016384	2661	2784	3070
MSTRG.16965.3	coding	0.239	0	930	5265
MSTRG.16965.5	coding	0.157	51	561	3710
MSTRG.16965.6	coding	0.245	0	813	3146
MSTRG.16965.7	coding	0.264	0	369	584
MSTRG.16966.1	coding	0.04	7929	8691	19197
MSTRG.16966.2	coding	0.107	7914	8265	15722
MSTRG.16967.1	noncoding	-0.014336	864	1035	1707
MSTRG.16968.1	noncoding	-0.0073728	228	762	1441
MSTRG.16963.5	noncoding	-0.045056	0	165	319
MSTRG.16971.1	coding	0.17	438	2403	2792
MSTRG.16971.2	coding	0.146	432	2256	2644

MSTRG.16971.3	coding	0.146	309	2133	2522
MSTRG.16971.4	coding	0.169	309	1383	2772
MSTRG.16971.6	coding	0.146	0	1983	2509
MSTRG.16971.8	coding	0.096	111	1506	1895
MSTRG.16971.10	coding	0.139	1263	2016	2405
MSTRG.16971.9	coding	0.131	99	1635	2024
MSTRG.16973.1	noncoding	-0.0139264	852	1023	1442
MSTRG.16974.2	coding	0.465	462	1110	1618
MSTRG.16974.3	coding	0.465	525	1173	1682
MSTRG.16978.1	coding	0.291	363	2319	3960
MSTRG.16978.2	coding	0.23	144	3702	5342
MSTRG.16978.3	coding	0.335	2250	3831	5468
MSTRG.16978.4	coding	0.225	0	3702	5337
MSTRG.16988.6	coding	0.043	5628	7983	10549
MSTRG.16988.4	coding	0.036	3378	8817	26720
MSTRG.16988.5	coding	0.036	4722	10161	28289
MSTRG.16988.3	coding	0.036	2262	7701	25827
MSTRG.16988.9	coding	0.001	141	411	898
MSTRG.16992.2	coding	0.191	0	1275	1496
MSTRG.16996.1	noncoding	-0.024576	33	123	438
MSTRG.16997.1	noncoding	-0.0233472	285	291	649
MSTRG.16997.2	noncoding	-0.0004096	0	0	279
MSTRG.16998.1	noncoding	-0.06144	753	861	2308
MSTRG.16999.1	noncoding	-0.0466944	972	1032	1187
MSTRG.17005.1	noncoding	-0.0475136	0	3	611
MSTRG.17004.6	noncoding	-0.0290816	438	624	826
MSTRG.17004.7	noncoding	-0.0290816	723	909	1111
MSTRG.17004.8	noncoding	-0.0290816	429	615	817
MSTRG.17008.1	noncoding	-0.024576	111	183	431
MSTRG.17011.2	coding	0.563	111	738	5041
MSTRG.17011.3	coding	0.556	111	750	1847
MSTRG.17011.5	coding	0.556	111	750	1162
MSTRG.17012.1	noncoding	-0.1429504	0	765	2325
MSTRG.17012.2	noncoding	-0.1429504	0	765	2195
MSTRG.17016.1	noncoding	-0.0004096	144	258	689
MSTRG.17053.2	noncoding	-0.1110016	0	180	689
MSTRG.17053.1	noncoding	-0.0528384	63	369	649
MSTRG.17054.1	coding	0.296	0	5796	6634
MSTRG.17054.2	coding	0.311	243	5235	7205
MSTRG.17055.1	noncoding	-0.0008192	588	834	1153
MSTRG.17054.3	coding	0.377	0	1947	4543
MSTRG.17060.1	noncoding	-0.004096	0	39	655
MSTRG.17061.1	noncoding	-0.0630784	27	300	5115
MSTRG.17061.2	noncoding	-0.0524288	81	462	3638
MSTRG.17067.1	noncoding	-0.0086016	465	600	753
MSTRG.17067.2	noncoding	-0.0274432	690	1659	1815
MSTRG.17066.2	coding	0.256	171	4098	4947
MSTRG.17069.1	coding	0.385	1230	2016	2315
MSTRG.17069.2	coding	0.439	0	684	980
MSTRG.17074.2	coding	0.428	468	1167	2302
MSTRG.17074.4	coding	0.505	0	1065	2070
MSTRG.17074.6	coding	0.496	0	945	1949
MSTRG.17076.1	coding	0.511	0	1488	6949
MSTRG.17076.4	coding	0.503	1497	2880	3056

MSTRG.17076.6	coding	0.343	24	2385	2559
MSTRG.17077.1	coding	0.533	18	1089	1310
MSTRG.17078.5	noncoding	-0.116736	972	1344	7995
MSTRG.17078.1	coding	0.21	4902	5736	6902
MSTRG.17079.1	noncoding	-0.0520192	8022	8421	9076
MSTRG.17078.6	coding	0.285	21	528	10917
MSTRG.17085.2	noncoding	-0.1081344	0	171	3101
MSTRG.17087.3	coding	0.341	5496	7374	9597
MSTRG.17087.4	coding	0.341	1425	3303	5501
MSTRG.17092.1	noncoding	-0.034816	141	957	1112
MSTRG.17093.1	noncoding	-0.0434176	366	984	1633
MSTRG.17093.2	noncoding	-0.0454656	1308	1788	1943
MSTRG.17093.3	noncoding	-0.059392	900	1131	1286
MSTRG.17093.4	noncoding	-0.0421888	1161	1623	1779
MSTRG.17093.5	noncoding	-0.0475136	189	777	1402
MSTRG.17096.2	coding	0.463	516	2961	3780
MSTRG.17096.3	coding	0.487	2010	3243	3416
MSTRG.17101.1	coding	0.431	0	3993	6844
MSTRG.17105.1	noncoding	-0.0344064	165	516	945
MSTRG.17109.4	coding	0.294	30	1176	1331
MSTRG.17109.2	coding	0.451	432	2391	2552
MSTRG.17109.5	coding	0.458	168	1293	1446
MSTRG.17111.3	coding	0.417	0	1332	1498
MSTRG.17114.1	noncoding	-0.0536576	6186	6762	6916
MSTRG.17114.2	noncoding	-0.0724992	1317	2070	2225
MSTRG.17115.2	coding	0.491	0	1254	1606
MSTRG.17110.3	noncoding	-0.0700416	0	3759	5070
MSTRG.17110.1	coding	0.36	0	603	1922
MSTRG.17110.2	noncoding	-0.1298432	0	1674	2993
MSTRG.17110.4	noncoding	-0.1118208	0	3159	4562
MSTRG.17110.5	noncoding	-0.11264	0	3339	4742
MSTRG.17110.6	noncoding	-0.1097728	0	3648	4790
MSTRG.17116.1	coding	0.453	1419	2301	2653
MSTRG.17117.1	coding	0.483	348	2334	2720
MSTRG.17117.3	coding	0.483	342	2328	2669
MSTRG.17119.2	coding	0.431	138	1548	2395
MSTRG.17119.3	coding	0.431	120	1530	2377
MSTRG.17119.5	coding	0.431	150	1560	2408
MSTRG.17131.1	coding	0.462	105	1464	1620
MSTRG.17138.2	noncoding	-0.0589824	153	786	1334
MSTRG.17138.3	coding	0.287	4821	6162	7216
MSTRG.17138.4	coding	0.324	6102	6981	7567
MSTRG.17138.6	coding	0.333	4497	5529	5902
MSTRG.17140.2	noncoding	-0.0290816	195	1224	1720
MSTRG.17134.1	coding	0.317	0	939	5919
MSTRG.17134.2	noncoding	-0.1712128	639	975	5079
MSTRG.17145.1	noncoding	-0.028672	69	150	327
MSTRG.17150.1	noncoding	-0.012288	432	567	722
MSTRG.17150.2	noncoding	-0.0172032	552	624	777
MSTRG.17151.4	coding	0.147	1059	2580	2852
MSTRG.17152.2	coding	0.259	60	333	1136
MSTRG.17155.1	noncoding	-0.079872	6780	7281	7438
MSTRG.17155.4	noncoding	-0.0516096	3423	3951	5601
MSTRG.17166.1	noncoding	-0.0180224	837	1053	2655

MSTRG.17166.2	noncoding	-0.016384	207	264	455
MSTRG.17168.2	noncoding	-0.1425408	0	417	4240
MSTRG.17168.3	coding	0.459	20556	22158	25115
MSTRG.17158.1	noncoding	-0.151552	207	273	533
MSTRG.17172.2	coding	0.123	21	495	1249
MSTRG.17172.3	coding	0.089	21	444	1394
MSTRG.17192.2	coding	0.213	1449	1779	2081
MSTRG.17192.1	coding	0.073	291	1962	2264
MSTRG.17192.3	coding	0.213	1614	1944	2244
MSTRG.17192.4	coding	0.073	765	2436	2736
MSTRG.17193.1	noncoding	-0.0466944	0	387	1141
MSTRG.17202.1	noncoding	-0.016384	1911	2001	4118
MSTRG.17207.1	noncoding	-0.126976	750	801	1630
MSTRG.17215.2	noncoding	-0.008192	1662	1800	2253
MSTRG.17225.1	noncoding	-0.0004096	117	312	836
MSTRG.17175.1	noncoding	-0.172032	144	183	452
MSTRG.17175.2	noncoding	-0.073728	144	222	612
MSTRG.17176.1	noncoding	-0.073728	21	51	264
MSTRG.17177.4	coding	0.532	3213	5448	6506
MSTRG.17177.3	coding	0.498	78	2790	3848
MSTRG.17177.2	coding	0.415	0	3975	5033
MSTRG.17177.5	coding	0.457	141	3087	4046
MSTRG.17177.6	coding	0.483	234	3057	3934
MSTRG.17178.1	coding	0.074	1998	2709	3289
MSTRG.17178.2	coding	0.105	12	306	2137
MSTRG.17177.7	coding	0.242	0	1692	2364
MSTRG.17179.1	noncoding	-0.135168	2730	2775	2931
MSTRG.17180.1	noncoding	-0.0069632	0	309	1053
MSTRG.17398.2	coding	0.067	168	765	1113
MSTRG.17399.1	noncoding	-0.0540672	297	507	660
MSTRG.17400.1	noncoding	-0.016384	834	933	1615
MSTRG.17401.1	noncoding	-0.1232896	0	369	526
MSTRG.17402.1	noncoding	-0.0585728	3849	4068	4223
MSTRG.17402.2	noncoding	-0.02048	264	354	1441
MSTRG.17402.3	noncoding	-0.004096	1656	1821	2095
MSTRG.17402.5	noncoding	-0.0049152	135	300	521
MSTRG.17402.6	noncoding	-0.0008192	138	285	561
MSTRG.17402.11	noncoding	-0.0098304	846	1110	7397
MSTRG.17403.1	noncoding	-0.065536	540	699	1793
MSTRG.17402.13	noncoding	-0.0688128	3057	3366	4354
MSTRG.17405.1	noncoding	-0.0356352	3612	3882	4446
MSTRG.17402.14	noncoding	-0.0315392	1131	1338	3957
MSTRG.17402.15	coding	0.455	129	2595	3766
MSTRG.17402.16	noncoding	-0.1462272	2094	2514	5070
MSTRG.17402.19	coding	0.138	201	1998	4523
MSTRG.17402.17	coding	0.215	201	663	1924
MSTRG.17402.20	coding	0.166	201	1038	3383
MSTRG.17402.21	coding	0.166	105	942	2352
MSTRG.17402.24	coding	0.156	3462	5073	5554
MSTRG.17402.23	coding	0.166	66	903	2102
MSTRG.17402.25	coding	0.166	63	900	1609
MSTRG.17402.29	coding	0.114	9	1542	2620
MSTRG.17402.27	coding	0.125	9	1650	3627
MSTRG.17402.31	coding	0.113	6	1542	2522

MSTRG.17402.32	coding	0.124	6	1656	2185
MSTRG.17406.1	noncoding	-0.053248	336	426	1355
MSTRG.17407.1	coding	0.176	84	795	949
MSTRG.17402.34	noncoding	-0.135168	1716	1758	2573
MSTRG.17402.35	noncoding	-0.0155648	510	534	1134
MSTRG.17402.36	noncoding	-0.0720896	1386	1443	1765
MSTRG.17402.37	coding	0.156	1158	2769	3091
MSTRG.17408.1	noncoding	-0.008192	1299	1314	1769
MSTRG.17409.1	noncoding	-0.02048	456	642	1938
MSTRG.17409.2	noncoding	-0.02048	507	693	1877
MSTRG.17409.3	noncoding	-0.02048	507	693	1878
MSTRG.17410.1	noncoding	-0.04096	93	174	329
MSTRG.17411.1	noncoding	-0.0004096	345	396	1032
MSTRG.17412.1	noncoding	-0.0004096	720	720	1498
MSTRG.17413.1	noncoding	-0.0577536	417	684	3384
MSTRG.17414.1	noncoding	-0.114688	21	33	601
MSTRG.17402.38	noncoding	-0.026624	111	327	2384
MSTRG.17415.1	noncoding	-0.057344	564	756	1624
MSTRG.17416.1	noncoding	-0.1232896	0	69	802
MSTRG.17417.1	noncoding	-0.0733184	666	699	1231
MSTRG.17407.2	coding	0.146	66	1065	2330
MSTRG.17407.3	coding	0.15	66	1050	5402
MSTRG.17407.4	coding	0.242	0	264	1409
MSTRG.17418.1	noncoding	-0.0233472	45	213	367
MSTRG.17419.1	noncoding	-0.2023424	0	114	967
MSTRG.17420.1	noncoding	-0.0827392	555	831	1153
MSTRG.17421.2	coding	0.245	45	624	1556
MSTRG.17421.4	coding	0.243	36	594	828
MSTRG.17422.1	coding	0.074	705	1725	1978
MSTRG.17400.2	noncoding	-0.0126976	609	762	917
MSTRG.17402.68	noncoding	-0.03072	2013	2037	2486
MSTRG.17402.75	coding	0.118	1212	2979	3134
MSTRG.17402.77	coding	0.1	210	627	911
MSTRG.17424.1	noncoding	-0.0970752	0	258	1125
MSTRG.17407.10	coding	0.144	1440	4071	4360
MSTRG.17407.12	coding	0.141	2178	4275	4588
MSTRG.17428.1	noncoding	-0.0741376	0	189	573
MSTRG.17421.5	coding	0.049	0	423	1331
MSTRG.17432.1	noncoding	-0.045056	93	204	359
MSTRG.17421.13	coding	0.145	117	1053	2442
MSTRG.17432.2	noncoding	-0.0987136	135	426	884
MSTRG.17187.1	noncoding	-0.02048	1593	1749	2644
MSTRG.17189.1	noncoding	-0.0794624	585	612	767
MSTRG.17189.2	noncoding	-0.1519616	816	831	984
MSTRG.17276.3	noncoding	-0.0151552	7122	7236	7515
MSTRG.17276.4	coding	0.115	9171	9447	21590
MSTRG.17276.5	coding	0.115	16914	17190	24725
MSTRG.17276.6	noncoding	-0.059392	7161	7413	7615
MSTRG.17276.7	noncoding	-0.0581632	159	330	838
MSTRG.17278.1	coding	0.259	210	756	1138
MSTRG.17278.3	coding	0.157	189	885	1260
MSTRG.17278.2	coding	0.157	183	879	1313
MSTRG.17278.4	coding	0.259	345	891	1124
MSTRG.17279.4	coding	0.191	2835	3723	4243

MSTRG.17279.3	coding	0.19	0	1362	1760
MSTRG.17280.5	coding	0.278	1029	2406	2560
MSTRG.17280.4	coding	0.259	0	2250	2539
MSTRG.17190.1	coding	0.132	192	4077	5497
MSTRG.17263.3	noncoding	-0.0770048	2433	2751	4501
MSTRG.17264.1	noncoding	-0.0540672	8853	9006	10507
MSTRG.17264.2	noncoding	-0.08192	3687	3768	4658
MSTRG.17265.1	noncoding	-0.024576	714	837	1006
MSTRG.17266.1	coding	0.34	0	864	9552
MSTRG.17266.3	coding	0.342	0	663	3855
MSTRG.17266.5	coding	0.337	5646	5982	9174
MSTRG.17266.6	coding	0.328	3288	3825	7004
MSTRG.17191.1	noncoding	-0.0008192	159	258	923
MSTRG.17270.1	noncoding	-0.1007616	3015	3753	3909
MSTRG.17269.4	coding	0.2	240	2595	3265
MSTRG.17271.1	noncoding	-0.0139264	1194	1410	3071
MSTRG.17292.1	noncoding	-0.0339968	279	597	981
MSTRG.17292.2	noncoding	-0.0339968	0	93	247
MSTRG.17294.1	coding	0.331	0	1626	8155
MSTRG.17294.2	coding	0.328	0	1614	7907
MSTRG.17294.3	coding	0.266	6813	7926	17591
MSTRG.17294.5	coding	0.302	0	948	14128
MSTRG.17294.4	coding	0.296	171	1620	2476
MSTRG.17294.6	coding	0.294	942	2439	3206
MSTRG.17294.7	noncoding	-0.0499712	9321	9849	10861
MSTRG.17294.9	coding	0.38	0	411	9252
MSTRG.17294.8	noncoding	-0.0876544	843	1056	9899
MSTRG.17294.10	coding	0.166	642	864	3553
MSTRG.17294.12	noncoding	-0.012288	1806	1818	2607
MSTRG.17294.13	noncoding	-0.016384	37776	37914	39055
MSTRG.17294.14	noncoding	-0.016384	27015	27153	28328
MSTRG.17291.1	noncoding	-0.028672	72	90	244
MSTRG.17299.2	coding	0.357	2127	4662	5653
MSTRG.17299.3	coding	0.357	4902	6846	7009
MSTRG.17301.3	coding	0.284	708	1503	1773
MSTRG.17297.2	coding	0.354	258	1110	2485
MSTRG.17307.1	noncoding	-0.0487424	33	423	1119
MSTRG.17308.3	coding	0.31	825	2745	7334
MSTRG.17308.6	coding	0.279	33	2706	7125
MSTRG.17304.1	noncoding	-0.0004096	0	0	371
MSTRG.17306.1	noncoding	-0.0012288	225	273	548
MSTRG.17309.2	coding	0.262	627	1905	3072
MSTRG.17310.1	noncoding	-0.08192	0	18	291
MSTRG.17310.2	noncoding	-0.094208	60	123	378
MSTRG.17311.2	coding	0.508	0	1713	3731
MSTRG.17313.1	noncoding	-0.016384	930	1521	1678
MSTRG.17313.2	noncoding	-0.0385024	132	309	523
MSTRG.17315.2	coding	0.302	0	1293	1581
MSTRG.17326.3	coding	0.184	144	1242	3877
MSTRG.17329.1	coding	0.098	1461	2769	9366
MSTRG.17333.3	coding	0.171	3228	3960	4116
MSTRG.17333.2	noncoding	-0.0475136	996	1344	1500
MSTRG.17334.2	coding	0.016	480	1596	1923
MSTRG.17337.1	noncoding	-0.0024576	4899	5049	5598

MSTRG.17339.1	noncoding	-0.02048	6	111	373
MSTRG.17336.6	noncoding	-0.008192	234	438	639
MSTRG.17336.7	noncoding	-0.0045056	108	246	571
MSTRG.17340.1	noncoding	-0.008192	1059	1260	1492
MSTRG.17341.2	coding	0.143	912	1656	2411
MSTRG.17341.6	coding	0.146	183	1044	1820
MSTRG.17343.1	noncoding	-0.0524288	126	294	475
MSTRG.17349.5	coding	0.245	0	2904	3610
MSTRG.17349.4	coding	0.247	0	2970	3125
MSTRG.17350.1	coding	0.044	141	372	810
MSTRG.17349.9	coding	0.227	72	2112	4173
MSTRG.17349.12	coding	0.228	72	2613	2897
MSTRG.17349.8	coding	0.226	72	3021	3177
MSTRG.17349.7	coding	0.226	72	3039	8565
MSTRG.17349.10	coding	0.262	72	3945	4099
MSTRG.17349.11	coding	0.261	72	3837	3991
MSTRG.17349.13	coding	0.231	54	2916	3073
MSTRG.17349.14	noncoding	-0.0139264	24	261	8082
MSTRG.17349.15	coding	0.222	30	2904	3618
MSTRG.17351.1	noncoding	-0.0139264	1785	1944	6525
MSTRG.17352.1	noncoding	-0.0360448	1395	1551	4985
MSTRG.17347.1	noncoding	-0.192512	294	354	509
MSTRG.17348.1	coding	0.297	567	3354	5986
MSTRG.17348.2	coding	0.297	318	3105	5735
MSTRG.17348.4	coding	0.297	579	3366	5997
MSTRG.17368.10	coding	0.035	18	948	3126
MSTRG.17368.1	coding	0.062	402	1485	5688
MSTRG.17368.4	coding	0.062	402	1485	5697
MSTRG.17368.5	coding	0.062	402	1485	5701
MSTRG.17368.7	coding	0.029	402	3345	5463
MSTRG.17368.12	coding	0.021	402	4329	5654
MSTRG.17369.1	noncoding	-0.018432	333	507	3053
MSTRG.17368.13	noncoding	-0.069632	762	768	924
MSTRG.17368.14	noncoding	-0.0544768	0	204	4113
MSTRG.17364.3	noncoding	-0.0659456	0	237	7128
MSTRG.17366.1	coding	0.165	534	2199	13229
MSTRG.17370.2	coding	0.253	0	777	4316
MSTRG.17371.2	coding	0.187	2076	3762	4896
MSTRG.17372.1	coding	0.108	10698	10974	11409
MSTRG.17370.3	noncoding	-0.043008	4338	4566	8577
MSTRG.17373.1	coding	0.043	1683	1869	3423
MSTRG.17374.1	noncoding	-0.0188416	6081	6255	6781
MSTRG.17370.4	noncoding	-0.043008	3432	3660	21768
MSTRG.17370.5	noncoding	-0.069632	429	507	7029
MSTRG.17370.6	coding	0.256	0	270	6187
MSTRG.17370.7	noncoding	-0.098304	1053	1134	4041
MSTRG.17370.8	noncoding	-0.012288	7557	7668	10424
MSTRG.17370.9	coding	0.043	13554	14121	16357
MSTRG.17375.1	noncoding	-0.0376832	141	276	432
MSTRG.17370.10	noncoding	-0.008192	2616	2739	8216
MSTRG.17379.1	coding	0.225	1824	2127	4752
MSTRG.17379.13	noncoding	-0.0364544	33	255	557
MSTRG.17379.14	coding	0.225	1119	1422	3794
MSTRG.17379.12	coding	0.225	477	780	3132

MSTRG.17379.8	coding	0.225	1824	2127	4913
MSTRG.17379.10	coding	0.225	1827	2130	4890
MSTRG.17379.11	coding	0.225	1821	2124	4955
MSTRG.17379.15	coding	0.225	1818	2121	8009
MSTRG.17380.1	noncoding	-0.032768	939	1047	2477
MSTRG.17381.1	coding	0.043	717	837	5431
MSTRG.17387.2	coding	0.506	0	132	857
MSTRG.17397.1	noncoding	-0.0131072	0	45	423
MSTRG.17438.3	coding	0.211	294	1878	2100
MSTRG.17438.6	coding	0.162	345	2814	3987
MSTRG.17438.7	coding	0.157	345	2826	3999
MSTRG.17438.8	coding	0.162	306	2877	4050
MSTRG.17438.9	coding	0.204	5190	6774	6976
MSTRG.17438.10	coding	0.18	630	2682	4136
MSTRG.17438.11	coding	0.098	1494	2526	2774
MSTRG.17437.1	noncoding	-0.04096	885	1215	1369
MSTRG.17441.1	coding	0.445	63	1500	2427
MSTRG.17441.2	coding	0.445	63	1500	2520
MSTRG.17441.3	noncoding	-0.0393216	384	423	578
MSTRG.17462.1	noncoding	-0.0446464	5331	5472	9025
MSTRG.17463.1	noncoding	-0.0548864	0	72	1602
MSTRG.17464.1	noncoding	-0.053248	3801	4434	4589
MSTRG.17464.4	noncoding	-0.06144	753	1209	1364
MSTRG.17465.1	noncoding	-0.0581632	2643	2721	3611
MSTRG.17467.1	noncoding	-0.0434176	300	393	844
MSTRG.17469.1	noncoding	-0.028672	1878	2043	4920
MSTRG.17470.1	noncoding	-0.0290816	2991	3165	5638
MSTRG.17471.1	noncoding	-0.18432	495	534	1726
MSTRG.17474.1	noncoding	-0.0483328	2883	2997	14941
MSTRG.17475.1	noncoding	-0.012288	4296	4458	23732
MSTRG.17475.2	noncoding	-0.012288	3990	4152	17717
MSTRG.17475.3	noncoding	-0.151552	9372	9414	12242
MSTRG.17476.1	noncoding	-0.0106496	291	450	5310
MSTRG.17477.1	noncoding	-0.0233472	750	948	5403
MSTRG.17478.1	noncoding	-0.0073728	1599	1713	1991
MSTRG.17464.12	coding	0.315	915	5754	5907
MSTRG.17464.11	coding	0.338	915	4155	5710
MSTRG.17464.10	coding	0.336	915	4077	6933
MSTRG.17464.14	coding	0.34	879	5160	9797
MSTRG.17464.16	coding	0.219	2475	3003	7265
MSTRG.17480.1	noncoding	-0.0602112	57	405	999
MSTRG.17481.1	coding	0.188	708	6174	7196
MSTRG.17481.4	coding	0.188	900	6369	7380
MSTRG.17481.5	coding	0.188	708	6177	7173
MSTRG.17481.7	coding	0.188	900	6366	7356
MSTRG.17481.6	coding	0.188	576	6045	7035
MSTRG.17446.1	noncoding	-0.008192	159	327	1393
MSTRG.17450.1	coding	0.08	270	4638	5202
MSTRG.17454.1	coding	0.265	0	1704	2662
MSTRG.17454.3	coding	0.261	114	1740	1951
MSTRG.17456.2	coding	0.167	153	2640	4339
MSTRG.17456.3	coding	0.167	69	2559	4257
MSTRG.17456.4	coding	0.172	0	2538	4236
MSTRG.17456.5	coding	0.17	363	2901	4601

MSTRG.17453.2	coding	0.167	18	834	1307
MSTRG.17458.3	coding	0.236	408	1527	1811
MSTRG.17524.1	coding	0.154	102	1350	5416
MSTRG.17528.1	coding	0.389	813	4263	7166
MSTRG.17528.3	coding	0.389	0	3468	4806
MSTRG.17528.2	coding	0.394	168	3531	4868
MSTRG.17528.5	coding	0.445	405	3003	6189
MSTRG.17529.1	noncoding	-0.0237568	0	180	360
MSTRG.17530.1	noncoding	-0.06144	2130	2199	2404
MSTRG.17531.1	noncoding	-0.04096	531	609	1186
MSTRG.17528.6	noncoding	-0.0294912	3609	3789	24162
MSTRG.17528.7	noncoding	-0.016384	1245	1395	7345
MSTRG.17528.8	noncoding	-0.0589824	13494	13761	25791
MSTRG.17528.9	noncoding	-0.0208896	5295	5424	8147
MSTRG.17532.1	noncoding	-0.0401408	7611	7797	28868
MSTRG.17532.2	noncoding	-0.004096	3753	3900	5147
MSTRG.17532.3	noncoding	-0.0294912	12009	12264	14815
MSTRG.17528.10	noncoding	-0.065536	1236	1284	1951
MSTRG.17528.11	noncoding	-0.0049152	8925	9027	10849
MSTRG.17533.1	noncoding	-0.008192	870	963	1735
MSTRG.17528.12	noncoding	-0.016384	10206	10473	15584
MSTRG.17528.13	noncoding	-0.0540672	0	243	22038
MSTRG.17528.14	noncoding	-0.016384	8037	8304	13416
MSTRG.17528.15	noncoding	-0.024576	3579	3699	6427
MSTRG.17528.16	noncoding	-0.024576	3582	3702	6429
MSTRG.17528.17	noncoding	-0.0540672	0	240	6569
MSTRG.17528.18	noncoding	-0.04096	7941	8031	10562
MSTRG.17535.1	noncoding	-0.0258048	615	693	898
MSTRG.17528.19	noncoding	-0.059392	0	252	2178
MSTRG.17528.20	noncoding	-0.0483328	0	219	851
MSTRG.17536.1	coding	0.082	156	306	2804
MSTRG.17528.21	noncoding	-0.0622592	0	261	3855
MSTRG.17459.2	coding	0.169	0	588	1531
MSTRG.17482.3	noncoding	-0.0065536	2517	2718	7118
MSTRG.17482.4	coding	0.113	1905	2202	7131
MSTRG.17490.2	coding	0.053	426	1620	1817
MSTRG.17490.5	coding	0.259	1233	1935	4306
MSTRG.17490.4	coding	0.239	402	1473	5795
MSTRG.17460.3	coding	0.043	294	459	796
MSTRG.17496.2	coding	0.213	141	864	1106
MSTRG.17498.2	coding	0.177	21	1200	2787
MSTRG.17498.3	coding	0.175	0	1065	2837
MSTRG.17539.1	coding	0.135	270	7890	10376
MSTRG.17539.2	coding	0.102	786	3924	4081
MSTRG.17541.1	noncoding	-0.0249856	1599	1791	4622
MSTRG.17541.2	coding	0.052	432	957	3619
MSTRG.17541.3	coding	0.05	504	1110	1385
MSTRG.17541.4	coding	0.05	903	1509	1785
MSTRG.17541.5	coding	0.05	1287	1893	2168
MSTRG.17541.6	coding	0.042	528	1047	2103
MSTRG.17541.7	coding	0.024	372	828	1330
MSTRG.17546.1	coding	0.087	1650	1926	5867
MSTRG.17541.8	coding	0.099	459	732	7860
MSTRG.17548.1	noncoding	-0.045056	627	753	1507

MSTRG.17550.1	noncoding	-0.0339968	2226	2394	2906
MSTRG.17551.1	coding	0.195	0	609	1199
MSTRG.17551.2	coding	0.384	0	270	850
MSTRG.17552.1	noncoding	-0.0180224	2184	2385	2667
MSTRG.17553.1	noncoding	-0.0045056	0	48	1773
MSTRG.17557.1	coding	0.034	0	3801	5490
MSTRG.17512.2	coding	0.238	243	1986	2200
MSTRG.17515.1	noncoding	-0.0004096	336	390	1675
MSTRG.17516.3	noncoding	-0.0425984	0	279	947
MSTRG.17516.4	coding	0.179	2580	4785	6225
MSTRG.17516.5	coding	0.168	7263	9138	10577
MSTRG.17523.6	coding	0.181	135	1335	2888
MSTRG.17523.4	coding	0.181	177	1377	4034
MSTRG.17523.7	coding	0.181	171	1371	4029
MSTRG.17523.5	coding	0.147	177	2514	3982
MSTRG.17523.8	coding	0.142	162	2655	4123
MSTRG.17559.1	coding	0.331	2715	4299	6360
MSTRG.17559.3	coding	0.241	0	1716	3036
MSTRG.17559.4	coding	0.209	1455	3156	4430
MSTRG.17559.5	coding	0.224	1707	3477	4745
MSTRG.17559.7	coding	0.225	933	2700	3933
MSTRG.17559.6	coding	0.224	3684	5454	6685
MSTRG.17559.8	coding	0.27	2085	2760	2924
MSTRG.17560.1	noncoding	-0.004096	3264	3351	4495
MSTRG.17560.2	noncoding	-0.06144	216	240	803
MSTRG.17566.3	noncoding	-0.0278528	4518	4920	5074
MSTRG.17566.5	noncoding	-0.0229376	75	291	2726
MSTRG.17567.1	coding	0.039	1119	1890	2162
MSTRG.17567.2	coding	0.144	1110	1332	8673
MSTRG.17568.1	noncoding	-0.012288	2955	3096	5666
MSTRG.17579.1	noncoding	-0.0299008	1413	1740	4300
MSTRG.17580.1	coding	0.336	711	6204	7189
MSTRG.17580.3	coding	0.339	2997	8478	8912
MSTRG.17580.2	coding	0.341	426	5634	6068
MSTRG.17580.4	coding	0.338	0	5382	5802
MSTRG.17580.6	coding	0.367	1509	4743	5148
MSTRG.17581.2	noncoding	-0.014336	30	144	298
MSTRG.17581.1	noncoding	-0.0135168	57	213	369
MSTRG.17581.3	noncoding	-0.0729088	0	93	411
MSTRG.17582.1	noncoding	-0.036864	26376	26661	34422
MSTRG.17582.2	noncoding	-0.034816	402	573	7429
MSTRG.17580.8	noncoding	-0.0483328	0	1215	23800
MSTRG.17580.9	noncoding	-0.110592	0	210	18411
MSTRG.17580.10	noncoding	-0.01024	6120	6354	7206
MSTRG.17580.11	noncoding	-0.0462848	0	1155	17221
MSTRG.17571.1	coding	0.263	0	3918	4414
MSTRG.17572.1	noncoding	-0.0159744	114	285	1154
MSTRG.17571.3	coding	0.247	120	3861	4495
MSTRG.17571.4	coding	0.25	15	3774	4408
MSTRG.17571.5	coding	0.256	15	3939	4625
MSTRG.17575.1	noncoding	-0.0004096	0	0	348
MSTRG.17577.1	noncoding	-0.03072	534	606	774
MSTRG.17577.2	noncoding	-0.0192512	516	582	751
MSTRG.17584.1	noncoding	-0.1470464	0	51	2647

MSTRG.17585.9	coding	0.099	3315	3534	25985
MSTRG.17585.8	noncoding	-0.0004096	3903	4380	5068
MSTRG.17585.5	noncoding	-0.1613824	0	309	5003
MSTRG.17585.12	noncoding	-0.0679936	0	345	4891
MSTRG.17585.15	coding	0.099	3246	3465	27415
MSTRG.17585.16	coding	0.099	3246	3465	31031
MSTRG.17585.13	noncoding	-0.1613824	0	309	5062
MSTRG.17585.14	noncoding	-0.1613824	0	309	5060
MSTRG.17585.17	noncoding	-0.0004096	3804	4281	4971
MSTRG.17585.18	noncoding	-0.0004096	2127	2604	5785
MSTRG.17590.2	coding	0.129	471	1779	2677
MSTRG.17590.3	coding	0.184	777	2592	3123
MSTRG.17590.6	coding	0.188	774	1860	2392
MSTRG.17592.1	noncoding	-0.0217088	1311	1410	5602
MSTRG.17597.2	noncoding	-0.1847296	0	78	1688
MSTRG.17602.1	noncoding	-0.0208896	0	129	695
MSTRG.17615.1	noncoding	-0.0376832	291	555	4698
MSTRG.17615.2	coding	0.125	2775	2991	3146
MSTRG.17615.15	coding	0.351	1326	3168	3644
MSTRG.17615.5	coding	0.363	414	2604	3081
MSTRG.17615.18	noncoding	-0.0897024	93	594	4643
MSTRG.17605.2	coding	0.271	0	468	805
MSTRG.17605.3	coding	0.164	177	594	931
MSTRG.17608.6	coding	0.499	0	4455	5062
MSTRG.17608.8	coding	0.497	0	4380	4987
MSTRG.17608.4	coding	0.495	0	4431	5039
MSTRG.17608.5	coding	0.497	0	4461	5069
MSTRG.17608.7	coding	0.498	0	4581	5189
MSTRG.17608.9	coding	0.497	378	4884	5492
MSTRG.17608.2	coding	0.503	0	4263	4870
MSTRG.17607.1	noncoding	-0.0118784	0	99	1368
MSTRG.17619.1	coding	0.274	135	2034	2660
MSTRG.17622.1	coding	0.249	141	2073	5750
MSTRG.17622.2	coding	0.273	0	1776	5436
MSTRG.17619.3	coding	0.235	420	711	2245
MSTRG.17622.8	coding	0.22	1419	3606	4604
MSTRG.17622.9	coding	0.22	1407	3585	4540
MSTRG.17622.11	coding	0.271	0	1779	2004
MSTRG.17628.2	coding	0.15	0	600	2625
MSTRG.17629.1	coding	0.296	516	870	1840
MSTRG.17631.2	coding	0.288	186	2382	2606
MSTRG.17631.3	coding	0.264	237	2583	2960
MSTRG.17631.4	coding	0.283	444	2700	3078
MSTRG.17624.1	coding	0.266	0	390	597
MSTRG.17635.2	coding	0.459	0	1674	3593
MSTRG.17635.1	coding	0.395	459	1671	3887
MSTRG.17635.3	coding	0.459	0	1662	3584
MSTRG.17636.1	noncoding	-0.0352256	675	882	1168
MSTRG.17635.4	coding	0.421	2190	3597	5522
MSTRG.17635.6	coding	0.421	996	2403	4301
MSTRG.17635.7	coding	0.421	996	2403	4301
MSTRG.17635.9	coding	0.421	153	1560	3457
MSTRG.17635.10	noncoding	-0.008192	567	729	886
MSTRG.17638.1	noncoding	-0.0159744	5328	5604	7044

MSTRG.17639.1	noncoding	-0.0331776	8184	8547	11009
MSTRG.17640.1	noncoding	-0.0536576	450	657	1131
MSTRG.17641.1	noncoding	-0.012288	951	1020	2221
MSTRG.17635.12	coding	0.179	7875	8262	10059
MSTRG.17632.2	coding	0.492	957	2970	4545
MSTRG.17632.3	coding	0.492	831	2844	4385
MSTRG.17643.2	coding	0.298	0	222	807
MSTRG.17643.3	coding	0.358	0	132	812
MSTRG.17645.3	coding	0.319	351	4035	4204
MSTRG.17645.7	coding	0.296	1278	1902	7376
MSTRG.17647.1	coding	0.29	0	6006	22062
MSTRG.17647.2	coding	0.29	0	5862	9160
MSTRG.17647.3	coding	0.291	0	5727	14478
MSTRG.17647.4	coding	0.291	0	5922	12912
MSTRG.17648.2	coding	0.155	0	2070	2303
MSTRG.17653.1	noncoding	-0.008192	105	207	974
MSTRG.17654.1	noncoding	-0.086016	345	441	2207
MSTRG.17660.1	coding	0.351	297	891	6360
MSTRG.17661.3	coding	0.302	102	576	741
MSTRG.17661.4	coding	0.308	66	492	656
MSTRG.17663.2	noncoding	-0.0147456	483	633	787
MSTRG.17665.2	noncoding	-0.0827392	0	237	428
MSTRG.17667.2	coding	0.346	306	711	945
MSTRG.17669.1	noncoding	-0.07168	0	3	364
MSTRG.17670.1	noncoding	-0.0446464	114	297	451
MSTRG.17672.1	noncoding	-0.24576	27	45	254
MSTRG.17674.2	noncoding	-0.0851968	183	408	1335
MSTRG.17674.1	noncoding	-0.088064	183	411	1463
MSTRG.17674.3	noncoding	-0.0700416	831	1053	1484
MSTRG.17677.1	coding	0.357	0	771	2821
MSTRG.17677.3	coding	0.37	0	921	2807
MSTRG.17679.2	coding	0.457	1011	1821	3204
MSTRG.17676.2	noncoding	-0.1257472	0	612	1758
MSTRG.17681.1	coding	0.331	186	1245	2375
MSTRG.17681.3	coding	0.343	138	1515	2640
MSTRG.17682.2	coding	0.566	402	1152	2322
MSTRG.17685.2	coding	0.35	0	1074	1775
MSTRG.17686.1	noncoding	-0.0618496	765	1086	1293
MSTRG.17706.6	coding	0.162	1431	8094	9190
MSTRG.17706.4	coding	0.137	2880	10824	11920
MSTRG.17706.5	coding	0.156	1638	8544	9639
MSTRG.17706.8	coding	0.156	1584	8490	9576
MSTRG.17706.7	coding	0.156	1557	8463	9548
MSTRG.17706.9	coding	0.156	2229	9135	10220
MSTRG.17706.12	coding	0.162	12675	19338	20361
MSTRG.17706.14	coding	0.156	1440	8346	9367
MSTRG.17706.11	coding	0.156	1554	8460	9481
MSTRG.17706.10	coding	0.133	30	8235	9257
MSTRG.17706.16	coding	0.162	1593	8256	9270
MSTRG.17706.18	coding	0.162	2778	9441	10450
MSTRG.17706.17	coding	0.156	1485	8391	9399
MSTRG.17706.19	coding	0.162	1308	7971	8957
MSTRG.17706.20	coding	0.156	1434	8340	9326
MSTRG.17706.22	coding	0.027	2154	3300	4089

MSTRG.17706.23	noncoding	-0.0008192	381	456	2798
MSTRG.17695.1	coding	0.248	1143	2025	2371
MSTRG.17697.1	noncoding	-0.0303104	24	189	1186
MSTRG.17698.2	coding	0.282	330	1377	2129
MSTRG.17698.3	coding	0.219	15	1221	1586
MSTRG.17701.2	coding	0.284	0	858	3349
MSTRG.17702.1	noncoding	-0.032768	30	66	233
MSTRG.17710.3	coding	0.384	0	3009	5568
MSTRG.17710.4	coding	0.383	0	3006	5565
MSTRG.17710.6	coding	0.372	0	3144	3429
MSTRG.17711.4	noncoding	-0.1138688	66	312	551
MSTRG.17711.2	coding	0.463	0	2901	3141
MSTRG.17709.1	noncoding	-0.0233472	147	264	426
MSTRG.17714.1	coding	0.347	2208	3642	11364
MSTRG.17714.4	coding	0.366	0	1425	3736
MSTRG.17718.2	coding	0.184	0	2334	5521
MSTRG.17718.3	noncoding	-0.0421888	264	501	2556
MSTRG.17730.1	noncoding	-0.045056	183	291	2805
MSTRG.17731.1	noncoding	-0.0045056	1638	2388	5834
MSTRG.17731.2	noncoding	-0.139264	651	693	1382
MSTRG.17729.6	noncoding	-0.0581632	9675	9912	16185
MSTRG.17729.4	coding	0.294	0	4722	8215
MSTRG.17729.5	coding	0.295	0	4890	8383
MSTRG.17729.7	coding	0.282	0	4497	4651
MSTRG.17729.8	coding	0.284	456	4839	4994
MSTRG.17729.9	noncoding	-0.043008	4107	4377	7939
MSTRG.17732.1	noncoding	-0.0323584	5364	5529	7373
MSTRG.17729.10	noncoding	-0.0974848	3753	3996	4150
MSTRG.17733.7	coding	0.251	966	2328	4345
MSTRG.17733.10	coding	0.161	0	2259	4276
MSTRG.17733.12	coding	0.219	0	2340	2656
MSTRG.17733.11	coding	0.182	153	2022	2339
MSTRG.17725.2	coding	0.291	114	501	3323
MSTRG.17728.1	noncoding	-0.0004096	147	147	392
MSTRG.17735.2	coding	0.178	396	591	1628
MSTRG.17740.2	coding	0.144	474	1434	1671
MSTRG.17741.4	noncoding	-0.0004096	1266	1329	2029
MSTRG.17741.5	noncoding	-0.057344	0	93	2679
MSTRG.17741.6	coding	0.048	3780	4032	4608
MSTRG.17744.1	noncoding	-0.01024	27	123	352
MSTRG.17751.2	coding	0.174	12	1032	1651
MSTRG.17755.2	noncoding	-0.057344	498	564	1671
MSTRG.17755.3	noncoding	-0.057344	507	573	1682
MSTRG.17747.1	noncoding	-0.045056	510	609	1166
MSTRG.17762.1	noncoding	-0.0004096	0	0	355
MSTRG.17775.2	noncoding	-0.0405504	4986	5169	5884
MSTRG.17776.2	coding	0.365	0	495	7123
MSTRG.17779.1	coding	0.196	1239	2280	32600
MSTRG.17779.2	coding	0.22	756	2472	14939
MSTRG.17779.4	coding	0.196	1362	2403	23835
MSTRG.17779.5	coding	0.213	699	2253	19781
MSTRG.17779.10	coding	0.222	4605	6198	18157
MSTRG.17779.11	coding	0.196	1662	2703	13056
MSTRG.17768.1	coding	0.591	0	1785	2757

MSTRG.17769.3	coding	0.332	87	774	1113
MSTRG.17770.2	noncoding	-0.0925696	462	1188	1669
MSTRG.17770.3	noncoding	-0.0954368	462	1068	1223
MSTRG.17773.1	noncoding	-0.043008	2592	3000	7413
MSTRG.17773.2	coding	0.131	0	4173	8526
MSTRG.17773.3	coding	0.108	10728	11187	15590
MSTRG.17774.1	noncoding	-0.0393216	63	84	680
MSTRG.17781.2	noncoding	-0.026624	7008	7179	7335
MSTRG.17781.3	coding	0.207	7323	8910	12244
MSTRG.17782.1	noncoding	-0.1024	1101	1185	4346
MSTRG.17784.3	coding	0.441	0	2667	3105
MSTRG.17785.2	coding	0.214	669	1140	1563
MSTRG.17785.3	noncoding	-0.024576	2835	3000	3156
MSTRG.17789.1	coding	0.139	231	1734	2368
MSTRG.17789.4	coding	0.139	321	1824	2458
MSTRG.17789.6	coding	0.144	231	1650	2284
MSTRG.17791.6	coding	0.313	996	2676	3349
MSTRG.17791.5	coding	0.313	1137	2817	3492
MSTRG.17791.4	coding	0.313	900	2580	3254
MSTRG.17792.1	noncoding	-0.036864	2019	2076	4311
MSTRG.17796.2	coding	0.405	1071	2934	3120
MSTRG.17794.1	coding	0.31	402	2643	2798
MSTRG.17802.1	coding	0.302	0	459	861
MSTRG.17807.1	coding	0.366	0	1932	4269
MSTRG.17806.1	noncoding	-0.0442368	0	282	622
MSTRG.17814.1	coding	0.356	111	825	1447
MSTRG.17814.2	noncoding	-0.0401408	0	315	14620
MSTRG.17816.1	noncoding	-0.0151552	1482	1800	2592
MSTRG.17814.6	coding	0.374	138	1314	3749
MSTRG.17814.4	coding	0.369	102	1188	3623
MSTRG.17814.5	coding	0.374	111	1326	3761
MSTRG.17817.2	noncoding	-0.0057344	840	1050	1204
MSTRG.17817.1	coding	0.113	0	840	1151
MSTRG.17817.3	coding	0.097	0	765	1313
MSTRG.17810.2	coding	0.361	99	1632	3128
MSTRG.17811.3	coding	0.415	588	1251	1406
MSTRG.17825.4	coding	0.365	117	2814	5991
MSTRG.17826.4	coding	0.564	0	1548	1719
MSTRG.17826.3	coding	0.489	0	1440	1613
MSTRG.17826.2	coding	0.486	0	1503	1679
MSTRG.17826.7	coding	0.478	72	732	904
MSTRG.17826.8	coding	0.469	309	1026	1197
MSTRG.17826.6	coding	0.468	180	897	1071
MSTRG.17826.9	coding	0.472	63	786	958
MSTRG.17828.6	coding	0.364	2013	2550	6311
MSTRG.17828.7	coding	0.349	4809	5508	9270
MSTRG.17828.3	coding	0.406	0	669	4348
MSTRG.17829.1	noncoding	-0.0008192	75	219	2377
MSTRG.17830.4	coding	0.314	18	651	2249
MSTRG.17830.5	coding	0.313	18	648	2246
MSTRG.17830.6	coding	0.319	2352	2901	4499
MSTRG.17832.2	coding	0.533	81	1392	1547
MSTRG.17833.1	noncoding	-0.0147456	0	18	284
MSTRG.17835.2	coding	0.368	0	903	1354

MSTRG.17836.2	coding	0.485	180	1827	2150
MSTRG.17836.3	coding	0.401	0	2235	2559
MSTRG.17837.3	coding	0.414	0	675	1662
MSTRG.17837.4	coding	0.494	0	315	1594
MSTRG.17839.4	coding	0.422	321	3843	4353
MSTRG.17819.1	noncoding	-0.0417792	588	720	1708
MSTRG.17821.2	coding	0.476	93	1623	2248
MSTRG.17841.2	coding	0.556	756	1761	1937
MSTRG.17841.3	coding	0.607	0	966	1140
MSTRG.17841.4	coding	0.557	162	1110	1286
MSTRG.17841.5	coding	0.556	831	1836	2012
MSTRG.17841.6	coding	0.557	237	1185	1361
MSTRG.17842.1	coding	0.455	1125	1749	2270
MSTRG.17842.4	coding	0.517	0	912	1432
MSTRG.17840.1	coding	0.533	120	1671	1828
MSTRG.17840.2	coding	0.526	15	1683	1846
MSTRG.17845.4	coding	0.331	1026	2280	2609
MSTRG.17847.1	coding	0.389	0	723	3200
MSTRG.17847.3	coding	0.357	408	1104	1657
MSTRG.17849.1	noncoding	-0.0176128	243	360	519
MSTRG.17848.5	coding	0.375	651	7251	7882
MSTRG.17856.1	coding	0.306	894	1491	1646
MSTRG.17863.4	coding	0.344	810	1653	1815
MSTRG.17863.2	coding	0.374	0	1395	1556
MSTRG.17863.3	coding	0.375	438	1635	1796
MSTRG.17863.5	coding	0.344	591	1434	1595
MSTRG.17873.4	coding	0.246	1077	1935	3236
MSTRG.17873.3	coding	0.298	0	1590	2890
MSTRG.17878.1	coding	0.386	0	2880	4346
MSTRG.17878.2	coding	0.412	0	2793	4259
MSTRG.17878.4	coding	0.395	0	2940	3776
MSTRG.17878.5	coding	0.372	0	1839	3475
MSTRG.17878.6	noncoding	-0.0335872	2889	3264	5275
MSTRG.17879.2	noncoding	-0.069632	240	933	1108
MSTRG.17878.7	noncoding	-0.0241664	3618	4062	4291
MSTRG.17865.1	noncoding	-0.012288	0	276	653
MSTRG.17866.1	noncoding	-0.0118784	255	321	1152
MSTRG.17867.1	noncoding	-0.0188416	414	561	1066
MSTRG.17891.1	coding	0.403	201	1536	3716
MSTRG.17892.2	coding	0.092	3759	4425	5342
MSTRG.17892.3	coding	0.067	447	600	3793
MSTRG.17892.4	noncoding	-0.045056	3942	4254	8565
MSTRG.17892.5	coding	0.092	3162	3828	4745
MSTRG.17892.7	noncoding	-0.012288	561	651	1754
MSTRG.17903.1	noncoding	-0.0176128	96	195	425
MSTRG.17904.6	coding	0.491	0	1383	1862
MSTRG.17904.5	coding	0.414	1767	3540	4021
MSTRG.17904.7	coding	0.502	549	1467	1915
MSTRG.17908.1	coding	0.27	0	690	1819
MSTRG.17906.1	noncoding	-0.0004096	0	0	579
MSTRG.17907.2	coding	0.528	0	402	1199
MSTRG.17912.2	noncoding	-0.0688128	87	369	524
MSTRG.17913.1	noncoding	-0.028672	420	543	1375
MSTRG.17921.1	coding	0.07	27	1197	2370

MSTRG.17921.5	coding	0.088	24	1347	1862
MSTRG.17923.1	coding	0.48	192	1185	1714
MSTRG.17924.1	coding	0.084	387	735	1275
MSTRG.17923.2	noncoding	-0.0708608	354	729	929
MSTRG.17923.3	coding	0.477	57	1017	1546
MSTRG.17923.4	coding	0.465	66	1086	2949
MSTRG.17923.5	noncoding	-0.024576	48	165	922
MSTRG.17918.1	noncoding	-0.008192	300	390	1291
MSTRG.17929.1	noncoding	-0.057344	240	291	1545
MSTRG.17919.1	noncoding	-0.0561152	0	153	324
MSTRG.17931.1	coding	0.246	0	891	1384
MSTRG.17931.3	noncoding	-0.0708608	54	360	616
MSTRG.17931.4	noncoding	-0.0700416	0	378	633
MSTRG.17941.1	coding	0.548	165	1680	4699
MSTRG.17942.3	coding	0.039	330	1662	3157
MSTRG.17943.4	noncoding	-0.04096	291	942	1133
MSTRG.17943.2	coding	0.197	75	1173	1362
MSTRG.17943.3	coding	0.202	441	1551	1740
MSTRG.17954.3	coding	0.201	3099	4179	5245
MSTRG.17952.2	coding	0.35	0	1191	3239
MSTRG.17958.1	coding	0.38	1749	2817	4559
MSTRG.17963.2	noncoding	-0.0004096	285	285	466
MSTRG.17963.1	noncoding	-0.380928	279	288	609
MSTRG.17972.5	coding	0.179	441	2574	5681
MSTRG.17972.7	coding	0.179	519	2652	5758
MSTRG.17972.6	coding	0.181	519	2631	12521
MSTRG.17972.10	coding	0.179	480	2613	5720
MSTRG.17972.8	coding	0.181	426	2538	5646
MSTRG.17972.9	coding	0.179	405	2538	5646
MSTRG.17972.11	coding	0.181	552	2664	3194
MSTRG.17973.1	noncoding	-0.02048	351	405	1308
MSTRG.17975.1	coding	0.465	0	1344	1681
MSTRG.17978.2	coding	0.468	234	1788	2136
MSTRG.17980.2	coding	0.411	381	2040	3925
MSTRG.17980.3	coding	0.414	1821	3690	5574
MSTRG.17981.1	noncoding	-0.004096	1578	1680	1837
MSTRG.17982.2	coding	0.297	135	1557	3146
MSTRG.17984.8	coding	0.367	219	2181	4842
MSTRG.17984.7	coding	0.337	219	1332	5734
MSTRG.17984.10	coding	0.387	1365	3501	6163
MSTRG.17985.1	noncoding	-0.0053248	864	1014	1663
MSTRG.17986.2	coding	0.243	0	4911	6739
MSTRG.17987.3	coding	0.527	819	2019	5109
MSTRG.17987.4	coding	0.527	855	2055	6452
MSTRG.17987.5	coding	0.507	1587	3342	7723
MSTRG.17988.1	noncoding	-0.139264	21	66	373
MSTRG.17989.1	noncoding	-0.0647168	0	321	1175
MSTRG.17999.2	coding	0.346	8976	12756	13261
MSTRG.17999.3	coding	0.327	9024	12240	14482
MSTRG.17999.5	coding	0.16	84	885	9524
MSTRG.17995.1	coding	0.381	0	333	521
MSTRG.17996.1	noncoding	-0.0131072	243	414	1033
MSTRG.17998.1	noncoding	-0.06144	162	243	521
MSTRG.18002.1	coding	0.335	123	558	817

MSTRG.18015.1	coding	0.375	3189	3834	4763
MSTRG.18019.1	noncoding	-0.032768	1044	1122	1605
MSTRG.18024.4	coding	0.251	27	1032	1835
MSTRG.18024.9	coding	0.431	351	1281	2608
MSTRG.18024.11	coding	0.408	261	1149	1588
MSTRG.18024.12	coding	0.304	408	951	1752
MSTRG.18024.13	coding	0.315	186	714	2059
MSTRG.18025.1	noncoding	-0.110592	2370	2433	3246
MSTRG.18026.2	coding	0.246	387	1005	1724
MSTRG.18023.2	coding	0.52	807	1980	2692
MSTRG.18031.3	coding	0.047	1275	2838	5411
MSTRG.18031.4	coding	0.047	1620	3183	5756
MSTRG.18032.1	noncoding	-0.012288	15	156	7259
MSTRG.18033.1	coding	0.104	1272	1464	5444
MSTRG.18037.2	coding	0.498	0	459	653
MSTRG.18038.3	coding	0.199	423	1680	2078
MSTRG.18038.2	coding	0.134	156	1506	1906
MSTRG.18040.7	noncoding	-0.0016384	1251	1494	2222
MSTRG.18040.10	coding	0.143	456	3519	4140
MSTRG.18040.9	coding	0.11	711	6738	7359
MSTRG.18040.12	coding	0.113	321	6081	6701
MSTRG.18040.13	coding	0.143	243	3306	3926
MSTRG.18040.4	coding	0.11	399	6459	7080
MSTRG.18040.8	coding	0.142	138	3237	3856
MSTRG.18046.1	noncoding	-0.0069632	180	423	874
MSTRG.18050.1	coding	0.107	0	918	1850
MSTRG.18051.1	noncoding	-0.02048	0	114	661
MSTRG.18054.3	coding	0.135	24	879	1081
MSTRG.18052.1	coding	0.128	72	948	1515
MSTRG.18063.1	coding	0.288	1959	3504	5196
MSTRG.18063.2	coding	0.295	285	1806	3456
MSTRG.18063.3	coding	0.288	1902	3447	5092
MSTRG.18063.5	coding	0.056	585	3366	5403
MSTRG.18064.2	coding	0.177	1071	1434	2219
MSTRG.18066.7	noncoding	-0.0978944	1605	1914	3359
MSTRG.18066.5	noncoding	-0.0770048	1665	1923	2455
MSTRG.18066.6	noncoding	-0.0815104	1605	1857	2389
MSTRG.18060.1	coding	0.307	0	2025	2199
MSTRG.18068.1	noncoding	-0.0487424	4482	4818	5040
MSTRG.18068.3	noncoding	-0.0487424	4575	4911	5133
MSTRG.18068.4	noncoding	-0.0487424	4461	4797	4995
MSTRG.18069.1	coding	0.474	918	3087	6546
MSTRG.18069.2	coding	0.403	8634	9072	9227
MSTRG.18070.1	noncoding	-0.049152	294	399	646
MSTRG.18069.4	coding	0.474	1341	3507	6966
MSTRG.18069.5	coding	0.417	537	3411	6869
MSTRG.18073.1	noncoding	-0.0569344	474	744	4657
MSTRG.18072.2	coding	0.447	0	1287	2974
MSTRG.18072.4	coding	0.427	1485	2799	2953
MSTRG.18072.8	coding	0.41	3162	4431	4586
MSTRG.18074.2	coding	0.35	141	1158	1398
MSTRG.18093.6	coding	0.189	1209	2313	3828
MSTRG.18093.2	coding	0.188	1002	1968	3481
MSTRG.18093.4	coding	0.186	207	1179	2694

MSTRG.18093.8	noncoding	-0.0188416	1743	1980	2388
MSTRG.18095.4	coding	0.136	960	1329	2310
MSTRG.18098.1	noncoding	-0.0462848	921	1074	2587
MSTRG.18099.2	coding	0.185	0	1767	5753
MSTRG.18101.1	noncoding	-0.028672	22284	22449	28351
MSTRG.18101.2	noncoding	-0.028672	10512	10677	12116
MSTRG.18102.3	coding	0.159	2130	2358	3086
MSTRG.18103.1	coding	0.051	3111	3282	8008
MSTRG.18105.1	noncoding	-0.065536	537	600	867
MSTRG.18106.1	noncoding	-0.012288	120	261	643
MSTRG.18083.2	coding	0.205	0	1053	2053
MSTRG.18083.3	noncoding	-0.1036288	8016	8328	24630
MSTRG.18085.2	noncoding	-0.0372736	21	213	368
MSTRG.18089.1	noncoding	-0.08192	696	780	2482
MSTRG.18091.3	noncoding	-0.063488	801	1134	1658
MSTRG.18089.2	noncoding	-0.0581632	1887	1917	2104
MSTRG.18122.1	noncoding	-0.0094208	0	81	510
MSTRG.18129.1	coding	0.607	0	3912	5864
MSTRG.18130.3	coding	0.437	198	3237	8799
MSTRG.18130.4	coding	0.437	282	3321	8832
MSTRG.18131.5	coding	0.385	4671	10152	10597
MSTRG.18131.4	coding	0.404	885	8940	9385
MSTRG.18131.3	coding	0.404	306	8361	8807
MSTRG.18131.6	coding	0.383	174	5256	7273
MSTRG.18132.1	noncoding	-0.0086016	0	120	785
MSTRG.18135.1	coding	0.472	87	3414	10626
MSTRG.18137.1	noncoding	-0.069632	90	165	516
MSTRG.18137.2	noncoding	-0.0139264	36	213	566
MSTRG.18138.2	noncoding	-0.0155648	654	825	981
MSTRG.18138.1	noncoding	-0.0155648	1059	1230	1386
MSTRG.18148.9	noncoding	-0.0176128	99	225	8654
MSTRG.18149.1	noncoding	-0.0065536	561	609	764
MSTRG.18151.1	noncoding	-0.0569344	225	471	3846
MSTRG.18152.2	coding	0.454	0	426	3924
MSTRG.18152.3	coding	0.253	0	978	2006
MSTRG.18152.4	coding	0.212	585	834	1928
MSTRG.18153.1	coding	0.303	1149	2574	5361
MSTRG.18153.2	coding	0.303	867	2292	4388
MSTRG.18142.3	coding	0.24	183	1572	1885
MSTRG.18147.2	coding	0.504	243	2316	2782
MSTRG.18140.1	noncoding	-0.045056	30	36	273
MSTRG.18140.2	noncoding	-0.045056	30	36	291
MSTRG.18155.1	noncoding	-0.0610304	2694	3021	16261
MSTRG.18155.2	noncoding	-0.0212992	3654	3858	4132
MSTRG.18156.1	noncoding	-0.0057344	4386	4518	8025
MSTRG.18157.1	noncoding	-0.0262144	2679	2793	6823
MSTRG.18158.3	noncoding	-0.012288	69	1434	1709
MSTRG.18159.1	noncoding	-0.0724992	0	477	2522
MSTRG.18161.4	coding	0.45	462	1338	2224
MSTRG.18162.2	coding	0.532	0	1344	4464
MSTRG.18164.1	coding	0.204	0	1314	1600
MSTRG.18169.1	coding	0.331	78	882	1154
MSTRG.18170.3	coding	0.488	0	528	1809
MSTRG.18174.1	coding	0.12	81	324	660

MSTRG.18174.5	noncoding	-0.0434176	48	333	626
MSTRG.18173.1	noncoding	-0.0827392	234	633	789
MSTRG.18173.2	noncoding	-0.0847872	234	651	807
MSTRG.18176.2	noncoding	-0.049152	84	201	413
MSTRG.18177.2	noncoding	-0.0208896	324	411	608
MSTRG.18183.4	coding	0.194	117	735	1002
MSTRG.18191.1	noncoding	-0.0786432	0	111	654
MSTRG.18191.2	noncoding	-0.0221184	429	585	969
MSTRG.18191.3	noncoding	-0.0151552	429	579	966
MSTRG.18191.4	noncoding	-0.0475136	0	129	672
MSTRG.18185.1	noncoding	-0.0323584	1422	1584	1947
MSTRG.18196.2	noncoding	-0.0118784	327	627	1074
MSTRG.18196.3	noncoding	-0.036864	198	270	631
MSTRG.18196.4	noncoding	-0.004096	54	183	711
MSTRG.18198.1	noncoding	-0.0176128	66	198	526
MSTRG.18200.2	noncoding	-0.0425984	105	546	929
MSTRG.18200.3	noncoding	-0.0425984	219	660	1041
MSTRG.18200.4	noncoding	-0.0425984	132	573	955
MSTRG.18200.5	noncoding	-0.0487424	0	171	697
MSTRG.18201.2	coding	0.365	0	1008	1294
MSTRG.18219.2	coding	0.434	708	4242	9443
MSTRG.18219.1	coding	0.448	162	3618	8819
MSTRG.18219.3	coding	0.448	135	3591	4989
MSTRG.18219.4	coding	0.449	0	3456	5252
MSTRG.18219.5	coding	0.466	0	3312	4984
MSTRG.18219.6	coding	0.446	135	3594	5348
MSTRG.18219.8	coding	0.458	405	3060	4705
MSTRG.18210.1	noncoding	-0.0352256	1266	1860	2013
MSTRG.18211.4	coding	0.275	360	1983	2415
MSTRG.18212.2	coding	0.166	2526	3165	3351
MSTRG.18214.1	noncoding	-0.331776	0	417	1397
MSTRG.18214.2	noncoding	-0.1908736	0	249	981
MSTRG.18215.1	noncoding	-0.0512	903	1107	3727
MSTRG.18220.2	coding	0.506	1167	2838	4160
MSTRG.18226.2	coding	0.733	0	264	1446
MSTRG.18229.2	coding	0.286	342	4089	5533
MSTRG.18229.1	coding	0.29	345	4017	5462
MSTRG.18229.5	coding	0.275	69	3963	5376
MSTRG.18234.1	noncoding	-0.024576	150	282	822
MSTRG.18240.1	coding	0.13	243	4440	7884
MSTRG.18240.8	coding	0.13	291	4491	5882
MSTRG.18240.7	coding	0.13	234	4431	6065
MSTRG.18240.9	coding	0.13	252	4449	7894
MSTRG.18240.10	coding	0.13	162	4359	7805
MSTRG.18240.11	coding	0.13	654	4854	6245
MSTRG.18241.1	coding	0.042	2151	2667	4038
MSTRG.18240.12	coding	0.13	2835	7032	8425
MSTRG.18242.2	coding	0.482	132	2724	4454
MSTRG.18242.4	coding	0.506	153	2235	3021
MSTRG.18245.1	noncoding	-0.012288	492	678	2441
MSTRG.18245.2	noncoding	-0.069632	378	438	593
MSTRG.18238.2	coding	0.309	501	5646	7298
MSTRG.18239.1	noncoding	-0.0720896	30	423	4900
MSTRG.18246.2	coding	0.068	432	1641	4000

MSTRG.18246.3	coding	0.031	1827	3861	4608
MSTRG.18252.2	coding	0.505	0	2199	2366
MSTRG.18253.2	coding	0.29	852	6420	7425
MSTRG.18251.1	noncoding	-0.0241664	141	360	1325
MSTRG.18259.2	coding	0.344	0	1122	3579
MSTRG.18261.1	noncoding	-0.065536	597	918	2329
MSTRG.18269.10	noncoding	-0.045056	1563	1650	1803
MSTRG.18269.8	noncoding	-0.053248	1596	1680	1835
MSTRG.18269.6	noncoding	-0.0004096	1599	1710	1866
MSTRG.18269.13	noncoding	-0.0344064	2598	2904	3147
MSTRG.18269.3	noncoding	-0.0557056	1599	1959	4915
MSTRG.18269.7	noncoding	-0.0278528	1794	1908	3631
MSTRG.18269.11	noncoding	-0.0004096	1407	1479	1674
MSTRG.18269.12	noncoding	-0.0888832	1821	2235	2389
MSTRG.18269.1	coding	0.503	0	738	3606
MSTRG.18269.14	coding	0.503	0	738	3027
MSTRG.18269.2	coding	0.503	0	738	5475
MSTRG.18269.4	coding	0.503	0	738	3603
MSTRG.18269.5	coding	0.503	0	738	3030
MSTRG.18269.9	coding	0.503	0	738	3454
MSTRG.18275.1	noncoding	-0.014336	5013	5196	7220
MSTRG.18276.4	noncoding	-0.0937984	0	189	5117
MSTRG.18276.3	noncoding	-0.063488	0	141	5010
MSTRG.18277.1	coding	0.454	294	3150	4451
MSTRG.18277.4	coding	0.268	21	2772	4069
MSTRG.18278.1	noncoding	-0.0110592	297	684	1028
MSTRG.18270.2	coding	0.528	0	960	2441
MSTRG.18274.1	noncoding	-0.0303104	69	261	688
MSTRG.18274.2	noncoding	-0.0344064	570	1656	1988
MSTRG.18274.3	noncoding	-0.0606208	1635	1896	2661
MSTRG.18274.4	noncoding	-0.0638976	321	555	1039
MSTRG.18273.2	coding	0.364	390	753	1332
MSTRG.18292.2	coding	0.518	0	3204	7125
MSTRG.18292.3	coding	0.52	0	3123	6464
MSTRG.18294.2	coding	0.373	9	738	1089
MSTRG.18294.5	coding	0.547	0	1422	1577
MSTRG.18329.1	noncoding	-0.0086016	2169	2364	2518
MSTRG.18346.1	noncoding	-0.0516096	2037	2388	2543
MSTRG.18346.2	noncoding	-0.106496	0	135	1702
MSTRG.18346.3	noncoding	-0.0925696	1068	1632	2094
MSTRG.18347.1	noncoding	-0.0503808	4992	5238	8711
MSTRG.18348.1	coding	0.397	726	3921	12025
MSTRG.18348.2	noncoding	-0.0913408	4572	4968	5680
MSTRG.18349.1	noncoding	-0.049152	23001	23325	35431
MSTRG.18350.1	noncoding	-0.057344	44856	45321	48418
MSTRG.18350.2	noncoding	-0.0323584	42198	42705	45333
MSTRG.18350.3	noncoding	-0.0397312	20793	21378	22085
MSTRG.18351.1	noncoding	-0.0262144	19650	19866	35960
MSTRG.18348.3	coding	0.362	231	3747	11824
MSTRG.18352.1	noncoding	-0.0516096	2562	3075	9884
MSTRG.18353.1	noncoding	-0.0360448	1197	1677	1834
MSTRG.18354.1	noncoding	-0.0217088	387	816	8440
MSTRG.18355.1	noncoding	-0.026624	183	372	783
MSTRG.18297.2	coding	0.418	474	3087	5448

MSTRG.18297.3	coding	0.427	570	3495	5558
MSTRG.18297.6	coding	0.35	0	2649	2805
MSTRG.18297.5	coding	0.407	351	2553	2709
MSTRG.18299.2	noncoding	-0.0032768	627	729	1395
MSTRG.18299.3	noncoding	-0.0483328	684	972	7226
MSTRG.18304.1	noncoding	-0.047104	174	285	489
MSTRG.18306.2	coding	0.122	1122	1374	1784
MSTRG.18306.3	coding	0.136	555	900	1057
MSTRG.18307.1	noncoding	-0.0536576	438	681	835
MSTRG.18321.1	noncoding	-0.0434176	117	312	2454
MSTRG.18369.2	coding	0.408	468	1266	1422
MSTRG.18369.5	coding	0.471	0	744	899
MSTRG.18369.4	coding	0.408	300	1098	1254
MSTRG.18369.3	coding	0.408	342	1140	1295
MSTRG.18371.2	noncoding	-0.0507904	0	639	1762
MSTRG.18373.1	noncoding	-0.12288	669	756	1884
MSTRG.18378.2	coding	0.44	528	1968	2185
MSTRG.18379.6	coding	0.532	696	2565	2795
MSTRG.18381.3	coding	0.314	660	2220	2990
MSTRG.18381.4	coding	0.365	0	2268	3034
MSTRG.18382.3	coding	0.428	7419	8493	8668
MSTRG.18382.4	coding	0.452	0	579	1445
MSTRG.18383.3	coding	0.292	0	1470	1640
MSTRG.18383.1	coding	0.279	888	2379	2751
MSTRG.18386.1	coding	0.411	0	822	977
MSTRG.18386.2	coding	0.397	0	690	845
MSTRG.18398.5	coding	0.522	0	1749	2072
MSTRG.18400.4	coding	0.457	2181	3996	4153
MSTRG.18400.3	coding	0.461	0	5271	5428
MSTRG.18400.5	coding	0.471	1266	6318	6473
MSTRG.18400.7	coding	0.472	33	5019	5173
MSTRG.18400.6	coding	0.468	87	5160	5316
MSTRG.18400.9	coding	0.462	33	4062	4215
MSTRG.18401.1	noncoding	-0.03072	3999	4209	5608
MSTRG.18405.1	noncoding	-0.0847872	1233	3078	3768
MSTRG.18406.1	coding	0.425	0	4875	5102
MSTRG.18408.1	noncoding	-0.0790528	0	1599	2274
MSTRG.18408.3	coding	0.2	0	1266	1521
MSTRG.18408.4	noncoding	-0.0724992	114	1341	1594
MSTRG.18413.2	coding	0.528	0	1950	3660
MSTRG.18413.3	coding	0.505	0	1752	3431
MSTRG.18414.1	coding	0.371	6	1668	2180
MSTRG.18416.4	coding	0.389	42	1920	2075
MSTRG.18416.5	coding	0.462	870	1857	2011
MSTRG.18416.6	coding	0.37	540	1659	1814
MSTRG.18417.1	coding	0.377	408	1842	2210
MSTRG.18417.3	coding	0.257	15	1236	1606
MSTRG.18433.6	coding	0.481	1905	3558	3746
MSTRG.18433.7	coding	0.496	0	1638	1824
MSTRG.18434.3	coding	0.554	0	4626	4921
MSTRG.18435.1	coding	0.633	315	1950	2107
MSTRG.18435.2	coding	0.59	315	1245	1967
MSTRG.18438.1	noncoding	-0.0249856	3855	4440	4595
MSTRG.18438.2	noncoding	-0.0561152	231	519	1179

MSTRG.18447.3	coding	0.489	2724	4578	4731
MSTRG.18448.1	noncoding	-0.0397312	0	543	697
MSTRG.18448.2	noncoding	-0.0774144	0	255	409
MSTRG.18447.5	coding	0.455	180	2319	2472
MSTRG.18424.4	coding	0.336	114	2070	2643
MSTRG.18424.3	coding	0.351	0	3195	3362
MSTRG.18424.2	coding	0.35	0	3150	3320
MSTRG.18425.1	noncoding	-0.1089536	1512	2565	5212
MSTRG.18425.2	noncoding	-0.0954368	588	2274	2933
MSTRG.18425.4	coding	0.266	402	1455	1996
MSTRG.18425.3	coding	0.246	117	1209	1751
MSTRG.18428.3	coding	0.44	0	1239	1585
MSTRG.18428.2	coding	0.414	2091	3318	3666
MSTRG.18428.4	noncoding	-0.0458752	2457	3417	4361
MSTRG.18428.5	noncoding	-0.0458752	2397	3357	4301
MSTRG.18452.2	noncoding	-0.028672	507	612	873
MSTRG.18449.1	noncoding	-0.0462848	0	96	627
MSTRG.18449.2	noncoding	-0.024576	162	267	506
MSTRG.18453.2	noncoding	-0.034816	357	1161	1897
MSTRG.18454.2	noncoding	-0.0339968	405	2223	3039
MSTRG.18454.1	noncoding	-0.0303104	123	2301	3119
MSTRG.18454.3	noncoding	-0.0294912	0	2172	2989
MSTRG.18459.1	noncoding	-0.0364544	858	7335	7520
MSTRG.18462.1	coding	0.474	129	2688	2843
MSTRG.18462.5	coding	0.488	750	3684	3838
MSTRG.18462.7	coding	0.434	249	3051	3204
MSTRG.18455.1	coding	0.51	0	2397	2553
MSTRG.18464.1	noncoding	-0.1978368	0	396	549
MSTRG.18466.1	noncoding	-0.0659456	201	645	1187
MSTRG.18465.3	coding	0.517	24	3861	4014
MSTRG.18465.2	coding	0.519	24	3894	4816
MSTRG.18465.5	coding	0.491	15	4062	4974
MSTRG.18465.4	coding	0.517	15	3891	5141
MSTRG.18468.2	coding	0.48	45	1323	1754
MSTRG.18470.4	coding	0.57	3315	5115	5591
MSTRG.18470.6	coding	0.302	2901	3183	4117
MSTRG.18470.8	noncoding	-0.0200704	252	663	1720
MSTRG.18470.9	coding	0.462	9852	12468	15568
MSTRG.18470.11	noncoding	-0.0724992	4818	5202	6589
MSTRG.18469.2	coding	0.425	0	3903	5325
MSTRG.18477.2	coding	0.352	243	3387	4439
MSTRG.18477.1	coding	0.351	273	3528	4582
MSTRG.18477.3	coding	0.361	237	3243	4299
MSTRG.18477.4	coding	0.304	231	2844	7177
MSTRG.18477.5	coding	0.351	231	3396	4448
MSTRG.18477.6	coding	0.348	222	3375	4429
MSTRG.18477.7	coding	0.351	468	3621	4675
MSTRG.18477.8	coding	0.361	216	3243	4296
MSTRG.18477.9	coding	0.36	426	3468	4521
MSTRG.18477.10	coding	0.342	225	3120	4175
MSTRG.18477.11	noncoding	-0.053248	39	117	768
MSTRG.18477.12	coding	0.389	2721	5241	6296
MSTRG.18478.1	noncoding	-0.0598016	3741	3966	6460
MSTRG.18477.14	coding	0.483	3996	4749	5802

MSTRG.18479.1	noncoding	-0.034816	69	420	836
MSTRG.18479.2	noncoding	-0.034816	69	420	974
MSTRG.18472.8	coding	0.257	0	3120	14759
MSTRG.18473.1	noncoding	-0.0339968	159	468	1321
MSTRG.18472.12	coding	0.473	396	1713	5410
MSTRG.18474.2	noncoding	-0.0126976	447	603	1254
MSTRG.18481.1	coding	0.457	411	3408	4077
MSTRG.18482.1	noncoding	-0.094208	114	183	1377
MSTRG.18483.2	noncoding	-0.0299008	564	717	986
MSTRG.18484.1	noncoding	-0.1212416	564	870	1521
MSTRG.18484.2	noncoding	-0.1212416	564	870	1625
MSTRG.18484.3	noncoding	-0.0454656	735	1152	1307
MSTRG.18480.2	coding	0.537	405	1191	2491
MSTRG.18485.1	noncoding	-0.032768	120	201	357
MSTRG.18487.1	noncoding	-0.0499712	759	954	1130
MSTRG.18487.2	noncoding	-0.0823296	3825	4200	4355
MSTRG.18492.2	coding	0.277	81	3651	3805
MSTRG.18490.1	noncoding	-0.0356352	0	153	380
MSTRG.18499.2	coding	0.193	0	918	1420
MSTRG.18500.1	noncoding	-0.008192	174	696	1521
MSTRG.18504.1	coding	0.089	933	1176	1778
MSTRG.18507.4	noncoding	-0.04096	4458	4569	10368
MSTRG.18507.5	noncoding	-0.110592	1176	1248	1520
MSTRG.18507.6	noncoding	-0.045056	1086	1197	3643
MSTRG.18508.2	noncoding	-0.0438272	171	1092	3386
MSTRG.18508.3	noncoding	-0.043008	174	576	2842
MSTRG.18511.1	coding	0.415	1014	2088	6354
MSTRG.18511.2	coding	0.415	984	2058	2626
MSTRG.18511.3	coding	0.415	1116	2190	2756
MSTRG.18511.4	coding	0.415	1068	2142	2710
MSTRG.18513.3	coding	0.309	0	897	2986
MSTRG.18513.2	coding	0.355	213	708	2799
MSTRG.18516.2	coding	0.096	483	3369	8897
MSTRG.18516.5	coding	0.108	285	3141	8620
MSTRG.18517.1	noncoding	-0.0217088	156	234	1208
MSTRG.18514.2	coding	0.195	432	1119	1449
MSTRG.18514.3	coding	0.152	432	1041	1371
MSTRG.18514.4	coding	0.22	0	1269	1598
MSTRG.18519.1	noncoding	-0.0004096	0	0	291
MSTRG.18524.1	coding	0.12	123	2550	3788
MSTRG.18524.2	coding	0.118	105	2430	4564
MSTRG.18524.4	coding	0.083	330	2364	3074
MSTRG.18527.1	coding	0.426	0	3324	3478
MSTRG.18527.2	noncoding	-0.0090112	237	339	823
MSTRG.18529.3	coding	0.137	0	1014	1765
MSTRG.18529.5	coding	0.058	306	1146	1705
MSTRG.18529.4	coding	0.074	243	1083	1715
MSTRG.18530.1	noncoding	-0.0258048	10491	10770	10927
MSTRG.18530.2	noncoding	-0.018432	5811	5937	10540
MSTRG.18531.1	noncoding	-0.094208	3789	3882	5922
MSTRG.18536.1	noncoding	-0.0004096	0	0	344
MSTRG.18538.1	noncoding	-0.176128	183	210	402
MSTRG.18540.1	noncoding	-0.086016	198	216	506
MSTRG.18600.2	noncoding	-0.018432	2664	2802	3561

MSTRG.18614.1	coding	0.117	282	474	1473
MSTRG.18615.1	noncoding	-0.02048	1773	1902	2606
MSTRG.18626.2	noncoding	-0.0004096	195	408	1870
MSTRG.18622.6	noncoding	-0.0253952	2271	2520	11406
MSTRG.18622.7	noncoding	-0.135168	2607	2655	5465
MSTRG.18631.2	noncoding	-0.0782336	7398	8520	12067
MSTRG.18631.3	noncoding	-0.311296	2652	2685	3117
MSTRG.18632.1	noncoding	-0.024576	1329	1431	4823
MSTRG.18631.4	noncoding	-0.0782336	4146	5268	15388
MSTRG.18633.1	noncoding	-0.0004096	0	0	392
MSTRG.18622.8	noncoding	-0.1441792	1278	2049	5055
MSTRG.18622.14	noncoding	-0.1425408	1329	2073	3281
MSTRG.18631.6	coding	0.239	5430	6189	6342
MSTRG.18631.7	noncoding	-0.06144	0	78	233
MSTRG.18622.16	noncoding	-0.0049152	12	153	308
MSTRG.18622.17	noncoding	-0.0372736	576	759	7428
MSTRG.18631.9	noncoding	-0.0782336	1407	2529	3402
MSTRG.18631.8	coding	0.231	525	2562	3981
MSTRG.18622.20	noncoding	-0.012288	768	888	4796
MSTRG.18622.21	noncoding	-0.012288	762	882	4790
MSTRG.18622.22	noncoding	-0.049152	684	723	1218
MSTRG.18631.10	coding	0.261	525	2070	2225
MSTRG.18622.34	noncoding	-0.1441792	4728	5499	6660
MSTRG.18638.1	noncoding	-0.057344	9	114	268
MSTRG.18637.1	noncoding	-0.004096	3	147	301
MSTRG.18542.3	coding	0.556	141	1131	4476
MSTRG.18546.4	coding	0.149	0	861	1014
MSTRG.18546.5	noncoding	-0.0401408	20637	20871	28286
MSTRG.18546.6	coding	0.109	1299	1479	38258
MSTRG.18546.7	coding	0.031	237	492	4448
MSTRG.18546.8	coding	0.047	237	642	22046
MSTRG.18546.9	coding	0.054	237	633	20728
MSTRG.18546.10	coding	0.101	0	537	13940
MSTRG.18546.11	coding	0.116	0	396	2401
MSTRG.18546.12	noncoding	-0.0356352	18822	19044	26496
MSTRG.18546.13	coding	0.101	0	537	8816
MSTRG.18546.14	coding	0.098	0	654	5964
MSTRG.18548.2	noncoding	-0.02048	12	192	356
MSTRG.18551.1	noncoding	-0.12288	5511	5577	7390
MSTRG.18552.1	noncoding	-0.0393216	7680	7968	12535
MSTRG.18552.2	noncoding	-0.034816	2982	3183	4090
MSTRG.18555.1	coding	0.313	180	2460	5472
MSTRG.18555.2	coding	0.313	432	2712	4808
MSTRG.18557.4	coding	0.444	0	198	1232
MSTRG.18558.1	noncoding	-0.0577536	396	414	2857
MSTRG.18559.1	noncoding	-0.0749568	0	660	3464
MSTRG.18559.4	coding	0.023	1242	1851	5866
MSTRG.18559.6	noncoding	-0.0749568	0	660	5119
MSTRG.18561.1	coding	0.102	18	2349	4210
MSTRG.18561.3	coding	0.045	93	456	3157
MSTRG.18562.1	coding	0.36	441	906	1327
MSTRG.18562.2	coding	0.36	525	990	1372
MSTRG.18562.3	coding	0.356	201	1113	1733
MSTRG.18563.1	noncoding	-0.012288	237	327	497

MSTRG.18564.1	coding	0.24	0	1794	7457
MSTRG.18564.2	coding	0.256	0	1383	6822
MSTRG.18565.1	coding	0.232	0	546	3362
MSTRG.18565.2	coding	0.21	66	1107	1565
MSTRG.18568.1	coding	0.099	75	4329	5095
MSTRG.18568.3	coding	0.273	0	897	2548
MSTRG.18568.6	coding	0.241	801	1296	2854
MSTRG.18572.1	coding	0.083	528	3108	4791
MSTRG.18572.4	coding	0.083	972	3552	5236
MSTRG.18574.4	coding	0.096	1647	2799	3179
MSTRG.18574.5	coding	0.096	1746	2898	3276
MSTRG.18574.6	coding	0.094	1860	3009	3389
MSTRG.18574.7	noncoding	-0.0172032	3900	4053	7799
MSTRG.18567.2	noncoding	-0.0176128	2007	2139	3083
MSTRG.18589.1	noncoding	-0.0004096	0	0	215
MSTRG.18590.1	noncoding	-0.0004096	0	168	327
MSTRG.18594.2	coding	0.192	0	540	1117
MSTRG.18594.3	coding	0.141	141	387	679
MSTRG.18594.5	coding	0.144	342	837	1130
MSTRG.18594.6	coding	0.163	264	468	1043
MSTRG.18594.7	coding	0.079	0	642	935
MSTRG.18654.3	noncoding	-0.0802816	5301	5637	5915
MSTRG.18654.4	coding	0.309	360	12819	15740
MSTRG.18656.1	noncoding	-0.024576	594	729	919
MSTRG.18645.1	noncoding	-0.04096	3666	3786	4533
MSTRG.18647.2	coding	0.348	0	570	980
MSTRG.18653.1	noncoding	-0.08192	27	30	387
MSTRG.18659.1	coding	0.306	0	852	1157
MSTRG.18659.5	coding	0.042	13380	13539	18100
MSTRG.18662.5	coding	0.261	291	4977	6008
MSTRG.18662.3	coding	0.245	0	5454	6485
MSTRG.18666.8	coding	0.099	855	1377	3171
MSTRG.18666.9	coding	0.069	897	1140	3286
MSTRG.18666.4	coding	0.158	0	405	2550
MSTRG.18666.5	coding	0.069	294	537	2681
MSTRG.18666.6	coding	0.082	171	513	2658
MSTRG.18666.7	coding	0.069	249	492	2638
MSTRG.18666.10	coding	0.101	720	1341	3084
MSTRG.18667.2	coding	0.239	501	1500	2438
MSTRG.18677.1	noncoding	-0.0151552	285	489	1917
MSTRG.18679.3	noncoding	-0.0135168	45	216	380
MSTRG.18681.1	noncoding	-0.016384	189	267	423
MSTRG.18684.2	coding	0.356	0	924	1376
MSTRG.18685.1	noncoding	-0.053248	0	45	632
MSTRG.18698.1	noncoding	-0.016384	3372	3447	3602
MSTRG.18697.4	coding	0.243	246	939	1663
MSTRG.18702.2	coding	0.208	5901	6615	8823
MSTRG.18703.1	noncoding	-0.0036864	1932	2082	3072
MSTRG.18704.1	noncoding	-0.0315392	3492	3615	5259
MSTRG.18705.1	noncoding	-0.110592	420	471	1209
MSTRG.18706.1	noncoding	-0.0536576	1536	1761	7080
MSTRG.18707.1	noncoding	-0.057344	51	66	8950
MSTRG.18709.2	coding	0.231	126	999	11646
MSTRG.18709.3	noncoding	-0.1056768	126	576	8951

MSTRG.18709.4	coding	0.339	123	522	12331
MSTRG.18711.4	coding	0.122	0	504	1738
MSTRG.18713.1	noncoding	-0.0208896	75	162	547
MSTRG.18715.2	coding	0.11	0	744	900
MSTRG.18715.4	coding	0.317	189	1794	1950
MSTRG.18722.1	noncoding	-0.0393216	0	489	898
MSTRG.18725.6	coding	0.171	2880	4086	6350
MSTRG.18725.7	coding	0.177	1278	2592	5857
MSTRG.18726.1	noncoding	-0.0028672	426	606	852
MSTRG.18718.1	noncoding	-0.004096	363	522	2496
MSTRG.18732.2	noncoding	-0.0258048	2616	2757	5817
MSTRG.18733.1	noncoding	-0.0565248	945	996	2266
MSTRG.18734.1	noncoding	-0.0401408	2949	3057	9700
MSTRG.18734.2	noncoding	-0.192512	432	450	4446
MSTRG.18741.3	coding	0.226	150	951	2597
MSTRG.18743.3	coding	0.096	471	1011	1395
MSTRG.18744.1	coding	0.206	45	807	2550
MSTRG.18749.1	coding	0.132	0	3810	5107
MSTRG.18749.2	coding	0.133	0	3822	6270
MSTRG.18749.3	coding	0.133	0	3822	8219
MSTRG.18750.1	noncoding	-0.073728	72	90	845
MSTRG.18751.2	coding	0.049	201	594	1318
MSTRG.18751.4	coding	0.049	195	588	805
MSTRG.18752.1	noncoding	-0.055296	2319	2520	2808
MSTRG.18752.2	noncoding	-0.1024	2319	2403	2558
MSTRG.18762.2	noncoding	-0.0180224	1461	1641	3761
MSTRG.18762.1	noncoding	-0.0180224	1461	1641	3664
MSTRG.18763.1	coding	0.106	810	4347	4697
MSTRG.18762.3	coding	0.01	687	1071	1738
MSTRG.18764.1	noncoding	-0.045056	1365	1458	4573
MSTRG.18762.4	noncoding	-0.0004096	861	936	1628
MSTRG.18763.2	coding	0.119	1743	4332	4683
MSTRG.18763.4	coding	0.106	171	3708	4058
MSTRG.18763.5	coding	0.106	171	3705	4055
MSTRG.18763.7	coding	0.105	1287	4293	4644
MSTRG.18768.2	coding	0.117	0	6201	7446
MSTRG.18756.1	coding	0.071	0	480	673
MSTRG.18761.1	coding	0.256	600	3096	5527
MSTRG.18769.5	coding	0.253	2550	6624	10337
MSTRG.18769.6	coding	0.256	327	4626	7945
MSTRG.18770.1	noncoding	-0.0397312	3132	3483	7025
MSTRG.18771.1	noncoding	-0.110592	1140	1185	1411
MSTRG.18772.1	noncoding	-0.1040384	0	69	487
MSTRG.18773.1	noncoding	-0.004096	453	777	1078
MSTRG.18776.1	noncoding	-0.1253376	0	285	552
MSTRG.18805.1	coding	0.053	15294	16533	18699
MSTRG.18805.2	noncoding	-0.0212992	7410	7572	7727
MSTRG.18816.4	noncoding	-0.094208	504	549	703
MSTRG.18816.5	noncoding	-0.0180224	624	783	1840
MSTRG.18818.2	coding	0.505	0	339	3219
MSTRG.18825.7	coding	0.102	366	1674	7942
MSTRG.18829.1	noncoding	-0.032768	3186	3276	8023
MSTRG.18821.1	noncoding	-0.08192	615	678	1410
MSTRG.18822.4	coding	0.024	1110	1341	1847

MSTRG.18830.2	noncoding	-0.06144	156	204	2776
MSTRG.18830.3	noncoding	-0.028672	108	141	298
MSTRG.18838.1	noncoding	-0.008192	0	261	414
MSTRG.18841.1	noncoding	-0.0847872	603	894	1212
MSTRG.18850.1	noncoding	-0.0004096	21	21	505
MSTRG.18852.1	coding	0.225	96	7134	10781
MSTRG.18861.1	noncoding	-0.045056	621	672	1167
MSTRG.18862.1	noncoding	-0.0159744	216	222	378
MSTRG.18872.1	noncoding	-0.0372736	0	36	218
MSTRG.18882.1	noncoding	-0.0196608	18	333	488
MSTRG.18894.1	coding	0.075	99	312	517
MSTRG.18894.4	coding	0.092	234	522	723
MSTRG.18894.6	coding	0.113	198	435	636
MSTRG.18894.7	coding	0.079	48	333	535
MSTRG.18894.3	coding	0.096	21	294	494
MSTRG.18894.5	coding	0.092	111	399	601
MSTRG.18906.1	noncoding	-0.0643072	4092	4218	9654
MSTRG.18907.1	noncoding	-0.0315392	2073	2208	3175
MSTRG.18908.1	noncoding	-0.106496	507	555	1050
MSTRG.18911.1	noncoding	-0.098304	39	99	252
MSTRG.18917.2	coding	0.257	636	2391	4023
MSTRG.18917.3	coding	0.257	1041	2796	4239
MSTRG.18958.1	noncoding	-0.028672	183	219	438
MSTRG.18967.1	noncoding	-0.0106496	81	279	1308
MSTRG.18967.3	coding	0.084	684	1269	5440
MSTRG.18955.1	noncoding	-0.135168	300	330	1416
MSTRG.18979.1	noncoding	-0.0438272	318	501	1019
MSTRG.18983.2	coding	0.035	30	3210	4150
MSTRG.18986.1	coding	0.065	606	798	1651
MSTRG.18987.1	coding	0.068	729	900	2778
MSTRG.18984.2	coding	0.014	75	243	524
MSTRG.18997.1	noncoding	-0.083968	0	351	896
MSTRG.18997.2	noncoding	-0.098304	0	372	1586
MSTRG.18999.1	noncoding	-0.0319488	315	576	733
MSTRG.18999.2	coding	0.168	375	1035	1191
MSTRG.19005.1	noncoding	-0.0180224	3201	3249	8811
MSTRG.19006.1	noncoding	-0.0086016	4596	4743	16484
MSTRG.19014.6	coding	0.035	0	2148	3409
MSTRG.19014.5	coding	0.042	444	2361	3624
MSTRG.19015.1	noncoding	-0.0659456	84	357	510
MSTRG.19015.2	noncoding	-0.32768	393	408	565
MSTRG.19015.3	noncoding	-0.032768	279	363	850
MSTRG.19009.1	noncoding	-0.0004096	0	0	234
MSTRG.19024.1	noncoding	-0.0004096	0	0	431
MSTRG.19041.1	noncoding	-0.028672	63	69	222
MSTRG.19049.1	coding	0.123	17598	17832	21591
MSTRG.19049.2	coding	0.077	135	633	17093
MSTRG.19049.3	noncoding	-0.0004096	0	0	526
MSTRG.19049.4	noncoding	-0.008192	3471	3660	4763
MSTRG.19052.2	noncoding	-0.0569344	1863	2616	3097
MSTRG.19034.1	noncoding	-0.0495616	765	1077	1232
MSTRG.19038.1	noncoding	-0.086016	639	672	994
MSTRG.19039.3	noncoding	-0.0724992	3723	4104	4312
MSTRG.19053.2	coding	0.127	129	1626	3056

MSTRG.19053.3	coding	0.119	702	1977	3467
MSTRG.19059.4	coding	0.088	336	8223	8794
MSTRG.19059.3	coding	0.086	336	8193	9864
MSTRG.19061.1	coding	0.368	123	1032	1186
MSTRG.19063.1	noncoding	-0.0610304	312	624	780
MSTRG.19066.1	noncoding	-0.024576	165	237	1248
MSTRG.19068.2	noncoding	-0.077824	288	300	586
MSTRG.19069.1	coding	0.201	405	1137	3423
MSTRG.19069.2	coding	0.201	102	834	3121
MSTRG.19069.4	coding	0.201	330	1062	3299
MSTRG.19069.6	noncoding	-0.0851968	24552	25065	25219
MSTRG.19072.1	coding	0.19	0	2199	2689
MSTRG.19072.2	coding	0.187	477	2916	3514
MSTRG.19072.3	coding	0.151	477	3174	3772
MSTRG.19072.4	coding	0.203	0	2019	2701
MSTRG.19073.3	noncoding	-0.098304	0	141	354
MSTRG.19075.1	coding	0.094	2778	2991	5466
MSTRG.19089.2	coding	0.213	7701	9384	10459
MSTRG.19089.3	coding	0.217	1212	3456	3718
MSTRG.19090.2	noncoding	-0.0126976	171	333	1897
MSTRG.19094.1	noncoding	-0.0937984	4269	4725	5027
MSTRG.19094.2	noncoding	-0.100352	4230	4617	4771
MSTRG.19094.3	noncoding	-0.100352	4137	4524	4679
MSTRG.19094.5	coding	0.054	771	1221	2793
MSTRG.19095.1	noncoding	-0.0602112	888	1254	1411
MSTRG.19096.1	coding	0.085	162	12387	14239
MSTRG.19099.1	noncoding	-0.0004096	0	0	901
MSTRG.19100.1	noncoding	-0.028672	195	306	460
MSTRG.19101.1	noncoding	-0.049152	429	510	1354
MSTRG.19078.2	coding	0.128	0	1335	2692
MSTRG.19078.3	coding	0.202	864	1536	2892
MSTRG.19079.1	coding	0.083	501	681	1995
MSTRG.19081.1	coding	0.116	2304	2673	3777
MSTRG.19083.1	noncoding	-0.02048	129	219	565
MSTRG.19084.3	noncoding	-0.1990656	0	141	1886
MSTRG.19085.1	noncoding	-0.057344	237	303	2065
MSTRG.19107.3	coding	0.355	870	3462	4155
MSTRG.19110.1	noncoding	-0.0098304	261	423	895
MSTRG.19113.4	coding	0.298	9	1062	2958
MSTRG.19115.4	coding	0.255	1299	2592	3072
MSTRG.19115.5	coding	0.255	141	1434	1912
MSTRG.19116.2	noncoding	-0.0454656	510	720	2930
MSTRG.19116.4	coding	0.107	636	906	1086
MSTRG.19117.2	coding	0.299	0	990	1262
MSTRG.19121.1	noncoding	-0.0057344	129	276	431
MSTRG.19122.1	noncoding	-0.077824	15	87	294
MSTRG.19126.2	coding	0.212	0	327	2963
MSTRG.19127.1	noncoding	-0.147456	303	321	493
MSTRG.19129.1	noncoding	-0.1024	0	174	4500
MSTRG.19129.2	noncoding	-0.0438272	2805	3021	8897
MSTRG.19129.4	coding	0.096	0	585	2181
MSTRG.19129.5	noncoding	-0.0929792	0	165	2380
MSTRG.19131.8	noncoding	-0.0278528	807	939	1094
MSTRG.19131.7	coding	0.122	342	780	4405

MSTRG.19131.4	coding	0.134	342	708	5493
MSTRG.19131.5	coding	0.107	222	453	5413
MSTRG.19131.6	coding	0.134	222	588	5375
MSTRG.19133.3	noncoding	-0.0618496	1476	1689	2325
MSTRG.19133.4	noncoding	-0.0618496	1473	1686	3756
MSTRG.19134.3	noncoding	-0.0995328	0	138	4262
MSTRG.19134.4	coding	0.113	0	477	3870
MSTRG.19136.1	noncoding	-0.0004096	864	900	1301
MSTRG.19138.1	coding	0.066	8010	13101	13531
MSTRG.19142.5	coding	0.22	558	1929	3739
MSTRG.19148.3	coding	0.246	0	153	2864
MSTRG.19148.4	coding	0.001	642	777	2094
MSTRG.19150.1	coding	0.202	1308	2622	7942
MSTRG.19150.2	coding	0.202	1440	2754	9381
MSTRG.19152.2	coding	0.322	0	546	1305
MSTRG.19144.1	noncoding	-0.0032768	363	525	679
MSTRG.19146.1	noncoding	-0.131072	570	618	774
MSTRG.19155.1	noncoding	-0.0139264	606	765	1129
MSTRG.19156.1	noncoding	-0.053248	0	6	203
MSTRG.19158.4	coding	0.438	945	1668	5488
MSTRG.19175.1	noncoding	-0.360448	294	309	971
MSTRG.19177.4	coding	0.01	5481	5736	6166
MSTRG.19177.1	coding	0.129	0	612	1666
MSTRG.19177.3	coding	0.077	150	462	1476
MSTRG.19177.5	coding	0.129	0	612	1495
MSTRG.19177.6	noncoding	-0.0274432	2346	2511	3466
MSTRG.19177.7	coding	0.081	753	1917	5191
MSTRG.19177.8	coding	0.092	576	1800	2641
MSTRG.19177.9	coding	0.129	0	612	3086
MSTRG.19177.10	coding	0.068	1812	2616	3869
MSTRG.19177.12	noncoding	-0.055296	234	561	2136
MSTRG.19177.13	coding	0.077	150	462	1809
MSTRG.19183.1	noncoding	-0.004096	549	627	1211
MSTRG.19183.2	noncoding	-0.004096	75	153	748
MSTRG.19189.2	noncoding	-0.096256	0	405	2016
MSTRG.19166.2	coding	0.024	1671	1821	3489
MSTRG.19166.3	noncoding	-0.088064	2697	3162	3317
MSTRG.19168.1	noncoding	-0.02048	213	348	2079
MSTRG.19169.4	coding	0.063	1338	1932	3344
MSTRG.19171.7	coding	0.079	1551	1734	1951
MSTRG.19171.2	coding	0.079	1491	1674	1893
MSTRG.19171.3	coding	0.079	1206	1389	1606
MSTRG.19171.4	coding	0.079	1407	1590	1808
MSTRG.19171.5	coding	0.079	1470	1653	1872
MSTRG.19171.6	coding	0.079	1341	1524	1743
MSTRG.19194.2	coding	0.053	255	492	926
MSTRG.19196.1	noncoding	-0.0188416	405	564	1021
MSTRG.19196.2	noncoding	-0.0258048	357	603	1064
MSTRG.19203.1	coding	0.169	96	1110	1541
MSTRG.19203.2	coding	0.175	156	450	648
MSTRG.19204.3	coding	0.25	1029	1491	2761
MSTRG.19205.1	noncoding	-0.0495616	399	564	1568
MSTRG.19205.2	noncoding	-0.0495616	681	846	1835
MSTRG.19205.3	noncoding	-0.004096	0	72	1056

MSTRG.19206.1	noncoding	-0.0425984	39	57	283
MSTRG.19207.2	noncoding	-0.004096	624	849	1239
MSTRG.19210.5	coding	0.073	864	1062	2670
MSTRG.19210.6	coding	0.092	7260	9438	10292
MSTRG.19210.4	coding	0.093	2808	5001	5853
MSTRG.19210.7	coding	0.085	6939	9141	9995
MSTRG.19210.3	coding	0.085	5697	7899	8751
MSTRG.19210.8	noncoding	-0.0671744	3786	4020	4176
MSTRG.19197.1	noncoding	-0.0049152	1509	1899	2171
MSTRG.19202.1	noncoding	-0.069632	336	417	696
MSTRG.19213.1	noncoding	-0.0155648	495	660	2107
MSTRG.19213.2	noncoding	-0.0335872	0	129	1668
MSTRG.19215.1	noncoding	-0.028672	174	459	2296
MSTRG.19217.4	coding	0.072	681	1722	3024
MSTRG.19217.2	coding	0.072	639	1680	2980
MSTRG.19217.5	coding	0.072	681	1716	2319
MSTRG.19218.1	noncoding	-0.02048	6	129	861
MSTRG.19318.1	noncoding	-0.0270336	3438	3669	5925
MSTRG.19319.3	coding	0.251	0	1749	2767
MSTRG.19323.1	noncoding	-0.0380928	192	279	1166
MSTRG.19319.4	noncoding	-0.0638976	9177	9441	9672
MSTRG.19319.5	noncoding	-0.0086016	822	963	3332
MSTRG.19319.6	noncoding	-0.0139264	5121	5289	5934
MSTRG.19325.1	noncoding	-0.049152	2796	2883	4401
MSTRG.19325.2	noncoding	-0.0032768	4410	4908	8539
MSTRG.19326.1	noncoding	-0.012288	573	726	8218
MSTRG.19328.1	noncoding	-0.004096	11388	11514	11791
MSTRG.19338.1	noncoding	-0.0249856	1356	1569	2537
MSTRG.19339.1	noncoding	-0.008192	6045	6183	8312
MSTRG.19340.1	noncoding	-0.02048	2235	2361	6707
MSTRG.19342.1	noncoding	-0.0352256	1542	1623	3415
MSTRG.19347.2	noncoding	-0.0270336	849	1071	12469
MSTRG.19339.2	coding	0.068	22185	22788	33253
MSTRG.19339.3	coding	0.087	5955	6285	10211
MSTRG.19339.4	coding	0.061	5085	5313	8541
MSTRG.19339.5	noncoding	-0.0663552	1521	1698	12283
MSTRG.19339.6	noncoding	-0.016384	2364	2490	6459
MSTRG.19339.7	noncoding	-0.016384	141	267	2642
MSTRG.19339.8	noncoding	-0.0028672	1551	1770	5934
MSTRG.19349.1	noncoding	-0.0741376	1647	1665	2906
MSTRG.19221.1	noncoding	-0.024576	5211	5334	9078
MSTRG.19221.2	noncoding	-0.022528	465	558	2513
MSTRG.19230.1	noncoding	-0.1056768	0	132	1059
MSTRG.19235.1	noncoding	-0.0499712	129	315	470
MSTRG.19249.1	noncoding	-0.002048	15	159	1854
MSTRG.19249.2	noncoding	-0.024576	924	1032	2062
MSTRG.19249.3	noncoding	-0.022528	354	522	744
MSTRG.19249.4	noncoding	-0.0356352	1299	1476	2565
MSTRG.19237.1	coding	0.057	636	978	3282
MSTRG.19252.4	noncoding	-0.0577536	186	645	1789
MSTRG.19253.1	noncoding	-0.1273856	336	678	1201
MSTRG.19253.4	coding	0.319	69	408	919
MSTRG.19253.5	noncoding	-0.0520192	207	384	895
MSTRG.19254.1	coding	0.117	2055	2310	5126

MSTRG.19258.1	noncoding	-0.0868352	0	75	3515
MSTRG.19258.2	noncoding	-0.004096	126	240	2337
MSTRG.19264.1	noncoding	-0.0516096	6912	7377	7782
MSTRG.19265.1	noncoding	-0.0626688	792	1296	1451
MSTRG.19266.2	noncoding	-0.02048	3339	3462	3618
MSTRG.19270.2	coding	0.295	213	1602	2275
MSTRG.19278.1	noncoding	-0.0380928	393	510	667
MSTRG.19281.2	coding	0.189	723	1320	2741
MSTRG.19285.2	coding	0.312	0	231	6074
MSTRG.19285.3	noncoding	-0.036864	471	564	6865
MSTRG.19285.4	coding	0.312	0	231	6468
MSTRG.19291.1	noncoding	-0.045056	3429	3513	4140
MSTRG.19291.2	noncoding	-0.036864	279	333	1617
MSTRG.19283.1	coding	0.149	345	1242	3141
MSTRG.19283.3	coding	0.149	378	1275	6305
MSTRG.19305.4	noncoding	-0.0913408	0	159	2958
MSTRG.19311.1	coding	0.003	1326	1944	3394
MSTRG.19311.2	coding	0.104	0	1695	13532
MSTRG.19311.4	noncoding	-0.0057344	189	291	538
MSTRG.19311.5	coding	0.034	3921	6684	9169
MSTRG.19311.6	coding	0.012	60	2955	5506
MSTRG.19312.1	noncoding	-0.026624	447	657	811
MSTRG.19314.1	noncoding	-0.053248	60	423	3001
MSTRG.19314.3	coding	0.101	84	864	3793
MSTRG.19314.4	coding	0.185	0	504	3058
MSTRG.19316.1	coding	0.091	2376	3003	3376
MSTRG.19314.6	coding	0.033	2490	2775	3486
MSTRG.19316.2	coding	0.091	2376	3003	5284
MSTRG.19316.3	coding	0.091	2376	3003	3645
MSTRG.19316.4	coding	0.091	2391	3018	3594
MSTRG.19317.1	noncoding	-0.0016384	1626	1731	1907
MSTRG.19313.1	coding	0.079	3444	3852	4792
MSTRG.19352.1	coding	0.359	0	1353	5773
MSTRG.19352.3	coding	0.357	0	1287	4163
MSTRG.19352.4	coding	0.232	1200	2028	2855
MSTRG.19352.5	coding	0.236	1782	2562	3389
MSTRG.19352.12	coding	0.227	666	1098	1374
MSTRG.19352.10	coding	0.237	6165	6507	6783
MSTRG.19352.13	coding	0.223	21	831	1108
MSTRG.19352.9	coding	0.218	21	891	1168
MSTRG.19352.8	coding	0.233	1782	2604	2879
MSTRG.19352.11	coding	0.252	2076	2820	3097
MSTRG.19352.6	coding	0.35	0	1386	1661
MSTRG.19354.1	noncoding	-0.0077824	1182	1302	3926
MSTRG.19359.2	coding	0.246	0	141	1426
MSTRG.19359.3	noncoding	-0.02048	1392	1434	4112
MSTRG.19359.4	coding	0.211	0	138	2305
MSTRG.19359.5	coding	0.259	0	123	1150
MSTRG.19361.1	noncoding	-0.0483328	5811	6021	6176
MSTRG.19361.2	noncoding	-0.0483328	4320	4530	4686
MSTRG.19361.3	noncoding	-0.055296	1668	1872	2027
MSTRG.19361.4	noncoding	-0.0483328	4086	4296	4453
MSTRG.19361.5	noncoding	-0.0483328	7572	7782	7937
MSTRG.19361.6	noncoding	-0.0483328	11769	11979	12135

MSTRG.19361.10	coding	0.03	1128	1269	1662
MSTRG.19361.7	noncoding	-0.0483328	3204	3414	3570
MSTRG.19361.8	noncoding	-0.0483328	2376	2586	2742
MSTRG.19361.9	noncoding	-0.0483328	3486	3696	3852
MSTRG.19361.11	noncoding	-0.0483328	4278	4488	4644
MSTRG.19361.12	noncoding	-0.0483328	13494	13704	13860
MSTRG.19361.13	noncoding	-0.0483328	8004	8214	8371
MSTRG.19361.14	noncoding	-0.0126976	2385	2514	7824
MSTRG.19363.1	noncoding	-0.016384	159	450	632
MSTRG.19401.1	noncoding	-0.0126976	2736	2805	3876
MSTRG.19402.1	noncoding	-0.0004096	0	0	426
MSTRG.19368.1	noncoding	-0.0004096	0	0	223
MSTRG.19369.1	noncoding	-0.0053248	315	573	728
MSTRG.19370.1	noncoding	-0.0004096	0	0	358
MSTRG.19407.1	noncoding	-0.151552	648	675	917
MSTRG.19408.1	noncoding	-0.1241088	1272	1794	1973
MSTRG.19414.1	coding	0.112	558	2031	2709
MSTRG.19414.2	coding	0.096	9	1956	2798
MSTRG.19415.1	coding	0.105	837	2130	2868
MSTRG.19415.3	coding	0.133	0	1620	1789
MSTRG.19415.4	coding	0.103	174	1464	1632
MSTRG.19416.1	noncoding	-0.0577536	3	309	2739
MSTRG.19416.2	coding	0.025	630	996	1834
MSTRG.19413.1	noncoding	-0.0278528	957	1299	1454
MSTRG.19420.2	coding	0.189	0	1560	9443
MSTRG.19422.1	coding	0.384	705	1449	1604
MSTRG.19422.3	coding	0.44	705	2124	4664
MSTRG.19422.2	coding	0.466	783	1926	2809
MSTRG.19422.4	coding	0.459	804	2001	2511
MSTRG.19422.5	coding	0.461	783	1926	2427
MSTRG.19423.1	noncoding	-0.0131072	1218	1374	3239
MSTRG.19425.1	coding	0.471	84	2805	4809
MSTRG.19425.4	coding	0.459	432	3228	8463
MSTRG.19428.1	coding	0.16	60	2310	2984
MSTRG.19432.1	coding	0.463	0	144	1062
MSTRG.19434.1	coding	0.034	99	2631	3328
MSTRG.19435.1	noncoding	-0.151552	273	309	1634
MSTRG.19436.1	noncoding	-0.0323584	975	1155	1445
MSTRG.19440.2	noncoding	-0.0139264	1902	2043	2207
MSTRG.19440.1	noncoding	-0.0004096	315	426	592
MSTRG.19441.1	noncoding	-0.059392	255	870	1025
MSTRG.19446.2	coding	0.065	1377	2037	6547
MSTRG.19464.1	coding	0.036	6663	9420	11294
MSTRG.19464.6	noncoding	-0.073728	30	78	812
MSTRG.19478.2	coding	0.116	0	1113	3199
MSTRG.19479.1	noncoding	-0.004096	837	972	1248
MSTRG.19448.1	noncoding	-0.053248	39	96	1542
MSTRG.19458.2	coding	0.429	0	66	2722
MSTRG.19458.4	noncoding	-0.0671744	0	42	2707
MSTRG.19458.3	noncoding	-0.004096	195	294	621
MSTRG.19458.5	coding	0.198	1731	4275	10278
MSTRG.19459.1	noncoding	-0.0405504	1965	2133	3968
MSTRG.19481.1	noncoding	-0.065536	5655	5733	9242
MSTRG.19480.2	coding	0.263	6411	6921	9592

MSTRG.19485.1	coding	0.076	342	639	6734
MSTRG.19485.2	noncoding	-0.0749568	174	852	6949
MSTRG.19485.3	noncoding	-0.0946176	0	483	872
MSTRG.19486.1	noncoding	-0.0241664	1431	1533	6701
MSTRG.19490.5	coding	0.118	0	3831	6139
MSTRG.19490.8	coding	0.135	456	1557	1810
MSTRG.19491.1	noncoding	-0.0438272	675	825	2296
MSTRG.19492.1	coding	0.073	567	810	10202
MSTRG.19493.1	noncoding	-0.0335872	264	300	2311
MSTRG.19494.1	noncoding	-0.053248	255	327	1757
MSTRG.19495.1	noncoding	-0.045056	3333	3438	6926
MSTRG.19496.1	noncoding	-0.069632	1284	1368	3187
MSTRG.19505.2	coding	0.023	1392	1890	2367
MSTRG.19505.3	coding	0.023	336	834	1313
MSTRG.19505.4	coding	0.023	1620	2118	2596
MSTRG.19506.1	noncoding	-0.0561152	387	474	1966
MSTRG.19507.4	coding	0.151	588	999	1289
MSTRG.19508.3	coding	0.461	0	189	802
MSTRG.19509.2	coding	0.212	0	1869	2204
MSTRG.19515.3	coding	0.098	258	546	936
MSTRG.19515.2	noncoding	-0.053248	9	198	3863
MSTRG.19515.4	coding	0.09	78	351	740
MSTRG.19521.1	coding	0.05	183	1110	1397
MSTRG.19521.2	coding	0.006	291	897	1891
MSTRG.19522.4	coding	0.098	330	990	3303
MSTRG.19526.1	noncoding	-0.0212992	186	201	355
MSTRG.19530.1	noncoding	-0.004096	150	294	5417
MSTRG.19531.1	coding	0.109	2394	2604	4855
MSTRG.19526.2	noncoding	-0.0372736	792	1155	7882
MSTRG.19532.1	noncoding	-0.0339968	180	417	749
MSTRG.19516.1	noncoding	-0.0098304	909	1080	2109
MSTRG.19533.5	noncoding	-0.043008	5613	5811	9803
MSTRG.19533.6	noncoding	-0.008192	0	135	427
MSTRG.19534.1	noncoding	-0.032768	2259	2304	3594
MSTRG.19533.7	coding	0.513	21	3270	5894
MSTRG.19535.1	noncoding	-0.0172032	1044	1191	1688
MSTRG.19538.1	noncoding	-0.0049152	126	267	2946
MSTRG.19540.1	noncoding	-0.0196608	2775	3177	5225
MSTRG.19543.4	coding	0.231	411	2874	3448
MSTRG.19543.5	coding	0.246	0	2304	2879
MSTRG.19543.6	coding	0.22	0	2730	3305
MSTRG.19544.1	noncoding	-0.0712704	207	249	1140
MSTRG.19547.1	noncoding	-0.0323584	630	795	6569
MSTRG.19551.2	coding	0.131	180	768	1092
MSTRG.19555.1	coding	0.145	360	1776	1961
MSTRG.19559.7	coding	0.321	96	1149	3728
MSTRG.19559.8	coding	0.327	96	1197	3776
MSTRG.19559.5	coding	0.326	540	1674	4252
MSTRG.19559.6	coding	0.321	540	1560	4138
MSTRG.19560.1	noncoding	-0.0630784	14493	14691	40849
MSTRG.19561.1	noncoding	-0.012288	7299	7413	18371
MSTRG.19562.2	noncoding	-0.0262144	2556	2757	39462
MSTRG.19562.1	noncoding	-0.036864	2460	2559	2896
MSTRG.19562.4	noncoding	-0.0262144	16011	16212	52749

MSTRG.19562.5	noncoding	-0.0262144	15642	15843	41335
MSTRG.19562.3	noncoding	-0.0262144	17601	17802	43292
MSTRG.19562.6	noncoding	-0.0262144	17601	17802	54484
MSTRG.19559.9	noncoding	-0.1560576	0	102	9553
MSTRG.19563.1	noncoding	-0.0520192	0	303	2389
MSTRG.19568.1	noncoding	-0.2772992	1407	2430	2587
MSTRG.19568.2	coding	0.507	303	909	1063
MSTRG.19568.3	coding	0.495	36	753	907
MSTRG.19568.4	coding	0.446	480	1011	1168
MSTRG.19572.9	coding	0.32	258	3693	4060
MSTRG.19572.8	coding	0.318	228	4023	4392
MSTRG.19572.5	coding	0.331	834	4566	4933
MSTRG.19572.7	coding	0.328	198	3948	4315
MSTRG.19572.6	coding	0.353	0	3726	7472
MSTRG.19573.1	noncoding	-0.0753664	642	744	3371
MSTRG.19572.10	noncoding	-0.032768	1836	1914	5410
MSTRG.19572.11	coding	0.57	0	207	8062
MSTRG.19574.1	noncoding	-0.049152	1011	1092	3027
MSTRG.19572.12	noncoding	-0.2121728	0	156	9794
MSTRG.19570.2	coding	0.216	0	669	2302
MSTRG.19570.3	coding	0.196	882	1599	9703
MSTRG.19570.4	coding	0.189	780	1473	4603
MSTRG.19571.2	coding	0.468	0	2658	3825
MSTRG.19577.2	coding	0.241	33	522	1384
MSTRG.19579.1	coding	0.443	441	2142	4562
MSTRG.19579.2	coding	0.413	456	1950	2383
MSTRG.19582.2	coding	0.421	1422	6033	8196
MSTRG.19582.4	coding	0.419	1419	6168	7523
MSTRG.19582.5	coding	0.419	1419	6078	7433
MSTRG.19582.6	coding	0.441	18	4251	5604
MSTRG.19582.3	coding	0.419	1419	6159	8324
MSTRG.19582.7	coding	0.421	1419	6021	8186
MSTRG.19581.1	noncoding	-0.01024	585	747	1221
MSTRG.19583.1	coding	0.199	24	2058	3004
MSTRG.19590.1	coding	0.235	108	711	1427
MSTRG.19590.2	noncoding	-0.028672	333	468	1007
MSTRG.19591.1	noncoding	-0.0282624	30	276	745
MSTRG.19591.2	noncoding	-0.0282624	27	273	916
MSTRG.19593.1	coding	0.187	147	687	1221
MSTRG.19596.4	coding	0.252	171	3174	3533
MSTRG.19598.3	coding	0.187	0	11928	16544
MSTRG.19598.5	coding	0.188	0	11772	14445
MSTRG.19598.6	coding	0.207	3396	10893	15510
MSTRG.19600.1	coding	0.285	0	1038	2682
MSTRG.19602.1	coding	0.473	0	4191	5496
MSTRG.19603.1	noncoding	-0.06144	45	360	800
MSTRG.19602.3	coding	0.484	0	3861	5144
MSTRG.19602.4	coding	0.453	162	4161	5466
MSTRG.19602.5	coding	0.541	4476	6174	7475
MSTRG.19602.6	coding	0.498	81	1611	2915
MSTRG.19604.6	coding	0.113	339	1680	6140
MSTRG.19604.4	coding	0.113	333	1674	6110
MSTRG.19604.8	coding	0.114	1194	2550	5765
MSTRG.19622.1	noncoding	-0.0208896	783	939	2606

MSTRG.19624.7	coding	0.522	582	3492	4878
MSTRG.19624.9	coding	0.521	582	3495	4881
MSTRG.19616.2	coding	0.16	24	2340	10178
MSTRG.19614.1	coding	0.45	0	1851	2890
MSTRG.19614.2	coding	0.455	0	1671	2681
MSTRG.19629.1	coding	0.385	273	2052	3810
MSTRG.19628.1	noncoding	-0.0188416	24	411	701
MSTRG.19628.2	noncoding	-0.0217088	33	318	951
MSTRG.19638.4	coding	0.158	0	5523	6246
MSTRG.19638.2	coding	0.158	0	5580	7402
MSTRG.19638.1	coding	0.158	0	5523	9495
MSTRG.19639.1	noncoding	-0.028672	690	705	3071
MSTRG.19640.1	noncoding	-0.016384	69	174	2527
MSTRG.19642.5	coding	0.1	267	1050	4865
MSTRG.19642.6	coding	0.126	546	1167	4982
MSTRG.19642.7	coding	0.126	552	1173	4986
MSTRG.19643.1	coding	0.461	1026	5514	6308
MSTRG.19648.1	coding	0.323	150	1830	6372
MSTRG.19649.3	coding	0.11	498	1911	2348
MSTRG.19650.1	noncoding	-0.0200704	0	69	233
MSTRG.19671.9	coding	0.473	0	1950	2133
MSTRG.19666.1	coding	0.251	471	2139	3900
MSTRG.19672.2	noncoding	-0.053248	807	1101	1267
MSTRG.19672.3	noncoding	-0.0536576	402	708	873
MSTRG.19672.4	noncoding	-0.0536576	393	699	864
MSTRG.19674.1	coding	0.519	120	1263	1418
MSTRG.19675.1	coding	0.118	282	1401	1841
MSTRG.19676.2	coding	0.088	843	1083	3252
MSTRG.19678.5	coding	0.392	126	4092	5508
MSTRG.19678.8	coding	0.466	171	1347	2728
MSTRG.19681.1	noncoding	-0.02048	1035	1053	5029
MSTRG.19682.1	noncoding	-0.0118784	669	855	1009
MSTRG.19683.1	coding	0.1	663	1197	1365
MSTRG.19684.1	coding	0.182	126	2412	2811
MSTRG.19684.2	coding	0.182	153	2439	2839
MSTRG.19701.1	noncoding	-0.077824	519	585	740
MSTRG.19707.2	coding	0.15	1092	2235	3704
MSTRG.19708.1	noncoding	-0.0004096	279	372	902
MSTRG.19707.3	noncoding	-0.016384	2703	2802	3552
MSTRG.19713.2	coding	0.069	480	888	1331
MSTRG.19718.1	noncoding	-0.221184	846	879	1945
MSTRG.19720.3	coding	0.359	0	405	6402
MSTRG.19721.1	noncoding	-0.012288	2841	2964	3667
MSTRG.19720.4	noncoding	-0.116736	1149	1743	9077
MSTRG.19720.5	noncoding	-0.098304	5271	5334	6695
MSTRG.19722.1	coding	0.365	0	2418	3682
MSTRG.19723.1	coding	0.124	102	1377	6516
MSTRG.19723.4	noncoding	-0.0479232	3543	3822	4375
MSTRG.19723.3	coding	0.091	216	1035	2909
MSTRG.19723.5	coding	0.124	102	1377	2326
MSTRG.19723.6	coding	0.124	102	1377	1893
MSTRG.19733.1	coding	0.199	69	2583	3484
MSTRG.19743.1	noncoding	-0.04096	144	231	528
MSTRG.19744.2	noncoding	-0.200704	0	45	1532

MSTRG.19744.4	noncoding	-0.057344	0	111	796
MSTRG.19744.5	noncoding	-0.131072	0	57	2721
MSTRG.19744.6	noncoding	-0.073728	0	102	1708
MSTRG.19744.7	noncoding	-0.098304	0	81	237
MSTRG.19744.8	noncoding	-0.008192	0	132	893
MSTRG.19744.9	noncoding	-0.098304	0	90	701
MSTRG.19744.10	noncoding	-0.0086016	0	219	620
MSTRG.19744.11	noncoding	-0.106496	0	78	300
MSTRG.19744.12	noncoding	-0.024576	0	120	275
MSTRG.19744.13	noncoding	-0.106496	0	78	368
MSTRG.19744.14	noncoding	-0.106496	0	87	476
MSTRG.19744.15	noncoding	-0.0004096	0	288	1142
MSTRG.19744.16	noncoding	-0.131072	0	57	213
MSTRG.19744.17	noncoding	-0.126976	0	66	223
MSTRG.19744.18	noncoding	-0.131072	0	57	213
MSTRG.19744.19	noncoding	-0.131072	0	54	211
MSTRG.19744.20	noncoding	-0.0212992	0	150	304
MSTRG.19744.21	noncoding	-0.131072	0	60	217
MSTRG.19744.22	noncoding	-0.126976	0	63	219
MSTRG.19744.23	noncoding	-0.098304	0	81	237
MSTRG.19744.24	noncoding	-0.126976	0	66	223
MSTRG.19744.25	noncoding	-0.126976	0	66	222
MSTRG.19744.26	noncoding	-0.200704	0	45	227
MSTRG.19744.27	noncoding	-0.073728	0	102	258
MSTRG.19744.28	noncoding	-0.131072	0	57	301
MSTRG.19744.29	noncoding	-0.131072	0	54	210
MSTRG.19744.30	noncoding	-0.1024	0	75	328
MSTRG.19744.31	noncoding	-0.167936	0	48	205
MSTRG.19744.32	noncoding	-0.200704	0	45	201
MSTRG.19739.1	noncoding	-0.108544	0	120	537
MSTRG.19751.1	noncoding	-0.0360448	7149	7854	8008
MSTRG.19752.1	noncoding	-0.008192	6	108	3109
MSTRG.19752.2	noncoding	-0.09216	420	495	1371
MSTRG.19751.2	noncoding	-0.0897024	1611	1872	2028
MSTRG.19751.3	noncoding	-0.0897024	318	579	772
MSTRG.19755.1	coding	0.103	501	4302	5202
MSTRG.19755.2	coding	0.107	330	2997	3897
MSTRG.19755.3	coding	0.109	462	3438	4338
MSTRG.19755.4	coding	0.11	0	2796	3697
MSTRG.19755.5	coding	0.105	231	2784	3685
MSTRG.19761.1	noncoding	-0.047104	717	798	2765
MSTRG.19765.1	noncoding	-0.04096	405	492	757
MSTRG.19766.1	noncoding	-0.0159744	2304	2403	2624
MSTRG.19767.3	coding	0.048	534	690	1701
MSTRG.19768.1	noncoding	-0.0425984	1158	1305	3589
MSTRG.19757.2	noncoding	-0.18432	165	753	1835
MSTRG.19774.1	coding	0.12	0	2853	3785
MSTRG.19779.2	coding	0.363	0	354	3988
MSTRG.19784.2	coding	0.22	81	1383	1830
MSTRG.19785.3	coding	0.138	306	498	818
MSTRG.19788.1	noncoding	-0.045056	5913	6012	10372
MSTRG.19791.1	noncoding	-0.03072	216	873	1315
MSTRG.19803.2	coding	0.287	105	2685	7967
MSTRG.19803.6	coding	0.298	201	2427	3049

MSTRG.19803.7	coding	0.291	207	2496	3117
MSTRG.19803.5	coding	0.291	9	2682	3303
MSTRG.19804.1	noncoding	-0.0069632	576	735	890
MSTRG.19805.1	coding	0.255	282	681	4168
MSTRG.19806.1	noncoding	-0.032768	381	465	996
MSTRG.19807.3	coding	0.192	390	1542	1698
MSTRG.19809.1	noncoding	-0.036864	84	99	268
MSTRG.19811.1	noncoding	-0.0929792	0	426	1381
MSTRG.19821.1	coding	0.219	1710	2448	2602
MSTRG.19822.1	noncoding	-0.04096	234	303	499
MSTRG.19821.2	noncoding	-0.04096	0	120	1386
MSTRG.19821.3	noncoding	-0.0466944	2934	3174	4928
MSTRG.19821.4	noncoding	-0.004096	129	270	2299
MSTRG.19821.5	noncoding	-0.0684032	13245	13599	19256
MSTRG.19821.7	noncoding	-0.0638976	12507	12861	15392
MSTRG.19821.6	noncoding	-0.0671744	4542	4896	7427
MSTRG.19821.8	noncoding	-0.0004096	1230	1371	2146
MSTRG.19821.9	noncoding	-0.08192	0	108	439
MSTRG.19825.1	coding	0.203	81	768	922
MSTRG.19827.1	coding	0.178	0	336	702
MSTRG.19831.2	coding	0.208	129	501	11021
MSTRG.19840.1	noncoding	-0.0172032	192	468	1172
MSTRG.19841.1	coding	0.34	141	8823	14445
MSTRG.19848.2	coding	0.294	240	1407	2350
MSTRG.19856.8	coding	0.279	255	2106	2261
MSTRG.19856.9	coding	0.279	240	2091	2246
MSTRG.19856.11	coding	0.285	1458	3573	11569
MSTRG.19857.2	coding	0.216	0	882	1038
MSTRG.19861.2	coding	0.159	222	1308	2469
MSTRG.19863.3	coding	0.288	30	1458	1901
MSTRG.19863.2	coding	0.292	381	1836	2279
MSTRG.19863.4	coding	0.285	876	2301	2739
MSTRG.19868.1	noncoding	-0.012288	84	204	775
MSTRG.19868.2	noncoding	-0.012288	84	204	635
MSTRG.19879.1	noncoding	-0.0004096	0	0	844
MSTRG.19884.1	noncoding	-0.0475136	3660	3873	7871
MSTRG.19884.2	noncoding	-0.1048576	15	30	464
MSTRG.19884.3	noncoding	-0.077824	933	969	2345
MSTRG.19884.4	noncoding	-0.077824	897	933	1492
MSTRG.19885.1	noncoding	-0.0253952	87	252	1973
MSTRG.19888.1	noncoding	-0.057344	138	141	394
MSTRG.19893.3	coding	0.306	171	1635	3466
MSTRG.19906.4	coding	0.318	0	2769	3838
MSTRG.19907.1	noncoding	-0.0897024	993	996	1151
MSTRG.19908.2	coding	0.511	0	669	1298
MSTRG.19909.5	coding	0.373	519	1284	2281
MSTRG.19909.7	coding	0.376	1593	2394	3391
MSTRG.19909.6	coding	0.381	189	930	1926
MSTRG.19904.1	coding	0.305	486	1257	1444
MSTRG.19904.2	coding	0.306	486	1275	1602
MSTRG.19904.3	coding	0.278	471	948	1121
MSTRG.19905.1	coding	0.452	75	1377	1634
MSTRG.19905.2	coding	0.359	450	1128	1382
MSTRG.19917.1	coding	0.168	1053	2253	3103

MSTRG.19918.3	coding	0.256	0	1065	5389
MSTRG.19912.3	coding	0.436	60	516	861
MSTRG.19912.4	coding	0.43	93	555	884
MSTRG.19913.2	coding	0.214	72	768	936
MSTRG.19914.2	coding	0.45	9	4113	4933
MSTRG.19923.1	noncoding	-0.0233472	201	309	462
MSTRG.19924.1	noncoding	-0.045056	411	519	673
MSTRG.19924.2	noncoding	-0.032768	264	450	831
MSTRG.19925.3	coding	0.46	0	4776	5379
MSTRG.19925.5	coding	0.488	69	2433	2992
MSTRG.19926.5	coding	0.432	0	582	1182
MSTRG.19926.4	coding	0.394	36	1092	1248
MSTRG.19926.3	coding	0.37	252	1458	1613
MSTRG.19930.1	noncoding	-0.036864	3297	3423	6170
MSTRG.19931.1	noncoding	-0.0421888	174	177	462
MSTRG.19937.1	noncoding	-0.0028672	204	339	1741
MSTRG.19938.1	noncoding	-0.0004096	0	0	1036
MSTRG.19939.4	noncoding	-0.0950272	63	306	1521
MSTRG.19941.1	coding	0.12	0	669	965
MSTRG.19951.1	noncoding	-0.0643072	105	348	911
MSTRG.19954.2	coding	0.346	120	978	1257
MSTRG.19958.1	noncoding	-0.049152	405	615	3883
MSTRG.19959.1	coding	0.117	306	6990	8118
MSTRG.19959.3	coding	0.117	306	6990	7615
MSTRG.19959.5	coding	0.116	306	7059	8083
MSTRG.19959.2	coding	0.117	303	6987	8013
MSTRG.19959.8	coding	0.168	4395	6699	7068
MSTRG.19959.9	coding	0.109	1941	7392	8160
MSTRG.19959.10	coding	0.168	4389	6693	7060
MSTRG.19959.11	coding	0.097	231	7671	8274
MSTRG.19959.12	coding	0.131	876	5829	6932
MSTRG.19959.13	coding	0.091	297	4935	6163
MSTRG.19942.1	coding	0.043	114	1239	5893
MSTRG.19945.1	noncoding	-0.126976	867	915	1319
MSTRG.19947.1	noncoding	-0.053248	360	429	859
MSTRG.19946.2	coding	0.204	156	1761	5399
MSTRG.19967.1	coding	0.174	273	1293	27195
MSTRG.19967.5	coding	0.174	171	1191	5841
MSTRG.19967.4	noncoding	-0.0253952	5760	5910	10227
MSTRG.19967.8	noncoding	-0.028672	861	945	1564
MSTRG.19965.2	coding	0.416	171	1677	4051
MSTRG.19978.1	coding	0.374	96	2259	2652
MSTRG.19978.3	coding	0.27	405	2553	2947
MSTRG.19978.2	coding	0.375	381	2586	2979
MSTRG.19978.4	coding	0.27	573	2721	3102
MSTRG.19978.5	coding	0.27	252	2400	2777
MSTRG.19979.1	noncoding	-0.0782336	1950	2370	4170
MSTRG.19980.1	coding	0.628	0	1281	1969
MSTRG.19983.1	coding	0.234	435	837	3799
MSTRG.19985.1	noncoding	-0.0294912	1170	1716	7334
MSTRG.19981.1	noncoding	-0.108544	0	60	595
MSTRG.19990.1	noncoding	-0.0065536	1170	1350	1885
MSTRG.19990.2	noncoding	-0.0139264	396	564	857
MSTRG.20000.1	noncoding	-0.0086016	156	327	1889

MSTRG.20003.1	noncoding	-0.0393216	72	597	4946
MSTRG.20006.1	noncoding	-0.14336	111	168	680
MSTRG.20008.1	coding	0.125	237	516	689
MSTRG.20013.4	coding	0.568	180	5985	7005
MSTRG.20013.5	noncoding	-0.0598016	351	537	5216
MSTRG.20015.3	coding	0.076	885	1326	2221
MSTRG.20015.5	coding	0.076	726	1167	2064
MSTRG.20015.6	noncoding	-0.036864	1377	1500	3755
MSTRG.20018.1	coding	0.243	0	933	1382
MSTRG.20018.2	coding	0.243	0	933	1252
MSTRG.20018.3	coding	0.269	0	708	1448
MSTRG.20027.1	coding	0.105	411	1398	4142
MSTRG.20033.1	noncoding	-0.0385024	1491	1548	1880
MSTRG.20037.1	noncoding	-0.0004096	273	387	856
MSTRG.20038.4	noncoding	-0.02048	18	63	1332
MSTRG.20047.1	noncoding	-0.008192	456	492	2863
MSTRG.20056.1	coding	0.038	558	735	1199
MSTRG.20056.4	noncoding	-0.02048	1383	1551	1870
MSTRG.20057.3	coding	0.128	108	618	1273
MSTRG.20057.4	coding	0.128	69	579	1052
MSTRG.20059.1	coding	0.074	723	5616	5773
MSTRG.20059.5	noncoding	-0.0069632	696	849	1884
MSTRG.20061.3	noncoding	-0.0299008	252	507	1684
MSTRG.20061.2	noncoding	-0.0299008	252	507	2311
MSTRG.20063.1	noncoding	-0.032768	381	465	848
MSTRG.20066.1	noncoding	-0.114688	570	582	975
MSTRG.20069.4	coding	0.215	117	390	974
MSTRG.20074.2	coding	0.323	0	1584	2763
MSTRG.20074.4	coding	0.334	0	1533	2712
MSTRG.20075.1	noncoding	-0.038912	90	249	2309
MSTRG.20079.1	coding	0.036	408	1872	4183
MSTRG.20086.5	coding	0.083	2652	4641	6108
MSTRG.20086.6	coding	0.083	2700	4689	8748
MSTRG.20086.9	coding	0.083	2916	4905	6370
MSTRG.20087.1	noncoding	-0.026624	372	570	6840
MSTRG.20086.10	coding	0.083	3267	5256	6721
MSTRG.20088.2	coding	0.099	513	942	1247
MSTRG.20090.1	coding	0.345	0	288	442
MSTRG.20092.2	coding	0.123	138	1716	2661
MSTRG.20094.2	coding	0.184	1443	1986	3840
MSTRG.20097.2	coding	0.12	90	1218	2119
MSTRG.20098.1	noncoding	-0.036864	468	648	6857
MSTRG.20107.1	noncoding	-0.0004096	336	411	565
MSTRG.20109.1	noncoding	-0.0110592	2724	2895	3131
MSTRG.20110.1	coding	0.257	423	1200	1587
MSTRG.20110.2	coding	0.248	294	978	1363
MSTRG.20110.3	coding	0.237	126	816	4983
MSTRG.20114.1	coding	0.135	225	1509	2317
MSTRG.20114.2	coding	0.125	168	1155	1600
MSTRG.20114.4	coding	0.128	369	1521	2328
MSTRG.20115.1	noncoding	-0.0356352	3909	4074	6913
MSTRG.20112.1	noncoding	-0.0057344	60	75	310
MSTRG.20118.1	coding	0.08	0	234	398
MSTRG.20117.3	coding	0.228	615	1665	2502

MSTRG.20121.2	noncoding	-0.0499712	843	1137	1462
MSTRG.20121.1	noncoding	-0.049152	1470	1557	1881
MSTRG.20119.1	noncoding	-0.0380928	282	558	872
MSTRG.20120.1	coding	0.345	375	2076	2688
MSTRG.20126.2	coding	0.471	342	1308	3901
MSTRG.20126.4	noncoding	-0.0761856	579	846	1126
MSTRG.20126.3	coding	0.471	126	918	3922
MSTRG.20128.2	noncoding	-0.0892928	147	567	1095
MSTRG.20132.3	coding	0.227	435	855	1058
MSTRG.20132.4	coding	0.227	855	1275	1479
MSTRG.20135.1	coding	0.18	243	1959	2710
MSTRG.20148.1	noncoding	-0.0417792	186	348	2016
MSTRG.20158.1	noncoding	-0.0004096	0	0	592
MSTRG.20158.2	noncoding	-0.0004096	0	0	626
MSTRG.20158.5	coding	0.146	0	1689	4113
MSTRG.20158.6	noncoding	-0.0200704	1503	1635	2146
MSTRG.20162.4	coding	0.201	0	3498	4436
MSTRG.20163.1	noncoding	-0.008192	60	141	1181
MSTRG.20164.1	noncoding	-0.073728	1173	1236	2273
MSTRG.20168.1	noncoding	-0.026624	9606	10374	11743
MSTRG.20154.2	coding	0.131	267	870	2884
MSTRG.20155.1	noncoding	-0.04096	4098	4200	7367
MSTRG.20156.1	noncoding	-0.0004096	6354	6492	7982
MSTRG.20157.1	coding	0.052	3780	4341	4496
MSTRG.20154.3	coding	0.114	945	1365	4790
MSTRG.20172.3	coding	0.109	594	1434	1668
MSTRG.20173.1	noncoding	-0.073728	2037	2109	2419
MSTRG.20177.2	coding	0.354	75	1257	1872
MSTRG.20179.1	noncoding	-0.0004096	87	222	635
MSTRG.20180.1	coding	0.127	0	195	385
MSTRG.20186.1	coding	0.467	0	1431	3226
MSTRG.20188.1	noncoding	-0.0376832	468	663	1239
MSTRG.20189.2	noncoding	-0.016384	15	138	336
MSTRG.20189.1	noncoding	-0.008192	15	153	395
MSTRG.20190.1	noncoding	-0.0712704	51	117	413
MSTRG.20193.2	noncoding	-0.0360448	3372	4158	19034
MSTRG.20193.3	noncoding	-0.0761856	0	105	26598
MSTRG.20198.2	coding	0.437	42	1830	4055
MSTRG.20198.1	coding	0.438	42	1854	5302
MSTRG.20199.1	noncoding	-0.0462848	147	414	571
MSTRG.20200.3	coding	0.457	0	2316	2515
MSTRG.20202.1	noncoding	-0.0794624	0	558	715
MSTRG.20203.2	coding	0.397	567	1548	2421
MSTRG.20210.4	coding	0.197	1575	3525	4147
MSTRG.20210.3	coding	0.196	807	2754	3375
MSTRG.20210.5	coding	0.132	15690	15942	25454
MSTRG.20210.6	noncoding	-0.08192	9150	9234	12562
MSTRG.20212.1	noncoding	-0.024576	3951	4059	4608
MSTRG.20212.2	noncoding	-0.024576	2322	2430	10213
MSTRG.20212.3	noncoding	-0.049152	372	408	1385
MSTRG.20212.4	noncoding	-0.0012288	3312	3363	3897
MSTRG.20210.7	noncoding	-0.0049152	54	201	903
MSTRG.20207.2	coding	0.444	927	2397	2685
MSTRG.20207.3	coding	0.442	0	1467	1621

MSTRG.20207.4	coding	0.443	15	1485	1772
MSTRG.20208.3	coding	0.486	2184	5043	5441
MSTRG.20213.5	coding	0.308	159	720	6732
MSTRG.20213.6	coding	0.327	63	663	4498
MSTRG.20214.1	noncoding	-0.0344064	3243	3381	4258
MSTRG.20215.1	noncoding	-0.014336	417	570	723
MSTRG.20216.1	noncoding	-0.045056	1227	1308	6665
MSTRG.20218.4	coding	0.153	201	2553	4149
MSTRG.20218.5	coding	0.149	303	2592	4189
MSTRG.20220.1	noncoding	-0.012288	885	3009	3381
MSTRG.20225.1	noncoding	-0.0745472	573	840	997
MSTRG.20225.2	noncoding	-0.0421888	267	570	793
MSTRG.20226.2	coding	0.556	0	1080	1307
MSTRG.20226.3	coding	0.55	0	1248	1602
MSTRG.20226.4	coding	0.461	0	801	1269
MSTRG.20227.6	coding	0.222	300	5805	6505
MSTRG.20227.5	coding	0.222	852	6885	7465
MSTRG.20227.3	coding	0.209	57	6696	7278
MSTRG.20227.4	coding	0.209	57	6693	7275
MSTRG.20227.8	coding	0.222	2799	8832	9411
MSTRG.20227.10	coding	0.223	864	6909	7457
MSTRG.20227.9	coding	0.224	282	6315	6865
MSTRG.20227.11	coding	0.222	1311	7347	7893
MSTRG.20227.12	coding	0.224	477	6507	7042
MSTRG.20228.1	noncoding	-0.1024	264	339	495
MSTRG.20229.1	coding	0.467	216	5679	7998
MSTRG.20222.3	coding	0.254	0	258	1400
MSTRG.20222.4	coding	0.392	108	828	1035
MSTRG.20223.1	coding	0.433	0	1497	7173
MSTRG.20223.2	coding	0.433	0	1497	4625
MSTRG.20241.1	coding	0.448	630	5262	5417
MSTRG.20241.2	coding	0.449	630	5220	5375
MSTRG.20241.3	coding	0.448	495	5127	5281
MSTRG.20241.5	coding	0.449	375	4965	5120
MSTRG.20241.4	coding	0.45	558	5316	6165
MSTRG.20241.6	coding	0.449	237	4881	5034
MSTRG.20231.1	noncoding	-0.0303104	195	1548	1703
MSTRG.20231.2	noncoding	-0.0352256	105	1176	1357
MSTRG.20232.1	noncoding	-0.0364544	147	285	507
MSTRG.20233.1	coding	0.202	0	1923	2172
MSTRG.20242.4	coding	0.246	0	1482	7675
MSTRG.20242.1	coding	0.243	0	1488	4547
MSTRG.20242.2	coding	0.247	0	1476	4535
MSTRG.20242.3	coding	0.23	378	1776	4835
MSTRG.20242.5	coding	0.239	378	1782	7976
MSTRG.20242.8	coding	0.235	240	1656	7846
MSTRG.20244.2	coding	0.349	411	1788	2794
MSTRG.20245.1	coding	0.336	1215	1989	2173
MSTRG.20246.1	coding	0.194	0	750	13824
MSTRG.20246.3	coding	0.2	0	780	1773
MSTRG.20249.4	coding	0.158	1140	3390	4189
MSTRG.20250.2	coding	0.186	0	1797	4516
MSTRG.20250.4	coding	0.183	0	1773	4754
MSTRG.20250.7	coding	0.149	0	2259	4953

MSTRG.20250.8	coding	0.183	0	1773	4008
MSTRG.20251.1	noncoding	-0.004096	444	549	966
MSTRG.20247.1	coding	0.264	0	3765	7325
MSTRG.20247.2	coding	0.266	0	3756	7402
MSTRG.20248.1	noncoding	-0.016384	801	924	1544
MSTRG.20257.1	coding	0.248	309	1170	1483
MSTRG.20257.2	coding	0.159	312	585	739
MSTRG.20269.2	noncoding	-0.0987136	2454	4305	4572
MSTRG.20270.1	noncoding	-0.1277952	0	96	822
MSTRG.20270.2	noncoding	-0.018432	426	900	1053
MSTRG.20270.3	noncoding	-0.0290816	399	813	966
MSTRG.20271.2	coding	0.603	759	2880	3419
MSTRG.20272.1	coding	0.551	0	1209	3735
MSTRG.20274.1	noncoding	-0.0098304	0	99	423
MSTRG.20274.5	coding	0.422	414	1917	2880
MSTRG.20274.4	coding	0.422	573	2076	3041
MSTRG.20274.6	noncoding	-0.0913408	570	1029	2543
MSTRG.20277.1	noncoding	-0.0733184	0	642	1921
MSTRG.20278.2	coding	0.236	132	510	1023
MSTRG.20279.1	coding	0.37	447	1998	2167
MSTRG.20281.2	coding	0.463	960	2280	2643
MSTRG.20281.4	coding	0.466	246	1572	1991
MSTRG.20287.3	coding	0.439	1275	3525	4625
MSTRG.20288.2	coding	0.297	495	1224	1845
MSTRG.20288.3	coding	0.276	144	858	1479
MSTRG.20290.1	coding	0.225	966	3012	6843
MSTRG.20291.3	coding	0.402	0	432	834
MSTRG.20292.1	coding	0.431	426	3234	4967
MSTRG.20292.2	coding	0.429	423	3249	4982
MSTRG.20292.3	coding	0.431	519	3327	5060
MSTRG.20292.4	coding	0.431	327	3135	4740
MSTRG.20292.7	coding	0.412	738	3384	5115
MSTRG.20293.1	noncoding	-0.0679936	939	1446	1600
MSTRG.20298.3	coding	0.278	645	1425	4786
MSTRG.20298.4	coding	0.318	948	2271	5632
MSTRG.20298.5	coding	0.318	0	1446	4807
MSTRG.20298.6	coding	0.317	0	1422	4782
MSTRG.20298.7	coding	0.318	564	1884	5245
MSTRG.20299.1	noncoding	-0.004096	1581	1806	2807
MSTRG.20315.2	coding	0.334	1515	6633	7731
MSTRG.20315.4	coding	0.377	0	3189	4281
MSTRG.20315.6	coding	0.331	144	2784	3016
MSTRG.20315.5	coding	0.41	309	3162	3393
MSTRG.20315.7	noncoding	-0.049152	180	282	1111
MSTRG.20315.11	coding	0.218	1554	3810	5507
MSTRG.20315.14	coding	0.213	2178	3846	5468
MSTRG.20315.16	coding	0.22	1098	2613	4234
MSTRG.20318.1	noncoding	-0.02048	246	318	581
MSTRG.20308.1	noncoding	-0.0323584	216	756	911
MSTRG.20310.1	coding	0.558	195	1029	3013
MSTRG.20313.1	coding	0.207	84	585	1071
MSTRG.20313.2	coding	0.303	519	1356	1844
MSTRG.20313.3	coding	0.353	135	1320	1806
MSTRG.20313.4	noncoding	-0.0393216	303	372	528

MSTRG.20313.5	coding	0.177	156	630	941
MSTRG.20334.1	coding	0.064	144	2484	4028
MSTRG.20335.3	coding	0.118	57	378	549
MSTRG.20338.1	noncoding	-0.0233472	12	198	3776
MSTRG.20338.2	noncoding	-0.04096	822	927	2480
MSTRG.20339.3	noncoding	-0.0581632	1086	1236	1393
MSTRG.20339.4	noncoding	-0.0581632	1239	1389	1546
MSTRG.20345.2	coding	0.41	0	507	5715
MSTRG.20346.2	coding	0.332	0	729	1005
MSTRG.20347.4	coding	0.413	630	1251	1701
MSTRG.20321.1	noncoding	-0.1273856	0	126	321
MSTRG.20328.4	noncoding	-0.1409024	0	150	1211
MSTRG.20328.5	noncoding	-0.1409024	0	150	980
MSTRG.20328.6	noncoding	-0.1409024	0	150	1240
MSTRG.20330.1	noncoding	-0.0438272	117	219	452
MSTRG.20332.6	noncoding	-0.114688	1248	1338	2976
MSTRG.20354.1	noncoding	-0.0004096	60	123	312
MSTRG.20361.1	noncoding	-0.0004096	0	0	327
MSTRG.20362.1	noncoding	-0.0004096	243	243	549
MSTRG.20362.2	noncoding	-0.053248	546	636	1804
MSTRG.20362.3	noncoding	-0.024576	78	177	1074
MSTRG.20366.1	noncoding	-0.032768	738	867	2941
MSTRG.20367.3	coding	0.161	72	510	2145
MSTRG.20367.4	coding	0.165	72	513	2148
MSTRG.20367.6	coding	0.217	42	471	877
MSTRG.20367.5	coding	0.172	42	456	2122
MSTRG.20376.4	coding	0.461	60	780	2229
MSTRG.20376.5	coding	0.482	675	1281	2731
MSTRG.20376.7	coding	0.478	57	1029	2478
MSTRG.20376.8	coding	0.482	591	1197	3691
MSTRG.20376.6	coding	0.482	342	948	12127
MSTRG.20376.9	noncoding	-0.0393216	291	915	13832
MSTRG.20376.10	coding	0.482	15216	15822	27002
MSTRG.20378.2	coding	0.243	93	1542	1800
MSTRG.20382.3	coding	0.504	798	4401	8719
MSTRG.20382.5	coding	0.5	789	4428	8752
MSTRG.20382.8	coding	0.509	543	4011	4164
MSTRG.20387.2	coding	0.157	999	3114	4997
MSTRG.20387.8	noncoding	-0.0008192	513	795	1072
MSTRG.20387.7	coding	0.157	4923	7038	8916
MSTRG.20388.1	noncoding	-0.0475136	195	570	948
MSTRG.20393.1	noncoding	-0.049152	360	462	1756
MSTRG.20394.1	coding	0.381	30	2889	3944
MSTRG.20395.1	coding	0.429	0	1308	6335
MSTRG.20395.2	coding	0.429	0	1308	6167
MSTRG.20395.3	coding	0.5	0	1086	5448
MSTRG.20395.4	coding	0.366	87	1236	5810
MSTRG.20395.14	coding	0.391	276	1470	2757
MSTRG.20395.10	coding	0.464	0	1257	2544
MSTRG.20395.11	coding	0.464	0	1257	2485
MSTRG.20395.7	coding	0.464	0	1257	2544
MSTRG.20395.8	coding	0.43	0	1302	2649
MSTRG.20395.12	coding	0.393	1185	2334	3014
MSTRG.20395.13	coding	0.306	0	1845	2593

MSTRG.20395.9	coding	0.429	0	1308	2367
MSTRG.20396.1	noncoding	-0.1110016	123	606	2277
MSTRG.20397.1	coding	0.398	636	1686	1884
MSTRG.20396.2	noncoding	-0.09216	489	762	2224
MSTRG.20396.3	noncoding	-0.09216	336	609	2070
MSTRG.20396.4	noncoding	-0.1052672	279	522	1824
MSTRG.20390.2	coding	0.184	0	2379	5109
MSTRG.20392.1	coding	0.447	0	5289	7055
MSTRG.20400.7	coding	0.111	0	276	657
MSTRG.20401.1	noncoding	-0.0012288	1122	1203	1732
MSTRG.20402.1	noncoding	-0.0004096	372	516	1375
MSTRG.20403.1	noncoding	-0.0094208	537	729	1203
MSTRG.20407.1	noncoding	-0.0487424	2364	3285	4555
MSTRG.20414.2	coding	0.428	0	2310	2845
MSTRG.20415.1	noncoding	-0.055296	780	1953	2112
MSTRG.20416.1	coding	0.51	0	966	4047
MSTRG.20421.1	coding	0.201	0	1104	2505
MSTRG.20421.2	coding	0.201	0	1104	1954
MSTRG.20421.3	coding	0.201	0	1104	5123
MSTRG.20424.1	noncoding	-0.0004096	888	888	1337
MSTRG.20426.1	coding	0.45	660	2328	4945
MSTRG.20426.2	coding	0.45	429	2097	4655
MSTRG.20429.2	coding	0.344	66	624	777
MSTRG.20430.1	noncoding	-0.0008192	42	48	204
MSTRG.20433.4	coding	0.441	426	3102	3560
MSTRG.20434.1	noncoding	-0.0053248	2745	2871	5323
MSTRG.20433.7	coding	0.34	237	1011	1312
MSTRG.20431.2	coding	0.307	3045	4341	4535
MSTRG.20431.3	coding	0.281	0	879	1576
MSTRG.20431.4	noncoding	-0.1142784	3657	3957	4388
MSTRG.20438.4	coding	0.225	0	399	1319
MSTRG.20439.1	coding	0.497	0	1119	15062
MSTRG.20439.2	coding	0.525	0	1029	5018
MSTRG.20439.3	noncoding	-0.2142208	0	180	3005
MSTRG.20440.1	noncoding	-0.0585728	10980	11313	12225
MSTRG.20440.2	coding	0.12	4296	4563	5872
MSTRG.20446.2	noncoding	-0.0512	765	1980	2186
MSTRG.20451.2	coding	0.351	36	1539	10263
MSTRG.20452.1	noncoding	-0.1355776	0	135	292
MSTRG.20453.1	coding	0.112	558	888	2197
MSTRG.20455.2	coding	0.091	678	840	1068
MSTRG.20457.2	coding	0.053	975	1605	3257
MSTRG.20457.4	coding	0.053	816	1446	4749
MSTRG.20457.7	coding	0.053	771	1401	3228
MSTRG.20457.6	coding	0.053	1446	2076	3904
MSTRG.20457.8	coding	0.035	1101	1251	2394
MSTRG.20469.1	coding	0.276	87	729	988
MSTRG.20469.2	coding	0.29	72	813	1075
MSTRG.20471.1	noncoding	-0.016384	2568	2649	3702
MSTRG.20471.2	noncoding	-0.0090112	1254	1419	1573
MSTRG.20473.2	noncoding	-0.0512	138	333	811
MSTRG.20474.1	coding	0.268	0	144	298
MSTRG.20476.1	noncoding	-0.008192	141	189	677
MSTRG.20482.6	noncoding	-0.073728	2169	2247	3990

MSTRG.20483.1	noncoding	-0.0380928	807	891	8118
MSTRG.20484.1	noncoding	-0.0045056	2817	3009	3180
MSTRG.20486.1	noncoding	-0.016384	159	246	1001
MSTRG.20479.1	noncoding	-0.008192	510	513	703
MSTRG.20489.1	noncoding	-0.0192512	405	549	2951
MSTRG.20504.2	noncoding	-0.0458752	417	834	991
MSTRG.20505.1	coding	0.068	0	1680	3616
MSTRG.20506.1	coding	0.072	357	879	1498
MSTRG.20512.1	noncoding	-0.03072	21	300	5212
MSTRG.20512.2	noncoding	-0.0569344	18	309	668
MSTRG.20512.3	noncoding	-0.0917504	933	1206	1900
MSTRG.20512.4	noncoding	-0.03072	6	285	1543
MSTRG.20512.5	noncoding	-0.0274432	1008	1245	3974
MSTRG.20512.6	noncoding	-0.0577536	102	117	1685
MSTRG.20514.1	noncoding	-0.004096	0	123	441
MSTRG.20516.1	noncoding	-0.0004096	0	0	222
MSTRG.20526.1	noncoding	-0.028672	1164	1194	2703
MSTRG.20528.1	coding	0.094	2139	3123	4369
MSTRG.20547.1	noncoding	-0.110592	894	966	1154
MSTRG.20552.1	noncoding	-0.2797568	0	495	650
MSTRG.20565.1	noncoding	-0.0004096	0	0	395
MSTRG.20566.1	noncoding	-0.057344	705	804	1251
MSTRG.20568.2	coding	0.086	1059	2700	3106
MSTRG.20569.1	noncoding	-0.1011712	81	87	486
MSTRG.20570.1	noncoding	-0.0110592	207	285	540
MSTRG.20571.1	coding	0.193	96	1515	1668
MSTRG.20574.1	noncoding	-0.02048	453	585	968
MSTRG.20577.2	coding	0.261	789	2067	3852
MSTRG.20577.3	noncoding	-0.032768	1626	1725	2433
MSTRG.20579.2	noncoding	-0.0270336	381	549	4341
MSTRG.20579.4	coding	0.17	63	963	4846
MSTRG.20580.1	noncoding	-0.0299008	342	372	1232
MSTRG.20581.1	noncoding	-0.0008192	1917	1974	2709
MSTRG.20583.1	coding	0.284	0	372	6247
MSTRG.20584.1	noncoding	-0.0004096	0	0	820
MSTRG.20593.2	coding	0.634	0	546	7179
MSTRG.20593.3	coding	0.634	0	546	3061
MSTRG.20608.7	coding	0.15	0	1452	1934
MSTRG.20608.6	coding	0.175	0	2376	4264
MSTRG.20609.1	noncoding	-0.0393216	5118	5697	8063
MSTRG.20610.1	coding	0.046	7140	7962	10253
MSTRG.20608.8	coding	0.202	2139	3942	5779
MSTRG.20611.1	noncoding	-0.069632	879	972	3372
MSTRG.20612.1	noncoding	-0.024576	426	555	5346
MSTRG.20608.9	coding	0.19	540	1524	10381
MSTRG.20603.1	noncoding	-0.02048	426	561	4484
MSTRG.20603.2	noncoding	-0.012288	699	801	3622
MSTRG.20603.3	coding	0.21	165	867	10394
MSTRG.20613.1	coding	0.437	18	564	1514
MSTRG.20613.2	coding	0.45	0	555	1424
MSTRG.20614.1	noncoding	-0.0004096	0	0	346
MSTRG.20616.1	noncoding	-0.094208	300	312	516
MSTRG.20618.2	coding	0.355	0	1137	2405
MSTRG.20620.2	noncoding	-0.028672	30	120	292

MSTRG.20619.4	coding	0.157	0	2112	3387
MSTRG.20621.1	coding	0.059	5145	5793	8279
MSTRG.20628.2	coding	0.485	0	1800	3761
MSTRG.20629.1	noncoding	-0.032768	63	165	322
MSTRG.20629.2	noncoding	-0.006144	159	213	368
MSTRG.20631.2	coding	0.114	378	963	2692
MSTRG.20631.3	noncoding	-0.0790528	1233	1491	5980
MSTRG.20634.1	coding	0.147	0	2586	3512
MSTRG.20634.2	coding	0.136	138	2517	2801
MSTRG.20634.3	coding	0.129	804	1140	1420
MSTRG.20637.3	coding	0.036	195	474	4275
MSTRG.20637.5	coding	0.036	243	522	2330
MSTRG.20637.4	coding	0.261	0	1044	2850
MSTRG.20638.1	coding	0.538	0	258	1547
MSTRG.20642.1	coding	0.142	432	747	1613
MSTRG.20654.1	noncoding	-0.0065536	0	81	252
MSTRG.20654.2	coding	0.042	8325	14001	14871
MSTRG.20654.4	noncoding	-0.0262144	2130	2316	2471
MSTRG.20654.3	noncoding	-0.016384	2253	2352	2508
MSTRG.20654.5	noncoding	-0.08192	1887	1959	2114
MSTRG.20654.12	coding	0.02	210	411	1238
MSTRG.20654.8	coding	0.11	3990	4197	7290
MSTRG.20654.10	coding	0.001	1824	2292	3026
MSTRG.20654.11	coding	0.058	7107	10191	12817
MSTRG.20654.9	coding	0.058	7050	10134	12761
MSTRG.20654.6	coding	0.042	7392	13068	13939
MSTRG.20654.7	coding	0.042	7755	13431	14302
MSTRG.20654.13	noncoding	-0.012288	729	864	4094
MSTRG.20656.1	noncoding	-0.0004096	3225	3318	8276
MSTRG.20657.1	noncoding	-0.0299008	480	570	2255
MSTRG.20658.1	noncoding	-0.0053248	720	912	1254
MSTRG.20654.14	coding	0.006	1434	1626	3684
MSTRG.20660.2	coding	0.117	99	1812	3786
MSTRG.20644.1	coding	0.154	0	1869	2774
MSTRG.20644.3	coding	0.099	147	1650	6915
MSTRG.20644.6	noncoding	-0.0446464	4797	5238	10352
MSTRG.20646.1	coding	0.315	417	1821	2133
MSTRG.20649.2	coding	0.407	1737	4368	5244
MSTRG.20649.3	coding	0.406	342	2967	3842
MSTRG.20662.1	noncoding	-0.0897024	0	147	425
MSTRG.20662.2	noncoding	-0.016384	288	369	873
MSTRG.20662.3	coding	0.193	78	420	1069
MSTRG.20662.4	noncoding	-0.0790528	204	546	1195
MSTRG.20662.5	noncoding	-0.067584	0	510	1159
MSTRG.20662.6	noncoding	-0.067584	0	603	1252
MSTRG.20664.1	coding	0.343	0	1497	4391
MSTRG.20664.2	coding	0.286	1242	2157	3915
MSTRG.20667.2	noncoding	-0.055296	522	1128	1673
MSTRG.20668.1	coding	0.223	249	705	1013
MSTRG.20669.2	coding	0.366	4140	5007	5350
MSTRG.20670.1	noncoding	-0.0151552	639	1101	10076
MSTRG.20672.6	coding	0.453	1044	2370	2803
MSTRG.20672.8	coding	0.404	2862	3714	4147
MSTRG.20674.1	coding	0.076	1764	6399	11126

MSTRG.20674.3	coding	0.082	1245	6381	10189
MSTRG.20674.5	coding	0.084	1764	6537	7206
MSTRG.20674.6	noncoding	-0.08192	162	273	2283
MSTRG.20678.2	coding	0.103	3837	5436	7076
MSTRG.20678.3	noncoding	-0.0626688	0	87	934
MSTRG.20678.4	coding	0.099	3543	5154	7086
MSTRG.20679.1	coding	0.052	222	429	1231
MSTRG.20677.1	noncoding	-0.0004096	4356	4530	7969
MSTRG.20681.1	noncoding	-0.049152	360	408	563
MSTRG.20682.1	noncoding	-0.012288	36	147	485
MSTRG.20682.2	noncoding	-0.0323584	36	162	507
MSTRG.20688.2	noncoding	-0.0770048	0	177	3126
MSTRG.20705.1	coding	0.349	33	525	821
MSTRG.20705.2	noncoding	-0.1396736	15	480	710
MSTRG.20710.3	noncoding	-0.0684032	63	285	4078
MSTRG.20710.5	noncoding	-0.04096	0	654	10269
MSTRG.20711.1	noncoding	-0.0442368	519	705	862
MSTRG.20713.1	noncoding	-0.032768	1263	1371	1731
MSTRG.20708.2	coding	0.157	0	651	2723
MSTRG.20709.1	noncoding	-0.0147456	33	327	628
MSTRG.20714.1	coding	0.473	624	1455	3158
MSTRG.20725.3	coding	0.14	10689	10923	15327
MSTRG.20725.5	noncoding	-0.0196608	1866	2022	2400
MSTRG.20725.4	coding	0.082	399	1446	3766
MSTRG.20725.6	coding	0.082	516	1563	3881
MSTRG.20725.7	noncoding	-0.0278528	18	81	275
MSTRG.20725.8	coding	0.082	564	1611	3930
MSTRG.20725.9	noncoding	-0.0602112	1221	1344	3970
MSTRG.20725.10	noncoding	-0.032768	4989	5073	9170
MSTRG.20725.11	coding	0.082	3873	4920	7240
MSTRG.20725.12	noncoding	-0.077824	2745	2781	4858
MSTRG.20725.14	noncoding	-0.0602112	1116	1239	2826
MSTRG.20726.1	noncoding	-0.0774144	2985	3039	5275
MSTRG.20727.1	noncoding	-0.0741376	477	489	3102
MSTRG.20728.1	noncoding	-0.036864	2955	3198	5172
MSTRG.20730.12	noncoding	-0.004096	1173	1293	1449
MSTRG.20730.6	coding	0.052	0	3405	3939
MSTRG.20730.8	coding	0.058	0	4131	5034
MSTRG.20730.11	coding	0.049	0	3354	5176
MSTRG.20730.10	coding	0.055	0	4500	6718
MSTRG.20730.7	coding	0.051	0	4539	6418
MSTRG.20730.4	coding	0.05	0	4485	6336
MSTRG.20730.5	coding	0.051	0	4509	6394
MSTRG.20730.9	coding	0.054	0	3429	5314
MSTRG.20730.13	coding	0.124	600	1551	3691
MSTRG.20730.14	coding	0.117	579	1407	3178
MSTRG.20718.1	noncoding	-0.12288	384	393	949
MSTRG.20720.2	noncoding	-0.0745472	0	147	435
MSTRG.20734.1	noncoding	-0.0004096	7620	7845	8159
MSTRG.20740.1	coding	0.265	0	1593	2858
MSTRG.20742.2	coding	0.125	348	1098	1536
MSTRG.20742.3	coding	0.116	291	1134	1756
MSTRG.20742.4	coding	0.102	54	861	1297
MSTRG.20743.1	coding	0.106	120	1428	2182

MSTRG.20746.1	noncoding	-0.065536	978	1056	2526
MSTRG.20737.1	noncoding	-0.0217088	693	873	1859
MSTRG.20737.2	coding	0.098	378	999	2578
MSTRG.20751.2	coding	0.026	615	1170	1643
MSTRG.20753.3	coding	0.188	0	2535	3306
MSTRG.20753.4	coding	0.225	1560	2898	3667
MSTRG.20750.3	coding	0.147	1191	3477	3983
MSTRG.20750.1	coding	0.149	435	2730	3236
MSTRG.20758.1	coding	0.183	90	1269	2198
MSTRG.20763.10	noncoding	-0.0167936	0	96	729
MSTRG.20763.8	coding	0.079	327	8433	8589
MSTRG.20763.13	coding	0.096	558	6198	7956
MSTRG.20774.1	coding	0.026	5136	5310	8406
MSTRG.20775.4	coding	0.227	6	1695	2342
MSTRG.20775.6	coding	0.212	6	1527	2267
MSTRG.20775.7	coding	0.226	6	2610	11435
MSTRG.20775.8	coding	0.212	6	1527	10311
MSTRG.20775.9	noncoding	-0.0139264	3204	3297	3477
MSTRG.20776.1	coding	0.01	2205	2337	6726
MSTRG.20776.2	coding	0.01	2205	2337	6581
MSTRG.20777.1	noncoding	-0.01024	3303	3456	7166
MSTRG.20778.1	noncoding	-0.0139264	2241	2394	3043
MSTRG.20780.1	noncoding	-0.02048	495	507	2250
MSTRG.20781.1	noncoding	-0.06144	255	312	2716
MSTRG.20782.1	coding	0.097	2838	3141	7794
MSTRG.20783.1	coding	0.078	21	165	3945
MSTRG.20781.2	noncoding	-0.016384	7137	7260	7510
MSTRG.20784.1	noncoding	-0.0098304	5547	5688	9598
MSTRG.20785.1	noncoding	-0.0012288	1890	2160	2678
MSTRG.20786.1	noncoding	-0.008192	2046	2178	3725
MSTRG.20775.10	coding	0.142	147	1233	25752
MSTRG.20787.1	coding	0.079	5673	6147	7313
MSTRG.20787.2	coding	0.079	10722	11196	18030
MSTRG.20792.1	noncoding	-0.008192	6723	6897	12543
MSTRG.20793.1	noncoding	-0.024576	3348	3531	16596
MSTRG.20794.1	coding	0.045	666	891	4076
MSTRG.20800.1	coding	0.098	1635	2940	3445
MSTRG.20800.2	noncoding	-0.0339968	75	237	774
MSTRG.20801.1	noncoding	-0.098304	756	768	921
MSTRG.20797.1	noncoding	-0.0049152	288	477	632
MSTRG.20798.4	coding	0.363	657	1710	2058
MSTRG.20819.4	coding	0.21	0	8253	14172
MSTRG.20819.8	coding	0.214	0	11532	11687
MSTRG.20822.1	noncoding	-0.0299008	24	39	437
MSTRG.20816.5	coding	0.341	192	1179	1894
MSTRG.20816.3	coding	0.303	189	1296	2013
MSTRG.20816.4	coding	0.345	354	1077	1792
MSTRG.20816.2	coding	0.317	126	1254	1970
MSTRG.20823.3	coding	0.461	2646	4812	6703
MSTRG.20823.4	coding	0.459	297	2514	4405
MSTRG.20823.2	coding	0.469	597	2904	4795
MSTRG.20823.1	coding	0.359	36	5826	7715
MSTRG.20817.2	coding	0.532	0	1770	1987
MSTRG.20817.1	coding	0.505	0	1944	2175

MSTRG.20828.4	coding	0.356	597	2490	2645
MSTRG.20828.5	noncoding	-0.028672	1029	1218	2916
MSTRG.20829.1	coding	0.414	0	2247	2804
MSTRG.20829.2	coding	0.461	297	2220	2777
MSTRG.20829.5	coding	0.424	168	1656	2586
MSTRG.20830.4	coding	0.204	117	4350	4619
MSTRG.20825.3	coding	0.29	48	396	1082
MSTRG.20825.4	coding	0.292	72	447	1133
MSTRG.20826.2	noncoding	-0.0512	0	291	1744
MSTRG.20831.1	coding	0.472	0	588	964
MSTRG.20832.3	coding	0.371	684	1590	1790
MSTRG.20836.2	coding	0.396	1755	8826	9878
MSTRG.20836.3	coding	0.396	708	7779	8849
MSTRG.20836.4	coding	0.399	795	8412	9462
MSTRG.20840.1	coding	0.433	45	1224	4183
MSTRG.20845.4	coding	0.289	57	5067	7832
MSTRG.20845.7	coding	0.289	4992	10002	12765
MSTRG.20845.6	coding	0.289	327	5337	8100
MSTRG.20845.9	noncoding	-0.0016384	3390	3561	4199
MSTRG.20844.2	coding	0.188	603	1929	2112
MSTRG.20850.1	coding	0.078	612	1053	18344
MSTRG.20850.3	noncoding	-0.008192	3282	3426	7520
MSTRG.20852.4	coding	0.461	0	2667	4059
MSTRG.20854.1	noncoding	-0.0974848	0	462	1013
MSTRG.20854.2	noncoding	-0.0790528	81	474	1025
MSTRG.20866.1	noncoding	-0.1024	75	150	303
MSTRG.20867.2	coding	0.297	132	1602	1893
MSTRG.20873.1	coding	0.462	0	1683	2075
MSTRG.20873.2	coding	0.473	0	1641	2033
MSTRG.20873.3	coding	0.507	42	1434	1827
MSTRG.20873.4	coding	0.51	105	1476	1869
MSTRG.20875.3	noncoding	-0.0741376	4368	4824	5173
MSTRG.20875.2	coding	0.425	501	5043	7772
MSTRG.20876.1	noncoding	-0.0483328	2691	2883	6541
MSTRG.20877.1	noncoding	-0.0192512	204	495	2282
MSTRG.20878.1	noncoding	-0.0397312	11229	11499	15239
MSTRG.20872.1	noncoding	-0.028672	60	99	488
MSTRG.20882.1	coding	0.13	1560	2883	8400
MSTRG.20883.1	noncoding	-0.02048	186	420	1278
MSTRG.20884.2	coding	0.271	12	2091	2745
MSTRG.20884.3	coding	0.39	0	2973	3627
MSTRG.20884.4	coding	0.441	0	1419	1958
MSTRG.20891.1	noncoding	-0.0233472	1851	2232	21809
MSTRG.20891.2	noncoding	-0.0241664	3162	3465	5664
MSTRG.20891.3	noncoding	-0.0241664	3162	3465	4270
MSTRG.20892.1	noncoding	-0.0045056	3903	4155	5183
MSTRG.20895.1	coding	0.225	159	2031	4557
MSTRG.20900.4	coding	0.455	123	1254	1410
MSTRG.20900.3	coding	0.455	3786	4920	5075
MSTRG.20912.2	coding	0.228	447	993	4130
MSTRG.20913.3	coding	0.569	1473	7275	10947
MSTRG.20913.4	coding	0.581	0	5673	5915
MSTRG.20913.5	coding	0.56	405	6279	9799
MSTRG.20915.1	noncoding	-0.083968	0	117	412

MSTRG.20919.1	coding	0.419	0	2247	2411
MSTRG.20914.1	noncoding	-0.0028672	285	393	712
MSTRG.20914.2	noncoding	-0.0753664	0	237	499
MSTRG.20914.3	noncoding	-0.0589824	0	213	633
MSTRG.20923.1	noncoding	-0.0462848	0	99	351
MSTRG.20921.1	noncoding	-0.0700416	15	255	409
MSTRG.20921.2	noncoding	-0.038912	15	171	800
MSTRG.20932.2	coding	0.279	123	861	1437
MSTRG.20934.1	noncoding	-0.08192	138	393	773
MSTRG.20930.1	coding	0.403	0	1413	1649
MSTRG.20943.1	noncoding	-0.0585728	756	951	1478
MSTRG.20944.1	noncoding	-0.0897024	0	282	1369
MSTRG.20940.1	noncoding	-0.0651264	0	240	510
MSTRG.20949.2	coding	0.509	0	1764	2362
MSTRG.20951.1	coding	0.418	0	996	2455
MSTRG.20952.2	noncoding	-0.0565248	0	1239	2037
MSTRG.20953.4	coding	0.309	87	759	6396
MSTRG.20953.2	coding	0.3	474	1026	2185
MSTRG.20953.5	noncoding	-0.1007616	441	729	5355
MSTRG.20950.2	coding	0.599	0	2370	4405
MSTRG.20955.3	coding	0.415	237	1623	1805
MSTRG.20955.5	coding	0.401	456	2112	2296
MSTRG.20955.4	coding	0.403	207	1866	2050
MSTRG.20955.1	coding	0.424	609	2118	2302
MSTRG.20957.1	noncoding	-0.0004096	648	792	2853
MSTRG.20959.1	noncoding	-0.606208	30	36	231
MSTRG.20967.3	coding	0.222	84	1974	5627
MSTRG.20967.7	coding	0.295	63	1740	5391
MSTRG.20967.9	coding	0.341	84	4662	5234
MSTRG.20967.10	coding	0.371	84	4104	4677
MSTRG.20967.8	coding	0.412	63	3870	4443
MSTRG.20967.11	coding	0.484	2112	4398	4969
MSTRG.20967.13	coding	0.484	510	2796	3371
MSTRG.20967.14	coding	0.484	66	2352	2924
MSTRG.20969.2	noncoding	-0.0651264	27	249	419
MSTRG.20963.4	coding	0.494	1440	3909	4066
MSTRG.20963.5	coding	0.496	1440	3897	4054
MSTRG.20963.8	coding	0.503	786	3699	3856
MSTRG.20963.6	coding	0.502	147	3072	3227
MSTRG.20975.2	coding	0.251	189	618	818
MSTRG.20979.2	coding	0.534	0	2007	3008
MSTRG.20981.2	coding	0.54	375	2151	2471
MSTRG.20977.2	coding	0.579	33	846	3508
MSTRG.20978.1	coding	0.454	0	1518	1717
MSTRG.20978.3	coding	0.442	24	1377	5329
MSTRG.20986.2	coding	0.329	18	2436	4056
MSTRG.20986.5	coding	0.457	0	1680	2229
MSTRG.20986.1	coding	0.348	60	2400	4020
MSTRG.20986.3	coding	0.347	99	2445	4064
MSTRG.20986.7	coding	0.33	60	2472	3822
MSTRG.20986.8	coding	0.458	60	1800	2690
MSTRG.20989.1	noncoding	-0.1097728	465	1278	1546
MSTRG.20990.1	noncoding	-0.1220608	0	372	1101
MSTRG.20990.2	noncoding	-0.1417216	612	1044	1199

MSTRG.20994.3	coding	0.385	0	6081	7406
MSTRG.20999.2	coding	0.187	0	7965	12702
MSTRG.20999.3	coding	0.189	2442	9222	13652
MSTRG.21000.1	noncoding	-0.0376832	609	801	1168
MSTRG.21002.1	noncoding	-0.0303104	1263	1986	5102
MSTRG.21011.4	coding	0.322	0	2415	2634
MSTRG.21017.1	noncoding	-0.0176128	111	291	446
MSTRG.21018.1	coding	0.449	0	1560	3721
MSTRG.21018.2	coding	0.453	0	1557	3718
MSTRG.21018.3	coding	0.524	0	990	1144
MSTRG.21024.5	coding	0.22	60	5007	9524
MSTRG.21024.6	coding	0.223	747	5274	9762
MSTRG.21024.7	coding	0.218	864	5853	10341
MSTRG.21022.1	noncoding	-0.0004096	0	0	350
MSTRG.21027.4	coding	0.132	0	1785	2335
MSTRG.21027.6	coding	0.137	0	1827	2375
MSTRG.21027.5	coding	0.148	12	1539	2088
MSTRG.21027.8	coding	0.154	0	1422	1972
MSTRG.21037.2	coding	0.242	468	936	2010
MSTRG.21038.2	coding	0.083	4722	5790	6349
MSTRG.21038.5	noncoding	-0.0684032	249	288	1040
MSTRG.21038.6	noncoding	-0.014336	4719	4863	6234
MSTRG.21039.2	coding	0.18	822	1530	2754
MSTRG.21048.3	coding	0.103	354	3585	11134
MSTRG.21048.5	coding	0.103	645	3876	4203
MSTRG.21049.2	noncoding	-0.0516096	8532	8709	10145
MSTRG.21061.1	coding	0.159	0	3336	8598
MSTRG.21061.2	coding	0.094	0	2520	11348
MSTRG.21062.1	coding	0.075	0	480	931
MSTRG.21061.3	coding	0.158	948	1746	2179
MSTRG.21066.1	coding	0.2	0	636	1181
MSTRG.21068.3	coding	0.196	0	1719	2915
MSTRG.21074.1	coding	0.048	3483	4218	5671
MSTRG.21076.4	coding	0.153	0	525	808
MSTRG.21079.1	noncoding	-0.026624	975	1089	1244
MSTRG.21080.3	noncoding	-0.110592	6279	6384	7557
MSTRG.21080.4	coding	0.135	129	225	887
MSTRG.21082.1	noncoding	-0.0507904	636	639	1104
MSTRG.21083.1	noncoding	-0.0364544	3219	3360	3955
MSTRG.21086.1	noncoding	-0.1134592	0	141	772
MSTRG.21087.3	coding	0.209	360	1347	4825
MSTRG.21090.1	coding	0.012	810	2805	2959
MSTRG.21089.3	noncoding	-0.0548864	2772	3258	3415
MSTRG.21096.4	coding	0.075	846	1545	2224
MSTRG.21092.2	coding	0.246	1695	2484	3581
MSTRG.21093.3	coding	0.093	300	3318	3807
MSTRG.21104.3	coding	0.157	807	2079	2923
MSTRG.21104.4	coding	0.157	771	2043	2543
MSTRG.21105.1	noncoding	-0.126976	2088	2145	2803
MSTRG.21106.5	noncoding	-0.104448	156	891	4608
MSTRG.21106.7	coding	0.17	156	1815	3624
MSTRG.21106.6	coding	0.164	156	1860	3686
MSTRG.21106.2	noncoding	-0.104448	156	891	4827
MSTRG.21106.4	noncoding	-0.104448	156	891	4936

MSTRG.21106.8	coding	0.13	465	1596	3405
MSTRG.21106.9	coding	0.13	96	1227	3035
MSTRG.21106.10	coding	0.122	516	1692	3501
MSTRG.21106.11	coding	0.126	456	1566	3216
MSTRG.21101.3	coding	0.115	264	1002	4866
MSTRG.21098.1	noncoding	-0.0823296	9123	9555	9710
MSTRG.21102.2	coding	0.177	0	810	1101
MSTRG.21107.2	coding	0.218	861	1830	7948
MSTRG.21107.3	coding	0.132	240	2205	2574
MSTRG.21108.1	noncoding	-0.0147456	4227	4338	7813
MSTRG.21109.1	noncoding	-0.032768	1026	1116	3910
MSTRG.21107.4	coding	0.218	336	1305	1673
MSTRG.21111.2	coding	0.319	51	3039	4669
MSTRG.21112.1	coding	0.26	375	1269	7031
MSTRG.21112.2	noncoding	-0.0249856	5022	5502	6343
MSTRG.21115.1	noncoding	-0.1236992	0	636	2035
MSTRG.21117.1	noncoding	-0.131072	156	195	350
MSTRG.21120.1	noncoding	-0.135168	240	285	457
MSTRG.21126.2	coding	0.235	303	2352	4508
MSTRG.21123.1	noncoding	-0.0331776	225	507	769
MSTRG.21130.3	coding	0.145	939	1494	4651
MSTRG.21127.1	coding	0.302	0	5226	8099
MSTRG.21127.2	coding	0.3	0	5184	8057
MSTRG.21128.1	noncoding	-0.1482752	81	90	322
MSTRG.21146.3	coding	0.379	36	963	2031
MSTRG.21147.1	noncoding	-0.008192	0	129	688
MSTRG.21146.4	coding	0.396	501	1353	1575
MSTRG.21146.6	coding	0.396	471	1323	1545
MSTRG.21146.5	coding	0.363	6915	7944	8164
MSTRG.21146.7	coding	0.369	888	1950	2172
MSTRG.21146.8	coding	0.406	120	786	956
MSTRG.21146.9	noncoding	-0.053248	3513	3612	4099
MSTRG.21146.10	noncoding	-0.0192512	759	930	7677
MSTRG.21146.11	noncoding	-0.0483328	0	180	9405
MSTRG.21146.12	noncoding	-0.1032192	0	369	1461
MSTRG.21148.10	coding	0.131	0	3201	3725
MSTRG.21148.12	coding	0.134	0	3252	3778
MSTRG.21148.6	coding	0.117	132	3339	3864
MSTRG.21148.8	coding	0.106	261	3318	3844
MSTRG.21148.9	coding	0.121	132	3489	4014
MSTRG.21134.1	coding	0.309	66	993	1525
MSTRG.21151.1	noncoding	-0.0466944	2268	2658	3100
MSTRG.21152.1	coding	0.121	0	2700	5069
MSTRG.21153.1	coding	0.304	0	2832	6740
MSTRG.21154.1	coding	0.063	186	951	3892
MSTRG.21154.2	coding	0.09	138	573	761
MSTRG.21155.1	noncoding	-0.0372736	108	240	786
MSTRG.21156.2	coding	0.115	429	1080	2069
MSTRG.21154.3	coding	0.158	0	345	1305
MSTRG.21154.4	coding	0.075	1665	3306	7780
MSTRG.21159.3	coding	0.068	717	1473	3241
MSTRG.21159.4	noncoding	-0.09216	201	633	3450
MSTRG.21159.5	noncoding	-0.0876544	198	816	4963
MSTRG.21159.6	noncoding	-0.09216	201	633	2673

MSTRG.21159.7	noncoding	-0.0376832	54	126	2474
MSTRG.21162.6	coding	0.083	474	1704	3027
MSTRG.21162.7	coding	0.102	612	2148	3471
MSTRG.21163.1	noncoding	-0.1241088	0	141	778
MSTRG.21163.2	noncoding	-0.053248	267	357	1878
MSTRG.21173.1	coding	0.116	0	9723	17106
MSTRG.21169.1	noncoding	-0.032768	12	117	1219
MSTRG.21176.1	noncoding	-0.04096	0	102	451
MSTRG.21176.2	noncoding	-0.0724992	0	78	410
MSTRG.21179.3	coding	0.154	153	9300	10168
MSTRG.21179.2	coding	0.154	165	9312	10181
MSTRG.21179.5	coding	0.154	441	9588	10444
MSTRG.21179.6	coding	0.154	147	9294	10130
MSTRG.21181.2	coding	0.396	381	3993	11119
MSTRG.21198.1	coding	0.254	0	1371	1935
MSTRG.21198.2	coding	0.211	648	1239	3647
MSTRG.21198.3	coding	0.275	0	1167	3571
MSTRG.21212.1	coding	0.094	195	2319	4467
MSTRG.21212.2	coding	0.085	195	1206	3428
MSTRG.21213.2	coding	0.219	1881	2850	3003
MSTRG.21216.2	noncoding	-0.016384	108	231	4481
MSTRG.21218.2	coding	0.132	0	615	1101
MSTRG.21218.3	noncoding	-0.0720896	537	720	873
MSTRG.21220.3	coding	0.134	0	774	1917
MSTRG.21220.4	noncoding	-0.045056	84	327	3431
MSTRG.21224.1	noncoding	-0.02048	126	192	748
MSTRG.21235.3	coding	0.338	402	2490	2921
MSTRG.21234.1	noncoding	-0.045056	111	117	311
MSTRG.21244.1	noncoding	-0.012288	99	303	674
MSTRG.21248.2	coding	0.26	51	633	900
MSTRG.21249.1	noncoding	-0.0495616	3879	4227	4384
MSTRG.21250.2	coding	0.208	0	939	2594
MSTRG.21251.1	noncoding	-0.0475136	150	462	813
MSTRG.21251.2	noncoding	-0.0110592	42	957	1121
MSTRG.21252.1	noncoding	-0.0086016	1407	2556	2756
MSTRG.21252.2	noncoding	-0.0278528	1758	1917	3717
MSTRG.21254.1	noncoding	-0.0249856	1299	1497	1821
MSTRG.21256.5	coding	0.265	0	165	3651
MSTRG.21256.6	noncoding	-0.0004096	186	303	1274
MSTRG.21257.1	noncoding	-0.008192	75	141	603
MSTRG.21260.1	coding	0.235	1110	1335	2338
MSTRG.21262.1	noncoding	-0.004096	189	249	928
MSTRG.21268.1	noncoding	-0.004096	891	1008	1621
MSTRG.21269.1	coding	0.236	0	4095	5929
MSTRG.21269.2	noncoding	-0.0540672	573	978	1443
MSTRG.21271.2	coding	0.296	0	1980	5184
MSTRG.21272.1	noncoding	-0.06144	363	480	852
MSTRG.21278.2	coding	0.254	408	954	3650
MSTRG.21301.3	coding	0.34	3	2571	4695
MSTRG.21302.1	noncoding	-0.0360448	3972	4131	6382
MSTRG.21308.1	coding	0.225	0	1614	4842
MSTRG.21309.2	coding	0.276	294	3855	4856
MSTRG.21310.3	coding	0.388	543	2331	2598
MSTRG.21315.2	coding	0.413	132	4320	7013

MSTRG.21316.1	noncoding	-0.012288	600	696	2625
MSTRG.21317.1	noncoding	-0.073728	126	204	627
MSTRG.21318.1	noncoding	-0.065536	1338	1392	1564
MSTRG.21315.4	noncoding	-0.1880064	0	321	1746
MSTRG.21314.1	coding	0.48	228	1350	1506
MSTRG.21319.1	coding	0.457	297	3060	3238
MSTRG.21323.1	coding	0.564	0	5847	8362
MSTRG.21323.5	coding	0.665	0	4758	7386
MSTRG.21323.3	coding	0.652	0	4986	7616
MSTRG.21323.8	coding	0.605	0	5328	5789
MSTRG.21323.7	coding	0.586	0	5571	5727
MSTRG.21323.9	coding	0.596	0	5526	5979
MSTRG.21321.1	coding	0.555	0	4329	5069
MSTRG.21321.2	coding	0.587	0	4044	4784
MSTRG.21321.3	coding	0.589	120	4068	4234
MSTRG.21324.2	coding	0.523	0	2223	4329
MSTRG.21324.3	coding	0.458	0	2547	2866
MSTRG.21326.1	noncoding	-0.0303104	732	1491	3755
MSTRG.21327.3	coding	0.424	648	1869	2647
MSTRG.21327.6	coding	0.425	903	2085	2864
MSTRG.21327.8	coding	0.432	165	735	2066
MSTRG.21330.2	coding	0.176	0	465	619
MSTRG.21333.4	coding	0.363	0	1851	2019
MSTRG.21334.5	coding	0.289	0	4809	6392
MSTRG.21334.6	coding	0.284	0	5016	6599
MSTRG.21334.7	coding	0.289	0	4878	6460
MSTRG.21334.8	coding	0.285	303	4893	6477
MSTRG.21334.9	coding	0.292	588	5037	6621
MSTRG.21334.10	coding	0.287	3111	7431	8186
MSTRG.21335.1	noncoding	-0.016384	5385	5487	8337
MSTRG.21336.1	noncoding	-0.0835584	0	279	4465
MSTRG.21336.2	noncoding	-0.0643072	1377	1614	4287
MSTRG.21336.3	noncoding	-0.0643072	1536	1773	3898
MSTRG.21338.4	coding	0.38	14076	14985	18924
MSTRG.21338.6	coding	0.376	405	2076	5771
MSTRG.21338.7	coding	0.428	87	2772	6460
MSTRG.21338.9	noncoding	-0.0241664	1107	1293	1446
MSTRG.21338.10	noncoding	-0.0536576	1248	1569	2120
MSTRG.21338.13	coding	0.525	0	1644	5147
MSTRG.21338.12	coding	0.503	0	1725	2067
MSTRG.21342.1	noncoding	-0.0479232	234	600	2531
MSTRG.21343.3	coding	0.242	339	846	1203
MSTRG.21348.4	coding	0.43	222	5352	5507
MSTRG.21348.1	coding	0.38	1596	7449	7606
MSTRG.21348.2	coding	0.381	1596	7452	7609
MSTRG.21349.1	coding	0.534	465	1530	2740
MSTRG.21351.1	noncoding	-0.0004096	141	282	436
MSTRG.21345.1	coding	0.181	0	267	3293
MSTRG.21371.3	coding	0.577	0	2982	3817
MSTRG.21371.5	coding	0.632	1977	3471	4187
MSTRG.21371.4	coding	0.521	90	5124	10724
MSTRG.21371.6	coding	0.406	5904	8307	9004
MSTRG.21371.7	coding	0.51	0	1527	4031
MSTRG.21371.9	coding	0.522	0	4572	10632

MSTRG.21371.10	coding	0.521	1596	6735	12793
MSTRG.21371.11	coding	0.523	1815	6939	12995
MSTRG.21371.13	coding	0.522	630	5745	11745
MSTRG.21371.12	coding	0.522	1881	6996	12997
MSTRG.21371.15	coding	0.523	78	5097	10983
MSTRG.21371.16	coding	0.523	639	5763	11651
MSTRG.21371.17	coding	0.507	210	4941	10706
MSTRG.21371.18	coding	0.522	1695	6810	9680
MSTRG.21372.1	noncoding	-0.0217088	7065	9243	9582
MSTRG.21373.1	noncoding	-0.0790528	468	708	863
MSTRG.21372.2	noncoding	-0.01024	99	4416	5892
MSTRG.21356.1	coding	0.646	54	1002	1251
MSTRG.21359.1	noncoding	-0.034816	1566	2085	2841
MSTRG.21361.1	coding	0.344	0	699	3362
MSTRG.21362.1	noncoding	-0.004096	1455	1599	1752
MSTRG.21363.1	coding	0.196	414	828	1968
MSTRG.21368.2	coding	0.162	873	1776	3197
MSTRG.21368.3	coding	0.145	1452	2361	3783
MSTRG.21369.1	noncoding	-0.04096	633	744	1755
MSTRG.21377.1	noncoding	-0.036864	0	21	238
MSTRG.21375.1	noncoding	-0.0139264	243	258	420
MSTRG.21384.1	noncoding	-0.126976	267	276	1669
MSTRG.21385.5	noncoding	-0.0004096	3405	3528	4682
MSTRG.21385.4	coding	0.047	168	1179	2042
MSTRG.21385.7	noncoding	-0.0004096	9519	9642	9977
MSTRG.21385.8	noncoding	-0.192512	1311	1347	1648
MSTRG.21385.9	coding	0.062	0	990	4380
MSTRG.21385.10	noncoding	-0.0425984	0	78	847
MSTRG.21395.3	coding	0.136	0	507	983
MSTRG.21396.3	coding	0.39	87	2316	5041
MSTRG.21396.4	coding	0.346	780	3567	3721
MSTRG.21406.1	noncoding	-0.0790528	3570	4857	5012
MSTRG.21406.2	noncoding	-0.0815104	1215	2271	2428
MSTRG.21400.1	noncoding	-0.077824	405	447	817
MSTRG.21402.1	noncoding	-0.004096	393	624	780
MSTRG.21403.1	noncoding	-0.03072	708	741	989
MSTRG.21414.1	noncoding	-0.02048	600	633	966
MSTRG.21415.1	noncoding	-0.0339968	2478	2655	10457
MSTRG.21416.1	noncoding	-0.0090112	201	378	3776
MSTRG.21433.1	coding	0.164	0	3054	7479
MSTRG.21433.4	coding	0.139	291	3216	5227
MSTRG.21433.3	coding	0.144	192	3144	7569
MSTRG.21433.5	coding	0.166	0	3042	12169
MSTRG.21433.6	coding	0.14	645	3789	24139
MSTRG.21433.7	coding	0.139	804	3729	5719
MSTRG.21433.8	coding	0.136	2487	5571	7582
MSTRG.21433.9	coding	0.048	4731	5484	17628
MSTRG.21434.1	coding	0.142	15	471	917
MSTRG.21433.10	coding	0.142	4215	7164	9120
MSTRG.21433.11	coding	0.052	9639	10635	25980
MSTRG.21433.13	coding	0.143	138	3099	5087
MSTRG.21433.14	coding	0.139	894	3927	5883
MSTRG.21433.15	coding	0.139	672	3705	5695
MSTRG.21433.16	coding	0.139	459	3384	5340

MSTRG.21433.18	coding	0.14	147	3081	5060
MSTRG.21433.19	coding	0.148	1839	3300	3691
MSTRG.21433.20	coding	0.141	2166	5106	7095
MSTRG.21433.22	noncoding	-0.0499712	948	1191	2199
MSTRG.21441.2	coding	0.26	0	531	715
MSTRG.21419.1	coding	0.082	435	8979	10069
MSTRG.21425.1	noncoding	-0.110592	123	138	292
MSTRG.21427.2	coding	0.06	564	921	1400
MSTRG.21427.1	coding	0.06	564	921	2889
MSTRG.21428.1	noncoding	-0.159744	123	177	330
MSTRG.21429.1	noncoding	-0.0086016	384	474	797
MSTRG.21432.1	noncoding	-0.008192	27	129	486
MSTRG.21449.1	coding	0.184	0	2643	8328
MSTRG.21449.4	coding	0.139	777	2739	2896
MSTRG.21450.1	coding	0.062	6561	6753	10910
MSTRG.21450.2	noncoding	-0.008192	654	810	2412
MSTRG.21451.1	noncoding	-0.0126976	105	303	1616
MSTRG.21449.5	noncoding	-0.0159744	5385	5622	12673
MSTRG.21452.1	noncoding	-0.0417792	1353	1674	15114
MSTRG.21453.1	noncoding	-0.0483328	9549	9738	26598
MSTRG.21454.1	noncoding	-0.0233472	4818	4995	11154
MSTRG.21454.2	coding	0.057	1245	1422	2837
MSTRG.21446.2	coding	0.107	1062	2550	2728
MSTRG.21447.4	noncoding	-0.053248	375	558	1946
MSTRG.21447.2	noncoding	-0.053248	375	558	2112
MSTRG.21471.1	coding	0.327	609	4641	7813
MSTRG.21471.3	coding	0.354	1848	4254	7437
MSTRG.21472.1	noncoding	-0.0118784	84	198	388
MSTRG.21470.3	coding	0.086	369	615	903
MSTRG.21474.3	coding	0.16	771	1209	1419
MSTRG.21484.4	coding	0.2	1278	3336	7211
MSTRG.21486.2	coding	0.099	240	624	1229
MSTRG.21502.2	coding	0.146	2847	4764	6716
MSTRG.21502.7	coding	0.125	1785	3522	4853
MSTRG.21502.4	coding	0.141	3120	4767	6099
MSTRG.21502.6	coding	0.15	1770	3678	5009
MSTRG.21502.5	coding	0.145	2862	4806	6137
MSTRG.21503.1	noncoding	-0.02048	0	192	905
MSTRG.21511.1	noncoding	-0.090112	1290	1365	2897
MSTRG.21520.1	noncoding	-0.0090112	360	393	2502
MSTRG.21526.1	coding	0.108	6	291	1250
MSTRG.21533.1	noncoding	-0.0630784	0	117	377
MSTRG.21534.1	noncoding	-0.004096	0	63	322
MSTRG.21532.2	coding	0.16	441	2667	3190
MSTRG.21535.1	noncoding	-0.0110592	5178	5331	10088
MSTRG.21532.3	coding	0.091	7734	8277	8554
MSTRG.21536.1	noncoding	-0.0004096	0	0	395
MSTRG.21537.1	noncoding	-0.0053248	15	102	2409
MSTRG.21532.4	noncoding	-0.012288	954	1095	6791
MSTRG.21538.1	noncoding	-0.024576	843	945	5785
MSTRG.21499.2	coding	0.232	210	663	1069
MSTRG.21499.3	coding	0.234	87	543	949
MSTRG.21552.1	noncoding	-0.0172032	2976	3120	6070
MSTRG.21553.1	coding	0.114	792	1023	8609

MSTRG.21555.1	noncoding	-0.0045056	2892	3039	4778
MSTRG.21556.1	noncoding	-0.0425984	5469	5631	6135
MSTRG.21569.1	noncoding	-0.0643072	282	561	857
MSTRG.21572.1	noncoding	-0.028672	348	633	939
MSTRG.21572.2	noncoding	-0.0688128	1014	1380	1617
MSTRG.21573.5	coding	0.181	1929	2343	10219
MSTRG.21573.6	coding	0.054	417	2058	3263
MSTRG.21573.7	coding	0.05	885	2529	4249
MSTRG.21573.9	coding	0.049	414	2094	3814
MSTRG.21573.14	coding	0.181	1929	2343	3576
MSTRG.21575.1	coding	0.4	42	1092	1996
MSTRG.21580.1	noncoding	-0.024576	153	282	5537
MSTRG.21580.2	coding	0.139	1746	1956	3122
MSTRG.21580.3	noncoding	-0.0192512	1176	1344	1571
MSTRG.21598.1	noncoding	-0.282624	99	135	292
MSTRG.21612.1	noncoding	-0.0012288	0	60	215
MSTRG.21649.1	noncoding	-0.086016	348	384	1114
MSTRG.21656.9	coding	0.422	9858	11472	13151
MSTRG.21657.1	noncoding	-0.012288	93	183	385
MSTRG.21670.1	noncoding	-0.0253952	339	450	1541
MSTRG.21670.2	noncoding	-0.073728	9	36	412
MSTRG.21695.4	coding	0.149	165	2499	3326
MSTRG.21696.1	noncoding	-0.008192	4065	4176	11003
MSTRG.21695.6	coding	0.14	159	2700	2855
MSTRG.21697.1	noncoding	-0.0008192	225	378	1758
MSTRG.21695.7	coding	0.056	231	1965	2866
MSTRG.21698.1	noncoding	-0.04096	1824	1881	2484
MSTRG.21695.10	coding	0.084	0	1065	2078
MSTRG.21708.1	noncoding	-0.0004096	0	0	209
MSTRG.21674.1	noncoding	-0.0057344	558	723	889
MSTRG.21676.1	noncoding	-0.0278528	753	867	1066
MSTRG.21680.1	noncoding	-0.016384	669	792	946
MSTRG.21729.1	noncoding	-0.221184	96	129	717
MSTRG.21725.1	noncoding	-0.0118784	720	936	1100
MSTRG.21739.1	noncoding	-0.08192	600	828	1603
MSTRG.21741.2	noncoding	-0.065536	258	357	907
MSTRG.21741.1	noncoding	-0.012288	543	681	1276
MSTRG.21749.1	coding	0.112	81	2682	4442
MSTRG.21749.4	coding	0.109	90	2397	2710
MSTRG.21749.5	noncoding	-0.045056	987	1191	14224
MSTRG.21749.6	noncoding	-0.045056	708	912	11715
MSTRG.21749.7	noncoding	-0.200704	108	153	358
MSTRG.21751.1	noncoding	-0.0376832	213	318	1860
MSTRG.21752.1	noncoding	-0.0004096	0	0	955
MSTRG.21749.8	noncoding	-0.028672	108	180	2194
MSTRG.21753.1	coding	0.079	1218	1875	5860
MSTRG.21754.1	noncoding	-0.08192	1728	1749	2362
MSTRG.21754.2	noncoding	-0.0294912	3936	4083	4388
MSTRG.21749.9	coding	0.102	300	2208	2924
MSTRG.21755.1	noncoding	-0.0229376	177	339	609
MSTRG.21755.2	noncoding	-0.016384	525	651	909
MSTRG.21760.1	noncoding	-0.0311296	468	648	3232
MSTRG.21771.6	coding	0.024	3741	8046	15724
MSTRG.21772.1	noncoding	-0.0360448	9837	9990	10782

MSTRG.21771.19	noncoding	-0.0585728	102	333	2786
MSTRG.21771.20	coding	0.075	87	726	4952
MSTRG.21771.21	coding	0.075	75	714	2525
MSTRG.21771.22	coding	0.075	117	753	3056
MSTRG.21771.23	noncoding	-0.0585728	60	291	1921
MSTRG.21773.1	noncoding	-0.0004096	159	288	2336
MSTRG.21775.1	noncoding	-0.0589824	702	1068	1223
MSTRG.21771.24	coding	0.04	3741	12441	15507
MSTRG.21771.25	coding	0.037	3741	12504	15832
MSTRG.21771.26	coding	0.037	3741	12453	15630
MSTRG.21771.27	coding	0.039	3741	12390	15167
MSTRG.21771.28	coding	0.062	1749	4377	5052
MSTRG.21771.29	noncoding	-0.032768	3102	3234	3389
MSTRG.21763.1	noncoding	-0.008192	45	141	391
MSTRG.21779.2	coding	0.262	0	4809	6932
MSTRG.21779.1	coding	0.267	0	4857	6981
MSTRG.21790.1	coding	0.177	426	891	3571
MSTRG.21791.2	noncoding	-0.0659456	0	132	1709
MSTRG.21795.1	noncoding	-0.0192512	1194	1404	1560
MSTRG.21795.3	noncoding	-0.026624	4092	4305	4460
MSTRG.21795.4	coding	0.023	528	705	1303
MSTRG.21795.5	noncoding	-0.026624	1440	1653	1808
MSTRG.21795.6	coding	0.05	552	843	1697
MSTRG.21796.1	noncoding	-0.032768	702	864	1019
MSTRG.21797.1	coding	0.267	276	1827	4461
MSTRG.21797.2	coding	0.267	291	1842	4862
MSTRG.21794.1	noncoding	-0.0004096	0	0	209
MSTRG.21806.2	coding	0.212	156	681	1158
MSTRG.21812.2	noncoding	-0.0434176	2703	2961	4081
MSTRG.21812.1	noncoding	-0.0208896	0	600	1507
MSTRG.21812.4	noncoding	-0.0253952	5433	5856	6295
MSTRG.21808.2	coding	0.185	0	615	3180
MSTRG.21815.1	noncoding	-0.0335872	618	672	826
MSTRG.21817.2	noncoding	-0.106496	0	138	610
MSTRG.21818.3	noncoding	-0.0114688	192	552	1034
MSTRG.21819.1	coding	0.377	0	909	2064
MSTRG.21820.1	noncoding	-0.0434176	7086	7272	11129
MSTRG.21820.2	coding	0.106	264	450	2074
MSTRG.21827.2	coding	0.416	0	984	1738
MSTRG.21827.3	coding	0.339	456	1293	2027
MSTRG.21829.2	coding	0.451	0	6822	8261
MSTRG.21829.3	coding	0.443	405	7371	8625
MSTRG.21831.1	coding	0.47	0	3234	4588
MSTRG.21831.10	coding	0.472	0	3162	4516
MSTRG.21831.11	coding	0.467	0	3264	4618
MSTRG.21831.3	coding	0.467	0	3336	4690
MSTRG.21831.5	coding	0.473	0	3267	4621
MSTRG.21831.7	coding	0.475	0	3198	4552
MSTRG.21831.9	coding	0.426	0	2496	3079
MSTRG.21831.12	noncoding	-0.055296	9678	11184	17445
MSTRG.21831.13	noncoding	-0.137216	11403	13809	13964
MSTRG.21831.14	noncoding	-0.0864256	780	3477	5269
MSTRG.21830.1	noncoding	-0.053248	264	324	773
MSTRG.21834.3	coding	0.493	0	1635	2757

MSTRG.21834.2	coding	0.474	138	1632	2754
MSTRG.21837.5	coding	0.392	0	9084	11865
MSTRG.21837.6	coding	0.396	0	8841	11622
MSTRG.21837.8	coding	0.393	0	9087	11868
MSTRG.21837.9	coding	0.391	0	8952	11701
MSTRG.21842.2	coding	0.317	378	2994	3303
MSTRG.21842.6	noncoding	-0.0909312	0	216	4546
MSTRG.21841.1	noncoding	-0.012288	177	288	578
MSTRG.21846.1	coding	0.296	168	2331	4511
MSTRG.21846.2	noncoding	-0.0335872	1287	2490	3091
MSTRG.21846.3	coding	0.318	84	1002	1775
MSTRG.21856.2	coding	0.147	690	1563	1977
MSTRG.21863.1	noncoding	-0.0405504	339	657	2314
MSTRG.21864.2	coding	0.058	819	1221	6719
MSTRG.21865.1	noncoding	-0.0442368	0	606	6173
MSTRG.21850.3	coding	0.164	156	783	2630
MSTRG.21850.2	coding	0.193	0	1002	2851
MSTRG.21867.1	coding	0.514	0	1611	1978
MSTRG.21870.1	noncoding	-0.110592	105	132	419
MSTRG.21874.1	coding	0.005	75	519	2866
MSTRG.21876.1	noncoding	-0.1867776	66	75	642
MSTRG.21880.1	coding	0.356	780	3879	8412
MSTRG.21880.2	coding	0.332	2139	4056	8587
MSTRG.21880.3	coding	0.328	267	2235	6757
MSTRG.21880.4	noncoding	-0.0258048	330	642	8243
MSTRG.21880.5	noncoding	-0.0421888	13569	13890	42836
MSTRG.21880.6	noncoding	-0.0180224	37491	37785	38687
MSTRG.21881.1	noncoding	-0.02048	0	63	258
MSTRG.21880.7	noncoding	-0.0339968	13788	13986	17189
MSTRG.21880.8	noncoding	-0.0114688	32355	32514	32669
MSTRG.21880.9	noncoding	-0.0180224	21753	22047	22949
MSTRG.21885.4	coding	0.289	177	900	1056
MSTRG.21885.3	coding	0.323	678	1281	1437
MSTRG.21886.1	noncoding	-0.0036864	2163	2265	2767
MSTRG.21887.1	coding	0.46	1599	5025	5437
MSTRG.21889.1	noncoding	-0.0544768	795	1242	1427
MSTRG.21890.2	coding	0.582	1416	4281	4894
MSTRG.21890.3	noncoding	-0.2056192	534	1389	1563
MSTRG.21890.4	coding	0.582	801	3666	3842
MSTRG.21890.5	noncoding	-0.229376	534	2301	2475
MSTRG.21890.6	coding	0.578	1353	4038	4214
MSTRG.21890.7	coding	0.57	534	2757	2931
MSTRG.21890.8	coding	0.533	483	2736	2912
MSTRG.21890.12	coding	0.486	27	726	880
MSTRG.21890.20	coding	0.535	27	1182	1336
MSTRG.21890.15	coding	0.476	27	798	954
MSTRG.21890.19	noncoding	-0.2367488	27	2655	2810
MSTRG.21890.10	coding	0.507	27	843	999
MSTRG.21890.21	noncoding	-0.2285568	597	2166	2319
MSTRG.21890.22	coding	0.49	126	831	984
MSTRG.21882.1	noncoding	-0.0004096	63	162	680
MSTRG.21883.1	noncoding	-0.0176128	105	336	1122
MSTRG.21897.10	coding	0.487	0	8532	9004
MSTRG.21897.11	coding	0.491	0	8364	8838

MSTRG.21897.12	coding	0.492	0	8370	8844
MSTRG.21897.13	coding	0.482	12	8517	8993
MSTRG.21897.15	coding	0.485	12	8412	8888
MSTRG.21897.16	coding	0.491	0	8364	8838
MSTRG.21897.17	coding	0.486	0	8529	9003
MSTRG.21897.18	coding	0.482	12	8460	8936
MSTRG.21897.3	coding	0.492	0	8370	8844
MSTRG.21897.4	coding	0.492	0	8367	8841
MSTRG.21897.5	coding	0.495	0	8229	8703
MSTRG.21897.6	coding	0.489	0	8418	8892
MSTRG.21897.7	coding	0.484	12	8409	8885
MSTRG.21897.8	coding	0.489	0	8418	8892
MSTRG.21897.9	coding	0.489	0	8421	8895
MSTRG.21898.2	noncoding	-0.0356352	696	873	1703
MSTRG.21898.1	noncoding	-0.0241664	0	108	1628
MSTRG.21899.1	noncoding	-0.14336	417	480	868
MSTRG.21897.19	coding	0.48	1644	8712	9187
MSTRG.21897.20	coding	0.472	840	7818	8292
MSTRG.21897.21	coding	0.472	11562	18384	18860
MSTRG.21897.22	coding	0.441	0	6891	7367
MSTRG.21897.23	coding	0.459	0	6468	6942
MSTRG.21897.24	coding	0.467	0	6291	6766
MSTRG.21897.25	coding	0.461	387	6393	6867
MSTRG.21897.26	coding	0.443	465	5475	5951
MSTRG.21902.1	noncoding	-0.196608	1020	1071	1226
MSTRG.21903.2	coding	0.096	1014	1656	4305
MSTRG.21903.5	noncoding	-0.0094208	888	1056	1211
MSTRG.21903.3	noncoding	-0.0606208	444	723	876
MSTRG.21903.4	noncoding	-0.0753664	3198	3414	3568
MSTRG.21903.7	noncoding	-0.114688	555	639	793
MSTRG.21905.2	noncoding	-0.108544	0	399	5067
MSTRG.21905.3	noncoding	-0.1163264	0	399	11573
MSTRG.21905.6	noncoding	-0.0446464	8880	9093	20743
MSTRG.21907.2	coding	0.316	0	1734	4936
MSTRG.21907.3	coding	0.236	0	2550	5461
MSTRG.21912.1	coding	0.413	0	2142	2934
MSTRG.21912.2	coding	0.335	0	1488	1658
MSTRG.21900.5	coding	0.312	0	1044	1198
MSTRG.21916.3	coding	0.113	1638	2817	5032
MSTRG.21917.1	noncoding	-0.012288	132	231	1741
MSTRG.21918.2	noncoding	-0.147456	0	429	1221
MSTRG.21919.4	coding	0.267	297	702	974
MSTRG.21919.5	noncoding	-0.098304	213	267	564
MSTRG.21920.7	coding	0.05	1014	3963	4507
MSTRG.21925.1	coding	0.564	96	3906	6705
MSTRG.21926.1	noncoding	-0.0073728	105	324	485
MSTRG.21925.2	coding	0.53	1629	3816	4075
MSTRG.21925.3	coding	0.564	1482	5292	8333
MSTRG.21927.1	noncoding	-0.012288	207	252	406
MSTRG.21927.2	noncoding	-0.0274432	99	339	685
MSTRG.21925.4	coding	0.564	351	4161	6940
MSTRG.21925.5	coding	0.564	717	4530	7570
MSTRG.21928.2	noncoding	-0.1757184	585	1038	1368
MSTRG.21921.2	coding	0.341	0	813	2554

MSTRG.21932.2	coding	0.175	0	1011	2628
MSTRG.21932.3	coding	0.221	1146	1896	2445
MSTRG.21934.2	noncoding	-0.04096	495	609	928
MSTRG.21935.2	coding	0.308	0	165	430
MSTRG.21941.1	coding	0.102	1209	6504	7981
MSTRG.21941.2	coding	0.102	1545	6840	8253
MSTRG.21942.1	noncoding	-0.0438272	1386	1914	2280
MSTRG.21942.5	noncoding	-0.0323584	1110	1776	2292
MSTRG.21939.2	coding	0.335	141	4269	5881
MSTRG.21940.1	noncoding	-0.0241664	672	795	1287
MSTRG.21939.3	coding	0.334	363	6561	8173
MSTRG.21939.4	coding	0.343	96	6492	8106
MSTRG.21943.1	coding	0.456	12	1950	2105
MSTRG.21943.2	coding	0.487	0	1911	2099
MSTRG.21949.1	coding	0.476	0	621	3714
MSTRG.21953.1	noncoding	-0.0004096	0	0	306
MSTRG.21955.1	noncoding	-0.0155648	570	708	910
MSTRG.21958.4	noncoding	-0.06144	183	528	683
MSTRG.21958.5	noncoding	-0.1028096	357	729	1010
MSTRG.21958.10	noncoding	-0.075776	561	795	1227
MSTRG.21958.3	coding	0.337	147	552	739
MSTRG.21958.6	noncoding	-0.09216	147	399	897
MSTRG.21958.7	noncoding	-0.09216	147	399	1124
MSTRG.21958.8	coding	0.33	147	534	966
MSTRG.21958.9	noncoding	-0.09216	147	399	1043
MSTRG.21959.1	noncoding	-0.004096	243	378	767
MSTRG.21961.2	coding	0.456	0	2400	3613
MSTRG.21961.3	coding	0.458	0	2409	3622
MSTRG.21961.4	coding	0.435	12	1533	2790
MSTRG.21961.5	coding	0.405	12	1659	2916
MSTRG.21963.3	coding	0.233	0	1350	1606
MSTRG.21977.2	coding	0.285	267	1257	1882
MSTRG.21978.2	coding	0.073	462	2301	2460
MSTRG.21980.1	noncoding	-0.049152	351	402	1715
MSTRG.21985.2	coding	0.204	0	3474	5931
MSTRG.21985.4	coding	0.232	0	3027	5483
MSTRG.21985.5	coding	0.219	783	3501	5957
MSTRG.21985.6	coding	0.232	0	2940	3743
MSTRG.21985.7	coding	0.232	0	3027	3953
MSTRG.21985.8	coding	0.261	4020	5262	9683
MSTRG.21987.2	coding	0.176	0	1746	7021
MSTRG.21987.3	noncoding	-0.004096	540	756	1700
MSTRG.21965.1	coding	0.244	0	711	2349
MSTRG.21965.2	coding	0.085	132	510	801
MSTRG.21966.1	noncoding	-0.208896	3	60	1157
MSTRG.21967.1	coding	0.043	192	519	1100
MSTRG.21967.2	coding	0.061	207	513	1094
MSTRG.21967.4	coding	0.061	135	441	1021
MSTRG.21967.5	coding	0.041	120	447	1027
MSTRG.21967.6	coding	0.043	192	519	1099
MSTRG.21968.3	coding	0.082	114	552	775
MSTRG.21971.1	coding	0.06	0	1683	8233
MSTRG.21971.2	coding	0.107	801	1731	7731
MSTRG.21972.1	coding	0.336	0	882	1269

MSTRG.21994.4	coding	0.383	0	2019	4981
MSTRG.21994.3	coding	0.267	0	1863	4984
MSTRG.21994.5	coding	0.47	0	963	3498
MSTRG.21996.12	coding	0.077	1017	2337	3135
MSTRG.21996.17	coding	0.055	7143	9690	10488
MSTRG.21996.14	coding	0.078	2268	4737	5533
MSTRG.21996.16	coding	0.149	216	885	3668
MSTRG.21996.9	coding	0.056	603	3426	4222
MSTRG.21996.10	coding	0.105	0	3270	4067
MSTRG.21996.13	coding	0.061	1674	4287	5085
MSTRG.21996.6	coding	0.113	0	3054	3851
MSTRG.21996.8	coding	0.098	0	3198	3995
MSTRG.21996.15	coding	0.058	0	3156	3953
MSTRG.21997.1	noncoding	-0.053248	42	111	3413
MSTRG.22002.2	coding	0.277	1698	2982	4157
MSTRG.22005.4	coding	0.274	609	3216	3814
MSTRG.22008.1	noncoding	-0.0311296	6411	6726	8009
MSTRG.22008.2	noncoding	-0.0311296	6438	6753	20159
MSTRG.22008.3	noncoding	-0.0311296	696	1011	1687
MSTRG.22010.3	coding	0.392	1689	7689	10129
MSTRG.22010.4	coding	0.442	1401	6162	8601
MSTRG.22010.5	coding	0.389	255	6300	8725
MSTRG.22021.1	noncoding	-0.0077824	975	1089	1502
MSTRG.22014.1	noncoding	-0.004096	291	546	701
MSTRG.22026.1	coding	0.308	0	1038	3283
MSTRG.22028.1	coding	0.075	9054	9291	10582
MSTRG.22027.4	noncoding	-0.065536	2895	2970	3447
MSTRG.22029.1	noncoding	-0.06144	123	138	1527
MSTRG.22027.6	coding	0.305	516	2673	3956
MSTRG.22034.2	coding	0.287	0	2007	6953
MSTRG.22034.5	coding	0.179	0	1392	4270
MSTRG.22030.3	coding	0.448	66	3174	8021
MSTRG.22030.2	coding	0.448	66	3174	9162
MSTRG.22030.4	coding	0.447	258	3309	9177
MSTRG.22038.3	coding	0.408	0	1935	3351
MSTRG.22041.3	coding	0.567	0	438	2411
MSTRG.22041.4	coding	0.441	1332	1674	1864
MSTRG.22042.1	noncoding	-0.057344	126	150	461
MSTRG.22043.1	coding	0.406	0	1797	3311
MSTRG.22043.3	coding	0.419	183	777	1309
MSTRG.22043.4	coding	0.372	0	1962	2263
MSTRG.22045.2	coding	0.371	54	573	3852
MSTRG.22046.2	coding	0.24	0	612	14928
MSTRG.22050.3	coding	0.171	525	7104	7878
MSTRG.22051.1	noncoding	-0.0004096	0	0	955
MSTRG.22051.2	noncoding	-0.0004096	0	0	966
MSTRG.22050.4	coding	0.118	843	1653	2428
MSTRG.22056.1	noncoding	-0.0495616	0	117	764
MSTRG.22058.1	noncoding	-0.04096	405	465	619
MSTRG.22063.3	coding	0.472	2235	4509	5109
MSTRG.22063.4	coding	0.525	45	2850	3448
MSTRG.22063.5	coding	0.446	912	1770	2369
MSTRG.22059.1	noncoding	-0.0442368	0	471	963
MSTRG.22059.2	noncoding	-0.057344	0	189	401

MSTRG.22064.3	coding	0.397	0	1965	2634
MSTRG.22064.4	coding	0.377	0	2094	2763
MSTRG.22065.2	coding	0.481	252	1146	1886
MSTRG.22068.1	coding	0.595	0	2583	3249
MSTRG.22071.2	coding	0.416	516	1524	1711
MSTRG.22072.1	coding	0.456	63	2562	5038
MSTRG.22072.3	coding	0.437	1224	1866	2021
MSTRG.22074.1	noncoding	-0.0274432	33	264	438
MSTRG.22070.1	coding	0.181	87	639	4589
MSTRG.22070.2	coding	0.256	0	564	4939
MSTRG.22075.2	coding	0.349	300	2406	4574
MSTRG.22075.3	coding	0.349	645	2751	4818
MSTRG.22075.4	coding	0.349	396	2502	4570
MSTRG.22076.1	noncoding	-0.1593344	75	105	260
MSTRG.22078.3	coding	0.219	33	1845	2721
MSTRG.22080.1	coding	0.227	90	888	1057
MSTRG.22080.6	coding	0.21	159	942	1110
MSTRG.22113.6	coding	0.392	7611	11316	11472
MSTRG.22113.5	coding	0.298	0	13392	15602
MSTRG.22113.7	coding	0.257	0	11496	11653
MSTRG.22114.1	noncoding	-0.0585728	3009	3186	3722
MSTRG.22113.9	coding	0.392	5898	9600	9757
MSTRG.22115.3	coding	0.203	417	2238	3088
MSTRG.22116.1	noncoding	-0.0446464	831	1173	1539
MSTRG.22117.1	noncoding	-0.0004096	87	87	534
MSTRG.22118.4	coding	0.106	0	2523	3186
MSTRG.22118.2	coding	0.144	0	2319	4982
MSTRG.22118.3	coding	0.158	0	1296	4271
MSTRG.22118.5	coding	0.132	1233	3345	3907
MSTRG.22126.1	coding	0.181	618	2610	3598
MSTRG.22126.2	coding	0.175	609	2607	3488
MSTRG.22126.3	coding	0.181	750	2754	6169
MSTRG.22126.4	coding	0.181	615	2607	3486
MSTRG.22126.5	coding	0.169	99	2031	3053
MSTRG.22126.10	coding	0.139	15	2562	3150
MSTRG.22082.3	coding	0.481	606	8553	9223
MSTRG.22082.5	coding	0.287	1329	7536	7693
MSTRG.22082.4	coding	0.486	0	7701	7858
MSTRG.22084.2	coding	0.351	6534	8958	10290
MSTRG.22086.1	coding	0.295	0	423	704
MSTRG.22089.3	coding	0.193	54	1383	3094
MSTRG.22089.4	coding	0.252	2355	3234	4943
MSTRG.22089.5	coding	0.16	54	1314	1469
MSTRG.22091.1	noncoding	-0.0647168	45	246	668
MSTRG.22091.3	coding	0.209	0	324	747
MSTRG.22098.2	coding	0.14	57	429	3015
MSTRG.22099.2	coding	0.329	297	1320	1477
MSTRG.22103.2	coding	0.588	0	627	4016
MSTRG.22139.5	coding	0.186	279	1365	2201
MSTRG.22139.6	coding	0.221	279	2178	3140
MSTRG.22141.1	noncoding	-0.0622592	852	1290	1740
MSTRG.22141.2	noncoding	-0.1294336	0	180	3380
MSTRG.22141.3	noncoding	-0.114688	0	153	780
MSTRG.22142.1	coding	0.46	366	924	1796

MSTRG.22145.2	coding	0.263	0	486	877
MSTRG.22146.1	coding	0.382	0	3297	7258
MSTRG.22146.2	coding	0.371	234	3654	7220
MSTRG.22146.3	coding	0.371	213	3633	6868
MSTRG.22147.2	coding	0.49	12	1002	3547
MSTRG.22147.3	coding	0.483	819	1776	4322
MSTRG.22155.1	coding	0.481	258	7101	8805
MSTRG.22155.2	coding	0.478	75	7002	8705
MSTRG.22155.4	coding	0.494	2595	7191	7651
MSTRG.22155.3	coding	0.494	2472	7068	7527
MSTRG.22155.7	coding	0.388	207	717	918
MSTRG.22150.1	coding	0.247	0	351	2240
MSTRG.22152.2	coding	0.209	0	3069	8043
MSTRG.22152.3	coding	0.386	1917	3090	8091
MSTRG.22152.5	coding	0.363	1002	2946	9084
MSTRG.22153.1	coding	0.298	9	1548	3803
MSTRG.22168.1	noncoding	-0.063488	5193	5349	5539
MSTRG.22179.1	noncoding	-0.065536	19914	20130	29808
MSTRG.22179.2	noncoding	-0.0679936	0	1050	7590
MSTRG.22179.3	noncoding	-0.0417792	10863	11064	12282
MSTRG.22179.4	noncoding	-0.065536	10878	11094	20255
MSTRG.22179.5	noncoding	-0.0602112	9912	10128	19288
MSTRG.22179.7	noncoding	-0.0299008	4383	4563	8547
MSTRG.22179.8	noncoding	-0.0004096	624	762	3450
MSTRG.22179.9	noncoding	-0.0004096	624	762	4625
MSTRG.22179.6	noncoding	-0.0299008	4191	4371	8815
MSTRG.22179.10	noncoding	-0.0299008	4377	4557	8974
MSTRG.22179.11	noncoding	-0.0299008	4188	4368	8785
MSTRG.22179.13	noncoding	-0.0299008	4377	4557	9001
MSTRG.22179.12	noncoding	-0.0004096	621	759	5255
MSTRG.22179.14	noncoding	-0.0520192	1464	1851	2654
MSTRG.22179.15	noncoding	-0.0393216	10515	10881	31423
MSTRG.22179.16	noncoding	-0.0393216	19632	19998	32402
MSTRG.22181.1	noncoding	-0.0167936	6594	6750	10550
MSTRG.22181.2	noncoding	-0.0950272	4365	4548	11045
MSTRG.22181.3	noncoding	-0.0385024	7422	7659	10692
MSTRG.22181.4	noncoding	-0.0167936	5802	5958	6918
MSTRG.22181.5	noncoding	-0.04096	26670	26901	36338
MSTRG.22181.6	noncoding	-0.04096	10437	10668	20101
MSTRG.22182.1	coding	0.186	1098	1986	3332
MSTRG.22182.3	coding	0.182	396	1278	2622
MSTRG.22181.7	noncoding	-0.04096	4368	4599	7829
MSTRG.22184.1	coding	0.325	540	5940	11115
MSTRG.22184.2	noncoding	-0.0663552	531	993	7108
MSTRG.22184.3	coding	0.325	579	5979	11155
MSTRG.22184.5	coding	0.325	7602	13002	18177
MSTRG.22184.6	coding	0.325	945	6345	11522
MSTRG.22185.1	noncoding	-0.0434176	2298	2631	3880
MSTRG.22170.2	coding	0.46	1110	1737	1892
MSTRG.22172.3	coding	0.221	0	777	1271
MSTRG.22175.1	coding	0.55	3840	7821	8156
MSTRG.22175.2	coding	0.55	3069	7050	7384
MSTRG.22189.2	noncoding	-0.0159744	705	849	1004
MSTRG.22190.1	noncoding	-0.0258048	882	1053	1841

MSTRG.22190.2	noncoding	-0.034816	2154	2460	4986
MSTRG.22191.2	noncoding	-0.063488	873	1164	4051
MSTRG.22192.3	coding	0.381	1098	3084	4241
MSTRG.22195.3	coding	0.62	267	1455	2014
MSTRG.22195.2	coding	0.606	366	1602	2159
MSTRG.22178.2	noncoding	-0.0581632	375	612	1259
MSTRG.22178.3	noncoding	-0.0270336	3066	3750	9445
MSTRG.22188.1	noncoding	-0.0180224	744	834	991
MSTRG.22197.1	noncoding	-0.0425984	0	75	347
MSTRG.22219.1	coding	0.331	2496	7521	10386
MSTRG.22220.1	noncoding	-0.026624	279	435	594
MSTRG.22219.2	coding	0.169	33	744	1398
MSTRG.22219.5	coding	0.155	2106	2796	4918
MSTRG.22219.3	coding	0.144	3870	4542	6664
MSTRG.22223.5	noncoding	-0.0483328	276	471	1672
MSTRG.22224.1	noncoding	-0.0004096	3858	4095	8487
MSTRG.22226.1	noncoding	-0.094208	7458	7545	19619
MSTRG.22228.1	coding	0.095	20553	21672	23063
MSTRG.22228.2	noncoding	-0.004096	1002	1023	1179
MSTRG.22223.6	coding	0.187	0	426	4163
MSTRG.22230.1	noncoding	-0.151552	1392	1401	2053
MSTRG.22199.1	noncoding	-0.0577536	0	198	492
MSTRG.22200.1	noncoding	-0.036864	2781	2883	3665
MSTRG.22203.2	coding	0.185	9	789	1009
MSTRG.22204.1	noncoding	-0.0045056	189	336	894
MSTRG.22205.1	coding	0.318	0	1170	1889
MSTRG.22205.2	coding	0.319	0	1017	1736
MSTRG.22205.4	coding	0.319	0	1017	2602
MSTRG.22207.3	coding	0.338	453	1551	1840
MSTRG.22207.7	coding	0.338	375	1473	1752
MSTRG.22207.9	coding	0.338	354	1452	1731
MSTRG.22208.4	coding	0.233	0	1686	1984
MSTRG.22211.5	coding	0.273	738	2226	2562
MSTRG.22212.2	coding	0.446	318	2292	2577
MSTRG.22212.3	coding	0.444	192	1974	2257
MSTRG.22213.4	coding	0.439	297	990	2745
MSTRG.22213.5	coding	0.439	657	1350	3106
MSTRG.22213.2	coding	0.439	1233	1926	3682
MSTRG.22213.3	coding	0.439	828	1521	3278
MSTRG.22214.4	noncoding	-0.0393216	1761	2667	3320
MSTRG.22216.7	coding	0.489	12	2205	2605
MSTRG.22217.1	noncoding	-0.0540672	0	375	1880
MSTRG.22238.4	noncoding	-0.0712704	8868	9477	9632
MSTRG.22238.6	noncoding	-0.0004096	4509	4665	9104
MSTRG.22238.7	noncoding	-0.012288	1539	1707	4116
MSTRG.22239.1	noncoding	-0.0237568	4704	4851	10653
MSTRG.22240.1	noncoding	-0.0004096	0	0	1754
MSTRG.22241.1	coding	0.19	261	1011	1271
MSTRG.22242.2	coding	0.514	348	1266	1734
MSTRG.22242.3	coding	0.537	18	972	1351
MSTRG.22242.4	coding	0.513	300	1176	1505
MSTRG.22245.2	coding	0.533	0	2397	2656
MSTRG.22246.3	coding	0.578	0	2088	2296
MSTRG.22251.3	coding	0.438	444	939	1787

MSTRG.22251.2	coding	0.482	156	1437	2287
MSTRG.22251.4	coding	0.465	72	1338	2187
MSTRG.22261.1	coding	0.172	369	1971	12174
MSTRG.22261.2	coding	0.425	339	924	3015
MSTRG.22262.2	coding	0.321	0	228	675
MSTRG.22262.3	coding	0.326	0	315	1147
MSTRG.22262.4	coding	0.315	0	345	1858
MSTRG.22262.5	coding	0.325	0	471	670
MSTRG.22265.1	noncoding	-0.0528384	0	111	398
MSTRG.22264.3	coding	0.571	0	2085	3543
MSTRG.22264.4	coding	0.571	258	2367	3815
MSTRG.22276.2	noncoding	-0.0344064	585	1239	4046
MSTRG.22277.1	coding	0.429	744	3834	4600
MSTRG.22277.3	coding	0.436	1143	4110	4342
MSTRG.22277.10	coding	0.443	0	2802	3031
MSTRG.22277.5	coding	0.456	0	2748	2977
MSTRG.22277.9	coding	0.423	2088	5094	5325
MSTRG.22277.7	coding	0.435	2184	5139	5369
MSTRG.22277.11	noncoding	-0.0253952	1875	2025	2179
MSTRG.22269.2	noncoding	-0.0602112	1545	2130	4455
MSTRG.22269.3	noncoding	-0.047104	63	246	2672
MSTRG.22271.1	noncoding	-0.0319488	402	618	870
MSTRG.22282.1	noncoding	-0.0356352	0	2412	4950
MSTRG.22282.2	noncoding	-0.0434176	9966	12240	12396
MSTRG.22282.3	noncoding	-0.0544768	9849	11595	12216
MSTRG.22283.3	coding	0.246	456	1479	9606
MSTRG.22284.2	coding	0.21	0	3033	3507
MSTRG.22285.1	coding	0.271	309	3897	4050
MSTRG.22286.3	coding	0.526	126	1236	2341
MSTRG.22286.6	coding	0.526	396	1506	2412
MSTRG.22301.1	noncoding	-0.004096	228	498	1076
MSTRG.22309.1	coding	0.27	0	384	562
MSTRG.22310.1	noncoding	-0.0135168	2595	2838	3687
MSTRG.22312.3	noncoding	-0.0212992	762	1119	1908
MSTRG.22312.2	noncoding	-0.0126976	786	1008	1548
MSTRG.22306.4	coding	0.521	0	447	659
MSTRG.22315.1	coding	0.313	0	264	418
MSTRG.22306.5	noncoding	-0.0565248	21	213	369
MSTRG.22306.7	coding	0.444	0	618	822
MSTRG.22306.8	coding	0.592	0	258	462
MSTRG.22306.6	coding	0.56	0	294	509
MSTRG.22306.9	coding	0.567	0	300	513
MSTRG.22306.11	coding	0.373	0	360	576
MSTRG.22295.1	coding	0.427	6	2103	5088
MSTRG.22295.3	noncoding	-0.129024	0	156	701
MSTRG.22299.3	coding	0.552	0	1128	1481
MSTRG.22300.1	coding	0.448	0	399	565
MSTRG.22300.3	coding	0.371	81	624	788
MSTRG.22300.4	noncoding	-0.0876544	198	438	604
MSTRG.22316.1	noncoding	-0.1335296	20550	21288	22121
MSTRG.22316.2	noncoding	-0.0380928	3243	5061	8466
MSTRG.22319.2	coding	0.583	0	546	838
MSTRG.22319.3	coding	0.408	0	888	1182
MSTRG.22332.7	coding	0.405	5172	7728	7882

MSTRG.22326.2	coding	0.381	1143	2604	2760
MSTRG.22327.1	coding	0.467	18	1512	2399
MSTRG.22327.3	coding	0.558	291	2331	2534
MSTRG.22325.1	noncoding	-0.0446464	0	141	2684
MSTRG.22335.2	coding	0.523	513	1497	1652
MSTRG.22335.3	coding	0.485	0	1161	1317
MSTRG.22341.2	noncoding	-0.0303104	0	678	2653
MSTRG.22341.3	noncoding	-0.0401408	462	1017	4994
MSTRG.22344.1	coding	0.576	738	1803	1956
MSTRG.22344.2	coding	0.522	240	1464	1618
MSTRG.22344.4	coding	0.575	492	1542	1699
MSTRG.22344.5	coding	0.362	126	1155	1322
MSTRG.22344.7	coding	0.369	117	903	1058
MSTRG.22344.9	coding	0.553	540	1713	1869
MSTRG.22346.1	noncoding	-0.0462848	0	105	261
MSTRG.22344.11	coding	0.284	12	510	665
MSTRG.22338.2	coding	0.293	0	2601	2822
MSTRG.22338.4	noncoding	-0.0872448	1386	1686	1840
MSTRG.22340.2	noncoding	-0.032768	1401	1479	1633
MSTRG.22348.3	coding	0.303	2643	3273	6237
MSTRG.22355.2	coding	0.333	0	492	2014
MSTRG.22356.1	coding	0.357	942	3636	3851
MSTRG.22356.4	coding	0.371	0	2445	2659
MSTRG.22356.5	coding	0.296	159	3681	3895
MSTRG.22356.3	coding	0.292	942	4500	4714
MSTRG.22351.2	coding	0.398	0	2058	2214
MSTRG.22351.3	coding	0.51	444	2025	2212
MSTRG.22358.2	noncoding	-0.0303104	0	264	742
MSTRG.22359.2	coding	0.568	0	6516	6741
MSTRG.22361.2	noncoding	-0.0913408	4782	6288	6656
MSTRG.22370.2	coding	0.587	156	2454	3217
MSTRG.22370.4	coding	0.572	0	1758	3168
MSTRG.22378.3	coding	0.539	855	3339	4216
MSTRG.22378.5	coding	0.558	0	2382	3258
MSTRG.22378.6	coding	0.534	579	3051	3928
MSTRG.22378.7	coding	0.533	2769	5271	6088
MSTRG.22367.3	noncoding	-0.217088	849	2646	3286
MSTRG.22367.4	noncoding	-0.2195456	7602	9291	9929
MSTRG.22367.5	coding	0.216	21	804	5417
MSTRG.22382.1	coding	0.41	5463	7827	9666
MSTRG.22382.4	coding	0.426	858	2811	4302
MSTRG.22382.5	coding	0.449	615	1953	3457
MSTRG.22382.8	coding	0.459	483	2256	3762
MSTRG.22382.6	coding	0.406	3822	5934	7439
MSTRG.22382.7	coding	0.406	783	2895	4401
MSTRG.22382.9	coding	0.406	564	2676	4150
MSTRG.22383.1	coding	0.638	69	1152	1418
MSTRG.22383.2	coding	0.638	69	1152	1418
MSTRG.22383.3	coding	0.638	69	1152	1418
MSTRG.22383.4	coding	0.634	69	1152	1418
MSTRG.22390.8	coding	0.456	4581	6564	7029
MSTRG.22390.7	coding	0.46	2862	4824	5288
MSTRG.22390.6	coding	0.465	534	3441	3904
MSTRG.22390.2	coding	0.462	438	3558	4023

MSTRG.22391.2	coding	0.407	66	558	807
MSTRG.22391.4	coding	0.345	666	1056	1305
MSTRG.22392.2	noncoding	-0.06144	2169	3771	3927
MSTRG.22392.4	noncoding	-0.0565248	183	564	720
MSTRG.22394.2	noncoding	-0.065536	300	621	1380
MSTRG.22394.3	coding	0.416	639	1455	1773
MSTRG.22385.2	coding	0.428	2484	4524	5355
MSTRG.22386.2	coding	0.567	0	1323	1702
MSTRG.22397.2	coding	0.445	0	1605	1884
MSTRG.22397.3	coding	0.471	0	1563	1840
MSTRG.22397.4	coding	0.447	1008	2559	2837
MSTRG.22398.1	coding	0.585	0	981	2501
MSTRG.22399.1	coding	0.503	0	3561	3779
MSTRG.22399.2	coding	0.545	0	3186	3342
MSTRG.22400.1	noncoding	-0.0753664	21	870	1992
MSTRG.22402.2	coding	0.543	0	480	1392
MSTRG.22402.3	coding	0.526	0	477	1152
MSTRG.22403.1	noncoding	-0.0376832	201	2679	4653
MSTRG.22406.2	coding	0.49	0	690	909
MSTRG.22406.5	coding	0.474	0	663	881
MSTRG.22411.2	coding	0.332	0	1395	2223
MSTRG.22411.1	coding	0.33	0	1344	3130
MSTRG.22407.1	coding	0.509	0	1512	1698
MSTRG.22407.2	coding	0.491	354	1878	2033
MSTRG.22408.1	noncoding	-0.0356352	0	1290	2032
MSTRG.22419.1	noncoding	-0.0077824	408	558	993
MSTRG.22420.1	noncoding	-0.135168	2052	2115	3383
MSTRG.22417.1	noncoding	-0.069632	174	237	951
MSTRG.22417.2	noncoding	-0.03072	348	657	1070
MSTRG.22426.1	noncoding	-0.036864	228	327	565
MSTRG.22428.2	coding	0.412	45	2850	3153
MSTRG.22430.2	coding	0.323	2886	5154	5697
MSTRG.22435.2	coding	0.439	138	3714	4477
MSTRG.22431.1	coding	0.37	813	1614	2398
MSTRG.22444.7	coding	0.437	0	1314	2290
MSTRG.22444.5	coding	0.438	0	1491	2467
MSTRG.22449.1	coding	0.43	273	3159	3572
MSTRG.22444.10	noncoding	-0.0004096	0	0	310
MSTRG.22449.3	coding	0.43	423	3309	3715
MSTRG.22449.4	coding	0.43	468	3354	3768
MSTRG.22449.5	coding	0.421	387	3360	3772
MSTRG.22449.9	coding	0.421	387	3360	3766
MSTRG.22449.10	coding	0.43	336	3222	3628
MSTRG.22451.3	coding	0.4	981	3630	7948
MSTRG.22451.8	coding	0.363	0	3126	7444
MSTRG.22451.5	coding	0.397	9	2730	7049
MSTRG.22451.9	coding	0.397	84	2733	6987
MSTRG.22451.13	coding	0.408	1011	1590	1759
MSTRG.22451.14	coding	0.397	1344	3999	4168
MSTRG.22451.11	coding	0.397	1332	3987	4157
MSTRG.22451.12	coding	0.399	468	3171	3339
MSTRG.22442.1	noncoding	-0.12288	1698	1770	2538
MSTRG.22459.3	coding	0.149	4485	5439	6146
MSTRG.22459.1	coding	0.168	861	2001	2707

MSTRG.22459.4	coding	0.177	1167	2325	3032
MSTRG.22459.5	coding	0.168	570	1710	2409
MSTRG.22459.6	coding	0.254	300	2694	3394
MSTRG.22460.1	noncoding	-0.067584	2847	3312	11914
MSTRG.22460.2	noncoding	-0.0311296	297	465	1679
MSTRG.22461.1	noncoding	-0.0458752	168	420	1146
MSTRG.22461.2	noncoding	-0.0397312	36	201	690
MSTRG.22461.4	noncoding	-0.0507904	168	432	1772
MSTRG.22461.5	noncoding	-0.012288	129	261	751
MSTRG.22461.6	noncoding	-0.0397312	435	702	1412
MSTRG.22454.1	coding	0.366	2139	2733	2889
MSTRG.22454.2	coding	0.352	1806	2349	2503
MSTRG.22455.3	coding	0.58	387	2397	3986
MSTRG.22464.1	noncoding	-0.090112	3612	3693	12812
MSTRG.22465.1	noncoding	-0.02048	2751	2874	5777
MSTRG.22466.1	noncoding	-0.0077824	2592	2784	7100
MSTRG.22478.3	coding	0.137	0	1755	2378
MSTRG.22478.2	coding	0.128	0	1719	5767
MSTRG.22478.4	coding	0.127	0	1761	2821
MSTRG.22478.5	coding	0.129	0	1719	5767
MSTRG.22478.7	coding	0.109	366	2259	6840
MSTRG.22478.6	coding	0.123	27	1908	6604
MSTRG.22478.10	coding	0.123	0	1881	3325
MSTRG.22478.11	coding	0.127	0	1761	2634
MSTRG.22480.1	coding	0.082	2706	3021	3488
MSTRG.22482.1	noncoding	-0.0004096	0	0	334
MSTRG.22490.1	coding	0.247	39	933	2110
MSTRG.22490.2	coding	0.217	39	585	1010
MSTRG.22490.3	coding	0.231	39	1038	2048
MSTRG.22490.4	coding	0.221	39	444	1534
MSTRG.22490.5	coding	0.204	591	1323	2159
MSTRG.22490.6	coding	0.224	327	870	1706
MSTRG.22490.7	coding	0.231	39	1257	2185
MSTRG.22490.9	coding	0.219	30	666	2022
MSTRG.22468.1	noncoding	-0.069632	9	33	407
MSTRG.22469.1	noncoding	-0.01024	1911	2076	2232
MSTRG.22476.1	noncoding	-0.1081344	27	66	576
MSTRG.22477.1	coding	0.181	45	1095	1250
MSTRG.22519.1	coding	0.498	129	1758	4111
MSTRG.22521.1	noncoding	-0.008192	360	495	1326
MSTRG.22517.1	noncoding	-0.055296	0	186	636
MSTRG.22517.2	noncoding	-0.0897024	0	717	2517
MSTRG.22517.3	noncoding	-0.0638976	1491	1644	1941
MSTRG.22524.1	noncoding	-0.16384	516	579	735
MSTRG.22526.1	coding	0.386	9	1020	2172
MSTRG.22539.1	noncoding	-0.1654784	0	135	371
MSTRG.22542.2	coding	0.445	60	1773	3403
MSTRG.22544.1	noncoding	-0.0004096	180	336	797
MSTRG.22544.2	noncoding	-0.0118784	180	429	646
MSTRG.22528.1	noncoding	-0.032768	63	159	316
MSTRG.22530.1	noncoding	-0.0126976	633	1017	1252
MSTRG.22549.1	noncoding	-0.0630784	297	780	4585
MSTRG.22549.2	noncoding	-0.0581632	309	786	4386
MSTRG.22551.3	noncoding	-0.045056	426	477	917

MSTRG.22551.2	noncoding	-0.0360448	6	594	1480
MSTRG.22551.4	noncoding	-0.0024576	378	465	619
MSTRG.22551.5	noncoding	-0.1150976	0	96	506
MSTRG.22552.2	coding	0.077	90	1023	1985
MSTRG.22550.2	coding	0.115	1263	2985	5057
MSTRG.22553.3	coding	0.16	153	1209	1791
MSTRG.22563.1	coding	0.45	0	1890	12310
MSTRG.22563.3	noncoding	-0.0319488	2505	2721	2984
MSTRG.22564.3	coding	0.348	366	1278	2747
MSTRG.22572.1	coding	0.524	249	4179	4616
MSTRG.22574.1	noncoding	-0.028672	2004	2340	2549
MSTRG.22572.2	coding	0.537	0	3900	5164
MSTRG.22572.3	coding	0.537	0	3885	4384
MSTRG.22576.1	noncoding	-0.016384	75	201	831
MSTRG.22580.1	noncoding	-0.024576	915	1029	1281
MSTRG.22579.3	noncoding	-0.0712704	6	306	686
MSTRG.22579.4	noncoding	-0.0126976	6	183	669
MSTRG.22583.2	coding	0.287	0	246	692
MSTRG.22583.5	coding	0.287	0	246	811
MSTRG.22592.2	coding	0.556	93	1653	2038
MSTRG.22592.3	coding	0.555	93	1656	2041
MSTRG.22592.4	coding	0.518	30	1752	2137
MSTRG.22585.2	coding	0.338	714	1050	1568
MSTRG.22585.1	coding	0.317	6	570	1099
MSTRG.22593.4	coding	0.366	897	4269	4684
MSTRG.22598.2	coding	0.456	0	1521	1825
MSTRG.22598.4	coding	0.47	0	1464	1705
MSTRG.22599.1	coding	0.292	642	1368	1915
MSTRG.22599.2	coding	0.295	120	825	1370
MSTRG.22599.3	coding	0.29	63	783	1325
MSTRG.22599.4	noncoding	-0.0393216	231	471	781
MSTRG.22599.5	coding	0.287	2796	3483	4030
MSTRG.22605.1	coding	0.283	105	1425	4860
MSTRG.22607.3	coding	0.584	1389	1983	6754
MSTRG.22609.1	coding	0.322	0	105	2601
MSTRG.22608.2	coding	0.317	1689	3147	5551
MSTRG.22610.1	noncoding	-0.0602112	4809	5061	5366
MSTRG.22610.2	noncoding	-0.0712704	1128	1206	4683
MSTRG.22611.1	noncoding	-0.0131072	2778	3060	3588
MSTRG.22612.1	noncoding	-0.0479232	3135	3408	6310
MSTRG.22608.4	coding	0.335	0	1563	3968
MSTRG.22617.3	noncoding	-0.016384	612	735	7384
MSTRG.22617.1	noncoding	-0.036864	87	255	7612
MSTRG.22617.4	noncoding	-0.016384	615	738	7251
MSTRG.22618.1	coding	0.262	1047	1476	1776
MSTRG.22627.2	coding	0.569	23721	27279	31057
MSTRG.22627.3	coding	0.569	702	4224	7940
MSTRG.22627.4	coding	0.575	81	3561	7056
MSTRG.22627.5	coding	0.581	0	3552	7180
MSTRG.22627.6	coding	0.499	174	4341	8083
MSTRG.22627.7	coding	0.569	78	3627	7196
MSTRG.22627.8	coding	0.575	51	3558	7298
MSTRG.22621.2	coding	0.207	303	753	1072
MSTRG.22624.2	coding	0.528	0	1314	2179

MSTRG.22633.4	coding	0.303	0	1167	1606
MSTRG.22634.3	noncoding	-0.0565248	2247	3063	3279
MSTRG.22644.1	noncoding	-0.055296	267	750	7457
MSTRG.22644.4	coding	0.146	0	405	780
MSTRG.22644.5	noncoding	-0.0155648	249	411	786
MSTRG.22644.3	noncoding	-0.0495616	216	894	1269
MSTRG.22646.1	noncoding	-0.1241088	0	309	2234
MSTRG.22646.2	noncoding	-0.1560576	0	213	2636
MSTRG.22646.4	coding	0.082	2745	2991	4578
MSTRG.22650.1	noncoding	-0.0278528	0	135	674
MSTRG.22650.5	noncoding	-0.0278528	0	135	885
MSTRG.22650.6	noncoding	-0.045056	948	1188	1818
MSTRG.22650.3	noncoding	-0.055296	669	861	1181
MSTRG.22650.4	noncoding	-0.0454656	51	453	933
MSTRG.22650.7	noncoding	-0.0352256	0	378	1063
MSTRG.22639.2	coding	0.422	69	1557	1931
MSTRG.22651.3	coding	0.437	0	1848	2311
MSTRG.22651.4	coding	0.261	0	2967	3430
MSTRG.22651.5	coding	0.256	0	3072	3535
MSTRG.22651.6	coding	0.258	0	3063	3526
MSTRG.22651.7	coding	0.26	0	2976	3439
MSTRG.22654.1	noncoding	-0.0262144	9	1044	1198
MSTRG.22656.1	noncoding	-0.0208896	1458	3264	7588
MSTRG.22660.2	noncoding	-0.0299008	30	1155	1926
MSTRG.22668.2	coding	0.384	351	1806	1961
MSTRG.22664.1	coding	0.136	0	4059	4550
MSTRG.22683.4	noncoding	-0.0872448	0	1353	1592
MSTRG.22684.1	noncoding	-0.0335872	0	2181	2351
MSTRG.22686.2	coding	0.472	522	8652	9024
MSTRG.22686.3	coding	0.472	552	8682	9052
MSTRG.22689.1	noncoding	-0.0761856	4659	5049	19557
MSTRG.22679.1	noncoding	-0.0929792	1353	2061	5395
MSTRG.22703.3	coding	0.508	0	825	1654
MSTRG.22703.2	coding	0.506	0	840	1169
MSTRG.22709.2	coding	0.371	636	1584	1969
MSTRG.22713.1	coding	0.475	3387	4917	5910
MSTRG.22713.5	coding	0.54	0	1743	2735
MSTRG.22713.4	coding	0.54	0	1734	2662
MSTRG.22714.1	noncoding	-0.0512	2382	2844	2999
MSTRG.22694.2	coding	0.443	102	1404	5430
MSTRG.22718.1	noncoding	-0.0561152	486	759	3277
MSTRG.22718.2	coding	0.331	0	360	9494
MSTRG.22718.3	noncoding	-0.045056	4662	4758	4915
MSTRG.22718.4	coding	0.331	0	360	2546
MSTRG.22721.1	noncoding	-0.057344	2217	2322	4358
MSTRG.22722.1	noncoding	-0.028672	996	1104	3188
MSTRG.22725.1	noncoding	-0.0729088	774	810	1044
MSTRG.22731.2	coding	0.481	0	1554	3884
MSTRG.22733.1	coding	0.165	84	429	1276
MSTRG.22733.2	coding	0.001	297	438	1285
MSTRG.22737.2	coding	0.118	3462	3807	5235
MSTRG.22739.1	noncoding	-0.1355776	0	21	1284
MSTRG.22740.1	noncoding	-0.1101824	792	1323	1479
MSTRG.22740.2	noncoding	-0.090112	1551	2043	2199

MSTRG.22740.4	coding	0.095	456	936	1212
MSTRG.22742.1	coding	0.108	324	2589	3386
MSTRG.22742.2	coding	0.088	165	1458	4054
MSTRG.22744.2	coding	0.249	171	3738	4964
MSTRG.22744.3	coding	0.247	2505	3972	5198
MSTRG.22744.5	coding	0.245	3330	5223	6019
MSTRG.22744.4	coding	0.255	183	3807	5019
MSTRG.22744.6	coding	0.234	174	2649	4956
MSTRG.22744.8	coding	0.242	183	1164	3401
MSTRG.22746.1	coding	0.269	18	3690	4818
MSTRG.22747.1	coding	0.201	0	948	1287
MSTRG.22747.3	coding	0.497	0	351	6294
MSTRG.22747.4	coding	0.233	0	2730	3031
MSTRG.22749.4	noncoding	-0.096256	0	237	6263
MSTRG.22750.5	coding	0.681	0	159	2168
MSTRG.22755.1	noncoding	-0.045056	0	111	693
MSTRG.22763.2	coding	0.214	24	2001	2472
MSTRG.22763.4	coding	0.229	0	1917	2354
MSTRG.22763.5	coding	0.199	0	1788	2226
MSTRG.22763.6	coding	0.257	0	1827	2259
MSTRG.22764.1	coding	0.184	0	1827	3322
MSTRG.22764.3	coding	0.124	216	1614	2221
MSTRG.22770.2	coding	0.05	534	2886	7626
MSTRG.22774.1	noncoding	-0.0004096	0	0	379
MSTRG.22776.1	coding	0.118	174	273	468
MSTRG.22779.1	noncoding	-0.073728	264	321	627
MSTRG.22780.1	noncoding	-0.036864	354	405	575
MSTRG.22790.3	coding	0.077	3486	8997	9246
MSTRG.22790.4	coding	0.06	1659	8733	10855
MSTRG.22790.5	coding	0.073	3336	8799	10917
MSTRG.22793.1	noncoding	-0.0073728	327	369	1416
MSTRG.22798.3	coding	0.108	9	2451	4261
MSTRG.22800.5	coding	0.143	708	1461	2303
MSTRG.22800.3	coding	0.143	327	1080	1920
MSTRG.22784.1	coding	0.134	138	3822	4548
MSTRG.22785.4	coding	0.228	1668	3027	8211
MSTRG.22785.5	coding	0.276	0	1056	3888
MSTRG.22785.6	noncoding	-0.0057344	4161	4548	11797
MSTRG.22786.1	noncoding	-0.069632	153	198	890
MSTRG.22787.1	noncoding	-0.0032768	2256	2313	2532
MSTRG.22789.2	coding	0.33	732	1158	1386
MSTRG.22803.1	noncoding	-0.016384	102	237	454
MSTRG.22804.1	coding	0.277	0	1311	1466
MSTRG.22804.3	coding	0.228	0	1695	1848
MSTRG.22804.4	coding	0.228	0	1698	1851
MSTRG.22806.2	noncoding	-0.0987136	0	108	318
MSTRG.22811.1	coding	0.265	0	2133	3664
MSTRG.22812.3	coding	0.503	654	1878	7447
MSTRG.22813.1	noncoding	-0.0331776	5280	5973	8471
MSTRG.22814.2	coding	0.265	261	2949	3102
MSTRG.22818.2	coding	0.349	171	2541	2843
MSTRG.22819.2	coding	0.231	0	1383	1726
MSTRG.22819.1	coding	0.231	0	1386	4231
MSTRG.22822.2	coding	0.397	279	597	967

MSTRG.22822.3	coding	0.43	48	426	740
MSTRG.22825.1	coding	0.288	153	912	3094
MSTRG.22825.7	coding	0.215	714	1119	1739
MSTRG.22825.5	coding	0.288	252	1011	1632
MSTRG.22825.6	coding	0.288	150	909	1531
MSTRG.22827.1	noncoding	-0.0196608	1386	1422	1971
MSTRG.22829.3	coding	0.156	351	615	1652
MSTRG.22829.4	coding	0.156	792	1056	2093
MSTRG.22829.2	coding	0.165	537	909	4336
MSTRG.22824.1	coding	0.241	171	1692	3760
MSTRG.22832.5	coding	0.273	81	651	1318
MSTRG.22832.6	coding	0.275	684	1575	2243
MSTRG.22832.4	coding	0.274	435	1332	1998
MSTRG.22833.2	coding	0.38	507	1302	2074
MSTRG.22833.3	coding	0.373	9	801	1572
MSTRG.22841.2	coding	0.469	516	2532	2909
MSTRG.22841.3	coding	0.469	510	2526	5537
MSTRG.22842.8	coding	0.604	666	2892	3683
MSTRG.22842.9	coding	0.579	249	2754	3564
MSTRG.22842.10	coding	0.547	60	2829	3621
MSTRG.22842.12	coding	0.584	210	2832	3591
MSTRG.22842.13	coding	0.567	75	2865	3555
MSTRG.22835.1	coding	0.364	309	2166	3887
MSTRG.22835.2	coding	0.378	120	2205	3234
MSTRG.22836.1	noncoding	-0.057344	693	1167	2432
MSTRG.22838.4	coding	0.337	42	600	849
MSTRG.22838.5	coding	0.353	0	585	832
MSTRG.22838.3	coding	0.359	15	606	1124
MSTRG.22838.2	coding	0.384	15	750	1225
MSTRG.22845.2	coding	0.22	171	945	5496
MSTRG.22847.3	coding	0.203	141	1443	1633
MSTRG.22857.1	coding	0.381	0	1314	2219
MSTRG.22857.2	coding	0.541	0	597	2029
MSTRG.22858.3	coding	0.113	243	7152	7688
MSTRG.22858.5	coding	0.113	348	7260	7797
MSTRG.22858.6	coding	0.113	348	7254	7791
MSTRG.22858.7	coding	0.113	348	7257	7794
MSTRG.22858.8	coding	0.113	288	7191	7727
MSTRG.22858.10	coding	0.113	435	7359	7897
MSTRG.22858.4	coding	0.113	297	7188	7724
MSTRG.22858.9	coding	0.113	438	7347	7883
MSTRG.22867.1	coding	0.236	798	2997	3852
MSTRG.22871.1	noncoding	-0.053248	225	285	1344
MSTRG.22871.2	noncoding	-0.028672	336	369	744
MSTRG.22881.2	coding	0.353	504	2817	3571
MSTRG.22881.3	coding	0.306	390	2076	2628
MSTRG.22892.2	coding	0.336	0	783	2611
MSTRG.22892.4	coding	0.336	0	783	2551
MSTRG.22892.5	noncoding	-0.0999424	0	198	870
MSTRG.22893.1	coding	0.115	0	237	453
MSTRG.22895.2	coding	0.163	63	588	863
MSTRG.22896.1	coding	0.03	1179	1887	4217
MSTRG.22896.4	coding	0.03	1179	1887	2800
MSTRG.22898.2	coding	0.081	0	1821	2131

MSTRG.22899.1	coding	0.371	0	915	1451
MSTRG.22909.2	coding	0.219	384	1077	1281
MSTRG.22911.3	coding	0.46	564	1941	2095
MSTRG.22912.1	noncoding	-0.0507904	579	1485	2392
MSTRG.22913.2	coding	0.404	0	1860	2030
MSTRG.22914.3	coding	0.218	246	1458	1951
MSTRG.22919.1	coding	0.316	159	3741	4509
MSTRG.22921.1	noncoding	-0.0004096	0	0	405
MSTRG.22922.1	coding	0.1	186	2280	3623
MSTRG.22922.3	coding	0.107	120	2037	3609
MSTRG.22924.1	coding	0.304	0	1287	2071
MSTRG.22922.6	noncoding	-0.0638976	1266	1605	1776
MSTRG.22924.3	coding	0.306	5286	5826	5981
MSTRG.22924.2	coding	0.287	387	1413	1566
MSTRG.22928.2	coding	0.305	243	4320	4662
MSTRG.22931.5	coding	0.213	1110	3273	4633
MSTRG.22931.6	coding	0.213	618	2775	4135
MSTRG.22933.1	coding	0.264	855	2313	2563
MSTRG.22935.1	coding	0.469	0	852	1199
MSTRG.22936.1	coding	0.271	39	963	2193
MSTRG.22937.2	coding	0.455	465	1638	2344
MSTRG.22937.3	coding	0.455	606	1779	2485
MSTRG.22941.4	coding	0.448	474	2313	2468
MSTRG.22943.1	coding	0.426	102	1083	1652
MSTRG.22944.2	coding	0.195	246	519	1028
MSTRG.22925.3	coding	0.413	69	4218	4696
MSTRG.22946.1	coding	0.331	528	1029	1315
MSTRG.22946.4	coding	0.312	423	873	1029
MSTRG.22947.2	coding	0.268	273	993	1149
MSTRG.22948.3	coding	0.457	1566	4680	5229
MSTRG.22948.4	coding	0.454	1416	4620	5167
MSTRG.22948.2	coding	0.461	702	3831	4379
MSTRG.22948.5	coding	0.396	0	3786	4333
MSTRG.22951.1	coding	0.268	189	2493	2854
MSTRG.22951.2	coding	0.388	507	1968	2329
MSTRG.22953.1	coding	0.154	504	828	5027
MSTRG.22953.2	noncoding	-0.1232896	4101	4422	4577
MSTRG.22953.8	coding	0.119	2964	3177	4303
MSTRG.22953.4	coding	0.154	2157	2481	5634
MSTRG.22953.5	coding	0.154	2157	2481	4164
MSTRG.22953.6	coding	0.202	2298	2583	5738
MSTRG.22953.7	coding	0.202	2298	2583	4496
MSTRG.22953.3	coding	0.154	834	1158	3978
MSTRG.22954.1	noncoding	-0.0282624	1113	1191	2633
MSTRG.22955.1	noncoding	-0.1523712	0	12	206
MSTRG.22965.3	noncoding	-0.0811008	0	315	1795
MSTRG.22965.5	noncoding	-0.0811008	0	315	1361
MSTRG.22965.4	coding	0.423	483	2922	3586
MSTRG.22965.6	coding	0.421	528	3024	3693
MSTRG.22965.8	coding	0.016	159	816	2424
MSTRG.22976.4	noncoding	-0.0774144	564	2124	2661
MSTRG.22977.2	coding	0.18	444	2412	2600
MSTRG.22979.2	coding	0.543	0	747	8403
MSTRG.22979.3	coding	0.245	114	1347	8366

MSTRG.22983.2	coding	0.339	597	1917	2134
MSTRG.22984.5	coding	0.31	0	420	1216
MSTRG.22984.1	coding	0.266	0	528	2952
MSTRG.22984.3	coding	0.266	0	528	2437
MSTRG.22984.4	coding	0.33	0	336	1985
MSTRG.22985.2	coding	0.139	468	1059	1345
MSTRG.22986.3	coding	0.307	0	1149	3101
MSTRG.22986.4	coding	0.307	0	1149	2409
MSTRG.22986.5	coding	0.283	0	1257	5029
MSTRG.22966.6	coding	0.188	0	750	5003
MSTRG.22966.2	coding	0.149	339	1116	12573
MSTRG.22966.3	coding	0.149	393	1170	12627
MSTRG.22966.4	noncoding	-0.0561152	393	864	12756
MSTRG.22966.5	noncoding	-0.0385024	8013	8244	8773
MSTRG.22967.1	noncoding	-0.069632	366	390	2020
MSTRG.22968.1	noncoding	-0.04096	3504	3588	6882
MSTRG.22967.2	noncoding	-0.0352256	2070	2214	4548
MSTRG.22966.7	coding	0.149	5322	6099	17554
MSTRG.22966.8	coding	0.142	93	900	12354
MSTRG.22966.9	coding	0.027	2691	4266	4935
MSTRG.22966.10	coding	0.027	8994	10569	15585
MSTRG.22972.1	coding	0.329	357	1737	2208
MSTRG.23003.1	noncoding	-0.073728	0	96	263
MSTRG.23003.2	noncoding	-0.008192	18	54	481
MSTRG.23003.3	noncoding	-0.008192	6	42	544
MSTRG.23015.1	coding	0.377	837	12318	16415
MSTRG.23015.2	coding	0.378	357	11991	16088
MSTRG.23016.1	noncoding	-0.0045056	1740	1896	2120
MSTRG.23015.3	coding	0.378	21	11613	15709
MSTRG.23017.1	noncoding	-0.08192	3909	4002	6471
MSTRG.23018.1	noncoding	-0.0503808	2178	2394	4647
MSTRG.23006.1	noncoding	-0.077824	192	225	462
MSTRG.23007.1	coding	0.367	597	3564	3959
MSTRG.23007.3	coding	0.362	405	3360	3757
MSTRG.23009.1	noncoding	-0.0397312	132	396	2242
MSTRG.23009.2	coding	0.051	387	705	2278
MSTRG.23009.3	noncoding	-0.0397312	132	396	2221
MSTRG.23049.1	coding	0.151	0	312	468
MSTRG.23050.1	noncoding	-0.053248	231	285	1238
MSTRG.23048.5	coding	0.229	147	1089	2198
MSTRG.23048.4	coding	0.221	297	1224	2335
MSTRG.23048.6	coding	0.223	336	1236	2346
MSTRG.23052.2	coding	0.239	0	1845	3211
MSTRG.23055.1	coding	0.111	81	2544	3858
MSTRG.23055.5	coding	0.115	333	2811	4124
MSTRG.23054.3	coding	0.194	744	2889	3536
MSTRG.23057.1	noncoding	-0.0987136	39	528	753
MSTRG.23058.1	noncoding	-0.077824	375	441	1327
MSTRG.23061.1	coding	0.037	4749	5667	8759
MSTRG.23061.2	noncoding	-0.028672	975	1098	1253
MSTRG.23071.1	coding	0.203	720	1956	2310
MSTRG.23080.2	noncoding	-0.0692224	14715	15279	16432
MSTRG.23080.1	noncoding	-0.0692224	219	783	1751
MSTRG.23082.1	coding	0.346	7641	15459	15613

MSTRG.23082.2	coding	0.154	561	1530	1736
MSTRG.23082.3	coding	0.346	7395	15213	15368
MSTRG.23082.4	coding	0.346	7326	15144	15297
MSTRG.23084.1	noncoding	-0.1609728	162	177	646
MSTRG.23072.2	noncoding	-0.043008	237	561	1049
MSTRG.23074.1	coding	0.116	183	1374	2329
MSTRG.23074.2	coding	0.115	0	1281	2234
MSTRG.23075.1	noncoding	-0.008192	276	567	5174
MSTRG.23076.1	noncoding	-0.14336	603	615	1567
MSTRG.23087.3	coding	0.452	918	4728	13173
MSTRG.23079.2	coding	0.506	225	3393	5101
MSTRG.23089.2	noncoding	-0.0053248	297	309	699
MSTRG.23089.1	noncoding	-0.0053248	312	324	788
MSTRG.23094.2	coding	0.297	1932	2283	6199
MSTRG.23094.4	noncoding	-0.018432	4434	4602	9437
MSTRG.23094.5	coding	0.314	807	1233	5150
MSTRG.23094.6	coding	0.314	4044	4470	8386
MSTRG.23101.1	coding	0.066	0	4221	6485
MSTRG.23101.2	coding	0.059	0	4185	6396
MSTRG.23106.3	coding	0.202	408	2526	2753
MSTRG.23106.4	coding	0.202	498	2613	2840
MSTRG.23106.5	coding	0.204	132	2250	2476
MSTRG.23108.1	coding	0.34	0	2109	3292
MSTRG.23105.2	coding	0.29	726	1077	3726
MSTRG.23110.2	coding	0.545	0	1473	1963
MSTRG.23110.3	coding	0.466	0	1824	2313
MSTRG.23122.1	coding	0.324	0	2133	2531
MSTRG.23122.2	coding	0.359	90	1938	2335
MSTRG.23122.3	coding	0.389	393	1908	2306
MSTRG.23127.1	noncoding	-0.012288	1113	1233	1808
MSTRG.23127.2	noncoding	-0.012288	8556	8676	9165
MSTRG.23128.1	coding	0.014	96	285	593
MSTRG.23130.1	noncoding	-0.1314816	0	153	1536
MSTRG.23134.1	noncoding	-0.0692224	1701	2049	5258
MSTRG.23139.2	coding	0.093	246	2352	2505
MSTRG.23139.4	coding	0.151	246	450	832
MSTRG.23139.5	coding	0.13	246	594	850
MSTRG.23139.6	coding	0.176	0	213	539
MSTRG.23139.7	coding	0.124	87	309	726
MSTRG.23142.2	noncoding	-0.0577536	4518	4755	5569
MSTRG.23142.1	noncoding	-0.0577536	4524	4761	5364
MSTRG.23145.1	coding	0.045	1038	1203	5297
MSTRG.23145.2	noncoding	-0.032768	2619	3036	4683
MSTRG.23146.1	coding	0.098	1575	1932	2085
MSTRG.23153.2	coding	0.428	12	2457	2741
MSTRG.23153.3	coding	0.434	0	2391	2675
MSTRG.23150.2	coding	0.44	0	909	1645
MSTRG.23151.1	noncoding	-0.004096	582	687	843
MSTRG.23154.1	coding	0.187	231	507	796
MSTRG.23159.3	coding	0.175	6	594	1341
MSTRG.23160.1	coding	0.342	156	1830	2058
MSTRG.23158.1	noncoding	-0.047104	237	471	626
MSTRG.23158.2	noncoding	-0.0503808	180	381	536
MSTRG.23166.1	noncoding	-0.0823296	0	279	503

MSTRG.23169.1	noncoding	-0.0651264	183	852	1174
MSTRG.23173.1	noncoding	-0.0311296	0	186	350
MSTRG.23175.1	coding	0.127	366	546	765
MSTRG.23175.2	coding	0.127	240	420	663
MSTRG.23174.1	coding	0.137	45	201	383
MSTRG.23178.1	noncoding	-0.088064	468	1353	1510
MSTRG.23182.1	coding	0.375	39	942	1505
MSTRG.23187.1	noncoding	-0.0262144	750	954	1366
MSTRG.23188.1	noncoding	-0.065536	441	864	1783
MSTRG.23188.2	noncoding	-0.065536	75	498	6252
MSTRG.23188.4	noncoding	-0.065536	75	498	6180
MSTRG.23188.3	noncoding	-0.065536	441	864	1371
MSTRG.23189.2	coding	0.326	0	7089	10301
MSTRG.23191.3	coding	0.333	81	612	902
MSTRG.23191.4	coding	0.346	312	897	1187
MSTRG.23191.2	coding	0.291	183	927	1218
MSTRG.23192.4	noncoding	-0.0356352	99	333	507
MSTRG.23186.2	coding	0.234	1314	3162	3319
MSTRG.23194.1	coding	0.307	0	3744	4284
MSTRG.23194.2	coding	0.308	0	3747	4287
MSTRG.23195.3	coding	0.342	408	1920	2075
MSTRG.23195.4	coding	0.345	408	2076	2231
MSTRG.23195.2	coding	0.342	144	1656	1813
MSTRG.23195.1	coding	0.345	462	2130	2286
MSTRG.23196.1	noncoding	-0.008192	9	153	1571
MSTRG.23193.1	coding	0.425	0	1980	2408
MSTRG.23197.2	noncoding	-0.1150976	0	489	643
MSTRG.23202.1	coding	0.345	15	765	976
MSTRG.23204.3	coding	0.412	303	4821	4980
MSTRG.23201.2	coding	0.513	0	501	860
MSTRG.23207.1	coding	0.335	666	1611	2004
MSTRG.23207.4	coding	0.283	114	879	1273
MSTRG.23212.1	noncoding	-0.034816	243	387	1018
MSTRG.23213.1	coding	0.001	717	1338	1960
MSTRG.23221.3	coding	0.114	240	1482	2426
MSTRG.23221.2	coding	0.101	69	1311	6786
MSTRG.23222.1	noncoding	-0.02048	156	360	551
MSTRG.23223.1	noncoding	-0.034816	3252	3570	3927
MSTRG.23223.2	coding	0.046	522	1332	2521
MSTRG.23223.3	coding	0.046	1005	1815	3804
MSTRG.23223.4	coding	0.046	309	1119	3116
MSTRG.23224.1	noncoding	-0.0372736	117	345	1216
MSTRG.23234.1	noncoding	-0.0401408	3	258	1322
MSTRG.23237.1	coding	0.062	2322	2958	8447
MSTRG.23237.3	coding	0.062	1941	2577	7903
MSTRG.23239.1	coding	0.027	192	1590	1810
MSTRG.23241.1	noncoding	-0.0385024	750	1317	5886
MSTRG.23253.1	noncoding	-0.0565248	2391	3246	4975
MSTRG.23255.2	coding	0.449	0	6264	8311
MSTRG.23255.4	coding	0.495	1599	7104	9080
MSTRG.23255.5	coding	0.495	411	5913	7961
MSTRG.23255.6	coding	0.43	0	4215	4746
MSTRG.23257.1	noncoding	-0.034816	2076	2226	2827
MSTRG.23255.7	coding	0.338	1023	3717	4247

MSTRG.23255.8	coding	0.408	381	2031	2768
MSTRG.23265.1	coding	0.468	87	2376	3280
MSTRG.23265.2	coding	0.464	84	2859	3016
MSTRG.23265.3	coding	0.396	75	1125	1662
MSTRG.23265.12	coding	0.468	156	2445	3072
MSTRG.23265.11	coding	0.394	51	3312	3468
MSTRG.23265.13	coding	0.466	156	3207	3362
MSTRG.23265.7	coding	0.464	156	3168	3323
MSTRG.23265.15	noncoding	-0.0856064	3174	3474	3766
MSTRG.23262.1	coding	0.288	255	1872	4633
MSTRG.23264.1	coding	0.325	522	1980	2250
MSTRG.23258.1	noncoding	-0.1929216	0	99	256
MSTRG.23259.1	noncoding	-0.08192	0	375	831
MSTRG.23268.1	coding	0.401	267	1020	1174
MSTRG.23272.1	noncoding	-0.0917504	4557	4920	5169
MSTRG.23272.2	noncoding	-0.0274432	2034	2766	4883
MSTRG.23272.3	noncoding	-0.0577536	939	1524	10160
MSTRG.23272.4	noncoding	-0.0888832	4260	5028	7282
MSTRG.23272.5	noncoding	-0.0872448	747	1266	1921
MSTRG.23271.3	coding	0.561	207	909	1247
MSTRG.23273.3	coding	0.424	0	783	2159
MSTRG.23275.3	coding	0.469	0	1032	1727
MSTRG.23280.2	coding	0.459	0	870	1741
MSTRG.23284.2	coding	0.314	12	828	1579
MSTRG.23285.3	coding	0.422	0	1758	1971
MSTRG.23285.4	coding	0.431	0	2394	2607
MSTRG.23285.5	coding	0.407	873	3417	3630
MSTRG.23285.6	coding	0.422	501	2898	3111
MSTRG.23285.8	coding	0.408	2442	4818	5032
MSTRG.23279.1	coding	0.397	0	402	1140
MSTRG.23291.2	coding	0.227	57	444	640
MSTRG.23291.3	coding	0.365	0	348	523
MSTRG.23292.3	coding	0.321	789	2316	3798
MSTRG.23292.4	coding	0.321	807	2334	3816
MSTRG.23292.5	coding	0.385	4971	7185	8665
MSTRG.23292.6	coding	0.391	138	2244	3623
MSTRG.23292.8	coding	0.385	5481	7695	9177
MSTRG.23292.9	coding	0.391	3795	5901	7281
MSTRG.23292.10	coding	0.386	2625	4779	6531
MSTRG.23297.4	coding	0.568	0	3165	3478
MSTRG.23297.3	coding	0.562	0	3060	3424
MSTRG.23295.1	coding	0.246	861	1941	2328
MSTRG.23296.2	coding	0.232	0	993	1721
MSTRG.23296.3	noncoding	-0.0978944	0	1077	1805
MSTRG.23301.1	coding	0.245	267	702	918
MSTRG.23307.1	coding	0.514	126	1932	7455
MSTRG.23307.2	coding	0.557	450	2094	4195
MSTRG.23307.3	coding	0.531	114	1875	6266
MSTRG.23307.7	coding	0.548	0	1737	2318
MSTRG.23318.4	coding	0.423	288	1875	2173
MSTRG.23318.6	coding	0.423	243	1860	2156
MSTRG.23318.7	coding	0.395	411	2205	2503
MSTRG.23318.8	coding	0.425	153	1776	2072
MSTRG.23318.9	coding	0.425	129	1752	2050

MSTRG.23321.3	coding	0.48	0	534	713
MSTRG.23321.2	coding	0.381	0	528	707
MSTRG.23322.1	noncoding	-0.1777664	0	435	1524
MSTRG.23322.4	noncoding	-0.0561152	234	441	1344
MSTRG.23322.5	noncoding	-0.0606208	693	933	1089
MSTRG.23322.3	coding	0.356	0	405	1533
MSTRG.23323.3	coding	0.418	264	1989	2238
MSTRG.23314.1	coding	0.359	0	3219	4974
MSTRG.23324.2	coding	0.402	0	1860	3179
MSTRG.23325.1	coding	0.44	237	1527	2660
MSTRG.23332.2	coding	0.465	0	858	4163
MSTRG.23333.1	coding	0.376	189	804	1268
MSTRG.23333.3	coding	0.367	765	1353	1815
MSTRG.23327.1	noncoding	-0.0176128	2775	2943	3525
MSTRG.23327.2	noncoding	-0.0176128	4536	4704	5287
MSTRG.23327.3	noncoding	-0.0176128	1734	1902	2485
MSTRG.23327.4	noncoding	-0.045056	567	891	3975
MSTRG.23339.3	coding	0.324	189	2262	3792
MSTRG.23347.2	coding	0.37	477	8901	9289
MSTRG.23347.3	coding	0.371	447	8979	10219
MSTRG.23348.2	noncoding	-0.0643072	354	1332	1555
MSTRG.23348.1	noncoding	-0.0765952	669	1737	1959
MSTRG.23349.1	coding	0.378	174	840	1012
MSTRG.23349.2	coding	0.395	0	651	818
MSTRG.23349.3	coding	0.366	3	684	838
MSTRG.23349.4	coding	0.414	0	759	929
MSTRG.23349.5	coding	0.449	0	720	881
MSTRG.23350.1	coding	0.418	0	4746	5521
MSTRG.23350.4	coding	0.424	444	8181	8957
MSTRG.23355.2	coding	0.432	1539	3072	3226
MSTRG.23357.1	coding	0.372	1599	3000	4044
MSTRG.23360.1	noncoding	-0.0749568	183	1146	2635
MSTRG.23360.2	noncoding	-0.1007616	393	861	2347
MSTRG.23360.3	noncoding	-0.0749568	177	1140	2488
MSTRG.23361.1	noncoding	-0.0851968	495	813	968
MSTRG.23352.3	coding	0.431	480	3195	3526
MSTRG.23352.4	coding	0.431	102	3051	3381
MSTRG.23352.5	coding	0.389	0	3162	3379
MSTRG.23365.2	noncoding	-0.0462848	267	1146	1325
MSTRG.23369.1	coding	0.059	465	999	1215
MSTRG.23369.9	coding	0.059	771	1305	1520
MSTRG.23369.10	coding	0.059	474	1008	1224
MSTRG.23367.1	noncoding	-0.049152	0	108	342
MSTRG.23367.3	coding	0.221	171	1008	1172
MSTRG.23370.3	coding	0.095	0	732	994
MSTRG.23373.2	noncoding	-0.1077248	216	945	1101
MSTRG.23374.3	coding	0.435	3	1932	2135
MSTRG.23377.2	noncoding	-0.0905216	183	834	1271
MSTRG.23377.3	noncoding	-0.0995328	12	711	1150
MSTRG.23379.1	noncoding	-0.0892928	480	723	2313
MSTRG.23380.1	coding	0.274	237	1779	2265
MSTRG.23379.3	noncoding	-0.1335296	333	867	1021
MSTRG.23380.2	coding	0.266	237	1923	2379
MSTRG.23380.3	coding	0.254	237	2103	2532

MSTRG.23380.4	coding	0.264	237	1995	2424
MSTRG.23379.4	noncoding	-0.14336	93	525	680
MSTRG.23380.5	coding	0.273	237	1851	2607
MSTRG.23380.6	coding	0.273	237	1815	2242
MSTRG.23380.7	coding	0.169	237	3156	3522
MSTRG.23380.8	coding	0.268	132	1854	2605
MSTRG.23382.4	coding	0.65	342	1776	1938
MSTRG.23384.2	coding	0.502	528	1872	2061
MSTRG.23387.2	coding	0.024	1278	1503	2008
MSTRG.23388.2	coding	0.302	189	435	998
MSTRG.23391.1	coding	0.066	75	2037	3904
MSTRG.23391.2	coding	0.093	0	150	688
MSTRG.23391.4	coding	0.08	1764	3987	4205
MSTRG.23392.1	coding	0.232	168	2514	3354
MSTRG.23396.1	noncoding	-0.0241664	36	195	480
MSTRG.23397.2	coding	0.416	162	1902	3201
MSTRG.23401.5	coding	0.425	0	1182	1531
MSTRG.23399.3	coding	0.595	0	1146	1340
MSTRG.23407.2	coding	0.46	0	1545	1698
MSTRG.23412.4	noncoding	-0.1650688	0	363	7096
MSTRG.23412.2	coding	0.481	0	1431	2792
MSTRG.23412.3	coding	0.481	0	1431	2864
MSTRG.23413.1	noncoding	-0.0864256	0	294	3482
MSTRG.23406.1	coding	0.42	495	1077	6753
MSTRG.23414.1	coding	0.247	39	624	1550
MSTRG.23415.1	coding	0.598	0	1785	2504
MSTRG.23415.2	coding	0.506	0	435	912
MSTRG.23415.3	coding	0.566	0	549	6458
MSTRG.23415.5	coding	0.38	360	759	2844
MSTRG.23415.4	coding	0.611	0	1890	3974
MSTRG.23416.1	coding	0.456	0	2532	5190
MSTRG.23416.2	coding	0.457	0	2463	3467
MSTRG.23420.1	coding	0.284	90	2307	3170
MSTRG.23430.2	coding	0.297	204	1485	2226
MSTRG.23433.1	coding	0.297	867	2655	3450
MSTRG.23433.2	coding	0.376	963	2079	2627
MSTRG.23435.1	noncoding	-0.077824	1710	2484	2653
MSTRG.23443.5	coding	0.614	186	1560	3007
MSTRG.23443.6	coding	0.574	516	2385	3834
MSTRG.23443.7	coding	0.614	84	1467	2858
MSTRG.23438.3	noncoding	-0.1122304	0	699	856
MSTRG.23451.2	coding	0.339	372	2181	2372
MSTRG.23451.3	coding	0.341	345	2154	2345
MSTRG.23454.2	coding	0.43	18	492	735
MSTRG.23455.1	noncoding	-0.0466944	18	288	1169
MSTRG.23456.1	coding	0.441	1113	3933	4141
MSTRG.23461.2	coding	0.517	438	1833	4181
MSTRG.23462.1	coding	0.443	504	2130	2840
MSTRG.23457.2	coding	0.517	0	663	818
MSTRG.23463.2	coding	0.342	6	378	534
MSTRG.23467.2	coding	0.48	417	4116	6537
MSTRG.23467.3	coding	0.41	1101	5670	5957
MSTRG.23467.5	coding	0.497	1635	5301	5541
MSTRG.23467.4	coding	0.481	279	4269	4509

MSTRG.23466.3	noncoding	-0.0720896	3	1167	1323
MSTRG.23470.1	coding	0.441	66	2106	2985
MSTRG.23470.2	coding	0.486	3	1968	2573
MSTRG.23474.2	coding	0.337	459	1440	1663
MSTRG.23479.8	coding	0.326	1098	4239	4828
MSTRG.23479.5	coding	0.331	2136	5091	5354
MSTRG.23479.7	coding	0.322	2145	5220	5483
MSTRG.23479.9	coding	0.247	687	3948	4203
MSTRG.23479.3	coding	0.32	1242	4302	4559
MSTRG.23479.10	coding	0.336	1128	4149	4705
MSTRG.23479.11	coding	0.336	1149	4170	4728
MSTRG.23482.3	coding	0.494	489	3300	3504
MSTRG.23482.4	coding	0.487	42	2862	3068
MSTRG.23484.1	coding	0.239	705	1296	1465
MSTRG.23485.2	coding	0.42	147	1419	2277
MSTRG.23486.1	coding	0.423	594	1419	1576
MSTRG.23488.2	coding	0.369	0	1164	1970
MSTRG.23488.4	coding	0.358	4440	5163	5971
MSTRG.23491.2	coding	0.351	117	702	856
MSTRG.23492.2	coding	0.583	0	1329	1483
MSTRG.23492.5	coding	0.399	402	1149	4212
MSTRG.23495.1	coding	0.319	0	675	1291
MSTRG.23505.2	coding	0.378	0	2013	2305
MSTRG.23510.2	coding	0.164	255	2511	2735
MSTRG.23515.4	coding	0.265	0	1968	2195
MSTRG.23520.1	coding	0.321	840	1458	1736
MSTRG.23520.2	coding	0.347	117	699	978
MSTRG.23520.3	coding	0.32	213	825	1102
MSTRG.23520.4	coding	0.318	174	771	1048
MSTRG.23513.1	coding	0.263	1479	2619	3961
MSTRG.23522.5	noncoding	-0.2727936	3480	4290	4503
MSTRG.23522.3	noncoding	-0.2658304	3078	3915	4161
MSTRG.23522.4	noncoding	-0.2727936	3078	3888	4134
MSTRG.23525.2	coding	0.508	0	3453	4355
MSTRG.23528.2	coding	0.424	5121	8253	8728
MSTRG.23529.9	coding	0.201	129	1806	2561
MSTRG.23530.1	coding	0.387	0	1335	1622
MSTRG.23531.2	coding	0.311	267	1296	1450
MSTRG.23531.1	coding	0.323	102	996	1372
MSTRG.23530.3	coding	0.398	0	1353	1914
MSTRG.23532.1	noncoding	-0.008192	0	69	360
MSTRG.23535.3	coding	0.377	0	1743	2646
MSTRG.23535.2	coding	0.366	102	1761	2670
MSTRG.23535.4	coding	0.385	318	1995	2905
MSTRG.23535.6	coding	0.423	24	1392	2486
MSTRG.23534.2	noncoding	-0.0905216	102	360	1600
MSTRG.23545.2	coding	0.421	1908	7866	8758
MSTRG.23550.1	coding	0.487	720	4347	4502
MSTRG.23550.3	coding	0.487	2610	6237	6394
MSTRG.23550.4	coding	0.487	477	4104	4260
MSTRG.23551.4	coding	0.267	231	1437	1607
MSTRG.23552.4	coding	0.189	0	495	675
MSTRG.23552.5	noncoding	-0.0671744	0	408	2934
MSTRG.23552.2	noncoding	-0.0671744	0	408	2127

MSTRG.23552.3	noncoding	-0.0671744	0	408	3990
MSTRG.23553.4	coding	0.432	0	1467	1698
MSTRG.23567.3	coding	0.6	0	3183	3812
MSTRG.23568.2	coding	0.364	372	1518	1857
MSTRG.23568.3	noncoding	-0.057344	432	522	1523
MSTRG.23572.3	coding	0.468	63	645	1444
MSTRG.23572.4	coding	0.436	72	591	1381
MSTRG.23574.4	coding	0.457	330	1863	2879
MSTRG.23575.2	coding	0.357	255	1164	1761
MSTRG.23559.4	coding	0.329	249	1350	1830
MSTRG.23559.5	coding	0.328	288	1440	1885
MSTRG.23560.2	coding	0.215	81	339	1656
MSTRG.23561.1	coding	0.231	0	2289	3779
MSTRG.23561.2	coding	0.325	0	519	924
MSTRG.23556.1	coding	0.277	114	792	968
MSTRG.23558.2	coding	0.501	0	1359	1847
MSTRG.23562.1	noncoding	-0.0905216	345	699	897
MSTRG.23577.2	coding	0.41	12	636	3322
MSTRG.23579.1	coding	0.33	1590	6609	8440
MSTRG.23580.1	coding	0.459	0	867	1362
MSTRG.23582.1	coding	0.264	57	945	1110
MSTRG.23583.2	coding	0.589	183	1068	1893
MSTRG.23585.4	coding	0.202	177	708	1074
MSTRG.23586.1	coding	0.326	213	1698	2713
MSTRG.23586.2	coding	0.326	189	1557	1803
MSTRG.23586.3	noncoding	-0.0782336	165	1419	3182
MSTRG.23585.6	noncoding	-0.0462848	123	1266	2193
MSTRG.23585.7	coding	0.279	60	399	1267
MSTRG.23585.8	coding	0.284	60	957	1323
MSTRG.23585.9	coding	0.303	60	447	1262
MSTRG.23585.10	coding	0.316	0	378	1296
MSTRG.23585.11	coding	0.316	0	426	1248
MSTRG.23587.1	coding	0.323	504	1074	1469
MSTRG.23587.2	coding	0.29	474	1002	1199
MSTRG.23592.1	noncoding	-0.012288	378	519	1111
MSTRG.23593.1	noncoding	-0.065536	630	657	2280
MSTRG.23594.1	noncoding	-0.0331776	363	540	2273
MSTRG.23594.2	noncoding	-0.0331776	498	675	1416
MSTRG.23594.3	noncoding	-0.0331776	492	669	4671
MSTRG.23594.4	noncoding	-0.002048	141	354	1078
MSTRG.23595.1	coding	0.178	525	1194	1744
MSTRG.23595.2	coding	0.178	360	1029	1355
MSTRG.23595.3	coding	0.164	720	1320	1866
MSTRG.23595.4	coding	0.185	117	735	1281
MSTRG.23594.5	noncoding	-0.065536	282	384	1203
MSTRG.23597.2	coding	0.34	39	789	2124
MSTRG.23597.3	coding	0.508	0	540	694
MSTRG.23598.1	coding	0.289	897	3597	5851
MSTRG.23598.2	coding	0.284	996	3762	4842
MSTRG.23598.3	coding	0.292	570	3270	4352
MSTRG.23598.4	coding	0.464	597	1257	1412
MSTRG.23603.2	coding	0.119	1353	1965	3267
MSTRG.23607.2	noncoding	-0.0012288	30	180	378
MSTRG.23608.2	noncoding	-0.0380928	3	726	883

MSTRG.23619.1	coding	0.127	225	837	1665
MSTRG.23621.5	coding	0.308	363	588	993
MSTRG.23621.4	coding	0.094	414	2361	2543
MSTRG.23621.1	coding	0.085	609	2544	5210
MSTRG.23621.6	coding	0.089	609	2376	5042
MSTRG.23621.3	coding	0.1	219	2187	5920
MSTRG.23622.1	noncoding	-0.0016384	213	318	1435
MSTRG.23621.7	coding	0.078	507	2343	2544
MSTRG.23621.9	coding	0.095	3129	4380	4562
MSTRG.23621.11	coding	0.095	2550	3801	7535
MSTRG.23621.17	noncoding	-0.08192	3384	3468	6404
MSTRG.23621.18	coding	0.095	4497	5301	5953
MSTRG.23623.1	noncoding	-0.006144	201	282	462
MSTRG.23629.1	noncoding	-0.0425984	1032	1287	2982
MSTRG.23630.1	noncoding	-0.049152	6162	6258	7160
MSTRG.23630.2	coding	0.027	1479	1641	2072
MSTRG.23630.3	coding	0.066	1332	1503	3009
MSTRG.23630.4	coding	0.066	798	969	6406
MSTRG.23631.1	noncoding	-0.004096	273	360	825
MSTRG.23616.2	noncoding	-0.002048	2769	2886	3150
MSTRG.23753.1	noncoding	-0.008192	921	1038	1602
MSTRG.23754.2	coding	0.032	714	2277	3214
MSTRG.23754.1	coding	0.031	597	1998	2941
MSTRG.23754.4	coding	0.026	945	2328	2482
MSTRG.23754.3	coding	0.031	936	2337	3278
MSTRG.23754.5	coding	0.049	264	3090	4031
MSTRG.23754.7	coding	0.034	264	1665	2606
MSTRG.23754.13	coding	0.058	1356	1812	3231
MSTRG.23754.14	coding	0.06	1092	2487	4775
MSTRG.23754.16	coding	0.06	1080	2397	3340
MSTRG.23758.1	noncoding	-0.0004096	489	570	766
MSTRG.23763.1	coding	0.026	0	99	3712
MSTRG.23769.5	coding	0.131	39	474	6828
MSTRG.23769.6	noncoding	-0.0557056	0	201	10400
MSTRG.23769.8	coding	0.089	99	498	5510
MSTRG.23770.2	coding	0.051	174	1767	5235
MSTRG.23770.1	coding	0.051	762	2355	5822
MSTRG.23770.3	coding	0.036	1158	2721	6189
MSTRG.23770.4	noncoding	-0.008192	1707	1833	11573
MSTRG.23770.5	coding	0.036	3240	4803	8144
MSTRG.23770.6	coding	0.052	375	1989	5329
MSTRG.23770.7	coding	0.051	2502	4095	7434
MSTRG.23770.8	coding	0.272	0	243	4176
MSTRG.23770.10	coding	0.051	1239	2832	5925
MSTRG.23770.9	coding	0.036	1155	2718	5811
MSTRG.23773.2	coding	0.049	273	2706	3946
MSTRG.23769.11	coding	0.179	0	204	2058
MSTRG.23765.13	noncoding	-0.0847872	159	810	1143
MSTRG.23765.15	noncoding	-0.0806912	159	765	1098
MSTRG.23765.10	noncoding	-0.0876544	213	762	1108
MSTRG.23765.12	noncoding	-0.0970752	213	810	1156
MSTRG.23765.9	noncoding	-0.0884736	213	750	1096
MSTRG.23765.8	noncoding	-0.07168	1047	1506	1841
MSTRG.23765.11	noncoding	-0.0974848	213	846	2262

MSTRG.23765.14	noncoding	-0.08192	201	807	2224
MSTRG.23765.4	noncoding	-0.0823296	1359	1959	2292
MSTRG.23765.7	noncoding	-0.0823296	1347	1947	2280
MSTRG.23765.5	noncoding	-0.069632	870	1281	1616
MSTRG.23765.6	noncoding	-0.0835584	1353	1845	2178
MSTRG.23770.13	coding	0.263	3	252	3048
MSTRG.23774.1	coding	0.036	252	1749	3839
MSTRG.23770.14	noncoding	-0.0004096	3075	3198	3408
MSTRG.23775.1	noncoding	-0.14336	351	399	650
MSTRG.23776.1	noncoding	-0.01024	816	1017	1413
MSTRG.23777.1	noncoding	-0.0618496	2097	2433	8755
MSTRG.23777.2	coding	0.08	84	1680	6548
MSTRG.23777.3	noncoding	-0.0618496	492	828	7064
MSTRG.23778.1	noncoding	-0.049152	321	414	1267
MSTRG.23778.2	noncoding	-0.0495616	1722	1890	6428
MSTRG.23779.1	coding	0.045	78	2304	4267
MSTRG.23779.2	coding	0.051	555	2889	3763
MSTRG.23779.3	coding	0.048	555	2805	3679
MSTRG.23783.1	noncoding	-0.0004096	0	0	391
MSTRG.23785.1	noncoding	-0.018432	339	435	590
MSTRG.23786.1	noncoding	-0.0028672	63	111	338
MSTRG.23788.2	coding	0.163	144	585	5322
MSTRG.23788.3	coding	0.186	51	492	2905
MSTRG.23788.4	coding	0.186	51	492	2372
MSTRG.23788.5	noncoding	-0.059392	147	408	5579
MSTRG.23788.7	coding	0.186	63	504	2296
MSTRG.23788.6	coding	0.158	147	516	4154
MSTRG.23788.8	coding	0.216	114	510	716
MSTRG.23788.9	noncoding	-0.059392	33	267	422
MSTRG.23789.1	coding	0.029	1407	1560	2276
MSTRG.23790.1	noncoding	-0.0397312	3	162	1379
MSTRG.23790.2	noncoding	-0.0090112	330	486	2431
MSTRG.23789.2	noncoding	-0.049152	3819	4092	4324
MSTRG.23789.3	noncoding	-0.049152	720	993	1283
MSTRG.23791.1	coding	0.083	2913	3435	7788
MSTRG.23791.3	noncoding	-0.0065536	1518	1668	4066
MSTRG.23791.5	coding	0.106	2853	3372	7723
MSTRG.23791.6	noncoding	-0.059392	2502	2703	7569
MSTRG.23791.4	noncoding	-0.0503808	2574	2757	7655
MSTRG.23791.7	noncoding	-0.0049152	129	288	4979
MSTRG.23791.8	coding	0.106	2886	3405	7757
MSTRG.23791.9	noncoding	-0.034816	4674	5250	7520
MSTRG.23791.11	noncoding	-0.0835584	2754	2952	7850
MSTRG.23791.10	coding	0.106	2754	3273	7659
MSTRG.23632.1	noncoding	-0.0466944	5532	5703	6217
MSTRG.23632.3	noncoding	-0.0151552	2067	2244	6274
MSTRG.23632.4	noncoding	-0.032768	63	174	2608
MSTRG.23632.5	noncoding	-0.032768	60	171	2680
MSTRG.23632.6	noncoding	-0.057344	60	165	2773
MSTRG.23632.7	coding	0.2	0	111	2045
MSTRG.23632.8	noncoding	-0.0151552	45	222	2071
MSTRG.23632.9	noncoding	-0.0221184	0	123	3464
MSTRG.23632.10	noncoding	-0.0466944	1848	2019	2352
MSTRG.23632.11	coding	0.01	1104	1401	3510

MSTRG.23635.2	coding	0.138	525	912	3009
MSTRG.23635.3	coding	0.142	471	849	2945
MSTRG.23639.1	noncoding	-0.0503808	5439	5778	5932
MSTRG.23651.1	coding	0.207	81	378	1470
MSTRG.23652.1	noncoding	-0.139264	48	111	275
MSTRG.23653.1	noncoding	-0.0262144	189	234	616
MSTRG.23654.1	coding	0.151	2997	3414	4824
MSTRG.23659.1	noncoding	-0.018432	453	561	1411
MSTRG.23651.2	noncoding	-0.02048	387	423	814
MSTRG.23660.1	noncoding	-0.0004096	213	543	1259
MSTRG.23651.3	noncoding	-0.0004096	0	0	613
MSTRG.23651.4	coding	0.159	3396	3777	8200
MSTRG.23651.5	noncoding	-0.028672	3	54	1602
MSTRG.23663.1	noncoding	-0.094208	114	126	695
MSTRG.23664.1	noncoding	-0.024576	363	438	994
MSTRG.23651.6	coding	0.189	867	1137	1878
MSTRG.23651.7	coding	0.169	1710	1986	7710
MSTRG.23651.8	noncoding	-0.02048	192	231	466
MSTRG.23666.1	noncoding	-0.165888	0	87	628
MSTRG.23667.1	noncoding	-0.090112	84	174	984
MSTRG.23668.1	noncoding	-0.0004096	138	138	616
MSTRG.23670.1	coding	0.238	153	456	4309
MSTRG.23670.2	coding	0.197	108	366	3774
MSTRG.23670.3	coding	0.197	108	366	3704
MSTRG.23671.1	noncoding	-0.1622016	0	90	257
MSTRG.23644.2	noncoding	-0.0585728	9	198	659
MSTRG.23644.1	noncoding	-0.0585728	9	195	352
MSTRG.23644.3	noncoding	-0.0585728	0	189	895
MSTRG.23644.4	noncoding	-0.0610304	0	180	874
MSTRG.23677.1	coding	0.459	0	1983	3020
MSTRG.23677.2	coding	0.453	0	1908	5778
MSTRG.23678.6	coding	0.556	117	1560	1740
MSTRG.23681.4	coding	0.556	468	1218	1710
MSTRG.23681.5	coding	0.445	0	1215	1707
MSTRG.23681.6	coding	0.422	0	1449	1941
MSTRG.23683.1	noncoding	-0.06144	2004	2601	4642
MSTRG.23683.2	noncoding	-0.0458752	786	1368	1525
MSTRG.23683.3	noncoding	-0.0200704	21	474	1352
MSTRG.23690.1	noncoding	-0.0045056	81	303	1153
MSTRG.23694.7	coding	0.44	0	2007	2162
MSTRG.23694.5	coding	0.514	0	3081	3236
MSTRG.23694.6	coding	0.429	0	2157	2311
MSTRG.23694.4	coding	0.489	0	3345	3499
MSTRG.23696.1	coding	0.423	573	2622	6456
MSTRG.23696.3	coding	0.421	276	2262	6104
MSTRG.23687.1	coding	0.286	354	843	1012
MSTRG.23688.1	noncoding	-0.0299008	1299	1692	1848
MSTRG.23698.3	coding	0.321	240	2817	3165
MSTRG.23701.3	coding	0.383	303	1521	1743
MSTRG.23706.1	coding	0.553	237	3102	4027
MSTRG.23706.3	coding	0.586	1308	4023	4938
MSTRG.23706.2	coding	0.556	189	3036	3960
MSTRG.23699.1	noncoding	-0.0790528	159	822	1721
MSTRG.23709.3	coding	0.316	93	3129	3285

MSTRG.23709.4	coding	0.48	0	1116	1986
MSTRG.23709.5	coding	0.512	0	960	1822
MSTRG.23709.6	coding	0.37	180	981	1162
MSTRG.23709.7	coding	0.389	378	1146	1327
MSTRG.23712.1	noncoding	-0.0581632	486	771	1433
MSTRG.23711.2	noncoding	-0.086016	642	855	1029
MSTRG.23716.1	coding	0.563	0	2325	2960
MSTRG.23716.2	coding	0.563	0	2322	2957
MSTRG.23716.6	coding	0.483	6849	8616	9252
MSTRG.23716.7	coding	0.466	9555	11376	12010
MSTRG.23717.2	noncoding	-0.0806912	1029	1548	1725
MSTRG.23717.3	noncoding	-0.0831488	246	762	946
MSTRG.23718.2	coding	0.439	0	1119	1595
MSTRG.23720.4	coding	0.441	0	2847	5505
MSTRG.23720.5	coding	0.512	135	2475	5134
MSTRG.23727.3	coding	0.347	0	1077	1663
MSTRG.23728.1	noncoding	-0.0851968	0	243	530
MSTRG.23728.2	coding	0.255	0	606	896
MSTRG.23731.2	coding	0.534	0	2427	2582
MSTRG.23732.2	coding	0.616	477	2076	2285
MSTRG.23732.3	coding	0.61	477	1986	2195
MSTRG.23736.1	coding	0.302	0	1023	1385
MSTRG.23743.2	coding	0.388	0	1338	1493
MSTRG.23744.2	coding	0.209	135	1521	2140
MSTRG.23744.3	coding	0.221	0	1119	2010
MSTRG.23797.2	noncoding	-0.0704512	0	648	9161
MSTRG.23801.4	coding	0.155	0	1443	2066
MSTRG.23801.3	coding	0.228	0	393	967
MSTRG.23801.5	noncoding	-0.0876544	0	219	1717
MSTRG.23809.2	coding	0.071	210	2616	2990
MSTRG.23794.1	coding	0.176	9	732	2464
MSTRG.23794.3	coding	0.198	105	1611	4535
MSTRG.23814.1	coding	0.406	195	1350	1533
MSTRG.23817.2	noncoding	-0.028672	4101	4410	4566
MSTRG.23841.3	coding	0.207	3	1314	1469
MSTRG.23841.2	coding	0.234	3	1992	4953
MSTRG.23846.1	noncoding	-0.0032768	1605	1749	6794
MSTRG.23846.2	noncoding	-0.0188416	2631	3138	12041
MSTRG.23846.3	noncoding	-0.0188416	2607	3114	11996
MSTRG.23847.1	coding	0.124	75	894	2656
MSTRG.23849.1	noncoding	-0.016384	1611	1677	1832
MSTRG.23855.2	noncoding	-0.0360448	135	990	2402
MSTRG.23857.1	coding	0.074	195	666	2007
MSTRG.23857.2	noncoding	-0.0118784	1410	1533	3050
MSTRG.23855.3	coding	0.138	516	1731	3073
MSTRG.23855.4	coding	0.35	57	1356	5963
MSTRG.23860.1	coding	0.192	0	1425	7580
MSTRG.23860.2	coding	0.188	0	1242	1471
MSTRG.23857.3	noncoding	-0.339968	1080	1089	1412
MSTRG.23855.9	coding	0.328	603	1935	3621
MSTRG.23824.1	noncoding	-0.0004096	315	450	642
MSTRG.23825.1	coding	0.154	2412	3198	10404
MSTRG.23825.3	coding	0.154	912	1698	2746
MSTRG.23825.4	coding	0.154	1362	2148	3196

MSTRG.23825.5	coding	0.093	246	1350	8372
MSTRG.23827.1	noncoding	-0.0004096	867	1284	8839
MSTRG.23828.1	noncoding	-0.0004096	489	564	841
MSTRG.23833.1	coding	0.115	558	894	3881
MSTRG.23834.1	coding	0.089	1614	2064	3344
MSTRG.23834.2	coding	0.14	33	732	3231
MSTRG.23834.3	noncoding	-0.0425984	2319	2682	5858
MSTRG.23834.4	noncoding	-0.079872	33	306	5403
MSTRG.23834.5	noncoding	-0.104448	4836	5085	6553
MSTRG.23868.2	noncoding	-0.0688128	48	309	4800
MSTRG.23868.4	noncoding	-0.0954368	96	303	4795
MSTRG.23872.6	coding	0.073	8133	9468	10966
MSTRG.23872.7	coding	0.087	930	2322	3819
MSTRG.23872.3	coding	0.087	1170	2562	4059
MSTRG.23872.1	coding	0.087	1350	2742	4240
MSTRG.23873.1	noncoding	-0.016384	624	702	1697
MSTRG.23872.8	noncoding	-0.0643072	1443	1704	4604
MSTRG.23872.9	coding	0.192	0	465	1252
MSTRG.23875.1	noncoding	-0.057344	501	582	1336
MSTRG.23872.10	coding	0.092	0	786	5678
MSTRG.23877.6	coding	0.092	3153	4611	9051
MSTRG.23878.2	noncoding	-0.045056	5910	6123	7808
MSTRG.23879.2	coding	0.06	0	1551	2650
MSTRG.23879.3	noncoding	-0.0536576	0	603	2738
MSTRG.23879.4	noncoding	-0.0851968	501	807	3751
MSTRG.23880.1	coding	0.239	57	1986	10158
MSTRG.23880.3	coding	0.233	81	1971	2810
MSTRG.23881.3	coding	0.238	414	1929	3036
MSTRG.23881.5	coding	0.238	702	2217	3299
MSTRG.23883.1	noncoding	-0.126976	144	180	549
MSTRG.23840.2	coding	0.526	126	738	891
MSTRG.23890.1	coding	0.121	1182	1452	3732
MSTRG.23894.3	coding	0.465	162	2013	2228
MSTRG.23898.2	coding	0.256	744	2511	2763
MSTRG.23927.1	noncoding	-0.012288	927	1017	1217
MSTRG.23929.1	noncoding	-0.0004096	0	0	372
MSTRG.23931.3	coding	0.124	597	1878	2644
MSTRG.23933.2	noncoding	-0.1191936	51	300	1095
MSTRG.23944.2	noncoding	-0.053248	270	276	885
MSTRG.23946.1	noncoding	-0.1060864	0	120	385
MSTRG.23947.1	coding	0.124	459	2835	3059
MSTRG.23947.2	noncoding	-0.0299008	87	294	11349
MSTRG.23949.1	coding	0.501	0	1806	6992
MSTRG.23949.2	coding	0.497	0	1863	5610
MSTRG.23950.1	noncoding	-0.008192	219	351	630
MSTRG.23950.2	noncoding	-0.004096	1494	1749	1975
MSTRG.23952.2	coding	0.096	243	1566	2607
MSTRG.23952.3	coding	0.103	525	1938	2979
MSTRG.23952.5	coding	0.107	201	1497	1650
MSTRG.23952.4	coding	0.1	243	1449	1602
MSTRG.23954.3	coding	0.393	87	1587	4033
MSTRG.23954.5	coding	0.393	198	1695	7173
MSTRG.23962.5	coding	0.276	0	387	2357
MSTRG.23962.8	coding	0.177	0	609	23170

MSTRG.23962.4	coding	0.272	0	357	9174
MSTRG.23962.6	coding	0.265	0	441	2413
MSTRG.23962.1	coding	0.282	0	708	11572
MSTRG.23962.3	coding	0.321	0	936	5889
MSTRG.23963.1	noncoding	-0.0638976	11472	11871	14072
MSTRG.23964.1	noncoding	-0.008192	2382	2511	8499
MSTRG.23962.11	noncoding	-0.0208896	8349	8538	9497
MSTRG.23962.12	noncoding	-0.0004096	3288	3429	5551
MSTRG.23965.1	noncoding	-0.0528384	7023	7410	17817
MSTRG.23966.1	coding	0.042	501	678	2803
MSTRG.23962.13	noncoding	-0.0335872	11019	11622	21803
MSTRG.23967.1	noncoding	-0.0868352	6858	7125	7279
MSTRG.23968.1	noncoding	-0.0663552	0	627	18607
MSTRG.23958.1	noncoding	-0.004096	267	393	1005
MSTRG.23970.2	coding	0.489	0	198	1319
MSTRG.23976.1	coding	0.417	0	1632	1988
MSTRG.23977.1	coding	0.525	18	1392	1602
MSTRG.23981.3	coding	0.259	24	4929	5736
MSTRG.23981.4	coding	0.246	0	1800	2693
MSTRG.23981.5	coding	0.121	102	1710	2597
MSTRG.23981.6	coding	0.26	0	4851	5735
MSTRG.23981.7	coding	0.254	573	5385	6147
MSTRG.23981.8	coding	0.351	2040	5808	8219
MSTRG.23983.4	coding	0.458	1896	3009	3240
MSTRG.23983.3	coding	0.459	1842	3288	3519
MSTRG.23998.5	coding	0.222	78	4509	4665
MSTRG.23998.6	coding	0.219	285	4740	4896
MSTRG.23998.7	coding	0.221	171	4611	4767
MSTRG.23999.11	noncoding	-0.04096	1368	1461	2041
MSTRG.24000.2	noncoding	-0.0540672	0	459	775
MSTRG.24004.1	noncoding	-0.090112	663	672	1019
MSTRG.23986.1	noncoding	-0.135168	0	156	1003
MSTRG.23986.2	noncoding	-0.1376256	0	141	498
MSTRG.23987.2	noncoding	-0.0741376	2367	2751	2905
MSTRG.23988.1	noncoding	-0.036864	2385	2448	4176
MSTRG.23988.2	coding	0.11	489	720	13815
MSTRG.23989.1	noncoding	-0.032768	1593	1749	3918
MSTRG.23988.3	coding	0.011	1833	2067	5680
MSTRG.23988.4	coding	0.116	3813	4056	11152
MSTRG.23990.1	coding	0.046	2376	2955	3808
MSTRG.23990.2	noncoding	-0.0397312	5076	5253	7354
MSTRG.23990.3	coding	0.074	5562	5775	9006
MSTRG.23991.1	coding	0.055	1038	1581	6206
MSTRG.23992.1	coding	0.109	828	1239	1559
MSTRG.23984.1	noncoding	-0.0106496	2331	2484	3352
MSTRG.23995.1	coding	0.107	0	2946	3919
MSTRG.23995.4	coding	0.066	786	1041	2296
MSTRG.23996.4	noncoding	-0.0475136	78	297	764
MSTRG.23997.1	noncoding	-0.004096	414	558	1196
MSTRG.24007.1	noncoding	-0.0458752	60	354	1015
MSTRG.24020.1	coding	0.156	144	5427	6384
MSTRG.24020.3	noncoding	-0.0028672	987	1140	1718
MSTRG.24020.4	noncoding	-0.0028672	987	1140	1586
MSTRG.24023.1	noncoding	-0.1609728	615	642	1156

MSTRG.24022.3	noncoding	-0.0180224	1797	1908	3388
MSTRG.24026.3	coding	0.105	1389	2328	5238
MSTRG.24026.1	coding	0.156	0	1203	1633
MSTRG.24026.4	coding	0.1	177	870	1284
MSTRG.24027.1	noncoding	-0.0438272	2247	2724	2879
MSTRG.24011.2	coding	0.054	0	3369	5687
MSTRG.24013.3	coding	0.14	282	2904	3883
MSTRG.24013.5	coding	0.16	282	3090	3782
MSTRG.24014.1	noncoding	-0.0004096	0	0	230
MSTRG.24015.1	noncoding	-0.0094208	5586	5742	6974
MSTRG.24016.2	coding	0.041	4227	4989	10263
MSTRG.24017.3	coding	0.255	0	1782	2321
MSTRG.24019.1	coding	0.124	81	2007	6849
MSTRG.24019.2	coding	0.036	597	720	1145
MSTRG.24031.2	coding	0.327	0	477	757
MSTRG.24035.1	coding	0.098	0	4719	6824
MSTRG.24035.3	noncoding	-0.08192	9	96	675
MSTRG.24036.1	noncoding	-0.086016	801	810	2215
MSTRG.24037.1	noncoding	-0.028672	516	618	1004
MSTRG.24049.1	coding	0.108	168	2400	3001
MSTRG.24049.2	coding	0.108	135	2427	3019
MSTRG.24049.8	coding	0.104	366	2610	3211
MSTRG.24049.9	coding	0.113	12	2316	2916
MSTRG.24049.10	coding	0.103	30	2283	2876
MSTRG.24049.12	coding	0.097	3237	4593	5192
MSTRG.24050.2	coding	0.379	144	741	1367
MSTRG.24050.3	coding	0.343	186	924	1570
MSTRG.24051.1	noncoding	-0.0585728	0	408	1062
MSTRG.24041.2	coding	0.319	0	1728	2404
MSTRG.24057.3	coding	0.185	48	1359	2033
MSTRG.24057.4	coding	0.185	612	1923	2660
MSTRG.24058.1	noncoding	-0.069632	1254	1275	1958
MSTRG.24058.2	noncoding	-0.053248	48	132	471
MSTRG.24062.1	coding	0.171	0	4032	6577
MSTRG.24062.5	coding	0.177	0	3801	4491
MSTRG.24062.6	coding	0.177	0	3798	4488
MSTRG.24062.8	coding	0.166	432	4353	4904
MSTRG.24062.9	coding	0.165	309	4239	4789
MSTRG.24059.1	coding	0.113	1515	1701	2434
MSTRG.24059.3	coding	0.113	1029	1215	1918
MSTRG.24059.4	coding	0.113	1077	1263	1822
MSTRG.24063.1	noncoding	-0.0937984	174	738	2051
MSTRG.24063.2	noncoding	-0.0954368	174	741	2054
MSTRG.24063.3	noncoding	-0.1028096	0	513	1976
MSTRG.24063.6	noncoding	-0.098304	537	909	2022
MSTRG.24071.2	coding	0.14	18	432	645
MSTRG.24071.1	coding	0.193	0	438	1028
MSTRG.24081.1	coding	0.444	999	1905	3311
MSTRG.24081.2	coding	0.445	999	1905	3311
MSTRG.24081.9	coding	0.44	981	1863	2029
MSTRG.24081.5	coding	0.442	999	1905	2070
MSTRG.24081.7	coding	0.208	258	2445	2611
MSTRG.24081.10	coding	0.44	954	1863	2021
MSTRG.24075.2	coding	0.489	0	369	1090

MSTRG.24082.1	coding	0.265	123	2865	5438
MSTRG.24082.4	coding	0.267	132	2784	3317
MSTRG.24082.3	coding	0.263	711	3408	3941
MSTRG.24086.1	coding	0.551	0	2682	3185
MSTRG.24093.3	coding	0.314	2190	2697	4143
MSTRG.24095.3	coding	0.38	1194	4125	4588
MSTRG.24095.2	coding	0.31	0	3813	4275
MSTRG.24098.3	coding	0.072	0	1392	1855
MSTRG.24098.4	coding	0.117	0	1038	1501
MSTRG.24099.1	noncoding	-0.0196608	549	585	867
MSTRG.24102.1	noncoding	-0.126976	534	579	881
MSTRG.24104.1	noncoding	-0.0094208	1245	1401	1590
MSTRG.24105.1	noncoding	-0.0176128	297	432	1049
MSTRG.24105.2	noncoding	-0.057344	228	270	532
MSTRG.24107.1	noncoding	-0.0004096	408	516	1110
MSTRG.24108.1	noncoding	-0.0004096	0	0	278
MSTRG.24111.2	coding	0.561	0	465	5173
MSTRG.24112.1	noncoding	-0.0172032	225	384	1533
MSTRG.24113.3	coding	0.436	0	696	1253
MSTRG.24114.2	coding	0.234	555	1500	3298
MSTRG.24115.1	coding	0.547	0	1119	2511
MSTRG.24115.3	coding	0.562	0	1071	1225
MSTRG.24117.2	coding	0.268	207	1191	2835
MSTRG.24117.3	coding	0.248	57	1356	2967
MSTRG.24125.1	noncoding	-0.055296	15	126	1231
MSTRG.24125.2	noncoding	-0.0004096	0	0	347
MSTRG.24123.1	coding	0.293	0	1122	2327
MSTRG.24127.1	coding	0.278	2013	3069	5831
MSTRG.24134.2	coding	0.144	399	1143	1487
MSTRG.24135.4	coding	0.037	804	1482	3663
MSTRG.24135.2	coding	0.043	0	2079	3207
MSTRG.24135.6	coding	0.037	1419	2097	2485
MSTRG.24136.8	coding	0.197	861	1197	1744
MSTRG.24136.9	coding	0.225	0	435	982
MSTRG.24139.1	noncoding	-0.026624	471	687	1321
MSTRG.24140.1	noncoding	-0.0090112	135	216	811
MSTRG.24147.2	coding	0.585	0	1785	7718
MSTRG.24173.1	coding	0.081	4383	9267	12993
MSTRG.24173.3	coding	0.076	0	8706	9716
MSTRG.24173.4	coding	0.073	1122	8637	12315
MSTRG.24173.2	coding	0.076	6	8895	12572
MSTRG.24173.6	coding	0.079	0	8703	12023
MSTRG.24173.7	coding	0.077	0	8766	9660
MSTRG.24173.5	coding	0.076	0	8868	9846
MSTRG.24173.10	coding	0.074	2727	10647	11525
MSTRG.24173.8	coding	0.076	69	8955	12586
MSTRG.24173.9	coding	0.077	69	8895	9774
MSTRG.24173.13	coding	0.076	0	8736	12042
MSTRG.24173.14	coding	0.073	714	8229	11534
MSTRG.24173.11	coding	0.078	0	8805	12108
MSTRG.24173.12	coding	0.076	0	8865	12168
MSTRG.24173.15	coding	0.071	1026	8505	12135
MSTRG.24173.16	coding	0.079	0	8496	9369
MSTRG.24173.17	coding	0.079	0	8556	8785

MSTRG.24175.1	noncoding	-0.0049152	246	393	763
MSTRG.24148.2	noncoding	-0.06144	2406	2556	2711
MSTRG.24152.1	noncoding	-0.0196608	312	513	1155
MSTRG.24162.1	noncoding	-0.106496	3	33	1183
MSTRG.24163.1	noncoding	-0.004096	4092	4179	4900
MSTRG.24164.1	noncoding	-0.06144	348	774	1064
MSTRG.24163.2	coding	0.064	1086	2448	3222
MSTRG.24163.4	noncoding	-0.0790528	13914	14310	15155
MSTRG.24163.5	noncoding	-0.0815104	210	630	1476
MSTRG.24163.6	noncoding	-0.004096	609	696	2223
MSTRG.24158.1	coding	0.474	144	1185	2160
MSTRG.24153.1	coding	0.419	0	732	900
MSTRG.24168.1	coding	0.081	60	690	874
MSTRG.24171.1	noncoding	-0.0323584	444	609	1898
MSTRG.24172.1	coding	0.296	0	6636	11485
MSTRG.24172.2	coding	0.297	0	6651	6808
MSTRG.24184.1	coding	0.164	231	723	1301
MSTRG.24184.3	noncoding	-0.0458752	561	783	938
MSTRG.24184.4	noncoding	-0.0815104	0	246	778
MSTRG.24185.3	coding	0.395	222	2199	2908
MSTRG.24187.1	coding	0.342	750	1569	1982
MSTRG.24187.2	coding	0.342	333	1152	1556
MSTRG.24187.3	coding	0.325	450	1167	1555
MSTRG.24187.4	coding	0.342	450	1269	1589
MSTRG.24187.5	coding	0.301	450	984	1350
MSTRG.24183.1	noncoding	-0.012288	306	342	497
MSTRG.24190.6	coding	0.295	4416	5220	6681
MSTRG.24195.1	noncoding	-0.0167936	129	165	497
MSTRG.24197.1	coding	0.094	0	210	3126
MSTRG.24198.1	coding	0.147	213	2880	12138
MSTRG.24198.2	coding	0.147	2385	5052	9187
MSTRG.24201.3	coding	0.461	222	2355	3323
MSTRG.24204.4	coding	0.512	0	1587	2528
MSTRG.24209.1	coding	0.148	3549	4662	5332
MSTRG.24209.3	coding	0.093	330	4341	5011
MSTRG.24209.4	coding	0.148	2751	3864	4532
MSTRG.24211.2	coding	0.126	0	1569	5590
MSTRG.24211.3	coding	0.044	2682	3681	6853
MSTRG.24212.1	noncoding	-0.0004096	0	0	2211
MSTRG.24207.5	coding	0.102	0	372	2783
MSTRG.24207.4	coding	0.104	0	423	3975
MSTRG.24215.1	noncoding	-0.0126976	1437	1659	2081
MSTRG.24214.3	coding	0.461	102	2529	4974
MSTRG.24214.4	coding	0.453	69	2160	4606
MSTRG.24216.1	coding	0.324	0	474	2303
MSTRG.24222.1	noncoding	-0.016384	735	840	1101
MSTRG.24223.1	noncoding	-0.0483328	2625	2787	4498
MSTRG.24224.1	coding	0.337	0	921	5024
MSTRG.24225.2	coding	0.405	216	3399	3607
MSTRG.24228.5	coding	0.328	0	711	1958
MSTRG.24228.6	coding	0.342	0	642	1887
MSTRG.24228.7	coding	0.346	0	411	566
MSTRG.24229.7	coding	0.35	285	2586	3441
MSTRG.24229.10	coding	0.317	201	573	2573

MSTRG.24218.3	coding	0.283	261	633	977
MSTRG.24230.2	coding	0.213	0	735	1731
MSTRG.24232.3	coding	0.191	180	615	9934
MSTRG.24233.3	coding	0.549	0	3390	4145
MSTRG.24236.3	coding	0.364	273	801	2182
MSTRG.24252.5	coding	0.47	1965	3429	4853
MSTRG.24252.6	coding	0.355	1344	5526	5721
MSTRG.24252.7	coding	0.27	105	426	1148
MSTRG.24252.8	coding	0.288	0	2856	6677
MSTRG.24252.9	coding	0.269	54	2916	6407
MSTRG.24250.1	coding	0.396	276	2064	4991
MSTRG.24250.3	coding	0.402	261	1173	4125
MSTRG.24250.5	coding	0.402	660	1572	4482
MSTRG.24250.8	coding	0.394	0	1653	4562
MSTRG.24250.4	coding	0.391	96	1758	4710
MSTRG.24250.2	coding	0.391	198	1860	4814
MSTRG.24250.7	coding	0.389	207	1827	4781
MSTRG.24257.5	noncoding	-0.0405504	0	528	4079
MSTRG.24257.4	coding	0.066	480	1647	5356
MSTRG.24257.3	coding	0.066	480	1647	5634
MSTRG.24258.5	coding	0.603	3	3996	5359
MSTRG.24258.4	coding	0.603	1497	5484	6847
MSTRG.24260.1	coding	0.271	0	582	737
MSTRG.24262.1	noncoding	-0.04096	24	99	386
MSTRG.24265.2	noncoding	-0.0831488	816	825	1476
MSTRG.24265.3	noncoding	-0.0831488	816	825	1401
MSTRG.24265.5	noncoding	-0.0831488	768	777	1409
MSTRG.24265.6	noncoding	-0.0831488	762	771	1261
MSTRG.24268.2	coding	0.276	129	1212	1682
MSTRG.24269.3	noncoding	-0.0200704	504	663	912
MSTRG.24269.2	coding	0.287	0	804	959
MSTRG.24273.1	coding	0.168	0	999	5113
MSTRG.24275.2	coding	0.404	0	1770	3846
MSTRG.24276.2	coding	0.55	0	3045	4376
MSTRG.24276.3	coding	0.562	0	2940	3807
MSTRG.24285.2	coding	0.334	498	5598	8166
MSTRG.24285.3	coding	0.365	2466	6015	8568
MSTRG.24286.2	noncoding	-0.038912	60	447	3504
MSTRG.24286.3	coding	0.198	30	720	5319
MSTRG.24288.2	coding	0.118	756	948	1414
MSTRG.24288.3	noncoding	-0.0131072	3153	3258	8437
MSTRG.24288.4	noncoding	-0.008192	9087	9192	12506
MSTRG.24283.3	coding	0.239	12	429	806
MSTRG.24283.5	coding	0.372	0	2313	3359
MSTRG.24283.6	coding	0.472	0	1776	1930
MSTRG.24282.2	coding	0.248	324	708	865
MSTRG.24289.3	coding	0.44	114	789	1646
MSTRG.24289.2	coding	0.222	225	1704	2562
MSTRG.24290.3	coding	0.328	66	1053	1603
MSTRG.24291.1	coding	0.175	96	909	1242
MSTRG.24296.1	noncoding	-0.1277952	102	504	3068
MSTRG.24297.2	noncoding	-0.0446464	360	660	1142
MSTRG.24297.1	coding	0.163	876	3471	4959
MSTRG.24297.3	coding	0.158	870	3555	5045

MSTRG.24297.6	coding	0.163	762	3357	4801
MSTRG.24297.9	coding	0.158	3078	5763	7251
MSTRG.24295.1	noncoding	-0.18432	606	654	809
MSTRG.24302.1	noncoding	-0.0135168	549	684	1210
MSTRG.24303.1	coding	0.446	132	2313	5891
MSTRG.24313.2	coding	0.179	840	3189	5874
MSTRG.24315.1	noncoding	-0.075776	4989	5175	5331
MSTRG.24314.2	coding	0.087	210	1626	3762
MSTRG.24318.1	coding	0.325	6	3693	5751
MSTRG.24318.2	coding	0.328	6	3621	5679
MSTRG.24318.3	coding	0.315	438	3432	5490
MSTRG.24318.4	coding	0.315	1509	4494	6554
MSTRG.24306.2	coding	0.359	675	2364	2535
MSTRG.24306.3	coding	0.435	0	1626	1796
MSTRG.24307.1	coding	0.41	0	447	621
MSTRG.24307.2	coding	0.357	534	1023	1361
MSTRG.24307.3	coding	0.353	414	942	1115
MSTRG.24307.4	coding	0.43	0	537	719
MSTRG.24309.1	coding	0.319	0	978	1573
MSTRG.24305.1	noncoding	-0.0958464	0	951	1498
MSTRG.24304.4	coding	0.378	48	633	846
MSTRG.24328.2	coding	0.458	270	3060	4983
MSTRG.24328.5	coding	0.123	3741	3963	4502
MSTRG.24328.7	noncoding	-0.0585728	4503	5139	6151
MSTRG.24328.8	coding	0.425	4035	7149	9078
MSTRG.24336.3	noncoding	-0.0815104	102	468	647
MSTRG.24338.6	coding	0.504	0	2355	2519
MSTRG.24339.1	coding	0.406	3	1890	2143
MSTRG.24340.1	noncoding	-0.0192512	228	735	955
MSTRG.24341.3	coding	0.359	0	2178	2848
MSTRG.24344.1	noncoding	-0.0151552	54	216	749
MSTRG.24346.1	coding	0.198	9	852	1167
MSTRG.24346.6	coding	0.203	0	930	1276
MSTRG.24346.2	coding	0.239	0	849	1237
MSTRG.24346.3	coding	0.195	456	1344	1733
MSTRG.24346.5	coding	0.168	462	1464	1853
MSTRG.24346.4	coding	0.183	462	1410	1809
MSTRG.24346.7	coding	0.184	441	1404	1802
MSTRG.24348.3	coding	0.237	291	909	1596
MSTRG.24350.3	coding	0.457	0	2820	5157
MSTRG.24350.4	coding	0.383	2655	5586	6076
MSTRG.24350.5	coding	0.44	0	3660	4777
MSTRG.24351.7	coding	0.198	0	1452	2538
MSTRG.24351.5	coding	0.192	0	1548	3022
MSTRG.24351.4	coding	0.198	0	1452	3165
MSTRG.24351.1	coding	0.198	0	1452	3218
MSTRG.24351.3	coding	0.249	0	4104	4259
MSTRG.24351.2	coding	0.357	2205	4539	4694
MSTRG.24352.1	noncoding	-0.0602112	1341	1872	2865
MSTRG.24356.3	noncoding	-0.1150976	261	918	1087
MSTRG.24357.1	coding	0.309	684	2169	2327
MSTRG.24357.3	coding	0.262	951	2424	2580
MSTRG.24358.5	coding	0.109	6	3789	3950
MSTRG.24364.1	coding	0.311	0	1071	1663

MSTRG.24365.4	coding	0.424	276	6270	6521
MSTRG.24365.8	coding	0.439	276	5892	6458
MSTRG.24365.9	coding	0.449	276	5787	6353
MSTRG.24365.10	coding	0.425	276	6279	6558
MSTRG.24365.11	coding	0.439	276	5892	6485
MSTRG.24365.6	coding	0.425	276	6300	6579
MSTRG.24365.3	coding	0.438	276	5913	6575
MSTRG.24365.5	coding	0.439	276	5892	6554
MSTRG.24365.1	coding	0.438	276	5892	6388
MSTRG.24365.12	coding	0.426	276	6207	6491
MSTRG.24365.13	coding	0.423	273	6267	6519
MSTRG.24365.14	coding	0.436	273	5997	6563
MSTRG.24366.1	noncoding	-0.0311296	201	228	408
MSTRG.24367.1	noncoding	-0.008192	0	66	235
MSTRG.24374.2	coding	0.3	564	1818	2439
MSTRG.24376.1	coding	0.202	363	963	1689
MSTRG.24376.2	coding	0.16	0	585	1308
MSTRG.24376.5	coding	0.223	336	846	1571
MSTRG.24376.6	coding	0.212	1350	1668	2394
MSTRG.24378.4	coding	0.198	48	570	4574
MSTRG.24378.5	coding	0.185	129	618	947
MSTRG.24379.1	coding	0.155	843	1716	1887
MSTRG.24379.2	coding	0.155	516	1389	1561
MSTRG.24380.1	noncoding	-0.0249856	249	450	858
MSTRG.24359.2	coding	0.41	0	765	4222
MSTRG.24359.3	coding	0.382	645	1494	4849
MSTRG.24361.2	coding	0.265	765	1623	1779
MSTRG.24382.5	coding	0.376	1755	6765	7775
MSTRG.24387.6	coding	0.204	249	504	679
MSTRG.24387.1	noncoding	-0.0884736	153	411	587
MSTRG.24387.3	coding	0.274	378	663	839
MSTRG.24387.4	noncoding	-0.1159168	0	198	400
MSTRG.24389.1	noncoding	-0.053248	2007	2085	2813
MSTRG.24387.7	noncoding	-0.057344	4125	4293	5430
MSTRG.24390.8	coding	0.421	9	5325	5982
MSTRG.24384.3	coding	0.389	123	3531	4252
MSTRG.24384.2	coding	0.397	3756	7446	8170
MSTRG.24397.1	noncoding	-0.0229376	0	114	2620
MSTRG.24398.1	coding	0.131	90	366	667
MSTRG.24397.3	noncoding	-0.028672	102	240	2533
MSTRG.24398.4	coding	0.381	0	405	706
MSTRG.24400.1	coding	0.361	8502	10941	11455
MSTRG.24405.1	noncoding	-0.0421888	273	774	2712
MSTRG.24405.3	coding	0.163	141	1044	2703
MSTRG.24405.4	noncoding	-0.073728	141	492	2824
MSTRG.24406.3	coding	0.247	120	591	758
MSTRG.24408.1	noncoding	-0.075776	1026	1419	1576
MSTRG.24408.2	noncoding	-0.0073728	420	564	1067
MSTRG.24410.3	coding	0.182	0	630	6512
MSTRG.24412.1	coding	0.574	618	4713	6272
MSTRG.24412.2	coding	0.466	0	6378	7935
MSTRG.24413.1	noncoding	-0.0679936	663	879	3676
MSTRG.24401.1	noncoding	-0.0290816	2577	2742	3282
MSTRG.24404.2	noncoding	-0.0647168	2382	3570	3862

MSTRG.24415.7	coding	0.133	42	483	913
MSTRG.24415.3	coding	0.219	189	828	1065
MSTRG.24415.5	coding	0.235	408	774	1010
MSTRG.24415.4	coding	0.244	189	546	784
MSTRG.24415.6	coding	0.231	108	753	990
MSTRG.24415.2	coding	0.27	0	414	652
MSTRG.24417.1	coding	0.442	0	1617	4684
MSTRG.24417.6	coding	0.424	1701	3012	9374
MSTRG.24417.5	coding	0.444	0	1602	7965
MSTRG.24419.1	noncoding	-0.0897024	1143	1380	3555
MSTRG.24419.2	noncoding	-0.04096	2718	2838	7764
MSTRG.24420.1	noncoding	-0.0331776	423	678	1604
MSTRG.24421.1	noncoding	-0.0843776	0	1065	2958
MSTRG.24424.2	coding	0.091	1767	2280	4290
MSTRG.24436.1	coding	0.606	0	597	1401
MSTRG.24442.2	coding	0.426	330	13326	13692
MSTRG.24443.1	coding	0.142	114	1032	3685
MSTRG.24429.7	noncoding	-0.118784	102	111	538
MSTRG.24429.5	noncoding	-0.0233472	6	330	1302
MSTRG.24429.6	noncoding	-0.0233472	6	330	1120
MSTRG.24429.1	noncoding	-0.0233472	9	333	970
MSTRG.24429.2	noncoding	-0.0233472	9	333	1353
MSTRG.24429.3	noncoding	-0.0233472	9	333	1335
MSTRG.24429.4	noncoding	-0.0233472	9	333	1213
MSTRG.24429.8	noncoding	-0.0233472	9	333	803
MSTRG.24454.1	noncoding	-0.036864	855	954	1674
MSTRG.24456.2	coding	0.219	111	699	1923
MSTRG.24458.1	noncoding	-0.036864	195	273	433
MSTRG.24460.1	noncoding	-0.2281472	657	1068	1922
MSTRG.24460.2	noncoding	-0.1552384	96	978	1831
MSTRG.24460.3	noncoding	-0.2187264	660	1032	1630
MSTRG.24460.4	noncoding	-0.1605632	657	900	1498
MSTRG.24460.5	coding	0.282	96	672	2830
MSTRG.24460.6	coding	0.202	96	429	3844
MSTRG.24461.1	noncoding	-0.086016	270	354	924
MSTRG.24462.1	coding	0.092	288	600	2509
MSTRG.24464.3	coding	0.272	114	2037	3428
MSTRG.24464.2	coding	0.271	147	1866	3257
MSTRG.24464.1	coding	0.244	90	4065	4248
MSTRG.24465.1	coding	0.098	831	1002	4219
MSTRG.24463.1	coding	0.02	708	2115	6640
MSTRG.24468.1	coding	0.179	393	2619	3121
MSTRG.24468.2	coding	0.186	0	2181	5078
MSTRG.24484.2	coding	0.544	0	684	916
MSTRG.24484.3	coding	0.498	0	456	688
MSTRG.24484.4	coding	0.539	0	684	916
MSTRG.24484.5	coding	0.532	0	684	916
MSTRG.24484.6	coding	0.426	174	1023	1253
MSTRG.24484.7	coding	0.551	0	684	916
MSTRG.24485.3	coding	0.227	249	528	919
MSTRG.24485.2	noncoding	-0.0626688	249	489	887
MSTRG.24486.1	coding	0.303	144	561	839
MSTRG.24487.10	coding	0.279	261	7554	8836
MSTRG.24487.3	coding	0.281	153	7422	8706

MSTRG.24487.4	coding	0.281	153	7437	8721
MSTRG.24487.5	coding	0.283	153	7335	8619
MSTRG.24487.6	coding	0.276	153	7314	8598
MSTRG.24487.7	coding	0.277	153	7260	8544
MSTRG.24487.9	coding	0.28	153	7398	8682
MSTRG.24487.8	coding	0.281	153	7386	10294
MSTRG.24487.11	coding	0.308	1740	7446	10576
MSTRG.24487.17	coding	0.28	216	7482	8764
MSTRG.24487.18	coding	0.281	216	7485	8767
MSTRG.24487.19	coding	0.279	216	7518	8800
MSTRG.24488.7	coding	0.265	0	843	1930
MSTRG.24488.10	coding	0.167	1272	1845	2092
MSTRG.24488.8	coding	0.359	0	966	1215
MSTRG.24488.14	noncoding	-0.032768	858	936	1112
MSTRG.24489.2	coding	0.228	0	330	1493
MSTRG.24472.3	coding	0.421	282	3492	6447
MSTRG.24472.5	coding	0.466	327	2643	4397
MSTRG.24477.1	coding	0.148	678	3042	3205
MSTRG.24477.10	coding	0.165	888	2523	2684
MSTRG.24477.8	coding	0.191	0	2595	2750
MSTRG.24477.7	coding	0.182	828	2937	4183
MSTRG.24477.9	coding	0.161	828	2604	4079
MSTRG.24478.1	coding	0.375	0	2181	3018
MSTRG.24495.3	coding	0.47	648	2781	2987
MSTRG.24510.1	coding	0.541	294	4575	4751
MSTRG.24503.1	noncoding	-0.0380928	687	1023	1178
MSTRG.24504.1	coding	0.547	0	225	382
MSTRG.24514.2	coding	0.484	528	933	1344
MSTRG.24516.2	noncoding	-0.036864	90	255	410
MSTRG.24521.6	noncoding	-0.0282624	2262	2679	3679
MSTRG.24523.3	coding	0.357	0	3639	3893
MSTRG.24523.4	coding	0.364	4704	8415	8669
MSTRG.24520.2	coding	0.376	0	750	1025
MSTRG.24520.3	coding	0.444	0	648	921
MSTRG.24520.4	coding	0.373	1047	1662	1937
MSTRG.24520.6	coding	0.378	288	879	1153
MSTRG.24528.4	coding	0.258	444	1074	1447
MSTRG.24528.3	coding	0.268	177	843	1215
MSTRG.24528.2	coding	0.262	1809	2469	2841
MSTRG.24528.5	coding	0.276	192	912	1285
MSTRG.24528.6	noncoding	-0.1314816	0	270	952
MSTRG.24531.2	coding	0.413	0	426	4620
MSTRG.24539.3	coding	0.417	318	1245	1604
MSTRG.24539.4	coding	0.419	6375	7335	7691
MSTRG.24541.2	coding	0.483	153	720	1054
MSTRG.24543.1	noncoding	-0.016384	996	1074	1560
MSTRG.24548.1	coding	0.072	3630	3855	10378
MSTRG.24548.3	coding	0.064	2391	2613	5713
MSTRG.24554.1	coding	0.171	0	3972	5364
MSTRG.24554.4	coding	0.778	0	321	4907
MSTRG.24554.7	coding	0.148	390	1968	4584
MSTRG.24554.9	coding	0.135	309	2394	4115
MSTRG.24555.1	noncoding	-0.0004096	0	0	1097
MSTRG.24553.1	coding	0.466	0	1848	3318

MSTRG.24557.1	coding	0.093	492	1593	3393
MSTRG.24557.3	noncoding	-0.0700416	1707	2016	4314
MSTRG.24557.4	coding	0.078	12	1254	2025
MSTRG.24557.5	coding	0.096	12	1359	1586
MSTRG.24566.2	coding	0.304	0	2805	2958
MSTRG.24566.3	coding	0.264	51	3102	3258
MSTRG.24568.2	coding	0.441	537	1167	2906
MSTRG.24571.1	coding	0.478	0	2547	3908
MSTRG.24574.4	noncoding	-0.0253952	450	618	915
MSTRG.24574.5	noncoding	-0.090112	15	228	525
MSTRG.24574.3	noncoding	-0.0700416	0	171	469
MSTRG.24574.2	noncoding	-0.0253952	0	147	446
MSTRG.24574.6	noncoding	-0.002048	549	651	944
MSTRG.24575.1	coding	0.339	27	630	821
MSTRG.24580.2	noncoding	-0.1060864	57	432	1528
MSTRG.24584.2	coding	0.191	0	1719	2921
MSTRG.24584.3	coding	0.144	405	1659	3022
MSTRG.24585.2	noncoding	-0.1097728	0	504	658
MSTRG.24586.1	coding	0.246	0	852	1154
MSTRG.24588.3	coding	0.445	207	1035	1192
MSTRG.24589.5	coding	0.224	87	1338	3608
MSTRG.24589.6	coding	0.228	87	1350	3688
MSTRG.24589.4	coding	0.228	87	1350	4507
MSTRG.24589.7	coding	0.248	87	1257	9977
MSTRG.24591.10	coding	0.537	348	1143	1434
MSTRG.24591.3	coding	0.368	216	1518	1673
MSTRG.24591.4	coding	0.439	321	1362	2017
MSTRG.24591.5	coding	0.439	348	1371	2027
MSTRG.24591.6	coding	0.439	207	1248	1905
MSTRG.24591.7	coding	0.439	459	1482	2139
MSTRG.24591.8	coding	0.429	216	1263	1555
MSTRG.24591.9	coding	0.534	321	1134	1424
MSTRG.24594.1	noncoding	-0.0434176	372	678	3895
MSTRG.24594.2	noncoding	-0.0434176	372	678	2048
MSTRG.24595.3	coding	0.271	129	1713	3162
MSTRG.24595.4	coding	0.291	891	2043	2199
MSTRG.24597.2	coding	0.418	285	2217	8805
MSTRG.24599.3	coding	0.369	0	984	3409
MSTRG.24599.4	coding	0.299	0	1371	2336
MSTRG.24599.5	coding	0.356	0	1080	3159
MSTRG.24604.2	noncoding	-0.0274432	156	441	4105
MSTRG.24604.1	coding	0.208	7287	9261	10714
MSTRG.24604.3	coding	0.222	7287	9774	11271
MSTRG.24602.1	noncoding	-0.06144	237	327	622
MSTRG.24609.7	coding	0.429	768	2982	4262
MSTRG.24619.1	noncoding	-0.0688128	195	219	584
MSTRG.24620.2	coding	0.108	87	2508	3870
MSTRG.24620.3	coding	0.113	354	2637	3908
MSTRG.24620.4	coding	0.113	501	2784	3998
MSTRG.24620.5	coding	0.116	168	2181	2444
MSTRG.24621.1	coding	0.264	0	708	1243
MSTRG.24623.3	coding	0.502	1755	3915	5450
MSTRG.24625.4	coding	0.396	12	1224	4400
MSTRG.24625.5	coding	0.396	6	1218	4459

MSTRG.24629.1	coding	0.456	0	1353	4852
MSTRG.24629.3	coding	0.456	0	1353	4705
MSTRG.24630.1	coding	0.255	885	2115	3890
MSTRG.24633.1	coding	0.462	0	192	498
MSTRG.24637.1	coding	0.487	6	900	3862
MSTRG.24637.3	coding	0.493	177	819	3779
MSTRG.24637.2	coding	0.5	198	897	3859
MSTRG.24637.4	coding	0.497	153	807	3769
MSTRG.24640.2	coding	0.367	486	1068	1258
MSTRG.24648.1	noncoding	-0.049152	2721	2832	4587
MSTRG.24649.1	noncoding	-0.0004096	0	0	1468
MSTRG.24651.1	coding	0.044	345	1719	2821
MSTRG.24651.2	coding	0.061	345	1791	2721
MSTRG.24642.6	coding	0.153	0	4398	8815
MSTRG.24642.7	coding	0.153	0	3651	8397
MSTRG.24644.1	coding	0.438	75	1497	4388
MSTRG.24656.3	coding	0.221	603	1989	7706
MSTRG.24659.3	coding	0.433	0	528	1156
MSTRG.24659.4	coding	0.346	216	780	1296
MSTRG.24662.2	coding	0.443	294	3273	3752
MSTRG.24663.2	coding	0.457	1104	2379	5656
MSTRG.24664.2	coding	0.395	66	2118	2426
MSTRG.24666.3	coding	0.076	51	609	1845
MSTRG.24661.3	coding	0.247	0	2001	3891
MSTRG.24661.4	coding	0.247	0	2001	3907
MSTRG.24670.1	noncoding	-0.0376832	306	636	862
MSTRG.24669.3	coding	0.463	45	1338	2810
MSTRG.24669.6	coding	0.293	42	417	968
MSTRG.24673.2	coding	0.107	135	4881	5594
MSTRG.24672.1	noncoding	-0.0270336	345	903	1086
MSTRG.24676.1	noncoding	-0.024576	330	462	617
MSTRG.24676.2	noncoding	-0.032768	354	483	637
MSTRG.24677.1	noncoding	-0.032768	267	336	980
MSTRG.24693.3	coding	0.312	177	1332	1713
MSTRG.24696.1	coding	0.291	0	510	2203
MSTRG.24696.2	noncoding	-0.1196032	0	414	2161
MSTRG.24697.2	coding	0.439	162	3432	4468
MSTRG.24697.5	coding	0.454	252	3441	3955
MSTRG.24724.1	coding	0.691	0	1275	3738
MSTRG.24724.6	coding	0.696	0	1266	3109
MSTRG.24724.5	coding	0.694	0	1272	3111
MSTRG.24724.7	coding	0.691	0	1275	3182
MSTRG.24724.2	coding	0.691	0	1275	3124
MSTRG.24724.3	coding	0.691	0	1275	3130
MSTRG.24724.4	coding	0.691	0	1275	2535
MSTRG.24725.1	coding	0.227	0	2820	8191
MSTRG.24725.2	coding	0.254	750	3186	7518
MSTRG.24725.3	coding	0.248	750	3078	8208
MSTRG.24725.4	coding	0.401	5037	6795	7078
MSTRG.24725.5	coding	0.254	126	2562	7635
MSTRG.24729.1	coding	0.37	87	2052	2207
MSTRG.24729.2	coding	0.365	336	2358	2513
MSTRG.24729.3	coding	0.3	1170	2199	2356
MSTRG.24729.4	coding	0.346	114	2220	2375

MSTRG.24729.5	coding	0.3	1146	2175	2330
MSTRG.24729.6	coding	0.365	285	2307	2462
MSTRG.24732.1	coding	0.401	3045	3957	4646
MSTRG.24732.2	coding	0.401	3036	3948	4639
MSTRG.24732.4	coding	0.234	666	1680	3510
MSTRG.24735.4	coding	0.369	0	2427	2922
MSTRG.24710.4	coding	0.305	753	2979	5220
MSTRG.24710.5	coding	0.305	342	2568	4761
MSTRG.24706.1	coding	0.233	0	582	870
MSTRG.24711.3	coding	0.328	0	3495	3937
MSTRG.24711.4	coding	0.346	0	2943	3389
MSTRG.24717.2	coding	0.177	48	462	1139
MSTRG.24721.1	coding	0.273	4458	6324	7328
MSTRG.24721.2	coding	0.23	243	1029	3548
MSTRG.24721.4	coding	0.23	357	1143	2586
MSTRG.24722.2	coding	0.327	0	1833	1987
MSTRG.24722.4	coding	0.343	0	2403	2702
MSTRG.24714.2	coding	0.281	381	855	1008
MSTRG.24715.2	coding	0.449	0	1752	3915
MSTRG.24741.2	coding	0.4	165	996	2503
MSTRG.24742.3	coding	0.276	189	591	3568
MSTRG.24743.2	coding	0.044	132	5187	5864
MSTRG.24743.3	coding	0.044	132	5184	5861
MSTRG.24745.1	noncoding	-0.0110592	63	240	1012
MSTRG.24745.2	noncoding	-0.0110592	63	240	993
MSTRG.24745.3	noncoding	-0.0135168	525	693	848
MSTRG.24748.1	coding	0.418	582	1152	1734
MSTRG.24763.1	coding	0.272	0	642	8495
MSTRG.24763.3	coding	0.312	0	528	1175
MSTRG.24768.3	coding	0.49	150	3399	3575
MSTRG.24768.7	coding	0.49	150	3396	3572
MSTRG.24769.1	noncoding	-0.0323584	48	294	838
MSTRG.24772.2	coding	0.316	540	1572	4165
MSTRG.24774.5	coding	0.501	0	2325	3029
MSTRG.24774.6	coding	0.517	126	2259	2961
MSTRG.24784.2	coding	0.412	1965	3702	6499
MSTRG.24784.3	coding	0.496	18	513	1759
MSTRG.24785.3	coding	0.318	630	1698	2167
MSTRG.24788.1	noncoding	-0.049152	3699	4149	4465
MSTRG.24789.1	noncoding	-0.012288	885	924	1458
MSTRG.24792.2	coding	0.178	429	2616	3819
MSTRG.24793.1	noncoding	-0.008192	3	129	284
MSTRG.24793.2	noncoding	-0.016384	0	147	314
MSTRG.24778.1	coding	0.295	255	1341	1943
MSTRG.24782.1	coding	0.437	0	3147	3551
MSTRG.24795.2	coding	0.531	672	1407	2483
MSTRG.24801.1	coding	0.318	0	1035	1316
MSTRG.24801.2	coding	0.383	111	951	1232
MSTRG.24805.2	coding	0.27	48	1125	1400
MSTRG.24807.1	noncoding	-0.073728	0	63	462
MSTRG.24809.1	coding	0.505	153	2913	4476
MSTRG.24810.1	noncoding	-0.0958464	0	423	597
MSTRG.24814.1	coding	0.134	0	7662	8097
MSTRG.24815.2	coding	0.144	0	696	1138

MSTRG.24816.1	coding	0.274	297	696	1141
MSTRG.24816.2	coding	0.286	69	408	879
MSTRG.24817.1	coding	0.44	114	996	1151
MSTRG.24817.2	coding	0.326	450	1308	3135
MSTRG.24820.2	coding	0.252	81	1743	3456
MSTRG.24821.1	noncoding	-0.0475136	0	9	450
MSTRG.24825.1	noncoding	-0.016384	1050	1173	1326
MSTRG.24826.4	coding	0.383	189	1191	1526
MSTRG.24826.6	coding	0.382	273	1293	1629
MSTRG.24826.2	coding	0.383	300	1407	1744
MSTRG.24826.3	coding	0.384	189	1278	1614
MSTRG.24826.5	coding	0.383	273	1380	1717
MSTRG.24826.7	coding	0.382	300	1320	1657
MSTRG.24826.8	coding	0.365	1803	2712	3047
MSTRG.24827.6	coding	0.185	384	3435	4644
MSTRG.24827.7	coding	0.26	339	1809	3014
MSTRG.24827.8	coding	0.258	414	2073	3259
MSTRG.24827.9	coding	0.26	366	1836	3023
MSTRG.24827.10	coding	0.237	306	2157	3282
MSTRG.24827.11	coding	0.301	3558	4746	5862
MSTRG.24828.1	noncoding	-0.0495616	2715	3093	5181
MSTRG.24830.1	noncoding	-0.0524288	411	489	800
MSTRG.24833.1	noncoding	-0.016384	999	1131	1370
MSTRG.24835.1	coding	0.369	108	2145	5471
MSTRG.24836.1	noncoding	-0.1544192	0	99	524
MSTRG.24837.1	coding	0.089	546	2490	2644
MSTRG.24837.2	noncoding	-0.024576	171	261	1256
MSTRG.24837.3	coding	0.097	705	4647	4802
MSTRG.24837.4	coding	0.084	702	4248	4635
MSTRG.24837.5	coding	0.079	69	2031	2188
MSTRG.24841.2	coding	0.136	0	5319	5572
MSTRG.24841.1	coding	0.134	0	5373	5644
MSTRG.24834.3	coding	0.108	213	1080	1802
MSTRG.24844.1	coding	0.119	372	2412	3702
MSTRG.24844.2	coding	0.106	372	2505	3795
MSTRG.24844.3	coding	0.116	0	2154	3444
MSTRG.24844.8	coding	0.127	0	2268	3558
MSTRG.24844.9	coding	0.113	0	2361	3651
MSTRG.24844.10	coding	0.101	150	2040	2832
MSTRG.24846.1	noncoding	-0.0147456	1836	2004	2165
MSTRG.24847.1	noncoding	-0.036864	417	450	606
MSTRG.24842.4	coding	0.149	0	627	932
MSTRG.24850.1	noncoding	-0.0299008	7101	7242	7635
MSTRG.24849.3	coding	0.149	0	159	1613
MSTRG.24849.2	coding	0.288	0	5961	11913
MSTRG.24852.1	noncoding	-0.024576	1503	1566	2157
MSTRG.24853.1	noncoding	-0.0565248	759	786	2406
MSTRG.24854.1	noncoding	-0.0098304	5826	5925	6835
MSTRG.24855.1	noncoding	-0.073728	1188	1278	7315
MSTRG.24856.1	noncoding	-0.032768	153	231	546
MSTRG.24858.2	coding	0.509	429	1245	3011
MSTRG.24858.3	coding	0.509	231	1047	2814
MSTRG.24858.4	noncoding	-0.006144	702	888	1041
MSTRG.24860.3	coding	0.487	3	2298	2452

MSTRG.24861.2	coding	0.456	0	2130	2898
MSTRG.24861.3	coding	0.456	0	2130	2801
MSTRG.24861.4	coding	0.456	0	2130	2870
MSTRG.24861.5	coding	0.456	0	2130	2456
MSTRG.24862.1	coding	0.42	948	2334	2503
MSTRG.24866.1	coding	0.35	9	2184	4554
MSTRG.24866.2	coding	0.341	9	2277	4647
MSTRG.24866.3	coding	0.347	333	2436	4795
MSTRG.24866.7	coding	0.373	756	2223	2804
MSTRG.24867.2	coding	0.37	0	534	1336
MSTRG.24867.4	coding	0.321	0	735	1531
MSTRG.24867.3	coding	0.391	138	1737	2531
MSTRG.24867.5	coding	0.385	264	1932	2684
MSTRG.24867.11	coding	0.381	288	1500	2268
MSTRG.24867.7	coding	0.392	141	1287	2053
MSTRG.24867.8	coding	0.381	732	1944	2711
MSTRG.24878.1	coding	0.382	147	1434	1599
MSTRG.24879.2	coding	0.43	153	1503	2451
MSTRG.24864.1	coding	0.639	366	4206	5744
MSTRG.24865.1	noncoding	-0.0372736	345	531	1096
MSTRG.24865.2	noncoding	-0.0667648	330	585	1003
MSTRG.24869.2	noncoding	-0.0864256	3	204	594
MSTRG.24869.4	noncoding	-0.0630784	3	168	719
MSTRG.24869.3	noncoding	-0.0475136	243	510	663
MSTRG.24871.2	coding	0.525	258	3066	7757
MSTRG.24890.1	noncoding	-0.0663552	63	396	1576
MSTRG.24892.1	coding	0.144	2241	2961	3231
MSTRG.24892.3	coding	0.08	0	411	1823
MSTRG.24892.4	noncoding	-0.032768	498	591	1986
MSTRG.24892.5	coding	0.152	1983	2418	2653
MSTRG.24892.6	coding	0.078	435	1431	1699
MSTRG.24893.3	coding	0.092	390	2490	4483
MSTRG.24885.7	coding	0.145	54	5883	8941
MSTRG.24885.6	coding	0.126	657	7347	11242
MSTRG.24885.5	coding	0.189	93	8838	12732
MSTRG.24885.3	coding	0.189	99	9018	12911
MSTRG.24885.10	coding	0.165	1089	4464	4715
MSTRG.24885.11	coding	0.145	27	5478	5731
MSTRG.24885.8	coding	0.143	531	6330	6583
MSTRG.24885.9	coding	0.192	0	8367	8618
MSTRG.24898.1	coding	0.219	486	1584	2126
MSTRG.24898.2	coding	0.179	3474	4260	8877
MSTRG.24898.3	coding	0.226	486	1443	8633
MSTRG.24898.4	coding	0.215	486	1425	4923
MSTRG.24898.5	coding	0.183	19071	19590	21385
MSTRG.24898.6	coding	0.197	486	1212	2587
MSTRG.24903.1	noncoding	-0.1134592	0	174	546
MSTRG.24913.2	coding	0.433	66	2118	2408
MSTRG.24914.2	noncoding	-0.0442368	618	1347	2124
MSTRG.24911.4	coding	0.241	225	726	1027
MSTRG.24920.1	noncoding	-0.0233472	1572	2199	2961
MSTRG.24922.1	noncoding	-0.024576	138	354	1031
MSTRG.24924.2	coding	0.396	375	1446	2969
MSTRG.24925.1	coding	0.219	0	1248	2589

MSTRG.24925.2	coding	0.265	0	963	2304
MSTRG.24930.1	noncoding	-0.0155648	231	318	707
MSTRG.24932.1	noncoding	-0.0356352	66	237	498
MSTRG.24936.1	coding	0.312	18	1494	2774
MSTRG.24936.5	coding	0.312	15	1494	3619
MSTRG.24936.4	coding	0.282	15	1773	2987
MSTRG.24936.7	coding	0.349	5244	6408	7426
MSTRG.24958.2	coding	0.393	342	1788	2108
MSTRG.24958.1	coding	0.393	93	1542	1909
MSTRG.24959.1	coding	0.474	0	1878	2064
MSTRG.24959.2	coding	0.475	0	1881	2065
MSTRG.24960.1	coding	0.372	0	1215	2421
MSTRG.24960.2	coding	0.401	0	801	1305
MSTRG.24960.4	coding	0.457	0	801	1904
MSTRG.24960.5	coding	0.387	0	1077	1709
MSTRG.24961.1	noncoding	-0.2138112	153	207	363
MSTRG.24962.2	coding	0.337	342	2628	3172
MSTRG.24963.1	noncoding	-0.1253376	96	417	943
MSTRG.24969.4	noncoding	-0.0507904	75	435	590
MSTRG.24971.1	noncoding	-0.0761856	654	1131	1390
MSTRG.24971.2	noncoding	-0.0761856	846	1323	1583
MSTRG.24971.3	noncoding	-0.0761856	825	1302	1584
MSTRG.24971.4	noncoding	-0.0761856	1575	2052	2375
MSTRG.24960.6	coding	0.556	0	1422	2295
MSTRG.24960.7	coding	0.532	801	2217	3091
MSTRG.24944.2	coding	0.341	288	1350	2368
MSTRG.24946.4	coding	0.512	294	2334	2998
MSTRG.24946.3	coding	0.483	0	2544	3208
MSTRG.24946.5	coding	0.504	225	2181	2844
MSTRG.24948.2	coding	0.253	0	510	5015
MSTRG.24954.3	coding	0.4	129	3612	4497
MSTRG.24954.6	coding	0.422	0	3090	4071
MSTRG.24976.3	coding	0.541	198	753	1727
MSTRG.24976.2	coding	0.347	678	3402	6384
MSTRG.24976.4	coding	0.482	225	996	4344
MSTRG.25009.1	coding	0.349	0	585	1824
MSTRG.25010.1	coding	0.308	0	1941	4267
MSTRG.25010.8	coding	0.261	9114	24165	26122
MSTRG.25010.10	coding	0.271	3852	13848	15805
MSTRG.25010.11	coding	0.276	2964	13032	14989
MSTRG.25010.12	coding	0.269	2445	15603	17561
MSTRG.25010.13	coding	0.27	7482	17499	19457
MSTRG.25010.6	coding	0.302	1836	3753	5698
MSTRG.25010.4	coding	0.279	0	3114	5072
MSTRG.25010.7	coding	0.259	0	4611	6570
MSTRG.25010.3	coding	0.281	0	8061	10018
MSTRG.25010.5	coding	0.287	0	11127	13084
MSTRG.25012.1	noncoding	-0.0548864	138	216	626
MSTRG.25010.15	coding	0.295	0	2874	3029
MSTRG.25010.16	coding	0.262	2436	5838	5993
MSTRG.24992.2	coding	0.442	294	3051	3891
MSTRG.24993.1	coding	0.483	0	3597	4227
MSTRG.24993.3	coding	0.461	0	3609	4218
MSTRG.24993.4	coding	0.477	0	3600	4209

MSTRG.24993.5	coding	0.455	0	3672	4260
MSTRG.24986.1	coding	0.376	33	1500	4634
MSTRG.24996.1	noncoding	-0.0421888	2166	2391	5368
MSTRG.25001.2	coding	0.568	0	1290	4738
MSTRG.25001.4	coding	0.497	147	1578	3523
MSTRG.25003.2	coding	0.382	0	516	3322
MSTRG.25006.1	coding	0.402	0	1263	3092
MSTRG.25006.3	noncoding	-0.1675264	0	120	1470
MSTRG.25020.1	coding	0.477	432	3039	6881
MSTRG.25020.4	coding	0.477	90	2697	6540
MSTRG.25020.5	coding	0.377	45	570	805
MSTRG.25021.1	coding	0.259	0	5748	8322
MSTRG.25021.2	coding	0.259	0	5847	8421
MSTRG.25021.3	coding	0.262	0	5631	8140
MSTRG.25027.4	coding	0.596	10377	11514	13261
MSTRG.25027.7	coding	0.555	0	1773	3509
MSTRG.25027.9	coding	0.562	0	1860	3148
MSTRG.25027.8	coding	0.558	0	1788	3010
MSTRG.25028.2	coding	0.394	90	2175	2643
MSTRG.25029.1	noncoding	-0.049152	192	279	644
MSTRG.25031.2	noncoding	-0.0278528	219	972	1261
MSTRG.25031.3	noncoding	-0.0466944	534	882	1172
MSTRG.25031.1	noncoding	-0.0425984	825	1293	1581
MSTRG.25031.4	noncoding	-0.028672	324	795	1080
MSTRG.25033.2	noncoding	-0.0466944	4329	5118	5271
MSTRG.25032.4	coding	0.576	0	288	1791
MSTRG.25039.3	coding	0.287	201	1236	2050
MSTRG.25041.2	noncoding	-0.049152	1170	1236	2329
MSTRG.25038.1	noncoding	-0.069632	108	192	347
MSTRG.25042.1	coding	0.423	846	1635	1789
MSTRG.25045.3	coding	0.495	0	789	17801
MSTRG.25049.1	noncoding	-0.0843776	0	291	946
MSTRG.25050.2	coding	0.299	135	4647	4803
MSTRG.25050.4	coding	0.29	135	4593	4748
MSTRG.25050.5	coding	0.286	135	4434	4588
MSTRG.25051.3	coding	0.381	0	516	671
MSTRG.25051.4	coding	0.429	0	549	705
MSTRG.25051.6	noncoding	-0.0733184	135	372	529
MSTRG.25053.6	coding	0.611	0	2109	3789
MSTRG.25058.1	coding	0.276	210	2427	6310
MSTRG.25058.3	coding	0.234	270	1773	5036
MSTRG.25059.1	coding	0.486	489	3852	4919
MSTRG.25055.1	noncoding	-0.0884736	606	1011	1166
MSTRG.25056.1	coding	0.532	0	1776	2202
MSTRG.25060.3	coding	0.343	69	690	1782
MSTRG.25063.2	noncoding	-0.1056768	918	1233	3017
MSTRG.25063.1	noncoding	-0.1056768	552	867	2085
MSTRG.25064.2	coding	0.444	513	1563	1718
MSTRG.25067.2	coding	0.404	0	264	4699
MSTRG.25067.3	coding	0.425	0	348	3767
MSTRG.25070.1	noncoding	-0.0524288	408	729	1230
MSTRG.25070.2	noncoding	-0.0524288	405	726	1226
MSTRG.25070.4	noncoding	-0.0524288	378	699	1103
MSTRG.25072.4	coding	0.375	408	1431	1876

MSTRG.25072.3	coding	0.445	0	1482	1927
MSTRG.25074.2	coding	0.497	1653	4716	5927
MSTRG.25074.3	coding	0.502	1182	4215	5395
MSTRG.25071.3	coding	0.199	0	501	2311
MSTRG.25078.3	coding	0.319	1491	4227	5819
MSTRG.25079.3	coding	0.304	192	849	1302
MSTRG.25079.2	coding	0.322	195	885	1337
MSTRG.25078.4	coding	0.401	657	2823	4415
MSTRG.25080.2	coding	0.347	507	2370	2569
MSTRG.25076.2	noncoding	-0.0888832	60	447	1596
MSTRG.25076.3	noncoding	-0.034816	819	1026	1395
MSTRG.25085.2	coding	0.441	0	384	915
MSTRG.25085.3	coding	0.458	0	816	976
MSTRG.25081.1	coding	0.487	15	1956	4825
MSTRG.25090.3	coding	0.25	66	447	627
MSTRG.25108.4	coding	0.607	0	627	2469
MSTRG.25108.7	coding	0.613	0	600	3781
MSTRG.25109.1	noncoding	-0.016384	111	219	556
MSTRG.25110.1	coding	0.494	309	2355	3299
MSTRG.25103.2	coding	0.513	33	1932	2692
MSTRG.25103.3	coding	0.517	240	2088	2849
MSTRG.25103.5	coding	0.513	234	1959	2673
MSTRG.25103.4	coding	0.513	90	1815	2530
MSTRG.25103.6	coding	0.547	0	510	1178
MSTRG.25105.1	coding	0.261	147	435	600
MSTRG.25104.1	noncoding	-0.139264	246	267	420
MSTRG.25107.1	noncoding	-0.065536	6	60	361
MSTRG.25116.1	coding	0.441	0	1422	2839
MSTRG.25116.3	coding	0.426	75	1515	2932
MSTRG.25115.1	noncoding	-0.0049152	141	252	531
MSTRG.25118.2	noncoding	-0.0671744	576	1389	3063
MSTRG.25120.1	coding	0.41	75	1092	1300
MSTRG.25123.1	noncoding	-0.008192	564	699	1882
MSTRG.25124.1	coding	0.551	0	1311	3395
MSTRG.25124.2	coding	0.633	0	1122	3212
MSTRG.25125.1	noncoding	-0.0581632	180	543	710
MSTRG.25143.1	noncoding	-0.1097728	5013	5343	12557
MSTRG.25143.2	noncoding	-0.075776	1674	1944	8309
MSTRG.25143.3	noncoding	-0.1097728	5079	5409	11757
MSTRG.25128.1	noncoding	-0.0131072	684	882	1347
MSTRG.25138.1	noncoding	-0.2048	0	117	335
MSTRG.25139.1	noncoding	-0.028672	123	171	355
MSTRG.25150.4	coding	0.392	585	1548	1968
MSTRG.25166.1	noncoding	-0.012288	681	885	1073
MSTRG.25167.1	coding	0.146	678	5271	5831
MSTRG.25167.3	coding	0.188	2094	3831	4062
MSTRG.25168.1	coding	0.259	3042	4896	12953
MSTRG.25164.2	coding	0.319	276	972	1353
MSTRG.25164.1	coding	0.311	276	1047	1616
MSTRG.25164.4	coding	0.319	0	678	962
MSTRG.25170.1	noncoding	-0.07168	6375	6606	11362
MSTRG.25174.2	coding	0.332	252	1275	1432
MSTRG.25174.3	coding	0.33	252	966	1229
MSTRG.25176.4	coding	0.332	396	861	1056

MSTRG.25176.5	coding	0.332	282	747	941
MSTRG.25181.1	coding	0.101	1602	2346	2664
MSTRG.25185.1	coding	0.303	267	960	1658
MSTRG.25195.1	noncoding	-0.0741376	6075	6567	6724
MSTRG.25195.2	noncoding	-0.0815104	3147	3432	3588
MSTRG.25196.3	coding	0.42	99	1830	2257
MSTRG.25196.4	coding	0.369	582	2988	3413
MSTRG.25196.5	coding	0.482	72	1068	1317
MSTRG.25196.6	coding	0.454	81	777	974
MSTRG.25198.1	noncoding	-0.075776	60	90	252
MSTRG.25190.3	coding	0.537	0	1953	2162
MSTRG.25205.4	coding	0.155	3330	8403	11675
MSTRG.25205.5	coding	0.155	2094	7167	10439
MSTRG.25206.1	noncoding	-0.0290816	690	777	6205
MSTRG.25205.6	coding	0.153	117	1275	4450
MSTRG.25207.1	noncoding	-0.0155648	78	474	5238
MSTRG.25207.2	noncoding	-0.0831488	7026	7437	7627
MSTRG.25207.3	noncoding	-0.0897024	0	702	6341
MSTRG.25207.4	noncoding	-0.0626688	351	504	3421
MSTRG.25208.1	noncoding	-0.073728	12	72	228
MSTRG.25211.1	noncoding	-0.008192	1836	1962	4934
MSTRG.25214.1	noncoding	-0.1052672	1182	1542	1695
MSTRG.25214.2	noncoding	-0.03072	0	105	1465
MSTRG.25217.1	coding	0.096	285	1368	2281
MSTRG.25232.1	noncoding	-0.032768	294	393	728
MSTRG.25248.1	noncoding	-0.028672	8400	8523	8683
MSTRG.25254.2	coding	0.188	54	1680	2374
MSTRG.25221.1	noncoding	-0.0004096	264	264	449
MSTRG.25224.3	noncoding	-0.0004096	192	297	453
MSTRG.25226.1	noncoding	-0.1171456	0	444	42879
MSTRG.25226.3	noncoding	-0.036864	17517	18192	24386
MSTRG.25226.5	noncoding	-0.0360448	21093	21456	29446
MSTRG.25226.4	noncoding	-0.0671744	4653	4914	5068
MSTRG.25226.8	noncoding	-0.0360448	9723	10086	18140
MSTRG.25255.4	coding	0.163	1995	2346	3569
MSTRG.25255.1	coding	0.154	2043	3435	3842
MSTRG.25256.1	noncoding	-0.016384	1191	1284	3715
MSTRG.25257.1	noncoding	-0.024576	1539	1620	2836
MSTRG.25258.1	noncoding	-0.0151552	3954	3981	4419
MSTRG.25255.6	coding	0.124	714	1134	1523
MSTRG.25268.6	coding	0.261	4575	5865	8427
MSTRG.25269.1	noncoding	-0.0004096	1848	1848	2351
MSTRG.25280.2	coding	0.252	0	354	711
MSTRG.25261.1	coding	0.135	4866	5160	8665
MSTRG.25262.1	noncoding	-0.073728	864	894	1090
MSTRG.25262.2	noncoding	-0.018432	2208	2328	2753
MSTRG.25265.1	noncoding	-0.002048	51	174	327
MSTRG.25281.1	noncoding	-0.18432	447	492	648
MSTRG.25285.2	coding	0.346	0	345	610
MSTRG.25290.3	coding	0.165	906	2805	3122
MSTRG.25297.2	noncoding	-0.16384	126	189	467
MSTRG.25298.1	noncoding	-0.045056	2580	2745	8269
MSTRG.25302.1	noncoding	-0.0053248	132	288	450
MSTRG.25310.1	noncoding	-0.036864	684	801	1409

MSTRG.25318.1	coding	0.136	510	1908	5283
MSTRG.25315.1	noncoding	-0.0438272	2595	2907	3060
MSTRG.25327.1	coding	0.199	297	5274	6625
MSTRG.25327.2	coding	0.203	0	4992	6227
MSTRG.25327.3	coding	0.195	252	5220	6422
MSTRG.25328.1	noncoding	-0.049152	1386	1479	1634
MSTRG.25329.1	noncoding	-0.008192	132	264	432
MSTRG.25335.1	coding	0.05	180	2787	4392
MSTRG.25335.2	noncoding	-0.0782336	390	714	924
MSTRG.25342.1	coding	0.128	0	2358	8478
MSTRG.25342.4	coding	0.113	18	2409	3121
MSTRG.25342.5	coding	0.129	1596	3555	4266
MSTRG.25343.1	coding	0.404	0	222	1942
MSTRG.25348.2	coding	0.334	0	7353	8093
MSTRG.25350.2	coding	0.129	3	672	1348
MSTRG.25351.2	coding	0.103	168	7476	8899
MSTRG.25351.3	coding	0.102	168	7488	8911
MSTRG.25351.4	coding	0.098	132	7125	8545
MSTRG.25356.2	noncoding	-0.0073728	39	186	529
MSTRG.25359.1	coding	0.072	1095	1701	1883
MSTRG.25359.4	noncoding	-0.0454656	558	864	2800
MSTRG.25353.1	coding	0.254	15	711	1332
MSTRG.25353.3	coding	0.263	213	840	1460
MSTRG.25354.1	noncoding	-0.04096	309	351	554
MSTRG.25354.2	noncoding	-0.04096	0	87	442
MSTRG.25361.1	coding	0.074	0	858	5129
MSTRG.25361.3	noncoding	-0.0520192	0	630	1558
MSTRG.25364.1	noncoding	-0.0237568	3651	3804	4927
MSTRG.25362.2	coding	0.186	0	912	1145
MSTRG.25372.1	noncoding	-0.0438272	0	360	1132
MSTRG.25384.1	noncoding	-0.0643072	2217	2988	6240
MSTRG.25384.2	noncoding	-0.0643072	1230	2001	5039
MSTRG.25384.3	coding	0.1	648	2085	4718
MSTRG.25384.6	noncoding	-0.1060864	0	198	3715
MSTRG.25388.1	coding	0.061	1137	2628	6357
MSTRG.25388.5	coding	0.061	1137	2628	4953
MSTRG.25388.6	coding	0.061	1674	3165	5056
MSTRG.25388.7	coding	0.023	0	285	2264
MSTRG.25389.1	coding	0.1	3624	3822	5097
MSTRG.25390.1	coding	0.132	3300	3552	11291
MSTRG.25389.2	noncoding	-0.008192	2835	2946	3937
MSTRG.25389.3	noncoding	-0.0024576	1221	1269	1855
MSTRG.25409.4	coding	0.008	150	987	3756
MSTRG.25409.5	coding	0.016	150	1005	2417
MSTRG.25409.7	noncoding	-0.036864	150	270	1142
MSTRG.25410.2	coding	0.039	0	738	3773
MSTRG.25413.2	coding	0.13	0	1164	2839
MSTRG.25413.3	coding	0.13	0	1164	2169
MSTRG.25413.4	coding	0.13	0	1164	2150
MSTRG.25413.5	coding	0.13	0	1164	2179
MSTRG.25413.6	coding	0.048	945	1419	1575
MSTRG.25413.7	coding	0.15	0	1218	1608
MSTRG.25415.1	coding	0.094	3486	5010	9823
MSTRG.25415.2	coding	0.051	11184	13092	18583

MSTRG.25415.3	noncoding	-0.0155648	354	513	2862
MSTRG.25415.4	coding	0.083	4326	5472	5627
MSTRG.25417.1	noncoding	-0.0004096	0	0	1202
MSTRG.25399.1	coding	0.053	2538	2751	2987
MSTRG.25391.1	noncoding	-0.0004096	0	0	301
MSTRG.25405.2	coding	0.199	957	1410	1895
MSTRG.25406.1	noncoding	-0.0479232	1470	1542	3551
MSTRG.25405.3	noncoding	-0.0004096	198	198	714
MSTRG.25433.2	coding	0.328	300	591	1874
MSTRG.25434.1	noncoding	-0.0004096	0	0	881
MSTRG.25435.3	coding	0.086	0	666	1455
MSTRG.25436.1	coding	0.071	0	2046	4578
MSTRG.25436.2	coding	0.089	0	2331	8276
MSTRG.25436.5	coding	0.069	0	2010	6502
MSTRG.25437.1	noncoding	-0.065536	186	267	422
MSTRG.25441.2	coding	0.262	0	741	1713
MSTRG.25447.2	coding	0.18	1992	4608	6968
MSTRG.25447.6	coding	0.184	2412	4902	7262
MSTRG.25447.7	coding	0.18	1425	3912	4417
MSTRG.25448.1	noncoding	-0.053248	6627	6729	14866
MSTRG.25444.1	noncoding	-0.079872	72	795	1474
MSTRG.25451.4	noncoding	-0.0094208	156	375	1430
MSTRG.25462.1	noncoding	-0.0299008	3	462	784
MSTRG.25463.1	coding	0.202	1332	2151	2357
MSTRG.25465.1	noncoding	-0.0004096	4626	4740	5133
MSTRG.25472.5	coding	0.103	2601	3174	4124
MSTRG.25472.6	coding	0.103	2130	2703	3652
MSTRG.25472.7	coding	0.103	1818	2391	3340
MSTRG.25472.8	coding	0.103	1812	2385	3336
MSTRG.25477.1	coding	0.109	513	2229	5869
MSTRG.25477.2	coding	0.12	0	1557	5231
MSTRG.25478.1	noncoding	-0.0004096	150	333	2937
MSTRG.25478.3	coding	0.02	390	531	1058
MSTRG.25478.5	coding	0.03	1161	1296	1449
MSTRG.25480.3	coding	0.076	4878	8700	11363
MSTRG.25480.4	coding	0.079	0	3711	6358
MSTRG.25483.1	noncoding	-0.0004096	0	0	307
MSTRG.25483.2	noncoding	-0.0004096	183	213	681
MSTRG.25484.2	coding	0.225	0	525	1189
MSTRG.25489.2	coding	0.158	540	771	2381
MSTRG.25489.3	coding	0.158	138	369	2832
MSTRG.25491.1	coding	0.085	0	1434	13597
MSTRG.25492.4	noncoding	-0.1134592	0	348	1368
MSTRG.25492.3	coding	0.278	0	528	3246
MSTRG.25492.5	coding	0.278	0	528	3072
MSTRG.25499.3	noncoding	-0.06144	2193	2397	4457
MSTRG.25499.2	coding	0.15	1887	2475	3603
MSTRG.25499.6	noncoding	-0.06144	3543	3747	5750
MSTRG.25499.7	noncoding	-0.06144	1644	1848	8983
MSTRG.25499.8	coding	0.126	1782	2193	2989
MSTRG.25499.10	noncoding	-0.024576	30	534	1487
MSTRG.25508.1	noncoding	-0.0167936	48	303	1788
MSTRG.25508.3	noncoding	-0.0311296	18894	19236	38923
MSTRG.25508.4	coding	0.199	0	231	8021

MSTRG.25508.5	coding	0.199	0	231	2699
MSTRG.25508.6	noncoding	-0.0311296	7833	8175	24803
MSTRG.25508.7	noncoding	-0.12288	1806	1863	2019
MSTRG.25509.1	noncoding	-0.004096	5406	5550	14736
MSTRG.25508.9	noncoding	-0.034816	12462	12765	16875
MSTRG.25508.8	noncoding	-0.034816	1440	1743	5854
MSTRG.25510.1	noncoding	-0.0004096	0	0	217
MSTRG.25508.10	noncoding	-0.0462848	6711	6882	36845
MSTRG.25508.11	noncoding	-0.0462848	3816	3987	36294
MSTRG.25508.12	noncoding	-0.0462848	6666	6837	20867
MSTRG.25508.13	noncoding	-0.135168	4728	4767	5482
MSTRG.25511.1	noncoding	-0.094208	1470	1548	2739
MSTRG.25512.1	noncoding	-0.0483328	4317	4539	15326
MSTRG.25512.2	noncoding	-0.032768	6960	7305	9003
MSTRG.25513.1	coding	0.018	648	828	2518
MSTRG.25514.1	noncoding	-0.077824	3258	3333	6360
MSTRG.25508.14	noncoding	-0.0278528	0	96	1988
MSTRG.25517.1	noncoding	-0.047104	219	369	3843
MSTRG.25517.3	noncoding	-0.047104	204	354	1079
MSTRG.25517.4	noncoding	-0.047104	204	354	4098
MSTRG.25518.2	coding	0.127	405	3795	5150
MSTRG.25528.2	coding	0.114	9090	11274	15915
MSTRG.25528.3	coding	0.123	0	2436	2998
MSTRG.25529.1	noncoding	-0.0004096	672	816	3827
MSTRG.25528.4	noncoding	-0.024576	1749	1881	8754
MSTRG.25530.1	coding	0.11	5868	6099	11380
MSTRG.25531.1	noncoding	-0.0004096	0	0	1806
MSTRG.25532.1	noncoding	-0.014336	2898	3075	3828
MSTRG.25537.2	noncoding	-0.063488	48	339	6941
MSTRG.25537.1	noncoding	-0.063488	48	339	7083
MSTRG.25537.3	noncoding	-0.063488	48	339	7415
MSTRG.25540.1	coding	0.234	327	1950	2205
MSTRG.25546.1	noncoding	-0.0057344	2928	3306	3534
MSTRG.25550.3	coding	0.166	0	1638	3671
MSTRG.25552.1	coding	0.134	285	1800	5168
MSTRG.25552.2	coding	0.131	225	1740	4593
MSTRG.25552.3	coding	0.144	339	1872	5240
MSTRG.25552.4	coding	0.131	177	1692	5059
MSTRG.25552.5	coding	0.131	168	1683	4325
MSTRG.25552.6	coding	0.128	120	1737	4220
MSTRG.25552.7	coding	0.128	18	1635	4796
MSTRG.25552.9	coding	0.135	81	1572	4939
MSTRG.25552.10	coding	0.135	60	1551	4404
MSTRG.25556.3	noncoding	-0.0012288	7350	7602	8588
MSTRG.25557.2	coding	0.179	345	1053	3311
MSTRG.25571.1	noncoding	-0.0724992	0	171	1412
MSTRG.25577.7	coding	0.206	318	4860	5034
MSTRG.25577.8	coding	0.207	318	4812	4986
MSTRG.25582.3	coding	0.036	189	1548	1726
MSTRG.25593.2	coding	0.112	603	2208	4104
MSTRG.25594.1	noncoding	-0.0929792	0	129	1113
MSTRG.25595.1	coding	0.138	0	768	7260
MSTRG.25601.2	coding	0.193	2277	3117	10029
MSTRG.25597.2	coding	0.291	1182	3117	3615

MSTRG.25597.3	coding	0.084	846	1086	2186
MSTRG.25598.2	noncoding	-0.045056	327	399	863
MSTRG.25598.1	noncoding	-0.002048	627	753	944
MSTRG.25599.1	noncoding	-0.139264	2079	2142	3121
MSTRG.25597.4	noncoding	-0.0413696	3834	4224	18623
MSTRG.25602.1	coding	0.141	4737	6750	6907
MSTRG.25602.2	coding	0.133	0	1881	2038
MSTRG.25602.4	coding	0.163	4716	5007	6243
MSTRG.25602.5	noncoding	-0.036864	0	159	1209
MSTRG.25604.3	coding	0.095	2454	3960	4743
MSTRG.25604.2	coding	0.195	12	570	6040
MSTRG.25615.2	coding	0.078	7971	10350	10947
MSTRG.25615.6	coding	0.027	3072	10044	10631
MSTRG.25615.7	coding	0.027	2958	9927	10511
MSTRG.25615.9	coding	0.027	2565	9624	10222
MSTRG.25615.10	coding	0.076	1485	3948	4545
MSTRG.25607.5	coding	0.044	1173	8253	8835
MSTRG.25608.1	noncoding	-0.008192	420	510	1340
MSTRG.25609.1	noncoding	-0.028672	270	348	5375
MSTRG.25610.1	noncoding	-0.008192	129	261	2379
MSTRG.25610.2	coding	0.046	3570	4215	4780
MSTRG.25611.1	noncoding	-0.0786432	63	726	2976
MSTRG.25614.1	noncoding	-0.0053248	48	180	335
MSTRG.25616.2	coding	0.389	0	516	1652
MSTRG.25616.3	coding	0.024	537	1176	3233
MSTRG.25620.2	noncoding	-0.0466944	54	282	6336
MSTRG.25626.1	noncoding	-0.036864	474	606	2003
MSTRG.25622.1	noncoding	-0.0004096	0	0	481
MSTRG.25622.2	noncoding	-0.0004096	0	0	375
MSTRG.25623.1	noncoding	-0.0110592	0	12	211
MSTRG.25630.1	noncoding	-0.0004096	168	168	420
MSTRG.25631.2	coding	0.061	654	1131	1467
MSTRG.25632.2	coding	0.121	618	1005	3216
MSTRG.25632.4	coding	0.11	534	906	1647
MSTRG.25635.1	coding	0.053	10572	10737	11265
MSTRG.25637.2	coding	0.235	51	2970	4834
MSTRG.25638.1	coding	0.132	1224	1464	4132
MSTRG.25638.2	coding	0.055	189	360	519
MSTRG.25642.1	noncoding	-0.0004096	0	0	408
MSTRG.25642.2	noncoding	-0.077824	246	276	504
MSTRG.25639.1	noncoding	-0.077824	18	72	393
MSTRG.25653.1	noncoding	-0.086016	1089	1143	2351
MSTRG.25654.2	coding	0.115	3	273	1395
MSTRG.25654.3	noncoding	-0.12288	0	219	1152
MSTRG.25657.6	coding	0.149	1950	3930	4201
MSTRG.25657.7	coding	0.101	885	1911	2841
MSTRG.25664.2	coding	0.258	159	1710	2497
MSTRG.25667.1	noncoding	-0.0012288	75	168	349
MSTRG.25711.1	noncoding	-0.065536	237	291	1141
MSTRG.25711.2	noncoding	-0.065536	606	660	1477
MSTRG.25711.3	noncoding	-0.065536	495	549	1356
MSTRG.25711.4	noncoding	-0.065536	204	258	1108
MSTRG.25712.1	coding	0.093	1722	1908	3786
MSTRG.25711.5	noncoding	-0.012288	1050	1152	2097

MSTRG.25711.6	coding	0.098	1152	1407	2046
MSTRG.25711.7	noncoding	-0.065536	2709	2763	3557
MSTRG.25707.1	noncoding	-0.012288	429	495	835
MSTRG.25718.1	noncoding	-0.1024	108	168	602
MSTRG.25723.2	coding	0.4	9	891	1365
MSTRG.25725.1	noncoding	-0.03072	345	456	671
MSTRG.25726.3	coding	0.32	687	1068	1252
MSTRG.25730.2	noncoding	-0.1572864	396	855	1010
MSTRG.25734.2	coding	0.29	399	1836	3012
MSTRG.25734.3	coding	0.296	6	1455	2636
MSTRG.25736.1	noncoding	-0.0659456	0	459	778
MSTRG.25734.4	coding	0.259	0	567	1746
MSTRG.25737.2	coding	0.164	0	987	2652
MSTRG.25747.4	coding	0.473	60	7011	9126
MSTRG.25747.2	coding	0.48	0	9231	9650
MSTRG.25748.1	noncoding	-0.069632	174	213	2419
MSTRG.25748.2	noncoding	-0.069632	102	141	3561
MSTRG.25739.1	noncoding	-0.053248	207	270	757
MSTRG.25749.1	noncoding	-0.167936	42	99	685
MSTRG.25750.1	noncoding	-0.024576	123	246	403
MSTRG.25750.2	noncoding	-0.0118784	9	183	340
MSTRG.25752.1	coding	0.267	288	564	2192
MSTRG.25760.4	coding	0.14	231	2601	5418
MSTRG.25757.2	coding	0.148	39	492	980
MSTRG.25771.2	coding	0.374	864	4020	11892
MSTRG.25772.1	noncoding	-0.04096	576	801	1502
MSTRG.25771.3	coding	0.385	228	3165	7875
MSTRG.25771.4	coding	0.331	141	2223	6934
MSTRG.25771.5	coding	0.294	0	318	7071
MSTRG.25778.1	coding	0.187	822	1971	2963
MSTRG.25779.2	coding	0.026	534	756	1130
MSTRG.25779.3	noncoding	-0.0090112	651	822	1851
MSTRG.25773.1	noncoding	-0.008192	0	72	500
MSTRG.25781.2	coding	0.254	81	2769	3337
MSTRG.25782.1	noncoding	-0.0131072	123	309	472
MSTRG.25782.2	noncoding	-0.004096	162	282	436
MSTRG.25784.1	noncoding	-0.0524288	11955	12423	16447
MSTRG.25785.2	coding	0.153	1155	1548	3735
MSTRG.25790.1	noncoding	-0.069632	153	243	668
MSTRG.25796.1	coding	0.281	1770	3603	4655
MSTRG.25796.2	coding	0.281	1470	3303	4335
MSTRG.25798.1	noncoding	-0.047104	2475	2673	3653
MSTRG.25799.1	noncoding	-0.0385024	819	1158	2572
MSTRG.25802.1	noncoding	-0.0110592	288	594	879
MSTRG.25802.2	noncoding	-0.0507904	324	576	1259
MSTRG.25803.1	noncoding	-0.045056	111	318	689
MSTRG.25808.1	noncoding	-0.0647168	0	576	1838
MSTRG.25809.1	noncoding	-0.0667648	858	1188	3260
MSTRG.25809.2	noncoding	-0.0667648	798	1128	2587
MSTRG.25809.3	noncoding	-0.0667648	858	1188	2629
MSTRG.25806.1	noncoding	-0.0589824	324	759	982
MSTRG.25817.2	coding	0.523	0	1314	2247
MSTRG.25817.3	coding	0.529	0	1257	2261
MSTRG.25828.1	coding	0.332	99	2163	2371

MSTRG.25824.4	coding	0.359	15	1734	2041
MSTRG.25847.1	noncoding	-0.110592	468	540	696
MSTRG.25848.1	noncoding	-0.0565248	369	816	1081
MSTRG.25848.2	noncoding	-0.0565248	306	753	1018
MSTRG.25849.1	noncoding	-0.0700416	60	264	421
MSTRG.25857.1	noncoding	-0.028672	1188	1272	6607
MSTRG.25858.1	noncoding	-0.06144	2127	2226	3482
MSTRG.25857.2	noncoding	-0.0299008	177	237	393
MSTRG.25861.1	noncoding	-0.0221184	363	558	847
MSTRG.25862.1	noncoding	-0.0221184	72	258	415
MSTRG.25869.1	noncoding	-0.1277952	21	51	205
MSTRG.25872.1	noncoding	-0.028672	123	207	406
MSTRG.25897.4	coding	0.143	90	558	1542
MSTRG.25897.6	coding	0.143	90	558	1436
MSTRG.25897.3	coding	0.143	3246	3714	4580
MSTRG.25897.7	coding	0.143	90	558	1440
MSTRG.25898.1	noncoding	-0.032768	420	465	2869
MSTRG.25897.11	noncoding	-0.118784	423	426	1765
MSTRG.25897.12	noncoding	-0.0065536	606	777	3440
MSTRG.25897.13	coding	0.03	234	546	4352
MSTRG.25897.14	noncoding	-0.016384	1479	1563	5061
MSTRG.25874.2	coding	0.377	348	4179	4964
MSTRG.25874.1	coding	0.42	150	4641	5426
MSTRG.25907.2	coding	0.204	0	531	1342
MSTRG.25907.3	coding	0.132	0	699	19924
MSTRG.25911.2	coding	0.145	585	1335	1834
MSTRG.25912.2	coding	0.045	1056	1995	2312
MSTRG.25916.1	coding	0.123	1155	1650	3794
MSTRG.25916.2	coding	0.123	1041	1536	3680
MSTRG.25917.1	noncoding	-0.086016	84	111	267
MSTRG.25923.1	noncoding	-0.0155648	936	1068	1748
MSTRG.25926.4	coding	0.231	1974	2682	3431
MSTRG.25926.5	coding	0.182	0	1152	1901
MSTRG.25927.1	noncoding	-0.0024576	633	672	3352
MSTRG.25928.1	noncoding	-0.131072	159	177	1857
MSTRG.25926.6	coding	0.129	0	228	1515
MSTRG.25929.1	noncoding	-0.036864	3618	3744	5050
MSTRG.25940.1	coding	0.189	198	1641	1795
MSTRG.25940.5	coding	0.214	282	1671	1844
MSTRG.25940.3	coding	0.197	282	1731	1974
MSTRG.25949.3	coding	0.531	5103	9267	15747
MSTRG.25950.1	noncoding	-0.0626688	762	1080	3394
MSTRG.25951.1	noncoding	-0.0446464	3	540	5369
MSTRG.25949.5	coding	0.501	144	3723	10204
MSTRG.25949.6	coding	0.47	2511	4941	11330
MSTRG.25949.7	coding	0.419	387	2454	17518
MSTRG.25949.8	coding	0.473	186	2391	2546
MSTRG.25956.1	noncoding	-0.0282624	213	378	532
MSTRG.25958.1	noncoding	-0.0299008	23496	23691	26829
MSTRG.25958.2	noncoding	-0.0069632	5913	6078	8176
MSTRG.25958.3	noncoding	-0.0299008	441	636	3776
MSTRG.25959.1	noncoding	-0.069632	7728	7974	8696
MSTRG.25959.2	noncoding	-0.069632	10080	10326	10958
MSTRG.25959.3	coding	0.113	3948	4458	6712

MSTRG.25959.5	noncoding	-0.069632	15651	15897	16774
MSTRG.25959.4	noncoding	-0.069632	16002	16248	17125
MSTRG.25959.6	noncoding	-0.02048	2016	2130	4839
MSTRG.25959.7	noncoding	-0.043008	279	771	13851
MSTRG.25959.8	noncoding	-0.0077824	9198	9348	11937
MSTRG.25960.1	coding	0.401	90	1380	3998
MSTRG.25972.1	noncoding	-0.028672	0	63	218
MSTRG.25973.2	coding	0.246	270	768	2112
MSTRG.25973.3	coding	0.192	714	1977	3226
MSTRG.25973.4	coding	0.189	390	1404	2653
MSTRG.25973.6	coding	0.24	390	888	2069
MSTRG.25977.1	noncoding	-0.0802816	9882	10131	10607
MSTRG.25977.2	noncoding	-0.0622592	13326	13575	16060
MSTRG.25977.3	noncoding	-0.0262144	6075	6279	7133
MSTRG.25978.2	noncoding	-0.0458752	17070	17472	18683
MSTRG.25975.1	noncoding	-0.0004096	93	156	313
MSTRG.25980.2	noncoding	-0.2592768	0	267	421
MSTRG.25997.5	coding	0.263	3564	9156	12110
MSTRG.25997.4	coding	0.288	2757	6441	9397
MSTRG.25997.1	coding	0.263	2619	8304	11258
MSTRG.25997.2	coding	0.263	4119	9702	12658
MSTRG.25997.6	coding	0.288	4113	7797	10680
MSTRG.25998.1	noncoding	-0.0270336	165	789	1930
MSTRG.25997.8	coding	0.265	60	5769	6298
MSTRG.25997.7	coding	0.263	963	6546	7077
MSTRG.25997.9	coding	0.263	2622	8202	8731
MSTRG.25997.10	coding	0.263	1110	6696	7215
MSTRG.25997.11	coding	0.264	2430	8121	8637
MSTRG.25997.12	coding	0.288	10287	13971	14487
MSTRG.25997.13	coding	0.15	558	933	5331
MSTRG.25997.14	coding	0.208	1485	2298	2870
MSTRG.25997.18	noncoding	-0.096256	873	1230	1386
MSTRG.25997.19	noncoding	-0.0069632	726	765	921
MSTRG.25997.20	noncoding	-0.032768	1113	1158	4207
MSTRG.25997.21	noncoding	-0.0004096	0	0	2487
MSTRG.25989.1	noncoding	-0.0303104	19002	19770	23672
MSTRG.25989.2	noncoding	-0.0405504	8823	9522	39319
MSTRG.25989.3	noncoding	-0.0405504	1257	1956	23588
MSTRG.25989.5	noncoding	-0.0405504	1254	1953	32246
MSTRG.25989.6	noncoding	-0.0303104	11310	12078	15457
MSTRG.25991.1	noncoding	-0.065536	6321	6894	20908
MSTRG.25991.2	noncoding	-0.065536	5874	6447	18247
MSTRG.25991.3	noncoding	-0.065536	8058	8631	20275
MSTRG.25991.4	noncoding	-0.065536	8001	8574	22100
MSTRG.25991.5	noncoding	-0.065536	7710	8283	19830
MSTRG.25985.2	coding	0.384	0	1254	5532
MSTRG.25986.1	noncoding	-0.0679936	6609	7077	7329
MSTRG.25992.2	coding	0.433	0	1821	3054
MSTRG.25993.1	noncoding	-0.0110592	3312	3501	6645
MSTRG.25994.1	noncoding	-0.0004096	3690	3822	4359
MSTRG.25994.2	noncoding	-0.016384	1860	1977	6254
MSTRG.25995.1	noncoding	-0.0196608	1365	1500	6918
MSTRG.25992.3	coding	0.433	1071	1575	13343
MSTRG.25992.4	coding	0.632	0	600	6482

MSTRG.26010.2	coding	0.329	915	3051	4100
MSTRG.26010.3	noncoding	-0.0135168	1911	2055	2682
MSTRG.26016.1	noncoding	-0.0978944	1488	1761	11793
MSTRG.26018.1	noncoding	-0.004096	75	213	1578
MSTRG.26021.1	noncoding	-0.339968	942	966	2418
MSTRG.26026.5	noncoding	-0.0512	51	249	805
MSTRG.26026.4	noncoding	-0.0393216	219	459	820
MSTRG.26026.2	noncoding	-0.0585728	327	528	1082
MSTRG.26026.3	noncoding	-0.0671744	42	270	824
MSTRG.26028.1	noncoding	-0.1318912	0	114	2305
MSTRG.26033.1	coding	0.27	174	1131	1293
MSTRG.26034.1	noncoding	-0.159744	135	177	331
MSTRG.26034.3	noncoding	-0.0217088	384	513	2331
MSTRG.26034.2	noncoding	-0.0253952	1884	2073	4955
MSTRG.26034.4	noncoding	-0.0217088	189	318	4973
MSTRG.26034.5	noncoding	-0.0217088	189	318	2173
MSTRG.26034.6	noncoding	-0.024576	267	333	1542
MSTRG.26034.7	noncoding	-0.0319488	2715	2841	4006
MSTRG.26031.2	coding	0.185	174	741	897
MSTRG.26031.3	noncoding	-0.0151552	123	288	442
MSTRG.26035.2	coding	0.26	0	6270	7070
MSTRG.26035.3	coding	0.251	0	6327	7160
MSTRG.26036.1	coding	0.315	327	1560	2325
MSTRG.26038.2	coding	0.332	741	2241	2412
MSTRG.26038.3	coding	0.332	738	2238	2409
MSTRG.26037.1	coding	0.451	153	1101	1309
MSTRG.26041.2	coding	0.478	99	3072	3647
MSTRG.26042.1	coding	0.393	0	1434	2141
MSTRG.26042.2	coding	0.396	0	1425	1876
MSTRG.26051.1	coding	0.592	36	4146	8774
MSTRG.26052.1	coding	0.442	1245	2874	4419
MSTRG.26053.3	noncoding	-0.07168	21	255	447
MSTRG.26053.4	coding	0.199	678	2661	2943
MSTRG.26045.2	coding	0.339	0	768	1229
MSTRG.26048.1	noncoding	-0.0802816	276	315	469
MSTRG.26047.2	coding	0.249	0	726	1448
MSTRG.26059.1	coding	0.407	3	3138	3971
MSTRG.26059.2	coding	0.456	24	2832	3667
MSTRG.26083.4	coding	0.372	450	4347	5228
MSTRG.26083.5	coding	0.369	249	4164	5044
MSTRG.26087.7	noncoding	-0.069632	9906	10134	10290
MSTRG.26087.6	noncoding	-0.139264	0	315	4148
MSTRG.26087.5	coding	0.22	0	2817	3517
MSTRG.26088.1	noncoding	-0.0217088	3996	4143	7531
MSTRG.26089.1	noncoding	-0.04096	5196	5370	6681
MSTRG.26091.1	coding	0.213	0	1803	7219
MSTRG.26091.4	coding	0.213	54	1758	3141
MSTRG.26091.2	coding	0.21	27	1863	3246
MSTRG.26093.1	noncoding	-0.1990656	0	90	520
MSTRG.26096.1	noncoding	-0.0094208	261	519	979
MSTRG.26102.2	coding	0.46	138	699	1474
MSTRG.26106.1	noncoding	-0.0335872	2379	3489	15798
MSTRG.26106.2	noncoding	-0.0335872	2379	3489	13290
MSTRG.26107.2	noncoding	-0.0290816	45	234	3511

MSTRG.26108.1	coding	0.185	0	345	540
MSTRG.26109.1	noncoding	-0.0360448	0	333	2726
MSTRG.26111.4	coding	0.222	1050	3819	3973
MSTRG.26112.2	noncoding	-0.083968	0	93	423
MSTRG.26112.3	coding	0.111	120	417	1127
MSTRG.26112.4	coding	0.065	93	360	860
MSTRG.26113.1	noncoding	-0.086016	885	921	1358
MSTRG.26118.3	noncoding	-0.0180224	1845	2454	4374
MSTRG.26118.11	coding	0.21	213	1350	6181
MSTRG.26118.12	coding	0.151	138	1398	9895
MSTRG.26118.13	coding	0.194	0	1425	4935
MSTRG.26120.1	noncoding	-0.0086016	3447	3618	4674
MSTRG.26121.1	noncoding	-0.0024576	2424	2625	3028
MSTRG.26123.1	coding	0.233	276	1344	3287
MSTRG.26123.2	coding	0.224	0	873	1408
MSTRG.26123.8	coding	0.212	0	849	1850
MSTRG.26123.10	coding	0.25	0	975	1335
MSTRG.26123.12	coding	0.201	243	969	1328
MSTRG.26123.11	coding	0.226	0	876	1235
MSTRG.26123.9	coding	0.19	285	990	1146
MSTRG.26123.14	coding	0.218	252	741	898
MSTRG.26123.13	coding	0.124	417	783	938
MSTRG.26124.2	noncoding	-0.094208	0	390	5382
MSTRG.26126.2	coding	0.361	0	2265	4109
MSTRG.26126.4	coding	0.126	1695	2112	3476
MSTRG.26122.2	coding	0.29	0	1056	1474
MSTRG.26138.1	noncoding	-0.024576	93	105	604
MSTRG.26139.1	noncoding	-0.110592	294	360	2457
MSTRG.26142.2	coding	0.34	27	633	1049
MSTRG.26142.3	coding	0.327	1461	2043	2460
MSTRG.26147.3	coding	0.222	0	1794	2064
MSTRG.26148.1	noncoding	-0.0012288	4551	4662	9818
MSTRG.26147.6	coding	0.3	6	1272	3220
MSTRG.26154.2	coding	0.328	708	1254	2253
MSTRG.26154.3	coding	0.367	573	1161	2162
MSTRG.26158.3	coding	0.172	270	720	1162
MSTRG.26163.1	noncoding	-0.045056	216	270	560
MSTRG.26171.1	coding	0.328	186	576	6146
MSTRG.26171.3	noncoding	-0.088064	1122	1455	7785
MSTRG.26171.4	noncoding	-0.0978944	2226	2724	3672
MSTRG.26171.5	noncoding	-0.0708608	39	882	1518
MSTRG.26180.1	coding	0.234	4104	4902	7134
MSTRG.26180.2	coding	0.271	1152	6900	8458
MSTRG.26180.5	coding	0.288	0	5187	6718
MSTRG.26180.6	coding	0.285	0	4971	6529
MSTRG.26180.13	coding	0.278	0	4782	7839
MSTRG.26181.1	noncoding	-0.0458752	174	390	3014
MSTRG.26180.14	coding	0.047	2004	2154	2527
MSTRG.26180.17	noncoding	-0.012288	732	870	2256
MSTRG.26180.18	noncoding	-0.2269184	0	186	1326
MSTRG.26180.19	noncoding	-0.2162688	0	204	9047
MSTRG.26180.20	noncoding	-0.114688	0	378	26612
MSTRG.26180.21	coding	0.088	1905	2826	8254
MSTRG.26184.2	coding	0.23	0	774	1338

MSTRG.26184.3	coding	0.221	0	636	963
MSTRG.26184.5	coding	0.199	0	585	1096
MSTRG.26185.1	coding	0.343	696	3441	4473
MSTRG.26185.3	coding	0.347	399	3180	4254
MSTRG.26186.1	noncoding	-0.1024	216	270	492
MSTRG.26187.1	noncoding	-0.126976	585	669	824
MSTRG.26182.2	coding	0.093	120	1251	1683
MSTRG.26192.1	noncoding	-0.049152	180	234	424
MSTRG.26194.17	noncoding	-0.0548864	588	927	1423
MSTRG.26189.2	coding	0.38	0	510	667
MSTRG.26190.1	noncoding	-0.0385024	126	162	1002
MSTRG.26203.1	noncoding	-0.0004096	0	0	246
MSTRG.26212.1	noncoding	-0.034816	3144	3453	3666
MSTRG.26213.1	noncoding	-0.1052672	0	261	1251
MSTRG.26213.2	noncoding	-0.104448	0	528	724
MSTRG.26213.3	noncoding	-0.11264	0	582	1158
MSTRG.26213.5	noncoding	-0.1196032	0	459	993
MSTRG.26213.6	noncoding	-0.1052672	0	261	1186
MSTRG.26214.3	coding	0.339	0	1149	2289
MSTRG.26215.3	coding	0.195	210	912	1320
MSTRG.26216.1	coding	0.451	0	504	786
MSTRG.26220.2	coding	0.109	0	501	2170
MSTRG.26224.1	coding	0.417	531	1914	2289
MSTRG.26224.2	coding	0.381	111	1836	2211
MSTRG.26224.3	coding	0.448	0	1500	1843
MSTRG.26224.4	coding	0.421	228	1623	1964
MSTRG.26224.5	coding	0.388	0	1704	2057
MSTRG.26226.1	coding	0.199	0	1242	3010
MSTRG.26226.2	noncoding	-0.0446464	1734	2214	2367
MSTRG.26227.1	noncoding	-0.059392	2625	2814	3151
MSTRG.26230.2	coding	0.066	0	660	917
MSTRG.26232.3	coding	0.161	429	4314	4501
MSTRG.26232.4	coding	0.161	489	4356	4543
MSTRG.26232.5	coding	0.161	765	4650	4836
MSTRG.26240.5	coding	0.23	717	5838	8868
MSTRG.26240.6	coding	0.23	750	5871	8902
MSTRG.26240.7	coding	0.23	837	5958	8984
MSTRG.26241.1	noncoding	-0.024576	1200	1320	2166
MSTRG.26242.1	noncoding	-0.045056	2802	2862	4045
MSTRG.26244.1	noncoding	-0.065536	2217	2304	7431
MSTRG.26244.2	noncoding	-0.065536	2802	2889	10588
MSTRG.26245.1	noncoding	-0.0221184	837	882	1493
MSTRG.26246.1	coding	0.077	1041	1227	2879
MSTRG.26240.8	noncoding	-0.08192	6957	7341	7701
MSTRG.26247.1	noncoding	-0.0004096	0	0	299
MSTRG.26247.2	noncoding	-0.045056	51	135	726
MSTRG.26249.1	noncoding	-0.0004096	0	0	301
MSTRG.26239.1	coding	0.702	0	1497	2552
MSTRG.26239.2	coding	0.593	0	2097	3114
MSTRG.26239.4	coding	0.572	21	2052	2929
MSTRG.26259.5	noncoding	-0.1081344	195	531	2217
MSTRG.26260.1	noncoding	-0.069632	1518	2451	2657
MSTRG.26257.1	noncoding	-0.0335872	471	669	1169
MSTRG.26257.2	noncoding	-0.0331776	144	345	676

MSTRG.26265.1	coding	0.565	0	537	803
MSTRG.26266.1	noncoding	-0.0401408	252	384	4380
MSTRG.26266.2	noncoding	-0.0110592	4314	4458	4687
MSTRG.26266.3	noncoding	-0.053248	2451	2676	8370
MSTRG.26281.1	noncoding	-0.0004096	207	312	579
MSTRG.26282.2	noncoding	-0.0585728	84	783	1077
MSTRG.26286.2	coding	0.385	321	1521	2082
MSTRG.26287.5	noncoding	-0.0131072	744	978	2347
MSTRG.26287.3	noncoding	-0.0131072	810	1044	2175
MSTRG.26287.4	coding	0.235	171	1056	2535
MSTRG.26278.1	noncoding	-0.012288	162	336	1762
MSTRG.26291.3	coding	0.334	126	1878	2085
MSTRG.26291.6	coding	0.351	1917	3546	3753
MSTRG.26291.4	coding	0.328	0	1695	1900
MSTRG.26292.1	noncoding	-0.0135168	396	513	793
MSTRG.26297.3	coding	0.398	192	1023	1622
MSTRG.26297.4	noncoding	-0.0221184	36	237	438
MSTRG.26299.1	noncoding	-0.03072	882	1278	1677
MSTRG.26301.1	noncoding	-0.0233472	261	435	654
MSTRG.26303.1	coding	0.486	12	2175	2450
MSTRG.26308.1	noncoding	-0.012288	330	372	528
MSTRG.26308.2	noncoding	-0.0004096	537	660	1069
MSTRG.26315.1	noncoding	-0.0139264	1749	1902	2055
MSTRG.26316.1	noncoding	-0.024576	384	453	959
MSTRG.26321.1	coding	0.134	219	1623	6211
MSTRG.26321.2	coding	0.1	219	1725	5536
MSTRG.26321.3	coding	0.119	96	1134	3454
MSTRG.26321.4	coding	0.114	618	1674	1941
MSTRG.26321.5	coding	0.12	456	618	901
MSTRG.26322.1	noncoding	-0.028672	4437	4542	5065
MSTRG.26323.1	noncoding	-0.04096	0	51	1334
MSTRG.26324.1	noncoding	-0.249856	903	939	1472
MSTRG.26325.1	noncoding	-0.176128	2481	2496	3553
MSTRG.26338.1	noncoding	-0.0724992	75	273	780
MSTRG.26340.1	coding	0.468	114	1077	2171
MSTRG.26340.2	coding	0.504	96	1626	2180
MSTRG.26337.1	noncoding	-0.0925696	729	999	1268
MSTRG.26337.2	noncoding	-0.0503808	0	21	565
MSTRG.26343.1	coding	0.195	0	2568	2952
MSTRG.26344.1	noncoding	-0.106496	7725	7770	10880
MSTRG.26345.1	noncoding	-0.0004096	0	0	499
MSTRG.26345.2	noncoding	-0.0126976	3801	3852	4968
MSTRG.26346.1	noncoding	-0.004096	303	339	6128
MSTRG.26347.1	noncoding	-0.045056	1869	1899	4033
MSTRG.26348.1	noncoding	-0.049152	2649	2748	3108
MSTRG.26349.1	noncoding	-0.028672	1365	1455	2223
MSTRG.26369.3	coding	0.08	816	933	3240
MSTRG.26369.2	coding	0.08	816	933	5484
MSTRG.26369.4	coding	0.08	660	777	3084
MSTRG.26369.5	coding	0.08	702	819	3124
MSTRG.26370.2	coding	0.097	993	1530	1928
MSTRG.26373.2	coding	0.149	0	1740	2225
MSTRG.26373.3	coding	0.162	0	1548	1934
MSTRG.26374.1	coding	0.125	207	1788	2953

MSTRG.26375.1	coding	0.108	1374	1608	4161
MSTRG.26376.1	noncoding	-0.065536	804	861	1015
MSTRG.26377.1	noncoding	-0.0585728	219	237	1177
MSTRG.26374.5	noncoding	-0.0933888	18	453	7315
MSTRG.26374.6	noncoding	-0.0933888	18	453	4190
MSTRG.26374.7	noncoding	-0.12288	861	939	6254
MSTRG.26374.8	noncoding	-0.1073152	18	588	3885
MSTRG.26375.2	noncoding	-0.090112	1074	1134	2554
MSTRG.26378.2	coding	0.175	210	531	6230
MSTRG.26379.7	coding	0.059	3642	7329	8244
MSTRG.26379.6	coding	0.038	0	6924	8762
MSTRG.26379.9	coding	0.058	3849	8556	9470
MSTRG.26386.1	noncoding	-0.0679936	0	261	3775
MSTRG.26391.2	coding	0.063	0	432	1948
MSTRG.26391.3	coding	0.061	0	921	2194
MSTRG.26391.4	coding	0.071	0	906	8553
MSTRG.26391.5	coding	0.078	2991	3327	6652
MSTRG.26394.3	coding	0.068	2733	4272	8019
MSTRG.26394.4	coding	0.065	2742	4278	7898
MSTRG.26394.5	coding	0.065	2721	4257	6297
MSTRG.26394.8	coding	0.068	2829	4368	6397
MSTRG.26395.1	coding	0.068	8817	9570	13016
MSTRG.26394.10	coding	0.009	135	438	1681
MSTRG.26394.9	coding	0.012	135	441	1684
MSTRG.26389.1	noncoding	-0.073728	279	312	467
MSTRG.26428.1	coding	0.167	3102	3405	4596
MSTRG.26428.4	noncoding	-0.036864	2310	2376	3000
MSTRG.26433.1	coding	0.068	1791	2346	7197
MSTRG.26434.1	noncoding	-0.0172032	510	678	2738
MSTRG.26433.2	coding	0.045	5193	5409	8570
MSTRG.26428.7	coding	0.127	0	375	1453
MSTRG.26399.8	noncoding	-0.0118784	417	570	867
MSTRG.26399.7	coding	0.064	99	939	1235
MSTRG.26405.1	noncoding	-0.09216	2265	2559	2714
MSTRG.26405.2	coding	0.118	0	624	2821
MSTRG.26405.5	coding	0.075	0	456	1461
MSTRG.26405.4	noncoding	-0.1220608	0	162	1348
MSTRG.26405.6	noncoding	-0.022528	1074	1221	1374
MSTRG.26411.4	coding	0.18	1863	2268	3345
MSTRG.26411.5	coding	0.087	321	3972	5026
MSTRG.26411.3	coding	0.094	153	4149	5204
MSTRG.26412.1	noncoding	-0.0004096	0	0	882
MSTRG.26413.1	noncoding	-0.12288	798	855	2210
MSTRG.26415.1	noncoding	-0.221184	186	234	701
MSTRG.26408.1	noncoding	-0.0188416	1473	1563	1728
MSTRG.26439.1	coding	0.23	0	729	2663
MSTRG.26448.3	noncoding	-0.0557056	0	519	7988
MSTRG.26448.1	coding	0.04	30	4944	9313
MSTRG.26448.2	coding	0.042	39	3579	7948
MSTRG.26448.5	coding	0.04	39	4953	9130
MSTRG.26448.6	coding	0.136	0	519	5474
MSTRG.26448.7	noncoding	-0.0401408	3	498	2807
MSTRG.26452.3	coding	0.426	0	249	1883
MSTRG.26453.1	noncoding	-0.065536	1785	1833	5024

MSTRG.26455.1	coding	0.016	1239	1440	3176
MSTRG.26446.4	coding	0.138	0	642	1044
MSTRG.26446.5	coding	0.069	246	777	1180
MSTRG.26477.1	noncoding	-0.34816	246	264	562
MSTRG.26491.1	noncoding	-0.004096	7287	7431	13411
MSTRG.26493.1	noncoding	-0.0065536	6078	6213	8540
MSTRG.26495.1	noncoding	-0.0004096	0	0	570
MSTRG.26498.1	noncoding	-0.0086016	480	627	2470
MSTRG.26498.2	coding	0.149	831	1173	2910
MSTRG.26461.4	coding	0.415	69	1032	6779
MSTRG.26461.2	coding	0.299	69	1668	4952
MSTRG.26461.3	coding	0.299	69	1668	4680
MSTRG.26461.5	coding	0.292	66	1689	5441
MSTRG.26469.2	coding	0.242	0	186	1226
MSTRG.26470.2	noncoding	-0.0110592	0	333	950
MSTRG.26470.3	coding	0.064	114	264	936
MSTRG.26470.4	noncoding	-0.036864	51	150	943
MSTRG.26515.1	noncoding	-0.0135168	54	69	542
MSTRG.26515.2	noncoding	-0.0135168	54	69	495
MSTRG.26515.3	noncoding	-0.04096	183	201	484
MSTRG.26521.1	noncoding	-0.098304	0	81	238
MSTRG.26533.1	noncoding	-0.0004096	0	0	1136
MSTRG.26541.1	noncoding	-0.028672	33	171	449
MSTRG.26567.1	noncoding	-0.0172032	333	339	669
MSTRG.26569.2	coding	0.232	414	2475	2828
MSTRG.26569.5	coding	0.225	414	2382	2735
MSTRG.26569.6	coding	0.226	414	2331	2684
MSTRG.26569.7	coding	0.231	414	2427	2780
MSTRG.26569.8	coding	0.193	414	1995	2348
MSTRG.26569.3	coding	0.177	435	2013	2368
MSTRG.26569.4	coding	0.217	435	2208	2563
MSTRG.26569.10	coding	0.231	417	2433	2762
MSTRG.26569.11	coding	0.216	435	2352	2682
MSTRG.26569.12	coding	0.2	435	2064	2394
MSTRG.26569.9	noncoding	-0.1445888	372	1548	2682
MSTRG.26570.1	noncoding	-0.016384	3195	3333	7061
MSTRG.26571.1	coding	0.068	24858	25440	33450
MSTRG.26571.2	noncoding	-0.0446464	9489	9684	14308
MSTRG.26573.1	coding	0.044	7767	8697	10341
MSTRG.26574.1	noncoding	-0.282624	663	714	2247
MSTRG.26574.2	noncoding	-0.110592	1908	1983	5220
MSTRG.26571.3	coding	0.107	12243	12576	18727
MSTRG.26572.4	noncoding	-0.0299008	2790	2910	3130
MSTRG.26572.5	noncoding	-0.06144	3027	3096	3284
MSTRG.26572.6	noncoding	-0.047104	3318	3510	9534
MSTRG.26572.7	noncoding	-0.0139264	1911	2070	2309
MSTRG.26572.8	noncoding	-0.028672	969	1020	2007
MSTRG.26572.9	noncoding	-0.0139264	822	981	1299
MSTRG.26572.10	noncoding	-0.0237568	0	153	8995
MSTRG.26572.11	noncoding	-0.028672	2886	2985	3965
MSTRG.26572.12	noncoding	-0.0073728	867	1083	5635
MSTRG.26572.13	noncoding	-0.0049152	468	612	1101
MSTRG.26572.14	coding	0.081	357	735	891
MSTRG.26572.15	noncoding	-0.036864	2424	2619	2886

MSTRG.26572.16	noncoding	-0.036864	1968	2163	2430
MSTRG.26572.17	noncoding	-0.114688	2328	2376	2618
MSTRG.26575.1	coding	0.01	513	657	1122
MSTRG.26542.4	coding	0.07	114	573	3006
MSTRG.26542.3	coding	0.07	114	573	3111
MSTRG.26542.6	noncoding	-0.0004096	1218	1356	2986
MSTRG.26552.3	coding	0.164	1125	3687	6140
MSTRG.26552.5	coding	0.203	207	2637	5089
MSTRG.26553.2	coding	0.269	1200	3204	3978
MSTRG.26553.1	coding	0.269	693	2697	5365
MSTRG.26554.2	coding	0.176	3063	7563	15592
MSTRG.26554.1	coding	0.175	633	5109	13137
MSTRG.26554.4	coding	0.171	0	4605	10289
MSTRG.26554.3	coding	0.168	537	5133	10817
MSTRG.26554.5	coding	0.172	990	4224	9845
MSTRG.26555.3	coding	0.378	0	9240	10721
MSTRG.26557.2	coding	0.288	867	4383	5570
MSTRG.26546.1	coding	0.292	651	2778	3161
MSTRG.26547.1	noncoding	-0.100352	2193	2637	2801
MSTRG.26547.2	coding	0.172	0	402	1189
MSTRG.26548.3	coding	0.237	51	1560	2002
MSTRG.26548.5	coding	0.26	0	1515	1961
MSTRG.26560.1	coding	0.034	408	1920	3969
MSTRG.26563.1	coding	0.538	0	6369	7566
MSTRG.26586.3	coding	0.123	132	3204	3880
MSTRG.26593.1	noncoding	-0.012288	2058	2193	3523
MSTRG.26585.2	noncoding	-0.0004096	381	525	3641
MSTRG.26594.3	coding	0.311	81	1416	2292
MSTRG.26595.1	coding	0.11	363	5817	8872
MSTRG.26596.1	noncoding	-0.057344	576	630	2139
MSTRG.26598.1	coding	0.16	420	753	5320
MSTRG.26602.2	coding	0.395	111	3690	4051
MSTRG.26602.3	coding	0.441	24	3234	3595
MSTRG.26601.2	coding	0.367	9	1266	1916
MSTRG.26611.2	noncoding	-0.043008	3357	3528	3685
MSTRG.26614.1	coding	0.127	1146	1302	2622
MSTRG.26615.1	noncoding	-0.0262144	9	63	507
MSTRG.26615.2	noncoding	-0.0262144	24	78	352
MSTRG.26616.1	noncoding	-0.0884736	9	273	630
MSTRG.26616.2	noncoding	-0.1114112	0	126	659
MSTRG.26617.2	coding	0.235	39	852	1034
MSTRG.26617.3	coding	0.233	10854	11850	12032
MSTRG.26617.4	coding	0.233	1920	2916	3096
MSTRG.26620.4	noncoding	-0.0864256	0	294	588
MSTRG.26624.4	coding	0.289	0	2784	2951
MSTRG.26623.3	coding	0.306	0	528	796
MSTRG.26630.3	coding	0.361	0	324	2431
MSTRG.26630.2	coding	0.35	0	345	5018
MSTRG.26630.4	coding	0.35	0	345	4532
MSTRG.26636.2	noncoding	-0.323584	4236	4266	6429
MSTRG.26637.4	coding	0.365	0	3048	10247
MSTRG.26637.5	coding	0.379	294	2805	3446
MSTRG.26644.1	coding	0.504	0	2955	5081
MSTRG.26645.1	noncoding	-0.0258048	1896	2130	3179

MSTRG.26644.2	coding	0.504	0	2955	5081
MSTRG.26644.5	coding	0.517	0	2811	10489
MSTRG.26642.1	coding	0.371	51	372	942
MSTRG.26643.1	noncoding	-0.0626688	309	468	3302
MSTRG.26646.1	coding	0.289	0	315	1512
MSTRG.26647.1	noncoding	-0.0274432	372	846	1053
MSTRG.26647.2	coding	0.434	0	369	1120
MSTRG.26651.1	noncoding	-0.012288	270	354	512
MSTRG.26652.1	coding	0.064	1584	2388	7735
MSTRG.26657.2	coding	0.165	270	630	2391
MSTRG.26658.2	coding	0.136	327	642	2534
MSTRG.26660.2	coding	0.181	3435	3813	5979
MSTRG.26660.1	coding	0.181	510	888	3056
MSTRG.26660.8	coding	0.181	492	870	2773
MSTRG.26660.6	coding	0.091	1083	1815	2622
MSTRG.26660.10	coding	0.181	1095	1473	3368
MSTRG.26660.11	noncoding	-0.012288	2400	2529	4747
MSTRG.26660.12	coding	0.181	1098	1476	10064
MSTRG.26665.1	noncoding	-0.0651264	1503	1845	2000
MSTRG.26662.1	noncoding	-0.016384	9	108	464
MSTRG.26667.1	noncoding	-0.004096	288	363	604
MSTRG.26668.1	noncoding	-0.086016	36	114	553
MSTRG.26671.1	noncoding	-0.0233472	360	579	871
MSTRG.26675.2	coding	0.342	225	1950	2951
MSTRG.26675.6	coding	0.342	291	2016	3007
MSTRG.26675.7	coding	0.388	1089	1830	2822
MSTRG.26675.5	coding	0.338	9	1917	2908
MSTRG.26675.8	coding	0.388	1455	2196	2778
MSTRG.26675.11	coding	0.21	534	1338	1880
MSTRG.26675.12	coding	0.195	174	891	7139
MSTRG.26677.2	noncoding	-0.014336	3447	3603	8769
MSTRG.26677.1	noncoding	-0.012288	339	375	888
MSTRG.26680.2	coding	0.229	0	1683	2512
MSTRG.26684.1	coding	0.319	0	1875	2340
MSTRG.26684.2	coding	0.378	2202	3462	3926
MSTRG.26684.4	coding	0.412	1941	3168	3633
MSTRG.26684.6	coding	0.38	405	1758	2223
MSTRG.26685.3	coding	0.758	0	741	6828
MSTRG.26685.2	coding	0.738	0	813	6100
MSTRG.26686.1	coding	0.593	426	1239	2589
MSTRG.26688.1	noncoding	-0.0520192	822	1359	1573
MSTRG.26707.1	noncoding	-0.0299008	0	30	291
MSTRG.26709.1	noncoding	-0.0733184	0	396	737
MSTRG.26710.1	coding	0.201	708	1815	2046
MSTRG.26710.3	coding	0.157	0	1608	1839
MSTRG.26710.11	coding	0.201	414	1521	1751
MSTRG.26710.12	coding	0.197	426	1905	2061
MSTRG.26711.1	noncoding	-0.028672	624	762	1567
MSTRG.26708.1	noncoding	-0.024576	183	651	1562
MSTRG.26715.1	noncoding	-0.0913408	474	1200	1488
MSTRG.26715.2	coding	0.223	144	870	2068
MSTRG.26715.3	coding	0.223	144	870	1650
MSTRG.26715.4	coding	0.146	75	1221	1546
MSTRG.26715.7	noncoding	-0.0913408	360	1086	1378

MSTRG.26715.8	coding	0.223	351	1077	1370
MSTRG.26715.9	noncoding	-0.1028096	504	891	1184
MSTRG.26720.2	coding	0.461	477	1323	2280
MSTRG.26720.5	coding	0.442	1644	2739	3617
MSTRG.26720.6	noncoding	-0.0004096	2286	2454	5043
MSTRG.26720.7	coding	0.436	1257	2421	3182
MSTRG.26712.4	coding	0.419	0	789	1156
MSTRG.26712.3	coding	0.232	33	828	1604
MSTRG.26712.2	coding	0.248	42	870	1872
MSTRG.26713.1	noncoding	-0.0434176	102	273	1712
MSTRG.26722.2	noncoding	-0.1236992	0	558	5158
MSTRG.26722.3	coding	0.213	1092	3180	3959
MSTRG.26722.4	coding	0.186	468	1311	2091
MSTRG.26725.5	coding	0.389	0	3822	6552
MSTRG.26725.6	coding	0.375	0	4566	5600
MSTRG.26725.8	coding	0.36	735	4782	7473
MSTRG.26726.2	coding	0.558	402	1290	4979
MSTRG.26726.1	coding	0.281	213	474	761
MSTRG.26727.1	noncoding	-0.0004096	2601	2844	3312
MSTRG.26727.2	noncoding	-0.0774144	0	1050	2825
MSTRG.26727.4	coding	0.239	186	2517	4725
MSTRG.26727.3	coding	0.239	189	2520	6225
MSTRG.26727.5	coding	0.28	0	1794	5053
MSTRG.26728.1	noncoding	-0.02048	1797	1887	7643
MSTRG.26728.2	noncoding	-0.0200704	6951	7116	13009
MSTRG.26729.1	coding	0.023	2523	3108	4199
MSTRG.26739.3	coding	0.363	186	1827	5892
MSTRG.26739.4	coding	0.496	2550	7572	8374
MSTRG.26739.5	coding	0.496	579	5601	6385
MSTRG.26741.1	noncoding	-0.053248	2739	2850	3175
MSTRG.26744.1	coding	0.579	111	2118	3671
MSTRG.26744.4	coding	0.507	18	2472	4193
MSTRG.26742.2	coding	0.71	0	606	1350
MSTRG.26742.3	coding	0.578	99	840	1564
MSTRG.26748.1	noncoding	-0.0774144	0	684	6922
MSTRG.26749.1	coding	0.156	525	1743	2172
MSTRG.26749.2	coding	0.157	411	1671	2322
MSTRG.26751.1	coding	0.175	0	2559	3083
MSTRG.26751.4	noncoding	-0.1695744	0	84	599
MSTRG.26751.6	noncoding	-0.026624	849	1032	3999
MSTRG.26751.5	coding	0.126	0	1845	6493
MSTRG.26751.7	coding	0.168	0	2211	2733
MSTRG.26751.3	coding	0.173	0	2229	2753
MSTRG.26751.8	noncoding	-0.065536	5562	5631	7139
MSTRG.26753.1	noncoding	-0.0053248	2412	2502	9522
MSTRG.26751.9	coding	0.142	4302	5727	9230
MSTRG.26754.1	coding	0.029	873	1041	3635
MSTRG.26757.2	coding	0.607	213	1473	1724
MSTRG.26773.20	noncoding	-0.047104	51	933	2750
MSTRG.26774.2	coding	0.378	729	1920	2517
MSTRG.26776.1	noncoding	-0.0999424	0	606	1291
MSTRG.26777.2	coding	0.423	645	3837	4830
MSTRG.26777.3	coding	0.436	150	3231	4222
MSTRG.26765.1	noncoding	-0.0770048	45	228	383

MSTRG.26770.1	noncoding	-0.0487424	84	948	1206
MSTRG.26770.2	noncoding	-0.0413696	0	972	1289
MSTRG.26790.1	coding	0.562	822	2322	2479
MSTRG.26790.3	coding	0.549	0	3897	4705
MSTRG.26788.2	coding	0.289	24	441	649
MSTRG.26786.1	coding	0.075	1440	1803	2795
MSTRG.26792.3	coding	0.43	0	6198	6502
MSTRG.26792.4	coding	0.434	0	6072	6376
MSTRG.26793.2	coding	0.713	192	11454	11611
MSTRG.26793.3	coding	0.715	0	11415	11569
MSTRG.26795.2	noncoding	-0.0876544	0	549	1252
MSTRG.26795.3	noncoding	-0.0815104	0	678	1381
MSTRG.26796.1	coding	0.328	1059	2151	3648
MSTRG.26796.2	coding	0.441	0	918	2195
MSTRG.26796.3	coding	0.361	78	945	2100
MSTRG.26798.2	coding	0.386	0	786	7218
MSTRG.26803.3	coding	0.586	0	2610	5115
MSTRG.26804.1	noncoding	-0.024576	96	1017	1397
MSTRG.26805.1	noncoding	-0.0724992	63	795	949
MSTRG.26806.7	noncoding	-0.0978944	1440	1932	2089
MSTRG.26806.8	noncoding	-0.1056768	1440	1716	2130
MSTRG.26806.4	noncoding	-0.0856064	0	336	1916
MSTRG.26806.5	noncoding	-0.06144	6873	8688	8957
MSTRG.26808.1	noncoding	-0.1056768	0	711	1283
MSTRG.26808.2	coding	0.29	0	804	1375
MSTRG.26821.1	coding	0.234	822	1089	3340
MSTRG.26821.3	coding	0.22	252	522	824
MSTRG.26821.4	coding	0.193	798	1059	1362
MSTRG.26821.5	coding	0.224	1002	1227	1531
MSTRG.26821.6	coding	0.109	57	528	831
MSTRG.26830.1	noncoding	-0.0892928	2274	2592	2762
MSTRG.26830.2	noncoding	-0.0892928	1377	1695	1895
MSTRG.26830.3	noncoding	-0.0303104	3600	3765	3920
MSTRG.26828.2	coding	0.322	0	3699	10021
MSTRG.26835.2	noncoding	-0.045056	285	372	612
MSTRG.26836.2	coding	0.15	1956	3492	4066
MSTRG.26836.3	noncoding	-0.0012288	762	906	1061
MSTRG.26837.2	coding	0.206	0	1440	7391
MSTRG.26837.4	coding	0.135	0	3420	4305
MSTRG.26837.5	coding	0.149	762	2127	2530
MSTRG.26841.1	noncoding	-0.069632	870	960	2359
MSTRG.26843.2	coding	0.154	15	924	5441
MSTRG.26844.1	coding	0.028	117	588	872
MSTRG.26845.1	noncoding	-0.0090112	1530	1602	2105
MSTRG.26853.1	coding	0.048	3729	4500	5206
MSTRG.26838.1	noncoding	-0.0585728	441	618	891
MSTRG.26855.1	noncoding	-0.0319488	96	246	635
MSTRG.26856.1	noncoding	-0.06144	141	231	561
MSTRG.26863.4	coding	0.153	732	1143	1795
MSTRG.26863.5	coding	0.152	702	1968	7534
MSTRG.26864.1	noncoding	-0.024576	72	132	2875
MSTRG.26865.1	noncoding	-0.0425984	2742	3015	6081
MSTRG.26866.1	noncoding	-0.008192	477	606	2174
MSTRG.26867.1	noncoding	-0.004096	3570	3777	5904

MSTRG.26868.1	noncoding	-0.0004096	792	936	2593
MSTRG.26868.2	noncoding	-0.0004096	885	1029	16993
MSTRG.26868.3	noncoding	-0.0008192	4740	4830	7457
MSTRG.26870.1	noncoding	-0.090112	837	882	1038
MSTRG.26858.1	noncoding	-0.0372736	0	9	236
MSTRG.26861.1	noncoding	-0.0086016	138	288	469
MSTRG.26861.2	noncoding	-0.024576	549	678	832
MSTRG.26889.8	coding	0.492	1236	2238	3603
MSTRG.26889.9	coding	0.504	438	1680	3044
MSTRG.26889.4	coding	0.522	96	1254	2618
MSTRG.26889.7	coding	0.421	12	1503	2869
MSTRG.26889.6	coding	0.418	78	2082	3448
MSTRG.26889.5	coding	0.504	4431	5673	7039
MSTRG.26889.3	coding	0.418	4146	6156	7520
MSTRG.26889.2	coding	0.465	2652	3990	5356
MSTRG.26889.10	noncoding	-0.0507904	891	1122	1278
MSTRG.26889.11	noncoding	-0.0544768	891	1320	1574
MSTRG.26890.1	noncoding	-0.0282624	108	198	794
MSTRG.26889.12	coding	0.228	78	924	2361
MSTRG.26891.1	coding	0.049	192	360	11176
MSTRG.26892.1	coding	0.094	666	948	1256
MSTRG.26894.1	noncoding	-0.0581632	3756	4035	4400
MSTRG.26879.2	coding	0.494	15	813	3704
MSTRG.26879.3	coding	0.403	1377	1758	4626
MSTRG.26880.2	noncoding	-0.1826816	0	318	10402
MSTRG.26898.1	noncoding	-0.0167936	1422	1584	2319
MSTRG.26903.2	coding	0.136	0	2199	2460
MSTRG.26904.1	coding	0.044	186	396	733
MSTRG.26902.1	coding	0.565	1122	3867	4022
MSTRG.26910.3	coding	0.342	12	372	918
MSTRG.26910.4	coding	0.311	147	483	1357
MSTRG.26910.5	coding	0.342	15	375	920
MSTRG.26919.8	coding	0.127	759	2754	5194
MSTRG.26919.5	coding	0.127	2388	4944	7384
MSTRG.26919.1	coding	0.121	0	4359	6800
MSTRG.26919.2	coding	0.12	0	4362	6803
MSTRG.26919.3	coding	0.116	0	4251	6692
MSTRG.26919.7	coding	0.12	0	4362	6500
MSTRG.26919.9	coding	0.12	0	4425	6680
MSTRG.26919.10	coding	0.113	0	4137	6360
MSTRG.26920.1	coding	0.046	225	381	1263
MSTRG.26932.1	coding	0.299	0	1473	2056
MSTRG.26932.4	coding	0.296	0	1251	2425
MSTRG.26932.5	coding	0.327	270	1446	2619
MSTRG.26932.7	coding	0.381	855	2028	3203
MSTRG.26932.6	coding	0.332	852	1923	3030
MSTRG.26932.8	coding	0.332	513	1584	2229
MSTRG.26932.9	coding	0.357	516	1515	2684
MSTRG.26932.10	coding	0.369	75	903	2101
MSTRG.26933.3	coding	0.266	102	2253	2508
MSTRG.26937.2	coding	0.392	177	9072	10567
MSTRG.26937.5	coding	0.389	198	9297	10584
MSTRG.26929.1	coding	0.405	267	2628	3108
MSTRG.26929.2	coding	0.373	0	2871	3435

MSTRG.26930.1	noncoding	-0.077824	738	771	1055
MSTRG.26941.9	coding	0.184	0	1320	1849
MSTRG.26941.8	coding	0.19	0	6849	7469
MSTRG.26941.10	coding	0.189	0	6921	7628
MSTRG.26941.11	coding	0.189	0	6945	7565
MSTRG.26941.13	coding	0.189	966	7458	8164
MSTRG.26941.14	coding	0.168	723	1626	2065
MSTRG.26941.15	noncoding	-0.0004096	645	816	2945
MSTRG.26942.1	coding	0.319	0	1050	5669
MSTRG.26943.1	noncoding	-0.0352256	3525	3996	7969
MSTRG.26942.3	coding	0.398	1299	1830	4697
MSTRG.26945.1	coding	0.512	0	2241	3118
MSTRG.26944.11	coding	0.354	1161	1830	2269
MSTRG.26953.3	coding	0.175	0	597	3517
MSTRG.26953.2	coding	0.049	6075	7389	10041
MSTRG.26954.1	noncoding	-0.090112	546	552	2264
MSTRG.26955.2	coding	0.213	1245	2874	3033
MSTRG.26955.1	coding	0.231	1245	3096	3351
MSTRG.26955.3	coding	0.209	531	1563	1817
MSTRG.26955.5	coding	0.223	138	1896	2049
MSTRG.26957.1	noncoding	-0.069632	3912	4410	9652
MSTRG.26962.1	noncoding	-0.086016	156	177	331
MSTRG.26974.1	coding	0.461	78	3972	4316
MSTRG.26988.1	coding	0.072	4734	4953	9158
MSTRG.26988.2	noncoding	-0.032768	885	963	1126
MSTRG.26990.1	coding	0.258	0	1083	4178
MSTRG.26990.2	coding	0.228	2088	3051	4062
MSTRG.26990.3	coding	0.213	324	1527	4117
MSTRG.26994.1	noncoding	-0.0446464	465	666	3115
MSTRG.26990.4	coding	0.23	102	1389	5581
MSTRG.26990.6	coding	0.119	93	303	779
MSTRG.26990.5	coding	0.23	333	1620	4722
MSTRG.26997.1	coding	0.166	0	1002	1156
MSTRG.26998.1	noncoding	-0.0458752	24	333	488
MSTRG.27003.4	coding	0.195	0	1233	3064
MSTRG.27006.6	coding	0.238	252	897	1098
MSTRG.27006.8	coding	0.238	147	795	995
MSTRG.27006.9	coding	0.25	219	873	1073
MSTRG.27006.7	coding	0.229	228	948	1148
MSTRG.27006.5	coding	0.21	81	1398	1600
MSTRG.27006.4	coding	0.199	78	1536	1738
MSTRG.27006.2	coding	0.135	126	4221	4422
MSTRG.27007.1	noncoding	-0.043008	336	576	1886
MSTRG.27008.1	noncoding	-0.0339968	417	528	884
MSTRG.27011.1	noncoding	-0.0004096	0	0	727
MSTRG.27002.4	coding	0.474	0	2478	5211
MSTRG.27014.1	noncoding	-0.07168	3102	3507	3662
MSTRG.27017.6	noncoding	-0.0258048	4338	4554	7266
MSTRG.27017.5	noncoding	-0.012288	2841	2952	3106
MSTRG.27017.7	coding	0.411	1458	1926	6089
MSTRG.27018.1	noncoding	-0.0274432	2904	3105	3262
MSTRG.27017.9	coding	0.107	2385	3897	6875
MSTRG.27017.10	noncoding	-0.196608	1695	1722	2286
MSTRG.27017.11	noncoding	-0.196608	1626	1653	2127

MSTRG.27012.3	coding	0.521	273	2499	5673
MSTRG.27012.2	coding	0.542	273	2613	5794
MSTRG.27019.2	coding	0.277	0	555	711
MSTRG.27021.2	noncoding	-0.008192	204	348	1125
MSTRG.27023.1	noncoding	-0.04096	1941	2376	2530
MSTRG.27027.1	noncoding	-0.1196032	0	72	460
MSTRG.27027.2	noncoding	-0.1441792	0	60	361
MSTRG.27029.1	noncoding	-0.004096	120	243	400
MSTRG.27031.2	coding	0.528	0	1959	2498
MSTRG.27035.4	noncoding	-0.034816	0	237	797
MSTRG.27035.6	noncoding	-0.0466944	0	474	1279
MSTRG.27035.3	coding	0.222	702	2670	3025
MSTRG.27035.5	coding	0.187	0	2457	2810
MSTRG.27035.7	noncoding	-0.0151552	1614	1755	2186
MSTRG.27038.2	coding	0.106	549	1275	2264
MSTRG.27038.3	coding	0.118	768	1038	2028
MSTRG.27038.4	coding	0.118	621	891	1879
MSTRG.27038.5	coding	0.136	792	1119	2109
MSTRG.27039.1	noncoding	-0.0356352	255	456	744
MSTRG.27039.2	noncoding	-0.016384	372	480	634
MSTRG.27069.1	noncoding	-0.0356352	2163	2316	3125
MSTRG.27069.2	coding	0.046	9591	9888	12338
MSTRG.27069.3	noncoding	-0.0356352	2838	2991	3801
MSTRG.27042.2	coding	0.053	603	1494	3301
MSTRG.27043.1	coding	0.198	630	1065	1407
MSTRG.27045.4	coding	0.079	303	1638	7059
MSTRG.27045.5	coding	0.084	0	1407	5385
MSTRG.27045.6	coding	0.079	216	1554	3909
MSTRG.27045.7	coding	0.078	183	1524	3576
MSTRG.27045.8	coding	0.081	468	1989	3097
MSTRG.27073.2	coding	0.172	0	4065	4762
MSTRG.27073.5	coding	0.204	1824	4782	5480
MSTRG.27073.6	coding	0.204	2676	5634	6330
MSTRG.27073.7	coding	0.173	420	4044	4486
MSTRG.27073.8	coding	0.191	1200	3834	4818
MSTRG.27073.9	coding	0.208	138	3072	3514
MSTRG.27051.1	noncoding	-0.0790528	0	123	419
MSTRG.27078.1	noncoding	-0.088064	534	1104	1398
MSTRG.27083.2	noncoding	-0.0602112	0	132	1285
MSTRG.27083.3	noncoding	-0.0659456	0	177	1271
MSTRG.27085.4	coding	0.144	585	4485	4875
MSTRG.27086.2	coding	0.126	45	909	1412
MSTRG.27098.1	coding	0.329	342	1062	1523
MSTRG.27098.2	coding	0.335	177	903	1344
MSTRG.27098.3	coding	0.346	195	948	1389
MSTRG.27099.1	noncoding	-0.028672	321	489	685
MSTRG.27099.2	noncoding	-0.045056	321	417	571
MSTRG.27101.3	coding	0.328	336	1893	3406
MSTRG.27101.2	coding	0.404	435	2973	3655
MSTRG.27101.1	coding	0.444	2631	4545	4991
MSTRG.27101.4	coding	0.403	414	2961	3338
MSTRG.27101.5	coding	0.442	2781	4710	5057
MSTRG.27101.10	coding	0.459	966	2259	2569
MSTRG.27101.11	coding	0.353	0	2283	2843

MSTRG.27101.12	coding	0.444	0	2274	2834
MSTRG.27101.8	coding	0.378	0	1293	2591
MSTRG.27101.14	coding	0.342	2574	4536	4877
MSTRG.27101.16	coding	0.402	414	2976	3275
MSTRG.27103.1	noncoding	-0.0053248	0	234	388
MSTRG.27104.1	coding	0.484	783	2094	2474
MSTRG.27104.2	coding	0.484	654	1965	2345
MSTRG.27104.3	coding	0.484	978	2289	2665
MSTRG.27104.4	coding	0.484	924	2229	2607
MSTRG.27104.5	coding	0.484	1992	3297	3682
MSTRG.27104.6	coding	0.484	960	2271	2655
MSTRG.27101.17	coding	0.287	702	1230	2177
MSTRG.27091.2	coding	0.328	177	1074	1386
MSTRG.27092.1	noncoding	-0.0671744	1308	1557	1714
MSTRG.27092.2	noncoding	-0.0536576	3309	4449	4639
MSTRG.27092.3	noncoding	-0.073728	1269	1479	1646
MSTRG.27092.4	noncoding	-0.067584	1257	1545	2467
MSTRG.27092.5	noncoding	-0.0663552	0	273	10772
MSTRG.27092.6	noncoding	-0.0663552	0	273	1754
MSTRG.27094.1	noncoding	-0.0700416	156	360	603
MSTRG.27092.7	noncoding	-0.012288	5955	6084	6301
MSTRG.27114.1	coding	0.117	780	4890	7032
MSTRG.27118.1	coding	0.078	1395	2061	2218
MSTRG.27118.2	noncoding	-0.028672	405	471	1548
MSTRG.27119.1	noncoding	-0.0421888	7602	7773	28383
MSTRG.27119.2	noncoding	-0.0110592	3057	3177	10814
MSTRG.27118.3	coding	0.093	1083	1740	2603
MSTRG.27118.4	coding	0.093	1053	1710	28225
MSTRG.27123.1	noncoding	-0.0466944	1578	1980	2253
MSTRG.27124.2	coding	0.492	660	2223	3816
MSTRG.27124.5	noncoding	-0.0114688	3621	3795	5672
MSTRG.27125.1	noncoding	-0.0385024	462	666	7874
MSTRG.27124.6	coding	0.439	1434	3924	8970
MSTRG.27124.7	noncoding	-0.0536576	1542	1821	2288
MSTRG.27106.1	coding	0.062	5493	5943	6759
MSTRG.27109.1	coding	0.25	1077	2193	3750
MSTRG.27129.1	noncoding	-0.0806912	45	51	593
MSTRG.27143.1	noncoding	-0.155648	21	45	288
MSTRG.27144.1	noncoding	-0.4096	297	324	1862
MSTRG.27150.1	noncoding	-0.0417792	2985	4227	13749
MSTRG.27156.1	noncoding	-0.053248	387	645	802
MSTRG.27157.2	noncoding	-0.0724992	1245	1473	1628
MSTRG.27179.1	noncoding	-0.0360448	0	102	2263
MSTRG.27191.1	coding	0.245	612	1230	2636
MSTRG.27192.1	coding	0.097	150	810	6349
MSTRG.27192.3	coding	0.155	1188	2289	2724
MSTRG.27192.2	coding	0.155	138	1239	2330
MSTRG.27194.1	noncoding	-0.0073728	399	543	992
MSTRG.27198.1	coding	0.079	198	1212	1462
MSTRG.27202.5	coding	0.112	1023	4347	7854
MSTRG.27217.1	noncoding	-0.073728	2154	2223	2791
MSTRG.27217.2	noncoding	-0.12288	0	180	2658
MSTRG.27212.1	noncoding	-0.090112	81	135	950
MSTRG.27221.2	coding	0.595	0	798	8526

MSTRG.27222.1	noncoding	-0.0442368	4233	4437	7667
MSTRG.27223.1	noncoding	-0.032768	237	516	1958
MSTRG.27226.1	coding	0.076	0	1188	3178
MSTRG.27226.4	coding	0.111	549	1167	1536
MSTRG.27226.5	coding	0.064	0	1611	1970
MSTRG.27230.2	coding	0.282	0	540	1130
MSTRG.27230.4	noncoding	-0.069632	249	306	848
MSTRG.27230.9	noncoding	-0.053248	42	528	17853
MSTRG.27230.16	noncoding	-0.002048	3759	3879	10102
MSTRG.27230.17	noncoding	-0.045056	2172	2271	3250
MSTRG.27239.1	noncoding	-0.0462848	1734	2052	8164
MSTRG.27239.2	noncoding	-0.0253952	123	156	1976
MSTRG.27282.1	noncoding	-0.0851968	1377	1572	2577
MSTRG.27282.3	noncoding	-0.0851968	5409	5604	6533
MSTRG.27282.11	noncoding	-0.024576	792	918	7752
MSTRG.27282.10	noncoding	-0.0512	5103	5304	15051
MSTRG.27282.12	noncoding	-0.012288	5250	5367	7715
MSTRG.27282.14	coding	0.082	1317	1590	5520
MSTRG.27282.15	noncoding	-0.0749568	1998	2154	7631
MSTRG.27285.1	coding	0.096	18096	19005	25719
MSTRG.27282.16	noncoding	-0.0323584	738	861	1301
MSTRG.27282.20	coding	0.453	0	96	2124
MSTRG.27282.21	noncoding	-0.126976	2760	3282	7448
MSTRG.27282.18	noncoding	-0.157696	2475	3627	6457
MSTRG.27288.1	noncoding	-0.0487424	0	267	1262
MSTRG.27291.1	coding	0.199	1005	2412	3885
MSTRG.27291.2	noncoding	-0.0663552	1431	1821	2177
MSTRG.27292.1	noncoding	-0.032768	366	489	3200
MSTRG.27293.1	noncoding	-0.0077824	474	477	1339
MSTRG.27291.7	coding	0.45	2103	2514	2669
MSTRG.27291.8	coding	0.437	2103	2499	2654
MSTRG.27247.3	coding	0.15	123	414	975
MSTRG.27247.2	coding	0.193	15	336	899
MSTRG.27248.1	coding	0.035	897	1239	1421
MSTRG.27250.1	noncoding	-0.016384	1338	1416	3336
MSTRG.27251.1	coding	0.089	165	1635	2096
MSTRG.27255.2	coding	0.217	825	1932	3016
MSTRG.27256.1	noncoding	-0.0192512	393	489	1233
MSTRG.27263.1	noncoding	-0.1323008	0	189	1574
MSTRG.27264.1	noncoding	-0.045056	1929	2016	3442
MSTRG.27263.2	noncoding	-0.004096	528	600	1435
MSTRG.27263.3	coding	0.449	210	3144	4661
MSTRG.27268.1	noncoding	-0.0045056	2460	2676	4142
MSTRG.27272.1	noncoding	-0.0004096	426	480	2533
MSTRG.27276.1	noncoding	-0.03072	2331	2457	5461
MSTRG.27275.2	noncoding	-0.0454656	30	279	6367
MSTRG.27304.3	coding	0.273	573	885	4010
MSTRG.27305.1	noncoding	-0.0004096	44361	44484	57734
MSTRG.27305.2	noncoding	-0.036864	1548	1737	16087
MSTRG.27305.3	noncoding	-0.0086016	7017	7068	9143
MSTRG.27305.4	noncoding	-0.0086016	6408	6459	8536
MSTRG.27305.5	noncoding	-0.004096	5997	6210	32178
MSTRG.27305.6	noncoding	-0.004096	5793	6006	6409
MSTRG.27304.6	noncoding	-0.065536	2256	2364	6100

MSTRG.27304.7	noncoding	-0.0192512	6555	6726	17641
MSTRG.27304.8	noncoding	-0.008192	16287	16479	20421
MSTRG.27304.9	noncoding	-0.008192	7485	7677	20171
MSTRG.27304.10	coding	0.27	1440	5508	8477
MSTRG.27304.11	coding	0.268	13701	17787	20758
MSTRG.27304.12	noncoding	-0.032768	2598	2703	5233
MSTRG.27307.1	noncoding	-0.069632	4944	4986	6486
MSTRG.27304.13	noncoding	-0.114688	4767	4845	7729
MSTRG.27308.1	noncoding	-0.0385024	3024	3207	10288
MSTRG.27308.2	noncoding	-0.0385024	4752	4935	5677
MSTRG.27304.14	noncoding	-0.016384	1488	1596	1955
MSTRG.27309.1	noncoding	-0.0004096	4077	4260	8059
MSTRG.27309.2	noncoding	-0.0004096	5763	5946	8394
MSTRG.27310.1	noncoding	-0.073728	759	762	915
MSTRG.27311.1	noncoding	-0.0339968	228	390	692
MSTRG.27277.1	noncoding	-0.090112	24	114	873
MSTRG.27278.1	noncoding	-0.0438272	276	522	3276
MSTRG.27300.3	coding	0.128	5373	8001	9520
MSTRG.27300.4	coding	0.119	0	4422	4749
MSTRG.27300.5	coding	0.423	5940	8358	8515
MSTRG.27302.1	noncoding	-0.0192512	231	393	3268
MSTRG.27303.5	noncoding	-0.0294912	3378	3582	10722
MSTRG.27312.1	coding	0.273	237	1179	1436
MSTRG.27314.2	coding	0.149	114	2622	4999
MSTRG.27314.3	noncoding	-0.0114688	123	159	400
MSTRG.27329.1	noncoding	-0.0380928	8832	9039	16212
MSTRG.27329.2	noncoding	-0.0126976	2793	3057	4998
MSTRG.27328.4	noncoding	-0.0667648	234	903	2138
MSTRG.27330.1	noncoding	-0.0188416	3252	3405	7332
MSTRG.27368.2	noncoding	-0.024576	321	438	594
MSTRG.27368.3	noncoding	-0.004096	57	372	526
MSTRG.27368.4	coding	0.082	5898	6513	20866
MSTRG.27368.5	coding	0.175	1029	1473	1842
MSTRG.27368.6	noncoding	-0.0032768	11574	11697	14287
MSTRG.27370.1	noncoding	-0.135168	2655	2718	5241
MSTRG.27368.7	noncoding	-0.028672	2739	2865	12502
MSTRG.27373.1	coding	0.066	9585	9789	12296
MSTRG.27337.1	noncoding	-0.06144	153	228	869
MSTRG.27338.3	coding	0.104	0	2091	2631
MSTRG.27338.4	coding	0.11	693	2349	2888
MSTRG.27339.1	noncoding	-0.581632	1497	1503	2449
MSTRG.27340.1	coding	0.171	7137	7356	8953
MSTRG.27343.3	noncoding	-0.1912832	4386	4827	7466
MSTRG.27343.4	coding	0.454	4203	4755	7393
MSTRG.27343.6	noncoding	-0.1880064	507	933	2961
MSTRG.27343.8	noncoding	-0.1855488	6339	6822	8850
MSTRG.27359.4	coding	0.187	882	1650	2280
MSTRG.27362.3	coding	0.229	1764	3159	3626
MSTRG.27391.4	coding	0.175	1071	1998	4063
MSTRG.27391.2	coding	0.172	588	1638	3705
MSTRG.27391.7	coding	0.148	156	2256	4094
MSTRG.27391.8	coding	0.202	201	1224	3061
MSTRG.27391.10	coding	0.152	81	2130	2457
MSTRG.27391.9	coding	0.165	1281	3570	3897

MSTRG.27393.3	coding	0.546	0	276	2074
MSTRG.27393.4	coding	0.052	561	816	3085
MSTRG.27400.3	coding	0.06	1452	10560	10824
MSTRG.27400.4	coding	0.027	345	3540	3804
MSTRG.27401.1	coding	0.109	0	1548	2447
MSTRG.27401.2	coding	0.109	0	1548	3599
MSTRG.27401.3	coding	0.109	0	1548	3028
MSTRG.27401.4	coding	0.081	57	1545	6109
MSTRG.27404.1	noncoding	-0.0466944	909	1122	1277
MSTRG.27411.2	noncoding	-0.032768	144	456	8317
MSTRG.27412.1	noncoding	-0.0045056	1359	1497	1784
MSTRG.27411.3	coding	0.239	138	1740	3199
MSTRG.27413.1	noncoding	-0.0135168	1644	2193	5844
MSTRG.27413.2	noncoding	-0.0380928	2634	2910	12636
MSTRG.27413.3	noncoding	-0.0380928	4470	4746	12713
MSTRG.27411.5	coding	0.24	1734	3087	4547
MSTRG.27378.6	coding	0.224	0	447	973
MSTRG.27378.7	coding	0.087	1347	3048	4668
MSTRG.27378.8	coding	0.057	3324	5040	8056
MSTRG.27378.9	coding	0.065	1335	1749	4764
MSTRG.27387.1	noncoding	-0.045056	36	153	665
MSTRG.27387.2	noncoding	-0.045056	36	153	502
MSTRG.27387.3	noncoding	-0.016384	0	39	610
MSTRG.27387.4	noncoding	-0.028672	0	42	261
MSTRG.27387.5	noncoding	-0.016384	0	39	259
MSTRG.27387.6	noncoding	-0.016384	0	39	264
MSTRG.27387.7	coding	0.061	114	462	946
MSTRG.27387.8	coding	0.061	96	444	826
MSTRG.27387.9	noncoding	-0.016384	0	39	261
MSTRG.27387.10	noncoding	-0.016384	0	39	259
MSTRG.27387.11	noncoding	-0.016384	0	39	364
MSTRG.27420.1	coding	0.169	0	564	1431
MSTRG.27420.2	coding	0.319	0	1992	3726
MSTRG.27431.1	noncoding	-0.110592	84	90	326
MSTRG.27435.3	coding	0.27	321	4275	9905
MSTRG.27436.1	noncoding	-0.0487424	2469	2616	2773
MSTRG.27440.1	noncoding	-0.0663552	39	45	343
MSTRG.27427.9	coding	0.382	309	2247	2432
MSTRG.27427.8	coding	0.425	0	2454	2641
MSTRG.27427.7	coding	0.409	114	2394	2579
MSTRG.27427.10	coding	0.379	552	2523	2687
MSTRG.27427.11	coding	0.378	426	2355	2509
MSTRG.27427.12	coding	0.403	153	2331	2485
MSTRG.27427.13	coding	0.433	141	2148	2441
MSTRG.27428.2	noncoding	-0.0438272	237	486	1166
MSTRG.27428.1	noncoding	-0.02048	114	279	912
MSTRG.27428.3	noncoding	-0.0507904	261	567	771
MSTRG.27428.4	noncoding	-0.0004096	552	675	1004
MSTRG.27445.2	noncoding	-0.0729088	0	864	1778
MSTRG.27446.1	noncoding	-0.086016	195	201	1135
MSTRG.27449.1	coding	0.021	3891	4053	5388
MSTRG.27454.3	coding	0.121	612	2754	4230
MSTRG.27454.2	coding	0.121	540	2682	4424
MSTRG.27459.1	noncoding	-0.1130496	138	1017	1556

MSTRG.27459.2	noncoding	-0.1191936	0	345	968
MSTRG.27458.8	coding	0.137	5154	6063	6626
MSTRG.27461.1	coding	0.188	0	4896	7612
MSTRG.27461.3	coding	0.188	273	4863	7580
MSTRG.27461.5	coding	0.189	3225	8136	10853
MSTRG.27461.6	coding	0.186	3225	7971	10688
MSTRG.27461.7	coding	0.193	1623	6117	8833
MSTRG.27461.10	coding	0.189	6	4947	7664
MSTRG.27461.11	coding	0.184	18	4713	7430
MSTRG.27462.1	noncoding	-0.0688128	30	417	599
MSTRG.27462.2	noncoding	-0.0557056	30	375	583
MSTRG.27462.3	noncoding	-0.0565248	3	366	1701
MSTRG.27457.3	coding	0.395	276	7833	8009
MSTRG.27474.4	coding	0.497	1899	6366	7045
MSTRG.27474.2	coding	0.548	5052	8091	8770
MSTRG.27474.6	coding	0.548	6480	9519	10199
MSTRG.27475.1	coding	0.482	0	564	799
MSTRG.27479.1	coding	0.447	0	2286	3429
MSTRG.27480.2	coding	0.588	783	13488	13645
MSTRG.27480.1	coding	0.588	999	13704	13861
MSTRG.27480.4	coding	0.589	999	13245	13401
MSTRG.27480.5	coding	0.528	78	2109	2748
MSTRG.27480.6	coding	0.588	567	13269	13425
MSTRG.27480.8	coding	0.592	177	5580	5737
MSTRG.27480.9	coding	0.588	915	8298	9693
MSTRG.27480.7	coding	0.556	273	13776	13932
MSTRG.27480.10	coding	0.437	102	1632	2154
MSTRG.27480.12	coding	0.437	252	1782	2301
MSTRG.27463.1	noncoding	-0.016384	21	87	314
MSTRG.27466.2	coding	0.335	345	1743	2421
MSTRG.27466.3	coding	0.348	0	1311	1986
MSTRG.27466.4	coding	0.359	648	1830	2502
MSTRG.27467.2	coding	0.495	0	1143	1607
MSTRG.27469.3	coding	0.313	696	2574	2835
MSTRG.27471.2	coding	0.363	0	1158	1340
MSTRG.27471.6	coding	0.352	0	864	2137
MSTRG.27473.1	noncoding	-0.049152	291	348	701
MSTRG.27471.7	coding	0.227	246	624	1330
MSTRG.27471.8	noncoding	-0.0589824	4929	5478	8675
MSTRG.27486.2	coding	0.343	261	723	946
MSTRG.27486.3	coding	0.343	360	822	1044
MSTRG.27487.2	coding	0.361	0	2271	2459
MSTRG.27489.4	coding	0.331	459	1857	2383
MSTRG.27489.5	coding	0.352	927	2043	2856
MSTRG.27489.6	noncoding	-0.0253952	6	243	1081
MSTRG.27489.7	coding	0.372	390	1416	2127
MSTRG.27483.1	coding	0.261	405	1089	1550
MSTRG.27507.2	noncoding	-0.0557056	3696	4056	4269
MSTRG.27507.3	coding	0.136	1221	1581	1792
MSTRG.27507.4	coding	0.136	978	1338	1549
MSTRG.27507.5	coding	0.136	1197	1557	1769
MSTRG.27507.7	noncoding	-0.0557056	1881	2241	2452
MSTRG.27507.8	coding	0.148	81	540	1212
MSTRG.27508.1	noncoding	-0.1024	0	426	702

MSTRG.27510.1	coding	0.338	5466	9483	12576
MSTRG.27512.12	coding	0.39	93	747	1129
MSTRG.27510.3	coding	0.338	1437	5454	5621
MSTRG.27510.4	coding	0.33	2313	6714	7896
MSTRG.27510.5	coding	0.322	2967	7314	7482
MSTRG.27510.6	noncoding	-0.0262144	495	645	2779
MSTRG.27514.1	coding	0.265	339	4869	5371
MSTRG.27514.2	coding	0.265	711	5241	5743
MSTRG.27515.1	noncoding	-0.0208896	93	252	904
MSTRG.27521.1	coding	0.36	0	6252	8781
MSTRG.27521.2	coding	0.368	231	6150	8676
MSTRG.27521.3	coding	0.367	504	6318	8808
MSTRG.27524.2	coding	0.413	618	1929	3311
MSTRG.27526.1	coding	0.274	597	1620	2324
MSTRG.27526.4	coding	0.299	729	1464	1946
MSTRG.27497.1	coding	0.498	0	1326	1630
MSTRG.27499.2	coding	0.319	111	1425	1939
MSTRG.27499.4	coding	0.309	369	1740	3618
MSTRG.27505.1	coding	0.17	354	3537	4184
MSTRG.27505.5	coding	0.12	2889	3228	9971
MSTRG.27502.1	coding	0.293	0	2934	3090
MSTRG.27533.4	coding	0.522	261	1650	1843
MSTRG.27540.1	noncoding	-0.0483328	165	342	3171
MSTRG.27572.2	coding	0.302	0	573	1881
MSTRG.27573.4	noncoding	-0.0884736	0	225	1644
MSTRG.27573.3	noncoding	-0.0790528	0	312	2289
MSTRG.27573.2	noncoding	-0.1138688	0	525	1789
MSTRG.27580.6	coding	0.134	840	1971	2637
MSTRG.27580.7	coding	0.135	303	1428	2094
MSTRG.27580.8	coding	0.134	639	1770	2437
MSTRG.27580.5	coding	0.134	462	1593	2259
MSTRG.27580.9	coding	0.083	2481	2673	7065
MSTRG.27581.3	coding	0.28	15324	16731	17085
MSTRG.27581.6	noncoding	-0.1081344	729	1191	3461
MSTRG.27581.5	coding	0.28	11388	12795	13149
MSTRG.27584.1	coding	0.436	0	4203	4358
MSTRG.27584.10	coding	0.44	0	4257	4587
MSTRG.27584.11	coding	0.425	0	4488	4818
MSTRG.27584.9	coding	0.438	0	4227	5467
MSTRG.27584.12	coding	0.441	0	4161	7281
MSTRG.27584.13	coding	0.433	0	4401	4731
MSTRG.27584.14	coding	0.45	195	4296	4627
MSTRG.27584.15	coding	0.449	9	4155	4487
MSTRG.27587.3	coding	0.502	0	258	644
MSTRG.27587.2	coding	0.302	783	1671	1945
MSTRG.27588.4	coding	0.325	0	1554	1748
MSTRG.27589.2	coding	0.373	0	3948	4229
MSTRG.27589.4	coding	0.433	0	1632	1889
MSTRG.27590.5	coding	0.313	363	1719	2132
MSTRG.27541.1	noncoding	-0.0475136	90	387	1086
MSTRG.27541.2	noncoding	-0.0135168	0	144	1252
MSTRG.27544.2	coding	0.346	0	1215	1371
MSTRG.27544.3	coding	0.336	14598	15801	15977
MSTRG.27547.1	coding	0.416	0	7242	11928

MSTRG.27548.2	coding	0.41	321	2553	2911
MSTRG.27550.1	noncoding	-0.0069632	81	114	354
MSTRG.27554.1	noncoding	-0.0098304	135	387	1651
MSTRG.27555.1	noncoding	-0.006144	84	324	729
MSTRG.27555.2	noncoding	-0.1183744	0	168	598
MSTRG.27555.3	noncoding	-0.1183744	0	168	532
MSTRG.27556.1	coding	0.31	630	1167	2223
MSTRG.27557.1	coding	0.175	0	2976	3711
MSTRG.27558.2	coding	0.446	0	6471	7161
MSTRG.27559.4	coding	0.242	96	492	998
MSTRG.27559.5	coding	0.264	246	618	1125
MSTRG.27560.1	noncoding	-0.0794624	459	792	1016
MSTRG.27563.1	coding	0.156	222	5325	7636
MSTRG.27563.10	coding	0.298	0	300	9780
MSTRG.27563.9	coding	0.165	0	5232	14221
MSTRG.27563.6	coding	0.246	14055	17277	19728
MSTRG.27563.7	coding	0.259	0	3339	5622
MSTRG.27563.8	coding	0.27	0	3018	5301
MSTRG.27563.11	coding	0.246	2409	5631	8082
MSTRG.27563.12	coding	0.249	9153	12423	14728
MSTRG.27563.13	coding	0.248	489	3678	3851
MSTRG.27563.14	noncoding	-0.137216	0	75	701
MSTRG.27563.15	coding	0.245	1437	4677	6982
MSTRG.27565.1	coding	0.06	2208	3264	5518
MSTRG.27563.16	coding	0.25	426	3582	5885
MSTRG.27563.17	coding	0.244	6702	9672	12124
MSTRG.27567.5	coding	0.549	351	3576	3734
MSTRG.27567.6	coding	0.536	471	3888	4046
MSTRG.27569.2	coding	0.211	657	3696	6188
MSTRG.27596.1	coding	0.48	600	4407	4625
MSTRG.27596.3	coding	0.47	0	3981	4201
MSTRG.27596.4	coding	0.469	0	3984	4204
MSTRG.27597.2	noncoding	-0.004096	921	1062	1446
MSTRG.27617.2	coding	0.457	321	1134	1481
MSTRG.27617.3	coding	0.453	984	1803	2150
MSTRG.27617.4	coding	0.453	642	1461	1809
MSTRG.27620.8	noncoding	-0.0786432	4668	5277	5434
MSTRG.27620.2	coding	0.16	3111	7779	8518
MSTRG.27620.3	coding	0.16	3177	7845	8583
MSTRG.27620.4	coding	0.16	5205	9873	10612
MSTRG.27620.5	coding	0.16	4632	9300	10037
MSTRG.27620.6	coding	0.16	3102	7770	8509
MSTRG.27620.7	coding	0.16	3114	7782	8521
MSTRG.27620.9	coding	0.141	4770	7842	10091
MSTRG.27620.10	coding	0.16	8388	13056	13489
MSTRG.27620.11	coding	0.16	2910	7578	8033
MSTRG.27620.12	noncoding	-0.06144	1920	1989	2289
MSTRG.27622.2	coding	0.432	0	8481	8649
MSTRG.27624.2	noncoding	-0.0864256	0	237	1217
MSTRG.27624.3	noncoding	-0.19456	24138	24639	24976
MSTRG.27598.2	coding	0.422	927	2634	2874
MSTRG.27598.3	coding	0.406	1617	3420	3662
MSTRG.27601.1	noncoding	-0.0720896	0	132	342
MSTRG.27602.1	noncoding	-0.073728	129	201	1103

MSTRG.27605.1	coding	0.093	57	708	1116
MSTRG.27605.5	coding	0.162	342	1140	1467
MSTRG.27605.2	coding	0.136	0	1452	1880
MSTRG.27605.4	coding	0.121	0	1401	1829
MSTRG.27606.1	coding	0.001	300	618	1136
MSTRG.27599.2	coding	0.143	24	426	1821
MSTRG.27630.1	noncoding	-0.0258048	885	1041	2206
MSTRG.27633.1	noncoding	-0.0765952	357	570	2605
MSTRG.27626.1	coding	0.519	546	4917	8761
MSTRG.27626.2	noncoding	-0.0700416	0	159	414
MSTRG.27626.3	noncoding	-0.06144	9	288	523
MSTRG.27627.1	noncoding	-0.006144	2286	2442	2598
MSTRG.27628.1	noncoding	-0.0004096	1701	1866	4170
MSTRG.27651.1	coding	0.509	1092	2811	3109
MSTRG.27651.2	coding	0.511	1932	3639	3939
MSTRG.27651.3	coding	0.386	546	1452	1751
MSTRG.27655.1	coding	0.269	282	984	5658
MSTRG.27655.2	coding	0.295	585	1068	1771
MSTRG.27666.2	coding	0.435	0	597	823
MSTRG.27667.1	noncoding	-0.0188416	738	906	1921
MSTRG.27668.6	coding	0.352	714	1959	4141
MSTRG.27668.7	coding	0.388	681	1056	3304
MSTRG.27668.8	coding	0.3	255	1236	2779
MSTRG.27668.9	coding	0.352	255	1500	3682
MSTRG.27668.10	noncoding	-0.067584	1383	2475	2676
MSTRG.27669.5	coding	0.326	0	267	710
MSTRG.27669.6	coding	0.326	0	267	1134
MSTRG.27669.7	noncoding	-0.0770048	873	1434	1588
MSTRG.27669.8	noncoding	-0.0503808	6	381	1714
MSTRG.27659.1	noncoding	-0.0008192	54	201	573
MSTRG.27673.1	noncoding	-0.0028672	4767	5067	5223
MSTRG.27676.3	coding	0.297	0	2127	2366
MSTRG.27676.4	coding	0.31	0	1968	2624
MSTRG.27676.6	coding	0.298	0	2112	2349
MSTRG.27676.7	coding	0.298	0	2109	2346
MSTRG.27676.8	coding	0.292	3	2139	2378
MSTRG.27676.10	coding	0.292	360	2511	2750
MSTRG.27680.1	coding	0.457	627	2550	6498
MSTRG.27680.3	coding	0.457	372	2295	6243
MSTRG.27680.5	coding	0.221	246	720	4957
MSTRG.27680.4	coding	0.457	624	2547	3388
MSTRG.27684.4	noncoding	-0.0966656	39	579	732
MSTRG.27684.3	noncoding	-0.0970752	153	690	845
MSTRG.27684.2	coding	0.349	198	1083	1236
MSTRG.27689.1	noncoding	-0.0098304	123	378	562
MSTRG.27690.2	coding	0.288	156	462	616
MSTRG.27695.1	coding	0.326	69	1590	2698
MSTRG.27695.3	coding	0.334	165	1467	1625
MSTRG.27704.1	noncoding	-0.0135168	1911	2046	2779
MSTRG.27706.1	noncoding	-0.0126976	1086	1203	4061
MSTRG.27707.2	noncoding	-0.04096	306	402	4113
MSTRG.27707.1	coding	0.274	2046	2307	2892
MSTRG.27707.4	noncoding	-0.0638976	288	600	2717
MSTRG.27708.2	coding	0.305	186	438	593

MSTRG.27708.3	coding	0.463	0	399	553
MSTRG.27717.5	coding	0.523	0	4326	5718
MSTRG.27717.1	coding	0.526	0	4458	5849
MSTRG.27717.2	coding	0.526	0	4458	5769
MSTRG.27720.1	noncoding	-0.126976	123	180	517
MSTRG.27722.1	noncoding	-0.016384	804	927	1604
MSTRG.27718.2	coding	0.493	0	1248	1827
MSTRG.27719.1	noncoding	-0.0270336	495	1284	1462
MSTRG.27724.4	coding	0.365	369	2397	5955
MSTRG.27724.5	coding	0.353	129	2070	5604
MSTRG.27730.2	coding	0.164	3	255	652
MSTRG.27731.3	coding	0.41	1575	4197	5376
MSTRG.27732.2	coding	0.336	0	1851	3353
MSTRG.27725.1	noncoding	-0.1343488	0	105	577
MSTRG.27737.2	coding	0.519	1545	6363	7787
MSTRG.27738.1	noncoding	-0.0516096	255	2436	3556
MSTRG.27747.1	coding	0.537	0	441	1720
MSTRG.27747.2	coding	0.599	0	3888	7226
MSTRG.27747.3	coding	0.563	0	4011	7221
MSTRG.27747.4	coding	0.573	0	4098	7388
MSTRG.27748.1	noncoding	-0.0827392	195	1170	8388
MSTRG.27748.2	noncoding	-0.083968	9552	10563	16981
MSTRG.27751.1	noncoding	-0.07168	0	357	511
MSTRG.27747.5	coding	0.533	1419	5136	8333
MSTRG.27752.1	noncoding	-0.1015808	0	336	492
MSTRG.27753.1	noncoding	-0.0786432	0	99	483
MSTRG.27747.6	coding	0.563	159	3894	7232
MSTRG.27747.8	coding	0.592	0	3612	6935
MSTRG.27754.1	noncoding	-0.0442368	0	2478	3724
MSTRG.27744.2	coding	0.447	0	1215	2182
MSTRG.27745.1	coding	0.395	18	3504	7312
MSTRG.27745.3	coding	0.339	3	4317	4471
MSTRG.27769.1	noncoding	-0.0262144	5391	6297	8995
MSTRG.27772.3	coding	0.441	0	882	1614
MSTRG.27772.4	coding	0.447	0	1008	1740
MSTRG.27772.6	coding	0.453	0	1005	1271
MSTRG.27778.2	noncoding	-0.1155072	0	231	3893
MSTRG.27781.2	coding	0.497	0	2229	2664
MSTRG.27755.1	coding	0.405	69	1818	3015
MSTRG.27757.1	coding	0.632	0	915	1076
MSTRG.27782.3	coding	0.228	0	2448	3303
MSTRG.27783.1	noncoding	-0.0557056	195	1017	1900
MSTRG.27784.1	coding	0.46	39	1863	2215
MSTRG.27784.3	coding	0.552	564	1776	2127
MSTRG.27784.2	coding	0.639	0	1389	1739
MSTRG.27786.1	coding	0.692	1188	4080	4700
MSTRG.27793.1	noncoding	-0.0569344	54	363	622
MSTRG.27794.1	noncoding	-0.036864	261	336	492
MSTRG.27795.1	noncoding	-0.0815104	4248	4536	6604
MSTRG.27796.4	coding	0.63	0	5787	5944
MSTRG.27798.3	noncoding	-0.1347584	0	804	1067
MSTRG.27800.1	noncoding	-0.1073152	0	792	1549
MSTRG.27802.2	coding	0.443	0	1953	2424
MSTRG.27802.3	coding	0.419	0	2064	2438

MSTRG.27802.4	coding	0.423	936	2589	3051
MSTRG.27802.5	coding	0.397	330	2025	2398
MSTRG.27802.6	coding	0.409	4254	6054	6452
MSTRG.27804.2	noncoding	-0.08192	57	426	686
MSTRG.27804.3	noncoding	-0.0638976	123	546	699
MSTRG.27801.1	coding	0.274	126	879	1916
MSTRG.27801.2	coding	0.452	0	558	1223
MSTRG.27807.2	coding	0.379	0	2280	3010
MSTRG.27808.4	coding	0.296	4164	6111	6668
MSTRG.27810.2	noncoding	-0.0442368	375	525	857
MSTRG.27814.1	coding	0.383	4008	5880	7588
MSTRG.27814.4	coding	0.378	3072	4395	7720
MSTRG.27814.5	coding	0.357	4302	5976	7684
MSTRG.27814.12	coding	0.368	1311	2247	4373
MSTRG.27814.14	coding	0.35	3993	5241	6948
MSTRG.27818.1	noncoding	-0.075776	339	714	1373
MSTRG.27814.15	noncoding	-0.0483328	1584	2298	2506
MSTRG.27814.16	noncoding	-0.047104	1584	2292	2496
MSTRG.27814.17	noncoding	-0.0610304	9	810	2519
MSTRG.27838.3	coding	0.198	306	1731	2210
MSTRG.27838.2	coding	0.189	156	1581	2061
MSTRG.27838.4	coding	0.18	0	1545	2026
MSTRG.27839.1	noncoding	-0.26624	108	141	295
MSTRG.27840.1	noncoding	-0.0233472	5127	5208	7854
MSTRG.27842.1	noncoding	-0.0540672	0	186	917
MSTRG.27842.2	noncoding	-0.0319488	0	255	661
MSTRG.27846.1	coding	0.468	0	708	1805
MSTRG.27846.2	coding	0.365	5802	6351	7446
MSTRG.27846.3	coding	0.365	5043	5592	6689
MSTRG.27846.4	coding	0.12	15	3228	3791
MSTRG.27846.5	coding	0.12	15	3228	6995
MSTRG.27846.7	coding	0.102	291	3273	4021
MSTRG.27846.9	coding	0.103	987	3912	4660
MSTRG.27846.8	coding	0.103	2232	5163	6889
MSTRG.27846.11	coding	0.123	2514	5664	7042
MSTRG.27846.12	coding	0.103	2292	5223	6603
MSTRG.27847.1	noncoding	-0.004096	2754	2892	4676
MSTRG.27848.1	noncoding	-0.008192	213	354	1235
MSTRG.27846.13	coding	0.124	12960	14706	15330
MSTRG.27846.15	coding	0.174	291	1794	5344
MSTRG.27844.2	noncoding	-0.0622592	867	1113	1683
MSTRG.27884.3	coding	0.363	0	16785	17671
MSTRG.27884.6	coding	0.306	0	22566	23452
MSTRG.27885.1	noncoding	-0.0278528	1092	1263	4564
MSTRG.27884.11	coding	0.36	393	16944	17097
MSTRG.27884.9	coding	0.359	393	16926	17079
MSTRG.27884.8	coding	0.357	393	17064	17950
MSTRG.27884.12	coding	0.373	3555	19248	20201
MSTRG.27884.14	coding	0.407	9309	21399	22287
MSTRG.27884.15	coding	0.387	1038	14985	15873
MSTRG.27884.16	coding	0.382	948	8841	9728
MSTRG.27884.17	coding	0.383	897	8763	8918
MSTRG.27884.18	coding	0.378	879	8883	9770
MSTRG.27884.19	coding	0.402	2802	7620	7774

MSTRG.27884.20	coding	0.355	1839	3930	4084
MSTRG.27854.1	noncoding	-0.1507328	0	57	234
MSTRG.27852.1	coding	0.082	168	663	908
MSTRG.27862.1	noncoding	-0.002048	408	525	904
MSTRG.27863.2	coding	0.298	561	1593	2865
MSTRG.27864.1	noncoding	-0.1024	177	246	707
MSTRG.27865.1	noncoding	-0.131072	0	135	1877
MSTRG.27870.1	coding	0.42	0	2241	3064
MSTRG.27870.3	coding	0.411	141	2340	2890
MSTRG.27870.4	coding	0.432	0	2082	2435
MSTRG.27870.5	coding	0.36	0	1575	10828
MSTRG.27875.2	noncoding	-0.0057344	159	279	1037
MSTRG.27876.1	coding	0.311	0	696	1064
MSTRG.27878.5	coding	0.163	0	3747	4495
MSTRG.27880.3	coding	0.282	486	4551	4885
MSTRG.27882.2	noncoding	-0.0528384	717	2034	2761
MSTRG.27882.4	noncoding	-0.0299008	0	162	668
MSTRG.27882.3	coding	0.4	0	381	887
MSTRG.27893.3	coding	0.291	945	3195	3797
MSTRG.27901.7	coding	0.365	0	2427	2763
MSTRG.27901.8	coding	0.393	0	2169	2478
MSTRG.27904.1	noncoding	-0.079872	2883	3765	4257
MSTRG.27905.5	coding	0.346	96	2292	2557
MSTRG.27908.4	coding	0.275	0	507	691
MSTRG.27908.5	coding	0.13	36	531	713
MSTRG.27909.1	coding	0.471	549	4374	4549
MSTRG.27909.4	coding	0.509	0	2679	2853
MSTRG.27909.3	coding	0.508	0	2643	4650
MSTRG.27909.5	coding	0.507	0	2637	2810
MSTRG.27910.1	noncoding	-0.018432	0	189	758
MSTRG.27896.1	coding	0.519	261	867	2209
MSTRG.27896.2	noncoding	-0.1892352	264	675	2316
MSTRG.27897.1	coding	0.428	99	720	947
MSTRG.27897.2	coding	0.434	3	612	846
MSTRG.27897.5	coding	0.429	18	639	874
MSTRG.27900.2	coding	0.59	0	756	7295
MSTRG.27922.2	noncoding	-0.0667648	17424	17961	29322
MSTRG.27913.2	noncoding	-0.0913408	0	585	886
MSTRG.27925.1	coding	0.289	21	573	7301
MSTRG.27925.3	coding	0.289	21	573	7156
MSTRG.27928.1	coding	0.229	0	714	6947
MSTRG.27934.2	coding	0.443	0	3615	5842
MSTRG.27935.1	noncoding	-0.0454656	5196	5499	17159
MSTRG.27936.1	noncoding	-0.036864	1728	2019	4645
MSTRG.27941.3	coding	0.357	96	1644	1898
MSTRG.27942.1	noncoding	-0.0782336	930	1401	1557
MSTRG.27942.4	noncoding	-0.0622592	204	624	780
MSTRG.27942.3	noncoding	-0.0577536	417	870	1026
MSTRG.27942.2	noncoding	-0.0421888	930	1194	1349
MSTRG.27943.2	noncoding	-0.0503808	1377	2136	4731
MSTRG.27943.1	noncoding	-0.0503808	1377	2136	4856
MSTRG.27943.3	noncoding	-0.0585728	2415	2787	4298
MSTRG.27945.3	coding	0.43	0	1911	2563
MSTRG.27945.5	coding	0.404	453	2112	2772

MSTRG.27940.2	coding	0.496	123	1656	3076
MSTRG.27949.1	coding	0.394	0	1890	2901
MSTRG.27949.2	coding	0.389	0	1974	2918
MSTRG.27950.3	coding	0.222	0	1905	4194
MSTRG.27950.4	coding	0.202	0	1926	3931
MSTRG.27951.1	noncoding	-0.065536	1695	1734	2237
MSTRG.27950.6	noncoding	-0.0528384	4158	4845	5000
MSTRG.27952.1	noncoding	-0.057344	3081	3186	5464
MSTRG.27953.1	coding	0.069	3783	3948	5733
MSTRG.27955.1	noncoding	-0.0356352	1152	1179	1333
MSTRG.27954.5	coding	0.352	1194	2172	2331
MSTRG.27956.1	coding	0.165	0	2010	4376
MSTRG.27956.5	coding	0.098	252	1836	2300
MSTRG.27957.4	coding	0.184	741	1749	2306
MSTRG.27957.3	coding	0.187	1167	2532	3089
MSTRG.27957.5	coding	0.164	0	2529	3086
MSTRG.27957.6	coding	0.141	0	2205	3135
MSTRG.27957.7	coding	0.149	0	2445	3002
MSTRG.27957.8	coding	0.144	0	2769	3226
MSTRG.27957.9	coding	0.156	0	2685	3142
MSTRG.27957.10	coding	0.15	0	2331	2486
MSTRG.27959.1	noncoding	-0.0831488	0	870	4215
MSTRG.27960.1	noncoding	-0.090112	612	702	5448
MSTRG.27960.2	noncoding	-0.049152	852	918	1509
MSTRG.27961.1	noncoding	-0.004096	948	1173	1908
MSTRG.27963.2	coding	0.406	0	3618	5748
MSTRG.27967.4	coding	0.188	324	1506	4858
MSTRG.27967.5	coding	0.188	408	1590	4942
MSTRG.27967.6	coding	0.188	999	2181	5531
MSTRG.27967.7	coding	0.188	954	2136	5487
MSTRG.27968.3	noncoding	-0.004096	1977	2121	3082
MSTRG.27968.4	noncoding	-0.0475136	5388	5547	5778
MSTRG.27964.2	noncoding	-0.0454656	2691	2871	3834
MSTRG.27969.3	coding	0.259	132	861	1112
MSTRG.27969.4	coding	0.268	207	918	1167
MSTRG.27970.2	coding	0.413	9	522	1565
MSTRG.27975.5	coding	0.095	9	459	3194
MSTRG.27975.4	coding	0.054	393	771	1288
MSTRG.27975.7	noncoding	-0.0495616	270	450	5769
MSTRG.27983.2	coding	0.334	393	864	1241
MSTRG.27985.1	noncoding	-0.0045056	33	177	1159
MSTRG.27988.3	coding	0.223	18	1491	2290
MSTRG.27995.1	noncoding	-0.0692224	0	1398	3523
MSTRG.27995.3	noncoding	-0.155648	4713	4788	4944
MSTRG.27996.1	noncoding	-0.0139264	14703	14787	15075
MSTRG.27996.2	noncoding	-0.057344	2463	2538	13288
MSTRG.27997.1	noncoding	-0.0446464	4644	4788	8904
MSTRG.28003.6	coding	0.148	159	2241	2855
MSTRG.28004.1	noncoding	-0.002048	0	147	302
MSTRG.28005.1	coding	0.119	14112	14319	16691
MSTRG.28005.2	coding	0.119	10806	11013	16701
MSTRG.28003.7	coding	0.192	0	1104	8408
MSTRG.28003.8	coding	0.138	657	1749	7743
MSTRG.28003.9	noncoding	-0.172032	3693	3744	3899

MSTRG.28007.2	coding	0.152	45	381	916
MSTRG.28008.1	coding	0.108	0	14553	14854
MSTRG.28008.2	coding	0.108	0	14544	14845
MSTRG.28008.3	coding	0.077	0	10989	11285
MSTRG.28009.1	noncoding	-0.0110592	81	327	1026
MSTRG.28011.1	coding	0.198	0	75	232
MSTRG.28012.5	coding	0.553	1464	4740	6731
MSTRG.28012.4	coding	0.55	1464	4767	7544
MSTRG.28013.3	coding	0.451	8343	10242	11023
MSTRG.28018.2	noncoding	-0.0557056	5343	5580	9463
MSTRG.28033.6	coding	0.228	399	1236	1907
MSTRG.28033.8	coding	0.228	531	1368	2039
MSTRG.28033.5	coding	0.22	312	1167	1839
MSTRG.28033.7	coding	0.228	312	1149	1821
MSTRG.28034.2	coding	0.165	213	4182	4339
MSTRG.28034.4	coding	0.142	780	4092	4248
MSTRG.28034.9	coding	0.141	1341	3135	4080
MSTRG.28034.5	coding	0.161	1659	3501	3656
MSTRG.28034.6	coding	0.161	1575	3417	3573
MSTRG.28025.1	noncoding	-0.0253952	168	432	2498
MSTRG.28024.4	coding	0.459	0	1572	3284
MSTRG.28027.1	noncoding	-0.0622592	0	156	505
MSTRG.28027.2	noncoding	-0.0622592	0	156	509
MSTRG.28040.1	coding	0.362	234	2202	5493
MSTRG.28040.2	coding	0.41	975	2634	6067
MSTRG.28040.4	coding	0.41	990	2649	5162
MSTRG.28045.3	coding	0.357	264	762	4464
MSTRG.28035.2	noncoding	-0.0700416	471	1446	1600
MSTRG.28055.1	noncoding	-0.0004096	1575	1716	5599
MSTRG.28058.1	noncoding	-0.0278528	1866	1956	4377
MSTRG.28059.1	noncoding	-0.0004096	0	0	792
MSTRG.28051.1	coding	0.143	10617	13728	16919
MSTRG.28051.4	noncoding	-0.0688128	1716	1905	9462
MSTRG.28051.7	noncoding	-0.0004096	8304	8448	10536
MSTRG.28062.1	noncoding	-0.0004096	12	423	1098
MSTRG.28064.1	coding	0.413	0	714	868
MSTRG.28070.1	coding	0.454	0	5268	6527
MSTRG.28072.3	coding	0.353	282	1035	1192
MSTRG.28076.1	noncoding	-0.0229376	0	201	355
MSTRG.28075.1	coding	0.541	0	756	911
MSTRG.28081.1	coding	0.388	0	2910	3653
MSTRG.28085.6	coding	0.486	273	2010	2711
MSTRG.28091.4	noncoding	-0.0376832	3390	3600	5149
MSTRG.28091.2	coding	0.119	612	2130	3394
MSTRG.28093.2	coding	0.456	93	1749	2665
MSTRG.28095.3	noncoding	-0.0688128	342	543	698
MSTRG.28095.2	coding	0.445	0	459	869
MSTRG.28097.2	noncoding	-0.0724992	0	273	778
MSTRG.28098.2	coding	0.373	159	642	3168
MSTRG.28098.1	coding	0.326	0	642	3382
MSTRG.28098.3	coding	0.185	132	696	1121
MSTRG.28099.3	coding	0.517	3	2535	2690
MSTRG.28100.1	coding	0.39	0	2286	2530
MSTRG.28100.5	coding	0.39	0	2271	2514

MSTRG.28100.6	coding	0.418	0	2145	2301
MSTRG.28100.7	coding	0.415	0	2064	2652
MSTRG.28100.8	coding	0.387	0	2190	2434
MSTRG.28105.5	coding	0.359	0	1635	2237
MSTRG.28105.6	coding	0.354	0	1533	2133
MSTRG.28107.1	coding	0.456	873	3075	3409
MSTRG.28109.3	coding	0.075	0	798	1260
MSTRG.28109.4	coding	0.17	0	498	1202
MSTRG.28109.2	coding	0.139	0	639	1105
MSTRG.28109.5	coding	0.113	0	801	1264
MSTRG.28121.1	noncoding	-0.028672	5010	5076	6396
MSTRG.28122.1	noncoding	-0.057344	264	315	477
MSTRG.28115.4	noncoding	-0.0004096	987	987	1353
MSTRG.28114.2	coding	0.387	0	1251	2157
MSTRG.28127.1	noncoding	-0.0004096	138	159	464
MSTRG.28132.2	coding	0.025	618	1485	1768
MSTRG.28137.1	noncoding	-0.0651264	666	981	1137
MSTRG.28131.3	noncoding	-0.0290816	426	588	742
MSTRG.28141.1	noncoding	-0.008192	30	141	455
MSTRG.28142.2	coding	0.347	219	2097	3367
MSTRG.28147.1	coding	0.113	321	2229	5258
MSTRG.28154.1	noncoding	-0.0847872	0	45	388
MSTRG.28160.1	coding	0.117	189	2169	2618
MSTRG.28160.5	coding	0.165	183	693	3450
MSTRG.28161.1	noncoding	-0.049152	96	186	763
MSTRG.28162.1	coding	0.062	192	696	1842
MSTRG.28168.3	coding	0.23	0	1368	1725
MSTRG.28174.1	noncoding	-0.0319488	231	465	1950
MSTRG.28174.2	noncoding	-0.0212992	75	264	1551
MSTRG.28175.1	noncoding	-0.0831488	330	534	1340
MSTRG.28177.1	noncoding	-0.0032768	1965	2052	2366
MSTRG.28178.1	coding	0.075	2082	3387	7084
MSTRG.28171.1	noncoding	-0.0053248	153	237	474
MSTRG.28186.1	noncoding	-0.086016	1158	1173	1378
MSTRG.28191.1	noncoding	-0.026624	102	261	928
MSTRG.28191.8	noncoding	-0.0700416	0	123	835
MSTRG.28192.3	coding	0.25	201	768	1374
MSTRG.28204.2	coding	0.249	39	261	1544
MSTRG.28207.1	noncoding	-0.0372736	0	117	353
MSTRG.28207.2	noncoding	-0.016384	360	498	738
MSTRG.28207.3	noncoding	-0.024576	0	48	219
MSTRG.28211.2	coding	0.304	396	1068	2122
MSTRG.28213.2	coding	0.248	0	333	926
MSTRG.28194.2	coding	0.378	0	579	735
MSTRG.28196.2	noncoding	-0.032768	429	438	649
MSTRG.28197.1	noncoding	-0.0438272	363	441	595
MSTRG.28214.1	noncoding	-0.02048	672	675	829
MSTRG.28220.1	coding	0.283	225	2565	3701
MSTRG.28220.2	coding	0.291	0	1872	8055
MSTRG.28223.2	noncoding	-0.0851968	369	600	1479
MSTRG.28224.2	coding	0.383	366	1203	2339
MSTRG.28217.1	noncoding	-0.012288	408	534	738
MSTRG.28217.2	noncoding	-0.02048	399	615	904
MSTRG.28225.1	coding	0.188	243	1065	1218

MSTRG.28227.3	coding	0.384	951	2103	2275
MSTRG.28249.2	coding	0.391	0	5172	5390
MSTRG.28249.3	coding	0.391	0	5172	5390
MSTRG.28249.4	coding	0.395	0	5073	5654
MSTRG.28249.5	coding	0.392	0	5172	5389
MSTRG.28249.7	coding	0.41	6	2799	3012
MSTRG.28249.8	coding	0.391	6	1938	3247
MSTRG.28249.6	coding	0.392	0	5172	5385
MSTRG.28249.9	coding	0.406	984	5055	5252
MSTRG.28249.11	coding	0.394	0	5136	5289
MSTRG.28250.1	coding	0.404	0	585	742
MSTRG.28251.1	coding	0.365	897	2955	4619
MSTRG.28251.2	coding	0.365	897	2955	4733
MSTRG.28251.5	coding	0.365	591	2649	4236
MSTRG.28251.6	noncoding	-0.1097728	588	882	1323
MSTRG.28249.13	coding	0.396	0	5184	5424
MSTRG.28232.2	coding	0.425	0	3588	3743
MSTRG.28233.2	coding	0.476	0	1029	1185
MSTRG.28233.3	coding	0.471	0	1149	1304
MSTRG.28233.4	coding	0.454	0	1032	1186
MSTRG.28236.2	coding	0.495	1566	3567	3790
MSTRG.28236.4	coding	0.495	1650	3651	3875
MSTRG.28229.1	coding	0.364	0	1125	1724
MSTRG.28239.1	coding	0.32	0	2403	2900
MSTRG.28244.2	coding	0.426	1542	5445	5623
MSTRG.28244.3	noncoding	-0.0004096	69	177	666
MSTRG.28244.6	coding	0.462	51	3480	3660
MSTRG.28246.2	coding	0.296	78	717	1029
MSTRG.28247.1	coding	0.222	576	888	3934
MSTRG.28247.3	coding	0.228	498	963	2780
MSTRG.28247.5	coding	0.199	30	702	2321
MSTRG.28247.2	coding	0.254	705	1089	2905
MSTRG.28241.1	noncoding	-0.024576	423	594	902
MSTRG.28242.1	coding	0.383	0	696	3788
MSTRG.28242.3	coding	0.383	0	696	3447
MSTRG.28242.5	coding	0.392	0	660	1997
MSTRG.28243.1	coding	0.186	384	1455	1677
MSTRG.28255.2	coding	0.249	993	1449	1639
MSTRG.28256.5	coding	0.311	18	3501	3657
MSTRG.28257.2	coding	0.262	108	6477	6635
MSTRG.28257.3	coding	0.26	708	7146	7300
MSTRG.28257.4	coding	0.26	108	6486	6641
MSTRG.28300.1	noncoding	-0.034816	576	735	889
MSTRG.28300.2	noncoding	-0.1015808	504	1002	2467
MSTRG.28301.1	noncoding	-0.045056	0	72	446
MSTRG.28300.3	coding	0.403	33	1086	2551
MSTRG.28300.4	coding	0.415	12	966	1125
MSTRG.28300.5	coding	0.41	12	969	1125
MSTRG.28300.6	coding	0.393	12	1074	3728
MSTRG.28300.8	coding	0.434	0	900	1056
MSTRG.28300.9	coding	0.443	0	996	2465
MSTRG.28300.10	coding	0.34	0	1029	3682
MSTRG.28308.1	coding	0.441	165	2880	3321
MSTRG.28308.6	coding	0.444	381	3114	3555

MSTRG.28312.1	coding	0.4	39	3129	4750
MSTRG.28312.3	coding	0.402	12	2955	4267
MSTRG.28312.2	coding	0.394	210	3285	4597
MSTRG.28312.4	coding	0.395	93	3159	4151
MSTRG.28315.1	noncoding	-0.0069632	12	126	561
MSTRG.28316.8	coding	0.46	0	1530	1894
MSTRG.28319.1	coding	0.185	3	4533	4686
MSTRG.28319.2	coding	0.185	3	4536	4689
MSTRG.28319.4	coding	0.188	123	4470	4623
MSTRG.28319.6	coding	0.178	123	5043	5196
MSTRG.28321.1	coding	0.285	0	2061	3003
MSTRG.28325.2	coding	0.191	900	1827	3658
MSTRG.28325.5	coding	0.151	108	1008	1324
MSTRG.28324.3	coding	0.301	366	3219	4350
MSTRG.28326.1	coding	0.55	252	2946	6011
MSTRG.28328.2	noncoding	-0.0589824	567	1584	2517
MSTRG.28329.3	coding	0.383	9	1875	6399
MSTRG.28332.2	noncoding	-0.0561152	195	738	972
MSTRG.28300.13	noncoding	-0.0147456	276	456	2768
MSTRG.28266.4	coding	0.453	0	1050	1655
MSTRG.28266.5	coding	0.459	0	921	1075
MSTRG.28266.7	coding	0.541	0	375	530
MSTRG.28267.1	noncoding	-0.0528384	612	939	1096
MSTRG.28267.2	noncoding	-0.028672	609	828	985
MSTRG.28266.17	coding	0.485	0	267	422
MSTRG.28274.1	coding	0.09	99	300	1731
MSTRG.28274.2	coding	0.259	0	231	1661
MSTRG.28270.1	coding	0.198	435	2040	5243
MSTRG.28271.1	coding	0.281	387	918	3405
MSTRG.28281.2	coding	0.441	0	2859	4216
MSTRG.28281.3	coding	0.423	0	2868	4224
MSTRG.28281.4	coding	0.424	0	2949	4305
MSTRG.28281.5	coding	0.436	0	2778	3952
MSTRG.28281.6	coding	0.461	0	2511	3868
MSTRG.28281.7	coding	0.462	0	2565	3020
MSTRG.28281.8	coding	0.433	201	2793	4149
MSTRG.28281.9	coding	0.407	3180	4215	6589
MSTRG.28281.10	coding	0.43	432	1302	1842
MSTRG.28294.2	coding	0.221	1200	2226	2590
MSTRG.28297.3	noncoding	-0.0618496	51	276	589
MSTRG.28335.1	coding	0.112	555	2316	5461
MSTRG.28336.1	noncoding	-0.0229376	36	108	270
MSTRG.28335.2	coding	0.117	0	1857	4649
MSTRG.28335.3	coding	0.116	0	1839	5611
MSTRG.28338.1	coding	0.136	216	2598	2777
MSTRG.28339.3	coding	0.193	4959	6297	7347
MSTRG.28341.2	coding	0.31	261	1035	2906
MSTRG.28341.4	coding	0.336	261	966	3780
MSTRG.28342.3	coding	0.121	273	1860	12054
MSTRG.28342.4	coding	0.121	378	1965	10774
MSTRG.28343.1	noncoding	-0.024576	210	246	540
MSTRG.28367.1	coding	0.472	3	366	522
MSTRG.28367.2	coding	0.505	0	345	500
MSTRG.28368.2	coding	0.593	0	348	505

MSTRG.28370.1	coding	0.492	30	375	529
MSTRG.28371.1	coding	0.247	12	1083	1612
MSTRG.28372.1	coding	0.365	192	477	633
MSTRG.28375.1	noncoding	-0.0053248	741	759	913
MSTRG.28377.1	coding	0.249	0	762	1079
MSTRG.28371.2	coding	0.374	27	318	472
MSTRG.28371.3	coding	0.304	0	597	2690
MSTRG.28378.1	coding	0.295	39	330	582
MSTRG.28379.1	coding	0.548	0	537	691
MSTRG.28380.1	coding	0.496	0	369	704
MSTRG.28386.3	coding	0.547	0	324	479
MSTRG.28386.2	coding	0.554	0	312	466
MSTRG.28388.1	coding	0.651	0	333	488
MSTRG.28386.4	coding	0.515	0	312	473
MSTRG.28388.2	noncoding	-0.2998272	0	318	568
MSTRG.28389.1	coding	0.425	2274	2889	5896
MSTRG.28379.2	coding	0.534	0	357	526
MSTRG.28388.3	coding	0.744	0	324	573
MSTRG.28371.4	coding	0.443	0	303	458
MSTRG.28359.7	noncoding	-0.0167936	1608	1785	2822
MSTRG.28359.6	coding	0.257	432	1884	2921
MSTRG.28359.3	coding	0.275	2709	4011	5049
MSTRG.28359.5	coding	0.261	1143	3075	4112
MSTRG.28359.8	coding	0.257	1518	2967	3999
MSTRG.28359.9	coding	0.26	396	2331	3363
MSTRG.28359.10	coding	0.065	3201	3453	4134
MSTRG.28361.1	coding	0.206	318	507	1772
MSTRG.28362.1	noncoding	-0.118784	4020	4044	4245
MSTRG.28359.11	noncoding	-0.0659456	13947	14151	17006
MSTRG.28363.1	noncoding	-0.0585728	23235	23448	38489
MSTRG.28363.2	noncoding	-0.053248	1950	2055	3541
MSTRG.28363.3	noncoding	-0.0319488	11220	11475	15908
MSTRG.28366.4	coding	0.284	201	1962	3051
MSTRG.28366.5	coding	0.28	3180	6114	7689
MSTRG.28366.6	coding	0.291	2451	4176	5266
MSTRG.28393.1	noncoding	-0.0360448	3159	3978	4287
MSTRG.28393.3	coding	0.108	2241	2799	4163
MSTRG.28393.4	coding	0.108	2241	2799	3939
MSTRG.28393.6	noncoding	-0.069632	282	660	4177
MSTRG.28393.2	coding	0.112	2241	2802	4076
MSTRG.28397.1	coding	0.135	0	1245	1946
MSTRG.28399.2	coding	0.295	0	1551	1843
MSTRG.28400.1	coding	0.272	0	1539	2159
MSTRG.28400.2	coding	0.271	0	1518	2138
MSTRG.28400.3	coding	0.277	0	1596	2217
MSTRG.28401.1	noncoding	-0.024576	270	384	643
MSTRG.28391.2	coding	0.309	0	543	816
MSTRG.28403.1	noncoding	-0.1445888	0	9	215
MSTRG.28404.1	coding	0.049	426	813	1086
MSTRG.28409.2	noncoding	-0.0475136	2994	3033	3257
MSTRG.28409.1	noncoding	-0.0475136	2505	3054	3208
MSTRG.28409.3	noncoding	-0.004096	528	570	793
MSTRG.28409.4	noncoding	-0.004096	387	429	653
MSTRG.28409.6	noncoding	-0.02048	2076	2181	2722

MSTRG.28409.5	noncoding	-0.065536	180	249	666
MSTRG.28409.7	noncoding	-0.004096	7032	7074	7273
MSTRG.28409.8	noncoding	-0.016384	198	276	825
MSTRG.28410.1	noncoding	-0.016384	6096	6162	6408
MSTRG.28409.9	noncoding	-0.0016384	1992	2118	2666
MSTRG.28409.10	noncoding	-0.167936	2238	2298	4942
MSTRG.28411.1	noncoding	-0.0004096	2166	2241	3550
MSTRG.28409.11	noncoding	-0.012288	90	156	506
MSTRG.28449.3	coding	0.513	99	1407	1567
MSTRG.28457.1	coding	0.093	390	1485	2702
MSTRG.28457.2	coding	0.093	390	1485	2447
MSTRG.28457.5	coding	0.089	2556	3864	4758
MSTRG.28457.6	coding	0.078	405	1695	2588
MSTRG.28459.1	noncoding	-0.008192	765	873	3355
MSTRG.28460.1	noncoding	-0.0098304	3675	4086	8758
MSTRG.28463.1	noncoding	-0.0118784	3462	3726	5114
MSTRG.28464.1	noncoding	-0.0270336	8985	9078	11933
MSTRG.28466.1	noncoding	-0.057344	615	723	1305
MSTRG.28467.1	noncoding	-0.110592	870	927	3366
MSTRG.28470.1	noncoding	-0.04096	0	126	7759
MSTRG.28470.2	noncoding	-0.04096	0	126	7489
MSTRG.28471.2	coding	0.148	5583	6510	9975
MSTRG.28471.3	coding	0.148	5412	6339	9745
MSTRG.28474.1	noncoding	-0.012288	1998	2121	2967
MSTRG.28471.4	noncoding	-0.124928	0	141	15005
MSTRG.28476.2	coding	0.18	342	807	1780
MSTRG.28430.1	noncoding	-0.0544768	1257	1431	1586
MSTRG.28430.2	noncoding	-0.0233472	0	204	1162
MSTRG.28432.1	coding	0.408	156	816	1044
MSTRG.28433.1	noncoding	-0.0139264	525	627	887
MSTRG.28439.1	coding	0.24	0	2496	4365
MSTRG.28443.1	noncoding	-0.02048	429	471	653
MSTRG.28442.2	coding	0.072	345	1146	3749
MSTRG.28445.1	coding	0.446	0	789	4639
MSTRG.28480.2	noncoding	-0.0098304	27	162	318
MSTRG.28480.1	noncoding	-0.0446464	318	363	518
MSTRG.28480.3	noncoding	-0.0065536	2127	2274	2535
MSTRG.28481.3	coding	0.342	66	1332	2932
MSTRG.28485.1	noncoding	-0.016384	18303	18408	26684
MSTRG.28485.2	noncoding	-0.016384	3375	3480	12020
MSTRG.28485.3	noncoding	-0.012288	2067	2193	9093
MSTRG.28485.4	noncoding	-0.08192	453	567	3683
MSTRG.28485.5	noncoding	-0.0331776	1602	1815	3185
MSTRG.28485.6	noncoding	-0.0118784	39	174	3047
MSTRG.28487.2	coding	0.206	111	1749	1993
MSTRG.28487.3	coding	0.24	96	1659	1903
MSTRG.28488.1	coding	0.412	258	3918	7823
MSTRG.28488.2	coding	0.413	795	4446	8242
MSTRG.28488.3	coding	0.413	1053	4704	8498
MSTRG.28488.4	coding	0.412	531	4191	5157
MSTRG.28488.5	coding	0.405	13920	17451	18427
MSTRG.28488.7	noncoding	-0.0712704	11277	11505	19246
MSTRG.28489.1	noncoding	-0.0290816	9000	9153	14934
MSTRG.28488.8	noncoding	-0.0159744	45654	46131	56960

MSTRG.28488.9	noncoding	-0.047104	1125	1287	41772
MSTRG.28488.10	noncoding	-0.0057344	8484	8613	36069
MSTRG.28488.11	noncoding	-0.0507904	10110	10374	15024
MSTRG.28491.1	noncoding	-0.0126976	438	810	2030
MSTRG.28492.1	noncoding	-0.049152	36	138	486
MSTRG.28495.1	noncoding	-0.004096	1923	2016	2523
MSTRG.28495.2	noncoding	-0.049152	1590	1632	2085
MSTRG.28499.3	coding	0.415	261	1014	2516
MSTRG.28501.3	coding	0.31	0	984	4205
MSTRG.28501.4	noncoding	-0.0233472	315	474	3967
MSTRG.28502.1	noncoding	-0.188416	78	99	255
MSTRG.28505.3	coding	0.338	228	1029	1598
MSTRG.28509.4	noncoding	-0.1073152	1122	1461	1680
MSTRG.28509.5	coding	0.359	1707	2004	2225
MSTRG.28509.2	coding	0.286	183	636	855
MSTRG.28509.3	noncoding	-0.1073152	3540	3879	4100
MSTRG.28510.1	noncoding	-0.06144	504	627	782
MSTRG.28514.5	coding	0.239	630	6786	6959
MSTRG.28515.1	coding	0.07	249	744	2086
MSTRG.28515.2	coding	0.07	336	831	1918
MSTRG.28515.3	coding	0.07	333	828	1222
MSTRG.28517.1	noncoding	-0.0098304	6	261	850
MSTRG.28517.2	noncoding	-0.0688128	0	312	565
MSTRG.28517.3	noncoding	-0.0049152	72	330	886
MSTRG.28517.4	noncoding	-0.0049152	198	441	795
MSTRG.28517.5	noncoding	-0.0098304	6	246	464
MSTRG.28520.2	noncoding	-0.0864256	1377	1809	1965
MSTRG.28522.2	coding	0.239	5949	6555	9588
MSTRG.28522.3	coding	0.207	0	1053	4100
MSTRG.28522.4	coding	0.198	0	930	3965
MSTRG.28522.6	coding	0.179	978	1791	4838
MSTRG.28522.8	coding	0.205	471	1257	4298
MSTRG.28525.2	coding	0.05	162	1275	2711
MSTRG.28525.3	coding	0.252	3447	3948	4257
MSTRG.28532.3	coding	0.127	372	774	2811
MSTRG.28532.5	noncoding	-0.0708608	189	381	2393
MSTRG.28533.1	noncoding	-0.0004096	2373	2571	3745
MSTRG.28538.2	coding	0.521	72	8496	9064
MSTRG.28546.1	coding	0.127	381	2445	2971
MSTRG.28546.2	noncoding	-0.0741376	1203	1485	1638
MSTRG.28546.3	coding	0.063	351	1515	2479
MSTRG.28546.4	noncoding	-0.0684032	2106	2352	2508
MSTRG.28546.12	coding	0.054	42	1074	3368
MSTRG.28546.10	coding	0.059	42	1086	2264
MSTRG.28546.8	coding	0.063	42	1206	1733
MSTRG.28539.2	coding	0.479	90	6438	9168
MSTRG.28539.3	coding	0.533	129	5550	8279
MSTRG.28539.5	coding	0.519	0	5604	8332
MSTRG.28539.6	coding	0.493	477	6459	8600
MSTRG.28539.7	coding	0.479	90	6435	8561
MSTRG.28539.8	coding	0.469	90	6597	7258
MSTRG.28550.3	noncoding	-0.0950272	0	474	4269
MSTRG.28551.1	noncoding	-0.0561152	2130	2811	4331
MSTRG.28552.1	noncoding	-0.0380928	408	993	1321

MSTRG.28553.1	noncoding	-0.038912	1353	1842	3225
MSTRG.28554.1	noncoding	-0.0843776	6543	6801	7008
MSTRG.28555.2	noncoding	-0.0638976	159	417	1134
MSTRG.28555.3	noncoding	-0.057344	159	450	879
MSTRG.28556.1	noncoding	-0.067584	354	564	2489
MSTRG.28557.1	noncoding	-0.04096	162	630	2533
MSTRG.28555.4	noncoding	-0.0372736	120	462	619
MSTRG.28559.1	noncoding	-0.0524288	372	561	922
MSTRG.28561.3	coding	0.191	477	1629	5542
MSTRG.28563.1	coding	0.539	153	1938	3331
MSTRG.28563.5	coding	0.555	417	2016	2171
MSTRG.28564.1	coding	0.123	1248	1491	5853
MSTRG.28565.1	noncoding	-0.0004096	396	522	895
MSTRG.28566.1	noncoding	-0.0233472	2415	2595	5343
MSTRG.28567.1	noncoding	-0.0004096	1842	1974	2589
MSTRG.28568.1	noncoding	-0.0331776	207	255	552
MSTRG.28571.2	noncoding	-0.0811008	1221	1818	2312
MSTRG.28571.3	noncoding	-0.0602112	15	294	1290
MSTRG.28572.1	noncoding	-0.0421888	75	504	659
MSTRG.28579.2	coding	0.533	0	1911	4670
MSTRG.28586.1	coding	0.656	0	1773	2010
MSTRG.28586.3	coding	0.686	0	1686	1919
MSTRG.28586.4	coding	0.698	0	1317	1552
MSTRG.28586.5	coding	0.703	0	1311	1548
MSTRG.28587.2	coding	0.301	57	642	1007
MSTRG.28587.3	coding	0.183	393	1143	1507
MSTRG.28588.1	coding	0.111	10527	11232	16784
MSTRG.28590.1	noncoding	-0.065536	0	36	205
MSTRG.28588.2	noncoding	-0.0618496	0	87	5470
MSTRG.28594.2	coding	0.277	270	1239	1395
MSTRG.28581.2	coding	0.524	0	1425	6430
MSTRG.28585.1	coding	0.426	39	486	640
MSTRG.28595.1	noncoding	-0.0462848	0	120	525
MSTRG.28600.2	coding	0.517	0	390	1644
MSTRG.28600.3	coding	0.555	0	750	2211
MSTRG.28600.6	coding	0.351	0	900	5828
MSTRG.28600.5	coding	0.401	0	1194	1975
MSTRG.28600.4	coding	0.425	0	1194	3107
MSTRG.28600.7	coding	0.334	4866	5250	5803
MSTRG.28600.8	coding	0.412	21	726	1459
MSTRG.28600.9	coding	0.452	0	996	1666
MSTRG.28602.1	noncoding	-0.0806912	51	63	229
MSTRG.28603.1	noncoding	-0.0577536	6354	6888	11523
MSTRG.28604.1	noncoding	-0.176128	198	264	625
MSTRG.28605.1	noncoding	-0.0897024	132	504	1377
MSTRG.28606.1	noncoding	-0.1093632	1674	2217	3136
MSTRG.28597.1	noncoding	-0.022528	714	1119	1274
MSTRG.28613.7	coding	0.216	0	1764	3875
MSTRG.28613.6	coding	0.227	0	3021	3177
MSTRG.28613.8	coding	0.226	0	2118	9251
MSTRG.28613.9	coding	0.226	0	2118	9449
MSTRG.28613.10	noncoding	-0.0057344	1731	1899	3194
MSTRG.28614.1	noncoding	-0.0651264	2166	2529	3257
MSTRG.28609.1	noncoding	-0.0651264	357	363	545

MSTRG.28618.1	noncoding	-0.0618496	153	465	1047
MSTRG.28620.4	coding	0.198	0	3315	4296
MSTRG.28620.3	coding	0.193	168	3411	4394
MSTRG.28623.2	coding	0.201	0	1203	2901
MSTRG.28625.1	coding	0.393	297	585	870
MSTRG.28625.2	coding	0.37	0	513	3363
MSTRG.28625.3	coding	0.438	0	507	794
MSTRG.28625.4	coding	0.393	291	579	864
MSTRG.28627.4	coding	0.512	651	3150	3796
MSTRG.28627.3	coding	0.513	15	2868	3514
MSTRG.28627.5	coding	0.505	0	2628	3272
MSTRG.28628.1	noncoding	-0.083968	0	681	836
MSTRG.28631.2	noncoding	-0.0638976	219	1239	1485
MSTRG.28631.3	noncoding	-0.0233472	6189	8097	19957
MSTRG.28633.1	noncoding	-0.008192	222	360	710
MSTRG.28642.2	coding	0.372	93	5733	6555
MSTRG.28642.8	coding	0.358	2025	5826	5980
MSTRG.28642.7	coding	0.355	4947	8790	8945
MSTRG.28642.5	coding	0.352	2952	7647	7803
MSTRG.28642.6	coding	0.35	4197	8979	9134
MSTRG.28642.3	coding	0.349	3186	7923	8079
MSTRG.28638.1	noncoding	-0.0004096	57	165	450
MSTRG.28638.2	noncoding	-0.0004096	57	156	795
MSTRG.28644.1	coding	0.017	7548	8034	10359
MSTRG.28645.2	coding	0.457	168	3408	7359
MSTRG.28646.3	noncoding	-0.13312	0	177	617
MSTRG.28646.4	noncoding	-0.0401408	186	357	512
MSTRG.28646.5	noncoding	-0.0917504	0	300	559
MSTRG.28647.1	noncoding	-0.04096	4356	4539	5219
MSTRG.28648.1	noncoding	-0.0864256	216	684	1029
MSTRG.28648.2	noncoding	-0.0864256	216	684	1213
MSTRG.28648.3	noncoding	-0.0221184	219	402	928
MSTRG.28650.1	noncoding	-0.04096	177	258	1046
MSTRG.28650.3	noncoding	-0.0241664	120	150	457
MSTRG.28650.2	noncoding	-0.04096	60	141	629
MSTRG.28652.1	noncoding	-0.0159744	18	96	369
MSTRG.28653.1	coding	0.593	0	3144	4627
MSTRG.28654.1	noncoding	-0.016384	1575	1590	1767
MSTRG.28656.1	noncoding	-0.0589824	45	96	252
MSTRG.28657.3	coding	0.292	9	6933	7770
MSTRG.28658.1	noncoding	-0.004096	768	900	1054
MSTRG.28662.4	coding	0.507	957	2637	3001
MSTRG.28662.3	coding	0.534	0	1623	1986
MSTRG.28661.1	noncoding	-0.014336	177	339	740
MSTRG.28661.2	noncoding	-0.0835584	0	147	405
MSTRG.28663.1	coding	0.096	981	1488	2607
MSTRG.28663.2	coding	0.096	1119	1626	2743
MSTRG.28669.2	noncoding	-0.0585728	1638	2457	2650
MSTRG.28672.1	noncoding	-0.0233472	42	327	859
MSTRG.28672.2	noncoding	-0.008192	219	360	593
MSTRG.28672.3	noncoding	-0.008192	261	402	637
MSTRG.28677.1	noncoding	-0.0090112	138	294	865
MSTRG.28677.2	noncoding	-0.032768	36	120	286
MSTRG.28682.2	coding	0.149	0	6447	6897

MSTRG.28688.2	coding	0.401	42	318	2314
MSTRG.28693.1	noncoding	-0.0004096	0	0	344
MSTRG.28702.1	noncoding	-0.008192	96	159	445
MSTRG.28705.1	noncoding	-0.0004096	0	0	395
MSTRG.28707.1	noncoding	-0.018432	39	354	1129
MSTRG.28712.2	noncoding	-0.1175552	0	279	433
MSTRG.28712.3	noncoding	-0.006144	69	192	347
MSTRG.28714.1	noncoding	-0.0004096	0	0	323
MSTRG.28716.1	noncoding	-0.012288	219	351	1318
MSTRG.28718.2	coding	0.076	9	2259	3307
MSTRG.28715.1	noncoding	-0.0217088	0	15	320
MSTRG.28720.1	coding	0.084	0	174	341
MSTRG.28731.1	noncoding	-0.1011712	0	3	676
MSTRG.28732.1	noncoding	-0.0196608	243	306	591
MSTRG.28742.4	coding	0.098	705	1839	1992
MSTRG.28743.1	noncoding	-0.1265664	81	102	866
MSTRG.28744.1	noncoding	-0.075776	2721	3255	4896
MSTRG.28745.1	coding	0.173	0	1047	1965
MSTRG.28745.3	coding	0.254	510	903	2595
MSTRG.28745.5	coding	0.227	3234	3900	5836
MSTRG.28746.1	coding	0.339	1035	4626	6484
MSTRG.28746.3	coding	0.341	1035	5031	5594
MSTRG.28746.4	noncoding	-0.1359872	0	219	669
MSTRG.28746.5	coding	0.428	1029	1677	2175
MSTRG.28737.2	coding	0.039	0	2016	2732
MSTRG.28737.3	coding	0.09	402	1734	2292
MSTRG.28739.3	coding	0.261	0	390	4338
MSTRG.28739.4	coding	0.261	0	390	1286
MSTRG.28739.5	noncoding	-0.02048	14292	14412	17791
MSTRG.28739.6	noncoding	-0.0118784	2313	2439	4435
MSTRG.28740.1	noncoding	-0.028672	306	426	1105
MSTRG.28755.1	noncoding	-0.036864	174	282	550
MSTRG.28765.1	noncoding	-0.0557056	2241	2460	2615
MSTRG.28770.1	coding	0.115	216	1149	8504
MSTRG.28770.3	coding	0.115	363	1296	8509
MSTRG.28780.1	coding	0.197	1011	2352	4045
MSTRG.28780.3	coding	0.197	255	1638	3330
MSTRG.28784.1	coding	0.192	555	7089	10879
MSTRG.28784.2	coding	0.192	69	6579	8358
MSTRG.28783.1	noncoding	-0.008192	3	48	1753
MSTRG.28790.1	noncoding	-0.057344	6	39	401
MSTRG.28790.2	noncoding	-0.1056768	165	237	444
MSTRG.28794.1	noncoding	-0.045056	279	288	444
MSTRG.28799.2	coding	0.212	48	3252	5167
MSTRG.28822.1	noncoding	-0.0139264	2916	3066	4929
MSTRG.28830.1	noncoding	-0.008192	75	147	429
MSTRG.28839.1	coding	0.144	0	2532	3217
MSTRG.28839.2	coding	0.144	0	2532	4138
MSTRG.28839.4	noncoding	-0.069632	21	723	2507
MSTRG.28842.1	noncoding	-0.0159744	3339	3489	6646
MSTRG.28844.6	coding	0.144	318	1452	3165
MSTRG.28844.5	coding	0.129	318	1347	4389
MSTRG.28846.3	noncoding	-0.1077248	3447	3795	3948
MSTRG.28853.2	coding	0.149	324	3099	5406

MSTRG.28853.3	coding	0.151	36	2922	10071
MSTRG.28853.5	coding	0.149	312	3087	3800
MSTRG.28853.6	coding	0.141	1725	1935	2602
MSTRG.28854.1	noncoding	-0.032768	120	159	597
MSTRG.28853.10	noncoding	-0.069632	450	504	1505
MSTRG.28853.9	coding	0.195	87	1710	3265
MSTRG.28856.1	noncoding	-0.0421888	3246	3474	6351
MSTRG.28857.6	coding	0.159	435	1356	1607
MSTRG.28857.5	coding	0.199	441	1674	1828
MSTRG.28857.8	coding	0.206	441	1593	2436
MSTRG.28857.7	coding	0.161	0	906	1158
MSTRG.28858.2	coding	0.238	0	714	4140
MSTRG.28859.1	noncoding	-0.0376832	5520	5895	13264
MSTRG.28862.2	coding	0.192	1854	10908	11292
MSTRG.28862.3	coding	0.195	3735	12648	12975
MSTRG.28862.4	coding	0.2	207	6507	6829
MSTRG.28862.5	coding	0.193	1059	10449	10742
MSTRG.28862.6	coding	0.208	4278	10509	10801
MSTRG.28862.7	coding	0.111	54	663	965
MSTRG.28860.2	coding	0.079	1980	2379	4033
MSTRG.28866.2	coding	0.066	231	2520	3185
MSTRG.28866.3	noncoding	-0.02048	201	252	887
MSTRG.28869.1	noncoding	-0.0004096	0	0	364
MSTRG.28879.1	coding	0.079	174	639	4309
MSTRG.28879.4	noncoding	-0.045056	510	639	878
MSTRG.28879.3	noncoding	-0.0671744	0	258	1167
MSTRG.28879.5	noncoding	-0.0925696	0	366	1041
MSTRG.28880.1	coding	0.206	19068	19518	23001
MSTRG.28880.3	noncoding	-0.090112	3075	3552	4098
MSTRG.28880.2	noncoding	-0.090112	5379	5856	6403
MSTRG.28880.4	coding	0.068	2064	2601	3798
MSTRG.28882.1	noncoding	-0.0229376	1740	1947	3915
MSTRG.28883.1	noncoding	-0.008192	2679	3087	6184
MSTRG.28884.1	noncoding	-0.0528384	945	987	1592
MSTRG.28885.2	noncoding	-0.0503808	279	555	709
MSTRG.28885.3	coding	0.053	105	465	636
MSTRG.28886.2	coding	0.136	453	2934	4773
MSTRG.28887.1	coding	0.107	327	1911	5270
MSTRG.28887.2	coding	0.083	417	1659	4620
MSTRG.28887.4	coding	0.083	678	1920	5281
MSTRG.28888.1	coding	0.08	171	2406	3733
MSTRG.28873.1	noncoding	-0.1376256	0	72	1034
MSTRG.28876.1	coding	0.132	240	1263	2310
MSTRG.28890.1	coding	0.326	0	930	2317
MSTRG.28890.5	coding	0.341	0	909	1237
MSTRG.28890.6	coding	0.342	0	900	1673
MSTRG.28890.4	coding	0.341	0	909	1336
MSTRG.28890.3	coding	0.341	0	909	3496
MSTRG.28893.1	noncoding	-0.032768	1533	1539	2316
MSTRG.28892.2	coding	0.078	0	879	4454
MSTRG.28894.1	noncoding	-0.14336	558	618	891
MSTRG.28894.2	noncoding	-0.0004096	0	0	288
MSTRG.28900.3	coding	0.039	594	846	2576
MSTRG.28905.2	coding	0.413	0	2646	5529

MSTRG.28907.1	coding	0.257	84	579	1026
MSTRG.28908.1	noncoding	-0.0258048	2757	2961	3116
MSTRG.28914.1	noncoding	-0.1552384	78	432	3311
MSTRG.28914.2	coding	0.446	843	3708	3862
MSTRG.28914.3	coding	0.38	243	1314	1910
MSTRG.28917.1	noncoding	-0.0012288	675	807	1673
MSTRG.28920.1	noncoding	-0.04096	3030	3129	4777
MSTRG.28923.4	coding	0.359	0	2550	3791
MSTRG.28923.3	coding	0.341	12	2883	4124
MSTRG.28923.5	coding	0.344	12	2829	4060
MSTRG.28923.7	coding	0.34	0	2778	3969
MSTRG.28923.6	coding	0.334	0	2973	4164
MSTRG.28923.8	coding	0.345	0	2607	3795
MSTRG.28934.1	noncoding	-0.0294912	4665	4824	7655
MSTRG.28935.1	noncoding	-0.014336	9939	10062	11437
MSTRG.28936.1	noncoding	-0.024576	2640	2769	8304
MSTRG.28937.1	noncoding	-0.192512	420	471	643
MSTRG.28933.3	noncoding	-0.0421888	264	456	1647
MSTRG.28941.1	noncoding	-0.0065536	2766	3339	5622
MSTRG.28942.1	noncoding	-0.0446464	642	1182	6076
MSTRG.28940.4	coding	0.361	129	2190	8013
MSTRG.28949.2	coding	0.088	0	750	1128
MSTRG.28949.4	coding	0.059	507	1071	1449
MSTRG.28951.2	noncoding	-0.0577536	114	990	4502
MSTRG.28951.1	coding	0.076	114	6912	9389
MSTRG.28951.6	coding	0.113	0	1107	2448
MSTRG.28951.5	coding	0.094	0	1176	3441
MSTRG.28952.1	noncoding	-0.02048	267	300	3212
MSTRG.28953.1	noncoding	-0.188416	1116	1155	1308
MSTRG.28947.1	coding	0.445	54	1995	3256
MSTRG.28961.6	coding	0.417	1362	4125	5066
MSTRG.28961.8	coding	0.429	1974	4068	5007
MSTRG.28961.4	coding	0.418	1893	4632	5572
MSTRG.28961.5	coding	0.418	1917	4683	5623
MSTRG.28961.3	coding	0.448	0	2778	3717
MSTRG.28961.7	coding	0.445	0	2856	3797
MSTRG.28961.10	coding	0.422	2460	5238	6118
MSTRG.28961.12	coding	0.417	1260	3996	4876
MSTRG.28961.9	coding	0.417	84	2943	3822
MSTRG.28961.13	noncoding	-0.008192	675	798	1867
MSTRG.28961.14	noncoding	-0.0004096	0	0	451
MSTRG.28961.15	noncoding	-0.0004096	0	0	308
MSTRG.28961.16	noncoding	-0.0004096	0	0	237
MSTRG.28963.1	coding	0.086	555	1083	1826
MSTRG.28967.1	noncoding	-0.0299008	471	723	1177
MSTRG.28975.1	noncoding	-0.0294912	69	234	522
MSTRG.28980.2	coding	0.112	25959	26358	26706
MSTRG.28976.2	coding	0.327	0	1104	1589
MSTRG.28976.3	coding	0.352	0	852	1336
MSTRG.28977.1	noncoding	-0.012288	0	183	978
MSTRG.28976.4	noncoding	-0.0503808	66	309	794
MSTRG.28987.2	coding	0.138	306	810	6176
MSTRG.28988.2	coding	0.312	33	2271	5079
MSTRG.28988.3	coding	0.256	2142	4128	5809

MSTRG.28990.1	coding	0.129	2730	3123	3735
MSTRG.28988.4	coding	0.396	33	801	1884
MSTRG.28988.5	coding	0.118	771	2031	3239
MSTRG.28988.6	coding	0.118	1482	2742	3950
MSTRG.28988.8	coding	0.123	693	1857	2375
MSTRG.28988.9	coding	0.123	1482	2646	3164
MSTRG.28988.10	coding	0.098	0	1893	2170
MSTRG.28988.11	coding	0.098	0	1917	2919
MSTRG.28988.12	coding	0.134	693	1773	2241
MSTRG.28993.2	coding	0.079	171	825	1110
MSTRG.28993.3	coding	0.069	528	1179	1505
MSTRG.29016.1	noncoding	-0.0888832	402	1137	1622
MSTRG.29017.8	coding	0.045	327	1074	3450
MSTRG.29017.9	coding	0.101	327	7551	9189
MSTRG.29017.10	coding	0.045	312	1059	2494
MSTRG.29017.12	coding	0.045	312	1059	1839
MSTRG.29017.13	coding	0.045	312	1059	2573
MSTRG.29017.11	coding	0.085	312	8652	9079
MSTRG.29000.1	noncoding	-0.0528384	207	426	1616
MSTRG.29008.1	noncoding	-0.0229376	6099	6240	11721
MSTRG.29009.1	noncoding	-0.0290816	9720	10155	17512
MSTRG.29013.3	noncoding	-0.0094208	5838	6060	6255
MSTRG.28996.1	coding	0.567	0	126	1064
MSTRG.29020.2	coding	0.265	222	5019	9197
MSTRG.29020.3	coding	0.248	1869	2721	2877
MSTRG.29021.1	noncoding	-0.012288	1338	1443	3831
MSTRG.29022.1	noncoding	-0.083968	0	84	330
MSTRG.29019.1	noncoding	-0.057344	270	330	570
MSTRG.29024.2	noncoding	-0.0192512	3501	4170	4604
MSTRG.29024.4	noncoding	-0.0192512	3501	4170	4835
MSTRG.29028.3	coding	0.166	201	7605	9290
MSTRG.29026.3	coding	0.234	1740	2247	3940
MSTRG.29027.1	noncoding	-0.032768	948	1125	1667
MSTRG.29029.1	coding	0.138	0	723	1403
MSTRG.29034.1	noncoding	-0.024576	1047	1155	2265
MSTRG.29035.1	coding	0.541	555	2835	5913
MSTRG.29037.1	noncoding	-0.0835584	0	351	1437
MSTRG.29041.1	noncoding	-0.0131072	7749	8100	9124
MSTRG.29041.5	noncoding	-0.0131072	966	1317	2906
MSTRG.29041.4	noncoding	-0.0167936	1290	1611	3343
MSTRG.29041.7	noncoding	-0.03072	27	387	2119
MSTRG.29043.1	noncoding	-0.0618496	0	1182	1357
MSTRG.29043.2	coding	0.195	0	990	1530
MSTRG.29043.3	noncoding	-0.135168	0	423	1690
MSTRG.29045.1	coding	0.267	102	1632	2259
MSTRG.29049.4	coding	0.186	318	678	1562
MSTRG.29051.2	noncoding	-0.0069632	132	279	547
MSTRG.29054.4	coding	0.097	0	1323	9789
MSTRG.29054.5	coding	0.076	147	1368	7184
MSTRG.29055.1	noncoding	-0.0315392	4857	5025	9395
MSTRG.29064.3	coding	0.448	0	1590	1750
MSTRG.29064.4	coding	0.414	327	1389	5357
MSTRG.29064.6	coding	0.43	87	1080	1241
MSTRG.29065.1	noncoding	-0.0520192	504	1167	2233

MSTRG.29065.2	noncoding	-0.004096	75	333	596
MSTRG.29068.1	noncoding	-0.047104	45	144	1459
MSTRG.29068.3	noncoding	-0.002048	987	1122	1277
MSTRG.29079.2	noncoding	-0.038912	0	150	449
MSTRG.29079.1	coding	0.536	54	5406	5703
MSTRG.29079.4	noncoding	-0.1024	396	495	651
MSTRG.29079.3	coding	0.536	0	5367	5718
MSTRG.29079.5	coding	0.533	0	5595	5864
MSTRG.29080.1	noncoding	-0.0335872	888	3150	4065
MSTRG.29086.2	coding	0.266	0	1488	3279
MSTRG.29086.3	coding	0.267	0	1275	3188
MSTRG.29082.3	coding	0.526	0	2196	2351
MSTRG.29082.4	coding	0.51	165	2292	2445
MSTRG.29083.1	noncoding	-0.047104	0	267	891
MSTRG.29085.1	coding	0.082	0	759	914
MSTRG.29092.2	coding	0.239	153	990	2760
MSTRG.29092.6	coding	0.239	291	1128	2898
MSTRG.29092.7	coding	0.239	582	1419	3187
MSTRG.29092.5	coding	0.239	390	1227	2996
MSTRG.29093.4	coding	0.068	720	1806	2179
MSTRG.29094.1	noncoding	-0.0823296	135	192	383
MSTRG.29099.2	coding	0.149	45	1173	3024
MSTRG.29101.1	coding	0.128	264	582	4052
MSTRG.29101.2	noncoding	-0.0139264	1665	1803	2917
MSTRG.29107.1	noncoding	-0.0057344	300	561	717
MSTRG.29108.1	noncoding	-0.0004096	0	0	276
MSTRG.29111.3	coding	0.436	0	663	2173
MSTRG.29111.4	coding	0.436	0	663	2297
MSTRG.29111.2	coding	0.395	0	741	2184
MSTRG.29120.3	noncoding	-0.0679936	2952	3144	3333
MSTRG.29120.4	noncoding	-0.0679936	1914	2106	2371
MSTRG.29121.1	noncoding	-0.0008192	4914	5190	8960
MSTRG.29121.3	coding	0.073	1869	3141	3914
MSTRG.29113.1	noncoding	-0.03072	66	249	1447
MSTRG.29115.1	noncoding	-0.0303104	66	105	854
MSTRG.29128.1	noncoding	-0.0069632	519	573	2216
MSTRG.29129.1	noncoding	-0.0090112	0	3	344
MSTRG.29129.2	noncoding	-0.012288	759	807	1069
MSTRG.29130.1	noncoding	-0.192512	249	309	2058
MSTRG.29122.1	noncoding	-0.26624	108	141	295
MSTRG.29126.1	noncoding	-0.0602112	0	99	256
MSTRG.29133.1	noncoding	-0.0421888	522	639	1023
MSTRG.29136.2	coding	0.206	207	4878	7348
MSTRG.29136.5	coding	0.213	0	5010	5244
MSTRG.29136.1	coding	0.213	207	4572	5822
MSTRG.29137.1	coding	0.011	279	444	653
MSTRG.29155.4	coding	0.273	402	3222	3418
MSTRG.29155.6	coding	0.273	564	3384	3580
MSTRG.29155.1	coding	0.271	468	3432	7941
MSTRG.29155.8	coding	0.272	390	3462	7971
MSTRG.29155.11	coding	0.265	387	2262	4079
MSTRG.29155.15	coding	0.314	672	1941	3083
MSTRG.29155.16	coding	0.273	399	3339	7850
MSTRG.29155.17	coding	0.27	114	3051	7521

MSTRG.29155.19	coding	0.328	0	1230	1490
MSTRG.29157.1	noncoding	-0.098304	4557	5001	5860
MSTRG.29155.22	coding	0.387	0	906	1166
MSTRG.29160.1	noncoding	-0.0425984	1806	1914	3346
MSTRG.29161.1	noncoding	-0.0004096	408	540	694
MSTRG.29142.1	noncoding	-0.004096	411	678	3268
MSTRG.29143.1	noncoding	-0.0724992	390	813	968
MSTRG.29144.3	coding	0.187	1278	2598	4915
MSTRG.29149.2	coding	0.469	489	4455	6079
MSTRG.29149.8	coding	0.467	0	3345	4298
MSTRG.29149.9	coding	0.194	696	1479	1825
MSTRG.29149.10	coding	0.46	114	888	2511
MSTRG.29149.11	coding	0.454	81	873	2496
MSTRG.29150.1	coding	0.269	0	1389	4833
MSTRG.29150.2	coding	0.27	0	1398	4001
MSTRG.29150.3	coding	0.27	0	1398	3878
MSTRG.29150.4	coding	0.257	0	1368	5515
MSTRG.29150.5	coding	0.27	0	1398	2356
MSTRG.29150.6	coding	0.269	0	1389	3858
MSTRG.29150.7	coding	0.27	0	1398	3080
MSTRG.29150.8	coding	0.27	0	1398	2011
MSTRG.29165.3	noncoding	-0.083968	180	786	993
MSTRG.29162.1	noncoding	-0.028672	255	354	668
MSTRG.29162.2	noncoding	-0.012288	99	213	626
MSTRG.29163.1	noncoding	-0.069632	249	267	626
MSTRG.29172.1	noncoding	-0.077824	21	54	220
MSTRG.29191.1	noncoding	-0.0782336	1809	2082	2448
MSTRG.29192.1	noncoding	-0.2412544	0	66	1190
MSTRG.29195.1	noncoding	-0.0479232	2238	2529	2685
MSTRG.29195.2	noncoding	-0.0294912	558	759	1213
MSTRG.29195.3	noncoding	-0.0376832	525	912	1066
MSTRG.29195.4	noncoding	-0.0569344	45	417	1071
MSTRG.29201.1	noncoding	-0.004096	42	54	232
MSTRG.29202.4	coding	0.215	174	1884	5813
MSTRG.29202.6	coding	0.142	0	1239	2782
MSTRG.29202.7	coding	0.142	0	1239	3698
MSTRG.29202.8	coding	0.05	363	1308	1591
MSTRG.29202.9	coding	0.05	1152	1344	2937
MSTRG.29196.1	noncoding	-0.114688	0	471	1362
MSTRG.29198.1	coding	0.345	0	4059	8020
MSTRG.29198.2	coding	0.323	0	2631	2786
MSTRG.29198.4	coding	0.682	0	642	1939
MSTRG.29207.2	coding	0.414	0	3891	4813
MSTRG.29207.3	coding	0.425	0	3816	4738
MSTRG.29208.1	noncoding	-0.0495616	24	27	221
MSTRG.29213.1	noncoding	-0.04096	0	309	465
MSTRG.29214.1	noncoding	-0.0131072	3186	3312	5338
MSTRG.29215.1	noncoding	-0.0118784	393	558	875
MSTRG.29219.1	noncoding	-0.012288	0	6	547
MSTRG.29231.1	noncoding	-0.0008192	2910	3042	3275
MSTRG.29231.2	noncoding	-0.0536576	3792	4053	4990
MSTRG.29229.16	noncoding	-0.0761856	606	633	1313
MSTRG.29232.1	noncoding	-0.0339968	36	186	1595
MSTRG.29232.2	noncoding	-0.0602112	0	75	1106

MSTRG.29221.2	noncoding	-0.0462848	219	489	643
MSTRG.29224.2	coding	0.383	93	1044	1198
MSTRG.29226.2	coding	0.512	0	390	559
MSTRG.29226.3	coding	0.512	0	390	559
MSTRG.29233.3	coding	0.243	456	1317	1581
MSTRG.29238.2	coding	0.499	60	783	1117
MSTRG.29239.3	coding	0.43	0	2169	2403
MSTRG.29240.1	coding	0.306	24	531	922
MSTRG.29242.2	coding	0.585	0	2256	3174
MSTRG.29242.3	coding	0.585	0	2373	3294
MSTRG.29242.5	coding	0.561	921	2748	3668
MSTRG.29242.6	coding	0.561	18	1710	2629
MSTRG.29246.3	coding	0.376	192	2520	3178
MSTRG.29257.4	coding	0.474	0	186	627
MSTRG.29257.2	noncoding	-0.0856064	99	531	800
MSTRG.29258.1	noncoding	-0.0925696	180	846	1089
MSTRG.29258.2	coding	0.246	0	594	838
MSTRG.29258.4	noncoding	-0.0790528	153	729	974
MSTRG.29258.5	noncoding	-0.0765952	147	699	943
MSTRG.29258.6	coding	0.193	0	216	436
MSTRG.29258.9	noncoding	-0.0733184	0	864	1017
MSTRG.29260.1	noncoding	-0.0753664	0	810	1089
MSTRG.29262.2	noncoding	-0.1093632	0	582	759
MSTRG.29254.1	coding	0.318	0	2055	2443
MSTRG.29255.4	coding	0.364	132	588	1200
MSTRG.29268.1	noncoding	-0.0630784	159	615	941
MSTRG.29271.3	noncoding	-0.0376832	228	2175	5891
MSTRG.29271.5	coding	0.316	306	963	3889
MSTRG.29276.1	coding	0.519	0	7566	7903
MSTRG.29276.3	coding	0.518	0	7533	7870
MSTRG.29278.1	coding	0.258	150	1197	1350
MSTRG.29278.2	coding	0.289	213	1092	1248
MSTRG.29278.3	coding	0.421	0	1014	1169
MSTRG.29279.2	coding	0.381	0	492	648
MSTRG.29281.3	coding	0.481	0	1989	4019
MSTRG.29282.2	coding	0.536	339	2349	2504
MSTRG.29283.2	noncoding	-0.0352256	3720	10968	11294
MSTRG.29283.3	noncoding	-0.0311296	0	2235	2391
MSTRG.29283.4	noncoding	-0.0282624	1125	8835	8989
MSTRG.29283.5	coding	0.083	3	8565	8719
MSTRG.29301.1	noncoding	-0.0978944	0	228	391
MSTRG.29302.3	coding	0.649	1011	3186	3634
MSTRG.29302.1	coding	0.525	0	4605	5053
MSTRG.29305.2	coding	0.403	369	4062	7460
MSTRG.29305.3	coding	0.385	2385	6363	9761
MSTRG.29307.3	noncoding	-0.0811008	0	882	3337
MSTRG.29308.4	coding	0.627	0	4134	5483
MSTRG.29308.5	coding	0.54	3246	6930	8278
MSTRG.29308.6	coding	0.581	618	3174	4652
MSTRG.29308.7	coding	0.581	525	3081	4429
MSTRG.29293.2	coding	0.366	369	1164	1345
MSTRG.29298.1	coding	0.331	516	1569	1828
MSTRG.29325.2	coding	0.55	0	2268	2500
MSTRG.29328.7	coding	0.249	0	1080	1924

MSTRG.29328.9	coding	0.224	573	1431	2275
MSTRG.29328.8	coding	0.224	2079	2937	3782
MSTRG.29333.2	coding	0.454	0	6159	6538
MSTRG.29334.1	coding	0.287	1359	6234	6630
MSTRG.29334.5	noncoding	-0.0319488	24	411	2525
MSTRG.29341.3	coding	0.493	252	1143	1347
MSTRG.29342.1	coding	0.265	1734	2730	7893
MSTRG.29342.2	coding	0.271	126	1821	6986
MSTRG.29343.1	noncoding	-0.0499712	1455	1683	8753
MSTRG.29342.4	coding	0.258	6417	8238	13566
MSTRG.29356.21	coding	0.255	1266	2193	7782
MSTRG.29356.18	coding	0.278	1545	3042	10127
MSTRG.29356.9	coding	0.263	405	1860	8945
MSTRG.29356.12	coding	0.416	3207	4092	12087
MSTRG.29356.13	coding	0.292	0	1599	8729
MSTRG.29356.14	coding	0.294	0	1596	8684
MSTRG.29356.16	coding	0.263	810	2265	9258
MSTRG.29356.5	coding	0.292	0	1599	8705
MSTRG.29356.6	coding	0.263	810	2265	9462
MSTRG.29356.8	coding	0.263	810	2265	9369
MSTRG.29356.15	coding	0.419	0	981	9090
MSTRG.29356.19	coding	0.295	0	1611	8780
MSTRG.29356.22	coding	0.416	3207	4092	12308
MSTRG.29356.23	coding	0.294	0	1596	8602
MSTRG.29356.24	coding	0.285	2580	4095	10937
MSTRG.29347.1	coding	0.5	420	2271	4742
MSTRG.29353.1	noncoding	-0.0806912	60	336	676
MSTRG.29354.1	coding	0.321	0	354	1051
MSTRG.29359.2	coding	0.372	771	1878	2708
MSTRG.29361.1	noncoding	-0.0557056	0	84	653
MSTRG.29363.1	noncoding	-0.004096	192	300	455
MSTRG.29364.1	noncoding	-0.02048	66	153	963
MSTRG.29367.2	noncoding	-0.13312	123	564	2772
MSTRG.29370.2	coding	0.162	261	1704	8243
MSTRG.29375.2	coding	0.269	102	636	2361
MSTRG.29376.3	noncoding	-0.0950272	0	789	946
MSTRG.29387.1	coding	0.34	354	5676	6727
MSTRG.29387.6	coding	0.34	252	5574	6132
MSTRG.29389.1	coding	0.504	0	2325	5873
MSTRG.29388.6	coding	0.275	204	675	1043
MSTRG.29378.1	noncoding	-0.028672	1317	1410	2275
MSTRG.29380.1	noncoding	-0.034816	11652	11820	13384
MSTRG.29379.3	coding	0.323	0	1698	2530
MSTRG.29393.3	noncoding	-0.0823296	138	858	1013
MSTRG.29393.2	noncoding	-0.0589824	168	777	1090
MSTRG.29393.4	noncoding	-0.1183744	168	672	825
MSTRG.29393.5	noncoding	-0.0782336	93	600	871
MSTRG.29393.6	noncoding	-0.0847872	90	456	612
MSTRG.29393.7	noncoding	-0.1036288	0	492	1349
MSTRG.29405.2	coding	0.503	0	2853	3530
MSTRG.29409.2	coding	0.411	87	780	934
MSTRG.29409.3	coding	0.408	1680	2277	2430
MSTRG.29411.1	noncoding	-0.07168	3228	4914	7708
MSTRG.29412.1	coding	0.37	1734	5175	5365

MSTRG.29417.3	coding	0.443	96	2112	2352
MSTRG.29417.4	coding	0.439	96	2121	2332
MSTRG.29418.4	coding	0.257	0	1752	2055
MSTRG.29420.2	coding	0.406	5442	5865	6723
MSTRG.29420.1	coding	0.406	837	1260	2118
MSTRG.29425.2	coding	0.276	0	2256	3912
MSTRG.29425.5	coding	0.252	0	2319	3972
MSTRG.29425.6	coding	0.216	0	2445	4099
MSTRG.29425.7	coding	0.315	1209	2682	4336
MSTRG.29426.3	coding	0.259	1401	3600	4251
MSTRG.29426.4	coding	0.294	0	3750	4400
MSTRG.29426.5	coding	0.263	1401	3258	4250
MSTRG.29426.6	coding	0.294	0	3753	4403
MSTRG.29395.2	coding	0.388	0	792	1461
MSTRG.29404.5	coding	0.44	153	927	1091
MSTRG.29404.3	coding	0.4	276	885	1053
MSTRG.29404.6	coding	0.421	318	1119	1287
MSTRG.29404.7	coding	0.455	0	1134	1289
MSTRG.29428.3	coding	0.375	189	1695	4848
MSTRG.29436.3	coding	0.356	927	1509	3543
MSTRG.29432.3	coding	0.285	114	3441	4215
MSTRG.29445.1	noncoding	-0.0888832	885	1137	1290
MSTRG.29447.1	noncoding	-0.0274432	822	1101	6658
MSTRG.29447.2	noncoding	-0.016384	1683	1761	1916
MSTRG.29449.1	noncoding	-0.012288	276	363	1952
MSTRG.29450.1	noncoding	-0.0004096	0	0	1174
MSTRG.29447.6	noncoding	-0.0221184	3213	3432	6613
MSTRG.29447.7	coding	0.124	735	1968	3291
MSTRG.29440.2	coding	0.546	651	1269	1472
MSTRG.29455.4	coding	0.449	0	2916	6563
MSTRG.29455.6	coding	0.448	15	2730	3327
MSTRG.29455.7	coding	0.428	3645	4605	7656
MSTRG.29454.1	noncoding	-0.0110592	195	348	643
MSTRG.29458.1	noncoding	-0.141312	174	591	746
MSTRG.29457.2	noncoding	-0.1024	0	414	607
MSTRG.29463.1	coding	0.259	0	468	3327
MSTRG.29463.7	coding	0.216	93	579	2007
MSTRG.29463.6	coding	0.202	141	612	2039
MSTRG.29466.1	coding	0.395	0	366	3247
MSTRG.29466.3	coding	0.425	0	411	3291
MSTRG.29467.2	coding	0.31	765	2640	2887
MSTRG.29467.3	coding	0.282	0	2502	2657
MSTRG.29467.4	noncoding	-0.049152	99	213	3401
MSTRG.29467.5	noncoding	-0.028672	258	465	1640
MSTRG.29481.2	coding	0.432	1218	2982	4901
MSTRG.29482.2	noncoding	-0.0487424	1218	1470	2091
MSTRG.29481.3	coding	0.425	33	1458	3375
MSTRG.29470.1	noncoding	-0.008192	375	438	1068
MSTRG.29470.2	noncoding	-0.110592	0	6	570
MSTRG.29471.1	coding	0.329	0	468	4589
MSTRG.29484.1	noncoding	-0.2883584	0	69	282
MSTRG.29495.2	coding	0.253	0	2082	2682
MSTRG.29495.3	coding	0.251	0	1932	3982
MSTRG.29497.1	coding	0.24	0	2373	3903

MSTRG.29499.4	coding	0.172	165	759	1198
MSTRG.29497.4	coding	0.206	6	2427	4076
MSTRG.29497.3	coding	0.219	24	2526	4177
MSTRG.29500.1	noncoding	-0.008192	756	924	1196
MSTRG.29504.3	coding	0.163	213	5175	7507
MSTRG.29504.4	coding	0.163	180	5142	7473
MSTRG.29504.2	coding	0.163	282	5244	7575
MSTRG.29506.3	noncoding	-0.0724992	189	417	2750
MSTRG.29502.2	coding	0.021	210	501	1118
MSTRG.29502.3	noncoding	-0.0004096	0	120	1021
MSTRG.29502.4	noncoding	-0.1314816	0	120	2295
MSTRG.29509.1	coding	0.502	495	4998	6538
MSTRG.29509.2	coding	0.473	495	5145	6685
MSTRG.29509.3	coding	0.512	0	4611	8797
MSTRG.29511.2	coding	0.363	546	1641	2007
MSTRG.29511.3	coding	0.37	339	1506	1873
MSTRG.29511.4	coding	0.381	354	1560	1926
MSTRG.29511.5	coding	0.361	330	1422	1790
MSTRG.29513.1	noncoding	-0.028672	63	120	603
MSTRG.29510.1	noncoding	-0.0004096	9	9	299
MSTRG.29512.1	noncoding	-0.233472	87	129	284
MSTRG.29521.2	coding	0.089	0	810	995
MSTRG.29521.3	noncoding	-0.0421888	0	120	2207
MSTRG.29521.4	coding	0.088	192	10947	13473
MSTRG.29524.1	noncoding	-0.0004096	0	0	847
MSTRG.29517.1	coding	0.432	432	2325	7027
MSTRG.29525.1	coding	0.324	0	567	894
MSTRG.29525.3	coding	0.291	561	1014	1288
MSTRG.29525.4	coding	0.323	171	639	903
MSTRG.29526.1	noncoding	-0.0090112	105	306	825
MSTRG.29532.5	coding	0.099	1779	2403	4681
MSTRG.29533.5	coding	0.13	660	1071	1225
MSTRG.29533.6	coding	0.107	357	807	961
MSTRG.29535.2	coding	0.199	0	669	1454
MSTRG.29535.3	coding	0.084	0	783	1570
MSTRG.29535.5	coding	0.259	0	438	1531
MSTRG.29535.4	noncoding	-0.0393216	456	654	1717
MSTRG.29536.1	coding	0.514	0	1125	2660
MSTRG.29530.1	coding	0.238	246	1950	2364
MSTRG.29530.3	coding	0.324	2610	3267	3637
MSTRG.29531.3	coding	0.197	357	1149	1513
MSTRG.29537.3	coding	0.073	21039	21897	22168
MSTRG.29537.4	coding	0.075	30	489	1782
MSTRG.29557.1	coding	0.141	3426	3891	21443
MSTRG.29557.2	coding	0.141	2544	3009	7315
MSTRG.29557.3	coding	0.141	2511	2976	6842
MSTRG.29557.4	coding	0.141	2661	3126	3905
MSTRG.29557.5	coding	0.141	3270	3735	4515
MSTRG.29557.6	coding	0.141	2640	3105	3884
MSTRG.29557.7	noncoding	-0.053248	0	132	2670
MSTRG.29557.8	noncoding	-0.053248	0	132	2630
MSTRG.29557.10	noncoding	-0.008192	57	192	2644
MSTRG.29558.5	coding	0.083	600	2409	2747
MSTRG.29558.6	noncoding	-0.065536	513	600	868

MSTRG.29558.7	coding	0.11	276	918	1255
MSTRG.29559.3	coding	0.214	396	1266	1567
MSTRG.29559.4	coding	0.214	531	1401	1702
MSTRG.29560.1	noncoding	-0.0319488	3027	3117	3813
MSTRG.29561.1	coding	0.106	525	666	2408
MSTRG.29565.1	coding	0.256	303	12417	12611
MSTRG.29566.1	noncoding	-0.004096	69	186	1395
MSTRG.29566.2	noncoding	-0.0520192	246	462	5706
MSTRG.29567.1	noncoding	-0.0241664	342	429	1554
MSTRG.29566.3	noncoding	-0.004096	180	297	4149
MSTRG.29544.1	coding	0.04	1734	1875	3448
MSTRG.29543.2	coding	0.026	480	4242	4964
MSTRG.29545.1	noncoding	-0.0004096	1026	1242	3196
MSTRG.29546.1	noncoding	-0.016384	324	387	1441
MSTRG.29550.1	coding	0.075	3285	4950	5107
MSTRG.29550.2	coding	0.075	3345	5010	6559
MSTRG.29551.1	noncoding	-0.0376832	7068	7284	11023
MSTRG.29552.2	coding	0.112	3588	4566	4901
MSTRG.29553.1	coding	0.075	51	750	1766
MSTRG.29553.2	coding	0.06	108	759	1810
MSTRG.29553.3	coding	0.069	204	570	1620
MSTRG.29553.4	coding	0.049	21	381	1432
MSTRG.29553.5	coding	0.069	204	570	1584
MSTRG.29553.6	coding	0.069	195	561	1612
MSTRG.29553.7	coding	0.06	108	759	1443
MSTRG.29554.2	noncoding	-0.1650688	0	90	1105
MSTRG.29556.1	coding	0.073	750	981	5550
MSTRG.29548.2	coding	0.323	327	2457	5539
MSTRG.29548.1	coding	0.199	291	4242	5382
MSTRG.29547.1	noncoding	-0.0364544	255	990	1310
MSTRG.29571.1	noncoding	-0.0585728	51	273	428
MSTRG.29576.3	coding	0.033	438	1620	1905
MSTRG.29576.4	noncoding	-0.0086016	738	888	2520
MSTRG.29578.3	coding	0.378	0	234	4561
MSTRG.29580.1	noncoding	-0.200704	177	207	373
MSTRG.29583.2	coding	0.285	0	3909	10572
MSTRG.29583.4	coding	0.281	0	3843	7415
MSTRG.29584.1	noncoding	-0.0004096	630	714	1181
MSTRG.29585.2	coding	0.384	93	1935	2152
MSTRG.29593.2	coding	0.394	0	1830	9687
MSTRG.29594.3	noncoding	-0.0339968	954	1221	4895
MSTRG.29596.1	noncoding	-0.0114688	42	105	772
MSTRG.29588.2	coding	0.461	21	1731	2092
MSTRG.29598.1	coding	0.55	0	492	1928
MSTRG.29600.2	coding	0.424	0	1935	9324
MSTRG.29601.1	noncoding	-0.0028672	3888	4053	4908
MSTRG.29599.1	noncoding	-0.147456	72	108	375
MSTRG.29599.2	noncoding	-0.147456	48	84	366
MSTRG.29603.3	coding	0.126	6684	9321	9476
MSTRG.29607.6	noncoding	-0.0434176	1440	1584	1749
MSTRG.29607.11	noncoding	-0.0462848	195	441	606
MSTRG.29607.10	coding	0.052	63	5055	6125
MSTRG.29607.5	coding	0.046	23415	28575	29646
MSTRG.29608.1	noncoding	-0.0192512	441	591	3633

MSTRG.29620.1	coding	0.18	771	2868	6844
MSTRG.29624.1	noncoding	-0.049152	15	198	455
MSTRG.29625.1	noncoding	-0.0012288	105	156	692
MSTRG.29625.2	noncoding	-0.073728	513	615	835
MSTRG.29627.1	noncoding	-0.016384	375	438	592
MSTRG.29633.2	noncoding	-0.028672	651	774	1494
MSTRG.29634.2	coding	0.448	939	6612	7222
MSTRG.29635.2	coding	0.365	0	5046	6815
MSTRG.29652.1	noncoding	-0.0503808	0	87	380
MSTRG.29654.1	coding	0.279	0	2382	2542
MSTRG.29654.4	coding	0.298	1470	3501	4387
MSTRG.29654.2	coding	0.295	159	2217	3104
MSTRG.29654.5	coding	0.287	156	2634	2991
MSTRG.29657.1	coding	0.407	0	3639	7411
MSTRG.29657.4	coding	0.355	0	6684	7299
MSTRG.29657.9	coding	0.307	0	2832	3342
MSTRG.29657.11	coding	0.532	0	2739	3145
MSTRG.29644.1	coding	0.284	0	2481	4036
MSTRG.29647.2	noncoding	-0.0548864	525	1812	5372
MSTRG.29647.3	noncoding	-0.0548864	1473	2760	6004
MSTRG.29663.2	coding	0.372	0	591	849
MSTRG.29664.2	coding	0.336	66	1011	1393
MSTRG.29666.5	coding	0.448	765	2049	2377
MSTRG.29667.1	coding	0.317	0	999	1283
MSTRG.29667.2	coding	0.309	0	1011	1293
MSTRG.29667.3	coding	0.307	0	1020	1285
MSTRG.29667.4	coding	0.268	810	1920	2126
MSTRG.29668.3	coding	0.306	198	996	1852
MSTRG.29672.2	coding	0.515	744	3000	5915
MSTRG.29673.1	coding	0.124	498	1137	1882
MSTRG.29673.3	coding	0.145	93	735	1021
MSTRG.29658.7	coding	0.236	156	420	823
MSTRG.29658.5	coding	0.21	156	345	919
MSTRG.29675.2	coding	0.15	0	3099	3404
MSTRG.29675.5	coding	0.227	0	1218	1768
MSTRG.29676.2	noncoding	-0.0745472	0	384	1425
MSTRG.29676.1	noncoding	-0.0671744	708	963	1483
MSTRG.29676.3	noncoding	-0.03072	711	1206	2555
MSTRG.29676.4	noncoding	-0.0462848	2289	2517	3128
MSTRG.29676.5	noncoding	-0.0462848	1671	1899	2513
MSTRG.29676.6	noncoding	-0.03072	714	1209	1802
MSTRG.29677.3	coding	0.453	0	315	1388
MSTRG.29677.6	coding	0.453	0	315	1315
MSTRG.29677.8	coding	0.46	0	1434	2514
MSTRG.29677.5	coding	0.417	0	1449	2528
MSTRG.29677.7	coding	0.399	0	1521	2601
MSTRG.29677.2	coding	0.386	0	1674	1843
MSTRG.29678.3	coding	0.342	483	2340	2542
MSTRG.29678.2	coding	0.345	507	2364	2567
MSTRG.29682.4	coding	0.353	117	1320	1493
MSTRG.29687.2	coding	0.419	51	1161	1463
MSTRG.29687.3	coding	0.42	9	1119	1306
MSTRG.29681.1	coding	0.232	132	1449	1777
MSTRG.29692.2	coding	0.326	0	1593	2193

MSTRG.29692.3	coding	0.37	0	759	1357
MSTRG.29693.2	noncoding	-0.0516096	135	600	1083
MSTRG.29695.2	coding	0.362	165	1395	3272
MSTRG.29696.1	coding	0.198	153	2217	2376
MSTRG.29696.2	noncoding	-0.0401408	2172	2343	2783
MSTRG.29696.3	noncoding	-0.0200704	87	243	397
MSTRG.29704.3	coding	0.24	24	9546	11205
MSTRG.29699.3	coding	0.51	0	243	584
MSTRG.29700.1	noncoding	-0.0733184	573	1062	1218
MSTRG.29700.2	noncoding	-0.0823296	165	636	1055
MSTRG.29701.3	noncoding	-0.0053248	6	93	295
MSTRG.29722.1	noncoding	-0.0413696	60	252	1717
MSTRG.29722.2	noncoding	-0.028672	249	291	685
MSTRG.29723.1	coding	0.47	0	708	1337
MSTRG.29723.3	noncoding	-0.008192	744	849	1652
MSTRG.29724.1	noncoding	-0.0446464	900	1050	2215
MSTRG.29725.1	noncoding	-0.012288	309	417	1492
MSTRG.29726.1	noncoding	-0.0299008	0	63	301
MSTRG.29724.4	noncoding	-0.0487424	1899	2838	3165
MSTRG.29724.5	noncoding	-0.1253376	2055	2418	4271
MSTRG.29724.6	coding	0.333	747	2808	3204
MSTRG.29727.1	noncoding	-0.049152	69	171	749
MSTRG.29724.7	coding	0.351	2025	4119	4515
MSTRG.29724.10	coding	0.39	957	2322	2717
MSTRG.29724.11	coding	0.29	9	2622	3019
MSTRG.29730.1	coding	0.234	759	1449	2363
MSTRG.29735.6	coding	0.295	297	5226	5866
MSTRG.29707.1	coding	0.397	108	1464	1801
MSTRG.29710.1	noncoding	-0.067584	681	1128	1527
MSTRG.29713.2	noncoding	-0.053248	159	495	1537
MSTRG.29715.3	coding	0.253	1305	2769	3070
MSTRG.29738.1	coding	0.281	0	1320	3005
MSTRG.29738.2	coding	0.281	0	1320	2928
MSTRG.29738.3	coding	0.281	0	1320	2614
MSTRG.29739.1	coding	0.136	975	1962	2149
MSTRG.29741.2	coding	0.165	639	1128	2462
MSTRG.29747.1	coding	0.49	99	951	1763
MSTRG.29748.1	noncoding	-0.0536576	780	1113	1268
MSTRG.29755.2	coding	0.473	0	1803	2231
MSTRG.29756.5	noncoding	-0.0483328	1158	1431	1779
MSTRG.29757.1	coding	0.481	30	645	800
MSTRG.29759.1	coding	0.176	288	888	4320
MSTRG.29758.1	coding	0.13	624	1617	2212
MSTRG.29763.1	coding	0.404	0	2373	4672
MSTRG.29763.4	coding	0.576	1476	2229	2878
MSTRG.29767.1	coding	0.295	0	729	1544
MSTRG.29767.2	coding	0.295	60	831	1317
MSTRG.29767.3	coding	0.295	0	729	1420
MSTRG.29767.4	coding	0.295	0	729	1151
MSTRG.29767.5	coding	0.28	0	774	2408
MSTRG.29773.2	noncoding	-0.004096	129	273	3358
MSTRG.29774.1	coding	0.388	612	936	1658
MSTRG.29774.4	coding	0.413	288	981	8208
MSTRG.29774.10	coding	0.416	1407	1788	11322

MSTRG.29774.9	coding	0.397	591	1260	8485
MSTRG.29774.7	coding	0.413	342	1035	8456
MSTRG.29774.12	coding	0.416	285	666	8031
MSTRG.29774.13	coding	0.381	549	1341	8499
MSTRG.29774.15	coding	0.416	558	1260	8486
MSTRG.29776.1	noncoding	-0.0233472	3288	3528	3838
MSTRG.29769.2	coding	0.379	0	1473	1896
MSTRG.29849.2	coding	0.374	177	4359	6255
MSTRG.29849.4	coding	0.376	177	4311	6207
MSTRG.29849.3	coding	0.375	975	5112	7006
MSTRG.29851.1	noncoding	-0.04096	5157	5286	6121
MSTRG.29852.1	noncoding	-0.0139264	1920	2097	3413
MSTRG.29853.1	noncoding	-0.0528384	4365	5007	21383
MSTRG.29854.1	noncoding	-0.159744	216	261	451
MSTRG.29856.1	noncoding	-0.086016	639	690	1699
MSTRG.29857.1	noncoding	-0.028672	210	315	43983
MSTRG.29858.1	noncoding	-0.0126976	1308	1455	10797
MSTRG.29860.1	noncoding	-0.024576	3447	3552	4139
MSTRG.29861.1	coding	0.049	1599	2037	6847
MSTRG.29861.2	coding	0.049	1764	2202	6865
MSTRG.29861.3	coding	0.049	1548	1986	3458
MSTRG.29849.5	noncoding	-0.086016	52668	52929	73817
MSTRG.29849.6	noncoding	-0.0323584	45006	45207	47030
MSTRG.29849.7	noncoding	-0.0155648	9630	9858	33376
MSTRG.29849.8	noncoding	-0.012288	17073	17184	31545
MSTRG.29849.9	noncoding	-0.0073728	27681	27828	29633
MSTRG.29849.10	noncoding	-0.008192	8412	8550	14733
MSTRG.29791.15	coding	0.089	591	1824	2519
MSTRG.29791.16	coding	0.101	690	2283	2828
MSTRG.29791.7	coding	0.101	690	2283	3113
MSTRG.29791.8	coding	0.136	582	3207	3901
MSTRG.29791.13	coding	0.139	2553	5511	6342
MSTRG.29791.10	coding	0.135	5649	8484	9027
MSTRG.29791.11	coding	0.148	0	3156	3701
MSTRG.29791.9	coding	0.134	0	3228	4058
MSTRG.29791.17	coding	0.146	4119	4896	6735
MSTRG.29791.18	coding	0.163	12	747	2072
MSTRG.29792.1	noncoding	-0.0888832	0	765	2588
MSTRG.29786.2	coding	0.505	48	2796	2952
MSTRG.29787.1	coding	0.641	483	2325	4482
MSTRG.29787.3	coding	0.633	483	2220	4291
MSTRG.29795.2	coding	0.295	48	717	871
MSTRG.29796.1	noncoding	-0.026624	18	198	880
MSTRG.29797.3	coding	0.306	147	873	1098
MSTRG.29799.1	noncoding	-0.2977792	0	681	2304
MSTRG.29799.2	coding	0.474	0	1128	1601
MSTRG.29799.3	noncoding	-0.0434176	309	621	1505
MSTRG.29810.3	coding	0.376	672	1722	2412
MSTRG.29810.4	coding	0.375	729	1800	2488
MSTRG.29810.7	coding	0.377	672	1725	2415
MSTRG.29810.8	coding	0.376	549	1599	2289
MSTRG.29810.6	coding	0.351	1524	1935	4969
MSTRG.29810.2	coding	0.374	561	1761	4861
MSTRG.29810.9	coding	0.35	0	1380	4846

MSTRG.29800.5	coding	0.265	1095	1548	2628
MSTRG.29800.6	noncoding	-0.0774144	555	843	1755
MSTRG.29801.3	noncoding	-0.008192	1329	1467	2339
MSTRG.29802.1	noncoding	-0.0405504	8442	8763	9400
MSTRG.29815.4	coding	0.227	0	3612	5401
MSTRG.29815.2	coding	0.216	72	3186	5235
MSTRG.29815.5	coding	0.226	0	3570	4963
MSTRG.29815.6	coding	0.096	1023	1275	1738
MSTRG.29821.4	coding	0.281	285	1179	1877
MSTRG.29821.5	coding	0.319	423	978	2123
MSTRG.29825.2	coding	0.223	105	693	892
MSTRG.29825.3	coding	0.243	261	840	1038
MSTRG.29827.1	noncoding	-0.0843776	144	2409	2862
MSTRG.29830.1	coding	0.511	0	819	2105
MSTRG.29830.2	coding	0.516	0	828	2101
MSTRG.29834.1	coding	0.54	48	2370	3029
MSTRG.29835.2	coding	0.335	27	336	552
MSTRG.29836.2	coding	0.425	0	2043	2199
MSTRG.29846.1	coding	0.518	18	2004	2904
MSTRG.29846.8	coding	0.45	18	4416	12831
MSTRG.29846.7	coding	0.425	534	5241	13656
MSTRG.29846.9	coding	0.415	1950	5406	13813
MSTRG.29846.10	coding	0.378	0	981	1134
MSTRG.29846.11	coding	0.438	75	3849	4003
MSTRG.29846.12	coding	0.143	9	465	10577
MSTRG.29846.13	noncoding	-0.0405504	4293	4800	16546
MSTRG.29846.14	noncoding	-0.0069632	6	201	6615
MSTRG.29847.1	noncoding	-0.0016384	3483	3630	10779
MSTRG.29845.1	coding	0.504	0	510	681
MSTRG.29868.1	coding	0.301	642	1506	3049
MSTRG.29869.1	coding	0.176	0	453	4785
MSTRG.29869.3	noncoding	-0.0364544	348	825	1172
MSTRG.29869.5	noncoding	-0.0651264	2619	2946	4653
MSTRG.29869.4	noncoding	-0.0364544	408	885	1230
MSTRG.29869.6	noncoding	-0.0651264	1617	1944	3361
MSTRG.29869.7	noncoding	-0.014336	2745	2946	5439
MSTRG.29866.2	noncoding	-0.028672	27	105	261
MSTRG.29867.1	coding	0.131	447	1674	1829
MSTRG.29867.2	noncoding	-0.0290816	393	678	1728
MSTRG.29879.4	coding	0.373	0	1776	2508
MSTRG.29879.6	coding	0.351	0	1251	1968
MSTRG.29879.7	coding	0.385	0	1728	1882
MSTRG.29879.3	coding	0.414	0	2040	2345
MSTRG.29880.2	coding	0.39	45	3189	3380
MSTRG.29880.3	coding	0.424	2109	4182	4374
MSTRG.29881.2	coding	0.302	81	1074	1295
MSTRG.29884.1	coding	0.367	0	501	943
MSTRG.29884.2	coding	0.369	0	483	971
MSTRG.29884.3	coding	0.354	102	531	897
MSTRG.29885.3	coding	0.482	1233	3474	5789
MSTRG.29890.4	coding	0.258	27	768	978
MSTRG.29891.1	coding	0.437	0	846	1287
MSTRG.29891.2	coding	0.335	501	1242	1682
MSTRG.29891.3	coding	0.306	0	1728	4529

MSTRG.29891.4	coding	0.314	0	1659	4460
MSTRG.29891.6	coding	0.306	0	1728	2123
MSTRG.29891.7	coding	0.319	321	1866	2261
MSTRG.29894.2	coding	0.414	0	1791	2198
MSTRG.29894.3	coding	0.363	483	2223	3463
MSTRG.29894.4	coding	0.356	462	2271	2649
MSTRG.29896.7	noncoding	-0.0577536	1275	1917	2710
MSTRG.29896.6	noncoding	-0.0663552	225	684	1530
MSTRG.29896.4	coding	0.24	0	1335	2180
MSTRG.29896.5	coding	0.249	0	1161	2006
MSTRG.29897.1	noncoding	-0.0126976	153	318	680
MSTRG.29897.2	noncoding	-0.0172032	378	771	1145
MSTRG.29897.3	noncoding	-0.0126976	135	300	730
MSTRG.29889.2	coding	0.314	387	915	1262
MSTRG.29900.1	coding	0.143	288	2163	2410
MSTRG.29903.3	coding	0.309	39	3990	4483
MSTRG.29903.1	coding	0.309	1032	5076	5568
MSTRG.29904.1	coding	0.325	477	2325	3264
MSTRG.29902.1	coding	0.305	0	306	1867
MSTRG.29902.2	coding	0.305	0	306	642
MSTRG.29902.3	noncoding	-0.118784	150	492	737
MSTRG.29910.3	coding	0.23	357	918	1657
MSTRG.29910.2	coding	0.265	711	1293	2033
MSTRG.29912.2	coding	0.281	444	1482	2340
MSTRG.29912.3	coding	0.282	987	2280	2553
MSTRG.29912.4	coding	0.282	1023	2316	2590
MSTRG.29912.5	coding	0.314	1125	1827	2687
MSTRG.29912.6	coding	0.332	582	1518	2377
MSTRG.29906.2	coding	0.308	654	2040	2592
MSTRG.29906.4	coding	0.317	993	2355	2742
MSTRG.29908.4	coding	0.289	6	1407	7721
MSTRG.29916.1	noncoding	-0.0004096	2118	2160	2489
MSTRG.29917.1	coding	0.163	120	1191	1459
MSTRG.29917.3	coding	0.164	78	1134	1404
MSTRG.29918.1	noncoding	-0.1024	177	267	592
MSTRG.29920.1	coding	0.407	453	2187	14284
MSTRG.29923.5	coding	0.232	750	1437	1690
MSTRG.29923.6	coding	0.206	519	1068	1258
MSTRG.29921.1	coding	0.01	219	615	1974
MSTRG.29921.4	coding	0.124	168	1302	1594
MSTRG.29921.5	coding	0.076	660	1662	1953
MSTRG.29926.3	coding	0.194	729	2613	4023
MSTRG.29926.2	coding	0.195	732	2619	4027
MSTRG.29927.3	coding	0.16	921	2343	2508
MSTRG.29934.4	coding	0.169	345	777	1045
MSTRG.29934.5	coding	0.169	225	657	925
MSTRG.29934.8	coding	0.171	303	717	872
MSTRG.29941.3	coding	0.368	0	2403	4967
MSTRG.29941.4	coding	0.382	0	2175	5365
MSTRG.29943.5	coding	0.587	519	1779	2549
MSTRG.29943.3	coding	0.53	0	9858	10627
MSTRG.29943.2	coding	0.522	981	11472	12241
MSTRG.29943.4	coding	0.52	981	11505	12274
MSTRG.29938.1	noncoding	-0.01024	225	372	527

MSTRG.29946.2	noncoding	-0.0790528	189	561	946
MSTRG.29947.1	noncoding	-0.063488	0	189	730
MSTRG.29949.1	noncoding	-0.1253376	1227	1833	1990
MSTRG.29948.2	noncoding	-0.0790528	0	1110	2075
MSTRG.29950.4	coding	0.381	588	4221	7468
MSTRG.29950.5	coding	0.312	4005	9243	12450
MSTRG.29950.6	coding	0.365	4845	8775	11943
MSTRG.29965.1	coding	0.198	153	2673	3938
MSTRG.29965.5	coding	0.198	177	2697	3004
MSTRG.29967.2	coding	0.401	552	1278	1524
MSTRG.29970.1	coding	0.294	1110	4782	5934
MSTRG.29970.2	coding	0.294	1266	4938	6090
MSTRG.29970.3	coding	0.294	540	4212	4730
MSTRG.29970.4	coding	0.29	804	4455	5606
MSTRG.29970.5	coding	0.29	786	4437	4953
MSTRG.29970.7	coding	0.29	228	3879	4395
MSTRG.29970.8	coding	0.294	609	4281	4797
MSTRG.29970.9	coding	0.348	120	3036	3553
MSTRG.29974.3	coding	0.488	0	1254	4192
MSTRG.29974.2	coding	0.552	0	1137	4075
MSTRG.29975.1	noncoding	-0.0536576	0	111	2736
MSTRG.29954.1	noncoding	-0.0147456	6	186	424
MSTRG.29955.1	noncoding	-0.0610304	1608	2529	2684
MSTRG.29957.2	coding	0.468	0	1962	4513
MSTRG.29961.2	noncoding	-0.0401408	12	657	1441
MSTRG.29980.5	noncoding	-0.0425984	2142	2316	14083
MSTRG.29978.3	noncoding	-0.0315392	5076	5268	7467
MSTRG.29978.1	noncoding	-0.032768	276	483	2765
MSTRG.29978.2	noncoding	-0.073728	60	87	876
MSTRG.29985.3	coding	0.249	222	3081	5414
MSTRG.29985.4	coding	0.249	1239	4098	6431
MSTRG.29990.1	coding	0.511	246	9834	10618
MSTRG.29990.2	coding	0.513	225	9660	10386
MSTRG.29990.5	coding	0.519	0	5592	7157
MSTRG.29990.6	coding	0.523	0	9144	10300
MSTRG.29990.7	coding	0.52	0	9159	10317
MSTRG.29990.8	coding	0.52	0	9255	9962
MSTRG.29990.9	coding	0.535	1353	9816	10440
MSTRG.29990.10	coding	0.469	603	10542	11327
MSTRG.29991.1	noncoding	-0.0323584	192	2193	2554
MSTRG.29991.2	noncoding	-0.0790528	219	1023	1586
MSTRG.29997.1	coding	0.262	0	3612	8506
MSTRG.29997.2	coding	0.27	0	3561	7373
MSTRG.30003.7	coding	0.555	45	2256	3127
MSTRG.30003.2	coding	0.575	183	3609	4479
MSTRG.30003.5	coding	0.547	132	5547	6417
MSTRG.30003.4	coding	0.518	468	6522	7393
MSTRG.30003.3	coding	0.535	123	6069	6938
MSTRG.30003.6	coding	0.541	0	5952	6821
MSTRG.30003.8	coding	0.577	279	3516	3795
MSTRG.30003.9	coding	0.282	144	1161	1348
MSTRG.29998.1	noncoding	-0.0446464	177	477	1114
MSTRG.29999.1	noncoding	-0.016384	282	609	1128
MSTRG.30009.10	coding	0.33	0	2166	2320

MSTRG.30009.11	coding	0.388	0	1746	1901
MSTRG.30009.6	coding	0.395	0	1848	2003
MSTRG.30009.7	coding	0.371	0	2103	2259
MSTRG.30009.15	coding	0.386	0	2079	4818
MSTRG.30009.8	coding	0.387	0	1914	4656
MSTRG.30009.17	coding	0.35	0	1839	1993
MSTRG.30009.18	coding	0.376	0	1929	2084
MSTRG.30009.19	coding	0.339	0	2124	4868
MSTRG.30009.20	coding	0.351	0	1815	1970
MSTRG.30009.21	coding	0.386	0	1947	2102
MSTRG.30009.22	coding	0.375	0	1758	1914
MSTRG.30009.23	coding	0.331	9	2250	2405
MSTRG.30009.24	noncoding	-0.1036288	231	1053	1800
MSTRG.30005.3	coding	0.51	0	1650	6579
MSTRG.30010.3	coding	0.418	837	2292	4354
MSTRG.30010.1	coding	0.489	0	2478	4540
MSTRG.30010.2	coding	0.51	0	2352	4414
MSTRG.30011.2	coding	0.337	60	2340	3377
MSTRG.30016.2	coding	0.246	561	3606	3968
MSTRG.30016.3	coding	0.248	141	3207	3853
MSTRG.30016.5	coding	0.562	168	1458	1621
MSTRG.30016.4	coding	0.249	915	3990	4638
MSTRG.30018.1	noncoding	-0.083968	0	495	733
MSTRG.30020.2	noncoding	-0.0864256	3576	3981	4229
MSTRG.30020.3	noncoding	-0.0487424	4380	4704	4860
MSTRG.30013.1	noncoding	-0.0323584	87	1911	2067
MSTRG.30014.1	noncoding	-0.0499712	30	345	774
MSTRG.30014.2	noncoding	-0.0364544	480	1026	1252
MSTRG.30014.3	noncoding	-0.0323584	21	771	1277
MSTRG.30014.4	noncoding	-0.026624	21	612	801
MSTRG.30024.3	coding	0.322	60	480	702
MSTRG.30033.1	noncoding	-0.0458752	579	1233	2272
MSTRG.30035.2	coding	0.416	1161	3069	3491
MSTRG.30036.2	coding	0.283	0	2241	2711
MSTRG.30036.3	coding	0.266	1923	4485	4953
MSTRG.30036.4	coding	0.354	543	2253	2622
MSTRG.30054.11	coding	0.297	2781	5046	6833
MSTRG.30054.13	coding	0.283	2781	5103	6892
MSTRG.30054.2	coding	0.302	2319	4491	6280
MSTRG.30054.7	coding	0.308	2301	4395	6183
MSTRG.30054.8	coding	0.3	0	2199	3988
MSTRG.30054.17	coding	0.304	2319	4596	6287
MSTRG.30054.19	coding	0.288	0	1230	1533
MSTRG.30054.21	coding	0.272	144	1722	1877
MSTRG.30054.20	coding	0.271	2244	4107	4261
MSTRG.30054.18	coding	0.266	2331	3987	4142
MSTRG.30040.2	coding	0.59	156	1392	2393
MSTRG.30040.3	coding	0.492	0	513	1514
MSTRG.30039.1	coding	0.455	0	669	2562
MSTRG.30046.1	noncoding	-0.09216	0	231	1041
MSTRG.30046.2	noncoding	-0.0630784	63	462	988
MSTRG.30049.3	coding	0.427	27	1029	2496
MSTRG.30049.2	coding	0.427	27	1029	2326
MSTRG.30055.1	noncoding	-0.0835584	738	1038	1222

MSTRG.30064.5	coding	0.027	123	366	726
MSTRG.30064.6	coding	0.058	93	273	622
MSTRG.30068.1	coding	0.091	129	309	554
MSTRG.30068.2	noncoding	-0.0495616	66	159	403
MSTRG.30087.1	noncoding	-0.0135168	30	96	249
MSTRG.30091.1	noncoding	-0.0196608	0	96	341
MSTRG.30114.1	coding	0.143	165	1965	2547
MSTRG.30114.3	noncoding	-0.1028096	450	1473	2299
MSTRG.30102.1	coding	0.097	246	921	1101
MSTRG.30102.2	coding	0.116	93	1866	2046
MSTRG.30102.3	coding	0.044	147	408	578
MSTRG.30126.5	coding	0.23	11121	12111	12551
MSTRG.30127.1	noncoding	-0.0397312	873	1284	1607
MSTRG.30128.1	noncoding	-0.004096	3132	3210	4195
MSTRG.30129.1	coding	0.08	3768	4272	7851
MSTRG.30130.1	coding	0.075	8532	9075	10413
MSTRG.30132.1	noncoding	-0.036864	231	297	1156
MSTRG.30121.2	coding	0.259	93	1089	2285
MSTRG.30122.3	noncoding	-0.1036288	0	18	350
MSTRG.30124.1	noncoding	-0.0004096	0	0	687
MSTRG.30123.2	coding	0.115	2856	3438	10065
MSTRG.30138.1	coding	0.185	0	696	1341
MSTRG.30143.1	noncoding	-0.0864256	0	87	1114
MSTRG.30133.1	noncoding	-0.0004096	0	0	326
MSTRG.30137.1	coding	0.104	0	696	871
MSTRG.30165.3	noncoding	-0.14336	312	375	675
MSTRG.30164.1	noncoding	-0.004096	513	657	1941
MSTRG.30164.2	noncoding	-0.0237568	1497	1563	1867
MSTRG.30175.1	noncoding	-0.012288	186	270	797
MSTRG.30176.1	noncoding	-0.032768	5310	5394	6522
MSTRG.30177.1	coding	0.371	432	2283	3353
MSTRG.30177.4	coding	0.309	0	753	3460
MSTRG.30178.1	noncoding	-0.012288	2883	3015	5060
MSTRG.30177.5	noncoding	-0.045056	2031	2211	2570
MSTRG.30177.6	noncoding	-0.004096	2781	3060	3215
MSTRG.30171.6	coding	0.079	588	1212	5871
MSTRG.30174.1	noncoding	-0.21504	303	309	553
MSTRG.30182.1	coding	0.177	0	591	1275
MSTRG.30186.3	coding	0.059	408	1011	4525
MSTRG.30190.2	coding	0.028	39	5802	7347
MSTRG.30198.1	coding	0.34	48	2076	7680
MSTRG.30198.3	coding	0.389	0	1407	1989
MSTRG.30198.4	coding	0.337	111	2133	2324
MSTRG.30199.2	noncoding	-0.0872448	6	231	5099
MSTRG.30199.3	noncoding	-0.0872448	3	228	4723
MSTRG.30204.1	noncoding	-0.0548864	3957	4158	6558
MSTRG.30204.2	noncoding	-0.0548864	3930	4131	7247
MSTRG.30189.2	coding	0.039	492	903	2432
MSTRG.30211.1	noncoding	-0.012288	1662	1677	2017
MSTRG.30212.3	coding	0.142	0	912	1591
MSTRG.30212.4	coding	0.074	417	1122	6604
MSTRG.30213.1	coding	0.056	1062	1218	1826
MSTRG.30214.1	noncoding	-0.0012288	7176	7332	11133
MSTRG.30215.2	coding	0.245	96	5310	6944

MSTRG.30215.4	coding	0.232	96	1080	1355
MSTRG.30216.1	noncoding	-0.016384	0	6	232
MSTRG.30217.2	coding	0.133	318	1518	2079
MSTRG.30219.1	coding	0.202	0	2109	2495
MSTRG.30227.1	noncoding	-0.0397312	0	132	449
MSTRG.30227.2	noncoding	-0.0032768	81	258	411
MSTRG.30240.2	coding	0.191	507	2910	4322
MSTRG.30241.1	noncoding	-0.0249856	1836	1920	4270
MSTRG.30245.1	noncoding	-0.008192	633	687	1186
MSTRG.30235.1	coding	0.208	246	933	1404
MSTRG.30239.1	noncoding	-0.0458752	441	657	1808
MSTRG.30248.3	coding	0.102	39	1293	1631
MSTRG.30248.2	coding	0.126	423	1317	1656
MSTRG.30248.4	noncoding	-0.098304	72	123	279
MSTRG.30248.5	coding	0.095	0	177	333
MSTRG.30248.7	coding	0.169	876	1524	1903
MSTRG.30248.9	coding	0.126	483	1377	1756
MSTRG.30248.8	coding	0.126	1773	2667	3045
MSTRG.30249.2	coding	0.231	117	2664	6093
MSTRG.30258.2	noncoding	-0.03072	3969	4725	5768
MSTRG.30258.3	coding	0.088	858	1455	3219
MSTRG.30258.4	noncoding	-0.03072	5436	6192	6504
MSTRG.30260.1	coding	0.043	612	759	1178
MSTRG.30258.5	coding	0.121	1305	1923	2678
MSTRG.30252.5	noncoding	-0.0438272	7266	7512	7731
MSTRG.30271.2	coding	0.057	414	738	5184
MSTRG.30271.3	coding	0.403	0	555	4773
MSTRG.30272.1	noncoding	-0.0221184	288	390	1284
MSTRG.30275.2	coding	0.158	4587	9351	13406
MSTRG.30275.6	coding	0.067	372	1740	10365
MSTRG.30275.7	coding	0.158	372	5136	15774
MSTRG.30275.8	coding	0.101	288	2058	2214
MSTRG.30275.9	noncoding	-0.0004096	2391	2688	2843
MSTRG.30275.12	noncoding	-0.036864	81	159	1683
MSTRG.30278.1	noncoding	-0.057344	2604	2673	4741
MSTRG.30264.3	coding	0.114	228	1563	5427
MSTRG.30265.1	noncoding	-0.0118784	1281	1440	1595
MSTRG.30281.1	coding	0.312	108	1095	2681
MSTRG.30281.2	coding	0.316	108	1143	3057
MSTRG.30282.2	coding	0.303	0	2862	3399
MSTRG.30282.3	coding	0.296	177	3045	3499
MSTRG.30287.2	coding	0.48	0	708	1405
MSTRG.30287.4	coding	0.468	0	588	1309
MSTRG.30288.2	coding	0.309	837	1203	1356
MSTRG.30290.1	coding	0.345	726	1590	3243
MSTRG.30291.5	coding	0.186	0	666	1215
MSTRG.30291.4	coding	0.149	252	474	904
MSTRG.30291.6	coding	0.138	0	504	935
MSTRG.30283.1	noncoding	-0.0536576	0	249	685
MSTRG.30302.5	coding	0.447	27	1116	1270
MSTRG.30315.3	coding	0.426	0	396	1407
MSTRG.30315.2	coding	0.36	0	528	1602
MSTRG.30315.4	coding	0.247	0	1296	1540
MSTRG.30315.5	coding	0.206	12	1200	1446

MSTRG.30320.1	coding	0.084	162	1053	4425
MSTRG.30321.3	noncoding	-0.0765952	0	276	1433
MSTRG.30321.4	coding	0.103	414	936	1560
MSTRG.30321.5	coding	0.114	369	942	1564
MSTRG.30321.6	coding	0.126	0	762	1385
MSTRG.30321.7	coding	0.07	162	540	1162
MSTRG.30312.1	noncoding	-0.200704	54	93	382
MSTRG.30313.1	noncoding	-0.2048	4251	4293	5234
MSTRG.30325.2	coding	0.104	0	1944	3654
MSTRG.30326.1	noncoding	-0.0352256	1257	1506	1662
MSTRG.30326.2	noncoding	-0.02048	1074	1146	1300
MSTRG.30322.3	coding	0.44	0	195	721
MSTRG.30329.2	coding	0.236	363	1344	5335
MSTRG.30331.2	coding	0.19	0	3171	4116
MSTRG.30332.2	coding	0.354	0	444	647
MSTRG.30332.3	coding	0.274	228	690	892
MSTRG.30343.1	coding	0.041	981	2163	6649
MSTRG.30343.6	noncoding	-0.0622592	4878	5070	5224
MSTRG.30343.4	noncoding	-0.0704512	6102	6285	6440
MSTRG.30344.1	coding	0.381	987	3501	4896
MSTRG.30345.1	noncoding	-0.0356352	0	462	684
MSTRG.30348.1	noncoding	-0.090112	12	60	310
MSTRG.30350.5	noncoding	-0.1773568	0	42	1171
MSTRG.30354.2	coding	0.352	0	681	1810
MSTRG.30357.2	coding	0.152	1020	3000	4310
MSTRG.30359.5	coding	0.158	960	1164	1319
MSTRG.30359.6	coding	0.14	0	1962	2115
MSTRG.30361.7	coding	0.295	201	1086	3033
MSTRG.30361.2	coding	0.295	330	1215	3163
MSTRG.30361.9	coding	0.291	264	1275	2542
MSTRG.30361.11	coding	0.289	315	1317	2907
MSTRG.30361.5	coding	0.297	2721	3615	5560
MSTRG.30361.4	coding	0.289	1164	2166	4114
MSTRG.30361.14	coding	0.338	0	933	2524
MSTRG.30361.15	coding	0.338	0	969	2239
MSTRG.30361.12	coding	0.341	0	984	2251
MSTRG.30361.6	coding	0.346	0	1017	2966
MSTRG.30361.16	coding	0.297	2721	3615	4860
MSTRG.30362.5	coding	0.217	60	5064	5351
MSTRG.30363.8	coding	0.315	1572	6579	7224
MSTRG.30363.3	coding	0.304	180	5847	6494
MSTRG.30363.5	coding	0.306	180	5748	6395
MSTRG.30363.6	coding	0.306	180	5751	6398
MSTRG.30363.2	coding	0.304	180	5862	6668
MSTRG.30363.4	coding	0.306	180	5760	6566
MSTRG.30363.7	coding	0.307	180	5763	6569
MSTRG.30365.1	coding	0.539	0	1257	1973
MSTRG.30367.1	coding	0.379	321	1479	1684
MSTRG.30367.4	coding	0.417	354	1077	1282
MSTRG.30368.7	coding	0.396	0	795	1038
MSTRG.30368.4	coding	0.35	0	948	1190
MSTRG.30368.9	coding	0.354	63	939	1181
MSTRG.30368.10	coding	0.351	27	918	1158
MSTRG.30375.1	coding	0.377	2487	4080	4672

MSTRG.30375.5	coding	0.378	738	2073	2666
MSTRG.30375.6	coding	0.352	738	1566	2649
MSTRG.30375.9	coding	0.378	2826	4161	4752
MSTRG.30375.7	coding	0.377	801	2394	2987
MSTRG.30375.8	coding	0.378	762	2373	2964
MSTRG.30375.12	coding	0.352	456	1290	2372
MSTRG.30375.11	coding	0.373	3981	5577	6169
MSTRG.30375.14	coding	0.38	231	1584	2175
MSTRG.30375.15	coding	0.376	396	1731	2322
MSTRG.30378.1	coding	0.364	846	1809	5077
MSTRG.30371.1	noncoding	-0.0786432	474	1188	1837
MSTRG.30371.2	noncoding	-0.0475136	0	273	814
MSTRG.30372.1	noncoding	-0.24576	237	264	418
MSTRG.30382.2	noncoding	-0.045056	225	264	419
MSTRG.30386.3	coding	0.285	495	1350	2171
MSTRG.30388.1	noncoding	-0.038912	0	87	327
MSTRG.30386.5	coding	0.327	81	1275	2087
MSTRG.30386.6	coding	0.298	81	1164	1434
MSTRG.30386.8	coding	0.29	441	1599	1771
MSTRG.30393.2	coding	0.185	279	1854	3221
MSTRG.30393.8	coding	0.176	198	1701	2552
MSTRG.30393.5	coding	0.181	168	1767	2616
MSTRG.30393.9	coding	0.236	1044	2082	2932
MSTRG.30393.7	coding	0.185	129	1704	2553
MSTRG.30393.3	coding	0.18	546	2025	2874
MSTRG.30394.1	noncoding	-0.0200704	1986	2088	3838
MSTRG.30395.1	noncoding	-0.049152	1005	1095	2231
MSTRG.30398.2	noncoding	-0.118784	249	327	990
MSTRG.30398.3	noncoding	-0.114688	57	126	830
MSTRG.30399.3	coding	0.359	51	5748	5959
MSTRG.30403.3	coding	0.487	15	3156	10408
MSTRG.30403.4	coding	0.496	0	3135	10384
MSTRG.30403.5	coding	0.483	24	3141	4679
MSTRG.30403.6	coding	0.475	2376	5325	6845
MSTRG.30404.1	noncoding	-0.1224704	0	18	559
MSTRG.30416.1	noncoding	-0.0086016	483	591	1001
MSTRG.30419.5	coding	0.054	2394	2553	3114
MSTRG.30409.1	coding	0.214	303	999	1180
MSTRG.30409.2	coding	0.212	153	837	1008
MSTRG.30410.1	noncoding	-0.065536	165	195	472
MSTRG.30411.3	coding	0.233	219	1029	1184
MSTRG.30411.4	coding	0.231	249	1044	1199
MSTRG.30411.2	coding	0.23	192	987	1144
MSTRG.30411.6	coding	0.23	81	882	1131
MSTRG.30411.5	coding	0.226	495	1299	1551
MSTRG.30408.1	noncoding	-0.0503808	21	636	1544
MSTRG.30436.1	noncoding	-0.0495616	1080	1962	2115
MSTRG.30437.1	noncoding	-0.0454656	138	504	1956
MSTRG.30438.1	coding	0.279	21	3075	5729
MSTRG.30439.1	coding	0.069	8055	8709	13213
MSTRG.30442.1	coding	0.45	0	2646	3485
MSTRG.30442.2	coding	0.294	69	459	614
MSTRG.30442.3	coding	0.296	18	321	476
MSTRG.30444.2	coding	0.375	2223	3234	3458

MSTRG.30445.2	coding	0.34	285	1737	2112
MSTRG.30448.1	noncoding	-0.0356352	36	3972	4915
MSTRG.30451.1	coding	0.481	0	492	783
MSTRG.30453.3	coding	0.492	0	3219	3387
MSTRG.30453.4	coding	0.567	510	3213	3383
MSTRG.30453.5	coding	0.58	975	3438	4710
MSTRG.30456.2	noncoding	-0.1040384	0	600	756
MSTRG.30456.1	coding	0.37	0	759	978
MSTRG.30456.7	noncoding	-0.0958464	0	867	1023
MSTRG.30456.4	noncoding	-0.0688128	60	1029	1183
MSTRG.30438.2	noncoding	-0.12288	516	579	974
MSTRG.30426.1	noncoding	-0.02048	39	60	462
MSTRG.30467.1	noncoding	-0.1335296	0	27	507
MSTRG.30469.1	noncoding	-0.1224704	0	21	321
MSTRG.30483.1	noncoding	-0.0131072	729	867	1501
MSTRG.30496.1	noncoding	-0.0827392	0	117	5874
MSTRG.30496.2	noncoding	-0.0651264	15	642	9911
MSTRG.30497.1	noncoding	-0.135168	639	726	3978
MSTRG.30497.2	coding	0.085	0	783	4088
MSTRG.30496.3	noncoding	-0.0057344	2148	2298	13586
MSTRG.30473.1	coding	0.053	396	582	852
MSTRG.30475.10	coding	0.147	10470	12474	13244
MSTRG.30475.11	coding	0.151	2580	4488	5260
MSTRG.30476.1	noncoding	-0.0618496	243	258	769
MSTRG.30479.1	noncoding	-0.0004096	480	612	1025
MSTRG.30481.1	coding	0.154	96	759	1750
MSTRG.30481.2	coding	0.154	96	759	3606
MSTRG.30481.3	coding	0.148	39	594	898
MSTRG.30477.2	coding	0.238	75	795	1893
MSTRG.30503.1	coding	0.332	984	4494	7301
MSTRG.30505.4	coding	0.084	468	1107	1358
MSTRG.30506.1	coding	0.095	444	627	3372
MSTRG.30506.4	noncoding	-0.0671744	0	828	2266
MSTRG.30507.2	coding	0.067	660	1446	4352
MSTRG.30518.1	coding	0.194	312	2346	2676
MSTRG.30518.2	coding	0.194	237	2271	2597
MSTRG.30518.6	coding	0.193	234	2289	2620
MSTRG.30517.2	coding	0.028	591	1038	2256
MSTRG.30522.5	noncoding	-0.0393216	4386	4725	7163
MSTRG.30522.6	coding	0.172	4794	5490	5643
MSTRG.30523.1	noncoding	-0.0417792	14763	15054	18491
MSTRG.30524.1	noncoding	-0.08192	2892	2958	5876
MSTRG.30525.1	coding	0.356	1746	6813	7015
MSTRG.30529.1	coding	0.135	0	1488	3864
MSTRG.30530.1	noncoding	-0.065536	1896	1968	5720
MSTRG.30530.2	noncoding	-0.065536	918	990	4390
MSTRG.30530.3	noncoding	-0.065536	918	990	1989
MSTRG.30531.3	coding	0.071	498	3309	7062
MSTRG.30536.1	noncoding	-0.131072	30	102	376
MSTRG.30539.1	noncoding	-0.0339968	0	168	1558
MSTRG.30534.2	coding	0.251	0	498	1345
MSTRG.30534.3	coding	0.266	0	714	1614
MSTRG.30534.4	noncoding	-0.0831488	0	165	4708
MSTRG.30547.1	coding	0.06	852	1221	1574

MSTRG.30547.2	noncoding	-0.0909312	8130	8766	8921
MSTRG.30547.6	coding	0.345	0	303	3403
MSTRG.30547.5	coding	0.345	0	303	3307
MSTRG.30547.7	noncoding	-0.0692224	3540	3783	6367
MSTRG.30548.1	noncoding	-0.004096	192	336	3924
MSTRG.30548.2	noncoding	-0.012288	3867	3984	8423
MSTRG.30548.3	noncoding	-0.04096	72	141	843
MSTRG.30549.1	noncoding	-0.0475136	4059	4260	4583
MSTRG.30550.1	noncoding	-0.0458752	597	738	3165
MSTRG.30551.1	noncoding	-0.3072	1800	1818	2364
MSTRG.30553.1	noncoding	-0.049152	1119	1233	2445
MSTRG.30554.1	noncoding	-0.131072	2193	2289	3351
MSTRG.30555.1	noncoding	-0.008192	759	891	1506
MSTRG.30556.1	coding	0.072	570	792	2391
MSTRG.30556.2	noncoding	-0.06144	1290	1383	1539
MSTRG.30545.1	noncoding	-0.049152	36	69	318
MSTRG.30559.1	coding	0.259	225	822	1248
MSTRG.30559.3	coding	0.259	90	687	1110
MSTRG.30561.2	coding	0.243	0	1554	2272
MSTRG.30561.3	coding	0.161	4827	6609	7329
MSTRG.30562.2	coding	0.149	117	1788	3414
MSTRG.30565.1	coding	0.144	207	1215	8182
MSTRG.30565.2	coding	0.689	0	831	9904
MSTRG.30565.4	coding	0.144	687	1695	3923
MSTRG.30566.1	noncoding	-0.098304	732	777	1267
MSTRG.30569.1	noncoding	-0.0352256	3261	3774	4089
MSTRG.30571.1	noncoding	-0.04096	93	108	465
MSTRG.30572.1	noncoding	-0.008192	57	120	736
MSTRG.30575.1	coding	0.118	624	1416	3916
MSTRG.30575.2	coding	0.087	207	1722	2462
MSTRG.30575.3	coding	0.048	774	936	1092
MSTRG.30575.5	noncoding	-0.032768	2046	2178	2332
MSTRG.30576.1	noncoding	-0.1024	483	516	684
MSTRG.30583.2	coding	0.194	180	1410	6872
MSTRG.30583.3	coding	0.194	180	1410	5066
MSTRG.30583.4	coding	0.191	180	1521	4960
MSTRG.30584.1	coding	0.059	1743	2481	4063
MSTRG.30595.3	noncoding	-0.0425984	3939	4656	4868
MSTRG.30595.5	noncoding	-0.024576	996	1281	1434
MSTRG.30595.4	noncoding	-0.0323584	1017	1308	1464
MSTRG.30596.1	coding	0.113	348	3138	4665
MSTRG.30597.1	coding	0.186	0	1896	2122
MSTRG.30597.3	coding	0.181	0	1890	2815
MSTRG.30597.5	coding	0.181	0	1884	2170
MSTRG.30599.4	coding	0.14	0	2826	12264
MSTRG.30614.8	coding	0.292	561	2814	3333
MSTRG.30616.7	coding	0.148	1878	2718	5825
MSTRG.30616.3	coding	0.126	651	2040	5148
MSTRG.30616.6	coding	0.131	651	2040	5148
MSTRG.30617.1	noncoding	-0.016384	3327	3429	5582
MSTRG.30616.8	coding	0.086	3	150	1400
MSTRG.30616.9	coding	0.03	11622	12081	15066
MSTRG.30616.10	coding	0.03	15510	15969	18157
MSTRG.30618.1	noncoding	-0.012288	180	306	518

MSTRG.30620.1	coding	0.256	0	1023	1501
MSTRG.30620.3	coding	0.256	0	1023	1221
MSTRG.30623.4	coding	0.428	0	2031	2520
MSTRG.30623.5	coding	0.37	0	1608	7750
MSTRG.30624.1	noncoding	-0.0344064	252	435	641
MSTRG.30625.1	coding	0.262	231	1731	1954
MSTRG.30625.2	coding	0.264	372	1875	2100
MSTRG.30625.3	coding	0.262	357	1857	2078
MSTRG.30625.4	coding	0.288	369	1203	2325
MSTRG.30625.5	coding	0.264	327	1830	2051
MSTRG.30625.6	coding	0.262	345	1845	2066
MSTRG.30628.1	noncoding	-0.028672	153	252	468
MSTRG.30631.2	noncoding	-0.0671744	354	558	900
MSTRG.30643.1	coding	0.128	63	1140	2407
MSTRG.30645.3	coding	0.048	105	849	1349
MSTRG.30646.1	coding	0.054	2526	3909	6960
MSTRG.30646.2	coding	0.046	2526	4530	9726
MSTRG.30637.1	noncoding	-0.0434176	18	255	783
MSTRG.30654.4	coding	0.297	12	999	3095
MSTRG.30657.1	noncoding	-0.002048	63	183	546
MSTRG.30658.2	coding	0.147	699	1926	2918
MSTRG.30658.3	coding	0.147	969	2196	3187
MSTRG.30658.4	coding	0.147	171	1398	2379
MSTRG.30669.1	noncoding	-0.098304	63	114	793
MSTRG.30668.5	coding	0.112	531	738	1621
MSTRG.30668.3	coding	0.109	810	1518	2752
MSTRG.30668.4	coding	0.08	810	2202	2882
MSTRG.30668.6	coding	0.095	531	2043	2397
MSTRG.30670.1	noncoding	-0.02048	132	207	1619
MSTRG.30671.10	noncoding	-0.0667648	2304	3087	3930
MSTRG.30676.4	coding	0.271	282	1065	1521
MSTRG.30678.1	coding	0.235	105	2943	6130
MSTRG.30685.1	coding	0.228	1158	2019	4701
MSTRG.30685.4	coding	0.262	1248	1971	4103
MSTRG.30685.5	coding	0.208	762	2691	4823
MSTRG.30685.6	coding	0.189	432	2580	4699
MSTRG.30685.7	coding	0.21	36	1779	5598
MSTRG.30685.8	noncoding	-0.0200704	309	807	2350
MSTRG.30685.9	coding	0.059	5331	6765	13262
MSTRG.30685.10	coding	0.059	12603	14037	17052
MSTRG.30685.11	coding	0.084	1194	1872	2224
MSTRG.30685.12	noncoding	-0.0364544	7407	7710	14699
MSTRG.30685.13	noncoding	-0.0503808	3642	4044	7253
MSTRG.30685.14	noncoding	-0.0135168	411	564	10175
MSTRG.30685.15	noncoding	-0.0135168	399	552	6773
MSTRG.30688.1	noncoding	-0.073728	249	321	787
MSTRG.30688.2	noncoding	-0.01024	396	570	2374
MSTRG.30693.1	coding	0.145	555	10266	11852
MSTRG.30693.6	coding	0.143	0	5052	6414
MSTRG.30693.2	coding	0.155	540	8427	9788
MSTRG.30693.3	coding	0.155	540	8427	9788
MSTRG.30693.4	coding	0.155	540	8427	9788
MSTRG.30693.7	coding	0.146	513	9876	11234
MSTRG.30693.9	coding	0.154	540	8079	9430

MSTRG.30695.1	noncoding	-0.0319488	102	270	432
MSTRG.30698.1	noncoding	-0.0139264	213	444	601
MSTRG.30698.2	noncoding	-0.0147456	60	240	395
MSTRG.30699.1	noncoding	-0.0610304	126	366	777
MSTRG.30701.1	noncoding	-0.038912	783	996	3092
MSTRG.30703.1	noncoding	-0.0290816	249	441	1717
MSTRG.30703.2	noncoding	-0.0290816	39	231	1337
MSTRG.30704.1	noncoding	-0.0249856	540	639	1820
MSTRG.30705.2	noncoding	-0.0651264	0	705	1065
MSTRG.30706.1	noncoding	-0.012288	84	219	732
MSTRG.30712.1	noncoding	-0.0299008	654	1422	1810
MSTRG.30712.2	noncoding	-0.0221184	90	1599	1974
MSTRG.30716.1	coding	0.534	132	2046	3488
MSTRG.30718.1	noncoding	-0.0569344	36	1374	10259
MSTRG.30718.2	noncoding	-0.0663552	6	1149	7699
MSTRG.30719.1	noncoding	-0.02048	150	459	819
MSTRG.30720.5	noncoding	-0.1069056	0	486	958
MSTRG.30720.4	coding	0.428	0	1587	2300
MSTRG.30720.3	coding	0.465	162	1575	2287
MSTRG.30720.2	coding	0.357	33	915	1389
MSTRG.30736.1	coding	0.481	0	8112	8267
MSTRG.30736.3	coding	0.496	0	4077	6788
MSTRG.30736.4	coding	0.51	0	6321	7575
MSTRG.30737.1	noncoding	-0.0208896	63	69	364
MSTRG.30725.2	coding	0.427	0	834	3463
MSTRG.30727.1	noncoding	-0.0704512	117	162	916
MSTRG.30728.1	noncoding	-0.0237568	0	843	2377
MSTRG.30740.1	coding	0.36	0	456	1429
MSTRG.30741.1	noncoding	-0.0458752	30	243	398
MSTRG.30743.6	noncoding	-0.0557056	0	417	1642
MSTRG.30743.4	noncoding	-0.063488	0	210	1269
MSTRG.30743.8	coding	0.3	297	960	1180
MSTRG.30743.9	coding	0.283	0	1155	2017
MSTRG.30743.10	coding	0.295	0	1530	2361
MSTRG.30743.7	coding	0.289	0	1554	3631
MSTRG.30747.5	coding	0.207	1836	2196	3266
MSTRG.30747.6	noncoding	-0.0905216	1299	1488	2529
MSTRG.30758.2	coding	0.205	0	1143	1520
MSTRG.30759.1	noncoding	-0.014336	306	477	835
MSTRG.30760.1	noncoding	-0.118784	852	1347	1583
MSTRG.30760.3	noncoding	-0.0274432	1935	2139	2292
MSTRG.30760.4	noncoding	-0.0610304	2991	3543	8813
MSTRG.30760.5	coding	0.487	750	2202	3059
MSTRG.30760.2	noncoding	-0.1052672	750	3585	7766
MSTRG.30762.1	noncoding	-0.016384	2589	2721	5143
MSTRG.30763.1	noncoding	-0.04096	2595	2763	4857
MSTRG.30760.7	noncoding	-0.0663552	14250	14670	18873
MSTRG.30760.8	noncoding	-0.0016384	195	339	2921
MSTRG.30765.1	noncoding	-0.0393216	1104	1767	2745
MSTRG.30768.1	noncoding	-0.06144	33	36	203
MSTRG.30771.1	noncoding	-0.032768	1113	1635	4301
MSTRG.30772.1	coding	0.403	0	438	5520
MSTRG.30772.2	coding	0.405	0	429	5349
MSTRG.30753.7	coding	0.463	297	2679	6106

MSTRG.30757.1	noncoding	-0.0188416	2409	2595	2997
MSTRG.30774.1	coding	0.313	330	3996	4151
MSTRG.30776.1	noncoding	-0.0647168	174	243	567
MSTRG.30787.1	coding	0.235	258	1698	3049
MSTRG.30791.1	noncoding	-0.1302528	0	9	239
MSTRG.30792.2	noncoding	-0.0237568	0	60	1084
MSTRG.30793.1	noncoding	-0.0065536	30	339	493
MSTRG.30792.3	noncoding	-0.19456	318	348	3017
MSTRG.30792.4	noncoding	-0.002048	3009	3156	3946
MSTRG.30792.5	noncoding	-0.008192	321	411	565
MSTRG.30792.6	noncoding	-0.0086016	285	291	448
MSTRG.30794.1	noncoding	-0.258048	294	315	472
MSTRG.30795.4	noncoding	-0.0479232	159	390	5310
MSTRG.30831.1	noncoding	-0.0135168	0	126	699
MSTRG.30832.2	noncoding	-0.147456	114	129	416
MSTRG.30798.1	noncoding	-0.053248	99	207	362
MSTRG.30801.1	coding	0.223	0	231	387
MSTRG.30802.1	noncoding	-0.012288	219	336	690
MSTRG.30803.2	noncoding	-0.0684032	138	501	656
MSTRG.30805.2	coding	0.21	513	1290	3344
MSTRG.30805.4	coding	0.24	0	927	2471
MSTRG.30806.1	coding	0.106	555	732	2282
MSTRG.30807.1	noncoding	-0.0126976	1425	1761	4266
MSTRG.30808.1	noncoding	-0.028672	7155	7320	16030
MSTRG.30808.2	coding	0.001	1620	1800	4553
MSTRG.30805.6	noncoding	-0.0057344	1752	1902	4459
MSTRG.30809.1	coding	0.053	351	516	841
MSTRG.30812.1	coding	0.158	342	1203	1674
MSTRG.30812.2	coding	0.073	0	3594	4325
MSTRG.30812.4	coding	0.105	1548	3588	4059
MSTRG.30815.2	coding	0.466	0	105	259
MSTRG.30817.1	noncoding	-0.0057344	6906	7101	7727
MSTRG.30826.2	coding	0.26	213	951	2112
MSTRG.30826.3	coding	0.242	213	741	2090
MSTRG.30826.5	coding	0.26	474	1212	1615
MSTRG.30826.6	coding	0.242	3462	3990	4581
MSTRG.30833.1	noncoding	-0.126976	234	330	629
MSTRG.30866.1	noncoding	-0.0499712	693	864	1323
MSTRG.30867.1	noncoding	-0.04096	2877	2994	5702
MSTRG.30866.2	noncoding	-0.057344	603	678	1064
MSTRG.30868.1	noncoding	-0.0229376	7023	7179	9456
MSTRG.30866.3	noncoding	-0.0335872	3171	3351	6508
MSTRG.30866.4	coding	0.048	225	780	2621
MSTRG.30866.5	noncoding	-0.0147456	6966	7314	16177
MSTRG.30866.6	noncoding	-0.0147456	6951	7299	9949
MSTRG.30866.7	coding	0.028	1401	1857	5214
MSTRG.30866.8	coding	0.028	7677	8133	10825
MSTRG.30835.1	noncoding	-0.0172032	1122	1275	2952
MSTRG.30836.1	noncoding	-0.032768	849	957	2331
MSTRG.30836.4	noncoding	-0.028672	2184	2409	2631
MSTRG.30836.3	noncoding	-0.02048	483	594	1943
MSTRG.30836.2	noncoding	-0.0004096	1461	1587	2936
MSTRG.30836.5	noncoding	-0.028672	1191	1416	1621
MSTRG.30836.6	noncoding	-0.032768	294	402	1722

MSTRG.30837.1	noncoding	-0.069632	3	81	1230
MSTRG.30889.5	coding	0.123	480	4098	4267
MSTRG.30889.7	coding	0.238	444	7800	8249
MSTRG.30889.10	noncoding	-0.028672	7419	7548	7831
MSTRG.30889.14	noncoding	-0.04096	1614	1641	4424
MSTRG.30892.1	noncoding	-0.045056	861	888	2922
MSTRG.30892.2	noncoding	-0.036864	753	819	3615
MSTRG.30892.3	noncoding	-0.045056	321	348	1501
MSTRG.30892.4	noncoding	-0.0831488	3546	3813	8105
MSTRG.30893.1	noncoding	-0.01024	1662	1803	7206
MSTRG.30894.1	noncoding	-0.0331776	23310	23568	24353
MSTRG.30889.16	noncoding	-0.032768	816	912	3650
MSTRG.30889.17	noncoding	-0.0499712	3216	4113	22243
MSTRG.30895.1	noncoding	-0.0446464	0	237	1144
MSTRG.30889.19	coding	0.203	0	429	1514
MSTRG.30889.18	coding	0.202	0	480	3863
MSTRG.30896.1	noncoding	-0.0421888	0	72	1605
MSTRG.30889.20	coding	0.099	723	969	3182
MSTRG.30897.1	noncoding	-0.0249856	1668	1836	2311
MSTRG.30889.21	coding	0.457	264	3180	3654
MSTRG.30871.1	noncoding	-0.0372736	336	513	1473
MSTRG.30874.1	noncoding	-0.0667648	3321	3933	4087
MSTRG.30874.2	noncoding	-0.073728	9354	9891	10047
MSTRG.30876.3	noncoding	-0.0999424	0	201	993
MSTRG.30877.4	coding	0.196	1296	3525	4475
MSTRG.30877.6	coding	0.188	267	2787	3738
MSTRG.30878.2	coding	0.25	57	2442	9166
MSTRG.30878.3	coding	0.266	2472	5121	11921
MSTRG.30880.2	coding	0.329	303	1359	5098
MSTRG.30883.2	coding	0.158	126	942	1098
MSTRG.30882.1	noncoding	-0.0192512	54	69	457
MSTRG.30888.2	coding	0.126	231	891	1181
MSTRG.30899.2	coding	0.37	702	1464	1902
MSTRG.30900.4	coding	0.071	981	1197	4355
MSTRG.30900.5	coding	0.071	1698	1914	5072
MSTRG.30900.6	coding	0.092	1560	3075	6234
MSTRG.30900.9	coding	0.071	891	1107	4118
MSTRG.30900.7	coding	0.126	0	1572	4582
MSTRG.30900.8	coding	0.09	627	2118	5150
MSTRG.30900.10	coding	0.099	192	1737	4766
MSTRG.30905.4	coding	0.204	1923	2499	3081
MSTRG.30906.1	noncoding	-0.0004096	0	0	345
MSTRG.30907.2	coding	0.071	720	1173	1599
MSTRG.30908.1	coding	0.017	528	1287	2061
MSTRG.30909.1	coding	0.033	1458	2418	3600
MSTRG.30909.2	coding	0.033	1428	2388	2893
MSTRG.30909.3	coding	0.033	1458	2418	2799
MSTRG.30909.4	coding	0.033	1458	2418	3887
MSTRG.30909.5	coding	0.033	1458	2418	3769
MSTRG.30909.6	coding	0.033	1458	2418	3727
MSTRG.30910.2	coding	0.084	678	2658	5075
MSTRG.30910.3	coding	0.109	972	2607	5002
MSTRG.30910.4	coding	0.106	1032	2610	4790
MSTRG.30910.5	coding	0.22	882	1485	9927

MSTRG.30910.8	coding	0.127	606	1566	7829
MSTRG.30910.9	coding	0.202	363	735	5143
MSTRG.30911.3	noncoding	-0.04096	426	429	735
MSTRG.30914.1	noncoding	-0.057344	51	69	224
MSTRG.30920.1	noncoding	-0.0434176	627	837	1010
MSTRG.30937.1	noncoding	-0.04096	843	972	1667
MSTRG.30937.2	coding	0.17	285	507	1217
MSTRG.30937.5	noncoding	-0.0315392	987	1146	1656
MSTRG.30937.6	noncoding	-0.0004096	2139	2241	5735
MSTRG.30937.7	noncoding	-0.0004096	6156	6552	9844
MSTRG.30937.8	noncoding	-0.008192	2115	2235	2392
MSTRG.30937.9	noncoding	-0.167936	3294	3354	3509
MSTRG.30937.10	noncoding	-0.167936	2667	2715	4147
MSTRG.30939.1	noncoding	-0.0274432	42	210	1046
MSTRG.30943.1	coding	0.222	27	1248	1402
MSTRG.30922.1	noncoding	-0.1232896	345	984	1139
MSTRG.30922.2	noncoding	-0.1253376	87	750	1088
MSTRG.30922.3	coding	0.31	0	1458	1758
MSTRG.30945.1	noncoding	-0.024576	1161	1296	1790
MSTRG.30948.11	coding	0.093	165	2472	2726
MSTRG.30948.8	coding	0.087	150	4842	5095
MSTRG.30948.18	coding	0.087	1056	5751	6004
MSTRG.30948.12	coding	0.079	243	6372	6626
MSTRG.30948.13	coding	0.084	0	6531	6786
MSTRG.30948.14	coding	0.081	0	6489	6744
MSTRG.30948.15	coding	0.107	0	3258	7654
MSTRG.30948.16	coding	0.077	243	6198	6452
MSTRG.30948.9	coding	0.079	234	6462	6715
MSTRG.30948.7	coding	0.082	66	6666	6921
MSTRG.30948.10	coding	0.076	399	6717	6971
MSTRG.30948.17	coding	0.081	144	6438	6693
MSTRG.30948.6	coding	0.085	144	6555	6810
MSTRG.30948.19	coding	0.002	1308	1602	2228
MSTRG.30949.1	noncoding	-0.159744	1026	1101	2839
MSTRG.30950.2	coding	0.134	1410	5376	5594
MSTRG.30951.1	noncoding	-0.0548864	0	3	246
MSTRG.30952.1	noncoding	-0.0331776	69	93	274
MSTRG.30950.5	coding	0.241	1485	3258	3476
MSTRG.30950.7	coding	0.274	1944	3363	3583
MSTRG.30929.3	coding	0.145	510	2538	3562
MSTRG.30961.3	coding	0.105	495	1224	2863
MSTRG.30961.4	coding	0.105	318	1047	2687
MSTRG.30965.1	coding	0.125	429	1980	3216
MSTRG.30965.2	coding	0.134	0	1413	3797
MSTRG.30965.3	coding	0.038	1647	2298	3530
MSTRG.30965.4	coding	0.058	1641	2361	3554
MSTRG.30965.6	coding	0.057	3174	3897	5089
MSTRG.30965.5	coding	0.372	0	441	5218
MSTRG.30966.1	noncoding	-0.0372736	534	558	731
MSTRG.30965.8	coding	0.372	0	441	1634
MSTRG.30965.9	noncoding	-0.0385024	2028	2148	4724
MSTRG.30965.10	noncoding	-0.11264	0	534	3680
MSTRG.30967.1	noncoding	-0.0643072	54	768	923
MSTRG.30967.3	noncoding	-0.0397312	900	1089	1327

MSTRG.30971.1	noncoding	-0.004096	186	450	607
MSTRG.30971.2	noncoding	-0.0073728	249	462	617
MSTRG.30968.1	noncoding	-0.004096	24	162	372
MSTRG.31004.1	coding	0.11	366	1011	1549
MSTRG.31004.2	coding	0.11	150	795	1365
MSTRG.31004.4	coding	0.11	150	795	1366
MSTRG.31004.5	coding	0.11	480	1125	1535
MSTRG.31006.1	noncoding	-0.0589824	1581	1611	2004
MSTRG.31007.1	noncoding	-0.0315392	2802	2991	3510
MSTRG.31011.1	noncoding	-0.0212992	468	606	1066
MSTRG.31011.2	noncoding	-0.0212992	468	606	915
MSTRG.31011.3	noncoding	-0.0212992	468	606	1065
MSTRG.31011.4	noncoding	-0.0212992	216	354	749
MSTRG.31011.5	noncoding	-0.0212992	216	354	982
MSTRG.31015.3	coding	0.405	16257	20547	20943
MSTRG.31015.4	coding	0.402	30021	34311	34467
MSTRG.31015.5	coding	0.403	7248	11529	11683
MSTRG.31015.6	coding	0.402	26142	30426	30581
MSTRG.31021.2	coding	0.115	0	1224	1918
MSTRG.31019.1	noncoding	-0.1544192	0	81	368
MSTRG.31027.1	noncoding	-0.0065536	135	423	2000
MSTRG.31029.2	coding	0.124	312	966	2100
MSTRG.31030.1	coding	0.096	0	342	3704
MSTRG.31035.1	coding	0.209	357	993	1623
MSTRG.31035.4	coding	0.284	0	282	937
MSTRG.31035.3	coding	0.284	0	282	1134
MSTRG.31035.5	coding	0.155	0	708	1338
MSTRG.31035.6	coding	0.226	435	915	1545
MSTRG.31035.7	coding	0.184	33	534	1165
MSTRG.31035.8	coding	0.196	165	711	1341
MSTRG.31031.1	noncoding	-0.02048	135	381	999
MSTRG.31036.2	coding	0.145	0	807	4852
MSTRG.31043.1	coding	0.114	0	813	1705
MSTRG.31044.1	noncoding	-0.04096	57	144	970
MSTRG.31045.13	coding	0.001	1305	1449	3164
MSTRG.31045.4	coding	0.076	6243	6807	9373
MSTRG.31045.7	coding	0.076	5661	6225	8792
MSTRG.31045.11	coding	0.076	5838	6402	8969
MSTRG.31045.15	coding	0.076	5925	6489	9055
MSTRG.31045.16	noncoding	-0.0937984	0	225	1549
MSTRG.31045.8	coding	0.076	5796	6360	8927
MSTRG.31045.14	noncoding	-0.0028672	294	441	1266
MSTRG.31045.12	coding	0.076	5547	6111	8679
MSTRG.31045.9	coding	0.076	5592	6156	8724
MSTRG.31045.10	coding	0.007	303	450	1276
MSTRG.31045.6	coding	0.076	5652	6216	8782
MSTRG.31046.1	noncoding	-0.06144	684	711	1535
MSTRG.31047.1	noncoding	-0.036864	1206	1314	5227
MSTRG.31051.1	noncoding	-0.057344	1044	1083	4830
MSTRG.31052.1	noncoding	-0.036864	4059	4125	8530
MSTRG.31053.5	coding	0.064	111	3762	3917
MSTRG.31058.1	coding	0.219	291	1248	4935
MSTRG.31071.1	noncoding	-0.0249856	417	525	940
MSTRG.31071.2	noncoding	-0.008192	276	432	792

MSTRG.31083.2	coding	0.207	1206	2865	3206
MSTRG.31105.3	coding	0.357	141	1806	4788
MSTRG.31105.4	coding	0.353	0	2283	5264
MSTRG.31111.2	coding	0.119	0	576	1002
MSTRG.31112.1	coding	0.144	0	828	1747
MSTRG.31116.2	coding	0.262	0	711	1800
MSTRG.31116.3	coding	0.227	486	4575	6591
MSTRG.31116.4	coding	0.231	4641	7002	11669
MSTRG.31116.5	coding	0.21	0	4785	5377
MSTRG.31117.1	noncoding	-0.036864	207	267	549
MSTRG.31117.2	noncoding	-0.126976	66	150	650
MSTRG.31116.6	coding	0.172	0	2529	2734
MSTRG.31119.2	coding	0.169	375	1431	2696
MSTRG.31120.8	coding	0.405	0	2703	2993
MSTRG.31121.1	noncoding	-0.0253952	510	759	914
MSTRG.31122.4	noncoding	-0.1089536	0	402	9044
MSTRG.31122.3	coding	0.36	0	2160	6752
MSTRG.31114.4	coding	0.29	201	1269	4849
MSTRG.31125.1	coding	0.183	0	294	3141
MSTRG.31125.2	noncoding	-0.0282624	1956	2073	2500
MSTRG.31128.3	coding	0.058	1269	1467	4121
MSTRG.31132.3	noncoding	-0.0442368	0	129	2298
MSTRG.31134.3	coding	0.119	0	771	1031
MSTRG.31134.4	noncoding	-0.057344	87	111	460
MSTRG.31134.5	coding	0.136	0	477	756
MSTRG.31134.6	noncoding	-0.012288	15	204	2108
MSTRG.31133.1	noncoding	-0.053248	201	288	549
MSTRG.31142.2	coding	0.294	0	699	2762
MSTRG.31143.2	coding	0.357	1146	2778	2942
MSTRG.31143.3	coding	0.319	1482	2769	2932
MSTRG.31146.1	coding	0.494	0	6000	7746
MSTRG.31146.2	noncoding	-0.0872448	0	684	1665
MSTRG.31146.4	noncoding	-0.0806912	0	774	1217
MSTRG.31146.3	noncoding	-0.0806912	0	681	1123
MSTRG.31146.5	noncoding	-0.0626688	63	1044	1197
MSTRG.31148.2	coding	0.335	318	6357	8408
MSTRG.31149.2	noncoding	-0.0262144	276	435	3886
MSTRG.31144.1	coding	0.335	0	1464	3336
MSTRG.31144.2	coding	0.351	0	1122	3129
MSTRG.31144.5	noncoding	-0.0024576	1596	1731	2388
MSTRG.31144.6	noncoding	-0.0147456	57	216	1170
MSTRG.31145.2	coding	0.343	0	1764	2478
MSTRG.31157.1	noncoding	-0.131072	15	93	551
MSTRG.31157.2	noncoding	-0.131072	0	69	511
MSTRG.31158.2	coding	0.134	621	1152	1365
MSTRG.31158.3	coding	0.13	381	1206	1417
MSTRG.31158.4	coding	0.134	498	1029	1241
MSTRG.31159.1	noncoding	-0.032768	555	651	2010
MSTRG.31160.2	coding	0.148	0	1224	1791
MSTRG.31160.3	coding	0.17	1737	2475	3042
MSTRG.31160.4	coding	0.176	210	963	1532
MSTRG.31161.1	noncoding	-0.0180224	0	204	809
MSTRG.31165.1	coding	0.389	0	264	2229
MSTRG.31165.3	coding	0.414	0	219	1617

MSTRG.31155.1	noncoding	-0.0090112	39	141	694
MSTRG.31155.2	noncoding	-0.0090112	30	132	463
MSTRG.31156.1	coding	0.202	783	1962	3312
MSTRG.31167.1	coding	0.455	222	789	3582
MSTRG.31167.2	coding	0.477	222	723	967
MSTRG.31169.5	coding	0.255	69	2130	3576
MSTRG.31169.6	coding	0.27	117	2067	3511
MSTRG.31169.7	coding	0.255	276	2343	11861
MSTRG.31171.1	coding	0.364	0	1848	3298
MSTRG.31172.3	coding	0.239	141	1950	2174
MSTRG.31175.2	coding	0.245	63	2967	7781
MSTRG.31175.3	coding	0.281	96	1671	4682
MSTRG.31174.1	coding	0.051	0	273	706
MSTRG.31187.3	coding	0.113	774	2403	3103
MSTRG.31188.4	coding	0.114	0	4779	5217
MSTRG.31189.2	coding	0.35	732	1452	2708
MSTRG.31189.3	coding	0.327	300	1794	3021
MSTRG.31189.4	coding	0.362	102	1248	2474
MSTRG.31193.1	noncoding	-0.0004096	189	306	551
MSTRG.31194.1	noncoding	-0.0315392	4671	4818	8619
MSTRG.31196.2	noncoding	-0.0004096	1824	1929	4254
MSTRG.31196.1	noncoding	-0.0004096	615	654	4525
MSTRG.31197.1	noncoding	-0.004096	1449	1596	2253
MSTRG.31201.1	noncoding	-0.12288	609	684	1046
MSTRG.31202.1	noncoding	-0.0073728	885	1125	1281
MSTRG.31205.1	noncoding	-0.004096	141	168	1415
MSTRG.31210.1	noncoding	-0.028672	636	744	1699
MSTRG.31211.1	coding	0.267	180	1776	6287
MSTRG.31215.1	coding	0.244	0	1668	5328
MSTRG.31215.2	coding	0.219	0	1932	5119
MSTRG.31218.2	coding	0.382	0	522	1156
MSTRG.31219.2	noncoding	-0.0544768	0	870	1201
MSTRG.31222.3	noncoding	-0.1437696	0	291	1270
MSTRG.31223.1	noncoding	-0.0200704	2343	2778	3164
MSTRG.31231.2	noncoding	-0.0393216	3015	3276	3843
MSTRG.31231.3	coding	0.24	174	447	655
MSTRG.31231.4	coding	0.277	252	615	823
MSTRG.31231.5	noncoding	-0.0823296	0	231	384
MSTRG.31231.6	noncoding	-0.077824	0	231	5954
MSTRG.31233.1	noncoding	-0.0512	5319	5853	8154
MSTRG.31235.1	noncoding	-0.0663552	3564	3867	4167
MSTRG.31236.1	noncoding	-0.0073728	8490	8625	9182
MSTRG.31237.1	noncoding	-0.0360448	24384	24723	35985
MSTRG.31238.1	noncoding	-0.0544768	1305	1539	9300
MSTRG.31231.7	noncoding	-0.03072	6018	7677	8261
MSTRG.31239.1	noncoding	-0.0299008	16026	16197	17044
MSTRG.31240.1	noncoding	-0.0413696	9600	10077	12417
MSTRG.31241.1	coding	0.131	459	825	2050
MSTRG.31242.1	noncoding	-0.0495616	1056	1734	5405
MSTRG.31240.2	noncoding	-0.0413696	6186	6663	9035
MSTRG.31244.1	noncoding	-0.0638976	5694	6330	7092
MSTRG.31244.2	noncoding	-0.0638976	2748	3384	4217
MSTRG.31245.2	noncoding	-0.0176128	1287	1455	1608
MSTRG.31230.1	noncoding	-0.0692224	0	57	212

MSTRG.31246.1	noncoding	-0.0270336	195	348	503
MSTRG.31246.2	noncoding	-0.0319488	219	447	657
MSTRG.31247.2	noncoding	-0.0872448	6	333	744
MSTRG.31251.1	coding	0.291	639	1716	4542
MSTRG.31251.3	coding	0.123	144	579	1007
MSTRG.31251.2	coding	0.229	144	1038	1353
MSTRG.31251.4	coding	0.296	0	1044	4079
MSTRG.31251.5	coding	0.108	0	315	657
MSTRG.31271.1	coding	0.163	138	354	1105
MSTRG.31271.2	noncoding	-0.02048	105	177	1808
MSTRG.31263.1	noncoding	-0.0290816	72	276	1641
MSTRG.31273.1	noncoding	-0.004096	3963	4074	4249
MSTRG.31278.3	coding	0.225	285	687	1376
MSTRG.31278.4	coding	0.222	186	603	1326
MSTRG.31278.5	coding	0.115	204	1125	4193
MSTRG.31279.1	noncoding	-0.147456	1947	1974	2147
MSTRG.31286.4	noncoding	-0.1130496	0	63	475
MSTRG.31287.3	coding	0.274	303	4701	13717
MSTRG.31285.1	noncoding	-0.057344	105	429	2974
MSTRG.31289.1	noncoding	-0.0479232	6183	6387	12891
MSTRG.31291.1	noncoding	-0.0544768	1272	1548	4125
MSTRG.31301.1	noncoding	-0.0528384	2376	2697	5283
MSTRG.31301.2	noncoding	-0.135168	0	147	807
MSTRG.31301.3	noncoding	-0.0528384	5313	5634	7351
MSTRG.31301.4	noncoding	-0.0528384	3171	3492	5211
MSTRG.31301.5	noncoding	-0.0528384	3180	3501	5218
MSTRG.31301.6	noncoding	-0.0454656	2121	2337	3614
MSTRG.31301.7	noncoding	-0.0454656	1683	1899	2431
MSTRG.31307.1	noncoding	-0.004096	45	159	857
MSTRG.31316.2	coding	0.423	0	1644	2461
MSTRG.31317.1	noncoding	-0.032768	225	279	702
MSTRG.31320.1	noncoding	-0.032768	1935	2301	3865
MSTRG.31320.3	noncoding	-0.0237568	1368	2106	3977
MSTRG.31320.2	noncoding	-0.0237568	2502	3240	5039
MSTRG.31320.7	noncoding	-0.0249856	1851	2196	2950
MSTRG.31320.4	noncoding	-0.0237568	1365	2103	4589
MSTRG.31320.5	noncoding	-0.0237568	1365	2103	4202
MSTRG.31320.6	noncoding	-0.0237568	1365	2103	4349
MSTRG.31320.8	noncoding	-0.0237568	594	1332	2928
MSTRG.31320.9	noncoding	-0.0237568	1368	2106	3683
MSTRG.31320.10	noncoding	-0.0237568	2502	3240	6217
MSTRG.31320.11	noncoding	-0.0339968	1974	2952	4238
MSTRG.31320.12	noncoding	-0.0339968	5682	6660	7971
MSTRG.31304.1	noncoding	-0.0004096	0	0	205
MSTRG.31324.2	coding	0.126	0	756	1348
MSTRG.31333.2	coding	0.132	165	1587	2341
MSTRG.31336.3	coding	0.483	0	1218	2751
MSTRG.31337.1	noncoding	-0.0045056	2457	2622	3423
MSTRG.31341.1	noncoding	-0.0077824	624	690	844
MSTRG.31347.1	coding	0.165	117	3261	3735
MSTRG.31348.1	noncoding	-0.0139264	330	414	766
MSTRG.31350.2	coding	0.347	6819	7392	16916
MSTRG.31351.3	coding	0.534	0	1293	2866
MSTRG.31363.1	noncoding	-0.0004096	153	156	386

MSTRG.31364.5	noncoding	-0.016384	3159	3291	10781
MSTRG.31364.6	noncoding	-0.008192	138	249	4661
MSTRG.31365.1	coding	0.076	282	639	1464
MSTRG.31367.2	coding	0.163	0	1668	2250
MSTRG.31371.1	noncoding	-0.0663552	186	231	1450
MSTRG.31372.2	coding	0.319	0	4194	5821
MSTRG.31374.1	noncoding	-0.028672	0	117	544
MSTRG.31375.1	noncoding	-0.053248	2763	2988	3496
MSTRG.31376.1	coding	0.155	3705	3972	4754
MSTRG.31378.4	coding	0.155	0	1320	1968
MSTRG.31379.1	noncoding	-0.0004096	0	0	1292
MSTRG.31380.2	coding	0.209	87	1014	6085
MSTRG.31380.5	coding	0.209	159	1086	6196
MSTRG.31381.1	coding	0.152	75	6471	7578
MSTRG.31383.1	noncoding	-0.053248	33	147	1177
MSTRG.31384.2	coding	0.287	2364	5253	10302
MSTRG.31385.1	coding	0.051	249	447	1172
MSTRG.31386.1	noncoding	-0.1323008	0	501	654
MSTRG.31390.1	noncoding	-0.0004096	0	0	363
MSTRG.31401.2	coding	0.306	492	2196	2869
MSTRG.31401.1	coding	0.306	735	2439	3116
MSTRG.31401.3	coding	0.306	591	2295	2901
MSTRG.31407.1	noncoding	-0.0987136	6006	6303	11417
MSTRG.31408.1	noncoding	-0.106496	1407	1458	2210
MSTRG.31407.2	noncoding	-0.0401408	246	444	1650
MSTRG.31419.1	noncoding	-0.0442368	7614	7857	25674
MSTRG.31421.1	noncoding	-0.0110592	3036	3240	4160
MSTRG.31426.1	coding	0.415	99	1407	3176
MSTRG.31426.2	coding	0.466	447	1587	3357
MSTRG.31431.1	noncoding	-0.022528	999	1155	1338
MSTRG.31435.6	coding	0.264	609	1761	2526
MSTRG.31435.10	coding	0.264	1719	2871	3634
MSTRG.31435.2	coding	0.264	549	1701	2466
MSTRG.31435.8	coding	0.264	345	1497	2261
MSTRG.31436.1	noncoding	-0.1081344	1191	1209	1726
MSTRG.31440.1	noncoding	-0.1306624	0	42	235
MSTRG.31459.1	coding	0.186	0	1074	1307
MSTRG.31474.1	coding	0.166	36	5475	7080
MSTRG.31474.2	coding	0.215	8862	9717	11042
MSTRG.31476.1	noncoding	-0.0831488	0	3	401
MSTRG.31477.1	noncoding	-0.012288	25653	25791	37340
MSTRG.31474.3	noncoding	-0.0241664	1203	1443	2725
MSTRG.31478.1	noncoding	-0.0483328	1734	2121	6027
MSTRG.31477.2	noncoding	-0.0004096	4701	4788	5456
MSTRG.31479.1	noncoding	-0.024576	3939	4209	6711
MSTRG.31474.4	noncoding	-0.06144	3774	3870	5362
MSTRG.31474.6	noncoding	-0.0098304	60	81	688
MSTRG.31474.7	noncoding	-0.008192	3255	3348	9005
MSTRG.31480.1	noncoding	-0.02048	162	363	799
MSTRG.31468.2	coding	0.251	243	2235	3828
MSTRG.31468.4	coding	0.191	117	369	2331
MSTRG.31468.5	coding	0.191	171	423	1398
MSTRG.31484.1	coding	0.26	225	3402	7529
MSTRG.31485.1	noncoding	-0.0565248	7956	8124	12014

MSTRG.31485.2	noncoding	-0.0565248	7944	8112	10950
MSTRG.31493.1	coding	0.09	0	840	5450
MSTRG.31493.2	coding	0.09	0	840	4665
MSTRG.31505.1	noncoding	-0.02048	5097	5190	5498
MSTRG.31506.1	coding	0.022	7773	8352	21630
MSTRG.31505.2	noncoding	-0.0004096	14541	14685	28971
MSTRG.31505.4	noncoding	-0.0016384	6570	6702	12678
MSTRG.31505.3	noncoding	-0.131072	7962	8040	11352
MSTRG.31507.1	coding	0.074	855	1410	17334
MSTRG.31505.5	noncoding	-0.0012288	2091	2223	7750
MSTRG.31495.1	noncoding	-0.0004096	1284	1368	2104
MSTRG.31511.1	noncoding	-0.032768	3783	3882	5144
MSTRG.31511.2	noncoding	-0.032768	2508	2607	3877
MSTRG.31512.1	coding	0.141	12594	12933	20274
MSTRG.31510.4	coding	0.065	1707	2109	2624
MSTRG.31510.5	coding	0.115	4755	4953	8312
MSTRG.31510.7	coding	0.115	4530	4728	6805
MSTRG.31510.8	noncoding	-0.0004096	2034	2247	12477
MSTRG.31510.9	noncoding	-0.0876544	0	186	1432
MSTRG.31513.1	noncoding	-0.002048	1557	1785	8926
MSTRG.31515.1	noncoding	-0.04096	1185	1272	4175
MSTRG.31517.1	noncoding	-0.0004096	0	0	302
MSTRG.31522.1	noncoding	-0.0004096	6	129	299
MSTRG.31525.1	coding	0.208	87	687	1192
MSTRG.31525.2	coding	0.223	243	1734	2238
MSTRG.31526.1	noncoding	-0.016384	75	78	232
MSTRG.31527.1	noncoding	-0.04096	243	345	695
MSTRG.31531.1	coding	0.037	2064	2199	2577
MSTRG.31531.2	coding	0.037	1902	2037	3701
MSTRG.31532.2	coding	0.481	0	423	5068
MSTRG.31531.4	coding	0.04	1887	3249	4124
MSTRG.31533.1	noncoding	-0.0004096	0	0	2073
MSTRG.31531.8	noncoding	-0.028672	3081	3243	3433
MSTRG.31536.1	noncoding	-0.034816	21	432	1075
MSTRG.31537.13	coding	0.106	699	1014	1396
MSTRG.31543.1	coding	0.128	3285	4839	10308
MSTRG.31544.3	noncoding	-0.0659456	582	945	1102
MSTRG.31546.5	coding	0.148	1677	3168	3606
MSTRG.31546.7	coding	0.16	1506	3636	4047
MSTRG.31548.1	coding	0.061	36216	37248	53509
MSTRG.31548.2	coding	0.061	36270	37302	53475
MSTRG.31548.3	coding	0.061	36087	37119	53284
MSTRG.31548.4	coding	0.069	36165	37239	44015
MSTRG.31548.5	coding	0.061	36084	37116	50303
MSTRG.31549.3	noncoding	-0.012288	12	126	866
MSTRG.31551.3	coding	0.313	0	768	3490
MSTRG.31551.2	coding	0.308	0	774	2315
MSTRG.31552.1	noncoding	-0.0065536	474	621	1663
MSTRG.31551.4	coding	0.18	4086	4932	5609
MSTRG.31559.1	coding	0.096	27	903	3015
MSTRG.31559.3	coding	0.095	75	669	2781
MSTRG.31559.4	coding	0.083	378	873	2983
MSTRG.31559.5	coding	0.064	0	825	2937
MSTRG.31559.6	coding	0.07	63	591	2703

MSTRG.31563.1	coding	0.429	111	1770	2568
MSTRG.31564.2	coding	0.435	744	3420	4298
MSTRG.31565.1	noncoding	-0.0004096	0	0	256
MSTRG.31566.2	coding	0.313	414	924	1911
MSTRG.31570.4	coding	0.501	0	1143	1521
MSTRG.31570.5	coding	0.358	0	3420	3798
MSTRG.31570.3	coding	0.413	519	2268	5368
MSTRG.31570.6	coding	0.355	0	3444	3796
MSTRG.31570.9	coding	0.439	4533	5646	5883
MSTRG.31570.8	coding	0.438	708	1542	1779
MSTRG.31570.7	coding	0.413	0	1749	5106
MSTRG.31571.2	noncoding	-0.0741376	1434	1902	2057
MSTRG.31571.1	noncoding	-0.0741376	7125	7593	7747
MSTRG.31571.3	noncoding	-0.0761856	7677	8103	8259
MSTRG.31571.4	noncoding	-0.077824	6018	6429	6586
MSTRG.31567.2	coding	0.517	57	648	2170
MSTRG.31576.1	noncoding	-0.004096	48	60	215
MSTRG.31581.4	coding	0.297	954	1869	4247
MSTRG.31586.5	noncoding	-0.0602112	42	225	520
MSTRG.31584.2	coding	0.357	690	1464	4338
MSTRG.31591.1	noncoding	-0.02048	582	666	840
MSTRG.31598.1	coding	0.465	669	2139	3943
MSTRG.31598.2	coding	0.465	51	1521	3183
MSTRG.31598.3	coding	0.465	51	1521	3181
MSTRG.31598.5	coding	0.465	300	1770	3437
MSTRG.31598.4	coding	0.465	459	1929	3595
MSTRG.31599.3	coding	0.204	117	1110	1951
MSTRG.31599.4	coding	0.204	117	1110	2342
MSTRG.31597.1	coding	0.401	0	786	1645
MSTRG.31597.2	coding	0.444	0	738	1635
MSTRG.31603.2	coding	0.444	0	1044	3012
MSTRG.31603.3	coding	0.45	0	1059	2522
MSTRG.31603.4	coding	0.451	0	1062	1391
MSTRG.31603.8	coding	0.44	0	1107	1615
MSTRG.31603.11	coding	0.357	138	1215	2042
MSTRG.31602.2	coding	0.065	48	675	4568
MSTRG.31606.1	coding	0.136	894	1644	3663
MSTRG.31606.2	coding	0.136	5169	5919	7939
MSTRG.31606.3	noncoding	-0.1024	1314	1386	1836
MSTRG.31606.4	noncoding	-0.086016	1545	1596	3722
MSTRG.31607.1	noncoding	-0.049152	618	681	2774
MSTRG.31606.6	noncoding	-0.0217088	17979	18192	26971
MSTRG.31606.7	coding	0.128	5088	6165	7016
MSTRG.31606.8	noncoding	-0.0622592	14223	14589	14742
MSTRG.31608.1	noncoding	-0.147456	2370	2433	6100
MSTRG.31606.10	coding	0.136	2208	2958	4978
MSTRG.31606.11	coding	0.136	2697	3447	5466
MSTRG.31617.1	coding	0.133	222	2133	2302
MSTRG.31618.1	coding	0.075	42	1494	2249
MSTRG.31618.4	noncoding	-0.0577536	1200	1533	2021
MSTRG.31618.5	noncoding	-0.0425984	1233	1758	2246
MSTRG.31619.1	coding	0.294	1116	1782	7157
MSTRG.31620.1	noncoding	-0.0344064	804	1215	1369
MSTRG.31622.2	coding	0.174	0	2376	3569

MSTRG.31623.4	coding	0.114	426	1692	5716
MSTRG.31625.1	noncoding	-0.077824	198	219	381
MSTRG.31626.3	coding	0.129	1245	5709	6867
MSTRG.31626.4	coding	0.129	1713	6177	7283
MSTRG.31626.5	coding	0.129	1323	5787	6892
MSTRG.31627.1	noncoding	-0.0217088	261	351	505
MSTRG.31613.2	coding	0.146	87	6012	6503
MSTRG.31613.3	coding	0.265	753	3978	5153
MSTRG.31614.1	noncoding	-0.1077248	369	1260	9850
MSTRG.31630.5	coding	0.323	0	5208	5363
MSTRG.31630.6	coding	0.321	0	4164	4332
MSTRG.31633.1	noncoding	-0.008192	3	99	1278
MSTRG.31633.2	noncoding	-0.008192	3	99	1265
MSTRG.31629.1	coding	0.185	42	780	1773
MSTRG.31636.5	coding	0.119	6	1140	2920
MSTRG.31637.2	coding	0.175	0	237	1421
MSTRG.31637.3	coding	0.181	0	219	1583
MSTRG.31638.1	noncoding	-0.02048	804	927	2185
MSTRG.31640.1	noncoding	-0.049152	2367	2445	3762
MSTRG.31641.1	noncoding	-0.180224	2334	2415	3551
MSTRG.31642.1	coding	0.048	2529	2724	3287
MSTRG.31643.1	noncoding	-0.0176128	153	315	4945
MSTRG.31637.6	coding	0.106	423	774	1595
MSTRG.31644.2	coding	0.156	609	1074	2448
MSTRG.31644.5	coding	0.188	495	6006	7352
MSTRG.31648.1	noncoding	-0.0114688	1620	1758	2052
MSTRG.31648.2	noncoding	-0.0704512	855	1059	2226
MSTRG.31648.3	coding	0.076	429	1092	4818
MSTRG.31648.5	coding	0.049	0	1014	5945
MSTRG.31650.1	noncoding	-0.0892928	57	63	263
MSTRG.31651.2	coding	0.168	0	996	1616
MSTRG.31661.1	noncoding	-0.008192	1983	2181	6610
MSTRG.31662.1	noncoding	-0.049152	1053	1158	1653
MSTRG.31672.2	coding	0.309	0	555	716
MSTRG.31672.3	coding	0.356	549	2028	2188
MSTRG.31673.1	noncoding	-0.0086016	750	870	1277
MSTRG.31678.2	noncoding	-0.0413696	3531	4146	29448
MSTRG.31678.1	noncoding	-0.0311296	4323	5247	41745
MSTRG.31679.2	coding	0.515	0	5466	7207
MSTRG.31682.1	noncoding	-0.008192	54	204	1206
MSTRG.31690.1	noncoding	-0.0192512	999	1149	1591
MSTRG.31691.1	coding	0.345	804	4308	7759
MSTRG.31691.2	coding	0.347	558	4044	7433
MSTRG.31692.1	noncoding	-0.0270336	63	219	654
MSTRG.31691.3	coding	0.327	81	3255	6788
MSTRG.31700.1	coding	0.137	1563	1878	2644
MSTRG.31703.2	noncoding	-0.0495616	1869	2301	2538
MSTRG.31694.1	coding	0.178	0	363	536
MSTRG.31721.4	coding	0.163	1983	2358	2651
MSTRG.31722.2	coding	0.088	120	450	864
MSTRG.31723.5	coding	0.288	180	681	1329
MSTRG.31723.6	coding	0.3	0	648	1210
MSTRG.31725.2	coding	0.384	87	882	2501
MSTRG.31725.3	coding	0.422	321	1071	2662

MSTRG.31725.4	coding	0.275	192	585	2205
MSTRG.31729.1	noncoding	-0.196608	72	138	490
MSTRG.31742.2	coding	0.054	177	1074	2488
MSTRG.31740.1	coding	0.438	0	2151	3866
MSTRG.31743.1	coding	0.302	180	948	1566
MSTRG.31743.2	coding	0.326	0	831	1450
MSTRG.31743.3	coding	0.257	75	564	1179
MSTRG.31745.3	coding	0.082	1665	2973	3542
MSTRG.31745.6	coding	0.066	684	1878	2446
MSTRG.31745.7	coding	0.077	1326	1725	2294
MSTRG.31746.1	noncoding	-0.024576	1005	1101	3944
MSTRG.31747.1	noncoding	-0.0032768	201	345	958
MSTRG.31745.10	noncoding	-0.098304	570	636	3650
MSTRG.31748.1	noncoding	-0.0086016	96	255	671
MSTRG.31748.2	noncoding	-0.1024	15	36	242
MSTRG.31748.3	noncoding	-0.0086016	93	252	535
MSTRG.31749.1	noncoding	-0.03072	69	261	446
MSTRG.31750.1	noncoding	-0.0028672	0	63	231
MSTRG.31753.1	coding	0.391	33	597	1056
MSTRG.31757.4	coding	0.114	660	4065	4738
MSTRG.31757.5	coding	0.114	396	3819	4493
MSTRG.31758.1	coding	0.337	438	2532	5088
MSTRG.31766.1	noncoding	-0.0294912	7008	7176	9708
MSTRG.31777.1	noncoding	-0.0004096	0	0	320
MSTRG.31763.1	noncoding	-0.0069632	378	672	1063
MSTRG.31786.3	noncoding	-0.008192	1023	1161	1317
MSTRG.31786.2	coding	0.233	0	2862	3548
MSTRG.31787.1	noncoding	-0.196608	96	111	267
MSTRG.31786.4	noncoding	-0.3497984	1197	1968	2542
MSTRG.31786.7	noncoding	-0.3497984	1173	1944	2516
MSTRG.31786.10	noncoding	-0.0516096	549	873	1026
MSTRG.31786.11	noncoding	-0.3837952	1146	1935	2458
MSTRG.31786.12	noncoding	-0.3743744	1191	1881	2405
MSTRG.31796.5	coding	0.07	507	1290	1592
MSTRG.31796.4	coding	0.078	507	1419	5954
MSTRG.31796.6	coding	0.083	0	1146	5680
MSTRG.31797.1	noncoding	-0.049152	1365	1473	7478
MSTRG.31799.1	coding	0.318	402	2745	3203
MSTRG.31801.1	noncoding	-0.0565248	1149	1833	2323
MSTRG.31801.2	noncoding	-0.083968	540	759	1477
MSTRG.31801.3	noncoding	-0.0999424	417	681	1230
MSTRG.31801.4	noncoding	-0.0622592	579	849	1333
MSTRG.31801.5	noncoding	-0.0729088	462	918	1404
MSTRG.31801.6	noncoding	-0.0643072	243	570	1059
MSTRG.31801.7	noncoding	-0.032768	207	348	832
MSTRG.31801.8	noncoding	-0.0372736	1284	1872	2362
MSTRG.31789.1	coding	0.359	444	1056	1212
MSTRG.31789.2	coding	0.334	450	1155	1549
MSTRG.31790.1	coding	0.31	138	1278	1772
MSTRG.31792.2	coding	0.26	0	1056	2397
MSTRG.31792.3	coding	0.241	21	879	1034
MSTRG.31793.1	noncoding	-0.0004096	0	0	569
MSTRG.31793.2	noncoding	-0.0004096	0	0	415
MSTRG.31792.4	coding	0.343	0	621	1539

MSTRG.31803.1	coding	0.237	0	4353	6831
MSTRG.31803.3	coding	0.144	117	1107	3313
MSTRG.31804.1	noncoding	-0.0319488	498	654	1186
MSTRG.31805.5	noncoding	-0.2781184	0	21	471
MSTRG.31805.3	coding	0.077	372	522	902
MSTRG.31805.1	coding	0.077	192	342	1557
MSTRG.31805.4	coding	0.077	192	342	1019
MSTRG.31806.1	coding	0.409	18	462	1182
MSTRG.31810.1	noncoding	-0.458752	606	618	953
MSTRG.31812.5	coding	0.157	708	2451	4139
MSTRG.31812.9	coding	0.157	609	2352	2619
MSTRG.31812.8	coding	0.157	561	2304	2571
MSTRG.31813.1	noncoding	-0.114688	27	36	702
MSTRG.31812.12	coding	0.111	264	618	803
MSTRG.31812.10	coding	0.107	297	600	785
MSTRG.31815.4	coding	0.234	615	3891	4227
MSTRG.31815.2	coding	0.235	615	3894	5231
MSTRG.31815.3	coding	0.235	615	3891	5228
MSTRG.31815.5	coding	0.187	1839	3198	3516
MSTRG.31818.2	coding	0.107	147	1311	2212
MSTRG.31818.3	coding	0.075	147	825	2637
MSTRG.31818.7	coding	0.1	363	894	3243
MSTRG.31820.2	coding	0.324	630	1221	4880
MSTRG.31820.3	coding	0.302	192	744	4176
MSTRG.31820.4	coding	0.324	192	783	4443
MSTRG.31820.5	coding	0.302	438	990	4422
MSTRG.31820.6	coding	0.324	216	807	4466
MSTRG.31820.7	coding	0.302	216	768	4199
MSTRG.31820.8	coding	0.324	228	819	4478
MSTRG.31820.9	coding	0.33	2130	2478	5911
MSTRG.31822.2	coding	0.534	1020	2127	2709
MSTRG.31824.1	noncoding	-0.0512	591	849	5058
MSTRG.31826.1	noncoding	-0.1191936	0	102	2974
MSTRG.31829.1	noncoding	-0.0086016	192	345	545
MSTRG.31833.4	coding	0.03	150	567	1145
MSTRG.31838.1	noncoding	-0.0475136	624	897	1435
MSTRG.31839.2	coding	0.388	249	1692	2222
MSTRG.31865.1	noncoding	-0.0167936	774	927	1259
MSTRG.31867.3	coding	0.144	285	1236	7656
MSTRG.31867.5	coding	0.115	99	969	7142
MSTRG.31867.7	coding	0.114	99	876	7049
MSTRG.31867.6	coding	0.113	303	1278	7450
MSTRG.31868.1	noncoding	-0.014336	1368	1437	2718
MSTRG.31867.8	noncoding	-0.008192	309	408	1486
MSTRG.31859.4	coding	0.051	30	1653	1903
MSTRG.31859.3	coding	0.058	42	1677	1929
MSTRG.31861.2	coding	0.154	1008	1230	4529
MSTRG.31875.1	noncoding	-0.0352256	0	201	1111
MSTRG.31883.1	noncoding	-0.028672	0	30	334
MSTRG.31885.3	coding	0.142	0	450	6688
MSTRG.31885.4	coding	0.01	2118	2232	4256
MSTRG.31886.1	noncoding	-0.0077824	768	915	2839
MSTRG.31886.2	noncoding	-0.0077824	2397	2544	4458
MSTRG.31897.1	coding	0.101	261	669	4101

MSTRG.31898.1	coding	0.083	165	372	3949
MSTRG.31897.2	noncoding	-0.090112	1470	1533	2914
MSTRG.31904.1	noncoding	-0.02048	573	651	857
MSTRG.31907.1	noncoding	-0.0028672	408	522	1504
MSTRG.31905.1	noncoding	-0.077824	105	147	592
MSTRG.31909.1	noncoding	-0.0401408	0	261	509
MSTRG.31910.1	coding	0.456	0	1071	1537
MSTRG.31911.2	coding	0.429	0	345	1110
MSTRG.31914.2	noncoding	-0.0319488	141	306	2075
MSTRG.31914.3	noncoding	-0.0380928	3693	3858	4257
MSTRG.31917.7	coding	0.306	0	261	414
MSTRG.31919.1	coding	0.092	0	555	4578
MSTRG.31920.6	coding	0.17	237	1347	1630
MSTRG.31920.8	coding	0.183	327	1263	1524
MSTRG.31920.9	coding	0.183	330	1266	1526
MSTRG.31923.1	noncoding	-0.0827392	306	540	1306
MSTRG.31923.2	noncoding	-0.0712704	213	432	980
MSTRG.31994.3	noncoding	-0.034816	3	273	850
MSTRG.31998.2	noncoding	-0.14336	783	843	997
MSTRG.31999.1	coding	0.059	1557	1986	2383
MSTRG.32003.1	noncoding	-0.0057344	3294	3534	5934
MSTRG.32003.2	noncoding	-0.0057344	3234	3474	5318
MSTRG.32016.1	noncoding	-0.028672	426	441	1108
MSTRG.32029.1	noncoding	-0.079872	717	762	2315
MSTRG.32030.1	noncoding	-0.0708608	1575	1635	1866
MSTRG.32051.1	noncoding	-0.090112	327	354	980
MSTRG.32052.1	coding	0.253	51	1110	5840
MSTRG.32052.2	coding	0.25	51	1152	5892
MSTRG.31935.1	coding	0.171	0	2988	11278
MSTRG.31935.2	coding	0.208	0	2403	3125
MSTRG.31935.6	coding	0.208	0	2403	5716
MSTRG.31935.8	noncoding	-0.1552384	0	924	2962
MSTRG.31935.9	noncoding	-0.1552384	0	924	5204
MSTRG.31935.10	noncoding	-0.155648	0	954	1732
MSTRG.31936.3	noncoding	-0.077824	102	183	1709
MSTRG.31939.1	noncoding	-0.0966656	0	561	1143
MSTRG.31939.2	noncoding	-0.1056768	0	474	1056
MSTRG.31939.3	noncoding	-0.090112	54	468	805
MSTRG.31939.4	noncoding	-0.0782336	252	600	756
MSTRG.31940.1	noncoding	-0.0192512	831	951	4532
MSTRG.31941.1	noncoding	-0.004096	12	153	1852
MSTRG.31941.2	noncoding	-0.0016384	2598	2742	9404
MSTRG.31941.3	noncoding	-0.0016384	5910	6054	13539
MSTRG.31941.4	noncoding	-0.0016384	5577	5721	12765
MSTRG.31942.4	noncoding	-0.0495616	2568	2691	5086
MSTRG.31928.2	coding	0.02	660	1572	3573
MSTRG.31930.1	coding	0.284	0	84	286
MSTRG.31930.2	coding	0.108	2256	3273	4716
MSTRG.31948.1	noncoding	-0.0004096	1767	1848	2219
MSTRG.31963.1	noncoding	-0.057344	9	102	341
MSTRG.31968.1	noncoding	-0.0028672	834	909	3981
MSTRG.31981.1	coding	0.094	2640	3084	4861
MSTRG.31990.1	noncoding	-0.053248	3540	3585	5008
MSTRG.31992.2	coding	0.481	480	8631	13441

MSTRG.32053.1	coding	0.09	81	1572	2150
MSTRG.32054.1	coding	0.104	57	285	775
MSTRG.32056.2	noncoding	-0.0159744	16341	16581	29923
MSTRG.32056.3	coding	0.393	7308	9240	9465
MSTRG.32058.1	noncoding	-0.0135168	690	834	8750
MSTRG.32059.1	coding	0.607	0	1194	2393
MSTRG.32068.2	coding	0.051	552	897	2468
MSTRG.32055.2	noncoding	-0.0053248	84	231	734
MSTRG.32069.2	noncoding	-0.028672	1518	1794	2740
MSTRG.32070.3	coding	0.1	198	414	574
MSTRG.32072.3	noncoding	-0.036864	1554	1683	7498
MSTRG.32076.5	coding	0.021	1044	2337	2791
MSTRG.32076.7	noncoding	-0.055296	1347	1623	2075
MSTRG.32076.3	coding	0.031	1044	2361	3083
MSTRG.32076.4	coding	0.064	1071	1893	2614
MSTRG.32076.6	coding	0.064	1383	2205	2926
MSTRG.32077.1	noncoding	-0.0004096	0	0	224
MSTRG.32078.1	coding	0.066	918	1803	2361
MSTRG.32084.1	noncoding	-0.0155648	258	270	820
MSTRG.32085.1	noncoding	-0.0004096	0	0	665
MSTRG.32086.1	noncoding	-0.0393216	594	834	2267
MSTRG.32091.1	noncoding	-0.04096	336	546	2587
MSTRG.32091.2	coding	0.06	141	369	1495
MSTRG.32104.3	coding	0.087	585	1080	1445
MSTRG.32105.1	noncoding	-0.016384	279	405	1530
MSTRG.32106.1	noncoding	-0.0004096	0	0	680
MSTRG.32107.1	noncoding	-0.036864	198	210	1395
MSTRG.32108.2	noncoding	-0.0004096	1017	1017	1284
MSTRG.32108.1	noncoding	-0.065536	978	1041	2596
MSTRG.32109.1	noncoding	-0.0004096	0	0	838
MSTRG.32110.1	noncoding	-0.057344	984	1116	4702
MSTRG.32104.4	noncoding	-0.0237568	5793	6063	6219
MSTRG.32111.1	coding	0.045	4137	4572	6273
MSTRG.32112.1	noncoding	-0.0249856	2634	2763	6911
MSTRG.32094.1	noncoding	-0.0004096	0	0	262
MSTRG.32094.2	noncoding	-0.0004096	0	0	238
MSTRG.32113.1	noncoding	-0.045056	348	423	580
MSTRG.32114.2	noncoding	-0.0221184	150	300	457
MSTRG.32115.1	noncoding	-0.0274432	432	672	826
MSTRG.32116.2	noncoding	-0.1060864	0	111	3504
MSTRG.32119.1	noncoding	-0.0561152	0	45	813
MSTRG.32123.1	noncoding	-0.0253952	0	42	241
MSTRG.32128.1	coding	0.051	105	237	1514
MSTRG.32129.1	noncoding	-0.016384	300	474	628
MSTRG.32130.2	noncoding	-0.0139264	849	1002	1175
MSTRG.32130.1	noncoding	-0.045056	2253	2481	2654
MSTRG.32130.3	noncoding	-0.047104	2643	2856	3013
MSTRG.32133.1	coding	0.106	324	462	4554
MSTRG.32134.1	coding	0.059	138	1335	7103
MSTRG.32134.2	coding	0.106	42	1353	6702
MSTRG.32134.4	noncoding	-0.0405504	1017	1176	11932
MSTRG.32137.1	coding	0.4	990	7479	10908
MSTRG.32137.2	coding	0.4	282	6771	9717
MSTRG.32137.3	coding	0.4	21	6483	9486

MSTRG.32136.1	noncoding	-0.0016384	279	423	687
MSTRG.32140.1	noncoding	-0.024576	447	561	716
MSTRG.32141.1	coding	0.164	231	2649	3215
MSTRG.32142.1	noncoding	-0.022528	384	696	853
MSTRG.32143.4	coding	0.092	603	816	2087
MSTRG.32146.1	coding	0.424	228	1899	3008
MSTRG.32154.2	coding	0.372	0	432	1714
MSTRG.32155.1	coding	0.174	114	1971	2691
MSTRG.32155.2	coding	0.173	21	1893	4135
MSTRG.32158.3	coding	0.173	201	723	879
MSTRG.32163.1	noncoding	-0.012288	330	396	666
MSTRG.32164.1	coding	0.109	267	1209	1972
MSTRG.32150.2	coding	0.073	615	1626	2140
MSTRG.32150.3	coding	0.108	0	1506	2007
MSTRG.32167.2	noncoding	-0.0208896	1227	1380	1632
MSTRG.32167.3	noncoding	-0.0167936	813	981	2232
MSTRG.32168.1	noncoding	-0.0004096	0	0	399
MSTRG.32169.2	coding	0.02	342	543	1112
MSTRG.32166.1	noncoding	-0.0262144	807	993	2347
MSTRG.32172.2	coding	0.242	570	1194	1558
MSTRG.32176.4	coding	0.231	0	1425	1985
MSTRG.32176.3	coding	0.215	237	1299	1859
MSTRG.32177.1	coding	0.178	87	444	719
MSTRG.32180.1	coding	0.199	27	1311	1706
MSTRG.32181.4	coding	0.415	285	1911	3009
MSTRG.32183.6	noncoding	-0.0356352	1260	1470	1912
MSTRG.32184.1	noncoding	-0.0188416	759	912	1254
MSTRG.32184.3	coding	0.25	21	396	1589
MSTRG.32199.1	noncoding	-0.0741376	8028	8442	8596
MSTRG.32199.2	noncoding	-0.063488	666	1152	1318
MSTRG.32199.3	noncoding	-0.0282624	1314	1503	2646
MSTRG.32201.1	noncoding	-0.0319488	126	294	561
MSTRG.32203.2	coding	0.317	0	1341	1766
MSTRG.32203.4	coding	0.285	99	1428	1853
MSTRG.32206.2	coding	0.249	147	1077	2023
MSTRG.32206.4	coding	0.252	537	1545	2493
MSTRG.32206.5	coding	0.256	255	1185	2133
MSTRG.32208.2	coding	0.288	81	1395	1770
MSTRG.32204.2	noncoding	-0.0393216	1968	3168	3322
MSTRG.32212.1	noncoding	-0.004096	234	369	2178
MSTRG.32225.1	noncoding	-0.0012288	0	117	329
MSTRG.32227.1	noncoding	-0.036864	138	258	424
MSTRG.32229.2	coding	0.379	273	732	2132
MSTRG.32232.1	coding	0.282	0	6306	10115
MSTRG.32232.2	coding	0.272	990	7155	8545
MSTRG.32231.1	noncoding	-0.0151552	9	15	213
MSTRG.32231.2	noncoding	-0.0151552	6	12	220
MSTRG.32237.2	coding	0.113	0	423	717
MSTRG.32238.1	noncoding	-0.0126976	147	297	681
MSTRG.32239.1	coding	0.303	9	468	673
MSTRG.32241.1	noncoding	-0.053248	282	342	602
MSTRG.32242.1	noncoding	-0.0274432	18	144	1014
MSTRG.32246.2	coding	0.331	1275	3081	7015
MSTRG.32246.3	coding	0.376	156	3198	4497

MSTRG.32249.1	coding	0.095	0	1812	4582
MSTRG.32249.5	coding	0.136	174	1887	5419
MSTRG.32250.1	noncoding	-0.08192	1605	1614	2562
MSTRG.32251.1	noncoding	-0.004096	336	420	1012
MSTRG.32251.2	coding	0.104	8163	8385	11842
MSTRG.32252.1	noncoding	-0.008192	303	426	724
MSTRG.32254.1	coding	0.303	0	3501	6197
MSTRG.32257.1	noncoding	-0.1572864	0	348	552
MSTRG.32260.2	coding	0.304	162	1032	2248
MSTRG.32265.1	coding	0.263	42	627	838
MSTRG.32266.1	coding	0.194	18	399	704
MSTRG.32267.1	coding	0.145	54	435	745
MSTRG.32270.2	coding	0.142	624	930	1306
MSTRG.32275.3	coding	0.258	234	948	2397
MSTRG.32275.1	coding	0.258	180	894	2344
MSTRG.32275.2	coding	0.258	405	1119	2568
MSTRG.32275.6	coding	0.258	168	882	2299
MSTRG.32275.7	coding	0.258	390	1104	2454
MSTRG.32276.4	coding	0.204	1605	2133	2953
MSTRG.32278.3	coding	0.333	0	1455	1694
MSTRG.32278.4	coding	0.334	0	1458	1667
MSTRG.32277.1	coding	0.591	0	1068	2677
MSTRG.32283.1	noncoding	-0.0528384	96	663	937
MSTRG.32283.2	noncoding	-0.0528384	96	663	1138
MSTRG.32285.1	noncoding	-0.04096	306	408	1457
MSTRG.32286.1	noncoding	-0.0704512	333	1209	1376
MSTRG.32289.1	coding	0.447	3	1932	2087
MSTRG.32291.2	noncoding	-0.0618496	177	387	603
MSTRG.32292.1	coding	0.583	108	1227	2032
MSTRG.32292.3	coding	0.578	243	1335	2138
MSTRG.32293.1	coding	0.313	0	1224	3601
MSTRG.32300.3	coding	0.439	675	1986	2810
MSTRG.32300.5	coding	0.441	672	2007	2821
MSTRG.32301.3	coding	0.31	264	1962	2426
MSTRG.32305.2	coding	0.259	186	1194	2344
MSTRG.32324.12	coding	0.319	0	10200	11089
MSTRG.32324.17	coding	0.309	0	10425	11314
MSTRG.32324.18	coding	0.336	0	10866	11755
MSTRG.32324.20	coding	0.332	0	9960	10849
MSTRG.32324.21	coding	0.32	0	11154	12043
MSTRG.32324.3	coding	0.285	0	6525	7414
MSTRG.32324.33	coding	0.314	0	10164	11053
MSTRG.32324.35	coding	0.319	0	9051	9940
MSTRG.32324.38	coding	0.326	0	9960	10849
MSTRG.32324.40	coding	0.32	0	9069	9958
MSTRG.32324.43	coding	0.321	0	10950	11839
MSTRG.32324.47	coding	0.329	0	12183	13072
MSTRG.32324.49	coding	0.327	0	10992	11881
MSTRG.32324.50	coding	0.327	0	11700	12589
MSTRG.32324.53	coding	0.309	0	8424	9313
MSTRG.32324.55	coding	0.324	0	10200	11089
MSTRG.32324.56	coding	0.325	0	12324	13213
MSTRG.32324.7	coding	0.326	0	9576	10465
MSTRG.32324.9	coding	0.331	0	11958	12847

MSTRG.32310.3	coding	0.352	0	2712	3356
MSTRG.32310.4	coding	0.343	0	2778	3422
MSTRG.32322.7	coding	0.331	0	1338	1547
MSTRG.32322.10	coding	0.315	426	1824	2033
MSTRG.32322.12	coding	0.312	426	1833	2042
MSTRG.32322.8	coding	0.348	951	2163	2372
MSTRG.32322.11	coding	0.345	1047	2268	2477
MSTRG.32322.3	coding	0.346	1047	2265	2474
MSTRG.32322.4	coding	0.329	378	1692	1900
MSTRG.32322.9	coding	0.329	561	1875	2082
MSTRG.32322.2	coding	0.329	795	2109	2317
MSTRG.32322.16	coding	0.329	447	1761	1968
MSTRG.32323.1	noncoding	-0.0667648	888	1128	1604
MSTRG.32320.3	coding	0.256	42	789	2022
MSTRG.32331.1	coding	0.251	30	1530	5244
MSTRG.32331.3	coding	0.258	51	1575	5017
MSTRG.32331.4	coding	0.254	48	1563	4976
MSTRG.32331.5	coding	0.251	0	1500	1897
MSTRG.32331.6	coding	0.252	117	1623	2018
MSTRG.32334.1	coding	0.322	2247	4851	5183
MSTRG.32334.2	coding	0.39	2205	4083	5180
MSTRG.32334.3	coding	0.323	507	3111	3443
MSTRG.32338.1	noncoding	-0.0561152	105	171	375
MSTRG.32339.2	noncoding	-0.0339968	687	861	1502
MSTRG.32339.1	noncoding	-0.0364544	2658	3309	4349
MSTRG.32339.3	noncoding	-0.018432	1359	1566	2200
MSTRG.32338.4	coding	0.253	360	1494	1657
MSTRG.32338.6	coding	0.235	318	1485	1649
MSTRG.32338.7	coding	0.249	258	1395	1559
MSTRG.32329.1	coding	0.314	426	1053	5111
MSTRG.32329.2	coding	0.316	720	1095	5117
MSTRG.32329.3	coding	0.322	921	1344	4409
MSTRG.32329.5	coding	0.316	900	1275	1962
MSTRG.32329.6	coding	0.259	1755	2217	2904
MSTRG.32329.7	coding	0.251	426	1143	1828
MSTRG.32340.2	coding	0.196	288	1344	6662
MSTRG.32340.4	coding	0.236	276	903	2113
MSTRG.32342.1	coding	0.242	75	813	3909
MSTRG.32343.2	coding	0.707	0	216	517
MSTRG.32344.1	noncoding	-0.1048576	0	744	898
MSTRG.32350.3	coding	0.376	0	2430	2585
MSTRG.32354.2	coding	0.348	0	1551	1813
MSTRG.32354.3	coding	0.567	0	681	1612
MSTRG.32356.1	coding	0.682	984	3117	3827
MSTRG.32358.2	coding	0.496	384	2022	2483
MSTRG.32358.3	coding	0.505	93	1773	2236
MSTRG.32358.4	coding	0.527	555	1638	2099
MSTRG.32364.3	coding	0.184	0	1215	1549
MSTRG.32365.1	noncoding	-0.0483328	1743	2382	2629
MSTRG.32365.2	noncoding	-0.057344	1149	1689	3229
MSTRG.32366.5	noncoding	-0.0331776	63	282	542
MSTRG.32369.4	noncoding	-0.0892928	165	462	976
MSTRG.32369.3	noncoding	-0.09216	462	936	1451
MSTRG.32371.2	coding	0.398	1326	2676	3005

MSTRG.32371.3	coding	0.402	429	1659	1989
MSTRG.32359.2	coding	0.262	24	876	1030
MSTRG.32372.1	noncoding	-0.26624	117	1536	2302
MSTRG.32377.1	coding	0.253	0	441	2374
MSTRG.32381.7	coding	0.532	0	1485	1831
MSTRG.32381.11	coding	0.548	141	4704	5051
MSTRG.32381.10	coding	0.563	0	3990	4338
MSTRG.32381.2	coding	0.546	0	4575	4921
MSTRG.32381.3	coding	0.503	0	4830	5176
MSTRG.32381.1	coding	0.504	0	4818	5164
MSTRG.32381.8	coding	0.545	0	4617	4963
MSTRG.32381.12	coding	0.553	225	4758	5106
MSTRG.32381.4	coding	0.584	0	5202	5548
MSTRG.32381.6	coding	0.465	5031	8190	8537
MSTRG.32381.14	noncoding	-0.0364544	3264	3579	5763
MSTRG.32378.1	noncoding	-0.024576	516	702	1216
MSTRG.32384.10	coding	0.225	39	1896	2822
MSTRG.32384.11	coding	0.238	0	2040	2967
MSTRG.32384.5	coding	0.235	0	2049	2972
MSTRG.32388.1	coding	0.638	684	1953	3387
MSTRG.32395.5	coding	0.326	0	1062	1341
MSTRG.32395.2	coding	0.296	228	1410	1689
MSTRG.32395.3	coding	0.353	0	1077	1357
MSTRG.32395.4	coding	0.376	327	1176	1457
MSTRG.32395.7	coding	0.414	213	936	1216
MSTRG.32394.2	coding	0.372	153	1089	1603
MSTRG.32397.2	coding	0.253	333	1467	1701
MSTRG.32397.3	coding	0.269	603	1956	2124
MSTRG.32397.4	coding	0.26	24	1362	1516
MSTRG.32397.5	coding	0.207	309	1155	3080
MSTRG.32397.6	coding	0.278	0	891	2785
MSTRG.32402.1	coding	0.37	60	1368	1648
MSTRG.32405.3	coding	0.319	249	549	1881
MSTRG.32407.1	noncoding	-0.106496	243	282	771
MSTRG.32411.2	coding	0.55	1446	2271	6968
MSTRG.32412.2	coding	0.42	0	1074	2556
MSTRG.32411.3	noncoding	-0.043008	7041	7356	7554
MSTRG.32411.4	noncoding	-0.0094208	7707	7908	8104
MSTRG.32411.5	noncoding	-0.0155648	414	576	7010
MSTRG.32415.1	coding	0.362	1662	2382	2999
MSTRG.32416.3	coding	0.513	0	2757	3090
MSTRG.32418.6	coding	0.488	0	5982	6138
MSTRG.32418.9	coding	0.488	0	5991	6147
MSTRG.32419.2	coding	0.533	0	3351	3511
MSTRG.32424.1	coding	0.269	0	2091	3486
MSTRG.32427.1	noncoding	-0.083968	528	1353	1508
MSTRG.32434.2	noncoding	-0.0565248	0	1395	2124
MSTRG.32441.1	coding	0.275	30	438	801
MSTRG.32441.2	coding	0.25	81	489	853
MSTRG.32441.3	coding	0.316	0	387	750
MSTRG.32444.1	noncoding	-0.0118784	738	891	1267
MSTRG.32444.2	noncoding	-0.1609728	0	384	2415
MSTRG.32456.3	coding	0.266	192	1563	2022
MSTRG.32456.2	coding	0.245	453	2013	2631

MSTRG.32447.2	coding	0.397	9	735	889
MSTRG.32451.2	coding	0.276	0	1995	2607
MSTRG.32457.1	noncoding	-0.0417792	378	594	986
MSTRG.32484.5	coding	0.387	435	1680	1851
MSTRG.32484.6	coding	0.369	606	1767	1938
MSTRG.32484.1	coding	0.373	0	2016	11148
MSTRG.32484.4	coding	0.335	306	1581	1939
MSTRG.32484.3	coding	0.425	390	933	1405
MSTRG.32484.2	coding	0.247	0	1908	2273
MSTRG.32484.7	coding	0.281	0	2025	10873
MSTRG.32484.8	coding	0.324	5499	7200	16179
MSTRG.32484.9	coding	0.351	270	1590	1948
MSTRG.32484.11	coding	0.386	96	555	743
MSTRG.32489.1	coding	0.159	141	1599	1821
MSTRG.32489.2	coding	0.16	141	1602	1824
MSTRG.32492.2	coding	0.444	0	3330	3564
MSTRG.32492.5	coding	0.67	0	1686	3847
MSTRG.32494.1	noncoding	-0.0155648	915	1092	1532
MSTRG.32468.1	coding	0.165	534	4317	7867
MSTRG.32468.4	coding	0.249	174	3837	6671
MSTRG.32468.5	coding	0.25	3	3714	6549
MSTRG.32468.6	noncoding	-0.02048	2826	2931	6203
MSTRG.32469.1	noncoding	-0.12288	216	276	6990
MSTRG.32474.2	noncoding	-0.0217088	396	1398	1553
MSTRG.32474.3	noncoding	-0.043008	117	315	877
MSTRG.32480.1	coding	0.399	0	756	991
MSTRG.32497.2	coding	0.241	807	1500	1998
MSTRG.32501.2	coding	0.372	1002	1626	1788
MSTRG.32501.3	coding	0.379	897	1422	1576
MSTRG.32507.4	coding	0.168	588	1659	2605
MSTRG.32507.3	coding	0.168	603	1467	2539
MSTRG.32507.6	coding	0.317	0	1368	1523
MSTRG.32509.1	coding	0.145	621	3171	3923
MSTRG.32509.3	coding	0.14	0	3087	3241
MSTRG.32513.1	noncoding	-0.0413696	261	561	716
MSTRG.32513.2	noncoding	-0.0630784	75	501	980
MSTRG.32514.4	coding	0.216	420	3027	4431
MSTRG.32514.5	coding	0.136	741	2511	8237
MSTRG.32515.1	noncoding	-0.036864	2979	3060	5393
MSTRG.32519.1	coding	0.368	0	630	4856
MSTRG.32519.3	coding	0.325	0	954	1828
MSTRG.32519.4	coding	0.368	0	630	1544
MSTRG.32519.5	coding	0.368	0	630	7329
MSTRG.32519.6	coding	0.351	0	603	3823
MSTRG.32519.7	coding	0.368	0	630	2433
MSTRG.32519.8	coding	0.368	0	630	2361
MSTRG.32519.9	coding	0.343	81	951	1824
MSTRG.32520.1	noncoding	-0.0319488	327	558	1729
MSTRG.32520.2	noncoding	-0.006144	57	204	1081
MSTRG.32520.3	noncoding	-0.006144	57	204	1241
MSTRG.32520.4	noncoding	-0.06144	0	114	474
MSTRG.32520.5	noncoding	-0.02048	57	162	566
MSTRG.32523.3	coding	0.307	129	516	703
MSTRG.32528.2	noncoding	-0.0380928	639	849	1075

MSTRG.32528.1	noncoding	-0.0561152	177	342	568
MSTRG.32534.11	coding	0.326	0	1086	4992
MSTRG.32534.10	coding	0.328	0	1191	8553
MSTRG.32534.3	coding	0.324	0	1212	10478
MSTRG.32534.4	coding	0.319	0	1353	11119
MSTRG.32534.6	coding	0.333	0	1164	9664
MSTRG.32534.7	coding	0.319	0	1353	10910
MSTRG.32534.13	coding	0.385	321	1380	2186
MSTRG.32534.5	coding	0.485	0	2763	3026
MSTRG.32534.12	coding	0.484	0	2751	3774
MSTRG.32534.8	coding	0.484	0	2751	3574
MSTRG.32542.1	coding	0.37	138	528	708
MSTRG.32542.3	coding	0.36	342	846	1026
MSTRG.32545.1	noncoding	-0.0200704	210	789	943
MSTRG.32546.2	coding	0.244	420	1155	2637
MSTRG.32547.4	coding	0.54	63	1581	2566
MSTRG.32547.5	coding	0.525	0	1710	2695
MSTRG.32547.6	coding	0.52	162	1566	2550
MSTRG.32549.1	noncoding	-0.0630784	7185	8244	8615
MSTRG.32549.2	noncoding	-0.0598016	4608	5175	5330
MSTRG.32549.3	noncoding	-0.0536576	294	882	6192
MSTRG.32550.1	noncoding	-0.0937984	597	1143	1313
MSTRG.32552.1	noncoding	-0.002048	0	135	561
MSTRG.32554.2	noncoding	-0.0008192	0	102	438
MSTRG.32554.1	noncoding	-0.0413696	189	405	909
MSTRG.32554.3	noncoding	-0.0671744	393	756	1212
MSTRG.32538.2	coding	0.258	0	411	669
MSTRG.32538.4	coding	0.264	0	267	624
MSTRG.32538.5	coding	0.253	96	1203	1456
MSTRG.32541.1	coding	0.458	0	1644	2916
MSTRG.32541.4	coding	0.45	0	1650	2916
MSTRG.32557.1	noncoding	-0.026624	216	348	735
MSTRG.32558.1	coding	0.544	0	2670	3536
MSTRG.32558.2	coding	0.551	0	2619	3483
MSTRG.32561.2	coding	0.504	1353	3921	4095
MSTRG.32561.3	coding	0.516	0	2532	2704
MSTRG.32561.4	coding	0.502	312	2595	2962
MSTRG.32561.5	coding	0.492	213	2493	3340
MSTRG.32564.1	noncoding	-0.057344	0	522	15108
MSTRG.32564.2	noncoding	-0.0581632	3627	3987	11888
MSTRG.32562.1	coding	0.641	0	399	748
MSTRG.32566.3	coding	0.02	4230	4494	4908
MSTRG.32566.4	noncoding	-0.0720896	1659	1965	5162
MSTRG.32566.5	noncoding	-0.0536576	2865	3636	15949
MSTRG.32568.3	noncoding	-0.118784	732	1875	2029
MSTRG.32575.3	coding	0.278	135	1227	4336
MSTRG.32575.4	coding	0.278	99	1191	2786
MSTRG.32575.5	coding	0.379	0	804	1185
MSTRG.32575.6	noncoding	-0.0618496	0	1419	3012
MSTRG.32575.8	coding	0.24	0	1155	2745
MSTRG.32575.10	coding	0.409	0	528	2133
MSTRG.32575.9	coding	0.409	0	528	3637
MSTRG.32575.11	coding	0.378	21	531	1934
MSTRG.32575.12	coding	0.378	21	531	2123

MSTRG.32574.3	noncoding	-0.1355776	1074	1536	1689
MSTRG.32576.2	coding	0.328	0	1563	1735
MSTRG.32582.1	coding	0.472	0	987	1292
MSTRG.32588.3	coding	0.504	417	2103	2518
MSTRG.32592.1	coding	0.322	0	1476	1631
MSTRG.32592.3	coding	0.396	483	1566	1720
MSTRG.32596.2	coding	0.534	0	1524	2046
MSTRG.32598.2	noncoding	-0.0679936	342	1014	1193
MSTRG.32606.2	coding	0.462	912	3225	3771
MSTRG.32606.4	coding	0.53	57	2370	2526
MSTRG.32606.3	coding	0.54	0	2352	2509
MSTRG.32607.2	coding	0.228	1215	1650	1821
MSTRG.32607.3	coding	0.294	0	1122	1292
MSTRG.32604.3	coding	0.349	0	3282	4603
MSTRG.32609.2	coding	0.36	870	3042	3243
MSTRG.32611.1	coding	0.399	825	1686	1840
MSTRG.32613.1	noncoding	-0.0004096	6	183	2628
MSTRG.32614.1	coding	0.531	0	1311	1478
MSTRG.32614.2	coding	0.447	357	2127	2295
MSTRG.32614.3	coding	0.431	648	2247	2415
MSTRG.32614.4	coding	0.447	648	2418	2586
MSTRG.32614.5	coding	0.543	0	1224	1391
MSTRG.32614.6	coding	0.536	18	1284	1452
MSTRG.32617.1	coding	0.447	0	441	594
MSTRG.32619.2	coding	0.43	0	450	643
MSTRG.32621.1	noncoding	-0.032768	135	138	406
MSTRG.32628.1	coding	0.059	180	1185	1617
MSTRG.32628.2	noncoding	-0.008192	150	291	447
MSTRG.32640.1	coding	0.271	0	2130	6983
MSTRG.32641.1	coding	0.134	120	5124	6747
MSTRG.32641.2	coding	0.134	120	5118	7398
MSTRG.32641.3	coding	0.139	96	4950	7211
MSTRG.32641.4	coding	0.132	5706	10614	12837
MSTRG.32634.1	coding	0.253	246	1416	7520
MSTRG.32634.2	coding	0.141	228	2763	5832
MSTRG.32646.2	noncoding	-0.0057344	0	36	293
MSTRG.32646.3	noncoding	-0.012288	0	54	325
MSTRG.32658.1	coding	0.402	6	585	1495
MSTRG.32658.2	coding	0.354	84	963	1575
MSTRG.32658.4	noncoding	-0.1150976	0	768	1303
MSTRG.32658.3	coding	0.318	45	927	1215
MSTRG.32660.1	noncoding	-0.1765376	777	786	1386
MSTRG.32661.2	coding	0.07	10269	10881	11167
MSTRG.32662.1	noncoding	-0.086016	249	306	842
MSTRG.32663.1	coding	0.342	126	1023	1365
MSTRG.32664.1	noncoding	-0.024576	609	711	1687
MSTRG.32665.1	noncoding	-0.02048	36	519	1407
MSTRG.32666.2	coding	0.081	12	867	1023
MSTRG.32656.1	coding	0.097	0	1206	8341
MSTRG.32656.3	coding	0.096	0	1176	2543
MSTRG.32668.5	coding	0.095	183	2844	5225
MSTRG.32668.6	coding	0.095	132	2793	3905
MSTRG.32674.2	coding	0.339	1137	2091	2852
MSTRG.32672.1	noncoding	-0.06144	195	291	1449

MSTRG.32681.3	coding	0.055	774	4191	7421
MSTRG.32683.1	noncoding	-0.180224	2493	2523	5760
MSTRG.32682.3	noncoding	-0.0004096	5253	5397	9485
MSTRG.32682.4	noncoding	-0.0004096	9027	9171	13246
MSTRG.32686.1	coding	0.094	177	3753	5802
MSTRG.32686.3	coding	0.094	438	4014	5624
MSTRG.32687.1	noncoding	-0.0004096	5397	5637	7373
MSTRG.32688.1	noncoding	-0.024576	243	366	1000
MSTRG.32689.1	noncoding	-0.04096	48	60	2697
MSTRG.32690.1	coding	0.222	114	2127	3595
MSTRG.32691.1	coding	0.386	72	600	1036
MSTRG.32692.1	coding	0.131	0	330	518
MSTRG.32697.1	noncoding	-0.0897024	0	114	270
MSTRG.32698.1	noncoding	-0.024576	348	354	832
MSTRG.32699.1	coding	0.486	0	1470	1853
MSTRG.32699.2	coding	0.48	0	1455	1824
MSTRG.32699.3	coding	0.482	0	1458	1825
MSTRG.32699.4	coding	0.505	0	1344	1500
MSTRG.32699.5	coding	0.506	0	1359	1515
MSTRG.32709.11	noncoding	-0.0098304	0	138	364
MSTRG.32712.1	coding	0.14	24	3348	12883
MSTRG.32719.2	coding	0.303	282	2208	6230
MSTRG.32720.1	noncoding	-0.069632	7410	7998	13803
MSTRG.32718.2	noncoding	-0.0172032	3	153	1389
MSTRG.32723.2	noncoding	-0.0446464	132	312	3800
MSTRG.32723.3	noncoding	-0.0446464	126	306	3957
MSTRG.32723.10	noncoding	-0.0446464	75	255	3619
MSTRG.32723.8	noncoding	-0.0770048	3453	3687	3843
MSTRG.32723.7	noncoding	-0.0704512	3615	3852	4006
MSTRG.32723.11	coding	0.07	312	474	3840
MSTRG.32723.9	noncoding	-0.0311296	165	330	3695
MSTRG.32723.12	noncoding	-0.0446464	132	312	3838
MSTRG.32723.14	noncoding	-0.0446464	132	312	3837
MSTRG.32723.16	noncoding	-0.0446464	132	312	3838
MSTRG.32723.13	coding	0.167	0	219	2354
MSTRG.32723.15	noncoding	-0.0647168	3444	3666	3822
MSTRG.32723.17	noncoding	-0.0446464	42	222	2802
MSTRG.32723.18	noncoding	-0.0638976	0	216	3856
MSTRG.32721.5	coding	0.107	378	807	1371
MSTRG.32721.6	coding	0.107	624	1053	1618
MSTRG.32730.1	coding	0.07	0	879	2079
MSTRG.32731.1	noncoding	-0.08192	33	78	236
MSTRG.32732.1	noncoding	-0.0004096	1059	1158	1787
MSTRG.32730.3	noncoding	-0.083968	1398	1809	1963
MSTRG.32738.1	coding	0.261	114	1638	2798
MSTRG.32738.2	coding	0.264	9	1584	2746
MSTRG.32738.4	coding	0.286	24	3333	4493
MSTRG.32738.5	coding	0.261	24	1614	2774
MSTRG.32738.6	coding	0.263	24	1605	2765
MSTRG.32738.7	coding	0.257	3603	5115	6276
MSTRG.32739.1	coding	0.152	0	3948	4380
MSTRG.32740.1	noncoding	-0.045056	63	96	805
MSTRG.32747.4	coding	0.121	753	1584	9127
MSTRG.32747.9	coding	0.111	33	834	1088

MSTRG.32749.1	noncoding	-0.04096	1719	1959	2113
MSTRG.32751.3	coding	0.14	2208	3117	3629
MSTRG.32751.2	coding	0.14	2247	3156	3670
MSTRG.32752.1	noncoding	-0.126976	81	141	294
MSTRG.32749.2	noncoding	-0.147456	345	405	892
MSTRG.32752.2	coding	0.188	264	693	916
MSTRG.32752.4	coding	0.188	195	624	843
MSTRG.32752.5	coding	0.236	0	327	548
MSTRG.32752.6	coding	0.236	0	327	548
MSTRG.32752.7	coding	0.284	0	303	524
MSTRG.32752.8	noncoding	-0.012288	159	300	524
MSTRG.32752.9	coding	0.316	0	405	675
MSTRG.32754.1	noncoding	-0.0073728	60	186	1623
MSTRG.32754.2	noncoding	-0.004096	282	321	841
MSTRG.32754.3	noncoding	-0.004096	162	201	468
MSTRG.32755.1	noncoding	-0.0073728	171	297	604
MSTRG.32754.4	noncoding	-0.0073728	144	270	428
MSTRG.32756.1	noncoding	-0.0004096	0	0	404
MSTRG.32757.1	noncoding	-0.028672	120	303	1863
MSTRG.32760.1	noncoding	-0.0110592	0	114	490
MSTRG.32765.1	noncoding	-0.0004096	1932	2118	3894
MSTRG.32773.1	coding	0.106	12489	12768	16420
MSTRG.32767.1	coding	0.175	99	711	1526
MSTRG.32781.1	noncoding	-0.0700416	0	918	1513
MSTRG.32782.1	coding	0.294	0	771	1415
MSTRG.32796.3	noncoding	-0.032768	408	513	669
MSTRG.32796.5	noncoding	-0.032768	408	513	669
MSTRG.32796.4	noncoding	-0.0098304	129	279	1025
MSTRG.32796.6	noncoding	-0.0221184	1083	1896	2637
MSTRG.32796.7	coding	0.196	990	1839	2793
MSTRG.32796.9	coding	0.159	867	1617	2283
MSTRG.32796.10	coding	0.196	867	1716	2668
MSTRG.32796.8	coding	0.196	867	1716	2668
MSTRG.32796.11	coding	0.196	846	1695	2601
MSTRG.32796.12	coding	0.197	858	2283	3188
MSTRG.32796.14	noncoding	-0.0589824	0	84	238
MSTRG.32796.13	coding	0.196	336	1185	2138
MSTRG.32796.15	coding	0.159	327	1077	2460
MSTRG.32796.16	coding	0.159	582	1332	1998
MSTRG.32796.17	coding	0.159	372	1122	2508
MSTRG.32796.18	coding	0.186	366	1215	2168
MSTRG.32796.19	coding	0.191	315	1164	2116
MSTRG.32796.20	coding	0.191	171	1020	1950
MSTRG.32798.3	coding	0.168	543	1455	1912
MSTRG.32798.4	coding	0.149	288	1254	1674
MSTRG.32798.5	coding	0.168	282	1194	1652
MSTRG.32798.2	coding	0.151	327	1293	1715
MSTRG.32798.6	coding	0.154	300	1266	1656
MSTRG.32798.8	coding	0.151	363	1329	2881
MSTRG.32789.1	coding	0.07	30	1134	3853
MSTRG.32792.1	noncoding	-0.0692224	210	285	535
MSTRG.32801.1	coding	0.168	0	2790	5015
MSTRG.32801.3	coding	0.173	0	2820	4849
MSTRG.32801.4	coding	0.141	9	2085	2661

MSTRG.32802.1	noncoding	-0.012288	135	261	416
MSTRG.32814.2	coding	0.204	246	1446	5178
MSTRG.32815.1	noncoding	-0.065536	93	126	280
MSTRG.32821.1	coding	0.105	1833	3708	6535
MSTRG.32821.2	coding	0.105	1452	3327	6154
MSTRG.32821.3	coding	0.105	1128	3003	5759
MSTRG.32821.4	coding	0.105	1089	2964	3633
MSTRG.32821.5	coding	0.105	1086	2961	3600
MSTRG.32819.1	noncoding	-0.0004096	0	0	301
MSTRG.32820.2	noncoding	-0.063488	0	576	1588
MSTRG.32827.1	coding	0.137	0	291	548
MSTRG.32828.1	noncoding	-0.0024576	231	291	511
MSTRG.32832.1	noncoding	-0.0004096	0	0	217
MSTRG.32832.2	noncoding	-0.0004096	0	0	382
MSTRG.32836.1	coding	0.215	1035	2784	6366
MSTRG.32837.1	noncoding	-0.155648	0	15	329
MSTRG.32841.1	noncoding	-0.0344064	198	543	1449
MSTRG.32842.7	noncoding	-0.018432	51	210	611
MSTRG.32842.8	noncoding	-0.018432	51	210	611
MSTRG.32842.6	noncoding	-0.018432	57	216	618
MSTRG.32842.2	noncoding	-0.018432	60	219	621
MSTRG.32839.1	noncoding	-0.069632	342	360	735
MSTRG.32839.2	noncoding	-0.069632	351	369	552
MSTRG.32839.3	noncoding	-0.069632	342	360	530
MSTRG.32840.1	noncoding	-0.016384	30	54	290
MSTRG.32849.1	noncoding	-0.069632	1437	1491	1681
MSTRG.32859.1	noncoding	-0.0569344	621	624	778
MSTRG.32860.1	noncoding	-0.0155648	2469	2631	25547
MSTRG.32862.1	noncoding	-0.0086016	3060	3273	3631
MSTRG.32859.2	noncoding	-0.045056	381	504	4443
MSTRG.32855.1	noncoding	-0.036864	135	147	374
MSTRG.32866.1	noncoding	-0.045056	195	297	2736
MSTRG.32867.1	noncoding	-0.0094208	1281	1401	2392
MSTRG.32867.2	noncoding	-0.077824	2031	2076	4336
MSTRG.32867.3	noncoding	-0.0376832	0	54	2616
MSTRG.32867.4	noncoding	-0.110592	2499	2562	7193
MSTRG.32867.5	coding	0.087	17226	17439	24017
MSTRG.32867.6	noncoding	-0.073728	1641	1659	2423
MSTRG.32867.7	noncoding	-0.0290816	1041	1212	6538
MSTRG.32863.5	coding	0.14	0	1740	2005
MSTRG.32863.6	coding	0.136	0	1776	3695
MSTRG.32864.2	noncoding	-0.057344	21	342	1040
MSTRG.32874.3	coding	0.242	0	4107	8577
MSTRG.32874.5	coding	0.223	4566	8394	12940
MSTRG.32874.6	coding	0.121	123	1383	2800
MSTRG.32876.1	noncoding	-0.059392	1356	1479	4440
MSTRG.32877.1	noncoding	-0.04096	13641	13830	14115
MSTRG.32869.1	coding	0.165	0	348	501
MSTRG.32880.1	noncoding	-0.0712704	30	378	532
MSTRG.32884.1	coding	0.229	300	726	1149
MSTRG.32888.1	noncoding	-0.0888832	135	489	643
MSTRG.32889.4	noncoding	-0.0462848	13569	16713	17154
MSTRG.32889.3	coding	0.204	288	3717	5202
MSTRG.32889.6	noncoding	-0.0454656	7506	10737	10893

MSTRG.32889.5	coding	0.199	0	2595	5248
MSTRG.32889.7	coding	0.176	0	3126	5800
MSTRG.32889.8	coding	0.184	0	2970	5383
MSTRG.32889.9	noncoding	-0.079872	7557	7980	8195
MSTRG.32892.1	coding	0.085	378	2061	3738
MSTRG.32892.5	noncoding	-0.047104	1500	1797	11240
MSTRG.32893.4	coding	0.179	3963	5829	6641
MSTRG.32895.3	coding	0.138	201	1746	1957
MSTRG.32897.1	noncoding	-0.004096	402	474	1024
MSTRG.32899.1	noncoding	-0.04096	6	105	261
MSTRG.32906.3	coding	0.271	705	1845	2342
MSTRG.32906.4	coding	0.252	0	1296	1737
MSTRG.32906.6	coding	0.251	894	2187	2546
MSTRG.32906.5	coding	0.252	105	1401	1759
MSTRG.32907.1	noncoding	-0.073728	93	171	326
MSTRG.32916.1	coding	0.099	0	606	985
MSTRG.32921.1	coding	0.078	0	168	737
MSTRG.32922.2	coding	0.114	0	1734	2001
MSTRG.32922.4	coding	0.091	246	1665	1931
MSTRG.32925.1	coding	0.038	201	342	667
MSTRG.32925.2	noncoding	-0.004096	1920	2064	2391
MSTRG.32927.1	noncoding	-0.0249856	228	387	6400
MSTRG.32929.1	coding	0.424	60	1473	2083
MSTRG.32930.2	coding	0.273	0	1944	2455
MSTRG.32933.1	coding	0.172	0	828	1150
MSTRG.32936.6	noncoding	-0.0032768	7437	7734	7889
MSTRG.32936.7	noncoding	-0.0602112	2052	2451	3654
MSTRG.32936.8	noncoding	-0.0602112	2052	2451	3715
MSTRG.32936.9	noncoding	-0.0602112	2052	2451	3750
MSTRG.32934.2	coding	0.295	90	2394	3558
MSTRG.32934.3	coding	0.295	90	2466	3396
MSTRG.32934.4	coding	0.284	90	2676	3606
MSTRG.32934.5	coding	0.287	90	2622	3550
MSTRG.32935.1	coding	0.188	177	1488	6297
MSTRG.32937.2	coding	0.445	0	1047	1329
MSTRG.32939.2	coding	0.437	0	1974	3393
MSTRG.32943.1	noncoding	-0.0438272	255	429	653
MSTRG.32945.2	coding	0.452	0	747	919
MSTRG.32946.1	coding	0.295	0	696	862
MSTRG.32951.4	coding	0.385	972	7317	8988
MSTRG.32953.1	coding	0.283	168	1899	2781
MSTRG.32957.5	coding	0.518	0	8184	8352
MSTRG.32957.3	coding	0.511	0	8247	8414
MSTRG.32954.2	noncoding	-0.0782336	267	627	1118
MSTRG.32961.2	coding	0.55	0	1335	2263
MSTRG.32964.1	coding	0.51	0	2409	2562
MSTRG.32968.2	coding	0.352	312	1833	2189
MSTRG.32984.2	noncoding	-0.0958464	2196	2697	3010
MSTRG.32984.4	noncoding	-0.0958464	48	549	873
MSTRG.32984.3	noncoding	-0.0970752	0	498	822
MSTRG.32984.5	noncoding	-0.0958464	2196	2697	3020
MSTRG.32981.1	coding	0.201	0	1104	1274
MSTRG.32988.1	noncoding	-0.0442368	0	12	413
MSTRG.32992.1	noncoding	-0.0004096	0	0	203

MSTRG.33013.3	coding	0.111	297	3324	5847
MSTRG.33013.4	coding	0.108	42	2991	4192
MSTRG.33014.1	coding	0.172	4146	4449	8443
MSTRG.33018.1	noncoding	-0.073728	1053	1134	8152
MSTRG.33003.1	noncoding	-0.108544	0	33	203
MSTRG.33021.1	coding	0.118	198	3432	9375
MSTRG.33021.2	coding	0.119	147	3333	3488
MSTRG.33021.3	coding	0.114	171	3009	8950
MSTRG.33023.1	noncoding	-0.0176128	3393	3474	5390
MSTRG.33024.1	noncoding	-0.069632	543	594	780
MSTRG.33025.1	noncoding	-0.045056	1599	1665	2600
MSTRG.33031.5	coding	0.116	492	1062	1628
MSTRG.33031.6	coding	0.116	153	723	1289
MSTRG.33033.2	coding	0.173	162	1644	2761
MSTRG.33036.1	noncoding	-0.0712704	2523	2586	4070
MSTRG.33036.3	noncoding	-0.006144	3807	3873	8516
MSTRG.33050.3	noncoding	-0.0004096	0	0	1108
MSTRG.33050.2	noncoding	-0.024576	1743	1806	2049
MSTRG.33050.5	noncoding	-0.0004096	0	0	784
MSTRG.33054.1	noncoding	-0.0045056	2136	2250	2466
MSTRG.33056.1	coding	0.176	0	1203	1952
MSTRG.33063.2	coding	0.173	180	1185	1912
MSTRG.33063.3	coding	0.168	312	1371	2021
MSTRG.33068.1	coding	0.251	78	954	1830
MSTRG.33069.1	coding	0.24	0	1305	1760
MSTRG.33083.6	coding	0.158	552	1236	1954
MSTRG.33088.1	coding	0.117	45	966	9613
MSTRG.33090.2	coding	0.127	4083	4779	11838
MSTRG.33090.3	noncoding	-0.0151552	1068	1122	3687
MSTRG.33097.2	coding	0.353	0	735	1967
MSTRG.33101.1	coding	0.098	225	4932	8176
MSTRG.33102.5	coding	0.089	1410	3246	3685
MSTRG.33102.4	coding	0.089	1308	3144	4919
MSTRG.33102.6	coding	0.089	1230	3066	4842
MSTRG.33114.1	coding	0.066	279	2052	4065
MSTRG.33114.2	coding	0.066	279	2052	4065
MSTRG.33114.3	coding	0.113	33	639	959
MSTRG.33114.4	coding	0.066	9	1782	3783
MSTRG.33114.5	coding	0.066	9	1782	3794
MSTRG.33114.6	coding	0.065	0	1767	3766
MSTRG.33114.7	coding	0.063	48	1707	3606
MSTRG.33114.8	coding	0.087	846	1467	3365
MSTRG.33114.9	coding	0.087	816	1437	3333
MSTRG.33109.1	noncoding	-0.0004096	0	0	630
MSTRG.33108.3	noncoding	-0.0036864	789	927	3198
MSTRG.33120.2	coding	0.112	0	909	1717
MSTRG.33124.3	noncoding	-0.126976	267	357	1751
MSTRG.33124.1	coding	0.172	1533	1950	2103
MSTRG.33127.4	coding	0.381	27	351	1696
MSTRG.33127.5	coding	0.522	0	756	2102
MSTRG.33128.1	coding	0.24	0	2088	6715
MSTRG.33131.1	noncoding	-0.004096	1677	1809	2137
MSTRG.33132.1	noncoding	-0.0032768	1686	1833	2405
MSTRG.33131.2	noncoding	-0.0344064	1911	2118	2446

MSTRG.33131.4	coding	0.323	240	996	1578
MSTRG.33133.1	noncoding	-0.1568768	8061	8349	8503
MSTRG.33129.2	coding	0.183	42	1014	2045
MSTRG.33135.1	coding	0.111	213	954	2720
MSTRG.33138.2	coding	0.366	0	489	679
MSTRG.33141.2	coding	0.091	69	507	2609
MSTRG.33144.1	coding	0.097	702	1971	3371
MSTRG.33144.2	coding	0.097	600	1869	3270
MSTRG.33150.2	coding	0.359	201	672	26017
MSTRG.33151.1	noncoding	-0.0258048	10491	10761	15596
MSTRG.33148.1	coding	0.155	147	1146	1919
MSTRG.33155.2	noncoding	-0.057344	1452	1500	1662
MSTRG.33158.1	coding	0.134	7260	9018	15961
MSTRG.33161.1	coding	0.051	1938	4215	5103
MSTRG.33161.2	coding	0.051	1338	3693	3914
MSTRG.33161.3	coding	0.053	540	3051	3274
MSTRG.33164.1	coding	0.16	420	771	1510
MSTRG.33165.1	coding	0.16	420	771	1510
MSTRG.33166.1	coding	0.144	711	1050	1705
MSTRG.33166.2	coding	0.144	729	1068	1793
MSTRG.33168.1	noncoding	-0.038912	0	60	256
MSTRG.33172.1	noncoding	-0.0004096	0	0	202
MSTRG.33179.1	coding	0.122	0	438	2434
MSTRG.33189.1	noncoding	-0.0237568	417	753	994
MSTRG.33224.2	coding	0.097	1284	2088	3813
MSTRG.33224.3	coding	0.097	771	1575	3298
MSTRG.33223.1	noncoding	-0.004096	0	81	239
MSTRG.33234.1	noncoding	-0.045056	111	114	364
MSTRG.33248.1	noncoding	-0.04096	225	288	824
MSTRG.33249.1	noncoding	-0.0270336	12	90	1255
MSTRG.33245.1	noncoding	-0.0159744	174	465	2609
MSTRG.33278.2	coding	0.247	639	1302	1489
MSTRG.33280.1	coding	0.38	0	879	3182
MSTRG.33282.4	noncoding	-0.0110592	543	669	5401
MSTRG.33284.1	coding	0.246	0	5277	5545
MSTRG.33287.3	coding	0.306	195	1476	2710
MSTRG.33301.2	coding	0.421	837	1485	2881
MSTRG.33306.3	noncoding	-0.024576	474	609	3817
MSTRG.33309.1	coding	0.249	870	1884	11481
MSTRG.33309.3	coding	0.304	1107	2577	3492
MSTRG.33319.1	noncoding	-0.04096	24	75	251
MSTRG.33322.3	coding	0.212	10677	13011	15183
MSTRG.33322.4	coding	0.212	10647	12981	15279
MSTRG.33322.5	noncoding	-0.0180224	6843	6969	12399
MSTRG.33323.1	noncoding	-0.0192512	14655	14772	16673
MSTRG.33325.2	coding	0.461	231	3162	3443
MSTRG.33325.4	coding	0.466	0	2901	3205
MSTRG.33325.5	coding	0.444	0	2700	3010
MSTRG.33333.1	coding	0.434	1215	2892	4153
MSTRG.33333.2	coding	0.411	1236	2958	3563
MSTRG.33333.5	coding	0.411	2997	4719	5324
MSTRG.33333.4	coding	0.43	2739	4281	7127
MSTRG.33328.1	noncoding	-0.090112	891	1182	4716
MSTRG.33329.1	coding	0.365	147	2172	2561

MSTRG.33329.2	coding	0.359	90	2553	2729
MSTRG.33329.4	coding	0.37	0	1959	2350
MSTRG.33329.5	coding	0.235	57	753	928
MSTRG.33338.1	noncoding	-0.0741376	183	597	2349
MSTRG.33338.3	coding	0.437	0	126	2387
MSTRG.33339.1	noncoding	-0.0237568	51	84	426
MSTRG.33348.1	noncoding	-0.036864	72	180	357
MSTRG.33367.7	coding	0.262	330	2829	3271
MSTRG.33373.2	coding	0.394	0	1434	3769
MSTRG.33373.3	coding	0.391	0	1605	3940
MSTRG.33373.5	coding	0.413	0	651	3569
MSTRG.33373.4	coding	0.316	216	1704	4039
MSTRG.33373.8	coding	0.348	453	1635	2053
MSTRG.33374.1	noncoding	-0.0585728	1191	1368	3449
MSTRG.33369.2	coding	0.375	207	1452	2929
MSTRG.33369.3	coding	0.383	0	1473	2946
MSTRG.33376.1	noncoding	-0.1232896	111	399	560
MSTRG.33386.1	noncoding	-0.0626688	228	246	706
MSTRG.33388.1	noncoding	-0.049152	462	468	969
MSTRG.33389.2	coding	0.176	96	2661	2860
MSTRG.33391.1	coding	0.152	0	3813	7427
MSTRG.33398.2	coding	0.449	0	540	2163
MSTRG.33398.1	coding	0.403	0	609	2243
MSTRG.33400.2	noncoding	-0.1523712	549	2256	3426
MSTRG.33400.1	noncoding	-0.1490944	606	2424	3266
MSTRG.33400.5	noncoding	-0.1490944	561	2379	2798
MSTRG.33403.5	coding	0.201	1194	3561	3916
MSTRG.33404.1	noncoding	-0.0106496	756	921	2238
MSTRG.33405.2	coding	0.365	345	2043	2457
MSTRG.33405.3	coding	0.361	120	1656	3297
MSTRG.33417.1	noncoding	-0.0315392	318	324	518
MSTRG.33419.13	coding	0.264	0	261	560
MSTRG.33419.20	coding	0.189	228	459	691
MSTRG.33419.23	coding	0.224	96	324	553
MSTRG.33419.24	coding	0.233	96	327	556
MSTRG.33408.3	coding	0.468	894	2199	2846
MSTRG.33423.3	coding	0.368	12	4515	4668
MSTRG.33423.4	coding	0.378	0	4641	4794
MSTRG.33424.1	coding	0.32	33	3861	5153
MSTRG.33424.2	coding	0.344	51	3738	4988
MSTRG.33424.3	coding	0.43	0	3771	4299
MSTRG.33443.13	coding	0.267	465	10239	10894
MSTRG.33443.9	coding	0.233	1014	13428	14085
MSTRG.33443.3	coding	0.229	486	13581	14237
MSTRG.33443.11	coding	0.228	387	13527	14184
MSTRG.33443.14	coding	0.228	507	13647	14304
MSTRG.33443.6	coding	0.228	321	13461	14118
MSTRG.33443.5	coding	0.228	222	13347	14003
MSTRG.33443.15	coding	0.228	26766	39906	40284
MSTRG.33448.8	coding	0.285	321	3435	5953
MSTRG.33450.1	coding	0.209	0	2550	4350
MSTRG.33451.1	noncoding	-0.02048	723	822	3584
MSTRG.33452.1	noncoding	-0.04096	1842	1899	4050
MSTRG.33453.1	coding	0.062	300	4911	9055

MSTRG.33453.2	coding	0.062	300	4911	9795
MSTRG.33453.3	coding	0.062	300	4911	9795
MSTRG.33453.4	coding	0.062	300	4911	8822
MSTRG.33453.5	coding	0.062	300	4911	13472
MSTRG.33453.6	coding	0.049	300	5010	5583
MSTRG.33430.1	noncoding	-0.036864	84	162	317
MSTRG.33439.2	coding	0.329	150	1902	2140
MSTRG.33439.3	coding	0.339	9	1293	3945
MSTRG.33444.1	noncoding	-0.069632	828	879	1359
MSTRG.33466.2	coding	0.135	729	1053	2050
MSTRG.33467.1	noncoding	-0.0004096	0	0	203
MSTRG.33482.1	coding	0.304	120	1695	2061
MSTRG.33482.3	coding	0.279	33	1794	2159
MSTRG.33483.1	coding	0.107	312	753	1243
MSTRG.33482.5	coding	0.319	0	2160	2524
MSTRG.33482.6	coding	0.343	0	1695	2059
MSTRG.33482.7	coding	0.338	0	1674	2038
MSTRG.33482.8	coding	0.346	0	1665	2029
MSTRG.33519.1	coding	0.231	201	2709	2898
MSTRG.33521.1	coding	0.218	0	2643	3684
MSTRG.33525.1	noncoding	-0.02048	0	60	447
MSTRG.33534.1	noncoding	-0.016384	261	393	1085
MSTRG.33534.2	noncoding	-0.016384	468	600	959
MSTRG.33535.4	noncoding	-0.0524288	2556	3219	6928
MSTRG.33535.5	noncoding	-0.0524288	2193	2856	5066
MSTRG.33535.6	coding	0.345	33	594	4549
MSTRG.33536.1	coding	0.183	96	321	480
MSTRG.33535.7	coding	0.356	0	549	4745
MSTRG.33537.1	coding	0.124	798	1059	1259
MSTRG.33535.8	noncoding	-0.1093632	0	375	1636
MSTRG.33535.9	coding	0.353	0	552	789
MSTRG.33546.1	coding	0.105	51	1986	4691
MSTRG.33546.3	coding	0.109	63	2031	4737
MSTRG.33548.4	coding	0.042	258	7842	9209
MSTRG.33553.1	coding	0.065	546	4539	6923
MSTRG.33553.2	coding	0.065	450	4443	6827
MSTRG.33553.3	coding	0.071	441	4302	6692
MSTRG.33555.1	noncoding	-0.0278528	2124	2361	4517
MSTRG.33553.8	coding	0.094	0	3915	4370
MSTRG.33550.2	coding	0.336	0	429	715
MSTRG.33559.4	coding	0.312	3144	5079	5313
MSTRG.33568.1	noncoding	-0.02048	243	270	452
MSTRG.33573.1	coding	0.241	1209	1854	11930
MSTRG.33577.2	coding	0.09	276	1194	8498
MSTRG.33577.1	coding	0.153	0	720	4437
MSTRG.33587.3	noncoding	-0.0188416	75	246	622
MSTRG.33609.2	coding	0.09	1998	2700	6822
MSTRG.33614.1	coding	0.271	45	1074	1675
MSTRG.33613.2	coding	0.216	0	1314	1630
MSTRG.33619.1	coding	0.217	225	1161	1655
MSTRG.33619.2	coding	0.228	252	1071	1566
MSTRG.33627.2	coding	0.209	192	1140	1814
MSTRG.33629.2	coding	0.453	300	2130	5266
MSTRG.33629.1	coding	0.45	300	2136	3709

MSTRG.33629.4	coding	0.454	291	2124	4470
MSTRG.33629.6	coding	0.452	291	2127	3510
MSTRG.33629.3	coding	0.45	291	2127	3714
MSTRG.33629.5	coding	0.453	291	2127	3702
MSTRG.33629.7	coding	0.453	285	2121	3707
MSTRG.33629.10	coding	0.455	282	2118	3514
MSTRG.33629.8	coding	0.455	282	2118	3502
MSTRG.33629.9	coding	0.451	282	2118	3514
MSTRG.33629.11	coding	0.454	222	2055	2518
MSTRG.33634.2	coding	0.168	66	4248	4834
MSTRG.33634.4	coding	0.154	519	1575	1973
MSTRG.33639.6	coding	0.174	1062	2241	2918
MSTRG.33639.7	noncoding	-0.075776	3303	3627	3782
MSTRG.33648.2	coding	0.063	225	903	1113
MSTRG.33648.5	coding	0.063	225	903	1162
MSTRG.33649.1	coding	0.281	114	1380	3453
MSTRG.33649.3	coding	0.281	87	1323	3208
MSTRG.33654.2	coding	0.07	5628	6165	14771
MSTRG.33658.1	coding	0.197	66	735	911
MSTRG.33662.3	coding	0.052	252	2319	6463
MSTRG.33662.4	coding	0.059	321	2373	7945
MSTRG.33661.1	coding	0.115	45	564	3954
MSTRG.33664.6	coding	0.311	0	2124	2617
MSTRG.33664.2	coding	0.342	0	1869	3685
MSTRG.33664.3	coding	0.318	0	2100	5684
MSTRG.33664.4	coding	0.334	0	1893	3709
MSTRG.33664.7	coding	0.356	0	1791	3607
MSTRG.33664.8	coding	0.334	54	1791	3606
MSTRG.33664.9	coding	0.331	9	1689	3504
MSTRG.33664.10	noncoding	-0.045056	2328	2529	4111
MSTRG.33666.1	coding	0.213	0	381	1828
MSTRG.33669.2	noncoding	-0.0278528	4335	4581	4736
MSTRG.33669.1	noncoding	-0.0278528	7659	7905	8060
MSTRG.33670.2	coding	0.336	0	3576	3912
MSTRG.33676.1	coding	0.103	903	2775	3330
MSTRG.33676.2	coding	0.103	966	2838	3353
MSTRG.33676.3	coding	0.103	381	2253	2768
MSTRG.33679.1	noncoding	-0.0700416	0	132	1241
MSTRG.33679.2	noncoding	-0.0036864	369	513	1186
MSTRG.33681.1	coding	0.104	204	2859	3116
MSTRG.33682.1	noncoding	-0.016384	363	405	1347
MSTRG.33683.1	coding	0.058	255	3801	7932
MSTRG.33683.2	coding	0.052	15	3471	7305
MSTRG.33683.3	coding	0.04	90	1746	5604
MSTRG.33686.2	coding	0.398	33	1713	1869
MSTRG.33687.3	coding	0.107	1332	1731	3167
MSTRG.33688.2	coding	0.156	3351	8022	11588
MSTRG.33690.1	noncoding	-0.028672	4803	5052	22728
MSTRG.33690.2	noncoding	-0.047104	4671	4869	8361
MSTRG.33692.1	noncoding	-0.0684032	19425	19653	27856
MSTRG.33692.2	noncoding	-0.0684032	14400	14628	39747
MSTRG.33692.3	noncoding	-0.0147456	13188	13335	30001
MSTRG.33709.1	noncoding	-0.0794624	312	1383	1551
MSTRG.33709.2	noncoding	-0.0692224	0	1332	1486

MSTRG.33709.3	noncoding	-0.0753664	216	1419	1574
MSTRG.33709.4	noncoding	-0.06144	411	1275	1448
MSTRG.33709.5	noncoding	-0.0794624	282	1353	1521
MSTRG.33709.6	noncoding	-0.0802816	213	1230	1386
MSTRG.33712.1	coding	0.145	207	1548	2127
MSTRG.33712.2	coding	0.033	147	1416	3609
MSTRG.33713.1	coding	0.098	189	825	1470
MSTRG.33714.1	noncoding	-0.049152	2697	2790	3659
MSTRG.33729.1	coding	0.285	114	1188	4859
MSTRG.33731.1	coding	0.173	483	2988	3205
MSTRG.33744.3	coding	0.15	0	1305	2106
MSTRG.33754.2	noncoding	-0.012288	96	363	592
MSTRG.33754.1	noncoding	-0.0098304	96	363	592
MSTRG.33754.4	noncoding	-0.0131072	96	363	592
MSTRG.33754.5	noncoding	-0.0131072	96	363	592
MSTRG.33754.3	noncoding	-0.0131072	165	432	663
MSTRG.33754.6	noncoding	-0.0196608	0	243	471
MSTRG.33756.2	coding	0.399	303	2298	2680
MSTRG.33760.1	noncoding	-0.294912	54	84	456
MSTRG.33760.2	noncoding	-0.0016384	360	453	681
MSTRG.33764.1	noncoding	-0.004096	42	312	535
MSTRG.33758.1	noncoding	-0.004096	48	321	552
MSTRG.33768.1	coding	0.327	69	1902	3776
MSTRG.33768.2	coding	0.327	12846	14679	20105
MSTRG.33768.3	coding	0.244	14283	15015	18637
MSTRG.33768.4	coding	0.417	0	1461	2571
MSTRG.33769.1	noncoding	-0.0585728	3240	3480	7810
MSTRG.33770.1	noncoding	-0.057344	5631	5871	6551
MSTRG.33768.5	noncoding	-0.0057344	2124	2403	3276
MSTRG.33768.6	noncoding	-0.0323584	3945	4212	5330
MSTRG.33771.1	noncoding	-0.026624	1014	1179	6720
MSTRG.33772.1	noncoding	-0.053248	3204	3321	4106
MSTRG.33773.1	noncoding	-0.0192512	2223	2781	7223
MSTRG.33787.1	noncoding	-0.0352256	387	603	1839
MSTRG.33789.1	noncoding	-0.0700416	0	180	777
MSTRG.33792.1	noncoding	-0.086016	474	537	696
MSTRG.33793.1	noncoding	-0.036864	12	135	945
MSTRG.33791.1	coding	0.178	27	540	883
MSTRG.33813.2	coding	0.523	0	1050	6279
MSTRG.33813.4	coding	0.443	0	1104	8526
MSTRG.33813.5	coding	0.418	1194	2061	9570
MSTRG.33813.6	coding	0.432	69	576	6574
MSTRG.33815.1	noncoding	-0.0319488	6549	6759	7704
MSTRG.33817.1	noncoding	-0.0270336	1617	1878	2034
MSTRG.33819.2	coding	0.256	0	762	1513
MSTRG.33820.1	coding	0.356	93	1740	5127
MSTRG.33820.2	coding	0.368	0	1407	4650
MSTRG.33820.4	coding	0.388	0	1479	4357
MSTRG.33822.1	coding	0.192	0	273	432
MSTRG.33824.1	noncoding	-0.12288	327	414	801
MSTRG.33817.2	coding	0.654	0	2478	4176
MSTRG.33817.4	noncoding	-0.0249856	1977	2559	4421
MSTRG.33804.1	noncoding	-0.036864	192	318	637
MSTRG.33803.1	noncoding	-0.0004096	0	0	224

MSTRG.33805.1	noncoding	-0.0319488	75	132	323
MSTRG.33806.1	noncoding	-0.0299008	60	117	366
MSTRG.33806.2	noncoding	-0.090112	282	351	505
MSTRG.33809.1	noncoding	-0.200704	237	306	462
MSTRG.33810.1	noncoding	-0.2048	24	48	1288
MSTRG.33811.1	noncoding	-0.114688	678	723	1104
MSTRG.33812.1	noncoding	-0.0200704	456	579	807
MSTRG.33812.2	noncoding	-0.016384	66	192	412
MSTRG.33846.1	noncoding	-0.0884736	1572	2862	5744
MSTRG.33829.1	noncoding	-0.0073728	1119	1308	2136
MSTRG.33831.1	noncoding	-0.055296	4530	5436	5593
MSTRG.33831.2	noncoding	-0.0360448	2646	3819	3972
MSTRG.33831.3	noncoding	-0.049152	1071	2067	2221
MSTRG.33831.4	noncoding	-0.0507904	792	1437	1592
MSTRG.33832.1	noncoding	-0.067584	669	2085	2571
MSTRG.33832.2	noncoding	-0.067584	150	1566	2761
MSTRG.33838.1	noncoding	-0.129024	0	72	336
MSTRG.33839.1	noncoding	-0.004096	138	273	429
MSTRG.33839.2	noncoding	-0.024576	123	210	364
MSTRG.33841.1	noncoding	-0.0176128	2346	2790	2961
MSTRG.33843.1	noncoding	-0.114688	2265	2355	4023
MSTRG.33851.1	noncoding	-0.0344064	6	198	405
MSTRG.33852.1	noncoding	-0.024576	168	519	1050
MSTRG.33858.1	noncoding	-0.012288	174	219	642
MSTRG.33860.1	noncoding	-0.0057344	72	606	1097
MSTRG.33861.1	noncoding	-0.0237568	951	1146	1783
MSTRG.33863.1	noncoding	-0.018432	405	552	890
MSTRG.33865.1	noncoding	-0.0077824	69	408	635
MSTRG.33868.1	noncoding	-0.032768	1746	2574	8432
MSTRG.33868.2	noncoding	-0.0557056	759	1179	5151
MSTRG.33869.1	noncoding	-0.192512	102	147	301
MSTRG.33869.2	noncoding	-0.192512	81	126	281
MSTRG.33875.1	noncoding	-0.1040384	1806	2643	3793
MSTRG.33875.2	noncoding	-0.0954368	1806	2415	5331
MSTRG.33875.4	coding	0.272	1053	1809	2959
MSTRG.33875.6	noncoding	-0.0360448	162	1035	3427
MSTRG.33874.1	noncoding	-0.0483328	0	180	334
MSTRG.33879.1	noncoding	-0.0032768	957	1101	1291
MSTRG.33882.1	coding	0.019	438	1461	2586
MSTRG.33883.1	noncoding	-0.016384	0	60	336
MSTRG.33886.1	noncoding	-0.004096	297	402	1217
MSTRG.33880.1	noncoding	-0.0176128	504	852	1008
MSTRG.33881.1	noncoding	-0.0004096	81	225	410
MSTRG.33896.2	coding	0.239	246	744	5352
MSTRG.33896.3	coding	0.187	2610	3060	6517
MSTRG.33896.1	coding	0.239	363	861	5839
MSTRG.33896.4	coding	0.239	483	981	3857
MSTRG.33896.5	coding	0.239	456	954	3330
MSTRG.33896.6	coding	0.183	2328	2805	3358
MSTRG.33894.2	noncoding	-0.0118784	585	762	1032
MSTRG.33898.1	noncoding	-0.12288	552	603	759
MSTRG.33898.2	noncoding	-0.12288	552	603	759
MSTRG.33898.3	noncoding	-0.03072	1758	1917	2105
MSTRG.33898.4	noncoding	-0.114688	177	216	371

MSTRG.33898.5	noncoding	-0.0425984	558	624	923
MSTRG.33898.6	noncoding	-0.0712704	168	192	871
MSTRG.33899.1	noncoding	-0.03072	420	579	1504
MSTRG.33899.2	noncoding	-0.036864	441	528	2090
MSTRG.33899.3	noncoding	-0.036864	420	507	2091
MSTRG.33899.4	noncoding	-0.036864	420	507	2091
MSTRG.33898.7	noncoding	-0.0425984	90	156	414
MSTRG.33901.1	noncoding	-0.0221184	0	57	839
MSTRG.33901.2	noncoding	-0.0380928	0	21	804
MSTRG.33901.3	noncoding	-0.073728	666	723	1772
MSTRG.33901.4	noncoding	-0.0237568	348	501	1373
MSTRG.33901.5	coding	0.171	162	264	832
MSTRG.33901.6	noncoding	-0.069632	141	198	368
MSTRG.33901.7	noncoding	-0.0004096	0	0	201
MSTRG.33901.8	noncoding	-0.024576	48	180	804
MSTRG.33901.9	noncoding	-0.077824	165	246	482
MSTRG.33903.1	coding	0.368	330	1302	2170
MSTRG.33910.1	noncoding	-0.09216	0	87	244
MSTRG.33910.2	noncoding	-0.0106496	42	168	322
MSTRG.33914.2	coding	0.369	93	1083	2269
MSTRG.33914.1	coding	0.37	108	1122	2309
MSTRG.33914.3	coding	0.386	0	984	1692
MSTRG.33917.1	noncoding	-0.11264	0	162	319
MSTRG.33911.1	noncoding	-0.012288	9	120	276
MSTRG.33920.2	noncoding	-0.036864	1980	2106	3013
MSTRG.33920.3	noncoding	-0.036864	1188	1314	2200
MSTRG.33921.2	noncoding	-0.0139264	1287	1566	1720
MSTRG.33921.3	noncoding	-0.002048	3294	3438	3594
MSTRG.33922.1	noncoding	-0.016384	402	522	719
MSTRG.33922.2	noncoding	-0.049152	195	276	432
MSTRG.33949.1	noncoding	-0.012288	21	66	449
MSTRG.33949.2	noncoding	-0.520192	189	210	363
MSTRG.33949.3	noncoding	-0.08192	390	453	609
MSTRG.33950.2	noncoding	-0.094208	474	777	4605
MSTRG.33950.5	noncoding	-0.026624	1437	1980	5598
MSTRG.33950.6	noncoding	-0.0425984	4845	5385	5540
MSTRG.33950.7	coding	0.125	429	1095	4713
MSTRG.33950.12	noncoding	-0.008192	849	1032	1186
MSTRG.33950.10	noncoding	-0.008192	3480	3666	3821
MSTRG.33950.13	noncoding	-0.0991232	408	783	12321
MSTRG.33950.14	coding	0.147	408	1170	12470
MSTRG.33950.9	coding	0.253	408	879	12387
MSTRG.33950.11	noncoding	-0.0581632	456	654	2288
MSTRG.33950.15	noncoding	-0.0180224	10179	10347	15199
MSTRG.33950.17	noncoding	-0.0045056	4410	4554	5081
MSTRG.33950.19	noncoding	-0.028672	81	132	868
MSTRG.33953.1	noncoding	-0.016384	96	228	1731
MSTRG.33931.1	noncoding	-0.0487424	2634	2814	4364
MSTRG.33932.1	noncoding	-0.032768	42	78	373
MSTRG.33931.2	noncoding	-0.004096	120	258	1808
MSTRG.33933.1	noncoding	-0.0126976	201	351	778
MSTRG.33934.1	noncoding	-0.024576	63	195	538
MSTRG.33928.1	noncoding	-0.0147456	48	111	331
MSTRG.33929.1	noncoding	-0.0012288	189	474	2631

MSTRG.33935.1	noncoding	-0.0442368	0	165	434
MSTRG.33941.2	noncoding	-0.073728	933	1023	1829
MSTRG.33944.1	noncoding	-0.0528384	279	459	909
MSTRG.33946.1	noncoding	-0.028672	330	441	596
MSTRG.33946.2	noncoding	-0.028672	330	441	596
MSTRG.33946.3	noncoding	-0.090112	72	111	360
MSTRG.33947.1	noncoding	-0.0229376	0	150	305
MSTRG.33960.1	noncoding	-0.012288	3315	3363	4133
MSTRG.33960.2	noncoding	-0.0258048	258	576	9154
MSTRG.33960.7	noncoding	-0.0258048	1083	1401	1725
MSTRG.33960.8	noncoding	-0.0258048	570	888	1213
MSTRG.33960.9	noncoding	-0.0258048	468	786	1108
MSTRG.33960.10	noncoding	-0.0335872	423	609	1301
MSTRG.33960.11	noncoding	-0.0372736	423	612	1304
MSTRG.33963.1	noncoding	-0.0311296	150	255	422
MSTRG.33964.1	noncoding	-0.006144	171	279	1989
MSTRG.33965.1	noncoding	-0.0036864	834	903	1194
MSTRG.33965.2	noncoding	-0.0036864	441	510	775
MSTRG.33966.1	noncoding	-0.024576	15	48	278
MSTRG.33962.1	noncoding	-0.0237568	75	156	311
MSTRG.33962.2	noncoding	-0.0180224	75	156	309
MSTRG.33967.1	noncoding	-0.0004096	24	114	268
MSTRG.33985.4	coding	0.153	516	1173	9208
MSTRG.33985.7	coding	0.138	516	1074	16711
MSTRG.33985.10	coding	0.153	498	1155	5833
MSTRG.33985.9	coding	0.158	498	1002	2634
MSTRG.33985.11	coding	0.176	405	876	16669
MSTRG.33979.1	noncoding	-0.0180224	0	513	846
MSTRG.33986.1	coding	0.065	87	303	1296
MSTRG.33986.2	noncoding	-0.0237568	39	192	625
MSTRG.33986.3	noncoding	-0.077824	213	288	451
MSTRG.33986.4	coding	0.058	285	480	700
MSTRG.33987.1	noncoding	-0.01024	261	363	696
MSTRG.33986.5	coding	0.058	285	480	665
MSTRG.33992.1	noncoding	-0.0823296	0	42	482
MSTRG.33993.1	noncoding	-0.077824	432	507	874
MSTRG.33993.2	noncoding	-0.016384	216	327	875
MSTRG.33998.1	noncoding	-0.0606208	0	204	359
MSTRG.33999.1	noncoding	-0.0475136	24	93	249
MSTRG.34000.2	noncoding	-0.0442368	861	1023	2935
MSTRG.34000.3	noncoding	-0.0512	1071	1440	5338
MSTRG.34000.1	noncoding	-0.036864	948	1158	2413
MSTRG.34000.4	noncoding	-0.0331776	2157	2709	6316
MSTRG.34000.5	noncoding	-0.0331776	2157	2709	6316
MSTRG.34000.6	noncoding	-0.0512	3078	3447	7346
MSTRG.34016.1	noncoding	-0.0155648	0	33	638
MSTRG.34016.2	noncoding	-0.0180224	0	27	524
MSTRG.34016.4	noncoding	-0.0495616	0	9	502
MSTRG.34016.5	noncoding	-0.0339968	780	1365	1640
MSTRG.34016.6	noncoding	-0.0339968	780	1365	1640
MSTRG.34016.3	noncoding	-0.118784	990	1044	1666
MSTRG.34016.7	noncoding	-0.0503808	0	6	504
MSTRG.34016.8	noncoding	-0.0610304	219	321	606
MSTRG.34016.9	noncoding	-0.1052672	0	321	732

MSTRG.34016.10	noncoding	-0.151552	1098	1146	1301
MSTRG.34016.11	noncoding	-0.0425984	561	627	936
MSTRG.34016.12	noncoding	-0.0425984	549	615	924
MSTRG.34016.13	noncoding	-0.0425984	54	120	431
MSTRG.34016.15	noncoding	-0.036864	912	999	1588
MSTRG.34016.14	noncoding	-0.024576	915	1008	1599
MSTRG.34016.16	noncoding	-0.036864	903	990	1580
MSTRG.34016.17	noncoding	-0.036864	438	525	1108
MSTRG.34016.18	noncoding	-0.036864	420	507	1097
MSTRG.34012.1	noncoding	-0.0417792	225	588	744
MSTRG.34013.1	noncoding	-0.0589824	0	162	350
MSTRG.34017.1	noncoding	-0.004096	48	171	867
MSTRG.34017.3	noncoding	-0.06144	66	153	308
MSTRG.34017.2	noncoding	-0.0339968	0	192	1438
MSTRG.34017.4	noncoding	-0.0528384	264	444	611
MSTRG.34017.5	noncoding	-0.0339968	0	192	1020
MSTRG.34018.1	noncoding	-0.016384	144	291	556
MSTRG.34026.2	noncoding	-0.0208896	108	249	402
MSTRG.34028.1	coding	0.111	0	252	476
MSTRG.34035.1	coding	0.136	0	795	952
MSTRG.34037.1	noncoding	-0.114688	0	57	213
MSTRG.34038.1	noncoding	-0.0630784	0	237	392
MSTRG.34046.1	noncoding	-0.1122304	0	354	1948
MSTRG.34046.2	noncoding	-0.110592	0	363	2047
MSTRG.34046.3	noncoding	-0.1122304	0	354	1724
MSTRG.34046.4	noncoding	-0.106496	0	321	5800
MSTRG.34046.5	noncoding	-0.1081344	0	324	2116
MSTRG.34046.6	noncoding	-0.110592	0	351	6142
MSTRG.34047.2	noncoding	-0.053248	195	303	456
MSTRG.34052.1	noncoding	-0.0106496	366	624	1149
MSTRG.34053.1	noncoding	-0.0712704	207	231	419
MSTRG.34053.2	noncoding	-0.0712704	207	231	397
MSTRG.34055.1	coding	0.147	123	612	843
MSTRG.34056.2	noncoding	-0.0516096	1725	2079	2800
MSTRG.34066.1	noncoding	-0.0192512	501	651	4438
MSTRG.34072.1	coding	0.23	27	153	323
MSTRG.34072.2	noncoding	-0.0380928	0	213	368
MSTRG.34072.3	noncoding	-0.0262144	0	168	322
MSTRG.34072.4	noncoding	-0.0282624	0	168	322
MSTRG.34072.5	noncoding	-0.0974848	0	120	276
MSTRG.34088.1	noncoding	-0.0004096	0	0	497
MSTRG.34090.1	noncoding	-0.0004096	0	0	295
MSTRG.34088.2	noncoding	-0.1114112	39	45	278
MSTRG.34084.1	noncoding	-0.0004096	0	0	243
MSTRG.34085.1	noncoding	-0.0004096	0	0	214
MSTRG.34093.1	coding	0.169	675	1164	1485
MSTRG.34094.1	coding	0.096	153	1137	1495
MSTRG.34101.1	noncoding	-0.0352256	3	60	975
MSTRG.34097.1	noncoding	-0.0004096	0	72	229
MSTRG.34108.2	coding	0.218	0	1209	1671
MSTRG.34110.1	noncoding	-0.03072	171	267	767
MSTRG.34110.2	noncoding	-0.2048	12	48	593
MSTRG.34129.1	noncoding	-0.028672	321	453	701
MSTRG.34130.3	noncoding	-0.090112	750	831	1076

MSTRG.34130.1	noncoding	-0.090112	750	831	1074
MSTRG.34130.2	noncoding	-0.090112	750	831	1075
MSTRG.34131.1	noncoding	-0.090112	294	375	620
MSTRG.34131.2	noncoding	-0.090112	192	273	519
MSTRG.34130.4	noncoding	-0.02048	669	801	2247
MSTRG.34130.5	noncoding	-0.090112	750	831	1076
MSTRG.34135.3	noncoding	-0.0364544	0	255	411
MSTRG.34135.1	noncoding	-0.038912	0	258	414
MSTRG.34135.2	noncoding	-0.038912	0	258	414
MSTRG.34135.4	noncoding	-0.034816	0	249	413
MSTRG.34136.1	noncoding	-0.0569344	399	564	1350
MSTRG.34135.5	noncoding	-0.0253952	0	303	457
MSTRG.34137.3	noncoding	-0.0712704	219	243	878
MSTRG.34137.1	noncoding	-0.0712704	219	243	870
MSTRG.34137.2	noncoding	-0.0712704	219	243	870
MSTRG.34135.6	noncoding	-0.0344064	0	459	615
MSTRG.34133.1	noncoding	-0.012288	117	162	377
MSTRG.34146.1	coding	0.101	0	3021	3239
MSTRG.34149.1	noncoding	-0.0344064	5223	5529	7834
MSTRG.34152.1	noncoding	-0.0004096	0	0	1043
MSTRG.34153.1	coding	0.205	0	144	305
MSTRG.34185.7	noncoding	-0.1196032	2973	3438	6469
MSTRG.34185.8	noncoding	-0.0651264	123	780	1067
MSTRG.34185.10	coding	0.084	156	453	1440
MSTRG.34185.9	noncoding	-0.038912	264	456	738
MSTRG.34185.11	noncoding	-0.0917504	0	588	1181
MSTRG.34185.12	noncoding	-0.0925696	0	588	1115
MSTRG.34185.13	noncoding	-0.0380928	111	303	617
MSTRG.34185.15	noncoding	-0.0733184	2532	3210	8522
MSTRG.34185.14	noncoding	-0.073728	2532	3162	3616
MSTRG.34185.16	noncoding	-0.0483328	258	450	723
MSTRG.34185.17	noncoding	-0.0733184	1875	2553	7866
MSTRG.34185.18	noncoding	-0.073728	6939	7569	8024
MSTRG.34185.19	noncoding	-0.043008	111	336	1079
MSTRG.34185.20	noncoding	-0.1929216	0	252	1010
MSTRG.34185.21	noncoding	-0.0733184	645	1323	1612
MSTRG.34185.22	noncoding	-0.073728	2424	3054	3509
MSTRG.34185.23	noncoding	-0.0241664	0	72	343
MSTRG.34154.1	noncoding	-0.065536	0	135	289
MSTRG.34155.1	noncoding	-0.053248	45	114	270
MSTRG.34154.2	noncoding	-0.0602112	0	108	264
MSTRG.34161.2	coding	0.213	639	1875	2453
MSTRG.34161.3	coding	0.236	0	1233	1687
MSTRG.34159.1	noncoding	-0.090112	672	753	1033
MSTRG.34164.1	noncoding	-0.0126976	63	213	1507
MSTRG.34164.2	coding	0.008	861	957	1639
MSTRG.34171.2	coding	0.149	1260	2259	2633
MSTRG.34171.5	coding	0.148	1272	2274	2704
MSTRG.34171.6	coding	0.147	333	1326	2472
MSTRG.34174.1	noncoding	-0.032768	0	99	253
MSTRG.34178.1	noncoding	-0.0598016	0	228	673
MSTRG.34183.2	coding	0.168	1239	2610	2766
MSTRG.34183.3	coding	0.172	15	1365	1519
MSTRG.34183.4	noncoding	-0.008192	249	252	658

MSTRG.34190.1	coding	0.143	72	3048	3216
MSTRG.34190.2	coding	0.143	72	3057	3225
MSTRG.34190.6	coding	0.125	780	2145	2437
MSTRG.34190.9	coding	0.123	78	1125	2044
MSTRG.34169.1	noncoding	-0.0610304	291	708	916
MSTRG.34196.1	noncoding	-0.0475136	237	528	3898
MSTRG.34198.3	coding	0.198	111	405	629
MSTRG.34198.4	coding	0.187	90	363	587
MSTRG.34206.1	noncoding	-0.098304	0	3	453
MSTRG.34202.1	noncoding	-0.0815104	870	936	2175
MSTRG.34209.1	noncoding	-0.004096	57	159	751
MSTRG.34209.2	noncoding	-0.0847872	207	285	475
MSTRG.34210.1	noncoding	-0.0139264	75	198	352
MSTRG.34210.2	noncoding	-0.008192	141	219	374
MSTRG.34219.1	noncoding	-0.1470464	0	30	283
MSTRG.34220.1	noncoding	-0.077824	27	108	305
MSTRG.34219.2	noncoding	-0.1470464	0	30	283
MSTRG.34220.2	noncoding	-0.077824	18	99	294
MSTRG.34220.3	noncoding	-0.04096	123	201	396
MSTRG.34219.3	noncoding	-0.0262144	156	192	374
MSTRG.34225.1	noncoding	-0.073728	474	552	730
MSTRG.34226.1	noncoding	-0.024576	189	288	802
MSTRG.34235.1	noncoding	-0.0004096	0	0	292
MSTRG.34238.1	noncoding	-0.0606208	0	390	544
MSTRG.34252.1	noncoding	-0.0233472	150	336	1253
MSTRG.34252.2	coding	0.062	0	246	1161
MSTRG.34252.3	noncoding	-0.0004096	1149	1335	1490
MSTRG.34252.4	noncoding	-0.0004096	1149	1335	1490
MSTRG.34272.1	noncoding	-0.0352256	12594	12807	14431
MSTRG.34272.2	noncoding	-0.0352256	12594	12807	14677
MSTRG.34272.4	noncoding	-0.0352256	12567	12780	15865
MSTRG.34272.3	noncoding	-0.0352256	12594	12807	13428
MSTRG.34272.6	noncoding	-0.0352256	12594	12807	13387
MSTRG.34272.5	noncoding	-0.0352256	13431	13644	14223
MSTRG.34272.7	noncoding	-0.0352256	10956	11169	11741
MSTRG.34272.8	noncoding	-0.0352256	12582	12795	13359
MSTRG.34272.9	noncoding	-0.0352256	12678	12891	13285
MSTRG.34293.1	noncoding	-0.0004096	0	0	1627
MSTRG.34294.1	noncoding	-0.0004096	342	342	776
MSTRG.34294.2	noncoding	-0.073728	258	291	651
MSTRG.34295.1	noncoding	-0.004096	6180	6291	9109
MSTRG.34295.2	noncoding	-0.0253952	3177	3255	4015
MSTRG.34295.4	noncoding	-0.094208	1266	1308	1465
MSTRG.34295.3	noncoding	-0.063488	549	609	1549
MSTRG.34295.5	noncoding	-0.147456	1383	1446	1600
MSTRG.34294.3	coding	0.108	3504	3972	21921
MSTRG.34294.4	noncoding	-0.0487424	16149	16329	17825
MSTRG.34294.6	noncoding	-0.0036864	2295	2511	15603
MSTRG.34294.5	noncoding	-0.0036864	2295	2511	15556
MSTRG.34295.6	noncoding	-0.0159744	1368	1482	4353
MSTRG.34294.8	noncoding	-0.016384	618	786	1131
MSTRG.34294.7	coding	0.078	12876	13182	14619
MSTRG.34294.9	noncoding	-0.024576	19203	19386	23293
MSTRG.34295.7	noncoding	-0.1155072	141	153	357

MSTRG.34294.10	coding	0.078	7236	7542	9109
MSTRG.34294.11	noncoding	-0.004096	858	996	1574
MSTRG.34298.1	noncoding	-0.006144	36	45	341
MSTRG.34298.2	noncoding	-0.006144	36	45	337
MSTRG.34299.1	noncoding	-0.077824	66	141	412
MSTRG.34282.1	noncoding	-0.0016384	48	174	1219
MSTRG.34283.1	noncoding	-0.016384	0	54	211
MSTRG.34284.1	noncoding	-0.008192	171	303	522
MSTRG.34287.1	noncoding	-0.0385024	18	78	389
MSTRG.34300.1	noncoding	-0.004096	93	213	368
MSTRG.34302.1	noncoding	-0.004096	378	525	681
MSTRG.34303.1	noncoding	-0.08192	225	273	430
MSTRG.34314.1	noncoding	-0.008192	180	219	387
MSTRG.34314.3	coding	0.058	264	459	3225
MSTRG.34314.4	noncoding	-0.0413696	4101	4311	4987
MSTRG.34314.2	noncoding	-0.0413696	4101	4311	5283
MSTRG.34315.1	coding	0.29	30495	31074	37600
MSTRG.34315.2	noncoding	-0.1101824	10557	11265	11420
MSTRG.34315.3	noncoding	-0.1404928	2337	3618	3774
MSTRG.34315.4	noncoding	-0.135168	10020	11235	11534
MSTRG.34317.2	noncoding	-0.1150976	5343	6174	7198
MSTRG.34317.3	noncoding	-0.0684032	594	1125	3456
MSTRG.34315.5	noncoding	-0.0794624	2475	3246	17952
MSTRG.34315.6	noncoding	-0.0221184	3	345	1939
MSTRG.34315.7	noncoding	-0.0106496	1992	2268	5982
MSTRG.34315.8	noncoding	-0.0782336	306	993	2503
MSTRG.34315.10	noncoding	-0.0892928	0	588	2078
MSTRG.34315.11	noncoding	-0.0794624	4893	5664	7155
MSTRG.34315.9	noncoding	-0.0794624	2349	3120	4610
MSTRG.34304.1	noncoding	-0.0004096	0	0	202
MSTRG.34305.1	noncoding	-0.012288	81	126	550
MSTRG.34306.2	noncoding	-0.0974848	648	693	857
MSTRG.34306.1	noncoding	-0.0974848	234	279	442
MSTRG.34307.1	noncoding	-0.0131072	33	75	450
MSTRG.34307.2	noncoding	-0.0131072	15	57	479
MSTRG.34308.1	noncoding	-0.0974848	327	372	591
MSTRG.34321.1	coding	0.085	1083	1512	1929
MSTRG.34328.1	noncoding	-0.0008192	696	795	951
MSTRG.34330.1	noncoding	-0.0335872	0	30	336
MSTRG.34327.1	coding	0.316	0	339	582
MSTRG.34334.2	coding	0.331	114	372	784
MSTRG.34346.1	noncoding	-0.0442368	390	552	767
MSTRG.34346.2	noncoding	-0.0442368	864	1026	1228
MSTRG.34346.3	noncoding	-0.0712704	225	249	414
MSTRG.34346.4	noncoding	-0.0712704	225	249	414
MSTRG.34342.1	noncoding	-0.065536	117	141	313
MSTRG.34342.2	noncoding	-0.065536	117	141	313
MSTRG.34351.1	coding	0.065	165	381	1515
MSTRG.34351.3	noncoding	-0.077824	408	483	803
MSTRG.34351.2	noncoding	-0.01024	372	597	754
MSTRG.34351.4	coding	0.065	207	423	1848
MSTRG.34351.5	noncoding	-0.01024	447	672	828
MSTRG.34351.6	noncoding	-0.0499712	168	171	325
MSTRG.34351.7	noncoding	-0.0004096	0	0	248

MSTRG.34351.8	noncoding	-0.0118784	102	129	283
MSTRG.34349.1	noncoding	-0.06144	18	93	384
MSTRG.34352.1	noncoding	-0.0311296	555	921	2105
MSTRG.34352.2	noncoding	-0.036864	0	249	548
MSTRG.34369.1	coding	0.168	8511	10032	11802
MSTRG.34369.2	coding	0.24	7554	8079	8795
MSTRG.34369.3	coding	0.246	8451	8946	12369
MSTRG.34369.4	coding	0.24	8352	8877	9595
MSTRG.34369.5	coding	0.246	6939	7434	8894
MSTRG.34369.7	coding	0.228	1422	1977	2694
MSTRG.34369.8	coding	0.145	4374	7008	7818
MSTRG.34369.9	coding	0.224	51	516	5634
MSTRG.34369.10	coding	0.135	45	510	1877
MSTRG.34369.11	coding	0.15	249	978	1749
MSTRG.34356.1	noncoding	-0.0024576	432	513	782
MSTRG.34356.2	noncoding	-0.008192	0	60	433
MSTRG.34357.1	noncoding	-0.0077824	201	447	836
MSTRG.34355.1	coding	0.307	0	273	1118
MSTRG.34363.2	coding	0.439	0	327	1640
MSTRG.34363.3	coding	0.446	150	1899	2409
MSTRG.34365.2	coding	0.166	0	612	3968
MSTRG.34365.3	noncoding	-0.0413696	0	297	4058
MSTRG.34365.4	noncoding	-0.0004096	0	114	267
MSTRG.34361.1	noncoding	-0.0712704	66	297	470
MSTRG.34373.1	noncoding	-0.0196608	0	321	706
MSTRG.34373.2	noncoding	-0.0221184	0	423	808
MSTRG.34373.3	noncoding	-0.0196608	0	321	706
MSTRG.34374.1	noncoding	-0.024576	294	351	506
MSTRG.34374.2	noncoding	-0.04096	60	87	445
MSTRG.34374.3	noncoding	-0.0004096	36	36	354
MSTRG.34375.1	noncoding	-0.0712704	618	642	1077
MSTRG.34375.2	noncoding	-0.0712704	621	645	1079
MSTRG.34373.4	noncoding	-0.0004096	501	639	1025
MSTRG.34373.5	noncoding	-0.0004096	501	639	1025
MSTRG.34376.5	noncoding	-0.0712704	360	384	1065
MSTRG.34376.2	noncoding	-0.0712704	381	405	571
MSTRG.34376.4	noncoding	-0.0712704	381	405	1051
MSTRG.34376.3	noncoding	-0.0712704	381	405	1076
MSTRG.34376.6	noncoding	-0.0565248	1356	1512	3404
MSTRG.34376.7	noncoding	-0.0712704	381	405	1076
MSTRG.34376.1	noncoding	-0.02048	870	969	1124
MSTRG.34383.1	noncoding	-0.106496	528	585	945
MSTRG.34383.2	noncoding	-0.106496	594	651	877
MSTRG.34383.3	noncoding	-0.106496	144	201	422
MSTRG.34384.1	noncoding	-0.12288	162	237	393
MSTRG.34389.1	noncoding	-0.0004096	195	279	435
MSTRG.34390.1	noncoding	-0.0401408	27	255	1138
MSTRG.34391.1	noncoding	-0.0262144	96	183	384
MSTRG.34393.1	noncoding	-0.0176128	255	414	6576
MSTRG.34395.2	coding	0.045	429	870	1394
MSTRG.34394.1	coding	0.123	0	564	1015
MSTRG.34394.3	coding	0.123	0	564	1819
MSTRG.34400.1	noncoding	-0.012288	459	612	769
MSTRG.34401.2	noncoding	-0.0004096	0	0	263

MSTRG.34401.1	noncoding	-0.0004096	0	0	254
MSTRG.34402.1	noncoding	-0.032768	183	201	357
MSTRG.34402.2	noncoding	-0.032768	183	201	357
MSTRG.34401.3	noncoding	-0.1273856	0	42	599
MSTRG.34402.3	noncoding	-0.032768	291	309	466
MSTRG.34400.2	noncoding	-0.012288	837	990	1146
MSTRG.34411.1	noncoding	-0.004096	15	123	459
MSTRG.34414.1	noncoding	-0.0892928	1866	3195	7020
MSTRG.34414.2	noncoding	-0.0892928	318	1608	4926
MSTRG.34414.3	noncoding	-0.0892928	318	1602	4923
MSTRG.34416.1	noncoding	-0.190464	0	366	600
MSTRG.34435.10	coding	0.184	0	1479	2830
MSTRG.34435.7	coding	0.163	0	1149	2816
MSTRG.34435.8	coding	0.172	0	1419	2770
MSTRG.34435.9	coding	0.184	0	1479	2830
MSTRG.34435.2	coding	0.184	3	1482	2833
MSTRG.34418.1	noncoding	-0.0004096	198	318	475
MSTRG.34420.1	noncoding	-0.049152	99	192	347
MSTRG.34421.1	noncoding	-0.0405504	0	108	261
MSTRG.34422.1	noncoding	-0.0151552	0	426	582
MSTRG.34427.1	noncoding	-0.0872448	54	1251	1510
MSTRG.34427.2	noncoding	-0.0806912	54	1344	1936
MSTRG.34427.3	noncoding	-0.0802816	54	1338	1921
MSTRG.34429.2	noncoding	-0.098304	93	111	267
MSTRG.34429.1	noncoding	-0.0180224	186	336	622
MSTRG.34438.1	noncoding	-0.0004096	132	132	286
MSTRG.34439.1	noncoding	-0.098304	189	207	362
MSTRG.34449.1	noncoding	-0.016384	138	171	325
MSTRG.34449.2	noncoding	-0.0196608	210	405	1991
MSTRG.34449.3	coding	0.059	753	930	1232
MSTRG.34449.4	noncoding	-0.077824	348	423	819
MSTRG.34449.5	noncoding	-0.0196608	210	405	2208
MSTRG.34449.6	noncoding	-0.0196608	201	396	2202
MSTRG.34449.7	noncoding	-0.0196608	201	396	2199
MSTRG.34449.9	noncoding	-0.0196608	195	390	2595
MSTRG.34449.8	noncoding	-0.0196608	201	396	2603
MSTRG.34449.10	coding	0.163	351	441	1380
MSTRG.34446.1	noncoding	-0.0036864	18	162	880
MSTRG.34446.2	noncoding	-0.08192	0	63	245
MSTRG.34452.1	noncoding	-0.0741376	0	237	760
MSTRG.34452.2	noncoding	-0.0925696	0	144	297
MSTRG.34458.1	noncoding	-0.0667648	444	558	961
MSTRG.34460.1	noncoding	-0.098304	231	300	541
MSTRG.34455.1	coding	0.09	0	99	418
MSTRG.34455.2	coding	0.159	0	54	354
MSTRG.34467.1	noncoding	-0.0786432	0	216	567
MSTRG.34471.1	noncoding	-0.0815104	123	246	443
MSTRG.34478.1	noncoding	-0.1060864	4239	4545	4713
MSTRG.34482.3	noncoding	-0.0090112	528	618	951
MSTRG.34481.1	noncoding	-0.012288	267	357	576
MSTRG.34486.1	noncoding	-0.057344	0	75	228
MSTRG.34489.1	noncoding	-0.03072	858	1059	1531
MSTRG.34501.1	noncoding	-0.049152	0	120	435
MSTRG.34505.1	coding	0.164	0	1677	2056

MSTRG.34511.1	noncoding	-0.0135168	849	1329	1484
MSTRG.34511.2	noncoding	-0.036864	153	225	670
MSTRG.34511.3	noncoding	-0.036864	153	225	670
MSTRG.34511.4	noncoding	-0.0290816	0	258	633
MSTRG.34512.1	noncoding	-0.0442368	288	450	638
MSTRG.34513.1	noncoding	-0.0712704	207	231	421
MSTRG.34514.1	noncoding	-0.0385024	60	99	283
MSTRG.34515.1	noncoding	-0.172032	210	255	411
MSTRG.34519.2	noncoding	-0.0712704	243	267	663
MSTRG.34519.1	noncoding	-0.03072	1086	1245	1553
MSTRG.34519.3	noncoding	-0.0712704	243	267	480
MSTRG.34519.4	noncoding	-0.0712704	243	267	480
MSTRG.34519.5	noncoding	-0.049152	0	48	1107
MSTRG.34519.6	noncoding	-0.049152	0	48	1109
MSTRG.34520.1	noncoding	-0.0446464	48	168	488
MSTRG.34521.1	noncoding	-0.0258048	48	51	205
MSTRG.34519.7	noncoding	-0.0712704	243	267	480
MSTRG.34524.1	noncoding	-0.0581632	0	123	757
MSTRG.34524.2	noncoding	-0.0356352	42	216	505
MSTRG.34524.3	noncoding	-0.0356352	42	216	617
MSTRG.34524.4	noncoding	-0.0356352	42	216	727
MSTRG.34524.5	noncoding	-0.0356352	42	216	726
MSTRG.34528.2	noncoding	-0.0606208	42	78	233
MSTRG.34528.1	noncoding	-0.0090112	1011	1158	1579
MSTRG.34529.1	noncoding	-0.034816	348	540	1064
MSTRG.34537.1	noncoding	-0.08192	7266	7356	7510
MSTRG.34537.3	noncoding	-0.016384	1251	1362	2932
MSTRG.34537.2	noncoding	-0.08192	525	615	769
MSTRG.34537.4	noncoding	-0.08192	6264	6354	6508
MSTRG.34532.1	noncoding	-0.055296	0	84	248
MSTRG.34554.1	coding	0.091	6	879	1236
MSTRG.34556.1	coding	0.051	147	720	873
MSTRG.34554.2	noncoding	-0.03072	57	660	3684
MSTRG.34557.2	noncoding	-0.014336	1020	1170	1375
MSTRG.34557.1	noncoding	-0.1335296	732	1032	1185
MSTRG.34557.4	coding	0.422	111	2127	3135
MSTRG.34550.1	noncoding	-0.0004096	492	633	789
MSTRG.34550.2	noncoding	-0.077824	57	147	302
MSTRG.34561.1	noncoding	-0.0843776	0	183	408
MSTRG.34561.2	noncoding	-0.0585728	0	48	203
MSTRG.34563.1	noncoding	-0.028672	501	612	786
MSTRG.34621.2	noncoding	-0.047104	2700	2973	3137
MSTRG.34621.1	coding	0.122	351	1854	2331
MSTRG.34621.3	noncoding	-0.0618496	8340	9114	11620
MSTRG.34621.4	noncoding	-0.0278528	981	1224	1377
MSTRG.34621.5	noncoding	-0.0520192	3369	3645	4034
MSTRG.34622.1	noncoding	-0.057344	0	1293	1446
MSTRG.34621.8	noncoding	-0.0581632	0	2625	2780
MSTRG.34621.6	noncoding	-0.0548864	0	2517	2710
MSTRG.34621.7	noncoding	-0.0475136	0	1821	1974
MSTRG.34621.11	noncoding	-0.0577536	0	2523	2677
MSTRG.34621.12	noncoding	-0.0438272	0	1920	2110
MSTRG.34621.10	noncoding	-0.0610304	0	2661	3296
MSTRG.34621.9	noncoding	-0.0602112	0	2805	2959

MSTRG.34621.13	noncoding	-0.0569344	0	3330	3484
MSTRG.34621.14	noncoding	-0.0602112	0	2601	2756
MSTRG.34621.15	noncoding	-0.0831488	0	1143	1300
MSTRG.34621.17	noncoding	-0.0831488	0	1140	1368
MSTRG.34621.16	noncoding	-0.0720896	0	465	620
MSTRG.34621.18	noncoding	-0.0811008	0	1314	1475
MSTRG.34622.3	noncoding	-0.0557056	0	2655	2857
MSTRG.34622.4	noncoding	-0.0434176	12	2319	2501
MSTRG.34622.5	noncoding	-0.0446464	12	2334	2516
MSTRG.34622.6	noncoding	-0.0499712	0	1275	1430
MSTRG.34622.7	noncoding	-0.0454656	6	1245	1413
MSTRG.34622.8	noncoding	-0.0548864	0	3078	3247
MSTRG.34622.10	noncoding	-0.0815104	633	2694	2849
MSTRG.34622.9	noncoding	-0.0831488	633	2475	2644
MSTRG.34622.11	noncoding	-0.0679936	0	2997	3152
MSTRG.34622.12	noncoding	-0.0622592	0	2661	2827
MSTRG.34622.13	noncoding	-0.0610304	0	2712	2865
MSTRG.34622.14	noncoding	-0.0679936	633	2712	2865
MSTRG.34622.15	noncoding	-0.0495616	0	2883	3047
MSTRG.34622.16	noncoding	-0.0524288	0	2574	2728
MSTRG.34568.1	coding	0.008	96	192	791
MSTRG.34568.2	noncoding	-0.06144	174	237	533
MSTRG.34569.1	noncoding	-0.0278528	105	222	570
MSTRG.34570.1	noncoding	-0.110592	117	147	307
MSTRG.34572.1	noncoding	-0.0610304	645	867	1029
MSTRG.34574.1	noncoding	-0.065536	0	81	241
MSTRG.34575.1	noncoding	-0.0004096	0	48	235
MSTRG.34576.1	noncoding	-0.0004096	0	0	276
MSTRG.34579.2	coding	0.246	144	927	1412
MSTRG.34580.1	noncoding	-0.196608	114	156	399
MSTRG.34583.1	noncoding	-0.065536	0	12	1030
MSTRG.34583.2	noncoding	-0.008192	957	1032	2698
MSTRG.34587.1	noncoding	-0.0790528	0	102	256
MSTRG.34592.2	noncoding	-0.045056	27	165	319
MSTRG.34592.1	noncoding	-0.0004096	6	111	267
MSTRG.34592.3	noncoding	-0.0004096	6	111	265
MSTRG.34592.4	noncoding	-0.0380928	42	105	261
MSTRG.34592.5	noncoding	-0.0131072	0	150	305
MSTRG.34595.7	noncoding	-0.0233472	45	351	555
MSTRG.34595.3	noncoding	-0.0233472	99	405	610
MSTRG.34595.5	noncoding	-0.0233472	99	405	610
MSTRG.34595.6	noncoding	-0.0233472	99	405	610
MSTRG.34595.8	coding	0.09	0	147	352
MSTRG.34595.2	noncoding	-0.028672	45	351	555
MSTRG.34595.4	coding	0.094	75	306	512
MSTRG.34595.9	noncoding	-0.0233472	99	405	608
MSTRG.34595.10	coding	0.094	0	156	362
MSTRG.34595.11	noncoding	-0.004096	21	156	390
MSTRG.34595.12	coding	0.094	75	306	512
MSTRG.34595.13	noncoding	-0.028672	45	351	553
MSTRG.34595.14	coding	0.08	48	234	434
MSTRG.34595.15	noncoding	-0.0282624	0	156	312
MSTRG.34595.16	noncoding	-0.004096	21	156	385
MSTRG.34600.1	noncoding	-0.04096	120	231	577

MSTRG.34601.2	noncoding	-0.0380928	0	111	640
MSTRG.34601.3	noncoding	-0.02048	180	228	396
MSTRG.34601.1	noncoding	-0.02048	180	228	401
MSTRG.34603.1	noncoding	-0.016384	0	120	495
MSTRG.34600.2	noncoding	-0.022528	2469	2619	3071
MSTRG.34600.3	noncoding	-0.024576	330	513	1480
MSTRG.34606.1	noncoding	-0.0126976	147	297	655
MSTRG.34600.5	noncoding	-0.024576	936	1119	2508
MSTRG.34600.4	coding	0.119	1674	1854	2253
MSTRG.34600.6	noncoding	-0.045056	18	180	593
MSTRG.34597.1	noncoding	-0.0147456	6	540	696
MSTRG.34612.1	noncoding	-0.069632	120	150	734
MSTRG.34613.1	noncoding	-0.0417792	168	210	536
MSTRG.34611.1	noncoding	-0.0417792	54	96	518
MSTRG.34616.1	noncoding	-0.0417792	12	213	367
MSTRG.34616.2	noncoding	-0.0417792	12	213	367
MSTRG.34616.3	noncoding	-0.0638976	0	177	332
MSTRG.34616.4	noncoding	-0.0417792	27	228	383
MSTRG.34616.5	noncoding	-0.0417792	12	213	367
MSTRG.34615.1	noncoding	-0.028672	0	81	237
MSTRG.34619.1	coding	0.163	0	228	383
MSTRG.34631.3	coding	0.276	0	819	1309
MSTRG.34631.2	coding	0.274	0	816	1957
MSTRG.34632.1	noncoding	-0.0786432	0	159	655
MSTRG.34633.1	noncoding	-0.0376832	0	243	488
MSTRG.34632.2	noncoding	-0.0946176	0	114	343
MSTRG.34638.2	coding	0.126	549	1659	5010
MSTRG.34638.1	coding	0.166	549	1182	1336
MSTRG.34637.2	coding	0.177	603	2604	3590
MSTRG.34637.4	coding	0.177	573	2574	3455
MSTRG.34653.1	coding	0.218	0	441	742
MSTRG.34655.2	noncoding	-0.0684032	1662	1836	2731
MSTRG.34655.1	noncoding	-0.0684032	1662	1836	3208
MSTRG.34656.2	noncoding	-0.0073728	414	426	732
MSTRG.34656.1	noncoding	-0.0073728	414	426	1160
MSTRG.34657.1	noncoding	-0.0004096	546	546	1316
MSTRG.34660.1	noncoding	-0.0004096	0	0	228
MSTRG.34661.1	noncoding	-0.8192	171	174	337
MSTRG.34653.2	noncoding	-0.006144	189	333	526
MSTRG.34664.1	noncoding	-0.0610304	345	543	713
MSTRG.34666.1	noncoding	-0.008192	45	90	519
MSTRG.34671.1	noncoding	-0.016384	141	282	2707
MSTRG.34646.1	noncoding	-0.425984	45	57	210
MSTRG.34647.3	coding	0.253	105	345	652
MSTRG.34647.1	coding	0.257	105	348	761
MSTRG.34675.3	coding	0.194	162	381	536
MSTRG.34675.2	coding	0.154	99	309	462
MSTRG.34675.4	noncoding	-0.0323584	69	264	419
MSTRG.34675.5	noncoding	-0.0258048	63	246	401
MSTRG.34681.2	coding	0.15	858	1827	5164
MSTRG.34681.4	coding	0.15	219	1188	1594
MSTRG.34681.3	coding	0.15	456	1425	3276
MSTRG.34682.7	coding	0.15	348	1314	1729
MSTRG.34682.9	coding	0.15	348	1314	1717

MSTRG.34682.11	coding	0.15	348	1314	1765
MSTRG.34682.12	coding	0.15	348	1314	1765
MSTRG.34683.1	noncoding	-0.012288	204	327	481
MSTRG.34707.1	noncoding	-0.0438272	0	36	350
MSTRG.34708.1	noncoding	-0.0004096	0	0	285
MSTRG.34712.1	noncoding	-0.069632	132	201	1006
MSTRG.34714.1	noncoding	-0.004096	462	537	765
MSTRG.34715.1	coding	0.34	108	1032	1186
MSTRG.34716.1	noncoding	-0.0004096	0	0	236
MSTRG.34717.1	noncoding	-0.094208	363	390	1523
MSTRG.34715.2	coding	0.374	93	1095	1519
MSTRG.34715.3	coding	0.37	93	981	1137
MSTRG.34718.1	noncoding	-0.012288	147	195	506
MSTRG.34719.1	noncoding	-0.126976	96	105	314
MSTRG.34720.1	noncoding	-0.0069632	234	366	1113
MSTRG.34721.1	noncoding	-0.012288	1437	1608	2316
MSTRG.34722.1	noncoding	-0.036864	39	105	587
MSTRG.34715.4	coding	0.366	93	1098	1937
MSTRG.34723.1	noncoding	-0.0077824	9	279	563
MSTRG.34723.2	noncoding	-0.0729088	648	705	984
MSTRG.34724.1	coding	0.301	105	1074	1689
MSTRG.34725.1	noncoding	-0.0131072	0	105	349
MSTRG.34719.2	noncoding	-0.0024576	0	60	1296
MSTRG.34717.2	noncoding	-0.094208	441	468	677
MSTRG.34717.3	noncoding	-0.094208	351	378	587
MSTRG.34726.1	coding	0.123	54	831	1296
MSTRG.34715.5	coding	0.308	72	642	798
MSTRG.34727.1	noncoding	-0.008192	258	357	510
MSTRG.34687.1	noncoding	-0.126976	162	171	414
MSTRG.34689.2	noncoding	-0.018432	57	216	545
MSTRG.34690.2	coding	0.194	1806	2769	3378
MSTRG.34692.1	noncoding	-0.02048	129	147	302
MSTRG.34701.1	noncoding	-0.0024576	192	252	461
MSTRG.34702.1	coding	0.198	0	255	481
MSTRG.34729.1	noncoding	-0.012288	72	345	776
MSTRG.34743.1	coding	0.165	0	210	852
MSTRG.34743.2	coding	0.264	0	315	955
MSTRG.34744.2	coding	0.083	0	1299	1673
MSTRG.34744.3	coding	0.085	105	1461	1673
MSTRG.34744.4	coding	0.086	12	1296	1529
MSTRG.34744.5	coding	0.067	1359	2859	5445
MSTRG.34751.1	coding	0.094	249	849	1174
MSTRG.34755.2	coding	0.264	897	2097	2744
MSTRG.34755.1	coding	0.202	897	2922	3534
MSTRG.34761.2	coding	0.087	336	753	906
MSTRG.34763.2	noncoding	-0.0462848	0	84	1399
MSTRG.34764.1	noncoding	-0.0221184	0	150	503
MSTRG.34771.9	noncoding	-0.0053248	96	372	605
MSTRG.34771.13	noncoding	-0.0053248	96	372	604
MSTRG.34778.1	noncoding	-0.0241664	99	405	615
MSTRG.34778.3	noncoding	-0.01024	42	276	431
MSTRG.34778.4	noncoding	-0.01024	42	276	431
MSTRG.34778.5	noncoding	-0.0004096	99	375	610
MSTRG.34778.6	noncoding	-0.0004096	99	375	610
