# Supplementary material for: Identification of Novel lncRNA and Differentially Expressed Genes (DEGs) of Testicular Tissues among Cattle, Yak, and Cattle-Yak Associated with Male Infertility
Source: Animals (Basel). 2021 Aug 17;11(8):2420. doi: 10.3390/ani11082420 (PMC8388754; doi:10.3390/ani11082420)
Supplement: Supplementary file 1 [file animals-11-02420-s001.zip › Table S2_cpc2_result.pdf]

#ID	peptide_length	Fickett_score	pI	ORF_integrity
MSTRG.12.1	128	0.34696	7.794006348	1
MSTRG.12.2	79	0.35564	10.80621338	1
MSTRG.12.3	79	0.36001	10.80621338	1
MSTRG.32.2	150	0.30796	6.080749512	1
MSTRG.33.1	64	0.40011	5.993103027	1
MSTRG.34.2	293	0.40653	8.83380127	1
MSTRG.35.1	84	0.44034	9.491638184	1
MSTRG.39.3	66	0.39042	7.87689209	1
MSTRG.39.6	489	0.34455	7.442321777	1
MSTRG.59.3	280	0.35659	5.304016113	1
MSTRG.60.1	134	0.34931	8.710632324	1
MSTRG.61.1	160	0.39483	10.48895264	-1
MSTRG.60.2	119	0.30919	8.764953613	1
MSTRG.63.2	620	0.39326	9.285705566	1
MSTRG.63.4	349	0.49018	5.421569824	1
MSTRG.63.6	349	0.35797	5.421569824	1
MSTRG.63.7	406	0.44125	10.0022583	1
MSTRG.63.5	349	0.42075	5.421569824	1
MSTRG.63.8	349	0.41566	5.421569824	1
MSTRG.63.3	620	0.33091	9.285705566	1
MSTRG.65.10	2291	0.43059	5.26348877	1
MSTRG.65.11	2304	0.42907	5.203430176	1
MSTRG.65.4	2401	0.43059	5.417785645	1
MSTRG.65.5	2412	0.43059	5.39276123	1
MSTRG.65.6	2408	0.44445	5.463562012	1
MSTRG.65.8	2445	0.43059	5.465393066	1
MSTRG.65.9	2301	0.42907	5.251525879	1
MSTRG.65.14	2084	0.40863	5.242980957	1
MSTRG.65.15	2074	0.40863	5.256164551	1
MSTRG.65.16	2077	0.40863	5.202697754	1
MSTRG.66.1	1010	0.4505	6.006164551	1
MSTRG.66.2	1010	0.45358	6.006164551	1
MSTRG.66.3	1010	0.46793	6.006164551	1
MSTRG.66.4	1010	0.45358	6.006164551	1
MSTRG.68.2	380	0.33126	9.426818848	1
MSTRG.42.1	560	0.37183	5.089416504	1
MSTRG.56.2	482	0.3765	5.250427246	1
MSTRG.56.4	485	0.3765	5.250427246	1
MSTRG.56.6	472	0.35788	5.09552002	1
MSTRG.56.7	483	0.43921	5.250427246	1
MSTRG.56.8	481	0.44245	5.250427246	1
MSTRG.52.1	1529	0.4455	6.603210449	1
MSTRG.52.2	1562	0.42187	8.266906738	1
MSTRG.53.1	290	0.43242	6.636657715	1
MSTRG.53.2	191	0.34974	8.406311035	1
MSTRG.70.1	194	0.29294	7.729309082	1
MSTRG.73.2	1120	0.45607	9.679748535	1
MSTRG.78.1	231	0.28338	9.469909668	1
MSTRG.79.2	187	0.32741	10.02825928	1
MSTRG.78.2	289	0.31516	9.847106934	1
MSTRG.80.1	56	0.4045	10.0479126	-1
MSTRG.81.1	107	0.37339	6.014465332	1
MSTRG.79.3	217	0.28167	9.250305176	1

MSTRG.82.1	64	0.41672	11.84906006	-1
MSTRG.79.4	74	0.28109	10.36798096	1
MSTRG.79.5	173	0.27274	9.09552002	1
MSTRG.79.6	173	0.33828	9.09552002	1
MSTRG.83.1	163	0.36521	4.596618652	1
MSTRG.79.7	143	0.28002	9.427307129	1
MSTRG.85.1	86	0.34296	6.89276123	1
MSTRG.86.1	94	0.28167	9.206481934	1
MSTRG.96.9	731	0.45637	5.741760254	1
MSTRG.96.10	826	0.47613	5.97076416	1
MSTRG.96.6	826	0.45737	5.97076416	1
MSTRG.96.8	731	0.46585	5.741760254	1
MSTRG.96.3	826	0.46585	5.97076416	1
MSTRG.97.1	100	0.35133	6.101745605	1
MSTRG.97.2	290	0.38392	5.312805176	1
MSTRG.91.1	106	0.33599	7.820251465	1
MSTRG.103.6	520	0.42403	5.34564209	1
MSTRG.103.2	991	0.34138	6.108581543	1
MSTRG.103.5	1561	0.42883	5.706481934	1
MSTRG.103.4	1567	0.46105	5.738098145	1
MSTRG.104.1	214	0.4362	5.018859863	1
MSTRG.104.3	192	0.45623	4.578063965	1
MSTRG.111.1	49	0.41714	9.496398926	1
MSTRG.117.6	454	0.47275	4.879577637	1
MSTRG.118.5	108	0.40454	9.298278809	1
MSTRG.123.1	53	0.39032	6.504333496	1
MSTRG.127.1	52	0.41895	10.15692139	1
MSTRG.131.1	43	0.40287	7.97845459	-1
MSTRG.132.1	115	0.30876	9.503234863	1
MSTRG.134.1	72	0.40375	9.300476074	1
MSTRG.151.1	84	0.28862	9.89642334	1
MSTRG.188.1	281	0.2921	9.54864502	1
MSTRG.189.1	97	0.28238	7.629333496	1
MSTRG.193.1	71	0.36569	5.99420166	1
MSTRG.196.1	83	0.45274	4.95135498	1
MSTRG.223.1	67	0.34413	9.745788574	1
MSTRG.241.1	250	0.30934	9.408630371	-1
MSTRG.158.1	668	0.38619	5.698181152	1
MSTRG.158.6	602	0.46477	5.969421387	1
MSTRG.158.5	695	0.48268	6.018859863	1
MSTRG.158.4	696	0.44483	6.017028809	1
MSTRG.158.8	1081	0.47243	5.22857666	1
MSTRG.158.7	639	0.46992	5.530334473	1
MSTRG.158.9	492	0.3843	5.348205566	1
MSTRG.160.1	52	0.36175	9.301452637	1
MSTRG.160.3	1156	0.39672	8.444396973	1
MSTRG.160.4	1156	0.36746	8.444396973	1
MSTRG.160.5	1156	0.39672	8.444396973	1
MSTRG.160.6	1156	0.38839	8.444396973	1
MSTRG.161.1	62	0.37378	9.249084473	1
MSTRG.162.1	162	0.31368	9.948791504	1
MSTRG.162.2	91	0.31048	9.821960449	1
MSTRG.160.7	93	0.31853	8.770690918	1
MSTRG.163.1	402	0.49562	5.159118652	1

MSTRG.163.3	359	0.37335	5.144470215	1
MSTRG.164.1	150	0.47021	5.06060791	-1
MSTRG.166.2	131	0.36085	8.848083496	1
MSTRG.168.2	79	0.45829	5.649841309	1
MSTRG.169.1	41	0.43347	10.59552002	1
MSTRG.175.3	1418	0.46244	6.03717041	1
MSTRG.175.4	63	0.3413	9.433654785	1
MSTRG.175.5	1274	0.43859	5.898132324	-1
MSTRG.175.6	728	0.43553	5.644470215	1
MSTRG.265.8	117	0.29059	9.020935059	1
MSTRG.265.9	210	0.28982	4.955871582	1
MSTRG.266.1	94	0.32986	4.952941895	1
MSTRG.267.1	252	0.36573	9.406188965	1
MSTRG.268.1	60	0.3118	10.2744751	1
MSTRG.269.1	54	0.3208	6.69708252	1
MSTRG.268.2	183	0.33313	10.02410889	1
MSTRG.270.1	114	0.29543	11.00006104	1
MSTRG.271.1	78	0.27831	5.60736084	1
MSTRG.272.1	113	0.27517	9.936218262	1
MSTRG.273.1	99	0.29374	10.41595459	1
MSTRG.274.1	74	0.28159	10.36431885	1
MSTRG.275.1	178	0.31513	9.945129395	1
MSTRG.276.1	59	0.3036	6.469055176	1
MSTRG.277.1	99	0.28644	10.61419678	1
MSTRG.277.2	123	0.31368	10.10919189	1
MSTRG.278.1	190	0.3	6.438049316	1
MSTRG.265.10	88	0.2921	8.03302002	1
MSTRG.265.11	180	0.28958	9.281433105	1
MSTRG.279.1	189	0.28334	11.04364014	1
MSTRG.265.12	82	0.2831	8.664978027	1
MSTRG.280.1	120	0.25957	9.045837402	1
MSTRG.281.1	140	0.26633	8.314880371	1
MSTRG.282.1	88	0.25588	6.468566895	1
MSTRG.283.1	81	0.29895	8.979431152	1
MSTRG.284.1	142	0.28178	10.0269165	1
MSTRG.284.2	160	0.29366	10.34259033	1
MSTRG.265.14	151	0.28982	9.914611816	1
MSTRG.285.1	228	0.30736	8.207458496	1
MSTRG.250.1	122	0.29003	7.864440918	1
MSTRG.255.1	160	0.30816	9.409973145	1
MSTRG.258.1	75	0.3118	8.664245605	1
MSTRG.261.1	121	0.38	10.97833252	1
MSTRG.262.1	56	0.28165	8.503356934	1
MSTRG.263.1	52	0.27893	8.358947754	1
MSTRG.297.1	69	0.38711	7.925109863	1
MSTRG.307.1	329	0.31852	5.797912598	1
MSTRG.308.1	75	0.3208	5.43939209	1
MSTRG.312.1	76	0.38478	8.740539551	-1
MSTRG.314.1	399	0.37001	5.410949707	1
MSTRG.314.3	917	0.40399	5.430114746	1
MSTRG.325.3	697	0.47843	6.514343262	1
MSTRG.325.4	698	0.47471	6.514343262	1
MSTRG.326.1	84	0.32641	10.20220947	1
MSTRG.327.1	177	0.32213	9.558044434	1

MSTRG.324.1	36	0.37762	9.515563965	-1
MSTRG.328.1	53	0.27446	6.696228027	1
MSTRG.328.2	83	0.30508	5.386657715	-1
MSTRG.332.1	147	0.33298	9.19744873	1
MSTRG.348.1	38	0.46701	4.830993652	1
MSTRG.351.1	429	0.46045	6.107849121	1
MSTRG.352.1	189	0.28662	10.53656006	1
MSTRG.353.1	110	0.35253	6.371520996	1
MSTRG.356.1	161	0.29575	10.07757568	1
MSTRG.361.1	33	0.40095	11.73394775	1
MSTRG.375.2	2848	0.49497	4.981262207	1
MSTRG.375.5	2705	0.47652	5.014831543	1
MSTRG.377.1	51	0.41182	4.944641113	1
MSTRG.377.2	62	0.39293	7.809875488	1
MSTRG.379.3	484	0.44779	7.216247559	1
MSTRG.379.5	461	0.45329	6.651062012	1
MSTRG.369.1	51	0.40426	9.301208496	1
MSTRG.370.1	44	0.39912	7.804748535	-1
MSTRG.371.1	253	0.33735	5.390808105	1
MSTRG.381.3	757	0.37186	6.36541748	1
MSTRG.396.1	911	0.41204	5.840270996	1
MSTRG.397.1	70	0.29387	10.87786865	1
MSTRG.396.3	893	0.46364	6.00592041	1
MSTRG.398.1	75	0.27433	4.272766113	1
MSTRG.399.1	90	0.31852	9.27142334	1
MSTRG.400.1	74	0.28982	7.694274902	1
MSTRG.401.1	128	0.29772	6.01348877	1
MSTRG.404.3	452	0.38695	5.367614746	1
MSTRG.404.4	286	0.39948	4.79473877	1
MSTRG.404.6	422	0.37853	5.937316895	1
MSTRG.404.5	321	0.32162	6.273132324	1
MSTRG.404.7	407	0.37853	5.933532715	1
MSTRG.404.8	104	0.26157	9.358947754	1
MSTRG.391.3	529	0.48067	6.99017334	1
MSTRG.392.2	120	0.31603	9.653625488	1
MSTRG.393.2	63	0.33825	4.763122559	1
MSTRG.405.1	397	0.43257	5.014343262	1
MSTRG.405.7	540	0.39299	7.558288574	1
MSTRG.405.3	397	0.44017	5.014343262	1
MSTRG.405.4	541	0.39299	7.558288574	1
MSTRG.405.5	396	0.44017	5.014343262	1
MSTRG.405.6	400	0.44017	4.944763184	1
MSTRG.405.8	464	0.41326	5.60369873	1
MSTRG.405.9	80	0.37786	10.03692627	1
MSTRG.407.1	447	0.47081	9.300109863	1
MSTRG.416.1	99	0.40312	10.01654053	1
MSTRG.423.2	57	0.35768	9.300598145	1
MSTRG.424.1	1052	0.40501	8.326965332	1
MSTRG.424.2	1052	0.42968	8.326965332	1
MSTRG.424.3	768	0.3873	8.452209473	1
MSTRG.426.1	204	0.32818	10.12713623	1
MSTRG.427.1	122	0.2785	6.376281738	1
MSTRG.429.1	873	0.46615	5.161560059	1
MSTRG.429.2	853	0.47791	5.047912598	1

MSTRG.449.1	20	0.36292	8.384094238	1
MSTRG.451.1	287	0.28982	8.590026855	1
MSTRG.451.2	114	0.33851	6.428161621	1
MSTRG.452.1	65	0.32632	10.61297607	1
MSTRG.457.1	129	0.28982	5.711730957	1
MSTRG.435.3	217	0.45123	5.806335449	1
MSTRG.439.1	99	0.26633	7.682067871	1
MSTRG.438.2	176	0.33061	8.774597168	1
MSTRG.440.1	108	0.28726	5.808776855	1
MSTRG.441.1	187	0.29754	9.255187988	1
MSTRG.467.6	84	0.32236	11.04364014	1
MSTRG.467.7	65	0.38461	9.831237793	1
MSTRG.468.1	103	0.29615	8.897155762	1
MSTRG.468.2	128	0.28982	11.64971924	1
MSTRG.468.3	63	0.29327	6.950012207	1
MSTRG.468.4	105	0.30328	9.689147949	1
MSTRG.468.5	203	0.3208	8.676940918	1
MSTRG.470.1	60	0.29433	8.289123535	1
MSTRG.467.10	912	0.36597	8.79510498	1
MSTRG.467.9	942	0.37806	9.00213623	1
MSTRG.467.11	749	0.3413	9.275695801	1
MSTRG.471.1	83	0.33194	4.641662598	1
MSTRG.472.1	168	0.3176	10.7074585	1
MSTRG.463.1	321	0.30303	8.047912598	1
MSTRG.463.4	310	0.39577	8.056335449	1
MSTRG.464.1	68	0.33929	6.692932129	1
MSTRG.474.1	53	0.35131	9.138000488	1
MSTRG.479.1	365	0.44333	5.661804199	1
MSTRG.481.2	38	0.36817	5.327941895	1
MSTRG.481.1	75	0.41569	9.52142334	-1
MSTRG.487.2	588	0.45793	5.556213379	1
MSTRG.488.3	470	0.41777	5.292419434	1
MSTRG.484.1	26	0.395	4.351989746	-1
MSTRG.493.1	68	0.39821	9.570983887	1
MSTRG.497.1	50	0.44249	12.01300049	-1
MSTRG.506.1	55	0.39236	4.258850098	1
MSTRG.508.3	334	0.38857	5.536071777	1
MSTRG.508.4	337	0.37471	5.518005371	1
MSTRG.508.2	549	0.44402	5.703186035	1
MSTRG.508.5	546	0.32762	5.711364746	1
MSTRG.509.1	106	0.33122	9.80267334	-1
MSTRG.512.2	52	0.33951	6.885559082	1
MSTRG.513.4	1116	0.38389	8.015441895	1
MSTRG.514.4	929	0.46219	8.68182373	1
MSTRG.514.3	1166	0.46079	7.158874512	1
MSTRG.514.5	1108	0.47924	7.812072754	1
MSTRG.515.1	119	0.33779	9.517028809	1
MSTRG.521.1	425	0.39376	6.828674316	1
MSTRG.521.3	425	0.37077	6.828674316	1
MSTRG.521.4	210	0.32322	8.860290527	1
MSTRG.521.6	209	0.3216	9.259338379	1
MSTRG.521.7	331	0.44183	6.305969238	1
MSTRG.526.3	262	0.34952	9.452209473	1
MSTRG.526.1	288	0.32465	9.356994629	1

MSTRG.526.2	156	0.29115	9.87701416	1
MSTRG.527.2	211	0.28794	9.761779785	1
MSTRG.533.2	23	0.46425	5.585144043	1
MSTRG.533.3	898	0.46629	6.141662598	1
MSTRG.535.1	169	0.36184	8.961730957	1
MSTRG.535.4	95	0.30419	8.565124512	1
MSTRG.538.1	618	0.40125	5.414978027	1
MSTRG.538.2	618	0.40643	5.414978027	1
MSTRG.538.3	618	0.41511	5.414978027	1
MSTRG.538.4	618	0.40643	5.414978027	1
MSTRG.539.1	86	0.29615	8.950378418	1
MSTRG.570.1	668	0.4502	6.054626465	1
MSTRG.570.15	668	0.30595	6.054626465	1
MSTRG.570.14	741	0.36618	6.100280762	1
MSTRG.570.16	741	0.33465	6.100280762	1
MSTRG.570.18	741	0.34583	6.100280762	1
MSTRG.570.17	741	0.36466	6.100280762	1
MSTRG.570.19	741	0.37265	6.100280762	1
MSTRG.570.20	668	0.36893	6.054626465	1
MSTRG.570.22	741	0.30595	6.100280762	1
MSTRG.570.21	668	0.34156	6.054626465	1
MSTRG.570.23	668	0.34583	6.054626465	1
MSTRG.571.1	189	0.3767	9.272155762	-1
MSTRG.572.1	146	0.29135	9.074035645	1
MSTRG.570.24	90	0.29543	7.11541748	1
MSTRG.573.1	31	0.25049	6.685852051	1
MSTRG.574.1	77	0.28819	9.772888184	-1
MSTRG.575.1	43	0.36347	9.49810791	1
MSTRG.576.1	73	0.31997	9.456359863	1
MSTRG.577.1	96	0.30513	10.59991455	1
MSTRG.578.1	132	0.2921	8.463562012	1
MSTRG.570.25	48	0.33414	11.00006104	1
MSTRG.579.1	148	0.30576	10.79571533	1
MSTRG.580.1	233	0.32389	9.083679199	-1
MSTRG.581.1	128	0.27289	9.783996582	1
MSTRG.582.1	120	0.2895	9.120666504	1
MSTRG.583.1	107	0.4019	9.183410645	-1
MSTRG.584.1	45	0.33659	4.414611816	1
MSTRG.546.1	155	0.46333	13.13702393	1
MSTRG.546.2	235	0.44993	4.574523926	1
MSTRG.547.1	309	0.44983	5.846252441	-1
MSTRG.560.4	329	0.33214	7.695129395	1
MSTRG.560.1	261	0.30829	7.63470459	1
MSTRG.560.6	329	0.38555	7.695129395	1
MSTRG.561.1	124	0.30276	7.061340332	-1
MSTRG.562.1	71	0.31732	4.681945801	1
MSTRG.564.1	39	0.29607	9.428894043	1
MSTRG.601.2	361	0.41625	8.960510254	1
MSTRG.615.6	319	0.43895	8.094543457	1
MSTRG.615.4	319	0.44166	8.094543457	1
MSTRG.615.5	400	0.48358	8.750793457	1
MSTRG.616.1	64	0.3173	9.742370605	1
MSTRG.617.1	73	0.35997	8.504089355	1
MSTRG.622.2	208	0.2921	6.577575684	1

MSTRG.622.3	208	0.28165	6.577575684	1
MSTRG.622.4	163	0.2921	8.125305176	1
MSTRG.623.1	161	0.31425	8.549987793	1
MSTRG.624.2	306	0.34351	5.458190918	1
MSTRG.624.3	306	0.34351	5.458190918	1
MSTRG.625.1	66	0.32811	9.63861084	1
MSTRG.627.1	65	0.35515	9.817443848	1
MSTRG.628.1	439	0.47499	4.695373535	1
MSTRG.628.2	438	0.46043	4.706481934	1
MSTRG.628.3	439	0.46364	4.695373535	1
MSTRG.628.6	438	0.46311	4.706481934	1
MSTRG.628.5	438	0.47423	4.706481934	1
MSTRG.639.1	79	0.30593	10.68621826	1
MSTRG.639.2	103	0.27517	7.626525879	1
MSTRG.645.1	102	0.31553	9.388244629	1
MSTRG.646.1	2416	0.46809	6.606872559	1
MSTRG.646.3	169	0.32417	10.19232178	1
MSTRG.646.4	191	0.29793	8.731994629	1
MSTRG.633.4	519	0.42152	9.567199707	1
MSTRG.633.5	598	0.44277	9.43548584	1
MSTRG.633.6	598	0.44277	9.43548584	1
MSTRG.633.7	519	0.42792	9.567199707	1
MSTRG.635.1	31	0.40674	4.054992676	1
MSTRG.635.2	27	0.39663	5.059875488	1
MSTRG.650.1	337	0.2921	4.809143066	1
MSTRG.665.2	100	0.31281	10.02423096	1
MSTRG.665.3	53	0.32061	7.928161621	1
MSTRG.671.6	1081	0.47526	5.653137207	1
MSTRG.671.9	489	0.44581	4.707946777	1
MSTRG.671.10	1165	0.4637	6.038024902	1
MSTRG.671.12	1165	0.4637	6.038024902	1
MSTRG.671.15	1153	0.4637	5.972717285	1
MSTRG.671.16	1153	0.4637	5.972717285	1
MSTRG.671.18	256	0.37886	8.882751465	1
MSTRG.672.1	431	0.32121	8.380065918	1
MSTRG.667.3	516	0.43437	8.685974121	1
MSTRG.691.1	2380	0.45015	5.735290527	1
MSTRG.691.2	2389	0.45015	5.74420166	1
MSTRG.691.5	1650	0.46286	5.72454834	1
MSTRG.691.6	1641	0.46438	5.713195801	1
MSTRG.691.3	2964	0.46681	5.71282959	1
MSTRG.691.7	26	0.3954	5.221862793	-1
MSTRG.691.8	2383	0.46521	5.623962402	1
MSTRG.691.9	248	0.28584	10.2164917	1
MSTRG.691.10	2281	0.46708	5.65045166	1
MSTRG.691.11	2384	0.45177	5.637145996	1
MSTRG.691.12	61	0.36041	9.812805176	1
MSTRG.693.1	74	0.29452	8.636535645	1
MSTRG.694.1	103	0.32858	9.779724121	1
MSTRG.691.13	2381	0.4749	5.623962402	-1
MSTRG.691.14	2289	0.44746	5.646057129	1
MSTRG.691.15	2282	0.45696	5.663391113	1
MSTRG.691.17	1748	0.3387	5.508361816	1
MSTRG.691.18	230	0.31958	8.908874512	1

MSTRG.695.1	81	0.29607	8.736755371	1
MSTRG.691.21	146	0.27911	8.557678223	1
MSTRG.691.20	743	0.40193	5.987365723	1
MSTRG.691.22	846	0.41457	5.862976074	1
MSTRG.696.1	180	0.28199	9.745178223	1
MSTRG.675.2	1343	0.33435	5.528503418	1
MSTRG.681.5	794	0.47505	6.031311035	1
MSTRG.681.4	794	0.46937	6.031311035	1
MSTRG.684.1	403	0.45484	6.352478027	1
MSTRG.684.4	433	0.48119	5.894714355	1
MSTRG.699.1	693	0.42257	7.237731934	1
MSTRG.699.3	692	0.3825	7.237731934	1
MSTRG.699.4	735	0.41881	7.644226074	1
MSTRG.702.1	110	0.3638	11.06561279	1
MSTRG.704.4	232	0.33698	8.985778809	1
MSTRG.704.6	73	0.30419	9.339660645	1
MSTRG.704.7	209	0.30191	10.69903564	1
MSTRG.685.2	251	0.27639	11.86846924	1
MSTRG.685.1	251	0.31158	11.86846924	1
MSTRG.685.3	251	0.30501	11.86846924	1
MSTRG.687.5	752	0.29976	5.675476074	1
MSTRG.687.6	286	0.35139	6.241760254	1
MSTRG.705.6	633	0.32865	8.004943848	1
MSTRG.705.3	1007	0.4491	6.965881348	1
MSTRG.705.4	1054	0.34885	7.155700684	1
MSTRG.705.5	980	0.39057	7.320129395	1
MSTRG.705.1	1039	0.39737	6.992004395	1
MSTRG.705.7	1054	0.38809	7.155700684	1
MSTRG.705.8	1039	0.39489	6.992004395	1
MSTRG.706.3	769	0.4216	5.954528809	1
MSTRG.706.4	133	0.28267	9.186950684	1
MSTRG.707.1	52	0.30265	6.81439209	1
MSTRG.708.1	367	0.3462	6.820251465	1
MSTRG.708.5	321	0.34706	6.481140137	1
MSTRG.708.4	384	0.44423	7.156921387	1
MSTRG.708.3	321	0.47081	6.481140137	1
MSTRG.710.3	258	0.3208	5.284240723	1
MSTRG.713.3	358	0.45098	6.363342285	1
MSTRG.714.1	107	0.37175	4.749816895	1
MSTRG.714.2	107	0.3638	4.749816895	1
MSTRG.715.1	150	0.29059	6.270324707	1
MSTRG.717.1	33	0.36569	9.102355957	1
MSTRG.718.1	55	0.28862	7.868835449	1
MSTRG.730.7	533	0.46866	5.51763916	1
MSTRG.731.1	44	0.41275	7.944641113	1
MSTRG.732.1	183	0.26969	11.35992432	1
MSTRG.733.1	72	0.41181	3.616638184	1
MSTRG.727.2	344	0.4676	8.890563965	1
MSTRG.727.3	344	0.43714	8.890563965	1
MSTRG.727.4	344	0.43838	8.890563965	1
MSTRG.728.1	52	0.35089	7.960266113	1
MSTRG.728.2	52	0.32779	7.960266113	1
MSTRG.728.3	52	0.32603	7.960266113	1
MSTRG.735.1	85	0.33282	4.855285645	1

MSTRG.739.1	60	0.3	9.493103027	1
MSTRG.740.1	75	0.29594	6.86895752	1
MSTRG.741.1	188	0.3046	10.64141846	1
MSTRG.743.2	216	0.44441	9.651306152	1
MSTRG.752.5	160	0.4253	5.147766113	1
MSTRG.752.6	160	0.41509	5.147766113	1
MSTRG.755.1	47	0.41138	8.493225098	1
MSTRG.756.1	123	0.29859	6.567443848	1
MSTRG.758.1	88	0.28923	10.8961792	1
MSTRG.774.1	170	0.32237	10.23602295	1
MSTRG.776.1	95	0.32237	9.162658691	1
MSTRG.777.1	63	0.31544	9.340393066	1
MSTRG.770.1	100	0.39032	6.039367676	-1
MSTRG.771.1	32	0.38859	9.894104004	1
MSTRG.771.2	16	0.46451	5.897644043	1
MSTRG.778.1	39	0.44532	5.934753418	1
MSTRG.782.2	451	0.28982	7.416442871	1
MSTRG.782.3	614	0.28298	7.443054199	1
MSTRG.782.4	451	0.41114	7.416442871	1
MSTRG.782.5	614	0.41539	7.443054199	1
MSTRG.784.1	557	0.40005	9.76385498	-1
MSTRG.784.2	534	0.37862	9.744934082	1
MSTRG.786.1	182	0.37294	6.895324707	1
MSTRG.794.2	124	0.38335	9.14263916	1
MSTRG.803.3	358	0.42046	5.920959473	1
MSTRG.804.1	55	0.29244	8.801330566	1
MSTRG.804.2	19	0.37487	11.71136475	1
MSTRG.805.1	170	0.40183	7.625793457	1
MSTRG.806.1	557	0.41355	9.036315918	1
MSTRG.806.4	178	0.39627	6.382995605	1
MSTRG.801.1	68	0.41736	8.508605957	-1
MSTRG.813.2	589	0.29691	9.571472168	1
MSTRG.817.1	595	0.33187	6.727111816	1
MSTRG.831.2	1426	0.45968	5.414245605	1
MSTRG.831.4	1152	0.47815	5.806335449	1
MSTRG.833.1	58	0.41473	9.034851074	-1
MSTRG.835.1	192	0.28033	4.813049316	1
MSTRG.836.2	485	0.41067	9.120666504	1
MSTRG.837.4	1312	0.4525	5.175109863	1
MSTRG.839.2	159	0.36592	6.426452637	1
MSTRG.840.3	364	0.43303	9.522155762	1
MSTRG.840.2	482	0.40185	9.373718262	1
MSTRG.841.3	1247	0.40062	6.034118652	1
MSTRG.841.2	1247	0.37065	6.034118652	1
MSTRG.854.3	682	0.43688	7.598327637	1
MSTRG.855.1	377	0.32179	9.790710449	1
MSTRG.855.2	188	0.30092	9.571838379	1
MSTRG.848.3	600	0.32771	6.024597168	1
MSTRG.848.5	600	0.37826	6.024597168	1
MSTRG.848.6	600	0.45266	6.024597168	1
MSTRG.848.8	600	0.45688	6.024597168	1
MSTRG.859.4	176	0.33379	6.37677002	1
MSTRG.859.6	62	0.3127	6.157165527	1
MSTRG.862.2	1098	0.4129	6.146911621	1

MSTRG.862.5	58	0.4178	5.562683105	1
MSTRG.862.7	58	0.31444	5.562683105	1
MSTRG.862.6	914	0.46623	6.296813965	1
MSTRG.862.3	1089	0.47345	6.308776855	1
MSTRG.862.8	293	0.41594	8.67755127	1
MSTRG.874.1	68	0.36296	4.450866699	1
MSTRG.892.3	569	0.36948	5.213806152	1
MSTRG.892.2	569	0.34386	5.213806152	1
MSTRG.892.1	863	0.37645	5.361999512	1
MSTRG.897.2	136	0.29844	8.97052002	1
MSTRG.914.4	267	0.37873	8.567443848	1
MSTRG.914.3	378	0.38378	9.805480957	1
MSTRG.914.5	309	0.39923	9.95513916	1
MSTRG.915.2	577	0.41362	4.975891113	1
MSTRG.915.3	577	0.41362	4.975891113	1
MSTRG.908.1	39	0.43484	5.939025879	-1
MSTRG.920.1	89	0.28066	4.514099121	1
MSTRG.921.1	51	0.42563	8.90838623	-1
MSTRG.945.1	137	0.47449	5.780212402	1
MSTRG.934.4	306	0.36827	8.300964355	1
MSTRG.951.3	896	0.45027	7.522521973	1
MSTRG.952.1	94	0.41656	9.513244629	-1
MSTRG.956.4	465	0.37823	6.826721191	1
MSTRG.957.1	127	0.28478	10.97186279	1
MSTRG.959.1	138	0.3508	7.150939941	1
MSTRG.965.1	74	0.29988	9.09967041	1
MSTRG.965.2	59	0.30008	6.69354248	1
MSTRG.965.3	53	0.37379	5.108093262	1
MSTRG.979.3	1074	0.34314	8.908508301	1
MSTRG.979.7	156	0.3119	4.898498535	1
MSTRG.980.3	131	0.27678	11.59063721	1
MSTRG.981.1	109	0.29772	5.004333496	1
MSTRG.980.5	118	0.28982	9.809875488	1
MSTRG.980.6	124	0.28982	9.028625488	1
MSTRG.980.7	1317	0.3926	7.024475098	1
MSTRG.985.1	60	0.35363	9.485168457	1
MSTRG.967.2	160	0.33654	6.518127441	1
MSTRG.972.2	64	0.31343	7.888000488	1
MSTRG.974.1	543	0.44936	4.831726074	1
MSTRG.975.1	70	0.36279	9.593688965	1
MSTRG.991.1	71	0.3637	9.570983887	1
MSTRG.991.2	71	0.32234	9.570983887	1
MSTRG.991.3	71	0.36923	9.570983887	1
MSTRG.994.1	551	0.4674	7.88079834	-1
MSTRG.994.2	544	0.46798	6.969787598	-1
MSTRG.994.3	537	0.49355	6.616882324	-1
MSTRG.997.1	609	0.35732	8.673522949	1
MSTRG.1000.1	1051	0.42576	6.28326416	1
MSTRG.1000.2	1039	0.44116	6.366760254	1
MSTRG.1005.5	125	0.32082	11.91070557	1
MSTRG.1005.6	548	0.47775	4.540710449	1
MSTRG.1002.3	280	0.37298	8.820861816	1
MSTRG.1002.4	267	0.35248	8.702453613	1
MSTRG.1008.4	195	0.46323	6.041564941	1

MSTRG.1013.4	837	0.45138	5.289855957	1
MSTRG.1013.7	721	0.47498	4.960510254	1
MSTRG.1013.8	720	0.47498	4.960510254	1
MSTRG.1013.9	252	0.33	7.982727051	1
MSTRG.1012.2	128	0.31169	6.241149902	1
MSTRG.1012.1	128	0.32887	6.241149902	1
MSTRG.1029.1	503	0.46928	7.208679199	1
MSTRG.1029.3	383	0.39463	5.975280762	1
MSTRG.1031.1	360	0.32661	5.57635498	1
MSTRG.1031.2	360	0.31588	5.57635498	1
MSTRG.1031.3	192	0.34642	5.195373535	1
MSTRG.1041.2	264	0.39192	6.41192627	1
MSTRG.1044.3	640	0.43473	8.186706543	1
MSTRG.1036.3	512	0.35998	5.446350098	1
MSTRG.1037.1	101	0.36873	8.59942627	1
MSTRG.1060.2	130	0.35813	5.903991699	1
MSTRG.1063.1	222	0.43782	5.299743652	1
MSTRG.1063.2	222	0.44602	5.299743652	1
MSTRG.1066.1	334	0.45541	11.08001709	1
MSTRG.1066.2	334	0.42556	11.08001709	1
MSTRG.1066.3	158	0.42583	11.95782471	1
MSTRG.1065.1	28	0.36272	7.975280762	-1
MSTRG.1070.1	71	0.4494	4.82623291	-1
MSTRG.1085.2	188	0.32257	10.11651611	1
MSTRG.1086.2	352	0.4184	9.405944824	1
MSTRG.1096.1	744	0.46663	6.718322754	1
MSTRG.1097.1	43	0.34287	6.792663574	1
MSTRG.1096.2	699	0.3723	6.446838379	1
MSTRG.1108.1	30	0.39577	9.52130127	1
MSTRG.1119.1	177	0.28992	9.833679199	1
MSTRG.1132.1	228	0.28958	9.857116699	1
MSTRG.1125.1	283	0.34356	9.096496582	1
MSTRG.1141.1	104	0.33358	6.011169434	1
MSTRG.1149.1	79	0.37847	9.73248291	1
MSTRG.1150.1	85	0.41682	5.994567871	1
MSTRG.1157.6	347	0.32389	9.062927246	1
MSTRG.1157.7	295	0.28562	9.052185059	1
MSTRG.1157.8	277	0.41275	8.929748535	1
MSTRG.1157.10	384	0.39677	8.923278809	1
MSTRG.1157.9	387	0.4219	9.161437988	1
MSTRG.1158.1	51	0.30651	10.168396	1
MSTRG.1158.2	51	0.26982	10.32086182	1
MSTRG.1159.1	94	0.28576	5.378967285	1
MSTRG.1173.1	117	0.30824	10.30865479	1
MSTRG.1174.1	103	0.35999	10.16790771	-1
MSTRG.1175.1	210	0.29387	9.206848145	1
MSTRG.1161.2	232	0.35128	6.616027832	1
MSTRG.1161.3	80	0.28567	7.805969238	1
MSTRG.1164.2	76	0.33639	6.360656738	1
MSTRG.1167.1	47	0.46486	5.221008301	-1
MSTRG.1184.1	101	0.30611	10.79107666	1
MSTRG.1189.2	149	0.43199	6.640197754	1
MSTRG.1190.1	28	0.42464	5.713684082	1
MSTRG.1194.2	80	0.33953	9.137756348	1

MSTRG.1196.2	287	0.33115	4.906311035	1
MSTRG.1207.1	33	0.39879	10.43267822	1
MSTRG.1209.1	60	0.4013	9.514221191	-1
MSTRG.1214.2	103	0.36906	8.078796387	1
MSTRG.1215.1	182	0.3122	9.927307129	1
MSTRG.1211.1	23	0.44884	8.000305176	1
MSTRG.1236.4	199	0.33577	10.2868042	1
MSTRG.1245.2	696	0.41602	9.074523926	-1
MSTRG.1250.2	191	0.34787	6.101013184	1
MSTRG.1251.1	96	0.38338	10.26959229	1
MSTRG.1252.4	295	0.46276	9.412536621	1
MSTRG.1253.1	128	0.44172	10.82330322	-1
MSTRG.1259.2	123	0.41444	8.618591309	1
MSTRG.1260.1	62	0.33362	7.776672363	1
MSTRG.1261.2	161	0.37709	9.390808105	1
MSTRG.1261.5	386	0.43928	5.488098145	1
MSTRG.1261.6	189	0.43023	9.56072998	1
MSTRG.1254.1	45	0.32386	5.376525879	1
MSTRG.1263.1	790	0.43132	7.009460449	1
MSTRG.1267.4	480	0.36081	9.171691895	1
MSTRG.1269.1	125	0.36883	7.65447998	-1
MSTRG.1272.1	154	0.28165	6.308166504	1
MSTRG.1272.4	154	0.33754	6.308166504	1
MSTRG.1272.6	154	0.36841	6.308166504	1
MSTRG.1272.7	154	0.27863	6.308166504	1
MSTRG.1278.2	134	0.37415	5.278625488	1
MSTRG.1278.3	182	0.30829	9.909362793	1
MSTRG.1279.2	522	0.40397	6.787414551	1
MSTRG.1288.3	1070	0.47624	6.506652832	1
MSTRG.1288.4	235	0.35956	7.002502441	1
MSTRG.1288.5	235	0.35415	7.002502441	1
MSTRG.1285.1	108	0.35621	10.15289307	1
MSTRG.1309.1	82	0.31027	9.51763916	1
MSTRG.1310.2	65	0.28167	8.636047363	1
MSTRG.1310.1	43	0.36931	11.88482666	1
MSTRG.1310.3	38	0.46918	11.88482666	1
MSTRG.1311.1	377	0.31852	6.230895996	1
MSTRG.1311.7	377	0.34899	6.230895996	1
MSTRG.1311.5	377	0.37681	6.230895996	1
MSTRG.1311.6	377	0.37681	6.230895996	1
MSTRG.1311.8	377	0.33321	6.230895996	1
MSTRG.1311.9	377	0.36981	6.230895996	1
MSTRG.1311.10	377	0.42985	6.230895996	1
MSTRG.1309.2	82	0.3708	9.51763916	1
MSTRG.1312.1	142	0.36042	10.54095459	1
MSTRG.1314.2	411	0.47887	6.600036621	1
MSTRG.1317.1	151	0.34041	10.23260498	1
MSTRG.1318.2	531	0.4688	5.473083496	1
MSTRG.1318.3	785	0.45349	5.805358887	1
MSTRG.1319.1	79	0.28385	6.520080566	1
MSTRG.1320.1	179	0.33921	9.215270996	1
MSTRG.1321.1	182	0.40856	11.20367432	1
MSTRG.1323.1	189	0.28301	9.344421387	1
MSTRG.1318.4	144	0.31209	7.63861084	1

MSTRG.1325.1	541	0.39981	6.10760498	1
MSTRG.1332.2	431	0.29088	6.020446777	1
MSTRG.1338.1	808	0.45629	6.106384277	1
MSTRG.1338.2	723	0.47985	5.911682129	1
MSTRG.1338.4	723	0.47271	5.911682129	1
MSTRG.1338.3	766	0.50874	6.227233887	1
MSTRG.1338.7	356	0.43422	6.134094238	1
MSTRG.1338.8	280	0.33743	6.750061035	1
MSTRG.1339.1	74	0.33323	4.038391113	1
MSTRG.1344.2	175	0.32687	4.918518066	1
MSTRG.1346.1	271	0.35232	9.462463379	1
MSTRG.1351.5	1521	0.49467	6.01776123	1
MSTRG.1351.6	1398	0.46494	6.094299316	1
MSTRG.1352.6	209	0.28243	5.321228027	1
MSTRG.1352.2	107	0.30816	4.880065918	1
MSTRG.1352.4	167	0.39607	4.885925293	1
MSTRG.1352.9	107	0.29526	4.880065918	1
MSTRG.1352.11	67	0.45858	9.518249512	1
MSTRG.1352.12	47	0.37665	9.989196777	1
MSTRG.1352.10	107	0.38751	4.880065918	1
MSTRG.1352.13	127	0.4275	5.02130127	1
MSTRG.1352.14	107	0.37665	4.880065918	1
MSTRG.1352.15	130	0.41751	9.79876709	1
MSTRG.1355.4	583	0.43333	8.696350098	1
MSTRG.1363.1	106	0.3344	9.751281738	1
MSTRG.1367.2	136	0.30981	9.623596191	1
MSTRG.1367.3	271	0.33863	9.367858887	1
MSTRG.1380.9	77	0.33011	7.762878418	1
MSTRG.1380.7	693	0.46014	5.59161377	1
MSTRG.1380.5	842	0.46721	6.162414551	1
MSTRG.1380.8	665	0.46056	5.933654785	1
MSTRG.1380.10	166	0.36566	5.200378418	1
MSTRG.1380.11	48	0.26594	10.01287842	1
MSTRG.1382.1	116	0.3018	9.427429199	1
MSTRG.1384.4	914	0.4226	9.548400879	1
MSTRG.1384.3	2134	0.44205	8.780578613	1
MSTRG.1384.5	59	0.31637	9.44708252	1
MSTRG.1387.1	354	0.46483	9.333312988	1
MSTRG.1391.1	270	0.37988	9.575378418	1
MSTRG.1395.10	670	0.46431	9.493713379	1
MSTRG.1395.11	670	0.45395	9.493713379	1
MSTRG.1395.12	670	0.44287	9.493713379	1
MSTRG.1395.13	719	0.44791	9.429016113	1
MSTRG.1395.14	670	0.45834	9.493713379	1
MSTRG.1395.15	670	0.46266	9.493713379	1
MSTRG.1395.17	498	0.3555	9.648986816	1
MSTRG.1395.18	719	0.45243	9.429016113	1
MSTRG.1395.19	581	0.45243	9.511779785	1
MSTRG.1395.20	498	0.3555	9.648986816	1
MSTRG.1395.21	533	0.43619	9.420227051	1
MSTRG.1395.22	533	0.44625	9.420227051	1
MSTRG.1395.23	581	0.45243	9.511779785	1
MSTRG.1395.24	581	0.45395	9.511779785	1
MSTRG.1396.1	137	0.34924	9.472717285	1

MSTRG.1396.2	137	0.37438	9.472717285	1
MSTRG.1394.1	17	0.42277	6.494689941	1
MSTRG.1398.1	465	0.46944	8.91595459	1
MSTRG.1398.2	234	0.4082	5.78338623	1
MSTRG.1407.1	61	0.32494	11.253479	1
MSTRG.1408.1	385	0.42754	7.049865723	1
MSTRG.1414.3	139	0.39345	5.361877441	1
MSTRG.1415.1	527	0.37164	8.403747559	1
MSTRG.1415.3	533	0.38953	8.190246582	1
MSTRG.1416.2	319	0.45057	5.274108887	1
MSTRG.1419.4	74	0.36321	9.367858887	1
MSTRG.1419.3	215	0.43354	5.492492676	1
MSTRG.1420.1	109	0.35473	7.777648926	1
MSTRG.1421.1	402	0.41675	5.269104004	1
MSTRG.1426.2	778	0.43946	7.291320801	1
MSTRG.1432.3	45	0.40129	4.305236816	1
MSTRG.1432.2	133	0.35614	9.005554199	1
MSTRG.1433.1	120	0.34324	9.436462402	1
MSTRG.1433.5	676	0.37858	9.900085449	1
MSTRG.1429.2	220	0.33265	5.803283691	1
MSTRG.1429.3	308	0.33485	6.554138184	1
MSTRG.1428.1	98	0.4433	6.279724121	1
MSTRG.1436.1	376	0.30736	9.096252441	1
MSTRG.1436.4	174	0.29514	6.465881348	1
MSTRG.1436.2	376	0.28426	9.096252441	1
MSTRG.1436.3	376	0.28122	9.096252441	1
MSTRG.1437.1	85	0.31605	9.793151855	1
MSTRG.1438.1	151	0.27495	9.722229004	1
MSTRG.1436.7	376	0.31604	9.096252441	1
MSTRG.1436.6	376	0.30736	9.096252441	1
MSTRG.1439.2	754	0.45694	5.390563965	1
MSTRG.1440.1	115	0.30868	5.378967285	1
MSTRG.1441.1	285	0.46537	9.215515137	1
MSTRG.1447.1	91	0.30955	6.232971191	1
MSTRG.1449.1	522	0.45468	8.37322998	1
MSTRG.1450.1	234	0.39802	9.016784668	1
MSTRG.1451.2	145	0.42045	8.696716309	1
MSTRG.1451.3	145	0.42789	8.696716309	1
MSTRG.1454.5	137	0.3071	4.459533691	1
MSTRG.1455.2	79	0.33585	6.796081543	1
MSTRG.1455.1	78	0.39483	5.975769043	1
MSTRG.1460.1	61	0.36738	7.640197754	1
MSTRG.1463.2	345	0.43258	9.202453613	1
MSTRG.1464.1	86	0.35352	6.870056152	1
MSTRG.1465.2	214	0.4133	6.76348877	1
MSTRG.1467.2	1330	0.409	5.598815918	1
MSTRG.1469.1	516	0.43382	9.32244873	1
MSTRG.1470.1	220	0.36364	9.839782715	1
MSTRG.1475.3	108	0.27678	7.741638184	1
MSTRG.1475.2	371	0.42983	7.67791748	1
MSTRG.1475.4	371	0.44682	7.67791748	1
MSTRG.1475.5	371	0.42055	7.67791748	1
MSTRG.1475.6	346	0.44682	6.460876465	1
MSTRG.1477.1	189	0.27763	9.367736816	1

MSTRG.1473.1	1027	0.44385	5.241394043	1
MSTRG.1473.2	1017	0.44943	5.269470215	1
MSTRG.1494.2	226	0.42721	8.938415527	1
MSTRG.1495.1	90	0.36631	6.942810059	1
MSTRG.1496.1	1979	0.44379	6.273254395	1
MSTRG.1497.1	518	0.35722	6.283996582	1
MSTRG.1497.2	718	0.45057	6.35748291	1
MSTRG.1499.1	164	0.35948	6.512390137	1
MSTRG.1500.3	315	0.43542	6.527404785	1
MSTRG.1501.5	3151	0.40596	5.27557373	1
MSTRG.1501.1	3169	0.40596	5.339050293	1
MSTRG.1501.2	3115	0.40596	5.313781738	1
MSTRG.1501.3	3109	0.40596	5.319396973	1
MSTRG.1501.4	3175	0.40596	5.333312988	1
MSTRG.1501.6	3205	0.40596	5.299743652	1
MSTRG.1501.9	3116	0.40596	5.320495605	1
MSTRG.1501.10	3176	0.40596	5.340148926	1
MSTRG.1501.11	3168	0.40596	5.332214355	1
MSTRG.1501.14	805	0.41021	8.164733887	1
MSTRG.1481.1	73	0.31168	10.11114502	1
MSTRG.1483.2	184	0.27705	6.662658691	1
MSTRG.1484.1	178	0.32191	10.43988037	-1
MSTRG.1512.6	440	0.41135	8.507995605	1
MSTRG.1512.5	127	0.39622	5.215148926	1
MSTRG.1512.7	199	0.28248	7.312805176	1
MSTRG.1512.10	468	0.28338	8.717956543	1
MSTRG.1512.8	468	0.28584	8.717956543	1
MSTRG.1512.9	199	0.28584	7.312805176	1
MSTRG.1512.11	468	0.28584	8.717956543	1
MSTRG.1512.12	214	0.28567	9.888122559	1
MSTRG.1512.13	183	0.27458	10.1494751	1
MSTRG.1512.14	214	0.28584	9.888122559	1
MSTRG.1512.15	214	0.28584	9.888122559	1
MSTRG.1512.16	175	0.29776	10.44549561	1
MSTRG.1512.17	175	0.30132	10.44549561	1
MSTRG.1512.18	214	0.27911	9.888122559	1
MSTRG.1512.19	183	0.28179	10.1494751	1
MSTRG.1514.1	169	0.38358	5.851745605	1
MSTRG.1509.3	2287	0.43796	5.78326416	1
MSTRG.1511.2	533	0.37533	8.447814941	1
MSTRG.1511.3	564	0.30871	7.584777832	1
MSTRG.1511.5	390	0.27327	9.238830566	1
MSTRG.1516.1	72	0.3901	10.71307373	-1
MSTRG.1518.1	77	0.37723	8.954406738	1
MSTRG.1519.1	69	0.31609	9.61151123	-1
MSTRG.1520.2	368	0.31857	5.995666504	1
MSTRG.1535.1	110	0.29795	6.030700684	1
MSTRG.1536.1	82	0.3392	6.481994629	1
MSTRG.1537.4	138	0.43241	6.722351074	1
MSTRG.1538.1	188	0.31309	9.677429199	1
MSTRG.1538.7	1397	0.45618	6.042053223	-1
MSTRG.1538.8	358	0.44908	8.557189941	1
MSTRG.1538.9	882	0.37723	8.831970215	1
MSTRG.1538.10	1209	0.49712	6.141052246	1

MSTRG.1538.12	373	0.32396	5.582092285	1
MSTRG.1538.13	418	0.32396	5.672546387	1
MSTRG.1526.2	277	0.42886	6.656433105	1
MSTRG.1531.6	65	0.36971	7.607116699	1
MSTRG.1531.5	101	0.29415	7.737243652	1
MSTRG.1531.4	725	0.38655	8.931091309	1
MSTRG.1531.7	811	0.29268	9.203796387	1
MSTRG.1532.1	95	0.3654	9.771057129	1
MSTRG.1533.1	70	0.32557	7.878356934	1
MSTRG.1531.8	725	0.36605	8.931091309	1
MSTRG.1531.9	734	0.38655	8.866271973	1
MSTRG.1534.1	167	0.32208	10.20904541	1
MSTRG.1531.10	734	0.3489	8.866271973	1
MSTRG.1528.1	116	0.28153	10.70916748	1
MSTRG.1528.3	65	0.27851	9.056945801	1
MSTRG.1530.1	118	0.32126	6.708068848	1
MSTRG.1544.1	486	0.35593	6.81072998	1
MSTRG.1544.2	486	0.38733	6.81072998	1
MSTRG.1545.1	50	0.45249	11.9151001	-1
MSTRG.1549.1	72	0.3546	5.519470215	1
MSTRG.1550.5	818	0.46195	6.337585449	1
MSTRG.1550.6	1118	0.44085	6.910827637	1
MSTRG.1550.7	147	0.3344	5.749694824	1
MSTRG.1554.2	451	0.42904	5.22869873	1
MSTRG.1555.1	49	0.34311	10.84307861	1
MSTRG.1556.1	165	0.26999	9.359802246	1
MSTRG.1562.3	117	0.41501	9.774475098	1
MSTRG.1562.4	249	0.32032	5.922424316	1
MSTRG.1562.5	117	0.42186	9.774475098	1
MSTRG.1563.1	207	0.3656	7.736999512	1
MSTRG.1563.3	252	0.27781	8.940490723	1
MSTRG.1558.2	88	0.29447	6.041320801	-1
MSTRG.1560.1	105	0.29509	7.616638184	1
MSTRG.1576.2	525	0.46362	5.85760498	1
MSTRG.1578.4	616	0.39945	6.336975098	1
MSTRG.1578.7	899	0.46679	5.659118652	1
MSTRG.1578.9	86	0.36627	9.353942871	1
MSTRG.1578.11	110	0.3287	8.883605957	1
MSTRG.1578.10	92	0.29835	9.466003418	1
MSTRG.1578.12	361	0.32642	8.74810791	1
MSTRG.1578.13	167	0.29772	10.27178955	1
MSTRG.1578.14	85	0.34975	10.8324585	1
MSTRG.1567.3	188	0.38106	6.655334473	1
MSTRG.1567.4	188	0.32237	6.655334473	1
MSTRG.1567.5	264	0.39787	9.167053223	1
MSTRG.1567.7	69	0.3076	6.237121582	1
MSTRG.1568.2	287	0.37689	6.198425293	1
MSTRG.1568.4	287	0.39429	6.198425293	1
MSTRG.1568.6	549	0.42441	5.326477051	1
MSTRG.1568.5	549	0.43008	5.326477051	1
MSTRG.1573.1	144	0.37894	5.371643066	1
MSTRG.1574.2	295	0.46887	8.90423584	1
MSTRG.1574.3	295	0.37598	8.90423584	1
MSTRG.1589.1	57	0.41405	9.62310791	1

MSTRG.1590.1	144	0.38169	9.47479248	1
MSTRG.1592.2	198	0.34572	4.531799316	1
MSTRG.1592.1	335	0.31693	6.54473877	1
MSTRG.1592.4	164	0.30265	9.261413574	1
MSTRG.1592.5	335	0.40228	5.510559082	1
MSTRG.1592.6	335	0.4495	5.510559082	1
MSTRG.1593.1	50	0.29908	7.742980957	1
MSTRG.1592.7	178	0.34706	8.362976074	1
MSTRG.1592.8	95	0.34852	7.694763184	1
MSTRG.1594.1	213	0.40746	8.693908691	1
MSTRG.1595.1	107	0.31567	8.680236816	1
MSTRG.1596.1	82	0.35619	10.14581299	1
MSTRG.1597.1	27	0.39474	8.907653809	1
MSTRG.1598.1	81	0.31341	4.316101074	1
MSTRG.1596.2	146	0.28066	8.434265137	1
MSTRG.1583.1	18	0.41203	6.49822998	1
MSTRG.1583.2	22	0.35829	7.83404541	1
MSTRG.1585.1	69	0.47123	9.79107666	1
MSTRG.1600.1	79	0.38917	11.8604126	1
MSTRG.1603.1	342	0.48966	8.10357666	-1
MSTRG.1604.1	41	0.43825	9.945495605	1
MSTRG.1606.1	69	0.3547	12.00262451	1
MSTRG.1610.1	103	0.43617	10.09527588	1
MSTRG.1610.3	259	0.35568	5.153991699	1
MSTRG.1610.4	340	0.46829	6.202697754	1
MSTRG.1613.3	80	0.28165	9.695739746	1
MSTRG.1614.1	418	0.38655	9.886047363	1
MSTRG.1614.2	388	0.37942	9.83770752	1
MSTRG.1614.3	160	0.42084	5.949157715	1
MSTRG.1614.4	632	0.36033	9.611755371	1
MSTRG.1618.1	813	0.35168	9.190002441	1
MSTRG.1618.2	86	0.34003	7.664733887	-1
MSTRG.1618.4	458	0.41221	9.071105957	1
MSTRG.1618.3	584	0.44693	9.310852051	1
MSTRG.1619.1	129	0.29223	12.10003662	1
MSTRG.1619.2	141	0.26837	11.63140869	1
MSTRG.1623.2	379	0.41443	6.688415527	1
MSTRG.1637.4	96	0.28432	9.688903809	1
MSTRG.1637.5	311	0.31588	4.809631348	1
MSTRG.1632.1	146	0.29607	9.614074707	1
MSTRG.1632.2	146	0.30816	9.614074707	1
MSTRG.1644.3	2795	0.37782	6.133483887	1
MSTRG.1642.3	360	0.34162	7.656677246	1
MSTRG.1640.1	62	0.30705	6.498352051	1
MSTRG.1647.3	180	0.39385	9.38861084	1
MSTRG.1650.1	215	0.28311	9.178039551	1
MSTRG.1650.2	215	0.28463	9.178039551	1
MSTRG.1650.3	215	0.28463	9.178039551	1
MSTRG.1653.1	195	0.33128	9.25579834	1
MSTRG.1654.1	222	0.31672	5.389831543	1
MSTRG.1654.2	221	0.31672	5.389831543	1
MSTRG.1654.3	275	0.32925	5.828308105	1
MSTRG.1657.1	59	0.36168	9.518859863	1
MSTRG.1659.1	77	0.389	9.019836426	1

MSTRG.1662.1	1057	0.34907	9.908752441	1
MSTRG.1662.4	1004	0.47537	9.967102051	1
MSTRG.1662.7	1024	0.47537	9.896179199	1
MSTRG.1662.9	1057	0.46102	9.908752441	1
MSTRG.1662.10	1028	0.47484	9.883850098	1
MSTRG.1662.12	998	0.45418	9.814880371	1
MSTRG.1662.13	720	0.48524	9.711730957	1
MSTRG.1663.1	310	0.32934	9.181335449	1
MSTRG.1663.5	164	0.46126	5.167419434	-1
MSTRG.1663.4	63	0.3708	8.589538574	1
MSTRG.1663.3	87	0.34305	4.743713379	1
MSTRG.1665.1	65	0.31821	4.685852051	1
MSTRG.1667.1	78	0.26785	5.062072754	1
MSTRG.1671.1	483	0.4257	11.45977783	1
MSTRG.1673.5	228	0.37451	12.20574951	1
MSTRG.1673.4	845	0.46832	4.533630371	1
MSTRG.1673.3	767	0.45246	4.379089355	1
MSTRG.1675.1	98	0.3	10.67669678	1
MSTRG.1676.1	31	0.38592	11.52288818	1
MSTRG.1677.1	43	0.34842	10.01678467	1
MSTRG.1678.1	87	0.35248	10.73040771	-1
MSTRG.1681.1	261	0.44449	5.505432129	1
MSTRG.1685.2	204	0.33747	4.794616699	1
MSTRG.1685.3	204	0.44666	4.794616699	1
MSTRG.1689.1	33	0.42348	6.690734863	1
MSTRG.1691.1	37	0.29543	5.86920166	1
MSTRG.1692.1	95	0.39212	4.832824707	1
MSTRG.1697.2	216	0.45241	7.694274902	1
MSTRG.1697.1	61	0.32396	6.695251465	1
MSTRG.1697.3	275	0.32798	7.780822754	1
MSTRG.1697.4	61	0.43573	6.695251465	1
MSTRG.1709.2	576	0.35079	8.442443848	1
MSTRG.1709.4	1077	0.43206	7.963806152	1
MSTRG.1709.6	1077	0.44642	7.963806152	1
MSTRG.1709.8	843	0.42522	7.07989502	1
MSTRG.1716.2	199	0.38769	5.531188965	1
MSTRG.1716.1	124	0.37862	4.734558105	1
MSTRG.1716.3	124	0.43176	4.734558105	1
MSTRG.1717.1	111	0.41833	4.929870605	1
MSTRG.1725.1	206	0.35567	10.67547607	1
MSTRG.1726.1	123	0.31615	7.659729004	1
MSTRG.1721.1	23	0.40873	9.788513184	-1
MSTRG.1721.2	47	0.44478	4.386413574	-1
MSTRG.1729.2	369	0.32553	8.907165527	1
MSTRG.1730.1	171	0.30223	8.78692627	1
MSTRG.1731.1	101	0.34889	8.350036621	1
MSTRG.1734.1	89	0.29904	10.05194092	1
MSTRG.1734.2	56	0.40026	9.743469238	1
MSTRG.1727.1	145	0.29367	9.421569824	1
MSTRG.1727.2	30	0.416	8.518981934	1
MSTRG.1740.3	277	0.44964	4.478088379	1
MSTRG.1740.2	221	0.38402	4.30279541	1
MSTRG.1745.1	4906	0.45865	6.246032715	1
MSTRG.1745.3	6941	0.44727	6.046447754	1

MSTRG.1753.1	45	0.40891	6.367004395	1
MSTRG.1753.2	53	0.42474	9.295959473	1
MSTRG.1768.1	327	0.43985	9.146179199	1
MSTRG.1768.2	310	0.46708	9.392150879	1
MSTRG.1768.3	179	0.38549	8.798278809	1
MSTRG.1770.2	81	0.39384	9.624572754	1
MSTRG.1772.1	793	0.46393	6.405334473	1
MSTRG.1772.3	793	0.46145	6.405334473	1
MSTRG.1772.4	316	0.4409	5.899719238	-1
MSTRG.1773.1	30	0.47878	9.500183105	1
MSTRG.1774.1	33	0.35394	6.524597168	1
MSTRG.1777.2	512	0.47004	7.052185059	1
MSTRG.1777.3	496	0.41922	6.244689941	1
MSTRG.1782.1	1237	0.47516	5.058288574	1
MSTRG.1782.5	1083	0.46853	5.16229248	1
MSTRG.1782.4	930	0.34959	5.067199707	1
MSTRG.1783.1	67	0.28334	10.02215576	1
MSTRG.1784.1	182	0.28518	10.22991943	1
MSTRG.1787.1	61	0.40957	9.730163574	1
MSTRG.1787.2	61	0.3833	9.730163574	1
MSTRG.1779.1	60	0.3819	11.75421143	-1
MSTRG.1780.1	89	0.27385	9.496643066	1
MSTRG.1791.2	520	0.47254	7.61126709	1
MSTRG.1791.3	598	0.45713	8.114929199	1
MSTRG.1798.1	68	0.29366	8.936096191	1
MSTRG.1799.1	207	0.35415	6.733093262	1
MSTRG.1799.2	207	0.32442	6.733093262	1
MSTRG.1799.3	207	0.37626	6.733093262	1
MSTRG.1799.4	207	0.39392	6.733093262	1
MSTRG.1799.7	82	0.32697	5.144470215	1
MSTRG.1799.6	207	0.36554	6.733093262	1
MSTRG.1798.2	68	0.29575	8.936096191	1
MSTRG.1798.3	81	0.2921	10.62042236	1
MSTRG.1799.8	83	0.30092	5.440979004	1
MSTRG.1805.1	243	0.35373	7.663513184	1
MSTRG.1805.3	369	0.39677	9.947692871	1
MSTRG.1801.1	41	0.34112	7.933776855	1
MSTRG.1801.2	16	0.36466	5.895202637	1
MSTRG.1801.3	51	0.32546	9.559753418	1
MSTRG.1804.1	550	0.44215	9.629577637	-1
MSTRG.1819.1	22220	0.44832	6.498962402	1
MSTRG.1811.3	123	0.42413	8.41583252	1
MSTRG.1814.1	928	0.37021	6.36541748	1
MSTRG.1814.3	928	0.40483	6.36541748	1
MSTRG.1814.4	928	0.43494	6.36541748	1
MSTRG.1814.2	928	0.43494	6.36541748	1
MSTRG.1814.7	928	0.42437	6.36541748	1
MSTRG.1814.8	69	0.33388	9.969177246	1
MSTRG.1818.1	474	0.37532	6.736022949	1
MSTRG.1818.3	475	0.37532	6.736022949	1
MSTRG.1832.1	97	0.30387	8.675354004	1
MSTRG.1832.3	238	0.32586	6.31817627	1
MSTRG.1833.2	84	0.30806	7.777038574	1
MSTRG.1833.1	78	0.37598	4.777526855	1

MSTRG.1833.3	78	0.32954	4.777526855	1
MSTRG.1834.1	183	0.35032	10.39398193	1
MSTRG.1840.1	56	0.31574	11.39654541	1
MSTRG.1829.1	95	0.31553	9.137512207	1
MSTRG.1841.5	209	0.48726	6.586608887	-1
MSTRG.1847.1	429	0.4694	5.07989502	1
MSTRG.1847.2	152	0.32948	9.618103027	1
MSTRG.1869.1	70	0.2921	9.742370605	-1
MSTRG.1868.11	186	0.29367	10.94952393	1
MSTRG.1868.12	110	0.30132	9.778747559	1
MSTRG.1868.13	60	0.32421	8.934020996	1
MSTRG.1873.1	49	0.34372	10.2164917	1
MSTRG.1874.1	218	0.29115	9.828674316	1
MSTRG.1874.2	112	0.28683	10.53094482	1
MSTRG.1849.4	447	0.45871	6.61126709	1
MSTRG.1850.1	178	0.3052	10.02545166	1
MSTRG.1856.4	781	0.45905	6.822814941	1
MSTRG.1857.1	422	0.33514	5.448425293	1
MSTRG.1860.1	396	0.44888	7.641418457	1
MSTRG.1860.5	158	0.28883	8.809753418	1
MSTRG.1860.6	129	0.34203	8.52557373	1
MSTRG.1860.3	396	0.45761	7.641418457	1
MSTRG.1860.4	396	0.48426	7.641418457	1
MSTRG.1862.1	54	0.41095	4.750061035	1
MSTRG.1860.7	238	0.3208	9.300231934	1
MSTRG.1864.1	86	0.32185	9.124328613	1
MSTRG.1865.1	73	0.33354	4.366027832	1
MSTRG.1866.1	88	0.28848	11.29168701	1
MSTRG.1867.2	732	0.46079	5.309631348	1
MSTRG.1867.3	733	0.47038	5.309631348	1
MSTRG.1891.2	456	0.31653	5.097961426	1
MSTRG.1895.2	392	0.39257	9.09552002	1
MSTRG.1895.3	62	0.33863	9.299377441	1
MSTRG.1895.5	121	0.36389	9.012878418	1
MSTRG.1895.4	351	0.4119	8.202087402	1
MSTRG.1900.3	413	0.47249	5.59564209	1
MSTRG.1900.4	413	0.46496	5.59564209	1
MSTRG.1900.5	419	0.47634	5.59564209	1
MSTRG.1900.6	88	0.39094	9.673278809	1
MSTRG.1902.4	612	0.48525	5.167297363	1
MSTRG.1906.1	96	0.34504	8.589294434	1
MSTRG.1907.2	303	0.42291	5.153991699	1
MSTRG.1907.3	442	0.41754	5.643859863	1
MSTRG.1907.4	442	0.43804	5.643859863	1
MSTRG.1911.1	105	0.36796	9.225402832	-1
MSTRG.1912.2	413	0.32015	9.700012207	1
MSTRG.1912.4	116	0.43047	5.855285645	1
MSTRG.1912.7	429	0.31677	9.614562988	1
MSTRG.1914.1	68	0.36412	9.742370605	1
MSTRG.1912.8	39	0.31928	9.184875488	1
MSTRG.1912.9	298	0.30387	6.594909668	1
MSTRG.1909.2	387	0.43035	4.449035645	1
MSTRG.1910.1	54	0.38503	8.47467041	1
MSTRG.1922.1	225	0.46175	4.391540527	1

MSTRG.1922.4	142	0.44938	3.890441895	1
MSTRG.1922.6	142	0.47398	3.890441895	1
MSTRG.1922.5	287	0.48419	4.634094238	1
MSTRG.1923.1	69	0.43794	6.223449707	-1
MSTRG.1924.1	58	0.38533	5.25213623	1
MSTRG.1924.2	58	0.40225	5.25213623	1
MSTRG.1928.3	1088	0.44355	8.612121582	1
MSTRG.1928.5	1088	0.4296	8.612121582	1
MSTRG.1928.7	797	0.39394	6.727111816	1
MSTRG.1928.8	81	0.36241	9.11517334	-1
MSTRG.1928.9	349	0.37218	5.066711426	1
MSTRG.1928.10	88	0.29595	8.272277832	1
MSTRG.1930.1	125	0.30576	9.868103027	1
MSTRG.1928.11	90	0.29919	10.30059814	1
MSTRG.1928.12	145	0.29387	9.441345215	1
MSTRG.1928.13	105	0.31035	6.002990723	1
MSTRG.1920.4	1888	0.46333	5.717468262	1
MSTRG.1920.5	1889	0.46581	5.717468262	1
MSTRG.1920.6	599	0.35421	6.294616699	1
MSTRG.1920.7	1888	0.48146	5.717468262	1
MSTRG.1921.1	128	0.3046	9.300720215	1
MSTRG.1918.3	213	0.33985	9.051086426	1
MSTRG.1940.1	195	0.30387	7.085144043	1
MSTRG.1940.2	558	0.34519	5.349182129	1
MSTRG.1940.5	424	0.32642	5.382751465	1
MSTRG.1940.4	415	0.32257	5.671936035	1
MSTRG.1941.1	74	0.29615	8.297912598	1
MSTRG.1943.1	116	0.35742	5.883728027	-1
MSTRG.1943.2	52	0.30112	6.50982666	-1
MSTRG.1943.4	241	0.46633	5.611633301	1
MSTRG.1945.2	290	0.46391	9.09564209	1
MSTRG.1948.1	57	0.28726	9.694641113	1
MSTRG.1949.1	151	0.35155	10.07977295	1
MSTRG.1951.1	918	0.46606	5.414001465	1
MSTRG.1934.1	4649	0.46495	5.043762207	1
MSTRG.1953.1	86	0.30301	10.20123291	1
MSTRG.1954.1	81	0.29475	6.815612793	1
MSTRG.1995.1	83	0.33863	5.114440918	1
MSTRG.2012.1	90	0.3152	7.764953613	1
MSTRG.2013.1	61	0.45497	5.986633301	1
MSTRG.2022.1	126	0.25765	4.935974121	1
MSTRG.2023.1	108	0.29367	10.44903564	1
MSTRG.2023.2	108	0.29367	10.44903564	1
MSTRG.2025.1	71	0.27517	9.868103027	1
MSTRG.2028.1	116	0.2765	8.70892334	1
MSTRG.1959.1	868	0.48038	6.190979004	1
MSTRG.1959.3	1287	0.47855	6.540344238	1
MSTRG.1960.1	423	0.40611	7.604797363	1
MSTRG.1962.1	36	0.42036	8.504333496	1
MSTRG.1962.2	36	0.41493	8.504333496	1
MSTRG.1968.3	1141	0.46599	6.717834473	1
MSTRG.1968.8	1272	0.43965	6.897888184	1
MSTRG.1972.2	430	0.36032	8.895812988	1
MSTRG.1972.6	252	0.30596	9.443908691	1

MSTRG.1972.4	182	0.36197	9.030700684	1
MSTRG.1972.3	472	0.3918	9.175354004	1
MSTRG.1972.8	233	0.37782	9.559387207	1
MSTRG.1972.10	249	0.35339	8.673522949	1
MSTRG.1972.9	154	0.30576	6.215515137	1
MSTRG.1972.11	220	0.38152	8.582702637	1
MSTRG.1970.1	12	0.32598	6.487976074	1
MSTRG.1989.1	46	0.37419	6.691589355	1
MSTRG.1989.2	43	0.38543	6.694030762	1
MSTRG.1990.1	95	0.39094	9.641906738	-1
MSTRG.2059.1	107	0.37264	6.480529785	-1
MSTRG.2062.3	244	0.44417	4.794128418	1
MSTRG.2062.4	227	0.3207	5.095397949	1
MSTRG.2062.6	455	0.37886	6.476745605	1
MSTRG.2062.5	437	0.4352	5.784729004	-1
MSTRG.2063.1	137	0.27151	10.49859619	-1
MSTRG.2060.1	50	0.43881	3.851257324	1
MSTRG.2068.4	373	0.47024	8.511291504	1
MSTRG.2068.5	370	0.46701	8.210021973	1
MSTRG.2068.6	373	0.47229	8.511291504	1
MSTRG.2068.7	404	0.4508	8.917419434	1
MSTRG.2068.9	401	0.4508	8.793273926	1
MSTRG.2068.8	400	0.3538	8.793273926	1
MSTRG.2070.1	34	0.37011	10.3180542	1
MSTRG.2071.1	106	0.33162	5.436950684	1
MSTRG.2067.1	53	0.41385	11.71990967	1
MSTRG.2067.2	88	0.40667	10.76116943	1
MSTRG.2066.1	35	0.33597	4.529724121	1
MSTRG.2072.2	582	0.33326	6.157165527	1
MSTRG.2079.1	81	0.35996	9.096862793	1
MSTRG.2079.2	15	0.30828	9.996276855	1
MSTRG.2085.1	138	0.44403	7.672058105	1
MSTRG.2085.2	138	0.37792	7.672058105	1
MSTRG.2086.1	45	0.37086	8.200256348	-1
MSTRG.2088.6	563	0.46701	5.938049316	1
MSTRG.2088.4	663	0.46483	6.253479004	1
MSTRG.2088.5	701	0.45836	6.674621582	1
MSTRG.2088.8	650	0.37667	8.171203613	1
MSTRG.2083.1	107	0.3182	11.43487549	1
MSTRG.2095.1	401	0.36372	9.725280762	1
MSTRG.2095.2	85	0.4015	9.729064941	1
MSTRG.2098.1	98	0.32485	6.245056152	1
MSTRG.2097.6	2142	0.4599	6.177429199	1
MSTRG.2097.7	1373	0.46426	6.179748535	1
MSTRG.2097.8	1730	0.45277	6.472961426	1
MSTRG.2110.8	1001	0.42027	9.365539551	1
MSTRG.2110.1	1121	0.4229	9.242126465	1
MSTRG.2110.5	1120	0.42495	9.242126465	1
MSTRG.2111.1	82	0.31899	9.342590332	1
MSTRG.2110.9	181	0.28959	5.257385254	1
MSTRG.2112.1	37	0.46271	4.571105957	1
MSTRG.2113.1	67	0.31941	9.256164551	1
MSTRG.2114.1	603	0.45647	8.316223145	1
MSTRG.2116.1	179	0.37869	9.675598145	-1

MSTRG.2114.2	603	0.45385	8.316223145	1
MSTRG.2108.1	75	0.3926	9.575500488	-1
MSTRG.2119.3	727	0.42908	6.602111816	1
MSTRG.2118.1	64	0.43813	9.514709473	-1
MSTRG.2126.1	106	0.46144	5.215270996	-1
MSTRG.2148.1	76	0.3583	9.414489746	1
MSTRG.2152.1	107	0.37661	6.38494873	1
MSTRG.2154.1	66	0.27415	7.889831543	1
MSTRG.2153.6	114	0.27517	8.558654785	1
MSTRG.2153.3	102	0.29594	6.011535645	1
MSTRG.2153.5	102	0.34514	6.011535645	1
MSTRG.2153.4	226	0.27843	5.940856934	1
MSTRG.2153.7	281	0.33238	10.07720947	1
MSTRG.2155.1	914	0.45277	7.16619873	1
MSTRG.2155.2	910	0.46952	8.139465332	1
MSTRG.2155.3	896	0.47937	7.39276123	1
MSTRG.2155.4	915	0.45631	8.139465332	1
MSTRG.2155.7	130	0.43076	8.686462402	1
MSTRG.2155.8	269	0.44566	4.729309082	1
MSTRG.2155.9	135	0.34585	11.24005127	1
MSTRG.2144.1	55	0.36155	4.55657959	1
MSTRG.2145.1	120	0.39711	5.092956543	1
MSTRG.2146.1	115	0.2903	8.66583252	1
MSTRG.2146.2	165	0.29111	6.418151855	1
MSTRG.2175.1	6075	0.45873	9.11505127	-1
MSTRG.2175.2	1470	0.45067	9.463684082	1
MSTRG.2177.2	2439	0.44609	5.251647949	1
MSTRG.2177.1	2439	0.46039	5.251647949	1
MSTRG.2177.6	2439	0.45606	5.251647949	1
MSTRG.2177.4	2465	0.45038	5.326477051	1
MSTRG.2177.7	1591	0.36822	5.00189209	1
MSTRG.2177.8	2439	0.44356	5.251647949	1
MSTRG.2177.10	2431	0.47107	5.261535645	1
MSTRG.2177.12	1617	0.41017	5.074401855	1
MSTRG.2177.11	1591	0.4459	5.00189209	1
MSTRG.2157.1	309	0.45197	5.086242676	1
MSTRG.2166.1	32	0.33095	8.885314941	1
MSTRG.2167.1	63	0.41318	6.88873291	1
MSTRG.2179.2	725	0.41061	5.946838379	1
MSTRG.2179.3	725	0.46334	5.946838379	1
MSTRG.2179.4	372	0.39179	9.221618652	1
MSTRG.2200.2	1498	0.41345	9.082702637	1
MSTRG.2200.3	1498	0.39371	9.082702637	1
MSTRG.2200.5	1542	0.41886	8.781433105	1
MSTRG.2201.1	58	0.32854	8.477966309	1
MSTRG.2202.1	69	0.35173	9.30267334	1
MSTRG.2203.1	72	0.29343	6.244445801	1
MSTRG.2210.3	96	0.28644	10.00469971	1
MSTRG.2211.1	75	0.34947	7.735778809	1
MSTRG.2210.4	110	0.30684	8.528137207	1
MSTRG.2212.1	266	0.34547	8.17010498	1
MSTRG.2213.3	180	0.36514	9.26361084	1
MSTRG.2196.1	15	0.31444	3.799377441	1
MSTRG.2219.1	96	0.29367	8.094543457	1

MSTRG.2220.2	1214	0.42871	5.910095215	1
MSTRG.2221.1	100	0.29367	9.811340332	1
MSTRG.2221.2	71	0.36201	8.297912598	1
MSTRG.2222.1	76	0.29367	8.557312012	1
MSTRG.2222.2	73	0.28683	7.945739746	1
MSTRG.2220.3	1079	0.34306	9.463439941	1
MSTRG.2220.4	769	0.40078	8.977233887	1
MSTRG.2223.3	37	0.40556	6.511291504	1
MSTRG.2223.4	190	0.29772	9.664611816	1
MSTRG.2223.5	138	0.27433	9.117248535	1
MSTRG.2223.6	190	0.30735	9.664611816	1
MSTRG.2224.1	51	0.36389	4.837219238	1
MSTRG.2225.1	124	0.29644	9.618469238	1
MSTRG.2230.1	29	0.32954	11.73394775	1
MSTRG.2240.1	54	0.39942	11.00860596	-1
MSTRG.2241.1	57	0.42528	9.101257324	-1
MSTRG.2248.2	413	0.43443	6.679626465	1
MSTRG.2248.4	413	0.35886	6.679626465	1
MSTRG.2248.6	409	0.43059	6.430236816	-1
MSTRG.2250.1	115	0.30544	6.500671387	1
MSTRG.2252.1	811	0.37963	7.216369629	-1
MSTRG.2257.2	329	0.38395	9.097106934	1
MSTRG.2257.3	349	0.40124	8.458190918	1
MSTRG.2265.1	157	0.31561	8.487854004	1
MSTRG.2265.2	157	0.33131	8.487854004	1
MSTRG.2265.3	157	0.29526	8.487854004	1
MSTRG.2265.4	157	0.39175	8.487854004	1
MSTRG.2265.6	157	0.33131	8.487854004	1
MSTRG.2265.5	157	0.3295	8.487854004	1
MSTRG.2262.2	473	0.40391	6.299621582	1
MSTRG.2268.4	828	0.42251	9.350769043	1
MSTRG.2268.3	1037	0.45786	8.868103027	1
MSTRG.2268.5	812	0.39187	9.351013184	1
MSTRG.2269.1	73	0.34138	10.57415771	1
MSTRG.2273.1	120	0.40051	7.672668457	1
MSTRG.2278.1	48	0.30303	7.549987793	1
MSTRG.2280.1	976	0.38943	5.399841309	1
MSTRG.2281.1	201	0.29848	10.04022217	1
MSTRG.2282.1	47	0.32545	5.95526123	1
MSTRG.2283.1	98	0.39047	9.832702637	1
MSTRG.2284.1	157	0.33075	6.260314941	1
MSTRG.2270.4	378	0.33194	9.682189941	1
MSTRG.2270.5	378	0.30552	9.682189941	1
MSTRG.2288.1	739	0.34756	8.554504395	1
MSTRG.2288.3	673	0.31638	8.499206543	1
MSTRG.2289.1	105	0.30396	8.661071777	1
MSTRG.2285.1	20	0.3524	6.704406738	-1
MSTRG.2311.1	38	0.40502	6.51348877	1
MSTRG.2350.1	341	0.37936	9.137878418	1
MSTRG.2352.2	806	0.47928	8.921813965	1
MSTRG.2352.3	358	0.40306	5.79888916	1
MSTRG.2352.4	327	0.35741	6.768249512	1
MSTRG.2355.1	26	0.38684	8.886169434	1
MSTRG.2361.1	933	0.42555	7.346130371	1

MSTRG.2361.2	926	0.37465	7.346618652	1
MSTRG.2361.4	694	0.36749	8.156311035	1
MSTRG.2361.5	720	0.34495	7.560974121	1
MSTRG.2361.3	694	0.32185	8.156311035	1
MSTRG.2362.1	125	0.3701	9.666931152	1
MSTRG.2364.1	120	0.32237	8.850524902	1
MSTRG.2364.4	429	0.34002	9.137268066	1
MSTRG.2364.6	455	0.31035	9.395935059	1
MSTRG.2365.1	47	0.27433	9.383972168	1
MSTRG.2364.7	525	0.34367	6.388977051	1
MSTRG.2364.8	141	0.28883	9.762634277	1
MSTRG.2364.9	114	0.46915	5.320617676	1
MSTRG.2366.1	217	0.3572	9.182678223	1
MSTRG.2364.11	96	0.29022	9.194641113	1
MSTRG.2371.1	1557	0.44527	7.014831543	1
MSTRG.2381.1	1473	0.38007	8.967712402	1
MSTRG.2381.10	1242	0.37931	9.030822754	1
MSTRG.2381.7	1242	0.38007	9.030822754	1
MSTRG.2381.4	1234	0.37931	8.996398926	1
MSTRG.2381.6	1234	0.37931	8.996398926	1
MSTRG.2381.5	1465	0.41648	8.937194824	1
MSTRG.2381.2	1464	0.39393	8.899597168	1
MSTRG.2381.3	1456	0.38007	8.865661621	1
MSTRG.2381.9	1473	0.37294	8.967712402	1
MSTRG.2381.13	1260	0.38007	8.54107666	1
MSTRG.2381.12	1260	0.37931	8.54107666	1
MSTRG.2381.15	1260	0.37931	8.54107666	1
MSTRG.2381.17	1225	0.37931	8.913757324	1
MSTRG.2381.16	884	0.37574	9.39276123	1
MSTRG.2381.14	1456	0.38007	8.865661621	1
MSTRG.2381.18	1456	0.37294	8.865661621	1
MSTRG.2381.19	863	0.39938	9.532531738	1
MSTRG.2387.1	43	0.43212	7.88470459	1
MSTRG.2385.1	109	0.43035	8.915100098	1
MSTRG.2391.1	28	0.34816	5.193908691	-1
MSTRG.2392.1	60	0.38289	9.514343262	-1
MSTRG.2398.5	228	0.41991	5.585754395	1
MSTRG.2412.1	115	0.33387	5.731750488	1
MSTRG.2412.2	122	0.33851	7.80255127	1
MSTRG.2412.3	136	0.29367	10.43878174	1
MSTRG.2415.1	237	0.36813	8.157409668	1
MSTRG.2412.4	166	0.31543	10.30926514	1
MSTRG.2417.1	95	0.35007	10.47076416	1
MSTRG.2417.2	90	0.38202	9.694152832	1
MSTRG.2418.1	215	0.30411	9.905822754	1
MSTRG.2419.2	352	0.41152	9.29888916	1
MSTRG.2419.5	145	0.37209	10.58868408	1
MSTRG.2419.6	354	0.41656	9.224304199	1
MSTRG.2419.4	354	0.32591	9.224304199	1
MSTRG.2403.1	81	0.31985	9.159362793	1
MSTRG.2407.1	49	0.40077	4.795227051	-1
MSTRG.2426.1	33	0.38905	10.66497803	1
MSTRG.2431.1	23	0.41684	12.00848389	1
MSTRG.2433.1	88	0.40496	9.896057129	-1

MSTRG.2438.1	78	0.36533	7.00970459	1
MSTRG.2439.4	264	0.44462	6.314147949	1
MSTRG.2435.3	372	0.46621	5.296325684	1
MSTRG.2446.3	415	0.41895	9.059753418	1
MSTRG.2447.1	153	0.29539	5.998840332	1
MSTRG.2475.4	564	0.44656	5.505554199	1
MSTRG.2475.5	440	0.44499	9.899597168	1
MSTRG.2476.1	240	0.40967	10.30682373	1
MSTRG.2476.2	71	0.44988	6.172790527	1
MSTRG.2477.1	83	0.43489	8.553161621	-1
MSTRG.2479.4	498	0.43104	5.314880371	1
MSTRG.2480.1	109	0.31899	8.977844238	1
MSTRG.2479.5	162	0.45688	5.249816895	1
MSTRG.2479.6	157	0.4483	5.018371582	1
MSTRG.2479.7	157	0.45388	5.018371582	1
MSTRG.2479.8	171	0.45428	5.903625488	1
MSTRG.2479.9	292	0.42524	6.652893066	1
MSTRG.2479.10	278	0.40907	5.94744873	1
MSTRG.2479.13	157	0.44422	5.018493652	1
MSTRG.2479.14	177	0.416	5.604309082	1
MSTRG.2479.15	310	0.39226	6.522888184	1
MSTRG.2479.16	136	0.44298	4.789001465	1
MSTRG.2479.17	50	0.37806	6.700134277	1
MSTRG.2485.2	997	0.4635	5.388366699	1
MSTRG.2485.3	1031	0.4635	5.583312988	1
MSTRG.2460.1	681	0.46948	6.112487793	1
MSTRG.2464.2	163	0.28584	8.202087402	1
MSTRG.2468.1	152	0.3287	7.733093262	1
MSTRG.2489.3	213	0.36783	9.352478027	1
MSTRG.2492.2	878	0.43415	6.88885498	1
MSTRG.2493.1	587	0.44716	7.825134277	1
MSTRG.2498.2	558	0.3208	9.095153809	1
MSTRG.2509.3	258	0.33406	7.294250488	1
MSTRG.2509.5	485	0.43577	8.914245605	1
MSTRG.2509.4	258	0.37345	7.294250488	1
MSTRG.2510.1	113	0.37743	11.60028076	1
MSTRG.2510.2	113	0.39035	11.60028076	1
MSTRG.2513.3	115	0.31105	5.624084473	1
MSTRG.2513.5	75	0.31553	8.741394043	1
MSTRG.2514.3	46	0.37064	7.857727051	1
MSTRG.2515.1	143	0.32974	11.67718506	1
MSTRG.2499.1	54	0.34571	10.30621338	-1
MSTRG.2508.1	418	0.33863	7.008483887	1
MSTRG.2517.2	656	0.44662	7.860534668	1
MSTRG.2517.3	590	0.45189	6.762512207	1
MSTRG.2519.3	862	0.39301	5.993103027	1
MSTRG.2519.4	863	0.39301	6.058532715	1
MSTRG.2529.1	1038	0.36749	5.913146973	1
MSTRG.2529.2	53	0.33783	5.661315918	1
MSTRG.2530.1	62	0.32485	10.00604248	1
MSTRG.2566.1	127	0.34564	12.60296631	1
MSTRG.2567.1	44	0.30625	10.25604248	1
MSTRG.2568.2	66	0.33285	3.929870605	1
MSTRG.2569.1	721	0.47188	5.285583496	1

MSTRG.2569.2	584	0.41997	6.164611816	1
MSTRG.2569.4	584	0.35549	6.164611816	1
MSTRG.2569.6	178	0.45893	9.29876709	1
MSTRG.2569.5	584	0.43642	6.164611816	1
MSTRG.2572.1	128	0.35406	10.02423096	1
MSTRG.2569.7	2693	0.39062	5.933166504	1
MSTRG.2569.8	2693	0.41258	5.933166504	1
MSTRG.2569.9	2693	0.47601	5.933166504	1
MSTRG.2575.1	58	0.29639	9.019592285	1
MSTRG.2576.4	89	0.45999	4.808166504	-1
MSTRG.2576.3	423	0.3231	5.793029785	1
MSTRG.2577.1	183	0.38161	10.98162842	1
MSTRG.2576.5	447	0.3888	6.53338623	1
MSTRG.2576.6	100	0.30807	8.859069824	1
MSTRG.2576.7	93	0.29615	10.00872803	1
MSTRG.2579.2	1314	0.42145	9.053039551	1
MSTRG.2579.3	671	0.47072	5.895446777	1
MSTRG.2541.2	76	0.42799	6.817687988	1
MSTRG.2542.1	66	0.3847	11.00006104	1
MSTRG.2543.1	42	0.34996	7.741516113	1
MSTRG.2544.1	49	0.30387	10.29510498	1
MSTRG.2545.1	76	0.26931	9.965026855	1
MSTRG.2536.1	53	0.40522	6.491271973	1
MSTRG.2540.1	82	0.3484	6.229309082	1
MSTRG.2561.4	225	0.45552	4.509338379	1
MSTRG.2564.3	428	0.42431	7.539855957	1
MSTRG.2582.2	302	0.31137	8.374572754	1
MSTRG.2582.3	302	0.31137	8.374572754	1
MSTRG.2588.1	85	0.31151	12.05914307	1
MSTRG.2589.2	341	0.32729	5.455749512	1
MSTRG.2589.3	91	0.30648	11.89239502	1
MSTRG.2589.4	312	0.45938	5.05291748	1
MSTRG.2590.2	64	0.38872	6.792175293	1
MSTRG.2590.1	64	0.38467	6.792175293	1
MSTRG.2590.3	64	0.37002	6.792175293	1
MSTRG.2591.5	84	0.3333	10.06829834	1
MSTRG.2591.6	39	0.41495	4.780456543	1
MSTRG.2594.1	414	0.48838	6.127624512	1
MSTRG.2596.4	1723	0.44252	5.932922363	1
MSTRG.2596.3	2042	0.43134	6.184875488	1
MSTRG.2597.2	37	0.33159	9.49810791	1
MSTRG.2597.1	37	0.35804	9.49810791	1
MSTRG.2598.1	37	0.44865	4.889099121	-1
MSTRG.2602.1	92	0.35111	7.852844238	1
MSTRG.2602.2	92	0.34825	7.852844238	1
MSTRG.2602.3	92	0.34648	5.951965332	1
MSTRG.2602.10	92	0.30576	7.852844238	1
MSTRG.2602.11	92	0.30576	7.852844238	1
MSTRG.2602.12	92	0.28683	7.852844238	1
MSTRG.2601.1	47	0.40387	11.21966553	1
MSTRG.2601.2	73	0.42428	4.493469238	1
MSTRG.2605.3	527	0.36459	9.527526855	1
MSTRG.2605.4	527	0.35591	9.527526855	1
MSTRG.2605.5	3096	0.46306	6.434997559	1

MSTRG.2605.6	527	0.35591	9.527526855	1
MSTRG.2605.7	3256	0.43802	6.577941895	1
MSTRG.2605.8	1383	0.45778	6.588439941	1
MSTRG.2605.10	2055	0.47443	6.002624512	1
MSTRG.2605.9	1445	0.4532	6.12701416	1
MSTRG.2605.11	680	0.44437	5.768371582	1
MSTRG.2609.7	987	0.37332	8.54876709	1
MSTRG.2609.8	975	0.37332	8.701599121	1
MSTRG.2609.9	987	0.39312	8.54876709	1
MSTRG.2610.2	83	0.27517	10.38800049	1
MSTRG.2611.1	188	0.30807	5.213684082	1
MSTRG.2607.3	399	0.43603	6.93157959	1
MSTRG.2682.1	163	0.34157	7.659973145	1
MSTRG.2614.1	35	0.41719	7.934143066	1
MSTRG.2632.1	33	0.38056	10.11859131	1
MSTRG.2633.1	60	0.40635	9.381652832	-1
MSTRG.2634.3	86	0.42453	10.4019165	1
MSTRG.2635.1	142	0.30191	7.838562012	1
MSTRG.2636.1	66	0.37248	10.16790771	1
MSTRG.2638.1	33	0.39497	7.935119629	1
MSTRG.2639.1	617	0.34763	9.293273926	1
MSTRG.2640.1	108	0.28157	5.668762207	1
MSTRG.2641.1	91	0.28178	8.592834473	1
MSTRG.2642.1	59	0.36459	7.895690918	1
MSTRG.2645.1	75	0.29452	12.23480225	1
MSTRG.2645.2	91	0.30981	10.01519775	1
MSTRG.2645.3	67	0.3	8.570007324	1
MSTRG.2647.1	28	0.31501	9.983947754	1
MSTRG.2647.2	104	0.39951	7.565612793	1
MSTRG.2649.1	78	0.35529	6.348815918	1
MSTRG.2668.3	1716	0.36918	7.540710449	1
MSTRG.2668.4	1709	0.36918	7.540710449	1
MSTRG.2668.5	1841	0.36298	8.284851074	1
MSTRG.2668.6	1260	0.36453	8.515319824	1
MSTRG.2668.7	1260	0.37773	8.515319824	1
MSTRG.2706.2	269	0.46006	6.405212402	1
MSTRG.2706.3	329	0.44072	5.968688965	1
MSTRG.2711.3	76	0.36065	10.11541748	1
MSTRG.2711.4	99	0.34376	9.410583496	1
MSTRG.2714.3	346	0.442	7.61529541	1
MSTRG.2714.4	405	0.31973	6.812316895	1
MSTRG.2715.1	1315	0.46485	5.638122559	1
MSTRG.2715.4	1315	0.45629	5.638122559	1
MSTRG.2715.5	728	0.46178	5.821228027	1
MSTRG.2717.2	331	0.3183	9.129455566	1
MSTRG.2720.1	74	0.36894	9.88470459	1
MSTRG.2721.2	144	0.44944	9.55267334	1
MSTRG.2721.3	144	0.38185	9.55267334	1
MSTRG.2724.3	371	0.34006	6.358093262	1
MSTRG.2740.1	720	0.42311	7.312194824	1
MSTRG.2742.1	259	0.40941	6.097473145	1
MSTRG.2742.4	305	0.43011	6.457214355	1
MSTRG.2742.5	305	0.43562	6.457214355	1
MSTRG.2730.2	433	0.45701	4.899108887	1

MSTRG.2732.1	325	0.34111	5.824645996	1
MSTRG.2732.2	278	0.37205	5.083190918	1
MSTRG.2732.5	327	0.39644	4.937438965	1
MSTRG.2743.1	194	0.3801	8.81842041	1
MSTRG.2744.1	59	0.386	6.81451416	1
MSTRG.2747.4	343	0.46801	6.34576416	1
MSTRG.2750.1	90	0.35017	6.674255371	1
MSTRG.2750.2	63	0.36174	6.886657715	1
MSTRG.2750.3	67	0.3593	9.245544434	1
MSTRG.2749.5	1025	0.44468	5.470397949	1
MSTRG.2757.1	87	0.41104	11.42242432	1
MSTRG.2761.1	45	0.4206	12.48284912	1
MSTRG.2762.1	53	0.36207	7.900085449	1
MSTRG.2766.2	495	0.45742	8.558410645	1
MSTRG.2770.1	71	0.39397	8.45123291	-1
MSTRG.2771.3	135	0.34685	9.496154785	1
MSTRG.2771.2	147	0.34952	9.788269043	1
MSTRG.2772.1	480	0.35342	7.202331543	1
MSTRG.2774.2	421	0.46951	9.514099121	1
MSTRG.2774.3	405	0.45747	9.499450684	1
MSTRG.2775.1	110	0.32314	7.803771973	1
MSTRG.2777.3	746	0.49464	8.119934082	1
MSTRG.2778.1	32	0.39345	10.29986572	1
MSTRG.2778.2	30	0.44977	10.29986572	1
MSTRG.2804.3	45	0.35417	11.73394775	1
MSTRG.2806.5	419	0.33998	9.541809082	1
MSTRG.2806.6	403	0.36179	9.492736816	1
MSTRG.2806.7	297	0.31	8.914489746	1
MSTRG.2806.8	59	0.35377	8.24786377	-1
MSTRG.2807.1	151	0.29367	11.00006104	1
MSTRG.2808.1	51	0.38785	9.299255371	1
MSTRG.2809.1	108	0.29367	5.897644043	1
MSTRG.2810.2	381	0.3564	8.919128418	1
MSTRG.2811.8	291	0.37508	7.843688965	1
MSTRG.2811.10	238	0.41424	6.23248291	1
MSTRG.2788.4	562	0.35599	8.818664551	1
MSTRG.2788.5	589	0.35019	8.717102051	1
MSTRG.2788.6	586	0.45645	8.765808105	1
MSTRG.2788.7	565	0.4524	8.773498535	1
MSTRG.2793.3	211	0.37329	7.035095215	1
MSTRG.2798.2	573	0.32446	5.002258301	1
MSTRG.2822.10	1061	0.41852	6.21295166	1
MSTRG.2822.6	2006	0.45981	8.804504395	1
MSTRG.2829.1	501	0.44114	6.491882324	1
MSTRG.2830.1	115	0.32589	6.63885498	1
MSTRG.2799.1	558	0.35898	9.890563965	1
MSTRG.2800.2	720	0.38204	8.819030762	1
MSTRG.2817.1	95	0.31209	9.780212402	1
MSTRG.2819.1	82	0.39781	9.741882324	1
MSTRG.2819.2	82	0.40978	9.741882324	1
MSTRG.2819.3	71	0.42144	8.573181152	1
MSTRG.2819.4	100	0.34408	8.83782959	1
MSTRG.2820.2	121	0.27807	12.04864502	1
MSTRG.2820.3	375	0.36864	6.299743652	1

MSTRG.2816.4	348	0.32801	9.072814941	1
MSTRG.2816.3	348	0.32801	9.072814941	1
MSTRG.2816.1	348	0.32801	9.072814941	1
MSTRG.2816.5	112	0.3143	9.078918457	1
MSTRG.2814.1	45	0.31165	4.649353027	1
MSTRG.2821.2	932	0.45136	5.131652832	1
MSTRG.2821.3	817	0.48087	5.60723877	1
MSTRG.2833.3	630	0.4176	6.282409668	1
MSTRG.2833.4	144	0.31153	9.97076416	1
MSTRG.2833.5	228	0.32686	9.949401855	1
MSTRG.2836.2	717	0.44731	4.614074707	1
MSTRG.2837.1	205	0.28567	5.309631348	1
MSTRG.2837.2	205	0.30877	5.309631348	1
MSTRG.2843.2	110	0.44524	3.701721191	1
MSTRG.2840.3	293	0.39437	5.698181152	1
MSTRG.2848.2	882	0.45436	5.882263184	1
MSTRG.2848.3	882	0.4806	5.882263184	1
MSTRG.2848.4	362	0.44585	5.476745605	-1
MSTRG.2848.6	690	0.39029	5.884338379	1
MSTRG.2848.7	44	0.4099	9.489196777	1
MSTRG.2845.1	191	0.37952	11.73248291	1
MSTRG.2845.2	164	0.36108	11.75469971	1
MSTRG.2850.5	145	0.29152	9.508605957	1
MSTRG.2850.4	239	0.29576	6.399475098	1
MSTRG.2850.6	88	0.31193	12.00384521	1
MSTRG.2850.7	247	0.33243	9.258605957	1
MSTRG.2850.8	236	0.30644	8.755432129	1
MSTRG.2846.1	72	0.3619	9.737121582	1
MSTRG.2857.2	572	0.40358	8.381530762	1
MSTRG.2857.4	273	0.39656	9.075378418	1
MSTRG.2852.4	416	0.28247	9.702087402	1
MSTRG.2852.3	416	0.28247	9.702087402	1
MSTRG.2858.2	241	0.3635	8.266906738	1
MSTRG.2859.1	102	0.37206	6.813781738	1
MSTRG.2865.3	583	0.37643	8.237365723	1
MSTRG.2866.8	422	0.48845	5.24798584	1
MSTRG.2871.1	424	0.40929	4.737976074	1
MSTRG.2873.1	167	0.28842	4.473449707	1
MSTRG.2870.1	441	0.41492	6.81048584	1
MSTRG.2879.1	258	0.40464	6.504455566	1
MSTRG.2879.3	194	0.4489	5.660461426	1
MSTRG.2884.3	453	0.414	5.220397949	1
MSTRG.2889.3	99	0.36389	8.493713379	1
MSTRG.2889.4	99	0.32504	8.493713379	1
MSTRG.2894.2	8	0.29335	5.58770752	-1
MSTRG.2897.1	449	0.40753	4.921691895	1
MSTRG.2898.2	1078	0.42979	8.762878418	1
MSTRG.2898.1	1095	0.42979	8.718078613	1
MSTRG.2899.1	135	0.33715	6.042663574	1
MSTRG.2899.3	132	0.38466	6.042663574	1
MSTRG.2899.2	135	0.34335	6.042663574	1
MSTRG.2898.4	445	0.42144	9.421813965	1
MSTRG.2900.4	261	0.41059	9.452819824	1
MSTRG.2900.5	92	0.44693	9.979797363	1

MSTRG.2906.4	1594	0.40947	7.442565918	1
MSTRG.2906.5	1064	0.41392	7.49822998	1
MSTRG.2907.3	516	0.37748	7.74810791	1
MSTRG.2909.1	133	0.39452	6.727844238	1
MSTRG.2909.2	133	0.45408	6.727844238	1
MSTRG.2910.1	241	0.30349	9.721618652	1
MSTRG.2910.4	241	0.35674	9.721618652	1
MSTRG.2912.2	346	0.47626	4.780212402	1
MSTRG.2913.1	40	0.3478	6.071960449	1
MSTRG.2914.1	264	0.45162	9.202331543	1
MSTRG.2914.2	264	0.4429	9.202331543	1
MSTRG.2921.1	102	0.39165	9.075622559	-1
MSTRG.2922.1	114	0.41582	8.804016113	1
MSTRG.2922.2	69	0.41288	9.098937988	1
MSTRG.2920.1	29	0.38359	4.649353027	1
MSTRG.2924.2	1302	0.45139	6.723083496	1
MSTRG.2924.3	824	0.41936	6.062805176	1
MSTRG.2927.3	370	0.312	11.98956299	1
MSTRG.2932.2	596	0.41155	6.041931152	1
MSTRG.2932.6	219	0.35426	5.437927246	1
MSTRG.2929.1	71	0.41734	12.39923096	1
MSTRG.2935.3	563	0.47855	4.759338379	1
MSTRG.2943.3	234	0.41207	4.485900879	1
MSTRG.2943.4	137	0.38114	4.104553223	1
MSTRG.2943.5	137	0.41354	4.104553223	1
MSTRG.2943.7	137	0.38519	4.104553223	1
MSTRG.2943.6	274	0.43369	4.694396973	1
MSTRG.2950.1	422	0.37652	7.163146973	1
MSTRG.2950.2	422	0.43795	7.163146973	1
MSTRG.2950.3	444	0.3663	8.52130127	1
MSTRG.2951.1	78	0.43961	9.149719238	1
MSTRG.2956.4	306	0.36935	5.550720215	1
MSTRG.2956.5	306	0.36935	5.550720215	1
MSTRG.2956.6	702	0.37087	6.405212402	1
MSTRG.2956.7	702	0.40836	6.405212402	1
MSTRG.2957.1	111	0.2765	9.807312012	1
MSTRG.2948.3	527	0.32531	4.97454834	1
MSTRG.2960.1	49	0.3294	5.99432373	1
MSTRG.2968.1	263	0.3556	9.345031738	1
MSTRG.2968.2	126	0.3556	8.375671387	1
MSTRG.2968.3	292	0.36769	9.389099121	1
MSTRG.2973.1	159	0.45771	7.12310791	1
MSTRG.2978.3	722	0.46117	6.556091309	1
MSTRG.2978.1	712	0.4033	7.147766113	1
MSTRG.2978.2	678	0.44883	6.846252441	1
MSTRG.2979.1	35	0.30501	10.90313721	1
MSTRG.2980.1	81	0.30857	9.968566895	-1
MSTRG.2983.1	121	0.31869	10.58453369	-1
MSTRG.2995.1	90	0.39726	9.997009277	1
MSTRG.2996.3	334	0.39832	6.836486816	1
MSTRG.2996.4	333	0.39612	6.836486816	1
MSTRG.3004.4	345	0.44482	6.493103027	1
MSTRG.3004.6	465	0.44451	5.344421387	1
MSTRG.3004.7	355	0.40694	6.935119629	1

MSTRG.3001.1	229	0.37623	4.848693848	1
MSTRG.3012.4	350	0.44906	8.426086426	1
MSTRG.3012.5	364	0.43342	8.919494629	1
MSTRG.3012.6	350	0.43054	8.426086426	1
MSTRG.3017.3	155	0.31065	10.10748291	1
MSTRG.3017.7	155	0.33547	10.10748291	1
MSTRG.3014.2	344	0.46762	5.023742676	1
MSTRG.3021.2	487	0.45443	8.923400879	1
MSTRG.3026.1	42	0.47084	9.382629395	1
MSTRG.3027.2	452	0.36523	9.999938965	1
MSTRG.3027.3	388	0.36523	9.294616699	1
MSTRG.3027.4	128	0.36147	6.557678223	1
MSTRG.3028.1	253	0.36364	5.596130371	1
MSTRG.3028.2	146	0.30576	5.931213379	1
MSTRG.3028.3	153	0.37649	7.702697754	1
MSTRG.3028.4	153	0.31786	7.702697754	1
MSTRG.3028.5	159	0.4495	8.968078613	1
MSTRG.3029.1	68	0.33125	9.221252441	1
MSTRG.3031.6	876	0.37739	11.89923096	1
MSTRG.3031.10	875	0.37793	11.91119385	1
MSTRG.3031.7	861	0.37739	11.90423584	1
MSTRG.3031.9	875	0.37739	11.91119385	1
MSTRG.3036.2	183	0.39019	4.349182129	1
MSTRG.3037.1	420	0.45151	8.062194824	1
MSTRG.3037.5	406	0.42331	8.391052246	1
MSTRG.3037.6	359	0.46452	8.788513184	1
MSTRG.3039.3	335	0.36695	9.762512207	1
MSTRG.3043.1	262	0.28443	11.26153564	1
MSTRG.3043.7	213	0.28596	11.45635986	1
MSTRG.3043.4	111	0.36813	4.392028809	1
MSTRG.3043.6	135	0.3684	10.45721436	1
MSTRG.3043.3	111	0.30078	4.392028809	1
MSTRG.3043.10	167	0.27234	11.75909424	1
MSTRG.3043.11	166	0.27234	11.75909424	1
MSTRG.3043.12	166	0.26982	11.75909424	1
MSTRG.3043.9	110	0.36978	4.392028809	1
MSTRG.3043.13	93	0.30186	4.514709473	1
MSTRG.3042.1	51	0.33808	4.93572998	1
MSTRG.3050.1	44	0.32889	10.40875244	1
MSTRG.3054.2	227	0.42639	8.187927246	1
MSTRG.3055.2	155	0.34559	10.25531006	1
MSTRG.3044.2	758	0.39965	9.644958496	1
MSTRG.3056.2	177	0.3985	9.639099121	1
MSTRG.3059.1	880	0.4559	6.199645996	1
MSTRG.3060.1	349	0.3208	9.405334473	1
MSTRG.3061.1	61	0.29637	9.146911621	1
MSTRG.3061.2	61	0.3663	9.146911621	1
MSTRG.3064.6	633	0.4129	8.225891113	1
MSTRG.3064.5	507	0.3439	5.236999512	1
MSTRG.3066.1	117	0.35145	11.0848999	1
MSTRG.3068.4	665	0.44454	7.594787598	1
MSTRG.3068.5	665	0.44262	7.594787598	1
MSTRG.3068.3	665	0.4513	7.594787598	1
MSTRG.3065.1	14	0.42858	10.85992432	1

MSTRG.3070.1	115	0.34694	5.242858887	1
MSTRG.3076.1	1237	0.43458	6.261901855	1
MSTRG.3085.2	3912	0.41878	5.864685059	1
MSTRG.3087.2	68	0.41415	11.53900146	1
MSTRG.3087.1	96	0.4187	10.38580322	1
MSTRG.3093.1	37	0.31465	4.625061035	1
MSTRG.3092.4	694	0.451	5.832580566	1
MSTRG.3092.5	220	0.31205	12.0022583	1
MSTRG.3088.2	524	0.44386	6.284729004	1
MSTRG.3088.3	524	0.4676	6.284729004	1
MSTRG.3113.1	113	0.28311	9.725769043	1
MSTRG.3114.1	1586	0.46961	5.254211426	1
MSTRG.3115.1	185	0.2857	9.988586426	1
MSTRG.3116.1	106	0.29615	5.995544434	1
MSTRG.3116.2	106	0.29111	5.995544434	1
MSTRG.3114.2	1585	0.47487	5.254211426	1
MSTRG.3114.4	1567	0.47487	5.23614502	1
MSTRG.3114.3	1578	0.47487	5.282531738	1
MSTRG.3114.5	1566	0.47487	5.23614502	1
MSTRG.3114.7	1585	0.46549	5.254211426	1
MSTRG.3114.13	1616	0.47487	5.417663574	1
MSTRG.3114.8	1577	0.47487	5.282531738	1
MSTRG.3114.9	1078	0.46266	5.471130371	1
MSTRG.3117.1	91	0.31188	7.700378418	1
MSTRG.3118.1	88	0.2921	9.057678223	1
MSTRG.3114.14	1596	0.47187	5.301574707	1
MSTRG.3114.15	1537	0.46185	5.174987793	1
MSTRG.3122.1	23	0.39649	7.728210449	1
MSTRG.3124.3	555	0.38695	9.679016113	1
MSTRG.3124.4	517	0.40675	9.761535645	1
MSTRG.3124.5	517	0.37955	9.761535645	1
MSTRG.3124.8	214	0.35581	9.788757324	1
MSTRG.3100.1	193	0.32944	11.10748291	-1
MSTRG.3101.1	1058	0.42931	5.243347168	1
MSTRG.3101.3	1053	0.42931	5.262756348	1
MSTRG.3101.2	1052	0.39187	5.262756348	1
MSTRG.3101.4	1052	0.42931	5.262756348	1
MSTRG.3101.5	212	0.4413	9.073059082	1
MSTRG.3104.1	79	0.28126	12.25701904	1
MSTRG.3101.6	460	0.46073	5.601501465	1
MSTRG.3096.2	177	0.36739	6.321960449	1
MSTRG.3106.3	1180	0.44852	6.500671387	1
MSTRG.3106.4	1202	0.44573	6.606872559	1
MSTRG.3106.5	1141	0.44573	6.779968262	1
MSTRG.3106.6	1080	0.46982	7.573913574	-1
MSTRG.3106.7	533	0.47387	8.991760254	-1
MSTRG.3106.8	53	0.29111	12.00177002	1
MSTRG.3109.1	151	0.3493	8.664123535	1
MSTRG.3111.1	92	0.3546	9.812805176	1
MSTRG.3125.3	176	0.33099	4.974914551	1
MSTRG.3126.2	99	0.36205	10.57086182	1
MSTRG.3127.3	284	0.46806	5.381896973	1
MSTRG.3127.4	260	0.46329	6.453796387	1
MSTRG.3130.1	265	0.41387	6.601013184	1

MSTRG.3130.2	336	0.35407	6.184265137	1
MSTRG.3130.3	336	0.38891	6.184265137	1
MSTRG.3132.1	239	0.4265	7.670715332	1
MSTRG.3132.3	192	0.40755	5.254699707	1
MSTRG.3133.1	1015	0.46295	8.723449707	1
MSTRG.3133.3	992	0.45233	7.359436035	1
MSTRG.3133.4	992	0.46196	7.359436035	1
MSTRG.3133.5	993	0.46196	7.121398926	1
MSTRG.3134.1	118	0.33	9.762390137	1
MSTRG.3144.2	5099	0.46783	5.722961426	1
MSTRG.3136.1	113	0.33716	8.935119629	1
MSTRG.3137.1	48	0.4064	10.60577393	1
MSTRG.3135.1	97	0.41464	11.8012085	1
MSTRG.3135.2	70	0.36452	7.824768066	1
MSTRG.3141.1	129	0.44018	11.53875732	1
MSTRG.3146.1	87	0.38674	4.49407959	1
MSTRG.3146.2	82	0.40089	9.789489746	1
MSTRG.3153.1	69	0.3254	5.160461426	1
MSTRG.3154.1	1237	0.45326	5.356262207	1
MSTRG.3154.4	1237	0.45326	5.356262207	1
MSTRG.3154.5	456	0.43361	9.173522949	1
MSTRG.3155.1	127	0.2825	9.721252441	-1
MSTRG.3151.1	4	0.39489	5.274963379	1
MSTRG.3152.1	75	0.36724	4.765686035	1
MSTRG.3157.2	223	0.40095	7.876403809	1
MSTRG.3157.3	223	0.41084	7.876403809	1
MSTRG.3157.5	264	0.40496	8.301574707	1
MSTRG.3163.1	197	0.34913	8.653869629	1
MSTRG.3166.3	100	0.27878	9.850158691	1
MSTRG.3166.6	86	0.32727	8.770568848	-1
MSTRG.3166.7	86	0.42637	8.770568848	-1
MSTRG.3159.4	585	0.33427	9.364318848	1
MSTRG.3179.7	209	0.28719	9.299499512	1
MSTRG.3179.9	121	0.38897	10.26641846	1
MSTRG.3179.10	168	0.33481	6.769958496	1
MSTRG.3179.11	111	0.32502	8.691833496	1
MSTRG.3183.1	369	0.34779	7.074890137	1
MSTRG.3173.1	170	0.32194	9.320983887	1
MSTRG.3176.1	137	0.28883	9.142028809	1
MSTRG.3177.1	43	0.39989	8.635070801	1
MSTRG.3178.3	62	0.28527	9.100280762	1
MSTRG.3185.1	528	0.33957	8.782043457	1
MSTRG.3185.2	528	0.31974	8.782043457	1
MSTRG.3186.1	280	0.31204	9.868225098	1
MSTRG.3185.3	151	0.28374	4.839782715	1
MSTRG.3186.6	165	0.28807	5.068664551	1
MSTRG.3186.4	472	0.34309	6.253845215	1
MSTRG.3186.9	472	0.34309	6.253845215	1
MSTRG.3186.10	570	0.43015	4.724914551	1
MSTRG.3186.11	325	0.46279	5.539733887	1
MSTRG.3186.12	371	0.3458	5.905456543	1
MSTRG.3187.1	107	0.32464	9.55657959	1
MSTRG.3188.1	154	0.28301	10.56500244	1
MSTRG.3189.1	227	0.33212	11.5569458	1

MSTRG.3190.2	105	0.31772	8.65057373	1
MSTRG.3190.3	105	0.31772	8.65057373	1
MSTRG.3197.1	181	0.40229	5.30267334	1
MSTRG.3198.1	71	0.35386	11.71722412	1
MSTRG.3214.1	60	0.31601	9.698059082	1
MSTRG.3221.1	107	0.35408	5.071472168	1
MSTRG.3226.1	104	0.3974	10.29095459	1
MSTRG.3228.2	609	0.37855	5.191955566	1
MSTRG.3215.1	193	0.40485	9.708557129	1
MSTRG.3217.1	59	0.41485	8.931213379	1
MSTRG.3218.1	136	0.33126	11.2522583	1
MSTRG.3220.1	42	0.37725	6.468566895	-1
MSTRG.3234.2	248	0.44766	7.726623535	1
MSTRG.3248.1	84	0.29268	6.369934082	1
MSTRG.3249.1	96	0.29206	8.332336426	1
MSTRG.3243.1	43	0.35005	9.647033691	-1
MSTRG.3244.1	45	0.43236	8.636047363	1
MSTRG.3245.1	29	0.44466	10.29498291	1
MSTRG.3252.1	93	0.28467	9.09942627	1
MSTRG.3252.3	93	0.28467	9.09942627	1
MSTRG.3252.4	93	0.29291	9.09942627	1
MSTRG.3252.5	93	0.32161	9.09942627	1
MSTRG.3254.1	85	0.48009	10.42144775	1
MSTRG.3259.3	505	0.43885	5.882019043	1
MSTRG.3259.4	410	0.38849	7.594543457	1
MSTRG.3261.1	189	0.44232	9.114685059	1
MSTRG.3263.1	193	0.28648	9.063903809	1
MSTRG.3264.1	35	0.35168	6.70526123	1
MSTRG.3267.2	578	0.46094	4.973327637	1
MSTRG.3267.4	564	0.46118	5.054138184	1
MSTRG.3269.1	186	0.3391	10.01849365	1
MSTRG.3270.2	656	0.37021	8.04876709	1
MSTRG.3270.3	325	0.28267	6.270690918	1
MSTRG.3271.1	191	0.30816	7.004821777	1
MSTRG.3279.1	69	0.32429	6.408752441	1
MSTRG.3282.2	150	0.33487	9.139221191	1
MSTRG.3282.3	116	0.33487	8.807434082	1
MSTRG.3282.4	135	0.30958	9.789611816	1
MSTRG.3282.6	180	0.31382	9.167541504	1
MSTRG.3282.5	132	0.36426	9.509338379	1
MSTRG.3282.7	173	0.36978	9.803039551	1
MSTRG.3294.1	66	0.39938	7.84552002	-1
MSTRG.3294.2	15	0.39744	9.992614746	-1
MSTRG.3296.4	167	0.39649	6.906555176	1
MSTRG.3296.3	167	0.3489	6.906555176	1
MSTRG.3297.5	318	0.43722	9.072814941	1
MSTRG.3300.1	373	0.3143	9.08380127	1
MSTRG.3300.2	182	0.33219	10.59527588	1
MSTRG.3287.1	34	0.38776	9.772888184	-1
MSTRG.3307.1	521	0.416	9.267150879	1
MSTRG.3307.4	353	0.37334	9.355041504	1
MSTRG.3307.2	521	0.34722	9.267150879	1
MSTRG.3307.3	522	0.42552	9.267150879	1
MSTRG.3307.6	427	0.40764	9.414367676	1

MSTRG.3312.1	591	0.32386	5.428649902	1
MSTRG.3314.1	104	0.28165	9.402648926	1
MSTRG.3317.2	552	0.44592	5.614807129	1
MSTRG.3317.3	433	0.44284	5.75567627	1
MSTRG.3317.4	517	0.42611	5.337341309	1
MSTRG.3317.5	882	0.45739	5.770935059	1
MSTRG.3317.6	654	0.45278	6.100891113	1
MSTRG.3320.1	169	0.30706	10.30743408	1
MSTRG.3321.4	199	0.4104	8.397155762	1
MSTRG.3321.3	241	0.42884	9.15045166	1
MSTRG.3321.6	199	0.39155	8.397155762	1
MSTRG.3333.1	92	0.29528	9.815979004	1
MSTRG.3339.3	410	0.35349	6.212219238	1
MSTRG.3339.4	540	0.43064	5.602478027	1
MSTRG.3340.1	115	0.28014	9.230163574	1
MSTRG.3342.2	106	0.38987	4.311462402	1
MSTRG.3342.1	106	0.37854	4.311462402	1
MSTRG.3343.1	61	0.36345	7.728088379	1
MSTRG.3343.2	103	0.33434	7.887512207	1
MSTRG.3346.1	35	0.38828	9.398742676	1
MSTRG.3354.3	91	0.41185	6.723327637	-1
MSTRG.3357.1	1510	0.46229	5.484436035	1
MSTRG.3357.2	1550	0.46229	5.516662598	1
MSTRG.3358.1	88	0.30961	6.90032959	1
MSTRG.3357.4	1108	0.47533	5.372619629	1
MSTRG.3359.6	81	0.42066	5.192443848	1
MSTRG.3364.1	216	0.45376	9.465148926	1
MSTRG.3364.3	216	0.44348	9.465148926	1
MSTRG.3364.4	207	0.35976	4.528137207	1
MSTRG.3364.5	207	0.39495	4.528137207	1
MSTRG.3364.6	207	0.39495	4.528137207	1
MSTRG.3364.7	88	0.30988	9.521057129	1
MSTRG.3371.1	408	0.46384	9.183532715	1
MSTRG.3373.2	1062	0.49657	9.572937012	1
MSTRG.3374.3	139	0.2667	6.221740723	1
MSTRG.3375.1	110	0.43856	11.37542725	1
MSTRG.3381.2	69	0.4726	9.65045166	1
MSTRG.3384.1	662	0.47316	6.097839355	1
MSTRG.3389.3	225	0.35673	4.544006348	1
MSTRG.3389.2	459	0.432	5.054870605	1
MSTRG.3389.4	423	0.41397	4.81842041	1
MSTRG.3390.2	181	0.39949	9.20501709	1
MSTRG.3415.2	221	0.44666	4.445007324	1
MSTRG.3415.3	330	0.40935	4.961853027	1
MSTRG.3418.1	397	0.44384	9.308654785	1
MSTRG.3420.6	2965	0.44056	9.422790527	1
MSTRG.3420.7	2766	0.41493	9.466491699	1
MSTRG.3420.8	2721	0.41245	9.42767334	1
MSTRG.3421.1	79	0.32215	7.727966309	1
MSTRG.3424.1	414	0.42954	4.95880127	-1
MSTRG.3400.2	499	0.33097	9.655700684	1
MSTRG.3400.6	272	0.35569	6.566711426	1
MSTRG.3400.3	499	0.33511	9.655700684	1
MSTRG.3400.4	347	0.38544	6.894836426	1

MSTRG.3401.2	50	0.30877	9.299377441	1
MSTRG.3402.2	327	0.46349	7.692199707	1
MSTRG.3427.2	494	0.45887	8.95904541	1
MSTRG.3429.1	538	0.42774	5.43548584	1
MSTRG.3434.8	478	0.35499	6.577575684	1
MSTRG.3435.1	825	0.42388	5.121032715	1
MSTRG.3435.3	787	0.42084	5.069885254	1
MSTRG.3438.1	191	0.34976	11.39141846	1
MSTRG.3444.1	1149	0.29664	8.925231934	1
MSTRG.3444.3	1125	0.29664	8.927429199	1
MSTRG.3444.5	1149	0.37871	8.925231934	1
MSTRG.3444.7	1149	0.35845	8.925231934	1
MSTRG.3444.12	1149	0.33075	8.925231934	1
MSTRG.3444.13	1038	0.35821	8.992980957	1
MSTRG.3441.3	351	0.46283	8.07623291	1
MSTRG.3441.4	370	0.44119	8.859558105	1
MSTRG.3446.1	63	0.34272	11.11114502	1
MSTRG.3470.3	261	0.40709	4.60345459	1
MSTRG.3471.3	1085	0.47046	6.070007324	1
MSTRG.3471.1	1085	0.47706	6.070007324	1
MSTRG.3471.5	1085	0.47477	6.070007324	1
MSTRG.3471.4	1087	0.4594	6.609924316	1
MSTRG.3471.10	1104	0.42558	6.522277832	1
MSTRG.3471.11	1104	0.4532	6.522277832	1
MSTRG.3471.12	1087	0.43238	6.609924316	1
MSTRG.3471.13	1087	0.47654	6.609924316	1
MSTRG.3471.14	1040	0.47548	6.276428223	1
MSTRG.3471.16	99	0.32266	4.811828613	1
MSTRG.3472.3	98	0.27911	4.959533691	1
MSTRG.3474.1	91	0.37078	10.21832275	1
MSTRG.3475.1	248	0.33716	4.748840332	1
MSTRG.3475.3	248	0.43664	4.748840332	1
MSTRG.3477.1	36	0.46018	10.46124268	1
MSTRG.3477.2	35	0.4121	10.85992432	1
MSTRG.3478.5	1879	0.42031	6.756286621	1
MSTRG.3478.7	1589	0.45078	6.34173584	1
MSTRG.3482.1	203	0.39934	9.342590332	1
MSTRG.3447.2	51	0.33115	9.93951416	1
MSTRG.3454.1	85	0.44566	6.897766113	1
MSTRG.3463.2	97	0.44744	4.680480957	1
MSTRG.3469.2	95	0.38637	8.514465332	1
MSTRG.3493.2	82	0.3681	7.687927246	1
MSTRG.3497.1	36	0.40186	11.39801025	1
MSTRG.3498.2	560	0.39114	4.944030762	1
MSTRG.3504.1	112	0.31398	9.896057129	1
MSTRG.3509.1	46	0.37426	10.21466064	1
MSTRG.3521.1	822	0.39353	6.126281738	1
MSTRG.3521.2	822	0.45953	6.126281738	1
MSTRG.3521.3	837	0.45953	5.960510254	1
MSTRG.3521.4	801	0.46261	6.011413574	1
MSTRG.3521.5	837	0.45953	5.960510254	1
MSTRG.3521.6	801	0.46261	6.011413574	1
MSTRG.3521.7	837	0.45953	5.960510254	1
MSTRG.3521.11	813	0.44272	5.898010254	1

MSTRG.3530.4	187	0.43433	10.69085693	1
MSTRG.3536.2	90	0.40456	4.422790527	1
MSTRG.3536.3	90	0.3388	4.422790527	1
MSTRG.3536.4	90	0.43656	4.422790527	1
MSTRG.3536.5	90	0.44832	4.422790527	1
MSTRG.3536.6	90	0.36411	4.422790527	1
MSTRG.3543.3	453	0.40771	5.176208496	1
MSTRG.3544.1	469	0.42268	4.843322754	1
MSTRG.3544.2	469	0.43157	4.843322754	1
MSTRG.3544.4	469	0.44492	4.843322754	1
MSTRG.3544.3	303	0.35568	4.952453613	1
MSTRG.3544.7	294	0.37258	5.015319824	1
MSTRG.3546.1	380	0.41368	9.049743652	1
MSTRG.3547.5	1254	0.45692	6.092102051	1
MSTRG.3547.4	1254	0.45692	6.092102051	1
MSTRG.3547.6	190	0.32174	10.09710693	-1
MSTRG.3542.4	742	0.31764	6.345397949	1
MSTRG.3550.3	156	0.34122	6.919128418	1
MSTRG.3548.1	102	0.32255	6.249572754	1
MSTRG.3557.2	1367	0.37984	6.432800293	1
MSTRG.3557.3	1393	0.37709	6.855651855	1
MSTRG.3558.1	89	0.31909	6.023010254	1
MSTRG.3559.1	198	0.29583	9.452453613	1
MSTRG.3561.4	419	0.50187	9.867248535	1
MSTRG.3561.5	530	0.46924	9.574279785	1
MSTRG.3561.6	699	0.47015	9.435974121	1
MSTRG.3561.7	548	0.44693	9.647399902	1
MSTRG.3563.3	297	0.39817	9.166564941	1
MSTRG.3563.4	385	0.36276	9.026184082	1
MSTRG.3565.1	167	0.42473	5.152160645	1
MSTRG.3562.1	263	0.3997	3.806213379	1
MSTRG.3566.3	237	0.47665	5.71307373	1
MSTRG.3566.2	237	0.47422	5.71307373	1
MSTRG.3570.2	751	0.33208	7.302062988	1
MSTRG.3580.1	126	0.3455	10.39373779	1
MSTRG.3580.2	147	0.3891	10.55804443	1
MSTRG.3588.6	355	0.481	8.874328613	1
MSTRG.3588.7	359	0.42327	9.60357666	1
MSTRG.3592.6	224	0.43642	5.179992676	1
MSTRG.3593.3	106	0.33872	9.505187988	1
MSTRG.3598.1	156	0.35524	10.01483154	1
MSTRG.3602.1	137	0.37248	11.15606689	1
MSTRG.3604.2	474	0.35259	6.419494629	1
MSTRG.3604.3	475	0.35259	6.273498535	1
MSTRG.3604.4	268	0.3355	5.921203613	1
MSTRG.3604.5	174	0.37665	5.500305176	1
MSTRG.3604.6	174	0.33874	5.500305176	1
MSTRG.3606.2	126	0.31823	9.725646973	1
MSTRG.3606.3	244	0.31747	9.957336426	1
MSTRG.3612.3	68	0.30239	9.651672363	1
MSTRG.3612.2	157	0.29227	6.06842041	1
MSTRG.3612.4	148	0.33579	6.721374512	1
MSTRG.3614.1	79	0.34477	9.663513184	1
MSTRG.3623.1	449	0.33863	9.36151123	1

MSTRG.3623.2	466	0.43629	9.113464355	1
MSTRG.3623.7	243	0.34128	9.693664551	1
MSTRG.3631.1	38	0.39808	9.304138184	-1
MSTRG.3633.4	172	0.29257	9.706237793	1
MSTRG.3642.2	632	0.43704	4.557556152	1
MSTRG.3642.3	559	0.42324	4.660949707	1
MSTRG.3650.1	133	0.40939	10.22979736	1
MSTRG.3651.1	157	0.36466	8.863586426	1
MSTRG.3652.1	725	0.38384	7.863952637	1
MSTRG.3654.1	63	0.35574	8.969421387	1
MSTRG.3658.1	29	0.36477	5.380187988	1
MSTRG.3659.2	266	0.45017	9.257873535	1
MSTRG.3663.1	113	0.34233	8.211486816	1
MSTRG.3664.1	42	0.43804	8.293762207	-1
MSTRG.3675.1	317	0.33851	8.754821777	1
MSTRG.3675.2	641	0.38914	8.095397949	1
MSTRG.3675.3	641	0.35655	8.095397949	1
MSTRG.3675.4	418	0.3644	7.276794434	1
MSTRG.3675.5	185	0.34098	5.721618652	1
MSTRG.3675.6	494	0.38828	7.062438965	1
MSTRG.3675.7	458	0.39142	7.699401855	1
MSTRG.3675.8	455	0.34103	6.839172363	1
MSTRG.3675.9	641	0.35395	8.095397949	1
MSTRG.3675.10	641	0.38223	8.095397949	1
MSTRG.3675.11	451	0.29343	7.73236084	1
MSTRG.3676.1	135	0.31861	6.931335449	1
MSTRG.3674.1	40	0.42652	5.425354004	1
MSTRG.3679.1	600	0.46001	8.400085449	1
MSTRG.3679.2	625	0.44721	8.25982666	1
MSTRG.3679.3	408	0.40187	8.318786621	1
MSTRG.3685.1	1194	0.42764	5.653991699	1
MSTRG.3685.2	1194	0.40969	5.653991699	1
MSTRG.3687.2	245	0.356	5.745178223	1
MSTRG.3694.1	111	0.46049	9.655212402	1
MSTRG.3702.2	579	0.38919	8.618347168	1
MSTRG.3702.3	672	0.41515	8.367858887	1
MSTRG.3704.1	84	0.26684	9.257385254	1
MSTRG.3707.2	935	0.41169	8.651550293	1
MSTRG.3707.3	4	0.30439	4.032043457	1
MSTRG.3708.2	62	0.30015	9.097961426	1
MSTRG.3710.1	717	0.37001	7.564147949	1
MSTRG.3712.1	64	0.26409	10.94000244	1
MSTRG.3710.4	734	0.36749	7.561706543	1
MSTRG.3710.5	305	0.32697	5.15814209	1
MSTRG.3716.2	1121	0.3488	8.329406738	1
MSTRG.3716.3	670	0.34715	7.565979004	1
MSTRG.3716.5	1232	0.37751	8.549621582	1
MSTRG.3716.6	1209	0.37999	8.484924316	1
MSTRG.3716.7	670	0.34467	7.565979004	1
MSTRG.3716.9	670	0.44993	7.565979004	1
MSTRG.3725.1	80	0.28648	11.71466064	1
MSTRG.3733.3	65	0.38677	9.496520996	1
MSTRG.3737.2	501	0.37626	8.241516113	1
MSTRG.3721.1	133	0.31065	8.794494629	1

MSTRG.3721.2	133	0.34827	9.117492676	1
MSTRG.3722.1	338	0.43754	5.86907959	1
MSTRG.3748.4	193	0.34009	6.200134277	1
MSTRG.3749.6	993	0.46299	9.147155762	1
MSTRG.3749.7	1046	0.47578	9.109558105	1
MSTRG.3749.3	1036	0.46291	9.109558105	1
MSTRG.3749.5	1003	0.45733	9.147033691	1
MSTRG.3755.1	81	0.32237	9.917297363	1
MSTRG.3764.2	184	0.29059	6.387268066	1
MSTRG.3765.1	101	0.33219	9.694763184	1
MSTRG.3765.2	143	0.30119	6.144470215	1
MSTRG.3767.1	73	0.30046	10.08197021	1
MSTRG.3768.1	52	0.40125	8.906555176	1
MSTRG.3769.1	90	0.38062	9.777770996	1
MSTRG.3770.1	59	0.27574	9.301940918	1
MSTRG.3773.2	205	0.38268	9.551574707	1
MSTRG.3773.3	207	0.34656	9.551574707	1
MSTRG.3773.4	292	0.31641	9.796936035	1
MSTRG.3777.2	58	0.35017	7.793762207	1
MSTRG.3778.1	465	0.45123	8.875549316	1
MSTRG.3756.2	243	0.37474	9.90032959	1
MSTRG.3780.4	797	0.45688	9.721374512	1
MSTRG.3780.5	68	0.37206	9.508850098	1
MSTRG.3784.1	50	0.34851	9.800476074	1
MSTRG.3788.1	56	0.36613	6.494689941	1
MSTRG.3789.1	962	0.35021	10.04766846	1
MSTRG.3789.3	958	0.34367	10.03753662	1
MSTRG.3790.1	74	0.33334	9.182678223	1
MSTRG.3800.1	276	0.46929	9.149597168	1
MSTRG.3802.2	218	0.43836	6.44744873	1
MSTRG.3802.3	218	0.45222	6.44744873	1
MSTRG.3802.4	218	0.43836	6.44744873	1
MSTRG.3802.5	218	0.45532	6.44744873	1
MSTRG.3804.1	826	0.46845	6.103210449	1
MSTRG.3807.1	70	0.37099	9.778625488	1
MSTRG.3813.1	141	0.34165	11.70721436	1
MSTRG.3817.3	1645	0.40257	6.209899902	1
MSTRG.3821.2	1013	0.34958	5.741027832	1
MSTRG.3821.1	1013	0.34019	5.741027832	1
MSTRG.3821.4	1013	0.37491	5.741027832	1
MSTRG.3831.5	542	0.43298	5.402526855	1
MSTRG.3832.3	681	0.47372	6.003356934	1
MSTRG.3832.4	681	0.47372	6.003356934	1
MSTRG.3832.5	681	0.47299	6.003356934	1
MSTRG.3833.1	78	0.39661	5.453918457	-1
MSTRG.3834.1	684	0.43996	5.812072754	1
MSTRG.3834.2	666	0.36349	5.707458496	1
MSTRG.3839.1	592	0.48373	8.288269043	1
MSTRG.3825.2	543	0.408	10.53607178	1
MSTRG.3825.1	543	0.408	10.53607178	1
MSTRG.3828.1	382	0.4474	5.09161377	1
MSTRG.3842.1	270	0.2994	6.595153809	1
MSTRG.3842.2	334	0.2994	8.988586426	1
MSTRG.3842.3	601	0.38384	8.714782715	1

MSTRG.3842.4	80	0.33911	6.000549316	1
MSTRG.3842.6	475	0.39465	7.969299316	1
MSTRG.3855.1	85	0.32774	9.053894043	1
MSTRG.3854.3	364	0.34306	5.946960449	1
MSTRG.3856.1	51	0.3432	7.761413574	1
MSTRG.3852.1	375	0.40929	5.280090332	1
MSTRG.3852.2	375	0.45288	5.280090332	1
MSTRG.3868.1	53	0.29928	7.978942871	-1
MSTRG.3969.1	205	0.29127	9.513977051	1
MSTRG.3970.1	68	0.3096	4.940734863	1
MSTRG.3971.1	97	0.32545	8.736022949	1
MSTRG.3975.1	79	0.35874	9.625305176	1
MSTRG.3976.1	195	0.43373	9.488220215	1
MSTRG.3977.1	107	0.27433	6.80291748	1
MSTRG.3978.1	200	0.27097	8.848815918	1
MSTRG.3979.1	124	0.34772	7.820495605	1
MSTRG.3980.1	10	0.41194	5.752990723	1
MSTRG.3972.5	81	0.2831	9.301330566	1
MSTRG.3981.1	53	0.35048	8.035949707	1
MSTRG.3982.1	81	0.32465	8.531188965	1
MSTRG.3983.1	57	0.31746	9.897888184	1
MSTRG.3983.2	53	0.34866	5.872009277	1
MSTRG.3984.1	106	0.31059	8.722595215	1
MSTRG.3984.2	53	0.28467	11.52288818	1
MSTRG.3985.1	375	0.28657	8.778259277	1
MSTRG.3985.2	55	0.26449	8.659973145	1
MSTRG.3972.6	116	0.29059	8.42767334	1
MSTRG.3986.1	98	0.33218	7.781677246	1
MSTRG.3987.1	110	0.30411	11.73065186	1
MSTRG.3987.2	86	0.30411	12.01251221	1
MSTRG.3987.3	86	0.30796	12.01251221	1
MSTRG.3987.4	26	0.32928	8.191589355	1
MSTRG.3989.1	161	0.34747	4.819519043	1
MSTRG.3972.9	90	0.36239	6.799865723	1
MSTRG.3991.1	31	0.33932	8.534973145	1
MSTRG.3877.1	75	0.3331	9.743225098	1
MSTRG.3883.1	13	0.40479	4.85333252	1
MSTRG.3889.2	330	0.38674	9.131408691	1
MSTRG.3890.1	739	0.49123	5.281433105	1
MSTRG.3893.1	113	0.35913	9.582580566	1
MSTRG.3895.3	574	0.38964	9.425231934	1
MSTRG.3895.4	355	0.38964	9.876159668	1
MSTRG.3895.6	285	0.40382	9.882629395	-1
MSTRG.3895.7	269	0.37739	9.836608887	-1
MSTRG.3896.1	55	0.40785	8.636047363	-1
MSTRG.3897.1	309	0.44423	6.890075684	1
MSTRG.3900.2	340	0.4198	8.739929199	1
MSTRG.3902.2	694	0.42747	8.213317871	1
MSTRG.3903.2	418	0.39853	6.296936035	1
MSTRG.3903.3	418	0.38466	6.296936035	1
MSTRG.3903.4	418	0.4205	6.296936035	1
MSTRG.3903.5	128	0.2921	8.399353027	1
MSTRG.3910.2	994	0.47293	6.364074707	1
MSTRG.3911.3	600	0.4606	8.026184082	1

MSTRG.3913.3	360	0.3406	9.471252441	1
MSTRG.3918.1	60	0.37607	7.663513184	1
MSTRG.3935.6	356	0.37649	6.118713379	1
MSTRG.3935.5	361	0.38694	6.118713379	1
MSTRG.3935.7	361	0.38858	6.118713379	1
MSTRG.3935.8	203	0.43435	9.098571777	1
MSTRG.3935.9	302	0.44573	6.906555176	1
MSTRG.3935.11	74	0.28963	7.911315918	1
MSTRG.3936.1	113	0.34392	9.099182129	-1
MSTRG.3937.1	77	0.30751	9.980041504	1
MSTRG.3930.1	90	0.35706	9.301330566	1
MSTRG.3934.1	80	0.3277	8.984436035	1
MSTRG.3939.2	156	0.38325	5.859069824	1
MSTRG.3939.4	53	0.33103	4.269470215	1
MSTRG.3940.3	263	0.31255	9.184143066	1
MSTRG.3940.6	84	0.25541	9.64251709	1
MSTRG.3940.7	84	0.31255	9.64251709	1
MSTRG.3941.1	30	0.3827	10.39080811	-1
MSTRG.3954.1	84	0.414	11.00006104	1
MSTRG.3959.2	589	0.47534	8.882873535	1
MSTRG.3960.1	102	0.3853	6.874084473	1
MSTRG.3963.3	1224	0.50301	6.171813965	1
MSTRG.3964.1	54	0.32552	8.43536377	1
MSTRG.3963.6	745	0.46923	6.304260254	1
MSTRG.3965.2	239	0.34775	6.588195801	1
MSTRG.3993.2	197	0.32916	10.01751709	1
MSTRG.3995.1	41	0.347	8.978942871	1
MSTRG.3997.3	117	0.28119	10.00262451	1
MSTRG.3997.2	261	0.28842	6.405090332	1
MSTRG.4000.1	183	0.33535	10.48175049	1
MSTRG.4003.1	466	0.40895	5.997131348	1
MSTRG.4003.3	371	0.35663	5.437316895	1
MSTRG.4003.4	459	0.35663	5.954772949	1
MSTRG.4004.1	189	0.31656	10.73638916	1
MSTRG.4006.1	176	0.26498	4.686340332	1
MSTRG.4006.2	176	0.37158	4.686340332	1
MSTRG.4010.2	1353	0.43188	5.498474121	1
MSTRG.4010.1	1456	0.45143	5.483093262	1
MSTRG.4010.3	1353	0.35972	5.498474121	1
MSTRG.4011.1	190	0.25765	6.869445801	1
MSTRG.4010.6	120	0.30469	5.521789551	1
MSTRG.4014.1	63	0.34035	4.555847168	1
MSTRG.4017.1	491	0.39228	8.775939941	1
MSTRG.4017.3	491	0.44806	8.775939941	1
MSTRG.4008.3	751	0.49682	5.638000488	1
MSTRG.4020.1	102	0.28035	8.987731934	1
MSTRG.4023.2	393	0.29852	5.187316895	1
MSTRG.4024.2	121	0.41422	9.097839355	1
MSTRG.4024.4	92	0.4116	9.299133301	1
MSTRG.4024.5	105	0.45795	8.521057129	-1
MSTRG.4026.1	935	0.47071	9.250915527	1
MSTRG.4026.2	935	0.47071	9.250915527	1
MSTRG.4026.3	935	0.47755	9.250915527	1
MSTRG.4026.4	935	0.44679	9.250915527	1

MSTRG.4026.5	935	0.45944	9.250915527	1
MSTRG.4026.6	935	0.45689	9.250915527	1
MSTRG.4028.5	625	0.3864	5.35723877	1
MSTRG.4028.6	814	0.44579	5.864318848	1
MSTRG.4028.7	788	0.42821	5.87713623	1
MSTRG.4028.8	786	0.36633	5.936950684	1
MSTRG.4028.9	816	0.43193	5.813049316	1
MSTRG.4030.3	576	0.44953	8.926452637	1
MSTRG.4036.1	1495	0.46527	6.70111084	1
MSTRG.4037.1	21	0.38305	11.10540771	1
MSTRG.4047.3	1302	0.46474	8.286804199	1
MSTRG.4047.4	45	0.3132	9.099182129	1
MSTRG.4047.5	1368	0.46474	8.660583496	1
MSTRG.4047.6	73	0.29615	7.733337402	1
MSTRG.4047.7	1289	0.46583	8.223205566	1
MSTRG.4047.8	1271	0.39748	8.403503418	1
MSTRG.4047.9	262	0.34193	9.658630371	1
MSTRG.4038.1	53	0.38356	7.698425293	1
MSTRG.4051.1	90	0.37421	5.110168457	1
MSTRG.4051.2	111	0.30191	8.411804199	1
MSTRG.4052.4	373	0.42551	4.834533691	1
MSTRG.4053.1	138	0.44256	6.030334473	1
MSTRG.4055.1	10	0.39413	6.690124512	1
MSTRG.4058.1	804	0.4458	6.199035645	1
MSTRG.4058.5	767	0.45412	6.480163574	1
MSTRG.4072.1	587	0.46586	6.293518066	1
MSTRG.4072.3	46	0.42002	10.46112061	1
MSTRG.4074.1	112	0.44512	5.060852051	-1
MSTRG.4079.1	116	0.36357	5.79083252	1
MSTRG.4080.1	70	0.37847	6.558898926	-1
MSTRG.4081.1	236	0.33243	5.18939209	1
MSTRG.4082.1	65	0.40225	7.788269043	1
MSTRG.4083.1	307	0.45318	5.940734863	1
MSTRG.4068.2	765	0.45917	6.684509277	1
MSTRG.4070.1	59	0.42317	8.706848145	-1
MSTRG.4092.3	336	0.27937	8.583190918	1
MSTRG.4092.4	262	0.29033	10.44329834	1
MSTRG.4093.1	189	0.28157	9.762390137	1
MSTRG.4093.2	75	0.36111	9.83380127	1
MSTRG.4094.1	73	0.29594	5.760803223	1
MSTRG.4095.1	154	0.33222	10.08575439	1
MSTRG.4096.1	126	0.30229	7.024475098	1
MSTRG.4092.5	129	0.32082	5.76776123	1
MSTRG.4092.6	129	0.33466	5.76776123	1
MSTRG.4092.7	65	0.39549	9.070983887	1
MSTRG.4092.8	108	0.37226	9.871887207	1
MSTRG.4099.1	131	0.3439	4.717224121	1
MSTRG.4104.1	75	0.37127	10.25469971	1
MSTRG.4108.1	601	0.46473	5.714416504	1
MSTRG.4108.2	548	0.45882	5.61114502	1
MSTRG.4108.3	545	0.42783	5.61114502	-1
MSTRG.4108.6	92	0.31484	6.484436035	1
MSTRG.4108.5	601	0.44552	5.714416504	1
MSTRG.4116.1	205	0.3618	9.062805176	1

MSTRG.4122.3	213	0.28683	6.709411621	1
MSTRG.4122.2	593	0.42364	5.928527832	1
MSTRG.4122.1	892	0.31793	5.43927002	1
MSTRG.4125.3	353	0.40597	9.532287598	1
MSTRG.4129.4	614	0.43524	6.308288574	1
MSTRG.4129.7	614	0.46094	6.308288574	1
MSTRG.4129.8	615	0.4042	6.308288574	1
MSTRG.4129.9	613	0.4042	6.308288574	1
MSTRG.4130.1	157	0.3	7.600280762	1
MSTRG.4143.2	164	0.37326	9.867858887	1
MSTRG.4143.4	29	0.32935	10.4463501	-1
MSTRG.4143.3	203	0.31723	5.101379395	1
MSTRG.4144.1	7	0.32134	6.491027832	1
MSTRG.4143.6	120	0.33228	6.411560059	1
MSTRG.4146.2	127	0.37398	10.66571045	1
MSTRG.4147.1	28	0.40193	8.500061035	1
MSTRG.4152.2	102	0.39387	5.52947998	1
MSTRG.4131.1	30	0.38121	4.426940918	1
MSTRG.4132.3	351	0.42594	8.786437988	1
MSTRG.4132.5	351	0.33902	8.786437988	1
MSTRG.4132.6	351	0.46564	8.786437988	1
MSTRG.4133.1	144	0.32237	10.28106689	1
MSTRG.4134.1	231	0.29367	9.368347168	1
MSTRG.4139.1	119	0.28301	9.124084473	1
MSTRG.4163.2	1163	0.38473	6.648376465	1
MSTRG.4163.1	1446	0.3992	5.868225098	1
MSTRG.4163.3	352	0.39645	5.845275879	1
MSTRG.4163.4	1446	0.47095	5.868225098	1
MSTRG.4163.5	1163	0.43053	6.648376465	1
MSTRG.4163.6	1123	0.36558	6.44732666	1
MSTRG.4163.7	1446	0.4605	5.868225098	1
MSTRG.4162.1	41	0.37882	9.224304199	1
MSTRG.4178.5	337	0.3	8.663513184	1
MSTRG.4178.3	336	0.29615	9.416809082	1
MSTRG.4178.6	337	0.46846	8.663513184	1
MSTRG.4178.4	222	0.45876	9.062683105	1
MSTRG.4178.8	222	0.43163	9.062683105	1
MSTRG.4178.7	337	0.42187	8.663513184	1
MSTRG.4183.1	122	0.33118	9.390075684	1
MSTRG.4186.1	187	0.28385	10.55657959	1
MSTRG.4186.2	193	0.32789	10.54388428	1
MSTRG.4186.3	63	0.32746	11.05340576	1
MSTRG.4187.1	97	0.33406	10.14508057	1
MSTRG.4203.3	181	0.37307	9.696716309	1
MSTRG.4203.5	189	0.41796	9.375061035	1
MSTRG.4203.2	297	0.35489	9.442077637	1
MSTRG.4203.6	335	0.4252	9.38861084	-1
MSTRG.4203.7	121	0.38619	9.404846191	1
MSTRG.4213.3	330	0.40903	9.990539551	1
MSTRG.4214.1	85	0.28683	10.04046631	1
MSTRG.4216.1	60	0.33402	6.497375488	1
MSTRG.4217.3	86	0.38478	8.448059082	1
MSTRG.4226.1	546	0.38851	6.460754395	1
MSTRG.4226.2	422	0.45237	5.837463379	1

MSTRG.4227.1	59	0.28234	6.501159668	1
MSTRG.4226.3	336	0.40642	4.909973145	1
MSTRG.4228.7	300	0.2906	10.92266846	1
MSTRG.4228.3	293	0.28808	11.01800537	1
MSTRG.4229.4	209	0.33518	5.830993652	1
MSTRG.4229.2	226	0.28826	7.67755127	1
MSTRG.4229.3	336	0.36207	8.926330566	1
MSTRG.4230.1	158	0.38612	10.00469971	1
MSTRG.4231.1	44	0.44866	9.520812988	-1
MSTRG.4219.1	51	0.42655	9.969177246	1
MSTRG.4246.3	151	0.433	9.911193848	1
MSTRG.4250.5	61	0.34723	9.303283691	1
MSTRG.4256.1	401	0.43027	8.61126709	1
MSTRG.4256.3	392	0.32819	8.415466309	1
MSTRG.4256.2	386	0.32819	8.424255371	1
MSTRG.4256.4	407	0.32819	8.602722168	1
MSTRG.4257.1	33	0.37054	10.14068604	1
MSTRG.4260.3	365	0.44825	4.312194824	1
MSTRG.4260.4	527	0.39939	4.329528809	1
MSTRG.4261.3	751	0.45085	5.261291504	1
MSTRG.4261.2	787	0.45108	5.485290527	1
MSTRG.4261.4	667	0.40702	5.17755127	1
MSTRG.4262.1	21	0.40217	5.30267334	1
MSTRG.4293.3	141	0.40397	9.653015137	-1
MSTRG.4293.4	141	0.35248	9.653015137	-1
MSTRG.4293.5	143	0.30068	9.653015137	-1
MSTRG.4293.6	143	0.35766	9.653015137	-1
MSTRG.4293.7	143	0.39873	9.653015137	-1
MSTRG.4296.1	54	0.42857	6.707580566	1
MSTRG.4301.2	562	0.42528	6.201965332	1
MSTRG.4301.1	562	0.42788	6.201965332	1
MSTRG.4301.3	562	0.43914	6.201965332	1
MSTRG.4301.4	562	0.45297	6.201965332	1
MSTRG.4301.5	562	0.47131	6.201965332	1
MSTRG.4301.6	562	0.44162	6.201965332	1
MSTRG.4302.1	251	0.36732	10.22979736	1
MSTRG.4303.2	108	0.30864	5.546813965	1
MSTRG.4304.2	96	0.27837	10.23626709	1
MSTRG.4297.1	44	0.39305	5.292297363	1
MSTRG.4298.1	74	0.40783	9.507019043	1
MSTRG.4312.5	246	0.41781	8.632141113	1
MSTRG.4312.4	1607	0.4184	6.986022949	1
MSTRG.4312.2	2098	0.46053	6.352111816	1
MSTRG.4312.1	2098	0.38953	6.352111816	1
MSTRG.4312.3	2098	0.4257	6.352111816	1
MSTRG.4310.2	185	0.30523	6.794128418	1
MSTRG.4317.2	509	0.28683	8.661560059	1
MSTRG.4319.1	269	0.2921	9.18145752	1
MSTRG.4319.2	269	0.29387	9.18145752	1
MSTRG.4319.3	195	0.3	8.752380371	1
MSTRG.4319.4	116	0.29615	10.01800537	1
MSTRG.4319.5	101	0.28828	5.652282715	1
MSTRG.4325.2	375	0.38344	6.03717041	1
MSTRG.4325.3	127	0.36731	9.950622559	1

MSTRG.4326.2	55	0.30961	7.79510498	1
MSTRG.4328.2	351	0.31303	8.957336426	1
MSTRG.4328.6	288	0.37568	7.747131348	1
MSTRG.4328.5	412	0.47691	6.473083496	-1
MSTRG.4328.7	288	0.46641	7.747131348	1
MSTRG.4328.11	295	0.28842	8.36932373	1
MSTRG.4329.3	728	0.41365	5.039611816	1
MSTRG.4330.1	17	0.31911	8.344665527	-1
MSTRG.4335.2	830	0.38211	5.149475098	1
MSTRG.4341.1	123	0.34664	9.508361816	1
MSTRG.4341.2	69	0.37883	9.697692871	1
MSTRG.4341.3	51	0.32247	10.04400635	1
MSTRG.4343.1	536	0.46586	5.761657715	1
MSTRG.4344.1	87	0.35745	7.86932373	1
MSTRG.4340.1	50	0.38142	7.770080566	1
MSTRG.4346.2	114	0.35415	9.75592041	1
MSTRG.4345.1	311	0.37303	9.340270996	1
MSTRG.4359.2	2616	0.43896	5.768127441	1
MSTRG.4357.1	462	0.41003	7.950256348	1
MSTRG.4358.2	298	0.36657	9.885559082	1
MSTRG.4360.2	475	0.37281	8.260070801	1
MSTRG.4361.1	343	0.35425	5.624084473	1
MSTRG.4361.3	253	0.37526	5.792175293	1
MSTRG.4363.1	621	0.43138	6.729675293	1
MSTRG.4363.2	593	0.35987	6.854187012	1
MSTRG.4363.3	560	0.46001	6.360168457	1
MSTRG.4367.4	205	0.26913	10.48443604	1
MSTRG.4367.5	136	0.27112	9.341247559	1
MSTRG.4369.1	241	0.26999	5.504699707	1
MSTRG.4365.2	105	0.45953	5.543029785	1
MSTRG.4371.1	193	0.4758	4.941711426	1
MSTRG.4371.2	192	0.46145	5.053527832	1
MSTRG.4375.1	59	0.3549	9.624694824	1
MSTRG.4372.2	560	0.38289	9.089172363	1
MSTRG.4382.1	37	0.37214	5.272399902	1
MSTRG.4388.1	770	0.34599	5.027038574	1
MSTRG.4388.3	911	0.33805	4.968688965	1
MSTRG.4388.9	783	0.31729	5.179382324	1
MSTRG.4384.2	92	0.40143	5.895568848	1
MSTRG.4390.1	91	0.33706	8.88092041	1
MSTRG.4390.2	91	0.39063	8.88092041	1
MSTRG.4390.3	70	0.38017	11.70526123	1
MSTRG.4399.1	47	0.34961	9.00579834	1
MSTRG.4400.2	435	0.458	8.791442871	1
MSTRG.4401.2	155	0.28584	6.495056152	1
MSTRG.4401.3	656	0.28584	8.308776855	1
MSTRG.4399.3	296	0.42881	9.405822754	1
MSTRG.4401.5	155	0.28584	6.495056152	1
MSTRG.4401.8	155	0.28584	6.495056152	1
MSTRG.4401.9	155	0.28419	6.495056152	1
MSTRG.4401.11	155	0.28584	6.495056152	1
MSTRG.4401.12	155	0.28584	6.495056152	1
MSTRG.4401.10	155	0.28338	6.495056152	1
MSTRG.4401.13	155	0.28419	6.495056152	1

MSTRG.4401.14	155	0.28419	6.495056152	1
MSTRG.4401.16	155	0.28186	6.495056152	1
MSTRG.4403.1	354	0.40917	8.815490723	1
MSTRG.4403.3	1665	0.47841	8.36920166	1
MSTRG.4404.1	192	0.28726	9.229919434	1
MSTRG.4408.1	71	0.30411	9.020324707	1
MSTRG.4409.2	863	0.44049	9.167175293	1
MSTRG.4409.3	720	0.43545	9.392150879	1
MSTRG.4411.1	546	0.36189	6.765563965	1
MSTRG.4411.2	546	0.35685	6.765563965	1
MSTRG.4412.1	1421	0.49693	4.875915527	1
MSTRG.4412.2	1362	0.49693	4.879455566	1
MSTRG.4412.3	1362	0.48942	4.879455566	1
MSTRG.4413.1	158	0.29871	5.956359863	1
MSTRG.4413.2	158	0.35162	5.956359863	1
MSTRG.4413.5	100	0.40466	4.350036621	1
MSTRG.4414.2	115	0.41617	4.695739746	1
MSTRG.4418.1	1163	0.4538	4.782775879	1
MSTRG.4420.9	521	0.3644	5.975891113	1
MSTRG.4420.10	737	0.33492	6.236755371	1
MSTRG.4420.11	566	0.34775	5.843444824	1
MSTRG.4421.1	109	0.27097	9.465148926	1
MSTRG.4422.1	14	0.36868	5.945983887	1
MSTRG.4422.2	24	0.4128	8.68170166	1
MSTRG.4423.4	789	0.48623	4.513366699	1
MSTRG.4423.5	903	0.47972	4.500549316	1
MSTRG.4424.1	50	0.42539	9.871643066	-1
MSTRG.4426.4	440	0.43408	8.518981934	1
MSTRG.4428.2	154	0.33061	4.641174316	1
MSTRG.4429.1	82	0.28711	10.71710205	1
MSTRG.4431.1	98	0.32443	6.398498535	1
MSTRG.4432.2	124	0.33128	6.284729004	1
MSTRG.4434.1	187	0.3307	9.239807129	1
MSTRG.4446.2	104	0.35804	5.278015137	1
MSTRG.4446.1	490	0.44356	6.862731934	1
MSTRG.4446.4	517	0.42962	7.554138184	1
MSTRG.4448.1	227	0.27918	6.330871582	1
MSTRG.4448.2	227	0.34497	6.330871582	1
MSTRG.4449.1	145	0.28165	8.950866699	1
MSTRG.4450.1	66	0.40215	6.809753418	1
MSTRG.4451.1	63	0.32613	5.780944824	1
MSTRG.4452.1	90	0.32097	10.35638428	1
MSTRG.4454.1	83	0.41163	9.127868652	1
MSTRG.4456.1	42	0.39955	6.70916748	-1
MSTRG.4443.3	247	0.38082	6.702331543	1
MSTRG.4445.1	126	0.30816	6.424743652	1
MSTRG.4462.1	150	0.3182	9.728088379	1
MSTRG.4462.2	150	0.2895	9.728088379	1
MSTRG.4463.1	74	0.30644	9.915588379	1
MSTRG.4464.1	122	0.30595	9.774230957	1
MSTRG.4465.1	83	0.38577	5.852355957	1
MSTRG.4467.4	266	0.37328	5.528747559	1
MSTRG.4470.3	177	0.31857	5.316345215	1
MSTRG.4470.4	177	0.31208	5.316345215	1

MSTRG.4470.5	177	0.34593	5.316345215	1
MSTRG.4473.3	324	0.37153	6.23638916	1
MSTRG.4471.2	54	0.30808	9.887756348	-1
MSTRG.4471.1	105	0.3487	5.356384277	1
MSTRG.4474.5	525	0.40511	5.718566895	1
MSTRG.4474.4	579	0.44491	7.797058105	1
MSTRG.4474.7	579	0.28338	7.797058105	1
MSTRG.4474.8	384	0.28584	8.864807129	1
MSTRG.4474.10	612	0.39926	9.554016113	1
MSTRG.4474.12	612	0.3869	9.554016113	1
MSTRG.4474.13	343	0.39961	10.40606689	1
MSTRG.4474.14	453	0.37366	9.964294434	1
MSTRG.4474.11	791	0.3869	9.632995605	1
MSTRG.4479.6	39	0.43386	5.053894043	1
MSTRG.4479.4	91	0.34512	4.45111084	1
MSTRG.4479.7	91	0.3792	4.45111084	1
MSTRG.4479.2	147	0.32962	4.785583496	1
MSTRG.4479.1	91	0.47718	4.45111084	1
MSTRG.4480.2	147	0.31665	5.737487793	1
MSTRG.4481.4	749	0.31649	8.76361084	1
MSTRG.4481.5	787	0.31325	8.949645996	1
MSTRG.4481.1	749	0.31573	8.76361084	1
MSTRG.4481.2	749	0.31974	8.76361084	1
MSTRG.4477.2	688	0.41769	6.696228027	1
MSTRG.4477.3	517	0.44688	5.93157959	1
MSTRG.4477.4	708	0.40383	6.718078613	1
MSTRG.4487.1	461	0.38439	5.825866699	1
MSTRG.4488.1	168	0.28165	10.24957275	1
MSTRG.4488.2	168	0.3	10.24957275	1
MSTRG.4488.3	168	0.40021	10.24957275	1
MSTRG.4494.1	247	0.28982	8.379821777	-1
MSTRG.4495.1	192	0.32762	5.95892334	1
MSTRG.4494.8	198	0.33219	5.737731934	-1
MSTRG.4494.9	208	0.36504	10.99237061	1
MSTRG.4496.1	83	0.33316	10.81134033	1
MSTRG.4494.10	224	0.28982	8.90435791	1
MSTRG.4494.11	75	0.2985	9.895935059	1
MSTRG.4498.1	183	0.29607	11.23468018	1
MSTRG.4494.12	474	0.3409	6.245788574	1
MSTRG.4494.13	54	0.32229	4.62677002	1
MSTRG.4499.3	345	0.41002	9.357971191	1
MSTRG.4483.6	721	0.46703	4.291564941	1
MSTRG.4483.4	483	0.46452	4.209289551	1
MSTRG.4483.5	777	0.46985	4.29486084	1
MSTRG.4483.3	755	0.46894	4.321716309	1
MSTRG.4503.1	55	0.37113	11.56085205	1
MSTRG.4504.5	573	0.35633	6.45880127	1
MSTRG.4506.1	77	0.37932	4.225891113	1
MSTRG.4515.1	1207	0.45036	6.046936035	1
MSTRG.4516.2	28	0.39993	4.078918457	1
MSTRG.4516.1	28	0.36309	4.078918457	1
MSTRG.4520.2	612	0.37294	7.481872559	1
MSTRG.4520.4	571	0.37294	7.834777832	1
MSTRG.4521.1	48	0.38811	7.679626465	1

MSTRG.4533.11	247	0.31516	4.840637207	1
MSTRG.4533.7	186	0.33899	4.793029785	1
MSTRG.4533.10	149	0.31053	11.90484619	1
MSTRG.4533.3	245	0.32002	4.864562988	1
MSTRG.4533.1	308	0.33799	6.082092285	1
MSTRG.4533.2	282	0.33799	5.866271973	1
MSTRG.4533.4	271	0.32851	5.010681152	1
MSTRG.4533.8	271	0.32416	5.010681152	1
MSTRG.4533.6	245	0.31207	4.864562988	1
MSTRG.4533.5	280	0.35811	5.38848877	1
MSTRG.4523.3	337	0.39274	8.33026123	1
MSTRG.4523.4	335	0.41074	8.33026123	1
MSTRG.4524.1	124	0.27194	7.716247559	1
MSTRG.4525.1	146	0.28126	8.864929199	1
MSTRG.4538.1	49	0.30425	5.362121582	-1
MSTRG.4540.1	184	0.37292	8.74041748	-1
MSTRG.4541.2	311	0.39152	8.012756348	1
MSTRG.4549.1	33	0.34644	9.580871582	1
MSTRG.4552.4	980	0.42068	5.388122559	1
MSTRG.4553.1	94	0.28307	10.23895264	1
MSTRG.4543.2	1789	0.46697	6.04901123	1
MSTRG.4543.3	1832	0.44852	6.090759277	1
MSTRG.4562.2	268	0.28311	6.454162598	1
MSTRG.4562.3	214	0.31656	8.824890137	1
MSTRG.4564.1	499	0.40675	9.219543457	1
MSTRG.4555.2	1300	0.46203	5.72869873	-1
MSTRG.4557.1	33	0.42542	5.966369629	1
MSTRG.4559.3	135	0.4252	8.927185059	1
MSTRG.4571.1	151	0.30415	6.780944824	1
MSTRG.4572.1	150	0.29272	10.39605713	1
MSTRG.4574.1	81	0.31662	5.122741699	1
MSTRG.4578.1	66	0.35801	9.20526123	1
MSTRG.4578.3	66	0.38532	9.20526123	1
MSTRG.4579.2	284	0.41866	5.02557373	1
MSTRG.4580.2	93	0.35668	6.067199707	1
MSTRG.4592.1	377	0.28586	9.953552246	1
MSTRG.4592.2	324	0.41323	9.874938965	1
MSTRG.4594.1	694	0.39417	5.473449707	1
MSTRG.4594.2	997	0.46446	5.656555176	1
MSTRG.4594.4	771	0.42469	6.128601074	1
MSTRG.4594.6	264	0.38788	7.10369873	1
MSTRG.4594.5	901	0.45377	5.884460449	1
MSTRG.4594.7	394	0.29308	6.196716309	1
MSTRG.4595.1	120	0.33888	6.863586426	1
MSTRG.4594.9	102	0.30804	7.583068848	1
MSTRG.4594.10	447	0.4662	5.479553223	1
MSTRG.4594.11	162	0.32234	6.069885254	1
MSTRG.4585.3	180	0.3231	9.31817627	1
MSTRG.4585.4	89	0.42795	7.804748535	1
MSTRG.4585.5	576	0.3594	6.601989746	1
MSTRG.4583.1	40	0.46182	9.883605957	1
MSTRG.4588.1	94	0.36928	11.49505615	1
MSTRG.4600.1	108	0.30394	6.282775879	1
MSTRG.4600.2	158	0.28718	9.805969238	1

MSTRG.4600.3	158	0.27251	9.805969238	1
MSTRG.4601.3	578	0.40513	9.59564209	1
MSTRG.4601.4	639	0.39941	9.361022949	1
MSTRG.4602.1	160	0.33228	9.70904541	1
MSTRG.4603.1	154	0.41434	10.76629639	-1
MSTRG.4601.8	136	0.28209	8.295471191	1
MSTRG.4601.9	136	0.36551	8.295471191	1
MSTRG.4604.1	380	0.34169	7.661560059	1
MSTRG.4604.3	380	0.41599	7.661560059	1
MSTRG.4605.7	335	0.44932	4.889465332	1
MSTRG.4605.8	335	0.39829	4.889465332	1
MSTRG.4605.4	335	0.48884	4.889465332	1
MSTRG.4605.6	335	0.45379	4.889465332	1
MSTRG.4605.9	297	0.47691	4.624328613	1
MSTRG.4605.10	88	0.31348	6.247619629	1
MSTRG.4608.3	38	0.34559	7.73248291	-1
MSTRG.4608.6	370	0.42631	5.065368652	1
MSTRG.4609.2	523	0.37593	8.928527832	1
MSTRG.4609.1	524	0.3426	8.928527832	1
MSTRG.4611.1	23	0.3783	12.00848389	1
MSTRG.4615.2	39	0.42741	5.463928223	1
MSTRG.4616.2	1696	0.47252	6.49395752	1
MSTRG.4617.1	91	0.29343	5.348327637	1
MSTRG.4618.1	103	0.29892	5.336364746	-1
MSTRG.4616.3	86	0.35922	4.602233887	1
MSTRG.4627.9	482	0.4324	8.27923584	1
MSTRG.4627.10	510	0.43359	8.717834473	1
MSTRG.4627.7	511	0.43359	8.717834473	1
MSTRG.4620.2	253	0.35915	6.833312988	1
MSTRG.4632.3	93	0.31476	5.832946777	1
MSTRG.4634.9	632	0.46988	9.54107666	1
MSTRG.4634.10	513	0.47801	9.624328613	-1
MSTRG.4634.13	500	0.45173	9.663513184	1
MSTRG.4706.9	4082	0.46703	5.106262207	1
MSTRG.4706.6	5359	0.46417	5.213928223	1
MSTRG.4706.4	5422	0.48089	5.23626709	1
MSTRG.4706.27	3062	0.46514	5.207092285	1
MSTRG.4706.18	4077	0.46672	5.150939941	1
MSTRG.4706.25	6579	0.4565	5.146057129	1
MSTRG.4706.19	7405	0.46173	5.174255371	1
MSTRG.4706.12	5380	0.44636	5.221496582	1
MSTRG.4706.14	5364	0.40652	5.231506348	1
MSTRG.4706.24	5401	0.46785	5.22869873	1
MSTRG.4706.16	7447	0.4565	5.184753418	1
MSTRG.4706.15	7447	0.4565	5.184753418	1
MSTRG.4706.30	94	0.29772	9.206604004	1
MSTRG.4706.31	127	0.29788	9.333557129	1
MSTRG.4706.32	126	0.27843	9.120666504	1
MSTRG.4706.33	204	0.28002	11.93414307	1
MSTRG.4648.1	227	0.32505	9.29864502	-1
MSTRG.4649.1	78	0.36965	10.54095459	1
MSTRG.4649.2	78	0.32312	10.54095459	1
MSTRG.4651.1	280	0.38233	5.66986084	1
MSTRG.4660.3	750	0.44436	5.351135254	1

MSTRG.4654.1	158	0.31589	9.82989502	1
MSTRG.4654.2	205	0.31702	10.45233154	1
MSTRG.4666.1	258	0.44741	8.72833252	1
MSTRG.4673.2	354	0.40648	5.948791504	1
MSTRG.4673.3	356	0.46773	5.963317871	1
MSTRG.4675.2	436	0.44777	8.039978027	1
MSTRG.4679.3	559	0.46024	6.031921387	1
MSTRG.4679.2	472	0.44819	6.080505371	1
MSTRG.4679.5	472	0.46544	6.080505371	1
MSTRG.4680.6	957	0.40525	10.15716553	1
MSTRG.4680.7	100	0.32404	8.407043457	1
MSTRG.4680.8	97	0.36948	8.914245605	1
MSTRG.4677.1	134	0.35965	4.810852051	1
MSTRG.4677.4	134	0.33478	4.810852051	1
MSTRG.4682.1	365	0.44534	4.783508301	1
MSTRG.4682.2	146	0.41508	4.215759277	1
MSTRG.4687.1	430	0.45977	5.932312012	1
MSTRG.4687.5	430	0.47276	5.932312012	1
MSTRG.4687.4	430	0.46554	5.932312012	1
MSTRG.4689.1	860	0.31	9.256896973	1
MSTRG.4689.2	857	0.3842	9.273742676	1
MSTRG.4691.1	48	0.30647	10.00848389	1
MSTRG.4692.2	721	0.44087	5.449890137	1
MSTRG.4698.1	72	0.27846	6.229553223	1
MSTRG.4700.1	121	0.32396	11.11956787	1
MSTRG.4701.1	87	0.29003	6.868347168	1
MSTRG.4703.2	1137	0.42375	8.483215332	1
MSTRG.4703.1	453	0.4091	8.484558105	1
MSTRG.4703.3	441	0.4202	8.749816895	1
MSTRG.4704.1	126	0.40062	9.63861084	1
MSTRG.4703.4	844	0.39004	8.33770752	1
MSTRG.4718.3	317	0.39385	8.95916748	1
MSTRG.4718.6	458	0.35576	9.218444824	1
MSTRG.4718.8	183	0.41366	8.883605957	1
MSTRG.4705.2	123	0.32373	8.879699707	1
MSTRG.4709.2	32	0.33258	10.78643799	-1
MSTRG.4709.1	65	0.28405	9.298522949	-1
MSTRG.4711.4	1054	0.38373	5.877380371	1
MSTRG.4711.5	1047	0.40683	5.936462402	1
MSTRG.4711.6	1047	0.38373	5.936462402	1
MSTRG.4710.1	127	0.4215	5.543518066	1
MSTRG.4725.1	1291	0.43094	5.474182129	1
MSTRG.4725.2	1291	0.41144	5.474182129	1
MSTRG.4725.12	28	0.3383	7.942687988	1
MSTRG.4725.10	1285	0.4448	5.473327637	1
MSTRG.4725.11	1288	0.40106	5.474182129	1
MSTRG.4725.9	1288	0.41144	5.474182129	1
MSTRG.4725.13	1284	0.40924	5.473327637	1
MSTRG.4725.16	1043	0.40444	5.280944824	1
MSTRG.4725.17	1043	0.43251	5.280944824	1
MSTRG.4725.18	1042	0.43251	5.280944824	1
MSTRG.4725.19	1040	0.43194	5.280944824	1
MSTRG.4725.20	1040	0.40884	5.280944824	1
MSTRG.4725.21	866	0.42158	5.151062012	1

MSTRG.4725.22	1043	0.43251	5.280944824	1
MSTRG.4722.3	147	0.30852	11.60797119	-1
MSTRG.4729.1	126	0.45578	6.829528809	1
MSTRG.4734.4	878	0.42805	7.123962402	1
MSTRG.4734.5	680	0.46218	5.89263916	1
MSTRG.4735.1	124	0.44973	7.00189209	1
MSTRG.4735.2	252	0.40893	6.592224121	1
MSTRG.4730.1	186	0.43877	9.350891113	-1
MSTRG.4731.1	35	0.30435	6.683898926	1
MSTRG.4738.1	38	0.42192	9.976623535	1
MSTRG.4741.2	110	0.28483	11.53643799	1
MSTRG.4744.1	96	0.27879	9.954284668	1
MSTRG.4746.1	47	0.32902	4.629699707	1
MSTRG.4747.3	1130	0.36277	9.117370605	1
MSTRG.4750.1	71	0.41818	10.02935791	-1
MSTRG.4751.1	119	0.35945	7.983337402	-1
MSTRG.4754.1	793	0.47418	9.249572754	1
MSTRG.4754.4	810	0.45007	9.171691895	-1
MSTRG.4754.5	413	0.46717	9.600646973	1
MSTRG.4754.6	287	0.45186	9.731506348	1
MSTRG.4754.7	287	0.46197	9.731506348	1
MSTRG.4752.1	362	0.40746	7.114074707	1
MSTRG.4757.1	108	0.27739	11.16522217	1
MSTRG.4758.2	160	0.36234	6.589294434	1
MSTRG.4758.3	124	0.34713	8.086853027	1
MSTRG.4761.1	2624	0.46788	8.67401123	1
MSTRG.4761.3	2624	0.45792	8.67401123	1
MSTRG.4761.5	2624	0.45709	8.67401123	1
MSTRG.4762.1	85	0.3205	9.409851074	1
MSTRG.4763.1	238	0.27194	9.538269043	1
MSTRG.4767.2	104	0.28267	9.414855957	1
MSTRG.4768.1	103	0.40305	9.138244629	-1
MSTRG.4767.6	411	0.44968	5.499084473	1
MSTRG.4769.1	111	0.2845	8.823547363	1
MSTRG.4767.7	799	0.37819	4.475036621	1
MSTRG.4772.2	1050	0.46381	5.480773926	1
MSTRG.4766.1	34	0.30112	7.90045166	1
MSTRG.4766.2	34	0.33982	7.90045166	1
MSTRG.4766.3	39	0.34768	9.213684082	1
MSTRG.4774.3	311	0.45616	6.843200684	1
MSTRG.4774.2	312	0.45475	6.843200684	1
MSTRG.4781.2	1208	0.44915	6.945983887	1
MSTRG.4785.3	641	0.48395	7.126037598	1
MSTRG.4785.6	390	0.34395	9.724304199	1
MSTRG.4782.1	132	0.28159	9.044006348	1
MSTRG.4782.3	132	0.27592	9.044006348	1
MSTRG.4787.1	1084	0.45248	6.411071777	1
MSTRG.4787.2	592	0.38809	5.243591309	1
MSTRG.4787.3	1089	0.44981	6.501037598	1
MSTRG.4788.1	209	0.34575	8.569519043	1
MSTRG.4789.1	171	0.41322	6.990661621	1
MSTRG.4796.1	429	0.36607	6.113464355	1
MSTRG.4800.2	402	0.44252	9.306945801	1
MSTRG.4811.1	69	0.27866	10.58502197	1

MSTRG.4811.3	69	0.28031	10.58502197	1
MSTRG.4813.1	1609	0.43202	5.757751465	1
MSTRG.4814.1	202	0.23717	11.10614014	1
MSTRG.4815.1	20	0.40123	12.00006104	1
MSTRG.4826.4	160	0.35718	4.914855957	1
MSTRG.4826.5	300	0.42337	5.612854004	1
MSTRG.4826.2	280	0.43575	9.507629395	1
MSTRG.4826.3	1255	0.45768	4.746765137	1
MSTRG.4826.10	1317	0.41857	4.837341309	1
MSTRG.4816.1	114	0.32522	9.588317871	1
MSTRG.4818.1	361	0.39241	6.145812988	1
MSTRG.4818.2	361	0.38268	6.145812988	1
MSTRG.4818.4	361	0.36855	6.145812988	1
MSTRG.4819.1	28	0.41346	5.957092285	1
MSTRG.4830.2	633	0.46224	8.973693848	1
MSTRG.4831.4	426	0.42764	6.370056152	1
MSTRG.4831.5	426	0.43491	6.370056152	1
MSTRG.4836.1	571	0.42077	7.511413574	1
MSTRG.4836.2	544	0.40691	7.131530762	1
MSTRG.4843.1	40	0.33117	9.297912598	1
MSTRG.4844.1	29	0.40777	11.34661865	-1
MSTRG.4848.1	61	0.36875	6.691955566	1
MSTRG.4849.1	204	0.33582	9.034973145	1
MSTRG.4860.1	142	0.33458	9.248962402	1
MSTRG.4861.1	384	0.44564	10.05523682	1
MSTRG.4861.2	1235	0.46121	9.081848145	1
MSTRG.4870.1	86	0.41921	4.494445801	1
MSTRG.4876.1	350	0.39256	9.135314941	1
MSTRG.4877.1	61	0.38517	9.26763916	1
MSTRG.4882.1	67	0.31164	8.034362793	1
MSTRG.4882.2	78	0.34662	5.875427246	-1
MSTRG.4873.1	93	0.34698	6.386413574	1
MSTRG.4886.1	318	0.39207	7.236633301	1
MSTRG.4887.1	1105	0.46443	8.817077637	1
MSTRG.4887.2	414	0.41435	8.064880371	-1
MSTRG.4895.2	249	0.31793	7.778137207	1
MSTRG.4903.1	80	0.41812	4.975646973	1
MSTRG.4903.2	397	0.32136	9.344543457	1
MSTRG.4921.2	3847	0.4681	5.028015137	1
MSTRG.4921.3	3889	0.45901	5.016662598	-1
MSTRG.4922.2	443	0.45824	8.643981934	1
MSTRG.4924.1	151	0.30925	11.31829834	1
MSTRG.4925.1	211	0.44355	9.218444824	1
MSTRG.4928.3	1091	0.4594	5.001159668	1
MSTRG.4928.4	1091	0.46584	5.001159668	1
MSTRG.4929.1	204	0.26605	8.128356934	1
MSTRG.4928.6	73	0.42635	6.253601074	-1
MSTRG.4930.1	183	0.34403	10.64532471	1
MSTRG.4928.7	556	0.41511	4.915466309	1
MSTRG.4912.2	301	0.42759	6.491394043	1
MSTRG.4913.1	221	0.40766	5.447937012	1
MSTRG.4913.3	221	0.34973	5.447937012	1
MSTRG.4913.4	221	0.35948	5.447937012	1
MSTRG.4920.1	46	0.44017	11.63653564	1

MSTRG.4942.1	82	0.30664	8.678039551	-1
MSTRG.4950.1	79	0.32124	9.73260498	-1
MSTRG.4951.2	125	0.29772	10.25799561	1
MSTRG.4953.1	127	0.4387	11.00579834	1
MSTRG.4961.1	1312	0.41088	5.403137207	1
MSTRG.4961.7	569	0.36837	5.40826416	1
MSTRG.4961.4	1312	0.41169	5.403137207	1
MSTRG.4961.5	1312	0.40301	5.403137207	1
MSTRG.4961.6	774	0.38046	6.304626465	1
MSTRG.4961.10	1294	0.40813	5.328186035	1
MSTRG.4961.8	1312	0.40813	5.403137207	1
MSTRG.4961.9	1312	0.40813	5.403137207	1
MSTRG.4961.12	472	0.47438	5.014953613	1
MSTRG.4961.11	1089	0.41088	5.059631348	1
MSTRG.4961.13	1312	0.41088	5.403137207	1
MSTRG.4961.15	1312	0.41088	5.403137207	1
MSTRG.4961.14	1312	0.41088	5.403137207	1
MSTRG.4962.1	51	0.34852	4.427185059	1
MSTRG.4963.1	86	0.35663	4.375915527	1
MSTRG.4964.1	114	0.28165	9.298522949	1
MSTRG.4965.1	114	0.27289	4.550598145	1
MSTRG.4965.2	174	0.26822	9.585998535	1
MSTRG.4965.3	114	0.26381	4.55645752	1
MSTRG.4966.1	12	0.43729	3.998840332	-1
MSTRG.4967.1	50	0.31769	10.27044678	1
MSTRG.4961.17	171	0.31899	6.96270752	1
MSTRG.4955.1	62	0.32361	11.00006104	1
MSTRG.4956.1	39	0.46058	8.211853027	1
MSTRG.4959.3	620	0.43581	8.837341309	1
MSTRG.4960.1	64	0.32514	7.733825684	1
MSTRG.4975.2	316	0.39367	9.211853027	-1
MSTRG.4980.10	561	0.43948	6.299255371	1
MSTRG.4980.4	561	0.40969	6.299255371	1
MSTRG.4980.9	561	0.47667	6.299255371	1
MSTRG.4980.8	561	0.45334	6.299255371	1
MSTRG.4980.5	561	0.46458	6.299255371	1
MSTRG.4980.2	561	0.45892	6.299255371	1
MSTRG.4981.2	368	0.3461	6.022033691	1
MSTRG.4985.6	467	0.39686	6.085876465	1
MSTRG.4985.8	410	0.4388	6.74432373	1
MSTRG.4990.3	140	0.28478	10.23858643	1
MSTRG.4990.4	40	0.3696	6.702941895	1
MSTRG.4991.1	114	0.27433	8.945739746	1
MSTRG.4998.3	337	0.31233	7.594543457	1
MSTRG.4998.4	345	0.30228	7.098937988	1
MSTRG.4998.5	344	0.30228	7.098937988	1
MSTRG.4998.6	162	0.34162	5.371520996	1
MSTRG.4998.7	84	0.36099	8.836730957	1
MSTRG.4988.6	459	0.46871	5.67779541	1
MSTRG.5003.1	54	0.35766	9.490905762	1
MSTRG.5020.1	20	0.39313	8.93951416	1
MSTRG.5021.2	275	0.28159	6.590515137	1
MSTRG.5046.1	40	0.32207	9.48236084	1
MSTRG.5052.1	64	0.28982	9.162536621	1

MSTRG.5077.2	477	0.43802	5.700378418	1
MSTRG.5077.3	477	0.43864	5.700378418	1
MSTRG.5078.1	62	0.35947	7.898010254	1
MSTRG.5106.1	62	0.47055	10.54571533	1
MSTRG.5095.2	152	0.46597	9.209899902	1
MSTRG.5137.1	334	0.30993	9.260437012	1
MSTRG.5138.1	21	0.35052	4.780578613	-1
MSTRG.5137.3	72	0.3351	9.696105957	1
MSTRG.5137.2	154	0.28683	9.554626465	1
MSTRG.5137.4	630	0.44054	5.111755371	1
MSTRG.5137.5	90	0.30897	9.20111084	1
MSTRG.5137.6	54	0.34159	9.416320801	1
MSTRG.5140.2	161	0.36912	10.21173096	-1
MSTRG.5137.7	630	0.43649	5.111755371	1
MSTRG.5137.8	630	0.43649	5.111755371	1
MSTRG.5137.10	27	0.34816	6.515563965	1
MSTRG.5133.1	957	0.47604	6.229553223	1
MSTRG.5133.3	932	0.37205	6.315124512	1
MSTRG.5133.4	114	0.34336	4.734802246	1
MSTRG.5134.1	114	0.38465	8.47833252	1
MSTRG.5135.1	55	0.3401	11.78594971	1
MSTRG.5160.1	174	0.28334	11.04571533	1
MSTRG.5192.2	583	0.4311	6.316833496	1
MSTRG.5192.1	809	0.45287	5.497619629	1
MSTRG.5192.3	161	0.4626	4.524230957	1
MSTRG.5192.4	54	0.38527	3.798522949	1
MSTRG.5196.1	56	0.39693	9.989807129	1
MSTRG.5201.1	85	0.39225	6.888000488	-1
MSTRG.5209.1	29	0.32803	5.999328613	1
MSTRG.5236.1	4227	0.46618	5.889831543	1
MSTRG.5236.3	3279	0.45148	6.166931152	1
MSTRG.5237.1	130	0.30289	9.800476074	1
MSTRG.5236.6	1102	0.42309	5.467468262	1
MSTRG.5238.5	431	0.39059	8.225524902	1
MSTRG.5217.3	810	0.36009	5.859558105	1
MSTRG.5217.4	583	0.4637	6.405456543	1
MSTRG.5218.1	81	0.27289	9.063781738	1
MSTRG.5226.1	198	0.31849	9.895080566	1
MSTRG.5247.1	521	0.34626	8.425109863	1
MSTRG.5249.2	313	0.35072	6.674743652	1
MSTRG.5249.3	276	0.47	5.718322754	1
MSTRG.5249.4	265	0.37122	5.57232666	1
MSTRG.5250.1	58	0.3447	5.410705566	1
MSTRG.5250.2	164	0.29607	7.656677246	1
MSTRG.5232.1	555	0.40612	6.399963379	1
MSTRG.5240.3	87	0.31608	6.876403809	1
MSTRG.5252.4	180	0.25937	10.25152588	1
MSTRG.5252.3	180	0.43543	10.25152588	1
MSTRG.5252.10	138	0.35695	9.677062988	1
MSTRG.5256.1	67	0.40047	8.445129395	1
MSTRG.5257.1	165	0.33102	10.09307861	1
MSTRG.5257.3	89	0.35108	11.42242432	1
MSTRG.5257.5	140	0.29615	10.04656982	1
MSTRG.5257.6	140	0.26594	10.04656982	1

MSTRG.5258.1	57	0.29319	4.49029541	1
MSTRG.5260.1	158	0.4329	9.895935059	1
MSTRG.5261.4	330	0.37705	5.920349121	1
MSTRG.5261.5	353	0.39263	9.561706543	1
MSTRG.5265.1	66	0.33455	10.04217529	1
MSTRG.5254.1	157	0.45356	8.643005371	1
MSTRG.5285.2	1063	0.43305	8.866882324	1
MSTRG.5285.3	658	0.44629	9.074035645	1
MSTRG.5285.4	777	0.45123	8.605651855	1
MSTRG.5286.1	52	0.42744	8.338317871	1
MSTRG.5285.5	913	0.45185	8.613342285	1
MSTRG.5288.2	344	0.35663	7.047058105	1
MSTRG.5289.1	145	0.29747	6.377502441	-1
MSTRG.5293.1	600	0.4745	6.515197754	1
MSTRG.5297.5	110	0.37002	3.683288574	1
MSTRG.5298.1	80	0.28923	9.897766113	1
MSTRG.5297.6	191	0.33254	5.451477051	1
MSTRG.5297.7	17	0.38481	9.522644043	1
MSTRG.5297.8	17	0.40865	9.522644043	1
MSTRG.5299.3	65	0.39301	6.68939209	1
MSTRG.5300.1	57	0.32293	8.705871582	1
MSTRG.5301.1	915	0.4294	5.379943848	1
MSTRG.5301.2	32	0.28362	3.584411621	1
MSTRG.5301.4	915	0.4254	5.379943848	1
MSTRG.5301.8	915	0.42811	5.379943848	1
MSTRG.5301.7	915	0.42925	5.379943848	1
MSTRG.5301.3	915	0.42925	5.379943848	1
MSTRG.5301.6	915	0.42815	5.379943848	1
MSTRG.5301.5	915	0.42956	5.379943848	1
MSTRG.5305.1	137	0.28982	6.882263184	1
MSTRG.5305.2	77	0.29191	7.669006348	1
MSTRG.5306.1	189	0.4099	9.876525879	1
MSTRG.5307.1	96	0.32176	5.303649902	1
MSTRG.5271.1	43	0.42336	9.69744873	-1
MSTRG.5275.1	145	0.31208	11.64227295	1
MSTRG.5275.2	145	0.30431	11.64227295	1
MSTRG.5276.2	755	0.4215	7.951477051	1
MSTRG.5283.2	573	0.29595	4.913024902	1
MSTRG.5283.3	117	0.36353	4.765563965	1
MSTRG.5284.1	95	0.33098	9.804016113	1
MSTRG.5310.2	664	0.40966	7.771789551	1
MSTRG.5310.3	519	0.45957	7.184387207	1
MSTRG.5312.1	80	0.35101	9.559020996	1
MSTRG.5317.1	86	0.30176	9.138000488	1
MSTRG.5324.1	353	0.40273	5.047790527	1
MSTRG.5324.2	1053	0.45193	5.545593262	1
MSTRG.5324.3	130	0.39682	4.334655762	1
MSTRG.5331.1	127	0.31869	9.844909668	1
MSTRG.5358.2	77	0.32472	6.480529785	-1
MSTRG.5359.2	301	0.43627	6.933044434	1
MSTRG.5359.3	213	0.39986	6.875427246	1
MSTRG.5363.2	307	0.34287	7.047180176	1
MSTRG.5363.4	307	0.35155	7.047180176	1
MSTRG.5363.5	307	0.32072	7.047180176	1

MSTRG.5365.2	384	0.43428	5.597595215	1
MSTRG.5354.2	3304	0.43732	5.479797363	1
MSTRG.5354.3	434	0.46174	5.036193848	1
MSTRG.5370.2	81	0.44445	5.718933105	1
MSTRG.5382.1	193	0.38455	9.466491699	1
MSTRG.5384.1	76	0.3118	7.944641113	1
MSTRG.5383.7	154	0.29343	5.958557129	1
MSTRG.5385.2	69	0.34692	4.817687988	1
MSTRG.5386.1	686	0.37065	4.89276123	1
MSTRG.5386.3	676	0.37933	5.364685059	1
MSTRG.5386.4	702	0.44752	4.835266113	1
MSTRG.5386.5	686	0.4469	4.89276123	1
MSTRG.5386.7	717	0.4546	5.00604248	1
MSTRG.5386.9	60	0.36013	9.381530762	1
MSTRG.5387.1	175	0.30387	9.75201416	1
MSTRG.5388.1	143	0.28498	6.255065918	1
MSTRG.5389.1	151	0.27639	9.434509277	1
MSTRG.5391.1	91	0.33102	9.23638916	1
MSTRG.5377.3	734	0.33519	10.0302124	1
MSTRG.5377.2	734	0.32651	10.0302124	1
MSTRG.5377.1	858	0.33519	9.818664551	1
MSTRG.5392.1	491	0.37416	6.721618652	1
MSTRG.5392.2	124	0.43242	9.274475098	1
MSTRG.5393.2	233	0.29054	11.67767334	1
MSTRG.5401.3	58	0.37915	10.37542725	1
MSTRG.5401.2	94	0.35115	9.659973145	1
MSTRG.5401.1	58	0.40428	10.37542725	1
MSTRG.5402.3	512	0.40724	5.813049316	1
MSTRG.5402.4	141	0.3351	4.329650879	1
MSTRG.5403.1	78	0.4221	6.715515137	1
MSTRG.5404.2	843	0.47729	5.801330566	1
MSTRG.5404.1	829	0.35496	5.818054199	1
MSTRG.5404.3	829	0.42123	5.818054199	1
MSTRG.5405.1	67	0.39411	5.509338379	1
MSTRG.5404.4	215	0.50678	6.434509277	1
MSTRG.5408.4	240	0.44189	9.597106934	1
MSTRG.5399.1	504	0.37276	8.422912598	1
MSTRG.5399.2	503	0.37276	8.562805176	1
MSTRG.5412.1	110	0.29332	5.04876709	1
MSTRG.5413.2	1786	0.41699	4.820129395	1
MSTRG.5414.1	46	0.37409	6.494812012	-1
MSTRG.5416.3	732	0.41173	9.176818848	1
MSTRG.5417.1	205	0.43078	9.336120605	-1
MSTRG.5419.7	588	0.46347	4.883239746	1
MSTRG.5419.6	433	0.36408	4.779602051	1
MSTRG.5419.8	324	0.38729	4.760437012	1
MSTRG.5440.1	58	0.44861	5.074645996	-1
MSTRG.5445.2	409	0.39109	8.480041504	1
MSTRG.5491.1	345	0.31802	10.1317749	1
MSTRG.5493.1	172	0.43283	7.735046387	1
MSTRG.5493.2	219	0.39787	9.377990723	1
MSTRG.5470.2	161	0.3076	8.198303223	1
MSTRG.5471.1	51	0.36157	5.601867676	-1
MSTRG.5478.3	451	0.46537	7.110900879	1

MSTRG.5480.1	455	0.46605	5.198059082	1
MSTRG.5514.1	1701	0.47176	6.831970215	1
MSTRG.5515.1	25	0.37087	5.897644043	1
MSTRG.5558.2	126	0.3	9.299865723	1
MSTRG.5560.1	192	0.29691	8.764831543	1
MSTRG.5561.1	56	0.31857	10.40789795	1
MSTRG.5561.2	74	0.38562	9.640930176	1
MSTRG.5562.1	125	0.29346	8.706726074	1
MSTRG.5563.1	63	0.33851	7.83795166	1
MSTRG.5564.1	50	0.31209	6.675720215	1
MSTRG.5565.1	64	0.30861	5.432922363	1
MSTRG.5558.3	74	0.40762	7.101135254	1
MSTRG.5532.1	223	0.30419	7.794494629	1
MSTRG.5533.1	395	0.44919	5.690124512	1
MSTRG.5533.2	93	0.32244	9.608947754	1
MSTRG.5539.1	126	0.42929	8.529724121	1
MSTRG.5540.1	76	0.29367	7.122497559	1
MSTRG.5538.4	121	0.36598	10.12298584	1
MSTRG.5543.1	576	0.2908	10.00823975	1
MSTRG.5543.4	341	0.34634	9.694885254	1
MSTRG.5543.2	322	0.32293	9.04083252	1
MSTRG.5543.3	276	0.34775	8.146057129	1
MSTRG.5543.7	276	0.34547	8.146057129	1
MSTRG.5543.8	258	0.32501	9.606262207	1
MSTRG.5543.9	152	0.30576	8.755310059	1
MSTRG.5549.1	43	0.44287	11.06878662	1
MSTRG.5550.2	461	0.35806	9.096252441	1
MSTRG.5552.1	65	0.33171	9.59942627	1
MSTRG.5555.4	402	0.3696	9.928039551	1
MSTRG.5555.2	551	0.34567	9.718200684	1
MSTRG.5555.3	748	0.42183	9.287536621	1
MSTRG.5570.2	473	0.44634	5.892272949	1
MSTRG.5570.4	570	0.48975	5.211975098	1
MSTRG.5572.2	128	0.3818	9.81072998	1
MSTRG.5574.5	381	0.46224	4.674499512	1
MSTRG.5574.6	432	0.43298	4.750061035	1
MSTRG.5574.7	86	0.41287	4.389709473	1
MSTRG.5575.3	623	0.38005	5.427429199	1
MSTRG.5575.4	623	0.42639	5.427429199	1
MSTRG.5584.1	61	0.30378	8.770324707	1
MSTRG.5591.1	485	0.32122	6.14276123	1
MSTRG.5591.2	512	0.3299	6.633850098	1
MSTRG.5595.3	377	0.42488	9.56854248	1
MSTRG.5594.1	87	0.33484	5.082458496	1
MSTRG.5605.2	238	0.33802	6.640930176	1
MSTRG.5606.2	93	0.42866	9.512756348	1
MSTRG.5608.1	91	0.34972	9.351989746	1
MSTRG.5610.3	501	0.34777	5.12322998	1
MSTRG.5615.1	138	0.28432	9.408630371	1
MSTRG.5618.3	307	0.47928	8.29486084	1
MSTRG.5612.1	473	0.42254	7.604187012	1
MSTRG.5627.1	196	0.29772	9.773864746	1
MSTRG.5628.1	54	0.33909	9.300598145	-1
MSTRG.5629.1	85	0.32826	9.352478027	1

MSTRG.5629.2	81	0.40011	5.079528809	1
MSTRG.5627.3	179	0.32886	9.335632324	1
MSTRG.5627.4	162	0.28982	10.48272705	1
MSTRG.5627.5	184	0.3	9.512634277	1
MSTRG.5627.6	118	0.29772	9.297302246	1
MSTRG.5627.7	179	0.2921	9.335632324	1
MSTRG.5627.8	160	0.29772	9.690124512	1
MSTRG.5627.9	160	0.29772	9.690124512	1
MSTRG.5627.10	112	0.28883	9.010559082	1
MSTRG.5627.11	112	0.28883	9.010559082	1
MSTRG.5627.12	112	0.29268	9.010559082	1
MSTRG.5627.13	130	0.29088	5.261779785	1
MSTRG.5627.14	196	0.28982	9.773864746	1
MSTRG.5631.1	85	0.29772	7.636779785	1
MSTRG.5632.1	121	0.28683	9.578796387	1
MSTRG.5633.1	105	0.28923	6.635681152	1
MSTRG.5633.2	95	0.28683	9.299743652	1
MSTRG.5634.1	186	0.37574	10.24713135	1
MSTRG.5632.2	175	0.29367	8.769714355	1
MSTRG.5634.2	186	0.29871	10.24713135	1
MSTRG.5635.3	817	0.48747	5.338195801	1
MSTRG.5636.1	125	0.29619	10.70489502	1
MSTRG.5621.2	301	0.32244	5.541687012	1
MSTRG.5624.1	316	0.29367	9.223449707	1
MSTRG.5624.2	118	0.32501	10.59783936	1
MSTRG.5638.1	49	0.3285	8.841491699	1
MSTRG.5643.3	183	0.38726	5.227111816	1
MSTRG.5642.4	397	0.28419	6.340148926	1
MSTRG.5642.5	167	0.29088	10.15155029	1
MSTRG.5661.1	105	0.33695	9.451721191	1
MSTRG.5668.3	82	0.38257	9.765441895	-1
MSTRG.5669.3	623	0.43863	6.628723145	1
MSTRG.5669.4	571	0.42886	6.11907959	1
MSTRG.5669.5	540	0.44636	5.823425293	1
MSTRG.5670.1	39	0.38269	6.680236816	1
MSTRG.5671.1	160	0.34719	10.44329834	1
MSTRG.5672.1	24	0.41413	6.697937012	1
MSTRG.5667.1	70	0.36168	9.384216309	1
MSTRG.5681.1	1015	0.32477	5.288513184	1
MSTRG.5681.5	762	0.45031	5.266174316	-1
MSTRG.5681.10	233	0.35675	6.996154785	1
MSTRG.5681.7	266	0.32477	8.158874512	1
MSTRG.5681.11	219	0.35312	8.752868652	1
MSTRG.5722.15	704	0.39702	9.221984863	1
MSTRG.5726.1	62	0.29519	7.739807129	1
MSTRG.5726.2	85	0.26797	9.18560791	1
MSTRG.5722.17	74	0.35792	4.657409668	1
MSTRG.5722.18	75	0.28592	4.477966309	1
MSTRG.5727.1	125	0.3458	8.245056152	1
MSTRG.5728.1	102	0.2921	5.138244629	1
MSTRG.5729.1	67	0.35123	9.333557129	1
MSTRG.5730.4	252	0.46059	8.485534668	1
MSTRG.5722.19	704	0.41957	9.221984863	1
MSTRG.5722.20	116	0.28982	8.958190918	1

MSTRG.5722.21	325	0.36467	9.287536621	1
MSTRG.5734.1	68	0.33425	6.683044434	1
MSTRG.5722.22	881	0.39702	9.183654785	1
MSTRG.5687.2	782	0.44958	7.487609863	1
MSTRG.5690.2	167	0.37031	8.719543457	1
MSTRG.5690.3	193	0.43244	9.317565918	1
MSTRG.5695.3	90	0.27112	8.957580566	1
MSTRG.5696.1	199	0.29603	8.864685059	1
MSTRG.5695.5	537	0.27217	7.010192871	1
MSTRG.5695.6	537	0.32849	7.010192871	1
MSTRG.5693.1	54	0.32101	8.371276855	1
MSTRG.5699.1	125	0.30709	10.14129639	1
MSTRG.5704.3	305	0.41067	6.721984863	1
MSTRG.5707.14	197	0.40633	4.709411621	1
MSTRG.5707.17	197	0.38052	4.709411621	1
MSTRG.5707.15	447	0.44158	5.187194824	1
MSTRG.5707.11	197	0.4573	4.709411621	1
MSTRG.5707.13	197	0.45655	4.709411621	1
MSTRG.5707.16	197	0.46221	4.709411621	1
MSTRG.5707.6	197	0.465	4.709411621	1
MSTRG.5707.2	197	0.46727	4.709411621	1
MSTRG.5707.5	197	0.49342	4.709411621	1
MSTRG.5707.9	197	0.47294	4.709411621	1
MSTRG.5711.3	224	0.42272	9.04498291	1
MSTRG.5713.3	934	0.43504	6.679138184	1
MSTRG.5713.4	922	0.41249	6.660095215	1
MSTRG.5713.6	934	0.35406	6.679138184	1
MSTRG.5713.5	828	0.35778	9.028625488	1
MSTRG.5717.2	248	0.33528	11.56634521	1
MSTRG.5717.1	754	0.44523	6.531677246	1
MSTRG.5717.3	211	0.35808	11.58416748	1
MSTRG.5717.4	1024	0.43912	8.352478027	1
MSTRG.5717.5	964	0.4638	7.375915527	1
MSTRG.5715.4	215	0.30985	5.08404541	1
MSTRG.5715.3	153	0.40133	5.278259277	1
MSTRG.5716.1	159	0.32397	5.481018066	1
MSTRG.5740.3	293	0.45231	8.543518066	1
MSTRG.5742.1	34	0.34723	8.49017334	-1
MSTRG.5745.3	181	0.34787	6.539367676	1
MSTRG.5745.2	197	0.31406	6.250671387	1
MSTRG.5746.8	135	0.41475	7.836608887	1
MSTRG.5746.9	135	0.37068	7.836608887	1
MSTRG.5747.1	294	0.32989	9.42388916	1
MSTRG.5746.10	135	0.31205	7.836608887	1
MSTRG.5782.1	81	0.30807	5.028259277	1
MSTRG.5757.2	58	0.35726	10.22772217	1
MSTRG.5757.1	58	0.355	10.22772217	1
MSTRG.5761.1	1587	0.36765	7.007995605	1
MSTRG.5761.2	1587	0.36765	7.007995605	1
MSTRG.5763.2	169	0.29343	10.35968018	1
MSTRG.5762.1	61	0.3973	7.707458496	1
MSTRG.5835.1	84	0.31553	6.933532715	1
MSTRG.5835.2	165	0.28338	9.438537598	1
MSTRG.5835.3	86	0.32642	6.253723145	1

MSTRG.5835.4	184	0.29202	9.74041748	1
MSTRG.5835.5	86	0.28982	6.202575684	1
MSTRG.5835.6	105	0.28982	8.078918457	1
MSTRG.5835.7	211	0.2921	5.667175293	1
MSTRG.5835.9	133	0.42315	9.834655762	1
MSTRG.5836.1	137	0.29772	9.685119629	1
MSTRG.5835.11	118	0.30816	10.06536865	1
MSTRG.5837.1	137	0.29772	9.362976074	1
MSTRG.5838.1	81	0.30247	6.66973877	1
MSTRG.5835.13	89	0.31553	6.808166504	1
MSTRG.5835.12	142	0.32762	9.668395996	1
MSTRG.5835.14	142	0.29892	9.668395996	1
MSTRG.5835.15	142	0.29892	9.668395996	1
MSTRG.5835.16	136	0.31917	9.957092285	-1
MSTRG.5835.17	164	0.33359	9.358703613	1
MSTRG.5835.18	130	0.31917	10.02996826	-1
MSTRG.5835.20	275	0.29595	9.262268066	1
MSTRG.5835.22	110	0.31852	9.097229004	1
MSTRG.5835.23	156	0.2921	5.530334473	1
MSTRG.5835.25	211	0.2921	5.667175293	1
MSTRG.5835.26	88	0.27234	8.80279541	1
MSTRG.5835.27	120	0.2921	9.79510498	1
MSTRG.5835.28	82	0.34787	7.72088623	1
MSTRG.5835.29	88	0.31653	9.170593262	1
MSTRG.5835.30	86	0.37681	8.639831543	1
MSTRG.5840.1	154	0.38415	5.213806152	1
MSTRG.5797.1	1452	0.37367	9.714660645	1
MSTRG.5797.6	837	0.46023	6.016784668	1
MSTRG.5797.4	897	0.43185	6.106628418	1
MSTRG.5797.3	897	0.45917	6.106628418	1
MSTRG.5797.5	1118	0.48132	8.854553223	1
MSTRG.5797.7	897	0.47683	6.106628418	1
MSTRG.5798.1	155	0.3208	9.380187988	1
MSTRG.5802.3	77	0.27639	8.797912598	1
MSTRG.5803.1	38	0.34721	4.316101074	1
MSTRG.5805.1	150	0.29607	4.617492676	1
MSTRG.5799.1	33	0.39249	10.44647217	-1
MSTRG.5815.1	63	0.48174	9.019226074	1
MSTRG.5832.1	4	0.36474	8.500061035	1
MSTRG.5834.1	418	0.46892	5.876037598	1
MSTRG.5845.3	931	0.43876	8.500183105	-1
MSTRG.5845.4	503	0.39554	8.629943848	1
MSTRG.5856.2	377	0.38833	7.619812012	1
MSTRG.5858.1	98	0.33166	8.809631348	1
MSTRG.5860.1	116	0.37877	7.818664551	1
MSTRG.5862.1	167	0.37847	5.238342285	1
MSTRG.5864.1	505	0.41689	8.045349121	1
MSTRG.5865.1	122	0.31553	9.691589355	1
MSTRG.5866.1	51	0.32359	6.809265137	1
MSTRG.5867.1	79	0.35094	12.15557861	1
MSTRG.5872.5	553	0.4464	8.787902832	1
MSTRG.5873.1	70	0.27918	6.385559082	1
MSTRG.5874.2	232	0.33013	8.75958252	1
MSTRG.5874.3	152	0.3797	9.483215332	1

MSTRG.5874.5	228	0.35996	8.553649902	1
MSTRG.5874.4	228	0.34079	8.553649902	1
MSTRG.5877.1	613	0.37757	8.41986084	1
MSTRG.5879.1	95	0.28683	6.555114746	1
MSTRG.5885.2	497	0.41885	8.64263916	1
MSTRG.5886.1	32	0.42351	12.30426025	1
MSTRG.5890.2	664	0.4152	9.546813965	1
MSTRG.5890.3	664	0.45918	9.546813965	1
MSTRG.5891.1	340	0.42006	6.831481934	1
MSTRG.5891.2	340	0.43291	6.831481934	1
MSTRG.5891.3	340	0.41983	6.831481934	1
MSTRG.5896.2	393	0.2785	9.126159668	1
MSTRG.5897.1	101	0.3345	6.00177002	1
MSTRG.5901.1	74	0.39138	6.804382324	1
MSTRG.5904.1	343	0.44321	9.45123291	1
MSTRG.5904.2	244	0.41409	9.553771973	1
MSTRG.5904.4	343	0.38122	9.45123291	1
MSTRG.5907.1	2720	0.42061	5.672058105	1
MSTRG.5908.1	136	0.32512	10.4442749	1
MSTRG.5908.2	117	0.42749	11.62445068	1
MSTRG.5907.3	1849	0.43447	5.960510254	1
MSTRG.5907.4	1876	0.42061	5.938415527	1
MSTRG.5909.2	451	0.39145	5.081604004	1
MSTRG.5909.3	436	0.39145	6.313171387	1
MSTRG.5909.4	138	0.29873	5.308532715	1
MSTRG.5910.1	246	0.41567	5.511291504	1
MSTRG.5915.1	570	0.40173	8.004577637	1
MSTRG.5915.4	145	0.40082	6.585388184	1
MSTRG.5915.3	404	0.40254	7.437927246	1
MSTRG.5916.1	250	0.29367	8.722717285	1
MSTRG.5917.2	266	0.46257	8.90838623	-1
MSTRG.5918.1	18	0.31588	6.693054199	1
MSTRG.5919.1	189	0.3439	6.100891113	1
MSTRG.5920.1	172	0.29772	10.54083252	1
MSTRG.5924.1	95	0.29963	9.125183105	1
MSTRG.5924.6	503	0.36968	4.939147949	1
MSTRG.5924.7	467	0.48086	5.175476074	1
MSTRG.5924.8	448	0.4825	5.756774902	1
MSTRG.5921.1	45	0.38632	9.497741699	1
MSTRG.5922.3	86	0.39389	4.672546387	1
MSTRG.5922.4	95	0.27937	6.528869629	1
MSTRG.5923.1	127	0.2921	8.530944824	1
MSTRG.5925.1	502	0.39357	5.383728027	1
MSTRG.5925.2	502	0.38889	5.383728027	1
MSTRG.5925.4	502	0.34908	5.383728027	1
MSTRG.5925.3	369	0.34544	5.645080566	1
MSTRG.5925.5	502	0.3378	5.383728027	1
MSTRG.5927.1	570	0.36813	9.456115723	1
MSTRG.5927.2	269	0.32453	9.328552246	1
MSTRG.5932.1	180	0.35381	9.27935791	1
MSTRG.5934.1	288	0.35144	6.858337402	1
MSTRG.5934.3	436	0.36697	6.766906738	1
MSTRG.5934.4	436	0.37441	6.766906738	1
MSTRG.5935.2	326	0.39628	9.473693848	1

MSTRG.5936.1	871	0.46486	5.640563965	1
MSTRG.5939.1	32	0.37628	10.02215576	1
MSTRG.5940.1	46	0.3934	4.415710449	-1
MSTRG.5940.2	45	0.33141	9.992980957	1
MSTRG.5939.2	21	0.40425	9.489196777	1
MSTRG.5940.4	34	0.4002	4.128234863	-1
MSTRG.5939.3	48	0.37009	5.144470215	1
MSTRG.5939.4	54	0.36496	10.28631592	1
MSTRG.5940.5	16	0.45484	7.999816895	-1
MSTRG.5942.1	48	0.32016	5.144470215	1
MSTRG.5944.1	48	0.32047	5.144470215	1
MSTRG.5945.1	48	0.31868	4.856506348	1
MSTRG.5946.1	36	0.2881	6.484802246	1
MSTRG.5945.2	48	0.30938	4.856506348	1
MSTRG.5949.2	131	0.34086	9.738586426	1
MSTRG.5966.1	94	0.29355	10.14202881	1
MSTRG.5965.5	576	0.30078	5.401550293	1
MSTRG.5969.1	64	0.28301	6.220031738	1
MSTRG.5965.6	198	0.40364	4.83026123	1
MSTRG.5965.7	105	0.3272	10.47601318	1
MSTRG.5965.8	322	0.29835	6.132873535	1
MSTRG.5970.1	41	0.28719	10.637146	1
MSTRG.5972.1	94	0.31177	8.855895996	1
MSTRG.5951.1	144	0.37107	9.058654785	1
MSTRG.5953.1	124	0.41997	7.585510254	1
MSTRG.5955.2	121	0.35812	8.965026855	1
MSTRG.5956.3	403	0.49063	5.681945801	1
MSTRG.5956.2	475	0.35655	6.192077637	1
MSTRG.5956.4	88	0.35072	9.559509277	1
MSTRG.5958.1	139	0.29687	8.195373535	1
MSTRG.5956.6	369	0.47143	5.403381348	1
MSTRG.5956.7	445	0.4732	5.431945801	1
MSTRG.5959.1	74	0.27433	9.846252441	1
MSTRG.5960.1	90	0.30028	10.12921143	1
MSTRG.5961.1	68	0.32072	8.738952637	1
MSTRG.5962.1	56	0.31778	9.299743652	1
MSTRG.5956.8	218	0.44566	6.164489746	1
MSTRG.5985.4	555	0.33099	9.344177246	1
MSTRG.5985.7	485	0.33202	9.012390137	-1
MSTRG.5985.5	764	0.33347	9.468811035	1
MSTRG.5985.8	208	0.34554	9.727478027	1
MSTRG.5986.1	108	0.32413	8.981262207	1
MSTRG.5989.1	75	0.35693	9.35736084	1
MSTRG.5990.2	188	0.41958	9.420959473	1
MSTRG.5989.4	153	0.36969	4.898742676	1
MSTRG.5984.1	87	0.33043	6.001525879	1
MSTRG.5991.1	365	0.47167	4.670959473	1
MSTRG.5991.3	87	0.37903	4.071960449	1
MSTRG.5992.1	609	0.33865	6.214782715	1
MSTRG.5994.1	67	0.33619	9.34979248	-1
MSTRG.5995.1	116	0.36273	6.168395996	-1
MSTRG.5997.1	43	0.33999	6.691467285	1
MSTRG.5999.1	115	0.37018	6.142028809	1
MSTRG.6004.5	639	0.41115	6.680236816	1

MSTRG.6004.4	710	0.41419	6.292785645	1
MSTRG.6005.1	111	0.27517	9.453186035	1
MSTRG.6006.1	53	0.2921	9.397766113	1
MSTRG.6004.7	572	0.37825	6.737609863	1
MSTRG.6007.1	60	0.29438	5.58013916	1
MSTRG.6008.1	117	0.26785	6.38494873	1
MSTRG.6008.2	82	0.46602	10.53179932	1
MSTRG.6009.3	1207	0.41408	6.075256348	1
MSTRG.6001.2	27	0.37319	9.819763184	1
MSTRG.6010.2	117	0.31126	4.771057129	1
MSTRG.6020.4	958	0.44289	6.550720215	1
MSTRG.6029.2	201	0.40692	5.179626465	1
MSTRG.6029.3	241	0.4102	5.627746582	1
MSTRG.6029.4	1106	0.43485	9.551818848	1
MSTRG.6030.1	38	0.35726	9.184509277	1
MSTRG.6033.2	157	0.43633	8.514709473	1
MSTRG.6035.1	132	0.28999	9.268615723	1
MSTRG.6036.2	391	0.41522	10.12371826	1
MSTRG.6036.3	391	0.41218	10.12371826	1
MSTRG.6037.1	105	0.2985	9.442932129	1
MSTRG.6040.1	1170	0.32846	8.747497559	1
MSTRG.6040.2	1197	0.32846	8.680480957	1
MSTRG.6040.3	1170	0.32522	8.747497559	1
MSTRG.6040.5	787	0.49289	8.953308105	1
MSTRG.6040.4	1170	0.49138	8.838684082	1
MSTRG.6040.6	563	0.42874	9.349304199	-1
MSTRG.6040.7	642	0.45056	9.277404785	-1
MSTRG.6040.8	698	0.46562	9.496154785	-1
MSTRG.6040.9	433	0.46594	8.969665527	1
MSTRG.6040.10	386	0.47209	8.882141113	1
MSTRG.6040.11	368	0.46238	8.91204834	1
MSTRG.6042.1	466	0.44553	6.810852051	1
MSTRG.6043.3	510	0.35665	9.175476074	1
MSTRG.6045.2	738	0.34787	8.485168457	1
MSTRG.6050.1	493	0.33273	5.040100098	1
MSTRG.6050.3	416	0.33121	5.028137207	1
MSTRG.6050.4	493	0.35199	5.040100098	1
MSTRG.6050.7	306	0.3927	8.214904785	1
MSTRG.6056.1	1022	0.46679	9.03704834	1
MSTRG.6058.1	124	0.30725	11.66778564	1
MSTRG.6058.2	105	0.32046	9.421325684	1
MSTRG.6060.3	112	0.28014	6.254211426	1
MSTRG.6060.2	108	0.34787	6.967834473	1
MSTRG.6061.2	766	0.32237	7.269592285	1
MSTRG.6061.4	690	0.34547	9.00177002	1
MSTRG.6061.5	50	0.3208	10.82440186	1
MSTRG.6061.6	757	0.31852	8.504943848	1
MSTRG.6061.7	726	0.38408	8.27557373	1
MSTRG.6061.10	697	0.4495	9.135681152	-1
MSTRG.6061.11	435	0.40728	9.262756348	-1
MSTRG.6061.12	686	0.44085	8.44354248	1
MSTRG.6061.9	641	0.4455	6.611022949	1
MSTRG.6062.1	116	0.29512	9.217346191	1
MSTRG.6063.1	85	0.37636	9.303527832	1

MSTRG.6055.2	104	0.38245	9.220153809	1
MSTRG.6055.3	104	0.42568	9.220153809	1
MSTRG.6065.2	136	0.38167	7.624938965	1
MSTRG.6066.1	1622	0.41744	4.452331543	1
MSTRG.6067.2	153	0.37921	5.35369873	1
MSTRG.6068.2	243	0.47114	5.390930176	1
MSTRG.6073.1	151	0.37679	6.26361084	1
MSTRG.6074.1	66	0.38648	11.45318604	-1
MSTRG.6075.1	65	0.35622	4.860900879	1
MSTRG.6076.1	55	0.46096	12.36810303	1
MSTRG.6077.1	14	0.42778	7.999816895	1
MSTRG.6077.2	55	0.49213	11.77850342	1
MSTRG.6088.1	214	0.40933	6.06463623	1
MSTRG.6088.5	234	0.44695	6.139953613	-1
MSTRG.6089.2	399	0.36735	7.040588379	1
MSTRG.6089.3	343	0.36487	6.804260254	1
MSTRG.6096.1	41	0.3693	6.891540527	-1
MSTRG.6100.1	112	0.28624	9.16986084	1
MSTRG.6104.1	62	0.32446	11.78668213	1
MSTRG.6105.1	49	0.36902	10.637146	1
MSTRG.6112.1	118	0.3415	5.558410645	1
MSTRG.6113.1	83	0.40285	6.491149902	1
MSTRG.6121.3	715	0.29772	7.162780762	1
MSTRG.6121.5	595	0.42865	5.698547363	1
MSTRG.6121.7	509	0.29772	8.757995605	1
MSTRG.6121.9	748	0.37422	6.53326416	1
MSTRG.6121.10	637	0.368	5.613708496	1
MSTRG.6119.1	408	0.33043	9.824401855	1
MSTRG.6119.3	408	0.30957	9.824401855	1
MSTRG.6124.3	430	0.39687	6.26763916	1
MSTRG.6125.2	582	0.40913	7.571350098	1
MSTRG.6125.3	464	0.38658	8.394714355	1
MSTRG.6125.4	453	0.38091	8.394714355	1
MSTRG.6130.4	629	0.38872	6.217956543	1
MSTRG.6127.1	30	0.42446	7.835266113	1
MSTRG.6133.1	18	0.3854	7.81060791	1
MSTRG.6133.2	18	0.38746	7.81060791	1
MSTRG.6134.1	151	0.37384	4.885192871	1
MSTRG.6136.1	56	0.33693	6.23614502	-1
MSTRG.6136.2	261	0.37218	4.912658691	1
MSTRG.6139.6	559	0.35842	10.04156494	1
MSTRG.6140.2	35	0.36374	8.532409668	1
MSTRG.6141.2	553	0.38963	11.08428955	1
MSTRG.6143.1	294	0.43121	5.432067871	1
MSTRG.6145.3	297	0.45374	7.481628418	1
MSTRG.6149.1	174	0.44836	5.106628418	1
MSTRG.6154.3	247	0.41031	9.179382324	1
MSTRG.6163.1	227	0.3926	9.797302246	1
MSTRG.6163.2	227	0.40856	9.797302246	1
MSTRG.6163.4	401	0.36594	9.671203613	1
MSTRG.6163.5	422	0.4003	9.875549316	1
MSTRG.6161.2	255	0.34177	4.740905762	1
MSTRG.6161.3	426	0.39211	6.835388184	1
MSTRG.6167.1	625	0.45149	6.797058105	1

MSTRG.6167.3	625	0.45892	6.797058105	1
MSTRG.6169.2	470	0.42896	9.356384277	1
MSTRG.6170.1	82	0.30661	7.826721191	1
MSTRG.6171.1	58	0.42313	9.190612793	1
MSTRG.6171.2	58	0.39563	9.415588379	1
MSTRG.6173.2	79	0.29022	5.76348877	1
MSTRG.6173.3	75	0.35568	6.053283691	1
MSTRG.6174.7	326	0.45141	6.227111816	1
MSTRG.6174.1	326	0.46746	6.227111816	1
MSTRG.6174.6	326	0.45721	6.227111816	1
MSTRG.6174.9	27	0.35438	3.998840332	1
MSTRG.6174.10	27	0.36786	3.998840332	1
MSTRG.6186.2	232	0.35674	9.00604248	1
MSTRG.6190.2	4959	0.42851	6.077087402	1
MSTRG.6190.1	4968	0.42851	6.093811035	1
MSTRG.6190.10	4487	0.44512	6.227966309	1
MSTRG.6190.11	4488	0.44512	6.227966309	1
MSTRG.6190.15	4400	0.43126	6.21673584	1
MSTRG.6190.12	4487	0.44512	6.227966309	1
MSTRG.6190.5	4480	0.44512	6.208068848	1
MSTRG.6190.14	4491	0.43187	6.288513184	1
MSTRG.6190.9	4889	0.44512	6.099914551	1
MSTRG.6190.6	4967	0.44512	6.093811035	1
MSTRG.6190.4	4960	0.44512	6.077087402	1
MSTRG.6190.8	4958	0.44512	6.077087402	1
MSTRG.6190.17	465	0.34787	6.992126465	1
MSTRG.6192.1	111	0.27241	5.471984863	1
MSTRG.6193.1	43	0.30578	10.04425049	1
MSTRG.6179.1	70	0.28027	9.644592285	1
MSTRG.6179.2	94	0.37915	10.14007568	1
MSTRG.6180.6	160	0.30633	4.871154785	1
MSTRG.6180.7	141	0.27844	5.587097168	1
MSTRG.6180.5	160	0.30633	4.657043457	1
MSTRG.6180.2	418	0.43372	5.614929199	1
MSTRG.6180.4	418	0.44802	5.614929199	1
MSTRG.6180.8	282	0.30837	7.558776855	1
MSTRG.6181.1	47	0.29681	11.64117432	1
MSTRG.6181.2	56	0.32234	6.520690918	1
MSTRG.6180.10	92	0.38429	9.45135498	1
MSTRG.6180.11	92	0.39859	9.45135498	1
MSTRG.6180.9	92	0.38939	9.45135498	1
MSTRG.6182.1	158	0.42677	6.544372559	1
MSTRG.6184.1	112	0.32792	9.82598877	1
MSTRG.6196.1	27	0.44131	8.020324707	-1
MSTRG.6201.1	368	0.42728	5.916442871	1
MSTRG.6204.2	737	0.46447	8.885437012	1
MSTRG.6204.3	891	0.45671	6.69720459	1
MSTRG.6206.1	26	0.42564	8.210510254	1
MSTRG.6212.1	1157	0.39433	6.584899902	1
MSTRG.6212.2	1158	0.39433	6.584899902	1
MSTRG.6212.3	55	0.3495	7.85736084	-1
MSTRG.6212.8	476	0.45558	10.59527588	1
MSTRG.6220.1	53	0.40899	9.431213379	1
MSTRG.6222.3	356	0.46606	8.990905762	1

MSTRG.6222.4	209	0.44887	7.637268066	1
MSTRG.6223.1	129	0.29624	9.166442871	1
MSTRG.6231.1	466	0.44054	9.190612793	1
MSTRG.6232.2	968	0.43959	5.688293457	1
MSTRG.6232.4	1013	0.44412	7.98248291	1
MSTRG.6233.1	84	0.29373	9.296325684	1
MSTRG.6233.2	84	0.29469	9.296325684	1
MSTRG.6235.5	259	0.4506	4.261169434	1
MSTRG.6235.4	318	0.39475	8.999084473	1
MSTRG.6235.6	259	0.3185	4.261169434	1
MSTRG.6235.7	142	0.29736	8.990539551	1
MSTRG.6235.8	259	0.31752	4.261169434	1
MSTRG.6235.9	318	0.30989	8.999084473	1
MSTRG.6238.2	895	0.44756	6.255187988	1
MSTRG.6240.1	209	0.35393	10.06060791	1
MSTRG.6238.3	76	0.27413	11.94329834	-1
MSTRG.6242.1	88	0.3724	9.776550293	-1
MSTRG.6237.2	75	0.2845	5.623840332	1
MSTRG.6237.3	442	0.4321	6.857116699	1
MSTRG.6237.4	773	0.46379	7.480041504	1
MSTRG.6237.5	1140	0.44999	7.18560791	1
MSTRG.6251.1	843	0.42249	7.309875488	1
MSTRG.6251.2	522	0.35597	5.418273926	1
MSTRG.6251.3	825	0.35997	7.049499512	1
MSTRG.6251.4	721	0.3174	6.656433105	1
MSTRG.6251.5	417	0.28119	9.372375488	1
MSTRG.6251.7	417	0.36306	9.372375488	1
MSTRG.6251.8	136	0.31477	10.42877197	1
MSTRG.6252.1	334	0.35052	9.082092285	1
MSTRG.6253.1	1066	0.42464	7.085754395	1
MSTRG.6253.2	1057	0.42826	7.087341309	1
MSTRG.6253.3	1114	0.41228	6.831604004	1
MSTRG.6253.5	1074	0.447	7.232116699	1
MSTRG.6248.1	55	0.4424	4.352355957	1
MSTRG.6267.3	227	0.33481	10.02825928	1
MSTRG.6271.1	1988	0.46866	8.468322754	1
MSTRG.6271.3	1570	0.46455	6.647155762	1
MSTRG.6272.1	219	0.29224	8.944274902	1
MSTRG.6273.2	279	0.37366	8.294372559	1
MSTRG.6273.3	230	0.35961	9.560241699	1
MSTRG.6274.2	124	0.41378	5.432922363	1
MSTRG.6285.1	69	0.34696	10.42608643	1
MSTRG.6288.1	71	0.47806	9.588806152	1
MSTRG.6312.1	370	0.29367	9.062316895	1
MSTRG.6312.2	571	0.39359	8.081604004	1
MSTRG.6314.1	183	0.3562	8.526794434	1
MSTRG.6312.8	272	0.31852	11.82220459	1
MSTRG.6315.1	133	0.30078	6.202697754	1
MSTRG.6316.1	170	0.27289	11.06658936	1
MSTRG.6314.2	183	0.28982	8.526794434	1
MSTRG.6314.3	107	0.27994	5.120056152	1
MSTRG.6314.4	107	0.28642	5.120056152	1
MSTRG.6317.1	101	0.29366	8.880310059	1
MSTRG.6316.2	201	0.2921	10.46990967	1

MSTRG.6318.1	81	0.29374	6.225769043	1
MSTRG.6316.3	93	0.2921	9.738586426	1
MSTRG.6316.4	44	0.36754	9.746398926	1
MSTRG.6316.5	176	0.2831	10.91204834	1
MSTRG.6316.6	151	0.2831	7.06842041	1
MSTRG.6319.1	58	0.40978	6.813293457	1
MSTRG.6319.2	33	0.45806	6.693664551	1
MSTRG.6319.3	58	0.41942	6.813293457	1
MSTRG.6320.1	45	0.30749	7.774475098	1
MSTRG.6323.2	114	0.31035	5.676818848	1
MSTRG.6308.4	187	0.29022	5.885681152	1
MSTRG.6327.1	889	0.46474	6.518127441	1
MSTRG.6328.1	53	0.40159	6.473449707	1
MSTRG.6332.2	266	0.45353	5.659606934	1
MSTRG.6332.3	302	0.40048	6.22052002	1
MSTRG.6333.1	98	0.41705	10.35186768	1
MSTRG.6334.2	193	0.3311	8.95098877	1
MSTRG.6348.1	45	0.34101	10.637146	1
MSTRG.6348.2	109	0.29676	7.631164551	1
MSTRG.6349.2	1708	0.42215	7.040710449	1
MSTRG.6349.1	1754	0.41361	6.689147949	1
MSTRG.6349.3	1760	0.41361	6.739807129	1
MSTRG.6349.4	1746	0.41361	6.624572754	1
MSTRG.6349.5	1715	0.4447	7.155334473	1
MSTRG.6349.6	1730	0.42139	7.453063965	1
MSTRG.6349.7	1708	0.42139	7.041809082	1
MSTRG.6349.8	1742	0.39821	6.944519043	1
MSTRG.6350.1	33	0.32345	4.493103027	1
MSTRG.6351.1	79	0.28411	6.395080566	1
MSTRG.6351.3	353	0.30804	5.357971191	1
MSTRG.6355.5	57	0.25541	6.942932129	1
MSTRG.6355.4	771	0.37862	5.539733887	1
MSTRG.6356.1	72	0.29583	10.51885986	1
MSTRG.6357.1	79	0.37546	9.492980957	1
MSTRG.6355.6	53	0.29374	9.014465332	1
MSTRG.6358.1	150	0.29343	9.136901855	1
MSTRG.6359.1	105	0.31852	9.855529785	1
MSTRG.6359.2	105	0.31851	6.24822998	1
MSTRG.6355.7	101	0.35808	9.427429199	1
MSTRG.6360.1	40	0.34743	5.303771973	1
MSTRG.6363.1	1039	0.43997	5.121276855	1
MSTRG.6364.1	232	0.37539	8.174621582	1
MSTRG.6384.1	97	0.31753	9.505065918	1
MSTRG.6385.1	58	0.26785	7.749450684	1
MSTRG.6386.1	125	0.32709	10.21307373	1
MSTRG.6387.1	78	0.29387	7.606750488	1
MSTRG.6388.1	62	0.309	6.810852051	1
MSTRG.6389.1	190	0.30595	10.11834717	1
MSTRG.6389.2	179	0.35469	9.445373535	1
MSTRG.6389.3	172	0.44874	8.512023926	1
MSTRG.6383.2	739	0.36893	8.965270996	1
MSTRG.6383.3	446	0.381	9.432800293	1
MSTRG.6383.4	80	0.36467	6.787780762	1
MSTRG.6379.1	37	0.35339	4.868225098	1

MSTRG.6391.1	292	0.3685	9.239807129	1
MSTRG.6404.2	1632	0.48747	5.714660645	1
MSTRG.6404.3	1667	0.45318	5.724304199	1
MSTRG.6433.11	469	0.41802	8.207214355	1
MSTRG.6433.12	485	0.45536	8.439147949	1
MSTRG.6433.15	446	0.42174	8.227233887	1
MSTRG.6433.3	415	0.45569	8.38861084	1
MSTRG.6433.4	422	0.40124	8.289123535	1
MSTRG.6433.5	461	0.43486	8.50201416	1
MSTRG.6433.6	445	0.40157	8.265808105	1
MSTRG.6433.8	438	0.40124	8.412414551	1
MSTRG.6433.13	461	0.39752	8.38861084	1
MSTRG.6433.10	399	0.41263	8.222351074	1
MSTRG.6433.14	431	0.43486	8.627868652	1
MSTRG.6433.16	392	0.40113	8.419616699	1
MSTRG.6433.7	477	0.39473	8.583312988	1
MSTRG.6433.9	376	0.41728	8.252502441	1
MSTRG.6435.2	2762	0.3992	5.633483887	1
MSTRG.6436.2	373	0.36056	6.97467041	1
MSTRG.6430.2	246	0.29059	5.067321777	1
MSTRG.6442.1	73	0.32642	4.247253418	1
MSTRG.6454.2	96	0.31851	4.575256348	1
MSTRG.6456.1	67	0.30084	6.699157715	1
MSTRG.6456.2	64	0.27674	6.528137207	1
MSTRG.6457.4	65	0.34008	9.23614502	1
MSTRG.6457.2	65	0.34008	9.23614502	1
MSTRG.6457.3	65	0.32022	9.839294434	1
MSTRG.6457.5	37	0.31138	5.477844238	1
MSTRG.6464.3	149	0.42399	6.843322754	1
MSTRG.6478.1	700	0.43089	6.079650879	1
MSTRG.6497.2	396	0.41003	5.403991699	1
MSTRG.6497.3	430	0.41625	5.299743652	1
MSTRG.6497.4	1433	0.42228	6.827453613	1
MSTRG.6497.5	1433	0.4248	6.827453613	1
MSTRG.6497.6	1434	0.44688	6.827453613	1
MSTRG.6497.7	899	0.3556	6.215148926	1
MSTRG.6497.9	92	0.36062	10.2366333	1
MSTRG.6497.10	88	0.28982	6.792175293	1
MSTRG.6497.11	899	0.39276	6.215148926	1
MSTRG.6501.1	820	0.47294	5.106994629	1
MSTRG.6501.2	627	0.46326	5.215881348	1
MSTRG.6501.3	627	0.4814	5.215881348	1
MSTRG.6501.4	785	0.47581	5.028015137	1
MSTRG.6501.5	627	0.46572	5.215881348	1
MSTRG.6501.6	116	0.32469	9.373962402	1
MSTRG.6501.7	270	0.48337	4.425720215	1
MSTRG.6501.8	106	0.40392	4.785461426	1
MSTRG.6502.4	795	0.43394	9.25958252	1
MSTRG.6503.1	477	0.49759	5.640441895	1
MSTRG.6487.2	709	0.40488	8.016296387	1
MSTRG.6487.1	709	0.42413	8.016296387	1
MSTRG.6487.3	709	0.42293	8.016296387	1
MSTRG.6489.1	37	0.35821	9.688293457	1
MSTRG.6494.1	66	0.36156	9.183288574	1

MSTRG.6496.2	167	0.36224	6.143737793	1
MSTRG.6505.2	125	0.43197	5.237487793	1
MSTRG.6507.1	347	0.377	10.07452393	1
MSTRG.6507.2	332	0.37462	9.99005127	1
MSTRG.6508.2	158	0.33022	6.024230957	1
MSTRG.6513.1	231	0.36832	9.467956543	1
MSTRG.6513.2	153	0.37116	9.728210449	1
MSTRG.6515.1	29	0.44261	11.39801025	1
MSTRG.6520.1	163	0.39655	7.505310059	1
MSTRG.6522.3	417	0.44657	6.270935059	1
MSTRG.6522.4	417	0.45317	6.270935059	1
MSTRG.6530.1	362	0.39004	6.330505371	1
MSTRG.6530.2	362	0.40632	6.330505371	1
MSTRG.6530.7	353	0.45994	6.603942871	1
MSTRG.6530.11	334	0.47414	8.171691895	1
MSTRG.6530.13	353	0.41959	6.603942871	1
MSTRG.6530.12	353	0.41278	6.603942871	1
MSTRG.6530.14	439	0.44977	8.695983887	1
MSTRG.6530.15	141	0.35093	9.596496582	1
MSTRG.6524.1	482	0.34584	5.678405762	1
MSTRG.6524.2	738	0.28096	8.288391113	1
MSTRG.6524.3	786	0.28096	6.940002441	1
MSTRG.6524.6	779	0.43264	7.610778809	1
MSTRG.6524.7	842	0.41856	7.580505371	1
MSTRG.6524.8	779	0.44773	7.610778809	1
MSTRG.6526.1	421	0.33582	8.855285645	-1
MSTRG.6527.2	218	0.4523	5.094055176	1
MSTRG.6527.3	218	0.44988	5.094055176	1
MSTRG.6538.3	512	0.44078	6.379821777	1
MSTRG.6544.1	1488	0.40862	8.735778809	1
MSTRG.6544.3	1409	0.40862	8.661804199	1
MSTRG.6540.2	200	0.36018	9.547058105	1
MSTRG.6546.1	351	0.44602	5.216003418	1
MSTRG.6550.2	238	0.44986	5.320739746	1
MSTRG.6550.3	238	0.43298	5.320739746	1
MSTRG.6550.5	238	0.44986	5.320739746	1
MSTRG.6550.6	238	0.45906	5.320739746	1
MSTRG.6551.1	138	0.32345	9.069030762	1
MSTRG.6551.2	110	0.31168	6.713195801	1
MSTRG.6551.3	142	0.3208	6.150085449	1
MSTRG.6554.1	598	0.42055	6.821228027	1
MSTRG.6555.1	83	0.45509	9.183532715	1
MSTRG.6562.1	1939	0.4367	5.774963379	1
MSTRG.6562.3	239	0.3281	8.691345215	1
MSTRG.6568.1	209	0.38068	10.3303833	1
MSTRG.6567.6	108	0.36869	12.00750732	1
MSTRG.6564.2	97	0.46228	4.206970215	1
MSTRG.6564.3	106	0.34849	7.773742676	1
MSTRG.6577.3	403	0.37169	4.72064209	1
MSTRG.6577.2	543	0.37151	4.699645996	1
MSTRG.6582.3	562	0.45976	5.510437012	1
MSTRG.6582.2	562	0.4374	5.510437012	1
MSTRG.6582.1	569	0.46479	5.392150879	1
MSTRG.6582.4	569	0.44277	5.392150879	1

MSTRG.6585.1	417	0.40978	9.50579834	1
MSTRG.6587.1	147	0.33015	11.22979736	1
MSTRG.6589.2	116	0.32204	8.164001465	1
MSTRG.6591.2	677	0.44975	6.218200684	1
MSTRG.6591.3	679	0.47016	6.218200684	1
MSTRG.6592.1	2641	0.45172	8.757263184	1
MSTRG.6592.2	2641	0.46158	8.789978027	1
MSTRG.6594.2	600	0.40522	6.09954834	1
MSTRG.6594.3	760	0.42788	7.577453613	1
MSTRG.6598.1	42	0.27937	7.804504395	1
MSTRG.6601.4	57	0.40668	9.80255127	1
MSTRG.6605.1	317	0.39875	5.00592041	1
MSTRG.6606.2	175	0.30285	9.452453613	1
MSTRG.6614.1	85	0.35306	8.313415527	1
MSTRG.6614.2	139	0.31483	5.641418457	1
MSTRG.6612.3	766	0.41648	8.57611084	1
MSTRG.6624.2	449	0.45737	4.926574707	1
MSTRG.6624.3	414	0.44001	4.939880371	1
MSTRG.6624.4	412	0.43374	4.986999512	-1
MSTRG.6624.5	445	0.45871	5.01348877	-1
MSTRG.6624.7	405	0.42354	5.225769043	-1
MSTRG.6624.6	405	0.45704	5.225769043	-1
MSTRG.6624.8	375	0.44801	5.899230957	1
MSTRG.6624.9	256	0.42272	5.399353027	1
MSTRG.6624.10	410	0.45438	5.078552246	-1
MSTRG.6624.11	409	0.43646	5.119445801	-1
MSTRG.6626.1	8	0.33761	8.521057129	1
MSTRG.6627.1	456	0.4166	5.144104004	1
MSTRG.6627.2	451	0.45686	4.942810059	1
MSTRG.6627.3	477	0.44636	4.750305176	1
MSTRG.6627.4	451	0.46456	4.942810059	1
MSTRG.6627.5	483	0.44357	4.678161621	1
MSTRG.6627.6	479	0.44357	4.731018066	1
MSTRG.6627.7	451	0.44661	4.942810059	1
MSTRG.6622.2	489	0.42711	8.204406738	1
MSTRG.6622.3	62	0.37408	6.257629395	1
MSTRG.6622.4	489	0.43822	8.204406738	1
MSTRG.6633.1	5621	0.45787	5.492980957	1
MSTRG.6633.2	5609	0.45787	5.492980957	1
MSTRG.6633.7	3831	0.46097	5.269104004	-1
MSTRG.6633.5	5600	0.45622	5.492980957	1
MSTRG.6634.2	244	0.37351	6.714660645	1
MSTRG.6629.2	181	0.44592	6.84173584	1
MSTRG.6636.3	308	0.38425	6.272888184	1
MSTRG.6636.4	308	0.42469	6.272888184	1
MSTRG.6636.5	308	0.38425	6.272888184	1
MSTRG.6636.6	308	0.38048	6.272888184	1
MSTRG.6638.2	328	0.44247	5.356506348	1
MSTRG.6639.2	112	0.33969	9.995788574	1
MSTRG.6639.3	65	0.35275	9.295959473	1
MSTRG.6640.3	551	0.45829	8.926330566	1
MSTRG.6640.4	551	0.31292	8.926330566	1
MSTRG.6644.4	297	0.37519	6.512145996	1
MSTRG.6644.6	263	0.35266	8.702209473	1

MSTRG.6644.7	297	0.37781	6.512145996	1
MSTRG.6644.8	257	0.33097	8.743225098	1
MSTRG.6652.1	272	0.35119	11.31658936	1
MSTRG.6660.1	288	0.44174	5.711853027	1
MSTRG.6660.2	288	0.4005	5.711853027	1
MSTRG.6660.5	288	0.33719	5.711853027	1
MSTRG.6661.5	850	0.44721	8.437927246	1
MSTRG.6661.7	850	0.44517	8.437927246	1
MSTRG.6661.3	850	0.45248	8.437927246	1
MSTRG.6662.6	645	0.34731	8.180358887	1
MSTRG.6662.7	543	0.34471	7.975036621	1
MSTRG.6657.1	1146	0.43763	7.524719238	1
MSTRG.6670.1	67	0.27607	10.13275146	1
MSTRG.6681.1	162	0.29607	9.917541504	1
MSTRG.6682.1	148	0.28683	7.070373535	1
MSTRG.6685.1	437	0.43306	8.900817871	1
MSTRG.6678.2	36	0.3936	4.950012207	1
MSTRG.6698.1	67	0.48377	8.031921387	-1
MSTRG.6705.1	336	0.2895	5.098693848	1
MSTRG.6708.1	99	0.31035	10.22515869	1
MSTRG.6709.1	103	0.31642	5.817077637	1
MSTRG.6710.1	105	0.31977	8.843688965	1
MSTRG.6707.3	1322	0.42656	8.90814209	1
MSTRG.6707.4	1306	0.43017	8.945983887	1
MSTRG.6711.1	160	0.31645	10.95001221	-1
MSTRG.6713.1	19	0.35793	11.70025635	1
MSTRG.6714.1	170	0.30644	9.738342285	1
MSTRG.6714.2	180	0.31225	10.29315186	1
MSTRG.6714.3	109	0.26785	10.18304443	1
MSTRG.6714.4	116	0.27622	10.57415771	1
MSTRG.6714.5	101	0.35493	9.733703613	1
MSTRG.6715.1	125	0.30788	6.577575684	1
MSTRG.6694.1	114	0.29666	6.282897949	1
MSTRG.6694.2	116	0.30816	5.998962402	1
MSTRG.6695.1	85	0.29234	9.778747559	1
MSTRG.6694.3	128	0.28683	6.731506348	1
MSTRG.6694.4	48	0.3084	6.664123535	1
MSTRG.6726.2	252	0.41888	6.521789551	1
MSTRG.6739.2	79	0.29772	8.209289551	1
MSTRG.6739.4	35	0.33323	6.52130127	1
MSTRG.6740.2	76	0.27289	9.777038574	1
MSTRG.6741.3	317	0.47579	6.049133301	1
MSTRG.6741.4	347	0.47233	7.210510254	1
MSTRG.6741.5	306	0.48947	8.737121582	1
MSTRG.6744.1	64	0.4246	4.721740723	1
MSTRG.6744.2	47	0.32844	7.69354248	1
MSTRG.6748.3	838	0.43832	5.25994873	-1
MSTRG.6750.2	387	0.39215	8.890808105	1
MSTRG.6756.1	193	0.28498	5.413635254	1
MSTRG.6757.1	272	0.34457	9.218200684	1
MSTRG.6760.1	78	0.3272	5.350036621	1
MSTRG.6762.1	108	0.2921	9.605773926	1
MSTRG.6763.1	93	0.35875	7.979064941	1
MSTRG.6761.4	471	0.36159	7.964904785	1

MSTRG.6761.5	453	0.35114	8.219055176	1
MSTRG.6761.6	470	0.386	7.964904785	1
MSTRG.6764.1	66	0.41178	10.09002686	1
MSTRG.6774.1	89	0.27234	9.100280762	1
MSTRG.6775.1	168	0.2765	10.10565186	1
MSTRG.6776.1	174	0.33359	9.563903809	1
MSTRG.6777.1	119	0.33706	6.051330566	1
MSTRG.6776.2	132	0.3513	8.209655762	1
MSTRG.6777.2	77	0.32515	7.858947754	1
MSTRG.6778.1	93	0.30276	9.235656738	1
MSTRG.6777.3	119	0.31027	6.051330566	1
MSTRG.6777.5	98	0.28982	5.083190918	1
MSTRG.6780.1	63	0.29476	10.30450439	1
MSTRG.6781.3	74	0.30361	8.714050293	1
MSTRG.6781.4	82	0.32105	9.473327637	1
MSTRG.6785.2	218	0.40881	7.743347168	1
MSTRG.6786.1	57	0.35492	8.65045166	1
MSTRG.6786.2	32	0.37873	9.033752441	1
MSTRG.6792.5	429	0.48162	4.540588379	1
MSTRG.6797.3	961	0.48255	5.200866699	1
MSTRG.6797.4	250	0.44606	6.75604248	1
MSTRG.6798.1	56	0.31718	7.894714355	1
MSTRG.6800.1	49	0.26498	9.456115723	1
MSTRG.6806.2	432	0.46818	9.104064941	1
MSTRG.6804.1	190	0.48234	9.038757324	1
MSTRG.6817.3	143	0.33747	9.265075684	1
MSTRG.6823.1	104	0.32858	7.643737793	1
MSTRG.6811.1	92	0.33078	10.53668213	1
MSTRG.6811.2	80	0.3141	8.773376465	1
MSTRG.6857.1	197	0.29187	9.921936035	1
MSTRG.6857.2	702	0.33976	5.347961426	1
MSTRG.6857.3	1114	0.42371	5.840637207	1
MSTRG.6857.4	701	0.343	5.347961426	1
MSTRG.6857.5	98	0.29615	10.90008545	1
MSTRG.6857.6	136	0.28982	8.289123535	1
MSTRG.6857.7	240	0.28982	8.599182129	1
MSTRG.6857.8	131	0.29595	8.49005127	1
MSTRG.6859.1	103	0.30894	7.078063965	1
MSTRG.6859.2	171	0.28923	5.96270752	1
MSTRG.6859.3	91	0.3	6.476379395	1
MSTRG.6859.4	171	0.29088	5.96270752	1
MSTRG.6860.1	125	0.28334	8.420715332	1
MSTRG.6861.1	202	0.29772	8.843688965	1
MSTRG.6861.2	202	0.29772	8.843688965	1
MSTRG.6857.9	151	0.28982	9.817199707	1
MSTRG.6857.10	120	0.28982	5.38104248	1
MSTRG.6857.11	110	0.29367	9.494812012	1
MSTRG.6857.12	110	0.29367	9.494812012	1
MSTRG.6857.13	101	0.28518	9.045227051	1
MSTRG.6857.14	53	0.31638	9.695983887	1
MSTRG.6857.15	188	0.28982	10.19903564	1
MSTRG.6863.1	149	0.29772	5.222839355	1
MSTRG.6864.1	183	0.28982	11.20245361	1
MSTRG.6864.2	73	0.30816	10.43280029	1

MSTRG.6857.16	92	0.28982	5.74407959	1
MSTRG.6865.1	105	0.32256	9.263977051	1
MSTRG.6857.17	1138	0.40728	5.546936035	1
MSTRG.6857.19	515	0.49541	6.469665527	1
MSTRG.6866.1	309	0.29904	6.171813965	-1
MSTRG.6857.20	641	0.35753	5.603088379	1
MSTRG.6857.21	689	0.39342	5.090270996	1
MSTRG.6857.22	640	0.44261	5.603088379	1
MSTRG.6857.23	576	0.41828	5.054626465	1
MSTRG.6827.2	135	0.31753	9.13494873	1
MSTRG.6837.1	88	0.3287	8.804870605	1
MSTRG.6838.1	157	0.32885	7.182067871	1
MSTRG.6839.1	71	0.36666	9.590026855	1
MSTRG.6845.1	99	0.47251	8.807556152	-1
MSTRG.6851.1	205	0.36649	9.727111816	1
MSTRG.6851.2	80	0.32973	9.79119873	1
MSTRG.6852.1	72	0.36952	9.889709473	1
MSTRG.6869.1	729	0.46987	6.278747559	1
MSTRG.6871.2	35	0.37671	10.25592041	1
MSTRG.6873.2	211	0.37955	6.73626709	1
MSTRG.6873.3	211	0.34777	6.73626709	1
MSTRG.6884.1	1085	0.36443	6.397399902	1
MSTRG.6885.1	188	0.28923	9.205993652	1
MSTRG.6886.1	346	0.34614	9.299255371	1
MSTRG.6887.1	108	0.31168	9.393127441	1
MSTRG.6888.1	111	0.28982	5.751525879	1
MSTRG.6901.1	54	0.32237	9.30267334	1
MSTRG.6906.7	499	0.26347	5.788635254	1
MSTRG.6906.8	518	0.40451	5.406799316	1
MSTRG.6906.9	445	0.30918	5.759460449	1
MSTRG.6906.10	280	0.42004	5.089904785	1
MSTRG.6907.1	157	0.30186	7.63458252	1
MSTRG.6902.1	50	0.37964	10.69744873	-1
MSTRG.6919.1	96	0.27517	7.754699707	1
MSTRG.6920.1	156	0.27289	9.729675293	1
MSTRG.6921.1	66	0.33674	7.668884277	1
MSTRG.6922.4	182	0.28338	6.047058105	1
MSTRG.6922.3	922	0.40857	6.148742676	1
MSTRG.6922.5	118	0.29607	6.473937988	1
MSTRG.6923.1	30	0.28923	6.685852051	1
MSTRG.6925.1	82	0.42163	9.792663574	1
MSTRG.6925.2	82	0.26292	9.792663574	1
MSTRG.6925.3	82	0.33395	9.792663574	1
MSTRG.6926.1	58	0.34018	7.998596191	-1
MSTRG.6934.1	210	0.32179	8.300476074	1
MSTRG.6939.3	119	0.30557	6.285217285	1
MSTRG.6941.1	316	0.44012	5.612731934	1
MSTRG.6941.2	316	0.44012	5.612731934	1
MSTRG.6943.1	168	0.37602	4.777404785	1
MSTRG.6943.2	168	0.34675	4.777404785	1
MSTRG.6943.4	168	0.34498	4.777404785	1
MSTRG.6945.2	658	0.43845	8.129577637	1
MSTRG.6947.3	989	0.42875	8.844909668	1
MSTRG.6947.4	957	0.44925	8.782409668	1

MSTRG.6947.6	989	0.42875	8.844909668	1
MSTRG.6948.1	201	0.3481	9.69329834	1
MSTRG.6947.7	989	0.41954	8.844909668	1
MSTRG.6947.8	543	0.45651	8.874328613	1
MSTRG.6956.3	707	0.33497	6.043518066	1
MSTRG.6962.4	142	0.46649	6.11126709	1
MSTRG.6962.5	252	0.45635	4.843078613	1
MSTRG.6966.4	1867	0.40086	6.33770752	1
MSTRG.6967.3	164	0.34144	5.841369629	1
MSTRG.6970.3	1201	0.4448	5.316833496	1
MSTRG.6973.5	1000	0.45251	5.781066895	-1
MSTRG.6974.1	182	0.45231	4.807556152	1
MSTRG.6975.1	400	0.3691	6.424133301	1
MSTRG.6985.1	1086	0.42455	6.043395996	1
MSTRG.6986.1	211	0.38242	9.172668457	1
MSTRG.6986.4	187	0.40292	9.198303223	1
MSTRG.6988.1	60	0.36342	6.040588379	1
MSTRG.6997.1	41	0.29654	10.01068115	1
MSTRG.6997.2	41	0.29727	10.01068115	1
MSTRG.6995.2	91	0.37095	6.020202637	1
MSTRG.6995.4	91	0.343	6.020202637	1
MSTRG.6995.3	91	0.36939	6.020202637	1
MSTRG.6995.5	91	0.40722	6.020202637	-1
MSTRG.6977.1	113	0.4146	10.99310303	1
MSTRG.6977.2	201	0.34673	10.33795166	1
MSTRG.6977.3	201	0.39393	10.33795166	1
MSTRG.6977.4	201	0.40221	10.33795166	1
MSTRG.6977.5	28	0.40769	9.00592041	1
MSTRG.6980.3	488	0.4166	5.356506348	1
MSTRG.6980.2	547	0.41474	5.884338379	1
MSTRG.6981.6	205	0.30544	8.370910645	1
MSTRG.6981.4	215	0.27674	8.294128418	1
MSTRG.6981.5	215	0.27674	8.294128418	1
MSTRG.7007.1	298	0.38078	8.793518066	1
MSTRG.7007.2	774	0.37294	6.848815918	1
MSTRG.7007.4	341	0.37152	8.995666504	1
MSTRG.7007.5	774	0.34584	6.848815918	1
MSTRG.7008.2	138	0.41701	9.729431152	1
MSTRG.7007.11	775	0.38035	6.398620605	1
MSTRG.6982.1	109	0.4635	9.81060791	1
MSTRG.6983.1	78	0.31962	9.776184082	1
MSTRG.6982.3	109	0.40264	9.81060791	1
MSTRG.7005.3	108	0.45406	10.53363037	1
MSTRG.7006.1	406	0.43947	8.184020996	1
MSTRG.7012.2	237	0.42986	7.864807129	1
MSTRG.7019.1	183	0.27415	10.18499756	1
MSTRG.7035.2	565	0.42641	8.943054199	1
MSTRG.7041.4	148	0.40266	10.18438721	1
MSTRG.7044.1	70	0.34162	10.04400635	1
MSTRG.7050.1	47	0.43093	10.02435303	1
MSTRG.7050.3	50	0.41982	9.511657715	1
MSTRG.7050.2	91	0.32485	9.718688965	1
MSTRG.7054.1	68	0.42458	7.779724121	-1
MSTRG.7075.4	266	0.42528	8.679016113	1

MSTRG.7075.5	76	0.32402	8.686096191	1
MSTRG.7079.1	406	0.45354	5.950622559	1
MSTRG.7083.1	75	0.3351	6.476257324	-1
MSTRG.7077.3	288	0.45623	4.917297363	1
MSTRG.7091.3	756	0.47251	7.883728027	1
MSTRG.7091.5	928	0.46923	8.452087402	1
MSTRG.7091.4	928	0.45566	8.452087402	1
MSTRG.7096.1	112	0.28711	9.255065918	1
MSTRG.7102.1	289	0.34183	6.066833496	1
MSTRG.7102.2	427	0.33379	7.055480957	1
MSTRG.7102.3	289	0.36233	6.066833496	1
MSTRG.7103.1	123	0.35136	9.695861816	1
MSTRG.7104.1	915	0.4561	6.097717285	1
MSTRG.7110.1	54	0.38945	6.697937012	1
MSTRG.7111.3	398	0.44254	7.198913574	1
MSTRG.7111.4	256	0.46621	8.141052246	1
MSTRG.7111.5	264	0.43633	8.13861084	1
MSTRG.7118.2	150	0.43275	4.518981934	1
MSTRG.7122.3	160	0.42095	9.56463623	1
MSTRG.7130.2	1683	0.37996	5.819152832	1
MSTRG.7130.3	1683	0.383	5.819152832	1
MSTRG.7130.4	324	0.41909	5.735900879	1
MSTRG.7135.3	94	0.34351	9.514709473	1
MSTRG.7135.4	69	0.29856	8.930480957	-1
MSTRG.7138.2	718	0.44598	8.620056152	1
MSTRG.7141.1	629	0.44277	8.52154541	1
MSTRG.7141.3	497	0.37046	6.074157715	1
MSTRG.7142.1	73	0.29793	8.121276855	1
MSTRG.7147.3	51	0.33117	5.293273926	1
MSTRG.7147.2	573	0.44488	5.807922363	1
MSTRG.7146.1	72	0.40201	9.65411377	-1
MSTRG.7154.2	662	0.45693	4.91973877	1
MSTRG.7154.5	662	0.45385	4.91973877	1
MSTRG.7154.6	139	0.30173	9.239807129	1
MSTRG.7156.1	411	0.32255	8.858337402	1
MSTRG.7161.1	652	0.39359	7.671813965	1
MSTRG.7161.2	643	0.31725	7.63104248	1
MSTRG.7162.1	193	0.38199	6.741516113	1
MSTRG.7164.1	216	0.45755	9.679260254	-1
MSTRG.7165.1	156	0.37967	6.614196777	1
MSTRG.7165.2	124	0.33214	9.546813965	1
MSTRG.7166.1	116	0.3644	9.301940918	1
MSTRG.7169.1	51	0.34492	9.506286621	1
MSTRG.7177.1	151	0.33204	10.53265381	1
MSTRG.7178.1	104	0.35741	6.88885498	1
MSTRG.7178.2	102	0.41081	7.597106934	1
MSTRG.7177.2	55	0.28779	11.36480713	1
MSTRG.7177.3	55	0.28567	11.36480713	1
MSTRG.7177.4	55	0.27911	11.36480713	1
MSTRG.7177.8	55	0.28779	11.36480713	1
MSTRG.7177.7	55	0.28931	11.36480713	1
MSTRG.7177.6	55	0.29435	11.36480713	1
MSTRG.7177.5	55	0.2971	11.36480713	1
MSTRG.7177.9	93	0.34343	8.992980957	1

MSTRG.7177.10	55	0.28779	11.36480713	1
MSTRG.7177.11	39	0.28063	7.846496582	1
MSTRG.7177.12	39	0.28063	7.846496582	1
MSTRG.7177.15	55	0.31649	11.36480713	1
MSTRG.7177.16	55	0.28959	11.36480713	1
MSTRG.7177.13	55	0.28931	11.36480713	1
MSTRG.7177.14	55	0.29435	11.36480713	1
MSTRG.7177.18	108	0.29772	5.823425293	1
MSTRG.7177.19	93	0.34314	8.992980957	1
MSTRG.7179.1	71	0.33478	6.037658691	1
MSTRG.7172.1	115	0.27794	6.379577637	1
MSTRG.7176.7	352	0.34209	7.157653809	1
MSTRG.7176.4	801	0.45787	5.944030762	1
MSTRG.7176.5	352	0.34149	7.157653809	1
MSTRG.7176.6	352	0.33383	7.157653809	1
MSTRG.7183.1	505	0.3594	6.766540527	1
MSTRG.7183.3	505	0.40385	6.766540527	1
MSTRG.7189.1	107	0.28648	8.719299316	1
MSTRG.7191.3	355	0.35107	9.012268066	1
MSTRG.7194.1	125	0.35633	8.281921387	1
MSTRG.7195.1	302	0.32346	9.282043457	1
MSTRG.7195.2	325	0.35361	9.325012207	1
MSTRG.7196.1	200	0.28648	9.516296387	1
MSTRG.7195.3	152	0.29135	9.161437988	1
MSTRG.7186.1	29	0.43737	11.01275635	1
MSTRG.7197.3	602	0.38942	9.389587402	1
MSTRG.7200.2	734	0.41842	8.471740723	1
MSTRG.7200.3	734	0.41842	8.471740723	1
MSTRG.7202.1	114	0.34452	6.785461426	1
MSTRG.7206.4	449	0.30197	6.095031738	1
MSTRG.7206.5	381	0.36815	8.477966309	1
MSTRG.7206.6	380	0.44503	8.477966309	1
MSTRG.7207.1	96	0.29772	9.743469238	1
MSTRG.7206.7	117	0.28883	9.924865723	1
MSTRG.7211.1	93	0.31471	8.976867676	1
MSTRG.7213.1	166	0.35513	8.437316895	1
MSTRG.7216.2	548	0.43904	5.785339355	1
MSTRG.7220.2	197	0.4014	6.215881348	1
MSTRG.7220.3	107	0.36305	5.039367676	1
MSTRG.7220.4	119	0.48858	5.49005127	1
MSTRG.7225.1	596	0.34769	5.464294434	1
MSTRG.7227.1	269	0.35283	6.05682373	1
MSTRG.7232.2	175	0.28571	8.957092285	1
MSTRG.7232.3	122	0.27327	11.89935303	1
MSTRG.7232.1	824	0.29514	7.783752441	1
MSTRG.7232.4	824	0.29762	7.783752441	1
MSTRG.7232.5	824	0.29514	7.783752441	1
MSTRG.7232.6	144	0.27075	6.67376709	1
MSTRG.7235.3	1027	0.45179	5.866149902	1
MSTRG.7235.4	852	0.42073	5.543395996	-1
MSTRG.7245.1	80	0.36151	7.807800293	1
MSTRG.7258.7	535	0.47104	7.18157959	1
MSTRG.7258.6	576	0.47571	6.882995605	1
MSTRG.7258.5	587	0.48315	7.183410645	1

MSTRG.7260.1	87	0.30303	9.096252441	1
MSTRG.7262.3	778	0.40163	5.855773926	1
MSTRG.7262.5	203	0.30993	4.700378418	1
MSTRG.7263.1	128	0.29367	10.49493408	1
MSTRG.7263.2	121	0.31852	8.818054199	1
MSTRG.7264.1	61	0.28951	8.907409668	1
MSTRG.7266.9	1723	0.42806	8.097229004	1
MSTRG.7266.12	90	0.3375	6.696594238	1
MSTRG.7268.1	69	0.39053	9.503479004	1
MSTRG.7269.5	94	0.39218	9.151672363	1
MSTRG.7269.7	112	0.4082	8.913635254	1
MSTRG.7269.3	112	0.34875	8.913635254	1
MSTRG.7269.4	117	0.32478	8.821472168	1
MSTRG.7269.6	108	0.2765	9.715148926	1
MSTRG.7269.8	240	0.31041	6.686584473	1
MSTRG.7269.9	94	0.30141	9.151672363	1
MSTRG.7270.1	146	0.3561	10.09588623	1
MSTRG.7271.1	97	0.34331	10.14825439	1
MSTRG.7277.1	126	0.30119	9.514709473	1
MSTRG.7278.1	151	0.3096	10.71783447	1
MSTRG.7279.1	79	0.28982	8.985046387	1
MSTRG.7276.3	190	0.30816	9.446594238	1
MSTRG.7276.4	570	0.30387	8.528503418	1
MSTRG.7281.1	70	0.41832	10.18243408	-1
MSTRG.7281.2	70	0.35834	10.18243408	-1
MSTRG.7286.10	819	0.4353	8.704406738	1
MSTRG.7286.3	986	0.47921	6.880310059	1
MSTRG.7286.5	966	0.47921	7.060119629	1
MSTRG.7286.7	884	0.46076	6.106262207	1
MSTRG.7286.8	478	0.42681	8.510681152	1
MSTRG.7286.9	925	0.4723	6.269958496	1
MSTRG.7286.11	985	0.49307	6.880310059	1
MSTRG.7286.17	158	0.37649	5.614929199	1
MSTRG.7292.1	500	0.47011	9.214660645	1
MSTRG.7292.3	292	0.45215	6.44708252	1
MSTRG.7293.3	338	0.32178	4.91204834	1
MSTRG.7296.1	506	0.38993	5.448059082	1
MSTRG.7297.3	981	0.43288	5.344055176	1
MSTRG.7297.5	981	0.43288	5.375549316	1
MSTRG.7298.1	76	0.42828	5.364929199	1
MSTRG.7300.1	83	0.29367	7.749816895	1
MSTRG.7297.6	100	0.41927	10.00872803	1
MSTRG.7297.7	129	0.29526	9.715148926	1
MSTRG.7295.2	96	0.31532	9.147888184	1
MSTRG.7303.1	184	0.29727	10.18499756	1
MSTRG.7306.1	144	0.34227	4.293395996	1
MSTRG.7306.2	41	0.47102	5.601623535	1
MSTRG.7316.1	275	0.36484	8.955749512	1
MSTRG.7317.1	150	0.29839	8.57244873	1
MSTRG.7322.4	419	0.43487	5.855773926	1
MSTRG.7322.5	419	0.4505	5.855773926	1
MSTRG.7323.1	70	0.3895	9.828552246	1
MSTRG.7323.2	47	0.38733	5.13104248	1
MSTRG.7325.1	48	0.35614	6.508483887	1

MSTRG.7320.1	42	0.39752	11.55377197	1
MSTRG.7320.2	123	0.35941	9.912658691	1
MSTRG.7321.1	33	0.29579	9.034240723	1
MSTRG.7333.5	428	0.4369	4.641418457	1
MSTRG.7335.2	715	0.46052	6.39630127	1
MSTRG.7335.4	715	0.46601	6.39630127	1
MSTRG.7335.5	715	0.45772	6.39630127	1
MSTRG.7335.8	715	0.46668	6.39630127	1
MSTRG.7335.9	750	0.46304	6.383972168	1
MSTRG.7335.10	561	0.46806	8.162414551	1
MSTRG.7335.11	167	0.4503	4.975646973	1
MSTRG.7335.12	167	0.4503	4.975646973	1
MSTRG.7335.13	167	0.42963	4.975646973	1
MSTRG.7340.2	234	0.31903	5.982971191	1
MSTRG.7340.3	57	0.25541	8.602722168	1
MSTRG.7340.4	53	0.25541	6.708312988	1
MSTRG.7341.1	49	0.46508	5.079162598	-1
MSTRG.7342.1	114	0.3277	9.576721191	1
MSTRG.7343.1	213	0.33438	6.893127441	1
MSTRG.7344.4	122	0.30085	9.687072754	1
MSTRG.7346.1	73	0.38982	8.534729004	1
MSTRG.7348.1	71	0.44107	9.343200684	-1
MSTRG.7350.1	296	0.42119	5.664611816	1
MSTRG.7354.2	666	0.35806	7.63458252	1
MSTRG.7359.1	84	0.44235	11.39508057	1
MSTRG.7370.1	106	0.34952	4.538757324	1
MSTRG.7371.1	103	0.30647	9.831726074	1
MSTRG.7369.3	146	0.41532	8.866760254	1
MSTRG.7372.1	194	0.28923	9.368347168	1
MSTRG.7373.1	136	0.33843	9.160583496	1
MSTRG.7374.1	157	0.28584	9.971862793	1
MSTRG.7366.2	1037	0.44646	7.175354004	1
MSTRG.7365.1	26	0.39206	9.772888184	-1
MSTRG.7390.1	43	0.3963	5.461242676	1
MSTRG.7392.1	83	0.30807	10.0112915	1
MSTRG.7393.1	35	0.42548	9.18560791	-1
MSTRG.7394.1	65	0.37728	10.59429932	1
MSTRG.7376.3	358	0.44332	5.460876465	1
MSTRG.7376.5	358	0.44403	5.460876465	1
MSTRG.7382.1	106	0.31696	6.24420166	1
MSTRG.7385.1	84	0.30684	9.160705566	1
MSTRG.7387.1	350	0.33345	4.987609863	1
MSTRG.7415.2	819	0.46615	7.309265137	1
MSTRG.7415.4	99	0.2921	9.40057373	1
MSTRG.7415.3	819	0.36212	7.309265137	1
MSTRG.7415.5	170	0.28165	7.617004395	1
MSTRG.7415.6	144	0.3	7.660095215	1
MSTRG.7415.7	819	0.41871	7.309265137	1
MSTRG.7421.3	200	0.44925	5.086853027	1
MSTRG.7423.4	238	0.43027	9.062805176	1
MSTRG.7423.2	238	0.45299	9.062805176	1
MSTRG.7423.3	238	0.44098	9.062805176	1
MSTRG.7426.1	958	0.45914	6.648132324	1
MSTRG.7431.1	248	0.39475	9.284973145	1

MSTRG.7431.2	284	0.39069	9.042907715	1
MSTRG.7432.1	474	0.46795	10.31158447	1
MSTRG.7432.9	88	0.31596	9.199645996	1
MSTRG.7432.5	1117	0.45669	4.562927246	1
MSTRG.7432.6	1243	0.43744	4.647155762	1
MSTRG.7432.7	1243	0.46489	4.647155762	1
MSTRG.7434.1	32	0.28663	8.496398926	1
MSTRG.7429.1	120	0.40374	10.17315674	1
MSTRG.7429.2	120	0.3581	10.17315674	1
MSTRG.7437.1	17	0.41006	4.207092285	1
MSTRG.7438.1	160	0.3319	7.637878418	1
MSTRG.7439.4	329	0.46325	7.945861816	1
MSTRG.7441.1	656	0.36152	4.94329834	1
MSTRG.7448.1	122	0.29283	9.950134277	1
MSTRG.7448.2	110	0.30464	6.571228027	1
MSTRG.7452.1	643	0.3492	7.549865723	1
MSTRG.7452.3	643	0.34584	7.549865723	1
MSTRG.7454.1	127	0.28015	7.097473145	1
MSTRG.7451.2	394	0.37289	7.631408691	1
MSTRG.7455.1	1516	0.46956	5.132263184	1
MSTRG.7455.2	1613	0.4272	5.114318848	1
MSTRG.7456.6	361	0.40089	6.847717285	1
MSTRG.7456.7	361	0.44855	6.847717285	1
MSTRG.7456.8	361	0.44931	6.847717285	1
MSTRG.7458.1	82	0.35853	8.382629395	1
MSTRG.7457.3	93	0.29755	9.512145996	1
MSTRG.7462.1	84	0.45352	10.53375244	1
MSTRG.7467.1	141	0.47671	11.39508057	1
MSTRG.7465.1	457	0.39263	7.915710449	1
MSTRG.7470.1	31	0.35998	9.819763184	1
MSTRG.7474.2	144	0.43084	8.980285645	1
MSTRG.7476.1	48	0.36751	7.78704834	1
MSTRG.7487.1	68	0.34045	4.406066895	1
MSTRG.7485.2	408	0.47176	5.111877441	1
MSTRG.7499.1	991	0.48309	9.257751465	1
MSTRG.7496.1	96	0.38148	4.867370605	1
MSTRG.7501.1	56	0.35273	6.88470459	1
MSTRG.7505.1	496	0.39052	5.41217041	1
MSTRG.7505.2	496	0.39052	5.41217041	1
MSTRG.7518.1	71	0.31806	9.638122559	1
MSTRG.7529.1	21	0.35956	4.746154785	1
MSTRG.7529.2	77	0.33098	9.57623291	1
MSTRG.7531.1	74	0.34981	11.08831787	1
MSTRG.7534.1	77	0.428	4.333190918	1
MSTRG.7537.4	640	0.43901	4.454772949	1
MSTRG.7538.1	324	0.46022	7.060119629	-1
MSTRG.7539.1	739	0.42846	5.995910645	1
MSTRG.7541.1	298	0.37948	4.658630371	1
MSTRG.7542.1	29	0.31081	7.999450684	1
MSTRG.7539.2	705	0.41741	5.867492676	1
MSTRG.7544.2	147	0.3973	9.687561035	1
MSTRG.7551.1	1183	0.43703	9.02935791	1
MSTRG.7551.2	1183	0.43208	9.02935791	1
MSTRG.7552.1	68	0.4146	10.04803467	-1

MSTRG.7552.3	66	0.43473	10.04803467	-1
MSTRG.7552.2	71	0.40313	10.04412842	-1
MSTRG.7556.2	822	0.46821	4.900817871	1
MSTRG.7557.1	265	0.37291	8.828796387	1
MSTRG.7557.2	284	0.37287	8.666564941	1
MSTRG.7557.3	305	0.43353	9.403625488	1
MSTRG.7557.4	84	0.38184	11.78631592	1
MSTRG.7557.5	301	0.37039	9.512145996	1
MSTRG.7557.6	322	0.42647	9.840515137	1
MSTRG.7560.3	88	0.46094	12.12713623	1
MSTRG.7562.6	529	0.42225	8.471130371	1
MSTRG.7562.7	575	0.44449	9.184753418	1
MSTRG.7562.8	498	0.45374	8.777648926	1
MSTRG.7563.1	245	0.34824	6.547546387	1
MSTRG.7563.2	168	0.35445	8.512268066	1
MSTRG.7563.4	239	0.41799	6.196594238	1
MSTRG.7573.4	1904	0.46114	5.600036621	1
MSTRG.7573.5	1899	0.46114	5.721008301	1
MSTRG.7585.1	89	0.32477	4.741027832	1
MSTRG.7572.1	349	0.36185	6.594543457	1
MSTRG.7572.2	245	0.39731	8.863708496	1
MSTRG.7580.2	460	0.40788	7.640075684	1
MSTRG.7588.2	159	0.42763	5.911804199	1
MSTRG.7591.2	2810	0.40048	5.377258301	1
MSTRG.7591.3	2810	0.40048	5.377258301	1
MSTRG.7591.4	218	0.36834	6.305358887	1
MSTRG.7592.1	390	0.43611	5.428405762	1
MSTRG.7594.1	54	0.37467	9.075866699	1
MSTRG.7597.2	57	0.44861	8.844909668	1
MSTRG.7597.1	24	0.41278	4.01751709	1
MSTRG.7600.1	849	0.48772	6.358459473	1
MSTRG.7604.3	431	0.28063	4.983093262	1
MSTRG.7604.4	431	0.27587	4.983093262	1
MSTRG.7604.5	127	0.27251	7.835510254	1
MSTRG.7606.1	289	0.34855	4.747253418	1
MSTRG.7608.2	248	0.2912	9.120788574	1
MSTRG.7611.1	239	0.40422	7.431335449	1
MSTRG.7617.2	505	0.39252	8.070983887	1
MSTRG.7618.3	241	0.37893	10.11590576	1
MSTRG.7618.4	241	0.40532	10.11590576	1
MSTRG.7619.3	743	0.45073	8.719177246	1
MSTRG.7621.1	208	0.41662	10.00140381	1
MSTRG.7615.2	381	0.38843	5.174621582	1
MSTRG.7615.1	444	0.44029	5.096984863	1
MSTRG.7615.3	381	0.45462	5.174621582	1
MSTRG.7615.4	277	0.41961	5.160583496	1
MSTRG.7615.5	430	0.43716	5.040100098	1
MSTRG.7616.2	53	0.35867	8.840270996	1
MSTRG.7625.1	904	0.44113	5.902282715	1
MSTRG.7625.2	926	0.46885	5.798522949	1
MSTRG.7626.1	170	0.39888	8.81829834	1
MSTRG.7626.2	170	0.40539	8.81829834	1
MSTRG.7630.2	496	0.34486	8.766052246	1
MSTRG.7630.3	1041	0.35782	6.051818848	1

MSTRG.7632.3	216	0.32442	5.461486816	1
MSTRG.7637.1	303	0.34353	10.62200928	1
MSTRG.7637.4	313	0.36549	10.56695557	1
MSTRG.7637.5	311	0.36322	10.54400635	1
MSTRG.7638.1	460	0.38137	6.589782715	1
MSTRG.7638.10	2350	0.47529	5.859191895	1
MSTRG.7638.11	2192	0.45465	5.875671387	1
MSTRG.7638.5	2350	0.48915	5.798522949	1
MSTRG.7638.6	2351	0.48915	5.798522949	1
MSTRG.7638.7	2378	0.48915	5.812805176	1
MSTRG.7638.8	2323	0.48915	5.845031738	1
MSTRG.7638.9	2099	0.45717	5.886169434	1
MSTRG.7639.1	359	0.4221	6.474914551	1
MSTRG.7638.12	1077	0.36555	6.30267334	1
MSTRG.7640.2	309	0.40989	7.684753418	1
MSTRG.7640.3	181	0.352	8.641296387	1
MSTRG.7642.1	101	0.34981	9.690856934	1
MSTRG.7642.2	220	0.28518	10.06549072	1
MSTRG.7642.3	120	0.28923	6.962097168	1
MSTRG.7642.4	86	0.31516	5.869689941	1
MSTRG.7634.2	312	0.33559	8.19342041	1
MSTRG.7635.1	386	0.33179	6.273620605	1
MSTRG.7635.2	386	0.3328	6.273620605	1
MSTRG.7635.3	386	0.37762	6.273620605	1
MSTRG.7635.5	386	0.28559	6.273620605	1
MSTRG.7636.1	193	0.33194	9.063415527	1
MSTRG.7636.2	188	0.40633	9.857849121	1
MSTRG.7650.1	1410	0.44027	6.054138184	1
MSTRG.7646.1	205	0.37904	6.519592285	1
MSTRG.7647.1	74	0.46904	8.801818848	-1
MSTRG.7659.8	290	0.46205	4.971740723	1
MSTRG.7660.3	193	0.37226	5.217102051	1
MSTRG.7660.5	192	0.37694	5.217102051	1
MSTRG.7666.1	1942	0.44236	5.468688965	1
MSTRG.7666.3	1952	0.47705	5.421203613	1
MSTRG.7666.4	1914	0.4627	5.450256348	1
MSTRG.7666.5	1960	0.45001	5.465270996	1
MSTRG.7666.6	163	0.30736	6.027038574	1
MSTRG.7665.1	361	0.36571	7.864440918	1
MSTRG.7668.2	625	0.40856	5.362365723	1
MSTRG.7668.3	642	0.43049	5.256774902	1
MSTRG.7668.4	418	0.43919	4.912658691	-1
MSTRG.7673.1	100	0.31615	9.294494629	1
MSTRG.7674.2	144	0.39429	10.1696167	1
MSTRG.7675.2	342	0.44317	6.097106934	1
MSTRG.7680.1	72	0.3631	9.670349121	1
MSTRG.7686.3	188	0.49342	6.432678223	1
MSTRG.7694.1	95	0.37341	6.260681152	1
MSTRG.7705.5	726	0.43771	6.120544434	1
MSTRG.7703.1	573	0.39398	12.2074585	1
MSTRG.7707.1	385	0.4366	9.528381348	1
MSTRG.7707.3	384	0.44113	9.496765137	1
MSTRG.7711.4	165	0.31028	9.066101074	1
MSTRG.7711.5	104	0.31028	4.582946777	1

MSTRG.7711.6	138	0.28433	6.858947754	1
MSTRG.7711.3	197	0.31303	9.614685059	1
MSTRG.7711.1	220	0.28305	9.640319824	1
MSTRG.7717.2	346	0.41521	9.019714355	1
MSTRG.7717.1	346	0.39773	9.019714355	1
MSTRG.7717.3	175	0.36796	9.474182129	1
MSTRG.7717.4	175	0.28121	9.474182129	1
MSTRG.7717.5	65	0.39331	10.31329346	1
MSTRG.7717.8	186	0.42287	6.808776855	1
MSTRG.7714.2	561	0.39686	6.202575684	1
MSTRG.7718.1	577	0.41467	9.311828613	1
MSTRG.7722.2	285	0.35101	4.870178223	1
MSTRG.7722.3	261	0.34584	5.750183105	1
MSTRG.7723.1	297	0.28291	11.78399658	1
MSTRG.7722.5	463	0.35173	8.313659668	1
MSTRG.7722.6	285	0.34305	4.870178223	1
MSTRG.7723.2	297	0.27663	11.78399658	1
MSTRG.7730.5	1779	0.35168	6.381896973	1
MSTRG.7730.6	1767	0.2971	6.492858887	1
MSTRG.7731.1	141	0.28584	10.07098389	1
MSTRG.7733.3	678	0.42498	4.894592285	1
MSTRG.7737.1	50	0.30493	6.24017334	1
MSTRG.7738.1	369	0.36401	5.21661377	1
MSTRG.7739.4	69	0.34339	9.096740723	1
MSTRG.7740.2	217	0.4613	6.617492676	1
MSTRG.7742.2	142	0.33285	8.448181152	1
MSTRG.7742.3	64	0.30544	9.409851074	-1
MSTRG.7735.1	48	0.34564	11.75726318	1
MSTRG.7744.2	2421	0.47666	8.762756348	1
MSTRG.7745.1	105	0.27433	9.058166504	1
MSTRG.7747.2	706	0.42711	6.22442627	1
MSTRG.7747.3	691	0.42091	6.364929199	1
MSTRG.7747.4	706	0.42091	6.22442627	1
MSTRG.7747.1	706	0.38916	6.22442627	1
MSTRG.7747.5	612	0.37028	5.80657959	1
MSTRG.7751.3	519	0.43793	4.605529785	1
MSTRG.7751.6	509	0.43844	4.435241699	1
MSTRG.7751.7	441	0.45189	4.480895996	1
MSTRG.7751.8	441	0.42417	4.480895996	1
MSTRG.7751.9	481	0.40927	4.48651123	-1
MSTRG.7752.1	137	0.29674	9.223937988	1
MSTRG.7752.2	116	0.30939	9.013244629	1
MSTRG.7755.1	284	0.34157	5.321350098	1
MSTRG.7755.3	231	0.32107	5.611755371	1
MSTRG.7743.1	345	0.37391	6.346496582	1
MSTRG.7758.1	45	0.45729	4.856628418	-1
MSTRG.7762.2	168	0.3759	4.868469238	1
MSTRG.7760.2	137	0.35045	8.705383301	1
MSTRG.7767.1	52	0.36168	9.694396973	1
MSTRG.7767.2	52	0.38067	9.694396973	1
MSTRG.7769.2	192	0.33733	8.966125488	1
MSTRG.7770.1	126	0.33922	11.27362061	1
MSTRG.7770.2	115	0.39477	12.1461792	1
MSTRG.7771.4	100	0.33901	10.27032471	1

MSTRG.7772.3	799	0.39581	8.776184082	1
MSTRG.7773.2	299	0.29705	5.604309082	1
MSTRG.7780.2	60	0.33744	9.787414551	1
MSTRG.7782.1	199	0.37785	5.691467285	1
MSTRG.7782.2	199	0.36341	5.527893066	-1
MSTRG.7782.3	199	0.4203	5.527893066	-1
MSTRG.7782.4	199	0.32925	5.527893066	-1
MSTRG.7782.5	199	0.30396	5.527893066	-1
MSTRG.7782.8	115	0.30119	10.15875244	1
MSTRG.7782.6	199	0.32057	5.527893066	-1
MSTRG.7782.7	199	0.31477	5.527893066	-1
MSTRG.7782.9	199	0.36341	5.527893066	-1
MSTRG.7782.10	199	0.36197	5.527893066	-1
MSTRG.7782.12	199	0.38195	5.527893066	-1
MSTRG.7782.13	199	0.3411	5.527893066	1
MSTRG.7782.14	124	0.43259	6.108703613	-1
MSTRG.7783.1	88	0.35912	5.53717041	-1
MSTRG.7787.1	104	0.34047	9.858825684	1
MSTRG.7790.3	296	0.37169	9.313415527	1
MSTRG.7791.1	61	0.33118	7.999572754	1
MSTRG.7794.3	83	0.39194	4.92010498	1
MSTRG.7795.1	170	0.29897	10.52545166	1
MSTRG.7796.3	355	0.45431	9.311828613	1
MSTRG.7796.5	355	0.29751	9.311828613	1
MSTRG.7796.6	107	0.27711	6.888000488	1
MSTRG.7796.7	107	0.2933	6.888000488	1
MSTRG.7797.1	109	0.36137	10.13226318	1
MSTRG.7799.2	507	0.3683	5.292419434	1
MSTRG.7800.1	50	0.43161	11.41766357	1
MSTRG.7801.2	101	0.30485	9.096862793	1
MSTRG.7801.1	101	0.30485	9.096862793	1
MSTRG.7803.1	182	0.2968	9.163757324	1
MSTRG.7804.2	149	0.41224	6.169372559	1
MSTRG.7805.1	285	0.39303	6.530090332	1
MSTRG.7805.3	147	0.4399	10.05401611	1
MSTRG.7805.4	159	0.33223	10.04754639	1
MSTRG.7805.5	204	0.37009	8.804016113	1
MSTRG.7809.1	251	0.29777	8.303405762	1
MSTRG.7812.1	132	0.29607	9.932556152	1
MSTRG.7815.1	350	0.45535	9.118713379	1
MSTRG.7815.3	627	0.39946	8.364807129	1
MSTRG.7814.1	260	0.42953	5.661315918	1
MSTRG.7818.2	480	0.4248	6.246520996	1
MSTRG.7818.3	46	0.3653	5.964660645	1
MSTRG.7819.1	87	0.39291	8.678039551	1
MSTRG.7821.2	61	0.37042	10.62286377	-1
MSTRG.7825.2	210	0.41296	5.950622559	1
MSTRG.7832.1	152	0.26913	8.07989502	1
MSTRG.7832.2	117	0.27703	8.722106934	1
MSTRG.7840.1	74	0.33367	5.639465332	1
MSTRG.7841.3	346	0.43197	5.772399902	1
MSTRG.7842.2	964	0.46292	6.227722168	1
MSTRG.7843.2	322	0.46843	8.495910645	1
MSTRG.7844.1	321	0.46553	4.710388184	1

MSTRG.7844.4	99	0.38681	5.870666504	1
MSTRG.7844.3	420	0.42802	5.120300293	1
MSTRG.7844.5	321	0.36684	4.710388184	1
MSTRG.7844.6	321	0.45837	4.710388184	1
MSTRG.7848.1	144	0.32686	9.363098145	1
MSTRG.7848.2	60	0.46179	10.17266846	-1
MSTRG.7854.5	166	0.26642	11.74371338	1
MSTRG.7859.2	2340	0.431	9.069885254	1
MSTRG.7859.3	2340	0.40318	9.069885254	1
MSTRG.7859.1	2340	0.40318	9.069885254	1
MSTRG.7859.4	2318	0.40783	9.102844238	1
MSTRG.7859.8	1591	0.43024	8.790466309	1
MSTRG.7859.9	1597	0.4336	8.906433105	1
MSTRG.7859.7	2070	0.42559	8.662536621	1
MSTRG.7859.5	2215	0.35621	9.123718262	1
MSTRG.7859.6	2340	0.42776	9.135681152	1
MSTRG.7859.10	2340	0.42776	9.069885254	1
MSTRG.7859.11	2322	0.42156	9.023742676	1
MSTRG.7859.12	2340	0.43866	9.057067871	1
MSTRG.7860.1	31	0.46923	11.00006104	1
MSTRG.7859.14	70	0.27274	7.86920166	1
MSTRG.7859.13	2322	0.44578	9.051940918	1
MSTRG.7859.15	885	0.45101	6.667907715	1
MSTRG.7859.16	2662	0.46331	8.909729004	1
MSTRG.7863.1	33	0.4575	4.313781738	1
MSTRG.7855.7	295	0.33158	8.373840332	1
MSTRG.7868.7	60	0.28237	10.75445557	1
MSTRG.7870.1	101	0.31123	10.47637939	1
MSTRG.7866.2	270	0.42517	8.510192871	1
MSTRG.7866.3	319	0.40192	7.852233887	1
MSTRG.7874.1	99	0.30451	8.187194824	1
MSTRG.7873.2	151	0.4023	6.062194824	1
MSTRG.7879.1	138	0.28586	6.905456543	1
MSTRG.7878.1	157	0.27379	7.773376465	1
MSTRG.7881.1	133	0.39849	8.438903809	1
MSTRG.7881.2	108	0.33731	9.342956543	-1
MSTRG.7884.1	94	0.34261	10.08184814	1
MSTRG.7891.1	56	0.38005	6.498352051	1
MSTRG.7898.2	433	0.42313	9.788391113	1
MSTRG.7889.1	276	0.26545	9.369812012	1
MSTRG.7903.1	939	0.32508	9.481140137	1
MSTRG.7903.2	331	0.29689	8.178649902	1
MSTRG.7903.3	224	0.33086	6.791442871	1
MSTRG.7903.4	305	0.26791	6.925598145	1
MSTRG.7905.6	666	0.38541	6.99005127	1
MSTRG.7905.7	331	0.29857	5.085754395	1
MSTRG.7915.10	282	0.45265	4.938415527	1
MSTRG.7915.11	918	0.44441	4.769104004	1
MSTRG.7915.5	917	0.45007	4.769104004	1
MSTRG.7915.6	907	0.45007	4.784362793	1
MSTRG.7915.7	932	0.45828	4.812927246	1
MSTRG.7915.9	922	0.45828	4.828918457	1
MSTRG.7915.12	394	0.38506	5.368835449	1
MSTRG.7917.3	1889	0.42903	6.949768066	1

MSTRG.7917.4	1817	0.42903	6.789978027	1
MSTRG.7913.1	88	0.32952	10.60662842	1
MSTRG.7910.3	89	0.45168	4.662658691	1
MSTRG.7921.1	771	0.39665	9.021911621	1
MSTRG.7921.2	771	0.41247	9.021911621	1
MSTRG.7921.4	771	0.43062	9.021911621	1
MSTRG.7923.1	1726	0.43314	8.749206543	1
MSTRG.7923.2	833	0.4051	6.453796387	1
MSTRG.7922.2	321	0.37338	9.352600098	1
MSTRG.7922.3	209	0.36482	9.00592041	1
MSTRG.7926.2	103	0.32827	11.01580811	1
MSTRG.7930.1	155	0.31225	8.946472168	1
MSTRG.7933.1	130	0.41907	5.759216309	1
MSTRG.7933.2	114	0.4181	5.573913574	1
MSTRG.7932.1	17	0.34546	8.189025879	1
MSTRG.7939.1	173	0.35031	9.95135498	1
MSTRG.7939.2	81	0.34134	7.636901855	1
MSTRG.7939.3	81	0.36769	7.636901855	1
MSTRG.7939.4	39	0.37909	7.957702637	1
MSTRG.7935.2	626	0.49807	6.168273926	1
MSTRG.7943.1	61	0.33067	8.885070801	1
MSTRG.7944.3	189	0.33007	3.765075684	1
MSTRG.7944.4	189	0.32878	3.765075684	1
MSTRG.7944.5	301	0.30357	6.308410645	1
MSTRG.7944.6	243	0.3287	6.368347168	-1
MSTRG.7945.1	154	0.33919	7.768005371	1
MSTRG.7944.7	124	0.35529	8.435241699	-1
MSTRG.7958.1	63	0.41085	6.355529785	-1
MSTRG.7962.1	167	0.26512	9.974914551	1
MSTRG.7984.1	69	0.32502	7.794372559	1
MSTRG.7985.1	78	0.4681	4.087219238	1
MSTRG.7990.1	83	0.31903	6.534118652	1
MSTRG.7995.1	318	0.31348	9.824279785	1
MSTRG.7996.1	268	0.32705	9.772155762	1
MSTRG.7996.2	157	0.33914	8.944763184	1
MSTRG.7997.1	111	0.30504	6.137512207	1
MSTRG.7998.1	108	0.29367	9.781555176	1
MSTRG.7988.15	218	0.35933	5.25213623	1
MSTRG.7988.13	271	0.36273	5.223449707	1
MSTRG.7988.12	301	0.43298	5.484924316	1
MSTRG.7988.14	302	0.44767	5.351379395	1
MSTRG.7988.11	823	0.40208	5.727722168	1
MSTRG.7988.16	259	0.42816	5.691345215	1
MSTRG.7988.18	239	0.39972	5.374938965	1
MSTRG.7988.20	128	0.34072	8.242248535	1
MSTRG.7988.22	118	0.3263	7.914611816	1
MSTRG.8002.1	56	0.33754	7.704040527	1
MSTRG.8002.2	77	0.30172	6.993103027	1
MSTRG.8016.1	156	0.34219	6.528381348	1
MSTRG.8019.1	97	0.25564	9.896789551	1
MSTRG.8020.1	1112	0.39699	8.039489746	1
MSTRG.8022.1	40	0.34382	4.830993652	1
MSTRG.8023.1	71	0.28191	10.35369873	1
MSTRG.8020.4	93	0.36815	9.507263184	-1

MSTRG.8020.5	88	0.32905	9.507263184	-1
MSTRG.8025.1	170	0.28971	10.21820068	1
MSTRG.8024.7	160	0.29791	9.297790527	1
MSTRG.8026.1	1188	0.37362	8.890075684	1
MSTRG.8027.1	49	0.42291	10.18756104	-1
MSTRG.8029.1	1056	0.4296	6.790344238	1
MSTRG.8029.2	1086	0.4532	6.585144043	1
MSTRG.8031.1	120	0.29251	10.19866943	1
MSTRG.8032.2	289	0.38833	8.862487793	1
MSTRG.8010.1	31	0.42196	4.944641113	1
MSTRG.8060.1	146	0.30443	8.679138184	1
MSTRG.8071.1	94	0.45246	5.316223145	-1
MSTRG.8074.1	83	0.3823	6.710266113	1
MSTRG.8096.1	2505	0.46119	4.557678223	1
MSTRG.8096.2	1887	0.44877	5.718688965	1
MSTRG.8096.3	1740	0.45409	5.742248535	1
MSTRG.8096.4	1475	0.41114	5.395446777	1
MSTRG.8096.11	1481	0.43992	5.272766113	1
MSTRG.8096.12	1876	0.3694	5.68548584	1
MSTRG.8096.6	1745	0.47254	5.75982666	1
MSTRG.8096.10	1745	0.46179	5.75982666	1
MSTRG.8096.5	1751	0.45409	5.627990723	1
MSTRG.8096.7	1756	0.46179	5.644836426	1
MSTRG.8096.8	1756	0.44759	5.644836426	1
MSTRG.8096.9	1902	0.47254	5.777404785	1
MSTRG.8096.13	1097	0.44849	4.904968262	1
MSTRG.8096.14	1876	0.49071	5.832458496	1
MSTRG.8097.1	65	0.34157	7.725280762	1
MSTRG.8098.1	119	0.29367	9.544372559	1
MSTRG.8098.2	119	0.29367	9.544372559	1
MSTRG.8099.1	119	0.26982	6.686096191	1
MSTRG.8098.3	118	0.28478	8.304016113	1
MSTRG.8100.1	236	0.2921	9.532287598	1
MSTRG.8096.15	174	0.28982	9.40814209	1
MSTRG.8096.16	142	0.2921	9.766174316	1
MSTRG.8096.17	142	0.2921	9.766174316	1
MSTRG.8096.18	142	0.2921	9.766174316	1
MSTRG.8096.19	120	0.27234	6.181213379	1
MSTRG.8096.20	93	0.28443	7.77142334	1
MSTRG.8096.21	70	0.27639	9.919250488	1
MSTRG.8096.22	185	0.31035	9.50994873	1
MSTRG.8083.1	183	0.33118	10.57025146	1
MSTRG.8086.1	78	0.36972	10.07611084	-1
MSTRG.8103.1	257	0.46233	8.981018066	1
MSTRG.8109.3	248	0.45612	6.72088623	1
MSTRG.8110.2	938	0.44997	5.841003418	1
MSTRG.8110.4	905	0.44997	5.679870605	1
MSTRG.8110.6	1206	0.47178	6.067687988	1
MSTRG.8110.5	969	0.46257	7.039611816	1
MSTRG.8113.1	27	0.36153	6.53302002	1
MSTRG.8119.1	118	0.27651	9.18170166	1
MSTRG.8119.2	129	0.31069	9.389587402	1
MSTRG.8130.1	217	0.33717	11.83905029	1
MSTRG.8131.3	271	0.39159	9.619812012	1

MSTRG.8131.4	256	0.37207	9.706115723	1
MSTRG.8131.5	314	0.37819	9.534240723	1
MSTRG.8131.6	329	0.41409	9.473571777	1
MSTRG.8131.7	318	0.404	9.425842285	1
MSTRG.8117.3	343	0.44795	6.679138184	1
MSTRG.8143.1	306	0.46588	7.539001465	1
MSTRG.8145.1	51	0.41634	9.511657715	1
MSTRG.8146.1	29	0.37099	5.811340332	-1
MSTRG.8147.2	420	0.4633	6.800842285	1
MSTRG.8160.2	41	0.43194	10.36712646	-1
MSTRG.8160.1	169	0.29607	7.683044434	1
MSTRG.8160.3	62	0.36227	6.375793457	1
MSTRG.8160.4	169	0.29607	7.683044434	1
MSTRG.8165.1	2661	0.41409	5.455627441	1
MSTRG.8165.3	2565	0.41409	5.382995605	1
MSTRG.8171.1	16	0.34055	10.30621338	1
MSTRG.8171.2	45	0.27911	11.8348999	1
MSTRG.8172.11	104	0.32396	9.029846191	1
MSTRG.8172.7	279	0.41881	5.457336426	1
MSTRG.8172.6	513	0.47687	6.720397949	1
MSTRG.8172.9	513	0.46401	6.720397949	1
MSTRG.8172.10	513	0.44456	6.720397949	1
MSTRG.8172.5	513	0.47687	6.720397949	1
MSTRG.8172.8	279	0.3	5.457336426	1
MSTRG.8172.4	513	0.47861	6.720397949	1
MSTRG.8172.3	513	0.47199	6.720397949	1
MSTRG.8172.13	52	0.35096	4.780456543	1
MSTRG.8172.12	279	0.33257	5.457336426	1
MSTRG.8172.14	213	0.29772	9.602600098	1
MSTRG.8172.15	282	0.34295	9.192565918	1
MSTRG.8185.2	147	0.37065	7.666687012	1
MSTRG.8185.1	147	0.35591	7.666687012	1
MSTRG.8185.3	147	0.35504	7.666687012	1
MSTRG.8195.1	969	0.46632	5.192810059	1
MSTRG.8195.2	968	0.47402	5.192810059	1
MSTRG.8190.2	56	0.35552	6.094787598	1
MSTRG.8198.1	22	0.31696	8.841491699	-1
MSTRG.8201.1	184	0.30168	10.03167725	1
MSTRG.8201.2	184	0.31209	10.03167725	1
MSTRG.8202.1	81	0.29772	9.128845215	1
MSTRG.8200.2	413	0.37308	5.907897949	1
MSTRG.8200.3	70	0.33343	8.382751465	1
MSTRG.8203.1	19	0.31393	11.00030518	1
MSTRG.8209.1	47	0.35736	9.25213623	1
MSTRG.8209.2	55	0.37866	8.492736816	1
MSTRG.8210.1	43	0.48697	10.43267822	-1
MSTRG.8218.1	776	0.44388	8.667663574	1
MSTRG.8219.1	89	0.46827	5.543518066	-1
MSTRG.8224.3	218	0.3373	9.546691895	1
MSTRG.8221.1	39	0.37154	8.27520752	1
MSTRG.8221.2	84	0.43764	6.82611084	1
MSTRG.8238.1	132	0.42822	6.725646973	1
MSTRG.8238.2	132	0.35641	6.725646973	1
MSTRG.8238.5	230	0.38982	6.242370605	1

MSTRG.8238.6	212	0.36004	5.993347168	1
MSTRG.8238.7	132	0.37337	6.725646973	1
MSTRG.8238.8	132	0.37896	6.725646973	1
MSTRG.8239.1	94	0.27517	8.289245605	1
MSTRG.8241.3	77	0.30664	8.572814941	1
MSTRG.8241.2	193	0.28142	7.61541748	1
MSTRG.8241.4	219	0.35441	4.605895996	1
MSTRG.8242.1	151	0.29187	5.228942871	1
MSTRG.8241.5	247	0.36005	4.409362793	1
MSTRG.8241.6	319	0.38609	4.721618652	1
MSTRG.8244.1	73	0.36464	9.873474121	1
MSTRG.8241.7	231	0.39021	4.409362793	1
MSTRG.8241.8	165	0.3582	4.785949707	1
MSTRG.8246.2	57	0.37981	7.747741699	1
MSTRG.8246.6	92	0.42111	10.29205322	1
MSTRG.8246.5	364	0.47312	5.301086426	1
MSTRG.8246.4	365	0.46542	5.301086426	1
MSTRG.8248.1	83	0.29967	6.682189941	1
MSTRG.8246.7	124	0.30664	4.809265137	1
MSTRG.8249.1	189	0.38192	10.09527588	1
MSTRG.8255.1	70	0.42399	10.5199585	-1
MSTRG.8283.3	595	0.38211	8.695617676	1
MSTRG.8283.5	595	0.37827	8.695617676	1
MSTRG.8283.6	595	0.38676	8.695617676	1
MSTRG.8283.7	479	0.34787	8.601623535	1
MSTRG.8283.8	100	0.47023	9.389953613	1
MSTRG.8283.9	100	0.32948	9.389953613	1
MSTRG.8283.10	60	0.32463	6.724182129	1
MSTRG.8283.12	125	0.28082	11.00006104	1
MSTRG.8283.13	183	0.28334	10.43255615	1
MSTRG.8285.3	975	0.42291	5.550231934	1
MSTRG.8395.1	64	0.4156	9.988098145	-1
MSTRG.8397.1	72	0.4517	6.543029785	1
MSTRG.8409.1	105	0.28695	6.895324707	1
MSTRG.8410.1	87	0.40291	4.575012207	1
MSTRG.8411.1	148	0.33514	6.73626709	1
MSTRG.8412.1	63	0.28982	5.637390137	1
MSTRG.8417.2	80	0.29153	9.019958496	1
MSTRG.8419.1	43	0.31692	8.231140137	1
MSTRG.8394.2	108	0.32464	4.374206543	1
MSTRG.8394.3	61	0.34287	5.871520996	1
MSTRG.8420.1	120	0.29594	6.002258301	1
MSTRG.8422.1	110	0.34727	8.981262207	1
MSTRG.8423.1	47	0.31929	12.309021	1
MSTRG.8424.1	154	0.2921	5.286071777	1
MSTRG.8425.1	183	0.34907	10.09356689	1
MSTRG.8394.4	95	0.34162	8.014099121	1
MSTRG.8394.5	109	0.28562	6.499816895	1
MSTRG.8394.6	125	0.2921	5.178649902	1
MSTRG.8394.7	72	0.26785	6.494445801	1
MSTRG.8394.8	50	0.32086	4.290222168	1
MSTRG.8394.9	125	0.31861	10.21307373	1
MSTRG.8282.1	17	0.36139	8.253845215	1
MSTRG.8309.1	797	0.41534	7.640441895	1

MSTRG.8315.1	85	0.39617	6.515686035	1
MSTRG.8316.1	53	0.25734	10.05450439	1
MSTRG.8315.2	156	0.32237	10.00592041	1
MSTRG.8324.3	41	0.44497	3.409484863	1
MSTRG.8324.4	41	0.46167	3.409484863	1
MSTRG.8333.2	202	0.42714	7.737609863	1
MSTRG.8333.3	153	0.2921	6.050109863	1
MSTRG.8333.4	194	0.2921	6.588928223	1
MSTRG.8333.5	194	0.25541	6.588684082	1
MSTRG.8337.1	60	0.47612	6.706970215	-1
MSTRG.8338.2	265	0.37554	8.45880127	1
MSTRG.8346.2	317	0.47681	5.816101074	1
MSTRG.8349.6	61	0.27794	4.677062988	1
MSTRG.8351.1	58	0.37815	11.06878662	1
MSTRG.8351.5	95	0.3208	5.845397949	1
MSTRG.8352.3	946	0.43601	5.537414551	1
MSTRG.8352.4	328	0.44742	5.820495605	1
MSTRG.8353.1	1819	0.38097	8.984924316	1
MSTRG.8353.2	1595	0.3756	9.330993652	1
MSTRG.8353.4	517	0.31678	8.298156738	1
MSTRG.8353.6	517	0.33654	8.298156738	1
MSTRG.8353.5	517	0.37579	8.298156738	1
MSTRG.8353.7	441	0.40445	9.096496582	1
MSTRG.8353.8	441	0.4091	9.096496582	1
MSTRG.8354.1	65	0.32294	11.33477783	1
MSTRG.8355.1	115	0.43012	5.656555176	1
MSTRG.8356.1	69	0.3616	9.61932373	1
MSTRG.8340.1	68	0.38999	11.49932861	-1
MSTRG.8343.1	89	0.40345	10.4463501	-1
MSTRG.8359.5	248	0.43892	9.694030762	1
MSTRG.8359.3	248	0.33204	9.694030762	1
MSTRG.8359.4	248	0.40309	9.694030762	1
MSTRG.8360.1	26	0.412	6.008850098	1
MSTRG.8365.1	57	0.32886	9.687805176	1
MSTRG.8358.1	45	0.32906	6.704772949	1
MSTRG.8386.1	1124	0.39503	8.699157715	1
MSTRG.8387.1	50	0.42662	5.523498535	1
MSTRG.8392.1	72	0.31061	9.571105957	1
MSTRG.8432.1	132	0.26189	6.20123291	-1
MSTRG.8430.1	53	0.35469	9.019592285	1
MSTRG.8444.1	412	0.33728	7.08416748	1
MSTRG.8444.2	412	0.46353	7.08416748	1
MSTRG.8444.3	357	0.42619	5.981750488	1
MSTRG.8444.7	387	0.34338	6.305969238	1
MSTRG.8444.5	487	0.40093	6.509216309	1
MSTRG.8444.6	487	0.42423	6.509216309	1
MSTRG.8444.8	429	0.35822	7.901916504	1
MSTRG.8444.9	429	0.37075	7.901916504	1
MSTRG.8444.10	429	0.38544	7.901916504	1
MSTRG.8444.11	429	0.39385	7.901916504	1
MSTRG.8445.1	808	0.47516	5.364196777	1
MSTRG.8445.3	397	0.39825	6.725036621	1
MSTRG.8437.1	708	0.44538	8.153747559	1
MSTRG.8439.2	55	0.38143	6.480407715	1

MSTRG.8446.5	486	0.36756	8.429260254	1
MSTRG.8446.7	445	0.43462	6.048522949	1
MSTRG.8460.1	55	0.37863	11.00860596	-1
MSTRG.8482.1	919	0.44745	6.23223877	1
MSTRG.8486.1	50	0.33777	10.03668213	1
MSTRG.8488.1	63	0.44732	6.694396973	1
MSTRG.8488.2	41	0.33443	5.709411621	1
MSTRG.8488.3	52	0.36625	8.907653809	-1
MSTRG.8488.4	63	0.33334	6.694396973	1
MSTRG.8495.1	52	0.35391	9.445007324	1
MSTRG.8494.5	951	0.44861	7.980285645	1
MSTRG.8493.1	55	0.44559	12.1831665	-1
MSTRG.8499.1	346	0.35493	9.43963623	1
MSTRG.8506.1	563	0.32237	4.557556152	1
MSTRG.8507.1	88	0.32445	6.85760498	1
MSTRG.8508.1	136	0.35878	10.88946533	1
MSTRG.8509.1	95	0.35102	5.376281738	1
MSTRG.8512.2	709	0.45097	5.948913574	1
MSTRG.8512.3	742	0.44229	6.172546387	1
MSTRG.8512.4	568	0.45097	6.515319824	1
MSTRG.8502.3	1004	0.41574	9.277282715	1
MSTRG.8515.3	192	0.37894	9.96295166	1
MSTRG.8517.2	242	0.4724	8.467590332	1
MSTRG.8520.1	128	0.34788	7.66998291	1
MSTRG.8521.1	121	0.3614	11.2822876	1
MSTRG.8522.1	40	0.37213	3.293762207	1
MSTRG.8524.1	155	0.32181	9.666564941	1
MSTRG.8526.2	1291	0.43668	8.077941895	1
MSTRG.8527.1	187	0.33827	6.110778809	1
MSTRG.8526.3	88	0.4439	4.466369629	-1
MSTRG.8528.1	98	0.35979	9.877624512	1
MSTRG.8529.1	1767	0.49139	5.031921387	1
MSTRG.8529.2	1767	0.45165	5.031921387	1
MSTRG.8529.3	1688	0.45935	5.051818848	1
MSTRG.8529.4	1767	0.4443	5.031921387	1
MSTRG.8529.5	1767	0.46863	5.031921387	1
MSTRG.8529.7	1767	0.45165	5.031921387	1
MSTRG.8534.9	522	0.42964	5.94317627	1
MSTRG.8534.10	591	0.40666	7.557067871	1
MSTRG.8534.11	590	0.46044	7.557067871	1
MSTRG.8536.1	129	0.28683	4.942077637	1
MSTRG.8536.2	128	0.32078	8.281433105	1
MSTRG.8538.5	273	0.45905	7.604675293	-1
MSTRG.8539.3	736	0.42378	5.658508301	1
MSTRG.8539.4	736	0.40458	5.658508301	1
MSTRG.8539.5	757	0.45375	5.718444824	1
MSTRG.8543.1	60	0.39425	5.836486816	1
MSTRG.8544.1	50	0.32504	9.429992676	1
MSTRG.8542.2	57	0.29368	5.013122559	1
MSTRG.8547.1	181	0.38756	10.01397705	1
MSTRG.8548.3	276	0.33091	5.316345215	1
MSTRG.8548.2	444	0.31137	5.885559082	1
MSTRG.8556.1	89	0.45764	6.695861816	-1
MSTRG.8557.3	490	0.3413	8.58770752	1

MSTRG.8557.2	970	0.3554	6.553039551	1
MSTRG.8557.5	807	0.34515	7.19354248	1
MSTRG.8554.1	54	0.39828	9.696594238	1
MSTRG.8555.1	64	0.36333	8.768493652	1
MSTRG.8565.1	35	0.35757	7.976989746	1
MSTRG.8565.2	57	0.34846	4.698059082	1
MSTRG.8566.1	39	0.3983	9.996154785	1
MSTRG.8566.2	37	0.33231	9.824523926	1
MSTRG.8585.1	72	0.41283	9.991882324	-1
MSTRG.8587.1	54	0.43874	6.467346191	1
MSTRG.8589.1	229	0.38062	10.36920166	1
MSTRG.8586.1	160	0.43736	10.6050415	-1
MSTRG.8591.2	198	0.366	5.403503418	1
MSTRG.8594.2	1299	0.45384	5.604309082	1
MSTRG.8595.1	92	0.37482	4.734680176	-1
MSTRG.8598.3	895	0.46793	8.811218262	1
MSTRG.8598.4	896	0.46793	8.811218262	1
MSTRG.8600.3	467	0.3269	10.12249756	1
MSTRG.8601.1	73	0.35785	6.47052002	1
MSTRG.8604.1	150	0.38947	10.92999268	1
MSTRG.8604.2	33	0.39181	7.999450684	1
MSTRG.8605.4	196	0.46306	6.311828613	1
MSTRG.8605.8	212	0.45207	6.064025879	1
MSTRG.8605.5	189	0.37998	7.012634277	1
MSTRG.8605.6	189	0.29374	7.012634277	1
MSTRG.8606.3	292	0.2921	5.580383301	1
MSTRG.8606.5	226	0.2921	5.613342285	1
MSTRG.8606.4	232	0.2921	5.292541504	1
MSTRG.8608.1	105	0.28802	9.120178223	1
MSTRG.8611.3	450	0.43238	9.031555176	1
MSTRG.8613.1	93	0.30472	4.734802246	1
MSTRG.8617.1	101	0.33167	8.461608887	1
MSTRG.8614.2	93	0.31065	9.582824707	1
MSTRG.8614.3	78	0.41291	10.42279053	1
MSTRG.8624.10	559	0.43308	5.29107666	1
MSTRG.8624.11	553	0.43308	5.298034668	1
MSTRG.8624.12	553	0.46029	5.298034668	1
MSTRG.8624.13	567	0.46029	5.29107666	1
MSTRG.8620.1	35	0.37645	10.62957764	-1
MSTRG.8629.1	41	0.33864	5.203674316	1
MSTRG.8630.1	213	0.34726	9.189758301	1
MSTRG.8630.2	84	0.28663	9.029602051	1
MSTRG.8632.1	31	0.4516	9.694030762	1
MSTRG.8645.5	719	0.39422	6.506164551	1
MSTRG.8645.4	844	0.40191	6.530578613	1
MSTRG.8645.6	649	0.41123	8.158752441	1
MSTRG.8646.1	115	0.34847	6.956726074	1
MSTRG.8642.2	194	0.42492	4.992980957	1
MSTRG.8647.1	879	0.3966	6.111633301	1
MSTRG.8647.3	879	0.47507	6.111633301	1
MSTRG.8648.1	558	0.36086	6.104797363	1
MSTRG.8650.2	1250	0.46752	5.546691895	1
MSTRG.8650.1	1300	0.44633	5.635925293	1
MSTRG.8650.4	51	0.36323	10.56707764	1

MSTRG.8650.3	1300	0.44695	5.635925293	1
MSTRG.8656.3	1034	0.47688	7.832824707	1
MSTRG.8657.1	735	0.41423	5.761901855	1
MSTRG.8657.2	735	0.45193	5.761901855	1
MSTRG.8658.1	379	0.31653	9.265441895	1
MSTRG.8658.3	379	0.36801	9.265441895	1
MSTRG.8657.3	735	0.43245	5.761901855	1
MSTRG.8657.4	683	0.43245	6.016784668	1
MSTRG.8657.6	425	0.34103	7.45904541	1
MSTRG.8657.7	425	0.41679	7.45904541	1
MSTRG.8659.2	74	0.27686	8.932678223	1
MSTRG.8660.2	636	0.43894	9.270568848	1
MSTRG.8660.3	476	0.44111	9.644958496	1
MSTRG.8660.4	476	0.40675	9.644958496	1
MSTRG.8660.10	264	0.33686	9.996643066	1
MSTRG.8660.11	476	0.43189	9.644958496	1
MSTRG.8660.8	446	0.46049	9.402160645	1
MSTRG.8660.9	606	0.45229	8.924865723	1
MSTRG.8652.1	457	0.4014	9.589050293	1
MSTRG.8652.2	297	0.34339	9.224914551	1
MSTRG.8674.2	1024	0.46635	6.152648926	1
MSTRG.8674.1	1006	0.46353	6.187072754	1
MSTRG.8674.3	1004	0.44853	6.151916504	1
MSTRG.8674.4	1004	0.46353	6.16973877	1
MSTRG.8674.8	339	0.34922	6.320007324	1
MSTRG.8674.7	1004	0.43047	6.151916504	1
MSTRG.8677.5	76	0.36456	9.031555176	1
MSTRG.8677.3	75	0.38378	9.031555176	1
MSTRG.8677.4	34	0.31977	6.50567627	1
MSTRG.8677.8	76	0.42661	6.516296387	1
MSTRG.8677.9	76	0.42661	9.031555176	1
MSTRG.8677.10	76	0.41606	9.031555176	1
MSTRG.8678.2	446	0.4618	9.471374512	1
MSTRG.8680.3	611	0.4681	5.754943848	1
MSTRG.8680.2	1126	0.47489	6.175476074	1
MSTRG.8686.1	29	0.44012	5.970153809	1
MSTRG.8701.1	1276	0.35567	6.016052246	1
MSTRG.8701.2	1289	0.32389	6.126525879	1
MSTRG.8716.1	471	0.40523	6.036437988	1
MSTRG.8713.1	66	0.41528	9.429260254	-1
MSTRG.8732.1	75	0.30474	8.590515137	1
MSTRG.8733.1	36	0.34085	9.098693848	1
MSTRG.8733.2	112	0.34691	9.039245605	1
MSTRG.8733.3	97	0.31432	9.258483887	1
MSTRG.8734.1	1057	0.46679	5.524963379	1
MSTRG.8729.1	131	0.4599	4.461364746	1
MSTRG.8742.1	685	0.45593	6.082946777	1
MSTRG.8742.3	743	0.46531	5.664733887	1
MSTRG.8742.6	551	0.38707	4.996276855	1
MSTRG.8742.7	533	0.38707	4.882751465	1
MSTRG.8742.8	685	0.47394	6.082946777	1
MSTRG.8742.9	667	0.47394	5.86505127	1
MSTRG.8764.1	67	0.31903	9.368103027	1
MSTRG.8765.1	79	0.33128	5.014709473	1

MSTRG.8765.2	79	0.3208	5.454040527	1
MSTRG.8766.1	113	0.2895	9.70904541	1
MSTRG.8765.4	57	0.38922	9.151062012	1
MSTRG.8748.4	640	0.46378	5.362609863	1
MSTRG.8748.6	564	0.42555	5.645446777	1
MSTRG.8768.3	176	0.29072	9.53717041	1
MSTRG.8769.1	174	0.27234	5.323669434	1
MSTRG.8785.1	186	0.35901	9.812561035	1
MSTRG.8785.2	340	0.40466	10.17010498	1
MSTRG.8785.3	173	0.29772	10.63360596	1
MSTRG.8786.7	2245	0.4688	6.480651855	1
MSTRG.8786.13	2466	0.46408	6.34197998	1
MSTRG.8786.8	2216	0.45874	6.57989502	1
MSTRG.8786.12	2308	0.44848	6.401916504	1
MSTRG.8786.10	2560	0.45871	6.079040527	1
MSTRG.8786.11	1438	0.50273	5.084777832	1
MSTRG.8786.9	2453	0.45	6.316955566	1
MSTRG.8786.15	107	0.31596	10.03118896	-1
MSTRG.8787.1	111	0.32477	9.073547363	1
MSTRG.8789.1	247	0.4302	8.415344238	1
MSTRG.8803.1	222	0.31337	9.288513184	1
MSTRG.8808.2	569	0.32082	8.762878418	1
MSTRG.8809.1	449	0.46111	5.272155762	1
MSTRG.8809.4	449	0.44953	5.272155762	1
MSTRG.8818.3	373	0.45945	5.828796387	1
MSTRG.8815.1	74	0.42103	9.994445801	1
MSTRG.8815.2	78	0.33492	9.871520996	1
MSTRG.8815.3	83	0.33087	9.871520996	1
MSTRG.8815.4	69	0.39134	9.994445801	1
MSTRG.8825.6	1061	0.46198	6.089782715	1
MSTRG.8825.7	664	0.43282	6.504943848	-1
MSTRG.8825.8	406	0.41331	5.882873535	1
MSTRG.8826.4	1883	0.4439	6.925964355	1
MSTRG.8823.1	67	0.41788	9.381896973	1
MSTRG.8833.1	235	0.44462	8.50592041	1
MSTRG.8833.2	377	0.40903	7.577331543	1
MSTRG.8833.3	345	0.43852	9.210021973	1
MSTRG.8833.4	377	0.39765	7.577331543	1
MSTRG.8833.5	377	0.39294	7.577331543	1
MSTRG.8833.6	377	0.31366	7.577331543	1
MSTRG.8833.7	338	0.37955	9.342834473	1
MSTRG.8833.8	377	0.33595	7.577331543	1
MSTRG.8830.1	83	0.46241	11.39349365	1
MSTRG.8835.1	80	0.29163	9.05670166	1
MSTRG.8836.1	197	0.32061	9.423522949	1
MSTRG.8838.1	197	0.38979	8.855407715	1
MSTRG.8839.1	248	0.41399	5.595153809	1
MSTRG.8842.1	262	0.45725	5.336120605	1
MSTRG.8846.3	1117	0.42267	5.952575684	1
MSTRG.8846.4	1042	0.46112	5.690124512	1
MSTRG.8846.5	1042	0.39524	5.690124512	1
MSTRG.8846.6	1005	0.39524	5.742980957	1
MSTRG.8849.1	16	0.42989	9.006652832	1
MSTRG.8844.1	229	0.44084	5.39642334	1

MSTRG.8844.2	229	0.3884	5.39642334	1
MSTRG.8853.1	36	0.39806	9.588562012	1
MSTRG.8853.2	40	0.42151	11.00653076	1
MSTRG.8853.3	36	0.38197	9.588562012	1
MSTRG.8853.4	36	0.33481	9.588562012	1
MSTRG.8857.1	38	0.38287	7.768127441	-1
MSTRG.8862.1	233	0.43313	5.632385254	1
MSTRG.8862.2	88	0.31849	5.87310791	1
MSTRG.8863.1	183	0.29772	5.969543457	1
MSTRG.8864.1	199	0.32125	9.454162598	1
MSTRG.8865.1	144	0.28301	10.97515869	1
MSTRG.8881.2	183	0.39602	5.112609863	1
MSTRG.8888.1	164	0.29772	10.76568604	1
MSTRG.8893.1	66	0.28338	9.898986816	1
MSTRG.8894.1	148	0.28338	5.506164551	1
MSTRG.8898.11	979	0.43525	5.847473145	1
MSTRG.8898.12	1181	0.48703	6.98223877	1
MSTRG.8898.13	1181	0.45976	6.98223877	1
MSTRG.8902.1	348	0.33887	8.941345215	1
MSTRG.8896.1	109	0.32722	6.062316895	1
MSTRG.8912.1	829	0.44279	8.784362793	1
MSTRG.8912.2	82	0.44587	4.750061035	1
MSTRG.8913.1	89	0.37967	9.586120605	1
MSTRG.8913.2	89	0.38843	9.586120605	1
MSTRG.8914.1	420	0.39495	7.18157959	1
MSTRG.8914.2	403	0.4559	6.440612793	1
MSTRG.8908.1	234	0.36374	11.92242432	1
MSTRG.8916.1	40	0.40313	9.236999512	1
MSTRG.8919.5	3484	0.45542	6.274963379	1
MSTRG.8919.3	3484	0.44492	6.274963379	1
MSTRG.8919.1	3499	0.45162	6.304504395	1
MSTRG.8919.2	3512	0.44697	6.290100098	1
MSTRG.8919.6	3527	0.44697	6.320007324	1
MSTRG.8919.7	3499	0.46064	6.304504395	1
MSTRG.8919.8	3512	0.46083	6.290100098	1
MSTRG.8919.9	3512	0.46083	6.290100098	1
MSTRG.8920.1	35	0.41058	7.899841309	-1
MSTRG.8920.2	34	0.4338	7.916931152	-1
MSTRG.8918.2	709	0.44712	6.297912598	1
MSTRG.8932.2	426	0.4465	6.387390137	1
MSTRG.8941.1	276	0.31852	9.039001465	1
MSTRG.8943.1	2044	0.41694	6.81439209	1
MSTRG.8943.2	1855	0.41694	6.746520996	1
MSTRG.8943.3	1829	0.44155	6.398498535	-1
MSTRG.8943.4	1650	0.44463	6.340759277	-1
MSTRG.8943.5	1834	0.44155	6.398620605	-1
MSTRG.8943.6	192	0.31197	4.987121582	1
MSTRG.8943.7	1821	0.40826	6.646179199	1
MSTRG.8943.8	2048	0.45516	6.869445801	-1
MSTRG.8943.9	1883	0.45516	6.921569824	1
MSTRG.8943.10	1862	0.46336	6.511291504	-1
MSTRG.8943.11	1860	0.45721	6.746520996	1
MSTRG.8943.12	1719	0.45597	7.343811035	-1
MSTRG.8943.13	1637	0.46165	6.585266113	1

MSTRG.8923.1	167	0.32654	5.174499512	1
MSTRG.8923.4	208	0.44039	5.754821777	1
MSTRG.8923.3	167	0.35689	5.174499512	1
MSTRG.8924.1	65	0.3516	11.70587158	1
MSTRG.8928.5	480	0.35168	7.09197998	1
MSTRG.8929.1	130	0.30092	9.89251709	1
MSTRG.8949.1	135	0.30308	10.42242432	1
MSTRG.8949.2	66	0.32255	4.622375488	-1
MSTRG.8951.2	706	0.4114	8.657043457	1
MSTRG.8952.1	26	0.44436	11.82440186	1
MSTRG.8954.1	110	0.27678	9.556335449	1
MSTRG.8955.1	20	0.35791	5.750793457	1
MSTRG.8957.1	67	0.39218	11.28594971	1
MSTRG.8961.2	109	0.33868	9.492980957	1
MSTRG.8962.2	69	0.34349	10.83953857	1
MSTRG.8962.3	207	0.31361	9.489929199	1
MSTRG.8966.1	91	0.31165	10.0881958	1
MSTRG.8967.3	382	0.46929	9.471862793	1
MSTRG.8971.2	621	0.46306	6.215881348	1
MSTRG.8972.2	1078	0.36761	9.478820801	1
MSTRG.8976.1	51	0.3523	5.715881348	-1
MSTRG.8981.1	59	0.40798	11.23065186	1
MSTRG.8994.1	1002	0.36387	8.300476074	1
MSTRG.8994.6	378	0.33381	9.450500488	1
MSTRG.8994.2	716	0.36682	6.549377441	1
MSTRG.8994.4	941	0.36387	8.425598145	1
MSTRG.8994.7	137	0.30191	9.585144043	1
MSTRG.8995.1	83	0.28726	11.49310303	1
MSTRG.8996.1	52	0.37942	3.711853027	-1
MSTRG.8996.2	62	0.31638	11.21246338	-1
MSTRG.8996.3	33	0.32894	4.677062988	-1
MSTRG.9002.2	555	0.43028	5.008972168	1
MSTRG.9000.2	38	0.37571	9.184143066	1
MSTRG.9000.3	553	0.39408	8.517150879	1
MSTRG.9000.4	553	0.36172	8.517150879	1
MSTRG.9003.2	196	0.29452	7.517028809	1
MSTRG.9010.1	676	0.37931	6.653869629	1
MSTRG.9010.2	728	0.35881	5.694763184	1
MSTRG.9010.3	728	0.40459	5.694763184	1
MSTRG.9010.4	728	0.40795	5.694763184	1
MSTRG.9010.5	728	0.4367	5.694763184	1
MSTRG.9010.6	728	0.35297	5.694763184	1
MSTRG.9010.7	728	0.4213	5.694763184	1
MSTRG.9010.9	306	0.28243	8.818664551	1
MSTRG.9010.10	106	0.27739	9.957580566	1
MSTRG.9010.11	97	0.27194	11.36541748	1
MSTRG.9010.12	97	0.26627	11.36541748	1
MSTRG.9010.13	91	0.35284	9.637878418	-1
MSTRG.9005.1	79	0.33756	6.796325684	-1
MSTRG.9008.1	89	0.36458	9.763122559	1
MSTRG.9011.1	187	0.37	6.604064941	1
MSTRG.9025.1	351	0.47371	6.313903809	-1
MSTRG.9027.2	75	0.35419	8.626037598	1
MSTRG.9029.3	189	0.27403	12.02545166	1

MSTRG.9029.4	1405	0.34799	4.718200684	1
MSTRG.9029.5	140	0.27241	8.57611084	1
MSTRG.9034.1	340	0.42431	8.998962402	1
MSTRG.9034.2	261	0.37011	9.542907715	1
MSTRG.9036.2	157	0.41346	6.53338623	1
MSTRG.9036.3	200	0.44419	6.545227051	1
MSTRG.9038.1	69	0.39486	8.506530762	1
MSTRG.9040.3	730	0.38965	8.921813965	1
MSTRG.9040.4	730	0.46894	8.921813965	1
MSTRG.9041.1	559	0.43872	8.110656738	1
MSTRG.9041.2	473	0.37141	8.261169434	1
MSTRG.9042.1	69	0.37044	5.955871582	1
MSTRG.9042.2	69	0.41253	5.955871582	1
MSTRG.9043.1	44	0.37596	10.7366333	1
MSTRG.9045.2	74	0.39016	10.0479126	1
MSTRG.9046.2	254	0.37842	5.179016113	1
MSTRG.9050.1	488	0.36905	6.480163574	1
MSTRG.9051.1	93	0.28854	11.65875244	1
MSTRG.9053.3	98	0.37055	12.25604248	1
MSTRG.9053.5	97	0.37055	12.25604248	1
MSTRG.9053.6	105	0.28775	11.87542725	1
MSTRG.9053.4	1145	0.3801	8.513366699	1
MSTRG.9053.7	81	0.3328	8.83026123	1
MSTRG.9054.1	118	0.32487	5.457946777	-1
MSTRG.9055.1	3185	0.44816	5.95904541	1
MSTRG.9055.4	3017	0.44793	5.841369629	1
MSTRG.9058.1	578	0.424	8.10357666	1
MSTRG.9058.2	486	0.41243	8.384216309	1
MSTRG.9058.3	485	0.3657	9.443786621	1
MSTRG.9058.4	325	0.32749	9.130554199	1
MSTRG.9058.8	372	0.31981	9.172058105	-1
MSTRG.9058.10	317	0.3678	9.002380371	1
MSTRG.9058.11	532	0.41556	7.957580566	1
MSTRG.9058.5	357	0.39942	9.374328613	1
MSTRG.9058.6	456	0.42724	8.77166748	1
MSTRG.9058.7	416	0.40277	9.233459473	-1
MSTRG.9058.9	548	0.43925	8.106018066	1
MSTRG.9058.12	456	0.42413	8.77166748	1
MSTRG.9058.14	567	0.39066	7.639587402	1
MSTRG.9059.3	67	0.39702	12.37322998	1
MSTRG.9059.2	675	0.39961	5.453430176	1
MSTRG.9062.6	627	0.40011	6.161437988	1
MSTRG.9056.2	175	0.30463	9.259338379	1
MSTRG.9081.6	89	0.31734	12.00042725	1
MSTRG.9081.7	1420	0.4941	6.145202637	1
MSTRG.9081.5	1070	0.3665	6.282165527	1
MSTRG.9081.3	1419	0.48422	6.145202637	1
MSTRG.9081.1	1419	0.4864	6.145202637	1
MSTRG.9081.4	1419	0.49038	6.145202637	1
MSTRG.9081.8	1433	0.4555	6.145202637	1
MSTRG.9081.9	1379	0.47393	6.074768066	1
MSTRG.9081.10	760	0.3736	8.097229004	1
MSTRG.9081.11	282	0.31203	4.625183105	1
MSTRG.9081.12	209	0.25717	12.00115967	-1

MSTRG.9082.1	373	0.28002	8.821350098	1
MSTRG.9082.2	181	0.34558	8.892883301	1
MSTRG.9083.1	112	0.39089	9.071716309	1
MSTRG.9084.1	194	0.27883	10.42315674	1
MSTRG.9083.10	186	0.334	7.578918457	1
MSTRG.9083.12	186	0.334	7.578918457	1
MSTRG.9083.13	186	0.334	7.578918457	1
MSTRG.9083.15	186	0.31829	7.578918457	1
MSTRG.9083.9	186	0.32265	7.578918457	1
MSTRG.9083.4	186	0.31829	7.578918457	1
MSTRG.9083.8	186	0.334	7.578918457	1
MSTRG.9083.7	186	0.32918	7.578918457	1
MSTRG.9083.16	74	0.31581	10.62286377	1
MSTRG.9085.2	74	0.38518	6.501525879	1
MSTRG.9072.1	83	0.41195	12.5647583	1
MSTRG.9075.2	112	0.33451	5.461853027	1
MSTRG.9089.1	161	0.34726	12.06231689	1
MSTRG.9090.2	398	0.46535	9.829650879	1
MSTRG.9090.3	476	0.43033	9.183410645	1
MSTRG.9091.4	1335	0.44331	9.009094238	-1
MSTRG.9091.5	638	0.3255	9.612976074	1
MSTRG.9091.6	638	0.32085	9.612976074	1
MSTRG.9091.8	209	0.28015	9.188415527	1
MSTRG.9092.1	179	0.30465	6.877624512	1
MSTRG.9093.4	810	0.42472	4.942810059	1
MSTRG.9094.2	311	0.41163	8.281188965	1
MSTRG.9097.1	268	0.24332	9.422668457	1
MSTRG.9097.2	46	0.30161	9.175964355	1
MSTRG.9103.5	676	0.34617	6.031677246	1
MSTRG.9103.2	676	0.33148	6.031677246	1
MSTRG.9104.3	348	0.43323	8.831604004	1
MSTRG.9104.4	348	0.48358	8.831604004	1
MSTRG.9104.5	348	0.46474	8.831604004	1
MSTRG.9104.7	348	0.46206	8.831604004	1
MSTRG.9104.6	348	0.47079	8.831604004	1
MSTRG.9104.8	338	0.2501	8.929992676	1
MSTRG.9100.2	58	0.34339	9.617492676	1
MSTRG.9107.8	430	0.44854	6.167785645	1
MSTRG.9107.6	430	0.41705	6.167785645	1
MSTRG.9108.1	88	0.35244	11.32647705	1
MSTRG.9111.1	59	0.39585	9.669006348	1
MSTRG.9112.2	231	0.40301	10.62469482	1
MSTRG.9113.1	130	0.34627	10.95245361	1
MSTRG.9110.1	100	0.35786	7.023132324	-1
MSTRG.9116.3	596	0.40578	8.942199707	1
MSTRG.9125.6	291	0.39893	6.746398926	1
MSTRG.9125.2	251	0.3926	8.879333496	1
MSTRG.9125.3	491	0.42672	6.425231934	1
MSTRG.9125.4	264	0.3926	8.284729004	1
MSTRG.9125.5	272	0.41288	8.540710449	1
MSTRG.9126.4	978	0.38019	5.115905762	1
MSTRG.9127.1	374	0.28813	9.945251465	1
MSTRG.9120.2	242	0.37837	8.535095215	1
MSTRG.9120.3	239	0.27879	8.711975098	1

MSTRG.9123.1	127	0.46253	11.80267334	1
MSTRG.9123.2	127	0.47455	11.80267334	1
MSTRG.9129.1	106	0.35039	10.94573975	1
MSTRG.9129.2	106	0.37317	10.94573975	1
MSTRG.9128.1	171	0.42462	11.21624756	1
MSTRG.9133.3	456	0.42325	7.611877441	-1
MSTRG.9136.1	56	0.37257	8.000793457	-1
MSTRG.9138.1	557	0.40364	6.77142334	1
MSTRG.9138.3	557	0.34795	6.77142334	1
MSTRG.9139.1	93	0.4059	7.022521973	1
MSTRG.9138.4	76	0.29755	6.710266113	-1
MSTRG.9140.1	226	0.34956	9.55682373	1
MSTRG.9140.3	128	0.35382	11.37469482	1
MSTRG.9142.1	18	0.30727	6.495666504	1
MSTRG.9145.3	675	0.45934	7.596862793	1
MSTRG.9145.5	444	0.46411	6.335021973	1
MSTRG.9145.2	675	0.45564	7.596862793	1
MSTRG.9145.4	607	0.45729	6.82220459	1
MSTRG.9145.6	390	0.44946	8.702209473	1
MSTRG.9147.1	103	0.34755	9.511169434	1
MSTRG.9146.4	275	0.39192	5.26361084	1
MSTRG.9146.5	190	0.35756	5.249938965	1
MSTRG.9144.3	231	0.4513	9.869689941	1
MSTRG.9144.2	301	0.32767	9.702697754	1
MSTRG.9157.3	356	0.3288	5.081848145	1
MSTRG.9157.4	249	0.33886	9.645446777	1
MSTRG.9153.3	593	0.45783	4.483093262	1
MSTRG.9148.1	55	0.30827	6.721984863	1
MSTRG.9150.1	245	0.42386	10.2946167	1
MSTRG.9150.2	244	0.42386	10.2946167	1
MSTRG.9175.2	248	0.44976	5.429138184	1
MSTRG.9175.4	449	0.46583	5.887390137	1
MSTRG.9175.10	238	0.48083	5.429138184	1
MSTRG.9175.9	248	0.4599	5.429138184	1
MSTRG.9175.8	238	0.46155	5.429138184	1
MSTRG.9167.2	1030	0.42833	6.324035645	1
MSTRG.9168.1	109	0.36116	8.237121582	1
MSTRG.9188.1	43	0.33167	10.01239014	1
MSTRG.9190.1	126	0.44002	10.31451416	1
MSTRG.9190.3	126	0.43752	10.31451416	1
MSTRG.9190.2	131	0.46354	10.31451416	1
MSTRG.9194.1	140	0.30373	8.73614502	1
MSTRG.9194.2	101	0.32466	11.07501221	1
MSTRG.9193.3	180	0.27089	5.472351074	1
MSTRG.9202.1	1022	0.46956	5.703796387	1
MSTRG.9203.1	125	0.44477	5.060852051	-1
MSTRG.9205.1	1593	0.43615	5.107727051	-1
MSTRG.9205.2	358	0.46886	5.682434082	-1
MSTRG.9206.1	105	0.34431	9.430969238	1
MSTRG.9209.2	165	0.37567	9.604431152	1
MSTRG.9209.3	287	0.3158	9.668762207	1
MSTRG.9211.3	111	0.40237	9.510681152	1
MSTRG.9211.4	160	0.36901	8.754577637	1
MSTRG.9217.2	164	0.34635	6.382995605	1

MSTRG.9230.1	58	0.28027	9.235412598	1
MSTRG.9231.1	601	0.4224	7.504333496	1
MSTRG.9231.3	572	0.39241	8.43145752	1
MSTRG.9233.1	351	0.39145	9.688537598	1
MSTRG.9233.5	351	0.39145	9.688537598	1
MSTRG.9235.3	236	0.41479	9.13470459	1
MSTRG.9235.4	273	0.36933	9.636901855	1
MSTRG.9239.6	639	0.34868	9.945373535	1
MSTRG.9239.9	607	0.44584	9.734680176	1
MSTRG.9239.8	614	0.44532	9.820007324	1
MSTRG.9240.1	117	0.35321	8.755065918	1
MSTRG.9249.4	441	0.4109	5.971496582	1
MSTRG.9249.5	468	0.45255	5.57611084	1
MSTRG.9250.6	1096	0.43594	6.022277832	1
MSTRG.9250.7	1096	0.40547	6.022277832	1
MSTRG.9250.8	1096	0.43799	6.022277832	1
MSTRG.9250.10	1096	0.4606	6.022277832	1
MSTRG.9250.9	1082	0.35511	6.071350098	1
MSTRG.9258.3	683	0.3874	4.34967041	1
MSTRG.9258.5	263	0.42087	4.535949707	1
MSTRG.9259.2	567	0.36401	5.422912598	1
MSTRG.9260.1	37	0.34849	8.683532715	1
MSTRG.9260.2	37	0.3974	8.683532715	1
MSTRG.9256.3	296	0.4558	5.942932129	1
MSTRG.9257.3	89	0.38075	5.29901123	1
MSTRG.9264.2	128	0.43031	9.870666504	1
MSTRG.9266.1	176	0.33906	4.941467285	1
MSTRG.9266.2	176	0.34191	4.941467285	1
MSTRG.9266.3	94	0.36877	9.75592041	1
MSTRG.9267.2	467	0.43508	5.188903809	1
MSTRG.9267.3	410	0.4566	4.84564209	1
MSTRG.9268.2	141	0.45457	11.81341553	1
MSTRG.9269.1	83	0.30656	12.47772217	1
MSTRG.9298.1	1549	0.45745	10.04180908	1
MSTRG.9298.18	3583	0.48053	9.521179199	1
MSTRG.9298.10	2162	0.40578	9.527038574	1
MSTRG.9298.17	1417	0.46057	9.617980957	1
MSTRG.9298.20	2515	0.48053	9.505065918	-1
MSTRG.9298.11	3757	0.47233	9.481750488	-1
MSTRG.9298.12	3613	0.48053	9.487365723	1
MSTRG.9298.13	1523	0.45738	10.04888916	1
MSTRG.9298.15	2409	0.47531	8.871154785	1
MSTRG.9298.16	3659	0.47233	9.453063965	1
MSTRG.9298.9	1549	0.45738	10.04180908	1
MSTRG.9298.24	1196	0.46882	9.708068848	1
MSTRG.9298.26	1358	0.45902	9.511901855	-1
MSTRG.9298.25	1060	0.44884	10.19610596	1
MSTRG.9298.30	368	0.4429	9.686950684	-1
MSTRG.9298.31	368	0.446	9.686950684	-1
MSTRG.9298.29	1874	0.43713	9.460266113	1
MSTRG.9298.33	1549	0.45738	10.04180908	1
MSTRG.9298.34	1523	0.45993	10.04901123	1
MSTRG.9298.35	767	0.44932	9.779846191	1
MSTRG.9298.36	757	0.44932	9.852111816	1

MSTRG.9278.2	162	0.43978	5.452941895	1
MSTRG.9281.1	100	0.36024	8.739318848	-1
MSTRG.9283.2	223	0.44044	9.516540527	1
MSTRG.9284.1	40	0.28536	9.984313965	1
MSTRG.9285.2	186	0.45734	10.82672119	1
MSTRG.9287.2	95	0.32542	9.819641113	1
MSTRG.9290.1	680	0.44239	6.889099121	1
MSTRG.9291.1	899	0.41294	5.814758301	1
MSTRG.9291.3	276	0.31123	8.489562988	1
MSTRG.9295.1	646	0.30898	8.828063965	1
MSTRG.9295.2	646	0.30898	8.828063965	1
MSTRG.9295.4	639	0.3939	8.897644043	1
MSTRG.9296.1	114	0.3736	9.504089355	1
MSTRG.9303.2	114	0.34505	9.536560059	1
MSTRG.9304.1	440	0.43621	6.439025879	1
MSTRG.9305.2	141	0.37457	12.12762451	1
MSTRG.9305.3	55	0.36745	7.810119629	1
MSTRG.9304.3	432	0.45687	6.542297363	1
MSTRG.9306.1	54	0.37633	9.589782715	1
MSTRG.9306.2	31	0.3927	8.885314941	1
MSTRG.9307.2	290	0.4364	6.968566895	1
MSTRG.9308.4	206	0.41997	9.931213379	1
MSTRG.9312.11	2141	0.43411	8.56072998	1
MSTRG.9312.12	2009	0.44436	8.597229004	1
MSTRG.9312.13	2135	0.44436	8.593688965	1
MSTRG.9312.14	2000	0.44436	8.204528809	1
MSTRG.9312.3	2125	0.44436	8.562927246	1
MSTRG.9312.4	2136	0.44436	8.593688965	1
MSTRG.9312.9	2133	0.44436	8.805114746	1
MSTRG.9312.15	2016	0.44436	8.202575684	1
MSTRG.9312.23	370	0.36085	8.953308105	1
MSTRG.9312.24	261	0.26913	9.666320801	1
MSTRG.9312.25	105	0.40625	10.03875732	-1
MSTRG.9310.2	1063	0.46034	5.432434082	1
MSTRG.9315.1	315	0.28584	9.580505371	1
MSTRG.9316.1	7	0.37018	4.529724121	-1
MSTRG.9315.3	315	0.28584	9.580505371	1
MSTRG.9321.3	160	0.27274	11.1852417	1
MSTRG.9321.4	160	0.27274	11.1852417	1
MSTRG.9322.1	89	0.28014	8.742370605	1
MSTRG.9325.2	105	0.28415	8.818054199	1
MSTRG.9327.4	676	0.39993	6.015075684	1
MSTRG.9335.3	335	0.27217	11.52093506	1
MSTRG.9335.4	119	0.2689	10.230896	1
MSTRG.9333.2	397	0.42206	6.585876465	1
MSTRG.9338.1	151	0.32848	10.39996338	1
MSTRG.9361.4	1095	0.42978	9.356018066	1
MSTRG.9361.6	1092	0.45245	9.41595459	1
MSTRG.9361.7	1073	0.45245	9.469177246	1
MSTRG.9361.8	983	0.40361	9.535583496	1
MSTRG.9361.9	968	0.40949	9.61138916	1
MSTRG.9361.10	968	0.45497	9.61138916	1
MSTRG.9361.11	968	0.44575	9.61138916	1
MSTRG.9361.13	850	0.43437	9.450378418	1

MSTRG.9361.14	850	0.44823	9.450378418	1
MSTRG.9365.1	53	0.38408	10.41351318	1
MSTRG.9364.12	184	0.3204	10.34197998	1
MSTRG.9364.13	361	0.33085	4.920593262	1
MSTRG.9367.2	722	0.28862	8.752380371	1
MSTRG.9367.3	1073	0.42944	9.121154785	1
MSTRG.9367.4	1243	0.4467	9.154724121	1
MSTRG.9367.6	928	0.45217	8.92767334	-1
MSTRG.9367.9	1001	0.41911	8.941467285	1
MSTRG.9367.10	722	0.35158	8.752380371	1
MSTRG.9368.1	160	0.28883	10.85662842	1
MSTRG.9367.12	967	0.31588	8.92767334	-1
MSTRG.9343.3	68	0.34809	11.48675537	1
MSTRG.9349.1	232	0.37371	9.362854004	1
MSTRG.9353.1	90	0.33032	9.063415527	1
MSTRG.9358.4	338	0.37361	8.288269043	1
MSTRG.9358.1	240	0.34553	9.094909668	1
MSTRG.9358.3	240	0.36241	9.094909668	1
MSTRG.9358.5	240	0.36573	9.094909668	1
MSTRG.9358.6	287	0.36683	9.046447754	1
MSTRG.9360.3	498	0.39798	5.671813965	1
MSTRG.9369.3	427	0.42848	5.46685791	1
MSTRG.9369.4	427	0.42848	5.46685791	1
MSTRG.9369.5	253	0.4038	5.921813965	1
MSTRG.9374.1	1467	0.42735	6.070617676	1
MSTRG.9374.2	1599	0.44127	6.968078613	1
MSTRG.9374.3	1436	0.43564	6.732849121	1
MSTRG.9374.5	1431	0.42177	6.733459473	1
MSTRG.9374.4	1485	0.4418	7.13494873	1
MSTRG.9374.7	1296	0.4297	6.355773926	1
MSTRG.9372.2	343	0.36394	8.538635254	1
MSTRG.9372.3	293	0.35788	8.289123535	1
MSTRG.9373.3	129	0.31299	4.158874512	1
MSTRG.9377.2	253	0.44136	10.53289795	1
MSTRG.9386.6	263	0.46048	8.03338623	-1
MSTRG.9386.4	975	0.44113	5.958557129	1
MSTRG.9379.1	57	0.32631	9.300231934	1
MSTRG.9382.1	99	0.45689	11.59649658	1
MSTRG.9385.2	400	0.46496	8.020935059	1
MSTRG.9385.3	400	0.45783	8.020935059	1
MSTRG.9396.1	532	0.40408	5.45098877	1
MSTRG.9396.2	532	0.40533	5.45098877	1
MSTRG.9396.3	532	0.39227	5.45098877	1
MSTRG.9398.4	703	0.46414	8.585266113	1
MSTRG.9398.3	676	0.47143	7.945251465	1
MSTRG.9394.1	441	0.34081	8.719421387	1
MSTRG.9394.2	461	0.36205	8.496398926	1
MSTRG.9394.3	433	0.32872	8.770812988	1
MSTRG.9394.4	400	0.37956	8.049499512	1
MSTRG.9393.1	168	0.31441	9.533874512	1
MSTRG.9405.6	131	0.47448	5.519226074	1
MSTRG.9407.3	299	0.44299	5.059265137	1
MSTRG.9410.1	703	0.403	5.281066895	1
MSTRG.9420.2	248	0.43988	4.918762207	1

MSTRG.9422.1	887	0.42886	4.855041504	1
MSTRG.9422.4	887	0.44015	4.855041504	1
MSTRG.9423.2	369	0.4694	5.096496582	1
MSTRG.9423.3	246	0.45748	4.923278809	1
MSTRG.9423.4	246	0.4759	4.923278809	1
MSTRG.9425.2	134	0.34456	4.457824707	1
MSTRG.9411.2	70	0.46268	8.588562012	1
MSTRG.9413.2	39	0.42648	4.950012207	-1
MSTRG.9428.2	385	0.32101	9.332458496	1
MSTRG.9436.1	5474	0.43724	5.058654785	1
MSTRG.9436.2	5830	0.45358	5.129089355	1
MSTRG.9436.3	6300	0.46302	5.343078613	1
MSTRG.9436.4	5718	0.43972	5.140563965	1
MSTRG.9436.5	3344	0.44177	5.39654541	1
MSTRG.9436.6	4041	0.47139	5.73614502	1
MSTRG.9436.10	3047	0.47658	5.953918457	1
MSTRG.9436.11	3158	0.48733	5.917297363	1
MSTRG.9436.15	2433	0.43673	5.527526855	1
MSTRG.9436.12	5922	0.48147	5.403747559	1
MSTRG.9436.13	5100	0.4511	5.233703613	1
MSTRG.9436.14	5504	0.48147	5.492126465	1
MSTRG.9436.9	5225	0.43724	5.200744629	1
MSTRG.9436.16	5474	0.45532	5.058654785	1
MSTRG.9436.17	5315	0.44712	5.04473877	1
MSTRG.9436.18	6556	0.47377	5.309753418	1
MSTRG.9436.19	6444	0.48147	5.324401855	1
MSTRG.9431.3	464	0.35998	9.614318848	1
MSTRG.9431.4	464	0.34475	9.614318848	1
MSTRG.9445.1	322	0.28048	9.118591309	-1
MSTRG.9446.2	57	0.28584	7.976257324	1
MSTRG.9446.3	17	0.32839	9.186218262	1
MSTRG.9446.4	81	0.34225	9.93560791	1
MSTRG.9446.6	35	0.34332	11.71258545	1
MSTRG.9449.1	582	0.39263	9.375915527	1
MSTRG.9449.2	124	0.49241	5.572692871	1
MSTRG.9449.4	132	0.34723	8.579284668	1
MSTRG.9449.3	540	0.39965	9.439147949	1
MSTRG.9449.5	32	0.37574	7.976745605	1
MSTRG.9445.3	235	0.2908	9.510437012	1
MSTRG.9445.4	143	0.33433	9.298278809	1
MSTRG.9445.6	87	0.31941	12.01116943	1
MSTRG.9453.1	73	0.34738	8.581359863	1
MSTRG.9453.2	73	0.34251	8.581359863	1
MSTRG.9453.3	73	0.33045	8.581359863	1
MSTRG.9434.2	72	0.33527	10.63983154	1
MSTRG.9437.2	1137	0.42138	5.901550293	1
MSTRG.9441.1	467	0.35982	9.056335449	1
MSTRG.9458.2	1604	0.44484	8.150939941	1
MSTRG.9458.4	1503	0.43397	8.247741699	1
MSTRG.9457.1	366	0.42634	6.717956543	1
MSTRG.9464.2	37	0.36797	6.066589355	-1
MSTRG.9464.1	108	0.31802	11.03643799	1
MSTRG.9464.3	104	0.33491	11.27471924	-1
MSTRG.9464.4	104	0.43563	11.27471924	-1

MSTRG.9464.5	167	0.431	10.61016846	-1
MSTRG.9460.1	438	0.45859	5.55267334	1
MSTRG.9471.5	610	0.41411	4.711486816	1
MSTRG.9471.6	694	0.46381	4.976745605	1
MSTRG.9471.7	587	0.47654	4.686828613	1
MSTRG.9471.8	421	0.46013	4.601501465	1
MSTRG.9473.1	624	0.35297	6.127380371	1
MSTRG.9473.2	587	0.4116	6.193908691	1
MSTRG.9473.3	624	0.35297	6.127380371	1
MSTRG.9473.4	624	0.43189	6.127380371	1
MSTRG.9473.5	624	0.41227	6.127380371	1
MSTRG.9468.2	359	0.385	8.956726074	1
MSTRG.9468.3	262	0.36123	6.380554199	1
MSTRG.9481.4	706	0.42876	7.953063965	1
MSTRG.9481.11	120	0.27654	6.487609863	1
MSTRG.9481.17	706	0.42876	7.953063965	1
MSTRG.9481.14	706	0.42876	7.953063965	1
MSTRG.9481.15	702	0.44161	7.628234863	1
MSTRG.9481.16	702	0.42876	7.628234863	1
MSTRG.9481.9	702	0.42876	7.628234863	1
MSTRG.9481.10	895	0.34816	8.855773926	1
MSTRG.9481.18	564	0.31772	9.548522949	1
MSTRG.9481.7	895	0.37071	8.855773926	1
MSTRG.9477.2	101	0.38304	11.30743408	1
MSTRG.9478.4	248	0.41854	8.362487793	1
MSTRG.9482.7	530	0.43752	6.77947998	1
MSTRG.9484.1	60	0.32807	11.52459717	1
MSTRG.9486.2	306	0.43306	6.13067627	1
MSTRG.9493.6	1003	0.44063	5.664978027	1
MSTRG.9493.7	1002	0.42453	5.664978027	1
MSTRG.9493.8	617	0.403	5.70111084	1
MSTRG.9496.4	1974	0.43958	6.495788574	1
MSTRG.9498.2	320	0.4415	5.790222168	1
MSTRG.9498.8	189	0.38425	8.694274902	1
MSTRG.9499.1	274	0.37744	5.948669434	1
MSTRG.9499.2	252	0.42769	8.290344238	1
MSTRG.9499.4	142	0.40278	11.93048096	1
MSTRG.9499.5	203	0.44196	8.542053223	1
MSTRG.9500.1	343	0.34174	9.636657715	1
MSTRG.9500.3	343	0.36397	9.636657715	1
MSTRG.9500.4	343	0.37783	9.636657715	1
MSTRG.9503.1	533	0.49106	4.358825684	1
MSTRG.9503.2	533	0.48362	4.358825684	1
MSTRG.9503.3	533	0.47261	4.358825684	1
MSTRG.9503.4	582	0.48539	4.391906738	1
MSTRG.9503.6	533	0.4772	4.358825684	1
MSTRG.9505.2	208	0.43289	8.837219238	1
MSTRG.9505.3	377	0.37685	5.888000488	1
MSTRG.9505.4	433	0.44395	5.533996582	1
MSTRG.9505.5	433	0.37008	5.533996582	1
MSTRG.9505.6	433	0.47697	5.533996582	1
MSTRG.9509.1	83	0.33037	8.240539551	1
MSTRG.9509.3	118	0.35909	5.273254395	-1
MSTRG.9516.4	883	0.44387	7.606262207	1

MSTRG.9516.6	139	0.35421	4.781188965	1
MSTRG.9516.8	61	0.29747	9.182067871	1
MSTRG.9516.7	992	0.46997	6.196350098	1
MSTRG.9518.2	1332	0.44852	7.743591309	1
MSTRG.9518.3	1332	0.43418	7.715393066	1
MSTRG.9522.1	855	0.45498	5.822937012	1
MSTRG.9523.2	67	0.45251	6.297790527	1
MSTRG.9526.2	561	0.44428	6.134460449	1
MSTRG.9532.1	57	0.39341	5.75982666	1
MSTRG.9534.1	466	0.3496	8.961242676	1
MSTRG.9535.1	102	0.3133	10.12030029	1
MSTRG.9535.2	91	0.27327	8.958068848	1
MSTRG.9535.3	189	0.33595	9.367736816	1
MSTRG.9536.1	1619	0.38529	4.686340332	1
MSTRG.9543.1	83	0.35011	11.61077881	1
MSTRG.9543.2	37	0.38784	8.020690918	1
MSTRG.9550.1	65	0.35683	4.589416504	1
MSTRG.9547.1	624	0.35772	8.017272949	1
MSTRG.9554.1	290	0.44225	9.014953613	-1
MSTRG.9555.1	57	0.31939	8.057800293	1
MSTRG.9556.1	70	0.31629	6.240905762	1
MSTRG.9556.2	101	0.34243	9.293884277	1
MSTRG.9556.3	68	0.31571	6.240905762	1
MSTRG.9563.1	421	0.45177	6.90826416	1
MSTRG.9563.2	421	0.46598	6.90826416	1
MSTRG.9564.2	547	0.44547	5.265563965	1
MSTRG.9565.4	594	0.42487	8.926696777	-1
MSTRG.9565.5	389	0.41554	7.158874512	1
MSTRG.9566.2	235	0.41781	6.477600098	1
MSTRG.9566.3	293	0.38515	8.764221191	1
MSTRG.9574.3	1040	0.39889	9.156799316	1
MSTRG.9574.4	667	0.38665	6.84588623	1
MSTRG.9574.5	703	0.34356	6.898498535	1
MSTRG.9574.6	702	0.36306	6.898498535	1
MSTRG.9574.7	667	0.36836	6.84588623	1
MSTRG.9576.1	517	0.45277	6.663024902	1
MSTRG.9576.2	517	0.46817	6.663024902	1
MSTRG.9576.3	517	0.42928	6.663024902	1
MSTRG.9567.2	73	0.29512	5.733825684	1
MSTRG.9567.3	131	0.36764	8.174865723	1
MSTRG.9567.4	73	0.33455	5.733825684	1
MSTRG.9568.2	140	0.45687	9.230163574	1
MSTRG.9571.1	67	0.39214	10.32305908	-1
MSTRG.9577.4	322	0.46606	6.191711426	1
MSTRG.9586.3	444	0.38647	4.711853027	1
MSTRG.9587.2	80	0.35376	10.72589111	1
MSTRG.9589.3	315	0.45189	8.422668457	1
MSTRG.9589.5	363	0.41745	5.270935059	1
MSTRG.9590.2	312	0.35821	9.52154541	1
MSTRG.9590.3	645	0.35244	8.846496582	1
MSTRG.9590.5	288	0.45052	5.027526855	-1
MSTRG.9590.7	467	0.37435	8.65057373	1
MSTRG.9590.6	467	0.38279	8.65057373	1
MSTRG.9591.1	764	0.40543	5.90020752	1

MSTRG.9591.3	850	0.39904	6.194519043	1
MSTRG.9598.10	209	0.29151	11.35223389	1
MSTRG.9598.3	111	0.28283	7.940856934	1
MSTRG.9598.4	111	0.33738	7.940856934	1
MSTRG.9598.6	92	0.32637	7.917053223	1
MSTRG.9598.7	153	0.33767	10.80609131	1
MSTRG.9598.8	92	0.32077	7.917053223	1
MSTRG.9598.9	153	0.33485	10.80609131	1
MSTRG.9599.1	118	0.31496	8.686096191	1
MSTRG.9599.3	118	0.38	8.686096191	1
MSTRG.9599.4	73	0.31579	6.087463379	1
MSTRG.9600.12	450	0.31603	10.05145264	1
MSTRG.9600.8	846	0.46783	5.355529785	1
MSTRG.9600.13	1145	0.44518	5.648498535	1
MSTRG.9600.7	901	0.46599	5.408752441	1
MSTRG.9600.9	901	0.46599	5.408752441	1
MSTRG.9600.16	107	0.35889	6.314025879	1
MSTRG.9602.2	495	0.36516	9.351989746	1
MSTRG.9594.1	335	0.40293	7.971130371	1
MSTRG.9594.2	196	0.3764	7.016662598	1
MSTRG.9594.3	323	0.40994	6.877502441	1
MSTRG.9594.5	359	0.42025	7.597473145	1
MSTRG.9597.1	1116	0.43577	6.965759277	1
MSTRG.9597.2	1082	0.43577	6.471374512	1
MSTRG.9597.3	1116	0.45091	6.965759277	1
MSTRG.9597.4	1082	0.45417	6.471374512	1
MSTRG.9604.3	793	0.44649	8.305358887	1
MSTRG.9604.4	786	0.43435	8.305358887	1
MSTRG.9605.1	56	0.36823	6.801574707	-1
MSTRG.9605.2	56	0.30723	6.801574707	-1
MSTRG.9606.1	208	0.40703	8.590759277	1
MSTRG.9610.1	47	0.35979	6.230163574	1
MSTRG.9610.2	72	0.41335	6.480773926	-1
MSTRG.9612.2	186	0.36764	9.668395996	1
MSTRG.9612.3	186	0.38608	9.668395996	1
MSTRG.9613.1	39	0.40588	10.3883667	-1
MSTRG.9612.4	78	0.28578	9.691467285	1
MSTRG.9603.1	82	0.3515	7.998718262	-1
MSTRG.9617.3	388	0.4309	6.153015137	1
MSTRG.9619.2	946	0.43995	5.59954834	1
MSTRG.9620.1	183	0.39911	8.836486816	1
MSTRG.9620.2	265	0.46554	9.410217285	1
MSTRG.9624.3	286	0.49415	5.38079834	1
MSTRG.9625.1	146	0.27547	8.272888184	1
MSTRG.9626.1	389	0.42117	6.902770996	1
MSTRG.9626.2	359	0.47225	7.221618652	1
MSTRG.9630.2	470	0.4657	5.325378418	1
MSTRG.9634.3	120	0.29395	9.385803223	1
MSTRG.9634.4	107	0.35064	9.581726074	1
MSTRG.9633.3	138	0.39342	9.672912598	1
MSTRG.9633.2	138	0.39342	9.672912598	1
MSTRG.9638.1	451	0.465	6.221984863	1
MSTRG.9640.4	480	0.40586	5.249694824	1
MSTRG.9649.4	493	0.42896	9.266052246	1

MSTRG.9643.1	350	0.40849	4.92401123	1
MSTRG.9651.6	284	0.27133	6.59197998	1
MSTRG.9650.1	27	0.4548	10.46124268	1
MSTRG.9653.1	301	0.40663	5.399963379	1
MSTRG.9653.2	343	0.38479	5.409118652	1
MSTRG.9655.3	744	0.47533	6.161071777	1
MSTRG.9657.1	152	0.43817	11.03106689	-1
MSTRG.9658.4	468	0.3263	8.994445801	1
MSTRG.9661.1	39	0.32547	6.510314941	1
MSTRG.9662.4	345	0.36264	4.935974121	1
MSTRG.9663.3	314	0.42231	6.077697754	1
MSTRG.9663.4	314	0.42963	6.077697754	1
MSTRG.9665.1	79	0.287	8.937316895	1
MSTRG.9665.2	71	0.287	11.8291626	1
MSTRG.9665.3	71	0.287	11.8291626	1
MSTRG.9665.5	83	0.287	11.36480713	1
MSTRG.9667.2	438	0.32912	5.43548584	1
MSTRG.9667.3	420	0.35439	5.45489502	1
MSTRG.9675.2	124	0.40628	5.274963379	1
MSTRG.9675.3	65	0.47624	12.00042725	1
MSTRG.9675.4	76	0.3941	11.95013428	1
MSTRG.9670.2	212	0.42248	12.22784424	1
MSTRG.9671.2	83	0.39303	10.42681885	1
MSTRG.9676.2	1713	0.44737	9.399597168	1
MSTRG.9676.3	174	0.40174	5.660705566	1
MSTRG.9685.1	59	0.30297	8.294250488	1
MSTRG.9689.1	217	0.3723	8.809020996	1
MSTRG.9689.3	217	0.37695	8.809020996	1
MSTRG.9696.2	702	0.37649	6.343444824	1
MSTRG.9696.5	662	0.45893	5.858093262	1
MSTRG.9692.1	322	0.36906	5.576965332	1
MSTRG.9693.2	101	0.33223	12.38128662	1
MSTRG.9691.2	245	0.30644	5.039978027	1
MSTRG.9706.1	535	0.42226	5.419494629	1
MSTRG.9706.5	533	0.43821	5.417297363	1
MSTRG.9708.1	83	0.30237	10.31671143	1
MSTRG.9719.1	235	0.44151	10.26824951	1
MSTRG.9728.1	109	0.35037	9.894226074	1
MSTRG.9740.1	43	0.39259	10.48040771	1
MSTRG.9742.1	690	0.46253	5.991394043	1
MSTRG.9742.2	697	0.46997	6.129699707	1
MSTRG.9742.3	697	0.45433	6.129699707	1
MSTRG.9742.4	697	0.46253	6.129699707	1
MSTRG.9742.8	697	0.46336	6.129699707	1
MSTRG.9742.9	690	0.46997	5.991394043	1
MSTRG.9742.13	690	0.41793	5.991394043	1
MSTRG.9742.15	393	0.33686	6.432922363	1
MSTRG.9742.17	182	0.41679	8.987976074	1
MSTRG.9742.18	375	0.42236	6.636047363	1
MSTRG.9743.1	181	0.37149	9.039123535	1
MSTRG.9743.2	300	0.44784	9.135070801	1
MSTRG.9745.1	1163	0.41437	5.566589355	1
MSTRG.9745.3	1060	0.39484	5.446716309	1
MSTRG.9745.6	692	0.3503	9.298156738	1

MSTRG.9732.2	84	0.29751	8.611022949	1
MSTRG.9734.2	37	0.35732	9.809387207	1
MSTRG.9734.5	103	0.45574	9.426940918	1
MSTRG.9734.4	32	0.34821	9.102355957	1
MSTRG.9735.1	119	0.47574	6.574279785	1
MSTRG.9738.1	684	0.40317	7.856506348	1
MSTRG.9747.1	49	0.36324	9.414855957	1
MSTRG.9753.1	99	0.35434	5.311584473	1
MSTRG.9756.1	60	0.37021	10.16595459	-1
MSTRG.9760.1	295	0.46547	4.916687012	1
MSTRG.9760.2	294	0.47037	4.952453613	1
MSTRG.9760.3	180	0.46914	7.485412598	-1
MSTRG.9773.1	511	0.39298	5.687561035	1
MSTRG.9774.1	433	0.36993	7.596008301	1
MSTRG.9774.2	432	0.39617	7.596008301	1
MSTRG.9774.4	440	0.41667	8.014465332	1
MSTRG.9775.1	152	0.28683	10.85614014	1
MSTRG.9780.1	75	0.38146	9.743225098	1
MSTRG.9798.1	28	0.43173	4.298156738	-1
MSTRG.9815.16	46	0.26545	9.493103027	1
MSTRG.9815.18	178	0.28334	10.11749268	1
MSTRG.9815.19	98	0.29292	10.19525146	1
MSTRG.9815.20	185	0.30576	9.083557129	1
MSTRG.9815.21	185	0.3126	9.083557129	1
MSTRG.9816.1	35	0.47637	6.014099121	-1
MSTRG.9815.22	85	0.30357	7.81842041	1
MSTRG.9815.23	99	0.30411	6.680603027	1
MSTRG.9815.24	52	0.3249	6.885681152	1
MSTRG.9815.25	112	0.28334	10.50921631	1
MSTRG.9815.26	109	0.28035	8.71282959	1
MSTRG.9815.27	94	0.28083	10.29095459	1
MSTRG.9817.1	151	0.29367	10.09881592	1
MSTRG.9817.2	33	0.38145	11.09698486	1
MSTRG.9811.1	53	0.41074	9.390808105	1
MSTRG.9812.2	414	0.39885	9.29107666	1
MSTRG.9814.2	987	0.4694	5.995788574	1
MSTRG.9827.1	456	0.39142	9.795349121	1
MSTRG.9827.3	108	0.42472	9.536193848	1
MSTRG.9849.1	333	0.31981	5.00189209	1
MSTRG.9854.3	3083	0.45541	5.84161377	1
MSTRG.9858.2	136	0.33794	6.063293457	1
MSTRG.9859.1	335	0.31925	9.572937012	1
MSTRG.9859.2	335	0.31905	9.572937012	1
MSTRG.9862.1	65	0.32485	8.498596191	1
MSTRG.9863.1	70	0.32346	4.956604004	1
MSTRG.9878.1	59	0.32265	9.352355957	1
MSTRG.9884.1	70	0.35264	5.102478027	1
MSTRG.9889.1	84	0.26974	10.14471436	1
MSTRG.9889.2	76	0.28515	5.519470215	1
MSTRG.9890.3	306	0.38423	5.600158691	1
MSTRG.9891.1	432	0.36092	6.349304199	1
MSTRG.9891.2	351	0.37089	6.055603027	1
MSTRG.9891.3	351	0.41296	6.055603027	1
MSTRG.9891.4	432	0.39521	6.349304199	1

MSTRG.9891.6	352	0.37089	6.055603027	1
MSTRG.9891.7	352	0.41296	6.055603027	1
MSTRG.9891.8	351	0.40455	6.055603027	1
MSTRG.9891.9	351	0.41723	6.055603027	1
MSTRG.9891.10	433	0.39948	6.349304199	1
MSTRG.9891.11	433	0.41409	6.349304199	1
MSTRG.9893.1	44	0.34982	9.989196777	1
MSTRG.9894.1	862	0.4429	4.452209473	1
MSTRG.9894.2	934	0.4429	4.465881348	1
MSTRG.9894.3	846	0.44818	4.472106934	1
MSTRG.9898.1	109	0.36501	5.383728027	-1
MSTRG.9898.2	116	0.4398	5.413879395	-1
MSTRG.9902.6	674	0.47579	5.265563965	1
MSTRG.9902.5	625	0.45044	5.195251465	1
MSTRG.9902.9	101	0.29919	8.931091309	1
MSTRG.9902.8	443	0.4643	5.700622559	1
MSTRG.9902.10	481	0.47715	5.684509277	1
MSTRG.9902.11	316	0.4724	4.989196777	1
MSTRG.9899.3	242	0.3337	9.583435059	1
MSTRG.9899.4	126	0.31913	9.365661621	1
MSTRG.9899.5	139	0.33639	8.291687012	1
MSTRG.9906.2	1233	0.36954	8.917297363	1
MSTRG.9913.2	458	0.36757	8.757629395	1
MSTRG.9913.6	222	0.39607	9.620544434	1
MSTRG.9915.2	496	0.46221	8.445129395	1
MSTRG.9918.1	65	0.42151	10.84307861	1
MSTRG.9919.2	325	0.44133	8.855895996	1
MSTRG.9919.3	213	0.45017	9.125427246	1
MSTRG.9919.4	189	0.37445	10.41900635	1
MSTRG.9924.3	332	0.43955	6.488220215	1
MSTRG.9929.1	481	0.37675	8.860900879	1
MSTRG.9929.6	481	0.4047	8.860900879	1
MSTRG.9929.5	301	0.39381	7.341369629	1
MSTRG.9934.3	447	0.4212	8.824890137	1
MSTRG.9941.2	605	0.44137	4.601867676	1
MSTRG.9944.1	317	0.33299	6.427307129	1
MSTRG.9944.2	246	0.34508	6.343322754	1
MSTRG.9944.3	317	0.43346	6.427307129	1
MSTRG.9948.1	116	0.29924	7.733703613	1
MSTRG.9961.1	42	0.45666	5.750061035	-1
MSTRG.9962.3	385	0.35713	5.562561035	1
MSTRG.9962.4	385	0.36608	5.562561035	1
MSTRG.9963.2	465	0.29492	9.985290527	1
MSTRG.9960.1	51	0.38039	7.964294434	1
MSTRG.9972.3	368	0.36153	5.135559082	1
MSTRG.9972.4	519	0.39854	4.57611084	1
MSTRG.9974.1	83	0.36344	12.00848389	1
MSTRG.9977.1	177	0.28862	8.031066895	1
MSTRG.9977.2	177	0.36568	8.031066895	1
MSTRG.9984.1	1221	0.44803	8.983459473	1
MSTRG.9984.2	1221	0.4554	8.983459473	1
MSTRG.9985.2	260	0.39081	5.64666748	1
MSTRG.9986.1	180	0.32788	8.329162598	1
MSTRG.9986.3	183	0.31927	8.629089355	1

MSTRG.9987.3	369	0.42398	4.88458252	1
MSTRG.9987.4	413	0.44032	4.880310059	1
MSTRG.9990.1	22	0.4036	5.897644043	1
MSTRG.9993.2	698	0.4383	8.437805176	-1
MSTRG.9994.1	289	0.33275	10.61553955	1
MSTRG.9997.1	242	0.37045	4.281188965	1
MSTRG.9997.2	242	0.37665	4.281188965	1
MSTRG.9997.3	591	0.44527	4.807189941	1
MSTRG.9997.4	336	0.38358	9.732971191	1
MSTRG.9997.5	366	0.44281	9.297912598	1
MSTRG.10000.1	287	0.432	6.198303223	1
MSTRG.10001.1	567	0.41682	4.980285645	1
MSTRG.10001.4	572	0.4246	4.96282959	1
MSTRG.10002.1	246	0.36087	6.940124512	1
MSTRG.10005.2	210	0.35813	5.692687988	1
MSTRG.10005.1	210	0.37385	5.692687988	1
MSTRG.10008.4	683	0.34351	8.796813965	1
MSTRG.10009.2	413	0.39665	5.114318848	1
MSTRG.10011.1	57	0.42917	11.91326904	1
MSTRG.10012.2	453	0.3941	9.648620605	1
MSTRG.10012.3	453	0.38548	9.648620605	1
MSTRG.10012.4	495	0.35509	9.972595215	1
MSTRG.10013.1	68	0.3847	10.51885986	1
MSTRG.10014.1	171	0.31873	9.51348877	1
MSTRG.10014.3	171	0.28995	9.51348877	1
MSTRG.10016.1	128	0.32937	7.658630371	1
MSTRG.10020.4	377	0.45389	8.231140137	1
MSTRG.10026.10	618	0.41377	9.16619873	1
MSTRG.10026.13	401	0.40983	6.801330566	1
MSTRG.10026.11	629	0.41887	8.933410645	1
MSTRG.10026.12	630	0.42445	9.01739502	1
MSTRG.10026.18	649	0.41422	8.628601074	-1
MSTRG.10024.2	216	0.30897	9.542541504	1
MSTRG.10024.3	240	0.30897	9.165588379	1
MSTRG.10025.1	71	0.33483	10.61419678	1
MSTRG.10031.1	64	0.37482	9.493469238	1
MSTRG.10041.2	1411	0.4429	6.406921387	1
MSTRG.10041.3	1091	0.48552	5.485412598	1
MSTRG.10041.7	1401	0.43267	6.406921387	1
MSTRG.10042.1	63	0.29377	5.083190918	1
MSTRG.10043.2	868	0.43036	9.366638184	1
MSTRG.10043.5	839	0.41407	9.401550293	-1
MSTRG.10043.6	1131	0.41407	8.950744629	-1
MSTRG.10045.2	105	0.3256	10.54388428	1
MSTRG.10037.3	246	0.43561	9.155090332	1
MSTRG.10038.1	180	0.33255	11.60137939	1
MSTRG.10038.2	91	0.31253	9.297790527	1
MSTRG.10049.1	346	0.32387	8.90423584	1
MSTRG.10050.2	77	0.3407	11.44537354	1
MSTRG.10052.2	785	0.35504	9.221130371	1
MSTRG.10053.1	242	0.37798	9.09576416	1
MSTRG.10051.2	44	0.41878	8.574035645	-1
MSTRG.10058.1	124	0.28999	5.911071777	1
MSTRG.10060.1	767	0.4361	5.702209473	1

MSTRG.10078.4	593	0.39681	9.367980957	1
MSTRG.10078.5	512	0.3705	9.331115723	1
MSTRG.10078.3	597	0.41758	9.377624512	1
MSTRG.10079.1	152	0.29772	4.91204834	1
MSTRG.10079.2	119	0.43084	4.540588379	1
MSTRG.10079.3	119	0.4545	4.540588379	1
MSTRG.10082.2	115	0.31432	9.671691895	1
MSTRG.10087.3	306	0.46399	6.338195801	1
MSTRG.10091.1	352	0.46738	6.117248535	1
MSTRG.10091.2	352	0.43356	6.117248535	1
MSTRG.10091.3	319	0.41127	5.653503418	1
MSTRG.10092.1	96	0.40178	8.93182373	1
MSTRG.10094.8	369	0.44299	9.83392334	1
MSTRG.10094.6	372	0.45066	9.801940918	1
MSTRG.10094.7	369	0.45724	9.83392334	1
MSTRG.10094.9	368	0.44299	9.81463623	1
MSTRG.10094.10	371	0.45066	9.781799316	1
MSTRG.10094.11	371	0.46115	9.781799316	1
MSTRG.10100.1	123	0.33114	9.387268066	1
MSTRG.10100.2	87	0.37975	8.557556152	1
MSTRG.10101.1	57	0.29434	5.538513184	1
MSTRG.10101.3	56	0.2997	9.479064941	1
MSTRG.10104.2	179	0.29772	9.885559082	1
MSTRG.10105.2	51	0.29871	6.514465332	1
MSTRG.10107.1	642	0.30961	6.211608887	1
MSTRG.10102.2	350	0.33565	6.065856934	1
MSTRG.10103.1	134	0.34956	9.445983887	1
MSTRG.10121.1	555	0.30356	5.331115723	1
MSTRG.10121.3	555	0.34935	5.331115723	1
MSTRG.10121.4	204	0.36318	5.493225098	-1
MSTRG.10122.1	805	0.42044	8.204650879	1
MSTRG.10122.2	763	0.43181	7.542663574	1
MSTRG.10122.3	764	0.46099	7.542663574	1
MSTRG.10122.4	569	0.3852	9.05267334	1
MSTRG.10123.1	221	0.28266	9.886291504	1
MSTRG.10122.5	624	0.41704	9.29864502	1
MSTRG.10122.6	342	0.39669	8.253967285	1
MSTRG.10122.7	570	0.41668	9.05267334	1
MSTRG.10122.8	605	0.44498	8.428894043	1
MSTRG.10133.9	56	0.28165	9.301940918	1
MSTRG.10133.3	884	0.49546	4.785827637	1
MSTRG.10133.6	916	0.41374	4.86138916	1
MSTRG.10133.5	906	0.41074	4.793151855	1
MSTRG.10133.11	916	0.48022	4.806213379	1
MSTRG.10133.12	884	0.43684	4.785827637	1
MSTRG.10112.2	51	0.36288	4.489318848	1
MSTRG.10112.3	1137	0.46328	4.515441895	1
MSTRG.10112.4	1222	0.45645	4.507995605	1
MSTRG.10112.5	1295	0.4611	4.530212402	1
MSTRG.10112.10	972	0.46238	4.625061035	1
MSTRG.10112.11	868	0.4611	4.545837402	1
MSTRG.10112.12	899	0.47628	4.593933105	1
MSTRG.10115.4	456	0.38854	5.774230957	1
MSTRG.10115.3	456	0.35092	5.774230957	1

MSTRG.10115.5	159	0.37746	6.897766113	1
MSTRG.10116.2	392	0.2899	8.452209473	1
MSTRG.10134.1	115	0.347	6.143005371	1
MSTRG.10138.3	12	0.37987	7.976013184	1
MSTRG.10138.2	906	0.46266	5.949279785	1
MSTRG.10139.1	107	0.30328	5.424377441	1
MSTRG.10138.7	588	0.48898	5.411560059	1
MSTRG.10142.1	52	0.33607	8.841491699	1
MSTRG.10146.6	506	0.43249	5.737609863	1
MSTRG.10146.5	462	0.47095	5.521057129	1
MSTRG.10147.1	125	0.36125	10.3706665	1
MSTRG.10148.1	849	0.47677	5.867736816	1
MSTRG.10148.2	843	0.47437	5.420837402	1
MSTRG.10148.3	897	0.47842	5.710876465	1
MSTRG.10148.7	897	0.47842	5.710876465	1
MSTRG.10148.9	897	0.47929	5.710876465	1
MSTRG.10148.8	897	0.45323	5.710876465	1
MSTRG.10148.6	849	0.35371	5.867736816	1
MSTRG.10148.4	897	0.44548	5.710876465	1
MSTRG.10143.2	185	0.46906	5.190979004	1
MSTRG.10143.3	205	0.46643	7.747253418	1
MSTRG.10155.1	83	0.36721	11.65460205	1
MSTRG.10159.1	780	0.40631	5.089660645	1
MSTRG.10160.1	645	0.40655	9.520690918	1
MSTRG.10162.1	32	0.26876	11.4196167	1
MSTRG.10172.2	116	0.39342	4.329528809	1
MSTRG.10172.5	322	0.37494	6.066345215	1
MSTRG.10172.6	25	0.34133	8.119934082	1
MSTRG.10173.1	78	0.29368	9.99395752	1
MSTRG.10173.2	78	0.30016	9.99395752	1
MSTRG.10172.8	103	0.36126	4.232849121	1
MSTRG.10172.9	103	0.33724	4.232849121	1
MSTRG.10172.10	103	0.36117	4.232849121	1
MSTRG.10172.11	104	0.3619	4.595031738	1
MSTRG.10175.1	122	0.3217	6.324768066	1
MSTRG.10175.2	189	0.27987	7.760314941	1
MSTRG.10175.3	174	0.35317	6.445617676	1
MSTRG.10175.4	122	0.46914	6.324768066	1
MSTRG.10175.5	122	0.45073	6.324768066	1
MSTRG.10178.6	2304	0.43531	5.62322998	1
MSTRG.10178.15	1842	0.47176	6.821228027	1
MSTRG.10178.7	2593	0.44247	5.485290527	1
MSTRG.10178.8	2380	0.44247	5.704162598	1
MSTRG.10178.12	1596	0.46201	8.469177246	1
MSTRG.10178.11	1655	0.46201	8.519348145	1
MSTRG.10178.9	2616	0.44247	5.54876709	1
MSTRG.10178.13	1655	0.47176	8.519348145	1
MSTRG.10178.1	2633	0.461	5.534851074	1
MSTRG.10178.14	2635	0.44714	5.534240723	1
MSTRG.10178.16	2616	0.45333	5.54876709	1
MSTRG.10178.3	2652	0.45333	5.520568848	1
MSTRG.10178.10	2633	0.46585	5.534851074	1
MSTRG.10178.5	1655	0.43368	8.519348145	1
MSTRG.10178.17	2446	0.47768	5.792663574	1

MSTRG.10178.18	2411	0.46694	5.573791504	1
MSTRG.10178.19	1733	0.47111	6.734191895	1
MSTRG.10178.20	1775	0.46959	6.891540527	1
MSTRG.10178.21	1792	0.42581	6.818054199	1
MSTRG.10178.22	1520	0.46105	8.234924316	1
MSTRG.10178.24	1792	0.44806	6.818054199	1
MSTRG.10178.23	1792	0.44699	6.818054199	1
MSTRG.10180.2	263	0.36374	7.019958496	1
MSTRG.10164.2	163	0.46333	5.137145996	1
MSTRG.10164.1	493	0.48436	5.60760498	1
MSTRG.10164.3	163	0.45499	5.137145996	1
MSTRG.10183.4	123	0.34639	8.32232666	1
MSTRG.10184.1	187	0.30151	10.293396	1
MSTRG.10185.1	59	0.34615	11.52838135	1
MSTRG.10195.1	109	0.4355	12.07061768	1
MSTRG.10198.1	777	0.45205	5.08795166	1
MSTRG.10197.5	59	0.40914	4.484436035	1
MSTRG.10197.6	153	0.36735	7.738830566	1
MSTRG.10208.1	115	0.26101	7.65411377	1
MSTRG.10209.1	139	0.40813	5.565246582	1
MSTRG.10197.7	84	0.43189	6.814147949	1
MSTRG.10197.8	46	0.37995	11.10540771	1
MSTRG.10197.9	212	0.39715	12.02020264	1
MSTRG.10213.1	352	0.42647	11.28619385	1
MSTRG.10214.1	63	0.38659	11.24835205	1
MSTRG.10215.1	51	0.39868	8.473083496	1
MSTRG.10230.1	78	0.35654	6.375549316	1
MSTRG.10231.1	931	0.37456	4.769470215	1
MSTRG.10231.2	932	0.46951	4.777770996	1
MSTRG.10232.1	5	0.34632	5.274963379	1
MSTRG.10234.1	88	0.3596	9.298278809	1
MSTRG.10234.2	52	0.41162	6.707580566	1
MSTRG.10231.4	1047	0.46581	5.431091309	1
MSTRG.10234.3	35	0.3751	6.52545166	1
MSTRG.10235.1	239	0.42422	9.620300293	1
MSTRG.10231.9	932	0.45699	4.702819824	1
MSTRG.10236.1	98	0.39689	8.591491699	1
MSTRG.10231.12	931	0.46901	4.915588379	1
MSTRG.10231.16	816	0.44423	4.59552002	1
MSTRG.10231.15	932	0.36634	4.74041748	1
MSTRG.10231.18	854	0.42151	4.61907959	1
MSTRG.10231.19	269	0.35517	4.556335449	-1
MSTRG.10237.1	67	0.31454	6.029602051	1
MSTRG.10238.1	183	0.28562	10.33294678	1
MSTRG.10239.3	1210	0.45496	5.251159668	1
MSTRG.10239.4	1295	0.44535	5.34954834	1
MSTRG.10222.1	189	0.28519	9.207946777	1
MSTRG.10219.1	22	0.42718	11.52288818	1
MSTRG.10219.2	22	0.38434	11.52288818	1
MSTRG.10219.3	22	0.44668	11.52288818	1
MSTRG.10243.7	474	0.32485	4.744934082	1
MSTRG.10243.8	527	0.28267	5.133483887	1
MSTRG.10255.1	55	0.37927	6.696228027	1
MSTRG.10256.3	386	0.35443	5.972351074	1

MSTRG.10259.1	87	0.28338	9.506530762	1
MSTRG.10260.1	205	0.28419	10.1651001	1
MSTRG.10261.1	81	0.32575	9.836975098	1
MSTRG.10266.2	648	0.3824	6.384460449	1
MSTRG.10268.1	63	0.32548	9.59942627	1
MSTRG.10270.1	44	0.40604	6.807189941	-1
MSTRG.10273.2	342	0.44709	10.23297119	1
MSTRG.10273.4	210	0.4448	10.21478271	1
MSTRG.10273.3	210	0.43188	10.21478271	1
MSTRG.10273.5	210	0.42845	10.21478271	1
MSTRG.10275.1	49	0.39028	8.309143066	1
MSTRG.10278.2	1058	0.40513	9.243469238	1
MSTRG.10281.7	973	0.42072	8.253356934	1
MSTRG.10281.9	1011	0.46116	8.880432129	1
MSTRG.10281.6	676	0.45207	5.434020996	1
MSTRG.10281.4	1013	0.45207	8.000427246	1
MSTRG.10281.5	1079	0.45207	8.471130371	1
MSTRG.10281.8	1034	0.45207	8.36541748	1
MSTRG.10282.2	288	0.41191	6.071838379	1
MSTRG.10282.3	432	0.42786	6.149963379	1
MSTRG.10282.4	431	0.43644	6.415100098	1
MSTRG.10284.1	684	0.37743	5.979309082	1
MSTRG.10284.4	534	0.45946	5.849060059	1
MSTRG.10290.1	87	0.39307	6.538879395	-1
MSTRG.10290.2	79	0.3787	6.513122559	1
MSTRG.10302.4	1113	0.46739	5.831481934	1
MSTRG.10305.1	1249	0.33006	5.977111816	1
MSTRG.10305.2	78	0.3	8.737609863	1
MSTRG.10306.1	46	0.3923	5.987487793	1
MSTRG.10319.2	734	0.35371	8.768981934	1
MSTRG.10322.2	118	0.31236	8.49407959	1
MSTRG.10322.1	100	0.33699	9.60357666	1
MSTRG.10317.1	76	0.46447	8.06451416	-1
MSTRG.10326.3	190	0.47259	7.64276123	1
MSTRG.10330.3	151	0.44466	10.07476807	1
MSTRG.10331.1	882	0.47061	8.093200684	1
MSTRG.10331.2	882	0.46546	8.093200684	1
MSTRG.10329.3	681	0.29514	9.218688965	1
MSTRG.10343.1	501	0.30862	5.815002441	1
MSTRG.10344.1	104	0.32665	9.766662598	1
MSTRG.10343.4	403	0.37021	7.920837402	1
MSTRG.10343.5	501	0.28432	5.815002441	1
MSTRG.10335.1	488	0.46588	7.656799316	1
MSTRG.10335.3	488	0.48069	7.656799316	1
MSTRG.10336.2	667	0.47133	5.700866699	1
MSTRG.10337.1	63	0.41735	8.886047363	1
MSTRG.10341.2	387	0.46374	8.282043457	1
MSTRG.10341.3	249	0.42285	6.80682373	1
MSTRG.10342.1	70	0.41048	9.161560059	1
MSTRG.10350.1	438	0.3752	7.968200684	1
MSTRG.10351.1	1305	0.47281	5.783508301	1
MSTRG.10351.2	926	0.47281	5.895935059	1
MSTRG.10351.3	2843	0.47609	5.037658691	1
MSTRG.10356.3	372	0.49456	4.928405762	1

MSTRG.10356.4	431	0.46058	5.217346191	1
MSTRG.10362.2	127	0.44119	9.434265137	1
MSTRG.10366.2	500	0.45838	4.784851074	1
MSTRG.10370.2	366	0.42916	8.525817871	1
MSTRG.10370.3	206	0.30193	8.104675293	1
MSTRG.10373.3	921	0.3288	8.854553223	1
MSTRG.10373.2	1115	0.33973	8.710266113	1
MSTRG.10373.5	324	0.28338	9.145446777	-1
MSTRG.10374.1	92	0.36609	9.055480957	1
MSTRG.10375.1	126	0.45202	9.440856934	1
MSTRG.10377.1	20	0.36723	3.998840332	1
MSTRG.10379.1	31	0.37205	10.43267822	1
MSTRG.10372.1	115	0.28415	6.631896973	1
MSTRG.10372.2	105	0.28415	6.69708252	1
MSTRG.10437.2	289	0.28923	9.139099121	1
MSTRG.10437.3	289	0.28518	9.139099121	1
MSTRG.10437.4	88	0.32477	9.617004395	1
MSTRG.10437.5	193	0.30132	9.492492676	1
MSTRG.10437.7	103	0.32386	7.669006348	1
MSTRG.10437.6	167	0.2985	9.671203613	1
MSTRG.10437.8	289	0.28982	9.139099121	1
MSTRG.10437.9	289	0.28923	9.139099121	1
MSTRG.10437.10	167	0.2951	9.671203613	1
MSTRG.10437.11	215	0.30467	8.953674316	1
MSTRG.10437.12	240	0.28982	9.244445801	1
MSTRG.10437.13	188	0.28982	9.249450684	1
MSTRG.10437.14	289	0.28982	9.139099121	1
MSTRG.10437.15	191	0.29146	9.492492676	1
MSTRG.10437.16	236	0.28982	10.04742432	1
MSTRG.10437.17	91	0.29543	9.011901855	-1
MSTRG.10439.1	85	0.35123	9.070617676	-1
MSTRG.10437.18	301	0.3	9.395812988	1
MSTRG.10440.1	107	0.2731	5.328186035	1
MSTRG.10437.19	331	0.30804	8.674865723	1
MSTRG.10437.20	79	0.2921	9.694519043	1
MSTRG.10441.1	324	0.3	6.762145996	1
MSTRG.10442.1	280	0.29343	9.736755371	1
MSTRG.10443.1	84	0.30387	8.206115723	1
MSTRG.10445.1	60	0.37021	8.589538574	1
MSTRG.10437.21	110	0.3782	6.967712402	-1
MSTRG.10447.1	122	0.32741	10.21307373	1
MSTRG.10437.22	148	0.31444	10.38092041	1
MSTRG.10381.1	66	0.36618	5.037902832	1
MSTRG.10381.3	66	0.43303	5.037902832	1
MSTRG.10381.4	86	0.43372	4.781066895	1
MSTRG.10381.5	86	0.41968	4.781066895	1
MSTRG.10381.6	86	0.36212	4.781066895	1
MSTRG.10386.1	93	0.35575	7.894592285	-1
MSTRG.10391.1	47	0.31029	4.957702637	1
MSTRG.10383.1	117	0.36327	4.409240723	1
MSTRG.10392.1	153	0.45176	9.707092285	1
MSTRG.10399.2	355	0.47105	9.274719238	1
MSTRG.10399.3	290	0.45022	10.22418213	1
MSTRG.10400.1	136	0.2765	9.880065918	1

MSTRG.10401.3	173	0.36523	5.199035645	1
MSTRG.10410.2	51	0.40453	9.655212402	1
MSTRG.10419.2	294	0.34467	9.426696777	1
MSTRG.10419.3	294	0.31597	9.426696777	1
MSTRG.10419.4	625	0.34707	9.149475098	1
MSTRG.10420.1	143	0.29387	5.780700684	1
MSTRG.10419.6	186	0.28165	9.617492676	1
MSTRG.10407.1	487	0.33045	9.56463623	1
MSTRG.10407.2	280	0.45769	9.531311035	1
MSTRG.10407.3	73	0.30241	6.383239746	1
MSTRG.10409.1	318	0.41946	6.065734863	1
MSTRG.10470.8	223	0.379	9.895690918	1
MSTRG.10470.10	115	0.34209	10.13885498	1
MSTRG.10470.17	132	0.29187	6.938171387	1
MSTRG.10470.18	81	0.33073	9.951721191	1
MSTRG.10470.20	88	0.29502	6.80682373	1
MSTRG.10470.22	407	0.28584	9.125793457	1
MSTRG.10470.23	407	0.28923	9.125793457	1
MSTRG.10470.24	92	0.29387	7.684020996	1
MSTRG.10421.2	127	0.39861	7.717590332	1
MSTRG.10424.2	55	0.37622	6.695617676	1
MSTRG.10424.3	227	0.35272	4.167175293	1
MSTRG.10428.1	94	0.31588	8.970153809	1
MSTRG.10427.3	132	0.34656	8.617980957	1
MSTRG.10430.2	321	0.42117	9.035095215	1
MSTRG.10455.1	586	0.44941	6.66998291	1
MSTRG.10455.2	586	0.43117	6.66998291	1
MSTRG.10455.4	586	0.47205	6.66998291	1
MSTRG.10456.1	202	0.43902	5.696594238	1
MSTRG.10456.3	202	0.4651	5.696594238	1
MSTRG.10456.5	202	0.4651	5.696594238	1
MSTRG.10456.6	154	0.45124	5.084411621	1
MSTRG.10456.7	218	0.41922	5.210998535	1
MSTRG.10456.4	201	0.28883	5.696594238	1
MSTRG.10464.1	1319	0.43201	6.040344238	1
MSTRG.10466.1	47	0.45691	10.19232178	1
MSTRG.10435.2	222	0.31524	8.97064209	1
MSTRG.10488.3	334	0.46725	6.64239502	1
MSTRG.10488.5	334	0.42168	6.64239502	1
MSTRG.10488.2	334	0.45516	6.64239502	1
MSTRG.10488.4	334	0.47983	6.64239502	1
MSTRG.10489.1	131	0.35058	10.18499756	1
MSTRG.10481.1	62	0.38862	9.991882324	-1
MSTRG.10491.1	54	0.39523	10.15570068	1
MSTRG.10507.1	2596	0.42016	6.278869629	1
MSTRG.10508.1	38	0.40313	10.20733643	1
MSTRG.10507.3	867	0.45673	5.54095459	1
MSTRG.10513.4	28	0.38686	10.76312256	1
MSTRG.10516.1	135	0.35736	5.515319824	1
MSTRG.10511.1	224	0.35015	8.232971191	1
MSTRG.10522.3	349	0.45126	6.155090332	1
MSTRG.10523.1	89	0.31603	8.522766113	1
MSTRG.10524.1	30	0.31833	6.476989746	-1
MSTRG.10525.1	125	0.30632	9.72869873	1

MSTRG.10526.3	235	0.40891	4.91192627	1
MSTRG.10526.5	234	0.35883	5.063659668	1
MSTRG.10539.3	49	0.28191	8.421081543	1
MSTRG.10539.4	100	0.28643	8.541809082	1
MSTRG.10542.1	48	0.32052	4.670471191	1
MSTRG.10542.2	107	0.34526	5.359680176	1
MSTRG.10543.1	41	0.36901	8.536071777	1
MSTRG.10573.1	63	0.36783	11.39508057	1
MSTRG.10577.1	44	0.36608	8.328552246	-1
MSTRG.10582.1	51	0.26931	7.769470215	1
MSTRG.10583.1	111	0.32513	9.995788574	1
MSTRG.10584.1	47	0.35163	8.738220215	1
MSTRG.10590.2	134	0.45537	5.596496582	1
MSTRG.10590.3	166	0.27994	10.42144775	1
MSTRG.10595.3	465	0.33994	8.780578613	1
MSTRG.10595.4	433	0.33994	8.687805176	1
MSTRG.10595.5	433	0.33035	8.687805176	1
MSTRG.10595.6	433	0.31965	8.687805176	1
MSTRG.10595.7	465	0.36072	8.780578613	1
MSTRG.10604.1	54	0.3724	6.356506348	1
MSTRG.10633.2	370	0.40125	5.961120605	1
MSTRG.10634.1	34	0.28213	5.622375488	1
MSTRG.10635.1	78	0.29166	8.679260254	1
MSTRG.10633.3	141	0.28035	8.582580566	1
MSTRG.10633.4	324	0.3261	5.463684082	1
MSTRG.10633.5	185	0.31553	10.03521729	1
MSTRG.10636.1	48	0.42023	6.955383301	1
MSTRG.10640.1	144	0.25609	10.03643799	1
MSTRG.10641.1	114	0.31044	6.999084473	1
MSTRG.10639.2	898	0.39821	6.789978027	1
MSTRG.10642.2	917	0.46865	8.908630371	1
MSTRG.10643.1	111	0.37149	6.808654785	1
MSTRG.10642.3	253	0.42005	9.467956543	1
MSTRG.10623.2	273	0.43699	6.223449707	1
MSTRG.10623.3	273	0.46063	6.223449707	1
MSTRG.10626.2	1563	0.47052	7.09161377	1
MSTRG.10629.2	225	0.4116	9.336120605	1
MSTRG.10629.3	225	0.44385	9.336120605	1
MSTRG.10646.1	87	0.39723	7.915466309	1
MSTRG.10646.2	347	0.45336	5.438659668	1
MSTRG.10652.1	814	0.45256	5.244445801	1
MSTRG.10653.1	170	0.35502	12.03594971	1
MSTRG.10652.5	304	0.43093	4.760192871	1
MSTRG.10654.1	935	0.4119	5.731872559	1
MSTRG.10659.1	104	0.33644	10.45159912	1
MSTRG.10659.2	87	0.45033	9.381286621	1
MSTRG.10665.1	448	0.33225	7.539367676	1
MSTRG.10667.4	1610	0.37265	7.483093262	1
MSTRG.10667.5	1710	0.45915	6.710754395	1
MSTRG.10668.1	94	0.30104	6.50970459	1
MSTRG.10668.2	94	0.30894	6.50970459	1
MSTRG.10680.1	47	0.42826	10.21820068	1
MSTRG.10680.2	47	0.31981	10.21820068	1
MSTRG.10680.3	82	0.35469	9.069274902	1

MSTRG.10680.4	159	0.29343	9.595153809	1
MSTRG.10685.1	42	0.34971	5.934875488	1
MSTRG.10688.1	226	0.42646	9.76385498	1
MSTRG.10700.2	106	0.35773	9.16986084	1
MSTRG.10700.3	729	0.46562	8.840759277	1
MSTRG.10700.4	515	0.34235	8.743469238	1
MSTRG.10701.1	190	0.33291	9.207702637	1
MSTRG.10700.5	277	0.33598	9.606140137	1
MSTRG.10703.1	904	0.41815	6.050109863	1
MSTRG.10703.13	798	0.45162	6.067687988	1
MSTRG.10713.1	521	0.45211	7.336975098	1
MSTRG.10713.5	374	0.44279	5.840881348	1
MSTRG.10713.4	521	0.47275	7.336975098	1
MSTRG.10713.6	374	0.40896	5.840881348	1
MSTRG.10715.1	84	0.35299	11.38140869	1
MSTRG.10707.1	55	0.34725	10.58660889	1
MSTRG.10712.2	76	0.27502	8.857727051	1
MSTRG.10718.1	238	0.37908	6.241760254	1
MSTRG.10725.1	81	0.29367	9.894226074	1
MSTRG.10725.2	81	0.28467	9.894226074	1
MSTRG.10761.1	33	0.31342	9.694152832	1
MSTRG.10764.1	2	0.30533	5.274963379	1
MSTRG.10766.1	148	0.35615	8.390686035	1
MSTRG.10766.2	148	0.35615	8.390686035	1
MSTRG.10767.1	34	0.46093	10.95318604	-1
MSTRG.10768.1	48	0.36695	7.709411621	1
MSTRG.10769.1	106	0.28267	9.147521973	1
MSTRG.10765.1	184	0.32638	9.20526123	1
MSTRG.10765.2	78	0.33929	9.547546387	1
MSTRG.10772.1	33	0.33301	11.82440186	1
MSTRG.10775.1	29	0.35853	9.496643066	1
MSTRG.10776.1	71	0.35422	4.973327637	1
MSTRG.10776.2	94	0.28199	8.354431152	1
MSTRG.10777.1	67	0.28027	9.487121582	1
MSTRG.10774.2	50	0.32473	9.88458252	1
MSTRG.10778.1	330	0.36597	8.813293457	-1
MSTRG.10774.3	196	0.34658	8.021057129	1
MSTRG.10774.4	479	0.2886	9.367614746	1
MSTRG.10774.5	422	0.33781	8.427185059	1
MSTRG.10779.1	113	0.34079	8.733337402	1
MSTRG.10774.6	110	0.29319	9.032775879	1
MSTRG.10780.1	104	0.3372	9.136535645	1
MSTRG.10782.1	44	0.386	8.633850098	1
MSTRG.10786.2	452	0.46548	5.705993652	1
MSTRG.10786.4	455	0.3404	8.500305176	1
MSTRG.10787.1	254	0.28982	8.520568848	1
MSTRG.10789.1	152	0.32453	9.363830566	1
MSTRG.10790.1	80	0.29776	9.020812988	1
MSTRG.10791.1	111	0.30191	8.635681152	1
MSTRG.10792.1	76	0.31873	9.333679199	1
MSTRG.10793.3	709	0.45808	4.297424316	1
MSTRG.10793.2	709	0.3961	4.297424316	1
MSTRG.10794.1	97	0.36792	9.846862793	1
MSTRG.10794.2	68	0.36836	8.019104004	1

MSTRG.10795.4	1096	0.33518	7.084777832	1
MSTRG.10795.5	1096	0.34103	7.084777832	1
MSTRG.10796.1	19	0.33786	7.999816895	1
MSTRG.10806.4	544	0.40569	7.971740723	1
MSTRG.10807.1	128	0.30956	8.270446777	1
MSTRG.10808.2	346	0.36932	9.183532715	1
MSTRG.10808.5	161	0.30581	9.848571777	1
MSTRG.10808.6	205	0.28982	6.142883301	1
MSTRG.10808.7	381	0.36932	7.734558105	1
MSTRG.10809.1	61	0.33779	5.285827637	1
MSTRG.10811.1	41	0.33294	6.04107666	-1
MSTRG.10812.1	95	0.44589	10.67352295	-1
MSTRG.10820.1	266	0.39195	4.750183105	1
MSTRG.10825.1	208	0.25541	5.607116699	1
MSTRG.10830.1	118	0.38464	9.726623535	1
MSTRG.10831.1	86	0.30492	7.785827637	1
MSTRG.10829.4	208	0.29539	4.467590332	1
MSTRG.10829.5	185	0.32743	10.29071045	1
MSTRG.10837.1	636	0.47042	7.922790527	1
MSTRG.10837.2	493	0.4529	6.058654785	1
MSTRG.10837.4	301	0.3896	6.549987793	1
MSTRG.10838.1	48	0.31996	9.69708252	1
MSTRG.10840.2	840	0.4323	8.957092285	1
MSTRG.10840.3	132	0.2785	7.54876709	-1
MSTRG.10843.5	176	0.29607	5.696594238	1
MSTRG.10844.2	343	0.38439	9.348083496	1
MSTRG.10844.1	342	0.32266	8.940979004	1
MSTRG.10845.1	153	0.32253	5.879089355	1
MSTRG.10847.1	109	0.38802	11.00006104	1
MSTRG.10848.2	351	0.40497	6.386535645	1
MSTRG.10848.3	390	0.39053	6.154724121	1
MSTRG.10848.4	324	0.44677	6.380554199	1
MSTRG.10851.3	400	0.37206	4.979309082	1
MSTRG.10852.1	32	0.43641	4.136901855	1
MSTRG.10853.1	67	0.42892	9.871765137	1
MSTRG.10857.1	41	0.36926	4.749206543	1
MSTRG.10867.3	383	0.32149	9.105163574	1
MSTRG.10876.7	360	0.42545	5.143249512	1
MSTRG.10876.8	360	0.42917	5.143249512	1
MSTRG.10876.6	401	0.46145	6.234680176	1
MSTRG.10880.3	726	0.4303	5.808898926	1
MSTRG.10882.1	130	0.34554	9.794494629	1
MSTRG.10882.2	755	0.42555	8.237243652	1
MSTRG.10882.4	772	0.40301	8.338806152	1
MSTRG.10882.5	684	0.46855	6.275939941	1
MSTRG.10882.6	701	0.43365	8.117492676	1
MSTRG.10882.7	755	0.41359	8.285827637	1
MSTRG.10882.8	54	0.2908	8.581604004	1
MSTRG.10888.2	268	0.43754	5.98626709	1
MSTRG.10888.4	421	0.47046	8.309875488	1
MSTRG.10889.1	107	0.28842	10.01641846	1
MSTRG.10889.2	131	0.39365	9.232727051	1
MSTRG.10890.1	71	0.4054	12.07952881	1
MSTRG.10889.5	172	0.27911	8.663024902	1

MSTRG.10889.7	168	0.28338	6.890197754	1
MSTRG.10889.8	241	0.2785	7.815612793	1
MSTRG.10889.9	233	0.36231	9.642150879	1
MSTRG.10899.1	250	0.31099	6.191833496	1
MSTRG.10901.1	150	0.30092	8.183776855	1
MSTRG.10902.1	123	0.29919	7.855773926	1
MSTRG.10903.1	145	0.3	8.033508301	1
MSTRG.10896.2	195	0.42773	8.069152832	1
MSTRG.10896.3	250	0.40797	8.688659668	1
MSTRG.10906.1	109	0.32799	8.769958496	1
MSTRG.10907.2	309	0.4684	8.928161621	1
MSTRG.10907.3	157	0.43219	9.264953613	1
MSTRG.10910.1	31	0.42051	8.842834473	1
MSTRG.10922.3	1556	0.42178	5.410705566	1
MSTRG.10922.5	1091	0.38135	5.172424316	1
MSTRG.10923.1	45	0.34504	6.704406738	1
MSTRG.10925.1	419	0.31285	5.805114746	1
MSTRG.10925.9	406	0.31361	5.846008301	1
MSTRG.10928.1	95	0.38211	7.618103027	1
MSTRG.10913.1	82	0.36956	10.26470947	1
MSTRG.10919.1	518	0.32294	8.854919434	1
MSTRG.10921.1	105	0.40877	9.056945801	1
MSTRG.10935.1	533	0.36655	8.786071777	1
MSTRG.10935.2	547	0.39249	8.98260498	1
MSTRG.10936.2	468	0.39819	7.179504395	1
MSTRG.10941.2	591	0.39077	9.453186035	1
MSTRG.10942.2	96	0.28584	5.077331543	1
MSTRG.10943.1	37	0.27911	10.33221436	1
MSTRG.10944.1	606	0.35792	5.963562012	1
MSTRG.10950.3	970	0.43245	9.54486084	1
MSTRG.10950.4	1037	0.48149	9.691223145	1
MSTRG.10956.1	69	0.33846	6.528137207	-1
MSTRG.10958.1	54	0.39022	9.851989746	1
MSTRG.10961.1	51	0.38995	9.895935059	1
MSTRG.10971.1	54	0.37929	6.800231934	-1
MSTRG.10976.1	50	0.4024	5.285705566	1
MSTRG.10976.2	47	0.38389	8.977111816	1
MSTRG.10976.3	45	0.39953	10.21563721	-1
MSTRG.10984.1	544	0.45309	5.539245605	1
MSTRG.10984.3	77	0.28682	4.394348145	1
MSTRG.10990.4	136	0.28584	8.88067627	1
MSTRG.10990.3	124	0.29772	8.765441895	1
MSTRG.10990.5	81	0.27794	6.513122559	1
MSTRG.11012.1	346	0.31849	9.158996582	1
MSTRG.11012.2	347	0.34159	9.158996582	1
MSTRG.11039.1	96	0.28982	9.216125488	1
MSTRG.11040.1	114	0.31985	10.84088135	1
MSTRG.11041.1	145	0.28394	9.274597168	1
MSTRG.11042.1	210	0.30957	9.637878418	1
MSTRG.11043.1	184	0.28068	10.39141846	1
MSTRG.11044.1	147	0.35814	5.088195801	1
MSTRG.11045.1	179	0.35266	10.01483154	1
MSTRG.11046.1	62	0.3768	9.486755371	1
MSTRG.11036.2	388	0.38439	5.77911377	1

MSTRG.11036.1	617	0.49086	5.918762207	1
MSTRG.11037.1	23	0.41658	9.387512207	1
MSTRG.11050.1	68	0.31251	10.72589111	1
MSTRG.11050.2	29	0.43719	5.380187988	1
MSTRG.11055.1	102	0.42713	9.074401855	1
MSTRG.11057.1	54	0.439	9.102355957	-1
MSTRG.11060.1	344	0.45909	7.767272949	-1
MSTRG.11071.2	1949	0.46888	6.069030762	1
MSTRG.11071.3	1587	0.46888	6.326965332	1
MSTRG.11071.4	1587	0.46888	6.326965332	1
MSTRG.11071.5	1009	0.35996	5.455871582	1
MSTRG.11072.1	113	0.28178	11.52728271	1
MSTRG.11073.2	58	0.38517	5.303649902	1
MSTRG.11063.1	447	0.46785	5.85333252	1
MSTRG.11063.3	447	0.4574	5.85333252	1
MSTRG.11076.2	319	0.36493	8.861877441	1
MSTRG.11100.1	127	0.29387	9.459411621	1
MSTRG.11101.1	120	0.29374	8.864807129	1
MSTRG.11090.1	108	0.34443	12.44207764	1
MSTRG.11091.5	530	0.4652	9.14630127	1
MSTRG.11091.6	530	0.4776	9.14630127	1
MSTRG.11096.2	355	0.44514	5.293273926	1
MSTRG.11098.1	638	0.3413	5.934020996	1
MSTRG.11098.2	411	0.44497	5.557922363	1
MSTRG.11103.2	265	0.34657	8.898010254	1
MSTRG.11103.3	111	0.28922	7.728820801	1
MSTRG.11109.1	51	0.37142	8.032653809	1
MSTRG.11154.2	1891	0.43772	6.275085449	1
MSTRG.11156.1	99	0.40618	11.40924072	-1
MSTRG.11158.1	91	0.28923	10.07830811	1
MSTRG.11161.1	109	0.38723	9.605651855	1
MSTRG.11161.4	69	0.39129	8.840759277	1
MSTRG.11170.1	174	0.27967	5.826599121	1
MSTRG.11171.1	61	0.32254	7.835876465	1
MSTRG.11169.3	316	0.37218	6.199768066	1
MSTRG.11177.1	914	0.46317	6.214782715	1
MSTRG.11163.2	58	0.30639	6.514831543	1
MSTRG.11185.1	2103	0.46944	9.234191895	1
MSTRG.11186.1	89	0.28971	10.42486572	1
MSTRG.11188.3	680	0.35595	7.084777832	1
MSTRG.11190.3	1423	0.40827	6.05255127	1
MSTRG.11191.1	272	0.33002	8.702941895	1
MSTRG.11194.2	101	0.42449	9.320861816	1
MSTRG.11199.1	130	0.28751	6.563049316	1
MSTRG.11194.4	155	0.39044	9.754333496	1
MSTRG.11230.1	57	0.31637	4.828430176	1
MSTRG.11235.1	337	0.47026	5.336120605	1
MSTRG.11235.2	308	0.32181	5.575378418	1
MSTRG.11243.1	458	0.29367	7.040588379	1
MSTRG.11243.3	777	0.31806	8.035095215	1
MSTRG.11243.6	44	0.40413	5.11932373	1
MSTRG.11243.8	777	0.37133	8.035095215	1
MSTRG.11219.1	120	0.33666	7.003967285	1
MSTRG.11226.1	736	0.38067	4.726623535	1

MSTRG.11226.3	674	0.41512	4.774963379	1
MSTRG.11221.1	38	0.32015	11.71136475	1
MSTRG.11221.2	40	0.45289	4.41229248	1
MSTRG.11248.3	1572	0.46515	6.68951416	1
MSTRG.11248.9	1478	0.4624	6.866516113	1
MSTRG.11248.8	1496	0.48139	6.77923584	1
MSTRG.11248.6	1496	0.47238	6.77923584	1
MSTRG.11248.7	1554	0.46515	6.756408691	1
MSTRG.11249.1	107	0.28923	7.704040527	1
MSTRG.11257.1	26	0.44079	6.492248535	1
MSTRG.11256.1	26	0.47946	10.4105835	1
MSTRG.11279.1	41	0.34996	6.223937988	-1
MSTRG.11283.1	398	0.47847	4.719909668	1
MSTRG.11283.2	398	0.49116	4.719909668	1
MSTRG.11284.1	104	0.34942	9.297302246	1
MSTRG.11292.1	931	0.44001	6.009094238	1
MSTRG.11293.1	62	0.2921	9.246032715	1
MSTRG.11294.2	429	0.3288	4.491760254	1
MSTRG.11294.3	98	0.27327	9.910827637	1
MSTRG.11294.4	275	0.33716	5.417907715	1
MSTRG.11300.3	270	0.41631	5.952697754	1
MSTRG.11301.2	134	0.2908	9.72869873	1
MSTRG.11301.1	125	0.31236	8.764343262	1
MSTRG.11301.3	44	0.33291	4.295837402	1
MSTRG.11301.4	134	0.30564	9.72869873	1
MSTRG.11301.5	84	0.31381	9.738830566	1
MSTRG.11302.1	134	0.31215	9.851379395	1
MSTRG.11302.2	165	0.31337	9.660217285	-1
MSTRG.11303.1	26	0.33538	4.522399902	1
MSTRG.11304.2	263	0.36221	5.48651123	1
MSTRG.11304.3	254	0.35845	5.59197998	1
MSTRG.11373.3	882	0.47052	8.968444824	1
MSTRG.11373.4	121	0.29178	9.368835449	1
MSTRG.11373.8	97	0.30266	9.144348145	1
MSTRG.11377.1	1172	0.37058	6.613586426	1
MSTRG.11377.2	1197	0.45526	6.540588379	1
MSTRG.11378.1	39	0.30297	6.491027832	1
MSTRG.11377.3	1566	0.46224	6.522033691	1
MSTRG.11377.4	1292	0.44174	6.079772949	1
MSTRG.11377.5	1197	0.4302	6.540588379	1
MSTRG.11377.7	1325	0.47081	6.371887207	-1
MSTRG.11377.6	1362	0.46697	6.372619629	-1
MSTRG.11377.8	370	0.29367	9.362609863	1
MSTRG.11377.9	290	0.28719	9.265075684	1
MSTRG.11377.10	92	0.429	5.77532959	1
MSTRG.11379.1	64	0.32225	10.94000244	1
MSTRG.11377.11	202	0.2895	10.08111572	1
MSTRG.11381.1	109	0.2831	6.716003418	1
MSTRG.11382.1	65	0.32462	9.570861816	1
MSTRG.11377.18	279	0.35631	9.510803223	1
MSTRG.11377.19	279	0.28982	9.510803223	1
MSTRG.11377.20	279	0.30576	9.510803223	1
MSTRG.11377.21	279	0.36624	9.510803223	1
MSTRG.11377.22	35	0.3036	7.957336426	1

MSTRG.11377.23	144	0.27289	6.946838379	1
MSTRG.11377.24	261	0.2921	9.149719238	1
MSTRG.11377.25	279	0.29367	9.510803223	1
MSTRG.11377.26	84	0.34523	9.820007324	1
MSTRG.11377.27	155	0.31273	10.35736084	1
MSTRG.11309.1	294	0.35677	6.090881348	1
MSTRG.11307.2	116	0.32422	10.16424561	1
MSTRG.11313.1	133	0.26797	8.860168457	1
MSTRG.11313.2	211	0.28165	8.864074707	1
MSTRG.11313.3	213	0.30981	8.864074707	1
MSTRG.11313.4	213	0.32906	8.864074707	1
MSTRG.11319.1	68	0.39965	8.95489502	1
MSTRG.11319.2	68	0.33261	10.13275146	1
MSTRG.11321.1	870	0.39001	8.658630371	1
MSTRG.11326.1	80	0.42429	6.713439941	1
MSTRG.11328.2	418	0.43253	8.941101074	1
MSTRG.11328.4	161	0.27661	9.204772949	1
MSTRG.11331.1	199	0.3892	5.007019043	1
MSTRG.11335.1	43	0.44009	9.840026855	1
MSTRG.11336.2	507	0.33785	7.033996582	1
MSTRG.11336.3	467	0.37265	6.075622559	1
MSTRG.11338.2	87	0.31973	6.878234863	1
MSTRG.11338.3	107	0.33812	11.6317749	1
MSTRG.11350.1	3068	0.39679	5.944152832	1
MSTRG.11350.5	1339	0.45206	5.452697754	1
MSTRG.11350.6	682	0.4305	5.795593262	1
MSTRG.11350.4	2861	0.45096	5.877380371	1
MSTRG.11350.7	1983	0.44615	6.281311035	1
MSTRG.11351.1	196	0.31512	8.584899902	1
MSTRG.11352.1	78	0.34067	10.15606689	1
MSTRG.11350.8	1225	0.42581	6.205505371	1
MSTRG.11357.1	141	0.39012	6.06439209	1
MSTRG.11358.1	150	0.29355	7.788879395	1
MSTRG.11359.1	98	0.29772	8.604919434	1
MSTRG.11360.1	109	0.29772	9.747619629	1
MSTRG.11360.2	68	0.29754	7.762634277	1
MSTRG.11344.2	148	0.32237	9.111755371	1
MSTRG.11345.1	91	0.29022	9.86114502	1
MSTRG.11345.2	189	0.29206	10.3793335	1
MSTRG.11346.1	78	0.38927	8.535949707	1
MSTRG.11347.1	109	0.27433	9.623596191	-1
MSTRG.11348.3	209	0.35655	10.18292236	1
MSTRG.11363.1	48	0.29415	6.216125488	1
MSTRG.11365.1	108	0.29772	7.008850098	1
MSTRG.11366.1	122	0.29003	9.260314941	1
MSTRG.11367.1	130	0.29088	9.382629395	1
MSTRG.11367.2	130	0.29268	9.382629395	1
MSTRG.11400.1	770	0.28338	6.789733887	1
MSTRG.11400.2	719	0.3458	6.520446777	1
MSTRG.11400.3	719	0.36979	6.520446777	1
MSTRG.11400.4	130	0.29772	9.097595215	1
MSTRG.11400.5	287	0.28338	6.594177246	1
MSTRG.11403.1	1306	0.46438	7.68548584	1
MSTRG.11404.1	1348	0.4517	7.208312988	1

MSTRG.11404.2	1526	0.45213	7.011535645	1
MSTRG.11408.1	1332	0.42232	7.786315918	1
MSTRG.11410.1	327	0.41516	9.176330566	1
MSTRG.11411.1	133	0.40986	9.516906738	-1
MSTRG.11411.2	72	0.40516	9.836486816	1
MSTRG.11413.3	286	0.30064	5.304870605	1
MSTRG.11413.4	201	0.29277	6.447570801	1
MSTRG.11406.3	348	0.39175	9.123962402	1
MSTRG.11414.1	38	0.27819	6.822814941	1
MSTRG.11417.1	87	0.37301	6.816223145	1
MSTRG.11417.2	91	0.28462	10.02484131	1
MSTRG.11417.3	86	0.31332	9.86541748	-1
MSTRG.11417.4	91	0.33842	10.02484131	1
MSTRG.11418.4	1131	0.44818	8.915222168	1
MSTRG.11427.1	2986	0.47657	4.746643066	1
MSTRG.11427.3	3035	0.47576	4.769714355	1
MSTRG.11427.4	2919	0.47657	4.761535645	1
MSTRG.11429.1	1167	0.44835	7.077941895	1
MSTRG.11430.1	79	0.33768	10.60577393	1
MSTRG.11431.2	1237	0.38518	6.701843262	1
MSTRG.11431.3	1237	0.36208	6.701843262	1
MSTRG.11432.1	548	0.39956	8.49041748	1
MSTRG.11433.1	37	0.39016	11.69903564	1
MSTRG.11436.1	123	0.36531	7.487487793	1
MSTRG.11436.2	103	0.35783	6.005310059	1
MSTRG.11437.3	250	0.43613	5.316589355	1
MSTRG.11438.1	284	0.42882	4.630187988	1
MSTRG.11439.3	2541	0.44677	5.805847168	1
MSTRG.11441.4	351	0.37068	5.28314209	1
MSTRG.11441.2	555	0.39304	5.505065918	1
MSTRG.11441.3	475	0.37254	6.602722168	1
MSTRG.11442.1	56	0.34012	9.680847168	1
MSTRG.11443.4	394	0.40135	5.909729004	1
MSTRG.11443.5	394	0.35378	5.909729004	1
MSTRG.11443.7	394	0.39683	5.909729004	1
MSTRG.11443.8	394	0.37428	5.909729004	1
MSTRG.11443.6	394	0.32812	5.909729004	1
MSTRG.11421.3	64	0.4404	11.78961182	1
MSTRG.11423.1	204	0.35034	7.113464355	1
MSTRG.11424.2	774	0.41622	8.833557129	1
MSTRG.11449.2	75	0.34181	4.51739502	1
MSTRG.11457.1	213	0.31806	4.442321777	1
MSTRG.11457.2	213	0.34184	4.442321777	1
MSTRG.11457.3	163	0.42886	4.340270996	1
MSTRG.11460.1	95	0.35505	6.784362793	1
MSTRG.11462.12	432	0.35516	7.38482666	1
MSTRG.11462.7	464	0.38186	7.393981934	1
MSTRG.11462.8	514	0.38116	7.406311035	1
MSTRG.11462.9	432	0.37166	7.38482666	1
MSTRG.11462.13	432	0.41303	7.38482666	1
MSTRG.11462.14	474	0.35633	7.394958496	1
MSTRG.11462.15	74	0.27558	9.506408691	1
MSTRG.11463.1	53	0.34857	9.694152832	1
MSTRG.11464.1	77	0.29367	6.55267334	1

MSTRG.11465.1	48	0.42299	6.467468262	1
MSTRG.11466.1	650	0.46534	8.789611816	1
MSTRG.11466.2	650	0.35257	8.789611816	1
MSTRG.11472.1	546	0.37678	8.071716309	1
MSTRG.11472.4	253	0.50282	4.364196777	1
MSTRG.11472.2	546	0.39404	8.071716309	1
MSTRG.11472.5	236	0.38176	4.376037598	1
MSTRG.11472.6	104	0.2962	5.83026123	1
MSTRG.11472.7	132	0.25541	8.867248535	1
MSTRG.11472.8	119	0.3136	4.867248535	1
MSTRG.11473.1	108	0.31432	6.955383301	1
MSTRG.11473.2	108	0.28982	6.955383301	1
MSTRG.11473.3	108	0.29615	6.955383301	1
MSTRG.11474.1	75	0.28555	10.13238525	1
MSTRG.11473.4	108	0.29374	6.955383301	1
MSTRG.11472.9	52	0.45468	9.199157715	1
MSTRG.11472.10	257	0.33446	9.895080566	1
MSTRG.11475.1	183	0.35869	10.1494751	1
MSTRG.11469.1	328	0.38339	8.453552246	1
MSTRG.11470.5	712	0.427	5.901306152	1
MSTRG.11476.3	497	0.33431	9.236999512	1
MSTRG.11476.2	907	0.37241	7.935119629	1
MSTRG.11476.4	479	0.33675	9.309753418	1
MSTRG.11476.5	479	0.33059	9.309753418	1
MSTRG.11477.1	1579	0.4639	5.049133301	1
MSTRG.11478.1	92	0.28338	9.917907715	1
MSTRG.11483.2	122	0.42728	8.878601074	1
MSTRG.11489.1	25	0.42591	8.230651855	-1
MSTRG.11490.5	1594	0.43295	5.824401855	1
MSTRG.11490.6	1295	0.49521	5.258361816	1
MSTRG.11496.2	762	0.44512	5.464294434	1
MSTRG.11499.2	287	0.33695	8.286682129	1
MSTRG.11499.4	205	0.34272	7.515808105	1
MSTRG.11492.7	447	0.44382	4.75970459	1
MSTRG.11492.6	447	0.44632	4.75970459	1
MSTRG.11492.9	491	0.46516	4.902282715	1
MSTRG.11492.11	447	0.4683	4.75970459	1
MSTRG.11492.12	491	0.43957	4.902282715	1
MSTRG.11492.5	447	0.4407	4.75970459	1
MSTRG.11492.8	447	0.44415	4.75970459	1
MSTRG.11492.10	447	0.44754	4.75970459	1
MSTRG.11492.4	447	0.44936	4.75970459	1
MSTRG.11505.1	261	0.49144	3.950866699	1
MSTRG.11505.2	182	0.4348	11.89837646	1
MSTRG.11507.1	122	0.31889	9.842712402	1
MSTRG.11507.6	284	0.29728	8.081970215	1
MSTRG.11507.8	167	0.31714	8.947692871	1
MSTRG.11507.3	284	0.36987	8.081970215	1
MSTRG.11507.7	167	0.31714	8.947692871	1
MSTRG.11507.2	284	0.28195	8.081970215	1
MSTRG.11507.4	284	0.32018	8.081970215	1
MSTRG.11507.9	167	0.32018	8.947692871	1
MSTRG.11508.1	525	0.37343	7.987609863	1
MSTRG.11508.4	215	0.28536	6.028137207	1

MSTRG.11508.5	531	0.49547	7.987609863	1
MSTRG.11510.1	94	0.32246	7.799865723	1
MSTRG.11513.1	416	0.4545	9.477600098	1
MSTRG.11514.1	37	0.3396	10.41986084	1
MSTRG.11514.2	42	0.35572	5.601501465	-1
MSTRG.11515.1	177	0.41488	5.95489502	-1
MSTRG.11520.1	1372	0.45825	4.757385254	1
MSTRG.11534.1	417	0.39133	5.106750488	1
MSTRG.11537.1	183	0.32896	10.16326904	1
MSTRG.11539.3	579	0.37251	9.706604004	1
MSTRG.11540.4	150	0.41152	10.04119873	1
MSTRG.11530.2	232	0.37042	9.46697998	1
MSTRG.11551.1	165	0.29096	11.47283936	1
MSTRG.11551.2	165	0.31173	11.47283936	1
MSTRG.11553.1	313	0.4687	6.582092285	1
MSTRG.11549.5	43	0.30706	10.8838501	1
MSTRG.11549.4	113	0.27739	10.91851807	1
MSTRG.11542.1	223	0.30085	8.751159668	1
MSTRG.11545.1	143	0.28085	7.527770996	1
MSTRG.11556.2	109	0.38122	10.10540771	1
MSTRG.11560.1	66	0.40038	9.156188965	1
MSTRG.11568.1	263	0.43938	9.141906738	1
MSTRG.11570.1	63	0.32324	4.196350098	1
MSTRG.11571.1	41	0.43325	6.542663574	1
MSTRG.11584.1	83	0.39939	12.12982178	1
MSTRG.11586.1	190	0.37593	11.08648682	1
MSTRG.11589.2	735	0.39602	5.885803223	1
MSTRG.11590.3	283	0.47127	5.149475098	1
MSTRG.11591.2	969	0.37874	9.149353027	1
MSTRG.11594.3	725	0.33182	9.043884277	1
MSTRG.11595.1	90	0.40194	6.820983887	1
MSTRG.11595.2	49	0.40194	5.366882324	1
MSTRG.11594.4	725	0.35344	9.043884277	1
MSTRG.11606.4	901	0.45683	5.18572998	1
MSTRG.11606.10	915	0.48287	5.121154785	1
MSTRG.11599.1	729	0.46206	6.446350098	1
MSTRG.11599.2	299	0.44096	9.166564941	1
MSTRG.11610.1	62	0.45998	12.14752197	1
MSTRG.11610.2	62	0.47635	12.14752197	1
MSTRG.11611.2	616	0.3606	8.442932129	1
MSTRG.11611.4	608	0.3088	8.442932129	1
MSTRG.11613.1	40	0.37611	3.895446777	1
MSTRG.11615.1	338	0.41273	9.405700684	1
MSTRG.11616.1	26	0.33907	5.222351074	1
MSTRG.11618.2	243	0.32858	9.109924316	1
MSTRG.11617.1	83	0.43829	6.707946777	1
MSTRG.11622.1	106	0.28248	8.810852051	1
MSTRG.11622.2	71	0.37374	5.25177002	-1
MSTRG.11636.2	581	0.43112	7.965393066	1
MSTRG.11643.2	83	0.30894	6.698913574	1
MSTRG.11643.3	447	0.40635	5.819396973	1
MSTRG.11643.4	288	0.39902	6.157775879	1
MSTRG.11645.1	22	0.36227	6.50567627	1
MSTRG.11646.1	219	0.33652	5.515808105	1

MSTRG.11649.4	428	0.38056	8.118591309	1
MSTRG.11656.3	538	0.47676	5.884460449	1
MSTRG.11657.2	260	0.3263	5.664001465	1
MSTRG.11662.1	100	0.34362	6.002502441	1
MSTRG.11663.4	152	0.38163	10.44671631	1
MSTRG.11663.3	115	0.41742	9.061462402	1
MSTRG.11664.2	36	0.3761	7.999572754	1
MSTRG.11666.1	190	0.31588	9.261657715	1
MSTRG.11666.5	185	0.31429	9.140686035	1
MSTRG.11666.3	185	0.35975	9.140686035	1
MSTRG.11666.4	309	0.41294	10.08624268	1
MSTRG.11659.1	60	0.35658	9.969055176	1
MSTRG.11659.2	71	0.34922	9.160827637	1
MSTRG.11659.3	55	0.38146	6.518981934	1
MSTRG.11659.4	195	0.28567	10.67230225	1
MSTRG.11674.2	60	0.39549	5.049377441	1
MSTRG.11675.1	219	0.44554	4.68170166	1
MSTRG.11675.3	152	0.46853	4.419616699	-1
MSTRG.11676.3	641	0.36337	9.090515137	1
MSTRG.11678.1	103	0.3345	9.811218262	1
MSTRG.11678.5	143	0.35997	5.573669434	1
MSTRG.11678.3	159	0.32797	6.046813965	1
MSTRG.11679.1	110	0.33731	9.09967041	1
MSTRG.11680.1	1115	0.38195	6.808044434	1
MSTRG.11680.3	1138	0.38347	6.589416504	1
MSTRG.11677.3	447	0.35031	9.141174316	1
MSTRG.11683.1	502	0.41373	5.04864502	1
MSTRG.11684.1	850	0.29075	5.723693848	1
MSTRG.11684.4	1429	0.36257	6.530212402	1
MSTRG.11687.2	266	0.37028	8.692565918	1
MSTRG.11696.1	104	0.36537	10.10748291	1
MSTRG.11697.1	187	0.39696	10.15863037	1
MSTRG.11697.2	241	0.4261	10.38018799	1
MSTRG.11702.1	1226	0.43552	7.800964355	1
MSTRG.11702.3	1254	0.4616	7.45892334	1
MSTRG.11703.1	44	0.40594	5.258850098	-1
MSTRG.11707.1	186	0.35033	9.115661621	1
MSTRG.11708.1	65	0.34597	4.11932373	1
MSTRG.11708.2	82	0.33114	9.609924316	1
MSTRG.11717.1	331	0.38091	5.292907715	1
MSTRG.11724.2	463	0.45095	5.324768066	1
MSTRG.11724.7	439	0.46184	5.601501465	1
MSTRG.11724.6	463	0.42872	5.324768066	1
MSTRG.11727.1	86	0.38679	10.72003174	1
MSTRG.11728.1	476	0.32265	6.00592041	1
MSTRG.11731.1	33	0.34725	4.669494629	-1
MSTRG.11738.1	725	0.47705	6.612976074	1
MSTRG.11738.3	338	0.40219	9.025085449	1
MSTRG.11738.4	160	0.27433	9.778503418	1
MSTRG.11738.6	128	0.30419	9.425476074	1
MSTRG.11738.7	338	0.25541	9.025085449	1
MSTRG.11738.8	548	0.41578	8.908508301	1
MSTRG.11738.9	424	0.40575	8.19317627	1
MSTRG.11734.3	405	0.40355	4.817199707	1

MSTRG.11737.2	76	0.42634	9.335754395	1
MSTRG.11739.1	129	0.37187	9.194641113	1
MSTRG.11745.7	861	0.44409	5.680480957	1
MSTRG.11745.8	74	0.32314	9.071105957	1
MSTRG.11746.1	165	0.41766	9.16998291	1
MSTRG.11747.1	78	0.33207	6.995300293	1
MSTRG.11763.3	67	0.35027	9.505065918	1
MSTRG.11763.4	533	0.41771	6.472229004	1
MSTRG.11764.1	97	0.34197	9.399597168	1
MSTRG.11763.5	67	0.37854	4.351989746	1
MSTRG.11765.1	84	0.34387	8.63458252	1
MSTRG.11763.7	78	0.39969	12.08441162	1
MSTRG.11756.2	1303	0.42714	5.951965332	1
MSTRG.11756.6	1336	0.46361	6.164001465	1
MSTRG.11761.1	64	0.32414	6.807067871	1
MSTRG.11782.1	189	0.27617	9.780700684	1
MSTRG.11783.1	138	0.3634	5.409606934	1
MSTRG.11783.4	88	0.35145	11.28814697	1
MSTRG.11783.3	75	0.39505	9.694885254	1
MSTRG.11792.1	453	0.33974	8.780334473	1
MSTRG.11792.3	416	0.37828	8.485168457	1
MSTRG.11792.2	491	0.38027	8.504699707	1
MSTRG.11792.4	492	0.46003	9.08013916	1
MSTRG.11792.13	86	0.29115	5.332946777	1
MSTRG.11793.1	46	0.35383	5.617248535	1
MSTRG.11794.1	114	0.32608	5.718444824	1
MSTRG.11785.1	113	0.31269	11.31427002	1
MSTRG.11786.2	155	0.47819	4.520568848	-1
MSTRG.11788.1	334	0.48136	6.552307129	1
MSTRG.11788.2	334	0.4539	6.552307129	1
MSTRG.11788.4	334	0.47812	6.552307129	1
MSTRG.11795.1	49	0.39421	7.941833496	1
MSTRG.11805.1	40	0.47316	7.761535645	-1
MSTRG.11806.1	1161	0.46835	6.590148926	1
MSTRG.11807.1	58	0.30529	8.552062988	-1
MSTRG.11810.2	299	0.36024	5.433898926	1
MSTRG.11814.4	412	0.44908	5.020935059	-1
MSTRG.11800.1	62	0.36742	11.43890381	-1
MSTRG.11802.3	117	0.39221	9.88494873	1
MSTRG.11802.4	145	0.39282	9.272277832	1
MSTRG.11802.5	120	0.35998	6.039245605	1
MSTRG.11820.3	488	0.39866	9.385681152	1
MSTRG.11819.1	78	0.37527	6.519592285	1
MSTRG.11834.2	411	0.47255	6.853942871	1
MSTRG.11834.3	228	0.44321	7.487487793	1
MSTRG.11838.1	188	0.32803	9.743225098	1
MSTRG.11841.4	47	0.32296	7.835510254	1
MSTRG.11842.3	1343	0.37818	9.376647949	1
MSTRG.11842.6	913	0.34503	9.391906738	1
MSTRG.11821.2	163	0.3231	5.704406738	1
MSTRG.11824.2	154	0.28415	9.599304199	1
MSTRG.11824.3	154	0.28415	9.599304199	1
MSTRG.11824.4	121	0.28415	10.43548584	1
MSTRG.11824.5	154	0.28923	9.599304199	1

MSTRG.11825.3	281	0.37818	8.861877441	1
MSTRG.11825.4	384	0.39169	8.701721191	1
MSTRG.11827.1	41	0.35506	11.71136475	1
MSTRG.11827.3	52	0.38056	7.998718262	1
MSTRG.11827.2	47	0.35789	9.102600098	1
MSTRG.11828.1	61	0.38676	10.16595459	-1
MSTRG.11847.2	462	0.45399	6.941955566	1
MSTRG.11852.1	294	0.34706	9.643005371	1
MSTRG.11852.2	191	0.39776	6.629333496	1
MSTRG.11852.3	339	0.41796	9.491149902	1
MSTRG.11852.4	110	0.30132	5.891174316	1
MSTRG.11852.5	110	0.30981	5.891174316	1
MSTRG.11860.1	67	0.37222	5.587097168	1
MSTRG.11861.1	368	0.46773	9.189880371	1
MSTRG.11854.1	92	0.3928	5.34979248	1
MSTRG.11867.3	131	0.34567	4.600769043	1
MSTRG.11867.4	53	0.43559	9.416687012	1
MSTRG.11867.5	41	0.3921	10.46624756	1
MSTRG.11872.1	315	0.36197	8.378845215	1
MSTRG.11873.2	180	0.43318	5.560119629	1
MSTRG.11873.4	223	0.36087	9.45489502	1
MSTRG.11873.3	500	0.39108	6.211853027	1
MSTRG.11873.7	375	0.32646	7.127868652	1
MSTRG.11873.8	233	0.355	8.974060059	1
MSTRG.11873.6	358	0.45346	5.222473145	1
MSTRG.11875.1	8	0.3461	3.998840332	1
MSTRG.11876.1	378	0.39221	6.266296387	1
MSTRG.11876.3	378	0.44855	6.266296387	1
MSTRG.11882.3	582	0.47972	9.090270996	1
MSTRG.11882.4	582	0.47972	9.090270996	1
MSTRG.11889.1	975	0.47915	5.70111084	1
MSTRG.11889.2	975	0.48335	5.70111084	1
MSTRG.11890.1	26	0.39058	11.71136475	1
MSTRG.11901.1	99	0.3684	9.722106934	1
MSTRG.11901.2	81	0.30517	10.47283936	1
MSTRG.11902.1	23	0.40579	5.750305176	-1
MSTRG.11923.1	74	0.41096	9.897888184	-1
MSTRG.11924.1	36	0.43707	6.496643066	-1
MSTRG.11929.1	18	0.36092	9.34967041	1
MSTRG.11950.1	84	0.41575	11.39508057	1
MSTRG.11951.1	93	0.3892	8.798034668	1
MSTRG.11960.1	75	0.36212	10.13238525	1
MSTRG.11968.1	147	0.29772	10.73638916	1
MSTRG.11969.1	115	0.31373	10.23907471	1
MSTRG.11970.1	189	0.28478	10.293396	1
MSTRG.11973.4	436	0.46976	5.351623535	1
MSTRG.11973.3	417	0.46976	5.411437988	1
MSTRG.11973.2	81	0.42689	4.93157959	1
MSTRG.11973.1	427	0.44891	5.507019043	1
MSTRG.11974.1	438	0.44755	6.286437988	1
MSTRG.11974.2	438	0.49495	6.286437988	1
MSTRG.11974.3	438	0.48109	6.286437988	1
MSTRG.11973.7	51	0.34519	9.492858887	1
MSTRG.11973.8	39	0.35002	10.26483154	1

MSTRG.11973.9	67	0.38238	9.972351074	1
MSTRG.11973.10	39	0.35734	11.34466553	1
MSTRG.11973.11	45	0.43315	6.535583496	1
MSTRG.11973.12	25	0.36451	10.66680908	1
MSTRG.11973.13	46	0.40191	10.94000244	1
MSTRG.11973.14	46	0.32638	10.94000244	1
MSTRG.11973.15	63	0.44646	9.183410645	1
MSTRG.11973.16	46	0.30018	10.94000244	1
MSTRG.11973.17	42	0.34842	9.382629395	-1
MSTRG.11973.18	51	0.43158	9.492858887	1
MSTRG.11973.19	17	0.3623	10.7366333	1
MSTRG.11973.20	16	0.42016	10.7366333	1
MSTRG.11973.21	16	0.41188	10.7366333	1
MSTRG.11973.22	16	0.34268	10.7366333	1
MSTRG.11973.23	17	0.38448	8.495544434	1
MSTRG.11973.24	35	0.40236	7.977355957	1
MSTRG.11958.1	480	0.34396	9.50201416	1
MSTRG.11958.2	483	0.34396	9.55279541	1
MSTRG.11972.1	130	0.28648	10.45098877	1
MSTRG.11978.2	200	0.4667	4.963439941	1
MSTRG.11978.1	1008	0.38912	4.934631348	1
MSTRG.11979.1	57	0.31729	5.999816895	1
MSTRG.11978.4	871	0.33623	5.233581543	1
MSTRG.11978.5	777	0.33375	4.940979004	1
MSTRG.11985.2	3115	0.45396	5.105407715	1
MSTRG.11986.4	364	0.39963	8.863952637	1
MSTRG.11987.1	90	0.332	10.11114502	1
MSTRG.11995.1	1840	0.46286	6.950134277	1
MSTRG.11996.1	113	0.29131	9.769836426	1
MSTRG.11995.6	1840	0.46644	6.950134277	1
MSTRG.11995.5	1250	0.35797	7.980041504	1
MSTRG.11995.7	1423	0.38051	6.230651855	1
MSTRG.11995.8	1423	0.44307	6.230651855	1
MSTRG.11995.9	1423	0.45282	6.230651855	1
MSTRG.11991.1	75	0.27097	5.85748291	1
MSTRG.11994.2	343	0.44991	8.470031738	1
MSTRG.11994.3	329	0.45828	7.651794434	1
MSTRG.12000.1	1487	0.45058	5.048156738	1
MSTRG.12004.5	465	0.3349	9.129577637	1
MSTRG.12004.6	465	0.31793	9.129577637	1
MSTRG.12004.7	240	0.39079	9.605529785	1
MSTRG.12004.8	264	0.39226	9.487121582	1
MSTRG.12004.9	454	0.45654	9.04864502	1
MSTRG.12004.10	465	0.46841	9.129577637	1
MSTRG.12005.1	77	0.31996	9.09942627	1
MSTRG.12004.11	339	0.44877	5.269958496	1
MSTRG.12006.4	395	0.38425	5.153625488	1
MSTRG.12007.1	63	0.31753	7.08416748	1
MSTRG.12008.1	113	0.3858	8.367370605	-1
MSTRG.12009.1	86	0.39993	8.449401855	-1
MSTRG.12019.2	82	0.36129	10.79107666	1
MSTRG.12019.3	42	0.36005	8.633361816	1
MSTRG.12027.2	189	0.36089	6.653503418	1
MSTRG.12023.1	326	0.33762	8.731140137	1

MSTRG.12030.1	301	0.41316	5.457458496	1
MSTRG.12030.3	300	0.40731	5.457458496	1
MSTRG.12034.4	407	0.45929	9.485656738	1
MSTRG.12035.1	1088	0.46986	6.245910645	1
MSTRG.12039.1	58	0.38367	7.813781738	1
MSTRG.12040.1	149	0.3024	10.40228271	1
MSTRG.12041.3	842	0.47167	6.54119873	1
MSTRG.12042.1	208	0.28964	9.778625488	1
MSTRG.12043.1	95	0.27763	8.588195801	1
MSTRG.12044.1	107	0.3024	10.12982178	1
MSTRG.12045.3	387	0.35587	9.975402832	1
MSTRG.12045.4	1432	0.37368	5.895690918	1
MSTRG.12047.3	349	0.35778	9.922424316	-1
MSTRG.12053.2	137	0.35373	4.689880371	1
MSTRG.12053.3	192	0.2984	10.47845459	1
MSTRG.12054.1	122	0.29999	8.884094238	1
MSTRG.12057.4	118	0.46076	8.881408691	1
MSTRG.12055.5	99	0.28186	9.686828613	1
MSTRG.12055.6	148	0.28338	8.604431152	1
MSTRG.12055.7	43	0.33911	7.836120605	1
MSTRG.12055.8	135	0.29772	10.23406982	1
MSTRG.12055.9	96	0.28027	8.851379395	1
MSTRG.12055.10	71	0.28683	6.141174316	1
MSTRG.12055.11	148	0.28948	8.604431152	1
MSTRG.12055.12	86	0.37442	9.805358887	1
MSTRG.12055.13	111	0.29187	7.741394043	1
MSTRG.12055.14	101	0.28923	7.651672363	1
MSTRG.12059.1	84	0.32278	10.01678467	1
MSTRG.12049.1	85	0.38173	8.822692871	1
MSTRG.12050.1	65	0.35741	6.54107666	1
MSTRG.12051.1	50	0.36469	11.84039307	1
MSTRG.12069.1	64	0.31714	9.684265137	1
MSTRG.12069.2	391	0.29404	5.575378418	1
MSTRG.12069.5	183	0.32127	4.711608887	1
MSTRG.12070.1	147	0.27911	9.543884277	1
MSTRG.12069.7	132	0.33194	8.491760254	1
MSTRG.12072.3	590	0.40857	6.394348145	1
MSTRG.12072.10	590	0.42417	6.394348145	1
MSTRG.12072.9	590	0.40854	6.394348145	1
MSTRG.12072.12	695	0.2948	6.964782715	1
MSTRG.12080.1	761	0.41938	9.11138916	1
MSTRG.12079.1	48	0.42795	10.61212158	1
MSTRG.12088.1	92	0.35228	10.76287842	1
MSTRG.12093.2	612	0.35881	4.799865723	1
MSTRG.12095.3	454	0.3955	6.107727051	1
MSTRG.12098.1	129	0.30989	4.815368652	1
MSTRG.12099.2	262	0.34695	9.773620605	1
MSTRG.12096.1	20	0.316	7.977966309	1
MSTRG.12100.1	375	0.32386	9.263122559	1
MSTRG.12100.2	852	0.41596	8.684631348	1
MSTRG.12100.3	689	0.33828	6.622619629	1
MSTRG.12102.1	45	0.35516	11.55670166	1
MSTRG.12108.4	2313	0.44824	5.452209473	1
MSTRG.12108.1	2334	0.46045	5.483825684	1

MSTRG.12108.9	2032	0.47431	5.248962402	1
MSTRG.12108.8	2313	0.47431	5.452209473	1
MSTRG.12108.12	178	0.40218	5.311584473	-1
MSTRG.12104.3	731	0.45286	5.487365723	1
MSTRG.12121.2	242	0.46947	9.79473877	1
MSTRG.12121.5	296	0.45646	9.369934082	-1
MSTRG.12123.5	412	0.45158	5.634094238	1
MSTRG.12123.2	412	0.41749	5.634094238	1
MSTRG.12123.4	412	0.42866	5.634094238	1
MSTRG.12123.1	412	0.37595	5.634094238	1
MSTRG.12123.3	412	0.41326	5.634094238	1
MSTRG.12123.6	14	0.32933	5.895690918	1
MSTRG.12124.1	99	0.42803	7.641296387	1
MSTRG.12124.2	178	0.39329	11.69940186	1
MSTRG.12125.1	131	0.42078	4.683288574	-1
MSTRG.12114.2	141	0.37831	9.038879395	1
MSTRG.12130.3	290	0.43286	6.508972168	1
MSTRG.12131.1	50	0.41243	9.815856934	1
MSTRG.12138.1	123	0.33121	10.20977783	1
MSTRG.12145.1	104	0.37388	10.78631592	1
MSTRG.12146.1	88	0.41415	9.550231934	1
MSTRG.12147.1	132	0.45507	9.264099121	-1
MSTRG.12152.1	50	0.31463	4.748718262	1
MSTRG.12153.2	108	0.34631	5.470031738	1
MSTRG.12157.3	80	0.29328	9.503967285	1
MSTRG.12157.4	80	0.34109	9.503967285	1
MSTRG.12157.2	158	0.34825	6.711730957	1
MSTRG.12157.5	120	0.39981	5.168273926	1
MSTRG.12159.2	95	0.3263	10.53863525	1
MSTRG.12159.1	50	0.43328	10.16790771	1
MSTRG.12159.3	61	0.38262	8.789611816	-1
MSTRG.12160.1	197	0.28186	9.420227051	1
MSTRG.12161.1	84	0.36594	7.695129395	1
MSTRG.12168.3	128	0.34122	6.88067627	1
MSTRG.12188.1	90	0.33248	9.787658691	1
MSTRG.12189.1	173	0.37955	9.181091309	1
MSTRG.12189.3	173	0.36617	9.181091309	1
MSTRG.12195.1	105	0.30303	7.580993652	1
MSTRG.12196.1	68	0.30101	9.838439941	1
MSTRG.12197.2	64	0.33931	4.528259277	1
MSTRG.12197.3	754	0.44796	6.308044434	1
MSTRG.12197.4	1191	0.47272	8.43560791	1
MSTRG.12197.5	212	0.31041	10.1963501	1
MSTRG.12198.1	149	0.38651	9.805480957	1
MSTRG.12202.1	30	0.41908	8.34197998	-1
MSTRG.12233.1	45	0.35933	8.211120605	1
MSTRG.12238.1	88	0.28642	5.107849121	1
MSTRG.12246.2	184	0.33828	7.688781738	1
MSTRG.12208.1	20	0.36121	7.888244629	1
MSTRG.12214.5	151	0.28982	10.83953857	1
MSTRG.12214.6	62	0.38454	7.782775879	1
MSTRG.12214.8	123	0.32032	5.574645996	1
MSTRG.12215.1	82	0.37253	11.1461792	1
MSTRG.12207.1	83	0.34852	4.307678223	1

MSTRG.12218.5	221	0.44418	6.530090332	1
MSTRG.12218.6	221	0.44046	6.530090332	1
MSTRG.12224.1	27	0.39275	9.809387207	1
MSTRG.12228.1	287	0.39007	10.09527588	-1
MSTRG.12274.1	18	0.41115	6.491638184	1
MSTRG.12277.1	144	0.29607	9.60736084	1
MSTRG.12278.2	76	0.37167	10.80596924	-1
MSTRG.12279.1	362	0.38328	7.258239746	1
MSTRG.12280.1	251	0.33386	6.452087402	1
MSTRG.12280.2	111	0.26913	5.658752441	1
MSTRG.12280.3	93	0.29783	5.086242676	1
MSTRG.12281.1	205	0.40601	8.090148926	1
MSTRG.12281.2	114	0.28855	8.76776123	1
MSTRG.12285.2	351	0.40978	6.75958252	1
MSTRG.12288.2	462	0.43393	9.103210449	1
MSTRG.12288.3	462	0.45559	9.103210449	1
MSTRG.12302.2	361	0.47651	8.701599121	1
MSTRG.12303.1	56	0.37267	11.71173096	1
MSTRG.12302.3	361	0.38034	8.701599121	1
MSTRG.12302.4	272	0.41292	8.808166504	1
MSTRG.12302.5	361	0.43051	8.701599121	1
MSTRG.12320.2	1133	0.44772	6.297302246	1
MSTRG.12320.4	963	0.4575	5.97869873	1
MSTRG.12320.5	1032	0.40315	6.658630371	1
MSTRG.12320.7	1037	0.44772	6.561218262	1
MSTRG.12322.1	1254	0.3996	8.763122559	1
MSTRG.12323.1	167	0.39741	11.23468018	-1
MSTRG.12322.3	129	0.38433	9.694885254	1
MSTRG.12347.5	1821	0.44153	9.077209473	1
MSTRG.12347.6	1266	0.37955	9.068786621	1
MSTRG.12347.7	797	0.30413	9.171203613	1
MSTRG.12347.8	863	0.40502	9.327575684	1
MSTRG.12351.1	53	0.30268	10.01629639	1
MSTRG.12352.1	65	0.32461	5.784851074	1
MSTRG.12340.2	684	0.46367	9.319274902	1
MSTRG.12340.4	684	0.46039	9.319274902	1
MSTRG.12340.3	367	0.44354	5.805236816	1
MSTRG.12341.1	156	0.30436	6.727233887	1
MSTRG.12342.1	104	0.36118	9.892028809	1
MSTRG.12353.1	100	0.43812	10.17266846	-1
MSTRG.12356.1	23	0.37895	4.225524902	1
MSTRG.12355.2	392	0.38544	5.860778809	1
MSTRG.12360.3	434	0.35432	5.084411621	1
MSTRG.12359.1	40	0.30796	10.44647217	-1
MSTRG.12416.1	72	0.27772	4.689147949	1
MSTRG.12425.1	171	0.39086	7.675109863	1
MSTRG.12426.2	165	0.28683	9.74432373	1
MSTRG.12433.1	107	0.28774	8.822692871	1
MSTRG.12434.4	2320	0.45866	5.667541504	1
MSTRG.12434.5	2337	0.44047	5.651184082	1
MSTRG.12434.7	2354	0.45866	5.612365723	1
MSTRG.12434.8	2141	0.48346	5.569641113	1
MSTRG.12435.2	542	0.47698	5.86114502	1
MSTRG.12439.2	496	0.45304	5.508850098	1

MSTRG.12440.1	125	0.28982	8.783630371	1
MSTRG.12441.1	171	0.30807	9.847229004	1
MSTRG.12442.1	82	0.30275	8.293395996	1
MSTRG.12442.2	138	0.26845	10.50933838	1
MSTRG.12441.2	132	0.33562	8.677429199	1
MSTRG.12439.3	141	0.2917	7.708190918	1
MSTRG.12439.4	141	0.33798	7.708190918	1
MSTRG.12384.2	870	0.45919	4.867004395	1
MSTRG.12384.1	814	0.47358	5.130432129	1
MSTRG.12384.3	327	0.432	5.577453613	1
MSTRG.12389.1	116	0.41811	10.02850342	1
MSTRG.12390.3	347	0.45872	5.167663574	1
MSTRG.12391.1	157	0.31849	8.743835449	1
MSTRG.12391.2	116	0.35371	5.555725098	-1
MSTRG.12392.1	80	0.32144	4.787658691	-1
MSTRG.12395.1	87	0.34618	10.73699951	1
MSTRG.12407.1	232	0.41461	9.173156738	1
MSTRG.12400.2	260	0.46145	8.349182129	1
MSTRG.12447.5	928	0.47437	5.316345215	1
MSTRG.12448.1	145	0.31831	10.71954346	1
MSTRG.12449.1	69	0.32421	7.708557129	1
MSTRG.12450.1	99	0.28478	9.640930176	1
MSTRG.12444.1	170	0.35165	10.66937256	1
MSTRG.12445.2	99	0.41215	4.240783691	1
MSTRG.12474.3	57	0.29583	6.109558105	1
MSTRG.12474.5	93	0.42792	4.561706543	1
MSTRG.12474.2	79	0.38804	4.945373535	1
MSTRG.12474.4	110	0.3538	9.658996582	1
MSTRG.12474.1	79	0.38432	4.945373535	1
MSTRG.12474.7	93	0.42889	4.561706543	1
MSTRG.12474.6	57	0.29583	6.109558105	1
MSTRG.12474.9	93	0.42386	4.561706543	1
MSTRG.12474.8	79	0.40401	4.945373535	1
MSTRG.12475.1	476	0.34668	6.021911621	1
MSTRG.12475.2	398	0.34028	5.228088379	1
MSTRG.12475.3	446	0.35073	5.460388184	1
MSTRG.12475.5	443	0.30647	5.460388184	1
MSTRG.12476.1	439	0.47006	4.722595215	1
MSTRG.12476.2	439	0.46286	4.692443848	1
MSTRG.12476.3	459	0.4631	4.593566895	1
MSTRG.12476.7	375	0.45965	5.02166748	1
MSTRG.12476.8	459	0.49167	4.593566895	1
MSTRG.12476.6	432	0.47701	5.159851074	1
MSTRG.12476.9	503	0.49279	4.660095215	1
MSTRG.12494.1	78	0.30387	7.767150879	1
MSTRG.12495.1	74	0.33206	9.890686035	1
MSTRG.12495.2	112	0.33235	9.098205566	-1
MSTRG.12504.1	64	0.471	9.919616699	1
MSTRG.12504.2	122	0.30828	9.300476074	1
MSTRG.12505.1	140	0.28982	9.607971191	1
MSTRG.12506.1	125	0.31484	11.00006104	1
MSTRG.12507.1	33	0.40549	7.710266113	1
MSTRG.12505.2	139	0.29367	10.43719482	1
MSTRG.12501.1	1116	0.47431	5.735046387	1

MSTRG.12501.3	141	0.43884	9.097595215	1
MSTRG.12509.1	119	0.3331	9.614440918	1
MSTRG.12510.2	676	0.43636	6.746520996	1
MSTRG.12510.3	703	0.28576	6.151916504	1
MSTRG.12510.4	573	0.43064	7.562194824	1
MSTRG.12517.3	407	0.49463	5.796203613	1
MSTRG.12521.1	37	0.41161	5.957702637	1
MSTRG.12525.2	827	0.41466	7.145812988	1
MSTRG.12525.1	853	0.4215	6.74822998	1
MSTRG.12525.3	827	0.3825	7.145812988	1
MSTRG.12528.3	229	0.48244	5.030578613	1
MSTRG.12537.2	432	0.38038	5.163757324	1
MSTRG.12553.1	169	0.31638	9.483581543	-1
MSTRG.12558.1	41	0.3413	5.366882324	1
MSTRG.12558.2	22	0.43501	10.4463501	1
MSTRG.12558.3	96	0.28923	10.01641846	1
MSTRG.12560.1	110	0.29202	8.625183105	1
MSTRG.12550.2	458	0.45708	5.481750488	1
MSTRG.12550.3	458	0.47344	5.481750488	1
MSTRG.12569.1	62	0.38934	4.512512207	1
MSTRG.12572.1	45	0.45357	5.304016113	1
MSTRG.12573.1	71	0.41503	10.62042236	-1
MSTRG.12576.3	80	0.36199	9.643371582	1
MSTRG.12576.4	80	0.35026	9.643371582	1
MSTRG.12577.7	217	0.37002	7.082092285	1
MSTRG.12577.5	214	0.39792	5.004211426	1
MSTRG.12577.4	217	0.38211	7.082092285	1
MSTRG.12578.4	304	0.39794	6.398620605	1
MSTRG.12588.1	3127	0.46946	8.743835449	1
MSTRG.12588.2	3049	0.47151	8.747375488	1
MSTRG.12588.3	3127	0.46946	8.743835449	1
MSTRG.12588.5	3055	0.45686	8.747375488	1
MSTRG.12589.1	57	0.41581	6.999328613	1
MSTRG.12589.2	83	0.30963	6.225158691	1
MSTRG.12589.3	116	0.32037	8.77130127	1
MSTRG.12589.4	103	0.36707	9.23614502	1
MSTRG.12591.1	181	0.28592	9.829284668	1
MSTRG.12595.2	160	0.41738	4.508361816	-1
MSTRG.12596.3	265	0.37229	7.553527832	1
MSTRG.12596.4	89	0.28982	9.29083252	1
MSTRG.12596.5	70	0.33842	7.790588379	1
MSTRG.12599.1	110	0.27838	5.668151855	1
MSTRG.12598.4	440	0.32642	8.791442871	1
MSTRG.12600.1	231	0.28848	9.399597168	1
MSTRG.12598.5	440	0.3556	8.791442871	1
MSTRG.12598.6	65	0.28662	9.150695801	1
MSTRG.12598.8	380	0.3477	7.249206543	1
MSTRG.12598.9	380	0.38674	7.249206543	1
MSTRG.12601.1	43	0.3819	9.354797363	1
MSTRG.12602.1	95	0.32851	9.938293457	1
MSTRG.12602.3	50	0.32201	6.04510498	1
MSTRG.12602.2	46	0.32018	8.329284668	1
MSTRG.12602.4	46	0.28586	8.329284668	1
MSTRG.12603.1	164	0.36589	6.573791504	1

MSTRG.12603.2	218	0.40182	5.550109863	1
MSTRG.12603.3	164	0.37295	6.573791504	1
MSTRG.12603.4	164	0.35203	6.573791504	1
MSTRG.12607.2	570	0.46333	5.877502441	1
MSTRG.12607.3	559	0.43736	6.014221191	1
MSTRG.12611.5	1782	0.45915	4.95489502	1
MSTRG.12611.3	1896	0.46595	4.941467285	1
MSTRG.12611.7	1896	0.46595	4.941467285	1
MSTRG.12612.1	54	0.40466	5.658996582	1
MSTRG.12612.2	82	0.3492	5.006164551	1
MSTRG.12617.2	423	0.46454	6.902404785	1
MSTRG.12617.3	208	0.33565	6.292419434	1
MSTRG.12619.1	49	0.29928	9.489196777	1
MSTRG.12618.2	132	0.33664	6.49407959	1
MSTRG.12618.3	135	0.29507	10.78704834	1
MSTRG.12619.2	49	0.30641	9.489196777	1
MSTRG.12619.3	49	0.30641	9.489196777	1
MSTRG.12642.1	129	0.33481	9.59197998	1
MSTRG.12643.1	122	0.35371	9.839660645	1
MSTRG.12646.3	74	0.33695	7.981994629	1
MSTRG.12647.1	164	0.28298	8.650695801	1
MSTRG.12642.2	96	0.33358	7.645324707	1
MSTRG.12648.1	62	0.28083	6.490905762	1
MSTRG.12646.5	492	0.47681	5.717346191	1
MSTRG.12646.4	492	0.46428	5.717346191	1
MSTRG.12646.6	225	0.47226	6.28302002	1
MSTRG.12652.3	404	0.4556	8.332092285	-1
MSTRG.12652.4	66	0.33643	9.587463379	1
MSTRG.12623.3	453	0.3088	5.16973877	1
MSTRG.12623.4	453	0.30453	5.16973877	1
MSTRG.12623.6	453	0.3034	5.16973877	1
MSTRG.12623.5	453	0.40262	5.16973877	1
MSTRG.12625.2	270	0.41331	9.025939941	1
MSTRG.12625.3	254	0.4287	8.839904785	1
MSTRG.12627.1	257	0.41079	6.239318848	1
MSTRG.12627.2	303	0.43316	7.710266113	1
MSTRG.12633.2	334	0.37937	7.016540527	1
MSTRG.12635.1	760	0.44793	5.20892334	1
MSTRG.12655.1	91	0.43989	9.16583252	1
MSTRG.12660.1	107	0.44654	10.0055542	1
MSTRG.12661.2	264	0.40132	9.791442871	1
MSTRG.12664.1	238	0.31421	9.336853027	1
MSTRG.12674.3	172	0.44929	7.768005371	1
MSTRG.12676.1	2028	0.43557	5.426818848	1
MSTRG.12677.2	225	0.34956	9.510803223	1
MSTRG.12680.1	74	0.33717	9.510314941	-1
MSTRG.12681.2	644	0.44363	5.71673584	1
MSTRG.12682.1	291	0.38395	6.500061035	1
MSTRG.12685.1	247	0.29772	8.936828613	1
MSTRG.12685.2	255	0.29607	9.11541748	1
MSTRG.12686.1	31	0.35371	4.035705566	1
MSTRG.12683.2	543	0.43739	6.160583496	1
MSTRG.12709.4	182	0.37028	9.684631348	1
MSTRG.12711.1	110	0.29595	10.57415771	1

MSTRG. 12711. 2	164	0. 2921	9. 236999512	1
MSTRG. 12711. 3	207	0. 29615	9. 831237793	1
MSTRG. 12709. 6	175	0. 32237	9. 207824707	1
MSTRG. 12713. 1	64	0. 28982	8. 535827637	1
MSTRG. 12715. 1	157	0. 32413	5. 961608887	1
MSTRG. 12701. 1	55	0. 46026	10. 25604248	-1
MSTRG. 12716. 1	844	0. 43248	6. 252990723	1
MSTRG. 12716. 2	523	0. 32386	7. 16204834	1
MSTRG. 12716. 3	618	0. 3799	6. 548156738	1
MSTRG. 12717. 1	69	0. 31061	6. 227722168	1
MSTRG. 12716. 4	130	0. 31653	6. 870300293	1
MSTRG. 12718. 1	79	0. 32697	9. 590026855	1
MSTRG. 12716. 5	42	0. 35814	7. 996887207	-1
MSTRG. 12705. 2	4	0. 29593	5. 274963379	1
MSTRG. 12705. 4	84	0. 37152	6. 571594238	1
MSTRG. 12705. 3	1217	0. 48589	6. 53717041	1
MSTRG. 12705. 5	813	0. 48098	6. 274841309	1
MSTRG. 12730. 2	66	0. 2944	9. 801330566	1
MSTRG. 12731. 3	101	0. 3608	4. 444519043	1
MSTRG. 12731. 2	814	0. 40525	10. 13067627	1
MSTRG. 12731. 4	101	0. 29374	4. 444519043	1
MSTRG. 12731. 6	441	0. 40237	11. 21905518	1
MSTRG. 12731. 7	441	0. 35592	11. 21905518	1
MSTRG. 12731. 8	407	0. 38309	4. 796569824	1
MSTRG. 12720. 2	147	0. 38089	6. 96697998	1
MSTRG. 12721. 3	446	0. 40999	5. 912902832	1
MSTRG. 12722. 1	37	0. 40553	4. 55645752	-1
MSTRG. 12725. 1	23	0. 30756	7. 978088379	1
MSTRG. 12725. 2	61	0. 34868	7. 853942871	1
MSTRG. 12738. 3	284	0. 37777	7. 787658691	1
MSTRG. 12746. 1	233	0. 39437	9. 441955566	1
MSTRG. 12759. 1	62	0. 38451	10. 45001221	-1
MSTRG. 12768. 1	45	0. 36822	9. 764099121	1
MSTRG. 12769. 1	48	0. 3694	8. 015441895	1
MSTRG. 12791. 1	1973	0. 4591	5. 979797363	1
MSTRG. 12791. 2	1973	0. 47755	5. 979797363	1
MSTRG. 12792. 1	5560	0. 4738	5. 404724121	1
MSTRG. 12792. 2	5560	0. 47542	5. 404724121	1
MSTRG. 12792. 3	5560	0. 47135	5. 404724121	1
MSTRG. 12792. 6	62	0. 2994	6. 001647949	1
MSTRG. 12793. 2	72	0. 31558	10. 18719482	1
MSTRG. 12794. 1	96	0. 32947	10. 30474854	1
MSTRG. 12794. 2	96	0. 41201	10. 30474854	1
MSTRG. 12777. 1	452	0. 39792	5. 185241699	1
MSTRG. 12778. 1	250	0. 38264	6. 068664551	1
MSTRG. 12784. 1	28	0. 36978	7. 728210449	1
MSTRG. 12785. 2	142	0. 3	9. 848815918	1
MSTRG. 12785. 3	142	0. 3231	9. 848815918	1
MSTRG. 12785. 4	142	0. 33519	9. 848815918	1
MSTRG. 12789. 3	451	0. 42948	6. 496887207	1
MSTRG. 12800. 1	313	0. 45473	4. 585876465	1
MSTRG. 12800. 2	317	0. 43423	4. 663024902	1
MSTRG. 12800. 3	317	0. 44632	4. 585876465	1
MSTRG. 12804. 2	155	0. 34568	8. 332702637	1

MSTRG.12805.3	230	0.43941	5.338562012	-1
MSTRG.12806.2	552	0.47366	8.473937988	1
MSTRG.12806.1	617	0.47954	8.00592041	1
MSTRG.12806.4	623	0.49159	6.823791504	1
MSTRG.12806.5	552	0.46355	8.473937988	1
MSTRG.12808.2	373	0.48686	9.385803223	1
MSTRG.12810.1	92	0.32257	9.855651855	1
MSTRG.12813.1	144	0.34141	6.30291748	1
MSTRG.12816.1	60	0.33747	6.801696777	1
MSTRG.12829.1	562	0.34212	7.175476074	1
MSTRG.12829.6	562	0.45007	7.175476074	1
MSTRG.12829.4	562	0.45352	7.175476074	1
MSTRG.12829.7	463	0.46767	9.190490723	1
MSTRG.12830.4	893	0.46867	5.83416748	1
MSTRG.12831.1	136	0.36613	10.78045654	1
MSTRG.12835.1	66	0.36918	8.280212402	-1
MSTRG.12826.1	66	0.32881	6.532287598	1
MSTRG.12826.2	66	0.34581	6.532287598	1
MSTRG.12827.1	22	0.43501	6.809387207	-1
MSTRG.12839.2	322	0.44307	7.679260254	1
MSTRG.12847.3	653	0.48005	5.245666504	1
MSTRG.12847.4	653	0.48005	5.245666504	1
MSTRG.12848.1	65	0.34839	6.216125488	1
MSTRG.12851.2	472	0.42394	9.231628418	1
MSTRG.12851.3	307	0.40367	9.322692871	1
MSTRG.12863.6	923	0.41697	5.15032959	1
MSTRG.12863.7	923	0.41697	5.15032959	1
MSTRG.12863.14	972	0.43219	5.172302246	1
MSTRG.12863.16	973	0.42517	5.172302246	1
MSTRG.12863.11	924	0.43491	5.15032959	1
MSTRG.12863.13	923	0.43815	5.15032959	1
MSTRG.12863.15	693	0.44178	6.048400879	1
MSTRG.12863.12	972	0.45612	5.172302246	1
MSTRG.12863.18	1024	0.46095	5.226989746	1
MSTRG.12863.17	692	0.45064	6.048400879	1
MSTRG.12863.19	165	0.34931	8.744934082	1
MSTRG.12864.1	69	0.26594	9.098327637	1
MSTRG.12863.20	12	0.37671	5.752990723	1
MSTRG.12888.1	95	0.28486	6.684265137	1
MSTRG.12890.2	186	0.33929	9.61114502	1
MSTRG.12890.4	69	0.29595	10.53009033	1
MSTRG.12907.3	682	0.50244	6.122497559	1
MSTRG.12907.2	629	0.41038	5.793640137	1
MSTRG.12907.4	438	0.46639	6.873474121	1
MSTRG.12907.5	91	0.29594	6.487976074	1
MSTRG.12907.6	397	0.3398	7.473937988	1
MSTRG.12912.3	1037	0.47097	6.582580566	-1
MSTRG.12912.4	663	0.45272	8.393005371	-1
MSTRG.12914.1	32	0.33865	9.579406738	1
MSTRG.12933.4	188	0.36461	5.045349121	1
MSTRG.12933.6	187	0.36201	5.045349121	1
MSTRG.12933.5	189	0.37422	5.129821777	1
MSTRG.12933.3	188	0.33281	5.129821777	1
MSTRG.12933.7	189	0.33281	5.129821777	1

MSTRG.12933.8	725	0.40617	9.588195801	1
MSTRG.12934.1	84	0.3492	10.1774292	1
MSTRG.12919.1	142	0.37391	10.09161377	1
MSTRG.12920.1	502	0.45167	4.719909668	1
MSTRG.12920.2	502	0.42019	4.719909668	1
MSTRG.12925.1	26	0.4138	10.34735107	1
MSTRG.12925.2	33	0.40801	8.044616699	1
MSTRG.12926.1	14	0.41546	8.841491699	1
MSTRG.12938.1	42	0.36094	9.297302246	1
MSTRG.12947.3	1561	0.37871	8.252380371	1
MSTRG.12947.2	1512	0.40008	7.917175293	1
MSTRG.12971.1	847	0.31512	9.462463379	1
MSTRG.12978.1	82	0.37807	8.59173584	1
MSTRG.12978.2	158	0.29367	10.00958252	1
MSTRG.12978.3	158	0.29343	10.00958252	1
MSTRG.12983.5	747	0.44569	5.559387207	1
MSTRG.12984.1	47	0.43185	4.584289551	1
MSTRG.12939.1	546	0.39234	8.889343262	1
MSTRG.12939.2	418	0.37534	9.028869629	1
MSTRG.12939.3	418	0.42599	9.028869629	1
MSTRG.12940.1	74	0.31268	7.814025879	1
MSTRG.12939.4	418	0.43205	9.028869629	1
MSTRG.12939.5	418	0.33211	9.028869629	1
MSTRG.12988.1	98	0.30517	9.842346191	1
MSTRG.13003.1	84	0.31287	10.42242432	1
MSTRG.13008.1	85	0.28891	6.346740723	1
MSTRG.13008.2	99	0.35952	9.85357666	1
MSTRG.13009.1	44	0.35496	7.713684082	1
MSTRG.13010.1	36	0.44866	11.72296143	-1
MSTRG.13012.1	67	0.38662	9.359069824	-1
MSTRG.13013.1	91	0.36241	10.08929443	1
MSTRG.13013.2	91	0.28982	6.476135254	1
MSTRG.13013.6	69	0.42797	10.04644775	1
MSTRG.13013.4	139	0.33281	10.1206665	1
MSTRG.13013.5	234	0.33698	7.666320801	1
MSTRG.13014.1	100	0.35581	9.834533691	1
MSTRG.13022.1	148	0.29607	9.259460449	1
MSTRG.13022.2	92	0.29202	9.206359863	1
MSTRG.13025.2	135	0.29526	9.988586426	1
MSTRG.13025.4	509	0.29772	6.473693848	1
MSTRG.13025.5	125	0.29367	10.02215576	1
MSTRG.13025.7	163	0.29607	9.734802246	1
MSTRG.13025.8	102	0.29772	10.86627197	1
MSTRG.13025.9	558	0.29367	5.82232666	1
MSTRG.13025.10	81	0.30576	9.097595215	1
MSTRG.13025.11	509	0.29367	6.473693848	1
MSTRG.13025.12	279	0.30297	9.69354248	1
MSTRG.13030.3	1039	0.47357	5.280212402	1
MSTRG.13030.1	3434	0.47422	5.229431152	1
MSTRG.13034.1	9	0.35569	5.840148926	-1
MSTRG.13034.2	11	0.39585	5.222595215	1
MSTRG.13034.3	61	0.39118	10.19378662	1
MSTRG.13040.4	1685	0.44343	6.960510254	1
MSTRG.13040.6	1685	0.46155	6.960510254	1

MSTRG.13039.1	32	0.43585	5.971618652	-1
MSTRG.13048.1	1116	0.3439	6.730529785	1
MSTRG.13049.1	97	0.32995	10.66534424	1
MSTRG.13055.1	45	0.31444	8.312561035	1
MSTRG.13053.1	92	0.36221	9.236022949	1
MSTRG.13056.2	410	0.35881	8.780212402	1
MSTRG.13061.1	693	0.42931	8.829528809	1
MSTRG.13061.3	693	0.35756	8.829528809	1
MSTRG.13062.1	125	0.34735	8.070617676	1
MSTRG.13061.5	150	0.31225	7.62322998	1
MSTRG.13060.1	76	0.35372	8.721496582	1
MSTRG.13063.1	66	0.30144	6.229431152	1
MSTRG.13065.2	210	0.45981	5.177185059	1
MSTRG.13066.3	354	0.45062	9.200256348	1
MSTRG.13068.1	21	0.44445	5.949157715	1
MSTRG.13077.4	227	0.45561	7.054504395	1
MSTRG.13078.1	114	0.36408	8.734558105	1
MSTRG.13079.2	363	0.40661	7.037536621	1
MSTRG.13082.3	41	0.45576	9.00982666	1
MSTRG.13082.4	81	0.36072	9.184143066	1
MSTRG.13082.2	210	0.30453	8.602600098	1
MSTRG.13087.1	1740	0.44659	4.300598145	1
MSTRG.13087.2	1740	0.44659	4.300598145	1
MSTRG.13087.4	1657	0.4562	4.320007324	1
MSTRG.13087.6	1631	0.42998	4.323059082	1
MSTRG.13091.2	172	0.30396	11.95062256	1
MSTRG.13096.2	322	0.35788	5.80255127	1
MSTRG.13106.2	8798	0.47931	5.395935059	1
MSTRG.13106.1	8781	0.47931	5.395690918	1
MSTRG.13106.3	8710	0.47931	5.383605957	1
MSTRG.13107.1	38	0.39615	9.692443848	1
MSTRG.13100.4	188	0.44669	9.564025879	1
MSTRG.13110.4	1268	0.43252	8.54498291	1
MSTRG.13110.3	1268	0.46773	8.54498291	1
MSTRG.13110.5	1268	0.43176	8.54498291	1
MSTRG.13110.6	723	0.4714	8.992980957	1
MSTRG.13110.7	1235	0.431	7.381164551	1
MSTRG.13111.1	55	0.38466	4.749694824	1
MSTRG.13112.1	79	0.28826	9.099914551	1
MSTRG.13115.1	1693	0.42084	6.878601074	1
MSTRG.13117.1	49	0.46543	8.938293457	1
MSTRG.13115.2	1693	0.44152	6.878601074	1
MSTRG.13118.2	153	0.41795	8.502868652	1
MSTRG.13119.1	130	0.27794	9.803894043	1
MSTRG.13120.1	72	0.36394	7.705627441	1
MSTRG.13118.4	191	0.32729	10.10150146	1
MSTRG.13118.5	99	0.30092	10.10003662	1
MSTRG.13143.4	1817	0.41709	6.012268066	1
MSTRG.13143.6	786	0.34283	8.840148926	1
MSTRG.13143.5	1854	0.44078	6.079040527	1
MSTRG.13143.7	1825	0.4079	5.94708252	1
MSTRG.13144.1	89	0.29534	9.692565918	1
MSTRG.13143.8	1829	0.39625	5.93145752	1
MSTRG.13143.9	1780	0.44242	5.863586426	1

MSTRG.13145.1	43	0.32241	9.992004395	1
MSTRG.13146.1	175	0.28982	9.111999512	1
MSTRG.13143.10	42	0.35596	4.21282959	1
MSTRG.13148.1	122	0.27653	6.007385254	1
MSTRG.13149.1	67	0.29852	9.36895752	1
MSTRG.13150.1	87	0.32181	7.63861084	1
MSTRG.13151.1	115	0.2921	9.295959473	1
MSTRG.13152.1	89	0.27794	7.683044434	1
MSTRG.13152.2	89	0.28199	7.683044434	1
MSTRG.13143.11	95	0.30191	8.382751465	1
MSTRG.13143.12	874	0.44978	5.266784668	1
MSTRG.13130.1	107	0.37471	8.549987793	1
MSTRG.13134.1	129	0.31697	10.80291748	1
MSTRG.13137.1	219	0.33523	11.90679932	1
MSTRG.13138.3	213	0.32671	9.573303223	1
MSTRG.13138.5	221	0.37837	9.481140137	1
MSTRG.13138.6	154	0.40312	9.169494629	1
MSTRG.13139.1	209	0.28186	10.05645752	1
MSTRG.13139.2	112	0.28779	7.651306152	1
MSTRG.13139.3	138	0.28698	10.34539795	1
MSTRG.13139.4	128	0.29772	9.713806152	1
MSTRG.13139.5	248	0.28186	9.45501709	1
MSTRG.13128.2	270	0.38512	6.206848145	1
MSTRG.13128.3	295	0.4069	6.682434082	1
MSTRG.13128.4	270	0.42634	6.206848145	1
MSTRG.13154.1	58	0.36612	5.992004395	1
MSTRG.13154.2	36	0.34411	5.497741699	1
MSTRG.13154.3	58	0.36252	5.992004395	1
MSTRG.13154.4	58	0.33701	5.992004395	1
MSTRG.13155.2	584	0.43939	7.09197998	1
MSTRG.13162.1	112	0.33046	5.89276123	1
MSTRG.13163.1	175	0.28706	8.931213379	1
MSTRG.13164.1	157	0.29022	8.500061035	1
MSTRG.13161.6	1035	0.4327	8.649108887	1
MSTRG.13161.4	1035	0.4579	8.649108887	1
MSTRG.13161.7	886	0.39988	8.32220459	1
MSTRG.13161.5	1380	0.37392	7.887512207	1
MSTRG.13165.1	57	0.44319	11.30865479	-1
MSTRG.13159.2	475	0.43423	7.584899902	1
MSTRG.13159.3	469	0.41656	7.974182129	1
MSTRG.13159.4	474	0.45562	8.233459473	1
MSTRG.13166.1	611	0.43383	9.473449707	1
MSTRG.13170.3	121	0.46124	5.674499512	1
MSTRG.13171.1	176	0.34719	7.808410645	1
MSTRG.13177.1	167	0.34648	12.003479	1
MSTRG.13177.4	93	0.3132	7.660583496	1
MSTRG.13177.5	45	0.33704	10.18243408	1
MSTRG.13174.1	2499	0.44718	5.617126465	1
MSTRG.13181.3	1355	0.45495	6.022277832	1
MSTRG.13181.4	1580	0.47682	5.977355957	1
MSTRG.13181.5	1327	0.46676	6.391296387	1
MSTRG.13179.1	99	0.33117	6.541931152	1
MSTRG.13179.3	62	0.38651	8.161315918	1
MSTRG.13179.2	98	0.40999	8.808532715	1

MSTRG.13180.1	90	0.32634	12.30352783	1
MSTRG.13184.1	460	0.35297	7.127868652	1
MSTRG.13184.2	460	0.41548	7.127868652	1
MSTRG.13199.1	257	0.46592	8.543151855	1
MSTRG.13199.2	269	0.46101	9.096374512	1
MSTRG.13201.1	54	0.44394	9.817443848	1
MSTRG.13183.1	59	0.42585	10.29486084	1
MSTRG.13206.2	0	0.40306	0	-1
MSTRG.13208.1	319	0.32237	6.93182373	1
MSTRG.13208.4	311	0.32948	6.93182373	1
MSTRG.13209.1	189	0.30576	9.869689941	1
MSTRG.13209.2	331	0.28478	9.277648926	1
MSTRG.13203.1	54	0.37141	10.65106201	1
MSTRG.13217.1	60	0.42514	9.819885254	1
MSTRG.13221.2	328	0.44213	8.424621582	1
MSTRG.13222.1	714	0.40988	6.215270996	1
MSTRG.13222.2	782	0.34583	6.124572754	1
MSTRG.13222.3	757	0.38544	6.096618652	1
MSTRG.13222.6	83	0.33146	8.57611084	1
MSTRG.13223.1	55	0.29615	10.29486084	1
MSTRG.13224.1	113	0.37511	7.682922363	1
MSTRG.13222.7	94	0.33878	9.854187012	1
MSTRG.13233.1	733	0.44704	8.883728027	1
MSTRG.13237.1	161	0.39083	10.21331787	1
MSTRG.13241.3	74	0.34807	4.904724121	1
MSTRG.13241.4	478	0.395	4.746643066	1
MSTRG.13241.5	347	0.39662	5.460144043	1
MSTRG.13241.6	211	0.321	4.405090332	1
MSTRG.13243.1	214	0.44411	7.037902832	1
MSTRG.13243.2	166	0.47427	6.246643066	1
MSTRG.13243.3	185	0.49272	7.113586426	1
MSTRG.13244.3	528	0.43479	9.178039551	1
MSTRG.13261.4	1768	0.49169	5.813049316	1
MSTRG.13261.5	1769	0.49169	5.787902832	1
MSTRG.13261.6	1768	0.49169	5.764465332	1
MSTRG.13261.7	1770	0.49169	5.764465332	1
MSTRG.13261.1	1771	0.45938	5.789001465	1
MSTRG.13261.8	1563	0.45773	5.704162598	1
MSTRG.13261.9	1570	0.45521	5.63092041	1
MSTRG.13261.10	1468	0.43242	5.611633301	1
MSTRG.13261.11	1468	0.45521	5.611633301	1
MSTRG.13261.12	1470	0.45521	5.611633301	1
MSTRG.13261.13	1475	0.44577	5.542053223	1
MSTRG.13261.14	1475	0.43866	5.542053223	1
MSTRG.13263.1	125	0.35869	9.028503418	1
MSTRG.13266.1	104	0.31209	10.36883545	1
MSTRG.13267.1	181	0.28061	6.881774902	1
MSTRG.13267.2	127	0.37575	11.0322876	1
MSTRG.13267.3	101	0.34109	11.70648193	1
MSTRG.13267.4	101	0.36035	11.70648193	1
MSTRG.13254.2	66	0.27732	10.2366333	1
MSTRG.13254.1	13	0.34347	5.222351074	1
MSTRG.13253.3	295	0.37867	9.096130371	1
MSTRG.13253.4	303	0.33853	8.862487793	1

MSTRG.13259.2	922	0.40939	4.532409668	1
MSTRG.13260.1	197	0.38141	9.550109863	1
MSTRG.13268.1	85	0.29427	9.445007324	1
MSTRG.13268.2	745	0.42546	7.484558105	1
MSTRG.13268.3	202	0.33097	9.84967041	1
MSTRG.13268.4	181	0.30917	8.665588379	1
MSTRG.13268.5	123	0.38855	9.84552002	1
MSTRG.13269.1	150	0.32603	8.987365723	1
MSTRG.13268.8	70	0.34463	9.930236816	1
MSTRG.13270.1	87	0.30877	10.78973389	1
MSTRG.13273.4	449	0.41466	6.413513184	1
MSTRG.13281.1	343	0.38169	9.201965332	1
MSTRG.13281.2	48	0.43849	6.815124512	1
MSTRG.13282.2	17	0.40922	5.218933105	1
MSTRG.13284.8	196	0.30709	6.298522949	1
MSTRG.13284.6	472	0.41325	7.886047363	1
MSTRG.13284.9	107	0.27763	6.223815918	1
MSTRG.13284.10	325	0.41993	8.544616699	1
MSTRG.13277.1	104	0.37637	5.415222168	1
MSTRG.13277.2	97	0.32717	6.820251465	1
MSTRG.13286.3	215	0.28823	9.047180176	1
MSTRG.13287.1	56	0.34479	11.4196167	1
MSTRG.13303.1	137	0.42616	5.282287598	1
MSTRG.13305.1	29	0.35962	4.55657959	1
MSTRG.13314.1	423	0.29772	7.955749512	1
MSTRG.13314.3	212	0.2903	9.683776855	1
MSTRG.13314.2	329	0.28671	9.105163574	1
MSTRG.13323.1	97	0.30488	10.48858643	1
MSTRG.13323.2	50	0.32138	9.691345215	1
MSTRG.13320.3	444	0.38998	10.06585693	1
MSTRG.13320.4	189	0.29547	9.861022949	1
MSTRG.13320.5	482	0.40384	10.12200928	1
MSTRG.13320.6	189	0.28478	9.861022949	1
MSTRG.13320.7	189	0.38685	9.861022949	1
MSTRG.13320.9	65	0.30661	8.804748535	1
MSTRG.13324.1	221	0.28478	9.90802002	1
MSTRG.13325.1	72	0.29615	6.001037598	1
MSTRG.13327.1	244	0.28338	8.70513916	1
MSTRG.13329.1	54	0.29861	5.563659668	1
MSTRG.13331.1	58	0.34643	9.25970459	1
MSTRG.13332.1	54	0.42061	9.605651855	-1
MSTRG.13332.2	35	0.38695	10.28643799	1
MSTRG.13332.3	87	0.31478	8.507507324	1
MSTRG.13334.1	68	0.34305	6.71295166	1
MSTRG.13334.4	70	0.28167	6.360534668	1
MSTRG.13318.1	661	0.43585	6.069763184	1
MSTRG.13345.1	76	0.39312	5.425231934	1
MSTRG.13356.1	617	0.41438	7.598449707	1
MSTRG.13357.1	145	0.32022	8.38458252	1
MSTRG.13356.3	453	0.40571	8.183288574	1
MSTRG.13356.4	617	0.35564	7.598449707	1
MSTRG.13356.5	191	0.28883	6.788513184	1
MSTRG.13364.2	140	0.36624	4.824279785	1
MSTRG.13365.1	33	0.38809	4.601013184	-1

MSTRG.13366.3	88	0.31204	8.977722168	1
MSTRG.13367.1	34	0.39413	8.843200684	-1
MSTRG.13367.2	34	0.36078	8.843200684	-1
MSTRG.13369.2	151	0.31369	8.712463379	1
MSTRG.13369.4	151	0.33363	8.712463379	1
MSTRG.13369.3	151	0.35673	8.712463379	1
MSTRG.13378.1	63	0.32238	9.573181152	1
MSTRG.13378.2	63	0.36507	9.573181152	1
MSTRG.13381.2	68	0.33563	9.149475098	1
MSTRG.13389.1	782	0.36837	6.723815918	1
MSTRG.13391.6	491	0.4618	8.933410645	1
MSTRG.13391.3	575	0.45413	7.023010254	1
MSTRG.13396.1	66	0.3118	5.053649902	1
MSTRG.13397.1	129	0.28982	10.02630615	1
MSTRG.13398.1	185	0.30664	8.52557373	1
MSTRG.13401.1	100	0.30972	8.422912598	1
MSTRG.13409.2	1548	0.47572	5.695739746	1
MSTRG.13410.1	157	0.30837	9.442932129	1
MSTRG.13410.2	157	0.32955	9.442932129	1
MSTRG.13409.3	1486	0.46957	5.686340332	1
MSTRG.13409.4	1233	0.46883	5.965637207	1
MSTRG.13409.5	1176	0.46883	5.92401123	1
MSTRG.13409.8	1069	0.4673	8.252258301	1
MSTRG.13409.6	1138	0.48742	7.557312012	1
MSTRG.13409.7	1069	0.4673	8.252258301	1
MSTRG.13409.9	1138	0.46947	7.557312012	1
MSTRG.13411.1	81	0.32237	9.426696777	1
MSTRG.13411.4	651	0.4714	4.893371582	1
MSTRG.13423.1	74	0.33179	9.055480957	-1
MSTRG.13425.1	179	0.28419	9.502502441	1
MSTRG.13427.1	149	0.31565	6.585510254	1
MSTRG.13427.2	238	0.30644	5.58782959	1
MSTRG.13427.4	238	0.35231	5.58782959	1
MSTRG.13427.3	149	0.43896	6.585510254	1
MSTRG.13427.6	188	0.36382	5.798034668	1
MSTRG.13427.8	197	0.34416	5.028015137	1
MSTRG.13427.9	45	0.39196	11.88165283	1
MSTRG.13427.10	66	0.37054	11.41766357	1
MSTRG.13427.11	18	0.4278	5.583068848	1
MSTRG.13428.1	189	0.28982	10.38995361	1
MSTRG.13428.2	125	0.27958	10.11029053	1
MSTRG.13429.1	143	0.28982	9.829650879	1
MSTRG.13430.1	84	0.37017	10.94671631	1
MSTRG.13431.1	140	0.27433	11.07843018	1
MSTRG.13431.2	128	0.3136	6.017272949	1
MSTRG.13432.1	100	0.29772	5.985534668	1
MSTRG.13413.2	134	0.43017	5.36505127	1
MSTRG.13414.1	200	0.29754	10.35028076	1
MSTRG.13418.1	93	0.26141	7.93170166	1
MSTRG.13420.1	286	0.31035	8.645202637	1
MSTRG.13421.2	228	0.35168	6.065368652	1
MSTRG.13419.1	30	0.43381	9.626525879	-1
MSTRG.13419.2	39	0.36949	10.05609131	1
MSTRG.13419.3	37	0.33815	5.821105957	1

MSTRG. 13433. 1	119	0. 36687	9. 722351074	1
MSTRG. 13437. 2	177	0. 37819	8. 38458252	1
MSTRG. 13440. 1	62	0. 29561	9. 68963623	1
MSTRG. 13449. 1	36	0. 35325	6. 045593262	1
MSTRG. 13450. 3	630	0. 4162	5. 380065918	1
MSTRG. 13450. 2	974	0. 3892	5. 90020752	1
MSTRG. 13451. 1	61	0. 30159	10. 01495361	1
MSTRG. 13454. 2	76	0. 33939	9. 895202637	1
MSTRG. 13456. 1	120	0. 29366	5. 695861816	1
MSTRG. 13456. 2	93	0. 26449	7. 543640137	1
MSTRG. 13457. 1	89	0. 28478	10. 28717041	1
MSTRG. 13459. 1	14	0. 45751	7. 976013184	1
MSTRG. 13461. 6	163	0. 3163	9. 657653809	1
MSTRG. 13461. 5	480	0. 37649	9. 335021973	1
MSTRG. 13462. 1	36	0. 33013	8. 295471191	1
MSTRG. 13481. 1	13	0. 4604	3. 998840332	1
MSTRG. 13486. 2	194	0. 34432	8. 945251465	1
MSTRG. 13492. 1	94	0. 35226	10. 4307251	1
MSTRG. 13506. 1	56	0. 36477	10. 54095459	1
MSTRG. 13506. 2	108	0. 39869	11. 54949951	1
MSTRG. 13506. 3	108	0. 40273	11. 54949951	1
MSTRG. 13506. 4	108	0. 41875	11. 54949951	1
MSTRG. 13510. 1	1907	0. 40013	6. 520812988	1
MSTRG. 13510. 2	1907	0. 46046	6. 520812988	1
MSTRG. 13510. 3	1906	0. 4673	6. 520812988	1
MSTRG. 13510. 4	1906	0. 44119	6. 520812988	1
MSTRG. 13510. 5	1431	0. 44793	8. 075744629	1
MSTRG. 13510. 6	1171	0. 46073	6. 487731934	1
MSTRG. 13497. 2	650	0. 42861	5. 74041748	1
MSTRG. 13504. 1	38	0. 37577	9. 304382324	1
MSTRG. 13512. 4	403	0. 41149	6. 27142334	1
MSTRG. 13512. 6	428	0. 48155	5. 742126465	1
MSTRG. 13512. 3	552	0. 48192	6. 105651855	1
MSTRG. 13512. 7	356	0. 44533	6. 094055176	1
MSTRG. 13512. 8	356	0. 4501	6. 094055176	1
MSTRG. 13512. 9	451	0. 4366	5. 785461426	1
MSTRG. 13518. 1	425	0. 3458	6. 729797363	1
MSTRG. 13518. 3	171	0. 29328	8. 684143066	1
MSTRG. 13519. 1	314	0. 35314	4. 526794434	1
MSTRG. 13520. 4	785	0. 46074	9. 434875488	1
MSTRG. 13531. 1	143	0. 28803	9. 330749512	1
MSTRG. 13532. 1	642	0. 42176	7. 076843262	1
MSTRG. 13532. 3	65	0. 42433	8. 760437012	1
MSTRG. 13532. 6	642	0. 42451	7. 076843262	1
MSTRG. 13538. 2	488	0. 47472	8. 333190918	1
MSTRG. 13540. 4	39	0. 46003	12. 847229	1
MSTRG. 13543. 1	75	0. 47243	6. 498596191	-1
MSTRG. 13554. 1	213	0. 28982	8. 543762207	1
MSTRG. 13555. 1	35	0. 3374	6. 530212402	1
MSTRG. 13560. 2	179	0. 41442	8. 130187988	1
MSTRG. 13560. 3	205	0. 38463	6. 627868652	1
MSTRG. 13562. 1	114	0. 428	4. 259460449	1
MSTRG. 13564. 1	772	0. 44283	6. 72454834	1
MSTRG. 13564. 2	769	0. 39517	6. 72454834	1

MSTRG.13563.1	161	0.28305	9.148254395	1
MSTRG.13563.2	50	0.35626	9.497619629	1
MSTRG.13566.3	1376	0.4661	6.336608887	1
MSTRG.13568.5	391	0.46974	8.442443848	1
MSTRG.13568.6	458	0.47097	6.741882324	1
MSTRG.13570.1	1017	0.30827	8.14239502	1
MSTRG.13578.1	1916	0.37291	9.117736816	1
MSTRG.13578.4	526	0.3582	7.468322754	1
MSTRG.13578.2	1845	0.37975	9.226135254	1
MSTRG.13578.3	1935	0.37291	9.088439941	1
MSTRG.13578.6	1895	0.37291	9.112731934	1
MSTRG.13578.7	1936	0.37291	9.088439941	1
MSTRG.13578.8	1864	0.37975	9.198181152	1
MSTRG.13578.9	1917	0.37975	9.117736816	1
MSTRG.13578.10	1153	0.3251	9.533630371	1
MSTRG.13578.11	1298	0.34512	9.617248535	1
MSTRG.13579.1	183	0.33783	11.46160889	1
MSTRG.13580.1	73	0.4179	5.980407715	1
MSTRG.13580.2	47	0.37998	5.303771973	1
MSTRG.13578.12	756	0.46587	6.667541504	1
MSTRG.13578.13	774	0.36467	6.584655762	1
MSTRG.13578.14	755	0.34547	6.667541504	1
MSTRG.13578.15	756	0.46359	6.667541504	1
MSTRG.13578.16	558	0.37142	6.281677246	1
MSTRG.13581.1	130	0.39812	11.269104	-1
MSTRG.13583.1	122	0.30708	10.22723389	1
MSTRG.13584.2	94	0.39842	9.61895752	1
MSTRG.13572.3	596	0.46477	8.199157715	1
MSTRG.13573.4	404	0.45897	5.900939941	1
MSTRG.13573.5	384	0.46717	5.68170166	1
MSTRG.13573.3	499	0.41403	6.900939941	1
MSTRG.13573.7	498	0.43712	6.900939941	1
MSTRG.13573.1	498	0.41707	6.900939941	1
MSTRG.13575.2	382	0.37041	8.770324707	1
MSTRG.13576.4	337	0.4321	5.674255371	1
MSTRG.13576.3	337	0.44468	5.674255371	1
MSTRG.13576.5	346	0.36759	5.33416748	1
MSTRG.13576.6	210	0.36759	8.785827637	1
MSTRG.13576.7	220	0.40668	7.675720215	1
MSTRG.13576.8	346	0.37647	5.33416748	1
MSTRG.13576.9	373	0.39492	5.772155762	1
MSTRG.13577.2	46	0.36457	3.434265137	1
MSTRG.13577.1	110	0.33069	3.940734863	1
MSTRG.13577.5	222	0.41408	4.917541504	1
MSTRG.13577.9	46	0.39643	3.434265137	1
MSTRG.13577.10	118	0.4384	5.037780762	1
MSTRG.13577.11	182	0.41654	4.518005371	1
MSTRG.13577.6	110	0.36879	3.940734863	1
MSTRG.13577.7	134	0.40981	6.278015137	1
MSTRG.13577.8	110	0.36879	3.940734863	1
MSTRG.13577.12	64	0.36604	4.328796387	1
MSTRG.13589.3	576	0.36364	8.702697754	1
MSTRG.13589.4	182	0.35925	9.85369873	1
MSTRG.13589.7	202	0.33506	4.132019043	1

MSTRG.13589.6	555	0.3351	4.968322754	1
MSTRG.13591.1	48	0.30656	9.516662598	1
MSTRG.13593.5	565	0.46638	7.859680176	1
MSTRG.13593.6	572	0.46815	7.863220215	1
MSTRG.13593.7	507	0.45182	7.531433105	1
MSTRG.13594.1	80	0.35756	7.026794434	1
MSTRG.13593.8	507	0.41088	7.531433105	1
MSTRG.13595.2	407	0.4095	5.66583252	1
MSTRG.13595.1	347	0.41202	5.152770996	1
MSTRG.13596.1	85	0.27767	6.823547363	1
MSTRG.13597.1	132	0.29848	10.11029053	1
MSTRG.13586.1	1453	0.42475	6.268249512	1
MSTRG.13586.2	1453	0.45492	6.268249512	1
MSTRG.13586.3	1433	0.41266	6.334411621	1
MSTRG.13587.1	67	0.26785	5.203430176	1
MSTRG.13598.2	104	0.36207	4.508483887	1
MSTRG.13599.1	355	0.34715	8.955627441	1
MSTRG.13599.2	282	0.42248	6.188659668	1
MSTRG.13599.3	282	0.40032	6.188659668	1
MSTRG.13599.4	153	0.34196	7.756896973	1
MSTRG.13602.1	315	0.43032	4.725158691	1
MSTRG.13602.3	315	0.40851	4.725158691	1
MSTRG.13602.5	315	0.41213	4.725158691	1
MSTRG.13600.1	161	0.32565	7.497009277	-1
MSTRG.13603.1	89	0.36983	8.635681152	1
MSTRG.13603.2	151	0.30697	10.57281494	1
MSTRG.13608.3	676	0.38017	7.274475098	1
MSTRG.13608.2	676	0.38106	7.274475098	1
MSTRG.13608.6	676	0.45676	7.274475098	1
MSTRG.13608.5	676	0.458	7.274475098	1
MSTRG.13605.1	901	0.37917	5.251525879	1
MSTRG.13612.2	130	0.36554	5.768981934	1
MSTRG.13614.2	587	0.45031	6.642883301	1
MSTRG.13618.3	127	0.44413	6.577575684	1
MSTRG.13618.1	222	0.36341	4.614074707	1
MSTRG.13618.2	222	0.34063	4.614074707	1
MSTRG.13627.2	1070	0.36123	6.113708496	1
MSTRG.13627.3	1070	0.38518	6.113708496	1
MSTRG.13630.1	435	0.45833	5.762390137	1
MSTRG.13637.2	358	0.36515	5.328796387	1
MSTRG.13657.4	193	0.31207	5.403747559	1
MSTRG.13658.8	539	0.41896	4.963562012	1
MSTRG.13666.4	102	0.39607	5.854919434	1
MSTRG.13666.3	142	0.4289	5.048278809	1
MSTRG.13666.5	102	0.4315	5.854919434	1
MSTRG.13666.2	102	0.40298	5.854919434	1
MSTRG.13667.1	1290	0.50382	5.597229004	1
MSTRG.13667.5	793	0.34385	4.929382324	1
MSTRG.13667.6	1249	0.50382	5.695617676	1
MSTRG.13667.13	559	0.3458	9.414001465	1
MSTRG.13667.12	229	0.32961	10.6696167	1
MSTRG.13652.1	289	0.37154	6.750427246	1
MSTRG.13652.2	289	0.41565	6.750427246	1
MSTRG.13652.3	25	0.37315	9.497009277	1

MSTRG.13653.2	336	0.38505	8.72845459	1
MSTRG.13653.3	160	0.38895	6.286560059	1
MSTRG.13669.2	540	0.44383	5.906433105	1
MSTRG.13671.1	72	0.33311	7.848327637	1
MSTRG.13672.6	213	0.46582	6.848205566	1
MSTRG.13672.5	213	0.46954	6.848205566	1
MSTRG.13672.4	213	0.45743	6.848205566	1
MSTRG.13682.1	68	0.37465	5.33392334	1
MSTRG.13684.1	90	0.45742	8.93548584	1
MSTRG.13687.2	39	0.44671	10.78057861	1
MSTRG.13690.1	161	0.39037	4.66229248	1
MSTRG.13690.2	177	0.38919	5.805480957	1
MSTRG.13690.5	120	0.37014	9.460388184	-1
MSTRG.13690.3	161	0.42039	4.66229248	1
MSTRG.13690.4	120	0.39471	9.460388184	-1
MSTRG.13711.1	2583	0.40572	6.026428223	1
MSTRG.13711.2	2304	0.48574	6.208312988	1
MSTRG.13711.3	2377	0.45855	6.080749512	1
MSTRG.13711.4	2583	0.48729	6.026428223	1
MSTRG.13711.5	2583	0.40572	6.026428223	1
MSTRG.13711.6	2244	0.46421	6.392883301	1
MSTRG.13713.1	601	0.45045	4.68560791	1
MSTRG.13713.3	611	0.4539	4.721862793	1
MSTRG.13713.2	612	0.4616	4.721862793	1
MSTRG.13706.1	293	0.4027	4.944519043	1
MSTRG.13706.18	307	0.44069	4.952697754	1
MSTRG.13706.20	293	0.42989	4.944519043	1
MSTRG.13706.17	306	0.44355	4.952697754	1
MSTRG.13709.1	113	0.35162	8.860168457	1
MSTRG.13706.22	293	0.30509	4.944519043	1
MSTRG.13727.1	357	0.36272	5.491149902	1
MSTRG.13727.2	357	0.44109	5.491149902	1
MSTRG.13727.3	357	0.43547	5.491149902	1
MSTRG.13727.4	357	0.45553	5.491149902	1
MSTRG.13725.4	303	0.30958	8.85369873	1
MSTRG.13725.6	109	0.32721	8.898498535	1
MSTRG.13725.7	272	0.33828	8.853820801	1
MSTRG.13725.10	119	0.38888	9.776428223	1
MSTRG.13725.12	272	0.27075	8.853820801	1
MSTRG.13725.13	272	0.3485	8.853820801	1
MSTRG.13729.1	63	0.40631	6.234436035	1
MSTRG.13714.3	167	0.47155	9.52935791	1
MSTRG.13717.1	29	0.30328	9.186218262	1
MSTRG.13723.1	210	0.28199	6.080383301	1
MSTRG.13738.1	287	0.39805	9.120666504	1
MSTRG.13746.1	60	0.35775	6.874572754	1
MSTRG.13746.2	68	0.32408	6.874938965	1
MSTRG.13755.2	58	0.38343	10.58660889	1
MSTRG.13755.1	1150	0.3618	6.604797363	1
MSTRG.13755.3	1150	0.39138	6.604797363	1
MSTRG.13755.5	1150	0.46343	6.604797363	1
MSTRG.13755.6	1135	0.46174	6.797302246	1
MSTRG.13756.1	177	0.31432	9.631164551	1
MSTRG.13758.2	296	0.28808	9.499206543	1

MSTRG.13758.3	117	0.28165	10.15020752	1
MSTRG.13758.4	80	0.34711	7.964050293	1
MSTRG.13750.1	29	0.4429	11.43719482	1
MSTRG.13760.1	157	0.36854	9.226135254	1
MSTRG.13760.2	47	0.29919	5.424865723	1
MSTRG.13759.2	224	0.43848	4.453430176	1
MSTRG.13761.2	2202	0.47438	5.268981934	1
MSTRG.13767.1	153	0.32324	9.300964355	1
MSTRG.13768.4	1419	0.42238	8.392028809	1
MSTRG.13768.3	1419	0.43624	8.392028809	1
MSTRG.13768.2	1419	0.4199	8.392028809	1
MSTRG.13768.5	632	0.40391	9.285949707	1
MSTRG.13769.1	183	0.33178	10.56329346	1
MSTRG.13768.7	350	0.39341	8.413391113	1
MSTRG.13765.1	933	0.4354	9.064758301	1
MSTRG.13765.2	926	0.41609	9.165344238	1
MSTRG.13764.1	377	0.48521	5.229431152	1
MSTRG.13780.2	51	0.34699	3.768493652	1
MSTRG.13782.1	209	0.31048	10.29351807	1
MSTRG.13780.3	75	0.30167	10.07830811	1
MSTRG.13783.1	103	0.3904	9.120666504	1
MSTRG.13784.1	55	0.28165	7.856384277	1
MSTRG.13808.1	239	0.30958	6.489929199	1
MSTRG.13808.3	281	0.37623	6.300231934	1
MSTRG.13808.4	281	0.34129	6.300231934	1
MSTRG.13808.2	281	0.34907	6.300231934	1
MSTRG.13808.5	115	0.29367	7.504211426	1
MSTRG.13809.1	69	0.34157	6.890930176	1
MSTRG.13811.1	99	0.36089	8.017883301	1
MSTRG.13819.3	218	0.49137	4.953674316	1
MSTRG.13820.1	36	0.34405	7.822692871	1
MSTRG.13813.1	35	0.33096	6.207336426	1
MSTRG.13822.1	53	0.34846	4.225524902	1
MSTRG.13829.1	43	0.36049	5.335266113	1
MSTRG.13831.2	89	0.37123	4.392028809	1
MSTRG.13831.3	462	0.42955	5.037414551	1
MSTRG.13832.1	79	0.37582	9.776062012	1
MSTRG.13831.4	322	0.42678	6.066101074	1
MSTRG.13836.1	445	0.39317	9.016906738	1
MSTRG.13837.1	99	0.29835	9.922912598	1
MSTRG.13838.5	1139	0.50132	5.715148926	1
MSTRG.13838.6	732	0.43561	8.280334473	1
MSTRG.13834.2	1048	0.47938	5.076965332	1
MSTRG.13844.2	477	0.26913	8.954162598	1
MSTRG.13844.4	775	0.36083	9.440002441	1
MSTRG.13845.1	182	0.33287	10.42034912	1
MSTRG.13846.1	356	0.45818	5.594543457	1
MSTRG.13846.2	264	0.44299	5.231140137	1
MSTRG.13854.3	482	0.44231	5.109680176	1
MSTRG.13849.2	167	0.35654	6.718811035	1
MSTRG.13858.2	1565	0.41327	9.363952637	1
MSTRG.13859.1	44	0.27781	10.20733643	1
MSTRG.13862.3	122	0.3363	7.57220459	1
MSTRG.13862.2	450	0.3799	6.444152832	1

MSTRG.13862.4	450	0.46956	6.444152832	1
MSTRG.13862.5	90	0.43937	9.061828613	-1
MSTRG.13867.1	462	0.39189	10.08355713	1
MSTRG.13870.4	1089	0.48204	6.057189941	1
MSTRG.13871.3	845	0.44645	5.207702637	1
MSTRG.13871.4	803	0.41774	6.035827637	1
MSTRG.13877.3	3004	0.47254	6.597351074	1
MSTRG.13877.4	2955	0.47254	6.425231934	1
MSTRG.13877.5	1525	0.41193	5.557556152	1
MSTRG.13884.4	1504	0.3943	6.38079834	1
MSTRG.13884.6	1504	0.4184	6.38079834	1
MSTRG.13884.5	1504	0.38965	6.38079834	1
MSTRG.13884.7	1504	0.4184	6.38079834	1
MSTRG.13884.8	1034	0.38965	5.67791748	1
MSTRG.13885.2	133	0.33109	8.230163574	1
MSTRG.13885.3	216	0.38556	5.170349121	1
MSTRG.13882.1	370	0.35942	5.618347168	1
MSTRG.13881.1	225	0.41572	4.981994629	1
MSTRG.13886.2	758	0.42249	6.483215332	1
MSTRG.13886.5	427	0.45936	8.251647949	1
MSTRG.13888.1	105	0.39405	7.690856934	-1
MSTRG.13900.1	76	0.44381	11.23284912	1
MSTRG.13902.1	127	0.33613	6.393737793	1
MSTRG.13903.3	1664	0.45206	6.590637207	1
MSTRG.13904.1	71	0.43616	9.588806152	1
MSTRG.13908.1	4465	0.39685	6.22869873	1
MSTRG.13908.2	3607	0.37607	5.873962402	1
MSTRG.13910.2	1195	0.4547	6.661804199	1
MSTRG.13910.5	1236	0.4205	6.542785645	1
MSTRG.13910.6	1150	0.43224	6.473205566	1
MSTRG.13913.3	1232	0.44684	5.670715332	1
MSTRG.13913.4	98	0.31268	6.710754395	1
MSTRG.13891.1	98	0.33184	8.986755371	1
MSTRG.13899.1	73	0.2787	9.382019043	1
MSTRG.13918.2	131	0.29879	10.43939209	1
MSTRG.13918.3	106	0.37994	10.5927124	1
MSTRG.13919.2	446	0.37033	8.341003418	1
MSTRG.13919.1	380	0.32846	7.136169434	1
MSTRG.13940.1	82	0.30492	9.585021973	1
MSTRG.13941.2	334	0.42716	7.280700684	1
MSTRG.13941.3	334	0.45511	7.280700684	1
MSTRG.13942.1	93	0.33158	6.057067871	1
MSTRG.13942.2	50	0.35915	6.706726074	1
MSTRG.13942.3	81	0.34	9.936340332	-1
MSTRG.13942.4	41	0.31427	10.59552002	1
MSTRG.13942.5	58	0.3291	10.28094482	-1
MSTRG.13942.6	67	0.38028	9.097717285	-1
MSTRG.13942.7	40	0.40224	10.59552002	-1
MSTRG.13942.10	45	0.36871	7.978942871	1
MSTRG.13942.11	16	0.35959	10.7366333	1
MSTRG.13942.12	16	0.42051	10.7366333	1
MSTRG.13942.8	16	0.3379	10.7366333	1
MSTRG.13942.9	16	0.41711	10.7366333	1
MSTRG.13942.13	16	0.40665	10.7366333	1

MSTRG.13942.17	16	0.35959	10.7366333	1
MSTRG.13942.18	16	0.37131	10.7366333	1
MSTRG.13942.19	20	0.32711	9.976501465	1
MSTRG.13942.20	16	0.42083	10.7366333	1
MSTRG.13942.14	16	0.36176	10.7366333	1
MSTRG.13942.15	16	0.41298	10.7366333	1
MSTRG.13942.16	50	0.37309	8.937194824	1
MSTRG.13942.21	16	0.36145	10.7366333	1
MSTRG.13942.22	16	0.35676	10.7366333	1
MSTRG.13942.25	21	0.3318	9.976501465	1
MSTRG.13942.27	16	0.33554	10.7366333	1
MSTRG.13942.28	16	0.36145	10.7366333	1
MSTRG.13942.29	30	0.37948	8.939025879	1
MSTRG.13942.30	16	0.38288	10.7366333	1
MSTRG.13942.31	16	0.41529	10.7366333	1
MSTRG.13942.23	16	0.42299	10.7366333	1
MSTRG.13942.24	16	0.34171	10.7366333	1
MSTRG.13942.26	16	0.35959	10.7366333	1
MSTRG.13942.32	16	0.41529	10.7366333	1
MSTRG.13942.33	31	0.36638	10.66680908	-1
MSTRG.13927.1	229	0.30394	4.810119629	1
MSTRG.13928.2	1256	0.45308	6.774597168	-1
MSTRG.13944.3	684	0.45193	7.84576416	1
MSTRG.13945.1	50	0.35526	12.31060791	1
MSTRG.13946.3	475	0.45816	5.86505127	1
MSTRG.13929.2	384	0.40285	9.11517334	1
MSTRG.13930.2	63	0.30901	6.227355957	1
MSTRG.13952.1	302	0.39501	5.094299316	1
MSTRG.13952.2	284	0.47004	4.837097168	1
MSTRG.13952.3	147	0.45086	5.240661621	1
MSTRG.13955.1	127	0.3767	9.713562012	1
MSTRG.13962.2	73	0.37513	11.46514893	1
MSTRG.13964.1	49	0.42745	5.727111816	1
MSTRG.13965.1	69	0.42004	9.355773926	1
MSTRG.13969.2	40	0.34289	5.49786377	1
MSTRG.13971.1	73	0.38287	9.488098145	1
MSTRG.13981.1	50	0.3462	8.931213379	1
MSTRG.13981.2	50	0.36295	8.931213379	1
MSTRG.13982.1	66	0.37805	5.64251709	1
MSTRG.13983.1	99	0.39032	4.902038574	1
MSTRG.13984.1	741	0.30721	8.646057129	1
MSTRG.13984.3	741	0.31369	8.646057129	1
MSTRG.13984.4	741	0.40204	8.646057129	1
MSTRG.13986.1	77	0.31035	9.020568848	1
MSTRG.13980.2	65	0.29343	7.960388184	1
MSTRG.14009.1	4855	0.45965	5.701843262	1
MSTRG.14009.2	4855	0.45461	5.718688965	1
MSTRG.14009.3	4841	0.45965	5.718566895	1
MSTRG.14009.4	4856	0.45965	5.727233887	1
MSTRG.14010.7	268	0.29793	7.566467285	1
MSTRG.14010.6	435	0.39288	8.505554199	1
MSTRG.14010.8	256	0.43599	7.965759277	1
MSTRG.14010.9	202	0.27643	8.649719238	1
MSTRG.13999.1	94	0.35231	9.510070801	1

MSTRG.14003.1	141	0.32573	9.659240723	1
MSTRG.14003.4	16	0.35989	12.30108643	1
MSTRG.14001.2	339	0.40654	8.755310059	1
MSTRG.14014.1	33	0.45113	4.780578613	-1
MSTRG.14034.2	82	0.34159	8.968811035	1
MSTRG.14035.1	112	0.35915	8.861022949	1
MSTRG.14036.1	124	0.29772	9.326843262	1
MSTRG.14037.1	208	0.32518	9.336242676	1
MSTRG.14036.2	89	0.28584	8.982727051	1
MSTRG.14038.1	68	0.3152	9.506286621	1
MSTRG.14038.2	76	0.34162	8.274230957	1
MSTRG.14039.1	163	0.29772	9.316345215	1
MSTRG.14039.2	201	0.29367	9.042419434	1
MSTRG.14041.1	146	0.28923	9.825134277	1
MSTRG.14034.3	386	0.3582	5.307312012	1
MSTRG.14034.5	85	0.29772	6.157409668	1
MSTRG.14034.6	468	0.40208	6.25189209	1
MSTRG.14043.1	62	0.35756	8.766784668	1
MSTRG.14026.1	105	0.38962	4.773620605	1
MSTRG.14029.2	76	0.38407	6.72064209	-1
MSTRG.14030.1	58	0.29176	9.945495605	1
MSTRG.14059.4	986	0.46535	6.757873535	1
MSTRG.14059.3	995	0.43617	6.805236816	1
MSTRG.14059.5	985	0.4374	6.757873535	1
MSTRG.14059.6	994	0.4374	6.713684082	1
MSTRG.14060.2	68	0.34328	4.66973877	1
MSTRG.14063.1	61	0.31176	6.008972168	1
MSTRG.14066.3	600	0.46372	10.34039307	1
MSTRG.14047.1	28	0.3345	5.939025879	1
MSTRG.14050.4	272	0.44118	5.628479004	1
MSTRG.14069.1	370	0.28883	6.340148926	1
MSTRG.14069.2	154	0.3319	10.41326904	1
MSTRG.14075.1	682	0.44835	6.515319824	1
MSTRG.14075.6	580	0.4072	7.116760254	1
MSTRG.14076.1	70	0.29849	11.32415771	1
MSTRG.14077.1	405	0.34437	6.769836426	1
MSTRG.14077.2	205	0.41804	5.985778809	1
MSTRG.14079.1	183	0.28026	10.96490479	1
MSTRG.14079.2	94	0.26101	9.989196777	1
MSTRG.14081.1	75	0.25685	5.722473145	1
MSTRG.14082.1	74	0.35594	6.528503418	1
MSTRG.14083.1	64	0.31903	7.866760254	1
MSTRG.14084.1	249	0.38643	9.864685059	1
MSTRG.14085.1	441	0.29615	9.058532715	1
MSTRG.14093.10	802	0.38486	9.156799316	1
MSTRG.14093.11	794	0.36226	9.183654785	1
MSTRG.14093.4	802	0.32102	9.156799316	1
MSTRG.14093.5	733	0.32318	9.225280762	1
MSTRG.14093.6	733	0.38109	9.225280762	1
MSTRG.14093.7	794	0.36226	9.183654785	1
MSTRG.14093.9	733	0.35854	9.225280762	1
MSTRG.14093.12	794	0.31501	9.183654785	1
MSTRG.14094.1	118	0.29308	11.44769287	1
MSTRG.14095.1	114	0.31293	5.071350098	1

MSTRG.14099.1	263	0.40013	8.796447754	-1
MSTRG.14100.2	273	0.42614	5.862854004	1
MSTRG.14089.2	270	0.30957	8.934753418	1
MSTRG.14109.1	34	0.32987	6.484436035	1
MSTRG.14115.3	768	0.38074	5.620300293	1
MSTRG.14126.1	63	0.36477	5.963928223	1
MSTRG.14127.1	158	0.36237	4.762634277	1
MSTRG.14130.1	124	0.35516	10.41107178	1
MSTRG.14141.6	1087	0.44901	7.350036621	1
MSTRG.14141.9	1087	0.46941	7.350036621	1
MSTRG.14141.7	974	0.43007	6.356750488	1
MSTRG.14141.11	79	0.27517	5.024230957	1
MSTRG.14142.3	1871	0.42119	8.55255127	1
MSTRG.14142.4	1846	0.49993	8.52911377	1
MSTRG.14143.1	20	0.34773	7.858581543	1
MSTRG.14144.2	254	0.33585	7.993835449	1
MSTRG.14147.1	168	0.2943	6.82208252	1
MSTRG.14147.2	60	0.31793	5.59552002	1
MSTRG.14153.2	160	0.44409	9.615539551	1
MSTRG.14153.3	160	0.41954	9.615539551	1
MSTRG.14157.1	137	0.28063	9.459533691	1
MSTRG.14157.2	137	0.28842	9.459533691	1
MSTRG.14157.4	137	0.28923	9.459533691	1
MSTRG.14157.3	183	0.28842	11.17718506	1
MSTRG.14157.5	183	0.28923	11.17718506	1
MSTRG.14167.1	122	0.32061	11.3706665	-1
MSTRG.14172.1	19	0.40378	5.937683105	1
MSTRG.14177.1	17	0.4132	12.00848389	1
MSTRG.14182.3	236	0.48367	8.69732666	1
MSTRG.14183.4	336	0.37723	8.610656738	1
MSTRG.14183.6	319	0.35712	8.143737793	1
MSTRG.14183.7	336	0.35371	8.610656738	1
MSTRG.14183.5	336	0.40512	8.610656738	1
MSTRG.14183.1	535	0.4414	8.543762207	1
MSTRG.14183.8	515	0.43865	8.547546387	1
MSTRG.14186.2	270	0.41662	6.116271973	1
MSTRG.14190.2	895	0.40611	5.68951416	1
MSTRG.14191.1	52	0.39805	4.911193848	1
MSTRG.14192.1	31	0.42788	10.15118408	-1
MSTRG.14193.1	374	0.37257	7.183532715	1
MSTRG.14193.2	158	0.29524	5.890808105	1
MSTRG.14193.3	630	0.34804	7.859191895	1
MSTRG.14194.3	191	0.44962	3.960266113	1
MSTRG.14194.2	191	0.42973	3.960266113	1
MSTRG.14189.2	1109	0.44042	5.858337402	1
MSTRG.14189.3	1165	0.44065	6.042663574	1
MSTRG.14198.1	116	0.28683	9.726745605	1
MSTRG.14198.2	73	0.34787	6.535827637	1
MSTRG.14198.3	64	0.28567	8.843322754	1
MSTRG.14198.4	73	0.30429	6.535827637	1
MSTRG.14198.5	95	0.31745	8.684020996	1
MSTRG.14199.3	104	0.4374	5.125793457	1
MSTRG.14201.4	245	0.44216	8.858947754	1
MSTRG.14201.5	406	0.45061	9.378845215	-1

MSTRG.14201.3	416	0.4145	9.278381348	1
MSTRG.14201.8	329	0.44876	6.461730957	1
MSTRG.14201.7	353	0.2906	6.91986084	1
MSTRG.14213.1	37	0.31989	9.383972168	1
MSTRG.14219.1	99	0.29927	7.548034668	1
MSTRG.14220.2	260	0.32237	6.480407715	1
MSTRG.14223.1	147	0.32599	8.402526855	1
MSTRG.14227.1	30	0.43543	10.05230713	1
MSTRG.14221.1	301	0.4282	7.762145996	1
MSTRG.14221.2	320	0.40669	8.884216309	1
MSTRG.14221.3	305	0.39715	8.681945801	-1
MSTRG.14233.2	38	0.27089	11.82391357	1
MSTRG.14233.3	38	0.3077	11.82391357	1
MSTRG.14233.1	120	0.34755	9.797668457	1
MSTRG.14233.4	38	0.3363	11.82391357	1
MSTRG.14235.1	48	0.39187	6.20904541	1
MSTRG.14251.3	108	0.35783	11.83673096	1
MSTRG.14251.4	183	0.28662	10.60968018	1
MSTRG.14251.5	55	0.34519	10.29986572	1
MSTRG.14257.1	178	0.29772	10.22735596	1
MSTRG.14249.6	206	0.44151	5.96697998	1
MSTRG.14249.7	204	0.39341	5.784484863	1
MSTRG.14249.5	207	0.42803	5.792175293	1
MSTRG.14249.8	198	0.37039	5.613830566	1
MSTRG.14248.2	143	0.32395	5.192810059	1
MSTRG.14266.3	1090	0.47549	5.629699707	1
MSTRG.14269.2	170	0.39126	5.948547363	1
MSTRG.14269.4	111	0.30664	6.478942871	1
MSTRG.14272.2	817	0.48244	9.09161377	1
MSTRG.14277.1	67	0.29395	6.809265137	1
MSTRG.14278.1	102	0.3136	6.378723145	-1
MSTRG.14287.6	1319	0.41368	5.697570801	1
MSTRG.14287.7	1329	0.41984	5.635437012	1
MSTRG.14287.8	1300	0.42754	5.628601074	1
MSTRG.14287.10	1300	0.43379	5.628601074	1
MSTRG.14287.11	1328	0.45989	5.570251465	1
MSTRG.14287.12	1300	0.43298	5.628601074	1
MSTRG.14287.9	1300	0.44321	5.628601074	1
MSTRG.14287.13	1328	0.46297	5.570251465	1
MSTRG.14288.1	110	0.35868	5.471252441	1
MSTRG.14287.14	595	0.4106	5.25201416	1
MSTRG.14280.1	138	0.37278	5.573547363	1
MSTRG.14281.1	53	0.37391	7.815612793	1
MSTRG.14281.3	116	0.35059	9.515075684	1
MSTRG.14281.4	53	0.33128	7.815612793	1
MSTRG.14281.5	72	0.32181	6.476989746	1
MSTRG.14282.1	97	0.28883	6.802062988	1
MSTRG.14282.2	70	0.29187	5.114440918	1
MSTRG.14283.1	92	0.29374	9.624450684	1
MSTRG.14284.1	64	0.29526	8.462219238	1
MSTRG.14285.1	83	0.30387	7.755432129	1
MSTRG.14290.1	258	0.29835	8.847839355	1
MSTRG.14290.4	112	0.30387	9.380554199	1
MSTRG.14290.5	336	0.33054	8.596984863	1

MSTRG.14290.6	427	0.32654	8.848327637	1
MSTRG.14290.3	336	0.33054	8.596984863	1
MSTRG.14290.7	336	0.33754	8.596984863	1
MSTRG.14291.1	69	0.43263	5.480529785	1
MSTRG.14304.1	28	0.43878	9.77911377	1
MSTRG.14304.2	98	0.3978	6.81842041	1
MSTRG.14308.1	171	0.3363	11.246521	1
MSTRG.14295.1	77	0.29607	8.538757324	-1
MSTRG.14296.2	756	0.47328	4.279724121	1
MSTRG.14297.1	90	0.45559	5.424255371	-1
MSTRG.14298.1	22	0.40733	11.00030518	1
MSTRG.14310.2	180	0.44808	4.992004395	1
MSTRG.14311.1	83	0.29367	10.71075439	1
MSTRG.14311.2	82	0.37644	10.71075439	1
MSTRG.14311.3	83	0.31596	10.71075439	1
MSTRG.14311.4	82	0.28726	10.71075439	1
MSTRG.14313.2	118	0.30082	7.735778809	1
MSTRG.14317.2	119	0.36158	9.56060791	1
MSTRG.14318.2	89	0.3245	6.714050293	1
MSTRG.14319.1	1413	0.42337	5.525085449	-1
MSTRG.14319.2	1448	0.40942	5.38494873	1
MSTRG.14319.4	1435	0.4181	5.452697754	-1
MSTRG.14319.3	1573	0.40942	5.550964355	1
MSTRG.14319.12	1398	0.41234	5.449523926	-1
MSTRG.14319.13	687	0.45957	4.41973877	1
MSTRG.14319.14	638	0.42158	4.44354248	-1
MSTRG.14320.1	113	0.40128	7.768371582	1
MSTRG.14324.1	121	0.28883	6.558776855	1
MSTRG.14325.1	497	0.39979	9.671691895	1
MSTRG.14325.2	372	0.45535	9.825866699	1
MSTRG.14326.1	11	0.3151	3.929626465	1
MSTRG.14330.1	32	0.36698	4.36907959	1
MSTRG.14331.1	829	0.33098	8.649597168	1
MSTRG.14331.3	391	0.32203	9.04473877	1
MSTRG.14334.2	321	0.372	5.96661377	1
MSTRG.14349.2	263	0.42904	9.316467285	1
MSTRG.14350.1	209	0.37526	6.693908691	1
MSTRG.14354.1	105	0.28963	6.809631348	1
MSTRG.14343.1	103	0.43519	11.59503174	1
MSTRG.14359.2	764	0.47055	4.871398926	1
MSTRG.14360.2	358	0.45837	9.25958252	1
MSTRG.14367.4	435	0.44085	6.146179199	1
MSTRG.14367.5	435	0.42909	6.146179199	1
MSTRG.14368.1	27	0.39388	9.129211426	1
MSTRG.14369.1	128	0.27433	10.08673096	1
MSTRG.14401.1	34	0.29668	6.493225098	1
MSTRG.14409.7	6895	0.46754	5.199768066	1
MSTRG.14409.8	6869	0.46754	5.19354248	1
MSTRG.14409.9	6895	0.46534	5.199768066	1
MSTRG.14409.10	62	0.35576	10.29498291	1
MSTRG.14409.11	5897	0.46754	5.166687012	1
MSTRG.14409.12	5423	0.47486	5.146789551	1
MSTRG.14409.13	4479	0.4671	5.119567871	1
MSTRG.14409.14	4478	0.4671	5.119567871	1

MSTRG.14411.3	587	0.43603	7.590393066	1
MSTRG.14411.4	593	0.43603	7.589050293	1
MSTRG.14411.5	587	0.43997	7.590393066	1
MSTRG.14411.6	593	0.43603	7.589050293	1
MSTRG.14412.1	909	0.43818	6.647155762	1
MSTRG.14376.1	644	0.43328	5.594543457	1
MSTRG.14376.2	713	0.33263	5.965637207	1
MSTRG.14379.1	45	0.37257	9.183898926	1
MSTRG.14376.3	25	0.41095	5.426086426	1
MSTRG.14379.2	61	0.32704	12.24310303	1
MSTRG.14380.1	598	0.42576	6.06439209	-1
MSTRG.14380.2	278	0.43205	8.526794434	1
MSTRG.14371.4	288	0.43745	9.639709473	1
MSTRG.14385.1	35	0.39038	10.45001221	1
MSTRG.14386.1	43	0.41011	10.25604248	-1
MSTRG.14387.1	34	0.40862	4.895812988	1
MSTRG.14395.1	144	0.31444	10.15386963	1
MSTRG.14398.1	280	0.37112	6.405212402	1
MSTRG.14399.1	188	0.29202	10.15655518	1
MSTRG.14419.1	91	0.29059	4.716003418	1
MSTRG.14421.1	118	0.40847	9.729064941	-1
MSTRG.14422.1	119	0.36928	10.11724854	-1
MSTRG.14424.1	65	0.38051	9.840026855	1
MSTRG.14424.2	103	0.2886	9.067443848	1
MSTRG.14426.2	37	0.3346	9.497009277	1
MSTRG.14426.3	44	0.33175	6.504943848	1
MSTRG.14427.3	641	0.4392	5.895324707	1
MSTRG.14427.4	675	0.45147	5.769470215	1
MSTRG.14428.1	132	0.3	10.31768799	-1
MSTRG.14429.1	215	0.37353	9.950134277	1
MSTRG.14414.1	94	0.28432	8.654602051	1
MSTRG.14434.1	1359	0.43646	8.43939209	1
MSTRG.14435.1	198	0.41321	5.938781738	1
MSTRG.14436.2	58	0.41659	12.55194092	1
MSTRG.14437.1	232	0.43047	9.36517334	1
MSTRG.14436.4	354	0.44789	5.899475098	1
MSTRG.14437.5	232	0.35992	9.36517334	1
MSTRG.14439.2	319	0.39432	9.428039551	1
MSTRG.14439.3	319	0.3721	9.428039551	1
MSTRG.14454.3	2092	0.4762	5.559997559	-1
MSTRG.14455.1	350	0.33787	5.610534668	1
MSTRG.14461.1	50	0.33732	9.558166504	1
MSTRG.14440.1	37	0.425	5.004699707	1
MSTRG.14441.2	338	0.31105	8.119445801	1
MSTRG.14442.1	892	0.4325	5.24822998	1
MSTRG.14442.3	867	0.41941	5.446105957	1
MSTRG.14442.4	827	0.43336	5.36541748	1
MSTRG.14442.5	872	0.41941	5.35345459	1
MSTRG.14442.6	822	0.44359	5.465759277	1
MSTRG.14445.2	20	0.34164	11.39801025	-1
MSTRG.14445.1	44	0.39506	6.235534668	1
MSTRG.14445.3	42	0.34566	9.002990723	1
MSTRG.14446.1	38	0.36993	8.017883301	1
MSTRG.14447.1	946	0.37405	5.452575684	1

MSTRG.14448.1	115	0.34952	6.045227051	1
MSTRG.14453.2	557	0.41589	9.297424316	1
MSTRG.14453.4	557	0.39491	9.297424316	1
MSTRG.14453.5	334	0.41285	9.237609863	1
MSTRG.14486.1	189	0.32663	8.03302002	1
MSTRG.14486.3	189	0.34973	8.03302002	1
MSTRG.14486.4	189	0.32663	8.03302002	1
MSTRG.14487.1	698	0.32514	10.70281982	1
MSTRG.14487.2	698	0.32514	10.70281982	1
MSTRG.14488.3	102	0.28167	10.09527588	1
MSTRG.14488.5	300	0.28429	5.078063965	1
MSTRG.14491.3	272	0.4593	11.58526611	1
MSTRG.14491.5	145	0.29415	12.11505127	1
MSTRG.14491.8	145	0.29415	12.11505127	1
MSTRG.14491.7	272	0.35608	11.58526611	1
MSTRG.14491.10	272	0.29919	11.58526611	1
MSTRG.14491.6	272	0.29202	11.58526611	1
MSTRG.14491.4	272	0.41232	11.58526611	1
MSTRG.14491.11	272	0.44188	11.58526611	1
MSTRG.14489.1	370	0.40705	8.31463623	1
MSTRG.14489.3	369	0.38209	8.31463623	1
MSTRG.14489.4	370	0.33293	8.31463623	1
MSTRG.14492.2	90	0.30475	7.81854248	1
MSTRG.14492.3	114	0.30191	7.64276123	1
MSTRG.14492.4	59	0.34079	6.22833252	1
MSTRG.14493.1	126	0.28695	5.342102051	1
MSTRG.14492.6	67	0.33151	6.258361816	1
MSTRG.14492.5	108	0.31292	9.038635254	1
MSTRG.14495.1	523	0.37229	6.460144043	1
MSTRG.14508.5	82	0.2951	10.38397217	1
MSTRG.14508.7	405	0.39459	7.276672363	1
MSTRG.14508.8	1445	0.46083	6.612976074	1
MSTRG.14508.4	789	0.38829	6.61151123	1
MSTRG.14508.6	1201	0.37395	6.309753418	1
MSTRG.14508.9	614	0.39649	6.445007324	1
MSTRG.14509.1	112	0.33836	9.051696777	1
MSTRG.14508.10	531	0.33778	8.272277832	1
MSTRG.14513.1	67	0.32072	8.769470215	1
MSTRG.14512.3	1784	0.37779	8.515686035	1
MSTRG.14512.4	1784	0.41348	8.515686035	1
MSTRG.14515.1	99	0.29583	9.032287598	1
MSTRG.14515.2	35	0.3892	9.77911377	1
MSTRG.14515.3	83	0.30576	8.502868652	1
MSTRG.14516.1	68	0.39144	11.70526123	-1
MSTRG.14528.1	183	0.28982	10.70281982	1
MSTRG.14528.3	88	0.32167	7.844177246	1
MSTRG.14528.5	65	0.37955	8.064147949	1
MSTRG.14529.1	134	0.29772	9.968078613	1
MSTRG.14529.2	98	0.28584	9.130310059	1
MSTRG.14530.1	123	0.29772	9.03704834	1
MSTRG.14529.3	163	0.29367	7.904968262	1
MSTRG.14529.4	69	0.3327	7.77947998	1
MSTRG.14529.5	56	0.26113	6.694641113	1
MSTRG.14531.1	183	0.28584	10.60235596	1

MSTRG.14531.2	48	0.37017	9.489562988	1
MSTRG.14533.1	62	0.30608	7.799987793	1
MSTRG.14534.1	187	0.29772	8.984924316	1
MSTRG.14534.2	114	0.29551	10.29095459	1
MSTRG.14534.3	87	0.32086	9.41595459	1
MSTRG.14534.4	104	0.28927	8.319519043	1
MSTRG.14533.2	103	0.29793	4.972106934	1
MSTRG.14535.1	73	0.43625	9.695007324	1
MSTRG.14517.3	347	0.3299	6.115661621	1
MSTRG.14517.4	209	0.27075	5.594177246	1
MSTRG.14518.1	197	0.38567	11.00958252	1
MSTRG.14519.1	44	0.3508	6.956604004	1
MSTRG.14526.3	777	0.37963	7.300598145	1
MSTRG.14536.1	72	0.37156	8.051330566	1
MSTRG.14538.2	208	0.28165	9.615783691	1
MSTRG.14538.3	72	0.37225	9.564025879	1
MSTRG.14538.4	300	0.25289	9.059387207	1
MSTRG.14538.5	89	0.28266	7.954406738	1
MSTRG.14539.5	468	0.45105	5.17376709	1
MSTRG.14539.7	474	0.48477	5.17376709	1
MSTRG.14540.1	518	0.40314	8.576965332	1
MSTRG.14540.11	457	0.40736	7.093566895	1
MSTRG.14540.9	509	0.3223	6.808532715	1
MSTRG.14540.12	642	0.45605	8.633728027	1
MSTRG.14540.13	593	0.4514	8.646911621	1
MSTRG.14540.10	593	0.41836	8.646911621	1
MSTRG.14540.4	642	0.44681	8.633728027	1
MSTRG.14540.3	593	0.43141	8.646911621	1
MSTRG.14540.6	593	0.43141	8.646911621	1
MSTRG.14540.7	370	0.36594	9.276062012	1
MSTRG.14540.2	642	0.40129	8.633728027	1
MSTRG.14540.8	642	0.40129	8.633728027	1
MSTRG.14540.14	226	0.29351	10.04022217	1
MSTRG.14541.1	64	0.33383	5.472595215	1
MSTRG.14543.5	1017	0.48309	8.936828613	1
MSTRG.14544.1	657	0.46087	6.098449707	1
MSTRG.14553.1	353	0.35412	4.861999512	1
MSTRG.14553.2	1102	0.41299	9.367248535	1
MSTRG.14553.3	353	0.33623	4.861999512	1
MSTRG.14558.2	351	0.43708	5.338317871	1
MSTRG.14560.3	338	0.41724	5.950012207	1
MSTRG.14560.7	79	0.32442	8.68560791	1
MSTRG.14563.6	428	0.31765	5.051330566	1
MSTRG.14563.4	428	0.35176	5.051330566	1
MSTRG.14563.5	428	0.35176	5.051330566	1
MSTRG.14564.6	95	0.32154	8.757751465	1
MSTRG.14564.4	518	0.44689	8.990539551	1
MSTRG.14564.5	374	0.42034	9.032409668	1
MSTRG.14564.2	407	0.43046	8.667297363	1
MSTRG.14565.1	457	0.46022	7.564880371	1
MSTRG.14564.7	53	0.39087	6.699035645	-1
MSTRG.14564.8	407	0.43126	8.804870605	1
MSTRG.14565.2	537	0.47981	8.285583496	1
MSTRG.14566.4	141	0.30483	5.777893066	1

MSTRG.14567.1	139	0.29615	9.891296387	1
MSTRG.14568.1	195	0.37617	9.049377441	1
MSTRG.14569.1	73	0.34716	11.39654541	1
MSTRG.14556.2	116	0.36453	6.579040527	1
MSTRG.14557.1	1934	0.37898	5.627258301	1
MSTRG.14574.4	764	0.32598	8.366027832	1
MSTRG.14576.2	109	0.40671	9.154846191	1
MSTRG.14579.4	2094	0.45421	6.060974121	1
MSTRG.14580.1	449	0.44832	9.01739502	1
MSTRG.14580.3	270	0.44094	9.135925293	1
MSTRG.14570.2	316	0.44679	8.881286621	1
MSTRG.14573.2	47	0.33595	9.765319824	1
MSTRG.14583.1	163	0.35927	9.296936035	1
MSTRG.14593.1	276	0.45722	5.412414551	1
MSTRG.14593.3	540	0.44434	6.153503418	1
MSTRG.14593.4	546	0.40057	6.195129395	1
MSTRG.14593.5	348	0.27251	8.386535645	1
MSTRG.14593.7	195	0.27251	4.644714355	1
MSTRG.14600.1	215	0.37269	5.731018066	1
MSTRG.14600.8	215	0.36029	5.709289551	1
MSTRG.14600.11	1021	0.42238	8.920837402	1
MSTRG.14600.5	1066	0.43199	9.210144043	1
MSTRG.14600.6	901	0.40698	8.996643066	1
MSTRG.14600.7	1034	0.4199	9.087097168	1
MSTRG.14600.2	1067	0.43199	9.210144043	1
MSTRG.14600.3	1033	0.4199	9.087097168	1
MSTRG.14600.9	934	0.4045	9.151062012	1
MSTRG.14600.12	627	0.33828	9.682067871	1
MSTRG.14601.1	55	0.36036	6.12689209	1
MSTRG.14600.13	75	0.28936	10.06011963	1
MSTRG.14600.14	75	0.30897	10.06011963	1
MSTRG.14603.2	255	0.3346	7.540710449	1
MSTRG.14605.3	102	0.32831	7.65423584	1
MSTRG.14605.4	131	0.31912	8.844665527	1
MSTRG.14605.2	116	0.41138	9.768127441	1
MSTRG.14605.5	79	0.27075	5.992614746	1
MSTRG.14591.4	350	0.32649	8.91595459	1
MSTRG.14591.5	375	0.3473	8.593566895	1
MSTRG.14589.1	42	0.37774	6.700744629	1
MSTRG.14590.1	78	0.43501	6.720397949	1
MSTRG.14592.1	433	0.43753	8.269470215	1
MSTRG.14592.2	476	0.43793	8.424377441	1
MSTRG.14608.1	423	0.28085	5.616149902	1
MSTRG.14608.2	423	0.28085	5.616149902	1
MSTRG.14608.5	423	0.32165	5.616149902	1
MSTRG.14608.4	423	0.29456	5.616149902	1
MSTRG.14610.1	86	0.31734	8.930969238	1
MSTRG.14608.7	158	0.28085	8.467956543	1
MSTRG.14612.1	85	0.32412	6.034240723	1
MSTRG.14615.1	318	0.28443	8.792663574	1
MSTRG.14615.2	318	0.28718	8.792663574	1
MSTRG.14615.3	216	0.32038	8.480773926	1
MSTRG.14615.5	318	0.28718	8.792663574	1
MSTRG.14619.3	787	0.39515	9.774230957	1

MSTRG.14619.2	752	0.41772	9.328063965	1
MSTRG.14619.4	787	0.39515	9.774230957	1
MSTRG.14620.1	216	0.46878	8.335998535	1
MSTRG.14620.2	216	0.46108	8.335998535	1
MSTRG.14620.3	161	0.40196	6.722473145	1
MSTRG.14620.4	216	0.449	8.335998535	1
MSTRG.14620.5	68	0.35004	8.058044434	1
MSTRG.14620.6	161	0.48509	6.722473145	1
MSTRG.14616.3	326	0.3072	4.663635254	1
MSTRG.14624.4	492	0.42886	6.963928223	1
MSTRG.14625.3	338	0.43771	5.425598145	1
MSTRG.14629.1	536	0.40635	9.524841309	1
MSTRG.14629.3	461	0.46924	9.602722168	1
MSTRG.14626.2	461	0.46789	6.415100098	1
MSTRG.14630.1	69	0.34022	9.301452637	1
MSTRG.14633.1	47	0.3381	10.79400635	1
MSTRG.14638.1	29	0.40424	7.99798584	1
MSTRG.14645.1	1130	0.47485	5.998596191	1
MSTRG.14646.1	123	0.35147	10.72283936	1
MSTRG.14648.2	114	0.37091	7.667297363	1
MSTRG.14648.3	612	0.40777	7.766662598	1
MSTRG.14651.1	376	0.47287	4.39654541	1
MSTRG.14651.3	376	0.45326	4.39654541	1
MSTRG.14651.4	337	0.44373	4.480407715	1
MSTRG.14647.1	25	0.40418	10.83441162	1
MSTRG.14658.1	115	0.35072	6.002746582	1
MSTRG.14656.1	34	0.33634	6.492004395	1
MSTRG.14672.1	75	0.45616	4.955871582	-1
MSTRG.14673.1	26	0.44576	10.93133545	1
MSTRG.14675.2	564	0.31035	5.906677246	1
MSTRG.14678.1	89	0.40194	10.51885986	1
MSTRG.14686.1	25	0.3903	6.491638184	1
MSTRG.14687.1	55	0.32919	10.02618408	1
MSTRG.14687.2	55	0.31922	10.02618408	1
MSTRG.14688.2	660	0.35881	8.112976074	1
MSTRG.14692.1	47	0.41703	9.822937012	1
MSTRG.14692.2	78	0.42221	11.16168213	1
MSTRG.14695.1	96	0.40102	10.18756104	-1
MSTRG.14696.5	151	0.29035	7.87713623	1
MSTRG.14696.6	151	0.28167	7.87713623	1
MSTRG.14697.1	120	0.38693	8.660217285	1
MSTRG.14699.2	216	0.27327	9.605407715	1
MSTRG.14699.1	216	0.27987	9.605407715	1
MSTRG.14699.3	216	0.27987	9.605407715	1
MSTRG.14699.4	216	0.27987	9.605407715	1
MSTRG.14699.5	216	0.27987	9.605407715	1
MSTRG.14699.6	216	0.27327	9.605407715	1
MSTRG.14701.8	108	0.32394	9.834533691	1
MSTRG.14701.4	539	0.41803	5.725402832	1
MSTRG.14701.5	515	0.41974	6.245666504	1
MSTRG.14701.7	631	0.41822	7.210632324	1
MSTRG.14701.9	275	0.41197	4.609802246	1
MSTRG.14702.1	1131	0.41174	8.382141113	1
MSTRG.14703.1	81	0.33105	9.265686035	1

MSTRG.14707.5	609	0.42903	8.300720215	1
MSTRG.14707.4	604	0.3942	8.190368652	1
MSTRG.14707.2	604	0.38132	8.190368652	1
MSTRG.14708.2	1454	0.33747	7.290100098	1
MSTRG.14708.6	461	0.32213	5.855041504	1
MSTRG.14708.7	312	0.39968	5.678894043	1
MSTRG.14709.1	248	0.34437	8.711730957	-1
MSTRG.14710.1	93	0.30808	7.997741699	1
MSTRG.14708.9	353	0.35972	6.630065918	1
MSTRG.14711.1	190	0.29305	9.420959473	1
MSTRG.14708.10	305	0.40309	7.551940918	1
MSTRG.14704.2	501	0.45064	6.410583496	1
MSTRG.14704.3	350	0.433	6.057434082	1
MSTRG.14704.4	455	0.43439	6.176330566	1
MSTRG.14706.1	90	0.33287	5.699645996	1
MSTRG.14715.1	458	0.32598	7.124450684	1
MSTRG.14715.6	458	0.44421	7.124450684	1
MSTRG.14715.7	458	0.44173	7.124450684	1
MSTRG.14715.8	458	0.44173	7.124450684	1
MSTRG.14716.1	73	0.32431	10.07489014	1
MSTRG.14717.1	185	0.27251	9.039978027	1
MSTRG.14718.1	109	0.3976	11.31658936	1
MSTRG.14719.1	85	0.2846	9.098449707	1
MSTRG.14720.1	99	0.29223	9.888977051	1
MSTRG.14721.1	184	0.2785	9.948791504	1
MSTRG.14722.1	130	0.29988	10.13641357	1
MSTRG.14723.1	63	0.29075	7.918640137	1
MSTRG.14727.1	50	0.32592	9.776062012	1
MSTRG.14727.2	46	0.35394	9.74786377	1
MSTRG.14730.1	133	0.32086	8.652893066	1
MSTRG.14731.2	612	0.3939	6.197570801	1
MSTRG.14734.2	367	0.44277	4.901550293	1
MSTRG.14735.2	925	0.45947	6.050842285	1
MSTRG.14735.3	992	0.45933	6.792907715	1
MSTRG.14736.1	243	0.27532	9.211975098	-1
MSTRG.14745.1	117	0.32466	9.841125488	1
MSTRG.14746.1	160	0.27883	11.32196045	1
MSTRG.14748.1	154	0.33926	11.71026611	1
MSTRG.14749.1	185	0.27469	9.69342041	1
MSTRG.14751.1	92	0.33952	9.586242676	1
MSTRG.14750.3	391	0.47474	6.676086426	1
MSTRG.14750.2	512	0.48204	6.296081543	1
MSTRG.14750.5	329	0.47611	8.317199707	-1
MSTRG.14750.4	450	0.46506	7.16217041	-1
MSTRG.14750.6	128	0.41229	9.784118652	1
MSTRG.14753.3	129	0.3199	11.34967041	1
MSTRG.14754.1	68	0.35273	4.66973877	1
MSTRG.14753.4	134	0.33768	9.096496582	1
MSTRG.14769.4	1784	0.45628	5.373596191	1
MSTRG.14771.1	135	0.30624	9.600036621	1
MSTRG.14772.1	265	0.36218	8.190734863	1
MSTRG.14772.2	535	0.45623	6.659362793	1
MSTRG.14772.4	265	0.36218	8.190734863	1
MSTRG.14773.1	114	0.31313	9.695861816	1

MSTRG.14773.2	157	0.27794	11.269104	1
MSTRG.14775.1	88	0.33578	7.864807129	1
MSTRG.14777.1	75	0.31092	10.37701416	1
MSTRG.14778.1	67	0.39006	6.969299316	1
MSTRG.14780.2	555	0.39577	5.817565918	1
MSTRG.14780.3	555	0.40931	5.991149902	1
MSTRG.14782.6	188	0.38868	5.76373291	1
MSTRG.14785.4	139	0.32002	9.293884277	1
MSTRG.14785.5	139	0.35266	9.293884277	1
MSTRG.14787.1	66	0.31745	9.506896973	1
MSTRG.14789.4	1048	0.45564	5.992248535	1
MSTRG.14790.3	667	0.47436	4.957946777	1
MSTRG.14790.4	517	0.45412	4.84161377	1
MSTRG.14791.2	49	0.43347	8.800964355	1
MSTRG.14792.2	145	0.43584	7.793884277	1
MSTRG.14793.2	282	0.40942	9.738342285	1
MSTRG.14796.1	237	0.42138	6.32232666	-1
MSTRG.14800.1	314	0.44915	6.604919434	1
MSTRG.14801.2	800	0.41632	8.214538574	1
MSTRG.14808.2	547	0.42756	6.247741699	1
MSTRG.14808.4	547	0.45054	6.247741699	1
MSTRG.14818.2	878	0.40198	9.297302246	1
MSTRG.14818.4	880	0.38413	9.297302246	1
MSTRG.14819.1	2057	0.43681	8.758239746	1
MSTRG.14821.2	261	0.37402	7.702087402	1
MSTRG.14821.3	261	0.37182	7.702087402	1
MSTRG.14821.5	144	0.40649	11.21038818	1
MSTRG.14814.1	41	0.42026	6.492248535	-1
MSTRG.14815.3	588	0.43691	6.503234863	1
MSTRG.14827.1	41	0.3376	4.944641113	1
MSTRG.14828.3	1178	0.46877	7.330505371	1
MSTRG.14828.4	1155	0.47756	7.151550293	1
MSTRG.14828.2	820	0.42495	5.583679199	1
MSTRG.14828.6	413	0.34284	9.471252441	1
MSTRG.14830.5	527	0.45414	5.951965332	1
MSTRG.14835.1	104	0.2735	7.77142334	1
MSTRG.14835.2	129	0.26837	9.095275879	1
MSTRG.14834.5	75	0.29147	9.509338379	1
MSTRG.14840.1	290	0.30191	6.152282715	1
MSTRG.14840.5	535	0.47256	6.528991699	1
MSTRG.14833.7	697	0.43008	5.653015137	1
MSTRG.14833.3	697	0.45635	5.653015137	1
MSTRG.14841.1	248	0.47332	8.182067871	1
MSTRG.14842.3	161	0.42783	10.01263428	1
MSTRG.14842.2	161	0.45645	10.01263428	1
MSTRG.14842.1	161	0.34035	10.01263428	1
MSTRG.14842.5	161	0.42766	10.01263428	1
MSTRG.14844.2	226	0.40409	6.044250488	1
MSTRG.14846.1	1253	0.45145	8.947937012	1
MSTRG.14846.3	1252	0.45266	8.947937012	1
MSTRG.14849.1	1245	0.35883	7.957336426	1
MSTRG.14849.2	1244	0.43734	7.957336426	1
MSTRG.14849.3	1270	0.43858	7.789245605	1
MSTRG.14852.1	143	0.28167	8.559509277	1

MSTRG.14852.2	210	0.28788	9.77935791	1
MSTRG.14853.1	51	0.46401	4.593933105	-1
MSTRG.14852.3	108	0.36172	7.034362793	1
MSTRG.14854.2	86	0.33248	9.79473877	1
MSTRG.14854.1	86	0.38081	9.79473877	1
MSTRG.14854.3	86	0.35594	8.928405762	1
MSTRG.14854.4	86	0.35594	8.928405762	1
MSTRG.14854.5	86	0.38512	8.928405762	1
MSTRG.14855.1	140	0.30894	9.081848145	1
MSTRG.14856.1	24	0.34421	10.7366333	-1
MSTRG.14864.1	23	0.36714	11.53900146	1
MSTRG.14866.2	322	0.40503	5.489318848	1
MSTRG.14866.3	322	0.40561	5.489318848	1
MSTRG.14867.5	103	0.31753	9.514831543	1
MSTRG.14867.6	158	0.28014	10.83587646	1
MSTRG.14865.2	295	0.46008	4.816711426	1
MSTRG.14869.2	1317	0.46942	7.009460449	1
MSTRG.14869.5	1144	0.48624	7.187927246	1
MSTRG.14869.1	1320	0.40577	6.909118652	1
MSTRG.14869.3	1256	0.40577	6.969665527	1
MSTRG.14868.1	400	0.41691	8.329284668	1
MSTRG.14871.2	46	0.31076	7.714172363	1
MSTRG.14871.1	47	0.36184	9.184997559	1
MSTRG.14871.3	69	0.33639	10.05450439	1
MSTRG.14872.2	302	0.38263	5.042175293	1
MSTRG.14872.3	246	0.32677	10.24237061	1
MSTRG.14873.2	305	0.36736	8.896179199	1
MSTRG.14873.3	478	0.39787	9.11907959	1
MSTRG.14875.1	32	0.41337	4.657043457	1
MSTRG.14883.1	56	0.32704	10.45587158	1
MSTRG.14884.1	237	0.29583	9.831604004	1
MSTRG.14885.1	60	0.39902	11.21246338	1
MSTRG.14877.1	46	0.34696	9.694030762	1
MSTRG.14878.1	105	0.35892	10.96820068	1
MSTRG.14890.2	232	0.4318	9.706481934	1
MSTRG.14892.2	47	0.44454	4.533996582	1
MSTRG.14905.1	410	0.44429	8.501525879	1
MSTRG.14907.2	54	0.33429	12.30267334	1
MSTRG.14909.2	303	0.4583	5.867614746	1
MSTRG.14909.3	140	0.35058	9.865661621	1
MSTRG.14909.4	76	0.41239	10.0479126	1
MSTRG.14909.5	255	0.47321	8.981506348	1
MSTRG.14911.1	334	0.46854	5.766662598	1
MSTRG.14913.2	1239	0.43408	5.324035645	1
MSTRG.14913.3	1246	0.43408	5.388977051	1
MSTRG.14921.1	99	0.29423	9.035705566	1
MSTRG.14920.2	344	0.38675	9.512878418	1
MSTRG.14914.2	224	0.36612	7.750061035	1
MSTRG.14915.1	83	0.45667	8.789489746	1
MSTRG.14915.3	108	0.35137	6.88104248	1
MSTRG.14915.4	83	0.41438	8.789489746	1
MSTRG.14930.4	181	0.40563	6.059020996	1
MSTRG.14930.5	100	0.32971	8.706115723	1
MSTRG.14930.6	99	0.43495	11.93792725	1

MSTRG.14931.1	85	0.30151	4.780334473	1
MSTRG.14932.1	59	0.3279	7.928894043	1
MSTRG.14933.1	41	0.40502	8.554138184	1
MSTRG.14933.2	70	0.40675	10.62286377	1
MSTRG.14939.1	59	0.38194	11.28924561	-1
MSTRG.14940.2	637	0.47587	9.189147949	1
MSTRG.14944.1	175	0.35489	6.911804199	1
MSTRG.14945.1	3621	0.47197	5.88861084	1
MSTRG.14945.5	3742	0.46942	5.852111816	1
MSTRG.14945.6	2247	0.47638	5.06048584	1
MSTRG.14946.1	80	0.36337	7.10345459	1
MSTRG.14947.1	76	0.26677	10.01495361	1
MSTRG.14945.7	3359	0.45853	5.63861084	1
MSTRG.14948.1	58	0.30862	10.14398193	1
MSTRG.14945.8	1109	0.44952	5.894348145	-1
MSTRG.14945.9	2326	0.45864	5.040466309	1
MSTRG.14942.2	72	0.33388	9.957214355	1
MSTRG.14942.3	74	0.36258	10.57366943	1
MSTRG.14942.6	87	0.34636	10.51983643	1
MSTRG.14942.5	269	0.42166	9.295227051	1
MSTRG.14942.4	147	0.27469	8.225524902	1
MSTRG.14958.1	59	0.35791	7.900817871	1
MSTRG.14957.2	287	0.36085	8.199035645	1
MSTRG.14959.1	311	0.39407	9.770202637	1
MSTRG.14962.2	431	0.37854	7.148620605	1
MSTRG.14960.1	18	0.36034	7.954650879	1
MSTRG.14965.4	451	0.36026	6.343566895	1
MSTRG.14965.3	1035	0.41776	5.164978027	1
MSTRG.14965.1	1528	0.44926	5.417297363	1
MSTRG.14965.2	1513	0.45174	5.318908691	1
MSTRG.14965.5	1514	0.44926	5.459533691	1
MSTRG.14966.1	85	0.2667	9.916564941	1
MSTRG.14968.7	1977	0.46009	5.846740723	1
MSTRG.14968.4	1455	0.38618	5.22479248	1
MSTRG.14968.5	1455	0.38618	5.22479248	1
MSTRG.14968.8	2108	0.40594	6.008728027	1
MSTRG.14969.1	55	0.32949	4.649230957	1
MSTRG.14970.1	25	0.3611	10.76312256	1
MSTRG.14975.1	37	0.38745	6.523498535	1
MSTRG.14986.2	130	0.27957	8.41204834	1
MSTRG.14986.3	130	0.3452	8.41204834	1
MSTRG.14987.2	1333	0.459	7.871154785	1
MSTRG.14989.1	297	0.36527	6.665344238	1
MSTRG.14989.2	170	0.36527	8.38494873	1
MSTRG.14989.3	170	0.30445	8.38494873	1
MSTRG.14990.1	178	0.3818	12.07440186	1
MSTRG.14992.2	364	0.4388	9.530578613	1
MSTRG.15000.1	27	0.37115	9.901550293	1
MSTRG.15001.1	153	0.36406	7.752746582	1
MSTRG.15007.4	4417	0.4884	5.770446777	1
MSTRG.15007.5	4454	0.47405	5.740905762	-1
MSTRG.15007.7	66	0.34294	6.52142334	1
MSTRG.15007.8	2969	0.4807	5.937561035	1
MSTRG.14997.1	32	0.42044	6.678649902	1

MSTRG.14998.1	51	0.36072	10.8147583	1
MSTRG.15017.1	62	0.38634	9.984069824	1
MSTRG.15018.1	90	0.29452	4.957458496	1
MSTRG.15021.1	780	0.3208	5.08782959	1
MSTRG.15021.4	677	0.427	4.977233887	1
MSTRG.15022.1	92	0.35836	10.6807251	1
MSTRG.15023.4	483	0.48148	6.281066895	1
MSTRG.15023.5	477	0.47093	6.171203613	-1
MSTRG.15033.1	890	0.40013	6.354431152	1
MSTRG.15033.2	890	0.39932	6.354431152	1
MSTRG.15033.5	890	0.40109	6.354431152	1
MSTRG.15026.1	106	0.37219	8.77166748	1
MSTRG.15028.1	211	0.28771	6.185852051	1
MSTRG.15038.2	122	0.461	4.917297363	1
MSTRG.15046.1	505	0.46954	6.862609863	1
MSTRG.15046.4	294	0.33851	5.737487793	1
MSTRG.15046.5	413	0.40078	8.819519043	1
MSTRG.15046.6	237	0.30989	8.355285645	1
MSTRG.15046.7	221	0.41437	8.372375488	1
MSTRG.15047.1	189	0.30084	10.01263428	1
MSTRG.15047.2	144	0.32072	6.680358887	1
MSTRG.15046.9	107	0.29202	6.678771973	1
MSTRG.15050.6	137	0.4981	9.843811035	1
MSTRG.15050.2	137	0.47965	9.843811035	1
MSTRG.15050.4	137	0.47493	9.843811035	1
MSTRG.15050.7	137	0.47188	9.843811035	1
MSTRG.15051.1	588	0.41605	7.247009277	1
MSTRG.15051.2	588	0.32477	7.247009277	1
MSTRG.15051.3	588	0.38835	7.247009277	1
MSTRG.15051.4	588	0.43372	7.247009277	1
MSTRG.15052.2	44	0.38199	9.984436035	1
MSTRG.15054.1	79	0.42364	6.157897949	1
MSTRG.15043.1	144	0.29543	9.300354004	1
MSTRG.15039.1	20	0.40727	6.501525879	1
MSTRG.15055.2	894	0.41195	7.006530762	1
MSTRG.15055.4	342	0.35564	8.875305176	1
MSTRG.15055.6	826	0.44668	7.163757324	1
MSTRG.15055.7	810	0.43759	8.230529785	1
MSTRG.15055.8	805	0.47841	6.995056152	1
MSTRG.15057.1	101	0.38162	10.28094482	-1
MSTRG.15058.1	71	0.3588	7.703796387	1
MSTRG.15045.1	41	0.33031	11.4196167	1
MSTRG.15068.1	56	0.32172	7.811218262	-1
MSTRG.15072.1	24	0.47872	5.19354248	1
MSTRG.15072.2	59	0.3593	10.28643799	1
MSTRG.15080.1	103	0.34956	10.00274658	1
MSTRG.15083.4	934	0.44615	5.803161621	1
MSTRG.15083.3	981	0.44512	5.924499512	1
MSTRG.15083.5	117	0.30817	7.902770996	1
MSTRG.15084.1	159	0.30191	10.28436279	1
MSTRG.15085.1	127	0.40426	10.36810303	1
MSTRG.15090.1	282	0.39503	8.936828613	1
MSTRG.15090.2	233	0.2887	6.236999512	1
MSTRG.15091.1	65	0.31696	9.783752441	1

MSTRG.15092.1	78	0.36556	6.829040527	1
MSTRG.15093.10	492	0.32507	6.623596191	1
MSTRG.15093.9	591	0.33375	9.020202637	1
MSTRG.15093.11	459	0.44525	8.194519043	1
MSTRG.15093.12	509	0.41412	8.463562012	1
MSTRG.15097.3	1417	0.44417	6.986877441	1
MSTRG.15097.4	273	0.30742	8.128479004	1
MSTRG.15100.2	1491	0.40237	7.485290527	1
MSTRG.15105.1	392	0.38439	5.101501465	1
MSTRG.15105.2	387	0.37124	5.056335449	1
MSTRG.15105.5	377	0.36156	5.649597168	1
MSTRG.15105.3	479	0.31369	4.979309082	1
MSTRG.15105.4	443	0.38439	5.829406738	1
MSTRG.15105.6	327	0.40524	5.101623535	1
MSTRG.15105.8	479	0.32723	4.979309082	1
MSTRG.15105.9	110	0.27398	7.63482666	1
MSTRG.15106.1	125	0.27661	10.62030029	1
MSTRG.15106.2	101	0.30868	10.07037354	1
MSTRG.15106.3	157	0.33264	9.886047363	1
MSTRG.15107.1	457	0.31793	4.784240723	1
MSTRG.15108.1	218	0.41106	9.464904785	1
MSTRG.15108.2	262	0.42801	9.013000488	1
MSTRG.15109.1	29	0.312	10.76287842	-1
MSTRG.15109.2	63	0.32531	8.653503418	-1
MSTRG.15105.11	69	0.2962	4.928527832	1
MSTRG.15124.5	572	0.38694	5.04901123	1
MSTRG.15126.1	46	0.36382	11.00006104	1
MSTRG.15127.5	150	0.45006	5.915588379	1
MSTRG.15127.4	180	0.29387	8.928649902	1
MSTRG.15127.3	176	0.28982	9.654602051	1
MSTRG.15127.1	176	0.2921	9.654602051	1
MSTRG.15129.1	82	0.3616	6.228210449	1
MSTRG.15131.1	67	0.29374	6.806091309	1
MSTRG.15132.1	68	0.32413	8.934387207	1
MSTRG.15118.3	159	0.43308	9.127258301	1
MSTRG.15119.1	42	0.40976	4.100524902	1
MSTRG.15141.1	149	0.2785	8.906555176	1
MSTRG.15141.2	870	0.45522	5.747741699	1
MSTRG.15141.4	646	0.39243	5.190856934	1
MSTRG.15138.1	110	0.31246	9.840026855	1
MSTRG.15138.2	41	0.34188	9.624694824	1
MSTRG.15145.1	205	0.33851	5.94354248	1
MSTRG.15146.4	56	0.26334	7.702819824	1
MSTRG.15146.5	219	0.30553	9.085510254	1
MSTRG.15146.6	230	0.36066	9.074157715	1
MSTRG.15146.7	237	0.33348	8.668395996	1
MSTRG.15146.10	124	0.35151	9.266296387	1
MSTRG.15146.13	124	0.31842	9.266296387	1
MSTRG.15146.11	219	0.34582	9.085510254	1
MSTRG.15146.15	219	0.34931	9.085510254	1
MSTRG.15146.8	219	0.32943	9.085510254	1
MSTRG.15146.14	227	0.36521	8.97479248	1
MSTRG.15146.12	124	0.37201	9.266296387	1
MSTRG.15147.2	81	0.39578	10.36700439	1

MSTRG.15149.1	536	0.32237	8.845275879	1
MSTRG.15151.1	126	0.44589	9.695861816	-1
MSTRG.15152.1	154	0.42436	7.673522949	-1
MSTRG.15143.1	31	0.3817	3.838806152	1
MSTRG.15156.3	667	0.43654	6.09942627	1
MSTRG.15156.4	236	0.36769	5.63861084	1
MSTRG.15153.1	314	0.44741	6.25189209	1
MSTRG.15153.2	314	0.4624	6.25189209	1
MSTRG.15167.2	74	0.38096	6.542419434	1
MSTRG.15174.3	864	0.45549	6.178649902	1
MSTRG.15175.1	99	0.31501	5.994934082	1
MSTRG.15176.1	104	0.30945	7.718811035	1
MSTRG.15174.4	110	0.3	6.71673584	1
MSTRG.15174.5	232	0.3287	9.20880127	1
MSTRG.15177.1	56	0.32821	5.997619629	1
MSTRG.15181.1	780	0.36604	6.493469238	1
MSTRG.15181.5	53	0.35887	6.68951416	1
MSTRG.15183.2	147	0.4498	3.862243652	1
MSTRG.15184.1	307	0.40171	6.098571777	1
MSTRG.15184.2	487	0.38703	8.694274902	1
MSTRG.15185.1	109	0.28648	9.207214355	1
MSTRG.15184.3	701	0.43829	8.489929199	1
MSTRG.15184.5	336	0.35025	7.941101074	1
MSTRG.15184.9	153	0.40617	8.537658691	1
MSTRG.15184.10	139	0.28567	10.03643799	1
MSTRG.15188.1	84	0.34993	4.621887207	1
MSTRG.15188.2	125	0.39224	9.652770996	-1
MSTRG.15190.1	583	0.41442	5.970031738	1
MSTRG.15190.6	156	0.33171	5.637023926	1
MSTRG.15191.1	83	0.28648	8.885559082	1
MSTRG.15192.1	93	0.28683	10.17938232	1
MSTRG.15194.1	147	0.28518	9.439025879	1
MSTRG.15219.1	1476	0.426	5.643005371	1
MSTRG.15219.2	323	0.4011	5.601013184	1
MSTRG.15219.3	1467	0.46376	5.642150879	1
MSTRG.15220.1	68	0.41749	10.27728271	-1
MSTRG.15221.1	56	0.44661	6.805480957	-1
MSTRG.15227.1	143	0.28831	10.11846924	1
MSTRG.15253.1	84	0.27893	10.95648193	1
MSTRG.15197.1	30	0.4341	8.34197998	-1
MSTRG.15199.1	119	0.46361	9.613952637	-1
MSTRG.15209.3	439	0.42545	5.143371582	1
MSTRG.15210.1	84	0.25541	9.353942871	1
MSTRG.15210.2	53	0.30831	4.206970215	1
MSTRG.15209.4	145	0.45442	6.18548584	-1
MSTRG.15212.1	55	0.40323	11.00860596	-1
MSTRG.15213.2	109	0.39332	6.719909668	1
MSTRG.15268.1	24	0.30546	8.345397949	1
MSTRG.15268.2	24	0.34625	8.345397949	1
MSTRG.15284.1	208	0.39897	8.842956543	1
MSTRG.15287.5	2363	0.45256	5.390319824	1
MSTRG.15287.6	2363	0.4647	5.390319824	1
MSTRG.15291.1	273	0.44103	6.497253418	1
MSTRG.15296.3	122	0.31035	4.90435791	1

MSTRG.15302.2	1840	0.45088	5.826477051	1
MSTRG.15312.1	157	0.27772	9.40826416	1
MSTRG.15304.1	123	0.3947	6.949035645	-1
MSTRG.15328.2	190	0.44625	5.66229248	1
MSTRG.15333.1	59	0.31889	4.657043457	1
MSTRG.15338.1	60	0.37555	6.151306152	1
MSTRG.15347.1	331	0.3283	4.983093262	1
MSTRG.15353.1	133	0.39367	5.774719238	1
MSTRG.15359.2	717	0.38713	6.33026123	1
MSTRG.15360.1	168	0.27415	7.653747559	1
MSTRG.15360.2	99	0.44223	5.570251465	-1
MSTRG.15362.1	74	0.3257	6.01385498	1
MSTRG.15357.1	48	0.31117	8.803405762	1
MSTRG.15368.2	487	0.4702	6.675720215	1
MSTRG.15368.3	487	0.48822	6.675720215	1
MSTRG.15375.2	3023	0.48143	5.53338623	1
MSTRG.15379.2	676	0.4461	5.493469238	1
MSTRG.15381.1	100	0.44099	6.292297363	1
MSTRG.15381.2	151	0.47275	6.073791504	1
MSTRG.15381.3	151	0.46066	6.073791504	1
MSTRG.15380.1	61	0.37176	9.648376465	1
MSTRG.15380.2	61	0.36342	9.648376465	1
MSTRG.15383.1	35	0.41037	7.918029785	1
MSTRG.15386.3	252	0.43116	9.066345215	1
MSTRG.15386.5	379	0.31588	7.265563965	1
MSTRG.15394.2	99	0.39061	6.934020996	1
MSTRG.15394.1	110	0.33322	6.985290527	1
MSTRG.15399.1	34	0.32076	6.057434082	1
MSTRG.15405.4	1064	0.42004	5.207214355	1
MSTRG.15405.6	496	0.39807	9.070495605	1
MSTRG.15402.1	76	0.41767	8.879699707	1
MSTRG.15416.3	37	0.43644	9.303161621	1
MSTRG.15416.4	257	0.33762	9.346496582	1
MSTRG.15421.1	607	0.41501	5.481994629	1
MSTRG.15421.2	571	0.35397	6.230773926	1
MSTRG.15422.1	61	0.37606	8.769470215	1
MSTRG.15423.1	195	0.33963	8.578186035	-1
MSTRG.15426.4	149	0.32106	8.969055176	1
MSTRG.15427.2	110	0.43438	11.12091064	1
MSTRG.15429.6	687	0.46207	6.971740723	1
MSTRG.15429.7	760	0.46672	6.260314941	1
MSTRG.15429.8	501	0.46672	5.574279785	1
MSTRG.15431.1	1609	0.45917	6.885314941	1
MSTRG.15431.3	1609	0.45917	6.885314941	1
MSTRG.15433.3	391	0.38919	8.960876465	1
MSTRG.15433.5	116	0.28842	9.510192871	1
MSTRG.15436.2	296	0.44559	8.596252441	1
MSTRG.15436.3	549	0.45789	8.968811035	1
MSTRG.15442.1	219	0.41175	5.655456543	1
MSTRG.15444.2	67	0.35209	9.896057129	1
MSTRG.15444.3	100	0.2908	4.975769043	1
MSTRG.15447.5	129	0.30989	9.546081543	1
MSTRG.15447.4	381	0.39091	7.699523926	1
MSTRG.15447.6	287	0.40246	9.376403809	1

MSTRG.15447.8	287	0.3886	9.376403809	1
MSTRG.15447.9	85	0.28338	9.235778809	1
MSTRG.15450.2	331	0.37197	9.888000488	1
MSTRG.15450.3	439	0.37983	9.761169434	1
MSTRG.15457.1	85	0.32573	10.75445557	1
MSTRG.15467.2	1802	0.46358	5.338439941	1
MSTRG.15469.1	32	0.444	3.929626465	1
MSTRG.15470.1	54	0.40072	6.355407715	1
MSTRG.15471.1	54	0.37824	11.00860596	-1
MSTRG.15486.2	601	0.46705	6.14263916	1
MSTRG.15486.4	180	0.44495	10.78131104	1
MSTRG.15490.1	131	0.29772	9.69342041	1
MSTRG.15491.1	115	0.32002	11.88800049	1
MSTRG.15492.1	118	0.29772	6.264709473	1
MSTRG.15492.2	105	0.29772	9.270202637	1
MSTRG.15493.1	116	0.29772	5.596252441	1
MSTRG.15494.1	86	0.31204	7.571838379	1
MSTRG.15486.5	396	0.32642	7.641296387	1
MSTRG.15494.2	66	0.29373	8.934875488	1
MSTRG.15497.1	274	0.29367	5.960021973	1
MSTRG.15499.1	91	0.29771	9.356750488	1
MSTRG.15499.2	103	0.37494	6.798156738	1
MSTRG.15500.1	73	0.29852	9.511901855	1
MSTRG.15502.1	227	0.33597	7.57208252	1
MSTRG.15503.1	166	0.33764	9.257629395	1
MSTRG.15502.2	233	0.38114	9.047058105	-1
MSTRG.15477.1	49	0.38747	8.958068848	1
MSTRG.15480.2	146	0.29636	9.695739746	1
MSTRG.15532.2	3547	0.47949	5.510070801	1
MSTRG.15532.1	3535	0.46893	5.524108887	1
MSTRG.15532.4	813	0.35567	6.069152832	1
MSTRG.15532.5	3348	0.48934	5.573425293	1
MSTRG.15532.3	3348	0.47949	5.573425293	1
MSTRG.15532.6	1757	0.47806	5.613952637	1
MSTRG.15533.1	101	0.33979	4.841125488	1
MSTRG.15532.8	1182	0.39591	5.191955566	1
MSTRG.15534.1	47	0.31108	5.302062988	1
MSTRG.15535.1	1071	0.47668	5.742858887	1
MSTRG.15536.1	268	0.29059	9.862731934	1
MSTRG.15535.3	1071	0.47799	5.742858887	1
MSTRG.15535.4	1071	0.48926	5.742858887	1
MSTRG.15538.1	84	0.30387	9.925598145	1
MSTRG.15517.1	133	0.28567	9.538757324	1
MSTRG.15518.4	574	0.39506	9.420471191	1
MSTRG.15518.5	574	0.38332	9.420471191	1
MSTRG.15518.6	461	0.37443	9.689147949	1
MSTRG.15518.8	443	0.41115	9.72076416	1
MSTRG.15518.7	416	0.41349	9.727478027	1
MSTRG.15519.1	106	0.34047	11.61700439	1
MSTRG.15521.2	157	0.2985	8.451721191	1
MSTRG.15521.3	97	0.31287	7.930603027	1
MSTRG.15528.3	59	0.32042	11.34661865	1
MSTRG.15540.2	1161	0.47357	5.677185059	1
MSTRG.15540.8	410	0.45457	5.156677246	1

MSTRG.15540.7	1043	0.46891	5.944641113	1
MSTRG.15547.1	103	0.28498	10.56072998	1
MSTRG.15547.2	88	0.33061	9.901184082	1
MSTRG.15547.3	92	0.32257	5.479431152	1
MSTRG.15548.1	209	0.33834	8.188415527	1
MSTRG.15548.2	153	0.44857	6.098937988	-1
MSTRG.15548.3	153	0.4495	6.098937988	-1
MSTRG.15549.1	124	0.40674	6.742248535	-1
MSTRG.15554.1	80	0.2982	12.01385498	1
MSTRG.15572.1	98	0.3783	9.74420166	1
MSTRG.15571.3	91	0.3208	8.61505127	1
MSTRG.15571.4	140	0.35757	8.97479248	1
MSTRG.15563.1	64	0.32213	6.948303223	1
MSTRG.15574.1	125	0.38938	5.090026855	1
MSTRG.15575.1	77	0.31641	8.403625488	1
MSTRG.15594.4	642	0.36009	5.797912598	1
MSTRG.15595.1	205	0.36481	5.931335449	1
MSTRG.15595.2	199	0.31637	6.597961426	1
MSTRG.15596.2	220	0.34522	6.691589355	1
MSTRG.15596.3	417	0.36111	6.258850098	1
MSTRG.15600.2	246	0.35214	5.971862793	1
MSTRG.15601.1	44	0.36594	8.843688965	1
MSTRG.15602.1	91	0.39036	4.96661377	1
MSTRG.15602.2	227	0.3369	8.907165527	1
MSTRG.15603.3	898	0.45097	8.850158691	1
MSTRG.15611.2	202	0.42546	8.570983887	1
MSTRG.15614.2	188	0.4715	4.901916504	1
MSTRG.15618.1	479	0.46468	5.239074707	-1
MSTRG.15619.2	529	0.38862	7.896911621	1
MSTRG.15619.3	292	0.45538	5.29473877	1
MSTRG.15619.4	282	0.31186	9.265563965	1
MSTRG.15625.1	1123	0.32947	8.032409668	1
MSTRG.15625.2	1124	0.45237	8.032409668	1
MSTRG.15628.2	286	0.36048	7.585144043	1
MSTRG.15630.3	123	0.38367	11.61651611	1
MSTRG.15630.2	190	0.32661	9.013977051	1
MSTRG.15638.2	574	0.36616	9.14666748	1
MSTRG.15638.3	574	0.35893	9.14666748	1
MSTRG.15638.4	414	0.42143	8.869689941	1
MSTRG.15638.8	86	0.36799	9.300842285	-1
MSTRG.15638.10	59	0.32157	6.875427246	1
MSTRG.15644.1	668	0.41192	5.810974121	1
MSTRG.15644.4	212	0.41045	9.435974121	1
MSTRG.15644.7	204	0.30297	7.637878418	1
MSTRG.15644.5	210	0.42803	9.32244873	1
MSTRG.15644.6	180	0.35877	9.068786621	1
MSTRG.15650.1	177	0.37376	10.51934814	-1
MSTRG.15651.1	225	0.35742	9.174377441	1
MSTRG.15656.1	104	0.30577	9.981994629	1
MSTRG.15671.1	45	0.43725	10.42486572	1
MSTRG.15654.1	644	0.47481	8.74005127	1
MSTRG.15654.2	649	0.48082	8.447692871	1
MSTRG.15677.1	473	0.38211	8.978210449	1
MSTRG.15678.2	327	0.32729	5.843078613	1

MSTRG.15679.1	65	0.31488	10.58868408	-1
MSTRG.15678.3	327	0.36997	5.843078613	1
MSTRG.15678.4	327	0.3508	5.843078613	1
MSTRG.15672.1	31	0.44244	5.410461426	-1
MSTRG.15674.1	295	0.48019	6.388122559	1
MSTRG.15675.1	49	0.34761	10.37982178	1
MSTRG.15681.2	56	0.44456	6.221008301	-1
MSTRG.15681.3	56	0.36024	6.221008301	-1
MSTRG.15681.5	383	0.41877	5.611022949	1
MSTRG.15681.4	383	0.42729	5.611022949	1
MSTRG.15682.1	112	0.32714	6.948547363	1
MSTRG.15683.1	55	0.27433	5.537414551	1
MSTRG.15681.6	52	0.41318	3.968322754	1
MSTRG.15684.1	252	0.45814	4.568908691	1
MSTRG.15681.8	86	0.36904	12.1260376	1
MSTRG.15685.2	740	0.41191	4.951721191	1
MSTRG.15692.1	26	0.43484	9.976501465	-1
MSTRG.15694.3	5931	0.40951	9.006286621	1
MSTRG.15694.1	6002	0.40951	9.02935791	1
MSTRG.15694.2	5994	0.40951	9.023376465	1
MSTRG.15694.4	5994	0.40951	9.01776123	1
MSTRG.15701.4	535	0.46684	5.023986816	1
MSTRG.15701.5	535	0.45	5.023986816	1
MSTRG.15703.5	171	0.4409	9.647644043	1
MSTRG.15703.4	202	0.4409	9.669616699	1
MSTRG.15703.6	202	0.4409	9.669616699	1
MSTRG.15703.7	171	0.4409	9.647644043	1
MSTRG.15705.3	493	0.48152	9.480895996	1
MSTRG.15707.2	150	0.3432	9.207214355	1
MSTRG.15707.4	150	0.35445	9.207214355	1
MSTRG.15714.3	161	0.33363	8.653991699	1
MSTRG.15714.4	157	0.38281	8.497253418	1
MSTRG.15715.1	304	0.3759	6.178527832	1
MSTRG.15715.5	372	0.39757	5.993103027	1
MSTRG.15715.6	304	0.3536	6.178527832	1
MSTRG.15719.2	69	0.30297	6.876403809	1
MSTRG.15720.1	638	0.43654	8.109069824	1
MSTRG.15720.2	638	0.43654	8.109069824	1
MSTRG.15720.3	638	0.43378	8.109069824	1
MSTRG.15708.1	79	0.31886	9.738708496	1
MSTRG.15709.1	398	0.3442	10.04608154	1
MSTRG.15711.3	163	0.44864	5.567810059	1
MSTRG.15722.4	581	0.3449	9.94732666	1
MSTRG.15722.10	507	0.33379	9.944152832	1
MSTRG.15722.9	796	0.40074	9.550354004	1
MSTRG.15724.2	622	0.46739	9.172668457	1
MSTRG.15726.5	281	0.4207	5.33392334	1
MSTRG.15726.6	266	0.42615	5.146911621	1
MSTRG.15723.2	564	0.43976	6.726379395	1
MSTRG.15723.3	344	0.49227	5.672424316	1
MSTRG.15747.3	1259	0.44166	8.442932129	1
MSTRG.15750.2	164	0.33229	5.002746582	1
MSTRG.15750.3	752	0.4006	7.867248535	1
MSTRG.15750.4	755	0.44906	8.021179199	1

MSTRG.15751.5	317	0.46255	5.76385498	1
MSTRG.15751.4	371	0.47152	5.743347168	1
MSTRG.15752.3	160	0.30981	8.257751465	1
MSTRG.15752.4	160	0.30981	8.257751465	1
MSTRG.15752.5	160	0.30981	8.257751465	1
MSTRG.15754.2	904	0.41165	5.874450684	1
MSTRG.15756.4	296	0.39473	8.559265137	1
MSTRG.15766.3	1399	0.46411	5.791442871	1
MSTRG.15766.4	1399	0.46411	5.791442871	1
MSTRG.15766.10	1440	0.46719	5.838439941	1
MSTRG.15766.8	1399	0.46411	5.791442871	1
MSTRG.15766.9	1441	0.46719	5.838439941	1
MSTRG.15767.1	54	0.28475	5.312683105	1
MSTRG.15766.11	122	0.28498	9.624328613	1
MSTRG.15766.12	66	0.44441	9.301086426	1
MSTRG.15766.13	85	0.33585	8.866271973	1
MSTRG.15768.1	127	0.36766	11.10809326	1
MSTRG.15769.1	169	0.33446	9.250305176	1
MSTRG.15773.1	121	0.28584	11.809021	1
MSTRG.15773.2	163	0.28584	11.70501709	1
MSTRG.15776.1	190	0.28567	7.683532715	1
MSTRG.15776.2	386	0.37874	9.38482666	1
MSTRG.15778.3	89	0.35312	9.097229004	1
MSTRG.15778.2	129	0.34103	8.957824707	1
MSTRG.15781.1	49	0.37964	10.75018311	1
MSTRG.15781.5	218	0.39726	11.3057251	1
MSTRG.15781.7	79	0.44236	8.88885498	1
MSTRG.15782.1	676	0.36527	5.762512207	1
MSTRG.15785.1	71	0.40793	9.972351074	1
MSTRG.15786.1	96	0.32651	9.875915527	1
MSTRG.15786.2	39	0.45266	8.939025879	1
MSTRG.15786.3	166	0.41979	9.899353027	1
MSTRG.15788.1	96	0.35923	9.804016113	1
MSTRG.15791.1	97	0.39071	10.06817627	1
MSTRG.15793.1	190	0.36775	7.707580566	1
MSTRG.15795.1	66	0.40004	7.835998535	1
MSTRG.15796.1	2081	0.46743	7.703063965	1
MSTRG.15796.2	164	0.26785	10.0166626	1
MSTRG.15796.3	164	0.30864	10.0166626	1
MSTRG.15797.4	125	0.43683	7.675231934	1
MSTRG.15797.6	144	0.34435	6.32232666	1
MSTRG.15798.1	49	0.32677	9.626037598	1
MSTRG.15804.2	362	0.40485	5.025817871	1
MSTRG.15804.3	362	0.44732	5.025817871	1
MSTRG.15804.5	362	0.4068	5.025817871	1
MSTRG.15800.1	147	0.30068	11.48297119	1
MSTRG.15801.1	196	0.40215	5.101867676	1
MSTRG.15809.10	802	0.44535	6.563171387	1
MSTRG.15810.1	13	0.38173	9.102233887	1
MSTRG.15811.1	188	0.34096	8.894348145	1
MSTRG.15812.1	88	0.25541	7.678039551	1
MSTRG.15813.2	186	0.29343	9.11114502	1
MSTRG.15813.1	171	0.29343	9.315368652	1
MSTRG.15813.3	171	0.29355	9.315368652	1

MSTRG.15813.4	171	0.29607	9.315368652	1
MSTRG.15809.11	265	0.33186	7.63470459	1
MSTRG.15806.1	99	0.33447	10.95648193	-1
MSTRG.15807.1	233	0.34081	6.102844238	1
MSTRG.15816.1	60	0.37562	6.042297363	1
MSTRG.15816.2	32	0.38282	9.302307129	1
MSTRG.15814.1	55	0.34587	5.987243652	1
MSTRG.15817.1	267	0.40701	9.768127441	1
MSTRG.15821.1	35	0.37457	4.129821777	1
MSTRG.15822.3	443	0.40305	6.45111084	1
MSTRG.15826.1	87	0.36711	8.572814941	1
MSTRG.15832.1	71	0.39599	8.507019043	1
MSTRG.15834.1	53	0.3689	8.590637207	1
MSTRG.15834.2	55	0.40902	9.036437988	1
MSTRG.15835.1	46	0.33362	9.984436035	1
MSTRG.15835.2	46	0.39885	9.984436035	1
MSTRG.15841.1	703	0.43288	6.417541504	1
MSTRG.15841.2	394	0.43968	6.623840332	-1
MSTRG.15843.2	137	0.35407	9.415222168	1
MSTRG.15846.6	134	0.37275	7.785583496	1
MSTRG.15846.5	134	0.37281	7.785583496	1
MSTRG.15847.1	92	0.37575	10.2321167	1
MSTRG.15848.1	290	0.4525	5.353088379	1
MSTRG.15848.2	290	0.44272	5.353088379	1
MSTRG.15848.3	290	0.43251	5.353088379	1
MSTRG.15858.1	109	0.29618	8.815002441	1
MSTRG.15858.2	259	0.34381	5.089416504	1
MSTRG.15858.3	369	0.41537	8.131286621	1
MSTRG.15868.1	1034	0.4392	6.370178223	1
MSTRG.15868.6	930	0.41174	6.216125488	1
MSTRG.15868.9	930	0.43932	6.216125488	1
MSTRG.15868.7	930	0.41174	6.216125488	1
MSTRG.15868.3	930	0.41882	6.216125488	1
MSTRG.15868.10	930	0.45349	6.216125488	1
MSTRG.15867.3	109	0.35235	8.976745605	1
MSTRG.15873.2	432	0.40259	4.910095215	1
MSTRG.15874.2	381	0.4718	6.639343262	1
MSTRG.15879.2	623	0.3672	7.916931152	1
MSTRG.15886.2	505	0.32755	8.637878418	1
MSTRG.15890.1	48	0.38057	10.55731201	-1
MSTRG.15891.2	245	0.42111	4.683410645	1
MSTRG.15896.2	136	0.37206	4.920837402	1
MSTRG.15902.5	836	0.4639	6.141174316	1
MSTRG.15902.3	890	0.49745	6.479919434	1
MSTRG.15902.4	1008	0.46518	6.253479004	1
MSTRG.15902.6	894	0.43251	6.699645996	1
MSTRG.15903.1	39	0.32365	9.186584473	1
MSTRG.15897.1	97	0.37349	6.506530762	1
MSTRG.15898.1	45	0.28683	9.098937988	1
MSTRG.15899.3	526	0.36494	9.023620605	1
MSTRG.15899.2	363	0.37405	8.081604004	1
MSTRG.15899.4	458	0.42486	8.565002441	1
MSTRG.15909.4	1752	0.40306	6.254577637	1
MSTRG.15909.1	1376	0.45068	6.76776123	1

MSTRG.15909.6	1752	0.43896	6.254577637	1
MSTRG.15907.1	27	0.37419	9.303649902	1
MSTRG.15917.2	293	0.29152	5.684631348	1
MSTRG.15917.3	223	0.29152	10.03424072	1
MSTRG.15917.6	209	0.30907	6.56817627	1
MSTRG.15917.7	293	0.4323	5.684631348	1
MSTRG.15917.5	293	0.39435	5.684631348	1
MSTRG.15917.8	293	0.38536	5.684631348	1
MSTRG.15917.9	209	0.36501	6.56817627	1
MSTRG.15917.10	127	0.27879	11.75250244	1
MSTRG.15914.2	447	0.41835	8.969665527	1
MSTRG.15913.1	84	0.35649	7.966125488	1
MSTRG.15921.1	55	0.37452	10.45001221	1
MSTRG.15929.1	1191	0.43746	7.534851074	1
MSTRG.15929.4	1112	0.45132	7.553771973	1
MSTRG.15930.1	117	0.28499	5.377380371	1
MSTRG.15929.5	1048	0.44995	6.352600098	1
MSTRG.15929.7	1026	0.40882	6.32244873	1
MSTRG.15929.9	1048	0.43137	6.324157715	1
MSTRG.15929.10	1070	0.40661	6.354309082	1
MSTRG.15929.11	1057	0.44428	6.45513916	1
MSTRG.15932.1	700	0.37174	9.071472168	1
MSTRG.15932.2	234	0.45344	8.72088623	1
MSTRG.15931.1	46	0.48281	10.08416748	1
MSTRG.15936.2	129	0.37195	8.496643066	1
MSTRG.15936.3	126	0.32485	9.016662598	1
MSTRG.15937.3	156	0.32099	5.350402832	1
MSTRG.15935.1	176	0.419	9.096618652	1
MSTRG.15940.1	262	0.46349	9.624328613	1
MSTRG.15940.4	190	0.49163	8.944763184	1
MSTRG.15943.1	41	0.38988	9.486877441	1
MSTRG.15961.2	454	0.31287	6.63861084	1
MSTRG.15962.3	1001	0.45968	5.229675293	1
MSTRG.15962.2	1069	0.4799	5.112365723	1
MSTRG.15962.7	1001	0.32453	5.229675293	1
MSTRG.15963.1	102	0.28883	6.879699707	1
MSTRG.15964.1	177	0.31852	6.814880371	1
MSTRG.15952.1	1006	0.46759	6.430114746	1
MSTRG.15955.1	79	0.3101	9.035217285	1
MSTRG.15956.1	152	0.31532	10.00384521	1
MSTRG.15957.1	187	0.35887	10.02130127	1
MSTRG.15958.1	64	0.3327	7.659484863	1
MSTRG.15951.1	81	0.43733	10.87799072	-1
MSTRG.15969.2	100	0.36815	5.625549316	1
MSTRG.15969.3	100	0.38024	5.625549316	1
MSTRG.15969.4	100	0.37838	5.625549316	1
MSTRG.15973.1	13	0.25693	11.69903564	1
MSTRG.15974.1	461	0.4697	7.818908691	1
MSTRG.15974.5	384	0.45898	7.179260254	1
MSTRG.15978.5	346	0.28584	5.269470215	1
MSTRG.15978.7	73	0.36207	9.381286621	1
MSTRG.15978.6	196	0.28982	9.08392334	1
MSTRG.15979.1	84	0.35764	10.54571533	1
MSTRG.15980.1	65	0.34059	10.17254639	1

MSTRG.15981.1	128	0.3674	4.785095215	1
MSTRG.15978.8	104	0.31512	9.139709473	1
MSTRG.15982.1	72	0.41791	6.571350098	1
MSTRG.15978.9	65	0.28165	10.02301025	1
MSTRG.15976.2	174	0.36426	9.738586426	1
MSTRG.15990.4	710	0.46265	5.384338379	1
MSTRG.15994.1	372	0.42911	5.360412598	1
MSTRG.15996.1	54	0.33842	4.641662598	1
MSTRG.15997.1	88	0.35222	9.59979248	1
MSTRG.15998.4	486	0.40383	8.455627441	1
MSTRG.15998.3	294	0.42147	7.688903809	1
MSTRG.15998.10	259	0.30648	8.957946777	1
MSTRG.15998.11	486	0.40961	8.455627441	1
MSTRG.15998.9	486	0.41713	8.455627441	1
MSTRG.15999.1	86	0.42054	9.690002441	1
MSTRG.16000.1	55	0.41275	11.8291626	1
MSTRG.16003.1	351	0.37314	8.421936035	1
MSTRG.16004.1	120	0.26101	8.54864502	1
MSTRG.16006.1	6	0.33248	7.999816895	1
MSTRG.16005.2	242	0.32599	10.01080322	-1
MSTRG.16012.1	45	0.37663	10.91619873	1
MSTRG.16013.2	1101	0.45549	8.089416504	1
MSTRG.16014.1	41	0.34236	7.869567871	1
MSTRG.16015.2	302	0.39792	9.686828613	1
MSTRG.16019.1	499	0.31285	7.728942871	1
MSTRG.16019.2	290	0.32263	9.046203613	1
MSTRG.16022.2	745	0.42598	5.671447754	1
MSTRG.16027.2	867	0.44463	7.65057373	1
MSTRG.16031.2	537	0.4512	5.737487793	1
MSTRG.16034.2	89	0.33599	11.25836182	1
MSTRG.16035.1	78	0.33791	4.862121582	1
MSTRG.16039.1	501	0.32504	8.561828613	1
MSTRG.16039.2	418	0.45263	8.769348145	1
MSTRG.16041.1	216	0.48104	9.019958496	1
MSTRG.16042.2	61	0.34609	11.40228271	1
MSTRG.16042.3	61	0.33909	11.40228271	1
MSTRG.16042.4	61	0.42345	11.40228271	1
MSTRG.16044.3	417	0.35788	5.932434082	1
MSTRG.16049.2	331	0.34153	8.841247559	1
MSTRG.16050.3	207	0.4641	6.44354248	1
MSTRG.16050.4	151	0.42983	9.557556152	1
MSTRG.16052.1	139	0.27327	10.44281006	1
MSTRG.16052.2	139	0.30431	10.44281006	1
MSTRG.16052.3	139	0.28989	10.44281006	1
MSTRG.16056.1	189	0.27987	10.16693115	1
MSTRG.16057.12	650	0.44781	7.530090332	1
MSTRG.16057.15	650	0.45795	7.530090332	1
MSTRG.16057.11	810	0.4901	6.997497559	1
MSTRG.16057.14	829	0.49134	7.244445801	1
MSTRG.16057.17	742	0.44519	6.48236084	1
MSTRG.16057.19	824	0.47619	6.769348145	1
MSTRG.16057.20	622	0.49462	5.945495605	1
MSTRG.16067.2	453	0.41306	5.744934082	1
MSTRG.16058.1	258	0.3636	4.312316895	1

MSTRG.16064.2	323	0.38767	4.823669434	1
MSTRG.16064.3	323	0.43096	4.823669434	1
MSTRG.16064.4	323	0.4411	4.823669434	1
MSTRG.16064.7	323	0.39355	4.823669434	1
MSTRG.16069.2	337	0.43885	5.575500488	1
MSTRG.16069.5	425	0.41551	6.083679199	1
MSTRG.16069.6	500	0.39775	6.085632324	1
MSTRG.16069.8	294	0.34913	9.017883301	1
MSTRG.16072.1	148	0.3935	11.29034424	-1
MSTRG.16079.2	1673	0.45463	6.258117676	1
MSTRG.16079.4	1479	0.48808	6.303771973	1
MSTRG.16074.1	593	0.44214	5.510192871	1
MSTRG.16074.3	239	0.3598	9.355041504	1
MSTRG.16081.1	583	0.44218	7.299255371	1
MSTRG.16082.2	324	0.39944	5.948059082	1
MSTRG.16082.3	289	0.38652	6.662658691	1
MSTRG.16082.4	161	0.32961	8.31817627	1
MSTRG.16076.2	348	0.39798	6.258117676	1
MSTRG.16083.1	106	0.28491	8.74029541	1
MSTRG.16083.2	105	0.28662	9.808410645	1
MSTRG.16083.3	76	0.35756	8.479431152	1
MSTRG.16088.2	223	0.34395	6.224182129	1
MSTRG.16088.4	135	0.35679	5.523254395	1
MSTRG.16085.1	421	0.41805	5.590026855	1
MSTRG.16092.3	912	0.41285	5.757995605	1
MSTRG.16093.3	275	0.33623	9.975280762	1
MSTRG.16104.1	696	0.34331	9.386169434	1
MSTRG.16104.4	696	0.34331	9.386169434	1
MSTRG.16104.5	896	0.38057	9.082214355	1
MSTRG.16102.1	269	0.36588	5.984069824	1
MSTRG.16109.4	2071	0.43417	8.158630371	1
MSTRG.16110.1	61	0.32619	8.636047363	1
MSTRG.16113.5	1948	0.36127	7.250671387	1
MSTRG.16113.6	1946	0.38059	7.250671387	1
MSTRG.16113.8	1945	0.38059	7.24798584	1
MSTRG.16113.7	2225	0.35274	8.455993652	1
MSTRG.16113.9	1667	0.38363	6.252990723	1
MSTRG.16113.11	1536	0.33785	6.732116699	1
MSTRG.16113.12	615	0.35182	5.938781738	1
MSTRG.16113.13	775	0.32412	6.026428223	1
MSTRG.16113.14	552	0.31639	5.641296387	1
MSTRG.16117.2	1146	0.47676	5.811950684	1
MSTRG.16117.3	401	0.42449	6.540588379	1
MSTRG.16118.1	75	0.35733	10.01971436	1
MSTRG.16123.1	130	0.32436	9.483215332	1
MSTRG.16128.1	2683	0.42094	7.926940918	1
MSTRG.16128.5	2662	0.42778	7.773376465	1
MSTRG.16128.6	2647	0.43987	7.934509277	1
MSTRG.16129.1	254	0.36854	8.834533691	1
MSTRG.16129.2	240	0.31209	8.973815918	1
MSTRG.16129.3	125	0.31849	9.301330566	1
MSTRG.16132.2	706	0.4663	9.719543457	1
MSTRG.16132.3	584	0.45841	9.246887207	1
MSTRG.16133.1	112	0.28002	5.311462402	1

MSTRG.16133.2	672	0.38809	6.885681152	1
MSTRG.16133.4	727	0.32076	6.288757324	1
MSTRG.16136.1	516	0.46366	6.68927002	1
MSTRG.16136.2	450	0.41941	6.61541748	1
MSTRG.16138.2	1021	0.31371	6.017883301	1
MSTRG.16138.5	880	0.31371	6.183776855	1
MSTRG.16138.6	1021	0.4621	6.017883301	1
MSTRG.16144.1	92	0.32431	10.00054932	1
MSTRG.16144.4	247	0.43741	8.764953613	1
MSTRG.16148.2	1563	0.442	6.36920166	1
MSTRG.16148.5	1563	0.42896	6.36920166	1
MSTRG.16149.1	81	0.28063	9.566955566	1
MSTRG.16159.1	996	0.40076	10.05169678	1
MSTRG.16159.2	956	0.42434	9.825378418	1
MSTRG.16159.3	895	0.40596	9.874084473	1
MSTRG.16159.4	858	0.40596	10.13214111	1
MSTRG.16159.5	935	0.36801	10.10443115	1
MSTRG.16162.1	169	0.28227	9.613830566	1
MSTRG.16163.5	1343	0.41764	6.74029541	1
MSTRG.16167.2	376	0.44192	5.97467041	1
MSTRG.16170.5	2328	0.41335	5.364929199	1
MSTRG.16174.2	175	0.36018	7.734802246	1
MSTRG.16191.3	599	0.43563	5.39251709	1
MSTRG.16191.4	599	0.44026	5.39251709	1
MSTRG.16191.5	477	0.42231	5.153991699	1
MSTRG.16182.2	295	0.35412	10.6630249	1
MSTRG.16196.2	839	0.44749	6.382141113	1
MSTRG.16198.1	722	0.45962	9.398986816	1
MSTRG.16198.2	722	0.4559	9.398986816	1
MSTRG.16199.1	139	0.31181	11.51580811	-1
MSTRG.16195.3	334	0.46549	8.540100098	1
MSTRG.16201.2	439	0.35475	7.916931152	1
MSTRG.16201.3	439	0.34487	7.916931152	1
MSTRG.16201.6	439	0.34487	7.916931152	1
MSTRG.16202.1	303	0.23179	8.297790527	1
MSTRG.16201.7	365	0.3244	7.906433105	1
MSTRG.16203.1	402	0.44072	6.646179199	1
MSTRG.16207.1	133	0.30317	11.50152588	1
MSTRG.16210.2	182	0.35871	9.229064941	1
MSTRG.16211.1	203	0.33986	8.781799316	1
MSTRG.16214.2	91	0.36993	11.04595947	1
MSTRG.16214.3	80	0.37576	7.795837402	1
MSTRG.16215.1	70	0.40558	5.077331543	1
MSTRG.16217.1	975	0.3054	9.457336426	1
MSTRG.16218.3	516	0.3808	7.436828613	1
MSTRG.16218.4	516	0.38388	7.436828613	1
MSTRG.16221.4	565	0.41878	5.641906738	1
MSTRG.16221.3	313	0.3565	5.881408691	1
MSTRG.16222.1	250	0.29145	11.44561768	1
MSTRG.16222.3	169	0.34609	10.22113037	1
MSTRG.16219.1	75	0.30919	7.002258301	1
MSTRG.16226.5	80	0.28953	6.170837402	1
MSTRG.16239.3	592	0.46111	9.296569824	1
MSTRG.16239.4	599	0.46882	9.535461426	1

MSTRG.16244.1	259	0.41808	6.259338379	1
MSTRG.16244.3	402	0.44088	8.313903809	1
MSTRG.16244.5	573	0.39187	9.335876465	1
MSTRG.16242.7	30	0.35245	5.999572754	1
MSTRG.16233.1	328	0.32989	10.38787842	1
MSTRG.16233.2	62	0.37648	4.418273926	1
MSTRG.16248.2	528	0.43702	6.29107666	1
MSTRG.16248.3	528	0.43702	6.29107666	1
MSTRG.16237.1	36	0.31947	11.53900146	1
MSTRG.16252.3	320	0.4314	9.261169434	1
MSTRG.16252.4	320	0.41444	9.261169434	1
MSTRG.16252.1	320	0.40102	9.261169434	1
MSTRG.16253.2	141	0.2728	11.66802979	1
MSTRG.16253.6	208	0.40648	9.535095215	1
MSTRG.16253.7	138	0.32646	11.82879639	1
MSTRG.16254.1	475	0.40196	6.295349121	1
MSTRG.16261.2	1323	0.45212	7.35369873	1
MSTRG.16261.6	1295	0.45238	7.342834473	1
MSTRG.16261.5	1323	0.44112	7.35369873	1
MSTRG.16263.5	367	0.45385	5.26751709	1
MSTRG.16263.3	233	0.3995	4.969421387	1
MSTRG.16263.4	233	0.45143	4.969421387	1
MSTRG.16263.2	367	0.31108	5.26751709	1
MSTRG.16258.1	79	0.29099	6.475402832	1
MSTRG.16258.2	79	0.31969	6.475402832	1
MSTRG.16264.6	829	0.37335	5.973815918	1
MSTRG.16265.1	100	0.29179	7.610412598	-1
MSTRG.16264.10	821	0.33713	6.073669434	1
MSTRG.16264.9	1246	0.46409	5.637512207	1
MSTRG.16264.11	60	0.40035	11.62371826	1
MSTRG.16269.1	120	0.28039	9.128479004	1
MSTRG.16269.2	120	0.32509	9.128479004	1
MSTRG.16269.3	120	0.28907	9.128479004	1
MSTRG.16270.1	563	0.42236	9.798156738	1
MSTRG.16270.2	563	0.4085	9.798156738	1
MSTRG.16270.3	563	0.40631	9.798156738	1
MSTRG.16273.1	32	0.32089	6.511901855	1
MSTRG.16274.2	467	0.42685	9.150695801	1
MSTRG.16275.1	78	0.42642	10.01641846	-1
MSTRG.16276.1	135	0.34642	10.4151001	1
MSTRG.16278.2	148	0.32823	9.204162598	1
MSTRG.16284.1	54	0.37111	10.54864502	-1
MSTRG.16288.1	225	0.39604	9.920959473	1
MSTRG.16291.1	126	0.34295	8.601867676	1
MSTRG.16296.1	53	0.31985	9.591125488	1
MSTRG.16299.1	69	0.3679	9.39666748	-1
MSTRG.16299.2	76	0.37932	9.69342041	1
MSTRG.16302.1	56	0.38721	6.49017334	1
MSTRG.16307.1	56	0.36208	5.667785645	1
MSTRG.16319.2	178	0.28165	6.20513916	1
MSTRG.16319.3	338	0.29751	5.606872559	1
MSTRG.16319.4	337	0.32906	5.606872559	1
MSTRG.16319.5	337	0.30092	5.606872559	1
MSTRG.16319.6	337	0.29387	5.606872559	1

MSTRG.16320.2	144	0.2782	9.961975098	1
MSTRG.16321.1	614	0.46362	5.073303223	1
MSTRG.16326.1	592	0.40569	7.776550293	1
MSTRG.16327.1	447	0.46039	8.999694824	1
MSTRG.16328.1	84	0.37685	11.00006104	1
MSTRG.16329.3	818	0.41013	7.762023926	1
MSTRG.16329.4	74	0.3058	6.547424316	1
MSTRG.16330.2	1899	0.3965	6.52130127	1
MSTRG.16332.1	994	0.44249	5.818054199	1
MSTRG.16334.5	1877	0.47866	5.168762207	1
MSTRG.16336.1	56	0.43939	9.624694824	-1
MSTRG.16337.1	100	0.38587	10.16778564	1
MSTRG.16339.2	85	0.3331	9.031799316	1
MSTRG.16349.1	80	0.32797	9.490661621	1
MSTRG.16345.2	100	0.2785	9.690002441	1
MSTRG.16345.3	100	0.3072	9.690002441	1
MSTRG.16343.1	44	0.34141	11.32427979	1
MSTRG.16340.1	35	0.42952	11.45159912	1
MSTRG.16351.1	65	0.32597	10.61419678	1
MSTRG.16351.2	53	0.36881	10.29205322	1
MSTRG.16352.4	90	0.29236	9.62677002	1
MSTRG.16353.1	76	0.27433	6.510559082	1
MSTRG.16354.1	55	0.244	8.023742676	1
MSTRG.16358.2	495	0.44741	9.483093262	1
MSTRG.16358.3	497	0.51011	9.161437988	1
MSTRG.16361.2	149	0.34706	8.367736816	1
MSTRG.16361.1	249	0.35409	9.241882324	1
MSTRG.16361.3	220	0.39142	9.384460449	1
MSTRG.16361.5	216	0.45249	9.248840332	1
MSTRG.16361.6	208	0.31793	10.71783447	1
MSTRG.16361.7	208	0.3756	10.16131592	1
MSTRG.16364.2	701	0.39276	6.032653809	1
MSTRG.16366.1	258	0.46288	6.684020996	1
MSTRG.16369.4	298	0.27151	9.45135498	1
MSTRG.16370.1	107	0.32081	5.065246582	1
MSTRG.16370.2	208	0.29657	9.954528809	1
MSTRG.16375.3	282	0.43941	6.49810791	1
MSTRG.16378.1	58	0.36519	5.464538574	1
MSTRG.16388.1	77	0.34696	10.29888916	1
MSTRG.16389.1	85	0.29387	9.131530762	1
MSTRG.16389.2	80	0.37031	9.466369629	1
MSTRG.16388.2	48	0.36206	9.035461426	1
MSTRG.16397.3	346	0.31674	9.223693848	1
MSTRG.16399.2	224	0.3652	5.19744873	1
MSTRG.16410.1	37	0.37246	10.01495361	1
MSTRG.16418.1	88	0.3908	11.80096436	1
MSTRG.16419.6	167	0.41987	10.35162354	1
MSTRG.16419.5	477	0.45016	5.857727051	1
MSTRG.16419.7	1134	0.43497	6.604309082	1
MSTRG.16419.8	777	0.43962	6.820495605	1
MSTRG.16419.10	1121	0.45045	6.526916504	1
MSTRG.16422.1	334	0.47636	7.096618652	1
MSTRG.16422.2	334	0.45471	7.096618652	1
MSTRG.16422.3	334	0.47071	7.096618652	1

MSTRG.16422.6	334	0.47693	7.096618652	1
MSTRG.16423.4	402	0.34787	9.026550293	1
MSTRG.16423.2	511	0.4116	8.065979004	1
MSTRG.16423.3	291	0.38054	6.716247559	1
MSTRG.16423.5	321	0.29283	8.657897949	1
MSTRG.16423.7	688	0.44251	7.40423584	1
MSTRG.16423.8	511	0.41054	8.065979004	1
MSTRG.16423.10	201	0.33775	9.914367676	1
MSTRG.16423.12	126	0.33856	4.740905762	1
MSTRG.16425.2	67	0.29607	5.785217285	1
MSTRG.16425.1	486	0.32921	6.117126465	1
MSTRG.16425.3	531	0.28739	6.082824707	1
MSTRG.16425.9	531	0.40572	6.082824707	1
MSTRG.16425.10	531	0.42352	6.082824707	1
MSTRG.16427.1	123	0.45932	9.06072998	-1
MSTRG.16417.1	74	0.41464	11.92144775	1
MSTRG.16429.2	28	0.29587	4.242858887	1
MSTRG.16431.3	407	0.44069	5.534851074	1
MSTRG.16431.4	407	0.44385	5.534851074	1
MSTRG.16470.1	450	0.29308	9.182556152	1
MSTRG.16472.1	104	0.31546	9.510681152	1
MSTRG.16473.2	35	0.35025	5.573669434	1
MSTRG.16446.1	32	0.35261	9.78326416	1
MSTRG.16448.5	128	0.35845	8.679138184	1
MSTRG.16448.6	49	0.39172	4.897644043	1
MSTRG.16448.2	400	0.43206	6.432067871	1
MSTRG.16448.4	400	0.39349	6.432067871	1
MSTRG.16448.8	158	0.27911	6.18572998	1
MSTRG.16448.9	176	0.3072	6.738830566	1
MSTRG.16452.3	1372	0.45859	6.355041504	1
MSTRG.16452.5	1417	0.4727	6.355529785	1
MSTRG.16452.6	1417	0.45859	6.355529785	1
MSTRG.16453.2	335	0.39079	5.948425293	1
MSTRG.16457.1	386	0.40643	6.745300293	1
MSTRG.16461.2	277	0.47123	8.350891113	1
MSTRG.16463.2	604	0.4125	6.571228027	1
MSTRG.16463.3	465	0.33311	8.233093262	1
MSTRG.16463.4	465	0.33869	8.233093262	1
MSTRG.16464.1	84	0.28883	9.17779541	1
MSTRG.16465.1	43	0.33322	5.380432129	1
MSTRG.16482.1	23	0.40187	10.17108154	1
MSTRG.16484.1	657	0.46508	5.662658691	1
MSTRG.16486.2	316	0.36954	5.587585449	1
MSTRG.16486.3	152	0.34556	5.690246582	1
MSTRG.16491.1	154	0.45071	4.948547363	1
MSTRG.16492.1	67	0.3788	4.64642334	1
MSTRG.16492.2	308	0.46362	5.591003418	1
MSTRG.16498.7	856	0.4572	8.892272949	1
MSTRG.16498.8	864	0.4572	8.785095215	1
MSTRG.16498.13	818	0.45133	8.418518066	1
MSTRG.16498.14	865	0.45443	8.785095215	1
MSTRG.16498.15	736	0.46256	8.819885254	1
MSTRG.16498.16	111	0.35946	9.99395752	1
MSTRG.16502.2	57	0.46791	9.873718262	1

MSTRG.16507.1	52	0.48943	4.885681152	1
MSTRG.16511.1	421	0.38782	5.72857666	1
MSTRG.16511.2	421	0.417	5.72857666	1
MSTRG.16511.7	421	0.44143	5.72857666	1
MSTRG.16514.1	668	0.48375	5.674377441	1
MSTRG.16514.2	693	0.47914	5.683166504	1
MSTRG.16515.3	360	0.43384	9.073791504	1
MSTRG.16539.4	843	0.40713	5.828186035	1
MSTRG.16539.5	739	0.41178	5.975524902	1
MSTRG.16539.2	2398	0.47684	5.703063965	1
MSTRG.16539.6	2468	0.46117	5.742248535	1
MSTRG.16539.7	1624	0.44784	5.721618652	1
MSTRG.16542.1	91	0.36115	9.512023926	1
MSTRG.16539.8	1307	0.45765	5.60369873	1
MSTRG.16565.1	63	0.34516	8.770080566	1
MSTRG.16584.1	47	0.40988	4.948303223	1
MSTRG.16584.2	53	0.32167	10.31939697	1
MSTRG.16516.1	356	0.47727	8.558898926	1
MSTRG.16520.1	67	0.35395	6.13104248	1
MSTRG.16535.6	241	0.44689	6.387756348	1
MSTRG.16535.7	77	0.30981	9.620544434	1
MSTRG.16537.1	94	0.29615	9.761779785	1
MSTRG.16590.2	673	0.36762	5.634338379	1
MSTRG.16590.4	573	0.35982	5.232849121	1
MSTRG.16590.3	673	0.38148	5.634338379	1
MSTRG.16590.6	99	0.29595	9.694763184	1
MSTRG.16597.2	2969	0.45649	5.585876465	1
MSTRG.16597.3	274	0.46824	4.922912598	1
MSTRG.16597.4	86	0.4412	9.68157959	-1
MSTRG.16597.5	18	0.34907	4.677062988	1
MSTRG.16597.6	23	0.34971	5.557922363	1
MSTRG.16613.1	59	0.35825	9.296813965	1
MSTRG.16616.1	150	0.2908	9.535949707	1
MSTRG.16616.2	150	0.27415	9.535949707	1
MSTRG.16616.3	290	0.2782	9.02947998	1
MSTRG.16619.1	76	0.27415	6.249572754	1
MSTRG.16620.1	160	0.28267	8.20526123	1
MSTRG.16616.5	192	0.42788	5.114196777	-1
MSTRG.16616.6	192	0.39544	5.114196777	-1
MSTRG.16616.7	189	0.35053	5.114196777	-1
MSTRG.16616.8	189	0.31261	5.114196777	-1
MSTRG.16620.2	217	0.28584	9.332580566	1
MSTRG.16620.3	148	0.28063	5.880310059	1
MSTRG.16620.4	148	0.27911	5.880310059	1
MSTRG.16620.5	160	0.28419	8.20526123	1
MSTRG.16620.6	160	0.28186	8.20526123	1
MSTRG.16622.1	98	0.28415	10.30999756	1
MSTRG.16587.1	83	0.37871	7.999084473	1
MSTRG.16601.1	67	0.32839	6.006164551	1
MSTRG.16602.1	161	0.29526	8.289001465	1
MSTRG.16603.3	128	0.3283	9.342956543	1
MSTRG.16603.4	336	0.4248	9.112243652	1
MSTRG.16604.2	176	0.38993	9.028137207	1
MSTRG.16629.2	215	0.43655	5.620666504	1

MSTRG.16629.3	215	0.4052	5.620666504	1
MSTRG.16630.1	94	0.30981	9.764709473	1
MSTRG.16631.3	136	0.28267	9.844665527	1
MSTRG.16629.4	215	0.41613	5.620666504	1
MSTRG.16629.5	215	0.35562	5.620666504	1
MSTRG.16632.1	53	0.403	11.00860596	-1
MSTRG.16642.1	629	0.38809	6.216247559	1
MSTRG.16642.2	382	0.35833	5.455383301	1
MSTRG.16644.1	351	0.36174	5.39276123	1
MSTRG.16645.2	168	0.38475	6.30291748	1
MSTRG.16653.5	816	0.48918	8.620788574	-1
MSTRG.16653.6	797	0.48918	8.56048584	-1
MSTRG.16653.7	877	0.46441	9.023986816	1
MSTRG.16653.8	823	0.46749	8.65838623	1
MSTRG.16653.9	659	0.41696	9.015930176	1
MSTRG.16653.10	365	0.36805	9.33782959	1
MSTRG.16654.1	93	0.27433	6.374938965	1
MSTRG.16652.1	682	0.37825	6.142883301	1
MSTRG.16660.1	338	0.31313	9.520080566	1
MSTRG.16660.3	205	0.31065	9.936462402	1
MSTRG.16660.4	151	0.30117	10.12249756	1
MSTRG.16660.5	151	0.3991	10.12249756	1
MSTRG.16665.3	249	0.32642	5.305847168	1
MSTRG.16665.4	249	0.35657	5.305847168	1
MSTRG.16671.5	48	0.40645	9.056091309	1
MSTRG.16672.2	667	0.37484	8.721618652	1
MSTRG.16672.5	669	0.36954	8.50579834	1
MSTRG.16672.7	667	0.39889	8.721618652	1
MSTRG.16669.1	57	0.292	6.10760498	1
MSTRG.16669.2	47	0.27991	8.95904541	1
MSTRG.16670.1	1171	0.47045	6.661071777	1
MSTRG.16674.1	138	0.30882	10.17364502	1
MSTRG.16676.1	385	0.45158	8.873352051	1
MSTRG.16676.3	325	0.35889	9.708312988	1
MSTRG.16677.4	445	0.36909	9.247253418	1
MSTRG.16677.3	604	0.36992	8.482971191	1
MSTRG.16678.1	26	0.32879	5.706726074	1
MSTRG.16679.2	120	0.28842	9.500427246	1
MSTRG.16679.1	128	0.4232	11.46002197	1
MSTRG.16679.3	120	0.28842	9.500427246	1
MSTRG.16679.4	43	0.38596	9.961608887	1
MSTRG.16679.5	43	0.28567	9.961608887	1
MSTRG.16685.2	492	0.37193	8.616882324	1
MSTRG.16687.1	139	0.31277	11.46185303	1
MSTRG.16684.1	78	0.45561	5.507263184	1
MSTRG.16691.1	126	0.35735	11.65924072	1
MSTRG.16694.5	114	0.27327	9.976379395	1
MSTRG.16692.1	67	0.4262	8.787658691	-1
MSTRG.16693.1	68	0.3762	12.30206299	-1
MSTRG.16702.1	130	0.31388	9.115905762	1
MSTRG.16702.4	119	0.29857	11.12677002	1
MSTRG.16702.5	35	0.3372	5.464782715	1
MSTRG.16704.3	30	0.41776	7.834899902	1
MSTRG.16702.10	538	0.33172	9.249938965	1

MSTRG.16702.12	320	0.33112	8.726013184	1
MSTRG.16702.13	130	0.28999	9.218322754	1
MSTRG.16709.4	1152	0.41425	4.977722168	1
MSTRG.16709.7	74	0.28648	9.730163574	1
MSTRG.16696.4	226	0.32246	4.807556152	1
MSTRG.16712.1	1375	0.35903	5.956115723	1
MSTRG.16712.2	1288	0.36068	6.266906738	1
MSTRG.16712.3	1375	0.37953	5.956115723	1
MSTRG.16712.5	1375	0.38118	5.956115723	1
MSTRG.16712.4	1375	0.42305	5.956115723	1
MSTRG.16712.6	1288	0.42224	6.266906738	1
MSTRG.16712.7	1288	0.39694	6.266906738	1
MSTRG.16712.8	1249	0.42011	6.31842041	1
MSTRG.16712.9	1336	0.45125	5.991394043	1
MSTRG.16712.10	1336	0.43075	5.991394043	1
MSTRG.16712.11	748	0.4184	8.058898926	1
MSTRG.16713.1	528	0.4702	8.184020996	1
MSTRG.16713.2	528	0.4683	8.184020996	1
MSTRG.16713.4	197	0.39012	5.071960449	1
MSTRG.16713.11	432	0.29059	5.84552002	1
MSTRG.16719.2	1791	0.46179	6.107849121	1
MSTRG.16722.1	1651	0.48599	5.434753418	1
MSTRG.16722.2	1549	0.50342	5.215393066	1
MSTRG.16722.3	1652	0.45878	5.434753418	1
MSTRG.16722.5	1073	0.43773	5.632629395	1
MSTRG.16722.6	841	0.43805	5.961853027	1
MSTRG.16726.1	38	0.37932	6.818664551	1
MSTRG.16733.1	132	0.37047	5.465759277	1
MSTRG.16733.3	301	0.46586	8.736633301	-1
MSTRG.16745.1	45	0.37975	9.849304199	1
MSTRG.16747.1	21	0.41195	11.00653076	1
MSTRG.16754.2	42	0.26963	5.15411377	1
MSTRG.16757.3	601	0.46376	5.516418457	1
MSTRG.16757.4	601	0.42968	5.516418457	1
MSTRG.16748.2	366	0.37425	8.920471191	1
MSTRG.16749.1	74	0.44064	8.721252441	-1
MSTRG.16749.2	32	0.37798	5.272766113	-1
MSTRG.16750.1	49	0.36742	10.26641846	1
MSTRG.16761.3	70	0.30788	8.986633301	1
MSTRG.16764.2	707	0.34431	6.401550293	1
MSTRG.16765.1	100	0.40619	5.863830566	1
MSTRG.16766.1	96	0.38904	6.002258301	1
MSTRG.16767.1	76	0.33851	4.650695801	1
MSTRG.16769.1	67	0.2785	8.781677246	1
MSTRG.16772.2	1280	0.44464	5.72454834	1
MSTRG.16772.3	1063	0.42414	8.566833496	1
MSTRG.16772.4	1050	0.44464	5.987365723	1
MSTRG.16772.5	1261	0.44464	5.995422363	1
MSTRG.16776.1	58	0.43599	5.376403809	1
MSTRG.16799.10	1012	0.42492	6.430358887	1
MSTRG.16799.7	1015	0.42492	6.430358887	1
MSTRG.16799.8	1012	0.42492	6.430358887	1
MSTRG.16799.9	1012	0.42573	6.430358887	1
MSTRG.16799.3	1015	0.42108	6.430358887	1

MSTRG.16799.2	3512	0.44237	6.776062012	1
MSTRG.16799.11	3601	0.44237	6.785217285	1
MSTRG.16799.4	3601	0.44237	6.785217285	1
MSTRG.16799.5	3637	0.44237	7.028869629	1
MSTRG.16799.1	3600	0.45105	7.075134277	1
MSTRG.16801.1	62	0.28508	8.541809082	1
MSTRG.16799.12	180	0.32567	6.899597168	1
MSTRG.16799.13	1439	0.47138	5.417663574	1
MSTRG.16802.1	110	0.34079	8.553161621	1
MSTRG.16803.1	67	0.3657	9.68951416	1
MSTRG.16780.1	30	0.32922	5.221862793	-1
MSTRG.16784.2	350	0.30846	9.277770996	1
MSTRG.16785.1	67	0.36113	9.696716309	-1
MSTRG.16787.1	23	0.40789	12.00848389	1
MSTRG.16789.1	47	0.27015	8.592346191	1
MSTRG.16823.10	888	0.43152	9.334411621	1
MSTRG.16823.8	888	0.43152	9.334411621	1
MSTRG.16823.1	993	0.41545	9.052062988	1
MSTRG.16823.5	980	0.41545	9.130432129	1
MSTRG.16823.6	983	0.41545	9.130432129	1
MSTRG.16823.7	1017	0.41545	9.143981934	1
MSTRG.16823.9	990	0.4545	9.46685791	1
MSTRG.16823.2	983	0.41545	9.130432129	1
MSTRG.16823.3	1007	0.41545	9.211486816	1
MSTRG.16823.11	1017	0.4404	9.143981934	1
MSTRG.16823.12	135	0.33953	7.02923584	1
MSTRG.16824.1	71	0.3578	10.1696167	1
MSTRG.16824.2	101	0.37675	9.821838379	1
MSTRG.16824.3	106	0.32427	8.737854004	1
MSTRG.16816.2	130	0.36366	9.946472168	1
MSTRG.16816.3	239	0.31596	4.695251465	1
MSTRG.16817.1	125	0.28174	10.43487549	1
MSTRG.16818.1	99	0.31437	6.713439941	1
MSTRG.16818.2	50	0.38363	5.048034668	1
MSTRG.16820.1	90	0.36771	10.5244751	1
MSTRG.16835.1	28	0.32076	7.728210449	1
MSTRG.16835.2	28	0.3356	7.728210449	1
MSTRG.16840.1	233	0.35039	9.00958252	1
MSTRG.16847.1	45	0.36462	9.300598145	1
MSTRG.16847.2	26	0.45531	11.00030518	1
MSTRG.16855.1	53	0.37241	10.08746338	1
MSTRG.16878.1	53	0.41412	6.824157715	-1
MSTRG.16879.1	59	0.44809	5.378723145	1
MSTRG.16884.1	89	0.32635	7.965026855	1
MSTRG.17027.1	595	0.3287	8.580871582	1
MSTRG.17027.4	466	0.29607	8.478210449	1
MSTRG.17027.5	483	0.31677	8.584289551	1
MSTRG.17027.3	533	0.36886	7.783874512	1
MSTRG.17027.6	610	0.32477	8.10369873	1
MSTRG.17028.1	114	0.35984	9.774230957	1
MSTRG.17027.8	387	0.2921	6.537414551	1
MSTRG.17027.12	399	0.34222	6.70135498	1
MSTRG.17027.13	459	0.37566	8.671203613	1
MSTRG.17027.20	317	0.29851	6.886047363	1

MSTRG.17027.16	325	0.29367	7.516418457	1
MSTRG.17027.17	325	0.31209	6.852722168	1
MSTRG.17027.21	336	0.29343	7.186462402	1
MSTRG.17027.22	253	0.2921	7.175842285	1
MSTRG.17027.23	139	0.3	6.479797363	1
MSTRG.17031.1	149	0.28982	8.842224121	1
MSTRG.17027.24	253	0.3208	7.175842285	1
MSTRG.17027.25	399	0.3373	6.70135498	1
MSTRG.17027.26	325	0.29343	7.516418457	1
MSTRG.17027.27	325	0.30804	7.516418457	1
MSTRG.17027.28	86	0.29895	8.503112793	1
MSTRG.17033.1	40	0.31463	9.511657715	1
MSTRG.17027.29	101	0.27517	6.219177246	1
MSTRG.17027.30	198	0.27517	9.444519043	1
MSTRG.17034.1	103	0.27517	6.92767334	1
MSTRG.17035.1	99	0.31901	9.101013184	1
MSTRG.17036.1	93	0.30387	7.715393066	1
MSTRG.17037.1	124	0.32072	11.30255127	1
MSTRG.17038.1	155	0.32557	10.03009033	1
MSTRG.17039.1	189	0.32237	9.577819824	1
MSTRG.17040.1	64	0.31901	8.571472168	1
MSTRG.17041.1	145	0.29366	10.0269165	1
MSTRG.17042.1	157	0.35839	11.00518799	1
MSTRG.17042.2	283	0.32238	9.183044434	1
MSTRG.17027.31	65	0.32237	8.890930176	1
MSTRG.17027.32	162	0.29367	8.300842285	1
MSTRG.17027.34	366	0.29595	8.651306152	1
MSTRG.17027.33	93	0.2921	9.956359863	1
MSTRG.17027.35	42	0.3703	5.25177002	1
MSTRG.17027.37	201	0.28562	8.841491699	1
MSTRG.17027.36	75	0.29343	4.909118652	1
MSTRG.17027.38	366	0.28982	8.651306152	1
MSTRG.17027.40	513	0.29387	9.349182129	1
MSTRG.17027.39	105	0.29374	6.695617676	1
MSTRG.17027.41	201	0.25074	8.841491699	1
MSTRG.17027.42	201	0.27435	8.841491699	1
MSTRG.17027.43	83	0.28165	9.102233887	1
MSTRG.17027.44	83	0.3118	9.102233887	1
MSTRG.17044.1	63	0.26963	11.16595459	1
MSTRG.17027.45	88	0.30135	10.54754639	1
MSTRG.17027.46	119	0.3118	8.598327637	1
MSTRG.17045.1	72	0.27025	5.43548584	1
MSTRG.17046.1	72	0.31204	9.256896973	1
MSTRG.17047.1	122	0.28644	9.740539551	1
MSTRG.17048.1	91	0.32389	8.987365723	1
MSTRG.17049.1	112	0.36019	6.892150879	1
MSTRG.17050.1	78	0.31368	10.46112061	1
MSTRG.17051.1	86	0.33902	9.216003418	1
MSTRG.16890.1	555	0.41932	5.307434082	1
MSTRG.16890.2	252	0.48436	4.719299316	-1
MSTRG.16899.2	1095	0.42799	7.899841309	1
MSTRG.16906.1	1104	0.46549	5.921936035	1
MSTRG.16906.3	197	0.44559	5.156677246	1
MSTRG.16907.3	212	0.41011	9.139099121	1

MSTRG.16907.1	261	0.33152	6.440246582	1
MSTRG.16907.2	85	0.27931	6.792175293	1
MSTRG.16907.4	85	0.27931	6.792175293	1
MSTRG.16907.5	98	0.29907	9.199157715	1
MSTRG.16907.6	184	0.36072	9.53314209	1
MSTRG.16907.8	261	0.4388	6.440246582	1
MSTRG.16907.7	85	0.28503	6.792175293	1
MSTRG.16907.9	81	0.3152	8.589294434	1
MSTRG.16911.1	65	0.35556	8.937927246	1
MSTRG.16912.1	281	0.31233	9.590270996	1
MSTRG.16915.1	992	0.47914	5.644348145	1
MSTRG.16918.2	184	0.34969	4.87689209	1
MSTRG.16918.1	150	0.31208	7.612609863	1
MSTRG.16912.2	520	0.4055	9.009460449	1
MSTRG.16912.3	520	0.454	9.009460449	1
MSTRG.16902.3	274	0.37229	8.926818848	1
MSTRG.16902.4	274	0.37229	8.926818848	1
MSTRG.16919.1	94	0.38917	9.588195801	-1
MSTRG.16920.1	68	0.32106	7.684631348	1
MSTRG.16922.1	38	0.38127	6.804748535	1
MSTRG.16930.6	869	0.46865	6.00579834	1
MSTRG.16927.1	364	0.41849	7.116760254	1
MSTRG.16928.1	60	0.45289	9.101257324	1
MSTRG.16935.7	379	0.3919	8.863708496	1
MSTRG.16936.1	128	0.29772	9.250793457	1
MSTRG.16935.8	373	0.4124	9.252624512	1
MSTRG.16938.1	169	0.30808	7.634460449	1
MSTRG.16940.1	117	0.33887	5.005187988	1
MSTRG.16940.2	117	0.33887	5.005187988	1
MSTRG.16940.3	142	0.34527	6.10369873	1
MSTRG.16940.4	132	0.32257	7.87286377	1
MSTRG.16945.1	1097	0.42712	4.827453613	1
MSTRG.16948.1	102	0.28584	9.195251465	1
MSTRG.16949.2	404	0.32625	6.575866699	1
MSTRG.16953.4	2062	0.47891	7.166320801	1
MSTRG.16954.1	123	0.30775	8.807678223	1
MSTRG.16959.1	224	0.38268	9.445251465	1
MSTRG.16959.2	224	0.38268	9.445251465	1
MSTRG.16961.1	90	0.34952	10.10809326	1
MSTRG.16962.1	71	0.3637	7.508239746	1
MSTRG.16965.1	207	0.39001	5.823181152	1
MSTRG.16965.2	207	0.38408	5.823181152	1
MSTRG.16965.4	122	0.30415	6.276184082	-1
MSTRG.16965.3	341	0.33091	8.16595459	1
MSTRG.16965.5	102	0.30415	6.513244629	1
MSTRG.16965.6	168	0.40074	5.409484863	1
MSTRG.16965.7	159	0.46093	8.562683105	1
MSTRG.16966.1	207	0.29526	8.92755127	1
MSTRG.16966.2	207	0.28338	8.79119873	1
MSTRG.16967.1	94	0.31951	6.007263184	1
MSTRG.16968.1	53	0.30145	4.316101074	1
MSTRG.16963.5	61	0.4615	7.854064941	-1
MSTRG.16971.1	646	0.46168	5.69354248	1
MSTRG.16971.2	599	0.455	5.558044434	1

MSTRG.16971.3	599	0.47004	5.558044434	1
MSTRG.16971.4	361	0.44958	5.868225098	1
MSTRG.16971.6	683	0.45452	6.675476074	1
MSTRG.16971.8	467	0.46581	5.370422363	1
MSTRG.16971.10	265	0.4697	5.550109863	1
MSTRG.16971.9	514	0.4779	5.527526855	1
MSTRG.16973.1	101	0.31765	7.848693848	1
MSTRG.16974.2	168	0.37383	6.229797363	1
MSTRG.16974.3	168	0.34429	6.229797363	1
MSTRG.16978.1	634	0.45837	8.628845215	1
MSTRG.16978.2	1194	0.4655	9.301574707	1
MSTRG.16978.3	630	0.35568	9.63067627	1
MSTRG.16978.4	1194	0.46345	9.301574707	1
MSTRG.16988.6	3484	0.44813	9.226745605	1
MSTRG.16988.4	8333	0.46503	9.02154541	1
MSTRG.16988.5	8308	0.46474	8.969299316	1
MSTRG.16988.3	8308	0.45068	8.967834473	1
MSTRG.16988.9	147	0.43345	6.702331543	1
MSTRG.16992.2	419	0.43369	9.270324707	1
MSTRG.16996.1	29	0.39634	6.888366699	1
MSTRG.16997.1	75	0.37095	6.038635254	1
MSTRG.16997.2	62	0.40747	6.054138184	1
MSTRG.16998.1	74	0.29727	9.097595215	1
MSTRG.16999.1	59	0.33348	9.510681152	1
MSTRG.17005.1	37	0.42865	4.55657959	1
MSTRG.17004.6	66	0.30827	6.23638916	1
MSTRG.17004.7	74	0.34072	10.04437256	-1
MSTRG.17004.8	49	0.29816	9.938537598	1
MSTRG.17008.1	73	0.42937	10.65814209	1
MSTRG.17011.2	234	0.33061	11.04632568	1
MSTRG.17011.3	217	0.33153	10.840271	1
MSTRG.17011.5	217	0.39248	10.840271	1
MSTRG.17012.1	133	0.42208	11.56195068	1
MSTRG.17012.2	133	0.35432	11.56195068	1
MSTRG.17016.1	63	0.41511	5.604675293	1
MSTRG.17053.2	8	0.34748	6.487731934	1
MSTRG.17053.1	19	0.31563	10.05218506	1
MSTRG.17054.1	1822	0.4689	7.563659668	1
MSTRG.17054.2	1685	0.45593	8.118713379	1
MSTRG.17055.1	156	0.33828	10.50616455	1
MSTRG.17054.3	468	0.42243	9.689025879	1
MSTRG.17060.1	52	0.3979	9.976623535	1
MSTRG.17061.1	126	0.27732	9.249328613	1
MSTRG.17061.2	103	0.28519	6.704040527	1
MSTRG.17067.1	56	0.34533	10.68951416	1
MSTRG.17067.2	142	0.35551	11.77984619	1
MSTRG.17066.2	1305	0.43794	9.002502441	1
MSTRG.17069.1	164	0.40601	4.891784668	1
MSTRG.17069.2	164	0.43741	4.891784668	1
MSTRG.17074.2	127	0.36706	9.428039551	1
MSTRG.17074.4	345	0.45199	8.192810059	1
MSTRG.17074.6	329	0.43123	8.769714355	1
MSTRG.17076.1	429	0.33186	6.471618652	1
MSTRG.17076.4	450	0.4461	6.359924316	1

MSTRG.17076.6	520	0.44059	7.045593262	1
MSTRG.17077.1	337	0.43796	9.625671387	1
MSTRG.17078.5	194	0.30092	5.483337402	1
MSTRG.17078.1	192	0.28731	5.018859863	1
MSTRG.17079.1	101	0.30961	6.567321777	1
MSTRG.17078.6	163	0.28167	5.744934082	1
MSTRG.17085.2	87	0.32575	11.75726318	1
MSTRG.17087.3	567	0.31175	7.58416748	1
MSTRG.17087.4	567	0.3721	7.58416748	1
MSTRG.17092.1	116	0.35103	11.04302979	1
MSTRG.17093.1	64	0.31765	10.71295166	1
MSTRG.17093.2	75	0.34952	12.00872803	1
MSTRG.17093.3	67	0.33803	10.65802002	1
MSTRG.17093.4	67	0.34084	10.65802002	1
MSTRG.17093.5	64	0.32573	10.71295166	1
MSTRG.17096.2	678	0.45841	6.741027832	1
MSTRG.17096.3	377	0.39123	6.47064209	1
MSTRG.17101.1	1128	0.45612	5.861999512	1
MSTRG.17105.1	159	0.3563	5.926452637	1
MSTRG.17109.4	203	0.35949	7.704406738	1
MSTRG.17109.2	686	0.38024	6.688415527	1
MSTRG.17109.5	239	0.2997	8.754333496	1
MSTRG.17111.3	399	0.42935	8.794006348	1
MSTRG.17114.1	205	0.31638	10.21112061	1
MSTRG.17114.2	82	0.31234	9.019226074	-1
MSTRG.17115.2	408	0.43287	8.377502441	1
MSTRG.17110.3	603	0.32791	9.786315918	1
MSTRG.17110.1	15	0.35303	11.70025635	-1
MSTRG.17110.2	501	0.39263	7.278503418	1
MSTRG.17110.4	926	0.33779	8.90447998	1
MSTRG.17110.5	986	0.31729	9.191589355	1
MSTRG.17110.6	1019	0.35634	9.079162598	1
MSTRG.17116.1	272	0.42347	6.092956543	1
MSTRG.17117.1	677	0.41395	6.433288574	1
MSTRG.17117.3	677	0.41091	6.433288574	1
MSTRG.17119.2	290	0.46851	4.826843262	1
MSTRG.17119.3	290	0.46851	4.826843262	1
MSTRG.17119.5	290	0.46975	4.826843262	1
MSTRG.17131.1	474	0.42213	5.867492676	-1
MSTRG.17138.2	114	0.29788	8.981018066	1
MSTRG.17138.3	474	0.34531	9.471862793	1
MSTRG.17138.4	287	0.33322	9.668273926	1
MSTRG.17138.6	375	0.3562	9.644226074	1
MSTRG.17140.2	107	0.28586	10.59515381	1
MSTRG.17134.1	314	0.3787	4.335754395	1
MSTRG.17134.2	216	0.3582	7.718444824	1
MSTRG.17145.1	53	0.37167	10.05609131	1
MSTRG.17150.1	78	0.35344	9.565246582	1
MSTRG.17150.2	78	0.36075	9.565246582	1
MSTRG.17151.4	533	0.39839	9.044616699	1
MSTRG.17152.2	94	0.37251	4.387268066	1
MSTRG.17155.1	271	0.27251	9.660583496	1
MSTRG.17155.4	271	0.28842	9.660583496	1
MSTRG.17166.1	85	0.32858	12.10455322	1

MSTRG.17166.2	51	0.35583	10.0401001	-1
MSTRG.17168.2	167	0.28779	6.43548584	1
MSTRG.17168.3	526	0.29408	7.807067871	1
MSTRG.17158.1	45	0.37291	9.185852051	1
MSTRG.17172.2	98	0.3364	6.040588379	1
MSTRG.17172.3	134	0.29328	6.991394043	1
MSTRG.17192.2	499	0.41257	9.616027832	1
MSTRG.17192.1	688	0.43377	9.381896973	1
MSTRG.17192.3	387	0.39579	9.27130127	1
MSTRG.17192.4	576	0.45496	8.92767334	1
MSTRG.17193.1	92	0.34834	7.892150879	1
MSTRG.17202.1	91	0.34998	9.382019043	1
MSTRG.17207.1	75	0.40275	9.797302246	1
MSTRG.17215.2	79	0.34996	9.127990723	1
MSTRG.17225.1	59	0.3222	10.73651123	1
MSTRG.17175.1	50	0.33329	3.692077637	1
MSTRG.17175.2	50	0.33398	3.692077637	1
MSTRG.17176.1	48	0.34332	4.406555176	-1
MSTRG.17177.4	731	0.43062	5.883361816	1
MSTRG.17177.3	828	0.4604	6.171447754	1
MSTRG.17177.2	1306	0.47306	6.254577637	1
MSTRG.17177.5	839	0.46264	6.288635254	1
MSTRG.17177.6	828	0.45247	6.171447754	1
MSTRG.17178.1	183	0.37355	9.083190918	1
MSTRG.17178.2	163	0.3409	9.29901123	1
MSTRG.17177.7	602	0.47087	6.200744629	1
MSTRG.17179.1	85	0.39463	9.389465332	1
MSTRG.17180.1	32	0.37102	4.529602051	1
MSTRG.17398.2	225	0.50425	9.716491699	1
MSTRG.17399.1	89	0.34939	9.483947754	1
MSTRG.17400.1	49	0.28246	5.262145996	1
MSTRG.17401.1	86	0.28959	12.25543213	1
MSTRG.17402.1	217	0.36157	8.23638916	1
MSTRG.17402.2	75	0.3771	11.00006104	1
MSTRG.17402.3	60	0.30704	4.581237793	1
MSTRG.17402.5	20	0.37813	9.186096191	-1
MSTRG.17402.6	89	0.35701	8.76361084	1
MSTRG.17402.11	114	0.28478	4.687927246	1
MSTRG.17403.1	94	0.31978	9.403137207	1
MSTRG.17402.13	137	0.31669	8.817443848	1
MSTRG.17405.1	77	0.28923	7.844787598	1
MSTRG.17402.14	79	0.32858	8.513244629	1
MSTRG.17402.15	563	0.47843	5.528503418	1
MSTRG.17402.16	144	0.30955	6.237976074	1
MSTRG.17402.19	705	0.44489	6.379821777	1
MSTRG.17402.17	178	0.33247	8.770080566	1
MSTRG.17402.20	403	0.35184	8.977111816	1
MSTRG.17402.21	418	0.39061	9.126525879	1
MSTRG.17402.24	732	0.34639	8.666809082	1
MSTRG.17402.23	403	0.45148	8.977111816	1
MSTRG.17402.25	418	0.43415	8.983947754	1
MSTRG.17402.29	732	0.43379	8.666809082	1
MSTRG.17402.27	610	0.38765	5.465148926	-1
MSTRG.17402.31	545	0.4432	7.849304199	1

MSTRG.17402.32	576	0.47766	6.926696777	1
MSTRG.17406.1	59	0.39106	9.699401855	1
MSTRG.17407.1	268	0.46367	8.944030762	-1
MSTRG.17402.34	85	0.2943	8.752990723	1
MSTRG.17402.35	65	0.33566	6.365661621	1
MSTRG.17402.36	57	0.34379	8.493103027	1
MSTRG.17402.37	705	0.46604	6.379821777	1
MSTRG.17408.1	50	0.3129	9.101501465	1
MSTRG.17409.1	110	0.34007	9.573181152	1
MSTRG.17409.2	110	0.31937	9.573181152	1
MSTRG.17409.3	110	0.35328	9.573181152	1
MSTRG.17410.1	60	0.37796	8.235046387	-1
MSTRG.17411.1	52	0.38808	5.381164551	1
MSTRG.17412.1	26	0.31738	5.570129395	1
MSTRG.17413.1	100	0.31233	9.056091309	1
MSTRG.17414.1	69	0.40107	10.70489502	-1
MSTRG.17402.38	179	0.32714	8.427429199	1
MSTRG.17415.1	80	0.31421	9.848937988	1
MSTRG.17416.1	67	0.30104	10.53375244	1
MSTRG.17417.1	56	0.33828	8.210388184	1
MSTRG.17407.2	357	0.43303	9.641052246	1
MSTRG.17407.3	357	0.38564	9.362365723	1
MSTRG.17407.4	83	0.44378	9.259338379	1
MSTRG.17418.1	36	0.36449	4.425720215	-1
MSTRG.17419.1	78	0.34624	9.887756348	1
MSTRG.17420.1	199	0.29517	10.83807373	-1
MSTRG.17421.2	225	0.43996	9.20892334	1
MSTRG.17421.4	216	0.48161	8.220397949	1
MSTRG.17422.1	409	0.44326	8.999694824	-1
MSTRG.17400.2	81	0.34876	6.944030762	1
MSTRG.17402.68	84	0.28922	10.01141357	1
MSTRG.17402.75	608	0.42956	5.424987793	-1
MSTRG.17402.77	184	0.44973	9.000305176	1
MSTRG.17424.1	67	0.37294	8.659973145	1
MSTRG.17407.10	920	0.44084	5.663879395	1
MSTRG.17407.12	758	0.45555	6.229309082	1
MSTRG.17428.1	89	0.4063	9.928283691	1
MSTRG.17421.5	71	0.3568	4.902038574	1
MSTRG.17432.1	61	0.43507	9.179504395	1
MSTRG.17421.13	338	0.42275	6.710144043	1
MSTRG.17432.2	104	0.33198	8.375183105	1
MSTRG.17187.1	58	0.32651	9.033752441	1
MSTRG.17189.1	42	0.37202	9.224304199	1
MSTRG.17189.2	60	0.35178	9.795959473	1
MSTRG.17276.3	143	0.28267	10.50213623	1
MSTRG.17276.4	275	0.28584	8.787902832	1
MSTRG.17276.5	275	0.29772	8.787902832	1
MSTRG.17276.6	156	0.34973	10.34539795	1
MSTRG.17276.7	67	0.39335	4.304626465	1
MSTRG.17278.1	218	0.42355	5.002502441	1
MSTRG.17278.3	172	0.42611	5.207092285	1
MSTRG.17278.2	172	0.40349	5.207092285	1
MSTRG.17278.4	218	0.43543	5.002502441	1
MSTRG.17279.4	335	0.35424	9.27142334	1

MSTRG.17279.3	465	0.4591	8.611755371	1
MSTRG.17280.5	512	0.37954	6.065490723	-1
MSTRG.17280.4	685	0.46823	5.785217285	1
MSTRG.17190.1	1271	0.47481	5.486755371	1
MSTRG.17263.3	153	0.28923	12.13970947	1
MSTRG.17264.1	145	0.28923	9.624450684	1
MSTRG.17264.2	103	0.29857	6.950378418	1
MSTRG.17265.1	88	0.34984	9.54095459	1
MSTRG.17266.1	300	0.30781	8.281555176	1
MSTRG.17266.3	233	0.35464	7.026550293	1
MSTRG.17266.5	149	0.29196	6.06427002	1
MSTRG.17266.6	169	0.31182	8.906066895	1
MSTRG.17191.1	40	0.32075	8.881652832	1
MSTRG.17270.1	244	0.3161	11.78961182	1
MSTRG.17269.4	831	0.41991	9.331970215	1
MSTRG.17271.1	97	0.3295	9.20489502	1
MSTRG.17292.1	127	0.3378	8.80291748	1
MSTRG.17292.2	26	0.46679	7.976257324	1
MSTRG.17294.1	519	0.33531	8.819763184	1
MSTRG.17294.2	515	0.33531	8.819763184	1
MSTRG.17294.3	409	0.28419	8.863830566	1
MSTRG.17294.5	293	0.27651	8.694396973	1
MSTRG.17294.4	520	0.44931	8.68963623	1
MSTRG.17294.6	522	0.43545	8.868347168	1
MSTRG.17294.7	136	0.27911	6.360046387	1
MSTRG.17294.9	163	0.27651	8.219543457	1
MSTRG.17294.8	163	0.27575	8.219543457	1
MSTRG.17294.10	123	0.32237	8.461730957	1
MSTRG.17294.12	58	0.2857	8.036804199	1
MSTRG.17294.13	152	0.28584	9.35723877	1
MSTRG.17294.14	133	0.28199	8.746765137	1
MSTRG.17291.1	17	0.38228	8.189025879	1
MSTRG.17299.2	779	0.42552	9.50567627	1
MSTRG.17299.3	681	0.39183	9.412658691	-1
MSTRG.17301.3	274	0.44609	9.157409668	1
MSTRG.17297.2	265	0.38015	7.122009277	1
MSTRG.17307.1	174	0.32317	10.75604248	1
MSTRG.17308.3	639	0.32022	8.25579834	1
MSTRG.17308.6	639	0.32456	8.25579834	1
MSTRG.17304.1	18	0.39874	11.69903564	1
MSTRG.17306.1	68	0.37151	5.29498291	1
MSTRG.17309.2	440	0.39474	6.33782959	1
MSTRG.17310.1	39	0.43791	7.99822998	-1
MSTRG.17310.2	56	0.44393	7.998596191	1
MSTRG.17311.2	500	0.40727	8.092834473	1
MSTRG.17313.1	69	0.34513	10.6293335	1
MSTRG.17313.2	29	0.4009	4.667419434	1
MSTRG.17315.2	401	0.45544	5.763000488	1
MSTRG.17326.3	500	0.3996	5.160827637	1
MSTRG.17329.1	656	0.29615	8.928649902	1
MSTRG.17333.3	245	0.32767	6.308532715	1
MSTRG.17333.2	115	0.41482	7.757141113	1
MSTRG.17334.2	407	0.41346	8.902893066	1
MSTRG.17337.1	113	0.30175	9.007873535	1

MSTRG.17339.1	121	0.39698	11.25592041	-1
MSTRG.17336.6	50	0.35977	7.732116699	1
MSTRG.17336.7	50	0.33623	7.732116699	1
MSTRG.17340.1	44	0.31442	4.96673584	1
MSTRG.17341.2	305	0.42543	5.434387207	1
MSTRG.17341.6	330	0.47156	5.625793457	1
MSTRG.17343.1	8	0.3697	5.753234863	1
MSTRG.17349.5	1005	0.46221	6.25592041	1
MSTRG.17349.4	1029	0.48232	6.258728027	1
MSTRG.17350.1	129	0.3155	9.556335449	1
MSTRG.17349.9	604	0.39821	7.30267334	1
MSTRG.17349.12	771	0.48057	6.14263916	1
MSTRG.17349.8	893	0.4735	6.74029541	1
MSTRG.17349.7	894	0.33959	6.74029541	1
MSTRG.17349.10	1216	0.46263	8.636413574	1
MSTRG.17349.11	1180	0.46346	8.47088623	1
MSTRG.17349.13	864	0.46846	6.422424316	1
MSTRG.17349.14	147	0.2921	9.636413574	1
MSTRG.17349.15	870	0.45694	6.738464355	1
MSTRG.17351.1	100	0.31171	6.952575684	1
MSTRG.17352.1	83	0.33658	9.78302002	1
MSTRG.17347.1	49	0.31265	10.94830322	1
MSTRG.17348.1	923	0.38993	5.56817627	1
MSTRG.17348.2	923	0.36683	5.56817627	1
MSTRG.17348.4	923	0.38993	5.56817627	1
MSTRG.17368.10	837	0.40657	8.123352051	1
MSTRG.17368.1	1165	0.45381	8.337097168	1
MSTRG.17368.4	1168	0.44611	8.467224121	1
MSTRG.17368.5	1162	0.44611	8.339782715	1
MSTRG.17368.7	1548	0.41057	8.659606934	1
MSTRG.17368.12	1612	0.41057	8.54510498	1
MSTRG.17369.1	129	0.34131	8.334289551	1
MSTRG.17368.13	102	0.31732	8.676574707	1
MSTRG.17368.14	226	0.35415	8.862854004	1
MSTRG.17364.3	180	0.28467	6.059143066	1
MSTRG.17366.1	529	0.28683	6.144104004	1
MSTRG.17370.2	270	0.32072	8.54107666	1
MSTRG.17371.2	582	0.40144	8.845397949	1
MSTRG.17372.1	182	0.27937	6.755310059	1
MSTRG.17370.3	123	0.30559	4.446350098	1
MSTRG.17373.1	151	0.33994	5.238220215	1
MSTRG.17374.1	109	0.29268	7.714904785	1
MSTRG.17370.4	192	0.29268	9.43182373	1
MSTRG.17370.5	192	0.31753	9.43182373	1
MSTRG.17370.6	120	0.29526	8.763366699	1
MSTRG.17370.7	68	0.35099	7.701599121	1
MSTRG.17370.8	116	0.32138	9.44342041	1
MSTRG.17370.9	212	0.29772	9.916931152	1
MSTRG.17375.1	53	0.45278	9.273986816	1
MSTRG.17370.10	75	0.28248	10.01678467	1
MSTRG.17379.1	1061	0.44491	8.562438965	1
MSTRG.17379.13	104	0.38386	6.860900879	1
MSTRG.17379.14	997	0.45846	8.529724121	1
MSTRG.17379.12	902	0.4778	8.470031738	1

MSTRG.17379.8	1046	0.44119	8.645202637	1
MSTRG.17379.10	1046	0.44119	8.645202637	1
MSTRG.17379.11	1059	0.44119	8.562438965	1
MSTRG.17379.15	940	0.36917	9.063415527	1
MSTRG.17380.1	74	0.30303	6.716125488	1
MSTRG.17381.1	91	0.28923	4.427185059	-1
MSTRG.17387.2	114	0.42535	4.771057129	1
MSTRG.17397.1	102	0.38551	9.85357666	-1
MSTRG.17438.3	557	0.46494	5.054504395	1
MSTRG.17438.6	868	0.46751	5.817443848	1
MSTRG.17438.7	872	0.47003	5.854675293	1
MSTRG.17438.8	871	0.48848	5.886901855	1
MSTRG.17438.9	557	0.35496	5.054504395	1
MSTRG.17438.10	730	0.43311	5.19732666	1
MSTRG.17438.11	394	0.44467	4.985412598	1
MSTRG.17437.1	71	0.35844	5.179870605	1
MSTRG.17441.1	481	0.43698	5.690002441	1
MSTRG.17441.2	481	0.46121	5.690002441	1
MSTRG.17441.3	42	0.35849	4.777893066	1
MSTRG.17462.1	98	0.29088	9.763122559	1
MSTRG.17463.1	59	0.26994	6.204772949	1
MSTRG.17464.1	129	0.29791	8.135559082	1
MSTRG.17464.4	53	0.3227	6.503234863	-1
MSTRG.17465.1	264	0.28151	9.925354004	1
MSTRG.17467.1	77	0.39661	5.376037598	1
MSTRG.17469.1	125	0.31873	9.301452637	1
MSTRG.17470.1	104	0.32237	4.818786621	1
MSTRG.17471.1	34	0.35823	9.698059082	1
MSTRG.17474.1	164	0.29367	8.946472168	1
MSTRG.17475.1	119	0.29187	9.450500488	1
MSTRG.17475.2	121	0.29268	7.769348145	1
MSTRG.17475.3	63	0.29772	10.38604736	1
MSTRG.17476.1	151	0.34787	9.751647949	1
MSTRG.17477.1	97	0.32396	9.019104004	1
MSTRG.17478.1	63	0.31161	10.28448486	1
MSTRG.17464.12	1620	0.48049	6.702087402	1
MSTRG.17464.11	1086	0.4127	9.046325684	1
MSTRG.17464.10	1060	0.42351	9.18572998	1
MSTRG.17464.14	1520	0.42714	6.27520752	1
MSTRG.17464.16	166	0.31437	5.016784668	1
MSTRG.17480.1	159	0.29703	10.52655029	1
MSTRG.17481.1	1951	0.43447	7.99786377	1
MSTRG.17481.4	1952	0.41907	7.99786377	1
MSTRG.17481.5	1952	0.43447	7.99786377	1
MSTRG.17481.7	1951	0.41907	7.99786377	1
MSTRG.17481.6	1952	0.45497	7.99786377	1
MSTRG.17446.1	82	0.38268	9.88848877	1
MSTRG.17450.1	1429	0.45154	8.947570801	1
MSTRG.17454.1	573	0.4758	5.160583496	1
MSTRG.17454.3	572	0.44331	5.160583496	1
MSTRG.17456.2	870	0.43108	8.744445801	1
MSTRG.17456.3	870	0.43356	8.744445801	1
MSTRG.17456.4	870	0.43356	8.744445801	1
MSTRG.17456.5	870	0.43642	8.744445801	1

MSTRG.17453.2	237	0.46502	7.211120605	1
MSTRG.17458.3	392	0.47395	5.645080566	1
MSTRG.17524.1	415	0.31641	9.487609863	1
MSTRG.17528.1	1041	0.38151	9.00579834	1
MSTRG.17528.3	1026	0.44572	9.040710449	1
MSTRG.17528.2	1026	0.44734	9.040710449	1
MSTRG.17528.5	922	0.38119	9.383361816	1
MSTRG.17529.1	96	0.42798	9.142150879	1
MSTRG.17530.1	96	0.37029	6.897399902	1
MSTRG.17531.1	90	0.37206	7.128479004	1
MSTRG.17528.6	182	0.29088	9.975646973	1
MSTRG.17528.7	132	0.29367	9.389221191	1
MSTRG.17528.8	177	0.28584	10.48040771	1
MSTRG.17528.9	94	0.29772	11.1272583	1
MSTRG.17532.1	150	0.32642	5.226257324	1
MSTRG.17532.2	97	0.34971	8.800720215	1
MSTRG.17532.3	152	0.29607	10.1428833	1
MSTRG.17528.10	122	0.30309	10.1104126	1
MSTRG.17528.11	123	0.28584	6.49005127	1
MSTRG.17533.1	67	0.32573	6.568664551	1
MSTRG.17528.12	140	0.29772	9.69342041	1
MSTRG.17528.13	140	0.29772	9.69342041	1
MSTRG.17528.14	140	0.29772	9.69342041	1
MSTRG.17528.15	98	0.29772	5.805847168	1
MSTRG.17528.16	98	0.29772	5.805847168	1
MSTRG.17528.17	122	0.32396	9.881774902	1
MSTRG.17528.18	177	0.28667	10.48040771	1
MSTRG.17535.1	75	0.3524	4.633850098	1
MSTRG.17528.19	122	0.27622	9.881774902	1
MSTRG.17528.20	115	0.40155	10.75445557	1
MSTRG.17536.1	167	0.40485	10.53594971	1
MSTRG.17528.21	122	0.38163	9.881774902	1
MSTRG.17459.2	131	0.47544	7.888977051	1
MSTRG.17482.3	181	0.30492	11.44927979	1
MSTRG.17482.4	188	0.32727	5.377990723	1
MSTRG.17490.2	386	0.47656	5.165710449	1
MSTRG.17490.5	266	0.32498	5.84954834	1
MSTRG.17490.4	360	0.2971	8.198669434	1
MSTRG.17460.3	88	0.3592	9.276550293	1
MSTRG.17496.2	282	0.45413	9.789367676	1
MSTRG.17498.2	345	0.40306	7.663146973	1
MSTRG.17498.3	321	0.40306	9.149841309	1
MSTRG.17539.1	2441	0.40356	5.307922363	1
MSTRG.17539.2	1209	0.45577	5.325378418	-1
MSTRG.17541.1	188	0.32413	10.15460205	1
MSTRG.17541.2	382	0.3076	6.42401123	1
MSTRG.17541.3	320	0.48161	8.40447998	1
MSTRG.17541.4	320	0.45318	8.40447998	1
MSTRG.17541.5	320	0.42735	8.40447998	1
MSTRG.17541.6	306	0.43171	7.61932373	1
MSTRG.17541.7	237	0.3995	6.428649902	1
MSTRG.17546.1	131	0.35655	9.815612793	1
MSTRG.17541.8	188	0.29595	7.673400879	1
MSTRG.17548.1	68	0.35161	10.00543213	1

MSTRG.17550.1	145	0.26969	10.18988037	1
MSTRG.17551.1	288	0.43994	9.29888916	1
MSTRG.17551.2	192	0.46256	8.975036621	1
MSTRG.17552.1	124	0.28808	9.250549316	1
MSTRG.17553.1	66	0.36259	9.438293457	1
MSTRG.17557.1	1455	0.43167	6.014831543	1
MSTRG.17512.2	593	0.44154	7.016418457	1
MSTRG.17515.1	69	0.30136	5.102478027	1
MSTRG.17516.3	64	0.42146	5.164367676	1
MSTRG.17516.4	742	0.37006	8.826477051	1
MSTRG.17516.5	596	0.29615	9.315368652	1
MSTRG.17523.6	431	0.36519	6.815124512	1
MSTRG.17523.4	431	0.34303	6.815124512	1
MSTRG.17523.7	431	0.34303	6.815124512	1
MSTRG.17523.5	793	0.42859	8.694152832	1
MSTRG.17523.8	845	0.43045	7.923522949	1
MSTRG.17559.1	495	0.38365	4.586608887	1
MSTRG.17559.3	629	0.44961	5.824035645	1
MSTRG.17559.4	613	0.39806	5.519104004	1
MSTRG.17559.5	411	0.34993	9.216247559	1
MSTRG.17559.7	411	0.386	9.216247559	1
MSTRG.17559.6	411	0.34055	9.216247559	1
MSTRG.17559.8	115	0.32478	9.015075684	1
MSTRG.17560.1	123	0.32044	5.690979004	1
MSTRG.17560.2	75	0.32296	8.932678223	1
MSTRG.17566.3	156	0.33078	9.04486084	1
MSTRG.17566.5	165	0.32429	9.206848145	1
MSTRG.17567.1	280	0.36783	9.589904785	1
MSTRG.17567.2	245	0.36004	9.691345215	1
MSTRG.17568.1	233	0.29772	8.082580566	1
MSTRG.17579.1	107	0.30197	6.000427246	1
MSTRG.17580.1	1792	0.45042	6.351135254	1
MSTRG.17580.3	1672	0.43622	7.547912598	1
MSTRG.17580.2	1773	0.46579	6.334899902	1
MSTRG.17580.4	1762	0.49816	6.307189941	1
MSTRG.17580.6	1114	0.46795	6.481750488	1
MSTRG.17581.2	21	0.44663	4.20916748	1
MSTRG.17581.1	21	0.44529	4.20916748	1
MSTRG.17581.3	49	0.46166	9.383239746	1
MSTRG.17582.1	195	0.29772	6.599914551	1
MSTRG.17582.2	75	0.30894	9.299255371	1
MSTRG.17580.8	132	0.28338	11.8682251	1
MSTRG.17580.9	98	0.29526	9.678771973	1
MSTRG.17580.10	87	0.28662	6.681335449	1
MSTRG.17580.11	183	0.28419	10.56842041	1
MSTRG.17571.1	1305	0.39917	7.657409668	1
MSTRG.17572.1	98	0.33107	9.299255371	1
MSTRG.17571.3	1248	0.39316	7.37677002	1
MSTRG.17571.4	1248	0.40951	7.37677002	1
MSTRG.17571.5	1305	0.38377	7.657409668	1
MSTRG.17575.1	27	0.39372	6.700866699	1
MSTRG.17577.1	49	0.3328	9.773132324	1
MSTRG.17577.2	49	0.31642	9.773132324	1
MSTRG.17584.1	49	0.28977	9.579040527	1

MSTRG.17585.9	368	0.2921	9.787414551	1
MSTRG.17585.8	646	0.35945	9.50201416	1
MSTRG.17585.5	601	0.37979	9.418640137	1
MSTRG.17585.12	543	0.33723	9.524841309	1
MSTRG.17585.15	368	0.29374	9.787414551	1
MSTRG.17585.16	368	0.28165	9.787414551	1
MSTRG.17585.13	601	0.40897	9.418640137	1
MSTRG.17585.14	601	0.36593	9.418640137	1
MSTRG.17585.17	646	0.37331	9.50201416	1
MSTRG.17585.18	471	0.3556	9.690734863	1
MSTRG.17590.2	404	0.41337	8.913146973	1
MSTRG.17590.3	569	0.45713	7.47454834	1
MSTRG.17590.6	396	0.44185	6.104064941	1
MSTRG.17592.1	135	0.3208	8.088195801	1
MSTRG.17597.2	94	0.34467	9.359802246	1
MSTRG.17602.1	80	0.45889	5.479431152	1
MSTRG.17615.1	130	0.30862	6.374328613	-1
MSTRG.17615.2	117	0.33487	5.720031738	-1
MSTRG.17615.15	590	0.40661	5.62677002	1
MSTRG.17615.5	757	0.44853	5.452087402	1
MSTRG.17615.18	116	0.28121	8.719177246	1
MSTRG.17605.2	148	0.46729	8.621276855	1
MSTRG.17605.3	148	0.48875	8.621276855	1
MSTRG.17608.6	1441	0.42729	8.981872559	1
MSTRG.17608.8	1411	0.42729	8.915710449	1
MSTRG.17608.4	1421	0.44115	8.939025879	1
MSTRG.17608.5	1431	0.44115	8.960998535	1
MSTRG.17608.7	1471	0.44115	9.038391113	1
MSTRG.17608.9	1509	0.43345	9.208190918	1
MSTRG.17608.2	1351	0.42729	8.738586426	1
MSTRG.17607.1	110	0.29961	8.258972168	1
MSTRG.17619.1	634	0.42449	9.089904785	1
MSTRG.17622.1	656	0.33883	7.946960449	1
MSTRG.17622.2	601	0.37319	7.802062988	1
MSTRG.17619.3	83	0.32097	5.096984863	1
MSTRG.17622.8	673	0.43419	7.950866699	1
MSTRG.17622.9	673	0.43419	7.950866699	1
MSTRG.17622.11	618	0.47078	7.810241699	1
MSTRG.17628.2	202	0.38695	8.729064941	1
MSTRG.17629.1	131	0.34116	5.015075684	1
MSTRG.17631.2	767	0.47468	6.663757324	1
MSTRG.17631.3	767	0.48024	6.663757324	1
MSTRG.17631.4	767	0.44865	6.663757324	1
MSTRG.17624.1	158	0.48815	9.971984863	1
MSTRG.17635.2	578	0.44496	4.731384277	1
MSTRG.17635.1	335	0.36759	4.82635498	1
MSTRG.17635.3	578	0.44496	4.731384277	1
MSTRG.17636.1	166	0.26594	8.840881348	1
MSTRG.17635.4	464	0.37351	4.649353027	1
MSTRG.17635.6	464	0.41467	4.649353027	1
MSTRG.17635.7	464	0.41467	4.649353027	1
MSTRG.17635.9	464	0.42701	4.649353027	1
MSTRG.17635.10	83	0.30446	7.54486084	1
MSTRG.17638.1	125	0.29332	8.361877441	1

MSTRG.17639.1	105	0.27911	8.834899902	1
MSTRG.17640.1	95	0.36792	10.40228271	1
MSTRG.17641.1	89	0.31641	9.836608887	1
MSTRG.17635.12	157	0.29059	11.60772705	1
MSTRG.17632.2	571	0.42886	6.633239746	1
MSTRG.17632.3	571	0.4473	6.633239746	1
MSTRG.17643.2	94	0.46298	5.363952637	1
MSTRG.17643.3	66	0.3926	4.499572754	1
MSTRG.17645.3	1221	0.47508	5.687072754	1
MSTRG.17645.7	254	0.31437	5.445129395	1
MSTRG.17647.1	1886	0.34584	5.917907715	1
MSTRG.17647.2	1873	0.46322	5.944641113	1
MSTRG.17647.3	1886	0.3802	5.917907715	1
MSTRG.17647.4	1886	0.40523	5.917907715	1
MSTRG.17648.2	728	0.44991	5.64630127	1
MSTRG.17653.1	70	0.31521	8.95904541	1
MSTRG.17654.1	107	0.30221	10.85491943	1
MSTRG.17660.1	220	0.27403	5.593811035	1
MSTRG.17661.3	135	0.46741	9.65814209	1
MSTRG.17661.4	135	0.46379	9.65814209	1
MSTRG.17663.2	74	0.40263	10.21832275	1
MSTRG.17665.2	91	0.46208	6.944763184	1
MSTRG.17667.2	145	0.40736	8.916687012	1
MSTRG.17669.1	64	0.31391	9.004211426	1
MSTRG.17670.1	37	0.43581	8.939147949	1
MSTRG.17672.1	47	0.36033	10.71795654	1
MSTRG.17674.2	202	0.3495	9.440002441	1
MSTRG.17674.1	202	0.33576	9.440002441	1
MSTRG.17674.3	202	0.33576	9.440002441	1
MSTRG.17677.1	288	0.32718	5.737731934	1
MSTRG.17677.3	343	0.37611	5.345275879	1
MSTRG.17679.2	348	0.34883	5.526306152	1
MSTRG.17676.2	218	0.35821	8.315124512	1
MSTRG.17681.1	266	0.41763	5.349182129	1
MSTRG.17681.3	456	0.43213	6.479797363	1
MSTRG.17682.2	239	0.40827	5.61138916	1
MSTRG.17685.2	370	0.38339	8.607116699	1
MSTRG.17686.1	151	0.33442	10.92132568	1
MSTRG.17706.6	2279	0.47742	5.753112793	1
MSTRG.17706.4	2655	0.42204	6.032409668	1
MSTRG.17706.5	2661	0.46452	6.049987793	1
MSTRG.17706.8	2394	0.45816	5.987243652	1
MSTRG.17706.7	2394	0.46452	5.987243652	1
MSTRG.17706.9	2394	0.41819	5.987243652	1
MSTRG.17706.12	2313	0.35797	5.731994629	1
MSTRG.17706.14	2382	0.45644	6.00604248	1
MSTRG.17706.11	2394	0.47661	5.987243652	1
MSTRG.17706.10	2629	0.45892	6.18939209	1
MSTRG.17706.16	2313	0.44276	5.731994629	1
MSTRG.17706.18	2313	0.44357	5.731994629	1
MSTRG.17706.17	2394	0.45892	5.987243652	1
MSTRG.17706.19	2301	0.45644	5.742492676	1
MSTRG.17706.20	2382	0.45568	6.00604248	1
MSTRG.17706.22	467	0.43113	9.765197754	1

MSTRG.17706.23	107	0.32653	6.005554199	1
MSTRG.17695.1	323	0.38122	7.649108887	1
MSTRG.17697.1	72	0.35165	10.3817749	-1
MSTRG.17698.2	254	0.42582	8.92755127	1
MSTRG.17698.3	254	0.4692	8.92755127	1
MSTRG.17701.2	267	0.32439	8.030700684	1
MSTRG.17702.1	19	0.40852	5.934875488	1
MSTRG.17710.3	1032	0.46824	6.043762207	1
MSTRG.17710.4	1031	0.46824	6.043762207	1
MSTRG.17710.6	908	0.46422	5.601379395	1
MSTRG.17711.4	93	0.40365	8.475524902	1
MSTRG.17711.2	832	0.43249	6.919372559	1
MSTRG.17709.1	85	0.455	7.799133301	1
MSTRG.17714.1	455	0.28119	6.247253418	1
MSTRG.17714.4	456	0.3669	6.247253418	1
MSTRG.17718.2	703	0.33921	8.948181152	1
MSTRG.17718.3	225	0.36678	8.408508301	-1
MSTRG.17730.1	97	0.33194	9.545471191	1
MSTRG.17731.1	97	0.28167	9.384216309	1
MSTRG.17731.2	36	0.38104	6.469787598	1
MSTRG.17729.6	186	0.27591	11.25775146	1
MSTRG.17729.4	1449	0.42121	6.609069824	1
MSTRG.17729.5	1539	0.42121	7.371765137	1
MSTRG.17729.7	1409	0.48595	6.550598145	1
MSTRG.17729.8	1449	0.47122	6.609069824	1
MSTRG.17729.9	119	0.27782	11.92156982	1
MSTRG.17732.1	128	0.2849	8.639709473	1
MSTRG.17729.10	139	0.38158	8.963806152	1
MSTRG.17733.7	445	0.3174	8.899841309	1
MSTRG.17733.10	527	0.37221	8.682067871	1
MSTRG.17733.12	548	0.38787	9.155090332	1
MSTRG.17733.11	527	0.41657	8.682067871	1
MSTRG.17725.2	178	0.34314	9.347595215	1
MSTRG.17728.1	86	0.37831	9.114440918	1
MSTRG.17735.2	41	0.32103	10.26641846	1
MSTRG.17740.2	333	0.39485	8.881652832	1
MSTRG.17741.4	58	0.30303	4.352478027	1
MSTRG.17741.5	210	0.36855	6.153869629	1
MSTRG.17741.6	189	0.3	5.953308105	1
MSTRG.17744.1	38	0.31573	9.414611816	1
MSTRG.17751.2	360	0.44005	5.414245605	1
MSTRG.17755.2	191	0.41018	9.320617676	1
MSTRG.17755.3	203	0.39664	9.165100098	1
MSTRG.17747.1	123	0.30725	9.030334473	1
MSTRG.17762.1	5	0.31261	5.274597168	1
MSTRG.17775.2	129	0.31213	9.804748535	1
MSTRG.17776.2	173	0.27403	9.851501465	1
MSTRG.17779.1	370	0.28923	6.605163574	1
MSTRG.17779.2	529	0.28718	5.974060059	1
MSTRG.17779.4	370	0.28923	6.605163574	1
MSTRG.17779.5	529	0.28648	5.974060059	1
MSTRG.17779.10	528	0.2785	6.06817627	1
MSTRG.17779.11	370	0.28443	6.605163574	1
MSTRG.17768.1	604	0.46661	4.467224121	1

MSTRG.17769.3	233	0.43487	9.233703613	1
MSTRG.17770.2	84	0.34148	8.967224121	1
MSTRG.17770.3	84	0.4054	8.967224121	1
MSTRG.17773.1	153	0.29607	9.458679199	1
MSTRG.17773.2	1415	0.36405	7.628723145	1
MSTRG.17773.3	191	0.29772	9.399475098	1
MSTRG.17774.1	34	0.3724	9.875427246	1
MSTRG.17781.2	97	0.3295	9.785705566	1
MSTRG.17781.3	665	0.30729	8.589172363	1
MSTRG.17782.1	93	0.29772	10.58758545	1
MSTRG.17784.3	869	0.46941	7.291809082	1
MSTRG.17785.2	109	0.37624	10.7276001	-1
MSTRG.17785.3	125	0.32473	9.831237793	1
MSTRG.17789.1	537	0.37639	6.757019043	1
MSTRG.17789.4	537	0.39689	6.757019043	1
MSTRG.17789.6	509	0.3736	6.928771973	1
MSTRG.17791.6	640	0.42076	8.826477051	1
MSTRG.17791.5	640	0.45666	8.826477051	1
MSTRG.17791.4	640	0.42827	8.826477051	1
MSTRG.17792.1	119	0.26749	8.573669434	1
MSTRG.17796.2	585	0.44906	6.466003418	1
MSTRG.17794.1	657	0.44733	5.657897949	1
MSTRG.17802.1	79	0.46102	8.73651123	1
MSTRG.17807.1	583	0.40392	9.433532715	1
MSTRG.17806.1	47	0.33123	5.477600098	1
MSTRG.17814.1	248	0.44877	4.910217285	1
MSTRG.17814.2	134	0.29526	9.948791504	1
MSTRG.17816.1	83	0.32217	9.300964355	1
MSTRG.17814.6	373	0.36267	5.719421387	1
MSTRG.17814.4	370	0.36432	5.84552002	1
MSTRG.17814.5	416	0.35058	8.042419434	1
MSTRG.17817.2	95	0.41221	4.999450684	1
MSTRG.17817.1	218	0.35559	6.640319824	1
MSTRG.17817.3	218	0.39178	6.640319824	1
MSTRG.17810.2	432	0.39815	9.232849121	1
MSTRG.17811.3	263	0.42323	10.62506104	1
MSTRG.17825.4	903	0.35844	9.809509277	1
MSTRG.17826.4	527	0.45784	7.245300293	1
MSTRG.17826.3	470	0.43771	9.309387207	1
MSTRG.17826.2	491	0.44554	9.152038574	1
MSTRG.17826.7	192	0.45023	5.191467285	1
MSTRG.17826.8	213	0.4197	5.20892334	1
MSTRG.17826.6	213	0.45623	5.20892334	1
MSTRG.17826.9	213	0.44899	5.20892334	1
MSTRG.17828.6	233	0.30813	6.075866699	1
MSTRG.17828.7	233	0.30899	6.075866699	1
MSTRG.17828.3	233	0.35749	6.075866699	1
MSTRG.17829.1	129	0.35369	9.050354004	1
MSTRG.17830.4	221	0.29889	5.995300293	1
MSTRG.17830.5	220	0.29889	5.995300293	1
MSTRG.17830.6	215	0.29328	5.78717041	1
MSTRG.17832.2	424	0.38538	8.567199707	1
MSTRG.17833.1	17	0.38425	12.30426025	-1
MSTRG.17835.2	260	0.41206	8.377258301	1

MSTRG.17836.2	513	0.43398	5.403259277	1
MSTRG.17836.3	513	0.42126	5.403259277	1
MSTRG.17837.3	201	0.39886	6.72857666	1
MSTRG.17837.4	132	0.35581	6.817932129	1
MSTRG.17839.4	1110	0.45163	8.229675293	1
MSTRG.17819.1	54	0.33744	8.988342285	-1
MSTRG.17821.2	458	0.46981	5.870056152	1
MSTRG.17841.2	333	0.46777	6.80657959	1
MSTRG.17841.3	333	0.42098	6.80657959	1
MSTRG.17841.4	341	0.44201	6.569152832	1
MSTRG.17841.5	333	0.46777	6.80657959	1
MSTRG.17841.6	341	0.45201	6.569152832	1
MSTRG.17842.1	193	0.35659	9.178039551	1
MSTRG.17842.4	332	0.40665	7.082092285	1
MSTRG.17840.1	463	0.44063	9.109802246	1
MSTRG.17840.2	463	0.44063	9.109802246	1
MSTRG.17845.4	417	0.35687	5.099304199	1
MSTRG.17847.1	228	0.3469	4.862731934	1
MSTRG.17847.3	228	0.46787	4.862731934	1
MSTRG.17849.1	103	0.36537	7.80291748	1
MSTRG.17848.5	2196	0.44059	8.147766113	1
MSTRG.17856.1	142	0.36907	9.213439941	1
MSTRG.17863.4	262	0.37597	9.426818848	1
MSTRG.17863.2	407	0.4485	4.795349121	1
MSTRG.17863.3	410	0.4632	4.909606934	1
MSTRG.17863.5	259	0.40692	9.316711426	1
MSTRG.17873.4	310	0.31975	9.787658691	1
MSTRG.17873.3	305	0.29887	9.821105957	1
MSTRG.17878.1	976	0.47685	9.995422363	1
MSTRG.17878.2	962	0.47685	9.722229004	1
MSTRG.17878.4	1016	0.44946	10.012146	1
MSTRG.17878.5	629	0.47669	9.858825684	1
MSTRG.17878.6	131	0.27515	8.403015137	1
MSTRG.17879.2	97	0.345	6.267028809	1
MSTRG.17878.7	108	0.30193	8.835876465	1
MSTRG.17865.1	68	0.43505	10.21820068	1
MSTRG.17866.1	92	0.33423	10.05499268	1
MSTRG.17867.1	84	0.35801	9.989196777	1
MSTRG.17891.1	299	0.3586	8.491516113	1
MSTRG.17892.2	1323	0.42174	8.832458496	1
MSTRG.17892.3	685	0.44535	7.75994873	1
MSTRG.17892.4	846	0.39885	9.079772949	1
MSTRG.17892.5	1330	0.43405	8.723327637	1
MSTRG.17892.7	191	0.38455	4.759094238	1
MSTRG.17903.1	25	0.44011	7.957824707	1
MSTRG.17904.6	394	0.4595	5.496765137	1
MSTRG.17904.5	394	0.35755	5.496765137	1
MSTRG.17904.7	270	0.40985	9.212341309	1
MSTRG.17908.1	194	0.36467	8.319641113	1
MSTRG.17906.1	18	0.39225	6.684265137	1
MSTRG.17907.2	180	0.41886	6.958312988	1
MSTRG.17912.2	64	0.34804	10.92218018	1
MSTRG.17913.1	73	0.34557	9.852844238	-1
MSTRG.17921.1	368	0.37768	7.960632324	1

MSTRG.17921.5	368	0.38254	7.960632324	1
MSTRG.17923.1	315	0.46239	7.662658691	1
MSTRG.17924.1	357	0.46804	9.360412598	1
MSTRG.17923.2	61	0.33197	5.852233887	1
MSTRG.17923.3	315	0.44007	7.662658691	1
MSTRG.17923.4	338	0.37989	6.968444824	1
MSTRG.17923.5	105	0.38554	6.621520996	1
MSTRG.17918.1	92	0.37792	6.632263184	1
MSTRG.17929.1	35	0.29202	5.74822998	1
MSTRG.17919.1	32	0.44044	8.537902832	1
MSTRG.17931.1	299	0.41071	8.983947754	1
MSTRG.17931.3	20	0.4111	6.507629395	1
MSTRG.17931.4	123	0.40601	8.850402832	1
MSTRG.17941.1	527	0.37473	5.917297363	1
MSTRG.17942.3	553	0.4039	5.132141113	1
MSTRG.17943.4	151	0.32974	6.03314209	1
MSTRG.17943.2	277	0.45317	5.51361084	1
MSTRG.17943.3	270	0.43509	5.402526855	1
MSTRG.17954.3	391	0.36188	7.292053223	1
MSTRG.17952.2	351	0.42063	6.012023926	1
MSTRG.17958.1	361	0.37657	8.915466309	1
MSTRG.17963.2	43	0.41692	9.467468262	1
MSTRG.17963.1	53	0.3737	11.57025146	1
MSTRG.17972.5	747	0.44567	9.245910645	1
MSTRG.17972.7	747	0.43883	9.245910645	1
MSTRG.17972.6	740	0.36517	9.272766113	1
MSTRG.17972.10	747	0.44567	9.245910645	1
MSTRG.17972.8	740	0.43883	9.272766113	1
MSTRG.17972.9	747	0.44567	9.245910645	1
MSTRG.17972.11	740	0.48163	9.272766113	1
MSTRG.17973.1	65	0.32018	7.815124512	1
MSTRG.17975.1	429	0.4467	8.485900879	1
MSTRG.17978.2	503	0.45639	6.821350098	1
MSTRG.17980.2	549	0.43277	6.288513184	1
MSTRG.17980.3	639	0.37055	6.216247559	1
MSTRG.17981.1	83	0.32709	5.347717285	1
MSTRG.17982.2	496	0.40562	8.946594238	1
MSTRG.17984.8	685	0.41605	9.214782715	1
MSTRG.17984.7	404	0.34844	9.699279785	1
MSTRG.17984.10	743	0.42466	8.063415527	1
MSTRG.17985.1	102	0.28536	12.00152588	1
MSTRG.17986.2	1535	0.42109	6.214050293	1
MSTRG.17987.3	412	0.33817	5.045837402	1
MSTRG.17987.4	412	0.33316	5.045837402	1
MSTRG.17987.5	544	0.33777	5.082458496	1
MSTRG.17988.1	13	0.30739	4.084533691	1
MSTRG.17989.1	91	0.33713	6.968933105	1
MSTRG.17999.2	1258	0.38358	8.972961426	1
MSTRG.17999.3	1059	0.35595	8.899841309	1
MSTRG.17999.5	268	0.31672	5.195617676	1
MSTRG.17995.1	100	0.43156	8.492370605	1
MSTRG.17996.1	202	0.37942	10.42877197	1
MSTRG.17998.1	71	0.29235	4.433654785	1
MSTRG.18002.1	169	0.43689	6.08013916	1

MSTRG.18015.1	149	0.36409	8.567810059	1
MSTRG.18019.1	72	0.40178	8.837585449	1
MSTRG.18024.4	184	0.32108	10.71331787	1
MSTRG.18024.9	299	0.38657	8.934997559	1
MSTRG.18024.11	283	0.47513	9.474304199	1
MSTRG.18024.12	184	0.32089	10.71331787	1
MSTRG.18024.13	181	0.34514	10.82928467	1
MSTRG.18025.1	189	0.30104	9.955749512	1
MSTRG.18026.2	328	0.44008	5.857971191	1
MSTRG.18023.2	334	0.41042	5.639831543	1
MSTRG.18031.3	862	0.35984	6.00201416	1
MSTRG.18031.4	862	0.39432	6.00201416	1
MSTRG.18032.1	120	0.28066	5.045593262	1
MSTRG.18033.1	125	0.31653	6.812072754	1
MSTRG.18037.2	64	0.40014	5.045715332	1
MSTRG.18038.3	433	0.43202	6.391540527	1
MSTRG.18038.2	205	0.44278	6.484436035	1
MSTRG.18040.7	135	0.35371	6.187683105	1
MSTRG.18040.10	1049	0.49596	9.483459473	1
MSTRG.18040.9	2037	0.50103	9.269592285	1
MSTRG.18040.12	1948	0.50544	9.208312988	1
MSTRG.18040.13	1049	0.47692	9.483459473	1
MSTRG.18040.4	2048	0.50544	9.329772949	1
MSTRG.18040.8	1061	0.4874	9.585510254	1
MSTRG.18046.1	49	0.39795	6.088928223	1
MSTRG.18050.1	276	0.36399	9.117858887	1
MSTRG.18051.1	20	0.37525	10.03692627	1
MSTRG.18054.3	279	0.44144	7.575256348	1
MSTRG.18052.1	288	0.33938	8.066101074	1
MSTRG.18063.1	612	0.40419	8.18548584	1
MSTRG.18063.2	537	0.39586	5.356872559	1
MSTRG.18063.3	612	0.42674	8.18548584	1
MSTRG.18063.5	953	0.44279	8.741638184	1
MSTRG.18064.2	145	0.35843	5.953552246	1
MSTRG.18066.7	367	0.39182	6.120544434	1
MSTRG.18066.5	367	0.43185	6.120544434	1
MSTRG.18066.6	367	0.43185	6.120544434	1
MSTRG.18060.1	690	0.46006	8.433654785	1
MSTRG.18068.1	104	0.32404	6.162902832	1
MSTRG.18068.3	104	0.32404	6.162902832	1
MSTRG.18068.4	104	0.34714	6.162902832	1
MSTRG.18069.1	731	0.37592	6.209655762	1
MSTRG.18069.2	177	0.2971	8.97454834	1
MSTRG.18070.1	94	0.37147	9.598083496	1
MSTRG.18069.4	731	0.37592	6.209655762	1
MSTRG.18069.5	990	0.38137	8.891418457	1
MSTRG.18073.1	129	0.35686	9.909118652	1
MSTRG.18072.2	285	0.42471	5.678771973	1
MSTRG.18072.4	285	0.4011	5.678771973	1
MSTRG.18072.8	285	0.31585	5.678771973	1
MSTRG.18074.2	184	0.46485	4.954528809	1
MSTRG.18093.6	375	0.3823	5.126159668	1
MSTRG.18093.2	346	0.39342	5.316955566	1
MSTRG.18093.4	335	0.40798	5.50982666	1

MSTRG.18093.8	66	0.34547	11.39801025	1
MSTRG.18095.4	144	0.29776	5.917907715	1
MSTRG.18098.1	75	0.28875	7.744445801	1
MSTRG.18099.2	608	0.33974	6.48248291	1
MSTRG.18101.1	184	0.29202	10.206604	1
MSTRG.18101.2	106	0.29607	11.49334717	1
MSTRG.18102.3	75	0.27097	4.96673584	1
MSTRG.18103.1	89	0.31437	9.250305176	1
MSTRG.18105.1	56	0.33627	9.872619629	1
MSTRG.18106.1	30	0.43233	9.692443848	1
MSTRG.18083.2	156	0.33226	5.91217041	1
MSTRG.18083.3	171	0.26913	9.415222168	1
MSTRG.18085.2	19	0.32743	4.780578613	-1
MSTRG.18089.1	125	0.30796	9.245300293	1
MSTRG.18091.3	105	0.33463	8.44720459	1
MSTRG.18089.2	125	0.31204	9.245300293	1
MSTRG.18122.1	62	0.33698	8.045593262	1
MSTRG.18129.1	1290	0.46213	5.69720459	1
MSTRG.18130.3	944	0.37956	5.773132324	1
MSTRG.18130.4	944	0.37956	5.773132324	1
MSTRG.18131.5	1805	0.40182	5.407653809	1
MSTRG.18131.4	2721	0.49286	5.871276855	1
MSTRG.18131.3	2721	0.47107	5.871276855	1
MSTRG.18131.6	1705	0.45773	5.668029785	1
MSTRG.18132.1	67	0.36486	9.891906738	-1
MSTRG.18135.1	975	0.36007	6.084777832	1
MSTRG.18137.1	71	0.36665	10.94671631	1
MSTRG.18137.2	84	0.42853	10.5178833	1
MSTRG.18138.2	41	0.28486	7.99810791	1
MSTRG.18138.1	57	0.32073	6.810852051	1
MSTRG.18148.9	116	0.28567	9.860046387	1
MSTRG.18149.1	64	0.42378	10.300354	1
MSTRG.18151.1	73	0.32005	11.20013428	1
MSTRG.18152.2	216	0.42047	12.08892822	1
MSTRG.18152.3	345	0.41999	11.47332764	1
MSTRG.18152.4	157	0.43856	11.94317627	1
MSTRG.18153.1	427	0.30721	4.866271973	1
MSTRG.18153.2	422	0.33031	8.102111816	1
MSTRG.18142.3	461	0.46253	5.399597168	1
MSTRG.18147.2	533	0.45511	5.683532715	1
MSTRG.18140.1	43	0.4472	9.690856934	1
MSTRG.18140.2	43	0.40971	9.690856934	1
MSTRG.18155.1	394	0.28338	9.419006348	1
MSTRG.18155.2	197	0.31588	6.057556152	1
MSTRG.18156.1	161	0.2886	8.112487793	1
MSTRG.18157.1	193	0.27651	9.324890137	1
MSTRG.18158.3	291	0.40868	8.063171387	1
MSTRG.18159.1	76	0.28195	5.763244629	1
MSTRG.18161.4	237	0.44087	8.738708496	1
MSTRG.18162.2	465	0.40383	6.64263916	1
MSTRG.18164.1	472	0.39318	7.455871582	1
MSTRG.18169.1	266	0.46138	9.647033691	1
MSTRG.18170.3	190	0.37825	8.578186035	1
MSTRG.18174.1	124	0.45869	9.503234863	1

MSTRG.18174.5	133	0.44162	9.382385254	1
MSTRG.18173.1	154	0.46058	10.24578857	1
MSTRG.18173.2	160	0.45853	10.18609619	1
MSTRG.18176.2	79	0.43732	9.253723145	1
MSTRG.18177.2	126	0.32935	8.851379395	1
MSTRG.18183.4	231	0.45133	9.180603027	1
MSTRG.18191.1	40	0.40869	11.54058838	-1
MSTRG.18191.2	42	0.37493	9.489562988	-1
MSTRG.18191.3	42	0.37493	9.489562988	-1
MSTRG.18191.4	40	0.42069	11.54058838	-1
MSTRG.18185.1	83	0.36375	9.075256348	1
MSTRG.18196.2	176	0.34594	9.191955566	1
MSTRG.18196.3	100	0.42497	10.08172607	1
MSTRG.18196.4	79	0.45621	9.605041504	1
MSTRG.18198.1	78	0.44141	8.980041504	1
MSTRG.18200.2	144	0.3974	4.77142334	1
MSTRG.18200.3	144	0.37825	4.77142334	1
MSTRG.18200.4	144	0.37395	4.77142334	1
MSTRG.18200.5	91	0.34575	7.882629395	1
MSTRG.18201.2	290	0.45689	9.455993652	1
MSTRG.18219.2	1129	0.32868	6.640441895	1
MSTRG.18219.1	1128	0.32113	6.640441895	1
MSTRG.18219.3	1128	0.39197	6.640441895	1
MSTRG.18219.4	1135	0.39197	6.640441895	1
MSTRG.18219.5	1129	0.38577	6.640441895	1
MSTRG.18219.6	1129	0.39197	6.640441895	1
MSTRG.18219.8	824	0.38292	7.31829834	1
MSTRG.18210.1	226	0.36982	8.435974121	1
MSTRG.18211.4	584	0.3868	9.592468262	1
MSTRG.18212.2	174	0.32883	6.137634277	1
MSTRG.18214.1	120	0.47125	10.45599365	1
MSTRG.18214.2	120	0.42641	10.45599365	1
MSTRG.18215.1	81	0.28948	6.885437012	1
MSTRG.18220.2	537	0.4132	5.895935059	1
MSTRG.18226.2	141	0.42671	5.412780762	1
MSTRG.18229.2	1173	0.41141	6.63470459	1
MSTRG.18229.1	1148	0.41141	6.710876465	1
MSTRG.18229.5	1168	0.43726	6.351379395	1
MSTRG.18234.1	33	0.32522	11.3336792	1
MSTRG.18240.1	1410	0.36082	7.142272949	1
MSTRG.18240.8	1411	0.44537	7.142272949	1
MSTRG.18240.7	1410	0.39687	7.142272949	1
MSTRG.18240.9	1410	0.36001	7.142272949	1
MSTRG.18240.10	1410	0.36001	7.142272949	1
MSTRG.18240.11	1411	0.41073	7.142272949	1
MSTRG.18241.1	174	0.30596	9.175354004	1
MSTRG.18240.12	1410	0.37291	7.142272949	1
MSTRG.18242.2	865	0.46099	9.324279785	1
MSTRG.18242.4	656	0.45994	9.27557373	1
MSTRG.18245.1	89	0.30846	9.494812012	1
MSTRG.18245.2	53	0.37568	10.04425049	1
MSTRG.18238.2	1591	0.46343	5.86505127	1
MSTRG.18239.1	133	0.28311	9.504577637	1
MSTRG.18246.2	550	0.37465	5.434265137	1

MSTRG.18246.3	620	0.43135	5.898620605	1
MSTRG.18252.2	781	0.4421	9.26348877	-1
MSTRG.18253.2	1918	0.43687	7.902770996	1
MSTRG.18251.1	96	0.369	9.841125488	1
MSTRG.18259.2	426	0.3644	8.507141113	1
MSTRG.18261.1	119	0.34732	10.98016357	-1
MSTRG.18269.10	142	0.27732	9.739929199	1
MSTRG.18269.8	74	0.33248	10.90313721	1
MSTRG.18269.6	63	0.32912	11.70526123	1
MSTRG.18269.13	108	0.28007	5.355651855	1
MSTRG.18269.3	108	0.27651	5.355651855	1
MSTRG.18269.7	105	0.35341	9.742248535	1
MSTRG.18269.11	58	0.30808	9.42791748	1
MSTRG.18269.12	105	0.30197	9.742248535	1
MSTRG.18269.1	271	0.37995	6.333068848	1
MSTRG.18269.14	271	0.37311	6.333068848	1
MSTRG.18269.2	271	0.30032	6.333068848	1
MSTRG.18269.4	271	0.37995	6.333068848	1
MSTRG.18269.5	271	0.37311	6.333068848	1
MSTRG.18269.9	271	0.39793	6.333068848	1
MSTRG.18275.1	165	0.28883	10.14678955	1
MSTRG.18276.4	75	0.27274	8.683898926	1
MSTRG.18276.3	75	0.27274	8.683898926	1
MSTRG.18277.1	947	0.46925	8.421813965	1
MSTRG.18277.4	509	0.43816	9.487487793	1
MSTRG.18278.1	123	0.41758	5.846984863	1
MSTRG.18270.2	140	0.39029	4.069763184	1
MSTRG.18274.1	112	0.39665	9.503234863	1
MSTRG.18274.2	241	0.33809	10.76031494	1
MSTRG.18274.3	128	0.30193	9.385437012	1
MSTRG.18274.4	161	0.38754	9.506530762	1
MSTRG.18273.2	129	0.35814	5.582458496	1
MSTRG.18292.2	951	0.45392	6.653259277	1
MSTRG.18292.3	994	0.4564	7.167175293	1
MSTRG.18294.2	109	0.40043	9.338684082	1
MSTRG.18294.5	392	0.4041	8.851989746	-1
MSTRG.18329.1	58	0.31637	4.655822754	1
MSTRG.18346.1	116	0.3143	9.831115723	1
MSTRG.18346.2	105	0.37122	10.27252197	1
MSTRG.18346.3	105	0.34819	10.27252197	1
MSTRG.18347.1	114	0.27911	10.07537842	1
MSTRG.18348.1	996	0.38051	8.026184082	1
MSTRG.18348.2	122	0.27587	8.777160645	1
MSTRG.18349.1	186	0.28419	6.499328613	1
MSTRG.18350.1	222	0.28419	4.843078613	1
MSTRG.18350.2	267	0.28419	4.775695801	1
MSTRG.18350.3	267	0.30661	4.714050293	1
MSTRG.18351.1	138	0.28267	10.23956299	1
MSTRG.18348.3	874	0.36206	9.001281738	1
MSTRG.18352.1	144	0.28267	8.35760498	1
MSTRG.18353.1	73	0.31768	9.565979004	1
MSTRG.18354.1	143	0.27587	8.92010498	1
MSTRG.18355.1	80	0.418	8.803527832	-1
MSTRG.18297.2	896	0.3692	8.757385254	1

MSTRG.18297.3	991	0.41425	8.832214355	1
MSTRG.18297.6	752	0.45613	8.124084473	-1
MSTRG.18297.5	752	0.43768	8.124084473	-1
MSTRG.18299.2	82	0.32037	10.03497314	1
MSTRG.18299.3	125	0.30221	9.852966309	1
MSTRG.18304.1	62	0.3884	10.60577393	1
MSTRG.18306.2	77	0.41634	4.110900879	1
MSTRG.18306.3	73	0.42228	4.270812988	-1
MSTRG.18307.1	46	0.39925	9.149719238	1
MSTRG.18321.1	82	0.33913	10.77105713	1
MSTRG.18369.2	257	0.36921	11.03509521	1
MSTRG.18369.5	257	0.43366	11.03509521	1
MSTRG.18369.4	257	0.42022	11.03509521	1
MSTRG.18369.3	257	0.4094	11.03509521	1
MSTRG.18371.2	111	0.37144	8.745666504	1
MSTRG.18373.1	127	0.37539	11.32806396	1
MSTRG.18378.2	493	0.43246	6.320983887	1
MSTRG.18379.6	515	0.3848	5.534973145	1
MSTRG.18381.3	616	0.29162	9.531677246	1
MSTRG.18381.4	462	0.42722	9.609924316	1
MSTRG.18382.3	274	0.33122	7.030090332	1
MSTRG.18382.4	82	0.38966	6.243103027	1
MSTRG.18383.3	448	0.43599	8.303771973	1
MSTRG.18383.1	446	0.40843	6.968200684	1
MSTRG.18386.1	192	0.4179	9.277648926	1
MSTRG.18386.2	148	0.42157	8.61932373	1
MSTRG.18398.5	545	0.43779	5.316345215	1
MSTRG.18400.4	356	0.42447	6.732849121	1
MSTRG.18400.3	1639	0.43361	6.308776855	1
MSTRG.18400.5	1630	0.43926	6.308288574	1
MSTRG.18400.7	1610	0.43493	6.307800293	1
MSTRG.18400.6	1639	0.44516	6.308776855	1
MSTRG.18400.9	1351	0.44351	6.148986816	-1
MSTRG.18401.1	143	0.30672	6.584533691	1
MSTRG.18405.1	171	0.28622	11.53839111	1
MSTRG.18406.1	1645	0.43405	6.855895996	1
MSTRG.18408.1	242	0.3331	6.940246582	1
MSTRG.18408.3	242	0.41342	6.940246582	1
MSTRG.18408.4	242	0.40568	6.940246582	1
MSTRG.18413.2	683	0.40643	5.97467041	1
MSTRG.18413.3	616	0.32864	6.184509277	1
MSTRG.18414.1	515	0.39709	9.379211426	1
MSTRG.18416.4	606	0.43179	7.879821777	1
MSTRG.18416.5	309	0.44711	9.015686035	1
MSTRG.18416.6	365	0.45689	9.094055176	1
MSTRG.18417.1	390	0.41696	4.929260254	1
MSTRG.18417.3	313	0.39839	10.28155518	1
MSTRG.18433.6	516	0.43868	5.176330566	1
MSTRG.18433.7	516	0.43738	5.176330566	1
MSTRG.18434.3	1541	0.40804	4.988464355	1
MSTRG.18435.1	570	0.41792	5.837585449	1
MSTRG.18435.2	347	0.41277	9.159606934	1
MSTRG.18438.1	140	0.29536	11.31536865	1
MSTRG.18438.2	140	0.41231	11.31536865	1

MSTRG.18447.3	637	0.36627	6.296081543	1
MSTRG.18448.1	76	0.33996	8.967468262	-1
MSTRG.18448.2	23	0.30775	9.369567871	1
MSTRG.18447.5	742	0.44385	6.242492676	1
MSTRG.18424.4	542	0.32613	11.44842529	1
MSTRG.18424.3	840	0.39683	10.99664307	1
MSTRG.18424.2	975	0.41069	10.95465088	1
MSTRG.18425.1	362	0.35387	9.03338623	1
MSTRG.18425.2	362	0.33471	9.03338623	1
MSTRG.18425.4	362	0.3462	9.03338623	1
MSTRG.18425.3	362	0.328	9.03338623	1
MSTRG.18428.3	387	0.3982	8.451721191	1
MSTRG.18428.2	433	0.40276	8.889709473	1
MSTRG.18428.4	139	0.34759	10.31976318	1
MSTRG.18428.5	139	0.3279	10.31976318	1
MSTRG.18452.2	165	0.35818	7.520080566	1
MSTRG.18449.1	119	0.40021	5.277648926	1
MSTRG.18449.2	100	0.3819	6.07208252	1
MSTRG.18453.2	102	0.35539	6.960144043	1
MSTRG.18454.2	193	0.28031	11.30584717	1
MSTRG.18454.1	184	0.33046	9.903991699	1
MSTRG.18454.3	184	0.30341	9.903991699	1
MSTRG.18459.1	214	0.33684	11.70098877	1
MSTRG.18462.1	859	0.42098	6.976379395	-1
MSTRG.18462.5	984	0.42819	7.203552246	-1
MSTRG.18462.7	859	0.43493	6.976379395	-1
MSTRG.18455.1	642	0.44803	9.149963379	1
MSTRG.18464.1	169	0.39861	6.175476074	-1
MSTRG.18466.1	85	0.33306	11.8805542	1
MSTRG.18465.3	1290	0.40615	8.152648926	1
MSTRG.18465.2	1287	0.44301	8.018859863	1
MSTRG.18465.5	1276	0.43743	7.681213379	1
MSTRG.18465.4	1289	0.43743	8.152648926	1
MSTRG.18468.2	383	0.35728	9.389465332	1
MSTRG.18470.4	678	0.38882	7.034484863	1
MSTRG.18470.6	131	0.29959	6.824157715	1
MSTRG.18470.8	77	0.31413	8.237243652	1
MSTRG.18470.9	718	0.32317	6.047790527	1
MSTRG.18470.11	143	0.26913	10.57684326	1
MSTRG.18469.2	1282	0.44853	6.504211426	1
MSTRG.18477.2	1052	0.46849	6.209533691	1
MSTRG.18477.1	1052	0.44643	6.209533691	1
MSTRG.18477.3	1006	0.46849	6.33795166	1
MSTRG.18477.4	932	0.40709	6.625305176	1
MSTRG.18477.5	1059	0.46849	6.160583496	1
MSTRG.18477.6	1055	0.46849	6.262023926	1
MSTRG.18477.7	1049	0.44643	6.473449707	1
MSTRG.18477.8	1013	0.46849	6.278991699	1
MSTRG.18477.9	1006	0.46601	6.33795166	1
MSTRG.18477.10	969	0.44481	6.610778809	1
MSTRG.18477.11	27	0.38705	8.418884277	1
MSTRG.18477.12	723	0.37068	5.942687988	1
MSTRG.18478.1	68	0.31588	10.55731201	1
MSTRG.18477.14	162	0.34332	8.85736084	1

MSTRG.18479.1	132	0.34417	10.01702881	1
MSTRG.18479.2	132	0.30863	10.01702881	1
MSTRG.18472.8	496	0.35107	9.290100098	1
MSTRG.18473.1	166	0.36591	9.394592285	1
MSTRG.18472.12	423	0.38667	9.280700684	1
MSTRG.18474.2	114	0.34671	4.84942627	1
MSTRG.18481.1	928	0.46012	6.41986084	1
MSTRG.18482.1	78	0.3696	10.67352295	1
MSTRG.18483.2	53	0.38837	9.296813965	1
MSTRG.18484.1	143	0.32934	9.546203613	1
MSTRG.18484.2	143	0.35561	9.546203613	1
MSTRG.18484.3	147	0.41046	7.003234863	1
MSTRG.18480.2	264	0.38767	9.137878418	1
MSTRG.18485.1	17	0.41691	4.207092285	1
MSTRG.18487.1	84	0.31402	8.811462402	1
MSTRG.18487.2	121	0.30317	9.726745605	1
MSTRG.18492.2	1236	0.48849	5.149841309	-1
MSTRG.18490.1	105	0.42735	10.03399658	-1
MSTRG.18499.2	293	0.43299	9.068786621	1
MSTRG.18500.1	77	0.34417	9.852722168	1
MSTRG.18504.1	107	0.27651	7.805358887	1
MSTRG.18507.4	103	0.29772	6.004211426	1
MSTRG.18507.5	70	0.3295	8.676330566	1
MSTRG.18507.6	52	0.32244	6.538391113	1
MSTRG.18508.2	105	0.32774	9.553405762	1
MSTRG.18508.3	105	0.32698	9.553405762	1
MSTRG.18511.1	339	0.35593	9.37713623	1
MSTRG.18511.2	339	0.45703	9.37713623	1
MSTRG.18511.3	339	0.46243	9.37713623	1
MSTRG.18511.4	339	0.42062	9.37713623	1
MSTRG.18513.3	162	0.32014	7.365539551	1
MSTRG.18513.2	162	0.31962	7.365539551	1
MSTRG.18516.2	1007	0.3889	6.496154785	1
MSTRG.18516.5	991	0.37681	6.809753418	1
MSTRG.18517.1	39	0.33605	7.958679199	1
MSTRG.18514.2	202	0.40065	8.870544434	1
MSTRG.18514.3	176	0.38679	7.675354004	1
MSTRG.18514.4	447	0.47294	5.424987793	1
MSTRG.18519.1	41	0.32701	11.73394775	1
MSTRG.18524.1	821	0.46029	6.198059082	1
MSTRG.18524.2	787	0.40643	6.234069824	1
MSTRG.18524.4	692	0.45764	6.261779785	1
MSTRG.18527.1	1157	0.41803	6.42010498	1
MSTRG.18527.2	53	0.34016	10.00860596	1
MSTRG.18529.3	178	0.43518	7.65814209	1
MSTRG.18529.5	324	0.43565	5.755310059	1
MSTRG.18529.4	324	0.43858	5.755310059	1
MSTRG.18530.1	122	0.28478	5.652893066	1
MSTRG.18530.2	122	0.29687	5.652893066	1
MSTRG.18531.1	103	0.35403	6.573059082	1
MSTRG.18536.1	20	0.34536	12.00848389	1
MSTRG.18538.1	30	0.38773	10.17108154	1
MSTRG.18540.1	95	0.41788	5.108703613	1
MSTRG.18600.2	84	0.29619	10.53216553	1

MSTRG.18614.1	120	0.3609	9.151184082	1
MSTRG.18615.1	83	0.35532	7.955627441	1
MSTRG.18626.2	80	0.34139	9.981750488	1
MSTRG.18622.6	91	0.29367	8.623962402	1
MSTRG.18622.7	60	0.29543	4.950012207	1
MSTRG.18631.2	152	0.34431	7.196594238	1
MSTRG.18631.3	74	0.30905	9.671813965	1
MSTRG.18632.1	135	0.30789	9.919372559	1
MSTRG.18631.4	152	0.30725	7.196594238	1
MSTRG.18633.1	33	0.29404	7.923034668	1
MSTRG.18622.8	165	0.44876	9.221008301	1
MSTRG.18622.14	165	0.45426	9.221008301	1
MSTRG.18631.6	269	0.34803	5.19342041	-1
MSTRG.18631.7	57	0.42172	3.794616699	1
MSTRG.18622.16	61	0.38348	6.50201416	-1
MSTRG.18622.17	124	0.31553	6.688903809	1
MSTRG.18631.9	152	0.40797	7.196594238	1
MSTRG.18631.8	516	0.35435	5.289245605	1
MSTRG.18622.20	81	0.28683	6.366638184	1
MSTRG.18622.21	81	0.28683	6.366638184	1
MSTRG.18622.22	46	0.37618	9.77923584	1
MSTRG.18631.10	557	0.3915	5.143493652	-1
MSTRG.18622.34	165	0.40019	9.221008301	1
MSTRG.18638.1	82	0.3325	8.770446777	-1
MSTRG.18637.1	48	0.36246	9.961730957	1
MSTRG.18542.3	363	0.35948	11.23760986	1
MSTRG.18546.4	248	0.48458	8.13104248	1
MSTRG.18546.5	98	0.29772	5.616271973	1
MSTRG.18546.6	129	0.28982	10.22515869	1
MSTRG.18546.7	141	0.29892	5.721496582	1
MSTRG.18546.8	189	0.29607	6.847473145	1
MSTRG.18546.9	189	0.29772	10.17498779	1
MSTRG.18546.10	189	0.29772	6.847473145	1
MSTRG.18546.11	134	0.27767	7.000061035	1
MSTRG.18546.12	117	0.2921	8.668151855	1
MSTRG.18546.13	191	0.27918	6.329040527	1
MSTRG.18546.14	191	0.32916	6.329040527	1
MSTRG.18548.2	2	0.33937	4.298156738	1
MSTRG.18551.1	150	0.32237	9.09576416	1
MSTRG.18552.1	115	0.28771	9.324035645	1
MSTRG.18552.2	89	0.28496	8.935119629	1
MSTRG.18555.1	758	0.35633	6.500061035	1
MSTRG.18555.2	758	0.36255	6.500061035	1
MSTRG.18557.4	250	0.40616	5.608215332	1
MSTRG.18558.1	72	0.31997	7.847595215	1
MSTRG.18559.1	665	0.38409	5.914367676	1
MSTRG.18559.4	604	0.36064	5.793640137	1
MSTRG.18559.6	665	0.38291	5.914367676	1
MSTRG.18561.1	838	0.4477	5.969543457	1
MSTRG.18561.3	199	0.35659	4.564025879	1
MSTRG.18562.1	107	0.40861	7.764709473	1
MSTRG.18562.2	156	0.44755	9.411193848	1
MSTRG.18562.3	336	0.47348	6.42791748	1
MSTRG.18563.1	41	0.27996	12.00567627	1

MSTRG.18564.1	355	0.32915	9.45501709	1
MSTRG.18564.2	355	0.36289	9.45501709	1
MSTRG.18565.1	207	0.35188	4.993469238	1
MSTRG.18565.2	207	0.36168	4.993469238	1
MSTRG.18568.1	1373	0.46494	6.039733887	1
MSTRG.18568.3	451	0.42455	6.332214355	1
MSTRG.18568.6	265	0.39797	5.204040527	1
MSTRG.18572.1	873	0.42948	5.713928223	1
MSTRG.18572.4	873	0.44176	5.713928223	1
MSTRG.18574.4	931	0.47994	8.668640137	1
MSTRG.18574.5	931	0.49839	8.668640137	1
MSTRG.18574.6	910	0.47622	8.799621582	1
MSTRG.18574.7	88	0.32477	10.22894287	1
MSTRG.18567.2	151	0.32246	9.908508301	1
MSTRG.18589.1	35	0.34427	7.957336426	-1
MSTRG.18590.1	32	0.33804	10.11859131	1
MSTRG.18594.2	227	0.38845	9.096740723	1
MSTRG.18594.3	115	0.3841	6.801452637	1
MSTRG.18594.5	167	0.37679	5.084777832	1
MSTRG.18594.6	116	0.36609	9.722229004	1
MSTRG.18594.7	133	0.39729	8.754699707	1
MSTRG.18654.3	113	0.33207	10.77484131	1
MSTRG.18654.4	4146	0.45867	6.80645752	1
MSTRG.18656.1	41	0.36243	6.901794434	1
MSTRG.18645.1	145	0.27803	7.792541504	1
MSTRG.18647.2	208	0.48731	5.37701416	1
MSTRG.18653.1	71	0.4478	4.55657959	1
MSTRG.18659.1	180	0.45569	9.09552002	1
MSTRG.18659.5	161	0.29387	5.143249512	1
MSTRG.18662.5	1589	0.44834	5.325256348	1
MSTRG.18662.3	1589	0.44902	5.325256348	1
MSTRG.18666.8	191	0.34812	6.279724121	1
MSTRG.18666.9	118	0.35072	7.700378418	1
MSTRG.18666.4	176	0.37421	8.642883301	1
MSTRG.18666.5	118	0.35371	7.700378418	1
MSTRG.18666.6	118	0.37421	7.700378418	1
MSTRG.18666.7	118	0.35576	7.700378418	1
MSTRG.18666.10	191	0.33711	6.279724121	1
MSTRG.18667.2	297	0.35894	10.14678955	1
MSTRG.18677.1	90	0.29059	7.613220215	1
MSTRG.18679.3	70	0.43627	9.151550293	1
MSTRG.18681.1	67	0.3263	6.22845459	1
MSTRG.18684.2	293	0.37697	8.363098145	1
MSTRG.18685.1	30	0.3626	10.65545654	1
MSTRG.18698.1	169	0.27622	5.483459473	1
MSTRG.18697.4	241	0.45816	8.807678223	1
MSTRG.18702.2	366	0.30816	9.870056152	1
MSTRG.18703.1	125	0.30467	10.24017334	1
MSTRG.18704.1	70	0.28334	10.05584717	1
MSTRG.18705.1	67	0.3261	6.695129395	1
MSTRG.18706.1	136	0.29367	8.635559082	1
MSTRG.18707.1	90	0.28562	5.457946777	1
MSTRG.18709.2	406	0.3287	9.256408691	1
MSTRG.18709.3	344	0.29526	9.362609863	1

MSTRG.18709.4	195	0.28883	6.410949707	1
MSTRG.18711.4	212	0.38059	6.080993652	1
MSTRG.18713.1	37	0.3291	9.648132324	1
MSTRG.18715.2	111	0.39651	5.038024902	-1
MSTRG.18715.4	550	0.4605	7.991149902	-1
MSTRG.18722.1	99	0.35251	7.955871582	1
MSTRG.18725.6	587	0.36389	6.93145752	1
MSTRG.18725.7	632	0.35371	6.741149902	1
MSTRG.18726.1	48	0.32015	9.057434082	1
MSTRG.18718.1	101	0.31587	5.807678223	1
MSTRG.18732.2	101	0.27517	7.82598877	1
MSTRG.18733.1	78	0.36365	6.527526855	1
MSTRG.18734.1	100	0.28982	9.302429199	1
MSTRG.18734.2	67	0.27937	9.260559082	1
MSTRG.18741.3	323	0.36626	8.992736816	1
MSTRG.18743.3	199	0.34444	5.622131348	1
MSTRG.18744.1	221	0.35161	4.287780762	1
MSTRG.18749.1	1223	0.46794	6.480407715	1
MSTRG.18749.2	1223	0.45648	6.480407715	1
MSTRG.18749.3	1223	0.39484	6.480407715	1
MSTRG.18750.1	34	0.34078	5.596496582	1
MSTRG.18751.2	177	0.3638	8.965637207	1
MSTRG.18751.4	177	0.4593	8.965637207	1
MSTRG.18752.1	681	0.46102	6.445617676	1
MSTRG.18752.2	681	0.47283	6.445617676	1
MSTRG.18762.2	315	0.3852	8.868713379	1
MSTRG.18762.1	271	0.3852	9.096740723	1
MSTRG.18763.1	1211	0.47647	5.990783691	1
MSTRG.18762.3	315	0.41695	8.868713379	1
MSTRG.18764.1	119	0.30303	9.358093262	1
MSTRG.18762.4	189	0.41702	6.20904541	1
MSTRG.18763.2	898	0.45114	6.017028809	1
MSTRG.18763.4	1211	0.45761	5.990783691	1
MSTRG.18763.5	1210	0.45761	5.990783691	1
MSTRG.18763.7	1034	0.48364	6.011779785	1
MSTRG.18768.2	1909	0.449	5.762756348	1
MSTRG.18756.1	194	0.49177	4.358093262	1
MSTRG.18761.1	868	0.34785	9.159973145	1
MSTRG.18769.5	1348	0.36918	6.282409668	1
MSTRG.18769.6	1465	0.41369	6.193664551	1
MSTRG.18770.1	123	0.27306	7.586730957	1
MSTRG.18771.1	74	0.32422	9.468566895	-1
MSTRG.18772.1	40	0.28966	5.086242676	1
MSTRG.18773.1	35	0.34229	8.938903809	1
MSTRG.18776.1	49	0.40621	6.699890137	1
MSTRG.18805.1	283	0.2895	9.19354248	1
MSTRG.18805.2	224	0.31917	9.553771973	1
MSTRG.18816.4	202	0.41984	9.623840332	-1
MSTRG.18816.5	75	0.32954	8.507019043	1
MSTRG.18818.2	102	0.31389	9.030944824	1
MSTRG.18825.7	556	0.29089	9.633361816	1
MSTRG.18829.1	83	0.31446	8.570007324	1
MSTRG.18821.1	50	0.31368	4.324645996	1
MSTRG.18822.4	112	0.38883	4.737243652	1

MSTRG.18830.2	157	0.30159	9.37286377	1
MSTRG.18830.3	36	0.45292	4.921447754	-1
MSTRG.18838.1	102	0.41288	10.5067749	-1
MSTRG.18841.1	99	0.34444	10.0413208	1
MSTRG.18850.1	24	0.41095	11.00860596	1
MSTRG.18852.1	2388	0.42024	6.279602051	1
MSTRG.18861.1	129	0.36658	6.472473145	1
MSTRG.18862.1	101	0.41634	5.88494873	-1
MSTRG.18872.1	40	0.3701	5.935241699	-1
MSTRG.18882.1	67	0.30055	8.63067627	1
MSTRG.18894.1	97	0.41788	5.14276123	1
MSTRG.18894.4	140	0.44918	5.530212402	1
MSTRG.18894.6	97	0.41844	5.14276123	1
MSTRG.18894.7	120	0.46224	7.735778809	1
MSTRG.18894.3	97	0.47602	5.14276123	1
MSTRG.18894.5	140	0.41451	5.530212402	1
MSTRG.18906.1	92	0.29187	10.92486572	1
MSTRG.18907.1	78	0.32971	9.662780762	1
MSTRG.18908.1	42	0.35831	9.620666504	1
MSTRG.18911.1	54	0.38524	7.948181152	1
MSTRG.18917.2	630	0.46653	4.54498291	1
MSTRG.18917.3	630	0.45184	4.54498291	1
MSTRG.18958.1	37	0.35781	8.887023926	1
MSTRG.18967.1	222	0.46849	6.128356934	1
MSTRG.18967.3	1695	0.46831	4.90423584	-1
MSTRG.18955.1	81	0.36493	10.31524658	1
MSTRG.18979.1	106	0.45971	11.51177979	1
MSTRG.18983.2	1090	0.45905	6.332214355	1
MSTRG.18986.1	147	0.29746	10.22747803	1
MSTRG.18987.1	293	0.349	8.61907959	1
MSTRG.18984.2	91	0.44753	9.098205566	1
MSTRG.18997.1	124	0.39435	6.412658691	1
MSTRG.18997.2	124	0.351	6.412658691	1
MSTRG.18999.1	77	0.33438	10.50933838	1
MSTRG.18999.2	306	0.4614	6.473571777	-1
MSTRG.19005.1	150	0.28498	7.717834473	1
MSTRG.19006.1	158	0.29367	6.359680176	1
MSTRG.19014.6	757	0.46866	5.215881348	1
MSTRG.19014.5	632	0.47271	5.261901855	1
MSTRG.19015.1	10	0.39517	4.36907959	1
MSTRG.19015.2	21	0.35748	7.976013184	1
MSTRG.19015.3	52	0.37628	10.01409912	1
MSTRG.19009.1	18	0.45234	9.524963379	1
MSTRG.19024.1	40	0.3962	5.425842285	1
MSTRG.19041.1	55	0.37105	9.742248535	-1
MSTRG.19049.1	150	0.29202	5.434509277	1
MSTRG.19049.2	150	0.29367	5.434509277	1
MSTRG.19049.3	31	0.37326	4.225524902	1
MSTRG.19049.4	69	0.31958	5.834289551	1
MSTRG.19052.2	184	0.3823	9.473571777	1
MSTRG.19034.1	84	0.34768	10.42938232	1
MSTRG.19038.1	69	0.39429	9.621276855	1
MSTRG.19039.3	280	0.38859	4.410949707	1
MSTRG.19053.2	467	0.37812	9.332214355	1

MSTRG.19053.3	467	0.36782	9.332214355	1
MSTRG.19059.4	2791	0.45295	5.598205566	1
MSTRG.19059.3	2781	0.4839	5.628112793	1
MSTRG.19061.1	334	0.46673	6.212097168	-1
MSTRG.19063.1	112	0.31867	9.779602051	1
MSTRG.19066.1	121	0.28612	5.64642334	1
MSTRG.19068.2	30	0.40805	10.04425049	1
MSTRG.19069.1	245	0.33245	4.726257324	1
MSTRG.19069.2	245	0.3272	4.726257324	1
MSTRG.19069.4	245	0.33851	4.726257324	1
MSTRG.19069.6	142	0.28165	12.19769287	1
MSTRG.19072.1	636	0.45909	9.515197754	1
MSTRG.19072.2	636	0.46442	9.515197754	1
MSTRG.19072.3	636	0.47408	9.515197754	1
MSTRG.19072.4	563	0.46883	9.658874512	1
MSTRG.19073.3	63	0.35565	5.768493652	1
MSTRG.19075.1	133	0.29772	10.0256958	1
MSTRG.19089.2	557	0.3195	8.228820801	1
MSTRG.19089.3	795	0.46678	8.508728027	1
MSTRG.19090.2	58	0.30419	9.265441895	1
MSTRG.19094.1	1266	0.44981	8.476501465	1
MSTRG.19094.2	1269	0.48853	8.288757324	1
MSTRG.19094.3	1322	0.4695	8.569885254	1
MSTRG.19094.5	523	0.44208	8.796081543	1
MSTRG.19095.1	58	0.42372	11.54656982	1
MSTRG.19096.1	3992	0.45158	6.10333252	1
MSTRG.19099.1	58	0.33559	7.636779785	1
MSTRG.19100.1	77	0.47759	4.465881348	-1
MSTRG.19101.1	107	0.29761	9.508239746	1
MSTRG.19078.2	418	0.42322	5.125793457	1
MSTRG.19078.3	260	0.27918	4.908508301	1
MSTRG.19079.1	82	0.29502	5.785095215	1
MSTRG.19081.1	211	0.3582	9.533752441	1
MSTRG.19083.1	62	0.36712	7.649475098	1
MSTRG.19084.3	255	0.43202	8.809997559	1
MSTRG.19085.1	107	0.3366	6.155456543	1
MSTRG.19107.3	939	0.40207	6.694641113	1
MSTRG.19110.1	57	0.33824	6.367004395	-1
MSTRG.19113.4	214	0.38971	7.855285645	1
MSTRG.19115.4	483	0.41248	5.829528809	1
MSTRG.19115.5	483	0.46879	5.829528809	1
MSTRG.19116.2	80	0.30104	10.14630127	1
MSTRG.19116.4	134	0.44369	8.622741699	1
MSTRG.19117.2	376	0.38208	8.331970215	1
MSTRG.19121.1	32	0.41338	4.239562988	1
MSTRG.19122.1	14	0.4316	5.58770752	1
MSTRG.19126.2	131	0.32237	4.888366699	1
MSTRG.19127.1	12	0.35712	9.700256348	1
MSTRG.19129.1	241	0.28419	9.573425293	1
MSTRG.19129.2	222	0.32642	9.536193848	1
MSTRG.19129.4	222	0.34378	9.536193848	1
MSTRG.19129.5	241	0.30605	9.573425293	1
MSTRG.19131.8	95	0.38965	4.710510254	1
MSTRG.19131.7	164	0.32663	4.112487793	1

MSTRG.19131.4	171	0.28883	5.855163574	1
MSTRG.19131.5	95	0.27838	4.710510254	1
MSTRG.19131.6	171	0.27838	5.855163574	1
MSTRG.19133.3	112	0.36161	8.330993652	1
MSTRG.19133.4	112	0.33347	8.330993652	1
MSTRG.19134.3	660	0.35421	8.058776855	1
MSTRG.19134.4	472	0.27234	8.336730957	1
MSTRG.19136.1	75	0.36021	10.3817749	1
MSTRG.19138.1	1614	0.40219	4.868713379	1
MSTRG.19142.5	538	0.37112	6.166931152	1
MSTRG.19148.3	933	0.46926	7.452575684	1
MSTRG.19148.4	193	0.37434	8.905334473	1
MSTRG.19150.1	984	0.38703	6.558410645	1
MSTRG.19150.2	984	0.36854	6.558410645	1
MSTRG.19152.2	188	0.44294	9.960266113	1
MSTRG.19144.1	25	0.36331	8.190246582	1
MSTRG.19146.1	28	0.38128	8.496154785	1
MSTRG.19155.1	38	0.3681	8.039978027	1
MSTRG.19156.1	59	0.35564	10.53375244	-1
MSTRG.19158.4	265	0.32661	6.018127441	1
MSTRG.19175.1	82	0.31273	10.53179932	1
MSTRG.19177.4	287	0.27517	9.667907715	1
MSTRG.19177.1	283	0.45957	5.646057129	1
MSTRG.19177.3	137	0.38615	4.710998535	1
MSTRG.19177.5	283	0.4589	5.646057129	1
MSTRG.19177.6	63	0.31226	9.33404541	1
MSTRG.19177.7	481	0.33029	8.643859863	1
MSTRG.19177.8	468	0.44225	8.93951416	1
MSTRG.19177.9	346	0.39641	9.29876709	1
MSTRG.19177.10	378	0.34002	9.289978027	1
MSTRG.19177.12	88	0.30981	9.504089355	1
MSTRG.19177.13	144	0.43545	4.722351074	1
MSTRG.19183.1	122	0.3438	9.44342041	1
MSTRG.19183.2	89	0.34135	8.958068848	1
MSTRG.19189.2	133	0.37362	9.570983887	1
MSTRG.19166.2	74	0.36487	10.36334229	1
MSTRG.19166.3	673	0.45254	6.388122559	1
MSTRG.19168.1	84	0.43011	10.01239014	1
MSTRG.19169.4	506	0.44341	7.984680176	1
MSTRG.19171.7	186	0.30777	7.623718262	1
MSTRG.19171.2	186	0.38722	7.623718262	1
MSTRG.19171.3	302	0.48326	6.756530762	1
MSTRG.19171.4	186	0.42363	7.623718262	1
MSTRG.19171.5	186	0.38722	7.623718262	1
MSTRG.19171.6	294	0.36594	7.186462402	1
MSTRG.19194.2	269	0.42175	9.442077637	1
MSTRG.19196.1	63	0.34713	4.135925293	1
MSTRG.19196.2	114	0.35739	6.820617676	1
MSTRG.19203.1	346	0.48131	8.932922363	1
MSTRG.19203.2	108	0.40899	6.707580566	1
MSTRG.19204.3	293	0.36161	6.164001465	1
MSTRG.19205.1	67	0.38792	9.664367676	1
MSTRG.19205.2	67	0.35645	9.664367676	1
MSTRG.19205.3	67	0.37162	9.664367676	1

MSTRG.19206.1	79	0.38961	10.13580322	-1
MSTRG.19207.2	121	0.37318	9.89251709	1
MSTRG.19210.5	143	0.29583	10.31854248	1
MSTRG.19210.6	810	0.28624	5.298278809	1
MSTRG.19210.4	509	0.3743	5.924865723	1
MSTRG.19210.7	509	0.34079	5.924865723	1
MSTRG.19210.3	921	0.33851	5.812805176	1
MSTRG.19210.8	87	0.33718	10.67205811	1
MSTRG.19197.1	167	0.34932	10.31707764	1
MSTRG.19202.1	59	0.37367	9.29107666	1
MSTRG.19213.1	100	0.36651	10.17657471	1
MSTRG.19213.2	55	0.36048	9.494445801	1
MSTRG.19215.1	209	0.38921	10.59783936	1
MSTRG.19217.4	473	0.45509	6.054992676	1
MSTRG.19217.2	595	0.47081	5.629699707	1
MSTRG.19217.5	470	0.45201	6.085876465	1
MSTRG.19218.1	37	0.30609	6.508361816	-1
MSTRG.19318.1	124	0.35631	11.1416626	1
MSTRG.19319.3	293	0.39473	7.69354248	1
MSTRG.19323.1	143	0.3701	10.97833252	1
MSTRG.19319.4	98	0.29178	7.785583496	1
MSTRG.19319.5	128	0.3	9.857116699	1
MSTRG.19319.6	107	0.32213	8.455871582	1
MSTRG.19325.1	74	0.29826	10.58843994	1
MSTRG.19325.2	113	0.2908	9.919006348	1
MSTRG.19326.1	139	0.2895	10.1116333	1
MSTRG.19328.1	149	0.29367	10.21502686	1
MSTRG.19338.1	107	0.34583	9.14251709	1
MSTRG.19339.1	186	0.28982	10.35345459	1
MSTRG.19340.1	113	0.29367	9.764709473	1
MSTRG.19342.1	94	0.29367	11.29364014	1
MSTRG.19347.2	111	0.28982	7.479431152	1
MSTRG.19339.2	233	0.29772	9.728210449	1
MSTRG.19339.3	145	0.29583	6.73651123	1
MSTRG.19339.4	109	0.3139	9.714782715	1
MSTRG.19339.5	183	0.29088	10.24163818	1
MSTRG.19339.6	183	0.3064	10.24163818	1
MSTRG.19339.7	75	0.36307	8.479797363	1
MSTRG.19339.8	110	0.31027	10.21661377	1
MSTRG.19349.1	65	0.31852	5.075866699	1
MSTRG.19221.1	101	0.3182	8.969055176	1
MSTRG.19221.2	91	0.40733	9.390441895	1
MSTRG.19230.1	61	0.37187	7.836608887	1
MSTRG.19235.1	19	0.39324	11.01275635	1
MSTRG.19249.1	105	0.37635	6.006408691	1
MSTRG.19249.2	91	0.34239	11.83258057	1
MSTRG.19249.3	80	0.35701	10.06292725	1
MSTRG.19249.4	73	0.29607	10.81915283	1
MSTRG.19237.1	314	0.35238	4.776794434	1
MSTRG.19252.4	60	0.34055	5.552062988	1
MSTRG.19253.1	89	0.4527	11.11590576	1
MSTRG.19253.4	45	0.42425	6.214416504	1
MSTRG.19253.5	45	0.37135	6.214416504	1
MSTRG.19254.1	72	0.32305	8.598449707	1

MSTRG.19258.1	188	0.32783	10.13641357	1
MSTRG.19258.2	108	0.36194	10.6038208	1
MSTRG.19264.1	215	0.26913	9.527526855	1
MSTRG.19265.1	178	0.41094	9.349304199	1
MSTRG.19266.2	495	0.38247	8.868103027	1
MSTRG.19270.2	456	0.45063	6.464782715	1
MSTRG.19278.1	77	0.36805	9.300354004	1
MSTRG.19281.2	238	0.38867	5.196838379	1
MSTRG.19285.2	141	0.29615	9.690856934	1
MSTRG.19285.3	77	0.31684	9.520935059	1
MSTRG.19285.4	142	0.3152	9.690856934	1
MSTRG.19291.1	96	0.27622	8.293518066	1
MSTRG.19291.2	84	0.37322	10.29205322	1
MSTRG.19283.1	345	0.42194	5.422546387	1
MSTRG.19283.3	345	0.32971	5.422546387	1
MSTRG.19305.4	95	0.31209	5.635681152	1
MSTRG.19311.1	738	0.38703	7.49798584	1
MSTRG.19311.2	3505	0.47369	6.241149902	1
MSTRG.19311.4	64	0.37854	4.593688965	1
MSTRG.19311.5	2674	0.48686	6.805725098	1
MSTRG.19311.6	1471	0.48688	6.092224121	1
MSTRG.19312.1	29	0.36215	11.8348999	1
MSTRG.19314.1	318	0.4327	7.832702637	1
MSTRG.19314.3	157	0.41142	9.835388184	1
MSTRG.19314.4	321	0.40646	8.53326416	1
MSTRG.19316.1	591	0.42293	5.717712402	1
MSTRG.19314.6	157	0.38399	9.835388184	1
MSTRG.19316.2	591	0.35972	5.717712402	1
MSTRG.19316.3	591	0.41888	5.717712402	1
MSTRG.19316.4	591	0.4029	5.717712402	1
MSTRG.19317.1	119	0.38387	9.88482666	1
MSTRG.19313.1	599	0.36573	6.105285645	1
MSTRG.19352.1	487	0.35673	4.891174316	1
MSTRG.19352.3	418	0.36875	5.033996582	1
MSTRG.19352.4	301	0.38587	4.525939941	1
MSTRG.19352.5	285	0.36141	4.422790527	1
MSTRG.19352.12	175	0.35645	3.984924316	1
MSTRG.19352.10	135	0.28662	9.775817871	1
MSTRG.19352.13	283	0.47101	4.37286377	1
MSTRG.19352.9	303	0.47198	4.525939941	1
MSTRG.19352.8	299	0.36385	4.473693848	1
MSTRG.19352.11	271	0.32149	4.660461426	1
MSTRG.19352.6	505	0.44791	5.065002441	1
MSTRG.19354.1	162	0.36432	10.24957275	1
MSTRG.19359.2	160	0.44025	5.719909668	1
MSTRG.19359.3	121	0.31204	10.68890381	1
MSTRG.19359.4	145	0.36597	4.766540527	1
MSTRG.19359.5	96	0.455	4.593566895	1
MSTRG.19361.1	549	0.34139	7.172790527	1
MSTRG.19361.2	748	0.40816	9.028869629	1
MSTRG.19361.3	170	0.34683	10.52044678	1
MSTRG.19361.4	549	0.38811	7.172790527	1
MSTRG.19361.5	596	0.30653	8.113098145	1
MSTRG.19361.6	596	0.28467	8.113098145	1

MSTRG.19361.10	138	0.3545	6.581237793	1
MSTRG.19361.7	350	0.39489	9.206604004	1
MSTRG.19361.8	350	0.42488	9.206604004	1
MSTRG.19361.9	549	0.40233	7.172790527	1
MSTRG.19361.11	199	0.27959	10.64349365	1
MSTRG.19361.12	106	0.27643	10.01202393	1
MSTRG.19361.13	84	0.28048	10.90643311	1
MSTRG.19361.14	73	0.28852	4.917663574	1
MSTRG.19363.1	33	0.44428	9.873474121	1
MSTRG.19401.1	129	0.30684	9.563537598	1
MSTRG.19402.1	16	0.37703	9.500061035	1
MSTRG.19368.1	7	0.40051	5.752990723	1
MSTRG.19369.1	37	0.3621	10.25616455	1
MSTRG.19370.1	65	0.40259	6.337219238	1
MSTRG.19407.1	62	0.33599	5.973571777	1
MSTRG.19408.1	181	0.3683	9.018493652	1
MSTRG.19414.1	571	0.4159	7.971252441	1
MSTRG.19414.2	643	0.41624	7.019958496	1
MSTRG.19415.1	350	0.41828	9.516296387	1
MSTRG.19415.3	498	0.46401	9.257019043	1
MSTRG.19415.4	349	0.47274	9.482727051	1
MSTRG.19416.1	95	0.35953	10.65985107	1
MSTRG.19416.2	95	0.35808	10.65985107	1
MSTRG.19413.1	159	0.35915	10.35247803	1
MSTRG.19420.2	492	0.28982	5.941345215	1
MSTRG.19422.1	296	0.47402	6.37286377	-1
MSTRG.19422.3	500	0.33046	8.925964355	1
MSTRG.19422.2	428	0.38733	8.724060059	1
MSTRG.19422.4	427	0.41512	8.953308105	1
MSTRG.19422.5	428	0.41394	8.827697754	1
MSTRG.19423.1	119	0.26749	6.346862793	1
MSTRG.19425.1	873	0.47402	6.710266113	1
MSTRG.19425.4	873	0.39433	6.710266113	1
MSTRG.19428.1	772	0.48136	9.025085449	1
MSTRG.19432.1	84	0.35293	6.289855957	1
MSTRG.19434.1	885	0.45448	8.523986816	1
MSTRG.19435.1	138	0.33671	10.50213623	1
MSTRG.19436.1	49	0.28301	5.013122559	1
MSTRG.19440.2	98	0.35972	9.942565918	1
MSTRG.19440.1	24	0.36033	4.529602051	1
MSTRG.19441.1	108	0.43927	7.834899902	-1
MSTRG.19446.2	519	0.36416	8.045471191	1
MSTRG.19464.1	3049	0.47527	6.434753418	1
MSTRG.19464.6	123	0.32659	7.054016113	1
MSTRG.19478.2	407	0.38722	6.198791504	1
MSTRG.19479.1	63	0.33798	10.21099854	1
MSTRG.19448.1	98	0.34336	10.10137939	1
MSTRG.19458.2	98	0.38765	9.693786621	1
MSTRG.19458.4	98	0.38537	9.693786621	1
MSTRG.19458.3	58	0.42582	5.361999512	1
MSTRG.19458.5	852	0.33105	6.550109863	1
MSTRG.19459.1	64	0.25541	9.148742676	1
MSTRG.19481.1	115	0.28165	5.60736084	1
MSTRG.19480.2	278	0.3	7.692687988	1

MSTRG.19485.1	86	0.3208	9.25604248	1
MSTRG.19485.2	186	0.34952	5.436950684	1
MSTRG.19485.3	145	0.46729	5.575500488	1
MSTRG.19486.1	87	0.27517	9.22052002	1
MSTRG.19490.5	1324	0.36594	9.196960449	1
MSTRG.19490.8	423	0.43977	9.110900879	1
MSTRG.19491.1	70	0.3091	5.079406738	1
MSTRG.19492.1	192	0.28683	8.569885254	1
MSTRG.19493.1	129	0.32249	6.221008301	1
MSTRG.19494.1	73	0.35862	9.992736816	1
MSTRG.19495.1	155	0.27502	8.06854248	1
MSTRG.19496.1	92	0.34547	11.04058838	1
MSTRG.19505.2	229	0.33202	9.133483887	1
MSTRG.19505.3	229	0.4134	9.133483887	1
MSTRG.19505.4	229	0.33558	9.133483887	1
MSTRG.19506.1	84	0.35292	6.740783691	1
MSTRG.19507.4	168	0.38627	9.077087402	1
MSTRG.19508.3	112	0.44192	9.395812988	1
MSTRG.19509.2	658	0.44128	5.058898926	1
MSTRG.19515.3	124	0.31593	8.913513184	1
MSTRG.19515.2	180	0.27433	6.971740723	1
MSTRG.19515.4	167	0.3346	6.48248291	1
MSTRG.19521.1	335	0.44282	5.327087402	1
MSTRG.19521.2	185	0.43383	5.140441895	1
MSTRG.19522.4	262	0.34947	9.665710449	1
MSTRG.19526.1	50	0.41161	6.54486084	1
MSTRG.19530.1	91	0.35339	4.856018066	1
MSTRG.19531.1	158	0.34201	10.33135986	1
MSTRG.19526.2	177	0.28535	8.859802246	1
MSTRG.19532.1	32	0.34936	6.137023926	1
MSTRG.19516.1	71	0.31437	10.15435791	1
MSTRG.19533.5	134	0.29526	7.616149902	1
MSTRG.19533.6	35	0.40893	9.496398926	1
MSTRG.19534.1	131	0.27639	8.772521973	1
MSTRG.19533.7	993	0.47757	4.750427246	1
MSTRG.19535.1	78	0.31401	4.437316895	1
MSTRG.19538.1	79	0.35573	7.998718262	1
MSTRG.19540.1	126	0.30813	9.332702637	1
MSTRG.19543.4	837	0.43856	5.046203613	1
MSTRG.19543.5	807	0.43772	4.968200684	1
MSTRG.19543.6	949	0.46803	5.159118652	1
MSTRG.19544.1	43	0.33959	7.73236084	1
MSTRG.19547.1	219	0.28786	7.678649902	1
MSTRG.19551.2	188	0.37132	6.09576416	1
MSTRG.19555.1	535	0.45023	9.265197754	1
MSTRG.19559.7	278	0.43427	4.945373535	1
MSTRG.19559.8	294	0.43427	4.934997559	1
MSTRG.19559.5	305	0.42495	4.891418457	1
MSTRG.19559.6	267	0.43427	4.995544434	1
MSTRG.19560.1	125	0.28982	9.146789551	1
MSTRG.19561.1	97	0.2921	9.495910645	1
MSTRG.19562.2	183	0.27042	10.57025146	1
MSTRG.19562.1	72	0.32305	9.663269043	1
MSTRG.19562.4	183	0.28302	10.57025146	1

MSTRG.19562.5	183	0.28302	10.39398193	1
MSTRG.19562.3	183	0.28302	10.39398193	1
MSTRG.19562.6	183	0.27042	10.57025146	1
MSTRG.19559.9	77	0.28165	9.212585449	1
MSTRG.19563.1	153	0.29152	8.849182129	1
MSTRG.19568.1	382	0.46015	5.736633301	-1
MSTRG.19568.2	240	0.45001	5.398132324	-1
MSTRG.19568.3	250	0.41636	5.879699707	-1
MSTRG.19568.4	215	0.46457	5.282165527	-1
MSTRG.19572.9	777	0.47576	6.908996582	1
MSTRG.19572.8	777	0.49323	6.908996582	1
MSTRG.19572.5	1246	0.50317	6.315246582	1
MSTRG.19572.7	1246	0.47888	6.315246582	1
MSTRG.19572.6	697	0.42725	9.185241699	1
MSTRG.19573.1	92	0.28805	8.29119873	1
MSTRG.19572.10	84	0.30807	6.00982666	1
MSTRG.19572.11	113	0.28248	6.852111816	1
MSTRG.19574.1	84	0.28306	11.39508057	1
MSTRG.19572.12	116	0.28478	9.077697754	1
MSTRG.19570.2	181	0.41714	6.376037598	1
MSTRG.19570.3	229	0.28584	5.568664551	1
MSTRG.19570.4	229	0.35108	5.568664551	1
MSTRG.19571.2	797	0.45059	6.702087402	1
MSTRG.19577.2	194	0.42188	8.882751465	1
MSTRG.19579.1	501	0.42891	5.312683105	1
MSTRG.19579.2	267	0.43657	5.152038574	1
MSTRG.19582.2	1431	0.42756	5.083190918	1
MSTRG.19582.4	1477	0.42294	5.066467285	1
MSTRG.19582.5	1447	0.42294	5.109436035	1
MSTRG.19582.6	1388	0.44132	5.229675293	1
MSTRG.19582.3	1474	0.42756	5.074768066	1
MSTRG.19582.7	1428	0.4137	5.09197998	1
MSTRG.19581.1	45	0.37687	6.798034668	1
MSTRG.19583.1	460	0.38761	8.191101074	1
MSTRG.19590.1	241	0.4075	8.308410645	1
MSTRG.19590.2	33	0.40854	8.496398926	1
MSTRG.19591.1	97	0.4031	11.80072021	1
MSTRG.19591.2	97	0.35695	11.80072021	1
MSTRG.19593.1	192	0.37258	7.554626465	1
MSTRG.19596.4	1040	0.46995	5.203918457	1
MSTRG.19598.3	3998	0.41403	9.232849121	1
MSTRG.19598.5	3710	0.43447	9.086364746	1
MSTRG.19598.6	2381	0.40306	6.273376465	1
MSTRG.19600.1	301	0.40411	4.369445801	1
MSTRG.19602.1	1322	0.45241	9.06072998	1
MSTRG.19603.1	122	0.41595	8.683044434	1
MSTRG.19602.3	1285	0.45408	8.981872559	1
MSTRG.19602.4	1202	0.44435	9.119567871	1
MSTRG.19602.5	758	0.3202	8.777160645	1
MSTRG.19602.6	446	0.47141	9.137878418	1
MSTRG.19604.6	483	0.3582	8.850280762	1
MSTRG.19604.4	483	0.3582	8.850280762	1
MSTRG.19604.8	483	0.3351	8.850280762	1
MSTRG.19622.1	105	0.35504	8.768859863	1

MSTRG.19624.7	1001	0.4761	5.264831543	1
MSTRG.19624.9	1002	0.4761	5.264831543	1
MSTRG.19616.2	754	0.27674	5.338317871	1
MSTRG.19614.1	637	0.45522	5.82635498	1
MSTRG.19614.2	595	0.44573	5.840515137	1
MSTRG.19629.1	606	0.33569	9.367980957	1
MSTRG.19628.1	20	0.38255	9.34967041	1
MSTRG.19628.2	76	0.35221	8.302307129	1
MSTRG.19638.4	1525	0.4515	5.458557129	1
MSTRG.19638.2	1544	0.45512	5.464294434	1
MSTRG.19638.1	1525	0.44601	5.458557129	1
MSTRG.19639.1	98	0.33906	10.0446167	1
MSTRG.19640.1	189	0.32905	10.96246338	1
MSTRG.19642.5	227	0.33125	5.360534668	1
MSTRG.19642.6	274	0.33125	5.20526123	1
MSTRG.19642.7	274	0.35175	5.20526123	1
MSTRG.19643.1	1490	0.3632	5.205505371	1
MSTRG.19648.1	516	0.34707	5.592712402	1
MSTRG.19649.3	364	0.38884	9.647888184	1
MSTRG.19650.1	68	0.37325	9.817565918	-1
MSTRG.19671.9	576	0.43128	5.243469238	1
MSTRG.19666.1	424	0.33725	6.867858887	1
MSTRG.19672.2	288	0.43012	6.125305176	1
MSTRG.19672.3	109	0.39892	10.25506592	-1
MSTRG.19672.4	109	0.39892	10.25506592	-1
MSTRG.19674.1	331	0.42791	8.960632324	1
MSTRG.19675.1	415	0.46535	5.120788574	1
MSTRG.19676.2	471	0.43668	5.928527832	1
MSTRG.19678.5	1221	0.45678	9.190368652	1
MSTRG.19678.8	408	0.41905	6.704528809	1
MSTRG.19681.1	104	0.29594	9.098327637	1
MSTRG.19682.1	83	0.37438	8.041931152	1
MSTRG.19683.1	196	0.4777	9.265075684	-1
MSTRG.19684.1	797	0.46357	7.973571777	1
MSTRG.19684.2	797	0.4614	7.973571777	1
MSTRG.19701.1	45	0.4376	5.60760498	1
MSTRG.19707.2	441	0.44019	4.630065918	1
MSTRG.19708.1	121	0.39818	10.42852783	1
MSTRG.19707.3	104	0.27988	6.707946777	1
MSTRG.19713.2	238	0.4807	6.248718262	1
MSTRG.19718.1	65	0.36152	5.464050293	1
MSTRG.19720.3	127	0.30303	8.462585449	1
MSTRG.19721.1	92	0.28529	8.633361816	1
MSTRG.19720.4	127	0.30221	8.462585449	1
MSTRG.19720.5	93	0.29883	8.084411621	1
MSTRG.19722.1	807	0.41449	5.778625488	1
MSTRG.19723.1	503	0.33863	9.166931152	1
MSTRG.19723.4	139	0.28385	9.823547363	1
MSTRG.19723.3	515	0.42437	9.31439209	1
MSTRG.19723.5	338	0.43415	6.920593262	1
MSTRG.19723.6	338	0.42664	6.920593262	1
MSTRG.19733.1	712	0.40329	8.871032715	1
MSTRG.19743.1	23	0.3726	10.69390869	1
MSTRG.19744.2	59	0.37002	11.45855713	1

MSTRG.19744.4	45	0.39295	8.255065918	1
MSTRG.19744.5	107	0.32969	6.255554199	1
MSTRG.19744.6	42	0.30268	7.769226074	1
MSTRG.19744.7	37	0.38801	9.29888916	-1
MSTRG.19744.8	133	0.35288	10.105896	1
MSTRG.19744.9	133	0.35108	10.105896	1
MSTRG.19744.10	144	0.39442	10.10137939	1
MSTRG.19744.11	34	0.41712	11.00006104	1
MSTRG.19744.12	50	0.37344	6.705993652	-1
MSTRG.19744.13	33	0.4204	9.968933105	1
MSTRG.19744.14	37	0.43432	9.890808105	1
MSTRG.19744.15	70	0.35191	10.98345947	1
MSTRG.19744.16	29	0.33755	10.66668701	-1
MSTRG.19744.17	32	0.33417	8.88482666	-1
MSTRG.19744.18	29	0.40137	9.968933105	-1
MSTRG.19744.19	28	0.35491	9.300231934	-1
MSTRG.19744.20	60	0.32134	7.812561035	-1
MSTRG.19744.21	30	0.34765	9.300598145	-1
MSTRG.19744.22	31	0.36623	10.66668701	-1
MSTRG.19744.23	37	0.38645	10.66668701	-1
MSTRG.19744.24	32	0.35319	9.300598145	-1
MSTRG.19744.25	32	0.38459	8.492858887	-1
MSTRG.19744.26	18	0.37092	11.53900146	1
MSTRG.19744.27	19	0.3863	9.976501465	1
MSTRG.19744.28	30	0.44986	8.211242676	-1
MSTRG.19744.29	28	0.4132	9.690734863	-1
MSTRG.19744.30	27	0.36664	9.522888184	-1
MSTRG.19744.31	26	0.36727	9.778991699	-1
MSTRG.19744.32	25	0.38987	9.489196777	-1
MSTRG.19739.1	89	0.43314	9.827941895	-1
MSTRG.19751.1	129	0.27794	10.65106201	1
MSTRG.19752.1	108	0.31421	5.264709473	1
MSTRG.19752.2	92	0.35626	5.966369629	1
MSTRG.19751.2	117	0.42206	5.565246582	1
MSTRG.19751.3	53	0.33303	11.32867432	1
MSTRG.19755.1	701	0.43785	6.294128418	1
MSTRG.19755.2	701	0.46267	6.294128418	1
MSTRG.19755.3	701	0.44441	6.294128418	1
MSTRG.19755.4	663	0.45597	6.168273926	1
MSTRG.19755.5	663	0.44934	6.168273926	1
MSTRG.19761.1	85	0.31177	9.303649902	1
MSTRG.19765.1	146	0.35819	10.40228271	1
MSTRG.19766.1	105	0.29072	9.023986816	1
MSTRG.19767.3	315	0.47294	9.509338379	1
MSTRG.19768.1	201	0.27794	9.971374512	1
MSTRG.19757.2	185	0.42718	5.230041504	1
MSTRG.19774.1	1038	0.47766	4.699279785	1
MSTRG.19779.2	141	0.30415	9.388000488	1
MSTRG.19784.2	333	0.42603	8.26348877	1
MSTRG.19785.3	61	0.41079	4.999450684	1
MSTRG.19788.1	137	0.28698	6.129211426	1
MSTRG.19791.1	115	0.31883	6.570495605	-1
MSTRG.19803.2	860	0.40401	6.083435059	1
MSTRG.19803.6	702	0.45441	8.070983887	1

MSTRG.19803.7	783	0.46071	6.610900879	1
MSTRG.19803.5	860	0.45598	6.083435059	1
MSTRG.19804.1	63	0.38874	9.099060059	-1
MSTRG.19805.1	285	0.30534	8.938537598	1
MSTRG.19806.1	96	0.37867	10.2901001	1
MSTRG.19807.3	352	0.45139	8.547668457	-1
MSTRG.19809.1	30	0.42025	5.971618652	-1
MSTRG.19811.1	114	0.43463	8.487487793	1
MSTRG.19821.1	288	0.4487	8.829406738	-1
MSTRG.19822.1	46	0.40193	10.0602417	1
MSTRG.19821.2	62	0.43835	4.164733887	1
MSTRG.19821.3	77	0.32646	6.791809082	1
MSTRG.19821.4	62	0.31561	4.164733887	1
MSTRG.19821.5	141	0.2895	4.583068848	1
MSTRG.19821.7	141	0.2895	4.583068848	1
MSTRG.19821.6	141	0.30475	4.583068848	1
MSTRG.19821.8	62	0.36377	4.164733887	1
MSTRG.19821.9	67	0.31003	4.787658691	1
MSTRG.19825.1	262	0.44643	6.502502441	1
MSTRG.19827.1	121	0.41188	9.660827637	1
MSTRG.19831.2	100	0.29607	9.885314941	1
MSTRG.19840.1	68	0.34229	9.415466309	1
MSTRG.19841.1	2984	0.44704	6.254333496	1
MSTRG.19848.2	388	0.3802	10.02923584	1
MSTRG.19856.8	656	0.46562	8.493103027	-1
MSTRG.19856.9	656	0.46562	8.493103027	-1
MSTRG.19856.11	673	0.31416	6.779968262	1
MSTRG.19857.2	329	0.43672	9.982727051	-1
MSTRG.19861.2	498	0.4364	6.062316895	1
MSTRG.19863.3	478	0.44347	7.259094238	1
MSTRG.19863.2	487	0.43651	6.832824707	1
MSTRG.19863.4	487	0.4087	6.832824707	1
MSTRG.19868.1	79	0.43787	8.656799316	1
MSTRG.19868.2	164	0.44919	7.159240723	1
MSTRG.19879.1	27	0.30561	10.7366333	1
MSTRG.19884.1	88	0.29595	9.237243652	1
MSTRG.19884.2	37	0.41247	9.301696777	1
MSTRG.19884.3	68	0.30564	9.880310059	1
MSTRG.19884.4	60	0.30289	9.680847168	1
MSTRG.19885.1	161	0.41602	9.546081543	1
MSTRG.19888.1	42	0.3484	9.492736816	1
MSTRG.19893.3	485	0.38993	6.211730957	1
MSTRG.19906.4	751	0.44791	6.149230957	1
MSTRG.19907.1	42	0.29428	8.858703613	1
MSTRG.19908.2	191	0.42366	8.40826416	1
MSTRG.19909.5	255	0.39793	7.609436035	1
MSTRG.19909.7	251	0.36219	8.101623535	1
MSTRG.19909.6	250	0.39903	8.66973877	1
MSTRG.19904.1	298	0.33469	6.390075684	1
MSTRG.19904.2	309	0.29635	6.657775879	1
MSTRG.19904.3	200	0.35788	5.203063965	1
MSTRG.19905.1	432	0.46904	6.125183105	1
MSTRG.19905.2	147	0.38924	8.967712402	1
MSTRG.19917.1	405	0.34071	8.25604248	1

MSTRG.19918.3	327	0.29527	6.599182129	1
MSTRG.19912.3	121	0.42985	3.468933105	1
MSTRG.19912.4	150	0.42943	3.667297363	1
MSTRG.19913.2	258	0.34503	6.539367676	1
MSTRG.19914.2	1264	0.46564	6.088928223	1
MSTRG.19923.1	26	0.39425	6.50982666	-1
MSTRG.19924.1	98	0.31878	8.933288574	-1
MSTRG.19924.2	53	0.36769	10.83428955	1
MSTRG.19925.3	1444	0.47031	5.88470459	1
MSTRG.19925.5	727	0.44683	6.063415527	1
MSTRG.19926.5	160	0.30226	5.627746582	1
MSTRG.19926.4	291	0.46936	6.664855957	1
MSTRG.19926.3	362	0.38205	9.075622559	1
MSTRG.19930.1	101	0.30235	9.555847168	1
MSTRG.19931.1	18	0.29764	12.00848389	1
MSTRG.19937.1	125	0.37334	9.454528809	1
MSTRG.19938.1	79	0.32995	10.46636963	1
MSTRG.19939.4	100	0.32255	6.026794434	1
MSTRG.19941.1	113	0.46782	5.157287598	1
MSTRG.19951.1	84	0.3901	6.405456543	1
MSTRG.19954.2	325	0.45553	9.065734863	1
MSTRG.19958.1	135	0.31982	6.900085449	1
MSTRG.19959.1	2345	0.44745	6.655700684	1
MSTRG.19959.3	2345	0.45954	6.655700684	1
MSTRG.19959.5	2368	0.45954	6.624938965	1
MSTRG.19959.2	2349	0.46774	6.422302246	1
MSTRG.19959.8	1422	0.4022	8.043518066	1
MSTRG.19959.9	1223	0.39549	6.304382324	1
MSTRG.19959.10	1426	0.40301	6.99395752	1
MSTRG.19959.11	1962	0.44745	6.786682129	1
MSTRG.19959.12	1557	0.42351	5.921813965	1
MSTRG.19959.13	1741	0.45006	7.308654785	1
MSTRG.19942.1	622	0.37782	6.037414551	1
MSTRG.19945.1	53	0.29988	6.809143066	1
MSTRG.19947.1	66	0.39558	9.50567627	1
MSTRG.19946.2	486	0.31	9.593933105	1
MSTRG.19967.1	352	0.27911	6.304748535	1
MSTRG.19967.5	352	0.33898	6.304748535	1
MSTRG.19967.4	128	0.31437	9.253234863	1
MSTRG.19967.8	78	0.36981	11.51898193	1
MSTRG.19965.2	523	0.40345	9.014953613	1
MSTRG.19978.1	526	0.44228	8.168640137	1
MSTRG.19978.3	568	0.38641	9.501037598	1
MSTRG.19978.2	580	0.444	6.929260254	1
MSTRG.19978.4	568	0.40399	9.501037598	1
MSTRG.19978.5	568	0.40027	9.501037598	1
MSTRG.19979.1	130	0.31702	11.10870361	1
MSTRG.19980.1	420	0.47451	9.215881348	1
MSTRG.19983.1	111	0.2912	10.03521729	1
MSTRG.19985.1	133	0.28415	6.73626709	1
MSTRG.19981.1	57	0.41641	8.035339355	1
MSTRG.19990.1	149	0.28387	8.765808105	1
MSTRG.19990.2	125	0.27883	8.838684082	1
MSTRG.20000.1	138	0.32499	9.622131348	1

MSTRG.20003.1	104	0.30091	7.010070801	1
MSTRG.20006.1	50	0.39242	9.782165527	1
MSTRG.20008.1	129	0.44412	8.070129395	1
MSTRG.20013.4	1942	0.42897	8.52935791	1
MSTRG.20013.5	195	0.30608	11.05535889	1
MSTRG.20015.3	301	0.32905	7.706481934	1
MSTRG.20015.5	386	0.38246	8.919372559	1
MSTRG.20015.6	167	0.32642	8.118347168	1
MSTRG.20018.1	351	0.43158	8.505187988	1
MSTRG.20018.2	367	0.39642	8.286682129	1
MSTRG.20018.3	267	0.40138	8.108947754	1
MSTRG.20027.1	407	0.39468	8.820617676	1
MSTRG.20033.1	68	0.34071	9.878723145	1
MSTRG.20037.1	78	0.41968	9.876281738	1
MSTRG.20038.4	92	0.28496	9.507263184	1
MSTRG.20047.1	78	0.33345	11.49432373	1
MSTRG.20056.1	260	0.45928	8.418518066	1
MSTRG.20056.4	260	0.41559	8.418518066	1
MSTRG.20057.3	168	0.39335	6.272277832	1
MSTRG.20057.4	168	0.36707	6.272277832	1
MSTRG.20059.1	1813	0.4801	9.168518066	1
MSTRG.20059.5	258	0.37903	8.213928223	1
MSTRG.20061.3	55	0.38505	8.801696777	1
MSTRG.20061.2	59	0.36364	8.179992676	1
MSTRG.20063.1	79	0.32813	9.135681152	1
MSTRG.20066.1	95	0.31833	5.756896973	1
MSTRG.20069.4	130	0.31489	8.527160645	1
MSTRG.20074.2	447	0.38852	9.180603027	1
MSTRG.20074.4	430	0.39317	9.188171387	1
MSTRG.20075.1	118	0.35425	8.550964355	1
MSTRG.20079.1	667	0.43658	5.17010498	1
MSTRG.20086.5	1590	0.43034	6.370788574	1
MSTRG.20086.6	1623	0.44304	6.412414551	1
MSTRG.20086.9	1623	0.44707	6.412414551	1
MSTRG.20087.1	119	0.34952	6.063171387	1
MSTRG.20086.10	1590	0.45083	6.370788574	1
MSTRG.20088.2	307	0.48146	5.24041748	1
MSTRG.20090.1	68	0.34301	9.494567871	-1
MSTRG.20092.2	567	0.41948	8.503845215	1
MSTRG.20094.2	815	0.4338	6.320861816	1
MSTRG.20097.2	356	0.46581	4.613464355	1
MSTRG.20098.1	162	0.2912	8.83013916	1
MSTRG.20107.1	79	0.40523	6.79510498	1
MSTRG.20109.1	143	0.28091	10.11834717	1
MSTRG.20110.1	300	0.37645	6.86517334	1
MSTRG.20110.2	213	0.31286	9.299133301	1
MSTRG.20110.3	213	0.31684	9.299133301	1
MSTRG.20114.1	441	0.4815	5.804504395	1
MSTRG.20114.2	353	0.43818	6.259094238	1
MSTRG.20114.4	243	0.47485	5.592102051	1
MSTRG.20115.1	70	0.31958	5.788757324	1
MSTRG.20112.1	22	0.43559	4.298156738	1
MSTRG.20118.1	97	0.46719	7.706481934	-1
MSTRG.20117.3	349	0.35881	5.672546387	1

MSTRG.20121.2	58	0.33153	10.09735107	1
MSTRG.20121.1	74	0.33576	9.416564941	1
MSTRG.20119.1	80	0.39836	11.36004639	1
MSTRG.20120.1	553	0.38581	8.49017334	1
MSTRG.20126.2	310	0.33311	4.794250488	1
MSTRG.20126.4	78	0.37165	11.88897705	1
MSTRG.20126.3	142	0.35648	4.597351074	1
MSTRG.20128.2	75	0.42097	6.530212402	-1
MSTRG.20132.3	242	0.45273	4.546203613	1
MSTRG.20132.4	242	0.45522	4.546203613	1
MSTRG.20135.1	640	0.4492	6.696594238	1
MSTRG.20148.1	147	0.36522	7.782165527	1
MSTRG.20158.1	22	0.33394	3.563415527	-1
MSTRG.20158.2	23	0.31953	3.563415527	1
MSTRG.20158.5	398	0.40643	8.945983887	1
MSTRG.20158.6	128	0.34059	9.89251709	1
MSTRG.20162.4	1198	0.46064	5.833679199	1
MSTRG.20163.1	89	0.36707	6.38494873	-1
MSTRG.20164.1	60	0.29966	6.519958496	1
MSTRG.20168.1	149	0.27251	8.504943848	1
MSTRG.20154.2	238	0.35127	9.601989746	1
MSTRG.20155.1	71	0.30191	9.585021973	1
MSTRG.20156.1	183	0.28883	9.329650879	1
MSTRG.20157.1	117	0.37438	5.104187012	1
MSTRG.20154.3	177	0.35151	9.748962402	1
MSTRG.20172.3	253	0.35925	8.938415527	1
MSTRG.20173.1	79	0.32575	6.793273926	1
MSTRG.20177.2	400	0.45759	5.680725098	1
MSTRG.20179.1	79	0.34052	11.87579346	1
MSTRG.20180.1	78	0.33799	6.026672363	1
MSTRG.20186.1	511	0.42159	7.57244873	1
MSTRG.20188.1	86	0.35108	6.385437012	1
MSTRG.20189.2	65	0.33008	7.738830566	1
MSTRG.20189.1	49	0.38252	6.365905762	1
MSTRG.20190.1	39	0.37553	9.862609863	1
MSTRG.20193.2	139	0.27089	9.47467041	1
MSTRG.20193.3	143	0.28338	9.814025879	1
MSTRG.20198.2	591	0.3928	8.832458496	1
MSTRG.20198.1	599	0.3904	8.832458496	1
MSTRG.20199.1	9	0.34813	11.69903564	1
MSTRG.20200.3	619	0.45115	5.047180176	1
MSTRG.20202.1	85	0.38487	11.24835205	1
MSTRG.20203.2	328	0.4105	9.834899902	1
MSTRG.20210.4	682	0.43992	9.521789551	1
MSTRG.20210.3	681	0.48263	9.497253418	1
MSTRG.20210.5	263	0.30419	8.061462402	1
MSTRG.20210.6	103	0.2921	9.342346191	1
MSTRG.20212.1	88	0.30463	9.85345459	1
MSTRG.20212.2	93	0.27289	6.681091309	1
MSTRG.20212.3	78	0.3145	9.741760254	1
MSTRG.20212.4	93	0.28385	6.681091309	1
MSTRG.20210.7	54	0.39965	6.066101074	1
MSTRG.20207.2	474	0.45106	5.347839355	1
MSTRG.20207.3	515	0.43741	5.670837402	-1

MSTRG.20207.4	474	0.46513	5.347839355	1
MSTRG.20208.3	808	0.43819	8.546691895	1
MSTRG.20213.5	124	0.27674	11.10101318	1
MSTRG.20213.6	124	0.27274	11.10101318	1
MSTRG.20214.1	94	0.31942	7.998352051	1
MSTRG.20215.1	78	0.31971	11.87835693	1
MSTRG.20216.1	91	0.32962	4.855285645	1
MSTRG.20218.4	517	0.37676	4.96661377	1
MSTRG.20218.5	480	0.4203	4.922180176	1
MSTRG.20220.1	142	0.32384	10.82818604	1
MSTRG.20225.1	65	0.3877	8.888244629	1
MSTRG.20225.2	25	0.41322	8.020935059	1
MSTRG.20226.2	180	0.42613	9.468933105	1
MSTRG.20226.3	243	0.41227	9.526184082	1
MSTRG.20226.4	177	0.44212	9.72442627	1
MSTRG.20227.6	1895	0.47047	8.040588379	1
MSTRG.20227.5	2031	0.46277	8.036804199	1
MSTRG.20227.3	2032	0.46791	8.036804199	1
MSTRG.20227.4	2031	0.46791	8.036804199	1
MSTRG.20227.8	2031	0.45785	8.036804199	1
MSTRG.20227.10	2032	0.46277	8.036804199	1
MSTRG.20227.9	2032	0.45523	8.036804199	1
MSTRG.20227.11	2031	0.45969	8.036804199	1
MSTRG.20227.12	2031	0.47129	8.036804199	1
MSTRG.20228.1	75	0.4057	8.22076416	1
MSTRG.20229.1	1761	0.38038	5.366760254	1
MSTRG.20222.3	112	0.36242	9.144958496	1
MSTRG.20222.4	229	0.47463	5.988464355	1
MSTRG.20223.1	520	0.31822	8.07611084	1
MSTRG.20223.2	520	0.35222	8.07611084	1
MSTRG.20241.1	1583	0.45114	5.931335449	1
MSTRG.20241.2	1569	0.45934	5.913269043	1
MSTRG.20241.3	1583	0.44835	5.931335449	1
MSTRG.20241.5	1569	0.46299	5.913269043	1
MSTRG.20241.4	1587	0.46705	5.931335449	1
MSTRG.20241.6	1587	0.46857	5.931335449	1
MSTRG.20231.1	242	0.31203	10.5055542	1
MSTRG.20231.2	117	0.29366	11.43206787	1
MSTRG.20232.1	55	0.35617	10.52874756	1
MSTRG.20233.1	329	0.43664	5.339172363	1
MSTRG.20242.4	486	0.29076	8.702697754	1
MSTRG.20242.1	486	0.31634	8.702697754	1
MSTRG.20242.2	482	0.31634	8.709289551	1
MSTRG.20242.3	486	0.31386	8.702697754	1
MSTRG.20242.5	482	0.27943	8.709289551	1
MSTRG.20242.8	486	0.27867	8.702697754	1
MSTRG.20244.2	499	0.43286	5.552429199	1
MSTRG.20245.1	248	0.34829	6.10723877	1
MSTRG.20246.1	259	0.28567	8.346008301	1
MSTRG.20246.3	314	0.36817	7.977111816	1
MSTRG.20249.4	803	0.34261	9.281555176	1
MSTRG.20250.2	625	0.35165	4.550598145	1
MSTRG.20250.4	616	0.46071	4.533630371	1
MSTRG.20250.7	597	0.36047	4.551330566	1

MSTRG.20250.8	616	0.43941	4.533630371	1
MSTRG.20251.1	55	0.31526	8.528137207	-1
MSTRG.20247.1	1283	0.40408	5.369567871	1
MSTRG.20247.2	1282	0.40408	5.369567871	1
MSTRG.20248.1	59	0.29871	6.208312988	1
MSTRG.20257.1	309	0.45917	9.219177246	1
MSTRG.20257.2	119	0.42641	5.075378418	-1
MSTRG.20269.2	309	0.28035	9.375549316	1
MSTRG.20270.1	62	0.343	7.740905762	1
MSTRG.20270.2	62	0.34459	7.740905762	1
MSTRG.20270.3	62	0.33802	7.740905762	1
MSTRG.20271.2	709	0.45538	6.448059082	1
MSTRG.20272.1	397	0.40982	5.751647949	1
MSTRG.20274.1	70	0.43047	11.28448486	1
MSTRG.20274.5	489	0.43903	8.955749512	1
MSTRG.20274.4	489	0.45264	8.955749512	1
MSTRG.20274.6	79	0.32762	10.00860596	1
MSTRG.20277.1	90	0.36769	5.891784668	1
MSTRG.20278.2	148	0.38921	8.764343262	1
MSTRG.20279.1	468	0.46048	6.279602051	1
MSTRG.20281.2	425	0.46184	6.197570801	1
MSTRG.20281.4	425	0.44573	6.197570801	1
MSTRG.20287.3	714	0.41894	9.147277832	1
MSTRG.20288.2	177	0.37862	9.924133301	1
MSTRG.20288.3	178	0.39071	9.893371582	1
MSTRG.20290.1	727	0.33144	4.969055176	1
MSTRG.20291.3	44	0.40943	9.09942627	1
MSTRG.20292.1	932	0.41532	6.411682129	1
MSTRG.20292.2	938	0.41532	6.340759277	1
MSTRG.20292.3	932	0.41656	6.411682129	1
MSTRG.20292.4	932	0.41532	6.411682129	1
MSTRG.20292.7	831	0.37643	6.710998535	1
MSTRG.20293.1	175	0.42213	10.50933838	1
MSTRG.20298.3	256	0.33323	7.969421387	1
MSTRG.20298.4	354	0.32858	8.566223145	1
MSTRG.20298.5	458	0.35156	6.348083496	1
MSTRG.20298.6	457	0.35156	6.348083496	1
MSTRG.20298.7	354	0.33323	8.566223145	1
MSTRG.20299.1	183	0.3216	10.77044678	1
MSTRG.20315.2	1460	0.43041	6.317565918	1
MSTRG.20315.4	681	0.46059	6.323547363	1
MSTRG.20315.6	614	0.41598	7.085510254	1
MSTRG.20315.5	681	0.46166	6.323547363	1
MSTRG.20315.7	87	0.31853	9.941833496	1
MSTRG.20315.11	696	0.38485	9.407409668	1
MSTRG.20315.14	567	0.30846	9.651062012	1
MSTRG.20315.16	547	0.34996	9.537902832	1
MSTRG.20318.1	38	0.38727	8.489807129	1
MSTRG.20308.1	212	0.39511	11.67718506	1
MSTRG.20310.1	258	0.36269	5.653991699	1
MSTRG.20313.1	168	0.46977	5.674865723	1
MSTRG.20313.2	235	0.42951	5.773742676	1
MSTRG.20313.3	375	0.49522	5.046936035	1
MSTRG.20313.4	55	0.42292	4.862976074	1

MSTRG.20313.5	168	0.4961	5.674865723	1
MSTRG.20334.1	861	0.37975	5.049255371	1
MSTRG.20335.3	142	0.497	4.374206543	1
MSTRG.20338.1	76	0.31089	6.88458252	1
MSTRG.20338.2	74	0.36711	10.07098389	1
MSTRG.20339.3	87	0.35019	6.174621582	1
MSTRG.20339.4	87	0.3407	6.174621582	1
MSTRG.20345.2	92	0.28247	6.825134277	1
MSTRG.20346.2	237	0.39156	8.291809082	1
MSTRG.20347.4	146	0.45047	8.38885498	1
MSTRG.20321.1	41	0.38487	9.148864746	1
MSTRG.20328.4	85	0.40879	4.545959473	1
MSTRG.20328.5	154	0.40879	5.315490723	1
MSTRG.20328.6	85	0.40879	4.545959473	1
MSTRG.20330.1	24	0.31455	9.34967041	1
MSTRG.20332.6	70	0.34554	7.659851074	1
MSTRG.20354.1	48	0.30368	9.381896973	1
MSTRG.20361.1	8	0.34377	5.272766113	1
MSTRG.20362.1	5	0.26606	5.272766113	1
MSTRG.20362.2	27	0.27884	6.682189941	1
MSTRG.20362.3	26	0.33102	5.816223145	1
MSTRG.20366.1	105	0.2782	8.763244629	1
MSTRG.20367.3	94	0.39759	10.00860596	1
MSTRG.20367.4	94	0.39573	10.00860596	1
MSTRG.20367.6	250	0.37223	9.277648926	1
MSTRG.20367.5	182	0.37976	9.585632324	1
MSTRG.20376.4	196	0.43319	10.15289307	1
MSTRG.20376.5	196	0.39385	10.15289307	1
MSTRG.20376.7	196	0.4425	10.15289307	1
MSTRG.20376.8	196	0.3969	10.15289307	1
MSTRG.20376.6	196	0.27437	10.15289307	1
MSTRG.20376.9	92	0.29526	9.239074707	1
MSTRG.20376.10	196	0.27379	10.15289307	1
MSTRG.20378.2	227	0.44312	7.012390137	1
MSTRG.20382.3	1195	0.43472	5.913024902	1
MSTRG.20382.5	1207	0.43472	6.067932129	1
MSTRG.20382.8	1163	0.44883	5.958312988	-1
MSTRG.20387.2	816	0.38938	9.084777832	1
MSTRG.20387.8	5	0.36354	8.500061035	1
MSTRG.20387.7	962	0.38046	6.592712402	1
MSTRG.20388.1	62	0.34219	9.913513184	1
MSTRG.20393.1	62	0.33695	10.28094482	1
MSTRG.20394.1	624	0.44435	6.069519043	1
MSTRG.20395.1	356	0.32513	8.052185059	1
MSTRG.20395.2	356	0.32513	8.052185059	1
MSTRG.20395.3	356	0.35383	8.052185059	1
MSTRG.20395.4	282	0.33063	7.164367676	1
MSTRG.20395.14	282	0.4461	7.164367676	1
MSTRG.20395.10	356	0.41461	8.052185059	1
MSTRG.20395.11	356	0.43006	8.052185059	1
MSTRG.20395.7	356	0.43096	8.052185059	1
MSTRG.20395.8	356	0.38163	8.052185059	1
MSTRG.20395.12	282	0.41038	7.164367676	1
MSTRG.20395.13	356	0.40013	8.052185059	1

MSTRG.20395.9	356	0.44242	8.052185059	1
MSTRG.20396.1	109	0.36839	5.323425293	1
MSTRG.20397.1	326	0.44329	5.064025879	1
MSTRG.20396.2	113	0.31098	6.704040527	1
MSTRG.20396.3	113	0.37478	6.704040527	1
MSTRG.20396.4	113	0.33221	6.704040527	1
MSTRG.20390.2	698	0.38079	4.495788574	1
MSTRG.20392.1	1634	0.45921	6.858947754	1
MSTRG.20400.7	127	0.35797	9.051208496	1
MSTRG.20401.1	84	0.35047	8.313293457	1
MSTRG.20402.1	58	0.35921	9.78326416	1
MSTRG.20403.1	125	0.38044	8.817810059	1
MSTRG.20407.1	121	0.34201	11.16351318	1
MSTRG.20414.2	797	0.4509	7.276306152	1
MSTRG.20415.1	106	0.28283	8.78704834	1
MSTRG.20416.1	299	0.35593	8.833312988	1
MSTRG.20421.1	432	0.44265	6.930725098	1
MSTRG.20421.2	432	0.44722	6.930725098	1
MSTRG.20421.3	432	0.38289	6.930725098	1
MSTRG.20424.1	34	0.33694	7.602478027	1
MSTRG.20426.1	514	0.38253	4.940368652	1
MSTRG.20426.2	514	0.39737	4.940368652	1
MSTRG.20429.2	200	0.435	5.524230957	1
MSTRG.20430.1	44	0.37863	11.73394775	-1
MSTRG.20433.4	914	0.4529	5.813049316	1
MSTRG.20434.1	89	0.28883	9.689147949	1
MSTRG.20433.7	140	0.39071	12.17779541	1
MSTRG.20431.2	429	0.37405	8.134460449	1
MSTRG.20431.3	319	0.44936	6.110534668	1
MSTRG.20431.4	133	0.30244	9.863464355	1
MSTRG.20438.4	219	0.29092	9.72052002	1
MSTRG.20439.1	307	0.29223	7.61517334	1
MSTRG.20439.2	307	0.36309	7.61517334	1
MSTRG.20439.3	159	0.36554	9.040710449	1
MSTRG.20440.1	205	0.27274	10.69366455	1
MSTRG.20440.2	205	0.27678	10.69366455	1
MSTRG.20446.2	388	0.40803	8.83795166	1
MSTRG.20451.2	517	0.29755	8.364562988	1
MSTRG.20452.1	28	0.43786	11.52459717	1
MSTRG.20453.1	552	0.48337	9.15423584	1
MSTRG.20455.2	193	0.46174	8.180603027	-1
MSTRG.20457.2	1049	0.4762	6.741394043	1
MSTRG.20457.4	810	0.45283	6.23236084	1
MSTRG.20457.7	810	0.46015	6.23236084	1
MSTRG.20457.6	810	0.44044	6.23236084	1
MSTRG.20457.8	351	0.40454	6.376403809	1
MSTRG.20469.1	250	0.42377	4.557800293	1
MSTRG.20469.2	283	0.43098	4.64666748	1
MSTRG.20471.1	83	0.28518	6.24005127	1
MSTRG.20471.2	98	0.28572	9.914367676	1
MSTRG.20473.2	135	0.37483	4.722106934	1
MSTRG.20474.1	58	0.43873	4.32598877	-1
MSTRG.20476.1	75	0.46202	7.781677246	1
MSTRG.20482.6	116	0.32413	6.229431152	1

MSTRG.20483.1	170	0.28334	8.910949707	1
MSTRG.20484.1	96	0.35545	6.818664551	1
MSTRG.20486.1	72	0.3307	7.702819824	1
MSTRG.20479.1	56	0.38915	8.737854004	-1
MSTRG.20489.1	78	0.40301	9.68951416	1
MSTRG.20504.2	123	0.36174	7.733093262	1
MSTRG.20505.1	538	0.44044	7.523864746	1
MSTRG.20506.1	205	0.4484	6.15435791	1
MSTRG.20512.1	78	0.28731	7.906555176	1
MSTRG.20512.2	61	0.37585	6.003112793	-1
MSTRG.20512.3	68	0.30521	4.457580566	1
MSTRG.20512.4	107	0.27587	8.960144043	1
MSTRG.20512.5	68	0.29919	4.457580566	1
MSTRG.20512.6	44	0.26381	10.27740479	1
MSTRG.20514.1	61	0.36826	4.87689209	1
MSTRG.20516.1	20	0.34385	9.782043457	-1
MSTRG.20526.1	75	0.33698	6.689758301	1
MSTRG.20528.1	337	0.34576	8.960876465	1
MSTRG.20547.1	74	0.32971	7.882873535	1
MSTRG.20552.1	46	0.41227	8.065002441	-1
MSTRG.20565.1	34	0.36178	9.05682373	1
MSTRG.20566.1	104	0.34606	8.440002441	1
MSTRG.20568.2	780	0.45607	6.658874512	1
MSTRG.20569.1	84	0.41014	10.29205322	1
MSTRG.20570.1	29	0.36341	10.7366333	1
MSTRG.20571.1	500	0.44686	8.723693848	1
MSTRG.20574.1	117	0.3819	11.57366943	1
MSTRG.20577.2	435	0.39616	6.643249512	1
MSTRG.20577.3	71	0.3333	7.90814209	1
MSTRG.20579.2	151	0.33514	10.47601318	1
MSTRG.20579.4	406	0.33698	7.774353027	1
MSTRG.20580.1	69	0.30638	10.10125732	1
MSTRG.20581.1	56	0.35496	9.099182129	1
MSTRG.20583.1	181	0.3	5.65838623	1
MSTRG.20584.1	29	0.30553	7.868469238	1
MSTRG.20593.2	240	0.29452	5.688415527	1
MSTRG.20593.3	259	0.39349	6.197937012	1
MSTRG.20608.7	524	0.45252	8.946350098	1
MSTRG.20608.6	804	0.39341	9.395324707	1
MSTRG.20609.1	212	0.28459	8.694641113	1
MSTRG.20610.1	204	0.30847	9.923522949	1
MSTRG.20608.8	632	0.31444	9.216247559	1
MSTRG.20611.1	67	0.32016	5.964538574	1
MSTRG.20612.1	63	0.31596	6.033752441	1
MSTRG.20608.9	371	0.36161	9.730895996	1
MSTRG.20603.1	98	0.31825	10.11541748	1
MSTRG.20603.2	98	0.34247	10.11541748	1
MSTRG.20603.3	235	0.31532	5.113342285	1
MSTRG.20613.1	177	0.41129	5.72454834	1
MSTRG.20613.2	177	0.43663	5.72454834	1
MSTRG.20614.1	62	0.38148	9.217834473	1
MSTRG.20616.1	88	0.36833	7.705383301	1
MSTRG.20618.2	330	0.37603	9.075744629	1
MSTRG.20620.2	7	0.32323	6.001647949	1

MSTRG.20619.4	763	0.48483	6.183166504	1
MSTRG.20621.1	141	0.2921	9.31829834	1
MSTRG.20628.2	524	0.41941	4.920715332	1
MSTRG.20629.1	33	0.45226	4.295837402	-1
MSTRG.20629.2	26	0.43922	12.30426025	1
MSTRG.20631.2	275	0.39192	9.944152832	1
MSTRG.20631.3	149	0.33906	9.369567871	1
MSTRG.20634.1	860	0.46336	6.393127441	1
MSTRG.20634.2	755	0.49724	6.178527832	1
MSTRG.20634.3	138	0.3056	8.91204834	1
MSTRG.20637.3	451	0.36389	5.711364746	1
MSTRG.20637.5	451	0.40765	5.711364746	1
MSTRG.20637.4	498	0.42864	5.768249512	1
MSTRG.20638.1	48	0.39423	10.05230713	1
MSTRG.20642.1	138	0.3363	6.868103027	1
MSTRG.20654.1	55	0.45679	6.808532715	-1
MSTRG.20654.2	4605	0.45229	6.225891113	1
MSTRG.20654.4	131	0.34561	9.636047363	1
MSTRG.20654.3	640	0.43859	7.779724121	1
MSTRG.20654.5	314	0.43128	9.307800293	1
MSTRG.20654.12	184	0.38791	9.657165527	1
MSTRG.20654.8	328	0.26107	9.333190918	1
MSTRG.20654.10	395	0.32053	6.325744629	1
MSTRG.20654.11	1593	0.45628	6.27166748	1
MSTRG.20654.9	2037	0.35925	6.395812988	1
MSTRG.20654.6	2799	0.38789	6.24041748	1
MSTRG.20654.7	2692	0.42048	6.186340332	1
MSTRG.20654.13	120	0.34358	10.55389404	1
MSTRG.20656.1	114	0.27289	7.047302246	1
MSTRG.20657.1	76	0.31525	7.813659668	1
MSTRG.20658.1	144	0.39852	10.17254639	1
MSTRG.20654.14	128	0.29257	5.770812988	1
MSTRG.20660.2	594	0.36612	9.326599121	1
MSTRG.20644.1	512	0.4033	7.076477051	1
MSTRG.20644.3	526	0.36221	7.727478027	1
MSTRG.20644.6	236	0.29615	8.430114746	1
MSTRG.20646.1	461	0.45141	8.089172363	1
MSTRG.20649.2	948	0.43692	8.222106934	1
MSTRG.20649.3	803	0.44549	7.670837402	1
MSTRG.20662.1	61	0.37436	10.61419678	1
MSTRG.20662.2	70	0.36702	10.04608154	1
MSTRG.20662.3	136	0.40288	11.26934814	1
MSTRG.20662.4	136	0.36698	11.26934814	1
MSTRG.20662.5	136	0.39357	11.26934814	1
MSTRG.20662.6	136	0.37691	11.26934814	1
MSTRG.20664.1	528	0.39212	5.077941895	1
MSTRG.20664.2	282	0.3481	4.863220215	1
MSTRG.20667.2	85	0.3164	9.424743652	1
MSTRG.20668.1	133	0.44329	5.546447754	1
MSTRG.20669.2	276	0.32284	9.32598877	1
MSTRG.20670.1	83	0.33266	6.25189209	1
MSTRG.20672.6	449	0.43033	4.513977051	1
MSTRG.20672.8	287	0.3417	4.460510254	1
MSTRG.20674.1	1569	0.38392	5.963806152	1

MSTRG.20674.3	1719	0.39438	6.097473145	1
MSTRG.20674.5	1615	0.4559	6.07635498	1
MSTRG.20674.6	65	0.36427	8.589782715	-1
MSTRG.20678.2	2273	0.47686	5.637268066	1
MSTRG.20678.3	166	0.45857	8.263122559	1
MSTRG.20678.4	1938	0.47758	5.934875488	1
MSTRG.20679.1	53	0.42178	9.430114746	1
MSTRG.20677.1	228	0.29305	8.920715332	1
MSTRG.20681.1	77	0.45513	9.596252441	-1
MSTRG.20682.1	86	0.39901	10.08966064	1
MSTRG.20682.2	97	0.42453	10.08966064	1
MSTRG.20688.2	82	0.35099	9.553649902	1
MSTRG.20705.1	153	0.37221	11.75811768	1
MSTRG.20705.2	153	0.38817	11.75811768	1
MSTRG.20710.3	91	0.34188	5.848449707	1
MSTRG.20710.5	164	0.30733	9.656433105	1
MSTRG.20711.1	62	0.36634	9.484069824	1
MSTRG.20713.1	115	0.35418	9.42401123	1
MSTRG.20708.2	233	0.46077	6.526550293	1
MSTRG.20709.1	138	0.39497	9.378356934	1
MSTRG.20714.1	231	0.33535	6.886047363	1
MSTRG.20725.3	208	0.27772	5.344787598	1
MSTRG.20725.5	176	0.36476	9.732971191	1
MSTRG.20725.4	418	0.39825	5.476989746	1
MSTRG.20725.6	418	0.39825	5.476989746	1
MSTRG.20725.7	25	0.42495	7.761657715	1
MSTRG.20725.8	418	0.39825	5.476989746	1
MSTRG.20725.9	116	0.34531	8.687927246	1
MSTRG.20725.10	129	0.30159	9.503601074	1
MSTRG.20725.11	418	0.35599	5.476989746	1
MSTRG.20725.12	129	0.33174	9.503601074	1
MSTRG.20725.14	153	0.34123	9.617126465	1
MSTRG.20726.1	62	0.34157	10.90802002	1
MSTRG.20727.1	114	0.32862	9.475646973	1
MSTRG.20728.1	101	0.29367	9.825134277	1
MSTRG.20730.12	68	0.30436	6.70135498	1
MSTRG.20730.6	1174	0.45157	6.79473877	1
MSTRG.20730.8	1426	0.44975	6.996887207	1
MSTRG.20730.11	1157	0.44713	6.989685059	1
MSTRG.20730.10	1549	0.46099	6.442077637	1
MSTRG.20730.7	1562	0.44285	7.482727051	1
MSTRG.20730.4	1534	0.44285	7.67755127	1
MSTRG.20730.5	1552	0.44285	7.672424316	1
MSTRG.20730.9	1192	0.44713	6.805603027	1
MSTRG.20730.13	310	0.35164	5.074401855	1
MSTRG.20730.14	259	0.35164	5.82598877	1
MSTRG.20718.1	54	0.34845	7.766174316	1
MSTRG.20720.2	91	0.42025	10.89581299	1
MSTRG.20734.1	96	0.32237	6.070861816	1
MSTRG.20740.1	303	0.33545	8.721252441	1
MSTRG.20742.2	312	0.47888	8.992248535	1
MSTRG.20742.3	377	0.46866	8.129943848	1
MSTRG.20742.4	288	0.46182	9.157897949	1
MSTRG.20743.1	510	0.45086	5.64642334	1

MSTRG.20746.1	98	0.30077	11.09698486	-1
MSTRG.20737.1	328	0.38809	6.046325684	1
MSTRG.20737.2	481	0.37476	5.33770752	1
MSTRG.20751.2	278	0.40482	5.423522949	1
MSTRG.20753.3	734	0.44905	5.262634277	1
MSTRG.20753.4	264	0.43179	4.439147949	1
MSTRG.20750.3	806	0.49005	4.973449707	1
MSTRG.20750.1	809	0.47758	4.937438965	1
MSTRG.20758.1	419	0.40795	7.031188965	1
MSTRG.20763.10	38	0.39717	9.019226074	1
MSTRG.20763.8	2775	0.44937	9.050964355	-1
MSTRG.20763.13	2277	0.42768	9.383361816	1
MSTRG.20774.1	150	0.30082	8.79083252	1
MSTRG.20775.4	566	0.46631	8.883605957	1
MSTRG.20775.6	498	0.48023	8.383850098	1
MSTRG.20775.7	858	0.34731	7.969177246	1
MSTRG.20775.8	498	0.32237	8.383850098	1
MSTRG.20775.9	105	0.28759	9.117858887	1
MSTRG.20776.1	116	0.30804	10.690979	1
MSTRG.20776.2	116	0.31209	10.690979	1
MSTRG.20777.1	86	0.28848	9.508728027	1
MSTRG.20778.1	153	0.33002	8.72454834	1
MSTRG.20780.1	69	0.34667	8.859802246	1
MSTRG.20781.1	189	0.30715	10.3394165	1
MSTRG.20782.1	159	0.35128	9.34161377	1
MSTRG.20783.1	82	0.30894	5.028991699	1
MSTRG.20781.2	162	0.27517	9.693664551	1
MSTRG.20784.1	164	0.33102	6.070861816	1
MSTRG.20785.1	172	0.33754	8.707580566	1
MSTRG.20786.1	188	0.32793	10.57025146	1
MSTRG.20775.10	186	0.28982	5.066833496	1
MSTRG.20787.1	206	0.27794	9.140075684	1
MSTRG.20787.2	206	0.27289	9.140075684	1
MSTRG.20792.1	94	0.28165	9.862365723	1
MSTRG.20793.1	120	0.2921	10.29486084	1
MSTRG.20794.1	205	0.31209	9.31829834	1
MSTRG.20800.1	262	0.33292	9.065856934	1
MSTRG.20800.2	209	0.45596	9.50970459	1
MSTRG.20801.1	52	0.33526	6.69732666	1
MSTRG.20797.1	51	0.33811	9.621032715	-1
MSTRG.20798.4	287	0.45304	8.81854248	1
MSTRG.20819.4	2796	0.37643	6.323913574	1
MSTRG.20819.8	3895	0.47649	6.184875488	-1
MSTRG.20822.1	55	0.33131	8.506896973	1
MSTRG.20816.5	341	0.37336	8.019226074	1
MSTRG.20816.3	381	0.38438	8.25579834	1
MSTRG.20816.4	282	0.38996	9.630187988	1
MSTRG.20816.2	381	0.41478	8.25579834	1
MSTRG.20823.3	661	0.37119	5.968200684	1
MSTRG.20823.4	704	0.42886	5.946716309	1
MSTRG.20823.2	757	0.44024	6.302429199	1
MSTRG.20823.1	757	0.4654	6.302429199	1
MSTRG.20817.2	316	0.40219	5.863830566	1
MSTRG.20817.1	634	0.42679	7.128112793	1

MSTRG.20828.4	586	0.4562	6.971008301	1
MSTRG.20828.5	142	0.29607	4.740539551	1
MSTRG.20829.1	655	0.46426	5.224060059	1
MSTRG.20829.2	655	0.46261	5.224060059	1
MSTRG.20829.5	510	0.40768	5.765319824	1
MSTRG.20830.4	1423	0.44444	11.53057861	1
MSTRG.20825.3	111	0.36397	5.475891113	1
MSTRG.20825.4	111	0.36769	5.475891113	1
MSTRG.20826.2	127	0.31065	9.252380371	1
MSTRG.20831.1	187	0.45481	6.052185059	1
MSTRG.20832.3	265	0.43445	7.059265137	1
MSTRG.20836.2	2323	0.49044	6.781066895	1
MSTRG.20836.3	2323	0.48784	6.781066895	1
MSTRG.20836.4	2560	0.47207	6.696960449	1
MSTRG.20840.1	374	0.42066	4.920349121	1
MSTRG.20845.4	1687	0.44621	5.783874512	1
MSTRG.20845.7	1687	0.38787	5.783874512	1
MSTRG.20845.6	1687	0.44621	5.783874512	1
MSTRG.20845.9	147	0.34287	9.431091309	1
MSTRG.20844.2	453	0.38452	5.270568848	1
MSTRG.20850.1	190	0.29772	4.93145752	1
MSTRG.20850.3	113	0.3152	10.56170654	-1
MSTRG.20852.4	839	0.44944	4.746887207	1
MSTRG.20854.1	173	0.39279	9.233581543	1
MSTRG.20854.2	181	0.36329	9.543029785	1
MSTRG.20866.1	25	0.38398	8.365661621	1
MSTRG.20867.2	501	0.44384	6.17388916	1
MSTRG.20873.1	489	0.47486	7.109802246	1
MSTRG.20873.2	475	0.47486	6.491882324	1
MSTRG.20873.3	434	0.44342	6.375061035	1
MSTRG.20873.4	434	0.44342	6.375061035	1
MSTRG.20875.3	77	0.30825	9.695007324	1
MSTRG.20875.2	1503	0.40858	7.449401855	1
MSTRG.20876.1	78	0.29772	7.565734863	1
MSTRG.20877.1	185	0.351	10.51885986	1
MSTRG.20878.1	103	0.28248	9.543029785	1
MSTRG.20872.1	149	0.3597	8.869567871	1
MSTRG.20882.1	475	0.32005	8.976257324	1
MSTRG.20883.1	44	0.33842	9.617980957	1
MSTRG.20884.2	729	0.46161	9.18170166	1
MSTRG.20884.3	1018	0.46368	6.242980957	1
MSTRG.20884.4	430	0.43258	4.50970459	1
MSTRG.20891.1	173	0.29712	8.213928223	1
MSTRG.20891.2	137	0.27763	6.685852051	1
MSTRG.20891.3	137	0.28218	6.685852051	1
MSTRG.20892.1	183	0.30624	10.2333374	1
MSTRG.20895.1	163	0.36356	9.852111816	1
MSTRG.20900.4	383	0.43192	9.075622559	1
MSTRG.20900.3	384	0.38228	9.075622559	1
MSTRG.20912.2	188	0.30813	5.854919434	1
MSTRG.20913.3	1937	0.4559	5.962463379	1
MSTRG.20913.4	1934	0.40867	6.046325684	1
MSTRG.20913.5	2043	0.45508	5.980773926	1
MSTRG.20915.1	85	0.36477	7.776794434	1

MSTRG.20919.1	730	0.45131	8.74395752	1
MSTRG.20914.1	52	0.39146	9.843200684	1
MSTRG.20914.2	31	0.40836	9.496276855	1
MSTRG.20914.3	24	0.39006	6.495788574	1
MSTRG.20923.1	14	0.41509	12.00006104	1
MSTRG.20921.1	103	0.38441	7.057312012	1
MSTRG.20921.2	78	0.31402	9.81072998	1
MSTRG.20932.2	234	0.34093	5.175964355	1
MSTRG.20934.1	69	0.43081	8.508239746	-1
MSTRG.20930.1	458	0.44423	6.77557373	1
MSTRG.20943.1	149	0.32558	9.800842285	1
MSTRG.20944.1	136	0.31479	10.96380615	1
MSTRG.20940.1	59	0.30829	8.528015137	-1
MSTRG.20949.2	615	0.4312	5.53326416	1
MSTRG.20951.1	324	0.4382	9.964782715	1
MSTRG.20952.2	196	0.38779	9.471252441	1
MSTRG.20953.4	276	0.29747	8.850646973	1
MSTRG.20953.2	205	0.30981	7.742370605	1
MSTRG.20953.5	109	0.30557	5.419372559	1
MSTRG.20950.2	729	0.46115	7.506286621	1
MSTRG.20955.3	405	0.42135	4.684143066	1
MSTRG.20955.5	409	0.40037	4.658874512	1
MSTRG.20955.4	409	0.3906	4.658874512	1
MSTRG.20955.1	405	0.4573	4.684143066	1
MSTRG.20957.1	97	0.29357	10.0112915	-1
MSTRG.20959.1	15	0.43351	7.83404541	1
MSTRG.20967.3	405	0.36022	8.469543457	1
MSTRG.20967.7	405	0.36022	8.469543457	1
MSTRG.20967.9	1112	0.45595	5.550842285	1
MSTRG.20967.10	1112	0.45991	5.550842285	1
MSTRG.20967.8	1112	0.45991	5.550842285	1
MSTRG.20967.11	747	0.42454	5.33782959	1
MSTRG.20967.13	747	0.45036	5.33782959	1
MSTRG.20967.14	747	0.45969	5.33782959	1
MSTRG.20969.2	89	0.42852	4.072570801	1
MSTRG.20963.4	821	0.3974	8.413513184	1
MSTRG.20963.5	817	0.3974	8.413513184	1
MSTRG.20963.8	955	0.45859	8.293640137	1
MSTRG.20963.6	959	0.41023	8.293640137	1
MSTRG.20975.2	142	0.39648	10.64227295	1
MSTRG.20979.2	643	0.39191	7.240539551	1
MSTRG.20981.2	570	0.46507	8.132263184	1
MSTRG.20977.2	191	0.36085	8.588684082	1
MSTRG.20978.1	355	0.45617	4.791442871	1
MSTRG.20978.3	355	0.32574	4.791442871	1
MSTRG.20986.2	406	0.42765	8.531311035	1
MSTRG.20986.5	566	0.43107	8.751037598	1
MSTRG.20986.1	557	0.41997	8.871032715	1
MSTRG.20986.3	557	0.42765	8.871032715	1
MSTRG.20986.7	406	0.42305	8.531311035	1
MSTRG.20986.8	589	0.41651	8.871643066	1
MSTRG.20989.1	249	0.40548	9.008728027	1
MSTRG.20990.1	47	0.3556	9.49029541	1
MSTRG.20990.2	103	0.39576	9.179138184	1

MSTRG.20994.3	2026	0.35912	5.838806152	1
MSTRG.20999.2	2328	0.4514	6.820617676	1
MSTRG.20999.3	1993	0.45041	6.206115723	1
MSTRG.21000.1	65	0.39898	8.841491699	1
MSTRG.21002.1	152	0.30553	10.13873291	1
MSTRG.21011.4	802	0.48044	6.004577637	1
MSTRG.21017.1	35	0.42583	3.905334473	1
MSTRG.21018.1	415	0.40726	6.63092041	1
MSTRG.21018.2	414	0.40726	6.310852051	1
MSTRG.21018.3	323	0.4369	8.340026855	-1
MSTRG.21024.5	1645	0.36388	8.31036377	1
MSTRG.21024.6	1458	0.36202	8.008605957	1
MSTRG.21024.7	1659	0.36464	8.301818848	1
MSTRG.21022.1	35	0.43296	6.507141113	1
MSTRG.21027.4	478	0.42689	6.202087402	1
MSTRG.21027.6	478	0.43154	6.202087402	1
MSTRG.21027.5	544	0.46074	6.16217041	1
MSTRG.21027.8	478	0.46193	6.202087402	1
MSTRG.21037.2	149	0.34268	5.526672363	1
MSTRG.21038.2	1126	0.40005	8.510192871	1
MSTRG.21038.5	168	0.34141	8.91607666	1
MSTRG.21038.6	267	0.2908	5.315979004	1
MSTRG.21039.2	261	0.39748	4.59197998	1
MSTRG.21048.3	1124	0.35836	6.753356934	1
MSTRG.21048.5	1124	0.46761	6.753356934	1
MSTRG.21049.2	245	0.28923	8.57208252	1
MSTRG.21061.1	1051	0.40125	5.292297363	1
MSTRG.21061.2	815	0.33902	5.214172363	1
MSTRG.21062.1	158	0.43589	9.051696777	1
MSTRG.21061.3	623	0.47442	5.132263184	1
MSTRG.21066.1	189	0.47759	8.648620605	1
MSTRG.21068.3	615	0.4149	6.41607666	1
MSTRG.21074.1	278	0.33573	10.00592041	1
MSTRG.21076.4	196	0.45928	8.547546387	1
MSTRG.21079.1	74	0.37736	5.327819824	1
MSTRG.21080.3	557	0.3208	8.023742676	1
MSTRG.21080.4	71	0.48264	4.412414551	1
MSTRG.21082.1	33	0.33399	9.489562988	1
MSTRG.21083.1	120	0.26189	10.54779053	1
MSTRG.21086.1	48	0.38293	6.803894043	1
MSTRG.21087.3	345	0.32561	8.729919434	1
MSTRG.21090.1	210	0.45618	9.656188965	1
MSTRG.21089.3	148	0.35461	5.803039551	1
MSTRG.21096.4	513	0.39466	9.077087402	1
MSTRG.21092.2	294	0.33747	8.730651855	1
MSTRG.21093.3	1039	0.449	6.204772949	1
MSTRG.21104.3	469	0.38051	4.991882324	1
MSTRG.21104.4	469	0.433	4.991882324	1
MSTRG.21105.1	53	0.3713	7.847106934	1
MSTRG.21106.5	444	0.29776	5.447814941	1
MSTRG.21106.7	585	0.379	6.399963379	1
MSTRG.21106.6	600	0.379	6.254699707	1
MSTRG.21106.2	413	0.34696	5.420349121	1
MSTRG.21106.4	413	0.29776	5.420349121	1

MSTRG.21106.8	413	0.38211	5.420349121	1
MSTRG.21106.9	451	0.38444	5.643859863	1
MSTRG.21106.10	428	0.35961	5.34552002	1
MSTRG.21106.11	398	0.36366	5.339782715	1
MSTRG.21101.3	219	0.34775	5.593078613	1
MSTRG.21098.1	92	0.28948	8.816467285	1
MSTRG.21102.2	225	0.46923	5.137390137	1
MSTRG.21107.2	342	0.32413	7.038146973	1
MSTRG.21107.3	669	0.45239	8.61932373	1
MSTRG.21108.1	156	0.3351	10.00372314	1
MSTRG.21109.1	115	0.31852	10.88446045	1
MSTRG.21107.4	342	0.42507	7.038146973	1
MSTRG.21111.2	1040	0.37761	8.679504395	1
MSTRG.21112.1	154	0.30121	5.040100098	1
MSTRG.21112.2	128	0.30121	7.742004395	1
MSTRG.21115.1	38	0.34196	9.836975098	1
MSTRG.21117.1	28	0.48755	10.43267822	1
MSTRG.21120.1	44	0.36076	9.773132324	-1
MSTRG.21126.2	723	0.43801	6.329650879	1
MSTRG.21123.1	107	0.35407	10.13885498	1
MSTRG.21130.3	244	0.33475	4.983947754	1
MSTRG.21127.1	1608	0.4016	5.00604248	1
MSTRG.21127.2	1594	0.4016	5.011779785	1
MSTRG.21128.1	48	0.34952	10.94000244	1
MSTRG.21146.3	239	0.41567	5.502502441	1
MSTRG.21147.1	71	0.46736	8.804870605	1
MSTRG.21146.4	377	0.43668	8.942321777	1
MSTRG.21146.6	367	0.42924	8.736755371	1
MSTRG.21146.5	307	0.31518	6.82220459	1
MSTRG.21146.7	348	0.44258	7.195739746	1
MSTRG.21146.8	186	0.43912	5.548522949	1
MSTRG.21146.9	182	0.29607	10.13519287	1
MSTRG.21146.10	153	0.33423	10.20977783	1
MSTRG.21146.11	108	0.29772	4.999328613	1
MSTRG.21146.12	108	0.39243	4.999328613	1
MSTRG.21148.10	1072	0.44408	8.569885254	1
MSTRG.21148.12	1073	0.44203	8.572814941	1
MSTRG.21148.6	1101	0.44261	8.709289551	1
MSTRG.21148.8	756	0.42172	7.744812012	1
MSTRG.21148.9	1151	0.43757	8.244689941	1
MSTRG.21134.1	304	0.47439	5.637878418	1
MSTRG.21151.1	138	0.27327	9.543884277	1
MSTRG.21152.1	931	0.44712	4.766052246	1
MSTRG.21153.1	933	0.42427	4.649597168	1
MSTRG.21154.1	277	0.34159	9.873596191	1
MSTRG.21154.2	243	0.44166	9.035827637	-1
MSTRG.21155.1	77	0.33531	5.563293457	1
MSTRG.21156.2	336	0.39192	6.383483887	1
MSTRG.21154.3	159	0.42059	7.77911377	1
MSTRG.21154.4	580	0.36239	9.189758301	1
MSTRG.21159.3	575	0.44808	8.756652832	1
MSTRG.21159.4	596	0.38922	8.954162598	1
MSTRG.21159.5	596	0.40643	8.954162598	1
MSTRG.21159.6	532	0.44032	8.754943848	1

MSTRG.21159.7	69	0.3484	6.698181152	1
MSTRG.21162.6	424	0.43527	5.585144043	1
MSTRG.21162.7	553	0.44655	5.432434082	1
MSTRG.21163.1	43	0.44264	7.976257324	1
MSTRG.21163.2	83	0.33187	9.687316895	1
MSTRG.21173.1	3143	0.44648	6.019226074	1
MSTRG.21169.1	58	0.31205	9.099914551	1
MSTRG.21176.1	25	0.46103	5.968444824	1
MSTRG.21176.2	25	0.36153	5.968444824	1
MSTRG.21179.3	3077	0.46674	4.995300293	1
MSTRG.21179.2	3077	0.46674	4.987121582	1
MSTRG.21179.5	3077	0.47444	4.987121582	1
MSTRG.21179.6	3077	0.46674	4.994689941	1
MSTRG.21181.2	1190	0.37781	8.373352051	1
MSTRG.21198.1	355	0.46146	10.09698486	1
MSTRG.21198.2	147	0.34583	10.45330811	1
MSTRG.21198.3	311	0.36466	10.19781494	1
MSTRG.21212.1	730	0.38695	8.968200684	1
MSTRG.21212.2	373	0.3582	8.257751465	1
MSTRG.21213.2	308	0.37244	7.047180176	1
MSTRG.21216.2	232	0.31418	4.649719238	1
MSTRG.21218.2	107	0.33767	5.437438965	1
MSTRG.21218.3	96	0.38511	7.756530762	1
MSTRG.21220.3	307	0.43136	7.188293457	1
MSTRG.21220.4	64	0.2921	9.167785645	1
MSTRG.21224.1	59	0.36286	8.617614746	1
MSTRG.21235.3	725	0.45997	5.799133301	1
MSTRG.21234.1	44	0.39633	5.617858887	1
MSTRG.21244.1	51	0.37712	6.809265137	1
MSTRG.21248.2	19	0.34907	11.69903564	1
MSTRG.21249.1	116	0.28855	9.453186035	1
MSTRG.21250.2	326	0.41507	6.355773926	1
MSTRG.21251.1	92	0.38023	8.906188965	1
MSTRG.21251.2	83	0.39307	7.979309082	1
MSTRG.21252.1	116	0.30753	10.37335205	1
MSTRG.21252.2	116	0.27883	10.37335205	1
MSTRG.21254.1	80	0.30055	8.934509277	1
MSTRG.21256.5	401	0.38012	8.012512207	1
MSTRG.21256.6	369	0.47113	6.097595215	1
MSTRG.21257.1	71	0.35159	8.80279541	1
MSTRG.21260.1	372	0.40786	5.571838379	1
MSTRG.21262.1	60	0.39613	9.512268066	1
MSTRG.21268.1	62	0.29628	6.699645996	1
MSTRG.21269.1	1049	0.33655	5.492614746	1
MSTRG.21269.2	65	0.37651	4.426940918	1
MSTRG.21271.2	559	0.35697	6.105773926	1
MSTRG.21272.1	59	0.35843	9.099060059	-1
MSTRG.21278.2	100	0.375	5.22479248	1
MSTRG.21301.3	903	0.4256	8.806335449	1
MSTRG.21302.1	188	0.28567	9.944152832	1
MSTRG.21308.1	502	0.37611	4.929992676	1
MSTRG.21309.2	1038	0.45889	6.496643066	1
MSTRG.21310.3	620	0.4418	8.153869629	1
MSTRG.21315.2	1448	0.42046	6.725891113	1

MSTRG.21316.1	183	0.33636	10.62164307	1
MSTRG.21317.1	60	0.31639	9.032897949	1
MSTRG.21318.1	110	0.35472	5.998596191	1
MSTRG.21315.4	169	0.38274	9.098083496	1
MSTRG.21314.1	385	0.41679	7.575134277	-1
MSTRG.21319.1	858	0.44516	6.842468262	1
MSTRG.21323.1	1619	0.43524	6.575500488	1
MSTRG.21323.5	1568	0.46379	6.643615723	1
MSTRG.21323.3	1616	0.46379	6.644714355	1
MSTRG.21323.8	1581	0.42896	6.452941895	1
MSTRG.21323.7	1616	0.43454	6.644714355	1
MSTRG.21323.9	1619	0.42896	6.575500488	1
MSTRG.21321.1	1337	0.45953	5.719177246	1
MSTRG.21321.2	1320	0.44518	5.684387207	1
MSTRG.21321.3	1276	0.42098	5.63848877	1
MSTRG.21324.2	604	0.37926	9.005065918	1
MSTRG.21324.3	604	0.37117	9.005065918	1
MSTRG.21326.1	150	0.30992	5.50201416	1
MSTRG.21327.3	375	0.45736	6.701843262	1
MSTRG.21327.6	375	0.39915	6.701843262	1
MSTRG.21327.8	297	0.40585	7.384216309	1
MSTRG.21330.2	159	0.4351	8.59576416	-1
MSTRG.21333.4	419	0.42214	8.76776123	1
MSTRG.21334.5	1272	0.4426	5.667541504	1
MSTRG.21334.6	1386	0.43795	5.987121582	1
MSTRG.21334.7	1386	0.44047	5.987121582	1
MSTRG.21334.8	1386	0.40702	5.987121582	1
MSTRG.21334.9	1386	0.40702	5.987121582	1
MSTRG.21334.10	1341	0.40537	5.816101074	1
MSTRG.21335.1	114	0.2921	6.379455566	1
MSTRG.21336.1	122	0.37942	9.010925293	1
MSTRG.21336.2	122	0.36357	9.010925293	1
MSTRG.21336.3	122	0.35501	9.010925293	1
MSTRG.21338.4	206	0.2689	9.515930176	1
MSTRG.21338.6	202	0.32188	8.683044434	1
MSTRG.21338.7	761	0.33965	6.532775879	1
MSTRG.21338.9	102	0.35247	6.515075684	1
MSTRG.21338.10	144	0.30424	9.53704834	1
MSTRG.21338.13	406	0.36075	6.03314209	1
MSTRG.21338.12	572	0.44177	5.50177002	1
MSTRG.21342.1	195	0.32917	6.69354248	1
MSTRG.21343.3	195	0.4144	8.413513184	1
MSTRG.21348.4	1709	0.39003	6.328063965	1
MSTRG.21348.1	1716	0.39558	6.261779785	1
MSTRG.21348.2	1717	0.39558	6.294616699	1
MSTRG.21349.1	339	0.40637	6.277038574	1
MSTRG.21351.1	27	0.38599	11.69903564	1
MSTRG.21345.1	133	0.34883	8.878967285	1
MSTRG.21371.3	1011	0.43793	4.643005371	1
MSTRG.21371.5	503	0.4678	4.450866699	1
MSTRG.21371.4	1599	0.42059	7.61932373	1
MSTRG.21371.6	503	0.39954	4.450866699	1
MSTRG.21371.7	551	0.45897	5.07989502	1
MSTRG.21371.9	1474	0.41894	8.851257324	1

MSTRG.21371.10	1599	0.41439	7.61932373	1
MSTRG.21371.11	1594	0.40519	7.816589355	1
MSTRG.21371.13	1591	0.41274	7.987976074	1
MSTRG.21371.12	1591	0.42059	7.987976074	1
MSTRG.21371.15	1594	0.41894	7.816589355	1
MSTRG.21371.16	1594	0.41274	7.816589355	1
MSTRG.21371.17	1482	0.41894	8.763000488	1
MSTRG.21371.18	1591	0.43142	7.987976074	1
MSTRG.21372.1	283	0.28832	6.339660645	1
MSTRG.21373.1	135	0.34145	12.25689697	1
MSTRG.21372.2	270	0.27586	8.496520996	1
MSTRG.21356.1	283	0.40681	5.27947998	1
MSTRG.21359.1	118	0.34107	5.90020752	1
MSTRG.21361.1	223	0.37565	4.703552246	1
MSTRG.21362.1	59	0.40282	5.114562988	1
MSTRG.21363.1	265	0.38837	7.704162598	1
MSTRG.21368.2	356	0.37428	8.880187988	1
MSTRG.21368.3	356	0.3755	8.880187988	1
MSTRG.21369.1	69	0.28929	6.802185059	1
MSTRG.21377.1	70	0.3754	9.817565918	-1
MSTRG.21375.1	82	0.36914	9.387268066	-1
MSTRG.21384.1	44	0.31325	9.513000488	1
MSTRG.21385.5	140	0.31172	11.53814697	1
MSTRG.21385.4	485	0.47401	7.55670166	1
MSTRG.21385.7	140	0.29343	11.53814697	1
MSTRG.21385.8	69	0.39363	6.144348145	1
MSTRG.21385.9	504	0.37524	7.90814209	1
MSTRG.21385.10	146	0.36095	9.865539551	-1
MSTRG.21395.3	194	0.43777	8.893615723	1
MSTRG.21396.3	744	0.41109	6.414978027	1
MSTRG.21396.4	744	0.45109	6.414978027	1
MSTRG.21406.1	88	0.28002	6.808898926	1
MSTRG.21406.2	54	0.31742	8.933898926	1
MSTRG.21400.1	54	0.34371	11.58099365	1
MSTRG.21402.1	60	0.36252	5.668029785	1
MSTRG.21403.1	59	0.30285	6.812683105	1
MSTRG.21414.1	60	0.33471	9.857971191	1
MSTRG.21415.1	118	0.29793	8.157775879	1
MSTRG.21416.1	129	0.30121	11.45855713	1
MSTRG.21433.1	1065	0.36153	8.739318848	1
MSTRG.21433.4	786	0.40576	8.619934082	1
MSTRG.21433.3	786	0.34103	8.619934082	1
MSTRG.21433.5	786	0.37771	8.619934082	1
MSTRG.21433.6	786	0.28243	8.619934082	1
MSTRG.21433.7	786	0.38321	8.619934082	1
MSTRG.21433.8	786	0.36706	8.619934082	1
MSTRG.21433.9	290	0.29607	7.95501709	1
MSTRG.21434.1	109	0.43457	5.153259277	1
MSTRG.21433.10	786	0.34103	8.619934082	1
MSTRG.21433.11	378	0.29772	9.478942871	1
MSTRG.21433.13	786	0.40576	8.619934082	1
MSTRG.21433.14	786	0.42116	8.619934082	1
MSTRG.21433.15	786	0.40576	8.619934082	1
MSTRG.21433.16	786	0.42116	8.619934082	1

MSTRG.21433.18	786	0.43502	8.619934082	1
MSTRG.21433.19	310	0.36347	9.819763184	1
MSTRG.21433.20	1030	0.36935	8.675842285	1
MSTRG.21433.22	96	0.33345	10.55145264	1
MSTRG.21441.2	214	0.47319	9.88848877	1
MSTRG.21419.1	2852	0.46336	5.261413574	1
MSTRG.21425.1	33	0.4494	9.186218262	1
MSTRG.21427.2	251	0.41056	9.77154541	1
MSTRG.21427.1	496	0.40638	9.99810791	1
MSTRG.21428.1	30	0.40738	11.33087158	1
MSTRG.21429.1	45	0.36455	6.810974121	1
MSTRG.21432.1	147	0.46081	11.25811768	1
MSTRG.21449.1	785	0.33623	7.07611084	1
MSTRG.21449.4	606	0.4382	8.034118652	-1
MSTRG.21450.1	150	0.28883	5.941101074	1
MSTRG.21450.2	102	0.3271	9.658996582	1
MSTRG.21451.1	89	0.27713	6.555114746	1
MSTRG.21449.5	94	0.27763	4.690856934	1
MSTRG.21452.1	214	0.29751	9.445495605	1
MSTRG.21453.1	172	0.29187	9.806945801	1
MSTRG.21454.1	162	0.27863	10.01617432	1
MSTRG.21454.2	98	0.29356	5.774230957	1
MSTRG.21446.2	490	0.43415	4.746765137	1
MSTRG.21447.4	220	0.35804	4.977844238	1
MSTRG.21447.2	220	0.41549	4.977844238	1
MSTRG.21471.1	1081	0.4216	6.385192871	1
MSTRG.21471.3	508	0.36143	5.94708252	1
MSTRG.21472.1	60	0.38933	11.36297607	-1
MSTRG.21470.3	130	0.35269	9.24786377	1
MSTRG.21474.3	289	0.46234	8.819274902	1
MSTRG.21484.4	724	0.33006	5.89239502	1
MSTRG.21486.2	260	0.41286	9.278015137	1
MSTRG.21502.2	591	0.30132	5.506164551	1
MSTRG.21502.7	485	0.34755	6.039123535	1
MSTRG.21502.4	455	0.33711	5.55279541	1
MSTRG.21502.6	591	0.36698	5.506164551	1
MSTRG.21502.5	591	0.31081	5.506164551	1
MSTRG.21503.1	67	0.37466	6.042663574	1
MSTRG.21511.1	84	0.34967	9.695251465	1
MSTRG.21520.1	90	0.33422	11.48028564	1
MSTRG.21526.1	75	0.37468	4.508605957	1
MSTRG.21533.1	30	0.41651	9.700744629	1
MSTRG.21534.1	80	0.4497	10.37542725	-1
MSTRG.21532.2	607	0.4677	5.425231934	1
MSTRG.21535.1	186	0.29687	8.734558105	1
MSTRG.21532.3	156	0.32082	4.982971191	1
MSTRG.21536.1	42	0.42024	4.796081543	1
MSTRG.21537.1	199	0.36942	10.1987915	1
MSTRG.21532.4	109	0.2831	8.867492676	1
MSTRG.21538.1	128	0.29771	8.969299316	1
MSTRG.21499.2	168	0.46471	4.03704834	1
MSTRG.21499.3	168	0.46333	4.03704834	1
MSTRG.21552.1	107	0.29187	9.097473145	1
MSTRG.21553.1	250	0.29526	9.636047363	1

MSTRG.21555.1	77	0.28338	6.233215332	1
MSTRG.21556.1	109	0.32477	5.020935059	1
MSTRG.21569.1	76	0.40607	9.989807129	1
MSTRG.21572.1	98	0.4612	6.486633301	1
MSTRG.21572.2	98	0.37591	6.486633301	1
MSTRG.21573.5	503	0.33694	9.459899902	1
MSTRG.21573.6	594	0.47443	6.273376465	1
MSTRG.21573.7	595	0.44809	6.823059082	1
MSTRG.21573.9	607	0.47954	6.596374512	1
MSTRG.21573.14	491	0.43962	9.536193848	1
MSTRG.21575.1	361	0.36898	5.519592285	1
MSTRG.21580.1	103	0.30981	9.592224121	1
MSTRG.21580.2	139	0.36161	9.499450684	1
MSTRG.21580.3	125	0.35235	10.12615967	1
MSTRG.21598.1	88	0.44791	6.805358887	-1
MSTRG.21612.1	8	0.45195	3.998840332	-1
MSTRG.21649.1	77	0.29435	5.607849121	1
MSTRG.21656.9	535	0.29755	5.121154785	1
MSTRG.21657.1	33	0.37704	9.301940918	1
MSTRG.21670.1	62	0.31454	6.80657959	1
MSTRG.21670.2	25	0.35674	9.992004395	1
MSTRG.21695.4	748	0.45297	5.43572998	1
MSTRG.21696.1	95	0.2727	9.438293457	1
MSTRG.21695.6	819	0.45406	5.347106934	-1
MSTRG.21697.1	118	0.31885	8.28302002	1
MSTRG.21695.7	601	0.44264	5.190979004	1
MSTRG.21698.1	66	0.36239	9.951477051	1
MSTRG.21695.10	331	0.45765	7.042541504	1
MSTRG.21708.1	42	0.37836	11.43719482	-1
MSTRG.21674.1	78	0.39939	10.81134033	1
MSTRG.21676.1	70	0.36294	10.35736084	1
MSTRG.21680.1	133	0.30905	9.729187012	1
MSTRG.21729.1	58	0.42886	5.086364746	1
MSTRG.21725.1	101	0.41683	10.28094482	-1
MSTRG.21739.1	58	0.3137	6.066955566	1
MSTRG.21741.2	40	0.36461	6.111877441	1
MSTRG.21741.1	77	0.38792	5.991027832	1
MSTRG.21749.1	886	0.47214	7.534851074	1
MSTRG.21749.4	774	0.46444	6.76739502	1
MSTRG.21749.5	189	0.32465	9.629577637	1
MSTRG.21749.6	81	0.32237	7.652893066	1
MSTRG.21749.7	49	0.36536	5.604309082	-1
MSTRG.21751.1	83	0.31924	7.002380371	1
MSTRG.21752.1	44	0.29895	8.737854004	1
MSTRG.21749.8	101	0.27994	8.330993652	1
MSTRG.21753.1	245	0.28982	9.702575684	1
MSTRG.21754.1	53	0.32538	6.700134277	1
MSTRG.21754.2	113	0.3208	9.296813965	1
MSTRG.21749.9	625	0.46875	7.553527832	1
MSTRG.21755.1	76	0.32392	6.786560059	-1
MSTRG.21755.2	76	0.35519	6.786560059	-1
MSTRG.21760.1	165	0.31356	10.82000732	1
MSTRG.21771.6	2550	0.43692	5.687927246	1
MSTRG.21772.1	150	0.29003	9.878845215	1

MSTRG.21771.19	513	0.43008	8.925476074	1
MSTRG.21771.20	577	0.41912	9.005432129	1
MSTRG.21771.21	577	0.4215	9.005432129	1
MSTRG.21771.22	554	0.39897	9.078430176	1
MSTRG.21771.23	448	0.46709	8.723083496	1
MSTRG.21773.1	84	0.3351	7.674987793	1
MSTRG.21775.1	55	0.38663	5.398864746	1
MSTRG.21771.24	4967	0.45739	6.126525879	1
MSTRG.21771.25	4988	0.46014	6.136657715	1
MSTRG.21771.26	4971	0.46014	6.156433105	1
MSTRG.21771.27	4942	0.45739	6.141418457	-1
MSTRG.21771.28	1493	0.44511	6.985534668	1
MSTRG.21771.29	949	0.46895	5.27923584	1
MSTRG.21763.1	59	0.44834	9.380065918	1
MSTRG.21779.2	1643	0.4474	6.140686035	1
MSTRG.21779.1	1642	0.44416	6.140686035	1
MSTRG.21790.1	232	0.27991	8.696105957	1
MSTRG.21791.2	56	0.31501	7.913879395	1
MSTRG.21795.1	339	0.45564	7.525939941	1
MSTRG.21795.3	348	0.34515	6.967224121	1
MSTRG.21795.4	91	0.30624	7.833679199	1
MSTRG.21795.5	232	0.44682	6.172668457	1
MSTRG.21795.6	91	0.33931	7.833679199	1
MSTRG.21796.1	69	0.35401	6.562194824	1
MSTRG.21797.1	519	0.36883	9.848693848	1
MSTRG.21797.2	519	0.37704	9.848693848	1
MSTRG.21794.1	38	0.39894	5.947814941	-1
MSTRG.21806.2	237	0.41999	6.704162598	1
MSTRG.21812.2	112	0.30396	4.707824707	1
MSTRG.21812.1	112	0.3794	4.707824707	1
MSTRG.21812.4	737	0.32105	9.202941895	1
MSTRG.21808.2	515	0.39766	9.170715332	1
MSTRG.21815.1	52	0.36757	8.932189941	1
MSTRG.21817.2	113	0.3432	8.640930176	1
MSTRG.21818.3	195	0.3769	8.641540527	1
MSTRG.21819.1	327	0.37596	6.988342285	1
MSTRG.21820.1	115	0.29251	9.741760254	1
MSTRG.21820.2	115	0.36184	9.741760254	1
MSTRG.21827.2	288	0.42857	8.802429199	1
MSTRG.21827.3	235	0.32289	9.798522949	1
MSTRG.21829.2	2245	0.47454	5.942321777	1
MSTRG.21829.3	2245	0.45636	5.942321777	1
MSTRG.21831.1	992	0.41844	9.624206543	1
MSTRG.21831.10	968	0.41968	9.557800293	1
MSTRG.21831.11	1002	0.43399	9.539733887	1
MSTRG.21831.3	1026	0.44785	9.706970215	1
MSTRG.21831.5	1003	0.43399	9.684509277	1
MSTRG.21831.7	980	0.43399	9.624328613	1
MSTRG.21831.9	745	0.43151	9.651184082	1
MSTRG.21831.12	175	0.27194	8.426086426	1
MSTRG.21831.13	602	0.28746	11.42657471	1
MSTRG.21831.14	461	0.35222	11.81658936	1
MSTRG.21830.1	53	0.29196	9.033508301	1
MSTRG.21834.3	557	0.46795	7.401306152	1

MSTRG.21834.2	497	0.45614	6.932556152	1
MSTRG.21837.5	3056	0.44651	9.340515137	1
MSTRG.21837.6	2975	0.44903	9.290466309	1
MSTRG.21837.8	3057	0.44651	9.340515137	1
MSTRG.21837.9	3012	0.45211	9.301696777	1
MSTRG.21842.2	868	0.43647	9.094665527	1
MSTRG.21842.6	78	0.31818	6.864196777	1
MSTRG.21841.1	115	0.41083	6.001403809	1
MSTRG.21846.1	605	0.42655	8.939025879	1
MSTRG.21846.2	108	0.30501	8.122741699	1
MSTRG.21846.3	260	0.46046	7.16595459	1
MSTRG.21856.2	295	0.4587	8.656311035	1
MSTRG.21863.1	50	0.31951	6.524597168	1
MSTRG.21864.2	211	0.30824	8.990783691	1
MSTRG.21865.1	115	0.28311	9.937683105	1
MSTRG.21850.3	148	0.29664	6.325134277	1
MSTRG.21850.2	327	0.37359	5.975280762	1
MSTRG.21867.1	524	0.4561	5.250183105	1
MSTRG.21870.1	61	0.35876	6.517150879	-1
MSTRG.21874.1	91	0.36721	7.768371582	1
MSTRG.21876.1	32	0.422	12.00042725	1
MSTRG.21880.1	1061	0.41806	4.766418457	1
MSTRG.21880.2	591	0.34844	5.042419434	1
MSTRG.21880.3	591	0.38587	5.042419434	1
MSTRG.21880.4	118	0.27526	9.160949707	1
MSTRG.21880.5	183	0.28248	9.983581543	1
MSTRG.21880.6	125	0.28002	6.070129395	1
MSTRG.21881.1	45	0.46247	8.491394043	1
MSTRG.21880.7	125	0.28002	6.070129395	1
MSTRG.21880.8	125	0.28503	6.070129395	1
MSTRG.21880.9	123	0.28002	8.480529785	1
MSTRG.21885.4	188	0.41159	8.491394043	1
MSTRG.21885.3	188	0.36327	8.491394043	1
MSTRG.21886.1	61	0.31934	9.625061035	1
MSTRG.21887.1	1060	0.46779	7.059509277	1
MSTRG.21889.1	91	0.30349	12.00140381	1
MSTRG.21890.2	918	0.45745	7.231872559	1
MSTRG.21890.3	310	0.44481	6.944274902	1
MSTRG.21890.4	918	0.4459	7.231872559	1
MSTRG.21890.5	614	0.43314	7.131286621	1
MSTRG.21890.6	918	0.43372	7.23223877	1
MSTRG.21890.7	766	0.4365	7.188049316	1
MSTRG.21890.8	710	0.45901	6.416442871	1
MSTRG.21890.12	269	0.42796	6.05291748	-1
MSTRG.21890.20	421	0.42796	6.319519043	-1
MSTRG.21890.15	293	0.42796	6.071228027	-1
MSTRG.21890.19	912	0.4213	7.23223877	-1
MSTRG.21890.10	308	0.42787	6.944274902	-1
MSTRG.21890.21	497	0.42429	6.406799316	-1
MSTRG.21890.22	269	0.42966	6.05291748	-1
MSTRG.21882.1	87	0.36519	9.648620605	1
MSTRG.21883.1	71	0.37274	10.85162354	1
MSTRG.21897.10	2577	0.46153	7.223693848	1
MSTRG.21897.11	2521	0.46153	7.297546387	1

MSTRG.21897.12	2523	0.46153	7.297546387	1
MSTRG.21897.13	2557	0.46153	7.301452637	1
MSTRG.21897.15	2522	0.46153	7.297546387	1
MSTRG.21897.16	2521	0.46153	7.297546387	1
MSTRG.21897.17	2576	0.46153	7.223693848	1
MSTRG.21897.18	2538	0.46153	7.219543457	1
MSTRG.21897.3	2523	0.46153	7.297546387	1
MSTRG.21897.4	2522	0.46153	7.297546387	1
MSTRG.21897.5	2476	0.46923	7.605163574	1
MSTRG.21897.6	2539	0.46153	7.219543457	1
MSTRG.21897.7	2521	0.46153	7.297546387	1
MSTRG.21897.8	2539	0.46153	7.219543457	1
MSTRG.21897.9	2540	0.46153	7.219543457	1
MSTRG.21898.2	100	0.30235	6.085510254	1
MSTRG.21898.1	80	0.33073	7.660949707	1
MSTRG.21899.1	56	0.34996	5.999328613	1
MSTRG.21897.19	2202	0.45385	6.966125488	1
MSTRG.21897.20	2086	0.44615	7.077819824	1
MSTRG.21897.21	2054	0.37894	6.574279785	1
MSTRG.21897.22	1973	0.44923	6.952087402	1
MSTRG.21897.23	1917	0.45743	7.00177002	1
MSTRG.21897.24	1872	0.46513	7.226379395	1
MSTRG.21897.25	1787	0.44923	6.859069824	1
MSTRG.21897.26	1326	0.45311	9.296325684	1
MSTRG.21902.1	59	0.31296	8.843322754	1
MSTRG.21903.2	257	0.28607	8.880187988	1
MSTRG.21903.5	100	0.39542	9.496398926	1
MSTRG.21903.3	99	0.4224	8.541442871	1
MSTRG.21903.4	93	0.30436	6.831359863	1
MSTRG.21903.7	93	0.36293	6.831359863	1
MSTRG.21905.2	112	0.29152	11.65509033	1
MSTRG.21905.3	123	0.27251	10.28253174	1
MSTRG.21905.6	141	0.28923	10.72283936	1
MSTRG.21907.2	664	0.40116	6.250915527	1
MSTRG.21907.3	854	0.44097	5.933898926	1
MSTRG.21912.1	372	0.45681	5.215393066	1
MSTRG.21912.2	181	0.39806	4.903259277	1
MSTRG.21900.5	365	0.43484	9.612731934	1
MSTRG.21916.3	876	0.43668	8.660461426	1
MSTRG.21917.1	90	0.33097	9.387390137	1
MSTRG.21918.2	158	0.38867	11.33319092	1
MSTRG.21919.4	172	0.3867	9.79888916	1
MSTRG.21919.5	44	0.3514	10.39080811	1
MSTRG.21920.7	875	0.4612	5.504455566	1
MSTRG.21925.1	1274	0.44432	6.75604248	1
MSTRG.21926.1	57	0.44934	9.301330566	1
MSTRG.21925.2	727	0.41032	5.329284668	1
MSTRG.21925.3	1274	0.4359	6.75604248	1
MSTRG.21927.1	44	0.3513	11.43719482	-1
MSTRG.21927.2	95	0.36017	9.828186035	1
MSTRG.21925.4	1274	0.45011	6.75604248	1
MSTRG.21925.5	1274	0.44951	6.75604248	1
MSTRG.21928.2	107	0.37029	5.739196777	1
MSTRG.21921.2	166	0.35099	10.33026123	1

MSTRG.21932.2	342	0.43218	6.108825684	1
MSTRG.21932.3	295	0.44074	6.446350098	1
MSTRG.21934.2	198	0.46452	5.321105957	1
MSTRG.21935.2	65	0.33648	4.889831543	1
MSTRG.21941.1	2204	0.48782	5.646179199	1
MSTRG.21941.2	2203	0.46937	5.627502441	1
MSTRG.21942.1	435	0.41555	11.3036499	1
MSTRG.21942.5	178	0.43242	8.57208252	1
MSTRG.21939.2	1394	0.46955	5.214782715	1
MSTRG.21940.1	53	0.39851	9.489807129	1
MSTRG.21939.3	2084	0.45565	5.218200684	1
MSTRG.21939.4	2150	0.46396	5.220031738	1
MSTRG.21943.1	540	0.42682	5.266906738	1
MSTRG.21943.2	638	0.42403	5.281066895	1
MSTRG.21949.1	210	0.34376	4.811828613	1
MSTRG.21953.1	16	0.40335	7.880432129	1
MSTRG.21955.1	62	0.37229	6.512390137	1
MSTRG.21958.4	89	0.46764	7.772644043	1
MSTRG.21958.5	96	0.39434	6.075378418	1
MSTRG.21958.10	96	0.40203	6.075378418	1
MSTRG.21958.3	135	0.40914	5.534851074	1
MSTRG.21958.6	114	0.45252	9.670227051	1
MSTRG.21958.7	114	0.3732	9.670227051	1
MSTRG.21958.8	135	0.41811	5.534851074	1
MSTRG.21958.9	114	0.39618	9.670227051	1
MSTRG.21959.1	91	0.43796	5.30657959	1
MSTRG.21961.2	776	0.45764	9.31829834	1
MSTRG.21961.3	779	0.45764	9.33380127	1
MSTRG.21961.4	402	0.43849	9.102233887	1
MSTRG.21961.5	402	0.42742	9.102233887	1
MSTRG.21963.3	479	0.48666	6.118225098	1
MSTRG.21977.2	316	0.4357	8.498962402	1
MSTRG.21978.2	662	0.46169	8.930969238	-1
MSTRG.21980.1	123	0.34734	10.690979	1
MSTRG.21985.2	1024	0.46224	5.409362793	1
MSTRG.21985.4	1024	0.46495	5.409362793	1
MSTRG.21985.5	899	0.46224	6.301940918	1
MSTRG.21985.6	997	0.47114	5.304016113	1
MSTRG.21985.7	1024	0.48139	5.409362793	1
MSTRG.21985.8	445	0.32417	4.848449707	1
MSTRG.21987.2	598	0.3405	5.514587402	1
MSTRG.21987.3	127	0.38464	9.593688965	1
MSTRG.21965.1	272	0.38051	4.647766113	1
MSTRG.21965.2	170	0.39831	8.785705566	1
MSTRG.21966.1	42	0.32943	9.786437988	1
MSTRG.21967.1	188	0.3445	9.51373291	1
MSTRG.21967.2	188	0.3445	9.51373291	1
MSTRG.21967.4	188	0.39149	9.51373291	1
MSTRG.21967.5	188	0.37099	9.51373291	1
MSTRG.21967.6	158	0.40238	10.26312256	1
MSTRG.21968.3	87	0.4839	5.147155762	1
MSTRG.21971.1	588	0.34463	7.752746582	1
MSTRG.21971.2	410	0.34463	8.669494629	1
MSTRG.21972.1	272	0.43977	7.144836426	1

MSTRG.21994.4	343	0.36439	5.521789551	1
MSTRG.21994.3	343	0.33135	5.521789551	1
MSTRG.21994.5	273	0.35768	7.663757324	1
MSTRG.21996.12	714	0.4518	9.612609863	1
MSTRG.21996.17	902	0.37222	8.38092041	1
MSTRG.21996.14	913	0.4072	7.319641113	1
MSTRG.21996.16	709	0.38423	9.671447754	1
MSTRG.21996.9	1031	0.46413	8.949401855	1
MSTRG.21996.10	1076	0.46446	9.049743652	1
MSTRG.21996.13	961	0.4476	8.075622559	1
MSTRG.21996.6	1004	0.48258	8.397155762	1
MSTRG.21996.8	1052	0.4665	8.760925293	1
MSTRG.21996.15	1049	0.46	8.960754395	1
MSTRG.21997.1	67	0.32089	6.143859863	1
MSTRG.22002.2	443	0.34198	5.485534668	1
MSTRG.22005.4	863	0.44965	6.989685059	1
MSTRG.22008.1	156	0.28396	9.891296387	1
MSTRG.22008.2	156	0.28648	9.891296387	1
MSTRG.22008.3	119	0.36843	10.95782471	1
MSTRG.22010.3	2016	0.45463	6.096374512	1
MSTRG.22010.4	1607	0.4508	6.36932373	1
MSTRG.22010.5	2016	0.49689	6.096374512	1
MSTRG.22021.1	84	0.32954	4.750305176	1
MSTRG.22014.1	124	0.42375	9.097595215	1
MSTRG.22026.1	331	0.36172	5.546813965	1
MSTRG.22028.1	189	0.30644	10.20318604	1
MSTRG.22027.4	112	0.28826	5.183776855	1
MSTRG.22029.1	60	0.35598	9.101013184	1
MSTRG.22027.6	728	0.44772	7.026184082	1
MSTRG.22034.2	347	0.34508	4.616394043	1
MSTRG.22034.5	194	0.29576	8.773864746	1
MSTRG.22030.3	988	0.37899	6.272277832	1
MSTRG.22030.2	988	0.37837	6.272277832	1
MSTRG.22030.4	981	0.37837	6.394958496	1
MSTRG.22038.3	620	0.444	5.848815918	1
MSTRG.22041.3	144	0.33634	6.727233887	1
MSTRG.22041.4	95	0.28111	9.297180176	1
MSTRG.22042.1	29	0.39381	9.514099121	1
MSTRG.22043.1	599	0.45171	5.421691895	1
MSTRG.22043.3	151	0.37837	6.742614746	1
MSTRG.22043.4	599	0.46137	5.421691895	1
MSTRG.22045.2	123	0.32018	6.062072754	1
MSTRG.22046.2	205	0.28167	8.344055176	1
MSTRG.22050.3	2075	0.42178	5.667297363	1
MSTRG.22051.1	81	0.35056	8.987976074	1
MSTRG.22051.2	83	0.34723	9.570983887	1
MSTRG.22050.4	277	0.40269	4.961242676	1
MSTRG.22056.1	82	0.35528	9.579406738	1
MSTRG.22058.1	58	0.42754	11.73919678	1
MSTRG.22063.3	743	0.41282	8.281433105	1
MSTRG.22063.4	923	0.46858	5.956970215	1
MSTRG.22063.5	282	0.3943	6.780456543	1
MSTRG.22059.1	103	0.36178	10.8270874	1
MSTRG.22059.2	48	0.35527	9.489196777	1

MSTRG.22064.3	457	0.40837	7.610656738	1
MSTRG.22064.4	500	0.40893	8.812683105	1
MSTRG.22065.2	310	0.4665	9.426208496	1
MSTRG.22068.1	876	0.43207	10.05255127	1
MSTRG.22071.2	330	0.4569	6.164001465	1
MSTRG.22072.1	816	0.41101	8.469543457	1
MSTRG.22072.3	163	0.41229	5.77947998	1
MSTRG.22074.1	130	0.38247	8.865539551	1
MSTRG.22070.1	183	0.31834	9.134094238	1
MSTRG.22070.2	210	0.30966	9.377624512	1
MSTRG.22075.2	700	0.38937	8.597229004	1
MSTRG.22075.3	700	0.36352	8.597229004	1
MSTRG.22075.4	700	0.33986	8.597229004	1
MSTRG.22076.1	65	0.45989	6.696594238	-1
MSTRG.22078.3	592	0.45962	6.718688965	1
MSTRG.22080.1	287	0.43675	10.68902588	1
MSTRG.22080.6	287	0.46686	10.68902588	1
MSTRG.22113.6	2492	0.43203	5.693786621	1
MSTRG.22113.5	4415	0.45961	5.713317871	1
MSTRG.22113.7	3825	0.48734	5.717346191	-1
MSTRG.22114.1	183	0.3064	9.75177002	1
MSTRG.22113.9	1982	0.43668	5.874328613	1
MSTRG.22115.3	581	0.39862	5.590148926	1
MSTRG.22116.1	32	0.33336	4.948425293	1
MSTRG.22117.1	52	0.32883	9.302307129	-1
MSTRG.22118.4	590	0.49517	6.671447754	1
MSTRG.22118.2	947	0.45711	6.205871582	1
MSTRG.22118.3	486	0.33958	7.588684082	1
MSTRG.22118.5	761	0.44656	5.886413574	1
MSTRG.22126.1	845	0.41752	5.675476074	1
MSTRG.22126.2	845	0.40234	5.649475098	1
MSTRG.22126.3	845	0.32999	5.649475098	1
MSTRG.22126.4	845	0.40234	5.675476074	1
MSTRG.22126.5	717	0.40572	5.851257324	1
MSTRG.22126.10	754	0.39693	6.195251465	1
MSTRG.22082.3	2473	0.43768	6.41583252	1
MSTRG.22082.5	983	0.47722	5.843200684	1
MSTRG.22082.4	2599	0.43768	7.180480957	-1
MSTRG.22084.2	424	0.29615	6.747375488	1
MSTRG.22086.1	23	0.46683	12.17852783	1
MSTRG.22089.3	224	0.32946	7.812561035	1
MSTRG.22089.4	213	0.34547	7.797180176	1
MSTRG.22089.5	224	0.3665	7.812561035	1
MSTRG.22091.1	89	0.40872	6.889587402	1
MSTRG.22091.3	89	0.42419	6.889587402	1
MSTRG.22098.2	71	0.32575	10.43939209	1
MSTRG.22099.2	211	0.41151	4.574157715	1
MSTRG.22103.2	216	0.2899	5.328918457	1
MSTRG.22139.5	470	0.46464	6.17779541	1
MSTRG.22139.6	713	0.4318	6.425964355	1
MSTRG.22141.1	70	0.33843	10.73651123	1
MSTRG.22141.2	118	0.27379	10.58892822	1
MSTRG.22141.3	51	0.30617	11.26971436	-1
MSTRG.22142.1	130	0.33287	8.81451416	1

MSTRG.22145.2	184	0.40334	9.535217285	1
MSTRG.22146.1	1068	0.4367	8.458435059	1
MSTRG.22146.2	1068	0.44272	8.458435059	1
MSTRG.22146.3	1068	0.42389	8.458435059	1
MSTRG.22147.2	323	0.35373	5.70501709	1
MSTRG.22147.3	312	0.31956	6.356872559	1
MSTRG.22155.1	2272	0.44221	6.061706543	1
MSTRG.22155.2	2273	0.43663	6.132263184	1
MSTRG.22155.4	1452	0.4418	6.223937988	1
MSTRG.22155.3	1452	0.44922	6.223937988	1
MSTRG.22155.7	189	0.47277	9.58416748	1
MSTRG.22150.1	83	0.313	4.761291504	1
MSTRG.22152.2	379	0.33988	9.116882324	1
MSTRG.22152.3	381	0.31986	5.288391113	1
MSTRG.22152.5	617	0.31337	5.566711426	1
MSTRG.22153.1	510	0.38207	6.061096191	1
MSTRG.22168.1	272	0.27957	9.716247559	1
MSTRG.22179.1	189	0.28002	6.525817871	1
MSTRG.22179.2	205	0.3016	12.17755127	1
MSTRG.22179.3	164	0.27251	10.75189209	1
MSTRG.22179.4	134	0.27251	5.756164551	1
MSTRG.22179.5	134	0.27251	5.756164551	1
MSTRG.22179.7	146	0.27651	9.493103027	1
MSTRG.22179.8	146	0.33464	9.493103027	1
MSTRG.22179.9	146	0.28195	9.493103027	1
MSTRG.22179.6	146	0.27651	9.493103027	1
MSTRG.22179.10	146	0.27651	9.493103027	1
MSTRG.22179.11	146	0.27651	9.493103027	1
MSTRG.22179.13	146	0.27651	9.493103027	1
MSTRG.22179.12	146	0.28195	9.493103027	1
MSTRG.22179.14	147	0.28324	9.675354004	1
MSTRG.22179.15	147	0.28923	9.675354004	1
MSTRG.22179.16	124	0.28567	8.937438965	1
MSTRG.22181.1	136	0.28923	9.92779541	1
MSTRG.22181.2	136	0.27911	9.92779541	1
MSTRG.22181.3	136	0.27992	9.92779541	1
MSTRG.22181.4	136	0.3331	9.92779541	1
MSTRG.22181.5	111	0.28923	9.97076416	1
MSTRG.22181.6	110	0.28518	9.509338379	1
MSTRG.22182.1	340	0.33897	9.361877441	1
MSTRG.22182.3	463	0.34523	9.108459473	1
MSTRG.22181.7	100	0.28518	7.069396973	1
MSTRG.22184.1	1800	0.38425	9.289611816	1
MSTRG.22184.2	124	0.28122	5.64666748	1
MSTRG.22184.3	1800	0.38425	9.289611816	1
MSTRG.22184.5	1800	0.31175	9.289611816	1
MSTRG.22184.6	1800	0.38198	9.289611816	1
MSTRG.22185.1	89	0.27379	9.09942627	1
MSTRG.22170.2	220	0.4354	6.500793457	1
MSTRG.22172.3	280	0.44723	7.969421387	1
MSTRG.22175.1	1295	0.44073	8.553405762	1
MSTRG.22175.2	1289	0.43303	5.953552246	1
MSTRG.22189.2	56	0.37351	8.88873291	1
MSTRG.22190.1	95	0.30557	8.735656738	1

MSTRG.22190.2	120	0.27437	11.02301025	1
MSTRG.22191.2	79	0.31988	11.54803467	1
MSTRG.22192.3	681	0.41285	6.173522949	1
MSTRG.22195.3	377	0.47277	9.358947754	1
MSTRG.22195.2	377	0.43284	9.358947754	1
MSTRG.22178.2	71	0.32486	6.889831543	1
MSTRG.22178.3	195	0.27987	11.50604248	1
MSTRG.22188.1	148	0.33419	9.098083496	1
MSTRG.22197.1	87	0.35185	7.767150879	-1
MSTRG.22219.1	1309	0.36087	6.219909668	1
MSTRG.22220.1	74	0.37287	9.684387207	-1
MSTRG.22219.2	271	0.40899	6.412536621	1
MSTRG.22219.5	198	0.32642	6.231384277	1
MSTRG.22219.3	198	0.32582	6.231384277	1
MSTRG.22223.5	94	0.34881	9.597961426	1
MSTRG.22224.1	193	0.30285	9.940612793	1
MSTRG.22226.1	183	0.28642	10.08807373	1
MSTRG.22228.1	530	0.2921	8.686584473	1
MSTRG.22228.2	37	0.30419	11.74432373	1
MSTRG.22223.6	195	0.33496	8.512023926	1
MSTRG.22230.1	53	0.2876	9.382629395	1
MSTRG.22199.1	87	0.44252	6.492004395	1
MSTRG.22200.1	109	0.27911	7.588317871	1
MSTRG.22203.2	180	0.45303	9.53692627	1
MSTRG.22204.1	71	0.40135	11.23431396	1
MSTRG.22205.1	469	0.40516	7.102478027	1
MSTRG.22205.2	402	0.40561	7.086364746	1
MSTRG.22205.4	402	0.37018	7.086364746	1
MSTRG.22207.3	386	0.39445	6.759094238	1
MSTRG.22207.7	386	0.40808	6.759094238	1
MSTRG.22207.9	386	0.41423	6.759094238	1
MSTRG.22208.4	584	0.41162	9.19732666	1
MSTRG.22211.5	223	0.44239	5.933532715	1
MSTRG.22212.2	683	0.46351	6.616394043	1
MSTRG.22212.3	619	0.47257	7.078674316	1
MSTRG.22213.4	149	0.39547	9.602600098	1
MSTRG.22213.5	149	0.36522	9.602600098	1
MSTRG.22213.2	153	0.3649	12.14300537	1
MSTRG.22213.3	153	0.38714	12.14300537	1
MSTRG.22214.4	103	0.31981	7.784606934	1
MSTRG.22216.7	633	0.46022	5.181335449	1
MSTRG.22217.1	77	0.28396	8.934509277	1
MSTRG.22238.4	183	0.28466	10.80096436	1
MSTRG.22238.6	180	0.34931	11.00238037	1
MSTRG.22238.7	183	0.30151	11.00213623	1
MSTRG.22239.1	104	0.2857	8.963317871	1
MSTRG.22240.1	94	0.2887	7.863708496	1
MSTRG.22241.1	354	0.46587	6.522644043	1
MSTRG.22242.2	217	0.39813	4.594543457	1
MSTRG.22242.3	276	0.42456	4.633483887	1
MSTRG.22242.4	216	0.38394	4.594543457	1
MSTRG.22245.2	793	0.44144	5.153381348	1
MSTRG.22246.3	661	0.41636	6.340393066	1
MSTRG.22251.3	133	0.37241	5.52947998	1

MSTRG.22251.2	400	0.4332	5.920959473	1
MSTRG.22251.4	346	0.45868	5.897155762	1
MSTRG.22261.1	188	0.2785	7.076721191	1
MSTRG.22261.2	188	0.34942	7.076721191	1
MSTRG.22262.2	93	0.32002	9.179504395	1
MSTRG.22262.3	147	0.41784	9.898132324	1
MSTRG.22262.4	140	0.35615	5.774597168	1
MSTRG.22262.5	156	0.46706	10.55426025	1
MSTRG.22265.1	32	0.31461	12.18109131	1
MSTRG.22264.3	693	0.47047	5.80657959	1
MSTRG.22264.4	693	0.46552	5.80657959	1
MSTRG.22276.2	147	0.35285	10.18865967	1
MSTRG.22277.1	1039	0.45057	8.547912598	1
MSTRG.22277.3	998	0.4534	8.007019043	1
MSTRG.22277.10	951	0.45571	8.749572754	1
MSTRG.22277.5	933	0.45875	8.565002441	1
MSTRG.22277.9	994	0.44909	8.922790527	1
MSTRG.22277.7	977	0.44909	8.789489746	1
MSTRG.22277.11	76	0.33058	5.096862793	1
MSTRG.22269.2	192	0.3227	6.08795166	1
MSTRG.22269.3	103	0.29456	9.97088623	1
MSTRG.22271.1	48	0.39435	4.049133301	1
MSTRG.22282.1	134	0.2857	10.27020264	1
MSTRG.22282.2	376	0.28561	11.08099365	1
MSTRG.22282.3	376	0.28561	11.08099365	1
MSTRG.22283.3	353	0.29395	9.15838623	1
MSTRG.22284.2	984	0.45253	8.309509277	1
MSTRG.22285.1	1250	0.42821	11.41143799	1
MSTRG.22286.3	388	0.43817	9.567565918	1
MSTRG.22286.6	388	0.45296	9.567565918	1
MSTRG.22301.1	87	0.37041	6.714416504	1
MSTRG.22309.1	162	0.45473	9.045227051	-1
MSTRG.22310.1	256	0.39612	8.741149902	1
MSTRG.22312.3	545	0.4638	8.121887207	1
MSTRG.22312.2	222	0.41295	8.806945801	1
MSTRG.22306.4	24	0.37633	9.349304199	1
MSTRG.22315.1	86	0.45175	9.577209473	1
MSTRG.22306.5	3	0.36156	3.799377441	1
MSTRG.22306.7	3	0.34503	3.799377441	1
MSTRG.22306.8	3	0.35889	3.799377441	1
MSTRG.22306.6	3	0.39961	3.799377441	1
MSTRG.22306.9	3	0.40577	3.799377441	1
MSTRG.22306.11	9	0.40751	5.972106934	1
MSTRG.22295.1	453	0.35136	9.048522949	1
MSTRG.22295.3	31	0.39004	8.023742676	1
MSTRG.22299.3	230	0.47344	4.72064209	1
MSTRG.22300.1	134	0.4291	4.739562988	1
MSTRG.22300.3	148	0.38829	4.781066895	1
MSTRG.22300.4	62	0.46036	7.777770996	-1
MSTRG.22316.1	210	0.27469	11.76422119	1
MSTRG.22316.2	180	0.30706	12.18670654	1
MSTRG.22319.2	181	0.45643	9.488830566	1
MSTRG.22319.3	122	0.48716	5.879943848	1
MSTRG.22332.7	561	0.29826	8.808532715	1

MSTRG.22326.2	352	0.35974	5.768249512	1
MSTRG.22327.1	377	0.35275	8.186950684	1
MSTRG.22327.3	568	0.38259	5.944763184	1
MSTRG.22325.1	152	0.31054	8.823669434	1
MSTRG.22335.2	294	0.38169	8.657531738	-1
MSTRG.22335.3	288	0.3567	8.379699707	-1
MSTRG.22341.2	167	0.31837	11.33660889	1
MSTRG.22341.3	234	0.22904	7.544372559	1
MSTRG.22344.1	340	0.44766	5.811828613	1
MSTRG.22344.2	340	0.44113	5.811828613	1
MSTRG.22344.4	340	0.43779	5.811828613	1
MSTRG.22344.5	295	0.45146	8.181091309	-1
MSTRG.22344.7	218	0.47409	5.632263184	-1
MSTRG.22344.9	379	0.43741	5.425964355	1
MSTRG.22346.1	47	0.33511	6.032043457	1
MSTRG.22344.11	45	0.38419	6.542541504	1
MSTRG.22338.2	696	0.38717	5.996154785	1
MSTRG.22338.4	110	0.38367	6.679504395	-1
MSTRG.22340.2	67	0.3215	8.886779785	1
MSTRG.22348.3	162	0.30124	5.725036621	1
MSTRG.22355.2	148	0.34639	8.575622559	1
MSTRG.22356.1	367	0.42537	10.41204834	1
MSTRG.22356.4	501	0.47193	8.793640137	1
MSTRG.22356.5	364	0.449	10.29193115	1
MSTRG.22356.3	376	0.41607	10.29193115	1
MSTRG.22351.2	513	0.42726	5.409362793	1
MSTRG.22351.3	513	0.42931	5.409362793	1
MSTRG.22358.2	108	0.34766	9.147399902	1
MSTRG.22359.2	2069	0.42098	6.506530762	1
MSTRG.22361.2	144	0.33498	7.866638184	1
MSTRG.22370.2	745	0.43511	5.878234863	1
MSTRG.22370.4	591	0.41921	6.01373291	1
MSTRG.22378.3	798	0.41037	8.410949707	1
MSTRG.22378.5	810	0.41925	8.764953613	1
MSTRG.22378.6	819	0.41692	8.40802002	1
MSTRG.22378.7	798	0.45337	8.410949707	1
MSTRG.22367.3	520	0.46367	9.24810791	1
MSTRG.22367.4	484	0.37201	9.16595459	1
MSTRG.22367.5	261	0.26902	10.45379639	1
MSTRG.22382.1	544	0.34856	5.148010254	1
MSTRG.22382.4	578	0.32323	5.55682373	1
MSTRG.22382.5	243	0.35844	4.735168457	1
MSTRG.22382.8	445	0.35414	5.042785645	1
MSTRG.22382.6	578	0.35011	5.55682373	1
MSTRG.22382.7	578	0.31376	5.55682373	1
MSTRG.22382.9	578	0.31438	5.55682373	1
MSTRG.22383.1	358	0.41398	8.938049316	1
MSTRG.22383.2	358	0.41398	8.938049316	1
MSTRG.22383.3	358	0.41398	8.938049316	1
MSTRG.22383.4	358	0.41398	9.016784668	1
MSTRG.22390.8	560	0.38689	8.729187012	1
MSTRG.22390.7	573	0.44117	8.471374512	1
MSTRG.22390.6	886	0.45596	8.52545166	1
MSTRG.22390.2	938	0.45596	8.628967285	1

MSTRG.22391.2	108	0.42557	12.10028076	1
MSTRG.22391.4	108	0.41043	12.10028076	1
MSTRG.22392.2	232	0.30359	9.485900879	1
MSTRG.22392.4	57	0.36805	12.22198486	1
MSTRG.22394.2	87	0.33648	5.515075684	1
MSTRG.22394.3	231	0.44709	4.907897949	1
MSTRG.22385.2	378	0.37057	6.212341309	1
MSTRG.22386.2	439	0.42544	10.0088501	1
MSTRG.22397.2	425	0.38719	6.38067627	1
MSTRG.22397.3	425	0.40769	6.38067627	1
MSTRG.22397.4	488	0.38663	6.62713623	1
MSTRG.22398.1	272	0.45064	5.610168457	1
MSTRG.22399.1	893	0.39208	6.411682129	1
MSTRG.22399.2	866	0.38464	6.295593262	1
MSTRG.22400.1	151	0.28628	10.4430542	1
MSTRG.22402.2	198	0.40134	9.233215332	1
MSTRG.22402.3	147	0.41832	9.270690918	1
MSTRG.22403.1	199	0.29566	11.56878662	1
MSTRG.22406.2	156	0.45939	6.356994629	1
MSTRG.22406.5	209	0.4624	4.85369873	1
MSTRG.22411.2	428	0.40751	6.415466309	1
MSTRG.22411.1	409	0.38277	7.488342285	1
MSTRG.22407.1	245	0.35419	9.184875488	1
MSTRG.22407.2	480	0.3669	9.241027832	1
MSTRG.22408.1	108	0.32907	11.2723999	1
MSTRG.22419.1	184	0.3432	9.969055176	-1
MSTRG.22420.1	53	0.33351	7.977600098	1
MSTRG.22417.1	41	0.3332	5.194885254	1
MSTRG.22417.2	72	0.36499	4.879699707	1
MSTRG.22426.1	73	0.38283	11.01019287	1
MSTRG.22428.2	695	0.45024	6.153137207	1
MSTRG.22430.2	650	0.33533	5.242614746	1
MSTRG.22435.2	1079	0.40691	6.207946777	1
MSTRG.22431.1	195	0.34857	9.226989746	1
MSTRG.22444.7	370	0.45869	8.036193848	1
MSTRG.22444.5	479	0.48432	6.085510254	1
MSTRG.22449.1	968	0.46516	5.304748535	1
MSTRG.22444.10	35	0.28167	11.699646	1
MSTRG.22449.3	968	0.49008	5.304748535	1
MSTRG.22449.4	968	0.47816	5.304748535	1
MSTRG.22449.5	968	0.48238	5.304748535	1
MSTRG.22449.9	968	0.49008	5.304748535	1
MSTRG.22449.10	968	0.47573	5.304748535	1
MSTRG.22451.3	878	0.3507	6.09185791	1
MSTRG.22451.8	878	0.3712	6.09185791	1
MSTRG.22451.5	878	0.37281	6.09185791	1
MSTRG.22451.9	878	0.35231	6.09185791	1
MSTRG.22451.13	186	0.43498	6.594665527	1
MSTRG.22451.14	878	0.46288	6.09185791	1
MSTRG.22451.11	878	0.46288	6.09185791	1
MSTRG.22451.12	878	0.4654	6.09185791	1
MSTRG.22442.1	73	0.34295	10.39910889	1
MSTRG.22459.3	228	0.28432	7.090881348	1
MSTRG.22459.1	388	0.29238	8.616638184	1

MSTRG.22459.4	431	0.28191	8.680725098	1
MSTRG.22459.5	388	0.29703	8.616638184	1
MSTRG.22459.6	917	0.40002	8.869445801	1
MSTRG.22460.1	130	0.29857	10.54547119	1
MSTRG.22460.2	130	0.32523	10.54547119	1
MSTRG.22461.1	151	0.34147	5.189758301	1
MSTRG.22461.2	71	0.37113	4.78692627	1
MSTRG.22461.4	82	0.34844	11.92462158	1
MSTRG.22461.5	71	0.37793	4.78692627	1
MSTRG.22461.6	141	0.31388	5.296813965	1
MSTRG.22454.1	128	0.32974	7.691223145	1
MSTRG.22454.2	95	0.39551	9.403015137	1
MSTRG.22455.3	638	0.36995	5.450134277	1
MSTRG.22464.1	94	0.29772	9.767272949	1
MSTRG.22465.1	181	0.26845	6.114196777	1
MSTRG.22466.1	79	0.31348	8.406433105	1
MSTRG.22478.3	592	0.47377	5.284362793	1
MSTRG.22478.2	580	0.3765	5.703308105	1
MSTRG.22478.4	594	0.48225	5.71282959	1
MSTRG.22478.5	580	0.37569	5.643615723	1
MSTRG.22478.7	596	0.37349	7.092224121	1
MSTRG.22478.6	634	0.39604	5.683654785	1
MSTRG.22478.10	634	0.47144	5.683654785	1
MSTRG.22478.11	594	0.46404	5.801818848	1
MSTRG.22480.1	122	0.32646	10.49517822	1
MSTRG.22482.1	21	0.33199	5.426330566	1
MSTRG.22490.1	339	0.46572	8.17755127	1
MSTRG.22490.2	265	0.46551	9.096984863	1
MSTRG.22490.3	378	0.48964	8.355651855	1
MSTRG.22490.4	175	0.38177	7.747253418	1
MSTRG.22490.5	271	0.45859	9.000793457	1
MSTRG.22490.6	187	0.46264	9.219299316	1
MSTRG.22490.7	451	0.48013	7.16973877	1
MSTRG.22490.9	253	0.42722	8.76361084	1
MSTRG.22468.1	58	0.38988	5.631774902	1
MSTRG.22469.1	104	0.34622	8.598693848	-1
MSTRG.22476.1	39	0.45359	9.496520996	1
MSTRG.22477.1	352	0.4861	9.196472168	-1
MSTRG.22519.1	548	0.4461	6.19329834	1
MSTRG.22521.1	69	0.28068	9.51739502	1
MSTRG.22517.1	42	0.34485	6.069152832	-1
MSTRG.22517.2	145	0.31974	9.313049316	1
MSTRG.22517.3	115	0.28415	7.530578613	1
MSTRG.22524.1	90	0.36185	9.659118652	1
MSTRG.22526.1	334	0.45751	4.919494629	1
MSTRG.22539.1	85	0.36495	9.075744629	1
MSTRG.22542.2	377	0.40971	6.486999512	1
MSTRG.22544.1	43	0.33976	4.512512207	1
MSTRG.22544.2	43	0.3485	4.512512207	1
MSTRG.22528.1	54	0.39917	9.29888916	-1
MSTRG.22530.1	74	0.40647	11.13287354	1
MSTRG.22549.1	139	0.28224	7.762023926	1
MSTRG.22549.2	139	0.28224	7.762023926	1
MSTRG.22551.3	49	0.4155	10.04400635	1

MSTRG.22551.2	74	0.35389	6.633361816	1
MSTRG.22551.4	55	0.33123	6.897521973	1
MSTRG.22551.5	44	0.34557	4.501037598	1
MSTRG.22552.2	323	0.47029	8.745788574	1
MSTRG.22550.2	595	0.40333	4.258972168	1
MSTRG.22553.3	396	0.37903	8.137268066	1
MSTRG.22563.1	597	0.32582	5.533630371	1
MSTRG.22563.3	92	0.31137	10.74359131	1
MSTRG.22564.3	223	0.39776	6.729309082	1
MSTRG.22572.1	1262	0.42465	6.389099121	1
MSTRG.22574.1	105	0.28096	9.256652832	1
MSTRG.22572.2	1262	0.43441	6.389099121	1
MSTRG.22572.3	1262	0.4263	6.389099121	1
MSTRG.22576.1	73	0.32442	9.37689209	1
MSTRG.22580.1	99	0.36086	8.676208496	1
MSTRG.22579.3	21	0.38336	11.71136475	1
MSTRG.22579.4	28	0.38339	9.500671387	1
MSTRG.22583.2	76	0.44926	4.144958496	1
MSTRG.22583.5	76	0.43771	4.144958496	1
MSTRG.22592.2	478	0.42166	6.309509277	1
MSTRG.22592.3	478	0.42166	6.309509277	1
MSTRG.22592.4	542	0.42166	6.554626465	1
MSTRG.22585.2	104	0.46227	9.414245605	1
MSTRG.22585.1	104	0.44076	6.405822754	1
MSTRG.22593.4	1052	0.45764	8.83026123	1
MSTRG.22598.2	541	0.42942	8.969055176	1
MSTRG.22598.4	507	0.40542	8.708557129	1
MSTRG.22599.1	211	0.37128	8.809875488	1
MSTRG.22599.2	211	0.34825	8.809875488	1
MSTRG.22599.3	211	0.37509	8.809875488	1
MSTRG.22599.4	60	0.35603	7.621643066	1
MSTRG.22599.5	211	0.29456	8.809875488	1
MSTRG.22605.1	431	0.34939	11.19647217	1
MSTRG.22607.3	138	0.29152	6.818786621	1
MSTRG.22609.1	82	0.30743	10.00189209	1
MSTRG.22608.2	479	0.30041	8.504699707	1
MSTRG.22610.1	186	0.30962	8.953918457	1
MSTRG.22610.2	107	0.29459	6.797790527	1
MSTRG.22611.1	143	0.31412	10.5848999	1
MSTRG.22612.1	139	0.27327	10.71954346	1
MSTRG.22608.4	479	0.35917	8.504699707	1
MSTRG.22617.3	82	0.30132	10.08648682	1
MSTRG.22617.1	82	0.29857	10.08648682	1
MSTRG.22617.4	82	0.30132	10.08648682	1
MSTRG.22618.1	99	0.34017	4.68157959	1
MSTRG.22627.2	1201	0.28746	7.347229004	1
MSTRG.22627.3	1214	0.44853	7.550720215	1
MSTRG.22627.4	1200	0.45973	7.551330566	1
MSTRG.22627.5	1200	0.45632	7.347229004	1
MSTRG.22627.6	1204	0.4498	7.552429199	1
MSTRG.22627.7	1203	0.45632	7.552429199	1
MSTRG.22627.8	1189	0.45018	7.553039551	1
MSTRG.22621.2	85	0.40072	4.826721191	1
MSTRG.22624.2	426	0.46078	6.378845215	1

MSTRG.22633.4	415	0.44409	8.457214355	1
MSTRG.22634.3	125	0.31824	7.707824707	1
MSTRG.22644.1	184	0.27274	10.00860596	1
MSTRG.22644.4	135	0.39207	9.719543457	1
MSTRG.22644.5	102	0.37538	9.892028809	1
MSTRG.22644.3	135	0.36171	9.719543457	1
MSTRG.22646.1	66	0.30132	5.160461426	1
MSTRG.22646.2	123	0.33911	11.33599854	1
MSTRG.22646.4	79	0.30981	9.645935059	1
MSTRG.22650.1	47	0.38378	6.222717285	1
MSTRG.22650.5	47	0.31824	6.222717285	1
MSTRG.22650.6	64	0.30988	11.15997314	1
MSTRG.22650.3	57	0.37477	8.657775879	-1
MSTRG.22650.4	57	0.36219	8.657775879	-1
MSTRG.22650.7	47	0.3376	6.222717285	1
MSTRG.22639.2	414	0.42016	4.636779785	1
MSTRG.22651.3	572	0.50404	5.648254395	1
MSTRG.22651.4	404	0.44616	6.496276855	1
MSTRG.22651.5	439	0.4323	6.885681152	1
MSTRG.22651.6	436	0.4323	6.885681152	1
MSTRG.22651.7	407	0.44616	6.496276855	1
MSTRG.22654.1	103	0.39104	5.180236816	1
MSTRG.22656.1	178	0.27869	8.59197998	1
MSTRG.22660.2	104	0.39986	8.949645996	-1
MSTRG.22668.2	318	0.38979	6.128723145	-1
MSTRG.22664.1	431	0.44239	6.625427246	1
MSTRG.22683.4	313	0.34806	8.994689941	1
MSTRG.22684.1	239	0.29946	11.11175537	1
MSTRG.22686.2	2565	0.44945	6.67755127	1
MSTRG.22686.3	2565	0.44597	6.67755127	1
MSTRG.22689.1	169	0.27439	11.25689697	1
MSTRG.22679.1	166	0.28305	12.034729	1
MSTRG.22703.3	196	0.47164	7.774475098	1
MSTRG.22703.2	196	0.45124	7.774475098	1
MSTRG.22709.2	348	0.3832	9.889221191	1
MSTRG.22713.1	514	0.32617	6.068908691	1
MSTRG.22713.5	608	0.4522	6.09967041	1
MSTRG.22713.4	608	0.4371	6.09967041	1
MSTRG.22714.1	111	0.35788	6.259216309	1
MSTRG.22694.2	390	0.3276	6.722961426	1
MSTRG.22718.1	87	0.30132	8.81829834	1
MSTRG.22718.2	240	0.27937	11.08868408	1
MSTRG.22718.3	81	0.2921	10.65106201	1
MSTRG.22718.4	142	0.3287	11.49676514	1
MSTRG.22721.1	86	0.31997	11.49310303	1
MSTRG.22722.1	137	0.33643	9.804016113	1
MSTRG.22725.1	56	0.30806	9.18536377	1
MSTRG.22731.2	523	0.40998	8.242126465	1
MSTRG.22733.1	88	0.38536	4.61138916	1
MSTRG.22733.2	88	0.35766	4.61138916	1
MSTRG.22737.2	223	0.32823	8.79095459	1
MSTRG.22739.1	64	0.31255	6.801940918	1
MSTRG.22740.1	271	0.38054	6.13104248	-1
MSTRG.22740.2	271	0.38512	6.13104248	-1

MSTRG.22740.4	152	0.40984	6.183898926	1
MSTRG.22742.1	722	0.48128	6.627258301	1
MSTRG.22742.2	398	0.38508	8.332214355	1
MSTRG.22744.2	782	0.45557	7.129577637	1
MSTRG.22744.3	507	0.43788	8.824157715	1
MSTRG.22744.5	637	0.39041	6.738952637	1
MSTRG.22744.4	1237	0.45633	8.994445801	1
MSTRG.22744.6	874	0.45819	8.853942871	1
MSTRG.22744.8	355	0.40365	10.17913818	1
MSTRG.22746.1	1032	0.45323	7.49798584	1
MSTRG.22747.1	228	0.46435	6.070495605	1
MSTRG.22747.3	87	0.29476	9.298522949	1
MSTRG.22747.4	693	0.46602	7.046203613	1
MSTRG.22749.4	304	0.28883	5.918029785	1
MSTRG.22750.5	77	0.34808	6.558288574	1
MSTRG.22755.1	70	0.33007	8.448181152	1
MSTRG.22763.2	607	0.37717	6.848205566	1
MSTRG.22763.4	659	0.40702	6.554870605	1
MSTRG.22763.5	580	0.4247	6.621032715	1
MSTRG.22763.6	639	0.40034	6.905700684	1
MSTRG.22764.1	651	0.43867	5.85760498	1
MSTRG.22764.3	482	0.47404	4.990661621	1
MSTRG.22770.2	953	0.3723	5.492614746	1
MSTRG.22774.1	38	0.4474	4.895812988	1
MSTRG.22776.1	71	0.33291	9.646911621	1
MSTRG.22779.1	56	0.3818	8.978820801	1
MSTRG.22780.1	79	0.43413	9.261901855	1
MSTRG.22790.3	2294	0.45366	7.012268066	1
MSTRG.22790.4	2866	0.47804	6.467468262	1
MSTRG.22790.5	2278	0.4462	6.76373291	1
MSTRG.22793.1	155	0.3726	11.02520752	1
MSTRG.22798.3	858	0.43565	5.698791504	1
MSTRG.22800.5	292	0.40365	9.337463379	1
MSTRG.22800.3	292	0.43167	9.337463379	1
MSTRG.22784.1	1270	0.4779	5.375671387	1
MSTRG.22785.4	491	0.32257	5.902282715	1
MSTRG.22785.5	238	0.37411	4.676940918	1
MSTRG.22785.6	128	0.29268	6.485656738	1
MSTRG.22786.1	53	0.37914	10.75018311	1
MSTRG.22787.1	83	0.35748	5.889343262	1
MSTRG.22789.2	64	0.35727	9.687805176	1
MSTRG.22803.1	116	0.35494	8.721374512	1
MSTRG.22804.1	453	0.45774	5.365661621	-1
MSTRG.22804.3	453	0.46823	5.365661621	-1
MSTRG.22804.4	453	0.45798	5.365661621	-1
MSTRG.22806.2	39	0.46685	7.366027832	1
MSTRG.22811.1	548	0.41803	6.001037598	1
MSTRG.22812.3	354	0.34386	5.615783691	1
MSTRG.22813.1	261	0.27379	8.720275879	1
MSTRG.22814.2	931	0.46467	6.077575684	1
MSTRG.22818.2	819	0.47404	5.499694824	1
MSTRG.22819.2	388	0.42922	5.327575684	1
MSTRG.22819.1	389	0.36196	5.327575684	1
MSTRG.22822.2	130	0.42228	9.158752441	1

MSTRG.22822.3	130	0.47619	9.158752441	1
MSTRG.22825.1	253	0.35156	5.204772949	1
MSTRG.22825.7	118	0.40413	4.774108887	1
MSTRG.22825.5	253	0.44465	5.204772949	1
MSTRG.22825.6	253	0.41096	5.204772949	1
MSTRG.22827.1	84	0.33578	11.00006104	1
MSTRG.22829.3	119	0.34455	4.522521973	1
MSTRG.22829.4	119	0.3277	4.522521973	1
MSTRG.22829.2	152	0.31056	4.309509277	1
MSTRG.22824.1	458	0.35832	6.989440918	1
MSTRG.22832.5	108	0.42811	4.768859863	1
MSTRG.22832.6	311	0.4104	5.226745605	1
MSTRG.22832.4	310	0.40041	5.226745605	1
MSTRG.22833.2	277	0.42469	4.88067627	1
MSTRG.22833.3	277	0.43813	4.88067627	1
MSTRG.22841.2	663	0.44189	7.808044434	1
MSTRG.22841.3	663	0.38598	7.808044434	1
MSTRG.22842.8	694	0.49342	6.313171387	1
MSTRG.22842.9	855	0.46521	7.621032715	1
MSTRG.22842.10	906	0.42995	8.397033691	1
MSTRG.22842.12	802	0.43101	6.324035645	1
MSTRG.22842.13	945	0.43101	8.111022949	1
MSTRG.22835.1	560	0.41084	7.964904785	1
MSTRG.22835.2	627	0.46717	7.07989502	1
MSTRG.22836.1	130	0.31285	10.29217529	1
MSTRG.22838.4	202	0.4253	7.535705566	1
MSTRG.22838.5	202	0.44597	7.535705566	1
MSTRG.22838.3	202	0.41744	7.535705566	1
MSTRG.22838.2	216	0.36274	9.194763184	1
MSTRG.22845.2	213	0.30051	6.487731934	1
MSTRG.22847.3	449	0.46376	8.56463623	1
MSTRG.22857.1	456	0.49129	4.931945801	1
MSTRG.22857.2	229	0.44677	9.257507324	1
MSTRG.22858.3	2377	0.48466	6.588439941	1
MSTRG.22858.5	2378	0.47231	6.588439941	1
MSTRG.22858.6	2376	0.48666	6.653259277	1
MSTRG.22858.7	2377	0.48666	6.588439941	1
MSTRG.22858.8	2375	0.48466	6.653259277	1
MSTRG.22858.10	2382	0.47696	6.653625488	1
MSTRG.22858.4	2371	0.48466	6.58795166	1
MSTRG.22858.9	2377	0.47696	6.588439941	1
MSTRG.22867.1	799	0.47819	4.566467285	1
MSTRG.22871.1	78	0.32553	9.443908691	1
MSTRG.22871.2	36	0.43394	10.02435303	1
MSTRG.22881.2	796	0.47802	4.743713379	1
MSTRG.22881.3	599	0.48159	5.186340332	1
MSTRG.22892.2	275	0.36693	8.84967041	1
MSTRG.22892.4	275	0.36693	8.84967041	1
MSTRG.22892.5	98	0.29527	9.429992676	1
MSTRG.22893.1	96	0.44672	9.696838379	1
MSTRG.22895.2	180	0.44574	9.600891113	1
MSTRG.22896.1	696	0.37818	7.649963379	1
MSTRG.22896.4	698	0.4664	7.236022949	1
MSTRG.22898.2	445	0.46546	5.197814941	1

MSTRG.22899.1	106	0.40844	12.17755127	1
MSTRG.22909.2	242	0.37229	5.938903809	1
MSTRG.22911.3	471	0.46574	9.213928223	1
MSTRG.22912.1	121	0.34043	9.676940918	1
MSTRG.22913.2	568	0.43894	5.396789551	1
MSTRG.22914.3	136	0.45849	6.309509277	1
MSTRG.22919.1	1196	0.46186	5.78326416	1
MSTRG.22921.1	66	0.3927	10.25604248	1
MSTRG.22922.1	632	0.37458	7.101135254	1
MSTRG.22922.3	642	0.39439	6.760314941	1
MSTRG.22924.1	368	0.37839	8.901550293	1
MSTRG.22922.6	69	0.412	4.950012207	1
MSTRG.22924.3	160	0.29328	9.338562012	1
MSTRG.22924.2	368	0.42173	8.901550293	1
MSTRG.22928.2	1326	0.46862	5.960754395	1
MSTRG.22931.5	727	0.413	6.508728027	1
MSTRG.22931.6	725	0.45076	6.508728027	1
MSTRG.22933.1	455	0.43287	5.684143066	1
MSTRG.22935.1	306	0.43486	10.21990967	1
MSTRG.22936.1	319	0.42056	5.290100098	1
MSTRG.22937.2	385	0.40876	5.938049316	1
MSTRG.22937.3	385	0.41496	5.938049316	1
MSTRG.22941.4	514	0.46383	5.196716309	-1
MSTRG.22943.1	303	0.45333	9.050354004	1
MSTRG.22944.2	127	0.33359	5.09588623	1
MSTRG.22925.3	1387	0.46513	5.602722168	1
MSTRG.22946.1	129	0.42862	9.096008301	1
MSTRG.22946.4	89	0.44042	4.376403809	1
MSTRG.22947.2	250	0.38838	5.964782715	1
MSTRG.22948.3	1034	0.43502	6.560241699	1
MSTRG.22948.4	1080	0.4586	6.237365723	1
MSTRG.22948.2	1055	0.42901	6.529968262	1
MSTRG.22948.5	1034	0.44082	6.560241699	1
MSTRG.22951.1	493	0.43006	6.739440918	1
MSTRG.22951.2	493	0.4567	6.739440918	1
MSTRG.22953.1	594	0.4144	6.370910645	1
MSTRG.22953.2	587	0.37772	6.282043457	1
MSTRG.22953.8	128	0.31852	8.666931152	1
MSTRG.22953.4	594	0.42227	6.370910645	1
MSTRG.22953.5	594	0.40045	6.370910645	1
MSTRG.22953.6	587	0.42723	6.282043457	1
MSTRG.22953.7	587	0.38419	6.282043457	1
MSTRG.22953.3	1073	0.4602	5.895935059	1
MSTRG.22954.1	59	0.30733	8.84197998	1
MSTRG.22955.1	55	0.41361	11.00860596	-1
MSTRG.22965.3	113	0.38657	5.300964355	1
MSTRG.22965.5	272	0.46888	9.210754395	-1
MSTRG.22965.4	631	0.49495	4.454040527	1
MSTRG.22965.6	879	0.48539	4.722717285	1
MSTRG.22965.8	380	0.41889	9.12677002	1
MSTRG.22976.4	512	0.4655	12.39862061	1
MSTRG.22977.2	686	0.44573	9.259094238	1
MSTRG.22979.2	297	0.32421	9.894470215	1
MSTRG.22979.3	403	0.32421	8.55657959	1

MSTRG.22983.2	426	0.46444	4.744934082	1
MSTRG.22984.5	189	0.43456	8.594543457	1
MSTRG.22984.1	217	0.36153	8.32635498	1
MSTRG.22984.3	217	0.37028	8.32635498	1
MSTRG.22984.4	153	0.41649	9.560974121	1
MSTRG.22985.2	247	0.37733	7.155334473	1
MSTRG.22986.3	425	0.36665	9.073059082	1
MSTRG.22986.4	425	0.41838	9.073059082	1
MSTRG.22986.5	469	0.32919	9.399475098	1
MSTRG.22966.6	1150	0.45261	4.961853027	1
MSTRG.22966.2	1150	0.32878	4.961853027	1
MSTRG.22966.3	1150	0.32878	4.961853027	1
MSTRG.22966.4	1150	0.32878	4.961853027	1
MSTRG.22966.5	166	0.27517	4.192565918	1
MSTRG.22967.1	48	0.30203	8.841125488	1
MSTRG.22968.1	134	0.28848	8.289855957	1
MSTRG.22967.2	154	0.28723	11.72576904	1
MSTRG.22966.7	1150	0.31596	4.961853027	1
MSTRG.22966.8	1149	0.32878	4.961853027	1
MSTRG.22966.9	1149	0.4431	4.961853027	1
MSTRG.22966.10	1096	0.34463	4.72076416	1
MSTRG.22972.1	413	0.45149	7.211730957	1
MSTRG.23003.1	46	0.39973	4.36895752	-1
MSTRG.23003.2	76	0.44608	10.84136963	1
MSTRG.23003.3	85	0.43183	10.84136963	1
MSTRG.23015.1	3704	0.43637	5.811828613	1
MSTRG.23015.2	3704	0.43637	5.811828613	1
MSTRG.23016.1	101	0.32837	8.856872559	1
MSTRG.23015.3	3704	0.44899	5.811828613	1
MSTRG.23017.1	105	0.27678	9.481384277	1
MSTRG.23018.1	155	0.28542	10.61785889	1
MSTRG.23006.1	42	0.34902	6.374694824	1
MSTRG.23007.1	1035	0.45824	6.14239502	1
MSTRG.23007.3	1020	0.47317	6.02923584	1
MSTRG.23009.1	52	0.31587	9.899719238	1
MSTRG.23009.2	149	0.32575	6.268371582	1
MSTRG.23009.3	106	0.3143	11.89666748	1
MSTRG.23049.1	126	0.43209	9.29901123	-1
MSTRG.23050.1	33	0.36201	9.186706543	1
MSTRG.23048.5	296	0.4464	4.635681152	1
MSTRG.23048.4	296	0.45144	4.635681152	1
MSTRG.23048.6	282	0.43386	4.716125488	1
MSTRG.23052.2	578	0.40088	6.383605957	1
MSTRG.23055.1	822	0.45006	6.356018066	1
MSTRG.23055.5	822	0.4362	6.356018066	1
MSTRG.23054.3	533	0.43207	4.795349121	1
MSTRG.23057.1	175	0.29059	10.18450928	1
MSTRG.23058.1	73	0.31885	6.88067627	1
MSTRG.23061.1	453	0.28923	8.821594238	1
MSTRG.23061.2	39	0.32983	10.91619873	1
MSTRG.23071.1	439	0.38235	5.979187012	1
MSTRG.23080.2	112	0.27274	10.64263916	1
MSTRG.23080.1	112	0.36273	10.64263916	1
MSTRG.23082.1	2629	0.44644	6.619689941	1

MSTRG.23082.2	329	0.38587	5.737243652	1
MSTRG.23082.3	2629	0.44644	6.619689941	1
MSTRG.23082.4	2629	0.44644	6.619689941	1
MSTRG.23084.1	43	0.35645	7.804504395	-1
MSTRG.23072.2	139	0.32075	9.907165527	1
MSTRG.23074.1	456	0.4736	5.073669434	1
MSTRG.23074.2	513	0.48253	5.136657715	1
MSTRG.23075.1	78	0.2725	9.376281738	1
MSTRG.23076.1	77	0.30242	9.299133301	1
MSTRG.23087.3	1300	0.36882	6.922790527	1
MSTRG.23079.2	1036	0.48753	6.81854248	1
MSTRG.23089.2	36	0.30935	10.18743896	1
MSTRG.23089.1	40	0.27991	4.957702637	1
MSTRG.23094.2	298	0.34162	8.57220459	1
MSTRG.23094.4	167	0.29115	8.256408691	1
MSTRG.23094.5	174	0.28334	7.716125488	1
MSTRG.23094.6	174	0.28982	7.716125488	1
MSTRG.23101.1	1300	0.42032	7.287780762	1
MSTRG.23101.2	1308	0.42032	7.420959473	1
MSTRG.23106.3	691	0.48037	5.639831543	1
MSTRG.23106.4	690	0.49121	5.639831543	1
MSTRG.23106.5	690	0.45752	5.639831543	1
MSTRG.23108.1	636	0.46286	5.716491699	1
MSTRG.23105.2	149	0.34079	9.941955566	1
MSTRG.23110.2	386	0.43442	5.461120605	1
MSTRG.23110.3	497	0.4432	5.661071777	1
MSTRG.23122.1	565	0.47014	7.346984863	1
MSTRG.23122.2	493	0.45361	6.771911621	1
MSTRG.23122.3	493	0.46875	6.771911621	1
MSTRG.23127.1	75	0.31099	11.18463135	1
MSTRG.23127.2	93	0.27678	8.797668457	1
MSTRG.23128.1	63	0.40909	9.168151855	1
MSTRG.23130.1	81	0.3366	4.239318848	1
MSTRG.23134.1	289	0.3462	8.270935059	1
MSTRG.23139.2	551	0.38812	8.849182129	1
MSTRG.23139.4	110	0.44464	3.83001709	1
MSTRG.23139.5	146	0.44955	4.135925293	1
MSTRG.23139.6	110	0.48895	3.83001709	1
MSTRG.23139.7	102	0.36918	4.33782959	1
MSTRG.23142.2	131	0.27327	9.958312988	1
MSTRG.23142.1	131	0.27327	9.958312988	1
MSTRG.23145.1	128	0.28927	9.011047363	1
MSTRG.23145.2	142	0.28243	6.877746582	1
MSTRG.23146.1	155	0.38473	4.922790527	-1
MSTRG.23153.2	681	0.4209	5.960021973	1
MSTRG.23153.3	673	0.40425	6.066711426	1
MSTRG.23150.2	237	0.42635	4.910583496	1
MSTRG.23151.1	68	0.42024	8.804016113	1
MSTRG.23154.1	92	0.3972	9.585144043	-1
MSTRG.23159.3	240	0.45382	5.663757324	1
MSTRG.23160.1	577	0.38162	7.111022949	1
MSTRG.23158.1	37	0.45932	7.88104248	1
MSTRG.23158.2	110	0.44554	11.94244385	1
MSTRG.23166.1	85	0.46526	5.285949707	1

MSTRG.23169.1	129	0.35008	9.920837402	1
MSTRG.23173.1	92	0.41703	4.895690918	1
MSTRG.23175.1	101	0.41631	10.17987061	1
MSTRG.23175.2	101	0.39683	10.17987061	1
MSTRG.23174.1	73	0.43383	8.683532715	1
MSTRG.23178.1	325	0.35135	5.261169434	1
MSTRG.23182.1	307	0.43165	6.429992676	1
MSTRG.23187.1	47	0.33119	9.300598145	1
MSTRG.23188.1	245	0.34938	9.610046387	1
MSTRG.23188.2	229	0.28948	9.727478027	1
MSTRG.23188.4	229	0.28948	9.727478027	1
MSTRG.23188.3	227	0.31759	9.152893066	1
MSTRG.23189.2	2448	0.36803	8.550109863	1
MSTRG.23191.3	150	0.32916	7.62701416	1
MSTRG.23191.4	258	0.35695	9.06842041	1
MSTRG.23191.2	232	0.36811	8.929992676	1
MSTRG.23192.4	94	0.38519	9.659851074	1
MSTRG.23186.2	655	0.40889	6.367370605	1
MSTRG.23194.1	1261	0.34876	9.083068848	1
MSTRG.23194.2	1262	0.34876	9.083068848	1
MSTRG.23195.3	544	0.41418	7.108581543	-1
MSTRG.23195.4	596	0.41623	7.787658691	-1
MSTRG.23195.2	544	0.38847	7.108581543	-1
MSTRG.23195.1	596	0.39432	7.787658691	-1
MSTRG.23196.1	139	0.34507	8.25604248	1
MSTRG.23193.1	645	0.47598	5.508972168	1
MSTRG.23197.2	97	0.39271	6.818908691	-1
MSTRG.23202.1	256	0.44586	6.230529785	1
MSTRG.23204.3	1500	0.49686	5.215393066	1
MSTRG.23201.2	183	0.37675	5.233337402	1
MSTRG.23207.1	263	0.43484	5.058654785	1
MSTRG.23207.4	156	0.39875	5.018615723	1
MSTRG.23212.1	82	0.33411	5.113952637	1
MSTRG.23213.1	539	0.39293	9.170227051	1
MSTRG.23221.3	420	0.36797	7.341369629	1
MSTRG.23221.2	420	0.29115	7.341369629	1
MSTRG.23222.1	54	0.32439	4.495178223	1
MSTRG.23223.1	528	0.34752	9.27923584	1
MSTRG.23223.2	425	0.38488	9.525695801	1
MSTRG.23223.3	425	0.3843	9.525695801	1
MSTRG.23223.4	425	0.37563	9.525695801	1
MSTRG.23224.1	49	0.31993	9.034973145	1
MSTRG.23234.1	94	0.32911	7.825866699	1
MSTRG.23237.1	821	0.31861	9.331115723	1
MSTRG.23237.3	812	0.31696	9.359436035	1
MSTRG.23239.1	474	0.42561	8.310241699	1
MSTRG.23241.1	124	0.34695	9.756530762	1
MSTRG.23253.1	87	0.45063	5.43939209	1
MSTRG.23255.2	1780	0.43693	8.660095215	1
MSTRG.23255.4	1781	0.4429	8.660095215	1
MSTRG.23255.5	1780	0.45743	8.660095215	1
MSTRG.23255.6	1210	0.46195	8.879333496	1
MSTRG.23257.1	56	0.30388	11.77276611	1
MSTRG.23255.7	744	0.3777	9.444396973	1

MSTRG.23255.8	547	0.43183	9.348083496	1
MSTRG.23265.1	794	0.44643	5.488952637	1
MSTRG.23265.2	968	0.41075	5.547790527	-1
MSTRG.23265.3	391	0.42122	5.678283691	1
MSTRG.23265.12	794	0.4348	5.488952637	1
MSTRG.23265.11	704	0.42621	5.525695801	-1
MSTRG.23265.13	1060	0.41938	5.51385498	-1
MSTRG.23265.7	1047	0.41938	5.520812988	-1
MSTRG.23265.15	90	0.32831	9.695007324	1
MSTRG.23262.1	379	0.29951	8.077453613	1
MSTRG.23264.1	383	0.36555	6.492126465	1
MSTRG.23258.1	53	0.41107	8.829772949	-1
MSTRG.23259.1	75	0.4563	4.962219238	1
MSTRG.23268.1	218	0.44163	4.807556152	1
MSTRG.23272.1	158	0.2884	11.23956299	1
MSTRG.23272.2	158	0.32014	11.23956299	1
MSTRG.23272.3	158	0.27067	11.23956299	1
MSTRG.23272.4	158	0.27067	10.04766846	1
MSTRG.23272.5	110	0.43037	9.557922363	1
MSTRG.23271.3	145	0.4391	4.364196777	1
MSTRG.23273.3	311	0.3672	8.907775879	1
MSTRG.23275.3	358	0.4748	5.872131348	1
MSTRG.23280.2	376	0.40439	9.355529785	1
MSTRG.23284.2	291	0.39305	8.993835449	1
MSTRG.23285.3	350	0.44803	9.252502441	1
MSTRG.23285.4	562	0.42958	9.773742676	1
MSTRG.23285.5	593	0.46826	9.796325684	1
MSTRG.23285.6	593	0.45477	9.796325684	1
MSTRG.23285.8	537	0.40196	9.703796387	1
MSTRG.23279.1	95	0.38128	10.35601807	1
MSTRG.23291.2	109	0.48638	5.875549316	1
MSTRG.23291.3	109	0.46056	5.875549316	1
MSTRG.23292.3	395	0.34057	9.081970215	1
MSTRG.23292.4	395	0.34243	9.081970215	1
MSTRG.23292.5	695	0.29024	6.338195801	1
MSTRG.23292.6	693	0.43691	6.434875488	1
MSTRG.23292.8	695	0.32983	6.338195801	1
MSTRG.23292.9	693	0.34768	6.434875488	1
MSTRG.23292.10	693	0.35072	6.365783691	1
MSTRG.23297.4	1066	0.48242	4.976135254	1
MSTRG.23297.3	1034	0.47219	5.065368652	1
MSTRG.23295.1	165	0.33223	12.15655518	1
MSTRG.23296.2	267	0.42912	11.3192749	1
MSTRG.23296.3	295	0.43098	11.73468018	1
MSTRG.23301.1	120	0.39726	5.120422363	1
MSTRG.23307.1	589	0.3586	9.24017334	1
MSTRG.23307.2	550	0.39937	9.266906738	1
MSTRG.23307.3	595	0.39328	9.213195801	1
MSTRG.23307.7	604	0.43105	9.356262207	1
MSTRG.23318.4	490	0.43049	6.192199707	1
MSTRG.23318.6	490	0.44062	6.192199707	1
MSTRG.23318.7	490	0.42366	6.192199707	1
MSTRG.23318.8	490	0.43444	6.192199707	1
MSTRG.23318.9	490	0.43136	6.192199707	1

MSTRG.23321.3	120	0.44677	6.782897949	1
MSTRG.23321.2	120	0.46242	6.782897949	1
MSTRG.23322.1	94	0.33418	10.61663818	1
MSTRG.23322.4	75	0.33799	10.78057861	1
MSTRG.23322.5	75	0.36441	10.78057861	1
MSTRG.23322.3	75	0.36828	10.78057861	1
MSTRG.23323.3	541	0.44189	6.870788574	1
MSTRG.23314.1	1103	0.35151	5.397399902	1
MSTRG.23324.2	554	0.40453	9.110656738	1
MSTRG.23325.1	367	0.45717	5.494812012	1
MSTRG.23332.2	282	0.35597	9.831604004	1
MSTRG.23333.1	235	0.40383	6.838562012	1
MSTRG.23333.3	76	0.36575	7.747253418	1
MSTRG.23327.1	108	0.29395	9.504699707	1
MSTRG.23327.2	108	0.32442	9.504699707	1
MSTRG.23327.3	108	0.30725	9.504699707	1
MSTRG.23327.4	156	0.34956	9.466491699	1
MSTRG.23339.3	605	0.43054	5.607849121	1
MSTRG.23347.2	2744	0.34942	6.370056152	1
MSTRG.23347.3	2789	0.36328	6.424499512	1
MSTRG.23348.2	363	0.42057	10.54229736	1
MSTRG.23348.1	363	0.32656	10.54229736	1
MSTRG.23349.1	232	0.46024	6.450378418	1
MSTRG.23349.2	232	0.43842	6.450378418	1
MSTRG.23349.3	232	0.44311	6.450378418	1
MSTRG.23349.4	232	0.43391	6.450378418	1
MSTRG.23349.5	232	0.41899	6.450378418	1
MSTRG.23350.1	1602	0.41933	8.802062988	1
MSTRG.23350.4	2508	0.43473	8.291320801	1
MSTRG.23355.2	477	0.36134	7.153747559	1
MSTRG.23357.1	359	0.35843	8.816711426	1
MSTRG.23360.1	130	0.3467	9.077941895	1
MSTRG.23360.2	130	0.34884	9.077941895	1
MSTRG.23360.3	130	0.33965	9.077941895	1
MSTRG.23361.1	145	0.44961	5.096862793	-1
MSTRG.23352.3	896	0.41314	8.330993652	1
MSTRG.23352.4	896	0.39775	8.330993652	1
MSTRG.23352.5	896	0.40285	8.330993652	1
MSTRG.23365.2	145	0.41884	10.31072998	1
MSTRG.23369.1	246	0.36983	9.040222168	1
MSTRG.23369.9	246	0.33568	9.040222168	1
MSTRG.23369.10	246	0.33954	9.040222168	1
MSTRG.23367.1	38	0.39999	4.51739502	1
MSTRG.23367.3	271	0.4284	8.755187988	1
MSTRG.23370.3	253	0.42192	6.082580566	1
MSTRG.23373.2	245	0.35908	9.643371582	1
MSTRG.23374.3	629	0.45359	6.207458496	1
MSTRG.23377.2	185	0.34804	9.363342285	1
MSTRG.23377.3	170	0.36422	8.967102051	1
MSTRG.23379.1	95	0.2827	5.886901855	1
MSTRG.23380.1	609	0.48618	11.87591553	1
MSTRG.23379.3	194	0.40441	4.110900879	1
MSTRG.23380.2	657	0.45102	11.86517334	1
MSTRG.23380.3	717	0.4567	11.88348389	1

MSTRG.23380.4	681	0.4567	11.8793335	1
MSTRG.23379.4	161	0.43021	4.065246582	-1
MSTRG.23380.5	633	0.48356	11.90057373	1
MSTRG.23380.6	621	0.46666	11.91571045	1
MSTRG.23380.7	693	0.47559	11.88360596	1
MSTRG.23380.8	669	0.47294	11.86785889	1
MSTRG.23382.4	465	0.43368	5.313659668	1
MSTRG.23384.2	480	0.43369	6.207214355	1
MSTRG.23387.2	497	0.37488	9.331970215	1
MSTRG.23388.2	103	0.44007	5.093688965	1
MSTRG.23391.1	678	0.37144	8.807678223	1
MSTRG.23391.2	70	0.47771	9.463928223	-1
MSTRG.23391.4	768	0.35239	8.756164551	1
MSTRG.23392.1	833	0.35723	8.908630371	1
MSTRG.23396.1	58	0.31603	9.487609863	1
MSTRG.23397.2	617	0.44905	5.613830566	1
MSTRG.23401.5	375	0.41262	8.957336426	1
MSTRG.23399.3	316	0.42078	5.674133301	1
MSTRG.23407.2	442	0.44356	5.490539551	1
MSTRG.23412.4	141	0.2707	11.56695557	1
MSTRG.23412.2	388	0.39025	6.194519043	1
MSTRG.23412.3	388	0.39765	6.194519043	1
MSTRG.23413.1	97	0.27313	10.29095459	1
MSTRG.23406.1	200	0.32854	4.97064209	1
MSTRG.23414.1	249	0.42236	8.768981934	1
MSTRG.23415.1	556	0.45925	9.372009277	1
MSTRG.23415.2	100	0.46636	11.43634033	1
MSTRG.23415.3	107	0.30249	10.92694092	1
MSTRG.23415.5	142	0.37694	9.290344238	1
MSTRG.23415.4	563	0.45672	9.171081543	1
MSTRG.23416.1	752	0.39834	9.698913574	1
MSTRG.23416.2	752	0.40158	9.698913574	1
MSTRG.23420.1	442	0.35635	5.013000488	1
MSTRG.23430.2	421	0.41315	8.684387207	1
MSTRG.23433.1	560	0.37938	8.753845215	1
MSTRG.23433.2	371	0.42568	5.409118652	1
MSTRG.23435.1	120	0.35559	10.85980225	1
MSTRG.23443.5	519	0.44739	8.696594238	1
MSTRG.23443.6	661	0.46121	8.495666504	1
MSTRG.23443.7	455	0.44414	8.972351074	1
MSTRG.23438.3	149	0.46814	5.264343262	-1
MSTRG.23451.2	502	0.41158	9.820251465	1
MSTRG.23451.3	502	0.43208	9.820251465	1
MSTRG.23454.2	133	0.38134	4.122131348	1
MSTRG.23455.1	68	0.39214	9.44317627	1
MSTRG.23456.1	878	0.46483	6.450622559	1
MSTRG.23461.2	352	0.36325	6.027893066	1
MSTRG.23462.1	539	0.44148	5.546936035	1
MSTRG.23457.2	122	0.41893	4.927429199	1
MSTRG.23463.2	129	0.4094	4.390441895	1
MSTRG.23467.2	1180	0.36518	6.135314941	1
MSTRG.23467.3	1243	0.394	5.969421387	1
MSTRG.23467.5	1254	0.40779	5.902893066	1
MSTRG.23467.4	1303	0.39033	5.815490723	1

MSTRG.23466.3	126	0.42095	11.64813232	1
MSTRG.23470.1	571	0.35788	5.036437988	1
MSTRG.23470.2	583	0.39583	5.10760498	1
MSTRG.23474.2	324	0.43264	8.826843262	1
MSTRG.23479.8	930	0.40959	9.084777832	1
MSTRG.23479.5	867	0.35212	9.262756348	1
MSTRG.23479.7	907	0.35298	9.217956543	1
MSTRG.23479.9	809	0.40794	9.446716309	1
MSTRG.23479.3	900	0.38209	9.217956543	1
MSTRG.23479.10	890	0.40959	9.121643066	1
MSTRG.23479.11	890	0.40959	9.121643066	1
MSTRG.23482.3	957	0.47888	5.820617676	1
MSTRG.23482.4	916	0.45001	5.866638184	1
MSTRG.23484.1	163	0.39511	7.571716309	1
MSTRG.23485.2	457	0.48025	9.107971191	1
MSTRG.23486.1	234	0.37106	7.756286621	1
MSTRG.23488.2	181	0.43412	11.65374756	1
MSTRG.23488.4	193	0.37209	8.872253418	1
MSTRG.23491.2	131	0.45186	4.446105957	1
MSTRG.23492.2	400	0.41938	5.432434082	1
MSTRG.23492.5	239	0.34451	4.938781738	1
MSTRG.23495.1	110	0.38777	4.852478027	1
MSTRG.23505.2	615	0.46278	5.295227051	1
MSTRG.23510.2	594	0.36612	10.09564209	1
MSTRG.23515.4	629	0.45542	4.393127441	1
MSTRG.23520.1	262	0.36505	8.925476074	1
MSTRG.23520.2	175	0.49409	5.885437012	1
MSTRG.23520.3	175	0.42743	5.885437012	1
MSTRG.23520.4	245	0.43963	7.145324707	1
MSTRG.23513.1	338	0.36116	9.757507324	1
MSTRG.23522.5	204	0.35272	11.75872803	1
MSTRG.23522.3	213	0.33594	11.72894287	1
MSTRG.23522.4	204	0.33594	11.75872803	1
MSTRG.23525.2	1185	0.4627	5.578552246	1
MSTRG.23528.2	1022	0.37786	8.000793457	1
MSTRG.23529.9	651	0.46811	5.873474121	1
MSTRG.23530.1	439	0.45467	5.912902832	1
MSTRG.23531.2	368	0.38687	5.06451416	1
MSTRG.23531.1	303	0.40204	7.48248291	1
MSTRG.23530.3	451	0.45466	6.057067871	1
MSTRG.23532.1	30	0.45674	10.637146	1
MSTRG.23535.3	485	0.46959	4.524597168	1
MSTRG.23535.2	485	0.47579	4.524597168	1
MSTRG.23535.4	526	0.45114	4.45526123	1
MSTRG.23535.6	438	0.43027	4.599304199	1
MSTRG.23534.2	104	0.37775	6.50982666	1
MSTRG.23545.2	1957	0.37766	8.176330566	1
MSTRG.23550.1	896	0.43421	10.34942627	1
MSTRG.23550.3	896	0.35337	10.34942627	1
MSTRG.23550.4	896	0.41371	10.34942627	1
MSTRG.23551.4	421	0.42277	4.992858887	1
MSTRG.23552.4	159	0.44461	4.967468262	1
MSTRG.23552.5	151	0.28426	5.358581543	1
MSTRG.23552.2	151	0.33128	5.358581543	1

MSTRG.23552.3	151	0.28122	5.358581543	1
MSTRG.23553.4	498	0.41704	6.38092041	1
MSTRG.23567.3	956	0.43995	7.750793457	1
MSTRG.23568.2	369	0.46366	5.631774902	1
MSTRG.23568.3	119	0.3332	9.910095215	1
MSTRG.23572.3	126	0.40112	4.452087402	1
MSTRG.23572.4	126	0.41321	4.452087402	1
MSTRG.23574.4	520	0.45023	5.286437988	1
MSTRG.23575.2	281	0.44727	4.874938965	1
MSTRG.23559.4	401	0.46954	8.359802246	1
MSTRG.23559.5	454	0.46178	8.819519043	1
MSTRG.23560.2	82	0.37607	4.460510254	1
MSTRG.23561.1	808	0.36125	9.135314941	1
MSTRG.23561.2	221	0.40248	9.391296387	1
MSTRG.23556.1	147	0.43069	9.068786621	1
MSTRG.23558.2	380	0.44844	8.878967285	1
MSTRG.23562.1	69	0.40894	10.16668701	1
MSTRG.23577.2	202	0.30129	5.81829834	1
MSTRG.23579.1	548	0.34172	8.519470215	1
MSTRG.23580.1	104	0.3752	5.709899902	1
MSTRG.23582.1	196	0.39065	9.437316895	1
MSTRG.23583.2	276	0.46025	6.926818848	1
MSTRG.23585.4	122	0.38368	10.09259033	1
MSTRG.23586.1	532	0.38218	9.313171387	1
MSTRG.23586.2	193	0.36714	5.408996582	1
MSTRG.23586.3	187	0.32578	8.775817871	1
MSTRG.23585.6	129	0.37236	8.816711426	1
MSTRG.23585.7	129	0.36856	8.816711426	1
MSTRG.23585.8	224	0.3956	9.29864502	1
MSTRG.23585.9	150	0.37135	8.691467285	1
MSTRG.23585.10	184	0.36338	9.054382324	1
MSTRG.23585.11	168	0.36613	8.949523926	1
MSTRG.23587.1	207	0.41161	4.509216309	1
MSTRG.23587.2	140	0.34673	4.604064941	1
MSTRG.23592.1	245	0.46874	8.094421387	-1
MSTRG.23593.1	255	0.32893	9.460144043	1
MSTRG.23594.1	211	0.36813	8.534240723	1
MSTRG.23594.2	76	0.3376	9.997497559	1
MSTRG.23594.3	294	0.35516	8.914978027	1
MSTRG.23594.4	76	0.32038	9.997497559	1
MSTRG.23595.1	200	0.38019	10.6050415	1
MSTRG.23595.2	200	0.42827	10.6050415	1
MSTRG.23595.3	200	0.31029	10.6050415	1
MSTRG.23595.4	200	0.38866	10.6050415	1
MSTRG.23594.5	95	0.40629	6.007873535	1
MSTRG.23597.2	109	0.38164	7.578186035	1
MSTRG.23597.3	210	0.41599	9.330749512	1
MSTRG.23598.1	869	0.36764	8.691223145	1
MSTRG.23598.2	869	0.41344	8.691223145	1
MSTRG.23598.3	912	0.39321	8.797302246	1
MSTRG.23598.4	218	0.43404	5.86505127	1
MSTRG.23603.2	582	0.37971	9.360656738	1
MSTRG.23607.2	42	0.41129	9.199523926	1
MSTRG.23608.2	263	0.28746	10.82794189	-1

MSTRG.23619.1	256	0.40058	6.623596191	1
MSTRG.23621.5	95	0.42061	5.381408691	1
MSTRG.23621.4	635	0.41839	9.34942627	1
MSTRG.23621.1	671	0.33337	9.27557373	1
MSTRG.23621.6	615	0.33337	9.221496582	1
MSTRG.23621.3	686	0.32469	9.215393066	1
MSTRG.23622.1	109	0.30082	10.00872803	1
MSTRG.23621.7	641	0.39476	9.187561035	1
MSTRG.23621.9	588	0.36289	9.32989502	1
MSTRG.23621.11	588	0.35339	9.32989502	1
MSTRG.23621.17	114	0.2849	9.184020996	1
MSTRG.23621.18	584	0.3582	8.903137207	1
MSTRG.23623.1	32	0.353	3.902160645	1
MSTRG.23629.1	99	0.28887	8.368469238	1
MSTRG.23630.1	312	0.28883	10.05914307	1
MSTRG.23630.2	312	0.3422	10.05914307	1
MSTRG.23630.3	312	0.38653	10.05828857	1
MSTRG.23630.4	316	0.3238	9.665222168	1
MSTRG.23631.1	37	0.38	4.25201416	1
MSTRG.23616.2	111	0.34312	8.987731934	1
MSTRG.23753.1	105	0.36711	9.66583252	1
MSTRG.23754.2	600	0.41306	9.194152832	1
MSTRG.23754.1	544	0.39511	9.168640137	1
MSTRG.23754.4	488	0.39339	8.979553223	-1
MSTRG.23754.3	544	0.37381	9.168640137	1
MSTRG.23754.5	1019	0.43393	9.22076416	1
MSTRG.23754.7	544	0.41548	9.165222168	1
MSTRG.23754.13	460	0.39491	8.935974121	1
MSTRG.23754.14	822	0.41836	9.192687988	1
MSTRG.23754.16	795	0.41653	9.253479004	1
MSTRG.23758.1	65	0.27139	9.99029541	1
MSTRG.23763.1	92	0.45308	8.804382324	1
MSTRG.23769.5	636	0.37983	8.676940918	1
MSTRG.23769.6	159	0.28982	9.097229004	1
MSTRG.23769.8	636	0.3713	8.676940918	1
MSTRG.23770.2	575	0.38226	8.814880371	1
MSTRG.23770.1	575	0.36612	8.814880371	1
MSTRG.23770.3	575	0.36137	8.814880371	1
MSTRG.23770.4	126	0.29772	9.182922363	1
MSTRG.23770.5	575	0.32145	8.814880371	1
MSTRG.23770.6	539	0.38226	9.012390137	1
MSTRG.23770.7	575	0.34934	8.814880371	1
MSTRG.23770.8	126	0.35631	9.182922363	1
MSTRG.23770.10	575	0.37975	8.814880371	1
MSTRG.23770.9	575	0.36813	8.814880371	1
MSTRG.23773.2	880	0.43136	9.112121582	1
MSTRG.23769.11	63	0.35598	4.534362793	1
MSTRG.23765.13	32	0.30587	6.703796387	-1
MSTRG.23765.15	32	0.30587	6.703796387	-1
MSTRG.23765.10	34	0.2991	6.703796387	-1
MSTRG.23765.12	34	0.2991	6.703796387	-1
MSTRG.23765.9	34	0.2991	6.703796387	-1
MSTRG.23765.8	55	0.29236	9.394104004	1
MSTRG.23765.11	55	0.26027	9.394104004	1

MSTRG.23765.14	55	0.24926	9.394104004	1
MSTRG.23765.4	55	0.27263	9.394104004	1
MSTRG.23765.7	55	0.28472	9.394104004	1
MSTRG.23765.5	55	0.26027	9.394104004	1
MSTRG.23765.6	55	0.28967	9.394104004	1
MSTRG.23770.13	126	0.39072	9.182922363	1
MSTRG.23774.1	893	0.47379	9.353210449	1
MSTRG.23770.14	93	0.31922	6.262878418	1
MSTRG.23775.1	71	0.30968	8.932434082	1
MSTRG.23776.1	72	0.32559	10.871521	1
MSTRG.23777.1	1372	0.47202	9.570251465	1
MSTRG.23777.2	1418	0.46622	9.513000488	1
MSTRG.23777.3	1372	0.47677	9.570251465	1
MSTRG.23778.1	64	0.34212	8.614807129	1
MSTRG.23778.2	721	0.38062	9.561462402	1
MSTRG.23779.1	824	0.41659	9.315734863	1
MSTRG.23779.2	1040	0.44421	9.304260254	1
MSTRG.23779.3	1012	0.42166	9.292297363	1
MSTRG.23783.1	25	0.28908	7.934020996	1
MSTRG.23785.1	125	0.39261	8.385681152	1
MSTRG.23786.1	29	0.28587	9.688293457	1
MSTRG.23788.2	973	0.47061	9.26739502	1
MSTRG.23788.3	753	0.45567	9.201599121	1
MSTRG.23788.4	585	0.454	9.052307129	1
MSTRG.23788.5	887	0.44719	9.466125488	1
MSTRG.23788.7	557	0.454	8.965148926	1
MSTRG.23788.6	949	0.47488	9.346740723	1
MSTRG.23788.8	196	0.45161	9.266540527	1
MSTRG.23788.9	98	0.47072	4.554748535	1
MSTRG.23789.1	198	0.34255	9.938903809	1
MSTRG.23790.1	78	0.38091	4.289367676	1
MSTRG.23790.2	66	0.3173	9.301330566	1
MSTRG.23789.2	154	0.31768	8.724182129	1
MSTRG.23789.3	139	0.36807	11.25762939	1
MSTRG.23791.1	611	0.33738	9.023010254	1
MSTRG.23791.3	531	0.37593	9.276062012	1
MSTRG.23791.5	611	0.31096	9.023010254	1
MSTRG.23791.6	582	0.33098	9.100036621	1
MSTRG.23791.4	531	0.30788	9.276062012	1
MSTRG.23791.7	610	0.32305	9.258728027	1
MSTRG.23791.8	611	0.36561	9.023010254	1
MSTRG.23791.9	611	0.33994	9.023010254	1
MSTRG.23791.11	415	0.32533	9.427062988	1
MSTRG.23791.10	611	0.32533	9.023010254	1
MSTRG.23632.1	418	0.29526	9.285705566	1
MSTRG.23632.3	602	0.33126	9.480529785	1
MSTRG.23632.4	382	0.39643	9.527160645	1
MSTRG.23632.5	382	0.36976	9.527160645	1
MSTRG.23632.6	418	0.39846	9.285705566	1
MSTRG.23632.7	566	0.4371	9.592834473	1
MSTRG.23632.8	574	0.43246	9.49041748	1
MSTRG.23632.9	549	0.38858	9.592956543	1
MSTRG.23632.10	59	0.2947	4.861022949	1
MSTRG.23632.11	418	0.35773	9.285705566	1

MSTRG.23635.2	541	0.37738	8.995666504	1
MSTRG.23635.3	541	0.35502	8.995666504	1
MSTRG.23639.1	396	0.30289	9.60369873	1
MSTRG.23651.1	174	0.41037	5.702819824	1
MSTRG.23652.1	57	0.36606	5.532409668	1
MSTRG.23653.1	75	0.3801	10.13238525	1
MSTRG.23654.1	169	0.28683	6.605407715	1
MSTRG.23659.1	120	0.29439	8.682067871	1
MSTRG.23651.2	47	0.37761	5.948425293	1
MSTRG.23660.1	38	0.37305	11.43719482	1
MSTRG.23651.3	36	0.36089	9.52142334	1
MSTRG.23651.4	174	0.29793	5.702819824	1
MSTRG.23651.5	63	0.34314	4.929260254	1
MSTRG.23663.1	85	0.34444	10.62957764	1
MSTRG.23664.1	58	0.39278	9.62286377	1
MSTRG.23651.6	174	0.34615	5.702819824	1
MSTRG.23651.7	174	0.28419	5.702819824	1
MSTRG.23651.8	55	0.33412	4.862854004	1
MSTRG.23666.1	65	0.36601	9.057312012	1
MSTRG.23667.1	161	0.40205	9.669006348	-1
MSTRG.23668.1	54	0.35035	9.235900879	1
MSTRG.23670.1	685	0.42944	9.731018066	1
MSTRG.23670.2	685	0.45185	9.731018066	1
MSTRG.23670.3	685	0.43799	9.731018066	1
MSTRG.23671.1	75	0.4681	5.608703613	-1
MSTRG.23644.2	58	0.35824	12.17852783	1
MSTRG.23644.1	58	0.44535	12.17852783	1
MSTRG.23644.3	61	0.29196	10.0401001	1
MSTRG.23644.4	58	0.36405	12.17852783	1
MSTRG.23677.1	563	0.45589	4.77947998	1
MSTRG.23677.2	538	0.38565	4.840881348	1
MSTRG.23678.6	371	0.42691	5.003234863	1
MSTRG.23681.4	223	0.39186	9.80657959	1
MSTRG.23681.5	269	0.42684	9.667297363	1
MSTRG.23681.6	278	0.43262	9.528381348	1
MSTRG.23683.1	221	0.27217	9.317687988	1
MSTRG.23683.2	148	0.31511	10.53387451	1
MSTRG.23683.3	157	0.31008	8.11151123	1
MSTRG.23690.1	137	0.36701	10.51983643	1
MSTRG.23694.7	638	0.42217	8.503845215	1
MSTRG.23694.5	1019	0.41296	8.812927246	1
MSTRG.23694.6	638	0.41758	8.503845215	1
MSTRG.23694.4	1030	0.39451	8.776062012	1
MSTRG.23696.1	641	0.34791	10.03851318	1
MSTRG.23696.3	620	0.34791	10.01068115	1
MSTRG.23687.1	202	0.42759	8.857727051	-1
MSTRG.23688.1	149	0.28373	8.081359863	-1
MSTRG.23698.3	785	0.46081	9.50579834	1
MSTRG.23701.3	342	0.3688	9.65057373	1
MSTRG.23706.1	863	0.41599	4.593200684	1
MSTRG.23706.3	863	0.44086	4.593200684	1
MSTRG.23706.2	807	0.41599	4.581359863	1
MSTRG.23699.1	126	0.35584	10.20391846	1
MSTRG.23709.3	677	0.46301	6.262512207	1

MSTRG.23709.4	373	0.39219	6.13873291	1
MSTRG.23709.5	301	0.38205	10.22564697	1
MSTRG.23709.6	296	0.431	10.32501221	1
MSTRG.23709.7	212	0.44696	9.869567871	1
MSTRG.23712.1	66	0.29143	11.21478271	1
MSTRG.23711.2	69	0.36022	6.708068848	-1
MSTRG.23716.1	764	0.42999	5.241149902	1
MSTRG.23716.2	763	0.42999	5.241149902	1
MSTRG.23716.6	546	0.36085	5.674865723	1
MSTRG.23716.7	564	0.33338	5.758239746	1
MSTRG.23717.2	44	0.36183	7.784240723	1
MSTRG.23717.3	23	0.41266	11.8291626	1
MSTRG.23718.2	403	0.38907	5.942321777	1
MSTRG.23720.4	819	0.33558	9.485168457	1
MSTRG.23720.5	820	0.3667	9.485168457	1
MSTRG.23727.3	289	0.35005	9.134338379	1
MSTRG.23728.1	17	0.40297	4.529602051	1
MSTRG.23728.2	46	0.44544	9.983825684	1
MSTRG.23731.2	744	0.43779	5.825378418	1
MSTRG.23732.2	499	0.45331	5.632751465	1
MSTRG.23732.3	469	0.45826	5.339172363	1
MSTRG.23736.1	232	0.44836	7.897644043	1
MSTRG.23743.2	315	0.38785	9.207214355	1
MSTRG.23744.2	461	0.3652	8.089172363	1
MSTRG.23744.3	381	0.39913	8.310119629	1
MSTRG.23797.2	610	0.30176	9.77911377	1
MSTRG.23801.4	517	0.42578	8.303894043	1
MSTRG.23801.3	138	0.47315	9.398376465	-1
MSTRG.23801.5	148	0.3296	9.56439209	1
MSTRG.23809.2	497	0.37751	8.898498535	1
MSTRG.23794.1	260	0.34284	4.855773926	1
MSTRG.23794.3	526	0.40958	5.942932129	1
MSTRG.23814.1	348	0.44488	6.069763184	1
MSTRG.23817.2	137	0.28846	7.590026855	1
MSTRG.23841.3	256	0.38464	8.167663574	-1
MSTRG.23841.2	626	0.3399	8.152648926	1
MSTRG.23846.1	82	0.29772	10.77105713	1
MSTRG.23846.2	119	0.29088	9.812194824	1
MSTRG.23846.3	119	0.29088	9.812194824	1
MSTRG.23847.1	260	0.40148	5.78326416	1
MSTRG.23849.1	56	0.29408	6.477478027	-1
MSTRG.23855.2	351	0.34287	9.528747559	1
MSTRG.23857.1	208	0.35823	7.633483887	1
MSTRG.23857.2	78	0.32501	9.222106934	1
MSTRG.23855.3	430	0.35483	8.700378418	1
MSTRG.23855.4	468	0.29896	8.734802246	1
MSTRG.23860.1	525	0.28546	9.445983887	1
MSTRG.23860.2	398	0.37351	9.398864746	1
MSTRG.23857.3	68	0.28679	4.973449707	1
MSTRG.23855.9	535	0.36691	9.279602051	1
MSTRG.23824.1	49	0.35728	4.352355957	1
MSTRG.23825.1	379	0.28567	9.275817871	1
MSTRG.23825.3	379	0.35269	9.275817871	1
MSTRG.23825.4	379	0.36954	9.275817871	1

MSTRG.23825.5	351	0.28567	9.312927246	1
MSTRG.23827.1	121	0.28982	7.72479248	1
MSTRG.23828.1	68	0.3159	7.825134277	-1
MSTRG.23833.1	291	0.29088	9.550842285	1
MSTRG.23834.1	565	0.42658	8.930480957	1
MSTRG.23834.2	658	0.38824	8.665100098	1
MSTRG.23834.3	536	0.29187	8.984436035	1
MSTRG.23834.4	536	0.3582	8.984436035	1
MSTRG.23834.5	536	0.29772	8.984436035	1
MSTRG.23868.2	420	0.37891	9.27154541	1
MSTRG.23868.4	420	0.37891	9.27154541	1
MSTRG.23872.6	444	0.28014	8.829040527	1
MSTRG.23872.7	486	0.3305	8.512512207	1
MSTRG.23872.3	486	0.29912	8.512512207	1
MSTRG.23872.1	486	0.29664	8.512512207	1
MSTRG.23873.1	173	0.34416	11.246521	1
MSTRG.23872.8	184	0.28338	8.605529785	1
MSTRG.23872.9	116	0.31989	10.31072998	1
MSTRG.23875.1	148	0.30808	10.57745361	1
MSTRG.23872.10	938	0.37816	9.321105957	1
MSTRG.23877.6	827	0.32514	9.081115723	1
MSTRG.23878.2	111	0.27911	10.95623779	1
MSTRG.23879.2	787	0.41011	7.905578613	-1
MSTRG.23879.3	651	0.40949	8.739196777	-1
MSTRG.23879.4	147	0.28002	3.983459473	1
MSTRG.23880.1	657	0.32401	7.703674316	1
MSTRG.23880.3	615	0.40626	8.363586426	1
MSTRG.23881.3	538	0.37636	9.211120605	1
MSTRG.23881.5	538	0.32817	9.211120605	1
MSTRG.23883.1	44	0.43511	4.74786377	1
MSTRG.23840.2	204	0.46041	9.733703613	1
MSTRG.23890.1	149	0.32714	6.308898926	1
MSTRG.23894.3	632	0.44564	9.01361084	1
MSTRG.23898.2	578	0.41754	6.074401855	1
MSTRG.23927.1	88	0.38495	6.81829834	1
MSTRG.23929.1	50	0.34353	6.219299316	1
MSTRG.23931.3	462	0.46739	4.962219238	1
MSTRG.23933.2	170	0.47037	4.609680176	1
MSTRG.23944.2	152	0.39449	6.807922363	1
MSTRG.23946.1	16	0.39033	11.00030518	1
MSTRG.23947.1	821	0.45263	8.378601074	1
MSTRG.23947.2	88	0.28923	8.842834473	1
MSTRG.23949.1	614	0.38292	8.307067871	1
MSTRG.23949.2	631	0.41456	8.559997559	1
MSTRG.23950.1	54	0.38825	8.683288574	1
MSTRG.23950.2	87	0.3509	9.913879395	1
MSTRG.23952.2	403	0.40097	9.12677002	1
MSTRG.23952.3	403	0.37723	9.12677002	1
MSTRG.23952.5	366	0.46047	9.337219238	-1
MSTRG.23952.4	366	0.46119	9.337219238	-1
MSTRG.23954.3	541	0.38846	4.777160645	1
MSTRG.23954.5	533	0.32961	4.706848145	1
MSTRG.23962.5	157	0.34147	9.843688965	1
MSTRG.23962.8	229	0.27251	7.743835449	1

MSTRG.23962.4	169	0.26837	7.205993652	1
MSTRG.23962.6	170	0.31772	9.325866699	1
MSTRG.23962.1	257	0.26913	8.567443848	1
MSTRG.23962.3	328	0.35012	8.914855957	1
MSTRG.23963.1	162	0.27264	9.370422363	1
MSTRG.23964.1	96	0.28904	8.954162598	1
MSTRG.23962.11	155	0.27429	9.20513916	1
MSTRG.23962.12	155	0.27591	9.20513916	1
MSTRG.23965.1	132	0.26837	9.875793457	1
MSTRG.23966.1	60	0.33747	5.995544434	1
MSTRG.23962.13	169	0.26837	7.205993652	1
MSTRG.23967.1	128	0.2785	11.33221436	1
MSTRG.23968.1	141	0.26913	10.33599854	1
MSTRG.23958.1	49	0.35892	4.713928223	1
MSTRG.23970.2	89	0.38169	6.813781738	1
MSTRG.23976.1	558	0.45674	5.878234863	1
MSTRG.23977.1	405	0.39303	10.53704834	1
MSTRG.23981.3	1197	0.41568	5.885192871	1
MSTRG.23981.4	639	0.41553	7.655090332	1
MSTRG.23981.5	380	0.39889	5.714904785	1
MSTRG.23981.6	1197	0.41568	5.885192871	1
MSTRG.23981.7	1197	0.41897	5.885192871	1
MSTRG.23981.8	1366	0.35131	7.821716309	1
MSTRG.23983.4	331	0.4082	6.247375488	1
MSTRG.23983.3	521	0.43849	10.01397705	1
MSTRG.23998.5	1525	0.46488	5.361755371	-1
MSTRG.23998.6	1525	0.46488	5.361755371	-1
MSTRG.23998.7	1525	0.46488	5.361755371	-1
MSTRG.23999.11	121	0.30415	8.504699707	1
MSTRG.24000.2	104	0.39016	5.818786621	1
MSTRG.24004.1	46	0.3312	4.734558105	1
MSTRG.23986.1	26	0.34997	10.30621338	1
MSTRG.23986.2	42	0.37365	6.520080566	-1
MSTRG.23987.2	54	0.31444	8.000793457	1
MSTRG.23988.1	109	0.26969	7.72052002	1
MSTRG.23988.2	182	0.32237	9.673034668	1
MSTRG.23989.1	88	0.27772	8.56817627	1
MSTRG.23988.3	116	0.31044	9.271789551	1
MSTRG.23988.4	183	0.29367	9.649230957	1
MSTRG.23990.1	240	0.33581	6.90423584	1
MSTRG.23990.2	143	0.29772	6.858459473	1
MSTRG.23990.3	143	0.29772	6.858459473	1
MSTRG.23991.1	173	0.29292	5.527160645	1
MSTRG.23992.1	176	0.45602	8.62701416	1
MSTRG.23984.1	151	0.34812	11.2555542	1
MSTRG.23995.1	1024	0.46382	7.528259277	1
MSTRG.23995.4	63	0.28742	8.890319824	1
MSTRG.23996.4	46	0.4116	9.35345459	1
MSTRG.23997.1	84	0.32947	9.513000488	1
MSTRG.24007.1	61	0.32883	9.588806152	-1
MSTRG.24020.1	1675	0.472	5.480895996	1
MSTRG.24020.3	47	0.34398	9.781799316	1
MSTRG.24020.4	47	0.3415	9.781799316	1
MSTRG.24023.1	155	0.37175	10.49542236	-1

MSTRG.24022.3	67	0.35776	9.217712402	1
MSTRG.24026.3	322	0.29343	8.824890137	1
MSTRG.24026.1	450	0.47683	6.390441895	1
MSTRG.24026.4	247	0.3731	9.199890137	1
MSTRG.24027.1	398	0.31917	8.355041504	1
MSTRG.24011.2	639	0.47521	5.424743652	1
MSTRG.24013.3	902	0.42735	7.150817871	1
MSTRG.24013.5	931	0.41246	6.248962402	1
MSTRG.24014.1	19	0.3836	5.940979004	1
MSTRG.24015.1	108	0.28478	9.273376465	1
MSTRG.24016.2	210	0.29835	8.999450684	1
MSTRG.24017.3	605	0.44534	9.25604248	1
MSTRG.24019.1	585	0.3787	6.439025879	1
MSTRG.24019.2	73	0.33422	8.976623535	1
MSTRG.24031.2	184	0.41737	10.22686768	1
MSTRG.24035.1	1564	0.44095	6.020202637	1
MSTRG.24035.3	87	0.37815	9.421325684	1
MSTRG.24036.1	91	0.29812	4.973327637	1
MSTRG.24037.1	109	0.32536	9.670349121	1
MSTRG.24049.1	718	0.45349	6.341247559	1
MSTRG.24049.2	738	0.45349	6.174377441	1
MSTRG.24049.8	735	0.40998	6.122741699	1
MSTRG.24049.9	738	0.42794	6.174377441	1
MSTRG.24049.10	738	0.45277	6.174377441	1
MSTRG.24049.12	512	0.36389	5.643371582	1
MSTRG.24050.2	181	0.43561	6.437561035	1
MSTRG.24050.3	250	0.40691	6.200134277	1
MSTRG.24051.1	103	0.36856	10.42279053	1
MSTRG.24041.2	324	0.47141	4.24822998	1
MSTRG.24057.3	443	0.45818	6.450744629	1
MSTRG.24057.4	443	0.42551	6.450744629	1
MSTRG.24058.1	67	0.33054	9.368591309	1
MSTRG.24058.2	67	0.42936	10.76287842	1
MSTRG.24062.1	1372	0.4238	4.926330566	1
MSTRG.24062.5	1295	0.47697	4.927307129	1
MSTRG.24062.6	1294	0.47697	4.927307129	1
MSTRG.24062.8	1346	0.47697	4.909729004	1
MSTRG.24062.9	1351	0.47917	4.909729004	1
MSTRG.24059.1	96	0.33738	6.580627441	1
MSTRG.24059.3	96	0.34767	6.580627441	1
MSTRG.24059.4	96	0.34542	6.580627441	1
MSTRG.24063.1	184	0.35137	7.16607666	1
MSTRG.24063.2	185	0.35137	7.16607666	1
MSTRG.24063.3	185	0.35578	7.16607666	1
MSTRG.24063.6	165	0.3431	6.96307373	1
MSTRG.24071.2	146	0.42131	9.149353027	1
MSTRG.24071.1	146	0.41515	9.149353027	1
MSTRG.24081.1	604	0.48253	7.572692871	1
MSTRG.24081.2	604	0.48473	8.030212402	1
MSTRG.24081.9	596	0.42619	8.033874512	1
MSTRG.24081.5	604	0.42495	7.572692871	1
MSTRG.24081.7	426	0.42144	9.344177246	1
MSTRG.24081.10	605	0.42619	7.572692871	1
MSTRG.24075.2	238	0.37431	9.814880371	1

MSTRG.24082.1	937	0.45427	5.223815918	1
MSTRG.24082.4	897	0.45602	5.064147949	1
MSTRG.24082.3	937	0.4734	5.223815918	1
MSTRG.24086.1	789	0.42753	6.94342041	1
MSTRG.24093.3	201	0.27251	8.468811035	1
MSTRG.24095.3	1002	0.44936	9.124206543	1
MSTRG.24095.2	1002	0.46664	9.124206543	1
MSTRG.24098.3	497	0.44737	8.463317871	1
MSTRG.24098.4	379	0.44137	8.408996582	1
MSTRG.24099.1	33	0.41208	9.842956543	1
MSTRG.24102.1	114	0.34431	6.04486084	1
MSTRG.24104.1	139	0.30788	9.45904541	1
MSTRG.24105.1	53	0.43394	5.934753418	1
MSTRG.24105.2	42	0.37298	11.16595459	1
MSTRG.24107.1	151	0.35342	9.276672363	1
MSTRG.24108.1	38	0.33798	10.90325928	1
MSTRG.24111.2	248	0.343	10.222229	1
MSTRG.24112.1	59	0.33363	11.90570068	1
MSTRG.24113.3	226	0.45347	4.742370605	1
MSTRG.24114.2	291	0.36315	10.17230225	1
MSTRG.24115.1	307	0.45963	9.438293457	1
MSTRG.24115.3	307	0.4347	9.438293457	1
MSTRG.24117.2	324	0.36298	6.355285645	1
MSTRG.24117.3	324	0.40306	6.355285645	1
MSTRG.24125.1	73	0.31301	9.207824707	1
MSTRG.24125.2	31	0.3135	5.060241699	-1
MSTRG.24123.1	418	0.36383	5.666320801	1
MSTRG.24127.1	305	0.293	9.65020752	1
MSTRG.24134.2	64	0.41923	6.078918457	1
MSTRG.24135.4	200	0.33513	5.02166748	1
MSTRG.24135.2	760	0.43588	9.051696777	1
MSTRG.24135.6	496	0.45316	7.590393066	1
MSTRG.24136.8	86	0.30172	4.14666748	1
MSTRG.24136.9	86	0.38209	4.14666748	1
MSTRG.24139.1	80	0.30945	10.21563721	1
MSTRG.24140.1	67	0.33794	6.218566895	1
MSTRG.24147.2	535	0.34699	4.760314941	1
MSTRG.24173.1	1667	0.43642	7.323669434	1
MSTRG.24173.3	2840	0.47053	7.106262207	1
MSTRG.24173.4	2581	0.42979	6.718078613	1
MSTRG.24173.2	2996	0.43179	7.366760254	1
MSTRG.24173.6	2819	0.45433	6.90411377	1
MSTRG.24173.7	2840	0.46901	7.106262207	1
MSTRG.24173.5	2996	0.46901	7.366760254	1
MSTRG.24173.10	2688	0.46253	7.027282715	1
MSTRG.24173.8	2995	0.45433	7.366760254	1
MSTRG.24173.9	2975	0.46901	7.10736084	1
MSTRG.24173.13	2841	0.45433	7.106262207	1
MSTRG.24173.14	2581	0.44047	6.718078613	1
MSTRG.24173.11	2975	0.45433	7.10736084	1
MSTRG.24173.12	2995	0.45433	7.366760254	1
MSTRG.24173.15	2541	0.42979	6.911193848	1
MSTRG.24173.16	2819	0.47328	6.90411377	1
MSTRG.24173.17	2845	0.47361	6.822937012	1

MSTRG.24175.1	26	0.38051	5.969055176	1
MSTRG.24148.2	244	0.36444	9.298400879	1
MSTRG.24152.1	76	0.33904	4.56817627	1
MSTRG.24162.1	53	0.32814	5.410461426	1
MSTRG.24163.1	100	0.31514	9.608459473	1
MSTRG.24164.1	82	0.40358	10.46258545	1
MSTRG.24163.2	527	0.38657	8.354064941	1
MSTRG.24163.4	153	0.31035	8.200256348	1
MSTRG.24163.5	237	0.41563	10.17584229	-1
MSTRG.24163.6	100	0.2675	9.608459473	1
MSTRG.24158.1	316	0.41477	7.98614502	1
MSTRG.24153.1	173	0.35065	10.59710693	1
MSTRG.24168.1	217	0.46626	6.690368652	1
MSTRG.24171.1	52	0.36517	5.012756348	1
MSTRG.24172.1	2232	0.4259	6.603088379	1
MSTRG.24172.2	2092	0.47541	6.446105957	1
MSTRG.24184.1	197	0.32436	6.750793457	1
MSTRG.24184.3	125	0.41524	10.21685791	1
MSTRG.24184.4	139	0.35497	10.3616333	1
MSTRG.24185.3	658	0.42681	5.808410645	1
MSTRG.24187.1	259	0.39572	8.708679199	1
MSTRG.24187.2	259	0.45353	8.708679199	1
MSTRG.24187.3	225	0.4258	8.92779541	1
MSTRG.24187.4	259	0.44124	8.708679199	1
MSTRG.24187.5	188	0.44332	12.19952393	1
MSTRG.24183.1	28	0.42212	8.80279541	1
MSTRG.24190.6	288	0.34434	9.753967285	1
MSTRG.24195.1	63	0.37264	10.45001221	1
MSTRG.24197.1	110	0.32022	9.300598145	1
MSTRG.24198.1	1001	0.33114	7.299133301	1
MSTRG.24198.2	1001	0.35371	7.299133301	1
MSTRG.24201.3	612	0.44912	6.40411377	1
MSTRG.24204.4	431	0.45238	5.463195801	1
MSTRG.24209.1	493	0.37537	4.835632324	1
MSTRG.24209.3	1488	0.45808	5.869567871	1
MSTRG.24209.4	850	0.36669	5.532043457	1
MSTRG.24211.2	559	0.32719	7.67779541	1
MSTRG.24211.3	201	0.32642	5.483581543	1
MSTRG.24212.1	80	0.33415	6.850036621	1
MSTRG.24207.5	170	0.30576	7.909484863	1
MSTRG.24207.4	187	0.27958	6.710144043	1
MSTRG.24215.1	106	0.39212	9.034240723	1
MSTRG.24214.3	743	0.47159	7.59979248	1
MSTRG.24214.4	670	0.44299	8.687927246	1
MSTRG.24216.1	200	0.29783	8.720275879	1
MSTRG.24222.1	45	0.36918	5.963562012	1
MSTRG.24223.1	98	0.32198	9.760681152	1
MSTRG.24224.1	318	0.3227	9.711364746	1
MSTRG.24225.2	1048	0.41597	5.406921387	1
MSTRG.24228.5	258	0.41079	9.549377441	1
MSTRG.24228.6	226	0.41141	9.647521973	1
MSTRG.24228.7	155	0.35238	8.692687988	-1
MSTRG.24229.7	709	0.46636	8.336608887	1
MSTRG.24229.10	128	0.37157	9.758850098	1

MSTRG.24218.3	144	0.37361	5.727233887	1
MSTRG.24230.2	255	0.44259	4.632507324	1
MSTRG.24232.3	105	0.34063	10.01812744	1
MSTRG.24233.3	1028	0.43512	9.32989502	1
MSTRG.24236.3	206	0.37413	5.497131348	1
MSTRG.24252.5	480	0.38971	4.799621582	1
MSTRG.24252.6	1412	0.44617	6.227355957	1
MSTRG.24252.7	150	0.40325	4.746276855	1
MSTRG.24252.8	1001	0.34069	9.231384277	1
MSTRG.24252.9	970	0.33883	9.231506348	1
MSTRG.24250.1	585	0.42108	8.785461426	1
MSTRG.24250.3	304	0.39307	6.427062988	1
MSTRG.24250.5	304	0.36401	6.427062988	1
MSTRG.24250.8	533	0.43537	8.813659668	1
MSTRG.24250.4	533	0.44263	8.813659668	1
MSTRG.24250.2	533	0.43715	8.813659668	1
MSTRG.24250.7	519	0.42329	8.003479004	1
MSTRG.24257.5	81	0.31454	9.238830566	1
MSTRG.24257.4	410	0.29772	6.968566895	1
MSTRG.24257.3	410	0.29772	6.968566895	1
MSTRG.24258.5	1330	0.46672	5.594055176	1
MSTRG.24258.4	1311	0.44851	5.693054199	1
MSTRG.24260.1	118	0.44224	9.723815918	-1
MSTRG.24262.1	62	0.40759	9.780212402	1
MSTRG.24265.2	364	0.40762	9.686584473	1
MSTRG.24265.3	364	0.39992	9.686584473	1
MSTRG.24265.5	364	0.39992	9.686584473	1
MSTRG.24265.6	227	0.42227	9.671936035	1
MSTRG.24268.2	294	0.45315	5.565124512	1
MSTRG.24269.3	90	0.27577	9.616638184	1
MSTRG.24269.2	278	0.42565	9.392028809	-1
MSTRG.24273.1	92	0.33287	6.793273926	1
MSTRG.24275.2	505	0.43737	5.89276123	1
MSTRG.24276.2	998	0.44673	5.320739746	1
MSTRG.24276.3	995	0.44793	5.318786621	1
MSTRG.24285.2	1583	0.4535	5.598327637	1
MSTRG.24285.3	1202	0.35907	5.902893066	1
MSTRG.24286.2	151	0.27327	8.237731934	1
MSTRG.24286.3	263	0.28387	6.95513916	1
MSTRG.24288.2	99	0.32533	5.216125488	1
MSTRG.24288.3	215	0.30191	10.67279053	1
MSTRG.24288.4	215	0.28982	10.67279053	1
MSTRG.24283.3	81	0.41507	7.707824707	1
MSTRG.24283.5	541	0.38939	9.204650879	1
MSTRG.24283.6	591	0.39754	9.227355957	-1
MSTRG.24282.2	133	0.45821	5.519226074	1
MSTRG.24289.3	225	0.40569	9.273620605	1
MSTRG.24289.2	85	0.3535	11.87872314	1
MSTRG.24290.3	355	0.41205	9.352478027	1
MSTRG.24291.1	297	0.3642	8.802307129	1
MSTRG.24296.1	167	0.28862	8.837097168	1
MSTRG.24297.2	122	0.34451	11.81317139	1
MSTRG.24297.1	910	0.37071	7.642883301	1
MSTRG.24297.3	940	0.39326	7.63885498	1

MSTRG.24297.6	910	0.39546	7.642883301	1
MSTRG.24297.9	940	0.34475	7.63885498	1
MSTRG.24295.1	59	0.34127	9.578308105	1
MSTRG.24302.1	92	0.30176	5.11895752	1
MSTRG.24303.1	705	0.42852	9.118835449	1
MSTRG.24313.2	778	0.43908	4.952819824	1
MSTRG.24315.1	243	0.2908	9.13470459	1
MSTRG.24314.2	507	0.3618	7.242370605	1
MSTRG.24318.1	1189	0.36391	7.001403809	1
MSTRG.24318.2	1165	0.38441	6.742004395	1
MSTRG.24318.3	1002	0.34996	6.056945801	1
MSTRG.24318.4	999	0.33172	6.08795166	1
MSTRG.24306.2	440	0.45263	8.334411621	1
MSTRG.24306.3	518	0.45135	6.845153809	1
MSTRG.24307.1	140	0.43625	8.461608887	1
MSTRG.24307.2	140	0.37207	8.461608887	1
MSTRG.24307.3	140	0.39389	8.461608887	1
MSTRG.24307.4	140	0.3956	8.461608887	1
MSTRG.24309.1	282	0.42643	9.774230957	1
MSTRG.24305.1	257	0.40559	7.865905762	1
MSTRG.24304.4	217	0.43035	9.57635498	1
MSTRG.24328.2	905	0.45884	4.166809082	1
MSTRG.24328.5	211	0.32066	8.543762207	1
MSTRG.24328.7	269	0.29212	10.74578857	1
MSTRG.24328.8	1068	0.39161	4.448303223	1
MSTRG.24336.3	135	0.39404	5.327697754	1
MSTRG.24338.6	664	0.48664	5.834777832	1
MSTRG.24339.1	651	0.45984	8.022766113	1
MSTRG.24340.1	133	0.37271	8.371765137	-1
MSTRG.24341.3	673	0.38753	5.638977051	1
MSTRG.24344.1	74	0.34961	7.755554199	1
MSTRG.24346.1	123	0.4387	4.605041504	1
MSTRG.24346.6	296	0.40534	4.989929199	1
MSTRG.24346.2	123	0.44564	4.605041504	1
MSTRG.24346.3	123	0.37628	4.605041504	1
MSTRG.24346.5	123	0.3738	4.605041504	1
MSTRG.24346.4	123	0.37628	4.605041504	1
MSTRG.24346.7	123	0.37628	4.605041504	1
MSTRG.24348.3	154	0.41626	5.074401855	1
MSTRG.24350.3	958	0.40325	5.493347168	1
MSTRG.24350.4	905	0.36609	5.402282715	1
MSTRG.24350.5	1269	0.44062	5.330749512	1
MSTRG.24351.7	520	0.33417	5.743103027	1
MSTRG.24351.5	552	0.32687	6.035949707	1
MSTRG.24351.4	520	0.34177	5.743103027	1
MSTRG.24351.1	520	0.32108	5.743103027	1
MSTRG.24351.3	741	0.45935	6.949157715	1
MSTRG.24351.2	741	0.38968	6.949157715	1
MSTRG.24352.1	109	0.37513	7.664123535	1
MSTRG.24356.3	219	0.44435	7.787536621	1
MSTRG.24357.1	491	0.37829	10.25958252	1
MSTRG.24357.3	328	0.31025	9.974182129	1
MSTRG.24358.5	1293	0.45721	8.461975098	1
MSTRG.24364.1	279	0.41151	5.925598145	1

MSTRG.24365.4	1984	0.45891	7.122497559	1
MSTRG.24365.8	1973	0.483	7.438781738	1
MSTRG.24365.9	1938	0.483	7.440734863	1
MSTRG.24365.10	1987	0.45891	7.122497559	1
MSTRG.24365.11	1973	0.483	7.438781738	1
MSTRG.24365.6	1994	0.44351	7.122253418	1
MSTRG.24365.3	1980	0.46914	7.438415527	1
MSTRG.24365.5	1973	0.483	7.438781738	1
MSTRG.24365.1	1973	0.483	7.311462402	1
MSTRG.24365.12	1963	0.45891	7.015441895	1
MSTRG.24365.13	1984	0.45891	7.246398926	1
MSTRG.24365.14	2009	0.46914	7.578552246	1
MSTRG.24366.1	61	0.33162	6.367004395	1
MSTRG.24367.1	0	0.41117	0	-1
MSTRG.24374.2	390	0.42013	5.398498535	1
MSTRG.24376.1	167	0.40693	9.64263916	1
MSTRG.24376.2	137	0.45808	10.02301025	1
MSTRG.24376.5	167	0.41941	9.64263916	1
MSTRG.24376.6	137	0.36483	10.02301025	1
MSTRG.24378.4	170	0.37349	5.351135254	1
MSTRG.24378.5	170	0.42629	5.351135254	1
MSTRG.24379.1	306	0.37643	5.668762207	1
MSTRG.24379.2	306	0.4001	5.668762207	1
MSTRG.24380.1	86	0.46981	8.736877441	-1
MSTRG.24359.2	264	0.32156	8.135192871	1
MSTRG.24359.3	262	0.29135	6.941955566	1
MSTRG.24361.2	278	0.38265	3.981140137	1
MSTRG.24382.5	1604	0.38138	8.902526855	1
MSTRG.24387.6	84	0.40368	10.73760986	-1
MSTRG.24387.1	84	0.43183	10.73760986	-1
MSTRG.24387.3	113	0.38363	5.804992676	1
MSTRG.24387.4	64	0.37576	6.070251465	1
MSTRG.24389.1	155	0.29236	8.915100098	1
MSTRG.24387.7	116	0.29099	6.090881348	1
MSTRG.24390.8	1810	0.462	6.653381348	1
MSTRG.24384.3	1170	0.39213	5.958068848	1
MSTRG.24384.2	1265	0.40025	5.633239746	1
MSTRG.24397.1	104	0.36638	5.355407715	1
MSTRG.24398.1	47	0.43808	10.08221436	1
MSTRG.24397.3	186	0.38247	10.01483154	1
MSTRG.24398.4	166	0.44863	4.805480957	1
MSTRG.24400.1	847	0.31385	8.250671387	1
MSTRG.24405.1	144	0.33119	5.526794434	1
MSTRG.24405.3	345	0.37162	8.659118652	1
MSTRG.24405.4	152	0.30069	9.43536377	1
MSTRG.24406.3	145	0.43362	10.5536499	1
MSTRG.24408.1	69	0.34295	10.29022217	1
MSTRG.24408.2	45	0.39873	11.32647705	1
MSTRG.24410.3	201	0.31164	4.807800293	1
MSTRG.24412.1	1343	0.47477	5.301086426	1
MSTRG.24412.2	1976	0.47183	5.436218262	1
MSTRG.24413.1	205	0.36831	9.024475098	1
MSTRG.24401.1	261	0.27599	9.56036377	1
MSTRG.24404.2	211	0.29492	9.845397949	1

MSTRG.24415.7	97	0.45173	5.397521973	1
MSTRG.24415.3	125	0.46475	4.621398926	1
MSTRG.24415.5	138	0.34349	4.863830566	1
MSTRG.24415.4	138	0.43888	4.863830566	1
MSTRG.24415.6	125	0.47256	4.621398926	1
MSTRG.24415.2	138	0.48329	4.863830566	1
MSTRG.24417.1	476	0.37154	6.915222168	1
MSTRG.24417.6	421	0.32507	7.671081543	1
MSTRG.24417.5	476	0.31117	6.915222168	1
MSTRG.24419.1	198	0.33119	7.557922363	1
MSTRG.24419.2	126	0.28711	9.273254395	1
MSTRG.24420.1	105	0.38979	9.16619873	1
MSTRG.24421.1	168	0.32985	10.77252197	1
MSTRG.24424.2	190	0.30781	5.565490723	1
MSTRG.24436.1	107	0.36297	10.05670166	1
MSTRG.24442.2	4292	0.43858	5.594665527	1
MSTRG.24443.1	363	0.30064	8.280334473	1
MSTRG.24429.7	87	0.37058	9.568054199	1
MSTRG.24429.5	47	0.34449	10.13238525	1
MSTRG.24429.6	47	0.38487	10.13238525	1
MSTRG.24429.1	76	0.33391	8.752624512	1
MSTRG.24429.2	131	0.30753	8.820983887	1
MSTRG.24429.3	137	0.30753	8.108215332	1
MSTRG.24429.4	47	0.3256	10.13238525	1
MSTRG.24429.8	77	0.35837	8.752624512	1
MSTRG.24454.1	82	0.39279	9.962585449	1
MSTRG.24456.2	160	0.42083	7.735656738	1
MSTRG.24458.1	65	0.36257	6.523254395	-1
MSTRG.24460.1	166	0.35297	11.82952881	1
MSTRG.24460.2	166	0.40716	11.82952881	1
MSTRG.24460.3	153	0.38661	11.65863037	1
MSTRG.24460.4	134	0.3901	10.30853271	1
MSTRG.24460.5	198	0.40068	10.21392822	1
MSTRG.24460.6	134	0.31964	10.20794678	1
MSTRG.24461.1	96	0.35272	10.88665771	1
MSTRG.24462.1	208	0.37519	8.291564941	1
MSTRG.24464.3	661	0.44583	9.227355957	1
MSTRG.24464.2	593	0.44583	9.245666504	1
MSTRG.24464.1	1084	0.45591	9.570983887	1
MSTRG.24465.1	175	0.3307	6.073669434	1
MSTRG.24463.1	796	0.37017	9.256530762	1
MSTRG.24468.1	771	0.36632	9.136047363	1
MSTRG.24468.2	757	0.34056	9.112487793	1
MSTRG.24484.2	229	0.41359	6.858825684	1
MSTRG.24484.3	153	0.41463	6.747131348	1
MSTRG.24484.4	229	0.41359	6.858825684	1
MSTRG.24484.5	229	0.41359	6.858825684	1
MSTRG.24484.6	229	0.42004	6.858825684	1
MSTRG.24484.7	229	0.41359	6.858825684	1
MSTRG.24485.3	75	0.38605	12.08331299	1
MSTRG.24485.2	75	0.43173	12.08331299	1
MSTRG.24486.1	102	0.3857	6.011169434	1
MSTRG.24487.10	2449	0.46111	6.70892334	1
MSTRG.24487.3	2441	0.46111	6.593811035	1

MSTRG.24487.4	2446	0.46111	6.849304199	1
MSTRG.24487.5	2412	0.46111	6.708435059	1
MSTRG.24487.6	2405	0.46111	6.578063965	1
MSTRG.24487.7	2387	0.46111	6.69342041	1
MSTRG.24487.9	2433	0.46111	6.616149902	1
MSTRG.24487.8	2449	0.43894	7.035705566	1
MSTRG.24487.11	1932	0.40458	6.52911377	1
MSTRG.24487.17	2440	0.46111	6.593811035	1
MSTRG.24487.18	2441	0.46111	6.593811035	1
MSTRG.24487.19	2452	0.46111	6.593811035	1
MSTRG.24488.7	268	0.38106	5.085266113	1
MSTRG.24488.10	130	0.35372	7.532165527	1
MSTRG.24488.8	352	0.44615	6.16192627	1
MSTRG.24488.14	54	0.28687	5.425842285	1
MSTRG.24489.2	71	0.32269	10.24700928	1
MSTRG.24472.3	1059	0.45352	5.82635498	1
MSTRG.24472.5	781	0.45578	5.365905762	1
MSTRG.24477.1	811	0.37343	9.025085449	-1
MSTRG.24477.10	562	0.38976	9.144104004	-1
MSTRG.24477.8	894	0.41665	9.212097168	-1
MSTRG.24477.7	724	0.34245	8.897766113	1
MSTRG.24477.9	606	0.30193	8.893859863	1
MSTRG.24478.1	384	0.40818	9.972473145	1
MSTRG.24495.3	642	0.3997	5.428283691	1
MSTRG.24510.1	1267	0.43456	5.758483887	1
MSTRG.24503.1	106	0.37017	11.04071045	1
MSTRG.24504.1	113	0.41094	5.412536621	-1
MSTRG.24514.2	89	0.43519	5.512512207	1
MSTRG.24516.2	31	0.42308	5.086242676	1
MSTRG.24521.6	152	0.29689	9.469543457	1
MSTRG.24523.3	734	0.40578	9.086486816	1
MSTRG.24523.4	1122	0.3676	9.094421387	1
MSTRG.24520.2	199	0.36028	9.119934082	1
MSTRG.24520.3	221	0.35914	9.621765137	1
MSTRG.24520.4	199	0.39242	9.119934082	1
MSTRG.24520.6	199	0.35227	9.119934082	1
MSTRG.24528.4	145	0.36997	5.456848145	1
MSTRG.24528.3	111	0.39839	6.027038574	1
MSTRG.24528.2	111	0.37924	6.027038574	1
MSTRG.24528.5	173	0.35526	6.122009277	1
MSTRG.24528.6	94	0.38209	11.82391357	1
MSTRG.24531.2	242	0.33322	11.54669189	1
MSTRG.24539.3	310	0.46195	9.246032715	1
MSTRG.24539.4	332	0.32396	9.215515137	1
MSTRG.24541.2	57	0.39606	11.19024658	1
MSTRG.24543.1	62	0.3387	11.331604	-1
MSTRG.24548.1	122	0.33674	8.713806152	1
MSTRG.24548.3	122	0.3443	8.713806152	1
MSTRG.24554.1	1307	0.48448	5.059265137	1
MSTRG.24554.4	157	0.33146	9.096740723	1
MSTRG.24554.7	652	0.44806	5.383239746	1
MSTRG.24554.9	839	0.48603	5.058044434	1
MSTRG.24555.1	42	0.36236	9.396057129	1
MSTRG.24553.1	574	0.37259	5.738464355	1

MSTRG.24557.1	415	0.34634	10.20379639	1
MSTRG.24557.3	311	0.31045	10.52471924	1
MSTRG.24557.4	432	0.40155	9.805114746	1
MSTRG.24557.5	491	0.42339	9.93182373	1
MSTRG.24566.2	946	0.45741	6.583435059	1
MSTRG.24566.3	791	0.4787	6.059875488	1
MSTRG.24568.2	159	0.36341	10.07879639	1
MSTRG.24571.1	864	0.44062	8.494445801	1
MSTRG.24574.4	85	0.39398	10.7991333	-1
MSTRG.24574.5	85	0.43033	10.7991333	-1
MSTRG.24574.3	85	0.45328	10.7991333	-1
MSTRG.24574.2	123	0.39469	10.03143311	-1
MSTRG.24574.6	83	0.3935	10.7991333	-1
MSTRG.24575.1	175	0.40762	12.44012451	1
MSTRG.24580.2	104	0.39447	6.014099121	1
MSTRG.24584.2	581	0.37829	8.912902832	1
MSTRG.24584.3	484	0.3618	8.488586426	1
MSTRG.24585.2	202	0.3619	10.07989502	-1
MSTRG.24586.1	225	0.46045	9.423400879	1
MSTRG.24588.3	272	0.44088	5.566833496	1
MSTRG.24589.5	441	0.39243	8.188903809	1
MSTRG.24589.6	442	0.37759	8.586364746	1
MSTRG.24589.4	442	0.34118	8.586364746	1
MSTRG.24589.7	428	0.28982	8.584655762	1
MSTRG.24591.10	263	0.35409	8.548156738	1
MSTRG.24591.3	456	0.44269	6.606018066	-1
MSTRG.24591.4	267	0.41213	7.071472168	1
MSTRG.24591.5	249	0.3676	6.707824707	1
MSTRG.24591.6	267	0.41705	7.071472168	1
MSTRG.24591.7	249	0.36294	6.707824707	1
MSTRG.24591.8	362	0.41564	8.961364746	1
MSTRG.24591.9	281	0.39762	8.64263916	1
MSTRG.24594.1	113	0.33167	6.472595215	1
MSTRG.24594.2	121	0.34457	8.859558105	1
MSTRG.24595.3	592	0.42539	7.985046387	1
MSTRG.24595.4	423	0.47672	8.813537598	-1
MSTRG.24597.2	576	0.32507	9.260314941	1
MSTRG.24599.3	377	0.40377	5.363586426	1
MSTRG.24599.4	503	0.48477	5.376281738	1
MSTRG.24599.5	384	0.43007	5.300231934	1
MSTRG.24604.2	55	0.28948	6.369689941	1
MSTRG.24604.1	571	0.31649	9.152282715	1
MSTRG.24604.3	847	0.31649	9.03338623	1
MSTRG.24602.1	33	0.33483	8.363220215	-1
MSTRG.24609.7	733	0.38571	4.577575684	1
MSTRG.24619.1	84	0.38582	11.62957764	1
MSTRG.24620.2	860	0.48804	5.303039551	1
MSTRG.24620.3	764	0.45551	5.544128418	1
MSTRG.24620.4	764	0.45995	5.544128418	1
MSTRG.24620.5	736	0.46988	5.21661377	1
MSTRG.24621.1	259	0.43188	7.703308105	1
MSTRG.24623.3	698	0.3564	9.528991699	1
MSTRG.24625.4	391	0.34628	6.207458496	1
MSTRG.24625.5	391	0.34628	6.207458496	1

MSTRG.24629.1	416	0.38981	6.901794434	1
MSTRG.24629.3	416	0.39353	6.901794434	1
MSTRG.24630.1	425	0.33624	10.16265869	1
MSTRG.24633.1	114	0.36654	9.500549316	1
MSTRG.24637.1	307	0.32598	9.166442871	1
MSTRG.24637.3	197	0.29542	5.012023926	1
MSTRG.24637.2	215	0.29542	5.136779785	1
MSTRG.24637.4	197	0.29704	5.012023926	1
MSTRG.24640.2	194	0.43875	11.47589111	1
MSTRG.24648.1	108	0.26413	9.620666504	1
MSTRG.24649.1	60	0.30503	7.67755127	1
MSTRG.24651.1	556	0.445	7.891052246	1
MSTRG.24651.2	616	0.46511	8.24005127	1
MSTRG.24642.6	1393	0.36266	9.391662598	1
MSTRG.24642.7	1263	0.37134	9.574035645	1
MSTRG.24644.1	448	0.35582	7.660583496	1
MSTRG.24656.3	452	0.30644	8.035339355	1
MSTRG.24659.3	153	0.36105	9.644958496	1
MSTRG.24659.4	206	0.39595	10.04290771	1
MSTRG.24662.2	989	0.43052	6.490539551	1
MSTRG.24663.2	410	0.33335	7.778625488	1
MSTRG.24664.2	605	0.41721	8.83013916	1
MSTRG.24666.3	198	0.36713	9.195861816	1
MSTRG.24661.3	703	0.46336	5.985656738	1
MSTRG.24661.4	703	0.46336	5.985656738	1
MSTRG.24670.1	47	0.33513	11.54058838	1
MSTRG.24669.3	414	0.45163	8.371276855	1
MSTRG.24669.6	24	0.44259	10.05230713	1
MSTRG.24673.2	1520	0.47739	8.786682129	1
MSTRG.24672.1	140	0.41686	6.09161377	1
MSTRG.24676.1	109	0.42597	4.628356934	1
MSTRG.24676.2	87	0.41263	4.43572998	1
MSTRG.24677.1	37	0.3629	9.102355957	1
MSTRG.24693.3	313	0.46856	5.823181152	1
MSTRG.24696.1	81	0.34975	11.18743896	1
MSTRG.24696.2	250	0.34781	8.057922363	-1
MSTRG.24697.2	1118	0.4553	6.97479248	1
MSTRG.24697.5	1091	0.47022	6.829162598	1
MSTRG.24724.1	390	0.46606	5.489074707	1
MSTRG.24724.6	390	0.45232	5.489074707	1
MSTRG.24724.5	390	0.45232	5.489074707	1
MSTRG.24724.7	390	0.46619	5.489074707	1
MSTRG.24724.2	390	0.45935	5.489074707	1
MSTRG.24724.3	390	0.46367	5.489074707	1
MSTRG.24724.4	390	0.48064	5.489074707	1
MSTRG.24725.1	832	0.34832	6.448791504	1
MSTRG.24725.2	832	0.39082	6.448791504	1
MSTRG.24725.3	796	0.37032	8.06829834	1
MSTRG.24725.4	832	0.35196	6.448791504	1
MSTRG.24725.5	832	0.39295	6.448791504	1
MSTRG.24729.1	562	0.47137	7.019714355	1
MSTRG.24729.2	562	0.47451	7.019714355	1
MSTRG.24729.3	294	0.41347	9.258117676	1
MSTRG.24729.4	562	0.48358	7.019714355	1

MSTRG.24729.5	294	0.39094	9.258117676	1
MSTRG.24729.6	562	0.48147	7.019714355	1
MSTRG.24732.1	369	0.37188	4.854064941	1
MSTRG.24732.2	369	0.39443	4.854064941	1
MSTRG.24732.4	357	0.36654	5.031677246	1
MSTRG.24735.4	702	0.38757	9.453430176	1
MSTRG.24710.4	747	0.35343	8.010437012	1
MSTRG.24710.5	747	0.34723	8.010437012	1
MSTRG.24706.1	177	0.41807	5.174255371	1
MSTRG.24711.3	606	0.42778	4.952453613	1
MSTRG.24711.4	997	0.42613	5.398498535	1
MSTRG.24717.2	141	0.39312	8.904602051	1
MSTRG.24721.1	697	0.33427	8.916564941	1
MSTRG.24721.2	254	0.33962	5.687194824	1
MSTRG.24721.4	254	0.36183	5.687194824	1
MSTRG.24722.2	623	0.46244	6.702819824	-1
MSTRG.24722.4	788	0.47559	5.918640137	1
MSTRG.24714.2	179	0.44992	4.919494629	-1
MSTRG.24715.2	613	0.44764	6.000671387	1
MSTRG.24741.2	259	0.41993	6.659118652	1
MSTRG.24742.3	171	0.27437	9.567565918	1
MSTRG.24743.2	1762	0.45994	5.581604004	1
MSTRG.24743.3	1761	0.45994	5.581604004	1
MSTRG.24745.1	75	0.35511	8.692199707	1
MSTRG.24745.2	88	0.41454	10.51483154	1
MSTRG.24745.3	71	0.34054	7.608459473	1
MSTRG.24748.1	200	0.44824	9.944274902	1
MSTRG.24763.1	191	0.28338	6.43939209	1
MSTRG.24763.3	195	0.42748	7.623962402	1
MSTRG.24768.3	1106	0.45872	5.77557373	1
MSTRG.24768.7	1106	0.45872	5.77557373	1
MSTRG.24769.1	163	0.3917	9.90802002	1
MSTRG.24772.2	347	0.33259	6.618347168	1
MSTRG.24774.5	720	0.4486	5.49810791	1
MSTRG.24774.6	720	0.4332	5.49810791	1
MSTRG.24784.2	574	0.33557	6.128601074	1
MSTRG.24784.3	110	0.38823	8.966125488	1
MSTRG.24785.3	359	0.44704	9.702697754	1
MSTRG.24788.1	98	0.28567	10.628479	1
MSTRG.24789.1	181	0.30016	10.17987061	1
MSTRG.24792.2	743	0.31878	9.402404785	1
MSTRG.24793.1	28	0.40448	11.8348999	1
MSTRG.24793.2	43	0.38135	11.43890381	1
MSTRG.24778.1	396	0.41864	6.924865723	1
MSTRG.24782.1	972	0.45797	4.850769043	1
MSTRG.24795.2	231	0.44898	9.217224121	1
MSTRG.24801.1	288	0.36937	7.715148926	1
MSTRG.24801.2	288	0.40388	7.715148926	1
MSTRG.24805.2	338	0.43572	10.70111084	1
MSTRG.24807.1	110	0.34686	10.00604248	1
MSTRG.24809.1	867	0.42061	5.38848877	1
MSTRG.24810.1	123	0.29762	11.11541748	1
MSTRG.24814.1	2556	0.46748	4.872497559	1
MSTRG.24815.2	137	0.42652	4.503601074	1

MSTRG.24816.1	148	0.35677	5.155334473	1
MSTRG.24816.2	164	0.37871	4.66619873	1
MSTRG.24817.1	290	0.38515	6.89263916	1
MSTRG.24817.2	170	0.32723	7.872375488	1
MSTRG.24820.2	587	0.46642	6.04486084	1
MSTRG.24821.1	25	0.35649	12.00006104	1
MSTRG.24825.1	67	0.31425	10.20587158	1
MSTRG.24826.4	341	0.45668	9.414855957	1
MSTRG.24826.6	341	0.45873	9.414855957	1
MSTRG.24826.2	370	0.44128	8.644470215	1
MSTRG.24826.3	370	0.46488	8.644470215	1
MSTRG.24826.5	370	0.44128	8.644470215	1
MSTRG.24826.7	341	0.46493	9.414855957	1
MSTRG.24826.8	371	0.3773	10.12042236	1
MSTRG.24827.6	1040	0.41151	8.885559082	1
MSTRG.24827.7	398	0.36733	8.628479004	1
MSTRG.24827.8	461	0.36733	8.972717285	1
MSTRG.24827.9	568	0.36919	8.731994629	1
MSTRG.24827.10	461	0.34683	8.972717285	1
MSTRG.24827.11	421	0.30003	9.59197998	1
MSTRG.24828.1	135	0.32789	8.058532715	1
MSTRG.24830.1	76	0.41647	6.003234863	1
MSTRG.24833.1	116	0.3769	11.4598999	1
MSTRG.24835.1	645	0.35581	5.213195801	1
MSTRG.24836.1	31	0.40274	6.473571777	1
MSTRG.24837.1	796	0.4508	5.614196777	-1
MSTRG.24837.2	192	0.34432	8.274719238	1
MSTRG.24837.3	1502	0.42245	5.199768066	-1
MSTRG.24837.4	1377	0.43873	5.346618652	1
MSTRG.24837.5	608	0.43873	5.015441895	-1
MSTRG.24841.2	1804	0.42431	5.975646973	1
MSTRG.24841.1	1822	0.42431	6.119812012	1
MSTRG.24834.3	130	0.44808	9.773986816	1
MSTRG.24844.1	718	0.44245	8.50970459	1
MSTRG.24844.2	750	0.4304	8.656188965	1
MSTRG.24844.3	750	0.44245	8.656188965	1
MSTRG.24844.8	765	0.40377	8.718688965	1
MSTRG.24844.9	796	0.40377	8.829040527	1
MSTRG.24844.10	713	0.43554	8.016662598	1
MSTRG.24846.1	40	0.28238	5.740783691	1
MSTRG.24847.1	42	0.38642	5.956970215	1
MSTRG.24842.4	239	0.36524	8.863586426	1
MSTRG.24850.1	148	0.27622	10.43084717	1
MSTRG.24849.3	91	0.41129	6.27557373	1
MSTRG.24849.2	2026	0.38592	8.530334473	1
MSTRG.24852.1	109	0.35372	7.06036377	1
MSTRG.24853.1	63	0.31209	10.00701904	1
MSTRG.24854.1	65	0.2921	4.668151855	1
MSTRG.24855.1	95	0.30419	9.623962402	1
MSTRG.24856.1	79	0.36944	9.256286621	1
MSTRG.24858.2	252	0.38309	8.867248535	1
MSTRG.24858.3	252	0.39771	8.867248535	1
MSTRG.24858.4	41	0.34237	7.87701416	1
MSTRG.24860.3	719	0.43493	6.170227051	1

MSTRG.24861.2	757	0.43894	5.844909668	1
MSTRG.24861.3	757	0.44704	5.844909668	1
MSTRG.24861.4	757	0.43894	5.844909668	1
MSTRG.24861.5	757	0.42124	5.844909668	1
MSTRG.24862.1	442	0.45765	8.157653809	1
MSTRG.24866.1	616	0.38372	9.287780762	1
MSTRG.24866.2	647	0.3831	9.396179199	1
MSTRG.24866.3	636	0.37752	8.417785645	1
MSTRG.24866.7	415	0.37665	9.149597168	1
MSTRG.24867.2	164	0.3409	11.23858643	1
MSTRG.24867.4	164	0.31122	11.23858643	1
MSTRG.24867.3	394	0.44432	5.845275879	1
MSTRG.24867.5	402	0.45037	5.925842285	1
MSTRG.24867.11	402	0.43732	5.925842285	1
MSTRG.24867.7	373	0.42926	6.001403809	1
MSTRG.24867.8	402	0.41229	5.925842285	1
MSTRG.24878.1	414	0.46634	7.110168457	1
MSTRG.24879.2	456	0.46751	9.471374512	1
MSTRG.24864.1	1265	0.42487	4.995422363	1
MSTRG.24865.1	87	0.33263	9.506896973	1
MSTRG.24865.2	97	0.39127	9.828430176	1
MSTRG.24869.2	109	0.46006	8.406433105	1
MSTRG.24869.4	89	0.43564	5.969177246	1
MSTRG.24869.3	81	0.43297	9.29473877	1
MSTRG.24871.2	857	0.4258	5.949035645	1
MSTRG.24890.1	317	0.35355	7.16595459	1
MSTRG.24892.1	319	0.38388	9.360900879	1
MSTRG.24892.3	207	0.33891	4.776428223	1
MSTRG.24892.4	75	0.31593	6.537658691	1
MSTRG.24892.5	294	0.35858	9.238952637	1
MSTRG.24892.6	396	0.41864	9.743713379	1
MSTRG.24893.3	506	0.38674	6.074768066	1
MSTRG.24885.7	1966	0.45981	8.741394043	1
MSTRG.24885.6	2123	0.45085	8.799255371	1
MSTRG.24885.5	2902	0.45549	5.868225098	1
MSTRG.24885.3	2960	0.45549	5.797424316	1
MSTRG.24885.10	1158	0.47808	9.61932373	1
MSTRG.24885.11	1840	0.46428	8.606994629	1
MSTRG.24885.8	1956	0.47814	8.717224121	1
MSTRG.24885.9	2839	0.48814	6.007995605	1
MSTRG.24898.1	268	0.38813	5.341247559	1
MSTRG.24898.2	278	0.30981	5.338195801	1
MSTRG.24898.3	270	0.28923	5.955383301	1
MSTRG.24898.4	231	0.31649	5.751525879	1
MSTRG.24898.5	199	0.28584	7.677307129	1
MSTRG.24898.6	162	0.35949	7.05279541	1
MSTRG.24903.1	40	0.31968	6.702697754	-1
MSTRG.24913.2	642	0.45073	7.018005371	1
MSTRG.24914.2	72	0.3234	11.83258057	1
MSTRG.24911.4	204	0.42091	6.294616699	1
MSTRG.24920.1	74	0.3205	9.742492676	1
MSTRG.24922.1	64	0.30061	6.054138184	1
MSTRG.24924.2	290	0.41074	6.155822754	1
MSTRG.24925.1	187	0.36085	6.516784668	1

MSTRG.24925.2	187	0.3809	6.516784668	1
MSTRG.24930.1	117	0.36125	8.594909668	1
MSTRG.24932.1	76	0.36637	6.803771973	1
MSTRG.24936.1	415	0.36588	9.766906738	1
MSTRG.24936.5	409	0.35593	9.66998291	1
MSTRG.24936.4	428	0.44502	9.94708252	1
MSTRG.24936.7	371	0.27867	10.04742432	1
MSTRG.24958.2	475	0.45804	8.832214355	1
MSTRG.24958.1	475	0.46369	8.832214355	1
MSTRG.24959.1	554	0.47831	5.862976074	1
MSTRG.24959.2	555	0.47831	5.862976074	1
MSTRG.24960.1	336	0.37654	9.151916504	1
MSTRG.24960.2	243	0.38433	6.642272949	1
MSTRG.24960.4	243	0.38129	6.642272949	1
MSTRG.24960.5	205	0.39328	5.82635498	1
MSTRG.24961.1	46	0.44007	6.218444824	-1
MSTRG.24962.2	456	0.482	5.986999512	1
MSTRG.24963.1	115	0.45231	6.804870605	1
MSTRG.24969.4	84	0.37674	7.992492676	1
MSTRG.24971.1	82	0.32164	10.19708252	1
MSTRG.24971.2	82	0.31296	10.19708252	1
MSTRG.24971.3	82	0.33606	10.19708252	1
MSTRG.24971.4	82	0.32546	10.19708252	1
MSTRG.24960.6	474	0.44998	4.797058105	1
MSTRG.24960.7	345	0.43449	4.633361816	1
MSTRG.24944.2	244	0.41429	9.569519043	1
MSTRG.24946.4	643	0.43916	5.60369873	1
MSTRG.24946.3	710	0.44904	5.805480957	1
MSTRG.24946.5	640	0.43916	5.598815918	1
MSTRG.24948.2	139	0.31065	6.707214355	1
MSTRG.24954.3	1081	0.45031	5.547058105	1
MSTRG.24954.6	992	0.45987	5.324890137	1
MSTRG.24976.3	115	0.4307	4.231384277	1
MSTRG.24976.2	931	0.36422	7.530090332	1
MSTRG.24976.4	228	0.36551	6.289733887	1
MSTRG.25009.1	215	0.32175	9.690734863	1
MSTRG.25010.1	683	0.43697	6.169372559	1
MSTRG.25010.8	5006	0.4541	6.203308105	1
MSTRG.25010.10	3251	0.41806	6.403503418	1
MSTRG.25010.11	3339	0.45782	6.364318848	1
MSTRG.25010.12	4403	0.46019	6.178527832	1
MSTRG.25010.13	3258	0.41806	6.384216309	1
MSTRG.25010.6	683	0.40345	6.169372559	1
MSTRG.25010.4	1027	0.45162	7.322937012	1
MSTRG.25010.7	1531	0.46343	6.760192871	1
MSTRG.25010.3	2730	0.46641	6.345397949	1
MSTRG.25010.5	3590	0.45959	6.324768066	1
MSTRG.25012.1	69	0.40621	4.39630127	1
MSTRG.25010.15	930	0.4563	6.666564941	-1
MSTRG.25010.16	1171	0.43852	6.803039551	-1
MSTRG.24992.2	937	0.44908	5.296447754	1
MSTRG.24993.1	1125	0.44089	5.246643066	1
MSTRG.24993.3	832	0.43785	4.994689941	1
MSTRG.24993.4	1125	0.43846	5.246643066	1

MSTRG.24993.5	832	0.43846	4.994689941	1
MSTRG.24986.1	445	0.35128	9.863220215	1
MSTRG.24996.1	124	0.35374	6.739074707	1
MSTRG.25001.2	401	0.36921	6.19732666	1
MSTRG.25001.4	457	0.38671	5.896179199	1
MSTRG.25003.2	143	0.3483	5.566101074	1
MSTRG.25006.1	397	0.42963	5.460266113	1
MSTRG.25006.3	61	0.37486	4.01751709	1
MSTRG.25020.1	811	0.40371	9.048278809	1
MSTRG.25020.4	811	0.40371	9.048278809	1
MSTRG.25020.5	171	0.44047	9.421813965	1
MSTRG.25021.1	1862	0.37052	6.994934082	1
MSTRG.25021.2	1895	0.3689	6.725646973	1
MSTRG.25021.3	1823	0.3689	6.88092041	1
MSTRG.25027.4	385	0.30712	7.713928223	1
MSTRG.25027.7	585	0.4724	9.079162598	1
MSTRG.25027.9	578	0.46885	7.216125488	1
MSTRG.25027.8	585	0.47053	9.079162598	1
MSTRG.25028.2	719	0.45302	6.064758301	1
MSTRG.25029.1	68	0.3588	10.37384033	1
MSTRG.25031.2	165	0.36232	10.03997803	1
MSTRG.25031.3	77	0.37252	11.32659912	1
MSTRG.25031.1	80	0.36508	8.099182129	1
MSTRG.25031.4	111	0.36132	10.47296143	1
MSTRG.25033.2	302	0.34164	4.729553223	1
MSTRG.25032.4	138	0.38475	5.532287598	1
MSTRG.25039.3	210	0.4418	9.492004395	1
MSTRG.25041.2	83	0.32346	9.89251709	1
MSTRG.25038.1	35	0.38292	7.880554199	-1
MSTRG.25042.1	207	0.41775	8.573791504	1
MSTRG.25045.3	289	0.27274	9.473083496	1
MSTRG.25049.1	90	0.33409	10.29205322	1
MSTRG.25050.2	1446	0.45232	6.06427002	-1
MSTRG.25050.4	1412	0.45067	5.992736816	-1
MSTRG.25050.5	1375	0.44505	6.257507324	-1
MSTRG.25051.3	166	0.4291	6.613220215	1
MSTRG.25051.4	163	0.4537	6.428771973	-1
MSTRG.25051.6	99	0.40736	7.92779541	-1
MSTRG.25053.6	707	0.46802	6.481506348	1
MSTRG.25058.1	660	0.37269	7.050964355	1
MSTRG.25058.3	426	0.3592	8.009460449	1
MSTRG.25059.1	1118	0.4724	5.727722168	1
MSTRG.25055.1	83	0.40935	10.26495361	1
MSTRG.25056.1	640	0.39724	6.07623291	1
MSTRG.25060.3	277	0.37985	10.94561768	1
MSTRG.25063.2	136	0.35259	11.26934814	1
MSTRG.25063.1	136	0.35226	11.26934814	1
MSTRG.25064.2	208	0.3987	6.242248535	1
MSTRG.25067.2	152	0.33265	9.526794434	1
MSTRG.25067.3	213	0.31203	8.04473877	1
MSTRG.25070.1	150	0.35501	10.29266357	1
MSTRG.25070.2	150	0.36888	10.29266357	1
MSTRG.25070.4	150	0.3726	10.29266357	1
MSTRG.25072.4	308	0.41083	6.198669434	1

MSTRG.25072.3	427	0.4647	7.582092285	1
MSTRG.25074.2	994	0.41128	5.94317627	1
MSTRG.25074.3	984	0.38356	5.905578613	1
MSTRG.25071.3	180	0.40109	7.007629395	1
MSTRG.25078.3	712	0.42109	5.422668457	1
MSTRG.25079.3	146	0.44074	10.10150146	1
MSTRG.25079.2	146	0.45067	10.10150146	1
MSTRG.25078.4	712	0.46748	5.422668457	1
MSTRG.25080.2	659	0.46312	5.603820801	1
MSTRG.25076.2	114	0.33744	12.2699585	1
MSTRG.25076.3	95	0.3568	9.503723145	1
MSTRG.25085.2	146	0.33767	4.932189941	1
MSTRG.25085.3	247	0.41122	5.499206543	1
MSTRG.25081.1	599	0.42266	8.122375488	1
MSTRG.25090.3	140	0.44512	5.929626465	1
MSTRG.25108.4	220	0.40474	10.0479126	1
MSTRG.25108.7	220	0.31047	10.0479126	1
MSTRG.25109.1	169	0.40865	8.512390137	-1
MSTRG.25110.1	596	0.46954	9.093688965	1
MSTRG.25103.2	616	0.45839	4.848571777	1
MSTRG.25103.3	616	0.47963	4.848571777	1
MSTRG.25103.5	575	0.46118	4.874450684	1
MSTRG.25103.4	575	0.48454	4.874450684	1
MSTRG.25103.6	173	0.44939	4.551086426	1
MSTRG.25105.1	70	0.44465	10.09820557	1
MSTRG.25104.1	20	0.36789	6.501525879	1
MSTRG.25107.1	29	0.37066	4.55657959	1
MSTRG.25116.1	347	0.44243	4.955627441	1
MSTRG.25116.3	347	0.45161	4.955627441	1
MSTRG.25115.1	60	0.33376	6.010314941	1
MSTRG.25118.2	156	0.27385	8.305725098	1
MSTRG.25120.1	334	0.41167	5.231872559	1
MSTRG.25123.1	132	0.3458	11.59124756	-1
MSTRG.25124.1	504	0.41151	9.436828613	1
MSTRG.25124.2	307	0.45847	5.804016113	1
MSTRG.25125.1	65	0.32634	11.43536377	1
MSTRG.25143.1	191	0.31454	9.171325684	1
MSTRG.25143.2	191	0.31	9.171325684	1
MSTRG.25143.3	191	0.31454	9.171325684	1
MSTRG.25128.1	61	0.2972	3.972106934	1
MSTRG.25138.1	48	0.37362	11.71258545	1
MSTRG.25139.1	73	0.48158	5.840026855	1
MSTRG.25150.4	265	0.45532	6.100646973	1
MSTRG.25166.1	77	0.34218	7.774963379	1
MSTRG.25167.1	1625	0.46229	8.773742676	1
MSTRG.25167.3	713	0.41207	6.833557129	1
MSTRG.25168.1	988	0.27911	6.69720459	1
MSTRG.25164.2	240	0.42518	9.975646973	1
MSTRG.25164.1	264	0.42009	10.13067627	1
MSTRG.25164.4	234	0.46834	10.07452393	1
MSTRG.25170.1	144	0.29637	11.03302002	1
MSTRG.25174.2	286	0.41653	10.26239014	1
MSTRG.25174.3	183	0.37868	9.126403809	1
MSTRG.25176.4	186	0.34111	9.012145996	1

MSTRG.25176.5	186	0.37141	9.012145996	1
MSTRG.25181.1	311	0.36499	7.558654785	-1
MSTRG.25185.1	159	0.38903	10.6104126	1
MSTRG.25195.1	362	0.30838	9.070495605	1
MSTRG.25195.2	362	0.3632	9.070495605	1
MSTRG.25196.3	586	0.45454	7.632751465	1
MSTRG.25196.4	752	0.45551	7.344543457	1
MSTRG.25196.5	339	0.47164	6.808532715	1
MSTRG.25196.6	230	0.47477	9.077087402	1
MSTRG.25198.1	33	0.46224	6.518981934	-1
MSTRG.25190.3	674	0.41362	6.612854004	1
MSTRG.25205.4	1701	0.36842	7.29888916	1
MSTRG.25205.5	1786	0.41692	7.796813965	1
MSTRG.25206.1	107	0.34162	10.12615967	1
MSTRG.25205.6	306	0.38786	7.138366699	1
MSTRG.25207.1	76	0.30289	5.420593262	1
MSTRG.25207.2	142	0.27651	9.296081543	1
MSTRG.25207.3	142	0.28588	9.296081543	1
MSTRG.25207.4	97	0.2908	10.93048096	1
MSTRG.25208.1	2	0.50083	4.596496582	-1
MSTRG.25211.1	69	0.28683	8.815734863	1
MSTRG.25214.1	90	0.37557	11.4944458	1
MSTRG.25214.2	72	0.35182	4.507141113	1
MSTRG.25217.1	371	0.44441	5.903747559	1
MSTRG.25232.1	79	0.35885	10.29205322	-1
MSTRG.25248.1	89	0.29367	9.338806152	1
MSTRG.25254.2	603	0.48387	5.551818848	1
MSTRG.25221.1	22	0.32646	12.30267334	-1
MSTRG.25224.3	74	0.46622	5.719665527	-1
MSTRG.25226.1	208	0.2785	6.07598877	1
MSTRG.25226.3	130	0.27251	10.23956299	1
MSTRG.25226.5	134	0.27327	8.464782715	1
MSTRG.25226.4	111	0.29443	6.532409668	1
MSTRG.25226.8	134	0.27385	8.464782715	1
MSTRG.25255.4	626	0.43152	5.516052246	1
MSTRG.25255.1	576	0.36757	5.512512207	1
MSTRG.25256.1	100	0.35732	9.81817627	1
MSTRG.25257.1	161	0.26101	10.79302979	1
MSTRG.25258.1	87	0.32469	7.065368652	1
MSTRG.25255.6	143	0.39192	5.136047363	1
MSTRG.25268.6	510	0.31264	6.738220215	1
MSTRG.25269.1	48	0.30014	10.17108154	1
MSTRG.25280.2	153	0.48401	6.170837402	1
MSTRG.25261.1	286	0.3208	9.052307129	1
MSTRG.25262.1	49	0.34027	7.877258301	1
MSTRG.25262.2	70	0.31027	5.769348145	1
MSTRG.25265.1	39	0.44768	6.199157715	-1
MSTRG.25281.1	45	0.41853	10.06292725	1
MSTRG.25285.2	108	0.43425	11.04962158	1
MSTRG.25290.3	639	0.43405	4.770080566	1
MSTRG.25297.2	142	0.38291	8.624450684	1
MSTRG.25298.1	110	0.3351	10.65802002	1
MSTRG.25302.1	112	0.47184	5.726501465	-1
MSTRG.25310.1	134	0.30649	9.707458496	1

MSTRG.25318.1	488	0.37649	4.472106934	1
MSTRG.25315.1	83	0.34675	4.715026855	1
MSTRG.25327.1	1703	0.43909	5.604553223	1
MSTRG.25327.2	1703	0.4338	5.604553223	1
MSTRG.25327.3	1699	0.43909	5.542541504	1
MSTRG.25328.1	122	0.34048	11.27008057	-1
MSTRG.25329.1	69	0.3345	10.24713135	1
MSTRG.25335.1	811	0.45382	6.610290527	1
MSTRG.25335.2	187	0.42446	9.053771973	1
MSTRG.25342.1	716	0.38518	4.922790527	1
MSTRG.25342.4	716	0.49863	4.922790527	1
MSTRG.25342.5	716	0.47604	4.922790527	1
MSTRG.25343.1	45	0.29403	9.58001709	1
MSTRG.25348.2	2463	0.45493	4.716003418	1
MSTRG.25350.2	220	0.38649	9.296936035	1
MSTRG.25351.2	2495	0.42996	4.811828613	1
MSTRG.25351.3	2499	0.42996	4.823547363	1
MSTRG.25351.4	2390	0.42996	4.861877441	1
MSTRG.25356.2	48	0.38503	10.34588623	1
MSTRG.25359.1	398	0.48956	6.23248291	1
MSTRG.25359.4	405	0.37539	6.359924316	1
MSTRG.25353.1	215	0.43474	5.461120605	1
MSTRG.25353.3	215	0.41873	5.461120605	1
MSTRG.25354.1	13	0.40706	5.823059082	1
MSTRG.25354.2	21	0.46366	9.69708252	1
MSTRG.25361.1	264	0.28851	8.435974121	1
MSTRG.25361.3	139	0.39569	11.48175049	1
MSTRG.25364.1	78	0.33387	7.674987793	1
MSTRG.25362.2	319	0.44631	6.010437012	1
MSTRG.25372.1	91	0.39353	9.032897949	1
MSTRG.25384.1	195	0.38914	5.041564941	1
MSTRG.25384.2	311	0.4382	5.104431152	1
MSTRG.25384.3	516	0.36082	10.35321045	1
MSTRG.25384.6	110	0.32057	9.828552246	1
MSTRG.25388.1	1412	0.45517	5.403259277	1
MSTRG.25388.5	1412	0.48982	5.403259277	1
MSTRG.25388.6	1372	0.46355	5.430236816	1
MSTRG.25388.7	404	0.44279	4.717956543	1
MSTRG.25389.1	123	0.33438	8.80255127	1
MSTRG.25390.1	149	0.3	7.75189209	1
MSTRG.25389.2	110	0.27886	6.558654785	1
MSTRG.25389.3	94	0.35728	8.885925293	1
MSTRG.25409.4	381	0.38223	6.019836426	1
MSTRG.25409.5	344	0.40694	5.222473145	1
MSTRG.25409.7	81	0.39027	4.749938965	1
MSTRG.25410.2	285	0.39334	6.156188965	1
MSTRG.25413.2	473	0.42994	8.723815918	1
MSTRG.25413.3	473	0.48362	8.723815918	1
MSTRG.25413.4	473	0.4577	8.723815918	1
MSTRG.25413.5	473	0.48362	8.723815918	1
MSTRG.25413.6	168	0.39846	7.09576416	1
MSTRG.25413.7	534	0.47147	6.380065918	-1
MSTRG.25415.1	488	0.34431	5.584289551	1
MSTRG.25415.2	973	0.34162	6.503967285	1

MSTRG.25415.3	53	0.25609	9.698425293	1
MSTRG.25415.4	468	0.34547	6.481384277	1
MSTRG.25417.1	44	0.29059	8.802429199	1
MSTRG.25399.1	158	0.3854	8.979064941	1
MSTRG.25391.1	79	0.42851	10.16094971	-1
MSTRG.25405.2	580	0.39265	9.062194824	1
MSTRG.25406.1	75	0.31209	8.295593262	-1
MSTRG.25405.3	122	0.40695	8.80291748	1
MSTRG.25433.2	94	0.41169	4.428649902	1
MSTRG.25434.1	56	0.27517	4.77166748	1
MSTRG.25435.3	285	0.46058	9.658752441	1
MSTRG.25436.1	706	0.4257	6.278503418	1
MSTRG.25436.2	706	0.35909	6.278503418	1
MSTRG.25436.5	706	0.41888	6.278503418	1
MSTRG.25437.1	44	0.3882	10.90325928	1
MSTRG.25441.2	225	0.44345	4.808166504	1
MSTRG.25447.2	782	0.3823	6.035583496	1
MSTRG.25447.6	782	0.3823	6.035583496	1
MSTRG.25447.7	782	0.45852	6.035583496	1
MSTRG.25448.1	125	0.27937	10.13580322	1
MSTRG.25444.1	284	0.35487	11.77044678	1
MSTRG.25451.4	304	0.46333	7.945373535	1
MSTRG.25462.1	89	0.39716	10.57415771	1
MSTRG.25463.1	306	0.30492	7.785339355	1
MSTRG.25465.1	95	0.32465	9.911437988	1
MSTRG.25472.5	302	0.32304	5.332092285	1
MSTRG.25472.6	473	0.43867	6.426818848	1
MSTRG.25472.7	473	0.43427	6.426818848	1
MSTRG.25472.8	473	0.42413	6.426818848	1
MSTRG.25477.1	523	0.39563	4.520202637	1
MSTRG.25477.2	523	0.38518	4.520202637	1
MSTRG.25478.1	90	0.28366	4.369812012	1
MSTRG.25478.3	90	0.42894	4.369812012	1
MSTRG.25478.5	281	0.44567	5.989196777	1
MSTRG.25480.3	1443	0.34952	7.624206543	1
MSTRG.25480.4	1345	0.4184	8.036315918	1
MSTRG.25483.1	43	0.36592	9.520935059	1
MSTRG.25483.2	56	0.37532	9.296813965	1
MSTRG.25484.2	269	0.42287	8.734680176	1
MSTRG.25489.2	546	0.39923	7.140075684	1
MSTRG.25489.3	546	0.42643	7.140075684	1
MSTRG.25491.1	311	0.29332	9.03692627	1
MSTRG.25492.4	222	0.28195	8.918884277	1
MSTRG.25492.3	216	0.35524	9.111022949	1
MSTRG.25492.5	215	0.35524	9.111022949	1
MSTRG.25499.3	212	0.3333	12.0269165	1
MSTRG.25499.2	253	0.35864	8.541931152	1
MSTRG.25499.6	212	0.33686	12.0269165	1
MSTRG.25499.7	101	0.29583	11.57684326	1
MSTRG.25499.8	212	0.36178	12.0269165	1
MSTRG.25499.10	172	0.36168	8.438537598	1
MSTRG.25508.1	55	0.34779	10.40594482	1
MSTRG.25508.3	272	0.29772	8.007385254	1
MSTRG.25508.4	169	0.28334	6.413391113	1

MSTRG.25508.5	182	0.32236	5.216369629	1
MSTRG.25508.6	272	0.29772	8.007385254	1
MSTRG.25508.7	127	0.33475	7.101135254	1
MSTRG.25509.1	175	0.32485	4.959289551	1
MSTRG.25508.9	155	0.2921	8.761901855	1
MSTRG.25508.8	144	0.30596	7.700622559	1
MSTRG.25510.1	38	0.38225	6.680236816	-1
MSTRG.25508.10	155	0.28982	8.761901855	1
MSTRG.25508.11	132	0.28982	10.2020874	1
MSTRG.25508.12	90	0.28982	9.58770752	1
MSTRG.25508.13	53	0.28686	8.682556152	1
MSTRG.25511.1	48	0.32389	9.744934082	1
MSTRG.25512.1	122	0.28982	9.552062988	1
MSTRG.25512.2	131	0.28971	9.753967285	1
MSTRG.25513.1	107	0.31742	6.155456543	1
MSTRG.25514.1	125	0.2765	10.88555908	1
MSTRG.25508.14	74	0.3708	8.768981934	-1
MSTRG.25517.1	96	0.34973	5.11895752	1
MSTRG.25517.3	96	0.34876	5.11895752	1
MSTRG.25517.4	96	0.35312	5.11895752	1
MSTRG.25518.2	1179	0.46832	5.66595459	1
MSTRG.25528.2	607	0.2921	9.574401855	1
MSTRG.25528.3	862	0.4248	8.47833252	1
MSTRG.25529.1	67	0.27517	8.934631348	1
MSTRG.25528.4	121	0.30596	8.77130127	1
MSTRG.25530.1	154	0.27661	8.530822754	1
MSTRG.25531.1	63	0.30268	8.710510254	1
MSTRG.25532.1	94	0.33419	9.740661621	1
MSTRG.25537.2	416	0.3439	6.800720215	1
MSTRG.25537.1	389	0.3208	6.382629395	1
MSTRG.25537.3	390	0.3208	6.382629395	1
MSTRG.25540.1	531	0.44095	9.070129395	1
MSTRG.25546.1	157	0.38517	10.93707275	-1
MSTRG.25550.3	487	0.42475	4.645690918	1
MSTRG.25552.1	520	0.38098	5.438049316	1
MSTRG.25552.2	547	0.40369	5.299865723	1
MSTRG.25552.3	553	0.38098	5.311828613	1
MSTRG.25552.4	547	0.37308	5.299865723	1
MSTRG.25552.5	547	0.39948	5.299865723	1
MSTRG.25552.6	520	0.42317	5.438049316	1
MSTRG.25552.7	520	0.40353	5.438049316	1
MSTRG.25552.9	520	0.38922	5.438049316	1
MSTRG.25552.10	520	0.40369	5.438049316	1
MSTRG.25556.3	100	0.28518	4.385437012	1
MSTRG.25557.2	344	0.39023	5.390441895	1
MSTRG.25571.1	387	0.44347	5.971374512	1
MSTRG.25577.7	1556	0.47668	6.094299316	1
MSTRG.25577.8	1540	0.47668	6.094055176	1
MSTRG.25582.3	439	0.47298	5.86505127	-1
MSTRG.25593.2	550	0.40812	5.772399902	1
MSTRG.25594.1	33	0.28784	8.253112793	1
MSTRG.25595.1	1708	0.42735	7.131286621	1
MSTRG.25601.2	613	0.31852	4.786193848	1
MSTRG.25597.2	648	0.33603	7.206848145	1

MSTRG.25597.3	96	0.31369	10.89093018	1
MSTRG.25598.2	36	0.35209	8.940490723	1
MSTRG.25598.1	36	0.38067	8.940490723	1
MSTRG.25599.1	142	0.28306	6.797058105	1
MSTRG.25597.4	179	0.28584	8.288879395	1
MSTRG.25602.1	717	0.33747	6.397521973	-1
MSTRG.25602.2	660	0.47803	5.898010254	-1
MSTRG.25602.4	151	0.29607	9.368347168	1
MSTRG.25602.5	85	0.41021	4.875671387	1
MSTRG.25604.3	790	0.44923	5.672424316	1
MSTRG.25604.2	880	0.36117	5.77130127	1
MSTRG.25615.2	1946	0.39342	6.40020752	1
MSTRG.25615.6	3231	0.49099	6.141784668	1
MSTRG.25615.7	3230	0.48847	6.157165527	1
MSTRG.25615.9	2864	0.4875	6.198303223	1
MSTRG.25615.10	1295	0.48598	6.307312012	1
MSTRG.25607.5	2370	0.48063	8.023620605	1
MSTRG.25608.1	60	0.26981	8.711364746	1
MSTRG.25609.1	110	0.30656	11.58636475	1
MSTRG.25610.1	138	0.34496	10.7276001	1
MSTRG.25610.2	190	0.31852	9.232849121	1
MSTRG.25611.1	55	0.27075	8.841125488	1
MSTRG.25614.1	71	0.41286	5.090148926	1
MSTRG.25616.2	279	0.36818	5.844909668	1
MSTRG.25616.3	69	0.28499	9.29888916	1
MSTRG.25620.2	695	0.35258	7.541320801	1
MSTRG.25626.1	74	0.33607	4.889221191	1
MSTRG.25622.1	50	0.36757	4.996765137	1
MSTRG.25622.2	37	0.38952	6.517028809	1
MSTRG.25623.1	61	0.36552	10.53375244	-1
MSTRG.25630.1	51	0.41611	9.588562012	1
MSTRG.25631.2	276	0.42977	9.739562988	1
MSTRG.25632.2	171	0.28982	4.498718262	1
MSTRG.25632.4	171	0.35178	4.498718262	1
MSTRG.25635.1	229	0.28683	11.03594971	1
MSTRG.25637.2	980	0.45331	5.929138184	1
MSTRG.25638.1	152	0.36161	9.070861816	-1
MSTRG.25638.2	86	0.40392	7.742736816	1
MSTRG.25642.1	55	0.38797	4.657043457	1
MSTRG.25642.2	55	0.399	4.657043457	1
MSTRG.25639.1	20	0.31301	10.00091553	-1
MSTRG.25653.1	53	0.39387	9.696228027	1
MSTRG.25654.2	127	0.37256	9.599060059	1
MSTRG.25654.3	127	0.31233	9.599060059	1
MSTRG.25657.6	582	0.47575	8.858093262	1
MSTRG.25657.7	283	0.38974	9.465393066	1
MSTRG.25664.2	459	0.4802	4.698791504	1
MSTRG.25667.1	45	0.36727	5.872009277	1
MSTRG.25711.1	19	0.30605	6.50567627	1
MSTRG.25711.2	49	0.3319	5.510192871	1
MSTRG.25711.3	26	0.30263	5.750305176	1
MSTRG.25711.4	49	0.33889	8.887023926	1
MSTRG.25712.1	103	0.29387	4.971496582	1
MSTRG.25711.5	83	0.33998	7.748596191	1

MSTRG.25711.6	211	0.35785	8.704040527	1
MSTRG.25711.7	127	0.29283	5.39666748	1
MSTRG.25707.1	57	0.34078	8.768493652	1
MSTRG.25718.1	25	0.29898	11.53900146	1
MSTRG.25723.2	277	0.43627	7.115783691	1
MSTRG.25725.1	84	0.29617	9.116271973	1
MSTRG.25726.3	89	0.4336	3.811950684	1
MSTRG.25730.2	255	0.35635	5.703186035	1
MSTRG.25734.2	445	0.43664	8.27520752	1
MSTRG.25734.3	445	0.45554	8.27520752	1
MSTRG.25736.1	194	0.32849	10.60455322	1
MSTRG.25734.4	134	0.39145	5.420471191	1
MSTRG.25737.2	354	0.38634	9.355773926	1
MSTRG.25747.4	2319	0.46397	5.86138916	1
MSTRG.25747.2	3105	0.41362	5.987121582	1
MSTRG.25748.1	79	0.32081	10.03619385	1
MSTRG.25748.2	79	0.32582	10.03619385	1
MSTRG.25739.1	149	0.38384	8.86907959	1
MSTRG.25749.1	46	0.46874	9.743469238	1
MSTRG.25750.1	39	0.39634	6.210998535	1
MSTRG.25750.2	39	0.39461	6.210998535	1
MSTRG.25752.1	82	0.34343	12.00360107	1
MSTRG.25760.4	842	0.42968	5.773620605	1
MSTRG.25757.2	104	0.43954	6.412414551	1
MSTRG.25771.2	1073	0.3295	6.882873535	1
MSTRG.25772.1	92	0.35485	6.554748535	1
MSTRG.25771.3	917	0.36241	7.164855957	1
MSTRG.25771.4	709	0.36378	7.346984863	1
MSTRG.25771.5	134	0.29507	6.496765137	1
MSTRG.25778.1	413	0.34197	7.963562012	1
MSTRG.25779.2	129	0.40979	6.952209473	1
MSTRG.25779.3	120	0.3799	6.022033691	1
MSTRG.25773.1	37	0.37253	5.302429199	-1
MSTRG.25781.2	778	0.45237	8.516784668	1
MSTRG.25782.1	28	0.46198	9.782287598	1
MSTRG.25782.2	28	0.48305	9.782287598	1
MSTRG.25784.1	210	0.26913	10.04803467	1
MSTRG.25785.2	179	0.28284	6.279968262	1
MSTRG.25790.1	36	0.34544	8.938415527	1
MSTRG.25796.1	670	0.32789	9.143249512	1
MSTRG.25796.2	670	0.37351	9.143249512	1
MSTRG.25798.1	130	0.3629	8.858459473	1
MSTRG.25799.1	131	0.31029	9.854553223	1
MSTRG.25802.1	66	0.37497	9.61151123	1
MSTRG.25802.2	90	0.4106	8.635925293	1
MSTRG.25803.1	118	0.44838	5.603088379	1
MSTRG.25808.1	178	0.35546	6.955871582	1
MSTRG.25809.1	134	0.2911	9.385192871	1
MSTRG.25809.2	184	0.30249	9.262268066	1
MSTRG.25809.3	184	0.30249	9.262268066	1
MSTRG.25806.1	94	0.35391	6.68951416	1
MSTRG.25817.2	451	0.36597	7.449401855	1
MSTRG.25817.3	422	0.39622	7.030944824	1
MSTRG.25828.1	601	0.44031	8.743469238	1

MSTRG.25824.4	576	0.46945	6.101867676	1
MSTRG.25847.1	107	0.38655	9.057800293	1
MSTRG.25848.1	173	0.41487	8.824401855	1
MSTRG.25848.2	173	0.33427	8.824401855	1
MSTRG.25849.1	108	0.34165	9.152038574	-1
MSTRG.25857.1	83	0.27965	8.818908691	1
MSTRG.25858.1	155	0.35069	10.34527588	1
MSTRG.25857.2	72	0.34331	6.797180176	1
MSTRG.25861.1	141	0.29673	6.570007324	1
MSTRG.25862.1	47	0.40719	12.22735596	1
MSTRG.25869.1	43	0.39134	11.73394775	-1
MSTRG.25872.1	79	0.40953	10.53375244	-1
MSTRG.25897.4	240	0.39657	11.19927979	1
MSTRG.25897.6	240	0.41083	11.19927979	1
MSTRG.25897.3	240	0.31674	11.19927979	1
MSTRG.25897.7	240	0.38165	11.19927979	1
MSTRG.25898.1	130	0.27994	8.685119629	1
MSTRG.25897.11	70	0.28013	7.741027832	1
MSTRG.25897.12	85	0.27398	4.104797363	1
MSTRG.25897.13	112	0.31048	8.20904541	1
MSTRG.25897.14	127	0.26785	11.32806396	1
MSTRG.25874.2	1183	0.45302	5.975280762	1
MSTRG.25874.1	1487	0.43284	6.703186035	1
MSTRG.25907.2	165	0.3663	9.87286377	1
MSTRG.25907.3	186	0.27794	8.971008301	1
MSTRG.25911.2	291	0.35356	6.892150879	1
MSTRG.25912.2	657	0.46422	5.907653809	1
MSTRG.25916.1	387	0.32485	9.11126709	1
MSTRG.25916.2	387	0.35355	9.11126709	1
MSTRG.25917.1	38	0.46198	4.497619629	-1
MSTRG.25923.1	72	0.27547	9.690856934	1
MSTRG.25926.4	407	0.33828	8.66595459	1
MSTRG.25926.5	395	0.4306	5.707702637	1
MSTRG.25927.1	88	0.27918	9.60369873	1
MSTRG.25928.1	114	0.33599	9.055603027	1
MSTRG.25926.6	103	0.37461	6.050476074	1
MSTRG.25929.1	187	0.34731	10.11187744	1
MSTRG.25940.1	446	0.43983	8.229309082	1
MSTRG.25940.5	446	0.46176	8.229309082	1
MSTRG.25940.3	446	0.46005	8.229309082	1
MSTRG.25949.3	1246	0.37533	5.61529541	1
MSTRG.25950.1	130	0.27193	10.03631592	1
MSTRG.25951.1	220	0.30317	10.95245361	1
MSTRG.25949.5	1177	0.39353	5.623840332	1
MSTRG.25949.6	625	0.35156	5.161682129	1
MSTRG.25949.7	586	0.2899	5.864685059	1
MSTRG.25949.8	625	0.44464	5.161682129	1
MSTRG.25956.1	64	0.36537	6.478820801	1
MSTRG.25958.1	145	0.28683	10.09710693	1
MSTRG.25958.2	145	0.29607	10.09710693	1
MSTRG.25958.3	132	0.27651	11.57366943	1
MSTRG.25959.1	130	0.27911	8.237976074	-1
MSTRG.25959.2	192	0.28648	8.956115723	1
MSTRG.25959.3	192	0.2782	8.956115723	1

MSTRG.25959.5	175	0.27992	9.781799316	1
MSTRG.25959.4	192	0.30132	8.956115723	1
MSTRG.25959.6	107	0.28567	9.81048584	1
MSTRG.25959.7	175	0.28267	9.781799316	1
MSTRG.25959.8	175	0.29892	9.781799316	1
MSTRG.25960.1	414	0.38219	8.656921387	1
MSTRG.25972.1	61	0.45646	9.819763184	-1
MSTRG.25973.2	184	0.38476	5.315856934	1
MSTRG.25973.3	416	0.41751	7.460632324	1
MSTRG.25973.4	255	0.42268	9.191345215	1
MSTRG.25973.6	184	0.40199	5.315856934	1
MSTRG.25977.1	116	0.27739	9.911315918	1
MSTRG.25977.2	185	0.27739	11.82928467	1
MSTRG.25977.3	121	0.30609	10.63214111	1
MSTRG.25978.2	165	0.28415	11.05487061	1
MSTRG.25975.1	45	0.45428	10.0524292	1
MSTRG.25980.2	113	0.44277	11.01202393	1
MSTRG.25997.5	1694	0.36271	8.585632324	1
MSTRG.25997.4	1108	0.38033	8.93548584	1
MSTRG.25997.1	1729	0.37984	8.543395996	1
MSTRG.25997.2	1792	0.36109	8.079406738	1
MSTRG.25997.6	1108	0.36427	8.93548584	1
MSTRG.25998.1	134	0.33925	8.754333496	1
MSTRG.25997.8	1827	0.45343	7.870056152	1
MSTRG.25997.7	1792	0.4338	8.079406738	1
MSTRG.25997.9	1694	0.40301	8.585632324	1
MSTRG.25997.10	1805	0.44198	8.06829834	1
MSTRG.25997.11	1840	0.40301	7.974060059	1
MSTRG.25997.12	1108	0.33905	8.93548584	1
MSTRG.25997.13	199	0.27863	9.352844238	1
MSTRG.25997.14	239	0.35792	9.570739746	1
MSTRG.25997.18	146	0.29227	11.72821045	1
MSTRG.25997.19	40	0.32731	7.978210449	1
MSTRG.25997.20	74	0.26409	5.813781738	1
MSTRG.25997.21	53	0.26498	11.55596924	1
MSTRG.25989.1	155	0.27987	6.497009277	1
MSTRG.25989.2	217	0.27883	10.96575928	1
MSTRG.25989.3	217	0.27883	10.96575928	1
MSTRG.25989.5	217	0.27547	10.96575928	1
MSTRG.25989.6	155	0.27851	6.497009277	1
MSTRG.25991.1	179	0.27274	9.694763184	1
MSTRG.25991.2	179	0.27526	9.694763184	1
MSTRG.25991.3	179	0.2785	9.694763184	1
MSTRG.25991.4	179	0.27526	9.694763184	1
MSTRG.25991.5	179	0.28002	9.694763184	1
MSTRG.25985.2	375	0.30712	9.49822998	1
MSTRG.25986.1	231	0.32507	9.79864502	1
MSTRG.25992.2	655	0.42148	6.46282959	1
MSTRG.25993.1	86	0.30735	10.909729	1
MSTRG.25994.1	83	0.28923	11.44610596	1
MSTRG.25994.2	108	0.28923	8.49017334	1
MSTRG.25995.1	219	0.2948	8.817199707	1
MSTRG.25992.3	152	0.28015	8.61126709	1
MSTRG.25992.4	232	0.34128	9.225891113	1

MSTRG.26010.2	683	0.44418	5.335021973	1
MSTRG.26010.3	128	0.29816	9.842712402	1
MSTRG.26016.1	103	0.27739	6.816101074	1
MSTRG.26018.1	105	0.33098	8.896789551	1
MSTRG.26021.1	69	0.35776	7.725036621	1
MSTRG.26026.5	221	0.33361	7.487121582	1
MSTRG.26026.4	222	0.32851	7.487121582	1
MSTRG.26026.2	205	0.30464	7.535095215	1
MSTRG.26026.3	206	0.30954	6.866760254	1
MSTRG.26028.1	151	0.43785	9.25958252	1
MSTRG.26033.1	325	0.39958	5.119445801	1
MSTRG.26034.1	34	0.38395	9.83380127	1
MSTRG.26034.3	83	0.39367	5.577819824	1
MSTRG.26034.2	599	0.36935	9.019348145	1
MSTRG.26034.4	68	0.29772	5.553894043	-1
MSTRG.26034.5	68	0.37811	5.553894043	-1
MSTRG.26034.6	56	0.34961	8.74017334	1
MSTRG.26034.7	56	0.29367	8.799865723	1
MSTRG.26031.2	224	0.49472	9.263366699	-1
MSTRG.26031.3	75	0.45389	10.32720947	1
MSTRG.26035.2	2096	0.46255	4.09564209	1
MSTRG.26035.3	2035	0.46255	4.094421387	1
MSTRG.26036.1	431	0.38982	6.804870605	1
MSTRG.26038.2	425	0.46109	6.240539551	1
MSTRG.26038.3	425	0.45985	6.240539551	1
MSTRG.26037.1	317	0.46358	4.992370605	1
MSTRG.26041.2	945	0.47711	6.572570801	1
MSTRG.26042.1	460	0.44326	5.616516113	1
MSTRG.26042.2	460	0.44182	5.616516113	1
MSTRG.26051.1	1349	0.44107	6.411071777	1
MSTRG.26052.1	516	0.4367	6.08380127	1
MSTRG.26053.3	119	0.45745	7.815246582	1
MSTRG.26053.4	694	0.46548	8.272033691	1
MSTRG.26045.2	297	0.35514	5.859436035	1
MSTRG.26048.1	31	0.47418	11.32427979	1
MSTRG.26047.2	94	0.44409	7.862365723	1
MSTRG.26059.1	823	0.44776	7.737609863	1
MSTRG.26059.2	823	0.44032	7.725158691	1
MSTRG.26083.4	1265	0.41724	8.238098145	1
MSTRG.26083.5	1265	0.43774	8.238098145	1
MSTRG.26087.7	173	0.28186	6.471618652	1
MSTRG.26087.6	114	0.35824	6.940490723	1
MSTRG.26087.5	753	0.47329	5.643005371	1
MSTRG.26088.1	123	0.30173	7.622009277	1
MSTRG.26089.1	118	0.29793	8.027770996	1
MSTRG.26091.1	561	0.30041	6.934265137	1
MSTRG.26091.4	486	0.30485	8.522155762	1
MSTRG.26091.2	562	0.30423	6.934265137	1
MSTRG.26093.1	56	0.35475	10.01239014	1
MSTRG.26096.1	82	0.37719	8.235046387	1
MSTRG.26102.2	199	0.44466	5.724914551	1
MSTRG.26106.1	116	0.27987	9.479797363	1
MSTRG.26106.2	116	0.28855	9.479797363	1
MSTRG.26107.2	71	0.32974	8.771057129	1

MSTRG.26108.1	126	0.45878	9.463439941	1
MSTRG.26109.1	77	0.32818	10.30609131	1
MSTRG.26111.4	849	0.45621	5.669494629	1
MSTRG.26112.2	55	0.33955	9.03302002	1
MSTRG.26112.3	157	0.41909	9.163757324	1
MSTRG.26112.4	147	0.37963	9.270568848	1
MSTRG.26113.1	80	0.33468	9.045715332	1
MSTRG.26118.3	224	0.34727	9.096130371	1
MSTRG.26118.11	278	0.27911	8.648376465	1
MSTRG.26118.12	375	0.293	8.232727051	1
MSTRG.26118.13	470	0.30663	7.076965332	1
MSTRG.26120.1	100	0.34624	4.675354004	1
MSTRG.26121.1	80	0.32738	11.05462646	1
MSTRG.26123.1	305	0.35113	7.699645996	1
MSTRG.26123.2	257	0.44278	6.191589355	1
MSTRG.26123.8	286	0.40534	6.419128418	1
MSTRG.26123.10	337	0.48594	6.938171387	1
MSTRG.26123.12	268	0.4162	6.378479004	1
MSTRG.26123.11	289	0.45621	5.956604004	1
MSTRG.26123.9	236	0.39099	6.870544434	1
MSTRG.26123.14	172	0.47114	5.239807129	-1
MSTRG.26123.13	121	0.47874	5.278259277	-1
MSTRG.26124.2	135	0.31056	9.979187012	1
MSTRG.26126.2	745	0.46582	6.468566895	1
MSTRG.26126.4	99	0.36016	9.939147949	1
MSTRG.26122.2	369	0.43373	5.371276855	1
MSTRG.26138.1	152	0.38566	8.441833496	-1
MSTRG.26139.1	76	0.33979	9.300109863	1
MSTRG.26142.2	136	0.33919	5.876403809	1
MSTRG.26142.3	219	0.3229	7.596862793	1
MSTRG.26147.3	569	0.42793	6.911682129	1
MSTRG.26148.1	103	0.29615	8.774108887	1
MSTRG.26147.6	453	0.3713	7.206848145	1
MSTRG.26154.2	165	0.34089	6.639709473	1
MSTRG.26154.3	181	0.38263	5.313903809	1
MSTRG.26158.3	163	0.42171	7.151550293	1
MSTRG.26163.1	87	0.43213	4.873474121	1
MSTRG.26171.1	124	0.31516	10.96380615	1
MSTRG.26171.3	229	0.28062	10.11322021	1
MSTRG.26171.4	99	0.31327	8.934020996	1
MSTRG.26171.5	106	0.40706	11.47564697	1
MSTRG.26180.1	261	0.33345	9.683166504	1
MSTRG.26180.2	1683	0.43488	6.29083252	1
MSTRG.26180.5	1762	0.44269	6.964782715	1
MSTRG.26180.6	1699	0.44889	7.105163574	1
MSTRG.26180.13	1624	0.43088	8.473937988	1
MSTRG.26181.1	83	0.33387	8.441589355	1
MSTRG.26180.14	98	0.37142	10.84747314	1
MSTRG.26180.17	160	0.31482	7.713562012	1
MSTRG.26180.18	160	0.31073	7.713562012	1
MSTRG.26180.19	180	0.27739	6.474182129	1
MSTRG.26180.20	293	0.28338	8.789978027	1
MSTRG.26180.21	293	0.32905	8.789978027	1
MSTRG.26184.2	182	0.33613	8.339172363	1

MSTRG.26184.3	148	0.36591	8.500305176	1
MSTRG.26184.5	129	0.38333	8.63079834	1
MSTRG.26185.1	826	0.36882	6.624938965	1
MSTRG.26185.3	826	0.36056	6.624938965	1
MSTRG.26186.1	60	0.43884	9.624816895	1
MSTRG.26187.1	71	0.33475	10.17254639	1
MSTRG.26182.2	121	0.41731	10.07244873	1
MSTRG.26192.1	25	0.44095	7.753845215	1
MSTRG.26194.17	134	0.39511	5.278625488	1
MSTRG.26189.2	208	0.45189	5.82989502	-1
MSTRG.26190.1	43	0.46236	4.749450684	-1
MSTRG.26203.1	53	0.44566	12.01251221	-1
MSTRG.26212.1	105	0.3133	8.808532715	1
MSTRG.26213.1	98	0.3849	7.569274902	1
MSTRG.26213.2	36	0.40727	8.001281738	1
MSTRG.26213.3	98	0.41072	7.569274902	1
MSTRG.26213.5	98	0.35467	7.569274902	1
MSTRG.26213.6	98	0.39317	7.569274902	1
MSTRG.26214.3	397	0.4688	7.905700684	1
MSTRG.26215.3	305	0.47225	5.161804199	1
MSTRG.26216.1	137	0.43143	5.302307129	1
MSTRG.26220.2	437	0.49235	6.040588379	1
MSTRG.26224.1	438	0.48991	6.259338379	1
MSTRG.26224.2	438	0.44266	6.259338379	1
MSTRG.26224.3	506	0.44195	6.049133301	1
MSTRG.26224.4	438	0.47126	6.259338379	1
MSTRG.26224.5	438	0.43476	6.259338379	1
MSTRG.26226.1	377	0.39079	4.934143066	1
MSTRG.26226.2	255	0.36769	4.708190918	1
MSTRG.26227.1	189	0.33889	9.47442627	1
MSTRG.26230.2	245	0.47576	9.388977051	1
MSTRG.26232.3	1406	0.46654	6.749206543	1
MSTRG.26232.4	1328	0.4705	6.296203613	1
MSTRG.26232.5	1406	0.49403	6.749206543	1
MSTRG.26240.5	1747	0.40698	6.541320801	1
MSTRG.26240.6	1747	0.41907	6.541320801	1
MSTRG.26240.7	1747	0.40698	6.541320801	1
MSTRG.26241.1	86	0.39604	8.858703613	1
MSTRG.26242.1	115	0.33061	10.98248291	1
MSTRG.26244.1	116	0.27146	10.65106201	1
MSTRG.26244.2	177	0.27794	10.25531006	1
MSTRG.26245.1	87	0.33407	9.604309082	1
MSTRG.26246.1	101	0.31558	9.101135254	1
MSTRG.26240.8	61	0.29772	9.298522949	1
MSTRG.26247.1	25	0.40027	3.665710449	-1
MSTRG.26247.2	159	0.42439	11.480896	-1
MSTRG.26249.1	37	0.39475	6.79095459	1
MSTRG.26239.1	451	0.41971	8.618469238	1
MSTRG.26239.2	614	0.41995	8.713684082	1
MSTRG.26239.4	620	0.40395	8.62677002	1
MSTRG.26259.5	132	0.33263	10.13446045	1
MSTRG.26260.1	233	0.29351	11.39532471	1
MSTRG.26257.1	174	0.31241	9.356384277	1
MSTRG.26257.2	147	0.37031	6.408508301	-1

MSTRG.26265.1	184	0.47646	9.542785645	1
MSTRG.26266.1	90	0.34981	6.707336426	1
MSTRG.26266.2	144	0.29435	7.669128418	1
MSTRG.26266.3	144	0.29776	7.669128418	1
MSTRG.26281.1	151	0.415	10.57220459	1
MSTRG.26282.2	77	0.38111	5.284606934	1
MSTRG.26286.2	403	0.45082	5.455627441	1
MSTRG.26287.5	86	0.32507	9.852355957	1
MSTRG.26287.3	86	0.36026	9.852355957	1
MSTRG.26287.4	340	0.39717	9.673400879	1
MSTRG.26278.1	105	0.41427	11.2555542	1
MSTRG.26291.3	490	0.38848	9.453186035	1
MSTRG.26291.6	490	0.32265	9.453186035	1
MSTRG.26291.4	544	0.38213	9.340637207	1
MSTRG.26292.1	94	0.41399	9.426452637	1
MSTRG.26297.3	282	0.41887	5.756530762	1
MSTRG.26297.4	38	0.3974	4.36907959	1
MSTRG.26299.1	152	0.3537	8.57220459	1
MSTRG.26301.1	38	0.34723	7.978088379	1
MSTRG.26303.1	391	0.42321	8.416442871	1
MSTRG.26308.1	52	0.37863	6.804016113	1
MSTRG.26308.2	46	0.38215	7.835510254	1
MSTRG.26315.1	110	0.33919	5.817077637	1
MSTRG.26316.1	113	0.35572	7.958190918	1
MSTRG.26321.1	507	0.39878	8.797302246	1
MSTRG.26321.2	385	0.41892	8.820495605	1
MSTRG.26321.3	385	0.44129	8.820495605	1
MSTRG.26321.4	385	0.47938	8.820495605	1
MSTRG.26321.5	85	0.39786	8.140930176	1
MSTRG.26322.1	91	0.37693	10.22003174	1
MSTRG.26323.1	68	0.28526	4.622375488	1
MSTRG.26324.1	64	0.30432	9.93548584	1
MSTRG.26325.1	39	0.27667	4.347595215	1
MSTRG.26338.1	45	0.37452	12.2432251	1
MSTRG.26340.1	302	0.4172	9.468688965	1
MSTRG.26340.2	546	0.46033	9.513000488	1
MSTRG.26337.1	84	0.3665	10.0680542	1
MSTRG.26337.2	27	0.36007	8.402282715	1
MSTRG.26343.1	833	0.46902	6.054748535	1
MSTRG.26344.1	114	0.29826	9.19317627	1
MSTRG.26345.1	91	0.36767	7.51373291	1
MSTRG.26345.2	87	0.28848	5.795959473	1
MSTRG.26346.1	101	0.31368	8.843566895	1
MSTRG.26347.1	55	0.33906	9.696105957	1
MSTRG.26348.1	58	0.31596	9.013244629	1
MSTRG.26349.1	83	0.33929	8.816589355	1
MSTRG.26369.3	476	0.39903	7.153381348	1
MSTRG.26369.2	703	0.33728	7.159240723	1
MSTRG.26369.4	476	0.42131	7.153381348	1
MSTRG.26369.5	476	0.39903	7.153381348	1
MSTRG.26370.2	269	0.42997	8.29486084	1
MSTRG.26373.2	550	0.45661	8.153869629	1
MSTRG.26373.3	550	0.48765	8.153869629	1
MSTRG.26374.1	713	0.49174	5.038024902	1

MSTRG.26375.1	344	0.30242	9.286315918	1
MSTRG.26376.1	71	0.35597	6.944030762	-1
MSTRG.26377.1	105	0.3036	9.549499512	1
MSTRG.26374.5	169	0.29835	7.864807129	1
MSTRG.26374.6	178	0.29479	8.966247559	1
MSTRG.26374.7	89	0.30952	5.76763916	1
MSTRG.26374.8	181	0.35889	6.973083496	1
MSTRG.26375.2	158	0.3018	11.39654541	1
MSTRG.26378.2	144	0.27918	5.06829834	1
MSTRG.26379.7	1785	0.4721	5.120178223	1
MSTRG.26379.6	2328	0.47857	5.222595215	1
MSTRG.26379.9	2610	0.47857	5.206726074	1
MSTRG.26386.1	168	0.30191	8.262268066	1
MSTRG.26391.2	213	0.35435	10.20928955	1
MSTRG.26391.3	356	0.3929	8.203430176	1
MSTRG.26391.4	313	0.30981	8.770324707	1
MSTRG.26391.5	114	0.29367	9.513244629	1
MSTRG.26394.3	1503	0.4363	6.18157959	1
MSTRG.26394.4	1502	0.4445	6.18157959	1
MSTRG.26394.5	1502	0.45426	6.18157959	1
MSTRG.26394.8	1513	0.46689	6.182067871	1
MSTRG.26395.1	325	0.33345	9.354797363	1
MSTRG.26394.10	279	0.44017	5.667663574	1
MSTRG.26394.9	280	0.44017	5.667663574	1
MSTRG.26389.1	57	0.41401	9.428894043	1
MSTRG.26428.1	125	0.30981	6.828796387	1
MSTRG.26428.4	78	0.31209	9.666442871	1
MSTRG.26433.1	219	0.2895	9.358581543	1
MSTRG.26434.1	100	0.32394	8.753112793	1
MSTRG.26433.2	252	0.27661	8.957214355	1
MSTRG.26428.7	84	0.43114	9.194885254	1
MSTRG.26399.8	210	0.44449	6.750793457	1
MSTRG.26399.7	221	0.43608	5.70501709	1
MSTRG.26405.1	97	0.30656	11.91522217	1
MSTRG.26405.2	149	0.36212	8.716003418	1
MSTRG.26405.5	149	0.40898	8.716003418	1
MSTRG.26405.4	58	0.31432	9.250427246	1
MSTRG.26405.6	52	0.34299	9.100524902	1
MSTRG.26411.4	483	0.45478	9.151062012	1
MSTRG.26411.5	1209	0.47732	5.820129395	1
MSTRG.26411.3	1209	0.48111	5.820129395	1
MSTRG.26412.1	44	0.2836	9.644836426	1
MSTRG.26413.1	75	0.32464	9.06463623	1
MSTRG.26415.1	39	0.36138	10.94000244	1
MSTRG.26408.1	92	0.33475	8.593444824	1
MSTRG.26439.1	211	0.38849	4.488464355	1
MSTRG.26448.3	1249	0.39432	5.464904785	1
MSTRG.26448.1	2495	0.48018	5.928771973	1
MSTRG.26448.2	2037	0.48018	5.718811035	1
MSTRG.26448.5	2306	0.47296	5.858459473	1
MSTRG.26448.6	844	0.45862	6.287414551	1
MSTRG.26448.7	86	0.31538	5.415100098	1
MSTRG.26452.3	158	0.37487	4.644592285	1
MSTRG.26453.1	130	0.26722	9.745178223	1

MSTRG.26455.1	107	0.33929	4.851867676	1
MSTRG.26446.4	284	0.45098	5.676940918	1
MSTRG.26446.5	86	0.4556	6.577453613	1
MSTRG.26477.1	26	0.38013	9.303649902	1
MSTRG.26491.1	133	0.2921	10.4899292	1
MSTRG.26493.1	173	0.28982	11.44927979	1
MSTRG.26495.1	52	0.37481	7.877624512	1
MSTRG.26498.1	164	0.2759	7.613098145	1
MSTRG.26498.2	166	0.30816	8.371276855	1
MSTRG.26461.4	721	0.29251	9.153137207	1
MSTRG.26461.2	1604	0.4799	9.319396973	-1
MSTRG.26461.3	1167	0.3892	9.576477051	1
MSTRG.26461.5	582	0.32005	9.734680176	1
MSTRG.26469.2	268	0.37119	6.354064941	1
MSTRG.26470.2	84	0.35316	9.643249512	1
MSTRG.26470.3	120	0.46181	9.637390137	1
MSTRG.26470.4	94	0.40128	9.719055176	1
MSTRG.26515.1	40	0.41667	9.008239746	1
MSTRG.26515.2	40	0.43014	9.008239746	1
MSTRG.26515.3	40	0.28419	9.008239746	1
MSTRG.26521.1	70	0.34935	9.098205566	-1
MSTRG.26533.1	27	0.29058	10.54852295	1
MSTRG.26541.1	63	0.42205	8.768859863	-1
MSTRG.26567.1	37	0.2849	10.93133545	1
MSTRG.26569.2	801	0.42111	4.03338623	1
MSTRG.26569.5	770	0.41859	4.058166504	1
MSTRG.26569.6	753	0.42011	4.074523926	1
MSTRG.26569.7	785	0.42111	4.045593262	1
MSTRG.26569.8	641	0.42383	4.198181152	1
MSTRG.26569.3	640	0.42383	4.207336426	1
MSTRG.26569.4	705	0.42259	4.116149902	1
MSTRG.26569.10	786	0.42111	4.04486084	1
MSTRG.26569.11	753	0.42111	4.081115723	1
MSTRG.26569.12	657	0.42383	4.176086426	1
MSTRG.26569.9	753	0.42111	4.073913574	1
MSTRG.26570.1	102	0.31292	6.954162598	1
MSTRG.26571.1	257	0.28982	7.585144043	1
MSTRG.26571.2	189	0.29772	9.235900879	1
MSTRG.26573.1	277	0.32477	6.659240723	1
MSTRG.26574.1	74	0.36698	10.09747314	1
MSTRG.26574.2	113	0.29236	9.36529541	1
MSTRG.26571.3	244	0.30191	9.913391113	1
MSTRG.26572.4	183	0.32595	9.940856934	1
MSTRG.26572.5	183	0.29725	9.940856934	1
MSTRG.26572.6	216	0.3	8.822937012	1
MSTRG.26572.7	48	0.36161	7.979064941	1
MSTRG.26572.8	86	0.36852	10.70220947	1
MSTRG.26572.9	74	0.41511	4.950134277	1
MSTRG.26572.10	183	0.33425	10.762146	1
MSTRG.26572.11	71	0.29751	9.098449707	1
MSTRG.26572.12	108	0.27794	7.66595459	1
MSTRG.26572.13	130	0.36092	7.75201416	1
MSTRG.26572.14	288	0.46864	7.48614502	-1
MSTRG.26572.15	190	0.32658	9.693054199	1

MSTRG.26572.16	190	0.30016	9.693054199	1
MSTRG.26572.17	190	0.32405	9.693054199	1
MSTRG.26575.1	344	0.44019	5.911804199	1
MSTRG.26542.4	421	0.41105	4.777038574	1
MSTRG.26542.3	421	0.39892	4.777038574	1
MSTRG.26542.6	107	0.3238	9.221252441	1
MSTRG.26552.3	690	0.34597	6.175720215	1
MSTRG.26552.5	849	0.36154	6.460876465	1
MSTRG.26553.2	666	0.42206	6.204040527	1
MSTRG.26553.1	666	0.42233	6.204040527	1
MSTRG.26554.2	1526	0.37006	4.394226074	1
MSTRG.26554.1	1521	0.41515	4.397277832	1
MSTRG.26554.4	1561	0.4352	4.42755127	1
MSTRG.26554.3	1561	0.44742	4.42755127	1
MSTRG.26554.5	1126	0.4198	4.472473145	1
MSTRG.26555.3	2856	0.46123	4.760803223	1
MSTRG.26557.2	1201	0.42996	5.198303223	1
MSTRG.26546.1	701	0.42169	5.05279541	1
MSTRG.26547.1	171	0.32322	11.09234619	1
MSTRG.26547.2	171	0.38126	11.09234619	1
MSTRG.26548.3	458	0.48067	6.554626465	1
MSTRG.26548.5	505	0.48015	6.38104248	1
MSTRG.26560.1	524	0.35777	6.743591309	1
MSTRG.26563.1	2005	0.43123	5.739196777	1
MSTRG.26586.3	553	0.4628	4.776916504	1
MSTRG.26593.1	1108	0.4603	6.163146973	-1
MSTRG.26585.2	490	0.35164	9.402893066	1
MSTRG.26594.3	448	0.35181	4.561340332	1
MSTRG.26595.1	1976	0.46313	5.269714355	1
MSTRG.26596.1	67	0.33678	10.0244751	1
MSTRG.26598.1	296	0.28159	4.757263184	1
MSTRG.26602.2	660	0.44555	7.956481934	1
MSTRG.26602.3	1036	0.43984	8.017272949	1
MSTRG.26601.2	454	0.45715	6.199401855	1
MSTRG.26611.2	127	0.26261	8.426696777	1
MSTRG.26614.1	83	0.31761	8.709899902	1
MSTRG.26615.1	47	0.3667	4.36895752	1
MSTRG.26615.2	47	0.42367	4.36895752	1
MSTRG.26616.1	148	0.38888	9.895690918	1
MSTRG.26616.2	35	0.32899	8.633850098	-1
MSTRG.26617.2	142	0.42079	9.415710449	1
MSTRG.26617.3	242	0.28091	9.25213623	1
MSTRG.26617.4	242	0.31917	9.25213623	1
MSTRG.26620.4	82	0.43362	5.589294434	-1
MSTRG.26624.4	852	0.45453	9.393127441	1
MSTRG.26623.3	175	0.44446	10.79656982	1
MSTRG.26630.3	104	0.32989	4.910095215	1
MSTRG.26630.2	766	0.40478	8.566345215	1
MSTRG.26630.4	812	0.42123	8.299499512	1
MSTRG.26636.2	81	0.28334	9.883728027	1
MSTRG.26637.4	1005	0.34896	4.846130371	1
MSTRG.26637.5	886	0.44285	4.754821777	1
MSTRG.26644.1	976	0.4758	5.843933105	1
MSTRG.26645.1	127	0.27251	7.052062988	1

MSTRG.26644.2	976	0.4758	5.843933105	1
MSTRG.26644.5	928	0.39381	5.729064941	1
MSTRG.26642.1	76	0.39926	10.02459717	1
MSTRG.26643.1	102	0.2911	5.66973877	1
MSTRG.26646.1	126	0.29719	6.40447998	1
MSTRG.26647.1	186	0.36055	9.75604248	1
MSTRG.26647.2	184	0.40656	9.75604248	-1
MSTRG.26651.1	36	0.34711	10.76312256	1
MSTRG.26652.1	241	0.34885	9.546691895	1
MSTRG.26657.2	139	0.41428	4.343566895	1
MSTRG.26658.2	78	0.40652	4.930603027	1
MSTRG.26660.2	739	0.38676	6.96282959	1
MSTRG.26660.1	739	0.4441	6.96282959	1
MSTRG.26660.8	511	0.41385	8.284118652	1
MSTRG.26660.6	672	0.41783	8.914123535	1
MSTRG.26660.10	739	0.42733	6.96282959	1
MSTRG.26660.11	51	0.26189	8.85736084	1
MSTRG.26660.12	745	0.3439	6.785461426	1
MSTRG.26665.1	63	0.28914	8.886047363	1
MSTRG.26662.1	39	0.36164	4.797058105	1
MSTRG.26667.1	90	0.43373	9.03338623	1
MSTRG.26668.1	78	0.38688	6.702575684	-1
MSTRG.26671.1	132	0.32014	10.55975342	1
MSTRG.26675.2	594	0.43301	5.604064941	1
MSTRG.26675.6	594	0.43878	5.604064941	1
MSTRG.26675.7	323	0.38791	5.96661377	1
MSTRG.26675.5	674	0.43716	6.52154541	1
MSTRG.26675.8	316	0.38604	5.809509277	1
MSTRG.26675.11	294	0.42127	7.01385498	1
MSTRG.26675.12	267	0.27598	8.516662598	1
MSTRG.26677.2	102	0.27863	6.252746582	1
MSTRG.26677.1	74	0.37812	6.951721191	1
MSTRG.26680.2	600	0.45709	8.601013184	1
MSTRG.26684.1	417	0.40583	8.909973145	1
MSTRG.26684.2	419	0.34299	8.704528809	1
MSTRG.26684.4	384	0.32983	8.990905762	1
MSTRG.26684.6	420	0.39453	9.189880371	1
MSTRG.26685.3	265	0.32686	5.019470215	1
MSTRG.26685.2	293	0.32663	4.722839355	1
MSTRG.26686.1	197	0.39749	11.80841064	1
MSTRG.26688.1	99	0.29075	11.8583374	1
MSTRG.26707.1	34	0.38898	8.023376465	1
MSTRG.26709.1	32	0.36092	4.797058105	1
MSTRG.26710.1	343	0.45647	6.026916504	1
MSTRG.26710.3	343	0.48434	6.026916504	1
MSTRG.26710.11	343	0.49931	6.026916504	1
MSTRG.26710.12	421	0.46051	6.262145996	1
MSTRG.26711.1	68	0.30193	9.48614502	1
MSTRG.26708.1	86	0.34332	8.46307373	1
MSTRG.26715.1	217	0.36735	9.501037598	1
MSTRG.26715.2	217	0.35902	9.501037598	1
MSTRG.26715.3	217	0.31195	9.501037598	1
MSTRG.26715.4	217	0.32345	9.501037598	1
MSTRG.26715.7	217	0.348	9.501037598	1

MSTRG.26715.8	217	0.36009	9.501037598	1
MSTRG.26715.9	88	0.35544	10.33087158	1
MSTRG.26720.2	324	0.37579	7.196716309	1
MSTRG.26720.5	408	0.32475	6.979553223	1
MSTRG.26720.6	116	0.28711	8.990539551	1
MSTRG.26720.7	443	0.37751	6.649475098	1
MSTRG.26712.4	178	0.46775	9.66192627	1
MSTRG.26712.3	249	0.36554	9.653015137	1
MSTRG.26712.2	237	0.36552	9.205505371	1
MSTRG.26713.1	98	0.34189	9.891906738	1
MSTRG.26722.2	124	0.26913	11.29119873	1
MSTRG.26722.3	619	0.32931	7.820983887	1
MSTRG.26722.4	176	0.33863	4.812683105	1
MSTRG.26725.5	1323	0.3838	5.543640137	1
MSTRG.26725.6	1349	0.4347	5.500061035	1
MSTRG.26725.8	718	0.37355	6.071350098	1
MSTRG.26726.2	266	0.31297	9.700744629	1
MSTRG.26726.1	115	0.41228	8.865905762	1
MSTRG.26727.1	95	0.3	9.222595215	1
MSTRG.26727.2	124	0.35525	5.534851074	1
MSTRG.26727.4	486	0.37676	6.559509277	1
MSTRG.26727.3	486	0.34072	6.559509277	1
MSTRG.26727.5	474	0.36579	6.559509277	1
MSTRG.26728.1	151	0.26818	8.37322998	1
MSTRG.26728.2	152	0.28883	5.602600098	1
MSTRG.26729.1	128	0.3046	6.74005127	1
MSTRG.26739.3	499	0.4159	9.162902832	1
MSTRG.26739.4	1644	0.46875	5.964416504	1
MSTRG.26739.5	1644	0.46179	5.964416504	1
MSTRG.26741.1	85	0.28096	5.461242676	1
MSTRG.26744.1	635	0.46071	6.41607666	1
MSTRG.26744.4	853	0.45499	7.017150879	1
MSTRG.26742.2	200	0.4544	5.608215332	1
MSTRG.26742.3	200	0.44506	5.608215332	1
MSTRG.26748.1	240	0.33623	11.324646	1
MSTRG.26749.1	429	0.46445	5.405212402	1
MSTRG.26749.2	429	0.44547	5.405212402	1
MSTRG.26751.1	729	0.39218	9.53338623	1
MSTRG.26751.4	28	0.41636	4.496887207	1
MSTRG.26751.6	127	0.2785	9.147888184	1
MSTRG.26751.5	608	0.34844	9.731750488	1
MSTRG.26751.7	722	0.42364	9.640930176	1
MSTRG.26751.3	728	0.42364	9.553649902	1
MSTRG.26751.8	74	0.2951	4.172180176	1
MSTRG.26753.1	86	0.32477	9.682312012	1
MSTRG.26751.9	509	0.28923	9.391174316	1
MSTRG.26754.1	99	0.31454	9.430969238	1
MSTRG.26757.2	381	0.40448	5.473083496	1
MSTRG.26773.20	127	0.31503	8.393005371	1
MSTRG.26774.2	136	0.3875	7.640075684	1
MSTRG.26776.1	70	0.33455	6.567810059	1
MSTRG.26777.2	1081	0.4558	6.827087402	1
MSTRG.26777.3	1081	0.47466	6.827087402	1
MSTRG.26765.1	60	0.296	11.60284424	1

MSTRG.26770.1	330	0.2545	8.663024902	1
MSTRG.26770.2	371	0.27753	8.995544434	1
MSTRG.26790.1	436	0.42138	5.090393066	-1
MSTRG.26790.3	1221	0.44803	5.40838623	1
MSTRG.26788.2	123	0.33402	5.627380371	1
MSTRG.26786.1	223	0.40518	9.298278809	1
MSTRG.26792.3	1613	0.42434	6.51776123	1
MSTRG.26792.4	1571	0.41608	6.337341309	1
MSTRG.26793.2	3721	0.41915	5.585998535	-1
MSTRG.26793.3	3813	0.41915	5.569763184	-1
MSTRG.26795.2	117	0.30643	9.857727051	1
MSTRG.26795.3	117	0.32953	9.857727051	1
MSTRG.26796.1	222	0.31609	4.645446777	1
MSTRG.26796.2	222	0.36263	4.645446777	1
MSTRG.26796.3	222	0.35277	4.645446777	1
MSTRG.26798.2	235	0.25474	9.393127441	1
MSTRG.26803.3	889	0.46983	6.028137207	1
MSTRG.26804.1	157	0.2704	10.14703369	1
MSTRG.26805.1	125	0.36313	9.400085449	1
MSTRG.26806.7	100	0.34848	7.794372559	1
MSTRG.26806.8	100	0.34848	7.794372559	1
MSTRG.26806.4	100	0.34571	7.794372559	1
MSTRG.26806.5	167	0.27721	10.84320068	1
MSTRG.26808.1	103	0.3383	11.5413208	1
MSTRG.26808.2	103	0.35582	11.5413208	1
MSTRG.26821.1	86	0.32729	9.297424316	1
MSTRG.26821.3	175	0.41757	9.342468262	1
MSTRG.26821.4	86	0.37079	9.297424316	1
MSTRG.26821.5	86	0.33137	9.297424316	1
MSTRG.26821.6	129	0.43258	9.725646973	1
MSTRG.26830.1	237	0.36203	9.024108887	1
MSTRG.26830.2	170	0.34449	5.391052246	-1
MSTRG.26830.3	127	0.2849	10.69659424	1
MSTRG.26828.2	1208	0.32763	6.844299316	1
MSTRG.26835.2	68	0.3922	10.01397705	1
MSTRG.26836.2	616	0.37183	6.063903809	1
MSTRG.26836.3	76	0.35936	10.91607666	1
MSTRG.26837.2	497	0.29615	8.827697754	1
MSTRG.26837.4	1118	0.45535	6.149597168	1
MSTRG.26837.5	464	0.44033	5.365905762	1
MSTRG.26841.1	65	0.33438	9.519348145	1
MSTRG.26843.2	235	0.36161	6.037780762	1
MSTRG.26844.1	248	0.41113	8.876647949	1
MSTRG.26845.1	68	0.31445	10.02618408	1
MSTRG.26853.1	130	0.32119	8.492126465	1
MSTRG.26838.1	65	0.32828	7.781311035	1
MSTRG.26855.1	79	0.39769	9.773376465	1
MSTRG.26856.1	50	0.38403	5.975402832	-1
MSTRG.26863.4	142	0.30627	9.301452637	1
MSTRG.26863.5	420	0.34547	8.163024902	1
MSTRG.26864.1	80	0.28096	9.334533691	1
MSTRG.26865.1	135	0.29772	8.494812012	1
MSTRG.26866.1	165	0.3556	10.76629639	1
MSTRG.26867.1	83	0.29343	11.46002197	1

MSTRG.26868.1	65	0.34162	9.138244629	1
MSTRG.26868.2	119	0.28982	9.300598145	1
MSTRG.26868.3	101	0.2765	9.605163574	1
MSTRG.26870.1	28	0.34358	4.867858887	-1
MSTRG.26858.1	54	0.35391	11.00860596	-1
MSTRG.26861.1	101	0.41786	7.007385254	1
MSTRG.26861.2	57	0.33424	11.41766357	-1
MSTRG.26889.8	333	0.38505	9.165344238	1
MSTRG.26889.9	412	0.45246	6.954162598	1
MSTRG.26889.4	371	0.43663	9.140686035	1
MSTRG.26889.7	427	0.44936	8.601989746	1
MSTRG.26889.6	628	0.45331	8.160949707	1
MSTRG.26889.5	412	0.38733	6.954162598	1
MSTRG.26889.3	630	0.37347	7.94342041	1
MSTRG.26889.2	424	0.37619	8.877380371	1
MSTRG.26889.10	99	0.28671	10.30279541	1
MSTRG.26889.11	76	0.31299	11.37139893	1
MSTRG.26890.1	42	0.34353	10.91619873	1
MSTRG.26889.12	244	0.35547	7.206481934	1
MSTRG.26891.1	88	0.28167	8.803771973	1
MSTRG.26892.1	125	0.3046	9.076965332	1
MSTRG.26894.1	105	0.30637	8.555603027	1
MSTRG.26879.2	210	0.36306	5.514587402	1
MSTRG.26879.3	117	0.29059	7.616271973	1
MSTRG.26880.2	158	0.28096	9.461853027	1
MSTRG.26898.1	112	0.36362	5.657897949	1
MSTRG.26903.2	642	0.46832	8.656555176	1
MSTRG.26904.1	122	0.36153	8.755065918	1
MSTRG.26902.1	831	0.38464	6.812927246	1
MSTRG.26910.3	71	0.47195	4.087463379	1
MSTRG.26910.4	106	0.41992	4.207824707	1
MSTRG.26910.5	71	0.47531	4.087463379	1
MSTRG.26919.8	666	0.38467	5.499328613	1
MSTRG.26919.5	1223	0.41852	8.645690918	1
MSTRG.26919.1	1491	0.45277	8.722229004	1
MSTRG.26919.2	1492	0.45277	8.765441895	1
MSTRG.26919.3	1455	0.45072	8.52923584	1
MSTRG.26919.7	1492	0.45072	8.765441895	1
MSTRG.26919.9	1513	0.45277	8.801574707	1
MSTRG.26919.10	1417	0.45072	8.629211426	1
MSTRG.26920.1	133	0.40366	7.777893066	1
MSTRG.26932.1	400	0.44223	5.373962402	1
MSTRG.26932.4	343	0.41097	6.128967285	1
MSTRG.26932.5	295	0.42103	5.156555176	1
MSTRG.26932.7	404	0.42588	5.09173584	1
MSTRG.26932.6	369	0.39023	5.063415527	1
MSTRG.26932.8	369	0.42276	5.063415527	1
MSTRG.26932.9	346	0.4234	5.092956543	1
MSTRG.26932.10	289	0.39621	5.519104004	1
MSTRG.26933.3	517	0.46302	5.235900879	1
MSTRG.26937.2	2969	0.46306	6.092834473	1
MSTRG.26937.5	3001	0.46327	6.083435059	1
MSTRG.26929.1	780	0.45507	9.53729248	1
MSTRG.26929.2	800	0.43959	9.56854248	1

MSTRG.26930.1	70	0.33675	5.944396973	1
MSTRG.26941.9	389	0.43167	5.878356934	1
MSTRG.26941.8	2221	0.45959	5.205383301	1
MSTRG.26941.10	2256	0.46238	5.194396973	1
MSTRG.26941.11	2254	0.46238	5.194396973	1
MSTRG.26941.13	2110	0.43675	5.187561035	1
MSTRG.26941.14	248	0.45173	5.644958496	1
MSTRG.26941.15	79	0.3205	5.973937988	1
MSTRG.26942.1	281	0.32598	5.120422363	1
MSTRG.26943.1	192	0.29776	9.831848145	1
MSTRG.26942.3	159	0.30496	5.08001709	1
MSTRG.26945.1	756	0.44679	6.273254395	1
MSTRG.26944.11	287	0.32573	5.910217285	1
MSTRG.26953.3	164	0.30918	5.489685059	1
MSTRG.26953.2	461	0.33914	6.256164551	1
MSTRG.26954.1	86	0.28529	8.684509277	1
MSTRG.26955.2	572	0.43966	5.65020752	1
MSTRG.26955.1	646	0.43196	5.64263916	1
MSTRG.26955.3	359	0.44038	5.639587402	1
MSTRG.26955.5	636	0.43274	5.898986816	-1
MSTRG.26957.1	141	0.27188	8.034973145	1
MSTRG.26962.1	35	0.38604	9.79473877	1
MSTRG.26974.1	1313	0.43	6.922058105	1
MSTRG.26988.1	232	0.36161	9.928161621	1
MSTRG.26988.2	46	0.34696	5.045715332	1
MSTRG.26990.1	380	0.34037	9.363342285	1
MSTRG.26990.2	303	0.29999	9.574890137	1
MSTRG.26990.3	419	0.33638	9.202087402	1
MSTRG.26994.1	104	0.33313	6.379455566	1
MSTRG.26990.4	475	0.37874	9.184265137	1
MSTRG.26990.6	112	0.42217	4.491149902	1
MSTRG.26990.5	450	0.33533	9.055358887	1
MSTRG.26997.1	366	0.42728	5.458190918	-1
MSTRG.26998.1	56	0.4627	10.73675537	1
MSTRG.27003.4	434	0.41776	6.316345215	1
MSTRG.27006.6	238	0.47449	4.865783691	1
MSTRG.27006.8	225	0.46462	4.915100098	1
MSTRG.27006.9	225	0.47341	4.915100098	1
MSTRG.27006.7	225	0.46207	4.915100098	1
MSTRG.27006.5	451	0.46806	5.413635254	1
MSTRG.27006.4	499	0.44346	5.014831543	1
MSTRG.27006.2	1274	0.47642	5.777282715	1
MSTRG.27007.1	136	0.29751	5.565490723	1
MSTRG.27008.1	57	0.33766	4.860900879	1
MSTRG.27011.1	56	0.39164	9.296447754	1
MSTRG.27002.4	526	0.41598	5.312561035	1
MSTRG.27014.1	187	0.28036	9.504455566	1
MSTRG.27017.6	134	0.29059	6.88848877	1
MSTRG.27017.5	134	0.33112	6.88848877	1
MSTRG.27017.7	1463	0.43971	10.07635498	1
MSTRG.27018.1	99	0.30152	9.351257324	-1
MSTRG.27017.9	1239	0.40618	10.03179932	1
MSTRG.27017.10	47	0.33996	6.022888184	1
MSTRG.27017.11	47	0.33285	6.022888184	1

MSTRG.27012.3	709	0.39343	6.410217285	1
MSTRG.27012.2	747	0.39591	5.378723145	1
MSTRG.27019.2	81	0.46079	7.886535645	-1
MSTRG.27021.2	93	0.35766	9.570983887	1
MSTRG.27023.1	50	0.3331	6.883117676	1
MSTRG.27027.1	32	0.39728	11.69903564	1
MSTRG.27027.2	50	0.39716	8.940856934	1
MSTRG.27029.1	103	0.45827	9.390563965	1
MSTRG.27031.2	580	0.44395	8.70880127	1
MSTRG.27035.4	94	0.32494	9.828308105	1
MSTRG.27035.6	94	0.37824	9.828308105	1
MSTRG.27035.3	660	0.4615	6.882141113	1
MSTRG.27035.5	660	0.46683	6.882141113	1
MSTRG.27035.7	58	0.35312	9.347595215	1
MSTRG.27038.2	203	0.38539	7.731628418	1
MSTRG.27038.3	139	0.35416	9.242858887	1
MSTRG.27038.4	111	0.34455	5.20123291	1
MSTRG.27038.5	196	0.35676	6.41595459	1
MSTRG.27039.1	43	0.35229	6.383117676	1
MSTRG.27039.2	33	0.42024	9.099182129	1
MSTRG.27069.1	108	0.30556	9.171081543	1
MSTRG.27069.2	112	0.29367	5.936096191	1
MSTRG.27069.3	73	0.28432	9.063049316	1
MSTRG.27042.2	221	0.32442	6.789489746	1
MSTRG.27043.1	201	0.36195	9.908630371	1
MSTRG.27045.4	535	0.3331	8.936462402	1
MSTRG.27045.5	535	0.35545	8.936462402	1
MSTRG.27045.6	535	0.39128	8.936462402	1
MSTRG.27045.7	535	0.39314	8.936462402	1
MSTRG.27045.8	593	0.3954	8.898132324	1
MSTRG.27073.2	1319	0.46012	5.663391113	1
MSTRG.27073.5	1031	0.46097	5.351745605	1
MSTRG.27073.6	1031	0.44791	5.351745605	1
MSTRG.27073.7	1256	0.46245	5.739440918	1
MSTRG.27073.8	923	0.46282	5.299865723	1
MSTRG.27073.9	1023	0.4788	5.351745605	1
MSTRG.27051.1	35	0.35241	9.382385254	1
MSTRG.27078.1	78	0.28622	9.233703613	1
MSTRG.27083.2	71	0.30594	10.97650146	1
MSTRG.27083.3	71	0.31061	10.97650146	1
MSTRG.27085.4	1307	0.46415	5.666931152	1
MSTRG.27086.2	156	0.46599	4.612854004	1
MSTRG.27098.1	237	0.45893	8.575866699	1
MSTRG.27098.2	237	0.45844	8.575866699	1
MSTRG.27098.3	237	0.46168	8.575866699	1
MSTRG.27099.1	43	0.4035	11.48028564	-1
MSTRG.27099.2	43	0.43537	11.48028564	-1
MSTRG.27101.3	462	0.38348	6.186462402	1
MSTRG.27101.2	789	0.47895	6.821105957	1
MSTRG.27101.1	699	0.38757	6.769714355	1
MSTRG.27101.4	806	0.46552	7.326965332	1
MSTRG.27101.5	694	0.38977	6.652893066	1
MSTRG.27101.10	360	0.44691	9.039733887	1
MSTRG.27101.11	360	0.4806	9.039733887	1

MSTRG.27101.12	788	0.47533	6.821105957	1
MSTRG.27101.8	447	0.406	6.551208496	1
MSTRG.27101.14	359	0.31772	8.979919434	1
MSTRG.27101.16	801	0.47005	7.121154785	1
MSTRG.27103.1	79	0.43874	6.006774902	1
MSTRG.27104.1	432	0.46215	5.90423584	1
MSTRG.27104.2	432	0.47278	5.90423584	1
MSTRG.27104.3	432	0.45228	5.90423584	1
MSTRG.27104.4	432	0.45583	5.90423584	1
MSTRG.27104.5	432	0.45192	5.90423584	1
MSTRG.27104.6	432	0.43043	5.90423584	1
MSTRG.27101.17	104	0.35967	11.90435791	1
MSTRG.27091.2	347	0.41963	8.702941895	1
MSTRG.27092.1	103	0.38976	6.553527832	1
MSTRG.27092.2	177	0.32283	11.85736084	1
MSTRG.27092.3	103	0.36686	6.553527832	1
MSTRG.27092.4	103	0.37301	6.553527832	1
MSTRG.27092.5	165	0.30906	7.789245605	1
MSTRG.27092.6	103	0.35248	6.553527832	1
MSTRG.27094.1	150	0.4668	7.04107666	-1
MSTRG.27092.7	165	0.28242	7.789245605	1
MSTRG.27114.1	1328	0.38277	9.055725098	1
MSTRG.27118.1	246	0.38605	7.049133301	-1
MSTRG.27118.2	64	0.30132	6.520690918	1
MSTRG.27119.1	216	0.29772	9.738220215	1
MSTRG.27119.2	160	0.29772	10.34588623	-1
MSTRG.27118.3	445	0.42353	8.961120605	1
MSTRG.27118.4	445	0.29526	8.961120605	1
MSTRG.27123.1	117	0.29476	5.986633301	1
MSTRG.27124.2	545	0.46466	7.914123535	1
MSTRG.27124.5	140	0.2785	9.340881348	1
MSTRG.27125.1	123	0.28887	8.723083496	1
MSTRG.27124.6	738	0.35483	5.594299316	1
MSTRG.27124.7	142	0.27437	6.696594238	1
MSTRG.27106.1	244	0.2782	8.112365723	1
MSTRG.27109.1	375	0.29827	8.53326416	1
MSTRG.27129.1	37	0.3151	5.221862793	1
MSTRG.27143.1	35	0.42317	7.847106934	1
MSTRG.27144.1	71	0.31225	9.696838379	1
MSTRG.27150.1	112	0.27587	8.986022949	1
MSTRG.27156.1	112	0.40156	8.753234863	-1
MSTRG.27157.2	114	0.37023	4.974182129	1
MSTRG.27179.1	101	0.26182	9.22454834	1
MSTRG.27191.1	412	0.36746	6.116516113	1
MSTRG.27192.1	142	0.32213	9.097351074	1
MSTRG.27192.3	434	0.40742	7.180969238	1
MSTRG.27192.2	434	0.42175	7.180969238	1
MSTRG.27194.1	76	0.30344	9.647277832	1
MSTRG.27198.1	430	0.47631	5.418395996	1
MSTRG.27202.5	1155	0.41142	5.193664551	1
MSTRG.27217.1	65	0.33899	6.237731934	1
MSTRG.27217.2	65	0.35591	6.237731934	1
MSTRG.27212.1	63	0.36508	10.44085693	1
MSTRG.27221.2	315	0.31942	8.625427246	1

MSTRG.27222.1	84	0.29772	10.30743408	1
MSTRG.27223.1	80	0.34727	4.749694824	1
MSTRG.27226.1	372	0.43907	4.69342041	1
MSTRG.27226.4	356	0.45419	4.407287598	1
MSTRG.27226.5	374	0.47619	4.46270752	1
MSTRG.27230.2	192	0.36305	4.609191895	1
MSTRG.27230.4	39	0.35705	5.302185059	1
MSTRG.27230.9	152	0.28923	9.796569824	1
MSTRG.27230.16	183	0.28186	10.44781494	1
MSTRG.27230.17	81	0.30132	9.152160645	1
MSTRG.27239.1	102	0.28466	9.43951416	1
MSTRG.27239.2	43	0.29194	6.807189941	1
MSTRG.27282.1	85	0.29654	6.823913574	1
MSTRG.27282.3	157	0.28243	6.998840332	1
MSTRG.27282.11	125	0.32511	10.690979	1
MSTRG.27282.10	125	0.28419	10.690979	1
MSTRG.27282.12	105	0.28584	8.735900879	1
MSTRG.27282.14	101	0.30289	6.722839355	1
MSTRG.27282.15	95	0.29772	6.62677002	1
MSTRG.27285.1	340	0.28982	9.523010254	1
MSTRG.27282.16	63	0.37038	9.185241699	1
MSTRG.27282.20	146	0.30668	8.408996582	1
MSTRG.27282.21	157	0.28931	6.998840332	1
MSTRG.27282.18	157	0.33091	6.998840332	1
MSTRG.27288.1	75	0.40329	9.922790527	1
MSTRG.27291.1	507	0.36461	8.162658691	1
MSTRG.27291.2	90	0.33194	10.50531006	1
MSTRG.27292.1	141	0.30972	5.698059082	1
MSTRG.27293.1	85	0.33996	5.988586426	1
MSTRG.27291.7	409	0.37218	8.227355957	1
MSTRG.27291.8	408	0.37218	8.227355957	1
MSTRG.27247.3	123	0.45589	9.644836426	1
MSTRG.27247.2	123	0.44097	9.644836426	1
MSTRG.27248.1	446	0.48833	5.401062012	1
MSTRG.27250.1	183	0.38395	9.61932373	1
MSTRG.27251.1	527	0.40461	6.866027832	1
MSTRG.27255.2	384	0.45124	5.321716309	1
MSTRG.27256.1	148	0.36826	11.00323486	1
MSTRG.27263.1	77	0.36957	9.380554199	1
MSTRG.27264.1	125	0.36597	10.42608643	1
MSTRG.27263.2	60	0.38204	4.246520996	1
MSTRG.27263.3	829	0.42944	6.097961426	1
MSTRG.27268.1	163	0.36019	9.638244629	1
MSTRG.27272.1	50	0.323	6.015319824	1
MSTRG.27276.1	100	0.31193	9.619934082	1
MSTRG.27275.2	175	0.32244	9.218322754	1
MSTRG.27304.3	177	0.322	8.657653809	1
MSTRG.27305.1	221	0.28883	9.822570801	1
MSTRG.27305.2	110	0.29387	9.77557373	1
MSTRG.27305.3	130	0.28982	9.363098145	1
MSTRG.27305.4	130	0.28982	9.363098145	1
MSTRG.27305.5	130	0.29387	9.363098145	1
MSTRG.27305.6	108	0.27433	9.943786621	1
MSTRG.27304.6	85	0.30191	6.552307129	1

MSTRG.27304.7	98	0.29387	7.605407715	1
MSTRG.27304.8	187	0.27937	10.77044678	1
MSTRG.27304.9	187	0.28982	10.77044678	1
MSTRG.27304.10	1244	0.38122	6.772399902	1
MSTRG.27304.11	1255	0.29772	6.872253418	1
MSTRG.27304.12	73	0.2857	6.507385254	1
MSTRG.27307.1	93	0.30839	6.944152832	1
MSTRG.27304.13	243	0.34952	10.49517822	1
MSTRG.27308.1	150	0.28982	6.918273926	1
MSTRG.27308.2	150	0.28199	6.918273926	1
MSTRG.27304.14	82	0.3453	9.217712402	1
MSTRG.27309.1	152	0.26413	11.42547607	1
MSTRG.27309.2	189	0.28706	9.780822754	1
MSTRG.27310.1	40	0.38847	8.634094238	1
MSTRG.27311.1	67	0.36288	9.817565918	-1
MSTRG.27277.1	68	0.42677	6.815246582	1
MSTRG.27278.1	109	0.31505	8.24798584	1
MSTRG.27300.3	921	0.36633	7.11907959	1
MSTRG.27300.4	1521	0.41877	7.276672363	1
MSTRG.27300.5	1937	0.41378	7.675476074	1
MSTRG.27302.1	56	0.36064	6.508361816	1
MSTRG.27303.5	124	0.2895	9.72467041	1
MSTRG.27312.1	340	0.50906	5.757385254	1
MSTRG.27314.2	854	0.34888	9.745178223	1
MSTRG.27314.3	14	0.38695	11.71136475	1
MSTRG.27329.1	101	0.29268	7.063781738	1
MSTRG.27329.2	68	0.35739	10.33258057	1
MSTRG.27328.4	90	0.31797	9.69354248	1
MSTRG.27330.1	77	0.32485	6.07598877	1
MSTRG.27368.2	54	0.38977	6.687072754	1
MSTRG.27368.3	42	0.47507	11.73394775	1
MSTRG.27368.4	249	0.29387	9.275695801	1
MSTRG.27368.5	149	0.38007	6.43939209	1
MSTRG.27368.6	95	0.31852	4.248352051	1
MSTRG.27370.1	91	0.29771	6.250183105	1
MSTRG.27368.7	141	0.3	8.942443848	1
MSTRG.27373.1	398	0.31292	9.847717285	1
MSTRG.27337.1	38	0.38695	5.967712402	-1
MSTRG.27338.3	598	0.44289	5.175231934	1
MSTRG.27338.4	687	0.43421	5.264343262	1
MSTRG.27339.1	50	0.25989	4.218811035	1
MSTRG.27340.1	146	0.31553	9.113586426	1
MSTRG.27343.3	159	0.29456	8.077331543	1
MSTRG.27343.4	184	0.28588	10.32550049	1
MSTRG.27343.6	144	0.33217	9.987854004	1
MSTRG.27343.8	181	0.28823	10.42987061	1
MSTRG.27359.4	272	0.39264	5.370910645	1
MSTRG.27362.3	337	0.40905	9.029724121	1
MSTRG.27391.4	318	0.31902	5.158752441	1
MSTRG.27391.2	359	0.40905	5.11895752	1
MSTRG.27391.7	710	0.45089	5.055480957	1
MSTRG.27391.8	318	0.42955	5.158752441	1
MSTRG.27391.10	693	0.47524	5.023742676	1
MSTRG.27391.9	787	0.45681	5.19708252	1

MSTRG.27393.3	120	0.37241	4.847473145	1
MSTRG.27393.4	235	0.32709	9.414855957	1
MSTRG.27400.3	3164	0.46484	5.829406738	1
MSTRG.27400.4	1093	0.49096	5.669250488	1
MSTRG.27401.1	708	0.48722	5.250427246	1
MSTRG.27401.2	708	0.44543	5.250427246	1
MSTRG.27401.3	708	0.43842	5.250427246	1
MSTRG.27401.4	672	0.36188	5.488708496	1
MSTRG.27404.1	175	0.28567	8.536804199	1
MSTRG.27411.2	91	0.29772	5.573913574	1
MSTRG.27412.1	114	0.31634	10.43707275	1
MSTRG.27411.3	527	0.39683	6.124694824	1
MSTRG.27413.1	92	0.27433	9.46270752	1
MSTRG.27413.2	135	0.28584	7.56048584	1
MSTRG.27413.3	135	0.30894	7.56048584	1
MSTRG.27411.5	459	0.33514	5.51385498	1
MSTRG.27378.6	196	0.43589	4.648376465	1
MSTRG.27378.7	792	0.46389	5.753967285	1
MSTRG.27378.8	733	0.30209	5.066589355	1
MSTRG.27378.9	227	0.28467	4.31829834	1
MSTRG.27387.1	33	0.45177	5.464904785	1
MSTRG.27387.2	23	0.36568	3.929626465	1
MSTRG.27387.3	23	0.40876	5.38067627	1
MSTRG.27387.4	18	0.42764	4.501037598	1
MSTRG.27387.5	18	0.46397	4.501037598	1
MSTRG.27387.6	18	0.42973	4.501037598	1
MSTRG.27387.7	204	0.37074	9.12298584	1
MSTRG.27387.8	174	0.42011	8.971008301	1
MSTRG.27387.9	20	0.46231	9.992614746	1
MSTRG.27387.10	18	0.39834	4.501037598	1
MSTRG.27387.11	27	0.42032	4.512512207	1
MSTRG.27420.1	182	0.3823	6.512268066	1
MSTRG.27420.2	604	0.42833	5.55657959	1
MSTRG.27431.1	17	0.33657	11.00030518	1
MSTRG.27435.3	1356	0.43224	6.13873291	1
MSTRG.27436.1	62	0.34952	4.245300293	-1
MSTRG.27440.1	45	0.418	10.46392822	-1
MSTRG.27427.9	559	0.44374	7.766296387	1
MSTRG.27427.8	674	0.41667	8.08026123	1
MSTRG.27427.7	685	0.41235	8.081359863	1
MSTRG.27427.10	570	0.42949	7.770202637	1
MSTRG.27427.11	561	0.42324	7.974060059	1
MSTRG.27427.12	676	0.42553	8.200622559	1
MSTRG.27427.13	674	0.41397	8.08026123	1
MSTRG.27428.2	73	0.29857	11.90545654	1
MSTRG.27428.1	73	0.36966	11.90545654	1
MSTRG.27428.3	40	0.40024	12.00006104	1
MSTRG.27428.4	109	0.35844	11.70367432	1
MSTRG.27445.2	135	0.33463	11.30377197	1
MSTRG.27446.1	65	0.3	9.162658691	-1
MSTRG.27449.1	134	0.31852	9.083190918	1
MSTRG.27454.3	588	0.39969	8.412780762	1
MSTRG.27454.2	588	0.38603	8.412780762	1
MSTRG.27459.1	103	0.32824	9.002380371	1

MSTRG.27459.2	102	0.37423	9.335388184	1
MSTRG.27458.8	401	0.32785	9.540222168	1
MSTRG.27461.1	1633	0.45184	6.429992676	1
MSTRG.27461.3	1581	0.43644	6.440856934	1
MSTRG.27461.5	1688	0.41594	6.480895996	1
MSTRG.27461.6	1633	0.41594	6.429992676	1
MSTRG.27461.7	1549	0.43031	6.240905762	1
MSTRG.27461.10	1677	0.46188	6.520324707	1
MSTRG.27461.11	1599	0.45184	6.68157959	1
MSTRG.27462.1	103	0.38394	10.6227417	1
MSTRG.27462.2	86	0.38734	10.90313721	1
MSTRG.27462.3	88	0.33514	10.87115479	1
MSTRG.27457.3	2524	0.34686	5.999816895	1
MSTRG.27474.4	1500	0.46309	5.120666504	1
MSTRG.27474.2	996	0.3947	4.831726074	1
MSTRG.27474.6	996	0.35164	4.831726074	1
MSTRG.27475.1	117	0.44977	3.981140137	1
MSTRG.27479.1	729	0.46976	6.359802246	1
MSTRG.27480.2	4266	0.42037	5.568786621	1
MSTRG.27480.1	4266	0.42037	5.568786621	1
MSTRG.27480.4	4113	0.42037	5.522399902	1
MSTRG.27480.5	695	0.4635	5.452575684	1
MSTRG.27480.6	4266	0.42037	5.568786621	1
MSTRG.27480.8	1769	0.42037	5.618713379	1
MSTRG.27480.9	2463	0.45727	5.66595459	1
MSTRG.27480.7	4266	0.42037	5.568786621	1
MSTRG.27480.10	523	0.43848	5.93145752	1
MSTRG.27480.12	523	0.43507	5.93145752	1
MSTRG.27463.1	69	0.37722	9.992004395	-1
MSTRG.27466.2	415	0.46206	5.102478027	1
MSTRG.27466.3	443	0.43842	5.333435059	1
MSTRG.27466.4	305	0.42485	5.243347168	1
MSTRG.27467.2	251	0.48504	7.75604248	1
MSTRG.27469.3	667	0.4692	8.054138184	1
MSTRG.27471.2	369	0.46492	8.106140137	1
MSTRG.27471.6	283	0.36551	8.856140137	1
MSTRG.27473.1	72	0.37225	4.47857666	1
MSTRG.27471.7	112	0.39176	5.272766113	1
MSTRG.27471.8	134	0.30087	9.011901855	1
MSTRG.27486.2	152	0.4213	11.80792236	1
MSTRG.27486.3	152	0.43794	11.80792236	1
MSTRG.27487.2	659	0.44023	6.100524902	1
MSTRG.27489.4	403	0.38642	8.416320801	1
MSTRG.27489.5	359	0.40155	6.791564941	1
MSTRG.27489.6	40	0.43097	9.984436035	1
MSTRG.27489.7	329	0.41396	5.99786377	1
MSTRG.27483.1	132	0.39265	5.74029541	1
MSTRG.27507.2	242	0.29452	9.470153809	1
MSTRG.27507.3	191	0.399	8.703918457	1
MSTRG.27507.4	167	0.43399	8.239318848	1
MSTRG.27507.5	191	0.44108	8.703918457	1
MSTRG.27507.7	197	0.38143	10.42974854	1
MSTRG.27507.8	197	0.40717	10.42974854	1
MSTRG.27508.1	146	0.39088	8.565002441	1

MSTRG.27510.1	1291	0.34832	5.974182129	1
MSTRG.27512.12	133	0.41901	4.29083252	1
MSTRG.27510.3	1291	0.46079	5.974182129	1
MSTRG.27510.4	1468	0.42369	5.757751465	1
MSTRG.27510.5	1291	0.39505	5.974182129	1
MSTRG.27510.6	99	0.31917	9.853210449	1
MSTRG.27514.1	1507	0.4589	4.926574707	1
MSTRG.27514.2	1507	0.48095	4.926574707	1
MSTRG.27515.1	116	0.34481	11.36932373	1
MSTRG.27521.1	2013	0.4593	6.727844238	1
MSTRG.27521.2	2053	0.45406	6.629089355	1
MSTRG.27521.3	2018	0.45654	6.902038574	1
MSTRG.27524.2	384	0.41316	7.576599121	1
MSTRG.27526.1	390	0.41308	5.373840332	1
MSTRG.27526.4	251	0.41719	5.003845215	1
MSTRG.27497.1	439	0.43654	5.96295166	1
MSTRG.27499.2	470	0.39495	6.742980957	1
MSTRG.27499.4	472	0.37894	7.066833496	1
MSTRG.27505.1	1138	0.43	5.929260254	1
MSTRG.27505.5	333	0.29751	9.361999512	1
MSTRG.27502.1	965	0.47661	6.197570801	1
MSTRG.27533.4	441	0.43137	6.81451416	1
MSTRG.27540.1	119	0.29901	9.47845459	1
MSTRG.27572.2	193	0.39204	5.760314941	1
MSTRG.27573.4	82	0.27313	5.004699707	1
MSTRG.27573.3	82	0.30468	5.004699707	1
MSTRG.27573.2	70	0.37049	8.249084473	1
MSTRG.27580.6	597	0.42079	8.832336426	1
MSTRG.27580.7	595	0.4025	8.832336426	1
MSTRG.27580.8	597	0.35993	8.832336426	1
MSTRG.27580.5	597	0.38229	8.832336426	1
MSTRG.27580.9	161	0.27918	8.425720215	1
MSTRG.27581.3	495	0.27327	11.58929443	1
MSTRG.27581.6	183	0.30939	9.08001709	1
MSTRG.27581.5	495	0.28396	11.58929443	1
MSTRG.27584.1	1324	0.45903	6.250061035	1
MSTRG.27584.10	1323	0.45599	6.250061035	1
MSTRG.27584.11	1326	0.45599	6.300476074	1
MSTRG.27584.9	1352	0.47141	6.60748291	1
MSTRG.27584.12	1304	0.43147	6.110290527	1
MSTRG.27584.13	1324	0.46673	6.250061035	1
MSTRG.27584.14	1324	0.46369	6.250061035	1
MSTRG.27584.15	1326	0.47189	6.300476074	1
MSTRG.27587.3	96	0.4128	10.74334717	1
MSTRG.27587.2	184	0.346	8.589294434	1
MSTRG.27588.4	276	0.4429	8.862731934	1
MSTRG.27589.2	1228	0.43056	6.660827637	1
MSTRG.27589.4	265	0.40448	6.170837402	1
MSTRG.27590.5	488	0.41067	6.119812012	1
MSTRG.27541.1	56	0.34479	9.150939941	1
MSTRG.27541.2	87	0.34446	6.042053223	1
MSTRG.27544.2	246	0.47937	5.469299316	1
MSTRG.27544.3	246	0.28338	5.469299316	1
MSTRG.27547.1	2439	0.37473	5.902526855	1

MSTRG.27548.2	742	0.43768	8.687438965	1
MSTRG.27550.1	43	0.33425	6.697570801	-1
MSTRG.27554.1	62	0.34372	7.816223145	1
MSTRG.27555.1	89	0.32203	7.855651855	1
MSTRG.27555.2	71	0.40057	10.01629639	1
MSTRG.27555.3	71	0.39753	10.01629639	1
MSTRG.27556.1	187	0.36219	4.93536377	1
MSTRG.27557.1	874	0.47223	7.05267334	1
MSTRG.27558.2	2135	0.46225	5.379211426	1
MSTRG.27559.4	141	0.41108	7.019836426	1
MSTRG.27559.5	141	0.37095	7.019836426	1
MSTRG.27560.1	126	0.33867	9.574035645	1
MSTRG.27563.1	1753	0.46099	4.844665527	1
MSTRG.27563.10	109	0.33686	9.254699707	1
MSTRG.27563.9	1761	0.40301	4.815979004	1
MSTRG.27563.6	1098	0.31929	4.929016113	1
MSTRG.27563.7	1097	0.45496	4.929016113	1
MSTRG.27563.8	990	0.46931	5.110534668	1
MSTRG.27563.11	1098	0.4198	4.929016113	1
MSTRG.27563.12	1097	0.36699	4.929016113	1
MSTRG.27563.13	1074	0.45669	4.93939209	1
MSTRG.27563.14	23	0.3633	5.949157715	1
MSTRG.27563.15	1097	0.42899	4.929016113	1
MSTRG.27565.1	289	0.3505	9.114807129	1
MSTRG.27563.16	1059	0.47799	4.835388184	1
MSTRG.27563.17	1049	0.38734	5.151428223	1
MSTRG.27567.5	1035	0.45072	8.035339355	1
MSTRG.27567.6	1155	0.44212	8.756774902	1
MSTRG.27569.2	996	0.45945	5.292785645	1
MSTRG.27596.1	1278	0.42012	6.69317627	1
MSTRG.27596.3	1278	0.41139	6.69317627	1
MSTRG.27596.4	1279	0.41139	6.783508301	1
MSTRG.27597.2	79	0.28571	10.45843506	-1
MSTRG.27617.2	295	0.46682	8.257019043	1
MSTRG.27617.3	247	0.43575	7.71685791	1
MSTRG.27617.4	247	0.44445	7.71685791	1
MSTRG.27620.8	521	0.33111	9.682067871	1
MSTRG.27620.2	1615	0.45274	5.504821777	1
MSTRG.27620.3	1615	0.46554	5.504821777	1
MSTRG.27620.4	1615	0.43768	5.504821777	1
MSTRG.27620.5	1615	0.43768	5.504821777	1
MSTRG.27620.6	1615	0.45274	5.504821777	1
MSTRG.27620.7	1615	0.45274	5.504821777	1
MSTRG.27620.9	1109	0.39087	5.18572998	1
MSTRG.27620.10	1615	0.39379	5.504821777	1
MSTRG.27620.11	1615	0.46821	5.504821777	1
MSTRG.27620.12	76	0.30532	10.04400635	-1
MSTRG.27622.2	1857	0.45298	5.726379395	1
MSTRG.27624.2	85	0.31801	7.707702637	1
MSTRG.27624.3	148	0.26913	6.524230957	1
MSTRG.27598.2	558	0.44687	8.620666504	1
MSTRG.27598.3	584	0.43112	8.27520752	1
MSTRG.27601.1	43	0.4694	6.50567627	1
MSTRG.27602.1	45	0.32402	5.981628418	1

MSTRG.27605.1	99	0.34785	5.102355957	1
MSTRG.27605.5	321	0.40773	4.764221191	1
MSTRG.27605.2	392	0.40525	6.082824707	1
MSTRG.27605.4	321	0.40745	4.764221191	1
MSTRG.27606.1	247	0.35894	7.521179199	1
MSTRG.27599.2	173	0.33163	8.733581543	1
MSTRG.27630.1	108	0.28711	11.31817627	1
MSTRG.27633.1	77	0.45313	4.726013184	1
MSTRG.27626.1	1454	0.45892	4.935974121	1
MSTRG.27626.2	65	0.40504	11.36663818	-1
MSTRG.27626.3	98	0.36812	9.872253418	-1
MSTRG.27627.1	84	0.32091	10.32659912	1
MSTRG.27628.1	82	0.28518	9.809631348	1
MSTRG.27651.1	543	0.42375	5.711120605	1
MSTRG.27651.2	563	0.43196	6.021057129	1
MSTRG.27651.3	208	0.4738	5.733581543	1
MSTRG.27655.1	236	0.31389	6.51373291	1
MSTRG.27655.2	177	0.34327	5.967834473	1
MSTRG.27666.2	212	0.46197	5.057800293	1
MSTRG.27667.1	109	0.36382	6.627502441	-1
MSTRG.27668.6	368	0.37012	6.537658691	1
MSTRG.27668.7	179	0.33073	6.039367676	1
MSTRG.27668.8	299	0.38647	8.540344238	1
MSTRG.27668.9	368	0.39292	6.537658691	1
MSTRG.27668.10	73	0.34201	6.252258301	1
MSTRG.27669.5	87	0.37028	8.857971191	1
MSTRG.27669.6	87	0.36646	8.857971191	1
MSTRG.27669.7	142	0.32888	10.94415283	1
MSTRG.27669.8	84	0.28085	9.381652832	1
MSTRG.27659.1	30	0.39259	6.698547363	1
MSTRG.27673.1	115	0.32646	9.354919434	1
MSTRG.27676.3	703	0.44541	5.813903809	1
MSTRG.27676.4	662	0.46578	6.148620605	1
MSTRG.27676.6	703	0.45361	5.813903809	1
MSTRG.27676.7	703	0.45361	5.813903809	1
MSTRG.27676.8	703	0.44541	5.813903809	1
MSTRG.27676.10	703	0.46207	5.813903809	1
MSTRG.27680.1	652	0.39647	6.201477051	1
MSTRG.27680.3	652	0.39323	6.201477051	1
MSTRG.27680.5	195	0.29155	6.310241699	1
MSTRG.27680.4	652	0.44059	6.201477051	1
MSTRG.27684.4	217	0.47245	10.52264404	1
MSTRG.27684.3	216	0.44812	10.52264404	1
MSTRG.27684.2	312	0.4556	10.90692139	1
MSTRG.27689.1	67	0.38791	4.721618652	1
MSTRG.27690.2	142	0.47533	9.578796387	-1
MSTRG.27695.1	405	0.39123	7.162414551	1
MSTRG.27695.3	405	0.41525	7.162414551	1
MSTRG.27704.1	183	0.37116	10.81610107	1
MSTRG.27706.1	49	0.2921	10.19720459	1
MSTRG.27707.2	251	0.33431	9.245788574	1
MSTRG.27707.1	102	0.34254	10.17254639	1
MSTRG.27707.4	152	0.3119	7.610290527	-1
MSTRG.27708.2	150	0.43054	9.227111816	1

MSTRG.27708.3	109	0.42076	7.689880371	1
MSTRG.27717.5	1452	0.46717	4.829772949	1
MSTRG.27717.1	1495	0.46717	4.909973145	1
MSTRG.27717.2	1495	0.46258	4.909973145	1
MSTRG.27720.1	65	0.39048	6.877868652	1
MSTRG.27722.1	70	0.32121	8.769104004	1
MSTRG.27718.2	476	0.45703	9.243347168	1
MSTRG.27719.1	89	0.35178	10.61212158	1
MSTRG.27724.4	620	0.41023	6.97845459	1
MSTRG.27724.5	620	0.37533	6.97845459	1
MSTRG.27730.2	70	0.45129	5.021179199	1
MSTRG.27731.3	691	0.39187	9.516540527	1
MSTRG.27732.2	365	0.41622	7.847839355	1
MSTRG.27725.1	25	0.28895	12.00848389	1
MSTRG.27737.2	1612	0.43484	6.324279785	1
MSTRG.27738.1	72	0.28882	7.806213379	1
MSTRG.27747.1	170	0.38691	8.879577637	1
MSTRG.27747.2	1189	0.45687	8.727233887	1
MSTRG.27747.3	1023	0.41752	7.366394043	1
MSTRG.27747.4	1204	0.43142	8.72442627	1
MSTRG.27748.1	123	0.27194	8.76739502	1
MSTRG.27748.2	148	0.27133	11.3550415	1
MSTRG.27751.1	141	0.38696	5.483337402	-1
MSTRG.27747.5	917	0.42829	7.593200684	1
MSTRG.27752.1	27	0.34589	12.00042725	1
MSTRG.27753.1	48	0.37627	11.32647705	1
MSTRG.27747.6	1112	0.45687	8.777038574	1
MSTRG.27747.8	1112	0.4593	8.36932373	1
MSTRG.27754.1	168	0.31051	10.78460693	1
MSTRG.27744.2	555	0.458	9.222595215	1
MSTRG.27745.1	1153	0.45611	9.215270996	1
MSTRG.27745.3	1295	0.46379	9.410949707	1
MSTRG.27769.1	160	0.27327	10.02398682	1
MSTRG.27772.3	312	0.44659	8.032165527	1
MSTRG.27772.4	354	0.43273	8.723815918	1
MSTRG.27772.6	349	0.42127	8.729675293	1
MSTRG.27778.2	118	0.28855	6.99798584	1
MSTRG.27781.2	435	0.43489	6.142272949	1
MSTRG.27755.1	480	0.38052	9.443664551	1
MSTRG.27757.1	231	0.34275	6.45904541	1
MSTRG.27782.3	313	0.35042	6.66619873	1
MSTRG.27783.1	286	0.30491	8.91619873	1
MSTRG.27784.1	328	0.37153	4.784484863	1
MSTRG.27784.3	412	0.37028	5.33392334	1
MSTRG.27784.2	470	0.37808	5.310119629	1
MSTRG.27786.1	902	0.4344	5.426940918	1
MSTRG.27793.1	26	0.38159	10.63983154	-1
MSTRG.27794.1	27	0.43496	11.70025635	-1
MSTRG.27795.1	210	0.27075	5.56072998	1
MSTRG.27796.4	1797	0.4192	6.785339355	-1
MSTRG.27798.3	199	0.37684	8.172790527	1
MSTRG.27800.1	115	0.38254	9.387878418	1
MSTRG.27802.2	459	0.43096	6.527893066	1
MSTRG.27802.3	486	0.4537	7.06829834	1

MSTRG.27802.4	459	0.43985	6.527893066	1
MSTRG.27802.5	486	0.43558	7.06829834	1
MSTRG.27802.6	459	0.36066	6.527893066	1
MSTRG.27804.2	134	0.45075	11.72540283	1
MSTRG.27804.3	88	0.42004	11.57659912	1
MSTRG.27801.1	124	0.32169	11.63812256	1
MSTRG.27801.2	124	0.33101	11.63812256	1
MSTRG.27807.2	591	0.37367	6.515319824	1
MSTRG.27808.4	504	0.3389	8.676940918	1
MSTRG.27810.2	77	0.40792	9.684753418	-1
MSTRG.27814.1	588	0.34638	6.204040527	1
MSTRG.27814.4	491	0.29603	6.546691895	1
MSTRG.27814.5	544	0.28029	6.193908691	1
MSTRG.27814.12	302	0.3538	6.18927002	1
MSTRG.27814.14	286	0.30267	6.275695801	1
MSTRG.27818.1	154	0.39366	4.319274902	1
MSTRG.27814.15	72	0.34969	6.718200684	1
MSTRG.27814.16	72	0.34101	6.718200684	1
MSTRG.27814.17	72	0.33485	6.718200684	1
MSTRG.27838.3	506	0.39349	7.653991699	1
MSTRG.27838.2	506	0.43033	7.653991699	1
MSTRG.27838.4	506	0.412	7.653991699	1
MSTRG.27839.1	90	0.45601	6.063415527	-1
MSTRG.27840.1	127	0.3	9.442932129	1
MSTRG.27842.1	63	0.38426	11.28363037	1
MSTRG.27842.2	24	0.33814	9.496276855	1
MSTRG.27846.1	211	0.45162	6.148742676	1
MSTRG.27846.2	153	0.28948	6.91204834	1
MSTRG.27846.3	153	0.28948	6.91204834	1
MSTRG.27846.4	1052	0.44015	6.560119629	1
MSTRG.27846.5	1052	0.36085	6.560119629	1
MSTRG.27846.7	915	0.42476	7.750183105	1
MSTRG.27846.9	892	0.3937	8.170593262	1
MSTRG.27846.8	895	0.35748	8.598571777	1
MSTRG.27846.11	1032	0.35745	7.641906738	1
MSTRG.27846.12	895	0.37294	8.598571777	1
MSTRG.27847.1	165	0.27433	8.714050293	1
MSTRG.27848.1	120	0.33114	8.89666748	1
MSTRG.27846.13	673	0.29892	8.159606934	1
MSTRG.27846.15	496	0.32005	6.584655762	1
MSTRG.27844.2	37	0.39165	8.632751465	1
MSTRG.27884.3	5642	0.47555	5.648864746	1
MSTRG.27884.6	7569	0.47555	5.07623291	1
MSTRG.27885.1	139	0.32396	4.798278809	1
MSTRG.27884.11	5562	0.46334	5.527038574	1
MSTRG.27884.9	5556	0.46334	5.507629395	1
MSTRG.27884.8	5599	0.47555	5.538269043	1
MSTRG.27884.12	5293	0.46726	5.448547363	1
MSTRG.27884.14	4009	0.43713	5.455627441	1
MSTRG.27884.15	4647	0.47555	5.469543457	1
MSTRG.27884.16	2652	0.46998	5.564758301	1
MSTRG.27884.17	2646	0.46281	5.564758301	1
MSTRG.27884.18	2689	0.46998	5.58782959	1
MSTRG.27884.19	1544	0.46736	5.91204834	1

MSTRG.27884.20	615	0.41248	9.930969238	1
MSTRG.27854.1	55	0.41695	6.245544434	-1
MSTRG.27852.1	223	0.4413	10.03900146	1
MSTRG.27862.1	80	0.38116	5.034484863	1
MSTRG.27863.2	268	0.40829	6.331359863	1
MSTRG.27864.1	63	0.28094	6.691223145	-1
MSTRG.27865.1	138	0.30505	7.827453613	1
MSTRG.27870.1	719	0.44926	6.105773926	1
MSTRG.27870.3	740	0.43894	6.50604248	1
MSTRG.27870.4	719	0.41654	6.320129395	1
MSTRG.27870.5	575	0.32858	9.58416748	1
MSTRG.27875.2	74	0.30515	6.95526123	1
MSTRG.27876.1	260	0.45831	4.430480957	1
MSTRG.27878.5	683	0.40137	8.717834473	1
MSTRG.27880.3	1365	0.39814	6.74420166	1
MSTRG.27882.2	469	0.41084	6.426330566	1
MSTRG.27882.4	78	0.45534	6.238098145	1
MSTRG.27882.3	163	0.4741	6.092346191	1
MSTRG.27893.3	665	0.37483	8.304992676	1
MSTRG.27901.7	748	0.48575	4.67376709	1
MSTRG.27901.8	748	0.45242	4.67376709	1
MSTRG.27904.1	236	0.28855	8.970153809	1
MSTRG.27905.5	724	0.44141	9.427307129	1
MSTRG.27908.4	177	0.4764	7.765441895	1
MSTRG.27908.5	64	0.4388	9.778991699	1
MSTRG.27909.1	1274	0.46221	9.02532959	1
MSTRG.27909.4	891	0.46481	6.602722168	1
MSTRG.27909.3	879	0.43897	6.686096191	1
MSTRG.27909.5	877	0.46481	6.602478027	1
MSTRG.27910.1	16	0.3515	8.500061035	1
MSTRG.27896.1	219	0.37517	11.88873291	1
MSTRG.27896.2	202	0.36025	11.96429443	1
MSTRG.27897.1	116	0.43248	4.561096191	1
MSTRG.27897.2	112	0.441	4.488098145	1
MSTRG.27897.5	206	0.42026	5.221008301	1
MSTRG.27900.2	310	0.34474	11.184021	1
MSTRG.27922.2	228	0.27599	9.260559082	1
MSTRG.27913.2	158	0.34889	11.29046631	-1
MSTRG.27925.1	172	0.30121	5.716125488	1
MSTRG.27925.3	172	0.32431	5.716125488	1
MSTRG.27928.1	216	0.30432	4.372741699	1
MSTRG.27934.2	1121	0.41628	5.944885254	1
MSTRG.27935.1	150	0.28503	6.233947754	1
MSTRG.27936.1	92	0.28338	6.386657715	1
MSTRG.27941.3	499	0.45922	4.979797363	1
MSTRG.27942.1	82	0.27217	8.690979004	1
MSTRG.27942.4	31	0.34957	4.973571777	1
MSTRG.27942.3	80	0.33017	9.186096191	1
MSTRG.27942.2	82	0.32559	8.690979004	1
MSTRG.27943.2	93	0.3053	9.637878418	1
MSTRG.27943.1	93	0.2766	9.637878418	1
MSTRG.27943.3	113	0.36053	10.07501221	1
MSTRG.27945.3	570	0.4724	5.698425293	1
MSTRG.27945.5	497	0.41151	5.898254395	1

MSTRG.27940.2	450	0.4388	6.910949707	1
MSTRG.27949.1	370	0.44252	9.355651855	1
MSTRG.27949.2	391	0.46486	9.590393066	1
MSTRG.27950.3	457	0.36589	5.89239502	1
MSTRG.27950.4	457	0.36589	5.89239502	1
MSTRG.27951.1	57	0.32161	4.100524902	1
MSTRG.27950.6	256	0.37351	8.600402832	1
MSTRG.27952.1	115	0.34079	8.931091309	1
MSTRG.27953.1	234	0.32974	9.054138184	1
MSTRG.27955.1	87	0.34339	5.649963379	1
MSTRG.27954.5	338	0.37635	7.939147949	1
MSTRG.27956.1	655	0.41346	5.739196777	1
MSTRG.27956.5	499	0.48053	5.821716309	1
MSTRG.27957.4	392	0.41675	4.934265137	1
MSTRG.27957.3	472	0.43018	4.870910645	1
MSTRG.27957.5	864	0.49723	5.277648926	1
MSTRG.27957.6	739	0.43641	7.77557373	1
MSTRG.27957.7	834	0.47058	5.545471191	1
MSTRG.27957.8	942	0.47445	6.108703613	1
MSTRG.27957.9	914	0.49785	5.368469238	1
MSTRG.27957.10	783	0.45726	6.424133301	-1
MSTRG.27959.1	224	0.31285	12.27398682	1
MSTRG.27960.1	134	0.29607	9.757019043	1
MSTRG.27960.2	100	0.37817	9.600524902	1
MSTRG.27961.1	157	0.32847	11.3069458	1
MSTRG.27963.2	627	0.4176	6.271911621	1
MSTRG.27967.4	419	0.31192	5.08380127	1
MSTRG.27967.5	419	0.32126	5.08380127	1
MSTRG.27967.6	419	0.31192	5.08380127	1
MSTRG.27967.7	419	0.32725	5.08380127	1
MSTRG.27968.3	172	0.36085	8.984924316	1
MSTRG.27968.4	123	0.36004	9.464416504	1
MSTRG.27964.2	150	0.37421	8.616516113	1
MSTRG.27969.3	263	0.41631	10.16168213	1
MSTRG.27969.4	263	0.41631	10.16168213	1
MSTRG.27970.2	161	0.36379	8.833679199	1
MSTRG.27975.5	138	0.28443	8.686950684	1
MSTRG.27975.4	168	0.36365	9.478942871	1
MSTRG.27975.7	161	0.27327	9.385559082	1
MSTRG.27983.2	184	0.43239	9.096984863	1
MSTRG.27985.1	62	0.40399	6.887145996	1
MSTRG.27988.3	490	0.45907	7.817321777	1
MSTRG.27995.1	227	0.29816	7.878356934	1
MSTRG.27995.3	74	0.29464	7.704284668	1
MSTRG.27996.1	132	0.32244	9.632141113	1
MSTRG.27996.2	132	0.32244	9.632141113	1
MSTRG.27997.1	137	0.28165	9.882873535	1
MSTRG.28003.6	708	0.41957	5.875427246	1
MSTRG.28004.1	35	0.39363	4.950378418	1
MSTRG.28005.1	152	0.29343	5.645935059	1
MSTRG.28005.2	152	0.29343	5.645935059	1
MSTRG.28003.7	385	0.34795	5.16217041	1
MSTRG.28003.8	403	0.33398	5.219787598	1
MSTRG.28003.9	113	0.30492	10.79803467	1

MSTRG.28007.2	142	0.34588	5.320495605	1
MSTRG.28008.1	4731	0.45833	5.725402832	1
MSTRG.28008.2	4731	0.45833	5.725402832	1
MSTRG.28008.3	3589	0.46542	5.617126465	1
MSTRG.28009.1	36	0.43264	9.498718262	1
MSTRG.28011.1	64	0.46348	9.51776123	-1
MSTRG.28012.5	1067	0.45689	6.505432129	1
MSTRG.28012.4	1076	0.46322	6.438537598	1
MSTRG.28013.3	639	0.3417	8.944030762	1
MSTRG.28018.2	112	0.29959	10.0894165	1
MSTRG.28033.6	312	0.42697	9.218688965	1
MSTRG.28033.8	312	0.431	9.218688965	1
MSTRG.28033.5	318	0.43771	9.218688965	1
MSTRG.28033.7	312	0.43771	9.218688965	1
MSTRG.28034.2	991	0.46317	8.530944824	-1
MSTRG.28034.4	991	0.44688	8.530944824	-1
MSTRG.28034.9	649	0.35168	7.409729004	1
MSTRG.28034.5	615	0.41107	8.356262207	-1
MSTRG.28034.6	615	0.45458	8.356262207	-1
MSTRG.28025.1	113	0.35081	11.18890381	1
MSTRG.28024.4	483	0.45182	6.025085449	1
MSTRG.28027.1	101	0.38489	10.12481689	-1
MSTRG.28027.2	101	0.39956	10.12481689	-1
MSTRG.28040.1	683	0.38993	9.412536621	1
MSTRG.28040.2	580	0.38518	9.757019043	1
MSTRG.28040.4	580	0.41248	9.757019043	1
MSTRG.28045.3	166	0.35824	5.341491699	1
MSTRG.28035.2	212	0.43006	9.296081543	-1
MSTRG.28055.1	49	0.29691	7.801330566	1
MSTRG.28058.1	107	0.28883	5.096618652	1
MSTRG.28059.1	23	0.32597	5.894958496	1
MSTRG.28051.1	1082	0.30801	6.298278809	1
MSTRG.28051.4	83	0.28248	8.50982666	1
MSTRG.28051.7	87	0.29367	6.162658691	1
MSTRG.28062.1	85	0.26751	8.591247559	1
MSTRG.28064.1	222	0.46056	8.663146973	1
MSTRG.28070.1	1701	0.4732	5.623596191	1
MSTRG.28072.3	261	0.43917	11.25604248	1
MSTRG.28076.1	43	0.40665	10.89508057	1
MSTRG.28075.1	280	0.4111	9.477233887	-1
MSTRG.28081.1	1037	0.41244	8.505187988	1
MSTRG.28085.6	548	0.47223	5.481628418	1
MSTRG.28091.4	143	0.2921	4.742004395	1
MSTRG.28091.2	559	0.28496	8.808654785	1
MSTRG.28093.2	514	0.46177	6.523010254	1
MSTRG.28095.3	122	0.37482	10.19622803	1
MSTRG.28095.2	179	0.42049	5.054748535	1
MSTRG.28097.2	61	0.42335	4.950012207	-1
MSTRG.28098.2	145	0.31548	10.76568604	1
MSTRG.28098.1	145	0.29703	10.76568604	1
MSTRG.28098.3	211	0.35012	9.410461426	1
MSTRG.28099.3	830	0.4381	7.853942871	1
MSTRG.28100.1	717	0.42465	5.555847168	1
MSTRG.28100.5	686	0.439	5.393981934	1

MSTRG.28100.6	752	0.42465	5.837097168	-1
MSTRG.28100.7	728	0.42217	5.811828613	1
MSTRG.28100.8	692	0.439	5.738708496	1
MSTRG.28105.5	456	0.43115	8.621398926	1
MSTRG.28105.6	456	0.41275	8.621398926	1
MSTRG.28107.1	724	0.46723	4.967224121	1
MSTRG.28109.3	271	0.45046	9.190856934	1
MSTRG.28109.4	205	0.37294	9.534118652	1
MSTRG.28109.2	217	0.43987	7.023986816	1
MSTRG.28109.5	271	0.41832	9.190856934	1
MSTRG.28121.1	77	0.30085	6.876403809	1
MSTRG.28122.1	77	0.35937	10.00750732	1
MSTRG.28115.4	54	0.2675	9.61932373	1
MSTRG.28114.2	252	0.38122	5.537414551	1
MSTRG.28127.1	68	0.43123	6.211853027	1
MSTRG.28132.2	366	0.44113	9.456115723	1
MSTRG.28137.1	63	0.27677	10.49578857	1
MSTRG.28131.3	129	0.37096	6.011169434	1
MSTRG.28141.1	36	0.30288	5.996154785	1
MSTRG.28142.2	649	0.4187	8.231384277	1
MSTRG.28147.1	590	0.43713	6.551940918	1
MSTRG.28154.1	21	0.31529	9.34967041	1
MSTRG.28160.1	709	0.47415	7.621276855	1
MSTRG.28160.5	279	0.34546	9.141784668	1
MSTRG.28161.1	67	0.41172	9.50982666	1
MSTRG.28162.1	142	0.4116	9.915466309	1
MSTRG.28168.3	543	0.46624	8.789001465	1
MSTRG.28174.1	103	0.34441	8.954772949	1
MSTRG.28174.2	103	0.36426	8.954772949	1
MSTRG.28175.1	163	0.37639	9.669006348	1
MSTRG.28177.1	121	0.33054	10.44329834	1
MSTRG.28178.1	243	0.36562	8.63848877	1
MSTRG.28171.1	46	0.3737	10.39801025	-1
MSTRG.28186.1	53	0.34678	5.052307129	1
MSTRG.28191.1	99	0.36088	10.78704834	1
MSTRG.28191.8	33	0.39671	12.31060791	1
MSTRG.28192.3	230	0.4633	8.782409668	1
MSTRG.28204.2	113	0.34566	10.04229736	1
MSTRG.28207.1	34	0.40149	4.723571777	1
MSTRG.28207.2	80	0.45973	10.06781006	1
MSTRG.28207.3	21	0.38466	11.71258545	1
MSTRG.28211.2	250	0.46275	9.300109863	1
MSTRG.28213.2	132	0.40042	5.192687988	1
MSTRG.28194.2	218	0.45604	5.574645996	-1
MSTRG.28196.2	42	0.31877	9.236633301	1
MSTRG.28197.1	44	0.43934	10.58258057	1
MSTRG.28214.1	49	0.38445	6.471130371	1
MSTRG.28220.1	439	0.41845	5.172302246	1
MSTRG.28220.2	430	0.35653	7.178649902	1
MSTRG.28223.2	94	0.37423	5.839172363	1
MSTRG.28224.2	287	0.40247	9.551330566	1
MSTRG.28217.1	42	0.33059	7.916687012	1
MSTRG.28217.2	42	0.37492	7.916687012	1
MSTRG.28225.1	316	0.47098	8.159973145	1

MSTRG.28227.3	379	0.43101	9.683044434	1
MSTRG.28249.2	1741	0.46385	7.213195801	1
MSTRG.28249.3	1741	0.46385	6.969299316	1
MSTRG.28249.4	1714	0.49217	7.065612793	1
MSTRG.28249.5	1741	0.4823	6.969177246	1
MSTRG.28249.7	909	0.45565	5.690734863	1
MSTRG.28249.8	609	0.45648	5.092224121	1
MSTRG.28249.6	1741	0.46385	7.081848145	1
MSTRG.28249.9	1397	0.46578	7.049133301	1
MSTRG.28249.11	1741	0.49	7.217956543	1
MSTRG.28250.1	146	0.44677	9.122497559	-1
MSTRG.28251.1	738	0.31824	8.343688965	1
MSTRG.28251.2	738	0.31581	8.343688965	1
MSTRG.28251.5	738	0.39049	8.343688965	1
MSTRG.28251.6	148	0.3481	9.500671387	1
MSTRG.28249.13	1749	0.46385	7.202453613	1
MSTRG.28232.2	1199	0.44816	5.982727051	1
MSTRG.28233.2	132	0.41355	4.63470459	1
MSTRG.28233.3	374	0.44509	9.495056152	1
MSTRG.28233.4	132	0.38604	4.63470459	1
MSTRG.28236.2	644	0.44062	5.13067627	1
MSTRG.28236.4	667	0.42055	5.945007324	1
MSTRG.28229.1	356	0.41171	5.739685059	1
MSTRG.28239.1	641	0.44424	5.89276123	1
MSTRG.28244.2	1319	0.46976	9.259094238	1
MSTRG.28244.3	42	0.37458	7.916564941	1
MSTRG.28244.6	969	0.49469	6.238952637	1
MSTRG.28246.2	169	0.4599	5.42767334	1
MSTRG.28247.1	99	0.31338	8.838562012	1
MSTRG.28247.3	234	0.3369	10.74676514	1
MSTRG.28247.5	94	0.33434	8.350524902	1
MSTRG.28247.2	94	0.29952	8.350524902	1
MSTRG.28241.1	157	0.38128	11.61444092	1
MSTRG.28242.1	215	0.35535	5.329040527	1
MSTRG.28242.3	215	0.37647	5.329040527	1
MSTRG.28242.5	215	0.4288	5.329040527	1
MSTRG.28243.1	351	0.36649	9.329528809	1
MSTRG.28255.2	116	0.3586	8.913269043	1
MSTRG.28256.5	1115	0.44842	5.400695801	1
MSTRG.28257.2	2143	0.44463	9.355285645	1
MSTRG.28257.3	2166	0.41625	9.456970215	1
MSTRG.28257.4	2147	0.44463	9.457092285	1
MSTRG.28300.1	51	0.3371	4.87689209	-1
MSTRG.28300.2	112	0.28195	7.903015137	1
MSTRG.28301.1	51	0.44945	6.047302246	1
MSTRG.28300.3	363	0.36816	5.561340332	1
MSTRG.28300.4	332	0.48281	5.341247559	1
MSTRG.28300.5	332	0.48281	5.184753418	1
MSTRG.28300.6	366	0.35018	5.363952637	1
MSTRG.28300.8	331	0.43366	5.191345215	-1
MSTRG.28300.9	363	0.39071	5.661804199	1
MSTRG.28300.10	363	0.37793	5.468322754	1
MSTRG.28308.1	777	0.43615	5.959777832	1
MSTRG.28308.6	801	0.44812	5.835510254	1

MSTRG.28312.1	915	0.43011	6.267150879	1
MSTRG.28312.3	915	0.42635	6.267150879	1
MSTRG.28312.2	915	0.40974	6.267150879	1
MSTRG.28312.4	915	0.42387	6.267150879	1
MSTRG.28315.1	27	0.44664	8.040588379	1
MSTRG.28316.8	348	0.48069	5.753967285	1
MSTRG.28319.1	1478	0.49621	5.696594238	1
MSTRG.28319.2	1479	0.49621	5.696594238	1
MSTRG.28319.4	1479	0.49456	5.696594238	1
MSTRG.28319.6	1670	0.49366	5.432678223	1
MSTRG.28321.1	608	0.37555	5.580749512	1
MSTRG.28325.2	280	0.3615	10.31939697	1
MSTRG.28325.5	326	0.42008	9.870666504	1
MSTRG.28324.3	924	0.41892	9.218322754	1
MSTRG.28326.1	844	0.4405	6.194274902	1
MSTRG.28328.2	187	0.34558	9.970031738	1
MSTRG.28329.3	469	0.36641	4.972106934	1
MSTRG.28332.2	147	0.44029	10.18511963	1
MSTRG.28300.13	76	0.3379	8.760681152	1
MSTRG.28266.4	373	0.46017	5.641906738	1
MSTRG.28266.5	357	0.45394	5.833679199	-1
MSTRG.28266.7	166	0.41329	4.944396973	-1
MSTRG.28267.1	130	0.39822	10.21966553	1
MSTRG.28267.2	93	0.43966	6.813537598	1
MSTRG.28266.17	130	0.41329	5.901672363	-1
MSTRG.28274.1	93	0.36121	8.73236084	1
MSTRG.28274.2	93	0.38328	8.73236084	1
MSTRG.28270.1	561	0.29912	9.564880371	1
MSTRG.28271.1	184	0.3808	7.716247559	1
MSTRG.28281.2	899	0.44018	8.496520996	1
MSTRG.28281.3	784	0.44018	8.27545166	1
MSTRG.28281.4	784	0.45558	8.27545166	1
MSTRG.28281.5	906	0.4572	8.496520996	1
MSTRG.28281.6	803	0.4396	8.379821777	1
MSTRG.28281.7	875	0.45082	8.469177246	1
MSTRG.28281.8	933	0.4396	8.486633301	1
MSTRG.28281.9	336	0.31201	8.273376465	1
MSTRG.28281.10	314	0.44226	9.09576416	1
MSTRG.28294.2	337	0.3658	5.25592041	1
MSTRG.28297.3	127	0.35748	9.49017334	1
MSTRG.28335.1	608	0.33667	6.70513916	1
MSTRG.28336.1	63	0.37539	10.45001221	1
MSTRG.28335.2	608	0.36813	6.70513916	1
MSTRG.28335.3	614	0.37681	6.843444824	1
MSTRG.28338.1	806	0.45249	5.009338379	1
MSTRG.28339.3	479	0.36444	8.585510254	1
MSTRG.28341.2	291	0.36682	5.702941895	1
MSTRG.28341.4	261	0.37002	5.416442871	1
MSTRG.28342.3	578	0.30235	7.041809082	1
MSTRG.28342.4	578	0.29551	7.041809082	1
MSTRG.28343.1	51	0.42619	4.800109863	1
MSTRG.28367.1	126	0.45365	10.31451416	1
MSTRG.28367.2	126	0.43979	10.31451416	1
MSTRG.28368.2	130	0.44573	10.90313721	1

MSTRG.28370.1	154	0.38986	11.54730225	1
MSTRG.28371.1	126	0.39984	10.31451416	1
MSTRG.28372.1	103	0.41335	11.362854	1
MSTRG.28375.1	90	0.33465	9.331237793	1
MSTRG.28377.1	39	0.47038	11.56756592	1
MSTRG.28371.2	126	0.41203	10.31451416	1
MSTRG.28371.3	126	0.37292	10.31451416	1
MSTRG.28378.1	128	0.46077	10.88311768	1
MSTRG.28379.1	218	0.42403	11.11212158	1
MSTRG.28380.1	126	0.4532	10.31451416	1
MSTRG.28386.3	126	0.44411	10.31451416	1
MSTRG.28386.2	126	0.45599	10.31451416	1
MSTRG.28388.1	136	0.42718	11.12701416	1
MSTRG.28386.4	131	0.38805	10.26434326	1
MSTRG.28388.2	136	0.48778	11.12701416	1
MSTRG.28389.1	221	0.32076	10.96539307	1
MSTRG.28379.2	136	0.47097	11.12701416	1
MSTRG.28388.3	136	0.49961	11.12701416	1
MSTRG.28371.4	126	0.43615	10.31451416	1
MSTRG.28359.7	101	0.30596	8.75958252	1
MSTRG.28359.6	509	0.35488	9.60760498	1
MSTRG.28359.3	459	0.32661	9.492858887	1
MSTRG.28359.5	697	0.40377	9.089172363	1
MSTRG.28359.8	508	0.34615	9.60760498	1
MSTRG.28359.9	698	0.42275	9.089172363	1
MSTRG.28359.10	89	0.29772	6.957946777	1
MSTRG.28361.1	81	0.38066	8.83026123	1
MSTRG.28362.1	102	0.32642	7.651062012	1
MSTRG.28359.11	139	0.29268	8.836120605	1
MSTRG.28363.1	146	0.28199	9.077087402	1
MSTRG.28363.2	146	0.30477	9.077087402	1
MSTRG.28363.3	144	0.28167	9.226135254	1
MSTRG.28366.4	610	0.44881	5.376281738	1
MSTRG.28366.5	1016	0.40054	5.175476074	1
MSTRG.28366.6	610	0.41295	5.376281738	1
MSTRG.28393.1	74	0.28799	8.609069824	1
MSTRG.28393.3	228	0.31274	8.767028809	1
MSTRG.28393.4	228	0.33291	8.767028809	1
MSTRG.28393.6	171	0.29116	6.236633301	1
MSTRG.28393.2	229	0.34144	8.915466309	1
MSTRG.28397.1	443	0.39616	7.666809082	1
MSTRG.28399.2	246	0.44808	9.016052246	1
MSTRG.28400.1	475	0.43756	5.456604004	1
MSTRG.28400.2	475	0.43756	5.456604004	1
MSTRG.28400.3	475	0.42408	5.456604004	1
MSTRG.28401.1	72	0.44139	9.019958496	-1
MSTRG.28391.2	154	0.43859	5.188903809	1
MSTRG.28403.1	7	0.43748	9.700256348	1
MSTRG.28404.1	125	0.34436	8.434143066	1
MSTRG.28409.2	108	0.32477	8.945617676	1
MSTRG.28409.1	91	0.3824	5.823669434	1
MSTRG.28409.3	28	0.35602	10.06011963	1
MSTRG.28409.4	53	0.38536	8.931335449	1
MSTRG.28409.6	76	0.33423	9.565734863	1

MSTRG.28409.5	41	0.39064	10.17108154	1
MSTRG.28409.7	124	0.29367	9.811340332	1
MSTRG.28409.8	115	0.3368	6.73236084	1
MSTRG.28410.1	95	0.29367	7.671325684	1
MSTRG.28409.9	107	0.34779	10.65545654	1
MSTRG.28409.10	164	0.30753	9.958312988	1
MSTRG.28411.1	148	0.34579	10.95001221	1
MSTRG.28409.11	43	0.41942	10.06292725	1
MSTRG.28449.3	379	0.39255	5.884216309	1
MSTRG.28457.1	571	0.42253	8.694885254	1
MSTRG.28457.2	571	0.45665	8.694885254	1
MSTRG.28457.5	464	0.39036	9.05291748	1
MSTRG.28457.6	578	0.46508	8.67364502	1
MSTRG.28459.1	84	0.3177	4.358825684	1
MSTRG.28460.1	105	0.29115	9.825378418	1
MSTRG.28463.1	127	0.3693	10.36334229	1
MSTRG.28464.1	148	0.29387	9.801452637	1
MSTRG.28466.1	81	0.28491	10.03948975	1
MSTRG.28467.1	87	0.30477	10.43707275	1
MSTRG.28470.1	79	0.28518	8.373596191	1
MSTRG.28470.2	79	0.28518	8.373596191	1
MSTRG.28471.2	224	0.29088	4.221618652	1
MSTRG.28471.3	224	0.28503	4.221618652	1
MSTRG.28474.1	126	0.36259	9.058654785	1
MSTRG.28471.4	139	0.28267	9.041564941	1
MSTRG.28476.2	151	0.37113	9.711486816	1
MSTRG.28430.1	60	0.34559	6.813049316	1
MSTRG.28430.2	61	0.30256	5.270324707	1
MSTRG.28432.1	164	0.37576	8.685119629	1
MSTRG.28433.1	57	0.34913	9.899719238	1
MSTRG.28439.1	820	0.40563	7.880310059	1
MSTRG.28443.1	39	0.34858	10.28094482	-1
MSTRG.28442.2	317	0.4184	5.692077637	1
MSTRG.28445.1	294	0.35217	5.608581543	1
MSTRG.28480.2	30	0.35828	9.304016113	-1
MSTRG.28480.1	64	0.33258	8.931945801	-1
MSTRG.28480.3	189	0.3582	10.02838135	1
MSTRG.28481.3	380	0.36378	7.503845215	1
MSTRG.28485.1	112	0.29367	9.183898926	1
MSTRG.28485.2	97	0.29772	6.395690918	1
MSTRG.28485.3	145	0.28923	9.368103027	1
MSTRG.28485.4	86	0.30958	5.721496582	1
MSTRG.28485.5	86	0.35421	5.721496582	1
MSTRG.28485.6	85	0.3076	9.067077637	1
MSTRG.28487.2	261	0.41541	4.328186035	1
MSTRG.28487.3	261	0.40921	4.328186035	1
MSTRG.28488.1	1236	0.43303	9.473693848	1
MSTRG.28488.2	1236	0.39288	9.473693848	1
MSTRG.28488.3	1236	0.40674	9.473693848	1
MSTRG.28488.4	1236	0.43861	9.473693848	1
MSTRG.28488.5	1212	0.32721	9.522033691	1
MSTRG.28488.7	186	0.28167	7.009216309	1
MSTRG.28489.1	173	0.27763	8.461242676	1
MSTRG.28488.8	126	0.27782	8.948181152	1

MSTRG.28488.9	126	0.27782	8.948181152	1
MSTRG.28488.10	126	0.28851	8.948181152	1
MSTRG.28488.11	119	0.27915	6.795471191	1
MSTRG.28491.1	101	0.35961	9.096618652	-1
MSTRG.28492.1	25	0.35927	3.998840332	1
MSTRG.28495.1	83	0.28648	10.37994385	1
MSTRG.28495.2	83	0.28648	10.37994385	1
MSTRG.28499.3	249	0.40265	6.500427246	1
MSTRG.28501.3	372	0.36166	10.00714111	1
MSTRG.28501.4	89	0.37067	8.890563965	1
MSTRG.28502.1	40	0.47196	4.601013184	-1
MSTRG.28505.3	310	0.40593	8.429382324	1
MSTRG.28509.4	81	0.35289	10.78729248	1
MSTRG.28509.5	129	0.40045	9.367248535	1
MSTRG.28509.2	235	0.40871	10.45196533	1
MSTRG.28509.3	86	0.30813	9.512268066	1
MSTRG.28510.1	68	0.36588	10.76495361	-1
MSTRG.28514.5	1926	0.446	8.650939941	1
MSTRG.28515.1	225	0.37871	9.55291748	1
MSTRG.28515.2	225	0.40865	9.55291748	1
MSTRG.28515.3	225	0.40717	9.55291748	1
MSTRG.28517.1	43	0.35902	9.300598145	1
MSTRG.28517.2	62	0.43954	6.520568848	1
MSTRG.28517.3	43	0.3553	9.300598145	1
MSTRG.28517.4	31	0.30449	10.02435303	1
MSTRG.28517.5	31	0.37221	10.02435303	1
MSTRG.28520.2	52	0.3376	6.546081543	1
MSTRG.28522.2	264	0.32831	9.483093262	1
MSTRG.28522.3	277	0.37176	9.602844238	1
MSTRG.28522.4	277	0.37046	9.602844238	1
MSTRG.28522.6	277	0.3624	9.602844238	1
MSTRG.28522.8	277	0.35584	9.602844238	1
MSTRG.28525.2	386	0.42128	9.369689941	1
MSTRG.28525.3	382	0.40182	9.369689941	1
MSTRG.28532.3	137	0.35643	6.409851074	1
MSTRG.28532.5	79	0.40125	8.330505371	1
MSTRG.28533.1	66	0.29319	9.438293457	1
MSTRG.28538.2	2818	0.41898	6.385437012	1
MSTRG.28546.1	767	0.4758	5.185974121	1
MSTRG.28546.2	186	0.39893	4.084289551	1
MSTRG.28546.3	459	0.44225	5.187927246	1
MSTRG.28546.4	180	0.39023	4.029724121	1
MSTRG.28546.12	372	0.39303	4.803161621	1
MSTRG.28546.10	374	0.44568	4.81427002	1
MSTRG.28546.8	467	0.4559	5.124938965	1
MSTRG.28539.2	1691	0.45482	6.294494629	1
MSTRG.28539.3	1691	0.47598	6.294494629	1
MSTRG.28539.5	1691	0.47598	6.294494629	1
MSTRG.28539.6	1691	0.45295	6.294494629	1
MSTRG.28539.7	1691	0.46065	6.294494629	1
MSTRG.28539.8	1745	0.44113	6.480651855	1
MSTRG.28550.3	131	0.27475	9.594421387	1
MSTRG.28551.1	132	0.29152	9.343444824	1
MSTRG.28552.1	181	0.28979	11.5368042	1

MSTRG.28553.1	111	0.28396	8.801696777	1
MSTRG.28554.1	87	0.31915	10.16680908	1
MSTRG.28555.2	69	0.35277	9.452331543	1
MSTRG.28555.3	40	0.41941	10.06085205	1
MSTRG.28556.1	60	0.28387	10.08209229	1
MSTRG.28557.1	66	0.31621	8.460388184	1
MSTRG.28555.4	54	0.3497	7.662902832	1
MSTRG.28559.1	117	0.35682	9.942077637	1
MSTRG.28561.3	303	0.30644	5.29083252	1
MSTRG.28563.1	567	0.46586	9.275085449	1
MSTRG.28563.5	412	0.45331	8.808044434	1
MSTRG.28564.1	102	0.30945	6.299499512	1
MSTRG.28565.1	47	0.32843	6.693786621	1
MSTRG.28566.1	133	0.28707	9.078918457	1
MSTRG.28567.1	150	0.28303	10.53863525	1
MSTRG.28568.1	50	0.31572	5.93536377	1
MSTRG.28571.2	221	0.40179	9.885925293	1
MSTRG.28571.3	143	0.42048	9.917663574	1
MSTRG.28572.1	119	0.33676	10.12969971	-1
MSTRG.28579.2	405	0.41686	8.684020996	1
MSTRG.28586.1	572	0.44254	4.910339355	1
MSTRG.28586.3	470	0.41794	4.824157715	1
MSTRG.28586.4	446	0.41794	4.709411621	1
MSTRG.28586.5	446	0.41794	4.709411621	1
MSTRG.28587.2	167	0.43874	8.746643066	1
MSTRG.28587.3	133	0.4097	6.433288574	1
MSTRG.28588.1	268	0.33	6.710632324	1
MSTRG.28590.1	51	0.34695	7.777404785	1
MSTRG.28588.2	84	0.32823	9.433898926	-1
MSTRG.28594.2	337	0.45872	4.906921387	-1
MSTRG.28581.2	392	0.40266	5.16192627	1
MSTRG.28585.1	39	0.4511	10.34735107	1
MSTRG.28595.1	40	0.44972	8.496398926	1
MSTRG.28600.2	118	0.37127	7.009216309	1
MSTRG.28600.3	267	0.39505	8.226989746	1
MSTRG.28600.6	188	0.28284	7.708679199	1
MSTRG.28600.5	283	0.45993	7.68572998	1
MSTRG.28600.4	293	0.42886	6.518981934	1
MSTRG.28600.7	100	0.31834	9.096740723	1
MSTRG.28600.8	188	0.39335	7.708679199	1
MSTRG.28600.9	283	0.4699	7.68572998	1
MSTRG.28602.1	23	0.39232	4.783508301	-1
MSTRG.28603.1	173	0.27251	9.253723145	1
MSTRG.28604.1	60	0.32421	8.682312012	1
MSTRG.28605.1	26	0.33194	7.998840332	1
MSTRG.28606.1	126	0.31581	9.230041504	1
MSTRG.28597.1	78	0.38463	9.21697998	1
MSTRG.28613.7	630	0.41107	8.664611816	1
MSTRG.28613.6	924	0.46175	6.666809082	1
MSTRG.28613.8	791	0.37415	6.738708496	1
MSTRG.28613.9	791	0.36029	6.738708496	1
MSTRG.28613.10	99	0.30573	9.66583252	1
MSTRG.28614.1	114	0.28751	9.643615723	1
MSTRG.28609.1	41	0.42275	9.186218262	1

MSTRG.28618.1	65	0.36306	9.101257324	-1
MSTRG.28620.4	1146	0.45477	9.108093262	1
MSTRG.28620.3	1146	0.45477	9.108093262	1
MSTRG.28623.2	292	0.35969	9.354553223	1
MSTRG.28625.1	73	0.32239	5.035339355	1
MSTRG.28625.2	142	0.355	5.531066895	1
MSTRG.28625.3	142	0.4326	5.531066895	1
MSTRG.28625.4	73	0.36473	5.035339355	1
MSTRG.28627.4	842	0.43565	5.269470215	1
MSTRG.28627.3	932	0.40843	5.29498291	1
MSTRG.28627.5	816	0.42817	5.119445801	1
MSTRG.28628.1	242	0.31822	10.9788208	1
MSTRG.28631.2	207	0.33059	10.54241943	1
MSTRG.28631.3	180	0.28338	10.12054443	1
MSTRG.28633.1	63	0.38195	9.521179199	1
MSTRG.28642.2	1841	0.4871	8.632263184	1
MSTRG.28642.8	1086	0.41744	8.57244873	-1
MSTRG.28642.7	1100	0.37404	8.46661377	-1
MSTRG.28642.5	1574	0.41633	8.643371582	-1
MSTRG.28642.6	1588	0.41616	8.589538574	-1
MSTRG.28642.3	1588	0.41323	8.589538574	-1
MSTRG.28638.1	30	0.39096	10.05230713	1
MSTRG.28638.2	85	0.33951	8.46673584	1
MSTRG.28644.1	119	0.27678	10.48980713	1
MSTRG.28645.2	1071	0.33224	9.403137207	1
MSTRG.28646.3	63	0.36459	11.88189697	-1
MSTRG.28646.4	63	0.28255	11.88189697	-1
MSTRG.28646.5	63	0.37345	11.88189697	-1
MSTRG.28647.1	172	0.27274	6.96270752	1
MSTRG.28648.1	114	0.33337	9.127502441	1
MSTRG.28648.2	114	0.33742	9.127502441	1
MSTRG.28648.3	149	0.30449	9.127624512	1
MSTRG.28650.1	67	0.32907	9.831115723	1
MSTRG.28650.3	58	0.41185	4.661437988	1
MSTRG.28650.2	58	0.32646	4.661437988	1
MSTRG.28652.1	17	0.28519	5.585388184	1
MSTRG.28653.1	821	0.47977	8.824157715	1
MSTRG.28654.1	120	0.32966	9.825256348	1
MSTRG.28656.1	26	0.46851	10.7366333	1
MSTRG.28657.3	2209	0.45118	6.220031738	1
MSTRG.28658.1	84	0.37841	7.662536621	1
MSTRG.28662.4	510	0.46717	5.41229248	1
MSTRG.28662.3	475	0.46114	5.597229004	1
MSTRG.28661.1	95	0.39407	9.363098145	1
MSTRG.28661.2	95	0.44959	9.363098145	1
MSTRG.28663.1	678	0.43462	9.653625488	1
MSTRG.28663.2	678	0.39821	9.653625488	1
MSTRG.28669.2	106	0.2997	10.21612549	1
MSTRG.28672.1	36	0.36277	11.45159912	1
MSTRG.28672.2	36	0.42557	11.45159912	1
MSTRG.28672.3	40	0.34478	8.842346191	1
MSTRG.28677.1	50	0.33182	11.54217529	1
MSTRG.28677.2	50	0.43069	11.54217529	1
MSTRG.28682.2	2228	0.48069	6.462585449	1

MSTRG.28688.2	112	0.32177	7.832336426	1
MSTRG.28693.1	51	0.36554	6.889465332	1
MSTRG.28702.1	46	0.34297	10.10577393	1
MSTRG.28705.1	10	0.36406	5.274963379	1
MSTRG.28707.1	63	0.40763	8.768249512	1
MSTRG.28712.2	86	0.43444	10.86114502	-1
MSTRG.28712.3	11	0.40733	12.480896	1
MSTRG.28714.1	22	0.32838	8.18939209	1
MSTRG.28716.1	107	0.31279	4.788513184	1
MSTRG.28718.2	788	0.46525	6.734680176	1
MSTRG.28715.1	54	0.29796	9.497253418	-1
MSTRG.28720.1	64	0.43974	9.032897949	1
MSTRG.28731.1	36	0.38801	4.698059082	1
MSTRG.28732.1	105	0.32346	4.821350098	1
MSTRG.28742.4	361	0.40446	8.85369873	1
MSTRG.28743.1	34	0.37641	9.188293457	1
MSTRG.28744.1	159	0.31437	11.6572876	1
MSTRG.28745.1	225	0.37015	8.912414551	1
MSTRG.28745.3	77	0.30312	7.725158691	1
MSTRG.28745.5	225	0.30421	8.912414551	1
MSTRG.28746.1	1201	0.4378	5.636901855	1
MSTRG.28746.3	1336	0.42084	5.602722168	1
MSTRG.28746.4	95	0.3062	6.707214355	1
MSTRG.28746.5	245	0.36806	4.555969238	1
MSTRG.28737.2	746	0.44876	5.77154541	1
MSTRG.28737.3	628	0.47104	5.123718262	1
MSTRG.28739.3	168	0.34157	8.465759277	1
MSTRG.28739.4	168	0.43035	8.465759277	1
MSTRG.28739.5	173	0.2921	9.833068848	1
MSTRG.28739.6	85	0.32072	6.127746582	1
MSTRG.28740.1	69	0.30934	9.428161621	1
MSTRG.28755.1	42	0.41594	10.50701904	1
MSTRG.28765.1	101	0.34544	9.144958496	1
MSTRG.28770.1	368	0.29772	5.826843262	1
MSTRG.28770.3	368	0.3	5.826843262	1
MSTRG.28780.1	460	0.32305	4.83026123	1
MSTRG.28780.3	460	0.35395	4.83026123	1
MSTRG.28784.1	2200	0.42351	5.642028809	1
MSTRG.28784.2	2200	0.44992	5.642028809	1
MSTRG.28783.1	70	0.36976	10.37384033	1
MSTRG.28790.1	31	0.384	8.274963379	1
MSTRG.28790.2	34	0.40078	4.96661377	1
MSTRG.28794.1	54	0.33004	6.520324707	1
MSTRG.28799.2	943	0.48731	4.943054199	1
MSTRG.28822.1	86	0.3351	10.09942627	-1
MSTRG.28830.1	44	0.37095	6.89666748	1
MSTRG.28839.1	864	0.46542	9.683166504	1
MSTRG.28839.2	864	0.48849	9.683166504	1
MSTRG.28839.4	251	0.4031	9.25567627	1
MSTRG.28842.1	85	0.30242	9.860290527	1
MSTRG.28844.6	363	0.38993	6.07244873	1
MSTRG.28844.5	328	0.37265	7.147644043	1
MSTRG.28846.3	255	0.39079	5.730651855	1
MSTRG.28853.2	950	0.43999	5.907409668	1

MSTRG.28853.3	950	0.34615	5.907409668	1
MSTRG.28853.5	950	0.46459	5.907409668	1
MSTRG.28853.6	134	0.3614	8.622131348	1
MSTRG.28854.1	40	0.32702	8.503845215	-1
MSTRG.28853.10	189	0.34904	10.23907471	1
MSTRG.28853.9	568	0.44304	5.527282715	1
MSTRG.28856.1	132	0.28096	9.097595215	1
MSTRG.28857.6	382	0.49172	8.257019043	1
MSTRG.28857.5	454	0.45874	4.879333496	1
MSTRG.28857.8	454	0.45926	4.879333496	1
MSTRG.28857.7	283	0.46567	5.60760498	1
MSTRG.28858.2	266	0.3787	8.402160645	1
MSTRG.28859.1	132	0.28338	8.282653809	1
MSTRG.28862.2	3005	0.39689	6.35760498	1
MSTRG.28862.3	2958	0.39255	6.294128418	1
MSTRG.28862.4	1899	0.43588	7.475646973	1
MSTRG.28862.5	3005	0.42164	6.35760498	1
MSTRG.28862.6	2115	0.39475	7.236999512	1
MSTRG.28862.7	219	0.37733	8.86907959	1
MSTRG.28860.2	244	0.3208	6.514831543	1
MSTRG.28866.2	889	0.47416	6.192199707	1
MSTRG.28866.3	80	0.41248	10.41558838	1
MSTRG.28869.1	19	0.42967	6.505310059	1
MSTRG.28879.1	119	0.3351	11.56414795	1
MSTRG.28879.4	112	0.40447	11.456604	1
MSTRG.28879.3	112	0.32366	11.456604	1
MSTRG.28879.5	112	0.41264	11.456604	1
MSTRG.28880.1	841	0.29607	9.229675293	1
MSTRG.28880.3	728	0.41761	9.252258301	-1
MSTRG.28880.2	702	0.32307	9.300231934	1
MSTRG.28880.4	728	0.43099	9.252258301	-1
MSTRG.28882.1	96	0.33467	9.770568848	1
MSTRG.28883.1	177	0.28199	10.02020264	1
MSTRG.28884.1	57	0.3443	10.02435303	1
MSTRG.28885.2	130	0.42218	6.691589355	1
MSTRG.28885.3	130	0.42988	6.691589355	1
MSTRG.28886.2	933	0.4431	6.131896973	1
MSTRG.28887.1	494	0.38676	5.150939941	1
MSTRG.28887.2	449	0.39252	5.16204834	1
MSTRG.28887.4	449	0.33686	5.16204834	1
MSTRG.28888.1	657	0.4021	4.892150879	1
MSTRG.28873.1	68	0.36752	10.42474365	1
MSTRG.28876.1	375	0.4076	4.68170166	1
MSTRG.28890.1	316	0.40331	5.870788574	1
MSTRG.28890.5	316	0.44798	5.870788574	1
MSTRG.28890.6	313	0.45238	6.003967285	1
MSTRG.28890.4	316	0.43165	5.870788574	1
MSTRG.28890.3	316	0.35893	5.870788574	1
MSTRG.28893.1	52	0.30237	9.221862793	1
MSTRG.28892.2	277	0.34426	9.833679199	1
MSTRG.28894.1	29	0.36539	8.211242676	1
MSTRG.28894.2	29	0.41298	6.695495605	1
MSTRG.28900.3	470	0.42005	6.830871582	1
MSTRG.28905.2	858	0.40758	5.846740723	1

MSTRG.28907.1	172	0.48147	4.671569824	1
MSTRG.28908.1	71	0.30653	9.69732666	1
MSTRG.28914.1	87	0.34711	5.344543457	1
MSTRG.28914.2	977	0.45807	6.749328613	1
MSTRG.28914.3	427	0.47206	5.141418457	1
MSTRG.28917.1	102	0.34028	6.248840332	1
MSTRG.28920.1	67	0.30816	6.475402832	1
MSTRG.28923.4	854	0.44594	5.03338623	1
MSTRG.28923.3	872	0.4352	5.022644043	1
MSTRG.28923.5	854	0.4352	5.03338623	1
MSTRG.28923.7	854	0.44906	5.03338623	1
MSTRG.28923.6	913	0.44906	5.02166748	1
MSTRG.28923.8	872	0.44594	5.022644043	1
MSTRG.28934.1	75	0.30396	10.418396	1
MSTRG.28935.1	118	0.31454	7.92755127	1
MSTRG.28936.1	163	0.29526	9.820495605	1
MSTRG.28937.1	58	0.42974	8.72454834	1
MSTRG.28933.3	141	0.33979	10.39129639	1
MSTRG.28941.1	106	0.32619	8.262145996	1
MSTRG.28942.1	144	0.28015	11.13897705	1
MSTRG.28940.4	705	0.35356	6.121276855	1
MSTRG.28949.2	242	0.46078	6.001281738	1
MSTRG.28949.4	152	0.45001	5.881896973	1
MSTRG.28951.2	177	0.28842	9.765686035	1
MSTRG.28951.1	1677	0.46624	6.957702637	1
MSTRG.28951.6	298	0.39742	8.832580566	1
MSTRG.28951.5	321	0.38511	8.274719238	1
MSTRG.28952.1	119	0.34547	8.370422363	1
MSTRG.28953.1	51	0.39159	9.649597168	1
MSTRG.28947.1	635	0.49189	5.69732666	1
MSTRG.28961.6	893	0.44207	4.760925293	1
MSTRG.28961.8	706	0.39436	4.780212402	1
MSTRG.28961.4	894	0.44574	4.760925293	1
MSTRG.28961.5	894	0.44574	4.760925293	1
MSTRG.28961.3	919	0.48974	4.655822754	1
MSTRG.28961.7	936	0.48974	4.61529541	1
MSTRG.28961.10	929	0.42718	4.690490723	1
MSTRG.28961.12	893	0.44263	4.760925293	1
MSTRG.28961.9	893	0.4829	4.760925293	1
MSTRG.28961.13	158	0.31241	10.4130249	-1
MSTRG.28961.14	56	0.34301	4.195983887	1
MSTRG.28961.15	40	0.31214	4.195983887	-1
MSTRG.28961.16	40	0.40304	4.195983887	-1
MSTRG.28963.1	204	0.35925	5.985290527	1
MSTRG.28967.1	124	0.391	9.653137207	1
MSTRG.28975.1	77	0.36473	9.980041504	-1
MSTRG.28980.2	349	0.2785	9.684631348	-1
MSTRG.28976.2	99	0.42347	5.674743652	1
MSTRG.28976.3	99	0.45443	5.674743652	1
MSTRG.28977.1	85	0.31138	8.714172363	1
MSTRG.28976.4	89	0.38843	10.77496338	1
MSTRG.28987.2	150	0.28964	8.976867676	1
MSTRG.28988.2	723	0.43019	8.69708252	1
MSTRG.28988.3	774	0.42916	8.967590332	1

MSTRG.28990.1	178	0.3598	8.626403809	1
MSTRG.28988.4	245	0.4215	4.875061035	1
MSTRG.28988.5	665	0.43646	5.957946777	1
MSTRG.28988.6	617	0.34711	6.053161621	1
MSTRG.28988.8	474	0.39802	6.108703613	1
MSTRG.28988.9	585	0.37021	6.102233887	1
MSTRG.28988.10	654	0.48543	6.106872559	1
MSTRG.28988.11	662	0.43881	6.190734863	1
MSTRG.28988.12	473	0.40143	6.040100098	1
MSTRG.28993.2	246	0.38772	6.109436035	1
MSTRG.28993.3	224	0.40379	6.432678223	1
MSTRG.29016.1	71	0.3519	12.00701904	1
MSTRG.29017.8	235	0.3616	8.530822754	1
MSTRG.29017.9	2699	0.47109	5.336120605	1
MSTRG.29017.10	235	0.3917	8.530822754	1
MSTRG.29017.12	296	0.39464	8.151428223	1
MSTRG.29017.13	239	0.33246	8.219421387	1
MSTRG.29017.11	2745	0.46801	5.350891113	1
MSTRG.29000.1	52	0.28085	11.61004639	1
MSTRG.29008.1	143	0.30092	8.586975098	1
MSTRG.29009.1	154	0.27678	6.41217041	1
MSTRG.29013.3	183	0.29367	10.831604	1
MSTRG.28996.1	79	0.36192	9.776062012	1
MSTRG.29020.2	1595	0.40707	9.751037598	1
MSTRG.29020.3	311	0.34908	6.137268066	-1
MSTRG.29021.1	134	0.28698	10.1651001	1
MSTRG.29022.1	27	0.41777	4.027648926	1
MSTRG.29019.1	26	0.38121	7.975524902	1
MSTRG.29024.2	123	0.28036	8.721252441	1
MSTRG.29024.4	123	0.28036	8.721252441	1
MSTRG.29028.3	2499	0.47075	5.889221191	1
MSTRG.29026.3	171	0.33531	5.484069824	1
MSTRG.29027.1	55	0.37842	10.02825928	1
MSTRG.29029.1	278	0.46455	5.327331543	1
MSTRG.29034.1	183	0.33031	9.465026855	1
MSTRG.29035.1	669	0.40235	5.359558105	1
MSTRG.29037.1	72	0.42248	11.46881104	1
MSTRG.29041.1	278	0.27433	5.872375488	1
MSTRG.29041.5	559	0.41098	8.57208252	1
MSTRG.29041.4	612	0.37692	8.208068848	1
MSTRG.29041.7	563	0.4334	8.656433105	1
MSTRG.29043.1	170	0.37965	8.807556152	1
MSTRG.29043.2	144	0.38295	8.621276855	1
MSTRG.29043.3	144	0.30898	8.621276855	1
MSTRG.29045.1	537	0.46669	7.211975098	1
MSTRG.29049.4	59	0.4022	5.34197998	1
MSTRG.29051.2	48	0.43789	6.892150879	1
MSTRG.29054.4	467	0.28165	6.133483887	1
MSTRG.29054.5	467	0.27937	6.133483887	1
MSTRG.29055.1	96	0.28165	6.472961426	1
MSTRG.29064.3	499	0.44803	6.885925293	1
MSTRG.29064.4	375	0.30721	7.044250488	1
MSTRG.29064.6	328	0.45512	9.699890137	1
MSTRG.29065.1	88	0.32799	6.569152832	1

MSTRG.29065.2	85	0.40951	11.65460205	1
MSTRG.29068.1	76	0.34519	9.405700684	1
MSTRG.29068.3	76	0.35913	9.405700684	1
MSTRG.29079.2	32	0.47297	4.129821777	1
MSTRG.29079.1	1828	0.41941	7.797424316	1
MSTRG.29079.4	69	0.32959	4.88079834	1
MSTRG.29079.3	1689	0.41941	8.456115723	1
MSTRG.29079.5	1820	0.41941	7.547302246	1
MSTRG.29080.1	154	0.33737	5.766662598	1
MSTRG.29086.2	511	0.48084	8.70501709	1
MSTRG.29086.3	445	0.45349	8.696228027	1
MSTRG.29082.3	610	0.45155	8.959289551	1
MSTRG.29082.4	619	0.45434	8.950256348	1
MSTRG.29083.1	82	0.35907	10.11505127	1
MSTRG.29085.1	250	0.48001	5.785339355	-1
MSTRG.29092.2	263	0.38698	9.93145752	1
MSTRG.29092.6	263	0.38264	9.93145752	1
MSTRG.29092.7	263	0.35673	9.93145752	1
MSTRG.29092.5	263	0.39202	9.93145752	1
MSTRG.29093.4	408	0.48588	5.562927246	1
MSTRG.29094.1	123	0.44496	10.80352783	-1
MSTRG.29099.2	372	0.35249	6.317199707	1
MSTRG.29101.1	224	0.30837	8.590637207	1
MSTRG.29101.2	73	0.29919	6.946838379	1
MSTRG.29107.1	44	0.28572	9.692443848	1
MSTRG.29108.1	79	0.40397	10.12921143	-1
MSTRG.29111.3	247	0.36937	11.35675049	1
MSTRG.29111.4	247	0.41591	11.35675049	1
MSTRG.29111.2	327	0.38323	11.16680908	1
MSTRG.29120.3	226	0.34647	9.401306152	1
MSTRG.29120.4	174	0.36805	9.081115723	1
MSTRG.29121.1	529	0.34952	8.34197998	1
MSTRG.29121.3	519	0.42553	8.046447754	1
MSTRG.29113.1	53	0.36833	9.880187988	1
MSTRG.29115.1	39	0.36153	6.530090332	1
MSTRG.29128.1	78	0.30824	9.138244629	1
MSTRG.29129.1	60	0.38066	11.28765869	1
MSTRG.29129.2	49	0.37367	5.464660645	1
MSTRG.29130.1	101	0.31109	10.43682861	1
MSTRG.29122.1	90	0.45907	6.068664551	-1
MSTRG.29126.1	20	0.43668	9.497619629	-1
MSTRG.29133.1	58	0.42613	9.817077637	1
MSTRG.29136.2	1454	0.42723	6.577819824	1
MSTRG.29136.5	1479	0.45087	6.638244629	1
MSTRG.29136.1	1454	0.4309	6.577819824	1
MSTRG.29137.1	87	0.43819	9.644836426	1
MSTRG.29155.4	750	0.44085	5.480285645	1
MSTRG.29155.6	750	0.43265	5.480285645	1
MSTRG.29155.1	834	0.33947	5.68182373	1
MSTRG.29155.8	834	0.33947	5.68182373	1
MSTRG.29155.11	582	0.39179	5.474914551	1
MSTRG.29155.15	370	0.37624	5.889099121	1
MSTRG.29155.16	834	0.33947	5.68182373	1
MSTRG.29155.17	834	0.38512	5.68182373	1

MSTRG.29155.19	370	0.46083	5.889099121	1
MSTRG.29157.1	338	0.35765	8.813049316	1
MSTRG.29155.22	345	0.46661	5.147644043	1
MSTRG.29160.1	92	0.35155	8.556945801	1
MSTRG.29161.1	50	0.34461	8.522399902	1
MSTRG.29142.1	125	0.34656	9.72442627	1
MSTRG.29143.1	58	0.41001	6.518615723	1
MSTRG.29144.3	475	0.33747	5.683898926	1
MSTRG.29149.2	1301	0.49349	8.972106934	1
MSTRG.29149.8	1159	0.46648	8.967102051	1
MSTRG.29149.9	265	0.35617	9.274841309	1
MSTRG.29149.10	244	0.41498	9.156921387	1
MSTRG.29149.11	250	0.39243	9.168029785	1
MSTRG.29150.1	515	0.37002	6.122619629	1
MSTRG.29150.2	518	0.37112	6.122619629	1
MSTRG.29150.3	518	0.39195	6.122619629	1
MSTRG.29150.4	496	0.37277	6.169006348	1
MSTRG.29150.5	518	0.4568	6.122619629	1
MSTRG.29150.6	515	0.36347	6.122619629	1
MSTRG.29150.7	518	0.41763	6.122619629	1
MSTRG.29150.8	518	0.4583	6.122619629	1
MSTRG.29165.3	237	0.4023	10.25323486	1
MSTRG.29162.1	88	0.40213	10.63983154	1
MSTRG.29162.2	98	0.30112	11.10479736	1
MSTRG.29163.1	20	0.39807	9.304016113	1
MSTRG.29172.1	19	0.34464	9.261535645	1
MSTRG.29191.1	171	0.36196	7.657653809	1
MSTRG.29192.1	81	0.34173	10.76495361	1
MSTRG.29195.1	135	0.33355	10.3180542	1
MSTRG.29195.2	95	0.32759	6.153503418	1
MSTRG.29195.3	44	0.32485	10.19195557	1
MSTRG.29195.4	44	0.35076	10.19195557	1
MSTRG.29201.1	6	0.46632	8.34576416	1
MSTRG.29202.4	593	0.32076	4.835876465	1
MSTRG.29202.6	438	0.41832	5.745544434	1
MSTRG.29202.7	438	0.34952	5.745544434	1
MSTRG.29202.8	282	0.46612	6.067687988	1
MSTRG.29202.9	129	0.30483	5.510437012	1
MSTRG.29196.1	83	0.38646	9.476867676	1
MSTRG.29198.1	1099	0.36649	6.47467041	1
MSTRG.29198.2	725	0.45713	6.026794434	-1
MSTRG.29198.4	310	0.38719	7.639099121	1
MSTRG.29207.2	1143	0.4638	5.49786377	1
MSTRG.29207.3	1111	0.47064	5.524230957	1
MSTRG.29208.1	43	0.39942	7.997741699	-1
MSTRG.29213.1	0	0.43827	0	-1
MSTRG.29214.1	155	0.30312	11.7288208	1
MSTRG.29215.1	66	0.35192	7.600524902	1
MSTRG.29219.1	64	0.41897	8.38092041	1
MSTRG.29231.1	65	0.32305	9.350402832	1
MSTRG.29231.2	172	0.30877	10.36273193	1
MSTRG.29229.16	72	0.37362	9.300598145	1
MSTRG.29232.1	57	0.32521	9.257019043	1
MSTRG.29232.2	38	0.29977	10.0223999	1

MSTRG.29221.2	61	0.35132	8.311340332	1
MSTRG.29224.2	355	0.39238	10.13421631	1
MSTRG.29226.2	142	0.42625	8.067443848	1
MSTRG.29226.3	142	0.42625	8.067443848	1
MSTRG.29233.3	285	0.39011	10.72808838	1
MSTRG.29238.2	205	0.45334	9.07623291	1
MSTRG.29239.3	636	0.43633	6.879577637	1
MSTRG.29240.1	117	0.39082	6.02130127	1
MSTRG.29242.2	684	0.43032	4.441833496	1
MSTRG.29242.3	653	0.4362	4.539489746	1
MSTRG.29242.5	507	0.44504	4.82208252	1
MSTRG.29242.6	462	0.42445	4.806945801	1
MSTRG.29246.3	581	0.36028	9.216125488	1
MSTRG.29257.4	103	0.37693	6.811218262	1
MSTRG.29257.2	148	0.34599	11.70819092	1
MSTRG.29258.1	149	0.36329	9.969299316	1
MSTRG.29258.2	149	0.40975	9.969299316	1
MSTRG.29258.4	149	0.43236	9.969299316	1
MSTRG.29258.5	149	0.42971	9.969299316	1
MSTRG.29258.6	99	0.32154	10.23321533	-1
MSTRG.29258.9	149	0.35698	9.969299316	1
MSTRG.29260.1	275	0.39727	9.976013184	1
MSTRG.29262.2	112	0.36555	8.220275879	1
MSTRG.29254.1	432	0.45066	6.42791748	1
MSTRG.29255.4	169	0.39024	6.129333496	1
MSTRG.29268.1	199	0.32206	10.87994385	1
MSTRG.29271.3	301	0.28401	10.68682861	1
MSTRG.29271.5	301	0.38919	10.68682861	1
MSTRG.29276.1	2345	0.41145	6.894104004	1
MSTRG.29276.3	2334	0.41145	6.841369629	1
MSTRG.29278.1	127	0.36829	10.88092041	1
MSTRG.29278.2	127	0.34237	10.88092041	1
MSTRG.29278.3	182	0.41362	8.540588379	1
MSTRG.29279.2	177	0.3924	4.695739746	1
MSTRG.29281.3	545	0.39219	8.180114746	1
MSTRG.29282.2	631	0.42076	5.395812988	1
MSTRG.29283.2	200	0.28331	11.77069092	1
MSTRG.29283.3	200	0.38929	11.64910889	1
MSTRG.29283.4	165	0.2756	9.486877441	1
MSTRG.29283.5	168	0.2756	10.25799561	1
MSTRG.29301.1	40	0.40795	4.192565918	1
MSTRG.29302.3	656	0.45791	6.185119629	1
MSTRG.29302.1	1457	0.4315	5.871520996	1
MSTRG.29305.2	1070	0.39248	7.282897949	1
MSTRG.29305.3	1256	0.37619	8.743591309	1
MSTRG.29307.3	183	0.30307	11.97467041	1
MSTRG.29308.4	1334	0.4379	5.289978027	1
MSTRG.29308.5	1075	0.40754	5.651306152	1
MSTRG.29308.6	819	0.43973	6.271057129	1
MSTRG.29308.7	819	0.44345	6.271057129	1
MSTRG.29293.2	293	0.43717	10.25030518	1
MSTRG.29298.1	388	0.41621	9.522766113	1
MSTRG.29325.2	781	0.41636	5.829162598	1
MSTRG.29328.7	136	0.39646	12.26800537	1

MSTRG.29328.9	305	0.37956	11.84637451	1
MSTRG.29328.8	305	0.34418	11.84637451	1
MSTRG.29333.2	1704	0.46388	6.771179199	1
MSTRG.29334.1	1777	0.46885	6.321838379	1
MSTRG.29334.5	119	0.28153	9.67364502	1
MSTRG.29341.3	173	0.41063	5.87298584	1
MSTRG.29342.1	321	0.28195	6.015930176	1
MSTRG.29342.2	553	0.31061	6.797058105	1
MSTRG.29343.1	141	0.29422	8.174255371	1
MSTRG.29342.4	530	0.27781	6.999206543	1
MSTRG.29356.21	1826	0.41236	12.04705811	1
MSTRG.29356.18	2727	0.4082	12.06695557	1
MSTRG.29356.9	2702	0.40114	12.08026123	1
MSTRG.29356.12	1956	0.41289	12.05340576	1
MSTRG.29356.13	2717	0.40114	12.05975342	1
MSTRG.29356.14	2702	0.40114	12.07855225	1
MSTRG.29356.16	2672	0.39087	12.05377197	1
MSTRG.29356.5	2709	0.40114	12.05084229	1
MSTRG.29356.6	2740	0.41137	12.07696533	1
MSTRG.29356.8	2709	0.41075	12.05084229	1
MSTRG.29356.15	1994	0.4302	12.04925537	1
MSTRG.29356.19	2730	0.40114	12.06842041	1
MSTRG.29356.22	1994	0.41597	12.04925537	1
MSTRG.29356.23	2739	0.40114	12.07525635	1
MSTRG.29356.24	2692	0.41522	12.07159424	1
MSTRG.29347.1	628	0.40564	7.167541504	1
MSTRG.29353.1	46	0.32687	6.006286621	1
MSTRG.29354.1	142	0.44759	4.450256348	1
MSTRG.29359.2	145	0.31993	4.437805176	1
MSTRG.29361.1	50	0.35473	4.93572998	1
MSTRG.29363.1	42	0.33569	4.651184082	1
MSTRG.29364.1	100	0.30552	5.715515137	1
MSTRG.29367.2	332	0.35238	9.33026123	1
MSTRG.29370.2	612	0.34947	6.744689941	1
MSTRG.29375.2	198	0.3013	6.538757324	1
MSTRG.29376.3	73	0.31493	11.47393799	1
MSTRG.29387.1	1754	0.43127	5.763977051	1
MSTRG.29387.6	1754	0.44761	5.763977051	1
MSTRG.29389.1	702	0.4338	6.660583496	1
MSTRG.29388.6	204	0.42086	4.85736084	1
MSTRG.29378.1	85	0.36277	6.882141113	1
MSTRG.29380.1	109	0.2785	8.787902832	1
MSTRG.29379.3	597	0.41828	8.982971191	1
MSTRG.29393.3	188	0.36374	10.25323486	1
MSTRG.29393.2	143	0.31879	11.25140381	1
MSTRG.29393.4	136	0.37889	10.31658936	1
MSTRG.29393.5	150	0.36432	10.41192627	1
MSTRG.29393.6	132	0.40754	8.320129395	1
MSTRG.29393.7	189	0.25528	11.05450439	1
MSTRG.29405.2	1011	0.44376	5.794250488	1
MSTRG.29409.2	153	0.43958	6.297546387	1
MSTRG.29409.3	153	0.42298	6.297546387	1
MSTRG.29411.1	155	0.29362	7.646911621	1
MSTRG.29412.1	1087	0.45748	5.917663574	1

MSTRG.29417.3	526	0.44643	5.84552002	1
MSTRG.29417.4	526	0.42718	5.84552002	1
MSTRG.29418.4	488	0.40597	4.760559082	1
MSTRG.29420.2	144	0.31389	4.580749512	1
MSTRG.29420.1	144	0.37085	4.580749512	1
MSTRG.29425.2	547	0.37894	9.333068848	1
MSTRG.29425.5	528	0.38256	9.228820801	1
MSTRG.29425.6	457	0.3721	8.577941895	1
MSTRG.29425.7	484	0.37918	9.008972168	1
MSTRG.29426.3	678	0.39756	9.55670166	1
MSTRG.29426.4	1006	0.42035	9.319274902	1
MSTRG.29426.5	558	0.40314	9.350158691	1
MSTRG.29426.6	1007	0.42035	9.319274902	1
MSTRG.29395.2	303	0.43107	8.391906738	1
MSTRG.29404.5	244	0.41099	5.042785645	1
MSTRG.29404.3	177	0.4882	5.328796387	1
MSTRG.29404.6	244	0.4589	5.042785645	1
MSTRG.29404.7	395	0.45542	4.896911621	1
MSTRG.29428.3	500	0.39291	7.931945801	1
MSTRG.29436.3	172	0.33265	7.004577637	1
MSTRG.29432.3	1137	0.49898	5.457946777	1
MSTRG.29445.1	78	0.31165	5.723815918	1
MSTRG.29447.1	180	0.28683	10.98162842	1
MSTRG.29447.2	180	0.33426	10.33795166	1
MSTRG.29449.1	79	0.34928	10.31097412	1
MSTRG.29450.1	55	0.32276	10.10870361	1
MSTRG.29447.6	137	0.29317	9.776672363	1
MSTRG.29447.7	654	0.45587	6.852111816	1
MSTRG.29440.2	217	0.46192	9.218933105	1
MSTRG.29455.4	939	0.41342	6.175476074	1
MSTRG.29455.6	880	0.439	5.930358887	1
MSTRG.29455.7	302	0.34996	5.03729248	1
MSTRG.29454.1	85	0.34243	5.037902832	1
MSTRG.29458.1	150	0.43202	11.82196045	1
MSTRG.29457.2	145	0.42823	11.17584229	1
MSTRG.29463.1	140	0.36194	9.008483887	1
MSTRG.29463.7	161	0.36706	5.992370605	1
MSTRG.29463.6	169	0.37314	6.008850098	1
MSTRG.29466.1	95	0.34722	11.34002686	1
MSTRG.29466.3	95	0.34908	11.34002686	1
MSTRG.29467.2	499	0.45186	5.416320801	1
MSTRG.29467.3	499	0.45969	5.416320801	1
MSTRG.29467.4	86	0.3486	8.625671387	1
MSTRG.29467.5	54	0.34567	9.890930176	1
MSTRG.29481.2	513	0.36035	5.488464355	1
MSTRG.29482.2	111	0.37506	9.620910645	1
MSTRG.29481.3	447	0.43483	6.019836426	1
MSTRG.29470.1	65	0.35006	5.160339355	1
MSTRG.29470.2	43	0.38267	9.819763184	1
MSTRG.29471.1	185	0.29476	9.635314941	1
MSTRG.29484.1	60	0.43219	8.040588379	1
MSTRG.29495.2	687	0.47383	7.82611084	1
MSTRG.29495.3	667	0.43564	8.640441895	1
MSTRG.29497.1	787	0.45759	5.251525879	1

MSTRG.29499.4	205	0.43964	5.44708252	1
MSTRG.29497.4	744	0.43164	5.467712402	1
MSTRG.29497.3	771	0.43164	5.309265137	1
MSTRG.29500.1	87	0.41476	7.683288574	1
MSTRG.29504.3	1732	0.44883	8.254333496	1
MSTRG.29504.4	1732	0.42833	8.254333496	1
MSTRG.29504.2	1732	0.44883	8.254333496	1
MSTRG.29506.3	109	0.27327	9.68572998	1
MSTRG.29502.2	174	0.45365	6.662414551	1
MSTRG.29502.3	82	0.41178	5.633850098	1
MSTRG.29502.4	96	0.32086	5.885437012	1
MSTRG.29509.1	1464	0.43946	8.164001465	1
MSTRG.29509.2	1204	0.43388	6.784851074	1
MSTRG.29509.3	1530	0.46854	7.692321777	1
MSTRG.29511.2	280	0.43571	8.410583496	1
MSTRG.29511.3	280	0.44957	8.410583496	1
MSTRG.29511.4	280	0.43881	8.410583496	1
MSTRG.29511.5	280	0.44142	8.410583496	1
MSTRG.29513.1	64	0.33247	8.797668457	1
MSTRG.29510.1	87	0.3869	10.01287842	-1
MSTRG.29512.1	90	0.41877	9.891784668	-1
MSTRG.29521.2	296	0.47312	9.679870605	1
MSTRG.29521.3	203	0.28584	9.379455566	1
MSTRG.29521.4	3646	0.47485	6.011291504	1
MSTRG.29524.1	69	0.30808	8.276550293	1
MSTRG.29517.1	532	0.36771	9.509460449	1
MSTRG.29525.1	217	0.43888	7.653869629	1
MSTRG.29525.3	179	0.36778	5.811340332	1
MSTRG.29525.4	201	0.46115	6.175598145	1
MSTRG.29526.1	86	0.39168	7.723205566	1
MSTRG.29532.5	1004	0.45426	8.837219238	1
MSTRG.29533.5	169	0.42604	6.992980957	-1
MSTRG.29533.6	169	0.45092	6.992980957	-1
MSTRG.29535.2	260	0.43209	6.173278809	1
MSTRG.29535.3	197	0.41018	8.850769043	1
MSTRG.29535.5	192	0.40899	5.023132324	1
MSTRG.29535.4	103	0.40812	7.750427246	1
MSTRG.29536.1	387	0.41307	7.555725098	1
MSTRG.29530.1	577	0.42585	7.862121582	1
MSTRG.29530.3	196	0.34471	5.840393066	1
MSTRG.29531.3	386	0.47452	5.489562988	1
MSTRG.29537.3	246	0.29367	7.665222168	1
MSTRG.29537.4	210	0.42752	9.738464355	1
MSTRG.29557.1	209	0.28982	6.840759277	1
MSTRG.29557.2	209	0.33159	6.840759277	1
MSTRG.29557.3	285	0.35209	9.220275879	1
MSTRG.29557.4	252	0.4037	9.192565918	1
MSTRG.29557.5	209	0.42839	6.840759277	1
MSTRG.29557.6	245	0.4037	9.312194824	1
MSTRG.29557.7	268	0.36032	9.288024902	1
MSTRG.29557.8	248	0.32453	9.244567871	1
MSTRG.29557.10	689	0.41794	9.158874512	1
MSTRG.29558.5	653	0.44441	8.523498535	1
MSTRG.29558.6	96	0.34052	8.771057129	1

MSTRG.29558.7	280	0.43007	8.031311035	1
MSTRG.29559.3	304	0.37405	5.04107666	1
MSTRG.29559.4	304	0.35989	5.04107666	1
MSTRG.29560.1	69	0.35435	7.866760254	1
MSTRG.29561.1	94	0.30595	9.001525879	1
MSTRG.29565.1	4069	0.47608	5.576599121	1
MSTRG.29566.1	89	0.31719	9.02935791	1
MSTRG.29566.2	119	0.35371	9.338806152	1
MSTRG.29567.1	91	0.3825	6.485168457	1
MSTRG.29566.3	117	0.33264	10.33673096	1
MSTRG.29544.1	125	0.33782	10.58660889	1
MSTRG.29543.2	1143	0.48462	6.354309082	1
MSTRG.29545.1	150	0.39099	9.21697998	1
MSTRG.29546.1	109	0.33149	9.555969238	1
MSTRG.29550.1	1667	0.50188	8.663635254	1
MSTRG.29550.2	1667	0.4612	8.663635254	1
MSTRG.29551.1	165	0.35371	9.973937988	1
MSTRG.29552.2	335	0.40813	6.113342285	1
MSTRG.29553.1	263	0.40996	9.337463379	1
MSTRG.29553.2	226	0.43642	7.77520752	1
MSTRG.29553.3	86	0.37648	7.822692871	1
MSTRG.29553.4	86	0.39169	7.822692871	1
MSTRG.29553.5	86	0.37648	7.822692871	1
MSTRG.29553.6	110	0.38113	10.53997803	1
MSTRG.29553.7	226	0.45029	7.77520752	1
MSTRG.29554.2	66	0.3926	10.55975342	1
MSTRG.29556.1	102	0.29507	6.942443848	1
MSTRG.29548.2	752	0.39128	6.128479004	1
MSTRG.29548.1	752	0.47444	6.128479004	1
MSTRG.29547.1	65	0.28572	5.17755127	1
MSTRG.29571.1	25	0.31424	12.30426025	1
MSTRG.29576.3	476	0.45719	8.562316895	1
MSTRG.29576.4	135	0.38494	7.710021973	1
MSTRG.29578.3	262	0.3058	8.89251709	1
MSTRG.29580.1	36	0.49343	11.16595459	1
MSTRG.29583.2	1347	0.3351	9.291442871	1
MSTRG.29583.4	1287	0.39788	9.132019043	1
MSTRG.29584.1	101	0.33622	5.658508301	1
MSTRG.29585.2	614	0.4666	5.290100098	1
MSTRG.29593.2	527	0.33959	8.903137207	1
MSTRG.29594.3	175	0.28559	9.202209473	1
MSTRG.29596.1	44	0.39623	5.968444824	1
MSTRG.29588.2	576	0.45775	9.258117676	1
MSTRG.29598.1	148	0.4096	4.666687012	1
MSTRG.29600.2	673	0.33443	6.59588623	1
MSTRG.29601.1	107	0.29187	6.424377441	1
MSTRG.29599.1	41	0.31305	5.961486816	-1
MSTRG.29599.2	33	0.39593	9.842956543	1
MSTRG.29603.3	1712	0.42375	9.66619873	1
MSTRG.29607.6	110	0.37543	9.061828613	1
MSTRG.29607.11	25	0.41606	12.30426025	1
MSTRG.29607.10	1696	0.41977	6.057556152	1
MSTRG.29607.5	1768	0.29387	6.221374512	1
MSTRG.29608.1	60	0.29374	10.18243408	1

MSTRG.29620.1	741	0.32858	7.496765137	1
MSTRG.29624.1	61	0.43141	9.292297363	1
MSTRG.29625.1	83	0.39938	9.36895752	1
MSTRG.29625.2	67	0.30378	10.42938232	1
MSTRG.29627.1	92	0.30804	9.198303223	1
MSTRG.29633.2	184	0.31772	6.27935791	1
MSTRG.29634.2	1801	0.4504	6.742980957	1
MSTRG.29635.2	1657	0.47884	5.16973877	1
MSTRG.29652.1	24	0.3798	7.999206543	-1
MSTRG.29654.1	683	0.39168	5.87677002	1
MSTRG.29654.4	670	0.31952	5.838195801	1
MSTRG.29654.2	670	0.32076	5.838195801	1
MSTRG.29654.5	683	0.39945	5.87677002	1
MSTRG.29657.1	1078	0.36761	8.700378418	1
MSTRG.29657.4	1047	0.48087	8.939758301	1
MSTRG.29657.9	959	0.47012	8.593566895	1
MSTRG.29657.11	912	0.46396	5.569519043	1
MSTRG.29644.1	343	0.44828	4.297912598	1
MSTRG.29647.2	178	0.28589	11.20794678	1
MSTRG.29647.3	178	0.31459	11.20794678	1
MSTRG.29663.2	44	0.43186	9.48651123	1
MSTRG.29664.2	261	0.40935	9.795837402	1
MSTRG.29666.5	445	0.46708	8.909729004	1
MSTRG.29667.1	244	0.4315	8.411804199	1
MSTRG.29667.2	244	0.42573	8.411804199	1
MSTRG.29667.3	244	0.41187	8.411804199	1
MSTRG.29667.4	244	0.36391	8.411804199	1
MSTRG.29668.3	271	0.38986	9.026672363	1
MSTRG.29672.2	771	0.39591	6.471862793	1
MSTRG.29673.1	254	0.30717	8.294372559	1
MSTRG.29673.3	239	0.36573	8.294372559	1
MSTRG.29658.7	120	0.42965	9.660827637	1
MSTRG.29658.5	90	0.35263	8.88873291	1
MSTRG.29675.2	399	0.40491	4.567687988	1
MSTRG.29675.5	386	0.4529	4.89276123	1
MSTRG.29676.2	114	0.38283	9.20123291	1
MSTRG.29676.1	99	0.37817	9.26385498	-1
MSTRG.29676.3	123	0.36208	11.32977295	1
MSTRG.29676.4	153	0.35928	11.06939697	1
MSTRG.29676.5	153	0.40386	11.06939697	1
MSTRG.29676.6	123	0.37596	11.32977295	1
MSTRG.29677.3	132	0.42122	4.352355957	1
MSTRG.29677.6	132	0.42076	4.352355957	1
MSTRG.29677.8	485	0.47377	9.122009277	1
MSTRG.29677.5	454	0.44912	9.36138916	1
MSTRG.29677.7	464	0.45837	9.315368652	1
MSTRG.29677.2	472	0.43778	9.315490723	1
MSTRG.29678.3	531	0.46214	9.488708496	1
MSTRG.29678.2	531	0.4556	9.488708496	1
MSTRG.29682.4	341	0.41426	4.417541504	1
MSTRG.29687.2	339	0.46244	5.321472168	1
MSTRG.29687.3	339	0.4675	5.321472168	1
MSTRG.29681.1	330	0.42965	5.564025879	1
MSTRG.29692.2	512	0.33597	8.385437012	1

MSTRG.29692.3	215	0.36677	10.0758667	1
MSTRG.29693.2	121	0.37519	8.779968262	1
MSTRG.29695.2	86	0.33099	10.34552002	1
MSTRG.29696.1	528	0.38901	10.51177979	1
MSTRG.29696.2	90	0.30825	10.07855225	1
MSTRG.29696.3	27	0.35203	3.916320801	-1
MSTRG.29704.3	3081	0.47441	5.447937012	1
MSTRG.29699.3	52	0.33908	11.69903564	1
MSTRG.29700.1	123	0.37483	9.85333252	1
MSTRG.29700.2	145	0.39724	10.49530029	1
MSTRG.29701.3	21	0.35833	9.992492676	-1
MSTRG.29722.1	100	0.33415	6.693908691	1
MSTRG.29722.2	47	0.36805	9.694396973	1
MSTRG.29723.1	136	0.41969	8.868835449	-1
MSTRG.29723.3	144	0.33547	6.273132324	1
MSTRG.29724.1	79	0.33491	10.55511475	1
MSTRG.29725.1	64	0.29956	8.528381348	1
MSTRG.29726.1	72	0.44871	8.803039551	1
MSTRG.29724.4	119	0.28989	11.26202393	1
MSTRG.29724.5	147	0.28396	7.054260254	1
MSTRG.29724.6	583	0.39888	9.430725098	1
MSTRG.29727.1	63	0.37002	6.687805176	1
MSTRG.29724.7	674	0.38396	9.41595459	1
MSTRG.29724.10	382	0.35532	9.659118652	1
MSTRG.29724.11	692	0.36546	9.371032715	1
MSTRG.29730.1	213	0.35729	6.581481934	1
MSTRG.29735.6	1682	0.38164	6.550476074	1
MSTRG.29707.1	412	0.44677	9.224304199	1
MSTRG.29710.1	53	0.33736	4.001647949	1
MSTRG.29713.2	131	0.32671	11.42071533	1
MSTRG.29715.3	331	0.45056	9.968566895	1
MSTRG.29738.1	462	0.44642	6.516540527	1
MSTRG.29738.2	462	0.4035	6.516540527	1
MSTRG.29738.3	462	0.45736	6.516540527	1
MSTRG.29739.1	443	0.47233	5.730163574	1
MSTRG.29741.2	448	0.37292	6.952697754	1
MSTRG.29747.1	281	0.45617	8.489685059	1
MSTRG.29748.1	66	0.3676	10.05462646	1
MSTRG.29755.2	575	0.46358	5.85723877	1
MSTRG.29756.5	106	0.36289	9.076599121	1
MSTRG.29757.1	218	0.4687	5.682067871	-1
MSTRG.29759.1	213	0.34161	6.66998291	1
MSTRG.29758.1	370	0.39604	9.196228027	1
MSTRG.29763.1	783	0.41864	6.184265137	1
MSTRG.29763.4	282	0.37223	7.135803223	1
MSTRG.29767.1	250	0.38307	8.90826416	1
MSTRG.29767.2	250	0.48087	8.90826416	1
MSTRG.29767.3	250	0.47796	8.90826416	1
MSTRG.29767.4	250	0.46059	8.90826416	1
MSTRG.29767.5	265	0.43833	8.956726074	1
MSTRG.29773.2	175	0.28567	8.917297363	1
MSTRG.29774.1	127	0.35453	4.378723145	1
MSTRG.29774.4	219	0.27651	4.760437012	1
MSTRG.29774.10	186	0.29607	4.799499512	1

MSTRG.29774.9	219	0.27651	4.760437012	1
MSTRG.29774.7	219	0.29961	4.760437012	1
MSTRG.29774.12	186	0.28567	4.799499512	1
MSTRG.29774.13	261	0.27651	4.784118652	1
MSTRG.29774.15	219	0.27651	4.760437012	1
MSTRG.29776.1	129	0.31849	10.03326416	1
MSTRG.29769.2	367	0.42128	7.611999512	1
MSTRG.29849.2	1293	0.44759	9.694763184	1
MSTRG.29849.4	1277	0.44294	9.678161621	1
MSTRG.29849.3	1278	0.41161	9.702819824	1
MSTRG.29851.1	181	0.30981	9.924987793	1
MSTRG.29852.1	170	0.27327	9.556945801	1
MSTRG.29853.1	137	0.27575	10.38739014	1
MSTRG.29854.1	69	0.35907	7.979309082	1
MSTRG.29856.1	113	0.35121	9.406799316	1
MSTRG.29857.1	130	0.28982	6.683898926	1
MSTRG.29858.1	160	0.2921	10.29205322	1
MSTRG.29860.1	49	0.3	9.200134277	1
MSTRG.29861.1	333	0.34162	9.633117676	1
MSTRG.29861.2	333	0.34063	9.633117676	1
MSTRG.29861.3	333	0.36345	9.633117676	1
MSTRG.29849.5	183	0.28883	11.19085693	1
MSTRG.29849.6	108	0.28883	9.812927246	1
MSTRG.29849.7	107	0.28883	9.885925293	1
MSTRG.29849.8	98	0.28883	7.582702637	1
MSTRG.29849.9	98	0.28883	7.582702637	1
MSTRG.29849.10	84	0.29534	10.8793335	1
MSTRG.29791.15	419	0.40755	9.517272949	1
MSTRG.29791.16	531	0.44317	9.118347168	1
MSTRG.29791.7	531	0.43816	9.118347168	1
MSTRG.29791.8	887	0.48083	6.690124512	1
MSTRG.29791.13	999	0.43793	6.08782959	1
MSTRG.29791.10	958	0.38223	8.066711426	1
MSTRG.29791.11	979	0.45009	6.379455566	1
MSTRG.29791.9	979	0.47313	6.379455566	1
MSTRG.29791.17	282	0.34367	4.894714355	1
MSTRG.29791.18	258	0.41501	5.096252441	1
MSTRG.29792.1	64	0.32617	11.53192139	1
MSTRG.29786.2	926	0.44983	6.649719238	1
MSTRG.29787.1	658	0.47632	6.12677002	1
MSTRG.29787.3	623	0.47384	6.219909668	1
MSTRG.29795.2	243	0.42313	9.500427246	1
MSTRG.29796.1	39	0.36849	10.28900146	1
MSTRG.29797.3	245	0.42666	5.335754395	1
MSTRG.29799.1	258	0.3579	4.838439941	1
MSTRG.29799.2	453	0.45615	5.305847168	1
MSTRG.29799.3	162	0.35587	11.39984131	1
MSTRG.29810.3	317	0.35965	8.899230957	1
MSTRG.29810.4	351	0.39091	8.934997559	1
MSTRG.29810.7	317	0.35965	8.899230957	1
MSTRG.29810.8	317	0.37413	8.899230957	1
MSTRG.29810.6	153	0.28195	7.724060059	1
MSTRG.29810.2	351	0.3277	8.934997559	1
MSTRG.29810.9	418	0.33106	9.459533691	1

MSTRG.29800.5	127	0.33006	9.568054199	1
MSTRG.29800.6	90	0.3849	6.799499512	-1
MSTRG.29801.3	74	0.34721	4.595031738	1
MSTRG.29802.1	141	0.31021	10.29022217	1
MSTRG.29815.4	1216	0.34996	10.53582764	1
MSTRG.29815.2	977	0.34123	10.019104	1
MSTRG.29815.5	1209	0.37306	10.53582764	1
MSTRG.29815.6	196	0.32429	8.97052002	1
MSTRG.29821.4	223	0.33527	9.55645752	1
MSTRG.29821.5	116	0.41456	10.06280518	1
MSTRG.29825.2	217	0.45783	6.649108887	1
MSTRG.29825.3	177	0.38415	7.718200684	1
MSTRG.29827.1	386	0.28084	12.21710205	1
MSTRG.29830.1	247	0.37428	4.800598145	1
MSTRG.29830.2	247	0.4387	4.800598145	1
MSTRG.29834.1	720	0.43202	7.547302246	1
MSTRG.29835.2	30	0.45403	4.749816895	1
MSTRG.29836.2	307	0.44347	6.374084473	1
MSTRG.29846.1	689	0.47174	5.27911377	1
MSTRG.29846.8	1443	0.37406	5.585754395	1
MSTRG.29846.7	1546	0.37406	5.793395996	1
MSTRG.29846.9	1034	0.35108	6.209655762	1
MSTRG.29846.10	363	0.45807	5.75567627	-1
MSTRG.29846.11	1292	0.44945	5.66973877	-1
MSTRG.29846.12	201	0.2785	8.783752441	1
MSTRG.29846.13	139	0.27327	10.55511475	1
MSTRG.29846.14	112	0.27327	8.834777832	1
MSTRG.29847.1	125	0.27526	5.581970215	1
MSTRG.29845.1	147	0.45814	4.61907959	1
MSTRG.29868.1	247	0.32565	6.320983887	1
MSTRG.29869.1	155	0.36279	5.328674316	1
MSTRG.29869.3	51	0.29944	7.770568848	1
MSTRG.29869.5	163	0.28247	9.00177002	1
MSTRG.29869.4	97	0.33205	8.302062988	1
MSTRG.29869.6	163	0.28283	9.00177002	1
MSTRG.29869.7	136	0.30961	8.704528809	1
MSTRG.29866.2	41	0.45437	4.812683105	-1
MSTRG.29867.1	223	0.44954	8.717712402	1
MSTRG.29867.2	108	0.35497	11.25714111	1
MSTRG.29879.4	527	0.41901	7.082702637	1
MSTRG.29879.6	365	0.37612	5.57611084	1
MSTRG.29879.7	525	0.44014	6.238342285	1
MSTRG.29879.3	613	0.43244	5.776794434	1
MSTRG.29880.2	645	0.44659	5.349182129	1
MSTRG.29880.3	700	0.41801	5.691833496	1
MSTRG.29881.2	352	0.40599	6.100280762	1
MSTRG.29884.1	140	0.41512	9.094177246	1
MSTRG.29884.2	140	0.40892	9.094177246	1
MSTRG.29884.3	167	0.41488	9.855773926	1
MSTRG.29885.3	733	0.40575	5.269470215	1
MSTRG.29890.4	221	0.41774	6.647521973	1
MSTRG.29891.1	100	0.42704	9.765441895	1
MSTRG.29891.2	117	0.41714	11.92645264	1
MSTRG.29891.3	569	0.38433	8.872131348	1

MSTRG.29891.4	546	0.38371	8.976379395	1
MSTRG.29891.6	569	0.3867	9.236022949	1
MSTRG.29891.7	319	0.38409	6.587219238	1
MSTRG.29894.2	635	0.44653	6.15447998	1
MSTRG.29894.3	227	0.40196	11.86065674	1
MSTRG.29894.4	143	0.44134	8.863464355	1
MSTRG.29896.7	206	0.32466	6.195251465	1
MSTRG.29896.6	142	0.32937	9.992492676	1
MSTRG.29896.4	488	0.3502	9.180969238	1
MSTRG.29896.5	430	0.3638	9.282409668	1
MSTRG.29897.1	62	0.39584	8.711486816	1
MSTRG.29897.2	62	0.33265	8.711486816	1
MSTRG.29897.3	62	0.38627	8.711486816	1
MSTRG.29889.2	204	0.44596	10.8057251	1
MSTRG.29900.1	633	0.45744	5.245422363	1
MSTRG.29903.3	1245	0.46303	5.58001709	1
MSTRG.29903.1	1421	0.43473	6.184265137	1
MSTRG.29904.1	578	0.40236	8.324401855	1
MSTRG.29902.1	99	0.35398	5.58013916	1
MSTRG.29902.2	99	0.4252	5.58013916	1
MSTRG.29902.3	48	0.41251	10.08197021	1
MSTRG.29910.3	213	0.40845	4.841247559	1
MSTRG.29910.2	121	0.35222	4.312194824	1
MSTRG.29912.2	238	0.39141	5.075500488	1
MSTRG.29912.3	227	0.38647	9.020324707	1
MSTRG.29912.4	227	0.37654	9.020324707	1
MSTRG.29912.5	226	0.33049	11.12347412	1
MSTRG.29912.6	286	0.39136	5.06060791	1
MSTRG.29906.2	471	0.35564	8.169616699	1
MSTRG.29906.4	471	0.37797	8.169616699	1
MSTRG.29908.4	490	0.28567	6.79095459	1
MSTRG.29916.1	61	0.28852	9.741271973	1
MSTRG.29917.1	390	0.47409	9.162658691	1
MSTRG.29917.3	390	0.46433	9.162658691	1
MSTRG.29918.1	17	0.40306	9.349304199	1
MSTRG.29920.1	546	0.29054	7.824645996	1
MSTRG.29923.5	226	0.44494	6.084655762	1
MSTRG.29923.6	180	0.37559	5.61505127	1
MSTRG.29921.1	571	0.44921	6.140075684	1
MSTRG.29921.4	402	0.39917	8.887634277	1
MSTRG.29921.5	324	0.37957	9.18939209	1
MSTRG.29926.3	579	0.33379	9.180603027	1
MSTRG.29926.2	579	0.33379	9.180603027	1
MSTRG.29927.3	523	0.34431	9.108947754	1
MSTRG.29934.4	144	0.36255	9.096496582	1
MSTRG.29934.5	144	0.39975	9.096496582	1
MSTRG.29934.8	144	0.37036	9.096496582	1
MSTRG.29941.3	731	0.42856	6.726623535	1
MSTRG.29941.4	728	0.42611	6.726623535	1
MSTRG.29943.5	428	0.4505	9.550476074	1
MSTRG.29943.3	3245	0.43267	8.539001465	1
MSTRG.29943.2	3499	0.4429	8.514953613	1
MSTRG.29943.4	3510	0.4429	8.53302002	1
MSTRG.29938.1	44	0.46372	6.699523926	1

MSTRG.29946.2	150	0.40619	10.51373291	1
MSTRG.29947.1	52	0.40085	5.17388916	1
MSTRG.29949.1	136	0.36439	11.44891357	1
MSTRG.29948.2	124	0.32195	10.78363037	1
MSTRG.29950.4	1087	0.37592	4.413513184	1
MSTRG.29950.5	1455	0.34927	4.493347168	1
MSTRG.29950.6	1263	0.35037	4.460510254	1
MSTRG.29965.1	871	0.43712	8.114685059	1
MSTRG.29965.5	871	0.4691	8.114685059	1
MSTRG.29967.2	242	0.45809	5.947570801	1
MSTRG.29970.1	1325	0.3778	8.729187012	1
MSTRG.29970.2	1325	0.40078	8.729187012	1
MSTRG.29970.3	1325	0.42849	8.729187012	1
MSTRG.29970.4	1318	0.40098	8.695373535	1
MSTRG.29970.5	1318	0.43533	8.695373535	1
MSTRG.29970.7	1318	0.44836	8.695373535	1
MSTRG.29970.8	1325	0.43114	8.729187012	1
MSTRG.29970.9	1030	0.43822	9.084655762	1
MSTRG.29974.3	351	0.40679	6.371398926	1
MSTRG.29974.2	400	0.39001	5.346618652	1
MSTRG.29975.1	84	0.27459	8.166564941	1
MSTRG.29954.1	13	0.38678	11.00030518	1
MSTRG.29955.1	198	0.27957	6.523986816	1
MSTRG.29957.2	669	0.43389	6.025085449	1
MSTRG.29961.2	88	0.30692	10.02972412	1
MSTRG.29980.5	119	0.31578	10.01446533	1
MSTRG.29978.3	331	0.35756	6.751525879	1
MSTRG.29978.1	108	0.3331	9.265930176	1
MSTRG.29978.2	31	0.29892	9.237976074	1
MSTRG.29985.3	869	0.39443	4.947692871	1
MSTRG.29985.4	889	0.36992	5.001647949	1
MSTRG.29990.1	2916	0.44278	8.591369629	1
MSTRG.29990.2	2865	0.44278	8.650695801	1
MSTRG.29990.5	1845	0.43756	8.315612793	1
MSTRG.29990.6	2874	0.44278	8.625305176	1
MSTRG.29990.7	2814	0.45664	8.887023926	1
MSTRG.29990.8	2917	0.43264	8.903747559	1
MSTRG.29990.9	2356	0.4428	8.799743652	1
MSTRG.29990.10	1209	0.44588	6.72076416	1
MSTRG.29991.1	109	0.32814	8.551818848	1
MSTRG.29991.2	86	0.32287	12.69952393	1
MSTRG.29997.1	588	0.36237	9.464294434	1
MSTRG.29997.2	588	0.39275	9.464294434	1
MSTRG.30003.7	456	0.44626	7.13104248	1
MSTRG.30003.2	1077	0.44626	6.052307129	1
MSTRG.30003.5	1678	0.435	5.614196777	1
MSTRG.30003.4	1922	0.44842	5.67755127	1
MSTRG.30003.3	1922	0.435	5.67755127	1
MSTRG.30003.6	1922	0.44058	5.67755127	1
MSTRG.30003.8	1064	0.44935	6.842834473	1
MSTRG.30003.9	87	0.39823	11.42340088	1
MSTRG.29998.1	42	0.31589	10.80657959	1
MSTRG.29999.1	111	0.3239	8.661560059	1
MSTRG.30009.10	591	0.44007	9.621765137	-1

MSTRG.30009.11	586	0.46011	8.257141113	1
MSTRG.30009.6	620	0.45241	8.238830566	1
MSTRG.30009.7	610	0.42336	7.131896973	1
MSTRG.30009.15	676	0.38037	8.403625488	1
MSTRG.30009.8	621	0.38998	8.238830566	1
MSTRG.30009.17	588	0.42024	9.65814209	-1
MSTRG.30009.18	652	0.46011	9.663635254	-1
MSTRG.30009.19	556	0.37789	6.805969238	1
MSTRG.30009.20	587	0.42024	9.621765137	-1
MSTRG.30009.21	664	0.46173	8.385437012	-1
MSTRG.30009.22	554	0.45084	6.805969238	-1
MSTRG.30009.23	687	0.46011	9.610656738	-1
MSTRG.30009.24	268	0.40411	5.941833496	1
MSTRG.30005.3	543	0.36886	5.294616699	1
MSTRG.30010.3	444	0.39518	9.467346191	1
MSTRG.30010.1	734	0.44693	9.352111816	1
MSTRG.30010.2	716	0.44997	9.268493652	1
MSTRG.30011.2	542	0.42704	6.422912598	1
MSTRG.30016.2	436	0.40969	8.750915527	1
MSTRG.30016.3	463	0.40504	9.23260498	1
MSTRG.30016.5	411	0.43958	9.07611084	1
MSTRG.30016.4	411	0.41982	9.07611084	1
MSTRG.30018.1	87	0.30078	9.585021973	-1
MSTRG.30020.2	90	0.27403	9.690246582	1
MSTRG.30020.3	90	0.3077	9.690246582	1
MSTRG.30013.1	100	0.30805	11.42144775	1
MSTRG.30014.1	35	0.34527	9.538269043	1
MSTRG.30014.2	46	0.29353	12.30108643	1
MSTRG.30014.3	46	0.2953	12.30108643	1
MSTRG.30014.4	35	0.36639	9.538269043	1
MSTRG.30024.3	144	0.44814	8.825134277	1
MSTRG.30033.1	46	0.30488	9.840026855	1
MSTRG.30035.2	354	0.37852	9.074523926	1
MSTRG.30036.2	331	0.35337	8.871276855	1
MSTRG.30036.3	439	0.40194	8.929626465	1
MSTRG.30036.4	618	0.3638	12.18341064	1
MSTRG.30054.11	729	0.36806	6.186828613	1
MSTRG.30054.13	751	0.34756	6.187683105	1
MSTRG.30054.2	729	0.36994	6.186828613	1
MSTRG.30054.7	679	0.37146	6.484802246	1
MSTRG.30054.8	679	0.39612	6.484802246	1
MSTRG.30054.17	740	0.36994	6.741516113	1
MSTRG.30054.19	382	0.44047	6.352111816	1
MSTRG.30054.21	509	0.44684	6.879333496	-1
MSTRG.30054.20	604	0.38404	8.232727051	-1
MSTRG.30054.18	559	0.37645	6.370788574	-1
MSTRG.30040.2	381	0.42182	5.373352051	1
MSTRG.30040.3	137	0.33039	5.140930176	1
MSTRG.30039.1	196	0.3871	8.534851074	1
MSTRG.30046.1	15	0.32159	11.06878662	1
MSTRG.30046.2	60	0.2968	11.63250732	1
MSTRG.30049.3	359	0.39062	5.242736816	1
MSTRG.30049.2	359	0.40457	5.242736816	1
MSTRG.30055.1	52	0.449	10.75018311	1

MSTRG.30064.5	111	0.44822	9.011779785	1
MSTRG.30064.6	65	0.43816	7.755310059	1
MSTRG.30068.1	42	0.33027	5.33392334	1
MSTRG.30068.2	32	0.42169	5.971618652	-1
MSTRG.30087.1	71	0.46457	6.89263916	1
MSTRG.30091.1	107	0.40574	10.13885498	-1
MSTRG.30114.1	612	0.43705	6.785339355	1
MSTRG.30114.3	602	0.42044	6.785339355	1
MSTRG.30102.1	238	0.43856	6.165588379	1
MSTRG.30102.2	629	0.46892	5.707092285	1
MSTRG.30102.3	144	0.38359	6.507019043	1
MSTRG.30126.5	426	0.29022	8.80279541	1
MSTRG.30127.1	114	0.3407	9.504211426	1
MSTRG.30128.1	151	0.3216	9.840881348	1
MSTRG.30129.1	270	0.3	8.106262207	1
MSTRG.30130.1	179	0.29754	9.565612793	1
MSTRG.30132.1	67	0.37535	6.80279541	1
MSTRG.30121.2	344	0.45647	6.281188965	1
MSTRG.30122.3	45	0.38407	4.779968262	1
MSTRG.30124.1	56	0.34419	7.655212402	1
MSTRG.30123.2	206	0.28807	4.804992676	1
MSTRG.30138.1	247	0.45175	6.232727051	1
MSTRG.30143.1	118	0.36681	9.460021973	1
MSTRG.30133.1	28	0.38871	8.495788574	1
MSTRG.30137.1	255	0.4663	6.211120605	1
MSTRG.30165.3	27	0.36499	10.94000244	1
MSTRG.30164.1	77	0.32417	6.477600098	1
MSTRG.30164.2	77	0.3088	6.477600098	1
MSTRG.30175.1	109	0.27398	6.161437988	1
MSTRG.30176.1	75	0.27433	6.22869873	1
MSTRG.30177.1	435	0.41072	9.055969238	1
MSTRG.30177.4	235	0.3795	9.433410645	1
MSTRG.30178.1	162	0.27794	8.363830566	1
MSTRG.30177.5	95	0.31113	8.768249512	1
MSTRG.30177.6	97	0.3285	6.478088379	1
MSTRG.30171.6	256	0.2921	6.327209473	1
MSTRG.30174.1	30	0.38415	3.912780762	1
MSTRG.30182.1	370	0.46104	8.358947754	1
MSTRG.30186.3	236	0.33267	8.645935059	1
MSTRG.30190.2	1940	0.47653	6.324157715	1
MSTRG.30198.1	683	0.33531	6.290100098	1
MSTRG.30198.3	504	0.43402	6.004699707	1
MSTRG.30198.4	683	0.42166	6.290100098	1
MSTRG.30199.2	170	0.30804	9.621643066	1
MSTRG.30199.3	123	0.3	7.504821777	1
MSTRG.30204.1	130	0.28096	9.793029785	1
MSTRG.30204.2	130	0.28096	9.793029785	1
MSTRG.30189.2	165	0.38082	6.166320801	1
MSTRG.30211.1	63	0.32761	9.347106934	1
MSTRG.30212.3	311	0.48189	6.535217285	1
MSTRG.30212.4	708	0.33194	5.527404785	1
MSTRG.30213.1	83	0.33826	7.749816895	1
MSTRG.30214.1	187	0.26101	10.12371826	1
MSTRG.30215.2	1738	0.46382	5.566833496	1

MSTRG.30215.4	300	0.46077	8.678894043	1
MSTRG.30216.1	62	0.46479	10.53375244	-1
MSTRG.30217.2	463	0.47809	6.870300293	1
MSTRG.30219.1	693	0.45935	5.528991699	1
MSTRG.30227.1	50	0.43779	5.644226074	1
MSTRG.30227.2	69	0.3458	10.909729	1
MSTRG.30240.2	805	0.40793	6.932189941	1
MSTRG.30241.1	95	0.30656	5.401184082	1
MSTRG.30245.1	80	0.28385	10.60577393	1
MSTRG.30235.1	334	0.48907	5.606262207	1
MSTRG.30239.1	137	0.28099	9.158752441	1
MSTRG.30248.3	421	0.46868	6.513000488	1
MSTRG.30248.2	340	0.42152	6.270935059	1
MSTRG.30248.4	57	0.42619	5.022888184	-1
MSTRG.30248.5	53	0.46408	4.351989746	-1
MSTRG.30248.7	254	0.40701	6.148620605	1
MSTRG.30248.9	340	0.40054	6.270935059	1
MSTRG.30248.8	340	0.37733	6.270935059	1
MSTRG.30249.2	862	0.37798	7.017028809	1
MSTRG.30258.2	1275	0.46325	5.872131348	1
MSTRG.30258.3	477	0.43883	5.943054199	1
MSTRG.30258.4	779	0.34302	5.261779785	1
MSTRG.30260.1	90	0.40463	9.386291504	1
MSTRG.30258.5	239	0.36837	9.680725098	1
MSTRG.30252.5	1482	0.438	9.336608887	1
MSTRG.30271.2	545	0.33364	7.465393066	1
MSTRG.30271.3	545	0.34692	7.465393066	1
MSTRG.30272.1	74	0.34928	5.398742676	1
MSTRG.30275.2	1552	0.3823	6.250427246	1
MSTRG.30275.6	433	0.3439	9.393249512	1
MSTRG.30275.7	1552	0.35312	6.250427246	1
MSTRG.30275.8	569	0.47198	5.973083496	-1
MSTRG.30275.9	93	0.30952	8.073425293	1
MSTRG.30275.12	107	0.33496	6.005187988	1
MSTRG.30278.1	122	0.2921	10.20465088	1
MSTRG.30264.3	483	0.33266	9.451477051	1
MSTRG.30265.1	89	0.44532	4.704284668	1
MSTRG.30281.1	361	0.34261	9.463806152	1
MSTRG.30281.2	377	0.32791	9.56036377	1
MSTRG.30282.2	871	0.49344	5.68548584	1
MSTRG.30282.3	871	0.47101	5.68548584	1
MSTRG.30287.2	227	0.38052	7.690002441	1
MSTRG.30287.4	186	0.40692	5.082214355	1
MSTRG.30288.2	123	0.44973	9.631164551	1
MSTRG.30290.1	236	0.37705	6.704162598	1
MSTRG.30291.5	146	0.3567	7.90447998	1
MSTRG.30291.4	114	0.35507	6.179016113	1
MSTRG.30291.6	114	0.37002	6.179016113	1
MSTRG.30283.1	26	0.3596	11.52288818	1
MSTRG.30302.5	379	0.4382	4.788757324	-1
MSTRG.30315.3	193	0.38647	9.230773926	1
MSTRG.30315.2	193	0.33163	6.581604004	1
MSTRG.30315.4	176	0.42703	9.480895996	1
MSTRG.30315.5	176	0.40993	9.480895996	1

MSTRG.30320.1	373	0.34103	9.769958496	1
MSTRG.30321.3	208	0.39939	8.260192871	1
MSTRG.30321.4	246	0.45849	5.855651855	1
MSTRG.30321.5	256	0.44264	5.855407715	1
MSTRG.30321.6	325	0.45955	6.33795166	1
MSTRG.30321.7	198	0.45808	8.33392334	1
MSTRG.30312.1	44	0.45015	9.696350098	1
MSTRG.30313.1	181	0.31345	10.1651001	1
MSTRG.30325.2	737	0.4307	7.292419434	1
MSTRG.30326.1	68	0.3708	9.983337402	1
MSTRG.30326.2	50	0.3972	6.711608887	1
MSTRG.30322.3	59	0.46869	6.181213379	1
MSTRG.30329.2	310	0.35595	9.459411621	1
MSTRG.30331.2	1058	0.45737	8.288024902	1
MSTRG.30332.2	151	0.41445	4.816101074	1
MSTRG.30332.3	166	0.38282	5.071472168	1
MSTRG.30343.1	1103	0.41888	8.88067627	1
MSTRG.30343.6	92	0.28385	10.44683838	1
MSTRG.30343.4	817	0.41755	6.440856934	1
MSTRG.30344.1	844	0.41229	8.104675293	1
MSTRG.30345.1	28	0.35049	8.519348145	1
MSTRG.30348.1	53	0.40179	7.730041504	1
MSTRG.30350.5	99	0.40777	7.921936035	1
MSTRG.30354.2	253	0.38854	8.908508301	1
MSTRG.30357.2	666	0.40108	4.734191895	1
MSTRG.30359.5	113	0.35639	5.883972168	-1
MSTRG.30359.6	620	0.49141	6.135192871	-1
MSTRG.30361.7	298	0.32269	9.073547363	1
MSTRG.30361.2	298	0.32455	9.073547363	1
MSTRG.30361.9	300	0.39827	9.063293457	1
MSTRG.30361.11	298	0.37046	9.073547363	1
MSTRG.30361.5	300	0.28305	9.063293457	1
MSTRG.30361.4	298	0.32411	9.073547363	1
MSTRG.30361.14	334	0.32848	9.069641113	1
MSTRG.30361.15	334	0.39827	9.069641113	1
MSTRG.30361.12	336	0.41367	9.061462402	1
MSTRG.30361.6	334	0.32759	9.069641113	1
MSTRG.30361.16	300	0.30615	9.063293457	1
MSTRG.30362.5	1619	0.47838	5.922424316	1
MSTRG.30363.8	1643	0.4473	5.554626465	1
MSTRG.30363.3	1890	0.46517	5.59161377	1
MSTRG.30363.5	1857	0.46517	5.551330566	1
MSTRG.30363.6	1858	0.46517	5.551330566	1
MSTRG.30363.2	1895	0.46269	5.573913574	1
MSTRG.30363.4	1861	0.46269	5.533874512	1
MSTRG.30363.7	1862	0.46269	5.533874512	1
MSTRG.30365.1	393	0.42288	9.982116699	1
MSTRG.30367.1	300	0.4464	6.752746582	1
MSTRG.30367.4	97	0.36263	10.5668335	1
MSTRG.30368.7	265	0.44245	4.909484863	1
MSTRG.30368.4	283	0.46767	4.831237793	1
MSTRG.30368.9	283	0.4496	4.831237793	1
MSTRG.30368.10	283	0.48042	4.831237793	1
MSTRG.30375.1	517	0.3945	8.210754395	1

MSTRG.30375.5	431	0.46235	8.312194824	1
MSTRG.30375.6	283	0.41137	7.10760498	1
MSTRG.30375.9	431	0.39616	8.312194824	1
MSTRG.30375.7	517	0.45465	8.210754395	1
MSTRG.30375.8	517	0.43171	8.210754395	1
MSTRG.30375.12	283	0.371	7.10760498	1
MSTRG.30375.11	517	0.37854	8.210754395	1
MSTRG.30375.14	431	0.43463	8.312194824	1
MSTRG.30375.15	431	0.42989	8.312194824	1
MSTRG.30378.1	308	0.37174	5.081726074	1
MSTRG.30371.1	187	0.3577	10.38824463	1
MSTRG.30371.2	85	0.43209	9.199890137	1
MSTRG.30372.1	52	0.33491	10.28448486	1
MSTRG.30382.2	47	0.38792	8.990783691	1
MSTRG.30386.3	249	0.34907	6.196350098	1
MSTRG.30388.1	34	0.36387	12.00286865	1
MSTRG.30386.5	424	0.46974	8.495666504	1
MSTRG.30386.6	386	0.47077	9.467224121	1
MSTRG.30386.8	377	0.45849	5.592224121	1
MSTRG.30393.2	560	0.42722	5.812072754	1
MSTRG.30393.8	369	0.45589	5.938659668	1
MSTRG.30393.5	568	0.46963	5.946105957	1
MSTRG.30393.9	369	0.43074	5.938659668	1
MSTRG.30393.7	560	0.47335	5.812072754	1
MSTRG.30393.3	369	0.41958	5.938659668	1
MSTRG.30394.1	171	0.30268	9.93572998	1
MSTRG.30395.1	113	0.2921	10.23260498	1
MSTRG.30398.2	58	0.39018	9.844299316	1
MSTRG.30398.3	58	0.37309	9.844299316	1
MSTRG.30399.3	1873	0.44112	9.088928223	1
MSTRG.30403.3	968	0.37339	9.022277832	1
MSTRG.30403.4	968	0.37339	9.022277832	1
MSTRG.30403.5	968	0.46079	9.022277832	1
MSTRG.30403.6	874	0.37489	9.137023926	1
MSTRG.30404.1	26	0.31294	8.401550293	1
MSTRG.30416.1	95	0.35889	11.246521	1
MSTRG.30419.5	147	0.37291	7.725036621	1
MSTRG.30409.1	241	0.44073	7.551330566	1
MSTRG.30409.2	213	0.4661	6.340759277	1
MSTRG.30410.1	34	0.35846	5.874450684	1
MSTRG.30411.3	244	0.37887	7.489196777	1
MSTRG.30411.4	244	0.35597	7.489196777	1
MSTRG.30411.2	244	0.34178	7.489196777	1
MSTRG.30411.6	244	0.33958	7.489196777	1
MSTRG.30411.5	244	0.32667	7.489196777	1
MSTRG.30408.1	123	0.32013	11.73895264	1
MSTRG.30436.1	162	0.31769	9.258239746	1
MSTRG.30437.1	88	0.32261	6.511047363	1
MSTRG.30438.1	956	0.4213	5.504455566	1
MSTRG.30439.1	266	0.29088	8.973205566	1
MSTRG.30442.1	831	0.38414	5.10369873	1
MSTRG.30442.2	151	0.4486	11.78155518	1
MSTRG.30442.3	123	0.40725	3.895812988	1
MSTRG.30444.2	265	0.3908	9.159973145	1

MSTRG.30445.2	382	0.44474	4.901428223	1
MSTRG.30448.1	330	0.25706	10.69549561	1
MSTRG.30451.1	20	0.44924	7.953430176	1
MSTRG.30453.3	905	0.43391	6.372375488	1
MSTRG.30453.4	905	0.43654	6.372375488	1
MSTRG.30453.5	878	0.45625	6.146179199	1
MSTRG.30456.2	193	0.37963	11.15997314	1
MSTRG.30456.1	291	0.43262	9.554870605	1
MSTRG.30456.7	229	0.36554	9.343444824	-1
MSTRG.30456.4	239	0.33621	9.421569824	-1
MSTRG.30438.2	55	0.44221	4.379333496	1
MSTRG.30426.1	31	0.42418	6.504577637	1
MSTRG.30467.1	61	0.43104	8.77154541	1
MSTRG.30469.1	19	0.44025	5.897644043	1
MSTRG.30483.1	128	0.3285	9.183776855	1
MSTRG.30496.1	171	0.35996	8.777526855	1
MSTRG.30496.2	211	0.29526	11.61395264	1
MSTRG.30497.1	68	0.31732	5.287414551	1
MSTRG.30497.2	305	0.34222	8.66998291	1
MSTRG.30496.3	143	0.29607	8.942932129	1
MSTRG.30473.1	104	0.38432	11.27032471	1
MSTRG.30475.10	706	0.32213	5.914367676	1
MSTRG.30475.11	674	0.39555	6.350524902	1
MSTRG.30476.1	47	0.33821	10.637146	1
MSTRG.30479.1	118	0.35829	9.659851074	1
MSTRG.30481.1	238	0.44823	6.777770996	1
MSTRG.30481.2	238	0.37645	6.777770996	1
MSTRG.30481.3	217	0.48638	6.859069824	1
MSTRG.30477.2	164	0.33175	5.927429199	1
MSTRG.30503.1	1081	0.3817	5.592956543	1
MSTRG.30505.4	260	0.40375	4.690856934	1
MSTRG.30506.1	69	0.3207	6.72076416	1
MSTRG.30506.4	69	0.31829	6.72076416	1
MSTRG.30507.2	582	0.3439	8.654846191	1
MSTRG.30518.1	683	0.46201	7.992370605	1
MSTRG.30518.2	683	0.47269	7.992370605	1
MSTRG.30518.6	683	0.49452	7.992370605	1
MSTRG.30517.2	413	0.35052	8.81463623	1
MSTRG.30522.5	126	0.28338	6.160827637	1
MSTRG.30522.6	176	0.31649	9.917297363	1
MSTRG.30523.1	130	0.28002	9.145690918	1
MSTRG.30524.1	65	0.31852	6.700378418	1
MSTRG.30525.1	1705	0.45573	9.29901123	1
MSTRG.30529.1	726	0.40238	5.535339355	1
MSTRG.30530.1	190	0.27517	8.549621582	1
MSTRG.30530.2	96	0.32697	5.479309082	1
MSTRG.30530.3	96	0.36035	5.479309082	1
MSTRG.30531.3	1007	0.34846	6.72064209	1
MSTRG.30536.1	49	0.40163	6.68939209	1
MSTRG.30539.1	65	0.32205	5.637390137	1
MSTRG.30534.2	215	0.38575	9.795959473	1
MSTRG.30534.3	281	0.43109	6.36529541	1
MSTRG.30534.4	78	0.29772	5.60760498	1
MSTRG.30547.1	444	0.47991	5.746643066	-1

MSTRG.30547.2	1234	0.34787	6.764953613	1
MSTRG.30547.6	726	0.47579	7.138977051	-1
MSTRG.30547.5	734	0.45529	6.519592285	-1
MSTRG.30547.7	1207	0.44083	6.507629395	1
MSTRG.30548.1	183	0.34082	10.77410889	1
MSTRG.30548.2	183	0.30159	10.38726807	1
MSTRG.30548.3	183	0.41352	11.00238037	1
MSTRG.30549.1	108	0.31852	10.27972412	1
MSTRG.30550.1	125	0.26113	10.2366333	1
MSTRG.30551.1	75	0.3443	9.899597168	1
MSTRG.30553.1	119	0.31458	7.546447754	1
MSTRG.30554.1	61	0.29884	10.01080322	1
MSTRG.30555.1	117	0.40288	6.883483887	1
MSTRG.30556.1	651	0.47269	6.20098877	-1
MSTRG.30556.2	123	0.34407	9.521057129	1
MSTRG.30545.1	54	0.48866	10.54864502	1
MSTRG.30559.1	235	0.29985	9.078674316	1
MSTRG.30559.3	235	0.38203	9.078674316	1
MSTRG.30561.2	552	0.44874	5.057189941	1
MSTRG.30561.3	401	0.30577	5.264709473	1
MSTRG.30562.2	568	0.43447	5.882019043	1
MSTRG.30565.1	405	0.35131	5.60369873	1
MSTRG.30565.2	405	0.3823	5.60369873	1
MSTRG.30565.4	405	0.38046	5.60369873	1
MSTRG.30566.1	85	0.40557	5.205627441	1
MSTRG.30569.1	116	0.28557	10.76849365	1
MSTRG.30571.1	10	0.39793	5.752990723	1
MSTRG.30572.1	47	0.34544	9.381896973	1
MSTRG.30575.1	284	0.34706	8.381652832	1
MSTRG.30575.2	511	0.42195	9.419372559	1
MSTRG.30575.3	83	0.37891	10.30389404	1
MSTRG.30575.5	219	0.37065	9.393249512	1
MSTRG.30576.1	39	0.40514	6.80267334	1
MSTRG.30583.2	366	0.32322	5.165710449	1
MSTRG.30583.3	366	0.32076	5.165710449	1
MSTRG.30583.4	403	0.34126	5.43560791	1
MSTRG.30584.1	276	0.32177	9.751525879	1
MSTRG.30595.3	336	0.28096	8.212219238	1
MSTRG.30595.5	292	0.40896	8.527160645	-1
MSTRG.30595.4	292	0.47176	8.527160645	-1
MSTRG.30596.1	1037	0.48142	6.205871582	1
MSTRG.30597.1	640	0.46641	5.573059082	1
MSTRG.30597.3	637	0.4569	5.631530762	1
MSTRG.30597.5	635	0.46525	5.573059082	1
MSTRG.30599.4	784	0.31712	5.269226074	1
MSTRG.30614.8	751	0.43298	5.872741699	1
MSTRG.30616.7	325	0.35788	8.993591309	1
MSTRG.30616.3	482	0.34638	8.718688965	1
MSTRG.30616.6	482	0.34638	7.992004395	1
MSTRG.30617.1	79	0.3	4.508850098	1
MSTRG.30616.8	70	0.35659	8.987976074	1
MSTRG.30616.9	179	0.28165	9.215759277	1
MSTRG.30616.10	179	0.28165	9.215759277	1
MSTRG.30618.1	55	0.42092	4.867858887	1

MSTRG.30620.1	382	0.43489	5.188537598	1
MSTRG.30620.3	382	0.46278	5.188537598	1
MSTRG.30623.4	657	0.45566	4.793762207	1
MSTRG.30623.5	520	0.33115	4.586364746	1
MSTRG.30624.1	112	0.41385	9.178894043	1
MSTRG.30625.1	506	0.46634	9.31439209	1
MSTRG.30625.2	507	0.45617	9.31439209	1
MSTRG.30625.3	506	0.47941	9.31439209	1
MSTRG.30625.4	305	0.40202	8.09942627	1
MSTRG.30625.5	507	0.44997	9.31439209	1
MSTRG.30625.6	506	0.45617	9.31439209	1
MSTRG.30628.1	47	0.37276	4.834655762	1
MSTRG.30631.2	78	0.35163	6.695617676	1
MSTRG.30643.1	403	0.35222	7.309509277	1
MSTRG.30645.3	244	0.46623	8.723693848	-1
MSTRG.30646.1	373	0.31793	8.532653809	1
MSTRG.30646.2	573	0.29607	9.090637207	1
MSTRG.30637.1	48	0.32558	10.58660889	1
MSTRG.30654.4	223	0.37647	5.116760254	1
MSTRG.30657.1	61	0.42257	10.78057861	1
MSTRG.30658.2	439	0.37162	6.239196777	1
MSTRG.30658.3	439	0.39417	6.239196777	1
MSTRG.30658.4	439	0.42976	6.239196777	1
MSTRG.30669.1	77	0.39108	10.75445557	1
MSTRG.30668.5	287	0.42129	8.655334473	1
MSTRG.30668.3	284	0.32989	8.71270752	1
MSTRG.30668.4	382	0.36496	4.886291504	1
MSTRG.30668.6	708	0.44744	5.481384277	1
MSTRG.30670.1	58	0.34755	4.39630127	1
MSTRG.30671.10	97	0.3014	9.943664551	1
MSTRG.30676.4	317	0.44298	8.616271973	1
MSTRG.30678.1	917	0.32951	8.862731934	1
MSTRG.30685.1	289	0.3329	8.418029785	1
MSTRG.30685.4	103	0.3061	10.77020264	1
MSTRG.30685.5	553	0.39659	9.084533691	1
MSTRG.30685.6	740	0.37404	8.897644043	1
MSTRG.30685.7	599	0.31649	8.524963379	1
MSTRG.30685.8	149	0.30396	9.361022949	1
MSTRG.30685.9	213	0.28923	8.739440918	1
MSTRG.30685.10	213	0.29387	8.739440918	1
MSTRG.30685.11	169	0.44156	8.213439941	1
MSTRG.30685.12	161	0.28167	9.43927002	1
MSTRG.30685.13	128	0.29059	10.43743896	1
MSTRG.30685.14	148	0.2785	8.932312012	1
MSTRG.30685.15	148	0.2846	8.932312012	1
MSTRG.30688.1	67	0.39622	8.50958252	1
MSTRG.30688.2	81	0.33387	12.18206787	1
MSTRG.30693.1	3354	0.45083	9.74005127	1
MSTRG.30693.6	1674	0.46683	9.764465332	1
MSTRG.30693.2	2746	0.45083	9.803405762	1
MSTRG.30693.3	2746	0.45083	9.796691895	1
MSTRG.30693.4	2746	0.45083	9.796691895	1
MSTRG.30693.7	3238	0.45083	9.747619629	1
MSTRG.30693.9	2630	0.42871	9.82220459	1

MSTRG.30695.1	121	0.38881	11.54022217	1
MSTRG.30698.1	20	0.38216	10.28900146	1
MSTRG.30698.2	20	0.37246	10.28900146	1
MSTRG.30699.1	120	0.34553	11.85113525	1
MSTRG.30701.1	80	0.33467	9.662414551	1
MSTRG.30703.1	97	0.33001	8.637878418	1
MSTRG.30703.2	84	0.31709	9.249816895	1
MSTRG.30704.1	194	0.34716	9.001037598	1
MSTRG.30705.2	142	0.31575	10.559021	1
MSTRG.30706.1	116	0.38527	9.784240723	1
MSTRG.30712.1	82	0.33637	8.58770752	1
MSTRG.30712.2	92	0.3388	9.547546387	1
MSTRG.30716.1	616	0.4291	9.304870605	1
MSTRG.30718.1	245	0.31535	9.415344238	1
MSTRG.30718.2	245	0.31532	9.415344238	1
MSTRG.30719.1	73	0.42045	10.52203369	1
MSTRG.30720.5	191	0.3278	11.63580322	-1
MSTRG.30720.4	410	0.36289	6.299499512	1
MSTRG.30720.3	410	0.39214	6.299499512	1
MSTRG.30720.2	227	0.40131	9.645080566	1
MSTRG.30736.1	2603	0.42592	6.222595215	1
MSTRG.30736.3	1245	0.46497	8.094421387	1
MSTRG.30736.4	2112	0.42727	6.251159668	1
MSTRG.30737.1	74	0.3947	11.46795654	-1
MSTRG.30725.2	166	0.35152	7.056335449	1
MSTRG.30727.1	109	0.38246	9.359436035	1
MSTRG.30728.1	128	0.34857	10.64825439	1
MSTRG.30740.1	154	0.37717	9.066955566	1
MSTRG.30741.1	73	0.31521	11.8583374	1
MSTRG.30743.6	140	0.39401	8.571838379	1
MSTRG.30743.4	66	0.31999	6.231506348	1
MSTRG.30743.8	179	0.44394	5.992370605	1
MSTRG.30743.9	363	0.41399	8.167419434	1
MSTRG.30743.10	505	0.4724	10.00726318	1
MSTRG.30743.7	476	0.45098	6.428527832	1
MSTRG.30747.5	76	0.27763	5.002990723	1
MSTRG.30747.6	106	0.29351	5.891052246	1
MSTRG.30758.2	185	0.45838	4.825744629	1
MSTRG.30759.1	118	0.36283	8.852722168	1
MSTRG.30760.1	94	0.32621	9.251281738	1
MSTRG.30760.3	61	0.35085	6.791564941	1
MSTRG.30760.4	163	0.27575	9.242004395	1
MSTRG.30760.5	517	0.35928	9.038146973	1
MSTRG.30760.2	517	0.29516	9.038146973	1
MSTRG.30762.1	125	0.26749	10.00872803	1
MSTRG.30763.1	144	0.33002	10.10809326	1
MSTRG.30760.7	240	0.27274	9.584777832	1
MSTRG.30760.8	148	0.27516	6.112243652	1
MSTRG.30765.1	203	0.32164	9.162536621	1
MSTRG.30768.1	15	0.41874	4.195983887	1
MSTRG.30771.1	187	0.34196	5.047668457	1
MSTRG.30772.1	166	0.30473	10.05682373	1
MSTRG.30772.2	166	0.32938	10.05682373	1
MSTRG.30753.7	790	0.423	6.513000488	1

MSTRG.30757.1	91	0.31208	9.496520996	1
MSTRG.30774.1	1248	0.40294	5.065856934	-1
MSTRG.30776.1	93	0.36193	8.340270996	1
MSTRG.30787.1	496	0.42057	5.518615723	1
MSTRG.30791.1	30	0.4485	9.122497559	1
MSTRG.30792.2	107	0.35172	8.448181152	1
MSTRG.30793.1	20	0.37946	3.799377441	1
MSTRG.30792.3	86	0.32667	10.22503662	1
MSTRG.30792.4	110	0.36342	9.77532959	1
MSTRG.30792.5	37	0.34855	8.138977051	1
MSTRG.30792.6	38	0.39749	8.906433105	1
MSTRG.30794.1	36	0.42056	7.93939209	1
MSTRG.30795.4	120	0.31673	4.90838623	1
MSTRG.30831.1	101	0.40617	9.23651123	1
MSTRG.30832.2	50	0.37283	5.146911621	1
MSTRG.30798.1	92	0.4663	9.216369629	1
MSTRG.30801.1	69	0.41623	9.300109863	1
MSTRG.30802.1	46	0.39932	7.998596191	1
MSTRG.30803.2	100	0.43746	9.851257324	1
MSTRG.30805.2	308	0.41483	8.508361816	1
MSTRG.30805.4	215	0.46506	5.805114746	1
MSTRG.30806.1	190	0.37434	7.566955566	1
MSTRG.30807.1	80	0.30664	4.59954834	1
MSTRG.30808.1	187	0.28662	9.023498535	1
MSTRG.30808.2	107	0.28307	6.153137207	1
MSTRG.30805.6	87	0.32642	6.553771973	1
MSTRG.30809.1	81	0.42996	9.751525879	1
MSTRG.30812.1	411	0.45878	5.08807373	1
MSTRG.30812.2	1283	0.45325	6.273986816	1
MSTRG.30812.4	772	0.33851	5.660705566	1
MSTRG.30815.2	64	0.39503	7.847473145	-1
MSTRG.30817.1	94	0.31204	9.684143066	1
MSTRG.30826.2	257	0.46093	8.447692871	1
MSTRG.30826.3	138	0.42051	6.960876465	1
MSTRG.30826.5	257	0.40194	8.447692871	1
MSTRG.30826.6	182	0.27931	10.71783447	1
MSTRG.30833.1	30	0.36429	9.864929199	1
MSTRG.30866.1	55	0.35915	8.492858887	1
MSTRG.30867.1	89	0.34763	9.29901123	1
MSTRG.30866.2	47	0.36459	10.83428955	1
MSTRG.30868.1	88	0.29367	4.911437988	1
MSTRG.30866.3	204	0.28334	9.649353027	1
MSTRG.30866.4	194	0.31193	8.542175293	1
MSTRG.30866.5	168	0.33674	9.480407715	1
MSTRG.30866.6	168	0.30792	9.480407715	1
MSTRG.30866.7	184	0.26072	5.320617676	1
MSTRG.30866.8	184	0.28048	5.320617676	1
MSTRG.30835.1	108	0.32785	10.37261963	1
MSTRG.30836.1	122	0.34701	9.12689209	1
MSTRG.30836.4	65	0.28027	7.816711426	1
MSTRG.30836.3	68	0.34662	8.537780762	1
MSTRG.30836.2	122	0.31674	9.12689209	1
MSTRG.30836.5	48	0.34761	4.749206543	1
MSTRG.30836.6	63	0.3259	3.93951416	-1

MSTRG.30837.1	98	0.31905	8.80670166	1
MSTRG.30889.5	1217	0.35483	5.938659668	1
MSTRG.30889.7	2463	0.408	5.851135254	1
MSTRG.30889.10	77	0.28334	9.178649902	1
MSTRG.30889.14	54	0.28082	9.236877441	1
MSTRG.30892.1	168	0.32762	9.16217041	1
MSTRG.30892.2	168	0.29011	9.16217041	1
MSTRG.30892.3	106	0.38074	6.379455566	-1
MSTRG.30892.4	144	0.30375	10.73236084	1
MSTRG.30893.1	77	0.28518	10.60296631	1
MSTRG.30894.1	183	0.29367	10.55560303	1
MSTRG.30889.16	102	0.32862	7.737243652	1
MSTRG.30889.17	165	0.28518	7.551818848	1
MSTRG.30895.1	91	0.35617	9.800842285	1
MSTRG.30889.19	177	0.36218	7.669250488	1
MSTRG.30889.18	219	0.28923	8.48626709	1
MSTRG.30896.1	72	0.33467	8.035827637	1
MSTRG.30889.20	113	0.30664	7.954406738	1
MSTRG.30897.1	88	0.34544	9.00201416	1
MSTRG.30889.21	883	0.44448	7.682922363	1
MSTRG.30871.1	52	0.3436	5.337219238	1
MSTRG.30874.1	115	0.33187	9.770812988	1
MSTRG.30874.2	146	0.29607	5.387756348	1
MSTRG.30876.3	125	0.4045	8.756896973	1
MSTRG.30877.4	771	0.41515	6.254089355	1
MSTRG.30877.6	872	0.47354	6.341491699	1
MSTRG.30878.2	801	0.33443	7.816589355	1
MSTRG.30878.3	863	0.31649	8.642272949	1
MSTRG.30880.2	357	0.28415	7.583557129	1
MSTRG.30883.2	240	0.4733	6.672180176	1
MSTRG.30882.1	50	0.38836	5.733581543	-1
MSTRG.30888.2	281	0.46806	6.107116699	1
MSTRG.30899.2	98	0.41188	9.77557373	1
MSTRG.30900.4	185	0.28165	10.83880615	1
MSTRG.30900.5	208	0.28982	10.8012085	1
MSTRG.30900.6	527	0.3208	9.006286621	1
MSTRG.30900.9	185	0.28165	10.83880615	1
MSTRG.30900.7	527	0.35724	9.006286621	1
MSTRG.30900.8	527	0.3351	9.006286621	1
MSTRG.30900.10	562	0.35599	9.065612793	1
MSTRG.30905.4	241	0.29272	8.690002441	1
MSTRG.30906.1	42	0.39006	6.692687988	-1
MSTRG.30907.2	361	0.46525	6.084289551	1
MSTRG.30908.1	309	0.3564	9.702697754	1
MSTRG.30909.1	852	0.45639	5.727355957	1
MSTRG.30909.2	760	0.45692	5.957458496	1
MSTRG.30909.3	838	0.45174	5.648254395	1
MSTRG.30909.4	860	0.45639	5.65045166	1
MSTRG.30909.5	846	0.48094	5.648254395	1
MSTRG.30909.6	832	0.48094	5.574523926	1
MSTRG.30910.2	674	0.37566	9.36920166	1
MSTRG.30910.3	664	0.37566	9.474060059	1
MSTRG.30910.4	645	0.37566	9.498352051	1
MSTRG.30910.5	235	0.29727	8.889099121	1

MSTRG.30910.8	335	0.28518	9.33807373	1
MSTRG.30910.9	154	0.32445	9.395080566	1
MSTRG.30911.3	148	0.44293	7.688781738	-1
MSTRG.30914.1	36	0.42609	4.800598145	-1
MSTRG.30920.1	68	0.35364	9.393493652	1
MSTRG.30937.1	62	0.45812	9.915710449	1
MSTRG.30937.2	39	0.36917	6.374694824	1
MSTRG.30937.5	129	0.33465	10.08978271	1
MSTRG.30937.6	85	0.38439	6.241271973	1
MSTRG.30937.7	209	0.3	9.774108887	1
MSTRG.30937.8	176	0.28662	10.59918213	1
MSTRG.30937.9	56	0.31768	9.570617676	1
MSTRG.30937.10	82	0.31035	9.510314941	1
MSTRG.30939.1	94	0.33964	7.675231934	1
MSTRG.30943.1	454	0.47929	5.642150879	1
MSTRG.30922.1	215	0.39386	10.0758667	1
MSTRG.30922.2	76	0.4318	12.22210693	1
MSTRG.30922.3	160	0.33139	9.603820801	1
MSTRG.30945.1	99	0.33254	5.745788574	1
MSTRG.30948.11	741	0.45266	4.632995605	1
MSTRG.30948.8	1527	0.42877	4.788757324	1
MSTRG.30948.18	1528	0.44263	4.788757324	1
MSTRG.30948.12	2081	0.439	4.95123291	1
MSTRG.30948.13	2158	0.42877	5.022888184	1
MSTRG.30948.14	2144	0.42877	5.013366699	1
MSTRG.30948.15	1426	0.44759	5.605529785	1
MSTRG.30948.16	2023	0.439	4.966491699	1
MSTRG.30948.9	2114	0.42877	4.984191895	1
MSTRG.30948.7	2177	0.439	5.047912598	1
MSTRG.30948.10	2144	0.439	5.013366699	1
MSTRG.30948.17	2075	0.439	5.026550293	1
MSTRG.30948.6	2114	0.439	4.984191895	1
MSTRG.30948.19	187	0.31501	9.622131348	1
MSTRG.30949.1	65	0.2921	6.218200684	1
MSTRG.30950.2	1306	0.44694	8.090026855	1
MSTRG.30951.1	37	0.40023	6.50201416	-1
MSTRG.30952.1	17	0.4053	12.00848389	1
MSTRG.30950.5	219	0.41013	6.346496582	1
MSTRG.30950.7	488	0.41515	6.601623535	1
MSTRG.30929.3	659	0.4517	6.34967041	1
MSTRG.30961.3	285	0.32485	4.770324707	1
MSTRG.30961.4	285	0.31035	4.770324707	1
MSTRG.30965.1	520	0.40647	6.379211426	1
MSTRG.30965.2	520	0.42403	6.379211426	1
MSTRG.30965.3	236	0.3208	5.481506348	1
MSTRG.30965.4	402	0.39355	5.755554199	1
MSTRG.30965.6	307	0.32464	6.686950684	1
MSTRG.30965.5	307	0.30419	6.686950684	1
MSTRG.30966.1	39	0.36844	5.967346191	1
MSTRG.30965.8	275	0.4744	5.45135498	1
MSTRG.30965.9	88	0.25198	7.667785645	1
MSTRG.30965.10	133	0.33006	8.632507324	1
MSTRG.30967.1	63	0.30218	11.87518311	1
MSTRG.30967.3	54	0.35269	4.777526855	-1

MSTRG.30971.1	42	0.37684	10.0758667	1
MSTRG.30971.2	42	0.36953	10.0758667	1
MSTRG.30968.1	66	0.4307	9.860290527	1
MSTRG.31004.1	263	0.36602	9.143249512	1
MSTRG.31004.2	263	0.36754	9.143249512	1
MSTRG.31004.4	263	0.36826	9.143249512	1
MSTRG.31004.5	293	0.42152	9.096374512	1
MSTRG.31006.1	42	0.30576	8.552062988	1
MSTRG.31007.1	87	0.28842	9.780212402	1
MSTRG.31011.1	47	0.3593	5.509338379	1
MSTRG.31011.2	47	0.35726	5.509338379	1
MSTRG.31011.3	47	0.34885	5.509338379	1
MSTRG.31011.4	47	0.38784	5.509338379	1
MSTRG.31011.5	51	0.38652	4.644348145	1
MSTRG.31015.3	1408	0.33443	7.080749512	1
MSTRG.31015.4	1408	0.28771	7.080749512	1
MSTRG.31015.5	1408	0.33615	7.080749512	1
MSTRG.31015.6	1408	0.29639	7.080749512	1
MSTRG.31021.2	271	0.41909	8.708557129	1
MSTRG.31019.1	35	0.37359	9.520812988	1
MSTRG.31027.1	133	0.282	6.635314941	1
MSTRG.31029.2	116	0.34062	6.140563965	1
MSTRG.31030.1	131	0.28982	4.601867676	1
MSTRG.31035.1	190	0.37112	6.092224121	1
MSTRG.31035.4	138	0.41634	5.604553223	1
MSTRG.31035.3	138	0.38022	5.604553223	1
MSTRG.31035.5	114	0.44388	11.49993896	1
MSTRG.31035.6	206	0.42972	7.598327637	1
MSTRG.31035.7	219	0.45829	6.06060791	1
MSTRG.31035.8	219	0.43863	6.946716309	1
MSTRG.31031.1	54	0.37634	8.492980957	1
MSTRG.31036.2	213	0.30188	9.465515137	1
MSTRG.31043.1	309	0.40932	5.201477051	1
MSTRG.31044.1	91	0.31209	9.946350098	1
MSTRG.31045.13	194	0.33351	9.537414551	1
MSTRG.31045.4	2805	0.46524	6.024963379	-1
MSTRG.31045.7	2805	0.46557	6.024963379	-1
MSTRG.31045.11	2805	0.4865	6.024963379	-1
MSTRG.31045.15	2805	0.46557	6.024963379	-1
MSTRG.31045.16	298	0.43091	9.498962402	1
MSTRG.31045.8	2805	0.4865	6.024963379	-1
MSTRG.31045.14	298	0.41657	9.498962402	1
MSTRG.31045.12	2805	0.4865	6.024963379	-1
MSTRG.31045.9	2805	0.48925	6.024963379	-1
MSTRG.31045.10	298	0.39379	9.498962402	1
MSTRG.31045.6	2805	0.48925	6.024963379	-1
MSTRG.31046.1	92	0.3136	10.16326904	1
MSTRG.31047.1	175	0.27842	9.619934082	1
MSTRG.31051.1	104	0.29771	9.301330566	1
MSTRG.31052.1	110	0.29543	9.442199707	1
MSTRG.31053.5	1253	0.49724	5.246765137	-1
MSTRG.31058.1	268	0.32762	5.605163574	1
MSTRG.31071.1	60	0.3636	8.432434082	1
MSTRG.31071.2	51	0.30378	9.619445801	1

MSTRG.31083.2	639	0.41558	6.698547363	1
MSTRG.31105.3	494	0.383	9.165588379	1
MSTRG.31105.4	494	0.40416	9.165588379	1
MSTRG.31111.2	61	0.45706	9.511657715	-1
MSTRG.31112.1	260	0.37444	4.676208496	1
MSTRG.31116.2	133	0.35632	9.20880127	1
MSTRG.31116.3	1387	0.35893	8.511169434	1
MSTRG.31116.4	797	0.28119	8.103088379	1
MSTRG.31116.5	1447	0.39801	8.56829834	1
MSTRG.31117.1	60	0.3223	10.84747314	1
MSTRG.31117.2	60	0.4534	10.84747314	1
MSTRG.31116.6	652	0.43854	9.123718262	1
MSTRG.31119.2	443	0.4278	9.603942871	1
MSTRG.31120.8	731	0.46891	6.217346191	1
MSTRG.31121.1	44	0.32181	8.942077637	1
MSTRG.31122.4	110	0.29206	10.69989014	1
MSTRG.31122.3	530	0.3999	5.322937012	1
MSTRG.31114.4	164	0.29547	6.430236816	1
MSTRG.31125.1	153	0.32237	5.252868652	1
MSTRG.31125.2	87	0.33105	6.797058105	1
MSTRG.31128.3	191	0.3582	4.99041748	1
MSTRG.31132.3	387	0.46864	6.457092285	1
MSTRG.31134.3	240	0.37801	9.435852051	1
MSTRG.31134.4	51	0.42378	9.570861816	1
MSTRG.31134.5	169	0.33231	9.255187988	1
MSTRG.31134.6	77	0.32346	9.056091309	1
MSTRG.31133.1	44	0.36402	5.194152832	-1
MSTRG.31142.2	189	0.38308	5.413391113	1
MSTRG.31143.2	396	0.41234	9.148010254	1
MSTRG.31143.3	261	0.36506	9.460510254	1
MSTRG.31146.1	1867	0.46946	5.873718262	1
MSTRG.31146.2	112	0.36033	10.61383057	1
MSTRG.31146.4	161	0.37576	10.51434326	1
MSTRG.31146.3	110	0.31633	10.62713623	1
MSTRG.31146.5	68	0.31644	10.28094482	1
MSTRG.31148.2	2011	0.41724	5.362854004	1
MSTRG.31149.2	76	0.31976	6.879333496	1
MSTRG.31144.1	418	0.42208	7.150085449	1
MSTRG.31144.2	303	0.38506	9.375793457	1
MSTRG.31144.5	41	0.27943	9.496887207	1
MSTRG.31144.6	79	0.30904	6.821838379	1
MSTRG.31145.2	567	0.40215	8.494689941	1
MSTRG.31157.1	31	0.37843	12.03204346	1
MSTRG.31157.2	31	0.42088	12.03204346	1
MSTRG.31158.2	181	0.37196	6.445251465	1
MSTRG.31158.3	181	0.45397	6.445251465	1
MSTRG.31158.4	181	0.40531	6.445251465	1
MSTRG.31159.1	92	0.33828	6.690979004	1
MSTRG.31160.2	283	0.37371	8.949279785	1
MSTRG.31160.3	283	0.3612	8.949279785	1
MSTRG.31160.4	293	0.40931	9.027282715	1
MSTRG.31161.1	43	0.35665	8.967102051	1
MSTRG.31165.1	107	0.37029	5.79107666	1
MSTRG.31165.3	73	0.35355	8.639587402	1

MSTRG.31155.1	69	0.41822	6.235412598	1
MSTRG.31155.2	50	0.3533	7.837341309	-1
MSTRG.31156.1	415	0.39814	6.022399902	1
MSTRG.31167.1	213	0.38063	8.778625488	1
MSTRG.31167.2	191	0.45012	9.086975098	1
MSTRG.31169.5	678	0.47461	5.248718262	1
MSTRG.31169.6	603	0.47542	5.25567627	1
MSTRG.31169.7	680	0.34998	5.248718262	1
MSTRG.31171.1	626	0.45428	8.867004395	1
MSTRG.31172.3	632	0.47397	8.256164551	1
MSTRG.31175.2	957	0.35673	6.261413574	1
MSTRG.31175.3	512	0.36837	9.019592285	1
MSTRG.31174.1	190	0.36723	9.981994629	1
MSTRG.31187.3	577	0.3892	6.260314941	1
MSTRG.31188.4	1593	0.4747	8.30291748	1
MSTRG.31189.2	246	0.39204	10.04876709	1
MSTRG.31189.3	511	0.42714	8.761047363	1
MSTRG.31189.4	409	0.47334	8.987976074	1
MSTRG.31193.1	39	0.43922	9.18560791	1
MSTRG.31194.1	86	0.31793	9.946594238	1
MSTRG.31196.2	94	0.34773	8.802429199	1
MSTRG.31196.1	111	0.27794	6.994689941	1
MSTRG.31197.1	89	0.31501	5.082214355	1
MSTRG.31201.1	64	0.36837	6.700866699	1
MSTRG.31202.1	25	0.37685	5.06060791	1
MSTRG.31205.1	122	0.2562	9.390808105	1
MSTRG.31210.1	79	0.3434	6.883972168	1
MSTRG.31211.1	288	0.34989	5.965637207	1
MSTRG.31215.1	578	0.32708	8.335021973	1
MSTRG.31215.2	503	0.33998	8.824157715	1
MSTRG.31218.2	204	0.36159	11.61578369	1
MSTRG.31219.2	137	0.36416	10.33599854	1
MSTRG.31222.3	25	0.33397	4.677062988	1
MSTRG.31223.1	97	0.34908	6.710510254	1
MSTRG.31231.2	83	0.33531	9.992126465	1
MSTRG.31231.3	92	0.44826	6.278015137	1
MSTRG.31231.4	137	0.38446	8.718322754	1
MSTRG.31231.5	20	0.36609	10.83428955	1
MSTRG.31231.6	91	0.28648	9.445251465	1
MSTRG.31233.1	133	0.28267	6.92376709	1
MSTRG.31235.1	284	0.29783	11.11029053	1
MSTRG.31236.1	85	0.28186	10.01409912	1
MSTRG.31237.1	157	0.29088	5.653015137	1
MSTRG.31238.1	135	0.28923	7.596862793	1
MSTRG.31231.7	168	0.28063	9.460754395	1
MSTRG.31239.1	116	0.28883	7.559509277	1
MSTRG.31240.1	192	0.29187	6.078308105	1
MSTRG.31241.1	219	0.43568	8.797790527	1
MSTRG.31242.1	169	0.28109	11.63995361	1
MSTRG.31240.2	145	0.28338	11.02813721	1
MSTRG.31244.1	130	0.28199	12.15594482	1
MSTRG.31244.2	130	0.28503	12.15594482	1
MSTRG.31245.2	78	0.24306	7.807922363	1
MSTRG.31230.1	63	0.38224	10.58843994	-1

MSTRG.31246.1	79	0.41362	5.918029785	-1
MSTRG.31246.2	71	0.4112	10.0112915	1
MSTRG.31247.2	74	0.32489	11.83990479	1
MSTRG.31251.1	334	0.38147	10.10162354	1
MSTRG.31251.3	97	0.38026	10.21710205	1
MSTRG.31251.2	270	0.46747	9.97845459	1
MSTRG.31251.4	344	0.41692	10.01983643	1
MSTRG.31251.5	107	0.41754	9.966369629	1
MSTRG.31271.1	114	0.39129	5.06451416	1
MSTRG.31271.2	105	0.34504	9.350158691	1
MSTRG.31263.1	146	0.34456	7.790344238	1
MSTRG.31273.1	96	0.31546	9.940124512	1
MSTRG.31278.3	151	0.38136	9.223327637	1
MSTRG.31278.4	211	0.39526	6.745788574	1
MSTRG.31278.5	219	0.28496	6.182800293	1
MSTRG.31279.1	61	0.37468	9.469055176	1
MSTRG.31286.4	52	0.41855	7.880554199	1
MSTRG.31287.3	1521	0.4087	6.392150879	1
MSTRG.31285.1	202	0.32421	9.680480957	1
MSTRG.31289.1	124	0.28635	9.349060059	1
MSTRG.31291.1	169	0.28842	8.966369629	1
MSTRG.31301.1	136	0.33699	5.926818848	1
MSTRG.31301.2	68	0.29631	7.81829834	1
MSTRG.31301.3	136	0.27327	5.926818848	1
MSTRG.31301.4	136	0.28195	5.926818848	1
MSTRG.31301.5	136	0.30505	5.926818848	1
MSTRG.31301.6	78	0.30069	6.139709473	1
MSTRG.31301.7	68	0.3199	7.81829834	1
MSTRG.31307.1	166	0.29897	8.766174316	-1
MSTRG.31316.2	608	0.38259	11.72369385	-1
MSTRG.31317.1	71	0.34348	11.51153564	1
MSTRG.31320.1	102	0.28195	10.85980225	1
MSTRG.31320.3	149	0.34584	8.918395996	1
MSTRG.31320.2	149	0.32534	8.918395996	1
MSTRG.31320.7	111	0.33213	5.130432129	1
MSTRG.31320.4	149	0.31065	8.918395996	1
MSTRG.31320.5	149	0.32274	8.918395996	1
MSTRG.31320.6	149	0.30813	8.918395996	1
MSTRG.31320.8	149	0.36992	8.918395996	1
MSTRG.31320.9	149	0.29404	8.918395996	1
MSTRG.31320.10	149	0.29196	8.918395996	1
MSTRG.31320.11	132	0.27758	5.374206543	1
MSTRG.31320.12	149	0.27327	8.918395996	1
MSTRG.31304.1	31	0.37744	12.01654053	-1
MSTRG.31324.2	282	0.3972	8.405822754	1
MSTRG.31333.2	487	0.39107	8.491760254	1
MSTRG.31336.3	420	0.37829	8.967346191	1
MSTRG.31337.1	108	0.31885	10.13885498	1
MSTRG.31341.1	57	0.3042	10.85980225	1
MSTRG.31347.1	1117	0.44823	5.555725098	1
MSTRG.31348.1	49	0.34073	10.83428955	1
MSTRG.31350.2	222	0.32237	8.730041504	1
MSTRG.31351.3	433	0.41499	9.434509277	1
MSTRG.31363.1	31	0.42679	10.7366333	1

MSTRG.31364.5	125	0.29615	9.661682129	1
MSTRG.31364.6	82	0.29251	9.721740723	1
MSTRG.31365.1	300	0.47462	5.90435791	1
MSTRG.31367.2	237	0.39513	4.925842285	1
MSTRG.31371.1	106	0.30884	9.300231934	1
MSTRG.31372.2	1282	0.39906	5.395202637	1
MSTRG.31374.1	74	0.3599	8.980041504	-1
MSTRG.31375.1	63	0.30191	6.520202637	1
MSTRG.31376.1	307	0.39236	8.487243652	1
MSTRG.31378.4	471	0.40377	8.208190918	1
MSTRG.31379.1	40	0.30324	5.617492676	1
MSTRG.31380.2	316	0.30958	7.68182373	1
MSTRG.31380.5	316	0.30958	7.68182373	1
MSTRG.31381.1	2142	0.47589	6.251403809	1
MSTRG.31383.1	112	0.38597	8.567810059	1
MSTRG.31384.2	999	0.3694	5.27142334	1
MSTRG.31385.1	170	0.34083	5.457336426	1
MSTRG.31386.1	96	0.39692	10.40374756	-1
MSTRG.31390.1	20	0.35674	7.975524902	-1
MSTRG.31401.2	629	0.41152	9.331970215	1
MSTRG.31401.1	629	0.41152	9.331970215	1
MSTRG.31401.3	629	0.38618	9.331970215	1
MSTRG.31407.1	173	0.28567	10.41546631	1
MSTRG.31408.1	63	0.27794	5.989807129	1
MSTRG.31407.2	45	0.32269	11.22784424	1
MSTRG.31419.1	245	0.28419	8.47052002	1
MSTRG.31421.1	173	0.30781	9.657287598	1
MSTRG.31426.1	390	0.4116	8.719299316	1
MSTRG.31426.2	344	0.38657	8.948791504	1
MSTRG.31431.1	103	0.43119	8.987365723	1
MSTRG.31435.6	414	0.35333	5.316833496	1
MSTRG.31435.10	414	0.3475	5.316833496	1
MSTRG.31435.2	414	0.33273	5.316833496	1
MSTRG.31435.8	414	0.41256	5.316833496	1
MSTRG.31436.1	48	0.32646	5.956481934	1
MSTRG.31440.1	33	0.45015	9.18560791	-1
MSTRG.31459.1	252	0.44023	6.757629395	1
MSTRG.31474.1	1721	0.4611	6.190124512	1
MSTRG.31474.2	319	0.31958	9.096618652	1
MSTRG.31476.1	43	0.37397	10.62042236	1
MSTRG.31477.1	186	0.28982	10.28631592	1
MSTRG.31474.3	104	0.38046	9.824279785	1
MSTRG.31478.1	169	0.28667	4.932922363	1
MSTRG.31477.2	80	0.28478	11.65460205	1
MSTRG.31479.1	117	0.34775	8.519714355	1
MSTRG.31474.4	95	0.28982	8.539855957	1
MSTRG.31474.6	37	0.36129	9.218322754	1
MSTRG.31474.7	107	0.28982	5.472717285	1
MSTRG.31480.1	104	0.39393	8.81829834	1
MSTRG.31468.2	698	0.41944	6.178771973	1
MSTRG.31468.4	140	0.39081	5.047302246	1
MSTRG.31468.5	140	0.39118	5.047302246	1
MSTRG.31484.1	1181	0.38182	8.923278809	1
MSTRG.31485.1	124	0.2921	7.72076416	1

MSTRG.31485.2	124	0.28982	7.72076416	1
MSTRG.31493.1	476	0.34547	6.228820801	1
MSTRG.31493.2	598	0.37806	8.520690918	1
MSTRG.31505.1	67	0.32237	10.39654541	1
MSTRG.31506.1	237	0.29088	8.174255371	1
MSTRG.31505.2	189	0.28982	9.813537598	1
MSTRG.31505.4	112	0.29607	9.182800293	1
MSTRG.31505.3	183	0.28982	10.39141846	1
MSTRG.31507.1	243	0.29595	8.597595215	1
MSTRG.31505.5	137	0.27289	11.03106689	1
MSTRG.31495.1	105	0.30524	10.8147583	1
MSTRG.31511.1	82	0.29892	8.858703613	1
MSTRG.31511.2	82	0.29367	8.858703613	1
MSTRG.31512.1	118	0.28562	4.714172363	1
MSTRG.31510.4	182	0.34427	6.167785645	1
MSTRG.31510.5	172	0.31512	11.20111084	1
MSTRG.31510.7	172	0.30644	11.20111084	1
MSTRG.31510.8	117	0.33061	8.732727051	1
MSTRG.31510.9	29	0.30375	8.345397949	1
MSTRG.31513.1	131	0.28165	9.453430176	1
MSTRG.31515.1	114	0.33466	10.03387451	1
MSTRG.31517.1	41	0.39337	8.38458252	-1
MSTRG.31522.1	70	0.45838	10.27728271	-1
MSTRG.31525.1	234	0.44456	9.449035645	1
MSTRG.31525.2	535	0.48034	6.563537598	1
MSTRG.31526.1	30	0.40187	5.971618652	-1
MSTRG.31527.1	81	0.33179	9.693664551	1
MSTRG.31531.1	144	0.42059	6.581848145	1
MSTRG.31531.2	81	0.32823	9.179992676	1
MSTRG.31532.2	557	0.45607	4.726501465	1
MSTRG.31531.4	987	0.50079	5.685241699	1
MSTRG.31533.1	55	0.30085	9.890686035	1
MSTRG.31531.8	89	0.36845	5.609558105	1
MSTRG.31536.1	53	0.35757	11.3682251	1
MSTRG.31537.13	177	0.43611	6.124938965	1
MSTRG.31543.1	1498	0.40547	7.163024902	1
MSTRG.31544.3	82	0.32037	9.567932129	1
MSTRG.31546.5	568	0.47883	9.96697998	1
MSTRG.31546.7	1149	0.46219	5.638122559	1
MSTRG.31548.1	385	0.29202	8.86126709	1
MSTRG.31548.2	385	0.29202	8.86126709	1
MSTRG.31548.3	385	0.29202	8.86126709	1
MSTRG.31548.4	488	0.29202	9.112121582	1
MSTRG.31548.5	385	0.28518	8.86126709	1
MSTRG.31549.3	81	0.47146	5.52935791	1
MSTRG.31551.3	293	0.41142	5.233581543	1
MSTRG.31551.2	331	0.42447	5.681335449	1
MSTRG.31552.1	90	0.32614	6.380065918	1
MSTRG.31551.4	300	0.3865	5.507019043	1
MSTRG.31559.1	348	0.38439	6.738830566	1
MSTRG.31559.3	302	0.35421	6.485046387	1
MSTRG.31559.4	302	0.3908	6.485046387	1
MSTRG.31559.5	302	0.3908	6.485046387	1
MSTRG.31559.6	302	0.36467	6.485046387	1

MSTRG.31563.1	463	0.44292	5.949035645	1
MSTRG.31564.2	921	0.45605	6.210510254	1
MSTRG.31565.1	17	0.37436	8.189025879	1
MSTRG.31566.2	172	0.37611	8.870178223	1
MSTRG.31570.4	373	0.43571	8.534606934	1
MSTRG.31570.5	568	0.45274	6.158752441	1
MSTRG.31570.3	568	0.33019	6.158752441	1
MSTRG.31570.6	568	0.43678	6.158752441	1
MSTRG.31570.9	384	0.38233	8.893859863	1
MSTRG.31570.8	296	0.42252	9.212097168	1
MSTRG.31570.7	568	0.29308	6.158752441	1
MSTRG.31571.2	73	0.31678	8.885803223	1
MSTRG.31571.1	132	0.28396	10.52593994	1
MSTRG.31571.3	132	0.2782	10.52593994	1
MSTRG.31571.4	132	0.28396	10.52593994	1
MSTRG.31567.2	184	0.45141	6.21685791	1
MSTRG.31576.1	33	0.44745	8.34197998	-1
MSTRG.31581.4	329	0.32863	9.436706543	1
MSTRG.31586.5	120	0.37079	6.564758301	-1
MSTRG.31584.2	268	0.33265	6.794250488	1
MSTRG.31591.1	56	0.40327	4.616027832	1
MSTRG.31598.1	497	0.3548	5.237243652	1
MSTRG.31598.2	424	0.36506	4.767272949	1
MSTRG.31598.3	424	0.36506	4.767272949	1
MSTRG.31598.5	424	0.36258	4.767272949	1
MSTRG.31598.4	424	0.3871	4.767272949	1
MSTRG.31599.3	357	0.45437	5.614562988	1
MSTRG.31599.4	357	0.45437	5.614562988	1
MSTRG.31597.1	229	0.41222	4.943054199	1
MSTRG.31597.2	229	0.40757	4.943054199	1
MSTRG.31603.2	375	0.36473	8.90838623	1
MSTRG.31603.3	399	0.43594	7.71697998	1
MSTRG.31603.4	400	0.43602	7.71697998	1
MSTRG.31603.8	410	0.45358	8.127868652	1
MSTRG.31603.11	205	0.3642	7.125305176	1
MSTRG.31602.2	246	0.28958	10.18389893	1
MSTRG.31606.1	312	0.31793	7.690124512	1
MSTRG.31606.2	312	0.33851	7.690124512	1
MSTRG.31606.3	49	0.31209	9.435241699	1
MSTRG.31606.4	84	0.36432	10.19232178	1
MSTRG.31607.1	57	0.33254	5.777038574	1
MSTRG.31606.6	120	0.28334	5.359558105	1
MSTRG.31606.7	351	0.33686	8.83404541	1
MSTRG.31606.8	286	0.30576	9.689758301	1
MSTRG.31608.1	108	0.27398	9.124450684	1
MSTRG.31606.10	312	0.32237	7.690124512	1
MSTRG.31606.11	312	0.32237	7.690124512	1
MSTRG.31617.1	663	0.45353	4.809631348	1
MSTRG.31618.1	509	0.42872	5.855651855	1
MSTRG.31618.4	304	0.40617	5.023498535	1
MSTRG.31618.5	304	0.40617	5.023498535	1
MSTRG.31619.1	240	0.33674	4.980529785	1
MSTRG.31620.1	80	0.3434	11.54803467	1
MSTRG.31622.2	888	0.47813	5.266052246	1

MSTRG.31623.4	463	0.37421	6.375671387	1
MSTRG.31625.1	46	0.41281	8.231018066	1
MSTRG.31626.3	1632	0.35669	7.792541504	1
MSTRG.31626.4	1632	0.33883	7.792541504	1
MSTRG.31626.5	1632	0.34283	7.792541504	1
MSTRG.31627.1	26	0.39513	9.992614746	1
MSTRG.31613.2	1094	0.42091	6.711364746	1
MSTRG.31613.3	1094	0.3931	6.711364746	1
MSTRG.31614.1	211	0.2972	9.475891113	1
MSTRG.31630.5	1702	0.45284	6.077087402	-1
MSTRG.31630.6	1400	0.44075	6.27911377	-1
MSTRG.31633.1	56	0.37619	11.43719482	1
MSTRG.31633.2	82	0.35768	9.731262207	1
MSTRG.31629.1	180	0.30377	5.879455566	1
MSTRG.31636.5	418	0.40422	6.471252441	1
MSTRG.31637.2	120	0.47659	5.041687012	1
MSTRG.31637.3	166	0.46339	5.44354248	1
MSTRG.31638.1	142	0.30816	10.27655029	1
MSTRG.31640.1	185	0.32157	10.52655029	1
MSTRG.31641.1	64	0.29217	4.441223145	1
MSTRG.31642.1	94	0.33081	5.089294434	1
MSTRG.31643.1	96	0.32197	5.973815918	1
MSTRG.31637.6	135	0.41827	4.917297363	1
MSTRG.31644.2	211	0.31137	9.728088379	1
MSTRG.31644.5	1871	0.43999	5.491394043	1
MSTRG.31648.1	198	0.34359	10.5512085	1
MSTRG.31648.2	99	0.39937	10.37493896	1
MSTRG.31648.3	447	0.31287	7.92364502	1
MSTRG.31648.5	415	0.32388	8.351257324	1
MSTRG.31650.1	17	0.41344	12.00848389	1
MSTRG.31651.2	294	0.41011	7.47467041	1
MSTRG.31661.1	84	0.31905	8.480773926	1
MSTRG.31662.1	58	0.33229	6.003601074	1
MSTRG.31672.2	105	0.46639	9.552307129	1
MSTRG.31672.3	462	0.46302	5.629577637	1
MSTRG.31673.1	97	0.36033	6.226379395	1
MSTRG.31678.2	160	0.28491	9.458068848	1
MSTRG.31678.1	160	0.28491	9.458068848	1
MSTRG.31679.2	1865	0.49139	6.952331543	1
MSTRG.31682.1	61	0.36353	10.32318115	1
MSTRG.31690.1	71	0.29948	9.692687988	1
MSTRG.31691.1	1072	0.39896	7.089050293	1
MSTRG.31691.2	1066	0.39896	7.090637207	1
MSTRG.31692.1	43	0.43892	8.938903809	1
MSTRG.31691.3	989	0.42475	6.932678223	1
MSTRG.31700.1	124	0.33015	8.433288574	-1
MSTRG.31703.2	169	0.40852	8.121276855	1
MSTRG.31694.1	162	0.45998	7.772521973	-1
MSTRG.31721.4	175	0.39222	4.474182129	1
MSTRG.31722.2	125	0.40841	4.910339355	1
MSTRG.31723.5	212	0.42426	9.000427246	1
MSTRG.31723.6	201	0.44828	9.164978027	1
MSTRG.31725.2	270	0.38145	5.815490723	1
MSTRG.31725.3	270	0.38083	5.815490723	1

MSTRG.31725.4	157	0.30736	8.113952637	1
MSTRG.31729.1	42	0.36183	5.983581543	1
MSTRG.31742.2	345	0.37248	9.872741699	1
MSTRG.31740.1	674	0.47328	6.26385498	1
MSTRG.31743.1	112	0.39908	9.913513184	1
MSTRG.31743.2	112	0.42364	9.913513184	1
MSTRG.31743.3	112	0.39851	9.913513184	1
MSTRG.31745.3	581	0.36078	5.808776855	1
MSTRG.31745.6	493	0.43627	7.327209473	1
MSTRG.31745.7	383	0.40569	8.365905762	1
MSTRG.31746.1	76	0.34554	9.557556152	1
MSTRG.31747.1	70	0.35537	7.999084473	1
MSTRG.31745.10	103	0.35024	10.65814209	1
MSTRG.31748.1	50	0.40543	4.319274902	1
MSTRG.31748.2	27	0.48193	10.27716064	1
MSTRG.31748.3	50	0.34544	4.319274902	1
MSTRG.31749.1	90	0.37937	8.373474121	1
MSTRG.31750.1	67	0.44076	9.987976074	1
MSTRG.31753.1	315	0.4345	8.504577637	-1
MSTRG.31757.4	1187	0.46897	5.319641113	1
MSTRG.31757.5	1193	0.50632	5.280700684	1
MSTRG.31758.1	748	0.36223	7.940734863	1
MSTRG.31766.1	128	0.28982	6.040100098	1
MSTRG.31777.1	39	0.34913	8.844543457	1
MSTRG.31763.1	74	0.38043	9.48614502	1
MSTRG.31786.3	70	0.33115	9.86541748	1
MSTRG.31786.2	989	0.47607	4.50177002	1
MSTRG.31787.1	63	0.36156	6.893981934	1
MSTRG.31786.4	746	0.42264	9.64239502	1
MSTRG.31786.7	505	0.38568	9.487365723	1
MSTRG.31786.10	103	0.40009	8.712585449	-1
MSTRG.31786.11	511	0.39388	9.280456543	1
MSTRG.31786.12	290	0.42264	9.033508301	1
MSTRG.31796.5	384	0.46801	7.127380371	1
MSTRG.31796.4	427	0.35599	6.155212402	1
MSTRG.31796.6	351	0.35599	5.344665527	1
MSTRG.31797.1	96	0.27433	9.299987793	1
MSTRG.31799.1	616	0.40418	6.504211426	1
MSTRG.31801.1	111	0.37951	10.0713501	1
MSTRG.31801.2	111	0.36366	10.0713501	1
MSTRG.31801.3	122	0.36607	10.46246338	1
MSTRG.31801.4	95	0.35913	10.98626709	1
MSTRG.31801.5	95	0.30911	10.98626709	1
MSTRG.31801.6	71	0.32951	10.17266846	1
MSTRG.31801.7	57	0.36411	6.67401123	1
MSTRG.31801.8	92	0.33447	7.856994629	1
MSTRG.31789.1	229	0.44259	9.72454834	1
MSTRG.31789.2	261	0.47241	9.791687012	1
MSTRG.31790.1	323	0.44587	6.739440918	1
MSTRG.31792.2	246	0.38485	9.963684082	1
MSTRG.31792.3	245	0.48552	9.963684082	-1
MSTRG.31793.1	45	0.30785	8.491271973	1
MSTRG.31793.2	45	0.28783	8.491271973	1
MSTRG.31792.4	219	0.43346	8.992370605	1

MSTRG.31803.1	1481	0.42466	8.49786377	1
MSTRG.31803.3	343	0.36682	9.299255371	1
MSTRG.31804.1	70	0.35705	11.19024658	1
MSTRG.31805.5	51	0.3143	6.516662598	1
MSTRG.31805.3	119	0.42723	5.86505127	1
MSTRG.31805.1	91	0.35742	10.03131104	1
MSTRG.31805.4	107	0.40116	9.758483887	1
MSTRG.31806.1	62	0.45479	10.10601807	1
MSTRG.31810.1	77	0.43947	9.12298584	1
MSTRG.31812.5	626	0.38664	5.540100098	1
MSTRG.31812.9	626	0.39604	5.540100098	1
MSTRG.31812.8	626	0.40619	5.540100098	1
MSTRG.31813.1	41	0.31402	7.680480957	-1
MSTRG.31812.12	40	0.34466	11.61004639	1
MSTRG.31812.10	40	0.33462	11.61004639	1
MSTRG.31815.4	1119	0.47913	4.727844238	1
MSTRG.31815.2	1120	0.46701	4.72088623	1
MSTRG.31815.3	1119	0.46701	4.72088623	1
MSTRG.31815.5	358	0.44509	5.946838379	1
MSTRG.31818.2	666	0.46024	8.748474121	-1
MSTRG.31818.3	392	0.44038	9.348815918	1
MSTRG.31818.7	392	0.42462	9.348815918	1
MSTRG.31820.2	289	0.29294	8.290710449	1
MSTRG.31820.3	200	0.3227	7.054138184	1
MSTRG.31820.4	289	0.29152	8.290710449	1
MSTRG.31820.5	200	0.29294	7.054138184	1
MSTRG.31820.6	289	0.29152	8.290710449	1
MSTRG.31820.7	200	0.294	7.054138184	1
MSTRG.31820.8	289	0.294	8.290710449	1
MSTRG.31820.9	73	0.27781	6.250793457	1
MSTRG.31822.2	472	0.44299	8.098449707	1
MSTRG.31824.1	120	0.2921	9.439880371	1
MSTRG.31826.1	73	0.33718	5.497619629	1
MSTRG.31829.1	41	0.37395	6.949890137	1
MSTRG.31833.4	273	0.41513	9.515441895	1
MSTRG.31838.1	40	0.33276	5.426086426	1
MSTRG.31839.2	476	0.46315	6.46697998	1
MSTRG.31865.1	315	0.47755	6.009338379	1
MSTRG.31867.3	2047	0.43852	5.614196777	1
MSTRG.31867.5	2320	0.45395	5.457214355	1
MSTRG.31867.7	2289	0.45395	5.528747559	1
MSTRG.31867.6	2325	0.44135	5.491149902	1
MSTRG.31868.1	76	0.39442	9.897399902	1
MSTRG.31867.8	52	0.3305	11.71258545	1
MSTRG.31859.4	591	0.45762	6.280456543	1
MSTRG.31859.3	589	0.45534	6.067443848	1
MSTRG.31861.2	292	0.34213	8.797790527	1
MSTRG.31875.1	53	0.31337	11.63653564	1
MSTRG.31883.1	54	0.44888	10.00872803	1
MSTRG.31885.3	180	0.33623	8.063781738	1
MSTRG.31885.4	144	0.34628	8.645812988	1
MSTRG.31886.1	88	0.30303	5.788024902	1
MSTRG.31886.2	88	0.29059	5.788024902	1
MSTRG.31897.1	169	0.32072	5.683288574	1

MSTRG.31898.1	163	0.35469	9.974060059	1
MSTRG.31897.2	208	0.34731	9.854553223	1
MSTRG.31904.1	68	0.36729	11.92462158	1
MSTRG.31907.1	156	0.34563	10.40887451	1
MSTRG.31905.1	69	0.39076	10.48516846	1
MSTRG.31909.1	109	0.38714	7.859069824	1
MSTRG.31910.1	375	0.40505	9.542907715	1
MSTRG.31911.2	133	0.41958	6.061828613	1
MSTRG.31914.2	90	0.27454	4.996765137	1
MSTRG.31914.3	84	0.30483	8.63458252	1
MSTRG.31917.7	45	0.35527	8.771057129	1
MSTRG.31919.1	303	0.33085	7.972839355	1
MSTRG.31920.6	413	0.46611	5.63861084	1
MSTRG.31920.8	315	0.47769	5.252990723	1
MSTRG.31920.9	315	0.47769	5.252990723	1
MSTRG.31923.1	72	0.39647	7.836486816	1
MSTRG.31923.2	100	0.44146	8.397644043	1
MSTRG.31994.3	216	0.4474	9.210754395	1
MSTRG.31998.2	144	0.46478	9.041931152	1
MSTRG.31999.1	178	0.3592	7.662658691	1
MSTRG.32003.1	231	0.36388	10.09539795	1
MSTRG.32003.2	231	0.36388	10.09539795	1
MSTRG.32016.1	183	0.38524	10.17498779	1
MSTRG.32029.1	70	0.35276	7.84576416	1
MSTRG.32030.1	66	0.32624	6.898742676	1
MSTRG.32051.1	31	0.35188	9.996276855	1
MSTRG.32052.1	352	0.36365	8.140808105	1
MSTRG.32052.2	366	0.36365	7.063049316	1
MSTRG.31935.1	527	0.35736	6.398010254	1
MSTRG.31935.2	528	0.45095	6.398254395	1
MSTRG.31935.6	522	0.38015	6.162658691	1
MSTRG.31935.8	519	0.39906	6.375061035	1
MSTRG.31935.9	410	0.42508	6.046081543	1
MSTRG.31935.10	64	0.4218	5.51348877	1
MSTRG.31936.3	48	0.36357	4.319274902	1
MSTRG.31939.1	65	0.38645	8.801208496	1
MSTRG.31939.2	65	0.40575	8.801208496	1
MSTRG.31939.3	60	0.38897	5.503723145	1
MSTRG.31939.4	30	0.39462	9.77911377	1
MSTRG.31940.1	100	0.30709	5.107727051	1
MSTRG.31941.1	84	0.33101	6.490661621	1
MSTRG.31941.2	72	0.29772	10.00872803	1
MSTRG.31941.3	72	0.29367	10.00872803	1
MSTRG.31941.4	72	0.29595	10.00872803	1
MSTRG.31942.4	94	0.28527	10.18756104	1
MSTRG.31928.2	699	0.43219	7.315734863	1
MSTRG.31930.1	86	0.43293	6.876403809	-1
MSTRG.31930.2	389	0.35209	8.35345459	1
MSTRG.31948.1	98	0.32993	6.48651123	1
MSTRG.31963.1	51	0.40364	9.996887207	1
MSTRG.31968.1	150	0.34428	10.94744873	1
MSTRG.31981.1	158	0.31287	9.319763184	1
MSTRG.31990.1	85	0.3231	7.634338379	1
MSTRG.31992.2	2696	0.42449	6.074645996	1

MSTRG.32053.1	478	0.41359	7.624938965	1
MSTRG.32054.1	146	0.45585	8.432678223	1
MSTRG.32056.2	110	0.29772	8.091247559	1
MSTRG.32056.3	570	0.29988	6.682067871	1
MSTRG.32058.1	170	0.3272	11.20367432	1
MSTRG.32059.1	328	0.4349	4.925720215	1
MSTRG.32068.2	438	0.39455	9.479064941	1
MSTRG.32055.2	72	0.3896	8.96270752	1
MSTRG.32069.2	74	0.3594	6.696228027	1
MSTRG.32070.3	122	0.37646	6.72857666	1
MSTRG.32072.3	76	0.3208	10.42791748	1
MSTRG.32076.5	459	0.35625	9.067810059	1
MSTRG.32076.7	320	0.3653	9.499816895	1
MSTRG.32076.3	464	0.3535	9.712585449	1
MSTRG.32076.4	563	0.37981	9.713317871	1
MSTRG.32076.6	377	0.35726	9.977111816	1
MSTRG.32077.1	26	0.43137	8.023376465	1
MSTRG.32078.1	389	0.35909	9.02911377	1
MSTRG.32084.1	102	0.30068	10.28485107	1
MSTRG.32085.1	54	0.3927	6.94744873	1
MSTRG.32086.1	107	0.3199	6.25567627	1
MSTRG.32091.1	123	0.38939	10.69378662	1
MSTRG.32091.2	139	0.44272	9.860656738	1
MSTRG.32104.3	138	0.43453	4.643249512	1
MSTRG.32105.1	85	0.3601	8.780822754	-1
MSTRG.32106.1	31	0.38819	5.823303223	1
MSTRG.32107.1	42	0.31977	6.88873291	1
MSTRG.32108.2	51	0.41042	8.837341309	1
MSTRG.32108.1	45	0.27818	9.222351074	1
MSTRG.32109.1	57	0.30925	5.573181152	1
MSTRG.32110.1	65	0.31122	9.09942627	1
MSTRG.32104.4	94	0.29343	7.58770752	1
MSTRG.32111.1	108	0.30918	8.321838379	1
MSTRG.32112.1	73	0.30804	4.63458252	1
MSTRG.32094.1	68	0.44626	9.468322754	-1
MSTRG.32094.2	68	0.44731	9.468322754	-1
MSTRG.32113.1	63	0.51241	8.010070801	-1
MSTRG.32114.2	37	0.44097	4.352355957	1
MSTRG.32115.1	30	0.29695	11.43719482	1
MSTRG.32116.2	75	0.30104	9.326843262	1
MSTRG.32119.1	52	0.31377	9.393615723	1
MSTRG.32123.1	27	0.32918	9.688171387	1
MSTRG.32128.1	87	0.35005	7.73638916	1
MSTRG.32129.1	56	0.39514	11.27325439	-1
MSTRG.32130.2	47	0.36755	9.494689941	-1
MSTRG.32130.1	63	0.39286	10.0479126	1
MSTRG.32130.3	76	0.36854	8.667053223	1
MSTRG.32133.1	152	0.32388	9.93572998	1
MSTRG.32134.1	360	0.35415	5.04107666	1
MSTRG.32134.2	460	0.37751	4.848327637	1
MSTRG.32134.4	75	0.2921	10.29888916	1
MSTRG.32137.1	2068	0.39136	8.884216309	1
MSTRG.32137.2	2068	0.40398	8.884216309	1
MSTRG.32137.3	2068	0.41661	8.884216309	1

MSTRG.32136.1	129	0.32544	10.06744385	1
MSTRG.32140.1	27	0.34933	11.82440186	1
MSTRG.32141.1	849	0.4559	8.472717285	1
MSTRG.32142.1	111	0.34081	9.690734863	1
MSTRG.32143.4	425	0.45543	8.957092285	1
MSTRG.32146.1	539	0.46206	4.865539551	1
MSTRG.32154.2	139	0.32508	9.500061035	1
MSTRG.32155.1	597	0.3961	9.596496582	1
MSTRG.32155.2	597	0.39341	9.596496582	1
MSTRG.32158.3	211	0.48983	5.902160645	-1
MSTRG.32163.1	58	0.45871	9.246643066	-1
MSTRG.32164.1	361	0.48349	8.845031738	1
MSTRG.32150.2	344	0.40946	8.951599121	1
MSTRG.32150.3	548	0.46239	8.884094238	1
MSTRG.32167.2	83	0.33234	9.688171387	1
MSTRG.32167.3	79	0.32305	9.198181152	1
MSTRG.32168.1	31	0.39551	6.009094238	1
MSTRG.32169.2	158	0.36406	7.001403809	1
MSTRG.32166.1	77	0.42773	9.694885254	1
MSTRG.32172.2	187	0.2996	4.725524902	1
MSTRG.32176.4	486	0.40811	8.632385254	1
MSTRG.32176.3	331	0.39282	8.296691895	1
MSTRG.32177.1	147	0.43999	4.88470459	1
MSTRG.32180.1	434	0.30835	6.903259277	1
MSTRG.32181.4	469	0.4185	9.10345459	1
MSTRG.32183.6	118	0.28739	11.67352295	1
MSTRG.32184.1	76	0.32914	6.695373535	1
MSTRG.32184.3	115	0.41021	9.032897949	1
MSTRG.32199.1	144	0.28432	10.76629639	1
MSTRG.32199.2	54	0.35469	9.300476074	1
MSTRG.32199.3	126	0.27691	7.883483887	1
MSTRG.32201.1	111	0.46622	9.181213379	1
MSTRG.32203.2	483	0.46265	6.139587402	1
MSTRG.32203.4	369	0.4627	8.487487793	1
MSTRG.32206.2	351	0.43808	6.149841309	1
MSTRG.32206.4	381	0.39474	6.651184082	1
MSTRG.32206.5	351	0.44188	6.149841309	1
MSTRG.32208.2	316	0.46268	5.637268066	1
MSTRG.32204.2	121	0.30515	8.092346191	1
MSTRG.32212.1	119	0.32785	9.162536621	1
MSTRG.32225.1	96	0.38037	8.461486816	1
MSTRG.32227.1	36	0.32131	7.999084473	-1
MSTRG.32229.2	142	0.3841	9.545837402	1
MSTRG.32232.1	2043	0.4119	6.68951416	1
MSTRG.32232.2	2082	0.41469	6.72467041	1
MSTRG.32231.1	65	0.43989	10.41998291	-1
MSTRG.32231.2	64	0.42125	10.41986084	-1
MSTRG.32237.2	154	0.44393	6.59954834	1
MSTRG.32238.1	72	0.41637	9.255310059	1
MSTRG.32239.1	181	0.42138	4.932189941	1
MSTRG.32241.1	39	0.40989	4.075378418	1
MSTRG.32242.1	101	0.41233	9.34967041	1
MSTRG.32246.2	503	0.28855	9.638122559	1
MSTRG.32246.3	1005	0.42308	6.576477051	1

MSTRG.32249.1	453	0.34971	5.886901855	1
MSTRG.32249.5	615	0.35391	5.64654541	1
MSTRG.32250.1	111	0.35224	9.546936035	1
MSTRG.32251.1	58	0.38928	11.55377197	1
MSTRG.32251.2	123	0.31793	5.195617676	1
MSTRG.32252.1	45	0.38871	5.042541504	1
MSTRG.32254.1	1057	0.43591	5.196716309	1
MSTRG.32257.1	50	0.33008	6.366760254	1
MSTRG.32260.2	281	0.40026	9.80255127	1
MSTRG.32265.1	235	0.43238	5.159729004	1
MSTRG.32266.1	127	0.37519	9.585144043	1
MSTRG.32267.1	127	0.38387	9.206604004	1
MSTRG.32270.2	154	0.34227	10.12969971	1
MSTRG.32275.3	242	0.33799	9.415344238	1
MSTRG.32275.1	242	0.31738	9.415344238	1
MSTRG.32275.2	242	0.34912	9.415344238	1
MSTRG.32275.6	242	0.36148	9.415344238	1
MSTRG.32275.7	242	0.34608	9.415344238	1
MSTRG.32276.4	254	0.35188	8.797424316	1
MSTRG.32278.3	479	0.405	8.67376709	1
MSTRG.32278.4	470	0.405	8.193908691	1
MSTRG.32277.1	292	0.36602	6.069763184	1
MSTRG.32283.1	269	0.40168	9.164489746	1
MSTRG.32283.2	257	0.40743	8.931335449	1
MSTRG.32285.1	65	0.29936	10.53009033	1
MSTRG.32286.1	211	0.33512	9.38861084	1
MSTRG.32289.1	466	0.4216	6.75592041	-1
MSTRG.32291.2	74	0.40342	8.085021973	1
MSTRG.32292.1	380	0.43467	8.683654785	1
MSTRG.32292.3	380	0.44034	8.683654785	1
MSTRG.32293.1	397	0.4151	9.460388184	1
MSTRG.32300.3	314	0.42022	6.393859863	1
MSTRG.32300.5	322	0.42792	6.394592285	1
MSTRG.32301.3	585	0.47768	6.074645996	1
MSTRG.32305.2	308	0.38079	7.001159668	1
MSTRG.32324.12	3350	0.44722	5.846496582	1
MSTRG.32324.17	3425	0.44722	5.829162598	1
MSTRG.32324.18	3572	0.44474	5.767883301	1
MSTRG.32324.20	3270	0.4395	5.839416504	1
MSTRG.32324.21	3668	0.44722	5.795593262	1
MSTRG.32324.3	2125	0.44004	6.083679199	1
MSTRG.32324.33	3338	0.44722	5.901428223	1
MSTRG.32324.35	2967	0.44438	5.890319824	1
MSTRG.32324.38	3270	0.4395	5.831237793	1
MSTRG.32324.40	2973	0.44438	5.873596191	1
MSTRG.32324.43	3600	0.44722	5.821960449	1
MSTRG.32324.47	4011	0.44102	5.815979004	1
MSTRG.32324.49	3614	0.44102	5.751525879	1
MSTRG.32324.50	3850	0.44102	5.742858887	1
MSTRG.32324.53	2758	0.44066	5.940979004	1
MSTRG.32324.55	3350	0.44102	5.854675293	1
MSTRG.32324.56	4058	0.44102	5.755432129	1
MSTRG.32324.7	3142	0.4457	5.863098145	1
MSTRG.32324.9	3936	0.44102	5.820495605	1

MSTRG.32310.3	944	0.44654	5.784118652	1
MSTRG.32310.4	966	0.44654	5.864807129	1
MSTRG.32322.7	396	0.46479	5.730285645	1
MSTRG.32322.10	394	0.44517	5.730285645	1
MSTRG.32322.12	397	0.44517	5.730285645	1
MSTRG.32322.8	394	0.42671	5.730285645	1
MSTRG.32322.11	397	0.42485	5.730285645	1
MSTRG.32322.3	396	0.42671	5.730285645	1
MSTRG.32322.4	460	0.44588	5.42791748	1
MSTRG.32322.9	460	0.47224	5.42791748	1
MSTRG.32322.2	460	0.43235	5.42791748	1
MSTRG.32322.16	460	0.47016	5.42791748	1
MSTRG.32323.1	75	0.29455	7.652038574	1
MSTRG.32320.3	191	0.37573	9.399963379	1
MSTRG.32331.1	513	0.34908	5.015197754	1
MSTRG.32331.3	513	0.34908	5.015197754	1
MSTRG.32331.4	513	0.37218	5.015197754	1
MSTRG.32331.5	513	0.4423	5.015197754	1
MSTRG.32331.6	531	0.43623	4.987731934	1
MSTRG.32334.1	619	0.40661	9.130432129	1
MSTRG.32334.2	619	0.40599	9.130432129	1
MSTRG.32334.3	619	0.45555	9.130432129	1
MSTRG.32338.1	22	0.35876	8.93963623	1
MSTRG.32339.2	62	0.40692	9.494934082	1
MSTRG.32339.1	134	0.2872	7.698425293	1
MSTRG.32339.3	113	0.34056	9.985534668	1
MSTRG.32338.4	385	0.41078	8.484802246	1
MSTRG.32338.6	385	0.36773	8.484802246	1
MSTRG.32338.7	385	0.39898	8.484802246	1
MSTRG.32329.1	160	0.33787	6.06072998	1
MSTRG.32329.2	179	0.30917	11.78814697	1
MSTRG.32329.3	94	0.30429	12.66937256	1
MSTRG.32329.5	94	0.36869	12.66937256	1
MSTRG.32329.6	108	0.32431	6.256164551	1
MSTRG.32329.7	94	0.40829	12.66937256	1
MSTRG.32340.2	368	0.29772	5.07635498	1
MSTRG.32340.4	226	0.4151	4.956848145	1
MSTRG.32342.1	237	0.36706	8.670349121	1
MSTRG.32343.2	75	0.43518	8.81842041	1
MSTRG.32344.1	46	0.39759	11.35064697	1
MSTRG.32350.3	732	0.46122	9.692321777	1
MSTRG.32354.2	241	0.43298	5.235412598	1
MSTRG.32354.3	129	0.43376	5.015075684	1
MSTRG.32356.1	677	0.40947	5.957214355	1
MSTRG.32358.2	429	0.45654	6.211364746	1
MSTRG.32358.3	550	0.4573	6.082824707	1
MSTRG.32358.4	368	0.4778	5.882873535	1
MSTRG.32364.3	150	0.38784	6.569885254	1
MSTRG.32365.1	127	0.34815	10.5947876	1
MSTRG.32365.2	127	0.33606	10.5947876	1
MSTRG.32366.5	111	0.34558	9.554016113	1
MSTRG.32369.4	157	0.28583	10.92559814	1
MSTRG.32369.3	168	0.3127	7.036682129	1
MSTRG.32371.2	395	0.33918	9.189025879	1

MSTRG.32371.3	395	0.36811	9.189025879	1
MSTRG.32359.2	326	0.41974	8.787658691	1
MSTRG.32372.1	465	0.43513	5.881164551	1
MSTRG.32377.1	183	0.36119	8.829772949	1
MSTRG.32381.7	417	0.41151	6.034973145	1
MSTRG.32381.11	1479	0.42505	5.550964355	1
MSTRG.32381.10	1247	0.41531	5.56048584	1
MSTRG.32381.2	1441	0.41217	5.330505371	1
MSTRG.32381.3	1171	0.4294	5.354797363	1
MSTRG.32381.1	1162	0.4294	5.353210449	1
MSTRG.32381.8	1450	0.41217	5.331970215	1
MSTRG.32381.12	1457	0.4135	5.335021973	1
MSTRG.32381.4	1686	0.39255	5.422180176	1
MSTRG.32381.6	810	0.3739	5.428771973	1
MSTRG.32381.14	167	0.30593	8.598449707	1
MSTRG.32378.1	56	0.39797	7.689025879	1
MSTRG.32384.10	456	0.36151	9.158752441	1
MSTRG.32384.11	453	0.41783	9.073425293	1
MSTRG.32384.5	433	0.43323	9.406311035	1
MSTRG.32388.1	482	0.47523	6.691955566	1
MSTRG.32395.5	226	0.3889	10.41217041	1
MSTRG.32395.2	226	0.36099	10.41217041	1
MSTRG.32395.3	226	0.40099	10.41217041	1
MSTRG.32395.4	226	0.36792	10.41217041	1
MSTRG.32395.7	226	0.40426	10.41217041	1
MSTRG.32394.2	79	0.42995	11.93658447	1
MSTRG.32397.2	258	0.41212	4.761291504	1
MSTRG.32397.3	463	0.43399	5.773498535	1
MSTRG.32397.4	451	0.465	5.513000488	1
MSTRG.32397.5	278	0.3417	9.329772949	1
MSTRG.32397.6	290	0.34635	9.447937012	1
MSTRG.32402.1	418	0.424	8.387268066	1
MSTRG.32405.3	76	0.35729	8.094421387	1
MSTRG.32407.1	52	0.37942	9.354553223	-1
MSTRG.32411.2	288	0.30415	5.20526123	1
MSTRG.32412.2	390	0.37931	5.821960449	1
MSTRG.32411.3	65	0.29919	9.839294434	1
MSTRG.32411.4	81	0.32022	10.84295654	1
MSTRG.32411.5	65	0.34797	9.839294434	1
MSTRG.32415.1	192	0.3761	4.976257324	1
MSTRG.32416.3	872	0.4388	5.632629395	1
MSTRG.32418.6	1861	0.41139	6.577209473	-1
MSTRG.32418.9	1864	0.41139	6.496398926	-1
MSTRG.32419.2	1041	0.41668	5.743103027	1
MSTRG.32424.1	267	0.37473	9.018615723	1
MSTRG.32427.1	129	0.36274	12.07440186	1
MSTRG.32434.2	197	0.37416	6.243225098	1
MSTRG.32441.1	115	0.36424	6.339172363	1
MSTRG.32441.2	115	0.38593	6.339172363	1
MSTRG.32441.3	115	0.39192	6.339172363	1
MSTRG.32444.1	87	0.35024	10.71405029	1
MSTRG.32444.2	78	0.40913	10.66558838	1
MSTRG.32456.3	256	0.41851	6.365661621	1
MSTRG.32456.2	256	0.38352	6.365661621	1

MSTRG.32447.2	150	0.42084	9.371887207	1
MSTRG.32451.2	337	0.44363	6.807678223	1
MSTRG.32457.1	82	0.30464	6.011779785	1
MSTRG.32484.5	362	0.42777	6.60736084	1
MSTRG.32484.6	362	0.37096	6.60736084	1
MSTRG.32484.1	669	0.33996	9.678894043	1
MSTRG.32484.4	362	0.42077	6.60736084	1
MSTRG.32484.3	143	0.40373	5.681091309	1
MSTRG.32484.2	362	0.39218	6.60736084	1
MSTRG.32484.7	551	0.31946	9.787780762	1
MSTRG.32484.8	551	0.27678	9.787780762	1
MSTRG.32484.9	362	0.44413	6.60736084	1
MSTRG.32484.11	143	0.40471	5.681091309	1
MSTRG.32489.1	566	0.44691	6.065612793	1
MSTRG.32489.2	567	0.44691	6.065612793	1
MSTRG.32492.2	930	0.42958	7.127502441	1
MSTRG.32492.5	563	0.45013	5.441467285	1
MSTRG.32494.1	68	0.28486	11.14312744	1
MSTRG.32468.1	765	0.34885	6.871398926	1
MSTRG.32468.4	980	0.35483	8.50213623	1
MSTRG.32468.5	980	0.36931	8.50213623	1
MSTRG.32468.6	126	0.32624	7.902160645	1
MSTRG.32469.1	88	0.30191	5.534118652	1
MSTRG.32474.2	87	0.3588	9.492492676	1
MSTRG.32474.3	32	0.396	6.804992676	1
MSTRG.32480.1	200	0.39509	5.938781738	1
MSTRG.32497.2	127	0.31915	9.513122559	1
MSTRG.32501.2	160	0.37485	5.416442871	1
MSTRG.32501.3	182	0.35123	10.17681885	1
MSTRG.32507.4	335	0.40164	5.695251465	1
MSTRG.32507.3	352	0.34832	8.051574707	1
MSTRG.32507.6	407	0.46578	6.236755371	1
MSTRG.32509.1	883	0.39869	8.894470215	1
MSTRG.32509.3	883	0.42333	8.894470215	1
MSTRG.32513.1	104	0.32626	8.640808105	1
MSTRG.32513.2	92	0.41202	10.44281006	1
MSTRG.32514.4	873	0.40554	4.474182129	1
MSTRG.32514.5	648	0.31208	5.576721191	1
MSTRG.32515.1	111	0.29967	9.330505371	1
MSTRG.32519.1	511	0.34372	5.36541748	1
MSTRG.32519.3	465	0.41958	5.66986084	-1
MSTRG.32519.4	478	0.44758	5.75579834	-1
MSTRG.32519.5	354	0.31208	4.787414551	1
MSTRG.32519.6	217	0.35948	4.669128418	1
MSTRG.32519.7	349	0.38252	4.987243652	1
MSTRG.32519.8	325	0.37928	4.741149902	1
MSTRG.32519.9	327	0.42143	4.987731934	1
MSTRG.32520.1	57	0.31506	8.282653809	1
MSTRG.32520.2	44	0.30823	7.977844238	1
MSTRG.32520.3	44	0.31341	7.977844238	1
MSTRG.32520.4	44	0.39239	7.977844238	1
MSTRG.32520.5	44	0.36703	7.977844238	1
MSTRG.32523.3	53	0.44157	12.3012085	1
MSTRG.32528.2	101	0.35263	6.719421387	1

MSTRG.32528.1	120	0.39713	9.931213379	1
MSTRG.32534.11	323	0.33569	9.067077637	1
MSTRG.32534.10	385	0.32231	8.779724121	1
MSTRG.32534.3	367	0.29456	9.255432129	1
MSTRG.32534.4	383	0.27379	9.206115723	1
MSTRG.32534.6	350	0.27379	8.746643066	1
MSTRG.32534.7	383	0.29456	9.206115723	1
MSTRG.32534.13	316	0.45417	8.452453613	1
MSTRG.32534.5	847	0.43059	8.885681152	1
MSTRG.32534.12	843	0.42517	8.88873291	1
MSTRG.32534.8	843	0.45182	8.88873291	1
MSTRG.32542.1	116	0.38198	5.829650879	1
MSTRG.32542.3	109	0.37496	5.820251465	1
MSTRG.32545.1	106	0.2535	10.6293335	1
MSTRG.32546.2	241	0.34599	8.895446777	1
MSTRG.32547.4	501	0.43251	5.132507324	1
MSTRG.32547.5	501	0.41069	5.132507324	1
MSTRG.32547.6	464	0.4266	5.111877441	1
MSTRG.32549.1	190	0.27089	11.28411865	1
MSTRG.32549.2	179	0.27739	11.19207764	1
MSTRG.32549.3	143	0.27739	10.74957275	1
MSTRG.32550.1	42	0.36867	5.446472168	1
MSTRG.32552.1	171	0.37256	9.733215332	-1
MSTRG.32554.2	70	0.41516	7.999694824	-1
MSTRG.32554.1	101	0.34274	6.392272949	1
MSTRG.32554.3	279	0.39866	10.34234619	1
MSTRG.32538.2	40	0.42835	6.011291504	1
MSTRG.32538.4	31	0.41754	6.011535645	1
MSTRG.32538.5	363	0.45565	5.429870605	1
MSTRG.32541.1	475	0.39059	8.733825684	1
MSTRG.32541.4	475	0.39514	8.733825684	1
MSTRG.32557.1	96	0.40282	9.692565918	1
MSTRG.32558.1	873	0.42214	6.655700684	1
MSTRG.32558.2	865	0.42214	6.497375488	1
MSTRG.32561.2	684	0.48064	5.892028809	1
MSTRG.32561.3	684	0.42592	5.892028809	1
MSTRG.32561.4	630	0.45826	5.758728027	1
MSTRG.32561.5	629	0.43905	5.858825684	1
MSTRG.32564.1	174	0.28002	9.127258301	1
MSTRG.32564.2	174	0.28338	9.127258301	1
MSTRG.32562.1	161	0.3886	10.35321045	1
MSTRG.32566.3	252	0.27415	9.703918457	1
MSTRG.32566.4	174	0.28796	11.02215576	1
MSTRG.32566.5	190	0.27385	9.096618652	1
MSTRG.32568.3	325	0.42389	5.304992676	1
MSTRG.32575.3	235	0.30184	8.960998535	1
MSTRG.32575.4	235	0.35973	8.960998535	1
MSTRG.32575.5	235	0.41378	8.960998535	1
MSTRG.32575.6	111	0.30673	9.750305176	1
MSTRG.32575.8	179	0.31798	9.109802246	1
MSTRG.32575.10	217	0.35085	8.822692871	1
MSTRG.32575.9	217	0.32261	8.822692871	1
MSTRG.32575.11	179	0.36309	9.109802246	1
MSTRG.32575.12	179	0.33901	9.109802246	1

MSTRG.32574.3	108	0.35221	10.17181396	1
MSTRG.32576.2	188	0.37385	9.437194824	1
MSTRG.32582.1	144	0.41912	6.140075684	1
MSTRG.32588.3	561	0.46309	5.227111816	1
MSTRG.32592.1	238	0.34654	6.674621582	1
MSTRG.32592.3	238	0.37414	6.674621582	1
MSTRG.32596.2	486	0.4086	6.827331543	1
MSTRG.32598.2	104	0.32844	11.94610596	1
MSTRG.32606.2	534	0.38728	9.112854004	1
MSTRG.32606.4	703	0.4006	7.070251465	1
MSTRG.32606.3	713	0.40648	7.065246582	1
MSTRG.32607.2	123	0.38941	4.96673584	1
MSTRG.32607.3	140	0.41384	5.048034668	1
MSTRG.32604.3	1080	0.37828	9.427307129	1
MSTRG.32609.2	708	0.45713	7.327941895	1
MSTRG.32611.1	170	0.40914	4.947814941	1
MSTRG.32613.1	134	0.30594	9.495788574	1
MSTRG.32614.1	399	0.38423	4.59161377	1
MSTRG.32614.2	456	0.39668	4.637756348	1
MSTRG.32614.3	399	0.43474	4.59161377	1
MSTRG.32614.4	456	0.42114	4.637756348	1
MSTRG.32614.5	399	0.39524	4.59161377	1
MSTRG.32614.6	399	0.39524	4.59161377	1
MSTRG.32617.1	146	0.4306	6.38494873	1
MSTRG.32619.2	124	0.42255	7.76751709	1
MSTRG.32621.1	80	0.41831	9.443664551	1
MSTRG.32628.1	443	0.42502	8.054626465	-1
MSTRG.32628.2	31	0.4587	12.00848389	1
MSTRG.32640.1	759	0.3463	6.814880371	1
MSTRG.32641.1	1685	0.44546	9.178649902	1
MSTRG.32641.2	1683	0.41828	9.178649902	1
MSTRG.32641.3	1635	0.41828	9.051452637	1
MSTRG.32641.4	1630	0.37087	9.053405762	1
MSTRG.32634.1	521	0.35655	9.067565918	1
MSTRG.32634.2	917	0.38635	9.247497559	1
MSTRG.32646.2	45	0.41273	12.40863037	1
MSTRG.32646.3	51	0.40798	12.55194092	1
MSTRG.32658.1	173	0.37446	4.73614502	1
MSTRG.32658.2	308	0.4324	5.256408691	1
MSTRG.32658.4	70	0.35542	12.47845459	1
MSTRG.32658.3	308	0.45364	5.559753418	1
MSTRG.32660.1	74	0.34927	5.496154785	-1
MSTRG.32661.2	715	0.26767	8.199890137	1
MSTRG.32662.1	96	0.46854	11.04364014	-1
MSTRG.32663.1	308	0.44781	5.331848145	1
MSTRG.32664.1	75	0.31425	10.22357178	1
MSTRG.32665.1	61	0.35309	6.692321777	1
MSTRG.32666.2	290	0.43123	9.512756348	-1
MSTRG.32656.1	407	0.32244	5.697570801	1
MSTRG.32656.3	406	0.39704	6.104797363	1
MSTRG.32668.5	918	0.43632	6.823303223	1
MSTRG.32668.6	918	0.46756	6.823303223	1
MSTRG.32674.2	274	0.40942	4.508728027	1
MSTRG.32672.1	82	0.29695	9.828552246	1

MSTRG.32681.3	1446	0.4437	8.344665527	1
MSTRG.32683.1	91	0.28165	9.482727051	1
MSTRG.32682.3	445	0.31204	5.900939941	1
MSTRG.32682.4	297	0.27517	6.503234863	1
MSTRG.32686.1	1231	0.45973	5.26776123	1
MSTRG.32686.3	1231	0.46461	5.26776123	1
MSTRG.32687.1	101	0.27661	5.444641113	1
MSTRG.32688.1	216	0.3609	10.15081787	1
MSTRG.32689.1	110	0.3279	8.215637207	1
MSTRG.32690.1	732	0.33102	5.976013184	1
MSTRG.32691.1	175	0.43216	5.281555176	1
MSTRG.32692.1	132	0.44549	5.79119873	1
MSTRG.32697.1	28	0.37444	11.69903564	-1
MSTRG.32698.1	56	0.33953	7.961364746	1
MSTRG.32699.1	504	0.43882	9.546447754	1
MSTRG.32699.2	499	0.42291	9.504089355	1
MSTRG.32699.3	499	0.42291	9.504089355	1
MSTRG.32699.4	468	0.41113	9.584533691	-1
MSTRG.32699.5	473	0.40618	9.390075684	-1
MSTRG.32709.11	30	0.32233	6.502746582	1
MSTRG.32712.1	1105	0.38046	8.600402832	1
MSTRG.32719.2	662	0.40374	5.457458496	1
MSTRG.32720.1	151	0.27327	6.949401855	1
MSTRG.32718.2	91	0.3937	9.327209473	1
MSTRG.32723.2	674	0.37814	8.458679199	1
MSTRG.32723.3	728	0.37814	8.425231934	1
MSTRG.32723.10	674	0.39354	8.458679199	1
MSTRG.32723.8	649	0.3963	8.629333496	1
MSTRG.32723.7	703	0.40002	8.59185791	1
MSTRG.32723.11	663	0.37442	8.526428223	1
MSTRG.32723.9	663	0.37442	8.526428223	1
MSTRG.32723.12	728	0.39354	8.425231934	1
MSTRG.32723.14	728	0.39354	8.425231934	1
MSTRG.32723.16	728	0.39354	8.425231934	1
MSTRG.32723.13	155	0.38902	4.824645996	1
MSTRG.32723.15	649	0.39878	8.629333496	1
MSTRG.32723.17	674	0.42437	8.458679199	1
MSTRG.32723.18	628	0.38728	8.313659668	1
MSTRG.32721.5	181	0.31497	9.551086426	1
MSTRG.32721.6	181	0.31345	9.551086426	1
MSTRG.32730.1	654	0.4455	8.854797363	-1
MSTRG.32731.1	15	0.36116	6.491394043	-1
MSTRG.32732.1	105	0.38784	10.28106689	-1
MSTRG.32730.3	340	0.39008	9.201965332	1
MSTRG.32738.1	500	0.43895	9.18548584	1
MSTRG.32738.2	500	0.44839	9.18548584	1
MSTRG.32738.4	1076	0.43751	4.195495605	1
MSTRG.32738.5	500	0.44086	9.18548584	1
MSTRG.32738.6	500	0.44086	9.18548584	1
MSTRG.32738.7	500	0.33219	9.18548584	1
MSTRG.32739.1	1337	0.441	5.681335449	1
MSTRG.32740.1	58	0.30553	4.460388184	1
MSTRG.32747.4	263	0.31204	9.070251465	1
MSTRG.32747.9	251	0.4372	9.140197754	1

MSTRG.32749.1	67	0.24856	9.773132324	-1
MSTRG.32751.3	896	0.43741	8.694641113	1
MSTRG.32751.2	896	0.42673	8.694641113	1
MSTRG.32752.1	43	0.41212	4.033508301	1
MSTRG.32749.2	37	0.39243	5.362121582	-1
MSTRG.32752.2	225	0.47481	8.977600098	1
MSTRG.32752.4	225	0.47543	8.977600098	1
MSTRG.32752.5	134	0.47654	8.608337402	1
MSTRG.32752.6	134	0.47654	8.608337402	1
MSTRG.32752.7	134	0.46336	8.591125488	1
MSTRG.32752.8	82	0.42659	9.625549316	1
MSTRG.32752.9	96	0.47239	10.22735596	1
MSTRG.32754.1	94	0.33285	9.571105957	1
MSTRG.32754.2	82	0.39606	8.52130127	1
MSTRG.32754.3	82	0.43536	8.52130127	1
MSTRG.32755.1	42	0.38238	7.807312012	1
MSTRG.32754.4	34	0.35689	7.794372559	-1
MSTRG.32756.1	63	0.421	5.968933105	1
MSTRG.32757.1	183	0.38433	11.17706299	1
MSTRG.32760.1	102	0.4578	10.84088135	-1
MSTRG.32765.1	82	0.32237	10.83416748	1
MSTRG.32773.1	298	0.2895	9.675842285	1
MSTRG.32767.1	245	0.40889	6.319885254	1
MSTRG.32781.1	97	0.41398	5.799499512	1
MSTRG.32782.1	113	0.38449	5.390686035	1
MSTRG.32796.3	51	0.31402	10.25604248	-1
MSTRG.32796.5	51	0.31402	10.25604248	-1
MSTRG.32796.4	90	0.37104	11.64190674	1
MSTRG.32796.6	145	0.28675	11.01409912	1
MSTRG.32796.7	521	0.34813	8.484558105	1
MSTRG.32796.9	296	0.37738	5.338806152	1
MSTRG.32796.10	521	0.34538	8.484558105	1
MSTRG.32796.8	521	0.34538	8.484558105	1
MSTRG.32796.11	521	0.35681	8.484558105	1
MSTRG.32796.12	713	0.39117	7.571350098	1
MSTRG.32796.14	10	0.25515	12.30108643	1
MSTRG.32796.13	521	0.37571	8.484558105	1
MSTRG.32796.15	296	0.38681	5.338806152	1
MSTRG.32796.16	296	0.36885	5.338806152	1
MSTRG.32796.17	296	0.38681	5.338806152	1
MSTRG.32796.18	521	0.36238	8.73626709	1
MSTRG.32796.19	521	0.38235	8.636413574	1
MSTRG.32796.20	521	0.39621	8.636413574	1
MSTRG.32798.3	350	0.3869	5.325622559	1
MSTRG.32798.4	360	0.4386	5.372619629	1
MSTRG.32798.5	350	0.38502	5.325622559	1
MSTRG.32798.2	360	0.41782	5.312805176	1
MSTRG.32798.6	360	0.4162	5.239562988	1
MSTRG.32798.8	360	0.33887	5.312805176	1
MSTRG.32789.1	416	0.37705	9.21295166	1
MSTRG.32792.1	38	0.42075	5.207336426	-1
MSTRG.32801.1	941	0.43606	5.764221191	1
MSTRG.32801.3	955	0.43525	5.641540527	1
MSTRG.32801.4	707	0.45082	5.396789551	1

MSTRG.32802.1	43	0.45198	9.519104004	1
MSTRG.32814.2	737	0.38558	6.02532959	1
MSTRG.32815.1	39	0.36957	8.736877441	-1
MSTRG.32821.1	859	0.39432	6.047546387	1
MSTRG.32821.2	859	0.4208	6.047546387	1
MSTRG.32821.3	859	0.42264	6.047546387	1
MSTRG.32821.4	859	0.44431	6.047546387	1
MSTRG.32821.5	859	0.44431	6.047546387	1
MSTRG.32819.1	55	0.34369	9.303894043	-1
MSTRG.32820.2	225	0.3543	11.27642822	-1
MSTRG.32827.1	131	0.43035	5.944763184	1
MSTRG.32828.1	95	0.42324	9.696105957	1
MSTRG.32832.1	17	0.36158	5.927185059	-1
MSTRG.32832.2	88	0.4015	5.79864502	-1
MSTRG.32836.1	607	0.36837	8.584533691	1
MSTRG.32837.1	54	0.45993	11.00860596	-1
MSTRG.32841.1	104	0.34908	6.487487793	1
MSTRG.32842.7	110	0.40879	12.56878662	1
MSTRG.32842.8	110	0.40879	12.56878662	1
MSTRG.32842.6	110	0.41203	12.56878662	1
MSTRG.32842.2	110	0.42589	12.56878662	1
MSTRG.32839.1	34	0.40712	4.628479004	1
MSTRG.32839.2	34	0.43638	4.895812988	1
MSTRG.32839.3	34	0.45867	4.895812988	1
MSTRG.32840.1	83	0.40768	8.504943848	1
MSTRG.32849.1	68	0.30894	6.228210449	1
MSTRG.32859.1	47	0.38601	5.098327637	1
MSTRG.32860.1	153	0.29367	11.19415283	1
MSTRG.32862.1	136	0.27218	9.50604248	1
MSTRG.32859.2	70	0.30277	9.625915527	1
MSTRG.32855.1	42	0.43444	4.027648926	1
MSTRG.32866.1	60	0.28958	4.295837402	-1
MSTRG.32867.1	63	0.32762	8.293395996	1
MSTRG.32867.2	100	0.30191	5.396057129	1
MSTRG.32867.3	73	0.28518	9.132019043	1
MSTRG.32867.4	189	0.32697	9.676330566	1
MSTRG.32867.5	144	0.2921	9.590393066	1
MSTRG.32867.6	100	0.31048	10.39251709	1
MSTRG.32867.7	144	0.2921	9.590393066	1
MSTRG.32863.5	550	0.45485	7.265686035	1
MSTRG.32863.6	562	0.41834	6.764465332	1
MSTRG.32864.2	281	0.34054	9.98223877	1
MSTRG.32874.3	1295	0.39173	8.61895752	1
MSTRG.32874.5	1219	0.34944	8.994934082	1
MSTRG.32874.6	372	0.3852	5.135070801	1
MSTRG.32876.1	189	0.30869	10.01495361	1
MSTRG.32877.1	211	0.29367	9.625793457	1
MSTRG.32869.1	147	0.45503	9.799621582	1
MSTRG.32880.1	36	0.46264	4.43572998	-1
MSTRG.32884.1	99	0.45077	10.02276611	1
MSTRG.32888.1	90	0.3676	9.000061035	1
MSTRG.32889.4	176	0.27075	9.817321777	1
MSTRG.32889.3	1025	0.40898	7.415588379	1
MSTRG.32889.6	176	0.29527	9.817321777	1

MSTRG.32889.5	727	0.36933	6.869812012	1
MSTRG.32889.7	727	0.34898	6.869812012	1
MSTRG.32889.8	685	0.34898	6.786437988	1
MSTRG.32889.9	176	0.29709	9.817321777	1
MSTRG.32892.1	620	0.37713	8.944885254	1
MSTRG.32892.5	155	0.30325	9.963684082	1
MSTRG.32893.4	593	0.31289	6.335510254	1
MSTRG.32895.3	620	0.46451	4.678405762	1
MSTRG.32897.1	52	0.36735	7.698425293	1
MSTRG.32899.1	66	0.33552	5.160339355	1
MSTRG.32906.3	369	0.39085	4.378845215	1
MSTRG.32906.4	369	0.43456	4.378845215	1
MSTRG.32906.6	369	0.38886	4.378845215	1
MSTRG.32906.5	369	0.43294	4.378845215	1
MSTRG.32907.1	56	0.40358	3.71661377	-1
MSTRG.32916.1	163	0.40758	8.97479248	1
MSTRG.32921.1	64	0.40021	9.812927246	1
MSTRG.32922.2	587	0.46544	7.602355957	1
MSTRG.32922.4	513	0.47867	8.280944824	1
MSTRG.32925.1	68	0.3592	8.118835449	1
MSTRG.32925.2	85	0.34959	8.625793457	1
MSTRG.32927.1	160	0.29526	8.490661621	1
MSTRG.32929.1	527	0.48875	8.938049316	1
MSTRG.32930.2	507	0.45749	9.636779785	1
MSTRG.32933.1	286	0.45245	6.447692871	1
MSTRG.32936.6	92	0.2785	9.379699707	1
MSTRG.32936.7	91	0.28218	5.066223145	1
MSTRG.32936.8	91	0.27943	5.066223145	1
MSTRG.32936.9	91	0.27943	5.066223145	1
MSTRG.32934.2	818	0.44001	4.268859863	1
MSTRG.32934.3	798	0.46417	4.224914551	1
MSTRG.32934.4	868	0.43933	4.162658691	1
MSTRG.32934.5	850	0.46754	4.181335449	1
MSTRG.32935.1	452	0.32274	9.164611816	1
MSTRG.32937.2	366	0.38495	4.98223877	1
MSTRG.32939.2	628	0.43966	5.684631348	1
MSTRG.32943.1	61	0.3466	4.08404541	1
MSTRG.32945.2	264	0.45095	9.02947998	1
MSTRG.32946.1	185	0.44467	5.49822998	1
MSTRG.32951.4	2005	0.46793	7.315368652	1
MSTRG.32953.1	596	0.46901	5.704528809	1
MSTRG.32957.5	2647	0.41757	5.737121582	1
MSTRG.32957.3	2647	0.42252	5.737121582	1
MSTRG.32954.2	172	0.33526	9.83416748	1
MSTRG.32961.2	425	0.43867	5.251647949	1
MSTRG.32964.1	844	0.40152	7.085510254	-1
MSTRG.32968.2	477	0.45318	8.791809082	1
MSTRG.32984.2	198	0.33642	6.430236816	1
MSTRG.32984.4	198	0.44393	6.430236816	1
MSTRG.32984.3	198	0.46511	6.430236816	1
MSTRG.32984.5	198	0.33642	6.430236816	1
MSTRG.32981.1	331	0.38265	8.343688965	1
MSTRG.32988.1	63	0.35772	8.841003418	1
MSTRG.32992.1	17	0.40294	6.693054199	1

MSTRG.33013.3	1038	0.46029	4.989196777	1
MSTRG.33013.4	1011	0.47647	4.925109863	1
MSTRG.33014.1	149	0.27289	7.663879395	1
MSTRG.33018.1	150	0.26969	10.54095459	1
MSTRG.33003.1	43	0.43802	9.382995605	1
MSTRG.33021.1	1101	0.40512	6.336608887	1
MSTRG.33021.2	1095	0.45751	6.046813965	-1
MSTRG.33021.3	981	0.40029	6.223449707	1
MSTRG.33023.1	95	0.31675	9.925964355	1
MSTRG.33024.1	84	0.39522	9.512878418	1
MSTRG.33025.1	84	0.30387	9.793029785	1
MSTRG.33031.5	245	0.44706	7.951965332	1
MSTRG.33031.6	245	0.47221	7.951965332	1
MSTRG.33033.2	510	0.3924	8.746398926	1
MSTRG.33036.1	80	0.29835	9.182678223	1
MSTRG.33036.3	112	0.3	8.01751709	1
MSTRG.33050.3	44	0.34862	4.830871582	1
MSTRG.33050.2	57	0.30391	8.739196777	1
MSTRG.33050.5	44	0.33857	4.830871582	1
MSTRG.33054.1	67	0.28723	4.636169434	1
MSTRG.33056.1	434	0.37179	5.60736084	1
MSTRG.33063.2	367	0.43561	5.801086426	1
MSTRG.33063.3	385	0.40536	5.981384277	1
MSTRG.33068.1	254	0.41602	3.991027832	1
MSTRG.33069.1	416	0.38997	4.196472168	1
MSTRG.33083.6	287	0.42258	9.750183105	1
MSTRG.33088.1	306	0.29551	9.729553223	1
MSTRG.33090.2	295	0.27517	4.80267334	1
MSTRG.33090.3	64	0.26498	7.928771973	1
MSTRG.33097.2	403	0.39925	5.771057129	1
MSTRG.33101.1	1577	0.46083	5.85369873	1
MSTRG.33102.5	694	0.43873	4.896179199	1
MSTRG.33102.4	694	0.40277	4.896179199	1
MSTRG.33102.6	694	0.41922	4.896179199	1
MSTRG.33114.1	856	0.42822	9.09588623	1
MSTRG.33114.2	856	0.42822	9.09588623	1
MSTRG.33114.3	216	0.45014	7.63494873	1
MSTRG.33114.4	856	0.47126	9.09588623	1
MSTRG.33114.5	855	0.47126	9.078674316	1
MSTRG.33114.6	856	0.47126	9.09588623	1
MSTRG.33114.7	822	0.42481	9.06427002	1
MSTRG.33114.8	631	0.41778	9.304748535	1
MSTRG.33114.9	630	0.39523	9.283874512	1
MSTRG.33109.1	42	0.40557	7.799743652	1
MSTRG.33108.3	144	0.28467	9.772277832	1
MSTRG.33120.2	334	0.43698	8.657409668	1
MSTRG.33124.3	75	0.36978	9.374328613	1
MSTRG.33124.1	259	0.46742	4.519958496	1
MSTRG.33127.4	77	0.36396	4.653137207	1
MSTRG.33127.5	200	0.39877	4.777893066	1
MSTRG.33128.1	685	0.36405	6.62713623	1
MSTRG.33131.1	71	0.32229	6.696960449	1
MSTRG.33132.1	111	0.31526	7.597106934	1
MSTRG.33131.2	71	0.32153	6.696960449	1

MSTRG.33131.4	383	0.40908	5.238952637	1
MSTRG.33133.1	103	0.31603	7.930480957	1
MSTRG.33129.2	342	0.45412	4.995544434	1
MSTRG.33135.1	398	0.38175	4.940368652	1
MSTRG.33138.2	180	0.45902	8.938537598	1
MSTRG.33141.2	142	0.33757	5.283752441	1
MSTRG.33144.1	432	0.42794	8.115783691	1
MSTRG.33144.2	432	0.42794	8.115783691	1
MSTRG.33150.2	157	0.28503	6.402160645	1
MSTRG.33151.1	104	0.29268	4.796691895	1
MSTRG.33148.1	348	0.31763	8.435241699	1
MSTRG.33155.2	149	0.31705	10.03729248	1
MSTRG.33158.1	544	0.32642	5.37689209	1
MSTRG.33161.1	669	0.45168	6.218688965	1
MSTRG.33161.2	840	0.47932	5.671447754	-1
MSTRG.33161.3	881	0.47143	5.861999512	-1
MSTRG.33164.1	153	0.3846	4.554626465	1
MSTRG.33165.1	153	0.3846	4.554626465	1
MSTRG.33166.1	176	0.39599	4.96685791	1
MSTRG.33166.2	176	0.39523	4.96685791	1
MSTRG.33168.1	53	0.44412	6.62701416	1
MSTRG.33172.1	54	0.445	10.29498291	-1
MSTRG.33179.1	109	0.30152	10.12298584	1
MSTRG.33189.1	225	0.35269	8.640441895	-1
MSTRG.33224.2	390	0.37608	6.22454834	1
MSTRG.33224.3	390	0.39557	6.22454834	1
MSTRG.33223.1	65	0.45351	10.54864502	1
MSTRG.33234.1	106	0.43778	11.31500244	-1
MSTRG.33248.1	76	0.29864	8.20513916	1
MSTRG.33249.1	52	0.35163	8.093200684	1
MSTRG.33245.1	476	0.42775	9.110778809	1
MSTRG.33278.2	256	0.39409	5.038635254	1
MSTRG.33280.1	187	0.38787	4.70135498	1
MSTRG.33282.4	82	0.28576	10.24664307	1
MSTRG.33284.1	1806	0.44965	5.108825684	1
MSTRG.33287.3	470	0.44604	9.009338379	1
MSTRG.33301.2	189	0.30446	8.831970215	1
MSTRG.33306.3	174	0.29708	9.207336426	1
MSTRG.33309.1	343	0.28683	9.096252441	1
MSTRG.33309.3	519	0.38314	4.86151123	1
MSTRG.33319.1	44	0.43898	6.688171387	1
MSTRG.33322.3	823	0.31852	6.42364502	1
MSTRG.33322.4	823	0.2985	6.42364502	1
MSTRG.33322.5	78	0.28982	6.542175293	1
MSTRG.33323.1	115	0.29387	6.554382324	1
MSTRG.33325.2	905	0.39709	5.868469238	1
MSTRG.33325.4	906	0.42586	5.868469238	1
MSTRG.33325.5	894	0.39792	8.337463379	1
MSTRG.33333.1	496	0.39029	7.22833252	1
MSTRG.33333.2	578	0.37351	8.267150879	1
MSTRG.33333.5	578	0.36197	8.267150879	1
MSTRG.33333.4	508	0.31941	6.915344238	1
MSTRG.33328.1	775	0.39263	9.022399902	1
MSTRG.33329.1	611	0.44249	9.282165527	1

MSTRG.33329.2	795	0.41425	9.318786621	1
MSTRG.33329.4	606	0.46666	9.282165527	1
MSTRG.33329.5	207	0.37979	8.465026855	1
MSTRG.33338.1	336	0.35244	9.241882324	1
MSTRG.33338.3	96	0.2912	9.760681152	1
MSTRG.33339.1	39	0.38772	8.462341309	1
MSTRG.33348.1	58	0.43429	9.804748535	1
MSTRG.33367.7	816	0.49163	5.20513916	1
MSTRG.33373.2	509	0.3276	5.868469238	1
MSTRG.33373.3	566	0.34908	6.101379395	1
MSTRG.33373.5	247	0.35315	7.624816895	1
MSTRG.33373.4	376	0.32522	5.786560059	1
MSTRG.33373.8	376	0.41495	5.786560059	1
MSTRG.33374.1	69	0.3271	6.51348877	1
MSTRG.33369.2	406	0.42271	5.774353027	1
MSTRG.33369.3	511	0.45564	5.230651855	1
MSTRG.33376.1	73	0.3802	12.52459717	1
MSTRG.33386.1	52	0.26294	5.343078613	1
MSTRG.33388.1	37	0.34348	7.922790527	1
MSTRG.33389.2	860	0.48053	6.305358887	1
MSTRG.33391.1	1226	0.37981	6.068664551	1
MSTRG.33398.2	164	0.39026	5.994567871	1
MSTRG.33398.1	164	0.38482	5.994567871	1
MSTRG.33400.2	714	0.41087	12.16278076	1
MSTRG.33400.1	651	0.4134	12.20172119	1
MSTRG.33400.5	651	0.4112	12.20172119	1
MSTRG.33403.5	785	0.43233	5.390441895	1
MSTRG.33404.1	91	0.36795	10.19732666	1
MSTRG.33405.2	558	0.4775	7.721862793	1
MSTRG.33405.3	496	0.40491	6.219787598	1
MSTRG.33417.1	41	0.39141	10.05596924	-1
MSTRG.33419.13	135	0.42576	3.923278809	1
MSTRG.33419.20	119	0.42817	3.839782715	1
MSTRG.33419.23	116	0.42303	3.867370605	1
MSTRG.33419.24	117	0.42303	3.867370605	1
MSTRG.33408.3	403	0.37406	3.911437988	1
MSTRG.33423.3	1513	0.45871	5.388122559	1
MSTRG.33423.4	1597	0.46175	5.335998535	1
MSTRG.33424.1	861	0.39528	8.244812012	1
MSTRG.33424.2	1257	0.37643	9.246398926	1
MSTRG.33424.3	1154	0.42526	6.517150879	1
MSTRG.33443.13	3215	0.47416	4.932678223	1
MSTRG.33443.9	4122	0.47134	5.036071777	1
MSTRG.33443.3	4402	0.47134	5.075500488	1
MSTRG.33443.11	4417	0.45699	5.09197998	1
MSTRG.33443.14	4417	0.45699	5.09197998	1
MSTRG.33443.6	4417	0.45699	5.09197998	1
MSTRG.33443.5	4412	0.45699	5.101501465	1
MSTRG.33443.15	4417	0.37186	5.09197998	1
MSTRG.33448.8	1024	0.38804	8.618835449	1
MSTRG.33450.1	994	0.42439	9.28704834	1
MSTRG.33451.1	128	0.33331	5.652770996	1
MSTRG.33452.1	96	0.32209	9.510803223	1
MSTRG.33453.1	1743	0.45195	5.289978027	1

MSTRG.33453.2	1743	0.44915	5.289978027	1
MSTRG.33453.3	1743	0.44915	5.289978027	1
MSTRG.33453.4	1743	0.4499	5.289978027	1
MSTRG.33453.5	1743	0.43368	5.289978027	1
MSTRG.33453.6	877	0.45891	5.77532959	1
MSTRG.33430.1	54	0.42649	4.794006348	-1
MSTRG.33439.2	439	0.49643	8.795715332	1
MSTRG.33439.3	434	0.38155	9.034484863	1
MSTRG.33444.1	58	0.3556	4.442565918	1
MSTRG.33466.2	206	0.4015	5.374572754	1
MSTRG.33467.1	20	0.37044	8.518737793	1
MSTRG.33482.1	536	0.46658	4.288879395	1
MSTRG.33482.3	569	0.47011	4.37310791	1
MSTRG.33483.1	309	0.43705	8.927185059	1
MSTRG.33482.5	766	0.45607	4.444763184	1
MSTRG.33482.6	611	0.47124	4.194885254	1
MSTRG.33482.7	604	0.47124	4.219299316	1
MSTRG.33482.8	601	0.47124	4.201721191	1
MSTRG.33519.1	846	0.3869	6.774841309	1
MSTRG.33521.1	826	0.37957	6.652526855	1
MSTRG.33525.1	78	0.45195	9.696105957	1
MSTRG.33534.1	114	0.27794	8.356262207	1
MSTRG.33534.2	114	0.33746	8.356262207	1
MSTRG.33535.4	1006	0.39213	8.281311035	1
MSTRG.33535.5	999	0.39662	7.851501465	1
MSTRG.33535.6	999	0.38243	7.851501465	1
MSTRG.33536.1	117	0.37792	5.900817871	-1
MSTRG.33535.7	196	0.32884	6.426330566	1
MSTRG.33537.1	222	0.37324	5.80291748	-1
MSTRG.33535.8	102	0.30088	6.570495605	1
MSTRG.33535.9	196	0.46783	6.426330566	1
MSTRG.33546.1	661	0.40871	6.731994629	1
MSTRG.33546.3	661	0.4079	6.731994629	1
MSTRG.33548.4	2554	0.48397	5.51751709	1
MSTRG.33553.1	1338	0.41228	8.926696777	1
MSTRG.33553.2	1338	0.41228	8.926696777	1
MSTRG.33553.3	1294	0.41228	8.891296387	1
MSTRG.33555.1	197	0.32181	5.684753418	1
MSTRG.33553.8	1318	0.45798	9.114562988	1
MSTRG.33550.2	171	0.39994	6.876647949	1
MSTRG.33559.4	676	0.372	5.298400879	1
MSTRG.33568.1	74	0.44088	10.29779053	-1
MSTRG.33573.1	207	0.29772	6.304992676	1
MSTRG.33577.2	288	0.32477	4.971374512	1
MSTRG.33577.1	218	0.33929	5.146179199	1
MSTRG.33587.3	98	0.37852	10.14666748	1
MSTRG.33609.2	717	0.33888	8.730285645	1
MSTRG.33614.1	337	0.40233	9.995300293	1
MSTRG.33613.2	436	0.43959	8.276428223	1
MSTRG.33619.1	281	0.43885	9.664611816	1
MSTRG.33619.2	281	0.45073	9.664611816	1
MSTRG.33627.2	340	0.44278	9.410827637	1
MSTRG.33629.2	607	0.46778	4.721740723	1
MSTRG.33629.1	614	0.4773	4.67791748	1

MSTRG.33629.4	589	0.45219	4.699645996	1
MSTRG.33629.6	614	0.45935	4.655090332	1
MSTRG.33629.3	614	0.46705	4.67791748	1
MSTRG.33629.5	614	0.46705	4.684387207	1
MSTRG.33629.7	614	0.46705	4.684387207	1
MSTRG.33629.10	614	0.45935	4.684387207	1
MSTRG.33629.8	614	0.45935	4.684387207	1
MSTRG.33629.9	614	0.45935	4.671203613	1
MSTRG.33629.11	589	0.45064	4.699645996	1
MSTRG.33634.2	1398	0.46081	5.475402832	1
MSTRG.33634.4	357	0.46063	4.651428223	1
MSTRG.33639.6	604	0.4483	8.296569824	1
MSTRG.33639.7	80	0.34931	8.987365723	1
MSTRG.33648.2	260	0.44197	5.739685059	1
MSTRG.33648.5	260	0.45471	5.608215332	1
MSTRG.33649.1	889	0.49692	9.01348877	1
MSTRG.33649.3	879	0.48581	8.965270996	1
MSTRG.33654.2	302	0.29178	6.870056152	1
MSTRG.33658.1	249	0.42783	7.860046387	1
MSTRG.33662.3	712	0.44652	6.680114746	1
MSTRG.33662.4	712	0.4129	6.680114746	1
MSTRG.33661.1	179	0.3439	8.297790527	1
MSTRG.33664.6	724	0.46914	9.036437988	1
MSTRG.33664.2	640	0.43403	9.362243652	1
MSTRG.33664.3	716	0.37819	9.058654785	1
MSTRG.33664.4	648	0.44789	9.342468262	1
MSTRG.33664.7	614	0.43775	9.370178223	1
MSTRG.33664.8	604	0.43555	9.323791504	1
MSTRG.33664.9	511	0.454	9.317321777	1
MSTRG.33664.10	92	0.2785	9.03729248	1
MSTRG.33666.1	151	0.31248	8.340148926	1
MSTRG.33669.2	131	0.34727	9.454650879	-1
MSTRG.33669.1	90	0.28567	9.467346191	1
MSTRG.33670.2	1221	0.4873	5.655822754	1
MSTRG.33676.1	658	0.41763	7.263122559	1
MSTRG.33676.2	658	0.37874	7.263122559	1
MSTRG.33676.3	658	0.42993	7.263122559	1
MSTRG.33679.1	70	0.30006	9.094543457	1
MSTRG.33679.2	55	0.27849	9.492736816	1
MSTRG.33681.1	914	0.3849	9.533874512	1
MSTRG.33682.1	183	0.38644	10.22991943	1
MSTRG.33683.1	1204	0.45478	4.30645752	1
MSTRG.33683.2	1174	0.45313	4.385925293	1
MSTRG.33683.3	634	0.39447	4.512756348	1
MSTRG.33686.2	563	0.44155	6.07598877	-1
MSTRG.33687.3	89	0.28711	12.17852783	1
MSTRG.33688.2	1526	0.37058	6.627624512	1
MSTRG.33690.1	163	0.28923	10.43084717	1
MSTRG.33690.2	157	0.28338	10.68353271	1
MSTRG.33692.1	144	0.28503	10.05474854	1
MSTRG.33692.2	149	0.28503	9.614196777	1
MSTRG.33692.3	198	0.28584	8.88494873	1
MSTRG.33709.1	216	0.36195	11.56365967	1
MSTRG.33709.2	216	0.4015	11.56365967	1

MSTRG.33709.3	216	0.3615	11.56365967	1
MSTRG.33709.4	129	0.36261	10.41766357	1
MSTRG.33709.5	216	0.40003	11.56365967	1
MSTRG.33709.6	216	0.40298	11.56365967	1
MSTRG.33712.1	502	0.44026	8.889099121	1
MSTRG.33712.2	485	0.39853	9.066345215	1
MSTRG.33713.1	224	0.38471	7.258483887	1
MSTRG.33714.1	139	0.36389	9.476257324	1
MSTRG.33729.1	246	0.33535	5.916320801	1
MSTRG.33731.1	851	0.49304	6.06829834	1
MSTRG.33744.3	302	0.4433	9.20135498	1
MSTRG.33754.2	114	0.38921	11.13922119	1
MSTRG.33754.1	114	0.38613	11.34991455	1
MSTRG.33754.4	114	0.39073	11.34991455	1
MSTRG.33754.5	114	0.39073	11.34991455	1
MSTRG.33754.3	114	0.35886	11.34991455	1
MSTRG.33754.6	114	0.43348	11.34991455	1
MSTRG.33756.2	666	0.45579	7.817687988	1
MSTRG.33760.1	100	0.45696	11.86322021	1
MSTRG.33760.2	54	0.37285	8.487243652	-1
MSTRG.33764.1	131	0.41834	10.5133667	1
MSTRG.33758.1	114	0.40192	11.58917236	1
MSTRG.33768.1	528	0.3791	5.404602051	1
MSTRG.33768.2	528	0.27867	5.404602051	1
MSTRG.33768.3	260	0.27327	6.57232666	1
MSTRG.33768.4	528	0.43284	5.404602051	1
MSTRG.33769.1	114	0.29637	8.438293457	1
MSTRG.33770.1	108	0.29551	9.676574707	1
MSTRG.33768.5	122	0.2846	8.743591309	-1
MSTRG.33768.6	130	0.32431	9.757019043	1
MSTRG.33771.1	125	0.28284	8.480773926	1
MSTRG.33772.1	92	0.27782	9.296813965	1
MSTRG.33773.1	156	0.26837	9.613098145	1
MSTRG.33787.1	99	0.32501	9.231262207	1
MSTRG.33789.1	133	0.39538	8.69329834	1
MSTRG.33792.1	74	0.37217	5.625427246	1
MSTRG.33793.1	68	0.36426	11.71173096	1
MSTRG.33791.1	174	0.5007	4.922058105	1
MSTRG.33813.2	347	0.35621	6.431945801	1
MSTRG.33813.4	310	0.2785	8.582458496	1
MSTRG.33813.5	310	0.2785	8.582458496	1
MSTRG.33813.6	173	0.29927	6.905700684	1
MSTRG.33815.1	179	0.28267	9.50213623	1
MSTRG.33817.1	64	0.31034	7.815124512	1
MSTRG.33819.2	289	0.42179	9.192565918	1
MSTRG.33820.1	425	0.35665	5.331481934	1
MSTRG.33820.2	389	0.36654	5.583312988	1
MSTRG.33820.4	425	0.36874	5.331481934	1
MSTRG.33822.1	143	0.45188	7.010681152	-1
MSTRG.33824.1	63	0.46978	7.782287598	1
MSTRG.33817.2	707	0.42539	5.918395996	1
MSTRG.33817.4	104	0.32831	5.833312988	1
MSTRG.33804.1	7	0.2525	5.272766113	1
MSTRG.33803.1	6	0.41142	6.507263184	1

MSTRG.33805.1	57	0.34128	8.018859863	1
MSTRG.33806.1	4	0.31617	5.274963379	1
MSTRG.33806.2	19	0.34647	6.492004395	1
MSTRG.33809.1	16	0.45483	6.50567627	1
MSTRG.33810.1	12	0.24946	6.487731934	1
MSTRG.33811.1	19	0.26503	6.492004395	1
MSTRG.33812.1	57	0.34559	8.842468262	1
MSTRG.33812.2	57	0.35463	8.842468262	1
MSTRG.33846.1	206	0.39917	6.28314209	1
MSTRG.33829.1	80	0.35677	5.458312988	1
MSTRG.33831.1	106	0.30388	8.950256348	1
MSTRG.33831.2	113	0.27437	8.373352051	1
MSTRG.33831.3	48	0.33528	10.08966064	1
MSTRG.33831.4	43	0.33296	10.09747314	1
MSTRG.33832.1	149	0.35031	7.624328613	1
MSTRG.33832.2	147	0.33309	10.86273193	1
MSTRG.33838.1	26	0.32742	8.210388184	1
MSTRG.33839.1	62	0.36515	6.546325684	-1
MSTRG.33839.2	48	0.36331	5.658508301	1
MSTRG.33841.1	88	0.31649	10.36431885	1
MSTRG.33843.1	77	0.25541	6.927062988	1
MSTRG.33851.1	42	0.41653	6.012878418	1
MSTRG.33852.1	92	0.34932	8.042419434	1
MSTRG.33858.1	102	0.28396	11.31890869	1
MSTRG.33860.1	67	0.28396	8.649841309	1
MSTRG.33861.1	85	0.25635	8.591247559	1
MSTRG.33863.1	85	0.31683	8.591247559	1
MSTRG.33865.1	52	0.44785	11.39508057	1
MSTRG.33868.1	196	0.27251	6.270568848	1
MSTRG.33868.2	196	0.27379	6.270568848	1
MSTRG.33869.1	44	0.31329	9.296569824	-1
MSTRG.33869.2	44	0.31753	9.296569824	-1
MSTRG.33875.1	164	0.30475	7.296691895	-1
MSTRG.33875.2	164	0.27466	7.296691895	-1
MSTRG.33875.4	164	0.37492	7.296691895	-1
MSTRG.33875.6	164	0.30866	7.296691895	-1
MSTRG.33874.1	84	0.42376	11.5803833	-1
MSTRG.33879.1	44	0.37651	7.655700684	1
MSTRG.33882.1	385	0.40862	9.650085449	1
MSTRG.33883.1	38	0.33963	9.09979248	1
MSTRG.33886.1	51	0.35423	6.803161621	1
MSTRG.33880.1	62	0.34809	9.737243652	1
MSTRG.33881.1	40	0.31811	9.976501465	1
MSTRG.33896.2	237	0.29988	6.086364746	1
MSTRG.33896.3	171	0.30781	8.347351074	1
MSTRG.33896.1	237	0.28338	6.086364746	1
MSTRG.33896.4	310	0.34234	5.798034668	1
MSTRG.33896.5	371	0.34283	6.464416504	1
MSTRG.33896.6	301	0.34393	8.14642334	1
MSTRG.33894.2	59	0.38606	7.737731934	1
MSTRG.33898.1	56	0.33294	8.840759277	1
MSTRG.33898.2	56	0.33294	8.840759277	1
MSTRG.33898.3	112	0.34984	9.659240723	1
MSTRG.33898.4	26	0.44028	9.782165527	1

MSTRG.33898.5	50	0.41334	8.63092041	1
MSTRG.33898.6	40	0.41129	7.999938965	1
MSTRG.33899.1	86	0.36263	9.434387207	1
MSTRG.33899.2	93	0.30966	9.245422363	1
MSTRG.33899.3	93	0.30966	9.245422363	1
MSTRG.33899.4	93	0.30966	9.245422363	1
MSTRG.33898.7	40	0.42793	9.492736816	1
MSTRG.33901.1	55	0.46631	9.510559082	1
MSTRG.33901.2	55	0.47189	9.510559082	1
MSTRG.33901.3	81	0.42423	9.063415527	1
MSTRG.33901.4	83	0.4406	9.250549316	1
MSTRG.33901.5	55	0.33589	10.69012451	1
MSTRG.33901.6	35	0.41371	5.332092285	-1
MSTRG.33901.7	25	0.44785	5.426330566	-1
MSTRG.33901.8	51	0.43786	10.01275635	1
MSTRG.33901.9	55	0.4555	10.16790771	1
MSTRG.33903.1	343	0.42461	6.430358887	1
MSTRG.33910.1	0	0.32015	0	-1
MSTRG.33910.2	0	0.33327	0	-1
MSTRG.33914.2	343	0.44251	6.779724121	1
MSTRG.33914.1	343	0.43786	6.779724121	1
MSTRG.33914.3	343	0.45869	6.779724121	1
MSTRG.33917.1	35	0.35421	10.94671631	-1
MSTRG.33911.1	43	0.32396	10.25469971	-1
MSTRG.33920.2	48	0.30644	5.751037598	1
MSTRG.33920.3	48	0.30328	5.751037598	1
MSTRG.33921.2	62	0.32414	10.21368408	-1
MSTRG.33921.3	55	0.30324	6.696960449	1
MSTRG.33922.1	104	0.41585	7.680847168	1
MSTRG.33922.2	32	0.41699	5.49822998	1
MSTRG.33949.1	39	0.45428	9.035583496	1
MSTRG.33949.2	32	0.39993	5.49822998	1
MSTRG.33949.3	47	0.43862	4.51739502	1
MSTRG.33950.2	137	0.36411	7.016784668	1
MSTRG.33950.5	195	0.36816	8.906555176	1
MSTRG.33950.6	137	0.3217	7.016784668	1
MSTRG.33950.7	120	0.38613	6.29876709	1
MSTRG.33950.12	67	0.42434	11.09954834	1
MSTRG.33950.10	67	0.33257	8.291320801	1
MSTRG.33950.13	158	0.3195	8.694152832	1
MSTRG.33950.14	249	0.3195	6.153259277	1
MSTRG.33950.9	158	0.3195	8.694152832	1
MSTRG.33950.11	94	0.33959	9.070983887	1
MSTRG.33950.15	98	0.28683	7.799377441	1
MSTRG.33950.17	76	0.3618	8.480895996	1
MSTRG.33950.19	67	0.33714	6.502502441	1
MSTRG.33953.1	95	0.35685	4.913513184	1
MSTRG.33931.1	83	0.28463	8.853088379	1
MSTRG.33932.1	38	0.44621	5.982849121	1
MSTRG.33931.2	63	0.37571	5.869812012	1
MSTRG.33933.1	83	0.38385	9.515563965	1
MSTRG.33934.1	32	0.40097	8.275817871	-1
MSTRG.33928.1	80	0.36914	9.566101074	1
MSTRG.33929.1	116	0.33439	8.864196777	1

MSTRG.33935.1	117	0.38476	6.692810059	-1
MSTRG.33941.2	95	0.30661	6.864318848	1
MSTRG.33944.1	90	0.34091	11.19415283	1
MSTRG.33946.1	62	0.30946	7.642028809	1
MSTRG.33946.2	62	0.30946	7.642028809	1
MSTRG.33946.3	25	0.28337	5.530822754	1
MSTRG.33947.1	49	0.33621	9.368591309	-1
MSTRG.33960.1	86	0.26413	8.845153809	1
MSTRG.33960.2	164	0.28432	5.336364746	1
MSTRG.33960.7	164	0.31325	5.336364746	1
MSTRG.33960.8	164	0.34875	5.336364746	1
MSTRG.33960.9	164	0.42033	5.336364746	1
MSTRG.33960.10	164	0.35732	5.336364746	1
MSTRG.33960.11	164	0.35732	5.336364746	1
MSTRG.33963.1	72	0.44937	3.94720459	1
MSTRG.33964.1	66	0.29307	7.864440918	1
MSTRG.33965.1	62	0.34431	10.01409912	1
MSTRG.33965.2	62	0.39718	9.743591309	1
MSTRG.33966.1	63	0.30431	6.24420166	-1
MSTRG.33962.1	52	0.3859	12.00421143	1
MSTRG.33962.2	52	0.37991	12.00421143	1
MSTRG.33967.1	52	0.4464	11.00006104	1
MSTRG.33985.4	299	0.28923	6.84564209	1
MSTRG.33985.7	211	0.29772	7.656188965	1
MSTRG.33985.10	318	0.29628	8.144348145	1
MSTRG.33985.9	192	0.33267	8.164978027	1
MSTRG.33985.11	174	0.29772	9.758483887	1
MSTRG.33979.1	57	0.30228	9.184265137	1
MSTRG.33986.1	91	0.38093	6.964538574	1
MSTRG.33986.2	83	0.40885	9.515563965	1
MSTRG.33986.3	55	0.4555	9.033996582	1
MSTRG.33986.4	91	0.47067	6.965637207	1
MSTRG.33987.1	55	0.472	9.510559082	1
MSTRG.33986.5	91	0.46738	6.965637207	1
MSTRG.33992.1	38	0.44245	5.982849121	1
MSTRG.33993.1	55	0.48036	9.510559082	1
MSTRG.33993.2	55	0.45892	9.510559082	1
MSTRG.33998.1	19	0.28743	12.00006104	1
MSTRG.33999.1	51	0.40806	12.00421143	-1
MSTRG.34000.2	154	0.28343	9.451477051	1
MSTRG.34000.3	112	0.30521	11.64703369	1
MSTRG.34000.1	135	0.30808	9.683044434	1
MSTRG.34000.4	112	0.27327	9.506164551	1
MSTRG.34000.5	112	0.27327	9.506164551	1
MSTRG.34000.6	112	0.27651	9.506164551	1
MSTRG.34016.1	82	0.3817	9.415466309	-1
MSTRG.34016.2	80	0.40822	9.415466309	-1
MSTRG.34016.4	74	0.40822	8.639465332	-1
MSTRG.34016.5	155	0.28428	11.81097412	1
MSTRG.34016.6	155	0.28428	11.81097412	1
MSTRG.34016.3	93	0.30801	9.245422363	1
MSTRG.34016.7	73	0.40741	8.639465332	-1
MSTRG.34016.8	82	0.40741	10.28094482	1
MSTRG.34016.9	155	0.35815	11.81097412	1

MSTRG.34016.10	56	0.36024	8.840759277	1
MSTRG.34016.11	50	0.41334	8.63092041	1
MSTRG.34016.12	50	0.41334	8.63092041	1
MSTRG.34016.13	50	0.42058	8.63092041	1
MSTRG.34016.15	114	0.34457	9.660217285	1
MSTRG.34016.14	115	0.37375	9.660217285	1
MSTRG.34016.16	115	0.34457	9.660217285	1
MSTRG.34016.17	53	0.36171	4.830871582	1
MSTRG.34016.18	53	0.38297	4.830871582	1
MSTRG.34012.1	44	0.35352	11.7119751	1
MSTRG.34013.1	88	0.36709	10.30267334	1
MSTRG.34017.1	90	0.34761	11.19415283	1
MSTRG.34017.3	41	0.37587	8.883239746	1
MSTRG.34017.2	52	0.35428	11.00006104	1
MSTRG.34017.4	90	0.37988	11.19415283	1
MSTRG.34017.5	52	0.37698	11.00006104	1
MSTRG.34018.1	35	0.39896	9.778991699	1
MSTRG.34026.2	42	0.46191	4.289367676	-1
MSTRG.34028.1	96	0.38572	9.695251465	1
MSTRG.34035.1	304	0.44635	6.69354248	1
MSTRG.34037.1	28	0.33533	11.34869385	-1
MSTRG.34038.1	47	0.33133	4.63885498	-1
MSTRG.34046.1	93	0.35772	8.817199707	1
MSTRG.34046.2	104	0.3202	9.228820801	1
MSTRG.34046.3	93	0.38163	8.817199707	1
MSTRG.34046.4	241	0.31674	8.840515137	1
MSTRG.34046.5	93	0.37062	9.336364746	1
MSTRG.34046.6	241	0.30816	8.840515137	1
MSTRG.34047.2	63	0.46132	3.800476074	1
MSTRG.34052.1	52	0.32184	11.00006104	1
MSTRG.34053.1	40	0.45446	7.999938965	1
MSTRG.34053.2	40	0.43558	7.999938965	1
MSTRG.34055.1	216	0.48286	10.84625244	1
MSTRG.34056.2	84	0.32228	7.70501709	1
MSTRG.34066.1	96	0.36318	9.625793457	1
MSTRG.34072.1	0	0.25855	0	-1
MSTRG.34072.2	0	0.3076	0	-1
MSTRG.34072.3	0	0.27064	0	-1
MSTRG.34072.4	0	0.27064	0	-1
MSTRG.34072.5	0	0.28165	0	-1
MSTRG.34088.1	2	0.2789	6.491394043	1
MSTRG.34090.1	19	0.40636	6.492004395	-1
MSTRG.34088.2	2	0.36273	6.491394043	1
MSTRG.34084.1	7	0.41378	6.491394043	-1
MSTRG.34085.1	2	0.38833	6.491394043	1
MSTRG.34093.1	202	0.45114	5.901550293	1
MSTRG.34094.1	466	0.46807	6.574279785	-1
MSTRG.34101.1	49	0.43214	7.746520996	1
MSTRG.34097.1	47	0.44804	6.711730957	-1
MSTRG.34108.2	411	0.46212	9.27947998	1
MSTRG.34110.1	35	0.37023	5.791320801	-1
MSTRG.34110.2	23	0.39754	11.52288818	1
MSTRG.34129.1	159	0.34166	10.50970459	1
MSTRG.34130.3	68	0.39587	4.734802246	1

MSTRG.34130.1	68	0.38047	4.734802246	1
MSTRG.34130.2	68	0.38047	4.734802246	1
MSTRG.34131.1	68	0.30031	4.734802246	1
MSTRG.34131.2	68	0.35583	4.734802246	1
MSTRG.34130.4	84	0.31781	10.99395752	1
MSTRG.34130.5	68	0.39587	4.734802246	1
MSTRG.34135.3	58	0.30682	8.93560791	-1
MSTRG.34135.1	59	0.30982	8.93560791	-1
MSTRG.34135.2	59	0.30982	8.93560791	-1
MSTRG.34135.4	59	0.33852	8.93560791	-1
MSTRG.34136.1	86	0.3889	9.434387207	1
MSTRG.34135.5	59	0.32851	8.93560791	-1
MSTRG.34137.3	78	0.41334	8.905578613	1
MSTRG.34137.1	78	0.41334	8.905578613	1
MSTRG.34137.2	78	0.41334	8.905578613	1
MSTRG.34135.6	70	0.34211	11.92120361	1
MSTRG.34133.1	38	0.28335	8.683166504	-1
MSTRG.34146.1	1066	0.46818	6.90435791	-1
MSTRG.34149.1	98	0.31037	8.18182373	1
MSTRG.34152.1	39	0.33783	4.192565918	1
MSTRG.34153.1	59	0.44773	10.20733643	-1
MSTRG.34185.7	149	0.31381	9.160461426	1
MSTRG.34185.8	64	0.38374	6.698425293	1
MSTRG.34185.10	77	0.31464	4.906188965	1
MSTRG.34185.9	55	0.42499	4.960144043	1
MSTRG.34185.11	55	0.39334	4.929382324	1
MSTRG.34185.12	55	0.38812	4.960144043	1
MSTRG.34185.13	55	0.43707	4.960144043	1
MSTRG.34185.15	168	0.37701	9.745178223	1
MSTRG.34185.14	168	0.43763	9.893981934	1
MSTRG.34185.16	55	0.42642	4.909240723	1
MSTRG.34185.17	168	0.35383	9.745178223	1
MSTRG.34185.18	168	0.36742	9.893981934	1
MSTRG.34185.19	168	0.4519	9.745178223	1
MSTRG.34185.20	55	0.41205	4.960144043	1
MSTRG.34185.21	168	0.39919	9.745178223	1
MSTRG.34185.22	168	0.38635	9.893981934	1
MSTRG.34185.23	40	0.44697	4.433776855	1
MSTRG.34154.1	69	0.38614	10.52496338	1
MSTRG.34155.1	15	0.41043	8.000305176	-1
MSTRG.34154.2	69	0.38437	10.52496338	1
MSTRG.34161.2	441	0.40383	9.561096191	1
MSTRG.34161.3	441	0.44985	8.90826416	1
MSTRG.34159.1	68	0.39587	4.734802246	1
MSTRG.34164.1	83	0.42025	9.914978027	1
MSTRG.34164.2	83	0.42228	9.250549316	1
MSTRG.34171.2	353	0.40583	5.367370605	1
MSTRG.34171.5	364	0.3889	5.377258301	1
MSTRG.34171.6	353	0.46128	5.367370605	1
MSTRG.34174.1	61	0.39289	11.85870361	-1
MSTRG.34178.1	32	0.37053	7.835021973	1
MSTRG.34183.2	489	0.4348	7.994934082	-1
MSTRG.34183.3	482	0.44419	8.249572754	-1
MSTRG.34183.4	21	0.35513	5.274719238	1

MSTRG.34190.1	1021	0.42644	6.416442871	1
MSTRG.34190.2	1024	0.42644	6.35369873	1
MSTRG.34190.6	415	0.48317	7.159240723	1
MSTRG.34190.9	357	0.43952	4.907287598	1
MSTRG.34169.1	119	0.2733	12.00469971	1
MSTRG.34196.1	84	0.28037	8.183410645	1
MSTRG.34198.3	144	0.47376	3.923278809	1
MSTRG.34198.4	135	0.42531	3.923278809	1
MSTRG.34206.1	36	0.34857	6.030822754	1
MSTRG.34202.1	69	0.34755	9.445739746	1
MSTRG.34209.1	83	0.37935	9.842712402	1
MSTRG.34209.2	59	0.40657	9.019592285	1
MSTRG.34210.1	89	0.36691	6.005432129	1
MSTRG.34210.2	62	0.39612	10.53192139	-1
MSTRG.34219.1	30	0.37528	8.020690918	-1
MSTRG.34220.1	5	0.37483	5.272766113	1
MSTRG.34219.2	30	0.37528	8.020690918	-1
MSTRG.34220.2	5	0.35399	5.272766113	1
MSTRG.34220.3	5	0.34305	5.272766113	1
MSTRG.34219.3	45	0.36485	6.22845459	-1
MSTRG.34225.1	19	0.27066	6.492004395	1
MSTRG.34226.1	53	0.33845	9.184265137	1
MSTRG.34235.1	86	0.47444	9.736999512	-1
MSTRG.34238.1	0	0.38655	0	-1
MSTRG.34252.1	161	0.33075	10.98529053	-1
MSTRG.34252.2	130	0.33861	9.686950684	-1
MSTRG.34252.3	86	0.32179	9.434387207	1
MSTRG.34252.4	86	0.32179	9.434387207	1
MSTRG.34272.1	120	0.29452	7.68572998	1
MSTRG.34272.2	120	0.29452	7.68572998	1
MSTRG.34272.4	120	0.29857	7.68572998	1
MSTRG.34272.3	120	0.29452	7.68572998	1
MSTRG.34272.6	120	0.29452	7.68572998	1
MSTRG.34272.5	120	0.29452	7.68572998	1
MSTRG.34272.7	120	0.3032	7.68572998	1
MSTRG.34272.8	120	0.29452	7.68572998	1
MSTRG.34272.9	120	0.29727	7.68572998	1
MSTRG.34293.1	127	0.32361	6.863586426	1
MSTRG.34294.1	190	0.36053	9.72857666	1
MSTRG.34294.2	190	0.34878	9.565002441	1
MSTRG.34295.1	132	0.29772	8.969177246	1
MSTRG.34295.2	62	0.29772	10.36273193	1
MSTRG.34295.4	61	0.3094	6.518371582	1
MSTRG.34295.3	61	0.31264	6.518371582	1
MSTRG.34295.5	66	0.35841	11.74737549	1
MSTRG.34294.3	227	0.28396	9.504699707	1
MSTRG.34294.4	122	0.28648	7.034973145	1
MSTRG.34294.6	122	0.28243	7.034973145	1
MSTRG.34294.5	122	0.28648	7.034973145	1
MSTRG.34295.6	119	0.31398	9.354919434	1
MSTRG.34294.8	114	0.34453	6.635437012	-1
MSTRG.34294.7	122	0.29857	7.034973145	1
MSTRG.34294.9	122	0.28648	7.034973145	1
MSTRG.34295.7	20	0.28914	12.00006104	1

MSTRG.34294.10	90	0.29705	9.588806152	1
MSTRG.34294.11	83	0.3319	8.890808105	1
MSTRG.34298.1	32	0.45841	4.957702637	-1
MSTRG.34298.2	31	0.47934	4.957702637	-1
MSTRG.34299.1	55	0.49052	9.510559082	1
MSTRG.34282.1	51	0.39215	4.655822754	1
MSTRG.34283.1	0	0.33168	0	-1
MSTRG.34284.1	52	0.42752	11.00006104	1
MSTRG.34287.1	47	0.326	8.508850098	1
MSTRG.34300.1	71	0.44791	8.028869629	-1
MSTRG.34302.1	83	0.41843	9.250549316	1
MSTRG.34303.1	58	0.4349	9.183410645	1
MSTRG.34314.1	40	0.45676	5.756652832	-1
MSTRG.34314.3	91	0.3777	6.965637207	1
MSTRG.34314.4	111	0.30309	10.54327393	1
MSTRG.34314.2	111	0.2952	10.54327393	1
MSTRG.34315.1	193	0.28419	9.358947754	1
MSTRG.34315.2	168	0.28063	12.2678833	1
MSTRG.34315.3	304	0.26247	12.37432861	1
MSTRG.34315.4	176	0.29498	12.10272217	1
MSTRG.34317.2	467	0.31593	9.078308105	1
MSTRG.34317.3	172	0.35719	5.820617676	1
MSTRG.34315.5	192	0.29639	9.953674316	1
MSTRG.34315.6	163	0.34964	8.441589355	1
MSTRG.34315.7	90	0.28584	5.089904785	1
MSTRG.34315.8	139	0.37867	8.836975098	1
MSTRG.34315.10	55	0.37578	4.633850098	1
MSTRG.34315.11	168	0.28719	9.667297363	1
MSTRG.34315.9	168	0.3568	9.667297363	1
MSTRG.34304.1	30	0.327	8.49420166	-1
MSTRG.34305.1	85	0.29189	8.591247559	1
MSTRG.34306.2	12	0.27327	5.270568848	1
MSTRG.34306.1	12	0.31335	5.270568848	1
MSTRG.34307.1	29	0.29995	4.944641113	1
MSTRG.34307.2	29	0.3039	5.478210449	1
MSTRG.34308.1	12	0.28932	6.487731934	1
MSTRG.34321.1	184	0.4404	9.085510254	1
MSTRG.34328.1	38	0.4194	5.982849121	1
MSTRG.34330.1	51	0.42396	10.01275635	1
MSTRG.34327.1	20	0.41137	9.692199707	1
MSTRG.34334.2	88	0.40545	10.01422119	1
MSTRG.34346.1	60	0.43167	7.869689941	1
MSTRG.34346.2	90	0.41215	10.65814209	1
MSTRG.34346.3	78	0.42612	8.905578613	1
MSTRG.34346.4	78	0.42612	8.905578613	1
MSTRG.34342.1	21	0.34561	8.537902832	-1
MSTRG.34342.2	21	0.34561	8.537902832	-1
MSTRG.34351.1	91	0.37409	6.964538574	1
MSTRG.34351.3	55	0.46724	9.510559082	1
MSTRG.34351.2	55	0.35429	9.033996582	1
MSTRG.34351.4	91	0.41161	6.964538574	1
MSTRG.34351.5	55	0.37739	9.033996582	1
MSTRG.34351.6	38	0.461	5.747497559	-1
MSTRG.34351.7	38	0.44738	5.747497559	-1

MSTRG.34351.8	52	0.47933	9.033996582	-1
MSTRG.34349.1	49	0.42481	9.972351074	1
MSTRG.34352.1	69	0.32184	10.52496338	1
MSTRG.34352.2	52	0.38985	11.00006104	1
MSTRG.34369.1	473	0.29772	6.590515137	1
MSTRG.34369.2	132	0.29772	6.942810059	1
MSTRG.34369.3	121	0.29772	6.807067871	1
MSTRG.34369.4	132	0.33851	6.942810059	1
MSTRG.34369.5	121	0.29772	6.807067871	1
MSTRG.34369.7	132	0.29867	6.942810059	1
MSTRG.34369.8	530	0.37705	6.670471191	1
MSTRG.34369.9	103	0.30981	6.272399902	1
MSTRG.34369.10	212	0.38771	9.481750488	1
MSTRG.34369.11	283	0.4575	6.18951416	1
MSTRG.34356.1	52	0.30709	10.27728271	1
MSTRG.34356.2	49	0.38986	9.972351074	1
MSTRG.34357.1	52	0.37443	10.36431885	1
MSTRG.34355.1	190	0.45916	6.587585449	1
MSTRG.34363.2	131	0.35191	9.934631348	1
MSTRG.34363.3	567	0.44116	7.680480957	1
MSTRG.34365.2	197	0.27911	7.911193848	1
MSTRG.34365.3	142	0.2785	4.808898926	1
MSTRG.34365.4	50	0.45023	12.47784424	-1
MSTRG.34361.1	84	0.35824	11.20684814	1
MSTRG.34373.1	135	0.38976	9.430114746	-1
MSTRG.34373.2	169	0.40197	9.440979004	-1
MSTRG.34373.3	135	0.38976	9.430114746	-1
MSTRG.34374.1	28	0.39636	9.624694824	1
MSTRG.34374.2	28	0.38427	9.624694824	1
MSTRG.34374.3	28	0.41002	9.624694824	1
MSTRG.34375.1	87	0.35024	9.39654541	1
MSTRG.34375.2	110	0.37814	9.957214355	1
MSTRG.34373.4	91	0.36265	9.161682129	1
MSTRG.34373.5	91	0.36265	9.161682129	1
MSTRG.34376.5	78	0.40121	8.905578613	1
MSTRG.34376.2	40	0.41869	7.999938965	1
MSTRG.34376.4	78	0.38319	8.905578613	1
MSTRG.34376.3	78	0.38319	8.905578613	1
MSTRG.34376.6	85	0.36035	9.39642334	1
MSTRG.34376.7	78	0.38319	8.905578613	1
MSTRG.34376.1	78	0.38967	8.905578613	1
MSTRG.34383.1	19	0.27231	6.492004395	1
MSTRG.34383.2	19	0.27934	6.492004395	1
MSTRG.34383.3	5	0.32348	5.272766113	1
MSTRG.34384.1	47	0.29919	6.812561035	-1
MSTRG.34389.1	52	0.43328	11.00006104	1
MSTRG.34390.1	67	0.33971	9.096984863	1
MSTRG.34391.1	52	0.3797	12.00421143	1
MSTRG.34393.1	141	0.31035	10.01812744	1
MSTRG.34395.2	373	0.46135	5.609436035	-1
MSTRG.34394.1	166	0.45465	4.936340332	1
MSTRG.34394.3	140	0.39511	5.385070801	1
MSTRG.34400.1	67	0.43452	9.969665527	-1
MSTRG.34401.2	53	0.38359	4.749694824	1

MSTRG.34401.1	22	0.40676	5.746154785	1
MSTRG.34402.1	29	0.36901	8.364440918	1
MSTRG.34402.2	29	0.36901	8.364440918	1
MSTRG.34401.3	44	0.40849	8.045227051	-1
MSTRG.34402.3	29	0.37995	8.364440918	1
MSTRG.34400.2	117	0.38188	9.453674316	1
MSTRG.34411.1	48	0.4605	8.27520752	1
MSTRG.34414.1	170	0.32495	6.72088623	1
MSTRG.34414.2	170	0.36036	6.72088623	1
MSTRG.34414.3	170	0.36036	6.72088623	1
MSTRG.34416.1	42	0.35932	10.83441162	1
MSTRG.34435.10	515	0.40399	8.298400879	1
MSTRG.34435.7	390	0.34404	6.793395996	1
MSTRG.34435.8	495	0.40399	8.192443848	1
MSTRG.34435.9	515	0.40399	8.298400879	1
MSTRG.34435.2	515	0.40399	8.298400879	1
MSTRG.34418.1	0	0.32013	0	-1
MSTRG.34420.1	30	0.33782	7.834777832	1
MSTRG.34421.1	50	0.30152	11.83428955	1
MSTRG.34422.1	0	0.30991	0	-1
MSTRG.34427.1	165	0.46781	4.566345215	1
MSTRG.34427.2	165	0.45907	4.566345215	1
MSTRG.34427.3	165	0.45907	4.566345215	1
MSTRG.34429.2	26	0.45877	11.12213135	1
MSTRG.34429.1	83	0.33901	9.726623535	1
MSTRG.34438.1	26	0.46454	11.75421143	1
MSTRG.34439.1	45	0.4817	6.810974121	-1
MSTRG.34449.1	26	0.45357	10.5647583	1
MSTRG.34449.2	83	0.32646	9.515563965	1
MSTRG.34449.3	111	0.38919	9.677307129	1
MSTRG.34449.4	55	0.47195	9.510559082	1
MSTRG.34449.5	83	0.30877	9.515563965	1
MSTRG.34449.6	83	0.37006	9.515563965	1
MSTRG.34449.7	83	0.37874	9.515563965	1
MSTRG.34449.9	83	0.31437	9.515563965	1
MSTRG.34449.8	83	0.31437	9.515563965	1
MSTRG.34449.10	83	0.36513	9.515563965	1
MSTRG.34446.1	44	0.40034	9.609558105	1
MSTRG.34446.2	0	0.41804	0	-1
MSTRG.34452.1	66	0.32927	10.66656494	1
MSTRG.34452.2	66	0.40041	10.66656494	1
MSTRG.34458.1	80	0.44838	7.847229004	1
MSTRG.34460.1	55	0.46463	9.510559082	1
MSTRG.34455.1	63	0.36476	5.585754395	1
MSTRG.34455.2	63	0.34541	5.585754395	1
MSTRG.34467.1	66	0.35544	9.940979004	1
MSTRG.34471.1	0	0.28	0	-1
MSTRG.34478.1	92	0.31037	7.81842041	1
MSTRG.34482.3	39	0.39297	9.983947754	1
MSTRG.34481.1	46	0.39044	9.894104004	1
MSTRG.34486.1	38	0.43098	4.075378418	-1
MSTRG.34489.1	95	0.34587	10.00079346	1
MSTRG.34501.1	17	0.37725	5.194152832	1
MSTRG.34505.1	574	0.46904	8.696838379	1

MSTRG.34511.1	86	0.32885	9.434387207	1
MSTRG.34511.2	86	0.33529	9.434387207	1
MSTRG.34511.3	86	0.33529	9.434387207	1
MSTRG.34511.4	134	0.37283	9.686950684	-1
MSTRG.34512.1	60	0.42305	7.869689941	1
MSTRG.34513.1	40	0.46496	7.999938965	1
MSTRG.34514.1	19	0.36665	3.877380371	-1
MSTRG.34515.1	32	0.26	3.503723145	1
MSTRG.34519.2	78	0.376	8.905578613	1
MSTRG.34519.1	87	0.35243	10.14666748	1
MSTRG.34519.3	78	0.43948	8.905578613	1
MSTRG.34519.4	78	0.43948	8.905578613	1
MSTRG.34519.5	109	0.38188	10.03448486	1
MSTRG.34519.6	87	0.37956	10.14666748	1
MSTRG.34520.1	67	0.32188	8.769714355	1
MSTRG.34521.1	23	0.33882	9.303649902	1
MSTRG.34519.7	78	0.43948	8.905578613	1
MSTRG.34524.1	19	0.38968	7.999816895	-1
MSTRG.34524.2	76	0.42411	11.6005249	1
MSTRG.34524.3	76	0.45092	11.6005249	1
MSTRG.34524.4	76	0.47764	11.6005249	1
MSTRG.34524.5	76	0.45105	11.6005249	1
MSTRG.34528.2	44	0.32513	11.31182861	-1
MSTRG.34528.1	100	0.30527	5.535827637	1
MSTRG.34529.1	49	0.35455	9.972351074	1
MSTRG.34537.1	121	0.31917	6.253234863	-1
MSTRG.34537.3	75	0.37206	8.969787598	1
MSTRG.34537.2	121	0.36753	6.253234863	-1
MSTRG.34537.4	121	0.2908	6.253234863	-1
MSTRG.34532.1	26	0.33845	9.693908691	1
MSTRG.34554.1	282	0.33743	8.810974121	1
MSTRG.34556.1	235	0.38027	6.42364502	-1
MSTRG.34554.2	256	0.33974	8.24029541	1
MSTRG.34557.2	91	0.38277	8.934509277	-1
MSTRG.34557.1	181	0.41631	9.647888184	1
MSTRG.34557.4	647	0.44673	8.623718262	1
MSTRG.34550.1	19	0.34323	6.492004395	1
MSTRG.34550.2	5	0.36939	5.272766113	1
MSTRG.34561.1	0	0.26014	0	-1
MSTRG.34561.2	0	0.33168	0	-1
MSTRG.34563.1	52	0.32714	6.012268066	1
MSTRG.34621.2	164	0.30245	9.79876709	1
MSTRG.34621.1	310	0.34247	10.50823975	1
MSTRG.34621.3	237	0.30253	10.24212646	1
MSTRG.34621.4	114	0.3166	10.14556885	1
MSTRG.34621.5	114	0.27911	9.382019043	1
MSTRG.34622.1	162	0.27814	11.92254639	1
MSTRG.34621.8	217	0.27516	11.80853271	1
MSTRG.34621.6	217	0.29392	11.80853271	1
MSTRG.34621.7	70	0.24708	10.80657959	1
MSTRG.34621.11	217	0.26958	11.80853271	1
MSTRG.34621.12	127	0.26522	11.64471436	-1
MSTRG.34621.10	217	0.30601	11.80853271	1
MSTRG.34621.9	217	0.24708	11.80853271	1

MSTRG.34621.13	314	0.27484	12.07952881	1
MSTRG.34621.14	217	0.30778	11.80853271	1
MSTRG.34621.15	217	0.33666	11.80853271	1
MSTRG.34621.17	217	0.31616	11.80853271	1
MSTRG.34621.16	84	0.26089	12.0010376	1
MSTRG.34621.18	217	0.33961	11.80853271	1
MSTRG.34622.3	327	0.25669	11.98602295	1
MSTRG.34622.4	311	0.26214	11.47894287	1
MSTRG.34622.5	316	0.27082	11.41802979	1
MSTRG.34622.6	222	0.28374	11.91766357	1
MSTRG.34622.7	218	0.26605	12.10345459	-1
MSTRG.34622.8	327	0.25669	11.98602295	1
MSTRG.34622.10	714	0.29392	11.78521729	-1
MSTRG.34622.9	640	0.2964	11.87762451	1
MSTRG.34622.11	325	0.2446	11.38092041	1
MSTRG.34622.12	325	0.25855	11.38092041	1
MSTRG.34622.13	325	0.24212	11.38092041	1
MSTRG.34622.14	535	0.2733	11.49066162	1
MSTRG.34622.15	327	0.28539	11.98602295	1
MSTRG.34622.16	410	0.2446	12.07513428	-1
MSTRG.34568.1	83	0.36855	9.250549316	1
MSTRG.34568.2	63	0.38143	6.15802002	1
MSTRG.34569.1	80	0.40181	8.712341309	1
MSTRG.34570.1	57	0.41493	5.519470215	1
MSTRG.34572.1	58	0.42625	7.656677246	1
MSTRG.34574.1	7	0.27885	5.272766113	1
MSTRG.34575.1	57	0.32608	9.184265137	1
MSTRG.34576.1	5	0.3488	5.272766113	1
MSTRG.34579.2	264	0.37964	4.017150879	1
MSTRG.34580.1	24	0.44866	3.665710449	-1
MSTRG.34583.1	84	0.33722	9.945129395	1
MSTRG.34583.2	106	0.30981	9.807922363	1
MSTRG.34587.1	0	0.28324	0	-1
MSTRG.34592.2	69	0.45347	12.31158447	1
MSTRG.34592.1	76	0.43069	12.65960693	-1
MSTRG.34592.3	76	0.43069	12.65960693	-1
MSTRG.34592.4	50	0.45358	12.70611572	-1
MSTRG.34592.5	74	0.39626	12.65899658	-1
MSTRG.34595.7	114	0.34111	11.06304932	1
MSTRG.34595.3	114	0.36821	11.06304932	1
MSTRG.34595.5	114	0.36821	11.06304932	1
MSTRG.34595.6	114	0.36821	11.06304932	1
MSTRG.34595.8	13	0.38181	11.52459717	1
MSTRG.34595.2	114	0.36981	10.87286377	1
MSTRG.34595.4	21	0.35775	11.09698486	1
MSTRG.34595.9	114	0.36821	11.06304932	1
MSTRG.34595.10	13	0.38367	11.52459717	1
MSTRG.34595.11	13	0.3976	11.52459717	1
MSTRG.34595.12	21	0.35775	11.09698486	1
MSTRG.34595.13	114	0.36981	10.87286377	1
MSTRG.34595.14	13	0.37093	11.52459717	1
MSTRG.34595.15	13	0.36981	11.52459717	1
MSTRG.34595.16	13	0.3899	11.52459717	1
MSTRG.34600.1	40	0.45021	4.779968262	1

MSTRG.34601.2	42	0.46225	6.226745605	-1
MSTRG.34601.3	42	0.45287	6.226745605	-1
MSTRG.34601.1	42	0.44419	6.226745605	-1
MSTRG.34603.1	21	0.42011	4.571960449	1
MSTRG.34600.2	83	0.32646	9.250549316	1
MSTRG.34600.3	83	0.33153	8.853088379	1
MSTRG.34606.1	83	0.37577	9.515563965	1
MSTRG.34600.5	83	0.39459	8.853088379	1
MSTRG.34600.4	83	0.33514	9.250549316	1
MSTRG.34600.6	80	0.46138	9.154846191	1
MSTRG.34597.1	52	0.41372	11.00006104	1
MSTRG.34612.1	116	0.33648	9.660217285	1
MSTRG.34613.1	53	0.37729	4.749694824	1
MSTRG.34611.1	53	0.35153	4.749694824	1
MSTRG.34616.1	114	0.42091	10.581604	-1
MSTRG.34616.2	114	0.42091	10.581604	-1
MSTRG.34616.3	66	0.39483	9.940979004	1
MSTRG.34616.4	114	0.38395	10.581604	-1
MSTRG.34616.5	114	0.42091	10.581604	-1
MSTRG.34615.1	14	0.36253	12.30108643	-1
MSTRG.34619.1	100	0.40039	11.362854	-1
MSTRG.34631.3	212	0.43263	3.995300293	1
MSTRG.34631.2	212	0.42738	4.032165527	1
MSTRG.34632.1	65	0.32603	6.551330566	1
MSTRG.34633.1	121	0.40194	11.58758545	1
MSTRG.34632.2	38	0.33354	11.29364014	-1
MSTRG.34638.2	188	0.37763	11.253479	1
MSTRG.34638.1	188	0.46988	11.26165771	1
MSTRG.34637.2	845	0.40366	5.612121582	1
MSTRG.34637.4	845	0.38993	5.612121582	1
MSTRG.34653.1	163	0.44375	3.953186035	1
MSTRG.34655.2	79	0.34566	4.628356934	1
MSTRG.34655.1	76	0.33423	8.480895996	1
MSTRG.34656.2	54	0.41586	9.694274902	1
MSTRG.34656.1	73	0.35701	6.373718262	1
MSTRG.34657.1	29	0.42956	5.823303223	1
MSTRG.34660.1	29	0.47323	8.555603027	-1
MSTRG.34661.1	35	0.41648	4.22052002	1
MSTRG.34653.2	76	0.36955	8.480895996	1
MSTRG.34664.1	121	0.41109	6.739562988	1
MSTRG.34666.1	41	0.29318	7.745544434	1
MSTRG.34671.1	141	0.34316	9.954162598	1
MSTRG.34646.1	34	0.36309	9.199523926	1
MSTRG.34647.3	121	0.46688	3.960021973	1
MSTRG.34647.1	124	0.48747	3.909973145	1
MSTRG.34675.3	111	0.42604	3.741027832	-1
MSTRG.34675.2	111	0.43171	3.741027832	-1
MSTRG.34675.4	109	0.42466	3.780334473	-1
MSTRG.34675.5	105	0.4259	3.766540527	-1
MSTRG.34681.2	360	0.30628	5.280822754	1
MSTRG.34681.4	360	0.44022	5.280822754	1
MSTRG.34681.3	360	0.35626	5.280822754	1
MSTRG.34682.7	360	0.39558	5.491516113	1
MSTRG.34682.9	360	0.40944	5.491516113	1

MSTRG.34682.11	360	0.41534	5.491516113	1
MSTRG.34682.12	360	0.41534	5.491516113	1
MSTRG.34683.1	57	0.37938	10.25604248	-1
MSTRG.34707.1	69	0.43772	6.893981934	1
MSTRG.34708.1	90	0.40232	7.614562988	-1
MSTRG.34712.1	53	0.40961	6.47857666	1
MSTRG.34714.1	45	0.38078	7.999084473	1
MSTRG.34715.1	334	0.46141	5.776916504	-1
MSTRG.34716.1	23	0.45165	9.187805176	1
MSTRG.34717.1	58	0.35682	10.01678467	1
MSTRG.34715.2	343	0.45388	6.737976074	1
MSTRG.34715.3	274	0.46137	6.031921387	1
MSTRG.34718.1	104	0.39582	4.55657959	1
MSTRG.34719.1	49	0.38671	8.292907715	1
MSTRG.34720.1	82	0.38312	5.690612793	1
MSTRG.34721.1	68	0.3814	9.79107666	1
MSTRG.34722.1	63	0.33063	9.68939209	1
MSTRG.34715.4	343	0.39402	6.126159668	1
MSTRG.34723.1	23	0.3667	8.044616699	1
MSTRG.34723.2	54	0.33783	7.901428223	1
MSTRG.34724.1	343	0.37887	9.24005127	1
MSTRG.34725.1	26	0.31899	3.584411621	1
MSTRG.34719.2	57	0.34146	9.149719238	1
MSTRG.34717.2	58	0.40257	10.01678467	1
MSTRG.34717.3	58	0.4715	10.01678467	1
MSTRG.34726.1	262	0.38392	6.368347168	1
MSTRG.34715.5	208	0.45794	5.624084473	-1
MSTRG.34727.1	63	0.35287	4.112365723	-1
MSTRG.34687.1	75	0.29998	9.897766113	1
MSTRG.34689.2	80	0.43012	10.293396	1
MSTRG.34690.2	500	0.34156	6.505187988	1
MSTRG.34692.1	16	0.43132	5.752258301	1
MSTRG.34701.1	95	0.39699	9.696105957	1
MSTRG.34702.1	131	0.43344	5.944763184	1
MSTRG.34729.1	138	0.41216	10.63775635	1
MSTRG.34743.1	96	0.34848	9.579040527	1
MSTRG.34743.2	96	0.40669	9.579040527	1
MSTRG.34744.2	297	0.47439	4.886047363	1
MSTRG.34744.3	307	0.47249	5.24420166	1
MSTRG.34744.4	291	0.48652	4.91583252	1
MSTRG.34744.5	570	0.44365	5.781799316	1
MSTRG.34751.1	238	0.31894	5.307434082	1
MSTRG.34755.2	539	0.43132	4.761413574	1
MSTRG.34755.1	669	0.45986	4.782653809	1
MSTRG.34761.2	184	0.47346	5.615661621	-1
MSTRG.34763.2	389	0.43512	6.708557129	1
MSTRG.34764.1	96	0.39361	9.384216309	1
MSTRG.34771.9	114	0.36821	10.87286377	1
MSTRG.34771.13	114	0.38367	10.87286377	1
MSTRG.34778.1	114	0.36821	10.69244385	1
MSTRG.34778.3	114	0.37921	10.87286377	1
MSTRG.34778.4	114	0.37921	10.87286377	1
MSTRG.34778.5	114	0.38367	10.69244385	1
MSTRG.34778.6	114	0.38367	10.69244385	1

coding_probability	label
0.43947	noncoding
0.083571	noncoding
0.0873591	noncoding
0.72401	coding
0.0694934	noncoding
0.999933	coding
0.279463	noncoding
0.0539043	noncoding
1	coding
0.999959	coding
0.477182	noncoding
0.208052	noncoding
0.19386	noncoding
1	coding
1	coding
0.999999	coding
1	coding
1	coding
1	coding
1	coding
1	coding
1	coding
1	coding
1	coding
1	coding
1	coding
1	coding
1	coding
1	coding
1	coding
1	coding
1	coding
1	coding
1	coding
0.999881	coding
1	coding
1	coding
1	coding
1	coding
1	coding
1	coding
1	coding
1	coding
0.999993	coding
0.934215	coding
0.829377	coding
1	coding
0.81872	coding
0.758752	coding
0.987051	coding
0.107095	noncoding
0.417751	noncoding
0.764007	coding

0.17939	noncoding
0.0461449	noncoding
0.434383	noncoding
0.779852	coding
0.980443	coding
0.24338	noncoding
0.0971334	noncoding
0.0715293	noncoding
1	coding
1	coding
1	coding
1	coding
1	coding
0.248777	noncoding
0.999992	coding
0.184304	noncoding
1	coding
1	coding
1	coding
1	coding
0.999765	coding
0.999369	coding
0.0395802	noncoding
1	coding
0.416003	noncoding
0.0287351	noncoding
0.0543857	noncoding
0.037944	noncoding
0.161793	noncoding
0.0989945	noncoding
0.0574046	noncoding
0.963923	coding
0.0917063	noncoding
0.071765	noncoding
0.403951	noncoding
0.0456876	noncoding
0.171913	noncoding
1	coding
1	coding
1	coding
1	coding
1	coding
1	coding
1	coding
0.0258648	noncoding
1	coding
1	coding
1	coding
1	coding
0.0443178	noncoding
0.488776	noncoding
0.0798721	noncoding
0.0886923	noncoding
1	coding

1	coding
0.153412	noncoding
0.495518	noncoding
0.264345	noncoding
0.0426876	noncoding
1	coding
0.0363683	noncoding
1	coding
1	coding
0.147613	noncoding
0.991349	coding
0.257069	noncoding
0.995866	coding
0.0325868	noncoding
0.0188313	noncoding
0.763092	coding
0.120017	noncoding
0.0989736	noncoding
0.111413	noncoding
0.0883135	noncoding
0.0461153	noncoding
0.628745	coding
0.0253381	noncoding
0.0841486	noncoding
0.203725	noncoding
0.923374	coding
0.0639435	noncoding
0.553244	coding
0.383154	noncoding
0.0497351	noncoding
0.13355	noncoding
0.257791	noncoding
0.11979	noncoding
0.0510246	noncoding
0.220091	noncoding
0.339634	noncoding
0.296214	noncoding
0.954191	coding
0.205336	noncoding
0.480624	noncoding
0.0430996	noncoding
0.396728	noncoding
0.0210919	noncoding
0.0184622	noncoding
0.0609049	noncoding
0.999976	coding
0.0800556	noncoding
0.0809752	noncoding
1	coding
1	coding
1	coding
1	coding
0.0747929	noncoding
0.695437	coding

0.0906376	noncoding
0.0230252	noncoding
0.0730396	noncoding
0.508634	coding
0.03042	noncoding
1	coding
0.465068	noncoding
0.344647	noncoding
0.375101	noncoding
0.020612	noncoding
1	coding
1	coding
0.0571582	noncoding
0.0446624	noncoding
1	coding
1	coding
0.0360806	noncoding
0.0366384	noncoding
0.999569	coding
1	coding
1	coding
0.0441862	noncoding
1	coding
0.215449	noncoding
0.0808647	noncoding
0.0392982	noncoding
0.459104	noncoding
1	coding
0.999997	coding
1	coding
0.999942	coding
1	coding
0.089138	noncoding
1	coding
0.2014	noncoding
0.0637003	noncoding
1	coding
1	coding
1	coding
1	coding
1	coding
1	coding
1	coding
0.109977	noncoding
1	coding
0.308134	noncoding
0.0312085	noncoding
1	coding
1	coding
1	coding
0.848232	coding
0.324932	noncoding
1	coding
1	coding

0.00452412	noncoding
0.986248	coding
0.342529	noncoding
0.042034	noncoding
0.51436	coding
0.999695	coding
0.0979853	noncoding
0.785475	coding
0.268127	noncoding
0.656273	coding
0.0714594	noncoding
0.062226	noncoding
0.102531	noncoding
0.12746	noncoding
0.0280254	noncoding
0.113526	noncoding
0.898031	coding
0.0230039	noncoding
1	coding
1	coding
1	coding
0.185276	noncoding
0.489705	noncoding
0.998817	coding
0.999977	coding
0.0406402	noncoding
0.0242707	noncoding
1	coding
0.0139527	noncoding
0.0947569	noncoding
1	coding
1	coding
0.0123454	noncoding
0.0800201	noncoding
0.174147	noncoding
0.0905306	noncoding
0.999999	coding
0.999999	coding
1	coding
1	coding
0.162985	noncoding
0.0175647	noncoding
1	coding
1	coding
1	coding
1	coding
0.259936	noncoding
1	coding
1	coding
0.919857	coding
0.894696	coding
1	coding
0.994569	coding
0.994516	coding

0.334742	noncoding
0.707798	coding
0.0082531	noncoding
1	coding
0.853175	coding
0.0846586	noncoding
1	coding
1	coding
1	coding
1	coding
0.0593129	noncoding
1	coding
1	coding
1	coding
1	coding
1	coding
1	coding
1	coding
1	coding
1	coding
1	coding
1	coding
0.195688	noncoding
0.312532	noncoding
0.0816812	noncoding
0.0162982	noncoding
0.171637	noncoding
0.0184777	noncoding
0.0458538	noncoding
0.0884985	noncoding
0.246959	noncoding
0.026273	noncoding
0.287214	noncoding
0.197059	noncoding
0.155696	noncoding
0.157407	noncoding
0.111855	noncoding
0.027599	noncoding
0.816715	coding
0.999967	coding
0.67427	coding
0.999873	coding
0.992605	coding
0.999991	coding
0.139817	noncoding
0.0961962	noncoding
0.0138468	noncoding
0.999999	coding
0.999997	coding
0.999998	coding
1	coding
0.0345441	noncoding
0.0576935	noncoding
0.95388	coding

0.943786	coding
0.537187	coding
0.588895	coding
0.999982	coding
0.999982	coding
0.0386276	noncoding
0.0467483	noncoding
1	coding
1	coding
1	coding
1	coding
1	coding
0.0569048	noncoding
0.111749	noncoding
0.117491	noncoding
1	coding
0.589986	coding
0.73793	coding
1	coding
1	coding
1	coding
1	coding
0.0266913	noncoding
0.0100626	noncoding
0.999989	coding
0.107194	noncoding
0.0172165	noncoding
1	coding
1	coding
1	coding
1	coding
1	coding
1	coding
0.998566	coding
0.999993	coding
1	coding
1	coding
1	coding
1	coding
1	coding
1	coding
0.0136874	noncoding
1	coding
0.823966	coding
1	coding
1	coding
0.0415551	noncoding
0.038795	noncoding
0.139776	noncoding
1	coding
1	coding
1	coding
1	coding
0.956188	coding

0.0497973	noncoding
0.305781	noncoding
1	coding
1	coding
0.464894	noncoding
1	coding
1	coding
1	coding
1	coding
1	coding
1	coding
1	coding
1	coding
0.236152	noncoding
0.977167	coding
0.0414243	noncoding
0.669674	coding
0.464473	noncoding
0.74293	coding
0.693499	coding
1	coding
0.999901	coding
1	coding
1	coding
1	coding
1	coding
1	coding
1	coding
1	coding
1	coding
0.205505	noncoding
0.0171795	noncoding
0.999997	coding
0.999977	coding
1	coding
1	coding
0.9995	coding
1	coding
0.599524	coding
0.567698	coding
0.653929	coding
0.0106292	noncoding
0.019031	noncoding
1	coding
0.0203822	noncoding
0.26412	noncoding
0.406223	noncoding
1	coding
0.999999	coding
0.999999	coding
0.0188475	noncoding
0.0168589	noncoding
0.0167652	noncoding
0.183266	noncoding

0.0274647	noncoding
0.0470864	noncoding
0.559428	coding
0.998307	coding
0.988761	coding
0.986822	coding
0.0262248	noncoding
0.32825	noncoding
0.0661331	noncoding
0.583011	coding
0.0994802	noncoding
0.0308343	noncoding
0.0473228	noncoding
0.014728	noncoding
0.00478487	noncoding
0.0213176	noncoding
0.999994	coding
1	coding
1	coding
1	coding
0.79152	coding
1	coding
0.972499	coding
0.516381	coding
1	coding
0.0205289	noncoding
0.0107343	noncoding
0.962997	coding
1	coding
0.985379	coding
0.0574145	noncoding
0.999998	coding
1	coding
1	coding
1	coding
0.0661847	noncoding
0.980567	coding
1	coding
1	coding
0.913015	coding
0.999999	coding
1	coding
1	coding
1	coding
1	coding
0.999597	coding
0.652853	coding
1	coding
1	coding
1	coding
1	coding
0.929129	coding
0.0314809	noncoding
1	coding

0.0681679	noncoding
0.0320039	noncoding
1	coding
1	coding
0.999959	coding
0.122006	noncoding
1	coding
1	coding
1	coding
0.270254	noncoding
0.999329	coding
0.999992	coding
0.999898	coding
1	coding
1	coding
0.0181393	noncoding
0.287094	noncoding
0.0554791	noncoding
0.942415	coding
0.999881	coding
1	coding
0.110093	noncoding
1	coding
0.143232	noncoding
0.630535	coding
0.0409434	noncoding
0.0242682	noncoding
0.0400068	noncoding
1	coding
0.904928	coding
0.124365	noncoding
0.400448	noncoding
0.141272	noncoding
0.177962	noncoding
1	coding
0.0356171	noncoding
0.845947	coding
0.026964	noncoding
1	coding
0.0595121	noncoding
0.0623908	noncoding
0.044066	noncoding
0.0660134	noncoding
0.983133	coding
0.987721	coding
0.991568	coding
1	coding
1	coding
1	coding
0.148166	noncoding
1	coding
0.999462	coding
0.997795	coding
0.998599	coding

1	coding
1	coding
1	coding
0.993642	coding
0.455207	noncoding
0.510026	coding
1	coding
1	coding
0.999998	coding
0.999996	coding
0.990954	coding
0.999907	coding
1	coding
1	coding
0.213382	noncoding
0.693981	coding
0.999829	coding
0.999844	coding
0.999994	coding
0.999972	coding
0.848814	coding
0.0496561	noncoding
0.0369127	noncoding
0.730955	coding
0.999997	coding
1	coding
0.0112912	noncoding
1	coding
0.0125583	noncoding
0.47851	noncoding
0.797488	coding
0.997798	coding
0.256914	noncoding
0.104028	noncoding
0.255982	noncoding
0.999567	coding
0.980252	coding
0.999868	coding
0.999999	coding
1	coding
0.0241072	noncoding
0.0283348	noncoding
0.201662	noncoding
0.160249	noncoding
0.160763	noncoding
0.791357	coding
0.997244	coding
0.0486179	noncoding
0.0644521	noncoding
0.0236762	noncoding
0.100014	noncoding
0.950618	coding
0.0100649	noncoding
0.0674144	noncoding

0.999955	coding
0.0191303	noncoding
0.0892858	noncoding
0.236148	noncoding
0.643045	coding
0.00832506	noncoding
0.845928	coding
0.972241	coding
0.980789	coding
0.214922	noncoding
0.99999	coding
0.217733	noncoding
0.660941	coding
0.026917	noncoding
0.841016	coding
1	coding
0.989125	coding
0.0172415	noncoding
1	coding
1	coding
0.0954955	noncoding
0.67167	coding
0.82198	coding
0.898991	coding
0.667427	coding
0.853176	coding
0.620405	coding
1	coding
1	coding
0.997599	coding
0.997111	coding
0.227071	noncoding
0.0587602	noncoding
0.0287253	noncoding
0.0235206	noncoding
0.0747009	noncoding
0.999996	coding
0.999999	coding
1	coding
1	coding
0.999998	coding
1	coding
1	coding
0.105608	noncoding
0.533209	coding
1	coding
0.527038	coding
1	coding
1	coding
0.0642904	noncoding
0.817444	coding
0.942029	coding
0.577732	coding
0.480623	noncoding

1	coding
0.999999	coding
1	coding
1	coding
1	coding
1	coding
1	coding
0.999592	coding
0.16764	noncoding
0.971353	coding
0.996686	coding
1	coding
1	coding
0.984991	coding
0.399391	noncoding
0.990255	coding
0.399307	noncoding
0.17408	noncoding
0.0271136	noncoding
0.640293	coding
0.916362	coding
0.597158	coding
0.739353	coding
1	coding
0.16496	noncoding
0.284622	noncoding
0.994345	coding
0.0517483	noncoding
1	coding
1	coding
1	coding
0.973536	coding
0.025293	noncoding
0.155002	noncoding
1	coding
1	coding
0.0273965	noncoding
1	coding
0.998867	coding
1	coding
1	coding
1	coding
1	coding
1	coding
1	coding
1	coding
1	coding
1	coding
1	coding
1	coding
1	coding
1	coding
1	coding
0.479654	noncoding

0.613345	coding
0.00411474	noncoding
1	coding
0.999787	coding
0.0377743	noncoding
1	coding
0.916143	coding
1	coding
1	coding
1	coding
0.0685899	noncoding
0.999659	coding
0.25786	noncoding
1	coding
1	coding
0.0523564	noncoding
0.489266	noncoding
0.288335	noncoding
1	coding
0.995319	coding
0.999913	coding
0.48398	noncoding
0.999584	coding
0.838023	coding
0.998248	coding
0.997897	coding
0.0691002	noncoding
0.259285	noncoding
0.99976	coding
0.999584	coding
1	coding
0.417823	noncoding
0.999985	coding
0.117198	noncoding
1	coding
0.997686	coding
0.881898	coding
0.89787	coding
0.825646	coding
0.066744	noncoding
0.144853	noncoding
0.0328603	noncoding
0.999998	coding
0.108415	noncoding
0.99863	coding
1	coding
1	coding
0.976524	coding
0.12778	noncoding
1	coding
1	coding
1	coding
1	coding
0.545138	coding

1	coding
1	coding
0.998695	coding
0.148823	noncoding
1	coding
1	coding
1	coding
0.919643	coding
0.999999	coding
1	coding
1	coding
1	coding
1	coding
1	coding
1	coding
1	coding
1	coding
1	coding
1	coding
0.0473583	noncoding
0.840936	coding
0.158923	noncoding
1	coding
0.855382	coding
0.863239	coding
0.999947	coding
0.999957	coding
0.871194	coding
0.999957	coding
0.697092	coding
0.40617	noncoding
0.698137	coding
0.698137	coding
0.449439	noncoding
0.470343	noncoding
0.656433	coding
0.443053	noncoding
0.976233	coding
1	coding
1	coding
1	coding
0.997248	coding
0.153046	noncoding
0.0869821	noncoding
0.161615	noncoding
0.999996	coding
0.26178	noncoding
0.0855756	noncoding
0.903198	coding
0.715187	coding
1	coding
1	coding
1	coding
1	coding

0.999999	coding
1	coding
0.999983	coding
0.0412297	noncoding
0.107328	noncoding
1	coding
1	coding
0.165734	noncoding
0.0368232	noncoding
1	coding
1	coding
0.559011	coding
1	coding
0.116895	noncoding
0.0302667	noncoding
0.311939	noncoding
1	coding
1	coding
0.17275	noncoding
0.0816644	noncoding
1	coding
1	coding
0.809396	coding
1	coding
0.0275697	noncoding
0.346555	noncoding
0.580584	coding
0.998359	coding
0.615677	coding
0.986025	coding
0.912367	coding
0.104325	noncoding
0.127508	noncoding
1	coding
1	coding
1	coding
0.116361	noncoding
0.180123	noncoding
0.0740462	noncoding
0.999854	coding
0.410709	noncoding
0.0958949	noncoding
0.986135	coding
0.938555	coding
0.999455	coding
0.0429363	noncoding
0.999967	coding
0.999983	coding
1	coding
1	coding
0.917574	coding
0.999993	coding
0.999758	coding
0.0583229	noncoding

0.722205	coding
0.996503	coding
0.999949	coding
0.496198	noncoding
1	coding
1	coding
0.0150444	noncoding
0.879259	coding
0.137141	noncoding
0.995565	coding
0.141785	noncoding
0.0934064	noncoding
0.00878003	noncoding
0.19242	noncoding
0.318756	noncoding
0.00409683	noncoding
0.00429283	noncoding
0.247779	noncoding
0.0965851	noncoding
0.766809	coding
0.0392353	noncoding
0.047258	noncoding
0.51333	coding
0.999874	coding
1	coding
0.0498012	noncoding
0.999999	coding
0.999993	coding
0.97904	coding
1	coding
1	coding
0.0887344	noncoding
1	coding
1	coding
0.109259	noncoding
0.13553	noncoding
1	coding
0.0769983	noncoding
0.999982	coding
0.302613	noncoding
0.351916	noncoding
1	coding
0.999984	coding
0.0286967	noncoding
0.954459	coding
0.768049	coding
0.775679	coding
0.775679	coding
0.870132	coding
0.995646	coding
0.995408	coding
0.999725	coding
0.0366361	noncoding
0.100529	noncoding

1	coding
1	coding
1	coding
1	coding
1	coding
1	coding
1	coding
0.998429	coding
0.177104	noncoding
0.0405542	noncoding
0.2283	noncoding
0.0705003	noncoding
0.160161	noncoding
1	coding
0.915361	coding
1	coding
1	coding
0.0892091	noncoding
0.0176734	noncoding
0.0195718	noncoding
0.172336	noncoding
0.999986	coding
0.996103	coding
0.999656	coding
0.0110718	noncoding
0.0113892	noncoding
0.477132	noncoding
0.999201	coding
0.0266379	noncoding
0.997949	coding
0.067908	noncoding
1	coding
1	coding
1	coding
1	coding
0.997325	coding
0.834231	coding
0.920986	coding
0.781975	coding
0.915826	coding
0.279096	noncoding
0.0738707	noncoding
0.0255031	noncoding
0.999864	coding
0.595968	coding
0.168743	noncoding
0.0699674	noncoding
0.0484136	noncoding
0.296375	noncoding
0.0108286	noncoding
0.999998	coding
0.999774	coding
1	coding
1	coding

0.0217308	noncoding
0.0506815	noncoding
0.999997	coding
0.999996	coding
0.949851	coding
0.135211	noncoding
1	coding
1	coding
0.673058	coding
0.0344735	noncoding
0.00706358	noncoding
1	coding
1	coding
1	coding
1	coding
1	coding
0.0368764	noncoding
0.446826	noncoding
0.0682051	noncoding
0.0506632	noncoding
0.178479	noncoding
0.0617802	noncoding
1	coding
1	coding
0.0324193	noncoding
0.989356	coding
0.975088	coding
0.994423	coding
0.996601	coding
0.133155	noncoding
0.992383	coding
0.0325145	noncoding
0.0565145	noncoding
0.11618	noncoding
0.996847	coding
0.999993	coding
0.0105868	noncoding
0.00307934	noncoding
0.0217116	noncoding
0.937518	coding
1	coding
0.703574	coding
1	coding
1	coding
1	coding
1	coding
1	coding
0.0469471	noncoding
1	coding
1	coding
0.0901768	noncoding
0.996424	coding
0.0601968	noncoding
0.200087	noncoding

0.133517	noncoding
0.818584	coding
0.0322477	noncoding
0.0929919	noncoding
0.278354	noncoding
1	coding
0.514003	coding
0.173674	noncoding
0.436631	noncoding
0.127953	noncoding
0.0266948	noncoding
0.0251467	noncoding
0.758998	coding
0.113352	noncoding
1	coding
0.559017	coding
1	coding
1	coding
1	coding
0.413119	noncoding
0.394	noncoding
1	coding
1	coding
0.0765488	noncoding
0.960285	coding
0.0717518	noncoding
0.132339	noncoding
0.0646373	noncoding
1	coding
1	coding
1	coding
0.999999	coding
0.0335109	noncoding
0.390432	noncoding
0.999999	coding
1	coding
1	coding
1	coding
0.174236	noncoding
1	coding
0.131637	noncoding
0.999999	coding
1	coding
1	coding
0.126419	noncoding
0.999889	coding
0.763235	coding
0.999929	coding
0.0566469	noncoding
0.0124351	noncoding
0.999435	coding
1	coding
0.0292171	noncoding
0.999944	coding

0.989297	coding
0.986908	coding
0.999999	coding
0.0302614	noncoding
0.0567085	noncoding
0.067903	noncoding
1	coding
1	coding
1	coding
0.111488	noncoding
1	coding
0.063739	noncoding
0.199552	noncoding
0.0726198	noncoding
0.296151	noncoding
0.229806	noncoding
1	coding
1	coding
1	coding
1	coding
0.225611	noncoding
0.953397	coding
0.909818	coding
1	coding
1	coding
1	coding
0.0384759	noncoding
0.0656254	noncoding
0.083148	noncoding
0.99995	coding
0.999989	coding
0.0262247	noncoding
0.602124	coding
1	coding
1	coding
0.0664242	noncoding
0.0614624	noncoding
0.152123	noncoding
0.0802541	noncoding
0.0870001	noncoding
0.69739	coding
0.109661	noncoding
0.109661	noncoding
0.0403922	noncoding
0.134572	noncoding
1	coding
1	coding
1	coding
0.015683	noncoding
0.0148949	noncoding
1	coding
1	coding
0.999999	coding
0.952954	coding

0.912519	coding
1	coding
0.99323	coding
0.99523	coding
0.738478	coding
0.993324	coding
0.00233927	noncoding
0.0161693	noncoding
0.014885	noncoding
0.123754	noncoding
0.0600459	noncoding
0.999977	coding
0.997787	coding
1	coding
0.9381	coding
0.112423	noncoding
0.134121	noncoding
1	coding
1	coding
1	coding
1	coding
1	coding
0.999995	coding
0.0161694	noncoding
0.339481	noncoding
0.0529435	noncoding
0.215265	noncoding
0.014481	noncoding
1	coding
0.0863819	noncoding
0.00741646	noncoding
0.898058	coding
0.714143	coding
0.0572479	noncoding
1	coding
1	coding
1	coding
1	coding
0.115477	noncoding
0.999991	coding
0.177536	noncoding
0.171908	noncoding
1	coding
1	coding
1	coding
1	coding
1	coding
1	coding
0.0617381	noncoding
0.951316	coding
0.0339922	noncoding
0.0358075	noncoding
1	coding
0.195981	noncoding

1	coding
0.106036	noncoding
1	coding
0.0811192	noncoding
0.067262	noncoding
0.0712303	noncoding
0.390841	noncoding
0.0305008	noncoding
0.12928	noncoding
0.195793	noncoding
0.262472	noncoding
0.985328	coding
0.990658	coding
1	coding
1	coding
1	coding
1	coding
0.799407	coding
0.999996	coding
0.334155	noncoding
0.0536832	noncoding
0.806552	coding
0.144324	noncoding
0.771116	coding
1	coding
1	coding
1	coding
1	coding
1	coding
1	coding
1	coding
1	coding
1	coding
1	coding
1	coding
1	coding
0.00866877	noncoding
0.060476	noncoding
1	coding
1	coding
0.999997	coding
1	coding
1	coding
1	coding
0.0233618	noncoding
0.0493861	noncoding
0.0506874	noncoding
0.0783472	noncoding
0.0556779	noncoding
0.145046	noncoding
0.997933	coding
0.905724	coding
0.0074484	noncoding
0.0849516	noncoding

1	coding
0.0908669	noncoding
0.0527844	noncoding
0.0413582	noncoding
0.0372039	noncoding
1	coding
1	coding
0.0126042	noncoding
0.6387	coding
0.217488	noncoding
0.696911	coding
0.0370734	noncoding
0.178992	noncoding
0.0153529	noncoding
0.150628	noncoding
0.0646692	noncoding
1	coding
1	coding
0.866685	coding
0.268171	noncoding
0.994083	coding
0.999963	coding
0.999997	coding
0.561698	coding
0.645148	coding
0.460153	noncoding
0.885254	coding
0.645148	coding
0.635609	coding
1	coding
1	coding
1	coding
1	coding
0.0601119	noncoding
0.585364	coding
0.013648	noncoding
1	coding
0.678945	coding
0.0156808	noncoding
0.253538	noncoding
0.829815	coding
0.999827	coding
0.999034	coding
1	coding
1	coding
0.118306	noncoding
0.0302345	noncoding
0.0133343	noncoding
0.999973	coding
1	coding
1	coding
0.999986	coding
0.00788725	noncoding
1	coding

1	coding
1	coding
1	coding
1	coding
0.449804	noncoding
0.231349	noncoding
0.999994	coding
0.999967	coding
0.0195298	noncoding
1	coding
0.242854	noncoding
0.81331	coding
0.976383	coding
0.0789318	noncoding
1	coding
1	coding
1	coding
1	coding
1	coding
1	coding
1	coding
1	coding
1	coding
1	coding
1	coding
1	coding
1	coding
1	coding
1	coding
1	coding
1	coding
1	coding
0.0229647	noncoding
0.54719	coding
0.0196102	noncoding
0.0998179	noncoding
0.999799	coding
0.422533	noncoding
0.335198	noncoding
0.207403	noncoding
0.996478	coding
0.501119	coding
0.136368	noncoding
0.168544	noncoding
0.805937	coding
0.999996	coding
0.624364	coding
0.999998	coding
0.999646	coding
0.0590566	noncoding
0.0191258	noncoding
0.0185878	noncoding
0.0149737	noncoding
0.124013	noncoding

0.0805681	noncoding
0.999979	coding
1	coding
1	coding
0.735186	coding
1	coding
1	coding
0.997681	coding
0.146776	noncoding
0.0659681	noncoding
1	coding
0.15488	noncoding
0.991848	coding
0.990048	coding
0.990225	coding
0.993305	coding
0.999993	coding
0.999985	coding
0.989819	coding
0.993912	coding
0.999994	coding
0.965018	coding
0.0209186	noncoding
1	coding
1	coding
1	coding
0.501521	coding
0.637631	coding
0.978559	coding
1	coding
1	coding
1	coding
0.997655	coding
1	coding
0.999478	coding
0.263379	noncoding
0.313784	noncoding
0.383021	noncoding
0.0443008	noncoding
0.0157419	noncoding
0.270781	noncoding
0.158649	noncoding
1	coding
1	coding
1	coding
1	coding
1	coding
1	coding
0.0243936	noncoding
0.0350153	noncoding
0.132959	noncoding
0.0199546	noncoding
0.117966	noncoding
1	coding

1	coding
1	coding
0.99134	coding
1	coding
0.391467	noncoding
1	coding
1	coding
1	coding
0.0234971	noncoding
0.055062	noncoding
1	coding
0.893797	coding
1	coding
0.102905	noncoding
0.0765799	noncoding
1	coding
1	coding
0.146389	noncoding
0.0696762	noncoding
0.0112209	noncoding
0.0233792	noncoding
0.0469003	noncoding
0.0332216	noncoding
0.0999501	noncoding
0.999939	coding
1	coding
0.997611	coding
0.997611	coding
0.0549245	noncoding
0.999995	coding
0.0646943	noncoding
1	coding
0.0504563	noncoding
0.0483906	noncoding
0.0418253	noncoding
0.0791598	noncoding
0.0300846	noncoding
1	coding
1	coding
1	coding
0.0127913	noncoding
0.0138423	noncoding
0.0194715	noncoding
0.122353	noncoding
0.118516	noncoding
0.175075	noncoding
0.0806754	noncoding
0.0806754	noncoding
0.0743714	noncoding
0.0396479	noncoding
0.290218	noncoding
1	coding
1	coding
1	coding

1	coding
1	coding
1	coding
1	coding
1	coding
1	coding
1	coding
1	coding
1	coding
0.0569365	noncoding
0.973938	coding
1	coding
0.802905	coding
0.0126599	noncoding
0.0155865	noncoding
0.0820712	noncoding
0.26025	noncoding
0.400958	noncoding
0.059733	noncoding
0.00944942	noncoding
1	coding
0.290998	noncoding
0.0661252	noncoding
0.0292856	noncoding
0.0420745	noncoding
0.0800041	noncoding
0.0304633	noncoding
0.0109375	noncoding
0.360153	noncoding
0.0840607	noncoding
1	coding
1	coding
1	coding
1	coding
1	coding
0.999987	coding
1	coding
0.0781392	noncoding
0.146186	noncoding
1	coding
0.999998	coding
1	coding
1	coding
1	coding
0.99881	coding
0.0772786	noncoding
0.928969	coding
0.720171	coding
0.999998	coding
1	coding
0.999944	coding
0.999998	coding
0.999998	coding
1	coding

0.999989	coding
0.99998	coding
1	coding
0.971707	coding
0.0369153	noncoding
1	coding
0.132448	noncoding
0.0362299	noncoding
0.0481991	noncoding
1	coding
0.199767	noncoding
0.0302333	noncoding
0.021243	noncoding
1	coding
0.0652283	noncoding
0.445533	noncoding
0.569166	coding
1	coding
1	coding
1	coding
0.186797	noncoding
1	coding
0.0170091	noncoding
0.0296319	noncoding
0.0245162	noncoding
0.999983	coding
0.999993	coding
0.994754	coding
0.0778674	noncoding
0.245839	noncoding
0.0306057	noncoding
0.256774	noncoding
0.999988	coding
0.999872	coding
0.999794	coding
1	coding
1	coding
1	coding
1	coding
0.994083	coding
1	coding
1	coding
1	coding
1	coding
0.298295	noncoding
1	coding
1	coding
0.0914673	noncoding
0.149771	noncoding
0.17522	noncoding
0.106118	noncoding
0.151657	noncoding
0.0920378	noncoding
1	coding

0.999669	coding
0.999669	coding
0.999669	coding
0.16104	noncoding
0.0254152	noncoding
1	coding
1	coding
1	coding
0.333818	noncoding
0.932709	coding
1	coding
0.982789	coding
0.988248	coding
0.92191	coding
0.999993	coding
1	coding
1	coding
0.853657	coding
1	coding
0.0283163	noncoding
0.858059	coding
0.57539	coding
0.284969	noncoding
0.990918	coding
0.0598551	noncoding
0.982358	coding
0.949829	coding
0.0651966	noncoding
1	coding
0.999659	coding
0.998529	coding
0.998529	coding
0.996347	coding
0.278194	noncoding
1	coding
1	coding
1	coding
0.955292	coding
1	coding
0.999904	coding
0.998697	coding
1	coding
0.186175	noncoding
0.118622	noncoding
0.0492912	noncoding
1	coding
1	coding
1	coding
0.657705	coding
0.795486	coding
0.681153	coding
1	coding
0.999537	coding
0.423958	noncoding

1	coding
1	coding
1	coding
0.781555	coding
0.897439	coding
0.912795	coding
0.988266	coding
1	coding
0.0109733	noncoding
0.999922	coding
0.999895	coding
0.10722	noncoding
0.547786	coding
0.0934183	noncoding
0.0126173	noncoding
1	coding
1	coding
0.973288	coding
1	coding
0.998124	coding
0.0810512	noncoding
1	coding
0.999944	coding
0.95378	coding
0.974617	coding
0.957358	coding
0.999997	coding
1	coding
1	coding
1	coding
0.204387	noncoding
0.999993	coding
0.999993	coding
1	coding
1	coding
0.107772	noncoding
1	coding
0.0172747	noncoding
0.996292	coding
0.427567	noncoding
0.999387	coding
0.977711	coding
1	coding
1	coding
1	coding
0.0181165	noncoding
0.170995	noncoding
0.164903	noncoding
0.207024	noncoding
0.999998	coding
0.999998	coding
1	coding
1	coding
1	coding

0.999703	coding
1	coding
1	coding
0.999999	coding
0.404038	noncoding
0.543915	coding
1	coding
1	coding
0.0558297	noncoding
0.999999	coding
0.999992	coding
0.606832	coding
0.999783	coding
0.698961	coding
0.844151	coding
0.599176	coding
0.968898	coding
0.0398055	noncoding
1	coding
1	coding
1	coding
1	coding
0.997584	coding
1	coding
1	coding
1	coding
0.999866	coding
0.717595	coding
0.454108	noncoding
0.702936	coding
0.513492	coding
0.545034	coding
0.187053	noncoding
0.184683	noncoding
0.180493	noncoding
0.69559	coding
0.290915	noncoding
0.0297736	noncoding
0.0209007	noncoding
0.999068	coding
0.592405	coding
1	coding
0.950221	coding
1	coding
0.999279	coding
0.0265496	noncoding
0.0388194	noncoding
1	coding
1	coding
0.242657	noncoding
1	coding
1	coding
1	coding
0.0115584	noncoding

0.540608	coding
1	coding
1	coding
0.100809	noncoding
0.339482	noncoding
0.0163477	noncoding
1	coding
0.555381	coding
1	coding
1	coding
0.118521	noncoding
1	coding
0.495233	noncoding
0.229276	noncoding
0.228461	noncoding
1	coding
1	coding
1	coding
1	coding
1	coding
1	coding
1	coding
1	coding
0.0822111	noncoding
0.0622376	noncoding
1	coding
1	coding
0.00526184	noncoding
1	coding
1	coding
1	coding
0.961774	coding
0.144002	noncoding
1	coding
1	coding
1	coding
1	coding
0.998041	coding
0.0467901	noncoding
1	coding
0.970276	coding
1	coding
1	coding
1	coding
0.999995	coding
0.973044	coding
0.0306742	noncoding
0.667385	coding
0.130519	noncoding
0.973709	coding
0.178271	noncoding
0.999998	coding
0.999976	coding
0.999949	coding

0.999995	coding
0.999999	coding
0.999642	coding
0.997937	coding
1	coding
1	coding
1	coding
1	coding
0.224928	noncoding
1	coding
0.220327	noncoding
0.0415553	noncoding
0.259865	noncoding
0.0505057	noncoding
0.759397	coding
0.388637	noncoding
0.156443	noncoding
0.0682139	noncoding
1	coding
1	coding
1	coding
0.152629	noncoding
0.00206017	noncoding
0.156655	noncoding
0.997867	coding
0.998412	coding
0.999759	coding
0.942906	coding
0.083404	noncoding
0.132767	noncoding
0.0752098	noncoding
1	coding
0.742118	coding
0.481667	noncoding
0.87377	coding
0.180332	noncoding
0.999996	coding
0.655222	coding
0.24286	noncoding
0.0195469	noncoding
0.027369	noncoding
1	coding
1	coding
0.979484	coding
0.866993	coding
0.913477	coding
1	coding
1	coding
1	coding
1	coding
0.999999	coding
0.152181	noncoding
0.253697	noncoding
0.823418	coding

0.135471	noncoding
0.135471	noncoding
0.995098	coding
0.0550361	noncoding
0.0297566	noncoding
0.476068	noncoding
0.33445	noncoding
1	coding
0.980281	coding
0.0563039	noncoding
0.279694	noncoding
0.026274	noncoding
0.999875	coding
0.0811816	noncoding
0.0822928	noncoding
0.125144	noncoding
0.0304544	noncoding
0.0259507	noncoding
0.0702386	noncoding
0.0702386	noncoding
0.0731115	noncoding
0.0918717	noncoding
0.541789	coding
1	coding
1	coding
0.99299	coding
0.657706	coding
0.0076795	noncoding
1	coding
1	coding
0.811301	coding
1	coding
0.999747	coding
0.907836	coding
0.0420584	noncoding
0.552852	coding
0.23811	noncoding
0.271298	noncoding
0.702963	coding
0.502886	coding
0.869486	coding
0.0473445	noncoding
0.0845872	noncoding
0.963173	coding
0.89419	coding
0.999994	coding
0.999705	coding
0.699575	coding
0.0923418	noncoding
1	coding
0.999973	coding
1	coding
1	coding
1	coding

1	coding
0.0941615	noncoding
1	coding
1	coding
1	coding
1	coding
1	coding
0.474443	noncoding
0.992196	coding
0.999408	coding
0.986656	coding
0.0735893	noncoding
1	coding
1	coding
0.126343	noncoding
0.728002	coding
0.686922	coding
0.0318144	noncoding
0.159723	noncoding
0.0145998	noncoding
0.0446396	noncoding
1	coding
1	coding
0.0832888	noncoding
1	coding
0.295216	noncoding
0.998799	coding
0.998355	coding
0.998619	coding
0.99947	coding
0.99947	coding
0.0712309	noncoding
1	coding
1	coding
0.527214	coding
0.570717	coding
0.243803	noncoding
1	coding
0.999497	coding
1	coding
1	coding
0.964729	coding
0.999922	coding
1	coding
1	coding
1	coding
1	coding
1	coding
0.0534268	noncoding
0.93735	coding
0.999998	coding
0.999748	coding
0.999999	coding
0.999999	coding

0.0190057	noncoding
0.999999	coding
1	coding
1	coding
1	coding
1	coding
1	coding
0.76199	coding
1	coding
1	coding
1	coding
1	coding
1	coding
1	coding
1	coding
1	coding
0.0438168	noncoding
0.999988	coding
1	coding
1	coding
1	coding
1	coding
1	coding
1	coding
1	coding
1	coding
1	coding
0.323941	noncoding
0.308239	noncoding
0.153717	noncoding
0.999712	coding
0.999982	coding
0.0505873	noncoding
0.0255046	noncoding
1	coding
1	coding
0.987582	coding
0.0241902	noncoding
0.263237	noncoding
0.67872	coding
0.208194	noncoding
0.094492	noncoding
0.0244281	noncoding
1	coding
0.154233	noncoding
0.0267328	noncoding
1	coding
1	coding
1	coding
1	coding
1	coding
1	coding
1	coding
1	coding

0.98448	coding
0.517284	coding
0.295987	noncoding
0.603252	coding
0.613456	coding
0.364805	noncoding
1	coding
1	coding
1	coding
1	coding
0.999993	coding
0.999993	coding
0.999999	coding
1	coding
1	coding
0.165605	noncoding
1	coding
0.79857	coding
0.199676	noncoding
1	coding
1	coding
0.120915	noncoding
0.705018	coding
1	coding
1	coding
1	coding
1	coding
0.9999	coding
0.999992	coding
0.992839	coding
0.999995	coding
0.999929	coding
0.999929	coding
1	coding
0.320729	noncoding
0.734319	coding
1	coding
0.999998	coding
0.999862	coding
0.174817	noncoding
0.673366	coding
0.495554	noncoding
1	coding
1	coding
0.999647	coding
0.983988	coding
0.961644	coding
0.242583	noncoding
0.94377	coding
0.0365936	noncoding
0.755011	coding
0.721344	coding
0.0717826	noncoding
0.999996	coding

1	coding
0.980527	coding
0.0702345	noncoding
0.468004	noncoding
1	coding
1	coding
0.72043	coding
0.784766	coding
1	coding
0.0375568	noncoding
0.00776664	noncoding
0.999925	coding
0.246499	noncoding
0.0358907	noncoding
0.999522	coding
1	coding
1	coding
1	coding
0.976261	coding
1	coding
1	coding
1	coding
1	coding
1	coding
0.999992	coding
0.476171	noncoding
0.0249329	noncoding
1	coding
1	coding
1	coding
1	coding
1	coding
0.999479	coding
0.729736	coding
1	coding
1	coding
0.0539472	noncoding
1	coding
0.00406678	noncoding
0.027338	noncoding
1	coding
0.0435795	noncoding
1	coding
0.999974	coding
1	coding
1	coding
1	coding
1	coding
1	coding
1	coding
0.0527521	noncoding
0.0604926	noncoding
1	coding
0.294831	noncoding

0.445455	noncoding
1	coding
0.977339	coding
1	coding
1	coding
1	coding
1	coding
0.0644277	noncoding
0.88977	coding
0.136708	noncoding
0.622245	coding
0.0448897	noncoding
0.0336083	noncoding
0.166164	noncoding
0.0266581	noncoding
0.979176	coding
0.939407	coding
0.989475	coding
0.0247669	noncoding
1	coding
0.994016	coding
1	coding
0.0592815	noncoding
0.0244906	noncoding
0.0274227	noncoding
1	coding
1	coding
0.0507385	noncoding
0.999978	coding
0.999488	coding
0.999583	coding
0.999488	coding
0.999597	coding
1	coding
0.0662338	noncoding
0.302828	noncoding
1	coding
1	coding
1	coding
1	coding
1	coding
1	coding
1	coding
1	coding
0.0318324	noncoding
1	coding
1	coding
1	coding
1	coding
1	coding
1	coding
0.997569	coding
0.997429	coding
1	coding

0.0901598	noncoding
1	coding
0.0726141	noncoding
0.999999	coding
0.0168851	noncoding
1	coding
1	coding
0.127317	noncoding
0.717731	coding
0.0725523	noncoding
0.10999	noncoding
0.0825299	noncoding
0.993012	coding
0.169022	noncoding
0.651194	coding
0.393427	noncoding
0.00286172	noncoding
0.0495806	noncoding
0.0198915	noncoding
0.0588782	noncoding
0.0281143	noncoding
0.0239785	noncoding
0.129777	noncoding
0.0326081	noncoding
0.998986	coding
0.0234523	noncoding
0.15396	noncoding
0.129231	noncoding
0.099244	noncoding
0.0556991	noncoding
0.0563452	noncoding
0.00551606	noncoding
0.961219	coding
0.146681	noncoding
0.00756622	noncoding
0.0562312	noncoding
0.00481194	noncoding
0.999968	coding
1	coding
0.282867	noncoding
1	coding
0.999982	coding
0.371919	noncoding
0.283588	noncoding
0.0556681	noncoding
0.999998	coding
0.999997	coding
1	coding
1	coding
1	coding
1	coding
0.223525	noncoding
1	coding
1	coding

0.999845	coding
0.0337973	noncoding
0.999999	coding
1	coding
1	coding
0.996012	coding
0.999997	coding
0.0386041	noncoding
0.141885	noncoding
0.0515863	noncoding
0.122011	noncoding
0.0597743	noncoding
0.948176	coding
0.0468204	noncoding
0.979496	coding
0.0578051	noncoding
0.0644112	noncoding
0.12168	noncoding
0.202266	noncoding
1	coding
0.317087	noncoding
1	coding
0.0193729	noncoding
1	coding
0.997923	coding
0.826392	coding
0.0135574	noncoding
0.126487	noncoding
0.995817	coding
0.73621	coding
1	coding
1	coding
1	coding
0.633571	coding
0.964845	coding
0.991867	coding
1	coding
1	coding
1	coding
0.820659	coding
0.452091	noncoding
0.0721297	noncoding
1	coding
1	coding
1	coding
0.0898851	noncoding
0.999999	coding
0.63086	coding
0.259927	noncoding
0.0851212	noncoding
1	coding
1	coding
1	coding
1	coding

1	coding
1	coding
1	coding
1	coding
1	coding
1	coding
1	coding
1	coding
1	coding
0.0116841	noncoding
1	coding
0.0151637	noncoding
1	coding
0.037691	noncoding
1	coding
1	coding
0.991216	coding
0.024989	noncoding
0.296529	noncoding
0.144873	noncoding
1	coding
0.934249	coding
0.00223582	noncoding
1	coding
1	coding
1	coding
0.0438856	noncoding
0.070081	noncoding
0.539238	coding
0.0377967	noncoding
0.998936	coding
0.0579077	noncoding
0.999999	coding
1	coding
0.0555493	noncoding
0.995754	coding
0.869514	coding
0.523332	coding
0.0737074	noncoding
0.0642421	noncoding
0.517371	coding
0.317907	noncoding
0.56445	coding
0.610253	coding
0.0621511	noncoding
0.281893	noncoding
0.807515	coding
0.0848922	noncoding
1	coding
1	coding
0.988738	coding
0.114693	noncoding
1	coding
0.967375	coding

0.953313	coding
1	coding
1	coding
0.999994	coding
1	coding
1	coding
1	coding
1	coding
0.571259	coding
0.82808	coding
0.175482	noncoding
0.991069	coding
0.00186658	noncoding
0.397749	noncoding
0.448099	noncoding
0.00861178	noncoding
0.48487	noncoding
0.0149511	noncoding
0.999999	coding
0.99989	coding
1	coding
0.366311	noncoding
0.871323	coding
0.14496	noncoding
1	coding
1	coding
1	coding
1	coding
1	coding
1	coding
1	coding
0.0173064	noncoding
0.998469	coding
0.995294	coding
0.999999	coding
0.999327	coding
0.998511	coding
0.999997	coding
0.263092	noncoding
0.43462	noncoding
0.752531	coding
0.0405324	noncoding
0.122345	noncoding
0.915652	coding
0.985285	coding
0.99905	coding
0.556233	coding
0.489043	noncoding
0.999976	coding
0.0592253	noncoding
0.0273597	noncoding
0.137944	noncoding
1	coding
1	coding

0.0285711	noncoding
1	coding
0.915375	coding
0.880837	coding
0.99188	coding
0.940443	coding
0.99993	coding
0.831981	coding
0.0649572	noncoding
0.0552252	noncoding
0.924208	coding
0.034203	noncoding
1	coding
0.999977	coding
0.99997	coding
0.999984	coding
0.0148598	noncoding
1	coding
1	coding
1	coding
1	coding
1	coding
0.00639986	noncoding
0.163307	noncoding
0.166628	noncoding
0.161406	noncoding
0.16701	noncoding
0.164861	noncoding
0.0419806	noncoding
1	coding
1	coding
1	coding
1	coding
1	coding
1	coding
0.992766	coding
0.309107	noncoding
0.0761217	noncoding
0.0255848	noncoding
0.11755	noncoding
0.999493	coding
1	coding
1	coding
1	coding
1	coding
0.890328	coding
0.999991	coding
0.96172	coding
0.964306	coding
0.772937	coding
0.140598	noncoding
0.225154	noncoding
1	coding
0.448837	noncoding

0.0183122	noncoding
0.999381	coding
0.999865	coding
0.926844	coding
0.999993	coding
0.991134	coding
1	coding
0.0998481	noncoding
1	coding
0.326457	noncoding
0.0682584	noncoding
0.0240586	noncoding
1	coding
0.10502	noncoding
0.0209772	noncoding
0.2719	noncoding
0.99981	coding
1	coding
1	coding
0.99923	coding
1	coding
0.999998	coding
0.999823	coding
1	coding
1	coding
1	coding
0.463053	noncoding
0.195027	noncoding
0.994915	coding
0.685038	coding
0.999117	coding
0.999071	coding
0.0354267	noncoding
1	coding
0.0138593	noncoding
1	coding
1	coding
1	coding
0.315616	noncoding
0.0993767	noncoding
0.187652	noncoding
0.0676777	noncoding
0.0179633	noncoding
1	coding
0.658372	coding
1	coding
0.999966	coding
0.658372	coding
0.658372	coding
0.654989	coding
0.658372	coding
0.658372	coding
0.653397	coding
0.654989	coding

0.654989	coding
0.650537	coding
0.999998	coding
1	coding
0.636699	coding
0.0372199	noncoding
1	coding
1	coding
1	coding
1	coding
1	coding
1	coding
1	coding
0.790764	coding
0.906003	coding
0.689417	coding
0.844747	coding
1	coding
1	coding
1	coding
1	coding
0.101718	noncoding
0.00274865	noncoding
0.00795437	noncoding
1	coding
1	coding
0.0872133	noncoding
1	coding
0.932749	coding
0.057385	noncoding
0.162696	noncoding
0.460573	noncoding
0.828124	coding
0.414104	noncoding
1	coding
1	coding
0.978864	coding
0.996495	coding
0.281926	noncoding
0.0648433	noncoding
0.0382257	noncoding
0.0860728	noncoding
0.176614	noncoding
0.0239162	noncoding
0.99953	coding
0.397721	noncoding
0.427188	noncoding
0.298694	noncoding
0.0464778	noncoding
0.186529	noncoding
0.177805	noncoding
0.999934	coding
0.958023	coding
0.953192	coding

0.976022	coding
0.999995	coding
0.173486	noncoding
0.385203	noncoding
1	coding
1	coding
1	coding
0.999111	coding
1	coding
1	coding
0.999967	coding
0.999999	coding
1	coding
0.0293281	noncoding
0.318566	noncoding
0.430499	noncoding
0.889882	coding
0.575923	coding
0.763171	coding
1	coding
1	coding
1	coding
1	coding
1	coding
1	coding
1	coding
1	coding
0.342016	noncoding
0.432009	noncoding
0.914386	coding
0.191301	noncoding
0.972996	coding
0.176988	noncoding
0.928453	coding
0.0769934	noncoding
0.864907	coding
0.0461829	noncoding
0.394063	noncoding
1	coding
0.0403341	noncoding
0.999994	coding
1	coding
1	coding
1	coding
1	coding
0.0384958	noncoding
1	coding
0.26497	noncoding
1	coding
0.0200176	noncoding
0.0137756	noncoding
1	coding
1	coding
0.0197966	noncoding

0.999353	coding
0.989315	coding
0.208504	noncoding
0.999351	coding
0.999956	coding
0.999859	coding
0.999863	coding
0.999841	coding
0.999194	coding
0.999957	coding
0.999992	coding
0.999996	coding
0.20649	noncoding
0.292235	noncoding
0.0529932	noncoding
0.17782	noncoding
0.999975	coding
0.011535	noncoding
1	coding
0.0738396	noncoding
1	coding
1	coding
0.996187	coding
0.916898	coding
1	coding
1	coding
0.0128879	noncoding
0.823073	coding
0.626428	coding
0.275013	noncoding
0.124089	noncoding
0.0453466	noncoding
0.0594811	noncoding
0.999997	coding
0.194946	noncoding
0.994981	coding
0.999977	coding
1	coding
1	coding
1	coding
0.99981	coding
1	coding
0.999994	coding
0.376316	noncoding
0.125113	noncoding
1	coding
0.859511	coding
0.741503	coding
0.259119	noncoding
1	coding
0.0529092	noncoding
0.140522	noncoding
0.224142	noncoding
0.335817	noncoding

0.28433	noncoding
1	coding
1	coding
0.599023	coding
0.225156	noncoding
0.260351	noncoding
0.601235	coding
0.999994	coding
1	coding
1	coding
1	coding
1	coding
1	coding
0.999999	coding
0.10361	noncoding
0.0606122	noncoding
1	coding
1	coding
1	coding
0.0118634	noncoding
0.0231066	noncoding
1	coding
0.175752	noncoding
0.0949241	noncoding
0.269591	noncoding
1	coding
1	coding
1	coding
0.999201	coding
0.153131	noncoding
1	coding
0.963612	coding
1	coding
1	coding
1	coding
1	coding
1	coding
1	coding
1	coding
1	coding
1	coding
1	coding
1	coding
1	coding
1	coding
0.0778489	noncoding
0.202567	noncoding
0.167944	noncoding
0.295103	noncoding
0.191112	noncoding
0.0941395	noncoding
0.0612002	noncoding
0.999976	coding
1	coding

0.476698	noncoding
0.770624	coding
0.999896	coding
1	coding
1	coding
1	coding
1	coding
1	coding
1	coding
1	coding
0.1223	noncoding
0.184338	noncoding
0.860987	coding
0.801685	coding
1	coding
0.984953	coding
1	coding
1	coding
1	coding
1	coding
1	coding
0.0211809	noncoding
1	coding
0.056471	noncoding
0.187064	noncoding
0.0774166	noncoding
1	coding
1	coding
1	coding
0.617987	coding
1	coding
0.999963	coding
0.999999	coding
0.980159	coding
0.257043	noncoding
0.182665	noncoding
0.172739	noncoding
1	coding
1	coding
1	coding
0.876506	coding
1	coding
1	coding
0.00570872	noncoding
1	coding
1	coding
1	coding
1	coding
1	coding
1	coding
1	coding
1	coding
1	coding
1	coding

1	coding
0.112502	noncoding
0.846519	coding
1	coding
1	coding
0.815131	coding
0.999868	coding
0.236499	noncoding
0.00798619	noncoding
0.0274463	noncoding
0.0930266	noncoding
0.075741	noncoding
0.0272516	noncoding
1	coding
0.112314	noncoding
0.104005	noncoding
1	coding
0.997747	coding
1	coding
0.999972	coding
0.999981	coding
1	coding
0.0930823	noncoding
0.903387	coding
0.37842	noncoding
1	coding
1	coding
1	coding
0.0697638	noncoding
0.786201	coding
0.0946634	noncoding
0.106439	noncoding
1	coding
0.120798	noncoding
1	coding
1	coding
0.00791443	noncoding
0.00753395	noncoding
0.0133105	noncoding
0.999999	coding
0.999999	coding
1	coding
1	coding
0.999948	coding
0.20285	noncoding
0.193398	noncoding
1	coding
1	coding
1	coding
0.963948	coding
0.978825	coding
1	coding
1	coding
0.04242	noncoding

0.0422686	noncoding
1	coding
0.271122	noncoding
0.0117147	noncoding
0.963718	coding
0.999998	coding
0.999934	coding
1	coding
1	coding
0.190147	noncoding
1	coding
1	coding
0.999999	coding
0.00862605	noncoding
1	coding
1	coding
1	coding
1	coding
1	coding
0.0135415	noncoding
0.145751	noncoding
0.0356809	noncoding
0.925138	coding
0.463959	noncoding
1	coding
1	coding
0.483634	noncoding
0.999991	coding
0.0475521	noncoding
0.0304585	noncoding
0.0460749	noncoding
0.159507	noncoding
0.999992	coding
1	coding
0.751074	coding
0.990222	coding
0.304422	noncoding
0.999892	coding
1	coding
1	coding
1	coding
0.27373	noncoding
0.997979	coding
1	coding
1	coding
0.750843	coding
0.031893	noncoding
0.767856	coding
1	coding
0.999997	coding
0.999629	coding
0.998072	coding
0.998561	coding
0.0606792	noncoding

0.147958	noncoding
0.162189	noncoding
0.174187	noncoding
0.772385	coding
1	coding
1	coding
1	coding
1	coding
1	coding
1	coding
1	coding
1	coding
1	coding
1	coding
1	coding
1	coding
1	coding
0.0410899	noncoding
0.294043	noncoding
0.123658	noncoding
0.6013	coding
0.376896	noncoding
0.636505	coding
0.0134013	noncoding
0.0241801	noncoding
0.835004	coding
0.03874	noncoding
0.0259354	noncoding
1	coding
0.0281638	noncoding
0.40651	noncoding
1	coding
1	coding
1	coding
1	coding
1	coding
1	coding
0.999999	coding
1	coding
1	coding
0.210751	noncoding
0.0111285	noncoding
0.122319	noncoding
0.99978	coding
0.999862	coding
0.999856	coding
0.937127	coding
0.0972568	noncoding
1	coding
0.0285595	noncoding
0.00612942	noncoding
0.996513	coding
0.0140954	noncoding
0.0292858	noncoding

1	coding
1	coding
0.0325684	noncoding
0.215306	noncoding
0.968102	coding
0.998036	coding
0.0149471	noncoding
0.0509476	noncoding
0.323906	noncoding
1	coding
0.0743261	noncoding
0.0253631	noncoding
0.18972	noncoding
1	coding
1	coding
0.00500455	noncoding
1	coding
1	coding
0.598626	coding
0.408629	noncoding
0.0313696	noncoding
0.309674	noncoding
1	coding
1	coding
0.994801	coding
0.106302	noncoding
0.0488491	noncoding
0.0455076	noncoding
0.00599769	noncoding
1	coding
1	coding
0.220347	noncoding
1	coding
1	coding
1	coding
1	coding
0.0487779	noncoding
0.78991	coding
1	coding
0.999963	coding
0.999995	coding
0.999921	coding
0.0366843	noncoding
0.617551	coding
1	coding
0.0829067	noncoding
0.320769	noncoding
0.982004	coding
0.522873	coding
0.0663986	noncoding
0.606871	coding
0.10016	noncoding
0.248198	noncoding
0.182759	noncoding

0.0619179	noncoding
0.946262	coding
0.999998	coding
0.999988	coding
0.0428304	noncoding
0.968476	coding
1	coding
1	coding
1	coding
0.0397532	noncoding
1	coding
0.999992	coding
0.154244	noncoding
1	coding
0.802804	coding
0.0515963	noncoding
0.983271	coding
0.00629083	noncoding
0.00720527	noncoding
0.0568426	noncoding
0.0226639	noncoding
1	coding
0.0385565	noncoding
1	coding
1	coding
1	coding
1	coding
1	coding
1	coding
0.417663	noncoding
0.0441629	noncoding
0.978127	coding
0.227632	noncoding
0.076241	noncoding
0.224161	noncoding
0.20243	noncoding
1	coding
1	coding
0.706364	coding
0.110678	noncoding
1	coding
1	coding
0.0786493	noncoding
0.061694	noncoding
1	coding
1	coding
0.935693	coding
0.247635	noncoding
0.0707787	noncoding
0.999996	coding
0.997784	coding
0.999891	coding
0.999927	coding
0.999698	coding

1	coding
1	coding
1	coding
0.276794	noncoding
0.966893	coding
0.0439851	noncoding
0.746572	coding
0.0910035	noncoding
1	coding
1	coding
1	coding
1	coding
1	coding
0.0367888	noncoding
0.553108	coding
0.571914	coding
0.277812	noncoding
0.0945077	noncoding
1	coding
1	coding
1	coding
1	coding
0.745669	coding
0.538599	coding
0.0470139	noncoding
0.134113	noncoding
0.0614851	noncoding
1	coding
0.900375	coding
0.156778	noncoding
1	coding
1	coding
1	coding
0.0939133	noncoding
0.999444	coding
0.999512	coding
1	coding
1	coding
0.406317	noncoding
1	coding
0.0286861	noncoding
1	coding
0.26412	noncoding
1	coding
1	coding
1	coding
0.0276066	noncoding
1	coding
0.997644	coding
0.982557	coding
0.994054	coding
0.584813	coding
0.0262534	noncoding
1	coding

1	coding
1	coding
0.0053967	noncoding
0.202699	noncoding
0.736674	coding
0.0300171	noncoding
0.0907945	noncoding
0.197773	noncoding
0.0290874	noncoding
0.015634	noncoding
0.0464257	noncoding
0.102819	noncoding
0.953532	coding
1	coding
0.0942324	noncoding
0.755902	coding
0.0463724	noncoding
0.374875	noncoding
0.999985	coding
0.999691	coding
0.998812	coding
0.998854	coding
0.998736	coding
0.979867	coding
0.442467	noncoding
0.0575819	noncoding
1	coding
0.0378547	noncoding
0.999992	coding
1	coding
1	coding
1	coding
1	coding
0.539549	coding
1	coding
1	coding
0.484933	noncoding
1	coding
1	coding
0.0252469	noncoding
1	coding
1	coding
0.999999	coding
0.182285	noncoding
0.996809	coding
0.338367	noncoding
0.116697	noncoding
1	coding
0.227741	noncoding
0.999998	coding
1	coding
0.669688	coding
0.126651	noncoding
0.0744764	noncoding

0.259227	noncoding
0.762612	coding
0.324466	noncoding
0.622887	coding
0.158674	noncoding
0.554331	coding
0.405389	noncoding
0.405389	noncoding
0.126173	noncoding
0.126173	noncoding
0.1302	noncoding
0.612276	coding
0.620722	coding
0.0609246	noncoding
0.151075	noncoding
0.167738	noncoding
0.075311	noncoding
0.921443	coding
0.585877	coding
0.553944	coding
1	coding
0.160995	noncoding
0.999937	coding
0.99176	coding
0.194383	noncoding
0.0171669	noncoding
0.994279	coding
0.999989	coding
0.385898	noncoding
0.165589	noncoding
0.12375	noncoding
1	coding
1	coding
1	coding
0.0115656	noncoding
0.630919	coding
0.00592106	noncoding
0.0571844	noncoding
1	coding
0.999881	coding
0.997071	coding
0.995149	coding
0.980093	coding
1	coding
0.0244952	noncoding
0.0550902	noncoding
0.142987	noncoding
0.158415	noncoding
0.37751	noncoding
0.300273	noncoding
0.0454751	noncoding
0.999906	coding
1	coding
0.143381	noncoding

0.999855	coding
0.0394453	noncoding
1	coding
1	coding
0.876272	coding
0.99227	coding
0.0627805	noncoding
0.76903	coding
1	coding
1	coding
0.0189083	noncoding
0.196989	noncoding
0.999995	coding
0.999066	coding
0.998246	coding
1	coding
0.999499	coding
0.999499	coding
0.999493	coding
0.999486	coding
0.999478	coding
0.999167	coding
0.999449	coding
0.99825	coding
1	coding
1	coding
1	coding
1	coding
0.905362	coding
1	coding
0.873361	coding
1	coding
1	coding
0.994588	coding
0.970783	coding
0.892247	coding
0.999989	coding
0.07939	noncoding
0.955899	coding
0.963814	coding
0.809614	coding
0.637502	coding
0.996402	coding
0.369034	noncoding
0.130054	noncoding
0.0384091	noncoding
0.0377806	noncoding
1	coding
1	coding
0.394037	noncoding
0.044113	noncoding
0.0714811	noncoding
0.393013	noncoding
0.100979	noncoding

0.551661	coding
0.095126	noncoding
0.113598	noncoding
0.982867	coding
0.790059	coding
0.248844	noncoding
0.167991	noncoding
0.259958	noncoding
0.0643081	noncoding
0.0917931	noncoding
0.408144	noncoding
0.284282	noncoding
0.284282	noncoding
0.167183	noncoding
0.666328	coding
0.167224	noncoding
0.971047	coding
0.158332	noncoding
0.819858	coding
0.982867	coding
0.0594314	noncoding
0.152038	noncoding
0.076027	noncoding
0.0737587	noncoding
0.126263	noncoding
0.963142	coding
1	coding
1	coding
1	coding
1	coding
1	coding
1	coding
0.508119	coding
0.0427319	noncoding
0.0200572	noncoding
0.886756	coding
0.118101	noncoding
0.17069	noncoding
0.00214538	noncoding
1	coding
0.999515	coding
1	coding
0.999999	coding
0.121978	noncoding
0.427262	noncoding
0.980802	coding
1	coding
0.210958	noncoding
0.0160723	noncoding
0.0571132	noncoding
1	coding
0.048448	noncoding
0.974487	coding
0.783332	coding

0.990721	coding
0.981716	coding
1	coding
0.118727	noncoding
1	coding
0.0208283	noncoding
1	coding
1	coding
1	coding
1	coding
0.999999	coding
0.998453	coding
0.229691	noncoding
0.0899565	noncoding
0.999998	coding
0.998968	coding
0.999969	coding
1	coding
0.310127	noncoding
0.55247	coding
1	coding
1	coding
1	coding
1	coding
0.700063	coding
0.999936	coding
1	coding
0.898742	coding
1	coding
0.953053	coding
0.474782	noncoding
0.00331561	noncoding
0.976306	coding
0.419392	noncoding
0.0813489	noncoding
1	coding
1	coding
1	coding
0.0238216	noncoding
0.368442	noncoding
0.122128	noncoding
0.212347	noncoding
1	coding
1	coding
1	coding
0.999999	coding
1	coding
1	coding
0.988955	coding
0.87354	coding
0.999833	coding
1	coding
1	coding
0.999967	coding

1	coding
0.0141818	noncoding
0.0174414	noncoding
0.0197671	noncoding
0.00844261	noncoding
0.0143944	noncoding
0.0280941	noncoding
0.0350724	noncoding
0.0210722	noncoding
0.0225081	noncoding
0.0224941	noncoding
0.0259508	noncoding
0.0100101	noncoding
0.0271008	noncoding
0.368464	noncoding
0.0776519	noncoding
1	coding
0.0387961	noncoding
0.99896	coding
0.141864	noncoding
0.999873	coding
0.0216097	noncoding
0.0865425	noncoding
0.686612	coding
0.718775	coding
0.366069	noncoding
1	coding
1	coding
0.106506	noncoding
0.328589	noncoding
1	coding
1	coding
0.043482	noncoding
0.0727411	noncoding
0.0348712	noncoding
0.023983	noncoding
0.999616	coding
1	coding
0.367012	noncoding
1	coding
0.934556	coding
0.158864	noncoding
0.0669679	noncoding
0.985011	coding
0.958337	coding
0.11888	noncoding
1	coding
0.436313	noncoding
1	coding
0.136089	noncoding
0.0666982	noncoding
0.0112916	noncoding
0.507133	coding
1	coding

1	coding
0.108932	noncoding
0.0216195	noncoding
1	coding
0.0386114	noncoding
0.285098	noncoding
0.41735	noncoding
1	coding
0.0105401	noncoding
0.556625	coding
1	coding
0.998909	coding
0.999887	coding
1	coding
0.013148	noncoding
0.955504	coding
0.213216	noncoding
0.999999	coding
0.999999	coding
0.109212	noncoding
1	coding
1	coding
1	coding
1	coding
1	coding
0.92776	coding
0.984385	coding
0.994463	coding
1	coding
1	coding
1	coding
1	coding
1	coding
1	coding
1	coding
1	coding
1	coding
0.999963	coding
1	coding
0.138609	noncoding
0.136464	noncoding
0.251524	noncoding
0.262672	noncoding
1	coding
1	coding
0.0267151	noncoding
1	coding
1	coding
0.991972	coding
0.696051	coding
1	coding
1	coding
0.147054	noncoding
0.125536	noncoding

0.281952	noncoding
0.460878	noncoding
0.710074	coding
1	coding
0.952051	coding
0.999962	coding
0.90183	coding
0.174796	noncoding
0.0770688	noncoding
0.105605	noncoding
0.0041041	noncoding
0.27573	noncoding
0.999071	coding
0.339396	noncoding
1	coding
0.999996	coding
0.0327321	noncoding
0.122205	noncoding
0.0363424	noncoding
0.0310625	noncoding
0.51505	coding
0.175616	noncoding
1	coding
1	coding
0.999996	coding
1	coding
1	coding
0.999923	coding
0.999662	coding
1	coding
1	coding
1	coding
1	coding
1	coding
0.009889	noncoding
0.0038087	noncoding
0.0038462	noncoding
0.95737	coding
0.0456852	noncoding
0.999951	coding
1	coding
0.0098489	noncoding
1	coding
0.999998	coding
0.999995	coding
0.996625	coding
0.999172	coding
0.994019	coding
0.996612	coding
0.999993	coding
1	coding
0.999837	coding
1	coding
1	coding

1	coding
1	coding
0.0549458	noncoding
0.0625698	noncoding
0.047396	noncoding
0.0863227	noncoding
0.0790621	noncoding
1	coding
1	coding
1	coding
0.0127738	noncoding
0.0141381	noncoding
0.988792	coding
1	coding
1	coding
1	coding
1	coding
1	coding
1	coding
1	coding
1	coding
1	coding
1	coding
1	coding
1	coding
1	coding
0.368282	noncoding
0.0183254	noncoding
0.0377712	noncoding
0.190232	noncoding
0.919077	coding
0.669741	coding
0.932511	coding
1	coding
1	coding
0.997216	coding
0.027058	noncoding
0.0214838	noncoding
0.185517	noncoding
0.222364	noncoding
0.197856	noncoding
0.970367	coding
0.182907	noncoding
0.0258817	noncoding
1	coding
1	coding
1	coding
0.00861689	noncoding
1	coding
1	coding
0.0685709	noncoding
1	coding
0.0432777	noncoding
1	coding

0.998705	coding
0.213871	noncoding
1	coding
1	coding
1	coding
0.0560075	noncoding
0.0562506	noncoding
0.999995	coding
0.999965	coding
0.999848	coding
0.308878	noncoding
0.999844	coding
0.99736	coding
1	coding
0.943105	coding
0.0988921	noncoding
0.133636	noncoding
0.0812323	noncoding
1	coding
1	coding
1	coding
1	coding
1	coding
1	coding
1	coding
0.999072	coding
0.999997	coding
0.271122	noncoding
0.999832	coding
1	coding
1	coding
1	coding
1	coding
0.128945	noncoding
0.944665	coding
1	coding
1	coding
0.850367	coding
0.999633	coding
0.984836	coding
0.848587	coding
0.0544088	noncoding
0.282557	noncoding
0.998852	coding
1	coding
0.917759	coding
0.869715	coding
0.494593	noncoding
0.254969	noncoding
0.657501	coding
0.37484	noncoding
0.364197	noncoding
0.0946115	noncoding
0.584734	coding

0.0754648	noncoding
0.0740239	noncoding
0.0211014	noncoding
0.33182	noncoding
0.521377	coding
0.0441653	noncoding
0.013483	noncoding
0.0484646	noncoding
0.01203	noncoding
0.362256	noncoding
0.934766	coding
1	coding
0.0322091	noncoding
0.99999	coding
0.999994	coding
0.354498	noncoding
0.875384	coding
0.0232727	noncoding
0.147839	noncoding
1	coding
1	coding
1	coding
1	coding
1	coding
1	coding
1	coding
1	coding
0.013384	noncoding
0.0673789	noncoding
0.999994	coding
0.0335772	noncoding
1	coding
0.0452322	noncoding
0.0982233	noncoding
0.0198925	noncoding
0.346116	noncoding
0.132464	noncoding
0.219277	noncoding
0.187135	noncoding
0.0139863	noncoding
1	coding
0.996441	coding
0.102083	noncoding
0.0246996	noncoding
0.251754	noncoding
0.0462815	noncoding
0.026746	noncoding
0.64319	coding
0.864757	coding
0.985487	coding
1	coding
1	coding
0.0921653	noncoding
0.0146161	noncoding

0.999479	coding
1	coding
1	coding
1	coding
1	coding
1	coding
1	coding
1	coding
1	coding
1	coding
1	coding
1	coding
1	coding
1	coding
1	coding
1	coding
1	coding
1	coding
0.999999	coding
0.998385	coding
0.137917	noncoding
0.318125	noncoding
0.0345337	noncoding
0.0368275	noncoding
0.0377525	noncoding
0.0377525	noncoding
0.0368387	noncoding
0.0113698	noncoding
0.939687	coding
1	coding
1	coding
1	coding
1	coding
1	coding
1	coding
1	coding
0.140753	noncoding
0.0825497	noncoding
1	coding
1	coding
1	coding
1	coding
1	coding
1	coding
0.202998	noncoding
0.999997	coding
0.699861	coding
1	coding
1	coding
1	coding
1	coding
1	coding
0.0146465	noncoding
0.0466737	noncoding

0.947783	coding
0.896097	coding
0.999935	coding
0.999871	coding
0.855794	coding
0.990015	coding
0.743785	coding
0.0301989	noncoding
0.941449	coding
1	coding
1	coding
1	coding
1	coding
1	coding
1	coding
1	coding
1	coding
1	coding
0.525406	coding
1	coding
1	coding
1	coding
1	coding
1	coding
1	coding
0.359872	noncoding
0.999834	coding
0.999832	coding
1	coding
1	coding
1	coding
0.947529	coding
1	coding
0.999948	coding
0.999936	coding
0.999948	coding
0.99995	coding
0.378325	noncoding
0.217029	noncoding
0.668046	coding
1	coding
0.310088	noncoding
1	coding
0.980247	coding
0.972722	coding
0.169734	noncoding
0.745535	coding
0.213833	noncoding
1	coding
1	coding
1	coding
1	coding
1	coding
1	coding

1	coding
0.348279	noncoding
0.217155	noncoding
1	coding
1	coding
1	coding
1	coding
1	coding
1	coding
0.0124909	noncoding
0.0552638	noncoding
0.999999	coding
0.574021	coding
0.0909378	noncoding
0.689119	coding
1	coding
1	coding
1	coding
0.9387	coding
0.976394	coding
0.907795	coding
0.944207	coding
1	coding
0.999974	coding
0.951376	coding
0.933596	coding
0.00270416	noncoding
1	coding
1	coding
1	coding
1	coding
1	coding
1	coding
1	coding
1	coding
0.0436618	noncoding
1	coding
1	coding
1	coding
1	coding
1	coding
0.999278	coding
0.994072	coding
0.999992	coding
0.999998	coding
0.999992	coding
0.999991	coding
1	coding
0.210029	noncoding
0.0421299	noncoding
1	coding
1	coding
0.999973	coding
0.99737	coding

0.999976	coding
0.991319	coding
0.981632	coding
0.999997	coding
0.999992	coding
0.999913	coding
1	coding
1	coding
1	coding
1	coding
1	coding
1	coding
0.0380901	noncoding
0.395439	noncoding
0.499838	noncoding
1	coding
0.0184186	noncoding
0.0425591	noncoding
0.99998	coding
0.101463	noncoding
0.236103	noncoding
0.1373	noncoding
1	coding
1	coding
0.142657	noncoding
0.0107085	noncoding
0.529374	coding
0.591847	coding
0.0980986	noncoding
0.114631	noncoding
0.179986	noncoding
0.367204	noncoding
0.270284	noncoding
0.338219	noncoding
0.0593395	noncoding
0.341114	noncoding
0.0143807	noncoding
0.999905	coding
0.0464172	noncoding
0.00750236	noncoding
0.0454317	noncoding
1	coding
1	coding
0.999998	coding
0.158884	noncoding
0.0131614	noncoding
0.999955	coding
0.999999	coding
0.966046	coding
0.996298	coding
0.0991233	noncoding
0.112076	noncoding
0.138437	noncoding
1	coding

1	coding
1	coding
0.0933011	noncoding
0.0610467	noncoding
0.332171	noncoding
0.734863	coding
0.444172	noncoding
0.485947	noncoding
0.049763	noncoding
0.078241	noncoding
0.369488	noncoding
0.269893	noncoding
0.034662	noncoding
0.040106	noncoding
0.0634792	noncoding
0.997897	coding
0.0270214	noncoding
0.0106217	noncoding
1	coding
1	coding
0.999931	coding
0.0193967	noncoding
0.0226947	noncoding
1	coding
0.997543	coding
0.488726	noncoding
0.161184	noncoding
0.100119	noncoding
0.0530504	noncoding
0.623722	coding
1	coding
1	coding
1	coding
0.0856275	noncoding
0.278628	noncoding
0.931996	coding
0.248364	noncoding
0.146498	noncoding
0.862056	coding
0.104548	noncoding
0.864271	coding
0.189985	noncoding
0.79683	coding
0.79683	coding
0.301371	noncoding
0.467715	noncoding
0.12054	noncoding
0.12054	noncoding
0.0891943	noncoding
0.0234389	noncoding
0.517393	coding
0.81319	coding
0.365142	noncoding
0.0480702	noncoding

0.150962	noncoding
0.140107	noncoding
1	coding
1	coding
0.387059	noncoding
1	coding
1	coding
1	coding
1	coding
0.32396	noncoding
0.0810842	noncoding
0.738498	coding
0.0644487	noncoding
0.0890434	noncoding
0.962127	coding
0.0655363	noncoding
0.0717325	noncoding
1	coding
0.0172017	noncoding
0.995961	coding
0.989587	coding
1	coding
0.620867	coding
0.999838	coding
0.136223	noncoding
0.304118	noncoding
0.0226414	noncoding
1	coding
1	coding
1	coding
0.999996	coding
0.575318	coding
0.146907	noncoding
0.0860006	noncoding
0.279249	noncoding
0.0329702	noncoding
0.899883	coding
1	coding
0.286328	noncoding
0.00733171	noncoding
0.206655	noncoding
0.0539722	noncoding
0.0730244	noncoding
0.0822488	noncoding
0.937095	coding
0.331391	noncoding
1	coding
1	coding
0.986926	coding
0.974653	coding
0.973689	coding
1	coding
1	coding
1	coding

1	coding
0.92249	coding
1	coding
1	coding
1	coding
0.95352	coding
0.999987	coding
1	coding
0.921488	coding
1	coding
0.999996	coding
0.998476	coding
1	coding
1	coding
0.986628	coding
0.976289	coding
0.0375883	noncoding
0.01745	noncoding
0.0174093	noncoding
0.210324	noncoding
0.157851	noncoding
0.206773	noncoding
0.0406726	noncoding
0.491169	noncoding
0.891229	coding
0.97494	coding
0.980839	coding
0.0105159	noncoding
1	coding
1	coding
0.876458	coding
0.830526	coding
0.830526	coding
0.999847	coding
1	coding
0.999964	coding
1	coding
0.809725	coding
1	coding
0.72578	coding
0.0563732	noncoding
0.418887	noncoding
0.677073	coding
1	coding
0.999594	coding
0.400567	noncoding
1	coding
0.819025	coding
0.0520977	noncoding
0.0488333	noncoding
0.0431647	noncoding
0.0906884	noncoding
0.0423038	noncoding
0.999868	coding

0.0485251	noncoding
1	coding
0.0607353	noncoding
0.999999	coding
1	coding
1	coding
1	coding
0.122296	noncoding
0.999897	coding
1	coding
0.999955	coding
0.341751	noncoding
1	coding
0.0291891	noncoding
1	coding
0.999941	coding
0.999922	coding
0.988053	coding
0.937842	coding
1	coding
1	coding
1	coding
0.122128	noncoding
0.158272	noncoding
1	coding
1	coding
1	coding
0.0371862	noncoding
0.0247965	noncoding
1	coding
0.102706	noncoding
1	coding
1	coding
0.291299	noncoding
0.999971	coding
1	coding
1	coding
0.989307	coding
0.351519	noncoding
0.915613	coding
0.278665	noncoding
0.33458	noncoding
0.0233591	noncoding
0.45554	noncoding
0.252803	noncoding
0.373416	noncoding
0.0330896	noncoding
0.0334373	noncoding
0.0347864	noncoding
0.0330896	noncoding
0.0328642	noncoding
0.0322491	noncoding
0.0319924	noncoding
0.115541	noncoding

0.0330896	noncoding
0.0110805	noncoding
0.0110805	noncoding
0.0314755	noncoding
0.0328248	noncoding
0.0328642	noncoding
0.0322491	noncoding
0.266649	noncoding
0.115162	noncoding
0.0547381	noncoding
0.262461	noncoding
0.999987	coding
1	coding
0.999987	coding
0.99998	coding
1	coding
1	coding
0.10971	noncoding
0.99994	coding
0.422582	noncoding
0.996812	coding
0.999729	coding
0.656715	coding
0.351287	noncoding
0.0271952	noncoding
1	coding
1	coding
1	coding
0.330717	noncoding
1	coding
0.999997	coding
1	coding
0.0836132	noncoding
0.13542	noncoding
0.0858713	noncoding
0.832615	coding
1	coding
0.996361	coding
0.515614	coding
0.822458	coding
1	coding
0.999799	coding
0.524965	coding
0.0974038	noncoding
1	coding
1	coding
1	coding
0.493847	noncoding
1	coding
0.99991	coding
0.0794988	noncoding
1	coding
1	coding
1	coding

0.0641483	noncoding
1	coding
0.993108	coding
0.172865	noncoding
0.228466	noncoding
0.0256132	noncoding
1	coding
0.116457	noncoding
0.0757331	noncoding
0.218707	noncoding
0.484752	noncoding
0.246311	noncoding
0.217216	noncoding
0.100618	noncoding
0.992543	coding
0.0798465	noncoding
0.578414	coding
0.136018	noncoding
0.200989	noncoding
0.32666	noncoding
0.04627	noncoding
0.72128	coding
1	coding
0.118405	noncoding
0.149384	noncoding
1	coding
1	coding
1	coding
1	coding
1	coding
1	coding
1	coding
0.954708	coding
1	coding
0.999997	coding
0.999996	coding
1	coding
1	coding
1	coding
0.226207	noncoding
0.0550621	noncoding
0.388547	noncoding
0.19851	noncoding
0.0960411	noncoding
0.537372	coding
0.926259	coding
0.0264085	noncoding
0.998919	coding
0.403913	noncoding
1	coding
1	coding
0.0817216	noncoding
0.0314774	noncoding
0.0163248	noncoding

0.0294045	noncoding
0.371211	noncoding
0.0101096	noncoding
1	coding
1	coding
1	coding
1	coding
1	coding
1	coding
1	coding
0.995136	coding
0.995136	coding
0.994195	coding
0.996058	coding
0.0273624	noncoding
0.0316025	noncoding
0.0253182	noncoding
0.196197	noncoding
0.984315	coding
0.177242	noncoding
0.0799444	noncoding
0.0801544	noncoding
0.999997	coding
1	coding
0.29793	noncoding
0.536139	coding
0.110207	noncoding
0.87325	coding
0.647582	coding
0.426459	noncoding
0.313981	noncoding
1	coding
0.0850288	noncoding
0.0229907	noncoding
0.0621972	noncoding
0.0541187	noncoding
0.0622182	noncoding
1	coding
1	coding
0.226087	noncoding
0.0597036	noncoding
0.999999	coding
1	coding
0.0872766	noncoding
1	coding
0.621548	coding
0.430388	noncoding
1	coding
0.999432	coding
0.999359	coding
0.999685	coding
0.999545	coding
1	coding
0.998516	coding

0.999757	coding
1	coding
0.0735124	noncoding
1	coding
1	coding
1	coding
0.0089912	noncoding
0.550666	coding
0.329768	noncoding
0.00974561	noncoding
0.740088	coding
0.999999	coding
1	coding
0.158806	noncoding
0.217812	noncoding
1	coding
1	coding
0.283896	noncoding
1	coding
1	coding
1	coding
1	coding
1	coding
1	coding
0.0847149	noncoding
0.0760065	noncoding
0.371102	noncoding
0.951625	coding
1	coding
0.0119122	noncoding
0.896508	coding
0.0169454	noncoding
0.103269	noncoding
1	coding
1	coding
0.445786	noncoding
0.0233051	noncoding
1	coding
1	coding
0.0433115	noncoding
0.00606901	noncoding
0.0582963	noncoding
0.0657444	noncoding
0.386087	noncoding
1	coding
0.67647	coding
1	coding
0.999997	coding
0.00627477	noncoding
1	coding
0.807385	coding
1	coding
1	coding
0.111804	noncoding

0.105471	noncoding
0.118095	noncoding
1	coding
0.998871	coding
0.999606	coding
0.999983	coding
0.107511	noncoding
0.999601	coding
0.999987	coding
0.383853	noncoding
1	coding
1	coding
1	coding
0.998573	coding
0.839936	coding
0.999829	coding
1	coding
1	coding
0.223973	noncoding
0.999997	coding
0.998765	coding
1	coding
0.980354	coding
1	coding
1	coding
0.997257	coding
1	coding
0.0298744	noncoding
0.0760185	noncoding
0.0182952	noncoding
1	coding
1	coding
1	coding
0.217208	noncoding
0.999983	coding
0.927563	coding
0.999404	coding
1	coding
0.993593	coding
0.997689	coding
1	coding
0.99271	coding
1	coding
1	coding
1	coding
0.999995	coding
1	coding
0.023961	noncoding
1	coding
1	coding
0.944166	coding
0.952422	coding
1	coding
1	coding

0.994714	coding
0.99574	coding
0.999121	coding
0.998956	coding
1	coding
1	coding
1	coding
1	coding
1	coding
1	coding
1	coding
1	coding
1	coding
1	coding
0.99999	coding
0.896246	coding
0.168953	noncoding
0.706718	coding
0.249118	noncoding
0.110304	noncoding
0.999605	coding
0.999999	coding
0.999999	coding
1	coding
0.999985	coding
0.872764	coding
0.974644	coding
1	coding
0.994999	coding
0.0644376	noncoding
0.999999	coding
0.99556	coding
0.995819	coding
1	coding
1	coding
1	coding
1	coding
0.837258	coding
0.999995	coding
1	coding
1	coding
0.951624	coding
0.110807	noncoding
0.753494	coding
1	coding
0.0654533	noncoding
0.99725	coding
0.240511	noncoding
1	coding
1	coding
1	coding
1	coding
0.563425	coding
0.414079	noncoding

0.420535	noncoding
0.778724	coding
0.744699	coding
0.999997	coding
0.999993	coding
0.885321	coding
0.451455	noncoding
0.0726658	noncoding
0.993616	coding
1	coding
1	coding
0.999977	coding
0.999702	coding
0.722554	coding
1	coding
0.999969	coding
0.669101	coding
1	coding
1	coding
0.223732	noncoding
1	coding
0.017572	noncoding
1	coding
0.0446245	noncoding
0.999561	coding
0.496337	noncoding
0.163813	noncoding
0.0261313	noncoding
1	coding
0.0950353	noncoding
1	coding
1	coding
1	coding
1	coding
1	coding
1	coding
1	coding
1	coding
1	coding
0.977484	coding
0.262018	noncoding
0.173343	noncoding
0.999941	coding
0.996995	coding
0.999999	coding
0.0238726	noncoding
0.985835	coding
0.517514	coding
0.0282137	noncoding
0.0328057	noncoding
0.889732	coding
0.2462	noncoding
0.275752	noncoding
0.141861	noncoding

1	coding
0.999804	coding
0.0336728	noncoding
0.996045	coding
0.16664	noncoding
0.206104	noncoding
0.179818	noncoding
0.206264	noncoding
0.142803	noncoding
0.187387	noncoding
0.193346	noncoding
0.16664	noncoding
0.166644	noncoding
0.171027	noncoding
0.990722	coding
0.0704495	noncoding
0.0428399	noncoding
0.166147	noncoding
0.999604	coding
0.0256495	noncoding
0.282677	noncoding
0.414492	noncoding
1	coding
0.997929	coding
0.162694	noncoding
0.166334	noncoding
0.250445	noncoding
1	coding
0.0644158	noncoding
0.102826	noncoding
0.102826	noncoding
0.622139	coding
0.945886	coding
0.999972	coding
0.920828	coding
0.565196	coding
0.976474	coding
0.971514	coding
0.209882	noncoding
1	coding
1	coding
0.999978	coding
1	coding
0.0175564	noncoding
0.160904	noncoding
0.15448	noncoding
0.998991	coding
0.366844	noncoding
0.138498	noncoding
0.0736702	noncoding
1	coding
1	coding
0.999999	coding
1	coding

0.368625	noncoding
1	coding
0.999999	coding
1	coding
0.437746	noncoding
0.103699	noncoding
0.176651	noncoding
1	coding
1	coding
1	coding
1	coding
1	coding
1	coding
1	coding
1	coding
1	coding
1	coding
1	coding
1	coding
0.0538166	noncoding
0.0353535	noncoding
1	coding
1	coding
1	coding
0.0316364	noncoding
0.99878	coding
0.0351808	noncoding
0.107141	noncoding
0.999904	coding
0.999991	coding
0.100237	noncoding
0.945985	coding
0.416915	noncoding
0.456403	noncoding
0.713793	coding
0.15027	noncoding
0.122206	noncoding
0.0310416	noncoding
1	coding
0.903226	coding
1	coding
0.998728	coding
0.990674	coding
0.9975	coding
1	coding
0.999982	coding
0.999998	coding
1	coding
1	coding
1	coding
1	coding
1	coding
1	coding
1	coding

1	coding
0.087382	noncoding
0.553728	coding
1	coding
1	coding
1	coding
1	coding
1	coding
0.999882	coding
0.9759	coding
0.127185	noncoding
0.490208	noncoding
0.879642	coding
0.739568	coding
0.00361842	noncoding
0.78607	coding
0.0685298	noncoding
0.0898727	noncoding
0.0117251	noncoding
1	coding
0.0284936	noncoding
0.995944	coding
0.995838	coding
0.99965	coding
0.255201	noncoding
0.705264	coding
0.122513	noncoding
0.0281366	noncoding
0.297622	noncoding
0.0351326	noncoding
0.447818	noncoding
0.0771331	noncoding
0.994778	coding
0.985379	coding
0.657754	coding
0.266005	noncoding
0.112901	noncoding
0.99856	coding
0.999949	coding
0.999999	coding
0.999999	coding
1	coding
0.999976	coding
0.999866	coding
0.388949	noncoding
0.252216	noncoding
0.0207358	noncoding
0.0497868	noncoding
0.832674	coding
0.0769398	noncoding
1	coding
0.016994	noncoding
0.0428645	noncoding
0.128917	noncoding

0.153283	noncoding
0.394081	noncoding
0.434628	noncoding
1	coding
0.10074	noncoding
1	coding
1	coding
0.147405	noncoding
0.999818	coding
0.0171137	noncoding
0.386823	noncoding
0.0510344	noncoding
0.132773	noncoding
1	coding
1	coding
1	coding
1	coding
1	coding
1	coding
1	coding
1	coding
1	coding
1	coding
1	coding
1	coding
1	coding
1	coding
0.0325257	noncoding
0.153516	noncoding
0.153516	noncoding
0.262059	noncoding
0.160036	noncoding
0.870788	coding
0.495189	noncoding
0.257853	noncoding
0.257853	noncoding
0.257853	noncoding
0.337256	noncoding
0.0777194	noncoding
0.0395955	noncoding
0.692346	coding
0.703204	coding
0.141433	noncoding
0.999922	coding
0.999934	coding
1	coding
1	coding
1	coding
1	coding
0.00520767	noncoding
0.134069	noncoding
0.247146	noncoding
0.758247	coding
0.999322	coding

0.996723	coding
0.999847	coding
0.999988	coding
0.999967	coding
1	coding
0.999998	coding
0.0435138	noncoding
0.0190587	noncoding
1	coding
0.0998757	noncoding
0.661269	coding
0.0379516	noncoding
0.661269	coding
1	coding
1	coding
0.00762953	noncoding
0.0299247	noncoding
0.138235	noncoding
0.999993	coding
1	coding
1	coding
1	coding
1	coding
0.999604	coding
1	coding
1	coding
0.0365233	noncoding
0.999872	coding
0.790539	coding
0.99744	coding
0.776941	coding
0.71629	coding
0.712445	coding
1	coding
1	coding
0.0277413	noncoding
0.124392	noncoding
0.58153	coding
0.64734	coding
0.0512492	noncoding
1	coding
0.0396567	noncoding
0.0114857	noncoding
0.0199353	noncoding
0.0290777	noncoding
0.0976885	noncoding
1	coding
0.0479165	noncoding
0.94634	coding
0.011821	noncoding
0.238996	noncoding
0.857082	coding
0.620081	coding
0.99933	coding

0.996134	coding
0.69265	coding
0.715042	coding
0.0737691	noncoding
0.0449584	noncoding
0.797269	coding
0.99917	coding
0.822149	coding
0.999899	coding
0.999999	coding
0.0708742	noncoding
0.99989	coding
0.976241	coding
0.0299697	noncoding
0.306996	noncoding
1	coding
1	coding
0.0696164	noncoding
0.635246	coding
0.945385	coding
0.132998	noncoding
1	coding
1	coding
1	coding
1	coding
0.627362	coding
0.128047	noncoding
0.0252904	noncoding
0.132905	noncoding
0.421653	noncoding
1	coding
0.10573	noncoding
0.145757	noncoding
0.151883	noncoding
0.432772	noncoding
0.71728	coding
0.0449551	noncoding
0.0481057	noncoding
0.0116242	noncoding
0.526517	coding
0.0358981	noncoding
0.366136	noncoding
0.225347	noncoding
0.0221462	noncoding
0.836227	coding
0.832794	coding
0.123827	noncoding
0.203623	noncoding
0.568588	coding
0.0566739	noncoding
0.0392206	noncoding
0.225614	noncoding
0.00373961	noncoding
1	coding

0.179538	noncoding
0.0312585	noncoding
0.483804	noncoding
0.110139	noncoding
0.103388	noncoding
0.996685	coding
0.721267	coding
0.916502	coding
0.873077	coding
0.0280517	noncoding
0.999217	coding
1	coding
0.0843872	noncoding
0.0479733	noncoding
0.171181	noncoding
1	coding
1	coding
1	coding
1	coding
1	coding
1	coding
1	coding
1	coding
1	coding
0.0416806	noncoding
0.770026	coding
0.056642	noncoding
0.175863	noncoding
0.149371	noncoding
0.999634	coding
0.97677	coding
0.99862	coding
0.00741114	noncoding
0.0282087	noncoding
0.012187	noncoding
1	coding
0.0452795	noncoding
0.0425846	noncoding
0.194955	noncoding
0.0241929	noncoding
0.999999	coding
1	coding
1	coding
0.999999	coding
1	coding
1	coding
1	coding
1	coding
1	coding
1	coding
1	coding
1	coding
1	coding
0.0298142	noncoding

1	coding
1	coding
0.161323	noncoding
1	coding
0.0244774	noncoding
0.0830042	noncoding
0.0123387	noncoding
0.0851642	noncoding
0.030519	noncoding
0.0253239	noncoding
1	coding
0.182037	noncoding
0.999885	coding
1	coding
0.0918715	noncoding
0.438567	noncoding
0.262144	noncoding
1	coding
1	coding
1	coding
1	coding
0.950311	coding
0.99987	coding
0.45116	noncoding
0.29117	noncoding
0.054208	noncoding
0.495128	noncoding
1	coding
0.969503	coding
0.0527953	noncoding
0.172227	noncoding
1	coding
1	coding
1	coding
1	coding
1	coding
1	coding
1	coding
1	coding
1	coding
0.663633	coding
0.308508	noncoding
0.469791	noncoding
1	coding
1	coding
1	coding
0.0546473	noncoding
0.0202543	noncoding
0.0450876	noncoding
0.936579	coding
0.999863	coding
1	coding
0.0444663	noncoding
1	coding

1	coding
1	coding
0.0427371	noncoding
0.0406825	noncoding
0.00847724	noncoding
0.0500781	noncoding
0.0226365	noncoding
0.0141292	noncoding
0.113014	noncoding
0.0471202	noncoding
0.988036	coding
0.250854	noncoding
0.995454	coding
1	coding
0.037219	noncoding
1	coding
1	coding
0.99998	coding
0.0643259	noncoding
0.733696	coding
0.00934464	noncoding
0.998505	coding
0.999503	coding
0.983425	coding
0.867438	coding
0.999685	coding
0.992084	coding
0.995886	coding
0.101689	noncoding
1	coding
0.257621	noncoding
0.137552	noncoding
0.0843382	noncoding
0.161951	noncoding
1	coding
1	coding
1	coding
1	coding
0.140011	noncoding
0.0149734	noncoding
0.960814	coding
0.0538031	noncoding
0.0258891	noncoding
1	coding
1	coding
1	coding
0.343001	noncoding
0.998965	coding
1	coding
1	coding
1	coding
1	coding
1	coding
0.0320151	noncoding

1	coding
1	coding
1	coding
1	coding
0.99974	coding
0.99999	coding
1	coding
1	coding
0.999999	coding
1	coding
0.0392836	noncoding
1	coding
1	coding
1	coding
0.986803	coding
1	coding
1	coding
1	coding
1	coding
0.998628	coding
1	coding
1	coding
1	coding
1	coding
0.999994	coding
1	coding
0.072896	noncoding
0.0863952	noncoding
0.00725052	noncoding
0.152863	noncoding
0.154332	noncoding
0.134588	noncoding
1	coding
1	coding
1	coding
0.0107825	noncoding
1	coding
1	coding
1	coding
0.0846346	noncoding
0.0414442	noncoding
0.0110929	noncoding
0.239531	noncoding
0.098525	noncoding
1	coding
0.962502	coding
1	coding
1	coding
1	coding
1	coding
1	coding
1	coding
0.0363638	noncoding
0.125175	noncoding

0.096816	noncoding
0.124766	noncoding
0.0400777	noncoding
1	coding
1	coding
0.503613	coding
0.922383	coding
0.894898	coding
0.999978	coding
0.416437	noncoding
1	coding
1	coding
1	coding
1	coding
1	coding
1	coding
1	coding
0.168077	noncoding
0.176155	noncoding
0.99971	coding
0.911354	coding
1	coding
1	coding
1	coding
1	coding
0.149178	noncoding
0.0643903	noncoding
0.0739116	noncoding
0.0815443	noncoding
1	coding
0.996921	coding
1	coding
1	coding
0.0948602	noncoding
0.999602	coding
1	coding
0.999999	coding
1	coding
1	coding
0.999966	coding
0.999962	coding
0.999991	coding
0.403502	noncoding
0.0482362	noncoding
0.828726	coding
0.981391	coding
0.999937	coding
0.99999	coding
1	coding
1	coding
1	coding
1	coding
0.00666215	noncoding
0.999888	coding

0.999645	coding
0.0174994	noncoding
0.0361671	noncoding
0.0155027	noncoding
0.0127165	noncoding
0.0391342	noncoding
0.999883	coding
0.122308	noncoding
0.924793	coding
0.83973	coding
0.192574	noncoding
0.995774	coding
0.347677	noncoding
0.0351859	noncoding
0.751912	coding
1	coding
1	coding
1	coding
0.999849	coding
0.291148	noncoding
1	coding
0.41736	noncoding
0.15657	noncoding
0.174788	noncoding
1	coding
1	coding
0.926367	coding
0.0201837	noncoding
1	coding
1	coding
1	coding
1	coding
1	coding
1	coding
1	coding
1	coding
0.0317165	noncoding
0.02793	noncoding
1	coding
1	coding
0.991277	coding
1	coding
1	coding
1	coding
1	coding
1	coding
0.984029	coding
1	coding
1	coding
1	coding
1	coding
1	coding
1	coding
1	coding

0.945154	coding
0.999398	coding
0.970233	coding
0.0452755	noncoding
1	coding
0.212879	noncoding
0.228162	noncoding
0.036735	noncoding
1	coding
0.0260521	noncoding
0.106548	noncoding
0.00393247	noncoding
0.076388	noncoding
0.19285	noncoding
0.0537519	noncoding
0.844047	coding
0.0814075	noncoding
1	coding
1	coding
1	coding
0.0299021	noncoding
0.068183	noncoding
1	coding
0.999887	coding
1	coding
1	coding
0.265425	noncoding
0.0573645	noncoding
0.0171939	noncoding
0.178901	noncoding
0.022358	noncoding
1	coding
0.0145401	noncoding
1	coding
1	coding
0.862602	coding
1	coding
1	coding
1	coding
1	coding
1	coding
1	coding
1	coding
0.987308	coding
0.0957172	noncoding
0.0747358	noncoding
0.0754482	noncoding
0.14234	noncoding
0.0671138	noncoding
0.131271	noncoding
0.981056	coding
0.820415	coding
0.0602246	noncoding
0.216594	noncoding

1	coding
0.250674	noncoding
0.999997	coding
0.997471	coding
0.960581	coding
0.998428	coding
0.0693734	noncoding
1	coding
1	coding
1	coding
1	coding
0.0683875	noncoding
0.10667	noncoding
0.0270398	noncoding
0.0998949	noncoding
0.999919	coding
1	coding
0.0674913	noncoding
0.113035	noncoding
0.109806	noncoding
0.0786768	noncoding
1	coding
0.0642253	noncoding
0.0817499	noncoding
1	coding
1	coding
1	coding
1	coding
1	coding
0.999092	coding
0.232415	noncoding
0.999864	coding
1	coding
0.999995	coding
1	coding
0.640908	coding
1	coding
1	coding
1	coding
0.0537589	noncoding
1	coding
1	coding
0.601733	coding
0.0629179	noncoding
1	coding
1	coding
1	coding
1	coding
1	coding
1	coding
1	coding
1	coding
0.99992	coding
0.018428	noncoding

0.998306	coding
0.867802	coding
0.404808	noncoding
0.463619	noncoding
0.914071	coding
0.914071	coding
0.914071	coding
0.875639	coding
0.887347	coding
0.875639	coding
0.914071	coding
0.903391	coding
0.05207	noncoding
0.0896583	noncoding
0.100541	noncoding
0.422484	noncoding
0.403188	noncoding
1	coding
1	coding
0.999991	coding
1	coding
1	coding
0.716361	coding
0.851835	coding
1	coding
0.999987	coding
0.787831	coding
0.0160885	noncoding
1	coding
1	coding
0.999999	coding
1	coding
1	coding
1	coding
1	coding
0.976233	coding
0.0313427	noncoding
1	coding
1	coding
0.10083	noncoding
0.0522837	noncoding
0.994365	coding
0.323269	noncoding
0.0703317	noncoding
1	coding
0.999981	coding
0.998914	coding
1	coding
0.999624	coding
0.999865	coding
1	coding
0.995312	coding
0.997628	coding
0.894774	coding

0.833237	coding
0.88322	coding
0.181748	noncoding
0.242991	noncoding
0.942587	coding
0.874094	coding
0.0567368	noncoding
1	coding
1	coding
0.262674	noncoding
0.1071	noncoding
0.97422	coding
0.303876	noncoding
0.00358074	noncoding
1	coding
1	coding
1	coding
1	coding
1	coding
0.176128	noncoding
0.999985	coding
0.991967	coding
0.999357	coding
0.995988	coding
0.999999	coding
0.983556	coding
1	coding
0.0197236	noncoding
0.998957	coding
0.998904	coding
0.999971	coding
1	coding
0.999941	coding
0.999972	coding
0.999946	coding
1	coding
0.266991	noncoding
0.0184995	noncoding
0.791219	coding
0.78062	coding
0.902014	coding
0.329196	noncoding
0.114895	noncoding
0.929976	coding
1	coding
0.0871116	noncoding
1	coding
0.865859	coding
0.181321	noncoding
0.852989	coding
0.98857	coding
0.443377	noncoding
0.828198	coding
0.901414	coding

0.0251204	noncoding
1	coding
1	coding
0.999984	coding
0.999984	coding
0.998762	coding
0.998362	coding
1	coding
1	coding
1	coding
0.30908	noncoding
1	coding
1	coding
1	coding
1	coding
1	coding
1	coding
1	coding
1	coding
0.999993	coding
1	coding
0.0105524	noncoding
0.0141207	noncoding
0.999998	coding
0.280136	noncoding
0.774047	coding
0.978366	coding
0.979657	coding
0.166526	noncoding
1	coding
1	coding
0.880937	coding
0.043182	noncoding
1	coding
1	coding
1	coding
1	coding
1	coding
1	coding
1	coding
1	coding
1	coding
1	coding
1	coding
1	coding
0.999995	coding
1	coding
0.699232	coding
0.711323	coding
1	coding
1	coding
1	coding
1	coding
1	coding

0.989659	coding
0.111827	noncoding
0.998746	coding
0.0177111	noncoding
0.991999	coding
0.104345	noncoding
1	coding
1	coding
0.992478	coding
1	coding
1	coding
1	coding
0.349299	noncoding
0.244728	noncoding
1	coding
0.388189	noncoding
0.0243426	noncoding
1	coding
0.0337973	noncoding
0.0105633	noncoding
0.999992	coding
0.992991	coding
1	coding
1	coding
1	coding
1	coding
1	coding
1	coding
1	coding
1	coding
0.999984	coding
0.848059	coding
0.144423	noncoding
1	coding
0.981769	coding
0.00951016	noncoding
0.981769	coding
0.211766	noncoding
0.211766	noncoding
0.0616227	noncoding
0.101088	noncoding
1	coding
0.815055	coding
0.120529	noncoding
1	coding
0.446383	noncoding
1	coding
1	coding
1	coding
1	coding
1	coding
1	coding
1	coding
1	coding

1	coding
0.0401596	noncoding
0.668166	coding
0.999999	coding
1	coding
1	coding
1	coding
0.999582	coding
1	coding
1	coding
0.281572	noncoding
0.472984	noncoding
0.0508799	noncoding
0.992628	coding
0.0897751	noncoding
0.999982	coding
0.987314	coding
0.993348	coding
0.994136	coding
0.999395	coding
1	coding
1	coding
1	coding
0.999919	coding
1	coding
1	coding
1	coding
1	coding
1	coding
1	coding
0.99997	coding
0.999637	coding
0.802622	coding
0.999591	coding
0.437874	noncoding
1	coding
0.0257349	noncoding
0.545303	coding
1	coding
1	coding
1	coding
1	coding
1	coding
1	coding
1	coding
0.999998	coding
1	coding
0.999993	coding
1	coding
0.57708	coding
0.923981	coding
0.999999	coding
1	coding
0.999979	coding

1	coding
1	coding
1	coding
0.999979	coding
0.999977	coding
0.861681	coding
0.167374	noncoding
0.0177238	noncoding
0.99983	coding
1	coding
1	coding
1	coding
1	coding
1	coding
1	coding
1	coding
1	coding
1	coding
1	coding
1	coding
1	coding
1	coding
1	coding
1	coding
1	coding
1	coding
0.999999	coding
0.999998	coding
0.12923	noncoding
0.0207731	noncoding
0.00524921	noncoding
0.0771156	noncoding
0.0178163	noncoding
1	coding
0.856076	coding
0.449473	noncoding
1	coding
0.00795724	noncoding
0.863764	coding
0.470353	noncoding
0.0605831	noncoding
0.0514942	noncoding
0.04931	noncoding
0.0447111	noncoding
0.0556004	noncoding
1	coding
1	coding
1	coding
1	coding
1	coding
0.0236792	noncoding
0.129211	noncoding
0.169506	noncoding
0.202995	noncoding

0.254026	noncoding
1	coding
1	coding
1	coding
1	coding
1	coding
1	coding
1	coding
1	coding
1	coding
1	coding
0.999993	coding
0.9997	coding
1	coding
0.292205	noncoding
1	coding
1	coding
1	coding
1	coding
1	coding
1	coding
1	coding
1	coding
0.219847	noncoding
0.999615	coding
1	coding
0.036339	noncoding
0.999998	coding
1	coding
1	coding
1	coding
1	coding
1	coding
0.969294	coding
0.999946	coding
0.999779	coding
0.605472	coding
0.997196	coding
0.999652	coding
0.999902	coding
0.999955	coding
1	coding
1	coding
1	coding
1	coding
1	coding
0.997087	coding
1	coding
1	coding
1	coding
1	coding
0.0665834	noncoding
0.0605648	noncoding
1	coding

0.885827	coding
0.0267299	noncoding
1	coding
1	coding
1	coding
1	coding
0.11481	noncoding
1	coding
0.0466317	noncoding
1	coding
0.113968	noncoding
0.0645958	noncoding
0.852551	coding
1	coding
0.0788726	noncoding
0.011327	noncoding
0.0898002	noncoding
1	coding
0.49455	noncoding
0.0205985	noncoding
0.0452776	noncoding
0.154272	noncoding
0.0410714	noncoding
1	coding
1	coding
1	coding
0.951256	coding
1	coding
0.999726	coding
0.999842	coding
1	coding
1	coding
1	coding
1	coding
1	coding
1	coding
1	coding
1	coding
0.0653156	noncoding
0.555725	coding
0.0677036	noncoding
0.925169	coding
0.132737	noncoding
1	coding
1	coding
0.0851575	noncoding
0.999997	coding
1	coding
0.999546	coding
1	coding
0.671326	coding
1	coding
1	coding
1	coding

1	coding
0.490005	noncoding
0.136919	noncoding
0.222701	noncoding
0.0942679	noncoding
0.479174	noncoding
0.0896701	noncoding
0.462967	noncoding
0.202094	noncoding
0.434311	noncoding
0.0550053	noncoding
0.999924	coding
1	coding
1	coding
1	coding
1	coding
0.333641	noncoding
1	coding
0.999996	coding
0.987639	coding
0.999998	coding
1	coding
1	coding
1	coding
1	coding
1	coding
1	coding
1	coding
0.0378602	noncoding
0.084917	noncoding
0.99435	coding
0.0167024	noncoding
0.0326352	noncoding
0.921186	coding
0.953081	coding
0.110409	noncoding
0.0474199	noncoding
0.0853727	noncoding
1	coding
1	coding
0.971078	coding
0.999949	coding
0.999997	coding
0.317927	noncoding
1	coding
1	coding
1	coding
0.160217	noncoding
0.209368	noncoding
0.712154	coding
0.712154	coding
1	coding
1	coding
1	coding

1	coding
0.996717	coding
0.0290396	noncoding
0.999998	coding
1	coding
1	coding
0.257553	noncoding
0.999997	coding
0.0092236	noncoding
1	coding
0.999999	coding
0.999999	coding
0.0458598	noncoding
0.0441345	noncoding
0.0441345	noncoding
0.0580481	noncoding
1	coding
1	coding
0.847189	coding
0.261257	noncoding
0.0891987	noncoding
0.972945	coding
0.150857	noncoding
1	coding
0.989775	coding
0.022129	noncoding
0.988223	coding
0.989913	coding
1	coding
1	coding
0.999997	coding
0.0750484	noncoding
0.998858	coding
1	coding
1	coding
0.0612602	noncoding
0.999123	coding
0.220453	noncoding
0.0285236	noncoding
1	coding
1	coding
1	coding
1	coding
1	coding
1	coding
1	coding
0.999999	coding
0.980215	coding
1	coding
0.928729	coding
0.999989	coding
1	coding
1	coding
1	coding

0.0553841	noncoding
0.0151077	noncoding
0.599567	coding
0.00953705	noncoding
0.800513	coding
1	coding
0.0235096	noncoding
0.327876	noncoding
0.136039	noncoding
0.999999	coding
0.999999	coding
0.183761	noncoding
1	coding
1	coding
1	coding
1	coding
0.238927	noncoding
0.0912876	noncoding
0.0162255	noncoding
0.0211142	noncoding
0.422418	noncoding
0.0858385	noncoding
0.703696	coding
0.740419	coding
0.0176998	noncoding
0.0609978	noncoding
0.136918	noncoding
0.0167602	noncoding
0.11078	noncoding
0.112488	noncoding
0.0733426	noncoding
0.30057	noncoding
0.0186682	noncoding
0.0437508	noncoding
1	coding
1	coding
1	coding
0.51407	coding
0.999994	coding
1	coding
0.670175	coding
0.998402	coding
0.998384	coding
0.0307686	noncoding
0.0792815	noncoding
0.0275767	noncoding
0.087168	noncoding
0.057302	noncoding
0.0891892	noncoding
0.999996	coding
1	coding
0.999999	coding
1	coding
1	coding

0.999999	coding
1	coding
1	coding
1	coding
1	coding
1	coding
0.0203387	noncoding
1	coding
1	coding
1	coding
0.0531513	noncoding
0.0679544	noncoding
1	coding
1	coding
0.0984078	noncoding
1	coding
1	coding
1	coding
0.974728	coding
0.253594	noncoding
0.489757	noncoding
1	coding
1	coding
0.993869	coding
1	coding
0.107884	noncoding
0.999997	coding
0.998544	coding
0.921838	coding
1	coding
1	coding
1	coding
0.999978	coding
1	coding
1	coding
0.999949	coding
0.998743	coding
0.999999	coding
0.186976	noncoding
0.0198451	noncoding
1	coding
1	coding
0.999767	coding
0.02234	noncoding
1	coding
1	coding
0.0770288	noncoding
0.658393	coding
0.928818	coding
1	coding
1	coding
0.99994	coding
0.828037	coding
0.792609	coding

1	coding
1	coding
0.00558444	noncoding
0.992682	coding
0.988311	coding
0.999914	coding
0.999929	coding
1	coding
0.999949	coding
1	coding
0.999994	coding
1	coding
1	coding
0.998798	coding
0.996437	coding
0.997731	coding
1	coding
1	coding
0.0737721	noncoding
1	coding
1	coding
0.999999	coding
0.075596	noncoding
0.63021	coding
0.463227	noncoding
0.373524	noncoding
1	coding
1	coding
1	coding
1	coding
1	coding
0.966954	coding
0.860933	coding
0.947916	coding
0.0536126	noncoding
0.0510462	noncoding
1	coding
1	coding
1	coding
0.0578554	noncoding
1	coding
0.988021	coding
0.999495	coding
0.138488	noncoding
0.999641	coding
0.541916	coding
0.0794491	noncoding
0.999625	coding
0.063644	noncoding
1	coding
0.996643	coding
0.0454223	noncoding
0.420556	noncoding
1	coding

1	coding
1	coding
1	coding
0.869748	coding
0.905793	coding
0.914552	coding
0.170848	noncoding
0.999999	coding
1	coding
1	coding
0.999999	coding
0.264074	noncoding
0.999999	coding
1	coding
1	coding
0.999999	coding
1	coding
1	coding
0.270719	noncoding
0.136267	noncoding
0.0341628	noncoding
0.024076	noncoding
0.534195	coding
0.0177301	noncoding
1	coding
0.999995	coding
0.450836	noncoding
1	coding
1	coding
0.176475	noncoding
1	coding
1	coding
1	coding
1	coding
0.718943	coding
1	coding
0.999995	coding
1	coding
1	coding
0.0238563	noncoding
1	coding
1	coding
1	coding
1	coding
1	coding
0.0445071	noncoding
1	coding
1	coding
1	coding
1	coding
1	coding
1	coding
1	coding
1	coding

0.914251	coding
0.999712	coding
0.414594	noncoding
0.00279871	noncoding
1	coding
0.310669	noncoding
1	coding
0.0200427	noncoding
1	coding
1	coding
0.382223	noncoding
1	coding
1	coding
1	coding
1	coding
1	coding
1	coding
1	coding
1	coding
0.998309	coding
0.998746	coding
0.0922836	noncoding
1	coding
1	coding
0.0261891	noncoding
0.844506	coding
0.999996	coding
0.00517161	noncoding
0.0499935	noncoding
0.0512817	noncoding
0.589486	coding
0.508425	coding
0.589138	coding
0.541451	coding
0.399044	noncoding
0.748775	coding
0.94706	coding
0.836315	coding
0.824745	coding
1	coding
1	coding
1	coding
1	coding
1	coding
1	coding
1	coding
1	coding
1	coding
1	coding
1	coding
1	coding
1	coding
1	coding
1	coding

1	coding
1	coding
1	coding
1	coding
1	coding
1	coding
1	coding
0.999543	coding
0.9929	coding
1	coding
0.992921	coding
0.35526	noncoding
0.573879	coding
0.0379179	noncoding
0.449246	noncoding
1	coding
0.119823	noncoding
0.812909	coding
0.161901	noncoding
0.925489	coding
0.228607	noncoding
0.0307335	noncoding
0.947022	coding
0.999985	coding
0.0620097	noncoding
0.0284871	noncoding
0.0844619	noncoding
1	coding
1	coding
0.00183943	noncoding
0.116162	noncoding
0.0319865	noncoding
1	coding
0.00885101	noncoding
0.999038	coding
1	coding
0.267068	noncoding
1	coding
1	coding
1	coding
1	coding
0.365432	noncoding
0.0419069	noncoding
0.444815	noncoding
1	coding
1	coding
0.605128	coding
0.0174615	noncoding
0.0124371	noncoding
0.0233431	noncoding
1	coding
1	coding
0.0281074	noncoding
1	coding

0.0596309	noncoding
0.597651	coding
0.0658156	noncoding
1	coding
0.0338981	noncoding
0.0244031	noncoding
0.999997	coding
0.997197	coding
0.995656	coding
0.99512	coding
0.0230158	noncoding
1	coding
1	coding
1	coding
1	coding
1	coding
1	coding
1	coding
0.999992	coding
1	coding
1	coding
1	coding
1	coding
0.0429307	noncoding
0.108618	noncoding
1	coding
1	coding
0.0456064	noncoding
0.0219607	noncoding
1	coding
0.20029	noncoding
0.139839	noncoding
0.0496535	noncoding
0.997093	coding
0.942211	coding
1	coding
1	coding
1	coding
1	coding
0.141117	noncoding
1	coding
1	coding
1	coding
1	coding
1	coding
0.0705998	noncoding
1	coding
0.999873	coding
0.096055	noncoding
1	coding
1	coding
1	coding
1	coding
1	coding

1	coding
0.80772	coding
1	coding
1	coding
0.886544	coding
1	coding
1	coding
0.133372	noncoding
0.145526	noncoding
0.835204	coding
0.00900152	noncoding
0.014816	noncoding
0.236059	noncoding
0.163124	noncoding
0.977873	coding
0.973682	coding
0.0814871	noncoding
0.698817	coding
0.147214	noncoding
0.4651	noncoding
0.978431	coding
0.977873	coding
0.446661	noncoding
0.878795	coding
0.895878	coding
0.619669	coding
0.978431	coding
0.625889	coding
0.814563	coding
0.16341	noncoding
0.120938	noncoding
0.988266	coding
0.351209	noncoding
0.998744	coding
0.0495871	noncoding
0.99975	coding
0.958694	coding
0.0573658	noncoding
0.0349432	noncoding
0.0667681	noncoding
0.233661	noncoding
0.352355	noncoding
0.0819884	noncoding
0.158066	noncoding
0.464209	noncoding
0.431995	noncoding
0.254311	noncoding
0.0861151	noncoding
0.0242836	noncoding
0.758075	coding
0.957821	coding
1	coding
0.999966	coding
0.18831	noncoding

0.982421	coding
0.0392828	noncoding
0.998248	coding
0.992896	coding
1	coding
0.667185	coding
0.5185	coding
0.999997	coding
0.99997	coding
0.0493506	noncoding
0.999999	coding
0.987647	coding
0.234164	noncoding
0.363757	noncoding
0.0693349	noncoding
0.0821841	noncoding
0.999375	coding
0.999503	coding
0.0779329	noncoding
0.668479	coding
0.0273417	noncoding
0.999662	coding
0.0898776	noncoding
0.444647	noncoding
0.999991	coding
1	coding
1	coding
1	coding
0.999127	coding
0.999276	coding
0.999276	coding
0.987372	coding
0.999717	coding
0.970325	coding
1	coding
0.0768475	noncoding
0.929712	coding
1	coding
0.999999	coding
1	coding
1	coding
0.396549	noncoding
0.117197	noncoding
0.0450938	noncoding
1	coding
0.0237713	noncoding
1	coding
0.0150839	noncoding
0.792057	coding
0.986877	coding
1	coding
0.0747834	noncoding
0.0525468	noncoding
0.203665	noncoding

0.999913	coding
0.999536	coding
0.0164801	noncoding
0.0896571	noncoding
0.0284372	noncoding
0.40139	noncoding
0.0136031	noncoding
0.0509279	noncoding
0.0632831	noncoding
0.0190984	noncoding
0.170042	noncoding
0.0170692	noncoding
0.936829	coding
0.311174	noncoding
0.999999	coding
0.999997	coding
0.999995	coding
0.999989	coding
1	coding
0.0266345	noncoding
1	coding
0.0131754	noncoding
0.044273	noncoding
0.273093	noncoding
0.999986	coding
0.675647	coding
0.0264644	noncoding
0.185592	noncoding
0.228632	noncoding
1	coding
1	coding
0.389482	noncoding
0.999505	coding
0.999987	coding
0.999991	coding
1	coding
0.997296	coding
0.999052	coding
0.167111	noncoding
1	coding
1	coding
0.523451	coding
1	coding
1	coding
0.153843	noncoding
0.350855	noncoding
1	coding
1	coding
1	coding
0.117217	noncoding
0.120429	noncoding
0.0491497	noncoding
0.0217848	noncoding
0.0848205	noncoding

0.383918	noncoding
0.0128036	noncoding
0.99812	coding
0.22266	noncoding
1	coding
1	coding
0.85458	coding
0.993898	coding
1	coding
1	coding
1	coding
1	coding
1	coding
1	coding
0.088635	noncoding
0.0337073	noncoding
0.0416632	noncoding
0.999423	coding
0.0538331	noncoding
0.0523947	noncoding
0.0117832	noncoding
0.00210648	noncoding
0.685118	coding
0.685118	coding
0.115669	noncoding
0.0167644	noncoding
0.101056	noncoding
0.793047	coding
0.064869	noncoding
0.0168395	noncoding
0.00980375	noncoding
0.0995377	noncoding
0.0740008	noncoding
0.0339555	noncoding
0.0225629	noncoding
0.364196	noncoding
0.951505	coding
0.999918	coding
0.999997	coding
0.2329	noncoding
0.123058	noncoding
0.161085	noncoding
0.0182651	noncoding
1	coding
0.999999	coding
0.959799	coding
0.501646	coding
0.0492253	noncoding
0.141542	noncoding
0.0498239	noncoding
1	coding
1	coding
0.183961	noncoding
0.047812	noncoding

1	coding
1	coding
0.00379008	noncoding
1	coding
0.272701	noncoding
0.999961	coding
0.443879	noncoding
0.962656	coding
0.999999	coding
0.0440195	noncoding
0.0387741	noncoding
0.168164	noncoding
0.999985	coding
0.973658	coding
0.440193	noncoding
0.0640552	noncoding
0.994862	coding
0.72262	coding
1	coding
1	coding
0.999988	coding
0.019861	noncoding
1	coding
0.17331	noncoding
0.915759	coding
0.999977	coding
0.999508	coding
0.813994	coding
0.318423	noncoding
1	coding
1	coding
1	coding
1	coding
0.0337522	noncoding
0.120138	noncoding
0.0222227	noncoding
0.999869	coding
1	coding
1	coding
1	coding
1	coding
0.378789	noncoding
1	coding
1	coding
1	coding
1	coding
1	coding
0.0192534	noncoding
0.999985	coding
1	coding
0.104746	noncoding
0.650008	coding
0.081676	noncoding
0.499516	noncoding

0.712107	coding
0.949346	coding
0.987337	coding
0.997221	coding
0.445684	noncoding
0.228286	noncoding
0.406315	noncoding
0.994441	coding
0.999413	coding
0.173567	noncoding
0.999997	coding
0.947079	coding
0.0131511	noncoding
1	coding
1	coding
0.0127564	noncoding
1	coding
0.999999	coding
0.204442	noncoding
0.108832	noncoding
1	coding
0.395864	noncoding
1	coding
1	coding
1	coding
1	coding
0.256748	noncoding
0.0190609	noncoding
1	coding
1	coding
1	coding
0.0558903	noncoding
0.040563	noncoding
0.0356119	noncoding
0.0332954	noncoding
0.040919	noncoding
0.0225865	noncoding
0.110024	noncoding
1	coding
0.180107	noncoding
0.239777	noncoding
0.198944	noncoding
0.0723444	noncoding
0.999309	coding
0.999827	coding
0.0787551	noncoding
0.158689	noncoding
0.272069	noncoding
0.829998	coding
0.417923	noncoding
0.914833	coding
0.829817	coding
0.0475895	noncoding
1	coding

1	coding
0.00996146	noncoding
0.043483	noncoding
0.0131135	noncoding
0.437855	noncoding
0.0601233	noncoding
0.715502	coding
1	coding
1	coding
1	coding
1	coding
0.0969097	noncoding
0.0552338	noncoding
1	coding
1	coding
0.999875	coding
0.191196	noncoding
0.168356	noncoding
0.0955229	noncoding
1	coding
1	coding
1	coding
1	coding
1	coding
0.996407	coding
0.148113	noncoding
0.0209126	noncoding
1	coding
0.190736	noncoding
0.0700185	noncoding
0.351615	noncoding
0.0694223	noncoding
0.881145	coding
0.0244393	noncoding
0.999993	coding
1	coding
0.0237532	noncoding
1	coding
0.0675715	noncoding
1	coding
1	coding
0.995325	coding
0.416998	noncoding
0.384108	noncoding
0.836829	coding
0.0429351	noncoding
1	coding
0.999954	coding
0.999998	coding
1	coding
0.0310439	noncoding
1	coding
0.356763	noncoding
1	coding

1	coding
0.0200288	noncoding
0.0479209	noncoding
1	coding
1	coding
1	coding
1	coding
1	coding
0.13011	noncoding
0.00808808	noncoding
0.0434202	noncoding
0.0324687	noncoding
1	coding
1	coding
0.186861	noncoding
1	coding
0.0278554	noncoding
1	coding
0.078133	noncoding
0.999871	coding
0.99998	coding
0.212653	noncoding
0.240868	noncoding
0.0276465	noncoding
0.253812	noncoding
0.0655056	noncoding
0.271538	noncoding
0.166688	noncoding
0.0085817	noncoding
0.999888	coding
0.999757	coding
1	coding
0.161336	noncoding
0.0887603	noncoding
1	coding
1	coding
0.00998937	noncoding
1	coding
1	coding
1	coding
1	coding
1	coding
0.998287	coding
0.973673	coding
0.406468	noncoding
0.0407544	noncoding
0.623223	coding
0.186261	noncoding
0.0360819	noncoding
0.997955	coding
0.959267	coding
0.979393	coding
0.998698	coding
0.00821354	noncoding

0.450604	noncoding
0.952035	coding
0.965286	coding
0.0878981	noncoding
0.396638	noncoding
0.999957	coding
0.191568	noncoding
0.193862	noncoding
0.771532	coding
0.893023	coding
0.940093	coding
0.074056	noncoding
0.045864	noncoding
1	coding
0.177761	noncoding
1	coding
0.355756	noncoding
0.998374	coding
0.0432835	noncoding
1	coding
1	coding
0.0846903	noncoding
0.134195	noncoding
1	coding
1	coding
1	coding
1	coding
1	coding
0.854407	coding
0.0693635	noncoding
1	coding
0.881001	coding
0.45353	noncoding
0.0894182	noncoding
0.120396	noncoding
0.0309495	noncoding
0.46626	noncoding
0.0697033	noncoding
0.517183	coding
0.100971	noncoding
0.15525	noncoding
0.944257	coding
0.0172818	noncoding
0.169027	noncoding
0.164624	noncoding
0.201665	noncoding
0.205593	noncoding
1	coding
1	coding
1	coding
0.225149	noncoding
0.998015	coding
1	coding
1	coding

1	coding
1	coding
0.99999	coding
0.14955	noncoding
0.108256	noncoding
0.999776	coding
0.944992	coding
0.99999	coding
0.0116085	noncoding
0.142546	noncoding
0.0684873	noncoding
0.168202	noncoding
0.105414	noncoding
1	coding
1	coding
1	coding
1	coding
1	coding
0.0709982	noncoding
1	coding
1	coding
1	coding
0.0222949	noncoding
0.477447	noncoding
0.310017	noncoding
0.999973	coding
0.999998	coding
1	coding
1	coding
1	coding
1	coding
0.0287849	noncoding
1	coding
1	coding
1	coding
1	coding
0.999999	coding
0.12465	noncoding
0.982075	coding
1	coding
0.141662	noncoding
0.997428	coding
0.998561	coding
0.995596	coding
0.171135	noncoding
1	coding
1	coding
1	coding
1	coding
1	coding
1	coding
0.0415733	noncoding
0.0269716	noncoding
0.0564182	noncoding

0.0288711	noncoding
1	coding
1	coding
1	coding
0.999979	coding
1	coding
0.999902	coding
0.22933	noncoding
0.179844	noncoding
0.568963	coding
0.191021	noncoding
0.166682	noncoding
0.170634	noncoding
0.0460472	noncoding
0.168933	noncoding
0.0702814	noncoding
0.982529	coding
0.86602	coding
0.999978	coding
1	coding
0.999999	coding
1	coding
0.999999	coding
0.999998	coding
1	coding
0.0696012	noncoding
0.702102	coding
0.0298379	noncoding
1	coding
1	coding
1	coding
0.998747	coding
0.972788	coding
1	coding
1	coding
1	coding
1	coding
1	coding
1	coding
1	coding
1	coding
1	coding
0.999994	coding
0.957924	coding
0.217223	noncoding
0.993152	coding
0.629734	coding
0.999718	coding
0.629734	coding
0.987265	coding
0.997465	coding
0.646744	coding
1	coding
0.976796	coding

1	coding
0.0997431	noncoding
1	coding
0.0168027	noncoding
0.0247619	noncoding
0.141742	noncoding
1	coding
1	coding
0.729042	coding
1	coding
0.865992	coding
0.991171	coding
0.255742	noncoding
0.341982	noncoding
0.999999	coding
0.0221939	noncoding
0.105152	noncoding
0.906463	coding
0.377559	noncoding
0.32189	noncoding
0.0699889	noncoding
0.999878	coding
0.0840277	noncoding
0.0199327	noncoding
0.111341	noncoding
0.897019	coding
1	coding
0.999998	coding
1	coding
1	coding
0.2276	noncoding
0.0368207	noncoding
1	coding
1	coding
1	coding
1	coding
0.999984	coding
0.152011	noncoding
0.224007	noncoding
1	coding
1	coding
0.0375548	noncoding
0.999992	coding
0.00632154	noncoding
0.978278	coding
0.232672	noncoding
0.103237	noncoding
0.0293819	noncoding
1	coding
0.0705945	noncoding
1	coding
0.999987	coding
0.00395	noncoding
0.99661	coding

1	coding
1	coding
0.999429	coding
0.238739	noncoding
0.747083	coding
0.568678	coding
0.0098809	noncoding
0.777257	coding
0.744718	coding
0.91515	coding
0.99994	coding
0.039713	noncoding
0.051413	noncoding
0.0295924	noncoding
0.478767	noncoding
0.0791249	noncoding
0.999888	coding
0.196325	noncoding
1	coding
0.14979	noncoding
0.860881	coding
0.855127	coding
0.198301	noncoding
1	coding
1	coding
0.999999	coding
1	coding
1	coding
1	coding
0.998912	coding
0.225106	noncoding
0.960708	coding
0.998768	coding
1	coding
1	coding
0.0182692	noncoding
0.896177	coding
0.107539	noncoding
0.0700998	noncoding
0.999999	coding
1	coding
1	coding
1	coding
0.153966	noncoding
1	coding
0.0176859	noncoding
1	coding
0.999991	coding
0.302379	noncoding
0.221543	noncoding
0.978627	coding
1	coding
1	coding
1	coding

0.160798	noncoding
0.517461	coding
1	coding
0.0463434	noncoding
0.951083	coding
0.0595354	noncoding
0.0463219	noncoding
1	coding
0.133232	noncoding
0.145395	noncoding
0.0798627	noncoding
0.0964851	noncoding
1	coding
1	coding
0.0302044	noncoding
0.490994	noncoding
0.845915	coding
0.100616	noncoding
0.106902	noncoding
0.999999	coding
1	coding
1	coding
1	coding
0.145368	noncoding
0.0181497	noncoding
0.38917	noncoding
0.12965	noncoding
0.206821	noncoding
1	coding
1	coding
1	coding
0.02263	noncoding
0.0261539	noncoding
1	coding
0.139106	noncoding
0.999987	coding
0.951044	coding
0.177953	noncoding
0.462603	noncoding
0.785527	coding
0.549556	coding
1	coding
0.0990563	noncoding
1	coding
0.999575	coding
0.78989	coding
0.0131768	noncoding
1	coding
1	coding
0.895033	coding
0.311427	noncoding
0.311427	noncoding
0.136389	noncoding
0.330541	noncoding

0.999578	coding
0.999999	coding
0.021735	noncoding
0.0236695	noncoding
0.0192773	noncoding
0.125278	noncoding
1	coding
0.998105	coding
0.992497	coding
0.999994	coding
0.280158	noncoding
0.289052	noncoding
0.0716584	noncoding
1	coding
0.352663	noncoding
0.825383	coding
0.0603153	noncoding
0.0259661	noncoding
0.999889	coding
0.996336	coding
0.981536	coding
1	coding
0.99999	coding
0.988784	coding
1	coding
0.00381501	noncoding
1	coding
1	coding
1	coding
1	coding
1	coding
1	coding
0.0146734	noncoding
0.19865	noncoding
0.0593537	noncoding
0.0139342	noncoding
0.111066	noncoding
0.0184998	noncoding
0.00576154	noncoding
0.194307	noncoding
0.199629	noncoding
0.0764523	noncoding
0.25695	noncoding
0.163096	noncoding
0.482736	noncoding
1	coding
1	coding
0.344882	noncoding
1	coding
1	coding
1	coding
1	coding
0.0233205	noncoding
0.0179298	noncoding

0.0674704	noncoding
0.0211147	noncoding
0.0256228	noncoding
0.0119842	noncoding
0.0372559	noncoding
0.0242165	noncoding
0.110312	noncoding
0.0246192	noncoding
0.115406	noncoding
0.0525795	noncoding
0.00891708	noncoding
0.0113067	noncoding
0.0104959	noncoding
0.00871837	noncoding
0.00432776	noncoding
0.0112592	noncoding
0.999999	coding
0.999999	coding
0.168502	noncoding
0.999487	coding
1	coding
0.0258121	noncoding
1	coding
1	coding
1	coding
0.999998	coding
0.0954845	noncoding
1	coding
0.12632	noncoding
1	coding
1	coding
1	coding
1	coding
1	coding
0.0834456	noncoding
0.999999	coding
0.999999	coding
1	coding
0.999998	coding
0.999992	coding
0.996917	coding
0.999156	coding
1	coding
1	coding
0.0508144	noncoding
1	coding
1	coding
0.0271309	noncoding
0.0956941	noncoding
0.0724958	noncoding
0.0982916	noncoding
0.0138487	noncoding
0.977784	coding
0.999669	coding

0.999998	coding
0.999998	coding
1	coding
1	coding
0.0328898	noncoding
0.305248	noncoding
1	coding
0.697891	coding
0.0742053	noncoding
0.117493	noncoding
0.999962	coding
1	coding
0.290786	noncoding
0.879689	coding
0.566258	coding
0.189489	noncoding
0.782743	coding
0.0823632	noncoding
0.329463	noncoding
0.0114124	noncoding
0.220021	noncoding
0.0758916	noncoding
0.0524507	noncoding
0.349835	noncoding
0.131928	noncoding
0.149936	noncoding
0.106837	noncoding
0.071795	noncoding
0.129514	noncoding
0.0412291	noncoding
0.0293363	noncoding
0.0341746	noncoding
0.999998	coding
0.983041	coding
0.257609	noncoding
0.382292	noncoding
1	coding
1	coding
1	coding
1	coding
1	coding
0.0549044	noncoding
0.124321	noncoding
1	coding
1	coding
0.698161	coding
0.992275	coding
0.0041721	noncoding
0.999812	coding
1	coding
1	coding
0.0252446	noncoding
1	coding
1	coding

1	coding
1	coding
0.142874	noncoding
1	coding
0.999814	coding
0.563756	coding
1	coding
1	coding
1	coding
1	coding
1	coding
0.00273832	noncoding
0.391184	noncoding
0.854106	coding
0.0861349	noncoding
0.69127	coding
0.999994	coding
0.0424624	noncoding
0.252152	noncoding
0.236715	noncoding
0.233444	noncoding
0.148305	noncoding
0.031019	noncoding
0.403009	noncoding
0.0503274	noncoding
0.0708453	noncoding
0.851026	coding
0.80539	coding
0.104638	noncoding
0.0604304	noncoding
0.0747774	noncoding
0.619834	coding
0.101428	noncoding
0.485265	noncoding
0.0949774	noncoding
0.913929	coding
0.880367	coding
0.135033	noncoding
0.037516	noncoding
0.0769613	noncoding
1	coding
1	coding
0.7969	coding
0.775923	coding
0.0347586	noncoding
0.0146155	noncoding
0.184716	noncoding
0.911124	coding
0.00394961	noncoding
0.244474	noncoding
0.0409357	noncoding
0.514338	coding
0.107056	noncoding
0.247561	noncoding

0.999597	coding
0.999572	coding
0.011676	noncoding
0.331146	noncoding
0.0040768	noncoding
0.289781	noncoding
0.165531	noncoding
0.999999	coding
0.998421	coding
0.341578	noncoding
0.216389	noncoding
0.994399	coding
0.136589	noncoding
1	coding
1	coding
1	coding
1	coding
0.0389849	noncoding
0.999994	coding
0.999841	coding
0.999999	coding
1	coding
1	coding
1	coding
1	coding
1	coding
0.226731	noncoding
0.567835	coding
1	coding
1	coding
1	coding
1	coding
0.0247247	noncoding
0.0421412	noncoding
1	coding
1	coding
1	coding
0.686207	coding
0.215021	noncoding
0.146222	noncoding
0.0105419	noncoding
1	coding
1	coding
0.187221	noncoding
0.136245	noncoding
0.954034	coding
0.385094	noncoding
0.109641	noncoding
1	coding
1	coding
1	coding
1	coding
1	coding
1	coding

0.188958	noncoding
0.537901	coding
0.0528313	noncoding
0.166865	noncoding
0.390897	noncoding
0.369123	noncoding
0.560288	coding
1	coding
1	coding
1	coding
0.581567	coding
1	coding
0.557827	coding
0.063572	noncoding
0.0467986	noncoding
0.0992546	noncoding
0.998431	coding
0.999945	coding
1	coding
0.325988	noncoding
0.0350148	noncoding
0.0835546	noncoding
0.716807	coding
0.717115	coding
0.0267197	noncoding
0.610106	coding
0.220815	noncoding
0.239836	noncoding
0.212015	noncoding
0.612214	coding
0.0267197	noncoding
0.600499	coding
0.26047	noncoding
1	coding
1	coding
1	coding
1	coding
1	coding
1	coding
1	coding
1	coding
1	coding
1	coding
1	coding
0.0465399	noncoding
0.0547133	noncoding
0.14866	noncoding
0.205298	noncoding
0.199603	noncoding
0.245813	noncoding
0.188724	noncoding
0.00988628	noncoding
0.221005	noncoding
1	coding

0.897041	coding
0.24332	noncoding
1	coding
1	coding
1	coding
1	coding
0.0150336	noncoding
1	coding
1	coding
1	coding
0.999912	coding
1	coding
0.170141	noncoding
0.0141236	noncoding
0.0166285	noncoding
0.0795537	noncoding
0.126173	noncoding
1	coding
1	coding
0.112821	noncoding
0.0403069	noncoding
0.140356	noncoding
0.0891038	noncoding
0.0786444	noncoding
0.995169	coding
0.999506	coding
0.996664	coding
0.999993	coding
1	coding
1	coding
1	coding
1	coding
0.0432401	noncoding
0.08241	noncoding
0.200354	noncoding
0.224865	noncoding
0.485045	noncoding
0.137964	noncoding
0.999544	coding
0.0640293	noncoding
0.0401488	noncoding
0.314117	noncoding
0.999994	coding
0.848007	coding
0.999999	coding
0.0302119	noncoding
0.999997	coding
1	coding
0.020159	noncoding
0.108001	noncoding
0.0178391	noncoding
0.0134347	noncoding
0.0143449	noncoding
0.927729	coding

0.999431	coding
0.938053	coding
0.902867	coding
1	coding
1	coding
1	coding
1	coding
1	coding
0.045327	noncoding
0.16618	noncoding
1	coding
0.987601	coding
0.0192646	noncoding
0.560548	coding
0.193516	noncoding
0.0192087	noncoding
0.0192087	noncoding
0.328947	noncoding
0.338824	noncoding
0.0476291	noncoding
0.45172	noncoding
0.122597	noncoding
0.032978	noncoding
1	coding
1	coding
0.99979	coding
0.837821	coding
0.0405014	noncoding
1	coding
1	coding
1	coding
1	coding
0.9998	coding
0.99975	coding
0.999931	coding
0.999994	coding
0.999995	coding
1	coding
0.34968	noncoding
0.62068	coding
0.999282	coding
0.948392	coding
0.987135	coding
1	coding
0.973843	coding
0.143166	noncoding
1	coding
0.999973	coding
0.947486	coding
0.951198	coding
0.015953	noncoding
1	coding
0.913124	coding
0.116121	noncoding

0.44272	noncoding
0.725071	coding
0.707435	coding
0.0271635	noncoding
0.839906	coding
0.102667	noncoding
1	coding
1	coding
1	coding
0.0430604	noncoding
0.416222	noncoding
0.0606447	noncoding
0.059316	noncoding
0.00273376	noncoding
0.127748	noncoding
1	coding
1	coding
0.0346551	noncoding
0.517678	coding
1	coding
0.408472	noncoding
0.999999	coding
0.999962	coding
1	coding
0.848621	coding
1	coding
0.0156938	noncoding
0.00494089	noncoding
0.0284203	noncoding
0.999841	coding
0.996536	coding
0.139452	noncoding
0.0222296	noncoding
0.0176255	noncoding
1	coding
1	coding
1	coding
1	coding
1	coding
0.0343505	noncoding
0.0472985	noncoding
0.112406	noncoding
0.311548	noncoding
1	coding
0.999785	coding
0.00598152	noncoding
0.28054	noncoding
0.377177	noncoding
0.438011	noncoding
1	coding
1	coding
1	coding
1	coding
0.707968	coding

0.357809	noncoding
1	coding
1	coding
1	coding
1	coding
1	coding
0.0920987	noncoding
0.744492	coding
0.0263569	noncoding
1	coding
1	coding
1	coding
1	coding
1	coding
0.48736	noncoding
0.0710508	noncoding
0.0358538	noncoding
0.0396908	noncoding
0.0159923	noncoding
0.999999	coding
1	coding
1	coding
0.0415182	noncoding
1	coding
0.999947	coding
1	coding
1	coding
1	coding
1	coding
1	coding
1	coding
1	coding
1	coding
1	coding
1	coding
0.788231	coding
0.0359519	noncoding
0.00263605	noncoding
0.113221	noncoding
0.836839	coding
0.0419277	noncoding
1	coding
1	coding
1	coding
0.103627	noncoding
0.999998	coding
0.999997	coding
0.993165	coding
0.0109094	noncoding
0.993671	coding
0.992813	coding
0.994992	coding
0.985167	coding
0.985969	coding

1	coding
0.0932741	noncoding
0.63685	coding
1	coding
1	coding
0.0154762	noncoding
0.0106713	noncoding
0.00496677	noncoding
0.0165104	noncoding
1	coding
1	coding
1	coding
0.1069	noncoding
0.350157	noncoding
0.349094	noncoding
1	coding
0.0645597	noncoding
1	coding
0.999999	coding
1	coding
0.0408487	noncoding
1	coding
0.999986	coding
0.0941837	noncoding
0.0676519	noncoding
0.0861715	noncoding
0.177757	noncoding
0.012675	noncoding
0.152908	noncoding
0.095686	noncoding
0.138852	noncoding
0.104277	noncoding
0.133169	noncoding
0.385016	noncoding
0.991027	coding
0.175699	noncoding
0.334657	noncoding
0.0706866	noncoding
0.221299	noncoding
1	coding
0.171102	noncoding
0.416432	noncoding
0.0951685	noncoding
1	coding
0.0533248	noncoding
1	coding
0.972752	coding
1	coding
1	coding
0.0177264	noncoding
0.00332013	noncoding
0.0590287	noncoding
1	coding
1	coding

0.0162096	noncoding
1	coding
0.114303	noncoding
0.0127966	noncoding
0.13988	noncoding
0.999998	coding
1	coding
1	coding
0.391468	noncoding
0.548555	coding
0.0631648	noncoding
0.0378189	noncoding
0.999701	coding
1	coding
0.00652095	noncoding
0.999701	coding
0.320057	noncoding
1	coding
0.0365365	noncoding
0.0877825	noncoding
0.880025	coding
1	coding
1	coding
1	coding
1	coding
0.271904	noncoding
0.999994	coding
1	coding
1	coding
1	coding
0.0195408	noncoding
0.992851	coding
1	coding
1	coding
1	coding
1	coding
1	coding
0.0613187	noncoding
0.0464619	noncoding
1	coding
0.0633289	noncoding
1	coding
0.91915	coding
0.16897	noncoding
0.0554221	noncoding
0.777253	coding
0.0935389	noncoding
1	coding
1	coding
1	coding
1	coding
0.0671802	noncoding
1	coding
1	coding

0.0180929	noncoding
0.531296	coding
0.0286347	noncoding
0.382372	noncoding
0.0335682	noncoding
0.0755007	noncoding
0.138191	noncoding
0.0686549	noncoding
0.0684709	noncoding
0.0839975	noncoding
1	coding
0.287675	noncoding
0.221796	noncoding
0.742675	coding
0.908499	coding
0.988922	coding
0.883027	coding
0.621667	coding
0.154825	noncoding
0.203556	noncoding
0.199139	noncoding
0.877317	coding
0.999933	coding
0.999989	coding
0.999981	coding
0.0349013	noncoding
0.0102214	noncoding
0.0338241	noncoding
0.0284919	noncoding
1	coding
0.352997	noncoding
0.534589	coding
0.436839	noncoding
1	coding
1	coding
1	coding
1	coding
0.15646	noncoding
1	coding
1	coding
1	coding
1	coding
0.857332	coding
0.892772	coding
0.454368	noncoding
0.0903435	noncoding
0.0210268	noncoding
1	coding
1	coding
1	coding
1	coding
0.171535	noncoding
0.0428523	noncoding
0.31293	noncoding

0.0587199	noncoding
1	coding
1	coding
0.999938	coding
0.999958	coding
0.0796285	noncoding
0.0848372	noncoding
0.00516634	noncoding
0.999862	coding
0.999854	coding
0.660423	coding
0.991419	coding
0.0384896	noncoding
0.0811652	noncoding
0.999998	coding
1	coding
1	coding
1	coding
0.0677617	noncoding
0.0278436	noncoding
0.37857	noncoding
0.116754	noncoding
1	coding
0.857982	coding
0.114795	noncoding
1	coding
1	coding
0.997401	coding
0.99918	coding
0.989769	coding
0.996535	coding
1	coding
1	coding
1	coding
1	coding
1	coding
1	coding
1	coding
1	coding
1	coding
1	coding
1	coding
1	coding
1	coding
0.410783	noncoding
0.117907	noncoding
0.804207	coding
0.429151	noncoding
0.115399	noncoding
0.144124	noncoding
0.0377052	noncoding
0.00298613	noncoding
0.999749	coding
0.999031	coding

1	coding
0.968722	coding
0.0584534	noncoding
1	coding
0.868002	coding
0.727323	coding
0.515583	coding
0.513001	coding
0.0526842	noncoding
0.0714057	noncoding
1	coding
0.999977	coding
0.0310584	noncoding
0.00540431	noncoding
0.954165	coding
1	coding
0.217006	noncoding
0.999995	coding
0.463794	noncoding
0.140745	noncoding
0.805698	coding
0.0348317	noncoding
0.945667	coding
0.0109165	noncoding
0.999975	coding
0.736254	coding
0.993211	coding
0.0910151	noncoding
0.0213532	noncoding
1	coding
0.596929	coding
1	coding
0.531281	coding
0.95694	coding
0.0295269	noncoding
0.729064	coding
0.0551938	noncoding
0.92115	coding
0.0282213	noncoding
0.0297846	noncoding
0.0810947	noncoding
0.0184911	noncoding
0.0686632	noncoding
0.0415618	noncoding
0.0478821	noncoding
1	coding
0.159206	noncoding
1	coding
0.469338	noncoding
1	coding
1	coding
0.885063	coding
0.909945	coding
0.0141348	noncoding

0.0704697	noncoding
0.056043	noncoding
0.0787689	noncoding
0.475387	noncoding
0.581494	coding
0.702024	coding
0.0331939	noncoding
0.0451607	noncoding
0.0406393	noncoding
1	coding
1	coding
1	coding
0.06143	noncoding
0.180399	noncoding
0.756412	coding
0.106412	noncoding
1	coding
0.453516	noncoding
0.573036	coding
1	coding
1	coding
1	coding
1	coding
1	coding
1	coding
1	coding
0.0616994	noncoding
1	coding
0.132682	noncoding
0.493552	noncoding
0.670915	coding
0.996955	coding
0.999242	coding
0.956028	coding
0.987997	coding
0.993837	coding
0.0292061	noncoding
0.057616	noncoding
0.00559014	noncoding
0.501968	coding
0.148198	noncoding
0.25405	noncoding
0.114684	noncoding
0.161757	noncoding
0.492775	noncoding
0.183564	noncoding
0.933961	coding
0.629843	coding
0.0781764	noncoding
0.993911	coding
0.997868	coding
0.0624703	noncoding
0.0186333	noncoding
0.00964159	noncoding

0.36897	noncoding
0.941335	coding
0.0302099	noncoding
0.00902809	noncoding
1	coding
1	coding
0.0313756	noncoding
0.0625129	noncoding
0.412263	noncoding
0.0846986	noncoding
0.0659905	noncoding
0.00512148	noncoding
0.535136	coding
1	coding
0.00879398	noncoding
0.00993816	noncoding
0.916447	coding
0.135661	noncoding
0.0390999	noncoding
0.311711	noncoding
0.329553	noncoding
0.410311	noncoding
1	coding
1	coding
1	coding
1	coding
1	coding
1	coding
1	coding
0.0150543	noncoding
1	coding
1	coding
1	coding
1	coding
1	coding
1	coding
1	coding
0.557863	coding
0.999998	coding
1	coding
0.269367	noncoding
1	coding
0.0833807	noncoding
1	coding
1	coding
0.0381518	noncoding
0.0355215	noncoding
0.846224	coding
0.0075228	noncoding
0.980369	coding
0.995394	coding
0.893886	coding
1	coding
1	coding

0.385213	noncoding
0.0239084	noncoding
1	coding
1	coding
1	coding
1	coding
1	coding
1	coding
1	coding
1	coding
1	coding
1	coding
1	coding
1	coding
1	coding
1	coding
0.624159	coding
0.139369	noncoding
0.0269646	noncoding
1	coding
1	coding
1	coding
1	coding
1	coding
0.204102	noncoding
0.180969	noncoding
0.241265	noncoding
1	coding
1	coding
1	coding
1	coding
1	coding
1	coding
0.999996	coding
1	coding
1	coding
0.999999	coding
0.981	coding
0.998077	coding
1	coding
1	coding
0.0652463	noncoding
0.648209	coding
0.999816	coding
0.0979879	noncoding
0.867505	coding
0.99827	coding
0.762781	coding
0.860039	coding
0.762781	coding
0.108186	noncoding
1	coding
0.876917	coding
0.997591	coding

1	coding
0.0187031	noncoding
1	coding
1	coding
1	coding
0.0819205	noncoding
1	coding
1	coding
1	coding
0.0751741	noncoding
0.211012	noncoding
1	coding
1	coding
1	coding
0.0938247	noncoding
0.55766	coding
0.99993	coding
0.99999	coding
0.999981	coding
0.708744	coding
1	coding
1	coding
1	coding
0.152957	noncoding
0.132291	noncoding
0.325751	noncoding
1	coding
1	coding
1	coding
1	coding
1	coding
0.737725	coding
1	coding
0.849144	coding
0.999466	coding
0.998963	coding
1	coding
1	coding
1	coding
1	coding
0.977641	coding
1	coding
0.449419	noncoding
0.967609	coding
0.565619	coding
0.475071	noncoding
1	coding
1	coding
1	coding
1	coding
0.906427	coding
0.999938	coding
0.999988	coding
0.0086404	noncoding

0.999983	coding
0.952929	coding
1	coding
0.0422661	noncoding
0.999401	coding
0.999419	coding
0.999345	coding
0.0869851	noncoding
0.39865	noncoding
0.049443	noncoding
0.986626	coding
0.987538	coding
0.143507	noncoding
0.99243	coding
0.135726	noncoding
1	coding
1	coding
1	coding
1	coding
1	coding
1	coding
1	coding
1	coding
1	coding
0.999998	coding
1	coding
0.999999	coding
1	coding
0.264564	noncoding
0.999916	coding
1	coding
1	coding
1	coding
1	coding
0.995965	coding
0.170943	noncoding
0.996329	coding
0.471759	noncoding
0.941566	coding
0.997657	coding
0.0647297	noncoding
0.988212	coding
0.0089232	noncoding
0.967776	coding
0.999838	coding
0.0299305	noncoding
0.0361415	noncoding
0.0499542	noncoding
1	coding
1	coding
1	coding
1	coding
0.643619	coding
0.973653	coding

0.125572	noncoding
0.0683417	noncoding
0.0303234	noncoding
0.786217	coding
0.0211986	noncoding
0.999932	coding
1	coding
0.507893	coding
1	coding
1	coding
1	coding
1	coding
0.707499	coding
0.999996	coding
1	coding
1	coding
1	coding
0.0604417	noncoding
0.772015	coding
0.0477601	noncoding
0.299558	noncoding
0.0197345	noncoding
0.993336	coding
0.999947	coding
0.999791	coding
0.999847	coding
0.183558	noncoding
0.0419324	noncoding
0.182824	noncoding
0.999786	coding
0.00827217	noncoding
0.00786888	noncoding
0.0519224	noncoding
0.0177285	noncoding
0.379983	noncoding
1	coding
0.101226	noncoding
0.999999	coding
1	coding
0.0916308	noncoding
1	coding
1	coding
1	coding
0.999813	coding
1	coding
0.721081	coding
1	coding
0.999991	coding
1	coding
0.918171	coding
1	coding
0.0219923	noncoding
0.339286	noncoding
1	coding

1	coding
0.0868504	noncoding
1	coding
1	coding
1	coding
1	coding
1	coding
1	coding
1	coding
1	coding
1	coding
1	coding
1	coding
1	coding
0.401667	noncoding
0.999294	coding
1	coding
0.999845	coding
1	coding
1	coding
0.0703054	noncoding
0.235874	noncoding
0.504896	coding
1	coding
0.152798	noncoding
1	coding
1	coding
1	coding
1	coding
1	coding
1	coding
0.136135	noncoding
0.122235	noncoding
0.0397034	noncoding
0.19746	noncoding
0.278652	noncoding
1	coding
0.999993	coding
0.0570968	noncoding
0.999999	coding
1	coding
0.155053	noncoding
0.0180425	noncoding
0.156639	noncoding
0.0196239	noncoding
0.170096	noncoding
0.0900071	noncoding
0.122595	noncoding
0.0149736	noncoding
0.00857535	noncoding
0.0113457	noncoding
0.00881641	noncoding
0.010984	noncoding
0.0100814	noncoding

0.00857535	noncoding
0.00866158	noncoding
0.00803472	noncoding
0.0113817	noncoding
0.00857959	noncoding
0.0105921	noncoding
0.0236911	noncoding
0.00857867	noncoding
0.00857759	noncoding
0.00824982	noncoding
0.00887473	noncoding
0.00857867	noncoding
0.00939423	noncoding
0.00891073	noncoding
0.0108051	noncoding
0.0116338	noncoding
0.00873614	noncoding
0.00857535	noncoding
0.0108051	noncoding
0.149023	noncoding
0.997836	coding
1	coding
1	coding
0.0237748	noncoding
1	coding
0.999999	coding
0.0323961	noncoding
0.999998	coding
0.999998	coding
0.97756	coding
0.504534	coding
0.0759876	noncoding
0.039337	noncoding
0.106925	noncoding
0.0127795	noncoding
0.0826597	noncoding
0.0197523	noncoding
0.0219365	noncoding
0.0703732	noncoding
0.522855	coding
1	coding
1	coding
1	coding
0.0473657	noncoding
0.0274482	noncoding
1	coding
1	coding
1	coding
1	coding
0.992351	coding
1	coding
0.999884	coding
0.71582	coding
0.135381	noncoding

0.39033	noncoding
0.00873216	noncoding
0.999995	coding
0.0187454	noncoding
0.0734206	noncoding
0.280687	noncoding
0.18672	noncoding
0.898354	coding
0.0624925	noncoding
0.0377257	noncoding
0.0545984	noncoding
0.456952	noncoding
0.753091	coding
0.269258	noncoding
1	coding
0.0915483	noncoding
1	coding
0.0353112	noncoding
0.636385	coding
0.0408787	noncoding
0.0282654	noncoding
1	coding
1	coding
1	coding
1	coding
0.090836	noncoding
0.0315018	noncoding
1	coding
0.00571301	noncoding
0.999992	coding
0.999971	coding
0.490204	noncoding
1	coding
1	coding
0.0446396	noncoding
1	coding
0.998724	coding
0.347703	noncoding
0.0714847	noncoding
0.114456	noncoding
0.0656274	noncoding
0.0274783	noncoding
0.997191	coding
0.999911	coding
1	coding
1	coding
1	coding
1	coding
1	coding
1	coding
1	coding
1	coding
0.116037	noncoding
0.463277	noncoding

0.320471	noncoding
0.999988	coding
0.985222	coding
0.0071554	noncoding
1	coding
0.0461315	noncoding
0.967637	coding
0.343923	noncoding
1	coding
1	coding
1	coding
0.152267	noncoding
1	coding
1	coding
0.00384177	noncoding
0.995341	coding
0.751607	coding
0.0348579	noncoding
0.964903	coding
0.935083	coding
0.212768	noncoding
0.230644	noncoding
0.232703	noncoding
0.361347	noncoding
0.365439	noncoding
0.149083	noncoding
0.00457736	noncoding
0.0113366	noncoding
0.999848	coding
0.999977	coding
0.999908	coding
0.999922	coding
0.999994	coding
1	coding
1	coding
0.999977	coding
1	coding
0.0539297	noncoding
0.0846237	noncoding
0.999999	coding
0.792861	coding
1	coding
0.999617	coding
0.999558	coding
1	coding
1	coding
0.131579	noncoding
0.0578663	noncoding
0.0281482	noncoding
0.047038	noncoding
0.0944581	noncoding
0.70392	coding
0.999727	coding
0.808019	coding

1	coding
1	coding
0.999864	coding
0.0122423	noncoding
0.106286	noncoding
0.998458	coding
0.518386	coding
0.0222072	noncoding
0.999991	coding
0.999984	coding
0.40403	noncoding
0.0297302	noncoding
0.02094	noncoding
0.297528	noncoding
0.0192313	noncoding
0.0231808	noncoding
0.160543	noncoding
0.420028	noncoding
0.0320479	noncoding
0.492632	noncoding
0.999216	coding
0.99792	coding
0.9992	coding
0.99518	coding
0.811341	coding
1	coding
0.979886	coding
0.23597	noncoding
1	coding
0.0341811	noncoding
0.0971136	noncoding
1	coding
1	coding
1	coding
1	coding
1	coding
1	coding
1	coding
1	coding
0.476296	noncoding
1	coding
0.855406	coding
0.0231603	noncoding
0.279443	noncoding
0.0176463	noncoding
0.0472982	noncoding
0.116902	noncoding
0.0804407	noncoding
0.0723534	noncoding
0.0269495	noncoding
0.0568271	noncoding
0.966839	coding
0.141537	noncoding
0.999726	coding

0.999989	coding
0.999726	coding
0.999813	coding
0.151618	noncoding
0.0190466	noncoding
0.310316	noncoding
0.560425	coding
0.1531	noncoding
1	coding
0.047443	noncoding
0.0139063	noncoding
0.997941	coding
0.059798	noncoding
0.117263	noncoding
0.0674166	noncoding
0.0573882	noncoding
0.202297	noncoding
0.349214	noncoding
0.0996563	noncoding
1	coding
1	coding
1	coding
1	coding
1	coding
1	coding
0.99899	coding
0.485571	noncoding
0.297495	noncoding
1	coding
1	coding
0.00546452	noncoding
0.0154026	noncoding
1	coding
0.999915	coding
0.999996	coding
0.999808	coding
0.995015	coding
0.157346	noncoding
0.446944	noncoding
1	coding
1	coding
1	coding
1	coding
0.00938452	noncoding
0.152056	noncoding
0.008334	noncoding
1	coding
1	coding
1	coding
0.0446391	noncoding
1	coding
1	coding
1	coding
1	coding

1	coding
1	coding
1	coding
1	coding
1	coding
1	coding
1	coding
0.0197776	noncoding
0.00841495	noncoding
0.0309941	noncoding
0.992862	coding
0.999951	coding
0.999956	coding
0.019643	noncoding
0.104831	noncoding
0.0192124	noncoding
0.337054	noncoding
0.999925	coding
0.535833	coding
0.253214	noncoding
0.142965	noncoding
0.164367	noncoding
0.0596481	noncoding
0.0964732	noncoding
0.0128521	noncoding
0.0119489	noncoding
1	coding
1	coding
0.152774	noncoding
0.978121	coding
0.0729842	noncoding
1	coding
0.997862	coding
0.044554	noncoding
0.998996	coding
1	coding
0.987728	coding
0.999951	coding
0.999851	coding
1	coding
0.999998	coding
0.0218861	noncoding
0.0250719	noncoding
0.999575	coding
1	coding
1	coding
1	coding
1	coding
1	coding
0.191616	noncoding
0.0186359	noncoding
0.0141734	noncoding
0.0105947	noncoding
1	coding

0.435636	noncoding
1	coding
1	coding
0.999992	coding
0.887995	coding
0.938439	coding
0.887995	coding
1	coding
1	coding
0.0887312	noncoding
0.999867	coding
0.999823	coding
0.142292	noncoding
0.142292	noncoding
0.97973	coding
0.798167	coding
0.747913	coding
0.998428	coding
0.9996	coding
0.999999	coding
0.999998	coding
0.999961	coding
0.074532	noncoding
0.182042	noncoding
0.0286299	noncoding
0.54871	coding
0.0409591	noncoding
0.139817	noncoding
1	coding
0.057824	noncoding
1	coding
1	coding
1	coding
1	coding
1	coding
0.215176	noncoding
1	coding
0.0336573	noncoding
1	coding
1	coding
0.0898466	noncoding
0.0164438	noncoding
0.0556835	noncoding
0.179153	noncoding
0.427156	noncoding
0.0771786	noncoding
0.0461518	noncoding
0.223292	noncoding
0.0818069	noncoding
0.187186	noncoding
0.568646	coding
0.0368316	noncoding
0.0317262	noncoding
0.416591	noncoding

0.0240432	noncoding
0.024429	noncoding
0.68359	coding
0.1303	noncoding
0.0750378	noncoding
0.105927	noncoding
0.333984	noncoding
0.170559	noncoding
0.999992	coding
0.976968	coding
0.943361	coding
0.0121729	noncoding
1	coding
0.0602111	noncoding
0.668648	coding
0.0710404	noncoding
0.937508	coding
0.0655871	noncoding
1	coding
1	coding
1	coding
1	coding
1	coding
1	coding
1	coding
1	coding
1	coding
1	coding
1	coding
0.999983	coding
1	coding
1	coding
0.790133	coding
0.0468222	noncoding
1	coding
1	coding
1	coding
1	coding
0.999999	coding
1	coding
1	coding
0.0547512	noncoding
1	coding
1	coding
1	coding
0.0982026	noncoding
1	coding
0.999999	coding
1	coding
1	coding
0.0291204	noncoding
1	coding
1	coding
0.666352	coding

0.248423	noncoding
0.966955	coding
0.059863	noncoding
0.450477	noncoding
1	coding
1	coding
0.438351	noncoding
1	coding
1	coding
0.999922	coding
0.999996	coding
0.0204073	noncoding
0.78967	coding
0.999996	coding
1	coding
1	coding
0.996846	coding
0.98585	coding
0.998207	coding
0.997466	coding
1	coding
1	coding
1	coding
1	coding
1	coding
1	coding
1	coding
1	coding
0.0269894	noncoding
0.0459009	noncoding
0.0493441	noncoding
0.996734	coding
0.148277	noncoding
0.306988	noncoding
0.549477	coding
0.0911941	noncoding
0.999722	coding
0.999982	coding
0.0132113	noncoding
0.175545	noncoding
1	coding
1	coding
0.999999	coding
0.999999	coding
1	coding
0.999999	coding
0.068326	noncoding
0.411	noncoding
0.102715	noncoding
0.992376	coding
0.993325	coding
0.942878	coding
0.993325	coding
1	coding

1	coding
1	coding
0.999332	coding
0.999215	coding
0.957448	coding
0.998963	coding
0.0404563	noncoding
0.984095	coding
0.999991	coding
1	coding
1	coding
1	coding
1	coding
1	coding
0.0447972	noncoding
0.025215	noncoding
0.00819083	noncoding
1	coding
0.305297	noncoding
0.374962	noncoding
1	coding
1	coding
1	coding
1	coding
0.0151027	noncoding
0.445438	noncoding
0.00716122	noncoding
0.0401548	noncoding
0.0265615	noncoding
1	coding
0.212013	noncoding
0.00545454	noncoding
0.028165	noncoding
0.0271502	noncoding
1	coding
0.0388222	noncoding
0.179602	noncoding
0.1442	noncoding
0.442208	noncoding
0.413227	noncoding
0.492752	noncoding
0.669455	coding
0.707986	coding
0.707986	coding
0.707986	coding
0.707986	coding
0.669455	coding
0.154424	noncoding
1	coding
1	coding
1	coding
0.999996	coding
1	coding
0.0655065	noncoding

1	coding
1	coding
1	coding
1	coding
1	coding
0.999998	coding
0.231692	noncoding
0.0838297	noncoding
0.999997	coding
0.635543	coding
0.999985	coding
1	coding
1	coding
1	coding
0.156819	noncoding
1	coding
1	coding
1	coding
1	coding
0.0509499	noncoding
0.527294	coding
0.337238	noncoding
0.0553146	noncoding
0.0875964	noncoding
0.452643	noncoding
0.20403	noncoding
0.0255573	noncoding
0.0220735	noncoding
0.021205	noncoding
0.338691	noncoding
1	coding
1	coding
1	coding
1	coding
0.129348	noncoding
0.203145	noncoding
0.215931	noncoding
0.377291	noncoding
0.46603	noncoding
0.108955	noncoding
1	coding
1	coding
0.704612	coding
0.9406	coding
0.695096	coding
0.200022	noncoding
0.0978381	noncoding
0.404946	noncoding
1	coding
0.266286	noncoding
0.998893	coding
1	coding
0.998893	coding
0.163499	noncoding

0.207962	noncoding
0.0874289	noncoding
0.0513791	noncoding
0.0589178	noncoding
1	coding
1	coding
0.993783	coding
0.362238	noncoding
0.526728	coding
0.0355956	noncoding
1	coding
1	coding
1	coding
0.0401796	noncoding
0.920208	coding
0.999798	coding
0.296411	noncoding
0.999999	coding
1	coding
1	coding
1	coding
1	coding
1	coding
1	coding
0.999407	coding
0.999355	coding
0.743957	coding
0.020457	noncoding
1	coding
0.0167472	noncoding
1	coding
1	coding
1	coding
0.999984	coding
1	coding
0.111192	noncoding
0.169478	noncoding
0.0431288	noncoding
0.999481	coding
1	coding
1	coding
1	coding
0.999916	coding
0.945979	coding
0.975044	coding
0.633764	coding
0.945738	coding
0.99951	coding
1	coding
1	coding
1	coding
1	coding
1	coding
0.291397	noncoding

0.699417	coding
0.0298403	noncoding
0.301188	noncoding
0.0827958	noncoding
0.142599	noncoding
0.100432	noncoding
0.100432	noncoding
0.141975	noncoding
0.332814	noncoding
0.171707	noncoding
0.0123513	noncoding
0.999999	coding
0.999999	coding
0.123806	noncoding
0.245998	noncoding
0.999999	coding
1	coding
1	coding
1	coding
1	coding
1	coding
0.0124431	noncoding
0.0201621	noncoding
0.0483313	noncoding
0.999997	coding
0.954797	coding
0.999778	coding
1	coding
0.0202487	noncoding
0.0311133	noncoding
0.866134	coding
0.0636675	noncoding
0.020199	noncoding
0.195651	noncoding
0.998889	coding
0.0698601	noncoding
1	coding
0.0256519	noncoding
0.999999	coding
0.500241	coding
0.144882	noncoding
0.999935	coding
1	coding
1	coding
1	coding
0.0888371	noncoding
0.999977	coding
0.994262	coding
0.295637	noncoding
0.277988	noncoding
0.177069	noncoding
0.991978	coding
0.128666	noncoding
0.36076	noncoding

0.181162	noncoding
0.0229826	noncoding
0.0180674	noncoding
0.107787	noncoding
0.169797	noncoding
1	coding
0.937374	coding
1	coding
1	coding
1	coding
0.086054	noncoding
0.0476048	noncoding
1	coding
0.0297362	noncoding
0.999999	coding
1	coding
0.052112	noncoding
0.0754697	noncoding
0.100416	noncoding
0.999819	coding
0.32747	noncoding
0.0277373	noncoding
0.999605	coding
0.999899	coding
1	coding
0.00361771	noncoding
1	coding
1	coding
1	coding
1	coding
1	coding
0.0577705	noncoding
1	coding
1	coding
1	coding
1	coding
0.0422086	noncoding
0.0121496	noncoding
0.010881	noncoding
0.212379	noncoding
0.424066	noncoding
1	coding
0.999953	coding
0.888717	coding
0.636152	coding
0.743663	coding
0.999999	coding
0.0107305	noncoding
0.800475	coding
1	coding
1	coding
0.0389823	noncoding
1	coding
0.0101849	noncoding

0.0322617	noncoding
0.0568785	noncoding
0.208027	noncoding
1	coding
1	coding
0.134337	noncoding
1	coding
0.976692	coding
1	coding
1	coding
1	coding
0.267817	noncoding
0.968995	coding
0.90808	coding
1	coding
0.999939	coding
1	coding
0.967128	coding
0.997979	coding
0.614716	coding
0.621936	coding
0.178454	noncoding
0.968284	coding
0.950366	coding
0.944339	coding
0.94008	coding
1	coding
1	coding
1	coding
1	coding
0.0247419	noncoding
0.191395	noncoding
0.301491	noncoding
0.00449426	noncoding
1	coding
0.999929	coding
1	coding
1	coding
1	coding
0.156511	noncoding
0.0501693	noncoding
0.0215633	noncoding
0.0952544	noncoding
0.00898571	noncoding
0.0408699	noncoding
0.179242	noncoding
1	coding
1	coding
0.202209	noncoding
0.37602	noncoding
0.626497	coding
0.999792	coding
0.987966	coding
0.0359242	noncoding

0.0833277	noncoding
1	coding
1	coding
1	coding
1	coding
1	coding
0.992763	coding
1	coding
1	coding
1	coding
1	coding
1	coding
1	coding
1	coding
1	coding
0.139528	noncoding
0.136599	noncoding
0.105943	noncoding
0.561395	coding
1	coding
0.995811	coding
0.999821	coding
0.194491	noncoding
0.121436	noncoding
0.0823735	noncoding
1	coding
0.027648	noncoding
0.972596	coding
0.612754	coding
0.487511	noncoding
0.500377	coding
0.114039	noncoding
0.0342311	noncoding
0.0364641	noncoding
0.954053	coding
0.0545844	noncoding
0.301796	noncoding
1	coding
1	coding
0.143549	noncoding
0.0159137	noncoding
0.989859	coding
0.0244226	noncoding
0.888593	coding
0.988944	coding
0.982668	coding
0.362038	noncoding
0.239667	noncoding
0.969626	coding
0.973033	coding
0.947328	coding
0.9898	coding
0.457515	noncoding
0.144632	noncoding

1	coding
0.157419	noncoding
0.117756	noncoding
0.0234074	noncoding
1	coding
0.999455	coding
0.999999	coding
0.999999	coding
0.0847566	noncoding
1	coding
0.185299	noncoding
0.133183	noncoding
0.202269	noncoding
0.964783	coding
0.0248312	noncoding
1	coding
0.0212535	noncoding
0.992616	coding
0.999996	coding
1	coding
0.112285	noncoding
1	coding
0.999954	coding
0.896193	coding
0.215174	noncoding
0.223392	noncoding
0.148066	noncoding
1	coding
0.877763	coding
0.0518583	noncoding
0.0730935	noncoding
0.280134	noncoding
1	coding
0.999999	coding
1	coding
0.121361	noncoding
0.0266139	noncoding
0.238161	noncoding
0.0597985	noncoding
0.0321035	noncoding
0.14963	noncoding
1	coding
0.0568252	noncoding
0.0515394	noncoding
0.105839	noncoding
0.149643	noncoding
0.455308	noncoding
0.00567969	noncoding
0.00526099	noncoding
0.992021	coding
1	coding
1	coding
0.999985	coding
0.596834	coding

1	coding
0.319384	noncoding
0.0725072	noncoding
0.998256	coding
0.0521647	noncoding
0.040723	noncoding
0.999995	coding
0.851234	coding
1	coding
0.572954	coding
0.0508658	noncoding
0.061246	noncoding
0.0158126	noncoding
1	coding
1	coding
1	coding
1	coding
0.510481	coding
0.974246	coding
0.973904	coding
0.0446463	noncoding
0.0414379	noncoding
0.0119052	noncoding
0.999713	coding
0.999982	coding
0.293679	noncoding
0.242686	noncoding
0.00782959	noncoding
1	coding
1	coding
0.13471	noncoding
0.0252757	noncoding
0.989788	coding
1	coding
1	coding
0.0398195	noncoding
0.185871	noncoding
0.477505	noncoding
0.563981	coding
1	coding
1	coding
1	coding
1	coding
1	coding
0.999999	coding
0.135362	noncoding
0.999989	coding
1	coding
0.999541	coding
0.0498323	noncoding
0.305617	noncoding
0.24074	noncoding
1	coding
0.999838	coding

0.999704	coding
0.0554978	noncoding
0.99986	coding
0.999999	coding
0.0768732	noncoding
1	coding
0.0402827	noncoding
0.0348131	noncoding
0.161166	noncoding
1	coding
0.983909	coding
0.215046	noncoding
0.119751	noncoding
0.312029	noncoding
0.108953	noncoding
0.383998	noncoding
0.0682499	noncoding
0.999993	coding
0.030323	noncoding
0.998841	coding
0.071227	noncoding
0.299957	noncoding
0.0414246	noncoding
0.98786	coding
0.712122	coding
0.246779	noncoding
0.0255887	noncoding
0.299811	noncoding
1	coding
1	coding
1	coding
1	coding
1	coding
1	coding
0.380303	noncoding
1	coding
0.0205576	noncoding
1	coding
0.928268	coding
1	coding
1	coding
0.0619941	noncoding
0.201533	noncoding
1	coding
1	coding
1	coding
1	coding
1	coding
0.135083	noncoding
0.478339	noncoding
0.102018	noncoding
0.0351912	noncoding
1	coding
1	coding

1	coding
0.0914977	noncoding
0.0875148	noncoding
0.177305	noncoding
0.962565	coding
0.117302	noncoding
0.117819	noncoding
0.0699604	noncoding
0.0491513	noncoding
0.217171	noncoding
0.0840535	noncoding
0.577386	coding
0.0293334	noncoding
0.834086	coding
0.0471375	noncoding
1	coding
0.99519	coding
0.959374	coding
0.993235	coding
1	coding
0.999303	coding
0.0165795	noncoding
0.385086	noncoding
0.973113	coding
1	coding
0.995375	coding
0.998845	coding
0.986252	coding
1	coding
0.999999	coding
0.988558	coding
1	coding
1	coding
0.999751	coding
0.368031	noncoding
0.841162	coding
1	coding
1	coding
1	coding
0.117172	noncoding
0.0233437	noncoding
1	coding
0.994255	coding
0.908894	coding
0.996487	coding
0.895499	coding
0.204884	noncoding
0.98358	coding
0.112337	noncoding
0.0530906	noncoding
1	coding
1	coding
1	coding
0.999981	coding

0.179127	noncoding
0.999997	coding
0.999994	coding
0.0159881	noncoding
0.999998	coding
0.0264056	noncoding
0.024744	noncoding
0.0340858	noncoding
1	coding
1	coding
0.248872	noncoding
0.0407059	noncoding
0.116154	noncoding
0.99999	coding
0.0841672	noncoding
1	coding
0.0720982	noncoding
1	coding
1	coding
1	coding
1	coding
1	coding
1	coding
0.978628	coding
0.995938	coding
0.995938	coding
0.978628	coding
1	coding
0.595424	coding
0.656502	coding
0.684702	coding
0.861286	coding
0.999988	coding
1	coding
0.999969	coding
0.0361487	noncoding
1	coding
1	coding
1	coding
0.0577467	noncoding
0.999941	coding
0.990308	coding
1	coding
0.999998	coding
1	coding
1	coding
0.999995	coding
0.99999	coding
1	coding
1	coding
1	coding
0.949259	coding
1	coding
1	coding

1	coding
1	coding
0.5807	coding
0.5807	coding
0.5807	coding
1	coding
0.999924	coding
1	coding
1	coding
1	coding
1	coding
1	coding
0.0371419	noncoding
0.151706	noncoding
0.127963	noncoding
0.0775948	noncoding
0.382298	noncoding
0.720741	coding
0.104129	noncoding
0.210268	noncoding
0.784923	coding
0.999996	coding
0.110875	noncoding
0.374607	noncoding
0.0339801	noncoding
0.979266	coding
0.212546	noncoding
1	coding
0.109111	noncoding
0.109117	noncoding
0.0305526	noncoding
0.948965	coding
0.159316	noncoding
0.245503	noncoding
0.968988	coding
0.0597456	noncoding
1	coding
0.288166	noncoding
0.470487	noncoding
0.779877	coding
0.753005	coding
0.020583	noncoding
1	coding
1	coding
1	coding
0.213661	noncoding
0.998429	coding
1	coding
0.00435301	noncoding
0.886402	coding
0.0745311	noncoding
0.639399	coding
0.499543	noncoding
0.500199	coding

0.514131	coding
0.997553	coding
0.17353	noncoding
0.997635	coding
0.0421103	noncoding
0.0118299	noncoding
0.0254186	noncoding
0.999517	coding
0.0230896	noncoding
1	coding
0.117222	noncoding
0.0776115	noncoding
0.0246723	noncoding
0.0438262	noncoding
0.0205849	noncoding
0.0314529	noncoding
1	coding
0.845762	coding
0.507574	coding
0.637771	coding
0.63805	coding
0.169474	noncoding
0.999998	coding
0.999998	coding
0.999998	coding
0.124335	noncoding
0.999823	coding
1	coding
1	coding
1	coding
1	coding
1	coding
1	coding
1	coding
0.231981	noncoding
1	coding
1	coding
1	coding
1	coding
0.139457	noncoding
0.999973	coding
0.891432	coding
1	coding
1	coding
1	coding
1	coding
0.0124736	noncoding
0.245747	noncoding
0.0159483	noncoding
1	coding
0.999996	coding
1	coding
1	coding
1	coding

1	coding
0.00891102	noncoding
0.999648	coding
0.764797	coding
0.970223	coding
0.999998	coding
0.999993	coding
0.99999	coding
0.993836	coding
0.112316	noncoding
1	coding
0.0903775	noncoding
0.0402646	noncoding
1	coding
1	coding
0.434547	noncoding
1	coding
1	coding
1	coding
1	coding
1	coding
1	coding
0.999645	coding
0.114171	noncoding
0.539052	coding
0.281107	noncoding
0.88126	coding
0.97368	coding
0.99993	coding
0.997945	coding
0.0202268	noncoding
1	coding
1	coding
1	coding
1	coding
0.137284	noncoding
0.879804	coding
1	coding
0.0508164	noncoding
0.411648	noncoding
0.890054	coding
0.0293037	noncoding
0.160586	noncoding
0.346258	noncoding
0.38914	noncoding
0.382339	noncoding
0.0268282	noncoding
1	coding
1	coding
0.999982	coding
0.0651059	noncoding
0.695267	coding
0.102338	noncoding
0.0438454	noncoding

0.834548	coding
0.12524	noncoding
0.111833	noncoding
0.0351052	noncoding
0.85904	coding
1	coding
1	coding
0.0411209	noncoding
0.108569	noncoding
1	coding
0.999984	coding
0.974574	coding
1	coding
1	coding
0.23716	noncoding
0.0547002	noncoding
0.999989	coding
0.145937	noncoding
0.00210001	noncoding
0.173521	noncoding
0.0286968	noncoding
1	coding
0.0105349	noncoding
0.99988	coding
1	coding
0.995765	coding
1	coding
1	coding
1	coding
0.089477	noncoding
0.133808	noncoding
1	coding
1	coding
0.99946	coding
0.041018	noncoding
0.0395692	noncoding
0.0901737	noncoding
1	coding
0.999758	coding
0.999236	coding
0.92169	coding
0.178419	noncoding
0.252132	noncoding
0.211192	noncoding
0.468716	noncoding
1	coding
1	coding
1	coding
1	coding
1	coding
1	coding
1	coding
1	coding
0.99996	coding

1	coding
1	coding
1	coding
1	coding
1	coding
1	coding
1	coding
0.999017	coding
0.211211	noncoding
1	coding
1	coding
1	coding
0.990991	coding
1	coding
0.999999	coding
0.999968	coding
0.687224	coding
1	coding
0.105273	noncoding
0.0988099	noncoding
0.0643975	noncoding
0.995891	coding
0.789429	coding
1	coding
1	coding
0.991112	coding
1	coding
1	coding
1	coding
0.999885	coding
1	coding
0.026762	noncoding
1	coding
1	coding
1	coding
1	coding
1	coding
1	coding
1	coding
1	coding
1	coding
1	coding
1	coding
0.0721751	noncoding
0.298661	noncoding
1	coding
1	coding
1	coding
0.997697	coding
0.958876	coding
0.246032	noncoding
1	coding
1	coding
0.395035	noncoding

1	coding
1	coding
1	coding
1	coding
1	coding
1	coding
1	coding
0.0940598	noncoding
0.999729	coding
1	coding
1	coding
0.0504994	noncoding
1	coding
1	coding
1	coding
1	coding
1	coding
0.401816	noncoding
1	coding
1	coding
1	coding
0.9199	coding
1	coding
1	coding
1	coding
0.996516	coding
1	coding
1	coding
1	coding
0.125044	noncoding
0.999999	coding
1	coding
1	coding
1	coding
0.953719	coding
0.999952	coding
1	coding
0.168267	noncoding
0.89844	coding
0.938356	coding
0.142811	noncoding
0.0931245	noncoding
0.154767	noncoding
1	coding
1	coding
1	coding
1	coding
0.999988	coding
0.680305	coding
0.716964	coding
0.0462046	noncoding
0.075032	noncoding
1	coding
1	coding

0.999949	coding
1	coding
1	coding
0.00647675	noncoding
0.997142	coding
0.102811	noncoding
1	coding
1	coding
0.019129	noncoding
0.999992	coding
0.999984	coding
0.999971	coding
0.137208	noncoding
0.991671	coding
0.217677	noncoding
1	coding
1	coding
1	coding
1	coding
1	coding
0.999867	coding
0.999946	coding
0.999998	coding
0.0634916	noncoding
0.0653744	noncoding
1	coding
0.147414	noncoding
1	coding
1	coding
0.0602189	noncoding
0.145868	noncoding
0.234052	noncoding
0.156669	noncoding
1	coding
1	coding
1	coding
0.00653963	noncoding
1	coding
0.116027	noncoding
0.400758	noncoding
0.492409	noncoding
0.148454	noncoding
0.993781	coding
0.363398	noncoding
0.0231018	noncoding
0.111094	noncoding
0.0924082	noncoding
0.0332984	noncoding
0.0336059	noncoding
0.864863	coding
0.999972	coding
0.999993	coding
0.999974	coding
0.999965	coding

0.223227	noncoding
1	coding
1	coding
1	coding
0.123271	noncoding
1	coding
0.049062	noncoding
1	coding
1	coding
1	coding
0.0788011	noncoding
0.255224	noncoding
0.0762874	noncoding
0.0627654	noncoding
0.0832553	noncoding
0.101651	noncoding
0.0238598	noncoding
0.0322205	noncoding
0.0419618	noncoding
0.0347185	noncoding
0.0677894	noncoding
0.0612694	noncoding
0.0313214	noncoding
1	coding
1	coding
0.651781	coding
0.993237	coding
0.992938	coding
0.998817	coding
0.763238	coding
0.96926	coding
1	coding
0.999969	coding
0.951751	coding
0.383865	noncoding
0.720349	coding
0.999992	coding
0.0420337	noncoding
0.0716047	noncoding
0.0572532	noncoding
0.0962673	noncoding
0.0203721	noncoding
0.999175	coding
0.999202	coding
0.0172168	noncoding
0.136875	noncoding
0.945199	coding
1	coding
1	coding
1	coding
1	coding
1	coding
1	coding
1	coding

1	coding
0.999991	coding
1	coding
0.999963	coding
0.996076	coding
1	coding
1	coding
0.884197	coding
0.738383	coding
0.0482034	noncoding
1	coding
1	coding
1	coding
1	coding
0.127132	noncoding
0.112318	noncoding
0.0155469	noncoding
1	coding
1	coding
0.999897	coding
0.124973	noncoding
0.00961025	noncoding
0.0119962	noncoding
0.454047	noncoding
0.0421606	noncoding
1	coding
1	coding
0.725349	coding
0.85257	coding
1	coding
1	coding
1	coding
0.999999	coding
1	coding
0.999984	coding
1	coding
0.999999	coding
1	coding
0.0545548	noncoding
0.015447	noncoding
0.0115388	noncoding
1	coding
0.999996	coding
0.878389	coding
0.988537	coding
0.122916	noncoding
0.999999	coding
1	coding
1	coding
1	coding
1	coding
1	coding
0.262085	noncoding
0.138846	noncoding

0.0549288	noncoding
1	coding
1	coding
1	coding
1	coding
1	coding
0.999999	coding
1	coding
1	coding
1	coding
1	coding
1	coding
0.134475	noncoding
1	coding
0.0334653	noncoding
0.0435382	noncoding
0.0271204	noncoding
1	coding
0.0495376	noncoding
0.999903	coding
0.050276	noncoding
0.0781493	noncoding
1	coding
1	coding
1	coding
0.0898303	noncoding
1	coding
0.999997	coding
0.107488	noncoding
0.00502141	noncoding
0.00482537	noncoding
0.034151	noncoding
0.31353	noncoding
0.261087	noncoding
0.969213	coding
0.0686958	noncoding
0.461474	noncoding
0.213111	noncoding
0.170455	noncoding
0.149331	noncoding
0.176112	noncoding
0.776757	coding
0.686271	coding
0.685297	coding
0.46739	noncoding
0.458387	noncoding
0.08165	noncoding
0.110949	noncoding
0.0436795	noncoding
0.51588	coding
0.301508	noncoding
0.999996	coding
0.944842	coding
0.999639	coding

0.999326	coding
0.086881	noncoding
0.200852	noncoding
0.999476	coding
0.997311	coding
0.148499	noncoding
1	coding
1	coding
0.999999	coding
0.967265	coding
0.999676	coding
0.999591	coding
1	coding
1	coding
1	coding
0.999979	coding
0.124453	noncoding
1	coding
0.998084	coding
0.784956	coding
0.329513	noncoding
0.828757	coding
0.999299	coding
0.99974	coding
0.0299092	noncoding
1	coding
1	coding
1	coding
0.0274922	noncoding
0.0170928	noncoding
1	coding
0.273352	noncoding
1	coding
0.999697	coding
1	coding
1	coding
0.00547312	noncoding
0.150428	noncoding
0.662526	coding
0.150428	noncoding
0.0242863	noncoding
0.0190545	noncoding
1	coding
0.216359	noncoding
0.262971	noncoding
0.273934	noncoding
0.112581	noncoding
0.0620198	noncoding
0.174349	noncoding
0.271843	noncoding
0.134132	noncoding
0.00963501	noncoding
0.00935342	noncoding
1	coding

0.999399	coding
0.20399	noncoding
1	coding
0.0426727	noncoding
0.998353	coding
1	coding
1	coding
1	coding
1	coding
1	coding
1	coding
1	coding
1	coding
1	coding
1	coding
1	coding
1	coding
1	coding
0.998084	coding
0.999999	coding
1	coding
1	coding
1	coding
1	coding
1	coding
1	coding
0.0105154	noncoding
0.80821	coding
0.59396	coding
0.0246363	noncoding
0.0138497	noncoding
0.0315385	noncoding
1	coding
1	coding
0.999992	coding
0.0631735	noncoding
0.0162355	noncoding
0.029103	noncoding
0.0363425	noncoding
1	coding
0.453851	noncoding
0.320417	noncoding
0.135671	noncoding
0.0312502	noncoding
1	coding
1	coding
1	coding
1	coding
0.0845799	noncoding
1	coding
1	coding
1	coding
1	coding
1	coding

1	coding
1	coding
1	coding
1	coding
1	coding
0.0255886	noncoding
0.904547	coding
1	coding
0.212068	noncoding
0.0547668	noncoding
0.0266138	noncoding
0.998763	coding
0.127236	noncoding
0.013792	noncoding
0.0173134	noncoding
1	coding
1	coding
1	coding
1	coding
1	coding
1	coding
1	coding
1	coding
1	coding
1	coding
0.553219	coding
0.063078	noncoding
0.23668	noncoding
0.150074	noncoding
0.462229	noncoding
0.999167	coding
0.145514	noncoding
0.143277	noncoding
0.0380008	noncoding
0.141733	noncoding
0.00556106	noncoding
0.00548045	noncoding
0.986426	coding
0.0193131	noncoding
0.0317676	noncoding
0.0345281	noncoding
0.0268154	noncoding
0.0941139	noncoding
0.0830204	noncoding
1	coding
0.999986	coding
0.999998	coding
1	coding
1	coding
0.291866	noncoding
0.999984	coding
0.999999	coding
1	coding
0.999571	coding

0.999156	coding
0.999847	coding
0.999646	coding
0.988087	coding
0.521257	coding
0.342935	noncoding
0.995578	coding
0.999999	coding
0.999147	coding
0.99958	coding
0.059792	noncoding
0.0141815	noncoding
0.178354	noncoding
0.585509	coding
0.140141	noncoding
0.110334	noncoding
0.0843518	noncoding
0.183342	noncoding
0.491729	noncoding
0.779942	coding
0.0289344	noncoding
0.268986	noncoding
0.614806	coding
0.993666	coding
0.0319297	noncoding
0.516661	coding
0.999335	coding
0.074588	noncoding
0.0186606	noncoding
0.732509	coding
0.112551	noncoding
0.999045	coding
0.999983	coding
0.165557	noncoding
0.571533	coding
0.675682	coding
0.0518233	noncoding
0.0594466	noncoding
0.0422015	noncoding
0.070116	noncoding
0.203096	noncoding
0.0953363	noncoding
0.0411492	noncoding
0.152102	noncoding
0.0871416	noncoding
0.353345	noncoding
0.0572674	noncoding
0.085128	noncoding
1	coding
0.614494	coding
1	coding
1	coding
0.999245	coding
0.994847	coding

0.998991	coding
0.0754239	noncoding
0.0754239	noncoding
0.0890645	noncoding
0.901681	coding
0.999968	coding
0.0738876	noncoding
0.05498	noncoding
0.0407475	noncoding
0.984625	coding
1	coding
0.989925	coding
0.54886	coding
1	coding
1	coding
0.999196	coding
0.999196	coding
0.121607	noncoding
0.0329917	noncoding
0.0106755	noncoding
1	coding
1	coding
0.101343	noncoding
0.999998	coding
0.209588	noncoding
0.999999	coding
0.717948	coding
0.580418	coding
0.580418	coding
0.758907	coding
0.376038	noncoding
1	coding
0.0917205	noncoding
0.999999	coding
1	coding
0.21426	noncoding
0.991874	coding
0.991874	coding
0.115329	noncoding
0.0528915	noncoding
0.998054	coding
0.997717	coding
0.122883	noncoding
0.999867	coding
0.163435	noncoding
0.987353	coding
0.975747	coding
0.80632	coding
0.764169	coding
0.152445	noncoding
0.0512142	noncoding
0.0373861	noncoding
1	coding
1	coding

1	coding
1	coding
1	coding
1	coding
0.99999	coding
1	coding
0.12523	noncoding
0.959907	coding
0.922878	coding
1	coding
1	coding
1	coding
1	coding
1	coding
1	coding
1	coding
1	coding
0.944131	coding
1	coding
0.00699834	noncoding
0.0927873	noncoding
0.0652667	noncoding
0.0405725	noncoding
0.0300494	noncoding
0.0336859	noncoding
0.0372746	noncoding
0.157795	noncoding
0.0215865	noncoding
0.16954	noncoding
0.896979	coding
0.861174	coding
0.982161	coding
0.692655	coding
0.321419	noncoding
0.0882768	noncoding
0.00178136	noncoding
0.00830591	noncoding
1	coding
1	coding
0.53719	coding
1	coding
0.0410312	noncoding
0.163719	noncoding
0.151375	noncoding
0.0348797	noncoding
0.362339	noncoding
1	coding
0.990286	coding
0.993924	coding
0.463517	noncoding
1	coding
0.999997	coding
1	coding
1	coding

1	coding
0.999997	coding
0.972074	coding
0.976872	coding
0.158274	noncoding
0.830538	coding
0.0689521	noncoding
1	coding
1	coding
0.237291	noncoding
0.0395219	noncoding
0.0540782	noncoding
0.0482167	noncoding
0.049158	noncoding
0.0409355	noncoding
1	coding
1	coding
1	coding
0.920174	coding
0.979745	coding
1	coding
0.943318	coding
1	coding
0.790547	coding
0.151464	noncoding
1	coding
1	coding
0.189384	noncoding
1	coding
1	coding
1	coding
1	coding
0.999983	coding
1	coding
1	coding
0.999999	coding
0.999999	coding
0.999999	coding
0.954011	coding
0.145147	noncoding
0.999999	coding
0.995008	coding
0.999961	coding
0.100318	noncoding
0.999999	coding
0.988997	coding
0.0341298	noncoding
0.0746082	noncoding
0.0805451	noncoding
1	coding
0.463437	noncoding
0.891381	coding
0.938758	coding
0.058587	noncoding

0.138946	noncoding
0.777315	coding
1	coding
0.0198353	noncoding
0.202624	noncoding
0.380679	noncoding
1	coding
1	coding
0.999999	coding
1	coding
0.118328	noncoding
0.117209	noncoding
0.119155	noncoding
0.0717205	noncoding
0.0347312	noncoding
0.0561409	noncoding
0.0562315	noncoding
0.0209026	noncoding
1	coding
1	coding
1	coding
1	coding
1	coding
0.937447	coding
0.701095	coding
1	coding
0.158466	noncoding
0.0145537	noncoding
0.999826	coding
0.108498	noncoding
0.0313207	noncoding
0.050609	noncoding
0.987458	coding
0.0897015	noncoding
0.0599063	noncoding
0.0701193	noncoding
0.114212	noncoding
0.53937	coding
0.0942679	noncoding
0.350769	noncoding
0.0433604	noncoding
0.0560449	noncoding
1	coding
0.642409	coding
1	coding
0.807539	coding
0.999994	coding
1	coding
1	coding
1	coding
1	coding
1	coding
0.990203	coding
1	coding

1	coding
0.0499189	noncoding
0.485349	noncoding
0.0566006	noncoding
0.0367204	noncoding
0.0244028	noncoding
1	coding
0.0182307	noncoding
0.202178	noncoding
0.157003	noncoding
0.238916	noncoding
0.061364	noncoding
0.0382617	noncoding
0.00596984	noncoding
0.106748	noncoding
0.146721	noncoding
0.812735	coding
0.0583104	noncoding
0.04017	noncoding
0.0217188	noncoding
0.999999	coding
0.999989	coding
0.272045	noncoding
0.0150863	noncoding
0.071598	noncoding
0.101873	noncoding
0.998974	coding
0.999473	coding
0.798903	coding
0.0799607	noncoding
0.0580417	noncoding
0.996704	coding
0.992467	coding
0.0505692	noncoding
1	coding
1	coding
0.223893	noncoding
0.105748	noncoding
0.0851065	noncoding
1	coding
0.15521	noncoding
0.0254006	noncoding
0.017315	noncoding
0.0371787	noncoding
0.208694	noncoding
0.97101	coding
0.982053	coding
0.618636	coding
0.177673	noncoding
0.999785	coding
0.994738	coding
0.99211	coding
0.999821	coding
0.99984	coding

1	coding
0.931465	coding
1	coding
1	coding
0.150514	noncoding
0.2723	noncoding
0.143414	noncoding
0.105347	noncoding
0.99721	coding
0.996782	coding
0.679643	coding
0.621161	coding
0.011896	noncoding
0.760597	coding
1	coding
0.115183	noncoding
0.345566	noncoding
0.0115137	noncoding
1	coding
1	coding
0.999563	coding
0.978268	coding
1	coding
1	coding
0.470891	noncoding
0.463125	noncoding
0.460427	noncoding
0.263933	noncoding
0.0215835	noncoding
0.318557	noncoding
0.219502	noncoding
0.00390816	noncoding
1	coding
0.895154	coding
0.999948	coding
0.999759	coding
0.564828	coding
1	coding
1	coding
0.0112541	noncoding
0.0856442	noncoding
1	coding
0.0304065	noncoding
0.0560952	noncoding
1	coding
0.0542197	noncoding
0.0147414	noncoding
1	coding
1	coding
1	coding
0.997671	coding
0.572234	coding
1	coding
0.145953	noncoding

0.197843	noncoding
0.017705	noncoding
0.0155591	noncoding
0.020148	noncoding
0.999999	coding
1	coding
0.00200987	noncoding
1	coding
1	coding
0.258022	noncoding
1	coding
1	coding
1	coding
1	coding
1	coding
1	coding
1	coding
0.293678	noncoding
1	coding
0.136959	noncoding
0.077518	noncoding
0.0262041	noncoding
1	coding
1	coding
1	coding
1	coding
1	coding
1	coding
1	coding
1	coding
1	coding
0.398562	noncoding
0.121269	noncoding
0.985132	coding
0.891837	coding
1	coding
0.993345	coding
1	coding
0.822945	coding
0.68833	coding
0.894668	coding
0.14157	noncoding
0.646815	coding
0.785312	coding
0.172874	noncoding
0.040406	noncoding
0.194121	noncoding
0.754335	coding
0.0738561	noncoding
0.0453097	noncoding
1	coding
0.338673	noncoding
1	coding
1	coding

1	coding
1	coding
1	coding
1	coding
0.0467779	noncoding
0.0775054	noncoding
0.843367	coding
0.139482	noncoding
1	coding
1	coding
1	coding
1	coding
1	coding
1	coding
1	coding
0.0935037	noncoding
1	coding
1	coding
0.0213171	noncoding
0.0843984	noncoding
0.03735	noncoding
0.255343	noncoding
0.0598801	noncoding
0.884621	coding
0.178227	noncoding
0.246767	noncoding
0.386744	noncoding
0.0129382	noncoding
0.474754	noncoding
0.151983	noncoding
0.207521	noncoding
0.0351825	noncoding
0.601461	coding
0.108287	noncoding
0.0357163	noncoding
1	coding
1	coding
1	coding
1	coding
0.938515	coding
0.334744	noncoding
1	coding
1	coding
1	coding
1	coding
1	coding
0.125116	noncoding
1	coding
1	coding
1	coding
1	coding
1	coding
1	coding
1	coding

0.999853	coding
1	coding
0.999902	coding
1	coding
1	coding
1	coding
1	coding
0.363746	noncoding
0.207158	noncoding
0.1538	noncoding
0.506022	coding
0.21844	noncoding
0.393742	noncoding
0.0773016	noncoding
0.865033	coding
0.144654	noncoding
0.313515	noncoding
0.174869	noncoding
0.322113	noncoding
0.0368633	noncoding
0.266598	noncoding
0.266598	noncoding
0.266598	noncoding
0.184136	noncoding
0.184136	noncoding
0.23089	noncoding
0.397991	noncoding
0.144889	noncoding
0.138112	noncoding
0.457512	noncoding
0.912765	coding
0.467767	noncoding
0.89686	coding
0.400704	noncoding
0.979184	coding
1	coding
0.999468	coding
0.999574	coding
0.115472	noncoding
0.999965	coding
0.999998	coding
0.999977	coding
1	coding
1	coding
0.736423	coding
0.999993	coding
0.999999	coding
0.999998	coding
0.999995	coding
0.999995	coding
0.999618	coding
0.44147	noncoding
0.809009	coding
0.0523117	noncoding

0.201956	noncoding
0.999968	coding
0.996555	coding
0.170598	noncoding
0.0490666	noncoding
1	coding
1	coding
0.0722067	noncoding
0.121743	noncoding
1	coding
0.999999	coding
1	coding
1	coding
1	coding
1	coding
1	coding
1	coding
1	coding
1	coding
0.999993	coding
0.999999	coding
0.999986	coding
0.201086	noncoding
0.49625	noncoding
0.0473616	noncoding
0.595768	coding
0.633062	coding
0.998765	coding
0.991467	coding
0.952873	coding
0.240366	noncoding
1	coding
1	coding
1	coding
1	coding
1	coding
0.0154955	noncoding
0.0154949	noncoding
0.0707175	noncoding
0.927097	coding
0.0451199	noncoding
0.119391	noncoding
0.0868639	noncoding
0.0827664	noncoding
0.411471	noncoding
1	coding
0.121579	noncoding
1	coding
1	coding
1	coding
0.0061632	noncoding
0.0217743	noncoding
0.0208166	noncoding
0.0200762	noncoding

0.996472	coding
1	coding
1	coding
1	coding
0.996808	coding
0.993404	coding
1	coding
1	coding
1	coding
0.999999	coding
1	coding
1	coding
1	coding
0.386692	noncoding
0.123158	noncoding
0.292728	noncoding
0.125971	noncoding
0.0770968	noncoding
1	coding
1	coding
0.138167	noncoding
0.959277	coding
0.971336	coding
1	coding
1	coding
1	coding
1	coding
1	coding
1	coding
1	coding
0.140004	noncoding
1	coding
1	coding
1	coding
0.139761	noncoding
1	coding
1	coding
1	coding
0.985024	coding
0.762565	coding
1	coding
1	coding
1	coding
0.987741	coding
1	coding
0.999999	coding
1	coding
0.384875	noncoding
1	coding
1	coding
1	coding
1	coding
0.0583701	noncoding
0.208663	noncoding

0.0984391	noncoding
0.169815	noncoding
0.0789702	noncoding
0.212111	noncoding
1	coding
1	coding
0.528763	coding
0.148314	noncoding
1	coding
0.999093	coding
1	coding
1	coding
1	coding
1	coding
1	coding
0.0375068	noncoding
0.111032	noncoding
0.986285	coding
0.924839	coding
0.918467	coding
0.11838	noncoding
0.372467	noncoding
0.84394	coding
0.0300444	noncoding
0.0222163	noncoding
0.0275176	noncoding
0.936003	coding
0.903573	coding
0.903573	coding
0.999867	coding
1	coding
0.999999	coding
0.986066	coding
0.999986	coding
1	coding
0.999867	coding
0.999997	coding
0.432672	noncoding
1	coding
1	coding
1	coding
1	coding
1	coding
1	coding
1	coding
1	coding
1	coding
1	coding
1	coding
1	coding
1	coding
1	coding
1	coding
1	coding

0.273922	noncoding
0.999985	coding
0.160989	noncoding
0.999712	coding
0.999925	coding
0.995761	coding
0.00473052	noncoding
1	coding
1	coding
1	coding
0.235887	noncoding
1	coding
0.279449	noncoding
0.999999	coding
1	coding
1	coding
0.220344	noncoding
0.119079	noncoding
0.077863	noncoding
0.00985415	noncoding
0.308512	noncoding
1	coding
1	coding
1	coding
1	coding
0.0937075	noncoding
0.200949	noncoding
0.688146	coding
0.999988	coding
1	coding
1	coding
1	coding
0.820586	coding
0.132253	noncoding
0.0181405	noncoding
0.999986	coding
0.0638211	noncoding
0.996151	coding
0.945394	coding
0.0128264	noncoding
1	coding
0.983667	coding
0.987404	coding
0.208106	noncoding
0.00223908	noncoding
0.240896	noncoding
0.371406	noncoding
0.999956	coding
1	coding
0.999956	coding
1	coding
1	coding
0.999944	coding
1	coding

0.99922	coding
0.079437	noncoding
0.167997	noncoding
0.3603	noncoding
1	coding
0.672637	coding
0.0145398	noncoding
0.116318	noncoding
1	coding
0.0778558	noncoding
1	coding
0.17566	noncoding
0.254144	noncoding
1	coding
1	coding
1	coding
1	coding
1	coding
1	coding
0.143597	noncoding
1	coding
1	coding
0.25921	noncoding
1	coding
0.0184935	noncoding
0.999981	coding
0.217418	noncoding
0.0654207	noncoding
1	coding
1	coding
0.999999	coding
0.520921	coding
0.994754	coding
0.998239	coding
1	coding
0.999439	coding
1	coding
1	coding
1	coding
1	coding
0.998946	coding
0.999611	coding
0.999749	coding
0.99974	coding
0.993791	coding
0.993938	coding
0.998655	coding
0.430718	noncoding
0.98707	coding
0.986441	coding
0.984315	coding
1	coding
0.174493	noncoding
0.999755	coding

1	coding
1	coding
0.995744	coding
0.608295	coding
1	coding
0.116496	noncoding
1	coding
1	coding
0.999999	coding
1	coding
1	coding
1	coding
0.932739	coding
0.999998	coding
1	coding
1	coding
1	coding
0.999218	coding
0.99993	coding
0.230883	noncoding
1	coding
0.64974	coding
0.998257	coding
1	coding
1	coding
0.999456	coding
0.995253	coding
0.983368	coding
1	coding
1	coding
1	coding
1	coding
0.212024	noncoding
0.19447	noncoding
0.126588	noncoding
0.144929	noncoding
0.104256	noncoding
0.101869	noncoding
0.999686	coding
1	coding
1	coding
1	coding
1	coding
0.997569	coding
0.00861748	noncoding
1	coding
1	coding
0.999743	coding
0.963721	coding
0.00357364	noncoding
0.989219	coding
0.0470353	noncoding
0.147865	noncoding
0.999998	coding

0.999998	coding
0.999999	coding
1	coding
0.0342273	noncoding
0.999998	coding
0.999997	coding
0.375679	noncoding
0.199577	noncoding
0.0112574	noncoding
0.0154687	noncoding
0.999954	coding
0.00460551	noncoding
0.619652	coding
1	coding
1	coding
0.801001	coding
0.999996	coding
0.999992	coding
0.999999	coding
1	coding
0.999991	coding
0.0288959	noncoding
0.0364982	noncoding
1	coding
1	coding
1	coding
1	coding
1	coding
1	coding
1	coding
0.0288027	noncoding
1	coding
1	coding
1	coding
1	coding
0.126821	noncoding
1	coding
1	coding
0.999979	coding
1	coding
0.0714836	noncoding
1	coding
1	coding
1	coding
1	coding
0.00622154	noncoding
0.114055	noncoding
1	coding
1	coding
0.999701	coding
0.418947	noncoding
0.959843	coding
0.126644	noncoding
0.989985	coding

0.724085	coding
0.0907133	noncoding
0.630787	coding
0.999874	coding
0.999986	coding
0.629553	coding
0.742466	coding
0.622014	coding
1	coding
1	coding
1	coding
1	coding
0.543221	coding
0.365208	noncoding
0.105564	noncoding
1	coding
0.998874	coding
0.703954	coding
1	coding
1	coding
1	coding
1	coding
1	coding
1	coding
0.0269305	noncoding
0.998788	coding
0.00838527	noncoding
0.99998	coding
0.99912	coding
1	coding
1	coding
1	coding
1	coding
0.840786	coding
1	coding
1	coding
1	coding
1	coding
0.225819	noncoding
0.225819	noncoding
0.276141	noncoding
1	coding
0.601466	coding
0.171513	noncoding
1	coding
1	coding
0.419155	noncoding
0.999995	coding
0.999991	coding
0.999776	coding
0.998493	coding
1	coding
1	coding
1	coding

0.0477139	noncoding
0.661916	coding
0.0406335	noncoding
1	coding
0.502556	coding
0.0926892	noncoding
0.143608	noncoding
0.0752643	noncoding
0.0292938	noncoding
0.0189227	noncoding
0.856684	coding
0.376649	noncoding
0.0199112	noncoding
0.217481	noncoding
0.165385	noncoding
0.228405	noncoding
0.027834	noncoding
1	coding
1	coding
1	coding
1	coding
1	coding
1	coding
1	coding
0.132124	noncoding
1	coding
0.0699397	noncoding
0.25651	noncoding
0.0116241	noncoding
0.0213757	noncoding
0.129166	noncoding
0.103361	noncoding
0.0515797	noncoding
0.978999	coding
0.999961	coding
0.89136	coding
1	coding
0.999997	coding
1	coding
1	coding
0.0458925	noncoding
0.028368	noncoding
0.998307	coding
0.97026	coding
0.504575	coding
0.572518	coding
0.999961	coding
0.0806987	noncoding
0.999572	coding
1	coding
1	coding
0.99994	coding
0.96683	coding
0.841054	coding

0.855269	coding
0.967103	coding
0.974705	coding
0.210628	noncoding
0.3017	noncoding
0.999487	coding
0.158576	noncoding
0.0948201	noncoding
0.0948201	noncoding
0.15459	noncoding
0.0978492	noncoding
0.827498	coding
0.416283	noncoding
0.288647	noncoding
0.203607	noncoding
0.962432	coding
0.945527	coding
0.940708	coding
0.110197	noncoding
0.999983	coding
1	coding
1	coding
1	coding
1	coding
1	coding
1	coding
1	coding
0.993211	coding
1	coding
0.925701	coding
0.863087	coding
0.663319	coding
0.0605959	noncoding
1	coding
0.954299	coding
1	coding
1	coding
1	coding
0.0172171	noncoding
1	coding
1	coding
1	coding
1	coding
1	coding
1	coding
0.608443	coding
1	coding
1	coding
1	coding
0.0726483	noncoding
0.0351996	noncoding
1	coding
0.197329	noncoding
1	coding

1	coding
0.995263	coding
1	coding
0.179884	noncoding
1	coding
0.175902	noncoding
0.220181	noncoding
0.0580667	noncoding
0.0383977	noncoding
0.342847	noncoding
0.349213	noncoding
0.202228	noncoding
0.0257323	noncoding
0.112023	noncoding
0.999914	coding
0.99989	coding
0.998325	coding
0.999914	coding
0.99989	coding
0.999954	coding
0.363675	noncoding
0.0404145	noncoding
0.0404145	noncoding
1	coding
1	coding
0.812076	coding
0.969127	coding
0.428765	noncoding
0.949063	coding
0.216641	noncoding
0.870701	coding
0.721945	coding
1	coding
1	coding
0.409877	noncoding
0.620646	coding
0.0497334	noncoding
0.174017	noncoding
0.248254	noncoding
0.185507	noncoding
0.115815	noncoding
1	coding
0.156253	noncoding
0.879282	coding
0.995511	coding
0.99956	coding
0.999763	coding
0.197708	noncoding
1	coding
0.31489	noncoding
0.0458344	noncoding
0.256759	noncoding
0.0731482	noncoding
1	coding

1	coding
0.998422	coding
0.996975	coding
0.0660755	noncoding
0.191621	noncoding
0.0619828	noncoding
0.400646	noncoding
0.034291	noncoding
0.0257341	noncoding
0.0788783	noncoding
0.989473	coding
0.999325	coding
0.998802	coding
0.998101	coding
0.3161	noncoding
0.397987	noncoding
1	coding
1	coding
0.999999	coding
1	coding
0.999062	coding
0.154717	noncoding
1	coding
1	coding
0.987759	coding
0.90143	coding
1	coding
1	coding
1	coding
1	coding
1	coding
1	coding
1	coding
0.57253	coding
0.255966	noncoding
1	coding
0.99603	coding
0.999724	coding
0.999655	coding
1	coding
1	coding
1	coding
1	coding
0.999994	coding
1	coding
1	coding
0.999879	coding
1	coding
1	coding
1	coding
1	coding
0.999996	coding
0.186817	noncoding
0.729645	coding

1	coding
0.122825	noncoding
0.00757333	noncoding
1	coding
0.999963	coding
1	coding
1	coding
0.999962	coding
0.99988	coding
0.99994	coding
0.999819	coding
1	coding
1	coding
0.449162	noncoding
0.351141	noncoding
0.876828	coding
0.783645	coding
0.343768	noncoding
0.224164	noncoding
0.351705	noncoding
0.764922	coding
0.605488	coding
0.766277	coding
0.999551	coding
0.999919	coding
0.99974	coding
1	coding
0.119941	noncoding
0.0661847	noncoding
1	coding
1	coding
1	coding
1	coding
0.999981	coding
1	coding
0.383715	noncoding
0.0462898	noncoding
1	coding
0.181038	noncoding
1	coding
1	coding
1	coding
1	coding
1	coding
1	coding
1	coding
1	coding
1	coding
1	coding
1	coding
0.00723009	noncoding
1	coding
0.0435455	noncoding
0.730302	coding

0.382484	noncoding
0.242157	noncoding
1	coding
0.832643	coding
1	coding
0.590465	coding
1	coding
0.0938949	noncoding
0.033827	noncoding
0.432072	noncoding
0.574456	coding
0.909976	coding
0.999197	coding
0.0103357	noncoding
0.0615632	noncoding
0.176623	noncoding
1	coding
0.14369	noncoding
0.999972	coding
0.0674544	noncoding
0.121765	noncoding
0.204515	noncoding
0.0396381	noncoding
0.0175772	noncoding
0.148075	noncoding
0.146762	noncoding
0.999863	coding
0.999999	coding
0.999999	coding
0.999995	coding
0.732256	coding
0.730114	coding
1	coding
1	coding
0.00954336	noncoding
0.98952	coding
0.9612	coding
1	coding
0.0212971	noncoding
1	coding
1	coding
1	coding
1	coding
0.0274906	noncoding
0.988619	coding
1	coding
1	coding
0.441595	noncoding
0.445708	noncoding
0.25237	noncoding
0.0110099	noncoding
0.0144579	noncoding
0.520352	coding
0.0615865	noncoding

0.363816	noncoding
0.085272	noncoding
0.0740344	noncoding
0.0690106	noncoding
0.0531077	noncoding
0.751931	coding
0.0456968	noncoding
0.26113	noncoding
0.595842	coding
0.00796255	noncoding
0.976986	coding
0.979854	coding
0.341021	noncoding
0.189891	noncoding
0.0320713	noncoding
0.363766	noncoding
0.92365	coding
1	coding
0.0730275	noncoding
0.0730275	noncoding
0.0246552	noncoding
0.984625	coding
0.924432	coding
0.126089	noncoding
0.0254398	noncoding
0.999529	coding
0.999931	coding
0.201556	noncoding
0.176009	noncoding
0.663699	coding
0.88565	coding
0.57692	coding
0.888983	coding
0.3533	noncoding
0.155494	noncoding
0.907464	coding
0.963782	coding
0.0022824	noncoding
0.486687	noncoding
0.132754	noncoding
0.0622824	noncoding
1	coding
1	coding
0.999931	coding
0.0388892	noncoding
1	coding
1	coding
1	coding
1	coding
0.997444	coding
0.43046	noncoding
0.960776	coding
1	coding
0.0277755	noncoding

0.999629	coding
0.99995	coding
0.997306	coding
0.997949	coding
1	coding
1	coding
0.999978	coding
1	coding
1	coding
1	coding
1	coding
1	coding
0.0836958	noncoding
0.447542	noncoding
0.0663495	noncoding
0.0129164	noncoding
0.995199	coding
0.49868	noncoding
0.982348	coding
0.335505	noncoding
0.700528	coding
0.181111	noncoding
1	coding
0.0110692	noncoding
0.360096	noncoding
0.999518	coding
0.279116	noncoding
0.991753	coding
0.893004	coding
1	coding
1	coding
0.978284	coding
0.342015	noncoding
0.926249	coding
0.353506	noncoding
0.439055	noncoding
0.361555	noncoding
0.971238	coding
0.998501	coding
0.0725654	noncoding
0.134596	noncoding
0.0403841	noncoding
0.999836	coding
0.0144336	noncoding
0.889863	coding
0.999788	coding
0.998449	coding
0.189143	noncoding
0.0400642	noncoding
0.0359316	noncoding
0.270723	noncoding
0.165181	noncoding
0.999959	coding
0.996412	coding

0.926415	coding
0.997716	coding
0.0133438	noncoding
0.0521965	noncoding
0.979309	coding
0.166232	noncoding
1	coding
1	coding
0.0169494	noncoding
0.218103	noncoding
0.0997535	noncoding
0.0872413	noncoding
0.0885357	noncoding
0.0329105	noncoding
0.99989	coding
0.990785	coding
0.999425	coding
1	coding
1	coding
1	coding
0.00876435	noncoding
0.898981	coding
0.991101	coding
1	coding
1	coding
0.999943	coding
0.999389	coding
1	coding
0.999986	coding
0.171883	noncoding
0.995785	coding
1	coding
1	coding
1	coding
1	coding
1	coding
0.99921	coding
1	coding
1	coding
1	coding
0.209567	noncoding
0.15243	noncoding
0.0198989	noncoding
0.0101761	noncoding
0.0254514	noncoding
0.97226	coding
0.920519	coding
0.255965	noncoding
0.0479418	noncoding
0.115958	noncoding
0.999989	coding
0.058967	noncoding
0.0395182	noncoding
0.736342	coding

0.423162	noncoding
0.0194566	noncoding
0.16211	noncoding
0.147517	noncoding
0.0156969	noncoding
1	coding
0.651002	coding
0.0472625	noncoding
0.023132	noncoding
0.0306647	noncoding
0.547817	coding
0.957332	coding
0.549493	coding
0.787841	coding
0.584856	coding
0.937858	coding
0.0729458	noncoding
0.0603248	noncoding
0.0177702	noncoding
0.0272878	noncoding
1	coding
1	coding
0.0115774	noncoding
0.99976	coding
1	coding
0.0996056	noncoding
0.653526	coding
1	coding
0.28411	noncoding
0.999269	coding
0.378819	noncoding
0.703346	coding
0.522789	coding
0.0646378	noncoding
0.643562	coding
0.431866	noncoding
0.725187	coding
1	coding
1	coding
0.00480539	noncoding
0.00420516	noncoding
0.0337063	noncoding
0.0119581	noncoding
0.019336	noncoding
0.11511	noncoding
0.786419	coding
0.788137	coding
0.0166622	noncoding
0.0500452	noncoding
0.946558	coding
0.092192	noncoding
0.0804881	noncoding
0.999995	coding
1	coding

1	coding
1	coding
1	coding
0.768443	coding
0.163892	noncoding
0.430987	noncoding
0.0163247	noncoding
0.999614	coding
0.999548	coding
0.99968	coding
0.116735	noncoding
1	coding
1	coding
1	coding
1	coding
0.0455508	noncoding
0.216468	noncoding
1	coding
1	coding
0.0247348	noncoding
1	coding
1	coding
1	coding
1	coding
0.0779724	noncoding
1	coding
0.022508	noncoding
0.0543128	noncoding
0.114604	noncoding
1	coding
0.999182	coding
0.0953371	noncoding
0.964862	coding
0.0345175	noncoding
0.999791	coding
0.282479	noncoding
1	coding
0.047359	noncoding
0.995098	coding
1	coding
1	coding
0.0552974	noncoding
0.866854	coding
0.999998	coding
0.0267001	noncoding
0.00436711	noncoding
0.731803	coding
0.00508191	noncoding
0.852898	coding
0.933662	coding
0.963321	coding
0.927385	coding
0.48912	noncoding
0.973913	coding

0.872304	coding
0.322657	noncoding
0.862039	coding
0.296874	noncoding
0.211901	noncoding
1	coding
0.999948	coding
0.0760621	noncoding
1	coding
1	coding
1	coding
0.96373	coding
1	coding
1	coding
0.991275	coding
0.00550373	noncoding
0.00752731	noncoding
0.0105218	noncoding
0.16029	noncoding
0.999365	coding
0.0640238	noncoding
0.932653	coding
0.999997	coding
0.931843	coding
0.999997	coding
0.0304137	noncoding
0.999999	coding
1	coding
0.99999	coding
0.999938	coding
0.071122	noncoding
0.979355	coding
0.307562	noncoding
0.0965722	noncoding
0.561268	coding
0.076768	noncoding
1	coding
0.256564	noncoding
1	coding
0.840936	coding
0.978116	coding
0.999998	coding
0.99107	coding
0.978116	coding
0.99991	coding
0.999803	coding
0.0961343	noncoding
0.373762	noncoding
1	coding
0.502518	coding
0.999959	coding
0.0687962	noncoding
0.0500792	noncoding
0.0578384	noncoding

0.133625	noncoding
0.41505	noncoding
0.25203	noncoding
1	coding
1	coding
1	coding
1	coding
0.0909556	noncoding
0.711537	coding
0.0389339	noncoding
0.199483	noncoding
0.0304472	noncoding
0.976084	coding
1	coding
1	coding
1	coding
0.0691163	noncoding
0.304946	noncoding
0.999955	coding
0.558771	coding
0.0945464	noncoding
0.201285	noncoding
0.15427	noncoding
0.0482056	noncoding
0.124742	noncoding
0.222065	noncoding
0.282624	noncoding
0.198749	noncoding
0.486461	noncoding
0.129069	noncoding
0.0743075	noncoding
0.158009	noncoding
0.867678	coding
0.540245	coding
0.142373	noncoding
0.485043	noncoding
0.58212	coding
0.0651372	noncoding
0.137381	noncoding
0.0572745	noncoding
0.117272	noncoding
0.238152	noncoding
0.0344559	noncoding
0.0111362	noncoding
0.401165	noncoding
0.0852861	noncoding
0.0871466	noncoding
0.04746	noncoding
0.999997	coding
0.0377525	noncoding
0.424111	noncoding
0.0255936	noncoding
0.016261	noncoding
0.0408265	noncoding

0.758508	coding
0.234779	noncoding
0.649848	coding
0.968751	coding
1	coding
1	coding
0.0813863	noncoding
0.999836	coding
0.26771	noncoding
0.0520088	noncoding
0.348224	noncoding
0.078571	noncoding
0.122495	noncoding
1	coding
0.999996	coding
0.180303	noncoding
1	coding
1	coding
0.106492	noncoding
1	coding
1	coding
0.0146546	noncoding
0.999997	coding
0.905179	coding
0.999988	coding
1	coding
0.824064	coding
1	coding
1	coding
1	coding
0.444682	noncoding
1	coding
1	coding
1	coding
0.999998	coding
0.999991	coding
0.99344	coding
0.206619	noncoding
0.999999	coding
1	coding
0.999996	coding
0.999886	coding
1	coding
0.755127	coding
0.986036	coding
0.176288	noncoding
0.934893	coding
0.67796	coding
1	coding
1	coding
0.703732	coding
1	coding
1	coding
1	coding

0.700309	coding
0.999992	coding
0.999998	coding
1	coding
0.469103	noncoding
0.0951791	noncoding
0.0597665	noncoding
0.10694	noncoding
0.0276464	noncoding
0.231425	noncoding
0.00578381	noncoding
0.00220501	noncoding
0.0173543	noncoding
0.0680611	noncoding
0.0352538	noncoding
0.92297	coding
1	coding
1	coding
0.999997	coding
1	coding
1	coding
0.150569	noncoding
0.147965	noncoding
0.697325	coding
1	coding
0.634185	coding
1	coding
1	coding
1	coding
1	coding
0.307928	noncoding
1	coding
1	coding
1	coding
0.11324	noncoding
1	coding
0.373701	noncoding
0.0356978	noncoding
0.172027	noncoding
0.00816789	noncoding
0.0705656	noncoding
1	coding
1	coding
0.352479	noncoding
1	coding
0.0403232	noncoding
0.140766	noncoding
0.244281	noncoding
0.237345	noncoding
0.079745	noncoding
1	coding
0.0345349	noncoding
0.372613	noncoding
0.994698	coding

0.071682	noncoding
0.985466	coding
0.969318	coding
0.0579798	noncoding
1	coding
1	coding
0.0744967	noncoding
0.706285	coding
0.503458	coding
0.067523	noncoding
0.407377	noncoding
0.111466	noncoding
0.966399	coding
0.998118	coding
0.970404	coding
0.100542	noncoding
0.911039	coding
0.649892	coding
1	coding
0.240134	noncoding
0.771914	coding
0.88598	coding
1	coding
0.998114	coding
0.993616	coding
0.0291263	noncoding
0.282696	noncoding
0.592236	coding
0.549524	coding
0.00697408	noncoding
0.0453843	noncoding
0.323405	noncoding
0.0178561	noncoding
0.198414	noncoding
1	coding
0.156103	noncoding
0.0711896	noncoding
0.22213	noncoding
1	coding
1	coding
1	coding
0.0112985	noncoding
0.922332	coding
0.987678	coding
1	coding
0.999997	coding
0.999999	coding
0.999999	coding
0.999993	coding
0.18026	noncoding
0.0824229	noncoding
0.346009	noncoding
0.0464349	noncoding
0.40509	noncoding

0.424054	noncoding
0.424054	noncoding
0.346009	noncoding
0.043748	noncoding
0.380565	noncoding
0.897699	coding
0.420729	noncoding
0.349226	noncoding
0.352757	noncoding
1	coding
1	coding
1	coding
1	coding
1	coding
0.0709089	noncoding
0.0934206	noncoding
0.202258	noncoding
0.0589941	noncoding
0.13607	noncoding
0.992284	coding
0.992635	coding
0.998696	coding
1	coding
0.991247	coding
1	coding
0.999993	coding
1	coding
1	coding
1	coding
1	coding
1	coding
1	coding
0.0154143	noncoding
1	coding
0.999205	coding
0.0119076	noncoding
0.218356	noncoding
0.118817	noncoding
0.977248	coding
1	coding
1	coding
1	coding
1	coding
0.999999	coding
1	coding
0.654377	coding
1	coding
1	coding
1	coding
1	coding
1	coding
1	coding
0.999999	coding
0.209076	noncoding

1	coding
1	coding
1	coding
1	coding
1	coding
1	coding
0.00674139	noncoding
0.0603862	noncoding
1	coding
1	coding
1	coding
0.133962	noncoding
0.684939	coding
0.997829	coding
0.999867	coding
0.999937	coding
1	coding
1	coding
0.999991	coding
0.123443	noncoding
1	coding
1	coding
0.999995	coding
0.157889	noncoding
0.157889	noncoding
0.999996	coding
1	coding
1	coding
1	coding
1	coding
0.104735	noncoding
0.105469	noncoding
0.305713	noncoding
1	coding
1	coding
0.0339907	noncoding
1	coding
0.523493	coding
0.157074	noncoding
0.999912	coding
0.0605976	noncoding
0.239561	noncoding
0.0688143	noncoding
0.237497	noncoding
0.0784759	noncoding
1	coding
1	coding
0.217861	noncoding
1	coding
1	coding
1	coding
1	coding
0.0113831	noncoding
0.0446652	noncoding

0.018894	noncoding
0.257515	noncoding
0.0107465	noncoding
0.0754387	noncoding
0.430791	noncoding
0.421703	noncoding
0.756958	coding
0.0261442	noncoding
0.0324892	noncoding
0.0209792	noncoding
0.0297879	noncoding
0.0591095	noncoding
0.175463	noncoding
0.107414	noncoding
0.0886224	noncoding
0.0992326	noncoding
0.0977081	noncoding
0.107983	noncoding
0.149156	noncoding
0.13465	noncoding
0.102769	noncoding
0.0508414	noncoding
0.010385	noncoding
0.00820168	noncoding
0.0285361	noncoding
0.0708573	noncoding
0.0962642	noncoding
0.106257	noncoding
0.0750004	noncoding
0.117264	noncoding
0.148447	noncoding
0.355638	noncoding
0.191929	noncoding
0.785382	coding
0.0306974	noncoding
1	coding
1	coding
1	coding
1	coding
1	coding
0.0645431	noncoding
0.569709	coding
0.104083	noncoding
0.999998	coding
0.557371	coding
0.997774	coding
1	coding
0.308371	noncoding
0.999998	coding
0.101198	noncoding
0.529602	coding
0.105083	noncoding
1	coding
1	coding

1	coding
1	coding
0.0825703	noncoding
0.989664	coding
0.202218	noncoding
0.703494	coding
0.0157127	noncoding
0.63116	coding
0.504652	coding
0.0329858	noncoding
0.215132	noncoding
0.0569483	noncoding
0.0820303	noncoding
0.833581	coding
0.833581	coding
0.839108	coding
0.104056	noncoding
0.0749838	noncoding
0.999973	coding
0.614902	coding
0.0925166	noncoding
0.0445275	noncoding
1	coding
0.999992	coding
0.994581	coding
0.994581	coding
1	coding
0.582087	coding
1	coding
1	coding
1	coding
1	coding
0.194316	noncoding
0.981671	coding
0.0139751	noncoding
0.0639004	noncoding
0.0192972	noncoding
0.0383285	noncoding
0.028608	noncoding
0.933438	coding
0.0163783	noncoding
1	coding
1	coding
0.0132212	noncoding
0.991397	coding
0.999674	coding
0.997912	coding
0.999202	coding
0.999878	coding
0.999497	coding
0.995788	coding
1	coding
0.796825	coding
0.999995	coding

0.999782	coding
0.966711	coding
0.994417	coding
0.999211	coding
1	coding
0.0187652	noncoding
0.148823	noncoding
0.0364592	noncoding
1	coding
1	coding
0.849493	coding
0.999997	coding
0.999992	coding
0.100237	noncoding
0.014995	noncoding
0.471358	noncoding
0.0663676	noncoding
0.19938	noncoding
0.817198	coding
0.16437	noncoding
0.999998	coding
0.484259	noncoding
1	coding
1	coding
1	coding
1	coding
1	coding
1	coding
1	coding
1	coding
1	coding
1	coding
1	coding
0.0181725	noncoding
0.0700467	noncoding
0.999984	coding
0.999904	coding
0.999995	coding
0.255276	noncoding
0.0839726	noncoding
1	coding
1	coding
1	coding
1	coding
1	coding
1	coding
0.211398	noncoding
1	coding
0.118288	noncoding
0.386449	noncoding
0.0443936	noncoding
0.327363	noncoding
0.170989	noncoding
0.362381	noncoding

0.147945	noncoding
0.0340056	noncoding
0.831422	coding
1	coding
0.557696	coding
0.99945	coding
0.999998	coding
0.740555	coding
0.999999	coding
0.999999	coding
0.999795	coding
1	coding
0.0470762	noncoding
0.171573	noncoding
0.0689141	noncoding
0.0615088	noncoding
0.999941	coding
0.999778	coding
0.973425	coding
0.952058	coding
1	coding
0.999174	coding
0.0325887	noncoding
0.0297917	noncoding
0.0585349	noncoding
0.175346	noncoding
0.295366	noncoding
1	coding
1	coding
0.327272	noncoding
1	coding
1	coding
1	coding
1	coding
0.489373	noncoding
1	coding
1	coding
0.135426	noncoding
1	coding
1	coding
1	coding
0.45564	noncoding
0.137035	noncoding
0.21924	noncoding
0.99997	coding
0.878741	coding
0.892042	coding
1	coding
1	coding
0.999956	coding
0.0535491	noncoding
0.015244	noncoding
0.0602466	noncoding
0.999999	coding

0.0320368	noncoding
0.0532695	noncoding
0.131973	noncoding
1	coding
0.99999	coding
0.0755983	noncoding
0.918579	coding
0.0340788	noncoding
0.99998	coding
0.99998	coding
1	coding
0.749002	coding
0.011095	noncoding
0.0129695	noncoding
1	coding
0.335433	noncoding
1	coding
0.0494234	noncoding
0.0265078	noncoding
0.313895	noncoding
0.984794	coding
0.0397559	noncoding
0.567379	coding
0.695931	coding
0.82798	coding
0.996249	coding
0.0622523	noncoding
1	coding
0.0596925	noncoding
0.0801146	noncoding
1	coding
0.119219	noncoding
0.0302275	noncoding
0.0217757	noncoding
0.0183865	noncoding
0.203485	noncoding
0.2367	noncoding
1	coding
1	coding
0.00799122	noncoding
1	coding
0.135204	noncoding
0.999979	coding
1	coding
1	coding
0.988409	coding
0.0980533	noncoding
0.0698375	noncoding
0.1096	noncoding
0.0541947	noncoding
0.105194	noncoding
0.0372973	noncoding
1	coding
0.984278	coding

1	coding
1	coding
0.124662	noncoding
0.121921	noncoding
0.0953302	noncoding
0.0516456	noncoding
0.236493	noncoding
1	coding
1	coding
0.322069	noncoding
0.0553763	noncoding
0.00702025	noncoding
0.980821	coding
0.998856	coding
0.984786	coding
1	coding
1	coding
1	coding
1	coding
1	coding
1	coding
1	coding
1	coding
1	coding
0.103755	noncoding
1	coding
0.28948	noncoding
0.99986	coding
1	coding
1	coding
1	coding
1	coding
1	coding
1	coding
1	coding
1	coding
0.894308	coding
0.11482	noncoding
0.0353797	noncoding
1	coding
0.999985	coding
0.999998	coding
0.999998	coding
0.999998	coding
0.999955	coding
0.999959	coding
1	coding
0.999191	coding
0.966025	coding
0.999941	coding
1	coding
1	coding
1	coding
1	coding

1	coding
0.125635	noncoding
1	coding
1	coding
0.027873	noncoding
0.999995	coding
0.0690653	noncoding
0.977329	coding
0.0285146	noncoding
0.0288291	noncoding
0.0276043	noncoding
1	coding
1	coding
0.148015	noncoding
1	coding
1	coding
0.0633819	noncoding
0.202484	noncoding
0.810571	coding
1	coding
1	coding
1	coding
1	coding
0.907461	coding
0.936417	coding
1	coding
0.0253795	noncoding
1	coding
1	coding
1	coding
1	coding
1	coding
0.962667	coding
0.995375	coding
0.999862	coding
1	coding
1	coding
0.999895	coding
0.619871	coding
1	coding
1	coding
1	coding
1	coding
0.0758798	noncoding
1	coding
0.999999	coding
1	coding
0.0131044	noncoding
0.96021	coding
0.999824	coding
0.992955	coding
0.999877	coding
1	coding
0.0846111	noncoding

0.990619	coding
1	coding
0.967024	coding
0.049719	noncoding
0.0770943	noncoding
0.132426	noncoding
0.121249	noncoding
0.0963468	noncoding
0.998325	coding
0.938071	coding
0.0177304	noncoding
0.425248	noncoding
0.97542	coding
0.425248	noncoding
0.00758538	noncoding
0.0425133	noncoding
0.0180896	noncoding
0.00219752	noncoding
0.00622365	noncoding
0.00748703	noncoding
0.00529623	noncoding
0.0986623	noncoding
0.24108	noncoding
0.235347	noncoding
0.996791	coding
0.934991	coding
0.991714	coding
0.971246	coding
0.99385	coding
0.973858	coding
0.482785	noncoding
0.072074	noncoding
0.479534	noncoding
0.999637	coding
1	coding
1	coding
1	coding
1	coding
0.0022532	noncoding
1	coding
0.0382582	noncoding
0.0390531	noncoding
1	coding
0.999917	coding
0.999917	coding
0.999984	coding
0.999261	coding
0.999988	coding
0.999999	coding
1	coding
1	coding
0.999996	coding
0.999968	coding
0.999999	coding

1	coding
0.522218	coding
1	coding
0.241853	noncoding
0.439129	noncoding
0.288751	noncoding
1	coding
1	coding
0.42909	noncoding
0.0846762	noncoding
0.0362017	noncoding
0.521492	coding
0.233175	noncoding
1	coding
0.103638	noncoding
0.999518	coding
1	coding
1	coding
1	coding
0.00718275	noncoding
1	coding
1	coding
0.999074	coding
0.176332	noncoding
1	coding
0.0649814	noncoding
0.459788	noncoding
1	coding
1	coding
0.234093	noncoding
0.774402	coding
0.997904	coding
0.999924	coding
0.796696	coding
0.455072	noncoding
0.478919	noncoding
1	coding
0.999999	coding
0.0265721	noncoding
1	coding
0.226558	noncoding
1	coding
1	coding
1	coding
1	coding
1	coding
0.999984	coding
0.999998	coding
0.0842414	noncoding
0.0820304	noncoding
0.904837	coding
0.0301062	noncoding
0.183147	noncoding
0.345037	noncoding

0.477253	noncoding
0.178394	noncoding
0.0416491	noncoding
0.0668086	noncoding
0.134579	noncoding
0.447098	noncoding
1	coding
0.999135	coding
0.0447295	noncoding
0.0296835	noncoding
0.0975498	noncoding
0.101671	noncoding
0.101648	noncoding
0.0255115	noncoding
0.0683467	noncoding
0.130293	noncoding
0.0566996	noncoding
0.999832	coding
0.0450726	noncoding
0.0382849	noncoding
0.0107833	noncoding
0.182452	noncoding
1	coding
0.196905	noncoding
0.0141478	noncoding
1	coding
0.314133	noncoding
1	coding
0.0404802	noncoding
0.477761	noncoding
0.999997	coding
0.0409088	noncoding
0.028114	noncoding
0.938284	coding
0.00627659	noncoding
0.995839	coding
0.999902	coding
1	coding
1	coding
0.80721	coding
0.768742	coding
1	coding
0.0432101	noncoding
0.0344063	noncoding
0.999963	coding
0.106604	noncoding
0.13926	noncoding
0.998319	coding
0.992724	coding
0.995215	coding
0.0475517	noncoding
0.12564	noncoding
0.999948	coding
0.00191882	noncoding

1	coding
0.269778	noncoding
1	coding
0.0213842	noncoding
0.0204883	noncoding
0.999294	coding
0.553298	coding
1	coding
1	coding
0.311559	noncoding
1	coding
1	coding
1	coding
0.0334252	noncoding
0.592958	coding
0.0262381	noncoding
1	coding
0.394155	noncoding
1	coding
0.999989	coding
0.951422	coding
0.969121	coding
0.999998	coding
1	coding
1	coding
1	coding
1	coding
0.26185	noncoding
0.192927	noncoding
0.0449582	noncoding
0.772083	coding
0.493814	noncoding
1	coding
1	coding
1	coding
0.943316	coding
1	coding
1	coding
1	coding
0.0517693	noncoding
0.0655963	noncoding
0.646037	coding
0.443144	noncoding
0.592808	coding
0.497624	noncoding
1	coding
0.99997	coding
0.0674921	noncoding
0.930413	coding
0.990872	coding
0.0914481	noncoding
1	coding
0.999982	coding
1	coding

1	coding
1	coding
0.0830878	noncoding
1	coding
0.98338	coding
1	coding
0.050455	noncoding
0.889376	coding
0.0948667	noncoding
0.182184	noncoding
0.377705	noncoding
0.0849362	noncoding
0.551124	coding
0.648035	coding
0.166523	noncoding
0.491596	noncoding
0.0431077	noncoding
0.284652	noncoding
0.999851	coding
0.727537	coding
0.99388	coding
0.982999	coding
0.871085	coding
1	coding
1	coding
0.00728678	noncoding
1	coding
0.272847	noncoding
0.21867	noncoding
1	coding
0.31892	noncoding
0.591003	coding
0.0422367	noncoding
0.199444	noncoding
0.0933819	noncoding
0.0359609	noncoding
1	coding
1	coding
1	coding
1	coding
1	coding
1	coding
1	coding
1	coding
0.999994	coding
0.999707	coding
0.0201235	noncoding
0.286164	noncoding
0.16515	noncoding
0.999011	coding
0.999998	coding
1	coding
0.999986	coding
1	coding

0.150558	noncoding
0.999998	coding
1	coding
0.99999	coding
1	coding
0.999995	coding
1	coding
1	coding
1	coding
0.0162639	noncoding
1	coding
1	coding
0.399152	noncoding
1	coding
1	coding
1	coding
1	coding
0.101408	noncoding
0.149124	noncoding
0.156048	noncoding
0.0589713	noncoding
0.581778	coding
0.0444119	noncoding
0.619529	coding
0.717528	coding
0.13606	noncoding
0.32215	noncoding
0.889446	coding
0.791471	coding
0.718006	coding
0.968249	coding
0.691193	coding
0.664135	coding
0.0726073	noncoding
0.145445	noncoding
0.839848	coding
0.991727	coding
0.998317	coding
0.017656	noncoding
0.139117	noncoding
0.999983	coding
1	coding
1	coding
0.0209319	noncoding
0.999988	coding
0.999999	coding
0.999568	coding
1	coding
1	coding
1	coding
1	coding
1	coding
0.999998	coding
1	coding

1	coding
0.821386	coding
1	coding
1	coding
1	coding
1	coding
0.511525	coding
0.52664	coding
0.237285	noncoding
0.997489	coding
0.999959	coding
1	coding
1	coding
1	coding
1	coding
1	coding
1	coding
1	coding
0.551	coding
1	coding
0.979157	coding
0.165351	noncoding
1	coding
0.937575	coding
0.896177	coding
0.00630757	noncoding
1	coding
1	coding
1	coding
1	coding
1	coding
0.0502363	noncoding
1	coding
0.0467435	noncoding
0.823617	coding
0.0918202	noncoding
0.689338	coding
0.999997	coding
0.0176656	noncoding
1	coding
1	coding
1	coding
0.643219	coding
0.426668	noncoding
0.43201	noncoding
0.581981	coding
0.781909	coding
1	coding
0.999997	coding
0.954279	coding
1	coding
1	coding
1	coding
0.104436	noncoding

1	coding
0.0374192	noncoding
0.0145095	noncoding
0.00512633	noncoding
0.0102169	noncoding
0.316172	noncoding
0.054407	noncoding
0.999091	coding
0.055597	noncoding
1	coding
0.453456	noncoding
0.242985	noncoding
0.135205	noncoding
1	coding
0.999992	coding
0.973837	coding
0.981506	coding
0.921503	coding
0.335371	noncoding
1	coding
1	coding
1	coding
1	coding
1	coding
0.165441	noncoding
0.00434792	noncoding
0.999998	coding
0.999998	coding
1	coding
1	coding
1	coding
1	coding
1	coding
1	coding
0.641131	coding
1	coding
1	coding
1	coding
1	coding
0.721395	coding
1	coding
1	coding
0.948108	coding
1	coding
0.999999	coding
1	coding
1	coding
1	coding
1	coding
1	coding
1	coding
0.999192	coding
0.0209341	noncoding
0.319594	noncoding

1	coding
1	coding
1	coding
0.062334	noncoding
0.354719	noncoding
1	coding
0.0462962	noncoding
1	coding
1	coding
0.575548	coding
1	coding
1	coding
1	coding
0.0136958	noncoding
1	coding
1	coding
1	coding
1	coding
0.865835	coding
1	coding
0.767089	coding
0.999221	coding
0.999985	coding
1	coding
1	coding
0.942334	coding
1	coding
1	coding
0.954979	coding
1	coding
0.997063	coding
1	coding
0.991636	coding
0.997193	coding
0.125388	noncoding
1	coding
0.239546	noncoding
0.0109579	noncoding
0.116922	noncoding
0.019204	noncoding
0.999711	coding
0.998366	coding
0.86475	coding
1	coding
0.998667	coding
1	coding
1	coding
1	coding
0.0227233	noncoding
1	coding
1	coding
1	coding
1	coding
0.999999	coding

1	coding
1	coding
1	coding
1	coding
0.997374	coding
0.0696345	noncoding
0.999893	coding
0.999953	coding
1	coding
0.558398	coding
0.158993	noncoding
1	coding
1	coding
0.871432	coding
0.277743	noncoding
0.0150309	noncoding
0.0579235	noncoding
0.120261	noncoding
1	coding
0.214352	noncoding
0.999495	coding
1	coding
1	coding
0.0275743	noncoding
0.9999	coding
0.196407	noncoding
1	coding
1	coding
0.9997	coding
1	coding
0.997735	coding
0.520859	coding
0.510963	coding
0.388991	noncoding
0.654501	coding
1	coding
1	coding
1	coding
1	coding
1	coding
0.999999	coding
0.200825	noncoding
1	coding
1	coding
0.994091	coding
0.360524	noncoding
0.0895835	noncoding
0.999999	coding
0.951996	coding
1	coding
1	coding
1	coding
1	coding
1	coding

0.0457074	noncoding
1	coding
1	coding
0.0258723	noncoding
0.0735874	noncoding
1	coding
0.0243659	noncoding
0.00863893	noncoding
0.00506962	noncoding
1	coding
1	coding
1	coding
1	coding
1	coding
0.999999	coding
0.504685	coding
0.999333	coding
1	coding
0.999993	coding
0.999973	coding
0.998781	coding
0.36772	noncoding
0.218731	noncoding
0.999996	coding
0.0293117	noncoding
0.0313938	noncoding
1	coding
0.0229458	noncoding
0.0216577	noncoding
0.0108725	noncoding
0.136065	noncoding
0.999999	coding
0.17213	noncoding
0.131466	noncoding
0.153972	noncoding
0.118413	noncoding
0.0495619	noncoding
1	coding
1	coding
0.0501964	noncoding
1	coding
0.0533734	noncoding
0.0280488	noncoding
1	coding
0.121443	noncoding
1	coding
0.102321	noncoding
0.425646	noncoding
1	coding
0.520553	coding
1	coding
1	coding
1	coding
1	coding

0.728183	coding
0.0262767	noncoding
0.391591	noncoding
0.906931	coding
0.716056	coding
1	coding
1	coding
1	coding
1	coding
1	coding
1	coding
1	coding
1	coding
1	coding
1	coding
1	coding
1	coding
0.804204	coding
1	coding
1	coding
0.999982	coding
0.15356	noncoding
1	coding
1	coding
1	coding
1	coding
1	coding
1	coding
1	coding
0.256693	noncoding
0.474984	noncoding
0.400139	noncoding
0.362256	noncoding
0.59424	coding
0.897077	coding
1	coding
0.241868	noncoding
0.323967	noncoding
1	coding
1	coding
0.961687	coding
0.991219	coding
1	coding
1	coding
1	coding
1	coding
0.014917	noncoding
0.456911	noncoding
1	coding
1	coding
1	coding
1	coding
1	coding
1	coding

1	coding
1	coding
1	coding
1	coding
1	coding
1	coding
1	coding
1	coding
0.998543	coding
0.195973	noncoding
0.962018	coding
0.999994	coding
0.464665	noncoding
0.999618	coding
0.0778562	noncoding
0.999722	coding
0.999987	coding
0.999988	coding
0.0379191	noncoding
0.123062	noncoding
0.117242	noncoding
0.0163199	noncoding
0.211471	noncoding
1	coding
0.169462	noncoding
0.0823371	noncoding
1	coding
0.173231	noncoding
0.9943	coding
1	coding
1	coding
0.0838367	noncoding
0.0206285	noncoding
0.0319969	noncoding
0.0424062	noncoding
0.0235074	noncoding
0.0335542	noncoding
0.181902	noncoding
0.15452	noncoding
1	coding
1	coding
1	coding
1	coding
1	coding
1	coding
1	coding
0.994972	coding
0.761713	coding
1	coding
0.998795	coding
1	coding
1	coding
1	coding
1	coding

1	coding
0.999495	coding
1	coding
0.116114	noncoding
0.99919	coding
1	coding
0.022966	noncoding
0.999065	coding
1	coding
0.020064	noncoding
0.0140698	noncoding
0.94218	coding
1	coding
0.982488	coding
0.704425	coding
0.133224	noncoding
0.0973339	noncoding
0.317829	noncoding
0.808715	coding
0.455252	noncoding
0.313081	noncoding
0.187286	noncoding
1	coding
0.998982	coding
0.999784	coding
1	coding
1	coding
0.169647	noncoding
0.430623	noncoding
0.999989	coding
1	coding
0.999598	coding
1	coding
1	coding
1	coding
1	coding
1	coding
0.0620505	noncoding
0.0913661	noncoding
0.0862594	noncoding
0.195207	noncoding
0.0159216	noncoding
0.13511	noncoding
1	coding
0.695819	coding
0.90896	coding
0.0378216	noncoding
0.9448	coding
0.112513	noncoding
0.216839	noncoding
0.997835	coding
0.997862	coding
0.110978	noncoding
0.915569	coding

0.0666345	noncoding
0.425503	noncoding
0.132387	noncoding
0.49774	noncoding
0.264912	noncoding
0.999999	coding
1	coding
1	coding
1	coding
1	coding
1	coding
0.114174	noncoding
0.566394	coding
0.35184	noncoding
0.0436465	noncoding
0.0143969	noncoding
0.0835187	noncoding
1	coding
0.0119863	noncoding
0.0268521	noncoding
0.00972953	noncoding
1	coding
0.0719295	noncoding
0.999957	coding
0.22074	noncoding
1	coding
0.0528611	noncoding
1	coding
0.170326	noncoding
0.133797	noncoding
0.0647273	noncoding
0.258461	noncoding
0.0936513	noncoding
0.150932	noncoding
0.0266111	noncoding
0.011891	noncoding
0.126613	noncoding
1	coding
1	coding
0.787517	coding
0.0586222	noncoding
0.0247201	noncoding
0.0687612	noncoding
0.0136063	noncoding
0.0921983	noncoding
0.878819	coding
0.0180482	noncoding
0.177249	noncoding
1	coding
0.0773733	noncoding
0.0533399	noncoding
0.431516	noncoding
1	coding
0.292835	noncoding

1	coding
1	coding
1	coding
1	coding
1	coding
0.0739049	noncoding
0.0448478	noncoding
1	coding
1	coding
1	coding
1	coding
1	coding
1	coding
0.0974119	noncoding
1	coding
1	coding
0.876443	coding
0.0193325	noncoding
1	coding
0.999989	coding
0.0780208	noncoding
0.999848	coding
0.103276	noncoding
0.0492761	noncoding
1	coding
1	coding
0.018641	noncoding
0.999732	coding
0.495553	noncoding
0.707601	coding
1	coding
1	coding
0.0249094	noncoding
0.241196	noncoding
0.972363	coding
0.999992	coding
0.134734	noncoding
0.308808	noncoding
0.999963	coding
0.944154	coding
1	coding
1	coding
1	coding
1	coding
1	coding
1	coding
1	coding
1	coding
1	coding
0.521051	coding
0.999563	coding
0.999905	coding
0.0200305	noncoding
1	coding

1	coding
1	coding
1	coding
1	coding
1	coding
1	coding
0.0561721	noncoding
0.683203	coding
1	coding
0.139444	noncoding
0.999964	coding
0.999992	coding
0.0159465	noncoding
0.872932	coding
0.122933	noncoding
0.637839	coding
0.999997	coding
1	coding
0.0402823	noncoding
0.141667	noncoding
0.0230543	noncoding
1	coding
1	coding
1	coding
0.133339	noncoding
0.463943	noncoding
0.402278	noncoding
0.0413581	noncoding
0.402278	noncoding
0.403087	noncoding
0.17347	noncoding
0.985712	coding
0.946376	coding
0.0307866	noncoding
1	coding
0.0615971	noncoding
1	coding
0.999998	coding
1	coding
1	coding
1	coding
1	coding
1	coding
0.438673	noncoding
0.887377	coding
0.533654	coding
0.999701	coding
0.538975	coding
0.957514	coding
0.442936	noncoding
0.121774	noncoding
0.0746786	noncoding
1	coding
1	coding

1	coding
1	coding
1	coding
1	coding
1	coding
1	coding
1	coding
1	coding
1	coding
1	coding
1	coding
1	coding
1	coding
0.177209	noncoding
0.0596897	noncoding
0.0274325	noncoding
1	coding
1	coding
1	coding
1	coding
1	coding
1	coding
1	coding
1	coding
0.0242226	noncoding
0.945037	coding
0.288084	noncoding
0.36953	noncoding
0.102399	noncoding
0.168617	noncoding
0.0968244	noncoding
0.132661	noncoding
0.206674	noncoding
1	coding
1	coding
1	coding
0.996172	coding
0.999999	coding
1	coding
0.0917576	noncoding
0.752252	coding
0.910976	coding
0.0223697	noncoding
1	coding
1	coding
0.0872863	noncoding
1	coding
1	coding
0.182987	noncoding
0.155534	noncoding
1	coding
1	coding
0.439808	noncoding
0.711797	coding

1	coding
0.999997	coding
0.999251	coding
0.0659139	noncoding
1	coding
1	coding
0.999999	coding
0.984939	coding
1	coding
0.0390794	noncoding
1	coding
1	coding
1	coding
1	coding
0.997637	coding
0.00380336	noncoding
0.0403495	noncoding
0.362323	noncoding
0.330357	noncoding
0.355314	noncoding
0.908746	coding
0.727809	coding
0.34561	noncoding
0.919894	coding
0.449476	noncoding
0.470638	noncoding
1	coding
1	coding
1	coding
1	coding
1	coding
0.999995	coding
0.992884	coding
0.290971	noncoding
1	coding
1	coding
1	coding
1	coding
1	coding
1	coding
1	coding
0.548935	coding
0.999986	coding
0.943829	coding
0.0167166	noncoding
0.871072	coding
0.871072	coding
0.965291	coding
0.937571	coding
0.875907	coding
0.389743	noncoding
1	coding
0.999995	coding
0.999975	coding

0.999999	coding
0.999996	coding
0.999413	coding
1	coding
1	coding
1	coding
1	coding
1	coding
1	coding
1	coding
1	coding
1	coding
1	coding
0.040744	noncoding
1	coding
1	coding
0.305908	noncoding
0.315278	noncoding
0.327747	noncoding
1	coding
1	coding
1	coding
0.181291	noncoding
0.709685	coding
0.999998	coding
0.630082	coding
0.40903	noncoding
0.0337631	noncoding
1	coding
0.999999	coding
0.743753	coding
1	coding
1	coding
1	coding
1	coding
0.67951	coding
0.073468	noncoding
0.0117649	noncoding
1	coding
0.880927	coding
1	coding
0.441	noncoding
0.791608	coding
1	coding
0.0773699	noncoding
0.0849444	noncoding
0.999993	coding
0.0891232	noncoding
0.0790755	noncoding
1	coding
1	coding
0.99996	coding
0.195501	noncoding
0.021787	noncoding

1	coding
1	coding
0.999996	coding
1	coding
1	coding
1	coding
0.981948	coding
0.592217	coding
0.751926	coding
0.849111	coding
1	coding
1	coding
1	coding
0.0303701	noncoding
1	coding
0.999898	coding
0.999973	coding
1	coding
1	coding
1	coding
0.6314	coding
1	coding
1	coding
0.00995906	noncoding
0.136895	noncoding
1	coding
1	coding
1	coding
1	coding
1	coding
1	coding
1	coding
1	coding
1	coding
1	coding
1	coding
1	coding
1	coding
0.999997	coding
0.0330556	noncoding
0.979645	coding
0.979945	coding
0.994117	coding
0.23032	noncoding
0.267631	noncoding
0.0498913	noncoding
0.999712	coding
0.990245	coding
1	coding
1	coding
0.0534735	noncoding
0.117528	noncoding
0.180441	noncoding
0.353689	noncoding

0.96982	coding
1	coding
1	coding
1	coding
0.999994	coding
0.999889	coding
1	coding
1	coding
1	coding
1	coding
0.996733	coding
0.164536	noncoding
0.999952	coding
0.999999	coding
1	coding
1	coding
0.913299	coding
0.884109	coding
0.360475	noncoding
0.254207	noncoding
0.56432	coding
0.56432	coding
0.247871	noncoding
0.491975	noncoding
0.262162	noncoding
0.247871	noncoding
0.247871	noncoding
0.247871	noncoding
0.247871	noncoding
0.262162	noncoding
0.263248	noncoding
0.281989	noncoding
0.173372	noncoding
0.212973	noncoding
0.191641	noncoding
0.193144	noncoding
0.369817	noncoding
0.116737	noncoding
0.112572	noncoding
0.999663	coding
0.999999	coding
0.119201	noncoding
1	coding
0.467291	noncoding
1	coding
1	coding
1	coding
0.061074	noncoding
0.999509	coding
0.99998	coding
1	coding
1	coding
0.0312959	noncoding
0.0851894	noncoding

0.116163	noncoding
0.057502	noncoding
1	coding
1	coding
1	coding
0.0416956	noncoding
0.32946	noncoding
0.530916	coding
0.0820689	noncoding
1	coding
0.121639	noncoding
0.999965	coding
0.974703	coding
0.974324	coding
0.130232	noncoding
0.663405	coding
0.47525	noncoding
0.999997	coding
0.0209486	noncoding
0.91279	coding
0.0217642	noncoding
0.299717	noncoding
0.138091	noncoding
0.990734	coding
0.100516	noncoding
1	coding
1	coding
1	coding
1	coding
1	coding
1	coding
1	coding
0.999753	coding
1	coding
1	coding
0.817935	coding
0.684083	coding
0.426671	noncoding
0.562904	coding
0.139206	noncoding
1	coding
0.0429094	noncoding
0.387855	noncoding
0.739719	coding
0.458406	noncoding
0.0980784	noncoding
0.080059	noncoding
1	coding
0.999729	coding
0.999997	coding
0.999578	coding
1	coding
1	coding
0.816795	coding

1	coding
1	coding
0.819756	coding
0.959337	coding
0.0906742	noncoding
0.870394	coding
0.808498	coding
0.974538	coding
0.0168571	noncoding
1	coding
1	coding
0.563841	coding
1	coding
1	coding
1	coding
1	coding
1	coding
1	coding
0.102922	noncoding
0.966525	coding
0.0990988	noncoding
0.0694471	noncoding
0.186815	noncoding
0.974017	coding
0.974017	coding
0.997754	coding
1	coding
1	coding
1	coding
1	coding
0.141509	noncoding
0.188982	noncoding
0.999325	coding
1	coding
0.997592	coding
0.00793823	noncoding
0.353453	noncoding
0.00328847	noncoding
0.00311759	noncoding
0.00324258	noncoding
0.00457985	noncoding
0.00486719	noncoding
0.00246972	noncoding
0.999999	coding
0.00831472	noncoding
0.999942	coding
0.956995	coding
0.964859	coding
0.0370093	noncoding
0.321334	noncoding
0.278131	noncoding
0.992052	coding
0.838998	coding
0.999999	coding

0.999999	coding
0.999993	coding
1	coding
0.461366	noncoding
0.346105	noncoding
0.298888	noncoding
0.408175	noncoding
0.886932	coding
1	coding
1	coding
1	coding
0.528703	coding
0.349953	noncoding
1	coding
0.015561	noncoding
0.0169163	noncoding
1	coding
0.061236	noncoding
0.0343221	noncoding
0.850535	coding
0.625752	coding
0.999997	coding
1	coding
0.999999	coding
0.999998	coding
1	coding
1	coding
0.210453	noncoding
1	coding
0.568471	coding
1	coding
1	coding
1	coding
1	coding
1	coding
1	coding
1	coding
1	coding
0.746547	coding
1	coding
1	coding
0.999807	coding
1	coding
1	coding
1	coding
1	coding
0.999998	coding
0.999998	coding
0.999998	coding
0.999998	coding
1	coding
1	coding
1	coding
1	coding

0.372465	noncoding
0.294887	noncoding
0.90152	coding
0.0324248	noncoding
0.151167	noncoding
0.99994	coding
1	coding
1	coding
1	coding
1	coding
1	coding
0.999993	coding
1	coding
1	coding
0.256384	noncoding
0.985589	coding
0.881247	coding
0.423535	noncoding
0.978745	coding
0.99975	coding
1	coding
1	coding
0.99227	coding
1	coding
0.138657	noncoding
0.184539	noncoding
0.0180545	noncoding
0.014894	noncoding
0.122639	noncoding
0.0888083	noncoding
1	coding
1	coding
1	coding
0.920496	coding
1	coding
1	coding
1	coding
0.0243578	noncoding
1	coding
1	coding
1	coding
1	coding
1	coding
1	coding
1	coding
1	coding
1	coding
0.995431	coding
1	coding
1	coding
1	coding
0.0604148	noncoding
0.962258	coding
0.999639	coding

0.999815	coding
0.999731	coding
1	coding
0.189393	noncoding
0.265338	noncoding
0.9007	coding
0.130288	noncoding
0.0676648	noncoding
0.140569	noncoding
0.758251	coding
0.372881	noncoding
0.239501	noncoding
1	coding
0.0790035	noncoding
0.876391	coding
0.050039	noncoding
1	coding
1	coding
1	coding
1	coding
1	coding
1	coding
1	coding
1	coding
0.222803	noncoding
0.00447831	noncoding
1	coding
0.999954	coding
1	coding
0.952453	coding
0.999961	coding
0.995317	coding
1	coding
0.999732	coding
0.0502121	noncoding
0.120448	noncoding
0.0381128	noncoding
0.794252	coding
1	coding
0.0360538	noncoding
0.0332766	noncoding
0.413541	noncoding
0.173367	noncoding
0.131382	noncoding
1	coding
0.107853	noncoding
1	coding
0.0234976	noncoding
0.0244168	noncoding
0.0786189	noncoding
0.122642	noncoding
0.322441	noncoding
0.322441	noncoding
0.0441364	noncoding

0.0628507	noncoding
0.0197274	noncoding
0.0256742	noncoding
0.999999	coding
1	coding
0.999999	coding
1	coding
0.0831749	noncoding
0.998829	coding
1	coding
0.096873	noncoding
1	coding
1	coding
0.0467806	noncoding
0.179055	noncoding
0.0117819	noncoding
0.0104532	noncoding
0.429087	noncoding
0.422276	noncoding
1	coding
1	coding
1	coding
0.644086	coding
0.565131	coding
1	coding
1	coding
1	coding
0.983733	coding
0.965686	coding
0.985618	coding
0.0283813	noncoding
0.83363	coding
0.999915	coding
0.440559	noncoding
0.0600681	noncoding
0.999994	coding
0.742619	coding
0.172317	noncoding
0.304213	noncoding
0.169949	noncoding
1	coding
0.0583332	noncoding
0.0575159	noncoding
0.0583332	noncoding
0.378924	noncoding
1	coding
1	coding
1	coding
1	coding
1	coding
1	coding
1	coding
0.351148	noncoding
1	coding

1	coding
0.300936	noncoding
0.417435	noncoding
0.673482	coding
0.240542	noncoding
0.514582	coding
0.0605296	noncoding
0.224382	noncoding
0.0537766	noncoding
0.0202387	noncoding
0.0146687	noncoding
0.0393807	noncoding
0.0734137	noncoding
0.0826113	noncoding
0.0149551	noncoding
1	coding
1	coding
1	coding
1	coding
1	coding
1	coding
0.543179	coding
0.554701	coding
0.101513	noncoding
0.521865	coding
1	coding
0.99956	coding
0.749034	coding
1	coding
1	coding
0.23989	noncoding
0.179557	noncoding
0.997951	coding
0.997133	coding
0.999965	coding
1	coding
1	coding
1	coding
0.387842	noncoding
0.999998	coding
0.0628619	noncoding
0.613523	coding
0.0565906	noncoding
0.28164	noncoding
0.0692969	noncoding
0.400059	noncoding
0.0228582	noncoding
1	coding
0.378983	noncoding
0.289157	noncoding
0.960069	coding
0.0294128	noncoding
0.347252	noncoding
0.353663	noncoding

0.952494	coding
1	coding
1	coding
1	coding
1	coding
1	coding
1	coding
1	coding
0.999988	coding
1	coding
0.999842	coding
0.0617373	noncoding
1	coding
0.999684	coding
0.0706955	noncoding
0.0389309	noncoding
1	coding
1	coding
1	coding
1	coding
1	coding
1	coding
1	coding
0.0312344	noncoding
0.0479702	noncoding
0.034335	noncoding
0.202073	noncoding
1	coding
1	coding
1	coding
0.677194	coding
1	coding
0.999884	coding
0.999964	coding
1	coding
1	coding
0.99985	coding
0.381076	noncoding
0.0397104	noncoding
0.128994	noncoding
0.0448038	noncoding
0.306211	noncoding
0.973872	coding
0.977204	coding
0.973962	coding
0.0217673	noncoding
1	coding
0.999999	coding
0.935178	coding
1	coding
1	coding
1	coding
1	coding
0.76636	coding

0.912145	coding
0.999781	coding
0.839037	coding
0.999982	coding
0.999966	coding
0.0800826	noncoding
0.705368	coding
0.658904	coding
0.927304	coding
1	coding
0.7873	coding
0.999999	coding
0.999999	coding
0.999996	coding
0.999997	coding
1	coding
1	coding
1	coding
1	coding
1	coding
1	coding
1	coding
1	coding
1	coding
0.234738	noncoding
0.996809	coding
0.997957	coding
0.996139	coding
0.979413	coding
0.97149	coding
1	coding
1	coding
0.999325	coding
1	coding
1	coding
1	coding
1	coding
1	coding
1	coding
1	coding
1	coding
1	coding
0.0569718	noncoding
0.0293106	noncoding
1	coding
1	coding
0.999089	coding
0.999089	coding
0.0866532	noncoding
0.467212	noncoding
0.988559	coding
1	coding
1	coding
1	coding

0.259005	noncoding
0.999566	coding
1	coding
0.281954	noncoding
1	coding
0.92591	coding
1	coding
0.0748866	noncoding
1	coding
1	coding
0.999994	coding
0.166645	noncoding
0.408957	noncoding
0.999999	coding
1	coding
1	coding
1	coding
1	coding
0.999971	coding
1	coding
1	coding
1	coding
0.992832	coding
0.999993	coding
0.682523	coding
1	coding
0.781024	coding
0.600508	coding
0.999837	coding
1	coding
1	coding
1	coding
1	coding
1	coding
1	coding
1	coding
1	coding
0.285971	noncoding
1	coding
1	coding
1	coding
1	coding
1	coding
0.0238828	noncoding
0.14595	noncoding
0.658714	coding
0.521896	coding
1	coding
1	coding
1	coding
1	coding
1	coding
0.99337	coding
0.999977	coding

1	coding
0.992318	coding
0.986825	coding
0.990123	coding
0.903285	coding
0.999247	coding
0.999999	coding
1	coding
0.999996	coding
1	coding
1	coding
1	coding
1	coding
0.965779	coding
0.0159705	noncoding
0.260926	noncoding
0.184744	noncoding
1	coding
1	coding
1	coding
1	coding
1	coding
0.0178406	noncoding
0.250399	noncoding
0.277225	noncoding
1	coding
1	coding
0.131116	noncoding
1	coding
0.0941588	noncoding
0.262672	noncoding
0.0115738	noncoding
1	coding
1	coding
0.0237064	noncoding
0.744449	coding
0.0931035	noncoding
0.134705	noncoding
0.0107332	noncoding
0.999999	coding
0.999999	coding
0.999998	coding
1	coding
1	coding
1	coding
1	coding
0.434859	noncoding
0.0446404	noncoding
0.999933	coding
0.0195386	noncoding
1	coding
0.103615	noncoding
0.265393	noncoding
1	coding

0.999999	coding
1	coding
1	coding
0.0563048	noncoding
0.339355	noncoding
1	coding
1	coding
0.0458859	noncoding
0.0466688	noncoding
1	coding
1	coding
0.0152847	noncoding
0.0255365	noncoding
0.999207	coding
0.555719	coding
0.649828	coding
0.675565	coding
1	coding
1	coding
1	coding
1	coding
1	coding
1	coding
0.530064	coding
1	coding
1	coding
1	coding
1	coding
1	coding
0.0521974	noncoding
0.0689855	noncoding
0.0672398	noncoding
0.214463	noncoding
0.999262	coding
1	coding
0.912776	coding
0.989833	coding
0.855231	coding
0.586048	coding
0.163116	noncoding
0.163116	noncoding
0.197607	noncoding
0.455604	noncoding
0.0979277	noncoding
1	coding
1	coding
0.999947	coding
0.0930003	noncoding
0.116731	noncoding
0.999942	coding
1	coding
0.0205887	noncoding
0.550461	coding
0.385485	noncoding

0.386115	noncoding
0.504216	coding
0.386468	noncoding
0.3032	noncoding
0.139046	noncoding
0.999997	coding
0.999998	coding
0.0178397	noncoding
0.987653	coding
0.814497	coding
0.814497	coding
0.939633	coding
1	coding
0.627373	coding
0.996413	coding
0.992912	coding
0.204178	noncoding
1	coding
1	coding
1	coding
0.958747	coding
0.968644	coding
0.926382	coding
0.942783	coding
0.535566	coding
1	coding
0.0518252	noncoding
0.999967	coding
1	coding
0.992563	coding
0.999991	coding
0.979183	coding
0.140525	noncoding
1	coding
1	coding
0.999984	coding
0.0433226	noncoding
1	coding
0.999999	coding
0.999999	coding
0.999999	coding
0.017512	noncoding
0.105841	noncoding
1	coding
1	coding
1	coding
0.330913	noncoding
0.401527	noncoding
1	coding
1	coding
1	coding
1	coding
0.0316433	noncoding
1	coding

1	coding
1	coding
0.999978	coding
1	coding
1	coding
0.999193	coding
0.999997	coding
0.999996	coding
0.0906603	noncoding
0.99985	coding
1	coding
0.0585382	noncoding
0.279798	noncoding
0.999861	coding
0.24109	noncoding
0.371408	noncoding
0.198216	noncoding
0.265451	noncoding
0.569345	coding
0.986116	coding
0.99983	coding
1	coding
0.999998	coding
0.999851	coding
0.999999	coding
1	coding
1	coding
1	coding
1	coding
0.201434	noncoding
0.68703	coding
0.711094	coding
0.999978	coding
0.99998	coding
1	coding
1	coding
1	coding
1	coding
1	coding
1	coding
1	coding
0.328562	noncoding
0.999324	coding
0.999713	coding
0.803894	coding
1	coding
1	coding
1	coding
1	coding
1	coding
1	coding
1	coding
1	coding
1	coding

0.776531	coding
0.798693	coding
0.10971	noncoding
0.0625784	noncoding
0.0795139	noncoding
0.0827502	noncoding
1	coding
1	coding
1	coding
1	coding
0.997606	coding
0.999484	coding
0.068705	noncoding
0.114216	noncoding
0.156886	noncoding
0.129543	noncoding
0.674756	coding
1	coding
1	coding
1	coding
0.999995	coding
0.998669	coding
0.999846	coding
0.999792	coding
0.999808	coding
0.999773	coding
0.999685	coding
1	coding
1	coding
1	coding
0.999974	coding
0.40712	noncoding
0.4173	noncoding
0.374601	noncoding
0.124192	noncoding
1	coding
1	coding
1	coding
0.851922	coding
0.996341	coding
0.986044	coding
0.988009	coding
0.0287193	noncoding
0.999907	coding
0.999942	coding
0.991413	coding
1	coding
0.875415	coding
0.866266	coding
0.1627	noncoding
1	coding
0.99934	coding
1	coding
1	coding

1	coding
0.179376	noncoding
1	coding
1	coding
1	coding
1	coding
1	coding
1	coding
1	coding
0.697985	coding
1	coding
0.0828882	noncoding
1	coding
1	coding
0.0266307	noncoding
1	coding
0.999999	coding
0.999999	coding
1	coding
0.140113	noncoding
1	coding
1	coding
0.0769083	noncoding
0.992793	coding
0.999605	coding
1	coding
0.636436	coding
0.110288	noncoding
0.686275	coding
1	coding
1	coding
1	coding
1	coding
1	coding
1	coding
1	coding
0.290753	noncoding
1	coding
1	coding
1	coding
0.141077	noncoding
1	coding
1	coding
0.940151	coding
0.0730724	noncoding
1	coding
0.999999	coding
1	coding
0.883025	coding
0.941862	coding
1	coding
1	coding
1	coding
1	coding

0.605026	coding
1	coding
1	coding
0.999996	coding
1	coding
1	coding
1	coding
1	coding
1	coding
1	coding
1	coding
1	coding
1	coding
0.938203	coding
1	coding
0.997115	coding
0.963325	coding
0.961761	coding
0.963552	coding
1	coding
0.999525	coding
0.688761	coding
1	coding
1	coding
1	coding
0.998069	coding
0.994035	coding
0.993078	coding
0.999865	coding
0.999838	coding
0.794741	coding
0.750429	coding
0.693242	coding
1	coding
1	coding
1	coding
1	coding
1	coding
0.999984	coding
1	coding
0.0367982	noncoding
1	coding
1	coding
1	coding
1	coding
0.343061	noncoding
1	coding
1	coding
1	coding
1	coding
1	coding
0.991458	coding
0.799869	coding
0.869066	coding

0.798916	coding
1	coding
1	coding
1	coding
0.238564	noncoding
0.916095	coding
0.930246	coding
1	coding
0.999998	coding
1	coding
1	coding
0.286101	noncoding
1	coding
0.995368	coding
0.91019	coding
1	coding
0.103466	noncoding
0.974287	coding
1	coding
0.418315	noncoding
0.976315	coding
0.999989	coding
0.471873	noncoding
1	coding
0.994014	coding
0.83512	coding
0.53074	coding
0.51162	coding
0.995184	coding
0.76034	coding
0.922004	coding
0.861738	coding
0.999648	coding
0.892021	coding
0.391663	noncoding
0.982813	coding
0.984195	coding
0.0621422	noncoding
0.999325	coding
0.0542782	noncoding
0.953168	coding
0.990314	coding
0.682134	coding
0.964238	coding
0.3614	noncoding
0.357523	noncoding
0.994876	coding
1	coding
1	coding
1	coding
0.999629	coding
1	coding
0.023968	noncoding
0.0665735	noncoding

0.999864	coding
0.485505	noncoding
1	coding
1	coding
1	coding
1	coding
0.122713	noncoding
1	coding
1	coding
1	coding
0.12773	noncoding
1	coding
0.018312	noncoding
0.0892513	noncoding
0.971739	coding
0.998203	coding
0.999818	coding
0.99727	coding
0.0256921	noncoding
0.22135	noncoding
0.240694	noncoding
1	coding
1	coding
0.741741	coding
1	coding
1	coding
1	coding
1	coding
1	coding
1	coding
0.0360446	noncoding
0.402509	noncoding
1	coding
0.403024	noncoding
1	coding
1	coding
1	coding
1	coding
0.200151	noncoding
1	coding
1	coding
1	coding
0.406704	noncoding
1	coding
1	coding
1	coding
0.0820776	noncoding
0.071773	noncoding
0.071773	noncoding
0.0813217	noncoding
0.0813217	noncoding
0.0813217	noncoding
0.0229856	noncoding
0.0273044	noncoding

0.0312418	noncoding
0.0246879	noncoding
0.0233625	noncoding
0.0273044	noncoding
0.0230831	noncoding
0.577152	coding
1	coding
0.132375	noncoding
0.0377221	noncoding
0.0522973	noncoding
1	coding
1	coding
1	coding
0.0334294	noncoding
1	coding
1	coding
1	coding
1	coding
0.00610733	noncoding
0.591691	coding
0.0117697	noncoding
1	coding
1	coding
1	coding
1	coding
1	coding
1	coding
0.996221	coding
0.692258	coding
0.886964	coding
0.273193	noncoding
0.0343986	noncoding
0.52507	coding
0.477924	noncoding
1	coding
1	coding
1	coding
1	coding
0.999998	coding
1	coding
1	coding
1	coding
0.999953	coding
1	coding
0.999706	coding
1	coding
0.999998	coding
0.999989	coding
1	coding
1	coding
1	coding
1	coding
0.0536293	noncoding
0.999997	coding

1	coding
1	coding
0.999428	coding
0.991165	coding
0.0388737	noncoding
0.0930169	noncoding
0.768187	coding
0.173217	noncoding
0.020672	noncoding
0.0216027	noncoding
0.0135166	noncoding
0.909471	coding
0.0600931	noncoding
0.0918769	noncoding
0.0478642	noncoding
0.962047	coding
0.896196	coding
0.038152	noncoding
0.0456264	noncoding
0.185745	noncoding
0.0257171	noncoding
1	coding
1	coding
1	coding
0.0370355	noncoding
0.0323139	noncoding
0.0928974	noncoding
0.0313202	noncoding
0.0333403	noncoding
1	coding
1	coding
1	coding
0.992546	coding
0.999815	coding
0.999916	coding
0.728503	coding
0.345143	noncoding
0.565241	coding
0.521912	coding
1	coding
1	coding
1	coding
1	coding
1	coding
1	coding
0.235495	noncoding
0.17375	noncoding
1	coding
0.999921	coding
1	coding
1	coding
1	coding
0.373773	noncoding
1	coding

1	coding
0.999562	coding
0.999938	coding
0.997922	coding
0.0409846	noncoding
0.0459497	noncoding
1	coding
1	coding
1	coding
1	coding
0.0132823	noncoding
0.0149939	noncoding
1	coding
1	coding
1	coding
0.998704	coding
0.00741982	noncoding
0.0570007	noncoding
1	coding
1	coding
1	coding
0.999651	coding
0.999933	coding
1	coding
0.999999	coding
0.999999	coding
1	coding
0.174104	noncoding
0.479837	noncoding
1	coding
0.999867	coding
1	coding
1	coding
0.335259	noncoding
0.282256	noncoding
1	coding
0.0593171	noncoding
0.1463	noncoding
0.1463	noncoding
0.999951	coding
0.0941809	noncoding
0.999792	coding
0.984291	coding
0.0554406	noncoding
0.999999	coding
0.999984	coding
0.999969	coding
0.999997	coding
0.0840081	noncoding
1	coding
0.0406759	noncoding
0.998122	coding
0.999974	coding
0.999991	coding

0.995356	coding
0.205624	noncoding
0.108445	noncoding
0.971694	coding
1	coding
1	coding
0.999995	coding
1	coding
0.999997	coding
0.999999	coding
0.999999	coding
0.999822	coding
1	coding
0.999995	coding
0.999993	coding
0.625949	coding
0.624285	coding
0.179135	noncoding
0.31132	noncoding
1	coding
1	coding
0.10163	noncoding
0.99523	coding
0.958766	coding
0.928537	coding
1	coding
1	coding
1	coding
1	coding
0.0488034	noncoding
0.99796	coding
0.744461	coding
1	coding
1	coding
0.171433	noncoding
0.0179848	noncoding
1	coding
0.996816	coding
0.914822	coding
0.00966858	noncoding
1	coding
0.0612324	noncoding
1	coding
1	coding
0.0312515	noncoding
0.104707	noncoding
1	coding
0.999998	coding
0.760069	coding
0.762274	coding
1	coding
1	coding
0.615989	coding
0.916772	coding

0.635794	coding
0.630408	coding
0.925787	coding
0.999812	coding
0.336008	noncoding
0.0785635	noncoding
0.308982	noncoding
0.31395	noncoding
0.162475	noncoding
0.0317219	noncoding
0.635794	coding
0.128563	noncoding
0.182158	noncoding
0.0336518	noncoding
0.173515	noncoding
1	coding
0.999996	coding
1	coding
1	coding
1	coding
1	coding
1	coding
1	coding
0.999999	coding
1	coding
1	coding
1	coding
1	coding
0.201282	noncoding
0.462398	noncoding
0.024423	noncoding
0.0110469	noncoding
0.027669	noncoding
0.0178635	noncoding
0.131697	noncoding
0.725286	coding
0.0599116	noncoding
0.172267	noncoding
0.563398	coding
0.996121	coding
0.506329	coding
0.506329	coding
0.913361	coding
0.990073	coding
0.470757	noncoding
1	coding
0.0273416	noncoding
0.0307781	noncoding
0.073559	noncoding
0.149491	noncoding
1	coding
0.0211823	noncoding
0.0209512	noncoding
0.191903	noncoding

0.0473217	noncoding
0.995571	coding
1	coding
0.996603	coding
0.99997	coding
1	coding
1	coding
1	coding
0.00400636	noncoding
0.107546	noncoding
0.829444	coding
1	coding
1	coding
0.04794	noncoding
0.974742	coding
1	coding
0.140456	noncoding
0.21194	noncoding
0.162874	noncoding
1	coding
1	coding
1	coding
1	coding
1	coding
1	coding
0.994264	coding
0.999885	coding
0.2228	noncoding
1	coding
1	coding
1	coding
0.038999	noncoding
0.132377	noncoding
1	coding
1	coding
1	coding
1	coding
1	coding
0.157421	noncoding
0.174208	noncoding
0.170254	noncoding
0.950398	coding
0.952814	coding
0.957878	coding
0.865756	coding
0.884419	coding
0.868854	coding
1	coding
1	coding
1	coding
1	coding
1	coding
1	coding
0.992817	coding

1	coding
1	coding
1	coding
1	coding
0.71357	coding
1	coding
1	coding
1	coding
1	coding
0.018513	noncoding
0.403265	noncoding
0.305008	noncoding
0.0491052	noncoding
0.0252114	noncoding
0.657546	coding
0.0190184	noncoding
0.97815	coding
0.033398	noncoding
0.999929	coding
0.998403	coding
0.999994	coding
0.999985	coding
0.999992	coding
0.999998	coding
0.042585	noncoding
0.0330288	noncoding
1	coding
0.981388	coding
0.0814889	noncoding
0.99354	coding
1	coding
1	coding
0.270213	noncoding
0.418186	noncoding
0.0582506	noncoding
0.042922	noncoding
1	coding
1	coding
1	coding
1	coding
1	coding
1	coding
1	coding
1	coding
1	coding
1	coding
1	coding
1	coding
1	coding
1	coding
1	coding
1	coding
1	coding
1	coding

0.00596969	noncoding
0.994183	coding
0.142349	noncoding
0.0261873	noncoding
0.109795	noncoding
0.16688	noncoding
1	coding
0.51784	coding
0.324403	noncoding
0.0808772	noncoding
0.999993	coding
0.739991	coding
0.999566	coding
0.0363909	noncoding
1	coding
1	coding
0.959135	coding
0.66715	coding
0.48628	noncoding
1	coding
0.999426	coding
0.999919	coding
0.998565	coding
0.999882	coding
0.967649	coding
0.0111775	noncoding
0.996959	coding
0.054485	noncoding
0.160156	noncoding
1	coding
1	coding
1	coding
1	coding
1	coding
1	coding
1	coding
1	coding
0.988456	coding
0.0683357	noncoding
0.688878	coding
0.856242	coding
0.338974	noncoding
1	coding
1	coding
0.796301	coding
0.017065	noncoding
0.110765	noncoding
0.997173	coding
1	coding
0.999432	coding
0.997008	coding
0.153968	noncoding
1	coding
0.487985	noncoding

0.88676	coding
0.999991	coding
0.171068	noncoding
1	coding
0.997573	coding
1	coding
1	coding
0.977501	coding
1	coding
1	coding
1	coding
0.999992	coding
0.999974	coding
1	coding
1	coding
1	coding
1	coding
0.0571334	noncoding
0.999992	coding
0.999992	coding
1	coding
1	coding
0.146669	noncoding
0.0703538	noncoding
0.999997	coding
0.999995	coding
0.999995	coding
0.998024	coding
0.999999	coding
0.0638904	noncoding
0.419122	noncoding
0.119495	noncoding
1	coding
1	coding
1	coding
1	coding
1	coding
0.353271	noncoding
0.991855	coding
0.273131	noncoding
0.708158	coding
0.625967	coding
0.15448	noncoding
1	coding
0.864442	coding
0.935684	coding
0.996793	coding
0.0785568	noncoding
0.999997	coding
0.999652	coding
0.484981	noncoding
0.195529	noncoding
1	coding
1	coding

1	coding
1	coding
0.0319123	noncoding
0.202682	noncoding
1	coding
1	coding
0.91386	coding
1	coding
1	coding
1	coding
1	coding
1	coding
1	coding
1	coding
0.889044	coding
0.67228	coding
0.766845	coding
0.773377	coding
0.999898	coding
0.999735	coding
0.997545	coding
1	coding
0.928897	coding
0.867399	coding
1	coding
0.897649	coding
1	coding
1	coding
0.118278	noncoding
1	coding
0.0531536	noncoding
0.92744	coding
0.999998	coding
0.929828	coding
0.833171	coding
0.826586	coding
0.833171	coding
0.833171	coding
0.981961	coding
1	coding
1	coding
1	coding
1	coding
1	coding
1	coding
1	coding
1	coding
1	coding
0.3299	noncoding
0.999188	coding
1	coding
0.994712	coding
1	coding
0.999987	coding

1	coding
1	coding
1	coding
1	coding
1	coding
1	coding
1	coding
1	coding
1	coding
1	coding
1	coding
1	coding
0.0293504	noncoding
0.00770471	noncoding
1	coding
0.936076	coding
0.916901	coding
0.953597	coding
0.539111	coding
0.980426	coding
0.993304	coding
0.999994	coding
0.999998	coding
0.0732051	noncoding
0.994081	coding
0.993401	coding
0.999996	coding
1	coding
0.1579	noncoding
0.156352	noncoding
0.576236	coding
0.0525093	noncoding
0.395621	noncoding
0.308295	noncoding
1	coding
1	coding
1	coding
0.433604	noncoding
0.0549375	noncoding
0.940903	coding
0.995417	coding
1	coding
0.798869	coding
0.999979	coding
0.374676	noncoding
0.889821	coding
0.0521173	noncoding
0.0343956	noncoding
0.991693	coding
1	coding
1	coding
0.97387	coding
0.885605	coding
0.739948	coding

0.574642	coding
0.936005	coding
0.849446	coding
0.966668	coding
0.931443	coding
0.960556	coding
1	coding
0.999997	coding
1	coding
0.946354	coding
0.177627	noncoding
0.318887	noncoding
0.558552	coding
0.967431	coding
0.241023	noncoding
1	coding
0.999655	coding
0.129264	noncoding
0.0230267	noncoding
0.0298435	noncoding
0.0527408	noncoding
0.268213	noncoding
0.353185	noncoding
0.0215994	noncoding
0.0693914	noncoding
0.142598	noncoding
0.955348	coding
0.040542	noncoding
0.524951	coding
0.831697	coding
0.655591	coding
0.62951	coding
0.978231	coding
0.285616	noncoding
0.139761	noncoding
0.986927	coding
1	coding
1	coding
1	coding
0.93478	coding
1	coding
1	coding
1	coding
0.999424	coding
0.946127	coding
0.999424	coding
0.999424	coding
0.999512	coding
0.999424	coding
0.0733339	noncoding
0.144448	noncoding
0.392111	noncoding
1	coding
1	coding

1	coding
1	coding
1	coding
1	coding
1	coding
1	coding
1	coding
1	coding
1	coding
1	coding
0.999972	coding
0.512534	coding
1	coding
0.03401	noncoding
0.0480962	noncoding
1	coding
1	coding
0.928285	coding
0.807498	coding
0.994781	coding
1	coding
1	coding
0.999998	coding
1	coding
1	coding
0.22956	noncoding
0.0561069	noncoding
0.404295	noncoding
0.0159722	noncoding
0.355778	noncoding
1	coding
1	coding
0.954356	coding
0.976604	coding
0.982803	coding
0.941938	coding
0.902581	coding
0.576996	coding
0.499326	noncoding
0.956515	coding
0.146453	noncoding
0.883246	coding
0.999996	coding
0.999081	coding
0.0547352	noncoding
0.181452	noncoding
0.296625	noncoding
0.325091	noncoding
1	coding
0.60609	coding
1	coding
1	coding
0.0170939	noncoding
1	coding

0.999966	coding
0.983714	coding
1	coding
1	coding
1	coding
1	coding
0.737723	coding
1	coding
0.16286	noncoding
0.160208	noncoding
0.164372	noncoding
0.16086	noncoding
0.161745	noncoding
0.790422	coding
0.45497	noncoding
1	coding
1	coding
0.206485	noncoding
0.999415	coding
0.999993	coding
1	coding
1	coding
0.999999	coding
0.9999	coding
0.997786	coding
0.938637	coding
0.999931	coding
0.999337	coding
0.999941	coding
0.999218	coding
0.999999	coding
0.999841	coding
0.306723	noncoding
0.313143	noncoding
1	coding
0.899496	coding
1	coding
1	coding
1	coding
1	coding
0.0235089	noncoding
1	coding
1	coding
0.0876576	noncoding
1	coding
0.119584	noncoding
1	coding
1	coding
1	coding
1	coding
0.999905	coding
1	coding
1	coding
1	coding

1	coding
1	coding
0.999954	coding
0.320782	noncoding
0.99793	coding
0.983624	coding
0.992432	coding
0.983979	coding
0.984795	coding
0.0969119	noncoding
0.0224909	noncoding
1	coding
1	coding
1	coding
1	coding
1	coding
0.999995	coding
0.698009	coding
0.983823	coding
1	coding
0.999997	coding
1	coding
0.959994	coding
1	coding
1	coding
0.0255885	noncoding
1	coding
0.0179411	noncoding
1	coding
0.917622	coding
0.811984	coding
0.49201	noncoding
0.0126073	noncoding
1	coding
0.0811299	noncoding
0.244448	noncoding
1	coding
1	coding
1	coding
1	coding
1	coding
1	coding
1	coding
1	coding
1	coding
1	coding
1	coding
1	coding
1	coding
1	coding
1	coding
1	coding
0.999935	coding
1	coding

0.999828	coding
1	coding
1	coding
1	coding
1	coding
1	coding
1	coding
1	coding
0.995806	coding
1	coding
1	coding
0.760928	coding
1	coding
0.999485	coding
0.999762	coding
0.996707	coding
1	coding
0.220437	noncoding
1	coding
0.999934	coding
0.384293	noncoding
1	coding
1	coding
0.061139	noncoding
0.24308	noncoding
0.0428629	noncoding
0.99611	coding
0.904023	coding
0.995217	coding
1	coding
1	coding
0.881512	coding
0.999986	coding
1	coding
1	coding
1	coding
0.369827	noncoding
0.999999	coding
0.0819401	noncoding
0.534294	coding
1	coding
0.0170598	noncoding
0.027263	noncoding
1	coding
1	coding
0.999443	coding
0.999828	coding
0.999957	coding
0.999991	coding
0.216336	noncoding
1	coding
0.144822	noncoding
1	coding
0.969921	coding

0.913378	coding
0.98564	coding
0.999961	coding
0.786456	coding
1	coding
0.0125092	noncoding
0.040336	noncoding
0.999999	coding
0.999999	coding
1	coding
1	coding
1	coding
0.999999	coding
0.999976	coding
1	coding
0.999998	coding
1	coding
1	coding
0.999999	coding
0.999693	coding
0.41894	noncoding
0.160831	noncoding
0.297398	noncoding
1	coding
0.00859308	noncoding
0.999906	coding
0.930818	coding
1	coding
1	coding
0.998143	coding
1	coding
1	coding
0.853915	coding
1	coding
1	coding
1	coding
1	coding
1	coding
1	coding
0.016436	noncoding
0.0164993	noncoding
0.994556	coding
0.217143	noncoding
0.29624	noncoding
1	coding
0.285276	noncoding
0.034233	noncoding
0.0827708	noncoding
0.0850993	noncoding
0.0895605	noncoding
0.99853	coding
0.999154	coding
0.010546	noncoding
1	coding

1	coding
1	coding
1	coding
1	coding
1	coding
1	coding
1	coding
1	coding
0.999999	coding
0.535898	coding
0.363809	noncoding
1	coding
1	coding
1	coding
1	coding
1	coding
1	coding
1	coding
1	coding
0.0843716	noncoding
0.247162	noncoding
0.660113	coding
0.354091	noncoding
0.218101	noncoding
1	coding
0.999955	coding
0.999923	coding
0.996903	coding
0.0525246	noncoding
0.999241	coding
1	coding
1	coding
1	coding
1	coding
1	coding
1	coding
1	coding
1	coding
1	coding
1	coding
0.999971	coding
0.999738	coding
0.998412	coding
0.996065	coding
0.838876	coding
0.885225	coding
0.0606025	noncoding
1	coding
0.0459714	noncoding
0.99841	coding
0.0493967	noncoding
0.0367765	noncoding
0.999992	coding
0.977254	coding

0.986458	coding
0.343329	noncoding
0.0761915	noncoding
0.999996	coding
0.99999	coding
1	coding
0.989712	coding
1	coding
1	coding
1	coding
1	coding
0.999959	coding
0.999496	coding
0.999444	coding
0.997972	coding
0.020917	noncoding
1	coding
0.725597	coding
0.11368	noncoding
0.0674241	noncoding
0.0631787	noncoding
0.0764226	noncoding
0.0695654	noncoding
1	coding
1	coding
0.998966	coding
1	coding
1	coding
1	coding
0.522135	coding
1	coding
1	coding
0.904644	coding
1	coding
0.998338	coding
0.894311	coding
1	coding
1	coding
1	coding
1	coding
1	coding
1	coding
1	coding
1	coding
1	coding
1	coding
1	coding
0.215137	noncoding
0.999971	coding
0.999998	coding
1	coding
1	coding
1	coding
1	coding

1	coding
0.999996	coding
0.499667	noncoding
1	coding
1	coding
0.832022	coding
1	coding
0.121968	noncoding
1	coding
1	coding
0.979112	coding
1	coding
1	coding
1	coding
0.999956	coding
1	coding
1	coding
1	coding
1	coding
0.0578278	noncoding
0.780594	coding
0.0873514	noncoding
0.0824783	noncoding
0.258434	noncoding
0.99999	coding
0.885114	coding
0.997571	coding
0.0693212	noncoding
0.0399035	noncoding
0.995662	coding
0.940847	coding
0.0977265	noncoding
1	coding
1	coding
1	coding
0.982061	coding
0.135601	noncoding
0.0673545	noncoding
1	coding
1	coding
1	coding
1	coding
0.187209	noncoding
1	coding
0.99725	coding
0.369919	noncoding
0.368338	noncoding
0.997989	coding
0.537615	coding
0.935627	coding
0.597774	coding
0.674905	coding
0.694532	coding
0.999997	coding

1	coding
1	coding
1	coding
0.98362	coding
1	coding
0.918138	coding
0.936168	coding
1	coding
1	coding
0.1148	noncoding
0.148246	noncoding
0.885544	coding
0.999934	coding
1	coding
0.945841	coding
0.993786	coding
0.846968	coding
0.15683	noncoding
1	coding
1	coding
1	coding
1	coding
1	coding
0.997708	coding
0.18084	noncoding
0.00360055	noncoding
0.01184	noncoding
1	coding
1	coding
0.0310468	noncoding
0.387308	noncoding
1	coding
0.163298	noncoding
1	coding
0.999999	coding
0.0418207	noncoding
0.783792	coding
0.761163	coding
0.783792	coding
0.100192	noncoding
0.0297531	noncoding
0.170469	noncoding
0.999985	coding
0.0571341	noncoding
1	coding
1	coding
1	coding
0.99894	coding
0.999578	coding
0.999673	coding
0.221338	noncoding
0.999817	coding
0.944813	coding
0.872751	coding

0.943429	coding
0.380731	noncoding
0.820515	coding
0.999374	coding
0.999977	coding
1	coding
1	coding
1	coding
0.999724	coding
0.0175732	noncoding
1	coding
1	coding
1	coding
0.183388	noncoding
0.999983	coding
0.0847513	noncoding
0.235287	noncoding
0.258592	noncoding
0.0810306	noncoding
0.0117825	noncoding
0.0329848	noncoding
0.133807	noncoding
0.131841	noncoding
1	coding
0.156102	noncoding
0.0653867	noncoding
1	coding
0.181265	noncoding
0.0356934	noncoding
0.963164	coding
0.153483	noncoding
0.223185	noncoding
0.219264	noncoding
0.224029	noncoding
1	coding
1	coding
0.179046	noncoding
0.215605	noncoding
0.0841194	noncoding
0.941737	coding
1	coding
0.0221391	noncoding
0.975649	coding
0.99458	coding
0.0152926	noncoding
0.0535787	noncoding
0.0187239	noncoding
0.0379531	noncoding
0.543927	coding
1	coding
0.736444	coding
0.176126	noncoding
0.0697464	noncoding
0.2572	noncoding

1	coding
0.197679	noncoding
1	coding
1	coding
1	coding
0.168711	noncoding
0.0487179	noncoding
1	coding
0.987496	coding
1	coding
1	coding
1	coding
0.0175668	noncoding
1	coding
0.991994	coding
1	coding
1	coding
1	coding
0.0324282	noncoding
1	coding
1	coding
0.999673	coding
0.999566	coding
0.00329718	noncoding
0.0180111	noncoding
0.972099	coding
0.607461	coding
0.0559271	noncoding
1	coding
0.196011	noncoding
0.99783	coding
1	coding
1	coding
0.157632	noncoding
1	coding
1	coding
1	coding
1	coding
0.294891	noncoding
0.472315	noncoding
0.205861	noncoding
0.140916	noncoding
1	coding
1	coding
0.274564	noncoding
0.999981	coding
1	coding
1	coding
1	coding
1	coding
0.963725	coding
0.990061	coding
1	coding
1	coding

0.029104	noncoding
1	coding
0.014191	noncoding
0.861503	coding
0.123424	noncoding
1	coding
0.129776	noncoding
0.61196	coding
0.60653	coding
0.0667214	noncoding
0.999978	coding
1	coding
1	coding
1	coding
0.0300779	noncoding
0.999915	coding
1	coding
1	coding
1	coding
0.147548	noncoding
0.9814	coding
0.999997	coding
0.198369	noncoding
0.998515	coding
0.103675	noncoding
0.999955	coding
1	coding
1	coding
1	coding
1	coding
1	coding
0.313969	noncoding
0.599757	coding
0.999995	coding
1	coding
1	coding
0.0188651	noncoding
0.0345001	noncoding
0.999875	coding
1	coding
1	coding
0.991882	coding
0.824102	coding
0.974571	coding
0.973436	coding
0.66257	coding
0.997059	coding
0.687806	coding
0.082153	noncoding
0.835311	coding
0.887361	coding
0.0330535	noncoding
0.990024	coding
0.786637	coding

0.972354	coding
0.990024	coding
0.425373	noncoding
0.969198	coding
0.405984	noncoding
0.449372	noncoding
0.025641	noncoding
0.39637	noncoding
0.188732	noncoding
0.0667972	noncoding
0.0191047	noncoding
0.0202794	noncoding
0.159751	noncoding
0.195101	noncoding
0.243409	noncoding
0.131435	noncoding
0.0892448	noncoding
0.301217	noncoding
0.298675	noncoding
0.31046	noncoding
1	coding
0.999998	coding
1	coding
0.032029	noncoding
0.198687	noncoding
0.359293	noncoding
0.0268735	noncoding
0.110675	noncoding
1	coding
0.999998	coding
0.999998	coding
1	coding
0.206645	noncoding
1	coding
1	coding
1	coding
1	coding
1	coding
1	coding
1	coding
1	coding
1	coding
1	coding
0.425647	noncoding
1	coding
1	coding
1	coding
1	coding
0.963016	coding
1	coding
0.008783	noncoding
1	coding
1	coding
1	coding

0.0931315	noncoding
0.0109699	noncoding
0.012718	noncoding
0.468838	noncoding
0.632262	coding
0.97279	coding
0.99926	coding
0.350819	noncoding
0.372763	noncoding
1	coding
1	coding
1	coding
1	coding
1	coding
1	coding
1	coding
1	coding
0.0261468	noncoding
0.106107	noncoding
0.398472	noncoding
0.79217	coding
0.0227162	noncoding
0.174302	noncoding
0.999949	coding
0.0348634	noncoding
1	coding
0.10625	noncoding
0.0332704	noncoding
0.011068	noncoding
0.154303	noncoding
0.0442033	noncoding
0.999881	coding
0.962584	coding
0.985062	coding
0.622538	coding
1	coding
0.157804	noncoding
0.173287	noncoding
0.0671664	noncoding
0.0760118	noncoding
0.176501	noncoding
0.0389416	noncoding
0.486596	noncoding
0.234115	noncoding
1	coding
0.999634	coding
1	coding
0.0172798	noncoding
0.00379275	noncoding
0.0203849	noncoding
0.00639527	noncoding
0.0180631	noncoding
0.336541	noncoding
0.0735777	noncoding

0.976101	coding
0.551059	coding
0.0250719	noncoding
0.016509	noncoding
0.999981	coding
0.0560204	noncoding
0.700852	coding
0.999724	coding
1	coding
1	coding
0.755798	coding
0.880253	coding
0.999987	coding
1	coding
1	coding
0.0602111	noncoding
0.062601	noncoding
0.795777	coding
0.0774109	noncoding
0.0139947	noncoding
0.0137924	noncoding
0.0628768	noncoding
1	coding
0.561695	coding
1	coding
0.157548	noncoding
1	coding
1	coding
0.449755	noncoding
0.999998	coding
0.773242	coding
0.635208	coding
0.0182205	noncoding
1	coding
0.0267696	noncoding
0.0385025	noncoding
0.549935	coding
0.863988	coding
0.0107193	noncoding
1	coding
1	coding
0.493714	noncoding
0.246129	noncoding
0.0568618	noncoding
0.227329	noncoding
0.830586	coding
0.945718	coding
0.223268	noncoding
0.66139	coding
0.66139	coding
0.165221	noncoding
1	coding
1	coding
1	coding

1	coding
0.328382	noncoding
0.96778	coding
0.777378	coding
0.142357	noncoding
0.0512475	noncoding
0.615186	coding
0.0501539	noncoding
0.522297	coding
0.031874	noncoding
0.17153	noncoding
0.14407	noncoding
0.992174	coding
0.995589	coding
0.84242	coding
0.98582	coding
0.201869	noncoding
0.0345074	noncoding
0.346266	noncoding
0.166607	noncoding
0.119976	noncoding
1	coding
1	coding
0.806761	coding
0.569949	coding
0.999866	coding
1	coding
0.999909	coding
0.999986	coding
0.0237516	noncoding
0.0403803	noncoding
0.999991	coding
1	coding
0.0609744	noncoding
0.223074	noncoding
0.361627	noncoding
0.845666	coding
1	coding
1	coding
1	coding
1	coding
0.156657	noncoding
0.703987	coding
1	coding
1	coding
1	coding
1	coding
0.0425118	noncoding
0.244839	noncoding
0.273934	noncoding
0.126745	noncoding
0.169923	noncoding
0.662211	coding
0.617907	coding

0.416782	noncoding
0.741524	coding
0.10333	noncoding
0.430386	noncoding
0.520581	coding
1	coding
0.0889787	noncoding
0.993855	coding
1	coding
0.999651	coding
0.995888	coding
0.121114	noncoding
0.240212	noncoding
0.165876	noncoding
0.271669	noncoding
0.0637916	noncoding
0.655704	coding
1	coding
1	coding
1	coding
1	coding
1	coding
0.425795	noncoding
1	coding
1	coding
1	coding
1	coding
1	coding
1	coding
0.622982	coding
0.988929	coding
0.171792	noncoding
0.0098702	noncoding
0.0912411	noncoding
0.0391805	noncoding
0.646722	coding
0.514767	coding
0.514767	coding
0.492737	noncoding
0.644512	coding
0.417221	noncoding
0.428986	noncoding
0.444868	noncoding
0.428986	noncoding
0.452595	noncoding
0.99925	coding
0.940841	coding
1	coding
0.0686449	noncoding
0.0577165	noncoding
0.117931	noncoding
0.869274	coding
0.348748	noncoding
0.977495	coding

1	coding
0.197337	noncoding
0.145757	noncoding
0.155648	noncoding
0.0450475	noncoding
0.983535	coding
0.981499	coding
0.921034	coding
0.957509	coding
0.93656	coding
1	coding
0.0153792	noncoding
0.215415	noncoding
1	coding
0.0751097	noncoding
0.029186	noncoding
0.0252679	noncoding
0.021196	noncoding
0.416605	noncoding
0.265567	noncoding
1	coding
1	coding
1	coding
1	coding
1	coding
1	coding
1	coding
1	coding
1	coding
1	coding
1	coding
0.766811	coding
1	coding
0.99997	coding
0.0629339	noncoding
0.371403	noncoding
1	coding
1	coding
1	coding
1	coding
0.804631	coding
0.367753	noncoding
1	coding
0.245864	noncoding
0.186183	noncoding
1	coding
0.999997	coding
1	coding
0.0336953	noncoding
0.104197	noncoding
0.998983	coding
0.126864	noncoding
0.135806	noncoding
0.0417667	noncoding

0.855379	coding
0.0609328	noncoding
1	coding
0.0240692	noncoding
0.929193	coding
0.747929	coding
0.0638965	noncoding
0.97648	coding
0.969905	coding
0.999678	coding
1	coding
0.411272	noncoding
0.0662408	noncoding
0.99984	coding
0.999968	coding
0.999984	coding
1	coding
0.999967	coding
0.999997	coding
0.999268	coding
0.205445	noncoding
0.0911809	noncoding
0.268104	noncoding
1	coding
0.179066	noncoding
1	coding
0.134061	noncoding
0.0587656	noncoding
0.698678	coding
0.972736	coding
1	coding
0.103177	noncoding
1	coding
0.879869	coding
0.992209	coding
0.969258	coding
0.461967	noncoding
0.186645	noncoding
0.720604	coding
0.104291	noncoding
0.338123	noncoding
0.986622	coding
1	coding
1	coding
1	coding
1	coding
0.0689544	noncoding
0.188271	noncoding
0.656165	coding
0.637197	coding
0.840531	coding
0.982041	coding
0.997806	coding
0.867531	coding

0.725662	coding
0.590912	coding
1	coding
1	coding
0.0947237	noncoding
0.0518648	noncoding
0.636349	coding
0.00826509	noncoding
0.89678	coding
0.268824	noncoding
0.0242901	noncoding
0.1772	noncoding
0.128395	noncoding
0.241573	noncoding
0.0124553	noncoding
0.320611	noncoding
0.169655	noncoding
0.265445	noncoding
1	coding
1	coding
0.948153	coding
1	coding
1	coding
1	coding
1	coding
1	coding
1	coding
1	coding
0.999934	coding
0.857986	coding
0.999883	coding
1	coding
1	coding
1	coding
1	coding
1	coding
1	coding
0.161783	noncoding
0.180516	noncoding
0.111204	noncoding
0.376869	noncoding
0.0861204	noncoding
0.113968	noncoding
0.0272893	noncoding
0.0121609	noncoding
0.257921	noncoding
0.0111722	noncoding
1	coding
1	coding
1	coding
0.325831	noncoding
0.623958	coding
0.630788	coding
0.0950082	noncoding

0.996064	coding
0.130896	noncoding
0.409727	noncoding
0.421429	noncoding
0.866183	coding
0.156722	noncoding
1	coding
0.0774508	noncoding
0.111851	noncoding
0.999981	coding
0.382109	noncoding
1	coding
0.999996	coding
1	coding
0.291067	noncoding
0.999993	coding
0.0307298	noncoding
0.70411	coding
0.00943442	noncoding
1	coding
0.0232319	noncoding
0.0171434	noncoding
0.361809	noncoding
0.295911	noncoding
1	coding
1	coding
1	coding
1	coding
0.153151	noncoding
0.165918	noncoding
0.106707	noncoding
0.0340536	noncoding
0.0386861	noncoding
0.0226676	noncoding
0.999957	coding
1	coding
0.112571	noncoding
0.00635593	noncoding
1	coding
0.143394	noncoding
0.145571	noncoding
0.120448	noncoding
0.112307	noncoding
0.027629	noncoding
0.0242581	noncoding
0.073924	noncoding
1	coding
1	coding
1	coding
1	coding
0.999923	coding
1	coding
1	coding
1	coding

0.997817	coding
0.0528599	noncoding
0.114173	noncoding
0.650637	coding
0.59769	coding
0.129974	noncoding
0.956853	coding
0.272068	noncoding
0.788855	coding
1	coding
1	coding
1	coding
0.616816	coding
0.947711	coding
0.999998	coding
0.997485	coding
0.134474	noncoding
1	coding
1	coding
1	coding
1	coding
0.999168	coding
0.999995	coding
0.999995	coding
0.0559424	noncoding
0.344915	noncoding
0.0529266	noncoding
0.802743	coding
0.120717	noncoding
0.907946	coding
0.238912	noncoding
0.999086	coding
0.999739	coding
0.0726174	noncoding
0.708131	coding
0.877691	coding
0.0252513	noncoding
0.0219518	noncoding
1	coding
1	coding
1	coding
0.0181165	noncoding
0.0486137	noncoding
0.0210541	noncoding
0.10027	noncoding
0.999536	coding
1	coding
1	coding
1	coding
1	coding
1	coding
0.135342	noncoding
0.977635	coding
0.157253	noncoding

0.462795	noncoding
0.999997	coding
0.301628	noncoding
0.00874233	noncoding
0.189467	noncoding
0.287263	noncoding
0.0222645	noncoding
0.548055	coding
0.619843	coding
1	coding
1	coding
1	coding
1	coding
0.999856	coding
0.0945449	noncoding
0.821605	coding
0.25113	noncoding
0.0216258	noncoding
0.0250768	noncoding
0.0135035	noncoding
0.115948	noncoding
0.0145394	noncoding
0.0598102	noncoding
0.0215172	noncoding
1	coding
1	coding
1	coding
1	coding
1	coding
1	coding
1	coding
1	coding
1	coding
1	coding
1	coding
0.149584	noncoding
0.98386	coding
0.674094	coding
0.999278	coding
0.0771546	noncoding
0.130428	noncoding
0.904478	coding
0.73087	coding
0.555694	coding
0.875046	coding
0.0166136	noncoding
0.123213	noncoding
0.223975	noncoding
0.700648	coding
0.0367698	noncoding
0.130591	noncoding
0.532504	coding
0.549161	coding
0.798457	coding

0.650777	coding
0.786119	coding
1	coding
1	coding
1	coding
0.151914	noncoding
1	coding
1	coding
1	coding
1	coding
1	coding
1	coding
1	coding
1	coding
1	coding
1	coding
1	coding
1	coding
0.499474	noncoding
0.827766	coding
1	coding
1	coding
1	coding
1	coding
1	coding
0.999999	coding
1	coding
1	coding
1	coding
0.0449652	noncoding
0.999892	coding
1	coding
1	coding
1	coding
0.179568	noncoding
0.0606628	noncoding
0.0388952	noncoding
0.0705975	noncoding
0.774437	coding
0.105124	noncoding
0.855775	coding
0.870477	coding
0.964613	coding
0.0368321	noncoding
1	coding
0.978952	coding
0.386459	noncoding
1	coding
1	coding
0.052211	noncoding
1	coding
1	coding
1	coding
0.283541	noncoding

1	coding
1	coding
0.121483	noncoding
0.230271	noncoding
0.377665	noncoding
0.901231	coding
0.220002	noncoding
0.0176578	noncoding
0.985749	coding
0.972845	coding
0.25588	noncoding
1	coding
1	coding
1	coding
1	coding
1	coding
0.0220188	noncoding
1	coding
0.0272793	noncoding
0.0180251	noncoding
0.309105	noncoding
0.0409868	noncoding
0.259491	noncoding
1	coding
1	coding
0.999998	coding
1	coding
0.999997	coding
0.99999	coding
0.957694	coding
0.179204	noncoding
0.0758299	noncoding
1	coding
1	coding
0.999996	coding
0.999937	coding
1	coding
0.999794	coding
0.99997	coding
0.927282	coding
0.0719338	noncoding
0.00971239	noncoding
0.0117092	noncoding
1	coding
1	coding
1	coding
1	coding
0.0356166	noncoding
0.0857912	noncoding
0.980373	coding
0.973897	coding
0.878761	coding
0.914752	coding
0.962012	coding

0.974835	coding
0.115115	noncoding
0.999989	coding
0.999998	coding
0.139763	noncoding
1	coding
0.992991	coding
0.994383	coding
0.992752	coding
0.138611	noncoding
0.115954	noncoding
1	coding
0.980467	coding
1	coding
1	coding
1	coding
0.972845	coding
0.544443	coding
0.0816591	noncoding
0.654393	coding
1	coding
1	coding
1	coding
0.328921	noncoding
0.775322	coding
0.373439	noncoding
1	coding
1	coding
1	coding
0.139562	noncoding
1	coding
1	coding
0.99919	coding
0.999127	coding
0.909608	coding
1	coding
1	coding
1	coding
0.0176498	noncoding
0.171692	noncoding
1	coding
1	coding
1	coding
0.166565	noncoding
0.0764248	noncoding
0.999968	coding
0.105619	noncoding
1	coding
0.276209	noncoding
0.732993	coding
0.0447508	noncoding
1	coding
1	coding
0.0356107	noncoding

0.982448	coding
0.997352	coding
0.945651	coding
1	coding
0.548157	coding
0.996342	coding
1	coding
1	coding
1	coding
1	coding
0.162946	noncoding
0.215787	noncoding
0.997981	coding
0.999436	coding
0.999245	coding
0.702494	coding
1	coding
0.254765	noncoding
0.422666	noncoding
0.167601	noncoding
0.167601	noncoding
0.162364	noncoding
0.274124	noncoding
0.125036	noncoding
0.153388	noncoding
0.0762306	noncoding
0.969558	coding
0.122304	noncoding
0.0792635	noncoding
0.793182	coding
0.992581	coding
0.12385	noncoding
0.150367	noncoding
1	coding
0.0790716	noncoding
1	coding
0.0777633	noncoding
0.999991	coding
1	coding
1	coding
0.0380176	noncoding
0.998986	coding
0.999352	coding
0.0406469	noncoding
0.31925	noncoding
0.02994	noncoding
0.132208	noncoding
0.0240429	noncoding
0.327998	noncoding
0.999999	coding
0.0481695	noncoding
0.281856	noncoding
0.690928	coding
0.0579468	noncoding

0.0375796	noncoding
0.151001	noncoding
0.0847396	noncoding
0.0184442	noncoding
0.174291	noncoding
0.407827	noncoding
0.18263	noncoding
0.999969	coding
1	coding
1	coding
1	coding
1	coding
1	coding
1	coding
1	coding
0.0847196	noncoding
0.0531013	noncoding
0.0219823	noncoding
0.997998	coding
0.0598808	noncoding
0.212727	noncoding
0.121802	noncoding
0.997353	coding
0.186947	noncoding
0.333786	noncoding
0.498744	noncoding
1	coding
0.396679	noncoding
1	coding
0.32129	noncoding
0.822999	coding
0.309868	noncoding
1	coding
1	coding
1	coding
1	coding
1	coding
1	coding
1	coding
1	coding
0.755582	coding
1	coding
1	coding
0.999998	coding
1	coding
1	coding
1	coding
1	coding
0.999993	coding
1	coding
1	coding
1	coding
1	coding
1	coding

0.054332	noncoding
1	coding
1	coding
1	coding
1	coding
1	coding
0.999966	coding
0.07763	noncoding
0.999902	coding
0.635931	coding
0.898043	coding
1	coding
0.999818	coding
0.89466	coding
1	coding
0.0567325	noncoding
1	coding
1	coding
1	coding
0.997131	coding
0.294119	noncoding
0.01615	noncoding
1	coding
0.983855	coding
0.0220691	noncoding
0.999939	coding
0.98688	coding
0.999987	coding
0.226146	noncoding
1	coding
0.849915	coding
0.999997	coding
0.827193	coding
0.14694	noncoding
1	coding
0.999962	coding
0.999912	coding
0.999906	coding
0.999912	coding
1	coding
1	coding
1	coding
0.631845	coding
0.0432532	noncoding
0.040321	noncoding
1	coding
0.530662	coding
0.387458	noncoding
0.518532	coding
1	coding
0.165021	noncoding
1	coding
0.0158314	noncoding
0.0155187	noncoding

1	coding
1	coding
0.0502577	noncoding
0.139353	noncoding
0.0155008	noncoding
0.0193655	noncoding
0.0293496	noncoding
0.610441	coding
1	coding
0.100526	noncoding
0.187748	noncoding
1	coding
1	coding
0.0317754	noncoding
0.990436	coding
0.5369	coding
0.480482	noncoding
0.985553	coding
0.0123996	noncoding
0.0158699	noncoding
0.128833	noncoding
0.289947	noncoding
0.0383027	noncoding
0.986839	coding
0.944017	coding
1	coding
1	coding
1	coding
1	coding
1	coding
1	coding
1	coding
1	coding
1	coding
1	coding
1	coding
0.0119966	noncoding
0.0453604	noncoding
0.0465231	noncoding
0.0472581	noncoding
1	coding
0.991983	coding
0.999753	coding
0.999749	coding
0.999769	coding
0.160232	noncoding
0.151383	noncoding
1	coding
1	coding
1	coding
1	coding
1	coding
1	coding
1	coding

1	coding
1	coding
0.999645	coding
1	coding
0.223101	noncoding
1	coding
1	coding
1	coding
1	coding
1	coding
1	coding
0.142599	noncoding
0.999998	coding
0.368772	noncoding
0.40907	noncoding
0.290934	noncoding
0.31064	noncoding
0.670423	coding
0.249561	noncoding
0.117409	noncoding
0.5557	coding
1	coding
0.265799	noncoding
0.0315895	noncoding
0.793455	coding
0.139608	noncoding
1	coding
0.99993	coding
0.334808	noncoding
1	coding
0.22745	noncoding
0.178651	noncoding
1	coding
0.472721	noncoding
0.941125	coding
0.999644	coding
0.0123105	noncoding
0.0131426	noncoding
0.0422752	noncoding
0.116296	noncoding
0.101276	noncoding
0.658929	coding
0.0834162	noncoding
1	coding
0.408289	noncoding
1	coding
1	coding
0.0472056	noncoding
1	coding
1	coding
0.0387231	noncoding
0.0438302	noncoding
0.0508326	noncoding
0.998775	coding

0.0613972	noncoding
0.168289	noncoding
1	coding
1	coding
1	coding
0.996534	coding
0.0138745	noncoding
0.306445	noncoding
0.412181	noncoding
0.0522446	noncoding
0.0906235	noncoding
0.0123154	noncoding
0.0723733	noncoding
0.590747	coding
0.228857	noncoding
0.143611	noncoding
0.10273	noncoding
0.145398	noncoding
0.116266	noncoding
0.993348	coding
0.0443811	noncoding
0.414632	noncoding
0.610227	coding
0.763223	coding
0.121545	noncoding
1	coding
0.0953667	noncoding
0.690042	coding
0.116697	noncoding
1	coding
0.999999	coding
0.82617	coding
0.771183	coding
1	coding
0.943957	coding
1	coding
1	coding
0.592443	coding
0.083487	noncoding
0.401924	noncoding
0.108529	noncoding
1	coding
0.777579	coding
0.0179923	noncoding
0.106329	noncoding
0.707044	coding
0.760702	coding
0.76243	coding
0.119341	noncoding
0.199913	noncoding
0.199913	noncoding
0.208773	noncoding
0.0988997	noncoding
0.0792002	noncoding

0.0986512	noncoding
0.385945	noncoding
0.443066	noncoding
1	coding
1	coding
0.0499513	noncoding
0.101056	noncoding
0.975162	coding
0.551039	coding
0.53127	coding
0.0777817	noncoding
0.163673	noncoding
0.553751	coding
0.0151812	noncoding
0.130666	noncoding
0.0929334	noncoding
0.156496	noncoding
1	coding
1	coding
1	coding
0.0261539	noncoding
0.164791	noncoding
1	coding
1	coding
0.0091595	noncoding
0.125729	noncoding
0.0569202	noncoding
0.0821236	noncoding
0.0691859	noncoding
0.029328	noncoding
0.105922	noncoding
0.928646	coding
0.888237	coding
0.35829	noncoding
0.113735	noncoding
0.313112	noncoding
0.999614	coding
0.0199164	noncoding
1	coding
1	coding
0.114836	noncoding
0.412525	noncoding
0.51515	coding
0.453463	noncoding
0.432871	noncoding
0.436339	noncoding
0.99998	coding
0.999993	coding
0.999982	coding
1	coding
1	coding
1	coding
1	coding
1	coding

0.761896	coding
0.962192	coding
1	coding
1	coding
1	coding
1	coding
1	coding
1	coding
0.569366	coding
0.1547	noncoding
0.160716	noncoding
1	coding
0.0667586	noncoding
0.314871	noncoding
0.379348	noncoding
1	coding
0.999429	coding
1	coding
1	coding
0.99819	coding
0.0170909	noncoding
0.0112326	noncoding
0.00741085	noncoding
0.00974822	noncoding
0.00968626	noncoding
0.00985869	noncoding
0.973543	coding
0.972333	coding
0.0191013	noncoding
0.00773505	noncoding
0.0168637	noncoding
0.982567	coding
1	coding
0.00978988	noncoding
1	coding
0.0233191	noncoding
0.112138	noncoding
1	coding
1	coding
1	coding
1	coding
1	coding
1	coding
1	coding
0.0441909	noncoding
0.0627428	noncoding
0.0250519	noncoding
0.176186	noncoding
0.281816	noncoding
0.162071	noncoding
0.321443	noncoding
1	coding
1	coding
0.140241	noncoding

0.23732	noncoding
0.999921	coding
1	coding
1	coding
1	coding
1	coding
1	coding
1	coding
1	coding
0.266496	noncoding
0.152492	noncoding
0.0907655	noncoding
1	coding
1	coding
1	coding
1	coding
0.937232	coding
1	coding
1	coding
1	coding
1	coding
1	coding
1	coding
1	coding
1	coding
1	coding
1	coding
1	coding
0.128508	noncoding
1	coding
1	coding
0.999999	coding
0.999945	coding
1	coding
1	coding
0.999334	coding
0.165036	noncoding
0.668152	coding
0.262307	noncoding
0.810388	coding
0.877288	coding
1	coding
1	coding
1	coding
0.0340957	noncoding
0.999999	coding
0.843408	coding
0.90244	coding
0.98161	coding
0.974477	coding
0.994042	coding
0.953678	coding
0.979477	coding
0.806039	coding

1	coding
0.964089	coding
1	coding
1	coding
1	coding
0.111044	noncoding
1	coding
1	coding
0.200735	noncoding
1	coding
1	coding
1	coding
1	coding
1	coding
0.999973	coding
1	coding
1	coding
1	coding
1	coding
0.99548	coding
1	coding
1	coding
0.163023	noncoding
0.995811	coding
0.178735	noncoding
0.138711	noncoding
0.0546339	noncoding
1	coding
1	coding
1	coding
1	coding
0.433744	noncoding
0.983386	coding
0.708813	coding
0.992916	coding
1	coding
1	coding
1	coding
1	coding
1	coding
1	coding
1	coding
1	coding
0.308196	noncoding
0.895158	coding
0.999955	coding
1	coding
0.999953	coding
1	coding
0.0265188	noncoding
0.13121	noncoding
0.999965	coding
0.996921	coding
1	coding

1	coding
0.0494443	noncoding
0.0287268	noncoding
0.0805949	noncoding
0.0997037	noncoding
0.0959804	noncoding
0.993507	coding
1	coding
1	coding
0.875994	coding
0.760293	coding
0.319313	noncoding
1	coding
0.189792	noncoding
1	coding
1	coding
1	coding
1	coding
1	coding
1	coding
1	coding
0.00456476	noncoding
1	coding
0.998752	coding
1	coding
1	coding
1	coding
1	coding
1	coding
1	coding
1	coding
1	coding
0.163819	noncoding
0.999994	coding
0.999823	coding
0.999858	coding
0.999999	coding
1	coding
1	coding
1	coding
1	coding
1	coding
1	coding
1	coding
1	coding
1	coding
0.173061	noncoding
1	coding
0.0670506	noncoding
0.560223	coding
1	coding
1	coding
0.0262519	noncoding
0.0140252	noncoding

0.336901	noncoding
1	coding
1	coding
1	coding
0.998088	coding
0.770836	coding
0.0948632	noncoding
0.342192	noncoding
1	coding
0.168394	noncoding
0.145195	noncoding
0.0717231	noncoding
0.0534536	noncoding
1	coding
1	coding
0.999499	coding
0.993098	coding
0.958386	coding
0.999761	coding
0.0669941	noncoding
0.999999	coding
0.947944	coding
0.999908	coding
1	coding
0.0595337	noncoding
0.123286	noncoding
0.117817	noncoding
0.335189	noncoding
0.0541193	noncoding
0.00731376	noncoding
0.201428	noncoding
1	coding
1	coding
1	coding
1	coding
1	coding
1	coding
1	coding
1	coding
0.935196	coding
1	coding
0.999176	coding
0.997915	coding
0.999985	coding
0.130774	noncoding
0.193822	noncoding
1	coding
1	coding
0.871041	coding
0.023287	noncoding
0.986152	coding
0.158263	noncoding
0.158131	noncoding
0.921577	coding

0.510543	coding
1	coding
1	coding
1	coding
0.053636	noncoding
0.0379449	noncoding
1	coding
0.113358	noncoding
1	coding
1	coding
0.210839	noncoding
1	coding
1	coding
0.0190269	noncoding
1	coding
0.0361155	noncoding
0.923968	coding
1	coding
1	coding
1	coding
0.15709	noncoding
0.166216	noncoding
0.0803519	noncoding
1	coding
0.0134813	noncoding
0.0320149	noncoding
1	coding
1	coding
0.438971	noncoding
1	coding
1	coding
1	coding
0.284022	noncoding
0.999997	coding
0.999999	coding
0.999998	coding
0.230035	noncoding
1	coding
1	coding
0.996619	coding
0.999963	coding
0.990038	coding
0.999999	coding
1	coding
1	coding
1	coding
0.132211	noncoding
0.146895	noncoding
0.97878	coding
1	coding
0.981353	coding
0.400426	noncoding
1	coding
1	coding

1	coding
1	coding
1	coding
0.832334	coding
0.227609	noncoding
0.165303	noncoding
0.185674	noncoding
1	coding
1	coding
0.10537	noncoding
1	coding
1	coding
1	coding
0.999965	coding
0.999291	coding
0.985488	coding
0.0536647	noncoding
0.0500321	noncoding
0.0479035	noncoding
1	coding
1	coding
1	coding
0.0444209	noncoding
0.205064	noncoding
0.0602535	noncoding
0.00771113	noncoding
0.999439	coding
0.582449	coding
0.582449	coding
1	coding
1	coding
1	coding
1	coding
1	coding
1	coding
1	coding
0.418761	noncoding
0.256632	noncoding
1	coding
1	coding
0.0133163	noncoding
1	coding
1	coding
0.823999	coding
1	coding
1	coding
1	coding
1	coding
1	coding
1	coding
1	coding
1	coding
1	coding
1	coding

1	coding
0.0242634	noncoding
0.998513	coding
0.206173	noncoding
0.99996	coding
0.123759	noncoding
0.373956	noncoding
1	coding
1	coding
1	coding
1	coding
0.0442925	noncoding
0.999995	coding
1	coding
1	coding
1	coding
0.21551	noncoding
0.988161	coding
1	coding
1	coding
1	coding
0.897632	coding
1	coding
0.993669	coding
0.118755	noncoding
1	coding
1	coding
1	coding
1	coding
0.00380956	noncoding
0.923545	coding
0.789705	coding
0.886638	coding
0.870157	coding
0.999382	coding
0.993838	coding
0.790991	coding
0.170077	noncoding
0.903979	coding
0.933284	coding
0.997366	coding
1	coding
0.649818	coding
0.114181	noncoding
1	coding
0.0499253	noncoding
0.00957043	noncoding
0.06215	noncoding
0.0621869	noncoding
0.0808232	noncoding
0.0691665	noncoding
0.28084	noncoding
1	coding
1	coding

1	coding
1	coding
1	coding
1	coding
1	coding
0.0644269	noncoding
0.99853	coding
0.246179	noncoding
0.971413	coding
0.150976	noncoding
0.999989	coding
1	coding
1	coding
1	coding
1	coding
1	coding
1	coding
1	coding
1	coding
1	coding
0.999775	coding
0.506223	coding
0.225084	noncoding
0.232583	noncoding
0.399989	noncoding
1	coding
1	coding
1	coding
1	coding
1	coding
0.86596	coding
0.385272	noncoding
0.775135	coding
0.999471	coding
0.999471	coding
0.813001	coding
0.257608	noncoding
0.833325	coding
0.330814	noncoding
0.987984	coding
0.0520286	noncoding
1	coding
0.949615	coding
0.0392437	noncoding
0.30236	noncoding
0.30236	noncoding
0.202055	noncoding
1	coding
0.0172818	noncoding
0.774458	coding
0.774458	coding
1	coding
1	coding
0.132289	noncoding

0.841325	coding
1	coding
1	coding
1	coding
0.024399	noncoding
0.0793784	noncoding
1	coding
1	coding
1	coding
0.130684	noncoding
0.999986	coding
0.999988	coding
0.999994	coding
0.999991	coding
0.999925	coding
0.999835	coding
1	coding
0.957366	coding
0.988189	coding
0.210812	noncoding
1	coding
0.149649	noncoding
0.145838	noncoding
1	coding
1	coding
1	coding
0.965205	coding
0.275825	noncoding
0.0145814	noncoding
0.365554	noncoding
0.00448843	noncoding
1	coding
0.051396	noncoding
0.0999891	noncoding
0.0552901	noncoding
0.999409	coding
1	coding
0.999463	coding
0.0343567	noncoding
0.383767	noncoding
1	coding
1	coding
0.827382	coding
0.999997	coding
1	coding
0.42408	noncoding
0.996809	coding
0.0256566	noncoding
0.310535	noncoding
0.243281	noncoding
0.956865	coding
1	coding
1	coding
1	coding

0.999464	coding
1	coding
1	coding
1	coding
1	coding
1	coding
0.999946	coding
0.971779	coding
0.99927	coding
0.999827	coding
0.0511203	noncoding
0.0794726	noncoding
0.0261554	noncoding
0.99988	coding
0.109455	noncoding
1	coding
0.0368292	noncoding
0.718554	coding
0.00971124	noncoding
1	coding
1	coding
0.00678333	noncoding
1	coding
0.997515	coding
0.0895024	noncoding
0.816778	coding
1	coding
0.16997	noncoding
0.217195	noncoding
0.841219	coding
0.230501	noncoding
0.996136	coding
0.136864	noncoding
0.0328967	noncoding
0.171687	noncoding
0.0165464	noncoding
0.999648	coding
0.232842	noncoding
0.0198458	noncoding
0.33901	noncoding
0.0118321	noncoding
0.999873	coding
0.899434	coding
0.327444	noncoding
0.0141155	noncoding
0.0538259	noncoding
0.0216829	noncoding
1	coding
1	coding
0.265887	noncoding
0.999815	coding
0.0107611	noncoding
0.013261	noncoding
0.999999	coding

1	coding
1	coding
1	coding
1	coding
1	coding
1	coding
1	coding
1	coding
1	coding
1	coding
0.159846	noncoding
1	coding
1	coding
1	coding
0.586766	coding
1	coding
1	coding
0.945067	coding
1	coding
0.913063	coding
1	coding
1	coding
1	coding
1	coding
1	coding
0.0132294	noncoding
1	coding
0.994315	coding
0.104548	noncoding
0.934207	coding
0.108299	noncoding
0.0799812	noncoding
0.665526	coding
0.997921	coding
0.998875	coding
0.999679	coding
0.999958	coding
0.316972	noncoding
1	coding
1	coding
1	coding
1	coding
0.0273137	noncoding
0.141976	noncoding
0.0452873	noncoding
1	coding
1	coding
1	coding
1	coding
0.765691	coding
1	coding
1	coding
1	coding
1	coding

1	coding
1	coding
1	coding
1	coding
0.0105141	noncoding
1	coding
1	coding
1	coding
1	coding
1	coding
1	coding
0.996907	coding
0.999985	coding
1	coding
1	coding
0.84707	coding
1	coding
0.920271	coding
0.0546175	noncoding
1	coding
0.833696	coding
0.134963	noncoding
0.633566	coding
0.362887	noncoding
0.0725188	noncoding
0.141387	noncoding
0.185178	noncoding
0.999996	coding
0.970005	coding
1	coding
1	coding
1	coding
1	coding
1	coding
1	coding
1	coding
0.999475	coding
0.999994	coding
0.999999	coding
0.414787	noncoding
1	coding
0.055934	noncoding
1	coding
1	coding
1	coding
1	coding
0.999978	coding
0.999911	coding
1	coding
1	coding
0.0701321	noncoding
0.843562	coding
0.790256	coding
0.830932	coding

0.69964	coding
0.59407	coding
0.348608	noncoding
0.0950697	noncoding
0.0839109	noncoding
0.658417	coding
0.451844	noncoding
0.87336	coding
0.993406	coding
0.841987	coding
0.807913	coding
0.851577	coding
0.785116	coding
0.588526	coding
0.959918	coding
0.814356	coding
0.931487	coding
0.973143	coding
0.774688	coding
0.104782	noncoding
1	coding
0.999991	coding
1	coding
1	coding
1	coding
0.0822141	noncoding
0.107127	noncoding
0.145544	noncoding
0.279415	noncoding
0.282534	noncoding
0.364967	noncoding
0.262005	noncoding
1	coding
1	coding
1	coding
0.0383842	noncoding
0.945381	coding
0.97263	coding
0.835948	coding
0.978861	coding
1	coding
0.999772	coding
1	coding
1	coding
1	coding
0.0701462	noncoding
0.985247	coding
0.00592715	noncoding
0.364421	noncoding
0.160269	noncoding
0.254874	noncoding
0.011211	noncoding
0.0303948	noncoding
0.0576956	noncoding

0.0241929	noncoding
0.170557	noncoding
0.321614	noncoding
0.0871876	noncoding
0.189236	noncoding
0.468868	noncoding
0.468291	noncoding
0.0348153	noncoding
1	coding
1	coding
1	coding
1	coding
1	coding
0.2154	noncoding
0.102033	noncoding
0.4415	noncoding
0.298346	noncoding
0.0531407	noncoding
0.0696377	noncoding
0.0452102	noncoding
0.0452102	noncoding
0.998249	coding
0.998136	coding
0.439806	noncoding
0.243359	noncoding
0.727039	coding
0.0276146	noncoding
0.0444457	noncoding
0.876433	coding
0.0330389	noncoding
1	coding
0.151987	noncoding
0.999999	coding
0.99997	coding
0.0967768	noncoding
0.12368	noncoding
0.895652	coding
0.999999	coding
0.129725	noncoding
0.137233	noncoding
0.283905	noncoding
0.115993	noncoding
0.15453	noncoding
0.0617203	noncoding
0.999993	coding
0.999992	coding
1	coding
1	coding
1	coding
1	coding
1	coding
0.822939	coding
0.524065	coding
0.171333	noncoding

0.171333	noncoding
0.187516	noncoding
0.249695	noncoding
0.124664	noncoding
0.0116385	noncoding
0.057632	noncoding
0.057632	noncoding
0.999823	coding
0.999949	coding
0.135018	noncoding
0.0251218	noncoding
0.99998	coding
0.087064	noncoding
0.657273	coding
0.996646	coding
0.0658765	noncoding
0.164213	noncoding
1	coding
0.990555	coding
0.996692	coding
0.99652	coding
0.0170908	noncoding
0.0762486	noncoding
0.0167552	noncoding
0.0126229	noncoding
0.0133128	noncoding
0.0182402	noncoding
0.987221	coding
0.998803	coding
0.998732	coding
0.998178	coding
0.997547	coding
1	coding
0.999999	coding
0.713854	coding
0.120626	noncoding
0.0325965	noncoding
1	coding
1	coding
0.998755	coding
1	coding
0.997843	coding
1	coding
1	coding
1	coding
1	coding
1	coding
1	coding
1	coding
1	coding
1	coding
0.172772	noncoding
0.214602	noncoding
0.31058	noncoding

0.120638	noncoding
0.0770483	noncoding
0.0509484	noncoding
0.0300605	noncoding
0.0310525	noncoding
0.0305787	noncoding
0.0201638	noncoding
0.303965	noncoding
0.999925	coding
1	coding
1	coding
0.181072	noncoding
0.0134793	noncoding
0.217972	noncoding
0.237328	noncoding
0.0185528	noncoding
0.993966	coding
0.853741	coding
0.169246	noncoding
1	coding
1	coding
1	coding
1	coding
1	coding
0.975145	coding
0.84251	coding
0.999026	coding
0.0172486	noncoding
0.150793	noncoding
0.856387	coding
1	coding
0.0485404	noncoding
0.0268257	noncoding
0.469308	noncoding
0.999719	coding
0.758671	coding
0.999989	coding
0.999995	coding
0.113354	noncoding
0.982673	coding
0.999991	coding
0.0122583	noncoding
0.418072	noncoding
0.0256243	noncoding
0.00526197	noncoding
0.246378	noncoding
0.10189	noncoding
1	coding
1	coding
1	coding
1	coding
0.0973445	noncoding
0.126401	noncoding
0.0256443	noncoding

0.101584	noncoding
1	coding
1	coding
0.999131	coding
0.0885229	noncoding
0.844782	coding
0.956853	coding
0.119255	noncoding
1	coding
1	coding
1	coding
0.879384	coding
0.840814	coding
0.435251	noncoding
0.0527164	noncoding
1	coding
0.99967	coding
0.995577	coding
0.999996	coding
0.999997	coding
0.999996	coding
0.0142876	noncoding
0.0791396	noncoding
0.123166	noncoding
1	coding
0.179474	noncoding
0.124736	noncoding
0.179738	noncoding
0.709401	coding
0.215154	noncoding
0.226306	noncoding
0.385956	noncoding
0.0415103	noncoding
0.103916	noncoding
0.0492459	noncoding
0.00570229	noncoding
1	coding
0.236066	noncoding
0.0387599	noncoding
1	coding
0.117249	noncoding
1	coding
1	coding
0.23484	noncoding
0.454602	noncoding
1	coding
1	coding
0.111229	noncoding
0.0193062	noncoding
0.0318469	noncoding
0.012413	noncoding
0.0277287	noncoding
0.0623464	noncoding
1	coding

0.197175	noncoding
0.019553	noncoding
0.0219086	noncoding
0.00254759	noncoding
0.0615719	noncoding
0.164689	noncoding
0.00722955	noncoding
0.0046227	noncoding
0.419178	noncoding
1	coding
0.171071	noncoding
0.102127	noncoding
0.0200233	noncoding
0.401654	noncoding
0.999998	coding
0.0120903	noncoding
0.296668	noncoding
0.990897	coding
0.0447686	noncoding
0.913266	coding
1	coding
1	coding
0.116367	noncoding
0.999897	coding
1	coding
1	coding
0.79289	coding
0.972782	coding
0.461537	noncoding
0.097472	noncoding
0.0375189	noncoding
0.0348755	noncoding
0.159923	noncoding
0.999991	coding
0.999992	coding
1	coding
1	coding
1	coding
1	coding
0.0693048	noncoding
0.00844644	noncoding
0.0171967	noncoding
0.0197825	noncoding
1	coding
0.164475	noncoding
0.0138209	noncoding
1	coding
1	coding
0.999085	coding
0.0629802	noncoding
1	coding
0.99999	coding
0.999911	coding
1	coding

1	coding
1	coding
0.542283	coding
0.104714	noncoding
0.854629	coding
1	coding
0.200046	noncoding
1	coding
1	coding
1	coding
0.999997	coding
0.99937	coding
0.237225	noncoding
1	coding
1	coding
1	coding
1	coding
1	coding
0.990698	coding
0.996257	coding
1	coding
0.174339	noncoding
0.00485826	noncoding
0.178916	noncoding
0.385858	noncoding
0.136891	noncoding
0.429063	noncoding
1	coding
0.977355	coding
1	coding
0.987052	coding
0.11914	noncoding
0.418269	noncoding
0.0327633	noncoding
0.830043	coding
0.845676	coding
1	coding
1	coding
1	coding
1	coding
1	coding
0.062943	noncoding
1	coding
0.999999	coding
1	coding
0.999999	coding
0.999999	coding
0.999991	coding
0.0199541	noncoding
0.99495	coding
0.0068135	noncoding
0.00799318	noncoding
1	coding
1	coding

0.996596	coding
0.0411395	noncoding
0.177785	noncoding
1	coding
1	coding
0.231743	noncoding
0.0363795	noncoding
1	coding
1	coding
1	coding
1	coding
1	coding
1	coding
0.0498594	noncoding
0.221833	noncoding
0.405877	noncoding
0.0613315	noncoding
0.421785	noncoding
0.157968	noncoding
0.179322	noncoding
1	coding
0.999939	coding
0.976318	coding
0.47659	noncoding
1	coding
0.999924	coding
0.999976	coding
0.308815	noncoding
0.0343261	noncoding
1	coding
1	coding
1	coding
1	coding
1	coding
1	coding
1	coding
1	coding
1	coding
1	coding
0.153947	noncoding
0.0607024	noncoding
0.0261187	noncoding
0.0163158	noncoding
0.993774	coding
0.544677	coding
0.14042	noncoding
0.0825787	noncoding
0.518083	coding
0.575492	coding
0.062391	noncoding
0.174306	noncoding
0.341289	noncoding
1	coding
1	coding

0.903623	coding
0.999967	coding
1	coding
1	coding
1	coding
1	coding
1	coding
1	coding
1	coding
0.999758	coding
0.999245	coding
0.0490633	noncoding
0.993823	coding
1	coding
0.997884	coding
0.999944	coding
0.987711	coding
1	coding
0.0329994	noncoding
0.352069	noncoding
0.646977	coding
0.433699	noncoding
0.0865694	noncoding
1	coding
0.426399	noncoding
0.192253	noncoding
0.0232215	noncoding
0.00595803	noncoding
0.166884	noncoding
0.166884	noncoding
1	coding
0.952204	coding
0.0391597	noncoding
0.999997	coding
0.788218	coding
1	coding
0.135972	noncoding
0.998512	coding
1	coding
1	coding
1	coding
0.911	coding
0.756109	coding
0.391958	noncoding
1	coding
0.0689959	noncoding
0.0307612	noncoding
1	coding
1	coding
0.128904	noncoding
1	coding
0.999974	coding
0.999998	coding
0.101607	noncoding

0.170355	noncoding
0.0625118	noncoding
0.0717735	noncoding
0.0290479	noncoding
1	coding
0.0796586	noncoding
1	coding
1	coding
0.867157	coding
1	coding
1	coding
1	coding
1	coding
0.096246	noncoding
0.485102	noncoding
0.998492	coding
0.998189	coding
0.994508	coding
0.998778	coding
1	coding
0.212074	noncoding
0.999999	coding
0.934553	coding
0.0423663	noncoding
0.0181577	noncoding
0.127502	noncoding
0.979151	coding
0.996939	coding
0.99959	coding
0.973627	coding
0.893323	coding
1	coding
1	coding
0.0321448	noncoding
0.0102113	noncoding
0.0489742	noncoding
0.0519963	noncoding
0.026744	noncoding
0.107213	noncoding
0.0447885	noncoding
0.0521119	noncoding
0.0746893	noncoding
1	coding
1	coding
1	coding
0.31031	noncoding
1	coding
1	coding
1	coding
1	coding
1	coding
1	coding
1	coding
1	coding

1	coding
0.999929	coding
1	coding
0.12078	noncoding
0.0179995	noncoding
0.339155	noncoding
0.0465895	noncoding
1	coding
1	coding
1	coding
0.996852	coding
0.999188	coding
0.998633	coding
1	coding
1	coding
1	coding
1	coding
1	coding
1	coding
1	coding
1	coding
0.996579	coding
0.203857	noncoding
0.0866289	noncoding
0.00719728	noncoding
0.00574535	noncoding
0.909884	coding
0.0782745	noncoding
0.345294	noncoding
0.158938	noncoding
0.0197567	noncoding
0.0214057	noncoding
0.00394166	noncoding
1	coding
1	coding
1	coding
0.999996	coding
0.568579	coding
0.132463	noncoding
1	coding
0.999597	coding
0.999977	coding
1	coding
1	coding
0.0392719	noncoding
0.0294346	noncoding
0.22873	noncoding
0.0369612	noncoding
0.0698179	noncoding
0.0344632	noncoding
0.501997	coding
0.0696734	noncoding
0.0253845	noncoding
0.0158608	noncoding

0.0301302	noncoding
0.99998	coding
0.885132	coding
0.885132	coding
0.99902	coding
0.997914	coding
1	coding
0.636676	coding
1	coding
1	coding
1	coding
1	coding
1	coding
0.305625	noncoding
0.370002	noncoding
0.655096	coding
0.856472	coding
0.914569	coding
0.908977	coding
0.170034	noncoding
0.620431	coding
0.999426	coding
0.313302	noncoding
1	coding
0.975437	coding
0.716085	coding
0.913585	coding
0.999507	coding
1	coding
1	coding
0.404229	noncoding
0.290512	noncoding
0.980909	coding
0.995315	coding
1	coding
1	coding
0.323657	noncoding
0.926768	coding
0.357026	noncoding
0.317378	noncoding
0.0441295	noncoding
1	coding
1	coding
1	coding
1	coding
0.311638	noncoding
1	coding
1	coding
1	coding
1	coding
0.999948	coding
0.999999	coding
1	coding
0.436846	noncoding

0.996957	coding
0.981483	coding
1	coding
1	coding
0.136324	noncoding
0.989457	coding
0.999786	coding
1	coding
0.338542	noncoding
1	coding
1	coding
1	coding
1	coding
1	coding
1	coding
1	coding
1	coding
1	coding
1	coding
1	coding
1	coding
1	coding
1	coding
1	coding
1	coding
1	coding
0.0146766	noncoding
0.982428	coding
0.894246	coding
0.0308294	noncoding
0.0204053	noncoding
0.209889	noncoding
0.999785	coding
1	coding
0.943017	coding
0.0490565	noncoding
1	coding
1	coding
1	coding
0.999497	coding
0.113723	noncoding
0.110563	noncoding
1	coding
0.89807	coding
0.273359	noncoding
0.589817	coding
0.641716	coding
0.74246	coding
0.27335	noncoding
1	coding
0.969878	coding
0.961973	coding
0.519493	coding
1	coding

1	coding
1	coding
1	coding
0.868786	coding
0.945591	coding
1	coding
1	coding
1	coding
1	coding
1	coding
1	coding
1	coding
1	coding
0.999989	coding
0.999948	coding
0.996083	coding
0.999973	coding
1	coding
1	coding
0.875981	coding
1	coding
0.0801743	noncoding
0.361903	noncoding
0.72266	coding
0.0785986	noncoding
0.0279197	noncoding
0.232782	noncoding
1	coding
0.999151	coding
1	coding
1	coding
0.999989	coding
0.178997	noncoding
0.842957	coding
0.848685	coding
0.599019	coding
0.940423	coding
0.966525	coding
0.117046	noncoding
0.119468	noncoding
1	coding
1	coding
0.0912071	noncoding
0.028736	noncoding
1	coding
0.323186	noncoding
1	coding
0.0629558	noncoding
0.022867	noncoding
0.586528	coding
0.062379	noncoding
1	coding
1	coding
1	coding

0.999411	coding
1	coding
1	coding
0.2059	noncoding
1	coding
1	coding
1	coding
0.101653	noncoding
0.992155	coding
0.240136	noncoding
0.175952	noncoding
1	coding
1	coding
1	coding
0.999964	coding
0.999977	coding
0.999968	coding
0.99997	coding
0.0321075	noncoding
0.135009	noncoding
0.122443	noncoding
0.999993	coding
0.688242	coding
1	coding
0.0328972	noncoding
1	coding
0.999014	coding
0.981441	coding
0.998982	coding
0.150296	noncoding
1	coding
0.131726	noncoding
0.144628	noncoding
0.999968	coding
0.98975	coding
0.998364	coding
0.373301	noncoding
1	coding
1	coding
0.987044	coding
1	coding
0.976509	coding
0.995861	coding
0.942329	coding
0.980013	coding
0.998492	coding
0.999181	coding
0.998618	coding
0.998743	coding
0.997673	coding
0.977041	coding
1	coding
1	coding
0.124962	noncoding

0.999965	coding
0.999996	coding
0.999994	coding
0.0437387	noncoding
0.0825259	noncoding
1	coding
0.0763476	noncoding
0.320806	noncoding
0.207989	noncoding
0.215407	noncoding
0.275078	noncoding
1	coding
0.819771	coding
0.176097	noncoding
1	coding
1	coding
0.743211	coding
1	coding
0.999602	coding
0.999364	coding
0.125054	noncoding
0.149686	noncoding
0.125054	noncoding
0.320395	noncoding
0.999537	coding
0.076381	noncoding
0.13657	noncoding
1	coding
1	coding
0.0657384	noncoding
0.0141762	noncoding
1	coding
0.707814	coding
0.977688	coding
0.125612	noncoding
1	coding
1	coding
0.265113	noncoding
1	coding
1	coding
0.499948	noncoding
0.0202668	noncoding
1	coding
0.978593	coding
1	coding
0.196945	noncoding
0.052167	noncoding
0.0160449	noncoding
1	coding
0.318079	noncoding
0.0144506	noncoding
1	coding
1	coding
0.0312788	noncoding

1	coding
0.0830919	noncoding
0.154411	noncoding
0.0399475	noncoding
0.0788476	noncoding
0.936521	coding
1	coding
1	coding
0.0392687	noncoding
1	coding
1	coding
1	coding
1	coding
1	coding
1	coding
1	coding
1	coding
1	coding
0.321405	noncoding
0.477885	noncoding
0.0366611	noncoding
0.999396	coding
1	coding
0.999669	coding
0.999606	coding
0.999385	coding
0.996597	coding
0.99952	coding
1	coding
0.982123	coding
0.996219	coding
0.690026	coding
0.113665	noncoding
1	coding
1	coding
0.392035	noncoding
0.118084	noncoding
0.304558	noncoding
0.577428	coding
0.811162	coding
0.364685	noncoding
0.960762	coding
0.960527	coding
1	coding
1	coding
1	coding
1	coding
1	coding
1	coding
1	coding
1	coding
1	coding
1	coding
1	coding

0.970477	coding
0.447227	noncoding
0.0834325	noncoding
1	coding
0.0768855	noncoding
0.0119964	noncoding
1	coding
0.0291988	noncoding
0.44591	noncoding
0.759534	coding
0.123391	noncoding
0.176097	noncoding
0.0238126	noncoding
0.128009	noncoding
0.72637	coding
0.0692993	noncoding
0.0271883	noncoding
0.16324	noncoding
0.120702	noncoding
0.484548	noncoding
1	coding
0.0403058	noncoding
1	coding
0.999968	coding
1	coding
0.993728	coding
1	coding
1	coding
0.0558673	noncoding
0.223024	noncoding
0.999997	coding
1	coding
1	coding
1	coding
1	coding
1	coding
0.999981	coding
0.0562333	noncoding
1	coding
0.23775	noncoding
0.339853	noncoding
0.989265	coding
0.999997	coding
1	coding
0.999873	coding
0.998332	coding
0.999929	coding
0.999924	coding
0.99988	coding
0.999897	coding
0.528823	coding
0.837032	coding
0.994929	coding
0.977235	coding

0.994929	coding
0.996232	coding
0.97514	coding
0.999315	coding
0.994929	coding
0.254719	noncoding
1	coding
1	coding
1	coding
1	coding
0.620747	coding
0.374279	noncoding
0.177082	noncoding
0.0458283	noncoding
0.257731	noncoding
0.371201	noncoding
0.336454	noncoding
0.0179527	noncoding
0.99948	coding
0.999451	coding
0.999845	coding
0.36164	noncoding
0.108472	noncoding
0.10547	noncoding
0.0973269	noncoding
0.0973269	noncoding
0.0616769	noncoding
1	coding
1	coding
1	coding
1	coding
1	coding
1	coding
1	coding
1	coding
0.999963	coding
0.99998	coding
0.0401969	noncoding
1	coding
1	coding
1	coding
0.999237	coding
0.019574	noncoding
0.999954	coding
0.999912	coding
1	coding
0.588386	coding
0.999814	coding
0.999993	coding
0.999814	coding
0.99991	coding
0.449807	noncoding
0.999746	coding
0.999971	coding

0.294192	noncoding
0.049165	noncoding
0.134292	noncoding
0.292116	noncoding
1	coding
1	coding
1	coding
0.865026	coding
0.952073	coding
0.562292	coding
0.999539	coding
0.960077	coding
0.834912	coding
0.999903	coding
0.99998	coding
1	coding
0.0198139	noncoding
0.999999	coding
1	coding
1	coding
1	coding
1	coding
0.85852	coding
1	coding
0.703337	coding
0.17505	noncoding
0.116819	noncoding
0.496802	noncoding
0.985685	coding
0.997725	coding
0.941221	coding
0.0156645	noncoding
0.409731	noncoding
0.119229	noncoding
0.411266	noncoding
0.319438	noncoding
0.02202	noncoding
0.99925	coding
0.190285	noncoding
1	coding
1	coding
1	coding
1	coding
1	coding
1	coding
1	coding
0.828472	coding
0.807682	coding
0.945222	coding
1	coding
0.99926	coding
0.423426	noncoding
0.443428	noncoding
1	coding

1	coding
1	coding
0.999994	coding
1	coding
0.978871	coding
0.912237	coding
0.982879	coding
0.400584	noncoding
1	coding
0.999999	coding
0.0507835	noncoding
0.029225	noncoding
0.0458657	noncoding
0.995238	coding
1	coding
1	coding
1	coding
1	coding
0.294588	noncoding
0.535735	coding
0.0409151	noncoding
0.999649	coding
0.782151	coding
0.999866	coding
0.995054	coding
0.993001	coding
0.863851	coding
0.999994	coding
1	coding
1	coding
1	coding
0.0296337	noncoding
1	coding
1	coding
0.00648998	noncoding
1	coding
0.999782	coding
0.987525	coding
1	coding
1	coding
0.999936	coding
1	coding
1	coding
1	coding
0.642099	coding
0.80768	coding
0.681662	coding
1	coding
1	coding
1	coding
1	coding
1	coding
1	coding
0.0276808	noncoding

0.832574	coding
0.0482298	noncoding
0.407248	noncoding
0.210817	noncoding
1	coding
1	coding
1	coding
1	coding
1	coding
0.99994	coding
1	coding
1	coding
1	coding
1	coding
1	coding
1	coding
1	coding
1	coding
1	coding
1	coding
0.0543571	noncoding
0.00842301	noncoding
0.917551	coding
1	coding
0.0716423	noncoding
0.189799	noncoding
0.999989	coding
0.175238	noncoding
0.00991276	noncoding
1	coding
1	coding
1	coding
1	coding
1	coding
1	coding
1	coding
1	coding
1	coding
1	coding
0.176071	noncoding
0.0411031	noncoding
1	coding
1	coding
1	coding
1	coding
1	coding
1	coding
1	coding
1	coding
1	coding
0.166115	noncoding
0.0210414	noncoding
0.178356	noncoding
0.955208	coding

1	coding
1	coding
1	coding
1	coding
1	coding
0.911486	coding
0.987575	coding
1	coding
0.912543	coding
0.994868	coding
0.986732	coding
0.991812	coding
0.999968	coding
1	coding
1	coding
1	coding
1	coding
1	coding
1	coding
1	coding
1	coding
1	coding
0.167966	noncoding
0.0639643	noncoding
0.075283	noncoding
0.0901725	noncoding
0.0123114	noncoding
0.0253034	noncoding
0.0249244	noncoding
0.0133401	noncoding
0.927256	coding
0.0190716	noncoding
0.999986	coding
0.999868	coding
1	coding
0.999999	coding
1	coding
1	coding
1	coding
1	coding
1	coding
1	coding
1	coding
0.970411	coding
0.90415	coding
0.949806	coding
1	coding
0.778093	coding
0.981257	coding
0.00997935	noncoding
0.0354739	noncoding
1	coding
1	coding
0.094801	noncoding

0.657268	coding
0.0852388	noncoding
0.0148518	noncoding
0.0162009	noncoding
0.141711	noncoding
0.150629	noncoding
1	coding
1	coding
0.999884	coding
1	coding
0.86001	coding
0.999851	coding
0.231911	noncoding
0.447138	noncoding
0.989212	coding
0.56402	coding
0.051813	noncoding
1	coding
0.0323727	noncoding
0.0214797	noncoding
0.990901	coding
0.999945	coding
0.363028	noncoding
0.00786152	noncoding
0.999972	coding
0.0136367	noncoding
0.0607557	noncoding
0.0575779	noncoding
0.242178	noncoding
0.066517	noncoding
1	coding
0.994609	coding
0.443921	noncoding
0.0891568	noncoding
0.156833	noncoding
0.995695	coding
0.0214461	noncoding
1	coding
0.981637	coding
1	coding
1	coding
1	coding
1	coding
0.548847	coding
0.249383	noncoding
0.1738	noncoding
0.1738	noncoding
0.960848	coding
0.0327994	noncoding
0.999999	coding
1	coding
0.0723994	noncoding
0.371847	noncoding
1	coding

0.999994	coding
0.124011	noncoding
1	coding
1	coding
0.0461508	noncoding
0.0546421	noncoding
1	coding
0.198873	noncoding
0.0542503	noncoding
1	coding
0.223804	noncoding
1	coding
1	coding
0.024149	noncoding
0.0336648	noncoding
0.999915	coding
0.999999	coding
0.999998	coding
1	coding
1	coding
1	coding
1	coding
0.221083	noncoding
0.992066	coding
1	coding
1	coding
1	coding
0.091422	noncoding
1	coding
0.999994	coding
1	coding
0.995809	coding
0.0841476	noncoding
0.29298	noncoding
0.153797	noncoding
0.999998	coding
0.544051	coding
0.99987	coding
0.999801	coding
1	coding
1	coding
0.997068	coding
0.997334	coding
0.804583	coding
0.998992	coding
0.694136	coding
0.427132	noncoding
0.487496	noncoding
0.0135971	noncoding
0.911418	coding
0.971319	coding
0.96318	coding
0.976861	coding
0.963277	coding

0.999869	coding
0.993824	coding
0.999957	coding
0.999974	coding
1	coding
0.997521	coding
0.0506656	noncoding
0.620163	coding
1	coding
0.0624073	noncoding
0.0248071	noncoding
0.074382	noncoding
0.999477	coding
1	coding
0.981635	coding
0.983799	coding
1	coding
0.070997	noncoding
1	coding
1	coding
0.00670882	noncoding
0.0297446	noncoding
0.317014	noncoding
0.998836	coding
1	coding
0.0637736	noncoding
0.998461	coding
0.996829	coding
0.997106	coding
0.999921	coding
0.999688	coding
0.980859	coding
0.997043	coding
0.999438	coding
0.999986	coding
0.999994	coding
0.999409	coding
0.993468	coding
1	coding
1	coding
1	coding
1	coding
1	coding
1	coding
1	coding
1	coding
1	coding
0.999997	coding
0.168288	noncoding
0.999994	coding
0.999998	coding
0.999998	coding
0.999998	coding
1	coding

1	coding
0.999972	coding
1	coding
1	coding
1	coding
0.999877	coding
1	coding
1	coding
1	coding
0.999997	coding
0.868272	coding
0.25222	noncoding
0.0272914	noncoding
0.0234541	noncoding
0.99919	coding
0.0166735	noncoding
1	coding
1	coding
1	coding
1	coding
1	coding
1	coding
1	coding
1	coding
1	coding
0.497784	noncoding
0.1238	noncoding
0.0485062	noncoding
0.0411031	noncoding
1	coding
1	coding
1	coding
1	coding
1	coding
0.00603817	noncoding
0.136644	noncoding
0.782379	coding
0.999792	coding
0.999514	coding
0.00861012	noncoding
0.998927	coding
0.997567	coding
0.995913	coding
0.995571	coding
0.992918	coding
0.152326	noncoding
0.563819	coding
0.0995941	noncoding
1	coding
0.962701	coding
1	coding
0.908112	coding
0.941841	coding
0.999316	coding

1	coding
0.851723	coding
0.00686615	noncoding
1	coding
1	coding
1	coding
0.912862	coding
0.999957	coding
0.22913	noncoding
0.204756	noncoding
0.126636	noncoding
0.0100298	noncoding
0.0738052	noncoding
0.00571093	noncoding
0.30658	noncoding
0.865298	coding
0.475018	noncoding
0.0601755	noncoding
0.999382	coding
0.314176	noncoding
0.247279	noncoding
1	coding
1	coding
0.0246929	noncoding
0.324176	noncoding
0.999855	coding
0.99903	coding
0.999578	coding
0.900235	coding
1	coding
0.999985	coding
0.0385161	noncoding
0.0382088	noncoding
1	coding
1	coding
1	coding
1	coding
0.999996	coding
0.398621	noncoding
0.623868	coding
0.18743	noncoding
0.0317785	noncoding
1	coding
1	coding
0.637599	coding
0.216553	noncoding
0.28724	noncoding
1	coding
0.0244379	noncoding
0.044406	noncoding
0.986698	coding
0.99999	coding
0.0860908	noncoding
0.970079	coding

1	coding
0.99935	coding
0.999431	coding
1	coding
0.737598	coding
0.535231	coding
0.957602	coding
0.141879	noncoding
0.131194	noncoding
0.0630125	noncoding
0.246582	noncoding
0.031238	noncoding
0.599785	coding
0.998811	coding
0.315849	noncoding
0.217787	noncoding
0.920664	coding
0.995977	coding
1	coding
1	coding
1	coding
1	coding
1	coding
1	coding
0.311092	noncoding
0.119467	noncoding
0.00262548	noncoding
0.0193023	noncoding
0.999014	coding
1	coding
0.12633	noncoding
0.98483	coding
0.0136834	noncoding
0.999999	coding
0.999999	coding
1	coding
0.98632	coding
0.997528	coding
0.40455	noncoding
0.575178	coding
1	coding
1	coding
1	coding
1	coding
1	coding
1	coding
0.999846	coding
1	coding
1	coding
0.163739	noncoding
0.0515519	noncoding
0.508985	coding
0.508985	coding
0.0830377	noncoding

1	coding
1	coding
1	coding
1	coding
0.511696	coding
1	coding
1	coding
1	coding
0.999976	coding
1	coding
1	coding
0.0316969	noncoding
0.0744062	noncoding
0.999999	coding
0.40416	noncoding
0.999886	coding
0.999999	coding
0.0244659	noncoding
0.999439	coding
0.0922923	noncoding
1	coding
1	coding
1	coding
0.116039	noncoding
0.999954	coding
0.997134	coding
1	coding
1	coding
0.0623618	noncoding
0.0887771	noncoding
0.999996	coding
1	coding
0.998447	coding
0.105306	noncoding
1	coding
1	coding
1	coding
0.370167	noncoding
0.827547	coding
0.845932	coding
0.980696	coding
0.357938	noncoding
0.168686	noncoding
0.293649	noncoding
0.31201	noncoding
0.0637515	noncoding
0.0529084	noncoding
1	coding
1	coding
1	coding
1	coding
1	coding
1	coding
1	coding

0.384698	noncoding
0.00932705	noncoding
0.0090223	noncoding
0.185433	noncoding
0.0676901	noncoding
0.115569	noncoding
0.064753	noncoding
0.921157	coding
0.305982	noncoding
0.419215	noncoding
0.0681206	noncoding
0.107927	noncoding
1	coding
0.959089	coding
0.959042	coding
0.147607	noncoding
0.118749	noncoding
1	coding
1	coding
0.995954	coding
1	coding
1	coding
1	coding
0.177161	noncoding
0.88763	coding
0.333346	noncoding
0.33728	noncoding
0.803456	coding
0.0455389	noncoding
0.764096	coding
0.0376644	noncoding
0.995449	coding
0.999999	coding
1	coding
1	coding
0.13184	noncoding
0.239744	noncoding
0.998746	coding
0.355806	noncoding
0.10013	noncoding
0.0302648	noncoding
0.363276	noncoding
1	coding
0.999993	coding
0.137855	noncoding
0.41471	noncoding
0.793761	coding
0.640614	coding
0.876498	coding
0.0092315	noncoding
0.988176	coding
0.46046	noncoding
0.608463	coding
1	coding

0.0798198	noncoding
1	coding
0.142231	noncoding
1	coding
0.0187193	noncoding
0.218538	noncoding
0.0114711	noncoding
0.0799516	noncoding
0.269745	noncoding
0.00930274	noncoding
0.0158048	noncoding
0.0138335	noncoding
0.580344	coding
0.337615	noncoding
0.0324735	noncoding
0.483696	noncoding
0.100765	noncoding
0.0202706	noncoding
0.477304	noncoding
0.999983	coding
0.999677	coding
0.975753	coding
0.158141	noncoding
0.618131	coding
0.221516	noncoding
0.0963729	noncoding
0.221611	noncoding
1	coding
1	coding
1	coding
0.0476995	noncoding
0.0884035	noncoding
0.999931	coding
0.876556	coding
0.999568	coding
0.365255	noncoding
0.0117594	noncoding
0.0247579	noncoding
0.105305	noncoding
0.0285161	noncoding
0.19752	noncoding
0.649098	coding
0.834835	coding
0.709833	coding
0.542942	coding
0.94952	coding
0.95156	coding
0.157055	noncoding
0.326584	noncoding
0.0286091	noncoding
0.04089	noncoding
0.224186	noncoding
0.0287666	noncoding
0.0263477	noncoding

0.106743	noncoding
1	coding
1	coding
0.0436667	noncoding
0.0222715	noncoding
0.681409	coding
0.47123	noncoding
0.0558523	noncoding
0.26211	noncoding
0.0506904	noncoding
0.466749	noncoding
0.147069	noncoding
0.597257	coding
0.128159	noncoding
0.93249	coding
0.874101	coding
0.0428511	noncoding
0.172523	noncoding
0.0972787	noncoding
1	coding
0.0268748	noncoding
0.210796	noncoding
0.763071	coding
0.639066	coding
1	coding
1	coding
1	coding
1	coding
0.999582	coding
0.999908	coding
0.0222451	noncoding
0.999995	coding
0.331456	noncoding
0.378942	noncoding
0.565347	coding
1	coding
0.378942	noncoding
1	coding
1	coding
1	coding
0.936118	coding
0.0253769	noncoding
1	coding
0.999325	coding
1	coding
1	coding
1	coding
1	coding
1	coding
1	coding
1	coding
1	coding
1	coding
0.923428	coding

0.992017	coding
0.518326	coding
0.114641	noncoding
0.0169465	noncoding
0.0488512	noncoding
0.151111	noncoding
0.0109639	noncoding
0.312268	noncoding
0.169957	noncoding
0.764686	coding
0.379942	noncoding
0.0253931	noncoding
0.058755	noncoding
0.119514	noncoding
1	coding
0.988358	coding
0.139681	noncoding
0.601024	coding
0.229039	noncoding
1	coding
1	coding
1	coding
1	coding
1	coding
1	coding
1	coding
1	coding
1	coding
1	coding
1	coding
1	coding
1	coding
0.723841	coding
0.0378062	noncoding
1	coding
0.020943	noncoding
0.0106773	noncoding
0.999179	coding
1	coding
1	coding
0.999947	coding
0.999914	coding
1	coding
1	coding
0.99797	coding
1	coding
0.999832	coding
0.999589	coding
0.0119374	noncoding
0.999995	coding
0.0773192	noncoding
0.378159	noncoding
0.0362393	noncoding
0.0235009	noncoding

0.0222866	noncoding
0.0212499	noncoding
0.117485	noncoding
0.997937	coding
0.998064	coding
0.998121	coding
0.999956	coding
0.0119716	noncoding
0.0617153	noncoding
0.0207515	noncoding
0.0204571	noncoding
0.0194281	noncoding
0.0266069	noncoding
0.0521269	noncoding
1	coding
1	coding
1	coding
1	coding
0.999874	coding
0.0137828	noncoding
0.398116	noncoding
0.40556	noncoding
0.745988	coding
0.989002	coding
0.928496	coding
0.870422	coding
0.638485	coding
0.997679	coding
0.999704	coding
0.999366	coding
0.0271041	noncoding
0.822719	coding
0.999999	coding
0.0813444	noncoding
0.85895	coding
1	coding
1	coding
1	coding
1	coding
0.99997	coding
1	coding
0.999945	coding
1	coding
1	coding
0.999849	coding
1	coding
0.0853993	noncoding
0.424303	noncoding
0.105648	noncoding
0.122719	noncoding
1	coding
0.999668	coding
0.0322375	noncoding
0.0211735	noncoding

1	coding
1	coding
1	coding
0.0767905	noncoding
0.999963	coding
0.483099	noncoding
1	coding
1	coding
1	coding
0.0361149	noncoding
0.148543	noncoding
1	coding
1	coding
1	coding
0.0141557	noncoding
0.111141	noncoding
1	coding
0.771626	coding
0.87425	coding
0.0936374	noncoding
0.994072	coding
1	coding
0.995484	coding
0.0484219	noncoding
0.709047	coding
0.0519153	noncoding
0.0208484	noncoding
0.994914	coding
1	coding
0.997052	coding
1	coding
0.258337	noncoding
0.783984	coding
0.141692	noncoding
0.0424322	noncoding
1	coding
0.0514868	noncoding
1	coding
0.99984	coding
0.0163799	noncoding
0.0573143	noncoding
1	coding
0.015573	noncoding
0.0215954	noncoding
0.977092	coding
0.995451	coding
0.989679	coding
0.128752	noncoding
0.999502	coding
0.99913	coding
0.999931	coding
0.0154579	noncoding
0.433058	noncoding
0.0548506	noncoding

0.103006	noncoding
0.0626383	noncoding
1	coding
0.98926	coding
0.994932	coding
1	coding
1	coding
1	coding
1	coding
1	coding
1	coding
1	coding
0.92349	coding
1	coding
1	coding
0.997077	coding
1	coding
1	coding
0.0280462	noncoding
0.0731553	noncoding
0.125651	noncoding
0.178599	noncoding
0.182628	noncoding
0.0418134	noncoding
0.00751938	noncoding
0.132775	noncoding
0.0698065	noncoding
0.99993	coding
1	coding
1	coding
0.856167	coding
0.518237	coding
0.00752509	noncoding
0.178715	noncoding
0.0776566	noncoding
0.396709	noncoding
0.687733	coding
0.0102734	noncoding
0.0672824	noncoding
0.359758	noncoding
0.893031	coding
0.058336	noncoding
0.809974	coding
0.319033	noncoding
0.400079	noncoding
0.181781	noncoding
0.939589	coding
0.998574	coding
0.222992	noncoding
0.19415	noncoding
0.101167	noncoding
0.102829	noncoding
0.062878	noncoding
0.147018	noncoding

0.0336787	noncoding
0.114457	noncoding
0.0484923	noncoding
0.999908	coding
0.214141	noncoding
0.999955	coding
0.999992	coding
0.466439	noncoding
0.723355	coding
0.18314	noncoding
0.644134	coding
0.0976155	noncoding
0.791072	coding
0.997396	coding
0.977089	coding
0.04445	noncoding
0.0337113	noncoding
1	coding
0.852032	coding
0.166067	noncoding
0.488615	noncoding
0.684279	coding
0.0308698	noncoding
0.550952	coding
0.551052	coding
0.584644	coding
0.067563	noncoding
0.0327555	noncoding
0.170743	noncoding
0.288487	noncoding
0.0537192	noncoding
0.0868756	noncoding
0.614893	coding
0.503319	coding
0.453323	noncoding
0.427701	noncoding
0.489445	noncoding
0.415582	noncoding
0.735862	coding
0.35414	noncoding
0.346088	noncoding
0.616688	coding
0.287018	noncoding
0.177864	noncoding
0.99987	coding
1	coding
0.999999	coding
0.143615	noncoding
0.0321089	noncoding
1	coding
0.0272988	noncoding
0.951228	coding
1	coding
0.024777	noncoding

0.182315	noncoding
0.0542814	noncoding
0.999999	coding
0.999889	coding
0.124632	noncoding
1	coding
0.104687	noncoding
0.0301495	noncoding
0.999955	coding
1	coding
0.0133502	noncoding
0.999304	coding
0.999304	coding
1	coding
0.387932	noncoding
1	coding
0.955852	coding
0.154197	noncoding
0.0505511	noncoding
1	coding
1	coding
1	coding
0.375286	noncoding
0.0433628	noncoding
0.0240303	noncoding
0.937181	coding
0.569305	coding
1	coding
0.999987	coding
0.468431	noncoding
1	coding
1	coding
1	coding
1	coding
0.0165177	noncoding
0.0481136	noncoding
0.999932	coding
1	coding
0.998309	coding
0.0252664	noncoding
0.494041	noncoding
0.274587	noncoding
0.936077	coding
0.0533182	noncoding
0.29381	noncoding
0.077572	noncoding
0.0129377	noncoding
0.303546	noncoding
0.322967	noncoding
1	coding
0.933129	coding
0.93358	coding
1	coding
0.229451	noncoding

0.225745	noncoding
1	coding
1	coding
0.0429523	noncoding
0.946217	coding
0.566976	coding
0.13257	noncoding
0.46248	noncoding
0.949693	coding
0.153857	noncoding
0.109261	noncoding
0.052421	noncoding
0.0512735	noncoding
0.582035	coding
0.964192	coding
0.4417	noncoding
0.391981	noncoding
0.234706	noncoding
0.00696806	noncoding
0.18682	noncoding
0.208555	noncoding
0.0481916	noncoding
0.119441	noncoding
0.999415	coding
1	coding
0.0164352	noncoding
0.0685403	noncoding
0.922991	coding
0.0634098	noncoding
1	coding
1	coding
0.0255354	noncoding
0.216577	noncoding
0.0338748	noncoding
0.99381	coding
1	coding
0.0636498	noncoding
1	coding
1	coding
0.999418	coding
0.999418	coding
0.999418	coding
0.999971	coding
0.999108	coding
0.29413	noncoding
0.999997	coding
1	coding
0.11633	noncoding
0.999995	coding
0.999999	coding
0.999953	coding
0.99999	coding
0.99999	coding
0.99997	coding

1	coding
1	coding
0.00379481	noncoding
0.909915	coding
1	coding
1	coding
1	coding
1	coding
0.999998	coding
0.999961	coding
1	coding
0.0419582	noncoding
0.169391	noncoding
0.160672	noncoding
0.169391	noncoding
0.996564	coding
0.0317535	noncoding
0.998987	coding
0.0715847	noncoding
0.999046	coding
0.0867246	noncoding
1	coding
1	coding
1	coding
1	coding
1	coding
1	coding
1	coding
0.999877	coding
0.999862	coding
0.999991	coding
1	coding
1	coding
1	coding
0.988603	coding
0.838825	coding
0.999442	coding
0.999795	coding
0.0190264	noncoding
0.110215	noncoding
0.0284901	noncoding
0.475036	noncoding
0.999868	coding
0.980809	coding
0.102385	noncoding
0.99955	coding
0.99955	coding
1	coding
1	coding
0.999999	coding
0.999999	coding
0.999404	coding
0.0693175	noncoding
1	coding

1	coding
0.0239158	noncoding
1	coding
1	coding
1	coding
0.0119914	noncoding
1	coding
1	coding
0.788845	coding
1	coding
1	coding
0.042323	noncoding
0.0925868	noncoding
0.926873	coding
1	coding
0.876731	coding
0.992981	coding
0.291283	noncoding
0.662927	coding
0.0909257	noncoding
0.242564	noncoding
0.170998	noncoding
0.947273	coding
0.83599	coding
1	coding
0.854553	coding
0.291346	noncoding
0.999997	coding
0.99999	coding
0.0113599	noncoding
0.99998	coding
0.063272	noncoding
0.0278511	noncoding
0.682565	coding
1	coding
0.230564	noncoding
0.362987	noncoding
0.362987	noncoding
1	coding
0.0459009	noncoding
0.0400318	noncoding
1	coding
1	coding
0.0321275	noncoding
1	coding
0.140289	noncoding
0.960862	coding
0.146318	noncoding
0.995625	coding
0.888747	coding
0.996836	coding
0.996938	coding
0.999948	coding
0.999947	coding

0.551644	coding
0.0135293	noncoding
0.999928	coding
1	coding
0.43671	noncoding
0.561874	coding
0.433956	noncoding
1	coding
1	coding
1	coding
0.0638043	noncoding
0.0464895	noncoding
0.173039	noncoding
0.0742855	noncoding
0.0455044	noncoding
0.0404458	noncoding
0.154173	noncoding
0.146268	noncoding
0.542107	coding
1	coding
1	coding
1	coding
0.444854	noncoding
0.0120793	noncoding
0.0839515	noncoding
0.0470503	noncoding
1	coding
0.0361374	noncoding
1	coding
1	coding
0.0928094	noncoding
1	coding
0.999952	coding
1	coding
1	coding
0.999999	coding
0.0740194	noncoding
1	coding
0.33528	noncoding
0.275176	noncoding
0.371782	noncoding
0.14401	noncoding
0.0866285	noncoding
0.0499296	noncoding
0.0275947	noncoding
0.102408	noncoding
0.999089	coding
0.999942	coding
0.999999	coding
0.996335	coding
0.51675	coding
0.0132416	noncoding
0.0140298	noncoding
0.998376	coding

1	coding
0.999942	coding
0.0606757	noncoding
0.0168241	noncoding
0.789774	coding
0.130766	noncoding
0.38861	noncoding
0.149283	noncoding
0.19423	noncoding
1	coding
1	coding
1	coding
0.0934736	noncoding
0.0209303	noncoding
0.0208206	noncoding
1	coding
1	coding
1	coding
1	coding
0.993591	coding
1	coding
1	coding
0.991625	coding
0.954624	coding
0.991103	coding
0.909099	coding
0.991103	coding
0.911023	coding
0.991997	coding
0.0586597	noncoding
1	coding
0.156582	noncoding
0.0758733	noncoding
0.0118995	noncoding
0.999783	coding
0.0128565	noncoding
1	coding
1	coding
1	coding
1	coding
1	coding
1	coding
0.113014	noncoding
0.0286608	noncoding
1	coding
1	coding
0.998764	coding
0.029522	noncoding
0.0903752	noncoding
0.870881	coding
0.577637	coding
0.122308	noncoding
0.124961	noncoding
0.919474	coding

0.73242	coding
0.934188	coding
0.0519711	noncoding
0.58939	coding
0.084258	noncoding
0.369149	noncoding
0.999998	coding
0.882972	coding
0.238318	noncoding
0.0575585	noncoding
0.0160177	noncoding
0.999398	coding
1	coding
1	coding
1	coding
0.0787493	noncoding
0.457208	noncoding
0.998641	coding
0.948207	coding
0.930965	coding
0.982824	coding
0.982824	coding
0.933981	coding
0.0452222	noncoding
0.0330296	noncoding
0.0122775	noncoding
0.999987	coding
0.999998	coding
1	coding
1	coding
1	coding
1	coding
1	coding
0.103344	noncoding
0.0413034	noncoding
0.0539228	noncoding
0.0670011	noncoding
0.0591431	noncoding
0.0135117	noncoding
0.280313	noncoding
0.0881585	noncoding
0.0428306	noncoding
0.0423851	noncoding
0.0426192	noncoding
0.0744191	noncoding
1	coding
0.0422454	noncoding
0.999995	coding
0.165306	noncoding
0.0418453	noncoding
0.4771	noncoding
0.495436	noncoding
0.0700663	noncoding
1	coding

1	coding
0.943678	coding
0.141289	noncoding
1	coding
0.501349	coding
1	coding
1	coding
0.0797763	noncoding
0.0653611	noncoding
0.57291	coding
0.0563865	noncoding
1	coding
0.999783	coding
0.999999	coding
1	coding
0.999948	coding
0.00862545	noncoding
0.999992	coding
0.100814	noncoding
0.0291621	noncoding
0.23875	noncoding
0.484872	noncoding
0.892715	coding
0.970912	coding
0.103997	noncoding
0.00890878	noncoding
0.0103101	noncoding
0.0346366	noncoding
0.0172574	noncoding
0.0306976	noncoding
0.0312844	noncoding
0.0852501	noncoding
0.141475	noncoding
0.110849	noncoding
0.0817881	noncoding
0.0817105	noncoding
0.0356035	noncoding
0.0408528	noncoding
0.0184981	noncoding
0.043582	noncoding
0.0209898	noncoding
0.0093062	noncoding
0.0975794	noncoding
0.163195	noncoding
0.103767	noncoding
0.0643641	noncoding
0.0733971	noncoding
0.462272	noncoding
1	coding
1	coding
0.0474218	noncoding
1	coding
1	coding
1	coding

0.277708	noncoding
0.0137769	noncoding
1	coding
0.209744	noncoding
1	coding
1	coding
0.376646	noncoding
1	coding
1	coding
0.312313	noncoding
0.0652412	noncoding
1	coding
0.999996	coding
1	coding
0.0739165	noncoding
0.0565016	noncoding
0.00894749	noncoding
0.875998	coding
0.180157	noncoding
0.980714	coding
1	coding
0.99999	coding
0.981498	coding
0.999999	coding
1	coding
0.104496	noncoding
0.056383	noncoding
0.534051	coding
0.205093	noncoding
0.0268458	noncoding
0.212402	noncoding
0.736996	coding
1	coding
1	coding
1	coding
1	coding
1	coding
1	coding
0.21648	noncoding
0.234423	noncoding
0.201639	noncoding
0.0924304	noncoding
0.711807	coding
1	coding
1	coding
0.121523	noncoding
0.123729	noncoding
0.969501	coding
0.115553	noncoding
0.997521	coding
0.0458789	noncoding
0.363953	noncoding
0.999932	coding
1	coding

1	coding
1	coding
0.243722	noncoding
0.0502357	noncoding
0.571972	coding
0.0294852	noncoding
1	coding
0.0166262	noncoding
0.999669	coding
0.999931	coding
0.500439	coding
0.554314	coding
0.573088	coding
0.980823	coding
0.957908	coding
0.987566	coding
0.992317	coding
0.986001	coding
0.995618	coding
1	coding
1	coding
0.999967	coding
0.999638	coding
0.999529	coding
0.0379481	noncoding
0.930917	coding
0.918123	coding
0.0952986	noncoding
1	coding
1	coding
1	coding
0.999998	coding
0.999999	coding
1	coding
0.999982	coding
1	coding
1	coding
1	coding
1	coding
1	coding
1	coding
1	coding
1	coding
1	coding
1	coding
1	coding
1	coding
1	coding
1	coding
1	coding
1	coding
1	coding
1	coding
1	coding

1	coding
1	coding
1	coding
1	coding
1	coding
1	coding
1	coding
1	coding
1	coding
1	coding
1	coding
1	coding
0.0410036	noncoding
0.954147	coding
1	coding
1	coding
1	coding
1	coding
1	coding
1	coding
1	coding
1	coding
0.0059171	noncoding
0.066315	noncoding
0.297644	noncoding
0.218971	noncoding
1	coding
0.999997	coding
0.999999	coding
0.882529	coding
0.356406	noncoding
0.0465758	noncoding
0.0721343	noncoding
0.256322	noncoding
0.125051	noncoding
0.999997	coding
0.999857	coding
0.995009	coding
0.15924	noncoding
0.0353423	noncoding
1	coding
0.999951	coding
0.930821	coding
1	coding
1	coding
1	coding
1	coding
0.902846	coding
0.330292	noncoding
0.282189	noncoding
0.22395	noncoding
0.252752	noncoding
0.786745	coding
0.999972	coding

0.999996	coding
0.999994	coding
1	coding
0.919928	coding
1	coding
1	coding
1	coding
1	coding
1	coding
1	coding
1	coding
1	coding
1	coding
1	coding
0.596885	coding
0.0337361	noncoding
1	coding
1	coding
1	coding
1	coding
0.989679	coding
0.971348	coding
0.993378	coding
0.977737	coding
0.994126	coding
0.172834	noncoding
0.999985	coding
1	coding
1	coding
0.996411	coding
0.998058	coding
1	coding
0.0630136	noncoding
0.091436	noncoding
0.999842	coding
1	coding
0.0341238	noncoding
0.0654898	noncoding
0.0442238	noncoding
0.996546	coding
1	coding
1	coding
1	coding
1	coding
0.998914	coding
0.258932	noncoding
0.992489	coding
0.459851	noncoding
0.551164	coding
0.575982	coding
0.103749	noncoding
0.15371	noncoding
0.999934	coding
0.99981	coding

0.901362	coding
1	coding
0.0850091	noncoding
1	coding
0.999999	coding
1	coding
1	coding
0.932254	coding
1	coding
0.999999	coding
0.999928	coding
1	coding
0.933374	coding
1	coding
1	coding
1	coding
1	coding
0.0431292	noncoding
1	coding
1	coding
1	coding
0.326719	noncoding
0.138082	noncoding
0.111964	noncoding
0.00837788	noncoding
0.997116	coding
0.257222	noncoding
0.963704	coding
0.831163	coding
1	coding
0.999973	coding
1	coding
1	coding
1	coding
0.143548	noncoding
0.271134	noncoding
1	coding
1	coding
0.132105	noncoding
1	coding
0.95056	coding
0.974534	coding
0.999998	coding
0.999137	coding
1	coding
0.999999	coding
1	coding
0.0208113	noncoding
0.0117497	noncoding
0.0116877	noncoding
0.0170441	noncoding
0.0140646	noncoding
0.0660343	noncoding
0.220278	noncoding

0.522303	coding
0.999409	coding
0.999926	coding
0.998449	coding
0.996894	coding
0.995557	coding
0.99914	coding
0.999998	coding
1	coding
1	coding
1	coding
0.610574	coding
0.477793	noncoding
0.0923196	noncoding
0.989475	coding
1	coding
1	coding
1	coding
0.301536	noncoding
0.293083	noncoding
0.18819	noncoding
0.0169803	noncoding
0.188456	noncoding
0.0484358	noncoding
0.217551	noncoding
0.999396	coding
0.0203071	noncoding
0.0105948	noncoding
1	coding
1	coding
1	coding
0.2753	noncoding
1	coding
1	coding
1	coding
1	coding
1	coding
1	coding
0.471516	noncoding
0.488077	noncoding
0.844473	coding
0.835659	coding
0.33187	noncoding
0.56386	coding
1	coding
0.93092	coding
0.991465	coding
0.998756	coding
0.139306	noncoding
0.722387	coding
0.975636	coding
0.938354	coding
0.901208	coding
0.822252	coding

0.215564	noncoding
0.943987	coding
0.935617	coding
1	coding
0.997543	coding
0.999035	coding
1	coding
0.0988619	noncoding
1	coding
1	coding
1	coding
0.828751	coding
0.954842	coding
1	coding
1	coding
0.993522	coding
0.262093	noncoding
1	coding
1	coding
1	coding
1	coding
1	coding
1	coding
0.945705	coding
0.721891	coding
0.174915	noncoding
0.839218	coding
0.0414625	noncoding
1	coding
1	coding
1	coding
1	coding
1	coding
1	coding
1	coding
0.0285677	noncoding
0.0307231	noncoding
0.990434	coding
0.999999	coding
0.0381011	noncoding
0.999999	coding
0.0386043	noncoding
1	coding
0.199541	noncoding
1	coding
0.0516656	noncoding
0.0312629	noncoding
0.467064	noncoding
1	coding
1	coding
1	coding
1	coding
0.999996	coding
0.0558555	noncoding

1	coding
0.0661728	noncoding
1	coding
0.998478	coding
1	coding
1	coding
0.265608	noncoding
0.963852	coding
0.187004	noncoding
1	coding
0.995797	coding
0.917205	coding
0.175649	noncoding
0.0213101	noncoding
1	coding
1	coding
1	coding
0.747589	coding
0.745764	coding
0.00589845	noncoding
1	coding
1	coding
0.519692	coding
0.19957	noncoding
1	coding
1	coding
1	coding
1	coding
1	coding
1	coding
1	coding
1	coding
1	coding
1	coding
0.976887	coding
1	coding
1	coding
1	coding
0.685585	coding
0.676695	coding
0.98733	coding
0.0230575	noncoding
0.157595	noncoding
0.999983	coding
1	coding
1	coding
1	coding
1	coding
1	coding
0.999999	coding
1	coding
0.0586192	noncoding
0.980825	coding
0.999744	coding

0.151682	noncoding
1	coding
1	coding
0.0625855	noncoding
0.0167907	noncoding
0.999634	coding
0.999639	coding
0.92237	coding
0.92237	coding
0.904208	coding
0.217441	noncoding
0.631161	coding
0.108544	noncoding
0.132138	noncoding
0.213032	noncoding
0.0138042	noncoding
0.051862	noncoding
0.0808917	noncoding
0.889722	coding
0.191577	noncoding
0.0684478	noncoding
0.972411	coding
0.999836	coding
0.440561	noncoding
0.637591	coding
0.17849	noncoding
0.17849	noncoding
0.120765	noncoding
0.202183	noncoding
1	coding
0.999993	coding
1	coding
1	coding
1	coding
1	coding
0.0314569	noncoding
1	coding
0.999995	coding
0.99999	coding
0.999995	coding
1	coding
1	coding
1	coding
1	coding
1	coding
1	coding
1	coding
1	coding
0.999999	coding
0.999999	coding
0.0169224	noncoding
1	coding
1	coding
1	coding

0.0465156	noncoding
1	coding
0.0704247	noncoding
1	coding
1	coding
1	coding
1	coding
1	coding
0.122443	noncoding
0.173514	noncoding
0.891191	coding
0.343252	noncoding
0.0187815	noncoding
0.0381493	noncoding
1	coding
0.139369	noncoding
0.255548	noncoding
0.224939	noncoding
0.224939	noncoding
0.237593	noncoding
0.30198	noncoding
0.0221883	noncoding
0.0232927	noncoding
0.0235621	noncoding
0.15941	noncoding
0.0410623	noncoding
0.0315708	noncoding
0.240315	noncoding
0.191638	noncoding
0.0388743	noncoding
0.0700687	noncoding
0.0527601	noncoding
0.02812	noncoding
0.242632	noncoding
0.0385721	noncoding
0.795498	coding
0.276995	noncoding
0.103931	noncoding
0.276995	noncoding
1	coding
1	coding
0.994147	coding
1	coding
1	coding
1	coding
0.664098	coding
0.755619	coding
0.946517	coding
0.0234175	noncoding
0.5398	coding
0.136317	noncoding
0.37867	noncoding
1	coding
0.503401	coding

1	coding
1	coding
1	coding
0.514122	coding
1	coding
0.376717	noncoding
1	coding
1	coding
0.0206827	noncoding
0.0605167	noncoding
1	coding
1	coding
1	coding
1	coding
0.0222786	noncoding
0.933596	coding
0.0708627	noncoding
1	coding
1	coding
0.0440428	noncoding
0.0883967	noncoding
0.487341	noncoding
1	coding
1	coding
0.999995	coding
0.0671815	noncoding
0.220018	noncoding
0.225419	noncoding
0.225419	noncoding
1	coding
1	coding
1	coding
1	coding
0.999991	coding
1	coding
1	coding
0.0883299	noncoding
0.999925	coding
0.997796	coding
1	coding
1	coding
1	coding
1	coding
0.709572	coding
1	coding
0.998615	coding
1	coding
0.977292	coding
0.998311	coding
0.998637	coding
0.977292	coding
0.999983	coding
0.037411	noncoding
0.00358371	noncoding

1	coding
1	coding
0.39746	noncoding
0.206623	noncoding
0.0365264	noncoding
1	coding
0.999999	coding
1	coding
0.0958307	noncoding
0.154649	noncoding
0.0613844	noncoding
0.999833	coding
0.999902	coding
1	coding
0.0499571	noncoding
0.155763	noncoding
0.0219577	noncoding
0.0217238	noncoding
0.0210587	noncoding
0.0983982	noncoding
1	coding
1	coding
1	coding
0.999992	coding
1	coding
0.999909	coding
0.982668	coding
0.99986	coding
0.0305827	noncoding
1	coding
1	coding
1	coding
1	coding
1	coding
1	coding
1	coding
0.999181	coding
1	coding
1	coding
1	coding
1	coding
1	coding
1	coding
0.0167119	noncoding
0.246919	noncoding
0.999998	coding
0.0768043	noncoding
0.999994	coding
0.178219	noncoding
0.999028	coding
1	coding
0.0428224	noncoding
0.185557	noncoding
0.0426892	noncoding

1	coding
0.131764	noncoding
1	coding
1	coding
0.992485	coding
0.824554	coding
1	coding
1	coding
0.690832	coding
0.380217	noncoding
0.999715	coding
0.394326	noncoding
1	coding
1	coding
0.999975	coding
0.999983	coding
0.97701	coding
0.97701	coding
0.994145	coding
0.994045	coding
0.0447819	noncoding
0.104714	noncoding
0.122891	noncoding
0.215391	noncoding
1	coding
1	coding
0.179558	noncoding
0.208019	noncoding
0.0416562	noncoding
0.0192275	noncoding
1	coding
0.999962	coding
0.997256	coding
0.0555929	noncoding
1	coding
1	coding
0.747745	coding
0.554395	coding
0.995719	coding
1	coding
0.0245846	noncoding
1	coding
1	coding
0.0596809	noncoding
0.249256	noncoding
1	coding
1	coding
1	coding
1	coding
1	coding
1	coding
1	coding
1	coding
1	coding

1	coding
1	coding
0.987077	coding
0.999836	coding
0.0800589	noncoding
0.0137639	noncoding
0.0836692	noncoding
1	coding
1	coding
1	coding
0.997442	coding
0.999999	coding
1	coding
0.0414396	noncoding
1	coding
1	coding
0.0490892	noncoding
0.0511468	noncoding
0.00880244	noncoding
1	coding
1	coding
0.969607	coding
0.965818	coding
1	coding
1	coding
1	coding
1	coding
0.148498	noncoding
1	coding
1	coding
0.105747	noncoding
0.979248	coding
0.945624	coding
0.928569	coding
0.933007	coding
1	coding
1	coding
1	coding
1	coding
1	coding
1	coding
1	coding
1	coding
1	coding
1	coding
1	coding
1	coding
1	coding
1	coding
1	coding
1	coding
0.609287	coding
0.10347	noncoding
1	coding

1	coding
1	coding
1	coding
1	coding
1	coding
0.0234268	noncoding
1	coding
1	coding
0.0647127	noncoding
0.998938	coding
0.00481813	noncoding
1	coding
1	coding
0.999991	coding
1	coding
1	coding
1	coding
1	coding
1	coding
1	coding
0.265257	noncoding
0.135389	noncoding
0.237733	noncoding
1	coding
1	coding
1	coding
0.0603945	noncoding
0.969513	coding
0.215526	noncoding
0.158049	noncoding
0.998469	coding
1	coding
1	coding
1	coding
1	coding
1	coding
1	coding
0.981001	coding
1	coding
0.973297	coding
1	coding
0.12436	noncoding
0.966084	coding
0.999942	coding
0.997674	coding
0.217221	noncoding
1	coding
0.999976	coding
1	coding
0.999939	coding
0.999961	coding
0.999998	coding
1	coding
1	coding

1	coding
1	coding
1	coding
1	coding
1	coding
1	coding
1	coding
1	coding
1	coding
1	coding
1	coding
1	coding
0.0732808	noncoding
0.999981	coding
0.999985	coding
1	coding
1	coding
0.998923	coding
0.99979	coding
1	coding
1	coding
0.877795	coding
1	coding
1	coding
1	coding
1	coding
1	coding
1	coding
1	coding
0.0669214	noncoding
0.496515	noncoding
0.158528	noncoding
0.0652423	noncoding
1	coding
1	coding
1	coding
1	coding
0.0358073	noncoding
0.0244191	noncoding
1	coding
0.934769	coding
1	coding
1	coding
1	coding
0.991293	coding
0.0547623	noncoding
1	coding
0.331621	noncoding
0.26129	noncoding
0.236629	noncoding
0.283145	noncoding
0.708998	coding
0.905146	coding
0.975677	coding

0.903765	coding
0.425068	noncoding
0.974348	coding
0.97695	coding
1	coding
1	coding
0.997681	coding
0.579607	coding
0.998833	coding
1	coding
0.999988	coding
0.360617	noncoding
0.32921	noncoding
0.349611	noncoding
0.349611	noncoding
0.229906	noncoding
0.586634	coding
1	coding
0.528832	coding
0.0680645	noncoding
0.733262	coding
0.377827	noncoding
1	coding
1	coding
0.991966	coding
1	coding
0.151501	noncoding
0.1151	noncoding
0.166009	noncoding
0.290423	noncoding
0.187164	noncoding
0.0669511	noncoding
0.273597	noncoding
0.117638	noncoding
0.693579	coding
0.103982	noncoding
0.0544648	noncoding
0.994455	coding
0.999997	coding
0.98967	coding
0.98967	coding
0.795239	coding
0.485424	noncoding
0.0267248	noncoding
0.999942	coding
1	coding
1	coding
1	coding
0.101353	noncoding
0.102014	noncoding
1	coding
0.263225	noncoding
0.0112508	noncoding
0.00193054	noncoding

0.0226632	noncoding
0.00204047	noncoding
0.00323993	noncoding
0.00446321	noncoding
0.0089764	noncoding
0.00745153	noncoding
0.0261928	noncoding
0.0278957	noncoding
0.997685	coding
0.130691	noncoding
0.120191	noncoding
0.129141	noncoding
0.0227991	noncoding
0.0189594	noncoding
0.715157	coding
0.399727	noncoding
0.0055529	noncoding
0.0384596	noncoding
0.021435	noncoding
0.078031	noncoding
0.0672295	noncoding
0.0210771	noncoding
0.118675	noncoding
0.0835054	noncoding
0.030554	noncoding
0.0583155	noncoding
0.0647549	noncoding
0.0939869	noncoding
0.922085	coding
0.923134	coding
0.151667	noncoding
0.147042	noncoding
0.170407	noncoding
0.191794	noncoding
0.124749	noncoding
0.167143	noncoding
0.188814	noncoding
0.0144885	noncoding
0.999999	coding
0.0120315	noncoding
0.0182219	noncoding
0.0387485	noncoding
0.0162271	noncoding
0.993534	coding
0.665833	coding
0.990359	coding
0.999977	coding
0.999998	coding
0.999582	coding
0.0353683	noncoding
0.0233149	noncoding
0.0233149	noncoding
0.243038	noncoding
0.0174901	noncoding

0.0323972	noncoding
0.0161723	noncoding
0.112111	noncoding
0.0826964	noncoding
0.0826964	noncoding
0.0826964	noncoding
0.0283704	noncoding
0.108804	noncoding
0.118929	noncoding
0.18883	noncoding
0.260782	noncoding
0.0321816	noncoding
0.01594	noncoding
0.0146142	noncoding
0.0656236	noncoding
0.11023	noncoding
1	coding
0.000402857	noncoding
0.000461091	noncoding
1	coding
1	coding
1	coding
0.170857	noncoding
0.173252	noncoding
0.0184957	noncoding
0.0188565	noncoding
0.171236	noncoding
0.0200546	noncoding
0.418115	noncoding
0.0134421	noncoding
0.0323437	noncoding
0.0117073	noncoding
0.0695611	noncoding
0.688903	coding
0.96039	coding
0.502819	coding
0.629102	coding
0.121182	noncoding
0.0343959	noncoding
0.576855	coding
0.997865	coding
0.576855	coding
0.115172	noncoding
0.0926347	noncoding
0.0672923	noncoding
0.0396806	noncoding
0.336317	noncoding
0.0516064	noncoding
0.0197784	noncoding
0.0530228	noncoding
0.12867	noncoding
0.0392631	noncoding
0.0958639	noncoding
0.235841	noncoding

0.0664591	noncoding
0.111223	noncoding
0.0976918	noncoding
0.0245746	noncoding
0.0245746	noncoding
0.00879805	noncoding
0.130566	noncoding
0.0569996	noncoding
0.883942	coding
0.911041	coding
0.952409	coding
0.989239	coding
0.960324	coding
0.960324	coding
0.401518	noncoding
0.0285732	noncoding
0.039451	noncoding
0.0617513	noncoding
0.0791762	noncoding
0.0343066	noncoding
0.03273	noncoding
0.0922626	noncoding
0.998685	coding
0.919592	coding
0.997929	coding
0.909819	coding
0.507866	coding
0.0234751	noncoding
0.183684	noncoding
0.176876	noncoding
0.079193	noncoding
0.382537	noncoding
0.119138	noncoding
0.379098	noncoding
0.0194879	noncoding
0.136234	noncoding
0.0968591	noncoding
0.0174739	noncoding
0.17494	noncoding
0.317511	noncoding
0.107279	noncoding
0.269733	noncoding
0.110283	noncoding
0.110283	noncoding
0.112146	noncoding
0.110343	noncoding
0.0967864	noncoding
0.0669604	noncoding
0.17471	noncoding
0.17471	noncoding
0.0815639	noncoding
0.0665646	noncoding
0.175355	noncoding
0.472838	noncoding

0.0278019	noncoding
0.0323972	noncoding
0.0323972	noncoding
0.0350672	noncoding
0.241849	noncoding
0.358364	noncoding
0.24931	noncoding
0.0410907	noncoding
0.0509154	noncoding
0.0238009	noncoding
0.131848	noncoding
0.104617	noncoding
0.0154847	noncoding
0.0325011	noncoding
0.152124	noncoding
0.0378384	noncoding
0.0177567	noncoding
0.0274777	noncoding
0.221654	noncoding
0.999998	coding
0.192094	noncoding
0.0256445	noncoding
0.135862	noncoding
0.132093	noncoding
0.1819	noncoding
0.96935	coding
0.161837	noncoding
0.958201	coding
0.277706	noncoding
0.028876	noncoding
0.0243863	noncoding
0.0203453	noncoding
0.999336	coding
0.0663277	noncoding
0.166803	noncoding
0.00110406	noncoding
0.000397128	noncoding
0.00073563	noncoding
0.00073563	noncoding
0.000553359	noncoding
0.00267832	noncoding
0.015648	noncoding
0.0012749	noncoding
0.0126291	noncoding
0.00135738	noncoding
0.999122	coding
0.964673	coding
0.0320068	noncoding
0.0227355	noncoding
1	coding
0.0206866	noncoding
0.0138272	noncoding
0.583338	coding
0.150198	noncoding

0.126117	noncoding
0.126117	noncoding
0.0852824	noncoding
0.0971174	noncoding
0.0694245	noncoding
0.150198	noncoding
0.149121	noncoding
0.146247	noncoding
0.146247	noncoding
0.115524	noncoding
0.154206	noncoding
0.126176	noncoding
0.140509	noncoding
0.140509	noncoding
0.140509	noncoding
0.0463722	noncoding
0.163979	noncoding
0.999997	coding
0.101297	noncoding
0.0220788	noncoding
0.104941	noncoding
0.428751	noncoding
0.0487107	noncoding
0.114518	noncoding
0.081286	noncoding
0.0610134	noncoding
0.056669	noncoding
0.087425	noncoding
0.868766	coding
0.971459	coding
0.0845443	noncoding
0.779603	coding
0.829507	coding
0.981107	coding
0.0728061	noncoding
0.923835	coding
0.890847	coding
0.047489	noncoding
0.0799962	noncoding
0.0263568	noncoding
0.0784148	noncoding
1	coding
1	coding
0.150198	noncoding
0.213417	noncoding
0.20536	noncoding
1	coding
1	coding
1	coding
0.178509	noncoding
0.00754453	noncoding
0.920908	coding
0.922488	coding
0.00477992	noncoding

1	coding
1	coding
1	coding
1	coding
0.0901847	noncoding
0.0539573	noncoding
0.988319	coding
0.979137	coding
0.00889107	noncoding
0.0486195	noncoding
0.124697	noncoding
0.0520025	noncoding
0.183924	noncoding
0.136749	noncoding
0.0441802	noncoding
0.00196189	noncoding
0.0441802	noncoding
0.00184247	noncoding
0.00184802	noncoding
0.0285074	noncoding
0.00653133	noncoding
0.022752	noncoding
0.112749	noncoding
0.00239394	noncoding
0.157786	noncoding
0.165076	noncoding
0.0731713	noncoding
0.0731713	noncoding
0.207868	noncoding
0.207868	noncoding
0.214423	noncoding
0.207868	noncoding
0.207868	noncoding
0.207868	noncoding
0.222862	noncoding
0.207868	noncoding
0.212235	noncoding
0.405914	noncoding
0.916279	coding
0.891035	coding
0.241544	noncoding
0.0341228	noncoding
0.0271877	noncoding
0.0271588	noncoding
0.0483438	noncoding
0.799849	coding
0.255756	noncoding
0.252038	noncoding
0.255756	noncoding
0.195085	noncoding
0.0822801	noncoding
0.272653	noncoding
0.255756	noncoding
0.017363	noncoding

0.069514	noncoding
0.0691951	noncoding
0.0184402	noncoding
0.0193157	noncoding
0.16018	noncoding
0.0551274	noncoding
0.000450717	noncoding
0.0673139	noncoding
0.014644	noncoding
0.0460821	noncoding
0.195295	noncoding
0.0728577	noncoding
0.0191612	noncoding
0.17696	noncoding
0.127496	noncoding
0.118436	noncoding
0.612041	coding
0.155893	noncoding
0.381111	noncoding
0.231104	noncoding
0.999992	coding
0.964121	coding
0.614182	coding
0.789612	coding
0.202952	noncoding
0.678533	coding
0.0594075	noncoding
0.413857	noncoding
0.797023	coding
0.100336	noncoding
0.0560092	noncoding
0.00685208	noncoding
0.00322932	noncoding
0.0105425	noncoding
0.00802926	noncoding
0.00339685	noncoding
0.990298	coding
0.0169005	noncoding
0.0538295	noncoding
0.00912665	noncoding
0.21378	noncoding
0.0607987	noncoding
0.251106	noncoding
0.16541	noncoding
0.16541	noncoding
0.0770353	noncoding
0.0770353	noncoding
0.169806	noncoding
0.110424	noncoding
0.0264339	noncoding
0.256961	noncoding
0.0317964	noncoding
0.0186651	noncoding
0.0181219	noncoding

0.0535198	noncoding
0.0489481	noncoding
0.0461163	noncoding
0.0423633	noncoding
1	coding
0.376027	noncoding
0.282697	noncoding
0.519052	coding
0.282697	noncoding
0.378324	noncoding
1	coding
0.19072	noncoding
0.987672	coding
0.999995	coding
0.0254357	noncoding
0.0330119	noncoding
0.0352139	noncoding
0.997437	coding
0.413722	noncoding
1	coding
0.780995	coding
0.813771	coding
0.18058	noncoding
0.0968213	noncoding
0.148992	noncoding
0.186869	noncoding
0.148992	noncoding
0.0118284	noncoding
0.0109427	noncoding
0.0131969	noncoding
0.100983	noncoding
0.321692	noncoding
0.134732	noncoding
0.134732	noncoding
0.120673	noncoding
0.0173127	noncoding
0.0970274	noncoding
0.0970274	noncoding
0.104657	noncoding
0.0970274	noncoding
0.104807	noncoding
0.00630033	noncoding
0.0054736	noncoding
0.00201876	noncoding
0.0908109	noncoding
0.0736648	noncoding
0.0399358	noncoding
0.0326794	noncoding
0.305475	noncoding
0.890015	coding
0.995005	coding
0.922028	coding
0.102867	noncoding
0.0536577	noncoding

0.00597706	noncoding
0.00719013	noncoding
0.00719013	noncoding
0.0379917	noncoding
0.00759567	noncoding
0.419784	noncoding
0.0448864	noncoding
0.863688	coding
0.935492	coding
0.935492	coding
0.0230418	noncoding
1	coding
0.999999	coding
1	coding
1	coding
1	coding
0.000402811	noncoding
0.00614537	noncoding
0.027904	noncoding
0.000394785	noncoding
0.995762	coding
0.995977	coding
0.995977	coding
0.0345781	noncoding
0.0790552	noncoding
0.0394863	noncoding
0.0219291	noncoding
0.0278584	noncoding
0.0690912	noncoding
0.381766	noncoding
0.119043	noncoding
0.0600822	noncoding
0.109042	noncoding
0.120903	noncoding
0.0625461	noncoding
0.0625461	noncoding
0.102985	noncoding
0.0265157	noncoding
0.0108638	noncoding
0.0442397	noncoding
0.0842058	noncoding
0.209379	noncoding
0.105946	noncoding
0.0530265	noncoding
0.0451978	noncoding
0.0496239	noncoding
0.000574412	noncoding
0.083482	noncoding
0.0215521	noncoding
0.0284717	noncoding
0.0202834	noncoding
0.131233	noncoding
0.00432254	noncoding
1	coding

0.0780824	noncoding
0.0831694	noncoding
0.0831694	noncoding
0.159834	noncoding
0.0554308	noncoding
0.026831	noncoding
0.00983627	noncoding
0.0918095	noncoding
0.0892465	noncoding
0.107306	noncoding
0.196297	noncoding
0.196297	noncoding
0.325619	noncoding
0.147801	noncoding
0.0338737	noncoding
0.00690008	noncoding
0.196297	noncoding
0.0334922	noncoding
0.157331	noncoding
0.252785	noncoding
0.416647	noncoding
0.2534	noncoding
0.188195	noncoding
0.227868	noncoding
0.0252988	noncoding
0.103689	noncoding
0.0751527	noncoding
0.0698126	noncoding
0.140356	noncoding
0.0089567	noncoding
0.9976	coding
0.242258	noncoding
0.995615	coding
0.0983312	noncoding
0.9749	coding
1	coding
0.0032374	noncoding
0.00191637	noncoding
0.00104104	noncoding
0.000450717	noncoding
0.0200411	noncoding
0.453051	noncoding
0.996904	coding
0.861124	coding
0.165489	noncoding
0.120827	noncoding
0.171496	noncoding
0.340544	noncoding
0.453911	noncoding
0.0564795	noncoding
0.314005	noncoding
0.0651806	noncoding
0.541624	coding
0.242684	noncoding

0.622609	coding
0.55495	coding
0.760951	coding
0.618247	coding
0.0613206	noncoding
0.778745	coding
0.524628	coding
0.675008	coding
0.77478	coding
0.385259	noncoding
0.0197683	noncoding
0.524628	coding
0.00143366	noncoding
0.999708	coding
0.600835	coding
0.714352	coding
0.581631	coding
0.993129	coding
0.786702	coding
0.00102927	noncoding
0.10487	noncoding
0.0511661	noncoding
0.130988	noncoding
0.0637058	noncoding
0.0503929	noncoding
0.00465241	noncoding
0.0251101	noncoding
0.00183841	noncoding
0.999989	coding
0.0212837	noncoding
0.0816635	noncoding
0.124391	noncoding
0.000534766	noncoding
0.158906	noncoding
0.18926	noncoding
0.18926	noncoding
0.179728	noncoding
0.171242	noncoding
0.197177	noncoding
0.279798	noncoding
0.279798	noncoding
0.279798	noncoding
0.00876626	noncoding
0.296608	noncoding
0.0110923	noncoding
0.279798	noncoding
0.00879981	noncoding
0.00923713	noncoding
0.0110923	noncoding
0.296608	noncoding
0.00867239	noncoding
0.00867214	noncoding
0.00895289	noncoding
0.0381461	noncoding

0.0199773	noncoding
0.019817	noncoding
0.0196661	noncoding
0.0108525	noncoding
0.0676191	noncoding
0.0687929	noncoding
0.116664	noncoding
0.139062	noncoding
0.0732742	noncoding
0.29543	noncoding
0.0554186	noncoding
0.231773	noncoding
0.0501026	noncoding
0.0395065	noncoding
0.178429	noncoding
0.178429	noncoding
0.0740637	noncoding
0.174161	noncoding
0.178429	noncoding
0.183905	noncoding
0.188787	noncoding
0.999896	coding
0.999884	coding
0.0335877	noncoding
0.447676	noncoding
0.189103	noncoding
0.883199	coding
0.994395	coding
1	coding
1	coding
0.997305	coding
0.168313	noncoding
0.0517984	noncoding
0.0523451	noncoding
0.0654281	noncoding
0.0106681	noncoding
0.0312414	noncoding
0.0339179	noncoding
0.0731065	noncoding
0.696862	coding
0.0107727	noncoding
0.465371	noncoding
0.0113259	noncoding
0.948057	coding
0.939123	coding
0.0801639	noncoding
0.086546	noncoding
0.0751375	noncoding
0.0713393	noncoding
0.999996	coding
1	coding
1	coding
1	coding
1	coding

1	coding
1	coding
0.13179	noncoding
0.107206	noncoding
0.056369	noncoding
0.0347182	noncoding
0.0164076	noncoding
0.789068	coding
0.0134923	noncoding
0.0370436	noncoding
1	coding
0.999993	coding
0.681278	coding
0.0222371	noncoding
0.174539	noncoding
0.0680724	noncoding
0.0354688	noncoding
0.999999	coding
0.00485279	noncoding
0.0191537	noncoding
0.999972	coding
0.0153236	noncoding
0.0270719	noncoding
0.0572872	noncoding
0.161779	noncoding
0.999868	coding
0.289232	noncoding
0.0224116	noncoding
0.0464559	noncoding
0.223766	noncoding
1	coding
0.00471072	noncoding
0.246527	noncoding
0.89476	coding
0.76133	coding
0.139355	noncoding
0.287942	noncoding
0.999999	coding
1	coding
0.999999	coding
1	coding
0.998401	coding
1	coding
1	coding
0.224365	noncoding
1	coding
0.242713	noncoding
0.290575	noncoding
0.35365	noncoding
0.299162	noncoding
0.334356	noncoding
0.334356	noncoding
0.363235	noncoding
0.363235	noncoding
