# Supplementary material for: Identification of Novel lncRNA and Differentially Expressed Genes (DEGs) of Testicular Tissues among Cattle, Yak, and Cattle-Yak Associated with Male Infertility
Source: Animals (Basel). 2021 Aug 17;11(8):2420. doi: 10.3390/ani11082420 (PMC8388754; doi:10.3390/ani11082420)
Supplement: Supplementary file 1 [file animals-11-02420-s001.zip › Table S3_pfam_result.pdf]

MSTRG.12.1	2283	coding	PF00517
MSTRG.12.2	676	noncoding	-
MSTRG.12.3	1262	noncoding	-
MSTRG.32.2	4334	noncoding	-
MSTRG.33.1	420	noncoding	-
MSTRG.34.2	1149	noncoding	-
MSTRG.35.1	867	coding	PF14981
MSTRG.39.3	1065	noncoding	-
MSTRG.39.6	10401	coding	PF00474
MSTRG.59.3	1764	coding	PF16617
MSTRG.60.1	2252	noncoding	-
MSTRG.61.1	1132	noncoding	-
MSTRG.60.2	4486	noncoding	-
MSTRG.63.2	4052	noncoding	-
MSTRG.63.4	3991	noncoding	-
MSTRG.63.6	6056	noncoding	-
MSTRG.63.7	3748	noncoding	-
MSTRG.63.5	3494	noncoding	-
MSTRG.63.8	3902	noncoding	-
MSTRG.63.3	4087	noncoding	-
MSTRG.65.10	8160	noncoding	-
MSTRG.65.11	8199	noncoding	-
MSTRG.65.4	8008	noncoding	-
MSTRG.65.5	8193	noncoding	-
MSTRG.65.6	8029	noncoding	-
MSTRG.65.8	8140	noncoding	-
MSTRG.65.9	8190	noncoding	-
MSTRG.65.14	7836	noncoding	-
MSTRG.65.15	7806	noncoding	-
MSTRG.65.16	7815	noncoding	-
MSTRG.66.1	4138	coding	PF01071
MSTRG.66.2	4105	coding	PF01071
MSTRG.66.3	4040	coding	PF01071
MSTRG.66.4	3995	coding	PF01071
MSTRG.68.2	3679	coding	PF09350
MSTRG.42.1	5454	noncoding	-
MSTRG.56.2	4773	noncoding	-
MSTRG.56.4	4711	noncoding	-
MSTRG.56.6	7508	noncoding	-
MSTRG.56.7	2812	noncoding	-
MSTRG.56.8	2870	noncoding	-
MSTRG.52.1	7093	coding	PF02383
MSTRG.52.2	6984	coding	PF02383
MSTRG.53.1	1555	coding	PF11069
MSTRG.53.2	1632	coding	PF11069
MSTRG.70.1	3716	noncoding	-
MSTRG.73.2	3795	noncoding	-
MSTRG.78.1	7956	noncoding	-
MSTRG.79.2	2529	noncoding	-
MSTRG.78.2	8273	noncoding	-
MSTRG.80.1	328	noncoding	-
MSTRG.81.1	1462	coding	PF00078
MSTRG.79.3	9939	noncoding	-
MSTRG.82.1	446	noncoding	-

MSTRG.79.4	3123	noncoding	-
MSTRG.79.5	6284	noncoding	-
MSTRG.79.6	3198	noncoding	-
MSTRG.83.1	3477	noncoding	-
MSTRG.79.7	9751	noncoding	-
MSTRG.85.1	1564	noncoding	-
MSTRG.86.1	6606	noncoding	-
MSTRG.96.9	3025	coding	PF00443
MSTRG.96.10	3187	coding	PF00443
MSTRG.96.6	4207	coding	PF00443
MSTRG.96.8	3423	coding	PF00443
MSTRG.96.3	3545	coding	PF00443
MSTRG.97.1	2424	noncoding	-
MSTRG.97.2	2332	noncoding	-
MSTRG.91.1	1940	noncoding	-
MSTRG.103.6	3212	noncoding	-
MSTRG.103.2	7991	noncoding	-
MSTRG.103.5	7127	noncoding	-
MSTRG.103.4	5797	noncoding	-
MSTRG.104.1	1278	noncoding	-
MSTRG.104.3	822	noncoding	-
MSTRG.111.1	462	noncoding	-
MSTRG.117.6	1795	coding	PF00178
MSTRG.118.5	509	coding	PF05511
MSTRG.123.1	386	noncoding	-
MSTRG.127.1	672	noncoding	-
MSTRG.131.1	226	noncoding	-
MSTRG.132.1	2022	noncoding	-
MSTRG.134.1	408	noncoding	-
MSTRG.151.1	2839	noncoding	-
MSTRG.188.1	18614	coding	PF00078
MSTRG.189.1	3356	coding	PF02994
MSTRG.193.1	669	noncoding	-
MSTRG.196.1	1087	coding	PF00078
MSTRG.223.1	670	noncoding	-
MSTRG.241.1	1743	noncoding	-
MSTRG.158.1	9706	noncoding	-
MSTRG.158.6	3741	noncoding	-
MSTRG.158.5	3712	noncoding	-
MSTRG.158.4	3958	noncoding	-
MSTRG.158.8	3405	noncoding	-
MSTRG.158.7	3554	noncoding	-
MSTRG.158.9	4805	coding	PF00443
MSTRG.160.1	1441	noncoding	-
MSTRG.160.3	7489	coding	PF15690
MSTRG.160.4	7684	coding	PF15690
MSTRG.160.5	6419	coding	PF15690
MSTRG.160.6	7735	coding	PF15690
MSTRG.161.1	806	noncoding	-
MSTRG.162.1	11244	noncoding	-
MSTRG.162.2	4935	noncoding	-
MSTRG.160.7	1232	noncoding	-
MSTRG.163.1	2378	coding	PF00012
MSTRG.163.3	4264	coding	PF00012

MSTRG.164.1	948	noncoding	-
MSTRG.166.2	1809	coding	PF00076
MSTRG.168.2	518	noncoding	-
MSTRG.169.1	333	noncoding	-
MSTRG.175.3	5067	coding	PF07679
MSTRG.175.4	3094	noncoding	-
MSTRG.175.5	3963	coding	PF07679
MSTRG.175.6	3343	coding	PF07679
MSTRG.265.8	4630	noncoding	-
MSTRG.265.9	16818	coding	PF00078
MSTRG.266.1	1593	noncoding	-
MSTRG.267.1	7874	noncoding	-
MSTRG.268.1	5779	noncoding	-
MSTRG.269.1	1804	noncoding	-
MSTRG.268.2	2022	noncoding	-
MSTRG.270.1	5102	noncoding	-
MSTRG.271.1	4432	noncoding	-
MSTRG.272.1	10685	noncoding	-
MSTRG.273.1	3854	noncoding	-
MSTRG.274.1	4757	noncoding	-
MSTRG.275.1	2568	noncoding	-
MSTRG.276.1	2355	noncoding	-
MSTRG.277.1	6352	noncoding	-
MSTRG.277.2	4614	noncoding	-
MSTRG.278.1	15264	noncoding	-
MSTRG.265.10	5365	noncoding	-
MSTRG.265.11	15534	noncoding	-
MSTRG.279.1	13704	noncoding	-
MSTRG.265.12	10450	noncoding	-
MSTRG.280.1	5462	noncoding	-
MSTRG.281.1	14521	noncoding	-
MSTRG.282.1	7437	noncoding	-
MSTRG.283.1	5415	noncoding	-
MSTRG.284.1	6419	noncoding	-
MSTRG.284.2	4997	noncoding	-
MSTRG.265.14	31168	coding	PF00078
MSTRG.285.1	6274	noncoding	-
MSTRG.250.1	3949	noncoding	-
MSTRG.255.1	3932	noncoding	-
MSTRG.258.1	7201	noncoding	-
MSTRG.261.1	1785	noncoding	-
MSTRG.262.1	4299	noncoding	-
MSTRG.263.1	2450	noncoding	-
MSTRG.297.1	987	noncoding	-
MSTRG.307.1	11283	noncoding	-
MSTRG.308.1	3366	noncoding	-
MSTRG.312.1	900	noncoding	-
MSTRG.314.1	2186	noncoding	-
MSTRG.314.3	5604	noncoding	-
MSTRG.325.3	2349	coding	PF01404
MSTRG.325.4	2546	coding	PF01404
MSTRG.326.1	4429	noncoding	-
MSTRG.327.1	5165	noncoding	-
MSTRG.324.1	377	noncoding	-

MSTRG.328.1	1297	noncoding	-
MSTRG.328.2	4067	noncoding	-
MSTRG.332.1	5526	noncoding	-
MSTRG.348.1	277	noncoding	-
MSTRG.351.1	2447	coding	PF00025
MSTRG.352.1	6301	noncoding	-
MSTRG.353.1	1004	noncoding	-
MSTRG.356.1	1752	noncoding	-
MSTRG.361.1	224	noncoding	-
MSTRG.375.2	11388	coding	PF00025
MSTRG.375.5	10605	coding	PF00030
MSTRG.377.1	1084	noncoding	-
MSTRG.377.2	1407	noncoding	-
MSTRG.379.3	1947	coding	PF08007
MSTRG.379.5	1795	coding	PF08007
MSTRG.369.1	921	noncoding	-
MSTRG.370.1	213	noncoding	-
MSTRG.371.1	1977	noncoding	-
MSTRG.381.3	5793	coding	PF00754
MSTRG.396.1	7683	noncoding	-
MSTRG.397.1	11618	noncoding	-
MSTRG.396.3	4020	noncoding	-
MSTRG.398.1	7300	noncoding	-
MSTRG.399.1	4279	noncoding	-
MSTRG.400.1	5086	noncoding	-
MSTRG.401.1	3548	noncoding	-
MSTRG.404.3	3800	noncoding	-
MSTRG.404.4	2391	noncoding	-
MSTRG.404.6	3203	noncoding	-
MSTRG.404.5	3481	noncoding	-
MSTRG.404.7	3160	noncoding	-
MSTRG.404.8	3547	noncoding	-
MSTRG.391.3	2350	noncoding	-
MSTRG.392.2	2025	coding	PF15419
MSTRG.393.2	822	noncoding	-
MSTRG.405.1	1840	noncoding	-
MSTRG.405.7	2317	noncoding	-
MSTRG.405.3	1940	noncoding	-
MSTRG.405.4	2331	noncoding	-
MSTRG.405.5	1937	noncoding	-
MSTRG.405.6	1949	noncoding	-
MSTRG.405.8	1920	noncoding	-
MSTRG.405.9	1719	noncoding	-
MSTRG.407.1	1465	noncoding	-
MSTRG.416.1	916	noncoding	-
MSTRG.423.2	2210	coding	PF15473
MSTRG.424.1	7023	coding	PF15131
MSTRG.424.2	6720	coding	PF15131
MSTRG.424.3	6242	noncoding	-
MSTRG.426.1	5075	noncoding	-
MSTRG.427.1	5367	coding	PF15131
MSTRG.429.1	3857	coding	PF14580
MSTRG.429.2	3432	coding	PF14580
MSTRG.449.1	301	noncoding	-

MSTRG.451.1	16139	noncoding	-
MSTRG.451.2	4912	noncoding	-
MSTRG.452.1	1063	noncoding	-
MSTRG.457.1	13686	noncoding	-
MSTRG.435.3	1434	noncoding	-
MSTRG.439.1	1614	noncoding	-
MSTRG.438.2	3811	coding	PF02761
MSTRG.440.1	8222	noncoding	-
MSTRG.441.1	5551	noncoding	-
MSTRG.467.6	8476	coding	PF00078
MSTRG.467.7	795	noncoding	-
MSTRG.468.1	28602	noncoding	-
MSTRG.468.2	21333	noncoding	-
MSTRG.468.3	4826	noncoding	-
MSTRG.468.4	2649	noncoding	-
MSTRG.468.5	9859	noncoding	-
MSTRG.470.1	1555	noncoding	-
MSTRG.467.10	8230	coding	PF09667
MSTRG.467.9	8202	coding	PF09667
MSTRG.467.11	8946	coding	PF09667
MSTRG.471.1	2752	noncoding	-
MSTRG.472.1	6260	noncoding	-
MSTRG.463.1	4928	coding	PF08204
MSTRG.463.4	2748	coding	PF08204
MSTRG.464.1	2608	noncoding	-
MSTRG.474.1	1811	noncoding	-
MSTRG.479.1	1304	coding	PF01576
MSTRG.481.2	533	coding	PF00063
MSTRG.481.1	513	coding	PF00063
MSTRG.487.2	3659	noncoding	-
MSTRG.488.3	2220	noncoding	-
MSTRG.484.1	310	noncoding	-
MSTRG.493.1	884	noncoding	-
MSTRG.497.1	311	noncoding	-
MSTRG.506.1	872	noncoding	-
MSTRG.508.3	3919	coding	PF08205
MSTRG.508.4	4202	coding	PF08205
MSTRG.508.2	2701	coding	PF08205
MSTRG.508.5	10720	coding	PF08205
MSTRG.509.1	1125	noncoding	-
MSTRG.512.2	761	noncoding	-
MSTRG.513.4	6094	noncoding	-
MSTRG.514.4	3432	noncoding	-
MSTRG.514.3	3938	noncoding	-
MSTRG.514.5	3707	noncoding	-
MSTRG.515.1	4307	noncoding	-
MSTRG.521.1	2518	noncoding	-
MSTRG.521.3	2203	noncoding	-
MSTRG.521.4	2292	coding	PF04142
MSTRG.521.6	1818	coding	PF04142
MSTRG.521.7	2428	noncoding	-
MSTRG.526.3	2484	noncoding	-
MSTRG.526.1	5409	noncoding	-
MSTRG.526.2	10942	noncoding	-

MSTRG.527.2	2549	noncoding	-
MSTRG.533.2	620	noncoding	-
MSTRG.533.3	2975	coding	PF15678
MSTRG.535.1	1805	coding	PF00583
MSTRG.535.4	2959	noncoding	-
MSTRG.538.1	4464	coding	PF00006
MSTRG.538.2	4857	coding	PF00006
MSTRG.538.3	4420	coding	PF00006
MSTRG.538.4	4694	coding	PF00006
MSTRG.539.1	2739	noncoding	-
MSTRG.570.1	4124	coding	PF00651
MSTRG.570.15	28296	coding	PF00651
MSTRG.570.14	10728	coding	PF00651
MSTRG.570.16	24886	coding	PF00651
MSTRG.570.18	11197	coding	PF00651
MSTRG.570.17	11343	coding	PF00651
MSTRG.570.19	11680	coding	PF00651
MSTRG.570.20	12149	coding	PF00651
MSTRG.570.22	28372	coding	PF00651
MSTRG.570.21	11567	coding	PF00651
MSTRG.570.23	11470	coding	PF00651
MSTRG.571.1	1869	noncoding	-
MSTRG.572.1	1690	noncoding	-
MSTRG.570.24	12937	noncoding	-
MSTRG.573.1	884	noncoding	-
MSTRG.574.1	1848	noncoding	-
MSTRG.575.1	265	noncoding	-
MSTRG.576.1	1934	noncoding	-
MSTRG.577.1	3572	noncoding	-
MSTRG.578.1	12679	coding	PF00078
MSTRG.570.25	2096	noncoding	-
MSTRG.579.1	6774	noncoding	-
MSTRG.580.1	3642	noncoding	-
MSTRG.581.1	9689	noncoding	-
MSTRG.582.1	7226	noncoding	-
MSTRG.583.1	792	noncoding	-
MSTRG.584.1	1597	noncoding	-
MSTRG.546.1	1478	coding	PF06614
MSTRG.546.2	1548	coding	PF06614
MSTRG.547.1	1414	coding	PF07679
MSTRG.560.4	3605	noncoding	-
MSTRG.560.1	3265	coding	PF07686
MSTRG.560.6	1888	noncoding	-
MSTRG.561.1	2009	noncoding	-
MSTRG.562.1	2221	noncoding	-
MSTRG.564.1	827	noncoding	-
MSTRG.601.2	1650	coding	PF10271
MSTRG.615.6	1420	coding	PF00069
MSTRG.615.4	1543	coding	PF00069
MSTRG.615.5	1544	coding	PF00069
MSTRG.616.1	1868	noncoding	-
MSTRG.617.1	2030	noncoding	-
MSTRG.622.2	6793	noncoding	-
MSTRG.622.3	5259	noncoding	-

MSTRG.622.4	6825	noncoding	-
MSTRG.623.1	1389	noncoding	-
MSTRG.624.2	3651	coding	PF07648
MSTRG.624.3	3503	coding	PF07648
MSTRG.625.1	1091	noncoding	-
MSTRG.627.1	2349	noncoding	-
MSTRG.628.1	2622	coding	PF11521
MSTRG.628.2	2721	coding	PF11521
MSTRG.628.3	1946	coding	PF11521
MSTRG.628.6	3441	coding	PF11521
MSTRG.628.5	1994	coding	PF11521
MSTRG.639.1	4455	noncoding	-
MSTRG.639.2	12639	noncoding	-
MSTRG.645.1	2423	noncoding	-
MSTRG.646.1	9004	coding	PF00476
MSTRG.646.3	3301	noncoding	-
MSTRG.646.4	4504	noncoding	-
MSTRG.633.4	2317	noncoding	-
MSTRG.633.5	2214	noncoding	-
MSTRG.633.6	2171	noncoding	-
MSTRG.633.7	2186	noncoding	-
MSTRG.635.1	810	noncoding	-
MSTRG.635.2	475	noncoding	-
MSTRG.650.1	8225	coding	PF16186
MSTRG.665.2	1579	coding	PF04387
MSTRG.665.3	1257	noncoding	-
MSTRG.671.6	5177	coding	PF00069
MSTRG.671.9	2301	coding	PF00069
MSTRG.671.10	3950	coding	PF00069
MSTRG.671.12	3950	coding	PF00069
MSTRG.671.15	3914	coding	PF00069
MSTRG.671.16	3914	coding	PF00069
MSTRG.671.18	1357	coding	PF07679
MSTRG.672.1	6181	coding	PF00078
MSTRG.667.3	2241	coding	PF15254
MSTRG.691.1	13843	coding	PF16609
MSTRG.691.2	13870	coding	PF16609
MSTRG.691.5	11375	coding	PF00621
MSTRG.691.6	12421	coding	PF00621
MSTRG.691.3	14952	coding	PF16609
MSTRG.691.7	341	noncoding	-
MSTRG.691.8	9053	coding	PF16609
MSTRG.691.9	23036	coding	PF00078
MSTRG.691.10	9449	coding	PF16609
MSTRG.691.11	13172	coding	PF16609
MSTRG.691.12	2639	noncoding	-
MSTRG.693.1	10826	noncoding	-
MSTRG.694.1	2144	noncoding	-
MSTRG.691.13	9745	coding	PF16609
MSTRG.691.14	15325	coding	PF16609
MSTRG.691.15	9401	coding	PF16609
MSTRG.691.17	30379	coding	PF16609
MSTRG.691.18	15278	coding	PF00078
MSTRG.695.1	5304	noncoding	-

MSTRG.691.21	14645	noncoding	-
MSTRG.691.20	5117	coding	PF16609
MSTRG.691.22	4118	coding	PF16609
MSTRG.696.1	6548	noncoding	-
MSTRG.675.2	10001	noncoding	-
MSTRG.681.5	2790	noncoding	-
MSTRG.681.4	3288	noncoding	-
MSTRG.684.1	1615	noncoding	-
MSTRG.684.4	1406	noncoding	-
MSTRG.699.1	2576	noncoding	-
MSTRG.699.3	4028	noncoding	-
MSTRG.699.4	2441	noncoding	-
MSTRG.702.1	3322	coding	PF01247
MSTRG.704.4	4609	noncoding	-
MSTRG.704.6	2959	noncoding	-
MSTRG.704.7	13606	noncoding	-
MSTRG.685.2	6877	coding	PF07078
MSTRG.685.1	5119	coding	PF07078
MSTRG.685.3	4736	coding	PF07078
MSTRG.687.5	5563	coding	PF13901
MSTRG.687.6	1552	noncoding	-
MSTRG.705.6	7076	noncoding	-
MSTRG.705.3	3897	coding	PF07714
MSTRG.705.4	10002	coding	PF07714
MSTRG.705.5	5311	coding	PF07714
MSTRG.705.1	5266	coding	PF07714
MSTRG.705.7	5212	coding	PF07714
MSTRG.705.8	5104	coding	PF07714
MSTRG.706.3	5291	coding	PF04389
MSTRG.706.4	5449	noncoding	-
MSTRG.707.1	653	noncoding	-
MSTRG.708.1	5127	coding	PF01467
MSTRG.708.5	3198	coding	PF01467
MSTRG.708.4	1556	coding	PF01467
MSTRG.708.3	1320	coding	PF01467
MSTRG.710.3	6744	coding	PF00789
MSTRG.713.3	1801	noncoding	-
MSTRG.714.1	1915	noncoding	-
MSTRG.714.2	2194	noncoding	-
MSTRG.715.1	3601	noncoding	-
MSTRG.717.1	516	noncoding	-
MSTRG.718.1	1535	noncoding	-
MSTRG.730.7	2727	coding	PF10608
MSTRG.731.1	719	noncoding	-
MSTRG.732.1	6110	noncoding	-
MSTRG.733.1	1395	noncoding	-
MSTRG.727.2	1672	coding	PF00106
MSTRG.727.3	1640	coding	PF00106
MSTRG.727.4	1826	coding	PF00106
MSTRG.728.1	2248	noncoding	-
MSTRG.728.2	1835	noncoding	-
MSTRG.728.3	1645	noncoding	-
MSTRG.735.1	1746	noncoding	-
MSTRG.739.1	4526	noncoding	-

MSTRG.740.1	3842	noncoding	-
MSTRG.741.1	3971	noncoding	-
MSTRG.743.2	1318	noncoding	-
MSTRG.752.5	972	coding	PF04970
MSTRG.752.6	918	coding	PF04970
MSTRG.755.1	518	noncoding	-
MSTRG.756.1	3083	noncoding	-
MSTRG.758.1	5125	noncoding	-
MSTRG.774.1	7359	noncoding	-
MSTRG.776.1	6943	noncoding	-
MSTRG.777.1	2218	noncoding	-
MSTRG.770.1	552	coding	PF00870
MSTRG.771.1	437	noncoding	-
MSTRG.771.2	228	noncoding	-
MSTRG.778.1	363	noncoding	-
MSTRG.782.2	18584	coding	PF00412
MSTRG.782.3	17253	coding	PF00412
MSTRG.782.4	2281	coding	PF00412
MSTRG.782.5	3474	coding	PF00412
MSTRG.784.1	1690	coding	PF13695
MSTRG.784.2	2254	coding	PF13695
MSTRG.786.1	2289	coding	PF00089
MSTRG.794.2	535	noncoding	-
MSTRG.803.3	1988	coding	PF00226
MSTRG.804.1	1257	coding	PF00226
MSTRG.804.2	404	coding	PF00226
MSTRG.805.1	2354	noncoding	-
MSTRG.806.1	3284	coding	PF07986
MSTRG.806.4	2048	coding	PF00030
MSTRG.801.1	800	noncoding	-
MSTRG.813.2	6871	coding	PF02902
MSTRG.817.1	5449	noncoding	-
MSTRG.831.2	5847	coding	PF12816
MSTRG.831.4	3823	coding	PF12816
MSTRG.833.1	236	noncoding	-
MSTRG.835.1	4143	coding	PF15010
MSTRG.836.2	3156	coding	PF00654
MSTRG.837.4	5318	coding	PF02854
MSTRG.839.2	1037	noncoding	-
MSTRG.840.3	1451	coding	PF05208
MSTRG.840.2	2211	coding	PF05208
MSTRG.841.3	4153	coding	PF13757
MSTRG.841.2	4479	coding	PF13757
MSTRG.854.3	4121	coding	PF00005
MSTRG.855.1	5141	noncoding	-
MSTRG.855.2	5732	noncoding	-
MSTRG.848.3	6790	coding	PF00651
MSTRG.848.5	4597	coding	PF00651
MSTRG.848.6	2444	coding	PF00651
MSTRG.848.8	2476	coding	PF00651
MSTRG.859.4	845	coding	PF03556
MSTRG.859.6	1525	noncoding	-
MSTRG.862.2	6923	coding	PF16212
MSTRG.862.5	1214	noncoding	-

MSTRG.862.7	1333	noncoding	-
MSTRG.862.6	4094	coding	PF16212
MSTRG.862.3	4270	coding	PF16212
MSTRG.862.8	1114	coding	PF16209
MSTRG.874.1	2596	noncoding	-
MSTRG.892.3	7443	noncoding	-
MSTRG.892.2	7254	noncoding	-
MSTRG.892.1	7720	noncoding	-
MSTRG.897.2	5388	coding	PF04799
MSTRG.914.4	2234	noncoding	-
MSTRG.914.3	6733	noncoding	-
MSTRG.914.5	2062	noncoding	-
MSTRG.915.2	2892	noncoding	-
MSTRG.915.3	2908	noncoding	-
MSTRG.908.1	232	noncoding	-
MSTRG.920.1	6148	noncoding	-
MSTRG.921.1	591	noncoding	-
MSTRG.945.1	629	coding	PF02800
MSTRG.934.4	1661	coding	PF15015
MSTRG.951.3	4108	coding	PF00621
MSTRG.952.1	342	noncoding	-
MSTRG.956.4	2429	noncoding	-
MSTRG.957.1	16602	noncoding	-
MSTRG.959.1	834	noncoding	-
MSTRG.965.1	926	noncoding	-
MSTRG.965.2	3161	noncoding	-
MSTRG.965.3	538	noncoding	-
MSTRG.979.3	11959	noncoding	-
MSTRG.979.7	2961	noncoding	-
MSTRG.980.3	4964	noncoding	-
MSTRG.981.1	9865	noncoding	-
MSTRG.980.5	25559	noncoding	-
MSTRG.980.6	19375	noncoding	-
MSTRG.980.7	9626	coding	PF00069
MSTRG.985.1	1485	noncoding	-
MSTRG.967.2	3871	coding	PF01287
MSTRG.972.2	1216	coding	PF01776
MSTRG.974.1	2327	coding	PF13906
MSTRG.975.1	2726	noncoding	-
MSTRG.991.1	873	noncoding	-
MSTRG.991.2	923	noncoding	-
MSTRG.991.3	656	noncoding	-
MSTRG.994.1	1949	noncoding	-
MSTRG.994.2	2431	noncoding	-
MSTRG.994.3	1925	noncoding	-
MSTRG.997.1	6877	coding	PF00651
MSTRG.1000.1	3712	noncoding	-
MSTRG.1000.2	3927	noncoding	-
MSTRG.1005.5	3684	noncoding	-
MSTRG.1005.6	4311	noncoding	-
MSTRG.1002.3	1227	noncoding	-
MSTRG.1002.4	1188	noncoding	-
MSTRG.1008.4	1081	coding	PF06840
MSTRG.1013.4	2651	noncoding	-

MSTRG.1013.7	2691	noncoding	-
MSTRG.1013.8	2687	noncoding	-
MSTRG.1013.9	2613	noncoding	-
MSTRG.1012.2	4015	noncoding	-
MSTRG.1012.1	2210	noncoding	-
MSTRG.1029.1	2592	coding	PF04981
MSTRG.1029.3	2629	coding	PF04981
MSTRG.1031.1	7550	coding	PF00481
MSTRG.1031.2	7277	coding	PF00481
MSTRG.1031.3	2575	noncoding	-
MSTRG.1041.2	4311	noncoding	-
MSTRG.1044.3	3261	noncoding	-
MSTRG.1036.3	3272	coding	PF13553
MSTRG.1037.1	1931	coding	PF03039
MSTRG.1060.2	1073	coding	PF06907
MSTRG.1063.1	1393	coding	PF06907
MSTRG.1063.2	1201	coding	PF06907
MSTRG.1066.1	1454	noncoding	-
MSTRG.1066.2	1498	noncoding	-
MSTRG.1066.3	962	noncoding	-
MSTRG.1065.1	227	noncoding	-
MSTRG.1070.1	473	noncoding	-
MSTRG.1085.2	4713	coding	PF07074
MSTRG.1086.2	2682	coding	PF00248
MSTRG.1096.1	3021	coding	PF00117
MSTRG.1097.1	2344	noncoding	-
MSTRG.1096.2	8562	coding	PF00117
MSTRG.1108.1	275	noncoding	-
MSTRG.1119.1	7339	noncoding	-
MSTRG.1132.1	13698	noncoding	-
MSTRG.1125.1	4084	noncoding	-
MSTRG.1141.1	1539	noncoding	-
MSTRG.1149.1	2740	noncoding	-
MSTRG.1150.1	457	noncoding	-
MSTRG.1157.6	5509	noncoding	-
MSTRG.1157.7	22857	noncoding	-
MSTRG.1157.8	3188	noncoding	-
MSTRG.1157.10	3019	noncoding	-
MSTRG.1157.9	3003	noncoding	-
MSTRG.1158.1	2676	noncoding	-
MSTRG.1158.2	3976	noncoding	-
MSTRG.1159.1	8625	noncoding	-
MSTRG.1173.1	3213	noncoding	-
MSTRG.1174.1	3382	noncoding	-
MSTRG.1175.1	21537	noncoding	-
MSTRG.1161.2	2093	coding	PF03145
MSTRG.1161.3	2927	noncoding	-
MSTRG.1164.2	1275	noncoding	-
MSTRG.1167.1	285	noncoding	-
MSTRG.1184.1	1977	noncoding	-
MSTRG.1189.2	815	noncoding	-
MSTRG.1190.1	677	noncoding	-
MSTRG.1194.2	1625	coding	PF14763
MSTRG.1196.2	2186	noncoding	-

MSTRG.1207.1	304	noncoding	-
MSTRG.1209.1	201	noncoding	-
MSTRG.1214.2	1041	coding	PF03803
MSTRG.1215.1	1241	noncoding	-
MSTRG.1211.1	360	noncoding	-
MSTRG.1236.4	5657	noncoding	-
MSTRG.1245.2	2239	coding	PF08344
MSTRG.1250.2	2835	noncoding	-
MSTRG.1251.1	1162	noncoding	-
MSTRG.1252.4	938	noncoding	-
MSTRG.1253.1	411	noncoding	-
MSTRG.1259.2	1089	coding	PF00370
MSTRG.1260.1	1804	noncoding	-
MSTRG.1261.2	1242	noncoding	-
MSTRG.1261.5	1450	coding	PF08781
MSTRG.1261.6	1541	coding	PF08781
MSTRG.1254.1	726	noncoding	-
MSTRG.1263.1	5495	noncoding	-
MSTRG.1267.4	4855	noncoding	-
MSTRG.1269.1	478	noncoding	-
MSTRG.1272.1	5637	coding	PF00153
MSTRG.1272.4	5009	coding	PF00153
MSTRG.1272.6	3588	coding	PF00153
MSTRG.1272.7	5724	coding	PF00153
MSTRG.1278.2	2526	noncoding	-
MSTRG.1278.3	5293	noncoding	-
MSTRG.1279.2	4337	coding	PF04053
MSTRG.1288.3	3937	coding	PF00613
MSTRG.1288.4	3521	coding	PF00454
MSTRG.1288.5	2809	coding	PF00454
MSTRG.1285.1	720	noncoding	-
MSTRG.1309.1	2948	noncoding	-
MSTRG.1310.2	1897	noncoding	-
MSTRG.1310.1	879	noncoding	-
MSTRG.1310.3	286	noncoding	-
MSTRG.1311.1	10779	coding	PF00017
MSTRG.1311.7	3115	coding	PF00017
MSTRG.1311.5	2926	coding	PF00017
MSTRG.1311.6	3055	coding	PF00017
MSTRG.1311.8	6596	coding	PF00017
MSTRG.1311.9	3104	coding	PF00017
MSTRG.1311.10	1873	coding	PF00017
MSTRG.1309.2	2817	noncoding	-
MSTRG.1312.1	1779	noncoding	-
MSTRG.1314.2	1478	noncoding	-
MSTRG.1317.1	1912	noncoding	-
MSTRG.1318.2	2861	coding	PF08514
MSTRG.1318.3	3458	coding	PF08514
MSTRG.1319.1	2063	noncoding	-
MSTRG.1320.1	3196	noncoding	-
MSTRG.1321.1	1464	noncoding	-
MSTRG.1323.1	10102	noncoding	-
MSTRG.1318.4	4913	noncoding	-
MSTRG.1325.1	5485	coding	PF01039

MSTRG.1332.2	10108	coding	PF16685
MSTRG.1338.1	3433	coding	PF17045
MSTRG.1338.2	3005	coding	PF17045
MSTRG.1338.4	3063	coding	PF17045
MSTRG.1338.3	2937	coding	PF17045
MSTRG.1338.7	2533	coding	PF17045
MSTRG.1338.8	2338	coding	PF17045
MSTRG.1339.1	1295	coding	PF05839
MSTRG.1344.2	3722	coding	PF00071
MSTRG.1346.1	4771	coding	PF09439
MSTRG.1351.5	5113	coding	PF12738
MSTRG.1351.6	5392	coding	PF12738
MSTRG.1352.6	2937	coding	PF15359
MSTRG.1352.2	2768	coding	PF15359
MSTRG.1352.4	2552	coding	PF15359
MSTRG.1352.9	2699	coding	PF15359
MSTRG.1352.11	1166	coding	PF15359
MSTRG.1352.12	1188	coding	PF15359
MSTRG.1352.10	939	coding	PF15359
MSTRG.1352.13	1222	coding	PF15359
MSTRG.1352.14	1044	coding	PF15359
MSTRG.1352.15	1326	coding	PF15359
MSTRG.1355.4	2229	coding	PF15759
MSTRG.1363.1	1382	noncoding	-
MSTRG.1367.2	6001	noncoding	-
MSTRG.1367.3	4006	noncoding	-
MSTRG.1380.9	2339	noncoding	-
MSTRG.1380.7	3406	coding	PF00122
MSTRG.1380.5	3330	coding	PF00122
MSTRG.1380.8	3368	coding	PF00122
MSTRG.1380.10	2202	coding	PF00122
MSTRG.1380.11	1353	noncoding	-
MSTRG.1382.1	1423	noncoding	-
MSTRG.1384.4	5851	coding	PF00439
MSTRG.1384.3	10228	coding	PF00439
MSTRG.1384.5	2261	noncoding	-
MSTRG.1387.1	1400	coding	PF01359
MSTRG.1391.1	2349	noncoding	-
MSTRG.1395.10	2814	coding	PF15619
MSTRG.1395.11	2407	coding	PF15619
MSTRG.1395.12	2343	coding	PF15619
MSTRG.1395.13	2648	coding	PF15619
MSTRG.1395.14	2249	coding	PF15619
MSTRG.1395.15	2501	coding	PF15619
MSTRG.1395.17	2380	coding	PF15619
MSTRG.1395.18	2484	coding	PF15619
MSTRG.1395.19	2452	coding	PF15619
MSTRG.1395.20	2538	coding	PF15619
MSTRG.1395.21	2375	coding	PF15619
MSTRG.1395.22	2306	coding	PF15619
MSTRG.1395.23	2458	coding	PF15619
MSTRG.1395.24	2453	coding	PF15619
MSTRG.1396.1	3276	noncoding	-
MSTRG.1396.2	2871	noncoding	-

MSTRG.1394.1	290	noncoding	-
MSTRG.1398.1	2247	noncoding	-
MSTRG.1398.2	1881	noncoding	-
MSTRG.1407.1	581	noncoding	-
MSTRG.1408.1	1263	noncoding	-
MSTRG.1414.3	1118	noncoding	-
MSTRG.1415.1	2427	noncoding	-
MSTRG.1415.3	2959	noncoding	-
MSTRG.1416.2	1785	coding	PF00233
MSTRG.1419.4	2481	noncoding	-
MSTRG.1419.3	1761	noncoding	-
MSTRG.1420.1	1157	noncoding	-
MSTRG.1421.1	2498	coding	PF16493
MSTRG.1426.2	2591	coding	PF00069
MSTRG.1432.3	766	noncoding	-
MSTRG.1432.2	1002	noncoding	-
MSTRG.1433.1	2566	coding	PF05997
MSTRG.1433.5	6677	coding	PF05997
MSTRG.1429.2	6349	coding	PF08543
MSTRG.1429.3	6732	coding	PF08543
MSTRG.1428.1	573	noncoding	-
MSTRG.1436.1	8252	noncoding	-
MSTRG.1436.4	5668	noncoding	-
MSTRG.1436.2	8206	noncoding	-
MSTRG.1436.3	11908	noncoding	-
MSTRG.1437.1	2673	noncoding	-
MSTRG.1438.1	8281	noncoding	-
MSTRG.1436.7	5744	noncoding	-
MSTRG.1436.6	8608	noncoding	-
MSTRG.1439.2	4070	coding	PF04003
MSTRG.1440.1	894	noncoding	-
MSTRG.1441.1	1392	noncoding	-
MSTRG.1447.1	2899	noncoding	-
MSTRG.1449.1	2207	noncoding	-
MSTRG.1450.1	2879	noncoding	-
MSTRG.1451.2	906	noncoding	-
MSTRG.1451.3	953	noncoding	-
MSTRG.1454.5	2016	coding	PF00179
MSTRG.1455.2	1196	noncoding	-
MSTRG.1455.1	992	noncoding	-
MSTRG.1460.1	1037	noncoding	-
MSTRG.1463.2	1501	coding	PF02137
MSTRG.1464.1	2525	noncoding	-
MSTRG.1465.2	2144	coding	PF10250
MSTRG.1467.2	4285	coding	PF06482
MSTRG.1469.1	2465	coding	PF01770
MSTRG.1470.1	1880	noncoding	-
MSTRG.1475.3	4992	noncoding	-
MSTRG.1475.2	1949	coding	PF00013
MSTRG.1475.4	1986	coding	PF00013
MSTRG.1475.5	2068	coding	PF00013
MSTRG.1475.6	1911	coding	PF00013
MSTRG.1477.1	10235	noncoding	-
MSTRG.1473.1	4010	coding	PF00092

MSTRG.1473.2	3939	coding	PF00092
MSTRG.1494.2	940	coding	PF15059
MSTRG.1495.1	927	noncoding	-
MSTRG.1496.1	6383	coding	PF16769
MSTRG.1497.1	4239	noncoding	-
MSTRG.1497.2	2352	noncoding	-
MSTRG.1499.1	4282	coding	PF02130
MSTRG.1500.3	1648	noncoding	-
MSTRG.1501.5	9914	noncoding	-
MSTRG.1501.1	9970	noncoding	-
MSTRG.1501.2	9811	noncoding	-
MSTRG.1501.3	9793	noncoding	-
MSTRG.1501.4	9991	noncoding	-
MSTRG.1501.6	10081	noncoding	-
MSTRG.1501.9	9808	noncoding	-
MSTRG.1501.10	9972	noncoding	-
MSTRG.1501.11	9948	noncoding	-
MSTRG.1501.14	6479	noncoding	-
MSTRG.1481.1	667	noncoding	-
MSTRG.1483.2	2973	coding	PF15683
MSTRG.1484.1	1225	noncoding	-
MSTRG.1512.6	1759	noncoding	-
MSTRG.1512.5	1484	noncoding	-
MSTRG.1512.7	35438	noncoding	-
MSTRG.1512.10	12550	coding	PF02093
MSTRG.1512.8	49985	coding	PF02093
MSTRG.1512.9	12905	noncoding	-
MSTRG.1512.11	33095	coding	PF02093
MSTRG.1512.12	16815	coding	PF02994
MSTRG.1512.13	20386	noncoding	-
MSTRG.1512.14	48143	coding	PF02994
MSTRG.1512.15	31165	coding	PF02994
MSTRG.1512.16	9064	coding	PF02994
MSTRG.1512.17	3323	noncoding	-
MSTRG.1512.18	6011	coding	PF00106
MSTRG.1512.19	9369	noncoding	-
MSTRG.1514.1	1464	noncoding	-
MSTRG.1509.3	7455	coding	PF04118
MSTRG.1511.2	3101	coding	PF15512
MSTRG.1511.3	6025	coding	PF15512
MSTRG.1511.5	15714	coding	PF15512
MSTRG.1516.1	675	noncoding	-
MSTRG.1518.1	1247	noncoding	-
MSTRG.1519.1	790	noncoding	-
MSTRG.1520.2	4414	coding	PF03099
MSTRG.1535.1	3533	noncoding	-
MSTRG.1536.1	469	noncoding	-
MSTRG.1537.4	626	coding	PF08510
MSTRG.1538.1	4020	noncoding	-
MSTRG.1538.7	8474	noncoding	-
MSTRG.1538.8	1819	noncoding	-
MSTRG.1538.9	5089	noncoding	-
MSTRG.1538.10	4773	noncoding	-
MSTRG.1538.12	6762	noncoding	-

MSTRG.1538.13	6981	noncoding	-
MSTRG.1526.2	2749	coding	PF03643
MSTRG.1531.6	2134	noncoding	-
MSTRG.1531.5	9026	noncoding	-
MSTRG.1531.4	5035	coding	PF00069
MSTRG.1531.7	17349	coding	PF00069
MSTRG.1532.1	1785	noncoding	-
MSTRG.1533.1	2264	noncoding	-
MSTRG.1531.8	5227	coding	PF00069
MSTRG.1531.9	5254	coding	PF00069
MSTRG.1534.1	4151	noncoding	-
MSTRG.1531.10	7302	coding	PF00069
MSTRG.1528.1	2444	noncoding	-
MSTRG.1528.3	3067	noncoding	-
MSTRG.1530.1	6984	coding	PF01007
MSTRG.1544.1	3160	coding	PF00178
MSTRG.1544.2	2859	coding	PF00178
MSTRG.1545.1	224	noncoding	-
MSTRG.1549.1	778	noncoding	-
MSTRG.1550.5	2949	noncoding	-
MSTRG.1550.6	4408	noncoding	-
MSTRG.1550.7	2473	noncoding	-
MSTRG.1554.2	2473	coding	PF05276
MSTRG.1555.1	1969	noncoding	-
MSTRG.1556.1	5429	noncoding	-
MSTRG.1562.3	1100	noncoding	-
MSTRG.1562.4	2307	noncoding	-
MSTRG.1562.5	1068	noncoding	-
MSTRG.1563.1	1491	coding	PF09816
MSTRG.1563.3	4021	coding	PF09816
MSTRG.1558.2	1568	noncoding	-
MSTRG.1560.1	2208	noncoding	-
MSTRG.1576.2	2053	noncoding	-
MSTRG.1578.4	4764	coding	PF12796
MSTRG.1578.7	5253	coding	PF12796
MSTRG.1578.9	2431	noncoding	-
MSTRG.1578.11	9741	noncoding	-
MSTRG.1578.10	5818	noncoding	-
MSTRG.1578.12	11419	noncoding	-
MSTRG.1578.13	15302	noncoding	-
MSTRG.1578.14	6310	noncoding	-
MSTRG.1567.3	2045	noncoding	-
MSTRG.1567.4	1952	noncoding	-
MSTRG.1567.5	1662	noncoding	-
MSTRG.1567.7	2342	noncoding	-
MSTRG.1568.2	2314	coding	PF15250
MSTRG.1568.4	2289	coding	PF15250
MSTRG.1568.6	2618	coding	PF15250
MSTRG.1568.5	2719	coding	PF15250
MSTRG.1573.1	4177	noncoding	-
MSTRG.1574.2	1227	coding	PF00076
MSTRG.1574.3	2708	coding	PF00076
MSTRG.1589.1	693	noncoding	-
MSTRG.1590.1	1556	noncoding	-

MSTRG.1592.2	5648	noncoding	-
MSTRG.1592.1	8452	noncoding	-
MSTRG.1592.4	2086	noncoding	-
MSTRG.1592.5	2898	noncoding	-
MSTRG.1592.6	1956	noncoding	-
MSTRG.1593.1	698	noncoding	-
MSTRG.1592.7	2751	noncoding	-
MSTRG.1592.8	1994	noncoding	-
MSTRG.1594.1	1661	noncoding	-
MSTRG.1595.1	2133	noncoding	-
MSTRG.1596.1	3306	noncoding	-
MSTRG.1597.1	942	noncoding	-
MSTRG.1598.1	3230	noncoding	-
MSTRG.1596.2	8377	coding	PF02994
MSTRG.1583.1	265	noncoding	-
MSTRG.1583.2	360	noncoding	-
MSTRG.1585.1	515	noncoding	-
MSTRG.1600.1	365	noncoding	-
MSTRG.1603.1	1210	noncoding	-
MSTRG.1604.1	345	noncoding	-
MSTRG.1606.1	1398	noncoding	-
MSTRG.1610.1	990	noncoding	-
MSTRG.1610.3	2587	noncoding	-
MSTRG.1610.4	1328	noncoding	-
MSTRG.1613.3	6703	noncoding	-
MSTRG.1614.1	1502	noncoding	-
MSTRG.1614.2	1548	noncoding	-
MSTRG.1614.3	1194	coding	PF00481
MSTRG.1614.4	2953	noncoding	-
MSTRG.1618.1	8654	coding	PF06466
MSTRG.1618.2	808	coding	PF06466
MSTRG.1618.4	3394	coding	PF06466
MSTRG.1618.3	3874	coding	PF06466
MSTRG.1619.1	2077	noncoding	-
MSTRG.1619.2	2461	noncoding	-
MSTRG.1623.2	2107	coding	PF02023
MSTRG.1637.4	5416	noncoding	-
MSTRG.1637.5	6652	coding	PF16186
MSTRG.1632.1	6583	noncoding	-
MSTRG.1632.2	5764	noncoding	-
MSTRG.1644.3	14784	coding	PF00632
MSTRG.1642.3	2996	coding	PF05653
MSTRG.1640.1	2030	noncoding	-
MSTRG.1647.3	1893	noncoding	-
MSTRG.1650.1	10151	noncoding	-
MSTRG.1650.2	10044	noncoding	-
MSTRG.1650.3	9992	noncoding	-
MSTRG.1653.1	3005	coding	PF14944
MSTRG.1654.1	3414	noncoding	-
MSTRG.1654.2	3411	noncoding	-
MSTRG.1654.3	3722	noncoding	-
MSTRG.1657.1	873	noncoding	-
MSTRG.1659.1	496	noncoding	-
MSTRG.1662.1	13359	coding	PF16014

MSTRG.1662.4	3972	coding	PF16014
MSTRG.1662.7	4011	coding	PF16014
MSTRG.1662.9	3889	coding	PF16014
MSTRG.1662.10	4658	coding	PF16014
MSTRG.1662.12	3849	coding	PF16014
MSTRG.1662.13	3192	coding	PF16014
MSTRG.1663.1	5349	coding	PF01871
MSTRG.1663.5	516	noncoding	-
MSTRG.1663.4	2251	noncoding	-
MSTRG.1663.3	1583	noncoding	-
MSTRG.1665.1	4198	noncoding	-
MSTRG.1667.1	3095	noncoding	-
MSTRG.1671.1	2883	coding	PF00001
MSTRG.1673.5	5206	noncoding	-
MSTRG.1673.4	4595	noncoding	-
MSTRG.1673.3	6046	noncoding	-
MSTRG.1675.1	8938	noncoding	-
MSTRG.1676.1	244	noncoding	-
MSTRG.1677.1	974	noncoding	-
MSTRG.1678.1	288	noncoding	-
MSTRG.1681.1	1599	coding	PF00089
MSTRG.1685.2	3059	noncoding	-
MSTRG.1685.3	1709	noncoding	-
MSTRG.1689.1	322	noncoding	-
MSTRG.1691.1	2246	coding	PF15127
MSTRG.1692.1	696	coding	PF15127
MSTRG.1697.2	2098	coding	PF00517
MSTRG.1697.1	812	noncoding	-
MSTRG.1697.3	6450	coding	PF00607
MSTRG.1697.4	1056	noncoding	-
MSTRG.1709.2	4127	coding	PF12796
MSTRG.1709.4	4327	coding	PF12796
MSTRG.1709.6	3986	coding	PF12796
MSTRG.1709.8	3246	coding	PF12796
MSTRG.1716.2	1647	coding	PF00640
MSTRG.1716.1	1475	coding	PF00640
MSTRG.1716.3	1110	coding	PF00640
MSTRG.1717.1	1816	noncoding	-
MSTRG.1725.1	4991	noncoding	-
MSTRG.1726.1	5290	noncoding	-
MSTRG.1721.1	321	noncoding	-
MSTRG.1721.2	395	noncoding	-
MSTRG.1729.2	1897	noncoding	-
MSTRG.1730.1	6301	coding	PF00078
MSTRG.1731.1	1819	noncoding	-
MSTRG.1734.1	5731	coding	PF10577
MSTRG.1734.2	1688	noncoding	-
MSTRG.1727.1	10124	coding	PF01198
MSTRG.1727.2	894	noncoding	-
MSTRG.1740.3	2155	noncoding	-
MSTRG.1740.2	1977	noncoding	-
MSTRG.1745.1	20894	coding	PF15783
MSTRG.1745.3	20967	coding	PF15783
MSTRG.1753.1	391	noncoding	-

MSTRG.1753.2	458	noncoding	-
MSTRG.1768.1	1135	coding	PF05172
MSTRG.1768.2	1541	coding	PF05172
MSTRG.1768.3	1370	noncoding	-
MSTRG.1770.2	624	coding	PF09735
MSTRG.1772.1	4283	coding	PF00226
MSTRG.1772.3	3975	coding	PF00226
MSTRG.1772.4	1532	coding	PF00226
MSTRG.1773.1	474	noncoding	-
MSTRG.1774.1	227	noncoding	-
MSTRG.1777.2	1734	coding	PF00233
MSTRG.1777.3	2956	coding	PF00233
MSTRG.1782.1	5481	coding	PF14723
MSTRG.1782.5	4910	coding	PF14723
MSTRG.1782.4	6376	coding	PF14723
MSTRG.1783.1	6921	noncoding	-
MSTRG.1784.1	11439	noncoding	-
MSTRG.1787.1	506	noncoding	-
MSTRG.1787.2	499	noncoding	-
MSTRG.1779.1	401	noncoding	-
MSTRG.1780.1	1963	noncoding	-
MSTRG.1791.2	2074	coding	PF00781
MSTRG.1791.3	1980	coding	PF00781
MSTRG.1798.1	4198	noncoding	-
MSTRG.1799.1	5681	coding	PF00179
MSTRG.1799.2	3875	coding	PF00179
MSTRG.1799.3	2052	coding	PF00179
MSTRG.1799.4	1095	coding	PF00179
MSTRG.1799.7	2938	coding	PF00179
MSTRG.1799.6	1423	coding	PF00179
MSTRG.1798.2	4969	noncoding	-
MSTRG.1798.3	10417	noncoding	-
MSTRG.1799.8	4560	noncoding	-
MSTRG.1805.1	3623	noncoding	-
MSTRG.1805.3	2674	noncoding	-
MSTRG.1801.1	616	noncoding	-
MSTRG.1801.2	580	noncoding	-
MSTRG.1801.3	1731	noncoding	-
MSTRG.1804.1	1837	coding	PF07679
MSTRG.1819.1	68416	coding	PF00069
MSTRG.1811.3	1003	coding	PF00254
MSTRG.1814.1	7597	coding	PF01237
MSTRG.1814.3	4405	coding	PF01237
MSTRG.1814.4	4501	coding	PF01237
MSTRG.1814.2	4586	coding	PF01237
MSTRG.1814.7	5234	coding	PF01237
MSTRG.1814.8	1507	noncoding	-
MSTRG.1818.1	4596	noncoding	-
MSTRG.1818.3	4599	noncoding	-
MSTRG.1832.1	7304	noncoding	-
MSTRG.1832.3	2076	noncoding	-
MSTRG.1833.2	2442	noncoding	-
MSTRG.1833.1	1146	noncoding	-
MSTRG.1833.3	1660	noncoding	-

MSTRG.1834.1	3092	noncoding	-
MSTRG.1840.1	1429	noncoding	-
MSTRG.1829.1	3310	noncoding	-
MSTRG.1841.5	940	noncoding	-
MSTRG.1847.1	2215	coding	PF10058
MSTRG.1847.2	3565	coding	PF10058
MSTRG.1869.1	3871	noncoding	-
MSTRG.1868.11	35981	noncoding	-
MSTRG.1868.12	7977	noncoding	-
MSTRG.1868.13	2349	noncoding	-
MSTRG.1873.1	1407	noncoding	-
MSTRG.1874.1	34390	noncoding	-
MSTRG.1874.2	16937	noncoding	-
MSTRG.1849.4	2149	noncoding	-
MSTRG.1850.1	2928	noncoding	-
MSTRG.1856.4	3473	noncoding	-
MSTRG.1857.1	6096	noncoding	-
MSTRG.1860.1	1638	coding	PF06071
MSTRG.1860.5	7782	coding	PF01926
MSTRG.1860.6	3087	coding	PF01926
MSTRG.1860.3	2209	coding	PF06071
MSTRG.1860.4	2013	coding	PF06071
MSTRG.1862.1	583	noncoding	-
MSTRG.1860.7	6323	coding	PF06071
MSTRG.1864.1	2720	noncoding	-
MSTRG.1865.1	1854	noncoding	-
MSTRG.1866.1	9772	noncoding	-
MSTRG.1867.2	3832	noncoding	-
MSTRG.1867.3	3887	noncoding	-
MSTRG.1891.2	6858	coding	PF07714
MSTRG.1895.2	5378	coding	PF10436
MSTRG.1895.3	551	noncoding	-
MSTRG.1895.5	1454	coding	PF10436
MSTRG.1895.4	1542	coding	PF10436
MSTRG.1900.3	1825	coding	PF10394
MSTRG.1900.4	1924	coding	PF10394
MSTRG.1900.5	1481	coding	PF10394
MSTRG.1900.6	1189	noncoding	-
MSTRG.1902.4	2255	noncoding	-
MSTRG.1906.1	1615	noncoding	-
MSTRG.1907.2	3629	coding	PF15802
MSTRG.1907.3	3639	coding	PF15802
MSTRG.1907.4	3563	coding	PF15802
MSTRG.1911.1	626	noncoding	-
MSTRG.1912.2	3853	noncoding	-
MSTRG.1912.4	1143	noncoding	-
MSTRG.1912.7	7046	coding	PF00069
MSTRG.1914.1	1316	noncoding	-
MSTRG.1912.8	1807	noncoding	-
MSTRG.1912.9	6839	coding	PF00069
MSTRG.1909.2	2261	coding	PF04495
MSTRG.1910.1	625	noncoding	-
MSTRG.1922.1	934	noncoding	-
MSTRG.1922.4	1635	noncoding	-

MSTRG.1922.6	1639	noncoding	-
MSTRG.1922.5	1018	noncoding	-
MSTRG.1923.1	346	noncoding	-
MSTRG.1924.1	938	noncoding	-
MSTRG.1924.2	796	noncoding	-
MSTRG.1928.3	5115	coding	PF00063
MSTRG.1928.5	5122	coding	PF00063
MSTRG.1928.7	4930	coding	PF00063
MSTRG.1928.8	1421	noncoding	-
MSTRG.1928.9	3890	coding	PF00063
MSTRG.1928.10	7864	coding	PF01359
MSTRG.1930.1	7548	noncoding	-
MSTRG.1928.11	4852	noncoding	-
MSTRG.1928.12	17946	noncoding	-
MSTRG.1928.13	10750	noncoding	-
MSTRG.1920.4	7991	coding	PF02207
MSTRG.1920.5	7974	coding	PF02207
MSTRG.1920.6	4192	noncoding	-
MSTRG.1920.7	6852	coding	PF02207
MSTRG.1921.1	3176	coding	PF00078
MSTRG.1918.3	2545	coding	PF05383
MSTRG.1940.1	10100	noncoding	-
MSTRG.1940.2	9950	coding	PF00651
MSTRG.1940.5	6731	coding	PF00651
MSTRG.1940.4	6856	coding	PF00651
MSTRG.1941.1	8733	noncoding	-
MSTRG.1943.1	3704	coding	PF00078
MSTRG.1943.2	1891	noncoding	-
MSTRG.1943.4	1308	coding	PF06888
MSTRG.1945.2	1047	noncoding	-
MSTRG.1948.1	1342	noncoding	-
MSTRG.1949.1	1361	noncoding	-
MSTRG.1951.1	3042	coding	PF07289
MSTRG.1934.1	15307	coding	PF00057
MSTRG.1953.1	2578	noncoding	-
MSTRG.1954.1	4334	noncoding	-
MSTRG.1995.1	3928	noncoding	-
MSTRG.2012.1	3912	noncoding	-
MSTRG.2013.1	371	noncoding	-
MSTRG.2022.1	3387	noncoding	-
MSTRG.2023.1	10824	noncoding	-
MSTRG.2023.2	5156	noncoding	-
MSTRG.2025.1	3127	noncoding	-
MSTRG.2028.1	1877	noncoding	-
MSTRG.1959.1	4793	noncoding	-
MSTRG.1959.3	4330	noncoding	-
MSTRG.1960.1	2707	coding	PF00652
MSTRG.1962.1	921	noncoding	-
MSTRG.1962.2	738	noncoding	-
MSTRG.1968.3	5275	coding	PF09469
MSTRG.1968.8	4030	coding	PF09469
MSTRG.1972.2	2627	coding	PF08947
MSTRG.1972.6	6478	noncoding	-
MSTRG.1972.4	2498	coding	PF08947

MSTRG.1972.3	1845	coding	PF08947
MSTRG.1972.8	2408	coding	PF08947
MSTRG.1972.10	1536	noncoding	-
MSTRG.1972.9	6594	noncoding	-
MSTRG.1972.11	1859	coding	PF08947
MSTRG.1970.1	222	noncoding	-
MSTRG.1989.1	745	noncoding	-
MSTRG.1989.2	500	noncoding	-
MSTRG.1990.1	558	noncoding	-
MSTRG.2059.1	629	coding	PF00078
MSTRG.2062.3	1854	coding	PF12845
MSTRG.2062.4	5070	coding	PF12845
MSTRG.2062.6	4410	coding	PF12845
MSTRG.2062.5	2511	coding	PF12845
MSTRG.2063.1	3876	noncoding	-
MSTRG.2060.1	400	noncoding	-
MSTRG.2068.4	1525	coding	PF00076
MSTRG.2068.5	1604	coding	PF00076
MSTRG.2068.6	1471	coding	PF00076
MSTRG.2068.7	1762	coding	PF00076
MSTRG.2068.9	1753	coding	PF00076
MSTRG.2068.8	4016	coding	PF00076
MSTRG.2070.1	368	noncoding	-
MSTRG.2071.1	2108	noncoding	-
MSTRG.2067.1	243	noncoding	-
MSTRG.2067.2	325	coding	PF01283
MSTRG.2066.1	647	noncoding	-
MSTRG.2072.2	2205	coding	PF00040
MSTRG.2079.1	3616	noncoding	-
MSTRG.2079.2	706	noncoding	-
MSTRG.2085.1	1067	noncoding	-
MSTRG.2085.2	2137	noncoding	-
MSTRG.2086.1	311	noncoding	-
MSTRG.2088.6	2335	coding	PF12906
MSTRG.2088.4	3021	coding	PF12906
MSTRG.2088.5	2412	coding	PF12906
MSTRG.2088.8	3585	coding	PF12906
MSTRG.2083.1	7792	noncoding	-
MSTRG.2095.1	5285	coding	PF00078
MSTRG.2095.2	1006	noncoding	-
MSTRG.2098.1	2846	noncoding	-
MSTRG.2097.6	8174	coding	PF00439
MSTRG.2097.7	5707	noncoding	-
MSTRG.2097.8	6896	coding	PF01429
MSTRG.2110.8	4183	noncoding	-
MSTRG.2110.1	3808	noncoding	-
MSTRG.2110.5	3805	noncoding	-
MSTRG.2111.1	1872	noncoding	-
MSTRG.2110.9	1546	noncoding	-
MSTRG.2112.1	751	noncoding	-
MSTRG.2113.1	1749	noncoding	-
MSTRG.2114.1	2395	noncoding	-
MSTRG.2116.1	1198	coding	PF02994
MSTRG.2114.2	2385	noncoding	-

MSTRG.2108.1	570	noncoding	-
MSTRG.2119.3	4434	coding	PF16901
MSTRG.2118.1	242	noncoding	-
MSTRG.2126.1	406	noncoding	-
MSTRG.2148.1	1296	noncoding	-
MSTRG.2152.1	910	noncoding	-
MSTRG.2154.1	2752	noncoding	-
MSTRG.2153.6	5155	coding	PF15062
MSTRG.2153.3	1857	coding	PF15062
MSTRG.2153.5	1836	coding	PF15062
MSTRG.2153.4	3044	coding	PF15062
MSTRG.2153.7	2620	coding	PF03467
MSTRG.2155.1	4273	coding	PF01846
MSTRG.2155.2	4613	coding	PF01846
MSTRG.2155.3	3697	coding	PF01846
MSTRG.2155.4	4106	coding	PF01846
MSTRG.2155.7	729	noncoding	-
MSTRG.2155.8	1625	noncoding	-
MSTRG.2155.9	1982	noncoding	-
MSTRG.2144.1	958	noncoding	-
MSTRG.2145.1	998	noncoding	-
MSTRG.2146.1	11452	noncoding	-
MSTRG.2146.2	20377	noncoding	-
MSTRG.2175.1	18820	noncoding	-
MSTRG.2175.2	4765	noncoding	-
MSTRG.2177.2	8891	coding	PF12231
MSTRG.2177.1	9062	coding	PF12231
MSTRG.2177.6	11614	coding	PF12231
MSTRG.2177.4	10896	coding	PF12231
MSTRG.2177.7	15564	coding	PF12231
MSTRG.2177.8	8421	coding	PF12231
MSTRG.2177.10	11306	coding	PF12231
MSTRG.2177.12	9858	noncoding	-
MSTRG.2177.11	10874	coding	PF12231
MSTRG.2157.1	1520	coding	PF07292
MSTRG.2166.1	752	noncoding	-
MSTRG.2167.1	385	noncoding	-
MSTRG.2179.2	6115	coding	PF00225
MSTRG.2179.3	2781	coding	PF00225
MSTRG.2179.4	5771	noncoding	-
MSTRG.2200.2	6842	noncoding	-
MSTRG.2200.3	6484	noncoding	-
MSTRG.2200.5	6527	noncoding	-
MSTRG.2201.1	2333	noncoding	-
MSTRG.2202.1	1955	noncoding	-
MSTRG.2203.1	3579	noncoding	-
MSTRG.2210.3	6201	noncoding	-
MSTRG.2211.1	4060	noncoding	-
MSTRG.2210.4	8054	noncoding	-
MSTRG.2212.1	6762	noncoding	-
MSTRG.2213.3	3989	noncoding	-
MSTRG.2196.1	467	noncoding	-
MSTRG.2219.1	9913	noncoding	-
MSTRG.2220.2	6033	noncoding	-

MSTRG.2221.1	17829	noncoding	-
MSTRG.2221.2	981	noncoding	-
MSTRG.2222.1	12317	noncoding	-
MSTRG.2222.2	6978	noncoding	-
MSTRG.2220.3	7010	noncoding	-
MSTRG.2220.4	4190	noncoding	-
MSTRG.2223.3	787	noncoding	-
MSTRG.2223.4	9652	coding	PF00078
MSTRG.2223.5	6259	noncoding	-
MSTRG.2223.6	5490	noncoding	-
MSTRG.2224.1	2448	noncoding	-
MSTRG.2225.1	1188	noncoding	-
MSTRG.2230.1	774	noncoding	-
MSTRG.2240.1	216	noncoding	-
MSTRG.2241.1	884	noncoding	-
MSTRG.2248.2	1849	coding	PF00651
MSTRG.2248.4	3828	coding	PF00651
MSTRG.2248.6	1641	coding	PF00651
MSTRG.2250.1	885	noncoding	-
MSTRG.2252.1	2612	noncoding	-
MSTRG.2257.2	2372	coding	PF00001
MSTRG.2257.3	2058	coding	PF00001
MSTRG.2265.1	6939	noncoding	-
MSTRG.2265.2	3522	noncoding	-
MSTRG.2265.3	9834	noncoding	-
MSTRG.2265.4	2553	noncoding	-
MSTRG.2265.6	3865	noncoding	-
MSTRG.2265.5	5727	noncoding	-
MSTRG.2262.2	2777	coding	PF00789
MSTRG.2268.4	3906	noncoding	-
MSTRG.2268.3	4306	noncoding	-
MSTRG.2268.5	4439	noncoding	-
MSTRG.2269.1	2404	noncoding	-
MSTRG.2273.1	706	noncoding	-
MSTRG.2278.1	1907	noncoding	-
MSTRG.2280.1	6410	coding	PF13890
MSTRG.2281.1	2766	noncoding	-
MSTRG.2282.1	558	noncoding	-
MSTRG.2283.1	2458	noncoding	-
MSTRG.2284.1	3430	noncoding	-
MSTRG.2270.4	5619	noncoding	-
MSTRG.2270.5	6037	noncoding	-
MSTRG.2288.1	7913	coding	PF15024
MSTRG.2288.3	8256	coding	PF15024
MSTRG.2289.1	7857	noncoding	-
MSTRG.2285.1	405	noncoding	-
MSTRG.2311.1	453	noncoding	-
MSTRG.2350.1	1445	coding	PF00002
MSTRG.2352.2	3569	noncoding	-
MSTRG.2352.3	2279	noncoding	-
MSTRG.2352.4	2186	noncoding	-
MSTRG.2355.1	418	noncoding	-
MSTRG.2361.1	5682	coding	PF00102
MSTRG.2361.2	6918	coding	PF00102

MSTRG.2361.4	7441	coding	PF00102
MSTRG.2361.5	10589	coding	PF00102
MSTRG.2361.3	10368	coding	PF00102
MSTRG.2362.1	1616	noncoding	-
MSTRG.2364.1	6197	coding	PF09379
MSTRG.2364.4	8154	coding	PF09380
MSTRG.2364.6	8973	coding	PF09380
MSTRG.2365.1	2277	noncoding	-
MSTRG.2364.7	8748	coding	PF09380
MSTRG.2364.8	12542	noncoding	-
MSTRG.2364.9	1331	noncoding	-
MSTRG.2366.1	3360	noncoding	-
MSTRG.2364.11	7645	noncoding	-
MSTRG.2371.1	6802	noncoding	-
MSTRG.2381.1	7775	noncoding	-
MSTRG.2381.10	7089	noncoding	-
MSTRG.2381.7	6821	noncoding	-
MSTRG.2381.4	7506	noncoding	-
MSTRG.2381.6	7774	noncoding	-
MSTRG.2381.5	7666	noncoding	-
MSTRG.2381.2	7886	noncoding	-
MSTRG.2381.3	8101	noncoding	-
MSTRG.2381.9	8389	noncoding	-
MSTRG.2381.13	6806	noncoding	-
MSTRG.2381.12	7775	noncoding	-
MSTRG.2381.15	7408	noncoding	-
MSTRG.2381.17	7402	noncoding	-
MSTRG.2381.16	11949	noncoding	-
MSTRG.2381.14	8418	noncoding	-
MSTRG.2381.18	8325	noncoding	-
MSTRG.2381.19	3846	noncoding	-
MSTRG.2387.1	464	noncoding	-
MSTRG.2385.1	444	noncoding	-
MSTRG.2391.1	209	noncoding	-
MSTRG.2392.1	275	noncoding	-
MSTRG.2398.5	823	noncoding	-
MSTRG.2412.1	4321	noncoding	-
MSTRG.2412.2	4315	noncoding	-
MSTRG.2412.3	7458	noncoding	-
MSTRG.2415.1	5963	noncoding	-
MSTRG.2412.4	6076	noncoding	-
MSTRG.2417.1	2620	noncoding	-
MSTRG.2417.2	2528	noncoding	-
MSTRG.2418.1	10784	noncoding	-
MSTRG.2419.2	1726	coding	PF04960
MSTRG.2419.5	1120	noncoding	-
MSTRG.2419.6	1767	coding	PF04960
MSTRG.2419.4	2081	coding	PF04960
MSTRG.2403.1	737	noncoding	-
MSTRG.2407.1	737	noncoding	-
MSTRG.2426.1	875	noncoding	-
MSTRG.2431.1	215	noncoding	-
MSTRG.2433.1	278	noncoding	-
MSTRG.2438.1	871	noncoding	-

MSTRG.2439.4	1377	coding	PF03028
MSTRG.2435.3	1312	coding	PF00069
MSTRG.2446.3	2700	noncoding	-
MSTRG.2447.1	3737	noncoding	-
MSTRG.2475.4	2264	noncoding	-
MSTRG.2475.5	1469	coding	PF00118
MSTRG.2476.1	873	coding	PF00166
MSTRG.2476.2	527	coding	PF00166
MSTRG.2477.1	561	noncoding	-
MSTRG.2479.4	2975	coding	PF15250
MSTRG.2480.1	7270	noncoding	-
MSTRG.2479.5	2012	coding	PF00076
MSTRG.2479.6	1967	coding	PF00076
MSTRG.2479.7	1990	coding	PF00076
MSTRG.2479.8	1738	coding	PF00076
MSTRG.2479.9	2134	coding	PF00076
MSTRG.2479.10	2669	coding	PF00076
MSTRG.2479.13	1516	coding	PF00076
MSTRG.2479.14	2826	coding	PF00076
MSTRG.2479.15	2538	coding	PF00076
MSTRG.2479.16	1149	coding	PF00076
MSTRG.2479.17	2256	coding	PF00076
MSTRG.2485.2	4145	coding	PF00388
MSTRG.2485.3	4180	coding	PF00388
MSTRG.2460.1	2196	coding	PF16534
MSTRG.2464.2	3126	coding	PF07837
MSTRG.2468.1	4664	coding	PF13621
MSTRG.2489.3	2551	noncoding	-
MSTRG.2492.2	4224	coding	PF00941
MSTRG.2493.1	3688	coding	PF02738
MSTRG.2498.2	6338	noncoding	-
MSTRG.2509.3	1601	coding	PF00069
MSTRG.2509.5	1585	coding	PF00069
MSTRG.2509.4	1993	coding	PF00069
MSTRG.2510.1	2183	noncoding	-
MSTRG.2510.2	1539	noncoding	-
MSTRG.2513.3	4638	noncoding	-
MSTRG.2513.5	6780	noncoding	-
MSTRG.2514.3	1158	noncoding	-
MSTRG.2515.1	4414	noncoding	-
MSTRG.2499.1	232	noncoding	-
MSTRG.2508.1	1821	noncoding	-
MSTRG.2517.2	1997	coding	PF15729
MSTRG.2517.3	1799	coding	PF15729
MSTRG.2519.3	6290	coding	PF02204
MSTRG.2519.4	6161	coding	PF02204
MSTRG.2529.1	10322	noncoding	-
MSTRG.2529.2	2077	noncoding	-
MSTRG.2530.1	1627	noncoding	-
MSTRG.2566.1	1724	noncoding	-
MSTRG.2567.1	2063	noncoding	-
MSTRG.2568.2	2112	coding	PF08154
MSTRG.2569.1	3309	coding	PF15299
MSTRG.2569.2	3411	coding	PF15299

MSTRG.2569.4	4865	coding	PF15299
MSTRG.2569.6	1160	coding	PF15299
MSTRG.2569.5	2831	coding	PF15299
MSTRG.2572.1	1443	noncoding	-
MSTRG.2569.7	17256	coding	PF16057
MSTRG.2569.8	15390	coding	PF16057
MSTRG.2569.9	8461	coding	PF16057
MSTRG.2575.1	1896	noncoding	-
MSTRG.2576.4	292	noncoding	-
MSTRG.2576.3	13110	coding	PF00018
MSTRG.2577.1	2095	noncoding	-
MSTRG.2576.5	5395	coding	PF07815
MSTRG.2576.6	8095	noncoding	-
MSTRG.2576.7	14455	coding	PF00018
MSTRG.2579.2	7772	noncoding	-
MSTRG.2579.3	2385	noncoding	-
MSTRG.2541.2	873	coding	PF12053
MSTRG.2542.1	370	noncoding	-
MSTRG.2543.1	419	noncoding	-
MSTRG.2544.1	1911	noncoding	-
MSTRG.2545.1	2925	noncoding	-
MSTRG.2536.1	447	noncoding	-
MSTRG.2540.1	649	noncoding	-
MSTRG.2561.4	870	coding	PF00736
MSTRG.2564.3	2567	noncoding	-
MSTRG.2582.2	7056	noncoding	-
MSTRG.2582.3	6873	noncoding	-
MSTRG.2588.1	1648	noncoding	-
MSTRG.2589.2	5827	noncoding	-
MSTRG.2589.3	1849	noncoding	-
MSTRG.2589.4	1265	noncoding	-
MSTRG.2590.2	1972	coding	PF10294
MSTRG.2590.1	1934	coding	PF10294
MSTRG.2590.3	2050	coding	PF10294
MSTRG.2591.5	5570	noncoding	-
MSTRG.2591.6	462	noncoding	-
MSTRG.2594.1	1834	noncoding	-
MSTRG.2596.4	9241	coding	PF00118
MSTRG.2596.3	9312	coding	PF00118
MSTRG.2597.2	910	noncoding	-
MSTRG.2597.1	797	noncoding	-
MSTRG.2598.1	233	noncoding	-
MSTRG.2602.1	677	noncoding	-
MSTRG.2602.2	505	noncoding	-
MSTRG.2602.3	502	noncoding	-
MSTRG.2602.10	5075	noncoding	-
MSTRG.2602.11	5067	noncoding	-
MSTRG.2602.12	1362	noncoding	-
MSTRG.2601.1	420	noncoding	-
MSTRG.2601.2	324	noncoding	-
MSTRG.2605.3	5675	noncoding	-
MSTRG.2605.4	5372	noncoding	-
MSTRG.2605.5	11145	coding	PF15778
MSTRG.2605.6	5565	noncoding	-

MSTRG.2605.7	13368	coding	PF15778
MSTRG.2605.8	4417	noncoding	-
MSTRG.2605.10	6774	noncoding	-
MSTRG.2605.9	6599	noncoding	-
MSTRG.2605.11	3327	noncoding	-
MSTRG.2609.7	4551	coding	PF15275
MSTRG.2609.8	4377	coding	PF15275
MSTRG.2609.9	4516	coding	PF15275
MSTRG.2610.2	6722	coding	PF15275
MSTRG.2611.1	4492	noncoding	-
MSTRG.2607.3	1595	coding	PF05147
MSTRG.2682.1	4907	coding	PF01359
MSTRG.2614.1	276	noncoding	-
MSTRG.2632.1	1097	noncoding	-
MSTRG.2633.1	513	noncoding	-
MSTRG.2634.3	1206	noncoding	-
MSTRG.2635.1	3104	noncoding	-
MSTRG.2636.1	1735	noncoding	-
MSTRG.2638.1	1017	noncoding	-
MSTRG.2639.1	6591	noncoding	-
MSTRG.2640.1	5197	noncoding	-
MSTRG.2641.1	1839	noncoding	-
MSTRG.2642.1	508	noncoding	-
MSTRG.2645.1	5407	noncoding	-
MSTRG.2645.2	8683	noncoding	-
MSTRG.2645.3	868	noncoding	-
MSTRG.2647.1	593	noncoding	-
MSTRG.2647.2	879	noncoding	-
MSTRG.2649.1	952	noncoding	-
MSTRG.2668.3	10210	coding	PF10409
MSTRG.2668.4	10189	coding	PF10409
MSTRG.2668.5	10313	coding	PF10409
MSTRG.2668.6	8973	coding	PF08416
MSTRG.2668.7	6121	coding	PF08416
MSTRG.2706.2	1067	noncoding	-
MSTRG.2706.3	1391	noncoding	-
MSTRG.2711.3	580	noncoding	-
MSTRG.2711.4	1481	coding	PF00168
MSTRG.2714.3	1566	noncoding	-
MSTRG.2714.4	3661	coding	PF05773
MSTRG.2715.1	4526	coding	PF00069
MSTRG.2715.4	4320	coding	PF00069
MSTRG.2715.5	3991	coding	PF00069
MSTRG.2717.2	1412	coding	PF00110
MSTRG.2720.1	1287	noncoding	-
MSTRG.2721.2	1019	coding	PF00178
MSTRG.2721.3	878	coding	PF00178
MSTRG.2724.3	2268	noncoding	-
MSTRG.2740.1	2897	noncoding	-
MSTRG.2742.1	1549	coding	PF00069
MSTRG.2742.4	1515	coding	PF00069
MSTRG.2742.5	1425	coding	PF00069
MSTRG.2730.2	2039	coding	PF03953
MSTRG.2732.1	2793	coding	PF00226

MSTRG.2732.2	1898	coding	PF00226
MSTRG.2732.5	1204	coding	PF00226
MSTRG.2743.1	3290	coding	PF16650
MSTRG.2744.1	798	noncoding	-
MSTRG.2747.4	1568	noncoding	-
MSTRG.2750.1	589	coding	PF07679
MSTRG.2750.2	576	coding	PF07679
MSTRG.2750.3	630	coding	PF07679
MSTRG.2749.5	7260	coding	PF07679
MSTRG.2757.1	386	noncoding	-
MSTRG.2761.1	256	noncoding	-
MSTRG.2762.1	435	noncoding	-
MSTRG.2766.2	2032	coding	PF03484
MSTRG.2770.1	315	noncoding	-
MSTRG.2771.3	819	coding	PF01127
MSTRG.2771.2	759	noncoding	-
MSTRG.2772.1	2986	noncoding	-
MSTRG.2774.2	2222	coding	PF00079
MSTRG.2774.3	2022	coding	PF00079
MSTRG.2775.1	2961	noncoding	-
MSTRG.2777.3	3112	coding	PF00888
MSTRG.2778.1	413	noncoding	-
MSTRG.2778.2	341	noncoding	-
MSTRG.2804.3	1492	noncoding	-
MSTRG.2806.5	8758	noncoding	-
MSTRG.2806.6	6113	noncoding	-
MSTRG.2806.7	11784	noncoding	-
MSTRG.2806.8	370	noncoding	-
MSTRG.2807.1	7118	noncoding	-
MSTRG.2808.1	1546	noncoding	-
MSTRG.2809.1	8207	noncoding	-
MSTRG.2810.2	7254	noncoding	-
MSTRG.2811.8	4207	coding	PF05644
MSTRG.2811.10	2314	coding	PF05644
MSTRG.2788.4	8272	noncoding	-
MSTRG.2788.5	9350	noncoding	-
MSTRG.2788.6	2494	noncoding	-
MSTRG.2788.7	2421	noncoding	-
MSTRG.2793.3	1619	coding	PF00400
MSTRG.2798.2	2979	noncoding	-
MSTRG.2822.10	5894	coding	PF00632
MSTRG.2822.6	6362	coding	PF00632
MSTRG.2829.1	3146	noncoding	-
MSTRG.2830.1	2173	noncoding	-
MSTRG.2799.1	2962	coding	PF03172
MSTRG.2800.2	2803	coding	PF03172
MSTRG.2817.1	4693	noncoding	-
MSTRG.2819.1	627	noncoding	-
MSTRG.2819.2	450	noncoding	-
MSTRG.2819.3	573	noncoding	-
MSTRG.2819.4	1800	noncoding	-
MSTRG.2820.2	3233	noncoding	-
MSTRG.2820.3	3960	coding	PF08569
MSTRG.2816.4	5290	noncoding	-

MSTRG.2816.3	5352	noncoding	-
MSTRG.2816.1	5252	noncoding	-
MSTRG.2816.5	4657	noncoding	-
MSTRG.2814.1	754	noncoding	-
MSTRG.2821.2	3094	noncoding	-
MSTRG.2821.3	3347	noncoding	-
MSTRG.2833.3	4202	noncoding	-
MSTRG.2833.4	2512	noncoding	-
MSTRG.2833.5	1843	noncoding	-
MSTRG.2836.2	2637	coding	PF00076
MSTRG.2837.1	12154	noncoding	-
MSTRG.2837.2	4995	noncoding	-
MSTRG.2843.2	1190	noncoding	-
MSTRG.2840.3	2248	noncoding	-
MSTRG.2848.2	3914	coding	PF00773
MSTRG.2848.3	3300	coding	PF00773
MSTRG.2848.4	1434	noncoding	-
MSTRG.2848.6	4513	coding	PF00773
MSTRG.2848.7	257	noncoding	-
MSTRG.2845.1	969	noncoding	-
MSTRG.2845.2	888	noncoding	-
MSTRG.2850.5	4449	coding	PF01652
MSTRG.2850.4	4335	coding	PF01652
MSTRG.2850.6	1901	noncoding	-
MSTRG.2850.7	8626	coding	PF01652
MSTRG.2850.8	7773	coding	PF01652
MSTRG.2846.1	1666	noncoding	-
MSTRG.2857.2	3734	coding	PF16616
MSTRG.2857.4	2339	coding	PF16616
MSTRG.2852.4	15093	noncoding	-
MSTRG.2852.3	15127	noncoding	-
MSTRG.2858.2	3215	coding	PF00406
MSTRG.2859.1	352	noncoding	-
MSTRG.2865.3	2754	coding	PF01485
MSTRG.2866.8	1980	coding	PF15427
MSTRG.2871.1	3691	coding	PF12265
MSTRG.2873.1	5655	coding	PF01951
MSTRG.2870.1	2085	coding	PF00651
MSTRG.2879.1	1530	coding	PF00069
MSTRG.2879.3	1148	coding	PF00069
MSTRG.2884.3	2631	coding	PF00850
MSTRG.2889.3	1395	coding	PF10639
MSTRG.2889.4	1127	coding	PF10639
MSTRG.2894.2	322	noncoding	-
MSTRG.2897.1	6919	coding	PF03953
MSTRG.2898.2	3611	coding	PF07500
MSTRG.2898.1	3814	coding	PF07500
MSTRG.2899.1	1920	noncoding	-
MSTRG.2899.3	1161	noncoding	-
MSTRG.2899.2	1098	noncoding	-
MSTRG.2898.4	1688	coding	PF07744
MSTRG.2900.4	1540	noncoding	-
MSTRG.2900.5	778	noncoding	-
MSTRG.2906.4	5090	noncoding	-

MSTRG.2906.5	3363	noncoding	-
MSTRG.2907.3	2392	coding	PF03348
MSTRG.2909.1	1347	coding	PF00061
MSTRG.2909.2	1005	coding	PF00061
MSTRG.2910.1	3603	noncoding	-
MSTRG.2910.4	2015	noncoding	-
MSTRG.2912.2	2245	coding	PF01034
MSTRG.2913.1	962	noncoding	-
MSTRG.2914.1	2420	coding	PF03821
MSTRG.2914.2	2202	coding	PF03821
MSTRG.2921.1	889	noncoding	-
MSTRG.2922.1	793	noncoding	-
MSTRG.2922.2	688	noncoding	-
MSTRG.2920.1	256	noncoding	-
MSTRG.2924.2	5218	coding	PF00102
MSTRG.2924.3	5295	coding	PF00102
MSTRG.2927.3	13020	noncoding	-
MSTRG.2932.2	5404	coding	PF05902
MSTRG.2932.6	2985	coding	PF05902
MSTRG.2929.1	781	coding	PF00001
MSTRG.2935.3	2542	coding	PF01342
MSTRG.2943.3	1010	noncoding	-
MSTRG.2943.4	1481	coding	PF00076
MSTRG.2943.5	1189	coding	PF00076
MSTRG.2943.7	1135	noncoding	-
MSTRG.2943.6	1022	coding	PF00076
MSTRG.2950.1	2659	noncoding	-
MSTRG.2950.2	2281	noncoding	-
MSTRG.2950.3	3184	noncoding	-
MSTRG.2951.1	903	noncoding	-
MSTRG.2956.4	3995	noncoding	-
MSTRG.2956.5	3839	noncoding	-
MSTRG.2956.6	6402	noncoding	-
MSTRG.2956.7	5042	noncoding	-
MSTRG.2957.1	4411	coding	PF01359
MSTRG.2948.3	5344	noncoding	-
MSTRG.2960.1	1130	noncoding	-
MSTRG.2968.1	3035	coding	PF16046
MSTRG.2968.2	3213	coding	PF16046
MSTRG.2968.3	3149	coding	PF16046
MSTRG.2973.1	669	coding	PF05283
MSTRG.2978.3	2669	noncoding	-
MSTRG.2978.1	3890	noncoding	-
MSTRG.2978.2	2610	noncoding	-
MSTRG.2979.1	440	noncoding	-
MSTRG.2980.1	2458	noncoding	-
MSTRG.2983.1	2242	noncoding	-
MSTRG.2995.1	1344	noncoding	-
MSTRG.2996.3	3132	coding	PF01255
MSTRG.2996.4	3075	coding	PF01255
MSTRG.3004.4	1414	coding	PF08059
MSTRG.3004.6	1864	coding	PF08059
MSTRG.3004.7	1650	coding	PF08059
MSTRG.3001.1	1820	noncoding	-

MSTRG.3012.4	1386	coding	PF03006
MSTRG.3012.5	1866	coding	PF03006
MSTRG.3012.6	1421	coding	PF03006
MSTRG.3017.3	3063	coding	PF05308
MSTRG.3017.7	2383	coding	PF05308
MSTRG.3014.2	4018	noncoding	-
MSTRG.3021.2	2606	coding	PF09726
MSTRG.3026.1	359	noncoding	-
MSTRG.3027.2	4023	coding	PF00853
MSTRG.3027.3	3739	coding	PF00853
MSTRG.3027.4	1394	coding	PF00853
MSTRG.3028.1	4390	noncoding	-
MSTRG.3028.2	4148	noncoding	-
MSTRG.3028.3	3934	noncoding	-
MSTRG.3028.4	4711	noncoding	-
MSTRG.3028.5	1527	noncoding	-
MSTRG.3029.1	1423	noncoding	-
MSTRG.3031.6	3947	coding	PF01480
MSTRG.3031.10	3718	coding	PF01480
MSTRG.3031.7	3895	coding	PF01480
MSTRG.3031.9	3937	coding	PF01480
MSTRG.3036.2	1293	coding	PF04847
MSTRG.3037.1	2104	noncoding	-
MSTRG.3037.5	2861	noncoding	-
MSTRG.3037.6	1928	noncoding	-
MSTRG.3039.3	2263	noncoding	-
MSTRG.3043.1	6325	coding	PF00076
MSTRG.3043.7	4368	noncoding	-
MSTRG.3043.4	2381	noncoding	-
MSTRG.3043.6	2371	noncoding	-
MSTRG.3043.3	6157	noncoding	-
MSTRG.3043.10	7064	noncoding	-
MSTRG.3043.11	7061	noncoding	-
MSTRG.3043.12	4314	noncoding	-
MSTRG.3043.9	2314	noncoding	-
MSTRG.3043.13	2190	noncoding	-
MSTRG.3042.1	1012	noncoding	-
MSTRG.3050.1	501	noncoding	-
MSTRG.3054.2	800	coding	PF02230
MSTRG.3055.2	1939	coding	PF06201
MSTRG.3044.2	4773	coding	PF06881
MSTRG.3056.2	601	coding	PF00673
MSTRG.3059.1	4040	coding	PF01412
MSTRG.3060.1	3533	coding	PF07500
MSTRG.3061.1	1907	noncoding	-
MSTRG.3061.2	1471	noncoding	-
MSTRG.3064.6	3070	coding	PF00076
MSTRG.3064.5	19187	coding	PF00076
MSTRG.3066.1	1184	noncoding	-
MSTRG.3068.4	3438	coding	PF01593
MSTRG.3068.5	3001	coding	PF01593
MSTRG.3068.3	3167	coding	PF01593
MSTRG.3065.1	464	noncoding	-
MSTRG.3070.1	2168	noncoding	-

MSTRG.3076.1	7673	coding	PF00651
MSTRG.3085.2	12360	coding	PF02210
MSTRG.3087.2	724	noncoding	-
MSTRG.3087.1	691	noncoding	-
MSTRG.3093.1	393	noncoding	-
MSTRG.3092.4	3274	coding	PF02145
MSTRG.3092.5	1709	noncoding	-
MSTRG.3088.2	2463	coding	PF00245
MSTRG.3088.3	2461	coding	PF00245
MSTRG.3113.1	3625	noncoding	-
MSTRG.3114.1	6502	coding	PF02854
MSTRG.3115.1	12912	noncoding	-
MSTRG.3116.1	17077	noncoding	-
MSTRG.3116.2	13185	noncoding	-
MSTRG.3114.2	5864	coding	PF02854
MSTRG.3114.4	5713	coding	PF02854
MSTRG.3114.3	5813	coding	PF02854
MSTRG.3114.5	5777	coding	PF02854
MSTRG.3114.7	6662	coding	PF02854
MSTRG.3114.13	5891	coding	PF02854
MSTRG.3114.8	5774	coding	PF02854
MSTRG.3114.9	6872	coding	PF02854
MSTRG.3117.1	3470	noncoding	-
MSTRG.3118.1	3973	noncoding	-
MSTRG.3114.14	7394	coding	PF02854
MSTRG.3114.15	6706	coding	PF02854
MSTRG.3122.1	546	noncoding	-
MSTRG.3124.3	4095	noncoding	-
MSTRG.3124.4	3422	noncoding	-
MSTRG.3124.5	3408	noncoding	-
MSTRG.3124.8	3718	noncoding	-
MSTRG.3100.1	2860	noncoding	-
MSTRG.3101.1	4100	coding	PF00225
MSTRG.3101.3	4036	coding	PF00225
MSTRG.3101.2	8631	coding	PF00225
MSTRG.3101.4	3884	coding	PF00225
MSTRG.3101.5	1515	noncoding	-
MSTRG.3104.1	1983	noncoding	-
MSTRG.3101.6	2023	coding	PF03345
MSTRG.3096.2	2463	coding	PF13920
MSTRG.3106.3	3892	coding	PF13768
MSTRG.3106.4	3752	coding	PF13768
MSTRG.3106.5	3629	coding	PF13768
MSTRG.3106.6	3730	coding	PF13768
MSTRG.3106.7	1690	coding	PF13768
MSTRG.3106.8	953	noncoding	-
MSTRG.3109.1	1417	coding	PF00068
MSTRG.3111.1	757	noncoding	-
MSTRG.3125.3	1736	coding	PF03045
MSTRG.3126.2	638	noncoding	-
MSTRG.3127.3	1366	coding	PF01115
MSTRG.3127.4	1404	coding	PF01115
MSTRG.3130.1	1064	coding	PF00248
MSTRG.3130.2	4167	coding	PF00248

MSTRG.3130.3	4060	coding	PF00248
MSTRG.3132.1	2769	noncoding	-
MSTRG.3132.3	1972	noncoding	-
MSTRG.3133.1	5271	coding	PF07774
MSTRG.3133.3	4412	coding	PF07774
MSTRG.3133.4	4326	coding	PF07774
MSTRG.3133.5	4329	coding	PF07774
MSTRG.3134.1	2278	noncoding	-
MSTRG.3144.2	15753	coding	PF13764
MSTRG.3136.1	2374	noncoding	-
MSTRG.3137.1	972	noncoding	-
MSTRG.3135.1	988	noncoding	-
MSTRG.3135.2	684	noncoding	-
MSTRG.3141.1	654	noncoding	-
MSTRG.3146.1	548	noncoding	-
MSTRG.3146.2	993	noncoding	-
MSTRG.3153.1	631	noncoding	-
MSTRG.3154.1	4439	coding	PF00621
MSTRG.3154.4	4416	coding	PF00621
MSTRG.3154.5	1961	coding	PF00621
MSTRG.3155.1	1717	noncoding	-
MSTRG.3151.1	473	noncoding	-
MSTRG.3152.1	927	coding	PF03068
MSTRG.3157.2	1490	coding	PF13085
MSTRG.3157.3	1360	coding	PF13085
MSTRG.3157.5	1313	coding	PF13085
MSTRG.3163.1	1505	noncoding	-
MSTRG.3166.3	7976	noncoding	-
MSTRG.3166.6	1214	noncoding	-
MSTRG.3166.7	1071	noncoding	-
MSTRG.3159.4	5642	noncoding	-
MSTRG.3179.7	9743	noncoding	-
MSTRG.3179.9	1972	noncoding	-
MSTRG.3179.10	10496	noncoding	-
MSTRG.3179.11	2632	coding	PF00078
MSTRG.3183.1	3120	coding	PF04666
MSTRG.3173.1	1416	noncoding	-
MSTRG.3176.1	7359	coding	PF04178
MSTRG.3177.1	417	noncoding	-
MSTRG.3178.3	3435	coding	PF01359
MSTRG.3185.1	4959	noncoding	-
MSTRG.3185.2	10303	coding	PF15761
MSTRG.3186.1	14103	coding	PF01359
MSTRG.3185.3	4189	noncoding	-
MSTRG.3186.6	9912	noncoding	-
MSTRG.3186.4	3402	noncoding	-
MSTRG.3186.9	3208	noncoding	-
MSTRG.3186.10	3156	noncoding	-
MSTRG.3186.11	3310	noncoding	-
MSTRG.3186.12	5425	noncoding	-
MSTRG.3187.1	5376	noncoding	-
MSTRG.3188.1	5010	noncoding	-
MSTRG.3189.1	1178	coding	PF03650
MSTRG.3190.2	1471	noncoding	-

MSTRG.3190.3	1468	noncoding	-
MSTRG.3197.1	1440	noncoding	-
MSTRG.3198.1	1153	noncoding	-
MSTRG.3214.1	4637	noncoding	-
MSTRG.3221.1	1186	noncoding	-
MSTRG.3226.1	1025	noncoding	-
MSTRG.3228.2	4508	coding	PF03184
MSTRG.3215.1	1290	noncoding	-
MSTRG.3217.1	769	noncoding	-
MSTRG.3218.1	4544	noncoding	-
MSTRG.3220.1	358	noncoding	-
MSTRG.3234.2	1164	noncoding	-
MSTRG.3248.1	7473	noncoding	-
MSTRG.3249.1	9651	noncoding	-
MSTRG.3243.1	833	noncoding	-
MSTRG.3244.1	430	noncoding	-
MSTRG.3245.1	282	noncoding	-
MSTRG.3252.1	2916	noncoding	-
MSTRG.3252.3	2874	noncoding	-
MSTRG.3252.4	4189	noncoding	-
MSTRG.3252.5	4043	noncoding	-
MSTRG.3254.1	801	coding	PF15768
MSTRG.3259.3	3403	noncoding	-
MSTRG.3259.4	3350	noncoding	-
MSTRG.3261.1	887	coding	PF01852
MSTRG.3263.1	11233	noncoding	-
MSTRG.3264.1	615	noncoding	-
MSTRG.3267.2	3207	coding	PF02191
MSTRG.3267.4	2866	coding	PF02191
MSTRG.3269.1	2428	noncoding	-
MSTRG.3270.2	6842	noncoding	-
MSTRG.3270.3	6005	coding	PF02252
MSTRG.3271.1	4799	noncoding	-
MSTRG.3279.1	4980	noncoding	-
MSTRG.3282.2	1064	coding	PF01127
MSTRG.3282.3	962	noncoding	-
MSTRG.3282.4	1019	noncoding	-
MSTRG.3282.6	2608	coding	PF01127
MSTRG.3282.5	763	coding	PF01127
MSTRG.3282.7	688	coding	PF01127
MSTRG.3294.1	634	noncoding	-
MSTRG.3294.2	293	noncoding	-
MSTRG.3296.4	1045	coding	PF08694
MSTRG.3296.3	1112	coding	PF08694
MSTRG.3297.5	1646	noncoding	-
MSTRG.3300.1	5175	noncoding	-
MSTRG.3300.2	1160	noncoding	-
MSTRG.3287.1	364	noncoding	-
MSTRG.3307.1	5616	coding	PF06638
MSTRG.3307.4	6048	coding	PF06638
MSTRG.3307.2	8418	coding	PF06638
MSTRG.3307.3	5456	coding	PF06638
MSTRG.3307.6	5089	coding	PF06638
MSTRG.3312.1	22351	coding	PF04614

MSTRG.3314.1	6724	noncoding	-
MSTRG.3317.2	3411	coding	PF00122
MSTRG.3317.3	2952	coding	PF00122
MSTRG.3317.4	2869	coding	PF00122
MSTRG.3317.5	2939	coding	PF00122
MSTRG.3317.6	2979	coding	PF00122
MSTRG.3320.1	7012	noncoding	-
MSTRG.3321.4	1447	coding	PF00307
MSTRG.3321.3	1536	coding	PF00307
MSTRG.3321.6	1564	coding	PF00307
MSTRG.3333.1	1564	noncoding	-
MSTRG.3339.3	9998	coding	PF13895
MSTRG.3339.4	2520	coding	PF13895
MSTRG.3340.1	4615	noncoding	-
MSTRG.3342.2	888	coding	PF00530
MSTRG.3342.1	1127	coding	PF00530
MSTRG.3343.1	1179	noncoding	-
MSTRG.3343.2	3455	noncoding	-
MSTRG.3346.1	298	noncoding	-
MSTRG.3354.3	274	coding	PF00178
MSTRG.3357.1	6946	coding	PF09128
MSTRG.3357.2	7066	coding	PF09128
MSTRG.3358.1	2728	noncoding	-
MSTRG.3357.4	6096	coding	PF09128
MSTRG.3359.6	798	noncoding	-
MSTRG.3364.1	2265	noncoding	-
MSTRG.3364.3	2303	noncoding	-
MSTRG.3364.4	2762	noncoding	-
MSTRG.3364.5	2340	noncoding	-
MSTRG.3364.6	2541	noncoding	-
MSTRG.3364.7	2039	noncoding	-
MSTRG.3371.1	1934	noncoding	-
MSTRG.3373.2	5028	coding	PF00616
MSTRG.3374.3	13965	noncoding	-
MSTRG.3375.1	682	noncoding	-
MSTRG.3381.2	987	noncoding	-
MSTRG.3384.1	3026	coding	PF01403
MSTRG.3389.3	7560	coding	PF00240
MSTRG.3389.2	3550	coding	PF00240
MSTRG.3389.4	4331	coding	PF00240
MSTRG.3390.2	847	noncoding	-
MSTRG.3415.2	1168	noncoding	-
MSTRG.3415.3	2842	noncoding	-
MSTRG.3418.1	1886	coding	PF10236
MSTRG.3420.6	10753	coding	PF00856
MSTRG.3420.7	10570	coding	PF00856
MSTRG.3420.8	10654	coding	PF00856
MSTRG.3421.1	2496	noncoding	-
MSTRG.3424.1	2586	coding	PF00348
MSTRG.3400.2	4137	coding	PF04144
MSTRG.3400.6	3937	coding	PF04144
MSTRG.3400.3	4013	coding	PF04144
MSTRG.3400.4	3928	coding	PF04144
MSTRG.3401.2	3141	noncoding	-

MSTRG.3402.2	1456	coding	PF11801
MSTRG.3427.2	1589	noncoding	-
MSTRG.3429.1	2351	coding	PF00651
MSTRG.3434.8	3248	coding	PF00640
MSTRG.3435.1	3689	noncoding	-
MSTRG.3435.3	4157	noncoding	-
MSTRG.3438.1	1707	noncoding	-
MSTRG.3444.1	12624	coding	PF02137
MSTRG.3444.3	12487	coding	PF02137
MSTRG.3444.5	6318	coding	PF02137
MSTRG.3444.7	6482	coding	PF02137
MSTRG.3444.12	8907	coding	PF02137
MSTRG.3444.13	5807	coding	PF02137
MSTRG.3441.3	1403	coding	PF00076
MSTRG.3441.4	1365	noncoding	-
MSTRG.3446.1	734	noncoding	-
MSTRG.3470.3	1175	noncoding	-
MSTRG.3471.3	4444	noncoding	-
MSTRG.3471.1	4073	noncoding	-
MSTRG.3471.5	3434	noncoding	-
MSTRG.3471.4	3661	noncoding	-
MSTRG.3471.10	4195	noncoding	-
MSTRG.3471.11	3599	noncoding	-
MSTRG.3471.12	4110	noncoding	-
MSTRG.3471.13	3907	noncoding	-
MSTRG.3471.14	4148	noncoding	-
MSTRG.3471.16	2112	noncoding	-
MSTRG.3472.3	1318	coding	PF07406
MSTRG.3474.1	1688	noncoding	-
MSTRG.3475.1	4985	coding	PF00261
MSTRG.3475.3	1867	coding	PF00261
MSTRG.3477.1	598	noncoding	-
MSTRG.3477.2	454	noncoding	-
MSTRG.3478.5	5822	noncoding	-
MSTRG.3478.7	6515	noncoding	-
MSTRG.3482.1	1162	coding	PF00071
MSTRG.3447.2	1238	coding	PF05439
MSTRG.3454.1	571	noncoding	-
MSTRG.3463.2	789	noncoding	-
MSTRG.3469.2	723	noncoding	-
MSTRG.3493.2	1320	noncoding	-
MSTRG.3497.1	374	noncoding	-
MSTRG.3498.2	2980	coding	PF00567
MSTRG.3504.1	4022	noncoding	-
MSTRG.3509.1	632	noncoding	-
MSTRG.3521.1	8349	coding	PF00454
MSTRG.3521.2	3747	coding	PF00454
MSTRG.3521.3	3716	coding	PF00454
MSTRG.3521.4	3608	coding	PF00454
MSTRG.3521.5	3716	coding	PF00454
MSTRG.3521.6	3617	coding	PF00454
MSTRG.3521.7	3706	coding	PF00454
MSTRG.3521.11	4437	coding	PF00454
MSTRG.3530.4	965	coding	PF15805

MSTRG.3536.2	924	noncoding	-
MSTRG.3536.3	833	noncoding	-
MSTRG.3536.4	765	noncoding	-
MSTRG.3536.5	689	noncoding	-
MSTRG.3536.6	869	noncoding	-
MSTRG.3543.3	4173	coding	PF02833
MSTRG.3544.1	2445	coding	PF04424
MSTRG.3544.2	2698	coding	PF04424
MSTRG.3544.4	2332	coding	PF04424
MSTRG.3544.3	3220	coding	PF04424
MSTRG.3544.7	2993	coding	PF04424
MSTRG.3546.1	2102	coding	PF03798
MSTRG.3547.5	4341	coding	PF05033
MSTRG.3547.4	4409	coding	PF05033
MSTRG.3547.6	2195	noncoding	-
MSTRG.3542.4	4597	noncoding	-
MSTRG.3550.3	3064	noncoding	-
MSTRG.3548.1	1087	noncoding	-
MSTRG.3557.2	8057	coding	PF04818
MSTRG.3557.3	8135	coding	PF04818
MSTRG.3558.1	2072	noncoding	-
MSTRG.3559.1	17056	coding	PF00078
MSTRG.3561.4	1878	coding	PF08572
MSTRG.3561.5	2637	coding	PF08572
MSTRG.3561.6	2741	coding	PF08572
MSTRG.3561.7	2314	coding	PF08572
MSTRG.3563.3	1301	coding	PF15673
MSTRG.3563.4	1367	coding	PF15673
MSTRG.3565.1	1991	coding	PF06105
MSTRG.3562.1	3427	noncoding	-
MSTRG.3566.3	1354	noncoding	-
MSTRG.3566.2	1400	noncoding	-
MSTRG.3570.2	5934	noncoding	-
MSTRG.3580.1	2561	noncoding	-
MSTRG.3580.2	2174	noncoding	-
MSTRG.3588.6	1497	noncoding	-
MSTRG.3588.7	1378	noncoding	-
MSTRG.3592.6	1280	noncoding	-
MSTRG.3593.3	1287	noncoding	-
MSTRG.3598.1	1311	noncoding	-
MSTRG.3602.1	1831	noncoding	-
MSTRG.3604.2	3733	coding	PF14954
MSTRG.3604.3	3736	coding	PF14954
MSTRG.3604.4	3697	coding	PF14954
MSTRG.3604.5	3239	noncoding	-
MSTRG.3604.6	3123	coding	PF00076
MSTRG.3606.2	1583	coding	PF05648
MSTRG.3606.3	1589	coding	PF05648
MSTRG.3612.3	2594	noncoding	-
MSTRG.3612.2	6649	coding	PF13639
MSTRG.3612.4	3023	coding	PF13639
MSTRG.3614.1	923	noncoding	-
MSTRG.3623.1	11680	coding	PF12537
MSTRG.3623.2	1563	coding	PF12537

MSTRG.3623.7	2479	coding	PF12430
MSTRG.3631.1	505	noncoding	-
MSTRG.3633.4	7667	noncoding	-
MSTRG.3642.2	3242	coding	PF06758
MSTRG.3642.3	3023	coding	PF06758
MSTRG.3650.1	2020	noncoding	-
MSTRG.3651.1	3572	coding	PF08516
MSTRG.3652.1	3048	coding	PF08516
MSTRG.3654.1	2208	noncoding	-
MSTRG.3658.1	355	noncoding	-
MSTRG.3659.2	1487	coding	PF00579
MSTRG.3663.1	1300	noncoding	-
MSTRG.3664.1	384	noncoding	-
MSTRG.3675.1	8445	coding	PF01532
MSTRG.3675.2	7898	coding	PF01532
MSTRG.3675.3	7318	coding	PF01532
MSTRG.3675.4	5368	coding	PF01532
MSTRG.3675.5	5608	coding	PF01532
MSTRG.3675.6	6134	coding	PF01532
MSTRG.3675.7	3627	coding	PF01532
MSTRG.3675.8	7054	coding	PF01532
MSTRG.3675.9	6708	coding	PF01532
MSTRG.3675.10	5238	coding	PF01532
MSTRG.3675.11	7489	coding	PF01532
MSTRG.3676.1	5543	noncoding	-
MSTRG.3674.1	255	noncoding	-
MSTRG.3679.1	2371	coding	PF00630
MSTRG.3679.2	2718	coding	PF00630
MSTRG.3679.3	2515	coding	PF00630
MSTRG.3685.1	6906	noncoding	-
MSTRG.3685.2	7582	noncoding	-
MSTRG.3687.2	4358	noncoding	-
MSTRG.3694.1	921	coding	PF01216
MSTRG.3702.2	6702	coding	PF05483
MSTRG.3702.3	5242	coding	PF05483
MSTRG.3704.1	593	noncoding	-
MSTRG.3707.2	5165	coding	PF02141
MSTRG.3707.3	346	noncoding	-
MSTRG.3708.2	982	noncoding	-
MSTRG.3710.1	7925	coding	PF00439
MSTRG.3712.1	4536	coding	PF01359
MSTRG.3710.4	7422	coding	PF00439
MSTRG.3710.5	7522	coding	PF00439
MSTRG.3716.2	7527	coding	PF00069
MSTRG.3716.3	7653	coding	PF00069
MSTRG.3716.5	8341	coding	PF00069
MSTRG.3716.6	8003	coding	PF00069
MSTRG.3716.7	7676	coding	PF00069
MSTRG.3716.9	3456	coding	PF00069
MSTRG.3725.1	8224	coding	PF01602
MSTRG.3733.3	919	noncoding	-
MSTRG.3737.2	3876	noncoding	-
MSTRG.3721.1	4753	coding	PF12020
MSTRG.3721.2	1845	coding	PF12020

MSTRG.3722.1	1873	noncoding	-
MSTRG.3748.4	2718	coding	PF00071
MSTRG.3749.6	3315	coding	PF13087
MSTRG.3749.7	3246	coding	PF13087
MSTRG.3749.3	3243	coding	PF13087
MSTRG.3749.5	3349	coding	PF13087
MSTRG.3755.1	3044	noncoding	-
MSTRG.3764.2	4984	coding	PF00071
MSTRG.3765.1	1041	noncoding	-
MSTRG.3765.2	2916	noncoding	-
MSTRG.3767.1	1140	noncoding	-
MSTRG.3768.1	1081	noncoding	-
MSTRG.3769.1	1333	noncoding	-
MSTRG.3770.1	925	noncoding	-
MSTRG.3773.2	1145	noncoding	-
MSTRG.3773.3	4940	noncoding	-
MSTRG.3773.4	6649	noncoding	-
MSTRG.3777.2	559	noncoding	-
MSTRG.3778.1	2126	coding	PF00704
MSTRG.3756.2	968	noncoding	-
MSTRG.3780.4	4142	coding	PF15741
MSTRG.3780.5	1362	noncoding	-
MSTRG.3784.1	776	noncoding	-
MSTRG.3788.1	2165	noncoding	-
MSTRG.3789.1	6286	coding	PF00076
MSTRG.3789.3	10170	coding	PF00076
MSTRG.3790.1	2168	noncoding	-
MSTRG.3800.1	1010	coding	PF00043
MSTRG.3802.2	1194	coding	PF00043
MSTRG.3802.3	1194	coding	PF00043
MSTRG.3802.4	1193	coding	PF00043
MSTRG.3802.5	1193	coding	PF00043
MSTRG.3804.1	4148	coding	PF00962
MSTRG.3807.1	1490	noncoding	-
MSTRG.3813.1	1521	noncoding	-
MSTRG.3817.3	6378	coding	PF16489
MSTRG.3821.2	4799	noncoding	-
MSTRG.3821.1	6604	noncoding	-
MSTRG.3821.4	3747	noncoding	-
MSTRG.3831.5	2136	coding	PF05934
MSTRG.3832.3	2852	noncoding	-
MSTRG.3832.4	3170	noncoding	-
MSTRG.3832.5	3779	noncoding	-
MSTRG.3833.1	985	noncoding	-
MSTRG.3834.1	2662	coding	PF12443
MSTRG.3834.2	6434	coding	PF12443
MSTRG.3839.1	2323	coding	PF00995
MSTRG.3825.2	3901	noncoding	-
MSTRG.3825.1	3823	noncoding	-
MSTRG.3828.1	1449	noncoding	-
MSTRG.3842.1	6432	coding	PF00153
MSTRG.3842.2	6580	coding	PF00153
MSTRG.3842.3	5386	coding	PF00153
MSTRG.3842.4	3144	noncoding	-

MSTRG.3842.6	1963	coding	PF00153
MSTRG.3855.1	4547	noncoding	-
MSTRG.3854.3	4598	coding	PF00055
MSTRG.3856.1	1055	noncoding	-
MSTRG.3852.1	1365	noncoding	-
MSTRG.3852.2	2109	noncoding	-
MSTRG.3868.1	1703	noncoding	-
MSTRG.3969.1	4659	noncoding	-
MSTRG.3970.1	3249	noncoding	-
MSTRG.3971.1	2968	noncoding	-
MSTRG.3975.1	3862	noncoding	-
MSTRG.3976.1	2004	coding	PF00078
MSTRG.3977.1	6824	noncoding	-
MSTRG.3978.1	5107	noncoding	-
MSTRG.3979.1	3486	noncoding	-
MSTRG.3980.1	457	noncoding	-
MSTRG.3972.5	11297	noncoding	-
MSTRG.3981.1	1480	noncoding	-
MSTRG.3982.1	1843	noncoding	-
MSTRG.3983.1	3232	noncoding	-
MSTRG.3983.2	1166	noncoding	-
MSTRG.3984.1	5506	noncoding	-
MSTRG.3984.2	3276	noncoding	-
MSTRG.3985.1	6073	noncoding	-
MSTRG.3985.2	4108	noncoding	-
MSTRG.3972.6	6526	noncoding	-
MSTRG.3986.1	1506	noncoding	-
MSTRG.3987.1	5423	noncoding	-
MSTRG.3987.2	4246	noncoding	-
MSTRG.3987.3	3742	noncoding	-
MSTRG.3987.4	919	noncoding	-
MSTRG.3989.1	5822	noncoding	-
MSTRG.3972.9	4446	noncoding	-
MSTRG.3991.1	1074	noncoding	-
MSTRG.3877.1	3647	noncoding	-
MSTRG.3883.1	322	noncoding	-
MSTRG.3889.2	3206	coding	PF09258
MSTRG.3890.1	2641	coding	PF13895
MSTRG.3893.1	3562	noncoding	-
MSTRG.3895.3	2062	coding	PF14671
MSTRG.3895.4	2362	coding	PF14671
MSTRG.3895.6	1415	noncoding	-
MSTRG.3895.7	2019	coding	PF14671
MSTRG.3896.1	339	noncoding	-
MSTRG.3897.1	1433	coding	PF01137
MSTRG.3900.2	3595	coding	PF05206
MSTRG.3902.2	3980	coding	PF16531
MSTRG.3903.2	2628	noncoding	-
MSTRG.3903.3	2503	noncoding	-
MSTRG.3903.4	2325	noncoding	-
MSTRG.3903.5	6908	noncoding	-
MSTRG.3910.2	4152	coding	PF06202
MSTRG.3911.3	2492	coding	PF02014
MSTRG.3913.3	4599	noncoding	-

MSTRG.3918.1	688	noncoding	-
MSTRG.3935.6	3045	noncoding	-
MSTRG.3935.5	3091	noncoding	-
MSTRG.3935.7	2975	noncoding	-
MSTRG.3935.8	1962	noncoding	-
MSTRG.3935.9	2073	noncoding	-
MSTRG.3935.11	2747	noncoding	-
MSTRG.3936.1	816	noncoding	-
MSTRG.3937.1	3504	noncoding	-
MSTRG.3930.1	858	noncoding	-
MSTRG.3934.1	2109	noncoding	-
MSTRG.3939.2	1098	noncoding	-
MSTRG.3939.4	660	noncoding	-
MSTRG.3940.3	5947	noncoding	-
MSTRG.3940.6	3306	noncoding	-
MSTRG.3940.7	3309	noncoding	-
MSTRG.3941.1	492	noncoding	-
MSTRG.3954.1	448	noncoding	-
MSTRG.3959.2	2156	coding	PF06472
MSTRG.3960.1	827	noncoding	-
MSTRG.3963.3	4101	coding	PF00620
MSTRG.3964.1	836	noncoding	-
MSTRG.3963.6	2716	coding	PF00620
MSTRG.3965.2	6797	noncoding	-
MSTRG.3993.2	5195	coding	PF08698
MSTRG.3995.1	469	noncoding	-
MSTRG.3997.3	4775	noncoding	-
MSTRG.3997.2	24021	noncoding	-
MSTRG.4000.1	1893	noncoding	-
MSTRG.4003.1	4423	noncoding	-
MSTRG.4003.3	5162	noncoding	-
MSTRG.4003.4	5696	noncoding	-
MSTRG.4004.1	5092	noncoding	-
MSTRG.4006.1	7922	coding	PF00808
MSTRG.4006.2	2182	coding	PF00808
MSTRG.4010.2	6017	noncoding	-
MSTRG.4010.1	5410	noncoding	-
MSTRG.4010.3	16470	noncoding	-
MSTRG.4011.1	1904	noncoding	-
MSTRG.4010.6	3121	noncoding	-
MSTRG.4014.1	3180	noncoding	-
MSTRG.4017.1	3984	coding	PF14061
MSTRG.4017.3	2570	coding	PF14061
MSTRG.4008.3	2598	coding	PF00566
MSTRG.4020.1	6337	noncoding	-
MSTRG.4023.2	3674	noncoding	-
MSTRG.4024.2	623	coding	PF15162
MSTRG.4024.4	568	coding	PF15162
MSTRG.4024.5	537	coding	PF15162
MSTRG.4026.1	3612	coding	PF17035
MSTRG.4026.2	3468	coding	PF17035
MSTRG.4026.3	3463	coding	PF17035
MSTRG.4026.4	4878	coding	PF17035
MSTRG.4026.5	3440	coding	PF17035

MSTRG.4026.6	3170	coding	PF17035
MSTRG.4028.5	6035	noncoding	-
MSTRG.4028.6	4436	noncoding	-
MSTRG.4028.7	4446	noncoding	-
MSTRG.4028.8	5876	noncoding	-
MSTRG.4028.9	4382	noncoding	-
MSTRG.4030.3	3434	noncoding	-
MSTRG.4036.1	6704	noncoding	-
MSTRG.4037.1	314	noncoding	-
MSTRG.4047.3	5667	noncoding	-
MSTRG.4047.4	1669	noncoding	-
MSTRG.4047.5	5624	noncoding	-
MSTRG.4047.6	4852	noncoding	-
MSTRG.4047.7	4202	noncoding	-
MSTRG.4047.8	8437	noncoding	-
MSTRG.4047.9	3825	noncoding	-
MSTRG.4038.1	1866	noncoding	-
MSTRG.4051.1	3991	noncoding	-
MSTRG.4051.2	11712	noncoding	-
MSTRG.4052.4	2075	coding	PF04988
MSTRG.4053.1	2594	coding	PF12534
MSTRG.4055.1	806	noncoding	-
MSTRG.4058.1	6582	coding	PF12534
MSTRG.4058.5	5031	coding	PF12534
MSTRG.4072.1	2120	coding	PF02263
MSTRG.4072.3	344	coding	PF02263
MSTRG.4074.1	369	coding	PF02841
MSTRG.4079.1	2367	coding	PF02841
MSTRG.4080.1	427	coding	PF02263
MSTRG.4081.1	3913	coding	PF02841
MSTRG.4082.1	611	coding	PF02263
MSTRG.4083.1	1718	coding	PF02263
MSTRG.4068.2	3405	coding	PF02185
MSTRG.4070.1	399	noncoding	-
MSTRG.4092.3	17295	coding	PF12001
MSTRG.4092.4	10095	noncoding	-
MSTRG.4093.1	3740	noncoding	-
MSTRG.4093.2	271	coding	PF00078
MSTRG.4094.1	4358	noncoding	-
MSTRG.4095.1	1479	noncoding	-
MSTRG.4096.1	4472	noncoding	-
MSTRG.4092.5	8876	noncoding	-
MSTRG.4092.6	8574	noncoding	-
MSTRG.4092.7	1377	noncoding	-
MSTRG.4092.8	2232	noncoding	-
MSTRG.4099.1	6881	coding	PF03114
MSTRG.4104.1	1411	noncoding	-
MSTRG.4108.1	2319	noncoding	-
MSTRG.4108.2	2106	noncoding	-
MSTRG.4108.3	4191	noncoding	-
MSTRG.4108.6	2034	noncoding	-
MSTRG.4108.5	2044	noncoding	-
MSTRG.4116.1	4131	noncoding	-
MSTRG.4122.3	4829	noncoding	-

MSTRG.4122.2	2987	noncoding	-
MSTRG.4122.1	8733	noncoding	-
MSTRG.4125.3	2518	noncoding	-
MSTRG.4129.4	2607	coding	PF11559
MSTRG.4129.7	2643	coding	PF11559
MSTRG.4129.8	3375	coding	PF11559
MSTRG.4129.9	3369	coding	PF11559
MSTRG.4130.1	9426	noncoding	-
MSTRG.4143.2	2137	coding	PF15743
MSTRG.4143.4	426	noncoding	-
MSTRG.4143.3	1895	coding	PF15743
MSTRG.4144.1	366	noncoding	-
MSTRG.4143.6	4200	noncoding	-
MSTRG.4146.2	1475	noncoding	-
MSTRG.4147.1	496	noncoding	-
MSTRG.4152.2	1494	noncoding	-
MSTRG.4131.1	496	noncoding	-
MSTRG.4132.3	3703	coding	PF00069
MSTRG.4132.5	3482	coding	PF00069
MSTRG.4132.6	1571	coding	PF00069
MSTRG.4133.1	9020	noncoding	-
MSTRG.4134.1	4348	noncoding	-
MSTRG.4139.1	5900	noncoding	-
MSTRG.4163.2	7000	coding	PF02354
MSTRG.4163.1	7061	coding	PF02354
MSTRG.4163.3	2609	coding	PF02354
MSTRG.4163.4	5810	coding	PF02354
MSTRG.4163.5	5763	coding	PF02354
MSTRG.4163.6	7637	coding	PF02354
MSTRG.4163.7	5138	coding	PF02354
MSTRG.4162.1	216	noncoding	-
MSTRG.4178.5	8196	coding	PF00226
MSTRG.4178.3	9969	coding	PF01556
MSTRG.4178.6	1220	coding	PF00226
MSTRG.4178.4	1221	coding	PF01556
MSTRG.4178.8	1283	coding	PF01556
MSTRG.4178.7	2033	coding	PF00226
MSTRG.4183.1	2082	noncoding	-
MSTRG.4186.1	7118	coding	PF01359
MSTRG.4186.2	3836	coding	PF00078
MSTRG.4186.3	1528	noncoding	-
MSTRG.4187.1	833	noncoding	-
MSTRG.4203.3	1252	noncoding	-
MSTRG.4203.5	830	noncoding	-
MSTRG.4203.2	2675	noncoding	-
MSTRG.4203.6	1142	noncoding	-
MSTRG.4203.7	2374	noncoding	-
MSTRG.4213.3	1406	noncoding	-
MSTRG.4214.1	2634	noncoding	-
MSTRG.4216.1	592	noncoding	-
MSTRG.4217.3	894	noncoding	-
MSTRG.4226.1	4110	coding	PF11904
MSTRG.4226.2	2213	coding	PF11904
MSTRG.4227.1	910	noncoding	-

MSTRG.4226.3	2215	coding	PF11904
MSTRG.4228.7	8390	noncoding	-
MSTRG.4228.3	7748	noncoding	-
MSTRG.4229.4	4684	noncoding	-
MSTRG.4229.2	5730	noncoding	-
MSTRG.4229.3	3002	coding	PF01359
MSTRG.4230.1	936	noncoding	-
MSTRG.4231.1	219	noncoding	-
MSTRG.4219.1	289	noncoding	-
MSTRG.4246.3	1489	noncoding	-
MSTRG.4250.5	2986	coding	PF00631
MSTRG.4256.1	3455	coding	PF04774
MSTRG.4256.3	7198	coding	PF04774
MSTRG.4256.2	7201	coding	PF04774
MSTRG.4256.4	7356	coding	PF04774
MSTRG.4257.1	378	noncoding	-
MSTRG.4260.3	2205	noncoding	-
MSTRG.4260.4	3833	noncoding	-
MSTRG.4261.3	4077	noncoding	-
MSTRG.4261.2	4018	noncoding	-
MSTRG.4261.4	3911	noncoding	-
MSTRG.4262.1	348	noncoding	-
MSTRG.4293.3	941	coding	PF00179
MSTRG.4293.4	1323	coding	PF00179
MSTRG.4293.5	1107	coding	PF00179
MSTRG.4293.6	1161	coding	PF00179
MSTRG.4293.7	1043	coding	PF00179
MSTRG.4296.1	1111	noncoding	-
MSTRG.4301.2	2969	coding	PF02878
MSTRG.4301.1	2822	coding	PF02878
MSTRG.4301.3	2924	coding	PF02878
MSTRG.4301.4	2483	coding	PF02878
MSTRG.4301.5	2032	coding	PF02878
MSTRG.4301.6	2860	coding	PF02878
MSTRG.4302.1	2460	noncoding	-
MSTRG.4303.2	4891	noncoding	-
MSTRG.4304.2	1468	coding	PF06729
MSTRG.4297.1	799	noncoding	-
MSTRG.4298.1	894	noncoding	-
MSTRG.4312.5	1450	noncoding	-
MSTRG.4312.4	7020	coding	PF06920
MSTRG.4312.2	9709	coding	PF06920
MSTRG.4312.1	15143	coding	PF06920
MSTRG.4312.3	11509	coding	PF06920
MSTRG.4310.2	2552	noncoding	-
MSTRG.4317.2	8555	coding	PF00859
MSTRG.4319.1	23271	noncoding	-
MSTRG.4319.2	60402	noncoding	-
MSTRG.4319.3	9501	coding	PF00078
MSTRG.4319.4	24222	noncoding	-
MSTRG.4319.5	1952	noncoding	-
MSTRG.4325.2	3448	coding	PF00067
MSTRG.4325.3	823	noncoding	-
MSTRG.4326.2	1696	noncoding	-

MSTRG.4328.2	8106	coding	PF00067
MSTRG.4328.6	5472	coding	PF00067
MSTRG.4328.5	1443	coding	PF00067
MSTRG.4328.7	1676	coding	PF00067
MSTRG.4328.11	7757	coding	PF00067
MSTRG.4329.3	5574	coding	PF05622
MSTRG.4330.1	519	noncoding	-
MSTRG.4335.2	6217	coding	PF04433
MSTRG.4341.1	1028	noncoding	-
MSTRG.4341.2	579	noncoding	-
MSTRG.4341.3	1079	noncoding	-
MSTRG.4343.1	3457	noncoding	-
MSTRG.4344.1	1772	coding	PF00078
MSTRG.4340.1	421	noncoding	-
MSTRG.4346.2	3365	noncoding	-
MSTRG.4345.1	3128	noncoding	-
MSTRG.4359.2	10549	coding	PF00443
MSTRG.4357.1	4134	noncoding	-
MSTRG.4358.2	1318	noncoding	-
MSTRG.4360.2	3906	noncoding	-
MSTRG.4361.1	4747	noncoding	-
MSTRG.4361.3	1797	noncoding	-
MSTRG.4363.1	3835	coding	PF10223
MSTRG.4363.2	8114	coding	PF10223
MSTRG.4363.3	2786	noncoding	-
MSTRG.4367.4	7030	noncoding	-
MSTRG.4367.5	40314	noncoding	-
MSTRG.4369.1	25459	noncoding	-
MSTRG.4365.2	523	noncoding	-
MSTRG.4371.1	1055	coding	PF12280
MSTRG.4371.2	1035	coding	PF12280
MSTRG.4375.1	971	noncoding	-
MSTRG.4372.2	4535	coding	PF09531
MSTRG.4382.1	942	noncoding	-
MSTRG.4388.1	7138	coding	PF00058
MSTRG.4388.3	7561	noncoding	-
MSTRG.4388.9	7177	coding	PF00058
MSTRG.4384.2	767	coding	PF02792
MSTRG.4390.1	2047	noncoding	-
MSTRG.4390.2	1610	noncoding	-
MSTRG.4390.3	1547	noncoding	-
MSTRG.4399.1	572	noncoding	-
MSTRG.4400.2	2298	coding	PF02036
MSTRG.4401.2	10257	noncoding	-
MSTRG.4401.3	10085	noncoding	-
MSTRG.4399.3	1451	coding	PF00378
MSTRG.4401.5	11743	noncoding	-
MSTRG.4401.8	11124	noncoding	-
MSTRG.4401.9	12847	noncoding	-
MSTRG.4401.11	10238	noncoding	-
MSTRG.4401.12	10301	noncoding	-
MSTRG.4401.10	12938	noncoding	-
MSTRG.4401.13	10437	noncoding	-
MSTRG.4401.14	10374	noncoding	-

MSTRG.4401.16	7520	noncoding	-
MSTRG.4403.1	2843	noncoding	-
MSTRG.4403.3	7094	noncoding	-
MSTRG.4404.1	2672	noncoding	-
MSTRG.4408.1	2370	noncoding	-
MSTRG.4409.2	3130	coding	PF01426
MSTRG.4409.3	2671	noncoding	-
MSTRG.4411.1	10320	noncoding	-
MSTRG.4411.2	8836	noncoding	-
MSTRG.4412.1	4766	coding	PF11979
MSTRG.4412.2	4582	coding	PF11979
MSTRG.4412.3	4386	coding	PF11979
MSTRG.4413.1	5657	coding	PF01849
MSTRG.4413.2	3466	coding	PF01849
MSTRG.4413.5	1108	noncoding	-
MSTRG.4414.2	1495	coding	PF13899
MSTRG.4418.1	4176	coding	PF16187
MSTRG.4420.9	5535	coding	PF01237
MSTRG.4420.10	11231	coding	PF01237
MSTRG.4420.11	5419	coding	PF01237
MSTRG.4421.1	5550	noncoding	-
MSTRG.4422.1	359	noncoding	-
MSTRG.4422.2	346	noncoding	-
MSTRG.4423.4	3173	coding	PF12763
MSTRG.4423.5	3930	coding	PF12763
MSTRG.4424.1	255	coding	PF05071
MSTRG.4426.4	2799	coding	PF10300
MSTRG.4428.2	2232	coding	PF13639
MSTRG.4429.1	1936	noncoding	-
MSTRG.4431.1	1648	noncoding	-
MSTRG.4432.2	1968	coding	PF12796
MSTRG.4434.1	1995	noncoding	-
MSTRG.4446.2	989	noncoding	-
MSTRG.4446.1	1676	noncoding	-
MSTRG.4446.4	1684	noncoding	-
MSTRG.4448.1	28325	coding	PF03184
MSTRG.4448.2	6046	coding	PF03184
MSTRG.4449.1	6784	noncoding	-
MSTRG.4450.1	835	noncoding	-
MSTRG.4451.1	2684	noncoding	-
MSTRG.4452.1	2549	noncoding	-
MSTRG.4454.1	938	noncoding	-
MSTRG.4456.1	248	noncoding	-
MSTRG.4443.3	1192	noncoding	-
MSTRG.4445.1	3568	noncoding	-
MSTRG.4462.1	10178	coding	PF02337
MSTRG.4462.2	12615	coding	PF02337
MSTRG.4463.1	4867	noncoding	-
MSTRG.4464.1	970	noncoding	-
MSTRG.4465.1	304	noncoding	-
MSTRG.4467.4	4463	noncoding	-
MSTRG.4470.3	3630	coding	PF06644
MSTRG.4470.4	3761	coding	PF06644
MSTRG.4470.5	2230	coding	PF06644

MSTRG.4473.3	2841	coding	PF00069
MSTRG.4471.2	570	noncoding	-
MSTRG.4471.1	840	noncoding	-
MSTRG.4474.5	4474	coding	PF01425
MSTRG.4474.4	3737	coding	PF01425
MSTRG.4474.7	15197	coding	PF01425
MSTRG.4474.8	9348	coding	PF01189
MSTRG.4474.10	4745	noncoding	-
MSTRG.4474.12	4098	noncoding	-
MSTRG.4474.13	3517	noncoding	-
MSTRG.4474.14	3899	noncoding	-
MSTRG.4474.11	4795	noncoding	-
MSTRG.4479.6	1018	coding	PF02320
MSTRG.4479.4	1576	coding	PF02320
MSTRG.4479.7	1330	coding	PF02320
MSTRG.4479.2	1879	coding	PF02320
MSTRG.4479.1	624	coding	PF02320
MSTRG.4480.2	2453	noncoding	-
MSTRG.4481.4	10153	coding	PF00176
MSTRG.4481.5	12175	coding	PF00176
MSTRG.4481.1	12065	coding	PF00176
MSTRG.4481.2	11335	coding	PF00176
MSTRG.4477.2	2997	coding	PF03071
MSTRG.4477.3	3189	coding	PF03071
MSTRG.4477.4	3050	coding	PF03071
MSTRG.4487.1	4772	coding	PF16454
MSTRG.4488.1	14126	noncoding	-
MSTRG.4488.2	7700	noncoding	-
MSTRG.4488.3	1556	noncoding	-
MSTRG.4494.1	15951	noncoding	-
MSTRG.4495.1	6450	noncoding	-
MSTRG.4494.8	3001	noncoding	-
MSTRG.4494.9	2030	noncoding	-
MSTRG.4496.1	2031	noncoding	-
MSTRG.4494.10	11302	noncoding	-
MSTRG.4494.11	7615	noncoding	-
MSTRG.4498.1	8408	noncoding	-
MSTRG.4494.12	2845	coding	PF15337
MSTRG.4494.13	1827	noncoding	-
MSTRG.4499.3	1754	noncoding	-
MSTRG.4483.6	3492	noncoding	-
MSTRG.4483.4	6414	noncoding	-
MSTRG.4483.5	3306	noncoding	-
MSTRG.4483.3	3710	noncoding	-
MSTRG.4503.1	546	noncoding	-
MSTRG.4504.5	5273	coding	PF07714
MSTRG.4506.1	473	noncoding	-
MSTRG.4515.1	4499	coding	PF12349
MSTRG.4516.2	509	noncoding	-
MSTRG.4516.1	562	noncoding	-
MSTRG.4520.2	5089	coding	PF00225
MSTRG.4520.4	4812	coding	PF00225
MSTRG.4521.1	853	noncoding	-
MSTRG.4533.11	2131	coding	PF15926

MSTRG.4533.7	2021	coding	PF15926
MSTRG.4533.10	1950	coding	PF15926
MSTRG.4533.3	1847	coding	PF15926
MSTRG.4533.1	2161	coding	PF15926
MSTRG.4533.2	2083	coding	PF15926
MSTRG.4533.4	2165	coding	PF15926
MSTRG.4533.8	1957	coding	PF15926
MSTRG.4533.6	1956	coding	PF15926
MSTRG.4533.5	1778	coding	PF15926
MSTRG.4523.3	1706	coding	PF00929
MSTRG.4523.4	1543	coding	PF00929
MSTRG.4524.1	6065	noncoding	-
MSTRG.4525.1	2100	noncoding	-
MSTRG.4538.1	492	noncoding	-
MSTRG.4540.1	781	coding	PF00209
MSTRG.4541.2	1454	coding	PF15669
MSTRG.4549.1	686	noncoding	-
MSTRG.4552.4	4189	coding	PF13832
MSTRG.4553.1	3148	noncoding	-
MSTRG.4543.2	7212	coding	PF00102
MSTRG.4543.3	8005	coding	PF00102
MSTRG.4562.2	6993	coding	PF10232
MSTRG.4562.3	4153	coding	PF10232
MSTRG.4564.1	2217	noncoding	-
MSTRG.4555.2	4100	noncoding	-
MSTRG.4557.1	348	noncoding	-
MSTRG.4559.3	1214	coding	PF14886
MSTRG.4571.1	2916	coding	PF13853
MSTRG.4572.1	6422	noncoding	-
MSTRG.4574.1	1177	noncoding	-
MSTRG.4578.1	673	coding	PF15674
MSTRG.4578.3	629	coding	PF15674
MSTRG.4579.2	1048	noncoding	-
MSTRG.4580.2	1306	coding	PF10504
MSTRG.4592.1	2223	coding	PF00160
MSTRG.4592.2	1786	coding	PF00160
MSTRG.4594.1	4929	coding	PF15742
MSTRG.4594.2	4526	coding	PF15742
MSTRG.4594.4	3902	coding	PF15742
MSTRG.4594.6	2323	coding	PF15742
MSTRG.4594.5	5073	coding	PF15742
MSTRG.4594.7	9802	coding	PF15742
MSTRG.4595.1	6305	coding	PF02994
MSTRG.4594.9	9913	noncoding	-
MSTRG.4594.10	2687	noncoding	-
MSTRG.4594.11	2205	noncoding	-
MSTRG.4585.3	1971	coding	PF00250
MSTRG.4585.4	526	noncoding	-
MSTRG.4585.5	4827	coding	PF00250
MSTRG.4583.1	571	coding	PF02058
MSTRG.4588.1	1024	noncoding	-
MSTRG.4600.1	9778	noncoding	-
MSTRG.4600.2	2909	noncoding	-
MSTRG.4600.3	3639	noncoding	-

MSTRG.4601.3	3312	coding	PF12140
MSTRG.4601.4	3455	coding	PF12140
MSTRG.4602.1	4597	noncoding	-
MSTRG.4603.1	1334	noncoding	-
MSTRG.4601.8	2596	noncoding	-
MSTRG.4601.9	1744	noncoding	-
MSTRG.4604.1	6088	noncoding	-
MSTRG.4604.3	1687	noncoding	-
MSTRG.4605.7	1428	noncoding	-
MSTRG.4605.8	4319	noncoding	-
MSTRG.4605.4	1691	noncoding	-
MSTRG.4605.6	2183	noncoding	-
MSTRG.4605.9	1302	noncoding	-
MSTRG.4605.10	4583	noncoding	-
MSTRG.4608.3	353	noncoding	-
MSTRG.4608.6	2166	noncoding	-
MSTRG.4609.2	3855	coding	PF01352
MSTRG.4609.1	6909	coding	PF01352
MSTRG.4611.1	229	noncoding	-
MSTRG.4615.2	458	coding	PF15844
MSTRG.4616.2	6152	noncoding	-
MSTRG.4617.1	16053	noncoding	-
MSTRG.4618.1	3389	noncoding	-
MSTRG.4616.3	2085	noncoding	-
MSTRG.4627.9	3008	coding	PF01213
MSTRG.4627.10	2657	coding	PF01213
MSTRG.4627.7	2660	coding	PF01213
MSTRG.4620.2	3127	coding	PF01715
MSTRG.4632.3	3531	noncoding	-
MSTRG.4634.9	3107	coding	PF00658
MSTRG.4634.10	1724	coding	PF00076
MSTRG.4634.13	1824	coding	PF00658
MSTRG.4706.9	13984	coding	PF02187
MSTRG.4706.6	17442	coding	PF02187
MSTRG.4706.4	17385	coding	PF02187
MSTRG.4706.27	11242	coding	PF02187
MSTRG.4706.18	15399	coding	PF02187
MSTRG.4706.25	20787	coding	PF02187
MSTRG.4706.19	23164	coding	PF02187
MSTRG.4706.12	30801	coding	PF02187
MSTRG.4706.14	44413	coding	PF02187
MSTRG.4706.24	18709	coding	PF02187
MSTRG.4706.16	23388	coding	PF02187
MSTRG.4706.15	23272	coding	PF02187
MSTRG.4706.30	13445	noncoding	-
MSTRG.4706.31	1658	noncoding	-
MSTRG.4706.32	6555	noncoding	-
MSTRG.4706.33	50619	noncoding	-
MSTRG.4648.1	1878	noncoding	-
MSTRG.4649.1	1117	noncoding	-
MSTRG.4649.2	1026	noncoding	-
MSTRG.4651.1	1673	noncoding	-
MSTRG.4660.3	3118	noncoding	-
MSTRG.4654.1	2845	coding	PF15855

MSTRG.4654.2	2986	coding	PF15855
MSTRG.4666.1	2699	coding	PF10211
MSTRG.4673.2	2171	coding	PF10188
MSTRG.4673.3	1590	coding	PF10188
MSTRG.4675.2	3169	coding	PF00069
MSTRG.4679.3	2291	coding	PF07653
MSTRG.4679.2	2309	coding	PF14604
MSTRG.4679.5	1825	noncoding	-
MSTRG.4680.6	4299	coding	PF15440
MSTRG.4680.7	9427	noncoding	-
MSTRG.4680.8	3350	noncoding	-
MSTRG.4677.1	1722	coding	PF04051
MSTRG.4677.4	1843	coding	PF04051
MSTRG.4682.1	1778	noncoding	-
MSTRG.4682.2	1206	noncoding	-
MSTRG.4687.1	2343	coding	PF03148
MSTRG.4687.5	2080	coding	PF03148
MSTRG.4687.4	2019	coding	PF03148
MSTRG.4689.1	8850	coding	PF02171
MSTRG.4689.2	8186	coding	PF02171
MSTRG.4691.1	432	noncoding	-
MSTRG.4692.2	3369	coding	PF05536
MSTRG.4698.1	1774	noncoding	-
MSTRG.4700.1	6070	noncoding	-
MSTRG.4701.1	4573	noncoding	-
MSTRG.4703.2	5559	coding	PF14291
MSTRG.4703.1	3961	noncoding	-
MSTRG.4703.3	2030	noncoding	-
MSTRG.4704.1	1738	noncoding	-
MSTRG.4703.4	5349	coding	PF14291
MSTRG.4718.3	1415	noncoding	-
MSTRG.4718.6	5826	noncoding	-
MSTRG.4718.8	2822	noncoding	-
MSTRG.4705.2	1279	coding	PF15990
MSTRG.4709.2	1028	noncoding	-
MSTRG.4709.1	799	noncoding	-
MSTRG.4711.4	3339	coding	PF02023
MSTRG.4711.5	3312	coding	PF02023
MSTRG.4711.6	3328	coding	PF02023
MSTRG.4710.1	2186	noncoding	-
MSTRG.4725.1	5869	coding	PF02213
MSTRG.4725.2	7017	coding	PF02213
MSTRG.4725.12	647	noncoding	-
MSTRG.4725.10	5628	coding	PF02213
MSTRG.4725.11	7325	coding	PF02213
MSTRG.4725.9	6870	coding	PF02213
MSTRG.4725.13	7147	coding	PF02213
MSTRG.4725.16	7054	coding	PF02213
MSTRG.4725.17	4943	coding	PF02213
MSTRG.4725.18	4985	coding	PF02213
MSTRG.4725.19	6163	coding	PF02213
MSTRG.4725.20	6226	coding	PF02213
MSTRG.4725.21	6053	coding	PF02213
MSTRG.4725.22	4961	coding	PF02213

MSTRG.4722.3	4744	noncoding	-
MSTRG.4729.1	854	coding	PF00201
MSTRG.4734.4	2962	noncoding	-
MSTRG.4734.5	3295	noncoding	-
MSTRG.4735.1	825	noncoding	-
MSTRG.4735.2	2390	noncoding	-
MSTRG.4730.1	596	noncoding	-
MSTRG.4731.1	772	noncoding	-
MSTRG.4738.1	569	noncoding	-
MSTRG.4741.2	7133	noncoding	-
MSTRG.4744.1	3928	noncoding	-
MSTRG.4746.1	799	noncoding	-
MSTRG.4747.3	11286	coding	PF01412
MSTRG.4750.1	377	noncoding	-
MSTRG.4751.1	668	noncoding	-
MSTRG.4754.1	2877	coding	PF00004
MSTRG.4754.4	2759	coding	PF00004
MSTRG.4754.5	1659	coding	PF00004
MSTRG.4754.6	2990	coding	PF00004
MSTRG.4754.7	1977	coding	PF00004
MSTRG.4752.1	1788	noncoding	-
MSTRG.4757.1	7550	noncoding	-
MSTRG.4758.2	1622	coding	PF10075
MSTRG.4758.3	1382	coding	PF10075
MSTRG.4761.1	9094	coding	PF00092
MSTRG.4761.3	8905	coding	PF00092
MSTRG.4761.5	8755	coding	PF00092
MSTRG.4762.1	765	noncoding	-
MSTRG.4763.1	10596	coding	PF00071
MSTRG.4767.2	5213	noncoding	-
MSTRG.4768.1	1469	noncoding	-
MSTRG.4767.6	2023	coding	PF09738
MSTRG.4769.1	3712	noncoding	-
MSTRG.4767.7	10996	coding	PF09738
MSTRG.4772.2	5680	noncoding	-
MSTRG.4766.1	711	noncoding	-
MSTRG.4766.2	816	noncoding	-
MSTRG.4766.3	673	noncoding	-
MSTRG.4774.3	1330	coding	PF00266
MSTRG.4774.2	1491	coding	PF00266
MSTRG.4781.2	6759	coding	PF12114
MSTRG.4785.3	2805	coding	PF10243
MSTRG.4785.6	5226	coding	PF10243
MSTRG.4782.1	7010	coding	PF12796
MSTRG.4782.3	6612	coding	PF12796
MSTRG.4787.1	8756	coding	PF00850
MSTRG.4787.2	6636	coding	PF00850
MSTRG.4787.3	4956	coding	PF00850
MSTRG.4788.1	1647	noncoding	-
MSTRG.4789.1	991	noncoding	-
MSTRG.4796.1	4545	coding	PF09240
MSTRG.4800.2	2094	noncoding	-
MSTRG.4811.1	2696	noncoding	-
MSTRG.4811.3	2461	noncoding	-

MSTRG.4813.1	7790	coding	PF00225
MSTRG.4814.1	7159	noncoding	-
MSTRG.4815.1	514	noncoding	-
MSTRG.4826.4	1552	noncoding	-
MSTRG.4826.5	1679	noncoding	-
MSTRG.4826.2	2136	noncoding	-
MSTRG.4826.3	5877	coding	PF00069
MSTRG.4826.10	4950	coding	PF00069
MSTRG.4816.1	1268	noncoding	-
MSTRG.4818.1	2993	coding	PF00735
MSTRG.4818.2	3696	coding	PF00735
MSTRG.4818.4	3321	coding	PF00735
MSTRG.4819.1	434	noncoding	-
MSTRG.4830.2	2244	coding	PF09380
MSTRG.4831.4	1735	coding	PF00069
MSTRG.4831.5	1671	coding	PF00069
MSTRG.4836.1	2638	coding	PF02913
MSTRG.4836.2	2535	coding	PF02913
MSTRG.4843.1	286	noncoding	-
MSTRG.4844.1	232	noncoding	-
MSTRG.4848.1	703	noncoding	-
MSTRG.4849.1	1968	noncoding	-
MSTRG.4860.1	1799	noncoding	-
MSTRG.4861.1	1499	coding	PF09469
MSTRG.4861.2	5086	coding	PF09469
MSTRG.4870.1	589	noncoding	-
MSTRG.4876.1	1728	coding	PF07354
MSTRG.4877.1	2621	noncoding	-
MSTRG.4882.1	1272	noncoding	-
MSTRG.4882.2	1288	noncoding	-
MSTRG.4873.1	917	noncoding	-
MSTRG.4886.1	2961	coding	PF00005
MSTRG.4887.1	4175	coding	PF00005
MSTRG.4887.2	2445	coding	PF00005
MSTRG.4895.2	3962	coding	PF00069
MSTRG.4903.1	662	noncoding	-
MSTRG.4903.2	2669	coding	PF02536
MSTRG.4921.2	12812	coding	PF10495
MSTRG.4921.3	11889	coding	PF10495
MSTRG.4922.2	1419	coding	PF00067
MSTRG.4924.1	1464	noncoding	-
MSTRG.4925.1	818	noncoding	-
MSTRG.4928.3	5461	noncoding	-
MSTRG.4928.4	3741	noncoding	-
MSTRG.4929.1	4115	noncoding	-
MSTRG.4928.6	505	noncoding	-
MSTRG.4930.1	5370	coding	PF00078
MSTRG.4928.7	4539	noncoding	-
MSTRG.4912.2	1343	noncoding	-
MSTRG.4913.1	3173	noncoding	-
MSTRG.4913.3	5062	noncoding	-
MSTRG.4913.4	5603	noncoding	-
MSTRG.4920.1	466	noncoding	-
MSTRG.4942.1	451	noncoding	-

MSTRG.4950.1	1196	noncoding	-
MSTRG.4951.2	3316	noncoding	-
MSTRG.4953.1	1402	coding	PF16297
MSTRG.4961.1	10354	coding	PF07647
MSTRG.4961.7	7744	coding	PF07647
MSTRG.4961.4	10120	coding	PF07647
MSTRG.4961.5	10100	coding	PF07647
MSTRG.4961.6	8455	coding	PF07647
MSTRG.4961.10	10337	coding	PF07647
MSTRG.4961.8	10393	coding	PF07647
MSTRG.4961.9	10391	coding	PF07647
MSTRG.4961.12	2572	noncoding	-
MSTRG.4961.11	9559	coding	PF07647
MSTRG.4961.13	10323	coding	PF07647
MSTRG.4961.15	10186	coding	PF07647
MSTRG.4961.14	10298	coding	PF07647
MSTRG.4962.1	2006	noncoding	-
MSTRG.4963.1	2256	noncoding	-
MSTRG.4964.1	8243	noncoding	-
MSTRG.4965.1	9619	noncoding	-
MSTRG.4965.2	4819	noncoding	-
MSTRG.4965.3	3053	noncoding	-
MSTRG.4966.1	225	noncoding	-
MSTRG.4967.1	1793	noncoding	-
MSTRG.4961.17	3919	noncoding	-
MSTRG.4955.1	1276	noncoding	-
MSTRG.4956.1	280	noncoding	-
MSTRG.4959.3	3051	coding	PF00153
MSTRG.4960.1	540	noncoding	-
MSTRG.4975.2	958	noncoding	-
MSTRG.4980.10	2051	coding	PF00733
MSTRG.4980.4	2788	coding	PF00733
MSTRG.4980.9	1935	coding	PF00733
MSTRG.4980.8	2277	coding	PF00733
MSTRG.4980.5	2127	coding	PF00733
MSTRG.4980.2	2306	coding	PF00733
MSTRG.4981.2	5990	noncoding	-
MSTRG.4985.6	4422	coding	PF17034
MSTRG.4985.8	1630	coding	PF17034
MSTRG.4990.3	16036	noncoding	-
MSTRG.4990.4	1043	noncoding	-
MSTRG.4991.1	7490	noncoding	-
MSTRG.4998.3	3796	coding	PF15388
MSTRG.4998.4	6751	coding	PF15388
MSTRG.4998.5	6748	coding	PF15388
MSTRG.4998.6	5448	coding	PF15388
MSTRG.4998.7	793	noncoding	-
MSTRG.4988.6	1661	coding	PF06456
MSTRG.5003.1	680	noncoding	-
MSTRG.5020.1	495	noncoding	-
MSTRG.5021.2	6109	coding	PF07092
MSTRG.5046.1	637	noncoding	-
MSTRG.5052.1	2744	noncoding	-
MSTRG.5077.2	2276	coding	PF04621

MSTRG.5077.3	2210	coding	PF04621
MSTRG.5078.1	448	noncoding	-
MSTRG.5106.1	410	noncoding	-
MSTRG.5095.2	931	noncoding	-
MSTRG.5137.1	11272	coding	PF00078
MSTRG.5138.1	569	noncoding	-
MSTRG.5137.3	2845	noncoding	-
MSTRG.5137.2	12163	noncoding	-
MSTRG.5137.4	3498	noncoding	-
MSTRG.5137.5	3280	noncoding	-
MSTRG.5137.6	1418	noncoding	-
MSTRG.5140.2	762	noncoding	-
MSTRG.5137.7	3315	noncoding	-
MSTRG.5137.8	3279	noncoding	-
MSTRG.5137.10	1259	noncoding	-
MSTRG.5133.1	3560	coding	PF08628
MSTRG.5133.3	6448	coding	PF08628
MSTRG.5133.4	2151	noncoding	-
MSTRG.5134.1	2177	noncoding	-
MSTRG.5135.1	3290	noncoding	-
MSTRG.5160.1	5343	noncoding	-
MSTRG.5192.2	2903	coding	PF00850
MSTRG.5192.1	2992	coding	PF00850
MSTRG.5192.3	807	coding	PF00850
MSTRG.5192.4	371	noncoding	-
MSTRG.5196.1	590	noncoding	-
MSTRG.5201.1	394	noncoding	-
MSTRG.5209.1	392	noncoding	-
MSTRG.5236.1	17583	coding	PF03028
MSTRG.5236.3	14323	coding	PF03028
MSTRG.5237.1	4180	noncoding	-
MSTRG.5236.6	9360	coding	PF03028
MSTRG.5238.5	1986	coding	PF10497
MSTRG.5217.3	6802	coding	PF00617
MSTRG.5217.4	2225	coding	PF00617
MSTRG.5218.1	3968	noncoding	-
MSTRG.5226.1	4973	noncoding	-
MSTRG.5247.1	4346	coding	PF09790
MSTRG.5249.2	4447	coding	PF00651
MSTRG.5249.3	1590	coding	PF00651
MSTRG.5249.4	4579	coding	PF00651
MSTRG.5250.1	620	noncoding	-
MSTRG.5250.2	7994	noncoding	-
MSTRG.5232.1	3443	noncoding	-
MSTRG.5240.3	2764	coding	PF02410
MSTRG.5252.4	7232	coding	PF00076
MSTRG.5252.3	1975	coding	PF00076
MSTRG.5252.10	2147	coding	PF00076
MSTRG.5256.1	1053	noncoding	-
MSTRG.5257.1	7802	coding	PF15027
MSTRG.5257.3	3649	noncoding	-
MSTRG.5257.5	7925	coding	PF15027
MSTRG.5257.6	3927	coding	PF15027
MSTRG.5258.1	1786	noncoding	-

MSTRG.5260.1	1940	coding	PF00078
MSTRG.5261.4	2263	noncoding	-
MSTRG.5261.5	1829	coding	PF07535
MSTRG.5265.1	1709	noncoding	-
MSTRG.5254.1	580	coding	PF00664
MSTRG.5285.2	3649	coding	PF00664
MSTRG.5285.3	3739	coding	PF00664
MSTRG.5285.4	3289	coding	PF00664
MSTRG.5286.1	1856	noncoding	-
MSTRG.5285.5	2911	coding	PF00664
MSTRG.5288.2	6939	coding	PF00005
MSTRG.5289.1	5178	noncoding	-
MSTRG.5293.1	2211	coding	PF00755
MSTRG.5297.5	2319	noncoding	-
MSTRG.5298.1	6027	noncoding	-
MSTRG.5297.6	5386	noncoding	-
MSTRG.5297.7	499	noncoding	-
MSTRG.5297.8	606	noncoding	-
MSTRG.5299.3	1136	noncoding	-
MSTRG.5300.1	1686	noncoding	-
MSTRG.5301.1	3737	noncoding	-
MSTRG.5301.2	1935	noncoding	-
MSTRG.5301.4	3762	noncoding	-
MSTRG.5301.8	4569	noncoding	-
MSTRG.5301.7	3540	noncoding	-
MSTRG.5301.3	3648	noncoding	-
MSTRG.5301.6	3696	noncoding	-
MSTRG.5301.5	4054	noncoding	-
MSTRG.5305.1	18955	noncoding	-
MSTRG.5305.2	2600	noncoding	-
MSTRG.5306.1	2876	noncoding	-
MSTRG.5307.1	1464	noncoding	-
MSTRG.5271.1	232	noncoding	-
MSTRG.5275.1	2697	noncoding	-
MSTRG.5275.2	2697	noncoding	-
MSTRG.5276.2	4794	coding	PF01403
MSTRG.5283.2	7622	coding	PF08473
MSTRG.5283.3	1245	noncoding	-
MSTRG.5284.1	2514	coding	PF00078
MSTRG.5310.2	3703	coding	PF00089
MSTRG.5310.3	2776	coding	PF00089
MSTRG.5312.1	1500	noncoding	-
MSTRG.5317.1	3712	noncoding	-
MSTRG.5324.1	2214	noncoding	-
MSTRG.5324.2	3815	coding	PF00595
MSTRG.5324.3	1255	noncoding	-
MSTRG.5331.1	6251	noncoding	-
MSTRG.5358.2	2462	noncoding	-
MSTRG.5359.2	1760	noncoding	-
MSTRG.5359.3	2634	noncoding	-
MSTRG.5363.2	6094	coding	PF04826
MSTRG.5363.4	6065	coding	PF04826
MSTRG.5363.5	16144	coding	PF04826
MSTRG.5365.2	3074	coding	PF00675

MSTRG.5354.2	10254	noncoding	-
MSTRG.5354.3	1868	noncoding	-
MSTRG.5370.2	1541	coding	PF14630
MSTRG.5382.1	1347	noncoding	-
MSTRG.5384.1	4441	noncoding	-
MSTRG.5383.7	4084	noncoding	-
MSTRG.5385.2	5402	noncoding	-
MSTRG.5386.1	7133	coding	PF00069
MSTRG.5386.3	5435	coding	PF00069
MSTRG.5386.4	2285	coding	PF00069
MSTRG.5386.5	2349	coding	PF00069
MSTRG.5386.7	2442	coding	PF00069
MSTRG.5386.9	652	noncoding	-
MSTRG.5387.1	6983	noncoding	-
MSTRG.5388.1	11313	noncoding	-
MSTRG.5389.1	6218	noncoding	-
MSTRG.5391.1	3290	noncoding	-
MSTRG.5377.3	5132	coding	PF08313
MSTRG.5377.2	5168	coding	PF08313
MSTRG.5377.1	5505	coding	PF08313
MSTRG.5392.1	4538	coding	PF04095
MSTRG.5392.2	3404	coding	PF04095
MSTRG.5393.2	4599	coding	PF15374
MSTRG.5401.3	1064	noncoding	-
MSTRG.5401.2	1317	noncoding	-
MSTRG.5401.1	604	noncoding	-
MSTRG.5402.3	3087	coding	PF08517
MSTRG.5402.4	3959	noncoding	-
MSTRG.5403.1	424	noncoding	-
MSTRG.5404.2	4952	noncoding	-
MSTRG.5404.1	10759	noncoding	-
MSTRG.5404.3	6024	noncoding	-
MSTRG.5405.1	699	noncoding	-
MSTRG.5404.4	812	noncoding	-
MSTRG.5408.4	1052	coding	PF05529
MSTRG.5399.1	2055	noncoding	-
MSTRG.5399.2	2029	noncoding	-
MSTRG.5412.1	3753	noncoding	-
MSTRG.5413.2	5623	coding	PF00055
MSTRG.5414.1	218	noncoding	-
MSTRG.5416.3	3812	noncoding	-
MSTRG.5417.1	745	noncoding	-
MSTRG.5419.7	2465	coding	PF00334
MSTRG.5419.6	7888	coding	PF00334
MSTRG.5419.8	3049	coding	PF00334
MSTRG.5440.1	483	noncoding	-
MSTRG.5445.2	1467	coding	PF04184
MSTRG.5491.1	2099	noncoding	-
MSTRG.5493.1	735	noncoding	-
MSTRG.5493.2	2658	noncoding	-
MSTRG.5470.2	3495	coding	PF01359
MSTRG.5471.1	1922	noncoding	-
MSTRG.5478.3	1606	coding	PF05004
MSTRG.5480.1	1546	coding	PF12756

MSTRG.5514.1	6000	coding	PF06920
MSTRG.5515.1	362	noncoding	-
MSTRG.5558.2	7634	noncoding	-
MSTRG.5560.1	2993	noncoding	-
MSTRG.5561.1	1509	noncoding	-
MSTRG.5561.2	1360	noncoding	-
MSTRG.5562.1	6840	noncoding	-
MSTRG.5563.1	4051	noncoding	-
MSTRG.5564.1	1691	noncoding	-
MSTRG.5565.1	857	noncoding	-
MSTRG.5558.3	699	noncoding	-
MSTRG.5532.1	8488	coding	PF00226
MSTRG.5533.1	1364	coding	PF05485
MSTRG.5533.2	2665	coding	PF05485
MSTRG.5539.1	834	noncoding	-
MSTRG.5540.1	3328	noncoding	-
MSTRG.5538.4	1946	noncoding	-
MSTRG.5543.1	9345	noncoding	-
MSTRG.5543.4	2693	noncoding	-
MSTRG.5543.2	3801	noncoding	-
MSTRG.5543.3	3384	noncoding	-
MSTRG.5543.7	3560	noncoding	-
MSTRG.5543.8	6256	noncoding	-
MSTRG.5543.9	7960	noncoding	-
MSTRG.5549.1	413	noncoding	-
MSTRG.5550.2	2442	coding	PF10034
MSTRG.5552.1	1037	noncoding	-
MSTRG.5555.4	1848	coding	PF10034
MSTRG.5555.2	4540	coding	PF10034
MSTRG.5555.3	3435	coding	PF10034
MSTRG.5570.2	2094	coding	PF14728
MSTRG.5570.4	2150	coding	PF14728
MSTRG.5572.2	998	noncoding	-
MSTRG.5574.5	2471	coding	PF00254
MSTRG.5574.6	3043	coding	PF00254
MSTRG.5574.7	1848	noncoding	-
MSTRG.5575.3	4179	coding	PF00651
MSTRG.5575.4	3647	coding	PF00651
MSTRG.5584.1	1956	noncoding	-
MSTRG.5591.1	6724	coding	PF00002
MSTRG.5591.2	6757	coding	PF00002
MSTRG.5595.3	1306	noncoding	-
MSTRG.5594.1	753	noncoding	-
MSTRG.5605.2	1771	coding	PF13639
MSTRG.5606.2	865	coding	PF01359
MSTRG.5608.1	796	noncoding	-
MSTRG.5610.3	6879	coding	PF08718
MSTRG.5615.1	2636	noncoding	-
MSTRG.5618.3	943	coding	PF00620
MSTRG.5612.1	2273	coding	PF00450
MSTRG.5627.1	73520	noncoding	-
MSTRG.5628.1	334	noncoding	-
MSTRG.5629.1	1526	noncoding	-
MSTRG.5629.2	768	noncoding	-

MSTRG.5627.3	3079	noncoding	-
MSTRG.5627.4	11190	noncoding	-
MSTRG.5627.5	14765	noncoding	-
MSTRG.5627.6	14449	noncoding	-
MSTRG.5627.7	9507	noncoding	-
MSTRG.5627.8	85626	noncoding	-
MSTRG.5627.9	75975	noncoding	-
MSTRG.5627.10	18506	noncoding	-
MSTRG.5627.11	18512	noncoding	-
MSTRG.5627.12	18468	noncoding	-
MSTRG.5627.13	15972	noncoding	-
MSTRG.5627.14	36838	noncoding	-
MSTRG.5631.1	6298	noncoding	-
MSTRG.5632.1	15990	noncoding	-
MSTRG.5633.1	31966	noncoding	-
MSTRG.5633.2	7531	noncoding	-
MSTRG.5634.1	2177	noncoding	-
MSTRG.5632.2	13911	noncoding	-
MSTRG.5634.2	5592	noncoding	-
MSTRG.5635.3	3862	coding	PF07888
MSTRG.5636.1	6166	noncoding	-
MSTRG.5621.2	6459	coding	PF03446
MSTRG.5624.1	50521	noncoding	-
MSTRG.5624.2	2221	noncoding	-
MSTRG.5638.1	1710	noncoding	-
MSTRG.5643.3	1729	coding	PF01393
MSTRG.5642.4	23956	noncoding	-
MSTRG.5642.5	10560	noncoding	-
MSTRG.5661.1	1077	noncoding	-
MSTRG.5668.3	1617	noncoding	-
MSTRG.5669.3	4153	coding	PF00625
MSTRG.5669.4	3403	coding	PF00625
MSTRG.5669.5	1956	coding	PF00625
MSTRG.5670.1	1085	noncoding	-
MSTRG.5671.1	4447	noncoding	-
MSTRG.5672.1	554	noncoding	-
MSTRG.5667.1	708	noncoding	-
MSTRG.5681.1	14479	coding	PF00567
MSTRG.5681.5	2802	coding	PF00567
MSTRG.5681.10	1236	coding	PF14753
MSTRG.5681.7	2554	coding	PF14753
MSTRG.5681.11	1974	coding	PF14753
MSTRG.5722.15	4345	noncoding	-
MSTRG.5726.1	1718	noncoding	-
MSTRG.5726.2	5275	noncoding	-
MSTRG.5722.17	2459	noncoding	-
MSTRG.5722.18	4420	noncoding	-
MSTRG.5727.1	4233	coding	PF01359
MSTRG.5728.1	7866	noncoding	-
MSTRG.5729.1	1601	noncoding	-
MSTRG.5730.4	1044	noncoding	-
MSTRG.5722.19	4134	noncoding	-
MSTRG.5722.20	17240	noncoding	-
MSTRG.5722.21	5335	noncoding	-

MSTRG.5734.1	1851	noncoding	-
MSTRG.5722.22	4816	noncoding	-
MSTRG.5687.2	2961	noncoding	-
MSTRG.5690.2	1083	coding	PF04005
MSTRG.5690.3	1047	coding	PF04005
MSTRG.5695.3	12963	noncoding	-
MSTRG.5696.1	6404	noncoding	-
MSTRG.5695.5	6752	coding	PF08416
MSTRG.5695.6	7448	coding	PF08416
MSTRG.5693.1	963	noncoding	-
MSTRG.5699.1	505	noncoding	-
MSTRG.5704.3	2521	coding	PF08368
MSTRG.5707.14	1420	coding	PF16545
MSTRG.5707.17	2950	coding	PF16545
MSTRG.5707.15	2164	coding	PF16545
MSTRG.5707.11	1740	coding	PF16545
MSTRG.5707.13	1590	coding	PF16545
MSTRG.5707.16	1678	coding	PF16545
MSTRG.5707.6	1852	coding	PF16545
MSTRG.5707.2	1929	coding	PF16545
MSTRG.5707.5	1836	coding	PF16545
MSTRG.5707.9	1986	coding	PF16545
MSTRG.5711.3	1124	noncoding	-
MSTRG.5713.3	4168	coding	PF02891
MSTRG.5713.4	4722	coding	PF02891
MSTRG.5713.6	9356	coding	PF02891
MSTRG.5713.5	4064	coding	PF02891
MSTRG.5717.2	1563	noncoding	-
MSTRG.5717.1	5213	coding	PF16870
MSTRG.5717.3	1061	noncoding	-
MSTRG.5717.4	4497	coding	PF16870
MSTRG.5717.5	3255	coding	PF16870
MSTRG.5715.4	3640	coding	PF14050
MSTRG.5715.3	989	coding	PF14050
MSTRG.5716.1	3269	coding	PF00957
MSTRG.5740.3	2266	coding	PF10391
MSTRG.5742.1	469	noncoding	-
MSTRG.5745.3	3582	coding	PF09166
MSTRG.5745.2	4354	coding	PF09166
MSTRG.5746.8	798	noncoding	-
MSTRG.5746.9	1195	noncoding	-
MSTRG.5747.1	5455	noncoding	-
MSTRG.5746.10	2650	noncoding	-
MSTRG.5782.1	2018	noncoding	-
MSTRG.5757.2	771	noncoding	-
MSTRG.5757.1	1036	noncoding	-
MSTRG.5761.1	8275	noncoding	-
MSTRG.5761.2	8710	noncoding	-
MSTRG.5763.2	4195	noncoding	-
MSTRG.5762.1	416	noncoding	-
MSTRG.5835.1	2548	noncoding	-
MSTRG.5835.2	9557	noncoding	-
MSTRG.5835.3	3582	noncoding	-
MSTRG.5835.4	19096	coding	PF02515

MSTRG.5835.5	15510	noncoding	-
MSTRG.5835.6	4542	coding	PF02515
MSTRG.5835.7	34385	noncoding	-
MSTRG.5835.9	1857	coding	PF02515
MSTRG.5836.1	3063	noncoding	-
MSTRG.5835.11	7172	coding	PF02515
MSTRG.5837.1	14643	noncoding	-
MSTRG.5838.1	3189	noncoding	-
MSTRG.5835.13	3935	noncoding	-
MSTRG.5835.12	7636	noncoding	-
MSTRG.5835.14	7511	noncoding	-
MSTRG.5835.15	7723	noncoding	-
MSTRG.5835.16	3272	noncoding	-
MSTRG.5835.17	3450	coding	PF02515
MSTRG.5835.18	3347	coding	PF02515
MSTRG.5835.20	16486	noncoding	-
MSTRG.5835.22	7240	noncoding	-
MSTRG.5835.23	27623	noncoding	-
MSTRG.5835.25	23947	noncoding	-
MSTRG.5835.26	6035	noncoding	-
MSTRG.5835.27	19207	noncoding	-
MSTRG.5835.28	3109	noncoding	-
MSTRG.5835.29	9740	noncoding	-
MSTRG.5835.30	2359	noncoding	-
MSTRG.5840.1	1854	noncoding	-
MSTRG.5797.1	8148	coding	PF00069
MSTRG.5797.6	4411	coding	PF00069
MSTRG.5797.4	6168	coding	PF00069
MSTRG.5797.3	4108	coding	PF00069
MSTRG.5797.5	3742	coding	PF00069
MSTRG.5797.7	3428	coding	PF00069
MSTRG.5798.1	8945	noncoding	-
MSTRG.5802.3	3805	noncoding	-
MSTRG.5803.1	1248	noncoding	-
MSTRG.5805.1	7041	noncoding	-
MSTRG.5799.1	381	noncoding	-
MSTRG.5815.1	362	noncoding	-
MSTRG.5832.1	395	noncoding	-
MSTRG.5834.1	2113	coding	PF12998
MSTRG.5845.3	3264	noncoding	-
MSTRG.5845.4	3108	noncoding	-
MSTRG.5856.2	2646	coding	PF13639
MSTRG.5858.1	820	noncoding	-
MSTRG.5860.1	1193	noncoding	-
MSTRG.5862.1	5740	noncoding	-
MSTRG.5864.1	3274	coding	PF00568
MSTRG.5865.1	5128	noncoding	-
MSTRG.5866.1	1632	noncoding	-
MSTRG.5867.1	947	noncoding	-
MSTRG.5872.5	1922	coding	PF01630
MSTRG.5873.1	4213	noncoding	-
MSTRG.5874.2	1725	coding	PF01630
MSTRG.5874.3	1054	coding	PF01630
MSTRG.5874.5	1451	coding	PF01630

MSTRG.5874.4	1761	coding	PF01630
MSTRG.5877.1	3070	noncoding	-
MSTRG.5879.1	2903	noncoding	-
MSTRG.5885.2	2345	coding	PF00003
MSTRG.5886.1	389	noncoding	-
MSTRG.5890.2	4046	coding	PF16624
MSTRG.5890.3	2755	coding	PF16624
MSTRG.5891.1	3537	coding	PF01465
MSTRG.5891.2	3316	coding	PF01465
MSTRG.5891.3	3273	coding	PF01465
MSTRG.5896.2	12861	noncoding	-
MSTRG.5897.1	11595	noncoding	-
MSTRG.5901.1	2102	coding	PF15220
MSTRG.5904.1	4055	coding	PF12480
MSTRG.5904.2	2038	coding	PF12480
MSTRG.5904.4	3504	coding	PF12480
MSTRG.5907.1	9154	coding	PF00630
MSTRG.5908.1	1826	noncoding	-
MSTRG.5908.2	1027	noncoding	-
MSTRG.5907.3	7435	coding	PF00630
MSTRG.5907.4	7489	coding	PF00630
MSTRG.5909.2	3119	noncoding	-
MSTRG.5909.3	3395	noncoding	-
MSTRG.5909.4	2392	noncoding	-
MSTRG.5910.1	2320	coding	PF00335
MSTRG.5915.1	5362	coding	PF05221
MSTRG.5915.4	1214	noncoding	-
MSTRG.5915.3	5067	coding	PF05221
MSTRG.5916.1	5224	noncoding	-
MSTRG.5917.2	1127	noncoding	-
MSTRG.5918.1	280	noncoding	-
MSTRG.5919.1	7350	noncoding	-
MSTRG.5920.1	11623	noncoding	-
MSTRG.5924.1	2104	noncoding	-
MSTRG.5924.6	6413	coding	PF10491
MSTRG.5924.7	1731	coding	PF10491
MSTRG.5924.8	2447	coding	PF10491
MSTRG.5921.1	1084	noncoding	-
MSTRG.5922.3	2427	coding	PF00179
MSTRG.5922.4	6628	noncoding	-
MSTRG.5923.1	9673	noncoding	-
MSTRG.5925.1	2215	coding	PF07967
MSTRG.5925.2	2393	coding	PF07967
MSTRG.5925.4	3197	coding	PF07967
MSTRG.5925.3	3067	coding	PF07967
MSTRG.5925.5	3183	coding	PF07967
MSTRG.5927.1	6777	coding	PF00078
MSTRG.5927.2	7821	noncoding	-
MSTRG.5932.1	998	coding	PF15468
MSTRG.5934.1	2419	coding	PF00246
MSTRG.5934.3	2157	coding	PF00246
MSTRG.5934.4	2205	coding	PF00246
MSTRG.5935.2	2342	coding	PF00561
MSTRG.5936.1	2978	coding	PF01602

MSTRG.5939.1	406	noncoding	-
MSTRG.5940.1	326	noncoding	-
MSTRG.5940.2	935	noncoding	-
MSTRG.5939.2	296	noncoding	-
MSTRG.5940.4	338	noncoding	-
MSTRG.5939.3	709	noncoding	-
MSTRG.5939.4	461	noncoding	-
MSTRG.5940.5	311	noncoding	-
MSTRG.5942.1	223	noncoding	-
MSTRG.5944.1	1044	noncoding	-
MSTRG.5945.1	263	noncoding	-
MSTRG.5946.1	844	noncoding	-
MSTRG.5945.2	247	noncoding	-
MSTRG.5949.2	733	coding	PF14994
MSTRG.5966.1	1444	noncoding	-
MSTRG.5965.5	8375	coding	PF06588
MSTRG.5969.1	1458	noncoding	-
MSTRG.5965.6	1433	coding	PF06588
MSTRG.5965.7	1645	noncoding	-
MSTRG.5965.8	6508	coding	PF06588
MSTRG.5970.1	741	noncoding	-
MSTRG.5972.1	3267	noncoding	-
MSTRG.5951.1	1613	noncoding	-
MSTRG.5953.1	1234	coding	PF08337
MSTRG.5955.2	1200	coding	PF05300
MSTRG.5956.3	2600	coding	PF04048
MSTRG.5956.2	6812	coding	PF04048
MSTRG.5956.4	2677	noncoding	-
MSTRG.5958.1	5275	noncoding	-
MSTRG.5956.6	2587	noncoding	-
MSTRG.5956.7	2984	noncoding	-
MSTRG.5959.1	6012	noncoding	-
MSTRG.5960.1	3483	noncoding	-
MSTRG.5961.1	4294	noncoding	-
MSTRG.5962.1	2202	noncoding	-
MSTRG.5956.8	1813	noncoding	-
MSTRG.5985.4	2562	noncoding	-
MSTRG.5985.7	2673	noncoding	-
MSTRG.5985.5	3406	noncoding	-
MSTRG.5985.8	3748	noncoding	-
MSTRG.5986.1	5464	coding	PF00078
MSTRG.5989.1	1382	noncoding	-
MSTRG.5990.2	1833	coding	PF14985
MSTRG.5989.4	1496	coding	PF15325
MSTRG.5984.1	2710	noncoding	-
MSTRG.5991.1	1480	noncoding	-
MSTRG.5991.3	522	noncoding	-
MSTRG.5992.1	6469	coding	PF00860
MSTRG.5994.1	1203	noncoding	-
MSTRG.5995.1	1073	noncoding	-
MSTRG.5997.1	287	noncoding	-
MSTRG.5999.1	503	noncoding	-
MSTRG.6004.5	2489	coding	PF14570
MSTRG.6004.4	2725	coding	PF14570

MSTRG.6005.1	5677	noncoding	-
MSTRG.6006.1	4577	noncoding	-
MSTRG.6004.7	2947	coding	PF14570
MSTRG.6007.1	1634	noncoding	-
MSTRG.6008.1	6249	noncoding	-
MSTRG.6008.2	639	noncoding	-
MSTRG.6009.3	6538	coding	PF11894
MSTRG.6001.2	413	coding	PF00827
MSTRG.6010.2	4061	coding	PF12796
MSTRG.6020.4	3737	coding	PF00439
MSTRG.6029.2	1093	noncoding	-
MSTRG.6029.3	1052	noncoding	-
MSTRG.6029.4	5935	coding	PF14075
MSTRG.6030.1	594	noncoding	-
MSTRG.6033.2	1130	noncoding	-
MSTRG.6035.1	4197	coding	PF01359
MSTRG.6036.2	2796	coding	PF03194
MSTRG.6036.3	2536	coding	PF03194
MSTRG.6037.1	5866	noncoding	-
MSTRG.6040.1	15070	coding	PF00069
MSTRG.6040.2	15005	coding	PF00069
MSTRG.6040.3	14792	coding	PF00069
MSTRG.6040.5	3077	noncoding	-
MSTRG.6040.4	3980	coding	PF00069
MSTRG.6040.6	2325	noncoding	-
MSTRG.6040.7	2343	noncoding	-
MSTRG.6040.8	2102	noncoding	-
MSTRG.6040.9	1921	coding	PF00069
MSTRG.6040.10	1536	coding	PF00069
MSTRG.6040.11	1614	coding	PF00069
MSTRG.6042.1	1899	coding	PF00067
MSTRG.6043.3	2827	noncoding	-
MSTRG.6045.2	4417	noncoding	-
MSTRG.6050.1	4752	coding	PF15815
MSTRG.6050.3	3263	coding	PF15815
MSTRG.6050.4	3533	coding	PF15815
MSTRG.6050.7	1320	noncoding	-
MSTRG.6056.1	4443	coding	PF02141
MSTRG.6058.1	3910	noncoding	-
MSTRG.6058.2	4101	noncoding	-
MSTRG.6060.3	4233	coding	PF14813
MSTRG.6060.2	4638	coding	PF14813
MSTRG.6061.2	9546	coding	PF07714
MSTRG.6061.4	7844	coding	PF07714
MSTRG.6061.5	4541	noncoding	-
MSTRG.6061.6	10573	coding	PF07714
MSTRG.6061.7	5589	coding	PF07714
MSTRG.6061.10	2109	coding	PF07714
MSTRG.6061.11	2051	coding	PF07714
MSTRG.6061.12	2490	coding	PF07714
MSTRG.6061.9	2476	coding	PF07714
MSTRG.6062.1	2702	noncoding	-
MSTRG.6063.1	1855	noncoding	-
MSTRG.6055.2	889	coding	PF00436

MSTRG.6055.3	714	coding	PF00436
MSTRG.6065.2	1249	noncoding	-
MSTRG.6066.1	6257	coding	PF01055
MSTRG.6067.2	1119	coding	PF00089
MSTRG.6068.2	1045	noncoding	-
MSTRG.6073.1	673	coding	PF07686
MSTRG.6074.1	500	noncoding	-
MSTRG.6075.1	1004	coding	PF00089
MSTRG.6076.1	797	coding	PF07654
MSTRG.6077.1	446	coding	PF07654
MSTRG.6077.2	746	coding	PF07654
MSTRG.6088.1	3446	coding	PF01323
MSTRG.6088.5	728	coding	PF01323
MSTRG.6089.2	3653	coding	PF00656
MSTRG.6089.3	4034	coding	PF00656
MSTRG.6096.1	268	noncoding	-
MSTRG.6100.1	7369	noncoding	-
MSTRG.6104.1	1311	noncoding	-
MSTRG.6105.1	818	noncoding	-
MSTRG.6112.1	4678	noncoding	-
MSTRG.6113.1	852	noncoding	-
MSTRG.6121.3	13239	coding	PF00856
MSTRG.6121.5	2559	coding	PF00856
MSTRG.6121.7	12292	coding	PF00856
MSTRG.6121.9	5283	coding	PF00856
MSTRG.6121.10	5436	coding	PF00856
MSTRG.6119.1	3877	noncoding	-
MSTRG.6119.3	3594	coding	PF01352
MSTRG.6124.3	2815	coding	PF01352
MSTRG.6125.2	4326	coding	PF01352
MSTRG.6125.3	4227	coding	PF01352
MSTRG.6125.4	4203	coding	PF01352
MSTRG.6130.4	3713	coding	PF01352
MSTRG.6127.1	378	noncoding	-
MSTRG.6133.1	373	noncoding	-
MSTRG.6133.2	311	noncoding	-
MSTRG.6134.1	479	noncoding	-
MSTRG.6136.1	663	noncoding	-
MSTRG.6136.2	1734	noncoding	-
MSTRG.6139.6	3237	noncoding	-
MSTRG.6140.2	661	noncoding	-
MSTRG.6141.2	2646	noncoding	-
MSTRG.6143.1	2011	coding	PF04548
MSTRG.6145.3	1752	coding	PF04548
MSTRG.6149.1	1921	coding	PF04548
MSTRG.6154.3	1028	noncoding	-
MSTRG.6163.1	1491	noncoding	-
MSTRG.6163.2	1545	noncoding	-
MSTRG.6163.4	2217	noncoding	-
MSTRG.6163.5	1856	noncoding	-
MSTRG.6161.2	2386	noncoding	-
MSTRG.6161.3	3165	coding	PF01412
MSTRG.6167.1	3616	coding	PF12848
MSTRG.6167.3	3328	coding	PF12848

MSTRG.6169.2	1712	coding	PF02201
MSTRG.6170.1	2052	noncoding	-
MSTRG.6171.1	981	noncoding	-
MSTRG.6171.2	879	noncoding	-
MSTRG.6173.2	6809	coding	PF00071
MSTRG.6173.3	2960	noncoding	-
MSTRG.6174.7	1912	noncoding	-
MSTRG.6174.1	1824	noncoding	-
MSTRG.6174.6	1819	noncoding	-
MSTRG.6174.9	316	noncoding	-
MSTRG.6174.10	325	noncoding	-
MSTRG.6186.2	1649	noncoding	-
MSTRG.6190.2	16909	coding	PF05965
MSTRG.6190.1	16949	coding	PF05965
MSTRG.6190.10	15065	coding	PF05965
MSTRG.6190.11	15388	coding	PF05965
MSTRG.6190.15	14784	coding	PF05965
MSTRG.6190.12	15047	coding	PF05965
MSTRG.6190.5	15034	coding	PF05965
MSTRG.6190.14	15452	coding	PF05965
MSTRG.6190.9	16113	coding	PF05965
MSTRG.6190.6	16370	coding	PF05965
MSTRG.6190.4	16371	coding	PF05965
MSTRG.6190.8	16365	coding	PF05965
MSTRG.6190.17	3994	coding	PF00628
MSTRG.6192.1	2744	noncoding	-
MSTRG.6193.1	3128	noncoding	-
MSTRG.6179.1	3253	noncoding	-
MSTRG.6179.2	738	noncoding	-
MSTRG.6180.6	4482	coding	PF00022
MSTRG.6180.7	6111	coding	PF00022
MSTRG.6180.5	4354	coding	PF00022
MSTRG.6180.2	2715	coding	PF00022
MSTRG.6180.4	2705	coding	PF00022
MSTRG.6180.8	5823	noncoding	-
MSTRG.6181.1	2674	noncoding	-
MSTRG.6181.2	3632	noncoding	-
MSTRG.6180.10	976	coding	PF00022
MSTRG.6180.11	1024	coding	PF00022
MSTRG.6180.9	1081	coding	PF00022
MSTRG.6182.1	1174	noncoding	-
MSTRG.6184.1	2402	noncoding	-
MSTRG.6196.1	246	noncoding	-
MSTRG.6201.1	2924	noncoding	-
MSTRG.6204.2	3491	coding	PF12738
MSTRG.6204.3	4807	coding	PF12738
MSTRG.6206.1	598	noncoding	-
MSTRG.6212.1	10373	noncoding	-
MSTRG.6212.2	9838	noncoding	-
MSTRG.6212.3	1080	noncoding	-
MSTRG.6212.8	4920	noncoding	-
MSTRG.6220.1	450	noncoding	-
MSTRG.6222.3	1349	noncoding	-
MSTRG.6222.4	1194	noncoding	-

MSTRG.6223.1	5618	noncoding	-
MSTRG.6231.1	2583	coding	PF02847
MSTRG.6232.2	4675	coding	PF00632
MSTRG.6232.4	4763	coding	PF00632
MSTRG.6233.1	1972	noncoding	-
MSTRG.6233.2	2418	noncoding	-
MSTRG.6235.5	1088	coding	PF00226
MSTRG.6235.4	1876	coding	PF00226
MSTRG.6235.6	8169	coding	PF00226
MSTRG.6235.7	7907	noncoding	-
MSTRG.6235.8	6304	coding	PF00226
MSTRG.6235.9	7384	coding	PF00226
MSTRG.6238.2	4774	coding	PF00102
MSTRG.6240.1	3461	noncoding	-
MSTRG.6238.3	834	coding	PF14948
MSTRG.6242.1	1273	noncoding	-
MSTRG.6237.2	3586	noncoding	-
MSTRG.6237.3	3004	noncoding	-
MSTRG.6237.4	3280	noncoding	-
MSTRG.6237.5	4800	coding	PF12422
MSTRG.6251.1	5923	coding	PF17047
MSTRG.6251.2	9067	coding	PF17047
MSTRG.6251.3	7187	coding	PF17047
MSTRG.6251.4	18747	coding	PF17047
MSTRG.6251.5	8424	coding	PF17047
MSTRG.6251.7	3287	coding	PF17047
MSTRG.6251.8	5815	coding	PF17047
MSTRG.6252.1	3487	noncoding	-
MSTRG.6253.1	5208	noncoding	-
MSTRG.6253.2	4854	noncoding	-
MSTRG.6253.3	5354	noncoding	-
MSTRG.6253.5	4087	noncoding	-
MSTRG.6248.1	773	noncoding	-
MSTRG.6267.3	1254	noncoding	-
MSTRG.6271.1	7105	noncoding	-
MSTRG.6271.3	6848	noncoding	-
MSTRG.6272.1	3658	coding	PF05485
MSTRG.6273.2	5670	noncoding	-
MSTRG.6273.3	6257	noncoding	-
MSTRG.6274.2	744	coding	PF12068
MSTRG.6285.1	1856	noncoding	-
MSTRG.6288.1	403	noncoding	-
MSTRG.6312.1	10245	coding	PF02214
MSTRG.6312.2	3656	coding	PF02214
MSTRG.6314.1	2004	noncoding	-
MSTRG.6312.8	6546	coding	PF02214
MSTRG.6315.1	11184	noncoding	-
MSTRG.6316.1	6954	noncoding	-
MSTRG.6314.2	11130	noncoding	-
MSTRG.6314.3	6799	noncoding	-
MSTRG.6314.4	6131	noncoding	-
MSTRG.6317.1	4392	noncoding	-
MSTRG.6316.2	15599	noncoding	-
MSTRG.6318.1	3067	noncoding	-

MSTRG.6316.3	2505	noncoding	-
MSTRG.6316.4	1528	noncoding	-
MSTRG.6316.5	20842	coding	PF00078
MSTRG.6316.6	8309	noncoding	-
MSTRG.6319.1	467	noncoding	-
MSTRG.6319.2	330	noncoding	-
MSTRG.6319.3	484	noncoding	-
MSTRG.6320.1	1277	noncoding	-
MSTRG.6323.2	13730	noncoding	-
MSTRG.6308.4	6026	noncoding	-
MSTRG.6327.1	3707	coding	PF01237
MSTRG.6328.1	945	noncoding	-
MSTRG.6332.2	1230	coding	PF12796
MSTRG.6332.3	4765	coding	PF12796
MSTRG.6333.1	566	noncoding	-
MSTRG.6334.2	1188	coding	PF00412
MSTRG.6348.1	1033	noncoding	-
MSTRG.6348.2	6248	noncoding	-
MSTRG.6349.2	6205	noncoding	-
MSTRG.6349.1	7983	noncoding	-
MSTRG.6349.3	8029	noncoding	-
MSTRG.6349.4	7935	noncoding	-
MSTRG.6349.5	6119	noncoding	-
MSTRG.6349.6	6282	noncoding	-
MSTRG.6349.7	6109	noncoding	-
MSTRG.6349.8	8513	noncoding	-
MSTRG.6350.1	689	noncoding	-
MSTRG.6351.1	7826	noncoding	-
MSTRG.6351.3	6332	noncoding	-
MSTRG.6355.5	2498	coding	PF15898
MSTRG.6355.4	9869	coding	PF15898
MSTRG.6356.1	1294	noncoding	-
MSTRG.6357.1	953	noncoding	-
MSTRG.6355.6	1396	noncoding	-
MSTRG.6358.1	3894	noncoding	-
MSTRG.6359.1	3620	noncoding	-
MSTRG.6359.2	5656	noncoding	-
MSTRG.6355.7	1799	noncoding	-
MSTRG.6360.1	1990	noncoding	-
MSTRG.6363.1	3563	noncoding	-
MSTRG.6364.1	1978	noncoding	-
MSTRG.6384.1	3449	noncoding	-
MSTRG.6385.1	2381	noncoding	-
MSTRG.6386.1	4194	noncoding	-
MSTRG.6387.1	4306	noncoding	-
MSTRG.6388.1	5356	noncoding	-
MSTRG.6389.1	9231	noncoding	-
MSTRG.6389.2	3648	noncoding	-
MSTRG.6389.3	1769	noncoding	-
MSTRG.6383.2	6647	coding	PF08409
MSTRG.6383.3	3488	coding	PF08409
MSTRG.6383.4	3037	noncoding	-
MSTRG.6379.1	548	noncoding	-
MSTRG.6391.1	5116	noncoding	-

MSTRG.6404.2	5754	noncoding	-
MSTRG.6404.3	6302	noncoding	-
MSTRG.6433.11	1954	coding	PF15663
MSTRG.6433.12	1937	coding	PF15663
MSTRG.6433.15	1820	coding	PF15663
MSTRG.6433.3	1695	coding	PF15663
MSTRG.6433.4	1872	coding	PF15663
MSTRG.6433.5	1989	coding	PF15663
MSTRG.6433.6	2006	coding	PF15663
MSTRG.6433.8	1920	coding	PF15663
MSTRG.6433.13	2245	coding	PF15663
MSTRG.6433.10	2246	coding	PF15663
MSTRG.6433.14	2277	coding	PF15663
MSTRG.6433.16	2993	coding	PF15663
MSTRG.6433.7	2331	coding	PF15663
MSTRG.6433.9	2112	coding	PF15663
MSTRG.6435.2	8595	coding	PF16574
MSTRG.6436.2	3048	coding	PF08409
MSTRG.6430.2	4769	coding	PF02404
MSTRG.6442.1	3290	noncoding	-
MSTRG.6454.2	1536	coding	PF07742
MSTRG.6456.1	606	noncoding	-
MSTRG.6456.2	593	noncoding	-
MSTRG.6457.4	2765	noncoding	-
MSTRG.6457.2	2873	noncoding	-
MSTRG.6457.3	3366	noncoding	-
MSTRG.6457.5	2866	noncoding	-
MSTRG.6464.3	1591	noncoding	-
MSTRG.6478.1	4657	noncoding	-
MSTRG.6497.2	2846	coding	PF00104
MSTRG.6497.3	2263	coding	PF00105
MSTRG.6497.4	5380	coding	PF00105
MSTRG.6497.5	5704	coding	PF00621
MSTRG.6497.6	5602	coding	PF00621
MSTRG.6497.7	11885	coding	PF00621
MSTRG.6497.9	2207	noncoding	-
MSTRG.6497.10	8200	noncoding	-
MSTRG.6497.11	6114	noncoding	-
MSTRG.6501.1	4041	coding	PF12632
MSTRG.6501.2	4126	coding	PF12632
MSTRG.6501.3	3130	coding	PF12632
MSTRG.6501.4	3711	coding	PF12632
MSTRG.6501.5	3913	coding	PF12632
MSTRG.6501.6	3167	noncoding	-
MSTRG.6501.7	1988	noncoding	-
MSTRG.6501.8	2637	noncoding	-
MSTRG.6502.4	3323	noncoding	-
MSTRG.6503.1	2171	noncoding	-
MSTRG.6487.2	4074	coding	PF00443
MSTRG.6487.1	3693	coding	PF00443
MSTRG.6487.3	3734	coding	PF00443
MSTRG.6489.1	1688	noncoding	-
MSTRG.6494.1	1562	coding	PF09735
MSTRG.6496.2	895	noncoding	-

MSTRG.6505.2	2484	coding	PF08441
MSTRG.6507.1	2289	noncoding	-
MSTRG.6507.2	2204	noncoding	-
MSTRG.6508.2	3635	noncoding	-
MSTRG.6513.1	1720	coding	PF00046
MSTRG.6513.2	1602	coding	PF00046
MSTRG.6515.1	423	noncoding	-
MSTRG.6520.1	1054	noncoding	-
MSTRG.6522.3	1994	noncoding	-
MSTRG.6522.4	1662	noncoding	-
MSTRG.6530.1	3211	coding	PF00069
MSTRG.6530.2	2704	coding	PF00013
MSTRG.6530.7	1857	coding	PF00013
MSTRG.6530.11	1273	coding	PF00013
MSTRG.6530.13	2025	coding	PF00013
MSTRG.6530.12	2160	coding	PF00013
MSTRG.6530.14	1683	coding	PF00013
MSTRG.6530.15	1019	noncoding	-
MSTRG.6524.1	4706	noncoding	-
MSTRG.6524.2	18420	noncoding	-
MSTRG.6524.3	17780	noncoding	-
MSTRG.6524.6	2893	noncoding	-
MSTRG.6524.7	3055	noncoding	-
MSTRG.6524.8	2897	noncoding	-
MSTRG.6526.1	2200	noncoding	-
MSTRG.6527.2	1184	coding	PF03690
MSTRG.6527.3	1004	coding	PF03690
MSTRG.6538.3	1867	coding	PF00282
MSTRG.6544.1	4951	coding	PF08416
MSTRG.6544.3	4992	coding	PF08416
MSTRG.6540.2	2129	noncoding	-
MSTRG.6546.1	1802	coding	PF00038
MSTRG.6550.2	1771	coding	PF00038
MSTRG.6550.3	1793	coding	PF00038
MSTRG.6550.5	1707	coding	PF00038
MSTRG.6550.6	1729	coding	PF00038
MSTRG.6551.1	3271	noncoding	-
MSTRG.6551.2	2445	noncoding	-
MSTRG.6551.3	2994	noncoding	-
MSTRG.6554.1	2557	coding	PF00105
MSTRG.6555.1	772	noncoding	-
MSTRG.6562.1	11808	coding	PF06512
MSTRG.6562.3	7926	noncoding	-
MSTRG.6568.1	1989	noncoding	-
MSTRG.6567.6	955	noncoding	-
MSTRG.6564.2	728	noncoding	-
MSTRG.6564.3	2083	noncoding	-
MSTRG.6577.3	3077	coding	PF16019
MSTRG.6577.2	4618	coding	PF16019
MSTRG.6582.3	3012	coding	PF01566
MSTRG.6582.2	3448	coding	PF01566
MSTRG.6582.1	2136	coding	PF01566
MSTRG.6582.4	3140	coding	PF01566
MSTRG.6585.1	1570	coding	PF00089

MSTRG.6587.1	2663	noncoding	-
MSTRG.6589.2	2073	noncoding	-
MSTRG.6591.2	2889	coding	PF05383
MSTRG.6591.3	2321	coding	PF05383
MSTRG.6592.1	8172	noncoding	-
MSTRG.6592.2	11450	noncoding	-
MSTRG.6594.2	3313	coding	PF00412
MSTRG.6594.3	3931	coding	PF00412
MSTRG.6598.1	2967	noncoding	-
MSTRG.6601.4	498	noncoding	-
MSTRG.6605.1	3084	coding	PF00858
MSTRG.6606.2	3036	coding	PF01359
MSTRG.6614.1	2293	noncoding	-
MSTRG.6614.2	5597	coding	PF06367
MSTRG.6612.3	2745	noncoding	-
MSTRG.6624.2	1566	coding	PF03953
MSTRG.6624.3	1853	coding	PF03953
MSTRG.6624.4	2003	coding	PF03953
MSTRG.6624.5	1440	coding	PF03953
MSTRG.6624.7	2064	coding	PF03953
MSTRG.6624.6	2084	coding	PF03953
MSTRG.6624.8	1398	coding	PF03953
MSTRG.6624.9	1256	coding	PF00091
MSTRG.6624.10	1800	coding	PF03953
MSTRG.6624.11	1894	coding	PF03953
MSTRG.6626.1	301	noncoding	-
MSTRG.6627.1	3234	coding	PF03953
MSTRG.6627.2	1714	coding	PF03953
MSTRG.6627.3	1792	coding	PF03953
MSTRG.6627.4	1633	coding	PF03953
MSTRG.6627.5	1722	coding	PF03953
MSTRG.6627.6	1710	coding	PF03953
MSTRG.6627.7	1626	coding	PF03953
MSTRG.6622.2	2011	noncoding	-
MSTRG.6622.3	408	noncoding	-
MSTRG.6622.4	1823	noncoding	-
MSTRG.6633.1	20646	coding	PF05965
MSTRG.6633.2	20610	coding	PF05965
MSTRG.6633.7	12236	coding	PF00628
MSTRG.6633.5	20093	coding	PF05965
MSTRG.6634.2	2538	noncoding	-
MSTRG.6629.2	888	coding	PF00025
MSTRG.6636.3	2680	coding	PF00625
MSTRG.6636.4	2784	coding	PF00625
MSTRG.6636.5	2764	coding	PF00625
MSTRG.6636.6	2536	coding	PF00625
MSTRG.6638.2	1730	coding	PF00211
MSTRG.6639.2	480	noncoding	-
MSTRG.6639.3	439	noncoding	-
MSTRG.6640.3	2291	noncoding	-
MSTRG.6640.4	13186	noncoding	-
MSTRG.6644.4	1912	coding	PF13891
MSTRG.6644.6	2067	coding	PF13891
MSTRG.6644.7	2114	coding	PF13891

MSTRG.6644.8	2070	coding	PF13891
MSTRG.6652.1	1024	noncoding	-
MSTRG.6660.1	2623	coding	PF15726
MSTRG.6660.2	2348	coding	PF12796
MSTRG.6660.5	6047	coding	PF12796
MSTRG.6661.5	3093	coding	PF00365
MSTRG.6661.7	3327	coding	PF00365
MSTRG.6661.3	3058	coding	PF00365
MSTRG.6662.6	4431	coding	PF02902
MSTRG.6662.7	4464	coding	PF02902
MSTRG.6657.1	3930	coding	PF01410
MSTRG.6670.1	2087	noncoding	-
MSTRG.6681.1	12823	noncoding	-
MSTRG.6682.1	8820	coding	PF00078
MSTRG.6685.1	2726	noncoding	-
MSTRG.6678.2	214	noncoding	-
MSTRG.6698.1	355	noncoding	-
MSTRG.6705.1	12135	coding	PF02994
MSTRG.6708.1	8962	noncoding	-
MSTRG.6709.1	6153	noncoding	-
MSTRG.6710.1	3506	noncoding	-
MSTRG.6707.3	5798	noncoding	-
MSTRG.6707.4	7136	noncoding	-
MSTRG.6711.1	2089	noncoding	-
MSTRG.6713.1	262	noncoding	-
MSTRG.6714.1	2288	noncoding	-
MSTRG.6714.2	1951	noncoding	-
MSTRG.6714.3	5183	noncoding	-
MSTRG.6714.4	3760	noncoding	-
MSTRG.6714.5	1478	noncoding	-
MSTRG.6715.1	4309	noncoding	-
MSTRG.6694.1	3255	noncoding	-
MSTRG.6694.2	1648	noncoding	-
MSTRG.6695.1	1786	noncoding	-
MSTRG.6694.3	8893	noncoding	-
MSTRG.6694.4	3408	noncoding	-
MSTRG.6726.2	3320	noncoding	-
MSTRG.6739.2	7327	noncoding	-
MSTRG.6739.4	643	noncoding	-
MSTRG.6740.2	5005	noncoding	-
MSTRG.6741.3	1362	noncoding	-
MSTRG.6741.4	1298	noncoding	-
MSTRG.6741.5	1559	noncoding	-
MSTRG.6744.1	1320	coding	PF00069
MSTRG.6744.2	1976	coding	PF00069
MSTRG.6748.3	3796	coding	PF08742
MSTRG.6750.2	2407	noncoding	-
MSTRG.6756.1	5811	coding	PF15089
MSTRG.6757.1	2848	noncoding	-
MSTRG.6760.1	5922	noncoding	-
MSTRG.6762.1	10197	noncoding	-
MSTRG.6763.1	1930	noncoding	-
MSTRG.6761.4	3628	noncoding	-
MSTRG.6761.5	3571	noncoding	-

MSTRG.6761.6	3434	noncoding	-
MSTRG.6764.1	451	noncoding	-
MSTRG.6774.1	5154	noncoding	-
MSTRG.6775.1	5694	noncoding	-
MSTRG.6776.1	2775	noncoding	-
MSTRG.6777.1	1765	noncoding	-
MSTRG.6776.2	1429	noncoding	-
MSTRG.6777.2	546	noncoding	-
MSTRG.6778.1	3076	noncoding	-
MSTRG.6777.3	3585	noncoding	-
MSTRG.6777.5	5860	noncoding	-
MSTRG.6780.1	2408	coding	PF02093
MSTRG.6781.3	946	noncoding	-
MSTRG.6781.4	1626	noncoding	-
MSTRG.6785.2	2176	coding	PF03366
MSTRG.6786.1	2240	noncoding	-
MSTRG.6786.2	708	noncoding	-
MSTRG.6792.5	1710	coding	PF13920
MSTRG.6797.3	3776	coding	PF04121
MSTRG.6797.4	1669	noncoding	-
MSTRG.6798.1	2354	noncoding	-
MSTRG.6800.1	5361	noncoding	-
MSTRG.6806.2	3331	coding	PF15501
MSTRG.6804.1	965	coding	PF14565
MSTRG.6817.3	5689	noncoding	-
MSTRG.6823.1	3707	noncoding	-
MSTRG.6811.1	1592	noncoding	-
MSTRG.6811.2	1500	noncoding	-
MSTRG.6857.1	17223	noncoding	-
MSTRG.6857.2	5758	coding	PF00595
MSTRG.6857.3	5066	coding	PF00595
MSTRG.6857.4	6051	coding	PF00595
MSTRG.6857.5	40847	noncoding	-
MSTRG.6857.6	19909	noncoding	-
MSTRG.6857.7	31708	noncoding	-
MSTRG.6857.8	80663	noncoding	-
MSTRG.6859.1	5162	noncoding	-
MSTRG.6859.2	10485	noncoding	-
MSTRG.6859.3	20457	noncoding	-
MSTRG.6859.4	18157	noncoding	-
MSTRG.6860.1	9631	noncoding	-
MSTRG.6861.1	15402	noncoding	-
MSTRG.6861.2	16720	noncoding	-
MSTRG.6857.9	45971	noncoding	-
MSTRG.6857.10	23811	noncoding	-
MSTRG.6857.11	16785	noncoding	-
MSTRG.6857.12	16837	noncoding	-
MSTRG.6857.13	6125	noncoding	-
MSTRG.6857.14	1269	noncoding	-
MSTRG.6857.15	41708	noncoding	-
MSTRG.6863.1	17379	noncoding	-
MSTRG.6864.1	19751	noncoding	-
MSTRG.6864.2	2902	noncoding	-
MSTRG.6857.16	3542	noncoding	-

MSTRG.6865.1	4823	noncoding	-
MSTRG.6857.17	6504	coding	PF00595
MSTRG.6857.19	1713	coding	PF00595
MSTRG.6866.1	5618	coding	PF00078
MSTRG.6857.20	3785	coding	PF00595
MSTRG.6857.21	4197	coding	PF00595
MSTRG.6857.22	2221	coding	PF00595
MSTRG.6857.23	3016	coding	PF00595
MSTRG.6827.2	2708	coding	PF01027
MSTRG.6837.1	2037	noncoding	-
MSTRG.6838.1	854	noncoding	-
MSTRG.6839.1	2214	noncoding	-
MSTRG.6845.1	305	noncoding	-
MSTRG.6851.1	2707	noncoding	-
MSTRG.6851.2	2155	noncoding	-
MSTRG.6852.1	2171	noncoding	-
MSTRG.6869.1	2475	coding	PF00069
MSTRG.6871.2	820	noncoding	-
MSTRG.6873.2	3154	coding	PF15087
MSTRG.6873.3	3009	coding	PF15087
MSTRG.6884.1	8610	coding	PF00620
MSTRG.6885.1	5434	noncoding	-
MSTRG.6886.1	5426	noncoding	-
MSTRG.6887.1	6580	noncoding	-
MSTRG.6888.1	12726	noncoding	-
MSTRG.6901.1	3211	coding	PF02994
MSTRG.6906.7	7343	coding	PF00443
MSTRG.6906.8	1999	coding	PF00443
MSTRG.6906.9	5712	coding	PF00443
MSTRG.6906.10	983	coding	PF14836
MSTRG.6907.1	3509	noncoding	-
MSTRG.6902.1	239	noncoding	-
MSTRG.6919.1	3718	noncoding	-
MSTRG.6920.1	5443	noncoding	-
MSTRG.6921.1	3224	noncoding	-
MSTRG.6922.4	4523	noncoding	-
MSTRG.6922.3	5210	coding	PF07679
MSTRG.6922.5	15303	noncoding	-
MSTRG.6923.1	739	noncoding	-
MSTRG.6925.1	1374	noncoding	-
MSTRG.6925.2	1452	noncoding	-
MSTRG.6925.3	910	noncoding	-
MSTRG.6926.1	560	noncoding	-
MSTRG.6934.1	1984	noncoding	-
MSTRG.6939.3	1378	noncoding	-
MSTRG.6941.1	1703	coding	PF04912
MSTRG.6941.2	1697	coding	PF04912
MSTRG.6943.1	2104	noncoding	-
MSTRG.6943.2	2396	noncoding	-
MSTRG.6943.4	1586	noncoding	-
MSTRG.6945.2	2695	coding	PF00620
MSTRG.6947.3	3884	noncoding	-
MSTRG.6947.4	3788	noncoding	-
MSTRG.6947.6	3889	noncoding	-

MSTRG.6948.1	6522	coding	PF00078
MSTRG.6947.7	4973	noncoding	-
MSTRG.6947.8	2488	noncoding	-
MSTRG.6956.3	6712	coding	PF00651
MSTRG.6962.4	808	coding	PF01849
MSTRG.6962.5	1019	coding	PF01849
MSTRG.6966.4	7943	coding	PF00439
MSTRG.6967.3	6244	coding	PF00076
MSTRG.6970.3	4230	coding	PF05029
MSTRG.6973.5	3270	coding	PF13423
MSTRG.6974.1	853	coding	PF11938
MSTRG.6975.1	2875	coding	PF00285
MSTRG.6985.1	8955	coding	PF12796
MSTRG.6986.1	1253	noncoding	-
MSTRG.6986.4	1047	noncoding	-
MSTRG.6988.1	1067	noncoding	-
MSTRG.6997.1	1503	noncoding	-
MSTRG.6997.2	1296	noncoding	-
MSTRG.6995.2	474	noncoding	-
MSTRG.6995.4	541	noncoding	-
MSTRG.6995.3	557	noncoding	-
MSTRG.6995.5	544	noncoding	-
MSTRG.6977.1	686	coding	PF01283
MSTRG.6977.2	1005	coding	PF01283
MSTRG.6977.3	957	coding	PF01283
MSTRG.6977.4	901	coding	PF01283
MSTRG.6977.5	786	coding	PF01283
MSTRG.6980.3	2490	coding	PF00174
MSTRG.6980.2	2777	coding	PF00174
MSTRG.6981.6	3244	coding	PF00071
MSTRG.6981.4	3878	coding	PF00071
MSTRG.6981.5	3937	coding	PF00071
MSTRG.7007.1	5009	coding	PF00069
MSTRG.7007.2	8271	coding	PF00069
MSTRG.7007.4	2445	coding	PF00069
MSTRG.7007.5	7614	coding	PF00069
MSTRG.7008.2	970	noncoding	-
MSTRG.7007.11	3410	coding	PF00609
MSTRG.6982.1	1452	noncoding	-
MSTRG.6983.1	1210	noncoding	-
MSTRG.6982.3	1305	noncoding	-
MSTRG.7005.3	948	noncoding	-
MSTRG.7006.1	3179	coding	PF00019
MSTRG.7012.2	1808	coding	PF00335
MSTRG.7019.1	3721	noncoding	-
MSTRG.7035.2	4148	coding	PF13863
MSTRG.7041.4	884	noncoding	-
MSTRG.7044.1	895	noncoding	-
MSTRG.7050.1	478	noncoding	-
MSTRG.7050.3	623	noncoding	-
MSTRG.7050.2	2291	noncoding	-
MSTRG.7054.1	225	noncoding	-
MSTRG.7075.4	2073	coding	PF00640
MSTRG.7075.5	1093	noncoding	-

MSTRG.7079.1	1826	coding	PF12796
MSTRG.7083.1	580	noncoding	-
MSTRG.7077.3	1556	coding	PF00022
MSTRG.7091.3	3934	noncoding	-
MSTRG.7091.5	4363	noncoding	-
MSTRG.7091.4	4575	noncoding	-
MSTRG.7096.1	2799	noncoding	-
MSTRG.7102.1	3395	noncoding	-
MSTRG.7102.2	3816	noncoding	-
MSTRG.7102.3	3245	noncoding	-
MSTRG.7103.1	1785	coding	PF00178
MSTRG.7104.1	3162	coding	PF07679
MSTRG.7110.1	405	noncoding	-
MSTRG.7111.3	1591	noncoding	-
MSTRG.7111.4	1557	noncoding	-
MSTRG.7111.5	1332	noncoding	-
MSTRG.7118.2	1004	noncoding	-
MSTRG.7122.3	796	coding	PF00049
MSTRG.7130.2	6180	coding	PF00193
MSTRG.7130.3	5984	coding	PF00193
MSTRG.7130.4	2144	coding	PF00193
MSTRG.7135.3	838	coding	PF14990
MSTRG.7135.4	451	coding	PF14990
MSTRG.7138.2	3340	coding	PF00808
MSTRG.7141.1	3478	coding	PF07992
MSTRG.7141.3	3425	coding	PF07992
MSTRG.7142.1	4798	noncoding	-
MSTRG.7147.3	1097	noncoding	-
MSTRG.7147.2	2980	noncoding	-
MSTRG.7146.1	255	noncoding	-
MSTRG.7154.2	2799	coding	PF16746
MSTRG.7154.5	2814	coding	PF16746
MSTRG.7154.6	3158	noncoding	-
MSTRG.7156.1	6242	coding	PF00069
MSTRG.7161.1	3931	coding	PF02257
MSTRG.7161.2	12280	coding	PF02257
MSTRG.7162.1	1811	noncoding	-
MSTRG.7164.1	1256	noncoding	-
MSTRG.7165.1	1867	noncoding	-
MSTRG.7165.2	3498	noncoding	-
MSTRG.7166.1	3716	coding	PF15475
MSTRG.7169.1	1097	noncoding	-
MSTRG.7177.1	3241	noncoding	-
MSTRG.7178.1	2058	noncoding	-
MSTRG.7178.2	641	noncoding	-
MSTRG.7177.2	3338	noncoding	-
MSTRG.7177.3	3196	noncoding	-
MSTRG.7177.4	3283	noncoding	-
MSTRG.7177.8	3178	noncoding	-
MSTRG.7177.7	3476	noncoding	-
MSTRG.7177.6	3875	noncoding	-
MSTRG.7177.5	3792	noncoding	-
MSTRG.7177.9	2502	noncoding	-
MSTRG.7177.10	3260	noncoding	-

MSTRG.7177.11	2717	noncoding	-
MSTRG.7177.12	2893	noncoding	-
MSTRG.7177.15	1682	noncoding	-
MSTRG.7177.16	1620	noncoding	-
MSTRG.7177.13	3279	noncoding	-
MSTRG.7177.14	3365	noncoding	-
MSTRG.7177.18	13479	noncoding	-
MSTRG.7177.19	4866	noncoding	-
MSTRG.7179.1	7196	noncoding	-
MSTRG.7172.1	9074	noncoding	-
MSTRG.7176.7	3360	noncoding	-
MSTRG.7176.4	3424	noncoding	-
MSTRG.7176.5	3192	noncoding	-
MSTRG.7176.6	3213	noncoding	-
MSTRG.7183.1	9818	coding	PF01139
MSTRG.7183.3	4823	coding	PF01139
MSTRG.7189.1	9899	noncoding	-
MSTRG.7191.3	3166	noncoding	-
MSTRG.7194.1	1975	noncoding	-
MSTRG.7195.1	4695	coding	PF00965
MSTRG.7195.2	2541	coding	PF00965
MSTRG.7196.1	13350	noncoding	-
MSTRG.7195.3	4367	coding	PF00965
MSTRG.7186.1	481	noncoding	-
MSTRG.7197.3	3426	coding	PF13775
MSTRG.7200.2	2438	coding	PF00493
MSTRG.7200.3	2541	coding	PF00493
MSTRG.7202.1	3279	noncoding	-
MSTRG.7206.4	5888	coding	PF00076
MSTRG.7206.5	3293	coding	PF00076
MSTRG.7206.6	1602	coding	PF00076
MSTRG.7207.1	4524	noncoding	-
MSTRG.7206.7	9169	noncoding	-
MSTRG.7211.1	1773	noncoding	-
MSTRG.7213.1	1418	coding	PF00085
MSTRG.7216.2	2031	coding	PF05091
MSTRG.7220.2	1202	noncoding	-
MSTRG.7220.3	864	coding	PF07653
MSTRG.7220.4	598	coding	PF07653
MSTRG.7225.1	4424	noncoding	-
MSTRG.7227.1	2806	coding	PF00386
MSTRG.7232.2	17882	noncoding	-
MSTRG.7232.3	13235	noncoding	-
MSTRG.7232.1	19555	coding	PF13855
MSTRG.7232.4	17949	coding	PF13855
MSTRG.7232.5	22517	coding	PF13855
MSTRG.7232.6	16380	noncoding	-
MSTRG.7235.3	4014	noncoding	-
MSTRG.7235.4	2668	noncoding	-
MSTRG.7245.1	700	noncoding	-
MSTRG.7258.7	4183	coding	PF01031
MSTRG.7258.6	2760	coding	PF01031
MSTRG.7258.5	2814	coding	PF01031
MSTRG.7260.1	3204	noncoding	-

MSTRG.7262.3	5589	coding	PF00621
MSTRG.7262.5	7545	noncoding	-
MSTRG.7263.1	13470	noncoding	-
MSTRG.7263.2	8104	noncoding	-
MSTRG.7264.1	2102	noncoding	-
MSTRG.7266.9	6237	coding	PF15395
MSTRG.7266.12	2219	noncoding	-
MSTRG.7268.1	452	noncoding	-
MSTRG.7269.5	782	noncoding	-
MSTRG.7269.7	718	noncoding	-
MSTRG.7269.3	1679	noncoding	-
MSTRG.7269.4	769	noncoding	-
MSTRG.7269.6	9114	noncoding	-
MSTRG.7269.8	3271	noncoding	-
MSTRG.7269.9	3608	noncoding	-
MSTRG.7270.1	1678	noncoding	-
MSTRG.7271.1	4198	noncoding	-
MSTRG.7277.1	1535	noncoding	-
MSTRG.7278.1	6771	coding	PF00078
MSTRG.7279.1	8112	noncoding	-
MSTRG.7276.3	11344	noncoding	-
MSTRG.7276.4	11063	coding	PF02759
MSTRG.7281.1	287	noncoding	-
MSTRG.7281.2	540	noncoding	-
MSTRG.7286.10	3536	coding	PF12287
MSTRG.7286.3	3495	coding	PF12287
MSTRG.7286.5	3435	coding	PF12287
MSTRG.7286.7	3229	coding	PF12287
MSTRG.7286.8	3285	coding	PF12287
MSTRG.7286.9	3312	coding	PF12287
MSTRG.7286.11	3381	coding	PF12287
MSTRG.7286.17	1623	coding	PF00386
MSTRG.7292.1	3316	coding	PF07993
MSTRG.7292.3	1301	coding	PF07993
MSTRG.7293.3	6223	noncoding	-
MSTRG.7296.1	6432	noncoding	-
MSTRG.7297.3	5024	noncoding	-
MSTRG.7297.5	5024	noncoding	-
MSTRG.7298.1	1752	coding	PF15480
MSTRG.7300.1	6445	noncoding	-
MSTRG.7297.6	1926	coding	PF01359
MSTRG.7297.7	6933	noncoding	-
MSTRG.7295.2	2327	noncoding	-
MSTRG.7303.1	5645	noncoding	-
MSTRG.7306.1	2926	noncoding	-
MSTRG.7306.2	1074	noncoding	-
MSTRG.7316.1	4327	coding	PF00078
MSTRG.7317.1	2818	noncoding	-
MSTRG.7322.4	2250	noncoding	-
MSTRG.7322.5	1946	noncoding	-
MSTRG.7323.1	475	noncoding	-
MSTRG.7323.2	880	noncoding	-
MSTRG.7325.1	402	noncoding	-
MSTRG.7320.1	436	noncoding	-

MSTRG.7320.2	628	noncoding	-
MSTRG.7321.1	305	noncoding	-
MSTRG.7333.5	2181	coding	PF15927
MSTRG.7335.2	2956	coding	PF00505
MSTRG.7335.4	2969	coding	PF00505
MSTRG.7335.5	3233	coding	PF00505
MSTRG.7335.8	3276	coding	PF00505
MSTRG.7335.9	3300	coding	PF00505
MSTRG.7335.10	2420	coding	PF00505
MSTRG.7335.11	1302	noncoding	-
MSTRG.7335.12	1323	noncoding	-
MSTRG.7335.13	1148	noncoding	-
MSTRG.7340.2	4856	coding	PF01633
MSTRG.7340.3	4604	noncoding	-
MSTRG.7340.4	4366	noncoding	-
MSTRG.7341.1	264	noncoding	-
MSTRG.7342.1	3974	noncoding	-
MSTRG.7343.1	3840	noncoding	-
MSTRG.7344.4	2252	noncoding	-
MSTRG.7346.1	550	noncoding	-
MSTRG.7348.1	258	noncoding	-
MSTRG.7350.1	1792	coding	PF05693
MSTRG.7354.2	3600	coding	PF03137
MSTRG.7359.1	383	noncoding	-
MSTRG.7370.1	5079	noncoding	-
MSTRG.7371.1	7826	noncoding	-
MSTRG.7369.3	1542	noncoding	-
MSTRG.7372.1	6608	noncoding	-
MSTRG.7373.1	3861	noncoding	-
MSTRG.7374.1	7923	noncoding	-
MSTRG.7366.2	3877	noncoding	-
MSTRG.7365.1	392	noncoding	-
MSTRG.7390.1	1094	noncoding	-
MSTRG.7392.1	4953	noncoding	-
MSTRG.7393.1	239	noncoding	-
MSTRG.7394.1	745	noncoding	-
MSTRG.7376.3	1902	coding	PF00388
MSTRG.7376.5	1825	coding	PF00388
MSTRG.7382.1	3073	noncoding	-
MSTRG.7385.1	1196	noncoding	-
MSTRG.7387.1	5962	coding	PF00400
MSTRG.7415.2	3930	coding	PF08416
MSTRG.7415.4	9285	coding	PF02994
MSTRG.7415.3	10971	coding	PF08416
MSTRG.7415.5	15571	noncoding	-
MSTRG.7415.6	11258	noncoding	-
MSTRG.7415.7	5828	coding	PF08416
MSTRG.7421.3	1146	coding	PF02115
MSTRG.7423.4	1467	coding	PF15047
MSTRG.7423.2	1701	coding	PF15047
MSTRG.7423.3	1430	coding	PF15047
MSTRG.7426.1	4384	coding	PF00211
MSTRG.7431.1	1589	coding	PF04916
MSTRG.7431.2	1839	coding	PF04916

MSTRG.7432.1	2181	noncoding	-
MSTRG.7432.9	6151	coding	PF16794
MSTRG.7432.5	8305	coding	PF16788
MSTRG.7432.6	9765	coding	PF16788
MSTRG.7432.7	8146	coding	PF16788
MSTRG.7434.1	582	noncoding	-
MSTRG.7429.1	1623	noncoding	-
MSTRG.7429.2	1949	noncoding	-
MSTRG.7437.1	266	noncoding	-
MSTRG.7438.1	2902	coding	PF00822
MSTRG.7439.4	1197	coding	PF07803
MSTRG.7441.1	5113	noncoding	-
MSTRG.7448.1	1726	noncoding	-
MSTRG.7448.2	767	noncoding	-
MSTRG.7452.1	6167	coding	PF00782
MSTRG.7452.3	5766	coding	PF00782
MSTRG.7454.1	3527	noncoding	-
MSTRG.7451.2	1980	noncoding	-
MSTRG.7455.1	5305	coding	PF14670
MSTRG.7455.2	10131	coding	PF14670
MSTRG.7456.6	2138	noncoding	-
MSTRG.7456.7	1788	noncoding	-
MSTRG.7456.8	1659	noncoding	-
MSTRG.7458.1	423	noncoding	-
MSTRG.7457.3	7432	noncoding	-
MSTRG.7462.1	381	noncoding	-
MSTRG.7467.1	1966	coding	PF00313
MSTRG.7465.1	2955	coding	PF07714
MSTRG.7470.1	298	noncoding	-
MSTRG.7474.2	808	coding	PF02991
MSTRG.7476.1	457	noncoding	-
MSTRG.7487.1	815	noncoding	-
MSTRG.7485.2	1625	coding	PF07678
MSTRG.7499.1	3933	noncoding	-
MSTRG.7496.1	855	noncoding	-
MSTRG.7501.1	2269	noncoding	-
MSTRG.7505.1	4735	coding	PF00083
MSTRG.7505.2	4892	coding	PF00083
MSTRG.7518.1	3186	noncoding	-
MSTRG.7529.1	703	coding	PF00530
MSTRG.7529.2	925	coding	PF00530
MSTRG.7531.1	990	coding	PF00530
MSTRG.7534.1	291	noncoding	-
MSTRG.7537.4	3230	noncoding	-
MSTRG.7538.1	1080	coding	PF00431
MSTRG.7539.1	2384	coding	PF00089
MSTRG.7541.1	1310	noncoding	-
MSTRG.7542.1	1271	noncoding	-
MSTRG.7539.2	2804	coding	PF00089
MSTRG.7544.2	1015	coding	PF03587
MSTRG.7551.1	4557	coding	PF03154
MSTRG.7551.2	4347	coding	PF03154
MSTRG.7552.1	729	coding	PF03154
MSTRG.7552.3	668	noncoding	-

MSTRG.7552.2	634	noncoding	-
MSTRG.7556.2	3799	noncoding	-
MSTRG.7557.1	2050	noncoding	-
MSTRG.7557.2	1906	noncoding	-
MSTRG.7557.3	1632	noncoding	-
MSTRG.7557.4	1397	noncoding	-
MSTRG.7557.5	1912	noncoding	-
MSTRG.7557.6	1638	noncoding	-
MSTRG.7560.3	1593	noncoding	-
MSTRG.7562.6	2828	noncoding	-
MSTRG.7562.7	3336	noncoding	-
MSTRG.7562.8	2533	noncoding	-
MSTRG.7563.1	2133	coding	PF12998
MSTRG.7563.2	1444	coding	PF12998
MSTRG.7563.4	933	coding	PF12998
MSTRG.7573.4	6126	coding	PF08074
MSTRG.7573.5	6111	coding	PF08074
MSTRG.7585.1	7673	coding	PF10244
MSTRG.7572.1	2161	coding	PF00858
MSTRG.7572.2	2125	coding	PF00858
MSTRG.7580.2	2347	noncoding	-
MSTRG.7588.2	1383	coding	PF00335
MSTRG.7591.2	8870	coding	PF16164
MSTRG.7591.3	8741	coding	PF16164
MSTRG.7591.4	1175	noncoding	-
MSTRG.7592.1	1415	coding	PF04547
MSTRG.7594.1	614	noncoding	-
MSTRG.7597.2	422	noncoding	-
MSTRG.7597.1	221	noncoding	-
MSTRG.7600.1	3060	coding	PF05716
MSTRG.7604.3	8045	coding	PF10154
MSTRG.7604.4	9449	coding	PF10154
MSTRG.7604.5	5692	noncoding	-
MSTRG.7606.1	6388	coding	PF00134
MSTRG.7608.2	4683	noncoding	-
MSTRG.7611.1	4131	coding	PF00335
MSTRG.7617.2	3403	coding	PF01167
MSTRG.7618.3	1443	coding	PF15319
MSTRG.7618.4	1610	coding	PF15319
MSTRG.7619.3	3627	coding	PF00250
MSTRG.7621.1	1193	coding	PF15046
MSTRG.7615.2	3262	coding	PF15907
MSTRG.7615.1	2728	coding	PF15907
MSTRG.7615.3	2048	coding	PF15907
MSTRG.7615.4	1906	coding	PF15907
MSTRG.7615.5	2115	coding	PF15907
MSTRG.7616.2	882	noncoding	-
MSTRG.7625.1	3886	coding	PF01369
MSTRG.7625.2	3939	coding	PF01369
MSTRG.7626.1	1809	coding	PF00209
MSTRG.7626.2	1929	coding	PF00209
MSTRG.7630.2	6315	noncoding	-
MSTRG.7630.3	9173	coding	PF08429
MSTRG.7632.3	1596	noncoding	-

MSTRG.7637.1	3105	noncoding	-
MSTRG.7637.4	3135	noncoding	-
MSTRG.7637.5	3274	noncoding	-
MSTRG.7638.1	3888	coding	PF00069
MSTRG.7638.10	9552	coding	PF00069
MSTRG.7638.11	9078	coding	PF00069
MSTRG.7638.5	9552	coding	PF00069
MSTRG.7638.6	9555	coding	PF00069
MSTRG.7638.7	9636	coding	PF00069
MSTRG.7638.8	9471	coding	PF00069
MSTRG.7638.9	8799	coding	PF00069
MSTRG.7639.1	1382	coding	PF00078
MSTRG.7638.12	8778	noncoding	-
MSTRG.7640.2	1893	coding	PF04098
MSTRG.7640.3	2097	coding	PF04098
MSTRG.7642.1	2291	noncoding	-
MSTRG.7642.2	27921	coding	PF01359
MSTRG.7642.3	12111	noncoding	-
MSTRG.7642.4	5571	noncoding	-
MSTRG.7634.2	1736	noncoding	-
MSTRG.7635.1	3925	coding	PF03006
MSTRG.7635.2	3466	coding	PF03006
MSTRG.7635.3	1820	coding	PF03006
MSTRG.7635.5	9004	coding	PF03006
MSTRG.7636.1	1923	noncoding	-
MSTRG.7636.2	1083	noncoding	-
MSTRG.7650.1	8326	coding	PF08763
MSTRG.7646.1	1947	coding	PF00962
MSTRG.7647.1	441	noncoding	-
MSTRG.7659.8	1169	noncoding	-
MSTRG.7660.3	1595	coding	PF06393
MSTRG.7660.5	1797	coding	PF06393
MSTRG.7666.1	11014	coding	PF12130
MSTRG.7666.3	8099	coding	PF00307
MSTRG.7666.4	7967	coding	PF12130
MSTRG.7666.5	6124	coding	PF12130
MSTRG.7666.6	5581	noncoding	-
MSTRG.7665.1	2049	noncoding	-
MSTRG.7668.2	5022	coding	PF00790
MSTRG.7668.3	3625	coding	PF00790
MSTRG.7668.4	1559	coding	PF00790
MSTRG.7673.1	1328	noncoding	-
MSTRG.7674.2	1408	noncoding	-
MSTRG.7675.2	1374	coding	PF00169
MSTRG.7680.1	1583	noncoding	-
MSTRG.7686.3	1972	coding	PF06456
MSTRG.7694.1	602	noncoding	-
MSTRG.7705.5	3867	coding	PF07738
MSTRG.7703.1	6273	coding	PF00354
MSTRG.7707.1	1499	coding	PF08210
MSTRG.7707.3	1496	coding	PF08210
MSTRG.7711.4	5275	noncoding	-
MSTRG.7711.5	3770	noncoding	-
MSTRG.7711.6	3897	noncoding	-

MSTRG.7711.3	4130	noncoding	-
MSTRG.7711.1	7521	noncoding	-
MSTRG.7717.2	1880	noncoding	-
MSTRG.7717.1	2393	noncoding	-
MSTRG.7717.3	4535	noncoding	-
MSTRG.7717.4	7281	noncoding	-
MSTRG.7717.5	630	noncoding	-
MSTRG.7717.8	1110	noncoding	-
MSTRG.7714.2	4349	noncoding	-
MSTRG.7718.1	4693	noncoding	-
MSTRG.7722.2	4410	noncoding	-
MSTRG.7722.3	4145	coding	PF13232
MSTRG.7723.1	8901	coding	PF13232
MSTRG.7722.5	4648	noncoding	-
MSTRG.7722.6	4663	noncoding	-
MSTRG.7723.2	10290	noncoding	-
MSTRG.7730.5	18041	coding	PF16608
MSTRG.7730.6	22672	coding	PF16608
MSTRG.7731.1	3676	noncoding	-
MSTRG.7733.3	3142	coding	PF02037
MSTRG.7737.1	1474	noncoding	-
MSTRG.7738.1	4079	noncoding	-
MSTRG.7739.4	314	noncoding	-
MSTRG.7740.2	804	coding	PF00226
MSTRG.7742.2	1486	coding	PF12678
MSTRG.7742.3	1515	coding	PF12678
MSTRG.7735.1	492	noncoding	-
MSTRG.7744.2	8776	coding	PF08214
MSTRG.7745.1	3654	noncoding	-
MSTRG.7747.2	5317	coding	PF02820
MSTRG.7747.3	4702	coding	PF02820
MSTRG.7747.4	4936	coding	PF02820
MSTRG.7747.1	5309	coding	PF02820
MSTRG.7747.5	4042	coding	PF02820
MSTRG.7751.3	3407	coding	PF07834
MSTRG.7751.6	3000	coding	PF07834
MSTRG.7751.7	1820	coding	PF07834
MSTRG.7751.8	1864	coding	PF07834
MSTRG.7751.9	1737	coding	PF07834
MSTRG.7752.1	1071	noncoding	-
MSTRG.7752.2	1973	noncoding	-
MSTRG.7755.1	3924	noncoding	-
MSTRG.7755.3	4132	noncoding	-
MSTRG.7743.1	4575	coding	PF07742
MSTRG.7758.1	300	noncoding	-
MSTRG.7762.2	1945	coding	PF05903
MSTRG.7760.2	1659	coding	PF01248
MSTRG.7767.1	735	noncoding	-
MSTRG.7767.2	634	noncoding	-
MSTRG.7769.2	3141	coding	PF15002
MSTRG.7770.1	1472	noncoding	-
MSTRG.7770.2	652	noncoding	-
MSTRG.7771.4	2557	noncoding	-
MSTRG.7772.3	6010	coding	PF00735

MSTRG.7773.2	4879	coding	PF10349
MSTRG.7780.2	547	coding	PF10161
MSTRG.7782.1	2742	noncoding	-
MSTRG.7782.2	3399	noncoding	-
MSTRG.7782.3	1117	noncoding	-
MSTRG.7782.4	7538	noncoding	-
MSTRG.7782.5	7009	noncoding	-
MSTRG.7782.8	3369	noncoding	-
MSTRG.7782.6	8524	noncoding	-
MSTRG.7782.7	5161	noncoding	-
MSTRG.7782.9	3657	noncoding	-
MSTRG.7782.10	1764	noncoding	-
MSTRG.7782.12	2429	noncoding	-
MSTRG.7782.13	2755	noncoding	-
MSTRG.7782.14	1493	noncoding	-
MSTRG.7783.1	659	noncoding	-
MSTRG.7787.1	2490	coding	PF12923
MSTRG.7790.3	3709	noncoding	-
MSTRG.7791.1	1678	noncoding	-
MSTRG.7794.3	1291	noncoding	-
MSTRG.7795.1	3855	noncoding	-
MSTRG.7796.3	1876	coding	PF04572
MSTRG.7796.5	13092	coding	PF04572
MSTRG.7796.6	8387	noncoding	-
MSTRG.7796.7	1809	noncoding	-
MSTRG.7797.1	2654	noncoding	-
MSTRG.7799.2	6380	coding	PF14604
MSTRG.7800.1	760	noncoding	-
MSTRG.7801.2	3619	noncoding	-
MSTRG.7801.1	4185	noncoding	-
MSTRG.7803.1	928	noncoding	-
MSTRG.7804.2	1022	coding	PF12201
MSTRG.7805.1	3200	coding	PF00698
MSTRG.7805.3	2034	noncoding	-
MSTRG.7805.4	1838	noncoding	-
MSTRG.7805.5	1940	noncoding	-
MSTRG.7809.1	4900	noncoding	-
MSTRG.7812.1	12473	noncoding	-
MSTRG.7815.1	1655	coding	PF08976
MSTRG.7815.3	4326	coding	PF08976
MSTRG.7814.1	2395	coding	PF00685
MSTRG.7818.2	1793	coding	PF01103
MSTRG.7818.3	293	noncoding	-
MSTRG.7819.1	523	noncoding	-
MSTRG.7821.2	623	noncoding	-
MSTRG.7825.2	1713	noncoding	-
MSTRG.7832.1	6796	noncoding	-
MSTRG.7832.2	3354	noncoding	-
MSTRG.7840.1	2164	noncoding	-
MSTRG.7841.3	2493	noncoding	-
MSTRG.7842.2	4268	coding	PF02463
MSTRG.7843.2	1419	coding	PF05914
MSTRG.7844.1	2562	coding	PF09759
MSTRG.7844.4	983	noncoding	-

MSTRG.7844.3	2511	coding	PF09759
MSTRG.7844.5	4637	coding	PF09759
MSTRG.7844.6	2733	coding	PF09759
MSTRG.7848.1	2378	noncoding	-
MSTRG.7848.2	301	noncoding	-
MSTRG.7854.5	5738	noncoding	-
MSTRG.7859.2	9682	coding	PF08016
MSTRG.7859.3	10841	coding	PF08016
MSTRG.7859.1	10908	coding	PF08016
MSTRG.7859.4	10580	coding	PF08016
MSTRG.7859.8	7859	coding	PF08016
MSTRG.7859.9	7877	coding	PF08016
MSTRG.7859.7	8982	coding	PF08016
MSTRG.7859.5	12084	coding	PF08016
MSTRG.7859.6	9490	coding	PF08016
MSTRG.7859.10	9086	coding	PF08016
MSTRG.7859.11	9102	coding	PF08016
MSTRG.7859.12	8374	coding	PF08016
MSTRG.7860.1	987	coding	PF08016
MSTRG.7859.14	630	noncoding	-
MSTRG.7859.13	7888	coding	PF08016
MSTRG.7859.15	4278	noncoding	-
MSTRG.7859.16	8371	coding	PF08016
MSTRG.7863.1	250	noncoding	-
MSTRG.7855.7	3794	noncoding	-
MSTRG.7868.7	1366	noncoding	-
MSTRG.7870.1	2837	noncoding	-
MSTRG.7866.2	3036	noncoding	-
MSTRG.7866.3	3229	noncoding	-
MSTRG.7874.1	508	noncoding	-
MSTRG.7873.2	1727	noncoding	-
MSTRG.7879.1	5207	noncoding	-
MSTRG.7878.1	4913	noncoding	-
MSTRG.7881.1	862	noncoding	-
MSTRG.7881.2	512	noncoding	-
MSTRG.7884.1	588	noncoding	-
MSTRG.7891.1	1315	noncoding	-
MSTRG.7898.2	2253	coding	PF03901
MSTRG.7889.1	3174	coding	PF00069
MSTRG.7903.1	10084	coding	PF03133
MSTRG.7903.2	9937	noncoding	-
MSTRG.7903.3	4344	noncoding	-
MSTRG.7903.4	11340	noncoding	-
MSTRG.7905.6	6280	coding	PF13087
MSTRG.7905.7	11256	coding	PF01466
MSTRG.7915.10	1543	coding	PF04499
MSTRG.7915.11	3380	coding	PF04499
MSTRG.7915.5	3917	coding	PF04499
MSTRG.7915.6	3749	coding	PF04499
MSTRG.7915.7	3913	coding	PF04499
MSTRG.7915.9	3883	coding	PF04499
MSTRG.7915.12	4830	coding	PF04499
MSTRG.7917.3	5894	coding	PF12335
MSTRG.7917.4	5654	coding	PF12335

MSTRG.7913.1	961	noncoding	-
MSTRG.7910.3	512	coding	PF15191
MSTRG.7921.1	5063	coding	PF00755
MSTRG.7921.2	4638	coding	PF00755
MSTRG.7921.4	2971	coding	PF00755
MSTRG.7923.1	7122	coding	PF07647
MSTRG.7923.2	4310	noncoding	-
MSTRG.7922.2	1311	coding	PF00089
MSTRG.7922.3	1281	coding	PF00089
MSTRG.7926.2	1027	noncoding	-
MSTRG.7930.1	3442	coding	PF00517
MSTRG.7933.1	620	coding	PF00210
MSTRG.7933.2	548	noncoding	-
MSTRG.7932.1	250	noncoding	-
MSTRG.7939.1	1196	coding	PF11176
MSTRG.7939.2	5918	coding	PF11176
MSTRG.7939.3	5673	coding	PF11176
MSTRG.7939.4	1923	noncoding	-
MSTRG.7935.2	2398	coding	PF00456
MSTRG.7943.1	884	noncoding	-
MSTRG.7944.3	4570	noncoding	-
MSTRG.7944.4	5544	coding	PF04410
MSTRG.7944.5	3951	coding	PF04410
MSTRG.7944.6	3457	coding	PF04410
MSTRG.7945.1	1819	noncoding	-
MSTRG.7944.7	2017	coding	PF01359
MSTRG.7958.1	1215	noncoding	-
MSTRG.7962.1	5490	noncoding	-
MSTRG.7984.1	1306	noncoding	-
MSTRG.7985.1	739	noncoding	-
MSTRG.7990.1	3483	noncoding	-
MSTRG.7995.1	15776	noncoding	-
MSTRG.7996.1	5064	noncoding	-
MSTRG.7996.2	4573	noncoding	-
MSTRG.7997.1	4484	noncoding	-
MSTRG.7998.1	6987	noncoding	-
MSTRG.7988.15	1070	noncoding	-
MSTRG.7988.13	2225	noncoding	-
MSTRG.7988.12	1308	noncoding	-
MSTRG.7988.14	1197	noncoding	-
MSTRG.7988.11	6916	coding	PF00233
MSTRG.7988.16	1366	noncoding	-
MSTRG.7988.18	1037	noncoding	-
MSTRG.7988.20	4479	noncoding	-
MSTRG.7988.22	7327	noncoding	-
MSTRG.8002.1	2927	noncoding	-
MSTRG.8002.2	7786	noncoding	-
MSTRG.8016.1	2624	noncoding	-
MSTRG.8019.1	4512	noncoding	-
MSTRG.8020.1	4920	noncoding	-
MSTRG.8022.1	1025	noncoding	-
MSTRG.8023.1	5107	noncoding	-
MSTRG.8020.4	906	noncoding	-
MSTRG.8020.5	785	noncoding	-

MSTRG.8025.1	5547	coding	PF05556
MSTRG.8024.7	2952	noncoding	-
MSTRG.8026.1	10249	noncoding	-
MSTRG.8027.1	623	noncoding	-
MSTRG.8029.1	3629	coding	PF04811
MSTRG.8029.2	3510	coding	PF04811
MSTRG.8031.1	4857	noncoding	-
MSTRG.8032.2	2866	coding	PF05063
MSTRG.8010.1	214	noncoding	-
MSTRG.8060.1	1416	noncoding	-
MSTRG.8071.1	334	noncoding	-
MSTRG.8074.1	1275	noncoding	-
MSTRG.8096.1	11359	noncoding	-
MSTRG.8096.2	8449	coding	PF12796
MSTRG.8096.3	8776	coding	PF12796
MSTRG.8096.4	8403	coding	PF12796
MSTRG.8096.11	8173	coding	PF12796
MSTRG.8096.12	17856	coding	PF12796
MSTRG.8096.6	7988	coding	PF12796
MSTRG.8096.10	8915	coding	PF12796
MSTRG.8096.5	8225	coding	PF12796
MSTRG.8096.7	8240	coding	PF12796
MSTRG.8096.8	9975	coding	PF12796
MSTRG.8096.9	8481	coding	PF12796
MSTRG.8096.13	4644	coding	PF00791
MSTRG.8096.14	7085	coding	PF12796
MSTRG.8097.1	5545	noncoding	-
MSTRG.8098.1	20310	noncoding	-
MSTRG.8098.2	19103	noncoding	-
MSTRG.8099.1	14009	noncoding	-
MSTRG.8098.3	3733	noncoding	-
MSTRG.8100.1	24349	noncoding	-
MSTRG.8096.15	25384	noncoding	-
MSTRG.8096.16	28985	noncoding	-
MSTRG.8096.17	26229	noncoding	-
MSTRG.8096.18	26274	noncoding	-
MSTRG.8096.19	22992	noncoding	-
MSTRG.8096.20	19100	noncoding	-
MSTRG.8096.21	8019	noncoding	-
MSTRG.8096.22	12057	noncoding	-
MSTRG.8083.1	3601	coding	PF00078
MSTRG.8086.1	528	noncoding	-
MSTRG.8103.1	1426	noncoding	-
MSTRG.8109.3	1797	coding	PF04678
MSTRG.8110.2	4061	coding	PF04811
MSTRG.8110.4	3938	coding	PF04811
MSTRG.8110.6	4454	coding	PF04811
MSTRG.8110.5	3992	coding	PF04811
MSTRG.8113.1	531	noncoding	-
MSTRG.8119.1	2749	noncoding	-
MSTRG.8119.2	18777	noncoding	-
MSTRG.8130.1	2360	noncoding	-
MSTRG.8131.3	2210	coding	PF08347
MSTRG.8131.4	2491	coding	PF08347

MSTRG.8131.5	2067	coding	PF08347
MSTRG.8131.6	2026	coding	PF08347
MSTRG.8131.7	1666	coding	PF08347
MSTRG.8117.3	1787	coding	PF01747
MSTRG.8143.1	1396	coding	PF01588
MSTRG.8145.1	792	noncoding	-
MSTRG.8146.1	370	noncoding	-
MSTRG.8147.2	1725	noncoding	-
MSTRG.8160.2	260	noncoding	-
MSTRG.8160.1	13637	noncoding	-
MSTRG.8160.3	2629	noncoding	-
MSTRG.8160.4	12349	noncoding	-
MSTRG.8165.1	8216	coding	PF00225
MSTRG.8165.3	7947	coding	PF00225
MSTRG.8171.1	352	noncoding	-
MSTRG.8171.2	475	noncoding	-
MSTRG.8172.11	4324	noncoding	-
MSTRG.8172.7	1350	noncoding	-
MSTRG.8172.6	2336	noncoding	-
MSTRG.8172.9	1821	noncoding	-
MSTRG.8172.10	2532	noncoding	-
MSTRG.8172.5	2168	noncoding	-
MSTRG.8172.8	15150	noncoding	-
MSTRG.8172.4	1847	noncoding	-
MSTRG.8172.3	2474	noncoding	-
MSTRG.8172.13	2683	noncoding	-
MSTRG.8172.12	11308	noncoding	-
MSTRG.8172.14	4432	noncoding	-
MSTRG.8172.15	7152	noncoding	-
MSTRG.8185.2	2412	coding	PF00179
MSTRG.8185.1	4507	coding	PF00179
MSTRG.8185.3	1764	coding	PF00179
MSTRG.8195.1	4468	coding	PF00554
MSTRG.8195.2	4620	coding	PF00554
MSTRG.8190.2	488	noncoding	-
MSTRG.8198.1	382	noncoding	-
MSTRG.8201.1	3043	noncoding	-
MSTRG.8201.2	6363	noncoding	-
MSTRG.8202.1	5647	noncoding	-
MSTRG.8200.2	4812	coding	PF00149
MSTRG.8200.3	1942	noncoding	-
MSTRG.8203.1	278	noncoding	-
MSTRG.8209.1	2319	noncoding	-
MSTRG.8209.2	746	noncoding	-
MSTRG.8210.1	324	noncoding	-
MSTRG.8218.1	3615	noncoding	-
MSTRG.8219.1	341	noncoding	-
MSTRG.8224.3	1400	coding	PF15256
MSTRG.8221.1	372	noncoding	-
MSTRG.8221.2	388	noncoding	-
MSTRG.8238.1	1860	coding	PF01652
MSTRG.8238.2	2445	coding	PF01652
MSTRG.8238.5	1707	coding	PF01652
MSTRG.8238.6	2354	coding	PF01652

MSTRG.8238.7	2052	coding	PF01652
MSTRG.8238.8	2385	coding	PF01652
MSTRG.8239.1	9995	noncoding	-
MSTRG.8241.3	10966	noncoding	-
MSTRG.8241.2	3177	coding	PF00335
MSTRG.8241.4	1305	coding	PF00335
MSTRG.8242.1	43372	noncoding	-
MSTRG.8241.5	2442	coding	PF00335
MSTRG.8241.6	1426	coding	PF00335
MSTRG.8244.1	828	noncoding	-
MSTRG.8241.7	1306	coding	PF00335
MSTRG.8241.8	2481	coding	PF00335
MSTRG.8246.2	933	noncoding	-
MSTRG.8246.6	1398	noncoding	-
MSTRG.8246.5	1778	noncoding	-
MSTRG.8246.4	1863	noncoding	-
MSTRG.8248.1	3355	noncoding	-
MSTRG.8246.7	1949	noncoding	-
MSTRG.8249.1	1079	noncoding	-
MSTRG.8255.1	281	noncoding	-
MSTRG.8283.3	4924	coding	PF00412
MSTRG.8283.5	4455	coding	PF00412
MSTRG.8283.6	4156	coding	PF00412
MSTRG.8283.7	4771	coding	PF00412
MSTRG.8283.8	662	coding	PF00595
MSTRG.8283.9	5221	coding	PF00595
MSTRG.8283.10	3112	noncoding	-
MSTRG.8283.12	17695	noncoding	-
MSTRG.8283.13	11259	noncoding	-
MSTRG.8285.3	5948	coding	PF00176
MSTRG.8395.1	231	noncoding	-
MSTRG.8397.1	649	noncoding	-
MSTRG.8409.1	6064	noncoding	-
MSTRG.8410.1	1072	noncoding	-
MSTRG.8411.1	7198	coding	PF00078
MSTRG.8412.1	1995	noncoding	-
MSTRG.8417.2	3330	noncoding	-
MSTRG.8419.1	2285	noncoding	-
MSTRG.8394.2	6244	noncoding	-
MSTRG.8394.3	644	noncoding	-
MSTRG.8420.1	7284	noncoding	-
MSTRG.8422.1	2437	noncoding	-
MSTRG.8423.1	1181	noncoding	-
MSTRG.8424.1	9786	noncoding	-
MSTRG.8425.1	2449	noncoding	-
MSTRG.8394.4	1662	noncoding	-
MSTRG.8394.5	5581	noncoding	-
MSTRG.8394.6	17583	noncoding	-
MSTRG.8394.7	2977	noncoding	-
MSTRG.8394.8	1181	noncoding	-
MSTRG.8394.9	3858	noncoding	-
MSTRG.8282.1	216	noncoding	-
MSTRG.8309.1	3274	noncoding	-
MSTRG.8315.1	613	noncoding	-

MSTRG.8316.1	1918	noncoding	-
MSTRG.8315.2	14760	noncoding	-
MSTRG.8324.3	733	coding	PF01387
MSTRG.8324.4	591	coding	PF01387
MSTRG.8333.2	1633	noncoding	-
MSTRG.8333.3	5997	coding	PF00481
MSTRG.8333.4	6342	coding	PF00481
MSTRG.8333.5	6861	coding	PF00481
MSTRG.8337.1	462	noncoding	-
MSTRG.8338.2	1279	noncoding	-
MSTRG.8346.2	2267	coding	PF00883
MSTRG.8349.6	6783	coding	PF11594
MSTRG.8351.1	848	noncoding	-
MSTRG.8351.5	6597	noncoding	-
MSTRG.8352.3	2983	coding	PF12719
MSTRG.8352.4	1266	coding	PF12719
MSTRG.8353.1	8096	coding	PF15090
MSTRG.8353.2	9030	coding	PF15090
MSTRG.8353.4	9057	noncoding	-
MSTRG.8353.6	8424	noncoding	-
MSTRG.8353.5	4570	noncoding	-
MSTRG.8353.7	2534	noncoding	-
MSTRG.8353.8	2467	noncoding	-
MSTRG.8354.1	3323	noncoding	-
MSTRG.8355.1	1092	noncoding	-
MSTRG.8356.1	2103	noncoding	-
MSTRG.8340.1	985	noncoding	-
MSTRG.8343.1	271	noncoding	-
MSTRG.8359.5	1147	coding	PF10274
MSTRG.8359.3	3819	coding	PF10274
MSTRG.8359.4	1708	coding	PF10274
MSTRG.8360.1	337	noncoding	-
MSTRG.8365.1	3590	noncoding	-
MSTRG.8358.1	638	noncoding	-
MSTRG.8386.1	4416	coding	PF13855
MSTRG.8387.1	455	noncoding	-
MSTRG.8392.1	714	noncoding	-
MSTRG.8432.1	12798	noncoding	-
MSTRG.8430.1	879	noncoding	-
MSTRG.8444.1	3691	coding	PF00454
MSTRG.8444.2	1883	coding	PF00454
MSTRG.8444.3	2364	coding	PF00454
MSTRG.8444.7	2536	coding	PF00454
MSTRG.8444.5	3291	coding	PF00454
MSTRG.8444.6	2538	coding	PF00454
MSTRG.8444.8	2745	coding	PF00454
MSTRG.8444.9	3256	coding	PF00454
MSTRG.8444.10	2509	coding	PF00454
MSTRG.8444.11	2482	coding	PF00454
MSTRG.8445.1	3167	coding	PF12896
MSTRG.8445.3	2091	coding	PF12896
MSTRG.8437.1	4008	noncoding	-
MSTRG.8439.2	1096	noncoding	-
MSTRG.8446.5	2670	coding	PF09270

MSTRG.8446.7	1494	coding	PF09270
MSTRG.8460.1	235	noncoding	-
MSTRG.8482.1	2937	noncoding	-
MSTRG.8486.1	733	noncoding	-
MSTRG.8488.1	480	noncoding	-
MSTRG.8488.2	615	noncoding	-
MSTRG.8488.3	941	noncoding	-
MSTRG.8488.4	548	noncoding	-
MSTRG.8495.1	762	noncoding	-
MSTRG.8494.5	3154	coding	PF00566
MSTRG.8493.1	225	noncoding	-
MSTRG.8499.1	4661	noncoding	-
MSTRG.8506.1	11951	coding	PF05334
MSTRG.8507.1	1901	noncoding	-
MSTRG.8508.1	3036	noncoding	-
MSTRG.8509.1	1725	noncoding	-
MSTRG.8512.2	3617	coding	PF07707
MSTRG.8512.3	3716	coding	PF07707
MSTRG.8512.4	3114	coding	PF07707
MSTRG.8502.3	8455	coding	PF08519
MSTRG.8515.3	1838	noncoding	-
MSTRG.8517.2	1264	coding	PF16881
MSTRG.8520.1	2123	noncoding	-
MSTRG.8521.1	1719	noncoding	-
MSTRG.8522.1	500	noncoding	-
MSTRG.8524.1	3197	noncoding	-
MSTRG.8526.2	8650	noncoding	-
MSTRG.8527.1	5316	noncoding	-
MSTRG.8526.3	1140	noncoding	-
MSTRG.8528.1	959	noncoding	-
MSTRG.8529.1	7129	coding	PF08590
MSTRG.8529.2	7231	coding	PF08590
MSTRG.8529.3	7061	coding	PF08590
MSTRG.8529.4	11546	coding	PF08590
MSTRG.8529.5	6557	coding	PF08590
MSTRG.8529.7	6889	coding	PF08590
MSTRG.8534.9	4551	coding	PF00076
MSTRG.8534.10	5129	coding	PF00076
MSTRG.8534.11	2801	coding	PF00076
MSTRG.8536.1	13322	noncoding	-
MSTRG.8536.2	4170	noncoding	-
MSTRG.8538.5	2111	coding	PF01189
MSTRG.8539.3	5492	coding	PF00640
MSTRG.8539.4	5570	coding	PF00640
MSTRG.8539.5	3609	coding	PF00640
MSTRG.8543.1	705	noncoding	-
MSTRG.8544.1	841	noncoding	-
MSTRG.8542.2	3250	noncoding	-
MSTRG.8547.1	1638	noncoding	-
MSTRG.8548.3	5560	noncoding	-
MSTRG.8548.2	5371	noncoding	-
MSTRG.8556.1	335	noncoding	-
MSTRG.8557.3	6111	coding	PF16212
MSTRG.8557.2	12584	coding	PF16212

MSTRG.8557.5	8576	coding	PF16212
MSTRG.8554.1	633	noncoding	-
MSTRG.8555.1	786	noncoding	-
MSTRG.8565.1	1716	noncoding	-
MSTRG.8565.2	1010	noncoding	-
MSTRG.8566.1	464	noncoding	-
MSTRG.8566.2	296	noncoding	-
MSTRG.8585.1	300	noncoding	-
MSTRG.8587.1	356	noncoding	-
MSTRG.8589.1	2917	coding	PF00078
MSTRG.8586.1	567	noncoding	-
MSTRG.8591.2	2986	noncoding	-
MSTRG.8594.2	5679	coding	PF16212
MSTRG.8595.1	322	noncoding	-
MSTRG.8598.3	3546	noncoding	-
MSTRG.8598.4	3549	noncoding	-
MSTRG.8600.3	6484	coding	PF15301
MSTRG.8601.1	924	noncoding	-
MSTRG.8604.1	1159	noncoding	-
MSTRG.8604.2	847	noncoding	-
MSTRG.8605.4	1167	coding	PF07051
MSTRG.8605.8	971	coding	PF07051
MSTRG.8605.5	1483	coding	PF07051
MSTRG.8605.6	4345	coding	PF07051
MSTRG.8606.3	4280	coding	PF03556
MSTRG.8606.5	4198	coding	PF03556
MSTRG.8606.4	4249	coding	PF03556
MSTRG.8608.1	7482	noncoding	-
MSTRG.8611.3	1859	coding	PF16026
MSTRG.8613.1	593	noncoding	-
MSTRG.8617.1	3792	noncoding	-
MSTRG.8614.2	2232	coding	PF00071
MSTRG.8614.3	1422	coding	PF00071
MSTRG.8624.10	4645	coding	PF05182
MSTRG.8624.11	4728	coding	PF05182
MSTRG.8624.12	4684	coding	PF05182
MSTRG.8624.13	4726	coding	PF05182
MSTRG.8620.1	413	noncoding	-
MSTRG.8629.1	902	noncoding	-
MSTRG.8630.1	1943	noncoding	-
MSTRG.8630.2	3983	noncoding	-
MSTRG.8632.1	420	noncoding	-
MSTRG.8645.5	3214	coding	PF14598
MSTRG.8645.4	3497	coding	PF14598
MSTRG.8645.6	3949	coding	PF14598
MSTRG.8646.1	1103	noncoding	-
MSTRG.8642.2	760	noncoding	-
MSTRG.8647.1	7171	coding	PF09763
MSTRG.8647.3	3307	coding	PF09763
MSTRG.8648.1	5178	noncoding	-
MSTRG.8650.2	5011	coding	PF15262
MSTRG.8650.1	6989	coding	PF15262
MSTRG.8650.4	652	noncoding	-
MSTRG.8650.3	6532	coding	PF15262

MSTRG.8656.3	3794	coding	PF00501
MSTRG.8657.1	5607	coding	PF01259
MSTRG.8657.2	3486	coding	PF01259
MSTRG.8658.1	9642	coding	PF00078
MSTRG.8658.3	5900	coding	PF00078
MSTRG.8657.3	5300	coding	PF01259
MSTRG.8657.4	5144	coding	PF01259
MSTRG.8657.6	5546	coding	PF01259
MSTRG.8657.7	2714	coding	PF01259
MSTRG.8659.2	1058	noncoding	-
MSTRG.8660.2	4024	noncoding	-
MSTRG.8660.3	3942	noncoding	-
MSTRG.8660.4	4308	noncoding	-
MSTRG.8660.10	5145	noncoding	-
MSTRG.8660.11	3501	noncoding	-
MSTRG.8660.8	3590	noncoding	-
MSTRG.8660.9	3672	noncoding	-
MSTRG.8652.1	2233	coding	PF14912
MSTRG.8652.2	1883	coding	PF14912
MSTRG.8674.2	5024	noncoding	-
MSTRG.8674.1	6975	noncoding	-
MSTRG.8674.3	7957	noncoding	-
MSTRG.8674.4	6969	noncoding	-
MSTRG.8674.8	2152	noncoding	-
MSTRG.8674.7	6522	noncoding	-
MSTRG.8677.5	628	noncoding	-
MSTRG.8677.3	751	coding	PF00050
MSTRG.8677.4	457	noncoding	-
MSTRG.8677.8	809	noncoding	-
MSTRG.8677.9	812	noncoding	-
MSTRG.8677.10	653	coding	PF00050
MSTRG.8678.2	2276	noncoding	-
MSTRG.8680.3	3626	coding	PF00562
MSTRG.8680.2	3654	coding	PF00562
MSTRG.8686.1	321	noncoding	-
MSTRG.8701.1	10370	coding	PF02354
MSTRG.8701.2	11309	coding	PF02354
MSTRG.8716.1	2466	coding	PF01404
MSTRG.8713.1	233	noncoding	-
MSTRG.8732.1	2657	noncoding	-
MSTRG.8733.1	511	noncoding	-
MSTRG.8733.2	2303	noncoding	-
MSTRG.8733.3	2130	noncoding	-
MSTRG.8734.1	3940	coding	PF10585
MSTRG.8729.1	635	coding	PF00956
MSTRG.8742.1	3023	coding	PF04146
MSTRG.8742.3	2660	coding	PF04146
MSTRG.8742.6	4582	coding	PF04146
MSTRG.8742.7	4528	coding	PF04146
MSTRG.8742.8	3534	coding	PF04146
MSTRG.8742.9	3480	coding	PF04146
MSTRG.8764.1	1789	noncoding	-
MSTRG.8765.1	5157	coding	PF15097
MSTRG.8765.2	17517	coding	PF15097

MSTRG.8766.1	6057	coding	PF15362
MSTRG.8765.4	1299	noncoding	-
MSTRG.8748.4	3499	coding	PF02759
MSTRG.8748.6	5390	coding	PF02759
MSTRG.8768.3	2314	noncoding	-
MSTRG.8769.1	6751	coding	PF03637
MSTRG.8785.1	2739	noncoding	-
MSTRG.8785.2	2575	noncoding	-
MSTRG.8785.3	2210	noncoding	-
MSTRG.8786.7	9533	coding	PF12796
MSTRG.8786.13	11629	coding	PF12796
MSTRG.8786.8	13769	coding	PF12796
MSTRG.8786.12	7937	coding	PF12796
MSTRG.8786.10	9110	coding	PF12796
MSTRG.8786.11	9067	coding	PF12796
MSTRG.8786.9	8765	coding	PF12796
MSTRG.8786.15	6405	noncoding	-
MSTRG.8787.1	11769	noncoding	-
MSTRG.8789.1	1276	noncoding	-
MSTRG.8803.1	3921	coding	PF05485
MSTRG.8808.2	6778	coding	PF00069
MSTRG.8809.1	1730	coding	PF02136
MSTRG.8809.4	3609	coding	PF02136
MSTRG.8818.3	1669	coding	PF01129
MSTRG.8815.1	494	noncoding	-
MSTRG.8815.2	679	noncoding	-
MSTRG.8815.3	694	noncoding	-
MSTRG.8815.4	418	noncoding	-
MSTRG.8825.6	3552	coding	PF15921
MSTRG.8825.7	3256	coding	PF15921
MSTRG.8825.8	2210	coding	PF15921
MSTRG.8826.4	5986	coding	PF08687
MSTRG.8823.1	1111	noncoding	-
MSTRG.8833.1	1772	coding	PF00134
MSTRG.8833.2	2463	coding	PF00134
MSTRG.8833.3	2047	coding	PF00134
MSTRG.8833.4	2389	coding	PF00134
MSTRG.8833.5	3484	coding	PF00134
MSTRG.8833.6	4438	coding	PF00134
MSTRG.8833.7	4886	coding	PF00134
MSTRG.8833.8	4451	coding	PF00134
MSTRG.8830.1	541	coding	PF03939
MSTRG.8835.1	2581	noncoding	-
MSTRG.8836.1	2358	noncoding	-
MSTRG.8838.1	2220	coding	PF00078
MSTRG.8839.1	1116	noncoding	-
MSTRG.8842.1	1350	coding	PF16184
MSTRG.8846.3	4404	coding	PF15282
MSTRG.8846.4	4204	coding	PF15282
MSTRG.8846.5	7556	coding	PF15282
MSTRG.8846.6	7445	coding	PF15282
MSTRG.8849.1	602	noncoding	-
MSTRG.8844.1	960	noncoding	-
MSTRG.8844.2	1384	noncoding	-

MSTRG.8853.1	491	noncoding	-
MSTRG.8853.2	422	noncoding	-
MSTRG.8853.3	660	noncoding	-
MSTRG.8853.4	420	noncoding	-
MSTRG.8857.1	207	noncoding	-
MSTRG.8862.1	1214	coding	PF14713
MSTRG.8862.2	2083	noncoding	-
MSTRG.8863.1	19986	noncoding	-
MSTRG.8864.1	2975	coding	PF00078
MSTRG.8865.1	2992	noncoding	-
MSTRG.8881.2	1297	coding	PF00957
MSTRG.8888.1	12999	noncoding	-
MSTRG.8893.1	4407	noncoding	-
MSTRG.8894.1	31076	coding	PF00078
MSTRG.8898.11	4591	noncoding	-
MSTRG.8898.12	4108	noncoding	-
MSTRG.8898.13	4535	noncoding	-
MSTRG.8902.1	5031	coding	PF03638
MSTRG.8896.1	981	noncoding	-
MSTRG.8912.1	4510	noncoding	-
MSTRG.8912.2	2039	noncoding	-
MSTRG.8913.1	674	coding	PF01084
MSTRG.8913.2	773	coding	PF01084
MSTRG.8914.1	2223	noncoding	-
MSTRG.8914.2	1643	noncoding	-
MSTRG.8908.1	2673	noncoding	-
MSTRG.8916.1	538	noncoding	-
MSTRG.8919.5	14067	coding	PF02138
MSTRG.8919.3	14499	coding	PF02138
MSTRG.8919.1	14812	coding	PF02138
MSTRG.8919.2	14750	coding	PF02138
MSTRG.8919.6	14795	coding	PF02138
MSTRG.8919.7	14022	coding	PF02138
MSTRG.8919.8	14162	coding	PF02138
MSTRG.8919.9	14110	coding	PF02138
MSTRG.8920.1	458	noncoding	-
MSTRG.8920.2	467	noncoding	-
MSTRG.8918.2	2660	coding	PF00620
MSTRG.8932.2	2445	coding	PF00069
MSTRG.8941.1	13154	noncoding	-
MSTRG.8943.1	10198	coding	PF16599
MSTRG.8943.2	9631	coding	PF16599
MSTRG.8943.3	5991	coding	PF16599
MSTRG.8943.4	5448	coding	PF16599
MSTRG.8943.5	5997	coding	PF16599
MSTRG.8943.6	3830	noncoding	-
MSTRG.8943.7	8069	coding	PF16599
MSTRG.8943.8	6863	coding	PF16599
MSTRG.8943.9	6392	coding	PF16599
MSTRG.8943.10	6301	coding	PF16599
MSTRG.8943.11	6404	coding	PF16599
MSTRG.8943.12	6635	coding	PF16599
MSTRG.8943.13	5607	coding	PF16599
MSTRG.8923.1	2703	coding	PF15022

MSTRG.8923.4	1192	coding	PF01758
MSTRG.8923.3	1633	coding	PF15022
MSTRG.8924.1	759	noncoding	-
MSTRG.8928.5	9773	coding	PF05110
MSTRG.8929.1	9545	noncoding	-
MSTRG.8949.1	2269	noncoding	-
MSTRG.8949.2	520	noncoding	-
MSTRG.8951.2	3728	noncoding	-
MSTRG.8952.1	315	noncoding	-
MSTRG.8954.1	5014	noncoding	-
MSTRG.8955.1	822	noncoding	-
MSTRG.8957.1	534	noncoding	-
MSTRG.8961.2	578	noncoding	-
MSTRG.8962.2	398	coding	PF10496
MSTRG.8962.3	7436	coding	PF10496
MSTRG.8966.1	1101	noncoding	-
MSTRG.8967.3	1483	noncoding	-
MSTRG.8971.2	2799	noncoding	-
MSTRG.8972.2	6772	noncoding	-
MSTRG.8976.1	297	noncoding	-
MSTRG.8981.1	410	noncoding	-
MSTRG.8994.1	7114	coding	PF16367
MSTRG.8994.6	5355	coding	PF16367
MSTRG.8994.2	6390	coding	PF16367
MSTRG.8994.4	6849	coding	PF16367
MSTRG.8994.7	1314	noncoding	-
MSTRG.8995.1	5347	noncoding	-
MSTRG.8996.1	696	noncoding	-
MSTRG.8996.2	759	noncoding	-
MSTRG.8996.3	718	noncoding	-
MSTRG.9002.2	3060	coding	PF12937
MSTRG.9000.2	851	noncoding	-
MSTRG.9000.3	2867	noncoding	-
MSTRG.9000.4	2892	noncoding	-
MSTRG.9003.2	4262	coding	PF02267
MSTRG.9010.1	4819	coding	PF05478
MSTRG.9010.2	5740	coding	PF05478
MSTRG.9010.3	3811	coding	PF05478
MSTRG.9010.4	3588	coding	PF05478
MSTRG.9010.5	3600	coding	PF05478
MSTRG.9010.6	4494	coding	PF05478
MSTRG.9010.7	3418	coding	PF05478
MSTRG.9010.9	6494	noncoding	-
MSTRG.9010.10	4923	noncoding	-
MSTRG.9010.11	1627	noncoding	-
MSTRG.9010.12	1900	noncoding	-
MSTRG.9010.13	866	noncoding	-
MSTRG.9005.1	395	noncoding	-
MSTRG.9008.1	515	noncoding	-
MSTRG.9011.1	1085	noncoding	-
MSTRG.9025.1	1200	noncoding	-
MSTRG.9027.2	1113	noncoding	-
MSTRG.9029.3	15751	noncoding	-
MSTRG.9029.4	8624	coding	PF15376

MSTRG.9029.5	7846	noncoding	-
MSTRG.9034.1	1477	noncoding	-
MSTRG.9034.2	3875	noncoding	-
MSTRG.9036.2	1900	coding	PF01025
MSTRG.9036.3	1686	coding	PF01025
MSTRG.9038.1	998	noncoding	-
MSTRG.9040.3	7476	noncoding	-
MSTRG.9040.4	2702	noncoding	-
MSTRG.9041.1	1854	coding	PF16182
MSTRG.9041.2	2137	coding	PF16182
MSTRG.9042.1	757	noncoding	-
MSTRG.9042.2	693	noncoding	-
MSTRG.9043.1	368	noncoding	-
MSTRG.9045.2	887	coding	PF01756
MSTRG.9046.2	1829	coding	PF07757
MSTRG.9050.1	2242	noncoding	-
MSTRG.9051.1	2858	noncoding	-
MSTRG.9053.3	498	noncoding	-
MSTRG.9053.5	495	noncoding	-
MSTRG.9053.6	1015	noncoding	-
MSTRG.9053.4	4312	coding	PF16613
MSTRG.9053.7	2002	noncoding	-
MSTRG.9054.1	1000	noncoding	-
MSTRG.9055.1	9922	noncoding	-
MSTRG.9055.4	10147	noncoding	-
MSTRG.9058.1	4231	coding	PF00069
MSTRG.9058.2	2835	coding	PF00069
MSTRG.9058.3	2966	noncoding	-
MSTRG.9058.4	4313	noncoding	-
MSTRG.9058.8	5725	noncoding	-
MSTRG.9058.10	1774	noncoding	-
MSTRG.9058.11	1764	coding	PF00069
MSTRG.9058.5	1427	noncoding	-
MSTRG.9058.6	1748	coding	PF00069
MSTRG.9058.7	1327	coding	PF00069
MSTRG.9058.9	1812	coding	PF00069
MSTRG.9058.12	1998	coding	PF00069
MSTRG.9058.14	1876	coding	PF00069
MSTRG.9059.3	768	coding	PF04147
MSTRG.9059.2	2931	coding	PF04147
MSTRG.9062.6	3778	coding	PF00596
MSTRG.9056.2	3908	noncoding	-
MSTRG.9081.6	3138	noncoding	-
MSTRG.9081.7	5036	coding	PF15914
MSTRG.9081.5	7858	coding	PF15914
MSTRG.9081.3	5084	coding	PF15914
MSTRG.9081.1	4800	coding	PF15914
MSTRG.9081.4	4914	coding	PF15914
MSTRG.9081.8	5954	coding	PF15914
MSTRG.9081.9	4610	coding	PF15914
MSTRG.9081.10	7584	noncoding	-
MSTRG.9081.11	3778	noncoding	-
MSTRG.9081.12	6322	noncoding	-
MSTRG.9082.1	14778	coding	PF00078

MSTRG.9082.2	4575	noncoding	-
MSTRG.9083.1	1162	noncoding	-
MSTRG.9084.1	4332	noncoding	-
MSTRG.9083.10	3135	noncoding	-
MSTRG.9083.12	3152	noncoding	-
MSTRG.9083.13	3155	noncoding	-
MSTRG.9083.15	3887	noncoding	-
MSTRG.9083.9	3530	noncoding	-
MSTRG.9083.4	3902	noncoding	-
MSTRG.9083.8	2867	noncoding	-
MSTRG.9083.7	3940	noncoding	-
MSTRG.9083.16	1128	noncoding	-
MSTRG.9085.2	1601	noncoding	-
MSTRG.9072.1	873	noncoding	-
MSTRG.9075.2	2387	noncoding	-
MSTRG.9089.1	753	coding	PF15161
MSTRG.9090.2	1987	noncoding	-
MSTRG.9090.3	2501	noncoding	-
MSTRG.9091.4	4230	coding	PF00856
MSTRG.9091.5	7831	coding	PF00855
MSTRG.9091.6	6947	coding	PF00855
MSTRG.9091.8	10980	coding	PF00078
MSTRG.9092.1	2954	noncoding	-
MSTRG.9093.4	3809	coding	PF05010
MSTRG.9094.2	2217	coding	PF10272
MSTRG.9097.1	3495	noncoding	-
MSTRG.9097.2	1076	coding	PF15242
MSTRG.9103.5	10161	coding	PF09740
MSTRG.9103.2	10023	coding	PF09740
MSTRG.9104.3	3751	coding	PF10607
MSTRG.9104.4	2113	coding	PF10607
MSTRG.9104.5	2388	coding	PF10607
MSTRG.9104.7	2604	coding	PF10607
MSTRG.9104.6	2315	coding	PF10607
MSTRG.9104.8	4429	noncoding	-
MSTRG.9100.2	659	noncoding	-
MSTRG.9107.8	2516	coding	PF02826
MSTRG.9107.6	2901	coding	PF02826
MSTRG.9108.1	610	noncoding	-
MSTRG.9111.1	504	noncoding	-
MSTRG.9112.2	1325	noncoding	-
MSTRG.9113.1	877	noncoding	-
MSTRG.9110.1	713	noncoding	-
MSTRG.9116.3	3091	coding	PF01229
MSTRG.9125.6	2594	coding	PF06736
MSTRG.9125.2	2621	coding	PF06736
MSTRG.9125.3	2700	coding	PF06736
MSTRG.9125.4	2660	coding	PF06736
MSTRG.9125.5	2804	coding	PF06736
MSTRG.9126.4	7735	coding	PF10409
MSTRG.9127.1	31197	noncoding	-
MSTRG.9120.2	1913	coding	PF16207
MSTRG.9120.3	5300	noncoding	-
MSTRG.9123.1	1171	noncoding	-

MSTRG.9123.2	1107	noncoding	-
MSTRG.9129.1	871	noncoding	-
MSTRG.9129.2	853	noncoding	-
MSTRG.9128.1	1701	noncoding	-
MSTRG.9133.3	2840	noncoding	-
MSTRG.9136.1	610	noncoding	-
MSTRG.9138.1	3197	noncoding	-
MSTRG.9138.3	4835	noncoding	-
MSTRG.9139.1	1099	noncoding	-
MSTRG.9138.4	2184	noncoding	-
MSTRG.9140.1	7114	noncoding	-
MSTRG.9140.3	1348	noncoding	-
MSTRG.9142.1	393	noncoding	-
MSTRG.9145.3	3158	coding	PF01380
MSTRG.9145.5	3007	coding	PF01380
MSTRG.9145.2	3315	coding	PF01380
MSTRG.9145.4	3142	coding	PF01380
MSTRG.9145.6	2552	coding	PF01380
MSTRG.9147.1	3501	noncoding	-
MSTRG.9146.4	3094	coding	PF00069
MSTRG.9146.5	4194	coding	PF00069
MSTRG.9144.3	1794	noncoding	-
MSTRG.9144.2	3881	noncoding	-
MSTRG.9157.3	2116	coding	PF16577
MSTRG.9157.4	2159	noncoding	-
MSTRG.9153.3	4248	coding	PF00262
MSTRG.9148.1	3397	coding	PF14645
MSTRG.9150.1	788	coding	PF14645
MSTRG.9150.2	785	coding	PF14645
MSTRG.9175.2	2479	coding	PF08080
MSTRG.9175.4	2324	coding	PF08080
MSTRG.9175.10	1781	coding	PF08080
MSTRG.9175.9	1811	coding	PF08080
MSTRG.9175.8	2033	coding	PF08080
MSTRG.9167.2	4115	coding	PF05986
MSTRG.9168.1	417	noncoding	-
MSTRG.9188.1	871	noncoding	-
MSTRG.9190.1	1189	noncoding	-
MSTRG.9190.3	619	noncoding	-
MSTRG.9190.2	440	noncoding	-
MSTRG.9194.1	4099	noncoding	-
MSTRG.9194.2	2088	noncoding	-
MSTRG.9193.3	10040	coding	PF13765
MSTRG.9202.1	3374	coding	PF00069
MSTRG.9203.1	423	coding	PF00069
MSTRG.9205.1	4867	coding	PF07679
MSTRG.9205.2	1315	coding	PF07679
MSTRG.9206.1	2617	noncoding	-
MSTRG.9209.2	1956	noncoding	-
MSTRG.9209.3	3649	noncoding	-
MSTRG.9211.3	780	coding	PF09776
MSTRG.9211.4	1720	coding	PF09776
MSTRG.9217.2	1068	coding	PF00089
MSTRG.9230.1	716	coding	PF07562

MSTRG.9231.1	4094	noncoding	-
MSTRG.9231.3	3571	noncoding	-
MSTRG.9233.1	4013	noncoding	-
MSTRG.9233.5	3897	noncoding	-
MSTRG.9235.3	1753	coding	PF03792
MSTRG.9235.4	2477	coding	PF03792
MSTRG.9239.6	5819	noncoding	-
MSTRG.9239.9	2881	noncoding	-
MSTRG.9239.8	3497	noncoding	-
MSTRG.9240.1	2203	coding	PF00193
MSTRG.9249.4	2584	noncoding	-
MSTRG.9249.5	2361	noncoding	-
MSTRG.9250.6	3909	coding	PF01585
MSTRG.9250.7	5883	coding	PF01585
MSTRG.9250.8	4003	coding	PF01585
MSTRG.9250.10	3714	coding	PF01585
MSTRG.9250.9	6784	noncoding	-
MSTRG.9258.3	2559	coding	PF05735
MSTRG.9258.5	2827	coding	PF05735
MSTRG.9259.2	6857	coding	PF12885
MSTRG.9260.1	646	noncoding	-
MSTRG.9260.2	485	noncoding	-
MSTRG.9256.3	1365	coding	PF12280
MSTRG.9257.3	748	coding	PF14966
MSTRG.9264.2	522	coding	PF00240
MSTRG.9266.1	2186	coding	PF10241
MSTRG.9266.2	1956	coding	PF10241
MSTRG.9266.3	1550	coding	PF10241
MSTRG.9267.2	1882	coding	PF00254
MSTRG.9267.3	1958	coding	PF00254
MSTRG.9268.2	928	coding	PF07303
MSTRG.9269.1	1562	noncoding	-
MSTRG.9298.1	5327	noncoding	-
MSTRG.9298.18	11096	noncoding	-
MSTRG.9298.10	9865	noncoding	-
MSTRG.9298.17	5064	noncoding	-
MSTRG.9298.20	7805	noncoding	-
MSTRG.9298.11	11530	noncoding	-
MSTRG.9298.12	11187	noncoding	-
MSTRG.9298.13	4924	noncoding	-
MSTRG.9298.15	10278	noncoding	-
MSTRG.9298.16	11332	noncoding	-
MSTRG.9298.9	5080	noncoding	-
MSTRG.9298.24	3985	noncoding	-
MSTRG.9298.26	4080	noncoding	-
MSTRG.9298.25	3274	noncoding	-
MSTRG.9298.30	1130	noncoding	-
MSTRG.9298.31	1130	noncoding	-
MSTRG.9298.29	5736	noncoding	-
MSTRG.9298.33	4903	noncoding	-
MSTRG.9298.34	5181	noncoding	-
MSTRG.9298.35	2407	noncoding	-
MSTRG.9298.36	2377	noncoding	-
MSTRG.9278.2	1007	coding	PF03227

MSTRG.9281.1	927	noncoding	-
MSTRG.9283.2	1100	coding	PF06244
MSTRG.9284.1	636	noncoding	-
MSTRG.9285.2	804	coding	PF01775
MSTRG.9287.2	811	noncoding	-
MSTRG.9290.1	5238	noncoding	-
MSTRG.9291.1	7747	coding	PF06292
MSTRG.9291.3	5338	coding	PF10540
MSTRG.9295.1	10724	coding	PF01182
MSTRG.9295.2	10643	coding	PF01182
MSTRG.9295.4	3550	noncoding	-
MSTRG.9296.1	1850	coding	PF13905
MSTRG.9303.2	1661	coding	PF10172
MSTRG.9304.1	1715	noncoding	-
MSTRG.9305.2	1476	noncoding	-
MSTRG.9305.3	866	noncoding	-
MSTRG.9304.3	2159	noncoding	-
MSTRG.9306.1	957	noncoding	-
MSTRG.9306.2	1043	coding	PF00105
MSTRG.9307.2	1626	coding	PF00104
MSTRG.9308.4	1321	coding	PF07303
MSTRG.9312.11	7435	coding	PF00063
MSTRG.9312.12	7456	coding	PF00063
MSTRG.9312.13	7417	coding	PF00063
MSTRG.9312.14	7429	coding	PF00063
MSTRG.9312.3	7387	coding	PF00063
MSTRG.9312.4	7420	coding	PF00063
MSTRG.9312.9	7411	coding	PF00063
MSTRG.9312.15	7605	coding	PF00063
MSTRG.9312.23	3838	coding	PF00063
MSTRG.9312.24	15200	noncoding	-
MSTRG.9312.25	836	noncoding	-
MSTRG.9310.2	4134	coding	PF07678
MSTRG.9315.1	13155	noncoding	-
MSTRG.9316.1	219	noncoding	-
MSTRG.9315.3	14832	noncoding	-
MSTRG.9321.3	14721	noncoding	-
MSTRG.9321.4	19190	noncoding	-
MSTRG.9322.1	6551	noncoding	-
MSTRG.9325.2	7606	noncoding	-
MSTRG.9327.4	3587	coding	PF15391
MSTRG.9335.3	8683	noncoding	-
MSTRG.9335.4	8252	noncoding	-
MSTRG.9333.2	3018	coding	PF00928
MSTRG.9338.1	1776	noncoding	-
MSTRG.9361.4	4447	noncoding	-
MSTRG.9361.6	4165	noncoding	-
MSTRG.9361.7	4166	noncoding	-
MSTRG.9361.8	4975	noncoding	-
MSTRG.9361.9	5131	noncoding	-
MSTRG.9361.10	4369	noncoding	-
MSTRG.9361.11	3862	noncoding	-
MSTRG.9361.13	3643	noncoding	-
MSTRG.9361.14	3538	noncoding	-

MSTRG.9365.1	525	noncoding	-
MSTRG.9364.12	3366	noncoding	-
MSTRG.9364.13	3123	coding	PF04988
MSTRG.9367.2	10602	coding	PF17035
MSTRG.9367.3	5963	coding	PF17035
MSTRG.9367.4	5532	coding	PF17035
MSTRG.9367.6	2933	coding	PF17035
MSTRG.9367.9	5709	coding	PF17035
MSTRG.9367.10	7623	coding	PF17035
MSTRG.9368.1	10623	coding	PF01359
MSTRG.9367.12	25987	coding	PF17035
MSTRG.9343.3	528	noncoding	-
MSTRG.9349.1	2549	coding	PF00517
MSTRG.9353.1	501	noncoding	-
MSTRG.9358.4	2411	coding	PF01556
MSTRG.9358.1	2348	coding	PF01556
MSTRG.9358.3	2563	coding	PF01556
MSTRG.9358.5	2396	coding	PF01556
MSTRG.9358.6	2078	coding	PF00226
MSTRG.9360.3	2413	coding	PF00069
MSTRG.9369.3	3683	coding	PF00270
MSTRG.9369.4	3514	coding	PF00270
MSTRG.9369.5	2075	coding	PF00270
MSTRG.9374.1	5218	coding	PF02354
MSTRG.9374.2	7393	coding	PF02191
MSTRG.9374.3	7914	coding	PF02354
MSTRG.9374.5	5102	coding	PF02354
MSTRG.9374.4	6853	coding	PF02191
MSTRG.9374.7	5107	coding	PF02354
MSTRG.9372.2	5343	coding	PF00069
MSTRG.9372.3	5004	coding	PF00069
MSTRG.9373.3	3708	coding	PF11771
MSTRG.9377.2	1330	noncoding	-
MSTRG.9386.6	1242	coding	PF04589
MSTRG.9386.4	4693	coding	PF04589
MSTRG.9379.1	738	noncoding	-
MSTRG.9382.1	765	noncoding	-
MSTRG.9385.2	1903	coding	PF04502
MSTRG.9385.3	1516	coding	PF04502
MSTRG.9396.1	3847	coding	PF00651
MSTRG.9396.2	3551	coding	PF00651
MSTRG.9396.3	3707	coding	PF00651
MSTRG.9398.4	2456	coding	PF02005
MSTRG.9398.3	2377	coding	PF02005
MSTRG.9394.1	5941	coding	PF00859
MSTRG.9394.2	5654	coding	PF00859
MSTRG.9394.3	5529	coding	PF00859
MSTRG.9394.4	4045	coding	PF00859
MSTRG.9393.1	2154	noncoding	-
MSTRG.9405.6	797	noncoding	-
MSTRG.9407.3	1387	coding	PF01351
MSTRG.9410.1	2509	coding	PF05622
MSTRG.9420.2	800	coding	PF02374
MSTRG.9422.1	7773	noncoding	-

MSTRG.9422.4	3257	noncoding	-
MSTRG.9423.2	1627	coding	PF01916
MSTRG.9423.3	1267	coding	PF01916
MSTRG.9423.4	1247	coding	PF01916
MSTRG.9425.2	1139	noncoding	-
MSTRG.9411.2	714	coding	PF03669
MSTRG.9413.2	586	coding	PF01352
MSTRG.9428.2	3386	coding	PF01352
MSTRG.9436.1	20970	coding	PF01390
MSTRG.9436.2	21306	coding	PF01390
MSTRG.9436.3	20731	coding	PF01390
MSTRG.9436.4	20970	coding	PF01390
MSTRG.9436.5	13848	coding	PF01390
MSTRG.9436.6	13954	coding	PF01390
MSTRG.9436.10	9779	coding	PF01390
MSTRG.9436.11	10112	coding	PF01390
MSTRG.9436.15	9922	coding	PF01390
MSTRG.9436.12	18799	coding	PF01390
MSTRG.9436.13	18318	coding	PF01390
MSTRG.9436.14	17545	coding	PF01390
MSTRG.9436.9	18693	coding	PF01390
MSTRG.9436.16	20048	coding	PF01390
MSTRG.9436.17	19571	coding	PF01390
MSTRG.9436.18	20770	coding	PF01390
MSTRG.9436.19	20434	coding	PF01390
MSTRG.9431.3	5175	coding	PF01352
MSTRG.9431.4	4146	coding	PF01352
MSTRG.9445.1	8490	coding	PF00078
MSTRG.9446.2	2297	noncoding	-
MSTRG.9446.3	709	noncoding	-
MSTRG.9446.4	700	noncoding	-
MSTRG.9446.6	601	noncoding	-
MSTRG.9449.1	3028	coding	PF01352
MSTRG.9449.2	551	coding	PF01352
MSTRG.9449.4	1612	coding	PF01352
MSTRG.9449.3	2779	coding	PF01352
MSTRG.9449.5	532	noncoding	-
MSTRG.9445.3	18147	coding	PF02994
MSTRG.9445.4	2291	coding	PF01352
MSTRG.9445.6	1269	noncoding	-
MSTRG.9453.1	759	noncoding	-
MSTRG.9453.2	744	noncoding	-
MSTRG.9453.3	2119	noncoding	-
MSTRG.9434.2	1218	coding	PF13616
MSTRG.9437.2	3489	noncoding	-
MSTRG.9441.1	2245	coding	PF00147
MSTRG.9458.2	5502	coding	PF12047
MSTRG.9458.4	6508	coding	PF12047
MSTRG.9457.1	2487	noncoding	-
MSTRG.9464.2	586	noncoding	-
MSTRG.9464.1	3160	noncoding	-
MSTRG.9464.3	3064	noncoding	-
MSTRG.9464.4	774	noncoding	-
MSTRG.9464.5	1718	noncoding	-

MSTRG.9460.1	3357	noncoding	-
MSTRG.9471.5	3902	coding	PF00233
MSTRG.9471.6	2525	coding	PF00233
MSTRG.9471.7	2511	coding	PF00233
MSTRG.9471.8	1841	coding	PF00233
MSTRG.9473.1	7883	coding	PF00651
MSTRG.9473.2	3020	coding	PF00651
MSTRG.9473.3	8243	coding	PF00651
MSTRG.9473.4	2542	coding	PF00651
MSTRG.9473.5	2308	coding	PF00651
MSTRG.9468.2	1609	coding	PF03416
MSTRG.9468.3	1674	coding	PF03416
MSTRG.9481.4	3794	coding	PF07528
MSTRG.9481.11	2101	noncoding	-
MSTRG.9481.17	3993	coding	PF07528
MSTRG.9481.14	3776	coding	PF07528
MSTRG.9481.15	3629	coding	PF07528
MSTRG.9481.16	3764	coding	PF07528
MSTRG.9481.9	3740	coding	PF07528
MSTRG.9481.10	6445	coding	PF07528
MSTRG.9481.18	6359	coding	PF07528
MSTRG.9481.7	6421	coding	PF07528
MSTRG.9477.2	1342	noncoding	-
MSTRG.9478.4	1406	coding	PF01702
MSTRG.9482.7	4155	coding	PF01031
MSTRG.9484.1	749	noncoding	-
MSTRG.9486.2	2652	noncoding	-
MSTRG.9493.6	6649	coding	PF00176
MSTRG.9493.7	4276	coding	PF00176
MSTRG.9493.8	2271	coding	PF00176
MSTRG.9496.4	6424	coding	PF06920
MSTRG.9498.2	1662	coding	PF00071
MSTRG.9498.8	914	coding	PF13664
MSTRG.9499.1	3160	noncoding	-
MSTRG.9499.2	2350	noncoding	-
MSTRG.9499.4	2634	noncoding	-
MSTRG.9499.5	1511	noncoding	-
MSTRG.9500.1	2472	noncoding	-
MSTRG.9500.3	2698	noncoding	-
MSTRG.9500.4	2739	noncoding	-
MSTRG.9503.1	2169	coding	PF12999
MSTRG.9503.2	2204	coding	PF12999
MSTRG.9503.3	2197	coding	PF12999
MSTRG.9503.4	2139	coding	PF12999
MSTRG.9503.6	2219	coding	PF12999
MSTRG.9505.2	2076	noncoding	-
MSTRG.9505.3	3292	noncoding	-
MSTRG.9505.4	3149	coding	PF06239
MSTRG.9505.5	4086	coding	PF06239
MSTRG.9505.6	1953	coding	PF06239
MSTRG.9509.1	1099	coding	PF05129
MSTRG.9509.3	631	coding	PF05129
MSTRG.9516.4	5786	coding	PF00621
MSTRG.9516.6	2380	noncoding	-

MSTRG.9516.8	1603	noncoding	-
MSTRG.9516.7	5061	coding	PF00621
MSTRG.9518.2	4566	coding	PF00027
MSTRG.9518.3	4298	coding	PF00027
MSTRG.9522.1	3639	coding	PF08683
MSTRG.9523.2	440	coding	PF09803
MSTRG.9526.2	2011	coding	PF00995
MSTRG.9532.1	583	noncoding	-
MSTRG.9534.1	5014	coding	PF00076
MSTRG.9535.1	6213	noncoding	-
MSTRG.9535.2	1584	noncoding	-
MSTRG.9535.3	6955	noncoding	-
MSTRG.9536.1	5671	coding	PF00683
MSTRG.9543.1	1065	noncoding	-
MSTRG.9543.2	865	noncoding	-
MSTRG.9550.1	1206	noncoding	-
MSTRG.9547.1	3993	coding	PF01562
MSTRG.9554.1	1343	coding	PF00076
MSTRG.9555.1	1054	coding	PF00076
MSTRG.9556.1	1310	noncoding	-
MSTRG.9556.2	1628	coding	PF16564
MSTRG.9556.3	1372	noncoding	-
MSTRG.9563.1	2165	noncoding	-
MSTRG.9563.2	2006	noncoding	-
MSTRG.9564.2	2842	noncoding	-
MSTRG.9565.4	1795	coding	PF06814
MSTRG.9565.5	1851	coding	PF06814
MSTRG.9566.2	1624	noncoding	-
MSTRG.9566.3	1828	noncoding	-
MSTRG.9574.3	5756	coding	PF00153
MSTRG.9574.4	3679	coding	PF00013
MSTRG.9574.5	4750	coding	PF00013
MSTRG.9574.6	4273	coding	PF00013
MSTRG.9574.7	3335	coding	PF00013
MSTRG.9576.1	1849	coding	PF05793
MSTRG.9576.2	1816	coding	PF05793
MSTRG.9576.3	1895	coding	PF05793
MSTRG.9567.2	451	noncoding	-
MSTRG.9567.3	625	noncoding	-
MSTRG.9567.4	656	noncoding	-
MSTRG.9568.2	1116	coding	PF00574
MSTRG.9571.1	977	noncoding	-
MSTRG.9577.4	1886	coding	PF00501
MSTRG.9586.3	3949	noncoding	-
MSTRG.9587.2	2032	noncoding	-
MSTRG.9589.3	2184	coding	PF01207
MSTRG.9589.5	1350	coding	PF15776
MSTRG.9590.2	3354	coding	PF15020
MSTRG.9590.3	5967	coding	PF15020
MSTRG.9590.5	1510	coding	PF15020
MSTRG.9590.7	2215	coding	PF15020
MSTRG.9590.6	2346	coding	PF15020
MSTRG.9591.1	2807	coding	PF05362
MSTRG.9591.3	3207	coding	PF05362

MSTRG.9598.10	2505	noncoding	-
MSTRG.9598.3	2211	noncoding	-
MSTRG.9598.4	1324	noncoding	-
MSTRG.9598.6	1287	noncoding	-
MSTRG.9598.7	1651	noncoding	-
MSTRG.9598.8	1255	noncoding	-
MSTRG.9598.9	2921	noncoding	-
MSTRG.9599.1	981	coding	PF15884
MSTRG.9599.3	836	coding	PF15884
MSTRG.9599.4	824	coding	PF15884
MSTRG.9600.12	8791	noncoding	-
MSTRG.9600.8	2864	noncoding	-
MSTRG.9600.13	4505	noncoding	-
MSTRG.9600.7	2931	noncoding	-
MSTRG.9600.9	2931	noncoding	-
MSTRG.9600.16	1980	noncoding	-
MSTRG.9602.2	2672	noncoding	-
MSTRG.9594.1	1203	coding	PF13639
MSTRG.9594.2	860	coding	PF13639
MSTRG.9594.3	1241	coding	PF13639
MSTRG.9594.5	1623	coding	PF13639
MSTRG.9597.1	5300	coding	PF13832
MSTRG.9597.2	5198	coding	PF13832
MSTRG.9597.3	5782	coding	PF13832
MSTRG.9597.4	5819	coding	PF13832
MSTRG.9604.3	4186	coding	PF12148
MSTRG.9604.4	2587	coding	PF12148
MSTRG.9605.1	454	noncoding	-
MSTRG.9605.2	313	noncoding	-
MSTRG.9606.1	999	noncoding	-
MSTRG.9610.1	581	noncoding	-
MSTRG.9610.2	718	noncoding	-
MSTRG.9612.2	3155	coding	PF05527
MSTRG.9612.3	2871	coding	PF05527
MSTRG.9613.1	570	noncoding	-
MSTRG.9612.4	849	noncoding	-
MSTRG.9603.1	640	noncoding	-
MSTRG.9617.3	1787	coding	PF09409
MSTRG.9619.2	3845	coding	PF15539
MSTRG.9620.1	958	noncoding	-
MSTRG.9620.2	922	noncoding	-
MSTRG.9624.3	1492	coding	PF04502
MSTRG.9625.1	2048	noncoding	-
MSTRG.9626.1	1764	coding	PF02146
MSTRG.9626.2	1646	coding	PF02146
MSTRG.9630.2	2132	coding	PF02891
MSTRG.9634.3	2339	noncoding	-
MSTRG.9634.4	1098	noncoding	-
MSTRG.9633.3	1496	noncoding	-
MSTRG.9633.2	1785	noncoding	-
MSTRG.9638.1	2344	noncoding	-
MSTRG.9640.4	3904	coding	PF01504
MSTRG.9649.4	1926	noncoding	-
MSTRG.9643.1	1238	noncoding	-

MSTRG.9651.6	17190	coding	PF00859
MSTRG.9650.1	340	noncoding	-
MSTRG.9653.1	3385	coding	PF00503
MSTRG.9653.2	5509	coding	PF00503
MSTRG.9655.3	2530	coding	PF03920
MSTRG.9657.1	1283	coding	PF01352
MSTRG.9658.4	4658	coding	PF01352
MSTRG.9661.1	1177	noncoding	-
MSTRG.9662.4	2825	coding	PF16546
MSTRG.9663.3	1636	noncoding	-
MSTRG.9663.4	1833	noncoding	-
MSTRG.9665.1	3453	coding	PF00631
MSTRG.9665.2	3938	coding	PF00631
MSTRG.9665.3	3451	coding	PF00631
MSTRG.9665.5	3403	coding	PF00631
MSTRG.9667.2	4386	coding	PF00038
MSTRG.9667.3	3983	coding	PF00038
MSTRG.9675.2	688	coding	PF01423
MSTRG.9675.3	739	coding	PF01423
MSTRG.9675.4	506	coding	PF01423
MSTRG.9670.2	3778	coding	PF07679
MSTRG.9671.2	1013	coding	PF02100
MSTRG.9676.2	7608	coding	PF08123
MSTRG.9676.3	1782	coding	PF08123
MSTRG.9685.1	1507	noncoding	-
MSTRG.9689.1	3011	coding	PF03637
MSTRG.9689.3	2752	coding	PF03637
MSTRG.9696.2	9125	coding	PF00225
MSTRG.9696.5	3139	coding	PF00225
MSTRG.9692.1	3016	coding	PF00605
MSTRG.9693.2	1457	coding	PF00605
MSTRG.9691.2	5984	noncoding	-
MSTRG.9706.1	2456	coding	PF08336
MSTRG.9706.5	2229	coding	PF08336
MSTRG.9708.1	1310	noncoding	-
MSTRG.9719.1	789	coding	PF16121
MSTRG.9728.1	4329	noncoding	-
MSTRG.9740.1	590	noncoding	-
MSTRG.9742.1	2788	coding	PF00501
MSTRG.9742.2	2743	coding	PF00501
MSTRG.9742.3	2812	coding	PF00501
MSTRG.9742.4	2745	coding	PF00501
MSTRG.9742.8	3674	coding	PF00501
MSTRG.9742.9	2600	coding	PF00501
MSTRG.9742.13	3947	coding	PF00501
MSTRG.9742.15	8157	noncoding	-
MSTRG.9742.17	995	noncoding	-
MSTRG.9742.18	1669	noncoding	-
MSTRG.9743.1	1285	noncoding	-
MSTRG.9743.2	991	noncoding	-
MSTRG.9745.1	4452	coding	PF14637
MSTRG.9745.3	7179	coding	PF14637
MSTRG.9745.6	4236	coding	PF00617
MSTRG.9732.2	3354	noncoding	-

MSTRG.9734.2	1724	noncoding	-
MSTRG.9734.5	462	noncoding	-
MSTRG.9734.4	662	noncoding	-
MSTRG.9735.1	541	coding	PF01230
MSTRG.9738.1	3166	coding	PF05679
MSTRG.9747.1	734	noncoding	-
MSTRG.9753.1	801	noncoding	-
MSTRG.9756.1	240	noncoding	-
MSTRG.9760.1	1148	noncoding	-
MSTRG.9760.2	950	noncoding	-
MSTRG.9760.3	596	noncoding	-
MSTRG.9773.1	4335	coding	PF00171
MSTRG.9774.1	3883	noncoding	-
MSTRG.9774.2	2871	noncoding	-
MSTRG.9774.4	2866	noncoding	-
MSTRG.9775.1	4464	noncoding	-
MSTRG.9780.1	628	coding	PF00078
MSTRG.9798.1	247	noncoding	-
MSTRG.9815.16	1463	noncoding	-
MSTRG.9815.18	14104	noncoding	-
MSTRG.9815.19	4246	noncoding	-
MSTRG.9815.20	12184	coding	PF00078
MSTRG.9815.21	10165	noncoding	-
MSTRG.9816.1	291	noncoding	-
MSTRG.9815.22	3871	noncoding	-
MSTRG.9815.23	9434	noncoding	-
MSTRG.9815.24	1198	noncoding	-
MSTRG.9815.25	19800	noncoding	-
MSTRG.9815.26	4764	noncoding	-
MSTRG.9815.27	4613	noncoding	-
MSTRG.9817.1	36278	noncoding	-
MSTRG.9817.2	537	noncoding	-
MSTRG.9811.1	450	noncoding	-
MSTRG.9812.2	4186	coding	PF00069
MSTRG.9814.2	4649	coding	PF12416
MSTRG.9827.1	2180	noncoding	-
MSTRG.9827.3	648	noncoding	-
MSTRG.9849.1	3284	noncoding	-
MSTRG.9854.3	11619	coding	PF12234
MSTRG.9858.2	2600	coding	PF03942
MSTRG.9859.1	3894	noncoding	-
MSTRG.9859.2	4656	noncoding	-
MSTRG.9862.1	2769	noncoding	-
MSTRG.9863.1	2198	noncoding	-
MSTRG.9878.1	2388	noncoding	-
MSTRG.9884.1	1221	noncoding	-
MSTRG.9889.1	5289	noncoding	-
MSTRG.9889.2	4037	noncoding	-
MSTRG.9890.3	1880	noncoding	-
MSTRG.9891.1	3892	coding	PF13639
MSTRG.9891.2	3269	coding	PF13639
MSTRG.9891.3	3225	coding	PF13639
MSTRG.9891.4	3289	coding	PF13639
MSTRG.9891.6	3272	coding	PF13639

MSTRG.9891.7	3228	coding	PF13639
MSTRG.9891.8	3199	coding	PF13639
MSTRG.9891.9	3102	coding	PF13639
MSTRG.9891.10	3139	coding	PF13639
MSTRG.9891.11	3165	coding	PF13639
MSTRG.9893.1	468	noncoding	-
MSTRG.9894.1	2900	coding	PF00028
MSTRG.9894.2	2943	coding	PF00028
MSTRG.9894.3	2679	coding	PF00028
MSTRG.9898.1	647	noncoding	-
MSTRG.9898.2	636	noncoding	-
MSTRG.9902.6	2220	noncoding	-
MSTRG.9902.5	2375	noncoding	-
MSTRG.9902.9	4998	noncoding	-
MSTRG.9902.8	1755	noncoding	-
MSTRG.9902.10	1686	noncoding	-
MSTRG.9902.11	1170	noncoding	-
MSTRG.9899.3	2158	noncoding	-
MSTRG.9899.4	2061	noncoding	-
MSTRG.9899.5	2183	noncoding	-
MSTRG.9906.2	9366	coding	PF00856
MSTRG.9913.2	2270	coding	PF00412
MSTRG.9913.6	1594	noncoding	-
MSTRG.9915.2	2448	coding	PF00270
MSTRG.9918.1	625	noncoding	-
MSTRG.9919.2	1518	coding	PF13733
MSTRG.9919.3	1455	coding	PF13733
MSTRG.9919.4	1962	coding	PF13733
MSTRG.9924.3	1698	coding	PF00076
MSTRG.9929.1	3319	coding	PF00069
MSTRG.9929.6	2320	coding	PF00069
MSTRG.9929.5	2142	coding	PF00069
MSTRG.9934.3	3167	coding	PF03071
MSTRG.9941.2	1894	coding	PF13765
MSTRG.9944.1	4973	coding	PF00129
MSTRG.9944.2	3660	coding	PF00129
MSTRG.9944.3	1937	coding	PF00129
MSTRG.9948.1	2131	noncoding	-
MSTRG.9961.1	508	noncoding	-
MSTRG.9962.3	3487	coding	PF05276
MSTRG.9962.4	3328	coding	PF05276
MSTRG.9963.2	4092	noncoding	-
MSTRG.9960.1	1375	noncoding	-
MSTRG.9972.3	2182	noncoding	-
MSTRG.9972.4	2972	noncoding	-
MSTRG.9974.1	522	noncoding	-
MSTRG.9977.1	2871	noncoding	-
MSTRG.9977.2	1286	noncoding	-
MSTRG.9984.1	5234	coding	PF00940
MSTRG.9984.2	5678	coding	PF00940
MSTRG.9985.2	1242	coding	PF13639
MSTRG.9986.1	1908	noncoding	-
MSTRG.9986.3	2022	noncoding	-
MSTRG.9987.3	3387	coding	PF03285

MSTRG.9987.4	3425	coding	PF03285
MSTRG.9990.1	333	noncoding	-
MSTRG.9993.2	2518	noncoding	-
MSTRG.9994.1	1185	coding	PF00089
MSTRG.9997.1	1721	noncoding	-
MSTRG.9997.2	1502	noncoding	-
MSTRG.9997.3	2915	coding	PF01388
MSTRG.9997.4	3169	coding	PF01388
MSTRG.9997.5	1352	coding	PF01388
MSTRG.10000.1	1154	coding	PF00060
MSTRG.10001.1	2520	coding	PF09746
MSTRG.10001.4	1968	coding	PF09746
MSTRG.10002.1	3107	coding	PF00307
MSTRG.10005.2	3161	coding	PF03871
MSTRG.10005.1	2905	coding	PF03871
MSTRG.10008.4	8009	coding	PF13872
MSTRG.10009.2	4950	coding	PF00069
MSTRG.10011.1	949	coding	PF02823
MSTRG.10012.2	3207	noncoding	-
MSTRG.10012.3	3717	noncoding	-
MSTRG.10012.4	3313	noncoding	-
MSTRG.10013.1	694	noncoding	-
MSTRG.10014.1	2227	coding	PF00076
MSTRG.10014.3	2614	coding	PF00076
MSTRG.10016.1	2112	coding	PF00812
MSTRG.10020.4	1327	coding	PF00076
MSTRG.10026.10	2456	coding	PF01483
MSTRG.10026.13	1627	coding	PF01483
MSTRG.10026.11	1966	coding	PF01483
MSTRG.10026.12	1969	coding	PF01483
MSTRG.10026.18	1979	coding	PF01483
MSTRG.10024.2	3349	coding	PF05986
MSTRG.10024.3	3295	coding	PF05986
MSTRG.10025.1	531	noncoding	-
MSTRG.10031.1	1170	noncoding	-
MSTRG.10041.2	4753	coding	PF16212
MSTRG.10041.3	4683	coding	PF16212
MSTRG.10041.7	4586	coding	PF16212
MSTRG.10042.1	1600	noncoding	-
MSTRG.10043.2	4476	coding	PF15870
MSTRG.10043.5	4269	coding	PF15870
MSTRG.10043.6	4213	coding	PF15870
MSTRG.10045.2	2657	noncoding	-
MSTRG.10037.3	2345	coding	PF00069
MSTRG.10038.1	4682	noncoding	-
MSTRG.10038.2	1904	noncoding	-
MSTRG.10049.1	2978	coding	PF00019
MSTRG.10050.2	703	coding	PF02939
MSTRG.10052.2	10686	coding	PF05110
MSTRG.10053.1	1559	noncoding	-
MSTRG.10051.2	637	noncoding	-
MSTRG.10058.1	3609	noncoding	-
MSTRG.10060.1	4786	noncoding	-
MSTRG.10078.4	3578	coding	PF00069

MSTRG.10078.5	3702	coding	PF00069
MSTRG.10078.3	4343	coding	PF00069
MSTRG.10079.1	8499	coding	PF00179
MSTRG.10079.2	723	coding	PF00179
MSTRG.10079.3	820	coding	PF00179
MSTRG.10082.2	3852	coding	PF00025
MSTRG.10087.3	2359	coding	PF00270
MSTRG.10091.1	1709	noncoding	-
MSTRG.10091.2	1663	noncoding	-
MSTRG.10091.3	1067	noncoding	-
MSTRG.10092.1	1187	noncoding	-
MSTRG.10094.8	1816	coding	PF01661
MSTRG.10094.6	1895	coding	PF01661
MSTRG.10094.7	1920	coding	PF01661
MSTRG.10094.9	1807	coding	PF01661
MSTRG.10094.10	1869	coding	PF01661
MSTRG.10094.11	2038	coding	PF01661
MSTRG.10100.1	964	noncoding	-
MSTRG.10100.2	766	noncoding	-
MSTRG.10101.1	898	noncoding	-
MSTRG.10101.3	909	noncoding	-
MSTRG.10104.2	13849	noncoding	-
MSTRG.10105.2	3218	noncoding	-
MSTRG.10107.1	11339	coding	PF02469
MSTRG.10102.2	7489	coding	PF03166
MSTRG.10103.1	1101	noncoding	-
MSTRG.10121.1	7841	coding	PF00651
MSTRG.10121.3	7955	coding	PF00651
MSTRG.10121.4	3190	noncoding	-
MSTRG.10122.1	4192	coding	PF07679
MSTRG.10122.2	3985	coding	PF07679
MSTRG.10122.3	3703	coding	PF07679
MSTRG.10122.4	3797	coding	PF07679
MSTRG.10123.1	8867	noncoding	-
MSTRG.10122.5	2586	coding	PF07679
MSTRG.10122.6	2417	coding	PF07679
MSTRG.10122.7	2426	coding	PF07679
MSTRG.10122.8	2256	coding	PF07679
MSTRG.10133.9	3905	noncoding	-
MSTRG.10133.3	4689	coding	PF00620
MSTRG.10133.6	9938	coding	PF00620
MSTRG.10133.5	11159	coding	PF00620
MSTRG.10133.11	3436	coding	PF00620
MSTRG.10133.12	9585	coding	PF00620
MSTRG.10112.2	398	noncoding	-
MSTRG.10112.3	4954	coding	PF00439
MSTRG.10112.4	4541	coding	PF00439
MSTRG.10112.5	4672	coding	PF00439
MSTRG.10112.10	3653	coding	PF00439
MSTRG.10112.11	3578	coding	PF00439
MSTRG.10112.12	3164	coding	PF00439
MSTRG.10115.4	2425	noncoding	-
MSTRG.10115.3	2690	noncoding	-
MSTRG.10115.5	1224	noncoding	-

MSTRG.10116.2	4722	coding	PF15242
MSTRG.10134.1	1505	noncoding	-
MSTRG.10138.3	313	noncoding	-
MSTRG.10138.2	3591	coding	PF01044
MSTRG.10139.1	5083	noncoding	-
MSTRG.10138.7	3161	coding	PF01044
MSTRG.10142.1	2050	noncoding	-
MSTRG.10146.6	1797	noncoding	-
MSTRG.10146.5	1994	noncoding	-
MSTRG.10147.1	1969	noncoding	-
MSTRG.10148.1	4006	noncoding	-
MSTRG.10148.2	4612	noncoding	-
MSTRG.10148.3	4135	noncoding	-
MSTRG.10148.7	3928	noncoding	-
MSTRG.10148.9	4180	noncoding	-
MSTRG.10148.8	4967	noncoding	-
MSTRG.10148.6	10450	noncoding	-
MSTRG.10148.4	7953	noncoding	-
MSTRG.10143.2	760	coding	PF15175
MSTRG.10143.3	979	coding	PF15175
MSTRG.10155.1	611	noncoding	-
MSTRG.10159.1	5098	coding	PF01369
MSTRG.10160.1	3172	coding	PF02158
MSTRG.10162.1	744	noncoding	-
MSTRG.10172.2	749	noncoding	-
MSTRG.10172.5	5665	coding	PF04845
MSTRG.10172.6	863	noncoding	-
MSTRG.10173.1	2906	noncoding	-
MSTRG.10173.2	2083	noncoding	-
MSTRG.10172.8	697	noncoding	-
MSTRG.10172.9	1127	noncoding	-
MSTRG.10172.10	580	noncoding	-
MSTRG.10172.11	870	noncoding	-
MSTRG.10175.1	4985	noncoding	-
MSTRG.10175.2	11334	noncoding	-
MSTRG.10175.3	1882	noncoding	-
MSTRG.10175.4	728	noncoding	-
MSTRG.10175.5	1089	noncoding	-
MSTRG.10178.6	7482	coding	PF12796
MSTRG.10178.15	8245	coding	PF12796
MSTRG.10178.7	8267	coding	PF12796
MSTRG.10178.8	7628	coding	PF12796
MSTRG.10178.12	8197	coding	PF12796
MSTRG.10178.11	8851	coding	PF12796
MSTRG.10178.9	8725	coding	PF12796
MSTRG.10178.13	9402	coding	PF12796
MSTRG.10178.1	9485	coding	PF12796
MSTRG.10178.14	9397	coding	PF12796
MSTRG.10178.16	13864	coding	PF12796
MSTRG.10178.3	13972	coding	PF12796
MSTRG.10178.10	12117	coding	PF07304
MSTRG.10178.5	13917	coding	PF12796
MSTRG.10178.17	9564	coding	PF12796
MSTRG.10178.18	9510	coding	PF12796

MSTRG.10178.19	7125	coding	PF12796
MSTRG.10178.20	7251	coding	PF12796
MSTRG.10178.21	12830	coding	PF12796
MSTRG.10178.22	7529	coding	PF12796
MSTRG.10178.24	7285	coding	PF12796
MSTRG.10178.23	7285	coding	PF12796
MSTRG.10180.2	3679	coding	PF07304
MSTRG.10164.2	1923	noncoding	-
MSTRG.10164.1	2507	noncoding	-
MSTRG.10164.3	1934	noncoding	-
MSTRG.10183.4	3320	noncoding	-
MSTRG.10184.1	3567	noncoding	-
MSTRG.10185.1	684	noncoding	-
MSTRG.10195.1	1377	coding	PF08266
MSTRG.10198.1	3340	coding	PF08266
MSTRG.10197.5	1081	coding	PF00028
MSTRG.10197.6	2360	coding	PF08266
MSTRG.10208.1	13561	noncoding	-
MSTRG.10209.1	1210	noncoding	-
MSTRG.10197.7	4428	coding	PF08266
MSTRG.10197.8	798	coding	PF08266
MSTRG.10197.9	1596	coding	PF00028
MSTRG.10213.1	4194	coding	PF08266
MSTRG.10214.1	1122	coding	PF08266
MSTRG.10215.1	957	noncoding	-
MSTRG.10230.1	3238	noncoding	-
MSTRG.10231.1	8229	coding	PF08266
MSTRG.10231.2	4702	coding	PF08266
MSTRG.10232.1	249	coding	PF16492
MSTRG.10234.1	2035	coding	PF08266
MSTRG.10234.2	2167	coding	PF00028
MSTRG.10231.4	5186	coding	PF08266
MSTRG.10234.3	1025	coding	PF08266
MSTRG.10235.1	2146	coding	PF08266
MSTRG.10231.9	4716	coding	PF08266
MSTRG.10236.1	4768	coding	PF08266
MSTRG.10231.12	4929	coding	PF08266
MSTRG.10231.16	7459	coding	PF08266
MSTRG.10231.15	9437	coding	PF08266
MSTRG.10231.18	5983	coding	PF08266
MSTRG.10231.19	4326	coding	PF00028
MSTRG.10237.1	4934	noncoding	-
MSTRG.10238.1	5164	noncoding	-
MSTRG.10239.3	5492	coding	PF02181
MSTRG.10239.4	5648	coding	PF02181
MSTRG.10222.1	6273	noncoding	-
MSTRG.10219.1	262	noncoding	-
MSTRG.10219.2	374	noncoding	-
MSTRG.10219.3	318	noncoding	-
MSTRG.10243.7	6884	coding	PF01485
MSTRG.10243.8	8910	coding	PF01485
MSTRG.10255.1	463	noncoding	-
MSTRG.10256.3	5088	coding	PF16746
MSTRG.10259.1	10772	noncoding	-

MSTRG.10260.1	8119	noncoding	-
MSTRG.10261.1	2742	noncoding	-
MSTRG.10266.2	2974	coding	PF02155
MSTRG.10268.1	548	noncoding	-
MSTRG.10270.1	276	noncoding	-
MSTRG.10273.2	1367	coding	PF14604
MSTRG.10273.4	1171	noncoding	-
MSTRG.10273.3	1424	noncoding	-
MSTRG.10273.5	1121	noncoding	-
MSTRG.10275.1	997	noncoding	-
MSTRG.10278.2	6314	noncoding	-
MSTRG.10281.7	3241	coding	PF01846
MSTRG.10281.9	3735	coding	PF01846
MSTRG.10281.6	4219	coding	PF01846
MSTRG.10281.4	4245	coding	PF01846
MSTRG.10281.5	4149	coding	PF01846
MSTRG.10281.8	4308	coding	PF01846
MSTRG.10282.2	1528	noncoding	-
MSTRG.10282.3	2432	noncoding	-
MSTRG.10282.4	2023	noncoding	-
MSTRG.10284.1	4977	noncoding	-
MSTRG.10284.4	2080	noncoding	-
MSTRG.10290.1	1204	coding	PF00050
MSTRG.10290.2	2820	noncoding	-
MSTRG.10302.4	4528	noncoding	-
MSTRG.10305.1	19064	noncoding	-
MSTRG.10305.2	5882	noncoding	-
MSTRG.10306.1	425	noncoding	-
MSTRG.10319.2	7856	coding	PF00916
MSTRG.10322.2	5525	coding	PF03184
MSTRG.10322.1	2569	coding	PF03184
MSTRG.10317.1	415	noncoding	-
MSTRG.10326.3	1518	coding	PF08831
MSTRG.10330.3	703	coding	PF00411
MSTRG.10331.1	4667	coding	PF12062
MSTRG.10331.2	5996	coding	PF12062
MSTRG.10329.3	12507	noncoding	-
MSTRG.10343.1	12577	noncoding	-
MSTRG.10344.1	1371	noncoding	-
MSTRG.10343.4	3966	noncoding	-
MSTRG.10343.5	12695	noncoding	-
MSTRG.10335.1	2554	noncoding	-
MSTRG.10335.3	2382	noncoding	-
MSTRG.10336.2	2464	coding	PF00191
MSTRG.10337.1	331	noncoding	-
MSTRG.10341.2	2198	noncoding	-
MSTRG.10341.3	1059	coding	PF01490
MSTRG.10342.1	693	noncoding	-
MSTRG.10350.1	5701	noncoding	-
MSTRG.10351.1	5292	coding	PF00028
MSTRG.10351.2	5377	coding	PF00028
MSTRG.10351.3	9775	coding	PF00028
MSTRG.10356.3	1872	coding	PF02136
MSTRG.10356.4	1684	coding	PF02136

MSTRG.10362.2	1382	coding	PF00060
MSTRG.10366.2	2236	coding	PF05334
MSTRG.10370.2	1943	coding	PF00535
MSTRG.10370.3	5627	coding	PF00652
MSTRG.10373.3	7640	coding	PF05383
MSTRG.10373.2	8269	coding	PF05383
MSTRG.10373.5	14684	noncoding	-
MSTRG.10374.1	2789	noncoding	-
MSTRG.10375.1	1156	coding	PF00237
MSTRG.10377.1	249	noncoding	-
MSTRG.10379.1	246	noncoding	-
MSTRG.10372.1	5914	noncoding	-
MSTRG.10372.2	3549	noncoding	-
MSTRG.10437.2	5515	coding	PF04790
MSTRG.10437.3	10034	coding	PF04790
MSTRG.10437.4	6047	noncoding	-
MSTRG.10437.5	7309	coding	PF04790
MSTRG.10437.7	1281	noncoding	-
MSTRG.10437.6	9005	noncoding	-
MSTRG.10437.8	21390	coding	PF04790
MSTRG.10437.9	5585	coding	PF04790
MSTRG.10437.10	6216	noncoding	-
MSTRG.10437.11	4189	noncoding	-
MSTRG.10437.12	52618	coding	PF00078
MSTRG.10437.13	37827	coding	PF00078
MSTRG.10437.14	20955	coding	PF04790
MSTRG.10437.15	12874	coding	PF04790
MSTRG.10437.16	27092	noncoding	-
MSTRG.10437.17	9419	noncoding	-
MSTRG.10439.1	2606	noncoding	-
MSTRG.10437.18	14501	noncoding	-
MSTRG.10440.1	4713	noncoding	-
MSTRG.10437.19	13019	noncoding	-
MSTRG.10437.20	6143	noncoding	-
MSTRG.10441.1	9486	noncoding	-
MSTRG.10442.1	12586	noncoding	-
MSTRG.10443.1	3694	noncoding	-
MSTRG.10445.1	1835	noncoding	-
MSTRG.10437.21	1153	coding	PF04790
MSTRG.10447.1	2833	noncoding	-
MSTRG.10437.22	4884	noncoding	-
MSTRG.10381.1	715	noncoding	-
MSTRG.10381.3	475	noncoding	-
MSTRG.10381.4	532	noncoding	-
MSTRG.10381.5	568	noncoding	-
MSTRG.10381.6	849	noncoding	-
MSTRG.10386.1	824	noncoding	-
MSTRG.10391.1	1472	noncoding	-
MSTRG.10383.1	2605	coding	PF12480
MSTRG.10392.1	647	noncoding	-
MSTRG.10399.2	2509	noncoding	-
MSTRG.10399.3	1150	noncoding	-
MSTRG.10400.1	3106	noncoding	-
MSTRG.10401.3	2189	coding	PF14413

MSTRG.10410.2	642	noncoding	-
MSTRG.10419.2	3306	coding	PF16423
MSTRG.10419.3	3904	coding	PF16423
MSTRG.10419.4	4450	coding	PF16422
MSTRG.10420.1	27797	noncoding	-
MSTRG.10419.6	15468	coding	PF16422
MSTRG.10407.1	4493	coding	PF00078
MSTRG.10407.2	1533	noncoding	-
MSTRG.10407.3	1371	noncoding	-
MSTRG.10409.1	2592	noncoding	-
MSTRG.10470.8	1677	noncoding	-
MSTRG.10470.10	1128	noncoding	-
MSTRG.10470.17	12285	noncoding	-
MSTRG.10470.18	1929	noncoding	-
MSTRG.10470.20	7485	noncoding	-
MSTRG.10470.22	10289	noncoding	-
MSTRG.10470.23	24071	noncoding	-
MSTRG.10470.24	13925	noncoding	-
MSTRG.10421.2	1468	noncoding	-
MSTRG.10424.2	430	noncoding	-
MSTRG.10424.3	6930	noncoding	-
MSTRG.10428.1	3964	noncoding	-
MSTRG.10427.3	5177	noncoding	-
MSTRG.10430.2	1188	coding	PF00386
MSTRG.10455.1	3957	coding	PF11708
MSTRG.10455.2	3777	coding	PF11708
MSTRG.10455.4	3217	coding	PF11708
MSTRG.10456.1	1381	coding	PF04856
MSTRG.10456.3	739	coding	PF04856
MSTRG.10456.5	748	coding	PF04856
MSTRG.10456.6	711	coding	PF04856
MSTRG.10456.7	1037	coding	PF04856
MSTRG.10456.4	5899	coding	PF04856
MSTRG.10464.1	4095	coding	PF16212
MSTRG.10466.1	447	noncoding	-
MSTRG.10435.2	3127	coding	PF02932
MSTRG.10488.3	1301	coding	PF04321
MSTRG.10488.5	2724	coding	PF04321
MSTRG.10488.2	1396	coding	PF04321
MSTRG.10488.4	1109	coding	PF04321
MSTRG.10489.1	891	noncoding	-
MSTRG.10481.1	219	noncoding	-
MSTRG.10491.1	519	noncoding	-
MSTRG.10507.1	9137	coding	PF06484
MSTRG.10508.1	507	noncoding	-
MSTRG.10507.3	3317	noncoding	-
MSTRG.10513.4	1305	noncoding	-
MSTRG.10516.1	4929	coding	PF00186
MSTRG.10511.1	1310	coding	PF00217
MSTRG.10522.3	1488	coding	PF04503
MSTRG.10523.1	1559	noncoding	-
MSTRG.10524.1	338	noncoding	-
MSTRG.10525.1	1987	noncoding	-
MSTRG.10526.3	1234	coding	PF03987

MSTRG.10526.5	1712	coding	PF03987
MSTRG.10539.3	4467	noncoding	-
MSTRG.10539.4	5398	noncoding	-
MSTRG.10542.1	1048	noncoding	-
MSTRG.10542.2	7456	noncoding	-
MSTRG.10543.1	443	noncoding	-
MSTRG.10573.1	446	coding	PF02935
MSTRG.10577.1	239	noncoding	-
MSTRG.10582.1	2107	noncoding	-
MSTRG.10583.1	2200	noncoding	-
MSTRG.10584.1	1810	noncoding	-
MSTRG.10590.2	1011	coding	PF10268
MSTRG.10590.3	3654	coding	PF10268
MSTRG.10595.3	3494	coding	PF00319
MSTRG.10595.4	3802	coding	PF00319
MSTRG.10595.5	3387	coding	PF00319
MSTRG.10595.6	3085	coding	PF00319
MSTRG.10595.7	2579	coding	PF00319
MSTRG.10604.1	366	noncoding	-
MSTRG.10633.2	4261	coding	PF09757
MSTRG.10634.1	1088	noncoding	-
MSTRG.10635.1	5796	noncoding	-
MSTRG.10633.3	3597	noncoding	-
MSTRG.10633.4	7406	coding	PF09757
MSTRG.10633.5	8181	coding	PF00078
MSTRG.10636.1	497	noncoding	-
MSTRG.10640.1	5382	noncoding	-
MSTRG.10641.1	1206	noncoding	-
MSTRG.10639.2	5691	coding	PF14906
MSTRG.10642.2	3018	coding	PF12796
MSTRG.10643.1	817	noncoding	-
MSTRG.10642.3	1376	noncoding	-
MSTRG.10623.2	1270	noncoding	-
MSTRG.10623.3	1160	noncoding	-
MSTRG.10626.2	5246	noncoding	-
MSTRG.10629.2	1110	coding	PF15824
MSTRG.10629.3	1066	coding	PF15824
MSTRG.10646.1	776	noncoding	-
MSTRG.10646.2	1393	coding	PF01483
MSTRG.10652.1	4685	coding	PF00748
MSTRG.10653.1	734	noncoding	-
MSTRG.10652.5	3269	noncoding	-
MSTRG.10654.1	5179	coding	PF01433
MSTRG.10659.1	2529	noncoding	-
MSTRG.10659.2	765	noncoding	-
MSTRG.10665.1	4865	coding	PF06534
MSTRG.10667.4	10819	coding	PF00176
MSTRG.10667.5	6475	coding	PF00176
MSTRG.10668.1	5796	noncoding	-
MSTRG.10668.2	5233	noncoding	-
MSTRG.10680.1	838	noncoding	-
MSTRG.10680.2	1042	noncoding	-
MSTRG.10680.3	3472	noncoding	-
MSTRG.10680.4	8626	noncoding	-

MSTRG.10685.1	360	noncoding	-
MSTRG.10688.1	2099	coding	PF00777
MSTRG.10700.2	1595	coding	PF03137
MSTRG.10700.3	2441	coding	PF03137
MSTRG.10700.4	2556	coding	PF03137
MSTRG.10701.1	5105	noncoding	-
MSTRG.10700.5	2996	noncoding	-
MSTRG.10703.1	3996	coding	PF03712
MSTRG.10703.13	2949	coding	PF03712
MSTRG.10713.1	3076	noncoding	-
MSTRG.10713.5	2250	noncoding	-
MSTRG.10713.4	2285	noncoding	-
MSTRG.10713.6	2412	noncoding	-
MSTRG.10715.1	1986	noncoding	-
MSTRG.10707.1	1025	noncoding	-
MSTRG.10712.2	4892	noncoding	-
MSTRG.10718.1	1151	noncoding	-
MSTRG.10725.1	6803	noncoding	-
MSTRG.10725.2	4339	noncoding	-
MSTRG.10761.1	1986	noncoding	-
MSTRG.10764.1	422	noncoding	-
MSTRG.10766.1	1627	coding	PF00812
MSTRG.10766.2	1629	coding	PF00812
MSTRG.10767.1	215	noncoding	-
MSTRG.10768.1	507	noncoding	-
MSTRG.10769.1	8278	noncoding	-
MSTRG.10765.1	2701	noncoding	-
MSTRG.10765.2	745	noncoding	-
MSTRG.10772.1	1162	noncoding	-
MSTRG.10775.1	1082	noncoding	-
MSTRG.10776.1	2408	noncoding	-
MSTRG.10776.2	5510	noncoding	-
MSTRG.10777.1	2631	noncoding	-
MSTRG.10774.2	2463	noncoding	-
MSTRG.10778.1	2662	noncoding	-
MSTRG.10774.3	2335	noncoding	-
MSTRG.10774.4	3602	noncoding	-
MSTRG.10774.5	2679	noncoding	-
MSTRG.10779.1	6090	noncoding	-
MSTRG.10774.6	8745	noncoding	-
MSTRG.10780.1	1067	noncoding	-
MSTRG.10782.1	808	noncoding	-
MSTRG.10786.2	1494	noncoding	-
MSTRG.10786.4	5126	coding	PF07714
MSTRG.10787.1	12714	noncoding	-
MSTRG.10789.1	8220	noncoding	-
MSTRG.10790.1	2951	noncoding	-
MSTRG.10791.1	7222	noncoding	-
MSTRG.10792.1	4062	noncoding	-
MSTRG.10793.3	3616	coding	PF13639
MSTRG.10793.2	5205	coding	PF13639
MSTRG.10794.1	557	noncoding	-
MSTRG.10794.2	409	noncoding	-
MSTRG.10795.4	8523	coding	PF01074

MSTRG.10795.5	8238	coding	PF01074
MSTRG.10796.1	333	noncoding	-
MSTRG.10806.4	2300	coding	PF15877
MSTRG.10807.1	1644	noncoding	-
MSTRG.10808.2	4471	noncoding	-
MSTRG.10808.5	2688	noncoding	-
MSTRG.10808.6	5805	noncoding	-
MSTRG.10808.7	4966	noncoding	-
MSTRG.10809.1	1525	noncoding	-
MSTRG.10811.1	1117	coding	PF15216
MSTRG.10812.1	298	noncoding	-
MSTRG.10820.1	4387	coding	PF00069
MSTRG.10825.1	5123	noncoding	-
MSTRG.10830.1	1723	noncoding	-
MSTRG.10831.1	3551	noncoding	-
MSTRG.10829.4	6549	noncoding	-
MSTRG.10829.5	2534	noncoding	-
MSTRG.10837.1	5018	coding	PF07679
MSTRG.10837.2	4511	coding	PF07679
MSTRG.10837.4	1716	coding	PF07679
MSTRG.10838.1	1738	noncoding	-
MSTRG.10840.2	4448	coding	PF14604
MSTRG.10840.3	7305	coding	PF00018
MSTRG.10843.5	9464	coding	PF01359
MSTRG.10844.2	4557	noncoding	-
MSTRG.10844.1	5163	noncoding	-
MSTRG.10845.1	2507	noncoding	-
MSTRG.10847.1	940	noncoding	-
MSTRG.10848.2	2269	coding	PF10228
MSTRG.10848.3	2386	coding	PF10228
MSTRG.10848.4	1504	coding	PF10228
MSTRG.10851.3	6743	coding	PF07679
MSTRG.10852.1	376	noncoding	-
MSTRG.10853.1	283	noncoding	-
MSTRG.10857.1	213	noncoding	-
MSTRG.10867.3	4574	coding	PF00652
MSTRG.10876.7	1985	coding	PF15007
MSTRG.10876.8	2123	coding	PF15007
MSTRG.10876.6	2259	coding	PF15007
MSTRG.10880.3	2507	coding	PF08516
MSTRG.10882.1	1642	coding	PF01562
MSTRG.10882.2	3206	coding	PF08516
MSTRG.10882.4	3263	coding	PF08516
MSTRG.10882.5	2372	coding	PF08516
MSTRG.10882.6	2432	coding	PF08516
MSTRG.10882.7	2933	coding	PF08516
MSTRG.10882.8	823	noncoding	-
MSTRG.10888.2	1943	noncoding	-
MSTRG.10888.4	1709	coding	PF00494
MSTRG.10889.1	14448	noncoding	-
MSTRG.10889.2	1671	coding	PF00320
MSTRG.10890.1	769	noncoding	-
MSTRG.10889.5	23935	noncoding	-
MSTRG.10889.7	18926	coding	PF05349

MSTRG.10889.8	7120	coding	PF05349
MSTRG.10889.9	2982	coding	PF05349
MSTRG.10899.1	7557	coding	PF09815
MSTRG.10901.1	13566	noncoding	-
MSTRG.10902.1	5507	noncoding	-
MSTRG.10903.1	16157	noncoding	-
MSTRG.10896.2	980	coding	PF00089
MSTRG.10896.3	1145	coding	PF00089
MSTRG.10906.1	498	noncoding	-
MSTRG.10907.2	1056	coding	PF00089
MSTRG.10907.3	874	coding	PF00089
MSTRG.10910.1	365	noncoding	-
MSTRG.10922.3	9147	coding	PF00225
MSTRG.10922.5	9212	coding	PF00225
MSTRG.10923.1	1318	noncoding	-
MSTRG.10925.1	3910	coding	PF04814
MSTRG.10925.9	4012	coding	PF04814
MSTRG.10928.1	4875	noncoding	-
MSTRG.10913.1	473	noncoding	-
MSTRG.10919.1	5118	coding	PF15997
MSTRG.10921.1	648	noncoding	-
MSTRG.10935.1	3387	coding	PF16199
MSTRG.10935.2	2877	coding	PF16199
MSTRG.10936.2	3643	coding	PF00530
MSTRG.10941.2	3254	coding	PF13878
MSTRG.10942.2	3952	noncoding	-
MSTRG.10943.1	2856	noncoding	-
MSTRG.10944.1	7261	noncoding	-
MSTRG.10950.3	4509	noncoding	-
MSTRG.10950.4	3502	noncoding	-
MSTRG.10956.1	548	noncoding	-
MSTRG.10958.1	1447	noncoding	-
MSTRG.10961.1	1055	noncoding	-
MSTRG.10971.1	1101	noncoding	-
MSTRG.10976.1	654	noncoding	-
MSTRG.10976.2	680	noncoding	-
MSTRG.10976.3	355	noncoding	-
MSTRG.10984.1	2347	noncoding	-
MSTRG.10984.3	3757	coding	PF15176
MSTRG.10990.4	26689	noncoding	-
MSTRG.10990.3	18650	noncoding	-
MSTRG.10990.5	7349	noncoding	-
MSTRG.11012.1	4066	coding	PF00076
MSTRG.11012.2	4046	coding	PF00076
MSTRG.11039.1	9137	noncoding	-
MSTRG.11040.1	6134	noncoding	-
MSTRG.11041.1	3811	noncoding	-
MSTRG.11042.1	8032	noncoding	-
MSTRG.11043.1	6260	noncoding	-
MSTRG.11044.1	2980	coding	PF00143
MSTRG.11045.1	1171	noncoding	-
MSTRG.11046.1	384	noncoding	-
MSTRG.11036.2	3769	coding	PF00651
MSTRG.11036.1	2571	coding	PF00651

MSTRG.11037.1	281	noncoding	-
MSTRG.11050.1	1316	noncoding	-
MSTRG.11050.2	287	noncoding	-
MSTRG.11055.1	495	coding	PF03366
MSTRG.11057.1	293	noncoding	-
MSTRG.11060.1	1261	coding	PF01699
MSTRG.11071.2	9194	coding	PF02141
MSTRG.11071.3	8777	coding	PF02141
MSTRG.11071.4	8726	coding	PF02141
MSTRG.11071.5	10014	noncoding	-
MSTRG.11072.1	4933	noncoding	-
MSTRG.11073.2	1631	noncoding	-
MSTRG.11063.1	1590	coding	PF03036
MSTRG.11063.3	1749	coding	PF03036
MSTRG.11076.2	3703	noncoding	-
MSTRG.11100.1	12271	noncoding	-
MSTRG.11101.1	3843	noncoding	-
MSTRG.11090.1	1086	noncoding	-
MSTRG.11091.5	3814	coding	PF11467
MSTRG.11091.6	3849	coding	PF11467
MSTRG.11096.2	1157	coding	PF12251
MSTRG.11098.1	9705	coding	PF10300
MSTRG.11098.2	2122	coding	PF10300
MSTRG.11103.2	1393	coding	PF01529
MSTRG.11103.3	1802	noncoding	-
MSTRG.11109.1	207	noncoding	-
MSTRG.11154.2	7557	coding	PF00102
MSTRG.11156.1	336	noncoding	-
MSTRG.11158.1	4158	coding	PF00041
MSTRG.11161.1	1191	coding	PF15055
MSTRG.11161.4	552	noncoding	-
MSTRG.11170.1	2986	coding	PF00078
MSTRG.11171.1	1597	noncoding	-
MSTRG.11169.3	2178	coding	PF02373
MSTRG.11177.1	3037	coding	PF02347
MSTRG.11163.2	2438	noncoding	-
MSTRG.11185.1	8646	noncoding	-
MSTRG.11186.1	2641	noncoding	-
MSTRG.11188.3	5994	coding	PF04389
MSTRG.11190.3	6759	coding	PF07064
MSTRG.11191.1	2751	noncoding	-
MSTRG.11194.2	702	noncoding	-
MSTRG.11199.1	2694	noncoding	-
MSTRG.11194.4	736	noncoding	-
MSTRG.11230.1	702	noncoding	-
MSTRG.11235.1	1416	noncoding	-
MSTRG.11235.2	3069	noncoding	-
MSTRG.11243.1	14645	noncoding	-
MSTRG.11243.3	7639	noncoding	-
MSTRG.11243.6	447	noncoding	-
MSTRG.11243.8	2668	noncoding	-
MSTRG.11219.1	1629	noncoding	-
MSTRG.11226.1	3182	coding	PF00057
MSTRG.11226.3	2683	coding	PF00057

MSTRG.11221.1	697	noncoding	-
MSTRG.11221.2	808	noncoding	-
MSTRG.11248.3	5795	coding	PF00176
MSTRG.11248.9	5538	coding	PF00176
MSTRG.11248.8	6030	coding	PF00176
MSTRG.11248.6	6412	coding	PF00176
MSTRG.11248.7	5799	coding	PF00176
MSTRG.11249.1	13478	noncoding	-
MSTRG.11257.1	420	noncoding	-
MSTRG.11256.1	304	noncoding	-
MSTRG.11279.1	649	noncoding	-
MSTRG.11283.1	2564	coding	PF02492
MSTRG.11283.2	1992	coding	PF02492
MSTRG.11284.1	1253	noncoding	-
MSTRG.11292.1	3846	coding	PF00595
MSTRG.11293.1	3027	noncoding	-
MSTRG.11294.2	6249	coding	PF00640
MSTRG.11294.3	4399	coding	PF00595
MSTRG.11294.4	5177	coding	PF00640
MSTRG.11300.3	1925	noncoding	-
MSTRG.11301.2	5142	noncoding	-
MSTRG.11301.1	6384	noncoding	-
MSTRG.11301.3	2428	noncoding	-
MSTRG.11301.4	7062	noncoding	-
MSTRG.11301.5	2483	noncoding	-
MSTRG.11302.1	1094	noncoding	-
MSTRG.11302.2	9992	coding	PF02994
MSTRG.11303.1	607	noncoding	-
MSTRG.11304.2	1993	coding	PF00629
MSTRG.11304.3	946	coding	PF00629
MSTRG.11373.3	3657	noncoding	-
MSTRG.11373.4	6336	noncoding	-
MSTRG.11373.8	1824	noncoding	-
MSTRG.11377.1	12541	coding	PF16519
MSTRG.11377.2	6866	coding	PF16519
MSTRG.11378.1	1192	noncoding	-
MSTRG.11377.3	7246	coding	PF16519
MSTRG.11377.4	7555	coding	PF16519
MSTRG.11377.5	10212	coding	PF16519
MSTRG.11377.7	4744	coding	PF16519
MSTRG.11377.6	5001	coding	PF16519
MSTRG.11377.8	17452	noncoding	-
MSTRG.11377.9	11104	noncoding	-
MSTRG.11377.10	1120	noncoding	-
MSTRG.11379.1	2736	noncoding	-
MSTRG.11377.11	9210	noncoding	-
MSTRG.11381.1	3044	noncoding	-
MSTRG.11382.1	2594	noncoding	-
MSTRG.11377.18	3619	noncoding	-
MSTRG.11377.19	26685	coding	PF02994
MSTRG.11377.20	15415	noncoding	-
MSTRG.11377.21	5204	noncoding	-
MSTRG.11377.22	1536	noncoding	-
MSTRG.11377.23	8068	noncoding	-

MSTRG.11377.24	34337	coding	PF02994
MSTRG.11377.25	25658	noncoding	-
MSTRG.11377.26	2255	noncoding	-
MSTRG.11377.27	3447	noncoding	-
MSTRG.11309.1	2806	coding	PF12695
MSTRG.11307.2	1210	coding	PF15851
MSTRG.11313.1	2700	noncoding	-
MSTRG.11313.2	15851	noncoding	-
MSTRG.11313.3	3975	noncoding	-
MSTRG.11313.4	4980	noncoding	-
MSTRG.11319.1	964	noncoding	-
MSTRG.11319.2	871	noncoding	-
MSTRG.11321.1	4637	coding	PF02816
MSTRG.11326.1	466	noncoding	-
MSTRG.11328.2	1758	coding	PF07942
MSTRG.11328.4	4590	coding	PF07942
MSTRG.11331.1	1046	noncoding	-
MSTRG.11335.1	335	noncoding	-
MSTRG.11336.2	5582	coding	PF16470
MSTRG.11336.3	4344	coding	PF01483
MSTRG.11338.2	3267	coding	PF01687
MSTRG.11338.3	3980	coding	PF01687
MSTRG.11350.1	22679	coding	PF16910
MSTRG.11350.5	5554	coding	PF16908
MSTRG.11350.6	5728	coding	PF16908
MSTRG.11350.4	9411	coding	PF16910
MSTRG.11350.7	10148	coding	PF16910
MSTRG.11351.1	4643	noncoding	-
MSTRG.11352.1	1777	noncoding	-
MSTRG.11350.8	5691	noncoding	-
MSTRG.11357.1	1754	noncoding	-
MSTRG.11358.1	5726	noncoding	-
MSTRG.11359.1	9327	noncoding	-
MSTRG.11360.1	20756	noncoding	-
MSTRG.11360.2	4492	noncoding	-
MSTRG.11344.2	4891	coding	PF00503
MSTRG.11345.1	8738	noncoding	-
MSTRG.11345.2	9873	noncoding	-
MSTRG.11346.1	661	noncoding	-
MSTRG.11347.1	17052	noncoding	-
MSTRG.11348.3	2738	noncoding	-
MSTRG.11363.1	1839	noncoding	-
MSTRG.11365.1	3861	noncoding	-
MSTRG.11366.1	4509	noncoding	-
MSTRG.11367.1	15468	noncoding	-
MSTRG.11367.2	14052	noncoding	-
MSTRG.11400.1	17998	coding	PF03920
MSTRG.11400.2	6963	coding	PF03920
MSTRG.11400.3	4969	coding	PF03920
MSTRG.11400.4	16586	noncoding	-
MSTRG.11400.5	18993	coding	PF03920
MSTRG.11403.1	4479	coding	PF14650
MSTRG.11404.1	4604	coding	PF14650
MSTRG.11404.2	4765	coding	PF14650

MSTRG.11408.1	6047	noncoding	-
MSTRG.11410.1	1702	coding	PF15371
MSTRG.11411.1	1278	noncoding	-
MSTRG.11411.2	705	noncoding	-
MSTRG.11413.3	5726	coding	PF16744
MSTRG.11413.4	6778	noncoding	-
MSTRG.11406.3	2623	coding	PF01556
MSTRG.11414.1	1865	noncoding	-
MSTRG.11417.1	1780	noncoding	-
MSTRG.11417.2	1994	noncoding	-
MSTRG.11417.3	2681	noncoding	-
MSTRG.11417.4	4220	noncoding	-
MSTRG.11418.4	3516	noncoding	-
MSTRG.11427.1	16481	coding	PF10540
MSTRG.11427.3	16774	coding	PF10540
MSTRG.11427.4	16621	coding	PF10540
MSTRG.11429.1	5799	coding	PF16212
MSTRG.11430.1	1710	noncoding	-
MSTRG.11431.2	4600	noncoding	-
MSTRG.11431.3	4750	noncoding	-
MSTRG.11432.1	2411	coding	PF00069
MSTRG.11433.1	695	noncoding	-
MSTRG.11436.1	1896	noncoding	-
MSTRG.11436.2	1807	noncoding	-
MSTRG.11437.3	1308	coding	PF00194
MSTRG.11438.1	1206	coding	PF00261
MSTRG.11439.3	8550	coding	PF08913
MSTRG.11441.4	3068	coding	PF04685
MSTRG.11441.2	3256	coding	PF04685
MSTRG.11441.3	3469	coding	PF04685
MSTRG.11442.1	1666	coding	PF04685
MSTRG.11443.4	2086	coding	PF08737
MSTRG.11443.5	2596	coding	PF08737
MSTRG.11443.7	2050	coding	PF08737
MSTRG.11443.8	2126	coding	PF08737
MSTRG.11443.6	2687	coding	PF08737
MSTRG.11421.3	551	coding	PF01230
MSTRG.11423.1	1646	coding	PF14753
MSTRG.11424.2	4732	coding	PF12036
MSTRG.11449.2	1809	noncoding	-
MSTRG.11457.1	6580	coding	PF01086
MSTRG.11457.2	4549	coding	PF01086
MSTRG.11457.3	1020	coding	PF01086
MSTRG.11460.1	1554	noncoding	-
MSTRG.11462.12	5737	coding	PF13639
MSTRG.11462.7	4362	coding	PF13639
MSTRG.11462.8	5154	coding	PF13639
MSTRG.11462.9	5318	coding	PF13639
MSTRG.11462.13	4581	coding	PF13639
MSTRG.11462.14	3499	coding	PF13639
MSTRG.11462.15	2030	noncoding	-
MSTRG.11463.1	1615	noncoding	-
MSTRG.11464.1	6437	noncoding	-
MSTRG.11465.1	371	noncoding	-

MSTRG.11466.1	2676	coding	PF00069
MSTRG.11466.2	5828	coding	PF00069
MSTRG.11472.1	3375	noncoding	-
MSTRG.11472.4	1060	noncoding	-
MSTRG.11472.2	2744	noncoding	-
MSTRG.11472.5	2638	noncoding	-
MSTRG.11472.6	6357	noncoding	-
MSTRG.11472.7	15536	noncoding	-
MSTRG.11472.8	7009	coding	PF00078
MSTRG.11473.1	6908	noncoding	-
MSTRG.11473.2	6347	noncoding	-
MSTRG.11473.3	5245	noncoding	-
MSTRG.11474.1	3861	noncoding	-
MSTRG.11473.4	4920	noncoding	-
MSTRG.11472.9	821	noncoding	-
MSTRG.11472.10	3647	noncoding	-
MSTRG.11475.1	1802	noncoding	-
MSTRG.11469.1	2258	coding	PF02826
MSTRG.11470.5	3565	coding	PF00651
MSTRG.11476.3	3562	coding	PF13229
MSTRG.11476.2	7352	coding	PF15178
MSTRG.11476.4	3272	coding	PF13229
MSTRG.11476.5	5715	coding	PF15178
MSTRG.11477.1	5048	noncoding	-
MSTRG.11478.1	4861	noncoding	-
MSTRG.11483.2	1145	coding	PF15985
MSTRG.11489.1	302	noncoding	-
MSTRG.11490.5	4847	coding	PF14643
MSTRG.11490.6	5071	coding	PF14643
MSTRG.11496.2	3430	coding	PF00567
MSTRG.11499.2	2860	coding	PF12368
MSTRG.11499.4	1457	noncoding	-
MSTRG.11492.7	1617	noncoding	-
MSTRG.11492.6	1832	noncoding	-
MSTRG.11492.9	1822	noncoding	-
MSTRG.11492.11	2299	noncoding	-
MSTRG.11492.12	1601	noncoding	-
MSTRG.11492.5	1611	noncoding	-
MSTRG.11492.8	1733	noncoding	-
MSTRG.11492.10	2036	noncoding	-
MSTRG.11492.4	1768	noncoding	-
MSTRG.11505.1	1767	coding	PF14580
MSTRG.11505.2	2443	coding	PF14580
MSTRG.11507.1	4790	noncoding	-
MSTRG.11507.6	5961	noncoding	-
MSTRG.11507.8	6725	noncoding	-
MSTRG.11507.3	4411	noncoding	-
MSTRG.11507.7	5284	noncoding	-
MSTRG.11507.2	6363	noncoding	-
MSTRG.11507.4	4746	noncoding	-
MSTRG.11507.9	6251	noncoding	-
MSTRG.11508.1	4806	coding	PF08953
MSTRG.11508.4	5301	coding	PF16300
MSTRG.11508.5	1765	coding	PF08953

MSTRG.11510.1	4684	noncoding	-
MSTRG.11513.1	1766	noncoding	-
MSTRG.11514.1	846	noncoding	-
MSTRG.11514.2	587	noncoding	-
MSTRG.11515.1	701	coding	PF12796
MSTRG.11520.1	5259	coding	PF06482
MSTRG.11534.1	3623	coding	PF13848
MSTRG.11537.1	1196	noncoding	-
MSTRG.11539.3	2639	coding	PF12796
MSTRG.11540.4	1356	noncoding	-
MSTRG.11530.2	1268	coding	PF00089
MSTRG.11551.1	5804	coding	PF14650
MSTRG.11551.2	4985	coding	PF14650
MSTRG.11553.1	1031	coding	PF00089
MSTRG.11549.5	890	noncoding	-
MSTRG.11549.4	12379	noncoding	-
MSTRG.11542.1	5586	coding	PF14650
MSTRG.11545.1	1131	noncoding	-
MSTRG.11556.2	1421	noncoding	-
MSTRG.11560.1	562	coding	PF01352
MSTRG.11568.1	1973	noncoding	-
MSTRG.11570.1	1108	noncoding	-
MSTRG.11571.1	435	noncoding	-
MSTRG.11584.1	1007	noncoding	-
MSTRG.11586.1	2866	noncoding	-
MSTRG.11589.2	3629	coding	PF01400
MSTRG.11590.3	1836	coding	PF05132
MSTRG.11591.2	7480	coding	PF02171
MSTRG.11594.3	7580	coding	PF02208
MSTRG.11595.1	666	noncoding	-
MSTRG.11595.2	521	noncoding	-
MSTRG.11594.4	4472	coding	PF02208
MSTRG.11606.4	3673	coding	PF14443
MSTRG.11606.10	3882	coding	PF14443
MSTRG.11599.1	3759	noncoding	-
MSTRG.11599.2	1600	noncoding	-
MSTRG.11610.1	1857	noncoding	-
MSTRG.11610.2	1778	noncoding	-
MSTRG.11611.2	5902	coding	PF01150
MSTRG.11611.4	5893	coding	PF01150
MSTRG.11613.1	328	noncoding	-
MSTRG.11615.1	3468	coding	PF00153
MSTRG.11616.1	484	noncoding	-
MSTRG.11618.2	3304	coding	PF00046
MSTRG.11617.1	531	noncoding	-
MSTRG.11622.1	4331	noncoding	-
MSTRG.11622.2	1220	noncoding	-
MSTRG.11636.2	2736	coding	PF15276
MSTRG.11643.2	4763	noncoding	-
MSTRG.11643.3	2083	noncoding	-
MSTRG.11643.4	2247	noncoding	-
MSTRG.11645.1	464	noncoding	-
MSTRG.11646.1	1373	coding	PF06553
MSTRG.11649.4	4197	noncoding	-

MSTRG.11656.3	2709	coding	PF07714
MSTRG.11657.2	3658	coding	PF13419
MSTRG.11662.1	901	noncoding	-
MSTRG.11663.4	2574	coding	PF04030
MSTRG.11663.3	1516	coding	PF04030
MSTRG.11664.2	745	noncoding	-
MSTRG.11666.1	5596	coding	PF11969
MSTRG.11666.5	3714	coding	PF11969
MSTRG.11666.3	1675	coding	PF11969
MSTRG.11666.4	1594	coding	PF11969
MSTRG.11659.1	1118	coding	PF00050
MSTRG.11659.2	1689	coding	PF00050
MSTRG.11659.3	936	coding	PF00050
MSTRG.11659.4	10398	noncoding	-
MSTRG.11674.2	1870	noncoding	-
MSTRG.11675.1	1010	coding	PF03357
MSTRG.11675.3	695	coding	PF03357
MSTRG.11676.3	3470	noncoding	-
MSTRG.11678.1	3631	coding	PF00179
MSTRG.11678.5	3486	coding	PF00179
MSTRG.11678.3	4324	coding	PF00179
MSTRG.11679.1	1559	noncoding	-
MSTRG.11680.1	5589	noncoding	-
MSTRG.11680.3	5523	noncoding	-
MSTRG.11677.3	4310	noncoding	-
MSTRG.11683.1	2688	noncoding	-
MSTRG.11684.1	6897	coding	PF00225
MSTRG.11684.4	8749	coding	PF00225
MSTRG.11687.2	1121	coding	PF15181
MSTRG.11696.1	1581	noncoding	-
MSTRG.11697.1	1473	coding	PF01388
MSTRG.11697.2	1260	coding	PF01388
MSTRG.11702.1	5242	noncoding	-
MSTRG.11702.3	4351	noncoding	-
MSTRG.11703.1	495	noncoding	-
MSTRG.11707.1	2183	noncoding	-
MSTRG.11708.1	700	noncoding	-
MSTRG.11708.2	1889	noncoding	-
MSTRG.11717.1	2199	coding	PF10343
MSTRG.11724.2	2238	coding	PF08067
MSTRG.11724.7	1895	coding	PF08067
MSTRG.11724.6	2658	coding	PF08067
MSTRG.11727.1	761	noncoding	-
MSTRG.11728.1	7105	coding	PF07679
MSTRG.11731.1	210	noncoding	-
MSTRG.11738.1	2638	coding	PF04112
MSTRG.11738.3	2407	coding	PF04112
MSTRG.11738.4	3864	coding	PF01359
MSTRG.11738.6	4305	noncoding	-
MSTRG.11738.7	5231	noncoding	-
MSTRG.11738.8	3691	noncoding	-
MSTRG.11738.9	2797	noncoding	-
MSTRG.11734.3	4234	noncoding	-
MSTRG.11737.2	657	noncoding	-

MSTRG.11739.1	1867	noncoding	-
MSTRG.11745.7	6734	coding	PF16631
MSTRG.11745.8	1098	noncoding	-
MSTRG.11746.1	2153	noncoding	-
MSTRG.11747.1	1439	noncoding	-
MSTRG.11763.3	1873	noncoding	-
MSTRG.11763.4	1905	noncoding	-
MSTRG.11764.1	1314	noncoding	-
MSTRG.11763.5	1799	coding	PF09127
MSTRG.11765.1	1815	noncoding	-
MSTRG.11763.7	1171	coding	PF09127
MSTRG.11756.2	8007	coding	PF12349
MSTRG.11756.6	4490	coding	PF12349
MSTRG.11761.1	863	noncoding	-
MSTRG.11782.1	10628	noncoding	-
MSTRG.11783.1	2871	noncoding	-
MSTRG.11783.4	1755	noncoding	-
MSTRG.11783.3	1578	noncoding	-
MSTRG.11792.1	7174	coding	PF14671
MSTRG.11792.3	4177	coding	PF14671
MSTRG.11792.2	3975	coding	PF14671
MSTRG.11792.4	1844	coding	PF14671
MSTRG.11792.13	8311	coding	PF00164
MSTRG.11793.1	618	noncoding	-
MSTRG.11794.1	4649	coding	PF02994
MSTRG.11785.1	2947	coding	PF13911
MSTRG.11786.2	570	coding	PF01352
MSTRG.11788.1	1434	coding	PF00112
MSTRG.11788.2	1602	coding	PF00112
MSTRG.11788.4	1226	coding	PF00112
MSTRG.11795.1	1049	noncoding	-
MSTRG.11805.1	262	noncoding	-
MSTRG.11806.1	4648	noncoding	-
MSTRG.11807.1	1580	noncoding	-
MSTRG.11810.2	2124	noncoding	-
MSTRG.11814.4	1363	coding	PF00093
MSTRG.11800.1	777	noncoding	-
MSTRG.11802.3	932	noncoding	-
MSTRG.11802.4	772	noncoding	-
MSTRG.11802.5	3396	noncoding	-
MSTRG.11820.3	3766	noncoding	-
MSTRG.11819.1	806	noncoding	-
MSTRG.11834.2	1411	noncoding	-
MSTRG.11834.3	1412	noncoding	-
MSTRG.11838.1	1533	noncoding	-
MSTRG.11841.4	2559	noncoding	-
MSTRG.11842.3	5576	coding	PF14650
MSTRG.11842.6	4023	coding	PF14650
MSTRG.11821.2	3285	coding	PF14650
MSTRG.11824.2	12045	coding	PF14650
MSTRG.11824.3	16861	coding	PF14650
MSTRG.11824.4	3269	noncoding	-
MSTRG.11824.5	22513	coding	PF14650
MSTRG.11825.3	1512	coding	PF00780

MSTRG.11825.4	1611	noncoding	-
MSTRG.11827.1	296	noncoding	-
MSTRG.11827.3	717	noncoding	-
MSTRG.11827.2	918	noncoding	-
MSTRG.11828.1	201	noncoding	-
MSTRG.11847.2	2074	coding	PF06529
MSTRG.11852.1	4016	coding	PF00378
MSTRG.11852.2	1320	coding	PF00378
MSTRG.11852.3	2077	coding	PF00378
MSTRG.11852.4	1477	coding	PF00378
MSTRG.11852.5	2667	coding	PF00378
MSTRG.11860.1	679	noncoding	-
MSTRG.11861.1	1840	noncoding	-
MSTRG.11854.1	386	noncoding	-
MSTRG.11867.3	4018	coding	PF02513
MSTRG.11867.4	592	noncoding	-
MSTRG.11867.5	507	noncoding	-
MSTRG.11872.1	2033	noncoding	-
MSTRG.11873.2	1594	noncoding	-
MSTRG.11873.4	1968	coding	PF00069
MSTRG.11873.3	5734	noncoding	-
MSTRG.11873.7	5405	noncoding	-
MSTRG.11873.8	2004	noncoding	-
MSTRG.11873.6	1716	noncoding	-
MSTRG.11875.1	453	noncoding	-
MSTRG.11876.1	3640	noncoding	-
MSTRG.11876.3	4195	coding	PF15237
MSTRG.11882.3	3069	noncoding	-
MSTRG.11882.4	3111	coding	PF01352
MSTRG.11889.1	4324	coding	PF13923
MSTRG.11889.2	5020	coding	PF13923
MSTRG.11890.1	633	noncoding	-
MSTRG.11901.1	3458	noncoding	-
MSTRG.11901.2	2615	noncoding	-
MSTRG.11902.1	490	noncoding	-
MSTRG.11923.1	332	noncoding	-
MSTRG.11924.1	688	noncoding	-
MSTRG.11929.1	447	noncoding	-
MSTRG.11950.1	366	noncoding	-
MSTRG.11951.1	1397	noncoding	-
MSTRG.11960.1	699	noncoding	-
MSTRG.11968.1	10591	noncoding	-
MSTRG.11969.1	6336	noncoding	-
MSTRG.11970.1	11970	noncoding	-
MSTRG.11973.4	1533	coding	PF00022
MSTRG.11973.3	1554	coding	PF00022
MSTRG.11973.2	558	coding	PF00022
MSTRG.11973.1	1512	coding	PF00022
MSTRG.11974.1	1698	coding	PF00022
MSTRG.11974.2	1560	coding	PF00022
MSTRG.11974.3	1519	coding	PF00022
MSTRG.11973.7	244	noncoding	-
MSTRG.11973.8	426	noncoding	-
MSTRG.11973.9	2213	noncoding	-

MSTRG.11973.10	276	noncoding	-
MSTRG.11973.11	364	noncoding	-
MSTRG.11973.12	211	noncoding	-
MSTRG.11973.13	600	noncoding	-
MSTRG.11973.14	608	noncoding	-
MSTRG.11973.15	754	noncoding	-
MSTRG.11973.16	497	noncoding	-
MSTRG.11973.17	254	noncoding	-
MSTRG.11973.18	399	noncoding	-
MSTRG.11973.19	222	noncoding	-
MSTRG.11973.20	355	noncoding	-
MSTRG.11973.21	390	noncoding	-
MSTRG.11973.22	367	noncoding	-
MSTRG.11973.23	338	noncoding	-
MSTRG.11973.24	329	noncoding	-
MSTRG.11958.1	4650	coding	PF09380
MSTRG.11958.2	4762	coding	PF09380
MSTRG.11972.1	5457	noncoding	-
MSTRG.11978.2	710	coding	PF03285
MSTRG.11978.1	6706	coding	PF03285
MSTRG.11979.1	497	noncoding	-
MSTRG.11978.4	7302	noncoding	-
MSTRG.11978.5	7481	noncoding	-
MSTRG.11985.2	10252	noncoding	-
MSTRG.11986.4	2530	coding	PF00001
MSTRG.11987.1	839	noncoding	-
MSTRG.11995.1	5816	coding	PF13001
MSTRG.11996.1	2981	noncoding	-
MSTRG.11995.6	6942	coding	PF13001
MSTRG.11995.5	10021	coding	PF13001
MSTRG.11995.7	8996	coding	PF13001
MSTRG.11995.8	5607	coding	PF13001
MSTRG.11995.9	5765	coding	PF13001
MSTRG.11991.1	6406	noncoding	-
MSTRG.11994.2	1610	coding	PF16884
MSTRG.11994.3	1483	coding	PF16884
MSTRG.12000.1	4986	noncoding	-
MSTRG.12004.5	10116	noncoding	-
MSTRG.12004.6	8900	coding	PF04177
MSTRG.12004.7	2631	noncoding	-
MSTRG.12004.8	1954	noncoding	-
MSTRG.12004.9	2363	noncoding	-
MSTRG.12004.10	1978	noncoding	-
MSTRG.12005.1	2940	noncoding	-
MSTRG.12004.11	1238	coding	PF04177
MSTRG.12006.4	2522	noncoding	-
MSTRG.12007.1	674	noncoding	-
MSTRG.12008.1	415	noncoding	-
MSTRG.12009.1	299	noncoding	-
MSTRG.12019.2	838	noncoding	-
MSTRG.12019.3	791	noncoding	-
MSTRG.12027.2	3763	coding	PF04145
MSTRG.12023.1	4083	noncoding	-
MSTRG.12030.1	2287	noncoding	-

MSTRG.12030.3	2584	noncoding	-
MSTRG.12034.4	1737	coding	PF15504
MSTRG.12035.1	4468	coding	PF00615
MSTRG.12039.1	936	noncoding	-
MSTRG.12040.1	4121	noncoding	-
MSTRG.12041.3	3364	noncoding	-
MSTRG.12042.1	5931	noncoding	-
MSTRG.12043.1	5105	noncoding	-
MSTRG.12044.1	4867	noncoding	-
MSTRG.12045.3	3373	noncoding	-
MSTRG.12045.4	5406	coding	PF12443
MSTRG.12047.3	1893	coding	PF00595
MSTRG.12053.2	2200	coding	PF14800
MSTRG.12053.3	6639	coding	PF14800
MSTRG.12054.1	4529	noncoding	-
MSTRG.12057.4	712	noncoding	-
MSTRG.12055.5	6420	noncoding	-
MSTRG.12055.6	9077	noncoding	-
MSTRG.12055.7	1064	noncoding	-
MSTRG.12055.8	12406	noncoding	-
MSTRG.12055.9	4767	coding	PF00229
MSTRG.12055.10	1898	noncoding	-
MSTRG.12055.11	2869	noncoding	-
MSTRG.12055.12	2418	noncoding	-
MSTRG.12055.13	9720	noncoding	-
MSTRG.12055.14	21455	coding	PF00229
MSTRG.12059.1	7323	noncoding	-
MSTRG.12049.1	696	noncoding	-
MSTRG.12050.1	923	noncoding	-
MSTRG.12051.1	587	noncoding	-
MSTRG.12069.1	2497	noncoding	-
MSTRG.12069.2	10380	noncoding	-
MSTRG.12069.5	2038	noncoding	-
MSTRG.12070.1	12775	noncoding	-
MSTRG.12069.7	3219	noncoding	-
MSTRG.12072.3	2273	noncoding	-
MSTRG.12072.10	2355	noncoding	-
MSTRG.12072.9	4114	noncoding	-
MSTRG.12072.12	11355	noncoding	-
MSTRG.12080.1	2763	noncoding	-
MSTRG.12079.1	445	noncoding	-
MSTRG.12088.1	647	noncoding	-
MSTRG.12093.2	6139	coding	PF00053
MSTRG.12095.3	2180	noncoding	-
MSTRG.12098.1	5531	coding	PF03091
MSTRG.12099.2	2889	coding	PF14061
MSTRG.12096.1	292	noncoding	-
MSTRG.12100.1	4972	coding	PF07678
MSTRG.12100.2	5002	coding	PF07678
MSTRG.12100.3	5160	coding	PF07678
MSTRG.12102.1	442	noncoding	-
MSTRG.12108.4	7468	coding	PF14580
MSTRG.12108.1	7646	coding	PF14580
MSTRG.12108.9	7721	coding	PF14580

MSTRG.12108.8	8044	coding	PF14580
MSTRG.12108.12	753	noncoding	-
MSTRG.12104.3	3254	coding	PF00626
MSTRG.12121.2	1452	coding	PF03607
MSTRG.12121.5	1256	coding	PF03607
MSTRG.12123.5	1614	coding	PF03561
MSTRG.12123.2	1873	coding	PF03561
MSTRG.12123.4	1854	coding	PF03561
MSTRG.12123.1	2410	coding	PF03561
MSTRG.12123.3	2058	coding	PF03561
MSTRG.12123.6	472	noncoding	-
MSTRG.12124.1	567	noncoding	-
MSTRG.12124.2	2175	coding	PF00059
MSTRG.12125.1	638	coding	PF00059
MSTRG.12114.2	2331	coding	PF03079
MSTRG.12130.3	3468	noncoding	-
MSTRG.12131.1	598	noncoding	-
MSTRG.12138.1	1258	noncoding	-
MSTRG.12145.1	1795	noncoding	-
MSTRG.12146.1	474	noncoding	-
MSTRG.12147.1	443	noncoding	-
MSTRG.12152.1	712	noncoding	-
MSTRG.12153.2	1671	noncoding	-
MSTRG.12157.3	2455	coding	PF01451
MSTRG.12157.4	1823	coding	PF01451
MSTRG.12157.2	2133	coding	PF01451
MSTRG.12157.5	672	coding	PF01451
MSTRG.12159.2	3165	coding	PF04366
MSTRG.12159.1	1877	coding	PF04366
MSTRG.12159.3	1134	noncoding	-
MSTRG.12160.1	7134	noncoding	-
MSTRG.12161.1	1487	noncoding	-
MSTRG.12168.3	3366	noncoding	-
MSTRG.12188.1	665	noncoding	-
MSTRG.12189.1	1567	noncoding	-
MSTRG.12189.3	2433	noncoding	-
MSTRG.12195.1	7929	noncoding	-
MSTRG.12196.1	2274	noncoding	-
MSTRG.12197.2	1760	noncoding	-
MSTRG.12197.3	3135	noncoding	-
MSTRG.12197.4	4138	coding	PF07500
MSTRG.12197.5	4186	noncoding	-
MSTRG.12198.1	3164	noncoding	-
MSTRG.12202.1	217	noncoding	-
MSTRG.12233.1	1742	noncoding	-
MSTRG.12238.1	4955	noncoding	-
MSTRG.12246.2	4677	noncoding	-
MSTRG.12208.1	523	noncoding	-
MSTRG.12214.5	9228	noncoding	-
MSTRG.12214.6	1700	noncoding	-
MSTRG.12214.8	4234	noncoding	-
MSTRG.12215.1	1417	noncoding	-
MSTRG.12207.1	1664	noncoding	-
MSTRG.12218.5	2335	coding	PF01412

MSTRG.12218.6	2217	coding	PF01412
MSTRG.12224.1	1025	noncoding	-
MSTRG.12228.1	1074	noncoding	-
MSTRG.12274.1	414	noncoding	-
MSTRG.12277.1	7243	coding	PF01359
MSTRG.12278.2	388	noncoding	-
MSTRG.12279.1	1774	noncoding	-
MSTRG.12280.1	4965	coding	PF00078
MSTRG.12280.2	8008	noncoding	-
MSTRG.12280.3	4293	noncoding	-
MSTRG.12281.1	2042	noncoding	-
MSTRG.12281.2	2919	noncoding	-
MSTRG.12285.2	2253	coding	PF01134
MSTRG.12288.2	1581	coding	PF00009
MSTRG.12288.3	2024	coding	PF00009
MSTRG.12302.2	1647	coding	PF03381
MSTRG.12303.1	1269	noncoding	-
MSTRG.12302.3	2913	coding	PF03381
MSTRG.12302.4	1201	coding	PF03381
MSTRG.12302.5	2178	coding	PF03381
MSTRG.12320.2	4462	coding	PF02902
MSTRG.12320.4	4338	coding	PF02902
MSTRG.12320.5	7520	coding	PF02902
MSTRG.12320.7	4101	coding	PF02902
MSTRG.12322.1	8462	coding	PF00063
MSTRG.12323.1	930	noncoding	-
MSTRG.12322.3	719	noncoding	-
MSTRG.12347.5	6775	coding	PF00439
MSTRG.12347.6	10564	coding	PF00439
MSTRG.12347.7	10374	coding	PF00439
MSTRG.12347.8	6116	coding	PF00439
MSTRG.12351.1	1882	noncoding	-
MSTRG.12352.1	963	noncoding	-
MSTRG.12340.2	3009	coding	PF15619
MSTRG.12340.4	2956	coding	PF15619
MSTRG.12340.3	3458	coding	PF15619
MSTRG.12341.1	4798	noncoding	-
MSTRG.12342.1	2550	noncoding	-
MSTRG.12353.1	408	noncoding	-
MSTRG.12356.1	587	noncoding	-
MSTRG.12355.2	2518	coding	PF02780
MSTRG.12360.3	5210	coding	PF07984
MSTRG.12359.1	277	noncoding	-
MSTRG.12416.1	2328	noncoding	-
MSTRG.12425.1	1161	noncoding	-
MSTRG.12426.2	14279	noncoding	-
MSTRG.12433.1	2024	noncoding	-
MSTRG.12434.4	7554	coding	PF04118
MSTRG.12434.5	9457	coding	PF04118
MSTRG.12434.7	7656	coding	PF04118
MSTRG.12434.8	8098	coding	PF04118
MSTRG.12435.2	2197	noncoding	-
MSTRG.12439.2	3322	coding	PF03949
MSTRG.12440.1	6524	noncoding	-

MSTRG.12441.1	8970	coding	PF02994
MSTRG.12442.1	5460	noncoding	-
MSTRG.12442.2	5923	noncoding	-
MSTRG.12441.2	7173	noncoding	-
MSTRG.12439.3	5173	noncoding	-
MSTRG.12439.4	6643	coding	PF00390
MSTRG.12384.2	4324	coding	PF07651
MSTRG.12384.1	4238	coding	PF07651
MSTRG.12384.3	2509	coding	PF01728
MSTRG.12389.1	1043	noncoding	-
MSTRG.12390.3	1349	noncoding	-
MSTRG.12391.1	5150	noncoding	-
MSTRG.12391.2	3116	noncoding	-
MSTRG.12392.1	353	noncoding	-
MSTRG.12395.1	427	coding	PF15510
MSTRG.12407.1	2049	noncoding	-
MSTRG.12400.2	1509	noncoding	-
MSTRG.12447.5	4009	coding	PF07534
MSTRG.12448.1	2203	noncoding	-
MSTRG.12449.1	5588	noncoding	-
MSTRG.12450.1	7779	noncoding	-
MSTRG.12444.1	1615	noncoding	-
MSTRG.12445.2	775	coding	PF13023
MSTRG.12474.3	1438	coding	PF02827
MSTRG.12474.5	1365	coding	PF02827
MSTRG.12474.2	1147	coding	PF02827
MSTRG.12474.4	1954	coding	PF02827
MSTRG.12474.1	1224	coding	PF02827
MSTRG.12474.7	1370	coding	PF02827
MSTRG.12474.6	1138	coding	PF02827
MSTRG.12474.9	1505	coding	PF02827
MSTRG.12474.8	1110	coding	PF02827
MSTRG.12475.1	2887	coding	PF03348
MSTRG.12475.2	2780	coding	PF03348
MSTRG.12475.3	2784	coding	PF03348
MSTRG.12475.5	5176	coding	PF03348
MSTRG.12476.1	2800	coding	PF00447
MSTRG.12476.2	2222	coding	PF00447
MSTRG.12476.3	3191	coding	PF00447
MSTRG.12476.7	2395	coding	PF00447
MSTRG.12476.8	2492	coding	PF00447
MSTRG.12476.6	2231	coding	PF00447
MSTRG.12476.9	2414	coding	PF00447
MSTRG.12494.1	2751	noncoding	-
MSTRG.12495.1	2235	noncoding	-
MSTRG.12495.2	3721	noncoding	-
MSTRG.12504.1	557	noncoding	-
MSTRG.12504.2	858	noncoding	-
MSTRG.12505.1	14178	noncoding	-
MSTRG.12506.1	5354	coding	PF00078
MSTRG.12507.1	449	noncoding	-
MSTRG.12505.2	4118	noncoding	-
MSTRG.12501.1	4254	coding	PF15665
MSTRG.12501.3	1242	noncoding	-

MSTRG.12509.1	1796	noncoding	-
MSTRG.12510.2	2764	noncoding	-
MSTRG.12510.3	6952	noncoding	-
MSTRG.12510.4	2507	noncoding	-
MSTRG.12517.3	1653	coding	PF00595
MSTRG.12521.1	554	noncoding	-
MSTRG.12525.2	3830	coding	PF02257
MSTRG.12525.1	4149	coding	PF02257
MSTRG.12525.3	4494	coding	PF02257
MSTRG.12528.3	1393	noncoding	-
MSTRG.12537.2	3109	coding	PF00956
MSTRG.12553.1	2358	noncoding	-
MSTRG.12558.1	1334	noncoding	-
MSTRG.12558.2	600	noncoding	-
MSTRG.12558.3	2613	noncoding	-
MSTRG.12560.1	18790	noncoding	-
MSTRG.12550.2	2197	coding	PF00850
MSTRG.12550.3	1947	coding	PF00850
MSTRG.12569.1	472	noncoding	-
MSTRG.12572.1	339	noncoding	-
MSTRG.12573.1	247	noncoding	-
MSTRG.12576.3	628	coding	PF14982
MSTRG.12576.4	648	coding	PF14982
MSTRG.12577.7	1497	coding	PF00091
MSTRG.12577.5	1641	coding	PF00091
MSTRG.12577.4	1492	coding	PF03953
MSTRG.12578.4	5308	coding	PF07714
MSTRG.12588.1	10685	coding	PF15735
MSTRG.12588.2	10813	coding	PF15735
MSTRG.12588.3	10652	coding	PF15735
MSTRG.12588.5	10379	coding	PF15735
MSTRG.12589.1	854	noncoding	-
MSTRG.12589.2	2802	noncoding	-
MSTRG.12589.3	1502	noncoding	-
MSTRG.12589.4	820	noncoding	-
MSTRG.12591.1	3693	noncoding	-
MSTRG.12595.2	620	noncoding	-
MSTRG.12596.3	2452	coding	PF01536
MSTRG.12596.4	9526	noncoding	-
MSTRG.12596.5	3536	noncoding	-
MSTRG.12599.1	4843	noncoding	-
MSTRG.12598.4	9348	coding	PF00069
MSTRG.12600.1	4065	noncoding	-
MSTRG.12598.5	6129	coding	PF00069
MSTRG.12598.6	1516	noncoding	-
MSTRG.12598.8	6392	coding	PF00069
MSTRG.12598.9	5338	coding	PF00069
MSTRG.12601.1	894	noncoding	-
MSTRG.12602.1	1752	noncoding	-
MSTRG.12602.3	896	noncoding	-
MSTRG.12602.2	494	noncoding	-
MSTRG.12602.4	761	noncoding	-
MSTRG.12603.1	1508	noncoding	-
MSTRG.12603.2	1389	noncoding	-

MSTRG.12603.3	1896	noncoding	-
MSTRG.12603.4	1277	noncoding	-
MSTRG.12607.2	3699	noncoding	-
MSTRG.12607.3	2563	noncoding	-
MSTRG.12611.5	6578	coding	PF00406
MSTRG.12611.3	6449	coding	PF00406
MSTRG.12611.7	6585	coding	PF00406
MSTRG.12612.1	1533	noncoding	-
MSTRG.12612.2	2525	noncoding	-
MSTRG.12617.2	1565	noncoding	-
MSTRG.12617.3	1569	noncoding	-
MSTRG.12619.1	1964	noncoding	-
MSTRG.12618.2	1129	noncoding	-
MSTRG.12618.3	7364	noncoding	-
MSTRG.12619.2	1867	noncoding	-
MSTRG.12619.3	1903	noncoding	-
MSTRG.12642.1	3471	noncoding	-
MSTRG.12643.1	4675	noncoding	-
MSTRG.12646.3	1136	coding	PF04636
MSTRG.12647.1	3628	noncoding	-
MSTRG.12642.2	3100	noncoding	-
MSTRG.12648.1	1757	noncoding	-
MSTRG.12646.5	1790	coding	PF04636
MSTRG.12646.4	2204	coding	PF04636
MSTRG.12646.6	1279	coding	PF04636
MSTRG.12652.3	2483	noncoding	-
MSTRG.12652.4	1353	noncoding	-
MSTRG.12623.3	7618	coding	PF16676
MSTRG.12623.4	7329	coding	PF16676
MSTRG.12623.6	9662	coding	PF16676
MSTRG.12623.5	2096	coding	PF16676
MSTRG.12625.2	1585	coding	PF03969
MSTRG.12625.3	1296	coding	PF03969
MSTRG.12627.1	1469	coding	PF00105
MSTRG.12627.2	2362	coding	PF00104
MSTRG.12633.2	3025	coding	PF09777
MSTRG.12635.1	4392	coding	PF00226
MSTRG.12655.1	468	noncoding	-
MSTRG.12660.1	1529	noncoding	-
MSTRG.12661.2	1142	noncoding	-
MSTRG.12664.1	2568	noncoding	-
MSTRG.12674.3	950	coding	PF13602
MSTRG.12676.1	8622	coding	PF00030
MSTRG.12677.2	2056	coding	PF04106
MSTRG.12680.1	318	noncoding	-
MSTRG.12681.2	2630	coding	PF02897
MSTRG.12682.1	1788	coding	PF04831
MSTRG.12685.1	6955	coding	PF00313
MSTRG.12685.2	5199	coding	PF00313
MSTRG.12686.1	362	noncoding	-
MSTRG.12683.2	2892	coding	PF00632
MSTRG.12709.4	2543	noncoding	-
MSTRG.12711.1	5705	coding	PF01359
MSTRG.12711.2	23852	coding	PF01359

MSTRG.12711.3	23540	coding	PF02994
MSTRG.12709.6	6235	noncoding	-
MSTRG.12713.1	7513	noncoding	-
MSTRG.12715.1	11289	noncoding	-
MSTRG.12701.1	224	noncoding	-
MSTRG.12716.1	4592	coding	PF00060
MSTRG.12716.2	4483	coding	PF00060
MSTRG.12716.3	4862	coding	PF10613
MSTRG.12717.1	2546	noncoding	-
MSTRG.12716.4	9199	noncoding	-
MSTRG.12718.1	3824	noncoding	-
MSTRG.12716.5	1502	noncoding	-
MSTRG.12705.2	265	noncoding	-
MSTRG.12705.4	1367	noncoding	-
MSTRG.12705.3	7677	coding	PF02889
MSTRG.12705.5	3704	coding	PF02889
MSTRG.12730.2	2060	noncoding	-
MSTRG.12731.3	3091	noncoding	-
MSTRG.12731.2	5635	coding	PF15996
MSTRG.12731.4	3291	noncoding	-
MSTRG.12731.6	6719	coding	PF15996
MSTRG.12731.7	6661	coding	PF15996
MSTRG.12731.8	5640	coding	PF15996
MSTRG.12720.2	1277	noncoding	-
MSTRG.12721.3	1897	noncoding	-
MSTRG.12722.1	330	noncoding	-
MSTRG.12725.1	420	noncoding	-
MSTRG.12725.2	425	noncoding	-
MSTRG.12738.3	1017	noncoding	-
MSTRG.12746.1	1569	coding	PF16317
MSTRG.12759.1	331	noncoding	-
MSTRG.12768.1	786	noncoding	-
MSTRG.12769.1	1218	noncoding	-
MSTRG.12791.1	7581	noncoding	-
MSTRG.12791.2	6889	noncoding	-
MSTRG.12792.1	18091	coding	PF07728
MSTRG.12792.2	18269	coding	PF07728
MSTRG.12792.3	18130	coding	PF07728
MSTRG.12792.6	1882	noncoding	-
MSTRG.12793.2	1978	coding	PF05347
MSTRG.12794.1	1021	noncoding	-
MSTRG.12794.2	648	noncoding	-
MSTRG.12777.1	4425	coding	PF04670
MSTRG.12778.1	1858	coding	PF00179
MSTRG.12784.1	596	noncoding	-
MSTRG.12785.2	5971	noncoding	-
MSTRG.12785.3	7237	noncoding	-
MSTRG.12785.4	5574	noncoding	-
MSTRG.12789.3	4282	coding	PF01331
MSTRG.12800.1	1198	noncoding	-
MSTRG.12800.2	1046	noncoding	-
MSTRG.12800.3	1050	noncoding	-
MSTRG.12804.2	2271	noncoding	-
MSTRG.12805.3	1798	coding	PF07034

MSTRG.12806.2	2497	coding	PF00750
MSTRG.12806.1	2882	coding	PF00750
MSTRG.12806.4	2584	coding	PF00750
MSTRG.12806.5	3273	coding	PF00750
MSTRG.12808.2	1597	coding	PF04142
MSTRG.12810.1	12164	coding	PF02994
MSTRG.12813.1	2631	noncoding	-
MSTRG.12816.1	1115	noncoding	-
MSTRG.12829.1	7464	coding	PF00076
MSTRG.12829.6	3597	coding	PF00076
MSTRG.12829.4	2295	coding	PF00076
MSTRG.12829.7	3136	coding	PF00076
MSTRG.12830.4	2860	coding	PF00787
MSTRG.12831.1	4084	noncoding	-
MSTRG.12835.1	293	noncoding	-
MSTRG.12826.1	1610	noncoding	-
MSTRG.12826.2	1844	noncoding	-
MSTRG.12827.1	329	noncoding	-
MSTRG.12839.2	2292	noncoding	-
MSTRG.12847.3	2294	coding	PF00102
MSTRG.12847.4	2288	coding	PF00102
MSTRG.12848.1	1419	noncoding	-
MSTRG.12851.2	2970	coding	PF00620
MSTRG.12851.3	2431	coding	PF00620
MSTRG.12863.6	6078	coding	PF05902
MSTRG.12863.7	6238	coding	PF05902
MSTRG.12863.14	5872	coding	PF05902
MSTRG.12863.16	6008	coding	PF05902
MSTRG.12863.11	4590	coding	PF05902
MSTRG.12863.13	4870	coding	PF05902
MSTRG.12863.15	4213	coding	PF05902
MSTRG.12863.12	4045	coding	PF05902
MSTRG.12863.18	3411	coding	PF05902
MSTRG.12863.17	3509	coding	PF05902
MSTRG.12863.19	7169	noncoding	-
MSTRG.12864.1	6043	noncoding	-
MSTRG.12863.20	465	noncoding	-
MSTRG.12888.1	3284	noncoding	-
MSTRG.12890.2	2959	coding	PF00352
MSTRG.12890.4	2282	coding	PF00352
MSTRG.12907.3	2814	coding	PF00009
MSTRG.12907.2	6797	coding	PF00009
MSTRG.12907.4	2580	coding	PF00009
MSTRG.12907.5	3373	noncoding	-
MSTRG.12907.6	5679	noncoding	-
MSTRG.12912.3	4084	coding	PF00018
MSTRG.12912.4	3583	coding	PF00018
MSTRG.12914.1	900	noncoding	-
MSTRG.12933.4	3603	noncoding	-
MSTRG.12933.6	4757	noncoding	-
MSTRG.12933.5	3609	noncoding	-
MSTRG.12933.3	7558	noncoding	-
MSTRG.12933.7	7684	noncoding	-
MSTRG.12933.8	3880	noncoding	-

MSTRG.12934.1	2128	noncoding	-
MSTRG.12919.1	1708	noncoding	-
MSTRG.12920.1	1791	coding	PF01108
MSTRG.12920.2	2140	coding	PF01108
MSTRG.12925.1	348	noncoding	-
MSTRG.12925.2	284	noncoding	-
MSTRG.12926.1	302	noncoding	-
MSTRG.12938.1	1043	noncoding	-
MSTRG.12947.3	6974	coding	PF15273
MSTRG.12947.2	7130	coding	PF15273
MSTRG.12971.1	12458	noncoding	-
MSTRG.12978.1	746	noncoding	-
MSTRG.12978.2	18716	noncoding	-
MSTRG.12978.3	12222	noncoding	-
MSTRG.12983.5	2596	coding	PF12763
MSTRG.12984.1	378	noncoding	-
MSTRG.12939.1	5109	coding	PF16002
MSTRG.12939.2	3184	coding	PF16002
MSTRG.12939.3	3418	coding	PF16002
MSTRG.12940.1	2957	noncoding	-
MSTRG.12939.4	3151	coding	PF16002
MSTRG.12939.5	5835	coding	PF16002
MSTRG.12988.1	5831	noncoding	-
MSTRG.13003.1	2893	noncoding	-
MSTRG.13008.1	2202	noncoding	-
MSTRG.13008.2	1978	noncoding	-
MSTRG.13009.1	1894	noncoding	-
MSTRG.13010.1	228	noncoding	-
MSTRG.13012.1	1092	noncoding	-
MSTRG.13013.1	3299	noncoding	-
MSTRG.13013.2	13443	noncoding	-
MSTRG.13013.6	853	noncoding	-
MSTRG.13013.4	8489	noncoding	-
MSTRG.13013.5	7190	coding	PF04750
MSTRG.13014.1	1225	noncoding	-
MSTRG.13022.1	27763	noncoding	-
MSTRG.13022.2	4485	noncoding	-
MSTRG.13025.2	24787	noncoding	-
MSTRG.13025.4	60730	noncoding	-
MSTRG.13025.5	12443	noncoding	-
MSTRG.13025.7	12028	noncoding	-
MSTRG.13025.8	12361	noncoding	-
MSTRG.13025.9	19710	noncoding	-
MSTRG.13025.10	6661	noncoding	-
MSTRG.13025.11	18149	noncoding	-
MSTRG.13025.12	7247	noncoding	-
MSTRG.13030.3	3967	coding	PF00307
MSTRG.13030.1	12150	coding	PF09069
MSTRG.13034.1	287	noncoding	-
MSTRG.13034.2	417	noncoding	-
MSTRG.13034.3	412	noncoding	-
MSTRG.13040.4	7808	noncoding	-
MSTRG.13040.6	5955	noncoding	-
MSTRG.13039.1	238	noncoding	-

MSTRG.13048.1	9162	coding	PF08366
MSTRG.13049.1	892	noncoding	-
MSTRG.13055.1	1968	noncoding	-
MSTRG.13053.1	565	noncoding	-
MSTRG.13056.2	3764	noncoding	-
MSTRG.13061.1	3057	noncoding	-
MSTRG.13061.3	10573	noncoding	-
MSTRG.13062.1	2397	noncoding	-
MSTRG.13061.5	3296	noncoding	-
MSTRG.13060.1	2265	coding	PF11977
MSTRG.13063.1	1895	noncoding	-
MSTRG.13065.2	1165	noncoding	-
MSTRG.13066.3	1696	coding	PF00004
MSTRG.13068.1	253	noncoding	-
MSTRG.13077.4	1830	coding	PF01135
MSTRG.13078.1	1142	noncoding	-
MSTRG.13079.2	1146	coding	PF07502
MSTRG.13082.3	688	noncoding	-
MSTRG.13082.4	1076	noncoding	-
MSTRG.13082.2	1313	noncoding	-
MSTRG.13087.1	8486	noncoding	-
MSTRG.13087.2	8382	noncoding	-
MSTRG.13087.4	10676	noncoding	-
MSTRG.13087.6	8355	noncoding	-
MSTRG.13091.2	9218	noncoding	-
MSTRG.13096.2	4805	coding	PF01937
MSTRG.13106.2	27569	noncoding	-
MSTRG.13106.1	27755	noncoding	-
MSTRG.13106.3	27747	noncoding	-
MSTRG.13107.1	1003	noncoding	-
MSTRG.13100.4	1024	coding	PF00472
MSTRG.13110.4	7654	noncoding	-
MSTRG.13110.3	5840	noncoding	-
MSTRG.13110.5	4920	noncoding	-
MSTRG.13110.6	3600	noncoding	-
MSTRG.13110.7	4504	noncoding	-
MSTRG.13111.1	1287	noncoding	-
MSTRG.13112.1	2596	noncoding	-
MSTRG.13115.1	7305	coding	PF00621
MSTRG.13117.1	442	noncoding	-
MSTRG.13115.2	6807	coding	PF00621
MSTRG.13118.2	1768	coding	PF00398
MSTRG.13119.1	9309	noncoding	-
MSTRG.13120.1	938	noncoding	-
MSTRG.13118.4	2585	coding	PF00398
MSTRG.13118.5	1808	coding	PF00398
MSTRG.13143.4	8153	coding	PF12031
MSTRG.13143.6	4991	coding	PF01388
MSTRG.13143.5	5581	coding	PF12031
MSTRG.13143.7	6870	coding	PF12031
MSTRG.13144.1	12965	noncoding	-
MSTRG.13143.8	7608	coding	PF12031
MSTRG.13143.9	5947	coding	PF12031
MSTRG.13145.1	972	noncoding	-

MSTRG.13146.1	4938	noncoding	-
MSTRG.13143.10	840	noncoding	-
MSTRG.13148.1	4702	noncoding	-
MSTRG.13149.1	7817	noncoding	-
MSTRG.13150.1	3081	noncoding	-
MSTRG.13151.1	5709	noncoding	-
MSTRG.13152.1	4471	noncoding	-
MSTRG.13152.2	5686	noncoding	-
MSTRG.13143.11	4039	noncoding	-
MSTRG.13143.12	3756	coding	PF12031
MSTRG.13130.1	416	noncoding	-
MSTRG.13134.1	2196	noncoding	-
MSTRG.13137.1	2571	noncoding	-
MSTRG.13138.3	2768	noncoding	-
MSTRG.13138.5	2030	coding	PF01529
MSTRG.13138.6	1079	noncoding	-
MSTRG.13139.1	9449	noncoding	-
MSTRG.13139.2	5368	noncoding	-
MSTRG.13139.3	13889	noncoding	-
MSTRG.13139.4	7270	noncoding	-
MSTRG.13139.5	22132	noncoding	-
MSTRG.13128.2	3036	coding	PF10456
MSTRG.13128.3	2743	coding	PF10456
MSTRG.13128.4	2829	coding	PF10456
MSTRG.13154.1	844	noncoding	-
MSTRG.13154.2	388	noncoding	-
MSTRG.13154.3	1007	noncoding	-
MSTRG.13154.4	1029	noncoding	-
MSTRG.13155.2	2395	noncoding	-
MSTRG.13162.1	3269	noncoding	-
MSTRG.13163.1	9280	noncoding	-
MSTRG.13164.1	23923	noncoding	-
MSTRG.13161.6	6216	coding	PF01167
MSTRG.13161.4	6057	coding	PF01167
MSTRG.13161.7	6196	coding	PF01167
MSTRG.13161.5	13916	coding	PF01167
MSTRG.13165.1	439	noncoding	-
MSTRG.13159.2	4674	coding	PF06664
MSTRG.13159.3	4633	coding	PF06664
MSTRG.13159.4	3551	coding	PF06664
MSTRG.13166.1	2756	coding	PF02318
MSTRG.13170.3	607	noncoding	-
MSTRG.13171.1	3596	coding	PF02777
MSTRG.13177.1	2146	coding	PF00118
MSTRG.13177.4	3666	noncoding	-
MSTRG.13177.5	1471	noncoding	-
MSTRG.13174.1	8993	coding	PF00878
MSTRG.13181.3	5365	coding	PF00069
MSTRG.13181.4	5282	coding	PF00069
MSTRG.13181.5	5112	coding	PF00069
MSTRG.13179.1	768	noncoding	-
MSTRG.13179.3	595	noncoding	-
MSTRG.13179.2	932	noncoding	-
MSTRG.13180.1	2305	noncoding	-

MSTRG.13184.1	3860	coding	PF00240
MSTRG.13184.2	1981	coding	PF00240
MSTRG.13199.1	1410	coding	PF10274
MSTRG.13199.2	1124	coding	PF10274
MSTRG.13201.1	894	noncoding	-
MSTRG.13183.1	891	noncoding	-
MSTRG.13206.2	526	noncoding	-
MSTRG.13208.1	13581	coding	PF16544
MSTRG.13208.4	7705	coding	PF16544
MSTRG.13209.1	12566	noncoding	-
MSTRG.13209.2	15529	noncoding	-
MSTRG.13203.1	785	noncoding	-
MSTRG.13217.1	202	noncoding	-
MSTRG.13221.2	1312	noncoding	-
MSTRG.13222.1	4573	coding	PF00233
MSTRG.13222.2	9868	coding	PF00233
MSTRG.13222.3	7950	coding	PF00233
MSTRG.13222.6	3946	noncoding	-
MSTRG.13223.1	3376	noncoding	-
MSTRG.13224.1	2284	noncoding	-
MSTRG.13222.7	3417	noncoding	-
MSTRG.13233.1	5182	coding	PF00069
MSTRG.13237.1	1690	noncoding	-
MSTRG.13241.3	1880	noncoding	-
MSTRG.13241.4	2412	coding	PF09398
MSTRG.13241.5	2405	coding	PF09398
MSTRG.13241.6	2794	noncoding	-
MSTRG.13243.1	1565	coding	PF07202
MSTRG.13243.2	741	coding	PF07202
MSTRG.13243.3	734	coding	PF07202
MSTRG.13244.3	1981	coding	PF03133
MSTRG.13261.4	7130	coding	PF00788
MSTRG.13261.5	7133	coding	PF00788
MSTRG.13261.6	7130	coding	PF00788
MSTRG.13261.7	7136	coding	PF00788
MSTRG.13261.1	7531	coding	PF00788
MSTRG.13261.8	7112	coding	PF00788
MSTRG.13261.9	6726	coding	PF00788
MSTRG.13261.10	7698	coding	PF00788
MSTRG.13261.11	6637	coding	PF00788
MSTRG.13261.12	6559	coding	PF00788
MSTRG.13261.13	7415	coding	PF00788
MSTRG.13261.14	7488	coding	PF00788
MSTRG.13263.1	1416	noncoding	-
MSTRG.13266.1	948	noncoding	-
MSTRG.13267.1	5126	noncoding	-
MSTRG.13267.2	1251	noncoding	-
MSTRG.13267.3	804	noncoding	-
MSTRG.13267.4	940	noncoding	-
MSTRG.13254.2	916	noncoding	-
MSTRG.13254.1	466	noncoding	-
MSTRG.13253.3	2497	coding	PF00086
MSTRG.13253.4	2687	coding	PF00086
MSTRG.13259.2	3093	coding	PF05735

MSTRG.13260.1	3742	coding	PF05735
MSTRG.13268.1	2438	noncoding	-
MSTRG.13268.2	2695	noncoding	-
MSTRG.13268.3	1339	noncoding	-
MSTRG.13268.4	7702	noncoding	-
MSTRG.13268.5	964	noncoding	-
MSTRG.13269.1	1957	noncoding	-
MSTRG.13268.8	3986	noncoding	-
MSTRG.13270.1	2279	noncoding	-
MSTRG.13273.4	1855	noncoding	-
MSTRG.13281.1	2797	noncoding	-
MSTRG.13281.2	271	noncoding	-
MSTRG.13282.2	231	noncoding	-
MSTRG.13284.8	4839	coding	PF13910
MSTRG.13284.6	2024	coding	PF13910
MSTRG.13284.9	4048	noncoding	-
MSTRG.13284.10	1895	noncoding	-
MSTRG.13277.1	901	noncoding	-
MSTRG.13277.2	996	noncoding	-
MSTRG.13286.3	3792	noncoding	-
MSTRG.13287.1	1077	noncoding	-
MSTRG.13303.1	554	noncoding	-
MSTRG.13305.1	243	noncoding	-
MSTRG.13314.1	15639	coding	PF05026
MSTRG.13314.3	7923	noncoding	-
MSTRG.13314.2	6102	noncoding	-
MSTRG.13323.1	2411	noncoding	-
MSTRG.13323.2	1901	noncoding	-
MSTRG.13320.3	3889	coding	PF09380
MSTRG.13320.4	9217	noncoding	-
MSTRG.13320.5	3430	coding	PF09380
MSTRG.13320.6	14926	noncoding	-
MSTRG.13320.7	1748	coding	PF00373
MSTRG.13320.9	2673	noncoding	-
MSTRG.13324.1	14321	noncoding	-
MSTRG.13325.1	2589	noncoding	-
MSTRG.13327.1	4484	noncoding	-
MSTRG.13329.1	2182	noncoding	-
MSTRG.13331.1	965	noncoding	-
MSTRG.13332.1	1041	noncoding	-
MSTRG.13332.2	977	noncoding	-
MSTRG.13332.3	2329	noncoding	-
MSTRG.13334.1	848	coding	PF11092
MSTRG.13334.4	2209	coding	PF11092
MSTRG.13318.1	2326	noncoding	-
MSTRG.13345.1	2559	noncoding	-
MSTRG.13356.1	3997	coding	PF12796
MSTRG.13357.1	3200	noncoding	-
MSTRG.13356.3	4237	coding	PF12796
MSTRG.13356.4	3824	coding	PF12796
MSTRG.13356.5	9374	coding	PF12796
MSTRG.13364.2	4031	coding	PF05995
MSTRG.13365.1	310	noncoding	-
MSTRG.13366.3	3279	noncoding	-

MSTRG.13367.1	440	noncoding	-
MSTRG.13367.2	426	noncoding	-
MSTRG.13369.2	1561	coding	PF14949
MSTRG.13369.4	1354	coding	PF14949
MSTRG.13369.3	1573	coding	PF14949
MSTRG.13378.1	1772	noncoding	-
MSTRG.13378.2	1099	noncoding	-
MSTRG.13381.2	970	noncoding	-
MSTRG.13389.1	5551	coding	PF15409
MSTRG.13391.6	2218	noncoding	-
MSTRG.13391.3	2607	noncoding	-
MSTRG.13396.1	3253	noncoding	-
MSTRG.13397.1	16732	noncoding	-
MSTRG.13398.1	6856	noncoding	-
MSTRG.13401.1	5315	noncoding	-
MSTRG.13409.2	5574	coding	PF00616
MSTRG.13410.1	2171	noncoding	-
MSTRG.13410.2	1976	noncoding	-
MSTRG.13409.3	6065	coding	PF00616
MSTRG.13409.4	4917	coding	PF00616
MSTRG.13409.5	4646	coding	PF00616
MSTRG.13409.8	4745	coding	PF00616
MSTRG.13409.6	4876	coding	PF00616
MSTRG.13409.7	4794	coding	PF00616
MSTRG.13409.9	4663	coding	PF00616
MSTRG.13411.1	5880	noncoding	-
MSTRG.13411.4	2341	noncoding	-
MSTRG.13423.1	236	noncoding	-
MSTRG.13425.1	19056	noncoding	-
MSTRG.13427.1	9357	noncoding	-
MSTRG.13427.2	2109	noncoding	-
MSTRG.13427.4	2215	noncoding	-
MSTRG.13427.3	2190	noncoding	-
MSTRG.13427.6	1710	noncoding	-
MSTRG.13427.8	2364	noncoding	-
MSTRG.13427.9	1410	noncoding	-
MSTRG.13427.10	1375	noncoding	-
MSTRG.13427.11	514	noncoding	-
MSTRG.13428.1	13897	noncoding	-
MSTRG.13428.2	7330	noncoding	-
MSTRG.13429.1	15878	noncoding	-
MSTRG.13430.1	2562	noncoding	-
MSTRG.13431.1	13439	coding	PF00078
MSTRG.13431.2	2707	noncoding	-
MSTRG.13432.1	8932	noncoding	-
MSTRG.13413.2	815	noncoding	-
MSTRG.13414.1	2412	noncoding	-
MSTRG.13418.1	2165	noncoding	-
MSTRG.13420.1	3499	coding	PF04144
MSTRG.13421.2	4973	coding	PF10242
MSTRG.13419.1	456	noncoding	-
MSTRG.13419.2	610	noncoding	-
MSTRG.13419.3	536	noncoding	-
MSTRG.13433.1	624	noncoding	-

MSTRG.13437.2	1836	noncoding	-
MSTRG.13440.1	2267	noncoding	-
MSTRG.13449.1	1500	noncoding	-
MSTRG.13450.3	4178	coding	PF15871
MSTRG.13450.2	8418	coding	PF15871
MSTRG.13451.1	4089	noncoding	-
MSTRG.13454.2	1270	coding	PF00568
MSTRG.13456.1	5479	noncoding	-
MSTRG.13456.2	7857	noncoding	-
MSTRG.13457.1	5339	noncoding	-
MSTRG.13459.1	233	noncoding	-
MSTRG.13461.6	1342	noncoding	-
MSTRG.13461.5	3032	coding	PF03828
MSTRG.13462.1	593	noncoding	-
MSTRG.13481.1	315	noncoding	-
MSTRG.13486.2	2853	coding	PF00071
MSTRG.13492.1	1622	noncoding	-
MSTRG.13506.1	689	noncoding	-
MSTRG.13506.2	2372	noncoding	-
MSTRG.13506.3	2147	noncoding	-
MSTRG.13506.4	1808	noncoding	-
MSTRG.13510.1	8978	coding	PF02141
MSTRG.13510.2	6152	coding	PF02141
MSTRG.13510.3	5873	coding	PF02141
MSTRG.13510.4	6337	coding	PF02141
MSTRG.13510.5	5935	coding	PF02141
MSTRG.13510.6	6125	coding	PF02141
MSTRG.13497.2	4025	coding	PF00773
MSTRG.13504.1	509	noncoding	-
MSTRG.13512.4	2063	coding	PF09817
MSTRG.13512.6	2083	coding	PF09817
MSTRG.13512.3	2311	coding	PF09817
MSTRG.13512.7	1993	coding	PF09817
MSTRG.13512.8	2111	coding	PF09817
MSTRG.13512.9	2333	coding	PF09817
MSTRG.13518.1	6002	coding	PF03166
MSTRG.13518.3	4004	coding	PF03166
MSTRG.13519.1	3161	noncoding	-
MSTRG.13520.4	2567	noncoding	-
MSTRG.13531.1	2193	noncoding	-
MSTRG.13532.1	2267	coding	PF14324
MSTRG.13532.3	1171	noncoding	-
MSTRG.13532.6	2342	coding	PF14324
MSTRG.13538.2	3369	coding	PF08953
MSTRG.13540.4	1091	noncoding	-
MSTRG.13543.1	1152	coding	PF15754
MSTRG.13554.1	11100	noncoding	-
MSTRG.13555.1	1242	coding	PF02994
MSTRG.13560.2	1004	coding	PF03006
MSTRG.13560.3	1240	coding	PF03006
MSTRG.13562.1	534	noncoding	-
MSTRG.13564.1	4119	coding	PF03920
MSTRG.13564.2	5158	coding	PF03920
MSTRG.13563.1	1750	noncoding	-

MSTRG.13563.2	1304	noncoding	-
MSTRG.13566.3	4799	coding	PF12796
MSTRG.13568.5	2777	noncoding	-
MSTRG.13568.6	1893	noncoding	-
MSTRG.13570.1	8162	coding	PF05986
MSTRG.13578.1	13202	coding	PF00063
MSTRG.13578.4	6728	coding	PF00620
MSTRG.13578.2	12801	coding	PF00063
MSTRG.13578.3	13071	coding	PF00063
MSTRG.13578.6	12951	coding	PF00063
MSTRG.13578.7	13074	coding	PF00063
MSTRG.13578.8	12821	coding	PF00063
MSTRG.13578.9	12980	coding	PF00063
MSTRG.13578.10	13508	coding	PF00620
MSTRG.13578.11	11930	coding	PF00620
MSTRG.13579.1	2688	noncoding	-
MSTRG.13580.1	577	noncoding	-
MSTRG.13580.2	391	noncoding	-
MSTRG.13578.12	3768	coding	PF00063
MSTRG.13578.13	6119	coding	PF00063
MSTRG.13578.14	9513	coding	PF00063
MSTRG.13578.15	3758	coding	PF00063
MSTRG.13578.16	4320	coding	PF00063
MSTRG.13581.1	398	noncoding	-
MSTRG.13583.1	4186	noncoding	-
MSTRG.13584.2	1192	noncoding	-
MSTRG.13572.3	2468	coding	PF00224
MSTRG.13573.4	2347	noncoding	-
MSTRG.13573.5	2287	noncoding	-
MSTRG.13573.3	2747	noncoding	-
MSTRG.13573.7	2593	noncoding	-
MSTRG.13573.1	2820	noncoding	-
MSTRG.13575.2	3081	coding	PF00076
MSTRG.13576.4	2187	coding	PF00728
MSTRG.13576.3	1957	coding	PF00728
MSTRG.13576.5	1855	coding	PF00728
MSTRG.13576.6	1946	coding	PF00728
MSTRG.13576.7	2110	coding	PF00728
MSTRG.13576.8	1864	coding	PF00728
MSTRG.13576.9	1711	coding	PF00728
MSTRG.13577.2	650	noncoding	-
MSTRG.13577.1	934	noncoding	-
MSTRG.13577.5	900	noncoding	-
MSTRG.13577.9	502	noncoding	-
MSTRG.13577.10	867	noncoding	-
MSTRG.13577.11	904	noncoding	-
MSTRG.13577.6	714	noncoding	-
MSTRG.13577.7	729	noncoding	-
MSTRG.13577.8	786	noncoding	-
MSTRG.13577.12	673	noncoding	-
MSTRG.13589.3	4795	coding	PF01485
MSTRG.13589.4	890	noncoding	-
MSTRG.13589.7	2452	coding	PF01485
MSTRG.13589.6	10320	coding	PF01485

MSTRG.13591.1	1840	noncoding	-
MSTRG.13593.5	2425	noncoding	-
MSTRG.13593.6	2912	noncoding	-
MSTRG.13593.7	2516	noncoding	-
MSTRG.13594.1	3405	noncoding	-
MSTRG.13593.8	4500	noncoding	-
MSTRG.13595.2	2175	coding	PF04587
MSTRG.13595.1	2231	coding	PF04587
MSTRG.13596.1	1064	noncoding	-
MSTRG.13597.1	1326	noncoding	-
MSTRG.13586.1	7327	coding	PF06583
MSTRG.13586.2	5258	coding	PF06583
MSTRG.13586.3	7014	coding	PF06583
MSTRG.13587.1	2988	noncoding	-
MSTRG.13598.2	1872	coding	PF15165
MSTRG.13599.1	3702	coding	PF15165
MSTRG.13599.2	1420	coding	PF07679
MSTRG.13599.3	1693	coding	PF07679
MSTRG.13599.4	1778	noncoding	-
MSTRG.13602.1	2754	noncoding	-
MSTRG.13602.3	2549	noncoding	-
MSTRG.13602.5	2552	noncoding	-
MSTRG.13600.1	2628	noncoding	-
MSTRG.13603.1	414	noncoding	-
MSTRG.13603.2	824	noncoding	-
MSTRG.13608.3	4939	coding	PF11977
MSTRG.13608.2	3797	coding	PF11977
MSTRG.13608.6	2332	coding	PF11977
MSTRG.13608.5	2405	coding	PF11977
MSTRG.13605.1	3198	noncoding	-
MSTRG.13612.2	807	coding	PF00106
MSTRG.13614.2	2257	coding	PF00868
MSTRG.13618.3	1428	coding	PF12689
MSTRG.13618.1	3203	coding	PF12689
MSTRG.13618.2	3793	coding	PF12689
MSTRG.13627.2	5816	coding	PF16678
MSTRG.13627.3	4930	coding	PF16678
MSTRG.13630.1	1526	coding	PF07002
MSTRG.13637.2	3378	noncoding	-
MSTRG.13657.4	3515	coding	PF00452
MSTRG.13658.8	2719	coding	PF11569
MSTRG.13666.4	1250	coding	PF14969
MSTRG.13666.3	1414	coding	PF14969
MSTRG.13666.5	1186	coding	PF14969
MSTRG.13666.2	1450	coding	PF14969
MSTRG.13667.1	4591	noncoding	-
MSTRG.13667.5	6670	noncoding	-
MSTRG.13667.6	4370	noncoding	-
MSTRG.13667.13	6776	noncoding	-
MSTRG.13667.12	5518	noncoding	-
MSTRG.13652.1	2000	coding	PF00227
MSTRG.13652.2	1752	coding	PF00227
MSTRG.13652.3	426	noncoding	-
MSTRG.13653.2	1263	coding	PF00227

MSTRG.13653.3	967	coding	PF00227
MSTRG.13669.2	2260	coding	PF15394
MSTRG.13671.1	1515	noncoding	-
MSTRG.13672.6	1711	coding	PF14735
MSTRG.13672.5	1771	coding	PF14735
MSTRG.13672.4	1592	coding	PF14735
MSTRG.13682.1	932	noncoding	-
MSTRG.13684.1	334	noncoding	-
MSTRG.13687.2	387	noncoding	-
MSTRG.13690.1	1717	coding	PF09291
MSTRG.13690.2	1900	coding	PF09291
MSTRG.13690.5	1380	coding	PF09291
MSTRG.13690.3	1394	coding	PF09291
MSTRG.13690.4	1353	coding	PF09291
MSTRG.13711.1	13519	coding	PF00176
MSTRG.13711.2	7460	coding	PF00176
MSTRG.13711.3	13601	coding	PF00176
MSTRG.13711.4	8387	coding	PF00176
MSTRG.13711.5	13491	coding	PF00176
MSTRG.13711.6	7295	coding	PF00176
MSTRG.13713.1	1930	noncoding	-
MSTRG.13713.3	1960	noncoding	-
MSTRG.13713.2	2137	noncoding	-
MSTRG.13706.1	1926	noncoding	-
MSTRG.13706.18	1643	noncoding	-
MSTRG.13706.20	2156	noncoding	-
MSTRG.13706.17	1918	noncoding	-
MSTRG.13709.1	5719	noncoding	-
MSTRG.13706.22	8201	noncoding	-
MSTRG.13727.1	4278	coding	PF03096
MSTRG.13727.2	2497	coding	PF03096
MSTRG.13727.3	2105	coding	PF03096
MSTRG.13727.4	2107	coding	PF03096
MSTRG.13725.4	14520	coding	PF09243
MSTRG.13725.6	3769	noncoding	-
MSTRG.13725.7	7428	coding	PF09243
MSTRG.13725.10	2491	noncoding	-
MSTRG.13725.12	2084	coding	PF09243
MSTRG.13725.13	2052	coding	PF09243
MSTRG.13729.1	579	noncoding	-
MSTRG.13714.3	822	coding	PF00074
MSTRG.13717.1	427	noncoding	-
MSTRG.13723.1	14612	coding	PF01048
MSTRG.13738.1	1309	coding	PF13853
MSTRG.13746.1	680	noncoding	-
MSTRG.13746.2	431	noncoding	-
MSTRG.13755.2	472	noncoding	-
MSTRG.13755.1	10666	noncoding	-
MSTRG.13755.3	7685	noncoding	-
MSTRG.13755.5	4895	noncoding	-
MSTRG.13755.6	5957	noncoding	-
MSTRG.13756.1	8521	noncoding	-
MSTRG.13758.2	4123	coding	PF13925
MSTRG.13758.3	12094	coding	PF03184

MSTRG.13758.4	2268	noncoding	-
MSTRG.13750.1	349	noncoding	-
MSTRG.13760.1	3236	noncoding	-
MSTRG.13760.2	2170	noncoding	-
MSTRG.13759.2	1190	noncoding	-
MSTRG.13761.2	8413	coding	PF06459
MSTRG.13767.1	2323	noncoding	-
MSTRG.13768.4	6001	coding	PF02181
MSTRG.13768.3	6243	coding	PF02181
MSTRG.13768.2	6228	coding	PF02181
MSTRG.13768.5	2957	noncoding	-
MSTRG.13769.1	1719	noncoding	-
MSTRG.13768.7	3006	coding	PF02181
MSTRG.13765.1	3803	coding	PF00620
MSTRG.13765.2	3416	coding	PF00620
MSTRG.13764.1	1380	coding	PF00022
MSTRG.13780.2	1546	coding	PF01902
MSTRG.13782.1	5994	noncoding	-
MSTRG.13780.3	5589	noncoding	-
MSTRG.13783.1	843	noncoding	-
MSTRG.13784.1	3620	noncoding	-
MSTRG.13808.1	9578	coding	PF14811
MSTRG.13808.3	1873	coding	PF14811
MSTRG.13808.4	1479	coding	PF14811
MSTRG.13808.2	5053	coding	PF14811
MSTRG.13808.5	30793	coding	PF14811
MSTRG.13809.1	3023	noncoding	-
MSTRG.13811.1	1608	noncoding	-
MSTRG.13819.3	1350	coding	PF14992
MSTRG.13820.1	633	noncoding	-
MSTRG.13813.1	437	noncoding	-
MSTRG.13822.1	1996	noncoding	-
MSTRG.13829.1	244	noncoding	-
MSTRG.13831.2	1564	noncoding	-
MSTRG.13831.3	3820	coding	PF15554
MSTRG.13832.1	1141	noncoding	-
MSTRG.13831.4	2074	coding	PF15554
MSTRG.13836.1	3442	noncoding	-
MSTRG.13837.1	6296	noncoding	-
MSTRG.13838.5	3930	coding	PF00069
MSTRG.13838.6	4526	noncoding	-
MSTRG.13834.2	3766	coding	PF08311
MSTRG.13844.2	17341	coding	PF01426
MSTRG.13844.4	4332	coding	PF01426
MSTRG.13845.1	2037	coding	PF14989
MSTRG.13846.1	1704	coding	PF00849
MSTRG.13846.2	1684	coding	PF00849
MSTRG.13854.3	2126	noncoding	-
MSTRG.13849.2	1795	noncoding	-
MSTRG.13858.2	6679	coding	PF00176
MSTRG.13859.1	2133	noncoding	-
MSTRG.13862.3	7126	noncoding	-
MSTRG.13862.2	4567	noncoding	-
MSTRG.13862.4	2203	coding	PF01612

MSTRG.13862.5	1135	noncoding	-
MSTRG.13867.1	2965	coding	PF16006
MSTRG.13870.4	3872	coding	PF08620
MSTRG.13871.3	4186	coding	PF07714
MSTRG.13871.4	3679	coding	PF07714
MSTRG.13877.3	11597	coding	PF00907
MSTRG.13877.4	11450	coding	PF00907
MSTRG.13877.5	11446	coding	PF00907
MSTRG.13884.4	6890	noncoding	-
MSTRG.13884.6	7299	noncoding	-
MSTRG.13884.5	7321	noncoding	-
MSTRG.13884.7	7317	noncoding	-
MSTRG.13884.8	5865	noncoding	-
MSTRG.13885.2	1285	coding	PF13621
MSTRG.13885.3	1238	coding	PF13621
MSTRG.13882.1	4432	noncoding	-
MSTRG.13881.1	2323	noncoding	-
MSTRG.13886.2	4768	coding	PF10366
MSTRG.13886.5	1860	coding	PF10366
MSTRG.13888.1	827	noncoding	-
MSTRG.13900.1	420	coding	PF01067
MSTRG.13902.1	1100	noncoding	-
MSTRG.13903.3	8137	noncoding	-
MSTRG.13904.1	375	noncoding	-
MSTRG.13908.1	17807	coding	PF00225
MSTRG.13908.2	16660	noncoding	-
MSTRG.13910.2	7793	coding	PF15296
MSTRG.13910.5	6489	noncoding	-
MSTRG.13910.6	4301	coding	PF00069
MSTRG.13913.3	5468	coding	PF02207
MSTRG.13913.4	5779	noncoding	-
MSTRG.13891.1	920	noncoding	-
MSTRG.13899.1	1414	noncoding	-
MSTRG.13918.2	1296	coding	PF00935
MSTRG.13918.3	558	coding	PF00935
MSTRG.13919.2	2782	coding	PF05060
MSTRG.13919.1	4283	coding	PF05060
MSTRG.13940.1	4225	noncoding	-
MSTRG.13941.2	2032	noncoding	-
MSTRG.13941.3	1812	noncoding	-
MSTRG.13942.1	1157	noncoding	-
MSTRG.13942.2	1709	noncoding	-
MSTRG.13942.3	727	noncoding	-
MSTRG.13942.4	1017	noncoding	-
MSTRG.13942.5	437	noncoding	-
MSTRG.13942.6	514	noncoding	-
MSTRG.13942.7	574	noncoding	-
MSTRG.13942.10	290	noncoding	-
MSTRG.13942.11	564	noncoding	-
MSTRG.13942.12	559	noncoding	-
MSTRG.13942.8	482	noncoding	-
MSTRG.13942.9	571	noncoding	-
MSTRG.13942.13	574	noncoding	-
MSTRG.13942.17	569	noncoding	-

MSTRG.13942.18	431	noncoding	-
MSTRG.13942.19	453	noncoding	-
MSTRG.13942.20	505	noncoding	-
MSTRG.13942.14	576	noncoding	-
MSTRG.13942.15	310	noncoding	-
MSTRG.13942.16	476	noncoding	-
MSTRG.13942.21	539	noncoding	-
MSTRG.13942.22	541	noncoding	-
MSTRG.13942.25	450	noncoding	-
MSTRG.13942.27	431	noncoding	-
MSTRG.13942.28	506	noncoding	-
MSTRG.13942.29	407	noncoding	-
MSTRG.13942.30	317	noncoding	-
MSTRG.13942.31	517	noncoding	-
MSTRG.13942.23	560	noncoding	-
MSTRG.13942.24	583	noncoding	-
MSTRG.13942.26	540	noncoding	-
MSTRG.13942.32	566	noncoding	-
MSTRG.13942.33	226	noncoding	-
MSTRG.13927.1	4329	coding	PF10294
MSTRG.13928.2	4413	coding	PF00617
MSTRG.13944.3	3163	noncoding	-
MSTRG.13945.1	1034	noncoding	-
MSTRG.13946.3	2206	coding	PF02263
MSTRG.13929.2	2321	noncoding	-
MSTRG.13930.2	3570	noncoding	-
MSTRG.13952.1	3172	noncoding	-
MSTRG.13952.2	1351	noncoding	-
MSTRG.13952.3	1141	noncoding	-
MSTRG.13955.1	703	noncoding	-
MSTRG.13962.2	842	noncoding	-
MSTRG.13964.1	515	noncoding	-
MSTRG.13965.1	573	noncoding	-
MSTRG.13969.2	941	noncoding	-
MSTRG.13971.1	564	noncoding	-
MSTRG.13981.1	957	noncoding	-
MSTRG.13981.2	1043	noncoding	-
MSTRG.13982.1	1761	coding	PF02100
MSTRG.13983.1	2196	noncoding	-
MSTRG.13984.1	8561	noncoding	-
MSTRG.13984.3	9352	noncoding	-
MSTRG.13984.4	5030	noncoding	-
MSTRG.13986.1	4276	noncoding	-
MSTRG.13980.2	2438	noncoding	-
MSTRG.14009.1	15198	coding	PF00632
MSTRG.14009.2	16435	coding	PF00632
MSTRG.14009.3	15522	coding	PF00632
MSTRG.14009.4	15160	coding	PF00632
MSTRG.14010.7	2779	coding	PF00443
MSTRG.14010.6	1679	coding	PF00443
MSTRG.14010.8	1337	coding	PF00443
MSTRG.14010.9	10060	coding	PF00443
MSTRG.13999.1	1012	noncoding	-
MSTRG.14003.1	862	coding	PF01667

MSTRG.14003.4	424	coding	PF01667
MSTRG.14001.2	1870	coding	PF00144
MSTRG.14014.1	250	noncoding	-
MSTRG.14034.2	3261	noncoding	-
MSTRG.14035.1	3195	noncoding	-
MSTRG.14036.1	24748	noncoding	-
MSTRG.14037.1	4259	noncoding	-
MSTRG.14036.2	13036	noncoding	-
MSTRG.14038.1	2409	noncoding	-
MSTRG.14038.2	6039	noncoding	-
MSTRG.14039.1	24583	noncoding	-
MSTRG.14039.2	14630	noncoding	-
MSTRG.14041.1	29071	noncoding	-
MSTRG.14034.3	5279	coding	PF00105
MSTRG.14034.5	6592	noncoding	-
MSTRG.14034.6	3602	coding	PF00105
MSTRG.14043.1	2018	noncoding	-
MSTRG.14026.1	1305	noncoding	-
MSTRG.14029.2	972	coding	PF02268
MSTRG.14030.1	2576	noncoding	-
MSTRG.14059.4	4551	coding	PF15303
MSTRG.14059.3	4782	coding	PF15303
MSTRG.14059.5	3374	coding	PF15303
MSTRG.14059.6	3345	coding	PF15303
MSTRG.14060.2	772	noncoding	-
MSTRG.14063.1	1197	noncoding	-
MSTRG.14066.3	3681	noncoding	-
MSTRG.14047.1	954	noncoding	-
MSTRG.14050.4	1466	noncoding	-
MSTRG.14069.1	5119	coding	PF15328
MSTRG.14069.2	2838	coding	PF15328
MSTRG.14075.1	3187	noncoding	-
MSTRG.14075.6	3938	noncoding	-
MSTRG.14076.1	1690	noncoding	-
MSTRG.14077.1	7032	coding	PF00078
MSTRG.14077.2	880	coding	PF00078
MSTRG.14079.1	4130	noncoding	-
MSTRG.14079.2	1810	noncoding	-
MSTRG.14081.1	1976	noncoding	-
MSTRG.14082.1	847	noncoding	-
MSTRG.14083.1	5587	noncoding	-
MSTRG.14084.1	5103	coding	PF00078
MSTRG.14085.1	20800	noncoding	-
MSTRG.14093.10	5357	coding	PF13836
MSTRG.14093.11	4620	coding	PF13836
MSTRG.14093.4	4843	coding	PF13836
MSTRG.14093.5	6027	coding	PF13836
MSTRG.14093.6	4442	coding	PF13836
MSTRG.14093.7	4479	coding	PF13836
MSTRG.14093.9	4760	coding	PF13836
MSTRG.14093.12	8766	coding	PF13836
MSTRG.14094.1	3934	noncoding	-
MSTRG.14095.1	2474	noncoding	-
MSTRG.14099.1	3720	noncoding	-

MSTRG.14100.2	1616	noncoding	-
MSTRG.14089.2	10189	noncoding	-
MSTRG.14109.1	562	noncoding	-
MSTRG.14115.3	5607	noncoding	-
MSTRG.14126.1	241	noncoding	-
MSTRG.14127.1	2463	noncoding	-
MSTRG.14130.1	1699	noncoding	-
MSTRG.14141.6	4597	coding	PF13889
MSTRG.14141.9	4057	coding	PF13889
MSTRG.14141.7	5012	coding	PF13889
MSTRG.14141.11	2697	noncoding	-
MSTRG.14142.3	13047	coding	PF00063
MSTRG.14142.4	7598	coding	PF00063
MSTRG.14143.1	431	noncoding	-
MSTRG.14144.2	2289	noncoding	-
MSTRG.14147.1	13995	coding	PF00429
MSTRG.14147.2	4757	noncoding	-
MSTRG.14153.2	1437	noncoding	-
MSTRG.14153.3	1417	noncoding	-
MSTRG.14157.1	18927	noncoding	-
MSTRG.14157.2	45615	noncoding	-
MSTRG.14157.4	43449	noncoding	-
MSTRG.14157.3	50292	noncoding	-
MSTRG.14157.5	46747	coding	PF00078
MSTRG.14167.1	1278	noncoding	-
MSTRG.14172.1	286	noncoding	-
MSTRG.14177.1	341	noncoding	-
MSTRG.14182.3	1018	noncoding	-
MSTRG.14183.4	2501	coding	PF14922
MSTRG.14183.6	2367	coding	PF14922
MSTRG.14183.7	2493	coding	PF14922
MSTRG.14183.5	1369	coding	PF14922
MSTRG.14183.1	2028	coding	PF14922
MSTRG.14183.8	1968	coding	PF14922
MSTRG.14186.2	1794	noncoding	-
MSTRG.14190.2	5941	coding	PF01248
MSTRG.14191.1	828	noncoding	-
MSTRG.14192.1	337	noncoding	-
MSTRG.14193.1	3897	coding	PF00640
MSTRG.14193.2	6207	noncoding	-
MSTRG.14193.3	7556	coding	PF00640
MSTRG.14194.3	1570	noncoding	-
MSTRG.14194.2	1445	noncoding	-
MSTRG.14189.2	5609	noncoding	-
MSTRG.14189.3	5753	noncoding	-
MSTRG.14198.1	12674	noncoding	-
MSTRG.14198.2	5207	noncoding	-
MSTRG.14198.3	6696	noncoding	-
MSTRG.14198.4	2098	noncoding	-
MSTRG.14198.5	3223	noncoding	-
MSTRG.14199.3	478	coding	PF00692
MSTRG.14201.4	1359	coding	PF00076
MSTRG.14201.5	1639	coding	PF00076
MSTRG.14201.3	1752	coding	PF00076

MSTRG.14201.8	1685	coding	PF00076
MSTRG.14201.7	10677	coding	PF00076
MSTRG.14213.1	554	noncoding	-
MSTRG.14219.1	3894	noncoding	-
MSTRG.14220.2	6195	coding	PF01545
MSTRG.14223.1	5656	noncoding	-
MSTRG.14227.1	470	noncoding	-
MSTRG.14221.1	1850	coding	PF10204
MSTRG.14221.2	1260	coding	PF10204
MSTRG.14221.3	1615	coding	PF10204
MSTRG.14233.2	656	noncoding	-
MSTRG.14233.3	629	noncoding	-
MSTRG.14233.1	2196	noncoding	-
MSTRG.14233.4	615	noncoding	-
MSTRG.14235.1	517	noncoding	-
MSTRG.14251.3	1364	noncoding	-
MSTRG.14251.4	9358	coding	PF00078
MSTRG.14251.5	1834	noncoding	-
MSTRG.14257.1	25364	noncoding	-
MSTRG.14249.6	825	coding	PF05706
MSTRG.14249.7	841	coding	PF05706
MSTRG.14249.5	1054	coding	PF05706
MSTRG.14249.8	873	coding	PF05706
MSTRG.14248.2	4165	coding	PF00241
MSTRG.14266.3	3944	coding	PF12341
MSTRG.14269.2	5223	coding	PF15822
MSTRG.14269.4	2971	noncoding	-
MSTRG.14272.2	2936	coding	PF03359
MSTRG.14277.1	2608	noncoding	-
MSTRG.14278.1	3387	noncoding	-
MSTRG.14287.6	4502	noncoding	-
MSTRG.14287.7	4658	noncoding	-
MSTRG.14287.8	4571	noncoding	-
MSTRG.14287.10	4680	noncoding	-
MSTRG.14287.11	5216	noncoding	-
MSTRG.14287.12	4730	noncoding	-
MSTRG.14287.9	4904	noncoding	-
MSTRG.14287.13	5129	noncoding	-
MSTRG.14288.1	2144	noncoding	-
MSTRG.14287.14	2364	noncoding	-
MSTRG.14280.1	1774	noncoding	-
MSTRG.14281.1	1162	coding	PF04710
MSTRG.14281.3	1064	coding	PF04710
MSTRG.14281.4	1171	coding	PF04710
MSTRG.14281.5	3737	coding	PF04710
MSTRG.14282.1	15650	noncoding	-
MSTRG.14282.2	2515	noncoding	-
MSTRG.14283.1	5451	noncoding	-
MSTRG.14284.1	6317	noncoding	-
MSTRG.14285.1	1923	noncoding	-
MSTRG.14290.1	5307	noncoding	-
MSTRG.14290.4	6505	noncoding	-
MSTRG.14290.5	3039	noncoding	-
MSTRG.14290.6	2304	noncoding	-

MSTRG.14290.3	2972	coding	PF11028
MSTRG.14290.7	3033	noncoding	-
MSTRG.14291.1	364	noncoding	-
MSTRG.14304.1	426	noncoding	-
MSTRG.14304.2	615	noncoding	-
MSTRG.14308.1	3005	noncoding	-
MSTRG.14295.1	4300	noncoding	-
MSTRG.14296.2	3109	coding	PF15767
MSTRG.14297.1	280	noncoding	-
MSTRG.14298.1	458	noncoding	-
MSTRG.14310.2	2276	coding	PF00227
MSTRG.14311.1	4007	noncoding	-
MSTRG.14311.2	1084	noncoding	-
MSTRG.14311.3	3777	noncoding	-
MSTRG.14311.4	3601	noncoding	-
MSTRG.14313.2	6880	noncoding	-
MSTRG.14317.2	609	coding	PF02064
MSTRG.14318.2	3950	coding	PF02064
MSTRG.14319.1	5317	coding	PF15324
MSTRG.14319.2	5301	coding	PF15324
MSTRG.14319.4	4797	coding	PF15324
MSTRG.14319.3	5310	coding	PF15324
MSTRG.14319.12	4396	coding	PF15324
MSTRG.14319.13	2845	coding	PF15324
MSTRG.14319.14	2068	coding	PF15324
MSTRG.14320.1	1962	noncoding	-
MSTRG.14324.1	14426	noncoding	-
MSTRG.14325.1	4783	noncoding	-
MSTRG.14325.2	2438	noncoding	-
MSTRG.14326.1	322	noncoding	-
MSTRG.14330.1	1057	noncoding	-
MSTRG.14331.1	5992	noncoding	-
MSTRG.14331.3	4301	noncoding	-
MSTRG.14334.2	2813	noncoding	-
MSTRG.14349.2	2733	coding	PF02475
MSTRG.14350.1	2845	noncoding	-
MSTRG.14354.1	3991	noncoding	-
MSTRG.14343.1	2574	coding	PF03381
MSTRG.14359.2	3999	coding	PF08778
MSTRG.14360.2	1335	coding	PF09808
MSTRG.14367.4	2031	coding	PF01603
MSTRG.14367.5	1973	coding	PF01603
MSTRG.14368.1	802	noncoding	-
MSTRG.14369.1	4955	noncoding	-
MSTRG.14401.1	631	noncoding	-
MSTRG.14409.7	21313	noncoding	-
MSTRG.14409.8	21225	noncoding	-
MSTRG.14409.9	21876	noncoding	-
MSTRG.14409.10	3372	noncoding	-
MSTRG.14409.11	18914	noncoding	-
MSTRG.14409.12	18692	noncoding	-
MSTRG.14409.13	14019	noncoding	-
MSTRG.14409.14	14466	noncoding	-
MSTRG.14411.3	3485	noncoding	-

MSTRG.14411.4	3497	noncoding	-
MSTRG.14411.5	4047	noncoding	-
MSTRG.14411.6	3502	noncoding	-
MSTRG.14412.1	3088	coding	PF01268
MSTRG.14376.1	2350	coding	PF00651
MSTRG.14376.2	6733	coding	PF00651
MSTRG.14379.1	1308	noncoding	-
MSTRG.14376.3	332	noncoding	-
MSTRG.14379.2	3153	noncoding	-
MSTRG.14380.1	2066	coding	PF00012
MSTRG.14380.2	1562	coding	PF00012
MSTRG.14371.4	1222	coding	PF14895
MSTRG.14385.1	841	noncoding	-
MSTRG.14386.1	207	noncoding	-
MSTRG.14387.1	490	noncoding	-
MSTRG.14395.1	4927	noncoding	-
MSTRG.14398.1	2245	coding	PF00078
MSTRG.14399.1	3945	noncoding	-
MSTRG.14419.1	5369	noncoding	-
MSTRG.14421.1	854	noncoding	-
MSTRG.14422.1	1271	noncoding	-
MSTRG.14424.1	677	noncoding	-
MSTRG.14424.2	920	noncoding	-
MSTRG.14426.2	826	noncoding	-
MSTRG.14426.3	1217	noncoding	-
MSTRG.14427.3	5468	coding	PF00625
MSTRG.14427.4	5179	coding	PF00625
MSTRG.14428.1	3854	noncoding	-
MSTRG.14429.1	804	noncoding	-
MSTRG.14414.1	4151	noncoding	-
MSTRG.14434.1	6569	coding	PF00784
MSTRG.14435.1	1126	coding	PF10181
MSTRG.14436.2	518	coding	PF00832
MSTRG.14437.1	1877	coding	PF12352
MSTRG.14436.4	1611	coding	PF00491
MSTRG.14437.5	1462	coding	PF12352
MSTRG.14439.2	2736	coding	PF00106
MSTRG.14439.3	2280	coding	PF00106
MSTRG.14454.3	6492	coding	PF01363
MSTRG.14455.1	3540	coding	PF08423
MSTRG.14461.1	2012	noncoding	-
MSTRG.14440.1	414	noncoding	-
MSTRG.14441.2	2828	coding	PF04553
MSTRG.14442.1	3001	coding	PF08726
MSTRG.14442.3	3075	coding	PF08726
MSTRG.14442.4	3239	coding	PF08726
MSTRG.14442.5	3148	coding	PF08726
MSTRG.14442.6	3282	coding	PF08726
MSTRG.14445.2	402	noncoding	-
MSTRG.14445.1	894	noncoding	-
MSTRG.14445.3	477	noncoding	-
MSTRG.14446.1	650	noncoding	-
MSTRG.14447.1	4432	noncoding	-
MSTRG.14448.1	5441	noncoding	-

MSTRG.14453.2	3957	coding	PF00652
MSTRG.14453.4	3943	coding	PF00652
MSTRG.14453.5	3527	coding	PF00652
MSTRG.14486.1	4917	coding	PF02535
MSTRG.14486.3	4875	coding	PF02535
MSTRG.14486.4	4882	coding	PF02535
MSTRG.14487.1	8511	noncoding	-
MSTRG.14487.2	8350	noncoding	-
MSTRG.14488.3	9289	noncoding	-
MSTRG.14488.5	5074	noncoding	-
MSTRG.14491.3	2099	coding	PF00078
MSTRG.14491.5	2617	coding	PF00076
MSTRG.14491.8	2564	coding	PF00076
MSTRG.14491.7	2824	coding	PF00076
MSTRG.14491.10	21114	coding	PF00076
MSTRG.14491.6	7990	coding	PF00076
MSTRG.14491.4	2147	coding	PF00078
MSTRG.14491.11	1186	coding	PF00076
MSTRG.14489.1	1641	coding	PF00086
MSTRG.14489.3	1795	coding	PF00086
MSTRG.14489.4	3136	coding	PF00086
MSTRG.14492.2	4294	noncoding	-
MSTRG.14492.3	8824	noncoding	-
MSTRG.14492.4	2634	noncoding	-
MSTRG.14493.1	6843	noncoding	-
MSTRG.14492.6	839	noncoding	-
MSTRG.14492.5	9320	noncoding	-
MSTRG.14495.1	2648	coding	PF01562
MSTRG.14508.5	4364	noncoding	-
MSTRG.14508.7	3758	noncoding	-
MSTRG.14508.8	4866	noncoding	-
MSTRG.14508.4	6299	noncoding	-
MSTRG.14508.6	7922	coding	PF05041
MSTRG.14508.9	3555	noncoding	-
MSTRG.14509.1	2599	noncoding	-
MSTRG.14508.10	3463	coding	PF05041
MSTRG.14513.1	4460	noncoding	-
MSTRG.14512.3	7666	coding	PF02145
MSTRG.14512.4	5992	coding	PF02145
MSTRG.14515.1	4293	noncoding	-
MSTRG.14515.2	603	noncoding	-
MSTRG.14515.3	2310	noncoding	-
MSTRG.14516.1	778	noncoding	-
MSTRG.14528.1	9916	noncoding	-
MSTRG.14528.3	3182	noncoding	-
MSTRG.14528.5	1511	noncoding	-
MSTRG.14529.1	12243	noncoding	-
MSTRG.14529.2	8271	noncoding	-
MSTRG.14530.1	11594	noncoding	-
MSTRG.14529.3	8467	noncoding	-
MSTRG.14529.4	2712	noncoding	-
MSTRG.14529.5	1519	noncoding	-
MSTRG.14531.1	20672	noncoding	-
MSTRG.14531.2	386	noncoding	-

MSTRG.14533.1	2795	noncoding	-
MSTRG.14534.1	58941	noncoding	-
MSTRG.14534.2	11168	noncoding	-
MSTRG.14534.3	2065	noncoding	-
MSTRG.14534.4	5919	noncoding	-
MSTRG.14533.2	4796	noncoding	-
MSTRG.14535.1	390	noncoding	-
MSTRG.14517.3	3035	coding	PF14051
MSTRG.14517.4	12112	noncoding	-
MSTRG.14518.1	1266	noncoding	-
MSTRG.14519.1	247	noncoding	-
MSTRG.14526.3	3609	coding	PF01363
MSTRG.14536.1	1459	noncoding	-
MSTRG.14538.2	5968	noncoding	-
MSTRG.14538.3	1144	noncoding	-
MSTRG.14538.4	7428	noncoding	-
MSTRG.14538.5	2350	noncoding	-
MSTRG.14539.5	2979	coding	PF01080
MSTRG.14539.7	2210	coding	PF01080
MSTRG.14540.1	3726	coding	PF06311
MSTRG.14540.11	3412	coding	PF06311
MSTRG.14540.9	14290	coding	PF06311
MSTRG.14540.12	3020	coding	PF06311
MSTRG.14540.13	2960	coding	PF06311
MSTRG.14540.10	3503	coding	PF06311
MSTRG.14540.4	3296	coding	PF06311
MSTRG.14540.3	3005	coding	PF06311
MSTRG.14540.6	3001	coding	PF06311
MSTRG.14540.7	5887	coding	PF06311
MSTRG.14540.2	6268	coding	PF06311
MSTRG.14540.8	6274	coding	PF06311
MSTRG.14540.14	6450	noncoding	-
MSTRG.14541.1	1348	noncoding	-
MSTRG.14543.5	3424	noncoding	-
MSTRG.14544.1	2498	coding	PF08007
MSTRG.14553.1	5934	coding	PF14893
MSTRG.14553.2	6734	noncoding	-
MSTRG.14553.3	3669	coding	PF14893
MSTRG.14558.2	2013	coding	PF16884
MSTRG.14560.3	2474	noncoding	-
MSTRG.14560.7	3460	noncoding	-
MSTRG.14563.6	6278	coding	PF01150
MSTRG.14563.4	5305	coding	PF01150
MSTRG.14563.5	5302	coding	PF01150
MSTRG.14564.6	1803	noncoding	-
MSTRG.14564.4	2885	coding	PF14988
MSTRG.14564.5	2685	coding	PF14988
MSTRG.14564.2	2745	coding	PF14988
MSTRG.14565.1	3277	coding	PF00171
MSTRG.14564.7	346	noncoding	-
MSTRG.14564.8	3097	coding	PF14988
MSTRG.14565.2	3297	coding	PF00171
MSTRG.14566.4	5616	coding	PF10044
MSTRG.14567.1	12577	noncoding	-

MSTRG.14568.1	5680	noncoding	-
MSTRG.14569.1	634	noncoding	-
MSTRG.14556.2	947	noncoding	-
MSTRG.14557.1	6683	coding	PF00683
MSTRG.14574.4	7166	coding	PF00632
MSTRG.14576.2	638	coding	PF04900
MSTRG.14579.4	7209	noncoding	-
MSTRG.14580.1	1937	coding	PF00198
MSTRG.14580.3	2639	coding	PF00198
MSTRG.14570.2	1404	coding	PF01008
MSTRG.14573.2	1102	noncoding	-
MSTRG.14583.1	1611	noncoding	-
MSTRG.14593.1	1682	noncoding	-
MSTRG.14593.3	2643	noncoding	-
MSTRG.14593.4	3804	noncoding	-
MSTRG.14593.5	12577	noncoding	-
MSTRG.14593.7	10330	noncoding	-
MSTRG.14600.1	1908	coding	PF03133
MSTRG.14600.8	2068	coding	PF03133
MSTRG.14600.11	4683	coding	PF03133
MSTRG.14600.5	4842	coding	PF03133
MSTRG.14600.6	4323	coding	PF03133
MSTRG.14600.7	4761	coding	PF03133
MSTRG.14600.2	4892	coding	PF03133
MSTRG.14600.3	4766	coding	PF03133
MSTRG.14600.9	4493	coding	PF03133
MSTRG.14600.12	11975	noncoding	-
MSTRG.14601.1	806	noncoding	-
MSTRG.14600.13	3647	noncoding	-
MSTRG.14600.14	2848	noncoding	-
MSTRG.14603.2	3303	coding	PF00019
MSTRG.14605.3	2886	noncoding	-
MSTRG.14605.4	4031	noncoding	-
MSTRG.14605.2	1210	coding	PF15305
MSTRG.14605.5	6920	coding	PF15305
MSTRG.14591.4	1483	noncoding	-
MSTRG.14591.5	1460	noncoding	-
MSTRG.14589.1	701	noncoding	-
MSTRG.14590.1	411	noncoding	-
MSTRG.14592.1	2061	coding	PF00105
MSTRG.14592.2	2253	coding	PF00105
MSTRG.14608.1	8090	noncoding	-
MSTRG.14608.2	7975	noncoding	-
MSTRG.14608.5	4089	noncoding	-
MSTRG.14608.4	5415	noncoding	-
MSTRG.14610.1	2742	noncoding	-
MSTRG.14608.7	5154	noncoding	-
MSTRG.14612.1	1006	noncoding	-
MSTRG.14615.1	7039	coding	PF15800
MSTRG.14615.2	7361	coding	PF15800
MSTRG.14615.3	4282	coding	PF15800
MSTRG.14615.5	5678	coding	PF15800
MSTRG.14619.3	3196	coding	PF02366
MSTRG.14619.2	3170	coding	PF02366

MSTRG.14619.4	3202	coding	PF02366
MSTRG.14620.1	1488	coding	PF02798
MSTRG.14620.2	1433	coding	PF02798
MSTRG.14620.3	1762	coding	PF02798
MSTRG.14620.4	1484	coding	PF13417
MSTRG.14620.5	993	noncoding	-
MSTRG.14620.6	1055	coding	PF02798
MSTRG.14616.3	8345	coding	PF13897
MSTRG.14624.4	2686	coding	PF09787
MSTRG.14625.3	1536	coding	PF09229
MSTRG.14629.1	3277	coding	PF02731
MSTRG.14629.3	2218	coding	PF02731
MSTRG.14626.2	2039	coding	PF03109
MSTRG.14630.1	649	noncoding	-
MSTRG.14633.1	975	noncoding	-
MSTRG.14638.1	372	noncoding	-
MSTRG.14645.1	5163	noncoding	-
MSTRG.14646.1	2073	noncoding	-
MSTRG.14648.2	937	noncoding	-
MSTRG.14648.3	2527	noncoding	-
MSTRG.14651.1	2910	coding	PF03153
MSTRG.14651.3	4399	coding	PF03153
MSTRG.14651.4	1695	coding	PF03153
MSTRG.14647.1	284	noncoding	-
MSTRG.14658.1	1916	noncoding	-
MSTRG.14656.1	570	noncoding	-
MSTRG.14672.1	359	noncoding	-
MSTRG.14673.1	364	noncoding	-
MSTRG.14675.2	5357	coding	PF04192
MSTRG.14678.1	849	noncoding	-
MSTRG.14686.1	633	noncoding	-
MSTRG.14687.1	1037	noncoding	-
MSTRG.14687.2	1342	noncoding	-
MSTRG.14688.2	8266	noncoding	-
MSTRG.14692.1	795	noncoding	-
MSTRG.14692.2	1002	noncoding	-
MSTRG.14695.1	758	noncoding	-
MSTRG.14696.5	6654	noncoding	-
MSTRG.14696.6	5272	noncoding	-
MSTRG.14697.1	1440	noncoding	-
MSTRG.14699.2	19685	noncoding	-
MSTRG.14699.1	30953	noncoding	-
MSTRG.14699.3	27471	noncoding	-
MSTRG.14699.4	34020	noncoding	-
MSTRG.14699.5	32094	noncoding	-
MSTRG.14699.6	19654	noncoding	-
MSTRG.14701.8	1005	coding	PF15244
MSTRG.14701.4	1911	coding	PF15244
MSTRG.14701.5	1839	coding	PF15244
MSTRG.14701.7	2187	coding	PF15244
MSTRG.14701.9	1494	coding	PF15244
MSTRG.14702.1	8441	coding	PF00102
MSTRG.14703.1	5121	noncoding	-
MSTRG.14707.5	2155	noncoding	-

MSTRG.14707.4	3013	noncoding	-
MSTRG.14707.2	3144	noncoding	-
MSTRG.14708.2	13168	coding	PF03451
MSTRG.14708.6	6512	coding	PF03451
MSTRG.14708.7	3935	coding	PF03451
MSTRG.14709.1	4068	noncoding	-
MSTRG.14710.1	2482	noncoding	-
MSTRG.14708.9	2459	coding	PF03451
MSTRG.14711.1	4558	coding	PF02994
MSTRG.14708.10	2320	noncoding	-
MSTRG.14704.2	2109	noncoding	-
MSTRG.14704.3	1965	noncoding	-
MSTRG.14704.4	2609	noncoding	-
MSTRG.14706.1	1678	noncoding	-
MSTRG.14715.1	7646	coding	PF00250
MSTRG.14715.6	3021	coding	PF00250
MSTRG.14715.7	3067	coding	PF00250
MSTRG.14715.8	3155	coding	PF00250
MSTRG.14716.1	1780	noncoding	-
MSTRG.14717.1	14685	noncoding	-
MSTRG.14718.1	1487	noncoding	-
MSTRG.14719.1	3967	noncoding	-
MSTRG.14720.1	5785	noncoding	-
MSTRG.14721.1	14929	noncoding	-
MSTRG.14722.1	4408	noncoding	-
MSTRG.14723.1	2156	noncoding	-
MSTRG.14727.1	1874	noncoding	-
MSTRG.14727.2	874	noncoding	-
MSTRG.14730.1	3175	noncoding	-
MSTRG.14731.2	2296	coding	PF06087
MSTRG.14734.2	1636	coding	PF00004
MSTRG.14735.2	4541	coding	PF08424
MSTRG.14735.3	4480	coding	PF08424
MSTRG.14736.1	2373	noncoding	-
MSTRG.14745.1	4225	noncoding	-
MSTRG.14746.1	2594	noncoding	-
MSTRG.14748.1	1975	noncoding	-
MSTRG.14749.1	2301	noncoding	-
MSTRG.14751.1	3353	noncoding	-
MSTRG.14750.3	1920	noncoding	-
MSTRG.14750.2	2187	noncoding	-
MSTRG.14750.5	1377	noncoding	-
MSTRG.14750.4	1541	noncoding	-
MSTRG.14750.6	1425	noncoding	-
MSTRG.14753.3	6965	coding	PF00069
MSTRG.14754.1	513	noncoding	-
MSTRG.14753.4	2913	noncoding	-
MSTRG.14769.4	7215	noncoding	-
MSTRG.14771.1	1584	coding	PF04321
MSTRG.14772.1	2085	coding	PF13765
MSTRG.14772.2	2176	coding	PF13765
MSTRG.14772.4	1909	coding	PF13765
MSTRG.14773.1	4384	noncoding	-
MSTRG.14773.2	6897	noncoding	-

MSTRG.14775.1	1412	noncoding	-
MSTRG.14777.1	6492	coding	PF01359
MSTRG.14778.1	1872	noncoding	-
MSTRG.14780.2	4929	coding	PF06814
MSTRG.14780.3	4747	coding	PF06814
MSTRG.14782.6	1653	noncoding	-
MSTRG.14785.4	874	coding	PF06773
MSTRG.14785.5	873	coding	PF06773
MSTRG.14787.1	834	noncoding	-
MSTRG.14789.4	3393	coding	PF08311
MSTRG.14790.3	2175	coding	PF00018
MSTRG.14790.4	2171	coding	PF00018
MSTRG.14791.2	545	noncoding	-
MSTRG.14792.2	869	noncoding	-
MSTRG.14793.2	1530	noncoding	-
MSTRG.14796.1	1065	noncoding	-
MSTRG.14800.1	1246	coding	PF01557
MSTRG.14801.2	3025	noncoding	-
MSTRG.14808.2	3592	noncoding	-
MSTRG.14808.4	3435	noncoding	-
MSTRG.14818.2	5405	noncoding	-
MSTRG.14818.4	4832	noncoding	-
MSTRG.14819.1	6523	coding	PF08150
MSTRG.14821.2	3639	noncoding	-
MSTRG.14821.3	3575	noncoding	-
MSTRG.14821.5	3293	noncoding	-
MSTRG.14814.1	315	noncoding	-
MSTRG.14815.3	2448	coding	PF14816
MSTRG.14827.1	824	noncoding	-
MSTRG.14828.3	4539	coding	PF15057
MSTRG.14828.4	4132	coding	PF15057
MSTRG.14828.2	4396	coding	PF15057
MSTRG.14828.6	4907	coding	PF15057
MSTRG.14830.5	4000	coding	PF16526
MSTRG.14835.1	2155	noncoding	-
MSTRG.14835.2	7155	noncoding	-
MSTRG.14834.5	2827	noncoding	-
MSTRG.14840.1	9392	coding	PF04666
MSTRG.14840.5	2196	coding	PF04666
MSTRG.14833.7	3158	noncoding	-
MSTRG.14833.3	2941	noncoding	-
MSTRG.14841.1	825	coding	PF16565
MSTRG.14842.3	934	noncoding	-
MSTRG.14842.2	903	noncoding	-
MSTRG.14842.1	1691	noncoding	-
MSTRG.14842.5	876	noncoding	-
MSTRG.14844.2	1834	noncoding	-
MSTRG.14846.1	4440	coding	PF16727
MSTRG.14846.3	4223	coding	PF16727
MSTRG.14849.1	9106	coding	PF05110
MSTRG.14849.2	5082	coding	PF05110
MSTRG.14849.3	5544	coding	PF05110
MSTRG.14852.1	13698	noncoding	-
MSTRG.14852.2	5867	coding	PF02337

MSTRG.14853.1	312	noncoding	-
MSTRG.14852.3	1522	noncoding	-
MSTRG.14854.2	1374	noncoding	-
MSTRG.14854.1	1560	noncoding	-
MSTRG.14854.3	3461	noncoding	-
MSTRG.14854.4	3461	noncoding	-
MSTRG.14854.5	2200	noncoding	-
MSTRG.14855.1	12426	coding	PF00517
MSTRG.14856.1	424	noncoding	-
MSTRG.14864.1	541	noncoding	-
MSTRG.14866.2	2620	coding	PF10155
MSTRG.14866.3	2530	coding	PF10155
MSTRG.14867.5	2545	noncoding	-
MSTRG.14867.6	9554	noncoding	-
MSTRG.14865.2	1086	noncoding	-
MSTRG.14869.2	4760	coding	PF00069
MSTRG.14869.5	4241	coding	PF00069
MSTRG.14869.1	7323	coding	PF00069
MSTRG.14869.3	7131	coding	PF00069
MSTRG.14868.1	1962	noncoding	-
MSTRG.14871.2	1666	noncoding	-
MSTRG.14871.1	1700	noncoding	-
MSTRG.14871.3	992	noncoding	-
MSTRG.14872.2	2112	noncoding	-
MSTRG.14872.3	3364	coding	PF16644
MSTRG.14873.2	3023	noncoding	-
MSTRG.14873.3	2771	noncoding	-
MSTRG.14875.1	583	noncoding	-
MSTRG.14883.1	1402	noncoding	-
MSTRG.14884.1	3068	noncoding	-
MSTRG.14885.1	632	noncoding	-
MSTRG.14877.1	425	noncoding	-
MSTRG.14878.1	1137	noncoding	-
MSTRG.14890.2	2428	coding	PF15323
MSTRG.14892.2	535	noncoding	-
MSTRG.14905.1	1752	noncoding	-
MSTRG.14907.2	820	noncoding	-
MSTRG.14909.2	1252	coding	PF11669
MSTRG.14909.3	1256	coding	PF11669
MSTRG.14909.4	1766	noncoding	-
MSTRG.14909.5	1174	noncoding	-
MSTRG.14911.1	1356	coding	PF15479
MSTRG.14913.2	4447	coding	PF12455
MSTRG.14913.3	4468	coding	PF12455
MSTRG.14921.1	3288	coding	PF00078
MSTRG.14920.2	5415	coding	PF00955
MSTRG.14914.2	1997	coding	PF03637
MSTRG.14915.1	471	noncoding	-
MSTRG.14915.3	2396	noncoding	-
MSTRG.14915.4	1327	noncoding	-
MSTRG.14930.4	946	coding	PF01712
MSTRG.14930.5	4714	coding	PF01712
MSTRG.14930.6	775	coding	PF01712
MSTRG.14931.1	3457	noncoding	-

MSTRG.14932.1	3170	noncoding	-
MSTRG.14933.1	812	coding	PF07114
MSTRG.14933.2	902	coding	PF07114
MSTRG.14939.1	591	noncoding	-
MSTRG.14940.2	2279	coding	PF15442
MSTRG.14944.1	2145	coding	PF08617
MSTRG.14945.1	11997	coding	PF15309
MSTRG.14945.5	11551	coding	PF15309
MSTRG.14945.6	9953	coding	PF15309
MSTRG.14946.1	1374	noncoding	-
MSTRG.14947.1	3467	noncoding	-
MSTRG.14945.7	16241	noncoding	-
MSTRG.14948.1	2904	noncoding	-
MSTRG.14945.8	7353	noncoding	-
MSTRG.14945.9	7532	noncoding	-
MSTRG.14942.2	1163	noncoding	-
MSTRG.14942.3	1192	noncoding	-
MSTRG.14942.6	1998	noncoding	-
MSTRG.14942.5	1025	noncoding	-
MSTRG.14942.4	4599	noncoding	-
MSTRG.14958.1	948	noncoding	-
MSTRG.14957.2	4368	coding	PF04091
MSTRG.14959.1	1848	noncoding	-
MSTRG.14962.2	5774	coding	PF00067
MSTRG.14960.1	336	noncoding	-
MSTRG.14965.4	5831	coding	PF16165
MSTRG.14965.3	5266	coding	PF08150
MSTRG.14965.1	6724	coding	PF08150
MSTRG.14965.2	7551	coding	PF08150
MSTRG.14965.5	6676	coding	PF08150
MSTRG.14966.1	8481	noncoding	-
MSTRG.14968.7	10701	noncoding	-
MSTRG.14968.4	4927	noncoding	-
MSTRG.14968.5	4937	noncoding	-
MSTRG.14968.8	6580	noncoding	-
MSTRG.14969.1	1312	noncoding	-
MSTRG.14970.1	506	noncoding	-
MSTRG.14975.1	649	noncoding	-
MSTRG.14986.2	2547	coding	PF05811
MSTRG.14986.3	2155	coding	PF05811
MSTRG.14987.2	4611	coding	PF02738
MSTRG.14989.1	1896	coding	PF01875
MSTRG.14989.2	1974	coding	PF01875
MSTRG.14989.3	1837	coding	PF01875
MSTRG.14990.1	1783	noncoding	-
MSTRG.14992.2	2248	coding	PF00004
MSTRG.15000.1	674	noncoding	-
MSTRG.15001.1	2500	noncoding	-
MSTRG.15007.4	13518	coding	PF12356
MSTRG.15007.5	13363	coding	PF12356
MSTRG.15007.7	3745	noncoding	-
MSTRG.15007.8	9776	coding	PF12356
MSTRG.14997.1	226	noncoding	-
MSTRG.14998.1	1079	noncoding	-

MSTRG.15017.1	1143	noncoding	-
MSTRG.15018.1	4151	noncoding	-
MSTRG.15021.1	13004	coding	PF08232
MSTRG.15021.4	6044	coding	PF08232
MSTRG.15022.1	1812	noncoding	-
MSTRG.15023.4	2468	noncoding	-
MSTRG.15023.5	2154	noncoding	-
MSTRG.15033.1	4243	coding	PF00069
MSTRG.15033.2	4336	coding	PF00069
MSTRG.15033.5	4086	coding	PF00069
MSTRG.15026.1	580	noncoding	-
MSTRG.15028.1	1938	noncoding	-
MSTRG.15038.2	669	noncoding	-
MSTRG.15046.1	2180	coding	PF13893
MSTRG.15046.4	2946	coding	PF13893
MSTRG.15046.5	2403	coding	PF13893
MSTRG.15046.6	2551	coding	PF13893
MSTRG.15046.7	2263	noncoding	-
MSTRG.15047.1	6794	noncoding	-
MSTRG.15047.2	5812	noncoding	-
MSTRG.15046.9	4736	noncoding	-
MSTRG.15050.6	1500	coding	PF00076
MSTRG.15050.2	1517	coding	PF00076
MSTRG.15050.4	1175	coding	PF00076
MSTRG.15050.7	1727	coding	PF00076
MSTRG.15051.1	2439	coding	PF10300
MSTRG.15051.2	9590	coding	PF10300
MSTRG.15051.3	3208	coding	PF10300
MSTRG.15051.4	3381	coding	PF10300
MSTRG.15052.2	448	noncoding	-
MSTRG.15054.1	687	noncoding	-
MSTRG.15043.1	1763	noncoding	-
MSTRG.15039.1	274	noncoding	-
MSTRG.15055.2	8891	coding	PF00069
MSTRG.15055.4	5129	coding	PF00069
MSTRG.15055.6	4326	coding	PF00069
MSTRG.15055.7	3679	coding	PF00069
MSTRG.15055.8	3060	coding	PF00069
MSTRG.15057.1	635	noncoding	-
MSTRG.15058.1	731	noncoding	-
MSTRG.15045.1	700	noncoding	-
MSTRG.15068.1	237	noncoding	-
MSTRG.15072.1	403	noncoding	-
MSTRG.15072.2	468	noncoding	-
MSTRG.15080.1	3362	noncoding	-
MSTRG.15083.4	3643	coding	PF03451
MSTRG.15083.3	4264	coding	PF03451
MSTRG.15083.5	3241	noncoding	-
MSTRG.15084.1	3290	noncoding	-
MSTRG.15085.1	878	noncoding	-
MSTRG.15090.1	2720	coding	PF02214
MSTRG.15090.2	17513	coding	PF02214
MSTRG.15091.1	2250	noncoding	-
MSTRG.15092.1	1958	noncoding	-

MSTRG.15093.10	4967	coding	PF01448
MSTRG.15093.9	5353	coding	PF01448
MSTRG.15093.11	2192	coding	PF01448
MSTRG.15093.12	1840	coding	PF01448
MSTRG.15097.3	5919	coding	PF10350
MSTRG.15097.4	2115	noncoding	-
MSTRG.15100.2	6492	coding	PF00784
MSTRG.15105.1	6746	coding	PF00481
MSTRG.15105.2	3127	coding	PF00481
MSTRG.15105.5	4445	coding	PF00481
MSTRG.15105.3	10841	coding	PF00481
MSTRG.15105.4	6437	coding	PF00481
MSTRG.15105.6	2499	coding	PF00481
MSTRG.15105.8	6933	coding	PF00481
MSTRG.15105.9	7848	noncoding	-
MSTRG.15106.1	10859	noncoding	-
MSTRG.15106.2	8542	noncoding	-
MSTRG.15106.3	3626	noncoding	-
MSTRG.15107.1	6926	coding	PF00102
MSTRG.15108.1	1574	noncoding	-
MSTRG.15108.2	1480	noncoding	-
MSTRG.15109.1	222	noncoding	-
MSTRG.15109.2	322	noncoding	-
MSTRG.15105.11	2225	noncoding	-
MSTRG.15124.5	4675	coding	PF00326
MSTRG.15126.1	898	noncoding	-
MSTRG.15127.5	803	noncoding	-
MSTRG.15127.4	3620	noncoding	-
MSTRG.15127.3	13780	noncoding	-
MSTRG.15127.1	23661	noncoding	-
MSTRG.15129.1	2971	noncoding	-
MSTRG.15131.1	7208	noncoding	-
MSTRG.15132.1	3050	noncoding	-
MSTRG.15118.3	1548	coding	PF12836
MSTRG.15119.1	290	noncoding	-
MSTRG.15141.1	6694	noncoding	-
MSTRG.15141.2	5737	coding	PF14598
MSTRG.15141.4	4945	coding	PF14598
MSTRG.15138.1	1887	coding	PF15444
MSTRG.15138.2	1284	noncoding	-
MSTRG.15145.1	3875	coding	PF00071
MSTRG.15146.4	5788	noncoding	-
MSTRG.15146.5	1914	coding	PF06699
MSTRG.15146.6	1297	coding	PF06699
MSTRG.15146.7	1154	coding	PF06699
MSTRG.15146.10	733	coding	PF06699
MSTRG.15146.13	1645	coding	PF06699
MSTRG.15146.11	1001	coding	PF06699
MSTRG.15146.15	1462	coding	PF06699
MSTRG.15146.8	1678	coding	PF06699
MSTRG.15146.14	755	coding	PF06699
MSTRG.15146.12	758	coding	PF06699
MSTRG.15147.2	1292	coding	PF10235
MSTRG.15149.1	4899	noncoding	-

MSTRG.15151.1	1019	noncoding	-
MSTRG.15152.1	1501	noncoding	-
MSTRG.15143.1	1047	noncoding	-
MSTRG.15156.3	2164	noncoding	-
MSTRG.15156.4	3230	noncoding	-
MSTRG.15153.1	1824	coding	PF00086
MSTRG.15153.2	1540	noncoding	-
MSTRG.15167.2	1059	noncoding	-
MSTRG.15174.3	4973	coding	PF12937
MSTRG.15175.1	3384	noncoding	-
MSTRG.15176.1	2558	noncoding	-
MSTRG.15174.4	14199	noncoding	-
MSTRG.15174.5	13283	noncoding	-
MSTRG.15177.1	2083	noncoding	-
MSTRG.15181.1	7800	coding	PF10212
MSTRG.15181.5	3333	noncoding	-
MSTRG.15183.2	886	coding	PF03153
MSTRG.15184.1	1996	noncoding	-
MSTRG.15184.2	3413	noncoding	-
MSTRG.15185.1	6646	noncoding	-
MSTRG.15184.3	2602	noncoding	-
MSTRG.15184.5	2028	noncoding	-
MSTRG.15184.9	768	noncoding	-
MSTRG.15184.10	11418	noncoding	-
MSTRG.15188.1	1409	noncoding	-
MSTRG.15188.2	1557	noncoding	-
MSTRG.15190.1	3920	coding	PF12369
MSTRG.15190.6	3421	noncoding	-
MSTRG.15191.1	7421	noncoding	-
MSTRG.15192.1	13063	noncoding	-
MSTRG.15194.1	14302	noncoding	-
MSTRG.15219.1	8178	coding	PF02210
MSTRG.15219.2	4185	coding	PF02210
MSTRG.15219.3	7295	coding	PF02210
MSTRG.15220.1	240	noncoding	-
MSTRG.15221.1	202	noncoding	-
MSTRG.15227.1	2725	noncoding	-
MSTRG.15253.1	1599	noncoding	-
MSTRG.15197.1	209	noncoding	-
MSTRG.15199.1	416	noncoding	-
MSTRG.15209.3	3046	coding	PF12796
MSTRG.15210.1	9596	coding	PF02994
MSTRG.15210.2	2612	coding	PF02994
MSTRG.15209.4	621	coding	PF12796
MSTRG.15212.1	231	noncoding	-
MSTRG.15213.2	417	noncoding	-
MSTRG.15268.1	922	noncoding	-
MSTRG.15268.2	923	noncoding	-
MSTRG.15284.1	1933	noncoding	-
MSTRG.15287.5	9132	coding	PF00435
MSTRG.15287.6	11326	coding	PF00435
MSTRG.15291.1	1586	noncoding	-
MSTRG.15296.3	2772	coding	PF13838
MSTRG.15302.2	8977	noncoding	-

MSTRG.15312.1	8711	noncoding	-
MSTRG.15304.1	1395	noncoding	-
MSTRG.15328.2	947	coding	PF09765
MSTRG.15333.1	658	noncoding	-
MSTRG.15338.1	922	noncoding	-
MSTRG.15347.1	9644	coding	PF16179
MSTRG.15353.1	2313	noncoding	-
MSTRG.15359.2	4802	coding	PF11822
MSTRG.15360.1	15509	noncoding	-
MSTRG.15360.2	497	noncoding	-
MSTRG.15362.1	3175	noncoding	-
MSTRG.15357.1	610	noncoding	-
MSTRG.15368.2	1992	coding	PF00735
MSTRG.15368.3	1815	coding	PF00735
MSTRG.15375.2	10062	coding	PF00638
MSTRG.15379.2	4215	coding	PF16704
MSTRG.15381.1	652	coding	PF00685
MSTRG.15381.2	700	coding	PF00685
MSTRG.15381.3	728	coding	PF00685
MSTRG.15380.1	990	noncoding	-
MSTRG.15380.2	851	noncoding	-
MSTRG.15383.1	431	noncoding	-
MSTRG.15386.3	1384	coding	PF01370
MSTRG.15386.5	7941	coding	PF01370
MSTRG.15394.2	599	coding	PF16860
MSTRG.15394.1	2656	coding	PF16860
MSTRG.15399.1	706	noncoding	-
MSTRG.15405.4	4925	coding	PF01369
MSTRG.15405.6	4521	coding	PF01369
MSTRG.15402.1	647	noncoding	-
MSTRG.15416.3	291	noncoding	-
MSTRG.15416.4	1929	noncoding	-
MSTRG.15421.1	2559	coding	PF13639
MSTRG.15421.2	3588	coding	PF13639
MSTRG.15422.1	654	noncoding	-
MSTRG.15423.1	710	noncoding	-
MSTRG.15426.4	4400	coding	PF03134
MSTRG.15427.2	909	noncoding	-
MSTRG.15429.6	2428	coding	PF09731
MSTRG.15429.7	2647	coding	PF09731
MSTRG.15429.8	2668	coding	PF09731
MSTRG.15431.1	6386	coding	PF00623
MSTRG.15431.3	6033	coding	PF00623
MSTRG.15433.3	2884	coding	PF00777
MSTRG.15433.5	10434	coding	PF00777
MSTRG.15436.2	2137	noncoding	-
MSTRG.15436.3	2096	noncoding	-
MSTRG.15442.1	3195	noncoding	-
MSTRG.15444.2	672	noncoding	-
MSTRG.15444.3	3217	noncoding	-
MSTRG.15447.5	2745	noncoding	-
MSTRG.15447.4	2462	noncoding	-
MSTRG.15447.6	2298	noncoding	-
MSTRG.15447.8	2307	noncoding	-

MSTRG.15447.9	2434	noncoding	-
MSTRG.15450.2	2965	noncoding	-
MSTRG.15450.3	2578	coding	PF08347
MSTRG.15457.1	3925	noncoding	-
MSTRG.15467.2	5517	coding	PF03028
MSTRG.15469.1	382	noncoding	-
MSTRG.15470.1	201	noncoding	-
MSTRG.15471.1	248	noncoding	-
MSTRG.15486.2	2612	coding	PF01044
MSTRG.15486.4	582	coding	PF01044
MSTRG.15490.1	4286	noncoding	-
MSTRG.15491.1	705	noncoding	-
MSTRG.15492.1	24385	coding	PF00078
MSTRG.15492.2	17879	coding	PF00078
MSTRG.15493.1	9873	noncoding	-
MSTRG.15494.1	4026	noncoding	-
MSTRG.15486.5	7387	coding	PF01044
MSTRG.15494.2	3078	noncoding	-
MSTRG.15497.1	17787	noncoding	-
MSTRG.15499.1	5518	noncoding	-
MSTRG.15499.2	965	noncoding	-
MSTRG.15500.1	5984	noncoding	-
MSTRG.15502.1	6800	noncoding	-
MSTRG.15503.1	3354	noncoding	-
MSTRG.15502.2	2046	noncoding	-
MSTRG.15477.1	468	noncoding	-
MSTRG.15480.2	3289	noncoding	-
MSTRG.15532.2	12713	noncoding	-
MSTRG.15532.1	12896	noncoding	-
MSTRG.15532.4	7549	noncoding	-
MSTRG.15532.5	11562	noncoding	-
MSTRG.15532.3	11739	noncoding	-
MSTRG.15532.6	12618	noncoding	-
MSTRG.15533.1	3794	noncoding	-
MSTRG.15532.8	6647	noncoding	-
MSTRG.15534.1	1701	noncoding	-
MSTRG.15535.1	3584	coding	PF08767
MSTRG.15536.1	6905	coding	PF01198
MSTRG.15535.3	4187	coding	PF08767
MSTRG.15535.4	4271	coding	PF08767
MSTRG.15538.1	6229	noncoding	-
MSTRG.15517.1	2687	noncoding	-
MSTRG.15518.4	2595	coding	PF10595
MSTRG.15518.5	3270	coding	PF10595
MSTRG.15518.6	4756	coding	PF10595
MSTRG.15518.8	2915	coding	PF10595
MSTRG.15518.7	2648	coding	PF10595
MSTRG.15519.1	1511	noncoding	-
MSTRG.15521.2	5932	coding	PF07258
MSTRG.15521.3	3976	coding	PF07258
MSTRG.15528.3	398	noncoding	-
MSTRG.15540.2	4033	coding	PF00307
MSTRG.15540.8	2323	noncoding	-
MSTRG.15540.7	3471	coding	PF00307

MSTRG.15547.1	6125	noncoding	-
MSTRG.15547.2	3745	noncoding	-
MSTRG.15547.3	4395	noncoding	-
MSTRG.15548.1	5981	noncoding	-
MSTRG.15548.2	1336	noncoding	-
MSTRG.15548.3	1165	noncoding	-
MSTRG.15549.1	373	noncoding	-
MSTRG.15554.1	1885	coding	PF02994
MSTRG.15572.1	1045	noncoding	-
MSTRG.15571.3	5703	coding	PF07928
MSTRG.15571.4	1151	noncoding	-
MSTRG.15563.1	4687	noncoding	-
MSTRG.15574.1	972	coding	PF00337
MSTRG.15575.1	1463	noncoding	-
MSTRG.15594.4	5511	noncoding	-
MSTRG.15595.1	1719	coding	PF00071
MSTRG.15595.2	2486	coding	PF00071
MSTRG.15596.2	4643	coding	PF05210
MSTRG.15596.3	4602	coding	PF05210
MSTRG.15600.2	2301	coding	PF16493
MSTRG.15601.1	858	noncoding	-
MSTRG.15602.1	1772	noncoding	-
MSTRG.15602.2	2131	noncoding	-
MSTRG.15603.3	2969	coding	PF15350
MSTRG.15611.2	1310	noncoding	-
MSTRG.15614.2	888	coding	PF13499
MSTRG.15618.1	1684	noncoding	-
MSTRG.15619.2	3075	coding	PF05586
MSTRG.15619.3	1343	coding	PF05587
MSTRG.15619.4	2340	coding	PF05586
MSTRG.15625.1	12625	coding	PF15282
MSTRG.15625.2	4594	coding	PF15282
MSTRG.15628.2	3037	noncoding	-
MSTRG.15630.3	547	noncoding	-
MSTRG.15630.2	4743	noncoding	-
MSTRG.15638.2	3725	coding	PF14952
MSTRG.15638.3	3611	coding	PF00076
MSTRG.15638.4	1852	coding	PF00076
MSTRG.15638.8	1044	coding	PF00076
MSTRG.15638.10	3082	noncoding	-
MSTRG.15644.1	2970	coding	PF00648
MSTRG.15644.4	1106	noncoding	-
MSTRG.15644.7	2051	noncoding	-
MSTRG.15644.5	1023	noncoding	-
MSTRG.15644.6	1205	noncoding	-
MSTRG.15650.1	787	noncoding	-
MSTRG.15651.1	2886	noncoding	-
MSTRG.15656.1	2368	noncoding	-
MSTRG.15671.1	870	noncoding	-
MSTRG.15654.1	2367	coding	PF01302
MSTRG.15654.2	3056	coding	PF01302
MSTRG.15677.1	2193	noncoding	-
MSTRG.15678.2	11983	coding	PF11357
MSTRG.15679.1	3739	noncoding	-

MSTRG.15678.3	2758	coding	PF00149
MSTRG.15678.4	3569	coding	PF00149
MSTRG.15672.1	260	noncoding	-
MSTRG.15674.1	1303	noncoding	-
MSTRG.15675.1	652	noncoding	-
MSTRG.15681.2	543	noncoding	-
MSTRG.15681.3	875	noncoding	-
MSTRG.15681.5	1679	coding	PF06113
MSTRG.15681.4	1921	coding	PF06113
MSTRG.15682.1	2250	noncoding	-
MSTRG.15683.1	6218	noncoding	-
MSTRG.15681.6	576	noncoding	-
MSTRG.15684.1	1064	coding	PF00294
MSTRG.15681.8	804	noncoding	-
MSTRG.15685.2	4566	coding	PF00498
MSTRG.15692.1	228	noncoding	-
MSTRG.15694.3	19246	noncoding	-
MSTRG.15694.1	18271	noncoding	-
MSTRG.15694.2	18251	noncoding	-
MSTRG.15694.4	18310	noncoding	-
MSTRG.15701.4	2350	noncoding	-
MSTRG.15701.5	2086	noncoding	-
MSTRG.15703.5	1604	noncoding	-
MSTRG.15703.4	1842	noncoding	-
MSTRG.15703.6	1839	noncoding	-
MSTRG.15703.7	1628	noncoding	-
MSTRG.15705.3	1646	coding	PF01008
MSTRG.15707.2	873	coding	PF04117
MSTRG.15707.4	851	coding	PF04117
MSTRG.15714.3	1658	coding	PF00226
MSTRG.15714.4	1587	coding	PF00226
MSTRG.15715.1	3912	noncoding	-
MSTRG.15715.5	3001	noncoding	-
MSTRG.15715.6	3051	noncoding	-
MSTRG.15719.2	957	noncoding	-
MSTRG.15720.1	3508	noncoding	-
MSTRG.15720.2	3709	noncoding	-
MSTRG.15720.3	3291	noncoding	-
MSTRG.15708.1	4083	noncoding	-
MSTRG.15709.1	2210	coding	PF15728
MSTRG.15711.3	575	noncoding	-
MSTRG.15722.4	2936	noncoding	-
MSTRG.15722.10	2434	noncoding	-
MSTRG.15722.9	2613	noncoding	-
MSTRG.15724.2	3112	coding	PF10151
MSTRG.15726.5	1683	coding	PF03271
MSTRG.15726.6	1594	coding	PF03271
MSTRG.15723.2	2288	noncoding	-
MSTRG.15723.3	1558	noncoding	-
MSTRG.15747.3	8162	coding	PF13919
MSTRG.15750.2	2918	noncoding	-
MSTRG.15750.3	5430	noncoding	-
MSTRG.15750.4	3437	noncoding	-
MSTRG.15751.5	1404	noncoding	-

MSTRG.15751.4	2642	noncoding	-
MSTRG.15752.3	4788	coding	PF00071
MSTRG.15752.4	8665	coding	PF00071
MSTRG.15752.5	9446	coding	PF00071
MSTRG.15754.2	3090	coding	PF00211
MSTRG.15756.4	2477	noncoding	-
MSTRG.15766.3	4807	coding	PF14598
MSTRG.15766.4	4939	coding	PF14598
MSTRG.15766.10	4530	coding	PF14598
MSTRG.15766.8	4587	coding	PF14598
MSTRG.15766.9	4533	coding	PF14598
MSTRG.15767.1	2904	noncoding	-
MSTRG.15766.11	4456	noncoding	-
MSTRG.15766.12	274	noncoding	-
MSTRG.15766.13	1054	noncoding	-
MSTRG.15768.1	1452	noncoding	-
MSTRG.15769.1	2978	noncoding	-
MSTRG.15773.1	6448	noncoding	-
MSTRG.15773.2	8359	noncoding	-
MSTRG.15776.1	5779	noncoding	-
MSTRG.15776.2	2522	noncoding	-
MSTRG.15778.3	2021	coding	PF00235
MSTRG.15778.2	2610	coding	PF00235
MSTRG.15781.1	1016	coding	PF00254
MSTRG.15781.5	1458	coding	PF00254
MSTRG.15781.7	638	coding	PF00254
MSTRG.15782.1	4182	coding	PF15390
MSTRG.15785.1	334	noncoding	-
MSTRG.15786.1	1213	noncoding	-
MSTRG.15786.2	242	noncoding	-
MSTRG.15786.3	1632	noncoding	-
MSTRG.15788.1	2033	noncoding	-
MSTRG.15791.1	917	noncoding	-
MSTRG.15793.1	2573	noncoding	-
MSTRG.15795.1	343	noncoding	-
MSTRG.15796.1	7244	coding	PF00567
MSTRG.15796.2	10375	noncoding	-
MSTRG.15796.3	5598	noncoding	-
MSTRG.15797.4	581	coding	PF10230
MSTRG.15797.6	1299	coding	PF10230
MSTRG.15798.1	970	noncoding	-
MSTRG.15804.2	3188	noncoding	-
MSTRG.15804.3	1588	noncoding	-
MSTRG.15804.5	3021	noncoding	-
MSTRG.15800.1	4106	noncoding	-
MSTRG.15801.1	2202	coding	PF00071
MSTRG.15809.10	5328	noncoding	-
MSTRG.15810.1	295	noncoding	-
MSTRG.15811.1	3224	noncoding	-
MSTRG.15812.1	2054	noncoding	-
MSTRG.15813.2	9314	noncoding	-
MSTRG.15813.1	9379	noncoding	-
MSTRG.15813.3	7099	noncoding	-
MSTRG.15813.4	7144	noncoding	-

MSTRG.15809.11	4260	noncoding	-
MSTRG.15806.1	2358	noncoding	-
MSTRG.15807.1	1665	noncoding	-
MSTRG.15816.1	635	noncoding	-
MSTRG.15816.2	547	noncoding	-
MSTRG.15814.1	1952	noncoding	-
MSTRG.15817.1	1937	noncoding	-
MSTRG.15821.1	225	noncoding	-
MSTRG.15822.3	1874	coding	PF06189
MSTRG.15826.1	953	noncoding	-
MSTRG.15832.1	351	noncoding	-
MSTRG.15834.1	483	noncoding	-
MSTRG.15834.2	565	noncoding	-
MSTRG.15835.1	970	noncoding	-
MSTRG.15835.2	458	noncoding	-
MSTRG.15841.1	3093	coding	PF15492
MSTRG.15841.2	1562	coding	PF15492
MSTRG.15843.2	2749	noncoding	-
MSTRG.15846.6	1120	coding	PF02146
MSTRG.15846.5	1274	coding	PF02146
MSTRG.15847.1	1425	noncoding	-
MSTRG.15848.1	1544	noncoding	-
MSTRG.15848.2	2131	noncoding	-
MSTRG.15848.3	1420	noncoding	-
MSTRG.15858.1	1678	noncoding	-
MSTRG.15858.2	2155	noncoding	-
MSTRG.15858.3	2179	coding	PF01399
MSTRG.15868.1	6431	coding	PF08235
MSTRG.15868.6	5891	coding	PF08235
MSTRG.15868.9	5553	coding	PF08235
MSTRG.15868.7	6195	coding	PF08235
MSTRG.15868.3	5457	coding	PF08235
MSTRG.15868.10	3431	coding	PF08235
MSTRG.15867.3	1749	noncoding	-
MSTRG.15873.2	2979	coding	PF00085
MSTRG.15874.2	1317	coding	PF03223
MSTRG.15879.2	2908	noncoding	-
MSTRG.15886.2	4308	noncoding	-
MSTRG.15890.1	320	noncoding	-
MSTRG.15891.2	1860	coding	PF00244
MSTRG.15896.2	2954	coding	PF00657
MSTRG.15902.5	3058	coding	PF01412
MSTRG.15902.3	3386	coding	PF01412
MSTRG.15902.4	3350	coding	PF01412
MSTRG.15902.6	3850	coding	PF01412
MSTRG.15903.1	722	noncoding	-
MSTRG.15897.1	2102	noncoding	-
MSTRG.15898.1	2839	noncoding	-
MSTRG.15899.3	3138	noncoding	-
MSTRG.15899.2	2197	noncoding	-
MSTRG.15899.4	2094	noncoding	-
MSTRG.15909.4	9162	coding	PF12796
MSTRG.15909.1	4613	coding	PF12796
MSTRG.15909.6	6799	coding	PF12796

MSTRG.15907.1	493	noncoding	-
MSTRG.15917.2	5884	noncoding	-
MSTRG.15917.3	5347	noncoding	-
MSTRG.15917.6	1931	noncoding	-
MSTRG.15917.7	1470	noncoding	-
MSTRG.15917.5	1787	noncoding	-
MSTRG.15917.8	2151	noncoding	-
MSTRG.15917.9	1705	noncoding	-
MSTRG.15917.10	3006	noncoding	-
MSTRG.15914.2	3332	noncoding	-
MSTRG.15913.1	994	noncoding	-
MSTRG.15921.1	513	noncoding	-
MSTRG.15929.1	6697	coding	PF12004
MSTRG.15929.4	6237	coding	PF12004
MSTRG.15930.1	2895	noncoding	-
MSTRG.15929.5	6830	coding	PF12004
MSTRG.15929.7	9739	coding	PF12004
MSTRG.15929.9	9805	coding	PF12004
MSTRG.15929.10	10504	coding	PF12004
MSTRG.15929.11	6720	coding	PF12004
MSTRG.15932.1	10762	coding	PF03133
MSTRG.15932.2	1034	noncoding	-
MSTRG.15931.1	359	noncoding	-
MSTRG.15936.2	915	noncoding	-
MSTRG.15936.3	768	noncoding	-
MSTRG.15937.3	754	noncoding	-
MSTRG.15935.1	1645	coding	PF00046
MSTRG.15940.1	1464	coding	PF01765
MSTRG.15940.4	689	coding	PF01765
MSTRG.15943.1	494	coding	PF13853
MSTRG.15961.2	8410	coding	PF00651
MSTRG.15962.3	4424	coding	PF00566
MSTRG.15962.2	4160	coding	PF00566
MSTRG.15962.7	15142	coding	PF00566
MSTRG.15963.1	5240	noncoding	-
MSTRG.15964.1	9389	noncoding	-
MSTRG.15952.1	3706	coding	PF02141
MSTRG.15955.1	3073	noncoding	-
MSTRG.15956.1	6436	noncoding	-
MSTRG.15957.1	3004	noncoding	-
MSTRG.15958.1	3338	noncoding	-
MSTRG.15951.1	426	noncoding	-
MSTRG.15969.2	1819	coding	PF00069
MSTRG.15969.3	1838	coding	PF00069
MSTRG.15969.4	2062	coding	PF00069
MSTRG.15973.1	311	noncoding	-
MSTRG.15974.1	3377	coding	PF00105
MSTRG.15974.5	2014	coding	PF00105
MSTRG.15978.5	14287	noncoding	-
MSTRG.15978.7	3523	coding	PF00105
MSTRG.15978.6	23994	noncoding	-
MSTRG.15979.1	2702	noncoding	-
MSTRG.15980.1	429	noncoding	-
MSTRG.15981.1	5408	noncoding	-

MSTRG.15978.8	8066	noncoding	-
MSTRG.15982.1	670	noncoding	-
MSTRG.15978.9	4319	noncoding	-
MSTRG.15976.2	1280	coding	PF00400
MSTRG.15990.4	3806	noncoding	-
MSTRG.15994.1	1538	noncoding	-
MSTRG.15996.1	430	noncoding	-
MSTRG.15997.1	946	noncoding	-
MSTRG.15998.4	2225	coding	PF16978
MSTRG.15998.3	1733	coding	PF16978
MSTRG.15998.10	8087	coding	PF16979
MSTRG.15998.11	2074	coding	PF16978
MSTRG.15998.9	2099	coding	PF16978
MSTRG.15999.1	580	noncoding	-
MSTRG.16000.1	609	noncoding	-
MSTRG.16003.1	1701	coding	PF03792
MSTRG.16004.1	2706	noncoding	-
MSTRG.16006.1	322	noncoding	-
MSTRG.16005.2	3557	noncoding	-
MSTRG.16012.1	627	noncoding	-
MSTRG.16013.2	3727	coding	PF02145
MSTRG.16014.1	1162	noncoding	-
MSTRG.16015.2	1837	coding	PF00083
MSTRG.16019.1	8210	noncoding	-
MSTRG.16019.2	3785	noncoding	-
MSTRG.16022.2	3750	noncoding	-
MSTRG.16027.2	3425	coding	PF00017
MSTRG.16031.2	2418	noncoding	-
MSTRG.16034.2	1329	noncoding	-
MSTRG.16035.1	778	noncoding	-
MSTRG.16039.1	4579	coding	PF00153
MSTRG.16039.2	2933	coding	PF00153
MSTRG.16041.1	876	coding	PF00061
MSTRG.16042.2	841	coding	PF03670
MSTRG.16042.3	708	coding	PF03670
MSTRG.16042.4	805	coding	PF03670
MSTRG.16044.3	4911	coding	PF02212
MSTRG.16049.2	4387	coding	PF01509
MSTRG.16050.3	1172	coding	PF05019
MSTRG.16050.4	1783	coding	PF05019
MSTRG.16052.1	7813	noncoding	-
MSTRG.16052.2	6044	noncoding	-
MSTRG.16052.3	5605	noncoding	-
MSTRG.16056.1	4868	noncoding	-
MSTRG.16057.12	2360	noncoding	-
MSTRG.16057.15	2355	noncoding	-
MSTRG.16057.11	4740	noncoding	-
MSTRG.16057.14	4797	noncoding	-
MSTRG.16057.17	5223	noncoding	-
MSTRG.16057.19	4724	noncoding	-
MSTRG.16057.20	3575	noncoding	-
MSTRG.16067.2	1427	noncoding	-
MSTRG.16058.1	1867	coding	PF00956
MSTRG.16064.2	3723	coding	PF00566

MSTRG.16064.3	2680	coding	PF00566
MSTRG.16064.4	1835	coding	PF00566
MSTRG.16064.7	3732	coding	PF00566
MSTRG.16069.2	2238	coding	PF02598
MSTRG.16069.5	1997	noncoding	-
MSTRG.16069.6	2997	noncoding	-
MSTRG.16069.8	2411	noncoding	-
MSTRG.16072.1	459	noncoding	-
MSTRG.16079.2	5458	noncoding	-
MSTRG.16079.4	5705	noncoding	-
MSTRG.16074.1	4290	coding	PF10265
MSTRG.16074.3	2819	coding	PF10265
MSTRG.16081.1	4134	coding	PF00755
MSTRG.16082.2	2817	coding	PF03095
MSTRG.16082.3	2480	coding	PF03095
MSTRG.16082.4	3498	coding	PF03095
MSTRG.16076.2	1363	coding	PF05760
MSTRG.16083.1	4056	noncoding	-
MSTRG.16083.2	1784	noncoding	-
MSTRG.16083.3	887	noncoding	-
MSTRG.16088.2	1103	coding	PF05891
MSTRG.16088.4	1238	coding	PF05891
MSTRG.16085.1	1852	coding	PF12796
MSTRG.16092.3	5902	coding	PF00443
MSTRG.16093.3	3717	noncoding	-
MSTRG.16104.1	9365	coding	PF07714
MSTRG.16104.4	7471	coding	PF07714
MSTRG.16104.5	9852	coding	PF07714
MSTRG.16102.1	4637	coding	PF00147
MSTRG.16109.4	7098	noncoding	-
MSTRG.16110.1	930	noncoding	-
MSTRG.16113.5	11038	coding	PF07001
MSTRG.16113.6	9130	coding	PF07001
MSTRG.16113.8	9127	coding	PF07001
MSTRG.16113.7	10949	coding	PF07001
MSTRG.16113.9	8596	coding	PF07001
MSTRG.16113.11	10106	noncoding	-
MSTRG.16113.12	5321	noncoding	-
MSTRG.16113.13	8280	noncoding	-
MSTRG.16113.14	7505	noncoding	-
MSTRG.16117.2	5646	coding	PF00617
MSTRG.16117.3	5436	coding	PF00617
MSTRG.16118.1	925	noncoding	-
MSTRG.16123.1	2218	noncoding	-
MSTRG.16128.1	13631	coding	PF13086
MSTRG.16128.5	11064	coding	PF13086
MSTRG.16128.6	11072	coding	PF13087
MSTRG.16129.1	7920	noncoding	-
MSTRG.16129.2	7779	noncoding	-
MSTRG.16129.3	3815	noncoding	-
MSTRG.16132.2	2530	coding	PF00270
MSTRG.16132.3	3012	coding	PF00270
MSTRG.16133.1	9437	noncoding	-
MSTRG.16133.2	7142	coding	PF12657

MSTRG.16133.4	13592	coding	PF12657
MSTRG.16136.1	1927	coding	PF00406
MSTRG.16136.2	1566	coding	PF00406
MSTRG.16138.2	7927	coding	PF04388
MSTRG.16138.5	7774	coding	PF04388
MSTRG.16138.6	3644	coding	PF04388
MSTRG.16144.1	1032	coding	PF03414
MSTRG.16144.4	1427	coding	PF03414
MSTRG.16148.2	5028	coding	PF08683
MSTRG.16148.5	4796	coding	PF08683
MSTRG.16149.1	1931	noncoding	-
MSTRG.16159.1	3634	noncoding	-
MSTRG.16159.2	3012	noncoding	-
MSTRG.16159.3	3403	noncoding	-
MSTRG.16159.4	3374	noncoding	-
MSTRG.16159.5	4061	noncoding	-
MSTRG.16162.1	1609	coding	PF05180
MSTRG.16163.5	5256	coding	PF00249
MSTRG.16167.2	2961	coding	PF00675
MSTRG.16170.5	8677	coding	PF12931
MSTRG.16174.2	1346	coding	PF01553
MSTRG.16191.3	3437	noncoding	-
MSTRG.16191.4	3793	noncoding	-
MSTRG.16191.5	2778	noncoding	-
MSTRG.16182.2	2155	noncoding	-
MSTRG.16196.2	4848	coding	PF00307
MSTRG.16198.1	5729	coding	PF17035
MSTRG.16198.2	5596	coding	PF17035
MSTRG.16199.1	1408	noncoding	-
MSTRG.16195.3	1574	coding	PF00400
MSTRG.16201.2	5415	coding	PF00104
MSTRG.16201.3	4956	coding	PF00104
MSTRG.16201.6	4636	coding	PF00104
MSTRG.16202.1	3148	noncoding	-
MSTRG.16201.7	4988	coding	PF00104
MSTRG.16203.1	2579	coding	PF02191
MSTRG.16207.1	3073	noncoding	-
MSTRG.16210.2	974	coding	PF14892
MSTRG.16211.1	2114	coding	PF00318
MSTRG.16214.2	889	coding	PF15110
MSTRG.16214.3	694	coding	PF15110
MSTRG.16215.1	391	noncoding	-
MSTRG.16217.1	10519	noncoding	-
MSTRG.16218.3	3879	coding	PF16673
MSTRG.16218.4	3780	coding	PF16673
MSTRG.16221.4	2409	coding	PF12937
MSTRG.16221.3	2373	coding	PF12937
MSTRG.16222.1	2518	coding	PF12937
MSTRG.16222.3	1112	noncoding	-
MSTRG.16219.1	682	noncoding	-
MSTRG.16226.5	825	coding	PF15384
MSTRG.16239.3	2680	coding	PF01532
MSTRG.16239.4	2597	coding	PF01532
MSTRG.16244.1	1808	coding	PF01532

MSTRG.16244.3	2193	coding	PF01532
MSTRG.16244.5	4077	coding	PF01532
MSTRG.16242.7	400	noncoding	-
MSTRG.16233.1	4446	coding	PF15195
MSTRG.16233.2	776	coding	PF15195
MSTRG.16248.2	2235	coding	PF00667
MSTRG.16248.3	2286	coding	PF00667
MSTRG.16237.1	847	noncoding	-
MSTRG.16252.3	1746	noncoding	-
MSTRG.16252.4	2039	noncoding	-
MSTRG.16252.1	1569	noncoding	-
MSTRG.16253.2	1779	noncoding	-
MSTRG.16253.6	1155	noncoding	-
MSTRG.16253.7	1208	noncoding	-
MSTRG.16254.1	3076	coding	PF06209
MSTRG.16261.2	9022	coding	PF00027
MSTRG.16261.6	4358	coding	PF00027
MSTRG.16261.5	4356	coding	PF00027
MSTRG.16263.5	1641	noncoding	-
MSTRG.16263.3	1891	noncoding	-
MSTRG.16263.4	1577	noncoding	-
MSTRG.16263.2	2132	noncoding	-
MSTRG.16258.1	1887	noncoding	-
MSTRG.16258.2	1985	noncoding	-
MSTRG.16264.6	8035	coding	PF00856
MSTRG.16265.1	1471	noncoding	-
MSTRG.16264.10	11663	coding	PF00856
MSTRG.16264.9	4402	coding	PF00856
MSTRG.16264.11	857	noncoding	-
MSTRG.16269.1	4053	noncoding	-
MSTRG.16269.2	2917	noncoding	-
MSTRG.16269.3	3957	noncoding	-
MSTRG.16270.1	6811	coding	PF08763
MSTRG.16270.2	6743	coding	PF08763
MSTRG.16270.3	7592	coding	PF08763
MSTRG.16273.1	758	noncoding	-
MSTRG.16274.2	1975	coding	PF00567
MSTRG.16275.1	416	noncoding	-
MSTRG.16276.1	1766	noncoding	-
MSTRG.16278.2	3228	noncoding	-
MSTRG.16284.1	220	noncoding	-
MSTRG.16288.1	4296	noncoding	-
MSTRG.16291.1	2298	noncoding	-
MSTRG.16296.1	894	noncoding	-
MSTRG.16299.1	234	noncoding	-
MSTRG.16299.2	272	noncoding	-
MSTRG.16302.1	680	noncoding	-
MSTRG.16307.1	1719	noncoding	-
MSTRG.16319.2	8888	coding	PF05002
MSTRG.16319.3	6248	coding	PF05002
MSTRG.16319.4	6127	coding	PF05002
MSTRG.16319.5	6230	coding	PF05002
MSTRG.16319.6	5999	coding	PF05002
MSTRG.16320.2	4243	noncoding	-

MSTRG.16321.1	5529	coding	PF00178
MSTRG.16326.1	2321	coding	PF00651
MSTRG.16327.1	2014	coding	PF00472
MSTRG.16328.1	1120	noncoding	-
MSTRG.16329.3	4686	coding	PF12569
MSTRG.16329.4	4811	noncoding	-
MSTRG.16330.2	11258	coding	PF07728
MSTRG.16332.1	4290	coding	PF00609
MSTRG.16334.5	8920	coding	PF05716
MSTRG.16336.1	212	noncoding	-
MSTRG.16337.1	1114	coding	PF15107
MSTRG.16339.2	2852	noncoding	-
MSTRG.16349.1	5659	noncoding	-
MSTRG.16345.2	6023	noncoding	-
MSTRG.16345.3	5904	noncoding	-
MSTRG.16343.1	645	noncoding	-
MSTRG.16340.1	450	noncoding	-
MSTRG.16351.1	1114	coding	PF06624
MSTRG.16351.2	916	coding	PF06624
MSTRG.16352.4	2813	coding	PF01166
MSTRG.16353.1	7663	noncoding	-
MSTRG.16354.1	2841	noncoding	-
MSTRG.16358.2	1869	coding	PF10453
MSTRG.16358.3	1572	coding	PF10453
MSTRG.16361.2	9125	noncoding	-
MSTRG.16361.1	4406	coding	PF02270
MSTRG.16361.3	1968	coding	PF02270
MSTRG.16361.5	1181	coding	PF02270
MSTRG.16361.6	8011	noncoding	-
MSTRG.16361.7	1617	noncoding	-
MSTRG.16364.2	4535	coding	PF04136
MSTRG.16366.1	1102	coding	PF03145
MSTRG.16369.4	3380	noncoding	-
MSTRG.16370.1	2701	noncoding	-
MSTRG.16370.2	1763	noncoding	-
MSTRG.16375.3	1633	coding	PF00756
MSTRG.16378.1	343	noncoding	-
MSTRG.16388.1	2009	noncoding	-
MSTRG.16389.1	14558	noncoding	-
MSTRG.16389.2	2125	noncoding	-
MSTRG.16388.2	852	noncoding	-
MSTRG.16397.3	3164	coding	PF01359
MSTRG.16399.2	2423	coding	PF10018
MSTRG.16410.1	278	noncoding	-
MSTRG.16418.1	2009	noncoding	-
MSTRG.16419.6	1730	noncoding	-
MSTRG.16419.5	1839	noncoding	-
MSTRG.16419.7	7045	coding	PF00041
MSTRG.16419.8	6795	coding	PF00041
MSTRG.16419.10	5663	coding	PF00041
MSTRG.16422.1	2017	coding	PF08569
MSTRG.16422.2	2060	coding	PF08569
MSTRG.16422.3	1702	coding	PF08569
MSTRG.16422.6	1998	coding	PF08569

MSTRG.16423.4	4109	coding	PF05033
MSTRG.16423.2	3173	coding	PF05033
MSTRG.16423.3	2705	noncoding	-
MSTRG.16423.5	7208	coding	PF05033
MSTRG.16423.7	2998	coding	PF05033
MSTRG.16423.8	4429	coding	PF05033
MSTRG.16423.10	4919	coding	PF13771
MSTRG.16423.12	3748	coding	PF13771
MSTRG.16425.2	5988	noncoding	-
MSTRG.16425.1	6193	coding	PF00415
MSTRG.16425.3	11317	coding	PF00415
MSTRG.16425.9	2839	coding	PF00415
MSTRG.16425.10	2054	coding	PF00415
MSTRG.16427.1	741	noncoding	-
MSTRG.16417.1	497	noncoding	-
MSTRG.16429.2	507	noncoding	-
MSTRG.16431.3	2022	noncoding	-
MSTRG.16431.4	1844	noncoding	-
MSTRG.16470.1	7901	noncoding	-
MSTRG.16472.1	3431	noncoding	-
MSTRG.16473.2	893	noncoding	-
MSTRG.16446.1	433	noncoding	-
MSTRG.16448.5	2791	noncoding	-
MSTRG.16448.6	1617	noncoding	-
MSTRG.16448.2	2030	noncoding	-
MSTRG.16448.4	2037	noncoding	-
MSTRG.16448.8	11671	noncoding	-
MSTRG.16448.9	8833	noncoding	-
MSTRG.16452.3	6509	coding	PF00122
MSTRG.16452.5	6194	coding	PF00122
MSTRG.16452.6	6928	coding	PF00122
MSTRG.16453.2	4004	coding	PF15924
MSTRG.16457.1	4206	coding	PF04157
MSTRG.16461.2	1098	coding	PF15433
MSTRG.16463.2	2908	coding	PF16675
MSTRG.16463.3	5542	coding	PF16675
MSTRG.16463.4	5541	coding	PF16675
MSTRG.16464.1	31071	noncoding	-
MSTRG.16465.1	1125	noncoding	-
MSTRG.16482.1	520	noncoding	-
MSTRG.16484.1	2703	coding	PF06419
MSTRG.16486.2	1861	noncoding	-
MSTRG.16486.3	2912	noncoding	-
MSTRG.16491.1	1209	coding	PF03671
MSTRG.16492.1	566	noncoding	-
MSTRG.16492.2	1406	coding	PF08344
MSTRG.16498.7	2732	coding	PF12090
MSTRG.16498.8	2756	coding	PF12090
MSTRG.16498.13	2775	coding	PF12090
MSTRG.16498.14	2879	coding	PF12090
MSTRG.16498.15	2734	coding	PF12090
MSTRG.16498.16	624	noncoding	-
MSTRG.16502.2	634	noncoding	-
MSTRG.16507.1	303	noncoding	-

MSTRG.16511.1	2990	coding	PF00134
MSTRG.16511.2	2853	coding	PF00134
MSTRG.16511.7	2158	coding	PF00134
MSTRG.16514.1	2858	coding	PF06911
MSTRG.16514.2	2815	coding	PF06911
MSTRG.16515.3	2247	noncoding	-
MSTRG.16539.4	5561	coding	PF02138
MSTRG.16539.5	4535	coding	PF02138
MSTRG.16539.2	9155	coding	PF02138
MSTRG.16539.6	10865	coding	PF02138
MSTRG.16539.7	5150	coding	PF15787
MSTRG.16542.1	652	noncoding	-
MSTRG.16539.8	4445	coding	PF15787
MSTRG.16565.1	1594	noncoding	-
MSTRG.16584.1	1663	noncoding	-
MSTRG.16584.2	3099	noncoding	-
MSTRG.16516.1	1339	coding	PF08542
MSTRG.16520.1	3030	noncoding	-
MSTRG.16535.6	1453	noncoding	-
MSTRG.16535.7	3516	noncoding	-
MSTRG.16537.1	4932	noncoding	-
MSTRG.16590.2	5784	noncoding	-
MSTRG.16590.4	4056	noncoding	-
MSTRG.16590.3	5732	noncoding	-
MSTRG.16590.6	4419	noncoding	-
MSTRG.16597.2	10377	coding	PF14222
MSTRG.16597.3	1332	noncoding	-
MSTRG.16597.4	927	noncoding	-
MSTRG.16597.5	585	noncoding	-
MSTRG.16597.6	914	noncoding	-
MSTRG.16613.1	1035	noncoding	-
MSTRG.16616.1	9321	noncoding	-
MSTRG.16616.2	14762	noncoding	-
MSTRG.16616.3	12960	noncoding	-
MSTRG.16619.1	6746	noncoding	-
MSTRG.16620.1	16497	coding	PF01359
MSTRG.16616.5	1407	noncoding	-
MSTRG.16616.6	2842	noncoding	-
MSTRG.16616.7	2342	noncoding	-
MSTRG.16616.8	4944	noncoding	-
MSTRG.16620.2	27710	noncoding	-
MSTRG.16620.3	41475	noncoding	-
MSTRG.16620.4	32042	noncoding	-
MSTRG.16620.5	57416	coding	PF01359
MSTRG.16620.6	54571	coding	PF01359
MSTRG.16622.1	3789	noncoding	-
MSTRG.16587.1	352	noncoding	-
MSTRG.16601.1	3069	noncoding	-
MSTRG.16602.1	5489	noncoding	-
MSTRG.16603.3	2137	noncoding	-
MSTRG.16603.4	1181	noncoding	-
MSTRG.16604.2	2095	noncoding	-
MSTRG.16629.2	2626	coding	PF09011
MSTRG.16629.3	3670	coding	PF09011

MSTRG.16630.1	8817	noncoding	-
MSTRG.16631.3	11110	noncoding	-
MSTRG.16629.4	2488	coding	PF09011
MSTRG.16629.5	4245	coding	PF09011
MSTRG.16632.1	219	noncoding	-
MSTRG.16642.1	7191	coding	PF13906
MSTRG.16642.2	6532	coding	PF13906
MSTRG.16644.1	3753	noncoding	-
MSTRG.16645.2	1179	coding	PF05348
MSTRG.16653.5	2448	noncoding	-
MSTRG.16653.6	2391	noncoding	-
MSTRG.16653.7	2781	noncoding	-
MSTRG.16653.8	2619	noncoding	-
MSTRG.16653.9	2777	noncoding	-
MSTRG.16653.10	4163	noncoding	-
MSTRG.16654.1	4363	noncoding	-
MSTRG.16652.1	4496	coding	PF00595
MSTRG.16660.1	5024	noncoding	-
MSTRG.16660.3	4138	noncoding	-
MSTRG.16660.4	2913	noncoding	-
MSTRG.16660.5	1499	noncoding	-
MSTRG.16665.3	5710	coding	PF00443
MSTRG.16665.4	6272	coding	PF00443
MSTRG.16671.5	855	noncoding	-
MSTRG.16672.2	2436	coding	PF13639
MSTRG.16672.5	3562	coding	PF13639
MSTRG.16672.7	2322	coding	PF13639
MSTRG.16669.1	3778	noncoding	-
MSTRG.16669.2	3032	noncoding	-
MSTRG.16670.1	3962	coding	PF16212
MSTRG.16674.1	6253	noncoding	-
MSTRG.16676.1	2031	coding	PF15967
MSTRG.16676.3	3759	coding	PF15967
MSTRG.16677.4	2188	coding	PF06602
MSTRG.16677.3	4097	coding	PF06602
MSTRG.16678.1	518	noncoding	-
MSTRG.16679.2	8231	noncoding	-
MSTRG.16679.1	1926	noncoding	-
MSTRG.16679.3	8541	noncoding	-
MSTRG.16679.4	1429	noncoding	-
MSTRG.16679.5	1138	noncoding	-
MSTRG.16685.2	5776	coding	PF00621
MSTRG.16687.1	1419	noncoding	-
MSTRG.16684.1	960	noncoding	-
MSTRG.16691.1	2010	noncoding	-
MSTRG.16694.5	3508	noncoding	-
MSTRG.16692.1	384	coding	PF00167
MSTRG.16693.1	555	noncoding	-
MSTRG.16702.1	10870	noncoding	-
MSTRG.16702.4	2394	noncoding	-
MSTRG.16702.5	832	noncoding	-
MSTRG.16704.3	547	noncoding	-
MSTRG.16702.10	3850	coding	PF01529
MSTRG.16702.12	4665	coding	PF01529

MSTRG.16702.13	3731	noncoding	-
MSTRG.16709.4	7931	coding	PF00069
MSTRG.16709.7	3733	noncoding	-
MSTRG.16696.4	9618	coding	PF10237
MSTRG.16712.1	10394	coding	PF12012
MSTRG.16712.2	10017	coding	PF12012
MSTRG.16712.3	8223	coding	PF12012
MSTRG.16712.5	9483	coding	PF12012
MSTRG.16712.4	6568	coding	PF12012
MSTRG.16712.6	5869	coding	PF12012
MSTRG.16712.7	6109	coding	PF12012
MSTRG.16712.8	4884	coding	PF12012
MSTRG.16712.9	4579	coding	PF12012
MSTRG.16712.10	4694	coding	PF12012
MSTRG.16712.11	3670	noncoding	-
MSTRG.16713.1	2620	coding	PF00076
MSTRG.16713.2	3174	coding	PF08075
MSTRG.16713.4	2642	noncoding	-
MSTRG.16713.11	13666	coding	PF08075
MSTRG.16719.2	6215	coding	PF08487
MSTRG.16722.1	5600	coding	PF00567
MSTRG.16722.2	5255	coding	PF00567
MSTRG.16722.3	5138	coding	PF00567
MSTRG.16722.5	6550	coding	PF00567
MSTRG.16722.6	6850	coding	PF00567
MSTRG.16726.1	1249	noncoding	-
MSTRG.16733.1	1337	noncoding	-
MSTRG.16733.3	1023	noncoding	-
MSTRG.16745.1	561	noncoding	-
MSTRG.16747.1	497	noncoding	-
MSTRG.16754.2	2039	coding	PF12554
MSTRG.16757.3	2735	noncoding	-
MSTRG.16757.4	3088	noncoding	-
MSTRG.16748.2	2837	noncoding	-
MSTRG.16749.1	274	noncoding	-
MSTRG.16749.2	335	noncoding	-
MSTRG.16750.1	691	noncoding	-
MSTRG.16761.3	1280	noncoding	-
MSTRG.16764.2	4190	coding	PF00566
MSTRG.16765.1	2168	noncoding	-
MSTRG.16766.1	1793	noncoding	-
MSTRG.16767.1	2482	noncoding	-
MSTRG.16769.1	2486	noncoding	-
MSTRG.16772.2	4586	coding	PF15949
MSTRG.16772.3	4650	coding	PF15949
MSTRG.16772.4	4583	coding	PF15949
MSTRG.16772.5	4617	coding	PF15949
MSTRG.16776.1	530	noncoding	-
MSTRG.16799.10	6535	noncoding	-
MSTRG.16799.7	6569	noncoding	-
MSTRG.16799.8	6848	noncoding	-
MSTRG.16799.9	6895	noncoding	-
MSTRG.16799.3	7089	noncoding	-
MSTRG.16799.2	14952	coding	PF08005

MSTRG.16799.11	14652	coding	PF08005
MSTRG.16799.4	14783	coding	PF08005
MSTRG.16799.5	14882	coding	PF08005
MSTRG.16799.1	14845	coding	PF08005
MSTRG.16801.1	2241	noncoding	-
MSTRG.16799.12	4549	noncoding	-
MSTRG.16799.13	5332	coding	PF08005
MSTRG.16802.1	1751	noncoding	-
MSTRG.16803.1	1257	noncoding	-
MSTRG.16780.1	235	noncoding	-
MSTRG.16784.2	2759	coding	PF15301
MSTRG.16785.1	230	noncoding	-
MSTRG.16787.1	214	noncoding	-
MSTRG.16789.1	278	noncoding	-
MSTRG.16823.10	6335	noncoding	-
MSTRG.16823.8	6346	noncoding	-
MSTRG.16823.1	7000	noncoding	-
MSTRG.16823.5	6961	noncoding	-
MSTRG.16823.6	6967	noncoding	-
MSTRG.16823.7	7069	noncoding	-
MSTRG.16823.9	5832	noncoding	-
MSTRG.16823.2	6971	noncoding	-
MSTRG.16823.3	7044	noncoding	-
MSTRG.16823.11	6265	noncoding	-
MSTRG.16823.12	3149	noncoding	-
MSTRG.16824.1	873	noncoding	-
MSTRG.16824.2	1614	noncoding	-
MSTRG.16824.3	1082	noncoding	-
MSTRG.16816.2	3154	noncoding	-
MSTRG.16816.3	3156	noncoding	-
MSTRG.16817.1	2135	noncoding	-
MSTRG.16818.1	2815	noncoding	-
MSTRG.16818.2	830	noncoding	-
MSTRG.16820.1	741	noncoding	-
MSTRG.16835.1	235	noncoding	-
MSTRG.16835.2	275	noncoding	-
MSTRG.16840.1	6151	noncoding	-
MSTRG.16847.1	1001	noncoding	-
MSTRG.16847.2	484	noncoding	-
MSTRG.16855.1	641	noncoding	-
MSTRG.16878.1	337	noncoding	-
MSTRG.16879.1	237	noncoding	-
MSTRG.16884.1	1462	noncoding	-
MSTRG.17027.1	8577	coding	PF01153
MSTRG.17027.4	12607	coding	PF01153
MSTRG.17027.5	8911	coding	PF01153
MSTRG.17027.3	3694	coding	PF01153
MSTRG.17027.6	10959	coding	PF01153
MSTRG.17028.1	3864	noncoding	-
MSTRG.17027.8	29632	coding	PF01153
MSTRG.17027.12	5170	coding	PF01153
MSTRG.17027.13	4339	coding	PF01153
MSTRG.17027.20	4431	coding	PF01153
MSTRG.17027.16	21252	coding	PF01153

MSTRG.17027.17	16054	coding	PF01153
MSTRG.17027.21	8258	coding	PF01153
MSTRG.17027.22	34059	coding	PF01153
MSTRG.17027.23	4062	noncoding	-
MSTRG.17031.1	14391	noncoding	-
MSTRG.17027.24	6689	coding	PF01153
MSTRG.17027.25	3631	coding	PF01153
MSTRG.17027.26	19184	noncoding	-
MSTRG.17027.27	14006	noncoding	-
MSTRG.17027.28	2641	noncoding	-
MSTRG.17033.1	1679	noncoding	-
MSTRG.17027.29	9257	noncoding	-
MSTRG.17027.30	7910	noncoding	-
MSTRG.17034.1	11327	noncoding	-
MSTRG.17035.1	3445	noncoding	-
MSTRG.17036.1	12077	noncoding	-
MSTRG.17037.1	2973	noncoding	-
MSTRG.17038.1	4062	noncoding	-
MSTRG.17039.1	7025	noncoding	-
MSTRG.17040.1	2726	noncoding	-
MSTRG.17041.1	9233	noncoding	-
MSTRG.17042.1	2598	coding	PF00078
MSTRG.17042.2	5725	coding	PF00078
MSTRG.17027.31	4277	noncoding	-
MSTRG.17027.32	25720	noncoding	-
MSTRG.17027.34	30300	coding	PF00078
MSTRG.17027.33	23637	noncoding	-
MSTRG.17027.35	489	noncoding	-
MSTRG.17027.37	25677	noncoding	-
MSTRG.17027.36	9163	noncoding	-
MSTRG.17027.38	12520	coding	PF00078
MSTRG.17027.40	9537	coding	PF00078
MSTRG.17027.39	9760	noncoding	-
MSTRG.17027.41	13188	noncoding	-
MSTRG.17027.42	7632	noncoding	-
MSTRG.17027.43	9410	noncoding	-
MSTRG.17027.44	4906	noncoding	-
MSTRG.17044.1	4513	noncoding	-
MSTRG.17027.45	9382	noncoding	-
MSTRG.17027.46	6387	noncoding	-
MSTRG.17045.1	5245	noncoding	-
MSTRG.17046.1	3780	noncoding	-
MSTRG.17047.1	12441	noncoding	-
MSTRG.17048.1	4629	noncoding	-
MSTRG.17049.1	891	noncoding	-
MSTRG.17050.1	3230	noncoding	-
MSTRG.17051.1	4105	noncoding	-
MSTRG.16890.1	3377	coding	PF01153
MSTRG.16890.2	896	coding	PF01153
MSTRG.16899.2	5677	coding	PF00005
MSTRG.16906.1	3590	coding	PF00005
MSTRG.16906.3	844	coding	PF00005
MSTRG.16907.3	2346	noncoding	-
MSTRG.16907.1	2065	noncoding	-

MSTRG.16907.2	7217	noncoding	-
MSTRG.16907.4	7214	noncoding	-
MSTRG.16907.5	5076	noncoding	-
MSTRG.16907.6	5504	noncoding	-
MSTRG.16907.8	2481	noncoding	-
MSTRG.16907.7	8708	noncoding	-
MSTRG.16907.9	3211	noncoding	-
MSTRG.16911.1	1209	noncoding	-
MSTRG.16912.1	4070	noncoding	-
MSTRG.16915.1	3860	coding	PF00005
MSTRG.16918.2	1088	noncoding	-
MSTRG.16918.1	1677	noncoding	-
MSTRG.16912.2	2157	coding	PF00005
MSTRG.16912.3	1915	coding	PF00005
MSTRG.16902.3	2260	noncoding	-
MSTRG.16902.4	2260	noncoding	-
MSTRG.16919.1	330	noncoding	-
MSTRG.16920.1	2288	noncoding	-
MSTRG.16922.1	530	noncoding	-
MSTRG.16930.6	3376	coding	PF13815
MSTRG.16927.1	1518	coding	PF00001
MSTRG.16928.1	291	noncoding	-
MSTRG.16935.7	2495	noncoding	-
MSTRG.16936.1	15963	noncoding	-
MSTRG.16935.8	2574	noncoding	-
MSTRG.16938.1	4193	noncoding	-
MSTRG.16940.1	2199	coding	PF00071
MSTRG.16940.2	2155	coding	PF00071
MSTRG.16940.3	3249	coding	PF00071
MSTRG.16940.4	5668	coding	PF00071
MSTRG.16945.1	8493	noncoding	-
MSTRG.16948.1	5733	noncoding	-
MSTRG.16949.2	5161	coding	PF00069
MSTRG.16953.4	7369	coding	PF06920
MSTRG.16954.1	2783	noncoding	-
MSTRG.16959.1	2474	noncoding	-
MSTRG.16959.2	2321	noncoding	-
MSTRG.16961.1	6532	noncoding	-
MSTRG.16962.1	827	noncoding	-
MSTRG.16965.1	2682	coding	PF03328
MSTRG.16965.2	2402	coding	PF03328
MSTRG.16965.4	3070	noncoding	-
MSTRG.16965.3	5265	coding	PF03328
MSTRG.16965.5	3710	coding	PF03328
MSTRG.16965.6	3146	coding	PF03328
MSTRG.16965.7	584	coding	PF03328
MSTRG.16966.1	19197	noncoding	-
MSTRG.16966.2	15722	noncoding	-
MSTRG.16967.1	1707	noncoding	-
MSTRG.16968.1	1441	noncoding	-
MSTRG.16963.5	319	noncoding	-
MSTRG.16971.1	2792	coding	PF02786
MSTRG.16971.2	2644	coding	PF02786
MSTRG.16971.3	2522	coding	PF02786

MSTRG.16971.4	2772	coding	PF02786
MSTRG.16971.6	2509	coding	PF02786
MSTRG.16971.8	1895	coding	PF02786
MSTRG.16971.10	2405	coding	PF02786
MSTRG.16971.9	2024	coding	PF02786
MSTRG.16973.1	1442	noncoding	-
MSTRG.16974.2	1618	coding	PF06094
MSTRG.16974.3	1682	coding	PF06094
MSTRG.16978.1	3960	noncoding	-
MSTRG.16978.2	5342	noncoding	-
MSTRG.16978.3	5468	noncoding	-
MSTRG.16978.4	5337	noncoding	-
MSTRG.16988.6	10549	coding	PF15804
MSTRG.16988.4	26720	coding	PF15804
MSTRG.16988.5	28289	coding	PF15804
MSTRG.16988.3	25827	coding	PF15804
MSTRG.16988.9	898	noncoding	-
MSTRG.16992.2	1496	coding	PF00867
MSTRG.16996.1	438	noncoding	-
MSTRG.16997.1	649	noncoding	-
MSTRG.16997.2	279	noncoding	-
MSTRG.16998.1	2308	noncoding	-
MSTRG.16999.1	1187	noncoding	-
MSTRG.17005.1	611	noncoding	-
MSTRG.17004.6	826	noncoding	-
MSTRG.17004.7	1111	noncoding	-
MSTRG.17004.8	817	noncoding	-
MSTRG.17008.1	431	noncoding	-
MSTRG.17011.2	5041	noncoding	-
MSTRG.17011.3	1847	noncoding	-
MSTRG.17011.5	1162	noncoding	-
MSTRG.17012.1	2325	coding	PF02994
MSTRG.17012.2	2195	coding	PF02994
MSTRG.17016.1	689	noncoding	-
MSTRG.17053.2	689	noncoding	-
MSTRG.17053.1	649	noncoding	-
MSTRG.17054.1	6634	coding	PF00063
MSTRG.17054.2	7205	coding	PF00063
MSTRG.17055.1	1153	noncoding	-
MSTRG.17054.3	4543	coding	PF15439
MSTRG.17060.1	655	noncoding	-
MSTRG.17061.1	5115	noncoding	-
MSTRG.17061.2	3638	noncoding	-
MSTRG.17067.1	753	noncoding	-
MSTRG.17067.2	1815	noncoding	-
MSTRG.17066.2	4947	coding	PF01413
MSTRG.17069.1	2315	noncoding	-
MSTRG.17069.2	980	noncoding	-
MSTRG.17074.2	2302	coding	PF01256
MSTRG.17074.4	2070	coding	PF01256
MSTRG.17074.6	1949	coding	PF01256
MSTRG.17076.1	6949	coding	PF01406
MSTRG.17076.4	3056	coding	PF01406
MSTRG.17076.6	2559	coding	PF01406

MSTRG.17077.1	1310	coding	PF12998
MSTRG.17078.5	7995	noncoding	-
MSTRG.17078.1	6902	coding	PF12796
MSTRG.17079.1	9076	noncoding	-
MSTRG.17078.6	10917	coding	PF12796
MSTRG.17085.2	3101	noncoding	-
MSTRG.17087.3	9597	coding	PF16523
MSTRG.17087.4	5501	coding	PF16523
MSTRG.17092.1	1112	noncoding	-
MSTRG.17093.1	1633	noncoding	-
MSTRG.17093.2	1943	noncoding	-
MSTRG.17093.3	1286	noncoding	-
MSTRG.17093.4	1779	noncoding	-
MSTRG.17093.5	1402	noncoding	-
MSTRG.17096.2	3780	coding	PF04130
MSTRG.17096.3	3416	coding	PF04130
MSTRG.17101.1	6844	coding	PF16212
MSTRG.17105.1	945	noncoding	-
MSTRG.17109.4	1331	coding	PF00089
MSTRG.17109.2	2552	coding	PF00089
MSTRG.17109.5	1446	coding	PF00089
MSTRG.17111.3	1498	noncoding	-
MSTRG.17114.1	6916	noncoding	-
MSTRG.17114.2	2225	noncoding	-
MSTRG.17115.2	1606	coding	PF01299
MSTRG.17110.3	5070	noncoding	-
MSTRG.17110.1	1922	noncoding	-
MSTRG.17110.2	2993	noncoding	-
MSTRG.17110.4	4562	noncoding	-
MSTRG.17110.5	4742	noncoding	-
MSTRG.17110.6	4790	noncoding	-
MSTRG.17116.1	2653	coding	PF03556
MSTRG.17117.1	2720	noncoding	-
MSTRG.17117.3	2669	noncoding	-
MSTRG.17119.2	2395	coding	PF08781
MSTRG.17119.3	2377	coding	PF08781
MSTRG.17119.5	2408	coding	PF08781
MSTRG.17131.1	1620	noncoding	-
MSTRG.17138.2	1334	noncoding	-
MSTRG.17138.3	7216	noncoding	-
MSTRG.17138.4	7567	noncoding	-
MSTRG.17138.6	5902	noncoding	-
MSTRG.17140.2	1720	noncoding	-
MSTRG.17134.1	5919	noncoding	-
MSTRG.17134.2	5079	noncoding	-
MSTRG.17145.1	327	noncoding	-
MSTRG.17150.1	722	noncoding	-
MSTRG.17150.2	777	noncoding	-
MSTRG.17151.4	2852	coding	PF12796
MSTRG.17152.2	1136	noncoding	-
MSTRG.17155.1	7438	coding	PF15744
MSTRG.17155.4	5601	coding	PF15744
MSTRG.17166.1	2655	noncoding	-
MSTRG.17166.2	455	noncoding	-

MSTRG.17168.2	4240	noncoding	-
MSTRG.17168.3	25115	coding	PF08005
MSTRG.17158.1	533	noncoding	-
MSTRG.17172.2	1249	noncoding	-
MSTRG.17172.3	1394	noncoding	-
MSTRG.17192.2	2081	noncoding	-
MSTRG.17192.1	2264	noncoding	-
MSTRG.17192.3	2244	noncoding	-
MSTRG.17192.4	2736	noncoding	-
MSTRG.17193.1	1141	noncoding	-
MSTRG.17202.1	4118	noncoding	-
MSTRG.17207.1	1630	noncoding	-
MSTRG.17215.2	2253	noncoding	-
MSTRG.17225.1	836	noncoding	-
MSTRG.17175.1	452	noncoding	-
MSTRG.17175.2	612	noncoding	-
MSTRG.17176.1	264	noncoding	-
MSTRG.17177.4	6506	coding	PF00787
MSTRG.17177.3	3848	coding	PF00787
MSTRG.17177.2	5033	coding	PF00225
MSTRG.17177.5	4046	coding	PF00787
MSTRG.17177.6	3934	coding	PF00787
MSTRG.17178.1	3289	noncoding	-
MSTRG.17178.2	2137	noncoding	-
MSTRG.17177.7	2364	coding	PF00225
MSTRG.17179.1	2931	noncoding	-
MSTRG.17180.1	1053	noncoding	-
MSTRG.17398.2	1113	noncoding	-
MSTRG.17399.1	660	noncoding	-
MSTRG.17400.1	1615	noncoding	-
MSTRG.17401.1	526	noncoding	-
MSTRG.17402.1	4223	coding	PF14915
MSTRG.17402.2	1441	noncoding	-
MSTRG.17402.3	2095	noncoding	-
MSTRG.17402.5	521	noncoding	-
MSTRG.17402.6	561	noncoding	-
MSTRG.17402.11	7397	coding	PF12001
MSTRG.17403.1	1793	noncoding	-
MSTRG.17402.13	4354	noncoding	-
MSTRG.17405.1	4446	noncoding	-
MSTRG.17402.14	3957	noncoding	-
MSTRG.17402.15	3766	noncoding	-
MSTRG.17402.16	5070	noncoding	-
MSTRG.17402.19	4523	coding	PF14915
MSTRG.17402.17	1924	coding	PF12796
MSTRG.17402.20	3383	coding	PF12796
MSTRG.17402.21	2352	coding	PF12796
MSTRG.17402.24	5554	coding	PF14915
MSTRG.17402.23	2102	coding	PF12796
MSTRG.17402.25	1609	coding	PF12796
MSTRG.17402.29	2620	coding	PF12796
MSTRG.17402.27	3627	coding	PF14915
MSTRG.17402.31	2522	coding	PF12796
MSTRG.17402.32	2185	coding	PF12796

MSTRG.17406.1	1355	noncoding	-
MSTRG.17407.1	949	coding	PF12796
MSTRG.17402.34	2573	noncoding	-
MSTRG.17402.35	1134	noncoding	-
MSTRG.17402.36	1765	noncoding	-
MSTRG.17402.37	3091	coding	PF14915
MSTRG.17408.1	1769	noncoding	-
MSTRG.17409.1	1938	noncoding	-
MSTRG.17409.2	1877	noncoding	-
MSTRG.17409.3	1878	noncoding	-
MSTRG.17410.1	329	noncoding	-
MSTRG.17411.1	1032	noncoding	-
MSTRG.17412.1	1498	noncoding	-
MSTRG.17413.1	3384	coding	PF12001
MSTRG.17414.1	601	noncoding	-
MSTRG.17402.38	2384	noncoding	-
MSTRG.17415.1	1624	noncoding	-
MSTRG.17416.1	802	noncoding	-
MSTRG.17417.1	1231	noncoding	-
MSTRG.17407.2	2330	coding	PF12796
MSTRG.17407.3	5402	coding	PF12796
MSTRG.17407.4	1409	coding	PF12796
MSTRG.17418.1	367	noncoding	-
MSTRG.17419.1	967	noncoding	-
MSTRG.17420.1	1153	noncoding	-
MSTRG.17421.2	1556	coding	PF12796
MSTRG.17421.4	828	coding	PF12796
MSTRG.17422.1	1978	noncoding	-
MSTRG.17400.2	917	noncoding	-
MSTRG.17402.68	2486	noncoding	-
MSTRG.17402.75	3134	coding	PF14915
MSTRG.17402.77	911	coding	PF12001
MSTRG.17424.1	1125	coding	PF12001
MSTRG.17407.10	4360	coding	PF14915
MSTRG.17407.12	4588	coding	PF14915
MSTRG.17428.1	573	noncoding	-
MSTRG.17421.5	1331	coding	PF12001
MSTRG.17432.1	359	noncoding	-
MSTRG.17421.13	2442	coding	PF12796
MSTRG.17432.2	884	noncoding	-
MSTRG.17187.1	2644	noncoding	-
MSTRG.17189.1	767	noncoding	-
MSTRG.17189.2	984	noncoding	-
MSTRG.17276.3	7515	noncoding	-
MSTRG.17276.4	21590	noncoding	-
MSTRG.17276.5	24725	noncoding	-
MSTRG.17276.6	7615	noncoding	-
MSTRG.17276.7	838	noncoding	-
MSTRG.17278.1	1138	noncoding	-
MSTRG.17278.3	1260	noncoding	-
MSTRG.17278.2	1313	noncoding	-
MSTRG.17278.4	1124	noncoding	-
MSTRG.17279.4	4243	noncoding	-
MSTRG.17279.3	1760	noncoding	-

MSTRG.17280.5	2560	coding	PF16870
MSTRG.17280.4	2539	coding	PF16870
MSTRG.17190.1	5497	coding	PF04050
MSTRG.17263.3	4501	noncoding	-
MSTRG.17264.1	10507	noncoding	-
MSTRG.17264.2	4658	noncoding	-
MSTRG.17265.1	1006	noncoding	-
MSTRG.17266.1	9552	coding	PF00378
MSTRG.17266.3	3855	coding	PF00378
MSTRG.17266.5	9174	coding	PF00378
MSTRG.17266.6	7004	coding	PF00378
MSTRG.17191.1	923	noncoding	-
MSTRG.17270.1	3909	noncoding	-
MSTRG.17269.4	3265	coding	PF00566
MSTRG.17271.1	3071	noncoding	-
MSTRG.17292.1	981	noncoding	-
MSTRG.17292.2	247	noncoding	-
MSTRG.17294.1	8155	coding	PF00076
MSTRG.17294.2	7907	coding	PF00076
MSTRG.17294.3	17591	coding	PF00076
MSTRG.17294.5	14128	coding	PF00076
MSTRG.17294.4	2476	coding	PF00076
MSTRG.17294.6	3206	coding	PF00076
MSTRG.17294.7	10861	noncoding	-
MSTRG.17294.9	9252	noncoding	-
MSTRG.17294.8	9899	coding	PF00076
MSTRG.17294.10	3553	coding	PF00076
MSTRG.17294.12	2607	noncoding	-
MSTRG.17294.13	39055	noncoding	-
MSTRG.17294.14	28328	noncoding	-
MSTRG.17291.1	244	noncoding	-
MSTRG.17299.2	5653	coding	PF00628
MSTRG.17299.3	7009	noncoding	-
MSTRG.17301.3	1773	coding	PF00231
MSTRG.17297.2	2485	coding	PF06668
MSTRG.17307.1	1119	noncoding	-
MSTRG.17308.3	7334	coding	PF12140
MSTRG.17308.6	7125	coding	PF12140
MSTRG.17304.1	371	noncoding	-
MSTRG.17306.1	548	noncoding	-
MSTRG.17309.2	3072	coding	PF00069
MSTRG.17310.1	291	noncoding	-
MSTRG.17310.2	378	noncoding	-
MSTRG.17311.2	3731	coding	PF01591
MSTRG.17313.1	1678	noncoding	-
MSTRG.17313.2	523	noncoding	-
MSTRG.17315.2	1581	noncoding	-
MSTRG.17326.3	3877	coding	PF12796
MSTRG.17329.1	9366	coding	PF01434
MSTRG.17333.3	4116	coding	PF00348
MSTRG.17333.2	1500	coding	PF00348
MSTRG.17334.2	1923	coding	PF02696
MSTRG.17337.1	5598	coding	PF00078
MSTRG.17339.1	373	noncoding	-

MSTRG.17336.6	639	noncoding	-
MSTRG.17336.7	571	noncoding	-
MSTRG.17340.1	1492	noncoding	-
MSTRG.17341.2	2411	coding	PF02173
MSTRG.17341.6	1820	coding	PF02173
MSTRG.17343.1	475	noncoding	-
MSTRG.17349.5	3610	coding	PF12053
MSTRG.17349.4	3125	coding	PF12053
MSTRG.17350.1	810	noncoding	-
MSTRG.17349.9	4173	coding	PF12053
MSTRG.17349.12	2897	coding	PF12053
MSTRG.17349.8	3177	coding	PF12053
MSTRG.17349.7	8565	coding	PF12053
MSTRG.17349.10	4099	coding	PF12053
MSTRG.17349.11	3991	coding	PF12053
MSTRG.17349.13	3073	coding	PF12053
MSTRG.17349.14	8082	noncoding	-
MSTRG.17349.15	3618	coding	PF12053
MSTRG.17351.1	6525	noncoding	-
MSTRG.17352.1	4985	noncoding	-
MSTRG.17347.1	509	noncoding	-
MSTRG.17348.1	5986	coding	PF00629
MSTRG.17348.2	5735	coding	PF00629
MSTRG.17348.4	5997	coding	PF00629
MSTRG.17368.10	3126	noncoding	-
MSTRG.17368.1	5688	coding	PF15368
MSTRG.17368.4	5697	coding	PF15368
MSTRG.17368.5	5701	coding	PF15368
MSTRG.17368.7	5463	coding	PF15368
MSTRG.17368.12	5654	coding	PF15368
MSTRG.17369.1	3053	coding	PF02994
MSTRG.17368.13	924	noncoding	-
MSTRG.17368.14	4113	coding	PF15368
MSTRG.17364.3	7128	coding	PF00025
MSTRG.17366.1	13229	noncoding	-
MSTRG.17370.2	4316	coding	PF14604
MSTRG.17371.2	4896	noncoding	-
MSTRG.17372.1	11409	noncoding	-
MSTRG.17370.3	8577	noncoding	-
MSTRG.17373.1	3423	coding	PF00078
MSTRG.17374.1	6781	noncoding	-
MSTRG.17370.4	21768	noncoding	-
MSTRG.17370.5	7029	noncoding	-
MSTRG.17370.6	6187	noncoding	-
MSTRG.17370.7	4041	noncoding	-
MSTRG.17370.8	10424	noncoding	-
MSTRG.17370.9	16357	noncoding	-
MSTRG.17375.1	432	noncoding	-
MSTRG.17370.10	8216	noncoding	-
MSTRG.17379.1	4752	coding	PF13832
MSTRG.17379.13	557	noncoding	-
MSTRG.17379.14	3794	coding	PF13832
MSTRG.17379.12	3132	coding	PF13832
MSTRG.17379.8	4913	coding	PF13832

MSTRG.17379.10	4890	coding	PF13832
MSTRG.17379.11	4955	coding	PF13832
MSTRG.17379.15	8009	coding	PF13832
MSTRG.17380.1	2477	noncoding	-
MSTRG.17381.1	5431	noncoding	-
MSTRG.17387.2	857	noncoding	-
MSTRG.17397.1	423	noncoding	-
MSTRG.17438.3	2100	noncoding	-
MSTRG.17438.6	3987	coding	PF14381
MSTRG.17438.7	3999	coding	PF14381
MSTRG.17438.8	4050	coding	PF14381
MSTRG.17438.9	6976	noncoding	-
MSTRG.17438.10	4136	noncoding	-
MSTRG.17438.11	2774	noncoding	-
MSTRG.17437.1	1369	noncoding	-
MSTRG.17441.1	2427	coding	PF02338
MSTRG.17441.2	2520	coding	PF02338
MSTRG.17441.3	578	noncoding	-
MSTRG.17462.1	9025	noncoding	-
MSTRG.17463.1	1602	noncoding	-
MSTRG.17464.1	4589	noncoding	-
MSTRG.17464.4	1364	noncoding	-
MSTRG.17465.1	3611	coding	PF00078
MSTRG.17467.1	844	noncoding	-
MSTRG.17469.1	4920	noncoding	-
MSTRG.17470.1	5638	noncoding	-
MSTRG.17471.1	1726	noncoding	-
MSTRG.17474.1	14941	noncoding	-
MSTRG.17475.1	23732	noncoding	-
MSTRG.17475.2	17717	noncoding	-
MSTRG.17475.3	12242	noncoding	-
MSTRG.17476.1	5310	noncoding	-
MSTRG.17477.1	5403	noncoding	-
MSTRG.17478.1	1991	noncoding	-
MSTRG.17464.12	5907	noncoding	-
MSTRG.17464.11	5710	noncoding	-
MSTRG.17464.10	6933	noncoding	-
MSTRG.17464.14	9797	noncoding	-
MSTRG.17464.16	7265	noncoding	-
MSTRG.17480.1	999	noncoding	-
MSTRG.17481.1	7196	coding	PF00620
MSTRG.17481.4	7380	coding	PF00620
MSTRG.17481.5	7173	coding	PF00620
MSTRG.17481.7	7356	coding	PF00620
MSTRG.17481.6	7035	coding	PF00620
MSTRG.17446.1	1393	noncoding	-
MSTRG.17450.1	5202	coding	PF00063
MSTRG.17454.1	2662	coding	PF11577
MSTRG.17454.3	1951	coding	PF11577
MSTRG.17456.2	4339	coding	PF09332
MSTRG.17456.3	4257	coding	PF09332
MSTRG.17456.4	4236	coding	PF09332
MSTRG.17456.5	4601	coding	PF09332
MSTRG.17453.2	1307	noncoding	-

MSTRG.17458.3	1811	noncoding	-
MSTRG.17524.1	5416	noncoding	-
MSTRG.17528.1	7166	coding	PF11819
MSTRG.17528.3	4806	coding	PF11819
MSTRG.17528.2	4868	coding	PF11819
MSTRG.17528.5	6189	coding	PF11819
MSTRG.17529.1	360	noncoding	-
MSTRG.17530.1	2404	noncoding	-
MSTRG.17531.1	1186	noncoding	-
MSTRG.17528.6	24162	noncoding	-
MSTRG.17528.7	7345	noncoding	-
MSTRG.17528.8	25791	noncoding	-
MSTRG.17528.9	8147	noncoding	-
MSTRG.17532.1	28868	noncoding	-
MSTRG.17532.2	5147	noncoding	-
MSTRG.17532.3	14815	noncoding	-
MSTRG.17528.10	1951	noncoding	-
MSTRG.17528.11	10849	noncoding	-
MSTRG.17533.1	1735	noncoding	-
MSTRG.17528.12	15584	noncoding	-
MSTRG.17528.13	22038	noncoding	-
MSTRG.17528.14	13416	noncoding	-
MSTRG.17528.15	6427	noncoding	-
MSTRG.17528.16	6429	noncoding	-
MSTRG.17528.17	6569	noncoding	-
MSTRG.17528.18	10562	noncoding	-
MSTRG.17535.1	898	noncoding	-
MSTRG.17528.19	2178	noncoding	-
MSTRG.17528.20	851	noncoding	-
MSTRG.17536.1	2804	noncoding	-
MSTRG.17528.21	3855	noncoding	-
MSTRG.17459.2	1531	coding	PF06625
MSTRG.17482.3	7118	noncoding	-
MSTRG.17482.4	7131	noncoding	-
MSTRG.17490.2	1817	coding	PF00012
MSTRG.17490.5	4306	coding	PF00012
MSTRG.17490.4	5795	coding	PF00012
MSTRG.17460.3	796	coding	PF15163
MSTRG.17496.2	1106	noncoding	-
MSTRG.17498.2	2787	coding	PF01233
MSTRG.17498.3	2837	coding	PF01233
MSTRG.17539.1	10376	coding	PF00431
MSTRG.17539.2	4081	coding	PF00431
MSTRG.17541.1	4622	noncoding	-
MSTRG.17541.2	3619	noncoding	-
MSTRG.17541.3	1385	coding	PF00145
MSTRG.17541.4	1785	coding	PF00145
MSTRG.17541.5	2168	coding	PF00777
MSTRG.17541.6	2103	coding	PF00145
MSTRG.17541.7	1330	coding	PF00777
MSTRG.17546.1	5867	noncoding	-
MSTRG.17541.8	7860	noncoding	-
MSTRG.17548.1	1507	noncoding	-
MSTRG.17550.1	2906	noncoding	-

MSTRG.17551.1	1199	coding	PF04387
MSTRG.17551.2	850	coding	PF04387
MSTRG.17552.1	2667	noncoding	-
MSTRG.17553.1	1773	noncoding	-
MSTRG.17557.1	5490	coding	PF00059
MSTRG.17512.2	2200	coding	PF00625
MSTRG.17515.1	1675	noncoding	-
MSTRG.17516.3	947	noncoding	-
MSTRG.17516.4	6225	coding	PF06752
MSTRG.17516.5	10577	coding	PF06752
MSTRG.17523.6	2888	coding	PF16618
MSTRG.17523.4	4034	coding	PF16618
MSTRG.17523.7	4029	coding	PF16618
MSTRG.17523.5	3982	coding	PF00620
MSTRG.17523.8	4123	coding	PF00620
MSTRG.17559.1	6360	noncoding	-
MSTRG.17559.3	3036	noncoding	-
MSTRG.17559.4	4430	noncoding	-
MSTRG.17559.5	4745	noncoding	-
MSTRG.17559.7	3933	noncoding	-
MSTRG.17559.6	6685	noncoding	-
MSTRG.17559.8	2924	noncoding	-
MSTRG.17560.1	4495	noncoding	-
MSTRG.17560.2	803	noncoding	-
MSTRG.17566.3	5074	coding	PF00078
MSTRG.17566.5	2726	noncoding	-
MSTRG.17567.1	2162	noncoding	-
MSTRG.17567.2	8673	coding	PF00078
MSTRG.17568.1	5666	noncoding	-
MSTRG.17579.1	4300	noncoding	-
MSTRG.17580.1	7189	coding	PF02209
MSTRG.17580.3	8912	coding	PF02209
MSTRG.17580.2	6068	coding	PF02209
MSTRG.17580.4	5802	coding	PF02209
MSTRG.17580.6	5148	coding	PF02209
MSTRG.17581.2	298	noncoding	-
MSTRG.17581.1	369	noncoding	-
MSTRG.17581.3	411	noncoding	-
MSTRG.17582.1	34422	noncoding	-
MSTRG.17582.2	7429	noncoding	-
MSTRG.17580.8	23800	noncoding	-
MSTRG.17580.9	18411	noncoding	-
MSTRG.17580.10	7206	noncoding	-
MSTRG.17580.11	17221	noncoding	-
MSTRG.17571.1	4414	coding	PF15351
MSTRG.17572.1	1154	noncoding	-
MSTRG.17571.3	4495	coding	PF15351
MSTRG.17571.4	4408	coding	PF15351
MSTRG.17571.5	4625	coding	PF15351
MSTRG.17575.1	348	noncoding	-
MSTRG.17577.1	774	noncoding	-
MSTRG.17577.2	751	noncoding	-
MSTRG.17584.1	2647	noncoding	-
MSTRG.17585.9	25985	noncoding	-

MSTRG.17585.8	5068	noncoding	-
MSTRG.17585.5	5003	noncoding	-
MSTRG.17585.12	4891	noncoding	-
MSTRG.17585.15	27415	noncoding	-
MSTRG.17585.16	31031	noncoding	-
MSTRG.17585.13	5062	noncoding	-
MSTRG.17585.14	5060	noncoding	-
MSTRG.17585.17	4971	noncoding	-
MSTRG.17585.18	5785	noncoding	-
MSTRG.17590.2	2677	noncoding	-
MSTRG.17590.3	3123	noncoding	-
MSTRG.17590.6	2392	noncoding	-
MSTRG.17592.1	5602	noncoding	-
MSTRG.17597.2	1688	coding	PF00071
MSTRG.17602.1	695	noncoding	-
MSTRG.17615.1	4698	noncoding	-
MSTRG.17615.2	3146	noncoding	-
MSTRG.17615.15	3644	coding	PF00209
MSTRG.17615.5	3081	coding	PF00209
MSTRG.17615.18	4643	noncoding	-
MSTRG.17605.2	805	coding	PF00241
MSTRG.17605.3	931	coding	PF00241
MSTRG.17608.6	5062	coding	PF05104
MSTRG.17608.8	4987	coding	PF05104
MSTRG.17608.4	5039	coding	PF05104
MSTRG.17608.5	5069	coding	PF05104
MSTRG.17608.7	5189	coding	PF05104
MSTRG.17608.9	5492	coding	PF05104
MSTRG.17608.2	4870	coding	PF05104
MSTRG.17607.1	1368	noncoding	-
MSTRG.17619.1	2660	coding	PF01352
MSTRG.17622.1	5750	noncoding	-
MSTRG.17622.2	5436	noncoding	-
MSTRG.17619.3	2245	coding	PF01352
MSTRG.17622.8	4604	noncoding	-
MSTRG.17622.9	4540	noncoding	-
MSTRG.17622.11	2004	noncoding	-
MSTRG.17628.2	2625	coding	PF05158
MSTRG.17629.1	1840	noncoding	-
MSTRG.17631.2	2606	coding	PF04811
MSTRG.17631.3	2960	coding	PF04811
MSTRG.17631.4	3078	coding	PF04811
MSTRG.17624.1	597	coding	PF14204
MSTRG.17635.2	3593	coding	PF01699
MSTRG.17635.1	3887	coding	PF01699
MSTRG.17635.3	3584	coding	PF01699
MSTRG.17636.1	1168	noncoding	-
MSTRG.17635.4	5522	coding	PF01699
MSTRG.17635.6	4301	coding	PF01699
MSTRG.17635.7	4301	coding	PF01699
MSTRG.17635.9	3457	coding	PF01699
MSTRG.17635.10	886	noncoding	-
MSTRG.17638.1	7044	noncoding	-
MSTRG.17639.1	11009	noncoding	-

MSTRG.17640.1	1131	noncoding	-
MSTRG.17641.1	2221	noncoding	-
MSTRG.17635.12	10059	coding	PF00078
MSTRG.17632.2	4545	coding	PF02204
MSTRG.17632.3	4385	coding	PF02204
MSTRG.17643.2	807	noncoding	-
MSTRG.17643.3	812	noncoding	-
MSTRG.17645.3	4204	coding	PF16092
MSTRG.17645.7	7376	coding	PF16092
MSTRG.17647.1	22062	coding	PF02145
MSTRG.17647.2	9160	coding	PF02145
MSTRG.17647.3	14478	coding	PF02145
MSTRG.17647.4	12912	coding	PF02145
MSTRG.17648.2	2303	noncoding	-
MSTRG.17653.1	974	noncoding	-
MSTRG.17654.1	2207	noncoding	-
MSTRG.17660.1	6360	coding	PF14938
MSTRG.17661.3	741	coding	PF00031
MSTRG.17661.4	656	coding	PF00031
MSTRG.17663.2	787	noncoding	-
MSTRG.17665.2	428	noncoding	-
MSTRG.17667.2	945	noncoding	-
MSTRG.17669.1	364	noncoding	-
MSTRG.17670.1	451	noncoding	-
MSTRG.17672.1	254	noncoding	-
MSTRG.17674.2	1335	noncoding	-
MSTRG.17674.1	1463	noncoding	-
MSTRG.17674.3	1484	noncoding	-
MSTRG.17677.1	2821	coding	PF03088
MSTRG.17677.3	2807	coding	PF03088
MSTRG.17679.2	3204	coding	PF13193
MSTRG.17676.2	1758	noncoding	-
MSTRG.17681.1	2375	noncoding	-
MSTRG.17681.3	2640	noncoding	-
MSTRG.17682.2	2322	coding	PF00343
MSTRG.17685.2	1775	noncoding	-
MSTRG.17686.1	1293	noncoding	-
MSTRG.17706.6	9190	coding	PF12509
MSTRG.17706.4	11920	coding	PF12509
MSTRG.17706.5	9639	coding	PF12509
MSTRG.17706.8	9576	coding	PF12509
MSTRG.17706.7	9548	coding	PF12509
MSTRG.17706.9	10220	coding	PF12509
MSTRG.17706.12	20361	coding	PF12509
MSTRG.17706.14	9367	coding	PF12509
MSTRG.17706.11	9481	coding	PF12509
MSTRG.17706.10	9257	coding	PF12509
MSTRG.17706.16	9270	coding	PF12509
MSTRG.17706.18	10450	coding	PF12509
MSTRG.17706.17	9399	coding	PF12509
MSTRG.17706.19	8957	coding	PF12509
MSTRG.17706.20	9326	coding	PF12509
MSTRG.17706.22	4089	coding	PF12509
MSTRG.17706.23	2798	coding	PF00078

MSTRG.17695.1	2371	noncoding	-
MSTRG.17697.1	1186	noncoding	-
MSTRG.17698.2	2129	noncoding	-
MSTRG.17698.3	1586	noncoding	-
MSTRG.17701.2	3349	noncoding	-
MSTRG.17702.1	233	noncoding	-
MSTRG.17710.3	5568	coding	PF08367
MSTRG.17710.4	5565	coding	PF08367
MSTRG.17710.6	3429	coding	PF08367
MSTRG.17711.4	551	noncoding	-
MSTRG.17711.2	3141	coding	PF00365
MSTRG.17709.1	426	noncoding	-
MSTRG.17714.1	11364	noncoding	-
MSTRG.17714.4	3736	noncoding	-
MSTRG.17718.2	5521	coding	PF05383
MSTRG.17718.3	2556	noncoding	-
MSTRG.17730.1	2805	noncoding	-
MSTRG.17731.1	5834	noncoding	-
MSTRG.17731.2	1382	noncoding	-
MSTRG.17729.6	16185	noncoding	-
MSTRG.17729.4	8215	noncoding	-
MSTRG.17729.5	8383	noncoding	-
MSTRG.17729.7	4651	noncoding	-
MSTRG.17729.8	4994	noncoding	-
MSTRG.17729.9	7939	noncoding	-
MSTRG.17732.1	7373	noncoding	-
MSTRG.17729.10	4150	noncoding	-
MSTRG.17733.7	4345	noncoding	-
MSTRG.17733.10	4276	noncoding	-
MSTRG.17733.12	2656	noncoding	-
MSTRG.17733.11	2339	noncoding	-
MSTRG.17725.2	3323	coding	PF00377
MSTRG.17728.1	392	noncoding	-
MSTRG.17735.2	1628	noncoding	-
MSTRG.17740.2	1671	coding	PF01148
MSTRG.17741.4	2029	noncoding	-
MSTRG.17741.5	2679	coding	PF03009
MSTRG.17741.6	4608	noncoding	-
MSTRG.17744.1	352	noncoding	-
MSTRG.17751.2	1651	coding	PF04189
MSTRG.17755.2	1671	noncoding	-
MSTRG.17755.3	1682	noncoding	-
MSTRG.17747.1	1166	noncoding	-
MSTRG.17762.1	355	noncoding	-
MSTRG.17775.2	5884	coding	PF13639
MSTRG.17776.2	7123	coding	PF03630
MSTRG.17779.1	32600	noncoding	-
MSTRG.17779.2	14939	noncoding	-
MSTRG.17779.4	23835	noncoding	-
MSTRG.17779.5	19781	noncoding	-
MSTRG.17779.10	18157	noncoding	-
MSTRG.17779.11	13056	noncoding	-
MSTRG.17768.1	2757	coding	PF09026
MSTRG.17769.3	1113	coding	PF06294

MSTRG.17770.2	1669	coding	PF15006
MSTRG.17770.3	1223	coding	PF15006
MSTRG.17773.1	7413	noncoding	-
MSTRG.17773.2	8526	coding	PF00059
MSTRG.17773.3	15590	coding	PF01597
MSTRG.17774.1	680	noncoding	-
MSTRG.17781.2	7335	noncoding	-
MSTRG.17781.3	12244	noncoding	-
MSTRG.17782.1	4346	noncoding	-
MSTRG.17784.3	3105	coding	PF00955
MSTRG.17785.2	1563	coding	PF01725
MSTRG.17785.3	3156	coding	PF00078
MSTRG.17789.1	2368	coding	PF04564
MSTRG.17789.4	2458	coding	PF04564
MSTRG.17789.6	2284	coding	PF04564
MSTRG.17791.6	3349	coding	PF00102
MSTRG.17791.5	3492	coding	PF00102
MSTRG.17791.4	3254	coding	PF00102
MSTRG.17792.1	4311	noncoding	-
MSTRG.17796.2	3120	coding	PF04841
MSTRG.17794.1	2798	coding	PF00246
MSTRG.17802.1	861	coding	PF00927
MSTRG.17807.1	4269	coding	PF07654
MSTRG.17806.1	622	noncoding	-
MSTRG.17814.1	1447	noncoding	-
MSTRG.17814.2	14620	coding	PF02093
MSTRG.17816.1	2592	noncoding	-
MSTRG.17814.6	3749	noncoding	-
MSTRG.17814.4	3623	noncoding	-
MSTRG.17814.5	3761	noncoding	-
MSTRG.17817.2	1204	noncoding	-
MSTRG.17817.1	1151	noncoding	-
MSTRG.17817.3	1313	noncoding	-
MSTRG.17810.2	3128	coding	PF00001
MSTRG.17811.3	1406	coding	PF15478
MSTRG.17825.4	5991	noncoding	-
MSTRG.17826.4	1719	coding	PF14681
MSTRG.17826.3	1613	coding	PF14681
MSTRG.17826.2	1679	coding	PF14681
MSTRG.17826.7	904	coding	PF14681
MSTRG.17826.8	1197	coding	PF14681
MSTRG.17826.6	1071	coding	PF14681
MSTRG.17826.9	958	coding	PF14681
MSTRG.17828.6	6311	coding	PF00226
MSTRG.17828.7	9270	coding	PF00226
MSTRG.17828.3	4348	coding	PF00226
MSTRG.17829.1	2377	noncoding	-
MSTRG.17830.4	2249	coding	PF04201
MSTRG.17830.5	2246	coding	PF04201
MSTRG.17830.6	4499	coding	PF04201
MSTRG.17832.2	1547	noncoding	-
MSTRG.17833.1	284	noncoding	-
MSTRG.17835.2	1354	noncoding	-
MSTRG.17836.2	2150	noncoding	-

MSTRG.17836.3	2559	noncoding	-
MSTRG.17837.3	1662	coding	PF00025
MSTRG.17837.4	1594	coding	PF00025
MSTRG.17839.4	4353	coding	PF06733
MSTRG.17819.1	1708	noncoding	-
MSTRG.17821.2	2248	coding	PF01342
MSTRG.17841.2	1937	noncoding	-
MSTRG.17841.3	1140	noncoding	-
MSTRG.17841.4	1286	noncoding	-
MSTRG.17841.5	2012	noncoding	-
MSTRG.17841.6	1361	noncoding	-
MSTRG.17842.1	2270	coding	PF07714
MSTRG.17842.4	1432	coding	PF07714
MSTRG.17840.1	1828	coding	PF00009
MSTRG.17840.2	1846	coding	PF00009
MSTRG.17845.4	2609	coding	PF01412
MSTRG.17847.1	3200	coding	PF10607
MSTRG.17847.3	1657	coding	PF10607
MSTRG.17849.1	519	noncoding	-
MSTRG.17848.5	7882	coding	PF07744
MSTRG.17856.1	1646	coding	PF01249
MSTRG.17863.4	1815	coding	PF04683
MSTRG.17863.2	1556	coding	PF04683
MSTRG.17863.3	1796	coding	PF04683
MSTRG.17863.5	1595	coding	PF04683
MSTRG.17873.4	3236	coding	PF09532
MSTRG.17873.3	2890	coding	PF12701
MSTRG.17878.1	4346	coding	PF05236
MSTRG.17878.2	4259	coding	PF05236
MSTRG.17878.4	3776	coding	PF05236
MSTRG.17878.5	3475	coding	PF05236
MSTRG.17878.6	5275	noncoding	-
MSTRG.17879.2	1108	noncoding	-
MSTRG.17878.7	4291	noncoding	-
MSTRG.17865.1	653	noncoding	-
MSTRG.17866.1	1152	noncoding	-
MSTRG.17867.1	1066	noncoding	-
MSTRG.17891.1	3716	coding	PF03370
MSTRG.17892.2	5342	noncoding	-
MSTRG.17892.3	3793	noncoding	-
MSTRG.17892.4	8565	noncoding	-
MSTRG.17892.5	4745	noncoding	-
MSTRG.17892.7	1754	noncoding	-
MSTRG.17903.1	425	coding	PF00503
MSTRG.17904.6	1862	coding	PF00503
MSTRG.17904.5	4021	coding	PF00503
MSTRG.17904.7	1915	noncoding	-
MSTRG.17908.1	1819	coding	PF00071
MSTRG.17906.1	579	noncoding	-
MSTRG.17907.2	1199	coding	PF12796
MSTRG.17912.2	524	coding	PF14945
MSTRG.17913.1	1375	noncoding	-
MSTRG.17921.1	2370	noncoding	-
MSTRG.17921.5	1862	noncoding	-

MSTRG.17923.1	1714	coding	PF00019
MSTRG.17924.1	1275	coding	PF04406
MSTRG.17923.2	929	noncoding	-
MSTRG.17923.3	1546	coding	PF00019
MSTRG.17923.4	2949	coding	PF00019
MSTRG.17923.5	922	noncoding	-
MSTRG.17918.1	1291	noncoding	-
MSTRG.17929.1	1545	noncoding	-
MSTRG.17919.1	324	noncoding	-
MSTRG.17931.1	1384	coding	PF04641
MSTRG.17931.3	616	noncoding	-
MSTRG.17931.4	633	noncoding	-
MSTRG.17941.1	4699	coding	PF15290
MSTRG.17942.3	3157	coding	PF04825
MSTRG.17943.4	1133	noncoding	-
MSTRG.17943.2	1362	coding	PF11566
MSTRG.17943.3	1740	noncoding	-
MSTRG.17954.3	5245	coding	PF00069
MSTRG.17952.2	3239	noncoding	-
MSTRG.17958.1	4559	coding	PF15397
MSTRG.17963.2	466	noncoding	-
MSTRG.17963.1	609	noncoding	-
MSTRG.17972.5	5681	coding	PF09041
MSTRG.17972.7	5758	coding	PF09041
MSTRG.17972.6	12521	coding	PF09041
MSTRG.17972.10	5720	coding	PF09041
MSTRG.17972.8	5646	coding	PF09041
MSTRG.17972.9	5646	coding	PF09041
MSTRG.17972.11	3194	coding	PF09041
MSTRG.17973.1	1308	noncoding	-
MSTRG.17975.1	1681	coding	PF03133
MSTRG.17978.2	2136	coding	PF07714
MSTRG.17980.2	3925	coding	PF02990
MSTRG.17980.3	5574	coding	PF02990
MSTRG.17981.1	1837	noncoding	-
MSTRG.17982.2	3146	noncoding	-
MSTRG.17984.8	4842	coding	PF00225
MSTRG.17984.7	5734	coding	PF00225
MSTRG.17984.10	6163	coding	PF00225
MSTRG.17985.1	1663	noncoding	-
MSTRG.17986.2	6739	coding	PF13919
MSTRG.17987.3	5109	noncoding	-
MSTRG.17987.4	6452	noncoding	-
MSTRG.17987.5	7723	noncoding	-
MSTRG.17988.1	373	noncoding	-
MSTRG.17989.1	1175	noncoding	-
MSTRG.17999.2	13261	noncoding	-
MSTRG.17999.3	14482	noncoding	-
MSTRG.17999.5	9524	noncoding	-
MSTRG.17995.1	521	noncoding	-
MSTRG.17996.1	1033	noncoding	-
MSTRG.17998.1	521	noncoding	-
MSTRG.18002.1	817	noncoding	-
MSTRG.18015.1	4763	coding	PF15318

MSTRG.18019.1	1605	noncoding	-
MSTRG.18024.4	1835	coding	PF00076
MSTRG.18024.9	2608	coding	PF00076
MSTRG.18024.11	1588	coding	PF00076
MSTRG.18024.12	1752	coding	PF00076
MSTRG.18024.13	2059	coding	PF00076
MSTRG.18025.1	3246	noncoding	-
MSTRG.18026.2	1724	coding	PF01873
MSTRG.18023.2	2692	coding	PF05221
MSTRG.18031.3	5411	coding	PF00632
MSTRG.18031.4	5756	coding	PF00632
MSTRG.18032.1	7259	coding	PF16906
MSTRG.18033.1	5444	noncoding	-
MSTRG.18037.2	653	coding	PF02991
MSTRG.18038.3	2078	coding	PF06728
MSTRG.18038.2	1906	coding	PF06728
MSTRG.18040.7	2222	coding	PF01359
MSTRG.18040.10	4140	coding	PF13820
MSTRG.18040.9	7359	coding	PF13820
MSTRG.18040.12	6701	coding	PF13820
MSTRG.18040.13	3926	coding	PF13820
MSTRG.18040.4	7080	coding	PF13820
MSTRG.18040.8	3856	coding	PF13820
MSTRG.18046.1	874	noncoding	-
MSTRG.18050.1	1850	coding	PF03981
MSTRG.18051.1	661	noncoding	-
MSTRG.18054.3	1081	coding	PF00777
MSTRG.18052.1	1515	noncoding	-
MSTRG.18063.1	5196	coding	PF07002
MSTRG.18063.2	3456	coding	PF07002
MSTRG.18063.3	5092	coding	PF07002
MSTRG.18063.5	5403	noncoding	-
MSTRG.18064.2	2219	coding	PF00266
MSTRG.18066.7	3359	coding	PF00076
MSTRG.18066.5	2455	coding	PF00076
MSTRG.18066.6	2389	coding	PF00076
MSTRG.18060.1	2199	coding	PF00027
MSTRG.18068.1	5040	noncoding	-
MSTRG.18068.3	5133	noncoding	-
MSTRG.18068.4	4995	noncoding	-
MSTRG.18069.1	6546	coding	PF09380
MSTRG.18069.2	9227	noncoding	-
MSTRG.18070.1	646	noncoding	-
MSTRG.18069.4	6966	coding	PF09380
MSTRG.18069.5	6869	coding	PF09380
MSTRG.18073.1	4657	noncoding	-
MSTRG.18072.2	2974	coding	PF03359
MSTRG.18072.4	2953	coding	PF03359
MSTRG.18072.8	4586	coding	PF03359
MSTRG.18074.2	1398	noncoding	-
MSTRG.18093.6	3828	coding	PF03096
MSTRG.18093.2	3481	coding	PF03096
MSTRG.18093.4	2694	coding	PF03096
MSTRG.18093.8	2388	noncoding	-

MSTRG.18095.4	2310	coding	PF08202
MSTRG.18098.1	2587	noncoding	-
MSTRG.18099.2	5753	noncoding	-
MSTRG.18101.1	28351	noncoding	-
MSTRG.18101.2	12116	noncoding	-
MSTRG.18102.3	3086	noncoding	-
MSTRG.18103.1	8008	noncoding	-
MSTRG.18105.1	867	noncoding	-
MSTRG.18106.1	643	coding	PF05817
MSTRG.18083.2	2053	coding	PF00017
MSTRG.18083.3	24630	noncoding	-
MSTRG.18085.2	368	noncoding	-
MSTRG.18089.1	2482	noncoding	-
MSTRG.18091.3	1658	noncoding	-
MSTRG.18089.2	2104	noncoding	-
MSTRG.18122.1	510	noncoding	-
MSTRG.18129.1	5864	coding	PF00388
MSTRG.18130.3	8799	coding	PF11569
MSTRG.18130.4	8832	coding	PF11569
MSTRG.18131.5	10597	coding	PF00176
MSTRG.18131.4	9385	coding	PF00176
MSTRG.18131.3	8807	coding	PF00176
MSTRG.18131.6	7273	coding	PF00271
MSTRG.18132.1	785	noncoding	-
MSTRG.18135.1	10626	coding	PF00102
MSTRG.18137.1	516	noncoding	-
MSTRG.18137.2	566	noncoding	-
MSTRG.18138.2	981	noncoding	-
MSTRG.18138.1	1386	noncoding	-
MSTRG.18148.9	8654	noncoding	-
MSTRG.18149.1	764	noncoding	-
MSTRG.18151.1	3846	noncoding	-
MSTRG.18152.2	3924	coding	PF00076
MSTRG.18152.3	2006	coding	PF00076
MSTRG.18152.4	1928	noncoding	-
MSTRG.18153.1	5361	coding	PF02820
MSTRG.18153.2	4388	coding	PF02820
MSTRG.18142.3	1885	noncoding	-
MSTRG.18147.2	2782	coding	PF09316
MSTRG.18140.1	273	noncoding	-
MSTRG.18140.2	291	noncoding	-
MSTRG.18155.1	16261	coding	PF00078
MSTRG.18155.2	4132	noncoding	-
MSTRG.18156.1	8025	noncoding	-
MSTRG.18157.1	6823	noncoding	-
MSTRG.18158.3	1709	coding	PF05604
MSTRG.18159.1	2522	noncoding	-
MSTRG.18161.4	2224	noncoding	-
MSTRG.18162.2	4464	coding	PF00105
MSTRG.18164.1	1600	coding	PF03348
MSTRG.18169.1	1154	coding	PF00658
MSTRG.18170.3	1809	noncoding	-
MSTRG.18174.1	660	noncoding	-
MSTRG.18174.5	626	noncoding	-

MSTRG.18173.1	789	noncoding	-
MSTRG.18173.2	807	noncoding	-
MSTRG.18176.2	413	noncoding	-
MSTRG.18177.2	608	noncoding	-
MSTRG.18183.4	1002	coding	PF15331
MSTRG.18191.1	654	coding	PF00014
MSTRG.18191.2	969	coding	PF00014
MSTRG.18191.3	966	coding	PF00014
MSTRG.18191.4	672	coding	PF00014
MSTRG.18185.1	1947	coding	PF00014
MSTRG.18196.2	1074	noncoding	-
MSTRG.18196.3	631	noncoding	-
MSTRG.18196.4	711	noncoding	-
MSTRG.18198.1	526	noncoding	-
MSTRG.18200.2	929	noncoding	-
MSTRG.18200.3	1041	noncoding	-
MSTRG.18200.4	955	noncoding	-
MSTRG.18200.5	697	noncoding	-
MSTRG.18201.2	1294	noncoding	-
MSTRG.18219.2	9443	coding	PF12237
MSTRG.18219.1	8819	coding	PF12237
MSTRG.18219.3	4989	noncoding	-
MSTRG.18219.4	5252	noncoding	-
MSTRG.18219.5	4984	noncoding	-
MSTRG.18219.6	5348	noncoding	-
MSTRG.18219.8	4705	noncoding	-
MSTRG.18210.1	2013	noncoding	-
MSTRG.18211.4	2415	noncoding	-
MSTRG.18212.2	3351	noncoding	-
MSTRG.18214.1	1397	coding	PF00028
MSTRG.18214.2	981	noncoding	-
MSTRG.18215.1	3727	noncoding	-
MSTRG.18220.2	4160	coding	PF16457
MSTRG.18226.2	1446	noncoding	-
MSTRG.18229.2	5533	coding	PF12064
MSTRG.18229.1	5462	coding	PF12064
MSTRG.18229.5	5376	coding	PF12064
MSTRG.18234.1	822	noncoding	-
MSTRG.18240.1	7884	coding	PF16279
MSTRG.18240.8	5882	coding	PF16279
MSTRG.18240.7	6065	coding	PF16279
MSTRG.18240.9	7894	coding	PF16279
MSTRG.18240.10	7805	coding	PF16279
MSTRG.18240.11	6245	coding	PF16279
MSTRG.18241.1	4038	noncoding	-
MSTRG.18240.12	8425	coding	PF16279
MSTRG.18242.2	4454	coding	PF12548
MSTRG.18242.4	3021	coding	PF12548
MSTRG.18245.1	2441	noncoding	-
MSTRG.18245.2	593	noncoding	-
MSTRG.18238.2	7298	coding	PF01369
MSTRG.18239.1	4900	noncoding	-
MSTRG.18246.2	4000	coding	PF08506
MSTRG.18246.3	4608	coding	PF03378

MSTRG.18252.2	2366	coding	PF00270
MSTRG.18253.2	7425	coding	PF13087
MSTRG.18251.1	1325	noncoding	-
MSTRG.18259.2	3579	coding	PF13733
MSTRG.18261.1	2329	noncoding	-
MSTRG.18269.10	1803	noncoding	-
MSTRG.18269.8	1835	noncoding	-
MSTRG.18269.6	1866	noncoding	-
MSTRG.18269.13	3147	noncoding	-
MSTRG.18269.3	4915	noncoding	-
MSTRG.18269.7	3631	noncoding	-
MSTRG.18269.11	1674	noncoding	-
MSTRG.18269.12	2389	coding	PF00179
MSTRG.18269.1	3606	coding	PF10520
MSTRG.18269.14	3027	coding	PF10520
MSTRG.18269.2	5475	coding	PF10520
MSTRG.18269.4	3603	coding	PF10520
MSTRG.18269.5	3030	coding	PF10520
MSTRG.18269.9	3454	coding	PF10520
MSTRG.18275.1	7220	noncoding	-
MSTRG.18276.4	5117	noncoding	-
MSTRG.18276.3	5010	noncoding	-
MSTRG.18277.1	4451	coding	PF15903
MSTRG.18277.4	4069	coding	PF15903
MSTRG.18278.1	1028	noncoding	-
MSTRG.18270.2	2441	noncoding	-
MSTRG.18274.1	688	noncoding	-
MSTRG.18274.2	1988	noncoding	-
MSTRG.18274.3	2661	noncoding	-
MSTRG.18274.4	1039	noncoding	-
MSTRG.18273.2	1332	noncoding	-
MSTRG.18292.2	7125	coding	PF16212
MSTRG.18292.3	6464	coding	PF16212
MSTRG.18294.2	1089	noncoding	-
MSTRG.18294.5	1577	noncoding	-
MSTRG.18329.1	2518	noncoding	-
MSTRG.18346.1	2543	noncoding	-
MSTRG.18346.2	1702	noncoding	-
MSTRG.18346.3	2094	noncoding	-
MSTRG.18347.1	8711	noncoding	-
MSTRG.18348.1	12025	noncoding	-
MSTRG.18348.2	5680	noncoding	-
MSTRG.18349.1	35431	noncoding	-
MSTRG.18350.1	48418	noncoding	-
MSTRG.18350.2	45333	noncoding	-
MSTRG.18350.3	22085	noncoding	-
MSTRG.18351.1	35960	noncoding	-
MSTRG.18348.3	11824	noncoding	-
MSTRG.18352.1	9884	noncoding	-
MSTRG.18353.1	1834	noncoding	-
MSTRG.18354.1	8440	noncoding	-
MSTRG.18355.1	783	noncoding	-
MSTRG.18297.2	5448	noncoding	-
MSTRG.18297.3	5558	noncoding	-

MSTRG.18297.6	2805	noncoding	-
MSTRG.18297.5	2709	noncoding	-
MSTRG.18299.2	1395	noncoding	-
MSTRG.18299.3	7226	noncoding	-
MSTRG.18304.1	489	noncoding	-
MSTRG.18306.2	1784	noncoding	-
MSTRG.18306.3	1057	noncoding	-
MSTRG.18307.1	835	noncoding	-
MSTRG.18321.1	2454	noncoding	-
MSTRG.18369.2	1422	coding	PF03947
MSTRG.18369.5	899	coding	PF03947
MSTRG.18369.4	1254	coding	PF03947
MSTRG.18369.3	1295	coding	PF03947
MSTRG.18371.2	1762	noncoding	-
MSTRG.18373.1	1884	noncoding	-
MSTRG.18378.2	2185	noncoding	-
MSTRG.18379.6	2795	coding	PF12796
MSTRG.18381.3	2990	coding	PF00225
MSTRG.18381.4	3034	coding	PF00225
MSTRG.18382.3	8668	noncoding	-
MSTRG.18382.4	1445	noncoding	-
MSTRG.18383.3	1640	coding	PF06237
MSTRG.18383.1	2751	coding	PF06237
MSTRG.18386.1	977	coding	PF15158
MSTRG.18386.2	845	coding	PF15158
MSTRG.18398.5	2072	coding	PF06546
MSTRG.18400.4	4153	noncoding	-
MSTRG.18400.3	5428	noncoding	-
MSTRG.18400.5	6473	noncoding	-
MSTRG.18400.7	5173	noncoding	-
MSTRG.18400.6	5316	noncoding	-
MSTRG.18400.9	4215	noncoding	-
MSTRG.18401.1	5608	noncoding	-
MSTRG.18405.1	3768	noncoding	-
MSTRG.18406.1	5102	noncoding	-
MSTRG.18408.1	2274	coding	PF16764
MSTRG.18408.3	1521	coding	PF16764
MSTRG.18408.4	1594	coding	PF16764
MSTRG.18413.2	3660	coding	PF02538
MSTRG.18413.3	3431	coding	PF02538
MSTRG.18414.1	2180	noncoding	-
MSTRG.18416.4	2075	coding	PF15059
MSTRG.18416.5	2011	coding	PF15059
MSTRG.18416.6	1814	coding	PF15059
MSTRG.18417.1	2210	noncoding	-
MSTRG.18417.3	1606	noncoding	-
MSTRG.18433.6	3746	coding	PF00076
MSTRG.18433.7	1824	coding	PF00076
MSTRG.18434.3	4921	coding	PF00595
MSTRG.18435.1	2107	coding	PF12796
MSTRG.18435.2	1967	coding	PF12796
MSTRG.18438.1	4595	noncoding	-
MSTRG.18438.2	1179	noncoding	-
MSTRG.18447.3	4731	coding	PF00736

MSTRG.18448.1	697	noncoding	-
MSTRG.18448.2	409	noncoding	-
MSTRG.18447.5	2472	coding	PF00736
MSTRG.18424.4	2643	noncoding	-
MSTRG.18424.3	3362	noncoding	-
MSTRG.18424.2	3320	noncoding	-
MSTRG.18425.1	5212	noncoding	-
MSTRG.18425.2	2933	noncoding	-
MSTRG.18425.4	1996	noncoding	-
MSTRG.18425.3	1751	noncoding	-
MSTRG.18428.3	1585	noncoding	-
MSTRG.18428.2	3666	noncoding	-
MSTRG.18428.4	4361	noncoding	-
MSTRG.18428.5	4301	noncoding	-
MSTRG.18452.2	873	noncoding	-
MSTRG.18449.1	627	noncoding	-
MSTRG.18449.2	506	noncoding	-
MSTRG.18453.2	1897	noncoding	-
MSTRG.18454.2	3039	noncoding	-
MSTRG.18454.1	3119	noncoding	-
MSTRG.18454.3	2989	noncoding	-
MSTRG.18459.1	7520	coding	PF01781
MSTRG.18462.1	2843	noncoding	-
MSTRG.18462.5	3838	noncoding	-
MSTRG.18462.7	3204	noncoding	-
MSTRG.18455.1	2553	coding	PF00002
MSTRG.18464.1	549	noncoding	-
MSTRG.18466.1	1187	noncoding	-
MSTRG.18465.3	4014	noncoding	-
MSTRG.18465.2	4816	noncoding	-
MSTRG.18465.5	4974	noncoding	-
MSTRG.18465.4	5141	noncoding	-
MSTRG.18468.2	1754	noncoding	-
MSTRG.18470.4	5591	noncoding	-
MSTRG.18470.6	4117	noncoding	-
MSTRG.18470.8	1720	noncoding	-
MSTRG.18470.9	15568	noncoding	-
MSTRG.18470.11	6589	noncoding	-
MSTRG.18469.2	5325	coding	PF02141
MSTRG.18477.2	4439	coding	PF07714
MSTRG.18477.1	4582	coding	PF07714
MSTRG.18477.3	4299	coding	PF07714
MSTRG.18477.4	7177	coding	PF07714
MSTRG.18477.5	4448	coding	PF07714
MSTRG.18477.6	4429	coding	PF07714
MSTRG.18477.7	4675	coding	PF07714
MSTRG.18477.8	4296	coding	PF07714
MSTRG.18477.9	4521	coding	PF07714
MSTRG.18477.10	4175	coding	PF07714
MSTRG.18477.11	768	noncoding	-
MSTRG.18477.12	6296	coding	PF07714
MSTRG.18478.1	6460	noncoding	-
MSTRG.18477.14	5802	coding	PF03623
MSTRG.18479.1	836	noncoding	-

MSTRG.18479.2	974	noncoding	-
MSTRG.18472.8	14759	coding	PF02171
MSTRG.18473.1	1321	noncoding	-
MSTRG.18472.12	5410	coding	PF02171
MSTRG.18474.2	1254	noncoding	-
MSTRG.18481.1	4077	noncoding	-
MSTRG.18482.1	1377	noncoding	-
MSTRG.18483.2	986	noncoding	-
MSTRG.18484.1	1521	noncoding	-
MSTRG.18484.2	1625	noncoding	-
MSTRG.18484.3	1307	noncoding	-
MSTRG.18480.2	2491	coding	PF07885
MSTRG.18485.1	357	noncoding	-
MSTRG.18487.1	1130	noncoding	-
MSTRG.18487.2	4355	noncoding	-
MSTRG.18492.2	3805	coding	PF12394
MSTRG.18490.1	380	noncoding	-
MSTRG.18499.2	1420	coding	PF16568
MSTRG.18500.1	1521	noncoding	-
MSTRG.18504.1	1778	noncoding	-
MSTRG.18507.4	10368	noncoding	-
MSTRG.18507.5	1520	noncoding	-
MSTRG.18507.6	3643	noncoding	-
MSTRG.18508.2	3386	noncoding	-
MSTRG.18508.3	2842	noncoding	-
MSTRG.18511.1	6354	coding	PF00777
MSTRG.18511.2	2626	coding	PF00777
MSTRG.18511.3	2756	coding	PF00777
MSTRG.18511.4	2710	coding	PF00777
MSTRG.18513.3	2986	noncoding	-
MSTRG.18513.2	2799	noncoding	-
MSTRG.18516.2	8897	coding	PF16660
MSTRG.18516.5	8620	coding	PF16660
MSTRG.18517.1	1208	noncoding	-
MSTRG.18514.2	1449	noncoding	-
MSTRG.18514.3	1371	noncoding	-
MSTRG.18514.4	1598	noncoding	-
MSTRG.18519.1	291	noncoding	-
MSTRG.18524.1	3788	noncoding	-
MSTRG.18524.2	4564	noncoding	-
MSTRG.18524.4	3074	noncoding	-
MSTRG.18527.1	3478	coding	PF00211
MSTRG.18527.2	823	coding	PF06327
MSTRG.18529.3	1765	coding	PF07159
MSTRG.18529.5	1705	coding	PF07159
MSTRG.18529.4	1715	coding	PF07159
MSTRG.18530.1	10927	noncoding	-
MSTRG.18530.2	10540	noncoding	-
MSTRG.18531.1	5922	noncoding	-
MSTRG.18536.1	344	noncoding	-
MSTRG.18538.1	402	noncoding	-
MSTRG.18540.1	506	noncoding	-
MSTRG.18600.2	3561	noncoding	-
MSTRG.18614.1	1473	noncoding	-

MSTRG.18615.1	2606	noncoding	-
MSTRG.18626.2	1870	noncoding	-
MSTRG.18622.6	11406	noncoding	-
MSTRG.18622.7	5465	noncoding	-
MSTRG.18631.2	12067	coding	PF00069
MSTRG.18631.3	3117	noncoding	-
MSTRG.18632.1	4823	noncoding	-
MSTRG.18631.4	15388	coding	PF00069
MSTRG.18633.1	392	noncoding	-
MSTRG.18622.8	5055	coding	PF00069
MSTRG.18622.14	3281	coding	PF00069
MSTRG.18631.6	6342	coding	PF00069
MSTRG.18631.7	233	noncoding	-
MSTRG.18622.16	308	noncoding	-
MSTRG.18622.17	7428	coding	PF00069
MSTRG.18631.9	3402	coding	PF00069
MSTRG.18631.8	3981	coding	PF00069
MSTRG.18622.20	4796	noncoding	-
MSTRG.18622.21	4790	noncoding	-
MSTRG.18622.22	1218	noncoding	-
MSTRG.18631.10	2225	coding	PF00069
MSTRG.18622.34	6660	coding	PF00069
MSTRG.18638.1	268	noncoding	-
MSTRG.18637.1	301	noncoding	-
MSTRG.18542.3	4476	coding	PF04970
MSTRG.18546.4	1014	noncoding	-
MSTRG.18546.5	28286	noncoding	-
MSTRG.18546.6	38258	noncoding	-
MSTRG.18546.7	4448	noncoding	-
MSTRG.18546.8	22046	noncoding	-
MSTRG.18546.9	20728	noncoding	-
MSTRG.18546.10	13940	noncoding	-
MSTRG.18546.11	2401	noncoding	-
MSTRG.18546.12	26496	noncoding	-
MSTRG.18546.13	8816	noncoding	-
MSTRG.18546.14	5964	noncoding	-
MSTRG.18548.2	356	noncoding	-
MSTRG.18551.1	7390	noncoding	-
MSTRG.18552.1	12535	noncoding	-
MSTRG.18552.2	4090	noncoding	-
MSTRG.18555.1	5472	coding	PF08397
MSTRG.18555.2	4808	coding	PF08397
MSTRG.18557.4	1232	coding	PF01026
MSTRG.18558.1	2857	noncoding	-
MSTRG.18559.1	3464	coding	PF13705
MSTRG.18559.4	5866	coding	PF13705
MSTRG.18559.6	5119	coding	PF13705
MSTRG.18561.1	4210	coding	PF14648
MSTRG.18561.3	3157	coding	PF14648
MSTRG.18562.1	1327	coding	PF00191
MSTRG.18562.2	1372	coding	PF00191
MSTRG.18562.3	1733	coding	PF00191
MSTRG.18563.1	497	noncoding	-
MSTRG.18564.1	7457	noncoding	-

MSTRG.18564.2	6822	noncoding	-
MSTRG.18565.1	3362	coding	PF09764
MSTRG.18565.2	1565	coding	PF09764
MSTRG.18568.1	5095	coding	PF00004
MSTRG.18568.3	2548	noncoding	-
MSTRG.18568.6	2854	noncoding	-
MSTRG.18572.1	4791	coding	PF11569
MSTRG.18572.4	5236	coding	PF11569
MSTRG.18574.4	3179	noncoding	-
MSTRG.18574.5	3276	noncoding	-
MSTRG.18574.6	3389	noncoding	-
MSTRG.18574.7	7799	noncoding	-
MSTRG.18567.2	3083	coding	PF04511
MSTRG.18589.1	215	noncoding	-
MSTRG.18590.1	327	noncoding	-
MSTRG.18594.2	1117	coding	PF00089
MSTRG.18594.3	679	noncoding	-
MSTRG.18594.5	1130	coding	PF00089
MSTRG.18594.6	1043	noncoding	-
MSTRG.18594.7	935	noncoding	-
MSTRG.18654.3	5915	coding	PF14951
MSTRG.18654.4	15740	coding	PF14950
MSTRG.18656.1	919	noncoding	-
MSTRG.18645.1	4533	coding	PF00179
MSTRG.18647.2	980	noncoding	-
MSTRG.18653.1	387	noncoding	-
MSTRG.18659.1	1157	noncoding	-
MSTRG.18659.5	18100	coding	PF01135
MSTRG.18662.5	6008	coding	PF10377
MSTRG.18662.3	6485	coding	PF10377
MSTRG.18666.8	3171	coding	PF02230
MSTRG.18666.9	3286	coding	PF02230
MSTRG.18666.4	2550	coding	PF02230
MSTRG.18666.5	2681	coding	PF02230
MSTRG.18666.6	2658	coding	PF02230
MSTRG.18666.7	2638	coding	PF02230
MSTRG.18666.10	3084	coding	PF02230
MSTRG.18667.2	2438	coding	PF00828
MSTRG.18677.1	1917	noncoding	-
MSTRG.18679.3	380	noncoding	-
MSTRG.18681.1	423	noncoding	-
MSTRG.18684.2	1376	coding	PF01160
MSTRG.18685.1	632	noncoding	-
MSTRG.18698.1	3602	noncoding	-
MSTRG.18697.4	1663	coding	PF00595
MSTRG.18702.2	8823	noncoding	-
MSTRG.18703.1	3072	noncoding	-
MSTRG.18704.1	5259	noncoding	-
MSTRG.18705.1	1209	noncoding	-
MSTRG.18706.1	7080	noncoding	-
MSTRG.18707.1	8950	noncoding	-
MSTRG.18709.2	11646	coding	PF00194
MSTRG.18709.3	8951	coding	PF00194
MSTRG.18709.4	12331	coding	PF00194

MSTRG.18711.4	1738	coding	PF00071
MSTRG.18713.1	547	noncoding	-
MSTRG.18715.2	900	noncoding	-
MSTRG.18715.4	1950	noncoding	-
MSTRG.18722.1	898	noncoding	-
MSTRG.18725.6	6350	coding	PF04146
MSTRG.18725.7	5857	coding	PF04146
MSTRG.18726.1	852	noncoding	-
MSTRG.18718.1	2496	noncoding	-
MSTRG.18732.2	5817	noncoding	-
MSTRG.18733.1	2266	noncoding	-
MSTRG.18734.1	9700	noncoding	-
MSTRG.18734.2	4446	noncoding	-
MSTRG.18741.3	2597	coding	PF05308
MSTRG.18743.3	1395	coding	PF00226
MSTRG.18744.1	2550	noncoding	-
MSTRG.18749.1	5107	noncoding	-
MSTRG.18749.2	6270	noncoding	-
MSTRG.18749.3	8219	noncoding	-
MSTRG.18750.1	845	noncoding	-
MSTRG.18751.2	1318	coding	PF00787
MSTRG.18751.4	805	coding	PF00787
MSTRG.18752.1	2808	noncoding	-
MSTRG.18752.2	2558	noncoding	-
MSTRG.18762.2	3761	noncoding	-
MSTRG.18762.1	3664	noncoding	-
MSTRG.18763.1	4697	noncoding	-
MSTRG.18762.3	1738	noncoding	-
MSTRG.18764.1	4573	noncoding	-
MSTRG.18762.4	1628	noncoding	-
MSTRG.18763.2	4683	noncoding	-
MSTRG.18763.4	4058	noncoding	-
MSTRG.18763.5	4055	noncoding	-
MSTRG.18763.7	4644	noncoding	-
MSTRG.18768.2	7446	coding	PF01369
MSTRG.18756.1	673	noncoding	-
MSTRG.18761.1	5527	coding	PF12548
MSTRG.18769.5	10337	coding	PF16665
MSTRG.18769.6	7945	coding	PF16665
MSTRG.18770.1	7025	noncoding	-
MSTRG.18771.1	1411	noncoding	-
MSTRG.18772.1	487	noncoding	-
MSTRG.18773.1	1078	noncoding	-
MSTRG.18776.1	552	noncoding	-
MSTRG.18805.1	18699	noncoding	-
MSTRG.18805.2	7727	noncoding	-
MSTRG.18816.4	703	noncoding	-
MSTRG.18816.5	1840	noncoding	-
MSTRG.18818.2	3219	noncoding	-
MSTRG.18825.7	7942	coding	PF16482
MSTRG.18829.1	8023	noncoding	-
MSTRG.18821.1	1410	noncoding	-
MSTRG.18822.4	1847	noncoding	-
MSTRG.18830.2	2776	noncoding	-

MSTRG.18830.3	298	noncoding	-
MSTRG.18838.1	414	noncoding	-
MSTRG.18841.1	1212	noncoding	-
MSTRG.18850.1	505	noncoding	-
MSTRG.18852.1	10781	coding	PF00046
MSTRG.18861.1	1167	noncoding	-
MSTRG.18862.1	378	noncoding	-
MSTRG.18872.1	218	noncoding	-
MSTRG.18882.1	488	noncoding	-
MSTRG.18894.1	517	noncoding	-
MSTRG.18894.4	723	coding	PF00061
MSTRG.18894.6	636	noncoding	-
MSTRG.18894.7	535	coding	PF00061
MSTRG.18894.3	494	noncoding	-
MSTRG.18894.5	601	coding	PF00061
MSTRG.18906.1	9654	noncoding	-
MSTRG.18907.1	3175	noncoding	-
MSTRG.18908.1	1050	noncoding	-
MSTRG.18911.1	252	noncoding	-
MSTRG.18917.2	4023	coding	PF04825
MSTRG.18917.3	4239	coding	PF04825
MSTRG.18958.1	438	noncoding	-
MSTRG.18967.1	1308	coding	PF00431
MSTRG.18967.3	5440	coding	PF00431
MSTRG.18955.1	1416	noncoding	-
MSTRG.18979.1	1019	noncoding	-
MSTRG.18983.2	4150	coding	PF10162
MSTRG.18986.1	1651	noncoding	-
MSTRG.18987.1	2778	noncoding	-
MSTRG.18984.2	524	noncoding	-
MSTRG.18997.1	896	noncoding	-
MSTRG.18997.2	1586	noncoding	-
MSTRG.18999.1	733	noncoding	-
MSTRG.18999.2	1191	noncoding	-
MSTRG.19005.1	8811	noncoding	-
MSTRG.19006.1	16484	noncoding	-
MSTRG.19014.6	3409	coding	PF07534
MSTRG.19014.5	3624	coding	PF07534
MSTRG.19015.1	510	noncoding	-
MSTRG.19015.2	565	noncoding	-
MSTRG.19015.3	850	noncoding	-
MSTRG.19009.1	234	noncoding	-
MSTRG.19024.1	431	noncoding	-
MSTRG.19041.1	222	noncoding	-
MSTRG.19049.1	21591	noncoding	-
MSTRG.19049.2	17093	noncoding	-
MSTRG.19049.3	526	noncoding	-
MSTRG.19049.4	4763	noncoding	-
MSTRG.19052.2	3097	coding	PF00153
MSTRG.19034.1	1232	noncoding	-
MSTRG.19038.1	994	noncoding	-
MSTRG.19039.3	4312	coding	PF02784
MSTRG.19053.2	3056	noncoding	-
MSTRG.19053.3	3467	noncoding	-

MSTRG.19059.4	8794	coding	PF00632
MSTRG.19059.3	9864	coding	PF00632
MSTRG.19061.1	1186	coding	PF04516
MSTRG.19063.1	780	noncoding	-
MSTRG.19066.1	1248	noncoding	-
MSTRG.19068.2	586	noncoding	-
MSTRG.19069.1	3423	coding	PF00244
MSTRG.19069.2	3121	coding	PF00244
MSTRG.19069.4	3299	coding	PF00244
MSTRG.19069.6	25219	coding	PF00244
MSTRG.19072.1	2689	coding	PF00658
MSTRG.19072.2	3514	coding	PF00658
MSTRG.19072.3	3772	coding	PF00658
MSTRG.19072.4	2701	coding	PF00076
MSTRG.19073.3	354	noncoding	-
MSTRG.19075.1	5466	noncoding	-
MSTRG.19089.2	10459	noncoding	-
MSTRG.19089.3	3718	noncoding	-
MSTRG.19090.2	1897	noncoding	-
MSTRG.19094.1	5027	coding	PF02937
MSTRG.19094.2	4771	noncoding	-
MSTRG.19094.3	4679	coding	PF02937
MSTRG.19094.5	2793	noncoding	-
MSTRG.19095.1	1411	noncoding	-
MSTRG.19096.1	14239	coding	PF16909
MSTRG.19099.1	901	noncoding	-
MSTRG.19100.1	460	noncoding	-
MSTRG.19101.1	1354	noncoding	-
MSTRG.19078.2	2692	coding	PF00069
MSTRG.19078.3	2892	coding	PF00069
MSTRG.19079.1	1995	noncoding	-
MSTRG.19081.1	3777	noncoding	-
MSTRG.19083.1	565	noncoding	-
MSTRG.19084.3	1886	coding	PF05653
MSTRG.19085.1	2065	coding	PF00078
MSTRG.19107.3	4155	coding	PF00092
MSTRG.19110.1	895	noncoding	-
MSTRG.19113.4	2958	coding	PF00956
MSTRG.19115.4	3072	noncoding	-
MSTRG.19115.5	1912	noncoding	-
MSTRG.19116.2	2930	coding	PF01034
MSTRG.19116.4	1086	coding	PF01034
MSTRG.19117.2	1262	coding	PF03034
MSTRG.19121.1	431	noncoding	-
MSTRG.19122.1	294	noncoding	-
MSTRG.19126.2	2963	coding	PF14996
MSTRG.19127.1	493	noncoding	-
MSTRG.19129.1	4500	coding	PF00494
MSTRG.19129.2	8897	noncoding	-
MSTRG.19129.4	2181	coding	PF00494
MSTRG.19129.5	2380	coding	PF00494
MSTRG.19131.8	1094	coding	PF14839
MSTRG.19131.7	4405	coding	PF14839
MSTRG.19131.4	5493	coding	PF14839

MSTRG.19131.5	5413	coding	PF14839
MSTRG.19131.6	5375	coding	PF14839
MSTRG.19133.3	2325	noncoding	-
MSTRG.19133.4	3756	noncoding	-
MSTRG.19134.3	4262	coding	PF10034
MSTRG.19134.4	3870	coding	PF10034
MSTRG.19136.1	1301	noncoding	-
MSTRG.19138.1	13531	coding	PF15912
MSTRG.19142.5	3739	coding	PF00481
MSTRG.19148.3	2864	coding	PF09773
MSTRG.19148.4	2094	coding	PF09773
MSTRG.19150.1	7942	noncoding	-
MSTRG.19150.2	9381	coding	PF06730
MSTRG.19152.2	1305	coding	PF06730
MSTRG.19144.1	679	noncoding	-
MSTRG.19146.1	774	noncoding	-
MSTRG.19155.1	1129	noncoding	-
MSTRG.19156.1	203	noncoding	-
MSTRG.19158.4	5488	coding	PF08788
MSTRG.19175.1	971	noncoding	-
MSTRG.19177.4	6166	noncoding	-
MSTRG.19177.1	1666	coding	PF02338
MSTRG.19177.3	1476	coding	PF02338
MSTRG.19177.5	1495	coding	PF02338
MSTRG.19177.6	3466	noncoding	-
MSTRG.19177.7	5191	coding	PF00078
MSTRG.19177.8	2641	coding	PF00078
MSTRG.19177.9	3086	coding	PF02338
MSTRG.19177.10	3869	noncoding	-
MSTRG.19177.12	2136	noncoding	-
MSTRG.19177.13	1809	coding	PF02338
MSTRG.19183.1	1211	noncoding	-
MSTRG.19183.2	748	noncoding	-
MSTRG.19189.2	2016	noncoding	-
MSTRG.19166.2	3489	coding	PF16508
MSTRG.19166.3	3317	coding	PF08599
MSTRG.19168.1	2079	noncoding	-
MSTRG.19169.4	3344	noncoding	-
MSTRG.19171.7	1951	noncoding	-
MSTRG.19171.2	1893	noncoding	-
MSTRG.19171.3	1606	coding	PF07714
MSTRG.19171.4	1808	coding	PF07714
MSTRG.19171.5	1872	noncoding	-
MSTRG.19171.6	1743	noncoding	-
MSTRG.19194.2	926	noncoding	-
MSTRG.19196.1	1021	noncoding	-
MSTRG.19196.2	1064	noncoding	-
MSTRG.19203.1	1541	coding	PF00027
MSTRG.19203.2	648	noncoding	-
MSTRG.19204.3	2761	coding	PF07002
MSTRG.19205.1	1568	coding	PF07002
MSTRG.19205.2	1835	coding	PF07002
MSTRG.19205.3	1056	coding	PF07002
MSTRG.19206.1	283	noncoding	-

MSTRG.19207.2	1239	noncoding	-
MSTRG.19210.5	2670	noncoding	-
MSTRG.19210.6	10292	coding	PF00632
MSTRG.19210.4	5853	coding	PF00632
MSTRG.19210.7	9995	coding	PF00632
MSTRG.19210.3	8751	coding	PF00632
MSTRG.19210.8	4176	noncoding	-
MSTRG.19197.1	2171	noncoding	-
MSTRG.19202.1	696	noncoding	-
MSTRG.19213.1	2107	coding	PF15679
MSTRG.19213.2	1668	noncoding	-
MSTRG.19215.1	2296	noncoding	-
MSTRG.19217.4	3024	noncoding	-
MSTRG.19217.2	2980	noncoding	-
MSTRG.19217.5	2319	noncoding	-
MSTRG.19218.1	861	noncoding	-
MSTRG.19318.1	5925	noncoding	-
MSTRG.19319.3	2767	noncoding	-
MSTRG.19323.1	1166	noncoding	-
MSTRG.19319.4	9672	noncoding	-
MSTRG.19319.5	3332	noncoding	-
MSTRG.19319.6	5934	noncoding	-
MSTRG.19325.1	4401	noncoding	-
MSTRG.19325.2	8539	noncoding	-
MSTRG.19326.1	8218	noncoding	-
MSTRG.19328.1	11791	coding	PF00078
MSTRG.19338.1	2537	noncoding	-
MSTRG.19339.1	8312	noncoding	-
MSTRG.19340.1	6707	noncoding	-
MSTRG.19342.1	3415	noncoding	-
MSTRG.19347.2	12469	noncoding	-
MSTRG.19339.2	33253	coding	PF00078
MSTRG.19339.3	10211	coding	PF00078
MSTRG.19339.4	8541	noncoding	-
MSTRG.19339.5	12283	coding	PF00078
MSTRG.19339.6	6459	noncoding	-
MSTRG.19339.7	2642	noncoding	-
MSTRG.19339.8	5934	noncoding	-
MSTRG.19349.1	2906	noncoding	-
MSTRG.19221.1	9078	noncoding	-
MSTRG.19221.2	2513	noncoding	-
MSTRG.19230.1	1059	noncoding	-
MSTRG.19235.1	470	noncoding	-
MSTRG.19249.1	1854	noncoding	-
MSTRG.19249.2	2062	noncoding	-
MSTRG.19249.3	744	noncoding	-
MSTRG.19249.4	2565	noncoding	-
MSTRG.19237.1	3282	coding	PF00787
MSTRG.19252.4	1789	noncoding	-
MSTRG.19253.1	1201	noncoding	-
MSTRG.19253.4	919	noncoding	-
MSTRG.19253.5	895	noncoding	-
MSTRG.19254.1	5126	noncoding	-
MSTRG.19258.1	3515	noncoding	-

MSTRG.19258.2	2337	noncoding	-
MSTRG.19264.1	7782	noncoding	-
MSTRG.19265.1	1451	coding	PF00572
MSTRG.19266.2	3618	coding	PF14919
MSTRG.19270.2	2275	coding	PF00083
MSTRG.19278.1	667	noncoding	-
MSTRG.19281.2	2741	coding	PF12796
MSTRG.19285.2	6074	noncoding	-
MSTRG.19285.3	6865	noncoding	-
MSTRG.19285.4	6468	noncoding	-
MSTRG.19291.1	4140	noncoding	-
MSTRG.19291.2	1617	noncoding	-
MSTRG.19283.1	3141	coding	PF13873
MSTRG.19283.3	6305	coding	PF13873
MSTRG.19305.4	2958	noncoding	-
MSTRG.19311.1	3394	coding	PF03028
MSTRG.19311.2	13532	coding	PF12774
MSTRG.19311.4	538	noncoding	-
MSTRG.19311.5	9169	coding	PF12774
MSTRG.19311.6	5506	coding	PF12774
MSTRG.19312.1	811	noncoding	-
MSTRG.19314.1	3001	noncoding	-
MSTRG.19314.3	3793	noncoding	-
MSTRG.19314.4	3058	noncoding	-
MSTRG.19316.1	3376	coding	PF00653
MSTRG.19314.6	3486	noncoding	-
MSTRG.19316.2	5284	coding	PF00653
MSTRG.19316.3	3645	coding	PF00653
MSTRG.19316.4	3594	coding	PF00653
MSTRG.19317.1	1907	noncoding	-
MSTRG.19313.1	4792	coding	PF00653
MSTRG.19352.1	5773	coding	PF00833
MSTRG.19352.3	4163	noncoding	-
MSTRG.19352.4	2855	noncoding	-
MSTRG.19352.5	3389	noncoding	-
MSTRG.19352.12	1374	noncoding	-
MSTRG.19352.10	6783	noncoding	-
MSTRG.19352.13	1108	noncoding	-
MSTRG.19352.9	1168	noncoding	-
MSTRG.19352.8	2879	noncoding	-
MSTRG.19352.11	3097	noncoding	-
MSTRG.19352.6	1661	noncoding	-
MSTRG.19354.1	3926	noncoding	-
MSTRG.19359.2	1426	coding	PF14926
MSTRG.19359.3	4112	coding	PF14926
MSTRG.19359.4	2305	coding	PF14926
MSTRG.19359.5	1150	coding	PF14926
MSTRG.19361.1	6176	coding	PF15352
MSTRG.19361.2	4686	coding	PF15352
MSTRG.19361.3	2027	coding	PF15352
MSTRG.19361.4	4453	coding	PF15352
MSTRG.19361.5	7937	coding	PF15352
MSTRG.19361.6	12135	coding	PF15352
MSTRG.19361.10	1662	coding	PF15352

MSTRG.19361.7	3570	coding	PF15352
MSTRG.19361.8	2742	coding	PF15352
MSTRG.19361.9	3852	coding	PF15352
MSTRG.19361.11	4644	coding	PF15352
MSTRG.19361.12	13860	coding	PF15352
MSTRG.19361.13	8371	coding	PF15352
MSTRG.19361.14	7824	coding	PF15352
MSTRG.19363.1	632	noncoding	-
MSTRG.19401.1	3876	noncoding	-
MSTRG.19402.1	426	noncoding	-
MSTRG.19368.1	223	noncoding	-
MSTRG.19369.1	728	noncoding	-
MSTRG.19370.1	358	noncoding	-
MSTRG.19407.1	917	noncoding	-
MSTRG.19408.1	1973	coding	PF09596
MSTRG.19414.1	2709	coding	PF06602
MSTRG.19414.2	2798	coding	PF06602
MSTRG.19415.1	2868	coding	PF14073
MSTRG.19415.3	1789	coding	PF14073
MSTRG.19415.4	1632	coding	PF14073
MSTRG.19416.1	2739	coding	PF16046
MSTRG.19416.2	1834	coding	PF16046
MSTRG.19413.1	1454	noncoding	-
MSTRG.19420.2	9443	coding	PF04636
MSTRG.19422.1	1604	coding	PF02373
MSTRG.19422.3	4664	coding	PF02373
MSTRG.19422.2	2809	coding	PF02373
MSTRG.19422.4	2511	coding	PF02373
MSTRG.19422.5	2427	coding	PF02373
MSTRG.19423.1	3239	noncoding	-
MSTRG.19425.1	4809	coding	PF12240
MSTRG.19425.4	8463	coding	PF12240
MSTRG.19428.1	2984	coding	PF02171
MSTRG.19432.1	1062	noncoding	-
MSTRG.19434.1	3328	coding	PF04676
MSTRG.19435.1	1634	noncoding	-
MSTRG.19436.1	1445	noncoding	-
MSTRG.19440.2	2207	noncoding	-
MSTRG.19440.1	592	noncoding	-
MSTRG.19441.1	1025	noncoding	-
MSTRG.19446.2	6547	coding	PF00888
MSTRG.19464.1	11294	coding	PF00454
MSTRG.19464.6	812	noncoding	-
MSTRG.19478.2	3199	coding	PF05686
MSTRG.19479.1	1248	noncoding	-
MSTRG.19448.1	1542	noncoding	-
MSTRG.19458.2	2722	noncoding	-
MSTRG.19458.4	2707	noncoding	-
MSTRG.19458.3	621	noncoding	-
MSTRG.19458.5	10278	coding	PF11977
MSTRG.19459.1	3968	noncoding	-
MSTRG.19481.1	9242	noncoding	-
MSTRG.19480.2	9592	coding	PF00769
MSTRG.19485.1	6734	noncoding	-

MSTRG.19485.2	6949	noncoding	-
MSTRG.19485.3	872	noncoding	-
MSTRG.19486.1	6701	noncoding	-
MSTRG.19490.5	6139	coding	PF00620
MSTRG.19490.8	1810	coding	PF00620
MSTRG.19491.1	2296	noncoding	-
MSTRG.19492.1	10202	noncoding	-
MSTRG.19493.1	2311	coding	PF02994
MSTRG.19494.1	1757	noncoding	-
MSTRG.19495.1	6926	coding	PF02994
MSTRG.19496.1	3187	noncoding	-
MSTRG.19505.2	2367	coding	PF07742
MSTRG.19505.3	1313	coding	PF07742
MSTRG.19505.4	2596	coding	PF07742
MSTRG.19506.1	1966	noncoding	-
MSTRG.19507.4	1289	noncoding	-
MSTRG.19508.3	802	coding	PF00069
MSTRG.19509.2	2204	noncoding	-
MSTRG.19515.3	936	coding	PF06608
MSTRG.19515.2	3863	coding	PF06608
MSTRG.19515.4	740	coding	PF06608
MSTRG.19521.1	1397	noncoding	-
MSTRG.19521.2	1891	noncoding	-
MSTRG.19522.4	3303	coding	PF15692
MSTRG.19526.1	355	noncoding	-
MSTRG.19530.1	5417	coding	PF01242
MSTRG.19531.1	4855	coding	PF02994
MSTRG.19526.2	7882	noncoding	-
MSTRG.19532.1	749	noncoding	-
MSTRG.19516.1	2109	noncoding	-
MSTRG.19533.5	9803	noncoding	-
MSTRG.19533.6	427	noncoding	-
MSTRG.19534.1	3594	noncoding	-
MSTRG.19533.7	5894	coding	PF07679
MSTRG.19535.1	1688	noncoding	-
MSTRG.19538.1	2946	noncoding	-
MSTRG.19540.1	5225	noncoding	-
MSTRG.19543.4	3448	coding	PF00443
MSTRG.19543.5	2879	coding	PF00443
MSTRG.19543.6	3305	coding	PF00443
MSTRG.19544.1	1140	noncoding	-
MSTRG.19547.1	6569	noncoding	-
MSTRG.19551.2	1092	coding	PF00929
MSTRG.19555.1	1961	noncoding	-
MSTRG.19559.7	3728	coding	PF08205
MSTRG.19559.8	3776	coding	PF08205
MSTRG.19559.5	4252	coding	PF08205
MSTRG.19559.6	4138	coding	PF08205
MSTRG.19560.1	40849	noncoding	-
MSTRG.19561.1	18371	noncoding	-
MSTRG.19562.2	39462	noncoding	-
MSTRG.19562.1	2896	noncoding	-
MSTRG.19562.4	52749	noncoding	-
MSTRG.19562.5	41335	noncoding	-

MSTRG.19562.3	43292	noncoding	-
MSTRG.19562.6	54484	noncoding	-
MSTRG.19559.9	9553	noncoding	-
MSTRG.19563.1	2389	noncoding	-
MSTRG.19568.1	2587	coding	PF01442
MSTRG.19568.2	1063	coding	PF01442
MSTRG.19568.3	907	coding	PF01442
MSTRG.19568.4	1168	coding	PF01442
MSTRG.19572.9	4060	noncoding	-
MSTRG.19572.8	4392	coding	PF00069
MSTRG.19572.5	4933	coding	PF00069
MSTRG.19572.7	4315	coding	PF00069
MSTRG.19572.6	7472	coding	PF00069
MSTRG.19573.1	3371	noncoding	-
MSTRG.19572.10	5410	noncoding	-
MSTRG.19572.11	8062	noncoding	-
MSTRG.19574.1	3027	noncoding	-
MSTRG.19572.12	9794	noncoding	-
MSTRG.19570.2	2302	noncoding	-
MSTRG.19570.3	9703	noncoding	-
MSTRG.19570.4	4603	noncoding	-
MSTRG.19571.2	3825	coding	PF13965
MSTRG.19577.2	1384	noncoding	-
MSTRG.19579.1	4562	noncoding	-
MSTRG.19579.2	2383	noncoding	-
MSTRG.19582.2	8196	noncoding	-
MSTRG.19582.4	7523	noncoding	-
MSTRG.19582.5	7433	noncoding	-
MSTRG.19582.6	5604	noncoding	-
MSTRG.19582.3	8324	noncoding	-
MSTRG.19582.7	8186	noncoding	-
MSTRG.19581.1	1221	noncoding	-
MSTRG.19583.1	3004	coding	PF00089
MSTRG.19590.1	1427	noncoding	-
MSTRG.19590.2	1007	noncoding	-
MSTRG.19591.1	745	noncoding	-
MSTRG.19591.2	916	noncoding	-
MSTRG.19593.1	1221	coding	PF16681
MSTRG.19596.4	3533	coding	PF10408
MSTRG.19598.3	16544	coding	PF00856
MSTRG.19598.5	14445	coding	PF00856
MSTRG.19598.6	15510	coding	PF00856
MSTRG.19600.1	2682	coding	PF12317
MSTRG.19602.1	5496	noncoding	-
MSTRG.19603.1	800	noncoding	-
MSTRG.19602.3	5144	noncoding	-
MSTRG.19602.4	5466	noncoding	-
MSTRG.19602.5	7475	noncoding	-
MSTRG.19602.6	2915	noncoding	-
MSTRG.19604.6	6140	coding	PF00270
MSTRG.19604.4	6110	coding	PF00270
MSTRG.19604.8	5765	coding	PF00270
MSTRG.19622.1	2606	noncoding	-
MSTRG.19624.7	4878	coding	PF00012

MSTRG.19624.9	4881	coding	PF00012
MSTRG.19616.2	10178	coding	PF02761
MSTRG.19614.1	2890	coding	PF08205
MSTRG.19614.2	2681	coding	PF08205
MSTRG.19629.1	3810	coding	PF00443
MSTRG.19628.1	701	noncoding	-
MSTRG.19628.2	951	noncoding	-
MSTRG.19638.4	6246	coding	PF09128
MSTRG.19638.2	7402	coding	PF09128
MSTRG.19638.1	9495	coding	PF09128
MSTRG.19639.1	3071	noncoding	-
MSTRG.19640.1	2527	noncoding	-
MSTRG.19642.5	4865	noncoding	-
MSTRG.19642.6	4982	noncoding	-
MSTRG.19642.7	4986	noncoding	-
MSTRG.19643.1	6308	coding	PF00094
MSTRG.19648.1	6372	coding	PF14604
MSTRG.19649.3	2348	coding	PF15261
MSTRG.19650.1	233	noncoding	-
MSTRG.19671.9	2133	coding	PF16016
MSTRG.19666.1	3900	coding	PF02023
MSTRG.19672.2	1267	coding	PF00415
MSTRG.19672.3	873	noncoding	-
MSTRG.19672.4	864	noncoding	-
MSTRG.19674.1	1418	coding	PF00005
MSTRG.19675.1	1841	noncoding	-
MSTRG.19676.2	3252	coding	PF00794
MSTRG.19678.5	5508	noncoding	-
MSTRG.19678.8	2728	noncoding	-
MSTRG.19681.1	5029	noncoding	-
MSTRG.19682.1	1009	noncoding	-
MSTRG.19683.1	1365	noncoding	-
MSTRG.19684.1	2811	coding	PF00505
MSTRG.19684.2	2839	coding	PF00505
MSTRG.19701.1	740	noncoding	-
MSTRG.19707.2	3704	coding	PF00233
MSTRG.19708.1	902	noncoding	-
MSTRG.19707.3	3552	noncoding	-
MSTRG.19713.2	1331	coding	PF00227
MSTRG.19718.1	1945	noncoding	-
MSTRG.19720.3	6402	coding	PF00071
MSTRG.19721.1	3667	noncoding	-
MSTRG.19720.4	9077	coding	PF00071
MSTRG.19720.5	6695	noncoding	-
MSTRG.19722.1	3682	coding	PF06468
MSTRG.19723.1	6516	coding	PF07993
MSTRG.19723.4	4375	coding	PF03015
MSTRG.19723.3	2909	coding	PF07993
MSTRG.19723.5	2326	coding	PF07993
MSTRG.19723.6	1893	coding	PF07993
MSTRG.19733.1	3484	coding	PF12130
MSTRG.19743.1	528	noncoding	-
MSTRG.19744.2	1532	noncoding	-
MSTRG.19744.4	796	noncoding	-

MSTRG.19744.5	2721	noncoding	-
MSTRG.19744.6	1708	noncoding	-
MSTRG.19744.7	237	noncoding	-
MSTRG.19744.8	893	noncoding	-
MSTRG.19744.9	701	noncoding	-
MSTRG.19744.10	620	noncoding	-
MSTRG.19744.11	300	noncoding	-
MSTRG.19744.12	275	noncoding	-
MSTRG.19744.13	368	noncoding	-
MSTRG.19744.14	476	noncoding	-
MSTRG.19744.15	1142	noncoding	-
MSTRG.19744.16	213	noncoding	-
MSTRG.19744.17	223	noncoding	-
MSTRG.19744.18	213	noncoding	-
MSTRG.19744.19	211	noncoding	-
MSTRG.19744.20	304	noncoding	-
MSTRG.19744.21	217	noncoding	-
MSTRG.19744.22	219	noncoding	-
MSTRG.19744.23	237	noncoding	-
MSTRG.19744.24	223	noncoding	-
MSTRG.19744.25	222	noncoding	-
MSTRG.19744.26	227	noncoding	-
MSTRG.19744.27	258	noncoding	-
MSTRG.19744.28	301	noncoding	-
MSTRG.19744.29	210	noncoding	-
MSTRG.19744.30	328	noncoding	-
MSTRG.19744.31	205	noncoding	-
MSTRG.19744.32	201	noncoding	-
MSTRG.19739.1	537	noncoding	-
MSTRG.19751.1	8008	noncoding	-
MSTRG.19752.1	3109	noncoding	-
MSTRG.19752.2	1371	noncoding	-
MSTRG.19751.2	2028	noncoding	-
MSTRG.19751.3	772	noncoding	-
MSTRG.19755.1	5202	coding	PF02854
MSTRG.19755.2	3897	coding	PF02854
MSTRG.19755.3	4338	coding	PF02854
MSTRG.19755.4	3697	coding	PF02854
MSTRG.19755.5	3685	coding	PF02854
MSTRG.19761.1	2765	noncoding	-
MSTRG.19765.1	757	noncoding	-
MSTRG.19766.1	2624	noncoding	-
MSTRG.19767.3	1701	coding	PF12335
MSTRG.19768.1	3589	noncoding	-
MSTRG.19757.2	1835	noncoding	-
MSTRG.19774.1	3785	coding	PF03810
MSTRG.19779.2	3988	noncoding	-
MSTRG.19784.2	1830	coding	PF15057
MSTRG.19785.3	818	noncoding	-
MSTRG.19788.1	10372	noncoding	-
MSTRG.19791.1	1315	noncoding	-
MSTRG.19803.2	7967	coding	PF02994
MSTRG.19803.6	3049	coding	PF07647
MSTRG.19803.7	3117	noncoding	-

MSTRG.19803.5	3303	noncoding	-
MSTRG.19804.1	890	noncoding	-
MSTRG.19805.1	4168	coding	PF02191
MSTRG.19806.1	996	noncoding	-
MSTRG.19807.3	1698	coding	PF00168
MSTRG.19809.1	268	noncoding	-
MSTRG.19811.1	1381	noncoding	-
MSTRG.19821.1	2602	coding	PF13853
MSTRG.19822.1	499	noncoding	-
MSTRG.19821.2	1386	coding	PF13853
MSTRG.19821.3	4928	noncoding	-
MSTRG.19821.4	2299	coding	PF13853
MSTRG.19821.5	19256	noncoding	-
MSTRG.19821.7	15392	noncoding	-
MSTRG.19821.6	7427	noncoding	-
MSTRG.19821.8	2146	coding	PF13853
MSTRG.19821.9	439	noncoding	-
MSTRG.19825.1	922	noncoding	-
MSTRG.19827.1	702	coding	PF01196
MSTRG.19831.2	11021	coding	PF01196
MSTRG.19840.1	1172	noncoding	-
MSTRG.19841.1	14445	noncoding	-
MSTRG.19848.2	2350	noncoding	-
MSTRG.19856.8	2261	coding	PF10257
MSTRG.19856.9	2246	coding	PF10257
MSTRG.19856.11	11569	coding	PF15668
MSTRG.19857.2	1038	coding	PF15668
MSTRG.19861.2	2469	coding	PF15227
MSTRG.19863.3	1901	coding	PF15227
MSTRG.19863.2	2279	coding	PF15227
MSTRG.19863.4	2739	coding	PF15227
MSTRG.19868.1	775	noncoding	-
MSTRG.19868.2	635	noncoding	-
MSTRG.19879.1	844	noncoding	-
MSTRG.19884.1	7871	coding	PF06817
MSTRG.19884.2	464	noncoding	-
MSTRG.19884.3	2345	noncoding	-
MSTRG.19884.4	1492	noncoding	-
MSTRG.19885.1	1973	coding	PF00517
MSTRG.19888.1	394	noncoding	-
MSTRG.19893.3	3466	coding	PF13765
MSTRG.19906.4	3838	coding	PF16533
MSTRG.19907.1	1151	noncoding	-
MSTRG.19908.2	1298	coding	PF00071
MSTRG.19909.5	2281	coding	PF10277
MSTRG.19909.7	3391	coding	PF10277
MSTRG.19909.6	1926	coding	PF10277
MSTRG.19904.1	1444	coding	PF01129
MSTRG.19904.2	1602	coding	PF01129
MSTRG.19904.3	1121	coding	PF01129
MSTRG.19905.1	1634	noncoding	-
MSTRG.19905.2	1382	noncoding	-
MSTRG.19917.1	3103	coding	PF01834
MSTRG.19918.3	5389	noncoding	-

MSTRG.19912.3	861	coding	PF15243
MSTRG.19912.4	884	coding	PF15243
MSTRG.19913.2	936	coding	PF03024
MSTRG.19914.2	4933	coding	PF07647
MSTRG.19923.1	462	noncoding	-
MSTRG.19924.1	673	noncoding	-
MSTRG.19924.2	831	noncoding	-
MSTRG.19925.3	5379	coding	PF00620
MSTRG.19925.5	2992	coding	PF00620
MSTRG.19926.5	1182	coding	PF01852
MSTRG.19926.4	1248	coding	PF01852
MSTRG.19926.3	1613	coding	PF01852
MSTRG.19930.1	6170	noncoding	-
MSTRG.19931.1	462	noncoding	-
MSTRG.19937.1	1741	noncoding	-
MSTRG.19938.1	1036	noncoding	-
MSTRG.19939.4	1521	noncoding	-
MSTRG.19941.1	965	coding	PF00338
MSTRG.19951.1	911	noncoding	-
MSTRG.19954.2	1257	coding	PF01556
MSTRG.19958.1	3883	noncoding	-
MSTRG.19959.1	8118	noncoding	-
MSTRG.19959.3	7615	noncoding	-
MSTRG.19959.5	8083	noncoding	-
MSTRG.19959.2	8013	noncoding	-
MSTRG.19959.8	7068	noncoding	-
MSTRG.19959.9	8160	noncoding	-
MSTRG.19959.10	7060	noncoding	-
MSTRG.19959.11	8274	noncoding	-
MSTRG.19959.12	6932	noncoding	-
MSTRG.19959.13	6163	noncoding	-
MSTRG.19942.1	5893	coding	PF02878
MSTRG.19945.1	1319	noncoding	-
MSTRG.19947.1	859	noncoding	-
MSTRG.19946.2	5399	noncoding	-
MSTRG.19967.1	27195	noncoding	-
MSTRG.19967.5	5841	noncoding	-
MSTRG.19967.4	10227	noncoding	-
MSTRG.19967.8	1564	noncoding	-
MSTRG.19965.2	4051	coding	PF03137
MSTRG.19978.1	2652	coding	PF03009
MSTRG.19978.3	2947	coding	PF03009
MSTRG.19978.2	2979	coding	PF03009
MSTRG.19978.4	3102	coding	PF03009
MSTRG.19978.5	2777	coding	PF03009
MSTRG.19979.1	4170	noncoding	-
MSTRG.19980.1	1969	coding	PF00079
MSTRG.19983.1	3799	noncoding	-
MSTRG.19985.1	7334	coding	PF03982
MSTRG.19981.1	595	noncoding	-
MSTRG.19990.1	1885	noncoding	-
MSTRG.19990.2	857	noncoding	-
MSTRG.20000.1	1889	noncoding	-
MSTRG.20003.1	4946	noncoding	-

MSTRG.20006.1	680	noncoding	-
MSTRG.20008.1	689	noncoding	-
MSTRG.20013.4	7005	coding	PF00063
MSTRG.20013.5	5216	noncoding	-
MSTRG.20015.3	2221	noncoding	-
MSTRG.20015.5	2064	noncoding	-
MSTRG.20015.6	3755	noncoding	-
MSTRG.20018.1	1382	noncoding	-
MSTRG.20018.2	1252	noncoding	-
MSTRG.20018.3	1448	noncoding	-
MSTRG.20027.1	4142	coding	PF01795
MSTRG.20033.1	1880	noncoding	-
MSTRG.20037.1	856	noncoding	-
MSTRG.20038.4	1332	noncoding	-
MSTRG.20047.1	2863	noncoding	-
MSTRG.20056.1	1199	coding	PF14949
MSTRG.20056.4	1870	coding	PF14949
MSTRG.20057.3	1273	noncoding	-
MSTRG.20057.4	1052	noncoding	-
MSTRG.20059.1	5773	noncoding	-
MSTRG.20059.5	1884	noncoding	-
MSTRG.20061.3	1684	noncoding	-
MSTRG.20061.2	2311	noncoding	-
MSTRG.20063.1	848	noncoding	-
MSTRG.20066.1	975	noncoding	-
MSTRG.20069.4	974	noncoding	-
MSTRG.20074.2	2763	coding	PF02165
MSTRG.20074.4	2712	coding	PF02165
MSTRG.20075.1	2309	noncoding	-
MSTRG.20079.1	4183	coding	PF15818
MSTRG.20086.5	6108	noncoding	-
MSTRG.20086.6	8748	noncoding	-
MSTRG.20086.9	6370	noncoding	-
MSTRG.20087.1	6840	noncoding	-
MSTRG.20086.10	6721	noncoding	-
MSTRG.20088.2	1247	noncoding	-
MSTRG.20090.1	442	noncoding	-
MSTRG.20092.2	2661	coding	PF05843
MSTRG.20094.2	3840	noncoding	-
MSTRG.20097.2	2119	coding	PF04379
MSTRG.20098.1	6857	noncoding	-
MSTRG.20107.1	565	noncoding	-
MSTRG.20109.1	3131	noncoding	-
MSTRG.20110.1	1587	coding	PF00178
MSTRG.20110.2	1363	coding	PF00178
MSTRG.20110.3	4983	coding	PF00178
MSTRG.20114.1	2317	coding	PF00198
MSTRG.20114.2	1600	coding	PF00198
MSTRG.20114.4	2328	coding	PF00198
MSTRG.20115.1	6913	noncoding	-
MSTRG.20112.1	310	noncoding	-
MSTRG.20118.1	398	noncoding	-
MSTRG.20117.3	2502	coding	PF00193
MSTRG.20121.2	1462	noncoding	-

MSTRG.20121.1	1881	noncoding	-
MSTRG.20119.1	872	noncoding	-
MSTRG.20120.1	2688	coding	PF00089
MSTRG.20126.2	3901	noncoding	-
MSTRG.20126.4	1126	noncoding	-
MSTRG.20126.3	3922	noncoding	-
MSTRG.20128.2	1095	coding	PF07258
MSTRG.20132.3	1058	noncoding	-
MSTRG.20132.4	1479	noncoding	-
MSTRG.20135.1	2710	coding	PF13855
MSTRG.20148.1	2016	coding	PF00517
MSTRG.20158.1	592	noncoding	-
MSTRG.20158.2	626	noncoding	-
MSTRG.20158.5	4113	coding	PF05918
MSTRG.20158.6	2146	noncoding	-
MSTRG.20162.4	4436	noncoding	-
MSTRG.20163.1	1181	noncoding	-
MSTRG.20164.1	2273	noncoding	-
MSTRG.20168.1	11743	noncoding	-
MSTRG.20154.2	2884	coding	PF00106
MSTRG.20155.1	7367	noncoding	-
MSTRG.20156.1	7982	noncoding	-
MSTRG.20157.1	4496	noncoding	-
MSTRG.20154.3	4790	coding	PF00106
MSTRG.20172.3	1668	noncoding	-
MSTRG.20173.1	2419	noncoding	-
MSTRG.20177.2	1872	coding	PF09258
MSTRG.20179.1	635	noncoding	-
MSTRG.20180.1	385	noncoding	-
MSTRG.20186.1	3226	coding	PF13873
MSTRG.20188.1	1239	noncoding	-
MSTRG.20189.2	336	noncoding	-
MSTRG.20189.1	395	noncoding	-
MSTRG.20190.1	413	noncoding	-
MSTRG.20193.2	19034	noncoding	-
MSTRG.20193.3	26598	coding	PF14915
MSTRG.20198.2	4055	coding	PF03441
MSTRG.20198.1	5302	coding	PF03441
MSTRG.20199.1	571	noncoding	-
MSTRG.20200.3	2515	coding	PF00640
MSTRG.20202.1	715	noncoding	-
MSTRG.20203.2	2421	coding	PF08610
MSTRG.20210.4	4147	coding	PF00628
MSTRG.20210.3	3375	coding	PF00628
MSTRG.20210.5	25454	noncoding	-
MSTRG.20210.6	12562	noncoding	-
MSTRG.20212.1	4608	noncoding	-
MSTRG.20212.2	10213	noncoding	-
MSTRG.20212.3	1385	noncoding	-
MSTRG.20212.4	3897	noncoding	-
MSTRG.20210.7	903	noncoding	-
MSTRG.20207.2	2685	noncoding	-
MSTRG.20207.3	1621	noncoding	-
MSTRG.20207.4	1772	noncoding	-

MSTRG.20208.3	5441	coding	PF00609
MSTRG.20213.5	6732	noncoding	-
MSTRG.20213.6	4498	noncoding	-
MSTRG.20214.1	4258	noncoding	-
MSTRG.20215.1	723	noncoding	-
MSTRG.20216.1	6665	noncoding	-
MSTRG.20218.4	4149	noncoding	-
MSTRG.20218.5	4189	noncoding	-
MSTRG.20220.1	3381	noncoding	-
MSTRG.20225.1	997	coding	PF09396
MSTRG.20225.2	793	noncoding	-
MSTRG.20226.2	1307	coding	PF00089
MSTRG.20226.3	1602	coding	PF00089
MSTRG.20226.4	1269	coding	PF00089
MSTRG.20227.6	6505	noncoding	-
MSTRG.20227.5	7465	noncoding	-
MSTRG.20227.3	7278	noncoding	-
MSTRG.20227.4	7275	noncoding	-
MSTRG.20227.8	9411	noncoding	-
MSTRG.20227.10	7457	noncoding	-
MSTRG.20227.9	6865	noncoding	-
MSTRG.20227.11	7893	noncoding	-
MSTRG.20227.12	7042	noncoding	-
MSTRG.20228.1	495	noncoding	-
MSTRG.20229.1	7998	coding	PF14670
MSTRG.20222.3	1400	noncoding	-
MSTRG.20222.4	1035	noncoding	-
MSTRG.20223.1	7173	coding	PF01412
MSTRG.20223.2	4625	coding	PF01412
MSTRG.20241.1	5417	coding	PF02141
MSTRG.20241.2	5375	coding	PF02141
MSTRG.20241.3	5281	coding	PF02141
MSTRG.20241.5	5120	coding	PF02141
MSTRG.20241.4	6165	coding	PF02141
MSTRG.20241.6	5034	coding	PF02141
MSTRG.20231.1	1703	noncoding	-
MSTRG.20231.2	1357	noncoding	-
MSTRG.20232.1	507	noncoding	-
MSTRG.20233.1	2172	coding	PF02535
MSTRG.20242.4	7675	coding	PF00076
MSTRG.20242.1	4547	coding	PF00076
MSTRG.20242.2	4535	coding	PF00076
MSTRG.20242.3	4835	coding	PF00076
MSTRG.20242.5	7976	coding	PF00076
MSTRG.20242.8	7846	coding	PF00076
MSTRG.20244.2	2794	coding	PF00651
MSTRG.20245.1	2173	coding	PF00329
MSTRG.20246.1	13824	noncoding	-
MSTRG.20246.3	1773	noncoding	-
MSTRG.20249.4	4189	noncoding	-
MSTRG.20250.2	4516	noncoding	-
MSTRG.20250.4	4754	noncoding	-
MSTRG.20250.7	4953	noncoding	-
MSTRG.20250.8	4008	noncoding	-

MSTRG.20251.1	966	noncoding	-
MSTRG.20247.1	7325	coding	PF00102
MSTRG.20247.2	7402	coding	PF00102
MSTRG.20248.1	1544	noncoding	-
MSTRG.20257.1	1483	coding	PF13853
MSTRG.20257.2	739	noncoding	-
MSTRG.20269.2	4572	coding	PF15327
MSTRG.20270.1	822	noncoding	-
MSTRG.20270.2	1053	noncoding	-
MSTRG.20270.3	966	noncoding	-
MSTRG.20271.2	3419	coding	PF03531
MSTRG.20272.1	3735	coding	PF00864
MSTRG.20274.1	423	noncoding	-
MSTRG.20274.5	2880	noncoding	-
MSTRG.20274.4	3041	noncoding	-
MSTRG.20274.6	2543	noncoding	-
MSTRG.20277.1	1921	noncoding	-
MSTRG.20278.2	1023	coding	PF00179
MSTRG.20279.1	2167	noncoding	-
MSTRG.20281.2	2643	coding	PF16575
MSTRG.20281.4	1991	coding	PF16575
MSTRG.20287.3	4625	coding	PF01529
MSTRG.20288.2	1845	coding	PF10278
MSTRG.20288.3	1479	coding	PF10278
MSTRG.20290.1	6843	coding	PF00651
MSTRG.20291.3	834	noncoding	-
MSTRG.20292.1	4967	noncoding	-
MSTRG.20292.2	4982	noncoding	-
MSTRG.20292.3	5060	noncoding	-
MSTRG.20292.4	4740	noncoding	-
MSTRG.20292.7	5115	noncoding	-
MSTRG.20293.1	1600	noncoding	-
MSTRG.20298.3	4786	noncoding	-
MSTRG.20298.4	5632	noncoding	-
MSTRG.20298.5	4807	noncoding	-
MSTRG.20298.6	4782	noncoding	-
MSTRG.20298.7	5245	noncoding	-
MSTRG.20299.1	2807	noncoding	-
MSTRG.20315.2	7731	coding	PF01237
MSTRG.20315.4	4281	coding	PF01237
MSTRG.20315.6	3016	coding	PF01237
MSTRG.20315.5	3393	coding	PF01237
MSTRG.20315.7	1111	noncoding	-
MSTRG.20315.11	5507	noncoding	-
MSTRG.20315.14	5468	noncoding	-
MSTRG.20315.16	4234	noncoding	-
MSTRG.20318.1	581	noncoding	-
MSTRG.20308.1	911	noncoding	-
MSTRG.20310.1	3013	coding	PF00804
MSTRG.20313.1	1071	coding	PF01122
MSTRG.20313.2	1844	coding	PF01122
MSTRG.20313.3	1806	coding	PF01122
MSTRG.20313.4	528	noncoding	-
MSTRG.20313.5	941	coding	PF01122

MSTRG.20334.1	4028	noncoding	-
MSTRG.20335.3	549	noncoding	-
MSTRG.20338.1	3776	noncoding	-
MSTRG.20338.2	2480	noncoding	-
MSTRG.20339.3	1393	noncoding	-
MSTRG.20339.4	1546	noncoding	-
MSTRG.20345.2	5715	coding	PF03643
MSTRG.20346.2	1005	coding	PF01878
MSTRG.20347.4	1701	coding	PF00441
MSTRG.20321.1	321	noncoding	-
MSTRG.20328.4	1211	noncoding	-
MSTRG.20328.5	980	noncoding	-
MSTRG.20328.6	1240	noncoding	-
MSTRG.20330.1	452	noncoding	-
MSTRG.20332.6	2976	noncoding	-
MSTRG.20354.1	312	noncoding	-
MSTRG.20361.1	327	noncoding	-
MSTRG.20362.1	549	noncoding	-
MSTRG.20362.2	1804	noncoding	-
MSTRG.20362.3	1074	noncoding	-
MSTRG.20366.1	2941	noncoding	-
MSTRG.20367.3	2145	coding	PF01359
MSTRG.20367.4	2148	coding	PF01359
MSTRG.20367.6	877	noncoding	-
MSTRG.20367.5	2122	coding	PF01359
MSTRG.20376.4	2229	noncoding	-
MSTRG.20376.5	2731	noncoding	-
MSTRG.20376.7	2478	coding	PF00001
MSTRG.20376.8	3691	noncoding	-
MSTRG.20376.6	12127	noncoding	-
MSTRG.20376.9	13832	noncoding	-
MSTRG.20376.10	27002	noncoding	-
MSTRG.20378.2	1800	coding	PF00704
MSTRG.20382.3	8719	coding	PF12424
MSTRG.20382.5	8752	coding	PF12424
MSTRG.20382.8	4164	coding	PF12424
MSTRG.20387.2	4997	coding	PF15663
MSTRG.20387.8	1072	noncoding	-
MSTRG.20387.7	8916	coding	PF15663
MSTRG.20388.1	948	noncoding	-
MSTRG.20393.1	1756	noncoding	-
MSTRG.20394.1	3944	coding	PF00505
MSTRG.20395.1	6335	coding	PF01633
MSTRG.20395.2	6167	coding	PF01633
MSTRG.20395.3	5448	coding	PF01633
MSTRG.20395.4	5810	coding	PF01633
MSTRG.20395.14	2757	coding	PF01633
MSTRG.20395.10	2544	coding	PF01633
MSTRG.20395.11	2485	coding	PF01633
MSTRG.20395.7	2544	coding	PF01633
MSTRG.20395.8	2649	coding	PF01633
MSTRG.20395.12	3014	coding	PF01633
MSTRG.20395.13	2593	coding	PF01633
MSTRG.20395.9	2367	coding	PF01633

MSTRG.20396.1	2277	coding	PF00026
MSTRG.20397.1	1884	coding	PF00026
MSTRG.20396.2	2224	coding	PF00026
MSTRG.20396.3	2070	coding	PF00026
MSTRG.20396.4	1824	coding	PF00026
MSTRG.20390.2	5109	coding	PF10472
MSTRG.20392.1	7055	coding	PF00613
MSTRG.20400.7	657	noncoding	-
MSTRG.20401.1	1732	noncoding	-
MSTRG.20402.1	1375	noncoding	-
MSTRG.20403.1	1203	noncoding	-
MSTRG.20407.1	4555	noncoding	-
MSTRG.20414.2	2845	coding	PF00069
MSTRG.20415.1	2112	noncoding	-
MSTRG.20416.1	4047	coding	PF00069
MSTRG.20421.1	2505	coding	PF00178
MSTRG.20421.2	1954	coding	PF00178
MSTRG.20421.3	5123	coding	PF00178
MSTRG.20424.1	1337	noncoding	-
MSTRG.20426.1	4945	noncoding	-
MSTRG.20426.2	4655	noncoding	-
MSTRG.20429.2	777	coding	PF00071
MSTRG.20430.1	204	noncoding	-
MSTRG.20433.4	3560	coding	PF00620
MSTRG.20434.1	5323	noncoding	-
MSTRG.20433.7	1312	noncoding	-
MSTRG.20431.2	4535	noncoding	-
MSTRG.20431.3	1576	noncoding	-
MSTRG.20431.4	4388	noncoding	-
MSTRG.20438.4	1319	coding	PF00069
MSTRG.20439.1	15062	coding	PF00069
MSTRG.20439.2	5018	coding	PF00069
MSTRG.20439.3	3005	noncoding	-
MSTRG.20440.1	12225	noncoding	-
MSTRG.20440.2	5872	noncoding	-
MSTRG.20446.2	2186	coding	PF07686
MSTRG.20451.2	10263	coding	PF01591
MSTRG.20452.1	292	noncoding	-
MSTRG.20453.1	2197	coding	PF00084
MSTRG.20455.2	1068	noncoding	-
MSTRG.20457.2	3257	noncoding	-
MSTRG.20457.4	4749	noncoding	-
MSTRG.20457.7	3228	noncoding	-
MSTRG.20457.6	3904	noncoding	-
MSTRG.20457.8	2394	noncoding	-
MSTRG.20469.1	988	noncoding	-
MSTRG.20469.2	1075	noncoding	-
MSTRG.20471.1	3702	noncoding	-
MSTRG.20471.2	1573	noncoding	-
MSTRG.20473.2	811	noncoding	-
MSTRG.20474.1	298	noncoding	-
MSTRG.20476.1	677	noncoding	-
MSTRG.20482.6	3990	noncoding	-
MSTRG.20483.1	8118	noncoding	-

MSTRG.20484.1	3180	noncoding	-
MSTRG.20486.1	1001	noncoding	-
MSTRG.20479.1	703	noncoding	-
MSTRG.20489.1	2951	noncoding	-
MSTRG.20504.2	991	noncoding	-
MSTRG.20505.1	3616	coding	PF05731
MSTRG.20506.1	1498	coding	PF01088
MSTRG.20512.1	5212	coding	PF09762
MSTRG.20512.2	668	noncoding	-
MSTRG.20512.3	1900	noncoding	-
MSTRG.20512.4	1543	noncoding	-
MSTRG.20512.5	3974	noncoding	-
MSTRG.20512.6	1685	noncoding	-
MSTRG.20514.1	441	noncoding	-
MSTRG.20516.1	222	noncoding	-
MSTRG.20526.1	2703	noncoding	-
MSTRG.20528.1	4369	noncoding	-
MSTRG.20547.1	1154	noncoding	-
MSTRG.20552.1	650	noncoding	-
MSTRG.20565.1	395	noncoding	-
MSTRG.20566.1	1251	noncoding	-
MSTRG.20568.2	3106	coding	PF02214
MSTRG.20569.1	486	noncoding	-
MSTRG.20570.1	540	noncoding	-
MSTRG.20571.1	1668	noncoding	-
MSTRG.20574.1	968	noncoding	-
MSTRG.20577.2	3852	coding	PF00105
MSTRG.20577.3	2433	noncoding	-
MSTRG.20579.2	4341	coding	PF01585
MSTRG.20579.4	4846	coding	PF01585
MSTRG.20580.1	1232	noncoding	-
MSTRG.20581.1	2709	noncoding	-
MSTRG.20583.1	6247	noncoding	-
MSTRG.20584.1	820	noncoding	-
MSTRG.20593.2	7179	noncoding	-
MSTRG.20593.3	3061	noncoding	-
MSTRG.20608.7	1934	coding	PF00069
MSTRG.20608.6	4264	coding	PF00069
MSTRG.20609.1	8063	coding	PF02994
MSTRG.20610.1	10253	noncoding	-
MSTRG.20608.8	5779	coding	PF00069
MSTRG.20611.1	3372	coding	PF02994
MSTRG.20612.1	5346	noncoding	-
MSTRG.20608.9	10381	coding	PF00069
MSTRG.20603.1	4484	noncoding	-
MSTRG.20603.2	3622	noncoding	-
MSTRG.20603.3	10394	coding	PF03476
MSTRG.20613.1	1514	coding	PF00782
MSTRG.20613.2	1424	coding	PF00782
MSTRG.20614.1	346	noncoding	-
MSTRG.20616.1	516	noncoding	-
MSTRG.20618.2	2405	noncoding	-
MSTRG.20620.2	292	noncoding	-
MSTRG.20619.4	3387	noncoding	-

MSTRG.20621.1	8279	coding	PF14186
MSTRG.20628.2	3761	coding	PF00648
MSTRG.20629.1	322	noncoding	-
MSTRG.20629.2	368	noncoding	-
MSTRG.20631.2	2692	noncoding	-
MSTRG.20631.3	5980	noncoding	-
MSTRG.20634.1	3512	coding	PF00004
MSTRG.20634.2	2801	coding	PF00004
MSTRG.20634.3	1420	noncoding	-
MSTRG.20637.3	4275	noncoding	-
MSTRG.20637.5	2330	coding	PF00400
MSTRG.20637.4	2850	coding	PF00400
MSTRG.20638.1	1547	noncoding	-
MSTRG.20642.1	1613	coding	PF03311
MSTRG.20654.1	252	noncoding	-
MSTRG.20654.2	14871	coding	PF03028
MSTRG.20654.4	2471	noncoding	-
MSTRG.20654.3	2508	noncoding	-
MSTRG.20654.5	2114	noncoding	-
MSTRG.20654.12	1238	noncoding	-
MSTRG.20654.8	7290	noncoding	-
MSTRG.20654.10	3026	noncoding	-
MSTRG.20654.11	12817	coding	PF12774
MSTRG.20654.9	12761	coding	PF12774
MSTRG.20654.6	13939	coding	PF03028
MSTRG.20654.7	14302	coding	PF03028
MSTRG.20654.13	4094	noncoding	-
MSTRG.20656.1	8276	noncoding	-
MSTRG.20657.1	2255	noncoding	-
MSTRG.20658.1	1254	noncoding	-
MSTRG.20654.14	3684	noncoding	-
MSTRG.20660.2	3786	coding	PF01222
MSTRG.20644.1	2774	coding	PF00568
MSTRG.20644.3	6915	coding	PF00568
MSTRG.20644.6	10352	coding	PF00568
MSTRG.20646.1	2133	coding	PF06441
MSTRG.20649.2	5244	noncoding	-
MSTRG.20649.3	3842	noncoding	-
MSTRG.20662.1	425	noncoding	-
MSTRG.20662.2	873	noncoding	-
MSTRG.20662.3	1069	coding	PF00125
MSTRG.20662.4	1195	coding	PF00125
MSTRG.20662.5	1159	coding	PF00125
MSTRG.20662.6	1252	coding	PF00125
MSTRG.20664.1	4391	coding	PF13897
MSTRG.20664.2	3915	coding	PF13897
MSTRG.20667.2	1673	noncoding	-
MSTRG.20668.1	1013	coding	PF13656
MSTRG.20669.2	5350	noncoding	-
MSTRG.20670.1	10076	noncoding	-
MSTRG.20672.6	2803	coding	PF01080
MSTRG.20672.8	4147	coding	PF01080
MSTRG.20674.1	11126	coding	PF00780
MSTRG.20674.3	10189	coding	PF00780

MSTRG.20674.5	7206	coding	PF00780
MSTRG.20674.6	2283	noncoding	-
MSTRG.20678.2	7076	coding	PF16687
MSTRG.20678.3	934	coding	PF16687
MSTRG.20678.4	7086	coding	PF16687
MSTRG.20679.1	1231	noncoding	-
MSTRG.20677.1	7969	noncoding	-
MSTRG.20681.1	563	noncoding	-
MSTRG.20682.1	485	noncoding	-
MSTRG.20682.2	507	noncoding	-
MSTRG.20688.2	3126	noncoding	-
MSTRG.20705.1	821	noncoding	-
MSTRG.20705.2	710	noncoding	-
MSTRG.20710.3	4078	coding	PF05903
MSTRG.20710.5	10269	noncoding	-
MSTRG.20711.1	862	noncoding	-
MSTRG.20713.1	1731	noncoding	-
MSTRG.20708.2	2723	coding	PF00709
MSTRG.20709.1	628	noncoding	-
MSTRG.20714.1	3158	coding	PF00651
MSTRG.20725.3	15327	noncoding	-
MSTRG.20725.5	2400	noncoding	-
MSTRG.20725.4	3766	coding	PF00069
MSTRG.20725.6	3881	coding	PF00069
MSTRG.20725.7	275	noncoding	-
MSTRG.20725.8	3930	coding	PF00069
MSTRG.20725.9	3970	coding	PF00078
MSTRG.20725.10	9170	noncoding	-
MSTRG.20725.11	7240	coding	PF00069
MSTRG.20725.12	4858	coding	PF01359
MSTRG.20725.14	2826	coding	PF00078
MSTRG.20726.1	5275	noncoding	-
MSTRG.20727.1	3102	noncoding	-
MSTRG.20728.1	5172	noncoding	-
MSTRG.20730.12	1449	noncoding	-
MSTRG.20730.6	3939	coding	PF15308
MSTRG.20730.8	5034	coding	PF15308
MSTRG.20730.11	5176	coding	PF15308
MSTRG.20730.10	6718	coding	PF15308
MSTRG.20730.7	6418	coding	PF15308
MSTRG.20730.4	6336	coding	PF15308
MSTRG.20730.5	6394	coding	PF15308
MSTRG.20730.9	5314	coding	PF15308
MSTRG.20730.13	3691	coding	PF15308
MSTRG.20730.14	3178	coding	PF15308
MSTRG.20718.1	949	noncoding	-
MSTRG.20720.2	435	noncoding	-
MSTRG.20734.1	8159	noncoding	-
MSTRG.20740.1	2858	coding	PF00287
MSTRG.20742.2	1536	noncoding	-
MSTRG.20742.3	1756	noncoding	-
MSTRG.20742.4	1297	noncoding	-
MSTRG.20743.1	2182	noncoding	-
MSTRG.20746.1	2526	noncoding	-

MSTRG.20737.1	1859	coding	PF00084
MSTRG.20737.2	2578	noncoding	-
MSTRG.20751.2	1643	noncoding	-
MSTRG.20753.3	3306	noncoding	-
MSTRG.20753.4	3667	noncoding	-
MSTRG.20750.3	3983	coding	PF05804
MSTRG.20750.1	3236	coding	PF05804
MSTRG.20758.1	2198	coding	PF00743
MSTRG.20763.10	729	noncoding	-
MSTRG.20763.8	8589	coding	PF07001
MSTRG.20763.13	7956	noncoding	-
MSTRG.20774.1	8406	noncoding	-
MSTRG.20775.4	2342	coding	PF01031
MSTRG.20775.6	2267	coding	PF01031
MSTRG.20775.7	11435	coding	PF01031
MSTRG.20775.8	10311	coding	PF01031
MSTRG.20775.9	3477	coding	PF00350
MSTRG.20776.1	6726	coding	PF00517
MSTRG.20776.2	6581	coding	PF00517
MSTRG.20777.1	7166	noncoding	-
MSTRG.20778.1	3043	noncoding	-
MSTRG.20780.1	2250	noncoding	-
MSTRG.20781.1	2716	noncoding	-
MSTRG.20782.1	7794	coding	PF00078
MSTRG.20783.1	3945	noncoding	-
MSTRG.20781.2	7510	noncoding	-
MSTRG.20784.1	9598	coding	PF03184
MSTRG.20785.1	2678	noncoding	-
MSTRG.20786.1	3725	noncoding	-
MSTRG.20775.10	25752	coding	PF02212
MSTRG.20787.1	7313	noncoding	-
MSTRG.20787.2	18030	coding	PF01359
MSTRG.20792.1	12543	noncoding	-
MSTRG.20793.1	16596	noncoding	-
MSTRG.20794.1	4076	noncoding	-
MSTRG.20800.1	3445	noncoding	-
MSTRG.20800.2	774	noncoding	-
MSTRG.20801.1	921	noncoding	-
MSTRG.20797.1	632	noncoding	-
MSTRG.20798.4	2058	coding	PF00106
MSTRG.20819.4	14172	coding	PF06650
MSTRG.20819.8	11687	coding	PF06650
MSTRG.20822.1	437	noncoding	-
MSTRG.20816.5	1894	coding	PF15734
MSTRG.20816.3	2013	coding	PF15734
MSTRG.20816.4	1792	coding	PF15734
MSTRG.20816.2	1970	coding	PF15734
MSTRG.20823.3	6703	coding	PF04799
MSTRG.20823.4	4405	coding	PF04799
MSTRG.20823.2	4795	coding	PF04799
MSTRG.20823.1	7715	coding	PF04799
MSTRG.20817.2	1987	coding	PF10222
MSTRG.20817.1	2175	coding	PF10222
MSTRG.20828.4	2645	noncoding	-

MSTRG.20828.5	2916	noncoding	-
MSTRG.20829.1	2804	coding	PF02219
MSTRG.20829.2	2777	coding	PF02219
MSTRG.20829.5	2586	coding	PF02219
MSTRG.20830.4	4619	coding	PF15736
MSTRG.20825.3	1082	coding	PF06396
MSTRG.20825.4	1133	coding	PF06396
MSTRG.20826.2	1744	noncoding	-
MSTRG.20831.1	964	noncoding	-
MSTRG.20832.3	1790	coding	PF04300
MSTRG.20836.2	9878	coding	PF02259
MSTRG.20836.3	8849	coding	PF02259
MSTRG.20836.4	9462	coding	PF02259
MSTRG.20840.1	4183	coding	PF04695
MSTRG.20845.4	7832	coding	PF00225
MSTRG.20845.7	12765	coding	PF00225
MSTRG.20845.6	8100	coding	PF00225
MSTRG.20845.9	4199	noncoding	-
MSTRG.20844.2	2112	noncoding	-
MSTRG.20850.1	18344	coding	PF06384
MSTRG.20850.3	7520	noncoding	-
MSTRG.20852.4	4059	noncoding	-
MSTRG.20854.1	1013	noncoding	-
MSTRG.20854.2	1025	noncoding	-
MSTRG.20866.1	303	noncoding	-
MSTRG.20867.2	1893	coding	PF00083
MSTRG.20873.1	2075	coding	PF00113
MSTRG.20873.2	2033	coding	PF00113
MSTRG.20873.3	1827	coding	PF00113
MSTRG.20873.4	1869	coding	PF00113
MSTRG.20875.3	5173	noncoding	-
MSTRG.20875.2	7772	coding	PF03154
MSTRG.20876.1	6541	noncoding	-
MSTRG.20877.1	2282	noncoding	-
MSTRG.20878.1	15239	noncoding	-
MSTRG.20872.1	488	noncoding	-
MSTRG.20882.1	8400	coding	PF12114
MSTRG.20883.1	1278	noncoding	-
MSTRG.20884.2	2745	noncoding	-
MSTRG.20884.3	3627	noncoding	-
MSTRG.20884.4	1958	noncoding	-
MSTRG.20891.1	21809	noncoding	-
MSTRG.20891.2	5664	noncoding	-
MSTRG.20891.3	4270	noncoding	-
MSTRG.20892.1	5183	noncoding	-
MSTRG.20895.1	4557	noncoding	-
MSTRG.20900.4	1410	noncoding	-
MSTRG.20900.3	5075	coding	PF00651
MSTRG.20912.2	4130	coding	PF04140
MSTRG.20913.3	10947	coding	PF08074
MSTRG.20913.4	5915	coding	PF08074
MSTRG.20913.5	9799	coding	PF08074
MSTRG.20915.1	412	noncoding	-
MSTRG.20919.1	2411	noncoding	-

MSTRG.20914.1	712	noncoding	-
MSTRG.20914.2	499	noncoding	-
MSTRG.20914.3	633	noncoding	-
MSTRG.20923.1	351	noncoding	-
MSTRG.20921.1	409	noncoding	-
MSTRG.20921.2	800	noncoding	-
MSTRG.20932.2	1437	coding	PF15772
MSTRG.20934.1	773	noncoding	-
MSTRG.20930.1	1649	noncoding	-
MSTRG.20943.1	1478	noncoding	-
MSTRG.20944.1	1369	noncoding	-
MSTRG.20940.1	510	noncoding	-
MSTRG.20949.2	2362	coding	PF03630
MSTRG.20951.1	2455	noncoding	-
MSTRG.20952.2	2037	coding	PF03248
MSTRG.20953.4	6396	noncoding	-
MSTRG.20953.2	2185	noncoding	-
MSTRG.20953.5	5355	noncoding	-
MSTRG.20950.2	4405	coding	PF02437
MSTRG.20955.3	1805	coding	PF00069
MSTRG.20955.5	2296	coding	PF00069
MSTRG.20955.4	2050	coding	PF00069
MSTRG.20955.1	2302	coding	PF00069
MSTRG.20957.1	2853	noncoding	-
MSTRG.20959.1	231	noncoding	-
MSTRG.20967.3	5627	coding	PF03151
MSTRG.20967.7	5391	coding	PF03151
MSTRG.20967.9	5234	coding	PF00069
MSTRG.20967.10	4677	coding	PF00069
MSTRG.20967.8	4443	coding	PF00069
MSTRG.20967.11	4969	coding	PF00069
MSTRG.20967.13	3371	coding	PF00069
MSTRG.20967.14	2924	coding	PF00069
MSTRG.20969.2	419	noncoding	-
MSTRG.20963.4	4066	coding	PF06701
MSTRG.20963.5	4054	coding	PF06701
MSTRG.20963.8	3856	coding	PF06701
MSTRG.20963.6	3227	coding	PF06701
MSTRG.20975.2	818	noncoding	-
MSTRG.20979.2	3008	coding	PF00778
MSTRG.20981.2	2471	coding	PF16661
MSTRG.20977.2	3508	noncoding	-
MSTRG.20978.1	1717	noncoding	-
MSTRG.20978.3	5329	noncoding	-
MSTRG.20986.2	4056	coding	PF03133
MSTRG.20986.5	2229	coding	PF03133
MSTRG.20986.1	4020	coding	PF03133
MSTRG.20986.3	4064	coding	PF03133
MSTRG.20986.7	3822	coding	PF03133
MSTRG.20986.8	2690	coding	PF03133
MSTRG.20989.1	1546	noncoding	-
MSTRG.20990.1	1101	noncoding	-
MSTRG.20990.2	1199	noncoding	-
MSTRG.20994.3	7406	coding	PF03146

MSTRG.20999.2	12702	coding	PF07744
MSTRG.20999.3	13652	coding	PF07744
MSTRG.21000.1	1168	noncoding	-
MSTRG.21002.1	5102	noncoding	-
MSTRG.21011.4	2634	coding	PF00498
MSTRG.21017.1	446	noncoding	-
MSTRG.21018.1	3721	noncoding	-
MSTRG.21018.2	3718	noncoding	-
MSTRG.21018.3	1144	noncoding	-
MSTRG.21024.5	9524	noncoding	-
MSTRG.21024.6	9762	noncoding	-
MSTRG.21024.7	10341	noncoding	-
MSTRG.21022.1	350	noncoding	-
MSTRG.21027.4	2335	noncoding	-
MSTRG.21027.6	2375	noncoding	-
MSTRG.21027.5	2088	noncoding	-
MSTRG.21027.8	1972	noncoding	-
MSTRG.21037.2	2010	coding	PF00578
MSTRG.21038.2	6349	noncoding	-
MSTRG.21038.5	1040	noncoding	-
MSTRG.21038.6	6234	noncoding	-
MSTRG.21039.2	2754	noncoding	-
MSTRG.21048.3	11134	noncoding	-
MSTRG.21048.5	4203	noncoding	-
MSTRG.21049.2	10145	noncoding	-
MSTRG.21061.1	8598	coding	PF00566
MSTRG.21061.2	11348	coding	PF00566
MSTRG.21062.1	931	noncoding	-
MSTRG.21061.3	2179	noncoding	-
MSTRG.21066.1	1181	coding	PF09032
MSTRG.21068.3	2915	coding	PF13923
MSTRG.21074.1	5671	noncoding	-
MSTRG.21076.4	808	coding	PF15079
MSTRG.21079.1	1244	noncoding	-
MSTRG.21080.3	7557	coding	PF00617
MSTRG.21080.4	887	noncoding	-
MSTRG.21082.1	1104	noncoding	-
MSTRG.21083.1	3955	noncoding	-
MSTRG.21086.1	772	noncoding	-
MSTRG.21087.3	4825	coding	PF06702
MSTRG.21090.1	2959	coding	PF00078
MSTRG.21089.3	3415	noncoding	-
MSTRG.21096.4	2224	coding	PF03062
MSTRG.21092.2	3581	coding	PF01145
MSTRG.21093.3	3807	coding	PF00567
MSTRG.21104.3	2923	coding	PF05609
MSTRG.21104.4	2543	coding	PF05609
MSTRG.21105.1	2803	noncoding	-
MSTRG.21106.5	4608	coding	PF05609
MSTRG.21106.7	3624	coding	PF05609
MSTRG.21106.6	3686	coding	PF05609
MSTRG.21106.2	4827	coding	PF05609
MSTRG.21106.4	4936	coding	PF05609
MSTRG.21106.8	3405	coding	PF05609

MSTRG.21106.9	3035	coding	PF05609
MSTRG.21106.10	3501	coding	PF05609
MSTRG.21106.11	3216	coding	PF05609
MSTRG.21101.3	4866	noncoding	-
MSTRG.21098.1	9710	noncoding	-
MSTRG.21102.2	1101	coding	PF00887
MSTRG.21107.2	7948	coding	PF03124
MSTRG.21107.3	2574	coding	PF03124
MSTRG.21108.1	7813	noncoding	-
MSTRG.21109.1	3910	noncoding	-
MSTRG.21107.4	1673	coding	PF03124
MSTRG.21111.2	4669	coding	PF15737
MSTRG.21112.1	7031	coding	PF09177
MSTRG.21112.2	6343	coding	PF09177
MSTRG.21115.1	2035	coding	PF05760
MSTRG.21117.1	350	noncoding	-
MSTRG.21120.1	457	noncoding	-
MSTRG.21126.2	4508	coding	PF12796
MSTRG.21123.1	769	noncoding	-
MSTRG.21130.3	4651	noncoding	-
MSTRG.21127.1	8099	coding	PF00055
MSTRG.21127.2	8057	coding	PF00055
MSTRG.21128.1	322	noncoding	-
MSTRG.21146.3	2031	noncoding	-
MSTRG.21147.1	688	noncoding	-
MSTRG.21146.4	1575	noncoding	-
MSTRG.21146.6	1545	noncoding	-
MSTRG.21146.5	8164	noncoding	-
MSTRG.21146.7	2172	noncoding	-
MSTRG.21146.8	956	noncoding	-
MSTRG.21146.9	4099	noncoding	-
MSTRG.21146.10	7677	noncoding	-
MSTRG.21146.11	9405	noncoding	-
MSTRG.21146.12	1461	noncoding	-
MSTRG.21148.10	3725	coding	PF10373
MSTRG.21148.12	3778	coding	PF10373
MSTRG.21148.6	3864	coding	PF10373
MSTRG.21148.8	3844	coding	PF10373
MSTRG.21148.9	4014	coding	PF10373
MSTRG.21134.1	1525	coding	PF01755
MSTRG.21151.1	3100	noncoding	-
MSTRG.21152.1	5069	coding	PF01532
MSTRG.21153.1	6740	noncoding	-
MSTRG.21154.1	3892	noncoding	-
MSTRG.21154.2	761	noncoding	-
MSTRG.21155.1	786	noncoding	-
MSTRG.21156.2	2069	coding	PF16207
MSTRG.21154.3	1305	noncoding	-
MSTRG.21154.4	7780	coding	PF00078
MSTRG.21159.3	3241	coding	PF02005
MSTRG.21159.4	3450	coding	PF02005
MSTRG.21159.5	4963	coding	PF02005
MSTRG.21159.6	2673	coding	PF02005
MSTRG.21159.7	2474	noncoding	-

MSTRG.21162.6	3027	coding	PF01344
MSTRG.21162.7	3471	coding	PF01344
MSTRG.21163.1	778	noncoding	-
MSTRG.21163.2	1878	noncoding	-
MSTRG.21173.1	17106	coding	PF07474
MSTRG.21169.1	1219	noncoding	-
MSTRG.21176.1	451	noncoding	-
MSTRG.21176.2	410	noncoding	-
MSTRG.21179.3	10168	coding	PF10481
MSTRG.21179.2	10181	coding	PF10481
MSTRG.21179.5	10444	coding	PF10481
MSTRG.21179.6	10130	coding	PF10481
MSTRG.21181.2	11119	coding	PF00102
MSTRG.21198.1	1935	coding	PF14822
MSTRG.21198.2	3647	coding	PF14822
MSTRG.21198.3	3571	coding	PF14822
MSTRG.21212.1	4467	noncoding	-
MSTRG.21212.2	3428	noncoding	-
MSTRG.21213.2	3003	noncoding	-
MSTRG.21216.2	4481	noncoding	-
MSTRG.21218.2	1101	noncoding	-
MSTRG.21218.3	873	noncoding	-
MSTRG.21220.3	1917	noncoding	-
MSTRG.21220.4	3431	noncoding	-
MSTRG.21224.1	748	noncoding	-
MSTRG.21235.3	2921	coding	PF06862
MSTRG.21234.1	311	noncoding	-
MSTRG.21244.1	674	noncoding	-
MSTRG.21248.2	900	noncoding	-
MSTRG.21249.1	4384	noncoding	-
MSTRG.21250.2	2594	coding	PF06365
MSTRG.21251.1	813	coding	PF06365
MSTRG.21251.2	1121	coding	PF06365
MSTRG.21252.1	2756	noncoding	-
MSTRG.21252.2	3717	noncoding	-
MSTRG.21254.1	1821	noncoding	-
MSTRG.21256.5	3651	coding	PF00084
MSTRG.21256.6	1274	coding	PF00084
MSTRG.21257.1	603	noncoding	-
MSTRG.21260.1	2338	coding	PF00084
MSTRG.21262.1	928	coding	PF00084
MSTRG.21268.1	1621	noncoding	-
MSTRG.21269.1	5929	coding	PF02210
MSTRG.21269.2	1443	noncoding	-
MSTRG.21271.2	5184	coding	PF02141
MSTRG.21272.1	852	noncoding	-
MSTRG.21278.2	3650	coding	PF00069
MSTRG.21301.3	4695	noncoding	-
MSTRG.21302.1	6382	noncoding	-
MSTRG.21308.1	4842	noncoding	-
MSTRG.21309.2	4856	coding	PF00225
MSTRG.21310.3	2598	coding	PF00270
MSTRG.21315.2	7013	coding	PF08683
MSTRG.21316.1	2625	noncoding	-

MSTRG.21317.1	627	noncoding	-
MSTRG.21318.1	1564	noncoding	-
MSTRG.21315.4	1746	noncoding	-
MSTRG.21314.1	1506	coding	PF11819
MSTRG.21319.1	3238	noncoding	-
MSTRG.21323.1	8362	coding	PF00225
MSTRG.21323.5	7386	coding	PF00225
MSTRG.21323.3	7616	coding	PF00225
MSTRG.21323.8	5789	coding	PF00225
MSTRG.21323.7	5727	coding	PF00225
MSTRG.21323.9	5979	coding	PF00225
MSTRG.21321.1	5069	coding	PF08763
MSTRG.21321.2	4784	coding	PF08763
MSTRG.21321.3	4234	coding	PF08763
MSTRG.21324.2	4329	noncoding	-
MSTRG.21324.3	2866	noncoding	-
MSTRG.21326.1	3755	noncoding	-
MSTRG.21327.3	2647	coding	PF03006
MSTRG.21327.6	2864	coding	PF03006
MSTRG.21327.8	2066	coding	PF03006
MSTRG.21330.2	619	coding	PF00651
MSTRG.21333.4	2019	coding	PF04666
MSTRG.21334.5	6392	coding	PF08429
MSTRG.21334.6	6599	coding	PF08429
MSTRG.21334.7	6460	coding	PF08429
MSTRG.21334.8	6477	coding	PF08429
MSTRG.21334.9	6621	coding	PF08429
MSTRG.21334.10	8186	coding	PF08429
MSTRG.21335.1	8337	noncoding	-
MSTRG.21336.1	4465	noncoding	-
MSTRG.21336.2	4287	noncoding	-
MSTRG.21336.3	3898	noncoding	-
MSTRG.21338.4	18924	coding	PF15898
MSTRG.21338.6	5771	coding	PF15898
MSTRG.21338.7	6460	coding	PF15898
MSTRG.21338.9	1446	noncoding	-
MSTRG.21338.10	2120	noncoding	-
MSTRG.21338.13	5147	coding	PF12796
MSTRG.21338.12	2067	coding	PF12796
MSTRG.21342.1	2531	noncoding	-
MSTRG.21343.3	1203	coding	PF00179
MSTRG.21348.4	5507	coding	PF00102
MSTRG.21348.1	7606	coding	PF00102
MSTRG.21348.2	7609	coding	PF00102
MSTRG.21349.1	2740	coding	PF00102
MSTRG.21351.1	436	noncoding	-
MSTRG.21345.1	3293	coding	PF02466
MSTRG.21371.3	3817	noncoding	-
MSTRG.21371.5	4187	noncoding	-
MSTRG.21371.4	10724	noncoding	-
MSTRG.21371.6	9004	coding	PF00078
MSTRG.21371.7	4031	noncoding	-
MSTRG.21371.9	10632	noncoding	-
MSTRG.21371.10	12793	noncoding	-

MSTRG.21371.11	12995	noncoding	-
MSTRG.21371.13	11745	noncoding	-
MSTRG.21371.12	12997	noncoding	-
MSTRG.21371.15	10983	noncoding	-
MSTRG.21371.16	11651	noncoding	-
MSTRG.21371.17	10706	noncoding	-
MSTRG.21371.18	9680	noncoding	-
MSTRG.21372.1	9582	noncoding	-
MSTRG.21373.1	863	noncoding	-
MSTRG.21372.2	5892	noncoding	-
MSTRG.21356.1	1251	noncoding	-
MSTRG.21359.1	2841	noncoding	-
MSTRG.21361.1	3362	coding	PF02460
MSTRG.21362.1	1752	noncoding	-
MSTRG.21363.1	1968	coding	PF14802
MSTRG.21368.2	3197	coding	PF00651
MSTRG.21368.3	3783	coding	PF07707
MSTRG.21369.1	1755	noncoding	-
MSTRG.21377.1	238	noncoding	-
MSTRG.21375.1	420	noncoding	-
MSTRG.21384.1	1669	noncoding	-
MSTRG.21385.5	4682	noncoding	-
MSTRG.21385.4	2042	coding	PF14709
MSTRG.21385.7	9977	noncoding	-
MSTRG.21385.8	1648	noncoding	-
MSTRG.21385.9	4380	coding	PF14709
MSTRG.21385.10	847	noncoding	-
MSTRG.21395.3	983	coding	PF03962
MSTRG.21396.3	5041	coding	PF00630
MSTRG.21396.4	3721	coding	PF00630
MSTRG.21406.1	5012	noncoding	-
MSTRG.21406.2	2428	noncoding	-
MSTRG.21400.1	817	noncoding	-
MSTRG.21402.1	780	noncoding	-
MSTRG.21403.1	989	noncoding	-
MSTRG.21414.1	966	noncoding	-
MSTRG.21415.1	10457	noncoding	-
MSTRG.21416.1	3776	noncoding	-
MSTRG.21433.1	7479	coding	PF00018
MSTRG.21433.4	5227	coding	PF00018
MSTRG.21433.3	7569	coding	PF00018
MSTRG.21433.5	12169	coding	PF00018
MSTRG.21433.6	24139	coding	PF01015
MSTRG.21433.7	5719	coding	PF00018
MSTRG.21433.8	7582	coding	PF00018
MSTRG.21433.9	17628	noncoding	-
MSTRG.21434.1	917	coding	PF00089
MSTRG.21433.10	9120	coding	PF00018
MSTRG.21433.11	25980	coding	PF00078
MSTRG.21433.13	5087	coding	PF00018
MSTRG.21433.14	5883	coding	PF00018
MSTRG.21433.15	5695	coding	PF00018
MSTRG.21433.16	5340	coding	PF00018
MSTRG.21433.18	5060	coding	PF00018

MSTRG.21433.19	3691	noncoding	-
MSTRG.21433.20	7095	coding	PF00018
MSTRG.21433.22	2199	noncoding	-
MSTRG.21441.2	715	coding	PF01015
MSTRG.21419.1	10069	coding	PF02138
MSTRG.21425.1	292	noncoding	-
MSTRG.21427.2	1400	noncoding	-
MSTRG.21427.1	2889	noncoding	-
MSTRG.21428.1	330	noncoding	-
MSTRG.21429.1	797	noncoding	-
MSTRG.21432.1	486	noncoding	-
MSTRG.21449.1	8328	coding	PF16746
MSTRG.21449.4	2896	coding	PF00620
MSTRG.21450.1	10910	noncoding	-
MSTRG.21450.2	2412	noncoding	-
MSTRG.21451.1	1616	noncoding	-
MSTRG.21449.5	12673	noncoding	-
MSTRG.21452.1	15114	noncoding	-
MSTRG.21453.1	26598	noncoding	-
MSTRG.21454.1	11154	noncoding	-
MSTRG.21454.2	2837	noncoding	-
MSTRG.21446.2	2728	noncoding	-
MSTRG.21447.4	1946	coding	PF03619
MSTRG.21447.2	2112	coding	PF03619
MSTRG.21471.1	7813	noncoding	-
MSTRG.21471.3	7437	noncoding	-
MSTRG.21472.1	388	noncoding	-
MSTRG.21470.3	903	coding	PF15849
MSTRG.21474.3	1419	coding	PF03308
MSTRG.21484.4	7211	noncoding	-
MSTRG.21486.2	1229	coding	PF03256
MSTRG.21502.2	6716	noncoding	-
MSTRG.21502.7	4853	noncoding	-
MSTRG.21502.4	6099	noncoding	-
MSTRG.21502.6	5009	noncoding	-
MSTRG.21502.5	6137	noncoding	-
MSTRG.21503.1	905	noncoding	-
MSTRG.21511.1	2897	noncoding	-
MSTRG.21520.1	2502	noncoding	-
MSTRG.21526.1	1250	noncoding	-
MSTRG.21533.1	377	noncoding	-
MSTRG.21534.1	322	noncoding	-
MSTRG.21532.2	3190	noncoding	-
MSTRG.21535.1	10088	noncoding	-
MSTRG.21532.3	8554	noncoding	-
MSTRG.21536.1	395	noncoding	-
MSTRG.21537.1	2409	noncoding	-
MSTRG.21532.4	6791	noncoding	-
MSTRG.21538.1	5785	noncoding	-
MSTRG.21499.2	1069	coding	PF13639
MSTRG.21499.3	949	coding	PF13639
MSTRG.21552.1	6070	noncoding	-
MSTRG.21553.1	8609	noncoding	-
MSTRG.21555.1	4778	noncoding	-

MSTRG.21556.1	6135	noncoding	-
MSTRG.21569.1	857	coding	PF15088
MSTRG.21572.1	939	noncoding	-
MSTRG.21572.2	1617	noncoding	-
MSTRG.21573.5	10219	coding	PF00178
MSTRG.21573.6	3263	coding	PF00178
MSTRG.21573.7	4249	coding	PF00178
MSTRG.21573.9	3814	coding	PF00178
MSTRG.21573.14	3576	coding	PF00178
MSTRG.21575.1	1996	noncoding	-
MSTRG.21580.1	5537	noncoding	-
MSTRG.21580.2	3122	noncoding	-
MSTRG.21580.3	1571	noncoding	-
MSTRG.21598.1	292	noncoding	-
MSTRG.21612.1	215	noncoding	-
MSTRG.21649.1	1114	noncoding	-
MSTRG.21656.9	13151	coding	PF13832
MSTRG.21657.1	385	noncoding	-
MSTRG.21670.1	1541	noncoding	-
MSTRG.21670.2	412	noncoding	-
MSTRG.21695.4	3326	coding	PF05383
MSTRG.21696.1	11003	coding	PF02994
MSTRG.21695.6	2855	coding	PF05383
MSTRG.21697.1	1758	noncoding	-
MSTRG.21695.7	2866	coding	PF05383
MSTRG.21698.1	2484	noncoding	-
MSTRG.21695.10	2078	coding	PF05383
MSTRG.21708.1	209	noncoding	-
MSTRG.21674.1	889	noncoding	-
MSTRG.21676.1	1066	noncoding	-
MSTRG.21680.1	946	noncoding	-
MSTRG.21729.1	717	noncoding	-
MSTRG.21725.1	1100	noncoding	-
MSTRG.21739.1	1603	noncoding	-
MSTRG.21741.2	907	noncoding	-
MSTRG.21741.1	1276	noncoding	-
MSTRG.21749.1	4442	coding	PF00004
MSTRG.21749.4	2710	coding	PF00004
MSTRG.21749.5	14224	noncoding	-
MSTRG.21749.6	11715	noncoding	-
MSTRG.21749.7	358	noncoding	-
MSTRG.21751.1	1860	noncoding	-
MSTRG.21752.1	955	noncoding	-
MSTRG.21749.8	2194	noncoding	-
MSTRG.21753.1	5860	noncoding	-
MSTRG.21754.1	2362	noncoding	-
MSTRG.21754.2	4388	noncoding	-
MSTRG.21749.9	2924	coding	PF00004
MSTRG.21755.1	609	noncoding	-
MSTRG.21755.2	909	coding	PF00004
MSTRG.21760.1	3232	coding	PF00167
MSTRG.21771.6	15724	coding	PF02137
MSTRG.21772.1	10782	noncoding	-
MSTRG.21771.19	2786	coding	PF02137

MSTRG.21771.20	4952	coding	PF02137
MSTRG.21771.21	2525	coding	PF02137
MSTRG.21771.22	3056	coding	PF02137
MSTRG.21771.23	1921	coding	PF02137
MSTRG.21773.1	2336	noncoding	-
MSTRG.21775.1	1223	coding	PF10479
MSTRG.21771.24	15507	coding	PF10479
MSTRG.21771.25	15832	coding	PF10479
MSTRG.21771.26	15630	coding	PF10479
MSTRG.21771.27	15167	coding	PF10479
MSTRG.21771.28	5052	noncoding	-
MSTRG.21771.29	3389	noncoding	-
MSTRG.21763.1	391	noncoding	-
MSTRG.21779.2	6932	coding	PF00617
MSTRG.21779.1	6981	coding	PF00617
MSTRG.21790.1	3571	coding	PF05187
MSTRG.21791.2	1709	noncoding	-
MSTRG.21795.1	1560	coding	PF07857
MSTRG.21795.3	4460	coding	PF07857
MSTRG.21795.4	1303	coding	PF07857
MSTRG.21795.5	1808	coding	PF07857
MSTRG.21795.6	1697	coding	PF07857
MSTRG.21796.1	1019	noncoding	-
MSTRG.21797.1	4461	coding	PF15051
MSTRG.21797.2	4862	coding	PF15051
MSTRG.21794.1	209	noncoding	-
MSTRG.21806.2	1158	coding	PF00211
MSTRG.21812.2	4081	coding	PF01352
MSTRG.21812.1	1507	coding	PF01352
MSTRG.21812.4	6295	coding	PF01352
MSTRG.21808.2	3180	coding	PF01352
MSTRG.21815.1	826	noncoding	-
MSTRG.21817.2	610	noncoding	-
MSTRG.21818.3	1034	coding	PF16564
MSTRG.21819.1	2064	noncoding	-
MSTRG.21820.1	11129	noncoding	-
MSTRG.21820.2	2074	noncoding	-
MSTRG.21827.2	1738	noncoding	-
MSTRG.21827.3	2027	coding	PF04117
MSTRG.21829.2	8261	coding	PF08490
MSTRG.21829.3	8625	coding	PF08490
MSTRG.21831.1	4588	coding	PF15336
MSTRG.21831.10	4516	coding	PF15336
MSTRG.21831.11	4618	coding	PF15336
MSTRG.21831.3	4690	coding	PF15336
MSTRG.21831.5	4621	coding	PF15336
MSTRG.21831.7	4552	coding	PF15336
MSTRG.21831.9	3079	coding	PF15336
MSTRG.21831.12	17445	noncoding	-
MSTRG.21831.13	13964	noncoding	-
MSTRG.21831.14	5269	noncoding	-
MSTRG.21830.1	773	noncoding	-
MSTRG.21834.3	2757	coding	PF03914
MSTRG.21834.2	2754	coding	PF03914

MSTRG.21837.5	11865	coding	PF15790
MSTRG.21837.6	11622	coding	PF15790
MSTRG.21837.8	11868	coding	PF15790
MSTRG.21837.9	11701	coding	PF15790
MSTRG.21842.2	3303	coding	PF09750
MSTRG.21842.6	4546	coding	PF01359
MSTRG.21841.1	578	noncoding	-
MSTRG.21846.1	4511	coding	PF00002
MSTRG.21846.2	3091	noncoding	-
MSTRG.21846.3	1775	noncoding	-
MSTRG.21856.2	1977	coding	PF00071
MSTRG.21863.1	2314	noncoding	-
MSTRG.21864.2	6719	coding	PF02171
MSTRG.21865.1	6173	noncoding	-
MSTRG.21850.3	2630	noncoding	-
MSTRG.21850.2	2851	noncoding	-
MSTRG.21867.1	1978	coding	PF16070
MSTRG.21870.1	419	noncoding	-
MSTRG.21874.1	2866	noncoding	-
MSTRG.21876.1	642	noncoding	-
MSTRG.21880.1	8412	coding	PF16070
MSTRG.21880.2	8587	coding	PF16070
MSTRG.21880.3	6757	coding	PF16070
MSTRG.21880.4	8243	noncoding	-
MSTRG.21880.5	42836	noncoding	-
MSTRG.21880.6	38687	noncoding	-
MSTRG.21881.1	258	noncoding	-
MSTRG.21880.7	17189	noncoding	-
MSTRG.21880.8	32669	noncoding	-
MSTRG.21880.9	22949	noncoding	-
MSTRG.21885.4	1056	coding	PF14965
MSTRG.21885.3	1437	coding	PF14965
MSTRG.21886.1	2767	noncoding	-
MSTRG.21887.1	5437	coding	PF07717
MSTRG.21889.1	1427	noncoding	-
MSTRG.21890.2	4894	coding	PF00240
MSTRG.21890.3	1563	coding	PF00240
MSTRG.21890.4	3842	coding	PF00240
MSTRG.21890.5	2475	coding	PF00240
MSTRG.21890.6	4214	coding	PF00240
MSTRG.21890.7	2931	coding	PF00240
MSTRG.21890.8	2912	coding	PF00240
MSTRG.21890.12	880	coding	PF00240
MSTRG.21890.20	1336	coding	PF00240
MSTRG.21890.15	954	coding	PF00240
MSTRG.21890.19	2810	coding	PF00240
MSTRG.21890.10	999	coding	PF00240
MSTRG.21890.21	2319	coding	PF00240
MSTRG.21890.22	984	coding	PF00240
MSTRG.21882.1	680	noncoding	-
MSTRG.21883.1	1122	noncoding	-
MSTRG.21897.10	9004	noncoding	-
MSTRG.21897.11	8838	noncoding	-
MSTRG.21897.12	8844	noncoding	-

MSTRG.21897.13	8993	noncoding	-
MSTRG.21897.15	8888	noncoding	-
MSTRG.21897.16	8838	noncoding	-
MSTRG.21897.17	9003	noncoding	-
MSTRG.21897.18	8936	noncoding	-
MSTRG.21897.3	8844	noncoding	-
MSTRG.21897.4	8841	noncoding	-
MSTRG.21897.5	8703	noncoding	-
MSTRG.21897.6	8892	noncoding	-
MSTRG.21897.7	8885	noncoding	-
MSTRG.21897.8	8892	noncoding	-
MSTRG.21897.9	8895	noncoding	-
MSTRG.21898.2	1703	noncoding	-
MSTRG.21898.1	1628	noncoding	-
MSTRG.21899.1	868	noncoding	-
MSTRG.21897.19	9187	noncoding	-
MSTRG.21897.20	8292	noncoding	-
MSTRG.21897.21	18860	noncoding	-
MSTRG.21897.22	7367	noncoding	-
MSTRG.21897.23	6942	noncoding	-
MSTRG.21897.24	6766	noncoding	-
MSTRG.21897.25	6867	noncoding	-
MSTRG.21897.26	5951	noncoding	-
MSTRG.21902.1	1226	noncoding	-
MSTRG.21903.2	4305	noncoding	-
MSTRG.21903.5	1211	noncoding	-
MSTRG.21903.3	876	noncoding	-
MSTRG.21903.4	3568	noncoding	-
MSTRG.21903.7	793	noncoding	-
MSTRG.21905.2	5067	noncoding	-
MSTRG.21905.3	11573	noncoding	-
MSTRG.21905.6	20743	noncoding	-
MSTRG.21907.2	4936	coding	PF01496
MSTRG.21907.3	5461	coding	PF01496
MSTRG.21912.1	2934	noncoding	-
MSTRG.21912.2	1658	noncoding	-
MSTRG.21900.5	1198	coding	PF00856
MSTRG.21916.3	5032	coding	PF13872
MSTRG.21917.1	1741	noncoding	-
MSTRG.21918.2	1221	coding	PF09806
MSTRG.21919.4	974	coding	PF00472
MSTRG.21919.5	564	noncoding	-
MSTRG.21920.7	4507	noncoding	-
MSTRG.21925.1	6705	coding	PF02121
MSTRG.21926.1	485	noncoding	-
MSTRG.21925.2	4075	coding	PF02121
MSTRG.21925.3	8333	coding	PF02121
MSTRG.21927.1	406	noncoding	-
MSTRG.21927.2	685	noncoding	-
MSTRG.21925.4	6940	coding	PF02121
MSTRG.21925.5	7570	coding	PF02121
MSTRG.21928.2	1368	coding	PF10500
MSTRG.21921.2	2554	noncoding	-
MSTRG.21932.2	2628	noncoding	-

MSTRG.21932.3	2445	noncoding	-
MSTRG.21934.2	928	noncoding	-
MSTRG.21935.2	430	coding	PF00240
MSTRG.21941.1	7981	coding	PF10493
MSTRG.21941.2	8253	coding	PF10493
MSTRG.21942.1	2280	noncoding	-
MSTRG.21942.5	2292	noncoding	-
MSTRG.21939.2	5881	coding	PF01302
MSTRG.21940.1	1287	noncoding	-
MSTRG.21939.3	8173	coding	PF01302
MSTRG.21939.4	8106	coding	PF01302
MSTRG.21943.1	2105	noncoding	-
MSTRG.21943.2	2099	noncoding	-
MSTRG.21949.1	3714	coding	PF04714
MSTRG.21953.1	306	noncoding	-
MSTRG.21955.1	910	noncoding	-
MSTRG.21958.4	683	noncoding	-
MSTRG.21958.5	1010	noncoding	-
MSTRG.21958.10	1227	noncoding	-
MSTRG.21958.3	739	noncoding	-
MSTRG.21958.6	897	noncoding	-
MSTRG.21958.7	1124	noncoding	-
MSTRG.21958.8	966	noncoding	-
MSTRG.21958.9	1043	noncoding	-
MSTRG.21959.1	767	coding	PF01242
MSTRG.21961.2	3613	coding	PF16866
MSTRG.21961.3	3622	coding	PF16866
MSTRG.21961.4	2790	noncoding	-
MSTRG.21961.5	2916	noncoding	-
MSTRG.21963.3	1606	coding	PF12862
MSTRG.21977.2	1882	coding	PF00864
MSTRG.21978.2	2460	noncoding	-
MSTRG.21980.1	1715	noncoding	-
MSTRG.21985.2	5931	coding	PF00122
MSTRG.21985.4	5483	coding	PF00122
MSTRG.21985.5	5957	coding	PF00122
MSTRG.21985.6	3743	coding	PF00122
MSTRG.21985.7	3953	coding	PF00122
MSTRG.21985.8	9683	coding	PF00689
MSTRG.21987.2	7021	noncoding	-
MSTRG.21987.3	1700	noncoding	-
MSTRG.21965.1	2349	coding	PF04062
MSTRG.21965.2	801	coding	PF04062
MSTRG.21966.1	1157	noncoding	-
MSTRG.21967.1	1100	coding	PF15107
MSTRG.21967.2	1094	coding	PF15107
MSTRG.21967.4	1021	coding	PF15107
MSTRG.21967.5	1027	coding	PF15107
MSTRG.21967.6	1099	coding	PF15107
MSTRG.21968.3	775	noncoding	-
MSTRG.21971.1	8233	coding	PF07773
MSTRG.21971.2	7731	coding	PF07773
MSTRG.21972.1	1269	coding	PF16799
MSTRG.21994.4	4981	coding	PF00017

MSTRG.21994.3	4984	coding	PF00017
MSTRG.21994.5	3498	coding	PF00017
MSTRG.21996.12	3135	noncoding	-
MSTRG.21996.17	10488	noncoding	-
MSTRG.21996.14	5533	coding	PF14438
MSTRG.21996.16	3668	coding	PF14438
MSTRG.21996.9	4222	coding	PF14438
MSTRG.21996.10	4067	coding	PF14438
MSTRG.21996.13	5085	coding	PF14438
MSTRG.21996.6	3851	coding	PF14438
MSTRG.21996.8	3995	coding	PF14438
MSTRG.21996.15	3953	coding	PF14438
MSTRG.21997.1	3413	noncoding	-
MSTRG.22002.2	4157	coding	PF07576
MSTRG.22005.4	3814	coding	PF00441
MSTRG.22008.1	8009	noncoding	-
MSTRG.22008.2	20159	noncoding	-
MSTRG.22008.3	1687	noncoding	-
MSTRG.22010.3	10129	coding	PF00069
MSTRG.22010.4	8601	coding	PF00069
MSTRG.22010.5	8725	coding	PF00069
MSTRG.22021.1	1502	noncoding	-
MSTRG.22014.1	701	noncoding	-
MSTRG.22026.1	3283	noncoding	-
MSTRG.22028.1	10582	noncoding	-
MSTRG.22027.4	3447	noncoding	-
MSTRG.22029.1	1527	noncoding	-
MSTRG.22027.6	3956	coding	PF00069
MSTRG.22034.2	6953	coding	PF00400
MSTRG.22034.5	4270	coding	PF00400
MSTRG.22030.3	8021	coding	PF02898
MSTRG.22030.2	9162	coding	PF02898
MSTRG.22030.4	9177	coding	PF02898
MSTRG.22038.3	3351	coding	PF08755
MSTRG.22041.3	2411	noncoding	-
MSTRG.22041.4	1864	noncoding	-
MSTRG.22042.1	461	noncoding	-
MSTRG.22043.1	3311	coding	PF12937
MSTRG.22043.3	1309	noncoding	-
MSTRG.22043.4	2263	coding	PF12937
MSTRG.22045.2	3852	noncoding	-
MSTRG.22046.2	14928	coding	PF10218
MSTRG.22050.3	7878	coding	PF11597
MSTRG.22051.1	955	noncoding	-
MSTRG.22051.2	966	noncoding	-
MSTRG.22050.4	2428	noncoding	-
MSTRG.22056.1	764	noncoding	-
MSTRG.22058.1	619	noncoding	-
MSTRG.22063.3	5109	coding	PF00076
MSTRG.22063.4	3448	coding	PF00076
MSTRG.22063.5	2369	coding	PF00076
MSTRG.22059.1	963	noncoding	-
MSTRG.22059.2	401	noncoding	-
MSTRG.22064.3	2634	coding	PF04916

MSTRG.22064.4	2763	coding	PF04916
MSTRG.22065.2	1886	noncoding	-
MSTRG.22068.1	3249	coding	PF00270
MSTRG.22071.2	1711	noncoding	-
MSTRG.22072.1	5038	noncoding	-
MSTRG.22072.3	2021	noncoding	-
MSTRG.22074.1	438	noncoding	-
MSTRG.22070.1	4589	coding	PF10421
MSTRG.22070.2	4939	coding	PF10421
MSTRG.22075.2	4574	coding	PF02318
MSTRG.22075.3	4818	coding	PF02318
MSTRG.22075.4	4570	coding	PF02318
MSTRG.22076.1	260	noncoding	-
MSTRG.22078.3	2721	coding	PF00102
MSTRG.22080.1	1057	coding	PF01159
MSTRG.22080.6	1110	coding	PF01159
MSTRG.22113.6	11472	noncoding	-
MSTRG.22113.5	15602	coding	PF00632
MSTRG.22113.7	11653	noncoding	-
MSTRG.22114.1	3722	noncoding	-
MSTRG.22113.9	9757	noncoding	-
MSTRG.22115.3	3088	noncoding	-
MSTRG.22116.1	1539	noncoding	-
MSTRG.22117.1	534	noncoding	-
MSTRG.22118.4	3186	coding	PF09797
MSTRG.22118.2	4982	coding	PF09797
MSTRG.22118.3	4271	coding	PF09797
MSTRG.22118.5	3907	coding	PF09797
MSTRG.22126.1	3598	coding	PF01421
MSTRG.22126.2	3488	coding	PF01421
MSTRG.22126.3	6169	coding	PF01421
MSTRG.22126.4	3486	coding	PF01421
MSTRG.22126.5	3053	coding	PF01421
MSTRG.22126.10	3150	coding	PF01421
MSTRG.22082.3	9223	noncoding	-
MSTRG.22082.5	7693	noncoding	-
MSTRG.22082.4	7858	noncoding	-
MSTRG.22084.2	10290	coding	PF03535
MSTRG.22086.1	704	coding	PF02146
MSTRG.22089.3	3094	noncoding	-
MSTRG.22089.4	4943	coding	PF00076
MSTRG.22089.5	1469	noncoding	-
MSTRG.22091.1	668	coding	PF01221
MSTRG.22091.3	747	coding	PF01221
MSTRG.22098.2	3015	coding	PF01900
MSTRG.22099.2	1477	coding	PF13499
MSTRG.22103.2	4016	coding	PF05351
MSTRG.22139.5	2201	coding	PF01412
MSTRG.22139.6	3140	coding	PF12205
MSTRG.22141.1	1740	noncoding	-
MSTRG.22141.2	3380	noncoding	-
MSTRG.22141.3	780	noncoding	-
MSTRG.22142.1	1796	coding	PF08718
MSTRG.22145.2	877	coding	PF01923

MSTRG.22146.1	7258	coding	PF00632
MSTRG.22146.2	7220	coding	PF00632
MSTRG.22146.3	6868	coding	PF00632
MSTRG.22147.2	3547	coding	PF02214
MSTRG.22147.3	4322	coding	PF02214
MSTRG.22155.1	8805	coding	PF08326
MSTRG.22155.2	8705	coding	PF08326
MSTRG.22155.4	7651	coding	PF08326
MSTRG.22155.3	7527	coding	PF08326
MSTRG.22155.7	918	coding	PF00289
MSTRG.22150.1	2240	noncoding	-
MSTRG.22152.2	8043	coding	PF00782
MSTRG.22152.3	8091	coding	PF00782
MSTRG.22152.5	9084	coding	PF00782
MSTRG.22153.1	3803	coding	PF08953
MSTRG.22168.1	5539	noncoding	-
MSTRG.22179.1	29808	coding	PF02994
MSTRG.22179.2	7590	coding	PF15229
MSTRG.22179.3	12282	noncoding	-
MSTRG.22179.4	20255	noncoding	-
MSTRG.22179.5	19288	noncoding	-
MSTRG.22179.7	8547	noncoding	-
MSTRG.22179.8	3450	noncoding	-
MSTRG.22179.9	4625	noncoding	-
MSTRG.22179.6	8815	noncoding	-
MSTRG.22179.10	8974	noncoding	-
MSTRG.22179.11	8785	noncoding	-
MSTRG.22179.13	9001	noncoding	-
MSTRG.22179.12	5255	noncoding	-
MSTRG.22179.14	2654	noncoding	-
MSTRG.22179.15	31423	noncoding	-
MSTRG.22179.16	32402	noncoding	-
MSTRG.22181.1	10550	noncoding	-
MSTRG.22181.2	11045	noncoding	-
MSTRG.22181.3	10692	noncoding	-
MSTRG.22181.4	6918	noncoding	-
MSTRG.22181.5	36338	coding	PF02171
MSTRG.22181.6	20101	coding	PF02171
MSTRG.22182.1	3332	coding	PF02171
MSTRG.22182.3	2622	coding	PF02171
MSTRG.22181.7	7829	coding	PF02171
MSTRG.22184.1	11115	coding	PF15327
MSTRG.22184.2	7108	noncoding	-
MSTRG.22184.3	11155	coding	PF15327
MSTRG.22184.5	18177	coding	PF15327
MSTRG.22184.6	11522	coding	PF15327
MSTRG.22185.1	3880	noncoding	-
MSTRG.22170.2	1892	coding	PF00030
MSTRG.22172.3	1271	coding	PF00069
MSTRG.22175.1	8156	noncoding	-
MSTRG.22175.2	7384	noncoding	-
MSTRG.22189.2	1004	noncoding	-
MSTRG.22190.1	1841	noncoding	-
MSTRG.22190.2	4986	noncoding	-

MSTRG.22191.2	4051	noncoding	-
MSTRG.22192.3	4241	noncoding	-
MSTRG.22195.3	2014	noncoding	-
MSTRG.22195.2	2159	noncoding	-
MSTRG.22178.2	1259	noncoding	-
MSTRG.22178.3	9445	noncoding	-
MSTRG.22188.1	991	noncoding	-
MSTRG.22197.1	347	noncoding	-
MSTRG.22219.1	10386	coding	PF02121
MSTRG.22220.1	594	noncoding	-
MSTRG.22219.2	1398	coding	PF02121
MSTRG.22219.5	4918	coding	PF02121
MSTRG.22219.3	6664	coding	PF02121
MSTRG.22223.5	1672	noncoding	-
MSTRG.22224.1	8487	noncoding	-
MSTRG.22226.1	19619	noncoding	-
MSTRG.22228.1	23063	noncoding	-
MSTRG.22228.2	1179	noncoding	-
MSTRG.22223.6	4163	noncoding	-
MSTRG.22230.1	2053	noncoding	-
MSTRG.22199.1	492	noncoding	-
MSTRG.22200.1	3665	noncoding	-
MSTRG.22203.2	1009	coding	PF15578
MSTRG.22204.1	894	noncoding	-
MSTRG.22205.1	1889	coding	PF00051
MSTRG.22205.2	1736	coding	PF01822
MSTRG.22205.4	2602	coding	PF01822
MSTRG.22207.3	1840	noncoding	-
MSTRG.22207.7	1752	noncoding	-
MSTRG.22207.9	1731	noncoding	-
MSTRG.22208.4	1984	noncoding	-
MSTRG.22211.5	2562	coding	PF09066
MSTRG.22212.2	2577	coding	PF09766
MSTRG.22212.3	2257	coding	PF09766
MSTRG.22213.4	2745	coding	PF07978
MSTRG.22213.5	3106	coding	PF07978
MSTRG.22213.2	3682	coding	PF07978
MSTRG.22213.3	3278	coding	PF07978
MSTRG.22214.4	3320	noncoding	-
MSTRG.22216.7	2605	noncoding	-
MSTRG.22217.1	1880	noncoding	-
MSTRG.22238.4	9632	noncoding	-
MSTRG.22238.6	9104	noncoding	-
MSTRG.22238.7	4116	noncoding	-
MSTRG.22239.1	10653	noncoding	-
MSTRG.22240.1	1754	noncoding	-
MSTRG.22241.1	1271	coding	PF02301
MSTRG.22242.2	1734	coding	PF13840
MSTRG.22242.3	1351	coding	PF13840
MSTRG.22242.4	1505	coding	PF13840
MSTRG.22245.2	2656	coding	PF01805
MSTRG.22246.3	2296	noncoding	-
MSTRG.22251.3	1787	noncoding	-
MSTRG.22251.2	2287	coding	PF00650

MSTRG.22251.4	2187	coding	PF00650
MSTRG.22261.1	12174	coding	PF00782
MSTRG.22261.2	3015	coding	PF00782
MSTRG.22262.2	675	noncoding	-
MSTRG.22262.3	1147	noncoding	-
MSTRG.22262.4	1858	noncoding	-
MSTRG.22262.5	670	noncoding	-
MSTRG.22265.1	398	noncoding	-
MSTRG.22264.3	3543	coding	PF01237
MSTRG.22264.4	3815	coding	PF01237
MSTRG.22276.2	4046	noncoding	-
MSTRG.22277.1	4600	coding	PF13589
MSTRG.22277.3	4342	coding	PF13589
MSTRG.22277.10	3031	coding	PF07496
MSTRG.22277.5	2977	coding	PF07496
MSTRG.22277.9	5325	coding	PF07496
MSTRG.22277.7	5369	coding	PF07496
MSTRG.22277.11	2179	noncoding	-
MSTRG.22269.2	4455	noncoding	-
MSTRG.22269.3	2672	noncoding	-
MSTRG.22271.1	870	noncoding	-
MSTRG.22282.1	4950	noncoding	-
MSTRG.22282.2	12396	coding	PF16637
MSTRG.22282.3	12216	coding	PF16637
MSTRG.22283.3	9606	coding	PF16897
MSTRG.22284.2	3507	coding	PF10477
MSTRG.22285.1	4050	noncoding	-
MSTRG.22286.3	2341	coding	PF02666
MSTRG.22286.6	2412	coding	PF02666
MSTRG.22301.1	1076	noncoding	-
MSTRG.22309.1	562	noncoding	-
MSTRG.22310.1	3687	noncoding	-
MSTRG.22312.3	1908	coding	PF13836
MSTRG.22312.2	1548	coding	PF13836
MSTRG.22306.4	659	coding	PF07654
MSTRG.22315.1	418	coding	PF07654
MSTRG.22306.5	369	noncoding	-
MSTRG.22306.7	822	coding	PF07654
MSTRG.22306.8	462	coding	PF07654
MSTRG.22306.6	509	coding	PF07654
MSTRG.22306.9	513	coding	PF07654
MSTRG.22306.11	576	coding	PF07654
MSTRG.22295.1	5088	noncoding	-
MSTRG.22295.3	701	noncoding	-
MSTRG.22299.3	1481	coding	PF04855
MSTRG.22300.1	565	coding	PF04511
MSTRG.22300.3	788	coding	PF04511
MSTRG.22300.4	604	noncoding	-
MSTRG.22316.1	22121	coding	PF01187
MSTRG.22316.2	8466	coding	PF01187
MSTRG.22319.2	838	coding	PF02798
MSTRG.22319.3	1182	noncoding	-
MSTRG.22332.7	7882	noncoding	-
MSTRG.22326.2	2760	coding	PF01019

MSTRG.22327.1	2399	coding	PF01019
MSTRG.22327.3	2534	coding	PF01019
MSTRG.22325.1	2684	noncoding	-
MSTRG.22335.2	1652	coding	PF00001
MSTRG.22335.3	1317	coding	PF00001
MSTRG.22341.2	2653	noncoding	-
MSTRG.22341.3	4994	noncoding	-
MSTRG.22344.1	1956	noncoding	-
MSTRG.22344.2	1618	noncoding	-
MSTRG.22344.4	1699	noncoding	-
MSTRG.22344.5	1322	coding	PF00503
MSTRG.22344.7	1058	noncoding	-
MSTRG.22344.9	1869	coding	PF00503
MSTRG.22346.1	261	noncoding	-
MSTRG.22344.11	665	noncoding	-
MSTRG.22338.2	2822	coding	PF00474
MSTRG.22338.4	1840	coding	PF00474
MSTRG.22340.2	1633	noncoding	-
MSTRG.22348.3	6237	coding	PF00069
MSTRG.22355.2	2014	noncoding	-
MSTRG.22356.1	3851	coding	PF00160
MSTRG.22356.4	2659	coding	PF00160
MSTRG.22356.5	3895	coding	PF00160
MSTRG.22356.3	4714	coding	PF00160
MSTRG.22351.2	2214	coding	PF15774
MSTRG.22351.3	2212	coding	PF15774
MSTRG.22358.2	742	noncoding	-
MSTRG.22359.2	6741	coding	PF00454
MSTRG.22361.2	6656	noncoding	-
MSTRG.22370.2	3217	coding	PF00651
MSTRG.22370.4	3168	coding	PF00651
MSTRG.22378.3	4216	coding	PF14724
MSTRG.22378.5	3258	coding	PF14724
MSTRG.22378.6	3928	coding	PF14724
MSTRG.22378.7	6088	coding	PF14724
MSTRG.22367.3	3286	coding	PF09606
MSTRG.22367.4	9929	coding	PF09606
MSTRG.22367.5	5417	coding	PF09606
MSTRG.22382.1	9666	coding	PF00069
MSTRG.22382.4	4302	coding	PF00069
MSTRG.22382.5	3457	noncoding	-
MSTRG.22382.8	3762	noncoding	-
MSTRG.22382.6	7439	coding	PF00069
MSTRG.22382.7	4401	coding	PF00069
MSTRG.22382.9	4150	coding	PF00069
MSTRG.22383.1	1418	coding	PF00069
MSTRG.22383.2	1418	coding	PF00069
MSTRG.22383.3	1418	coding	PF00069
MSTRG.22383.4	1418	coding	PF00069
MSTRG.22390.8	7029	coding	PF07569
MSTRG.22390.7	5288	coding	PF07569
MSTRG.22390.6	3904	coding	PF07569
MSTRG.22390.2	4023	coding	PF07569
MSTRG.22391.2	807	coding	PF09812

MSTRG.22391.4	1305	coding	PF09812
MSTRG.22392.2	3927	noncoding	-
MSTRG.22392.4	720	noncoding	-
MSTRG.22394.2	1380	noncoding	-
MSTRG.22394.3	1773	coding	PF03152
MSTRG.22385.2	5355	coding	PF00735
MSTRG.22386.2	1702	coding	PF00907
MSTRG.22397.2	1884	coding	PF07992
MSTRG.22397.3	1840	coding	PF07992
MSTRG.22397.4	2837	coding	PF07992
MSTRG.22398.1	2501	noncoding	-
MSTRG.22399.1	3779	noncoding	-
MSTRG.22399.2	3342	noncoding	-
MSTRG.22400.1	1992	noncoding	-
MSTRG.22402.2	1392	coding	PF05742
MSTRG.22402.3	1152	coding	PF05742
MSTRG.22403.1	4653	noncoding	-
MSTRG.22406.2	909	coding	PF00638
MSTRG.22406.5	881	coding	PF00638
MSTRG.22411.2	2223	noncoding	-
MSTRG.22411.1	3130	noncoding	-
MSTRG.22407.1	1698	coding	PF13855
MSTRG.22407.2	2033	coding	PF13855
MSTRG.22408.1	2032	noncoding	-
MSTRG.22419.1	993	noncoding	-
MSTRG.22420.1	3383	noncoding	-
MSTRG.22417.1	951	noncoding	-
MSTRG.22417.2	1070	noncoding	-
MSTRG.22426.1	565	noncoding	-
MSTRG.22428.2	3153	coding	PF12755
MSTRG.22430.2	5697	coding	PF10433
MSTRG.22435.2	4477	coding	PF07959
MSTRG.22431.1	2398	coding	PF00777
MSTRG.22444.7	2290	coding	PF00271
MSTRG.22444.5	2467	coding	PF00271
MSTRG.22449.1	3572	coding	PF01411
MSTRG.22444.10	310	noncoding	-
MSTRG.22449.3	3715	coding	PF01411
MSTRG.22449.4	3768	coding	PF01411
MSTRG.22449.5	3772	coding	PF01411
MSTRG.22449.9	3766	coding	PF01411
MSTRG.22449.10	3628	coding	PF01411
MSTRG.22451.3	7948	coding	PF01571
MSTRG.22451.8	7444	coding	PF01571
MSTRG.22451.5	7049	coding	PF01571
MSTRG.22451.9	6987	coding	PF01571
MSTRG.22451.13	1759	coding	PF01571
MSTRG.22451.14	4168	coding	PF01571
MSTRG.22451.11	4157	coding	PF01571
MSTRG.22451.12	3339	coding	PF01571
MSTRG.22442.1	2538	noncoding	-
MSTRG.22459.3	6146	coding	PF17034
MSTRG.22459.1	2707	coding	PF17034
MSTRG.22459.4	3032	coding	PF17034

MSTRG.22459.5	2409	coding	PF17034
MSTRG.22459.6	3394	coding	PF17034
MSTRG.22460.1	11914	noncoding	-
MSTRG.22460.2	1679	noncoding	-
MSTRG.22461.1	1146	noncoding	-
MSTRG.22461.2	690	coding	PF13639
MSTRG.22461.4	1772	noncoding	-
MSTRG.22461.5	751	coding	PF13639
MSTRG.22461.6	1412	noncoding	-
MSTRG.22454.1	2889	coding	PF00089
MSTRG.22454.2	2503	coding	PF00089
MSTRG.22455.3	3986	coding	PF12026
MSTRG.22464.1	12812	noncoding	-
MSTRG.22465.1	5777	noncoding	-
MSTRG.22466.1	7100	noncoding	-
MSTRG.22478.3	2378	noncoding	-
MSTRG.22478.2	5767	noncoding	-
MSTRG.22478.4	2821	noncoding	-
MSTRG.22478.5	5767	noncoding	-
MSTRG.22478.7	6840	coding	PF03184
MSTRG.22478.6	6604	coding	PF03184
MSTRG.22478.10	3325	noncoding	-
MSTRG.22478.11	2634	noncoding	-
MSTRG.22480.1	3488	noncoding	-
MSTRG.22482.1	334	noncoding	-
MSTRG.22490.1	2110	coding	PF00009
MSTRG.22490.2	1010	coding	PF00009
MSTRG.22490.3	2048	coding	PF00009
MSTRG.22490.4	1534	coding	PF00009
MSTRG.22490.5	2159	coding	PF00009
MSTRG.22490.6	1706	coding	PF00009
MSTRG.22490.7	2185	coding	PF00009
MSTRG.22490.9	2022	coding	PF00009
MSTRG.22468.1	407	noncoding	-
MSTRG.22469.1	2232	noncoding	-
MSTRG.22476.1	576	noncoding	-
MSTRG.22477.1	1250	noncoding	-
MSTRG.22519.1	4111	coding	PF03164
MSTRG.22521.1	1326	noncoding	-
MSTRG.22517.1	636	noncoding	-
MSTRG.22517.2	2517	noncoding	-
MSTRG.22517.3	1941	noncoding	-
MSTRG.22524.1	735	noncoding	-
MSTRG.22526.1	2172	noncoding	-
MSTRG.22539.1	371	noncoding	-
MSTRG.22542.2	3403	coding	PF03131
MSTRG.22544.1	797	noncoding	-
MSTRG.22544.2	646	noncoding	-
MSTRG.22528.1	316	noncoding	-
MSTRG.22530.1	1252	noncoding	-
MSTRG.22549.1	4585	noncoding	-
MSTRG.22549.2	4386	noncoding	-
MSTRG.22551.3	917	noncoding	-
MSTRG.22551.2	1480	coding	PF08583

MSTRG.22551.4	619	noncoding	-
MSTRG.22551.5	506	noncoding	-
MSTRG.22552.2	1985	coding	PF05238
MSTRG.22550.2	5057	noncoding	-
MSTRG.22553.3	1791	coding	PF15032
MSTRG.22563.1	12310	coding	PF07707
MSTRG.22563.3	2984	noncoding	-
MSTRG.22564.3	2747	noncoding	-
MSTRG.22572.1	4616	coding	PF00388
MSTRG.22574.1	2549	noncoding	-
MSTRG.22572.2	5164	coding	PF00388
MSTRG.22572.3	4384	coding	PF00388
MSTRG.22576.1	831	noncoding	-
MSTRG.22580.1	1281	noncoding	-
MSTRG.22579.3	686	noncoding	-
MSTRG.22579.4	669	noncoding	-
MSTRG.22583.2	692	coding	PF06825
MSTRG.22583.5	811	coding	PF06825
MSTRG.22592.2	2038	noncoding	-
MSTRG.22592.3	2041	noncoding	-
MSTRG.22592.4	2137	noncoding	-
MSTRG.22585.2	1568	noncoding	-
MSTRG.22585.1	1099	noncoding	-
MSTRG.22593.4	4684	coding	PF00082
MSTRG.22598.2	1825	coding	PF02137
MSTRG.22598.4	1705	coding	PF02137
MSTRG.22599.1	1915	noncoding	-
MSTRG.22599.2	1370	noncoding	-
MSTRG.22599.3	1325	noncoding	-
MSTRG.22599.4	781	noncoding	-
MSTRG.22599.5	4030	noncoding	-
MSTRG.22605.1	4860	coding	PF07534
MSTRG.22607.3	6754	noncoding	-
MSTRG.22609.1	2601	noncoding	-
MSTRG.22608.2	5551	noncoding	-
MSTRG.22610.1	5366	noncoding	-
MSTRG.22610.2	4683	coding	PF01140
MSTRG.22611.1	3588	coding	PF02093
MSTRG.22612.1	6310	noncoding	-
MSTRG.22608.4	3968	coding	PF03815
MSTRG.22617.3	7384	noncoding	-
MSTRG.22617.1	7612	noncoding	-
MSTRG.22617.4	7251	noncoding	-
MSTRG.22618.1	1776	coding	PF06730
MSTRG.22627.2	31057	coding	PF12540
MSTRG.22627.3	7940	coding	PF12540
MSTRG.22627.4	7056	coding	PF12540
MSTRG.22627.5	7180	coding	PF12540
MSTRG.22627.6	8083	coding	PF12540
MSTRG.22627.7	7196	coding	PF12540
MSTRG.22627.8	7298	coding	PF12540
MSTRG.22621.2	1072	coding	PF03665
MSTRG.22624.2	2179	coding	PF10401
MSTRG.22633.4	1606	noncoding	-

MSTRG.22634.3	3279	noncoding	-
MSTRG.22644.1	7457	coding	PF15132
MSTRG.22644.4	780	coding	PF15132
MSTRG.22644.5	786	coding	PF15132
MSTRG.22644.3	1269	coding	PF15132
MSTRG.22646.1	2234	coding	PF02991
MSTRG.22646.2	2636	coding	PF02991
MSTRG.22646.4	4578	coding	PF02991
MSTRG.22650.1	674	noncoding	-
MSTRG.22650.5	885	noncoding	-
MSTRG.22650.6	1818	noncoding	-
MSTRG.22650.3	1181	noncoding	-
MSTRG.22650.4	933	noncoding	-
MSTRG.22650.7	1063	noncoding	-
MSTRG.22639.2	1931	noncoding	-
MSTRG.22651.3	2311	noncoding	-
MSTRG.22651.4	3430	noncoding	-
MSTRG.22651.5	3535	noncoding	-
MSTRG.22651.6	3526	noncoding	-
MSTRG.22651.7	3439	noncoding	-
MSTRG.22654.1	1198	noncoding	-
MSTRG.22656.1	7588	noncoding	-
MSTRG.22660.2	1926	noncoding	-
MSTRG.22668.2	1961	noncoding	-
MSTRG.22664.1	4550	coding	PF00884
MSTRG.22683.4	1592	noncoding	-
MSTRG.22684.1	2351	noncoding	-
MSTRG.22686.2	9024	coding	PF12796
MSTRG.22686.3	9052	coding	PF12796
MSTRG.22689.1	19557	noncoding	-
MSTRG.22679.1	5395	coding	PF01294
MSTRG.22703.3	1654	noncoding	-
MSTRG.22703.2	1169	noncoding	-
MSTRG.22709.2	1969	noncoding	-
MSTRG.22713.1	5910	coding	PF16474
MSTRG.22713.5	2735	coding	PF16474
MSTRG.22713.4	2662	coding	PF16474
MSTRG.22714.1	2999	noncoding	-
MSTRG.22694.2	5430	coding	PF13901
MSTRG.22718.1	3277	noncoding	-
MSTRG.22718.2	9494	noncoding	-
MSTRG.22718.3	4915	noncoding	-
MSTRG.22718.4	2546	noncoding	-
MSTRG.22721.1	4358	noncoding	-
MSTRG.22722.1	3188	noncoding	-
MSTRG.22725.1	1044	noncoding	-
MSTRG.22731.2	3884	noncoding	-
MSTRG.22733.1	1276	coding	PF01359
MSTRG.22733.2	1285	coding	PF01359
MSTRG.22737.2	5235	noncoding	-
MSTRG.22739.1	1284	noncoding	-
MSTRG.22740.1	1479	coding	PF00723
MSTRG.22740.2	2199	coding	PF00723
MSTRG.22740.4	1212	coding	PF00723

MSTRG.22742.1	3386	noncoding	-
MSTRG.22742.2	4054	noncoding	-
MSTRG.22744.2	4964	coding	PF00005
MSTRG.22744.3	5198	coding	PF00005
MSTRG.22744.5	6019	coding	PF00005
MSTRG.22744.4	5019	coding	PF00005
MSTRG.22744.6	4956	coding	PF00005
MSTRG.22744.8	3401	coding	PF02994
MSTRG.22746.1	4818	coding	PF00005
MSTRG.22747.1	1287	coding	PF05362
MSTRG.22747.3	6294	noncoding	-
MSTRG.22747.4	3031	coding	PF05362
MSTRG.22749.4	6263	coding	PF03145
MSTRG.22750.5	2168	noncoding	-
MSTRG.22755.1	693	noncoding	-
MSTRG.22763.2	2472	coding	PF03828
MSTRG.22763.4	2354	coding	PF03828
MSTRG.22763.5	2226	coding	PF03828
MSTRG.22763.6	2259	coding	PF03828
MSTRG.22764.1	3322	coding	PF12024
MSTRG.22764.3	2221	coding	PF12024
MSTRG.22770.2	7626	coding	PF16607
MSTRG.22774.1	379	noncoding	-
MSTRG.22776.1	468	noncoding	-
MSTRG.22779.1	627	noncoding	-
MSTRG.22780.1	575	noncoding	-
MSTRG.22790.3	9246	coding	PF00176
MSTRG.22790.4	10855	coding	PF00176
MSTRG.22790.5	10917	coding	PF00176
MSTRG.22793.1	1416	noncoding	-
MSTRG.22798.3	4261	coding	PF01858
MSTRG.22800.5	2303	coding	PF00179
MSTRG.22800.3	1920	coding	PF00179
MSTRG.22784.1	4548	coding	PF11618
MSTRG.22785.4	8211	coding	PF12933
MSTRG.22785.5	3888	coding	PF12933
MSTRG.22785.6	11797	noncoding	-
MSTRG.22786.1	890	noncoding	-
MSTRG.22787.1	2532	noncoding	-
MSTRG.22789.2	1386	coding	PF00045
MSTRG.22803.1	454	noncoding	-
MSTRG.22804.1	1466	coding	PF00135
MSTRG.22804.3	1848	coding	PF00135
MSTRG.22804.4	1851	coding	PF00135
MSTRG.22806.2	318	noncoding	-
MSTRG.22811.1	3664	coding	PF13639
MSTRG.22812.3	7447	coding	PF00503
MSTRG.22813.1	8471	noncoding	-
MSTRG.22814.2	3102	coding	PF00135
MSTRG.22818.2	2843	coding	PF04097
MSTRG.22819.2	1726	noncoding	-
MSTRG.22819.1	4231	noncoding	-
MSTRG.22822.2	967	coding	PF07002
MSTRG.22822.3	740	coding	PF07002

MSTRG.22825.1	3094	coding	PF10187
MSTRG.22825.7	1739	coding	PF10187
MSTRG.22825.5	1632	coding	PF10187
MSTRG.22825.6	1531	coding	PF10187
MSTRG.22827.1	1971	noncoding	-
MSTRG.22829.3	1652	coding	PF11527
MSTRG.22829.4	2093	coding	PF11527
MSTRG.22829.2	4336	coding	PF11527
MSTRG.22824.1	3760	coding	PF00048
MSTRG.22832.5	1318	coding	PF05093
MSTRG.22832.6	2243	coding	PF05093
MSTRG.22832.4	1998	coding	PF05093
MSTRG.22833.2	2074	coding	PF08511
MSTRG.22833.3	1572	coding	PF08511
MSTRG.22841.2	2909	coding	PF13925
MSTRG.22841.3	5537	coding	PF13925
MSTRG.22842.8	3683	coding	PF00225
MSTRG.22842.9	3564	coding	PF00225
MSTRG.22842.10	3621	coding	PF00225
MSTRG.22842.12	3591	coding	PF00225
MSTRG.22842.13	3555	coding	PF00225
MSTRG.22835.1	3887	coding	PF00027
MSTRG.22835.2	3234	coding	PF00027
MSTRG.22836.1	2432	noncoding	-
MSTRG.22838.4	849	noncoding	-
MSTRG.22838.5	832	noncoding	-
MSTRG.22838.3	1124	noncoding	-
MSTRG.22838.2	1225	noncoding	-
MSTRG.22845.2	5496	noncoding	-
MSTRG.22847.3	1633	noncoding	-
MSTRG.22857.1	2219	coding	PF04054
MSTRG.22857.2	2029	noncoding	-
MSTRG.22858.3	7688	coding	PF04054
MSTRG.22858.5	7797	coding	PF04054
MSTRG.22858.6	7791	coding	PF04054
MSTRG.22858.7	7794	coding	PF04054
MSTRG.22858.8	7727	coding	PF04054
MSTRG.22858.10	7897	coding	PF04054
MSTRG.22858.4	7724	coding	PF04054
MSTRG.22858.9	7883	coding	PF04054
MSTRG.22867.1	3852	coding	PF01049
MSTRG.22871.1	1344	noncoding	-
MSTRG.22871.2	744	noncoding	-
MSTRG.22881.2	3571	coding	PF01049
MSTRG.22881.3	2628	coding	PF00028
MSTRG.22892.2	2611	coding	PF01712
MSTRG.22892.4	2551	coding	PF01712
MSTRG.22892.5	870	noncoding	-
MSTRG.22893.1	453	noncoding	-
MSTRG.22895.2	863	noncoding	-
MSTRG.22896.1	4217	noncoding	-
MSTRG.22896.4	2800	noncoding	-
MSTRG.22898.2	2131	noncoding	-
MSTRG.22899.1	1451	coding	PF00194

MSTRG.22909.2	1281	noncoding	-
MSTRG.22911.3	2095	noncoding	-
MSTRG.22912.1	2392	noncoding	-
MSTRG.22913.2	2030	coding	PF06046
MSTRG.22914.3	1951	coding	PF02319
MSTRG.22919.1	4509	noncoding	-
MSTRG.22921.1	405	noncoding	-
MSTRG.22922.1	3623	noncoding	-
MSTRG.22922.3	3609	noncoding	-
MSTRG.22924.1	2071	coding	PF01529
MSTRG.22922.6	1776	noncoding	-
MSTRG.22924.3	5981	noncoding	-
MSTRG.22924.2	1566	coding	PF01529
MSTRG.22928.2	4662	coding	PF15903
MSTRG.22931.5	4633	noncoding	-
MSTRG.22931.6	4135	noncoding	-
MSTRG.22933.1	2563	coding	PF10341
MSTRG.22935.1	1199	noncoding	-
MSTRG.22936.1	2193	coding	PF15762
MSTRG.22937.2	2344	noncoding	-
MSTRG.22937.3	2485	noncoding	-
MSTRG.22941.4	2468	coding	PF15739
MSTRG.22943.1	1652	coding	PF05485
MSTRG.22944.2	1028	coding	PF02136
MSTRG.22925.3	4696	coding	PF16529
MSTRG.22946.1	1315	coding	PF00227
MSTRG.22946.4	1029	coding	PF00227
MSTRG.22947.2	1149	coding	PF02450
MSTRG.22948.3	5229	noncoding	-
MSTRG.22948.4	5167	noncoding	-
MSTRG.22948.2	4379	noncoding	-
MSTRG.22948.5	4333	noncoding	-
MSTRG.22951.1	2854	coding	PF01207
MSTRG.22951.2	2329	coding	PF01207
MSTRG.22953.1	5027	noncoding	-
MSTRG.22953.2	4577	noncoding	-
MSTRG.22953.8	4303	noncoding	-
MSTRG.22953.4	5634	noncoding	-
MSTRG.22953.5	4164	noncoding	-
MSTRG.22953.6	5738	noncoding	-
MSTRG.22953.7	4496	noncoding	-
MSTRG.22953.3	3978	noncoding	-
MSTRG.22954.1	2633	noncoding	-
MSTRG.22955.1	206	noncoding	-
MSTRG.22965.3	1795	coding	PF01352
MSTRG.22965.5	1361	coding	PF01359
MSTRG.22965.4	3586	coding	PF01352
MSTRG.22965.6	3693	coding	PF01352
MSTRG.22965.8	2424	coding	PF01359
MSTRG.22976.4	2661	noncoding	-
MSTRG.22977.2	2600	noncoding	-
MSTRG.22979.2	8403	coding	PF00595
MSTRG.22979.3	8366	coding	PF00595
MSTRG.22983.2	2134	coding	PF04124

MSTRG.22984.5	1216	noncoding	-
MSTRG.22984.1	2952	noncoding	-
MSTRG.22984.3	2437	noncoding	-
MSTRG.22984.4	1985	noncoding	-
MSTRG.22985.2	1345	noncoding	-
MSTRG.22986.3	3101	noncoding	-
MSTRG.22986.4	2409	noncoding	-
MSTRG.22986.5	5029	coding	PF16772
MSTRG.22966.6	5003	coding	PF16179
MSTRG.22966.2	12573	coding	PF16179
MSTRG.22966.3	12627	coding	PF16179
MSTRG.22966.4	12756	coding	PF16179
MSTRG.22966.5	8773	noncoding	-
MSTRG.22967.1	2020	noncoding	-
MSTRG.22968.1	6882	noncoding	-
MSTRG.22967.2	4548	noncoding	-
MSTRG.22966.7	17554	coding	PF16179
MSTRG.22966.8	12354	coding	PF16179
MSTRG.22966.9	4935	coding	PF16179
MSTRG.22966.10	15585	coding	PF16179
MSTRG.22972.1	2208	coding	PF08772
MSTRG.23003.1	263	noncoding	-
MSTRG.23003.2	481	noncoding	-
MSTRG.23003.3	544	noncoding	-
MSTRG.23015.1	16415	coding	PF00046
MSTRG.23015.2	16088	coding	PF00046
MSTRG.23016.1	2120	noncoding	-
MSTRG.23015.3	15709	coding	PF00046
MSTRG.23017.1	6471	noncoding	-
MSTRG.23018.1	4647	noncoding	-
MSTRG.23006.1	462	noncoding	-
MSTRG.23007.1	3959	noncoding	-
MSTRG.23007.3	3757	noncoding	-
MSTRG.23009.1	2242	coding	PF02966
MSTRG.23009.2	2278	coding	PF02966
MSTRG.23009.3	2221	coding	PF02966
MSTRG.23049.1	468	coding	PF08016
MSTRG.23050.1	1238	noncoding	-
MSTRG.23048.5	2198	coding	PF03398
MSTRG.23048.4	2335	coding	PF03398
MSTRG.23048.6	2346	coding	PF03398
MSTRG.23052.2	3211	coding	PF08517
MSTRG.23055.1	3858	coding	PF01602
MSTRG.23055.5	4124	coding	PF01602
MSTRG.23054.3	3536	coding	PF00481
MSTRG.23057.1	753	noncoding	-
MSTRG.23058.1	1327	noncoding	-
MSTRG.23061.1	8759	coding	PF01352
MSTRG.23061.2	1253	noncoding	-
MSTRG.23071.1	2310	noncoding	-
MSTRG.23080.2	16432	noncoding	-
MSTRG.23080.1	1751	noncoding	-
MSTRG.23082.1	15613	noncoding	-
MSTRG.23082.2	1736	noncoding	-

MSTRG.23082.3	15368	noncoding	-
MSTRG.23082.4	15297	noncoding	-
MSTRG.23084.1	646	noncoding	-
MSTRG.23072.2	1049	coding	PF01868
MSTRG.23074.1	2329	noncoding	-
MSTRG.23074.2	2234	noncoding	-
MSTRG.23075.1	5174	noncoding	-
MSTRG.23076.1	1567	noncoding	-
MSTRG.23087.3	13173	coding	PF16606
MSTRG.23079.2	5101	noncoding	-
MSTRG.23089.2	699	noncoding	-
MSTRG.23089.1	788	noncoding	-
MSTRG.23094.2	6199	coding	PF10034
MSTRG.23094.4	9437	noncoding	-
MSTRG.23094.5	5150	coding	PF10034
MSTRG.23094.6	8386	coding	PF10034
MSTRG.23101.1	6485	noncoding	-
MSTRG.23101.2	6396	noncoding	-
MSTRG.23106.3	2753	noncoding	-
MSTRG.23106.4	2840	noncoding	-
MSTRG.23106.5	2476	noncoding	-
MSTRG.23108.1	3292	coding	PF03097
MSTRG.23105.2	3726	noncoding	-
MSTRG.23110.2	1963	coding	PF00557
MSTRG.23110.3	2313	coding	PF00557
MSTRG.23122.1	2531	coding	PF00342
MSTRG.23122.2	2335	coding	PF00342
MSTRG.23122.3	2306	coding	PF00342
MSTRG.23127.1	1808	noncoding	-
MSTRG.23127.2	9165	noncoding	-
MSTRG.23128.1	593	noncoding	-
MSTRG.23130.1	1536	coding	PF09252
MSTRG.23134.1	5258	coding	PF02093
MSTRG.23139.2	2505	coding	PF01352
MSTRG.23139.4	832	coding	PF01352
MSTRG.23139.5	850	coding	PF01352
MSTRG.23139.6	539	coding	PF01352
MSTRG.23139.7	726	coding	PF01352
MSTRG.23142.2	5569	noncoding	-
MSTRG.23142.1	5364	noncoding	-
MSTRG.23145.1	5297	noncoding	-
MSTRG.23145.2	4683	noncoding	-
MSTRG.23146.1	2085	noncoding	-
MSTRG.23153.2	2741	coding	PF16016
MSTRG.23153.3	2675	coding	PF16016
MSTRG.23150.2	1645	noncoding	-
MSTRG.23151.1	843	noncoding	-
MSTRG.23154.1	796	coding	PF02038
MSTRG.23159.3	1341	noncoding	-
MSTRG.23160.1	2058	noncoding	-
MSTRG.23158.1	626	noncoding	-
MSTRG.23158.2	536	noncoding	-
MSTRG.23166.1	503	noncoding	-
MSTRG.23169.1	1174	noncoding	-

MSTRG.23173.1	350	noncoding	-
MSTRG.23175.1	765	noncoding	-
MSTRG.23175.2	663	noncoding	-
MSTRG.23174.1	383	noncoding	-
MSTRG.23178.1	1510	coding	PF00178
MSTRG.23182.1	1505	noncoding	-
MSTRG.23187.1	1366	noncoding	-
MSTRG.23188.1	1783	noncoding	-
MSTRG.23188.2	6252	noncoding	-
MSTRG.23188.4	6180	noncoding	-
MSTRG.23188.3	1371	noncoding	-
MSTRG.23189.2	10301	coding	PF00856
MSTRG.23191.3	902	noncoding	-
MSTRG.23191.4	1187	noncoding	-
MSTRG.23191.2	1218	noncoding	-
MSTRG.23192.4	507	coding	PF10251
MSTRG.23186.2	3319	noncoding	-
MSTRG.23194.1	4284	coding	PF00620
MSTRG.23194.2	4287	coding	PF00620
MSTRG.23195.3	2075	noncoding	-
MSTRG.23195.4	2231	noncoding	-
MSTRG.23195.2	1813	noncoding	-
MSTRG.23195.1	2286	noncoding	-
MSTRG.23196.1	1571	noncoding	-
MSTRG.23193.1	2408	coding	PF12925
MSTRG.23197.2	643	noncoding	-
MSTRG.23202.1	976	noncoding	-
MSTRG.23204.3	4980	noncoding	-
MSTRG.23201.2	860	coding	PF14560
MSTRG.23207.1	2004	noncoding	-
MSTRG.23207.4	1273	noncoding	-
MSTRG.23212.1	1018	coding	PF01352
MSTRG.23213.1	1960	noncoding	-
MSTRG.23221.3	2426	coding	PF01352
MSTRG.23221.2	6786	coding	PF01352
MSTRG.23222.1	551	noncoding	-
MSTRG.23223.1	3927	coding	PF01352
MSTRG.23223.2	2521	noncoding	-
MSTRG.23223.3	3804	coding	PF01352
MSTRG.23223.4	3116	noncoding	-
MSTRG.23224.1	1216	noncoding	-
MSTRG.23234.1	1322	noncoding	-
MSTRG.23237.1	8447	coding	PF01352
MSTRG.23237.3	7903	coding	PF01352
MSTRG.23239.1	1810	noncoding	-
MSTRG.23241.1	5886	noncoding	-
MSTRG.23253.1	4975	noncoding	-
MSTRG.23255.2	8311	coding	PF02145
MSTRG.23255.4	9080	coding	PF02145
MSTRG.23255.5	7961	coding	PF02145
MSTRG.23255.6	4746	coding	PF02145
MSTRG.23257.1	2827	noncoding	-
MSTRG.23255.7	4247	coding	PF11881
MSTRG.23255.8	2768	coding	PF11881

MSTRG.23265.1	3280	coding	PF15064
MSTRG.23265.2	3016	coding	PF15064
MSTRG.23265.3	1662	coding	PF15064
MSTRG.23265.12	3072	coding	PF15064
MSTRG.23265.11	3468	coding	PF15064
MSTRG.23265.13	3362	coding	PF15064
MSTRG.23265.7	3323	coding	PF15064
MSTRG.23265.15	3766	coding	PF15064
MSTRG.23262.1	4633	coding	PF05210
MSTRG.23264.1	2250	coding	PF00130
MSTRG.23258.1	256	coding	PF01365
MSTRG.23259.1	831	noncoding	-
MSTRG.23268.1	1174	coding	PF10075
MSTRG.23272.1	5169	noncoding	-
MSTRG.23272.2	4883	noncoding	-
MSTRG.23272.3	10160	coding	PF00435
MSTRG.23272.4	7282	coding	PF00435
MSTRG.23272.5	1921	noncoding	-
MSTRG.23271.3	1247	coding	PF08726
MSTRG.23273.3	2159	coding	PF13893
MSTRG.23275.3	1727	coding	PF02146
MSTRG.23280.2	1741	noncoding	-
MSTRG.23284.2	1579	coding	PF04300
MSTRG.23285.3	1971	coding	PF00069
MSTRG.23285.4	2607	coding	PF00069
MSTRG.23285.5	3630	coding	PF00069
MSTRG.23285.6	3111	coding	PF00069
MSTRG.23285.8	5032	coding	PF00069
MSTRG.23279.1	1140	coding	PF04300
MSTRG.23291.2	640	coding	PF00241
MSTRG.23291.3	523	coding	PF00241
MSTRG.23292.3	3798	noncoding	-
MSTRG.23292.4	3816	noncoding	-
MSTRG.23292.5	8665	noncoding	-
MSTRG.23292.6	3623	noncoding	-
MSTRG.23292.8	9177	noncoding	-
MSTRG.23292.9	7281	noncoding	-
MSTRG.23292.10	6531	noncoding	-
MSTRG.23297.4	3478	coding	PF03439
MSTRG.23297.3	3424	coding	PF03439
MSTRG.23295.1	2328	noncoding	-
MSTRG.23296.2	1721	noncoding	-
MSTRG.23296.3	1805	noncoding	-
MSTRG.23301.1	918	coding	PF00337
MSTRG.23307.1	7455	coding	PF00069
MSTRG.23307.2	4195	coding	PF00069
MSTRG.23307.3	6266	coding	PF00069
MSTRG.23307.7	2318	coding	PF00069
MSTRG.23318.4	2173	coding	PF13918
MSTRG.23318.6	2156	coding	PF13918
MSTRG.23318.7	2503	coding	PF13918
MSTRG.23318.8	2072	coding	PF13918
MSTRG.23318.9	2050	coding	PF13918
MSTRG.23321.3	713	noncoding	-

MSTRG.23321.2	707	noncoding	-
MSTRG.23322.1	1524	coding	PF15410
MSTRG.23322.4	1344	noncoding	-
MSTRG.23322.5	1089	noncoding	-
MSTRG.23322.3	1533	coding	PF15410
MSTRG.23323.3	2238	coding	PF02214
MSTRG.23314.1	4974	noncoding	-
MSTRG.23324.2	3179	coding	PF06311
MSTRG.23325.1	2660	coding	PF03109
MSTRG.23332.2	4163	noncoding	-
MSTRG.23333.1	1268	coding	PF00071
MSTRG.23333.3	1815	coding	PF00071
MSTRG.23327.1	3525	noncoding	-
MSTRG.23327.2	5287	noncoding	-
MSTRG.23327.3	2485	noncoding	-
MSTRG.23327.4	3975	noncoding	-
MSTRG.23339.3	3792	coding	PF07714
MSTRG.23347.2	9289	coding	PF00431
MSTRG.23347.3	10219	coding	PF00431
MSTRG.23348.2	1555	coding	PF15455
MSTRG.23348.1	1959	coding	PF15455
MSTRG.23349.1	1012	noncoding	-
MSTRG.23349.2	818	noncoding	-
MSTRG.23349.3	838	noncoding	-
MSTRG.23349.4	929	noncoding	-
MSTRG.23349.5	881	noncoding	-
MSTRG.23350.1	5521	coding	PF00505
MSTRG.23350.4	8957	coding	PF16090
MSTRG.23355.2	3226	coding	PF00178
MSTRG.23357.1	4044	coding	PF00069
MSTRG.23360.1	2635	noncoding	-
MSTRG.23360.2	2347	noncoding	-
MSTRG.23360.3	2488	noncoding	-
MSTRG.23361.1	968	noncoding	-
MSTRG.23352.3	3526	noncoding	-
MSTRG.23352.4	3381	noncoding	-
MSTRG.23352.5	3379	noncoding	-
MSTRG.23365.2	1325	coding	PF01090
MSTRG.23369.1	1215	coding	PF00021
MSTRG.23369.9	1520	coding	PF00021
MSTRG.23369.10	1224	coding	PF00021
MSTRG.23367.1	342	noncoding	-
MSTRG.23367.3	1172	noncoding	-
MSTRG.23370.3	994	noncoding	-
MSTRG.23373.2	1101	noncoding	-
MSTRG.23374.3	2135	coding	PF01834
MSTRG.23377.2	1271	noncoding	-
MSTRG.23377.3	1150	noncoding	-
MSTRG.23379.1	2313	noncoding	-
MSTRG.23380.1	2265	noncoding	-
MSTRG.23379.3	1021	noncoding	-
MSTRG.23380.2	2379	noncoding	-
MSTRG.23380.3	2532	noncoding	-
MSTRG.23380.4	2424	noncoding	-

MSTRG.23379.4	680	noncoding	-
MSTRG.23380.5	2607	noncoding	-
MSTRG.23380.6	2242	noncoding	-
MSTRG.23380.7	3522	noncoding	-
MSTRG.23380.8	2605	noncoding	-
MSTRG.23382.4	1938	coding	PF05049
MSTRG.23384.2	2061	coding	PF05049
MSTRG.23387.2	2008	coding	PF01352
MSTRG.23388.2	998	coding	PF01352
MSTRG.23391.1	3904	noncoding	-
MSTRG.23391.2	688	noncoding	-
MSTRG.23391.4	4205	coding	PF01352
MSTRG.23392.1	3354	coding	PF01352
MSTRG.23396.1	480	noncoding	-
MSTRG.23397.2	3201	coding	PF08205
MSTRG.23401.5	1531	noncoding	-
MSTRG.23399.3	1340	coding	PF01442
MSTRG.23407.2	1698	coding	PF16181
MSTRG.23412.4	7096	noncoding	-
MSTRG.23412.2	2792	noncoding	-
MSTRG.23412.3	2864	noncoding	-
MSTRG.23413.1	3482	noncoding	-
MSTRG.23406.1	6753	coding	PF15753
MSTRG.23414.1	1550	coding	PF00106
MSTRG.23415.1	2504	noncoding	-
MSTRG.23415.2	912	noncoding	-
MSTRG.23415.3	6458	noncoding	-
MSTRG.23415.5	2844	noncoding	-
MSTRG.23415.4	3974	noncoding	-
MSTRG.23416.1	5190	coding	PF00069
MSTRG.23416.2	3467	coding	PF00069
MSTRG.23420.1	3170	noncoding	-
MSTRG.23430.2	2226	noncoding	-
MSTRG.23433.1	3450	noncoding	-
MSTRG.23433.2	2627	noncoding	-
MSTRG.23435.1	2653	coding	PF16878
MSTRG.23443.5	3007	coding	PF12295
MSTRG.23443.6	3834	coding	PF12295
MSTRG.23443.7	2858	coding	PF12295
MSTRG.23438.3	856	noncoding	-
MSTRG.23451.2	2372	coding	PF14893
MSTRG.23451.3	2345	coding	PF14893
MSTRG.23454.2	735	noncoding	-
MSTRG.23455.1	1169	noncoding	-
MSTRG.23456.1	4141	coding	PF00069
MSTRG.23461.2	4181	coding	PF04991
MSTRG.23462.1	2840	coding	PF00375
MSTRG.23457.2	818	coding	PF01217
MSTRG.23463.2	534	noncoding	-
MSTRG.23467.2	6537	noncoding	-
MSTRG.23467.3	5957	noncoding	-
MSTRG.23467.5	5541	noncoding	-
MSTRG.23467.4	4509	noncoding	-
MSTRG.23466.3	1323	coding	PF15826

MSTRG.23470.1	2985	coding	PF15266
MSTRG.23470.2	2573	coding	PF15266
MSTRG.23474.2	1663	coding	PF14938
MSTRG.23479.8	4828	noncoding	-
MSTRG.23479.5	5354	noncoding	-
MSTRG.23479.7	5483	noncoding	-
MSTRG.23479.9	4203	noncoding	-
MSTRG.23479.3	4559	noncoding	-
MSTRG.23479.10	4705	noncoding	-
MSTRG.23479.11	4728	noncoding	-
MSTRG.23482.3	3504	coding	PF01068
MSTRG.23482.4	3068	coding	PF01068
MSTRG.23484.1	1465	coding	PF00822
MSTRG.23485.2	2277	noncoding	-
MSTRG.23486.1	1576	noncoding	-
MSTRG.23488.2	1970	coding	PF00810
MSTRG.23488.4	5971	coding	PF00810
MSTRG.23491.2	856	coding	PF01007
MSTRG.23492.2	1483	coding	PF01369
MSTRG.23492.5	4212	coding	PF01369
MSTRG.23495.1	1291	noncoding	-
MSTRG.23505.2	2305	coding	PF01843
MSTRG.23510.2	2735	noncoding	-
MSTRG.23515.4	2195	coding	PF10488
MSTRG.23520.1	1736	coding	PF00210
MSTRG.23520.2	978	coding	PF00210
MSTRG.23520.3	1102	coding	PF00210
MSTRG.23520.4	1048	coding	PF00210
MSTRG.23513.1	3961	coding	PF00007
MSTRG.23522.5	4503	coding	PF00076
MSTRG.23522.3	4161	coding	PF00076
MSTRG.23522.4	4134	coding	PF00076
MSTRG.23525.2	4355	noncoding	-
MSTRG.23528.2	8728	noncoding	-
MSTRG.23529.9	2561	coding	PF00209
MSTRG.23530.1	1622	coding	PF01285
MSTRG.23531.2	1450	noncoding	-
MSTRG.23531.1	1372	noncoding	-
MSTRG.23530.3	1914	coding	PF01285
MSTRG.23532.1	360	noncoding	-
MSTRG.23535.3	2646	coding	PF14662
MSTRG.23535.2	2670	coding	PF14662
MSTRG.23535.4	2905	coding	PF14662
MSTRG.23535.6	2486	coding	PF14662
MSTRG.23534.2	1600	noncoding	-
MSTRG.23545.2	8758	coding	PF13926
MSTRG.23550.1	4502	noncoding	-
MSTRG.23550.3	6394	noncoding	-
MSTRG.23550.4	4260	noncoding	-
MSTRG.23551.4	1607	coding	PF00605
MSTRG.23552.4	675	noncoding	-
MSTRG.23552.5	2934	noncoding	-
MSTRG.23552.2	2127	noncoding	-
MSTRG.23552.3	3990	noncoding	-

MSTRG.23553.4	1698	coding	PF15358
MSTRG.23567.3	3812	coding	PF01602
MSTRG.23568.2	1857	noncoding	-
MSTRG.23568.3	1523	noncoding	-
MSTRG.23572.3	1444	coding	PF15798
MSTRG.23572.4	1381	coding	PF15798
MSTRG.23574.4	2879	coding	PF05064
MSTRG.23575.2	1761	noncoding	-
MSTRG.23559.4	1830	noncoding	-
MSTRG.23559.5	1885	noncoding	-
MSTRG.23560.2	1656	noncoding	-
MSTRG.23561.1	3779	coding	PF15005
MSTRG.23561.2	924	coding	PF15005
MSTRG.23556.1	968	coding	PF00026
MSTRG.23558.2	1847	noncoding	-
MSTRG.23562.1	897	coding	PF00178
MSTRG.23577.2	3322	coding	PF15876
MSTRG.23579.1	8440	coding	PF07647
MSTRG.23580.1	1362	noncoding	-
MSTRG.23582.1	1110	noncoding	-
MSTRG.23583.2	1893	noncoding	-
MSTRG.23585.4	1074	noncoding	-
MSTRG.23586.1	2713	noncoding	-
MSTRG.23586.2	1803	noncoding	-
MSTRG.23586.3	3182	noncoding	-
MSTRG.23585.6	2193	noncoding	-
MSTRG.23585.7	1267	noncoding	-
MSTRG.23585.8	1323	noncoding	-
MSTRG.23585.9	1262	noncoding	-
MSTRG.23585.10	1296	noncoding	-
MSTRG.23585.11	1248	noncoding	-
MSTRG.23587.1	1469	coding	PF00089
MSTRG.23587.2	1199	coding	PF00089
MSTRG.23592.1	1111	coding	PF01352
MSTRG.23593.1	2280	noncoding	-
MSTRG.23594.1	2273	coding	PF01352
MSTRG.23594.2	1416	coding	PF01352
MSTRG.23594.3	4671	coding	PF01352
MSTRG.23594.4	1078	coding	PF01352
MSTRG.23595.1	1744	noncoding	-
MSTRG.23595.2	1355	noncoding	-
MSTRG.23595.3	1866	noncoding	-
MSTRG.23595.4	1281	noncoding	-
MSTRG.23594.5	1203	noncoding	-
MSTRG.23597.2	2124	noncoding	-
MSTRG.23597.3	694	coding	PF13895
MSTRG.23598.1	5851	noncoding	-
MSTRG.23598.2	4842	noncoding	-
MSTRG.23598.3	4352	noncoding	-
MSTRG.23598.4	1412	coding	PF01012
MSTRG.23603.2	3267	noncoding	-
MSTRG.23607.2	378	noncoding	-
MSTRG.23608.2	883	noncoding	-
MSTRG.23619.1	1665	coding	PF01352

MSTRG.23621.5	993	coding	PF01352
MSTRG.23621.4	2543	coding	PF01352
MSTRG.23621.1	5210	coding	PF01352
MSTRG.23621.6	5042	coding	PF01352
MSTRG.23621.3	5920	coding	PF01352
MSTRG.23622.1	1435	coding	PF00078
MSTRG.23621.7	2544	coding	PF01352
MSTRG.23621.9	4562	coding	PF01352
MSTRG.23621.11	7535	noncoding	-
MSTRG.23621.17	6404	noncoding	-
MSTRG.23621.18	5953	coding	PF01352
MSTRG.23623.1	462	noncoding	-
MSTRG.23629.1	2982	noncoding	-
MSTRG.23630.1	7160	noncoding	-
MSTRG.23630.2	2072	noncoding	-
MSTRG.23630.3	3009	noncoding	-
MSTRG.23630.4	6406	coding	PF03402
MSTRG.23631.1	825	noncoding	-
MSTRG.23616.2	3150	noncoding	-
MSTRG.23753.1	1602	noncoding	-
MSTRG.23754.2	3214	coding	PF01352
MSTRG.23754.1	2941	coding	PF01352
MSTRG.23754.4	2482	coding	PF01352
MSTRG.23754.3	3278	coding	PF01352
MSTRG.23754.5	4031	coding	PF01352
MSTRG.23754.7	2606	coding	PF01352
MSTRG.23754.13	3231	coding	PF01352
MSTRG.23754.14	4775	coding	PF01352
MSTRG.23754.16	3340	coding	PF01352
MSTRG.23758.1	766	noncoding	-
MSTRG.23763.1	3712	noncoding	-
MSTRG.23769.5	6828	coding	PF01352
MSTRG.23769.6	10400	coding	PF01352
MSTRG.23769.8	5510	coding	PF01352
MSTRG.23770.2	5235	coding	PF01352
MSTRG.23770.1	5822	coding	PF01352
MSTRG.23770.3	6189	coding	PF01352
MSTRG.23770.4	11573	coding	PF01352
MSTRG.23770.5	8144	coding	PF01352
MSTRG.23770.6	5329	coding	PF01352
MSTRG.23770.7	7434	coding	PF01352
MSTRG.23770.8	4176	coding	PF01352
MSTRG.23770.10	5925	coding	PF01352
MSTRG.23770.9	5811	coding	PF01352
MSTRG.23773.2	3946	coding	PF01352
MSTRG.23769.11	2058	coding	PF01352
MSTRG.23765.13	1143	noncoding	-
MSTRG.23765.15	1098	noncoding	-
MSTRG.23765.10	1108	noncoding	-
MSTRG.23765.12	1156	noncoding	-
MSTRG.23765.9	1096	noncoding	-
MSTRG.23765.8	1841	noncoding	-
MSTRG.23765.11	2262	noncoding	-
MSTRG.23765.14	2224	noncoding	-

MSTRG.23765.4	2292	noncoding	-
MSTRG.23765.7	2280	noncoding	-
MSTRG.23765.5	1616	noncoding	-
MSTRG.23765.6	2178	noncoding	-
MSTRG.23770.13	3048	coding	PF01352
MSTRG.23774.1	3839	coding	PF01352
MSTRG.23770.14	3408	noncoding	-
MSTRG.23775.1	650	noncoding	-
MSTRG.23776.1	1413	noncoding	-
MSTRG.23777.1	8755	coding	PF01352
MSTRG.23777.2	6548	coding	PF01352
MSTRG.23777.3	7064	coding	PF01352
MSTRG.23778.1	1267	noncoding	-
MSTRG.23778.2	6428	noncoding	-
MSTRG.23779.1	4267	coding	PF01352
MSTRG.23779.2	3763	coding	PF01352
MSTRG.23779.3	3679	coding	PF01352
MSTRG.23783.1	391	noncoding	-
MSTRG.23785.1	590	noncoding	-
MSTRG.23786.1	338	noncoding	-
MSTRG.23788.2	5322	coding	PF01352
MSTRG.23788.3	2905	coding	PF01352
MSTRG.23788.4	2372	coding	PF01352
MSTRG.23788.5	5579	coding	PF01352
MSTRG.23788.7	2296	coding	PF01352
MSTRG.23788.6	4154	coding	PF01352
MSTRG.23788.8	716	coding	PF01352
MSTRG.23788.9	422	coding	PF01352
MSTRG.23789.1	2276	noncoding	-
MSTRG.23790.1	1379	coding	PF01352
MSTRG.23790.2	2431	noncoding	-
MSTRG.23789.2	4324	coding	PF00517
MSTRG.23789.3	1283	coding	PF01352
MSTRG.23791.1	7788	coding	PF01352
MSTRG.23791.3	4066	coding	PF01352
MSTRG.23791.5	7723	coding	PF01352
MSTRG.23791.6	7569	coding	PF01352
MSTRG.23791.4	7655	coding	PF01352
MSTRG.23791.7	4979	coding	PF01352
MSTRG.23791.8	7757	coding	PF01352
MSTRG.23791.9	7520	coding	PF01352
MSTRG.23791.11	7850	coding	PF01352
MSTRG.23791.10	7659	coding	PF01352
MSTRG.23632.1	6217	coding	PF01352
MSTRG.23632.3	6274	coding	PF01352
MSTRG.23632.4	2608	coding	PF01352
MSTRG.23632.5	2680	coding	PF01352
MSTRG.23632.6	2773	coding	PF01352
MSTRG.23632.7	2045	coding	PF01352
MSTRG.23632.8	2071	coding	PF01352
MSTRG.23632.9	3464	noncoding	-
MSTRG.23632.10	2352	coding	PF01352
MSTRG.23632.11	3510	coding	PF01352
MSTRG.23635.2	3009	coding	PF01352

MSTRG.23635.3	2945	coding	PF01352
MSTRG.23639.1	5932	coding	PF01352
MSTRG.23651.1	1470	coding	PF01352
MSTRG.23652.1	275	noncoding	-
MSTRG.23653.1	616	noncoding	-
MSTRG.23654.1	4824	coding	PF01352
MSTRG.23659.1	1411	noncoding	-
MSTRG.23651.2	814	noncoding	-
MSTRG.23660.1	1259	noncoding	-
MSTRG.23651.3	613	noncoding	-
MSTRG.23651.4	8200	coding	PF01352
MSTRG.23651.5	1602	noncoding	-
MSTRG.23663.1	695	noncoding	-
MSTRG.23664.1	994	noncoding	-
MSTRG.23651.6	1878	coding	PF01352
MSTRG.23651.7	7710	coding	PF01352
MSTRG.23651.8	466	noncoding	-
MSTRG.23666.1	628	noncoding	-
MSTRG.23667.1	984	noncoding	-
MSTRG.23668.1	616	noncoding	-
MSTRG.23670.1	4309	coding	PF01352
MSTRG.23670.2	3774	coding	PF01352
MSTRG.23670.3	3704	coding	PF01352
MSTRG.23671.1	257	noncoding	-
MSTRG.23644.2	659	noncoding	-
MSTRG.23644.1	352	noncoding	-
MSTRG.23644.3	895	noncoding	-
MSTRG.23644.4	874	noncoding	-
MSTRG.23677.1	3020	coding	PF01417
MSTRG.23677.2	5778	coding	PF01417
MSTRG.23678.6	1740	coding	PF00076
MSTRG.23681.4	1710	coding	PF15794
MSTRG.23681.5	1707	coding	PF15794
MSTRG.23681.6	1941	coding	PF15794
MSTRG.23683.1	4642	noncoding	-
MSTRG.23683.2	1525	noncoding	-
MSTRG.23683.3	1352	noncoding	-
MSTRG.23690.1	1153	noncoding	-
MSTRG.23694.7	2162	noncoding	-
MSTRG.23694.5	3236	noncoding	-
MSTRG.23694.6	2311	noncoding	-
MSTRG.23694.4	3499	noncoding	-
MSTRG.23696.1	6456	coding	PF13908
MSTRG.23696.3	6104	coding	PF13908
MSTRG.23687.1	1012	coding	PF15431
MSTRG.23688.1	1848	noncoding	-
MSTRG.23698.3	3165	coding	PF12480
MSTRG.23701.3	1743	noncoding	-
MSTRG.23706.1	4027	coding	PF04499
MSTRG.23706.3	4938	coding	PF04499
MSTRG.23706.2	3960	coding	PF04499
MSTRG.23699.1	1721	noncoding	-
MSTRG.23709.3	3285	coding	PF14740
MSTRG.23709.4	1986	coding	PF14740

MSTRG.23709.5	1822	coding	PF14740
MSTRG.23709.6	1162	coding	PF00992
MSTRG.23709.7	1327	coding	PF00992
MSTRG.23712.1	1433	noncoding	-
MSTRG.23711.2	1029	noncoding	-
MSTRG.23716.1	2960	coding	PF12796
MSTRG.23716.2	2957	coding	PF12796
MSTRG.23716.6	9252	coding	PF12796
MSTRG.23716.7	12010	coding	PF12796
MSTRG.23717.2	1725	noncoding	-
MSTRG.23717.3	946	noncoding	-
MSTRG.23718.2	1595	coding	PF04906
MSTRG.23720.4	5505	coding	PF03399
MSTRG.23720.5	5134	coding	PF03399
MSTRG.23727.3	1663	noncoding	-
MSTRG.23728.1	530	coding	PF07810
MSTRG.23728.2	896	coding	PF07810
MSTRG.23731.2	2582	coding	PF04065
MSTRG.23732.2	2285	coding	PF01798
MSTRG.23732.3	2195	coding	PF01798
MSTRG.23736.1	1385	noncoding	-
MSTRG.23743.2	1493	coding	PF02023
MSTRG.23744.2	2140	coding	PF02023
MSTRG.23744.3	2010	coding	PF02023
MSTRG.23797.2	9161	coding	PF01352
MSTRG.23801.4	2066	coding	PF01352
MSTRG.23801.3	967	coding	PF01352
MSTRG.23801.5	1717	coding	PF01352
MSTRG.23809.2	2990	coding	PF01352
MSTRG.23794.1	2464	coding	PF02023
MSTRG.23794.3	4535	coding	PF02023
MSTRG.23814.1	1533	coding	PF02023
MSTRG.23817.2	4566	noncoding	-
MSTRG.23841.3	1469	noncoding	-
MSTRG.23841.2	4953	coding	PF01352
MSTRG.23846.1	6794	noncoding	-
MSTRG.23846.2	12041	noncoding	-
MSTRG.23846.3	11996	noncoding	-
MSTRG.23847.1	2656	coding	PF01352
MSTRG.23849.1	1832	noncoding	-
MSTRG.23855.2	2402	noncoding	-
MSTRG.23857.1	2007	noncoding	-
MSTRG.23857.2	3050	noncoding	-
MSTRG.23855.3	3073	coding	PF01352
MSTRG.23855.4	5963	coding	PF01352
MSTRG.23860.1	7580	coding	PF01352
MSTRG.23860.2	1471	coding	PF01352
MSTRG.23857.3	1412	noncoding	-
MSTRG.23855.9	3621	coding	PF01352
MSTRG.23824.1	642	noncoding	-
MSTRG.23825.1	10404	coding	PF01352
MSTRG.23825.3	2746	coding	PF01352
MSTRG.23825.4	3196	noncoding	-
MSTRG.23825.5	8372	noncoding	-

MSTRG.23827.1	8839	coding	PF03402
MSTRG.23828.1	841	noncoding	-
MSTRG.23833.1	3881	noncoding	-
MSTRG.23834.1	3344	noncoding	-
MSTRG.23834.2	3231	coding	PF01352
MSTRG.23834.3	5858	coding	PF01352
MSTRG.23834.4	5403	coding	PF01352
MSTRG.23834.5	6553	noncoding	-
MSTRG.23868.2	4800	coding	PF01352
MSTRG.23868.4	4795	coding	PF01352
MSTRG.23872.6	10966	coding	PF01352
MSTRG.23872.7	3819	coding	PF01352
MSTRG.23872.3	4059	coding	PF01352
MSTRG.23872.1	4240	coding	PF01352
MSTRG.23873.1	1697	noncoding	-
MSTRG.23872.8	4604	noncoding	-
MSTRG.23872.9	1252	coding	PF01352
MSTRG.23875.1	1336	noncoding	-
MSTRG.23872.10	5678	coding	PF01352
MSTRG.23877.6	9051	coding	PF01352
MSTRG.23878.2	7808	noncoding	-
MSTRG.23879.2	2650	coding	PF01352
MSTRG.23879.3	2738	coding	PF01352
MSTRG.23879.4	3751	coding	PF01352
MSTRG.23880.1	10158	coding	PF01352
MSTRG.23880.3	2810	coding	PF01352
MSTRG.23881.3	3036	noncoding	-
MSTRG.23881.5	3299	noncoding	-
MSTRG.23883.1	549	noncoding	-
MSTRG.23840.2	891	coding	PF00177
MSTRG.23890.1	3732	noncoding	-
MSTRG.23894.3	2228	coding	PF03184
MSTRG.23898.2	2763	coding	PF02023
MSTRG.23927.1	1217	noncoding	-
MSTRG.23929.1	372	noncoding	-
MSTRG.23931.3	2644	coding	PF00790
MSTRG.23933.2	1095	coding	PF04442
MSTRG.23944.2	885	noncoding	-
MSTRG.23946.1	385	noncoding	-
MSTRG.23947.1	3059	noncoding	-
MSTRG.23947.2	11349	noncoding	-
MSTRG.23949.1	6992	noncoding	-
MSTRG.23949.2	5610	noncoding	-
MSTRG.23950.1	630	noncoding	-
MSTRG.23950.2	1975	noncoding	-
MSTRG.23952.2	2607	coding	PF15862
MSTRG.23952.3	2979	coding	PF15862
MSTRG.23952.5	1650	noncoding	-
MSTRG.23952.4	1602	coding	PF15862
MSTRG.23954.3	4033	noncoding	-
MSTRG.23954.5	7173	noncoding	-
MSTRG.23962.5	2357	coding	PF00076
MSTRG.23962.8	23170	noncoding	-
MSTRG.23962.4	9174	coding	PF00076

MSTRG.23962.6	2413	coding	PF00076
MSTRG.23962.1	11572	coding	PF00076
MSTRG.23962.3	5889	coding	PF00076
MSTRG.23963.1	14072	noncoding	-
MSTRG.23964.1	8499	noncoding	-
MSTRG.23962.11	9497	noncoding	-
MSTRG.23962.12	5551	noncoding	-
MSTRG.23965.1	17817	noncoding	-
MSTRG.23966.1	2803	noncoding	-
MSTRG.23962.13	21803	noncoding	-
MSTRG.23967.1	7279	noncoding	-
MSTRG.23968.1	18607	noncoding	-
MSTRG.23958.1	1005	noncoding	-
MSTRG.23970.2	1319	coding	PF01221
MSTRG.23976.1	1988	coding	PF07162
MSTRG.23977.1	1602	coding	PF03098
MSTRG.23981.3	5736	coding	PF06602
MSTRG.23981.4	2693	coding	PF00447
MSTRG.23981.5	2597	noncoding	-
MSTRG.23981.6	5735	coding	PF06602
MSTRG.23981.7	6147	coding	PF06602
MSTRG.23981.8	8219	coding	PF06602
MSTRG.23983.4	3240	coding	PF00735
MSTRG.23983.3	3519	coding	PF15548
MSTRG.23998.5	4665	noncoding	-
MSTRG.23998.6	4896	noncoding	-
MSTRG.23998.7	4767	noncoding	-
MSTRG.23999.11	2041	noncoding	-
MSTRG.24000.2	775	coding	PF10172
MSTRG.24004.1	1019	noncoding	-
MSTRG.23986.1	1003	noncoding	-
MSTRG.23986.2	498	noncoding	-
MSTRG.23987.2	2905	noncoding	-
MSTRG.23988.1	4176	noncoding	-
MSTRG.23988.2	13815	noncoding	-
MSTRG.23989.1	3918	noncoding	-
MSTRG.23988.3	5680	noncoding	-
MSTRG.23988.4	11152	noncoding	-
MSTRG.23990.1	3808	noncoding	-
MSTRG.23990.2	7354	noncoding	-
MSTRG.23990.3	9006	noncoding	-
MSTRG.23991.1	6206	noncoding	-
MSTRG.23992.1	1559	noncoding	-
MSTRG.23984.1	3352	noncoding	-
MSTRG.23995.1	3919	coding	PF10220
MSTRG.23995.4	2296	noncoding	-
MSTRG.23996.4	764	noncoding	-
MSTRG.23997.1	1196	noncoding	-
MSTRG.24007.1	1015	noncoding	-
MSTRG.24020.1	6384	coding	PF13838
MSTRG.24020.3	1718	coding	PF01981
MSTRG.24020.4	1586	coding	PF01981
MSTRG.24023.1	1156	noncoding	-
MSTRG.24022.3	3388	noncoding	-

MSTRG.24026.3	5238	coding	PF00069
MSTRG.24026.1	1633	coding	PF00069
MSTRG.24026.4	1284	coding	PF00069
MSTRG.24027.1	2879	noncoding	-
MSTRG.24011.2	5687	coding	PF14750
MSTRG.24013.3	3883	noncoding	-
MSTRG.24013.5	3782	noncoding	-
MSTRG.24014.1	230	noncoding	-
MSTRG.24015.1	6974	noncoding	-
MSTRG.24016.2	10263	noncoding	-
MSTRG.24017.3	2321	coding	PF00481
MSTRG.24019.1	6849	noncoding	-
MSTRG.24019.2	1145	noncoding	-
MSTRG.24031.2	757	noncoding	-
MSTRG.24035.1	6824	coding	PF00443
MSTRG.24035.3	675	noncoding	-
MSTRG.24036.1	2215	noncoding	-
MSTRG.24037.1	1004	noncoding	-
MSTRG.24049.1	3001	noncoding	-
MSTRG.24049.2	3019	noncoding	-
MSTRG.24049.8	3211	noncoding	-
MSTRG.24049.9	2916	noncoding	-
MSTRG.24049.10	2876	noncoding	-
MSTRG.24049.12	5192	noncoding	-
MSTRG.24050.2	1367	noncoding	-
MSTRG.24050.3	1570	noncoding	-
MSTRG.24051.1	1062	noncoding	-
MSTRG.24041.2	2404	coding	PF08164
MSTRG.24057.3	2033	noncoding	-
MSTRG.24057.4	2660	noncoding	-
MSTRG.24058.1	1958	noncoding	-
MSTRG.24058.2	471	noncoding	-
MSTRG.24062.1	6577	noncoding	-
MSTRG.24062.5	4491	noncoding	-
MSTRG.24062.6	4488	noncoding	-
MSTRG.24062.8	4904	noncoding	-
MSTRG.24062.9	4789	noncoding	-
MSTRG.24059.1	2434	noncoding	-
MSTRG.24059.3	1918	noncoding	-
MSTRG.24059.4	1822	noncoding	-
MSTRG.24063.1	2051	coding	PF04812
MSTRG.24063.2	2054	coding	PF04812
MSTRG.24063.3	1976	coding	PF04812
MSTRG.24063.6	2022	coding	PF04812
MSTRG.24071.2	645	coding	PF00048
MSTRG.24071.1	1028	coding	PF00048
MSTRG.24081.1	3311	noncoding	-
MSTRG.24081.2	3311	noncoding	-
MSTRG.24081.9	2029	noncoding	-
MSTRG.24081.5	2070	noncoding	-
MSTRG.24081.7	2611	noncoding	-
MSTRG.24081.10	2021	noncoding	-
MSTRG.24075.2	1090	noncoding	-
MSTRG.24082.1	5438	coding	PF01602

MSTRG.24082.4	3317	coding	PF01602
MSTRG.24082.3	3941	coding	PF01602
MSTRG.24086.1	3185	noncoding	-
MSTRG.24093.3	4143	noncoding	-
MSTRG.24095.3	4588	coding	PF01068
MSTRG.24095.2	4275	coding	PF01068
MSTRG.24098.3	1855	coding	PF00118
MSTRG.24098.4	1501	coding	PF00118
MSTRG.24099.1	867	noncoding	-
MSTRG.24102.1	881	noncoding	-
MSTRG.24104.1	1590	noncoding	-
MSTRG.24105.1	1049	noncoding	-
MSTRG.24105.2	532	noncoding	-
MSTRG.24107.1	1110	noncoding	-
MSTRG.24108.1	278	noncoding	-
MSTRG.24111.2	5173	noncoding	-
MSTRG.24112.1	1533	noncoding	-
MSTRG.24113.3	1253	noncoding	-
MSTRG.24114.2	3298	coding	PF06017
MSTRG.24115.1	2511	coding	PF03261
MSTRG.24115.3	1225	coding	PF03261
MSTRG.24117.2	2835	coding	PF01399
MSTRG.24117.3	2967	coding	PF01399
MSTRG.24125.1	1231	noncoding	-
MSTRG.24125.2	347	noncoding	-
MSTRG.24123.1	2327	noncoding	-
MSTRG.24127.1	5831	coding	PF01412
MSTRG.24134.2	1487	noncoding	-
MSTRG.24135.4	3663	coding	PF09733
MSTRG.24135.2	3207	coding	PF09733
MSTRG.24135.6	2485	coding	PF09733
MSTRG.24136.8	1744	coding	PF15340
MSTRG.24136.9	982	coding	PF15340
MSTRG.24139.1	1321	noncoding	-
MSTRG.24140.1	811	noncoding	-
MSTRG.24147.2	7718	noncoding	-
MSTRG.24173.1	12993	coding	PF00616
MSTRG.24173.3	9716	noncoding	-
MSTRG.24173.4	12315	coding	PF00616
MSTRG.24173.2	12572	noncoding	-
MSTRG.24173.6	12023	coding	PF00616
MSTRG.24173.7	9660	noncoding	-
MSTRG.24173.5	9846	noncoding	-
MSTRG.24173.10	11525	noncoding	-
MSTRG.24173.8	12586	noncoding	-
MSTRG.24173.9	9774	coding	PF00616
MSTRG.24173.13	12042	noncoding	-
MSTRG.24173.14	11534	coding	PF00616
MSTRG.24173.11	12108	coding	PF00616
MSTRG.24173.12	12168	noncoding	-
MSTRG.24173.15	12135	noncoding	-
MSTRG.24173.16	9369	coding	PF00616
MSTRG.24173.17	8785	coding	PF00616
MSTRG.24175.1	763	noncoding	-

MSTRG.24148.2	2711	noncoding	-
MSTRG.24152.1	1155	noncoding	-
MSTRG.24162.1	1183	noncoding	-
MSTRG.24163.1	4900	noncoding	-
MSTRG.24164.1	1064	noncoding	-
MSTRG.24163.2	3222	coding	PF00069
MSTRG.24163.4	15155	noncoding	-
MSTRG.24163.5	1476	noncoding	-
MSTRG.24163.6	2223	noncoding	-
MSTRG.24158.1	2160	coding	PF02214
MSTRG.24153.1	900	noncoding	-
MSTRG.24168.1	874	coding	PF00274
MSTRG.24171.1	1898	noncoding	-
MSTRG.24172.1	11485	coding	PF00069
MSTRG.24172.2	6808	coding	PF10351
MSTRG.24184.1	1301	noncoding	-
MSTRG.24184.3	938	noncoding	-
MSTRG.24184.4	778	noncoding	-
MSTRG.24185.3	2908	coding	PF14633
MSTRG.24187.1	1982	coding	PF00071
MSTRG.24187.2	1556	coding	PF00071
MSTRG.24187.3	1555	coding	PF00071
MSTRG.24187.4	1589	coding	PF00071
MSTRG.24187.5	1350	coding	PF00071
MSTRG.24183.1	497	noncoding	-
MSTRG.24190.6	6681	noncoding	-
MSTRG.24195.1	497	noncoding	-
MSTRG.24197.1	3126	noncoding	-
MSTRG.24198.1	12138	coding	PF00069
MSTRG.24198.2	9187	coding	PF00069
MSTRG.24201.3	3323	coding	PF11904
MSTRG.24204.4	2528	coding	PF08953
MSTRG.24209.1	5332	noncoding	-
MSTRG.24209.3	5011	noncoding	-
MSTRG.24209.4	4532	noncoding	-
MSTRG.24211.2	5590	coding	PF09745
MSTRG.24211.3	6853	noncoding	-
MSTRG.24212.1	2211	noncoding	-
MSTRG.24207.5	2783	noncoding	-
MSTRG.24207.4	3975	noncoding	-
MSTRG.24215.1	2081	noncoding	-
MSTRG.24214.3	4974	coding	PF00620
MSTRG.24214.4	4606	coding	PF00620
MSTRG.24216.1	2303	coding	PF02466
MSTRG.24222.1	1101	noncoding	-
MSTRG.24223.1	4498	noncoding	-
MSTRG.24224.1	5024	coding	PF00588
MSTRG.24225.2	3607	noncoding	-
MSTRG.24228.5	1958	noncoding	-
MSTRG.24228.6	1887	noncoding	-
MSTRG.24228.7	566	noncoding	-
MSTRG.24229.7	3441	coding	PF04100
MSTRG.24229.10	2573	noncoding	-
MSTRG.24218.3	977	noncoding	-

MSTRG.24230.2	1731	coding	PF00244
MSTRG.24232.3	9934	coding	PF07653
MSTRG.24233.3	4145	coding	PF00063
MSTRG.24236.3	2182	noncoding	-
MSTRG.24252.5	4853	coding	PF13638
MSTRG.24252.6	5721	noncoding	-
MSTRG.24252.7	1148	noncoding	-
MSTRG.24252.8	6677	coding	PF10373
MSTRG.24252.9	6407	coding	PF10373
MSTRG.24250.1	4991	noncoding	-
MSTRG.24250.3	4125	noncoding	-
MSTRG.24250.5	4482	noncoding	-
MSTRG.24250.8	4562	noncoding	-
MSTRG.24250.4	4710	noncoding	-
MSTRG.24250.2	4814	noncoding	-
MSTRG.24250.7	4781	noncoding	-
MSTRG.24257.5	4079	noncoding	-
MSTRG.24257.4	5356	coding	PF00400
MSTRG.24257.3	5634	noncoding	-
MSTRG.24258.5	5359	coding	PF13236
MSTRG.24258.4	6847	coding	PF13236
MSTRG.24260.1	737	noncoding	-
MSTRG.24262.1	386	noncoding	-
MSTRG.24265.2	1476	noncoding	-
MSTRG.24265.3	1401	noncoding	-
MSTRG.24265.5	1409	noncoding	-
MSTRG.24265.6	1261	noncoding	-
MSTRG.24268.2	1682	noncoding	-
MSTRG.24269.3	912	noncoding	-
MSTRG.24269.2	959	noncoding	-
MSTRG.24273.1	5113	noncoding	-
MSTRG.24275.2	3846	coding	PF00069
MSTRG.24276.2	4376	coding	PF00122
MSTRG.24276.3	3807	coding	PF00122
MSTRG.24285.2	8166	noncoding	-
MSTRG.24285.3	8568	noncoding	-
MSTRG.24286.2	3504	noncoding	-
MSTRG.24286.3	5319	noncoding	-
MSTRG.24288.2	1414	coding	PF00179
MSTRG.24288.3	8437	noncoding	-
MSTRG.24288.4	12506	noncoding	-
MSTRG.24283.3	806	noncoding	-
MSTRG.24283.5	3359	noncoding	-
MSTRG.24283.6	1930	noncoding	-
MSTRG.24282.2	865	coding	PF03148
MSTRG.24289.3	1646	coding	PF00307
MSTRG.24289.2	2562	coding	PF00307
MSTRG.24290.3	1603	noncoding	-
MSTRG.24291.1	1242	noncoding	-
MSTRG.24296.1	3068	noncoding	-
MSTRG.24297.2	1142	coding	PF05669
MSTRG.24297.1	4959	coding	PF15718
MSTRG.24297.3	5045	coding	PF15718
MSTRG.24297.6	4801	coding	PF15718

MSTRG.24297.9	7251	coding	PF15718
MSTRG.24295.1	809	noncoding	-
MSTRG.24302.1	1210	noncoding	-
MSTRG.24303.1	5891	coding	PF07717
MSTRG.24313.2	5874	coding	PF03528
MSTRG.24315.1	5331	noncoding	-
MSTRG.24314.2	3762	noncoding	-
MSTRG.24318.1	5751	coding	PF03859
MSTRG.24318.2	5679	coding	PF03859
MSTRG.24318.3	5490	coding	PF03859
MSTRG.24318.4	6554	coding	PF03859
MSTRG.24306.2	2535	coding	PF00113
MSTRG.24306.3	1796	coding	PF00113
MSTRG.24307.1	621	coding	PF00235
MSTRG.24307.2	1361	coding	PF00235
MSTRG.24307.3	1115	coding	PF00235
MSTRG.24307.4	719	coding	PF00235
MSTRG.24309.1	1573	noncoding	-
MSTRG.24305.1	1498	noncoding	-
MSTRG.24304.4	846	coding	PF02932
MSTRG.24328.2	4983	coding	PF08166
MSTRG.24328.5	4502	noncoding	-
MSTRG.24328.7	6151	noncoding	-
MSTRG.24328.8	9078	coding	PF08166
MSTRG.24336.3	647	noncoding	-
MSTRG.24338.6	2519	coding	PF00625
MSTRG.24339.1	2143	coding	PF00441
MSTRG.24340.1	955	noncoding	-
MSTRG.24341.3	2848	coding	PF00778
MSTRG.24344.1	749	noncoding	-
MSTRG.24346.1	1167	noncoding	-
MSTRG.24346.6	1276	noncoding	-
MSTRG.24346.2	1237	noncoding	-
MSTRG.24346.3	1733	noncoding	-
MSTRG.24346.5	1853	noncoding	-
MSTRG.24346.4	1809	noncoding	-
MSTRG.24346.7	1802	noncoding	-
MSTRG.24348.3	1596	coding	PF01287
MSTRG.24350.3	5157	coding	PF07177
MSTRG.24350.4	6076	coding	PF07177
MSTRG.24350.5	4777	coding	PF07177
MSTRG.24351.7	2538	coding	PF04979
MSTRG.24351.5	3022	coding	PF04979
MSTRG.24351.4	3165	coding	PF04979
MSTRG.24351.1	3218	coding	PF04979
MSTRG.24351.3	4259	coding	PF01412
MSTRG.24351.2	4694	coding	PF01412
MSTRG.24352.1	2865	coding	PF02214
MSTRG.24356.3	1087	noncoding	-
MSTRG.24357.1	2327	coding	PF15670
MSTRG.24357.3	2580	coding	PF15670
MSTRG.24358.5	3950	noncoding	-
MSTRG.24364.1	1663	noncoding	-
MSTRG.24365.4	6521	coding	PF04997

MSTRG.24365.8	6458	coding	PF04997
MSTRG.24365.9	6353	coding	PF04997
MSTRG.24365.10	6558	coding	PF04997
MSTRG.24365.11	6485	coding	PF04997
MSTRG.24365.6	6579	coding	PF04997
MSTRG.24365.3	6575	coding	PF04997
MSTRG.24365.5	6554	coding	PF04997
MSTRG.24365.1	6388	coding	PF04997
MSTRG.24365.12	6491	coding	PF04997
MSTRG.24365.13	6519	coding	PF04997
MSTRG.24365.14	6563	coding	PF04997
MSTRG.24366.1	408	noncoding	-
MSTRG.24367.1	235	noncoding	-
MSTRG.24374.2	2439	coding	PF00270
MSTRG.24376.1	1689	noncoding	-
MSTRG.24376.2	1308	noncoding	-
MSTRG.24376.5	1571	noncoding	-
MSTRG.24376.6	2394	noncoding	-
MSTRG.24378.4	4574	noncoding	-
MSTRG.24378.5	947	noncoding	-
MSTRG.24379.1	1887	coding	PF00054
MSTRG.24379.2	1561	coding	PF00054
MSTRG.24380.1	858	noncoding	-
MSTRG.24359.2	4222	coding	PF00287
MSTRG.24359.3	4849	coding	PF00287
MSTRG.24361.2	1779	noncoding	-
MSTRG.24382.5	7775	coding	PF02373
MSTRG.24387.6	679	noncoding	-
MSTRG.24387.1	587	noncoding	-
MSTRG.24387.3	839	noncoding	-
MSTRG.24387.4	400	noncoding	-
MSTRG.24389.1	2813	noncoding	-
MSTRG.24387.7	5430	noncoding	-
MSTRG.24390.8	5982	coding	PF08074
MSTRG.24384.3	4252	coding	PF12114
MSTRG.24384.2	8170	coding	PF12114
MSTRG.24397.1	2620	noncoding	-
MSTRG.24398.1	667	coding	PF04603
MSTRG.24397.3	2533	noncoding	-
MSTRG.24398.4	706	coding	PF04603
MSTRG.24400.1	11455	noncoding	-
MSTRG.24405.1	2712	noncoding	-
MSTRG.24405.3	2703	noncoding	-
MSTRG.24405.4	2824	noncoding	-
MSTRG.24406.3	758	coding	PF16906
MSTRG.24408.1	1576	noncoding	-
MSTRG.24408.2	1067	noncoding	-
MSTRG.24410.3	6512	coding	PF04880
MSTRG.24412.1	6272	coding	PF01576
MSTRG.24412.2	7935	coding	PF01576
MSTRG.24413.1	3676	coding	PF00063
MSTRG.24401.1	3282	noncoding	-
MSTRG.24404.2	3862	noncoding	-
MSTRG.24415.7	913	noncoding	-

MSTRG.24415.3	1065	noncoding	-
MSTRG.24415.5	1010	noncoding	-
MSTRG.24415.4	784	noncoding	-
MSTRG.24415.6	990	noncoding	-
MSTRG.24415.2	652	noncoding	-
MSTRG.24417.1	4684	coding	PF16623
MSTRG.24417.6	9374	coding	PF16623
MSTRG.24417.5	7965	coding	PF16623
MSTRG.24419.1	3555	noncoding	-
MSTRG.24419.2	7764	noncoding	-
MSTRG.24420.1	1604	noncoding	-
MSTRG.24421.1	2958	noncoding	-
MSTRG.24424.2	4290	coding	PF02630
MSTRG.24436.1	1401	coding	PF13908
MSTRG.24442.2	13692	coding	PF03028
MSTRG.24443.1	3685	coding	PF00069
MSTRG.24429.7	538	noncoding	-
MSTRG.24429.5	1302	noncoding	-
MSTRG.24429.6	1120	noncoding	-
MSTRG.24429.1	970	noncoding	-
MSTRG.24429.2	1353	noncoding	-
MSTRG.24429.3	1335	noncoding	-
MSTRG.24429.4	1213	noncoding	-
MSTRG.24429.8	803	noncoding	-
MSTRG.24454.1	1674	noncoding	-
MSTRG.24456.2	1923	coding	PF00822
MSTRG.24458.1	433	noncoding	-
MSTRG.24460.1	1922	coding	PF15213
MSTRG.24460.2	1831	coding	PF15213
MSTRG.24460.3	1630	coding	PF15213
MSTRG.24460.4	1498	coding	PF15213
MSTRG.24460.5	2830	coding	PF15213
MSTRG.24460.6	3844	coding	PF15213
MSTRG.24461.1	924	noncoding	-
MSTRG.24462.1	2509	coding	PF05832
MSTRG.24464.3	3428	noncoding	-
MSTRG.24464.2	3257	noncoding	-
MSTRG.24464.1	4248	noncoding	-
MSTRG.24465.1	4219	noncoding	-
MSTRG.24463.1	6640	coding	PF01352
MSTRG.24468.1	3121	coding	PF02023
MSTRG.24468.2	5078	coding	PF02023
MSTRG.24484.2	916	coding	PF00240
MSTRG.24484.3	688	coding	PF00240
MSTRG.24484.4	916	coding	PF00240
MSTRG.24484.5	916	coding	PF00240
MSTRG.24484.6	1253	coding	PF00240
MSTRG.24484.7	916	coding	PF00240
MSTRG.24485.3	919	noncoding	-
MSTRG.24485.2	887	noncoding	-
MSTRG.24486.1	839	noncoding	-
MSTRG.24487.10	8836	noncoding	-
MSTRG.24487.3	8706	noncoding	-
MSTRG.24487.4	8721	noncoding	-

MSTRG.24487.5	8619	noncoding	-
MSTRG.24487.6	8598	noncoding	-
MSTRG.24487.7	8544	noncoding	-
MSTRG.24487.9	8682	noncoding	-
MSTRG.24487.8	10294	noncoding	-
MSTRG.24487.11	10576	noncoding	-
MSTRG.24487.17	8764	noncoding	-
MSTRG.24487.18	8767	noncoding	-
MSTRG.24487.19	8800	noncoding	-
MSTRG.24488.7	1930	noncoding	-
MSTRG.24488.10	2092	noncoding	-
MSTRG.24488.8	1215	noncoding	-
MSTRG.24488.14	1112	noncoding	-
MSTRG.24489.2	1493	noncoding	-
MSTRG.24472.3	6447	coding	PF00307
MSTRG.24472.5	4397	noncoding	-
MSTRG.24477.1	3205	coding	PF00069
MSTRG.24477.10	2684	coding	PF00069
MSTRG.24477.8	2750	coding	PF00069
MSTRG.24477.7	4183	coding	PF00069
MSTRG.24477.9	4079	coding	PF00069
MSTRG.24478.1	3018	noncoding	-
MSTRG.24495.3	2987	coding	PF00069
MSTRG.24510.1	4751	noncoding	-
MSTRG.24503.1	1178	noncoding	-
MSTRG.24504.1	382	noncoding	-
MSTRG.24514.2	1344	noncoding	-
MSTRG.24516.2	410	noncoding	-
MSTRG.24521.6	3679	noncoding	-
MSTRG.24523.3	3893	noncoding	-
MSTRG.24523.4	8669	noncoding	-
MSTRG.24520.2	1025	coding	PF04191
MSTRG.24520.3	921	coding	PF04191
MSTRG.24520.4	1937	coding	PF04191
MSTRG.24520.6	1153	coding	PF04191
MSTRG.24528.4	1447	noncoding	-
MSTRG.24528.3	1215	noncoding	-
MSTRG.24528.2	2841	noncoding	-
MSTRG.24528.5	1285	noncoding	-
MSTRG.24528.6	952	noncoding	-
MSTRG.24531.2	4620	coding	PF13091
MSTRG.24539.3	1604	coding	PF00106
MSTRG.24539.4	7691	coding	PF00106
MSTRG.24541.2	1054	coding	PF14972
MSTRG.24543.1	1560	noncoding	-
MSTRG.24548.1	10378	noncoding	-
MSTRG.24548.3	5713	noncoding	-
MSTRG.24554.1	5364	coding	PF09744
MSTRG.24554.4	4907	coding	PF09744
MSTRG.24554.7	4584	coding	PF16471
MSTRG.24554.9	4115	coding	PF16471
MSTRG.24555.1	1097	noncoding	-
MSTRG.24553.1	3318	coding	PF00014
MSTRG.24557.1	3393	coding	PF03194

MSTRG.24557.3	4314	coding	PF03194
MSTRG.24557.4	2025	coding	PF03194
MSTRG.24557.5	1586	coding	PF03194
MSTRG.24566.2	2958	coding	PF14646
MSTRG.24566.3	3258	coding	PF14646
MSTRG.24568.2	2906	noncoding	-
MSTRG.24571.1	3908	coding	PF12529
MSTRG.24574.4	915	noncoding	-
MSTRG.24574.5	525	noncoding	-
MSTRG.24574.3	469	noncoding	-
MSTRG.24574.2	446	noncoding	-
MSTRG.24574.6	944	noncoding	-
MSTRG.24575.1	821	noncoding	-
MSTRG.24580.2	1528	noncoding	-
MSTRG.24584.2	2921	coding	PF01853
MSTRG.24584.3	3022	coding	PF01853
MSTRG.24585.2	658	coding	PF15388
MSTRG.24586.1	1154	noncoding	-
MSTRG.24588.3	1192	noncoding	-
MSTRG.24589.5	3608	noncoding	-
MSTRG.24589.6	3688	noncoding	-
MSTRG.24589.4	4507	noncoding	-
MSTRG.24589.7	9977	noncoding	-
MSTRG.24591.10	1434	coding	PF06888
MSTRG.24591.3	1673	coding	PF06888
MSTRG.24591.4	2017	coding	PF06888
MSTRG.24591.5	2027	coding	PF06888
MSTRG.24591.6	1905	coding	PF06888
MSTRG.24591.7	2139	coding	PF06888
MSTRG.24591.8	1555	coding	PF06888
MSTRG.24591.9	1424	coding	PF06888
MSTRG.24594.1	3895	noncoding	-
MSTRG.24594.2	2048	noncoding	-
MSTRG.24595.3	3162	noncoding	-
MSTRG.24595.4	2199	noncoding	-
MSTRG.24597.2	8805	noncoding	-
MSTRG.24599.3	3409	coding	PF07888
MSTRG.24599.4	2336	coding	PF07888
MSTRG.24599.5	3159	coding	PF07888
MSTRG.24604.2	4105	noncoding	-
MSTRG.24604.1	10714	coding	PF03133
MSTRG.24604.3	11271	coding	PF03133
MSTRG.24602.1	622	noncoding	-
MSTRG.24609.7	4262	noncoding	-
MSTRG.24619.1	584	noncoding	-
MSTRG.24620.2	3870	coding	PF01433
MSTRG.24620.3	3908	coding	PF01433
MSTRG.24620.4	3998	coding	PF01433
MSTRG.24620.5	2444	coding	PF01433
MSTRG.24621.1	1243	noncoding	-
MSTRG.24623.3	5450	coding	PF00620
MSTRG.24625.4	4400	noncoding	-
MSTRG.24625.5	4459	noncoding	-
MSTRG.24629.1	4852	coding	PF01504

MSTRG.24629.3	4705	coding	PF01504
MSTRG.24630.1	3890	coding	PF12542
MSTRG.24633.1	498	coding	PF15075
MSTRG.24637.1	3862	coding	PF14604
MSTRG.24637.3	3779	coding	PF14604
MSTRG.24637.2	3859	coding	PF14604
MSTRG.24637.4	3769	coding	PF14604
MSTRG.24640.2	1258	coding	PF01280
MSTRG.24648.1	4587	noncoding	-
MSTRG.24649.1	1468	noncoding	-
MSTRG.24651.1	2821	coding	PF10744
MSTRG.24651.2	2721	coding	PF10744
MSTRG.24642.6	8815	coding	PF00069
MSTRG.24642.7	8397	coding	PF00069
MSTRG.24644.1	4388	coding	PF09470
MSTRG.24656.3	7706	noncoding	-
MSTRG.24659.3	1156	coding	PF04061
MSTRG.24659.4	1296	coding	PF04061
MSTRG.24662.2	3752	coding	PF11277
MSTRG.24663.2	5656	coding	PF00105
MSTRG.24664.2	2426	coding	PF00105
MSTRG.24666.3	1845	noncoding	-
MSTRG.24661.3	3891	coding	PF09405
MSTRG.24661.4	3907	coding	PF09405
MSTRG.24670.1	862	noncoding	-
MSTRG.24669.3	2810	coding	PF00105
MSTRG.24669.6	968	noncoding	-
MSTRG.24673.2	5594	coding	PF16898
MSTRG.24672.1	1086	noncoding	-
MSTRG.24676.1	617	noncoding	-
MSTRG.24676.2	637	noncoding	-
MSTRG.24677.1	980	noncoding	-
MSTRG.24693.3	1713	coding	PF05822
MSTRG.24696.1	2203	coding	PF00651
MSTRG.24696.2	2161	coding	PF00651
MSTRG.24697.2	4468	coding	PF16114
MSTRG.24697.5	3955	coding	PF16114
MSTRG.24724.1	3738	coding	PF15237
MSTRG.24724.6	3109	coding	PF15237
MSTRG.24724.5	3111	coding	PF15237
MSTRG.24724.7	3182	coding	PF15237
MSTRG.24724.2	3124	coding	PF15237
MSTRG.24724.3	3130	coding	PF15237
MSTRG.24724.4	2535	coding	PF15237
MSTRG.24725.1	8191	coding	PF01496
MSTRG.24725.2	7518	coding	PF01496
MSTRG.24725.3	8208	coding	PF01496
MSTRG.24725.4	7078	coding	PF01496
MSTRG.24725.5	7635	coding	PF01496
MSTRG.24729.1	2207	coding	PF01121
MSTRG.24729.2	2513	coding	PF01121
MSTRG.24729.3	2356	coding	PF01121
MSTRG.24729.4	2375	coding	PF01121
MSTRG.24729.5	2330	coding	PF01121

MSTRG.24729.6	2462	coding	PF01121
MSTRG.24732.1	4646	coding	PF07106
MSTRG.24732.2	4639	coding	PF07106
MSTRG.24732.4	3510	noncoding	-
MSTRG.24735.4	2922	noncoding	-
MSTRG.24710.4	5220	coding	PF00856
MSTRG.24710.5	4761	coding	PF00856
MSTRG.24706.1	870	coding	PF04901
MSTRG.24711.3	3937	coding	PF00069
MSTRG.24711.4	3389	coding	PF00069
MSTRG.24717.2	1139	noncoding	-
MSTRG.24721.1	7328	coding	PF02252
MSTRG.24721.2	3548	coding	PF02252
MSTRG.24721.4	2586	coding	PF02252
MSTRG.24722.2	1987	coding	PF01179
MSTRG.24722.4	2702	coding	PF01179
MSTRG.24714.2	1008	noncoding	-
MSTRG.24715.2	3915	noncoding	-
MSTRG.24741.2	2503	coding	PF13602
MSTRG.24742.3	3568	coding	PF00071
MSTRG.24743.2	5864	coding	PF12820
MSTRG.24743.3	5861	coding	PF12820
MSTRG.24745.1	1012	noncoding	-
MSTRG.24745.2	993	noncoding	-
MSTRG.24745.3	848	noncoding	-
MSTRG.24748.1	1734	coding	PF00025
MSTRG.24763.1	8495	coding	PF09793
MSTRG.24763.3	1175	coding	PF09793
MSTRG.24768.3	3575	coding	PF00850
MSTRG.24768.7	3572	coding	PF00850
MSTRG.24769.1	838	noncoding	-
MSTRG.24772.2	4165	noncoding	-
MSTRG.24774.5	3029	coding	PF14887
MSTRG.24774.6	2961	coding	PF14887
MSTRG.24784.2	6499	coding	PF01421
MSTRG.24784.3	1759	noncoding	-
MSTRG.24785.3	2167	coding	PF00153
MSTRG.24788.1	4465	noncoding	-
MSTRG.24789.1	1458	noncoding	-
MSTRG.24792.2	3819	coding	PF07535
MSTRG.24793.1	284	noncoding	-
MSTRG.24793.2	314	noncoding	-
MSTRG.24778.1	1943	coding	PF00029
MSTRG.24782.1	3551	coding	PF16004
MSTRG.24795.2	2483	coding	PF01121
MSTRG.24801.1	1316	coding	PF15313
MSTRG.24801.2	1232	coding	PF15313
MSTRG.24805.2	1400	noncoding	-
MSTRG.24807.1	462	coding	PF15310
MSTRG.24809.1	4476	coding	PF00620
MSTRG.24810.1	597	noncoding	-
MSTRG.24814.1	8097	coding	PF14914
MSTRG.24815.2	1138	noncoding	-
MSTRG.24816.1	1141	coding	PF00062

MSTRG.24816.2	879	coding	PF00062
MSTRG.24817.1	1151	coding	PF00110
MSTRG.24817.2	3135	coding	PF00110
MSTRG.24820.2	3456	coding	PF00004
MSTRG.24821.1	450	noncoding	-
MSTRG.24825.1	1326	noncoding	-
MSTRG.24826.4	1526	noncoding	-
MSTRG.24826.6	1629	noncoding	-
MSTRG.24826.2	1744	noncoding	-
MSTRG.24826.3	1614	noncoding	-
MSTRG.24826.5	1717	noncoding	-
MSTRG.24826.7	1657	noncoding	-
MSTRG.24826.8	3047	noncoding	-
MSTRG.24827.6	4644	coding	PF15275
MSTRG.24827.7	3014	coding	PF15275
MSTRG.24827.8	3259	coding	PF15275
MSTRG.24827.9	3023	coding	PF15275
MSTRG.24827.10	3282	coding	PF15275
MSTRG.24827.11	5862	coding	PF15275
MSTRG.24828.1	5181	noncoding	-
MSTRG.24830.1	800	noncoding	-
MSTRG.24833.1	1370	noncoding	-
MSTRG.24835.1	5471	coding	PF00362
MSTRG.24836.1	524	noncoding	-
MSTRG.24837.1	2644	noncoding	-
MSTRG.24837.2	1256	noncoding	-
MSTRG.24837.3	4802	noncoding	-
MSTRG.24837.4	4635	noncoding	-
MSTRG.24837.5	2188	noncoding	-
MSTRG.24841.2	5572	noncoding	-
MSTRG.24841.1	5644	noncoding	-
MSTRG.24834.3	1802	noncoding	-
MSTRG.24844.1	3702	coding	PF00069
MSTRG.24844.2	3795	coding	PF00069
MSTRG.24844.3	3444	coding	PF00069
MSTRG.24844.8	3558	coding	PF00069
MSTRG.24844.9	3651	coding	PF00069
MSTRG.24844.10	2832	coding	PF00069
MSTRG.24846.1	2165	noncoding	-
MSTRG.24847.1	606	noncoding	-
MSTRG.24842.4	932	noncoding	-
MSTRG.24850.1	7635	noncoding	-
MSTRG.24849.3	1613	noncoding	-
MSTRG.24849.2	11913	coding	PF12796
MSTRG.24852.1	2157	noncoding	-
MSTRG.24853.1	2406	noncoding	-
MSTRG.24854.1	6835	noncoding	-
MSTRG.24855.1	7315	noncoding	-
MSTRG.24856.1	546	noncoding	-
MSTRG.24858.2	3011	coding	PF03188
MSTRG.24858.3	2814	coding	PF03188
MSTRG.24858.4	1041	noncoding	-
MSTRG.24860.3	2452	coding	PF01401
MSTRG.24861.2	2898	coding	PF01401

MSTRG.24861.3	2801	coding	PF01401
MSTRG.24861.4	2870	coding	PF01401
MSTRG.24861.5	2456	coding	PF01401
MSTRG.24862.1	2503	coding	PF00027
MSTRG.24866.1	4554	coding	PF00069
MSTRG.24866.2	4647	coding	PF00069
MSTRG.24866.3	4795	coding	PF00069
MSTRG.24866.7	2804	coding	PF00069
MSTRG.24867.2	1336	noncoding	-
MSTRG.24867.4	1531	noncoding	-
MSTRG.24867.3	2531	noncoding	-
MSTRG.24867.5	2684	noncoding	-
MSTRG.24867.11	2268	noncoding	-
MSTRG.24867.7	2053	noncoding	-
MSTRG.24867.8	2711	noncoding	-
MSTRG.24878.1	1599	coding	PF00004
MSTRG.24879.2	2451	coding	PF02201
MSTRG.24864.1	5744	coding	PF06512
MSTRG.24865.1	1096	noncoding	-
MSTRG.24865.2	1003	noncoding	-
MSTRG.24869.2	594	noncoding	-
MSTRG.24869.4	719	noncoding	-
MSTRG.24869.3	663	noncoding	-
MSTRG.24871.2	7757	coding	PF06479
MSTRG.24890.1	1576	noncoding	-
MSTRG.24892.1	3231	noncoding	-
MSTRG.24892.3	1823	noncoding	-
MSTRG.24892.4	1986	noncoding	-
MSTRG.24892.5	2653	noncoding	-
MSTRG.24892.6	1699	noncoding	-
MSTRG.24893.3	4483	coding	PF00632
MSTRG.24885.7	8941	coding	PF00439
MSTRG.24885.6	11242	coding	PF00439
MSTRG.24885.5	12732	coding	PF00439
MSTRG.24885.3	12911	coding	PF00439
MSTRG.24885.10	4715	coding	PF00439
MSTRG.24885.11	5731	coding	PF00439
MSTRG.24885.8	6583	coding	PF00439
MSTRG.24885.9	8618	coding	PF00439
MSTRG.24898.1	2126	coding	PF02121
MSTRG.24898.2	8877	coding	PF02121
MSTRG.24898.3	8633	coding	PF02121
MSTRG.24898.4	4923	coding	PF02121
MSTRG.24898.5	21385	coding	PF02121
MSTRG.24898.6	2587	coding	PF02121
MSTRG.24903.1	546	noncoding	-
MSTRG.24913.2	2408	coding	PF12612
MSTRG.24914.2	2124	noncoding	-
MSTRG.24911.4	1027	coding	PF03881
MSTRG.24920.1	2961	noncoding	-
MSTRG.24922.1	1031	noncoding	-
MSTRG.24924.2	2969	coding	PF02906
MSTRG.24925.1	2589	coding	PF15169
MSTRG.24925.2	2304	coding	PF15169

MSTRG.24930.1	707	noncoding	-
MSTRG.24932.1	498	noncoding	-
MSTRG.24936.1	2774	noncoding	-
MSTRG.24936.5	3619	noncoding	-
MSTRG.24936.4	2987	noncoding	-
MSTRG.24936.7	7426	coding	PF00069
MSTRG.24958.2	2108	coding	PF01207
MSTRG.24958.1	1909	coding	PF01207
MSTRG.24959.1	2064	coding	PF10602
MSTRG.24959.2	2065	coding	PF10602
MSTRG.24960.1	2421	coding	PF02434
MSTRG.24960.2	1305	coding	PF02434
MSTRG.24960.4	1904	coding	PF02434
MSTRG.24960.5	1709	coding	PF02434
MSTRG.24961.1	363	noncoding	-
MSTRG.24962.2	3172	noncoding	-
MSTRG.24963.1	943	coding	PF09415
MSTRG.24969.4	590	coding	PF12861
MSTRG.24971.1	1390	noncoding	-
MSTRG.24971.2	1583	noncoding	-
MSTRG.24971.3	1584	noncoding	-
MSTRG.24971.4	2375	noncoding	-
MSTRG.24960.6	2295	coding	PF13848
MSTRG.24960.7	3091	coding	PF13848
MSTRG.24944.2	2368	noncoding	-
MSTRG.24946.4	2998	coding	PF12210
MSTRG.24946.3	3208	coding	PF12210
MSTRG.24946.5	2844	coding	PF12210
MSTRG.24948.2	5015	coding	PF00025
MSTRG.24954.3	4497	noncoding	-
MSTRG.24954.6	4071	noncoding	-
MSTRG.24976.3	1727	noncoding	-
MSTRG.24976.2	6384	coding	PF14538
MSTRG.24976.4	4344	noncoding	-
MSTRG.25009.1	1824	coding	PF04493
MSTRG.25010.1	4267	noncoding	-
MSTRG.25010.8	26122	noncoding	-
MSTRG.25010.10	15805	coding	PF00097
MSTRG.25010.11	14989	coding	PF00097
MSTRG.25010.12	17561	noncoding	-
MSTRG.25010.13	19457	noncoding	-
MSTRG.25010.6	5698	noncoding	-
MSTRG.25010.4	5072	noncoding	-
MSTRG.25010.7	6570	coding	PF00097
MSTRG.25010.3	10018	coding	PF00097
MSTRG.25010.5	13084	coding	PF00097
MSTRG.25012.1	626	noncoding	-
MSTRG.25010.15	3029	noncoding	-
MSTRG.25010.16	5993	noncoding	-
MSTRG.24992.2	3891	coding	PF01055
MSTRG.24993.1	4227	noncoding	-
MSTRG.24993.3	4218	noncoding	-
MSTRG.24993.4	4209	noncoding	-
MSTRG.24993.5	4260	noncoding	-

MSTRG.24986.1	4634	noncoding	-
MSTRG.24996.1	5368	noncoding	-
MSTRG.25001.2	4738	coding	PF06079
MSTRG.25001.4	3523	coding	PF06079
MSTRG.25003.2	3322	coding	PF00965
MSTRG.25006.1	3092	coding	PF01369
MSTRG.25006.3	1470	noncoding	-
MSTRG.25020.1	6881	coding	PF07810
MSTRG.25020.4	6540	coding	PF07810
MSTRG.25020.5	805	noncoding	-
MSTRG.25021.1	8322	coding	PF16608
MSTRG.25021.2	8421	coding	PF16608
MSTRG.25021.3	8140	coding	PF16608
MSTRG.25027.4	13261	coding	PF00735
MSTRG.25027.7	3509	coding	PF00735
MSTRG.25027.9	3148	coding	PF00735
MSTRG.25027.8	3010	coding	PF00735
MSTRG.25028.2	2643	coding	PF04707
MSTRG.25029.1	644	noncoding	-
MSTRG.25031.2	1261	coding	PF15024
MSTRG.25031.3	1172	noncoding	-
MSTRG.25031.1	1581	noncoding	-
MSTRG.25031.4	1080	noncoding	-
MSTRG.25033.2	5271	coding	PF05978
MSTRG.25032.4	1791	noncoding	-
MSTRG.25039.3	2050	coding	PF00777
MSTRG.25041.2	2329	noncoding	-
MSTRG.25038.1	347	noncoding	-
MSTRG.25042.1	1789	noncoding	-
MSTRG.25045.3	17801	noncoding	-
MSTRG.25049.1	946	noncoding	-
MSTRG.25050.2	4803	coding	PF16043
MSTRG.25050.4	4748	coding	PF16043
MSTRG.25050.5	4588	coding	PF16043
MSTRG.25051.3	671	noncoding	-
MSTRG.25051.4	705	noncoding	-
MSTRG.25051.6	529	noncoding	-
MSTRG.25053.6	3789	coding	PF03081
MSTRG.25058.1	6310	coding	PF01756
MSTRG.25058.3	5036	coding	PF01756
MSTRG.25059.1	4919	noncoding	-
MSTRG.25055.1	1166	coding	PF00097
MSTRG.25056.1	2202	noncoding	-
MSTRG.25060.3	1782	noncoding	-
MSTRG.25063.2	3017	coding	PF00125
MSTRG.25063.1	2085	coding	PF00125
MSTRG.25064.2	1718	coding	PF10509
MSTRG.25067.2	4699	noncoding	-
MSTRG.25067.3	3767	noncoding	-
MSTRG.25070.1	1230	noncoding	-
MSTRG.25070.2	1226	noncoding	-
MSTRG.25070.4	1103	noncoding	-
MSTRG.25072.4	1876	coding	PF12928
MSTRG.25072.3	1927	coding	PF12928

MSTRG.25074.2	5927	noncoding	-
MSTRG.25074.3	5395	noncoding	-
MSTRG.25071.3	2311	coding	PF14604
MSTRG.25078.3	5819	coding	PF00790
MSTRG.25079.3	1302	coding	PF00177
MSTRG.25079.2	1337	coding	PF00177
MSTRG.25078.4	4415	coding	PF00790
MSTRG.25080.2	2569	coding	PF07575
MSTRG.25076.2	1596	coding	PF11976
MSTRG.25076.3	1395	coding	PF11976
MSTRG.25085.2	915	noncoding	-
MSTRG.25085.3	976	coding	PF06941
MSTRG.25081.1	4825	coding	PF15227
MSTRG.25090.3	627	coding	PF05873
MSTRG.25108.4	2469	noncoding	-
MSTRG.25108.7	3781	noncoding	-
MSTRG.25109.1	556	noncoding	-
MSTRG.25110.1	3299	noncoding	-
MSTRG.25103.2	2692	noncoding	-
MSTRG.25103.3	2849	noncoding	-
MSTRG.25103.5	2673	noncoding	-
MSTRG.25103.4	2530	noncoding	-
MSTRG.25103.6	1178	noncoding	-
MSTRG.25105.1	600	coding	PF01781
MSTRG.25104.1	420	noncoding	-
MSTRG.25107.1	361	noncoding	-
MSTRG.25116.1	2839	noncoding	-
MSTRG.25116.3	2932	noncoding	-
MSTRG.25115.1	531	noncoding	-
MSTRG.25118.2	3063	coding	PF15434
MSTRG.25120.1	1300	noncoding	-
MSTRG.25123.1	1882	noncoding	-
MSTRG.25124.1	3395	coding	PF12444
MSTRG.25124.2	3212	noncoding	-
MSTRG.25125.1	710	noncoding	-
MSTRG.25143.1	12557	noncoding	-
MSTRG.25143.2	8309	noncoding	-
MSTRG.25143.3	11757	noncoding	-
MSTRG.25128.1	1347	noncoding	-
MSTRG.25138.1	335	noncoding	-
MSTRG.25139.1	355	noncoding	-
MSTRG.25150.4	1968	noncoding	-
MSTRG.25166.1	1073	noncoding	-
MSTRG.25167.1	5831	coding	PF00005
MSTRG.25167.3	4062	coding	PF00005
MSTRG.25168.1	12953	coding	PF00005
MSTRG.25164.2	1353	noncoding	-
MSTRG.25164.1	1616	noncoding	-
MSTRG.25164.4	962	noncoding	-
MSTRG.25170.1	11362	noncoding	-
MSTRG.25174.2	1432	noncoding	-
MSTRG.25174.3	1229	noncoding	-
MSTRG.25176.4	1056	noncoding	-
MSTRG.25176.5	941	noncoding	-

MSTRG.25181.1	2664	noncoding	-
MSTRG.25185.1	1658	coding	PF00778
MSTRG.25195.1	6724	noncoding	-
MSTRG.25195.2	3588	noncoding	-
MSTRG.25196.3	2257	noncoding	-
MSTRG.25196.4	3413	coding	PF14846
MSTRG.25196.5	1317	noncoding	-
MSTRG.25196.6	974	noncoding	-
MSTRG.25198.1	252	noncoding	-
MSTRG.25190.3	2162	coding	PF00069
MSTRG.25205.4	11675	coding	PF13087
MSTRG.25205.5	10439	coding	PF13087
MSTRG.25206.1	6205	noncoding	-
MSTRG.25205.6	4450	coding	PF13087
MSTRG.25207.1	5238	noncoding	-
MSTRG.25207.2	7627	noncoding	-
MSTRG.25207.3	6341	noncoding	-
MSTRG.25207.4	3421	noncoding	-
MSTRG.25208.1	228	noncoding	-
MSTRG.25211.1	4934	noncoding	-
MSTRG.25214.1	1695	noncoding	-
MSTRG.25214.2	1465	noncoding	-
MSTRG.25217.1	2281	noncoding	-
MSTRG.25232.1	728	noncoding	-
MSTRG.25248.1	8683	noncoding	-
MSTRG.25254.2	2374	noncoding	-
MSTRG.25221.1	449	noncoding	-
MSTRG.25224.3	453	noncoding	-
MSTRG.25226.1	42879	noncoding	-
MSTRG.25226.3	24386	noncoding	-
MSTRG.25226.5	29446	noncoding	-
MSTRG.25226.4	5068	noncoding	-
MSTRG.25226.8	18140	noncoding	-
MSTRG.25255.4	3569	noncoding	-
MSTRG.25255.1	3842	noncoding	-
MSTRG.25256.1	3715	noncoding	-
MSTRG.25257.1	2836	noncoding	-
MSTRG.25258.1	4419	noncoding	-
MSTRG.25255.6	1523	noncoding	-
MSTRG.25268.6	8427	coding	PF12125
MSTRG.25269.1	2351	noncoding	-
MSTRG.25280.2	711	noncoding	-
MSTRG.25261.1	8665	noncoding	-
MSTRG.25262.1	1090	noncoding	-
MSTRG.25262.2	2753	noncoding	-
MSTRG.25265.1	327	noncoding	-
MSTRG.25281.1	648	noncoding	-
MSTRG.25285.2	610	coding	PF16906
MSTRG.25290.3	3122	noncoding	-
MSTRG.25297.2	467	noncoding	-
MSTRG.25298.1	8269	noncoding	-
MSTRG.25302.1	450	noncoding	-
MSTRG.25310.1	1409	noncoding	-
MSTRG.25318.1	5283	noncoding	-

MSTRG.25315.1	3060	noncoding	-
MSTRG.25327.1	6625	noncoding	-
MSTRG.25327.2	6227	noncoding	-
MSTRG.25327.3	6422	noncoding	-
MSTRG.25328.1	1634	noncoding	-
MSTRG.25329.1	432	noncoding	-
MSTRG.25335.1	4392	coding	PF10291
MSTRG.25335.2	924	noncoding	-
MSTRG.25342.1	8478	noncoding	-
MSTRG.25342.4	3121	noncoding	-
MSTRG.25342.5	4266	noncoding	-
MSTRG.25343.1	1942	noncoding	-
MSTRG.25348.2	8093	noncoding	-
MSTRG.25350.2	1348	coding	PF01039
MSTRG.25351.2	8899	coding	PF15963
MSTRG.25351.3	8911	coding	PF15963
MSTRG.25351.4	8545	coding	PF15963
MSTRG.25356.2	529	noncoding	-
MSTRG.25359.1	1883	coding	PF04056
MSTRG.25359.4	2800	coding	PF04056
MSTRG.25353.1	1332	coding	PF07303
MSTRG.25353.3	1460	coding	PF07303
MSTRG.25354.1	554	noncoding	-
MSTRG.25354.2	442	noncoding	-
MSTRG.25361.1	5129	coding	PF02291
MSTRG.25361.3	1558	coding	PF13238
MSTRG.25364.1	4927	noncoding	-
MSTRG.25362.2	1145	coding	PF00134
MSTRG.25372.1	1132	coding	PF16454
MSTRG.25384.1	6240	noncoding	-
MSTRG.25384.2	5039	noncoding	-
MSTRG.25384.3	4718	noncoding	-
MSTRG.25384.6	3715	noncoding	-
MSTRG.25388.1	6357	noncoding	-
MSTRG.25388.5	4953	noncoding	-
MSTRG.25388.6	5056	noncoding	-
MSTRG.25388.7	2264	noncoding	-
MSTRG.25389.1	5097	noncoding	-
MSTRG.25390.1	11291	noncoding	-
MSTRG.25389.2	3937	noncoding	-
MSTRG.25389.3	1855	noncoding	-
MSTRG.25409.4	3756	coding	PF06159
MSTRG.25409.5	2417	coding	PF06159
MSTRG.25409.7	1142	noncoding	-
MSTRG.25410.2	3773	coding	PF00025
MSTRG.25413.2	2839	coding	PF00160
MSTRG.25413.3	2169	coding	PF00160
MSTRG.25413.4	2150	coding	PF00160
MSTRG.25413.5	2179	coding	PF00160
MSTRG.25413.6	1575	coding	PF00160
MSTRG.25413.7	1608	coding	PF00160
MSTRG.25415.1	9823	coding	PF11802
MSTRG.25415.2	18583	coding	PF11802
MSTRG.25415.3	2862	noncoding	-

MSTRG.25415.4	5627	coding	PF11802
MSTRG.25417.1	1202	noncoding	-
MSTRG.25399.1	2987	noncoding	-
MSTRG.25391.1	301	noncoding	-
MSTRG.25405.2	1895	noncoding	-
MSTRG.25406.1	3551	noncoding	-
MSTRG.25405.3	714	noncoding	-
MSTRG.25433.2	1874	noncoding	-
MSTRG.25434.1	881	noncoding	-
MSTRG.25435.3	1455	coding	PF00398
MSTRG.25436.1	4578	coding	PF00225
MSTRG.25436.2	8276	coding	PF00225
MSTRG.25436.5	6502	coding	PF00225
MSTRG.25437.1	422	noncoding	-
MSTRG.25441.2	1713	noncoding	-
MSTRG.25447.2	6968	noncoding	-
MSTRG.25447.6	7262	noncoding	-
MSTRG.25447.7	4417	noncoding	-
MSTRG.25448.1	14866	noncoding	-
MSTRG.25444.1	1474	noncoding	-
MSTRG.25451.4	1430	noncoding	-
MSTRG.25462.1	784	noncoding	-
MSTRG.25463.1	2357	noncoding	-
MSTRG.25465.1	5133	noncoding	-
MSTRG.25472.5	4124	coding	PF15337
MSTRG.25472.6	3652	coding	PF15337
MSTRG.25472.7	3340	coding	PF15337
MSTRG.25472.8	3336	coding	PF15337
MSTRG.25477.1	5869	noncoding	-
MSTRG.25477.2	5231	noncoding	-
MSTRG.25478.1	2937	noncoding	-
MSTRG.25478.3	1058	noncoding	-
MSTRG.25478.5	1449	noncoding	-
MSTRG.25480.3	11363	coding	PF00069
MSTRG.25480.4	6358	coding	PF00069
MSTRG.25483.1	307	noncoding	-
MSTRG.25483.2	681	noncoding	-
MSTRG.25484.2	1189	noncoding	-
MSTRG.25489.2	2381	noncoding	-
MSTRG.25489.3	2832	noncoding	-
MSTRG.25491.1	13597	coding	PF10482
MSTRG.25492.4	1368	noncoding	-
MSTRG.25492.3	3246	noncoding	-
MSTRG.25492.5	3072	noncoding	-
MSTRG.25499.3	4457	noncoding	-
MSTRG.25499.2	3603	noncoding	-
MSTRG.25499.6	5750	noncoding	-
MSTRG.25499.7	8983	coding	PF00255
MSTRG.25499.8	2989	noncoding	-
MSTRG.25499.10	1487	noncoding	-
MSTRG.25508.1	1788	coding	PF00025
MSTRG.25508.3	38923	coding	PF02544
MSTRG.25508.4	8021	coding	PF00025
MSTRG.25508.5	2699	coding	PF00025

MSTRG.25508.6	24803	coding	PF02544
MSTRG.25508.7	2019	noncoding	-
MSTRG.25509.1	14736	noncoding	-
MSTRG.25508.9	16875	coding	PF00025
MSTRG.25508.8	5854	coding	PF00025
MSTRG.25510.1	217	noncoding	-
MSTRG.25508.10	36845	noncoding	-
MSTRG.25508.11	36294	noncoding	-
MSTRG.25508.12	20867	noncoding	-
MSTRG.25508.13	5482	noncoding	-
MSTRG.25511.1	2739	noncoding	-
MSTRG.25512.1	15326	noncoding	-
MSTRG.25512.2	9003	noncoding	-
MSTRG.25513.1	2518	coding	PF00078
MSTRG.25514.1	6360	noncoding	-
MSTRG.25508.14	1988	noncoding	-
MSTRG.25517.1	3843	noncoding	-
MSTRG.25517.3	1079	noncoding	-
MSTRG.25517.4	4098	noncoding	-
MSTRG.25518.2	5150	coding	PF00092
MSTRG.25528.2	15915	noncoding	-
MSTRG.25528.3	2998	noncoding	-
MSTRG.25529.1	3827	noncoding	-
MSTRG.25528.4	8754	noncoding	-
MSTRG.25530.1	11380	noncoding	-
MSTRG.25531.1	1806	noncoding	-
MSTRG.25532.1	3828	noncoding	-
MSTRG.25537.2	6941	noncoding	-
MSTRG.25537.1	7083	noncoding	-
MSTRG.25537.3	7415	noncoding	-
MSTRG.25540.1	2205	noncoding	-
MSTRG.25546.1	3534	noncoding	-
MSTRG.25550.3	3671	coding	PF15025
MSTRG.25552.1	5168	coding	PF08540
MSTRG.25552.2	4593	coding	PF08540
MSTRG.25552.3	5240	coding	PF08540
MSTRG.25552.4	5059	coding	PF08540
MSTRG.25552.5	4325	coding	PF08540
MSTRG.25552.6	4220	coding	PF08540
MSTRG.25552.7	4796	coding	PF08540
MSTRG.25552.9	4939	coding	PF08540
MSTRG.25552.10	4404	coding	PF08540
MSTRG.25556.3	8588	noncoding	-
MSTRG.25557.2	3311	coding	PF00651
MSTRG.25571.1	1412	noncoding	-
MSTRG.25577.7	5034	noncoding	-
MSTRG.25577.8	4986	noncoding	-
MSTRG.25582.3	1726	coding	PF00069
MSTRG.25593.2	4104	noncoding	-
MSTRG.25594.1	1113	noncoding	-
MSTRG.25595.1	7260	coding	PF14664
MSTRG.25601.2	10029	noncoding	-
MSTRG.25597.2	3615	coding	PF00054
MSTRG.25597.3	2186	noncoding	-

MSTRG.25598.2	863	noncoding	-
MSTRG.25598.1	944	noncoding	-
MSTRG.25599.1	3121	noncoding	-
MSTRG.25597.4	18623	noncoding	-
MSTRG.25602.1	6907	noncoding	-
MSTRG.25602.2	2038	noncoding	-
MSTRG.25602.4	6243	noncoding	-
MSTRG.25602.5	1209	noncoding	-
MSTRG.25604.3	4743	coding	PF08801
MSTRG.25604.2	6040	coding	PF08801
MSTRG.25615.2	10947	coding	PF15392
MSTRG.25615.6	10631	coding	PF15392
MSTRG.25615.7	10511	coding	PF15392
MSTRG.25615.9	10222	coding	PF15392
MSTRG.25615.10	4545	coding	PF15392
MSTRG.25607.5	8835	coding	PF12830
MSTRG.25608.1	1340	noncoding	-
MSTRG.25609.1	5375	noncoding	-
MSTRG.25610.1	2379	noncoding	-
MSTRG.25610.2	4780	noncoding	-
MSTRG.25611.1	2976	noncoding	-
MSTRG.25614.1	335	noncoding	-
MSTRG.25616.2	1652	noncoding	-
MSTRG.25616.3	3233	noncoding	-
MSTRG.25620.2	6336	coding	PF04791
MSTRG.25626.1	2003	noncoding	-
MSTRG.25622.1	481	noncoding	-
MSTRG.25622.2	375	noncoding	-
MSTRG.25623.1	211	noncoding	-
MSTRG.25630.1	420	noncoding	-
MSTRG.25631.2	1467	coding	PF04427
MSTRG.25632.2	3216	coding	PF02144
MSTRG.25632.4	1647	coding	PF02144
MSTRG.25635.1	11265	noncoding	-
MSTRG.25637.2	4834	coding	PF12796
MSTRG.25638.1	4132	noncoding	-
MSTRG.25638.2	519	noncoding	-
MSTRG.25642.1	408	noncoding	-
MSTRG.25642.2	504	noncoding	-
MSTRG.25639.1	393	noncoding	-
MSTRG.25653.1	2351	noncoding	-
MSTRG.25654.2	1395	coding	PF02229
MSTRG.25654.3	1152	coding	PF02229
MSTRG.25657.6	4201	coding	PF07528
MSTRG.25657.7	2841	coding	PF07528
MSTRG.25664.2	2497	coding	PF00028
MSTRG.25667.1	349	noncoding	-
MSTRG.25711.1	1141	noncoding	-
MSTRG.25711.2	1477	noncoding	-
MSTRG.25711.3	1356	noncoding	-
MSTRG.25711.4	1108	noncoding	-
MSTRG.25712.1	3786	noncoding	-
MSTRG.25711.5	2097	noncoding	-
MSTRG.25711.6	2046	noncoding	-

MSTRG.25711.7	3557	noncoding	-
MSTRG.25707.1	835	noncoding	-
MSTRG.25718.1	602	noncoding	-
MSTRG.25723.2	1365	coding	PF00063
MSTRG.25725.1	671	noncoding	-
MSTRG.25726.3	1252	noncoding	-
MSTRG.25730.2	1010	coding	PF12906
MSTRG.25734.2	3012	coding	PF07260
MSTRG.25734.3	2636	coding	PF07260
MSTRG.25736.1	778	noncoding	-
MSTRG.25734.4	1746	noncoding	-
MSTRG.25737.2	2652	coding	PF16218
MSTRG.25747.4	9126	coding	PF16609
MSTRG.25747.2	9650	coding	PF16609
MSTRG.25748.1	2419	noncoding	-
MSTRG.25748.2	3561	noncoding	-
MSTRG.25739.1	757	noncoding	-
MSTRG.25749.1	685	noncoding	-
MSTRG.25750.1	403	noncoding	-
MSTRG.25750.2	340	noncoding	-
MSTRG.25752.1	2192	noncoding	-
MSTRG.25760.4	5418	coding	PF12906
MSTRG.25757.2	980	coding	PF01738
MSTRG.25771.2	11892	coding	PF01403
MSTRG.25772.1	1502	noncoding	-
MSTRG.25771.3	7875	coding	PF01403
MSTRG.25771.4	6934	coding	PF01403
MSTRG.25771.5	7071	noncoding	-
MSTRG.25778.1	2963	coding	PF00667
MSTRG.25779.2	1130	coding	PF08368
MSTRG.25779.3	1851	coding	PF08373
MSTRG.25773.1	500	noncoding	-
MSTRG.25781.2	3337	coding	PF00211
MSTRG.25782.1	472	noncoding	-
MSTRG.25782.2	436	noncoding	-
MSTRG.25784.1	16447	noncoding	-
MSTRG.25785.2	3735	coding	PF02544
MSTRG.25790.1	668	noncoding	-
MSTRG.25796.1	4655	coding	PF05986
MSTRG.25796.2	4335	coding	PF05986
MSTRG.25798.1	3653	noncoding	-
MSTRG.25799.1	2572	noncoding	-
MSTRG.25802.1	879	noncoding	-
MSTRG.25802.2	1259	noncoding	-
MSTRG.25803.1	689	noncoding	-
MSTRG.25808.1	1838	noncoding	-
MSTRG.25809.1	3260	noncoding	-
MSTRG.25809.2	2587	noncoding	-
MSTRG.25809.3	2629	noncoding	-
MSTRG.25806.1	982	noncoding	-
MSTRG.25817.2	2247	noncoding	-
MSTRG.25817.3	2261	noncoding	-
MSTRG.25828.1	2371	coding	PF01529
MSTRG.25824.4	2041	noncoding	-

MSTRG.25847.1	696	noncoding	-
MSTRG.25848.1	1081	noncoding	-
MSTRG.25848.2	1018	noncoding	-
MSTRG.25849.1	421	noncoding	-
MSTRG.25857.1	6607	noncoding	-
MSTRG.25858.1	3482	noncoding	-
MSTRG.25857.2	393	noncoding	-
MSTRG.25861.1	847	noncoding	-
MSTRG.25862.1	415	noncoding	-
MSTRG.25869.1	205	noncoding	-
MSTRG.25872.1	406	noncoding	-
MSTRG.25897.4	1542	coding	PF07192
MSTRG.25897.6	1436	coding	PF07192
MSTRG.25897.3	4580	coding	PF07192
MSTRG.25897.7	1440	coding	PF07192
MSTRG.25898.1	2869	noncoding	-
MSTRG.25897.11	1765	noncoding	-
MSTRG.25897.12	3440	noncoding	-
MSTRG.25897.13	4352	noncoding	-
MSTRG.25897.14	5061	noncoding	-
MSTRG.25874.2	4964	coding	PF16212
MSTRG.25874.1	5426	coding	PF16212
MSTRG.25907.2	1342	coding	PF06936
MSTRG.25907.3	19924	coding	PF06936
MSTRG.25911.2	1834	coding	PF12796
MSTRG.25912.2	2312	coding	PF14694
MSTRG.25916.1	3794	coding	PF03798
MSTRG.25916.2	3680	coding	PF03798
MSTRG.25917.1	267	noncoding	-
MSTRG.25923.1	1748	noncoding	-
MSTRG.25926.4	3431	noncoding	-
MSTRG.25926.5	1901	noncoding	-
MSTRG.25927.1	3352	noncoding	-
MSTRG.25928.1	1857	noncoding	-
MSTRG.25926.6	1515	noncoding	-
MSTRG.25929.1	5050	noncoding	-
MSTRG.25940.1	1795	noncoding	-
MSTRG.25940.5	1844	noncoding	-
MSTRG.25940.3	1974	noncoding	-
MSTRG.25949.3	15747	coding	PF07714
MSTRG.25950.1	3394	noncoding	-
MSTRG.25951.1	5369	noncoding	-
MSTRG.25949.5	10204	coding	PF07714
MSTRG.25949.6	11330	coding	PF07714
MSTRG.25949.7	17518	coding	PF07714
MSTRG.25949.8	2546	coding	PF07714
MSTRG.25956.1	532	noncoding	-
MSTRG.25958.1	26829	noncoding	-
MSTRG.25958.2	8176	noncoding	-
MSTRG.25958.3	3776	noncoding	-
MSTRG.25959.1	8696	noncoding	-
MSTRG.25959.2	10958	noncoding	-
MSTRG.25959.3	6712	noncoding	-
MSTRG.25959.5	16774	noncoding	-

MSTRG.25959.4	17125	noncoding	-
MSTRG.25959.6	4839	noncoding	-
MSTRG.25959.7	13851	noncoding	-
MSTRG.25959.8	11937	noncoding	-
MSTRG.25960.1	3998	coding	PF00105
MSTRG.25972.1	218	noncoding	-
MSTRG.25973.2	2112	noncoding	-
MSTRG.25973.3	3226	coding	PF00168
MSTRG.25973.4	2653	coding	PF00168
MSTRG.25973.6	2069	noncoding	-
MSTRG.25977.1	10607	noncoding	-
MSTRG.25977.2	16060	noncoding	-
MSTRG.25977.3	7133	noncoding	-
MSTRG.25978.2	18683	noncoding	-
MSTRG.25975.1	313	noncoding	-
MSTRG.25980.2	421	noncoding	-
MSTRG.25997.5	12110	coding	PF00176
MSTRG.25997.4	9397	coding	PF00176
MSTRG.25997.1	11258	coding	PF00176
MSTRG.25997.2	12658	coding	PF00176
MSTRG.25997.6	10680	coding	PF00176
MSTRG.25998.1	1930	noncoding	-
MSTRG.25997.8	6298	coding	PF00176
MSTRG.25997.7	7077	coding	PF00176
MSTRG.25997.9	8731	coding	PF00176
MSTRG.25997.10	7215	coding	PF00176
MSTRG.25997.11	8637	coding	PF00176
MSTRG.25997.12	14487	coding	PF00176
MSTRG.25997.13	5331	noncoding	-
MSTRG.25997.14	2870	coding	PF00176
MSTRG.25997.18	1386	noncoding	-
MSTRG.25997.19	921	noncoding	-
MSTRG.25997.20	4207	noncoding	-
MSTRG.25997.21	2487	noncoding	-
MSTRG.25989.1	23672	noncoding	-
MSTRG.25989.2	39319	noncoding	-
MSTRG.25989.3	23588	noncoding	-
MSTRG.25989.5	32246	noncoding	-
MSTRG.25989.6	15457	noncoding	-
MSTRG.25991.1	20908	noncoding	-
MSTRG.25991.2	18247	noncoding	-
MSTRG.25991.3	20275	noncoding	-
MSTRG.25991.4	22100	noncoding	-
MSTRG.25991.5	19830	noncoding	-
MSTRG.25985.2	5532	coding	PF00777
MSTRG.25986.1	7329	coding	PF00777
MSTRG.25992.2	3054	coding	PF03137
MSTRG.25993.1	6645	noncoding	-
MSTRG.25994.1	4359	noncoding	-
MSTRG.25994.2	6254	noncoding	-
MSTRG.25995.1	6918	noncoding	-
MSTRG.25992.3	13343	coding	PF03137
MSTRG.25992.4	6482	coding	PF03137
MSTRG.26010.2	4100	noncoding	-

MSTRG.26010.3	2682	noncoding	-
MSTRG.26016.1	11793	noncoding	-
MSTRG.26018.1	1578	noncoding	-
MSTRG.26021.1	2418	noncoding	-
MSTRG.26026.5	805	coding	PF09737
MSTRG.26026.4	820	coding	PF09737
MSTRG.26026.2	1082	coding	PF09737
MSTRG.26026.3	824	coding	PF09737
MSTRG.26028.1	2305	coding	PF00517
MSTRG.26033.1	1293	noncoding	-
MSTRG.26034.1	331	noncoding	-
MSTRG.26034.3	2331	coding	PF00077
MSTRG.26034.2	4955	coding	PF00607
MSTRG.26034.4	4973	coding	PF00077
MSTRG.26034.5	2173	coding	PF00077
MSTRG.26034.6	1542	noncoding	-
MSTRG.26034.7	4006	noncoding	-
MSTRG.26031.2	897	coding	PF14204
MSTRG.26031.3	442	noncoding	-
MSTRG.26035.2	7070	coding	PF00193
MSTRG.26035.3	7160	coding	PF00193
MSTRG.26036.1	2325	coding	PF00754
MSTRG.26038.2	2412	noncoding	-
MSTRG.26038.3	2409	noncoding	-
MSTRG.26037.1	1309	noncoding	-
MSTRG.26041.2	3647	noncoding	-
MSTRG.26042.1	2141	coding	PF00909
MSTRG.26042.2	1876	coding	PF00909
MSTRG.26051.1	8774	coding	PF00225
MSTRG.26052.1	4419	coding	PF03036
MSTRG.26053.3	447	noncoding	-
MSTRG.26053.4	2943	noncoding	-
MSTRG.26045.2	1229	noncoding	-
MSTRG.26048.1	469	noncoding	-
MSTRG.26047.2	1448	coding	PF01217
MSTRG.26059.1	3971	coding	PF01403
MSTRG.26059.2	3667	coding	PF01403
MSTRG.26083.4	5228	noncoding	-
MSTRG.26083.5	5044	noncoding	-
MSTRG.26087.7	10290	noncoding	-
MSTRG.26087.6	4148	noncoding	-
MSTRG.26087.5	3517	coding	PF00233
MSTRG.26088.1	7531	noncoding	-
MSTRG.26089.1	6681	noncoding	-
MSTRG.26091.1	7219	coding	PF16368
MSTRG.26091.4	3141	coding	PF16368
MSTRG.26091.2	3246	coding	PF16368
MSTRG.26093.1	520	noncoding	-
MSTRG.26096.1	979	noncoding	-
MSTRG.26102.2	1474	noncoding	-
MSTRG.26106.1	15798	coding	PF02093
MSTRG.26106.2	13290	coding	PF02093
MSTRG.26107.2	3511	noncoding	-
MSTRG.26108.1	540	coding	PF02594

MSTRG.26109.1	2726	noncoding	-
MSTRG.26111.4	3973	coding	PF00009
MSTRG.26112.2	423	noncoding	-
MSTRG.26112.3	1127	noncoding	-
MSTRG.26112.4	860	noncoding	-
MSTRG.26113.1	1358	noncoding	-
MSTRG.26118.3	4374	noncoding	-
MSTRG.26118.11	6181	noncoding	-
MSTRG.26118.12	9895	noncoding	-
MSTRG.26118.13	4935	noncoding	-
MSTRG.26120.1	4674	noncoding	-
MSTRG.26121.1	3028	noncoding	-
MSTRG.26123.1	3287	coding	PF03114
MSTRG.26123.2	1408	coding	PF03114
MSTRG.26123.8	1850	coding	PF03114
MSTRG.26123.10	1335	coding	PF03114
MSTRG.26123.12	1328	coding	PF03114
MSTRG.26123.11	1235	coding	PF03114
MSTRG.26123.9	1146	coding	PF03114
MSTRG.26123.14	898	coding	PF03114
MSTRG.26123.13	938	coding	PF03114
MSTRG.26124.2	5382	noncoding	-
MSTRG.26126.2	4109	noncoding	-
MSTRG.26126.4	3476	noncoding	-
MSTRG.26122.2	1474	coding	PF00651
MSTRG.26138.1	604	noncoding	-
MSTRG.26139.1	2457	noncoding	-
MSTRG.26142.2	1049	coding	PF00112
MSTRG.26142.3	2460	coding	PF00112
MSTRG.26147.3	2064	coding	PF09298
MSTRG.26148.1	9818	noncoding	-
MSTRG.26147.6	3220	coding	PF09298
MSTRG.26154.2	2253	coding	PF10185
MSTRG.26154.3	2162	coding	PF10185
MSTRG.26158.3	1162	noncoding	-
MSTRG.26163.1	560	noncoding	-
MSTRG.26171.1	6146	noncoding	-
MSTRG.26171.3	7785	noncoding	-
MSTRG.26171.4	3672	noncoding	-
MSTRG.26171.5	1518	noncoding	-
MSTRG.26180.1	7134	coding	PF00595
MSTRG.26180.2	8458	coding	PF00791
MSTRG.26180.5	6718	coding	PF00791
MSTRG.26180.6	6529	coding	PF00791
MSTRG.26180.13	7839	coding	PF00791
MSTRG.26181.1	3014	coding	PF00791
MSTRG.26180.14	2527	noncoding	-
MSTRG.26180.17	2256	noncoding	-
MSTRG.26180.18	1326	noncoding	-
MSTRG.26180.19	9047	noncoding	-
MSTRG.26180.20	26612	noncoding	-
MSTRG.26180.21	8254	noncoding	-
MSTRG.26184.2	1338	noncoding	-
MSTRG.26184.3	963	noncoding	-

MSTRG.26184.5	1096	noncoding	-
MSTRG.26185.1	4473	coding	PF01483
MSTRG.26185.3	4254	coding	PF01483
MSTRG.26186.1	492	noncoding	-
MSTRG.26187.1	824	noncoding	-
MSTRG.26182.2	1683	coding	PF14580
MSTRG.26192.1	424	noncoding	-
MSTRG.26194.17	1423	coding	PF02093
MSTRG.26189.2	667	coding	PF02931
MSTRG.26190.1	1002	noncoding	-
MSTRG.26203.1	246	noncoding	-
MSTRG.26212.1	3666	noncoding	-
MSTRG.26213.1	1251	noncoding	-
MSTRG.26213.2	724	noncoding	-
MSTRG.26213.3	1158	noncoding	-
MSTRG.26213.5	993	noncoding	-
MSTRG.26213.6	1186	noncoding	-
MSTRG.26214.3	2289	coding	PF00226
MSTRG.26215.3	1320	coding	PF00400
MSTRG.26216.1	786	noncoding	-
MSTRG.26220.2	2170	coding	PF00330
MSTRG.26224.1	2289	coding	PF02931
MSTRG.26224.2	2211	coding	PF02931
MSTRG.26224.3	1843	coding	PF02931
MSTRG.26224.4	1964	coding	PF02931
MSTRG.26224.5	2057	coding	PF02931
MSTRG.26226.1	3010	noncoding	-
MSTRG.26226.2	2367	noncoding	-
MSTRG.26227.1	3151	noncoding	-
MSTRG.26230.2	917	coding	PF01012
MSTRG.26232.3	4501	coding	PF16501
MSTRG.26232.4	4543	coding	PF16501
MSTRG.26232.5	4836	coding	PF16501
MSTRG.26240.5	8868	noncoding	-
MSTRG.26240.6	8902	noncoding	-
MSTRG.26240.7	8984	noncoding	-
MSTRG.26241.1	2166	noncoding	-
MSTRG.26242.1	4045	noncoding	-
MSTRG.26244.1	7431	noncoding	-
MSTRG.26244.2	10588	noncoding	-
MSTRG.26245.1	1493	noncoding	-
MSTRG.26246.1	2879	noncoding	-
MSTRG.26240.8	7701	noncoding	-
MSTRG.26247.1	299	noncoding	-
MSTRG.26247.2	726	noncoding	-
MSTRG.26249.1	301	noncoding	-
MSTRG.26239.1	2552	coding	PF07679
MSTRG.26239.2	3114	coding	PF07679
MSTRG.26239.4	2929	coding	PF07679
MSTRG.26259.5	2217	noncoding	-
MSTRG.26260.1	2657	noncoding	-
MSTRG.26257.1	1169	noncoding	-
MSTRG.26257.2	676	noncoding	-
MSTRG.26265.1	803	noncoding	-

MSTRG.26266.1	4380	noncoding	-
MSTRG.26266.2	4687	noncoding	-
MSTRG.26266.3	8370	noncoding	-
MSTRG.26281.1	579	noncoding	-
MSTRG.26282.2	1077	coding	PF02284
MSTRG.26286.2	2082	coding	PF01238
MSTRG.26287.5	2347	noncoding	-
MSTRG.26287.3	2175	noncoding	-
MSTRG.26287.4	2535	coding	PF04144
MSTRG.26278.1	1762	noncoding	-
MSTRG.26291.3	2085	noncoding	-
MSTRG.26291.6	3753	noncoding	-
MSTRG.26291.4	1900	noncoding	-
MSTRG.26292.1	793	noncoding	-
MSTRG.26297.3	1622	noncoding	-
MSTRG.26297.4	438	noncoding	-
MSTRG.26299.1	1677	noncoding	-
MSTRG.26301.1	654	noncoding	-
MSTRG.26303.1	2450	coding	PF01186
MSTRG.26308.1	528	noncoding	-
MSTRG.26308.2	1069	noncoding	-
MSTRG.26315.1	2055	noncoding	-
MSTRG.26316.1	959	noncoding	-
MSTRG.26321.1	6211	coding	PF00013
MSTRG.26321.2	5536	coding	PF00013
MSTRG.26321.3	3454	coding	PF00013
MSTRG.26321.4	1941	coding	PF00013
MSTRG.26321.5	901	noncoding	-
MSTRG.26322.1	5065	noncoding	-
MSTRG.26323.1	1334	noncoding	-
MSTRG.26324.1	1472	noncoding	-
MSTRG.26325.1	3553	noncoding	-
MSTRG.26338.1	780	noncoding	-
MSTRG.26340.1	2171	coding	PF00250
MSTRG.26340.2	2180	coding	PF00250
MSTRG.26337.1	1268	noncoding	-
MSTRG.26337.2	565	noncoding	-
MSTRG.26343.1	2952	coding	PF00069
MSTRG.26344.1	10880	noncoding	-
MSTRG.26345.1	499	noncoding	-
MSTRG.26345.2	4968	noncoding	-
MSTRG.26346.1	6128	noncoding	-
MSTRG.26347.1	4033	noncoding	-
MSTRG.26348.1	3108	noncoding	-
MSTRG.26349.1	2223	noncoding	-
MSTRG.26369.3	3240	coding	PF13771
MSTRG.26369.2	5484	coding	PF13771
MSTRG.26369.4	3084	coding	PF13771
MSTRG.26369.5	3124	coding	PF13771
MSTRG.26370.2	1928	noncoding	-
MSTRG.26373.2	2225	coding	PF00092
MSTRG.26373.3	1934	coding	PF00092
MSTRG.26374.1	2953	coding	PF08232
MSTRG.26375.1	4161	noncoding	-

MSTRG.26376.1	1015	noncoding	-
MSTRG.26377.1	1177	noncoding	-
MSTRG.26374.5	7315	coding	PF08232
MSTRG.26374.6	4190	coding	PF08232
MSTRG.26374.7	6254	coding	PF08232
MSTRG.26374.8	3885	noncoding	-
MSTRG.26375.2	2554	noncoding	-
MSTRG.26378.2	6230	coding	PF01217
MSTRG.26379.7	8244	coding	PF00632
MSTRG.26379.6	8762	coding	PF00632
MSTRG.26379.9	9470	coding	PF00632
MSTRG.26386.1	3775	noncoding	-
MSTRG.26391.2	1948	coding	PF10609
MSTRG.26391.3	2194	coding	PF10609
MSTRG.26391.4	8553	coding	PF10609
MSTRG.26391.5	6652	noncoding	-
MSTRG.26394.3	8019	coding	PF00620
MSTRG.26394.4	7898	coding	PF00620
MSTRG.26394.5	6297	coding	PF00620
MSTRG.26394.8	6397	coding	PF00620
MSTRG.26395.1	13016	coding	PF00078
MSTRG.26394.10	1681	coding	PF00620
MSTRG.26394.9	1684	coding	PF00620
MSTRG.26389.1	467	noncoding	-
MSTRG.26428.1	4596	noncoding	-
MSTRG.26428.4	3000	noncoding	-
MSTRG.26433.1	7197	noncoding	-
MSTRG.26434.1	2738	noncoding	-
MSTRG.26433.2	8570	noncoding	-
MSTRG.26428.7	1453	noncoding	-
MSTRG.26399.8	867	coding	PF10238
MSTRG.26399.7	1235	coding	PF10238
MSTRG.26405.1	2714	noncoding	-
MSTRG.26405.2	2821	coding	PF00241
MSTRG.26405.5	1461	coding	PF00241
MSTRG.26405.4	1348	noncoding	-
MSTRG.26405.6	1374	noncoding	-
MSTRG.26411.4	3345	coding	PF00439
MSTRG.26411.5	5026	coding	PF10537
MSTRG.26411.3	5204	coding	PF10537
MSTRG.26412.1	882	noncoding	-
MSTRG.26413.1	2210	noncoding	-
MSTRG.26415.1	701	noncoding	-
MSTRG.26408.1	1728	noncoding	-
MSTRG.26439.1	2663	coding	PF14774
MSTRG.26448.3	7988	coding	PF02145
MSTRG.26448.1	9313	coding	PF02145
MSTRG.26448.2	7948	coding	PF02145
MSTRG.26448.5	9130	coding	PF02145
MSTRG.26448.6	5474	noncoding	-
MSTRG.26448.7	2807	noncoding	-
MSTRG.26452.3	1883	noncoding	-
MSTRG.26453.1	5024	coding	PF01359
MSTRG.26455.1	3176	coding	PF00078

MSTRG.26446.4	1044	noncoding	-
MSTRG.26446.5	1180	noncoding	-
MSTRG.26477.1	562	noncoding	-
MSTRG.26491.1	13411	noncoding	-
MSTRG.26493.1	8540	noncoding	-
MSTRG.26495.1	570	noncoding	-
MSTRG.26498.1	2470	noncoding	-
MSTRG.26498.2	2910	noncoding	-
MSTRG.26461.4	6779	noncoding	-
MSTRG.26461.2	4952	noncoding	-
MSTRG.26461.3	4680	noncoding	-
MSTRG.26461.5	5441	noncoding	-
MSTRG.26469.2	1226	coding	PF04938
MSTRG.26470.2	950	coding	PF04051
MSTRG.26470.3	936	coding	PF04051
MSTRG.26470.4	943	coding	PF04051
MSTRG.26515.1	542	noncoding	-
MSTRG.26515.2	495	noncoding	-
MSTRG.26515.3	484	noncoding	-
MSTRG.26521.1	238	noncoding	-
MSTRG.26533.1	1136	noncoding	-
MSTRG.26541.1	449	noncoding	-
MSTRG.26567.1	669	noncoding	-
MSTRG.26569.2	2828	noncoding	-
MSTRG.26569.5	2735	noncoding	-
MSTRG.26569.6	2684	noncoding	-
MSTRG.26569.7	2780	noncoding	-
MSTRG.26569.8	2348	noncoding	-
MSTRG.26569.3	2368	noncoding	-
MSTRG.26569.4	2563	noncoding	-
MSTRG.26569.10	2762	noncoding	-
MSTRG.26569.11	2682	noncoding	-
MSTRG.26569.12	2394	noncoding	-
MSTRG.26569.9	2682	noncoding	-
MSTRG.26570.1	7061	noncoding	-
MSTRG.26571.1	33450	noncoding	-
MSTRG.26571.2	14308	noncoding	-
MSTRG.26573.1	10341	coding	PF00078
MSTRG.26574.1	2247	noncoding	-
MSTRG.26574.2	5220	noncoding	-
MSTRG.26571.3	18727	noncoding	-
MSTRG.26572.4	3130	noncoding	-
MSTRG.26572.5	3284	noncoding	-
MSTRG.26572.6	9534	noncoding	-
MSTRG.26572.7	2309	noncoding	-
MSTRG.26572.8	2007	noncoding	-
MSTRG.26572.9	1299	noncoding	-
MSTRG.26572.10	8995	coding	PF00078
MSTRG.26572.11	3965	noncoding	-
MSTRG.26572.12	5635	noncoding	-
MSTRG.26572.13	1101	noncoding	-
MSTRG.26572.14	891	noncoding	-
MSTRG.26572.15	2886	noncoding	-
MSTRG.26572.16	2430	noncoding	-

MSTRG.26572.17	2618	noncoding	-
MSTRG.26575.1	1122	noncoding	-
MSTRG.26542.4	3006	noncoding	-
MSTRG.26542.3	3111	noncoding	-
MSTRG.26542.6	2986	noncoding	-
MSTRG.26552.3	6140	coding	PF02023
MSTRG.26552.5	5089	coding	PF02023
MSTRG.26553.2	3978	noncoding	-
MSTRG.26553.1	5365	noncoding	-
MSTRG.26554.2	15592	coding	PF09038
MSTRG.26554.1	13137	coding	PF09038
MSTRG.26554.4	10289	coding	PF09038
MSTRG.26554.3	10817	coding	PF09038
MSTRG.26554.5	9845	coding	PF09038
MSTRG.26555.3	10721	noncoding	-
MSTRG.26557.2	5570	coding	PF00328
MSTRG.26546.1	3161	noncoding	-
MSTRG.26547.1	2801	noncoding	-
MSTRG.26547.2	1189	noncoding	-
MSTRG.26548.3	2002	coding	PF00085
MSTRG.26548.5	1961	coding	PF00085
MSTRG.26560.1	3969	noncoding	-
MSTRG.26563.1	7566	coding	PF05622
MSTRG.26586.3	3880	coding	PF04802
MSTRG.26593.1	3523	coding	PF15149
MSTRG.26585.2	3641	noncoding	-
MSTRG.26594.3	2292	coding	PF14670
MSTRG.26595.1	8872	noncoding	-
MSTRG.26596.1	2139	noncoding	-
MSTRG.26598.1	5320	coding	PF02099
MSTRG.26602.2	4051	coding	PF02204
MSTRG.26602.3	3595	coding	PF02204
MSTRG.26601.2	1916	coding	PF01650
MSTRG.26611.2	3685	coding	PF15387
MSTRG.26614.1	2622	noncoding	-
MSTRG.26615.1	507	noncoding	-
MSTRG.26615.2	352	noncoding	-
MSTRG.26616.1	630	coding	PF02285
MSTRG.26616.2	659	noncoding	-
MSTRG.26617.2	1034	coding	PF15238
MSTRG.26617.3	12032	coding	PF15238
MSTRG.26617.4	3096	coding	PF15238
MSTRG.26620.4	588	noncoding	-
MSTRG.26624.4	2951	coding	PF00270
MSTRG.26623.3	796	coding	PF06140
MSTRG.26630.3	2431	noncoding	-
MSTRG.26630.2	5018	noncoding	-
MSTRG.26630.4	4532	noncoding	-
MSTRG.26636.2	6429	noncoding	-
MSTRG.26637.4	10247	noncoding	-
MSTRG.26637.5	3446	noncoding	-
MSTRG.26644.1	5081	noncoding	-
MSTRG.26645.1	3179	noncoding	-
MSTRG.26644.2	5081	noncoding	-

MSTRG.26644.5	10489	noncoding	-
MSTRG.26642.1	942	coding	PF00462
MSTRG.26643.1	3302	noncoding	-
MSTRG.26646.1	1512	coding	PF01840
MSTRG.26647.1	1053	noncoding	-
MSTRG.26647.2	1120	coding	PF01840
MSTRG.26651.1	512	noncoding	-
MSTRG.26652.1	7735	noncoding	-
MSTRG.26657.2	2391	coding	PF05303
MSTRG.26658.2	2534	noncoding	-
MSTRG.26660.2	5979	coding	PF04928
MSTRG.26660.1	3056	coding	PF04928
MSTRG.26660.8	2773	coding	PF04928
MSTRG.26660.6	2622	coding	PF04928
MSTRG.26660.10	3368	coding	PF04928
MSTRG.26660.11	4747	noncoding	-
MSTRG.26660.12	10064	coding	PF04928
MSTRG.26665.1	2000	noncoding	-
MSTRG.26662.1	464	noncoding	-
MSTRG.26667.1	604	noncoding	-
MSTRG.26668.1	553	noncoding	-
MSTRG.26671.1	871	noncoding	-
MSTRG.26675.2	2951	coding	PF09273
MSTRG.26675.6	3007	coding	PF09273
MSTRG.26675.7	2822	coding	PF09273
MSTRG.26675.5	2908	coding	PF09273
MSTRG.26675.8	2778	coding	PF09273
MSTRG.26675.11	1880	noncoding	-
MSTRG.26675.12	7139	noncoding	-
MSTRG.26677.2	8769	noncoding	-
MSTRG.26677.1	888	noncoding	-
MSTRG.26680.2	2512	noncoding	-
MSTRG.26684.1	2340	coding	PF00568
MSTRG.26684.2	3926	coding	PF00568
MSTRG.26684.4	3633	coding	PF00568
MSTRG.26684.6	2223	coding	PF00568
MSTRG.26685.3	6828	noncoding	-
MSTRG.26685.2	6100	noncoding	-
MSTRG.26686.1	2589	coding	PF00153
MSTRG.26688.1	1573	noncoding	-
MSTRG.26707.1	291	noncoding	-
MSTRG.26709.1	737	noncoding	-
MSTRG.26710.1	2046	coding	PF01603
MSTRG.26710.3	1839	coding	PF01603
MSTRG.26710.11	1751	coding	PF01603
MSTRG.26710.12	2061	coding	PF01603
MSTRG.26711.1	1567	noncoding	-
MSTRG.26708.1	1562	noncoding	-
MSTRG.26715.1	1488	noncoding	-
MSTRG.26715.2	2068	noncoding	-
MSTRG.26715.3	1650	noncoding	-
MSTRG.26715.4	1546	noncoding	-
MSTRG.26715.7	1378	noncoding	-
MSTRG.26715.8	1370	noncoding	-

MSTRG.26715.9	1184	noncoding	-
MSTRG.26720.2	2280	noncoding	-
MSTRG.26720.5	3617	noncoding	-
MSTRG.26720.6	5043	noncoding	-
MSTRG.26720.7	3182	noncoding	-
MSTRG.26712.4	1156	coding	PF00069
MSTRG.26712.3	1604	coding	PF00069
MSTRG.26712.2	1872	coding	PF00069
MSTRG.26713.1	1712	noncoding	-
MSTRG.26722.2	5158	noncoding	-
MSTRG.26722.3	3959	coding	PF15961
MSTRG.26722.4	2091	coding	PF15961
MSTRG.26725.5	6552	noncoding	-
MSTRG.26725.6	5600	noncoding	-
MSTRG.26725.8	7473	noncoding	-
MSTRG.26726.2	4979	noncoding	-
MSTRG.26726.1	761	noncoding	-
MSTRG.26727.1	3312	noncoding	-
MSTRG.26727.2	2825	noncoding	-
MSTRG.26727.4	4725	noncoding	-
MSTRG.26727.3	6225	noncoding	-
MSTRG.26727.5	5053	noncoding	-
MSTRG.26728.1	7643	noncoding	-
MSTRG.26728.2	13009	noncoding	-
MSTRG.26729.1	4199	noncoding	-
MSTRG.26739.3	5892	coding	PF00780
MSTRG.26739.4	8374	coding	PF00780
MSTRG.26739.5	6385	coding	PF00780
MSTRG.26741.1	3175	noncoding	-
MSTRG.26744.1	3671	coding	PF06046
MSTRG.26744.4	4193	coding	PF06046
MSTRG.26742.2	1350	coding	PF00071
MSTRG.26742.3	1564	coding	PF00071
MSTRG.26748.1	6922	noncoding	-
MSTRG.26749.1	2172	coding	PF01873
MSTRG.26749.2	2322	coding	PF01873
MSTRG.26751.1	3083	coding	PF00069
MSTRG.26751.4	599	noncoding	-
MSTRG.26751.6	3999	noncoding	-
MSTRG.26751.5	6493	coding	PF00069
MSTRG.26751.7	2733	coding	PF00069
MSTRG.26751.3	2753	coding	PF00069
MSTRG.26751.8	7139	noncoding	-
MSTRG.26753.1	9522	noncoding	-
MSTRG.26751.9	9230	coding	PF00069
MSTRG.26754.1	3635	noncoding	-
MSTRG.26757.2	1724	coding	PF00217
MSTRG.26773.20	2750	noncoding	-
MSTRG.26774.2	2517	noncoding	-
MSTRG.26776.1	1291	noncoding	-
MSTRG.26777.2	4830	coding	PF12796
MSTRG.26777.3	4222	coding	PF12796
MSTRG.26765.1	383	noncoding	-
MSTRG.26770.1	1206	noncoding	-

MSTRG.26770.2	1289	noncoding	-
MSTRG.26790.1	2479	coding	PF06367
MSTRG.26790.3	4705	noncoding	-
MSTRG.26788.2	649	coding	PF05458
MSTRG.26786.1	2795	noncoding	-
MSTRG.26792.3	6502	coding	PF15308
MSTRG.26792.4	6376	coding	PF15308
MSTRG.26793.2	11611	noncoding	-
MSTRG.26793.3	11569	noncoding	-
MSTRG.26795.2	1252	coding	PF15045
MSTRG.26795.3	1381	coding	PF15045
MSTRG.26796.1	3648	coding	PF06031
MSTRG.26796.2	2195	coding	PF06031
MSTRG.26796.3	2100	coding	PF06031
MSTRG.26798.2	7218	noncoding	-
MSTRG.26803.3	5115	coding	PF10254
MSTRG.26804.1	1397	noncoding	-
MSTRG.26805.1	949	coding	PF10254
MSTRG.26806.7	2089	noncoding	-
MSTRG.26806.8	2130	noncoding	-
MSTRG.26806.4	1916	noncoding	-
MSTRG.26806.5	8957	noncoding	-
MSTRG.26808.1	1283	coding	PF00412
MSTRG.26808.2	1375	coding	PF00412
MSTRG.26821.1	3340	coding	PF14955
MSTRG.26821.3	824	coding	PF14955
MSTRG.26821.4	1362	coding	PF14955
MSTRG.26821.5	1531	coding	PF14955
MSTRG.26821.6	831	coding	PF14955
MSTRG.26830.1	2762	noncoding	-
MSTRG.26830.2	1895	noncoding	-
MSTRG.26830.3	3920	coding	PF00078
MSTRG.26828.2	10021	coding	PF07714
MSTRG.26835.2	612	noncoding	-
MSTRG.26836.2	4066	coding	PF00069
MSTRG.26836.3	1061	noncoding	-
MSTRG.26837.2	7391	coding	PF00955
MSTRG.26837.4	4305	coding	PF00955
MSTRG.26837.5	2530	coding	PF07565
MSTRG.26841.1	2359	noncoding	-
MSTRG.26843.2	5441	coding	PF12845
MSTRG.26844.1	872	noncoding	-
MSTRG.26845.1	2105	noncoding	-
MSTRG.26853.1	5206	coding	PF02994
MSTRG.26838.1	891	noncoding	-
MSTRG.26855.1	635	noncoding	-
MSTRG.26856.1	561	noncoding	-
MSTRG.26863.4	1795	noncoding	-
MSTRG.26863.5	7534	coding	PF00076
MSTRG.26864.1	2875	noncoding	-
MSTRG.26865.1	6081	noncoding	-
MSTRG.26866.1	2174	noncoding	-
MSTRG.26867.1	5904	noncoding	-
MSTRG.26868.1	2593	noncoding	-

MSTRG.26868.2	16993	noncoding	-
MSTRG.26868.3	7457	noncoding	-
MSTRG.26870.1	1038	noncoding	-
MSTRG.26858.1	236	noncoding	-
MSTRG.26861.1	469	noncoding	-
MSTRG.26861.2	832	noncoding	-
MSTRG.26889.8	3603	coding	PF01237
MSTRG.26889.9	3044	coding	PF01237
MSTRG.26889.4	2618	coding	PF01237
MSTRG.26889.7	2869	coding	PF01237
MSTRG.26889.6	3448	coding	PF01237
MSTRG.26889.5	7039	coding	PF01237
MSTRG.26889.3	7520	coding	PF01237
MSTRG.26889.2	5356	coding	PF01237
MSTRG.26889.10	1278	noncoding	-
MSTRG.26889.11	1574	noncoding	-
MSTRG.26890.1	794	noncoding	-
MSTRG.26889.12	2361	noncoding	-
MSTRG.26891.1	11176	noncoding	-
MSTRG.26892.1	1256	noncoding	-
MSTRG.26894.1	4400	noncoding	-
MSTRG.26879.2	3704	coding	PF07479
MSTRG.26879.3	4626	coding	PF07479
MSTRG.26880.2	10402	noncoding	-
MSTRG.26898.1	2319	noncoding	-
MSTRG.26903.2	2460	noncoding	-
MSTRG.26904.1	733	noncoding	-
MSTRG.26902.1	4022	coding	PF00630
MSTRG.26910.3	918	noncoding	-
MSTRG.26910.4	1357	noncoding	-
MSTRG.26910.5	920	noncoding	-
MSTRG.26919.8	5194	noncoding	-
MSTRG.26919.5	7384	coding	PF12348
MSTRG.26919.1	6800	coding	PF12348
MSTRG.26919.2	6803	coding	PF12348
MSTRG.26919.3	6692	coding	PF12348
MSTRG.26919.7	6500	coding	PF12348
MSTRG.26919.9	6680	coding	PF12348
MSTRG.26919.10	6360	coding	PF12348
MSTRG.26920.1	1263	noncoding	-
MSTRG.26932.1	2056	coding	PF09738
MSTRG.26932.4	2425	coding	PF09738
MSTRG.26932.5	2619	coding	PF09738
MSTRG.26932.7	3203	coding	PF09738
MSTRG.26932.6	3030	coding	PF09738
MSTRG.26932.8	2229	coding	PF09738
MSTRG.26932.9	2684	coding	PF09738
MSTRG.26932.10	2101	coding	PF09738
MSTRG.26933.3	2508	coding	PF16413
MSTRG.26937.2	10567	noncoding	-
MSTRG.26937.5	10584	noncoding	-
MSTRG.26929.1	3108	coding	PF00069
MSTRG.26929.2	3435	coding	PF00069
MSTRG.26930.1	1055	noncoding	-

MSTRG.26941.9	1849	noncoding	-
MSTRG.26941.8	7469	noncoding	-
MSTRG.26941.10	7628	noncoding	-
MSTRG.26941.11	7565	noncoding	-
MSTRG.26941.13	8164	noncoding	-
MSTRG.26941.14	2065	noncoding	-
MSTRG.26941.15	2945	noncoding	-
MSTRG.26942.1	5669	coding	PF03031
MSTRG.26943.1	7969	noncoding	-
MSTRG.26942.3	4697	coding	PF03031
MSTRG.26945.1	3118	coding	PF00388
MSTRG.26944.11	2269	noncoding	-
MSTRG.26953.3	3517	coding	PF00069
MSTRG.26953.2	10041	coding	PF00069
MSTRG.26954.1	2264	noncoding	-
MSTRG.26955.2	3033	noncoding	-
MSTRG.26955.1	3351	noncoding	-
MSTRG.26955.3	1817	noncoding	-
MSTRG.26955.5	2049	noncoding	-
MSTRG.26957.1	9652	noncoding	-
MSTRG.26962.1	331	noncoding	-
MSTRG.26974.1	4316	noncoding	-
MSTRG.26988.1	9158	noncoding	-
MSTRG.26988.2	1126	noncoding	-
MSTRG.26990.1	4178	coding	PF01352
MSTRG.26990.2	4062	coding	PF01352
MSTRG.26990.3	4117	coding	PF01352
MSTRG.26994.1	3115	noncoding	-
MSTRG.26990.4	5581	coding	PF01352
MSTRG.26990.6	779	coding	PF01352
MSTRG.26990.5	4722	coding	PF01352
MSTRG.26997.1	1156	coding	PF00118
MSTRG.26998.1	488	noncoding	-
MSTRG.27003.4	3064	noncoding	-
MSTRG.27006.6	1098	noncoding	-
MSTRG.27006.8	995	noncoding	-
MSTRG.27006.9	1073	noncoding	-
MSTRG.27006.7	1148	noncoding	-
MSTRG.27006.5	1600	noncoding	-
MSTRG.27006.4	1738	noncoding	-
MSTRG.27006.2	4422	coding	PF00069
MSTRG.27007.1	1886	noncoding	-
MSTRG.27008.1	884	noncoding	-
MSTRG.27011.1	727	noncoding	-
MSTRG.27002.4	5211	coding	PF04849
MSTRG.27014.1	3662	noncoding	-
MSTRG.27017.6	7266	coding	PF00160
MSTRG.27017.5	3106	coding	PF00160
MSTRG.27017.7	6089	coding	PF00160
MSTRG.27018.1	3262	noncoding	-
MSTRG.27017.9	6875	coding	PF00160
MSTRG.27017.10	2286	noncoding	-
MSTRG.27017.11	2127	noncoding	-
MSTRG.27012.3	5673	coding	PF00651

MSTRG.27012.2	5794	coding	PF00651
MSTRG.27019.2	711	noncoding	-
MSTRG.27021.2	1125	coding	PF04588
MSTRG.27023.1	2530	noncoding	-
MSTRG.27027.1	460	noncoding	-
MSTRG.27027.2	361	noncoding	-
MSTRG.27029.1	400	noncoding	-
MSTRG.27031.2	2498	noncoding	-
MSTRG.27035.4	797	noncoding	-
MSTRG.27035.6	1279	noncoding	-
MSTRG.27035.3	3025	coding	PF04547
MSTRG.27035.5	2810	coding	PF04547
MSTRG.27035.7	2186	noncoding	-
MSTRG.27038.2	2264	coding	PF00561
MSTRG.27038.3	2028	coding	PF00561
MSTRG.27038.4	1879	coding	PF00561
MSTRG.27038.5	2109	noncoding	-
MSTRG.27039.1	744	noncoding	-
MSTRG.27039.2	634	noncoding	-
MSTRG.27069.1	3125	noncoding	-
MSTRG.27069.2	12338	noncoding	-
MSTRG.27069.3	3801	noncoding	-
MSTRG.27042.2	3301	coding	PF14688
MSTRG.27043.1	1407	noncoding	-
MSTRG.27045.4	7059	noncoding	-
MSTRG.27045.5	5385	noncoding	-
MSTRG.27045.6	3909	noncoding	-
MSTRG.27045.7	3576	noncoding	-
MSTRG.27045.8	3097	noncoding	-
MSTRG.27073.2	4762	coding	PF00225
MSTRG.27073.5	5480	coding	PF00225
MSTRG.27073.6	6330	coding	PF00225
MSTRG.27073.7	4486	coding	PF00225
MSTRG.27073.8	4818	coding	PF15908
MSTRG.27073.9	3514	coding	PF15908
MSTRG.27051.1	419	noncoding	-
MSTRG.27078.1	1398	noncoding	-
MSTRG.27083.2	1285	noncoding	-
MSTRG.27083.3	1271	noncoding	-
MSTRG.27085.4	4875	coding	PF14631
MSTRG.27086.2	1412	coding	PF01956
MSTRG.27098.1	1523	coding	PF02017
MSTRG.27098.2	1344	coding	PF02017
MSTRG.27098.3	1389	coding	PF02017
MSTRG.27099.1	685	coding	PF02017
MSTRG.27099.2	571	coding	PF02017
MSTRG.27101.3	3406	coding	PF03133
MSTRG.27101.2	3655	coding	PF03133
MSTRG.27101.1	4991	coding	PF05856
MSTRG.27101.4	3338	coding	PF03133
MSTRG.27101.5	5057	coding	PF05856
MSTRG.27101.10	2569	coding	PF03133
MSTRG.27101.11	2843	coding	PF03133
MSTRG.27101.12	2834	coding	PF03133

MSTRG.27101.8	2591	coding	PF03133
MSTRG.27101.14	4877	coding	PF05856
MSTRG.27101.16	3275	coding	PF03133
MSTRG.27103.1	388	noncoding	-
MSTRG.27104.1	2474	coding	PF10198
MSTRG.27104.2	2345	coding	PF10198
MSTRG.27104.3	2665	coding	PF10198
MSTRG.27104.4	2607	coding	PF10198
MSTRG.27104.5	3682	coding	PF10198
MSTRG.27104.6	2655	coding	PF10198
MSTRG.27101.17	2177	coding	PF05856
MSTRG.27091.2	1386	coding	PF07934
MSTRG.27092.1	1714	noncoding	-
MSTRG.27092.2	4639	noncoding	-
MSTRG.27092.3	1646	noncoding	-
MSTRG.27092.4	2467	noncoding	-
MSTRG.27092.5	10772	noncoding	-
MSTRG.27092.6	1754	noncoding	-
MSTRG.27094.1	603	coding	PF07002
MSTRG.27092.7	6301	noncoding	-
MSTRG.27114.1	7032	noncoding	-
MSTRG.27118.1	2218	noncoding	-
MSTRG.27118.2	1548	noncoding	-
MSTRG.27119.1	28383	noncoding	-
MSTRG.27119.2	10814	noncoding	-
MSTRG.27118.3	2603	noncoding	-
MSTRG.27118.4	28225	noncoding	-
MSTRG.27123.1	2253	noncoding	-
MSTRG.27124.2	3816	coding	PF00620
MSTRG.27124.5	5672	noncoding	-
MSTRG.27125.1	7874	noncoding	-
MSTRG.27124.6	8970	coding	PF00620
MSTRG.27124.7	2288	noncoding	-
MSTRG.27106.1	6759	noncoding	-
MSTRG.27109.1	3750	noncoding	-
MSTRG.27129.1	593	noncoding	-
MSTRG.27143.1	288	noncoding	-
MSTRG.27144.1	1862	noncoding	-
MSTRG.27150.1	13749	noncoding	-
MSTRG.27156.1	802	coding	PF03781
MSTRG.27157.2	1628	coding	PF00856
MSTRG.27179.1	2263	noncoding	-
MSTRG.27191.1	2636	noncoding	-
MSTRG.27192.1	6349	noncoding	-
MSTRG.27192.3	2724	coding	PF01743
MSTRG.27192.2	2330	coding	PF01743
MSTRG.27194.1	992	noncoding	-
MSTRG.27198.1	1462	coding	PF00041
MSTRG.27202.5	7854	coding	PF13882
MSTRG.27217.1	2791	noncoding	-
MSTRG.27217.2	2658	noncoding	-
MSTRG.27212.1	950	noncoding	-
MSTRG.27221.2	8526	noncoding	-
MSTRG.27222.1	7667	noncoding	-

MSTRG.27223.1	1958	noncoding	-
MSTRG.27226.1	3178	coding	PF09184
MSTRG.27226.4	1536	coding	PF09184
MSTRG.27226.5	1970	coding	PF09184
MSTRG.27230.2	1130	noncoding	-
MSTRG.27230.4	848	noncoding	-
MSTRG.27230.9	17853	noncoding	-
MSTRG.27230.16	10102	noncoding	-
MSTRG.27230.17	3250	noncoding	-
MSTRG.27239.1	8164	noncoding	-
MSTRG.27239.2	1976	noncoding	-
MSTRG.27282.1	2577	coding	PF06607
MSTRG.27282.3	6533	coding	PF06607
MSTRG.27282.11	7752	noncoding	-
MSTRG.27282.10	15051	noncoding	-
MSTRG.27282.12	7715	noncoding	-
MSTRG.27282.14	5520	noncoding	-
MSTRG.27282.15	7631	noncoding	-
MSTRG.27285.1	25719	coding	PF00078
MSTRG.27282.16	1301	coding	PF06607
MSTRG.27282.20	2124	coding	PF06607
MSTRG.27282.21	7448	coding	PF06607
MSTRG.27282.18	6457	coding	PF01652
MSTRG.27288.1	1262	coding	PF00078
MSTRG.27291.1	3885	coding	PF16159
MSTRG.27291.2	2177	noncoding	-
MSTRG.27292.1	3200	noncoding	-
MSTRG.27293.1	1339	noncoding	-
MSTRG.27291.7	2669	coding	PF16159
MSTRG.27291.8	2654	coding	PF16159
MSTRG.27247.3	975	noncoding	-
MSTRG.27247.2	899	noncoding	-
MSTRG.27248.1	1421	coding	PF00899
MSTRG.27250.1	3336	noncoding	-
MSTRG.27251.1	2096	noncoding	-
MSTRG.27255.2	3016	coding	PF08442
MSTRG.27256.1	1233	noncoding	-
MSTRG.27263.1	1574	noncoding	-
MSTRG.27264.1	3442	noncoding	-
MSTRG.27263.2	1435	noncoding	-
MSTRG.27263.3	4661	coding	PF07679
MSTRG.27268.1	4142	coding	PF02994
MSTRG.27272.1	2533	noncoding	-
MSTRG.27276.1	5461	noncoding	-
MSTRG.27275.2	6367	noncoding	-
MSTRG.27304.3	4010	noncoding	-
MSTRG.27305.1	57734	noncoding	-
MSTRG.27305.2	16087	noncoding	-
MSTRG.27305.3	9143	noncoding	-
MSTRG.27305.4	8536	noncoding	-
MSTRG.27305.5	32178	noncoding	-
MSTRG.27305.6	6409	noncoding	-
MSTRG.27304.6	6100	noncoding	-
MSTRG.27304.7	17641	noncoding	-

MSTRG.27304.8	20421	coding	PF00078
MSTRG.27304.9	20171	coding	PF00078
MSTRG.27304.10	8477	coding	PF16666
MSTRG.27304.11	20758	coding	PF16666
MSTRG.27304.12	5233	noncoding	-
MSTRG.27307.1	6486	noncoding	-
MSTRG.27304.13	7729	noncoding	-
MSTRG.27308.1	10288	noncoding	-
MSTRG.27308.2	5677	noncoding	-
MSTRG.27304.14	1955	noncoding	-
MSTRG.27309.1	8059	noncoding	-
MSTRG.27309.2	8394	noncoding	-
MSTRG.27310.1	915	noncoding	-
MSTRG.27311.1	692	noncoding	-
MSTRG.27277.1	873	noncoding	-
MSTRG.27278.1	3276	coding	PF02093
MSTRG.27300.3	9520	coding	PF05986
MSTRG.27300.4	4749	coding	PF05986
MSTRG.27300.5	8515	coding	PF08685
MSTRG.27302.1	3268	noncoding	-
MSTRG.27303.5	10722	noncoding	-
MSTRG.27312.1	1436	coding	PF10602
MSTRG.27314.2	4999	coding	PF08313
MSTRG.27314.3	400	noncoding	-
MSTRG.27329.1	16212	noncoding	-
MSTRG.27329.2	4998	noncoding	-
MSTRG.27328.4	2138	noncoding	-
MSTRG.27330.1	7332	noncoding	-
MSTRG.27368.2	594	noncoding	-
MSTRG.27368.3	526	noncoding	-
MSTRG.27368.4	20866	coding	PF02994
MSTRG.27368.5	1842	coding	PF01230
MSTRG.27368.6	14287	noncoding	-
MSTRG.27370.1	5241	noncoding	-
MSTRG.27368.7	12502	noncoding	-
MSTRG.27373.1	12296	noncoding	-
MSTRG.27337.1	869	noncoding	-
MSTRG.27338.3	2631	noncoding	-
MSTRG.27338.4	2888	noncoding	-
MSTRG.27339.1	2449	noncoding	-
MSTRG.27340.1	8953	noncoding	-
MSTRG.27343.3	7466	coding	PF06625
MSTRG.27343.4	7393	coding	PF06625
MSTRG.27343.6	2961	coding	PF06625
MSTRG.27343.8	8850	coding	PF06625
MSTRG.27359.4	2280	noncoding	-
MSTRG.27362.3	3626	noncoding	-
MSTRG.27391.4	4063	noncoding	-
MSTRG.27391.2	3705	noncoding	-
MSTRG.27391.7	4094	noncoding	-
MSTRG.27391.8	3061	noncoding	-
MSTRG.27391.10	2457	noncoding	-
MSTRG.27391.9	3897	coding	PF00498
MSTRG.27393.3	2074	noncoding	-

MSTRG.27393.4	3085	noncoding	-
MSTRG.27400.3	10824	coding	PF03028
MSTRG.27400.4	3804	coding	PF03028
MSTRG.27401.1	2447	coding	PF16746
MSTRG.27401.2	3599	coding	PF16746
MSTRG.27401.3	3028	coding	PF16746
MSTRG.27401.4	6109	coding	PF16746
MSTRG.27404.1	1277	coding	PF00046
MSTRG.27411.2	8317	noncoding	-
MSTRG.27412.1	1784	noncoding	-
MSTRG.27411.3	3199	coding	PF00621
MSTRG.27413.1	5844	noncoding	-
MSTRG.27413.2	12636	noncoding	-
MSTRG.27413.3	12713	noncoding	-
MSTRG.27411.5	4547	coding	PF00621
MSTRG.27378.6	973	noncoding	-
MSTRG.27378.7	4668	noncoding	-
MSTRG.27378.8	8056	noncoding	-
MSTRG.27378.9	4764	noncoding	-
MSTRG.27387.1	665	noncoding	-
MSTRG.27387.2	502	noncoding	-
MSTRG.27387.3	610	noncoding	-
MSTRG.27387.4	261	noncoding	-
MSTRG.27387.5	259	noncoding	-
MSTRG.27387.6	264	noncoding	-
MSTRG.27387.7	946	noncoding	-
MSTRG.27387.8	826	noncoding	-
MSTRG.27387.9	261	noncoding	-
MSTRG.27387.10	259	noncoding	-
MSTRG.27387.11	364	noncoding	-
MSTRG.27420.1	1431	noncoding	-
MSTRG.27420.2	3726	coding	PF08399
MSTRG.27431.1	326	noncoding	-
MSTRG.27435.3	9905	coding	PF00520
MSTRG.27436.1	2773	noncoding	-
MSTRG.27440.1	343	noncoding	-
MSTRG.27427.9	2432	coding	PF00069
MSTRG.27427.8	2641	coding	PF00069
MSTRG.27427.7	2579	coding	PF00069
MSTRG.27427.10	2687	coding	PF00069
MSTRG.27427.11	2509	coding	PF00069
MSTRG.27427.12	2485	coding	PF00069
MSTRG.27427.13	2441	coding	PF00069
MSTRG.27428.2	1166	noncoding	-
MSTRG.27428.1	912	noncoding	-
MSTRG.27428.3	771	noncoding	-
MSTRG.27428.4	1004	noncoding	-
MSTRG.27445.2	1778	noncoding	-
MSTRG.27446.1	1135	noncoding	-
MSTRG.27449.1	5388	noncoding	-
MSTRG.27454.3	4230	coding	PF12140
MSTRG.27454.2	4424	coding	PF12140
MSTRG.27459.1	1556	coding	PF06645
MSTRG.27459.2	968	coding	PF06645

MSTRG.27458.8	6626	coding	PF01501
MSTRG.27461.1	7612	coding	PF01426
MSTRG.27461.3	7580	coding	PF01426
MSTRG.27461.5	10853	coding	PF01426
MSTRG.27461.6	10688	coding	PF01426
MSTRG.27461.7	8833	coding	PF01426
MSTRG.27461.10	7664	coding	PF01426
MSTRG.27461.11	7430	coding	PF01426
MSTRG.27462.1	599	coding	PF15114
MSTRG.27462.2	583	coding	PF15114
MSTRG.27462.3	1701	coding	PF15114
MSTRG.27457.3	8009	coding	PF00193
MSTRG.27474.4	7045	coding	PF00787
MSTRG.27474.2	8770	coding	PF00787
MSTRG.27474.6	10199	coding	PF00787
MSTRG.27475.1	799	coding	PF13499
MSTRG.27479.1	3429	coding	PF01088
MSTRG.27480.2	13645	coding	PF03028
MSTRG.27480.1	13861	coding	PF03028
MSTRG.27480.4	13401	coding	PF03028
MSTRG.27480.5	2748	coding	PF03028
MSTRG.27480.6	13425	coding	PF03028
MSTRG.27480.8	5737	coding	PF03028
MSTRG.27480.9	9693	coding	PF03028
MSTRG.27480.7	13932	coding	PF03028
MSTRG.27480.10	2154	coding	PF13660
MSTRG.27480.12	2301	coding	PF13660
MSTRG.27463.1	314	noncoding	-
MSTRG.27466.2	2421	noncoding	-
MSTRG.27466.3	1986	noncoding	-
MSTRG.27466.4	2502	noncoding	-
MSTRG.27467.2	1607	coding	PF00241
MSTRG.27469.3	2835	coding	PF09029
MSTRG.27471.2	1340	coding	PF00400
MSTRG.27471.6	2137	coding	PF00400
MSTRG.27473.1	701	noncoding	-
MSTRG.27471.7	1330	noncoding	-
MSTRG.27471.8	8675	noncoding	-
MSTRG.27486.2	946	coding	PF01779
MSTRG.27486.3	1044	coding	PF01779
MSTRG.27487.2	2459	coding	PF01546
MSTRG.27489.4	2383	coding	PF00013
MSTRG.27489.5	2856	coding	PF00013
MSTRG.27489.6	1081	noncoding	-
MSTRG.27489.7	2127	coding	PF00013
MSTRG.27483.1	1550	coding	PF00644
MSTRG.27507.2	4269	noncoding	-
MSTRG.27507.3	1792	noncoding	-
MSTRG.27507.4	1549	noncoding	-
MSTRG.27507.5	1769	noncoding	-
MSTRG.27507.7	2452	coding	PF01251
MSTRG.27507.8	1212	noncoding	-
MSTRG.27508.1	702	noncoding	-
MSTRG.27510.1	12576	noncoding	-

MSTRG.27512.12	1129	noncoding	-
MSTRG.27510.3	5621	noncoding	-
MSTRG.27510.4	7896	noncoding	-
MSTRG.27510.5	7482	noncoding	-
MSTRG.27510.6	2779	noncoding	-
MSTRG.27514.1	5371	noncoding	-
MSTRG.27514.2	5743	noncoding	-
MSTRG.27515.1	904	noncoding	-
MSTRG.27521.1	8781	coding	PF16172
MSTRG.27521.2	8676	coding	PF16172
MSTRG.27521.3	8808	coding	PF16172
MSTRG.27524.2	3311	coding	PF00069
MSTRG.27526.1	2324	noncoding	-
MSTRG.27526.4	1946	noncoding	-
MSTRG.27497.1	1630	noncoding	-
MSTRG.27499.2	1939	coding	PF01630
MSTRG.27499.4	3618	coding	PF01630
MSTRG.27505.1	4184	noncoding	-
MSTRG.27505.5	9971	noncoding	-
MSTRG.27502.1	3090	coding	PF00022
MSTRG.27533.4	1843	coding	PF03770
MSTRG.27540.1	3171	noncoding	-
MSTRG.27572.2	1881	noncoding	-
MSTRG.27573.4	1644	noncoding	-
MSTRG.27573.3	2289	noncoding	-
MSTRG.27573.2	1789	coding	PF15128
MSTRG.27580.6	2637	coding	PF15771
MSTRG.27580.7	2094	coding	PF15771
MSTRG.27580.8	2437	coding	PF15771
MSTRG.27580.5	2259	coding	PF15771
MSTRG.27580.9	7065	coding	PF15771
MSTRG.27581.3	17085	coding	PF15374
MSTRG.27581.6	3461	noncoding	-
MSTRG.27581.5	13149	coding	PF15374
MSTRG.27584.1	4358	coding	PF00443
MSTRG.27584.10	4587	coding	PF16602
MSTRG.27584.11	4818	coding	PF00443
MSTRG.27584.9	5467	coding	PF04558
MSTRG.27584.12	7281	coding	PF00749
MSTRG.27584.13	4731	coding	PF00443
MSTRG.27584.14	4627	coding	PF00443
MSTRG.27584.15	4487	coding	PF00443
MSTRG.27587.3	644	noncoding	-
MSTRG.27587.2	1945	coding	PF04430
MSTRG.27588.4	1748	noncoding	-
MSTRG.27589.2	4229	noncoding	-
MSTRG.27589.4	1889	noncoding	-
MSTRG.27590.5	2132	coding	PF01485
MSTRG.27541.1	1086	noncoding	-
MSTRG.27541.2	1252	noncoding	-
MSTRG.27544.2	1371	coding	PF03770
MSTRG.27544.3	15977	coding	PF03770
MSTRG.27547.1	11928	coding	PF00028
MSTRG.27548.2	2911	coding	PF00916

MSTRG.27550.1	354	noncoding	-
MSTRG.27554.1	1651	noncoding	-
MSTRG.27555.1	729	noncoding	-
MSTRG.27555.2	598	noncoding	-
MSTRG.27555.3	532	noncoding	-
MSTRG.27556.1	2223	noncoding	-
MSTRG.27557.1	3711	noncoding	-
MSTRG.27558.2	7161	coding	PF08337
MSTRG.27559.4	998	coding	PF00334
MSTRG.27559.5	1125	coding	PF00334
MSTRG.27560.1	1016	noncoding	-
MSTRG.27563.1	7636	noncoding	-
MSTRG.27563.10	9780	noncoding	-
MSTRG.27563.9	14221	noncoding	-
MSTRG.27563.6	19728	noncoding	-
MSTRG.27563.7	5622	noncoding	-
MSTRG.27563.8	5301	noncoding	-
MSTRG.27563.11	8082	noncoding	-
MSTRG.27563.12	14728	noncoding	-
MSTRG.27563.13	3851	noncoding	-
MSTRG.27563.14	701	noncoding	-
MSTRG.27563.15	6982	noncoding	-
MSTRG.27565.1	5518	noncoding	-
MSTRG.27563.16	5885	noncoding	-
MSTRG.27563.17	12124	noncoding	-
MSTRG.27567.5	3734	noncoding	-
MSTRG.27567.6	4046	noncoding	-
MSTRG.27569.2	6188	coding	PF16496
MSTRG.27596.1	4625	coding	PF12349
MSTRG.27596.3	4201	coding	PF12349
MSTRG.27596.4	4204	coding	PF12349
MSTRG.27597.2	1446	noncoding	-
MSTRG.27617.2	1481	noncoding	-
MSTRG.27617.3	2150	noncoding	-
MSTRG.27617.4	1809	noncoding	-
MSTRG.27620.8	5434	noncoding	-
MSTRG.27620.2	8518	coding	PF00856
MSTRG.27620.3	8583	coding	PF00856
MSTRG.27620.4	10612	coding	PF00856
MSTRG.27620.5	10037	coding	PF00856
MSTRG.27620.6	8509	coding	PF00856
MSTRG.27620.7	8521	coding	PF00856
MSTRG.27620.9	10091	coding	PF00856
MSTRG.27620.10	13489	coding	PF00856
MSTRG.27620.11	8033	coding	PF00856
MSTRG.27620.12	2289	noncoding	-
MSTRG.27622.2	8649	coding	PF02138
MSTRG.27624.2	1217	noncoding	-
MSTRG.27624.3	24976	coding	PF08315
MSTRG.27598.2	2874	coding	PF00002
MSTRG.27598.3	3662	coding	PF00002
MSTRG.27601.1	342	noncoding	-
MSTRG.27602.1	1103	noncoding	-
MSTRG.27605.1	1116	coding	PF00089

MSTRG.27605.5	1467	coding	PF00089
MSTRG.27605.2	1880	coding	PF00089
MSTRG.27605.4	1829	coding	PF00089
MSTRG.27606.1	1136	coding	PF00089
MSTRG.27599.2	1821	coding	PF00089
MSTRG.27630.1	2206	noncoding	-
MSTRG.27633.1	2605	coding	PF00001
MSTRG.27626.1	8761	coding	PF10168
MSTRG.27626.2	414	coding	PF10168
MSTRG.27626.3	523	coding	PF10168
MSTRG.27627.1	2598	noncoding	-
MSTRG.27628.1	4170	noncoding	-
MSTRG.27651.1	3109	coding	PF00209
MSTRG.27651.2	3939	coding	PF00209
MSTRG.27651.3	1751	coding	PF00209
MSTRG.27655.1	5658	coding	PF00412
MSTRG.27655.2	1771	coding	PF00412
MSTRG.27666.2	823	noncoding	-
MSTRG.27667.1	1921	noncoding	-
MSTRG.27668.6	4141	coding	PF01529
MSTRG.27668.7	3304	coding	PF01529
MSTRG.27668.8	2779	coding	PF01529
MSTRG.27668.9	3682	coding	PF01529
MSTRG.27668.10	2676	noncoding	-
MSTRG.27669.5	710	noncoding	-
MSTRG.27669.6	1134	noncoding	-
MSTRG.27669.7	1588	noncoding	-
MSTRG.27669.8	1714	noncoding	-
MSTRG.27659.1	573	noncoding	-
MSTRG.27673.1	5223	noncoding	-
MSTRG.27676.3	2366	coding	PF16420
MSTRG.27676.4	2624	coding	PF16420
MSTRG.27676.6	2349	coding	PF16420
MSTRG.27676.7	2346	coding	PF16420
MSTRG.27676.8	2378	coding	PF16420
MSTRG.27676.10	2750	coding	PF16420
MSTRG.27680.1	6498	coding	PF10267
MSTRG.27680.3	6243	coding	PF10267
MSTRG.27680.5	4957	noncoding	-
MSTRG.27680.4	3388	coding	PF10267
MSTRG.27684.4	732	noncoding	-
MSTRG.27684.3	845	noncoding	-
MSTRG.27684.2	1236	coding	PF00538
MSTRG.27689.1	562	noncoding	-
MSTRG.27690.2	616	noncoding	-
MSTRG.27695.1	2698	noncoding	-
MSTRG.27695.3	1625	noncoding	-
MSTRG.27704.1	2779	noncoding	-
MSTRG.27706.1	4061	noncoding	-
MSTRG.27707.2	4113	coding	PF00607
MSTRG.27707.1	2892	coding	PF00517
MSTRG.27707.4	2717	coding	PF00517
MSTRG.27708.2	593	noncoding	-
MSTRG.27708.3	553	noncoding	-

MSTRG.27717.5	5718	noncoding	-
MSTRG.27717.1	5849	noncoding	-
MSTRG.27717.2	5769	noncoding	-
MSTRG.27720.1	517	noncoding	-
MSTRG.27722.1	1604	noncoding	-
MSTRG.27718.2	1827	coding	PF00595
MSTRG.27719.1	1462	noncoding	-
MSTRG.27724.4	5955	coding	PF00209
MSTRG.27724.5	5604	coding	PF00209
MSTRG.27730.2	652	coding	PF01423
MSTRG.27731.3	5376	coding	PF10405
MSTRG.27732.2	3353	coding	PF07787
MSTRG.27725.1	577	noncoding	-
MSTRG.27737.2	7787	noncoding	-
MSTRG.27738.1	3556	noncoding	-
MSTRG.27747.1	1720	noncoding	-
MSTRG.27747.2	7226	coding	PF16453
MSTRG.27747.3	7221	coding	PF16453
MSTRG.27747.4	7388	coding	PF16453
MSTRG.27748.1	8388	noncoding	-
MSTRG.27748.2	16981	noncoding	-
MSTRG.27751.1	511	noncoding	-
MSTRG.27747.5	8333	coding	PF16453
MSTRG.27752.1	492	noncoding	-
MSTRG.27753.1	483	noncoding	-
MSTRG.27747.6	7232	coding	PF16453
MSTRG.27747.8	6935	coding	PF16453
MSTRG.27754.1	3724	noncoding	-
MSTRG.27744.2	2182	coding	PF02771
MSTRG.27745.1	7312	coding	PF15084
MSTRG.27745.3	4471	coding	PF15084
MSTRG.27769.1	8995	noncoding	-
MSTRG.27772.3	1614	coding	PF02586
MSTRG.27772.4	1740	coding	PF02586
MSTRG.27772.6	1271	coding	PF02586
MSTRG.27778.2	3893	noncoding	-
MSTRG.27781.2	2664	coding	PF04597
MSTRG.27755.1	3015	coding	PF00320
MSTRG.27757.1	1076	coding	PF00226
MSTRG.27782.3	3303	coding	PF12146
MSTRG.27783.1	1900	noncoding	-
MSTRG.27784.1	2215	coding	PF00651
MSTRG.27784.3	2127	coding	PF00651
MSTRG.27784.2	1739	coding	PF00651
MSTRG.27786.1	4700	coding	PF00493
MSTRG.27793.1	622	noncoding	-
MSTRG.27794.1	492	noncoding	-
MSTRG.27795.1	6604	noncoding	-
MSTRG.27796.4	5944	coding	PF08337
MSTRG.27798.3	1067	coding	PF05300
MSTRG.27800.1	1549	noncoding	-
MSTRG.27802.2	2424	coding	PF07992
MSTRG.27802.3	2438	coding	PF07992
MSTRG.27802.4	3051	coding	PF07992

MSTRG.27802.5	2398	coding	PF07992
MSTRG.27802.6	6452	coding	PF07992
MSTRG.27804.2	686	noncoding	-
MSTRG.27804.3	699	noncoding	-
MSTRG.27801.1	1916	coding	PF01175
MSTRG.27801.2	1223	coding	PF01175
MSTRG.27807.2	3010	coding	PF15909
MSTRG.27808.4	6668	noncoding	-
MSTRG.27810.2	857	noncoding	-
MSTRG.27814.1	7588	noncoding	-
MSTRG.27814.4	7720	noncoding	-
MSTRG.27814.5	7684	noncoding	-
MSTRG.27814.12	4373	noncoding	-
MSTRG.27814.14	6948	noncoding	-
MSTRG.27818.1	1373	noncoding	-
MSTRG.27814.15	2506	noncoding	-
MSTRG.27814.16	2496	noncoding	-
MSTRG.27814.17	2519	noncoding	-
MSTRG.27838.3	2210	coding	PF04104
MSTRG.27838.2	2061	coding	PF04104
MSTRG.27838.4	2026	coding	PF04104
MSTRG.27839.1	295	noncoding	-
MSTRG.27840.1	7854	noncoding	-
MSTRG.27842.1	917	noncoding	-
MSTRG.27842.2	661	noncoding	-
MSTRG.27846.1	1805	noncoding	-
MSTRG.27846.2	7446	noncoding	-
MSTRG.27846.3	6689	noncoding	-
MSTRG.27846.4	3791	noncoding	-
MSTRG.27846.5	6995	noncoding	-
MSTRG.27846.7	4021	noncoding	-
MSTRG.27846.9	4660	noncoding	-
MSTRG.27846.8	6889	noncoding	-
MSTRG.27846.11	7042	noncoding	-
MSTRG.27846.12	6603	noncoding	-
MSTRG.27847.1	4676	noncoding	-
MSTRG.27848.1	1235	noncoding	-
MSTRG.27846.13	15330	noncoding	-
MSTRG.27846.15	5344	noncoding	-
MSTRG.27844.2	1683	noncoding	-
MSTRG.27884.3	17671	coding	PF02187
MSTRG.27884.6	23452	coding	PF02187
MSTRG.27885.1	4564	noncoding	-
MSTRG.27884.11	17097	coding	PF02187
MSTRG.27884.9	17079	coding	PF02187
MSTRG.27884.8	17950	coding	PF02187
MSTRG.27884.12	20201	coding	PF02187
MSTRG.27884.14	22287	coding	PF02187
MSTRG.27884.15	15873	coding	PF02187
MSTRG.27884.16	9728	coding	PF02187
MSTRG.27884.17	8918	coding	PF02187
MSTRG.27884.18	9770	coding	PF02187
MSTRG.27884.19	7774	coding	PF02187
MSTRG.27884.20	4084	coding	PF02187

MSTRG.27854.1	234	noncoding	-
MSTRG.27852.1	908	coding	PF03827
MSTRG.27862.1	904	noncoding	-
MSTRG.27863.2	2865	noncoding	-
MSTRG.27864.1	707	noncoding	-
MSTRG.27865.1	1877	noncoding	-
MSTRG.27870.1	3064	coding	PF00005
MSTRG.27870.3	2890	coding	PF00005
MSTRG.27870.4	2435	coding	PF00005
MSTRG.27870.5	10828	coding	PF02093
MSTRG.27875.2	1037	noncoding	-
MSTRG.27876.1	1064	coding	PF07654
MSTRG.27878.5	4495	coding	PF17035
MSTRG.27880.3	4885	coding	PF01410
MSTRG.27882.2	2761	coding	PF02535
MSTRG.27882.4	668	coding	PF00106
MSTRG.27882.3	887	coding	PF00106
MSTRG.27893.3	3797	coding	PF00617
MSTRG.27901.7	2763	coding	PF03344
MSTRG.27901.8	2478	coding	PF03344
MSTRG.27904.1	4257	noncoding	-
MSTRG.27905.5	2557	coding	PF00225
MSTRG.27908.4	691	coding	PF03091
MSTRG.27908.5	713	coding	PF03091
MSTRG.27909.1	4549	coding	PF12004
MSTRG.27909.4	2853	coding	PF12004
MSTRG.27909.3	4650	coding	PF12004
MSTRG.27909.5	2810	coding	PF12004
MSTRG.27910.1	758	noncoding	-
MSTRG.27896.1	2209	coding	PF00452
MSTRG.27896.2	2316	coding	PF00452
MSTRG.27897.1	947	coding	PF14836
MSTRG.27897.2	846	coding	PF14836
MSTRG.27897.5	874	coding	PF14836
MSTRG.27900.2	7295	noncoding	-
MSTRG.27922.2	29322	noncoding	-
MSTRG.27913.2	886	noncoding	-
MSTRG.27925.1	7301	noncoding	-
MSTRG.27925.3	7156	noncoding	-
MSTRG.27928.1	6947	coding	PF16158
MSTRG.27934.2	5842	coding	PF12796
MSTRG.27935.1	17159	noncoding	-
MSTRG.27936.1	4645	noncoding	-
MSTRG.27941.3	1898	coding	PF05794
MSTRG.27942.1	1557	noncoding	-
MSTRG.27942.4	780	noncoding	-
MSTRG.27942.3	1026	noncoding	-
MSTRG.27942.2	1349	noncoding	-
MSTRG.27943.2	4731	noncoding	-
MSTRG.27943.1	4856	noncoding	-
MSTRG.27943.3	4298	coding	PF07699
MSTRG.27945.3	2563	noncoding	-
MSTRG.27945.5	2772	noncoding	-
MSTRG.27940.2	3076	coding	PF00105

MSTRG.27949.1	2901	coding	PF01285
MSTRG.27949.2	2918	coding	PF01285
MSTRG.27950.3	4194	coding	PF00254
MSTRG.27950.4	3931	coding	PF00254
MSTRG.27951.1	2237	noncoding	-
MSTRG.27950.6	5000	coding	PF00254
MSTRG.27952.1	5464	noncoding	-
MSTRG.27953.1	5733	noncoding	-
MSTRG.27955.1	1333	noncoding	-
MSTRG.27954.5	2331	noncoding	-
MSTRG.27956.1	4376	noncoding	-
MSTRG.27956.5	2300	coding	PF00069
MSTRG.27957.4	2306	noncoding	-
MSTRG.27957.3	3089	noncoding	-
MSTRG.27957.5	3086	noncoding	-
MSTRG.27957.6	3135	noncoding	-
MSTRG.27957.7	3002	noncoding	-
MSTRG.27957.8	3226	noncoding	-
MSTRG.27957.9	3142	noncoding	-
MSTRG.27957.10	2486	noncoding	-
MSTRG.27959.1	4215	noncoding	-
MSTRG.27960.1	5448	noncoding	-
MSTRG.27960.2	1509	noncoding	-
MSTRG.27961.1	1908	noncoding	-
MSTRG.27963.2	5748	coding	PF13832
MSTRG.27967.4	4858	coding	PF16017
MSTRG.27967.5	4942	coding	PF16017
MSTRG.27967.6	5531	coding	PF16017
MSTRG.27967.7	5487	coding	PF16017
MSTRG.27968.3	3082	coding	PF00069
MSTRG.27968.4	5778	noncoding	-
MSTRG.27964.2	3834	coding	PF00076
MSTRG.27969.3	1112	coding	PF00900
MSTRG.27969.4	1167	coding	PF00900
MSTRG.27970.2	1565	coding	PF02234
MSTRG.27975.5	3194	noncoding	-
MSTRG.27975.4	1288	noncoding	-
MSTRG.27975.7	5769	noncoding	-
MSTRG.27983.2	1241	coding	PF15049
MSTRG.27985.1	1159	noncoding	-
MSTRG.27988.3	2290	noncoding	-
MSTRG.27995.1	3523	noncoding	-
MSTRG.27995.3	4944	noncoding	-
MSTRG.27996.1	15075	coding	PF01359
MSTRG.27996.2	13288	coding	PF01359
MSTRG.27997.1	8904	noncoding	-
MSTRG.28003.6	2855	coding	PF00651
MSTRG.28004.1	302	noncoding	-
MSTRG.28005.1	16691	noncoding	-
MSTRG.28005.2	16701	noncoding	-
MSTRG.28003.7	8408	coding	PF00651
MSTRG.28003.8	7743	coding	PF00651
MSTRG.28003.9	3899	noncoding	-
MSTRG.28007.2	916	noncoding	-

MSTRG.28008.1	14854	coding	PF03028
MSTRG.28008.2	14845	coding	PF03028
MSTRG.28008.3	11285	coding	PF08385
MSTRG.28009.1	1026	noncoding	-
MSTRG.28011.1	232	noncoding	-
MSTRG.28012.5	6731	coding	PF02181
MSTRG.28012.4	7544	coding	PF02181
MSTRG.28013.3	11023	coding	PF01967
MSTRG.28018.2	9463	noncoding	-
MSTRG.28033.6	1907	coding	PF02045
MSTRG.28033.8	2039	coding	PF02045
MSTRG.28033.5	1839	coding	PF02045
MSTRG.28033.7	1821	coding	PF02045
MSTRG.28034.2	4339	noncoding	-
MSTRG.28034.4	4248	noncoding	-
MSTRG.28034.9	4080	noncoding	-
MSTRG.28034.5	3656	noncoding	-
MSTRG.28034.6	3573	noncoding	-
MSTRG.28025.1	2498	noncoding	-
MSTRG.28024.4	3284	coding	PF16159
MSTRG.28027.1	505	coding	PF16159
MSTRG.28027.2	509	coding	PF16159
MSTRG.28040.1	5493	coding	PF00443
MSTRG.28040.2	6067	coding	PF00443
MSTRG.28040.4	5162	coding	PF00443
MSTRG.28045.3	4464	coding	PF07524
MSTRG.28035.2	1600	noncoding	-
MSTRG.28055.1	5599	noncoding	-
MSTRG.28058.1	4377	noncoding	-
MSTRG.28059.1	792	noncoding	-
MSTRG.28051.1	16919	coding	PF15249
MSTRG.28051.4	9462	noncoding	-
MSTRG.28051.7	10536	noncoding	-
MSTRG.28062.1	1098	noncoding	-
MSTRG.28064.1	868	noncoding	-
MSTRG.28070.1	6527	coding	PF11515
MSTRG.28072.3	1192	coding	PF03947
MSTRG.28076.1	355	noncoding	-
MSTRG.28075.1	911	noncoding	-
MSTRG.28081.1	3653	coding	PF00005
MSTRG.28085.6	2711	coding	PF15453
MSTRG.28091.4	5149	noncoding	-
MSTRG.28091.2	3394	coding	PF00817
MSTRG.28093.2	2665	noncoding	-
MSTRG.28095.3	698	noncoding	-
MSTRG.28095.2	869	noncoding	-
MSTRG.28097.2	778	noncoding	-
MSTRG.28098.2	3168	coding	PF00238
MSTRG.28098.1	3382	coding	PF00238
MSTRG.28098.3	1121	noncoding	-
MSTRG.28099.3	2690	noncoding	-
MSTRG.28100.1	2530	coding	PF00648
MSTRG.28100.5	2514	coding	PF00648
MSTRG.28100.6	2301	coding	PF00648

MSTRG.28100.7	2652	coding	PF00648
MSTRG.28100.8	2434	coding	PF00648
MSTRG.28105.5	2237	coding	PF01733
MSTRG.28105.6	2133	coding	PF01733
MSTRG.28107.1	3409	coding	PF00183
MSTRG.28109.3	1260	coding	PF15160
MSTRG.28109.4	1202	coding	PF15160
MSTRG.28109.2	1105	coding	PF15160
MSTRG.28109.5	1264	coding	PF15160
MSTRG.28121.1	6396	noncoding	-
MSTRG.28122.1	477	noncoding	-
MSTRG.28115.4	1353	noncoding	-
MSTRG.28114.2	2157	noncoding	-
MSTRG.28127.1	464	noncoding	-
MSTRG.28132.2	1768	noncoding	-
MSTRG.28137.1	1137	noncoding	-
MSTRG.28131.3	742	coding	PF03403
MSTRG.28141.1	455	noncoding	-
MSTRG.28142.2	3367	noncoding	-
MSTRG.28147.1	5258	coding	PF07653
MSTRG.28154.1	388	noncoding	-
MSTRG.28160.1	2618	coding	PF01642
MSTRG.28160.5	3450	coding	PF01642
MSTRG.28161.1	763	coding	PF01642
MSTRG.28162.1	1842	noncoding	-
MSTRG.28168.3	1725	coding	PF00162
MSTRG.28174.1	1950	coding	PF03299
MSTRG.28174.2	1551	coding	PF03299
MSTRG.28175.1	1340	coding	PF03299
MSTRG.28177.1	2366	coding	PF00078
MSTRG.28178.1	7084	coding	PF00078
MSTRG.28171.1	474	noncoding	-
MSTRG.28186.1	1378	noncoding	-
MSTRG.28191.1	928	coding	PF03647
MSTRG.28191.8	835	noncoding	-
MSTRG.28192.3	1374	coding	PF00043
MSTRG.28204.2	1544	noncoding	-
MSTRG.28207.1	353	noncoding	-
MSTRG.28207.2	738	noncoding	-
MSTRG.28207.3	219	noncoding	-
MSTRG.28211.2	2122	coding	PF01151
MSTRG.28213.2	926	coding	PF07654
MSTRG.28194.2	735	coding	PF00969
MSTRG.28196.2	649	noncoding	-
MSTRG.28197.1	595	noncoding	-
MSTRG.28214.1	829	noncoding	-
MSTRG.28220.1	3701	coding	PF12796
MSTRG.28220.2	8055	coding	PF03792
MSTRG.28223.2	1479	noncoding	-
MSTRG.28224.2	2339	coding	PF01553
MSTRG.28217.1	738	noncoding	-
MSTRG.28217.2	904	noncoding	-
MSTRG.28225.1	1218	noncoding	-
MSTRG.28227.3	2275	noncoding	-

MSTRG.28249.2	5390	coding	PF07678
MSTRG.28249.3	5390	coding	PF07678
MSTRG.28249.4	5654	coding	PF07678
MSTRG.28249.5	5389	coding	PF07678
MSTRG.28249.7	3012	coding	PF07678
MSTRG.28249.8	3247	coding	PF07678
MSTRG.28249.6	5385	coding	PF07678
MSTRG.28249.9	5252	coding	PF07678
MSTRG.28249.11	5289	coding	PF07678
MSTRG.28250.1	742	noncoding	-
MSTRG.28251.1	4619	coding	PF07678
MSTRG.28251.2	4733	coding	PF07678
MSTRG.28251.5	4236	coding	PF07678
MSTRG.28251.6	1323	noncoding	-
MSTRG.28249.13	5424	coding	PF07678
MSTRG.28232.2	3743	coding	PF08148
MSTRG.28233.2	1185	noncoding	-
MSTRG.28233.3	1304	noncoding	-
MSTRG.28233.4	1186	noncoding	-
MSTRG.28236.2	3790	coding	PF00856
MSTRG.28236.4	3875	coding	PF00856
MSTRG.28229.1	1724	coding	PF13088
MSTRG.28239.1	2900	coding	PF00012
MSTRG.28244.2	5623	coding	PF00133
MSTRG.28244.3	666	noncoding	-
MSTRG.28244.6	3660	coding	PF00133
MSTRG.28246.2	1029	noncoding	-
MSTRG.28247.1	3934	coding	PF15096
MSTRG.28247.3	2780	coding	PF15096
MSTRG.28247.5	2321	coding	PF15096
MSTRG.28247.2	2905	coding	PF15096
MSTRG.28241.1	902	noncoding	-
MSTRG.28242.1	3788	coding	PF01214
MSTRG.28242.3	3447	coding	PF01214
MSTRG.28242.5	1997	coding	PF01214
MSTRG.28243.1	1677	coding	PF01585
MSTRG.28255.2	1639	coding	PF11032
MSTRG.28256.5	3657	coding	PF12057
MSTRG.28257.2	6635	coding	PF07001
MSTRG.28257.3	7300	coding	PF07001
MSTRG.28257.4	6641	coding	PF07001
MSTRG.28300.1	889	coding	PF07654
MSTRG.28300.2	2467	coding	PF00129
MSTRG.28301.1	446	noncoding	-
MSTRG.28300.3	2551	coding	PF00129
MSTRG.28300.4	1125	coding	PF00129
MSTRG.28300.5	1125	coding	PF00129
MSTRG.28300.6	3728	coding	PF00129
MSTRG.28300.8	1056	coding	PF00129
MSTRG.28300.9	2465	coding	PF00129
MSTRG.28300.10	3682	coding	PF00129
MSTRG.28308.1	3321	coding	PF07111
MSTRG.28308.6	3555	coding	PF07111
MSTRG.28312.1	4750	coding	PF07714

MSTRG.28312.3	4267	coding	PF07714
MSTRG.28312.2	4597	coding	PF07714
MSTRG.28312.4	4151	coding	PF07714
MSTRG.28315.1	561	noncoding	-
MSTRG.28316.8	1894	noncoding	-
MSTRG.28319.1	4686	noncoding	-
MSTRG.28319.2	4689	noncoding	-
MSTRG.28319.4	4623	noncoding	-
MSTRG.28319.6	5196	noncoding	-
MSTRG.28321.1	3003	coding	PF13914
MSTRG.28325.2	3658	coding	PF05301
MSTRG.28325.5	1324	coding	PF01084
MSTRG.28324.3	4350	noncoding	-
MSTRG.28326.1	6011	noncoding	-
MSTRG.28328.2	2517	noncoding	-
MSTRG.28329.3	6399	noncoding	-
MSTRG.28332.2	972	coding	PF04032
MSTRG.28300.13	2768	noncoding	-
MSTRG.28266.4	1655	coding	PF00129
MSTRG.28266.5	1075	coding	PF00129
MSTRG.28266.7	530	coding	PF00129
MSTRG.28267.1	1096	coding	PF00129
MSTRG.28267.2	985	coding	PF00129
MSTRG.28266.17	422	coding	PF00129
MSTRG.28274.1	1731	noncoding	-
MSTRG.28274.2	1661	noncoding	-
MSTRG.28270.1	5243	coding	PF01352
MSTRG.28271.1	3405	noncoding	-
MSTRG.28281.2	4216	noncoding	-
MSTRG.28281.3	4224	noncoding	-
MSTRG.28281.4	4305	noncoding	-
MSTRG.28281.5	3952	noncoding	-
MSTRG.28281.6	3868	noncoding	-
MSTRG.28281.7	3020	noncoding	-
MSTRG.28281.8	4149	noncoding	-
MSTRG.28281.9	6589	noncoding	-
MSTRG.28281.10	1842	noncoding	-
MSTRG.28294.2	2590	coding	PF00622
MSTRG.28297.3	589	coding	PF00009
MSTRG.28335.1	5461	coding	PF02023
MSTRG.28336.1	270	noncoding	-
MSTRG.28335.2	4649	coding	PF02023
MSTRG.28335.3	5611	coding	PF02023
MSTRG.28338.1	2777	coding	PF13843
MSTRG.28339.3	7347	coding	PF02023
MSTRG.28341.2	2906	coding	PF02023
MSTRG.28341.4	3780	coding	PF02023
MSTRG.28342.3	12054	coding	PF02023
MSTRG.28342.4	10774	coding	PF02023
MSTRG.28343.1	540	noncoding	-
MSTRG.28367.1	522	noncoding	-
MSTRG.28367.2	500	noncoding	-
MSTRG.28368.2	505	noncoding	-
MSTRG.28370.1	529	noncoding	-

MSTRG.28371.1	1612	noncoding	-
MSTRG.28372.1	633	noncoding	-
MSTRG.28375.1	913	noncoding	-
MSTRG.28377.1	1079	coding	PF05577
MSTRG.28371.2	472	noncoding	-
MSTRG.28371.3	2690	noncoding	-
MSTRG.28378.1	582	noncoding	-
MSTRG.28379.1	691	coding	PF00125
MSTRG.28380.1	704	noncoding	-
MSTRG.28386.3	479	noncoding	-
MSTRG.28386.2	466	noncoding	-
MSTRG.28388.1	488	coding	PF00125
MSTRG.28386.4	473	noncoding	-
MSTRG.28388.2	568	coding	PF00125
MSTRG.28389.1	5896	coding	PF00538
MSTRG.28379.2	526	coding	PF00125
MSTRG.28388.3	573	coding	PF00125
MSTRG.28371.4	458	noncoding	-
MSTRG.28359.7	2822	noncoding	-
MSTRG.28359.6	2921	coding	PF16000
MSTRG.28359.3	5049	coding	PF16000
MSTRG.28359.5	4112	coding	PF16000
MSTRG.28359.8	3999	coding	PF16000
MSTRG.28359.9	3363	coding	PF16000
MSTRG.28359.10	4134	coding	PF16000
MSTRG.28361.1	1772	noncoding	-
MSTRG.28362.1	4245	noncoding	-
MSTRG.28359.11	17006	noncoding	-
MSTRG.28363.1	38489	noncoding	-
MSTRG.28363.2	3541	noncoding	-
MSTRG.28363.3	15908	noncoding	-
MSTRG.28366.4	3051	coding	PF15903
MSTRG.28366.5	7689	coding	PF15903
MSTRG.28366.6	5266	coding	PF15903
MSTRG.28393.1	4287	coding	PF15130
MSTRG.28393.3	4163	coding	PF15130
MSTRG.28393.4	3939	coding	PF15130
MSTRG.28393.6	4177	coding	PF15130
MSTRG.28393.2	4076	coding	PF15130
MSTRG.28397.1	1946	noncoding	-
MSTRG.28399.2	1843	coding	PF00171
MSTRG.28400.1	2159	coding	PF15354
MSTRG.28400.2	2138	coding	PF15354
MSTRG.28400.3	2217	coding	PF15354
MSTRG.28401.1	643	coding	PF14927
MSTRG.28391.2	816	noncoding	-
MSTRG.28403.1	215	noncoding	-
MSTRG.28404.1	1086	coding	PF00103
MSTRG.28409.2	3257	noncoding	-
MSTRG.28409.1	3208	coding	PF00855
MSTRG.28409.3	793	noncoding	-
MSTRG.28409.4	653	noncoding	-
MSTRG.28409.6	2722	noncoding	-
MSTRG.28409.5	666	noncoding	-

MSTRG.28409.7	7273	noncoding	-
MSTRG.28409.8	825	noncoding	-
MSTRG.28410.1	6408	noncoding	-
MSTRG.28409.9	2666	noncoding	-
MSTRG.28409.10	4942	coding	PF00078
MSTRG.28411.1	3550	noncoding	-
MSTRG.28409.11	506	noncoding	-
MSTRG.28449.3	1567	coding	PF00505
MSTRG.28457.1	2702	coding	PF00919
MSTRG.28457.2	2447	coding	PF00919
MSTRG.28457.5	4758	noncoding	-
MSTRG.28457.6	2588	coding	PF00919
MSTRG.28459.1	3355	noncoding	-
MSTRG.28460.1	8758	noncoding	-
MSTRG.28463.1	5114	noncoding	-
MSTRG.28464.1	11933	noncoding	-
MSTRG.28466.1	1305	noncoding	-
MSTRG.28467.1	3366	noncoding	-
MSTRG.28470.1	7759	noncoding	-
MSTRG.28470.2	7489	noncoding	-
MSTRG.28471.2	9975	coding	PF02319
MSTRG.28471.3	9745	coding	PF02319
MSTRG.28474.1	2967	noncoding	-
MSTRG.28471.4	15005	noncoding	-
MSTRG.28476.2	1780	coding	PF03062
MSTRG.28430.1	1586	noncoding	-
MSTRG.28430.2	1162	noncoding	-
MSTRG.28432.1	1044	noncoding	-
MSTRG.28433.1	887	noncoding	-
MSTRG.28439.1	4365	noncoding	-
MSTRG.28443.1	653	noncoding	-
MSTRG.28442.2	3749	coding	PF10599
MSTRG.28445.1	4639	noncoding	-
MSTRG.28480.2	318	noncoding	-
MSTRG.28480.1	518	noncoding	-
MSTRG.28480.3	2535	noncoding	-
MSTRG.28481.3	2932	coding	PF09379
MSTRG.28485.1	26684	noncoding	-
MSTRG.28485.2	12020	noncoding	-
MSTRG.28485.3	9093	noncoding	-
MSTRG.28485.4	3683	noncoding	-
MSTRG.28485.5	3185	noncoding	-
MSTRG.28485.6	3047	noncoding	-
MSTRG.28487.2	1993	coding	PF04440
MSTRG.28487.3	1903	coding	PF04440
MSTRG.28488.1	7823	coding	PF02373
MSTRG.28488.2	8242	coding	PF02373
MSTRG.28488.3	8498	coding	PF02373
MSTRG.28488.4	5157	coding	PF02373
MSTRG.28488.5	18427	coding	PF02373
MSTRG.28488.7	19246	noncoding	-
MSTRG.28489.1	14934	noncoding	-
MSTRG.28488.8	56960	noncoding	-
MSTRG.28488.9	41772	noncoding	-

MSTRG.28488.10	36069	noncoding	-
MSTRG.28488.11	15024	noncoding	-
MSTRG.28491.1	2030	noncoding	-
MSTRG.28492.1	486	noncoding	-
MSTRG.28495.1	2523	noncoding	-
MSTRG.28495.2	2085	noncoding	-
MSTRG.28499.3	2516	noncoding	-
MSTRG.28501.3	4205	coding	PF07798
MSTRG.28501.4	3967	coding	PF07798
MSTRG.28502.1	255	noncoding	-
MSTRG.28505.3	1598	coding	PF02146
MSTRG.28509.4	1680	noncoding	-
MSTRG.28509.5	2225	noncoding	-
MSTRG.28509.2	855	noncoding	-
MSTRG.28509.3	4100	noncoding	-
MSTRG.28510.1	782	noncoding	-
MSTRG.28514.5	6959	noncoding	-
MSTRG.28515.1	2086	coding	PF04750
MSTRG.28515.2	1918	coding	PF04750
MSTRG.28515.3	1222	coding	PF04750
MSTRG.28517.1	850	noncoding	-
MSTRG.28517.2	565	noncoding	-
MSTRG.28517.3	886	noncoding	-
MSTRG.28517.4	795	noncoding	-
MSTRG.28517.5	464	noncoding	-
MSTRG.28520.2	1965	noncoding	-
MSTRG.28522.2	9588	coding	PF01151
MSTRG.28522.3	4100	coding	PF01151
MSTRG.28522.4	3965	coding	PF01151
MSTRG.28522.6	4838	coding	PF01151
MSTRG.28522.8	4298	coding	PF01151
MSTRG.28525.2	2711	coding	PF00069
MSTRG.28525.3	4257	coding	PF00069
MSTRG.28532.3	2811	coding	PF14942
MSTRG.28532.5	2393	noncoding	-
MSTRG.28533.1	3745	noncoding	-
MSTRG.28538.2	9064	coding	PF00681
MSTRG.28546.1	2971	coding	PF15066
MSTRG.28546.2	1638	coding	PF15066
MSTRG.28546.3	2479	coding	PF15066
MSTRG.28546.4	2508	coding	PF15066
MSTRG.28546.12	3368	coding	PF15066
MSTRG.28546.10	2264	coding	PF15066
MSTRG.28546.8	1733	coding	PF15066
MSTRG.28539.2	9168	noncoding	-
MSTRG.28539.3	8279	noncoding	-
MSTRG.28539.5	8332	noncoding	-
MSTRG.28539.6	8600	noncoding	-
MSTRG.28539.7	8561	noncoding	-
MSTRG.28539.8	7258	noncoding	-
MSTRG.28550.3	4269	noncoding	-
MSTRG.28551.1	4331	noncoding	-
MSTRG.28552.1	1321	noncoding	-
MSTRG.28553.1	3225	noncoding	-

MSTRG.28554.1	7008	coding	PF01409
MSTRG.28555.2	1134	noncoding	-
MSTRG.28555.3	879	coding	PF05347
MSTRG.28556.1	2489	noncoding	-
MSTRG.28557.1	2533	noncoding	-
MSTRG.28555.4	619	noncoding	-
MSTRG.28559.1	922	noncoding	-
MSTRG.28561.3	5542	coding	PF08584
MSTRG.28563.1	3331	noncoding	-
MSTRG.28563.5	2171	noncoding	-
MSTRG.28564.1	5853	noncoding	-
MSTRG.28565.1	895	noncoding	-
MSTRG.28566.1	5343	noncoding	-
MSTRG.28567.1	2589	noncoding	-
MSTRG.28568.1	552	noncoding	-
MSTRG.28571.2	2312	coding	PF15023
MSTRG.28571.3	1290	coding	PF15023
MSTRG.28572.1	659	noncoding	-
MSTRG.28579.2	4670	noncoding	-
MSTRG.28586.1	2010	coding	PF03953
MSTRG.28586.3	1919	coding	PF03953
MSTRG.28586.4	1552	coding	PF03953
MSTRG.28586.5	1548	coding	PF03953
MSTRG.28587.2	1007	noncoding	-
MSTRG.28587.3	1507	noncoding	-
MSTRG.28588.1	16784	noncoding	-
MSTRG.28590.1	205	noncoding	-
MSTRG.28588.2	5470	noncoding	-
MSTRG.28594.2	1395	coding	PF00079
MSTRG.28581.2	6430	coding	PF12002
MSTRG.28585.1	640	coding	PF00069
MSTRG.28595.1	525	noncoding	-
MSTRG.28600.2	1644	coding	PF16363
MSTRG.28600.3	2211	coding	PF16363
MSTRG.28600.6	5828	coding	PF16363
MSTRG.28600.5	1975	coding	PF16363
MSTRG.28600.4	3107	coding	PF16363
MSTRG.28600.7	5803	coding	PF16363
MSTRG.28600.8	1459	coding	PF16363
MSTRG.28600.9	1666	coding	PF16363
MSTRG.28602.1	229	noncoding	-
MSTRG.28603.1	11523	noncoding	-
MSTRG.28604.1	625	noncoding	-
MSTRG.28605.1	1377	noncoding	-
MSTRG.28606.1	3136	noncoding	-
MSTRG.28597.1	1274	noncoding	-
MSTRG.28613.7	3875	coding	PF15469
MSTRG.28613.6	3177	coding	PF15469
MSTRG.28613.8	9251	coding	PF15469
MSTRG.28613.9	9449	coding	PF15469
MSTRG.28613.10	3194	noncoding	-
MSTRG.28614.1	3257	noncoding	-
MSTRG.28609.1	545	noncoding	-
MSTRG.28618.1	1047	noncoding	-

MSTRG.28620.4	4296	noncoding	-
MSTRG.28620.3	4394	noncoding	-
MSTRG.28623.2	2901	noncoding	-
MSTRG.28625.1	870	coding	PF02966
MSTRG.28625.2	3363	coding	PF02966
MSTRG.28625.3	794	coding	PF02966
MSTRG.28625.4	864	coding	PF02966
MSTRG.28627.4	3796	coding	PF09309
MSTRG.28627.3	3514	coding	PF09309
MSTRG.28627.5	3272	coding	PF09309
MSTRG.28628.1	836	noncoding	-
MSTRG.28631.2	1485	noncoding	-
MSTRG.28631.3	19957	noncoding	-
MSTRG.28633.1	710	noncoding	-
MSTRG.28642.2	6555	noncoding	-
MSTRG.28642.8	5980	noncoding	-
MSTRG.28642.7	8945	noncoding	-
MSTRG.28642.5	7803	noncoding	-
MSTRG.28642.6	9134	noncoding	-
MSTRG.28642.3	8079	noncoding	-
MSTRG.28638.1	450	noncoding	-
MSTRG.28638.2	795	noncoding	-
MSTRG.28644.1	10359	noncoding	-
MSTRG.28645.2	7359	noncoding	-
MSTRG.28646.3	617	noncoding	-
MSTRG.28646.4	512	noncoding	-
MSTRG.28646.5	559	noncoding	-
MSTRG.28647.1	5219	noncoding	-
MSTRG.28648.1	1029	noncoding	-
MSTRG.28648.2	1213	noncoding	-
MSTRG.28648.3	928	noncoding	-
MSTRG.28650.1	1046	noncoding	-
MSTRG.28650.3	457	noncoding	-
MSTRG.28650.2	629	noncoding	-
MSTRG.28652.1	369	noncoding	-
MSTRG.28653.1	4627	noncoding	-
MSTRG.28654.1	1767	noncoding	-
MSTRG.28656.1	252	noncoding	-
MSTRG.28657.3	7770	noncoding	-
MSTRG.28658.1	1054	noncoding	-
MSTRG.28662.4	3001	noncoding	-
MSTRG.28662.3	1986	noncoding	-
MSTRG.28661.1	740	noncoding	-
MSTRG.28661.2	405	noncoding	-
MSTRG.28663.1	2607	coding	PF15813
MSTRG.28663.2	2743	coding	PF15813
MSTRG.28669.2	2650	coding	PF08294
MSTRG.28672.1	859	noncoding	-
MSTRG.28672.2	593	noncoding	-
MSTRG.28672.3	637	noncoding	-
MSTRG.28677.1	865	noncoding	-
MSTRG.28677.2	286	noncoding	-
MSTRG.28682.2	6897	coding	PF14726
MSTRG.28688.2	2314	noncoding	-

MSTRG.28693.1	344	noncoding	-
MSTRG.28702.1	445	noncoding	-
MSTRG.28705.1	395	noncoding	-
MSTRG.28707.1	1129	noncoding	-
MSTRG.28712.2	433	noncoding	-
MSTRG.28712.3	347	coding	PF01159
MSTRG.28714.1	323	noncoding	-
MSTRG.28716.1	1318	noncoding	-
MSTRG.28718.2	3307	coding	PF00792
MSTRG.28715.1	320	noncoding	-
MSTRG.28720.1	341	coding	PF00338
MSTRG.28731.1	676	noncoding	-
MSTRG.28732.1	591	noncoding	-
MSTRG.28742.4	1992	coding	PF15369
MSTRG.28743.1	866	noncoding	-
MSTRG.28744.1	4896	noncoding	-
MSTRG.28745.1	1965	noncoding	-
MSTRG.28745.3	2595	noncoding	-
MSTRG.28745.5	5836	noncoding	-
MSTRG.28746.1	6484	coding	PF02181
MSTRG.28746.3	5594	coding	PF02181
MSTRG.28746.4	669	noncoding	-
MSTRG.28746.5	2175	noncoding	-
MSTRG.28737.2	2732	noncoding	-
MSTRG.28737.3	2292	noncoding	-
MSTRG.28739.3	4338	coding	PF16566
MSTRG.28739.4	1286	coding	PF16566
MSTRG.28739.5	17791	coding	PF16566
MSTRG.28739.6	4435	noncoding	-
MSTRG.28740.1	1105	noncoding	-
MSTRG.28755.1	550	noncoding	-
MSTRG.28765.1	2615	noncoding	-
MSTRG.28770.1	8504	coding	PF02023
MSTRG.28770.3	8509	coding	PF02023
MSTRG.28780.1	4045	noncoding	-
MSTRG.28780.3	3330	noncoding	-
MSTRG.28784.1	10879	coding	PF13919
MSTRG.28784.2	8358	coding	PF13919
MSTRG.28783.1	1753	noncoding	-
MSTRG.28790.1	401	noncoding	-
MSTRG.28790.2	444	noncoding	-
MSTRG.28794.1	444	noncoding	-
MSTRG.28799.2	5167	coding	PF00028
MSTRG.28822.1	4929	noncoding	-
MSTRG.28830.1	429	noncoding	-
MSTRG.28839.1	3217	coding	PF05236
MSTRG.28839.2	4138	coding	PF05236
MSTRG.28839.4	2507	noncoding	-
MSTRG.28842.1	6646	noncoding	-
MSTRG.28844.6	3165	coding	PF05030
MSTRG.28844.5	4389	coding	PF05030
MSTRG.28846.3	3948	coding	PF01205
MSTRG.28853.2	5406	coding	PF01237
MSTRG.28853.3	10071	coding	PF01237

MSTRG.28853.5	3800	coding	PF01237
MSTRG.28853.6	2602	noncoding	-
MSTRG.28854.1	597	noncoding	-
MSTRG.28853.10	1505	noncoding	-
MSTRG.28853.9	3265	coding	PF01237
MSTRG.28856.1	6351	noncoding	-
MSTRG.28857.6	1607	noncoding	-
MSTRG.28857.5	1828	noncoding	-
MSTRG.28857.8	2436	noncoding	-
MSTRG.28857.7	1158	noncoding	-
MSTRG.28858.2	4140	coding	PF10300
MSTRG.28859.1	13264	noncoding	-
MSTRG.28862.2	11292	coding	PF06008
MSTRG.28862.3	12975	coding	PF06008
MSTRG.28862.4	6829	coding	PF06008
MSTRG.28862.5	10742	coding	PF06008
MSTRG.28862.6	10801	coding	PF06008
MSTRG.28862.7	965	coding	PF00055
MSTRG.28860.2	4033	coding	PF01163
MSTRG.28866.2	3185	coding	PF10482
MSTRG.28866.3	887	noncoding	-
MSTRG.28869.1	364	noncoding	-
MSTRG.28879.1	4309	coding	PF01423
MSTRG.28879.4	878	coding	PF01423
MSTRG.28879.3	1167	coding	PF01423
MSTRG.28879.5	1041	coding	PF01423
MSTRG.28880.1	23001	coding	PF15782
MSTRG.28880.3	4098	coding	PF13878
MSTRG.28880.2	6403	coding	PF13880
MSTRG.28880.4	3798	coding	PF13878
MSTRG.28882.1	3915	noncoding	-
MSTRG.28883.1	6184	noncoding	-
MSTRG.28884.1	1592	noncoding	-
MSTRG.28885.2	709	noncoding	-
MSTRG.28885.3	636	noncoding	-
MSTRG.28886.2	4773	coding	PF08912
MSTRG.28887.1	5270	noncoding	-
MSTRG.28887.2	4620	noncoding	-
MSTRG.28887.4	5281	noncoding	-
MSTRG.28888.1	3733	coding	PF11957
MSTRG.28873.1	1034	noncoding	-
MSTRG.28876.1	2310	coding	PF01093
MSTRG.28890.1	2317	coding	PF00303
MSTRG.28890.5	1237	coding	PF00303
MSTRG.28890.6	1673	coding	PF00303
MSTRG.28890.4	1336	coding	PF00303
MSTRG.28890.3	3496	coding	PF00303
MSTRG.28893.1	2316	noncoding	-
MSTRG.28892.2	4454	noncoding	-
MSTRG.28894.1	891	noncoding	-
MSTRG.28894.2	288	noncoding	-
MSTRG.28900.3	2576	coding	PF05063
MSTRG.28905.2	5529	coding	PF08235
MSTRG.28907.1	1026	noncoding	-

MSTRG.28908.1	3116	noncoding	-
MSTRG.28914.1	3311	coding	PF03359
MSTRG.28914.2	3862	coding	PF03359
MSTRG.28914.3	1910	coding	PF03359
MSTRG.28917.1	1673	noncoding	-
MSTRG.28920.1	4777	noncoding	-
MSTRG.28923.4	3791	coding	PF05902
MSTRG.28923.3	4124	coding	PF05902
MSTRG.28923.5	4060	coding	PF05902
MSTRG.28923.7	3969	coding	PF05902
MSTRG.28923.6	4164	coding	PF05902
MSTRG.28923.8	3795	coding	PF05902
MSTRG.28934.1	7655	noncoding	-
MSTRG.28935.1	11437	noncoding	-
MSTRG.28936.1	8304	noncoding	-
MSTRG.28937.1	643	noncoding	-
MSTRG.28933.3	1647	noncoding	-
MSTRG.28941.1	5622	noncoding	-
MSTRG.28942.1	6076	noncoding	-
MSTRG.28940.4	8013	coding	PF14818
MSTRG.28949.2	1128	coding	PF01257
MSTRG.28949.4	1449	coding	PF01257
MSTRG.28951.2	4502	noncoding	-
MSTRG.28951.1	9389	coding	PF12796
MSTRG.28951.6	2448	coding	PF12796
MSTRG.28951.5	3441	coding	PF12796
MSTRG.28952.1	3212	noncoding	-
MSTRG.28953.1	1308	noncoding	-
MSTRG.28947.1	3256	coding	PF00620
MSTRG.28961.6	5066	noncoding	-
MSTRG.28961.8	5007	noncoding	-
MSTRG.28961.4	5572	noncoding	-
MSTRG.28961.5	5623	noncoding	-
MSTRG.28961.3	3717	noncoding	-
MSTRG.28961.7	3797	noncoding	-
MSTRG.28961.10	6118	noncoding	-
MSTRG.28961.12	4876	noncoding	-
MSTRG.28961.9	3822	noncoding	-
MSTRG.28961.13	1867	noncoding	-
MSTRG.28961.14	451	noncoding	-
MSTRG.28961.15	308	noncoding	-
MSTRG.28961.16	237	noncoding	-
MSTRG.28963.1	1826	coding	PF00078
MSTRG.28967.1	1177	noncoding	-
MSTRG.28975.1	522	noncoding	-
MSTRG.28980.2	26706	noncoding	-
MSTRG.28976.2	1589	coding	PF00459
MSTRG.28976.3	1336	coding	PF00459
MSTRG.28977.1	978	noncoding	-
MSTRG.28976.4	794	coding	PF00459
MSTRG.28987.2	6176	coding	PF04707
MSTRG.28988.2	5079	coding	PF16474
MSTRG.28988.3	5809	coding	PF15627
MSTRG.28990.1	3735	noncoding	-

MSTRG.28988.4	1884	coding	PF16474
MSTRG.28988.5	3239	coding	PF15627
MSTRG.28988.6	3950	coding	PF15627
MSTRG.28988.8	2375	coding	PF15627
MSTRG.28988.9	3164	coding	PF15627
MSTRG.28988.10	2170	coding	PF15627
MSTRG.28988.11	2919	coding	PF15627
MSTRG.28988.12	2241	coding	PF15627
MSTRG.28993.2	1110	coding	PF09754
MSTRG.28993.3	1505	coding	PF09754
MSTRG.29016.1	1622	noncoding	-
MSTRG.29017.8	3450	noncoding	-
MSTRG.29017.9	9189	noncoding	-
MSTRG.29017.10	2494	noncoding	-
MSTRG.29017.12	1839	noncoding	-
MSTRG.29017.13	2573	noncoding	-
MSTRG.29017.11	9079	noncoding	-
MSTRG.29000.1	1616	noncoding	-
MSTRG.29008.1	11721	noncoding	-
MSTRG.29009.1	17512	noncoding	-
MSTRG.29013.3	6255	noncoding	-
MSTRG.28996.1	1064	noncoding	-
MSTRG.29020.2	9197	noncoding	-
MSTRG.29020.3	2877	noncoding	-
MSTRG.29021.1	3831	noncoding	-
MSTRG.29022.1	330	noncoding	-
MSTRG.29019.1	570	noncoding	-
MSTRG.29024.2	4604	noncoding	-
MSTRG.29024.4	4835	noncoding	-
MSTRG.29028.3	9290	noncoding	-
MSTRG.29026.3	3940	noncoding	-
MSTRG.29027.1	1667	noncoding	-
MSTRG.29029.1	1403	noncoding	-
MSTRG.29034.1	2265	noncoding	-
MSTRG.29035.1	5913	coding	PF01477
MSTRG.29037.1	1437	noncoding	-
MSTRG.29041.1	9124	coding	PF02891
MSTRG.29041.5	2906	coding	PF14324
MSTRG.29041.4	3343	coding	PF14324
MSTRG.29041.7	2119	coding	PF14324
MSTRG.29043.1	1357	coding	PF00164
MSTRG.29043.2	1530	coding	PF00164
MSTRG.29043.3	1690	coding	PF00164
MSTRG.29045.1	2259	coding	PF00004
MSTRG.29049.4	1562	coding	PF13242
MSTRG.29051.2	547	noncoding	-
MSTRG.29054.4	9789	coding	PF03166
MSTRG.29054.5	7184	coding	PF03166
MSTRG.29055.1	9395	noncoding	-
MSTRG.29064.3	1750	noncoding	-
MSTRG.29064.4	5357	noncoding	-
MSTRG.29064.6	1241	noncoding	-
MSTRG.29065.1	2233	noncoding	-
MSTRG.29065.2	596	noncoding	-

MSTRG.29068.1	1459	noncoding	-
MSTRG.29068.3	1277	noncoding	-
MSTRG.29079.2	449	noncoding	-
MSTRG.29079.1	5703	coding	PF00063
MSTRG.29079.4	651	noncoding	-
MSTRG.29079.3	5718	coding	PF00063
MSTRG.29079.5	5864	coding	PF00063
MSTRG.29080.1	4065	noncoding	-
MSTRG.29086.2	3279	noncoding	-
MSTRG.29086.3	3188	noncoding	-
MSTRG.29082.3	2351	coding	PF12269
MSTRG.29082.4	2445	coding	PF12269
MSTRG.29083.1	891	noncoding	-
MSTRG.29085.1	914	coding	PF07160
MSTRG.29092.2	2760	noncoding	-
MSTRG.29092.6	2898	noncoding	-
MSTRG.29092.7	3187	noncoding	-
MSTRG.29092.5	2996	noncoding	-
MSTRG.29093.4	2179	coding	PF03949
MSTRG.29094.1	383	noncoding	-
MSTRG.29099.2	3024	noncoding	-
MSTRG.29101.1	4052	noncoding	-
MSTRG.29101.2	2917	noncoding	-
MSTRG.29107.1	717	noncoding	-
MSTRG.29108.1	276	noncoding	-
MSTRG.29111.3	2173	coding	PF14048
MSTRG.29111.4	2297	coding	PF14048
MSTRG.29111.2	2184	coding	PF14048
MSTRG.29120.3	3333	noncoding	-
MSTRG.29120.4	2371	noncoding	-
MSTRG.29121.1	8960	coding	PF15792
MSTRG.29121.3	3914	coding	PF15792
MSTRG.29113.1	1447	coding	PF09749
MSTRG.29115.1	854	noncoding	-
MSTRG.29128.1	2216	noncoding	-
MSTRG.29129.1	344	noncoding	-
MSTRG.29129.2	1069	noncoding	-
MSTRG.29130.1	2058	noncoding	-
MSTRG.29122.1	295	noncoding	-
MSTRG.29126.1	256	noncoding	-
MSTRG.29133.1	1023	noncoding	-
MSTRG.29136.2	7348	noncoding	-
MSTRG.29136.5	5244	noncoding	-
MSTRG.29136.1	5822	noncoding	-
MSTRG.29137.1	653	noncoding	-
MSTRG.29155.4	3418	coding	PF00632
MSTRG.29155.6	3580	coding	PF00632
MSTRG.29155.1	7941	coding	PF00632
MSTRG.29155.8	7971	coding	PF00632
MSTRG.29155.11	4079	coding	PF00632
MSTRG.29155.15	3083	coding	PF00168
MSTRG.29155.16	7850	coding	PF00632
MSTRG.29155.17	7521	coding	PF00632
MSTRG.29155.19	1490	coding	PF00168

MSTRG.29157.1	5860	noncoding	-
MSTRG.29155.22	1166	noncoding	-
MSTRG.29160.1	3346	noncoding	-
MSTRG.29161.1	694	noncoding	-
MSTRG.29142.1	3268	noncoding	-
MSTRG.29143.1	968	noncoding	-
MSTRG.29144.3	4915	coding	PF13895
MSTRG.29149.2	6079	noncoding	-
MSTRG.29149.8	4298	noncoding	-
MSTRG.29149.9	1825	noncoding	-
MSTRG.29149.10	2511	noncoding	-
MSTRG.29149.11	2496	noncoding	-
MSTRG.29150.1	4833	coding	PF03388
MSTRG.29150.2	4001	coding	PF03388
MSTRG.29150.3	3878	coding	PF03388
MSTRG.29150.4	5515	coding	PF03388
MSTRG.29150.5	2356	coding	PF03388
MSTRG.29150.6	3858	coding	PF03388
MSTRG.29150.7	3080	coding	PF03388
MSTRG.29150.8	2011	coding	PF03388
MSTRG.29165.3	993	noncoding	-
MSTRG.29162.1	668	noncoding	-
MSTRG.29162.2	626	noncoding	-
MSTRG.29163.1	626	noncoding	-
MSTRG.29172.1	220	noncoding	-
MSTRG.29191.1	2448	noncoding	-
MSTRG.29192.1	1190	noncoding	-
MSTRG.29195.1	2685	noncoding	-
MSTRG.29195.2	1213	noncoding	-
MSTRG.29195.3	1066	noncoding	-
MSTRG.29195.4	1071	noncoding	-
MSTRG.29201.1	232	noncoding	-
MSTRG.29202.4	5813	noncoding	-
MSTRG.29202.6	2782	noncoding	-
MSTRG.29202.7	3698	noncoding	-
MSTRG.29202.8	1591	noncoding	-
MSTRG.29202.9	2937	noncoding	-
MSTRG.29196.1	1362	noncoding	-
MSTRG.29198.1	8020	noncoding	-
MSTRG.29198.2	2786	noncoding	-
MSTRG.29198.4	1939	noncoding	-
MSTRG.29207.2	4813	coding	PF00481
MSTRG.29207.3	4738	coding	PF00481
MSTRG.29208.1	221	noncoding	-
MSTRG.29213.1	465	noncoding	-
MSTRG.29214.1	5338	noncoding	-
MSTRG.29215.1	875	noncoding	-
MSTRG.29219.1	547	noncoding	-
MSTRG.29231.1	3275	noncoding	-
MSTRG.29231.2	4990	noncoding	-
MSTRG.29229.16	1313	coding	PF13804
MSTRG.29232.1	1595	noncoding	-
MSTRG.29232.2	1106	noncoding	-
MSTRG.29221.2	643	coding	PF01096

MSTRG.29224.2	1198	coding	PF02245
MSTRG.29226.2	559	coding	PF00042
MSTRG.29226.3	559	coding	PF00042
MSTRG.29233.3	1581	coding	PF03194
MSTRG.29238.2	1117	noncoding	-
MSTRG.29239.3	2403	coding	PF12036
MSTRG.29240.1	922	coding	PF00334
MSTRG.29242.2	3174	coding	PF09457
MSTRG.29242.3	3294	coding	PF09457
MSTRG.29242.5	3668	coding	PF09457
MSTRG.29242.6	2629	coding	PF09457
MSTRG.29246.3	3178	coding	PF05024
MSTRG.29257.4	627	coding	PF06080
MSTRG.29257.2	800	coding	PF06080
MSTRG.29258.1	1089	coding	PF14799
MSTRG.29258.2	838	coding	PF14799
MSTRG.29258.4	974	coding	PF14799
MSTRG.29258.5	943	coding	PF14799
MSTRG.29258.6	436	noncoding	-
MSTRG.29258.9	1017	coding	PF14799
MSTRG.29260.1	1089	noncoding	-
MSTRG.29262.2	759	noncoding	-
MSTRG.29254.1	2443	coding	PF14739
MSTRG.29255.4	1200	coding	PF16123
MSTRG.29268.1	941	noncoding	-
MSTRG.29271.3	5891	coding	PF06762
MSTRG.29271.5	3889	coding	PF06762
MSTRG.29276.1	7903	coding	PF00520
MSTRG.29276.3	7870	coding	PF00520
MSTRG.29278.1	1350	noncoding	-
MSTRG.29278.2	1248	noncoding	-
MSTRG.29278.3	1169	coding	PF00168
MSTRG.29279.2	648	coding	PF04034
MSTRG.29281.3	4019	noncoding	-
MSTRG.29282.2	2504	coding	PF15450
MSTRG.29283.2	11294	noncoding	-
MSTRG.29283.3	2391	noncoding	-
MSTRG.29283.4	8989	noncoding	-
MSTRG.29283.5	8719	noncoding	-
MSTRG.29301.1	391	noncoding	-
MSTRG.29302.3	3634	noncoding	-
MSTRG.29302.1	5053	noncoding	-
MSTRG.29305.2	7460	noncoding	-
MSTRG.29305.3	9761	noncoding	-
MSTRG.29307.3	3337	noncoding	-
MSTRG.29308.4	5483	coding	PF09744
MSTRG.29308.5	8278	coding	PF09744
MSTRG.29308.6	4652	noncoding	-
MSTRG.29308.7	4429	noncoding	-
MSTRG.29293.2	1345	coding	PF00333
MSTRG.29298.1	1828	noncoding	-
MSTRG.29325.2	2500	noncoding	-
MSTRG.29328.7	1924	coding	PF00076
MSTRG.29328.9	2275	coding	PF00076

MSTRG.29328.8	3782	coding	PF00076
MSTRG.29333.2	6538	coding	PF00005
MSTRG.29334.1	6630	coding	PF00005
MSTRG.29334.5	2525	noncoding	-
MSTRG.29341.3	1347	coding	PF07969
MSTRG.29342.1	7893	coding	PF14593
MSTRG.29342.2	6986	coding	PF00069
MSTRG.29343.1	8753	noncoding	-
MSTRG.29342.4	13566	coding	PF00069
MSTRG.29356.21	7782	noncoding	-
MSTRG.29356.18	10127	noncoding	-
MSTRG.29356.9	8945	noncoding	-
MSTRG.29356.12	12087	noncoding	-
MSTRG.29356.13	8729	noncoding	-
MSTRG.29356.14	8684	noncoding	-
MSTRG.29356.16	9258	noncoding	-
MSTRG.29356.5	8705	noncoding	-
MSTRG.29356.6	9462	noncoding	-
MSTRG.29356.8	9369	noncoding	-
MSTRG.29356.15	9090	noncoding	-
MSTRG.29356.19	8780	noncoding	-
MSTRG.29356.22	12308	noncoding	-
MSTRG.29356.23	8602	noncoding	-
MSTRG.29356.24	10937	noncoding	-
MSTRG.29347.1	4742	coding	PF15423
MSTRG.29353.1	676	noncoding	-
MSTRG.29354.1	1051	coding	PF15225
MSTRG.29359.2	2708	coding	PF02023
MSTRG.29361.1	653	noncoding	-
MSTRG.29363.1	455	noncoding	-
MSTRG.29364.1	963	noncoding	-
MSTRG.29367.2	2772	coding	PF02023
MSTRG.29370.2	8243	coding	PF02023
MSTRG.29375.2	2361	noncoding	-
MSTRG.29376.3	946	coding	PF15486
MSTRG.29387.1	6727	noncoding	-
MSTRG.29387.6	6132	noncoding	-
MSTRG.29389.1	5873	noncoding	-
MSTRG.29388.6	1043	noncoding	-
MSTRG.29378.1	2275	noncoding	-
MSTRG.29380.1	13384	noncoding	-
MSTRG.29379.3	2530	coding	PF00211
MSTRG.29393.3	1013	coding	PF03656
MSTRG.29393.2	1090	coding	PF03656
MSTRG.29393.4	825	coding	PF03656
MSTRG.29393.5	871	coding	PF03656
MSTRG.29393.6	612	coding	PF03656
MSTRG.29393.7	1349	noncoding	-
MSTRG.29405.2	3530	coding	PF16300
MSTRG.29409.2	934	coding	PF05368
MSTRG.29409.3	2430	coding	PF05368
MSTRG.29411.1	7708	coding	PF01126
MSTRG.29412.1	5365	coding	PF16043
MSTRG.29417.3	2352	coding	PF12796

MSTRG.29417.4	2332	coding	PF12796
MSTRG.29418.4	2055	coding	PF15773
MSTRG.29420.2	6723	coding	PF02023
MSTRG.29420.1	2118	coding	PF02023
MSTRG.29425.2	3912	coding	PF03446
MSTRG.29425.5	3972	coding	PF03446
MSTRG.29425.6	4099	coding	PF03446
MSTRG.29425.7	4336	coding	PF03446
MSTRG.29426.3	4251	coding	PF14075
MSTRG.29426.4	4400	coding	PF14075
MSTRG.29426.5	4250	coding	PF14075
MSTRG.29426.6	4403	coding	PF14075
MSTRG.29395.2	1461	noncoding	-
MSTRG.29404.5	1091	coding	PF10294
MSTRG.29404.3	1053	noncoding	-
MSTRG.29404.6	1287	coding	PF10294
MSTRG.29404.7	1289	coding	PF10294
MSTRG.29428.3	4848	coding	PF00202
MSTRG.29436.3	3543	noncoding	-
MSTRG.29432.3	4215	coding	PF12436
MSTRG.29445.1	1290	noncoding	-
MSTRG.29447.1	6658	noncoding	-
MSTRG.29447.2	1916	noncoding	-
MSTRG.29449.1	1952	noncoding	-
MSTRG.29450.1	1174	noncoding	-
MSTRG.29447.6	6613	noncoding	-
MSTRG.29447.7	3291	coding	PF16794
MSTRG.29440.2	1472	coding	PF03148
MSTRG.29455.4	6563	coding	PF09758
MSTRG.29455.6	3327	coding	PF09758
MSTRG.29455.7	7656	noncoding	-
MSTRG.29454.1	643	noncoding	-
MSTRG.29458.1	746	coding	PF01254
MSTRG.29457.2	607	noncoding	-
MSTRG.29463.1	3327	coding	PF10601
MSTRG.29463.7	2007	coding	PF10601
MSTRG.29463.6	2039	coding	PF10601
MSTRG.29466.1	3247	noncoding	-
MSTRG.29466.3	3291	noncoding	-
MSTRG.29467.2	2887	noncoding	-
MSTRG.29467.3	2657	noncoding	-
MSTRG.29467.4	3401	noncoding	-
MSTRG.29467.5	1640	noncoding	-
MSTRG.29481.2	4901	coding	PF00787
MSTRG.29482.2	2091	noncoding	-
MSTRG.29481.3	3375	coding	PF00787
MSTRG.29470.1	1068	noncoding	-
MSTRG.29470.2	570	noncoding	-
MSTRG.29471.1	4589	noncoding	-
MSTRG.29484.1	282	noncoding	-
MSTRG.29495.2	2682	coding	PF15125
MSTRG.29495.3	3982	coding	PF15125
MSTRG.29497.1	3903	noncoding	-
MSTRG.29499.4	1198	coding	PF14736

MSTRG.29497.4	4076	noncoding	-
MSTRG.29497.3	4177	noncoding	-
MSTRG.29500.1	1196	noncoding	-
MSTRG.29504.3	7507	coding	PF11608
MSTRG.29504.4	7473	coding	PF11608
MSTRG.29504.2	7575	coding	PF11608
MSTRG.29506.3	2750	noncoding	-
MSTRG.29502.2	1118	noncoding	-
MSTRG.29502.3	1021	noncoding	-
MSTRG.29502.4	2295	noncoding	-
MSTRG.29509.1	6538	noncoding	-
MSTRG.29509.2	6685	noncoding	-
MSTRG.29509.3	8797	noncoding	-
MSTRG.29511.2	2007	coding	PF12529
MSTRG.29511.3	1873	coding	PF12529
MSTRG.29511.4	1926	coding	PF12529
MSTRG.29511.5	1790	coding	PF12529
MSTRG.29513.1	603	noncoding	-
MSTRG.29510.1	299	noncoding	-
MSTRG.29512.1	284	noncoding	-
MSTRG.29521.2	995	coding	PF00410
MSTRG.29521.3	2207	noncoding	-
MSTRG.29521.4	13473	coding	PF15785
MSTRG.29524.1	847	noncoding	-
MSTRG.29517.1	7027	noncoding	-
MSTRG.29525.1	894	coding	PF03232
MSTRG.29525.3	1288	coding	PF03232
MSTRG.29525.4	903	coding	PF03232
MSTRG.29526.1	825	noncoding	-
MSTRG.29532.5	4681	coding	PF16025
MSTRG.29533.5	1225	noncoding	-
MSTRG.29533.6	961	noncoding	-
MSTRG.29535.2	1454	noncoding	-
MSTRG.29535.3	1570	noncoding	-
MSTRG.29535.5	1531	noncoding	-
MSTRG.29535.4	1717	noncoding	-
MSTRG.29536.1	2660	noncoding	-
MSTRG.29530.1	2364	noncoding	-
MSTRG.29530.3	3637	coding	PF13193
MSTRG.29531.3	1513	noncoding	-
MSTRG.29537.3	22168	coding	PF02093
MSTRG.29537.4	1782	noncoding	-
MSTRG.29557.1	21443	noncoding	-
MSTRG.29557.2	7315	coding	PF00501
MSTRG.29557.3	6842	coding	PF00929
MSTRG.29557.4	3905	coding	PF00929
MSTRG.29557.5	4515	coding	PF00929
MSTRG.29557.6	3884	coding	PF00929
MSTRG.29557.7	2670	coding	PF00929
MSTRG.29557.8	2630	coding	PF00929
MSTRG.29557.10	2644	coding	PF00929
MSTRG.29558.5	2747	noncoding	-
MSTRG.29558.6	868	noncoding	-
MSTRG.29558.7	1255	noncoding	-

MSTRG.29559.3	1567	coding	PF03556
MSTRG.29559.4	1702	coding	PF03556
MSTRG.29560.1	3813	noncoding	-
MSTRG.29561.1	2408	noncoding	-
MSTRG.29565.1	12611	coding	PF03028
MSTRG.29566.1	1395	noncoding	-
MSTRG.29566.2	5706	noncoding	-
MSTRG.29567.1	1554	noncoding	-
MSTRG.29566.3	4149	noncoding	-
MSTRG.29544.1	3448	noncoding	-
MSTRG.29543.2	4964	coding	PF00005
MSTRG.29545.1	3196	noncoding	-
MSTRG.29546.1	1441	noncoding	-
MSTRG.29550.1	5107	coding	PF00005
MSTRG.29550.2	6559	coding	PF00005
MSTRG.29551.1	11023	coding	PF08079
MSTRG.29552.2	4901	coding	PF00675
MSTRG.29553.1	1766	noncoding	-
MSTRG.29553.2	1810	noncoding	-
MSTRG.29553.3	1620	noncoding	-
MSTRG.29553.4	1432	noncoding	-
MSTRG.29553.5	1584	noncoding	-
MSTRG.29553.6	1612	noncoding	-
MSTRG.29553.7	1443	noncoding	-
MSTRG.29554.2	1105	noncoding	-
MSTRG.29556.1	5550	noncoding	-
MSTRG.29548.2	5539	coding	PF13768
MSTRG.29548.1	5382	coding	PF13768
MSTRG.29547.1	1310	noncoding	-
MSTRG.29571.1	428	noncoding	-
MSTRG.29576.3	1905	noncoding	-
MSTRG.29576.4	2520	noncoding	-
MSTRG.29578.3	4561	coding	PF05219
MSTRG.29580.1	373	noncoding	-
MSTRG.29583.2	10572	coding	PF00443
MSTRG.29583.4	7415	coding	PF00443
MSTRG.29584.1	1181	noncoding	-
MSTRG.29585.2	2152	coding	PF10191
MSTRG.29593.2	9687	coding	PF00749
MSTRG.29594.3	4895	noncoding	-
MSTRG.29596.1	772	noncoding	-
MSTRG.29588.2	2092	coding	PF00069
MSTRG.29598.1	1928	noncoding	-
MSTRG.29600.2	9324	coding	PF00069
MSTRG.29601.1	4908	noncoding	-
MSTRG.29599.1	375	noncoding	-
MSTRG.29599.2	366	noncoding	-
MSTRG.29603.3	9476	coding	PF08783
MSTRG.29607.6	1749	noncoding	-
MSTRG.29607.11	606	noncoding	-
MSTRG.29607.10	6125	coding	PF16608
MSTRG.29607.5	29646	coding	PF16608
MSTRG.29608.1	3633	noncoding	-
MSTRG.29620.1	6844	coding	PF02023

MSTRG.29624.1	455	noncoding	-
MSTRG.29625.1	692	noncoding	-
MSTRG.29625.2	835	noncoding	-
MSTRG.29627.1	592	noncoding	-
MSTRG.29633.2	1494	noncoding	-
MSTRG.29634.2	7222	noncoding	-
MSTRG.29635.2	6815	coding	PF14652
MSTRG.29652.1	380	noncoding	-
MSTRG.29654.1	2542	coding	PF08916
MSTRG.29654.4	4387	coding	PF08916
MSTRG.29654.2	3104	coding	PF08916
MSTRG.29654.5	2991	coding	PF08916
MSTRG.29657.1	7411	coding	PF05470
MSTRG.29657.4	7299	coding	PF05470
MSTRG.29657.9	3342	coding	PF14438
MSTRG.29657.11	3145	coding	PF05470
MSTRG.29644.1	4036	noncoding	-
MSTRG.29647.2	5372	coding	PF01722
MSTRG.29647.3	6004	noncoding	-
MSTRG.29663.2	849	noncoding	-
MSTRG.29664.2	1393	coding	PF00907
MSTRG.29666.5	2377	coding	PF00274
MSTRG.29667.1	1283	noncoding	-
MSTRG.29667.2	1293	noncoding	-
MSTRG.29667.3	1285	noncoding	-
MSTRG.29667.4	2126	noncoding	-
MSTRG.29668.3	1852	noncoding	-
MSTRG.29672.2	5915	coding	PF00069
MSTRG.29673.1	1882	noncoding	-
MSTRG.29673.3	1021	noncoding	-
MSTRG.29658.7	823	noncoding	-
MSTRG.29658.5	919	noncoding	-
MSTRG.29675.2	3404	coding	PF15364
MSTRG.29675.5	1768	noncoding	-
MSTRG.29676.2	1425	noncoding	-
MSTRG.29676.1	1483	noncoding	-
MSTRG.29676.3	2555	noncoding	-
MSTRG.29676.4	3128	noncoding	-
MSTRG.29676.5	2513	noncoding	-
MSTRG.29676.6	1802	noncoding	-
MSTRG.29677.3	1388	noncoding	-
MSTRG.29677.6	1315	noncoding	-
MSTRG.29677.8	2514	noncoding	-
MSTRG.29677.5	2528	noncoding	-
MSTRG.29677.7	2601	noncoding	-
MSTRG.29677.2	1843	noncoding	-
MSTRG.29678.3	2542	coding	PF00225
MSTRG.29678.2	2567	coding	PF00225
MSTRG.29682.4	1493	coding	PF02213
MSTRG.29687.2	1463	coding	PF00735
MSTRG.29687.3	1306	coding	PF00735
MSTRG.29681.1	1777	noncoding	-
MSTRG.29692.2	2193	noncoding	-
MSTRG.29692.3	1357	noncoding	-

MSTRG.29693.2	1083	noncoding	-
MSTRG.29695.2	3272	noncoding	-
MSTRG.29696.1	2376	coding	PF15386
MSTRG.29696.2	2783	noncoding	-
MSTRG.29696.3	397	noncoding	-
MSTRG.29704.3	11205	coding	PF00176
MSTRG.29699.3	584	noncoding	-
MSTRG.29700.1	1218	noncoding	-
MSTRG.29700.2	1055	noncoding	-
MSTRG.29701.3	295	noncoding	-
MSTRG.29722.1	1717	noncoding	-
MSTRG.29722.2	685	noncoding	-
MSTRG.29723.1	1337	noncoding	-
MSTRG.29723.3	1652	noncoding	-
MSTRG.29724.1	2215	noncoding	-
MSTRG.29725.1	1492	noncoding	-
MSTRG.29726.1	301	noncoding	-
MSTRG.29724.4	3165	noncoding	-
MSTRG.29724.5	4271	noncoding	-
MSTRG.29724.6	3204	coding	PF16866
MSTRG.29727.1	749	noncoding	-
MSTRG.29724.7	4515	coding	PF16866
MSTRG.29724.10	2717	coding	PF16866
MSTRG.29724.11	3019	coding	PF16866
MSTRG.29730.1	2363	coding	PF07856
MSTRG.29735.6	5866	noncoding	-
MSTRG.29707.1	1801	coding	PF10436
MSTRG.29710.1	1527	noncoding	-
MSTRG.29713.2	1537	noncoding	-
MSTRG.29715.3	3070	noncoding	-
MSTRG.29738.1	3005	coding	PF04884
MSTRG.29738.2	2928	coding	PF04884
MSTRG.29738.3	2614	coding	PF04884
MSTRG.29739.1	2149	coding	PF00735
MSTRG.29741.2	2462	coding	PF01352
MSTRG.29747.1	1763	coding	PF00069
MSTRG.29748.1	1268	noncoding	-
MSTRG.29755.2	2231	coding	PF02836
MSTRG.29756.5	1779	noncoding	-
MSTRG.29757.1	800	coding	PF02836
MSTRG.29759.1	4320	coding	PF03874
MSTRG.29758.1	2212	noncoding	-
MSTRG.29763.1	4672	coding	PF02204
MSTRG.29763.4	2878	coding	PF02214
MSTRG.29767.1	1544	coding	PF01172
MSTRG.29767.2	1317	coding	PF01172
MSTRG.29767.3	1420	coding	PF01172
MSTRG.29767.4	1151	coding	PF01172
MSTRG.29767.5	2408	coding	PF01172
MSTRG.29773.2	3358	noncoding	-
MSTRG.29774.1	1658	coding	PF13499
MSTRG.29774.4	8208	coding	PF13499
MSTRG.29774.10	11322	coding	PF13499
MSTRG.29774.9	8485	coding	PF13499

MSTRG.29774.7	8456	coding	PF13499
MSTRG.29774.12	8031	coding	PF13499
MSTRG.29774.13	8499	coding	PF13499
MSTRG.29774.15	8486	coding	PF13499
MSTRG.29776.1	3838	noncoding	-
MSTRG.29769.2	1896	coding	PF00535
MSTRG.29849.2	6255	coding	PF15336
MSTRG.29849.4	6207	coding	PF15336
MSTRG.29849.3	7006	coding	PF15336
MSTRG.29851.1	6121	noncoding	-
MSTRG.29852.1	3413	noncoding	-
MSTRG.29853.1	21383	noncoding	-
MSTRG.29854.1	451	noncoding	-
MSTRG.29856.1	1699	noncoding	-
MSTRG.29857.1	43983	noncoding	-
MSTRG.29858.1	10797	noncoding	-
MSTRG.29860.1	4139	noncoding	-
MSTRG.29861.1	6847	coding	PF00078
MSTRG.29861.2	6865	coding	PF00078
MSTRG.29861.3	3458	coding	PF00078
MSTRG.29849.5	73817	noncoding	-
MSTRG.29849.6	47030	noncoding	-
MSTRG.29849.7	33376	noncoding	-
MSTRG.29849.8	31545	noncoding	-
MSTRG.29849.9	29633	noncoding	-
MSTRG.29849.10	14733	noncoding	-
MSTRG.29791.15	2519	coding	PF02946
MSTRG.29791.16	2828	coding	PF02946
MSTRG.29791.7	3113	coding	PF02946
MSTRG.29791.8	3901	coding	PF02946
MSTRG.29791.13	6342	coding	PF02946
MSTRG.29791.10	9027	coding	PF02946
MSTRG.29791.11	3701	coding	PF02946
MSTRG.29791.9	4058	coding	PF02946
MSTRG.29791.17	6735	coding	PF02946
MSTRG.29791.18	2072	coding	PF02946
MSTRG.29792.1	2588	noncoding	-
MSTRG.29786.2	2952	coding	PF02946
MSTRG.29787.1	4482	noncoding	-
MSTRG.29787.3	4291	noncoding	-
MSTRG.29795.2	871	coding	PF15164
MSTRG.29796.1	880	noncoding	-
MSTRG.29797.3	1098	coding	PF08241
MSTRG.29799.1	2304	coding	PF00804
MSTRG.29799.2	1601	coding	PF00804
MSTRG.29799.3	1505	noncoding	-
MSTRG.29810.3	2412	noncoding	-
MSTRG.29810.4	2488	noncoding	-
MSTRG.29810.7	2415	noncoding	-
MSTRG.29810.8	2289	noncoding	-
MSTRG.29810.6	4969	noncoding	-
MSTRG.29810.2	4861	noncoding	-
MSTRG.29810.9	4846	noncoding	-
MSTRG.29800.5	2628	noncoding	-

MSTRG.29800.6	1755	noncoding	-
MSTRG.29801.3	2339	noncoding	-
MSTRG.29802.1	9400	coding	PF00439
MSTRG.29815.4	5401	coding	PF15229
MSTRG.29815.2	5235	coding	PF15229
MSTRG.29815.5	4963	coding	PF15229
MSTRG.29815.6	1738	noncoding	-
MSTRG.29821.4	1877	noncoding	-
MSTRG.29821.5	2123	noncoding	-
MSTRG.29825.2	892	coding	PF00782
MSTRG.29825.3	1038	coding	PF00782
MSTRG.29827.1	2862	noncoding	-
MSTRG.29830.1	2105	coding	PF00244
MSTRG.29830.2	2101	coding	PF00244
MSTRG.29834.1	3029	coding	PF00616
MSTRG.29835.2	552	coding	PF13656
MSTRG.29836.2	2199	noncoding	-
MSTRG.29846.1	2904	coding	PF08172
MSTRG.29846.8	12831	coding	PF02376
MSTRG.29846.7	13656	coding	PF02376
MSTRG.29846.9	13813	coding	PF02376
MSTRG.29846.10	1134	coding	PF02376
MSTRG.29846.11	4003	coding	PF02376
MSTRG.29846.12	10577	noncoding	-
MSTRG.29846.13	16546	noncoding	-
MSTRG.29846.14	6615	noncoding	-
MSTRG.29847.1	10779	noncoding	-
MSTRG.29845.1	681	noncoding	-
MSTRG.29868.1	3049	noncoding	-
MSTRG.29869.1	4785	noncoding	-
MSTRG.29869.3	1172	noncoding	-
MSTRG.29869.5	4653	noncoding	-
MSTRG.29869.4	1230	noncoding	-
MSTRG.29869.6	3361	noncoding	-
MSTRG.29869.7	5439	noncoding	-
MSTRG.29866.2	261	noncoding	-
MSTRG.29867.1	1829	coding	PF03982
MSTRG.29867.2	1728	coding	PF03982
MSTRG.29879.4	2508	coding	PF00135
MSTRG.29879.6	1968	coding	PF00135
MSTRG.29879.7	1882	coding	PF00135
MSTRG.29879.3	2345	coding	PF00135
MSTRG.29880.2	3380	coding	PF04959
MSTRG.29880.3	4374	coding	PF04959
MSTRG.29881.2	1295	coding	PF00412
MSTRG.29884.1	943	noncoding	-
MSTRG.29884.2	971	noncoding	-
MSTRG.29884.3	897	coding	PF12328
MSTRG.29885.3	5789	coding	PF02213
MSTRG.29890.4	978	noncoding	-
MSTRG.29891.1	1287	coding	PF00431
MSTRG.29891.2	1682	coding	PF00431
MSTRG.29891.3	4529	coding	PF00431
MSTRG.29891.4	4460	coding	PF00431

MSTRG.29891.6	2123	noncoding	-
MSTRG.29891.7	2261	noncoding	-
MSTRG.29894.2	2198	noncoding	-
MSTRG.29894.3	3463	noncoding	-
MSTRG.29894.4	2649	noncoding	-
MSTRG.29896.7	2710	noncoding	-
MSTRG.29896.6	1530	noncoding	-
MSTRG.29896.4	2180	noncoding	-
MSTRG.29896.5	2006	noncoding	-
MSTRG.29897.1	680	noncoding	-
MSTRG.29897.2	1145	noncoding	-
MSTRG.29897.3	730	noncoding	-
MSTRG.29889.2	1262	coding	PF15775
MSTRG.29900.1	2410	coding	PF07496
MSTRG.29903.3	4483	coding	PF08514
MSTRG.29903.1	5568	coding	PF01153
MSTRG.29904.1	3264	coding	PF01153
MSTRG.29902.1	1867	coding	PF15806
MSTRG.29902.2	642	noncoding	-
MSTRG.29902.3	737	noncoding	-
MSTRG.29910.3	1657	coding	PF11938
MSTRG.29910.2	2033	coding	PF11938
MSTRG.29912.2	2340	coding	PF00928
MSTRG.29912.3	2553	coding	PF00928
MSTRG.29912.4	2590	coding	PF00928
MSTRG.29912.5	2687	coding	PF00928
MSTRG.29912.6	2377	coding	PF00928
MSTRG.29906.2	2592	coding	PF02023
MSTRG.29906.4	2742	coding	PF02023
MSTRG.29908.4	7721	coding	PF02023
MSTRG.29916.1	2489	noncoding	-
MSTRG.29917.1	1459	coding	PF00067
MSTRG.29917.3	1404	coding	PF00067
MSTRG.29918.1	592	noncoding	-
MSTRG.29920.1	14284	coding	PF02023
MSTRG.29923.5	1690	noncoding	-
MSTRG.29923.6	1258	noncoding	-
MSTRG.29921.1	1974	noncoding	-
MSTRG.29921.4	1594	coding	PF01352
MSTRG.29921.5	1953	coding	PF01352
MSTRG.29926.3	4023	coding	PF02023
MSTRG.29926.2	4027	coding	PF02023
MSTRG.29927.3	2508	noncoding	-
MSTRG.29934.4	1045	coding	PF01125
MSTRG.29934.5	925	coding	PF01125
MSTRG.29934.8	872	coding	PF01125
MSTRG.29941.3	4967	coding	PF00632
MSTRG.29941.4	5365	coding	PF00632
MSTRG.29943.5	2549	noncoding	-
MSTRG.29943.3	10627	noncoding	-
MSTRG.29943.2	12241	noncoding	-
MSTRG.29943.4	12274	noncoding	-
MSTRG.29938.1	527	noncoding	-
MSTRG.29946.2	946	coding	PF10164

MSTRG.29947.1	730	noncoding	-
MSTRG.29949.1	1990	noncoding	-
MSTRG.29948.2	2075	noncoding	-
MSTRG.29950.4	7468	noncoding	-
MSTRG.29950.5	12450	coding	PF07714
MSTRG.29950.6	11943	coding	PF07714
MSTRG.29965.1	3938	noncoding	-
MSTRG.29965.5	3004	noncoding	-
MSTRG.29967.2	1524	noncoding	-
MSTRG.29970.1	5934	coding	PF00443
MSTRG.29970.2	6090	coding	PF00443
MSTRG.29970.3	4730	coding	PF00443
MSTRG.29970.4	5606	coding	PF00443
MSTRG.29970.5	4953	coding	PF00443
MSTRG.29970.7	4395	coding	PF00443
MSTRG.29970.8	4797	coding	PF00443
MSTRG.29970.9	3553	coding	PF00443
MSTRG.29974.3	4192	coding	PF01369
MSTRG.29974.2	4075	coding	PF01369
MSTRG.29975.1	2736	noncoding	-
MSTRG.29954.1	424	noncoding	-
MSTRG.29955.1	2684	coding	PF15118
MSTRG.29957.2	4513	noncoding	-
MSTRG.29961.2	1441	noncoding	-
MSTRG.29980.5	14083	coding	PF01352
MSTRG.29978.3	7467	coding	PF00607
MSTRG.29978.1	2765	coding	PF00517
MSTRG.29978.2	876	noncoding	-
MSTRG.29985.3	5414	noncoding	-
MSTRG.29985.4	6431	noncoding	-
MSTRG.29990.1	10618	noncoding	-
MSTRG.29990.2	10386	noncoding	-
MSTRG.29990.5	7157	noncoding	-
MSTRG.29990.6	10300	noncoding	-
MSTRG.29990.7	10317	noncoding	-
MSTRG.29990.8	9962	noncoding	-
MSTRG.29990.9	10440	noncoding	-
MSTRG.29990.10	11327	noncoding	-
MSTRG.29991.1	2554	noncoding	-
MSTRG.29991.2	1586	noncoding	-
MSTRG.29997.1	8506	coding	PF00250
MSTRG.29997.2	7373	coding	PF00250
MSTRG.30003.7	3127	coding	PF00041
MSTRG.30003.2	4479	coding	PF00041
MSTRG.30003.5	6417	coding	PF07679
MSTRG.30003.4	7393	coding	PF07679
MSTRG.30003.3	6938	coding	PF07679
MSTRG.30003.6	6821	coding	PF07679
MSTRG.30003.8	3795	coding	PF00041
MSTRG.30003.9	1348	noncoding	-
MSTRG.29998.1	1114	noncoding	-
MSTRG.29999.1	1128	noncoding	-
MSTRG.30009.10	2320	noncoding	-
MSTRG.30009.11	1901	noncoding	-

MSTRG.30009.6	2003	noncoding	-
MSTRG.30009.7	2259	noncoding	-
MSTRG.30009.15	4818	noncoding	-
MSTRG.30009.8	4656	noncoding	-
MSTRG.30009.17	1993	noncoding	-
MSTRG.30009.18	2084	noncoding	-
MSTRG.30009.19	4868	noncoding	-
MSTRG.30009.20	1970	noncoding	-
MSTRG.30009.21	2102	noncoding	-
MSTRG.30009.22	1914	noncoding	-
MSTRG.30009.23	2405	noncoding	-
MSTRG.30009.24	1800	noncoding	-
MSTRG.30005.3	6579	coding	PF04906
MSTRG.30010.3	4354	coding	PF00225
MSTRG.30010.1	4540	coding	PF00225
MSTRG.30010.2	4414	coding	PF00225
MSTRG.30011.2	3377	coding	PF08662
MSTRG.30016.2	3968	coding	PF00787
MSTRG.30016.3	3853	coding	PF00787
MSTRG.30016.5	1621	coding	PF00787
MSTRG.30016.4	4638	coding	PF00787
MSTRG.30018.1	733	noncoding	-
MSTRG.30020.2	4229	coding	PF01728
MSTRG.30020.3	4860	coding	PF01728
MSTRG.30013.1	2067	noncoding	-
MSTRG.30014.1	774	noncoding	-
MSTRG.30014.2	1252	noncoding	-
MSTRG.30014.3	1277	noncoding	-
MSTRG.30014.4	801	noncoding	-
MSTRG.30024.3	702	coding	PF10178
MSTRG.30033.1	2272	noncoding	-
MSTRG.30035.2	3491	noncoding	-
MSTRG.30036.2	2711	noncoding	-
MSTRG.30036.3	4953	noncoding	-
MSTRG.30036.4	2622	noncoding	-
MSTRG.30054.11	6833	coding	PF09387
MSTRG.30054.13	6892	coding	PF09387
MSTRG.30054.2	6280	coding	PF09387
MSTRG.30054.7	6183	coding	PF09387
MSTRG.30054.8	3988	coding	PF09387
MSTRG.30054.17	6287	coding	PF07738
MSTRG.30054.19	1533	coding	PF09387
MSTRG.30054.21	1877	coding	PF09387
MSTRG.30054.20	4261	coding	PF09387
MSTRG.30054.18	4142	coding	PF09387
MSTRG.30040.2	2393	coding	PF00027
MSTRG.30040.3	1514	coding	PF00027
MSTRG.30039.1	2562	coding	PF04692
MSTRG.30046.1	1041	noncoding	-
MSTRG.30046.2	988	noncoding	-
MSTRG.30049.3	2496	coding	PF02460
MSTRG.30049.2	2326	coding	PF02460
MSTRG.30055.1	1222	noncoding	-
MSTRG.30064.5	726	noncoding	-

MSTRG.30064.6	622	noncoding	-
MSTRG.30068.1	554	noncoding	-
MSTRG.30068.2	403	noncoding	-
MSTRG.30087.1	249	noncoding	-
MSTRG.30091.1	341	noncoding	-
MSTRG.30114.1	2547	coding	PF00076
MSTRG.30114.3	2299	coding	PF00076
MSTRG.30102.1	1101	coding	PF04734
MSTRG.30102.2	2046	coding	PF04734
MSTRG.30102.3	578	coding	PF17048
MSTRG.30126.5	12551	noncoding	-
MSTRG.30127.1	1607	noncoding	-
MSTRG.30128.1	4195	noncoding	-
MSTRG.30129.1	7851	noncoding	-
MSTRG.30130.1	10413	noncoding	-
MSTRG.30132.1	1156	noncoding	-
MSTRG.30121.2	2285	coding	PF01747
MSTRG.30122.3	350	noncoding	-
MSTRG.30124.1	687	noncoding	-
MSTRG.30123.2	10065	coding	PF10409
MSTRG.30138.1	1341	noncoding	-
MSTRG.30143.1	1114	noncoding	-
MSTRG.30133.1	326	noncoding	-
MSTRG.30137.1	871	coding	PF01398
MSTRG.30165.3	675	noncoding	-
MSTRG.30164.1	1941	noncoding	-
MSTRG.30164.2	1867	noncoding	-
MSTRG.30175.1	797	noncoding	-
MSTRG.30176.1	6522	coding	PF01359
MSTRG.30177.1	3353	coding	PF00001
MSTRG.30177.4	3460	noncoding	-
MSTRG.30178.1	5060	noncoding	-
MSTRG.30177.5	2570	noncoding	-
MSTRG.30177.6	3215	noncoding	-
MSTRG.30171.6	5871	noncoding	-
MSTRG.30174.1	553	noncoding	-
MSTRG.30182.1	1275	noncoding	-
MSTRG.30186.3	4525	noncoding	-
MSTRG.30190.2	7347	coding	PF12054
MSTRG.30198.1	7680	coding	PF16367
MSTRG.30198.3	1989	coding	PF16367
MSTRG.30198.4	2324	coding	PF16367
MSTRG.30199.2	5099	coding	PF12906
MSTRG.30199.3	4723	coding	PF12906
MSTRG.30204.1	6558	noncoding	-
MSTRG.30204.2	7247	noncoding	-
MSTRG.30189.2	2432	coding	PF13931
MSTRG.30211.1	2017	noncoding	-
MSTRG.30212.3	1591	noncoding	-
MSTRG.30212.4	6604	coding	PF04091
MSTRG.30213.1	1826	noncoding	-
MSTRG.30214.1	11133	noncoding	-
MSTRG.30215.2	6944	coding	PF08150
MSTRG.30215.4	1355	coding	PF16165

MSTRG.30216.1	232	noncoding	-
MSTRG.30217.2	2079	noncoding	-
MSTRG.30219.1	2495	coding	PF00233
MSTRG.30227.1	449	noncoding	-
MSTRG.30227.2	411	noncoding	-
MSTRG.30240.2	4322	coding	PF00387
MSTRG.30241.1	4270	coding	PF00078
MSTRG.30245.1	1186	noncoding	-
MSTRG.30235.1	1404	coding	PF00067
MSTRG.30239.1	1808	noncoding	-
MSTRG.30248.3	1631	coding	PF00067
MSTRG.30248.2	1656	coding	PF00067
MSTRG.30248.4	279	noncoding	-
MSTRG.30248.5	333	coding	PF00067
MSTRG.30248.7	1903	coding	PF00067
MSTRG.30248.9	1756	coding	PF00067
MSTRG.30248.8	3045	coding	PF00067
MSTRG.30249.2	6093	coding	PF02208
MSTRG.30258.2	5768	coding	PF15625
MSTRG.30258.3	3219	noncoding	-
MSTRG.30258.4	6504	coding	PF15625
MSTRG.30260.1	1178	coding	PF00078
MSTRG.30258.5	2678	coding	PF01451
MSTRG.30252.5	7731	noncoding	-
MSTRG.30271.2	5184	coding	PF02990
MSTRG.30271.3	4773	coding	PF02990
MSTRG.30272.1	1284	noncoding	-
MSTRG.30275.2	13406	coding	PF15090
MSTRG.30275.6	10365	noncoding	-
MSTRG.30275.7	15774	coding	PF15090
MSTRG.30275.8	2214	noncoding	-
MSTRG.30275.9	2843	noncoding	-
MSTRG.30275.12	1683	coding	PF00078
MSTRG.30278.1	4741	noncoding	-
MSTRG.30264.3	5427	coding	PF00620
MSTRG.30265.1	1595	noncoding	-
MSTRG.30281.1	2681	coding	PF01529
MSTRG.30281.2	3057	coding	PF01529
MSTRG.30282.2	3399	coding	PF14500
MSTRG.30282.3	3499	coding	PF14500
MSTRG.30287.2	1405	coding	PF16455
MSTRG.30287.4	1309	coding	PF16455
MSTRG.30288.2	1356	coding	PF12796
MSTRG.30290.1	3243	noncoding	-
MSTRG.30291.5	1215	noncoding	-
MSTRG.30291.4	904	noncoding	-
MSTRG.30291.6	935	noncoding	-
MSTRG.30283.1	685	noncoding	-
MSTRG.30302.5	1270	noncoding	-
MSTRG.30315.3	1407	coding	PF00153
MSTRG.30315.2	1602	coding	PF00153
MSTRG.30315.4	1540	coding	PF00153
MSTRG.30315.5	1446	coding	PF00153
MSTRG.30320.1	4425	coding	PF02628

MSTRG.30321.3	1433	coding	PF03932
MSTRG.30321.4	1560	coding	PF03932
MSTRG.30321.5	1564	coding	PF03932
MSTRG.30321.6	1385	coding	PF03932
MSTRG.30321.7	1162	coding	PF03932
MSTRG.30312.1	382	noncoding	-
MSTRG.30313.1	5234	noncoding	-
MSTRG.30325.2	3654	coding	PF00069
MSTRG.30326.1	1662	coding	PF04677
MSTRG.30326.2	1300	noncoding	-
MSTRG.30322.3	721	noncoding	-
MSTRG.30329.2	5335	noncoding	-
MSTRG.30331.2	4116	noncoding	-
MSTRG.30332.2	647	coding	PF05821
MSTRG.30332.3	892	coding	PF05821
MSTRG.30343.1	6649	coding	PF14816
MSTRG.30343.6	5224	noncoding	-
MSTRG.30343.4	6440	coding	PF14816
MSTRG.30344.1	4896	coding	PF01403
MSTRG.30345.1	684	noncoding	-
MSTRG.30348.1	310	noncoding	-
MSTRG.30350.5	1171	noncoding	-
MSTRG.30354.2	1810	noncoding	-
MSTRG.30357.2	4310	coding	PF07555
MSTRG.30359.5	1319	coding	PF08427
MSTRG.30359.6	2115	coding	PF08427
MSTRG.30361.7	3033	noncoding	-
MSTRG.30361.2	3163	noncoding	-
MSTRG.30361.9	2542	noncoding	-
MSTRG.30361.11	2907	noncoding	-
MSTRG.30361.5	5560	noncoding	-
MSTRG.30361.4	4114	noncoding	-
MSTRG.30361.14	2524	noncoding	-
MSTRG.30361.15	2239	noncoding	-
MSTRG.30361.12	2251	noncoding	-
MSTRG.30361.6	2966	noncoding	-
MSTRG.30361.16	4860	noncoding	-
MSTRG.30362.5	5351	noncoding	-
MSTRG.30363.8	7224	coding	PF01369
MSTRG.30363.3	6494	coding	PF01369
MSTRG.30363.5	6395	coding	PF01369
MSTRG.30363.6	6398	coding	PF01369
MSTRG.30363.2	6668	coding	PF01369
MSTRG.30363.4	6566	coding	PF01369
MSTRG.30363.7	6569	coding	PF01369
MSTRG.30365.1	1973	coding	PF15410
MSTRG.30367.1	1684	noncoding	-
MSTRG.30367.4	1282	noncoding	-
MSTRG.30368.7	1038	noncoding	-
MSTRG.30368.4	1190	noncoding	-
MSTRG.30368.9	1181	noncoding	-
MSTRG.30368.10	1158	noncoding	-
MSTRG.30375.1	4672	noncoding	-
MSTRG.30375.5	2666	noncoding	-

MSTRG.30375.6	2649	noncoding	-
MSTRG.30375.9	4752	noncoding	-
MSTRG.30375.7	2987	noncoding	-
MSTRG.30375.8	2964	noncoding	-
MSTRG.30375.12	2372	noncoding	-
MSTRG.30375.11	6169	noncoding	-
MSTRG.30375.14	2175	noncoding	-
MSTRG.30375.15	2322	noncoding	-
MSTRG.30378.1	5077	coding	PF12470
MSTRG.30371.1	1837	noncoding	-
MSTRG.30371.2	814	noncoding	-
MSTRG.30372.1	418	noncoding	-
MSTRG.30382.2	419	noncoding	-
MSTRG.30386.3	2171	coding	PF00067
MSTRG.30388.1	327	noncoding	-
MSTRG.30386.5	2087	coding	PF00067
MSTRG.30386.6	1434	coding	PF00067
MSTRG.30386.8	1771	coding	PF00067
MSTRG.30393.2	3221	coding	PF05761
MSTRG.30393.8	2552	coding	PF05761
MSTRG.30393.5	2616	coding	PF05761
MSTRG.30393.9	2932	coding	PF05761
MSTRG.30393.7	2553	coding	PF05761
MSTRG.30393.3	2874	coding	PF05761
MSTRG.30394.1	3838	noncoding	-
MSTRG.30395.1	2231	noncoding	-
MSTRG.30398.2	990	coding	PF14960
MSTRG.30398.3	830	coding	PF14960
MSTRG.30399.3	5959	noncoding	-
MSTRG.30403.3	10408	noncoding	-
MSTRG.30403.4	10384	noncoding	-
MSTRG.30403.5	4679	noncoding	-
MSTRG.30403.6	6845	noncoding	-
MSTRG.30404.1	559	noncoding	-
MSTRG.30416.1	1001	noncoding	-
MSTRG.30419.5	3114	noncoding	-
MSTRG.30409.1	1180	coding	PF13417
MSTRG.30409.2	1008	coding	PF13417
MSTRG.30410.1	472	noncoding	-
MSTRG.30411.3	1184	coding	PF13417
MSTRG.30411.4	1199	coding	PF13417
MSTRG.30411.2	1144	coding	PF13417
MSTRG.30411.6	1131	coding	PF13417
MSTRG.30411.5	1551	coding	PF13417
MSTRG.30408.1	1544	noncoding	-
MSTRG.30436.1	2115	coding	PF00530
MSTRG.30437.1	1956	noncoding	-
MSTRG.30438.1	5729	coding	PF15902
MSTRG.30439.1	13213	noncoding	-
MSTRG.30442.1	3485	coding	PF00530
MSTRG.30442.2	614	noncoding	-
MSTRG.30442.3	476	noncoding	-
MSTRG.30444.2	3458	noncoding	-
MSTRG.30445.2	2112	coding	PF01593

MSTRG.30448.1	4915	coding	PF15314
MSTRG.30451.1	783	coding	PF00046
MSTRG.30453.3	3387	coding	PF04130
MSTRG.30453.4	3383	coding	PF04130
MSTRG.30453.5	4710	coding	PF04130
MSTRG.30456.2	756	noncoding	-
MSTRG.30456.1	978	coding	PF06294
MSTRG.30456.7	1023	noncoding	-
MSTRG.30456.4	1183	noncoding	-
MSTRG.30438.2	974	noncoding	-
MSTRG.30426.1	462	noncoding	-
MSTRG.30467.1	507	noncoding	-
MSTRG.30469.1	321	noncoding	-
MSTRG.30483.1	1501	noncoding	-
MSTRG.30496.1	5874	noncoding	-
MSTRG.30496.2	9911	noncoding	-
MSTRG.30497.1	3978	noncoding	-
MSTRG.30497.2	4088	coding	PF00078
MSTRG.30496.3	13586	noncoding	-
MSTRG.30473.1	852	noncoding	-
MSTRG.30475.10	13244	coding	PF00596
MSTRG.30475.11	5260	coding	PF00596
MSTRG.30476.1	769	noncoding	-
MSTRG.30479.1	1025	noncoding	-
MSTRG.30481.1	1750	coding	PF06003
MSTRG.30481.2	3606	coding	PF06003
MSTRG.30481.3	898	coding	PF06003
MSTRG.30477.2	1893	coding	PF00782
MSTRG.30503.1	7301	noncoding	-
MSTRG.30505.4	1358	noncoding	-
MSTRG.30506.1	3372	noncoding	-
MSTRG.30506.4	2266	coding	PF14777
MSTRG.30507.2	4352	noncoding	-
MSTRG.30518.1	2676	coding	PF00501
MSTRG.30518.2	2597	coding	PF00501
MSTRG.30518.6	2620	coding	PF00501
MSTRG.30517.2	2256	coding	PF01529
MSTRG.30522.5	7163	noncoding	-
MSTRG.30522.6	5643	coding	PF08347
MSTRG.30523.1	18491	noncoding	-
MSTRG.30524.1	5876	noncoding	-
MSTRG.30525.1	7015	noncoding	-
MSTRG.30529.1	3864	noncoding	-
MSTRG.30530.1	5720	noncoding	-
MSTRG.30530.2	4390	noncoding	-
MSTRG.30530.3	1989	noncoding	-
MSTRG.30531.3	7062	noncoding	-
MSTRG.30536.1	376	noncoding	-
MSTRG.30539.1	1558	noncoding	-
MSTRG.30534.2	1345	coding	PF01509
MSTRG.30534.3	1614	coding	PF01509
MSTRG.30534.4	4708	noncoding	-
MSTRG.30547.1	1574	coding	PF00431
MSTRG.30547.2	8921	coding	PF00059

MSTRG.30547.6	3403	coding	PF00059
MSTRG.30547.5	3307	coding	PF00059
MSTRG.30547.7	6367	coding	PF00059
MSTRG.30548.1	3924	noncoding	-
MSTRG.30548.2	8423	noncoding	-
MSTRG.30548.3	843	noncoding	-
MSTRG.30549.1	4583	noncoding	-
MSTRG.30550.1	3165	noncoding	-
MSTRG.30551.1	2364	noncoding	-
MSTRG.30553.1	2445	noncoding	-
MSTRG.30554.1	3351	noncoding	-
MSTRG.30555.1	1506	noncoding	-
MSTRG.30556.1	2391	coding	PF00226
MSTRG.30556.2	1539	coding	PF00226
MSTRG.30545.1	318	noncoding	-
MSTRG.30559.1	1248	noncoding	-
MSTRG.30559.3	1110	noncoding	-
MSTRG.30561.2	2272	noncoding	-
MSTRG.30561.3	7329	noncoding	-
MSTRG.30562.2	3414	noncoding	-
MSTRG.30565.1	8182	noncoding	-
MSTRG.30565.2	9904	noncoding	-
MSTRG.30565.4	3923	noncoding	-
MSTRG.30566.1	1267	noncoding	-
MSTRG.30569.1	4089	noncoding	-
MSTRG.30571.1	465	noncoding	-
MSTRG.30572.1	736	noncoding	-
MSTRG.30575.1	3916	coding	PF09457
MSTRG.30575.2	2462	coding	PF09457
MSTRG.30575.3	1092	coding	PF09457
MSTRG.30575.5	2332	coding	PF09457
MSTRG.30576.1	684	noncoding	-
MSTRG.30583.2	6872	noncoding	-
MSTRG.30583.3	5066	noncoding	-
MSTRG.30583.4	4960	noncoding	-
MSTRG.30584.1	4063	noncoding	-
MSTRG.30595.3	4868	coding	PF00076
MSTRG.30595.5	1434	coding	PF00076
MSTRG.30595.4	1464	coding	PF00076
MSTRG.30596.1	4665	coding	PF02383
MSTRG.30597.1	2122	coding	PF09739
MSTRG.30597.3	2815	coding	PF09739
MSTRG.30597.5	2170	coding	PF09739
MSTRG.30599.4	12264	coding	PF02862
MSTRG.30614.8	3333	coding	PF07714
MSTRG.30616.7	5825	coding	PF04377
MSTRG.30616.3	5148	coding	PF04377
MSTRG.30616.6	5148	coding	PF04377
MSTRG.30617.1	5582	noncoding	-
MSTRG.30616.8	1400	coding	PF04377
MSTRG.30616.9	15066	noncoding	-
MSTRG.30616.10	18157	noncoding	-
MSTRG.30618.1	518	noncoding	-
MSTRG.30620.1	1501	coding	PF08743

MSTRG.30620.3	1221	coding	PF08743
MSTRG.30623.4	2520	coding	PF05010
MSTRG.30623.5	7750	coding	PF05010
MSTRG.30624.1	641	noncoding	-
MSTRG.30625.1	1954	noncoding	-
MSTRG.30625.2	2100	noncoding	-
MSTRG.30625.3	2078	noncoding	-
MSTRG.30625.4	2325	noncoding	-
MSTRG.30625.5	2051	noncoding	-
MSTRG.30625.6	2066	noncoding	-
MSTRG.30628.1	468	noncoding	-
MSTRG.30631.2	900	noncoding	-
MSTRG.30643.1	2407	noncoding	-
MSTRG.30645.3	1349	noncoding	-
MSTRG.30646.1	6960	coding	PF00078
MSTRG.30646.2	9726	noncoding	-
MSTRG.30637.1	783	noncoding	-
MSTRG.30654.4	3095	noncoding	-
MSTRG.30657.1	546	noncoding	-
MSTRG.30658.2	2918	coding	PF00202
MSTRG.30658.3	3187	coding	PF00202
MSTRG.30658.4	2379	coding	PF00202
MSTRG.30669.1	793	noncoding	-
MSTRG.30668.5	1621	noncoding	-
MSTRG.30668.3	2752	noncoding	-
MSTRG.30668.4	2882	coding	PF02338
MSTRG.30668.6	2397	coding	PF02338
MSTRG.30670.1	1619	noncoding	-
MSTRG.30671.10	3930	noncoding	-
MSTRG.30676.4	1521	coding	PF12796
MSTRG.30678.1	6130	coding	PF01421
MSTRG.30685.1	4701	coding	PF15309
MSTRG.30685.4	4103	coding	PF15309
MSTRG.30685.5	4823	coding	PF15309
MSTRG.30685.6	4699	coding	PF15309
MSTRG.30685.7	5598	coding	PF15309
MSTRG.30685.8	2350	noncoding	-
MSTRG.30685.9	13262	noncoding	-
MSTRG.30685.10	17052	noncoding	-
MSTRG.30685.11	2224	noncoding	-
MSTRG.30685.12	14699	noncoding	-
MSTRG.30685.13	7253	noncoding	-
MSTRG.30685.14	10175	noncoding	-
MSTRG.30685.15	6773	noncoding	-
MSTRG.30688.1	787	noncoding	-
MSTRG.30688.2	2374	noncoding	-
MSTRG.30693.1	11852	coding	PF08065
MSTRG.30693.6	6414	coding	PF08065
MSTRG.30693.2	9788	coding	PF08065
MSTRG.30693.3	9788	coding	PF08065
MSTRG.30693.4	9788	coding	PF08065
MSTRG.30693.7	11234	coding	PF08065
MSTRG.30693.9	9430	coding	PF08065
MSTRG.30695.1	432	noncoding	-

MSTRG.30698.1	601	noncoding	-
MSTRG.30698.2	395	noncoding	-
MSTRG.30699.1	777	noncoding	-
MSTRG.30701.1	3092	noncoding	-
MSTRG.30703.1	1717	noncoding	-
MSTRG.30703.2	1337	noncoding	-
MSTRG.30704.1	1820	noncoding	-
MSTRG.30705.2	1065	noncoding	-
MSTRG.30706.1	732	noncoding	-
MSTRG.30712.1	1810	noncoding	-
MSTRG.30712.2	1974	noncoding	-
MSTRG.30716.1	3488	coding	PF00617
MSTRG.30718.1	10259	noncoding	-
MSTRG.30718.2	7699	noncoding	-
MSTRG.30719.1	819	noncoding	-
MSTRG.30720.5	958	noncoding	-
MSTRG.30720.4	2300	noncoding	-
MSTRG.30720.3	2287	noncoding	-
MSTRG.30720.2	1389	noncoding	-
MSTRG.30736.1	8267	noncoding	-
MSTRG.30736.3	6788	noncoding	-
MSTRG.30736.4	7575	noncoding	-
MSTRG.30737.1	364	noncoding	-
MSTRG.30725.2	3463	noncoding	-
MSTRG.30727.1	916	noncoding	-
MSTRG.30728.1	2377	noncoding	-
MSTRG.30740.1	1429	coding	PF06553
MSTRG.30741.1	398	noncoding	-
MSTRG.30743.6	1642	noncoding	-
MSTRG.30743.4	1269	noncoding	-
MSTRG.30743.8	1180	noncoding	-
MSTRG.30743.9	2017	noncoding	-
MSTRG.30743.10	2361	noncoding	-
MSTRG.30743.7	3631	noncoding	-
MSTRG.30747.5	3266	coding	PF01534
MSTRG.30747.6	2529	coding	PF01534
MSTRG.30758.2	1520	noncoding	-
MSTRG.30759.1	835	noncoding	-
MSTRG.30760.1	1583	noncoding	-
MSTRG.30760.3	2292	noncoding	-
MSTRG.30760.4	8813	noncoding	-
MSTRG.30760.5	3059	coding	PF03359
MSTRG.30760.2	7766	coding	PF03359
MSTRG.30762.1	5143	coding	PF00078
MSTRG.30763.1	4857	noncoding	-
MSTRG.30760.7	18873	noncoding	-
MSTRG.30760.8	2921	noncoding	-
MSTRG.30765.1	2745	noncoding	-
MSTRG.30768.1	203	noncoding	-
MSTRG.30771.1	4301	noncoding	-
MSTRG.30772.1	5520	noncoding	-
MSTRG.30772.2	5349	noncoding	-
MSTRG.30753.7	6106	coding	PF00621
MSTRG.30757.1	2997	noncoding	-

MSTRG.30774.1	4151	coding	PF00431
MSTRG.30776.1	567	noncoding	-
MSTRG.30787.1	3049	coding	PF00147
MSTRG.30791.1	239	noncoding	-
MSTRG.30792.2	1084	coding	PF05324
MSTRG.30793.1	493	noncoding	-
MSTRG.30792.3	3017	noncoding	-
MSTRG.30792.4	3946	noncoding	-
MSTRG.30792.5	565	noncoding	-
MSTRG.30792.6	448	noncoding	-
MSTRG.30794.1	472	noncoding	-
MSTRG.30795.4	5310	coding	PF01352
MSTRG.30831.1	699	noncoding	-
MSTRG.30832.2	416	noncoding	-
MSTRG.30798.1	362	noncoding	-
MSTRG.30801.1	387	noncoding	-
MSTRG.30802.1	690	noncoding	-
MSTRG.30803.2	656	coding	PF00711
MSTRG.30805.2	3344	coding	PF01275
MSTRG.30805.4	2471	coding	PF01275
MSTRG.30806.1	2282	noncoding	-
MSTRG.30807.1	4266	noncoding	-
MSTRG.30808.1	16030	noncoding	-
MSTRG.30808.2	4553	coding	PF00078
MSTRG.30805.6	4459	noncoding	-
MSTRG.30809.1	841	noncoding	-
MSTRG.30812.1	1674	noncoding	-
MSTRG.30812.2	4325	noncoding	-
MSTRG.30812.4	4059	noncoding	-
MSTRG.30815.2	259	noncoding	-
MSTRG.30817.1	7727	noncoding	-
MSTRG.30826.2	2112	coding	PF01112
MSTRG.30826.3	2090	coding	PF01112
MSTRG.30826.5	1615	coding	PF01112
MSTRG.30826.6	4581	coding	PF01112
MSTRG.30833.1	629	noncoding	-
MSTRG.30866.1	1323	noncoding	-
MSTRG.30867.1	5702	noncoding	-
MSTRG.30866.2	1064	noncoding	-
MSTRG.30868.1	9456	noncoding	-
MSTRG.30866.3	6508	noncoding	-
MSTRG.30866.4	2621	noncoding	-
MSTRG.30866.5	16177	noncoding	-
MSTRG.30866.6	9949	noncoding	-
MSTRG.30866.7	5214	coding	PF00078
MSTRG.30866.8	10825	coding	PF00078
MSTRG.30835.1	2952	noncoding	-
MSTRG.30836.1	2331	noncoding	-
MSTRG.30836.4	2631	noncoding	-
MSTRG.30836.3	1943	noncoding	-
MSTRG.30836.2	2936	noncoding	-
MSTRG.30836.5	1621	noncoding	-
MSTRG.30836.6	1722	noncoding	-
MSTRG.30837.1	1230	noncoding	-

MSTRG.30889.5	4267	coding	PF06484
MSTRG.30889.7	8249	coding	PF06484
MSTRG.30889.10	7831	noncoding	-
MSTRG.30889.14	4424	noncoding	-
MSTRG.30892.1	2922	noncoding	-
MSTRG.30892.2	3615	noncoding	-
MSTRG.30892.3	1501	noncoding	-
MSTRG.30892.4	8105	noncoding	-
MSTRG.30893.1	7206	noncoding	-
MSTRG.30894.1	24353	noncoding	-
MSTRG.30889.16	3650	noncoding	-
MSTRG.30889.17	22243	noncoding	-
MSTRG.30895.1	1144	noncoding	-
MSTRG.30889.19	1514	coding	PF06484
MSTRG.30889.18	3863	coding	PF06484
MSTRG.30896.1	1605	noncoding	-
MSTRG.30889.20	3182	coding	PF06484
MSTRG.30897.1	2311	noncoding	-
MSTRG.30889.21	3654	coding	PF15636
MSTRG.30871.1	1473	noncoding	-
MSTRG.30874.1	4087	noncoding	-
MSTRG.30874.2	10047	noncoding	-
MSTRG.30876.3	993	noncoding	-
MSTRG.30877.4	4475	coding	PF11817
MSTRG.30877.6	3738	coding	PF11817
MSTRG.30878.2	9166	coding	PF10264
MSTRG.30878.3	11921	coding	PF10264
MSTRG.30880.2	5098	coding	PF01663
MSTRG.30883.2	1098	coding	PF00605
MSTRG.30882.1	457	noncoding	-
MSTRG.30888.2	1181	coding	PF13097
MSTRG.30899.2	1902	coding	PF00153
MSTRG.30900.4	4355	noncoding	-
MSTRG.30900.5	5072	noncoding	-
MSTRG.30900.6	6234	noncoding	-
MSTRG.30900.9	4118	noncoding	-
MSTRG.30900.7	4582	noncoding	-
MSTRG.30900.8	5150	noncoding	-
MSTRG.30900.10	4766	noncoding	-
MSTRG.30905.4	3081	coding	PF08628
MSTRG.30906.1	345	noncoding	-
MSTRG.30907.2	1599	coding	PF07910
MSTRG.30908.1	2061	coding	PF15239
MSTRG.30909.1	3600	noncoding	-
MSTRG.30909.2	2893	noncoding	-
MSTRG.30909.3	2799	noncoding	-
MSTRG.30909.4	3887	noncoding	-
MSTRG.30909.5	3769	noncoding	-
MSTRG.30909.6	3727	noncoding	-
MSTRG.30910.2	5075	coding	PF02208
MSTRG.30910.3	5002	coding	PF02208
MSTRG.30910.4	4790	coding	PF02208
MSTRG.30910.5	9927	coding	PF02208
MSTRG.30910.8	7829	coding	PF02208

MSTRG.30910.9	5143	noncoding	-
MSTRG.30911.3	735	noncoding	-
MSTRG.30914.1	224	noncoding	-
MSTRG.30920.1	1010	coding	PF01200
MSTRG.30937.1	1667	noncoding	-
MSTRG.30937.2	1217	noncoding	-
MSTRG.30937.5	1656	noncoding	-
MSTRG.30937.6	5735	noncoding	-
MSTRG.30937.7	9844	noncoding	-
MSTRG.30937.8	2392	noncoding	-
MSTRG.30937.9	3509	noncoding	-
MSTRG.30937.10	4147	noncoding	-
MSTRG.30939.1	1046	noncoding	-
MSTRG.30943.1	1402	coding	PF13765
MSTRG.30922.1	1139	noncoding	-
MSTRG.30922.2	1088	noncoding	-
MSTRG.30922.3	1758	noncoding	-
MSTRG.30945.1	1790	noncoding	-
MSTRG.30948.11	2726	coding	PF15717
MSTRG.30948.8	5095	coding	PF15717
MSTRG.30948.18	6004	coding	PF15717
MSTRG.30948.12	6626	coding	PF15717
MSTRG.30948.13	6786	coding	PF15717
MSTRG.30948.14	6744	coding	PF15717
MSTRG.30948.15	7654	coding	PF15717
MSTRG.30948.16	6452	coding	PF15717
MSTRG.30948.9	6715	coding	PF15717
MSTRG.30948.7	6921	coding	PF15717
MSTRG.30948.10	6971	coding	PF15717
MSTRG.30948.17	6693	coding	PF15717
MSTRG.30948.6	6810	coding	PF15717
MSTRG.30948.19	2228	coding	PF15717
MSTRG.30949.1	2839	noncoding	-
MSTRG.30950.2	5594	coding	PF00147
MSTRG.30951.1	246	noncoding	-
MSTRG.30952.1	274	noncoding	-
MSTRG.30950.5	3476	noncoding	-
MSTRG.30950.7	3583	noncoding	-
MSTRG.30929.3	3562	coding	PF13906
MSTRG.30961.3	2863	noncoding	-
MSTRG.30961.4	2687	noncoding	-
MSTRG.30965.1	3216	noncoding	-
MSTRG.30965.2	3797	noncoding	-
MSTRG.30965.3	3530	noncoding	-
MSTRG.30965.4	3554	noncoding	-
MSTRG.30965.6	5089	noncoding	-
MSTRG.30965.5	5218	noncoding	-
MSTRG.30966.1	731	noncoding	-
MSTRG.30965.8	1634	noncoding	-
MSTRG.30965.9	4724	noncoding	-
MSTRG.30965.10	3680	noncoding	-
MSTRG.30967.1	923	noncoding	-
MSTRG.30967.3	1327	noncoding	-
MSTRG.30971.1	607	noncoding	-

MSTRG.30971.2	617	noncoding	-
MSTRG.30968.1	372	noncoding	-
MSTRG.31004.1	1549	coding	PF15379
MSTRG.31004.2	1365	coding	PF15379
MSTRG.31004.4	1366	coding	PF15379
MSTRG.31004.5	1535	coding	PF15379
MSTRG.31006.1	2004	noncoding	-
MSTRG.31007.1	3510	noncoding	-
MSTRG.31011.1	1066	noncoding	-
MSTRG.31011.2	915	noncoding	-
MSTRG.31011.3	1065	noncoding	-
MSTRG.31011.4	749	noncoding	-
MSTRG.31011.5	982	noncoding	-
MSTRG.31015.3	20943	coding	PF00078
MSTRG.31015.4	34467	noncoding	-
MSTRG.31015.5	11683	noncoding	-
MSTRG.31015.6	30581	noncoding	-
MSTRG.31021.2	1918	noncoding	-
MSTRG.31019.1	368	noncoding	-
MSTRG.31027.1	2000	noncoding	-
MSTRG.31029.2	2100	coding	PF06682
MSTRG.31030.1	3704	coding	PF04133
MSTRG.31035.1	1623	noncoding	-
MSTRG.31035.4	937	noncoding	-
MSTRG.31035.3	1134	noncoding	-
MSTRG.31035.5	1338	noncoding	-
MSTRG.31035.6	1545	noncoding	-
MSTRG.31035.7	1165	noncoding	-
MSTRG.31035.8	1341	noncoding	-
MSTRG.31031.1	999	noncoding	-
MSTRG.31036.2	4852	coding	PF02186
MSTRG.31043.1	1705	coding	PF00149
MSTRG.31044.1	970	noncoding	-
MSTRG.31045.13	3164	coding	PF12509
MSTRG.31045.4	9373	coding	PF15326
MSTRG.31045.7	8792	coding	PF15326
MSTRG.31045.11	8969	coding	PF15326
MSTRG.31045.15	9055	coding	PF15326
MSTRG.31045.16	1549	coding	PF12509
MSTRG.31045.8	8927	coding	PF15326
MSTRG.31045.14	1266	coding	PF12509
MSTRG.31045.12	8679	coding	PF15326
MSTRG.31045.9	8724	coding	PF15326
MSTRG.31045.10	1276	coding	PF12509
MSTRG.31045.6	8782	coding	PF15326
MSTRG.31046.1	1535	noncoding	-
MSTRG.31047.1	5227	noncoding	-
MSTRG.31051.1	4830	noncoding	-
MSTRG.31052.1	8530	noncoding	-
MSTRG.31053.5	3917	coding	PF09382
MSTRG.31058.1	4935	coding	PF02158
MSTRG.31071.1	940	noncoding	-
MSTRG.31071.2	792	noncoding	-
MSTRG.31083.2	3206	coding	PF03493

MSTRG.31105.3	4788	coding	PF09457
MSTRG.31105.4	5264	coding	PF09457
MSTRG.31111.2	1002	noncoding	-
MSTRG.31112.1	1747	noncoding	-
MSTRG.31116.2	1800	noncoding	-
MSTRG.31116.3	6591	coding	PF00856
MSTRG.31116.4	11669	coding	PF00856
MSTRG.31116.5	5377	coding	PF00856
MSTRG.31117.1	549	noncoding	-
MSTRG.31117.2	650	noncoding	-
MSTRG.31116.6	2734	noncoding	-
MSTRG.31119.2	2696	coding	PF07766
MSTRG.31120.8	2993	coding	PF07714
MSTRG.31121.1	914	noncoding	-
MSTRG.31122.4	9044	noncoding	-
MSTRG.31122.3	6752	coding	PF05010
MSTRG.31114.4	4849	noncoding	-
MSTRG.31125.1	3141	noncoding	-
MSTRG.31125.2	2500	noncoding	-
MSTRG.31128.3	4121	coding	PF00200
MSTRG.31132.3	2298	coding	PF01421
MSTRG.31134.3	1031	noncoding	-
MSTRG.31134.4	460	noncoding	-
MSTRG.31134.5	756	noncoding	-
MSTRG.31134.6	2108	noncoding	-
MSTRG.31133.1	549	noncoding	-
MSTRG.31142.2	2762	coding	PF16922
MSTRG.31143.2	2942	noncoding	-
MSTRG.31143.3	2932	noncoding	-
MSTRG.31146.1	7746	coding	PF12796
MSTRG.31146.2	1665	noncoding	-
MSTRG.31146.4	1217	noncoding	-
MSTRG.31146.3	1123	noncoding	-
MSTRG.31146.5	1197	noncoding	-
MSTRG.31148.2	8408	coding	PF01853
MSTRG.31149.2	3886	noncoding	-
MSTRG.31144.1	3336	coding	PF00928
MSTRG.31144.2	3129	coding	PF00928
MSTRG.31144.5	2388	noncoding	-
MSTRG.31144.6	1170	noncoding	-
MSTRG.31145.2	2478	coding	PF00089
MSTRG.31157.1	551	noncoding	-
MSTRG.31157.2	511	noncoding	-
MSTRG.31158.2	1365	coding	PF14792
MSTRG.31158.3	1417	coding	PF14792
MSTRG.31158.4	1241	coding	PF14792
MSTRG.31159.1	2010	noncoding	-
MSTRG.31160.2	1791	coding	PF01459
MSTRG.31160.3	3042	coding	PF01459
MSTRG.31160.4	1532	coding	PF01459
MSTRG.31161.1	809	noncoding	-
MSTRG.31165.1	2229	coding	PF15117
MSTRG.31165.3	1617	coding	PF15117
MSTRG.31155.1	694	noncoding	-

MSTRG.31155.2	463	noncoding	-
MSTRG.31156.1	3312	coding	PF02931
MSTRG.31167.1	3582	coding	PF05485
MSTRG.31167.2	967	coding	PF05485
MSTRG.31169.5	3576	coding	PF05622
MSTRG.31169.6	3511	coding	PF05622
MSTRG.31169.7	11861	coding	PF05622
MSTRG.31171.1	3298	noncoding	-
MSTRG.31172.3	2174	noncoding	-
MSTRG.31175.2	7781	coding	PF01369
MSTRG.31175.3	4682	coding	PF01369
MSTRG.31174.1	706	noncoding	-
MSTRG.31187.3	3103	coding	PF04721
MSTRG.31188.4	5217	coding	PF00521
MSTRG.31189.2	2708	coding	PF00104
MSTRG.31189.3	3021	coding	PF00105
MSTRG.31189.4	2474	coding	PF00104
MSTRG.31193.1	551	noncoding	-
MSTRG.31194.1	8619	noncoding	-
MSTRG.31196.2	4254	noncoding	-
MSTRG.31196.1	4525	noncoding	-
MSTRG.31197.1	2253	noncoding	-
MSTRG.31201.1	1046	noncoding	-
MSTRG.31202.1	1281	noncoding	-
MSTRG.31205.1	1415	noncoding	-
MSTRG.31210.1	1699	noncoding	-
MSTRG.31211.1	6287	coding	PF00105
MSTRG.31215.1	5328	coding	PF00105
MSTRG.31215.2	5119	coding	PF00105
MSTRG.31218.2	1156	coding	PF00827
MSTRG.31219.2	1201	noncoding	-
MSTRG.31222.3	1270	coding	PF00179
MSTRG.31223.1	3164	noncoding	-
MSTRG.31231.2	3843	noncoding	-
MSTRG.31231.3	655	coding	PF00179
MSTRG.31231.4	823	coding	PF00179
MSTRG.31231.5	384	noncoding	-
MSTRG.31231.6	5954	noncoding	-
MSTRG.31233.1	8154	noncoding	-
MSTRG.31235.1	4167	noncoding	-
MSTRG.31236.1	9182	noncoding	-
MSTRG.31237.1	35985	noncoding	-
MSTRG.31238.1	9300	noncoding	-
MSTRG.31231.7	8261	noncoding	-
MSTRG.31239.1	17044	noncoding	-
MSTRG.31240.1	12417	noncoding	-
MSTRG.31241.1	2050	noncoding	-
MSTRG.31242.1	5405	noncoding	-
MSTRG.31240.2	9035	noncoding	-
MSTRG.31244.1	7092	noncoding	-
MSTRG.31244.2	4217	noncoding	-
MSTRG.31245.2	1608	noncoding	-
MSTRG.31230.1	212	noncoding	-
MSTRG.31246.1	503	noncoding	-

MSTRG.31246.2	657	noncoding	-
MSTRG.31247.2	744	noncoding	-
MSTRG.31251.1	4542	noncoding	-
MSTRG.31251.3	1007	noncoding	-
MSTRG.31251.2	1353	noncoding	-
MSTRG.31251.4	4079	noncoding	-
MSTRG.31251.5	657	noncoding	-
MSTRG.31271.1	1105	noncoding	-
MSTRG.31271.2	1808	noncoding	-
MSTRG.31263.1	1641	coding	PF00517
MSTRG.31273.1	4249	noncoding	-
MSTRG.31278.3	1376	coding	PF00071
MSTRG.31278.4	1326	coding	PF00071
MSTRG.31278.5	4193	coding	PF00071
MSTRG.31279.1	2147	noncoding	-
MSTRG.31286.4	475	noncoding	-
MSTRG.31287.3	13717	coding	PF10441
MSTRG.31285.1	2974	coding	PF00517
MSTRG.31289.1	12891	noncoding	-
MSTRG.31291.1	4125	noncoding	-
MSTRG.31301.1	5283	coding	PF00069
MSTRG.31301.2	807	noncoding	-
MSTRG.31301.3	7351	coding	PF00069
MSTRG.31301.4	5211	coding	PF00069
MSTRG.31301.5	5218	coding	PF00069
MSTRG.31301.6	3614	noncoding	-
MSTRG.31301.7	2431	noncoding	-
MSTRG.31307.1	857	noncoding	-
MSTRG.31316.2	2461	noncoding	-
MSTRG.31317.1	702	noncoding	-
MSTRG.31320.1	3865	noncoding	-
MSTRG.31320.3	3977	coding	PF00069
MSTRG.31320.2	5039	coding	PF00069
MSTRG.31320.7	2950	noncoding	-
MSTRG.31320.4	4589	coding	PF00069
MSTRG.31320.5	4202	coding	PF00069
MSTRG.31320.6	4349	coding	PF00069
MSTRG.31320.8	2928	coding	PF00069
MSTRG.31320.9	3683	coding	PF00069
MSTRG.31320.10	6217	coding	PF00069
MSTRG.31320.11	4238	noncoding	-
MSTRG.31320.12	7971	coding	PF00069
MSTRG.31304.1	205	noncoding	-
MSTRG.31324.2	1348	coding	PF04161
MSTRG.31333.2	2341	coding	PF10263
MSTRG.31336.3	2751	noncoding	-
MSTRG.31337.1	3423	noncoding	-
MSTRG.31341.1	844	noncoding	-
MSTRG.31347.1	3735	noncoding	-
MSTRG.31348.1	766	noncoding	-
MSTRG.31350.2	16916	coding	PF07714
MSTRG.31351.3	2866	coding	PF11261
MSTRG.31363.1	386	noncoding	-
MSTRG.31364.5	10781	noncoding	-

MSTRG.31364.6	4661	noncoding	-
MSTRG.31365.1	1464	coding	PF00348
MSTRG.31367.2	2250	coding	PF01762
MSTRG.31371.1	1450	noncoding	-
MSTRG.31372.2	5821	coding	PF07474
MSTRG.31374.1	544	noncoding	-
MSTRG.31375.1	3496	noncoding	-
MSTRG.31376.1	4754	noncoding	-
MSTRG.31378.4	1968	coding	PF04137
MSTRG.31379.1	1292	noncoding	-
MSTRG.31380.2	6085	coding	PF00337
MSTRG.31380.5	6196	coding	PF00337
MSTRG.31381.1	7578	coding	PF08146
MSTRG.31383.1	1177	coding	PF08726
MSTRG.31384.2	10302	coding	PF02965
MSTRG.31385.1	1172	noncoding	-
MSTRG.31386.1	654	noncoding	-
MSTRG.31390.1	363	noncoding	-
MSTRG.31401.2	2869	coding	PF00001
MSTRG.31401.1	3116	coding	PF00001
MSTRG.31401.3	2901	coding	PF00001
MSTRG.31407.1	11417	noncoding	-
MSTRG.31408.1	2210	noncoding	-
MSTRG.31407.2	1650	noncoding	-
MSTRG.31419.1	25674	coding	PF02994
MSTRG.31421.1	4160	noncoding	-
MSTRG.31426.1	3176	coding	PF00617
MSTRG.31426.2	3357	coding	PF00617
MSTRG.31431.1	1338	noncoding	-
MSTRG.31435.6	2526	coding	PF08080
MSTRG.31435.10	3634	coding	PF08080
MSTRG.31435.2	2466	coding	PF08080
MSTRG.31435.8	2261	coding	PF08080
MSTRG.31436.1	1726	noncoding	-
MSTRG.31440.1	235	noncoding	-
MSTRG.31459.1	1307	noncoding	-
MSTRG.31474.1	7080	coding	PF12796
MSTRG.31474.2	11042	coding	PF12796
MSTRG.31476.1	401	noncoding	-
MSTRG.31477.1	37340	noncoding	-
MSTRG.31474.3	2725	noncoding	-
MSTRG.31478.1	6027	noncoding	-
MSTRG.31477.2	5456	noncoding	-
MSTRG.31479.1	6711	noncoding	-
MSTRG.31474.4	5362	noncoding	-
MSTRG.31474.6	688	noncoding	-
MSTRG.31474.7	9005	noncoding	-
MSTRG.31480.1	799	noncoding	-
MSTRG.31468.2	3828	noncoding	-
MSTRG.31468.4	2331	noncoding	-
MSTRG.31468.5	1398	noncoding	-
MSTRG.31484.1	7529	coding	PF01388
MSTRG.31485.1	12014	noncoding	-
MSTRG.31485.2	10950	noncoding	-

MSTRG.31493.1	5450	coding	PF08174
MSTRG.31493.2	4665	coding	PF08174
MSTRG.31505.1	5498	noncoding	-
MSTRG.31506.1	21630	noncoding	-
MSTRG.31505.2	28971	noncoding	-
MSTRG.31505.4	12678	noncoding	-
MSTRG.31505.3	11352	noncoding	-
MSTRG.31507.1	17334	noncoding	-
MSTRG.31505.5	7750	noncoding	-
MSTRG.31495.1	2104	noncoding	-
MSTRG.31511.1	5144	noncoding	-
MSTRG.31511.2	3877	noncoding	-
MSTRG.31512.1	20274	noncoding	-
MSTRG.31510.4	2624	noncoding	-
MSTRG.31510.5	8312	noncoding	-
MSTRG.31510.7	6805	noncoding	-
MSTRG.31510.8	12477	noncoding	-
MSTRG.31510.9	1432	noncoding	-
MSTRG.31513.1	8926	noncoding	-
MSTRG.31515.1	4175	noncoding	-
MSTRG.31517.1	302	noncoding	-
MSTRG.31522.1	299	noncoding	-
MSTRG.31525.1	1192	coding	PF01044
MSTRG.31525.2	2238	coding	PF01044
MSTRG.31526.1	232	noncoding	-
MSTRG.31527.1	695	noncoding	-
MSTRG.31531.1	2577	noncoding	-
MSTRG.31531.2	3701	noncoding	-
MSTRG.31532.2	5068	coding	PF02146
MSTRG.31531.4	4124	coding	PF00632
MSTRG.31533.1	2073	noncoding	-
MSTRG.31531.8	3433	noncoding	-
MSTRG.31536.1	1075	noncoding	-
MSTRG.31537.13	1396	noncoding	-
MSTRG.31543.1	10308	coding	PF12851
MSTRG.31544.3	1102	noncoding	-
MSTRG.31546.5	3606	coding	PF14444
MSTRG.31546.7	4047	coding	PF14443
MSTRG.31548.1	53509	coding	PF10264
MSTRG.31548.2	53475	coding	PF10264
MSTRG.31548.3	53284	coding	PF10264
MSTRG.31548.4	44015	coding	PF10264
MSTRG.31548.5	50303	coding	PF10264
MSTRG.31549.3	866	noncoding	-
MSTRG.31551.3	3490	coding	PF12309
MSTRG.31551.2	2315	coding	PF12309
MSTRG.31552.1	1663	noncoding	-
MSTRG.31551.4	5609	coding	PF12309
MSTRG.31559.1	3015	coding	PF03643
MSTRG.31559.3	2781	coding	PF03643
MSTRG.31559.4	2983	coding	PF03643
MSTRG.31559.5	2937	coding	PF03643
MSTRG.31559.6	2703	coding	PF03643
MSTRG.31563.1	2568	coding	PF03727

MSTRG.31564.2	4298	coding	PF03727
MSTRG.31565.1	256	noncoding	-
MSTRG.31566.2	1911	noncoding	-
MSTRG.31570.4	1521	noncoding	-
MSTRG.31570.5	3798	noncoding	-
MSTRG.31570.3	5368	noncoding	-
MSTRG.31570.6	3796	noncoding	-
MSTRG.31570.9	5883	noncoding	-
MSTRG.31570.8	1779	noncoding	-
MSTRG.31570.7	5106	noncoding	-
MSTRG.31571.2	2057	noncoding	-
MSTRG.31571.1	7747	noncoding	-
MSTRG.31571.3	8259	noncoding	-
MSTRG.31571.4	6586	noncoding	-
MSTRG.31567.2	2170	noncoding	-
MSTRG.31576.1	215	noncoding	-
MSTRG.31581.4	4247	noncoding	-
MSTRG.31586.5	520	noncoding	-
MSTRG.31584.2	4338	coding	PF01733
MSTRG.31591.1	840	noncoding	-
MSTRG.31598.1	3943	coding	PF10591
MSTRG.31598.2	3183	coding	PF10591
MSTRG.31598.3	3181	coding	PF10591
MSTRG.31598.5	3437	coding	PF10591
MSTRG.31598.4	3595	coding	PF10591
MSTRG.31599.3	1951	coding	PF10469
MSTRG.31599.4	2342	coding	PF10469
MSTRG.31597.1	1645	coding	PF07809
MSTRG.31597.2	1635	coding	PF07809
MSTRG.31603.2	3012	coding	PF09320
MSTRG.31603.3	2522	coding	PF00226
MSTRG.31603.4	1391	coding	PF09320
MSTRG.31603.8	1615	coding	PF00226
MSTRG.31603.11	2042	coding	PF09320
MSTRG.31602.2	4568	noncoding	-
MSTRG.31606.1	3663	coding	PF04678
MSTRG.31606.2	7939	coding	PF04678
MSTRG.31606.3	1836	noncoding	-
MSTRG.31606.4	3722	noncoding	-
MSTRG.31607.1	2774	noncoding	-
MSTRG.31606.6	26971	coding	PF00078
MSTRG.31606.7	7016	coding	PF04678
MSTRG.31606.8	14742	noncoding	-
MSTRG.31608.1	6100	noncoding	-
MSTRG.31606.10	4978	coding	PF04678
MSTRG.31606.11	5466	coding	PF04678
MSTRG.31617.1	2302	coding	PF07093
MSTRG.31618.1	2249	coding	PF12516
MSTRG.31618.4	2021	coding	PF12516
MSTRG.31618.5	2246	coding	PF12516
MSTRG.31619.1	7157	coding	PF00226
MSTRG.31620.1	1369	noncoding	-
MSTRG.31622.2	3569	noncoding	-
MSTRG.31623.4	5716	coding	PF00191

MSTRG.31625.1	381	noncoding	-
MSTRG.31626.3	6867	noncoding	-
MSTRG.31626.4	7283	noncoding	-
MSTRG.31626.5	6892	noncoding	-
MSTRG.31627.1	505	noncoding	-
MSTRG.31613.2	6503	coding	PF04811
MSTRG.31613.3	5153	coding	PF04811
MSTRG.31614.1	9850	coding	PF00852
MSTRG.31630.5	5363	noncoding	-
MSTRG.31630.6	4332	noncoding	-
MSTRG.31633.1	1278	noncoding	-
MSTRG.31633.2	1265	noncoding	-
MSTRG.31629.1	1773	coding	PF00089
MSTRG.31636.5	2920	coding	PF00928
MSTRG.31637.2	1421	noncoding	-
MSTRG.31637.3	1583	noncoding	-
MSTRG.31638.1	2185	noncoding	-
MSTRG.31640.1	3762	noncoding	-
MSTRG.31641.1	3551	noncoding	-
MSTRG.31642.1	3287	noncoding	-
MSTRG.31643.1	4945	noncoding	-
MSTRG.31637.6	1595	noncoding	-
MSTRG.31644.2	2448	noncoding	-
MSTRG.31644.5	7352	coding	PF01853
MSTRG.31648.1	2052	noncoding	-
MSTRG.31648.2	2226	coding	PF00782
MSTRG.31648.3	4818	coding	PF14360
MSTRG.31648.5	5945	coding	PF14360
MSTRG.31650.1	263	noncoding	-
MSTRG.31651.2	1616	coding	PF01459
MSTRG.31661.1	6610	noncoding	-
MSTRG.31662.1	1653	noncoding	-
MSTRG.31672.2	716	noncoding	-
MSTRG.31672.3	2188	noncoding	-
MSTRG.31673.1	1277	noncoding	-
MSTRG.31678.2	29448	noncoding	-
MSTRG.31678.1	41745	coding	PF16610
MSTRG.31679.2	7207	coding	PF04822
MSTRG.31682.1	1206	noncoding	-
MSTRG.31690.1	1591	noncoding	-
MSTRG.31691.1	7759	coding	PF02891
MSTRG.31691.2	7433	coding	PF02891
MSTRG.31692.1	654	noncoding	-
MSTRG.31691.3	6788	coding	PF02891
MSTRG.31700.1	2644	coding	PF02093
MSTRG.31703.2	2538	coding	PF14934
MSTRG.31694.1	536	noncoding	-
MSTRG.31721.4	2651	noncoding	-
MSTRG.31722.2	864	noncoding	-
MSTRG.31723.5	1329	noncoding	-
MSTRG.31723.6	1210	noncoding	-
MSTRG.31725.2	2501	coding	PF00335
MSTRG.31725.3	2662	coding	PF00335
MSTRG.31725.4	2205	coding	PF00335

MSTRG.31729.1	490	noncoding	-
MSTRG.31742.2	2488	noncoding	-
MSTRG.31740.1	3866	coding	PF00028
MSTRG.31743.1	1566	noncoding	-
MSTRG.31743.2	1450	noncoding	-
MSTRG.31743.3	1179	noncoding	-
MSTRG.31745.3	3542	noncoding	-
MSTRG.31745.6	2446	noncoding	-
MSTRG.31745.7	2294	noncoding	-
MSTRG.31746.1	3944	noncoding	-
MSTRG.31747.1	958	noncoding	-
MSTRG.31745.10	3650	noncoding	-
MSTRG.31748.1	671	noncoding	-
MSTRG.31748.2	242	noncoding	-
MSTRG.31748.3	535	noncoding	-
MSTRG.31749.1	446	noncoding	-
MSTRG.31750.1	231	noncoding	-
MSTRG.31753.1	1056	noncoding	-
MSTRG.31757.4	4738	coding	PF07814
MSTRG.31757.5	4493	coding	PF07814
MSTRG.31758.1	5088	coding	PF00412
MSTRG.31766.1	9708	noncoding	-
MSTRG.31777.1	320	noncoding	-
MSTRG.31763.1	1063	noncoding	-
MSTRG.31786.3	1317	noncoding	-
MSTRG.31786.2	3548	coding	PF05587
MSTRG.31787.1	267	noncoding	-
MSTRG.31786.4	2542	coding	PF05587
MSTRG.31786.7	2516	coding	PF05587
MSTRG.31786.10	1026	noncoding	-
MSTRG.31786.11	2458	coding	PF05587
MSTRG.31786.12	2405	noncoding	-
MSTRG.31796.5	1592	coding	PF00069
MSTRG.31796.4	5954	coding	PF00069
MSTRG.31796.6	5680	coding	PF00069
MSTRG.31797.1	7478	noncoding	-
MSTRG.31799.1	3203	coding	PF00620
MSTRG.31801.1	2323	noncoding	-
MSTRG.31801.2	1477	noncoding	-
MSTRG.31801.3	1230	noncoding	-
MSTRG.31801.4	1333	noncoding	-
MSTRG.31801.5	1404	noncoding	-
MSTRG.31801.6	1059	noncoding	-
MSTRG.31801.7	832	noncoding	-
MSTRG.31801.8	2362	noncoding	-
MSTRG.31789.1	1212	noncoding	-
MSTRG.31789.2	1549	noncoding	-
MSTRG.31790.1	1772	noncoding	-
MSTRG.31792.2	2397	noncoding	-
MSTRG.31792.3	1034	noncoding	-
MSTRG.31793.1	569	noncoding	-
MSTRG.31793.2	415	noncoding	-
MSTRG.31792.4	1539	noncoding	-
MSTRG.31803.1	6831	coding	PF00176

MSTRG.31803.3	3313	coding	PF00176
MSTRG.31804.1	1186	noncoding	-
MSTRG.31805.5	471	noncoding	-
MSTRG.31805.3	902	coding	PF15092
MSTRG.31805.1	1557	coding	PF15092
MSTRG.31805.4	1019	coding	PF15092
MSTRG.31806.1	1182	coding	PF16870
MSTRG.31810.1	953	noncoding	-
MSTRG.31812.5	4139	coding	PF05825
MSTRG.31812.9	2619	coding	PF12489
MSTRG.31812.8	2571	coding	PF12489
MSTRG.31813.1	702	noncoding	-
MSTRG.31812.12	803	coding	PF05825
MSTRG.31812.10	785	coding	PF05825
MSTRG.31815.4	4227	coding	PF15255
MSTRG.31815.2	5231	coding	PF15255
MSTRG.31815.3	5228	coding	PF15255
MSTRG.31815.5	3516	coding	PF15255
MSTRG.31818.2	2212	noncoding	-
MSTRG.31818.3	2637	coding	PF00240
MSTRG.31818.7	3243	noncoding	-
MSTRG.31820.2	4880	coding	PF12906
MSTRG.31820.3	4176	coding	PF12906
MSTRG.31820.4	4443	coding	PF12906
MSTRG.31820.5	4422	coding	PF12906
MSTRG.31820.6	4466	coding	PF12906
MSTRG.31820.7	4199	coding	PF12906
MSTRG.31820.8	4478	coding	PF12906
MSTRG.31820.9	5911	coding	PF12906
MSTRG.31822.2	2709	coding	PF00305
MSTRG.31824.1	5058	noncoding	-
MSTRG.31826.1	2974	noncoding	-
MSTRG.31829.1	545	noncoding	-
MSTRG.31833.4	1145	noncoding	-
MSTRG.31838.1	1435	noncoding	-
MSTRG.31839.2	2222	noncoding	-
MSTRG.31865.1	1259	coding	PF08925
MSTRG.31867.3	7656	noncoding	-
MSTRG.31867.5	7142	noncoding	-
MSTRG.31867.7	7049	noncoding	-
MSTRG.31867.6	7450	noncoding	-
MSTRG.31868.1	2718	noncoding	-
MSTRG.31867.8	1486	noncoding	-
MSTRG.31859.4	1903	coding	PF17045
MSTRG.31859.3	1929	coding	PF17045
MSTRG.31861.2	4529	coding	PF01490
MSTRG.31875.1	1111	noncoding	-
MSTRG.31883.1	334	noncoding	-
MSTRG.31885.3	6688	coding	PF04968
MSTRG.31885.4	4256	coding	PF04968
MSTRG.31886.1	2839	noncoding	-
MSTRG.31886.2	4458	noncoding	-
MSTRG.31897.1	4101	coding	PF00264
MSTRG.31898.1	3949	noncoding	-

MSTRG.31897.2	2914	coding	PF00264
MSTRG.31904.1	857	noncoding	-
MSTRG.31907.1	1504	noncoding	-
MSTRG.31905.1	592	noncoding	-
MSTRG.31909.1	509	noncoding	-
MSTRG.31910.1	1537	noncoding	-
MSTRG.31911.2	1110	coding	PF03949
MSTRG.31914.2	2075	coding	PF05603
MSTRG.31914.3	4257	coding	PF05603
MSTRG.31917.7	414	noncoding	-
MSTRG.31919.1	4578	noncoding	-
MSTRG.31920.6	1630	noncoding	-
MSTRG.31920.8	1524	noncoding	-
MSTRG.31920.9	1526	noncoding	-
MSTRG.31923.1	1306	noncoding	-
MSTRG.31923.2	980	noncoding	-
MSTRG.31994.3	850	coding	PF07114
MSTRG.31998.2	997	coding	PF07114
MSTRG.31999.1	2383	coding	PF10608
MSTRG.32003.1	5934	noncoding	-
MSTRG.32003.2	5318	noncoding	-
MSTRG.32016.1	1108	noncoding	-
MSTRG.32029.1	2315	noncoding	-
MSTRG.32030.1	1866	noncoding	-
MSTRG.32051.1	980	noncoding	-
MSTRG.32052.1	5840	coding	PF00625
MSTRG.32052.2	5892	coding	PF00625
MSTRG.31935.1	11278	coding	PF12796
MSTRG.31935.2	3125	coding	PF12796
MSTRG.31935.6	5716	coding	PF12796
MSTRG.31935.8	2962	coding	PF12796
MSTRG.31935.9	5204	coding	PF12796
MSTRG.31935.10	1732	coding	PF12796
MSTRG.31936.3	1709	noncoding	-
MSTRG.31939.1	1143	noncoding	-
MSTRG.31939.2	1056	noncoding	-
MSTRG.31939.3	805	noncoding	-
MSTRG.31939.4	756	noncoding	-
MSTRG.31940.1	4532	noncoding	-
MSTRG.31941.1	1852	noncoding	-
MSTRG.31941.2	9404	noncoding	-
MSTRG.31941.3	13539	noncoding	-
MSTRG.31941.4	12765	noncoding	-
MSTRG.31942.4	5086	noncoding	-
MSTRG.31928.2	3573	noncoding	-
MSTRG.31930.1	286	noncoding	-
MSTRG.31930.2	4716	coding	PF00078
MSTRG.31948.1	2219	noncoding	-
MSTRG.31963.1	341	noncoding	-
MSTRG.31968.1	3981	noncoding	-
MSTRG.31981.1	4861	coding	PF02994
MSTRG.31990.1	5008	noncoding	-
MSTRG.31992.2	13441	coding	PF06484
MSTRG.32053.1	2150	noncoding	-

MSTRG.32054.1	775	noncoding	-
MSTRG.32056.2	29923	noncoding	-
MSTRG.32056.3	9465	noncoding	-
MSTRG.32058.1	8750	noncoding	-
MSTRG.32059.1	2393	coding	PF00443
MSTRG.32068.2	2468	coding	PF03155
MSTRG.32055.2	734	coding	PF06374
MSTRG.32069.2	2740	noncoding	-
MSTRG.32070.3	574	coding	PF04430
MSTRG.32072.3	7498	noncoding	-
MSTRG.32076.5	2791	coding	PF03009
MSTRG.32076.7	2075	noncoding	-
MSTRG.32076.3	3083	coding	PF03009
MSTRG.32076.4	2614	noncoding	-
MSTRG.32076.6	2926	noncoding	-
MSTRG.32077.1	224	noncoding	-
MSTRG.32078.1	2361	noncoding	-
MSTRG.32084.1	820	noncoding	-
MSTRG.32085.1	665	noncoding	-
MSTRG.32086.1	2267	noncoding	-
MSTRG.32091.1	2587	noncoding	-
MSTRG.32091.2	1495	noncoding	-
MSTRG.32104.3	1445	noncoding	-
MSTRG.32105.1	1530	noncoding	-
MSTRG.32106.1	680	noncoding	-
MSTRG.32107.1	1395	noncoding	-
MSTRG.32108.2	1284	noncoding	-
MSTRG.32108.1	2596	noncoding	-
MSTRG.32109.1	838	noncoding	-
MSTRG.32110.1	4702	noncoding	-
MSTRG.32104.4	6219	noncoding	-
MSTRG.32111.1	6273	noncoding	-
MSTRG.32112.1	6911	noncoding	-
MSTRG.32094.1	262	noncoding	-
MSTRG.32094.2	238	noncoding	-
MSTRG.32113.1	580	noncoding	-
MSTRG.32114.2	457	noncoding	-
MSTRG.32115.1	826	noncoding	-
MSTRG.32116.2	3504	noncoding	-
MSTRG.32119.1	813	noncoding	-
MSTRG.32123.1	241	noncoding	-
MSTRG.32128.1	1514	noncoding	-
MSTRG.32129.1	628	coding	PF00093
MSTRG.32130.2	1175	noncoding	-
MSTRG.32130.1	2654	noncoding	-
MSTRG.32130.3	3013	coding	PF00209
MSTRG.32133.1	4554	noncoding	-
MSTRG.32134.1	7103	noncoding	-
MSTRG.32134.2	6702	noncoding	-
MSTRG.32134.4	11932	noncoding	-
MSTRG.32137.1	10908	noncoding	-
MSTRG.32137.2	9717	noncoding	-
MSTRG.32137.3	9486	noncoding	-
MSTRG.32136.1	687	noncoding	-

MSTRG.32140.1	716	noncoding	-
MSTRG.32141.1	3215	coding	PF02319
MSTRG.32142.1	853	coding	PF00412
MSTRG.32143.4	2087	coding	PF12796
MSTRG.32146.1	3008	coding	PF00102
MSTRG.32154.2	1714	noncoding	-
MSTRG.32155.1	2691	noncoding	-
MSTRG.32155.2	4135	noncoding	-
MSTRG.32158.3	879	coding	PF00056
MSTRG.32163.1	666	noncoding	-
MSTRG.32164.1	1972	coding	PF00056
MSTRG.32150.2	2140	coding	PF08567
MSTRG.32150.3	2007	coding	PF08567
MSTRG.32167.2	1632	noncoding	-
MSTRG.32167.3	2232	noncoding	-
MSTRG.32168.1	399	noncoding	-
MSTRG.32169.2	1112	noncoding	-
MSTRG.32166.1	2347	coding	PF13853
MSTRG.32172.2	1558	coding	PF05964
MSTRG.32176.4	1985	coding	PF03629
MSTRG.32176.3	1859	coding	PF03629
MSTRG.32177.1	719	noncoding	-
MSTRG.32180.1	1706	noncoding	-
MSTRG.32181.4	3009	coding	PF13895
MSTRG.32183.6	1912	noncoding	-
MSTRG.32184.1	1254	noncoding	-
MSTRG.32184.3	1589	noncoding	-
MSTRG.32199.1	8596	noncoding	-
MSTRG.32199.2	1318	noncoding	-
MSTRG.32199.3	2646	noncoding	-
MSTRG.32201.1	561	noncoding	-
MSTRG.32203.2	1766	coding	PF00271
MSTRG.32203.4	1853	coding	PF00271
MSTRG.32206.2	2023	noncoding	-
MSTRG.32206.4	2493	noncoding	-
MSTRG.32206.5	2133	noncoding	-
MSTRG.32208.2	1770	coding	PF01266
MSTRG.32204.2	3322	noncoding	-
MSTRG.32212.1	2178	noncoding	-
MSTRG.32225.1	329	noncoding	-
MSTRG.32227.1	424	noncoding	-
MSTRG.32229.2	2132	coding	PF00178
MSTRG.32232.1	10115	coding	PF00620
MSTRG.32232.2	8545	coding	PF00620
MSTRG.32231.1	213	noncoding	-
MSTRG.32231.2	220	noncoding	-
MSTRG.32237.2	717	coding	PF15212
MSTRG.32238.1	681	noncoding	-
MSTRG.32239.1	673	coding	PF07679
MSTRG.32241.1	602	noncoding	-
MSTRG.32242.1	1014	noncoding	-
MSTRG.32246.2	7015	coding	PF16638
MSTRG.32246.3	4497	coding	PF16638
MSTRG.32249.1	4582	coding	PF00651

MSTRG.32249.5	5419	coding	PF00651
MSTRG.32250.1	2562	noncoding	-
MSTRG.32251.1	1012	noncoding	-
MSTRG.32251.2	11842	noncoding	-
MSTRG.32252.1	724	noncoding	-
MSTRG.32254.1	6197	coding	PF00787
MSTRG.32257.1	552	noncoding	-
MSTRG.32260.2	2248	noncoding	-
MSTRG.32265.1	838	coding	PF00026
MSTRG.32266.1	704	coding	PF00026
MSTRG.32267.1	745	coding	PF00026
MSTRG.32270.2	1306	coding	PF00026
MSTRG.32275.3	2397	coding	PF03188
MSTRG.32275.1	2344	coding	PF03188
MSTRG.32275.2	2568	coding	PF03188
MSTRG.32275.6	2299	coding	PF03188
MSTRG.32275.7	2454	coding	PF03188
MSTRG.32276.4	2953	coding	PF14935
MSTRG.32278.3	1694	coding	PF15691
MSTRG.32278.4	1667	coding	PF15691
MSTRG.32277.1	2677	noncoding	-
MSTRG.32283.1	937	noncoding	-
MSTRG.32283.2	1138	noncoding	-
MSTRG.32285.1	1457	noncoding	-
MSTRG.32286.1	1376	noncoding	-
MSTRG.32289.1	2087	noncoding	-
MSTRG.32291.2	603	coding	PF05251
MSTRG.32292.1	2032	coding	PF00867
MSTRG.32292.3	2138	coding	PF00867
MSTRG.32293.1	3601	coding	PF00173
MSTRG.32300.3	2810	noncoding	-
MSTRG.32300.5	2821	noncoding	-
MSTRG.32301.3	2426	coding	PF01062
MSTRG.32305.2	2344	coding	PF01112
MSTRG.32324.12	11089	noncoding	-
MSTRG.32324.17	11314	noncoding	-
MSTRG.32324.18	11755	noncoding	-
MSTRG.32324.20	10849	noncoding	-
MSTRG.32324.21	12043	noncoding	-
MSTRG.32324.3	7414	noncoding	-
MSTRG.32324.33	11053	noncoding	-
MSTRG.32324.35	9940	noncoding	-
MSTRG.32324.38	10849	noncoding	-
MSTRG.32324.40	9958	noncoding	-
MSTRG.32324.43	11839	noncoding	-
MSTRG.32324.47	13072	noncoding	-
MSTRG.32324.49	11881	noncoding	-
MSTRG.32324.50	12589	noncoding	-
MSTRG.32324.53	9313	noncoding	-
MSTRG.32324.55	11089	noncoding	-
MSTRG.32324.56	13213	noncoding	-
MSTRG.32324.7	10465	noncoding	-
MSTRG.32324.9	12847	noncoding	-
MSTRG.32310.3	3356	coding	PF01055

MSTRG.32310.4	3422	coding	PF01055
MSTRG.32322.7	1547	coding	PF06775
MSTRG.32322.10	2033	coding	PF06775
MSTRG.32322.12	2042	coding	PF06775
MSTRG.32322.8	2372	coding	PF06775
MSTRG.32322.11	2477	coding	PF06775
MSTRG.32322.3	2474	coding	PF06775
MSTRG.32322.4	1900	coding	PF06775
MSTRG.32322.9	2082	coding	PF06775
MSTRG.32322.2	2317	coding	PF06775
MSTRG.32322.16	1968	coding	PF06775
MSTRG.32323.1	1604	coding	PF00631
MSTRG.32320.3	2022	noncoding	-
MSTRG.32331.1	5244	coding	PF00651
MSTRG.32331.3	5017	coding	PF00651
MSTRG.32331.4	4976	coding	PF00651
MSTRG.32331.5	1897	coding	PF00651
MSTRG.32331.6	2018	coding	PF00651
MSTRG.32334.1	5183	coding	PF02969
MSTRG.32334.2	5180	coding	PF02969
MSTRG.32334.3	3443	coding	PF02969
MSTRG.32338.1	375	noncoding	-
MSTRG.32339.2	1502	noncoding	-
MSTRG.32339.1	4349	noncoding	-
MSTRG.32339.3	2200	noncoding	-
MSTRG.32338.4	1657	noncoding	-
MSTRG.32338.6	1649	noncoding	-
MSTRG.32338.7	1559	noncoding	-
MSTRG.32329.1	5111	coding	PF04970
MSTRG.32329.2	5117	coding	PF04970
MSTRG.32329.3	4409	coding	PF04970
MSTRG.32329.5	1962	coding	PF04970
MSTRG.32329.6	2904	coding	PF04970
MSTRG.32329.7	1828	coding	PF04970
MSTRG.32340.2	6662	coding	PF02263
MSTRG.32340.4	2113	coding	PF02263
MSTRG.32342.1	3909	coding	PF02453
MSTRG.32343.2	517	noncoding	-
MSTRG.32344.1	898	noncoding	-
MSTRG.32350.3	2585	coding	PF00069
MSTRG.32354.2	1813	coding	PF10275
MSTRG.32354.3	1612	coding	PF10275
MSTRG.32356.1	3827	noncoding	-
MSTRG.32358.2	2483	noncoding	-
MSTRG.32358.3	2236	noncoding	-
MSTRG.32358.4	2099	noncoding	-
MSTRG.32364.3	1549	coding	PF00341
MSTRG.32365.1	2629	coding	PF00341
MSTRG.32365.2	3229	coding	PF00341
MSTRG.32366.5	542	coding	PF00254
MSTRG.32369.4	976	coding	PF10514
MSTRG.32369.3	1451	coding	PF10514
MSTRG.32371.2	3005	noncoding	-
MSTRG.32371.3	1989	noncoding	-

MSTRG.32359.2	1030	noncoding	-
MSTRG.32372.1	2302	noncoding	-
MSTRG.32377.1	2374	noncoding	-
MSTRG.32381.7	1831	coding	PF02210
MSTRG.32381.11	5051	coding	PF02210
MSTRG.32381.10	4338	coding	PF02210
MSTRG.32381.2	4921	coding	PF02210
MSTRG.32381.3	5176	coding	PF02210
MSTRG.32381.1	5164	coding	PF02210
MSTRG.32381.8	4963	coding	PF02210
MSTRG.32381.12	5106	coding	PF02210
MSTRG.32381.4	5548	coding	PF02210
MSTRG.32381.6	8537	coding	PF02210
MSTRG.32381.14	5763	noncoding	-
MSTRG.32378.1	1216	noncoding	-
MSTRG.32384.10	2822	coding	PF16275
MSTRG.32384.11	2967	coding	PF16275
MSTRG.32384.5	2972	coding	PF16275
MSTRG.32388.1	3387	coding	PF12763
MSTRG.32395.5	1341	coding	PF00025
MSTRG.32395.2	1689	coding	PF00025
MSTRG.32395.3	1357	coding	PF00025
MSTRG.32395.4	1457	coding	PF00025
MSTRG.32395.7	1216	coding	PF00025
MSTRG.32394.2	1603	coding	PF04212
MSTRG.32397.2	1701	noncoding	-
MSTRG.32397.3	2124	noncoding	-
MSTRG.32397.4	1516	noncoding	-
MSTRG.32397.5	3080	noncoding	-
MSTRG.32397.6	2785	noncoding	-
MSTRG.32402.1	1648	coding	PF01222
MSTRG.32405.3	1881	coding	PF05046
MSTRG.32407.1	771	noncoding	-
MSTRG.32411.2	6968	coding	PF01067
MSTRG.32412.2	2556	coding	PF14051
MSTRG.32411.3	7554	noncoding	-
MSTRG.32411.4	8104	noncoding	-
MSTRG.32411.5	7010	noncoding	-
MSTRG.32415.1	2999	coding	PF06677
MSTRG.32416.3	3090	coding	PF00307
MSTRG.32418.6	6138	coding	PF05041
MSTRG.32418.9	6147	coding	PF05041
MSTRG.32419.2	3511	coding	PF02145
MSTRG.32424.1	3486	noncoding	-
MSTRG.32427.1	1508	noncoding	-
MSTRG.32434.2	2124	noncoding	-
MSTRG.32441.1	801	coding	PF02961
MSTRG.32441.2	853	coding	PF02961
MSTRG.32441.3	750	coding	PF02961
MSTRG.32444.1	1267	noncoding	-
MSTRG.32444.2	2415	coding	PF06990
MSTRG.32456.3	2022	coding	PF00071
MSTRG.32456.2	2631	coding	PF00071
MSTRG.32447.2	889	coding	PF03878

MSTRG.32451.2	2607	coding	PF01733
MSTRG.32457.1	986	noncoding	-
MSTRG.32484.5	1851	coding	PF00076
MSTRG.32484.6	1938	coding	PF00076
MSTRG.32484.1	11148	coding	PF00076
MSTRG.32484.4	1939	coding	PF00076
MSTRG.32484.3	1405	coding	PF00076
MSTRG.32484.2	2273	coding	PF00076
MSTRG.32484.7	10873	coding	PF00076
MSTRG.32484.8	16179	coding	PF00076
MSTRG.32484.9	1948	coding	PF00076
MSTRG.32484.11	743	coding	PF00076
MSTRG.32489.1	1821	coding	PF15091
MSTRG.32489.2	1824	coding	PF15091
MSTRG.32492.2	3564	coding	PF02436
MSTRG.32492.5	3847	coding	PF02436
MSTRG.32494.1	1532	noncoding	-
MSTRG.32468.1	7867	coding	PF16866
MSTRG.32468.4	6671	coding	PF16866
MSTRG.32468.5	6549	coding	PF16866
MSTRG.32468.6	6203	noncoding	-
MSTRG.32469.1	6990	noncoding	-
MSTRG.32474.2	1553	coding	PF04081
MSTRG.32474.3	877	coding	PF04081
MSTRG.32480.1	991	coding	PF05915
MSTRG.32497.2	1998	coding	PF09806
MSTRG.32501.2	1788	noncoding	-
MSTRG.32501.3	1576	noncoding	-
MSTRG.32507.4	2605	coding	PF01633
MSTRG.32507.3	2539	coding	PF01633
MSTRG.32507.6	1523	coding	PF01633
MSTRG.32509.1	3923	noncoding	-
MSTRG.32509.3	3241	noncoding	-
MSTRG.32513.1	716	noncoding	-
MSTRG.32513.2	980	noncoding	-
MSTRG.32514.4	4431	coding	PF04499
MSTRG.32514.5	8237	coding	PF04499
MSTRG.32515.1	5393	noncoding	-
MSTRG.32519.1	4856	coding	PF03638
MSTRG.32519.3	1828	coding	PF03638
MSTRG.32519.4	1544	coding	PF03638
MSTRG.32519.5	7329	noncoding	-
MSTRG.32519.6	3823	noncoding	-
MSTRG.32519.7	2433	noncoding	-
MSTRG.32519.8	2361	noncoding	-
MSTRG.32519.9	1824	noncoding	-
MSTRG.32520.1	1729	coding	PF05392
MSTRG.32520.2	1081	coding	PF05392
MSTRG.32520.3	1241	coding	PF05392
MSTRG.32520.4	474	coding	PF05392
MSTRG.32520.5	566	coding	PF05392
MSTRG.32523.3	703	coding	PF00829
MSTRG.32528.2	1075	noncoding	-
MSTRG.32528.1	568	noncoding	-

MSTRG.32534.11	4992	coding	PF16178
MSTRG.32534.10	8553	coding	PF16178
MSTRG.32534.3	10478	coding	PF16178
MSTRG.32534.4	11119	coding	PF16178
MSTRG.32534.6	9664	coding	PF16178
MSTRG.32534.7	10910	coding	PF16178
MSTRG.32534.13	2186	coding	PF16178
MSTRG.32534.5	3026	coding	PF16178
MSTRG.32534.12	3774	coding	PF16178
MSTRG.32534.8	3574	coding	PF16178
MSTRG.32542.1	708	noncoding	-
MSTRG.32542.3	1026	noncoding	-
MSTRG.32545.1	943	noncoding	-
MSTRG.32546.2	2637	noncoding	-
MSTRG.32547.4	2566	coding	PF02218
MSTRG.32547.5	2695	coding	PF02218
MSTRG.32547.6	2550	coding	PF02218
MSTRG.32549.1	8615	noncoding	-
MSTRG.32549.2	5330	noncoding	-
MSTRG.32549.3	6192	noncoding	-
MSTRG.32550.1	1313	coding	PF07647
MSTRG.32552.1	561	noncoding	-
MSTRG.32554.2	438	noncoding	-
MSTRG.32554.1	909	noncoding	-
MSTRG.32554.3	1212	noncoding	-
MSTRG.32538.2	669	coding	PF00022
MSTRG.32538.4	624	coding	PF00022
MSTRG.32538.5	1456	coding	PF00022
MSTRG.32541.1	2916	coding	PF01222
MSTRG.32541.4	2916	coding	PF01222
MSTRG.32557.1	735	noncoding	-
MSTRG.32558.1	3536	coding	PF01237
MSTRG.32558.2	3483	coding	PF01237
MSTRG.32561.2	4095	coding	PF01406
MSTRG.32561.3	2704	coding	PF01406
MSTRG.32561.4	2962	coding	PF01406
MSTRG.32561.5	3340	coding	PF01406
MSTRG.32564.1	15108	coding	PF00078
MSTRG.32564.2	11888	coding	PF00078
MSTRG.32562.1	748	noncoding	-
MSTRG.32566.3	4908	noncoding	-
MSTRG.32566.4	5162	noncoding	-
MSTRG.32566.5	15949	noncoding	-
MSTRG.32568.3	2029	coding	PF15264
MSTRG.32575.3	4336	coding	PF08365
MSTRG.32575.4	2786	coding	PF08365
MSTRG.32575.5	1185	coding	PF08365
MSTRG.32575.6	3012	coding	PF08365
MSTRG.32575.8	2745	coding	PF08365
MSTRG.32575.10	2133	coding	PF08365
MSTRG.32575.9	3637	coding	PF08365
MSTRG.32575.11	1934	coding	PF08365
MSTRG.32575.12	2123	coding	PF08365
MSTRG.32574.3	1689	noncoding	-

MSTRG.32576.2	1735	coding	PF02029
MSTRG.32582.1	1292	coding	PF03637
MSTRG.32588.3	2518	coding	PF02296
MSTRG.32592.1	1631	coding	PF00335
MSTRG.32592.3	1720	coding	PF00335
MSTRG.32596.2	2046	noncoding	-
MSTRG.32598.2	1193	noncoding	-
MSTRG.32606.2	3771	coding	PF08416
MSTRG.32606.4	2526	coding	PF08416
MSTRG.32606.3	2509	coding	PF08416
MSTRG.32607.2	1821	coding	PF09799
MSTRG.32607.3	1292	coding	PF09799
MSTRG.32604.3	4603	coding	PF00628
MSTRG.32609.2	3243	noncoding	-
MSTRG.32611.1	1840	coding	PF00071
MSTRG.32613.1	2628	noncoding	-
MSTRG.32614.1	1478	noncoding	-
MSTRG.32614.2	2295	noncoding	-
MSTRG.32614.3	2415	noncoding	-
MSTRG.32614.4	2586	noncoding	-
MSTRG.32614.5	1391	noncoding	-
MSTRG.32614.6	1452	noncoding	-
MSTRG.32617.1	594	coding	PF04505
MSTRG.32619.2	643	coding	PF04505
MSTRG.32621.1	406	noncoding	-
MSTRG.32628.1	1617	coding	PF10482
MSTRG.32628.2	447	coding	PF10482
MSTRG.32640.1	6983	noncoding	-
MSTRG.32641.1	6747	noncoding	-
MSTRG.32641.2	7398	noncoding	-
MSTRG.32641.3	7211	noncoding	-
MSTRG.32641.4	12837	noncoding	-
MSTRG.32634.1	7520	noncoding	-
MSTRG.32634.2	5832	noncoding	-
MSTRG.32646.2	293	noncoding	-
MSTRG.32646.3	325	coding	PF00832
MSTRG.32658.1	1495	noncoding	-
MSTRG.32658.2	1575	coding	PF00046
MSTRG.32658.4	1303	noncoding	-
MSTRG.32658.3	1215	coding	PF00046
MSTRG.32660.1	1386	noncoding	-
MSTRG.32661.2	11167	coding	PF15783
MSTRG.32662.1	842	noncoding	-
MSTRG.32663.1	1365	coding	PF00046
MSTRG.32664.1	1687	noncoding	-
MSTRG.32665.1	1407	noncoding	-
MSTRG.32666.2	1023	noncoding	-
MSTRG.32656.1	8341	coding	PF01299
MSTRG.32656.3	2543	coding	PF01299
MSTRG.32668.5	5225	coding	PF00888
MSTRG.32668.6	3905	coding	PF00888
MSTRG.32674.2	2852	noncoding	-
MSTRG.32672.1	1449	noncoding	-
MSTRG.32681.3	7421	coding	PF11262

MSTRG.32683.1	5760	noncoding	-
MSTRG.32682.3	9485	coding	PF00653
MSTRG.32682.4	13246	coding	PF00653
MSTRG.32686.1	5802	coding	PF08514
MSTRG.32686.3	5624	coding	PF08514
MSTRG.32687.1	7373	noncoding	-
MSTRG.32688.1	1000	noncoding	-
MSTRG.32689.1	2697	noncoding	-
MSTRG.32690.1	3595	coding	PF15186
MSTRG.32691.1	1036	noncoding	-
MSTRG.32692.1	518	noncoding	-
MSTRG.32697.1	270	noncoding	-
MSTRG.32698.1	832	noncoding	-
MSTRG.32699.1	1853	noncoding	-
MSTRG.32699.2	1824	noncoding	-
MSTRG.32699.3	1825	noncoding	-
MSTRG.32699.4	1500	noncoding	-
MSTRG.32699.5	1515	noncoding	-
MSTRG.32709.11	364	noncoding	-
MSTRG.32712.1	12883	coding	PF00176
MSTRG.32719.2	6230	coding	PF00178
MSTRG.32720.1	13803	noncoding	-
MSTRG.32718.2	1389	noncoding	-
MSTRG.32723.2	3800	coding	PF13836
MSTRG.32723.3	3957	coding	PF13836
MSTRG.32723.10	3619	coding	PF13836
MSTRG.32723.8	3843	coding	PF13836
MSTRG.32723.7	4006	coding	PF13836
MSTRG.32723.11	3840	coding	PF13836
MSTRG.32723.9	3695	coding	PF13836
MSTRG.32723.12	3838	coding	PF13836
MSTRG.32723.14	3837	coding	PF13836
MSTRG.32723.16	3838	coding	PF13836
MSTRG.32723.13	2354	noncoding	-
MSTRG.32723.15	3822	coding	PF13836
MSTRG.32723.17	2802	coding	PF13836
MSTRG.32723.18	3856	coding	PF13836
MSTRG.32721.5	1371	coding	PF00153
MSTRG.32721.6	1618	coding	PF00153
MSTRG.32730.1	2079	noncoding	-
MSTRG.32731.1	236	noncoding	-
MSTRG.32732.1	1787	coding	PF02994
MSTRG.32730.3	1963	noncoding	-
MSTRG.32738.1	2798	coding	PF00620
MSTRG.32738.2	2746	coding	PF00620
MSTRG.32738.4	4493	coding	PF00620
MSTRG.32738.5	2774	coding	PF00620
MSTRG.32738.6	2765	coding	PF00620
MSTRG.32738.7	6276	coding	PF00620
MSTRG.32739.1	4380	noncoding	-
MSTRG.32740.1	805	noncoding	-
MSTRG.32747.4	9127	noncoding	-
MSTRG.32747.9	1088	noncoding	-
MSTRG.32749.1	2113	noncoding	-

MSTRG.32751.3	3629	coding	PF16674
MSTRG.32751.2	3670	coding	PF16674
MSTRG.32752.1	294	noncoding	-
MSTRG.32749.2	892	noncoding	-
MSTRG.32752.2	916	noncoding	-
MSTRG.32752.4	843	noncoding	-
MSTRG.32752.5	548	noncoding	-
MSTRG.32752.6	548	noncoding	-
MSTRG.32752.7	524	noncoding	-
MSTRG.32752.8	524	noncoding	-
MSTRG.32752.9	675	noncoding	-
MSTRG.32754.1	1623	noncoding	-
MSTRG.32754.2	841	noncoding	-
MSTRG.32754.3	468	noncoding	-
MSTRG.32755.1	604	noncoding	-
MSTRG.32754.4	428	noncoding	-
MSTRG.32756.1	404	noncoding	-
MSTRG.32757.1	1863	noncoding	-
MSTRG.32760.1	490	noncoding	-
MSTRG.32765.1	3894	noncoding	-
MSTRG.32773.1	16420	noncoding	-
MSTRG.32767.1	1526	coding	PF00156
MSTRG.32781.1	1513	coding	PF16297
MSTRG.32782.1	1415	coding	PF16297
MSTRG.32796.3	669	noncoding	-
MSTRG.32796.5	669	noncoding	-
MSTRG.32796.4	1025	noncoding	-
MSTRG.32796.6	2637	noncoding	-
MSTRG.32796.7	2793	coding	PF02023
MSTRG.32796.9	2283	coding	PF02023
MSTRG.32796.10	2668	coding	PF02023
MSTRG.32796.8	2668	coding	PF02023
MSTRG.32796.11	2601	coding	PF02023
MSTRG.32796.12	3188	coding	PF02023
MSTRG.32796.14	238	noncoding	-
MSTRG.32796.13	2138	coding	PF02023
MSTRG.32796.15	2460	coding	PF02023
MSTRG.32796.16	1998	coding	PF02023
MSTRG.32796.17	2508	coding	PF02023
MSTRG.32796.18	2168	coding	PF02023
MSTRG.32796.19	2116	coding	PF02023
MSTRG.32796.20	1950	coding	PF02023
MSTRG.32798.3	1912	coding	PF02023
MSTRG.32798.4	1674	coding	PF02023
MSTRG.32798.5	1652	coding	PF02023
MSTRG.32798.2	1715	coding	PF02023
MSTRG.32798.6	1656	coding	PF02023
MSTRG.32798.8	2881	coding	PF02023
MSTRG.32789.1	3853	coding	PF15300
MSTRG.32792.1	535	noncoding	-
MSTRG.32801.1	5015	noncoding	-
MSTRG.32801.3	4849	noncoding	-
MSTRG.32801.4	2661	noncoding	-
MSTRG.32802.1	416	noncoding	-

MSTRG.32814.2	5178	coding	PF00621
MSTRG.32815.1	280	noncoding	-
MSTRG.32821.1	6535	coding	PF16212
MSTRG.32821.2	6154	coding	PF16212
MSTRG.32821.3	5759	coding	PF16212
MSTRG.32821.4	3633	coding	PF16212
MSTRG.32821.5	3600	coding	PF16212
MSTRG.32819.1	301	noncoding	-
MSTRG.32820.2	1588	noncoding	-
MSTRG.32827.1	548	noncoding	-
MSTRG.32828.1	511	noncoding	-
MSTRG.32832.1	217	noncoding	-
MSTRG.32832.2	382	noncoding	-
MSTRG.32836.1	6366	coding	PF13855
MSTRG.32837.1	329	noncoding	-
MSTRG.32841.1	1449	noncoding	-
MSTRG.32842.7	611	noncoding	-
MSTRG.32842.8	611	noncoding	-
MSTRG.32842.6	618	noncoding	-
MSTRG.32842.2	621	noncoding	-
MSTRG.32839.1	735	noncoding	-
MSTRG.32839.2	552	noncoding	-
MSTRG.32839.3	530	noncoding	-
MSTRG.32840.1	290	noncoding	-
MSTRG.32849.1	1681	noncoding	-
MSTRG.32859.1	778	noncoding	-
MSTRG.32860.1	25547	noncoding	-
MSTRG.32862.1	3631	noncoding	-
MSTRG.32859.2	4443	noncoding	-
MSTRG.32855.1	374	noncoding	-
MSTRG.32866.1	2736	noncoding	-
MSTRG.32867.1	2392	noncoding	-
MSTRG.32867.2	4336	noncoding	-
MSTRG.32867.3	2616	noncoding	-
MSTRG.32867.4	7193	coding	PF01359
MSTRG.32867.5	24017	noncoding	-
MSTRG.32867.6	2423	noncoding	-
MSTRG.32867.7	6538	noncoding	-
MSTRG.32863.5	2005	coding	PF12235
MSTRG.32863.6	3695	coding	PF12235
MSTRG.32864.2	1040	noncoding	-
MSTRG.32874.3	8577	coding	PF05110
MSTRG.32874.5	12940	coding	PF05110
MSTRG.32874.6	2800	coding	PF05110
MSTRG.32876.1	4440	noncoding	-
MSTRG.32877.1	14115	noncoding	-
MSTRG.32869.1	501	noncoding	-
MSTRG.32880.1	532	noncoding	-
MSTRG.32884.1	1149	noncoding	-
MSTRG.32888.1	643	noncoding	-
MSTRG.32889.4	17154	noncoding	-
MSTRG.32889.3	5202	noncoding	-
MSTRG.32889.6	10893	noncoding	-
MSTRG.32889.5	5248	noncoding	-

MSTRG.32889.7	5800	noncoding	-
MSTRG.32889.8	5383	noncoding	-
MSTRG.32889.9	8195	noncoding	-
MSTRG.32892.1	3738	coding	PF06602
MSTRG.32892.5	11240	noncoding	-
MSTRG.32893.4	6641	coding	PF06602
MSTRG.32895.3	1957	noncoding	-
MSTRG.32897.1	1024	noncoding	-
MSTRG.32899.1	261	noncoding	-
MSTRG.32906.3	2342	noncoding	-
MSTRG.32906.4	1737	noncoding	-
MSTRG.32906.6	2546	noncoding	-
MSTRG.32906.5	1759	noncoding	-
MSTRG.32907.1	326	noncoding	-
MSTRG.32916.1	985	coding	PF13774
MSTRG.32921.1	737	coding	PF06071
MSTRG.32922.2	2001	coding	PF07651
MSTRG.32922.4	1931	coding	PF07651
MSTRG.32925.1	667	coding	PF08991
MSTRG.32925.2	2391	coding	PF01840
MSTRG.32927.1	6400	noncoding	-
MSTRG.32929.1	2083	coding	PF00625
MSTRG.32930.2	2455	coding	PF08068
MSTRG.32933.1	1150	noncoding	-
MSTRG.32936.6	7889	noncoding	-
MSTRG.32936.7	3654	noncoding	-
MSTRG.32936.8	3715	noncoding	-
MSTRG.32936.9	3750	noncoding	-
MSTRG.32934.2	3558	coding	PF14893
MSTRG.32934.3	3396	coding	PF14893
MSTRG.32934.4	3606	coding	PF14893
MSTRG.32934.5	3550	coding	PF14893
MSTRG.32935.1	6297	noncoding	-
MSTRG.32937.2	1329	noncoding	-
MSTRG.32939.2	3393	coding	PF00209
MSTRG.32943.1	653	noncoding	-
MSTRG.32945.2	919	coding	PF00180
MSTRG.32946.1	862	coding	PF05404
MSTRG.32951.4	8988	noncoding	-
MSTRG.32953.1	2781	coding	PF02779
MSTRG.32957.5	8352	coding	PF00630
MSTRG.32957.3	8414	coding	PF00630
MSTRG.32954.2	1118	noncoding	-
MSTRG.32961.2	2263	coding	PF00996
MSTRG.32964.1	2562	coding	PF01437
MSTRG.32968.2	2189	coding	PF01758
MSTRG.32984.2	3010	noncoding	-
MSTRG.32984.4	873	noncoding	-
MSTRG.32984.3	822	noncoding	-
MSTRG.32984.5	3020	noncoding	-
MSTRG.32981.1	1274	noncoding	-
MSTRG.32988.1	413	noncoding	-
MSTRG.32992.1	203	noncoding	-
MSTRG.33013.3	5847	coding	PF08374

MSTRG.33013.4	4192	coding	PF08374
MSTRG.33014.1	8443	noncoding	-
MSTRG.33018.1	8152	noncoding	-
MSTRG.33003.1	203	noncoding	-
MSTRG.33021.1	9375	coding	PF02181
MSTRG.33021.2	3488	coding	PF02181
MSTRG.33021.3	8950	coding	PF02181
MSTRG.33023.1	5390	noncoding	-
MSTRG.33024.1	780	noncoding	-
MSTRG.33025.1	2600	noncoding	-
MSTRG.33031.5	1628	coding	PF00335
MSTRG.33031.6	1289	coding	PF00335
MSTRG.33033.2	2761	coding	PF09162
MSTRG.33036.1	4070	noncoding	-
MSTRG.33036.3	8516	noncoding	-
MSTRG.33050.3	1108	noncoding	-
MSTRG.33050.2	2049	noncoding	-
MSTRG.33050.5	784	noncoding	-
MSTRG.33054.1	2466	noncoding	-
MSTRG.33056.1	1952	coding	PF16499
MSTRG.33063.2	1912	coding	PF00025
MSTRG.33063.3	2021	coding	PF00025
MSTRG.33068.1	1830	noncoding	-
MSTRG.33069.1	1760	noncoding	-
MSTRG.33083.6	1954	coding	PF05712
MSTRG.33088.1	9613	noncoding	-
MSTRG.33090.2	11838	coding	PF15814
MSTRG.33090.3	3687	noncoding	-
MSTRG.33097.2	1967	coding	PF15186
MSTRG.33101.1	8176	coding	PF00069
MSTRG.33102.5	3685	noncoding	-
MSTRG.33102.4	4919	noncoding	-
MSTRG.33102.6	4842	noncoding	-
MSTRG.33114.1	4065	noncoding	-
MSTRG.33114.2	4065	noncoding	-
MSTRG.33114.3	959	noncoding	-
MSTRG.33114.4	3783	noncoding	-
MSTRG.33114.5	3794	noncoding	-
MSTRG.33114.6	3766	noncoding	-
MSTRG.33114.7	3606	noncoding	-
MSTRG.33114.8	3365	noncoding	-
MSTRG.33114.9	3333	noncoding	-
MSTRG.33109.1	630	noncoding	-
MSTRG.33108.3	3198	noncoding	-
MSTRG.33120.2	1717	noncoding	-
MSTRG.33124.3	1751	noncoding	-
MSTRG.33124.1	2103	coding	PF05064
MSTRG.33127.4	1696	coding	PF01166
MSTRG.33127.5	2102	coding	PF01166
MSTRG.33128.1	6715	noncoding	-
MSTRG.33131.1	2137	noncoding	-
MSTRG.33132.1	2405	noncoding	-
MSTRG.33131.2	2446	noncoding	-
MSTRG.33131.4	1578	coding	PF15186

MSTRG.33133.1	8503	coding	PF15186
MSTRG.33129.2	2045	noncoding	-
MSTRG.33135.1	2720	coding	PF03416
MSTRG.33138.2	679	noncoding	-
MSTRG.33141.2	2609	noncoding	-
MSTRG.33144.1	3371	noncoding	-
MSTRG.33144.2	3270	noncoding	-
MSTRG.33150.2	26017	coding	PF14808
MSTRG.33151.1	15596	noncoding	-
MSTRG.33148.1	1919	coding	PF00093
MSTRG.33155.2	1662	noncoding	-
MSTRG.33158.1	15961	coding	PF00069
MSTRG.33161.1	5103	coding	PF04101
MSTRG.33161.2	3914	noncoding	-
MSTRG.33161.3	3274	noncoding	-
MSTRG.33164.1	1510	noncoding	-
MSTRG.33165.1	1510	noncoding	-
MSTRG.33166.1	1705	noncoding	-
MSTRG.33166.2	1793	noncoding	-
MSTRG.33168.1	256	noncoding	-
MSTRG.33172.1	202	noncoding	-
MSTRG.33179.1	2434	noncoding	-
MSTRG.33189.1	994	noncoding	-
MSTRG.33224.2	3813	coding	PF07984
MSTRG.33224.3	3298	coding	PF07984
MSTRG.33223.1	239	noncoding	-
MSTRG.33234.1	364	noncoding	-
MSTRG.33248.1	824	noncoding	-
MSTRG.33249.1	1255	noncoding	-
MSTRG.33245.1	2609	coding	PF00517
MSTRG.33278.2	1489	coding	PF04487
MSTRG.33280.1	3182	noncoding	-
MSTRG.33282.4	5401	coding	PF01359
MSTRG.33284.1	5545	coding	PF12157
MSTRG.33287.3	2710	coding	PF08075
MSTRG.33301.2	2881	coding	PF00812
MSTRG.33306.3	3817	noncoding	-
MSTRG.33309.1	11481	noncoding	-
MSTRG.33309.3	3492	coding	PF02166
MSTRG.33319.1	251	noncoding	-
MSTRG.33322.3	15183	coding	PF13832
MSTRG.33322.4	15279	coding	PF13832
MSTRG.33322.5	12399	noncoding	-
MSTRG.33323.1	16673	noncoding	-
MSTRG.33325.2	3443	coding	PF01585
MSTRG.33325.4	3205	coding	PF01585
MSTRG.33325.5	3010	coding	PF01585
MSTRG.33333.1	4153	coding	PF00069
MSTRG.33333.2	3563	coding	PF00069
MSTRG.33333.5	5324	coding	PF00069
MSTRG.33333.4	7127	coding	PF00069
MSTRG.33328.1	4716	coding	PF01352
MSTRG.33329.1	2561	noncoding	-
MSTRG.33329.2	2729	coding	PF00965

MSTRG.33329.4	2350	noncoding	-
MSTRG.33329.5	928	coding	PF00965
MSTRG.33338.1	2349	noncoding	-
MSTRG.33338.3	2387	coding	PF01352
MSTRG.33339.1	426	noncoding	-
MSTRG.33348.1	357	noncoding	-
MSTRG.33367.7	3271	coding	PF00850
MSTRG.33373.2	3769	noncoding	-
MSTRG.33373.3	3940	noncoding	-
MSTRG.33373.5	3569	noncoding	-
MSTRG.33373.4	4039	noncoding	-
MSTRG.33373.8	2053	noncoding	-
MSTRG.33374.1	3449	noncoding	-
MSTRG.33369.2	2929	noncoding	-
MSTRG.33369.3	2946	noncoding	-
MSTRG.33376.1	560	noncoding	-
MSTRG.33386.1	706	noncoding	-
MSTRG.33388.1	969	noncoding	-
MSTRG.33389.2	2860	coding	PF05716
MSTRG.33391.1	7427	coding	PF00609
MSTRG.33398.2	2163	noncoding	-
MSTRG.33398.1	2243	noncoding	-
MSTRG.33400.2	3426	noncoding	-
MSTRG.33400.1	3266	noncoding	-
MSTRG.33400.5	2798	noncoding	-
MSTRG.33403.5	3916	noncoding	-
MSTRG.33404.1	2238	noncoding	-
MSTRG.33405.2	2457	noncoding	-
MSTRG.33405.3	3297	noncoding	-
MSTRG.33417.1	518	noncoding	-
MSTRG.33419.13	560	coding	PF05831
MSTRG.33419.20	691	coding	PF05831
MSTRG.33419.23	553	coding	PF05831
MSTRG.33419.24	556	coding	PF05831
MSTRG.33408.3	2846	noncoding	-
MSTRG.33423.3	4668	coding	PF02373
MSTRG.33423.4	4794	coding	PF02373
MSTRG.33424.1	5153	coding	PF16453
MSTRG.33424.2	4988	coding	PF16453
MSTRG.33424.3	4299	coding	PF16453
MSTRG.33443.13	10894	coding	PF00632
MSTRG.33443.9	14085	coding	PF00632
MSTRG.33443.3	14237	coding	PF00632
MSTRG.33443.11	14184	coding	PF00632
MSTRG.33443.14	14304	coding	PF00632
MSTRG.33443.6	14118	coding	PF00632
MSTRG.33443.5	14003	coding	PF00632
MSTRG.33443.15	40284	coding	PF00632
MSTRG.33448.8	5953	noncoding	-
MSTRG.33450.1	4350	noncoding	-
MSTRG.33451.1	3584	noncoding	-
MSTRG.33452.1	4050	noncoding	-
MSTRG.33453.1	9055	coding	PF00069
MSTRG.33453.2	9795	coding	PF00069

MSTRG.33453.3	9795	coding	PF00069
MSTRG.33453.4	8822	coding	PF00069
MSTRG.33453.5	13472	coding	PF00069
MSTRG.33453.6	5583	coding	PF00069
MSTRG.33430.1	317	noncoding	-
MSTRG.33439.2	2140	coding	PF01926
MSTRG.33439.3	3945	coding	PF01926
MSTRG.33444.1	1359	noncoding	-
MSTRG.33466.2	2050	coding	PF04670
MSTRG.33467.1	203	noncoding	-
MSTRG.33482.1	2061	noncoding	-
MSTRG.33482.3	2159	coding	PF04031
MSTRG.33483.1	1243	noncoding	-
MSTRG.33482.5	2524	coding	PF04031
MSTRG.33482.6	2059	noncoding	-
MSTRG.33482.7	2038	noncoding	-
MSTRG.33482.8	2029	noncoding	-
MSTRG.33519.1	2898	coding	PF15909
MSTRG.33521.1	3684	coding	PF15909
MSTRG.33525.1	447	noncoding	-
MSTRG.33534.1	1085	noncoding	-
MSTRG.33534.2	959	noncoding	-
MSTRG.33535.4	6928	noncoding	-
MSTRG.33535.5	5066	noncoding	-
MSTRG.33535.6	4549	coding	PF00782
MSTRG.33536.1	480	noncoding	-
MSTRG.33535.7	4745	coding	PF00782
MSTRG.33537.1	1259	noncoding	-
MSTRG.33535.8	1636	noncoding	-
MSTRG.33535.9	789	coding	PF00782
MSTRG.33546.1	4691	coding	PF00270
MSTRG.33546.3	4737	coding	PF00270
MSTRG.33548.4	9209	coding	PF00443
MSTRG.33553.1	6923	coding	PF08638
MSTRG.33553.2	6827	coding	PF08638
MSTRG.33553.3	6692	coding	PF08638
MSTRG.33555.1	4517	noncoding	-
MSTRG.33553.8	4370	coding	PF08638
MSTRG.33550.2	715	coding	PF03650
MSTRG.33559.4	5313	coding	PF15808
MSTRG.33568.1	452	noncoding	-
MSTRG.33573.1	11930	coding	PF05147
MSTRG.33577.2	8498	coding	PF02319
MSTRG.33577.1	4437	noncoding	-
MSTRG.33587.3	622	noncoding	-
MSTRG.33609.2	6822	noncoding	-
MSTRG.33614.1	1675	noncoding	-
MSTRG.33613.2	1630	noncoding	-
MSTRG.33619.1	1655	noncoding	-
MSTRG.33619.2	1566	noncoding	-
MSTRG.33627.2	1814	coding	PF12440
MSTRG.33629.2	5266	noncoding	-
MSTRG.33629.1	3709	noncoding	-
MSTRG.33629.4	4470	noncoding	-

MSTRG.33629.6	3510	noncoding	-
MSTRG.33629.3	3714	noncoding	-
MSTRG.33629.5	3702	noncoding	-
MSTRG.33629.7	3707	noncoding	-
MSTRG.33629.10	3514	noncoding	-
MSTRG.33629.8	3502	noncoding	-
MSTRG.33629.9	3514	noncoding	-
MSTRG.33629.11	2518	noncoding	-
MSTRG.33634.2	4834	coding	PF00136
MSTRG.33634.4	1973	noncoding	-
MSTRG.33639.6	2918	coding	PF04704
MSTRG.33639.7	3782	noncoding	-
MSTRG.33648.2	1113	coding	PF00578
MSTRG.33648.5	1162	coding	PF00578
MSTRG.33649.1	3453	coding	PF00270
MSTRG.33649.3	3208	coding	PF00270
MSTRG.33654.2	14771	noncoding	-
MSTRG.33658.1	911	coding	PF00335
MSTRG.33662.3	6463	coding	PF00069
MSTRG.33662.4	7945	coding	PF00069
MSTRG.33661.1	3954	coding	PF01176
MSTRG.33664.6	2617	coding	PF05672
MSTRG.33664.2	3685	coding	PF05672
MSTRG.33664.3	5684	coding	PF05672
MSTRG.33664.4	3709	coding	PF05672
MSTRG.33664.7	3607	coding	PF05672
MSTRG.33664.8	3606	coding	PF05672
MSTRG.33664.9	3504	coding	PF05672
MSTRG.33664.10	4111	noncoding	-
MSTRG.33666.1	1828	coding	PF14949
MSTRG.33669.2	4736	noncoding	-
MSTRG.33669.1	8060	noncoding	-
MSTRG.33670.2	3912	coding	PF00723
MSTRG.33676.1	3330	noncoding	-
MSTRG.33676.2	3353	noncoding	-
MSTRG.33676.3	2768	noncoding	-
MSTRG.33679.1	1241	noncoding	-
MSTRG.33679.2	1186	noncoding	-
MSTRG.33681.1	3116	coding	PF00069
MSTRG.33682.1	1347	noncoding	-
MSTRG.33683.1	7932	noncoding	-
MSTRG.33683.2	7305	noncoding	-
MSTRG.33683.3	5604	noncoding	-
MSTRG.33686.2	1869	noncoding	-
MSTRG.33687.3	3167	noncoding	-
MSTRG.33688.2	11588	coding	PF15273
MSTRG.33690.1	22728	noncoding	-
MSTRG.33690.2	8361	noncoding	-
MSTRG.33692.1	27856	noncoding	-
MSTRG.33692.2	39747	noncoding	-
MSTRG.33692.3	30001	noncoding	-
MSTRG.33709.1	1551	noncoding	-
MSTRG.33709.2	1486	noncoding	-
MSTRG.33709.3	1574	noncoding	-

MSTRG.33709.4	1448	noncoding	-
MSTRG.33709.5	1521	noncoding	-
MSTRG.33709.6	1386	noncoding	-
MSTRG.33712.1	2127	coding	PF08288
MSTRG.33712.2	3609	coding	PF08288
MSTRG.33713.1	1470	coding	PF12796
MSTRG.33714.1	3659	noncoding	-
MSTRG.33729.1	4859	coding	PF01275
MSTRG.33731.1	3205	noncoding	-
MSTRG.33744.3	2106	coding	PF05712
MSTRG.33754.2	592	noncoding	-
MSTRG.33754.1	592	noncoding	-
MSTRG.33754.4	592	noncoding	-
MSTRG.33754.5	592	noncoding	-
MSTRG.33754.3	663	noncoding	-
MSTRG.33754.6	471	noncoding	-
MSTRG.33756.2	2680	coding	PF00654
MSTRG.33760.1	456	noncoding	-
MSTRG.33760.2	681	noncoding	-
MSTRG.33764.1	535	noncoding	-
MSTRG.33758.1	552	noncoding	-
MSTRG.33768.1	3776	coding	PF02101
MSTRG.33768.2	20105	noncoding	-
MSTRG.33768.3	18637	noncoding	-
MSTRG.33768.4	2571	coding	PF00400
MSTRG.33769.1	7810	noncoding	-
MSTRG.33770.1	6551	noncoding	-
MSTRG.33768.5	3276	noncoding	-
MSTRG.33768.6	5330	noncoding	-
MSTRG.33771.1	6720	noncoding	-
MSTRG.33772.1	4106	noncoding	-
MSTRG.33773.1	7223	noncoding	-
MSTRG.33787.1	1839	noncoding	-
MSTRG.33789.1	777	noncoding	-
MSTRG.33792.1	696	noncoding	-
MSTRG.33793.1	945	noncoding	-
MSTRG.33791.1	883	noncoding	-
MSTRG.33813.2	6279	coding	PF00069
MSTRG.33813.4	8526	coding	PF00069
MSTRG.33813.5	9570	coding	PF00069
MSTRG.33813.6	6574	coding	PF00069
MSTRG.33815.1	7704	noncoding	-
MSTRG.33817.1	2034	noncoding	-
MSTRG.33819.2	1513	coding	PF00884
MSTRG.33820.1	5127	noncoding	-
MSTRG.33820.2	4650	noncoding	-
MSTRG.33820.4	4357	noncoding	-
MSTRG.33822.1	432	noncoding	-
MSTRG.33824.1	801	noncoding	-
MSTRG.33817.2	4176	coding	PF00106
MSTRG.33817.4	4421	noncoding	-
MSTRG.33804.1	637	noncoding	-
MSTRG.33803.1	224	noncoding	-
MSTRG.33805.1	323	noncoding	-

MSTRG.33806.1	366	noncoding	-
MSTRG.33806.2	505	noncoding	-
MSTRG.33809.1	462	noncoding	-
MSTRG.33810.1	1288	noncoding	-
MSTRG.33811.1	1104	noncoding	-
MSTRG.33812.1	807	noncoding	-
MSTRG.33812.2	412	noncoding	-
MSTRG.33846.1	5744	coding	PF00069
MSTRG.33829.1	2136	noncoding	-
MSTRG.33831.1	5593	noncoding	-
MSTRG.33831.2	3972	noncoding	-
MSTRG.33831.3	2221	noncoding	-
MSTRG.33831.4	1592	noncoding	-
MSTRG.33832.1	2571	noncoding	-
MSTRG.33832.2	2761	noncoding	-
MSTRG.33838.1	336	noncoding	-
MSTRG.33839.1	429	noncoding	-
MSTRG.33839.2	364	noncoding	-
MSTRG.33841.1	2961	noncoding	-
MSTRG.33843.1	4023	coding	PF00510
MSTRG.33851.1	405	noncoding	-
MSTRG.33852.1	1050	noncoding	-
MSTRG.33858.1	642	noncoding	-
MSTRG.33860.1	1097	noncoding	-
MSTRG.33861.1	1783	noncoding	-
MSTRG.33863.1	890	noncoding	-
MSTRG.33865.1	635	noncoding	-
MSTRG.33868.1	8432	noncoding	-
MSTRG.33868.2	5151	noncoding	-
MSTRG.33869.1	301	noncoding	-
MSTRG.33869.2	281	noncoding	-
MSTRG.33875.1	3793	noncoding	-
MSTRG.33875.2	5331	noncoding	-
MSTRG.33875.4	2959	noncoding	-
MSTRG.33875.6	3427	noncoding	-
MSTRG.33874.1	334	noncoding	-
MSTRG.33879.1	1291	noncoding	-
MSTRG.33882.1	2586	noncoding	-
MSTRG.33883.1	336	noncoding	-
MSTRG.33886.1	1217	noncoding	-
MSTRG.33880.1	1008	noncoding	-
MSTRG.33881.1	410	noncoding	-
MSTRG.33896.2	5352	coding	PF09162
MSTRG.33896.3	6517	coding	PF09162
MSTRG.33896.1	5839	coding	PF09162
MSTRG.33896.4	3857	coding	PF09162
MSTRG.33896.5	3330	coding	PF09162
MSTRG.33896.6	3358	coding	PF09162
MSTRG.33894.2	1032	noncoding	-
MSTRG.33898.1	759	noncoding	-
MSTRG.33898.2	759	noncoding	-
MSTRG.33898.3	2105	noncoding	-
MSTRG.33898.4	371	noncoding	-
MSTRG.33898.5	923	noncoding	-

MSTRG.33898.6	871	noncoding	-
MSTRG.33899.1	1504	noncoding	-
MSTRG.33899.2	2090	noncoding	-
MSTRG.33899.3	2091	noncoding	-
MSTRG.33899.4	2091	noncoding	-
MSTRG.33898.7	414	noncoding	-
MSTRG.33901.1	839	noncoding	-
MSTRG.33901.2	804	noncoding	-
MSTRG.33901.3	1772	noncoding	-
MSTRG.33901.4	1373	coding	PF00069
MSTRG.33901.5	832	noncoding	-
MSTRG.33901.6	368	noncoding	-
MSTRG.33901.7	201	noncoding	-
MSTRG.33901.8	804	noncoding	-
MSTRG.33901.9	482	noncoding	-
MSTRG.33903.1	2170	noncoding	-
MSTRG.33910.1	244	noncoding	-
MSTRG.33910.2	322	noncoding	-
MSTRG.33914.2	2269	noncoding	-
MSTRG.33914.1	2309	noncoding	-
MSTRG.33914.3	1692	noncoding	-
MSTRG.33917.1	319	noncoding	-
MSTRG.33911.1	276	noncoding	-
MSTRG.33920.2	3013	noncoding	-
MSTRG.33920.3	2200	noncoding	-
MSTRG.33921.2	1720	noncoding	-
MSTRG.33921.3	3594	noncoding	-
MSTRG.33922.1	719	noncoding	-
MSTRG.33922.2	432	noncoding	-
MSTRG.33949.1	449	noncoding	-
MSTRG.33949.2	363	noncoding	-
MSTRG.33949.3	609	noncoding	-
MSTRG.33950.2	4605	noncoding	-
MSTRG.33950.5	5598	noncoding	-
MSTRG.33950.6	5540	noncoding	-
MSTRG.33950.7	4713	noncoding	-
MSTRG.33950.12	1186	noncoding	-
MSTRG.33950.10	3821	noncoding	-
MSTRG.33950.13	12321	noncoding	-
MSTRG.33950.14	12470	noncoding	-
MSTRG.33950.9	12387	noncoding	-
MSTRG.33950.11	2288	noncoding	-
MSTRG.33950.15	15199	coding	PF02093
MSTRG.33950.17	5081	noncoding	-
MSTRG.33950.19	868	noncoding	-
MSTRG.33953.1	1731	noncoding	-
MSTRG.33931.1	4364	noncoding	-
MSTRG.33932.1	373	noncoding	-
MSTRG.33931.2	1808	noncoding	-
MSTRG.33933.1	778	noncoding	-
MSTRG.33934.1	538	coding	PF00069
MSTRG.33928.1	331	noncoding	-
MSTRG.33929.1	2631	noncoding	-
MSTRG.33935.1	434	noncoding	-

MSTRG.33941.2	1829	noncoding	-
MSTRG.33944.1	909	noncoding	-
MSTRG.33946.1	596	noncoding	-
MSTRG.33946.2	596	noncoding	-
MSTRG.33946.3	360	noncoding	-
MSTRG.33947.1	305	noncoding	-
MSTRG.33960.1	4133	noncoding	-
MSTRG.33960.2	9154	coding	PF02337
MSTRG.33960.7	1725	noncoding	-
MSTRG.33960.8	1213	noncoding	-
MSTRG.33960.9	1108	noncoding	-
MSTRG.33960.10	1301	noncoding	-
MSTRG.33960.11	1304	noncoding	-
MSTRG.33963.1	422	noncoding	-
MSTRG.33964.1	1989	noncoding	-
MSTRG.33965.1	1194	noncoding	-
MSTRG.33965.2	775	noncoding	-
MSTRG.33966.1	278	noncoding	-
MSTRG.33962.1	311	noncoding	-
MSTRG.33962.2	309	noncoding	-
MSTRG.33967.1	268	noncoding	-
MSTRG.33985.4	9208	noncoding	-
MSTRG.33985.7	16711	noncoding	-
MSTRG.33985.10	5833	noncoding	-
MSTRG.33985.9	2634	noncoding	-
MSTRG.33985.11	16669	noncoding	-
MSTRG.33979.1	846	noncoding	-
MSTRG.33986.1	1296	coding	PF00069
MSTRG.33986.2	625	coding	PF00069
MSTRG.33986.3	451	noncoding	-
MSTRG.33986.4	700	noncoding	-
MSTRG.33987.1	696	noncoding	-
MSTRG.33986.5	665	noncoding	-
MSTRG.33992.1	482	coding	PF00069
MSTRG.33993.1	874	noncoding	-
MSTRG.33993.2	875	noncoding	-
MSTRG.33998.1	359	noncoding	-
MSTRG.33999.1	249	noncoding	-
MSTRG.34000.2	2935	noncoding	-
MSTRG.34000.3	5338	noncoding	-
MSTRG.34000.1	2413	noncoding	-
MSTRG.34000.4	6316	noncoding	-
MSTRG.34000.5	6316	noncoding	-
MSTRG.34000.6	7346	noncoding	-
MSTRG.34016.1	638	noncoding	-
MSTRG.34016.2	524	noncoding	-
MSTRG.34016.4	502	noncoding	-
MSTRG.34016.5	1640	noncoding	-
MSTRG.34016.6	1640	noncoding	-
MSTRG.34016.3	1666	noncoding	-
MSTRG.34016.7	504	noncoding	-
MSTRG.34016.8	606	noncoding	-
MSTRG.34016.9	732	noncoding	-
MSTRG.34016.10	1301	noncoding	-

MSTRG.34016.11	936	noncoding	-
MSTRG.34016.12	924	noncoding	-
MSTRG.34016.13	431	noncoding	-
MSTRG.34016.15	1588	noncoding	-
MSTRG.34016.14	1599	noncoding	-
MSTRG.34016.16	1580	noncoding	-
MSTRG.34016.17	1108	noncoding	-
MSTRG.34016.18	1097	noncoding	-
MSTRG.34012.1	744	noncoding	-
MSTRG.34013.1	350	noncoding	-
MSTRG.34017.1	867	noncoding	-
MSTRG.34017.3	308	noncoding	-
MSTRG.34017.2	1438	noncoding	-
MSTRG.34017.4	611	noncoding	-
MSTRG.34017.5	1020	noncoding	-
MSTRG.34018.1	556	noncoding	-
MSTRG.34026.2	402	noncoding	-
MSTRG.34028.1	476	noncoding	-
MSTRG.34035.1	952	coding	PF14915
MSTRG.34037.1	213	noncoding	-
MSTRG.34038.1	392	noncoding	-
MSTRG.34046.1	1948	noncoding	-
MSTRG.34046.2	2047	noncoding	-
MSTRG.34046.3	1724	noncoding	-
MSTRG.34046.4	5800	noncoding	-
MSTRG.34046.5	2116	noncoding	-
MSTRG.34046.6	6142	noncoding	-
MSTRG.34047.2	456	noncoding	-
MSTRG.34052.1	1149	noncoding	-
MSTRG.34053.1	419	noncoding	-
MSTRG.34053.2	397	noncoding	-
MSTRG.34055.1	843	noncoding	-
MSTRG.34056.2	2800	noncoding	-
MSTRG.34066.1	4438	noncoding	-
MSTRG.34072.1	323	noncoding	-
MSTRG.34072.2	368	noncoding	-
MSTRG.34072.3	322	noncoding	-
MSTRG.34072.4	322	noncoding	-
MSTRG.34072.5	276	noncoding	-
MSTRG.34088.1	497	noncoding	-
MSTRG.34090.1	295	noncoding	-
MSTRG.34088.2	278	noncoding	-
MSTRG.34084.1	243	noncoding	-
MSTRG.34085.1	214	noncoding	-
MSTRG.34093.1	1485	coding	PF00005
MSTRG.34094.1	1495	coding	PF00005
MSTRG.34101.1	975	noncoding	-
MSTRG.34097.1	229	noncoding	-
MSTRG.34108.2	1671	noncoding	-
MSTRG.34110.1	767	noncoding	-
MSTRG.34110.2	593	noncoding	-
MSTRG.34129.1	701	noncoding	-
MSTRG.34130.3	1076	noncoding	-
MSTRG.34130.1	1074	noncoding	-

MSTRG.34130.2	1075	noncoding	-
MSTRG.34131.1	620	noncoding	-
MSTRG.34131.2	519	noncoding	-
MSTRG.34130.4	2247	noncoding	-
MSTRG.34130.5	1076	noncoding	-
MSTRG.34135.3	411	noncoding	-
MSTRG.34135.1	414	noncoding	-
MSTRG.34135.2	414	noncoding	-
MSTRG.34135.4	413	noncoding	-
MSTRG.34136.1	1350	noncoding	-
MSTRG.34135.5	457	noncoding	-
MSTRG.34137.3	878	noncoding	-
MSTRG.34137.1	870	noncoding	-
MSTRG.34137.2	870	noncoding	-
MSTRG.34135.6	615	noncoding	-
MSTRG.34133.1	377	noncoding	-
MSTRG.34146.1	3239	coding	PF00005
MSTRG.34149.1	7834	coding	PF02994
MSTRG.34152.1	1043	noncoding	-
MSTRG.34153.1	305	noncoding	-
MSTRG.34185.7	6469	coding	PF00069
MSTRG.34185.8	1067	noncoding	-
MSTRG.34185.10	1440	coding	PF00069
MSTRG.34185.9	738	noncoding	-
MSTRG.34185.11	1181	noncoding	-
MSTRG.34185.12	1115	noncoding	-
MSTRG.34185.13	617	noncoding	-
MSTRG.34185.15	8522	coding	PF00069
MSTRG.34185.14	3616	coding	PF00069
MSTRG.34185.16	723	noncoding	-
MSTRG.34185.17	7866	coding	PF00069
MSTRG.34185.18	8024	coding	PF00069
MSTRG.34185.19	1079	noncoding	-
MSTRG.34185.20	1010	noncoding	-
MSTRG.34185.21	1612	noncoding	-
MSTRG.34185.22	3509	coding	PF00069
MSTRG.34185.23	343	coding	PF00069
MSTRG.34154.1	289	noncoding	-
MSTRG.34155.1	270	noncoding	-
MSTRG.34154.2	264	noncoding	-
MSTRG.34161.2	2453	noncoding	-
MSTRG.34161.3	1687	noncoding	-
MSTRG.34159.1	1033	noncoding	-
MSTRG.34164.1	1507	coding	PF00069
MSTRG.34164.2	1639	noncoding	-
MSTRG.34171.2	2633	coding	PF14915
MSTRG.34171.5	2704	coding	PF14915
MSTRG.34171.6	2472	coding	PF14915
MSTRG.34174.1	253	noncoding	-
MSTRG.34178.1	673	noncoding	-
MSTRG.34183.2	2766	coding	PF12796
MSTRG.34183.3	1519	coding	PF12796
MSTRG.34183.4	658	noncoding	-
MSTRG.34190.1	3216	coding	PF14915

MSTRG.34190.2	3225	coding	PF14915
MSTRG.34190.6	2437	coding	PF14915
MSTRG.34190.9	2044	coding	PF12796
MSTRG.34169.1	916	noncoding	-
MSTRG.34196.1	3898	coding	PF02994
MSTRG.34198.3	629	coding	PF05831
MSTRG.34198.4	587	coding	PF05831
MSTRG.34206.1	453	noncoding	-
MSTRG.34202.1	2175	noncoding	-
MSTRG.34209.1	751	noncoding	-
MSTRG.34209.2	475	noncoding	-
MSTRG.34210.1	352	noncoding	-
MSTRG.34210.2	374	noncoding	-
MSTRG.34219.1	283	noncoding	-
MSTRG.34220.1	305	noncoding	-
MSTRG.34219.2	283	noncoding	-
MSTRG.34220.2	294	noncoding	-
MSTRG.34220.3	396	noncoding	-
MSTRG.34219.3	374	noncoding	-
MSTRG.34225.1	730	noncoding	-
MSTRG.34226.1	802	noncoding	-
MSTRG.34235.1	292	noncoding	-
MSTRG.34238.1	544	noncoding	-
MSTRG.34252.1	1253	noncoding	-
MSTRG.34252.2	1161	noncoding	-
MSTRG.34252.3	1490	noncoding	-
MSTRG.34252.4	1490	noncoding	-
MSTRG.34272.1	14431	noncoding	-
MSTRG.34272.2	14677	noncoding	-
MSTRG.34272.4	15865	noncoding	-
MSTRG.34272.3	13428	noncoding	-
MSTRG.34272.6	13387	noncoding	-
MSTRG.34272.5	14223	noncoding	-
MSTRG.34272.7	11741	noncoding	-
MSTRG.34272.8	13359	noncoding	-
MSTRG.34272.9	13285	noncoding	-
MSTRG.34293.1	1627	noncoding	-
MSTRG.34294.1	776	noncoding	-
MSTRG.34294.2	651	noncoding	-
MSTRG.34295.1	9109	noncoding	-
MSTRG.34295.2	4015	noncoding	-
MSTRG.34295.4	1465	noncoding	-
MSTRG.34295.3	1549	noncoding	-
MSTRG.34295.5	1600	noncoding	-
MSTRG.34294.3	21921	noncoding	-
MSTRG.34294.4	17825	noncoding	-
MSTRG.34294.6	15603	noncoding	-
MSTRG.34294.5	15556	noncoding	-
MSTRG.34295.6	4353	noncoding	-
MSTRG.34294.8	1131	noncoding	-
MSTRG.34294.7	14619	noncoding	-
MSTRG.34294.9	23293	noncoding	-
MSTRG.34295.7	357	noncoding	-
MSTRG.34294.10	9109	noncoding	-

MSTRG.34294.11	1574	noncoding	-
MSTRG.34298.1	341	noncoding	-
MSTRG.34298.2	337	noncoding	-
MSTRG.34299.1	412	noncoding	-
MSTRG.34282.1	1219	noncoding	-
MSTRG.34283.1	211	noncoding	-
MSTRG.34284.1	522	noncoding	-
MSTRG.34287.1	389	noncoding	-
MSTRG.34300.1	368	coding	PF00069
MSTRG.34302.1	681	coding	PF00069
MSTRG.34303.1	430	noncoding	-
MSTRG.34314.1	387	noncoding	-
MSTRG.34314.3	3225	noncoding	-
MSTRG.34314.4	4987	noncoding	-
MSTRG.34314.2	5283	noncoding	-
MSTRG.34315.1	37600	coding	PF00069
MSTRG.34315.2	11420	coding	PF00069
MSTRG.34315.3	3774	noncoding	-
MSTRG.34315.4	11534	coding	PF02994
MSTRG.34317.2	7198	noncoding	-
MSTRG.34317.3	3456	noncoding	-
MSTRG.34315.5	17952	noncoding	-
MSTRG.34315.6	1939	noncoding	-
MSTRG.34315.7	5982	noncoding	-
MSTRG.34315.8	2503	noncoding	-
MSTRG.34315.10	2078	noncoding	-
MSTRG.34315.11	7155	coding	PF00069
MSTRG.34315.9	4610	noncoding	-
MSTRG.34304.1	202	noncoding	-
MSTRG.34305.1	550	noncoding	-
MSTRG.34306.2	857	noncoding	-
MSTRG.34306.1	442	noncoding	-
MSTRG.34307.1	450	noncoding	-
MSTRG.34307.2	479	noncoding	-
MSTRG.34308.1	591	noncoding	-
MSTRG.34321.1	1929	noncoding	-
MSTRG.34328.1	951	noncoding	-
MSTRG.34330.1	336	noncoding	-
MSTRG.34327.1	582	noncoding	-
MSTRG.34334.2	784	noncoding	-
MSTRG.34346.1	767	noncoding	-
MSTRG.34346.2	1228	noncoding	-
MSTRG.34346.3	414	noncoding	-
MSTRG.34346.4	414	noncoding	-
MSTRG.34342.1	313	noncoding	-
MSTRG.34342.2	313	noncoding	-
MSTRG.34351.1	1515	coding	PF00069
MSTRG.34351.3	803	noncoding	-
MSTRG.34351.2	754	coding	PF00069
MSTRG.34351.4	1848	coding	PF00069
MSTRG.34351.5	828	coding	PF00069
MSTRG.34351.6	325	noncoding	-
MSTRG.34351.7	248	noncoding	-
MSTRG.34351.8	283	noncoding	-

MSTRG.34349.1	384	noncoding	-
MSTRG.34352.1	2105	noncoding	-
MSTRG.34352.2	548	noncoding	-
MSTRG.34369.1	11802	coding	PF14915
MSTRG.34369.2	8795	coding	PF12001
MSTRG.34369.3	12369	coding	PF12001
MSTRG.34369.4	9595	coding	PF12001
MSTRG.34369.5	8894	coding	PF12001
MSTRG.34369.7	2694	noncoding	-
MSTRG.34369.8	7818	coding	PF14915
MSTRG.34369.9	5634	noncoding	-
MSTRG.34369.10	1877	noncoding	-
MSTRG.34369.11	1749	coding	PF14915
MSTRG.34356.1	782	noncoding	-
MSTRG.34356.2	433	noncoding	-
MSTRG.34357.1	836	noncoding	-
MSTRG.34355.1	1118	noncoding	-
MSTRG.34363.2	1640	noncoding	-
MSTRG.34363.3	2409	noncoding	-
MSTRG.34365.2	3968	noncoding	-
MSTRG.34365.3	4058	noncoding	-
MSTRG.34365.4	267	noncoding	-
MSTRG.34361.1	470	noncoding	-
MSTRG.34373.1	706	noncoding	-
MSTRG.34373.2	808	noncoding	-
MSTRG.34373.3	706	noncoding	-
MSTRG.34374.1	506	noncoding	-
MSTRG.34374.2	445	noncoding	-
MSTRG.34374.3	354	noncoding	-
MSTRG.34375.1	1077	noncoding	-
MSTRG.34375.2	1079	noncoding	-
MSTRG.34373.4	1025	noncoding	-
MSTRG.34373.5	1025	noncoding	-
MSTRG.34376.5	1065	noncoding	-
MSTRG.34376.2	571	noncoding	-
MSTRG.34376.4	1051	noncoding	-
MSTRG.34376.3	1076	noncoding	-
MSTRG.34376.6	3404	noncoding	-
MSTRG.34376.7	1076	noncoding	-
MSTRG.34376.1	1124	noncoding	-
MSTRG.34383.1	945	noncoding	-
MSTRG.34383.2	877	noncoding	-
MSTRG.34383.3	422	noncoding	-
MSTRG.34384.1	393	noncoding	-
MSTRG.34389.1	435	noncoding	-
MSTRG.34390.1	1138	noncoding	-
MSTRG.34391.1	384	noncoding	-
MSTRG.34393.1	6576	noncoding	-
MSTRG.34395.2	1394	noncoding	-
MSTRG.34394.1	1015	noncoding	-
MSTRG.34394.3	1819	noncoding	-
MSTRG.34400.1	769	noncoding	-
MSTRG.34401.2	263	noncoding	-
MSTRG.34401.1	254	noncoding	-

MSTRG.34402.1	357	noncoding	-
MSTRG.34402.2	357	noncoding	-
MSTRG.34401.3	599	noncoding	-
MSTRG.34402.3	466	noncoding	-
MSTRG.34400.2	1146	noncoding	-
MSTRG.34411.1	459	coding	PF02994
MSTRG.34414.1	7020	coding	PF00069
MSTRG.34414.2	4926	coding	PF00069
MSTRG.34414.3	4923	coding	PF00069
MSTRG.34416.1	600	noncoding	-
MSTRG.34435.10	2830	coding	PF09162
MSTRG.34435.7	2816	coding	PF09162
MSTRG.34435.8	2770	coding	PF09162
MSTRG.34435.9	2830	coding	PF09162
MSTRG.34435.2	2833	coding	PF09162
MSTRG.34418.1	475	noncoding	-
MSTRG.34420.1	347	noncoding	-
MSTRG.34421.1	261	noncoding	-
MSTRG.34422.1	582	noncoding	-
MSTRG.34427.1	1510	noncoding	-
MSTRG.34427.2	1936	coding	PF00069
MSTRG.34427.3	1921	coding	PF00069
MSTRG.34429.2	267	noncoding	-
MSTRG.34429.1	622	coding	PF00069
MSTRG.34438.1	286	noncoding	-
MSTRG.34439.1	362	noncoding	-
MSTRG.34449.1	325	noncoding	-
MSTRG.34449.2	1991	noncoding	-
MSTRG.34449.3	1232	coding	PF00069
MSTRG.34449.4	819	noncoding	-
MSTRG.34449.5	2208	noncoding	-
MSTRG.34449.6	2202	noncoding	-
MSTRG.34449.7	2199	noncoding	-
MSTRG.34449.9	2595	noncoding	-
MSTRG.34449.8	2603	noncoding	-
MSTRG.34449.10	1380	noncoding	-
MSTRG.34446.1	880	noncoding	-
MSTRG.34446.2	245	noncoding	-
MSTRG.34452.1	760	noncoding	-
MSTRG.34452.2	297	noncoding	-
MSTRG.34458.1	961	noncoding	-
MSTRG.34460.1	541	noncoding	-
MSTRG.34455.1	418	coding	PF00069
MSTRG.34455.2	354	noncoding	-
MSTRG.34467.1	567	noncoding	-
MSTRG.34471.1	443	noncoding	-
MSTRG.34478.1	4713	noncoding	-
MSTRG.34482.3	951	noncoding	-
MSTRG.34481.1	576	noncoding	-
MSTRG.34486.1	228	noncoding	-
MSTRG.34489.1	1531	noncoding	-
MSTRG.34501.1	435	coding	PF00164
MSTRG.34505.1	2056	coding	PF00005
MSTRG.34511.1	1484	noncoding	-

MSTRG.34511.2	670	noncoding	-
MSTRG.34511.3	670	noncoding	-
MSTRG.34511.4	633	noncoding	-
MSTRG.34512.1	638	noncoding	-
MSTRG.34513.1	421	noncoding	-
MSTRG.34514.1	283	noncoding	-
MSTRG.34515.1	411	noncoding	-
MSTRG.34519.2	663	noncoding	-
MSTRG.34519.1	1553	noncoding	-
MSTRG.34519.3	480	noncoding	-
MSTRG.34519.4	480	noncoding	-
MSTRG.34519.5	1107	noncoding	-
MSTRG.34519.6	1109	noncoding	-
MSTRG.34520.1	488	noncoding	-
MSTRG.34521.1	205	noncoding	-
MSTRG.34519.7	480	noncoding	-
MSTRG.34524.1	757	coding	PF00069
MSTRG.34524.2	505	coding	PF00069
MSTRG.34524.3	617	coding	PF00069
MSTRG.34524.4	727	coding	PF00069
MSTRG.34524.5	726	coding	PF00069
MSTRG.34528.2	233	noncoding	-
MSTRG.34528.1	1579	noncoding	-
MSTRG.34529.1	1064	noncoding	-
MSTRG.34537.1	7510	coding	PF00069
MSTRG.34537.3	2932	noncoding	-
MSTRG.34537.2	769	coding	PF00069
MSTRG.34537.4	6508	coding	PF00069
MSTRG.34532.1	248	noncoding	-
MSTRG.34554.1	1236	noncoding	-
MSTRG.34556.1	873	noncoding	-
MSTRG.34554.2	3684	noncoding	-
MSTRG.34557.2	1375	noncoding	-
MSTRG.34557.1	1185	noncoding	-
MSTRG.34557.4	3135	noncoding	-
MSTRG.34550.1	789	noncoding	-
MSTRG.34550.2	302	noncoding	-
MSTRG.34561.1	408	noncoding	-
MSTRG.34561.2	203	noncoding	-
MSTRG.34563.1	786	noncoding	-
MSTRG.34621.2	3137	noncoding	-
MSTRG.34621.1	2331	noncoding	-
MSTRG.34621.3	11620	noncoding	-
MSTRG.34621.4	1377	noncoding	-
MSTRG.34621.5	4034	noncoding	-
MSTRG.34622.1	1446	noncoding	-
MSTRG.34621.8	2780	noncoding	-
MSTRG.34621.6	2710	noncoding	-
MSTRG.34621.7	1974	noncoding	-
MSTRG.34621.11	2677	noncoding	-
MSTRG.34621.12	2110	noncoding	-
MSTRG.34621.10	3296	noncoding	-
MSTRG.34621.9	2959	noncoding	-
MSTRG.34621.13	3484	noncoding	-

MSTRG.34621.14	2756	noncoding	-
MSTRG.34621.15	1300	noncoding	-
MSTRG.34621.17	1368	noncoding	-
MSTRG.34621.16	620	noncoding	-
MSTRG.34621.18	1475	noncoding	-
MSTRG.34622.3	2857	noncoding	-
MSTRG.34622.4	2501	noncoding	-
MSTRG.34622.5	2516	noncoding	-
MSTRG.34622.6	1430	noncoding	-
MSTRG.34622.7	1413	noncoding	-
MSTRG.34622.8	3247	noncoding	-
MSTRG.34622.10	2849	noncoding	-
MSTRG.34622.9	2644	noncoding	-
MSTRG.34622.11	3152	noncoding	-
MSTRG.34622.12	2827	noncoding	-
MSTRG.34622.13	2865	noncoding	-
MSTRG.34622.14	2865	noncoding	-
MSTRG.34622.15	3047	noncoding	-
MSTRG.34622.16	2728	noncoding	-
MSTRG.34568.1	791	noncoding	-
MSTRG.34568.2	533	noncoding	-
MSTRG.34569.1	570	noncoding	-
MSTRG.34570.1	307	coding	PF07714
MSTRG.34572.1	1029	noncoding	-
MSTRG.34574.1	241	noncoding	-
MSTRG.34575.1	235	noncoding	-
MSTRG.34576.1	276	noncoding	-
MSTRG.34579.2	1412	noncoding	-
MSTRG.34580.1	399	noncoding	-
MSTRG.34583.1	1030	noncoding	-
MSTRG.34583.2	2698	noncoding	-
MSTRG.34587.1	256	noncoding	-
MSTRG.34592.2	319	noncoding	-
MSTRG.34592.1	267	noncoding	-
MSTRG.34592.3	265	noncoding	-
MSTRG.34592.4	261	noncoding	-
MSTRG.34592.5	305	noncoding	-
MSTRG.34595.7	555	noncoding	-
MSTRG.34595.3	610	noncoding	-
MSTRG.34595.5	610	noncoding	-
MSTRG.34595.6	610	noncoding	-
MSTRG.34595.8	352	noncoding	-
MSTRG.34595.2	555	noncoding	-
MSTRG.34595.4	512	noncoding	-
MSTRG.34595.9	608	noncoding	-
MSTRG.34595.10	362	noncoding	-
MSTRG.34595.11	390	noncoding	-
MSTRG.34595.12	512	noncoding	-
MSTRG.34595.13	553	noncoding	-
MSTRG.34595.14	434	noncoding	-
MSTRG.34595.15	312	noncoding	-
MSTRG.34595.16	385	noncoding	-
MSTRG.34600.1	577	noncoding	-
MSTRG.34601.2	640	noncoding	-

MSTRG.34601.3	396	noncoding	-
MSTRG.34601.1	401	noncoding	-
MSTRG.34603.1	495	coding	PF00069
MSTRG.34600.2	3071	coding	PF00069
MSTRG.34600.3	1480	noncoding	-
MSTRG.34606.1	655	coding	PF00069
MSTRG.34600.5	2508	noncoding	-
MSTRG.34600.4	2253	noncoding	-
MSTRG.34600.6	593	noncoding	-
MSTRG.34597.1	696	noncoding	-
MSTRG.34612.1	734	noncoding	-
MSTRG.34613.1	536	noncoding	-
MSTRG.34611.1	518	noncoding	-
MSTRG.34616.1	367	noncoding	-
MSTRG.34616.2	367	noncoding	-
MSTRG.34616.3	332	noncoding	-
MSTRG.34616.4	383	noncoding	-
MSTRG.34616.5	367	noncoding	-
MSTRG.34615.1	237	noncoding	-
MSTRG.34619.1	383	noncoding	-
MSTRG.34631.3	1309	noncoding	-
MSTRG.34631.2	1957	noncoding	-
MSTRG.34632.1	655	noncoding	-
MSTRG.34633.1	488	noncoding	-
MSTRG.34632.2	343	noncoding	-
MSTRG.34638.2	5010	coding	PF01421
MSTRG.34638.1	1336	coding	PF01421
MSTRG.34637.2	3590	coding	PF01421
MSTRG.34637.4	3455	coding	PF01421
MSTRG.34653.1	742	noncoding	-
MSTRG.34655.2	2731	noncoding	-
MSTRG.34655.1	3208	noncoding	-
MSTRG.34656.2	732	noncoding	-
MSTRG.34656.1	1160	noncoding	-
MSTRG.34657.1	1316	noncoding	-
MSTRG.34660.1	228	noncoding	-
MSTRG.34661.1	337	noncoding	-
MSTRG.34653.2	526	noncoding	-
MSTRG.34664.1	713	noncoding	-
MSTRG.34666.1	519	noncoding	-
MSTRG.34671.1	2707	noncoding	-
MSTRG.34646.1	210	noncoding	-
MSTRG.34647.3	652	coding	PF05831
MSTRG.34647.1	761	coding	PF05831
MSTRG.34675.3	536	coding	PF05831
MSTRG.34675.2	462	coding	PF05831
MSTRG.34675.4	419	coding	PF05831
MSTRG.34675.5	401	coding	PF05831
MSTRG.34681.2	5164	coding	PF02023
MSTRG.34681.4	1594	coding	PF02023
MSTRG.34681.3	3276	coding	PF02023
MSTRG.34682.7	1729	coding	PF02023
MSTRG.34682.9	1717	coding	PF02023
MSTRG.34682.11	1765	coding	PF02023

MSTRG.34682.12	1765	coding	PF02023
MSTRG.34683.1	481	noncoding	-
MSTRG.34707.1	350	noncoding	-
MSTRG.34708.1	285	noncoding	-
MSTRG.34712.1	1006	noncoding	-
MSTRG.34714.1	765	noncoding	-
MSTRG.34715.1	1186	noncoding	-
MSTRG.34716.1	236	noncoding	-
MSTRG.34717.1	1523	noncoding	-
MSTRG.34715.2	1519	noncoding	-
MSTRG.34715.3	1137	noncoding	-
MSTRG.34718.1	506	noncoding	-
MSTRG.34719.1	314	noncoding	-
MSTRG.34720.1	1113	noncoding	-
MSTRG.34721.1	2316	noncoding	-
MSTRG.34722.1	587	noncoding	-
MSTRG.34715.4	1937	noncoding	-
MSTRG.34723.1	563	noncoding	-
MSTRG.34723.2	984	noncoding	-
MSTRG.34724.1	1689	noncoding	-
MSTRG.34725.1	349	noncoding	-
MSTRG.34719.2	1296	noncoding	-
MSTRG.34717.2	677	noncoding	-
MSTRG.34717.3	587	noncoding	-
MSTRG.34726.1	1296	noncoding	-
MSTRG.34715.5	798	noncoding	-
MSTRG.34727.1	510	noncoding	-
MSTRG.34687.1	414	noncoding	-
MSTRG.34689.2	545	noncoding	-
MSTRG.34690.2	3378	coding	PF13855
MSTRG.34692.1	302	noncoding	-
MSTRG.34701.1	461	noncoding	-
MSTRG.34702.1	481	noncoding	-
MSTRG.34729.1	776	noncoding	-
MSTRG.34743.1	852	coding	PF11976
MSTRG.34743.2	955	coding	PF11976
MSTRG.34744.2	1673	noncoding	-
MSTRG.34744.3	1673	noncoding	-
MSTRG.34744.4	1529	noncoding	-
MSTRG.34744.5	5445	coding	PF16212
MSTRG.34751.1	1174	noncoding	-
MSTRG.34755.2	2744	noncoding	-
MSTRG.34755.1	3534	noncoding	-
MSTRG.34761.2	906	coding	PF15300
MSTRG.34763.2	1399	noncoding	-
MSTRG.34764.1	503	noncoding	-
MSTRG.34771.9	605	noncoding	-
MSTRG.34771.13	604	noncoding	-
MSTRG.34778.1	615	noncoding	-
MSTRG.34778.3	431	noncoding	-
MSTRG.34778.4	431	noncoding	-
MSTRG.34778.5	610	noncoding	-
MSTRG.34778.6	610	noncoding	-
