# Supplementary material for: Identification of Novel lncRNA and Differentially Expressed Genes (DEGs) of Testicular Tissues among Cattle, Yak, and Cattle-Yak Associated with Male Infertility
Source: Animals (Basel). 2021 Aug 17;11(8):2420. doi: 10.3390/ani11082420 (PMC8388754; doi:10.3390/ani11082420)
Supplement: Supplementary file 1 [file animals-11-02420-s001.zip › Table S4_plek_result.pdf]

MSTRG.12.1	0.600758	coding
MSTRG.12.2	-0.953233	noncoding
MSTRG.12.3	0.336613	coding
MSTRG.32.2	0.736719	coding
MSTRG.33.1	-1.47646	noncoding
MSTRG.34.2	1.01813	coding
MSTRG.35.1	-1.1028	noncoding
MSTRG.39.3	-2.26072	noncoding
MSTRG.39.6	1.22803	coding
MSTRG.59.3	0.93239	coding
MSTRG.60.1	-1.03311	noncoding
MSTRG.61.1	-0.38279	noncoding
MSTRG.60.2	-0.299877	noncoding
MSTRG.63.2	2.19406	coding
MSTRG.63.4	2.18397	coding
MSTRG.63.6	1.99462	coding
MSTRG.63.7	2.10208	coding
MSTRG.63.5	1.62219	coding
MSTRG.63.8	2.19354	coding
MSTRG.63.3	2.10572	coding
MSTRG.65.10	1.91514	coding
MSTRG.65.11	1.79222	coding
MSTRG.65.4	1.81497	coding
MSTRG.65.5	1.79537	coding
MSTRG.65.6	1.90983	coding
MSTRG.65.8	1.7945	coding
MSTRG.65.9	1.85825	coding
MSTRG.65.14	1.85715	coding
MSTRG.65.15	1.87842	coding
MSTRG.65.16	1.81366	coding
MSTRG.66.1	1.28082	coding
MSTRG.66.2	1.27372	coding
MSTRG.66.3	1.28684	coding
MSTRG.66.4	1.33317	coding
MSTRG.68.2	0.333751	coding
MSTRG.42.1	1.62891	coding
MSTRG.56.2	2.48424	coding
MSTRG.56.4	2.62509	coding
MSTRG.56.6	2.43996	coding
MSTRG.56.7	2.8558	coding
MSTRG.56.8	2.95323	coding
MSTRG.52.1	1.73654	coding
MSTRG.52.2	1.77941	coding
MSTRG.53.1	1.1575	coding
MSTRG.53.2	0.16988	coding
MSTRG.70.1	0.534174	coding
MSTRG.73.2	0.760232	coding
MSTRG.78.1	0.697796	coding
MSTRG.79.2	0.701041	coding
MSTRG.78.2	0.928397	coding
MSTRG.80.1	-2.46119	noncoding
MSTRG.81.1	-2.16966	noncoding
MSTRG.79.3	0.520742	coding
MSTRG.82.1	-1.90665	noncoding

MSTRG.79.4	-0.6246	noncoding
MSTRG.79.5	0.280853	coding
MSTRG.79.6	-0.132703	noncoding
MSTRG.83.1	0.378672	coding
MSTRG.79.7	-0.440016	noncoding
MSTRG.85.1	0.544405	coding
MSTRG.86.1	0.170831	coding
MSTRG.96.9	2.7665	coding
MSTRG.96.10	2.97586	coding
MSTRG.96.6	2.52525	coding
MSTRG.96.8	2.87884	coding
MSTRG.96.3	2.7532	coding
MSTRG.97.1	0.494862	coding
MSTRG.97.2	0.717318	coding
MSTRG.91.1	-0.700066	noncoding
MSTRG.103.6	2.00318	coding
MSTRG.103.2	2.44298	coding
MSTRG.103.5	2.71819	coding
MSTRG.103.4	3.45832	coding
MSTRG.104.1	0.209428	coding
MSTRG.104.3	0.664894	coding
MSTRG.111.1	-1.4473	noncoding
MSTRG.117.6	1.37478	coding
MSTRG.118.5	-0.94778	noncoding
MSTRG.123.1	-1.55434	noncoding
MSTRG.127.1	-1.75338	noncoding
MSTRG.131.1	-2.12335	noncoding
MSTRG.132.1	-1.40244	noncoding
MSTRG.134.1	-2.28285	noncoding
MSTRG.151.1	-1.05069	noncoding
MSTRG.188.1	0.00003	coding
MSTRG.189.1	-1.25433	noncoding
MSTRG.193.1	-1.76131	noncoding
MSTRG.196.1	1.35332	coding
MSTRG.223.1	-1.18267	noncoding
MSTRG.241.1	-0.862553	noncoding
MSTRG.158.1	1.78356	coding
MSTRG.158.6	1.36339	coding
MSTRG.158.5	1.4761	coding
MSTRG.158.4	2.08287	coding
MSTRG.158.8	1.16037	coding
MSTRG.158.7	1.77877	coding
MSTRG.158.9	1.72368	coding
MSTRG.160.1	0.31039	coding
MSTRG.160.3	1.80305	coding
MSTRG.160.4	1.75262	coding
MSTRG.160.5	0.993379	coding
MSTRG.160.6	0.81964	coding
MSTRG.161.1	-1.11556	noncoding
MSTRG.162.1	0.84792	coding
MSTRG.162.2	0.087269	coding
MSTRG.160.7	0.826628	coding
MSTRG.163.1	1.51286	coding
MSTRG.163.3	2.1411	coding

MSTRG.164.1	0.612102	coding
MSTRG.166.2	0.89045	coding
MSTRG.168.2	-0.440224	noncoding
MSTRG.169.1	-2.35918	noncoding
MSTRG.175.3	2.39172	coding
MSTRG.175.4	0.44992	coding
MSTRG.175.5	2.13964	coding
MSTRG.175.6	1.70182	coding
MSTRG.265.8	0.246941	coding
MSTRG.265.9	0.284625	coding
MSTRG.266.1	-1.93636	noncoding
MSTRG.267.1	1.38845	coding
MSTRG.268.1	-0.165034	noncoding
MSTRG.269.1	-0.122026	noncoding
MSTRG.268.2	0.63255	coding
MSTRG.270.1	-0.350686	noncoding
MSTRG.271.1	0.072162	coding
MSTRG.272.1	0.527137	coding
MSTRG.273.1	-0.20225	noncoding
MSTRG.274.1	0.673108	coding
MSTRG.275.1	-0.403531	noncoding
MSTRG.276.1	-1.56678	noncoding
MSTRG.277.1	0.220668	coding
MSTRG.277.2	0.449083	coding
MSTRG.278.1	0.579922	coding
MSTRG.265.10	-0.364923	noncoding
MSTRG.265.11	0.330961	coding
MSTRG.279.1	-0.210703	noncoding
MSTRG.265.12	0.627157	coding
MSTRG.280.1	-0.791325	noncoding
MSTRG.281.1	-0.026799	noncoding
MSTRG.282.1	-0.263031	noncoding
MSTRG.283.1	-1.13039	noncoding
MSTRG.284.1	-0.061492	noncoding
MSTRG.284.2	-0.265482	noncoding
MSTRG.265.14	0.136595	coding
MSTRG.285.1	-0.731737	noncoding
MSTRG.250.1	-0.500563	noncoding
MSTRG.255.1	0.284118	coding
MSTRG.258.1	0.089416	coding
MSTRG.261.1	-1.04678	noncoding
MSTRG.262.1	-0.949817	noncoding
MSTRG.263.1	-1.22275	noncoding
MSTRG.297.1	-1.78404	noncoding
MSTRG.307.1	1.47198	coding
MSTRG.308.1	-1.91936	noncoding
MSTRG.312.1	-1.42255	noncoding
MSTRG.314.1	2.13355	coding
MSTRG.314.3	1.71728	coding
MSTRG.325.3	2.30679	coding
MSTRG.325.4	2.1774	coding
MSTRG.326.1	-0.037483	noncoding
MSTRG.327.1	-0.042033	noncoding
MSTRG.324.1	-1.80101	noncoding

MSTRG.328.1	-1.29973	noncoding
MSTRG.328.2	0.603325	coding
MSTRG.332.1	0.845598	coding
MSTRG.348.1	-2.15213	noncoding
MSTRG.351.1	1.84647	coding
MSTRG.352.1	-0.393894	noncoding
MSTRG.353.1	-1.85046	noncoding
MSTRG.356.1	-0.728365	noncoding
MSTRG.361.1	-2.27956	noncoding
MSTRG.375.2	1.18909	coding
MSTRG.375.5	1.2496	coding
MSTRG.377.1	-1.91754	noncoding
MSTRG.377.2	-1.27699	noncoding
MSTRG.379.3	0.783254	coding
MSTRG.379.5	0.852872	coding
MSTRG.369.1	-2.29029	noncoding
MSTRG.370.1	-2.21644	noncoding
MSTRG.371.1	0.804592	coding
MSTRG.381.3	1.8541	coding
MSTRG.396.1	-0.412469	noncoding
MSTRG.397.1	0.602873	coding
MSTRG.396.3	0.212855	coding
MSTRG.398.1	-0.688465	noncoding
MSTRG.399.1	0.800982	coding
MSTRG.400.1	-1.14582	noncoding
MSTRG.401.1	0.268959	coding
MSTRG.404.3	2.20421	coding
MSTRG.404.4	1.72885	coding
MSTRG.404.6	1.71791	coding
MSTRG.404.5	1.39812	coding
MSTRG.404.7	1.60239	coding
MSTRG.404.8	-0.079951	noncoding
MSTRG.391.3	2.40272	coding
MSTRG.392.2	0.358125	coding
MSTRG.393.2	-0.634363	noncoding
MSTRG.405.1	1.63237	coding
MSTRG.405.7	1.74228	coding
MSTRG.405.3	2.02436	coding
MSTRG.405.4	1.77925	coding
MSTRG.405.5	2.01252	coding
MSTRG.405.6	1.98351	coding
MSTRG.405.8	2.20561	coding
MSTRG.405.9	-0.555047	noncoding
MSTRG.407.1	-0.185521	noncoding
MSTRG.416.1	-1.08389	noncoding
MSTRG.423.2	1.4724	coding
MSTRG.424.1	1.88048	coding
MSTRG.424.2	2.2125	coding
MSTRG.424.3	1.03772	coding
MSTRG.426.1	0.367243	coding
MSTRG.427.1	0.474269	coding
MSTRG.429.1	1.62043	coding
MSTRG.429.2	1.52683	coding
MSTRG.449.1	-2.529	noncoding

MSTRG.451.1	0.796103	coding
MSTRG.451.2	0.764155	coding
MSTRG.452.1	-1.67087	noncoding
MSTRG.457.1	-0.197596	noncoding
MSTRG.435.3	-1.17573	noncoding
MSTRG.439.1	-1.43247	noncoding
MSTRG.438.2	0.852584	coding
MSTRG.440.1	0.310851	coding
MSTRG.441.1	0.94304	coding
MSTRG.467.6	0.658156	coding
MSTRG.467.7	-1.65261	noncoding
MSTRG.468.1	0.848488	coding
MSTRG.468.2	0.506827	coding
MSTRG.468.3	-0.288589	noncoding
MSTRG.468.4	0.242605	coding
MSTRG.468.5	1.27702	coding
MSTRG.470.1	-0.691017	noncoding
MSTRG.467.10	1.8731	coding
MSTRG.467.9	1.83605	coding
MSTRG.467.11	1.88007	coding
MSTRG.471.1	0.348136	coding
MSTRG.472.1	-0.781097	noncoding
MSTRG.463.1	1.57428	coding
MSTRG.463.4	1.88277	coding
MSTRG.464.1	-0.83669	noncoding
MSTRG.474.1	-0.642923	noncoding
MSTRG.479.1	1.23057	coding
MSTRG.481.2	-0.479506	noncoding
MSTRG.481.1	0.060946	coding
MSTRG.487.2	0.865002	coding
MSTRG.488.3	1.25823	coding
MSTRG.484.1	-2.26518	noncoding
MSTRG.493.1	-2.54975	noncoding
MSTRG.497.1	-2.0426	noncoding
MSTRG.506.1	-1.37227	noncoding
MSTRG.508.3	2.93655	coding
MSTRG.508.4	3.28679	coding
MSTRG.508.2	2.84809	coding
MSTRG.508.5	1.58392	coding
MSTRG.509.1	-0.863191	noncoding
MSTRG.512.2	-0.524706	noncoding
MSTRG.513.4	1.78877	coding
MSTRG.514.4	0.885465	coding
MSTRG.514.3	0.83877	coding
MSTRG.514.5	0.936291	coding
MSTRG.515.1	0.067384	coding
MSTRG.521.1	0.69446	coding
MSTRG.521.3	0.391328	coding
MSTRG.521.4	0.035521	coding
MSTRG.521.6	-0.062332	noncoding
MSTRG.521.7	-0.147811	noncoding
MSTRG.526.3	0.989455	coding
MSTRG.526.1	1.25446	coding
MSTRG.526.2	0.720692	coding

MSTRG.527.2	-1.00617	noncoding
MSTRG.533.2	-2.33076	noncoding
MSTRG.533.3	1.03282	coding
MSTRG.535.1	0.495203	coding
MSTRG.535.4	0.039936	coding
MSTRG.538.1	1.60855	coding
MSTRG.538.2	1.73026	coding
MSTRG.538.3	1.63175	coding
MSTRG.538.4	1.74364	coding
MSTRG.539.1	0.339391	coding
MSTRG.570.1	2.26177	coding
MSTRG.570.15	1.16958	coding
MSTRG.570.14	1.53179	coding
MSTRG.570.16	0.820487	coding
MSTRG.570.18	1.48619	coding
MSTRG.570.17	1.43161	coding
MSTRG.570.19	1.64245	coding
MSTRG.570.20	1.45599	coding
MSTRG.570.22	1.14607	coding
MSTRG.570.21	1.46669	coding
MSTRG.570.23	1.57726	coding
MSTRG.571.1	0.353842	coding
MSTRG.572.1	-1.27294	noncoding
MSTRG.570.24	-0.273488	noncoding
MSTRG.573.1	-2.14353	noncoding
MSTRG.574.1	-0.787371	noncoding
MSTRG.575.1	-2.55826	noncoding
MSTRG.576.1	-1.08335	noncoding
MSTRG.577.1	0.050838	coding
MSTRG.578.1	0.275684	coding
MSTRG.570.25	-0.193884	noncoding
MSTRG.579.1	0.223553	coding
MSTRG.580.1	1.43039	coding
MSTRG.581.1	0.133742	coding
MSTRG.582.1	0.502565	coding
MSTRG.583.1	-0.755775	noncoding
MSTRG.584.1	-0.748713	noncoding
MSTRG.546.1	1.76924	coding
MSTRG.546.2	1.58587	coding
MSTRG.547.1	1.16308	coding
MSTRG.560.4	0.238652	coding
MSTRG.560.1	0.270135	coding
MSTRG.560.6	0.766502	coding
MSTRG.561.1	-1.99049	noncoding
MSTRG.562.1	-2.10007	noncoding
MSTRG.564.1	-1.54195	noncoding
MSTRG.601.2	1.25624	coding
MSTRG.615.6	1.57696	coding
MSTRG.615.4	0.993034	coding
MSTRG.615.5	1.00484	coding
MSTRG.616.1	-0.738509	noncoding
MSTRG.617.1	-1.76368	noncoding
MSTRG.622.2	0.878392	coding
MSTRG.622.3	0.920123	coding

MSTRG.622.4	0.901071	coding
MSTRG.623.1	-1.14109	noncoding
MSTRG.624.2	0.935841	coding
MSTRG.624.3	0.753874	coding
MSTRG.625.1	-3.23083	noncoding
MSTRG.627.1	1.02847	coding
MSTRG.628.1	1.65063	coding
MSTRG.628.2	2.45356	coding
MSTRG.628.3	1.89362	coding
MSTRG.628.6	3.04325	coding
MSTRG.628.5	2.00609	coding
MSTRG.639.1	1.53151	coding
MSTRG.639.2	1.02924	coding
MSTRG.645.1	-1.2699	noncoding
MSTRG.646.1	2.13715	coding
MSTRG.646.3	1.20114	coding
MSTRG.646.4	0.97177	coding
MSTRG.633.4	0.515954	coding
MSTRG.633.5	0.324474	coding
MSTRG.633.6	0.092462	coding
MSTRG.633.7	0.056921	coding
MSTRG.635.1	-2.9502	noncoding
MSTRG.635.2	-2.50744	noncoding
MSTRG.650.1	2.22198	coding
MSTRG.665.2	0.252018	coding
MSTRG.665.3	-0.338907	noncoding
MSTRG.671.6	3.29029	coding
MSTRG.671.9	3.16576	coding
MSTRG.671.10	2.31004	coding
MSTRG.671.12	2.29989	coding
MSTRG.671.15	2.3806	coding
MSTRG.671.16	2.37556	coding
MSTRG.671.18	0.790107	coding
MSTRG.672.1	-0.185684	noncoding
MSTRG.667.3	0.75816	coding
MSTRG.691.1	1.53216	coding
MSTRG.691.2	1.5308	coding
MSTRG.691.5	0.91635	coding
MSTRG.691.6	0.915461	coding
MSTRG.691.3	2.33263	coding
MSTRG.691.7	-2.73723	noncoding
MSTRG.691.8	2.47287	coding
MSTRG.691.9	-0.159959	noncoding
MSTRG.691.10	2.492	coding
MSTRG.691.11	1.50206	coding
MSTRG.691.12	-1.78811	noncoding
MSTRG.693.1	-0.660582	noncoding
MSTRG.694.1	-0.480645	noncoding
MSTRG.691.13	1.78931	coding
MSTRG.691.14	1.45595	coding
MSTRG.691.15	2.23401	coding
MSTRG.691.17	0.78361	coding
MSTRG.691.18	0.376876	coding
MSTRG.695.1	-1.0327	noncoding

MSTRG.691.21	-0.833422	noncoding
MSTRG.691.20	1.22385	coding
MSTRG.691.22	1.42324	coding
MSTRG.696.1	0.240281	coding
MSTRG.675.2	1.24447	coding
MSTRG.681.5	1.30288	coding
MSTRG.681.4	0.792939	coding
MSTRG.684.1	1.34724	coding
MSTRG.684.4	1.22603	coding
MSTRG.699.1	0.748281	coding
MSTRG.699.3	1.48598	coding
MSTRG.699.4	0.770212	coding
MSTRG.702.1	-0.345746	noncoding
MSTRG.704.4	0.36904	coding
MSTRG.704.6	0.120321	coding
MSTRG.704.7	0.544058	coding
MSTRG.685.2	1.43078	coding
MSTRG.685.1	1.8961	coding
MSTRG.685.3	1.77279	coding
MSTRG.687.5	1.85349	coding
MSTRG.687.6	0.210626	coding
MSTRG.705.6	0.490373	coding
MSTRG.705.3	1.3632	coding
MSTRG.705.4	0.837383	coding
MSTRG.705.5	0.952254	coding
MSTRG.705.1	0.869207	coding
MSTRG.705.7	0.837782	coding
MSTRG.705.8	0.897382	coding
MSTRG.706.3	1.23545	coding
MSTRG.706.4	-0.492212	noncoding
MSTRG.707.1	-1.66416	noncoding
MSTRG.708.1	0.899934	coding
MSTRG.708.5	2.06792	coding
MSTRG.708.4	1.9214	coding
MSTRG.708.3	1.80098	coding
MSTRG.710.3	-0.017192	noncoding
MSTRG.713.3	1.10218	coding
MSTRG.714.1	0.749928	coding
MSTRG.714.2	2.18374	coding
MSTRG.715.1	-0.048453	noncoding
MSTRG.717.1	-2.065	noncoding
MSTRG.718.1	-1.82103	noncoding
MSTRG.730.7	2.20569	coding
MSTRG.731.1	-1.76607	noncoding
MSTRG.732.1	-0.038128	noncoding
MSTRG.733.1	-0.665368	noncoding
MSTRG.727.2	1.47998	coding
MSTRG.727.3	1.57181	coding
MSTRG.727.4	1.79508	coding
MSTRG.728.1	-0.021427	noncoding
MSTRG.728.2	0.053911	coding
MSTRG.728.3	0.238816	coding
MSTRG.735.1	-0.841465	noncoding
MSTRG.739.1	-0.348441	noncoding

MSTRG.740.1	0.372994	coding
MSTRG.741.1	-0.197254	noncoding
MSTRG.743.2	0.976871	coding
MSTRG.752.5	-0.636379	noncoding
MSTRG.752.6	-0.633437	noncoding
MSTRG.755.1	-1.70624	noncoding
MSTRG.756.1	-0.701009	noncoding
MSTRG.758.1	-0.124062	noncoding
MSTRG.774.1	0.352252	coding
MSTRG.776.1	0.159978	coding
MSTRG.777.1	-1.66477	noncoding
MSTRG.770.1	-1.8313	noncoding
MSTRG.771.1	-1.45513	noncoding
MSTRG.771.2	-1.93686	noncoding
MSTRG.778.1	-1.5957	noncoding
MSTRG.782.2	0.831911	coding
MSTRG.782.3	1.16269	coding
MSTRG.782.4	0.677465	coding
MSTRG.782.5	1.52802	coding
MSTRG.784.1	-1.38382	noncoding
MSTRG.784.2	-0.495699	noncoding
MSTRG.786.1	0.193921	coding
MSTRG.794.2	-0.260115	noncoding
MSTRG.803.3	1.06096	coding
MSTRG.804.1	-1.19533	noncoding
MSTRG.804.2	-2.39059	noncoding
MSTRG.805.1	-0.81035	noncoding
MSTRG.806.1	1.34456	coding
MSTRG.806.4	0.919859	coding
MSTRG.801.1	-2.16044	noncoding
MSTRG.813.2	1.78644	coding
MSTRG.817.1	1.26792	coding
MSTRG.831.2	2.62109	coding
MSTRG.831.4	3.06024	coding
MSTRG.833.1	-2.34554	noncoding
MSTRG.835.1	0.552866	coding
MSTRG.836.2	2.95476	coding
MSTRG.837.4	2.52939	coding
MSTRG.839.2	0.154752	coding
MSTRG.840.3	1.628	coding
MSTRG.840.2	2.11115	coding
MSTRG.841.3	1.42645	coding
MSTRG.841.2	1.29863	coding
MSTRG.854.3	1.85737	coding
MSTRG.855.1	0.684083	coding
MSTRG.855.2	0.565796	coding
MSTRG.848.3	1.75957	coding
MSTRG.848.5	1.50343	coding
MSTRG.848.6	1.85598	coding
MSTRG.848.8	2.10647	coding
MSTRG.859.4	0.73603	coding
MSTRG.859.6	-2.2428	noncoding
MSTRG.862.2	2.48709	coding
MSTRG.862.5	0.153281	coding

MSTRG.862.7	0.357898	coding
MSTRG.862.6	2.88512	coding
MSTRG.862.3	3.16523	coding
MSTRG.862.8	2.25732	coding
MSTRG.874.1	-0.465662	noncoding
MSTRG.892.3	1.60599	coding
MSTRG.892.2	1.98749	coding
MSTRG.892.1	2.14128	coding
MSTRG.897.2	0.407851	coding
MSTRG.914.4	0.792099	coding
MSTRG.914.3	0.329088	coding
MSTRG.914.5	0.665753	coding
MSTRG.915.2	2.01229	coding
MSTRG.915.3	1.94307	coding
MSTRG.908.1	-2.27793	noncoding
MSTRG.920.1	-0.597937	noncoding
MSTRG.921.1	-1.20989	noncoding
MSTRG.945.1	1.13763	coding
MSTRG.934.4	1.00341	coding
MSTRG.951.3	1.69642	coding
MSTRG.952.1	-1.91054	noncoding
MSTRG.956.4	0.314145	coding
MSTRG.957.1	0.230111	coding
MSTRG.959.1	-2.00301	noncoding
MSTRG.965.1	-0.553804	noncoding
MSTRG.965.2	-0.267554	noncoding
MSTRG.965.3	-1.85033	noncoding
MSTRG.979.3	0.586289	coding
MSTRG.979.7	0.880606	coding
MSTRG.980.3	-0.772946	noncoding
MSTRG.981.1	-0.271318	noncoding
MSTRG.980.5	0.324483	coding
MSTRG.980.6	0.5855	coding
MSTRG.980.7	1.39632	coding
MSTRG.985.1	-0.541734	noncoding
MSTRG.967.2	1.12731	coding
MSTRG.972.2	0.454305	coding
MSTRG.974.1	2.79453	coding
MSTRG.975.1	0.200349	coding
MSTRG.991.1	1.01722	coding
MSTRG.991.2	1.04265	coding
MSTRG.991.3	0.647331	coding
MSTRG.994.1	0.20977	coding
MSTRG.994.2	0.296685	coding
MSTRG.994.3	0.588759	coding
MSTRG.997.1	1.01878	coding
MSTRG.1000.1	1.20816	coding
MSTRG.1000.2	0.828614	coding
MSTRG.1005.5	0.821924	coding
MSTRG.1005.6	2.68696	coding
MSTRG.1002.3	0.587507	coding
MSTRG.1002.4	0.568282	coding
MSTRG.1008.4	0.705132	coding
MSTRG.1013.4	1.75895	coding

MSTRG.1013.7	1.24786	coding
MSTRG.1013.8	1.2323	coding
MSTRG.1013.9	1.36901	coding
MSTRG.1012.2	0.090647	coding
MSTRG.1012.1	-0.427274	noncoding
MSTRG.1029.1	2.01757	coding
MSTRG.1029.3	1.27975	coding
MSTRG.1031.1	1.91507	coding
MSTRG.1031.2	1.56884	coding
MSTRG.1031.3	0.203722	coding
MSTRG.1041.2	1.95716	coding
MSTRG.1044.3	2.13433	coding
MSTRG.1036.3	2.14651	coding
MSTRG.1037.1	0.073674	coding
MSTRG.1060.2	-0.916475	noncoding
MSTRG.1063.1	0.56527	coding
MSTRG.1063.2	0.613836	coding
MSTRG.1066.1	0.686655	coding
MSTRG.1066.2	0.744178	coding
MSTRG.1066.3	-1.00086	noncoding
MSTRG.1065.1	-2.52177	noncoding
MSTRG.1070.1	-1.14505	noncoding
MSTRG.1085.2	0.832254	coding
MSTRG.1086.2	0.594186	coding
MSTRG.1096.1	1.40451	coding
MSTRG.1097.1	-0.079633	noncoding
MSTRG.1096.2	1.29058	coding
MSTRG.1108.1	-2.65863	noncoding
MSTRG.1119.1	-0.905173	noncoding
MSTRG.1132.1	0.52526	coding
MSTRG.1125.1	0.230137	coding
MSTRG.1141.1	-0.331025	noncoding
MSTRG.1149.1	-0.769584	noncoding
MSTRG.1150.1	-1.53711	noncoding
MSTRG.1157.6	1.28497	coding
MSTRG.1157.7	0.562276	coding
MSTRG.1157.8	1.27631	coding
MSTRG.1157.10	0.711357	coding
MSTRG.1157.9	0.692592	coding
MSTRG.1158.1	-0.721942	noncoding
MSTRG.1158.2	-0.228206	noncoding
MSTRG.1159.1	-0.065887	noncoding
MSTRG.1173.1	-0.11044	noncoding
MSTRG.1174.1	-1.09491	noncoding
MSTRG.1175.1	0.839327	coding
MSTRG.1161.2	0.641289	coding
MSTRG.1161.3	-0.130888	noncoding
MSTRG.1164.2	-0.362428	noncoding
MSTRG.1167.1	-1.814	noncoding
MSTRG.1184.1	-0.498331	noncoding
MSTRG.1189.2	-0.04496	noncoding
MSTRG.1190.1	-1.32048	noncoding
MSTRG.1194.2	0.247013	coding
MSTRG.1196.2	0.161753	coding

MSTRG.1207.1	-1.43881	noncoding
MSTRG.1209.1	-2.08471	noncoding
MSTRG.1214.2	0.313168	coding
MSTRG.1215.1	-1.52496	noncoding
MSTRG.1211.1	-2.05384	noncoding
MSTRG.1236.4	1.08634	coding
MSTRG.1245.2	2.55323	coding
MSTRG.1250.2	-0.304754	noncoding
MSTRG.1251.1	-1.06212	noncoding
MSTRG.1252.4	0.961225	coding
MSTRG.1253.1	-1.70333	noncoding
MSTRG.1259.2	-0.515417	noncoding
MSTRG.1260.1	-1.67606	noncoding
MSTRG.1261.2	-0.888588	noncoding
MSTRG.1261.5	0.814529	coding
MSTRG.1261.6	0.617425	coding
MSTRG.1254.1	-1.02185	noncoding
MSTRG.1263.1	2.21713	coding
MSTRG.1267.4	1.17309	coding
MSTRG.1269.1	-1.41014	noncoding
MSTRG.1272.1	2.09403	coding
MSTRG.1272.4	2.31067	coding
MSTRG.1272.6	2.13595	coding
MSTRG.1272.7	1.33395	coding
MSTRG.1278.2	0.858553	coding
MSTRG.1278.3	-0.056862	noncoding
MSTRG.1279.2	1.66305	coding
MSTRG.1288.3	3.29877	coding
MSTRG.1288.4	0.630854	coding
MSTRG.1288.5	0.900293	coding
MSTRG.1285.1	-1.28861	noncoding
MSTRG.1309.1	-0.374711	noncoding
MSTRG.1310.2	-0.691938	noncoding
MSTRG.1310.1	-0.720016	noncoding
MSTRG.1310.3	-1.52496	noncoding
MSTRG.1311.1	1.07869	coding
MSTRG.1311.7	1.42721	coding
MSTRG.1311.5	1.23849	coding
MSTRG.1311.6	1.48982	coding
MSTRG.1311.8	1.42124	coding
MSTRG.1311.9	1.35478	coding
MSTRG.1311.10	1.89605	coding
MSTRG.1309.2	-0.582773	noncoding
MSTRG.1312.1	-0.614272	noncoding
MSTRG.1314.2	1.63253	coding
MSTRG.1317.1	-2.08922	noncoding
MSTRG.1318.2	3.18925	coding
MSTRG.1318.3	3.03194	coding
MSTRG.1319.1	0.32939	coding
MSTRG.1320.1	1.41807	coding
MSTRG.1321.1	-0.919888	noncoding
MSTRG.1323.1	-0.082651	noncoding
MSTRG.1318.4	1.34397	coding
MSTRG.1325.1	1.21174	coding

MSTRG.1332.2	1.37941	coding
MSTRG.1338.1	0.294413	coding
MSTRG.1338.2	-0.208022	noncoding
MSTRG.1338.4	0.536619	coding
MSTRG.1338.3	0.509872	coding
MSTRG.1338.7	0.371936	coding
MSTRG.1338.8	0.670686	coding
MSTRG.1339.1	-1.2156	noncoding
MSTRG.1344.2	0.939435	coding
MSTRG.1346.1	0.137314	coding
MSTRG.1351.5	2.75983	coding
MSTRG.1351.6	2.69092	coding
MSTRG.1352.6	1.5365	coding
MSTRG.1352.2	0.544037	coding
MSTRG.1352.4	0.290516	coding
MSTRG.1352.9	0.510755	coding
MSTRG.1352.11	-0.642627	noncoding
MSTRG.1352.12	-0.667835	noncoding
MSTRG.1352.10	-0.359975	noncoding
MSTRG.1352.13	-0.907416	noncoding
MSTRG.1352.14	-0.944057	noncoding
MSTRG.1352.15	0.014206	coding
MSTRG.1355.4	1.91381	coding
MSTRG.1363.1	-1.11661	noncoding
MSTRG.1367.2	0.572143	coding
MSTRG.1367.3	0.624197	coding
MSTRG.1380.9	-0.004224	noncoding
MSTRG.1380.7	2.55527	coding
MSTRG.1380.5	2.59376	coding
MSTRG.1380.8	1.65806	coding
MSTRG.1380.10	0.949136	coding
MSTRG.1380.11	-0.538612	noncoding
MSTRG.1382.1	-1.53253	noncoding
MSTRG.1384.4	1.31553	coding
MSTRG.1384.3	1.99846	coding
MSTRG.1384.5	-1.1664	noncoding
MSTRG.1387.1	-0.271379	noncoding
MSTRG.1391.1	1.10167	coding
MSTRG.1395.10	0.579323	coding
MSTRG.1395.11	1.01637	coding
MSTRG.1395.12	0.801116	coding
MSTRG.1395.13	0.955692	coding
MSTRG.1395.14	0.865089	coding
MSTRG.1395.15	0.965663	coding
MSTRG.1395.17	0.6054	coding
MSTRG.1395.18	0.8114	coding
MSTRG.1395.19	0.775087	coding
MSTRG.1395.20	0.811095	coding
MSTRG.1395.21	0.993873	coding
MSTRG.1395.22	1.20425	coding
MSTRG.1395.23	0.741609	coding
MSTRG.1395.24	1.21376	coding
MSTRG.1396.1	0.626127	coding
MSTRG.1396.2	0.456453	coding

MSTRG.1394.1	-2.68868	noncoding
MSTRG.1398.1	3.20123	coding
MSTRG.1398.2	2.03854	coding
MSTRG.1407.1	-1.68942	noncoding
MSTRG.1408.1	0.495622	coding
MSTRG.1414.3	-1.70894	noncoding
MSTRG.1415.1	1.31133	coding
MSTRG.1415.3	1.17722	coding
MSTRG.1416.2	1.27864	coding
MSTRG.1419.4	0.277415	coding
MSTRG.1419.3	1.12581	coding
MSTRG.1420.1	-0.353819	noncoding
MSTRG.1421.1	0.431086	coding
MSTRG.1426.2	1.87346	coding
MSTRG.1432.3	-0.117956	noncoding
MSTRG.1432.2	0.568303	coding
MSTRG.1433.1	-0.325644	noncoding
MSTRG.1433.5	1.24507	coding
MSTRG.1429.2	0.571538	coding
MSTRG.1429.3	0.411873	coding
MSTRG.1428.1	-1.3115	noncoding
MSTRG.1436.1	0.840214	coding
MSTRG.1436.4	0.883931	coding
MSTRG.1436.2	0.886513	coding
MSTRG.1436.3	0.858685	coding
MSTRG.1437.1	-1.56307	noncoding
MSTRG.1438.1	-0.004428	noncoding
MSTRG.1436.7	1.20064	coding
MSTRG.1436.6	1.21967	coding
MSTRG.1439.2	2.13096	coding
MSTRG.1440.1	-1.56174	noncoding
MSTRG.1441.1	0.230153	coding
MSTRG.1447.1	-1.84573	noncoding
MSTRG.1449.1	-0.165458	noncoding
MSTRG.1450.1	0.373446	coding
MSTRG.1451.2	0.381681	coding
MSTRG.1451.3	0.294668	coding
MSTRG.1454.5	0.308045	coding
MSTRG.1455.2	-1.1972	noncoding
MSTRG.1455.1	-1.20052	noncoding
MSTRG.1460.1	-1.24987	noncoding
MSTRG.1463.2	0.929971	coding
MSTRG.1464.1	-0.794555	noncoding
MSTRG.1465.2	1.60139	coding
MSTRG.1467.2	1.14718	coding
MSTRG.1469.1	2.94371	coding
MSTRG.1470.1	-0.329547	noncoding
MSTRG.1475.3	-0.629636	noncoding
MSTRG.1475.2	0.491629	coding
MSTRG.1475.4	0.529637	coding
MSTRG.1475.5	0.298902	coding
MSTRG.1475.6	0.424949	coding
MSTRG.1477.1	0.529145	coding
MSTRG.1473.1	2.14745	coding

MSTRG.1473.2	2.18527	coding
MSTRG.1494.2	1.52361	coding
MSTRG.1495.1	-1.70376	noncoding
MSTRG.1496.1	2.05463	coding
MSTRG.1497.1	1.39511	coding
MSTRG.1497.2	1.00689	coding
MSTRG.1499.1	0.477212	coding
MSTRG.1500.3	0.163552	coding
MSTRG.1501.5	1.67973	coding
MSTRG.1501.1	1.62381	coding
MSTRG.1501.2	1.69655	coding
MSTRG.1501.3	1.69024	coding
MSTRG.1501.4	1.63682	coding
MSTRG.1501.6	1.62281	coding
MSTRG.1501.9	1.69327	coding
MSTRG.1501.10	1.62006	coding
MSTRG.1501.11	1.61013	coding
MSTRG.1501.14	1.66205	coding
MSTRG.1481.1	-1.29907	noncoding
MSTRG.1483.2	0.781196	coding
MSTRG.1484.1	-1.30594	noncoding
MSTRG.1512.6	0.654393	coding
MSTRG.1512.5	0.47873	coding
MSTRG.1512.7	0.271982	coding
MSTRG.1512.10	0.317585	coding
MSTRG.1512.8	0.532591	coding
MSTRG.1512.9	0.310355	coding
MSTRG.1512.11	0.661093	coding
MSTRG.1512.12	0.37009	coding
MSTRG.1512.13	0.287751	coding
MSTRG.1512.14	0.580099	coding
MSTRG.1512.15	0.5079	coding
MSTRG.1512.16	0.216015	coding
MSTRG.1512.17	-0.731453	noncoding
MSTRG.1512.18	0.110586	coding
MSTRG.1512.19	0.345141	coding
MSTRG.1514.1	-1.08145	noncoding
MSTRG.1509.3	2.66445	coding
MSTRG.1511.2	0.163996	coding
MSTRG.1511.3	0.150935	coding
MSTRG.1511.5	0.21063	coding
MSTRG.1516.1	-0.9636	noncoding
MSTRG.1518.1	-0.764718	noncoding
MSTRG.1519.1	-0.881909	noncoding
MSTRG.1520.2	0.595391	coding
MSTRG.1535.1	0.199894	coding
MSTRG.1536.1	-1.71962	noncoding
MSTRG.1537.4	-0.725007	noncoding
MSTRG.1538.1	0.892671	coding
MSTRG.1538.7	1.82434	coding
MSTRG.1538.8	0.382472	coding
MSTRG.1538.9	1.79623	coding
MSTRG.1538.10	1.28795	coding
MSTRG.1538.12	0.628081	coding

MSTRG.1538.13	0.68117	coding
MSTRG.1526.2	0.180214	coding
MSTRG.1531.6	-0.434054	noncoding
MSTRG.1531.5	1.23118	coding
MSTRG.1531.4	2.29101	coding
MSTRG.1531.7	1.63233	coding
MSTRG.1532.1	-0.021561	noncoding
MSTRG.1533.1	-0.376891	noncoding
MSTRG.1531.8	2.05557	coding
MSTRG.1531.9	2.04043	coding
MSTRG.1534.1	-0.010212	noncoding
MSTRG.1531.10	1.45584	coding
MSTRG.1528.1	-1.09999	noncoding
MSTRG.1528.3	-2.07973	noncoding
MSTRG.1530.1	-0.303176	noncoding
MSTRG.1544.1	2.24011	coding
MSTRG.1544.2	1.78728	coding
MSTRG.1545.1	-2.30765	noncoding
MSTRG.1549.1	-1.99346	noncoding
MSTRG.1550.5	1.02861	coding
MSTRG.1550.6	1.42704	coding
MSTRG.1550.7	0.097473	coding
MSTRG.1554.2	2.37158	coding
MSTRG.1555.1	-1.39787	noncoding
MSTRG.1556.1	0.221989	coding
MSTRG.1562.3	0.51413	coding
MSTRG.1562.4	0.667035	coding
MSTRG.1562.5	0.916161	coding
MSTRG.1563.1	0.953414	coding
MSTRG.1563.3	0.391569	coding
MSTRG.1558.2	0.669982	coding
MSTRG.1560.1	-0.094179	noncoding
MSTRG.1576.2	1.1322	coding
MSTRG.1578.4	1.0305	coding
MSTRG.1578.7	1.31232	coding
MSTRG.1578.9	-0.482674	noncoding
MSTRG.1578.11	0.469137	coding
MSTRG.1578.10	0.651419	coding
MSTRG.1578.12	0.852764	coding
MSTRG.1578.13	1.1511	coding
MSTRG.1578.14	0.871486	coding
MSTRG.1567.3	-0.298278	noncoding
MSTRG.1567.4	-0.387195	noncoding
MSTRG.1567.5	-0.331137	noncoding
MSTRG.1567.7	-0.016693	noncoding
MSTRG.1568.2	1.77281	coding
MSTRG.1568.4	1.81165	coding
MSTRG.1568.6	1.98117	coding
MSTRG.1568.5	1.82542	coding
MSTRG.1573.1	0.058994	coding
MSTRG.1574.2	-0.34013	noncoding
MSTRG.1574.3	0.410733	coding
MSTRG.1589.1	-2.3919	noncoding
MSTRG.1590.1	-1.49011	noncoding

MSTRG.1592.2	-0.139679	noncoding
MSTRG.1592.1	0.054074	coding
MSTRG.1592.4	-0.614113	noncoding
MSTRG.1592.5	0.04329	coding
MSTRG.1592.6	0.411376	coding
MSTRG.1593.1	-0.83151	noncoding
MSTRG.1592.7	0.876776	coding
MSTRG.1592.8	-0.333679	noncoding
MSTRG.1594.1	1.44266	coding
MSTRG.1595.1	-1.00197	noncoding
MSTRG.1596.1	-1.0317	noncoding
MSTRG.1597.1	-1.58362	noncoding
MSTRG.1598.1	-0.746457	noncoding
MSTRG.1596.2	0.966303	coding
MSTRG.1583.1	-2.16436	noncoding
MSTRG.1583.2	-2.9131	noncoding
MSTRG.1585.1	-1.43585	noncoding
MSTRG.1600.1	-1.92693	noncoding
MSTRG.1603.1	0.181755	coding
MSTRG.1604.1	-2.35956	noncoding
MSTRG.1606.1	-2.06027	noncoding
MSTRG.1610.1	-0.081393	noncoding
MSTRG.1610.3	0.511567	coding
MSTRG.1610.4	0.910595	coding
MSTRG.1613.3	0.477775	coding
MSTRG.1614.1	-0.200215	noncoding
MSTRG.1614.2	-0.067636	noncoding
MSTRG.1614.3	-0.465363	noncoding
MSTRG.1614.4	0.215289	coding
MSTRG.1618.1	2.31768	coding
MSTRG.1618.2	-0.759513	noncoding
MSTRG.1618.4	-2.03176	noncoding
MSTRG.1618.3	2.36698	coding
MSTRG.1619.1	-1.43185	noncoding
MSTRG.1619.2	-1.16404	noncoding
MSTRG.1623.2	0.904189	coding
MSTRG.1637.4	-0.144593	noncoding
MSTRG.1637.5	1.03706	coding
MSTRG.1632.1	0.377549	coding
MSTRG.1632.2	0.595899	coding
MSTRG.1644.3	1.79796	coding
MSTRG.1642.3	1.91494	coding
MSTRG.1640.1	-1.96996	noncoding
MSTRG.1647.3	0.704486	coding
MSTRG.1650.1	0.125146	coding
MSTRG.1650.2	0.13415	coding
MSTRG.1650.3	0.190468	coding
MSTRG.1653.1	0.962465	coding
MSTRG.1654.1	0.677285	coding
MSTRG.1654.2	0.678188	coding
MSTRG.1654.3	0.761325	coding
MSTRG.1657.1	-0.221708	noncoding
MSTRG.1659.1	-2.15378	noncoding
MSTRG.1662.1	2.09284	coding

MSTRG.1662.4	2.15139	coding
MSTRG.1662.7	2.05676	coding
MSTRG.1662.9	2.04286	coding
MSTRG.1662.10	2.04811	coding
MSTRG.1662.12	2.20482	coding
MSTRG.1662.13	1.92053	coding
MSTRG.1663.1	1.19413	coding
MSTRG.1663.5	-1.63332	noncoding
MSTRG.1663.4	0.774621	coding
MSTRG.1663.3	0.423853	coding
MSTRG.1665.1	0.586097	coding
MSTRG.1667.1	-0.930455	noncoding
MSTRG.1671.1	1.2887	coding
MSTRG.1673.5	-0.084498	noncoding
MSTRG.1673.4	1.31165	coding
MSTRG.1673.3	1.90456	coding
MSTRG.1675.1	1.35378	coding
MSTRG.1676.1	-2.30778	noncoding
MSTRG.1677.1	-2.05935	noncoding
MSTRG.1678.1	-1.98726	noncoding
MSTRG.1681.1	-0.469726	noncoding
MSTRG.1685.2	-0.090168	noncoding
MSTRG.1685.3	-0.612696	noncoding
MSTRG.1689.1	-2.31749	noncoding
MSTRG.1691.1	-0.427548	noncoding
MSTRG.1692.1	-1.15662	noncoding
MSTRG.1697.2	0.418973	coding
MSTRG.1697.1	-1.38625	noncoding
MSTRG.1697.3	0.922436	coding
MSTRG.1697.4	-0.246487	noncoding
MSTRG.1709.2	2.13781	coding
MSTRG.1709.4	2.27603	coding
MSTRG.1709.6	2.19633	coding
MSTRG.1709.8	1.96805	coding
MSTRG.1716.2	0.022149	coding
MSTRG.1716.1	-0.360795	noncoding
MSTRG.1716.3	0.360931	coding
MSTRG.1717.1	0.357343	coding
MSTRG.1725.1	0.007284	coding
MSTRG.1726.1	-0.102481	noncoding
MSTRG.1721.1	-2.75598	noncoding
MSTRG.1721.2	-2.34478	noncoding
MSTRG.1729.2	2.20781	coding
MSTRG.1730.1	0.934804	coding
MSTRG.1731.1	0.438952	coding
MSTRG.1734.1	0.480795	coding
MSTRG.1734.2	-1.21231	noncoding
MSTRG.1727.1	0.086397	coding
MSTRG.1727.2	-1.09362	noncoding
MSTRG.1740.3	1.42789	coding
MSTRG.1740.2	1.55176	coding
MSTRG.1745.1	1.85229	coding
MSTRG.1745.3	1.86673	coding
MSTRG.1753.1	-2.47582	noncoding

MSTRG.1753.2	-1.75251	noncoding
MSTRG.1768.1	-0.286343	noncoding
MSTRG.1768.2	-0.209518	noncoding
MSTRG.1768.3	-0.120645	noncoding
MSTRG.1770.2	-0.150913	noncoding
MSTRG.1772.1	2.46726	coding
MSTRG.1772.3	2.68673	coding
MSTRG.1772.4	2.79414	coding
MSTRG.1773.1	-1.69268	noncoding
MSTRG.1774.1	-2.23287	noncoding
MSTRG.1777.2	0.562151	coding
MSTRG.1777.3	0.581574	coding
MSTRG.1782.1	2.96902	coding
MSTRG.1782.5	2.12118	coding
MSTRG.1782.4	1.95051	coding
MSTRG.1783.1	-1.32343	noncoding
MSTRG.1784.1	-0.22578	noncoding
MSTRG.1787.1	-1.00998	noncoding
MSTRG.1787.2	-0.908015	noncoding
MSTRG.1779.1	-2.14306	noncoding
MSTRG.1780.1	-0.948681	noncoding
MSTRG.1791.2	1.14343	coding
MSTRG.1791.3	2.07022	coding
MSTRG.1798.1	-0.165527	noncoding
MSTRG.1799.1	0.352982	coding
MSTRG.1799.2	1.49575	coding
MSTRG.1799.3	0.65637	coding
MSTRG.1799.4	1.43345	coding
MSTRG.1799.7	0.524168	coding
MSTRG.1799.6	1.06232	coding
MSTRG.1798.2	-0.406529	noncoding
MSTRG.1798.3	0.247967	coding
MSTRG.1799.8	1.61954	coding
MSTRG.1805.1	0.415192	coding
MSTRG.1805.3	1.0941	coding
MSTRG.1801.1	-2.03398	noncoding
MSTRG.1801.2	-1.99419	noncoding
MSTRG.1801.3	-0.948106	noncoding
MSTRG.1804.1	0.149947	coding
MSTRG.1819.1	2.46364	coding
MSTRG.1811.3	-0.100429	noncoding
MSTRG.1814.1	2.05085	coding
MSTRG.1814.3	2.12299	coding
MSTRG.1814.4	2.24496	coding
MSTRG.1814.2	2.23186	coding
MSTRG.1814.7	1.95007	coding
MSTRG.1814.8	-1.09228	noncoding
MSTRG.1818.1	2.21969	coding
MSTRG.1818.3	2.2333	coding
MSTRG.1832.1	-0.17295	noncoding
MSTRG.1832.3	0.78868	coding
MSTRG.1833.2	0.761245	coding
MSTRG.1833.1	0.179197	coding
MSTRG.1833.3	-0.871497	noncoding

MSTRG.1834.1	-1.11716	noncoding
MSTRG.1840.1	-0.047301	noncoding
MSTRG.1829.1	0.599586	coding
MSTRG.1841.5	-0.322239	noncoding
MSTRG.1847.1	1.65562	coding
MSTRG.1847.2	2.03262	coding
MSTRG.1869.1	-1.43151	noncoding
MSTRG.1868.11	0.097858	coding
MSTRG.1868.12	-1.55146	noncoding
MSTRG.1868.13	-1.43181	noncoding
MSTRG.1873.1	-1.15826	noncoding
MSTRG.1874.1	0.199506	coding
MSTRG.1874.2	-0.074393	noncoding
MSTRG.1849.4	1.92033	coding
MSTRG.1850.1	-0.809249	noncoding
MSTRG.1856.4	1.35136	coding
MSTRG.1857.1	0.890819	coding
MSTRG.1860.1	1.16869	coding
MSTRG.1860.5	0.719999	coding
MSTRG.1860.6	2.13919	coding
MSTRG.1860.3	0.943677	coding
MSTRG.1860.4	1.13259	coding
MSTRG.1862.1	-2.0911	noncoding
MSTRG.1860.7	0.511989	coding
MSTRG.1864.1	-0.931302	noncoding
MSTRG.1865.1	-0.190664	noncoding
MSTRG.1866.1	0.264753	coding
MSTRG.1867.2	3.14724	coding
MSTRG.1867.3	3.21956	coding
MSTRG.1891.2	1.33513	coding
MSTRG.1895.2	1.37614	coding
MSTRG.1895.3	-1.45506	noncoding
MSTRG.1895.5	0.383321	coding
MSTRG.1895.4	1.13363	coding
MSTRG.1900.3	1.37523	coding
MSTRG.1900.4	1.08601	coding
MSTRG.1900.5	1.01172	coding
MSTRG.1900.6	-1.0617	noncoding
MSTRG.1902.4	2.31461	coding
MSTRG.1906.1	-0.781463	noncoding
MSTRG.1907.2	1.17208	coding
MSTRG.1907.3	1.28221	coding
MSTRG.1907.4	1.12659	coding
MSTRG.1911.1	-1.53349	noncoding
MSTRG.1912.2	0.680318	coding
MSTRG.1912.4	-0.050715	noncoding
MSTRG.1912.7	1.29663	coding
MSTRG.1914.1	-1.40816	noncoding
MSTRG.1912.8	-1.13941	noncoding
MSTRG.1912.9	0.755177	coding
MSTRG.1909.2	0.434055	coding
MSTRG.1910.1	-1.70889	noncoding
MSTRG.1922.1	0.145425	coding
MSTRG.1922.4	0.712303	coding

MSTRG.1922.6	0.685627	coding
MSTRG.1922.5	0.102901	coding
MSTRG.1923.1	-1.94804	noncoding
MSTRG.1924.1	-0.797943	noncoding
MSTRG.1924.2	-0.896077	noncoding
MSTRG.1928.3	1.55722	coding
MSTRG.1928.5	1.75688	coding
MSTRG.1928.7	1.9013	coding
MSTRG.1928.8	-0.874194	noncoding
MSTRG.1928.9	1.51444	coding
MSTRG.1928.10	0.582672	coding
MSTRG.1930.1	0.105147	coding
MSTRG.1928.11	-0.426399	noncoding
MSTRG.1928.12	-0.077736	noncoding
MSTRG.1928.13	-0.063241	noncoding
MSTRG.1920.4	2.92373	coding
MSTRG.1920.5	2.97374	coding
MSTRG.1920.6	0.797919	coding
MSTRG.1920.7	3.04062	coding
MSTRG.1921.1	-0.801788	noncoding
MSTRG.1918.3	1.82764	coding
MSTRG.1940.1	0.084926	coding
MSTRG.1940.2	0.374479	coding
MSTRG.1940.5	0.163557	coding
MSTRG.1940.4	0.07756	coding
MSTRG.1941.1	0.327021	coding
MSTRG.1943.1	1.62303	coding
MSTRG.1943.2	0.947959	coding
MSTRG.1943.4	1.05067	coding
MSTRG.1945.2	0.784419	coding
MSTRG.1948.1	-2.39875	noncoding
MSTRG.1949.1	0.119965	coding
MSTRG.1951.1	2.5393	coding
MSTRG.1934.1	2.46373	coding
MSTRG.1953.1	-0.708678	noncoding
MSTRG.1954.1	-0.064914	noncoding
MSTRG.1995.1	-0.288222	noncoding
MSTRG.2012.1	0.156077	coding
MSTRG.2013.1	-1.41342	noncoding
MSTRG.2022.1	-0.457682	noncoding
MSTRG.2023.1	-0.305981	noncoding
MSTRG.2023.2	-0.522156	noncoding
MSTRG.2025.1	-0.947779	noncoding
MSTRG.2028.1	0.515562	coding
MSTRG.1959.1	2.50252	coding
MSTRG.1959.3	2.64294	coding
MSTRG.1960.1	2.20252	coding
MSTRG.1962.1	-1.45019	noncoding
MSTRG.1962.2	-1.2273	noncoding
MSTRG.1968.3	0.615308	coding
MSTRG.1968.8	0.342038	coding
MSTRG.1972.2	1.09973	coding
MSTRG.1972.6	0.438685	coding
MSTRG.1972.4	0.765103	coding

MSTRG.1972.3	0.834629	coding
MSTRG.1972.8	0.618753	coding
MSTRG.1972.10	0.401994	coding
MSTRG.1972.9	0.616331	coding
MSTRG.1972.11	0.346042	coding
MSTRG.1970.1	-2.47353	noncoding
MSTRG.1989.1	-1.89895	noncoding
MSTRG.1989.2	-1.94979	noncoding
MSTRG.1990.1	-0.843945	noncoding
MSTRG.2059.1	-2.70176	noncoding
MSTRG.2062.3	0.817441	coding
MSTRG.2062.4	0.655937	coding
MSTRG.2062.6	0.49992	coding
MSTRG.2062.5	0.604057	coding
MSTRG.2063.1	0.174878	coding
MSTRG.2060.1	-1.95582	noncoding
MSTRG.2068.4	1.11068	coding
MSTRG.2068.5	1.91067	coding
MSTRG.2068.6	1.66011	coding
MSTRG.2068.7	2.11881	coding
MSTRG.2068.9	2.09691	coding
MSTRG.2068.8	3.60914	coding
MSTRG.2070.1	-2.63938	noncoding
MSTRG.2071.1	-0.819456	noncoding
MSTRG.2067.1	-2.24929	noncoding
MSTRG.2067.2	-1.52367	noncoding
MSTRG.2066.1	-1.65734	noncoding
MSTRG.2072.2	1.50423	coding
MSTRG.2079.1	0.038554	coding
MSTRG.2079.2	-1.52207	noncoding
MSTRG.2085.1	0.17947	coding
MSTRG.2085.2	-0.012505	noncoding
MSTRG.2086.1	-2.27747	noncoding
MSTRG.2088.6	0.008047	coding
MSTRG.2088.4	1.51708	coding
MSTRG.2088.5	1.12954	coding
MSTRG.2088.8	1.50181	coding
MSTRG.2083.1	-0.093	noncoding
MSTRG.2095.1	0.734472	coding
MSTRG.2095.2	-0.273915	noncoding
MSTRG.2098.1	-0.291888	noncoding
MSTRG.2097.6	2.51824	coding
MSTRG.2097.7	2.22013	coding
MSTRG.2097.8	2.42955	coding
MSTRG.2110.8	1.80311	coding
MSTRG.2110.1	1.78973	coding
MSTRG.2110.5	1.80039	coding
MSTRG.2111.1	0.164665	coding
MSTRG.2110.9	-1.39146	noncoding
MSTRG.2112.1	-0.790733	noncoding
MSTRG.2113.1	-0.306393	noncoding
MSTRG.2114.1	1.27839	coding
MSTRG.2116.1	-1.55485	noncoding
MSTRG.2114.2	0.753562	coding

MSTRG.2108.1	-1.72067	noncoding
MSTRG.2119.3	1.63703	coding
MSTRG.2118.1	-1.74983	noncoding
MSTRG.2126.1	-1.45063	noncoding
MSTRG.2148.1	0.108312	coding
MSTRG.2152.1	-1.43904	noncoding
MSTRG.2154.1	-0.642738	noncoding
MSTRG.2153.6	0.26302	coding
MSTRG.2153.3	0.498065	coding
MSTRG.2153.5	0.540381	coding
MSTRG.2153.4	0.652727	coding
MSTRG.2153.7	1.37633	coding
MSTRG.2155.1	3.04522	coding
MSTRG.2155.2	2.66184	coding
MSTRG.2155.3	2.63937	coding
MSTRG.2155.4	2.63736	coding
MSTRG.2155.7	0.067409	coding
MSTRG.2155.8	0.360209	coding
MSTRG.2155.9	1.06313	coding
MSTRG.2144.1	-0.740578	noncoding
MSTRG.2145.1	-2.43324	noncoding
MSTRG.2146.1	1.01613	coding
MSTRG.2146.2	0.649528	coding
MSTRG.2175.1	2.01153	coding
MSTRG.2175.2	1.68409	coding
MSTRG.2177.2	1.72635	coding
MSTRG.2177.1	1.75408	coding
MSTRG.2177.6	1.61923	coding
MSTRG.2177.4	1.76042	coding
MSTRG.2177.7	1.23569	coding
MSTRG.2177.8	1.86699	coding
MSTRG.2177.10	1.64269	coding
MSTRG.2177.12	0.794292	coding
MSTRG.2177.11	1.47135	coding
MSTRG.2157.1	1.27157	coding
MSTRG.2166.1	-1.28836	noncoding
MSTRG.2167.1	-1.39225	noncoding
MSTRG.2179.2	2.46957	coding
MSTRG.2179.3	2.19244	coding
MSTRG.2179.4	1.19899	coding
MSTRG.2200.2	1.12042	coding
MSTRG.2200.3	1.22736	coding
MSTRG.2200.5	0.969373	coding
MSTRG.2201.1	-0.392142	noncoding
MSTRG.2202.1	-0.338	noncoding
MSTRG.2203.1	0.787197	coding
MSTRG.2210.3	0.752033	coding
MSTRG.2211.1	0.246592	coding
MSTRG.2210.4	0.114543	coding
MSTRG.2212.1	1.51947	coding
MSTRG.2213.3	1.11967	coding
MSTRG.2196.1	-2.5497	noncoding
MSTRG.2219.1	-0.01522	noncoding
MSTRG.2220.2	1.38447	coding

MSTRG.2221.1	0.732128	coding
MSTRG.2221.2	-0.637765	noncoding
MSTRG.2222.1	0.236936	coding
MSTRG.2222.2	-0.028349	noncoding
MSTRG.2220.3	1.62339	coding
MSTRG.2220.4	0.829685	coding
MSTRG.2223.3	-0.673784	noncoding
MSTRG.2223.4	1.3716	coding
MSTRG.2223.5	1.2157	coding
MSTRG.2223.6	0.308657	coding
MSTRG.2224.1	-0.065953	noncoding
MSTRG.2225.1	-0.602053	noncoding
MSTRG.2230.1	-1.50559	noncoding
MSTRG.2240.1	-2.60305	noncoding
MSTRG.2241.1	-2.2317	noncoding
MSTRG.2248.2	1.98116	coding
MSTRG.2248.4	2.10882	coding
MSTRG.2248.6	1.33175	coding
MSTRG.2250.1	-1.07816	noncoding
MSTRG.2252.1	0.338981	coding
MSTRG.2257.2	1.3618	coding
MSTRG.2257.3	1.55061	coding
MSTRG.2265.1	0.034001	coding
MSTRG.2265.2	-0.321622	noncoding
MSTRG.2265.3	0.243754	coding
MSTRG.2265.4	0.201339	coding
MSTRG.2265.6	-0.367207	noncoding
MSTRG.2265.5	0.11727	coding
MSTRG.2262.2	1.45357	coding
MSTRG.2268.4	1.11508	coding
MSTRG.2268.3	1.52495	coding
MSTRG.2268.5	1.44142	coding
MSTRG.2269.1	-0.125858	noncoding
MSTRG.2273.1	-1.28406	noncoding
MSTRG.2278.1	-0.336996	noncoding
MSTRG.2280.1	1.12062	coding
MSTRG.2281.1	-0.889139	noncoding
MSTRG.2282.1	-1.77933	noncoding
MSTRG.2283.1	1.26499	coding
MSTRG.2284.1	-0.102079	noncoding
MSTRG.2270.4	0.045441	coding
MSTRG.2270.5	0.055379	coding
MSTRG.2288.1	1.30852	coding
MSTRG.2288.3	1.07742	coding
MSTRG.2289.1	0.73878	coding
MSTRG.2285.1	-1.95938	noncoding
MSTRG.2311.1	-2.45984	noncoding
MSTRG.2350.1	1.16309	coding
MSTRG.2352.2	2.28999	coding
MSTRG.2352.3	2.35741	coding
MSTRG.2352.4	2.2854	coding
MSTRG.2355.1	-2.28219	noncoding
MSTRG.2361.1	2.12777	coding
MSTRG.2361.2	2.03053	coding

MSTRG.2361.4	1.75624	coding
MSTRG.2361.5	1.30353	coding
MSTRG.2361.3	1.29225	coding
MSTRG.2362.1	-0.112112	noncoding
MSTRG.2364.1	1.25917	coding
MSTRG.2364.4	0.967333	coding
MSTRG.2364.6	0.936509	coding
MSTRG.2365.1	-1.3507	noncoding
MSTRG.2364.7	0.527278	coding
MSTRG.2364.8	-0.241706	noncoding
MSTRG.2364.9	-1.26848	noncoding
MSTRG.2366.1	-0.524646	noncoding
MSTRG.2364.11	0.524556	coding
MSTRG.2371.1	1.75574	coding
MSTRG.2381.1	2.18593	coding
MSTRG.2381.10	2.18249	coding
MSTRG.2381.7	2.1488	coding
MSTRG.2381.4	1.99068	coding
MSTRG.2381.6	2.01666	coding
MSTRG.2381.5	2.22076	coding
MSTRG.2381.2	2.15514	coding
MSTRG.2381.3	2.0982	coding
MSTRG.2381.9	2.22581	coding
MSTRG.2381.13	2.20941	coding
MSTRG.2381.12	2.04001	coding
MSTRG.2381.15	2.03005	coding
MSTRG.2381.17	1.95578	coding
MSTRG.2381.16	1.49795	coding
MSTRG.2381.14	2.0867	coding
MSTRG.2381.18	2.25386	coding
MSTRG.2381.19	1.63015	coding
MSTRG.2387.1	-2.21597	noncoding
MSTRG.2385.1	-1.28231	noncoding
MSTRG.2391.1	-2.33113	noncoding
MSTRG.2392.1	-1.6936	noncoding
MSTRG.2398.5	-0.738216	noncoding
MSTRG.2412.1	-1.16059	noncoding
MSTRG.2412.2	-0.835942	noncoding
MSTRG.2412.3	0.492239	coding
MSTRG.2415.1	-0.286848	noncoding
MSTRG.2412.4	-0.644758	noncoding
MSTRG.2417.1	-0.174292	noncoding
MSTRG.2417.2	-0.410292	noncoding
MSTRG.2418.1	-0.284555	noncoding
MSTRG.2419.2	2.95024	coding
MSTRG.2419.5	0.215466	coding
MSTRG.2419.6	2.3659	coding
MSTRG.2419.4	2.85225	coding
MSTRG.2403.1	-0.560829	noncoding
MSTRG.2407.1	-0.050486	noncoding
MSTRG.2426.1	-1.52915	noncoding
MSTRG.2431.1	-2.57656	noncoding
MSTRG.2433.1	-1.94012	noncoding
MSTRG.2438.1	-2.19884	noncoding

MSTRG.2439.4	1.20241	coding
MSTRG.2435.3	1.09503	coding
MSTRG.2446.3	0.942936	coding
MSTRG.2447.1	0.595466	coding
MSTRG.2475.4	1.44702	coding
MSTRG.2475.5	0.497961	coding
MSTRG.2476.1	-0.688925	noncoding
MSTRG.2476.2	-0.745466	noncoding
MSTRG.2477.1	-0.732203	noncoding
MSTRG.2479.4	1.52089	coding
MSTRG.2480.1	-0.354749	noncoding
MSTRG.2479.5	0.688648	coding
MSTRG.2479.6	0.736942	coding
MSTRG.2479.7	0.552069	coding
MSTRG.2479.8	0.600103	coding
MSTRG.2479.9	0.808518	coding
MSTRG.2479.10	0.667014	coding
MSTRG.2479.13	0.775361	coding
MSTRG.2479.14	1.02674	coding
MSTRG.2479.15	1.46527	coding
MSTRG.2479.16	0.520337	coding
MSTRG.2479.17	1.03122	coding
MSTRG.2485.2	1.80414	coding
MSTRG.2485.3	1.72658	coding
MSTRG.2460.1	1.65678	coding
MSTRG.2464.2	0.249333	coding
MSTRG.2468.1	0.695887	coding
MSTRG.2489.3	0.633371	coding
MSTRG.2492.2	1.47495	coding
MSTRG.2493.1	1.66352	coding
MSTRG.2498.2	2.50744	coding
MSTRG.2509.3	0.829059	coding
MSTRG.2509.5	0.390865	coding
MSTRG.2509.4	1.33762	coding
MSTRG.2510.1	-0.099405	noncoding
MSTRG.2510.2	-0.677302	noncoding
MSTRG.2513.3	1.22167	coding
MSTRG.2513.5	0.749377	coding
MSTRG.2514.3	-1.43544	noncoding
MSTRG.2515.1	-0.020083	noncoding
MSTRG.2499.1	-2.85501	noncoding
MSTRG.2508.1	1.04362	coding
MSTRG.2517.2	1.45874	coding
MSTRG.2517.3	1.12895	coding
MSTRG.2519.3	1.32714	coding
MSTRG.2519.4	1.25677	coding
MSTRG.2529.1	1.73898	coding
MSTRG.2529.2	0.552054	coding
MSTRG.2530.1	0.923229	coding
MSTRG.2566.1	-2.45442	noncoding
MSTRG.2567.1	-0.842062	noncoding
MSTRG.2568.2	1.08929	coding
MSTRG.2569.1	1.20245	coding
MSTRG.2569.2	1.55163	coding

MSTRG.2569.4	1.66874	coding
MSTRG.2569.6	-0.249746	noncoding
MSTRG.2569.5	0.92996	coding
MSTRG.2572.1	-1.015	noncoding
MSTRG.2569.7	2.18263	coding
MSTRG.2569.8	2.07713	coding
MSTRG.2569.9	2.45741	coding
MSTRG.2575.1	-0.884633	noncoding
MSTRG.2576.4	-1.14615	noncoding
MSTRG.2576.3	1.42901	coding
MSTRG.2577.1	-0.556349	noncoding
MSTRG.2576.5	2.05263	coding
MSTRG.2576.6	0.348941	coding
MSTRG.2576.7	1.00311	coding
MSTRG.2579.2	1.84545	coding
MSTRG.2579.3	1.12955	coding
MSTRG.2541.2	0.612138	coding
MSTRG.2542.1	-2.24261	noncoding
MSTRG.2543.1	-1.53768	noncoding
MSTRG.2544.1	-0.651382	noncoding
MSTRG.2545.1	-1.24144	noncoding
MSTRG.2536.1	-1.13174	noncoding
MSTRG.2540.1	-1.79027	noncoding
MSTRG.2561.4	0.739409	coding
MSTRG.2564.3	1.55401	coding
MSTRG.2582.2	1.13006	coding
MSTRG.2582.3	0.963645	coding
MSTRG.2588.1	-0.86576	noncoding
MSTRG.2589.2	1.36491	coding
MSTRG.2589.3	0.56826	coding
MSTRG.2589.4	0.592862	coding
MSTRG.2590.2	0.696214	coding
MSTRG.2590.1	0.723296	coding
MSTRG.2590.3	0.534706	coding
MSTRG.2591.5	0.25484	coding
MSTRG.2591.6	-1.89245	noncoding
MSTRG.2594.1	1.65249	coding
MSTRG.2596.4	2.36911	coding
MSTRG.2596.3	2.48201	coding
MSTRG.2597.2	-2.19286	noncoding
MSTRG.2597.1	-2.41881	noncoding
MSTRG.2598.1	-2.08816	noncoding
MSTRG.2602.1	-1.2448	noncoding
MSTRG.2602.2	-1.92357	noncoding
MSTRG.2602.3	-1.9492	noncoding
MSTRG.2602.10	0.176321	coding
MSTRG.2602.11	0.196212	coding
MSTRG.2602.12	-0.718817	noncoding
MSTRG.2601.1	-2.44298	noncoding
MSTRG.2601.2	-2.76485	noncoding
MSTRG.2605.3	0.993748	coding
MSTRG.2605.4	0.682518	coding
MSTRG.2605.5	1.74913	coding
MSTRG.2605.6	0.775077	coding

MSTRG.2605.7	1.99893	coding
MSTRG.2605.8	2.43676	coding
MSTRG.2605.10	1.79194	coding
MSTRG.2605.9	1.82345	coding
MSTRG.2605.11	2.27944	coding
MSTRG.2609.7	1.59969	coding
MSTRG.2609.8	1.56596	coding
MSTRG.2609.9	1.68928	coding
MSTRG.2610.2	-0.119085	noncoding
MSTRG.2611.1	-0.136812	noncoding
MSTRG.2607.3	1.01476	coding
MSTRG.2682.1	0.231083	coding
MSTRG.2614.1	-1.93779	noncoding
MSTRG.2632.1	-0.917415	noncoding
MSTRG.2633.1	-1.62086	noncoding
MSTRG.2634.3	0.208969	coding
MSTRG.2635.1	-1.01667	noncoding
MSTRG.2636.1	-0.858586	noncoding
MSTRG.2638.1	-2.68973	noncoding
MSTRG.2639.1	1.28064	coding
MSTRG.2640.1	0.600123	coding
MSTRG.2641.1	-0.446852	noncoding
MSTRG.2642.1	-1.6455	noncoding
MSTRG.2645.1	-0.388777	noncoding
MSTRG.2645.2	0.654929	coding
MSTRG.2645.3	-1.47236	noncoding
MSTRG.2647.1	-0.863232	noncoding
MSTRG.2647.2	-1.50314	noncoding
MSTRG.2649.1	-2.29624	noncoding
MSTRG.2668.3	2.26246	coding
MSTRG.2668.4	2.25346	coding
MSTRG.2668.5	2.34856	coding
MSTRG.2668.6	1.8626	coding
MSTRG.2668.7	1.94186	coding
MSTRG.2706.2	1.62629	coding
MSTRG.2706.3	1.67949	coding
MSTRG.2711.3	-1.67176	noncoding
MSTRG.2711.4	-0.455885	noncoding
MSTRG.2714.3	0.115553	coding
MSTRG.2714.4	0.437001	coding
MSTRG.2715.1	2.04744	coding
MSTRG.2715.4	1.95174	coding
MSTRG.2715.5	1.52303	coding
MSTRG.2717.2	1.78112	coding
MSTRG.2720.1	-1.40484	noncoding
MSTRG.2721.2	0.755307	coding
MSTRG.2721.3	0.400267	coding
MSTRG.2724.3	1.5564	coding
MSTRG.2740.1	0.783354	coding
MSTRG.2742.1	0.12693	coding
MSTRG.2742.4	0.455765	coding
MSTRG.2742.5	0.437598	coding
MSTRG.2730.2	2.1319	coding
MSTRG.2732.1	1.37994	coding

MSTRG.2732.2	1.30374	coding
MSTRG.2732.5	1.17644	coding
MSTRG.2743.1	1.68909	coding
MSTRG.2744.1	-1.36339	noncoding
MSTRG.2747.4	0.264132	coding
MSTRG.2750.1	-0.702145	noncoding
MSTRG.2750.2	-1.2254	noncoding
MSTRG.2750.3	-1.24398	noncoding
MSTRG.2749.5	1.70966	coding
MSTRG.2757.1	-2.37883	noncoding
MSTRG.2761.1	-2.25284	noncoding
MSTRG.2762.1	-2.6265	noncoding
MSTRG.2766.2	1.04336	coding
MSTRG.2770.1	-2.84841	noncoding
MSTRG.2771.3	-0.759007	noncoding
MSTRG.2771.2	-0.784664	noncoding
MSTRG.2772.1	1.06379	coding
MSTRG.2774.2	3.19602	coding
MSTRG.2774.3	2.15948	coding
MSTRG.2775.1	0.451357	coding
MSTRG.2777.3	1.79738	coding
MSTRG.2778.1	-0.984315	noncoding
MSTRG.2778.2	-1.7707	noncoding
MSTRG.2804.3	-0.351168	noncoding
MSTRG.2806.5	0.599536	coding
MSTRG.2806.6	0.378039	coding
MSTRG.2806.7	0.297972	coding
MSTRG.2806.8	-2.13185	noncoding
MSTRG.2807.1	-0.388992	noncoding
MSTRG.2808.1	-0.315431	noncoding
MSTRG.2809.1	0.996067	coding
MSTRG.2810.2	1.1954	coding
MSTRG.2811.8	1.37776	coding
MSTRG.2811.10	0.534692	coding
MSTRG.2788.4	2.22037	coding
MSTRG.2788.5	2.32861	coding
MSTRG.2788.6	1.63191	coding
MSTRG.2788.7	1.56687	coding
MSTRG.2793.3	0.80211	coding
MSTRG.2798.2	2.11751	coding
MSTRG.2822.10	2.60994	coding
MSTRG.2822.6	2.20596	coding
MSTRG.2829.1	1.07604	coding
MSTRG.2830.1	-1.43118	noncoding
MSTRG.2799.1	1.04743	coding
MSTRG.2800.2	0.737059	coding
MSTRG.2817.1	1.81166	coding
MSTRG.2819.1	-1.23759	noncoding
MSTRG.2819.2	-1.24202	noncoding
MSTRG.2819.3	-1.62841	noncoding
MSTRG.2819.4	-0.230658	noncoding
MSTRG.2820.2	0.174257	coding
MSTRG.2820.3	3.80647	coding
MSTRG.2816.4	0.616645	coding

MSTRG.2816.3	0.768192	coding
MSTRG.2816.1	0.79706	coding
MSTRG.2816.5	-0.038527	noncoding
MSTRG.2814.1	-2.48717	noncoding
MSTRG.2821.2	2.61218	coding
MSTRG.2821.3	2.39679	coding
MSTRG.2833.3	1.45721	coding
MSTRG.2833.4	0.033929	coding
MSTRG.2833.5	0.49158	coding
MSTRG.2836.2	3.03774	coding
MSTRG.2837.1	0.544682	coding
MSTRG.2837.2	0.548552	coding
MSTRG.2843.2	2.02452	coding
MSTRG.2840.3	0.614219	coding
MSTRG.2848.2	2.72626	coding
MSTRG.2848.3	2.82219	coding
MSTRG.2848.4	1.30541	coding
MSTRG.2848.6	2.44461	coding
MSTRG.2848.7	-1.82282	noncoding
MSTRG.2845.1	-0.756897	noncoding
MSTRG.2845.2	-1.11309	noncoding
MSTRG.2850.5	-0.226619	noncoding
MSTRG.2850.4	0.421845	coding
MSTRG.2850.6	-0.798227	noncoding
MSTRG.2850.7	0.115278	coding
MSTRG.2850.8	0.622238	coding
MSTRG.2846.1	-1.12154	noncoding
MSTRG.2857.2	1.1943	coding
MSTRG.2857.4	1.28707	coding
MSTRG.2852.4	0.475967	coding
MSTRG.2852.3	0.470297	coding
MSTRG.2858.2	1.44604	coding
MSTRG.2859.1	-2.51638	noncoding
MSTRG.2865.3	1.95262	coding
MSTRG.2866.8	1.38342	coding
MSTRG.2871.1	0.401521	coding
MSTRG.2873.1	1.82569	coding
MSTRG.2870.1	0.575481	coding
MSTRG.2879.1	0.928565	coding
MSTRG.2879.3	1.153	coding
MSTRG.2884.3	2.14159	coding
MSTRG.2889.3	0.043	coding
MSTRG.2889.4	-0.632207	noncoding
MSTRG.2894.2	-1.65558	noncoding
MSTRG.2897.1	1.41476	coding
MSTRG.2898.2	0.800998	coding
MSTRG.2898.1	0.777942	coding
MSTRG.2899.1	-0.298002	noncoding
MSTRG.2899.3	0.157523	coding
MSTRG.2899.2	0.004018	coding
MSTRG.2898.4	0.766933	coding
MSTRG.2900.4	1.37352	coding
MSTRG.2900.5	-0.622623	noncoding
MSTRG.2906.4	1.12091	coding

MSTRG.2906.5	0.808081	coding
MSTRG.2907.3	1.94099	coding
MSTRG.2909.1	-0.946762	noncoding
MSTRG.2909.2	-0.322492	noncoding
MSTRG.2910.1	-0.270833	noncoding
MSTRG.2910.4	-0.690453	noncoding
MSTRG.2912.2	1.54451	coding
MSTRG.2913.1	-1.27277	noncoding
MSTRG.2914.1	0.670698	coding
MSTRG.2914.2	0.843918	coding
MSTRG.2921.1	-0.558429	noncoding
MSTRG.2922.1	-2.2192	noncoding
MSTRG.2922.2	-1.72176	noncoding
MSTRG.2920.1	-1.97016	noncoding
MSTRG.2924.2	2.69102	coding
MSTRG.2924.3	2.80482	coding
MSTRG.2927.3	1.66418	coding
MSTRG.2932.2	1.40994	coding
MSTRG.2932.6	0.899537	coding
MSTRG.2929.1	-1.28765	noncoding
MSTRG.2935.3	0.811807	coding
MSTRG.2943.3	0.594759	coding
MSTRG.2943.4	0.518226	coding
MSTRG.2943.5	0.769696	coding
MSTRG.2943.7	0.72154	coding
MSTRG.2943.6	0.197758	coding
MSTRG.2950.1	1.12609	coding
MSTRG.2950.2	1.27168	coding
MSTRG.2950.3	1.36586	coding
MSTRG.2951.1	-0.620497	noncoding
MSTRG.2956.4	1.53175	coding
MSTRG.2956.5	1.66472	coding
MSTRG.2956.6	1.39877	coding
MSTRG.2956.7	1.22478	coding
MSTRG.2957.1	0.048781	coding
MSTRG.2948.3	1.81101	coding
MSTRG.2960.1	-0.825577	noncoding
MSTRG.2968.1	1.83487	coding
MSTRG.2968.2	1.88662	coding
MSTRG.2968.3	2.08668	coding
MSTRG.2973.1	-0.177158	noncoding
MSTRG.2978.3	1.94091	coding
MSTRG.2978.1	1.59046	coding
MSTRG.2978.2	2.08006	coding
MSTRG.2979.1	-2.6748	noncoding
MSTRG.2980.1	0.429035	coding
MSTRG.2983.1	0.556693	coding
MSTRG.2995.1	1.86189	coding
MSTRG.2996.3	-0.520043	noncoding
MSTRG.2996.4	-0.450099	noncoding
MSTRG.3004.4	1.19581	coding
MSTRG.3004.6	1.41477	coding
MSTRG.3004.7	1.19976	coding
MSTRG.3001.1	1.54396	coding

MSTRG.3012.4	2.4149	coding
MSTRG.3012.5	2.62044	coding
MSTRG.3012.6	2.27819	coding
MSTRG.3017.3	1.01198	coding
MSTRG.3017.7	0.170898	coding
MSTRG.3014.2	2.20282	coding
MSTRG.3021.2	1.72303	coding
MSTRG.3026.1	-2.15148	noncoding
MSTRG.3027.2	1.56131	coding
MSTRG.3027.3	1.91917	coding
MSTRG.3027.4	-0.128346	noncoding
MSTRG.3028.1	1.64529	coding
MSTRG.3028.2	1.82957	coding
MSTRG.3028.3	1.58797	coding
MSTRG.3028.4	1.53475	coding
MSTRG.3028.5	0.341001	coding
MSTRG.3029.1	-1.09609	noncoding
MSTRG.3031.6	0.784924	coding
MSTRG.3031.10	0.743389	coding
MSTRG.3031.7	0.760576	coding
MSTRG.3031.9	0.723797	coding
MSTRG.3036.2	1.2444	coding
MSTRG.3037.1	1.51195	coding
MSTRG.3037.5	1.89927	coding
MSTRG.3037.6	1.5192	coding
MSTRG.3039.3	0.507079	coding
MSTRG.3043.1	1.49047	coding
MSTRG.3043.7	1.31326	coding
MSTRG.3043.4	2.14097	coding
MSTRG.3043.6	2.14356	coding
MSTRG.3043.3	1.42264	coding
MSTRG.3043.10	0.843943	coding
MSTRG.3043.11	0.854456	coding
MSTRG.3043.12	1.16426	coding
MSTRG.3043.9	2.03907	coding
MSTRG.3043.13	0.657541	coding
MSTRG.3042.1	-0.42888	noncoding
MSTRG.3050.1	-2.08998	noncoding
MSTRG.3054.2	0.407504	coding
MSTRG.3055.2	1.0975	coding
MSTRG.3044.2	1.99469	coding
MSTRG.3056.2	0.379114	coding
MSTRG.3059.1	1.47477	coding
MSTRG.3060.1	1.08824	coding
MSTRG.3061.1	-1.0632	noncoding
MSTRG.3061.2	-0.784515	noncoding
MSTRG.3064.6	1.65221	coding
MSTRG.3064.5	0.911377	coding
MSTRG.3066.1	-0.777366	noncoding
MSTRG.3068.4	0.846831	coding
MSTRG.3068.5	1.07018	coding
MSTRG.3068.3	1.39711	coding
MSTRG.3065.1	-1.78494	noncoding
MSTRG.3070.1	0.388168	coding

MSTRG.3076.1	1.53791	coding
MSTRG.3085.2	1.54277	coding
MSTRG.3087.2	-1.70543	noncoding
MSTRG.3087.1	-1.56406	noncoding
MSTRG.3093.1	-1.36466	noncoding
MSTRG.3092.4	1.3449	coding
MSTRG.3092.5	-0.317794	noncoding
MSTRG.3088.2	1.6737	coding
MSTRG.3088.3	1.42937	coding
MSTRG.3113.1	0.484695	coding
MSTRG.3114.1	2.33419	coding
MSTRG.3115.1	0.995886	coding
MSTRG.3116.1	0.581526	coding
MSTRG.3116.2	0.525884	coding
MSTRG.3114.2	2.84012	coding
MSTRG.3114.4	2.89453	coding
MSTRG.3114.3	2.85622	coding
MSTRG.3114.5	2.81811	coding
MSTRG.3114.7	2.59586	coding
MSTRG.3114.13	2.79426	coding
MSTRG.3114.8	2.86967	coding
MSTRG.3114.9	2.75526	coding
MSTRG.3117.1	0.430014	coding
MSTRG.3118.1	0.612677	coding
MSTRG.3114.14	2.78165	coding
MSTRG.3114.15	2.90854	coding
MSTRG.3122.1	-2.38867	noncoding
MSTRG.3124.3	1.47513	coding
MSTRG.3124.4	1.34748	coding
MSTRG.3124.5	1.22955	coding
MSTRG.3124.8	0.641633	coding
MSTRG.3100.1	-0.374214	noncoding
MSTRG.3101.1	2.67348	coding
MSTRG.3101.3	2.68875	coding
MSTRG.3101.2	2.54308	coding
MSTRG.3101.4	2.70949	coding
MSTRG.3101.5	1.05956	coding
MSTRG.3104.1	-1.19497	noncoding
MSTRG.3101.6	2.02974	coding
MSTRG.3096.2	0.796934	coding
MSTRG.3106.3	2.46285	coding
MSTRG.3106.4	2.54853	coding
MSTRG.3106.5	2.58126	coding
MSTRG.3106.6	2.58898	coding
MSTRG.3106.7	2.345	coding
MSTRG.3106.8	-2.07034	noncoding
MSTRG.3109.1	-0.031634	noncoding
MSTRG.3111.1	-1.67055	noncoding
MSTRG.3125.3	-0.200482	noncoding
MSTRG.3126.2	-1.78185	noncoding
MSTRG.3127.3	1.02799	coding
MSTRG.3127.4	0.996734	coding
MSTRG.3130.1	1.15575	coding
MSTRG.3130.2	1.21849	coding

MSTRG.3130.3	1.4215	coding
MSTRG.3132.1	0.600055	coding
MSTRG.3132.3	0.0481	coding
MSTRG.3133.1	1.95317	coding
MSTRG.3133.3	1.51617	coding
MSTRG.3133.4	1.64659	coding
MSTRG.3133.5	1.60777	coding
MSTRG.3134.1	1.26635	coding
MSTRG.3144.2	2.68291	coding
MSTRG.3136.1	-0.814796	noncoding
MSTRG.3137.1	-2.26794	noncoding
MSTRG.3135.1	-1.12187	noncoding
MSTRG.3135.2	-0.956904	noncoding
MSTRG.3141.1	-0.599945	noncoding
MSTRG.3146.1	-1.72876	noncoding
MSTRG.3146.2	-0.628379	noncoding
MSTRG.3153.1	-1.19683	noncoding
MSTRG.3154.1	2.65008	coding
MSTRG.3154.4	2.30552	coding
MSTRG.3154.5	1.41355	coding
MSTRG.3155.1	-0.484766	noncoding
MSTRG.3151.1	-1.77585	noncoding
MSTRG.3152.1	0.961507	coding
MSTRG.3157.2	1.59658	coding
MSTRG.3157.3	1.67734	coding
MSTRG.3157.5	1.77218	coding
MSTRG.3163.1	-0.979538	noncoding
MSTRG.3166.3	0.126703	coding
MSTRG.3166.6	-0.163117	noncoding
MSTRG.3166.7	0.157977	coding
MSTRG.3159.4	0.686031	coding
MSTRG.3179.7	0.65215	coding
MSTRG.3179.9	-1.27189	noncoding
MSTRG.3179.10	-0.058742	noncoding
MSTRG.3179.11	0.240459	coding
MSTRG.3183.1	-0.197861	noncoding
MSTRG.3173.1	-0.138633	noncoding
MSTRG.3176.1	0.591157	coding
MSTRG.3177.1	-1.77353	noncoding
MSTRG.3178.3	0.832769	coding
MSTRG.3185.1	1.27965	coding
MSTRG.3185.2	1.09835	coding
MSTRG.3186.1	0.966993	coding
MSTRG.3185.3	0.078081	coding
MSTRG.3186.6	0.216992	coding
MSTRG.3186.4	2.23308	coding
MSTRG.3186.9	2.1048	coding
MSTRG.3186.10	1.29913	coding
MSTRG.3186.11	2.00119	coding
MSTRG.3186.12	1.30746	coding
MSTRG.3187.1	0.506561	coding
MSTRG.3188.1	-0.73724	noncoding
MSTRG.3189.1	-0.605071	noncoding
MSTRG.3190.2	-0.666768	noncoding

MSTRG.3190.3	-0.691714	noncoding
MSTRG.3197.1	0.509664	coding
MSTRG.3198.1	0.457464	coding
MSTRG.3214.1	0.539973	coding
MSTRG.3221.1	-1.36318	noncoding
MSTRG.3226.1	-1.51496	noncoding
MSTRG.3228.2	1.80767	coding
MSTRG.3215.1	-0.294816	noncoding
MSTRG.3217.1	-0.183085	noncoding
MSTRG.3218.1	1.01336	coding
MSTRG.3220.1	-2.48673	noncoding
MSTRG.3234.2	2.2068	coding
MSTRG.3248.1	0.004738	coding
MSTRG.3249.1	0.240493	coding
MSTRG.3243.1	-1.52362	noncoding
MSTRG.3244.1	-1.59351	noncoding
MSTRG.3245.1	-2.00643	noncoding
MSTRG.3252.1	-2.22262	noncoding
MSTRG.3252.3	-2.17932	noncoding
MSTRG.3252.4	-1.22637	noncoding
MSTRG.3252.5	-1.45828	noncoding
MSTRG.3254.1	-2.36627	noncoding
MSTRG.3259.3	1.50425	coding
MSTRG.3259.4	1.4452	coding
MSTRG.3261.1	0.1589	coding
MSTRG.3263.1	-0.246388	noncoding
MSTRG.3264.1	-1.44356	noncoding
MSTRG.3267.2	2.73308	coding
MSTRG.3267.4	3.29514	coding
MSTRG.3269.1	-0.956704	noncoding
MSTRG.3270.2	1.26492	coding
MSTRG.3270.3	0.937374	coding
MSTRG.3271.1	1.32679	coding
MSTRG.3279.1	-0.256482	noncoding
MSTRG.3282.2	-0.869851	noncoding
MSTRG.3282.3	-0.932769	noncoding
MSTRG.3282.4	-0.942315	noncoding
MSTRG.3282.6	0.026039	coding
MSTRG.3282.5	-1.09041	noncoding
MSTRG.3282.7	-1.05373	noncoding
MSTRG.3294.1	-1.50424	noncoding
MSTRG.3294.2	-2.34864	noncoding
MSTRG.3296.4	-0.191933	noncoding
MSTRG.3296.3	0.988128	coding
MSTRG.3297.5	1.61385	coding
MSTRG.3300.1	0.008935	coding
MSTRG.3300.2	-0.025525	noncoding
MSTRG.3287.1	-1.84481	noncoding
MSTRG.3307.1	1.92912	coding
MSTRG.3307.4	0.914449	coding
MSTRG.3307.2	1.40479	coding
MSTRG.3307.3	1.87691	coding
MSTRG.3307.6	1.24447	coding
MSTRG.3312.1	0.300063	coding

MSTRG.3314.1	-0.672906	noncoding
MSTRG.3317.2	2.39884	coding
MSTRG.3317.3	2.36706	coding
MSTRG.3317.4	2.15322	coding
MSTRG.3317.5	1.91341	coding
MSTRG.3317.6	2.13424	coding
MSTRG.3320.1	0.083776	coding
MSTRG.3321.4	0.769431	coding
MSTRG.3321.3	0.874618	coding
MSTRG.3321.6	1.56535	coding
MSTRG.3333.1	0.804462	coding
MSTRG.3339.3	1.08527	coding
MSTRG.3339.4	2.1922	coding
MSTRG.3340.1	0.022342	coding
MSTRG.3342.2	-0.174614	noncoding
MSTRG.3342.1	0.154282	coding
MSTRG.3343.1	-1.34078	noncoding
MSTRG.3343.2	-0.501603	noncoding
MSTRG.3346.1	-2.71826	noncoding
MSTRG.3354.3	-1.75061	noncoding
MSTRG.3357.1	2.44251	coding
MSTRG.3357.2	2.51598	coding
MSTRG.3358.1	-0.856048	noncoding
MSTRG.3357.4	1.71436	coding
MSTRG.3359.6	-0.278586	noncoding
MSTRG.3364.1	1.49661	coding
MSTRG.3364.3	1.6759	coding
MSTRG.3364.4	1.21209	coding
MSTRG.3364.5	1.31828	coding
MSTRG.3364.6	1.21271	coding
MSTRG.3364.7	1.19776	coding
MSTRG.3371.1	1.61661	coding
MSTRG.3373.2	3.11345	coding
MSTRG.3374.3	-0.24274	noncoding
MSTRG.3375.1	-1.41484	noncoding
MSTRG.3381.2	0.26029	coding
MSTRG.3384.1	1.23054	coding
MSTRG.3389.3	0.370951	coding
MSTRG.3389.2	0.210681	coding
MSTRG.3389.4	-0.227486	noncoding
MSTRG.3390.2	-0.528478	noncoding
MSTRG.3415.2	0.020837	coding
MSTRG.3415.3	0.705907	coding
MSTRG.3418.1	0.640693	coding
MSTRG.3420.6	2.84412	coding
MSTRG.3420.7	2.8176	coding
MSTRG.3420.8	2.80678	coding
MSTRG.3421.1	0.092741	coding
MSTRG.3424.1	1.98844	coding
MSTRG.3400.2	2.38377	coding
MSTRG.3400.6	2.39421	coding
MSTRG.3400.3	2.34292	coding
MSTRG.3400.4	2.40567	coding
MSTRG.3401.2	-0.64634	noncoding

MSTRG.3402.2	1.88055	coding
MSTRG.3427.2	1.92031	coding
MSTRG.3429.1	2.09499	coding
MSTRG.3434.8	1.84285	coding
MSTRG.3435.1	1.21943	coding
MSTRG.3435.3	1.03054	coding
MSTRG.3438.1	-1.0367	noncoding
MSTRG.3444.1	0.667495	coding
MSTRG.3444.3	0.559306	coding
MSTRG.3444.5	0.927128	coding
MSTRG.3444.7	0.420667	coding
MSTRG.3444.12	0.98082	coding
MSTRG.3444.13	0.881854	coding
MSTRG.3441.3	0.596515	coding
MSTRG.3441.4	0.85329	coding
MSTRG.3446.1	-0.660319	noncoding
MSTRG.3470.3	-0.893213	noncoding
MSTRG.3471.3	1.10792	coding
MSTRG.3471.1	0.871998	coding
MSTRG.3471.5	1.25349	coding
MSTRG.3471.4	1.33552	coding
MSTRG.3471.10	0.923157	coding
MSTRG.3471.11	1.09645	coding
MSTRG.3471.12	0.935538	coding
MSTRG.3471.13	1.2416	coding
MSTRG.3471.14	0.98115	coding
MSTRG.3471.16	0.61711	coding
MSTRG.3472.3	1.88632	coding
MSTRG.3474.1	-1.53697	noncoding
MSTRG.3475.1	1.47601	coding
MSTRG.3475.3	1.39671	coding
MSTRG.3477.1	-1.11196	noncoding
MSTRG.3477.2	-2.00834	noncoding
MSTRG.3478.5	2.04968	coding
MSTRG.3478.7	1.90401	coding
MSTRG.3482.1	0.984597	coding
MSTRG.3447.2	-1.63481	noncoding
MSTRG.3454.1	-1.45499	noncoding
MSTRG.3463.2	0.081476	coding
MSTRG.3469.2	-0.797222	noncoding
MSTRG.3493.2	-0.198538	noncoding
MSTRG.3497.1	-2.05956	noncoding
MSTRG.3498.2	0.346033	coding
MSTRG.3504.1	0.575131	coding
MSTRG.3509.1	-2.06863	noncoding
MSTRG.3521.1	1.3903	coding
MSTRG.3521.2	1.96907	coding
MSTRG.3521.3	2.04797	coding
MSTRG.3521.4	2.00326	coding
MSTRG.3521.5	1.99065	coding
MSTRG.3521.6	2.09979	coding
MSTRG.3521.7	2.15013	coding
MSTRG.3521.11	1.53872	coding
MSTRG.3530.4	0.743449	coding

MSTRG.3536.2	0.665671	coding
MSTRG.3536.3	0.46536	coding
MSTRG.3536.4	0.499527	coding
MSTRG.3536.5	0.513589	coding
MSTRG.3536.6	0.299809	coding
MSTRG.3543.3	1.20208	coding
MSTRG.3544.1	1.75968	coding
MSTRG.3544.2	1.43533	coding
MSTRG.3544.4	1.74319	coding
MSTRG.3544.3	1.40854	coding
MSTRG.3544.7	1.62311	coding
MSTRG.3546.1	2.04025	coding
MSTRG.3547.5	2.97038	coding
MSTRG.3547.4	3.00975	coding
MSTRG.3547.6	0.574029	coding
MSTRG.3542.4	1.1693	coding
MSTRG.3550.3	-0.007686	noncoding
MSTRG.3548.1	-0.146307	noncoding
MSTRG.3557.2	1.68579	coding
MSTRG.3557.3	1.70391	coding
MSTRG.3558.1	0.191091	coding
MSTRG.3559.1	0.825675	coding
MSTRG.3561.4	0.301311	coding
MSTRG.3561.5	1.01	coding
MSTRG.3561.6	0.77155	coding
MSTRG.3561.7	1.42642	coding
MSTRG.3563.3	-0.192264	noncoding
MSTRG.3563.4	-0.107734	noncoding
MSTRG.3565.1	0.646412	coding
MSTRG.3562.1	1.79671	coding
MSTRG.3566.3	1.76564	coding
MSTRG.3566.2	1.79108	coding
MSTRG.3570.2	1.94045	coding
MSTRG.3580.1	1.0393	coding
MSTRG.3580.2	0.318098	coding
MSTRG.3588.6	0.79622	coding
MSTRG.3588.7	0.594295	coding
MSTRG.3592.6	-0.889851	noncoding
MSTRG.3593.3	-1.86227	noncoding
MSTRG.3598.1	1.41427	coding
MSTRG.3602.1	1.81244	coding
MSTRG.3604.2	1.31367	coding
MSTRG.3604.3	1.31067	coding
MSTRG.3604.4	0.572263	coding
MSTRG.3604.5	0.546833	coding
MSTRG.3604.6	0.585862	coding
MSTRG.3606.2	0.377969	coding
MSTRG.3606.3	0.003118	coding
MSTRG.3612.3	-0.524867	noncoding
MSTRG.3612.2	1.69977	coding
MSTRG.3612.4	0.911861	coding
MSTRG.3614.1	-0.743716	noncoding
MSTRG.3623.1	0.773371	coding
MSTRG.3623.2	2.71832	coding

MSTRG.3623.7	2.48361	coding
MSTRG.3631.1	-1.78278	noncoding
MSTRG.3633.4	-1.47041	noncoding
MSTRG.3642.2	0.88656	coding
MSTRG.3642.3	0.663394	coding
MSTRG.3650.1	0.65615	coding
MSTRG.3651.1	-0.164988	noncoding
MSTRG.3652.1	0.969276	coding
MSTRG.3654.1	-0.651676	noncoding
MSTRG.3658.1	-1.40639	noncoding
MSTRG.3659.2	1.0158	coding
MSTRG.3663.1	-1.92571	noncoding
MSTRG.3664.1	-1.55922	noncoding
MSTRG.3675.1	2.39574	coding
MSTRG.3675.2	2.37406	coding
MSTRG.3675.3	2.35395	coding
MSTRG.3675.4	1.78512	coding
MSTRG.3675.5	1.18495	coding
MSTRG.3675.6	1.85665	coding
MSTRG.3675.7	1.77779	coding
MSTRG.3675.8	2.32279	coding
MSTRG.3675.9	2.18955	coding
MSTRG.3675.10	2.02199	coding
MSTRG.3675.11	0.449121	coding
MSTRG.3676.1	0.730096	coding
MSTRG.3674.1	-2.29706	noncoding
MSTRG.3679.1	0.385562	coding
MSTRG.3679.2	0.823065	coding
MSTRG.3679.3	0.621122	coding
MSTRG.3685.1	2.87976	coding
MSTRG.3685.2	2.95887	coding
MSTRG.3687.2	0.253478	coding
MSTRG.3694.1	-0.209221	noncoding
MSTRG.3702.2	0.856961	coding
MSTRG.3702.3	1.0057	coding
MSTRG.3704.1	-1.79521	noncoding
MSTRG.3707.2	2.65136	coding
MSTRG.3707.3	-1.71084	noncoding
MSTRG.3708.2	-0.503384	noncoding
MSTRG.3710.1	1.62906	coding
MSTRG.3712.1	-0.15082	noncoding
MSTRG.3710.4	1.0379	coding
MSTRG.3710.5	1.08396	coding
MSTRG.3716.2	2.2332	coding
MSTRG.3716.3	2.21811	coding
MSTRG.3716.5	2.31301	coding
MSTRG.3716.6	2.25557	coding
MSTRG.3716.7	2.12569	coding
MSTRG.3716.9	1.19273	coding
MSTRG.3725.1	-1.17633	noncoding
MSTRG.3733.3	-0.78123	noncoding
MSTRG.3737.2	1.41725	coding
MSTRG.3721.1	-0.547974	noncoding
MSTRG.3721.2	-0.248525	noncoding

MSTRG.3722.1	1.56075	coding
MSTRG.3748.4	1.30293	coding
MSTRG.3749.6	2.37128	coding
MSTRG.3749.7	2.47892	coding
MSTRG.3749.3	2.52375	coding
MSTRG.3749.5	2.5523	coding
MSTRG.3755.1	-0.215885	noncoding
MSTRG.3764.2	1.10592	coding
MSTRG.3765.1	-1.67861	noncoding
MSTRG.3765.2	-0.85614	noncoding
MSTRG.3767.1	-1.21406	noncoding
MSTRG.3768.1	0.278511	coding
MSTRG.3769.1	-0.053121	noncoding
MSTRG.3770.1	-0.793641	noncoding
MSTRG.3773.2	-1.33135	noncoding
MSTRG.3773.3	-0.06676	noncoding
MSTRG.3773.4	0.32968	coding
MSTRG.3777.2	-1.59454	noncoding
MSTRG.3778.1	1.70211	coding
MSTRG.3756.2	-1.38626	noncoding
MSTRG.3780.4	1.77893	coding
MSTRG.3780.5	-1.5056	noncoding
MSTRG.3784.1	-1.44144	noncoding
MSTRG.3788.1	-0.937192	noncoding
MSTRG.3789.1	2.45198	coding
MSTRG.3789.3	2.76364	coding
MSTRG.3790.1	-0.423039	noncoding
MSTRG.3800.1	0.068602	coding
MSTRG.3802.2	1.55905	coding
MSTRG.3802.3	1.58367	coding
MSTRG.3802.4	1.80003	coding
MSTRG.3802.5	1.68407	coding
MSTRG.3804.1	2.68439	coding
MSTRG.3807.1	-0.062186	noncoding
MSTRG.3813.1	-0.264144	noncoding
MSTRG.3817.3	2.62877	coding
MSTRG.3821.2	1.63731	coding
MSTRG.3821.1	1.1357	coding
MSTRG.3821.4	1.75314	coding
MSTRG.3831.5	1.26357	coding
MSTRG.3832.3	1.2282	coding
MSTRG.3832.4	0.969792	coding
MSTRG.3832.5	1.07251	coding
MSTRG.3833.1	-0.002201	noncoding
MSTRG.3834.1	0.990112	coding
MSTRG.3834.2	0.861023	coding
MSTRG.3839.1	1.32655	coding
MSTRG.3825.2	1.39926	coding
MSTRG.3825.1	1.42528	coding
MSTRG.3828.1	0.733844	coding
MSTRG.3842.1	-0.406936	noncoding
MSTRG.3842.2	-0.440268	noncoding
MSTRG.3842.3	-0.182714	noncoding
MSTRG.3842.4	0.105806	coding

MSTRG.3842.6	1.87494	coding
MSTRG.3855.1	0.28715	coding
MSTRG.3854.3	0.904129	coding
MSTRG.3856.1	-1.82124	noncoding
MSTRG.3852.1	0.768961	coding
MSTRG.3852.2	1.36541	coding
MSTRG.3868.1	-0.744265	noncoding
MSTRG.3969.1	0.83064	coding
MSTRG.3970.1	-1.01305	noncoding
MSTRG.3971.1	-0.344109	noncoding
MSTRG.3975.1	-0.263415	noncoding
MSTRG.3976.1	-0.121082	noncoding
MSTRG.3977.1	0.326171	coding
MSTRG.3978.1	-1.08475	noncoding
MSTRG.3979.1	0.428327	coding
MSTRG.3980.1	-2.1696	noncoding
MSTRG.3972.5	0.104045	coding
MSTRG.3981.1	-2.10345	noncoding
MSTRG.3982.1	-2.22779	noncoding
MSTRG.3983.1	-0.075169	noncoding
MSTRG.3983.2	-1.17849	noncoding
MSTRG.3984.1	1.10908	coding
MSTRG.3984.2	-0.486267	noncoding
MSTRG.3985.1	0.244617	coding
MSTRG.3985.2	-0.591264	noncoding
MSTRG.3972.6	-0.003195	noncoding
MSTRG.3986.1	-0.931049	noncoding
MSTRG.3987.1	0.281332	coding
MSTRG.3987.2	-0.36531	noncoding
MSTRG.3987.3	-0.559593	noncoding
MSTRG.3987.4	-1.46223	noncoding
MSTRG.3989.1	0.825648	coding
MSTRG.3972.9	-0.053585	noncoding
MSTRG.3991.1	-1.24934	noncoding
MSTRG.3877.1	-0.840552	noncoding
MSTRG.3883.1	-2.34485	noncoding
MSTRG.3889.2	0.480678	coding
MSTRG.3890.1	0.620506	coding
MSTRG.3893.1	-0.02938	noncoding
MSTRG.3895.3	2.39097	coding
MSTRG.3895.4	2.56986	coding
MSTRG.3895.6	1.90667	coding
MSTRG.3895.7	2.462	coding
MSTRG.3896.1	-2.21738	noncoding
MSTRG.3897.1	1.49787	coding
MSTRG.3900.2	2.19971	coding
MSTRG.3902.2	1.51651	coding
MSTRG.3903.2	1.10108	coding
MSTRG.3903.3	1.49273	coding
MSTRG.3903.4	0.769623	coding
MSTRG.3903.5	0.330267	coding
MSTRG.3910.2	2.37559	coding
MSTRG.3911.3	1.16866	coding
MSTRG.3913.3	1.46935	coding

MSTRG.3918.1	-1.59359	noncoding
MSTRG.3935.6	2.1556	coding
MSTRG.3935.5	2.32491	coding
MSTRG.3935.7	2.20903	coding
MSTRG.3935.8	1.47348	coding
MSTRG.3935.9	1.07404	coding
MSTRG.3935.11	-0.256709	noncoding
MSTRG.3936.1	-1.56547	noncoding
MSTRG.3937.1	-0.320434	noncoding
MSTRG.3930.1	-1.60177	noncoding
MSTRG.3934.1	-1.03969	noncoding
MSTRG.3939.2	0.759215	coding
MSTRG.3939.4	0.333658	coding
MSTRG.3940.3	0.91309	coding
MSTRG.3940.6	0.156548	coding
MSTRG.3940.7	0.9388	coding
MSTRG.3941.1	-1.49179	noncoding
MSTRG.3954.1	-2.78486	noncoding
MSTRG.3959.2	2.09589	coding
MSTRG.3960.1	-1.20843	noncoding
MSTRG.3963.3	1.91171	coding
MSTRG.3964.1	-1.82015	noncoding
MSTRG.3963.6	1.4468	coding
MSTRG.3965.2	0.841054	coding
MSTRG.3993.2	1.33723	coding
MSTRG.3995.1	-2.73079	noncoding
MSTRG.3997.3	-0.30947	noncoding
MSTRG.3997.2	0.68139	coding
MSTRG.4000.1	0.405986	coding
MSTRG.4003.1	1.76572	coding
MSTRG.4003.3	0.741419	coding
MSTRG.4003.4	0.957713	coding
MSTRG.4004.1	0.53597	coding
MSTRG.4006.1	0.557788	coding
MSTRG.4006.2	0.380799	coding
MSTRG.4010.2	1.3383	coding
MSTRG.4010.1	1.56595	coding
MSTRG.4010.3	1.67786	coding
MSTRG.4011.1	-0.504316	noncoding
MSTRG.4010.6	-0.494415	noncoding
MSTRG.4014.1	0.646897	coding
MSTRG.4017.1	1.12692	coding
MSTRG.4017.3	0.97911	coding
MSTRG.4008.3	0.935262	coding
MSTRG.4020.1	1.60039	coding
MSTRG.4023.2	1.72796	coding
MSTRG.4024.2	-0.365183	noncoding
MSTRG.4024.4	-0.708995	noncoding
MSTRG.4024.5	-0.318533	noncoding
MSTRG.4026.1	2.29217	coding
MSTRG.4026.2	2.41287	coding
MSTRG.4026.3	2.14682	coding
MSTRG.4026.4	1.52216	coding
MSTRG.4026.5	2.26719	coding

MSTRG.4026.6	2.50926	coding
MSTRG.4028.5	2.2669	coding
MSTRG.4028.6	1.77139	coding
MSTRG.4028.7	1.71133	coding
MSTRG.4028.8	2.33312	coding
MSTRG.4028.9	1.71974	coding
MSTRG.4030.3	1.3066	coding
MSTRG.4036.1	2.13438	coding
MSTRG.4037.1	-2.4925	noncoding
MSTRG.4047.3	2.73649	coding
MSTRG.4047.4	-0.101306	noncoding
MSTRG.4047.5	2.86662	coding
MSTRG.4047.6	-0.523809	noncoding
MSTRG.4047.7	2.73432	coding
MSTRG.4047.8	2.20736	coding
MSTRG.4047.9	0.332666	coding
MSTRG.4038.1	-1.05612	noncoding
MSTRG.4051.1	1.69175	coding
MSTRG.4051.2	1.4774	coding
MSTRG.4052.4	0.812383	coding
MSTRG.4053.1	-0.62422	noncoding
MSTRG.4055.1	-1.8026	noncoding
MSTRG.4058.1	1.72862	coding
MSTRG.4058.5	2.27747	coding
MSTRG.4072.1	1.72519	coding
MSTRG.4072.3	-1.60963	noncoding
MSTRG.4074.1	-1.83574	noncoding
MSTRG.4079.1	-0.357215	noncoding
MSTRG.4080.1	-3.02362	noncoding
MSTRG.4081.1	0.302636	coding
MSTRG.4082.1	-1.64944	noncoding
MSTRG.4083.1	0.88502	coding
MSTRG.4068.2	2.52057	coding
MSTRG.4070.1	-1.10909	noncoding
MSTRG.4092.3	0.93335	coding
MSTRG.4092.4	0.729776	coding
MSTRG.4093.1	0.115234	coding
MSTRG.4093.2	-2.35468	noncoding
MSTRG.4094.1	0.111405	coding
MSTRG.4095.1	-1.76559	noncoding
MSTRG.4096.1	-0.988987	noncoding
MSTRG.4092.5	1.49565	coding
MSTRG.4092.6	1.64542	coding
MSTRG.4092.7	0.225506	coding
MSTRG.4092.8	0.666875	coding
MSTRG.4099.1	0.917284	coding
MSTRG.4104.1	0.254359	coding
MSTRG.4108.1	0.574912	coding
MSTRG.4108.2	0.420262	coding
MSTRG.4108.3	1.21961	coding
MSTRG.4108.6	-0.19355	noncoding
MSTRG.4108.5	0.546092	coding
MSTRG.4116.1	1.18902	coding
MSTRG.4122.3	0.833695	coding

MSTRG.4122.2	0.99701	coding
MSTRG.4122.1	1.19092	coding
MSTRG.4125.3	0.750205	coding
MSTRG.4129.4	1.45094	coding
MSTRG.4129.7	1.57338	coding
MSTRG.4129.8	1.80216	coding
MSTRG.4129.9	1.73751	coding
MSTRG.4130.1	0.547798	coding
MSTRG.4143.2	0.21271	coding
MSTRG.4143.4	-2.19275	noncoding
MSTRG.4143.3	0.88501	coding
MSTRG.4144.1	-2.51708	noncoding
MSTRG.4143.6	0.433975	coding
MSTRG.4146.2	0.682587	coding
MSTRG.4147.1	-1.57789	noncoding
MSTRG.4152.2	0.704433	coding
MSTRG.4131.1	-2.87223	noncoding
MSTRG.4132.3	2.10684	coding
MSTRG.4132.5	2.17581	coding
MSTRG.4132.6	1.82285	coding
MSTRG.4133.1	1.25451	coding
MSTRG.4134.1	-0.593358	noncoding
MSTRG.4139.1	-0.069854	noncoding
MSTRG.4163.2	2.51021	coding
MSTRG.4163.1	2.39263	coding
MSTRG.4163.3	1.05469	coding
MSTRG.4163.4	2.31586	coding
MSTRG.4163.5	2.43062	coding
MSTRG.4163.6	1.77807	coding
MSTRG.4163.7	1.99445	coding
MSTRG.4162.1	-2.45303	noncoding
MSTRG.4178.5	1.83665	coding
MSTRG.4178.3	1.89236	coding
MSTRG.4178.6	1.00968	coding
MSTRG.4178.4	1.09731	coding
MSTRG.4178.8	1.06433	coding
MSTRG.4178.7	1.39522	coding
MSTRG.4183.1	-1.03098	noncoding
MSTRG.4186.1	0.139226	coding
MSTRG.4186.2	-0.121341	noncoding
MSTRG.4186.3	-0.460351	noncoding
MSTRG.4187.1	-2.21381	noncoding
MSTRG.4203.3	1.03392	coding
MSTRG.4203.5	0.137707	coding
MSTRG.4203.2	0.39556	coding
MSTRG.4203.6	-0.082951	noncoding
MSTRG.4203.7	0.834617	coding
MSTRG.4213.3	0.861598	coding
MSTRG.4214.1	-0.277802	noncoding
MSTRG.4216.1	-2.55118	noncoding
MSTRG.4217.3	-1.31317	noncoding
MSTRG.4226.1	1.76699	coding
MSTRG.4226.2	1.4794	coding
MSTRG.4227.1	-1.79526	noncoding

MSTRG.4226.3	0.376841	coding
MSTRG.4228.7	1.27672	coding
MSTRG.4228.3	1.11214	coding
MSTRG.4229.4	-0.388673	noncoding
MSTRG.4229.2	-0.127818	noncoding
MSTRG.4229.3	1.40407	coding
MSTRG.4230.1	-1.04799	noncoding
MSTRG.4231.1	-2.00913	noncoding
MSTRG.4219.1	-2.22072	noncoding
MSTRG.4246.3	-2.39846	noncoding
MSTRG.4250.5	-1.05442	noncoding
MSTRG.4256.1	2.10798	coding
MSTRG.4256.3	1.64726	coding
MSTRG.4256.2	1.66218	coding
MSTRG.4256.4	1.65037	coding
MSTRG.4257.1	-1.82246	noncoding
MSTRG.4260.3	0.585608	coding
MSTRG.4260.4	0.701415	coding
MSTRG.4261.3	2.0719	coding
MSTRG.4261.2	2.13633	coding
MSTRG.4261.4	1.31685	coding
MSTRG.4262.1	-2.18561	noncoding
MSTRG.4293.3	-0.143629	noncoding
MSTRG.4293.4	-0.099429	noncoding
MSTRG.4293.5	0.150087	coding
MSTRG.4293.6	-0.506036	noncoding
MSTRG.4293.7	-0.315846	noncoding
MSTRG.4296.1	-1.36494	noncoding
MSTRG.4301.2	2.07165	coding
MSTRG.4301.1	2.08896	coding
MSTRG.4301.3	2.05176	coding
MSTRG.4301.4	1.82295	coding
MSTRG.4301.5	1.99589	coding
MSTRG.4301.6	1.98356	coding
MSTRG.4302.1	-0.053261	noncoding
MSTRG.4303.2	-0.181193	noncoding
MSTRG.4304.2	-0.353615	noncoding
MSTRG.4297.1	-1.27557	noncoding
MSTRG.4298.1	-1.23024	noncoding
MSTRG.4312.5	0.590012	coding
MSTRG.4312.4	2.94368	coding
MSTRG.4312.2	2.65824	coding
MSTRG.4312.1	1.7396	coding
MSTRG.4312.3	2.66351	coding
MSTRG.4310.2	0.264522	coding
MSTRG.4317.2	1.51525	coding
MSTRG.4319.1	0.960225	coding
MSTRG.4319.2	0.803084	coding
MSTRG.4319.3	0.975058	coding
MSTRG.4319.4	0.568997	coding
MSTRG.4319.5	-0.920489	noncoding
MSTRG.4325.2	0.717322	coding
MSTRG.4325.3	-0.82007	noncoding
MSTRG.4326.2	-0.712253	noncoding

MSTRG.4328.2	-0.016709	noncoding
MSTRG.4328.6	-0.098551	noncoding
MSTRG.4328.5	1.03357	coding
MSTRG.4328.7	0.786511	coding
MSTRG.4328.11	0.29232	coding
MSTRG.4329.3	2.95168	coding
MSTRG.4330.1	-3.0683	noncoding
MSTRG.4335.2	2.27192	coding
MSTRG.4341.1	-1.33285	noncoding
MSTRG.4341.2	-0.13067	noncoding
MSTRG.4341.3	-0.516055	noncoding
MSTRG.4343.1	2.54145	coding
MSTRG.4344.1	-1.52357	noncoding
MSTRG.4340.1	-1.75154	noncoding
MSTRG.4346.2	-2.12191	noncoding
MSTRG.4345.1	0.878596	coding
MSTRG.4359.2	3.18023	coding
MSTRG.4357.1	0.808856	coding
MSTRG.4358.2	1.00901	coding
MSTRG.4360.2	0.537314	coding
MSTRG.4361.1	0.391302	coding
MSTRG.4361.3	-0.491708	noncoding
MSTRG.4363.1	1.57486	coding
MSTRG.4363.2	1.01531	coding
MSTRG.4363.3	2.39177	coding
MSTRG.4367.4	0.381567	coding
MSTRG.4367.5	0.152614	coding
MSTRG.4369.1	0.105907	coding
MSTRG.4365.2	0.149187	coding
MSTRG.4371.1	-0.290093	noncoding
MSTRG.4371.2	-0.167226	noncoding
MSTRG.4375.1	-1.80575	noncoding
MSTRG.4372.2	1.8685	coding
MSTRG.4382.1	-0.92923	noncoding
MSTRG.4388.1	2.01755	coding
MSTRG.4388.3	2.4788	coding
MSTRG.4388.9	2.0136	coding
MSTRG.4384.2	0.469128	coding
MSTRG.4390.1	-0.447752	noncoding
MSTRG.4390.2	-1.91495	noncoding
MSTRG.4390.3	-2.47456	noncoding
MSTRG.4399.1	-2.19656	noncoding
MSTRG.4400.2	1.72116	coding
MSTRG.4401.2	0.685048	coding
MSTRG.4401.3	0.748979	coding
MSTRG.4399.3	1.16795	coding
MSTRG.4401.5	0.518322	coding
MSTRG.4401.8	0.443913	coding
MSTRG.4401.9	0.243368	coding
MSTRG.4401.11	0.383795	coding
MSTRG.4401.12	0.358501	coding
MSTRG.4401.10	0.289719	coding
MSTRG.4401.13	0.368859	coding
MSTRG.4401.14	0.39337	coding

MSTRG.4401.16	0.046778	coding
MSTRG.4403.1	0.852512	coding
MSTRG.4403.3	1.91135	coding
MSTRG.4404.1	-0.645869	noncoding
MSTRG.4408.1	0.206093	coding
MSTRG.4409.2	1.29191	coding
MSTRG.4409.3	0.935975	coding
MSTRG.4411.1	1.07962	coding
MSTRG.4411.2	1.31771	coding
MSTRG.4412.1	1.26512	coding
MSTRG.4412.2	1.31073	coding
MSTRG.4412.3	0.815577	coding
MSTRG.4413.1	0.751696	coding
MSTRG.4413.2	0.927477	coding
MSTRG.4413.5	0.669004	coding
MSTRG.4414.2	0.685811	coding
MSTRG.4418.1	3.12611	coding
MSTRG.4420.9	0.394898	coding
MSTRG.4420.10	0.493005	coding
MSTRG.4420.11	0.517473	coding
MSTRG.4421.1	0.078973	coding
MSTRG.4422.1	-2.02479	noncoding
MSTRG.4422.2	-1.85344	noncoding
MSTRG.4423.4	1.35963	coding
MSTRG.4423.5	1.31431	coding
MSTRG.4424.1	-2.14333	noncoding
MSTRG.4426.4	2.37133	coding
MSTRG.4428.2	1.67058	coding
MSTRG.4429.1	-1.08558	noncoding
MSTRG.4431.1	-0.581797	noncoding
MSTRG.4432.2	0.266359	coding
MSTRG.4434.1	0.353896	coding
MSTRG.4446.2	0.342284	coding
MSTRG.4446.1	1.68354	coding
MSTRG.4446.4	1.58756	coding
MSTRG.4448.1	0.097135	coding
MSTRG.4448.2	-0.709718	noncoding
MSTRG.4449.1	-0.36788	noncoding
MSTRG.4450.1	-1.02935	noncoding
MSTRG.4451.1	-0.319796	noncoding
MSTRG.4452.1	-1.07287	noncoding
MSTRG.4454.1	-0.127637	noncoding
MSTRG.4456.1	-2.48812	noncoding
MSTRG.4443.3	0.346934	coding
MSTRG.4445.1	-0.15993	noncoding
MSTRG.4462.1	0.23308	coding
MSTRG.4462.2	0.41068	coding
MSTRG.4463.1	-1.52139	noncoding
MSTRG.4464.1	-1.34233	noncoding
MSTRG.4465.1	-2.62557	noncoding
MSTRG.4467.4	0.869763	coding
MSTRG.4470.3	1.77665	coding
MSTRG.4470.4	1.76417	coding
MSTRG.4470.5	1.61758	coding

MSTRG.4473.3	1.1901	coding
MSTRG.4471.2	-0.970134	noncoding
MSTRG.4471.1	-0.998812	noncoding
MSTRG.4474.5	1.72684	coding
MSTRG.4474.4	2.66315	coding
MSTRG.4474.7	1.10229	coding
MSTRG.4474.8	0.405757	coding
MSTRG.4474.10	0.861798	coding
MSTRG.4474.12	0.723222	coding
MSTRG.4474.13	0.882241	coding
MSTRG.4474.14	0.844484	coding
MSTRG.4474.11	0.804488	coding
MSTRG.4479.6	-1.57178	noncoding
MSTRG.4479.4	-0.151699	noncoding
MSTRG.4479.7	0.083984	coding
MSTRG.4479.2	-0.015055	noncoding
MSTRG.4479.1	-0.680447	noncoding
MSTRG.4480.2	0.481393	coding
MSTRG.4481.4	0.445926	coding
MSTRG.4481.5	0.340038	coding
MSTRG.4481.1	0.36219	coding
MSTRG.4481.2	0.389515	coding
MSTRG.4477.2	1.86023	coding
MSTRG.4477.3	1.82452	coding
MSTRG.4477.4	1.89699	coding
MSTRG.4487.1	1.96092	coding
MSTRG.4488.1	0.038143	coding
MSTRG.4488.2	0.028399	coding
MSTRG.4488.3	-0.879282	noncoding
MSTRG.4494.1	0.960461	coding
MSTRG.4495.1	-0.058049	noncoding
MSTRG.4494.8	0.934053	coding
MSTRG.4494.9	0.583965	coding
MSTRG.4496.1	-1.05032	noncoding
MSTRG.4494.10	1.02235	coding
MSTRG.4494.11	0.40016	coding
MSTRG.4498.1	-0.138639	noncoding
MSTRG.4494.12	1.0207	coding
MSTRG.4494.13	-0.580343	noncoding
MSTRG.4499.3	1.25813	coding
MSTRG.4483.6	2.06589	coding
MSTRG.4483.4	1.5552	coding
MSTRG.4483.5	2.15062	coding
MSTRG.4483.3	2.53546	coding
MSTRG.4503.1	-2.02165	noncoding
MSTRG.4504.5	1.30285	coding
MSTRG.4506.1	-1.01925	noncoding
MSTRG.4515.1	2.51597	coding
MSTRG.4516.2	-1.51265	noncoding
MSTRG.4516.1	-1.56256	noncoding
MSTRG.4520.2	1.75028	coding
MSTRG.4520.4	1.21727	coding
MSTRG.4521.1	-2.04995	noncoding
MSTRG.4533.11	-0.054355	noncoding

MSTRG.4533.7	1.07218	coding
MSTRG.4533.10	1.11155	coding
MSTRG.4533.3	1.28597	coding
MSTRG.4533.1	1.61664	coding
MSTRG.4533.2	1.63501	coding
MSTRG.4533.4	1.67002	coding
MSTRG.4533.8	0.997163	coding
MSTRG.4533.6	0.886635	coding
MSTRG.4533.5	0.557942	coding
MSTRG.4523.3	0.470538	coding
MSTRG.4523.4	0.318355	coding
MSTRG.4524.1	-0.286679	noncoding
MSTRG.4525.1	0.259254	coding
MSTRG.4538.1	-0.588602	noncoding
MSTRG.4540.1	-0.221776	noncoding
MSTRG.4541.2	0.126384	coding
MSTRG.4549.1	-1.39767	noncoding
MSTRG.4552.4	1.80895	coding
MSTRG.4553.1	-0.101877	noncoding
MSTRG.4543.2	2.73612	coding
MSTRG.4543.3	2.85694	coding
MSTRG.4562.2	0.031635	coding
MSTRG.4562.3	0.61317	coding
MSTRG.4564.1	2.13567	coding
MSTRG.4555.2	3.15169	coding
MSTRG.4557.1	-1.67236	noncoding
MSTRG.4559.3	-0.779799	noncoding
MSTRG.4571.1	0.370712	coding
MSTRG.4572.1	-1.14894	noncoding
MSTRG.4574.1	-0.769366	noncoding
MSTRG.4578.1	0.002777	coding
MSTRG.4578.3	0.109823	coding
MSTRG.4579.2	0.198696	coding
MSTRG.4580.2	-0.449019	noncoding
MSTRG.4592.1	1.96486	coding
MSTRG.4592.2	1.44529	coding
MSTRG.4594.1	1.33704	coding
MSTRG.4594.2	2.30909	coding
MSTRG.4594.4	2.33633	coding
MSTRG.4594.6	1.20866	coding
MSTRG.4594.5	1.45894	coding
MSTRG.4594.7	0.039477	coding
MSTRG.4595.1	-2.14296	noncoding
MSTRG.4594.9	0.046998	coding
MSTRG.4594.10	1.30743	coding
MSTRG.4594.11	0.286492	coding
MSTRG.4585.3	1.38666	coding
MSTRG.4585.4	-1.92196	noncoding
MSTRG.4585.5	0.970397	coding
MSTRG.4583.1	0.63783	coding
MSTRG.4588.1	-1.34932	noncoding
MSTRG.4600.1	-0.389643	noncoding
MSTRG.4600.2	-0.69714	noncoding
MSTRG.4600.3	-0.523204	noncoding

MSTRG.4601.3	1.1764	coding
MSTRG.4601.4	1.34127	coding
MSTRG.4602.1	0.468075	coding
MSTRG.4603.1	0.294809	coding
MSTRG.4601.8	1.62989	coding
MSTRG.4601.9	1.58918	coding
MSTRG.4604.1	0.759302	coding
MSTRG.4604.3	0.289091	coding
MSTRG.4605.7	0.689192	coding
MSTRG.4605.8	0.605531	coding
MSTRG.4605.4	1.39719	coding
MSTRG.4605.6	1.34539	coding
MSTRG.4605.9	0.938843	coding
MSTRG.4605.10	-0.227945	noncoding
MSTRG.4608.3	-1.65155	noncoding
MSTRG.4608.6	0.085448	coding
MSTRG.4609.2	1.32859	coding
MSTRG.4609.1	1.93812	coding
MSTRG.4611.1	-2.18027	noncoding
MSTRG.4615.2	-1.48425	noncoding
MSTRG.4616.2	2.1377	coding
MSTRG.4617.1	1.06063	coding
MSTRG.4618.1	1.32139	coding
MSTRG.4616.3	0.86137	coding
MSTRG.4627.9	-0.299875	noncoding
MSTRG.4627.10	0.121214	coding
MSTRG.4627.7	0.135624	coding
MSTRG.4620.2	1.52804	coding
MSTRG.4632.3	-0.341528	noncoding
MSTRG.4634.9	2.34224	coding
MSTRG.4634.10	1.80336	coding
MSTRG.4634.13	2.07646	coding
MSTRG.4706.9	1.9806	coding
MSTRG.4706.6	2.51	coding
MSTRG.4706.4	2.35225	coding
MSTRG.4706.27	2.2944	coding
MSTRG.4706.18	1.84023	coding
MSTRG.4706.25	2.00625	coding
MSTRG.4706.19	2.32523	coding
MSTRG.4706.12	1.6132	coding
MSTRG.4706.14	1.85062	coding
MSTRG.4706.24	2.24459	coding
MSTRG.4706.16	2.26273	coding
MSTRG.4706.15	2.20472	coding
MSTRG.4706.30	0.978328	coding
MSTRG.4706.31	-0.500495	noncoding
MSTRG.4706.32	-0.141559	noncoding
MSTRG.4706.33	0.243293	coding
MSTRG.4648.1	-0.576621	noncoding
MSTRG.4649.1	0.097845	coding
MSTRG.4649.2	0.355712	coding
MSTRG.4651.1	0.338787	coding
MSTRG.4660.3	1.1991	coding
MSTRG.4654.1	0.150309	coding

MSTRG.4654.2	0.128869	coding
MSTRG.4666.1	0.448119	coding
MSTRG.4673.2	1.11489	coding
MSTRG.4673.3	0.572206	coding
MSTRG.4675.2	2.75646	coding
MSTRG.4679.3	1.18655	coding
MSTRG.4679.2	1.25657	coding
MSTRG.4679.5	1.37859	coding
MSTRG.4680.6	0.83761	coding
MSTRG.4680.7	1.14708	coding
MSTRG.4680.8	-0.157789	noncoding
MSTRG.4677.1	0.116966	coding
MSTRG.4677.4	0.309889	coding
MSTRG.4682.1	1.54543	coding
MSTRG.4682.2	-0.215371	noncoding
MSTRG.4687.1	1.10546	coding
MSTRG.4687.5	1.00057	coding
MSTRG.4687.4	1.28466	coding
MSTRG.4689.1	2.72849	coding
MSTRG.4689.2	2.2502	coding
MSTRG.4691.1	-1.54241	noncoding
MSTRG.4692.2	1.73777	coding
MSTRG.4698.1	-2.16214	noncoding
MSTRG.4700.1	0.171194	coding
MSTRG.4701.1	0.21414	coding
MSTRG.4703.2	2.295	coding
MSTRG.4703.1	1.23315	coding
MSTRG.4703.3	0.461392	coding
MSTRG.4704.1	-0.763853	noncoding
MSTRG.4703.4	2.0905	coding
MSTRG.4718.3	0.995214	coding
MSTRG.4718.6	1.40213	coding
MSTRG.4718.8	0.582164	coding
MSTRG.4705.2	-0.078826	noncoding
MSTRG.4709.2	-1.77845	noncoding
MSTRG.4709.1	-1.03651	noncoding
MSTRG.4711.4	1.93453	coding
MSTRG.4711.5	1.89426	coding
MSTRG.4711.6	1.93628	coding
MSTRG.4710.1	0.897197	coding
MSTRG.4725.1	2.64337	coding
MSTRG.4725.2	2.44989	coding
MSTRG.4725.12	-1.38386	noncoding
MSTRG.4725.10	2.79762	coding
MSTRG.4725.11	2.45123	coding
MSTRG.4725.9	2.54536	coding
MSTRG.4725.13	2.63768	coding
MSTRG.4725.16	2.27259	coding
MSTRG.4725.17	2.55605	coding
MSTRG.4725.18	2.66425	coding
MSTRG.4725.19	2.38221	coding
MSTRG.4725.20	2.41886	coding
MSTRG.4725.21	2.3925	coding
MSTRG.4725.22	2.64393	coding

MSTRG.4722.3	-0.583516	noncoding
MSTRG.4729.1	0.785401	coding
MSTRG.4734.4	1.62752	coding
MSTRG.4734.5	1.77138	coding
MSTRG.4735.1	-0.679474	noncoding
MSTRG.4735.2	0.641249	coding
MSTRG.4730.1	-0.96324	noncoding
MSTRG.4731.1	-1.11986	noncoding
MSTRG.4738.1	-1.67828	noncoding
MSTRG.4741.2	0.484965	coding
MSTRG.4744.1	-1.39702	noncoding
MSTRG.4746.1	-0.63869	noncoding
MSTRG.4747.3	1.98525	coding
MSTRG.4750.1	-2.50059	noncoding
MSTRG.4751.1	-2.24383	noncoding
MSTRG.4754.1	2.86679	coding
MSTRG.4754.4	2.88571	coding
MSTRG.4754.5	1.95299	coding
MSTRG.4754.6	2.11531	coding
MSTRG.4754.7	2.07235	coding
MSTRG.4752.1	2.4376	coding
MSTRG.4757.1	-0.745369	noncoding
MSTRG.4758.2	0.21273	coding
MSTRG.4758.3	-0.202972	noncoding
MSTRG.4761.1	2.45532	coding
MSTRG.4761.3	2.30894	coding
MSTRG.4761.5	2.30319	coding
MSTRG.4762.1	-2.39848	noncoding
MSTRG.4763.1	0.347754	coding
MSTRG.4767.2	0.832545	coding
MSTRG.4768.1	-0.191071	noncoding
MSTRG.4767.6	0.381806	coding
MSTRG.4769.1	-0.241183	noncoding
MSTRG.4767.7	0.740562	coding
MSTRG.4772.2	0.994402	coding
MSTRG.4766.1	-1.68955	noncoding
MSTRG.4766.2	-1.50023	noncoding
MSTRG.4766.3	-1.80951	noncoding
MSTRG.4774.3	0.131663	coding
MSTRG.4774.2	0.077816	coding
MSTRG.4781.2	1.87477	coding
MSTRG.4785.3	2.5961	coding
MSTRG.4785.6	0.307631	coding
MSTRG.4782.1	1.1777	coding
MSTRG.4782.3	1.23142	coding
MSTRG.4787.1	1.56263	coding
MSTRG.4787.2	1.10543	coding
MSTRG.4787.3	1.60789	coding
MSTRG.4788.1	-0.488041	noncoding
MSTRG.4789.1	-1.26243	noncoding
MSTRG.4796.1	1.09648	coding
MSTRG.4800.2	1.30503	coding
MSTRG.4811.1	-0.576452	noncoding
MSTRG.4811.3	-0.469825	noncoding

MSTRG.4813.1	2.44498	coding
MSTRG.4814.1	-0.186429	noncoding
MSTRG.4815.1	-1.27386	noncoding
MSTRG.4826.4	-0.154944	noncoding
MSTRG.4826.5	0.598648	coding
MSTRG.4826.2	0.672151	coding
MSTRG.4826.3	1.90937	coding
MSTRG.4826.10	1.87271	coding
MSTRG.4816.1	-1.2214	noncoding
MSTRG.4818.1	1.80188	coding
MSTRG.4818.2	1.33836	coding
MSTRG.4818.4	1.39447	coding
MSTRG.4819.1	-1.54917	noncoding
MSTRG.4830.2	1.17884	coding
MSTRG.4831.4	1.56463	coding
MSTRG.4831.5	1.63313	coding
MSTRG.4836.1	0.638346	coding
MSTRG.4836.2	0.666351	coding
MSTRG.4843.1	-2.29902	noncoding
MSTRG.4844.1	-2.19693	noncoding
MSTRG.4848.1	-0.287935	noncoding
MSTRG.4849.1	-0.785135	noncoding
MSTRG.4860.1	-1.56581	noncoding
MSTRG.4861.1	0.639023	coding
MSTRG.4861.2	0.878339	coding
MSTRG.4870.1	-1.21003	noncoding
MSTRG.4876.1	1.48621	coding
MSTRG.4877.1	-0.818489	noncoding
MSTRG.4882.1	-0.95241	noncoding
MSTRG.4882.2	-0.699251	noncoding
MSTRG.4873.1	-1.84404	noncoding
MSTRG.4886.1	-0.297773	noncoding
MSTRG.4887.1	2.08446	coding
MSTRG.4887.2	1.61345	coding
MSTRG.4895.2	0.495702	coding
MSTRG.4903.1	-1.7642	noncoding
MSTRG.4903.2	0.750749	coding
MSTRG.4921.2	2.45435	coding
MSTRG.4921.3	2.3763	coding
MSTRG.4922.2	0.944074	coding
MSTRG.4924.1	-0.976263	noncoding
MSTRG.4925.1	-1.3242	noncoding
MSTRG.4928.3	3.04929	coding
MSTRG.4928.4	2.10955	coding
MSTRG.4929.1	-0.193848	noncoding
MSTRG.4928.6	-0.506619	noncoding
MSTRG.4930.1	0.469602	coding
MSTRG.4928.7	2.59757	coding
MSTRG.4912.2	2.2039	coding
MSTRG.4913.1	1.08697	coding
MSTRG.4913.3	0.753725	coding
MSTRG.4913.4	0.40008	coding
MSTRG.4920.1	-2.08779	noncoding
MSTRG.4942.1	-1.58488	noncoding

MSTRG.4950.1	-0.827621	noncoding
MSTRG.4951.2	-0.894962	noncoding
MSTRG.4953.1	-1.7354	noncoding
MSTRG.4961.1	2.46204	coding
MSTRG.4961.7	2.37195	coding
MSTRG.4961.4	2.50215	coding
MSTRG.4961.5	2.50048	coding
MSTRG.4961.6	2.38802	coding
MSTRG.4961.10	2.59437	coding
MSTRG.4961.8	2.57847	coding
MSTRG.4961.9	2.57812	coding
MSTRG.4961.12	1.67335	coding
MSTRG.4961.11	2.61511	coding
MSTRG.4961.13	2.57827	coding
MSTRG.4961.15	2.42657	coding
MSTRG.4961.14	2.45845	coding
MSTRG.4962.1	0.71304	coding
MSTRG.4963.1	-0.313426	noncoding
MSTRG.4964.1	-0.348267	noncoding
MSTRG.4965.1	0.272236	coding
MSTRG.4965.2	-0.800346	noncoding
MSTRG.4965.3	-1.06203	noncoding
MSTRG.4966.1	-1.8204	noncoding
MSTRG.4967.1	-0.071802	noncoding
MSTRG.4961.17	0.392404	coding
MSTRG.4955.1	-0.405962	noncoding
MSTRG.4956.1	-2.31549	noncoding
MSTRG.4959.3	1.88043	coding
MSTRG.4960.1	-1.8024	noncoding
MSTRG.4975.2	0.588088	coding
MSTRG.4980.10	1.56138	coding
MSTRG.4980.4	1.59954	coding
MSTRG.4980.9	1.5867	coding
MSTRG.4980.8	1.40023	coding
MSTRG.4980.5	1.34674	coding
MSTRG.4980.2	1.38077	coding
MSTRG.4981.2	1.17096	coding
MSTRG.4985.6	0.893932	coding
MSTRG.4985.8	1.48684	coding
MSTRG.4990.3	0.465858	coding
MSTRG.4990.4	-1.11752	noncoding
MSTRG.4991.1	-0.277312	noncoding
MSTRG.4998.3	3.07381	coding
MSTRG.4998.4	1.71917	coding
MSTRG.4998.5	1.70501	coding
MSTRG.4998.6	0.315122	coding
MSTRG.4998.7	-2.43907	noncoding
MSTRG.4988.6	1.44197	coding
MSTRG.5003.1	-0.898296	noncoding
MSTRG.5020.1	-1.78351	noncoding
MSTRG.5021.2	0.717924	coding
MSTRG.5046.1	-2.50636	noncoding
MSTRG.5052.1	-1.17627	noncoding
MSTRG.5077.2	1.49542	coding

MSTRG.5077.3	1.75177	coding
MSTRG.5078.1	-0.98832	noncoding
MSTRG.5106.1	-2.18026	noncoding
MSTRG.5095.2	-0.831139	noncoding
MSTRG.5137.1	1.63744	coding
MSTRG.5138.1	-1.25893	noncoding
MSTRG.5137.3	0.478507	coding
MSTRG.5137.2	1.18023	coding
MSTRG.5137.4	2.43088	coding
MSTRG.5137.5	0.136862	coding
MSTRG.5137.6	-0.616466	noncoding
MSTRG.5140.2	-0.962928	noncoding
MSTRG.5137.7	2.23186	coding
MSTRG.5137.8	2.29789	coding
MSTRG.5137.10	-0.642907	noncoding
MSTRG.5133.1	3.05708	coding
MSTRG.5133.3	2.26903	coding
MSTRG.5133.4	-1.76662	noncoding
MSTRG.5134.1	0.330835	coding
MSTRG.5135.1	-0.605569	noncoding
MSTRG.5160.1	0.21432	coding
MSTRG.5192.2	1.05297	coding
MSTRG.5192.1	1.17252	coding
MSTRG.5192.3	-1.10684	noncoding
MSTRG.5192.4	-1.19094	noncoding
MSTRG.5196.1	-2.58327	noncoding
MSTRG.5201.1	-1.48568	noncoding
MSTRG.5209.1	-2.05909	noncoding
MSTRG.5236.1	2.56434	coding
MSTRG.5236.3	3.00685	coding
MSTRG.5237.1	0.047593	coding
MSTRG.5236.6	2.27158	coding
MSTRG.5238.5	0.532506	coding
MSTRG.5217.3	1.22527	coding
MSTRG.5217.4	1.36096	coding
MSTRG.5218.1	-0.177317	noncoding
MSTRG.5226.1	-0.619982	noncoding
MSTRG.5247.1	0.907751	coding
MSTRG.5249.2	1.10304	coding
MSTRG.5249.3	1.27554	coding
MSTRG.5249.4	1.05552	coding
MSTRG.5250.1	-1.11365	noncoding
MSTRG.5250.2	0.471657	coding
MSTRG.5232.1	0.896916	coding
MSTRG.5240.3	-0.12767	noncoding
MSTRG.5252.4	0.442984	coding
MSTRG.5252.3	1.68339	coding
MSTRG.5252.10	-0.124387	noncoding
MSTRG.5256.1	-3.18703	noncoding
MSTRG.5257.1	1.67708	coding
MSTRG.5257.3	0.242962	coding
MSTRG.5257.5	1.07996	coding
MSTRG.5257.6	1.21498	coding
MSTRG.5258.1	-1.67864	noncoding

MSTRG.5260.1	0.691982	coding
MSTRG.5261.4	0.720267	coding
MSTRG.5261.5	1.14226	coding
MSTRG.5265.1	-1.89995	noncoding
MSTRG.5254.1	-0.948682	noncoding
MSTRG.5285.2	1.91529	coding
MSTRG.5285.3	1.85622	coding
MSTRG.5285.4	2.22641	coding
MSTRG.5286.1	-2.00227	noncoding
MSTRG.5285.5	1.96519	coding
MSTRG.5288.2	0.999292	coding
MSTRG.5289.1	-0.627237	noncoding
MSTRG.5293.1	2.93573	coding
MSTRG.5297.5	-0.67287	noncoding
MSTRG.5298.1	0.132147	coding
MSTRG.5297.6	0.569839	coding
MSTRG.5297.7	-1.94266	noncoding
MSTRG.5297.8	-2.03835	noncoding
MSTRG.5299.3	-0.447095	noncoding
MSTRG.5300.1	-0.745447	noncoding
MSTRG.5301.1	1.20468	coding
MSTRG.5301.2	-0.067373	noncoding
MSTRG.5301.4	0.745241	coding
MSTRG.5301.8	0.721923	coding
MSTRG.5301.7	0.911727	coding
MSTRG.5301.3	0.810148	coding
MSTRG.5301.6	0.923672	coding
MSTRG.5301.5	1.35408	coding
MSTRG.5305.1	0.315207	coding
MSTRG.5305.2	-0.266721	noncoding
MSTRG.5306.1	-0.844227	noncoding
MSTRG.5307.1	-0.802801	noncoding
MSTRG.5271.1	-2.03547	noncoding
MSTRG.5275.1	-0.058496	noncoding
MSTRG.5275.2	-0.565762	noncoding
MSTRG.5276.2	1.71664	coding
MSTRG.5283.2	2.47291	coding
MSTRG.5283.3	-0.368121	noncoding
MSTRG.5284.1	-0.745116	noncoding
MSTRG.5310.2	1.95889	coding
MSTRG.5310.3	1.98286	coding
MSTRG.5312.1	-1.2227	noncoding
MSTRG.5317.1	-0.244775	noncoding
MSTRG.5324.1	0.612172	coding
MSTRG.5324.2	1.2346	coding
MSTRG.5324.3	-0.444068	noncoding
MSTRG.5331.1	0.259695	coding
MSTRG.5358.2	-1.28919	noncoding
MSTRG.5359.2	0.256599	coding
MSTRG.5359.3	-0.220586	noncoding
MSTRG.5363.2	1.08786	coding
MSTRG.5363.4	1.11149	coding
MSTRG.5363.5	0.070036	coding
MSTRG.5365.2	1.65263	coding

MSTRG.5354.2	2.92261	coding
MSTRG.5354.3	0.830786	coding
MSTRG.5370.2	-0.958257	noncoding
MSTRG.5382.1	-0.124813	noncoding
MSTRG.5384.1	-1.03573	noncoding
MSTRG.5383.7	1.49317	coding
MSTRG.5385.2	-0.617408	noncoding
MSTRG.5386.1	1.41225	coding
MSTRG.5386.3	1.42742	coding
MSTRG.5386.4	2.03925	coding
MSTRG.5386.5	2.08668	coding
MSTRG.5386.7	2.12298	coding
MSTRG.5386.9	-0.965783	noncoding
MSTRG.5387.1	0.458991	coding
MSTRG.5388.1	0.22796	coding
MSTRG.5389.1	0.143306	coding
MSTRG.5391.1	0.208096	coding
MSTRG.5377.3	1.33432	coding
MSTRG.5377.2	1.30244	coding
MSTRG.5377.1	1.60416	coding
MSTRG.5392.1	1.05324	coding
MSTRG.5392.2	1.23237	coding
MSTRG.5393.2	2.36161	coding
MSTRG.5401.3	-1.1393	noncoding
MSTRG.5401.2	-1.07199	noncoding
MSTRG.5401.1	-1.77963	noncoding
MSTRG.5402.3	2.33004	coding
MSTRG.5402.4	0.137703	coding
MSTRG.5403.1	-3.18703	noncoding
MSTRG.5404.2	1.76398	coding
MSTRG.5404.1	1.21355	coding
MSTRG.5404.3	1.91038	coding
MSTRG.5405.1	-1.89122	noncoding
MSTRG.5404.4	-0.895976	noncoding
MSTRG.5408.4	0.851652	coding
MSTRG.5399.1	1.26623	coding
MSTRG.5399.2	1.18149	coding
MSTRG.5412.1	0.866101	coding
MSTRG.5413.2	2.60761	coding
MSTRG.5414.1	-2.22326	noncoding
MSTRG.5416.3	1.22749	coding
MSTRG.5417.1	-0.231196	noncoding
MSTRG.5419.7	0.94061	coding
MSTRG.5419.6	0.65531	coding
MSTRG.5419.8	0.778468	coding
MSTRG.5440.1	-0.76562	noncoding
MSTRG.5445.2	-0.163065	noncoding
MSTRG.5491.1	-0.341199	noncoding
MSTRG.5493.1	0.31383	coding
MSTRG.5493.2	1.29377	coding
MSTRG.5470.2	1.78596	coding
MSTRG.5471.1	-1.10087	noncoding
MSTRG.5478.3	2.5486	coding
MSTRG.5480.1	0.384913	coding

MSTRG.5514.1	2.28915	coding
MSTRG.5515.1	-2.23597	noncoding
MSTRG.5558.2	0.62193	coding
MSTRG.5560.1	-0.797004	noncoding
MSTRG.5561.1	-1.09249	noncoding
MSTRG.5561.2	-0.572512	noncoding
MSTRG.5562.1	0.175331	coding
MSTRG.5563.1	0.315476	coding
MSTRG.5564.1	-0.704821	noncoding
MSTRG.5565.1	-2.07214	noncoding
MSTRG.5558.3	-1.27894	noncoding
MSTRG.5532.1	0.511852	coding
MSTRG.5533.1	1.00184	coding
MSTRG.5533.2	1.29348	coding
MSTRG.5539.1	-1.0099	noncoding
MSTRG.5540.1	0.232227	coding
MSTRG.5538.4	-1.16249	noncoding
MSTRG.5543.1	0.434707	coding
MSTRG.5543.4	0.448784	coding
MSTRG.5543.2	0.828744	coding
MSTRG.5543.3	0.85739	coding
MSTRG.5543.7	1.64345	coding
MSTRG.5543.8	0.554965	coding
MSTRG.5543.9	0.738707	coding
MSTRG.5549.1	-2.2504	noncoding
MSTRG.5550.2	1.9999	coding
MSTRG.5552.1	-1.15396	noncoding
MSTRG.5555.4	1.66496	coding
MSTRG.5555.2	1.22515	coding
MSTRG.5555.3	1.92392	coding
MSTRG.5570.2	1.87653	coding
MSTRG.5570.4	1.58917	coding
MSTRG.5572.2	0.047024	coding
MSTRG.5574.5	0.589872	coding
MSTRG.5574.6	0.667876	coding
MSTRG.5574.7	-0.668536	noncoding
MSTRG.5575.3	1.65448	coding
MSTRG.5575.4	1.08522	coding
MSTRG.5584.1	0.088022	coding
MSTRG.5591.1	1.48394	coding
MSTRG.5591.2	1.61285	coding
MSTRG.5595.3	0.21965	coding
MSTRG.5594.1	-1.43966	noncoding
MSTRG.5605.2	0.20406	coding
MSTRG.5606.2	-1.70286	noncoding
MSTRG.5608.1	-2.12021	noncoding
MSTRG.5610.3	1.92952	coding
MSTRG.5615.1	0.139098	coding
MSTRG.5618.3	0.332239	coding
MSTRG.5612.1	1.54827	coding
MSTRG.5627.1	0.463229	coding
MSTRG.5628.1	-2.06809	noncoding
MSTRG.5629.1	-0.260036	noncoding
MSTRG.5629.2	-0.846277	noncoding

MSTRG.5627.3	1.12864	coding
MSTRG.5627.4	-0.290779	noncoding
MSTRG.5627.5	0.266685	coding
MSTRG.5627.6	-0.144927	noncoding
MSTRG.5627.7	0.253887	coding
MSTRG.5627.8	0.245193	coding
MSTRG.5627.9	0.147403	coding
MSTRG.5627.10	-0.084388	noncoding
MSTRG.5627.11	-0.089462	noncoding
MSTRG.5627.12	-0.101658	noncoding
MSTRG.5627.13	-0.165727	noncoding
MSTRG.5627.14	0.151397	coding
MSTRG.5631.1	0.666924	coding
MSTRG.5632.1	0.56425	coding
MSTRG.5633.1	0.118913	coding
MSTRG.5633.2	-0.186515	noncoding
MSTRG.5634.1	-0.866138	noncoding
MSTRG.5632.2	1.04055	coding
MSTRG.5634.2	-0.706813	noncoding
MSTRG.5635.3	1.88616	coding
MSTRG.5636.1	-0.259439	noncoding
MSTRG.5621.2	0.339493	coding
MSTRG.5624.1	-0.024809	noncoding
MSTRG.5624.2	-0.732706	noncoding
MSTRG.5638.1	-0.238119	noncoding
MSTRG.5643.3	1.32765	coding
MSTRG.5642.4	0.479446	coding
MSTRG.5642.5	-0.211907	noncoding
MSTRG.5661.1	0.395401	coding
MSTRG.5668.3	-0.639251	noncoding
MSTRG.5669.3	2.64457	coding
MSTRG.5669.4	1.21538	coding
MSTRG.5669.5	2.20714	coding
MSTRG.5670.1	-1.12055	noncoding
MSTRG.5671.1	-0.199888	noncoding
MSTRG.5672.1	-2.82071	noncoding
MSTRG.5667.1	-0.884786	noncoding
MSTRG.5681.1	1.32926	coding
MSTRG.5681.5	1.54757	coding
MSTRG.5681.10	0.225224	coding
MSTRG.5681.7	1.1443	coding
MSTRG.5681.11	-0.509072	noncoding
MSTRG.5722.15	1.02697	coding
MSTRG.5726.1	-1.0034	noncoding
MSTRG.5726.2	-0.035273	noncoding
MSTRG.5722.17	-2.27885	noncoding
MSTRG.5722.18	-0.4714	noncoding
MSTRG.5727.1	0.441304	coding
MSTRG.5728.1	0.047445	coding
MSTRG.5729.1	-1.33542	noncoding
MSTRG.5730.4	-0.37706	noncoding
MSTRG.5722.19	1.27504	coding
MSTRG.5722.20	0.673064	coding
MSTRG.5722.21	1.01093	coding

MSTRG.5734.1	-0.344201	noncoding
MSTRG.5722.22	0.928892	coding
MSTRG.5687.2	2.14848	coding
MSTRG.5690.2	-0.077181	noncoding
MSTRG.5690.3	-0.171	noncoding
MSTRG.5695.3	0.452546	coding
MSTRG.5696.1	-0.839665	noncoding
MSTRG.5695.5	0.809823	coding
MSTRG.5695.6	0.829719	coding
MSTRG.5693.1	-1.07488	noncoding
MSTRG.5699.1	-1.21682	noncoding
MSTRG.5704.3	1.43794	coding
MSTRG.5707.14	0.876979	coding
MSTRG.5707.17	0.403488	coding
MSTRG.5707.15	1.42379	coding
MSTRG.5707.11	1.54339	coding
MSTRG.5707.13	1.32613	coding
MSTRG.5707.16	1.55437	coding
MSTRG.5707.6	1.83165	coding
MSTRG.5707.2	1.82546	coding
MSTRG.5707.5	1.67268	coding
MSTRG.5707.9	1.86309	coding
MSTRG.5711.3	0.7369	coding
MSTRG.5713.3	1.52576	coding
MSTRG.5713.4	1.79964	coding
MSTRG.5713.6	1.36129	coding
MSTRG.5713.5	1.76496	coding
MSTRG.5717.2	0.676783	coding
MSTRG.5717.1	2.67447	coding
MSTRG.5717.3	0.614792	coding
MSTRG.5717.4	3.07013	coding
MSTRG.5717.5	2.55714	coding
MSTRG.5715.4	0.889713	coding
MSTRG.5715.3	0.573449	coding
MSTRG.5716.1	0.846041	coding
MSTRG.5740.3	1.15943	coding
MSTRG.5742.1	-1.75697	noncoding
MSTRG.5745.3	0.423439	coding
MSTRG.5745.2	0.992124	coding
MSTRG.5746.8	0.490379	coding
MSTRG.5746.9	0.36331	coding
MSTRG.5747.1	0.303013	coding
MSTRG.5746.10	1.79443	coding
MSTRG.5782.1	-0.869068	noncoding
MSTRG.5757.2	-0.741757	noncoding
MSTRG.5757.1	-0.445258	noncoding
MSTRG.5761.1	1.38589	coding
MSTRG.5761.2	1.42733	coding
MSTRG.5763.2	0.765747	coding
MSTRG.5762.1	-1.59533	noncoding
MSTRG.5835.1	-0.504264	noncoding
MSTRG.5835.2	-0.080399	noncoding
MSTRG.5835.3	0.534263	coding
MSTRG.5835.4	0.206524	coding

MSTRG.5835.5	0.51429	coding
MSTRG.5835.6	0.706678	coding
MSTRG.5835.7	0.597038	coding
MSTRG.5835.9	-0.563663	noncoding
MSTRG.5836.1	-0.151222	noncoding
MSTRG.5835.11	0.43907	coding
MSTRG.5837.1	0.158341	coding
MSTRG.5838.1	-0.709228	noncoding
MSTRG.5835.13	-0.979762	noncoding
MSTRG.5835.12	-0.281543	noncoding
MSTRG.5835.14	-0.307646	noncoding
MSTRG.5835.15	-0.265341	noncoding
MSTRG.5835.16	-0.144055	noncoding
MSTRG.5835.17	0.112509	coding
MSTRG.5835.18	-0.154395	noncoding
MSTRG.5835.20	0.041199	coding
MSTRG.5835.22	-0.135561	noncoding
MSTRG.5835.23	0.426861	coding
MSTRG.5835.25	0.390773	coding
MSTRG.5835.26	0.446906	coding
MSTRG.5835.27	0.396558	coding
MSTRG.5835.28	-0.568941	noncoding
MSTRG.5835.29	0.699365	coding
MSTRG.5835.30	0.494213	coding
MSTRG.5840.1	0.059089	coding
MSTRG.5797.1	2.55222	coding
MSTRG.5797.6	1.87657	coding
MSTRG.5797.4	2.26468	coding
MSTRG.5797.3	2.28294	coding
MSTRG.5797.5	2.29601	coding
MSTRG.5797.7	1.99481	coding
MSTRG.5798.1	0.785622	coding
MSTRG.5802.3	-0.114973	noncoding
MSTRG.5803.1	-0.313423	noncoding
MSTRG.5805.1	0.969503	coding
MSTRG.5799.1	-2.49953	noncoding
MSTRG.5815.1	-1.58523	noncoding
MSTRG.5832.1	-2.12448	noncoding
MSTRG.5834.1	2.29024	coding
MSTRG.5845.3	2.35714	coding
MSTRG.5845.4	1.56492	coding
MSTRG.5856.2	0.908192	coding
MSTRG.5858.1	-1.57777	noncoding
MSTRG.5860.1	-0.474872	noncoding
MSTRG.5862.1	0.859244	coding
MSTRG.5864.1	2.96157	coding
MSTRG.5865.1	-0.120419	noncoding
MSTRG.5866.1	-1.59679	noncoding
MSTRG.5867.1	-0.811984	noncoding
MSTRG.5872.5	0.985301	coding
MSTRG.5873.1	-1.3264	noncoding
MSTRG.5874.2	0.969184	coding
MSTRG.5874.3	0.244579	coding
MSTRG.5874.5	0.588331	coding

MSTRG.5874.4	1.04195	coding
MSTRG.5877.1	1.80699	coding
MSTRG.5879.1	-0.3614	noncoding
MSTRG.5885.2	1.69121	coding
MSTRG.5886.1	-2.13852	noncoding
MSTRG.5890.2	2.18674	coding
MSTRG.5890.3	1.30881	coding
MSTRG.5891.1	1.8728	coding
MSTRG.5891.2	1.96477	coding
MSTRG.5891.3	1.88114	coding
MSTRG.5896.2	0.430766	coding
MSTRG.5897.1	0.536012	coding
MSTRG.5901.1	0.143689	coding
MSTRG.5904.1	1.32856	coding
MSTRG.5904.2	0.539359	coding
MSTRG.5904.4	1.46636	coding
MSTRG.5907.1	1.87411	coding
MSTRG.5908.1	-1.07975	noncoding
MSTRG.5908.2	-1.38417	noncoding
MSTRG.5907.3	1.52947	coding
MSTRG.5907.4	1.49683	coding
MSTRG.5909.2	2.00317	coding
MSTRG.5909.3	2.01699	coding
MSTRG.5909.4	0.026369	coding
MSTRG.5910.1	1.32136	coding
MSTRG.5915.1	1.48367	coding
MSTRG.5915.4	0.006288	coding
MSTRG.5915.3	1.81812	coding
MSTRG.5916.1	0.911205	coding
MSTRG.5917.2	1.10401	coding
MSTRG.5918.1	-2.70897	noncoding
MSTRG.5919.1	0.574522	coding
MSTRG.5920.1	0.567176	coding
MSTRG.5924.1	-1.06316	noncoding
MSTRG.5924.6	1.17662	coding
MSTRG.5924.7	0.401897	coding
MSTRG.5924.8	1.51542	coding
MSTRG.5921.1	-1.44236	noncoding
MSTRG.5922.3	-0.781375	noncoding
MSTRG.5922.4	0.038071	coding
MSTRG.5923.1	0.29305	coding
MSTRG.5925.1	0.64266	coding
MSTRG.5925.2	0.366808	coding
MSTRG.5925.4	0.550971	coding
MSTRG.5925.3	0.613038	coding
MSTRG.5925.5	0.341599	coding
MSTRG.5927.1	0.605252	coding
MSTRG.5927.2	0.358486	coding
MSTRG.5932.1	-0.264882	noncoding
MSTRG.5934.1	0.892325	coding
MSTRG.5934.3	2.08179	coding
MSTRG.5934.4	2.09018	coding
MSTRG.5935.2	0.879689	coding
MSTRG.5936.1	1.73125	coding

MSTRG.5939.1	-1.53688	noncoding
MSTRG.5940.1	-1.56392	noncoding
MSTRG.5940.2	-0.750676	noncoding
MSTRG.5939.2	-1.69487	noncoding
MSTRG.5940.4	-1.26472	noncoding
MSTRG.5939.3	-0.736114	noncoding
MSTRG.5939.4	-1.33175	noncoding
MSTRG.5940.5	-1.4436	noncoding
MSTRG.5942.1	-1.94901	noncoding
MSTRG.5944.1	-0.275771	noncoding
MSTRG.5945.1	-2.15655	noncoding
MSTRG.5946.1	-0.796347	noncoding
MSTRG.5945.2	-2.12813	noncoding
MSTRG.5949.2	-0.02672	noncoding
MSTRG.5966.1	-1.87108	noncoding
MSTRG.5965.5	1.26354	coding
MSTRG.5969.1	-0.632531	noncoding
MSTRG.5965.6	0.232923	coding
MSTRG.5965.7	-0.302402	noncoding
MSTRG.5965.8	1.1112	coding
MSTRG.5970.1	-2.12634	noncoding
MSTRG.5972.1	-1.01761	noncoding
MSTRG.5951.1	-0.965524	noncoding
MSTRG.5953.1	-1.47868	noncoding
MSTRG.5955.2	0.953614	coding
MSTRG.5956.3	0.675295	coding
MSTRG.5956.2	0.385045	coding
MSTRG.5956.4	-1.13788	noncoding
MSTRG.5958.1	0.476349	coding
MSTRG.5956.6	1.57154	coding
MSTRG.5956.7	1.1392	coding
MSTRG.5959.1	0.251207	coding
MSTRG.5960.1	-0.649132	noncoding
MSTRG.5961.1	-0.427081	noncoding
MSTRG.5962.1	0.333415	coding
MSTRG.5956.8	0.489383	coding
MSTRG.5985.4	1.22611	coding
MSTRG.5985.7	1.58041	coding
MSTRG.5985.5	1.34871	coding
MSTRG.5985.8	0.450851	coding
MSTRG.5986.1	-0.084625	noncoding
MSTRG.5989.1	-0.302737	noncoding
MSTRG.5990.2	0.42311	coding
MSTRG.5989.4	-0.022483	noncoding
MSTRG.5984.1	-0.239835	noncoding
MSTRG.5991.1	1.17933	coding
MSTRG.5991.3	-0.838248	noncoding
MSTRG.5992.1	0.738039	coding
MSTRG.5994.1	-1.01408	noncoding
MSTRG.5995.1	0.214792	coding
MSTRG.5997.1	-2.18521	noncoding
MSTRG.5999.1	-1.11885	noncoding
MSTRG.6004.5	0.38378	coding
MSTRG.6004.4	0.4536	coding

MSTRG.6005.1	0.439392	coding
MSTRG.6006.1	-0.266169	noncoding
MSTRG.6004.7	0.711434	coding
MSTRG.6007.1	-0.214203	noncoding
MSTRG.6008.1	0.400186	coding
MSTRG.6008.2	-1.11376	noncoding
MSTRG.6009.3	2.24458	coding
MSTRG.6001.2	-1.34603	noncoding
MSTRG.6010.2	2.22589	coding
MSTRG.6020.4	2.30702	coding
MSTRG.6029.2	-0.204686	noncoding
MSTRG.6029.3	-0.477374	noncoding
MSTRG.6029.4	1.19155	coding
MSTRG.6030.1	-0.687513	noncoding
MSTRG.6033.2	-0.773159	noncoding
MSTRG.6035.1	0.597853	coding
MSTRG.6036.2	2.46682	coding
MSTRG.6036.3	2.1541	coding
MSTRG.6037.1	-0.379999	noncoding
MSTRG.6040.1	1.65239	coding
MSTRG.6040.2	1.63115	coding
MSTRG.6040.3	1.64413	coding
MSTRG.6040.5	2.32945	coding
MSTRG.6040.4	2.32239	coding
MSTRG.6040.6	1.65966	coding
MSTRG.6040.7	1.21093	coding
MSTRG.6040.8	1.40868	coding
MSTRG.6040.9	0.987621	coding
MSTRG.6040.10	0.876255	coding
MSTRG.6040.11	1.10564	coding
MSTRG.6042.1	1.2817	coding
MSTRG.6043.3	2.43853	coding
MSTRG.6045.2	2.93279	coding
MSTRG.6050.1	1.89844	coding
MSTRG.6050.3	1.37503	coding
MSTRG.6050.4	1.81902	coding
MSTRG.6050.7	0.542826	coding
MSTRG.6056.1	1.71067	coding
MSTRG.6058.1	0.32911	coding
MSTRG.6058.2	0.404734	coding
MSTRG.6060.3	-0.134515	noncoding
MSTRG.6060.2	0.277832	coding
MSTRG.6061.2	1.40945	coding
MSTRG.6061.4	0.958642	coding
MSTRG.6061.5	-0.021499	noncoding
MSTRG.6061.6	1.24793	coding
MSTRG.6061.7	1.6787	coding
MSTRG.6061.10	2.00624	coding
MSTRG.6061.11	1.99829	coding
MSTRG.6061.12	1.98047	coding
MSTRG.6061.9	1.92672	coding
MSTRG.6062.1	-1.8502	noncoding
MSTRG.6063.1	0.205299	coding
MSTRG.6055.2	-0.715843	noncoding

MSTRG.6055.3	-0.928853	noncoding
MSTRG.6065.2	-1.09628	noncoding
MSTRG.6066.1	-0.94164	noncoding
MSTRG.6067.2	0.996758	coding
MSTRG.6068.2	1.01549	coding
MSTRG.6073.1	-0.43795	noncoding
MSTRG.6074.1	-2.74804	noncoding
MSTRG.6075.1	1.63544	coding
MSTRG.6076.1	0.754453	coding
MSTRG.6077.1	-0.259194	noncoding
MSTRG.6077.2	0.121446	coding
MSTRG.6088.1	0.738905	coding
MSTRG.6088.5	0.12214	coding
MSTRG.6089.2	0.539915	coding
MSTRG.6089.3	0.752847	coding
MSTRG.6096.1	-2.1787	noncoding
MSTRG.6100.1	0.327074	coding
MSTRG.6104.1	-0.266972	noncoding
MSTRG.6105.1	-0.552272	noncoding
MSTRG.6112.1	0.177377	coding
MSTRG.6113.1	-1.64635	noncoding
MSTRG.6121.3	2.6703	coding
MSTRG.6121.5	2.46617	coding
MSTRG.6121.7	2.60851	coding
MSTRG.6121.9	2.40239	coding
MSTRG.6121.10	2.37265	coding
MSTRG.6119.1	1.04604	coding
MSTRG.6119.3	1.04533	coding
MSTRG.6124.3	1.80167	coding
MSTRG.6125.2	1.17518	coding
MSTRG.6125.3	1.22583	coding
MSTRG.6125.4	1.13608	coding
MSTRG.6130.4	1.74223	coding
MSTRG.6127.1	-1.90045	noncoding
MSTRG.6133.1	-1.33551	noncoding
MSTRG.6133.2	-1.59262	noncoding
MSTRG.6134.1	-1.28898	noncoding
MSTRG.6136.1	-0.916014	noncoding
MSTRG.6136.2	0.312462	coding
MSTRG.6139.6	2.42935	coding
MSTRG.6140.2	-0.028136	noncoding
MSTRG.6141.2	1.69483	coding
MSTRG.6143.1	1.28492	coding
MSTRG.6145.3	0.162567	coding
MSTRG.6149.1	0.384631	coding
MSTRG.6154.3	0.748785	coding
MSTRG.6163.1	1.29246	coding
MSTRG.6163.2	1.20229	coding
MSTRG.6163.4	1.63249	coding
MSTRG.6163.5	1.5757	coding
MSTRG.6161.2	1.80841	coding
MSTRG.6161.3	2.43408	coding
MSTRG.6167.1	1.91345	coding
MSTRG.6167.3	1.74294	coding

MSTRG.6169.2	1.94394	coding
MSTRG.6170.1	-0.392894	noncoding
MSTRG.6171.1	-0.02957	noncoding
MSTRG.6171.2	-0.314228	noncoding
MSTRG.6173.2	-0.032328	noncoding
MSTRG.6173.3	0.440092	coding
MSTRG.6174.7	1.34675	coding
MSTRG.6174.1	1.16727	coding
MSTRG.6174.6	1.24723	coding
MSTRG.6174.9	-1.753	noncoding
MSTRG.6174.10	-1.93162	noncoding
MSTRG.6186.2	1.7835	coding
MSTRG.6190.2	2.44363	coding
MSTRG.6190.1	2.44944	coding
MSTRG.6190.10	2.09571	coding
MSTRG.6190.11	2.00454	coding
MSTRG.6190.15	2.0006	coding
MSTRG.6190.12	2.02623	coding
MSTRG.6190.5	2.03464	coding
MSTRG.6190.14	2.18802	coding
MSTRG.6190.9	2.30087	coding
MSTRG.6190.6	2.31423	coding
MSTRG.6190.4	2.32204	coding
MSTRG.6190.8	2.31728	coding
MSTRG.6190.17	2.61813	coding
MSTRG.6192.1	0.066605	coding
MSTRG.6193.1	0.360664	coding
MSTRG.6179.1	-0.703733	noncoding
MSTRG.6179.2	-0.453159	noncoding
MSTRG.6180.6	0.577757	coding
MSTRG.6180.7	0.903988	coding
MSTRG.6180.5	0.514986	coding
MSTRG.6180.2	0.4952	coding
MSTRG.6180.4	0.551409	coding
MSTRG.6180.8	0.587699	coding
MSTRG.6181.1	-0.058554	noncoding
MSTRG.6181.2	0.334089	coding
MSTRG.6180.10	0.802078	coding
MSTRG.6180.11	0.874901	coding
MSTRG.6180.9	0.876294	coding
MSTRG.6182.1	-1.17263	noncoding
MSTRG.6184.1	-0.295844	noncoding
MSTRG.6196.1	-2.37372	noncoding
MSTRG.6201.1	0.901595	coding
MSTRG.6204.2	2.33045	coding
MSTRG.6204.3	1.21353	coding
MSTRG.6206.1	-1.91943	noncoding
MSTRG.6212.1	0.915892	coding
MSTRG.6212.2	1.04225	coding
MSTRG.6212.3	0.497558	coding
MSTRG.6212.8	0.402664	coding
MSTRG.6220.1	-2.30845	noncoding
MSTRG.6222.3	0.535584	coding
MSTRG.6222.4	0.208722	coding

MSTRG.6223.1	-0.066404	noncoding
MSTRG.6231.1	1.72557	coding
MSTRG.6232.2	2.23166	coding
MSTRG.6232.4	1.76865	coding
MSTRG.6233.1	-1.41113	noncoding
MSTRG.6233.2	-0.837419	noncoding
MSTRG.6235.5	0.360247	coding
MSTRG.6235.4	0.771564	coding
MSTRG.6235.6	0.362116	coding
MSTRG.6235.7	0.912828	coding
MSTRG.6235.8	0.985332	coding
MSTRG.6235.9	1.13573	coding
MSTRG.6238.2	2.6337	coding
MSTRG.6240.1	-0.862211	noncoding
MSTRG.6238.3	-0.506529	noncoding
MSTRG.6242.1	0.525897	coding
MSTRG.6237.2	-0.333081	noncoding
MSTRG.6237.3	0.389652	coding
MSTRG.6237.4	0.905507	coding
MSTRG.6237.5	0.950207	coding
MSTRG.6251.1	1.08496	coding
MSTRG.6251.2	1.18039	coding
MSTRG.6251.3	0.66163	coding
MSTRG.6251.4	0.420791	coding
MSTRG.6251.5	0.792577	coding
MSTRG.6251.7	1.49475	coding
MSTRG.6251.8	0.170933	coding
MSTRG.6252.1	0.783956	coding
MSTRG.6253.1	1.16307	coding
MSTRG.6253.2	1.16251	coding
MSTRG.6253.3	1.15731	coding
MSTRG.6253.5	1.46737	coding
MSTRG.6248.1	-0.113327	noncoding
MSTRG.6267.3	-0.70239	noncoding
MSTRG.6271.1	3.53306	coding
MSTRG.6271.3	3.63338	coding
MSTRG.6272.1	0.821437	coding
MSTRG.6273.2	1.03076	coding
MSTRG.6273.3	1.37076	coding
MSTRG.6274.2	-0.978854	noncoding
MSTRG.6285.1	-1.78036	noncoding
MSTRG.6288.1	-1.65036	noncoding
MSTRG.6312.1	1.68874	coding
MSTRG.6312.2	2.22574	coding
MSTRG.6314.1	-0.611442	noncoding
MSTRG.6312.8	1.04405	coding
MSTRG.6315.1	0.527548	coding
MSTRG.6316.1	-0.388743	noncoding
MSTRG.6314.2	0.183051	coding
MSTRG.6314.3	-0.284595	noncoding
MSTRG.6314.4	-0.374624	noncoding
MSTRG.6317.1	-1.37643	noncoding
MSTRG.6316.2	0.428965	coding
MSTRG.6318.1	-0.318532	noncoding

MSTRG.6316.3	-1.45958	noncoding
MSTRG.6316.4	-1.70551	noncoding
MSTRG.6316.5	0.084375	coding
MSTRG.6316.6	-0.401493	noncoding
MSTRG.6319.1	-2.26498	noncoding
MSTRG.6319.2	-2.24943	noncoding
MSTRG.6319.3	-2.58374	noncoding
MSTRG.6320.1	-1.32894	noncoding
MSTRG.6323.2	0.513986	coding
MSTRG.6308.4	1.74087	coding
MSTRG.6327.1	2.06099	coding
MSTRG.6328.1	-0.40463	noncoding
MSTRG.6332.2	0.309458	coding
MSTRG.6332.3	1.02682	coding
MSTRG.6333.1	-1.17044	noncoding
MSTRG.6334.2	0.482219	coding
MSTRG.6348.1	-1.85982	noncoding
MSTRG.6348.2	-0.708157	noncoding
MSTRG.6349.2	1.30268	coding
MSTRG.6349.1	1.92175	coding
MSTRG.6349.3	1.93679	coding
MSTRG.6349.4	1.93812	coding
MSTRG.6349.5	1.34811	coding
MSTRG.6349.6	1.41678	coding
MSTRG.6349.7	1.3803	coding
MSTRG.6349.8	1.92842	coding
MSTRG.6350.1	-0.007752	noncoding
MSTRG.6351.1	0.616627	coding
MSTRG.6351.3	2.59558	coding
MSTRG.6355.5	0.633525	coding
MSTRG.6355.4	2.12213	coding
MSTRG.6356.1	0.404853	coding
MSTRG.6357.1	-0.920992	noncoding
MSTRG.6355.6	-0.138233	noncoding
MSTRG.6358.1	1.06975	coding
MSTRG.6359.1	0.481943	coding
MSTRG.6359.2	0.038041	coding
MSTRG.6355.7	1.66688	coding
MSTRG.6360.1	-0.674269	noncoding
MSTRG.6363.1	2.4411	coding
MSTRG.6364.1	0.611842	coding
MSTRG.6384.1	-0.49442	noncoding
MSTRG.6385.1	-0.921763	noncoding
MSTRG.6386.1	-0.701485	noncoding
MSTRG.6387.1	0.814409	coding
MSTRG.6388.1	0.45678	coding
MSTRG.6389.1	0.794306	coding
MSTRG.6389.2	0.637921	coding
MSTRG.6389.3	-0.057978	noncoding
MSTRG.6383.2	1.80621	coding
MSTRG.6383.3	1.57196	coding
MSTRG.6383.4	0.160243	coding
MSTRG.6379.1	-1.78191	noncoding
MSTRG.6391.1	-0.010082	noncoding

MSTRG.6404.2	1.50325	coding
MSTRG.6404.3	1.31312	coding
MSTRG.6433.11	1.59009	coding
MSTRG.6433.12	1.15	coding
MSTRG.6433.15	1.31298	coding
MSTRG.6433.3	1.68691	coding
MSTRG.6433.4	0.949154	coding
MSTRG.6433.5	0.757214	coding
MSTRG.6433.6	1.21805	coding
MSTRG.6433.8	1.02063	coding
MSTRG.6433.13	1.28419	coding
MSTRG.6433.10	1.55239	coding
MSTRG.6433.14	1.31864	coding
MSTRG.6433.16	2.02042	coding
MSTRG.6433.7	1.31188	coding
MSTRG.6433.9	1.36479	coding
MSTRG.6435.2	1.54057	coding
MSTRG.6436.2	0.629455	coding
MSTRG.6430.2	1.10798	coding
MSTRG.6442.1	0.409711	coding
MSTRG.6454.2	1.44596	coding
MSTRG.6456.1	-1.71581	noncoding
MSTRG.6456.2	-1.60895	noncoding
MSTRG.6457.4	1.31882	coding
MSTRG.6457.2	1.04505	coding
MSTRG.6457.3	1.35158	coding
MSTRG.6457.5	1.04715	coding
MSTRG.6464.3	0.590184	coding
MSTRG.6478.1	1.38427	coding
MSTRG.6497.2	1.09685	coding
MSTRG.6497.3	1.89727	coding
MSTRG.6497.4	2.98274	coding
MSTRG.6497.5	2.47832	coding
MSTRG.6497.6	2.50675	coding
MSTRG.6497.7	2.09189	coding
MSTRG.6497.9	0.284349	coding
MSTRG.6497.10	0.433011	coding
MSTRG.6497.11	1.65007	coding
MSTRG.6501.1	0.986191	coding
MSTRG.6501.2	0.967411	coding
MSTRG.6501.3	0.145153	coding
MSTRG.6501.4	1.06848	coding
MSTRG.6501.5	1.07554	coding
MSTRG.6501.6	0.32428	coding
MSTRG.6501.7	-0.604668	noncoding
MSTRG.6501.8	0.87352	coding
MSTRG.6502.4	1.38397	coding
MSTRG.6503.1	2.21364	coding
MSTRG.6487.2	1.95324	coding
MSTRG.6487.1	2.06353	coding
MSTRG.6487.3	2.26411	coding
MSTRG.6489.1	-0.565874	noncoding
MSTRG.6494.1	0.371593	coding
MSTRG.6496.2	-0.283234	noncoding

MSTRG.6505.2	0.333403	coding
MSTRG.6507.1	-0.115707	noncoding
MSTRG.6507.2	-0.11598	noncoding
MSTRG.6508.2	0.207148	coding
MSTRG.6513.1	0.30012	coding
MSTRG.6513.2	0.222016	coding
MSTRG.6515.1	-0.192041	noncoding
MSTRG.6520.1	0.321128	coding
MSTRG.6522.3	0.692181	coding
MSTRG.6522.4	0.829096	coding
MSTRG.6530.1	0.112548	coding
MSTRG.6530.2	0.46359	coding
MSTRG.6530.7	0.845646	coding
MSTRG.6530.11	0.620463	coding
MSTRG.6530.13	-0.253929	noncoding
MSTRG.6530.12	0.684224	coding
MSTRG.6530.14	1.88793	coding
MSTRG.6530.15	-0.431624	noncoding
MSTRG.6524.1	1.21101	coding
MSTRG.6524.2	1.63362	coding
MSTRG.6524.3	1.5721	coding
MSTRG.6524.6	1.29732	coding
MSTRG.6524.7	1.65658	coding
MSTRG.6524.8	0.930089	coding
MSTRG.6526.1	0.542896	coding
MSTRG.6527.2	1.13903	coding
MSTRG.6527.3	0.677385	coding
MSTRG.6538.3	1.53583	coding
MSTRG.6544.1	2.56784	coding
MSTRG.6544.3	2.54835	coding
MSTRG.6540.2	0.949008	coding
MSTRG.6546.1	2.28159	coding
MSTRG.6550.2	1.01058	coding
MSTRG.6550.3	1.07462	coding
MSTRG.6550.5	0.957156	coding
MSTRG.6550.6	1.01675	coding
MSTRG.6551.1	0.239759	coding
MSTRG.6551.2	0.551979	coding
MSTRG.6551.3	0.081962	coding
MSTRG.6554.1	1.70194	coding
MSTRG.6555.1	-1.41316	noncoding
MSTRG.6562.1	3.13665	coding
MSTRG.6562.3	0.895554	coding
MSTRG.6568.1	-1.48673	noncoding
MSTRG.6567.6	-1.35132	noncoding
MSTRG.6564.2	-0.89377	noncoding
MSTRG.6564.3	-0.011093	noncoding
MSTRG.6577.3	2.64678	coding
MSTRG.6577.2	2.76425	coding
MSTRG.6582.3	1.72786	coding
MSTRG.6582.2	1.50526	coding
MSTRG.6582.1	1.79045	coding
MSTRG.6582.4	1.95624	coding
MSTRG.6585.1	-0.823185	noncoding

MSTRG.6587.1	-1.68926	noncoding
MSTRG.6589.2	0.58708	coding
MSTRG.6591.2	1.21736	coding
MSTRG.6591.3	1.03515	coding
MSTRG.6592.1	-0.550624	noncoding
MSTRG.6592.2	-0.230462	noncoding
MSTRG.6594.2	0.477605	coding
MSTRG.6594.3	0.7239	coding
MSTRG.6598.1	-1.26854	noncoding
MSTRG.6601.4	-0.96825	noncoding
MSTRG.6605.1	1.84107	coding
MSTRG.6606.2	1.68915	coding
MSTRG.6614.1	-0.479571	noncoding
MSTRG.6614.2	0.293598	coding
MSTRG.6612.3	1.38338	coding
MSTRG.6624.2	1.86848	coding
MSTRG.6624.3	2.15985	coding
MSTRG.6624.4	1.08227	coding
MSTRG.6624.5	1.41387	coding
MSTRG.6624.7	1.59267	coding
MSTRG.6624.6	1.18459	coding
MSTRG.6624.8	1.79537	coding
MSTRG.6624.9	1.22721	coding
MSTRG.6624.10	1.44514	coding
MSTRG.6624.11	2.38355	coding
MSTRG.6626.1	-2.42587	noncoding
MSTRG.6627.1	2.00234	coding
MSTRG.6627.2	2.03923	coding
MSTRG.6627.3	1.55782	coding
MSTRG.6627.4	1.89859	coding
MSTRG.6627.5	1.68043	coding
MSTRG.6627.6	1.7041	coding
MSTRG.6627.7	2.08628	coding
MSTRG.6622.2	2.44436	coding
MSTRG.6622.3	-1.65105	noncoding
MSTRG.6622.4	2.34908	coding
MSTRG.6633.1	2.43056	coding
MSTRG.6633.2	2.4239	coding
MSTRG.6633.7	1.91815	coding
MSTRG.6633.5	2.46429	coding
MSTRG.6634.2	0.6147	coding
MSTRG.6629.2	0.807152	coding
MSTRG.6636.3	0.715586	coding
MSTRG.6636.4	0.941334	coding
MSTRG.6636.5	0.882415	coding
MSTRG.6636.6	0.700787	coding
MSTRG.6638.2	1.83938	coding
MSTRG.6639.2	-1.1219	noncoding
MSTRG.6639.3	-1.34462	noncoding
MSTRG.6640.3	0.159626	coding
MSTRG.6640.4	0.9552	coding
MSTRG.6644.4	0.918768	coding
MSTRG.6644.6	1.02071	coding
MSTRG.6644.7	1.07719	coding

MSTRG.6644.8	0.852893	coding
MSTRG.6652.1	-0.744089	noncoding
MSTRG.6660.1	1.33609	coding
MSTRG.6660.2	0.329415	coding
MSTRG.6660.5	0.919631	coding
MSTRG.6661.5	2.18309	coding
MSTRG.6661.7	2.09954	coding
MSTRG.6661.3	2.15107	coding
MSTRG.6662.6	0.833704	coding
MSTRG.6662.7	0.932678	coding
MSTRG.6657.1	1.19296	coding
MSTRG.6670.1	-0.115383	noncoding
MSTRG.6681.1	-0.212097	noncoding
MSTRG.6682.1	1.04837	coding
MSTRG.6685.1	0.550905	coding
MSTRG.6678.2	-2.32728	noncoding
MSTRG.6698.1	-1.1016	noncoding
MSTRG.6705.1	0.354709	coding
MSTRG.6708.1	-0.517417	noncoding
MSTRG.6709.1	-1.54759	noncoding
MSTRG.6710.1	-0.655943	noncoding
MSTRG.6707.3	1.87838	coding
MSTRG.6707.4	1.87717	coding
MSTRG.6711.1	-0.805489	noncoding
MSTRG.6713.1	-2.54251	noncoding
MSTRG.6714.1	-0.345371	noncoding
MSTRG.6714.2	-2.0128	noncoding
MSTRG.6714.3	-0.621024	noncoding
MSTRG.6714.4	-1.16919	noncoding
MSTRG.6714.5	-1.89418	noncoding
MSTRG.6715.1	-0.228466	noncoding
MSTRG.6694.1	-1.18958	noncoding
MSTRG.6694.2	0.095025	coding
MSTRG.6695.1	-0.25673	noncoding
MSTRG.6694.3	-0.282142	noncoding
MSTRG.6694.4	-0.64711	noncoding
MSTRG.6726.2	1.91553	coding
MSTRG.6739.2	0.628515	coding
MSTRG.6739.4	-1.77935	noncoding
MSTRG.6740.2	0.261069	coding
MSTRG.6741.3	0.891049	coding
MSTRG.6741.4	0.636773	coding
MSTRG.6741.5	0.706367	coding
MSTRG.6744.1	0.03652	coding
MSTRG.6744.2	-0.262455	noncoding
MSTRG.6748.3	0.15125	coding
MSTRG.6750.2	0.697005	coding
MSTRG.6756.1	-0.288279	noncoding
MSTRG.6757.1	0.802618	coding
MSTRG.6760.1	-0.100738	noncoding
MSTRG.6762.1	1.23916	coding
MSTRG.6763.1	-1.5417	noncoding
MSTRG.6761.4	0.645552	coding
MSTRG.6761.5	0.607144	coding

MSTRG.6761.6	0.822942	coding
MSTRG.6764.1	-1.58839	noncoding
MSTRG.6774.1	-0.069479	noncoding
MSTRG.6775.1	-0.162982	noncoding
MSTRG.6776.1	1.33226	coding
MSTRG.6777.1	-0.260642	noncoding
MSTRG.6776.2	-1.75548	noncoding
MSTRG.6777.2	-0.829226	noncoding
MSTRG.6778.1	-0.25949	noncoding
MSTRG.6777.3	-0.055461	noncoding
MSTRG.6777.5	-0.225472	noncoding
MSTRG.6780.1	0.335117	coding
MSTRG.6781.3	-0.510136	noncoding
MSTRG.6781.4	0.093436	coding
MSTRG.6785.2	0.1677	coding
MSTRG.6786.1	-0.355862	noncoding
MSTRG.6786.2	-0.981284	noncoding
MSTRG.6792.5	0.76191	coding
MSTRG.6797.3	1.76357	coding
MSTRG.6797.4	-0.418127	noncoding
MSTRG.6798.1	-1.0838	noncoding
MSTRG.6800.1	0.848927	coding
MSTRG.6806.2	1.56159	coding
MSTRG.6804.1	0.310209	coding
MSTRG.6817.3	0.825293	coding
MSTRG.6823.1	-0.358174	noncoding
MSTRG.6811.1	-1.3696	noncoding
MSTRG.6811.2	-1.58486	noncoding
MSTRG.6857.1	1.56537	coding
MSTRG.6857.2	2.67687	coding
MSTRG.6857.3	2.28708	coding
MSTRG.6857.4	2.85156	coding
MSTRG.6857.5	0.705579	coding
MSTRG.6857.6	0.539274	coding
MSTRG.6857.7	0.64819	coding
MSTRG.6857.8	0.58677	coding
MSTRG.6859.1	0.274916	coding
MSTRG.6859.2	0.710765	coding
MSTRG.6859.3	0.069606	coding
MSTRG.6859.4	0.573583	coding
MSTRG.6860.1	0.614329	coding
MSTRG.6861.1	1.10824	coding
MSTRG.6861.2	1.14036	coding
MSTRG.6857.9	0.351179	coding
MSTRG.6857.10	0.331187	coding
MSTRG.6857.11	0.21356	coding
MSTRG.6857.12	0.254212	coding
MSTRG.6857.13	-0.39053	noncoding
MSTRG.6857.14	-0.793178	noncoding
MSTRG.6857.15	0.430042	coding
MSTRG.6863.1	0.369018	coding
MSTRG.6864.1	0.163434	coding
MSTRG.6864.2	-1.2868	noncoding
MSTRG.6857.16	0.679779	coding

MSTRG.6865.1	0.010134	coding
MSTRG.6857.17	2.11401	coding
MSTRG.6857.19	1.50927	coding
MSTRG.6866.1	1.39394	coding
MSTRG.6857.20	1.69072	coding
MSTRG.6857.21	1.9457	coding
MSTRG.6857.22	1.20064	coding
MSTRG.6857.23	1.42822	coding
MSTRG.6827.2	0.923337	coding
MSTRG.6837.1	-0.135435	noncoding
MSTRG.6838.1	-0.739817	noncoding
MSTRG.6839.1	-0.129738	noncoding
MSTRG.6845.1	-1.70801	noncoding
MSTRG.6851.1	0.950956	coding
MSTRG.6851.2	-0.827743	noncoding
MSTRG.6852.1	-1.37676	noncoding
MSTRG.6869.1	1.09778	coding
MSTRG.6871.2	-0.773581	noncoding
MSTRG.6873.2	1.40572	coding
MSTRG.6873.3	1.42385	coding
MSTRG.6884.1	1.22457	coding
MSTRG.6885.1	-0.546633	noncoding
MSTRG.6886.1	0.386386	coding
MSTRG.6887.1	0.08728	coding
MSTRG.6888.1	1.04407	coding
MSTRG.6901.1	-0.613264	noncoding
MSTRG.6906.7	1.22649	coding
MSTRG.6906.8	0.956742	coding
MSTRG.6906.9	0.074285	coding
MSTRG.6906.10	-0.129606	noncoding
MSTRG.6907.1	0.440213	coding
MSTRG.6902.1	-2.11079	noncoding
MSTRG.6919.1	0.081676	coding
MSTRG.6920.1	-0.157667	noncoding
MSTRG.6921.1	-0.356746	noncoding
MSTRG.6922.4	0.236232	coding
MSTRG.6922.3	1.78438	coding
MSTRG.6922.5	-0.115181	noncoding
MSTRG.6923.1	-1.86786	noncoding
MSTRG.6925.1	-1.17838	noncoding
MSTRG.6925.2	-1.61797	noncoding
MSTRG.6925.3	-1.47218	noncoding
MSTRG.6926.1	-1.33681	noncoding
MSTRG.6934.1	0.059246	coding
MSTRG.6939.3	-0.705044	noncoding
MSTRG.6941.1	1.02143	coding
MSTRG.6941.2	0.998829	coding
MSTRG.6943.1	0.632839	coding
MSTRG.6943.2	0.86213	coding
MSTRG.6943.4	1.44615	coding
MSTRG.6945.2	2.43976	coding
MSTRG.6947.3	1.70357	coding
MSTRG.6947.4	1.58911	coding
MSTRG.6947.6	1.6954	coding

MSTRG.6948.1	-0.578242	noncoding
MSTRG.6947.7	1.44339	coding
MSTRG.6947.8	0.405282	coding
MSTRG.6956.3	1.99551	coding
MSTRG.6962.4	1.15179	coding
MSTRG.6962.5	2.14406	coding
MSTRG.6966.4	2.58683	coding
MSTRG.6967.3	0.955217	coding
MSTRG.6970.3	2.96839	coding
MSTRG.6973.5	1.63879	coding
MSTRG.6974.1	0.833753	coding
MSTRG.6975.1	1.1398	coding
MSTRG.6985.1	2.02109	coding
MSTRG.6986.1	1.23153	coding
MSTRG.6986.4	0.675644	coding
MSTRG.6988.1	-1.59495	noncoding
MSTRG.6997.1	-0.129056	noncoding
MSTRG.6997.2	0.047379	coding
MSTRG.6995.2	-1.70775	noncoding
MSTRG.6995.4	-1.14954	noncoding
MSTRG.6995.3	-1.09281	noncoding
MSTRG.6995.5	-1.1565	noncoding
MSTRG.6977.1	-0.624553	noncoding
MSTRG.6977.2	-1.0609	noncoding
MSTRG.6977.3	-0.74853	noncoding
MSTRG.6977.4	-0.552882	noncoding
MSTRG.6977.5	-1.67984	noncoding
MSTRG.6980.3	0.753671	coding
MSTRG.6980.2	0.809378	coding
MSTRG.6981.6	0.690016	coding
MSTRG.6981.4	0.477621	coding
MSTRG.6981.5	0.428922	coding
MSTRG.7007.1	0.237437	coding
MSTRG.7007.2	1.39287	coding
MSTRG.7007.4	1.01747	coding
MSTRG.7007.5	1.43534	coding
MSTRG.7008.2	0.254283	coding
MSTRG.7007.11	1.79538	coding
MSTRG.6982.1	1.03883	coding
MSTRG.6983.1	-2.3294	noncoding
MSTRG.6982.3	1.12204	coding
MSTRG.7005.3	0.349063	coding
MSTRG.7006.1	1.85474	coding
MSTRG.7012.2	1.33037	coding
MSTRG.7019.1	-0.262535	noncoding
MSTRG.7035.2	1.00406	coding
MSTRG.7041.4	-0.670071	noncoding
MSTRG.7044.1	-1.62016	noncoding
MSTRG.7050.1	-1.47612	noncoding
MSTRG.7050.3	-1.72507	noncoding
MSTRG.7050.2	-0.200387	noncoding
MSTRG.7054.1	-2.14328	noncoding
MSTRG.7075.4	0.300869	coding
MSTRG.7075.5	-1.0104	noncoding

MSTRG.7079.1	0.502869	coding
MSTRG.7083.1	-2.6881	noncoding
MSTRG.7077.3	0.306241	coding
MSTRG.7091.3	1.91893	coding
MSTRG.7091.5	1.93044	coding
MSTRG.7091.4	1.64649	coding
MSTRG.7096.1	-0.071645	noncoding
MSTRG.7102.1	0.163928	coding
MSTRG.7102.2	-0.002608	noncoding
MSTRG.7102.3	0.243243	coding
MSTRG.7103.1	0.883776	coding
MSTRG.7104.1	1.83429	coding
MSTRG.7110.1	-1.83249	noncoding
MSTRG.7111.3	1.83278	coding
MSTRG.7111.4	1.84903	coding
MSTRG.7111.5	1.37698	coding
MSTRG.7118.2	0.582927	coding
MSTRG.7122.3	1.25713	coding
MSTRG.7130.2	2.59089	coding
MSTRG.7130.3	2.7026	coding
MSTRG.7130.4	1.95053	coding
MSTRG.7135.3	-0.114896	noncoding
MSTRG.7135.4	-1.66602	noncoding
MSTRG.7138.2	1.1656	coding
MSTRG.7141.1	1.70128	coding
MSTRG.7141.3	1.56057	coding
MSTRG.7142.1	-0.231504	noncoding
MSTRG.7147.3	-0.792651	noncoding
MSTRG.7147.2	1.57451	coding
MSTRG.7146.1	-2.03943	noncoding
MSTRG.7154.2	1.14667	coding
MSTRG.7154.5	1.22783	coding
MSTRG.7154.6	-0.598627	noncoding
MSTRG.7156.1	1.01674	coding
MSTRG.7161.1	2.82579	coding
MSTRG.7161.2	0.385169	coding
MSTRG.7162.1	-0.921056	noncoding
MSTRG.7164.1	-1.4473	noncoding
MSTRG.7165.1	0.000309	coding
MSTRG.7165.2	0.930326	coding
MSTRG.7166.1	1.28264	coding
MSTRG.7169.1	-0.7778	noncoding
MSTRG.7177.1	-0.480454	noncoding
MSTRG.7178.1	-0.897557	noncoding
MSTRG.7178.2	-1.6509	noncoding
MSTRG.7177.2	-1.03677	noncoding
MSTRG.7177.3	-0.815564	noncoding
MSTRG.7177.4	-0.858016	noncoding
MSTRG.7177.8	-0.759571	noncoding
MSTRG.7177.7	-0.804337	noncoding
MSTRG.7177.6	-0.56932	noncoding
MSTRG.7177.5	-0.497514	noncoding
MSTRG.7177.9	-0.020123	noncoding
MSTRG.7177.10	-0.795712	noncoding

MSTRG.7177.11	-0.556918	noncoding
MSTRG.7177.12	-0.446598	noncoding
MSTRG.7177.15	-1.71226	noncoding
MSTRG.7177.16	-1.78563	noncoding
MSTRG.7177.13	-0.848501	noncoding
MSTRG.7177.14	-0.839865	noncoding
MSTRG.7177.18	-0.283318	noncoding
MSTRG.7177.19	0.106826	coding
MSTRG.7179.1	-0.98228	noncoding
MSTRG.7172.1	-0.545805	noncoding
MSTRG.7176.7	1.89773	coding
MSTRG.7176.4	1.20134	coding
MSTRG.7176.5	1.27978	coding
MSTRG.7176.6	1.25347	coding
MSTRG.7183.1	0.611351	coding
MSTRG.7183.3	1.20061	coding
MSTRG.7189.1	0.719113	coding
MSTRG.7191.3	1.03706	coding
MSTRG.7194.1	-0.631798	noncoding
MSTRG.7195.1	1.07122	coding
MSTRG.7195.2	1.52805	coding
MSTRG.7196.1	-0.513901	noncoding
MSTRG.7195.3	0.470762	coding
MSTRG.7186.1	-2.54005	noncoding
MSTRG.7197.3	2.52159	coding
MSTRG.7200.2	2.4007	coding
MSTRG.7200.3	2.38888	coding
MSTRG.7202.1	-0.442126	noncoding
MSTRG.7206.4	1.12621	coding
MSTRG.7206.5	0.97998	coding
MSTRG.7206.6	-0.294664	noncoding
MSTRG.7207.1	0.263025	coding
MSTRG.7206.7	0.98745	coding
MSTRG.7211.1	0.012336	coding
MSTRG.7213.1	0.465795	coding
MSTRG.7216.2	2.0739	coding
MSTRG.7220.2	1.69624	coding
MSTRG.7220.3	1.34853	coding
MSTRG.7220.4	0.777095	coding
MSTRG.7225.1	0.981007	coding
MSTRG.7227.1	1.06638	coding
MSTRG.7232.2	-0.109944	noncoding
MSTRG.7232.3	-0.371293	noncoding
MSTRG.7232.1	0.989711	coding
MSTRG.7232.4	1.05064	coding
MSTRG.7232.5	0.722624	coding
MSTRG.7232.6	-0.095779	noncoding
MSTRG.7235.3	1.55714	coding
MSTRG.7235.4	1.55931	coding
MSTRG.7245.1	0.167227	coding
MSTRG.7258.7	1.75619	coding
MSTRG.7258.6	1.32178	coding
MSTRG.7258.5	1.44922	coding
MSTRG.7260.1	-0.484441	noncoding

MSTRG.7262.3	1.29258	coding
MSTRG.7262.5	-0.640073	noncoding
MSTRG.7263.1	0.815442	coding
MSTRG.7263.2	0.517016	coding
MSTRG.7264.1	-0.675791	noncoding
MSTRG.7266.9	1.20446	coding
MSTRG.7266.12	0.441473	coding
MSTRG.7268.1	-0.87001	noncoding
MSTRG.7269.5	-0.847533	noncoding
MSTRG.7269.7	-1.11405	noncoding
MSTRG.7269.3	-1.41447	noncoding
MSTRG.7269.4	-1.42488	noncoding
MSTRG.7269.6	0.28349	coding
MSTRG.7269.8	-0.552817	noncoding
MSTRG.7269.9	-0.182111	noncoding
MSTRG.7270.1	-0.577458	noncoding
MSTRG.7271.1	-0.875319	noncoding
MSTRG.7277.1	-0.18641	noncoding
MSTRG.7278.1	-0.828409	noncoding
MSTRG.7279.1	0.607546	coding
MSTRG.7276.3	0.883436	coding
MSTRG.7276.4	1.49259	coding
MSTRG.7281.1	-2.37381	noncoding
MSTRG.7281.2	-1.80771	noncoding
MSTRG.7286.10	1.9252	coding
MSTRG.7286.3	2.05933	coding
MSTRG.7286.5	2.10574	coding
MSTRG.7286.7	1.72757	coding
MSTRG.7286.8	1.71269	coding
MSTRG.7286.9	1.97585	coding
MSTRG.7286.11	1.96375	coding
MSTRG.7286.17	0.601316	coding
MSTRG.7292.1	1.70664	coding
MSTRG.7292.3	0.965243	coding
MSTRG.7293.3	0.48429	coding
MSTRG.7296.1	2.25918	coding
MSTRG.7297.3	3.08892	coding
MSTRG.7297.5	3.09132	coding
MSTRG.7298.1	0.432454	coding
MSTRG.7300.1	-0.576528	noncoding
MSTRG.7297.6	0.315007	coding
MSTRG.7297.7	0.119406	coding
MSTRG.7295.2	0.201712	coding
MSTRG.7303.1	-0.035973	noncoding
MSTRG.7306.1	0.619657	coding
MSTRG.7306.2	-0.774161	noncoding
MSTRG.7316.1	0.258669	coding
MSTRG.7317.1	0.248125	coding
MSTRG.7322.4	1.30932	coding
MSTRG.7322.5	1.27862	coding
MSTRG.7323.1	-2.28737	noncoding
MSTRG.7323.2	-1.9643	noncoding
MSTRG.7325.1	-1.48321	noncoding
MSTRG.7320.1	-1.48886	noncoding

MSTRG.7320.2	-0.644539	noncoding
MSTRG.7321.1	-2.36191	noncoding
MSTRG.7333.5	1.01907	coding
MSTRG.7335.2	1.31159	coding
MSTRG.7335.4	1.19795	coding
MSTRG.7335.5	1.58419	coding
MSTRG.7335.8	1.99403	coding
MSTRG.7335.9	1.82992	coding
MSTRG.7335.10	1.21179	coding
MSTRG.7335.11	0.325388	coding
MSTRG.7335.12	0.369521	coding
MSTRG.7335.13	0.66737	coding
MSTRG.7340.2	1.62748	coding
MSTRG.7340.3	0.173883	coding
MSTRG.7340.4	0.236679	coding
MSTRG.7341.1	-1.89308	noncoding
MSTRG.7342.1	1.58971	coding
MSTRG.7343.1	1.15071	coding
MSTRG.7344.4	0.430713	coding
MSTRG.7346.1	-1.63704	noncoding
MSTRG.7348.1	-1.88257	noncoding
MSTRG.7350.1	0.443764	coding
MSTRG.7354.2	1.57558	coding
MSTRG.7359.1	-2.07994	noncoding
MSTRG.7370.1	0.069709	coding
MSTRG.7371.1	-0.392857	noncoding
MSTRG.7369.3	1.45689	coding
MSTRG.7372.1	0.494336	coding
MSTRG.7373.1	-0.247868	noncoding
MSTRG.7374.1	-0.055362	noncoding
MSTRG.7366.2	1.92995	coding
MSTRG.7365.1	-1.23383	noncoding
MSTRG.7390.1	-1.33381	noncoding
MSTRG.7392.1	0.175024	coding
MSTRG.7393.1	-1.77602	noncoding
MSTRG.7394.1	-1.35357	noncoding
MSTRG.7376.3	1.03069	coding
MSTRG.7376.5	1.00803	coding
MSTRG.7382.1	-0.305709	noncoding
MSTRG.7385.1	-1.54681	noncoding
MSTRG.7387.1	1.60992	coding
MSTRG.7415.2	2.05026	coding
MSTRG.7415.4	0.232856	coding
MSTRG.7415.3	0.961457	coding
MSTRG.7415.5	1.0267	coding
MSTRG.7415.6	0.725491	coding
MSTRG.7415.7	1.54985	coding
MSTRG.7421.3	0.314039	coding
MSTRG.7423.4	1.02288	coding
MSTRG.7423.2	1.36822	coding
MSTRG.7423.3	1.3511	coding
MSTRG.7426.1	1.80492	coding
MSTRG.7431.1	0.725623	coding
MSTRG.7431.2	1.07196	coding

MSTRG.7432.1	0.52372	coding
MSTRG.7432.9	0.776969	coding
MSTRG.7432.5	2.03441	coding
MSTRG.7432.6	2.16972	coding
MSTRG.7432.7	2.07635	coding
MSTRG.7434.1	-0.966267	noncoding
MSTRG.7429.1	1.63411	coding
MSTRG.7429.2	1.0332	coding
MSTRG.7437.1	-2.21481	noncoding
MSTRG.7438.1	0.933612	coding
MSTRG.7439.4	0.454692	coding
MSTRG.7441.1	1.51056	coding
MSTRG.7448.1	-1.34626	noncoding
MSTRG.7448.2	-1.29152	noncoding
MSTRG.7452.1	1.40463	coding
MSTRG.7452.3	1.11991	coding
MSTRG.7454.1	0.628074	coding
MSTRG.7451.2	-0.203597	noncoding
MSTRG.7455.1	2.00882	coding
MSTRG.7455.2	2.91276	coding
MSTRG.7456.6	0.576978	coding
MSTRG.7456.7	0.327362	coding
MSTRG.7456.8	0.438703	coding
MSTRG.7458.1	-1.91937	noncoding
MSTRG.7457.3	0.022329	coding
MSTRG.7462.1	-2.27891	noncoding
MSTRG.7467.1	1.94895	coding
MSTRG.7465.1	0.354438	coding
MSTRG.7470.1	-2.36143	noncoding
MSTRG.7474.2	0.329253	coding
MSTRG.7476.1	-1.64997	noncoding
MSTRG.7487.1	-1.71989	noncoding
MSTRG.7485.2	1.05243	coding
MSTRG.7499.1	1.20277	coding
MSTRG.7496.1	-0.725125	noncoding
MSTRG.7501.1	-1.0169	noncoding
MSTRG.7505.1	0.669243	coding
MSTRG.7505.2	0.749614	coding
MSTRG.7518.1	-0.205569	noncoding
MSTRG.7529.1	-0.718915	noncoding
MSTRG.7529.2	-0.987827	noncoding
MSTRG.7531.1	-0.612678	noncoding
MSTRG.7534.1	-1.81768	noncoding
MSTRG.7537.4	2.66878	coding
MSTRG.7538.1	0.313257	coding
MSTRG.7539.1	2.8869	coding
MSTRG.7541.1	1.02685	coding
MSTRG.7542.1	-0.715536	noncoding
MSTRG.7539.2	3.12473	coding
MSTRG.7544.2	0.426912	coding
MSTRG.7551.1	2.03533	coding
MSTRG.7551.2	2.05034	coding
MSTRG.7552.1	-1.42457	noncoding
MSTRG.7552.3	-1.54517	noncoding

MSTRG.7552.2	-1.64826	noncoding
MSTRG.7556.2	1.61221	coding
MSTRG.7557.1	-0.517332	noncoding
MSTRG.7557.2	-0.178392	noncoding
MSTRG.7557.3	-0.524016	noncoding
MSTRG.7557.4	-0.8527	noncoding
MSTRG.7557.5	-0.107931	noncoding
MSTRG.7557.6	-0.562066	noncoding
MSTRG.7560.3	0.986589	coding
MSTRG.7562.6	0.330926	coding
MSTRG.7562.7	0.637504	coding
MSTRG.7562.8	0.586286	coding
MSTRG.7563.1	0.914452	coding
MSTRG.7563.2	0.969655	coding
MSTRG.7563.4	0.536965	coding
MSTRG.7573.4	3.13629	coding
MSTRG.7573.5	3.13308	coding
MSTRG.7585.1	0.308629	coding
MSTRG.7572.1	1.73218	coding
MSTRG.7572.2	1.821	coding
MSTRG.7580.2	1.23807	coding
MSTRG.7588.2	2.04446	coding
MSTRG.7591.2	1.5127	coding
MSTRG.7591.3	1.48445	coding
MSTRG.7591.4	-0.380796	noncoding
MSTRG.7592.1	1.1967	coding
MSTRG.7594.1	-0.854707	noncoding
MSTRG.7597.2	-2.18777	noncoding
MSTRG.7597.1	-2.68047	noncoding
MSTRG.7600.1	1.24525	coding
MSTRG.7604.3	0.835155	coding
MSTRG.7604.4	0.823435	coding
MSTRG.7604.5	-0.408433	noncoding
MSTRG.7606.1	2.14512	coding
MSTRG.7608.2	0.807252	coding
MSTRG.7611.1	0.936721	coding
MSTRG.7617.2	0.228409	coding
MSTRG.7618.3	0.250187	coding
MSTRG.7618.4	-0.012111	noncoding
MSTRG.7619.3	1.84107	coding
MSTRG.7621.1	1.53038	coding
MSTRG.7615.2	0.652424	coding
MSTRG.7615.1	0.066266	coding
MSTRG.7615.3	0.847593	coding
MSTRG.7615.4	0.64141	coding
MSTRG.7615.5	0.212764	coding
MSTRG.7616.2	-1.67214	noncoding
MSTRG.7625.1	2.20125	coding
MSTRG.7625.2	2.23382	coding
MSTRG.7626.1	1.6418	coding
MSTRG.7626.2	1.39282	coding
MSTRG.7630.2	2.09749	coding
MSTRG.7630.3	2.79424	coding
MSTRG.7632.3	-0.561005	noncoding

MSTRG.7637.1	0.197291	coding
MSTRG.7637.4	1.01013	coding
MSTRG.7637.5	0.126055	coding
MSTRG.7638.1	1.06591	coding
MSTRG.7638.10	2.13372	coding
MSTRG.7638.11	2.13009	coding
MSTRG.7638.5	2.17432	coding
MSTRG.7638.6	2.18054	coding
MSTRG.7638.7	2.12655	coding
MSTRG.7638.8	2.18823	coding
MSTRG.7638.9	2.21633	coding
MSTRG.7639.1	1.53555	coding
MSTRG.7638.12	1.55824	coding
MSTRG.7640.2	-0.133331	noncoding
MSTRG.7640.3	-0.34128	noncoding
MSTRG.7642.1	0.901591	coding
MSTRG.7642.2	0.679052	coding
MSTRG.7642.3	0.059997	coding
MSTRG.7642.4	-0.325366	noncoding
MSTRG.7634.2	1.00249	coding
MSTRG.7635.1	1.00464	coding
MSTRG.7635.2	1.01205	coding
MSTRG.7635.3	0.652957	coding
MSTRG.7635.5	0.497765	coding
MSTRG.7636.1	-2.00733	noncoding
MSTRG.7636.2	-1.78422	noncoding
MSTRG.7650.1	3.27636	coding
MSTRG.7646.1	0.591033	coding
MSTRG.7647.1	-1.5236	noncoding
MSTRG.7659.8	1.02771	coding
MSTRG.7660.3	1.31781	coding
MSTRG.7660.5	1.43848	coding
MSTRG.7666.1	2.14135	coding
MSTRG.7666.3	2.07963	coding
MSTRG.7666.4	2.04057	coding
MSTRG.7666.5	2.37835	coding
MSTRG.7666.6	-0.270448	noncoding
MSTRG.7665.1	1.50165	coding
MSTRG.7668.2	1.90507	coding
MSTRG.7668.3	2.23179	coding
MSTRG.7668.4	2.2413	coding
MSTRG.7673.1	-0.383865	noncoding
MSTRG.7674.2	0.659192	coding
MSTRG.7675.2	1.06323	coding
MSTRG.7680.1	-1.07788	noncoding
MSTRG.7686.3	1.30771	coding
MSTRG.7694.1	-1.76344	noncoding
MSTRG.7705.5	1.55381	coding
MSTRG.7703.1	1.79571	coding
MSTRG.7707.1	0.590235	coding
MSTRG.7707.3	0.584071	coding
MSTRG.7711.4	1.0732	coding
MSTRG.7711.5	0.961818	coding
MSTRG.7711.6	1.05228	coding

MSTRG.7711.3	1.2587	coding
MSTRG.7711.1	0.625965	coding
MSTRG.7717.2	1.64568	coding
MSTRG.7717.1	1.57049	coding
MSTRG.7717.3	0.829377	coding
MSTRG.7717.4	0.402176	coding
MSTRG.7717.5	-0.992575	noncoding
MSTRG.7717.8	1.27416	coding
MSTRG.7714.2	1.42681	coding
MSTRG.7718.1	2.55885	coding
MSTRG.7722.2	1.51527	coding
MSTRG.7722.3	2.00414	coding
MSTRG.7723.1	0.039034	coding
MSTRG.7722.5	1.45073	coding
MSTRG.7722.6	1.65066	coding
MSTRG.7723.2	0.01947	coding
MSTRG.7730.5	1.23027	coding
MSTRG.7730.6	1.19105	coding
MSTRG.7731.1	-0.289208	noncoding
MSTRG.7733.3	1.22264	coding
MSTRG.7737.1	-1.68443	noncoding
MSTRG.7738.1	1.26238	coding
MSTRG.7739.4	-2.22303	noncoding
MSTRG.7740.2	-0.780001	noncoding
MSTRG.7742.2	-0.010481	noncoding
MSTRG.7742.3	-0.089888	noncoding
MSTRG.7735.1	-2.04016	noncoding
MSTRG.7744.2	2.11641	coding
MSTRG.7745.1	-0.097076	noncoding
MSTRG.7747.2	2.14568	coding
MSTRG.7747.3	2.338	coding
MSTRG.7747.4	2.29386	coding
MSTRG.7747.1	2.11313	coding
MSTRG.7747.5	2.26119	coding
MSTRG.7751.3	2.16383	coding
MSTRG.7751.6	2.0752	coding
MSTRG.7751.7	1.19526	coding
MSTRG.7751.8	1.22352	coding
MSTRG.7751.9	1.05822	coding
MSTRG.7752.1	-1.19714	noncoding
MSTRG.7752.2	-0.635668	noncoding
MSTRG.7755.1	1.11165	coding
MSTRG.7755.3	0.879715	coding
MSTRG.7743.1	2.86328	coding
MSTRG.7758.1	-1.86338	noncoding
MSTRG.7762.2	-0.129484	noncoding
MSTRG.7760.2	0.474661	coding
MSTRG.7767.1	-1.75738	noncoding
MSTRG.7767.2	-1.82872	noncoding
MSTRG.7769.2	0.685713	coding
MSTRG.7770.1	-1.15453	noncoding
MSTRG.7770.2	-1.44843	noncoding
MSTRG.7771.4	-0.567235	noncoding
MSTRG.7772.3	1.39501	coding

MSTRG.7773.2	-0.406203	noncoding
MSTRG.7780.2	-0.665341	noncoding
MSTRG.7782.1	0.080682	coding
MSTRG.7782.2	0.288178	coding
MSTRG.7782.3	-0.28974	noncoding
MSTRG.7782.4	0.691982	coding
MSTRG.7782.5	0.692022	coding
MSTRG.7782.8	-0.129854	noncoding
MSTRG.7782.6	0.42669	coding
MSTRG.7782.7	0.120497	coding
MSTRG.7782.9	0.276041	coding
MSTRG.7782.10	0.268462	coding
MSTRG.7782.12	0.108116	coding
MSTRG.7782.13	0.228706	coding
MSTRG.7782.14	-0.602348	noncoding
MSTRG.7783.1	-1.15214	noncoding
MSTRG.7787.1	1.17291	coding
MSTRG.7790.3	0.499959	coding
MSTRG.7791.1	-1.16946	noncoding
MSTRG.7794.3	0.419657	coding
MSTRG.7795.1	-0.338355	noncoding
MSTRG.7796.3	1.69286	coding
MSTRG.7796.5	1.26445	coding
MSTRG.7796.6	0.631371	coding
MSTRG.7796.7	-1.38555	noncoding
MSTRG.7797.1	-0.201621	noncoding
MSTRG.7799.2	0.940547	coding
MSTRG.7800.1	-1.88012	noncoding
MSTRG.7801.2	-1.54161	noncoding
MSTRG.7801.1	-1.60605	noncoding
MSTRG.7803.1	-0.428817	noncoding
MSTRG.7804.2	1.67899	coding
MSTRG.7805.1	0.719121	coding
MSTRG.7805.3	0.481815	coding
MSTRG.7805.4	0.248496	coding
MSTRG.7805.5	0.581752	coding
MSTRG.7809.1	-0.359244	noncoding
MSTRG.7812.1	0.899393	coding
MSTRG.7815.1	1.2833	coding
MSTRG.7815.3	1.70383	coding
MSTRG.7814.1	1.30817	coding
MSTRG.7818.2	0.646922	coding
MSTRG.7818.3	-1.77086	noncoding
MSTRG.7819.1	-1.26501	noncoding
MSTRG.7821.2	-1.21705	noncoding
MSTRG.7825.2	1.72286	coding
MSTRG.7832.1	0.516157	coding
MSTRG.7832.2	0.199112	coding
MSTRG.7840.1	0.007167	coding
MSTRG.7841.3	1.79232	coding
MSTRG.7842.2	2.03357	coding
MSTRG.7843.2	2.12357	coding
MSTRG.7844.1	1.30737	coding
MSTRG.7844.4	-0.29335	noncoding

MSTRG.7844.3	1.63928	coding
MSTRG.7844.5	1.17494	coding
MSTRG.7844.6	1.20635	coding
MSTRG.7848.1	1.20311	coding
MSTRG.7848.2	-1.80737	noncoding
MSTRG.7854.5	-0.448071	noncoding
MSTRG.7859.2	1.59713	coding
MSTRG.7859.3	1.41974	coding
MSTRG.7859.1	1.4367	coding
MSTRG.7859.4	1.37802	coding
MSTRG.7859.8	1.36544	coding
MSTRG.7859.9	1.34312	coding
MSTRG.7859.7	1.44918	coding
MSTRG.7859.5	1.66347	coding
MSTRG.7859.6	1.38205	coding
MSTRG.7859.10	1.43318	coding
MSTRG.7859.11	1.36868	coding
MSTRG.7859.12	1.63387	coding
MSTRG.7860.1	-1.32449	noncoding
MSTRG.7859.14	-1.94004	noncoding
MSTRG.7859.13	1.68971	coding
MSTRG.7859.15	1.69342	coding
MSTRG.7859.16	2.08064	coding
MSTRG.7863.1	-2.1992	noncoding
MSTRG.7855.7	-0.568334	noncoding
MSTRG.7868.7	-0.288792	noncoding
MSTRG.7870.1	-0.252287	noncoding
MSTRG.7866.2	1.02686	coding
MSTRG.7866.3	1.0073	coding
MSTRG.7874.1	-0.448754	noncoding
MSTRG.7873.2	1.01507	coding
MSTRG.7879.1	-0.629445	noncoding
MSTRG.7878.1	-0.589115	noncoding
MSTRG.7881.1	-2.11517	noncoding
MSTRG.7881.2	-1.90589	noncoding
MSTRG.7884.1	-1.73535	noncoding
MSTRG.7891.1	-1.70949	noncoding
MSTRG.7898.2	1.12643	coding
MSTRG.7889.1	0.026132	coding
MSTRG.7903.1	1.62143	coding
MSTRG.7903.2	0.313857	coding
MSTRG.7903.3	0.426425	coding
MSTRG.7903.4	0.135165	coding
MSTRG.7905.6	0.937915	coding
MSTRG.7905.7	1.64331	coding
MSTRG.7915.10	1.05887	coding
MSTRG.7915.11	1.80423	coding
MSTRG.7915.5	1.87092	coding
MSTRG.7915.6	1.86967	coding
MSTRG.7915.7	1.86498	coding
MSTRG.7915.9	1.88276	coding
MSTRG.7915.12	-0.092057	noncoding
MSTRG.7917.3	1.98894	coding
MSTRG.7917.4	2.05373	coding

MSTRG.7913.1	-0.709948	noncoding
MSTRG.7910.3	-0.247059	noncoding
MSTRG.7921.1	2.27152	coding
MSTRG.7921.2	2.11669	coding
MSTRG.7921.4	1.93206	coding
MSTRG.7923.1	2.3586	coding
MSTRG.7923.2	2.20027	coding
MSTRG.7922.2	0.536896	coding
MSTRG.7922.3	0.730793	coding
MSTRG.7926.2	-0.832394	noncoding
MSTRG.7930.1	0.721804	coding
MSTRG.7933.1	-0.221716	noncoding
MSTRG.7933.2	-0.503907	noncoding
MSTRG.7932.1	-2.4099	noncoding
MSTRG.7939.1	2.35771	coding
MSTRG.7939.2	1.21674	coding
MSTRG.7939.3	0.913743	coding
MSTRG.7939.4	0.193153	coding
MSTRG.7935.2	2.56319	coding
MSTRG.7943.1	-2.40315	noncoding
MSTRG.7944.3	0.278694	coding
MSTRG.7944.4	0.450513	coding
MSTRG.7944.5	0.897498	coding
MSTRG.7944.6	0.422953	coding
MSTRG.7945.1	-3.15128	noncoding
MSTRG.7944.7	-0.310006	noncoding
MSTRG.7958.1	-2.33989	noncoding
MSTRG.7962.1	-0.439425	noncoding
MSTRG.7984.1	0.405041	coding
MSTRG.7985.1	-1.59507	noncoding
MSTRG.7990.1	-0.337558	noncoding
MSTRG.7995.1	1.12257	coding
MSTRG.7996.1	-0.06815	noncoding
MSTRG.7996.2	-0.155405	noncoding
MSTRG.7997.1	-1.05755	noncoding
MSTRG.7998.1	0.323189	coding
MSTRG.7988.15	-1.02031	noncoding
MSTRG.7988.13	-0.216923	noncoding
MSTRG.7988.12	-0.57638	noncoding
MSTRG.7988.14	-0.927252	noncoding
MSTRG.7988.11	1.84086	coding
MSTRG.7988.16	-0.054168	noncoding
MSTRG.7988.18	-0.52573	noncoding
MSTRG.7988.20	1.12426	coding
MSTRG.7988.22	-0.835311	noncoding
MSTRG.8002.1	1.33075	coding
MSTRG.8002.2	0.276672	coding
MSTRG.8016.1	-0.024836	noncoding
MSTRG.8019.1	0.044465	coding
MSTRG.8020.1	1.53472	coding
MSTRG.8022.1	-1.07462	noncoding
MSTRG.8023.1	0.612635	coding
MSTRG.8020.4	-0.614068	noncoding
MSTRG.8020.5	-0.63453	noncoding

MSTRG.8025.1	0.308051	coding
MSTRG.8024.7	-0.917227	noncoding
MSTRG.8026.1	1.03341	coding
MSTRG.8027.1	-2.20999	noncoding
MSTRG.8029.1	1.75795	coding
MSTRG.8029.2	1.78703	coding
MSTRG.8031.1	-0.602847	noncoding
MSTRG.8032.2	2.93734	coding
MSTRG.8010.1	-2.42489	noncoding
MSTRG.8060.1	-0.71092	noncoding
MSTRG.8071.1	-1.13167	noncoding
MSTRG.8074.1	-0.218631	noncoding
MSTRG.8096.1	1.55287	coding
MSTRG.8096.2	2.56953	coding
MSTRG.8096.3	2.57526	coding
MSTRG.8096.4	2.52668	coding
MSTRG.8096.11	2.62667	coding
MSTRG.8096.12	1.77717	coding
MSTRG.8096.6	2.61918	coding
MSTRG.8096.10	2.68278	coding
MSTRG.8096.5	2.67931	coding
MSTRG.8096.7	2.68627	coding
MSTRG.8096.8	2.62124	coding
MSTRG.8096.9	2.82528	coding
MSTRG.8096.13	2.50282	coding
MSTRG.8096.14	2.40009	coding
MSTRG.8097.1	0.597059	coding
MSTRG.8098.1	0.749424	coding
MSTRG.8098.2	0.734032	coding
MSTRG.8099.1	-0.16868	noncoding
MSTRG.8098.3	0.561587	coding
MSTRG.8100.1	0.089263	coding
MSTRG.8096.15	0.269971	coding
MSTRG.8096.16	0.623255	coding
MSTRG.8096.17	0.71282	coding
MSTRG.8096.18	0.702527	coding
MSTRG.8096.19	0.322884	coding
MSTRG.8096.20	0.44184	coding
MSTRG.8096.21	0.547955	coding
MSTRG.8096.22	-0.179609	noncoding
MSTRG.8083.1	-0.120427	noncoding
MSTRG.8086.1	-2.5095	noncoding
MSTRG.8103.1	1.38338	coding
MSTRG.8109.3	1.67749	coding
MSTRG.8110.2	1.49045	coding
MSTRG.8110.4	1.49914	coding
MSTRG.8110.6	1.82858	coding
MSTRG.8110.5	2.44928	coding
MSTRG.8113.1	-2.32832	noncoding
MSTRG.8119.1	-0.769762	noncoding
MSTRG.8119.2	0.479711	coding
MSTRG.8130.1	-0.10562	noncoding
MSTRG.8131.3	1.10442	coding
MSTRG.8131.4	0.64351	coding

MSTRG.8131.5	0.639612	coding
MSTRG.8131.6	0.606926	coding
MSTRG.8131.7	0.651684	coding
MSTRG.8117.3	1.55985	coding
MSTRG.8143.1	0.894293	coding
MSTRG.8145.1	-1.58489	noncoding
MSTRG.8146.1	-1.85977	noncoding
MSTRG.8147.2	0.460171	coding
MSTRG.8160.2	-2.02806	noncoding
MSTRG.8160.1	-0.157251	noncoding
MSTRG.8160.3	0.798669	coding
MSTRG.8160.4	-0.245551	noncoding
MSTRG.8165.1	0.987811	coding
MSTRG.8165.3	1.04698	coding
MSTRG.8171.1	-2.26469	noncoding
MSTRG.8171.2	-1.51419	noncoding
MSTRG.8172.11	-0.663951	noncoding
MSTRG.8172.7	-1.92833	noncoding
MSTRG.8172.6	0.291544	coding
MSTRG.8172.9	0.679276	coding
MSTRG.8172.10	0.280041	coding
MSTRG.8172.5	0.030048	coding
MSTRG.8172.8	0.542854	coding
MSTRG.8172.4	0.802206	coding
MSTRG.8172.3	1.39188	coding
MSTRG.8172.13	0.773363	coding
MSTRG.8172.12	0.823	coding
MSTRG.8172.14	1.24768	coding
MSTRG.8172.15	0.191489	coding
MSTRG.8185.2	0.969508	coding
MSTRG.8185.1	0.72836	coding
MSTRG.8185.3	0.760894	coding
MSTRG.8195.1	1.73692	coding
MSTRG.8195.2	1.19607	coding
MSTRG.8190.2	-2.0544	noncoding
MSTRG.8198.1	-2.41741	noncoding
MSTRG.8201.1	0.079517	coding
MSTRG.8201.2	0.516686	coding
MSTRG.8202.1	0.905703	coding
MSTRG.8200.2	2.61858	coding
MSTRG.8200.3	-1.57811	noncoding
MSTRG.8203.1	-2.58755	noncoding
MSTRG.8209.1	-0.06287	noncoding
MSTRG.8209.2	-0.242937	noncoding
MSTRG.8210.1	-1.29754	noncoding
MSTRG.8218.1	0.811753	coding
MSTRG.8219.1	-2.03466	noncoding
MSTRG.8224.3	-0.117555	noncoding
MSTRG.8221.1	-1.30189	noncoding
MSTRG.8221.2	-1.31189	noncoding
MSTRG.8238.1	1.48146	coding
MSTRG.8238.2	1.24026	coding
MSTRG.8238.5	1.56419	coding
MSTRG.8238.6	1.57705	coding

MSTRG.8238.7	1.33624	coding
MSTRG.8238.8	0.417051	coding
MSTRG.8239.1	0.105232	coding
MSTRG.8241.3	0.180209	coding
MSTRG.8241.2	2.27291	coding
MSTRG.8241.4	2.51428	coding
MSTRG.8242.1	0.297817	coding
MSTRG.8241.5	1.28185	coding
MSTRG.8241.6	1.98841	coding
MSTRG.8244.1	-1.20982	noncoding
MSTRG.8241.7	1.93769	coding
MSTRG.8241.8	1.83552	coding
MSTRG.8246.2	-1.99606	noncoding
MSTRG.8246.6	0.103902	coding
MSTRG.8246.5	0.187074	coding
MSTRG.8246.4	0.403493	coding
MSTRG.8248.1	-0.642466	noncoding
MSTRG.8246.7	-0.744557	noncoding
MSTRG.8249.1	-0.655247	noncoding
MSTRG.8255.1	-1.94531	noncoding
MSTRG.8283.3	1.9931	coding
MSTRG.8283.5	1.78265	coding
MSTRG.8283.6	1.79275	coding
MSTRG.8283.7	1.36017	coding
MSTRG.8283.8	-1.04491	noncoding
MSTRG.8283.9	0.263873	coding
MSTRG.8283.10	-0.500839	noncoding
MSTRG.8283.12	0.30984	coding
MSTRG.8283.13	0.70847	coding
MSTRG.8285.3	2.00767	coding
MSTRG.8395.1	-2.20589	noncoding
MSTRG.8397.1	-0.314488	noncoding
MSTRG.8409.1	0.341125	coding
MSTRG.8410.1	-0.760059	noncoding
MSTRG.8411.1	0.684466	coding
MSTRG.8412.1	-0.38138	noncoding
MSTRG.8417.2	0.228371	coding
MSTRG.8419.1	-1.37936	noncoding
MSTRG.8394.2	0.665428	coding
MSTRG.8394.3	-0.497897	noncoding
MSTRG.8420.1	0.18978	coding
MSTRG.8422.1	-0.744486	noncoding
MSTRG.8423.1	-1.12465	noncoding
MSTRG.8424.1	0.661418	coding
MSTRG.8425.1	-0.141543	noncoding
MSTRG.8394.4	0.485092	coding
MSTRG.8394.5	1.00754	coding
MSTRG.8394.6	0.75944	coding
MSTRG.8394.7	-1.43155	noncoding
MSTRG.8394.8	-0.368712	noncoding
MSTRG.8394.9	-0.823126	noncoding
MSTRG.8282.1	-2.40519	noncoding
MSTRG.8309.1	0.226173	coding
MSTRG.8315.1	-2.14223	noncoding

MSTRG.8316.1	-1.40698	noncoding
MSTRG.8315.2	0.446016	coding
MSTRG.8324.3	-0.708787	noncoding
MSTRG.8324.4	-1.02197	noncoding
MSTRG.8333.2	-0.323451	noncoding
MSTRG.8333.3	0.807214	coding
MSTRG.8333.4	1.27941	coding
MSTRG.8333.5	0.737363	coding
MSTRG.8337.1	-1.02325	noncoding
MSTRG.8338.2	-0.705157	noncoding
MSTRG.8346.2	2.51525	coding
MSTRG.8349.6	0.683718	coding
MSTRG.8351.1	-1.7468	noncoding
MSTRG.8351.5	0.599809	coding
MSTRG.8352.3	3.01057	coding
MSTRG.8352.4	0.654626	coding
MSTRG.8353.1	1.81791	coding
MSTRG.8353.2	2.16497	coding
MSTRG.8353.4	1.34733	coding
MSTRG.8353.6	1.3068	coding
MSTRG.8353.5	2.34142	coding
MSTRG.8353.7	2.42894	coding
MSTRG.8353.8	2.35899	coding
MSTRG.8354.1	0.452145	coding
MSTRG.8355.1	-0.87432	noncoding
MSTRG.8356.1	-0.230987	noncoding
MSTRG.8340.1	-1.56652	noncoding
MSTRG.8343.1	-2.00651	noncoding
MSTRG.8359.5	0.804346	coding
MSTRG.8359.3	1.94809	coding
MSTRG.8359.4	2.3227	coding
MSTRG.8360.1	-2.28813	noncoding
MSTRG.8365.1	-0.880857	noncoding
MSTRG.8358.1	-1.21447	noncoding
MSTRG.8386.1	2.36246	coding
MSTRG.8387.1	-1.85367	noncoding
MSTRG.8392.1	-2.06194	noncoding
MSTRG.8432.1	0.955546	coding
MSTRG.8430.1	-1.23656	noncoding
MSTRG.8444.1	1.755	coding
MSTRG.8444.2	1.09868	coding
MSTRG.8444.3	1.365	coding
MSTRG.8444.7	0.758727	coding
MSTRG.8444.5	2.46848	coding
MSTRG.8444.6	2.21097	coding
MSTRG.8444.8	1.14988	coding
MSTRG.8444.9	1.6493	coding
MSTRG.8444.10	1.38436	coding
MSTRG.8444.11	1.45142	coding
MSTRG.8445.1	2.25253	coding
MSTRG.8445.3	0.758132	coding
MSTRG.8437.1	1.98963	coding
MSTRG.8439.2	-0.108289	noncoding
MSTRG.8446.5	1.5579	coding

MSTRG.8446.7	1.2422	coding
MSTRG.8460.1	-2.66629	noncoding
MSTRG.8482.1	1.29459	coding
MSTRG.8486.1	-1.29278	noncoding
MSTRG.8488.1	-1.4971	noncoding
MSTRG.8488.2	-1.22916	noncoding
MSTRG.8488.3	-1.55716	noncoding
MSTRG.8488.4	-2.06438	noncoding
MSTRG.8495.1	-1.46669	noncoding
MSTRG.8494.5	2.00131	coding
MSTRG.8493.1	-2.35016	noncoding
MSTRG.8499.1	2.01607	coding
MSTRG.8506.1	0.317055	coding
MSTRG.8507.1	-0.269917	noncoding
MSTRG.8508.1	-0.435284	noncoding
MSTRG.8509.1	-1.66412	noncoding
MSTRG.8512.2	2.72362	coding
MSTRG.8512.3	2.59537	coding
MSTRG.8512.4	2.40185	coding
MSTRG.8502.3	1.64561	coding
MSTRG.8515.3	1.16209	coding
MSTRG.8517.2	1.04001	coding
MSTRG.8520.1	-0.253113	noncoding
MSTRG.8521.1	-0.408571	noncoding
MSTRG.8522.1	-1.12911	noncoding
MSTRG.8524.1	-0.62776	noncoding
MSTRG.8526.2	2.03204	coding
MSTRG.8527.1	1.31704	coding
MSTRG.8526.3	-0.111759	noncoding
MSTRG.8528.1	-0.507129	noncoding
MSTRG.8529.1	2.20631	coding
MSTRG.8529.2	2.29052	coding
MSTRG.8529.3	2.14099	coding
MSTRG.8529.4	1.07688	coding
MSTRG.8529.5	1.74229	coding
MSTRG.8529.7	2.17459	coding
MSTRG.8534.9	3.99231	coding
MSTRG.8534.10	3.65354	coding
MSTRG.8534.11	2.66091	coding
MSTRG.8536.1	0.39107	coding
MSTRG.8536.2	-0.006866	noncoding
MSTRG.8538.5	2.24343	coding
MSTRG.8539.3	2.11221	coding
MSTRG.8539.4	2.24197	coding
MSTRG.8539.5	1.54071	coding
MSTRG.8543.1	-0.946549	noncoding
MSTRG.8544.1	-0.709396	noncoding
MSTRG.8542.2	-0.592126	noncoding
MSTRG.8547.1	-1.14274	noncoding
MSTRG.8548.3	2.04057	coding
MSTRG.8548.2	2.10979	coding
MSTRG.8556.1	-1.57854	noncoding
MSTRG.8557.3	1.06532	coding
MSTRG.8557.2	1.05092	coding

MSTRG.8557.5	1.06034	coding
MSTRG.8554.1	-0.917618	noncoding
MSTRG.8555.1	-1.29666	noncoding
MSTRG.8565.1	-1.83003	noncoding
MSTRG.8565.2	-0.960523	noncoding
MSTRG.8566.1	-2.1832	noncoding
MSTRG.8566.2	-2.2685	noncoding
MSTRG.8585.1	-1.65412	noncoding
MSTRG.8587.1	-2.26605	noncoding
MSTRG.8589.1	-0.787686	noncoding
MSTRG.8586.1	-1.69581	noncoding
MSTRG.8591.2	1.64047	coding
MSTRG.8594.2	2.50377	coding
MSTRG.8595.1	-2.14052	noncoding
MSTRG.8598.3	2.24818	coding
MSTRG.8598.4	2.26505	coding
MSTRG.8600.3	1.09234	coding
MSTRG.8601.1	-1.27621	noncoding
MSTRG.8604.1	-1.29021	noncoding
MSTRG.8604.2	-1.5632	noncoding
MSTRG.8605.4	0.341465	coding
MSTRG.8605.8	-0.342488	noncoding
MSTRG.8605.5	0.896364	coding
MSTRG.8605.6	0.838327	coding
MSTRG.8606.3	0.987903	coding
MSTRG.8606.5	0.692724	coding
MSTRG.8606.4	0.759789	coding
MSTRG.8608.1	1.53635	coding
MSTRG.8611.3	-0.638024	noncoding
MSTRG.8613.1	-2.71408	noncoding
MSTRG.8617.1	0.266996	coding
MSTRG.8614.2	0.962554	coding
MSTRG.8614.3	0.115869	coding
MSTRG.8624.10	2.00031	coding
MSTRG.8624.11	2.07122	coding
MSTRG.8624.12	2.04268	coding
MSTRG.8624.13	2.07463	coding
MSTRG.8620.1	-2.11025	noncoding
MSTRG.8629.1	-0.83278	noncoding
MSTRG.8630.1	0.147638	coding
MSTRG.8630.2	-0.804858	noncoding
MSTRG.8632.1	-1.87525	noncoding
MSTRG.8645.5	0.666843	coding
MSTRG.8645.4	1.19736	coding
MSTRG.8645.6	0.185631	coding
MSTRG.8646.1	-1.42418	noncoding
MSTRG.8642.2	0.689415	coding
MSTRG.8647.1	0.697939	coding
MSTRG.8647.3	2.13407	coding
MSTRG.8648.1	2.698	coding
MSTRG.8650.2	1.9566	coding
MSTRG.8650.1	1.75718	coding
MSTRG.8650.4	-0.773008	noncoding
MSTRG.8650.3	1.78056	coding

MSTRG.8656.3	2.09097	coding
MSTRG.8657.1	1.28028	coding
MSTRG.8657.2	1.40815	coding
MSTRG.8658.1	1.5284	coding
MSTRG.8658.3	1.01084	coding
MSTRG.8657.3	1.01219	coding
MSTRG.8657.4	0.901311	coding
MSTRG.8657.6	1.10966	coding
MSTRG.8657.7	0.817842	coding
MSTRG.8659.2	-1.37482	noncoding
MSTRG.8660.2	1.15995	coding
MSTRG.8660.3	1.25741	coding
MSTRG.8660.4	1.39646	coding
MSTRG.8660.10	0.761944	coding
MSTRG.8660.11	1.00358	coding
MSTRG.8660.8	0.798081	coding
MSTRG.8660.9	0.703485	coding
MSTRG.8652.1	0.31171	coding
MSTRG.8652.2	-0.225701	noncoding
MSTRG.8674.2	1.65145	coding
MSTRG.8674.1	2.62858	coding
MSTRG.8674.3	2.783	coding
MSTRG.8674.4	2.65305	coding
MSTRG.8674.8	0.620927	coding
MSTRG.8674.7	1.14591	coding
MSTRG.8677.5	-0.604491	noncoding
MSTRG.8677.3	-0.601025	noncoding
MSTRG.8677.4	-0.968136	noncoding
MSTRG.8677.8	-0.925883	noncoding
MSTRG.8677.9	-0.877348	noncoding
MSTRG.8677.10	-1.15849	noncoding
MSTRG.8678.2	2.51479	coding
MSTRG.8680.3	2.88646	coding
MSTRG.8680.2	2.90686	coding
MSTRG.8686.1	-2.3618	noncoding
MSTRG.8701.1	1.72905	coding
MSTRG.8701.2	1.68218	coding
MSTRG.8716.1	0.756891	coding
MSTRG.8713.1	-2.0732	noncoding
MSTRG.8732.1	0.017505	coding
MSTRG.8733.1	-1.57295	noncoding
MSTRG.8733.2	0.23309	coding
MSTRG.8733.3	0.15318	coding
MSTRG.8734.1	1.9088	coding
MSTRG.8729.1	0.393996	coding
MSTRG.8742.1	2.01117	coding
MSTRG.8742.3	1.30696	coding
MSTRG.8742.6	2.36664	coding
MSTRG.8742.7	2.28733	coding
MSTRG.8742.8	2.07863	coding
MSTRG.8742.9	1.99404	coding
MSTRG.8764.1	-0.533281	noncoding
MSTRG.8765.1	0.318557	coding
MSTRG.8765.2	0.133426	coding

MSTRG.8766.1	-0.021418	noncoding
MSTRG.8765.4	-0.834037	noncoding
MSTRG.8748.4	1.50574	coding
MSTRG.8748.6	0.957635	coding
MSTRG.8768.3	-0.355299	noncoding
MSTRG.8769.1	1.18061	coding
MSTRG.8785.1	1.29842	coding
MSTRG.8785.2	1.32877	coding
MSTRG.8785.3	1.29913	coding
MSTRG.8786.7	1.4554	coding
MSTRG.8786.13	1.42939	coding
MSTRG.8786.8	1.55094	coding
MSTRG.8786.12	1.94593	coding
MSTRG.8786.10	1.44416	coding
MSTRG.8786.11	1.47937	coding
MSTRG.8786.9	1.38972	coding
MSTRG.8786.15	0.548578	coding
MSTRG.8787.1	0.423175	coding
MSTRG.8789.1	1.74771	coding
MSTRG.8803.1	1.16695	coding
MSTRG.8808.2	1.28384	coding
MSTRG.8809.1	1.19068	coding
MSTRG.8809.4	2.26022	coding
MSTRG.8818.3	0.463621	coding
MSTRG.8815.1	-1.57949	noncoding
MSTRG.8815.2	-2.00048	noncoding
MSTRG.8815.3	-2.01368	noncoding
MSTRG.8815.4	-1.47278	noncoding
MSTRG.8825.6	1.71156	coding
MSTRG.8825.7	1.62107	coding
MSTRG.8825.8	1.11367	coding
MSTRG.8826.4	2.47474	coding
MSTRG.8823.1	-0.305688	noncoding
MSTRG.8833.1	0.61873	coding
MSTRG.8833.2	1.10779	coding
MSTRG.8833.3	0.623875	coding
MSTRG.8833.4	1.07054	coding
MSTRG.8833.5	0.611849	coding
MSTRG.8833.6	1.08487	coding
MSTRG.8833.7	0.79766	coding
MSTRG.8833.8	0.696893	coding
MSTRG.8830.1	-0.640799	noncoding
MSTRG.8835.1	-0.567909	noncoding
MSTRG.8836.1	-0.383114	noncoding
MSTRG.8838.1	1.73619	coding
MSTRG.8839.1	1.41332	coding
MSTRG.8842.1	-0.033953	noncoding
MSTRG.8846.3	2.21988	coding
MSTRG.8846.4	2.43935	coding
MSTRG.8846.5	1.86503	coding
MSTRG.8846.6	1.88295	coding
MSTRG.8849.1	-2.00572	noncoding
MSTRG.8844.1	-0.235657	noncoding
MSTRG.8844.2	0.579006	coding

MSTRG.8853.1	-1.49789	noncoding
MSTRG.8853.2	-1.6642	noncoding
MSTRG.8853.3	-1.55549	noncoding
MSTRG.8853.4	-2.15092	noncoding
MSTRG.8857.1	-2.36961	noncoding
MSTRG.8862.1	-0.867222	noncoding
MSTRG.8862.2	1.25603	coding
MSTRG.8863.1	0.514909	coding
MSTRG.8864.1	0.689099	coding
MSTRG.8865.1	-0.455311	noncoding
MSTRG.8881.2	0.014193	coding
MSTRG.8888.1	1.18493	coding
MSTRG.8893.1	-0.411258	noncoding
MSTRG.8894.1	0.265534	coding
MSTRG.8898.11	2.0798	coding
MSTRG.8898.12	2.06613	coding
MSTRG.8898.13	1.90789	coding
MSTRG.8902.1	2.46257	coding
MSTRG.8896.1	-0.395955	noncoding
MSTRG.8912.1	1.9617	coding
MSTRG.8912.2	0.955409	coding
MSTRG.8913.1	-1.30195	noncoding
MSTRG.8913.2	-1.33708	noncoding
MSTRG.8914.1	1.362	coding
MSTRG.8914.2	1.53393	coding
MSTRG.8908.1	-1.11765	noncoding
MSTRG.8916.1	-1.36464	noncoding
MSTRG.8919.5	2.47167	coding
MSTRG.8919.3	2.50163	coding
MSTRG.8919.1	2.41857	coding
MSTRG.8919.2	2.37319	coding
MSTRG.8919.6	2.3566	coding
MSTRG.8919.7	2.35514	coding
MSTRG.8919.8	2.35274	coding
MSTRG.8919.9	2.22578	coding
MSTRG.8920.1	-1.29359	noncoding
MSTRG.8920.2	-1.32139	noncoding
MSTRG.8918.2	1.91708	coding
MSTRG.8932.2	0.864634	coding
MSTRG.8941.1	0.7516	coding
MSTRG.8943.1	1.28488	coding
MSTRG.8943.2	1.14937	coding
MSTRG.8943.3	2.14189	coding
MSTRG.8943.4	1.99052	coding
MSTRG.8943.5	2.19839	coding
MSTRG.8943.6	1.04359	coding
MSTRG.8943.7	1.44475	coding
MSTRG.8943.8	2.05049	coding
MSTRG.8943.9	1.83501	coding
MSTRG.8943.10	2.02993	coding
MSTRG.8943.11	1.72498	coding
MSTRG.8943.12	1.77719	coding
MSTRG.8943.13	1.786	coding
MSTRG.8923.1	0.193471	coding

MSTRG.8923.4	-0.291642	noncoding
MSTRG.8923.3	-0.367275	noncoding
MSTRG.8924.1	-1.80412	noncoding
MSTRG.8928.5	1.45833	coding
MSTRG.8929.1	0.758089	coding
MSTRG.8949.1	-1.18922	noncoding
MSTRG.8949.2	-2.21795	noncoding
MSTRG.8951.2	2.42619	coding
MSTRG.8952.1	-1.60996	noncoding
MSTRG.8954.1	0.175516	coding
MSTRG.8955.1	-1.66683	noncoding
MSTRG.8957.1	-1.78399	noncoding
MSTRG.8961.2	-1.06473	noncoding
MSTRG.8962.2	-1.2257	noncoding
MSTRG.8962.3	-0.146182	noncoding
MSTRG.8966.1	-0.34099	noncoding
MSTRG.8967.3	0.922738	coding
MSTRG.8971.2	1.39544	coding
MSTRG.8972.2	0.833067	coding
MSTRG.8976.1	-2.57796	noncoding
MSTRG.8981.1	-2.08348	noncoding
MSTRG.8994.1	3.13992	coding
MSTRG.8994.6	3.34244	coding
MSTRG.8994.2	3.781	coding
MSTRG.8994.4	3.33601	coding
MSTRG.8994.7	-0.061703	noncoding
MSTRG.8995.1	0.516229	coding
MSTRG.8996.1	-1.50288	noncoding
MSTRG.8996.2	-0.798678	noncoding
MSTRG.8996.3	-1.63038	noncoding
MSTRG.9002.2	1.30058	coding
MSTRG.9000.2	-0.000319	noncoding
MSTRG.9000.3	1.99667	coding
MSTRG.9000.4	1.64793	coding
MSTRG.9003.2	1.0045	coding
MSTRG.9010.1	0.991468	coding
MSTRG.9010.2	1.29367	coding
MSTRG.9010.3	1.19292	coding
MSTRG.9010.4	1.20457	coding
MSTRG.9010.5	1.497	coding
MSTRG.9010.6	1.09037	coding
MSTRG.9010.7	1.79905	coding
MSTRG.9010.9	1.30569	coding
MSTRG.9010.10	1.03157	coding
MSTRG.9010.11	0.285664	coding
MSTRG.9010.12	0.269555	coding
MSTRG.9010.13	0.151306	coding
MSTRG.9005.1	-2.12029	noncoding
MSTRG.9008.1	-1.14837	noncoding
MSTRG.9011.1	-0.341597	noncoding
MSTRG.9025.1	0.840388	coding
MSTRG.9027.2	-1.2563	noncoding
MSTRG.9029.3	0.979651	coding
MSTRG.9029.4	1.79822	coding

MSTRG.9029.5	0.186451	coding
MSTRG.9034.1	1.60654	coding
MSTRG.9034.2	2.93458	coding
MSTRG.9036.2	0.428613	coding
MSTRG.9036.3	0.165575	coding
MSTRG.9038.1	-2.08068	noncoding
MSTRG.9040.3	1.53	coding
MSTRG.9040.4	2.16441	coding
MSTRG.9041.1	1.36067	coding
MSTRG.9041.2	1.44959	coding
MSTRG.9042.1	-1.04505	noncoding
MSTRG.9042.2	-0.953372	noncoding
MSTRG.9043.1	-1.9842	noncoding
MSTRG.9045.2	-0.633101	noncoding
MSTRG.9046.2	1.1118	coding
MSTRG.9050.1	1.19034	coding
MSTRG.9051.1	-0.771817	noncoding
MSTRG.9053.3	-1.36291	noncoding
MSTRG.9053.5	-1.36576	noncoding
MSTRG.9053.6	-0.509689	noncoding
MSTRG.9053.4	1.45916	coding
MSTRG.9053.7	0.358794	coding
MSTRG.9054.1	-0.877837	noncoding
MSTRG.9055.1	1.68896	coding
MSTRG.9055.4	1.61391	coding
MSTRG.9058.1	2.05573	coding
MSTRG.9058.2	1.36395	coding
MSTRG.9058.3	1.70114	coding
MSTRG.9058.4	0.531828	coding
MSTRG.9058.8	1.37317	coding
MSTRG.9058.10	1.5615	coding
MSTRG.9058.11	1.49122	coding
MSTRG.9058.5	1.14541	coding
MSTRG.9058.6	1.42791	coding
MSTRG.9058.7	0.955921	coding
MSTRG.9058.9	1.54782	coding
MSTRG.9058.12	1.67454	coding
MSTRG.9058.14	1.57788	coding
MSTRG.9059.3	-1.57942	noncoding
MSTRG.9059.2	2.41354	coding
MSTRG.9062.6	0.766271	coding
MSTRG.9056.2	0.011028	coding
MSTRG.9081.6	-0.408016	noncoding
MSTRG.9081.7	2.53388	coding
MSTRG.9081.5	1.9988	coding
MSTRG.9081.3	2.55117	coding
MSTRG.9081.1	2.48562	coding
MSTRG.9081.4	2.50917	coding
MSTRG.9081.8	2.49385	coding
MSTRG.9081.9	2.34544	coding
MSTRG.9081.10	1.52105	coding
MSTRG.9081.11	0.563614	coding
MSTRG.9081.12	0.100691	coding
MSTRG.9082.1	1.10705	coding

MSTRG.9082.2	0.965177	coding
MSTRG.9083.1	-1.11893	noncoding
MSTRG.9084.1	-0.743349	noncoding
MSTRG.9083.10	0.758824	coding
MSTRG.9083.12	0.704095	coding
MSTRG.9083.13	0.726153	coding
MSTRG.9083.15	0.283149	coding
MSTRG.9083.9	-0.101192	noncoding
MSTRG.9083.4	0.249578	coding
MSTRG.9083.8	0.280139	coding
MSTRG.9083.7	0.368525	coding
MSTRG.9083.16	-2.005	noncoding
MSTRG.9085.2	-0.641054	noncoding
MSTRG.9072.1	-0.917985	noncoding
MSTRG.9075.2	0.739901	coding
MSTRG.9089.1	0.198686	coding
MSTRG.9090.2	1.22789	coding
MSTRG.9090.3	1.24736	coding
MSTRG.9091.4	2.04847	coding
MSTRG.9091.5	0.083842	coding
MSTRG.9091.6	0.308809	coding
MSTRG.9091.8	1.37394	coding
MSTRG.9092.1	0.411001	coding
MSTRG.9093.4	1.41155	coding
MSTRG.9094.2	1.06236	coding
MSTRG.9097.1	0.293591	coding
MSTRG.9097.2	0.349256	coding
MSTRG.9103.5	1.38114	coding
MSTRG.9103.2	1.19659	coding
MSTRG.9104.3	1.25507	coding
MSTRG.9104.4	1.56041	coding
MSTRG.9104.5	1.45504	coding
MSTRG.9104.7	1.22519	coding
MSTRG.9104.6	1.32517	coding
MSTRG.9104.8	-0.078906	noncoding
MSTRG.9100.2	-1.80338	noncoding
MSTRG.9107.8	0.984229	coding
MSTRG.9107.6	1.09643	coding
MSTRG.9108.1	-1.08245	noncoding
MSTRG.9111.1	-0.599947	noncoding
MSTRG.9112.2	-0.759219	noncoding
MSTRG.9113.1	0.119748	coding
MSTRG.9110.1	-0.421955	noncoding
MSTRG.9116.3	1.89185	coding
MSTRG.9125.6	0.89638	coding
MSTRG.9125.2	1.8223	coding
MSTRG.9125.3	1.79955	coding
MSTRG.9125.4	1.83183	coding
MSTRG.9125.5	1.86301	coding
MSTRG.9126.4	1.51954	coding
MSTRG.9127.1	-0.133177	noncoding
MSTRG.9120.2	1.09687	coding
MSTRG.9120.3	-0.119739	noncoding
MSTRG.9123.1	0.665777	coding

MSTRG.9123.2	0.751909	coding
MSTRG.9129.1	-1.12062	noncoding
MSTRG.9129.2	-0.234019	noncoding
MSTRG.9128.1	-0.284802	noncoding
MSTRG.9133.3	2.47361	coding
MSTRG.9136.1	-1.81421	noncoding
MSTRG.9138.1	1.45112	coding
MSTRG.9138.3	2.16125	coding
MSTRG.9139.1	-0.458502	noncoding
MSTRG.9138.4	0.762164	coding
MSTRG.9140.1	1.09356	coding
MSTRG.9140.3	-0.144776	noncoding
MSTRG.9142.1	-2.67636	noncoding
MSTRG.9145.3	1.41494	coding
MSTRG.9145.5	1.59314	coding
MSTRG.9145.2	1.53494	coding
MSTRG.9145.4	1.46117	coding
MSTRG.9145.6	1.68077	coding
MSTRG.9147.1	-0.607172	noncoding
MSTRG.9146.4	2.14159	coding
MSTRG.9146.5	1.33784	coding
MSTRG.9144.3	2.20986	coding
MSTRG.9144.2	0.539107	coding
MSTRG.9157.3	-0.219118	noncoding
MSTRG.9157.4	0.163765	coding
MSTRG.9153.3	3.23174	coding
MSTRG.9148.1	-0.070829	noncoding
MSTRG.9150.1	0.597823	coding
MSTRG.9150.2	0.5957	coding
MSTRG.9175.2	1.5139	coding
MSTRG.9175.4	1.31899	coding
MSTRG.9175.10	0.641915	coding
MSTRG.9175.9	0.591567	coding
MSTRG.9175.8	1.24457	coding
MSTRG.9167.2	1.74553	coding
MSTRG.9168.1	-2.39498	noncoding
MSTRG.9188.1	-0.753054	noncoding
MSTRG.9190.1	-0.211294	noncoding
MSTRG.9190.3	-0.996653	noncoding
MSTRG.9190.2	-1.29467	noncoding
MSTRG.9194.1	-0.32077	noncoding
MSTRG.9194.2	-0.236834	noncoding
MSTRG.9193.3	0.869569	coding
MSTRG.9202.1	1.53241	coding
MSTRG.9203.1	-0.07278	noncoding
MSTRG.9205.1	2.18252	coding
MSTRG.9205.2	1.84255	coding
MSTRG.9206.1	-0.468082	noncoding
MSTRG.9209.2	1.14429	coding
MSTRG.9209.3	1.44132	coding
MSTRG.9211.3	0.235331	coding
MSTRG.9211.4	1.0877	coding
MSTRG.9217.2	-0.900055	noncoding
MSTRG.9230.1	-1.60096	noncoding

MSTRG.9231.1	2.07364	coding
MSTRG.9231.3	1.95572	coding
MSTRG.9233.1	1.41673	coding
MSTRG.9233.5	1.52491	coding
MSTRG.9235.3	1.3705	coding
MSTRG.9235.4	1.64001	coding
MSTRG.9239.6	1.01715	coding
MSTRG.9239.9	1.36867	coding
MSTRG.9239.8	1.26991	coding
MSTRG.9240.1	-0.286673	noncoding
MSTRG.9249.4	1.95586	coding
MSTRG.9249.5	1.30849	coding
MSTRG.9250.6	1.87256	coding
MSTRG.9250.7	1.38622	coding
MSTRG.9250.8	1.87034	coding
MSTRG.9250.10	1.85044	coding
MSTRG.9250.9	0.656363	coding
MSTRG.9258.3	0.4034	coding
MSTRG.9258.5	0.307	coding
MSTRG.9259.2	1.00728	coding
MSTRG.9260.1	-0.302446	noncoding
MSTRG.9260.2	-0.560708	noncoding
MSTRG.9256.3	1.12738	coding
MSTRG.9257.3	0.322051	coding
MSTRG.9264.2	0.891129	coding
MSTRG.9266.1	0.362714	coding
MSTRG.9266.2	1.09216	coding
MSTRG.9266.3	1.0098	coding
MSTRG.9267.2	1.76374	coding
MSTRG.9267.3	1.69921	coding
MSTRG.9268.2	-0.314328	noncoding
MSTRG.9269.1	-0.960229	noncoding
MSTRG.9298.1	1.32517	coding
MSTRG.9298.18	0.85821	coding
MSTRG.9298.10	1.09834	coding
MSTRG.9298.17	1.20755	coding
MSTRG.9298.20	0.976196	coding
MSTRG.9298.11	0.799144	coding
MSTRG.9298.12	0.787271	coding
MSTRG.9298.13	1.42719	coding
MSTRG.9298.15	0.585266	coding
MSTRG.9298.16	0.785374	coding
MSTRG.9298.9	1.41436	coding
MSTRG.9298.24	0.624338	coding
MSTRG.9298.26	0.53904	coding
MSTRG.9298.25	0.79416	coding
MSTRG.9298.30	-1.07076	noncoding
MSTRG.9298.31	-1.04593	noncoding
MSTRG.9298.29	0.062921	coding
MSTRG.9298.33	1.43489	coding
MSTRG.9298.34	1.40311	coding
MSTRG.9298.35	-1.06363	noncoding
MSTRG.9298.36	-0.982984	noncoding
MSTRG.9278.2	-0.350958	noncoding

MSTRG.9281.1	-1.05698	noncoding
MSTRG.9283.2	1.18871	coding
MSTRG.9284.1	-1.01564	noncoding
MSTRG.9285.2	0.339366	coding
MSTRG.9287.2	1.18409	coding
MSTRG.9290.1	1.98081	coding
MSTRG.9291.1	1.62276	coding
MSTRG.9291.3	0.510821	coding
MSTRG.9295.1	1.98263	coding
MSTRG.9295.2	2.0171	coding
MSTRG.9295.4	2.75427	coding
MSTRG.9296.1	-0.367107	noncoding
MSTRG.9303.2	-0.626308	noncoding
MSTRG.9304.1	1.22015	coding
MSTRG.9305.2	-0.59722	noncoding
MSTRG.9305.3	-1.39747	noncoding
MSTRG.9304.3	1.1322	coding
MSTRG.9306.1	-0.332984	noncoding
MSTRG.9306.2	-0.193418	noncoding
MSTRG.9307.2	1.75213	coding
MSTRG.9308.4	1.35179	coding
MSTRG.9312.11	2.80254	coding
MSTRG.9312.12	2.64761	coding
MSTRG.9312.13	2.75278	coding
MSTRG.9312.14	2.77164	coding
MSTRG.9312.3	2.78765	coding
MSTRG.9312.4	2.75429	coding
MSTRG.9312.9	2.66177	coding
MSTRG.9312.15	2.80532	coding
MSTRG.9312.23	1.02908	coding
MSTRG.9312.24	0.194668	coding
MSTRG.9312.25	0.084911	coding
MSTRG.9310.2	1.82475	coding
MSTRG.9315.1	0.13384	coding
MSTRG.9316.1	-2.00097	noncoding
MSTRG.9315.3	0.734124	coding
MSTRG.9321.3	0.188619	coding
MSTRG.9321.4	0.449642	coding
MSTRG.9322.1	0.166763	coding
MSTRG.9325.2	0.443907	coding
MSTRG.9327.4	1.35018	coding
MSTRG.9335.3	-0.12707	noncoding
MSTRG.9335.4	0.507138	coding
MSTRG.9333.2	1.49799	coding
MSTRG.9338.1	-0.855988	noncoding
MSTRG.9361.4	1.6821	coding
MSTRG.9361.6	1.87032	coding
MSTRG.9361.7	1.88496	coding
MSTRG.9361.8	1.60533	coding
MSTRG.9361.9	1.779	coding
MSTRG.9361.10	1.68446	coding
MSTRG.9361.11	1.84297	coding
MSTRG.9361.13	1.89148	coding
MSTRG.9361.14	1.8172	coding

MSTRG.9365.1	-2.35805	noncoding
MSTRG.9364.12	-0.270245	noncoding
MSTRG.9364.13	1.98411	coding
MSTRG.9367.2	2.31886	coding
MSTRG.9367.3	2.93188	coding
MSTRG.9367.4	3.10914	coding
MSTRG.9367.6	2.46361	coding
MSTRG.9367.9	2.92273	coding
MSTRG.9367.10	1.88889	coding
MSTRG.9368.1	0.960683	coding
MSTRG.9367.12	0.555036	coding
MSTRG.9343.3	-1.00729	noncoding
MSTRG.9349.1	-0.116048	noncoding
MSTRG.9353.1	-2.11201	noncoding
MSTRG.9358.4	1.63945	coding
MSTRG.9358.1	1.54183	coding
MSTRG.9358.3	1.53137	coding
MSTRG.9358.5	1.49642	coding
MSTRG.9358.6	1.43481	coding
MSTRG.9360.3	0.999076	coding
MSTRG.9369.3	1.81216	coding
MSTRG.9369.4	1.88839	coding
MSTRG.9369.5	1.43041	coding
MSTRG.9374.1	3.33253	coding
MSTRG.9374.2	3.47074	coding
MSTRG.9374.3	3.65438	coding
MSTRG.9374.5	3.36063	coding
MSTRG.9374.4	3.68783	coding
MSTRG.9374.7	3.38179	coding
MSTRG.9372.2	1.22343	coding
MSTRG.9372.3	1.60779	coding
MSTRG.9373.3	1.51811	coding
MSTRG.9377.2	-0.531529	noncoding
MSTRG.9386.6	-0.119604	noncoding
MSTRG.9386.4	2.61462	coding
MSTRG.9379.1	-0.901999	noncoding
MSTRG.9382.1	-0.57382	noncoding
MSTRG.9385.2	1.69442	coding
MSTRG.9385.3	1.64496	coding
MSTRG.9396.1	2.09428	coding
MSTRG.9396.2	1.77006	coding
MSTRG.9396.3	1.95849	coding
MSTRG.9398.4	0.791484	coding
MSTRG.9398.3	0.445856	coding
MSTRG.9394.1	1.26144	coding
MSTRG.9394.2	1.31365	coding
MSTRG.9394.3	1.1944	coding
MSTRG.9394.4	1.4993	coding
MSTRG.9393.1	-0.418291	noncoding
MSTRG.9405.6	0.803116	coding
MSTRG.9407.3	0.456368	coding
MSTRG.9410.1	1.51942	coding
MSTRG.9420.2	0.49683	coding
MSTRG.9422.1	2.10727	coding

MSTRG.9422.4	2.2363	coding
MSTRG.9423.2	1.57035	coding
MSTRG.9423.3	0.658408	coding
MSTRG.9423.4	0.841356	coding
MSTRG.9425.2	0.888897	coding
MSTRG.9411.2	0.452813	coding
MSTRG.9413.2	-1.40294	noncoding
MSTRG.9428.2	1.57043	coding
MSTRG.9436.1	1.47982	coding
MSTRG.9436.2	1.46974	coding
MSTRG.9436.3	1.56151	coding
MSTRG.9436.4	1.47493	coding
MSTRG.9436.5	1.54951	coding
MSTRG.9436.6	1.60518	coding
MSTRG.9436.10	1.4501	coding
MSTRG.9436.11	1.40849	coding
MSTRG.9436.15	1.3568	coding
MSTRG.9436.12	1.5824	coding
MSTRG.9436.13	1.56676	coding
MSTRG.9436.14	1.68806	coding
MSTRG.9436.9	1.58308	coding
MSTRG.9436.16	1.50005	coding
MSTRG.9436.17	1.53614	coding
MSTRG.9436.18	1.53976	coding
MSTRG.9436.19	1.54834	coding
MSTRG.9431.3	0.876618	coding
MSTRG.9431.4	1.89143	coding
MSTRG.9445.1	1.00424	coding
MSTRG.9446.2	-1.23012	noncoding
MSTRG.9446.3	-1.72494	noncoding
MSTRG.9446.4	-1.67364	noncoding
MSTRG.9446.6	-1.90555	noncoding
MSTRG.9449.1	1.36153	coding
MSTRG.9449.2	-0.29445	noncoding
MSTRG.9449.4	-1.07867	noncoding
MSTRG.9449.3	1.1698	coding
MSTRG.9449.5	-2.1932	noncoding
MSTRG.9445.3	-0.164051	noncoding
MSTRG.9445.4	-0.416159	noncoding
MSTRG.9445.6	-1.7714	noncoding
MSTRG.9453.1	-1.88808	noncoding
MSTRG.9453.2	-1.80224	noncoding
MSTRG.9453.3	-0.850514	noncoding
MSTRG.9434.2	0.953146	coding
MSTRG.9437.2	0.560594	coding
MSTRG.9441.1	2.47258	coding
MSTRG.9458.2	3.3437	coding
MSTRG.9458.4	2.92362	coding
MSTRG.9457.1	0.909975	coding
MSTRG.9464.2	-0.727774	noncoding
MSTRG.9464.1	-0.590993	noncoding
MSTRG.9464.3	-0.643688	noncoding
MSTRG.9464.4	-0.609671	noncoding
MSTRG.9464.5	-0.548756	noncoding

MSTRG.9460.1	2.12772	coding
MSTRG.9471.5	2.13904	coding
MSTRG.9471.6	3.08689	coding
MSTRG.9471.7	3.08492	coding
MSTRG.9471.8	2.74577	coding
MSTRG.9473.1	2.3016	coding
MSTRG.9473.2	1.38344	coding
MSTRG.9473.3	1.98898	coding
MSTRG.9473.4	1.42083	coding
MSTRG.9473.5	1.48112	coding
MSTRG.9468.2	1.62622	coding
MSTRG.9468.3	1.68212	coding
MSTRG.9481.4	1.83647	coding
MSTRG.9481.11	-0.150157	noncoding
MSTRG.9481.17	1.51253	coding
MSTRG.9481.14	1.78599	coding
MSTRG.9481.15	1.73147	coding
MSTRG.9481.16	1.81534	coding
MSTRG.9481.9	1.80375	coding
MSTRG.9481.10	2.00464	coding
MSTRG.9481.18	1.93689	coding
MSTRG.9481.7	1.99923	coding
MSTRG.9477.2	-0.528688	noncoding
MSTRG.9478.4	1.68683	coding
MSTRG.9482.7	2.07478	coding
MSTRG.9484.1	-1.38432	noncoding
MSTRG.9486.2	1.8136	coding
MSTRG.9493.6	3.56657	coding
MSTRG.9493.7	2.68056	coding
MSTRG.9493.8	2.01106	coding
MSTRG.9496.4	2.943	coding
MSTRG.9498.2	1.59107	coding
MSTRG.9498.8	-0.164732	noncoding
MSTRG.9499.1	0.861892	coding
MSTRG.9499.2	1.061	coding
MSTRG.9499.4	0.487367	coding
MSTRG.9499.5	0.697245	coding
MSTRG.9500.1	2.08605	coding
MSTRG.9500.3	2.27616	coding
MSTRG.9500.4	2.15996	coding
MSTRG.9503.1	1.40933	coding
MSTRG.9503.2	1.65434	coding
MSTRG.9503.3	1.65104	coding
MSTRG.9503.4	1.46304	coding
MSTRG.9503.6	1.61345	coding
MSTRG.9505.2	1.85619	coding
MSTRG.9505.3	1.92895	coding
MSTRG.9505.4	2.40709	coding
MSTRG.9505.5	2.02816	coding
MSTRG.9505.6	1.96033	coding
MSTRG.9509.1	-0.924922	noncoding
MSTRG.9509.3	-0.866985	noncoding
MSTRG.9516.4	1.04969	coding
MSTRG.9516.6	-0.451002	noncoding

MSTRG.9516.8	0.307712	coding
MSTRG.9516.7	1.07016	coding
MSTRG.9518.2	2.74867	coding
MSTRG.9518.3	2.44767	coding
MSTRG.9522.1	2.55994	coding
MSTRG.9523.2	-1.44333	noncoding
MSTRG.9526.2	1.80711	coding
MSTRG.9532.1	-1.78395	noncoding
MSTRG.9534.1	0.963802	coding
MSTRG.9535.1	0.060261	coding
MSTRG.9535.2	0.657324	coding
MSTRG.9535.3	0.966776	coding
MSTRG.9536.1	1.07542	coding
MSTRG.9543.1	-0.472425	noncoding
MSTRG.9543.2	-0.873531	noncoding
MSTRG.9550.1	-0.735247	noncoding
MSTRG.9547.1	1.36779	coding
MSTRG.9554.1	0.989049	coding
MSTRG.9555.1	-1.99073	noncoding
MSTRG.9556.1	0.302169	coding
MSTRG.9556.2	-0.42017	noncoding
MSTRG.9556.3	0.571722	coding
MSTRG.9563.1	0.281731	coding
MSTRG.9563.2	0.260593	coding
MSTRG.9564.2	1.36619	coding
MSTRG.9565.4	2.33276	coding
MSTRG.9565.5	2.26503	coding
MSTRG.9566.2	1.13601	coding
MSTRG.9566.3	0.732486	coding
MSTRG.9574.3	2.2579	coding
MSTRG.9574.4	1.52164	coding
MSTRG.9574.5	1.69962	coding
MSTRG.9574.6	1.57766	coding
MSTRG.9574.7	2.29166	coding
MSTRG.9576.1	2.77197	coding
MSTRG.9576.2	2.85071	coding
MSTRG.9576.3	2.80244	coding
MSTRG.9567.2	-1.11432	noncoding
MSTRG.9567.3	0.275066	coding
MSTRG.9567.4	0.310314	coding
MSTRG.9568.2	0.660142	coding
MSTRG.9571.1	-0.418747	noncoding
MSTRG.9577.4	1.29857	coding
MSTRG.9586.3	0.586593	coding
MSTRG.9587.2	0.513253	coding
MSTRG.9589.3	2.70617	coding
MSTRG.9589.5	1.26188	coding
MSTRG.9590.2	1.727	coding
MSTRG.9590.3	1.57932	coding
MSTRG.9590.5	1.45198	coding
MSTRG.9590.7	1.06526	coding
MSTRG.9590.6	0.79324	coding
MSTRG.9591.1	1.98046	coding
MSTRG.9591.3	1.93049	coding

MSTRG.9598.10	0.604026	coding
MSTRG.9598.3	0.913325	coding
MSTRG.9598.4	0.353123	coding
MSTRG.9598.6	0.192168	coding
MSTRG.9598.7	0.694605	coding
MSTRG.9598.8	0.10177	coding
MSTRG.9598.9	0.516284	coding
MSTRG.9599.1	0.204322	coding
MSTRG.9599.3	0.042852	coding
MSTRG.9599.4	0.086403	coding
MSTRG.9600.12	1.44947	coding
MSTRG.9600.8	1.79598	coding
MSTRG.9600.13	2.23858	coding
MSTRG.9600.7	1.58861	coding
MSTRG.9600.9	1.59181	coding
MSTRG.9600.16	0.585663	coding
MSTRG.9602.2	1.11013	coding
MSTRG.9594.1	0.802023	coding
MSTRG.9594.2	0.02236	coding
MSTRG.9594.3	0.927318	coding
MSTRG.9594.5	0.855252	coding
MSTRG.9597.1	2.40871	coding
MSTRG.9597.2	2.39019	coding
MSTRG.9597.3	2.39184	coding
MSTRG.9597.4	2.08201	coding
MSTRG.9604.3	1.30023	coding
MSTRG.9604.4	0.055567	coding
MSTRG.9605.1	-1.84929	noncoding
MSTRG.9605.2	-1.69946	noncoding
MSTRG.9606.1	0.478537	coding
MSTRG.9610.1	-1.39796	noncoding
MSTRG.9610.2	-2.17063	noncoding
MSTRG.9612.2	1.26711	coding
MSTRG.9612.3	1.42964	coding
MSTRG.9613.1	-1.41121	noncoding
MSTRG.9612.4	-1.05477	noncoding
MSTRG.9603.1	-0.516484	noncoding
MSTRG.9617.3	0.968441	coding
MSTRG.9619.2	2.06603	coding
MSTRG.9620.1	0.303062	coding
MSTRG.9620.2	-0.115228	noncoding
MSTRG.9624.3	1.26013	coding
MSTRG.9625.1	-0.303445	noncoding
MSTRG.9626.1	0.124187	coding
MSTRG.9626.2	0.188015	coding
MSTRG.9630.2	3.01032	coding
MSTRG.9634.3	-0.02173	noncoding
MSTRG.9634.4	-0.439583	noncoding
MSTRG.9633.3	0.992573	coding
MSTRG.9633.2	0.321087	coding
MSTRG.9638.1	2.05456	coding
MSTRG.9640.4	1.79671	coding
MSTRG.9649.4	1.61064	coding
MSTRG.9643.1	0.297496	coding

MSTRG.9651.6	0.081222	coding
MSTRG.9650.1	-1.5241	noncoding
MSTRG.9653.1	2.42133	coding
MSTRG.9653.2	1.54462	coding
MSTRG.9655.3	1.38622	coding
MSTRG.9657.1	-0.583736	noncoding
MSTRG.9658.4	0.741143	coding
MSTRG.9661.1	-0.919607	noncoding
MSTRG.9662.4	0.982122	coding
MSTRG.9663.3	1.53513	coding
MSTRG.9663.4	1.64308	coding
MSTRG.9665.1	0.485329	coding
MSTRG.9665.2	0.540247	coding
MSTRG.9665.3	0.482366	coding
MSTRG.9665.5	0.465035	coding
MSTRG.9667.2	1.27912	coding
MSTRG.9667.3	0.95451	coding
MSTRG.9675.2	0.765239	coding
MSTRG.9675.3	0.620407	coding
MSTRG.9675.4	-0.110302	noncoding
MSTRG.9670.2	2.07066	coding
MSTRG.9671.2	-0.32085	noncoding
MSTRG.9676.2	2.3903	coding
MSTRG.9676.3	1.21124	coding
MSTRG.9685.1	0.065772	coding
MSTRG.9689.1	2.35184	coding
MSTRG.9689.3	2.42213	coding
MSTRG.9696.2	1.52987	coding
MSTRG.9696.5	1.84038	coding
MSTRG.9692.1	1.91652	coding
MSTRG.9693.2	0.113863	coding
MSTRG.9691.2	1.11217	coding
MSTRG.9706.1	2.16066	coding
MSTRG.9706.5	1.82238	coding
MSTRG.9708.1	-1.15022	noncoding
MSTRG.9719.1	-0.413642	noncoding
MSTRG.9728.1	-1.33242	noncoding
MSTRG.9740.1	-1.87514	noncoding
MSTRG.9742.1	2.07019	coding
MSTRG.9742.2	1.9551	coding
MSTRG.9742.3	1.9132	coding
MSTRG.9742.4	2.04011	coding
MSTRG.9742.8	2.25854	coding
MSTRG.9742.9	2.38248	coding
MSTRG.9742.13	1.63872	coding
MSTRG.9742.15	0.366256	coding
MSTRG.9742.17	-1.92331	noncoding
MSTRG.9742.18	0.20513	coding
MSTRG.9743.1	-0.173389	noncoding
MSTRG.9743.2	-0.737074	noncoding
MSTRG.9745.1	2.66216	coding
MSTRG.9745.3	2.61516	coding
MSTRG.9745.6	1.19001	coding
MSTRG.9732.2	0.618493	coding

MSTRG.9734.2	-0.202316	noncoding
MSTRG.9734.5	-1.42948	noncoding
MSTRG.9734.4	-0.40229	noncoding
MSTRG.9735.1	-0.24173	noncoding
MSTRG.9738.1	1.69563	coding
MSTRG.9747.1	-3.0908	noncoding
MSTRG.9753.1	-0.384454	noncoding
MSTRG.9756.1	-2.1721	noncoding
MSTRG.9760.1	-0.671311	noncoding
MSTRG.9760.2	-0.654603	noncoding
MSTRG.9760.3	-1.54876	noncoding
MSTRG.9773.1	2.0276	coding
MSTRG.9774.1	1.60364	coding
MSTRG.9774.2	1.43702	coding
MSTRG.9774.4	1.43777	coding
MSTRG.9775.1	-0.363213	noncoding
MSTRG.9780.1	-2.87617	noncoding
MSTRG.9798.1	-1.9271	noncoding
MSTRG.9815.16	-1.39184	noncoding
MSTRG.9815.18	0.444255	coding
MSTRG.9815.19	0.052847	coding
MSTRG.9815.20	0.874229	coding
MSTRG.9815.21	0.859773	coding
MSTRG.9816.1	-1.90282	noncoding
MSTRG.9815.22	0.186443	coding
MSTRG.9815.23	-1.26264	noncoding
MSTRG.9815.24	-2.20316	noncoding
MSTRG.9815.25	0.503692	coding
MSTRG.9815.26	-0.107012	noncoding
MSTRG.9815.27	0.53349	coding
MSTRG.9817.1	0.209733	coding
MSTRG.9817.2	-1.7131	noncoding
MSTRG.9811.1	-1.85706	noncoding
MSTRG.9812.2	2.53886	coding
MSTRG.9814.2	3.1005	coding
MSTRG.9827.1	0.937545	coding
MSTRG.9827.3	-0.735495	noncoding
MSTRG.9849.1	1.32108	coding
MSTRG.9854.3	2.79524	coding
MSTRG.9858.2	1.28283	coding
MSTRG.9859.1	1.38385	coding
MSTRG.9859.2	1.46171	coding
MSTRG.9862.1	-0.506078	noncoding
MSTRG.9863.1	-0.998856	noncoding
MSTRG.9878.1	0.049089	coding
MSTRG.9884.1	-1.20877	noncoding
MSTRG.9889.1	-0.831654	noncoding
MSTRG.9889.2	-0.494231	noncoding
MSTRG.9890.3	0.407155	coding
MSTRG.9891.1	1.86693	coding
MSTRG.9891.2	1.46716	coding
MSTRG.9891.3	1.55065	coding
MSTRG.9891.4	1.52818	coding
MSTRG.9891.6	1.43016	coding

MSTRG.9891.7	1.51353	coding
MSTRG.9891.8	1.55718	coding
MSTRG.9891.9	1.62784	coding
MSTRG.9891.10	1.57603	coding
MSTRG.9891.11	1.56531	coding
MSTRG.9893.1	-2.38621	noncoding
MSTRG.9894.1	1.64114	coding
MSTRG.9894.2	1.62943	coding
MSTRG.9894.3	1.68539	coding
MSTRG.9898.1	-1.0393	noncoding
MSTRG.9898.2	-0.95645	noncoding
MSTRG.9902.6	0.118838	coding
MSTRG.9902.5	0.996076	coding
MSTRG.9902.9	-0.14858	noncoding
MSTRG.9902.8	0.629068	coding
MSTRG.9902.10	0.321863	coding
MSTRG.9902.11	0.611395	coding
MSTRG.9899.3	-1.12753	noncoding
MSTRG.9899.4	-1.17901	noncoding
MSTRG.9899.5	-1.00233	noncoding
MSTRG.9906.2	1.49402	coding
MSTRG.9913.2	1.59142	coding
MSTRG.9913.6	2.07066	coding
MSTRG.9915.2	1.99776	coding
MSTRG.9918.1	-1.66929	noncoding
MSTRG.9919.2	1.66851	coding
MSTRG.9919.3	1.5774	coding
MSTRG.9919.4	0.920532	coding
MSTRG.9924.3	2.13034	coding
MSTRG.9929.1	1.96777	coding
MSTRG.9929.6	2.99324	coding
MSTRG.9929.5	1.86172	coding
MSTRG.9934.3	2.23982	coding
MSTRG.9941.2	1.54766	coding
MSTRG.9944.1	0.535941	coding
MSTRG.9944.2	0.026215	coding
MSTRG.9944.3	0.636842	coding
MSTRG.9948.1	0.556617	coding
MSTRG.9961.1	-2.25541	noncoding
MSTRG.9962.3	1.22862	coding
MSTRG.9962.4	1.41899	coding
MSTRG.9963.2	1.50296	coding
MSTRG.9960.1	0.828887	coding
MSTRG.9972.3	1.1762	coding
MSTRG.9972.4	1.30701	coding
MSTRG.9974.1	-1.30909	noncoding
MSTRG.9977.1	-0.765442	noncoding
MSTRG.9977.2	-1.32795	noncoding
MSTRG.9984.1	1.41974	coding
MSTRG.9984.2	1.4652	coding
MSTRG.9985.2	0.840829	coding
MSTRG.9986.1	1.79208	coding
MSTRG.9986.3	1.74247	coding
MSTRG.9987.3	0.162709	coding

MSTRG.9987.4	0.458317	coding
MSTRG.9990.1	-1.09651	noncoding
MSTRG.9993.2	1.06654	coding
MSTRG.9994.1	0.239736	coding
MSTRG.9997.1	1.58525	coding
MSTRG.9997.2	1.5253	coding
MSTRG.9997.3	3.28576	coding
MSTRG.9997.4	1.99527	coding
MSTRG.9997.5	1.60797	coding
MSTRG.10000.1	0.859878	coding
MSTRG.10001.1	3.25541	coding
MSTRG.10001.4	2.82554	coding
MSTRG.10002.1	0.581695	coding
MSTRG.10005.2	1.1012	coding
MSTRG.10005.1	1.31508	coding
MSTRG.10008.4	1.79501	coding
MSTRG.10009.2	1.2741	coding
MSTRG.10011.1	-0.056219	noncoding
MSTRG.10012.2	1.11263	coding
MSTRG.10012.3	1.32101	coding
MSTRG.10012.4	1.10711	coding
MSTRG.10013.1	-1.77986	noncoding
MSTRG.10014.1	0.695682	coding
MSTRG.10014.3	0.592222	coding
MSTRG.10016.1	1.20229	coding
MSTRG.10020.4	0.427224	coding
MSTRG.10026.10	0.903657	coding
MSTRG.10026.13	1.67401	coding
MSTRG.10026.11	1.52622	coding
MSTRG.10026.12	1.54046	coding
MSTRG.10026.18	1.29764	coding
MSTRG.10024.2	1.52897	coding
MSTRG.10024.3	1.72043	coding
MSTRG.10025.1	-1.83949	noncoding
MSTRG.10031.1	-0.595926	noncoding
MSTRG.10041.2	2.48903	coding
MSTRG.10041.3	2.535	coding
MSTRG.10041.7	2.46382	coding
MSTRG.10042.1	-1.31594	noncoding
MSTRG.10043.2	2.26534	coding
MSTRG.10043.5	1.90771	coding
MSTRG.10043.6	1.82566	coding
MSTRG.10045.2	0.406363	coding
MSTRG.10037.3	1.83329	coding
MSTRG.10038.1	-0.441637	noncoding
MSTRG.10038.2	-0.749895	noncoding
MSTRG.10049.1	0.115314	coding
MSTRG.10050.2	-1.4462	noncoding
MSTRG.10052.2	1.20716	coding
MSTRG.10053.1	0.212072	coding
MSTRG.10051.2	-1.42594	noncoding
MSTRG.10058.1	0.706373	coding
MSTRG.10060.1	1.4613	coding
MSTRG.10078.4	1.63013	coding

MSTRG.10078.5	1.72568	coding
MSTRG.10078.3	2.01104	coding
MSTRG.10079.1	0.28019	coding
MSTRG.10079.2	-0.544159	noncoding
MSTRG.10079.3	-0.14916	noncoding
MSTRG.10082.2	0.505433	coding
MSTRG.10087.3	1.50628	coding
MSTRG.10091.1	0.744898	coding
MSTRG.10091.2	0.905065	coding
MSTRG.10091.3	0.658457	coding
MSTRG.10092.1	-1.67053	noncoding
MSTRG.10094.8	1.48693	coding
MSTRG.10094.6	1.85957	coding
MSTRG.10094.7	1.609	coding
MSTRG.10094.9	1.40404	coding
MSTRG.10094.10	1.65371	coding
MSTRG.10094.11	1.48285	coding
MSTRG.10100.1	-1.19253	noncoding
MSTRG.10100.2	-1.51981	noncoding
MSTRG.10101.1	-0.548834	noncoding
MSTRG.10101.3	0.271318	coding
MSTRG.10104.2	1.50009	coding
MSTRG.10105.2	-0.813822	noncoding
MSTRG.10107.1	1.95251	coding
MSTRG.10102.2	1.94594	coding
MSTRG.10103.1	-1.73631	noncoding
MSTRG.10121.1	2.35203	coding
MSTRG.10121.3	1.43465	coding
MSTRG.10121.4	0.524305	coding
MSTRG.10122.1	1.40643	coding
MSTRG.10122.2	1.41364	coding
MSTRG.10122.3	1.315	coding
MSTRG.10122.4	1.15305	coding
MSTRG.10123.1	-0.411344	noncoding
MSTRG.10122.5	0.702502	coding
MSTRG.10122.6	1.02258	coding
MSTRG.10122.7	0.438916	coding
MSTRG.10122.8	0.830982	coding
MSTRG.10133.9	0.314902	coding
MSTRG.10133.3	2.32272	coding
MSTRG.10133.6	1.425	coding
MSTRG.10133.5	1.70708	coding
MSTRG.10133.11	2.57501	coding
MSTRG.10133.12	1.89684	coding
MSTRG.10112.2	-1.99205	noncoding
MSTRG.10112.3	1.40019	coding
MSTRG.10112.4	1.46527	coding
MSTRG.10112.5	1.23437	coding
MSTRG.10112.10	1.08433	coding
MSTRG.10112.11	1.25715	coding
MSTRG.10112.12	0.961431	coding
MSTRG.10115.4	1.01672	coding
MSTRG.10115.3	0.885317	coding
MSTRG.10115.5	0.167107	coding

MSTRG.10116.2	0.530616	coding
MSTRG.10134.1	-0.66217	noncoding
MSTRG.10138.3	-2.10696	noncoding
MSTRG.10138.2	2.02375	coding
MSTRG.10139.1	-0.333764	noncoding
MSTRG.10138.7	2.13926	coding
MSTRG.10142.1	-1.75813	noncoding
MSTRG.10146.6	1.97261	coding
MSTRG.10146.5	1.6035	coding
MSTRG.10147.1	-0.939306	noncoding
MSTRG.10148.1	2.24687	coding
MSTRG.10148.2	2.38772	coding
MSTRG.10148.3	2.19322	coding
MSTRG.10148.7	2.20345	coding
MSTRG.10148.9	2.64223	coding
MSTRG.10148.8	1.59161	coding
MSTRG.10148.6	1.38134	coding
MSTRG.10148.4	1.98579	coding
MSTRG.10143.2	0.477286	coding
MSTRG.10143.3	1.24315	coding
MSTRG.10155.1	-1.75747	noncoding
MSTRG.10159.1	1.54869	coding
MSTRG.10160.1	1.20837	coding
MSTRG.10162.1	-1.63357	noncoding
MSTRG.10172.2	-0.035495	noncoding
MSTRG.10172.5	3.61226	coding
MSTRG.10172.6	-1.32819	noncoding
MSTRG.10173.1	0.055267	coding
MSTRG.10173.2	-0.165861	noncoding
MSTRG.10172.8	-0.312051	noncoding
MSTRG.10172.9	-0.392045	noncoding
MSTRG.10172.10	-0.418559	noncoding
MSTRG.10172.11	-0.857007	noncoding
MSTRG.10175.1	0.034306	coding
MSTRG.10175.2	-0.093081	noncoding
MSTRG.10175.3	0.2789	coding
MSTRG.10175.4	-0.744779	noncoding
MSTRG.10175.5	-0.330027	noncoding
MSTRG.10178.6	1.52314	coding
MSTRG.10178.15	1.34002	coding
MSTRG.10178.7	1.38601	coding
MSTRG.10178.8	1.39272	coding
MSTRG.10178.12	1.56806	coding
MSTRG.10178.11	1.52949	coding
MSTRG.10178.9	1.45013	coding
MSTRG.10178.13	1.4381	coding
MSTRG.10178.1	1.42434	coding
MSTRG.10178.14	1.37655	coding
MSTRG.10178.16	1.20291	coding
MSTRG.10178.3	1.23021	coding
MSTRG.10178.10	1.19231	coding
MSTRG.10178.5	1.24675	coding
MSTRG.10178.17	1.04937	coding
MSTRG.10178.18	1.28197	coding

MSTRG.10178.19	1.27788	coding
MSTRG.10178.20	1.26143	coding
MSTRG.10178.21	1.08023	coding
MSTRG.10178.22	1.05556	coding
MSTRG.10178.24	1.16266	coding
MSTRG.10178.23	1.23013	coding
MSTRG.10180.2	1.11285	coding
MSTRG.10164.2	-0.044989	noncoding
MSTRG.10164.1	0.589331	coding
MSTRG.10164.3	0.58586	coding
MSTRG.10183.4	0.928681	coding
MSTRG.10184.1	0.282497	coding
MSTRG.10185.1	-1.22532	noncoding
MSTRG.10195.1	-1.98695	noncoding
MSTRG.10198.1	1.8019	coding
MSTRG.10197.5	-0.625929	noncoding
MSTRG.10197.6	-0.3529	noncoding
MSTRG.10208.1	-0.425862	noncoding
MSTRG.10209.1	1.26156	coding
MSTRG.10197.7	0.402901	coding
MSTRG.10197.8	-0.929614	noncoding
MSTRG.10197.9	-1.56759	noncoding
MSTRG.10213.1	-0.535455	noncoding
MSTRG.10214.1	-1.43644	noncoding
MSTRG.10215.1	-0.676555	noncoding
MSTRG.10230.1	0.515794	coding
MSTRG.10231.1	1.27316	coding
MSTRG.10231.2	1.62628	coding
MSTRG.10232.1	-1.82546	noncoding
MSTRG.10234.1	-0.644136	noncoding
MSTRG.10234.2	-0.821022	noncoding
MSTRG.10231.4	1.44833	coding
MSTRG.10234.3	-1.75562	noncoding
MSTRG.10235.1	-0.597518	noncoding
MSTRG.10231.9	1.46841	coding
MSTRG.10236.1	-0.552943	noncoding
MSTRG.10231.12	1.22993	coding
MSTRG.10231.16	2.2575	coding
MSTRG.10231.15	1.95113	coding
MSTRG.10231.18	0.996508	coding
MSTRG.10231.19	1.7552	coding
MSTRG.10237.1	-0.151994	noncoding
MSTRG.10238.1	0.491114	coding
MSTRG.10239.3	1.85895	coding
MSTRG.10239.4	2.31051	coding
MSTRG.10222.1	-0.161552	noncoding
MSTRG.10219.1	-2.85567	noncoding
MSTRG.10219.2	-2.92667	noncoding
MSTRG.10219.3	-2.28811	noncoding
MSTRG.10243.7	0.957968	coding
MSTRG.10243.8	-0.034208	noncoding
MSTRG.10255.1	-2.24518	noncoding
MSTRG.10256.3	1.06986	coding
MSTRG.10259.1	0.638754	coding

MSTRG.10260.1	0.451699	coding
MSTRG.10261.1	-0.870923	noncoding
MSTRG.10266.2	1.56366	coding
MSTRG.10268.1	-2.29967	noncoding
MSTRG.10270.1	-2.10488	noncoding
MSTRG.10273.2	-0.036281	noncoding
MSTRG.10273.4	-0.896187	noncoding
MSTRG.10273.3	-0.75692	noncoding
MSTRG.10273.5	-0.707395	noncoding
MSTRG.10275.1	-0.235422	noncoding
MSTRG.10278.2	2.58442	coding
MSTRG.10281.7	2.31079	coding
MSTRG.10281.9	2.25694	coding
MSTRG.10281.6	2.41646	coding
MSTRG.10281.4	2.49841	coding
MSTRG.10281.5	2.49186	coding
MSTRG.10281.8	2.49339	coding
MSTRG.10282.2	1.24622	coding
MSTRG.10282.3	0.582102	coding
MSTRG.10282.4	0.580715	coding
MSTRG.10284.1	2.01081	coding
MSTRG.10284.4	2.45856	coding
MSTRG.10290.1	0.423871	coding
MSTRG.10290.2	0.077575	coding
MSTRG.10302.4	1.1996	coding
MSTRG.10305.1	-0.075349	noncoding
MSTRG.10305.2	-1.43724	noncoding
MSTRG.10306.1	-2.39191	noncoding
MSTRG.10319.2	1.26073	coding
MSTRG.10322.2	1.04139	coding
MSTRG.10322.1	1.1633	coding
MSTRG.10317.1	-1.65097	noncoding
MSTRG.10326.3	1.04797	coding
MSTRG.10330.3	-1.23924	noncoding
MSTRG.10331.1	2.58528	coding
MSTRG.10331.2	2.22714	coding
MSTRG.10329.3	0.877441	coding
MSTRG.10343.1	1.10029	coding
MSTRG.10344.1	0.457718	coding
MSTRG.10343.4	0.836331	coding
MSTRG.10343.5	1.04612	coding
MSTRG.10335.1	2.25957	coding
MSTRG.10335.3	2.01528	coding
MSTRG.10336.2	2.28024	coding
MSTRG.10337.1	-2.20625	noncoding
MSTRG.10341.2	1.16179	coding
MSTRG.10341.3	-0.22484	noncoding
MSTRG.10342.1	-0.839069	noncoding
MSTRG.10350.1	0.894287	coding
MSTRG.10351.1	1.23604	coding
MSTRG.10351.2	1.23138	coding
MSTRG.10351.3	2.15146	coding
MSTRG.10356.3	0.786729	coding
MSTRG.10356.4	0.965342	coding

MSTRG.10362.2	-0.844467	noncoding
MSTRG.10366.2	1.34543	coding
MSTRG.10370.2	1.84475	coding
MSTRG.10370.3	0.196762	coding
MSTRG.10373.3	2.1518	coding
MSTRG.10373.2	2.28487	coding
MSTRG.10373.5	0.64985	coding
MSTRG.10374.1	0.941793	coding
MSTRG.10375.1	-0.047262	noncoding
MSTRG.10377.1	-2.44899	noncoding
MSTRG.10379.1	-2.21259	noncoding
MSTRG.10372.1	0.104062	coding
MSTRG.10372.2	-0.318091	noncoding
MSTRG.10437.2	1.09937	coding
MSTRG.10437.3	0.112103	coding
MSTRG.10437.4	0.181409	coding
MSTRG.10437.5	-0.886392	noncoding
MSTRG.10437.7	-1.21519	noncoding
MSTRG.10437.6	-0.130633	noncoding
MSTRG.10437.8	0.36396	coding
MSTRG.10437.9	0.314434	coding
MSTRG.10437.10	-0.284254	noncoding
MSTRG.10437.11	-0.622858	noncoding
MSTRG.10437.12	0.306751	coding
MSTRG.10437.13	0.439175	coding
MSTRG.10437.14	0.13674	coding
MSTRG.10437.15	0.061717	coding
MSTRG.10437.16	0.24796	coding
MSTRG.10437.17	0.776643	coding
MSTRG.10439.1	-1.45383	noncoding
MSTRG.10437.18	0.403021	coding
MSTRG.10440.1	0.106975	coding
MSTRG.10437.19	0.62565	coding
MSTRG.10437.20	0.306205	coding
MSTRG.10441.1	-0.368442	noncoding
MSTRG.10442.1	0.259222	coding
MSTRG.10443.1	-0.526486	noncoding
MSTRG.10445.1	0.14824	coding
MSTRG.10437.21	-1.13938	noncoding
MSTRG.10447.1	-1.01038	noncoding
MSTRG.10437.22	-0.665213	noncoding
MSTRG.10381.1	-0.924252	noncoding
MSTRG.10381.3	-1.43624	noncoding
MSTRG.10381.4	-1.31907	noncoding
MSTRG.10381.5	-1.30949	noncoding
MSTRG.10381.6	-1.50503	noncoding
MSTRG.10386.1	-1.82715	noncoding
MSTRG.10391.1	0.287161	coding
MSTRG.10383.1	0.640204	coding
MSTRG.10392.1	0.698819	coding
MSTRG.10399.2	1.10478	coding
MSTRG.10399.3	-0.000461	noncoding
MSTRG.10400.1	0.206565	coding
MSTRG.10401.3	1.56684	coding

MSTRG.10410.2	-1.21538	noncoding
MSTRG.10419.2	1.43346	coding
MSTRG.10419.3	1.16511	coding
MSTRG.10419.4	2.68036	coding
MSTRG.10420.1	0.299401	coding
MSTRG.10419.6	1.07191	coding
MSTRG.10407.1	0.799804	coding
MSTRG.10407.2	0.487371	coding
MSTRG.10407.3	-1.75401	noncoding
MSTRG.10409.1	1.68584	coding
MSTRG.10470.8	1.11359	coding
MSTRG.10470.10	1.62232	coding
MSTRG.10470.17	0.876957	coding
MSTRG.10470.18	0.226126	coding
MSTRG.10470.20	-0.745782	noncoding
MSTRG.10470.22	1.0326	coding
MSTRG.10470.23	0.325062	coding
MSTRG.10470.24	-0.304691	noncoding
MSTRG.10421.2	0.638925	coding
MSTRG.10424.2	-1.28614	noncoding
MSTRG.10424.3	1.90685	coding
MSTRG.10428.1	-0.350511	noncoding
MSTRG.10427.3	0.206555	coding
MSTRG.10430.2	1.14202	coding
MSTRG.10455.1	2.4943	coding
MSTRG.10455.2	2.70376	coding
MSTRG.10455.4	2.60692	coding
MSTRG.10456.1	1.10678	coding
MSTRG.10456.3	0.387705	coding
MSTRG.10456.5	0.329616	coding
MSTRG.10456.6	0.287402	coding
MSTRG.10456.7	1.44676	coding
MSTRG.10456.4	0.814588	coding
MSTRG.10464.1	1.79616	coding
MSTRG.10466.1	-1.43721	noncoding
MSTRG.10435.2	0.491305	coding
MSTRG.10488.3	0.360155	coding
MSTRG.10488.5	0.456134	coding
MSTRG.10488.2	0.638223	coding
MSTRG.10488.4	0.726895	coding
MSTRG.10489.1	0.343354	coding
MSTRG.10481.1	-2.16828	noncoding
MSTRG.10491.1	-1.17315	noncoding
MSTRG.10507.1	3.29545	coding
MSTRG.10508.1	-2.16976	noncoding
MSTRG.10507.3	2.4354	coding
MSTRG.10513.4	-0.582692	noncoding
MSTRG.10516.1	0.796018	coding
MSTRG.10511.1	1.20432	coding
MSTRG.10522.3	-0.041187	noncoding
MSTRG.10523.1	-0.666478	noncoding
MSTRG.10524.1	-2.71469	noncoding
MSTRG.10525.1	-0.651438	noncoding
MSTRG.10526.3	0.565741	coding

MSTRG.10526.5	0.858439	coding
MSTRG.10539.3	0.354665	coding
MSTRG.10539.4	-0.614861	noncoding
MSTRG.10542.1	-1.49076	noncoding
MSTRG.10542.2	0.26474	coding
MSTRG.10543.1	-1.41904	noncoding
MSTRG.10573.1	-1.55767	noncoding
MSTRG.10577.1	-2.0739	noncoding
MSTRG.10582.1	-1.72248	noncoding
MSTRG.10583.1	-0.748757	noncoding
MSTRG.10584.1	-0.97682	noncoding
MSTRG.10590.2	0.583716	coding
MSTRG.10590.3	-0.726608	noncoding
MSTRG.10595.3	2.01146	coding
MSTRG.10595.4	1.51437	coding
MSTRG.10595.5	1.90081	coding
MSTRG.10595.6	1.86336	coding
MSTRG.10595.7	2.18169	coding
MSTRG.10604.1	-2.27449	noncoding
MSTRG.10633.2	1.91081	coding
MSTRG.10634.1	-0.312943	noncoding
MSTRG.10635.1	0.555893	coding
MSTRG.10633.3	-0.159438	noncoding
MSTRG.10633.4	0.010187	coding
MSTRG.10633.5	-0.305749	noncoding
MSTRG.10636.1	-0.866497	noncoding
MSTRG.10640.1	-0.918976	noncoding
MSTRG.10641.1	-0.356117	noncoding
MSTRG.10639.2	2.20052	coding
MSTRG.10642.2	1.08655	coding
MSTRG.10643.1	-0.327945	noncoding
MSTRG.10642.3	-0.006962	noncoding
MSTRG.10623.2	1.41659	coding
MSTRG.10623.3	1.1727	coding
MSTRG.10626.2	0.736257	coding
MSTRG.10629.2	-0.242172	noncoding
MSTRG.10629.3	-0.222516	noncoding
MSTRG.10646.1	-0.597859	noncoding
MSTRG.10646.2	-0.182895	noncoding
MSTRG.10652.1	0.91172	coding
MSTRG.10653.1	-1.91916	noncoding
MSTRG.10652.5	0.574114	coding
MSTRG.10654.1	1.57429	coding
MSTRG.10659.1	-0.802364	noncoding
MSTRG.10659.2	-1.35665	noncoding
MSTRG.10665.1	2.01151	coding
MSTRG.10667.4	2.06656	coding
MSTRG.10667.5	2.87649	coding
MSTRG.10668.1	0.334037	coding
MSTRG.10668.2	0.599315	coding
MSTRG.10680.1	-0.684311	noncoding
MSTRG.10680.2	-0.573272	noncoding
MSTRG.10680.3	2.17114	coding
MSTRG.10680.4	-0.131033	noncoding

MSTRG.10685.1	-2.65198	noncoding
MSTRG.10688.1	0.009564	coding
MSTRG.10700.2	0.295425	coding
MSTRG.10700.3	1.73815	coding
MSTRG.10700.4	1.75905	coding
MSTRG.10701.1	-0.136875	noncoding
MSTRG.10700.5	0.075121	coding
MSTRG.10703.1	1.70444	coding
MSTRG.10703.13	2.16411	coding
MSTRG.10713.1	2.32783	coding
MSTRG.10713.5	3.11795	coding
MSTRG.10713.4	3.14159	coding
MSTRG.10713.6	2.13892	coding
MSTRG.10715.1	-0.738852	noncoding
MSTRG.10707.1	-0.859368	noncoding
MSTRG.10712.2	-0.058879	noncoding
MSTRG.10718.1	0.368835	coding
MSTRG.10725.1	0.327028	coding
MSTRG.10725.2	-0.289229	noncoding
MSTRG.10761.1	-0.619853	noncoding
MSTRG.10764.1	-1.99397	noncoding
MSTRG.10766.1	0.020062	coding
MSTRG.10766.2	0.003277	coding
MSTRG.10767.1	-2.23819	noncoding
MSTRG.10768.1	-2.00064	noncoding
MSTRG.10769.1	-0.11919	noncoding
MSTRG.10765.1	0.602233	coding
MSTRG.10765.2	-1.08019	noncoding
MSTRG.10772.1	-0.389017	noncoding
MSTRG.10775.1	-2.03466	noncoding
MSTRG.10776.1	-1.44211	noncoding
MSTRG.10776.2	0.589256	coding
MSTRG.10777.1	-0.907244	noncoding
MSTRG.10774.2	-0.560505	noncoding
MSTRG.10778.1	0.791893	coding
MSTRG.10774.3	0.379282	coding
MSTRG.10774.4	1.8736	coding
MSTRG.10774.5	0.622362	coding
MSTRG.10779.1	0.689824	coding
MSTRG.10774.6	-0.301508	noncoding
MSTRG.10780.1	-0.213545	noncoding
MSTRG.10782.1	-1.12625	noncoding
MSTRG.10786.2	0.305929	coding
MSTRG.10786.4	-0.122763	noncoding
MSTRG.10787.1	1.20376	coding
MSTRG.10789.1	1.46252	coding
MSTRG.10790.1	-1.08	noncoding
MSTRG.10791.1	-0.620542	noncoding
MSTRG.10792.1	-0.733218	noncoding
MSTRG.10793.3	1.14702	coding
MSTRG.10793.2	0.938058	coding
MSTRG.10794.1	-2.24466	noncoding
MSTRG.10794.2	-1.78734	noncoding
MSTRG.10795.4	2.26288	coding

MSTRG.10795.5	2.26435	coding
MSTRG.10796.1	-2.22699	noncoding
MSTRG.10806.4	1.23074	coding
MSTRG.10807.1	-0.119315	noncoding
MSTRG.10808.2	1.89911	coding
MSTRG.10808.5	1.79339	coding
MSTRG.10808.6	2.16379	coding
MSTRG.10808.7	1.456	coding
MSTRG.10809.1	-0.388938	noncoding
MSTRG.10811.1	-1.56181	noncoding
MSTRG.10812.1	-2.00829	noncoding
MSTRG.10820.1	1.09463	coding
MSTRG.10825.1	1.01321	coding
MSTRG.10830.1	-0.161061	noncoding
MSTRG.10831.1	-0.493783	noncoding
MSTRG.10829.4	-0.102602	noncoding
MSTRG.10829.5	-0.357306	noncoding
MSTRG.10837.1	0.903004	coding
MSTRG.10837.2	0.625274	coding
MSTRG.10837.4	0.193411	coding
MSTRG.10838.1	-0.827792	noncoding
MSTRG.10840.2	2.19429	coding
MSTRG.10840.3	0.834069	coding
MSTRG.10843.5	0.840022	coding
MSTRG.10844.2	1.71087	coding
MSTRG.10844.1	1.86847	coding
MSTRG.10845.1	-0.962371	noncoding
MSTRG.10847.1	-1.48816	noncoding
MSTRG.10848.2	0.368409	coding
MSTRG.10848.3	0.358898	coding
MSTRG.10848.4	1.18171	coding
MSTRG.10851.3	0.828657	coding
MSTRG.10852.1	-0.684853	noncoding
MSTRG.10853.1	-2.06505	noncoding
MSTRG.10857.1	-2.62689	noncoding
MSTRG.10867.3	2.56408	coding
MSTRG.10876.7	2.02693	coding
MSTRG.10876.8	1.97467	coding
MSTRG.10876.6	0.999289	coding
MSTRG.10880.3	0.552408	coding
MSTRG.10882.1	-0.351619	noncoding
MSTRG.10882.2	0.567572	coding
MSTRG.10882.4	0.178646	coding
MSTRG.10882.5	0.711843	coding
MSTRG.10882.6	0.059241	coding
MSTRG.10882.7	0.691079	coding
MSTRG.10882.8	-0.723928	noncoding
MSTRG.10888.2	0.532314	coding
MSTRG.10888.4	0.41849	coding
MSTRG.10889.1	0.881605	coding
MSTRG.10889.2	-0.950651	noncoding
MSTRG.10890.1	-0.246254	noncoding
MSTRG.10889.5	0.532295	coding
MSTRG.10889.7	0.349724	coding

MSTRG.10889.8	0.660816	coding
MSTRG.10889.9	1.48495	coding
MSTRG.10899.1	1.77309	coding
MSTRG.10901.1	0.105679	coding
MSTRG.10902.1	0.127588	coding
MSTRG.10903.1	1.30963	coding
MSTRG.10896.2	-1.08817	noncoding
MSTRG.10896.3	-1.26717	noncoding
MSTRG.10906.1	-1.0748	noncoding
MSTRG.10907.2	0.353088	coding
MSTRG.10907.3	0.376779	coding
MSTRG.10910.1	-1.55683	noncoding
MSTRG.10922.3	1.22107	coding
MSTRG.10922.5	1.16147	coding
MSTRG.10923.1	-0.454847	noncoding
MSTRG.10925.1	0.856575	coding
MSTRG.10925.9	1.02542	coding
MSTRG.10928.1	-0.056727	noncoding
MSTRG.10913.1	-1.17105	noncoding
MSTRG.10919.1	0.840439	coding
MSTRG.10921.1	-1.54183	noncoding
MSTRG.10935.1	0.967191	coding
MSTRG.10935.2	1.48246	coding
MSTRG.10936.2	1.24766	coding
MSTRG.10941.2	1.76004	coding
MSTRG.10942.2	0.026726	coding
MSTRG.10943.1	-0.394997	noncoding
MSTRG.10944.1	1.49996	coding
MSTRG.10950.3	2.33883	coding
MSTRG.10950.4	2.04388	coding
MSTRG.10956.1	-0.831686	noncoding
MSTRG.10958.1	-2.03152	noncoding
MSTRG.10961.1	-0.835356	noncoding
MSTRG.10971.1	-0.755609	noncoding
MSTRG.10976.1	-1.10221	noncoding
MSTRG.10976.2	-1.2165	noncoding
MSTRG.10976.3	-2.09806	noncoding
MSTRG.10984.1	0.403972	coding
MSTRG.10984.3	-0.167425	noncoding
MSTRG.10990.4	-0.134254	noncoding
MSTRG.10990.3	0.010711	coding
MSTRG.10990.5	0.448381	coding
MSTRG.11012.1	2.64581	coding
MSTRG.11012.2	2.63512	coding
MSTRG.11039.1	0.264664	coding
MSTRG.11040.1	0.335933	coding
MSTRG.11041.1	0.519258	coding
MSTRG.11042.1	0.525407	coding
MSTRG.11043.1	0.386245	coding
MSTRG.11044.1	-1.42134	noncoding
MSTRG.11045.1	-1.3046	noncoding
MSTRG.11046.1	-1.85252	noncoding
MSTRG.11036.2	1.52784	coding
MSTRG.11036.1	0.49015	coding

MSTRG.11037.1	-2.21722	noncoding
MSTRG.11050.1	-1.69229	noncoding
MSTRG.11050.2	-1.82363	noncoding
MSTRG.11055.1	-0.875228	noncoding
MSTRG.11057.1	-1.95398	noncoding
MSTRG.11060.1	1.40389	coding
MSTRG.11071.2	2.00087	coding
MSTRG.11071.3	2.27771	coding
MSTRG.11071.4	2.27527	coding
MSTRG.11071.5	1.37504	coding
MSTRG.11072.1	-0.535767	noncoding
MSTRG.11073.2	-1.36152	noncoding
MSTRG.11063.1	1.40358	coding
MSTRG.11063.3	1.58476	coding
MSTRG.11076.2	0.760293	coding
MSTRG.11100.1	0.693234	coding
MSTRG.11101.1	0.391094	coding
MSTRG.11090.1	-0.497297	noncoding
MSTRG.11091.5	1.82611	coding
MSTRG.11091.6	1.82673	coding
MSTRG.11096.2	0.589092	coding
MSTRG.11098.1	1.43938	coding
MSTRG.11098.2	1.74244	coding
MSTRG.11103.2	0.884002	coding
MSTRG.11103.3	0.991158	coding
MSTRG.11109.1	-2.23617	noncoding
MSTRG.11154.2	2.98588	coding
MSTRG.11156.1	-1.68154	noncoding
MSTRG.11158.1	0.91611	coding
MSTRG.11161.1	-0.975401	noncoding
MSTRG.11161.4	-1.81663	noncoding
MSTRG.11170.1	0.388909	coding
MSTRG.11171.1	-0.732672	noncoding
MSTRG.11169.3	0.787146	coding
MSTRG.11177.1	0.914533	coding
MSTRG.11163.2	-0.286594	noncoding
MSTRG.11185.1	2.17782	coding
MSTRG.11186.1	-0.828326	noncoding
MSTRG.11188.3	2.15459	coding
MSTRG.11190.3	2.51476	coding
MSTRG.11191.1	0.537937	coding
MSTRG.11194.2	-0.339263	noncoding
MSTRG.11199.1	-0.534108	noncoding
MSTRG.11194.4	-1.0561	noncoding
MSTRG.11230.1	-0.791504	noncoding
MSTRG.11235.1	1.40926	coding
MSTRG.11235.2	2.34639	coding
MSTRG.11243.1	0.24273	coding
MSTRG.11243.3	1.37816	coding
MSTRG.11243.6	-2.04549	noncoding
MSTRG.11243.8	1.46163	coding
MSTRG.11219.1	0.026197	coding
MSTRG.11226.1	1.41114	coding
MSTRG.11226.3	1.21309	coding

MSTRG.11221.1	-2.51501	noncoding
MSTRG.11221.2	-0.503708	noncoding
MSTRG.11248.3	3.5236	coding
MSTRG.11248.9	3.69466	coding
MSTRG.11248.8	3.53646	coding
MSTRG.11248.6	3.45734	coding
MSTRG.11248.7	3.56443	coding
MSTRG.11249.1	0.415312	coding
MSTRG.11257.1	-2.69572	noncoding
MSTRG.11256.1	-1.4242	noncoding
MSTRG.11279.1	-1.33253	noncoding
MSTRG.11283.1	1.16954	coding
MSTRG.11283.2	0.95414	coding
MSTRG.11284.1	-0.191808	noncoding
MSTRG.11292.1	1.84892	coding
MSTRG.11293.1	-0.880332	noncoding
MSTRG.11294.2	2.37554	coding
MSTRG.11294.3	0.620473	coding
MSTRG.11294.4	1.28777	coding
MSTRG.11300.3	0.334912	coding
MSTRG.11301.2	0.760578	coding
MSTRG.11301.1	0.121935	coding
MSTRG.11301.3	-1.09047	noncoding
MSTRG.11301.4	0.512564	coding
MSTRG.11301.5	-1.19296	noncoding
MSTRG.11302.1	-1.73137	noncoding
MSTRG.11302.2	-0.098254	noncoding
MSTRG.11303.1	-1.71565	noncoding
MSTRG.11304.2	1.20894	coding
MSTRG.11304.3	0.2239	coding
MSTRG.11373.3	2.10736	coding
MSTRG.11373.4	0.216165	coding
MSTRG.11373.8	-0.02072	noncoding
MSTRG.11377.1	0.315251	coding
MSTRG.11377.2	1.45746	coding
MSTRG.11378.1	-0.343366	noncoding
MSTRG.11377.3	2.40207	coding
MSTRG.11377.4	1.74833	coding
MSTRG.11377.5	2.40075	coding
MSTRG.11377.7	2.40351	coding
MSTRG.11377.6	2.33124	coding
MSTRG.11377.8	0.60759	coding
MSTRG.11377.9	-0.293063	noncoding
MSTRG.11377.10	-1.08761	noncoding
MSTRG.11379.1	-0.829725	noncoding
MSTRG.11377.11	-0.801041	noncoding
MSTRG.11381.1	0.528443	coding
MSTRG.11382.1	0.155895	coding
MSTRG.11377.18	0.445957	coding
MSTRG.11377.19	0.412783	coding
MSTRG.11377.20	0.408698	coding
MSTRG.11377.21	1.10889	coding
MSTRG.11377.22	-0.685545	noncoding
MSTRG.11377.23	-0.051182	noncoding

MSTRG.11377.24	0.766175	coding
MSTRG.11377.25	0.680679	coding
MSTRG.11377.26	-0.923703	noncoding
MSTRG.11377.27	-0.28672	noncoding
MSTRG.11309.1	0.950243	coding
MSTRG.11307.2	-1.17029	noncoding
MSTRG.11313.1	-0.394056	noncoding
MSTRG.11313.2	0.720641	coding
MSTRG.11313.3	2.19987	coding
MSTRG.11313.4	2.16905	coding
MSTRG.11319.1	-0.244397	noncoding
MSTRG.11319.2	-2.07322	noncoding
MSTRG.11321.1	1.03751	coding
MSTRG.11326.1	-0.071554	noncoding
MSTRG.11328.2	2.48693	coding
MSTRG.11328.4	0.749418	coding
MSTRG.11331.1	-0.07937	noncoding
MSTRG.11335.1	-1.7728	noncoding
MSTRG.11336.2	2.83388	coding
MSTRG.11336.3	1.66091	coding
MSTRG.11338.2	0.855891	coding
MSTRG.11338.3	1.93029	coding
MSTRG.11350.1	2.14039	coding
MSTRG.11350.5	2.44974	coding
MSTRG.11350.6	2.46422	coding
MSTRG.11350.4	2.76372	coding
MSTRG.11350.7	2.48386	coding
MSTRG.11351.1	-0.017117	noncoding
MSTRG.11352.1	-0.987445	noncoding
MSTRG.11350.8	1.77273	coding
MSTRG.11357.1	-0.505311	noncoding
MSTRG.11358.1	0.453432	coding
MSTRG.11359.1	-0.635126	noncoding
MSTRG.11360.1	0.259475	coding
MSTRG.11360.2	-0.84422	noncoding
MSTRG.11344.2	0.153229	coding
MSTRG.11345.1	-0.633973	noncoding
MSTRG.11345.2	-0.406746	noncoding
MSTRG.11346.1	-1.99343	noncoding
MSTRG.11347.1	0.29508	coding
MSTRG.11348.3	-0.272941	noncoding
MSTRG.11363.1	-0.370964	noncoding
MSTRG.11365.1	-0.673506	noncoding
MSTRG.11366.1	-0.213579	noncoding
MSTRG.11367.1	0.733041	coding
MSTRG.11367.2	0.628607	coding
MSTRG.11400.1	1.98618	coding
MSTRG.11400.2	0.833123	coding
MSTRG.11400.3	1.06586	coding
MSTRG.11400.4	0.779561	coding
MSTRG.11400.5	1.16972	coding
MSTRG.11403.1	1.08765	coding
MSTRG.11404.1	0.382462	coding
MSTRG.11404.2	0.590051	coding

MSTRG.11408.1	0.805573	coding
MSTRG.11410.1	-0.078776	noncoding
MSTRG.11411.1	-0.537628	noncoding
MSTRG.11411.2	-1.76954	noncoding
MSTRG.11413.3	0.526605	coding
MSTRG.11413.4	0.127871	coding
MSTRG.11406.3	1.86364	coding
MSTRG.11414.1	-0.181574	noncoding
MSTRG.11417.1	-1.09016	noncoding
MSTRG.11417.2	-0.189438	noncoding
MSTRG.11417.3	-0.345475	noncoding
MSTRG.11417.4	-0.50563	noncoding
MSTRG.11418.4	2.26129	coding
MSTRG.11427.1	1.19124	coding
MSTRG.11427.3	1.32641	coding
MSTRG.11427.4	1.16619	coding
MSTRG.11429.1	2.29913	coding
MSTRG.11430.1	0.048686	coding
MSTRG.11431.2	2.00436	coding
MSTRG.11431.3	2.16449	coding
MSTRG.11432.1	1.55608	coding
MSTRG.11433.1	-2.00245	noncoding
MSTRG.11436.1	0.807921	coding
MSTRG.11436.2	0.715609	coding
MSTRG.11437.3	0.268554	coding
MSTRG.11438.1	1.10236	coding
MSTRG.11439.3	2.47229	coding
MSTRG.11441.4	2.43372	coding
MSTRG.11441.2	2.46806	coding
MSTRG.11441.3	2.33245	coding
MSTRG.11442.1	-1.02359	noncoding
MSTRG.11443.4	0.146537	coding
MSTRG.11443.5	0.334919	coding
MSTRG.11443.7	0.029196	coding
MSTRG.11443.8	0.093232	coding
MSTRG.11443.6	0.383439	coding
MSTRG.11421.3	-0.509216	noncoding
MSTRG.11423.1	-0.475898	noncoding
MSTRG.11424.2	1.39159	coding
MSTRG.11449.2	1.0742	coding
MSTRG.11457.1	1.6078	coding
MSTRG.11457.2	0.777134	coding
MSTRG.11457.3	1.2529	coding
MSTRG.11460.1	-1.0565	noncoding
MSTRG.11462.12	1.30884	coding
MSTRG.11462.7	1.48693	coding
MSTRG.11462.8	1.89533	coding
MSTRG.11462.9	1.7209	coding
MSTRG.11462.13	1.71371	coding
MSTRG.11462.14	0.445803	coding
MSTRG.11462.15	1.79508	coding
MSTRG.11463.1	-0.985983	noncoding
MSTRG.11464.1	0.418925	coding
MSTRG.11465.1	-2.62352	noncoding

MSTRG.11466.1	1.39616	coding
MSTRG.11466.2	0.662017	coding
MSTRG.11472.1	1.83916	coding
MSTRG.11472.4	-0.675106	noncoding
MSTRG.11472.2	1.85407	coding
MSTRG.11472.5	0.854006	coding
MSTRG.11472.6	-0.336169	noncoding
MSTRG.11472.7	0.051981	coding
MSTRG.11472.8	-0.607384	noncoding
MSTRG.11473.1	-0.137954	noncoding
MSTRG.11473.2	-0.332405	noncoding
MSTRG.11473.3	-0.956285	noncoding
MSTRG.11474.1	-0.785677	noncoding
MSTRG.11473.4	-0.443042	noncoding
MSTRG.11472.9	-0.205831	noncoding
MSTRG.11472.10	1.10687	coding
MSTRG.11475.1	-0.457904	noncoding
MSTRG.11469.1	0.868085	coding
MSTRG.11470.5	1.59868	coding
MSTRG.11476.3	0.65838	coding
MSTRG.11476.2	1.23313	coding
MSTRG.11476.4	0.531557	coding
MSTRG.11476.5	0.639125	coding
MSTRG.11477.1	1.78761	coding
MSTRG.11478.1	-0.217049	noncoding
MSTRG.11483.2	0.571016	coding
MSTRG.11489.1	-1.80702	noncoding
MSTRG.11490.5	2.3942	coding
MSTRG.11490.6	2.42956	coding
MSTRG.11496.2	1.9589	coding
MSTRG.11499.2	1.38818	coding
MSTRG.11499.4	0.531074	coding
MSTRG.11492.7	0.715048	coding
MSTRG.11492.6	0.217784	coding
MSTRG.11492.9	0.339308	coding
MSTRG.11492.11	0.101264	coding
MSTRG.11492.12	0.633155	coding
MSTRG.11492.5	0.52257	coding
MSTRG.11492.8	0.357421	coding
MSTRG.11492.10	0.652459	coding
MSTRG.11492.4	0.229581	coding
MSTRG.11505.1	2.40971	coding
MSTRG.11505.2	2.19897	coding
MSTRG.11507.1	-0.756886	noncoding
MSTRG.11507.6	0.267645	coding
MSTRG.11507.8	0.054156	coding
MSTRG.11507.3	0.790835	coding
MSTRG.11507.7	0.303093	coding
MSTRG.11507.2	0.360018	coding
MSTRG.11507.4	0.566863	coding
MSTRG.11507.9	0.420523	coding
MSTRG.11508.1	1.18848	coding
MSTRG.11508.4	1.30213	coding
MSTRG.11508.5	2.66859	coding

MSTRG.11510.1	-0.120671	noncoding
MSTRG.11513.1	1.46549	coding
MSTRG.11514.1	-2.21787	noncoding
MSTRG.11514.2	-1.33001	noncoding
MSTRG.11515.1	0.171214	coding
MSTRG.11520.1	1.89485	coding
MSTRG.11534.1	0.270844	coding
MSTRG.11537.1	-0.696219	noncoding
MSTRG.11539.3	-0.113541	noncoding
MSTRG.11540.4	0.088212	coding
MSTRG.11530.2	0.758317	coding
MSTRG.11551.1	0.20776	coding
MSTRG.11551.2	0.869725	coding
MSTRG.11553.1	0.724594	coding
MSTRG.11549.5	-1.98015	noncoding
MSTRG.11549.4	-0.813721	noncoding
MSTRG.11542.1	0.113097	coding
MSTRG.11545.1	-2.05069	noncoding
MSTRG.11556.2	-0.595926	noncoding
MSTRG.11560.1	-0.729206	noncoding
MSTRG.11568.1	1.75982	coding
MSTRG.11570.1	-0.190495	noncoding
MSTRG.11571.1	-1.18752	noncoding
MSTRG.11584.1	0.34364	coding
MSTRG.11586.1	1.3122	coding
MSTRG.11589.2	2.82609	coding
MSTRG.11590.3	1.14993	coding
MSTRG.11591.2	0.343424	coding
MSTRG.11594.3	0.946956	coding
MSTRG.11595.1	-1.65601	noncoding
MSTRG.11595.2	-2.07817	noncoding
MSTRG.11594.4	1.514	coding
MSTRG.11606.4	2.39139	coding
MSTRG.11606.10	2.16442	coding
MSTRG.11599.1	2.39844	coding
MSTRG.11599.2	2.02784	coding
MSTRG.11610.1	1.88321	coding
MSTRG.11610.2	1.91378	coding
MSTRG.11611.2	1.35248	coding
MSTRG.11611.4	1.4728	coding
MSTRG.11613.1	-1.5322	noncoding
MSTRG.11615.1	1.94645	coding
MSTRG.11616.1	-2.14057	noncoding
MSTRG.11618.2	1.48639	coding
MSTRG.11617.1	-2.42372	noncoding
MSTRG.11622.1	-0.314499	noncoding
MSTRG.11622.2	-0.236354	noncoding
MSTRG.11636.2	0.728929	coding
MSTRG.11643.2	0.712486	coding
MSTRG.11643.3	1.16492	coding
MSTRG.11643.4	1.12923	coding
MSTRG.11645.1	-1.55575	noncoding
MSTRG.11646.1	-0.453517	noncoding
MSTRG.11649.4	1.32162	coding

MSTRG.11656.3	1.91139	coding
MSTRG.11657.2	0.772059	coding
MSTRG.11662.1	-1.68034	noncoding
MSTRG.11663.4	1.30465	coding
MSTRG.11663.3	1.50317	coding
MSTRG.11664.2	-1.2392	noncoding
MSTRG.11666.1	-0.111632	noncoding
MSTRG.11666.5	-0.392419	noncoding
MSTRG.11666.3	-1.37332	noncoding
MSTRG.11666.4	-1.49406	noncoding
MSTRG.11659.1	-0.377006	noncoding
MSTRG.11659.2	0.027048	coding
MSTRG.11659.3	-0.656237	noncoding
MSTRG.11659.4	-0.6966	noncoding
MSTRG.11674.2	0.492767	coding
MSTRG.11675.1	-0.121939	noncoding
MSTRG.11675.3	-0.172823	noncoding
MSTRG.11676.3	0.773172	coding
MSTRG.11678.1	1.10117	coding
MSTRG.11678.5	1.72263	coding
MSTRG.11678.3	1.73296	coding
MSTRG.11679.1	1.12658	coding
MSTRG.11680.1	1.2602	coding
MSTRG.11680.3	1.67439	coding
MSTRG.11677.3	1.17767	coding
MSTRG.11683.1	1.77305	coding
MSTRG.11684.1	0.945062	coding
MSTRG.11684.4	0.788071	coding
MSTRG.11687.2	-0.006613	noncoding
MSTRG.11696.1	-1.97171	noncoding
MSTRG.11697.1	0.73486	coding
MSTRG.11697.2	1.25593	coding
MSTRG.11702.1	0.274851	coding
MSTRG.11702.3	0.141253	coding
MSTRG.11703.1	-2.84902	noncoding
MSTRG.11707.1	-0.03954	noncoding
MSTRG.11708.1	-1.03095	noncoding
MSTRG.11708.2	-0.145162	noncoding
MSTRG.11717.1	0.013901	coding
MSTRG.11724.2	2.07195	coding
MSTRG.11724.7	1.54362	coding
MSTRG.11724.6	1.0673	coding
MSTRG.11727.1	0.070118	coding
MSTRG.11728.1	1.16789	coding
MSTRG.11731.1	-2.26721	noncoding
MSTRG.11738.1	3.21468	coding
MSTRG.11738.3	3.22291	coding
MSTRG.11738.4	-0.024814	noncoding
MSTRG.11738.6	0.263068	coding
MSTRG.11738.7	1.55337	coding
MSTRG.11738.8	1.99622	coding
MSTRG.11738.9	2.39223	coding
MSTRG.11734.3	1.64994	coding
MSTRG.11737.2	-0.837629	noncoding

MSTRG.11739.1	0.357074	coding
MSTRG.11745.7	2.32487	coding
MSTRG.11745.8	-2.67018	noncoding
MSTRG.11746.1	0.972983	coding
MSTRG.11747.1	-0.26036	noncoding
MSTRG.11763.3	0.403403	coding
MSTRG.11763.4	0.62111	coding
MSTRG.11764.1	-1.71015	noncoding
MSTRG.11763.5	0.575412	coding
MSTRG.11765.1	-0.15168	noncoding
MSTRG.11763.7	0.97362	coding
MSTRG.11756.2	2.91797	coding
MSTRG.11756.6	2.67783	coding
MSTRG.11761.1	-1.59907	noncoding
MSTRG.11782.1	0.127015	coding
MSTRG.11783.1	1.68438	coding
MSTRG.11783.4	0.87105	coding
MSTRG.11783.3	1.46885	coding
MSTRG.11792.1	0.284429	coding
MSTRG.11792.3	0.826479	coding
MSTRG.11792.2	0.978463	coding
MSTRG.11792.4	2.16325	coding
MSTRG.11792.13	1.33944	coding
MSTRG.11793.1	-1.95863	noncoding
MSTRG.11794.1	-0.463169	noncoding
MSTRG.11785.1	1.78102	coding
MSTRG.11786.2	-0.891487	noncoding
MSTRG.11788.1	1.538	coding
MSTRG.11788.2	1.28537	coding
MSTRG.11788.4	1.18841	coding
MSTRG.11795.1	-1.89396	noncoding
MSTRG.11805.1	-2.46695	noncoding
MSTRG.11806.1	1.2163	coding
MSTRG.11807.1	-1.41231	noncoding
MSTRG.11810.2	0.441132	coding
MSTRG.11814.4	0.83372	coding
MSTRG.11800.1	-0.81398	noncoding
MSTRG.11802.3	0.141212	coding
MSTRG.11802.4	-0.222356	noncoding
MSTRG.11802.5	0.024165	coding
MSTRG.11820.3	1.4511	coding
MSTRG.11819.1	0.019563	coding
MSTRG.11834.2	0.819376	coding
MSTRG.11834.3	-0.589939	noncoding
MSTRG.11838.1	-1.01559	noncoding
MSTRG.11841.4	-1.34832	noncoding
MSTRG.11842.3	1.11773	coding
MSTRG.11842.6	0.254006	coding
MSTRG.11821.2	0.116496	coding
MSTRG.11824.2	-0.10009	noncoding
MSTRG.11824.3	0.131071	coding
MSTRG.11824.4	0.416429	coding
MSTRG.11824.5	0.449862	coding
MSTRG.11825.3	-0.317754	noncoding

MSTRG.11825.4	0.612211	coding
MSTRG.11827.1	-2.15603	noncoding
MSTRG.11827.3	-1.95992	noncoding
MSTRG.11827.2	-1.75331	noncoding
MSTRG.11828.1	-2.20738	noncoding
MSTRG.11847.2	1.4339	coding
MSTRG.11852.1	2.263	coding
MSTRG.11852.2	0.551971	coding
MSTRG.11852.3	0.748853	coding
MSTRG.11852.4	0.04449	coding
MSTRG.11852.5	0.247744	coding
MSTRG.11860.1	-0.89513	noncoding
MSTRG.11861.1	0.956823	coding
MSTRG.11854.1	-2.52424	noncoding
MSTRG.11867.3	0.940313	coding
MSTRG.11867.4	-0.084349	noncoding
MSTRG.11867.5	-0.534418	noncoding
MSTRG.11872.1	1.05476	coding
MSTRG.11873.2	0.52753	coding
MSTRG.11873.4	1.62961	coding
MSTRG.11873.3	0.902631	coding
MSTRG.11873.7	-0.055616	noncoding
MSTRG.11873.8	0.584692	coding
MSTRG.11873.6	2.1396	coding
MSTRG.11875.1	-1.77325	noncoding
MSTRG.11876.1	2.23737	coding
MSTRG.11876.3	2.93394	coding
MSTRG.11882.3	2.81856	coding
MSTRG.11882.4	2.85296	coding
MSTRG.11889.1	1.90861	coding
MSTRG.11889.2	2.2471	coding
MSTRG.11890.1	-0.427074	noncoding
MSTRG.11901.1	-0.939359	noncoding
MSTRG.11901.2	-0.768769	noncoding
MSTRG.11902.1	-0.0987	noncoding
MSTRG.11923.1	-1.43898	noncoding
MSTRG.11924.1	-0.540168	noncoding
MSTRG.11929.1	-2.18002	noncoding
MSTRG.11950.1	-2.56833	noncoding
MSTRG.11951.1	-0.694943	noncoding
MSTRG.11960.1	-2.24619	noncoding
MSTRG.11968.1	0.017362	coding
MSTRG.11969.1	-0.256413	noncoding
MSTRG.11970.1	0.01134	coding
MSTRG.11973.4	1.68474	coding
MSTRG.11973.3	1.61969	coding
MSTRG.11973.2	-0.313798	noncoding
MSTRG.11973.1	1.54962	coding
MSTRG.11974.1	0.854185	coding
MSTRG.11974.2	1.04358	coding
MSTRG.11974.3	1.20451	coding
MSTRG.11973.7	-2.07887	noncoding
MSTRG.11973.8	-1.5	noncoding
MSTRG.11973.9	0.241994	coding

MSTRG.11973.10	-1.94634	noncoding
MSTRG.11973.11	-1.94529	noncoding
MSTRG.11973.12	-2.09684	noncoding
MSTRG.11973.13	-1.46479	noncoding
MSTRG.11973.14	-1.10834	noncoding
MSTRG.11973.15	-1.20677	noncoding
MSTRG.11973.16	-1.57034	noncoding
MSTRG.11973.17	-1.83903	noncoding
MSTRG.11973.18	-1.68178	noncoding
MSTRG.11973.19	-2.0925	noncoding
MSTRG.11973.20	-1.89897	noncoding
MSTRG.11973.21	-1.70674	noncoding
MSTRG.11973.22	-1.93203	noncoding
MSTRG.11973.23	-1.88203	noncoding
MSTRG.11973.24	-1.82197	noncoding
MSTRG.11958.1	1.29034	coding
MSTRG.11958.2	1.15901	coding
MSTRG.11972.1	-0.814012	noncoding
MSTRG.11978.2	-0.035667	noncoding
MSTRG.11978.1	1.54309	coding
MSTRG.11979.1	-2.32698	noncoding
MSTRG.11978.4	1.62868	coding
MSTRG.11978.5	1.57599	coding
MSTRG.11985.2	1.84576	coding
MSTRG.11986.4	1.55977	coding
MSTRG.11987.1	-1.58605	noncoding
MSTRG.11995.1	2.31485	coding
MSTRG.11996.1	-0.530007	noncoding
MSTRG.11995.6	2.08097	coding
MSTRG.11995.5	1.86465	coding
MSTRG.11995.7	2.00352	coding
MSTRG.11995.8	2.31748	coding
MSTRG.11995.9	2.21243	coding
MSTRG.11991.1	-0.557283	noncoding
MSTRG.11994.2	0.810388	coding
MSTRG.11994.3	0.662544	coding
MSTRG.12000.1	2.66385	coding
MSTRG.12004.5	1.114	coding
MSTRG.12004.6	2.16129	coding
MSTRG.12004.7	1.70408	coding
MSTRG.12004.8	2.09613	coding
MSTRG.12004.9	1.89981	coding
MSTRG.12004.10	0.995269	coding
MSTRG.12005.1	0.518171	coding
MSTRG.12004.11	1.67006	coding
MSTRG.12006.4	-0.538272	noncoding
MSTRG.12007.1	-1.20071	noncoding
MSTRG.12008.1	-1.66761	noncoding
MSTRG.12009.1	-2.04626	noncoding
MSTRG.12019.2	0.34641	coding
MSTRG.12019.3	0.045737	coding
MSTRG.12027.2	1.17471	coding
MSTRG.12023.1	-0.388573	noncoding
MSTRG.12030.1	0.25946	coding

MSTRG.12030.3	0.270463	coding
MSTRG.12034.4	-0.424655	noncoding
MSTRG.12035.1	2.19661	coding
MSTRG.12039.1	-1.21667	noncoding
MSTRG.12040.1	0.030776	coding
MSTRG.12041.3	2.47857	coding
MSTRG.12042.1	0.47891	coding
MSTRG.12043.1	-0.155726	noncoding
MSTRG.12044.1	-0.629114	noncoding
MSTRG.12045.3	-0.031412	noncoding
MSTRG.12045.4	1.49855	coding
MSTRG.12047.3	0.675659	coding
MSTRG.12053.2	1.65201	coding
MSTRG.12053.3	1.06142	coding
MSTRG.12054.1	0.994646	coding
MSTRG.12057.4	-0.715777	noncoding
MSTRG.12055.5	0.664883	coding
MSTRG.12055.6	0.014542	coding
MSTRG.12055.7	-0.176606	noncoding
MSTRG.12055.8	0.456393	coding
MSTRG.12055.9	-0.536459	noncoding
MSTRG.12055.10	-0.323953	noncoding
MSTRG.12055.11	-1.03966	noncoding
MSTRG.12055.12	-0.58468	noncoding
MSTRG.12055.13	0.284513	coding
MSTRG.12055.14	-0.355895	noncoding
MSTRG.12059.1	0.243014	coding
MSTRG.12049.1	-1.37252	noncoding
MSTRG.12050.1	-1.88328	noncoding
MSTRG.12051.1	-2.36325	noncoding
MSTRG.12069.1	-1.47932	noncoding
MSTRG.12069.2	0.004981	coding
MSTRG.12069.5	-0.280216	noncoding
MSTRG.12070.1	-0.894587	noncoding
MSTRG.12069.7	-0.120061	noncoding
MSTRG.12072.3	1.80744	coding
MSTRG.12072.10	1.76748	coding
MSTRG.12072.9	1.70845	coding
MSTRG.12072.12	1.19165	coding
MSTRG.12080.1	1.75602	coding
MSTRG.12079.1	-2.41468	noncoding
MSTRG.12088.1	-0.825156	noncoding
MSTRG.12093.2	2.26779	coding
MSTRG.12095.3	1.03424	coding
MSTRG.12098.1	1.69436	coding
MSTRG.12099.2	1.3166	coding
MSTRG.12096.1	-1.73554	noncoding
MSTRG.12100.1	2.11713	coding
MSTRG.12100.2	2.02856	coding
MSTRG.12100.3	1.90644	coding
MSTRG.12102.1	-1.03606	noncoding
MSTRG.12108.4	2.23051	coding
MSTRG.12108.1	2.24126	coding
MSTRG.12108.9	2.26562	coding

MSTRG.12108.8	2.16603	coding
MSTRG.12108.12	-1.74837	noncoding
MSTRG.12104.3	1.79676	coding
MSTRG.12121.2	1.2821	coding
MSTRG.12121.5	1.12245	coding
MSTRG.12123.5	-0.044921	noncoding
MSTRG.12123.2	0.051	coding
MSTRG.12123.4	0.09536	coding
MSTRG.12123.1	0.390743	coding
MSTRG.12123.3	0.28911	coding
MSTRG.12123.6	-2.25615	noncoding
MSTRG.12124.1	-0.336635	noncoding
MSTRG.12124.2	0.026647	coding
MSTRG.12125.1	-0.107019	noncoding
MSTRG.12114.2	0.482905	coding
MSTRG.12130.3	0.809729	coding
MSTRG.12131.1	-2.14041	noncoding
MSTRG.12138.1	-0.925489	noncoding
MSTRG.12145.1	-1.48003	noncoding
MSTRG.12146.1	-1.48361	noncoding
MSTRG.12147.1	-1.84803	noncoding
MSTRG.12152.1	-1.6225	noncoding
MSTRG.12153.2	0.170762	coding
MSTRG.12157.3	1.00578	coding
MSTRG.12157.4	0.871809	coding
MSTRG.12157.2	0.276974	coding
MSTRG.12157.5	0.475439	coding
MSTRG.12159.2	1.12821	coding
MSTRG.12159.1	1.86706	coding
MSTRG.12159.3	0.724563	coding
MSTRG.12160.1	0.472711	coding
MSTRG.12161.1	-1.61142	noncoding
MSTRG.12168.3	1.32405	coding
MSTRG.12188.1	-1.8168	noncoding
MSTRG.12189.1	1.13753	coding
MSTRG.12189.3	2.07931	coding
MSTRG.12195.1	0.603817	coding
MSTRG.12196.1	0.281851	coding
MSTRG.12197.2	-0.036532	noncoding
MSTRG.12197.3	2.77276	coding
MSTRG.12197.4	2.42048	coding
MSTRG.12197.5	0.117312	coding
MSTRG.12198.1	0.483862	coding
MSTRG.12202.1	-2.02018	noncoding
MSTRG.12233.1	-1.32251	noncoding
MSTRG.12238.1	-0.819525	noncoding
MSTRG.12246.2	-0.465686	noncoding
MSTRG.12208.1	-2.50346	noncoding
MSTRG.12214.5	0.100725	coding
MSTRG.12214.6	-1.09203	noncoding
MSTRG.12214.8	0.104859	coding
MSTRG.12215.1	-1.12008	noncoding
MSTRG.12207.1	-0.619596	noncoding
MSTRG.12218.5	1.83711	coding

MSTRG.12218.6	1.61783	coding
MSTRG.12224.1	-0.38787	noncoding
MSTRG.12228.1	-0.152161	noncoding
MSTRG.12274.1	-2.57281	noncoding
MSTRG.12277.1	1.34093	coding
MSTRG.12278.2	-1.76602	noncoding
MSTRG.12279.1	-0.314604	noncoding
MSTRG.12280.1	0.072568	coding
MSTRG.12280.2	-1.32751	noncoding
MSTRG.12280.3	-1.0081	noncoding
MSTRG.12281.1	-0.364617	noncoding
MSTRG.12281.2	-0.995367	noncoding
MSTRG.12285.2	2.13202	coding
MSTRG.12288.2	0.784252	coding
MSTRG.12288.3	1.06652	coding
MSTRG.12302.2	1.98174	coding
MSTRG.12303.1	-1.49704	noncoding
MSTRG.12302.3	2.0339	coding
MSTRG.12302.4	1.12714	coding
MSTRG.12302.5	1.26455	coding
MSTRG.12320.2	3.63143	coding
MSTRG.12320.4	3.83724	coding
MSTRG.12320.5	2.12904	coding
MSTRG.12320.7	2.8543	coding
MSTRG.12322.1	3.01986	coding
MSTRG.12323.1	-0.100981	noncoding
MSTRG.12322.3	-0.666188	noncoding
MSTRG.12347.5	2.85769	coding
MSTRG.12347.6	3.10657	coding
MSTRG.12347.7	1.59628	coding
MSTRG.12347.8	1.56419	coding
MSTRG.12351.1	-0.58807	noncoding
MSTRG.12352.1	-1.92225	noncoding
MSTRG.12340.2	0.35565	coding
MSTRG.12340.4	0.825573	coding
MSTRG.12340.3	1.02443	coding
MSTRG.12341.1	0.004138	coding
MSTRG.12342.1	-0.476239	noncoding
MSTRG.12353.1	-1.48284	noncoding
MSTRG.12356.1	-1.72739	noncoding
MSTRG.12355.2	1.95023	coding
MSTRG.12360.3	3.37066	coding
MSTRG.12359.1	-2.27835	noncoding
MSTRG.12416.1	0.292854	coding
MSTRG.12425.1	-1.13775	noncoding
MSTRG.12426.2	0.511977	coding
MSTRG.12433.1	-1.64664	noncoding
MSTRG.12434.4	2.57503	coding
MSTRG.12434.5	2.40022	coding
MSTRG.12434.7	2.51971	coding
MSTRG.12434.8	2.74053	coding
MSTRG.12435.2	2.23606	coding
MSTRG.12439.2	2.23085	coding
MSTRG.12440.1	0.967029	coding

MSTRG.12441.1	0.559619	coding
MSTRG.12442.1	-0.970423	noncoding
MSTRG.12442.2	-0.826881	noncoding
MSTRG.12441.2	-0.376391	noncoding
MSTRG.12439.3	-0.779732	noncoding
MSTRG.12439.4	-0.255672	noncoding
MSTRG.12384.2	1.73507	coding
MSTRG.12384.1	1.79359	coding
MSTRG.12384.3	1.57867	coding
MSTRG.12389.1	-0.84801	noncoding
MSTRG.12390.3	0.413036	coding
MSTRG.12391.1	0.998413	coding
MSTRG.12391.2	0.17565	coding
MSTRG.12392.1	-1.91181	noncoding
MSTRG.12395.1	-1.44117	noncoding
MSTRG.12407.1	1.00185	coding
MSTRG.12400.2	0.989362	coding
MSTRG.12447.5	2.6082	coding
MSTRG.12448.1	-1.53519	noncoding
MSTRG.12449.1	0.925093	coding
MSTRG.12450.1	0.489575	coding
MSTRG.12444.1	-1.8081	noncoding
MSTRG.12445.2	-0.397876	noncoding
MSTRG.12474.3	-0.166697	noncoding
MSTRG.12474.5	0.351488	coding
MSTRG.12474.2	-0.229903	noncoding
MSTRG.12474.4	0.046421	coding
MSTRG.12474.1	-0.432674	noncoding
MSTRG.12474.7	0.200358	coding
MSTRG.12474.6	-0.315251	noncoding
MSTRG.12474.9	0.27825	coding
MSTRG.12474.8	-0.316489	noncoding
MSTRG.12475.1	1.92186	coding
MSTRG.12475.2	1.70059	coding
MSTRG.12475.3	1.8488	coding
MSTRG.12475.5	1.3898	coding
MSTRG.12476.1	1.2282	coding
MSTRG.12476.2	1.51121	coding
MSTRG.12476.3	1.26972	coding
MSTRG.12476.7	1.27768	coding
MSTRG.12476.8	1.24529	coding
MSTRG.12476.6	0.951637	coding
MSTRG.12476.9	0.781148	coding
MSTRG.12494.1	-0.017672	noncoding
MSTRG.12495.1	-0.385054	noncoding
MSTRG.12495.2	-0.019491	noncoding
MSTRG.12504.1	-2.17399	noncoding
MSTRG.12504.2	-1.12204	noncoding
MSTRG.12505.1	0.067196	coding
MSTRG.12506.1	-0.454122	noncoding
MSTRG.12507.1	-2.12276	noncoding
MSTRG.12505.2	-1.0486	noncoding
MSTRG.12501.1	1.42755	coding
MSTRG.12501.3	0.029018	coding

MSTRG.12509.1	0.77454	coding
MSTRG.12510.2	1.3902	coding
MSTRG.12510.3	1.29375	coding
MSTRG.12510.4	0.711183	coding
MSTRG.12517.3	1.58174	coding
MSTRG.12521.1	-1.4086	noncoding
MSTRG.12525.2	1.65036	coding
MSTRG.12525.1	1.36293	coding
MSTRG.12525.3	1.35212	coding
MSTRG.12528.3	1.42858	coding
MSTRG.12537.2	-0.05806	noncoding
MSTRG.12553.1	-0.813027	noncoding
MSTRG.12558.1	-1.22402	noncoding
MSTRG.12558.2	-2.06803	noncoding
MSTRG.12558.3	-1.55783	noncoding
MSTRG.12560.1	-0.324914	noncoding
MSTRG.12550.2	2.04751	coding
MSTRG.12550.3	2.11634	coding
MSTRG.12569.1	-2.16651	noncoding
MSTRG.12572.1	-1.82019	noncoding
MSTRG.12573.1	-2.24027	noncoding
MSTRG.12576.3	-0.28531	noncoding
MSTRG.12576.4	-0.278045	noncoding
MSTRG.12577.7	1.26799	coding
MSTRG.12577.5	1.66089	coding
MSTRG.12577.4	1.05292	coding
MSTRG.12578.4	1.82663	coding
MSTRG.12588.1	3.33315	coding
MSTRG.12588.2	3.32157	coding
MSTRG.12588.3	3.34329	coding
MSTRG.12588.5	2.92258	coding
MSTRG.12589.1	-0.78515	noncoding
MSTRG.12589.2	-1.33857	noncoding
MSTRG.12589.3	0.72548	coding
MSTRG.12589.4	-0.840601	noncoding
MSTRG.12591.1	-0.172656	noncoding
MSTRG.12595.2	-0.225293	noncoding
MSTRG.12596.3	1.01723	coding
MSTRG.12596.4	0.457041	coding
MSTRG.12596.5	0.52756	coding
MSTRG.12599.1	-0.573472	noncoding
MSTRG.12598.4	1.20513	coding
MSTRG.12600.1	-1.49923	noncoding
MSTRG.12598.5	1.12042	coding
MSTRG.12598.6	-0.149511	noncoding
MSTRG.12598.8	1.44602	coding
MSTRG.12598.9	1.26634	coding
MSTRG.12601.1	-1.45829	noncoding
MSTRG.12602.1	-0.782664	noncoding
MSTRG.12602.3	-0.851907	noncoding
MSTRG.12602.2	-1.00556	noncoding
MSTRG.12602.4	-1.2748	noncoding
MSTRG.12603.1	0.674313	coding
MSTRG.12603.2	0.699255	coding

MSTRG.12603.3	0.539987	coding
MSTRG.12603.4	0.341585	coding
MSTRG.12607.2	1.10774	coding
MSTRG.12607.3	0.446156	coding
MSTRG.12611.5	2.51168	coding
MSTRG.12611.3	2.43568	coding
MSTRG.12611.7	2.49335	coding
MSTRG.12612.1	0.282206	coding
MSTRG.12612.2	0.314968	coding
MSTRG.12617.2	0.888961	coding
MSTRG.12617.3	0.904078	coding
MSTRG.12619.1	-0.365414	noncoding
MSTRG.12618.2	0.376228	coding
MSTRG.12618.3	0.118836	coding
MSTRG.12619.2	-0.112687	noncoding
MSTRG.12619.3	-0.189926	noncoding
MSTRG.12642.1	0.290468	coding
MSTRG.12643.1	0.467251	coding
MSTRG.12646.3	0.052299	coding
MSTRG.12647.1	-0.414586	noncoding
MSTRG.12642.2	0.497976	coding
MSTRG.12648.1	-2.10361	noncoding
MSTRG.12646.5	1.97066	coding
MSTRG.12646.4	1.18913	coding
MSTRG.12646.6	-0.089417	noncoding
MSTRG.12652.3	1.48637	coding
MSTRG.12652.4	-1.20143	noncoding
MSTRG.12623.3	1.7573	coding
MSTRG.12623.4	1.51764	coding
MSTRG.12623.6	0.529059	coding
MSTRG.12623.5	0.603587	coding
MSTRG.12625.2	0.860019	coding
MSTRG.12625.3	1.2938	coding
MSTRG.12627.1	-0.0476	noncoding
MSTRG.12627.2	0.040774	coding
MSTRG.12633.2	2.6277	coding
MSTRG.12635.1	2.48456	coding
MSTRG.12655.1	-0.737566	noncoding
MSTRG.12660.1	-0.087352	noncoding
MSTRG.12661.2	0.287286	coding
MSTRG.12664.1	0.702434	coding
MSTRG.12674.3	0.453166	coding
MSTRG.12676.1	1.44068	coding
MSTRG.12677.2	1.2621	coding
MSTRG.12680.1	-1.89125	noncoding
MSTRG.12681.2	1.96385	coding
MSTRG.12682.1	0.832324	coding
MSTRG.12685.1	1.09778	coding
MSTRG.12685.2	1.52547	coding
MSTRG.12686.1	-1.81228	noncoding
MSTRG.12683.2	2.56893	coding
MSTRG.12709.4	-0.07386	noncoding
MSTRG.12711.1	0.418033	coding
MSTRG.12711.2	0.40742	coding

MSTRG.12711.3	0.565153	coding
MSTRG.12709.6	0.559522	coding
MSTRG.12713.1	-0.347082	noncoding
MSTRG.12715.1	0.49267	coding
MSTRG.12701.1	-2.49281	noncoding
MSTRG.12716.1	3.16725	coding
MSTRG.12716.2	3.10058	coding
MSTRG.12716.3	1.02343	coding
MSTRG.12717.1	0.314732	coding
MSTRG.12716.4	0.837974	coding
MSTRG.12718.1	0.457517	coding
MSTRG.12716.5	0.237639	coding
MSTRG.12705.2	-1.95434	noncoding
MSTRG.12705.4	0.154558	coding
MSTRG.12705.3	3.12958	coding
MSTRG.12705.5	2.45567	coding
MSTRG.12730.2	0.182311	coding
MSTRG.12731.3	1.27158	coding
MSTRG.12731.2	1.94387	coding
MSTRG.12731.4	0.608967	coding
MSTRG.12731.6	1.77081	coding
MSTRG.12731.7	1.72328	coding
MSTRG.12731.8	1.93315	coding
MSTRG.12720.2	1.57306	coding
MSTRG.12721.3	0.346684	coding
MSTRG.12722.1	-2.45365	noncoding
MSTRG.12725.1	-2.54069	noncoding
MSTRG.12725.2	-2.60012	noncoding
MSTRG.12738.3	0.303429	coding
MSTRG.12746.1	0.606335	coding
MSTRG.12759.1	-2.61337	noncoding
MSTRG.12768.1	-0.868587	noncoding
MSTRG.12769.1	-2.13713	noncoding
MSTRG.12791.1	2.08945	coding
MSTRG.12791.2	1.48561	coding
MSTRG.12792.1	2.12183	coding
MSTRG.12792.2	2.14241	coding
MSTRG.12792.3	2.04469	coding
MSTRG.12792.6	0.542217	coding
MSTRG.12793.2	-0.26237	noncoding
MSTRG.12794.1	-0.403471	noncoding
MSTRG.12794.2	-1.18937	noncoding
MSTRG.12777.1	2.28365	coding
MSTRG.12778.1	2.23854	coding
MSTRG.12784.1	-2.44226	noncoding
MSTRG.12785.2	1.36185	coding
MSTRG.12785.3	1.31962	coding
MSTRG.12785.4	1.48688	coding
MSTRG.12789.3	2.40784	coding
MSTRG.12800.1	0.103747	coding
MSTRG.12800.2	-0.238928	noncoding
MSTRG.12800.3	-0.173402	noncoding
MSTRG.12804.2	1.25017	coding
MSTRG.12805.3	0.701589	coding

MSTRG.12806.2	1.71785	coding
MSTRG.12806.1	1.71423	coding
MSTRG.12806.4	2.33299	coding
MSTRG.12806.5	1.41528	coding
MSTRG.12808.2	1.4014	coding
MSTRG.12810.1	0.095834	coding
MSTRG.12813.1	0.372467	coding
MSTRG.12816.1	-0.478852	noncoding
MSTRG.12829.1	2.03464	coding
MSTRG.12829.6	2.34104	coding
MSTRG.12829.4	1.55947	coding
MSTRG.12829.7	2.63588	coding
MSTRG.12830.4	2.72532	coding
MSTRG.12831.1	-1.47175	noncoding
MSTRG.12835.1	-1.71835	noncoding
MSTRG.12826.1	-1.12915	noncoding
MSTRG.12826.2	-0.081771	noncoding
MSTRG.12827.1	-1.98911	noncoding
MSTRG.12839.2	0.529748	coding
MSTRG.12847.3	2.34865	coding
MSTRG.12847.4	2.35498	coding
MSTRG.12848.1	-1.0502	noncoding
MSTRG.12851.2	1.32989	coding
MSTRG.12851.3	0.829318	coding
MSTRG.12863.6	2.13119	coding
MSTRG.12863.7	2.09332	coding
MSTRG.12863.14	1.97929	coding
MSTRG.12863.16	2.04324	coding
MSTRG.12863.11	2.18021	coding
MSTRG.12863.13	2.06165	coding
MSTRG.12863.15	2.18605	coding
MSTRG.12863.12	2.42576	coding
MSTRG.12863.18	2.07973	coding
MSTRG.12863.17	1.95414	coding
MSTRG.12863.19	-0.292743	noncoding
MSTRG.12864.1	0.541629	coding
MSTRG.12863.20	-1.866	noncoding
MSTRG.12888.1	0.022044	coding
MSTRG.12890.2	0.924593	coding
MSTRG.12890.4	0.710816	coding
MSTRG.12907.3	2.69938	coding
MSTRG.12907.2	2.16908	coding
MSTRG.12907.4	1.26636	coding
MSTRG.12907.5	-0.755917	noncoding
MSTRG.12907.6	1.35739	coding
MSTRG.12912.3	2.88236	coding
MSTRG.12912.4	3.02694	coding
MSTRG.12914.1	-1.00407	noncoding
MSTRG.12933.4	1.78672	coding
MSTRG.12933.6	1.57017	coding
MSTRG.12933.5	1.16899	coding
MSTRG.12933.3	1.31951	coding
MSTRG.12933.7	1.43462	coding
MSTRG.12933.8	2.06774	coding

MSTRG.12934.1	-0.522127	noncoding
MSTRG.12919.1	-0.585335	noncoding
MSTRG.12920.1	0.3654	coding
MSTRG.12920.2	0.494972	coding
MSTRG.12925.1	-2.41943	noncoding
MSTRG.12925.2	-2.07676	noncoding
MSTRG.12926.1	-2.08975	noncoding
MSTRG.12938.1	-1.58166	noncoding
MSTRG.12947.3	1.48851	coding
MSTRG.12947.2	1.59811	coding
MSTRG.12971.1	0.976636	coding
MSTRG.12978.1	-1.61982	noncoding
MSTRG.12978.2	-0.172705	noncoding
MSTRG.12978.3	0.116221	coding
MSTRG.12983.5	1.96119	coding
MSTRG.12984.1	-1.82639	noncoding
MSTRG.12939.1	3.00978	coding
MSTRG.12939.2	1.55436	coding
MSTRG.12939.3	1.7772	coding
MSTRG.12940.1	-0.391377	noncoding
MSTRG.12939.4	2.88559	coding
MSTRG.12939.5	0.914726	coding
MSTRG.12988.1	0.216093	coding
MSTRG.13003.1	1.42938	coding
MSTRG.13008.1	-0.513418	noncoding
MSTRG.13008.2	-0.335648	noncoding
MSTRG.13009.1	0.073105	coding
MSTRG.13010.1	-2.17693	noncoding
MSTRG.13012.1	0.498827	coding
MSTRG.13013.1	-1.00229	noncoding
MSTRG.13013.2	0.900322	coding
MSTRG.13013.6	-0.571279	noncoding
MSTRG.13013.4	0.783354	coding
MSTRG.13013.5	0.725962	coding
MSTRG.13014.1	-1.00401	noncoding
MSTRG.13022.1	0.056237	coding
MSTRG.13022.2	0.461399	coding
MSTRG.13025.2	0.020736	coding
MSTRG.13025.4	0.549216	coding
MSTRG.13025.5	0.226978	coding
MSTRG.13025.7	-0.138035	noncoding
MSTRG.13025.8	-0.005309	noncoding
MSTRG.13025.9	0.780544	coding
MSTRG.13025.10	0.280547	coding
MSTRG.13025.11	0.624651	coding
MSTRG.13025.12	0.09214	coding
MSTRG.13030.3	2.55701	coding
MSTRG.13030.1	3.00561	coding
MSTRG.13034.1	-1.89353	noncoding
MSTRG.13034.2	-1.57434	noncoding
MSTRG.13034.3	-1.19212	noncoding
MSTRG.13040.4	2.74121	coding
MSTRG.13040.6	2.52157	coding
MSTRG.13039.1	-2.02151	noncoding

MSTRG.13048.1	2.5491	coding
MSTRG.13049.1	-2.26781	noncoding
MSTRG.13055.1	-0.455234	noncoding
MSTRG.13053.1	-2.54877	noncoding
MSTRG.13056.2	0.516322	coding
MSTRG.13061.1	1.41573	coding
MSTRG.13061.3	1.0874	coding
MSTRG.13062.1	-1.20713	noncoding
MSTRG.13061.5	0.615492	coding
MSTRG.13060.1	0.722573	coding
MSTRG.13063.1	-0.311999	noncoding
MSTRG.13065.2	0.974429	coding
MSTRG.13066.3	1.28974	coding
MSTRG.13068.1	-2.34685	noncoding
MSTRG.13077.4	1.4481	coding
MSTRG.13078.1	0.560036	coding
MSTRG.13079.2	0.593573	coding
MSTRG.13082.3	-0.604242	noncoding
MSTRG.13082.4	-0.909128	noncoding
MSTRG.13082.2	0.014768	coding
MSTRG.13087.1	1.84023	coding
MSTRG.13087.2	1.84193	coding
MSTRG.13087.4	1.58446	coding
MSTRG.13087.6	1.67743	coding
MSTRG.13091.2	1.46489	coding
MSTRG.13096.2	1.14083	coding
MSTRG.13106.2	2.59567	coding
MSTRG.13106.1	2.59627	coding
MSTRG.13106.3	2.59887	coding
MSTRG.13107.1	-1.94279	noncoding
MSTRG.13100.4	1.70971	coding
MSTRG.13110.4	2.28055	coding
MSTRG.13110.3	1.12646	coding
MSTRG.13110.5	1.38303	coding
MSTRG.13110.6	1.76974	coding
MSTRG.13110.7	1.041	coding
MSTRG.13111.1	-1.08114	noncoding
MSTRG.13112.1	-0.73546	noncoding
MSTRG.13115.1	1.60818	coding
MSTRG.13117.1	-2.04258	noncoding
MSTRG.13115.2	1.76783	coding
MSTRG.13118.2	0.134462	coding
MSTRG.13119.1	0.024114	coding
MSTRG.13120.1	-2.01443	noncoding
MSTRG.13118.4	-0.381365	noncoding
MSTRG.13118.5	-0.008911	noncoding
MSTRG.13143.4	3.28995	coding
MSTRG.13143.6	0.954713	coding
MSTRG.13143.5	2.00664	coding
MSTRG.13143.7	2.92208	coding
MSTRG.13144.1	0.692037	coding
MSTRG.13143.8	2.01397	coding
MSTRG.13143.9	1.86837	coding
MSTRG.13145.1	-2.14847	noncoding

MSTRG.13146.1	1.21688	coding
MSTRG.13143.10	-0.722436	noncoding
MSTRG.13148.1	0.037761	coding
MSTRG.13149.1	0.330767	coding
MSTRG.13150.1	-0.99526	noncoding
MSTRG.13151.1	-0.5082	noncoding
MSTRG.13152.1	-0.216107	noncoding
MSTRG.13152.2	0.252319	coding
MSTRG.13143.11	-0.077347	noncoding
MSTRG.13143.12	2.28017	coding
MSTRG.13130.1	-1.38866	noncoding
MSTRG.13134.1	-0.231815	noncoding
MSTRG.13137.1	-0.951021	noncoding
MSTRG.13138.3	0.756763	coding
MSTRG.13138.5	-0.140766	noncoding
MSTRG.13138.6	-1.17314	noncoding
MSTRG.13139.1	-0.251638	noncoding
MSTRG.13139.2	-0.91419	noncoding
MSTRG.13139.3	0.396291	coding
MSTRG.13139.4	0.699081	coding
MSTRG.13139.5	0.655295	coding
MSTRG.13128.2	0.687429	coding
MSTRG.13128.3	1.03138	coding
MSTRG.13128.4	1.1168	coding
MSTRG.13154.1	-0.667285	noncoding
MSTRG.13154.2	-1.44807	noncoding
MSTRG.13154.3	-1.18088	noncoding
MSTRG.13154.4	-1.3949	noncoding
MSTRG.13155.2	1.63172	coding
MSTRG.13162.1	-1.52728	noncoding
MSTRG.13163.1	1.20086	coding
MSTRG.13164.1	0.587417	coding
MSTRG.13161.6	3.06476	coding
MSTRG.13161.4	3.01894	coding
MSTRG.13161.7	3.11039	coding
MSTRG.13161.5	2.15194	coding
MSTRG.13165.1	-1.34069	noncoding
MSTRG.13159.2	1.71287	coding
MSTRG.13159.3	1.91095	coding
MSTRG.13159.4	0.588816	coding
MSTRG.13166.1	1.06834	coding
MSTRG.13170.3	-1.42569	noncoding
MSTRG.13171.1	1.83535	coding
MSTRG.13177.1	-0.962759	noncoding
MSTRG.13177.4	-0.040194	noncoding
MSTRG.13177.5	-1.37291	noncoding
MSTRG.13174.1	2.32947	coding
MSTRG.13181.3	2.37392	coding
MSTRG.13181.4	2.32202	coding
MSTRG.13181.5	2.25222	coding
MSTRG.13179.1	-0.421152	noncoding
MSTRG.13179.3	-0.415876	noncoding
MSTRG.13179.2	0.16693	coding
MSTRG.13180.1	-0.2431	noncoding

MSTRG.13184.1	0.910948	coding
MSTRG.13184.2	1.42532	coding
MSTRG.13199.1	0.167143	coding
MSTRG.13199.2	-0.010832	noncoding
MSTRG.13201.1	-0.96721	noncoding
MSTRG.13183.1	-2.38764	noncoding
MSTRG.13206.2	-1.40428	noncoding
MSTRG.13208.1	1.67706	coding
MSTRG.13208.4	2.12928	coding
MSTRG.13209.1	0.158276	coding
MSTRG.13209.2	1.21444	coding
MSTRG.13203.1	-1.02991	noncoding
MSTRG.13217.1	-2.15266	noncoding
MSTRG.13221.2	-0.490852	noncoding
MSTRG.13222.1	2.00466	coding
MSTRG.13222.2	1.79028	coding
MSTRG.13222.3	1.80007	coding
MSTRG.13222.6	1.37404	coding
MSTRG.13223.1	-0.151363	noncoding
MSTRG.13224.1	0.183544	coding
MSTRG.13222.7	-0.749058	noncoding
MSTRG.13233.1	2.45622	coding
MSTRG.13237.1	0.12056	coding
MSTRG.13241.3	2.29764	coding
MSTRG.13241.4	1.22829	coding
MSTRG.13241.5	1.23136	coding
MSTRG.13241.6	1.36844	coding
MSTRG.13243.1	1.27528	coding
MSTRG.13243.2	0.301428	coding
MSTRG.13243.3	0.122352	coding
MSTRG.13244.3	0.993954	coding
MSTRG.13261.4	2.23731	coding
MSTRG.13261.5	2.22773	coding
MSTRG.13261.6	2.24379	coding
MSTRG.13261.7	2.23713	coding
MSTRG.13261.1	2.11142	coding
MSTRG.13261.8	2.02946	coding
MSTRG.13261.9	1.96507	coding
MSTRG.13261.10	1.93057	coding
MSTRG.13261.11	2.07857	coding
MSTRG.13261.12	2.04243	coding
MSTRG.13261.13	1.96401	coding
MSTRG.13261.14	1.86072	coding
MSTRG.13263.1	-0.766721	noncoding
MSTRG.13266.1	-1.52711	noncoding
MSTRG.13267.1	-0.348342	noncoding
MSTRG.13267.2	-0.452917	noncoding
MSTRG.13267.3	-1.60555	noncoding
MSTRG.13267.4	-1.30417	noncoding
MSTRG.13254.2	-1.56468	noncoding
MSTRG.13254.1	-2.18078	noncoding
MSTRG.13253.3	0.535965	coding
MSTRG.13253.4	0.704502	coding
MSTRG.13259.2	0.85495	coding

MSTRG.13260.1	-0.551164	noncoding
MSTRG.13268.1	-0.703673	noncoding
MSTRG.13268.2	0.606839	coding
MSTRG.13268.3	-1.15081	noncoding
MSTRG.13268.4	0.291183	coding
MSTRG.13268.5	-0.352125	noncoding
MSTRG.13269.1	-0.632614	noncoding
MSTRG.13268.8	-0.349318	noncoding
MSTRG.13270.1	-0.315675	noncoding
MSTRG.13273.4	0.997174	coding
MSTRG.13281.1	0.435851	coding
MSTRG.13281.2	-2.49618	noncoding
MSTRG.13282.2	-2.2718	noncoding
MSTRG.13284.8	0.745565	coding
MSTRG.13284.6	1.13375	coding
MSTRG.13284.9	0.243154	coding
MSTRG.13284.10	-0.912384	noncoding
MSTRG.13277.1	-0.865292	noncoding
MSTRG.13277.2	-0.569134	noncoding
MSTRG.13286.3	-1.60818	noncoding
MSTRG.13287.1	-1.34884	noncoding
MSTRG.13303.1	-0.821123	noncoding
MSTRG.13305.1	-2.12333	noncoding
MSTRG.13314.1	0.890907	coding
MSTRG.13314.3	1.11981	coding
MSTRG.13314.2	1.34888	coding
MSTRG.13323.1	-1.38231	noncoding
MSTRG.13323.2	-1.80375	noncoding
MSTRG.13320.3	1.70257	coding
MSTRG.13320.4	0.167447	coding
MSTRG.13320.5	1.84532	coding
MSTRG.13320.6	0.601205	coding
MSTRG.13320.7	0.846721	coding
MSTRG.13320.9	-0.818745	noncoding
MSTRG.13324.1	-0.02483	noncoding
MSTRG.13325.1	-0.562241	noncoding
MSTRG.13327.1	0.470108	coding
MSTRG.13329.1	0.058297	coding
MSTRG.13331.1	-0.521367	noncoding
MSTRG.13332.1	-2.49729	noncoding
MSTRG.13332.2	-2.56778	noncoding
MSTRG.13332.3	-2.35278	noncoding
MSTRG.13334.1	-1.24128	noncoding
MSTRG.13334.4	0.432019	coding
MSTRG.13318.1	0.716008	coding
MSTRG.13345.1	-0.145537	noncoding
MSTRG.13356.1	0.97439	coding
MSTRG.13357.1	-0.24593	noncoding
MSTRG.13356.3	1.85697	coding
MSTRG.13356.4	1.24838	coding
MSTRG.13356.5	0.74655	coding
MSTRG.13364.2	1.65384	coding
MSTRG.13365.1	-2.43354	noncoding
MSTRG.13366.3	-0.020122	noncoding

MSTRG.13367.1	-1.89179	noncoding
MSTRG.13367.2	-1.97109	noncoding
MSTRG.13369.2	-0.142629	noncoding
MSTRG.13369.4	0.24194	coding
MSTRG.13369.3	0.359245	coding
MSTRG.13378.1	-0.635698	noncoding
MSTRG.13378.2	-0.457427	noncoding
MSTRG.13381.2	-0.794153	noncoding
MSTRG.13389.1	2.51634	coding
MSTRG.13391.6	1.39602	coding
MSTRG.13391.3	0.63515	coding
MSTRG.13396.1	-1.33072	noncoding
MSTRG.13397.1	0.368945	coding
MSTRG.13398.1	-0.002611	noncoding
MSTRG.13401.1	-0.79706	noncoding
MSTRG.13409.2	2.92744	coding
MSTRG.13410.1	-1.74059	noncoding
MSTRG.13410.2	-1.71717	noncoding
MSTRG.13409.3	2.74187	coding
MSTRG.13409.4	2.84059	coding
MSTRG.13409.5	3.19953	coding
MSTRG.13409.8	2.56458	coding
MSTRG.13409.6	2.67717	coding
MSTRG.13409.7	2.72338	coding
MSTRG.13409.9	2.51265	coding
MSTRG.13411.1	-0.158229	noncoding
MSTRG.13411.4	2.6643	coding
MSTRG.13423.1	-1.97244	noncoding
MSTRG.13425.1	0.933564	coding
MSTRG.13427.1	0.810894	coding
MSTRG.13427.2	1.90117	coding
MSTRG.13427.4	1.99253	coding
MSTRG.13427.3	1.50702	coding
MSTRG.13427.6	1.64642	coding
MSTRG.13427.8	1.72188	coding
MSTRG.13427.9	0.088388	coding
MSTRG.13427.10	0.413059	coding
MSTRG.13427.11	-1.32323	noncoding
MSTRG.13428.1	-0.296902	noncoding
MSTRG.13428.2	-0.072951	noncoding
MSTRG.13429.1	1.18349	coding
MSTRG.13430.1	-0.837632	noncoding
MSTRG.13431.1	0.607288	coding
MSTRG.13431.2	-0.754701	noncoding
MSTRG.13432.1	0.697134	coding
MSTRG.13413.2	0.33803	coding
MSTRG.13414.1	0.198486	coding
MSTRG.13418.1	0.197134	coding
MSTRG.13420.1	1.70613	coding
MSTRG.13421.2	1.09998	coding
MSTRG.13419.1	-1.43602	noncoding
MSTRG.13419.2	-1.33272	noncoding
MSTRG.13419.3	-1.45538	noncoding
MSTRG.13433.1	-1.02453	noncoding

MSTRG.13437.2	-0.091915	noncoding
MSTRG.13440.1	0.037099	coding
MSTRG.13449.1	-0.886386	noncoding
MSTRG.13450.3	2.98693	coding
MSTRG.13450.2	2.89578	coding
MSTRG.13451.1	-0.289867	noncoding
MSTRG.13454.2	0.845609	coding
MSTRG.13456.1	0.241379	coding
MSTRG.13456.2	-0.243467	noncoding
MSTRG.13457.1	-0.586893	noncoding
MSTRG.13459.1	-2.23534	noncoding
MSTRG.13461.6	1.17396	coding
MSTRG.13461.5	2.71664	coding
MSTRG.13462.1	-1.46638	noncoding
MSTRG.13481.1	-1.98993	noncoding
MSTRG.13486.2	0.129124	coding
MSTRG.13492.1	-0.679473	noncoding
MSTRG.13506.1	-0.821812	noncoding
MSTRG.13506.2	-0.285129	noncoding
MSTRG.13506.3	-0.842388	noncoding
MSTRG.13506.4	-0.97837	noncoding
MSTRG.13510.1	1.47393	coding
MSTRG.13510.2	1.80169	coding
MSTRG.13510.3	1.94153	coding
MSTRG.13510.4	2.15021	coding
MSTRG.13510.5	2.08049	coding
MSTRG.13510.6	2.0594	coding
MSTRG.13497.2	1.78931	coding
MSTRG.13504.1	-1.90489	noncoding
MSTRG.13512.4	1.39709	coding
MSTRG.13512.6	1.1512	coding
MSTRG.13512.3	1.91213	coding
MSTRG.13512.7	1.56185	coding
MSTRG.13512.8	1.72798	coding
MSTRG.13512.9	1.73675	coding
MSTRG.13518.1	1.40888	coding
MSTRG.13518.3	0.40507	coding
MSTRG.13519.1	1.33252	coding
MSTRG.13520.4	1.41954	coding
MSTRG.13531.1	-0.550872	noncoding
MSTRG.13532.1	1.95838	coding
MSTRG.13532.3	-0.992363	noncoding
MSTRG.13532.6	2.1883	coding
MSTRG.13538.2	1.40541	coding
MSTRG.13540.4	0.010211	coding
MSTRG.13543.1	0.541687	coding
MSTRG.13554.1	0.319337	coding
MSTRG.13555.1	-2.36444	noncoding
MSTRG.13560.2	1.27208	coding
MSTRG.13560.3	1.65391	coding
MSTRG.13562.1	-0.442443	noncoding
MSTRG.13564.1	1.36696	coding
MSTRG.13564.2	1.58567	coding
MSTRG.13563.1	-0.571993	noncoding

MSTRG.13563.2	-0.831573	noncoding
MSTRG.13566.3	0.965168	coding
MSTRG.13568.5	2.41216	coding
MSTRG.13568.6	1.90718	coding
MSTRG.13570.1	1.33115	coding
MSTRG.13578.1	2.1108	coding
MSTRG.13578.4	1.50868	coding
MSTRG.13578.2	2.19987	coding
MSTRG.13578.3	2.15339	coding
MSTRG.13578.6	2.16476	coding
MSTRG.13578.7	2.15796	coding
MSTRG.13578.8	2.15047	coding
MSTRG.13578.9	2.16679	coding
MSTRG.13578.10	1.84504	coding
MSTRG.13578.11	1.80231	coding
MSTRG.13579.1	-0.094996	noncoding
MSTRG.13580.1	-2.39704	noncoding
MSTRG.13580.2	-2.82322	noncoding
MSTRG.13578.12	2.35529	coding
MSTRG.13578.13	1.62587	coding
MSTRG.13578.14	1.62048	coding
MSTRG.13578.15	2.40306	coding
MSTRG.13578.16	0.571054	coding
MSTRG.13581.1	-2.08024	noncoding
MSTRG.13583.1	-0.124536	noncoding
MSTRG.13584.2	-0.68634	noncoding
MSTRG.13572.3	2.5192	coding
MSTRG.13573.4	1.36487	coding
MSTRG.13573.5	1.41456	coding
MSTRG.13573.3	1.55252	coding
MSTRG.13573.7	1.62008	coding
MSTRG.13573.1	1.58694	coding
MSTRG.13575.2	1.73738	coding
MSTRG.13576.4	1.046	coding
MSTRG.13576.3	1.7856	coding
MSTRG.13576.5	2.06389	coding
MSTRG.13576.6	1.9849	coding
MSTRG.13576.7	2.04328	coding
MSTRG.13576.8	1.86537	coding
MSTRG.13576.9	1.54085	coding
MSTRG.13577.2	-1.11371	noncoding
MSTRG.13577.1	-0.74135	noncoding
MSTRG.13577.5	-0.606227	noncoding
MSTRG.13577.9	-1.17034	noncoding
MSTRG.13577.10	-0.846115	noncoding
MSTRG.13577.11	-0.688982	noncoding
MSTRG.13577.6	-0.693925	noncoding
MSTRG.13577.7	-0.914408	noncoding
MSTRG.13577.8	-0.818341	noncoding
MSTRG.13577.12	-0.818755	noncoding
MSTRG.13589.3	0.795146	coding
MSTRG.13589.4	-1.20668	noncoding
MSTRG.13589.7	1.97813	coding
MSTRG.13589.6	1.85021	coding

MSTRG.13591.1	-1.06844	noncoding
MSTRG.13593.5	2.32686	coding
MSTRG.13593.6	2.1262	coding
MSTRG.13593.7	2.16353	coding
MSTRG.13594.1	-0.748474	noncoding
MSTRG.13593.8	1.61121	coding
MSTRG.13595.2	1.119	coding
MSTRG.13595.1	1.01684	coding
MSTRG.13596.1	-0.513728	noncoding
MSTRG.13597.1	-0.416976	noncoding
MSTRG.13586.1	2.3025	coding
MSTRG.13586.2	2.29649	coding
MSTRG.13586.3	2.38732	coding
MSTRG.13587.1	-1.01799	noncoding
MSTRG.13598.2	0.742097	coding
MSTRG.13599.1	1.33496	coding
MSTRG.13599.2	1.12061	coding
MSTRG.13599.3	0.77476	coding
MSTRG.13599.4	-0.057307	noncoding
MSTRG.13602.1	0.544797	coding
MSTRG.13602.3	0.846808	coding
MSTRG.13602.5	0.871529	coding
MSTRG.13600.1	0.250876	coding
MSTRG.13603.1	-1.64262	noncoding
MSTRG.13603.2	-0.602297	noncoding
MSTRG.13608.3	0.894361	coding
MSTRG.13608.2	1.39659	coding
MSTRG.13608.6	0.937091	coding
MSTRG.13608.5	0.977903	coding
MSTRG.13605.1	1.58529	coding
MSTRG.13612.2	-0.362461	noncoding
MSTRG.13614.2	1.31226	coding
MSTRG.13618.3	-0.216109	noncoding
MSTRG.13618.1	0.39695	coding
MSTRG.13618.2	0.454057	coding
MSTRG.13627.2	1.97336	coding
MSTRG.13627.3	2.09388	coding
MSTRG.13630.1	1.70072	coding
MSTRG.13637.2	1.38684	coding
MSTRG.13657.4	0.067452	coding
MSTRG.13658.8	1.75978	coding
MSTRG.13666.4	1.27181	coding
MSTRG.13666.3	1.10803	coding
MSTRG.13666.5	1.43394	coding
MSTRG.13666.2	1.34886	coding
MSTRG.13667.1	2.08806	coding
MSTRG.13667.5	1.89497	coding
MSTRG.13667.6	2.04809	coding
MSTRG.13667.13	1.52116	coding
MSTRG.13667.12	1.46305	coding
MSTRG.13652.1	1.1687	coding
MSTRG.13652.2	1.55951	coding
MSTRG.13652.3	-1.21052	noncoding
MSTRG.13653.2	1.13517	coding

MSTRG.13653.3	0.424548	coding
MSTRG.13669.2	1.61943	coding
MSTRG.13671.1	-0.11942	noncoding
MSTRG.13672.6	1.00918	coding
MSTRG.13672.5	1.08493	coding
MSTRG.13672.4	1.00647	coding
MSTRG.13682.1	-0.901034	noncoding
MSTRG.13684.1	-2.32488	noncoding
MSTRG.13687.2	-1.86619	noncoding
MSTRG.13690.1	0.50194	coding
MSTRG.13690.2	0.469771	coding
MSTRG.13690.5	0.478251	coding
MSTRG.13690.3	0.749333	coding
MSTRG.13690.4	0.622907	coding
MSTRG.13711.1	3.05784	coding
MSTRG.13711.2	2.70696	coding
MSTRG.13711.3	3.00719	coding
MSTRG.13711.4	2.87527	coding
MSTRG.13711.5	3.08397	coding
MSTRG.13711.6	2.81368	coding
MSTRG.13713.1	1.45148	coding
MSTRG.13713.3	1.35768	coding
MSTRG.13713.2	1.40766	coding
MSTRG.13706.1	2.58924	coding
MSTRG.13706.18	1.35111	coding
MSTRG.13706.20	1.71899	coding
MSTRG.13706.17	1.68565	coding
MSTRG.13709.1	0.171149	coding
MSTRG.13706.22	1.34208	coding
MSTRG.13727.1	0.24751	coding
MSTRG.13727.2	1.05551	coding
MSTRG.13727.3	1.16998	coding
MSTRG.13727.4	1.12424	coding
MSTRG.13725.4	1.38426	coding
MSTRG.13725.6	1.428	coding
MSTRG.13725.7	1.56565	coding
MSTRG.13725.10	-0.33817	noncoding
MSTRG.13725.12	1.39348	coding
MSTRG.13725.13	1.46058	coding
MSTRG.13729.1	-1.34927	noncoding
MSTRG.13714.3	1.02452	coding
MSTRG.13717.1	-1.73565	noncoding
MSTRG.13723.1	-0.190462	noncoding
MSTRG.13738.1	1.53637	coding
MSTRG.13746.1	-1.85609	noncoding
MSTRG.13746.2	-2.19796	noncoding
MSTRG.13755.2	-1.58792	noncoding
MSTRG.13755.1	2.56965	coding
MSTRG.13755.3	2.53986	coding
MSTRG.13755.5	2.11986	coding
MSTRG.13755.6	2.52535	coding
MSTRG.13756.1	0.193381	coding
MSTRG.13758.2	1.27366	coding
MSTRG.13758.3	0.587608	coding

MSTRG.13758.4	-0.726941	noncoding
MSTRG.13750.1	-2.77959	noncoding
MSTRG.13760.1	0.348282	coding
MSTRG.13760.2	-0.284054	noncoding
MSTRG.13759.2	0.581957	coding
MSTRG.13761.2	3.32114	coding
MSTRG.13767.1	-0.278601	noncoding
MSTRG.13768.4	1.09644	coding
MSTRG.13768.3	1.07864	coding
MSTRG.13768.2	1.05432	coding
MSTRG.13768.5	0.71906	coding
MSTRG.13769.1	0.174468	coding
MSTRG.13768.7	0.771217	coding
MSTRG.13765.1	0.572552	coding
MSTRG.13765.2	0.660637	coding
MSTRG.13764.1	1.8487	coding
MSTRG.13780.2	-1.29024	noncoding
MSTRG.13782.1	-0.474239	noncoding
MSTRG.13780.3	-0.379432	noncoding
MSTRG.13783.1	-0.433181	noncoding
MSTRG.13784.1	-0.238498	noncoding
MSTRG.13808.1	0.644715	coding
MSTRG.13808.3	0.591319	coding
MSTRG.13808.4	0.659265	coding
MSTRG.13808.2	-0.427222	noncoding
MSTRG.13808.5	0.815437	coding
MSTRG.13809.1	0.387918	coding
MSTRG.13811.1	-0.580505	noncoding
MSTRG.13819.3	-0.716007	noncoding
MSTRG.13820.1	-2.34096	noncoding
MSTRG.13813.1	-3.05068	noncoding
MSTRG.13822.1	-1.68299	noncoding
MSTRG.13829.1	-2.12851	noncoding
MSTRG.13831.2	0.740349	coding
MSTRG.13831.3	0.964342	coding
MSTRG.13832.1	-1.33807	noncoding
MSTRG.13831.4	1.81747	coding
MSTRG.13836.1	2.52309	coding
MSTRG.13837.1	-0.689885	noncoding
MSTRG.13838.5	2.76902	coding
MSTRG.13838.6	1.87842	coding
MSTRG.13834.2	1.34717	coding
MSTRG.13844.2	1.15262	coding
MSTRG.13844.4	1.76957	coding
MSTRG.13845.1	-0.04031	noncoding
MSTRG.13846.1	1.53628	coding
MSTRG.13846.2	0.949349	coding
MSTRG.13854.3	1.80606	coding
MSTRG.13849.2	1.0266	coding
MSTRG.13858.2	2.11241	coding
MSTRG.13859.1	-2.08085	noncoding
MSTRG.13862.3	-1.08133	noncoding
MSTRG.13862.2	0.905146	coding
MSTRG.13862.4	1.812	coding

MSTRG.13862.5	0.15159	coding
MSTRG.13867.1	0.882532	coding
MSTRG.13870.4	1.64847	coding
MSTRG.13871.3	1.79362	coding
MSTRG.13871.4	1.32196	coding
MSTRG.13877.3	1.51682	coding
MSTRG.13877.4	1.55	coding
MSTRG.13877.5	1.55968	coding
MSTRG.13884.4	2.11741	coding
MSTRG.13884.6	1.79232	coding
MSTRG.13884.5	1.75168	coding
MSTRG.13884.7	1.77441	coding
MSTRG.13884.8	1.95731	coding
MSTRG.13885.2	0.682648	coding
MSTRG.13885.3	0.78118	coding
MSTRG.13882.1	1.10735	coding
MSTRG.13881.1	0.361358	coding
MSTRG.13886.2	0.522967	coding
MSTRG.13886.5	0.887259	coding
MSTRG.13888.1	-0.433336	noncoding
MSTRG.13900.1	-1.84092	noncoding
MSTRG.13902.1	0.288244	coding
MSTRG.13903.3	1.17592	coding
MSTRG.13904.1	-2.23563	noncoding
MSTRG.13908.1	0.991782	coding
MSTRG.13908.2	0.890562	coding
MSTRG.13910.2	1.91115	coding
MSTRG.13910.5	1.17666	coding
MSTRG.13910.6	1.21482	coding
MSTRG.13913.3	1.56032	coding
MSTRG.13913.4	-0.092586	noncoding
MSTRG.13891.1	-0.975451	noncoding
MSTRG.13899.1	-0.409074	noncoding
MSTRG.13918.2	0.208967	coding
MSTRG.13918.3	-0.471089	noncoding
MSTRG.13919.2	1.78965	coding
MSTRG.13919.1	0.595874	coding
MSTRG.13940.1	-0.195273	noncoding
MSTRG.13941.2	1.02987	coding
MSTRG.13941.3	1.62108	coding
MSTRG.13942.1	-0.16245	noncoding
MSTRG.13942.2	-0.207067	noncoding
MSTRG.13942.3	-1.23554	noncoding
MSTRG.13942.4	-1.63878	noncoding
MSTRG.13942.5	-1.98014	noncoding
MSTRG.13942.6	-2.0113	noncoding
MSTRG.13942.7	-1.95805	noncoding
MSTRG.13942.10	-2.01101	noncoding
MSTRG.13942.11	-1.92827	noncoding
MSTRG.13942.12	-1.91359	noncoding
MSTRG.13942.8	-1.93557	noncoding
MSTRG.13942.9	-1.86139	noncoding
MSTRG.13942.13	-1.97682	noncoding
MSTRG.13942.17	-1.9502	noncoding

MSTRG.13942.18	-2.13555	noncoding
MSTRG.13942.19	-1.93667	noncoding
MSTRG.13942.20	-1.9751	noncoding
MSTRG.13942.14	-1.9524	noncoding
MSTRG.13942.15	-1.98146	noncoding
MSTRG.13942.16	-1.94347	noncoding
MSTRG.13942.21	-1.98044	noncoding
MSTRG.13942.22	-1.86222	noncoding
MSTRG.13942.25	-1.91269	noncoding
MSTRG.13942.27	-2.01838	noncoding
MSTRG.13942.28	-1.94525	noncoding
MSTRG.13942.29	-1.98661	noncoding
MSTRG.13942.30	-2.11926	noncoding
MSTRG.13942.31	-1.99313	noncoding
MSTRG.13942.23	-1.96208	noncoding
MSTRG.13942.24	-1.93415	noncoding
MSTRG.13942.26	-1.96561	noncoding
MSTRG.13942.32	-1.96302	noncoding
MSTRG.13942.33	-2.11035	noncoding
MSTRG.13927.1	0.186873	coding
MSTRG.13928.2	3.53265	coding
MSTRG.13944.3	1.74994	coding
MSTRG.13945.1	-1.90478	noncoding
MSTRG.13946.3	1.38359	coding
MSTRG.13929.2	1.95594	coding
MSTRG.13930.2	0.341914	coding
MSTRG.13952.1	1.24265	coding
MSTRG.13952.2	0.270612	coding
MSTRG.13952.3	-0.019954	noncoding
MSTRG.13955.1	-1.47466	noncoding
MSTRG.13962.2	-1.24449	noncoding
MSTRG.13964.1	-1.86665	noncoding
MSTRG.13965.1	-1.98911	noncoding
MSTRG.13969.2	0.032684	coding
MSTRG.13971.1	-1.65146	noncoding
MSTRG.13981.1	-0.891072	noncoding
MSTRG.13981.2	-0.880772	noncoding
MSTRG.13982.1	0.215292	coding
MSTRG.13983.1	-1.4307	noncoding
MSTRG.13984.1	1.97998	coding
MSTRG.13984.3	1.76847	coding
MSTRG.13984.4	1.60734	coding
MSTRG.13986.1	0.19292	coding
MSTRG.13980.2	1.15851	coding
MSTRG.14009.1	2.49788	coding
MSTRG.14009.2	2.29475	coding
MSTRG.14009.3	2.42127	coding
MSTRG.14009.4	2.51714	coding
MSTRG.14010.7	0.277556	coding
MSTRG.14010.6	0.47764	coding
MSTRG.14010.8	0.438319	coding
MSTRG.14010.9	-0.599357	noncoding
MSTRG.13999.1	-1.45816	noncoding
MSTRG.14003.1	-0.44106	noncoding

MSTRG.14003.4	-1.55676	noncoding
MSTRG.14001.2	1.97917	coding
MSTRG.14014.1	-2.12599	noncoding
MSTRG.14034.2	0.36504	coding
MSTRG.14035.1	0.229977	coding
MSTRG.14036.1	0.583706	coding
MSTRG.14037.1	0.036995	coding
MSTRG.14036.2	0.767331	coding
MSTRG.14038.1	0.261646	coding
MSTRG.14038.2	0.188471	coding
MSTRG.14039.1	0.389039	coding
MSTRG.14039.2	0.303878	coding
MSTRG.14041.1	-0.162613	noncoding
MSTRG.14034.3	1.41607	coding
MSTRG.14034.5	0.793513	coding
MSTRG.14034.6	1.98523	coding
MSTRG.14043.1	-1.55524	noncoding
MSTRG.14026.1	0.435045	coding
MSTRG.14029.2	-0.577019	noncoding
MSTRG.14030.1	-1.20216	noncoding
MSTRG.14059.4	0.799232	coding
MSTRG.14059.3	0.797406	coding
MSTRG.14059.5	0.675406	coding
MSTRG.14059.6	0.66488	coding
MSTRG.14060.2	-2.43248	noncoding
MSTRG.14063.1	-1.45896	noncoding
MSTRG.14066.3	2.59074	coding
MSTRG.14047.1	-0.859822	noncoding
MSTRG.14050.4	1.8591	coding
MSTRG.14069.1	1.25132	coding
MSTRG.14069.2	0.001489	coding
MSTRG.14075.1	1.5564	coding
MSTRG.14075.6	0.958191	coding
MSTRG.14076.1	-0.310667	noncoding
MSTRG.14077.1	0.927469	coding
MSTRG.14077.2	0.712531	coding
MSTRG.14079.1	-1.27915	noncoding
MSTRG.14079.2	-2.03207	noncoding
MSTRG.14081.1	-0.614618	noncoding
MSTRG.14082.1	-2.36917	noncoding
MSTRG.14083.1	0.855616	coding
MSTRG.14084.1	0.569555	coding
MSTRG.14085.1	1.97114	coding
MSTRG.14093.10	1.66771	coding
MSTRG.14093.11	1.27455	coding
MSTRG.14093.4	1.19678	coding
MSTRG.14093.5	1.87907	coding
MSTRG.14093.6	1.34706	coding
MSTRG.14093.7	1.30523	coding
MSTRG.14093.9	1.21315	coding
MSTRG.14093.12	0.304992	coding
MSTRG.14094.1	0.869713	coding
MSTRG.14095.1	-0.467692	noncoding
MSTRG.14099.1	0.552485	coding

MSTRG.14100.2	0.294924	coding
MSTRG.14089.2	1.40726	coding
MSTRG.14109.1	-2.02802	noncoding
MSTRG.14115.3	1.95571	coding
MSTRG.14126.1	-2.44803	noncoding
MSTRG.14127.1	0.290701	coding
MSTRG.14130.1	-1.16305	noncoding
MSTRG.14141.6	1.61779	coding
MSTRG.14141.9	1.66379	coding
MSTRG.14141.7	1.55789	coding
MSTRG.14141.11	-0.343797	noncoding
MSTRG.14142.3	1.40483	coding
MSTRG.14142.4	2.23597	coding
MSTRG.14143.1	-1.59904	noncoding
MSTRG.14144.2	0.238825	coding
MSTRG.14147.1	-0.08852	noncoding
MSTRG.14147.2	0.657497	coding
MSTRG.14153.2	0.448455	coding
MSTRG.14153.3	0.405453	coding
MSTRG.14157.1	-0.085801	noncoding
MSTRG.14157.2	0.054141	coding
MSTRG.14157.4	0.0628	coding
MSTRG.14157.3	-0.10574	noncoding
MSTRG.14157.5	-0.145818	noncoding
MSTRG.14167.1	-1.42631	noncoding
MSTRG.14172.1	-2.40402	noncoding
MSTRG.14177.1	-2.09674	noncoding
MSTRG.14182.3	1.22321	coding
MSTRG.14183.4	1.23646	coding
MSTRG.14183.6	-0.364821	noncoding
MSTRG.14183.7	-0.381902	noncoding
MSTRG.14183.5	0.75515	coding
MSTRG.14183.1	0.75642	coding
MSTRG.14183.8	0.645729	coding
MSTRG.14186.2	1.16437	coding
MSTRG.14190.2	1.87438	coding
MSTRG.14191.1	-1.23516	noncoding
MSTRG.14192.1	-1.97284	noncoding
MSTRG.14193.1	0.759739	coding
MSTRG.14193.2	0.402528	coding
MSTRG.14193.3	0.914058	coding
MSTRG.14194.3	2.23419	coding
MSTRG.14194.2	2.27096	coding
MSTRG.14189.2	1.53718	coding
MSTRG.14189.3	1.73137	coding
MSTRG.14198.1	0.315616	coding
MSTRG.14198.2	0.140782	coding
MSTRG.14198.3	-0.194623	noncoding
MSTRG.14198.4	-0.246317	noncoding
MSTRG.14198.5	-0.810215	noncoding
MSTRG.14199.3	0.549748	coding
MSTRG.14201.4	0.301311	coding
MSTRG.14201.5	0.337026	coding
MSTRG.14201.3	0.579355	coding

MSTRG.14201.8	0.904182	coding
MSTRG.14201.7	0.924188	coding
MSTRG.14213.1	-2.35068	noncoding
MSTRG.14219.1	2.14133	coding
MSTRG.14220.2	1.93504	coding
MSTRG.14223.1	1.30148	coding
MSTRG.14227.1	-1.81362	noncoding
MSTRG.14221.1	-0.242296	noncoding
MSTRG.14221.2	-0.199389	noncoding
MSTRG.14221.3	0.014209	coding
MSTRG.14233.2	-1.53691	noncoding
MSTRG.14233.3	-1.62978	noncoding
MSTRG.14233.1	-0.461331	noncoding
MSTRG.14233.4	-1.85548	noncoding
MSTRG.14235.1	-1.09989	noncoding
MSTRG.14251.3	-0.148875	noncoding
MSTRG.14251.4	-0.29468	noncoding
MSTRG.14251.5	0.404224	coding
MSTRG.14257.1	0.858626	coding
MSTRG.14249.6	-0.23524	noncoding
MSTRG.14249.7	0.625403	coding
MSTRG.14249.5	0.789947	coding
MSTRG.14249.8	0.719934	coding
MSTRG.14248.2	0.357692	coding
MSTRG.14266.3	2.28366	coding
MSTRG.14269.2	0.93128	coding
MSTRG.14269.4	-0.033022	noncoding
MSTRG.14272.2	2.15838	coding
MSTRG.14277.1	0.437647	coding
MSTRG.14278.1	-0.930868	noncoding
MSTRG.14287.6	1.68894	coding
MSTRG.14287.7	1.81237	coding
MSTRG.14287.8	1.8316	coding
MSTRG.14287.10	1.97941	coding
MSTRG.14287.11	1.93794	coding
MSTRG.14287.12	1.94076	coding
MSTRG.14287.9	2.0522	coding
MSTRG.14287.13	1.96436	coding
MSTRG.14288.1	0.776161	coding
MSTRG.14287.14	0.691489	coding
MSTRG.14280.1	-1.23754	noncoding
MSTRG.14281.1	-1.32331	noncoding
MSTRG.14281.3	0.074404	coding
MSTRG.14281.4	-1.43777	noncoding
MSTRG.14281.5	0.720339	coding
MSTRG.14282.1	0.613829	coding
MSTRG.14282.2	-0.076388	noncoding
MSTRG.14283.1	0.336482	coding
MSTRG.14284.1	-0.484273	noncoding
MSTRG.14285.1	-1.57044	noncoding
MSTRG.14290.1	0.654321	coding
MSTRG.14290.4	0.885446	coding
MSTRG.14290.5	2.12119	coding
MSTRG.14290.6	1.86818	coding

MSTRG.14290.3	1.9476	coding
MSTRG.14290.7	1.85583	coding
MSTRG.14291.1	-0.940311	noncoding
MSTRG.14304.1	-1.53284	noncoding
MSTRG.14304.2	-1.61767	noncoding
MSTRG.14308.1	0.551398	coding
MSTRG.14295.1	-0.230168	noncoding
MSTRG.14296.2	1.51934	coding
MSTRG.14297.1	-2.13	noncoding
MSTRG.14298.1	-1.99465	noncoding
MSTRG.14310.2	1.25907	coding
MSTRG.14311.1	1.4184	coding
MSTRG.14311.2	0.617824	coding
MSTRG.14311.3	-0.20166	noncoding
MSTRG.14311.4	-0.366571	noncoding
MSTRG.14313.2	1.22989	coding
MSTRG.14317.2	-0.706958	noncoding
MSTRG.14318.2	0.315927	coding
MSTRG.14319.1	2.07233	coding
MSTRG.14319.2	2.24447	coding
MSTRG.14319.4	2.13916	coding
MSTRG.14319.3	2.37074	coding
MSTRG.14319.12	1.92245	coding
MSTRG.14319.13	1.44578	coding
MSTRG.14319.14	1.32415	coding
MSTRG.14320.1	-0.044024	noncoding
MSTRG.14324.1	0.304016	coding
MSTRG.14325.1	2.15694	coding
MSTRG.14325.2	2.43265	coding
MSTRG.14326.1	-2.48062	noncoding
MSTRG.14330.1	-0.00501	noncoding
MSTRG.14331.1	2.49049	coding
MSTRG.14331.3	0.996442	coding
MSTRG.14334.2	1.03235	coding
MSTRG.14349.2	1.22189	coding
MSTRG.14350.1	1.46494	coding
MSTRG.14354.1	-0.380976	noncoding
MSTRG.14343.1	-0.302937	noncoding
MSTRG.14359.2	2.05259	coding
MSTRG.14360.2	1.26967	coding
MSTRG.14367.4	1.819	coding
MSTRG.14367.5	1.64201	coding
MSTRG.14368.1	-1.73835	noncoding
MSTRG.14369.1	-0.432628	noncoding
MSTRG.14401.1	-0.969981	noncoding
MSTRG.14409.7	2.16951	coding
MSTRG.14409.8	2.16397	coding
MSTRG.14409.9	2.15406	coding
MSTRG.14409.10	-0.194094	noncoding
MSTRG.14409.11	2.05437	coding
MSTRG.14409.12	2.11159	coding
MSTRG.14409.13	2.121	coding
MSTRG.14409.14	2.22173	coding
MSTRG.14411.3	1.0234	coding

MSTRG.14411.4	0.902467	coding
MSTRG.14411.5	0.957937	coding
MSTRG.14411.6	0.980728	coding
MSTRG.14412.1	1.72489	coding
MSTRG.14376.1	1.5268	coding
MSTRG.14376.2	1.28476	coding
MSTRG.14379.1	-1.50805	noncoding
MSTRG.14376.3	-1.70636	noncoding
MSTRG.14379.2	-1.31197	noncoding
MSTRG.14380.1	1.24369	coding
MSTRG.14380.2	0.481041	coding
MSTRG.14371.4	0.363498	coding
MSTRG.14385.1	-0.636216	noncoding
MSTRG.14386.1	-2.36862	noncoding
MSTRG.14387.1	-2.85763	noncoding
MSTRG.14395.1	1.10298	coding
MSTRG.14398.1	0.386174	coding
MSTRG.14399.1	0.312987	coding
MSTRG.14419.1	-1.20272	noncoding
MSTRG.14421.1	-0.553365	noncoding
MSTRG.14422.1	-0.639399	noncoding
MSTRG.14424.1	0.152392	coding
MSTRG.14424.2	-0.175387	noncoding
MSTRG.14426.2	-0.988834	noncoding
MSTRG.14426.3	0.09562	coding
MSTRG.14427.3	1.78037	coding
MSTRG.14427.4	1.97424	coding
MSTRG.14428.1	1.37002	coding
MSTRG.14429.1	-1.21419	noncoding
MSTRG.14414.1	-0.190034	noncoding
MSTRG.14434.1	2.64141	coding
MSTRG.14435.1	0.061916	coding
MSTRG.14436.2	-1.54894	noncoding
MSTRG.14437.1	1.20754	coding
MSTRG.14436.4	1.02201	coding
MSTRG.14437.5	1.45953	coding
MSTRG.14439.2	0.710078	coding
MSTRG.14439.3	0.385321	coding
MSTRG.14454.3	2.36242	coding
MSTRG.14455.1	0.813099	coding
MSTRG.14461.1	0.111403	coding
MSTRG.14440.1	-1.77872	noncoding
MSTRG.14441.2	2.21643	coding
MSTRG.14442.1	2.05194	coding
MSTRG.14442.3	2.85228	coding
MSTRG.14442.4	2.82296	coding
MSTRG.14442.5	2.96853	coding
MSTRG.14442.6	2.88094	coding
MSTRG.14445.2	-2.24591	noncoding
MSTRG.14445.1	-1.06935	noncoding
MSTRG.14445.3	-2.09385	noncoding
MSTRG.14446.1	-0.477994	noncoding
MSTRG.14447.1	1.94201	coding
MSTRG.14448.1	0.734544	coding

MSTRG.14453.2	1.17559	coding
MSTRG.14453.4	1.35854	coding
MSTRG.14453.5	1.098	coding
MSTRG.14486.1	0.317168	coding
MSTRG.14486.3	0.257791	coding
MSTRG.14486.4	0.260877	coding
MSTRG.14487.1	1.26336	coding
MSTRG.14487.2	1.37032	coding
MSTRG.14488.3	0.115339	coding
MSTRG.14488.5	1.08539	coding
MSTRG.14491.3	2.11627	coding
MSTRG.14491.5	0.496306	coding
MSTRG.14491.8	0.587315	coding
MSTRG.14491.7	1.18095	coding
MSTRG.14491.10	0.26025	coding
MSTRG.14491.6	0.186889	coding
MSTRG.14491.4	2.06762	coding
MSTRG.14491.11	-0.13694	noncoding
MSTRG.14489.1	1.57071	coding
MSTRG.14489.3	1.34525	coding
MSTRG.14489.4	1.2804	coding
MSTRG.14492.2	0.93484	coding
MSTRG.14492.3	0.774535	coding
MSTRG.14492.4	0.790961	coding
MSTRG.14493.1	0.84166	coding
MSTRG.14492.6	-1.83446	noncoding
MSTRG.14492.5	0.159118	coding
MSTRG.14495.1	0.030535	coding
MSTRG.14508.5	0.43629	coding
MSTRG.14508.7	1.56828	coding
MSTRG.14508.8	2.13407	coding
MSTRG.14508.4	2.14773	coding
MSTRG.14508.6	3.11253	coding
MSTRG.14508.9	0.827355	coding
MSTRG.14509.1	0.399392	coding
MSTRG.14508.10	2.46537	coding
MSTRG.14513.1	-0.064696	noncoding
MSTRG.14512.3	2.16738	coding
MSTRG.14512.4	1.85576	coding
MSTRG.14515.1	0.921073	coding
MSTRG.14515.2	-1.25103	noncoding
MSTRG.14515.3	-0.756839	noncoding
MSTRG.14516.1	-2.35043	noncoding
MSTRG.14528.1	0.116463	coding
MSTRG.14528.3	-0.381015	noncoding
MSTRG.14528.5	-1.16043	noncoding
MSTRG.14529.1	0.499052	coding
MSTRG.14529.2	0.093391	coding
MSTRG.14530.1	1.13661	coding
MSTRG.14529.3	0.234952	coding
MSTRG.14529.4	-0.187294	noncoding
MSTRG.14529.5	-2.19518	noncoding
MSTRG.14531.1	0.011037	coding
MSTRG.14531.2	-2.92191	noncoding

MSTRG.14533.1	-0.86051	noncoding
MSTRG.14534.1	0.055326	coding
MSTRG.14534.2	0.43255	coding
MSTRG.14534.3	-1.03468	noncoding
MSTRG.14534.4	-0.428096	noncoding
MSTRG.14533.2	-0.199402	noncoding
MSTRG.14535.1	-1.86377	noncoding
MSTRG.14517.3	1.59771	coding
MSTRG.14517.4	0.671623	coding
MSTRG.14518.1	-1.00537	noncoding
MSTRG.14519.1	-2.05839	noncoding
MSTRG.14526.3	2.03054	coding
MSTRG.14536.1	-0.166036	noncoding
MSTRG.14538.2	0.48482	coding
MSTRG.14538.3	0.636758	coding
MSTRG.14538.4	0.665874	coding
MSTRG.14538.5	0.946569	coding
MSTRG.14539.5	1.01968	coding
MSTRG.14539.7	1.20821	coding
MSTRG.14540.1	0.925815	coding
MSTRG.14540.11	1.18316	coding
MSTRG.14540.9	0.342232	coding
MSTRG.14540.12	0.790514	coding
MSTRG.14540.13	0.98651	coding
MSTRG.14540.10	1.1754	coding
MSTRG.14540.4	1.17812	coding
MSTRG.14540.3	0.843065	coding
MSTRG.14540.6	0.856946	coding
MSTRG.14540.7	0.351981	coding
MSTRG.14540.2	1.18116	coding
MSTRG.14540.8	1.17959	coding
MSTRG.14540.14	0.24674	coding
MSTRG.14541.1	0.119593	coding
MSTRG.14543.5	1.86875	coding
MSTRG.14544.1	0.413362	coding
MSTRG.14553.1	0.906924	coding
MSTRG.14553.2	1.79214	coding
MSTRG.14553.3	1.1572	coding
MSTRG.14558.2	1.10591	coding
MSTRG.14560.3	0.839152	coding
MSTRG.14560.7	-0.107582	noncoding
MSTRG.14563.6	0.766506	coding
MSTRG.14563.4	0.640244	coding
MSTRG.14563.5	0.63816	coding
MSTRG.14564.6	-0.665714	noncoding
MSTRG.14564.4	1.42765	coding
MSTRG.14564.5	1.14246	coding
MSTRG.14564.2	1.51398	coding
MSTRG.14565.1	1.3664	coding
MSTRG.14564.7	-2.13148	noncoding
MSTRG.14564.8	1.00561	coding
MSTRG.14565.2	2.06402	coding
MSTRG.14566.4	-0.259019	noncoding
MSTRG.14567.1	0.165558	coding

MSTRG.14568.1	0.302182	coding
MSTRG.14569.1	-1.89417	noncoding
MSTRG.14556.2	0.031974	coding
MSTRG.14557.1	1.94173	coding
MSTRG.14574.4	1.21965	coding
MSTRG.14576.2	0.139944	coding
MSTRG.14579.4	2.49072	coding
MSTRG.14580.1	0.331114	coding
MSTRG.14580.3	0.003024	coding
MSTRG.14570.2	1.24601	coding
MSTRG.14573.2	-0.064949	noncoding
MSTRG.14583.1	1.83607	coding
MSTRG.14593.1	0.824889	coding
MSTRG.14593.3	1.2256	coding
MSTRG.14593.4	0.580432	coding
MSTRG.14593.5	0.103356	coding
MSTRG.14593.7	-0.126763	noncoding
MSTRG.14600.1	1.38259	coding
MSTRG.14600.8	1.29725	coding
MSTRG.14600.11	1.53221	coding
MSTRG.14600.5	1.4703	coding
MSTRG.14600.6	1.5883	coding
MSTRG.14600.7	1.48818	coding
MSTRG.14600.2	1.45348	coding
MSTRG.14600.3	1.49102	coding
MSTRG.14600.9	1.52698	coding
MSTRG.14600.12	0.402327	coding
MSTRG.14601.1	-2.20241	noncoding
MSTRG.14600.13	0.26399	coding
MSTRG.14600.14	-0.433859	noncoding
MSTRG.14603.2	0.117583	coding
MSTRG.14605.3	-0.918473	noncoding
MSTRG.14605.4	0.106259	coding
MSTRG.14605.2	1.08231	coding
MSTRG.14605.5	-0.024082	noncoding
MSTRG.14591.4	-1.07943	noncoding
MSTRG.14591.5	0.267866	coding
MSTRG.14589.1	-0.65979	noncoding
MSTRG.14590.1	-1.76283	noncoding
MSTRG.14592.1	1.72211	coding
MSTRG.14592.2	1.7029	coding
MSTRG.14608.1	0.824853	coding
MSTRG.14608.2	0.774304	coding
MSTRG.14608.5	1.54667	coding
MSTRG.14608.4	1.36685	coding
MSTRG.14610.1	-0.636299	noncoding
MSTRG.14608.7	-0.349729	noncoding
MSTRG.14612.1	-0.618678	noncoding
MSTRG.14615.1	0.946732	coding
MSTRG.14615.2	0.594923	coding
MSTRG.14615.3	1.38243	coding
MSTRG.14615.5	0.391304	coding
MSTRG.14619.3	1.25545	coding
MSTRG.14619.2	1.6202	coding

MSTRG.14619.4	1.24618	coding
MSTRG.14620.1	0.671939	coding
MSTRG.14620.2	0.735446	coding
MSTRG.14620.3	0.397617	coding
MSTRG.14620.4	0.736314	coding
MSTRG.14620.5	-1.92815	noncoding
MSTRG.14620.6	0.092812	coding
MSTRG.14616.3	0.853079	coding
MSTRG.14624.4	0.483952	coding
MSTRG.14625.3	0.162668	coding
MSTRG.14629.1	1.74639	coding
MSTRG.14629.3	1.49015	coding
MSTRG.14626.2	1.23603	coding
MSTRG.14630.1	-1.23328	noncoding
MSTRG.14633.1	-2.19754	noncoding
MSTRG.14638.1	-2.025	noncoding
MSTRG.14645.1	2.10241	coding
MSTRG.14646.1	-0.896712	noncoding
MSTRG.14648.2	-1.28669	noncoding
MSTRG.14648.3	2.08709	coding
MSTRG.14651.1	2.40765	coding
MSTRG.14651.3	2.83134	coding
MSTRG.14651.4	2.58109	coding
MSTRG.14647.1	-2.21121	noncoding
MSTRG.14658.1	-0.753692	noncoding
MSTRG.14656.1	-1.8014	noncoding
MSTRG.14672.1	-1.23406	noncoding
MSTRG.14673.1	-2.06975	noncoding
MSTRG.14675.2	1.64886	coding
MSTRG.14678.1	-1.56666	noncoding
MSTRG.14686.1	-2.18301	noncoding
MSTRG.14687.1	0.016372	coding
MSTRG.14687.2	-0.763647	noncoding
MSTRG.14688.2	1.98945	coding
MSTRG.14692.1	-1.70182	noncoding
MSTRG.14692.2	-1.60971	noncoding
MSTRG.14695.1	-0.760036	noncoding
MSTRG.14696.5	0.298622	coding
MSTRG.14696.6	-0.025294	noncoding
MSTRG.14697.1	-0.243139	noncoding
MSTRG.14699.2	0.019418	coding
MSTRG.14699.1	0.199584	coding
MSTRG.14699.3	0.062176	coding
MSTRG.14699.4	0.056028	coding
MSTRG.14699.5	0.120742	coding
MSTRG.14699.6	-0.16798	noncoding
MSTRG.14701.8	0.074636	coding
MSTRG.14701.4	0.854864	coding
MSTRG.14701.5	0.727321	coding
MSTRG.14701.7	0.8509	coding
MSTRG.14701.9	0.790859	coding
MSTRG.14702.1	2.35434	coding
MSTRG.14703.1	0.92038	coding
MSTRG.14707.5	0.892198	coding

MSTRG.14707.4	1.36887	coding
MSTRG.14707.2	0.806948	coding
MSTRG.14708.2	1.80313	coding
MSTRG.14708.6	1.48111	coding
MSTRG.14708.7	1.1379	coding
MSTRG.14709.1	1.79537	coding
MSTRG.14710.1	-1.26212	noncoding
MSTRG.14708.9	1.00394	coding
MSTRG.14711.1	-0.471106	noncoding
MSTRG.14708.10	0.156945	coding
MSTRG.14704.2	1.56334	coding
MSTRG.14704.3	1.3637	coding
MSTRG.14704.4	1.8146	coding
MSTRG.14706.1	-1.33174	noncoding
MSTRG.14715.1	1.90994	coding
MSTRG.14715.6	1.50564	coding
MSTRG.14715.7	1.46406	coding
MSTRG.14715.8	1.2936	coding
MSTRG.14716.1	-0.072142	noncoding
MSTRG.14717.1	0.441451	coding
MSTRG.14718.1	-1.71332	noncoding
MSTRG.14719.1	-0.748132	noncoding
MSTRG.14720.1	0.047226	coding
MSTRG.14721.1	0.73043	coding
MSTRG.14722.1	0.239493	coding
MSTRG.14723.1	-1.03622	noncoding
MSTRG.14727.1	-0.654193	noncoding
MSTRG.14727.2	-1.36783	noncoding
MSTRG.14730.1	-0.281915	noncoding
MSTRG.14731.2	0.441578	coding
MSTRG.14734.2	1.5467	coding
MSTRG.14735.2	2.11161	coding
MSTRG.14735.3	2.26386	coding
MSTRG.14736.1	0.510286	coding
MSTRG.14745.1	-0.2921	noncoding
MSTRG.14746.1	-0.587521	noncoding
MSTRG.14748.1	-1.33746	noncoding
MSTRG.14749.1	-1.41861	noncoding
MSTRG.14751.1	-0.557841	noncoding
MSTRG.14750.3	-0.023872	noncoding
MSTRG.14750.2	0.701991	coding
MSTRG.14750.5	-0.323101	noncoding
MSTRG.14750.4	0.987739	coding
MSTRG.14750.6	-0.116621	noncoding
MSTRG.14753.3	0.362556	coding
MSTRG.14754.1	-1.942	noncoding
MSTRG.14753.4	-0.142084	noncoding
MSTRG.14769.4	1.79957	coding
MSTRG.14771.1	2.2032	coding
MSTRG.14772.1	0.356423	coding
MSTRG.14772.2	0.219882	coding
MSTRG.14772.4	0.932929	coding
MSTRG.14773.1	0.455717	coding
MSTRG.14773.2	0.257933	coding

MSTRG.14775.1	-1.45274	noncoding
MSTRG.14777.1	0.558251	coding
MSTRG.14778.1	-0.328901	noncoding
MSTRG.14780.2	2.58151	coding
MSTRG.14780.3	2.62503	coding
MSTRG.14782.6	0.204846	coding
MSTRG.14785.4	1.20718	coding
MSTRG.14785.5	0.820274	coding
MSTRG.14787.1	-1.1896	noncoding
MSTRG.14789.4	1.2127	coding
MSTRG.14790.3	1.96976	coding
MSTRG.14790.4	2.10335	coding
MSTRG.14791.2	-0.683376	noncoding
MSTRG.14792.2	-0.731117	noncoding
MSTRG.14793.2	0.541464	coding
MSTRG.14796.1	0.368375	coding
MSTRG.14800.1	0.738672	coding
MSTRG.14801.2	1.76861	coding
MSTRG.14808.2	1.34982	coding
MSTRG.14808.4	1.69881	coding
MSTRG.14818.2	0.98486	coding
MSTRG.14818.4	1.00698	coding
MSTRG.14819.1	2.68686	coding
MSTRG.14821.2	1.36794	coding
MSTRG.14821.3	1.38335	coding
MSTRG.14821.5	0.833401	coding
MSTRG.14814.1	-2.07357	noncoding
MSTRG.14815.3	1.70665	coding
MSTRG.14827.1	-1.82091	noncoding
MSTRG.14828.3	0.829875	coding
MSTRG.14828.4	1.17152	coding
MSTRG.14828.2	0.912079	coding
MSTRG.14828.6	-0.548698	noncoding
MSTRG.14830.5	2.97983	coding
MSTRG.14835.1	-0.334815	noncoding
MSTRG.14835.2	-0.530253	noncoding
MSTRG.14834.5	-0.577782	noncoding
MSTRG.14840.1	1.75951	coding
MSTRG.14840.5	3.11589	coding
MSTRG.14833.7	0.65405	coding
MSTRG.14833.3	0.605616	coding
MSTRG.14841.1	-0.454685	noncoding
MSTRG.14842.3	-0.78436	noncoding
MSTRG.14842.2	-0.184258	noncoding
MSTRG.14842.1	-0.20377	noncoding
MSTRG.14842.5	-0.238736	noncoding
MSTRG.14844.2	1.08837	coding
MSTRG.14846.1	1.63827	coding
MSTRG.14846.3	1.69795	coding
MSTRG.14849.1	2.51091	coding
MSTRG.14849.2	1.762	coding
MSTRG.14849.3	1.70308	coding
MSTRG.14852.1	1.36851	coding
MSTRG.14852.2	0.248965	coding

MSTRG.14853.1	-1.9553	noncoding
MSTRG.14852.3	-0.482629	noncoding
MSTRG.14854.2	0.010896	coding
MSTRG.14854.1	0.00291	coding
MSTRG.14854.3	-0.177755	noncoding
MSTRG.14854.4	-0.177755	noncoding
MSTRG.14854.5	-0.998243	noncoding
MSTRG.14855.1	1.82382	coding
MSTRG.14856.1	-2.07523	noncoding
MSTRG.14864.1	-1.34031	noncoding
MSTRG.14866.2	0.922957	coding
MSTRG.14866.3	0.856251	coding
MSTRG.14867.5	0.317617	coding
MSTRG.14867.6	0.853465	coding
MSTRG.14865.2	0.254942	coding
MSTRG.14869.2	2.90298	coding
MSTRG.14869.5	3.05847	coding
MSTRG.14869.1	2.72784	coding
MSTRG.14869.3	2.7974	coding
MSTRG.14868.1	0.390749	coding
MSTRG.14871.2	-1.24876	noncoding
MSTRG.14871.1	-0.682782	noncoding
MSTRG.14871.3	-1.13626	noncoding
MSTRG.14872.2	1.55882	coding
MSTRG.14872.3	2.06405	coding
MSTRG.14873.2	1.52094	coding
MSTRG.14873.3	1.15069	coding
MSTRG.14875.1	-0.755618	noncoding
MSTRG.14883.1	-0.414502	noncoding
MSTRG.14884.1	-0.139743	noncoding
MSTRG.14885.1	-2.09746	noncoding
MSTRG.14877.1	-1.982	noncoding
MSTRG.14878.1	-1.14755	noncoding
MSTRG.14890.2	-0.614819	noncoding
MSTRG.14892.2	-1.87263	noncoding
MSTRG.14905.1	0.710394	coding
MSTRG.14907.2	-0.588795	noncoding
MSTRG.14909.2	0.691548	coding
MSTRG.14909.3	0.704382	coding
MSTRG.14909.4	-0.320908	noncoding
MSTRG.14909.5	0.414857	coding
MSTRG.14911.1	1.208	coding
MSTRG.14913.2	2.19897	coding
MSTRG.14913.3	2.23858	coding
MSTRG.14921.1	-0.654593	noncoding
MSTRG.14920.2	0.573118	coding
MSTRG.14914.2	1.21804	coding
MSTRG.14915.1	-0.806496	noncoding
MSTRG.14915.3	0.378432	coding
MSTRG.14915.4	0.032228	coding
MSTRG.14930.4	-0.196258	noncoding
MSTRG.14930.5	0.828544	coding
MSTRG.14930.6	-0.460525	noncoding
MSTRG.14931.1	-0.510173	noncoding

MSTRG.14932.1	0.085254	coding
MSTRG.14933.1	-0.685343	noncoding
MSTRG.14933.2	-0.620564	noncoding
MSTRG.14939.1	-1.65753	noncoding
MSTRG.14940.2	0.736399	coding
MSTRG.14944.1	1.7044	coding
MSTRG.14945.1	1.01914	coding
MSTRG.14945.5	1.0013	coding
MSTRG.14945.6	1.04221	coding
MSTRG.14946.1	-1.4844	noncoding
MSTRG.14947.1	-0.765999	noncoding
MSTRG.14945.7	1.22199	coding
MSTRG.14948.1	-0.632799	noncoding
MSTRG.14945.8	0.452624	coding
MSTRG.14945.9	0.662717	coding
MSTRG.14942.2	0.238199	coding
MSTRG.14942.3	0.322932	coding
MSTRG.14942.6	-0.819149	noncoding
MSTRG.14942.5	-0.379581	noncoding
MSTRG.14942.4	-0.374148	noncoding
MSTRG.14958.1	-0.38045	noncoding
MSTRG.14957.2	-0.045244	noncoding
MSTRG.14959.1	-1.40167	noncoding
MSTRG.14962.2	1.10282	coding
MSTRG.14960.1	-2.72728	noncoding
MSTRG.14965.4	0.777475	coding
MSTRG.14965.3	2.75123	coding
MSTRG.14965.1	2.85434	coding
MSTRG.14965.2	2.93349	coding
MSTRG.14965.5	2.86373	coding
MSTRG.14966.1	-0.618212	noncoding
MSTRG.14968.7	1.80201	coding
MSTRG.14968.4	2.34569	coding
MSTRG.14968.5	2.25662	coding
MSTRG.14968.8	2.59591	coding
MSTRG.14969.1	-0.77086	noncoding
MSTRG.14970.1	-1.83579	noncoding
MSTRG.14975.1	-1.11975	noncoding
MSTRG.14986.2	0.990836	coding
MSTRG.14986.3	0.876592	coding
MSTRG.14987.2	2.37744	coding
MSTRG.14989.1	1.2165	coding
MSTRG.14989.2	1.41744	coding
MSTRG.14989.3	1.44048	coding
MSTRG.14990.1	-0.833042	noncoding
MSTRG.14992.2	1.46543	coding
MSTRG.15000.1	-1.14869	noncoding
MSTRG.15001.1	-0.814932	noncoding
MSTRG.15007.4	2.47808	coding
MSTRG.15007.5	2.52069	coding
MSTRG.15007.7	-0.214891	noncoding
MSTRG.15007.8	2.01023	coding
MSTRG.14997.1	-2.4725	noncoding
MSTRG.14998.1	-2.23508	noncoding

MSTRG.15017.1	-1.98276	noncoding
MSTRG.15018.1	-0.604447	noncoding
MSTRG.15021.1	1.12833	coding
MSTRG.15021.4	1.02971	coding
MSTRG.15022.1	-0.444106	noncoding
MSTRG.15023.4	1.46552	coding
MSTRG.15023.5	1.13191	coding
MSTRG.15033.1	2.47949	coding
MSTRG.15033.2	3.24905	coding
MSTRG.15033.5	3.1953	coding
MSTRG.15026.1	-2.34727	noncoding
MSTRG.15028.1	1.43818	coding
MSTRG.15038.2	-0.259782	noncoding
MSTRG.15046.1	1.69012	coding
MSTRG.15046.4	2.37803	coding
MSTRG.15046.5	0.495325	coding
MSTRG.15046.6	0.665947	coding
MSTRG.15046.7	1.5578	coding
MSTRG.15047.1	-0.337514	noncoding
MSTRG.15047.2	-0.249063	noncoding
MSTRG.15046.9	0.029389	coding
MSTRG.15050.6	0.087556	coding
MSTRG.15050.2	0.130416	coding
MSTRG.15050.4	0.206084	coding
MSTRG.15050.7	0.538027	coding
MSTRG.15051.1	1.81021	coding
MSTRG.15051.2	0.6625	coding
MSTRG.15051.3	0.727199	coding
MSTRG.15051.4	0.64481	coding
MSTRG.15052.2	-2.41765	noncoding
MSTRG.15054.1	-0.874755	noncoding
MSTRG.15043.1	-0.610209	noncoding
MSTRG.15039.1	-2.14384	noncoding
MSTRG.15055.2	0.983241	coding
MSTRG.15055.4	0.068239	coding
MSTRG.15055.6	1.83914	coding
MSTRG.15055.7	1.11239	coding
MSTRG.15055.8	1.01254	coding
MSTRG.15057.1	-1.49033	noncoding
MSTRG.15058.1	-1.10681	noncoding
MSTRG.15045.1	-2.96154	noncoding
MSTRG.15068.1	-2.64341	noncoding
MSTRG.15072.1	-2.16864	noncoding
MSTRG.15072.2	-2.4043	noncoding
MSTRG.15080.1	0.283234	coding
MSTRG.15083.4	2.25323	coding
MSTRG.15083.3	2.42709	coding
MSTRG.15083.5	1.63228	coding
MSTRG.15084.1	-0.067699	noncoding
MSTRG.15085.1	-0.474898	noncoding
MSTRG.15090.1	1.09567	coding
MSTRG.15090.2	1.8228	coding
MSTRG.15091.1	-0.387293	noncoding
MSTRG.15092.1	-0.7079	noncoding

MSTRG.15093.10	0.454719	coding
MSTRG.15093.9	0.545766	coding
MSTRG.15093.11	0.712599	coding
MSTRG.15093.12	1.81238	coding
MSTRG.15097.3	1.40346	coding
MSTRG.15097.4	0.816471	coding
MSTRG.15100.2	1.71815	coding
MSTRG.15105.1	1.84104	coding
MSTRG.15105.2	1.48829	coding
MSTRG.15105.5	1.81211	coding
MSTRG.15105.3	1.7485	coding
MSTRG.15105.4	1.9047	coding
MSTRG.15105.6	2.55618	coding
MSTRG.15105.8	1.12951	coding
MSTRG.15105.9	0.454548	coding
MSTRG.15106.1	0.370577	coding
MSTRG.15106.2	0.047007	coding
MSTRG.15106.3	-0.618473	noncoding
MSTRG.15107.1	1.22928	coding
MSTRG.15108.1	1.5915	coding
MSTRG.15108.2	0.221864	coding
MSTRG.15109.1	-2.25265	noncoding
MSTRG.15109.2	-2.02244	noncoding
MSTRG.15105.11	-0.591375	noncoding
MSTRG.15124.5	1.24253	coding
MSTRG.15126.1	-1.76551	noncoding
MSTRG.15127.5	-0.480917	noncoding
MSTRG.15127.4	0.79252	coding
MSTRG.15127.3	0.632006	coding
MSTRG.15127.1	0.7557	coding
MSTRG.15129.1	0.221446	coding
MSTRG.15131.1	0.013188	coding
MSTRG.15132.1	-0.822662	noncoding
MSTRG.15118.3	2.28067	coding
MSTRG.15119.1	-2.40261	noncoding
MSTRG.15141.1	-0.216756	noncoding
MSTRG.15141.2	2.84124	coding
MSTRG.15141.4	2.99502	coding
MSTRG.15138.1	-0.361472	noncoding
MSTRG.15138.2	-1.28169	noncoding
MSTRG.15145.1	2.76837	coding
MSTRG.15146.4	0.220855	coding
MSTRG.15146.5	-0.337912	noncoding
MSTRG.15146.6	-0.479169	noncoding
MSTRG.15146.7	-0.185645	noncoding
MSTRG.15146.10	-0.820472	noncoding
MSTRG.15146.13	-0.814109	noncoding
MSTRG.15146.11	-0.696852	noncoding
MSTRG.15146.15	-0.967879	noncoding
MSTRG.15146.8	-0.970332	noncoding
MSTRG.15146.14	-0.656888	noncoding
MSTRG.15146.12	-0.438488	noncoding
MSTRG.15147.2	1.12092	coding
MSTRG.15149.1	1.71526	coding

MSTRG.15151.1	0.34095	coding
MSTRG.15152.1	-0.183256	noncoding
MSTRG.15143.1	-0.837167	noncoding
MSTRG.15156.3	1.67413	coding
MSTRG.15156.4	0.494601	coding
MSTRG.15153.1	1.18431	coding
MSTRG.15153.2	1.2104	coding
MSTRG.15167.2	-0.312614	noncoding
MSTRG.15174.3	3.10976	coding
MSTRG.15175.1	-0.18616	noncoding
MSTRG.15176.1	-1.63105	noncoding
MSTRG.15174.4	1.26675	coding
MSTRG.15174.5	0.827505	coding
MSTRG.15177.1	-1.79574	noncoding
MSTRG.15181.1	2.01838	coding
MSTRG.15181.5	-0.347846	noncoding
MSTRG.15183.2	-0.356659	noncoding
MSTRG.15184.1	-0.845516	noncoding
MSTRG.15184.2	1.11434	coding
MSTRG.15185.1	-0.02509	noncoding
MSTRG.15184.3	1.44153	coding
MSTRG.15184.5	1.58553	coding
MSTRG.15184.9	-1.57969	noncoding
MSTRG.15184.10	-0.294096	noncoding
MSTRG.15188.1	-1.91135	noncoding
MSTRG.15188.2	-2.04896	noncoding
MSTRG.15190.1	1.09243	coding
MSTRG.15190.6	-0.211459	noncoding
MSTRG.15191.1	-0.79977	noncoding
MSTRG.15192.1	-0.365846	noncoding
MSTRG.15194.1	-0.294439	noncoding
MSTRG.15219.1	2.98296	coding
MSTRG.15219.2	2.05104	coding
MSTRG.15219.3	2.91708	coding
MSTRG.15220.1	-2.05743	noncoding
MSTRG.15221.1	-1.93876	noncoding
MSTRG.15227.1	-0.297682	noncoding
MSTRG.15253.1	-0.137352	noncoding
MSTRG.15197.1	-2.0987	noncoding
MSTRG.15199.1	-1.34573	noncoding
MSTRG.15209.3	2.14285	coding
MSTRG.15210.1	-0.600217	noncoding
MSTRG.15210.2	-1.85973	noncoding
MSTRG.15209.4	-0.740702	noncoding
MSTRG.15212.1	-2.53582	noncoding
MSTRG.15213.2	-0.993266	noncoding
MSTRG.15268.1	-1.78465	noncoding
MSTRG.15268.2	-1.82421	noncoding
MSTRG.15284.1	1.01177	coding
MSTRG.15287.5	2.81091	coding
MSTRG.15287.6	2.94253	coding
MSTRG.15291.1	1.75244	coding
MSTRG.15296.3	0.805884	coding
MSTRG.15302.2	1.88292	coding

MSTRG.15312.1	0.451334	coding
MSTRG.15304.1	-0.623268	noncoding
MSTRG.15328.2	0.592983	coding
MSTRG.15333.1	-1.18869	noncoding
MSTRG.15338.1	-0.731429	noncoding
MSTRG.15347.1	0.260517	coding
MSTRG.15353.1	0.639427	coding
MSTRG.15359.2	1.72698	coding
MSTRG.15360.1	0.361614	coding
MSTRG.15360.2	-1.32635	noncoding
MSTRG.15362.1	0.46278	coding
MSTRG.15357.1	-0.678647	noncoding
MSTRG.15368.2	1.08563	coding
MSTRG.15368.3	1.06848	coding
MSTRG.15375.2	3.09903	coding
MSTRG.15379.2	2.79828	coding
MSTRG.15381.1	-0.44179	noncoding
MSTRG.15381.2	-0.2388	noncoding
MSTRG.15381.3	-0.131454	noncoding
MSTRG.15380.1	-0.027075	noncoding
MSTRG.15380.2	-0.455838	noncoding
MSTRG.15383.1	-1.91402	noncoding
MSTRG.15386.3	1.66255	coding
MSTRG.15386.5	0.265344	coding
MSTRG.15394.2	0.348725	coding
MSTRG.15394.1	1.1928	coding
MSTRG.15399.1	-1.40114	noncoding
MSTRG.15405.4	1.0351	coding
MSTRG.15405.6	1.02748	coding
MSTRG.15402.1	-0.288229	noncoding
MSTRG.15416.3	-2.24464	noncoding
MSTRG.15416.4	-0.758014	noncoding
MSTRG.15421.1	2.43866	coding
MSTRG.15421.2	1.52925	coding
MSTRG.15422.1	-1.69656	noncoding
MSTRG.15423.1	-0.560839	noncoding
MSTRG.15426.4	1.35429	coding
MSTRG.15427.2	-0.303304	noncoding
MSTRG.15429.6	0.923986	coding
MSTRG.15429.7	1.13931	coding
MSTRG.15429.8	1.34197	coding
MSTRG.15431.1	1.9197	coding
MSTRG.15431.3	1.98501	coding
MSTRG.15433.3	1.40507	coding
MSTRG.15433.5	0.478734	coding
MSTRG.15436.2	1.69431	coding
MSTRG.15436.3	1.44874	coding
MSTRG.15442.1	0.277168	coding
MSTRG.15444.2	-0.215074	noncoding
MSTRG.15444.3	-0.371632	noncoding
MSTRG.15447.5	-0.007248	noncoding
MSTRG.15447.4	-0.424563	noncoding
MSTRG.15447.6	-0.452399	noncoding
MSTRG.15447.8	-0.511679	noncoding

MSTRG.15447.9	-0.248163	noncoding
MSTRG.15450.2	0.606787	coding
MSTRG.15450.3	1.4401	coding
MSTRG.15457.1	0.648679	coding
MSTRG.15467.2	2.40407	coding
MSTRG.15469.1	-1.82887	noncoding
MSTRG.15470.1	-2.31963	noncoding
MSTRG.15471.1	-2.55498	noncoding
MSTRG.15486.2	2.66525	coding
MSTRG.15486.4	-1.07453	noncoding
MSTRG.15490.1	-0.014547	noncoding
MSTRG.15491.1	-0.867237	noncoding
MSTRG.15492.1	0.506565	coding
MSTRG.15492.2	0.535374	coding
MSTRG.15493.1	0.727124	coding
MSTRG.15494.1	0.535114	coding
MSTRG.15486.5	0.694683	coding
MSTRG.15494.2	-0.406412	noncoding
MSTRG.15497.1	0.200827	coding
MSTRG.15499.1	0.045683	coding
MSTRG.15499.2	-1.89783	noncoding
MSTRG.15500.1	-0.188861	noncoding
MSTRG.15502.1	0.265913	coding
MSTRG.15503.1	0.84049	coding
MSTRG.15502.2	0.554386	coding
MSTRG.15477.1	-1.04633	noncoding
MSTRG.15480.2	-0.313459	noncoding
MSTRG.15532.2	2.82992	coding
MSTRG.15532.1	2.84089	coding
MSTRG.15532.4	1.40396	coding
MSTRG.15532.5	2.49245	coding
MSTRG.15532.3	2.82569	coding
MSTRG.15532.6	2.4869	coding
MSTRG.15533.1	-0.514628	noncoding
MSTRG.15532.8	2.90616	coding
MSTRG.15534.1	-0.103302	noncoding
MSTRG.15535.1	2.46913	coding
MSTRG.15536.1	0.539888	coding
MSTRG.15535.3	2.86619	coding
MSTRG.15535.4	2.68745	coding
MSTRG.15538.1	0.409081	coding
MSTRG.15517.1	0.520795	coding
MSTRG.15518.4	1.89335	coding
MSTRG.15518.5	1.82583	coding
MSTRG.15518.6	0.746909	coding
MSTRG.15518.8	0.957518	coding
MSTRG.15518.7	0.765044	coding
MSTRG.15519.1	-0.527005	noncoding
MSTRG.15521.2	1.18044	coding
MSTRG.15521.3	0.174262	coding
MSTRG.15528.3	-1.57138	noncoding
MSTRG.15540.2	2.19482	coding
MSTRG.15540.8	0.637648	coding
MSTRG.15540.7	1.48596	coding

MSTRG.15547.1	-0.31537	noncoding
MSTRG.15547.2	-0.097162	noncoding
MSTRG.15547.3	-0.312464	noncoding
MSTRG.15548.1	1.09264	coding
MSTRG.15548.2	-0.089368	noncoding
MSTRG.15548.3	-0.134974	noncoding
MSTRG.15549.1	-1.76549	noncoding
MSTRG.15554.1	0.338683	coding
MSTRG.15572.1	-0.691456	noncoding
MSTRG.15571.3	0.010673	coding
MSTRG.15571.4	0.270377	coding
MSTRG.15563.1	-0.584402	noncoding
MSTRG.15574.1	-0.340118	noncoding
MSTRG.15575.1	-0.34725	noncoding
MSTRG.15594.4	0.040466	coding
MSTRG.15595.1	1.31932	coding
MSTRG.15595.2	0.377263	coding
MSTRG.15596.2	1.01304	coding
MSTRG.15596.3	0.65674	coding
MSTRG.15600.2	0.609591	coding
MSTRG.15601.1	-1.4837	noncoding
MSTRG.15602.1	-1.00792	noncoding
MSTRG.15602.2	-0.320485	noncoding
MSTRG.15603.3	1.85199	coding
MSTRG.15611.2	-0.142391	noncoding
MSTRG.15614.2	-1.24215	noncoding
MSTRG.15618.1	0.733377	coding
MSTRG.15619.2	0.525119	coding
MSTRG.15619.3	-0.081909	noncoding
MSTRG.15619.4	-0.033394	noncoding
MSTRG.15625.1	1.061	coding
MSTRG.15625.2	1.28661	coding
MSTRG.15628.2	3.08402	coding
MSTRG.15630.3	-2.00119	noncoding
MSTRG.15630.2	0.586146	coding
MSTRG.15638.2	1.57499	coding
MSTRG.15638.3	2.00416	coding
MSTRG.15638.4	0.967736	coding
MSTRG.15638.8	-1.03634	noncoding
MSTRG.15638.10	1.32819	coding
MSTRG.15644.1	1.59278	coding
MSTRG.15644.4	0.216051	coding
MSTRG.15644.7	-0.038444	noncoding
MSTRG.15644.5	0.01903	coding
MSTRG.15644.6	0.202958	coding
MSTRG.15650.1	-0.878565	noncoding
MSTRG.15651.1	-1.1562	noncoding
MSTRG.15656.1	-0.372942	noncoding
MSTRG.15671.1	-0.31419	noncoding
MSTRG.15654.1	1.00364	coding
MSTRG.15654.2	0.846073	coding
MSTRG.15677.1	1.20103	coding
MSTRG.15678.2	1.27608	coding
MSTRG.15679.1	-1.52546	noncoding

MSTRG.15678.3	2.32354	coding
MSTRG.15678.4	1.7854	coding
MSTRG.15672.1	-1.76612	noncoding
MSTRG.15674.1	0.067176	coding
MSTRG.15675.1	-0.862515	noncoding
MSTRG.15681.2	-1.70903	noncoding
MSTRG.15681.3	-2.33593	noncoding
MSTRG.15681.5	1.03241	coding
MSTRG.15681.4	0.878573	coding
MSTRG.15682.1	0.463499	coding
MSTRG.15683.1	0.086404	coding
MSTRG.15681.6	-0.846753	noncoding
MSTRG.15684.1	0.362946	coding
MSTRG.15681.8	0.731709	coding
MSTRG.15685.2	1.19034	coding
MSTRG.15692.1	-2.12045	noncoding
MSTRG.15694.3	0.898551	coding
MSTRG.15694.1	0.878237	coding
MSTRG.15694.2	0.872827	coding
MSTRG.15694.4	0.882972	coding
MSTRG.15701.4	1.34371	coding
MSTRG.15701.5	1.60356	coding
MSTRG.15703.5	0.439408	coding
MSTRG.15703.4	0.633283	coding
MSTRG.15703.6	0.620166	coding
MSTRG.15703.7	0.5536	coding
MSTRG.15705.3	1.94859	coding
MSTRG.15707.2	-0.299546	noncoding
MSTRG.15707.4	-0.269009	noncoding
MSTRG.15714.3	0.063557	coding
MSTRG.15714.4	0.340863	coding
MSTRG.15715.1	0.134147	coding
MSTRG.15715.5	1.02652	coding
MSTRG.15715.6	1.00022	coding
MSTRG.15719.2	0.307587	coding
MSTRG.15720.1	1.5469	coding
MSTRG.15720.2	1.41051	coding
MSTRG.15720.3	1.56654	coding
MSTRG.15708.1	-0.320349	noncoding
MSTRG.15709.1	1.10451	coding
MSTRG.15711.3	-0.354201	noncoding
MSTRG.15722.4	1.06188	coding
MSTRG.15722.10	1.06208	coding
MSTRG.15722.9	1.12138	coding
MSTRG.15724.2	1.98438	coding
MSTRG.15726.5	0.878258	coding
MSTRG.15726.6	1.02208	coding
MSTRG.15723.2	1.34646	coding
MSTRG.15723.3	1.06917	coding
MSTRG.15747.3	0.234352	coding
MSTRG.15750.2	0.690826	coding
MSTRG.15750.3	3.18952	coding
MSTRG.15750.4	2.45781	coding
MSTRG.15751.5	2.06676	coding

MSTRG.15751.4	2.62263	coding
MSTRG.15752.3	1.49244	coding
MSTRG.15752.4	1.10791	coding
MSTRG.15752.5	0.160338	coding
MSTRG.15754.2	2.38651	coding
MSTRG.15756.4	-0.430557	noncoding
MSTRG.15766.3	1.27634	coding
MSTRG.15766.4	1.28338	coding
MSTRG.15766.10	1.03655	coding
MSTRG.15766.8	1.06414	coding
MSTRG.15766.9	1.03659	coding
MSTRG.15767.1	-1.26618	noncoding
MSTRG.15766.11	0.00171	coding
MSTRG.15766.12	-2.19638	noncoding
MSTRG.15766.13	-1.64261	noncoding
MSTRG.15768.1	-0.593223	noncoding
MSTRG.15769.1	0.506226	coding
MSTRG.15773.1	0.581841	coding
MSTRG.15773.2	0.319627	coding
MSTRG.15776.1	0.414394	coding
MSTRG.15776.2	-0.468186	noncoding
MSTRG.15778.3	-0.177603	noncoding
MSTRG.15778.2	0.42288	coding
MSTRG.15781.1	-0.713182	noncoding
MSTRG.15781.5	0.132069	coding
MSTRG.15781.7	-1.708	noncoding
MSTRG.15782.1	0.863647	coding
MSTRG.15785.1	-2.09605	noncoding
MSTRG.15786.1	-0.291817	noncoding
MSTRG.15786.2	-2.09384	noncoding
MSTRG.15786.3	-0.723677	noncoding
MSTRG.15788.1	-1.27113	noncoding
MSTRG.15791.1	-2.34473	noncoding
MSTRG.15793.1	-0.109647	noncoding
MSTRG.15795.1	-2.28782	noncoding
MSTRG.15796.1	1.98386	coding
MSTRG.15796.2	-0.518641	noncoding
MSTRG.15796.3	-0.568685	noncoding
MSTRG.15797.4	-1.16796	noncoding
MSTRG.15797.6	0.316926	coding
MSTRG.15798.1	-0.584232	noncoding
MSTRG.15804.2	0.705723	coding
MSTRG.15804.3	1.04766	coding
MSTRG.15804.5	0.741588	coding
MSTRG.15800.1	-0.162896	noncoding
MSTRG.15801.1	2.51288	coding
MSTRG.15809.10	2.02552	coding
MSTRG.15810.1	-2.07973	noncoding
MSTRG.15811.1	0.008372	coding
MSTRG.15812.1	-1.33267	noncoding
MSTRG.15813.2	1.65127	coding
MSTRG.15813.1	1.70131	coding
MSTRG.15813.3	1.85262	coding
MSTRG.15813.4	1.91453	coding

MSTRG.15809.11	1.17893	coding
MSTRG.15806.1	0.526343	coding
MSTRG.15807.1	1.00564	coding
MSTRG.15816.1	-2.4225	noncoding
MSTRG.15816.2	-1.07881	noncoding
MSTRG.15814.1	-1.17752	noncoding
MSTRG.15817.1	0.163518	coding
MSTRG.15821.1	-2.4191	noncoding
MSTRG.15822.3	0.472864	coding
MSTRG.15826.1	-1.07695	noncoding
MSTRG.15832.1	-2.19705	noncoding
MSTRG.15834.1	-1.26703	noncoding
MSTRG.15834.2	-1.10182	noncoding
MSTRG.15835.1	-0.723695	noncoding
MSTRG.15835.2	-1.47147	noncoding
MSTRG.15841.1	2.54826	coding
MSTRG.15841.2	1.61245	coding
MSTRG.15843.2	0.050686	coding
MSTRG.15846.6	0.350402	coding
MSTRG.15846.5	0.643638	coding
MSTRG.15847.1	-1.65268	noncoding
MSTRG.15848.1	0.137337	coding
MSTRG.15848.2	0.314474	coding
MSTRG.15848.3	0.409175	coding
MSTRG.15858.1	1.94193	coding
MSTRG.15858.2	2.8302	coding
MSTRG.15858.3	2.54715	coding
MSTRG.15868.1	1.64323	coding
MSTRG.15868.6	2.11485	coding
MSTRG.15868.9	2.23264	coding
MSTRG.15868.7	1.85849	coding
MSTRG.15868.3	2.05176	coding
MSTRG.15868.10	1.69912	coding
MSTRG.15867.3	-0.341822	noncoding
MSTRG.15873.2	1.37445	coding
MSTRG.15874.2	1.32467	coding
MSTRG.15879.2	1.9769	coding
MSTRG.15886.2	1.67532	coding
MSTRG.15890.1	-1.48846	noncoding
MSTRG.15891.2	1.71683	coding
MSTRG.15896.2	0.20019	coding
MSTRG.15902.5	1.44224	coding
MSTRG.15902.3	1.59906	coding
MSTRG.15902.4	2.0203	coding
MSTRG.15902.6	1.95096	coding
MSTRG.15903.1	-2.10515	noncoding
MSTRG.15897.1	-0.565151	noncoding
MSTRG.15898.1	0.595693	coding
MSTRG.15899.3	2.88181	coding
MSTRG.15899.2	2.6513	coding
MSTRG.15899.4	2.975	coding
MSTRG.15909.4	1.72738	coding
MSTRG.15909.1	2.23147	coding
MSTRG.15909.6	1.78151	coding

MSTRG.15907.1	-2.44021	noncoding
MSTRG.15917.2	0.551704	coding
MSTRG.15917.3	1.05056	coding
MSTRG.15917.6	0.083231	coding
MSTRG.15917.7	0.085175	coding
MSTRG.15917.5	0.127239	coding
MSTRG.15917.8	-0.155961	noncoding
MSTRG.15917.9	0.226943	coding
MSTRG.15917.10	0.082402	coding
MSTRG.15914.2	1.48599	coding
MSTRG.15913.1	0.455565	coding
MSTRG.15921.1	-1.6911	noncoding
MSTRG.15929.1	2.33365	coding
MSTRG.15929.4	2.39209	coding
MSTRG.15930.1	-0.581274	noncoding
MSTRG.15929.5	2.41039	coding
MSTRG.15929.7	1.35673	coding
MSTRG.15929.9	1.39902	coding
MSTRG.15929.10	1.19733	coding
MSTRG.15929.11	2.00086	coding
MSTRG.15932.1	0.359974	coding
MSTRG.15932.2	0.25236	coding
MSTRG.15931.1	-0.967318	noncoding
MSTRG.15936.2	-0.042841	noncoding
MSTRG.15936.3	0.144714	coding
MSTRG.15937.3	-0.340017	noncoding
MSTRG.15935.1	0.43454	coding
MSTRG.15940.1	0.295541	coding
MSTRG.15940.4	-0.24912	noncoding
MSTRG.15943.1	-1.49105	noncoding
MSTRG.15961.2	0.620295	coding
MSTRG.15962.3	1.90856	coding
MSTRG.15962.2	2.52356	coding
MSTRG.15962.7	0.768593	coding
MSTRG.15963.1	-0.400133	noncoding
MSTRG.15964.1	0.295302	coding
MSTRG.15952.1	1.7963	coding
MSTRG.15955.1	-1.04857	noncoding
MSTRG.15956.1	1.25931	coding
MSTRG.15957.1	0.437855	coding
MSTRG.15958.1	0.168913	coding
MSTRG.15951.1	-1.98244	noncoding
MSTRG.15969.2	0.243707	coding
MSTRG.15969.3	0.355628	coding
MSTRG.15969.4	0.551493	coding
MSTRG.15973.1	-2.02703	noncoding
MSTRG.15974.1	2.07815	coding
MSTRG.15974.5	1.97648	coding
MSTRG.15978.5	1.70284	coding
MSTRG.15978.7	1.33546	coding
MSTRG.15978.6	0.858884	coding
MSTRG.15979.1	-0.313711	noncoding
MSTRG.15980.1	-0.627534	noncoding
MSTRG.15981.1	-0.564085	noncoding

MSTRG.15978.8	-0.175233	noncoding
MSTRG.15982.1	-2.04971	noncoding
MSTRG.15978.9	2.40206	coding
MSTRG.15976.2	0.879332	coding
MSTRG.15990.4	0.104945	coding
MSTRG.15994.1	-1.1391	noncoding
MSTRG.15996.1	-1.87585	noncoding
MSTRG.15997.1	-1.37743	noncoding
MSTRG.15998.4	0.948801	coding
MSTRG.15998.3	1.03792	coding
MSTRG.15998.10	1.15734	coding
MSTRG.15998.11	1.42139	coding
MSTRG.15998.9	1.30977	coding
MSTRG.15999.1	-2.55298	noncoding
MSTRG.16000.1	-1.36351	noncoding
MSTRG.16003.1	1.72407	coding
MSTRG.16004.1	-0.875951	noncoding
MSTRG.16006.1	-2.0934	noncoding
MSTRG.16005.2	0.308938	coding
MSTRG.16012.1	-1.60494	noncoding
MSTRG.16013.2	2.10791	coding
MSTRG.16014.1	-1.27488	noncoding
MSTRG.16015.2	1.18591	coding
MSTRG.16019.1	0.447961	coding
MSTRG.16019.2	0.627595	coding
MSTRG.16022.2	2.05822	coding
MSTRG.16027.2	1.40362	coding
MSTRG.16031.2	1.828	coding
MSTRG.16034.2	0.861779	coding
MSTRG.16035.1	-1.34741	noncoding
MSTRG.16039.1	1.81266	coding
MSTRG.16039.2	1.42697	coding
MSTRG.16041.1	1.07574	coding
MSTRG.16042.2	-0.96646	noncoding
MSTRG.16042.3	-0.588302	noncoding
MSTRG.16042.4	-0.335359	noncoding
MSTRG.16044.3	1.11782	coding
MSTRG.16049.2	0.722551	coding
MSTRG.16050.3	-0.076649	noncoding
MSTRG.16050.4	0.308249	coding
MSTRG.16052.1	-0.7433	noncoding
MSTRG.16052.2	-1.0338	noncoding
MSTRG.16052.3	-0.905593	noncoding
MSTRG.16056.1	1.15326	coding
MSTRG.16057.12	2.0381	coding
MSTRG.16057.15	2.00771	coding
MSTRG.16057.11	1.79314	coding
MSTRG.16057.14	1.79801	coding
MSTRG.16057.17	1.58735	coding
MSTRG.16057.19	1.87665	coding
MSTRG.16057.20	1.86158	coding
MSTRG.16067.2	0.393075	coding
MSTRG.16058.1	2.42464	coding
MSTRG.16064.2	1.69737	coding

MSTRG.16064.3	1.49707	coding
MSTRG.16064.4	1.94483	coding
MSTRG.16064.7	1.73439	coding
MSTRG.16069.2	1.69273	coding
MSTRG.16069.5	1.2785	coding
MSTRG.16069.6	1.11895	coding
MSTRG.16069.8	1.05349	coding
MSTRG.16072.1	-1.02201	noncoding
MSTRG.16079.2	2.20534	coding
MSTRG.16079.4	2.15353	coding
MSTRG.16074.1	1.48482	coding
MSTRG.16074.3	0.464495	coding
MSTRG.16081.1	1.33732	coding
MSTRG.16082.2	1.25108	coding
MSTRG.16082.3	1.38314	coding
MSTRG.16082.4	0.021479	coding
MSTRG.16076.2	0.787221	coding
MSTRG.16083.1	-0.969307	noncoding
MSTRG.16083.2	-0.635928	noncoding
MSTRG.16083.3	-1.2828	noncoding
MSTRG.16088.2	0.542685	coding
MSTRG.16088.4	0.843603	coding
MSTRG.16085.1	1.13001	coding
MSTRG.16092.3	1.62332	coding
MSTRG.16093.3	0.802151	coding
MSTRG.16104.1	1.30622	coding
MSTRG.16104.4	1.77987	coding
MSTRG.16104.5	1.388	coding
MSTRG.16102.1	0.613005	coding
MSTRG.16109.4	1.26799	coding
MSTRG.16110.1	-0.317779	noncoding
MSTRG.16113.5	1.48524	coding
MSTRG.16113.6	1.3905	coding
MSTRG.16113.8	1.38433	coding
MSTRG.16113.7	1.46882	coding
MSTRG.16113.9	1.31294	coding
MSTRG.16113.11	1.32147	coding
MSTRG.16113.12	0.598514	coding
MSTRG.16113.13	0.521608	coding
MSTRG.16113.14	0.604658	coding
MSTRG.16117.2	2.16571	coding
MSTRG.16117.3	1.46484	coding
MSTRG.16118.1	-1.29928	noncoding
MSTRG.16123.1	0.057737	coding
MSTRG.16128.1	1.59983	coding
MSTRG.16128.5	1.69643	coding
MSTRG.16128.6	1.72292	coding
MSTRG.16129.1	0.180882	coding
MSTRG.16129.2	0.282063	coding
MSTRG.16129.3	-0.182426	noncoding
MSTRG.16132.2	1.00265	coding
MSTRG.16132.3	0.378191	coding
MSTRG.16133.1	0.579662	coding
MSTRG.16133.2	0.840445	coding

MSTRG.16133.4	1.28854	coding
MSTRG.16136.1	1.11297	coding
MSTRG.16136.2	1.0556	coding
MSTRG.16138.2	1.13293	coding
MSTRG.16138.5	1.13579	coding
MSTRG.16138.6	1.64924	coding
MSTRG.16144.1	-0.251395	noncoding
MSTRG.16144.4	0.564461	coding
MSTRG.16148.2	2.26028	coding
MSTRG.16148.5	2.28557	coding
MSTRG.16149.1	0.391159	coding
MSTRG.16159.1	0.665733	coding
MSTRG.16159.2	0.783356	coding
MSTRG.16159.3	1.11384	coding
MSTRG.16159.4	1.09465	coding
MSTRG.16159.5	1.30946	coding
MSTRG.16162.1	1.56263	coding
MSTRG.16163.5	0.988947	coding
MSTRG.16167.2	0.934496	coding
MSTRG.16170.5	1.40025	coding
MSTRG.16174.2	1.12801	coding
MSTRG.16191.3	2.08659	coding
MSTRG.16191.4	1.99171	coding
MSTRG.16191.5	1.2678	coding
MSTRG.16182.2	0.623508	coding
MSTRG.16196.2	2.78948	coding
MSTRG.16198.1	2.59518	coding
MSTRG.16198.2	2.53891	coding
MSTRG.16199.1	-1.26953	noncoding
MSTRG.16195.3	0.959399	coding
MSTRG.16201.2	1.05991	coding
MSTRG.16201.3	1.18504	coding
MSTRG.16201.6	1.28332	coding
MSTRG.16202.1	-0.01645	noncoding
MSTRG.16201.7	1.3011	coding
MSTRG.16203.1	2.65246	coding
MSTRG.16207.1	0.385126	coding
MSTRG.16210.2	-0.452964	noncoding
MSTRG.16211.1	2.09743	coding
MSTRG.16214.2	-1.18571	noncoding
MSTRG.16214.3	-1.03002	noncoding
MSTRG.16215.1	-1.49708	noncoding
MSTRG.16217.1	2.54693	coding
MSTRG.16218.3	1.58911	coding
MSTRG.16218.4	1.6166	coding
MSTRG.16221.4	1.63657	coding
MSTRG.16221.3	1.59829	coding
MSTRG.16222.1	0.468418	coding
MSTRG.16222.3	0.088106	coding
MSTRG.16219.1	-2.06848	noncoding
MSTRG.16226.5	0.259413	coding
MSTRG.16239.3	1.25152	coding
MSTRG.16239.4	1.45058	coding
MSTRG.16244.1	0.41903	coding

MSTRG.16244.3	0.942278	coding
MSTRG.16244.5	1.47681	coding
MSTRG.16242.7	-0.970139	noncoding
MSTRG.16233.1	0.989557	coding
MSTRG.16233.2	-0.263433	noncoding
MSTRG.16248.2	2.45457	coding
MSTRG.16248.3	2.57566	coding
MSTRG.16237.1	-0.568165	noncoding
MSTRG.16252.3	-0.750186	noncoding
MSTRG.16252.4	-0.714172	noncoding
MSTRG.16252.1	-0.362533	noncoding
MSTRG.16253.2	0.646438	coding
MSTRG.16253.6	0.71582	coding
MSTRG.16253.7	0.851324	coding
MSTRG.16254.1	2.06298	coding
MSTRG.16261.2	2.2699	coding
MSTRG.16261.6	2.32922	coding
MSTRG.16261.5	2.60836	coding
MSTRG.16263.5	0.778327	coding
MSTRG.16263.3	1.38474	coding
MSTRG.16263.4	1.63072	coding
MSTRG.16263.2	1.5861	coding
MSTRG.16258.1	-0.024123	noncoding
MSTRG.16258.2	0.014734	coding
MSTRG.16264.6	1.51033	coding
MSTRG.16265.1	-1.50221	noncoding
MSTRG.16264.10	1.1628	coding
MSTRG.16264.9	1.9555	coding
MSTRG.16264.11	-1.81563	noncoding
MSTRG.16269.1	-0.831453	noncoding
MSTRG.16269.2	-0.751131	noncoding
MSTRG.16269.3	-0.981106	noncoding
MSTRG.16270.1	2.13164	coding
MSTRG.16270.2	2.11943	coding
MSTRG.16270.3	1.5791	coding
MSTRG.16273.1	-2.74166	noncoding
MSTRG.16274.2	1.91092	coding
MSTRG.16275.1	-1.8119	noncoding
MSTRG.16276.1	0.175898	coding
MSTRG.16278.2	0.058973	coding
MSTRG.16284.1	-2.30413	noncoding
MSTRG.16288.1	0.08111	coding
MSTRG.16291.1	-1.34282	noncoding
MSTRG.16296.1	-1.17949	noncoding
MSTRG.16299.1	-2.12231	noncoding
MSTRG.16299.2	-2.02756	noncoding
MSTRG.16302.1	-0.740138	noncoding
MSTRG.16307.1	-1.02368	noncoding
MSTRG.16319.2	1.29123	coding
MSTRG.16319.3	1.84632	coding
MSTRG.16319.4	1.8786	coding
MSTRG.16319.5	1.83109	coding
MSTRG.16319.6	1.98864	coding
MSTRG.16320.2	-0.15921	noncoding

MSTRG.16321.1	2.16962	coding
MSTRG.16326.1	2.34271	coding
MSTRG.16327.1	2.22354	coding
MSTRG.16328.1	-1.63793	noncoding
MSTRG.16329.3	2.1834	coding
MSTRG.16329.4	0.645604	coding
MSTRG.16330.2	2.15294	coding
MSTRG.16332.1	2.65992	coding
MSTRG.16334.5	1.99867	coding
MSTRG.16336.1	-2.38844	noncoding
MSTRG.16337.1	-0.710291	noncoding
MSTRG.16339.2	0.826976	coding
MSTRG.16349.1	0.360028	coding
MSTRG.16345.2	0.546319	coding
MSTRG.16345.3	0.101354	coding
MSTRG.16343.1	-1.9165	noncoding
MSTRG.16340.1	-1.51266	noncoding
MSTRG.16351.1	0.402617	coding
MSTRG.16351.2	0.553642	coding
MSTRG.16352.4	0.080557	coding
MSTRG.16353.1	0.703641	coding
MSTRG.16354.1	-0.843335	noncoding
MSTRG.16358.2	1.58559	coding
MSTRG.16358.3	1.20183	coding
MSTRG.16361.2	1.70259	coding
MSTRG.16361.1	1.13508	coding
MSTRG.16361.3	1.42324	coding
MSTRG.16361.5	0.344555	coding
MSTRG.16361.6	0.295719	coding
MSTRG.16361.7	-0.086413	noncoding
MSTRG.16364.2	2.28119	coding
MSTRG.16366.1	0.658431	coding
MSTRG.16369.4	-0.430175	noncoding
MSTRG.16370.1	-1.04332	noncoding
MSTRG.16370.2	-1.16987	noncoding
MSTRG.16375.3	2.00228	coding
MSTRG.16378.1	-2.22535	noncoding
MSTRG.16388.1	-0.109228	noncoding
MSTRG.16389.1	0.497076	coding
MSTRG.16389.2	0.20451	coding
MSTRG.16388.2	-1.14729	noncoding
MSTRG.16397.3	1.5601	coding
MSTRG.16399.2	0.378207	coding
MSTRG.16410.1	-2.09504	noncoding
MSTRG.16418.1	-1.16808	noncoding
MSTRG.16419.6	0.577679	coding
MSTRG.16419.5	0.694598	coding
MSTRG.16419.7	1.54954	coding
MSTRG.16419.8	1.52303	coding
MSTRG.16419.10	1.74094	coding
MSTRG.16422.1	2.37337	coding
MSTRG.16422.2	2.08943	coding
MSTRG.16422.3	2.12598	coding
MSTRG.16422.6	1.75516	coding

MSTRG.16423.4	1.42634	coding
MSTRG.16423.2	2.24202	coding
MSTRG.16423.3	2.06189	coding
MSTRG.16423.5	1.13684	coding
MSTRG.16423.7	2.32123	coding
MSTRG.16423.8	1.77393	coding
MSTRG.16423.10	0.296651	coding
MSTRG.16423.12	-0.640904	noncoding
MSTRG.16425.2	0.179738	coding
MSTRG.16425.1	0.720457	coding
MSTRG.16425.3	1.29478	coding
MSTRG.16425.9	2.22868	coding
MSTRG.16425.10	0.664515	coding
MSTRG.16427.1	-1.55922	noncoding
MSTRG.16417.1	-0.81785	noncoding
MSTRG.16429.2	-2.14394	noncoding
MSTRG.16431.3	0.659614	coding
MSTRG.16431.4	0.715227	coding
MSTRG.16470.1	0.688833	coding
MSTRG.16472.1	-0.127626	noncoding
MSTRG.16473.2	-0.64808	noncoding
MSTRG.16446.1	-2.02568	noncoding
MSTRG.16448.5	1.46868	coding
MSTRG.16448.6	0.3015	coding
MSTRG.16448.2	1.258	coding
MSTRG.16448.4	1.22463	coding
MSTRG.16448.8	0.839289	coding
MSTRG.16448.9	0.848782	coding
MSTRG.16452.3	1.86497	coding
MSTRG.16452.5	1.74058	coding
MSTRG.16452.6	1.77734	coding
MSTRG.16453.2	2.36389	coding
MSTRG.16457.1	3.02673	coding
MSTRG.16461.2	-0.161518	noncoding
MSTRG.16463.2	2.41691	coding
MSTRG.16463.3	3.15951	coding
MSTRG.16463.4	3.18617	coding
MSTRG.16464.1	0.637161	coding
MSTRG.16465.1	-0.49299	noncoding
MSTRG.16482.1	-2.31984	noncoding
MSTRG.16484.1	1.94039	coding
MSTRG.16486.2	0.862985	coding
MSTRG.16486.3	1.1436	coding
MSTRG.16491.1	0.872204	coding
MSTRG.16492.1	-1.23142	noncoding
MSTRG.16492.2	0.363946	coding
MSTRG.16498.7	1.6086	coding
MSTRG.16498.8	1.68968	coding
MSTRG.16498.13	2.13031	coding
MSTRG.16498.14	2.04786	coding
MSTRG.16498.15	1.2824	coding
MSTRG.16498.16	-1.1963	noncoding
MSTRG.16502.2	0.546661	coding
MSTRG.16507.1	-2.41421	noncoding

MSTRG.16511.1	0.739701	coding
MSTRG.16511.2	0.728762	coding
MSTRG.16511.7	0.740608	coding
MSTRG.16514.1	1.7454	coding
MSTRG.16514.2	1.91913	coding
MSTRG.16515.3	1.47016	coding
MSTRG.16539.4	2.19142	coding
MSTRG.16539.5	2.81276	coding
MSTRG.16539.2	2.94879	coding
MSTRG.16539.6	3.23293	coding
MSTRG.16539.7	2.22138	coding
MSTRG.16542.1	-1.20459	noncoding
MSTRG.16539.8	1.95319	coding
MSTRG.16565.1	-0.629258	noncoding
MSTRG.16584.1	-0.537765	noncoding
MSTRG.16584.2	-0.318468	noncoding
MSTRG.16516.1	1.3925	coding
MSTRG.16520.1	0.267821	coding
MSTRG.16535.6	1.25079	coding
MSTRG.16535.7	1.05313	coding
MSTRG.16537.1	0.212353	coding
MSTRG.16590.2	2.45649	coding
MSTRG.16590.4	1.21715	coding
MSTRG.16590.3	2.47924	coding
MSTRG.16590.6	-0.07496	noncoding
MSTRG.16597.2	3.08138	coding
MSTRG.16597.3	-0.030337	noncoding
MSTRG.16597.4	-0.09191	noncoding
MSTRG.16597.5	-0.818397	noncoding
MSTRG.16597.6	0.503555	coding
MSTRG.16613.1	-1.17423	noncoding
MSTRG.16616.1	0.853885	coding
MSTRG.16616.2	0.597927	coding
MSTRG.16616.3	0.423984	coding
MSTRG.16619.1	-0.916253	noncoding
MSTRG.16620.1	0.030328	coding
MSTRG.16616.5	0.91762	coding
MSTRG.16616.6	0.283827	coding
MSTRG.16616.7	1.767	coding
MSTRG.16616.8	1.02283	coding
MSTRG.16620.2	0.415804	coding
MSTRG.16620.3	0.015308	coding
MSTRG.16620.4	0.007334	coding
MSTRG.16620.5	0.140242	coding
MSTRG.16620.6	0.099062	coding
MSTRG.16622.1	-1.01212	noncoding
MSTRG.16587.1	-1.78697	noncoding
MSTRG.16601.1	-0.72305	noncoding
MSTRG.16602.1	-0.521549	noncoding
MSTRG.16603.3	0.273028	coding
MSTRG.16603.4	0.822689	coding
MSTRG.16604.2	2.72316	coding
MSTRG.16629.2	2.49112	coding
MSTRG.16629.3	1.56749	coding

MSTRG.16630.1	0.052976	coding
MSTRG.16631.3	0.122967	coding
MSTRG.16629.4	1.97036	coding
MSTRG.16629.5	1.90345	coding
MSTRG.16632.1	-2.64024	noncoding
MSTRG.16642.1	0.91347	coding
MSTRG.16642.2	0.872238	coding
MSTRG.16644.1	1.41151	coding
MSTRG.16645.2	-0.854303	noncoding
MSTRG.16653.5	1.80528	coding
MSTRG.16653.6	1.80149	coding
MSTRG.16653.7	1.57964	coding
MSTRG.16653.8	1.63736	coding
MSTRG.16653.9	1.82549	coding
MSTRG.16653.10	-0.038097	noncoding
MSTRG.16654.1	-0.881267	noncoding
MSTRG.16652.1	2.47474	coding
MSTRG.16660.1	-0.094668	noncoding
MSTRG.16660.3	-0.212298	noncoding
MSTRG.16660.4	-0.105387	noncoding
MSTRG.16660.5	0.019434	coding
MSTRG.16665.3	1.21598	coding
MSTRG.16665.4	1.32604	coding
MSTRG.16671.5	0.092641	coding
MSTRG.16672.2	0.215651	coding
MSTRG.16672.5	0.897539	coding
MSTRG.16672.7	0.481522	coding
MSTRG.16669.1	-0.311804	noncoding
MSTRG.16669.2	0.005688	coding
MSTRG.16670.1	1.93319	coding
MSTRG.16674.1	-0.562343	noncoding
MSTRG.16676.1	1.70237	coding
MSTRG.16676.3	2.06112	coding
MSTRG.16677.4	2.36872	coding
MSTRG.16677.3	1.46639	coding
MSTRG.16678.1	-1.84116	noncoding
MSTRG.16679.2	0.122794	coding
MSTRG.16679.1	-0.34728	noncoding
MSTRG.16679.3	0.244942	coding
MSTRG.16679.4	-0.549668	noncoding
MSTRG.16679.5	-0.252152	noncoding
MSTRG.16685.2	1.88374	coding
MSTRG.16687.1	-2.70688	noncoding
MSTRG.16684.1	-1.15278	noncoding
MSTRG.16691.1	-0.660237	noncoding
MSTRG.16694.5	0.300238	coding
MSTRG.16692.1	-1.53263	noncoding
MSTRG.16693.1	-1.81231	noncoding
MSTRG.16702.1	0.553627	coding
MSTRG.16702.4	2.03256	coding
MSTRG.16702.5	-1.51533	noncoding
MSTRG.16704.3	-1.10443	noncoding
MSTRG.16702.10	2.47091	coding
MSTRG.16702.12	1.95577	coding

MSTRG.16702.13	1.65857	coding
MSTRG.16709.4	2.03806	coding
MSTRG.16709.7	1.17372	coding
MSTRG.16696.4	0.527786	coding
MSTRG.16712.1	1.98728	coding
MSTRG.16712.2	1.90329	coding
MSTRG.16712.3	2.19742	coding
MSTRG.16712.5	1.36543	coding
MSTRG.16712.4	2.29107	coding
MSTRG.16712.6	2.08716	coding
MSTRG.16712.7	2.32024	coding
MSTRG.16712.8	2.27395	coding
MSTRG.16712.9	1.77037	coding
MSTRG.16712.10	1.90169	coding
MSTRG.16712.11	0.477681	coding
MSTRG.16713.1	1.23315	coding
MSTRG.16713.2	2.06157	coding
MSTRG.16713.4	0.189727	coding
MSTRG.16713.11	1.83122	coding
MSTRG.16719.2	1.77894	coding
MSTRG.16722.1	1.85789	coding
MSTRG.16722.2	2.20337	coding
MSTRG.16722.3	1.99105	coding
MSTRG.16722.5	1.26401	coding
MSTRG.16722.6	0.710934	coding
MSTRG.16726.1	-1.00075	noncoding
MSTRG.16733.1	-0.404642	noncoding
MSTRG.16733.3	-0.133937	noncoding
MSTRG.16745.1	-2.29686	noncoding
MSTRG.16747.1	-2.35166	noncoding
MSTRG.16754.2	1.05842	coding
MSTRG.16757.3	2.04795	coding
MSTRG.16757.4	2.13116	coding
MSTRG.16748.2	0.948825	coding
MSTRG.16749.1	-2.22357	noncoding
MSTRG.16749.2	-1.97804	noncoding
MSTRG.16750.1	-2.15663	noncoding
MSTRG.16761.3	-0.896027	noncoding
MSTRG.16764.2	1.18697	coding
MSTRG.16765.1	-0.254488	noncoding
MSTRG.16766.1	-0.315962	noncoding
MSTRG.16767.1	0.674286	coding
MSTRG.16769.1	-0.06077	noncoding
MSTRG.16772.2	2.38556	coding
MSTRG.16772.3	2.15502	coding
MSTRG.16772.4	2.28273	coding
MSTRG.16772.5	2.43738	coding
MSTRG.16776.1	-3.16791	noncoding
MSTRG.16799.10	2.13115	coding
MSTRG.16799.7	2.14503	coding
MSTRG.16799.8	1.99579	coding
MSTRG.16799.9	2.18123	coding
MSTRG.16799.3	2.19292	coding
MSTRG.16799.2	2.56187	coding

MSTRG.16799.11	2.70021	coding
MSTRG.16799.4	2.7339	coding
MSTRG.16799.5	2.72915	coding
MSTRG.16799.1	2.676	coding
MSTRG.16801.1	-1.30885	noncoding
MSTRG.16799.12	0.444043	coding
MSTRG.16799.13	1.76614	coding
MSTRG.16802.1	0.1739	coding
MSTRG.16803.1	-1.76412	noncoding
MSTRG.16780.1	-2.37669	noncoding
MSTRG.16784.2	0.785913	coding
MSTRG.16785.1	-2.162	noncoding
MSTRG.16787.1	-2.12398	noncoding
MSTRG.16789.1	-2.2165	noncoding
MSTRG.16823.10	1.93381	coding
MSTRG.16823.8	1.93052	coding
MSTRG.16823.1	2.48303	coding
MSTRG.16823.5	2.45106	coding
MSTRG.16823.6	2.4361	coding
MSTRG.16823.7	2.48852	coding
MSTRG.16823.9	2.19659	coding
MSTRG.16823.2	2.43947	coding
MSTRG.16823.3	2.44563	coding
MSTRG.16823.11	2.23566	coding
MSTRG.16823.12	-0.698951	noncoding
MSTRG.16824.1	-1.56989	noncoding
MSTRG.16824.2	-1.35179	noncoding
MSTRG.16824.3	-1.00197	noncoding
MSTRG.16816.2	1.50219	coding
MSTRG.16816.3	1.62895	coding
MSTRG.16817.1	-1.34879	noncoding
MSTRG.16818.1	0.037324	coding
MSTRG.16818.2	-2.31262	noncoding
MSTRG.16820.1	-1.41545	noncoding
MSTRG.16835.1	-2.33424	noncoding
MSTRG.16835.2	-2.19896	noncoding
MSTRG.16840.1	1.05322	coding
MSTRG.16847.1	-1.24601	noncoding
MSTRG.16847.2	-1.96882	noncoding
MSTRG.16855.1	-1.54843	noncoding
MSTRG.16878.1	-2.50609	noncoding
MSTRG.16879.1	-1.47519	noncoding
MSTRG.16884.1	-2.08638	noncoding
MSTRG.17027.1	1.74783	coding
MSTRG.17027.4	1.03923	coding
MSTRG.17027.5	1.09932	coding
MSTRG.17027.3	1.48525	coding
MSTRG.17027.6	0.973804	coding
MSTRG.17028.1	1.12017	coding
MSTRG.17027.8	0.779299	coding
MSTRG.17027.12	0.716526	coding
MSTRG.17027.13	0.787544	coding
MSTRG.17027.20	0.148579	coding
MSTRG.17027.16	0.887922	coding

MSTRG.17027.17	0.04174	coding
MSTRG.17027.21	1.0188	coding
MSTRG.17027.22	0.305962	coding
MSTRG.17027.23	-0.450842	noncoding
MSTRG.17031.1	0.588099	coding
MSTRG.17027.24	0.956864	coding
MSTRG.17027.25	1.72982	coding
MSTRG.17027.26	0.557783	coding
MSTRG.17027.27	0.886527	coding
MSTRG.17027.28	0.234006	coding
MSTRG.17033.1	-0.838596	noncoding
MSTRG.17027.29	-0.051422	noncoding
MSTRG.17027.30	-0.226524	noncoding
MSTRG.17034.1	-0.202917	noncoding
MSTRG.17035.1	-1.00568	noncoding
MSTRG.17036.1	-0.022257	noncoding
MSTRG.17037.1	-1.30722	noncoding
MSTRG.17038.1	0.415622	coding
MSTRG.17039.1	-0.747651	noncoding
MSTRG.17040.1	-0.54971	noncoding
MSTRG.17041.1	0.674928	coding
MSTRG.17042.1	-0.929674	noncoding
MSTRG.17042.2	-0.36494	noncoding
MSTRG.17027.31	0.262469	coding
MSTRG.17027.32	0.411881	coding
MSTRG.17027.34	0.688113	coding
MSTRG.17027.33	0.719252	coding
MSTRG.17027.35	-1.37716	noncoding
MSTRG.17027.37	0.635711	coding
MSTRG.17027.36	0.700264	coding
MSTRG.17027.38	0.820232	coding
MSTRG.17027.40	0.636413	coding
MSTRG.17027.39	0.600351	coding
MSTRG.17027.41	0.310533	coding
MSTRG.17027.42	0.662102	coding
MSTRG.17027.43	0.182456	coding
MSTRG.17027.44	-0.59804	noncoding
MSTRG.17044.1	-0.972796	noncoding
MSTRG.17027.45	-0.1163	noncoding
MSTRG.17027.46	0.628328	coding
MSTRG.17045.1	-0.596851	noncoding
MSTRG.17046.1	-1.19392	noncoding
MSTRG.17047.1	0.46902	coding
MSTRG.17048.1	0.048031	coding
MSTRG.17049.1	-0.284376	noncoding
MSTRG.17050.1	0.314449	coding
MSTRG.17051.1	-0.554025	noncoding
MSTRG.16890.1	0.61673	coding
MSTRG.16890.2	0.324662	coding
MSTRG.16899.2	2.3863	coding
MSTRG.16906.1	1.92546	coding
MSTRG.16906.3	-0.112736	noncoding
MSTRG.16907.3	-0.556725	noncoding
MSTRG.16907.1	0.695285	coding

MSTRG.16907.2	-1.04942	noncoding
MSTRG.16907.4	-1.00512	noncoding
MSTRG.16907.5	-0.667049	noncoding
MSTRG.16907.6	0.676467	coding
MSTRG.16907.8	0.787881	coding
MSTRG.16907.7	-0.866641	noncoding
MSTRG.16907.9	0.138307	coding
MSTRG.16911.1	-1.874	noncoding
MSTRG.16912.1	0.338499	coding
MSTRG.16915.1	2.05018	coding
MSTRG.16918.2	-0.673792	noncoding
MSTRG.16918.1	-1.40583	noncoding
MSTRG.16912.2	1.26052	coding
MSTRG.16912.3	1.4533	coding
MSTRG.16902.3	-0.184893	noncoding
MSTRG.16902.4	-0.079981	noncoding
MSTRG.16919.1	-2.38251	noncoding
MSTRG.16920.1	-0.470248	noncoding
MSTRG.16922.1	-1.04856	noncoding
MSTRG.16930.6	1.76521	coding
MSTRG.16927.1	1.04596	coding
MSTRG.16928.1	-1.60185	noncoding
MSTRG.16935.7	0.683543	coding
MSTRG.16936.1	0.172694	coding
MSTRG.16935.8	1.18716	coding
MSTRG.16938.1	0.019759	coding
MSTRG.16940.1	2.43471	coding
MSTRG.16940.2	2.50414	coding
MSTRG.16940.3	2.773	coding
MSTRG.16940.4	1.78192	coding
MSTRG.16945.1	2.0046	coding
MSTRG.16948.1	0.314885	coding
MSTRG.16949.2	0.506979	coding
MSTRG.16953.4	2.85864	coding
MSTRG.16954.1	-0.55541	noncoding
MSTRG.16959.1	1.42894	coding
MSTRG.16959.2	1.26119	coding
MSTRG.16961.1	-0.312529	noncoding
MSTRG.16962.1	-1.74856	noncoding
MSTRG.16965.1	1.00445	coding
MSTRG.16965.2	0.807489	coding
MSTRG.16965.4	0.302524	coding
MSTRG.16965.3	0.788749	coding
MSTRG.16965.5	1.1102	coding
MSTRG.16965.6	0.721494	coding
MSTRG.16965.7	-0.632828	noncoding
MSTRG.16966.1	0.719638	coding
MSTRG.16966.2	0.409043	coding
MSTRG.16967.1	-2.32799	noncoding
MSTRG.16968.1	-1.24798	noncoding
MSTRG.16963.5	-1.7324	noncoding
MSTRG.16971.1	1.87373	coding
MSTRG.16971.2	1.82784	coding
MSTRG.16971.3	2.26389	coding

MSTRG.16971.4	2.30068	coding
MSTRG.16971.6	1.83863	coding
MSTRG.16971.8	1.71085	coding
MSTRG.16971.10	1.67992	coding
MSTRG.16971.9	1.95524	coding
MSTRG.16973.1	-0.808201	noncoding
MSTRG.16974.2	0.570417	coding
MSTRG.16974.3	0.299327	coding
MSTRG.16978.1	2.64305	coding
MSTRG.16978.2	3.58001	coding
MSTRG.16978.3	3.40447	coding
MSTRG.16978.4	3.37229	coding
MSTRG.16988.6	1.09177	coding
MSTRG.16988.4	0.940611	coding
MSTRG.16988.5	0.964564	coding
MSTRG.16988.3	0.982623	coding
MSTRG.16988.9	0.423658	coding
MSTRG.16992.2	0.379402	coding
MSTRG.16996.1	-1.79805	noncoding
MSTRG.16997.1	-0.976658	noncoding
MSTRG.16997.2	-2.02393	noncoding
MSTRG.16998.1	-0.779282	noncoding
MSTRG.16999.1	-0.723642	noncoding
MSTRG.17005.1	-1.32154	noncoding
MSTRG.17004.6	-1.62604	noncoding
MSTRG.17004.7	-0.494596	noncoding
MSTRG.17004.8	-0.965179	noncoding
MSTRG.17008.1	-1.92359	noncoding
MSTRG.17011.2	2.4253	coding
MSTRG.17011.3	1.57071	coding
MSTRG.17011.5	0.092705	coding
MSTRG.17012.1	0.177342	coding
MSTRG.17012.2	-0.028784	noncoding
MSTRG.17016.1	-0.488433	noncoding
MSTRG.17053.2	-0.996912	noncoding
MSTRG.17053.1	-0.640112	noncoding
MSTRG.17054.1	2.12713	coding
MSTRG.17054.2	1.69555	coding
MSTRG.17055.1	-0.596291	noncoding
MSTRG.17054.3	0.091922	coding
MSTRG.17060.1	-2.01604	noncoding
MSTRG.17061.1	0.008289	coding
MSTRG.17061.2	0.291795	coding
MSTRG.17067.1	-1.69772	noncoding
MSTRG.17067.2	0.013681	coding
MSTRG.17066.2	0.254854	coding
MSTRG.17069.1	0.651865	coding
MSTRG.17069.2	0.188919	coding
MSTRG.17074.2	0.274784	coding
MSTRG.17074.4	0.524933	coding
MSTRG.17074.6	0.493112	coding
MSTRG.17076.1	0.474214	coding
MSTRG.17076.4	1.28595	coding
MSTRG.17076.6	0.915891	coding

MSTRG.17077.1	0.379335	coding
MSTRG.17078.5	1.36778	coding
MSTRG.17078.1	1.42479	coding
MSTRG.17079.1	-0.716151	noncoding
MSTRG.17078.6	0.978699	coding
MSTRG.17085.2	0.269312	coding
MSTRG.17087.3	-0.077155	noncoding
MSTRG.17087.4	0.558253	coding
MSTRG.17092.1	-1.88759	noncoding
MSTRG.17093.1	-0.675037	noncoding
MSTRG.17093.2	-0.403172	noncoding
MSTRG.17093.3	0.45113	coding
MSTRG.17093.4	0.006914	coding
MSTRG.17093.5	-0.730393	noncoding
MSTRG.17096.2	2.20141	coding
MSTRG.17096.3	1.4497	coding
MSTRG.17101.1	1.66154	coding
MSTRG.17105.1	-1.27466	noncoding
MSTRG.17109.4	1.41314	coding
MSTRG.17109.2	1.37105	coding
MSTRG.17109.5	1.65236	coding
MSTRG.17111.3	2.59838	coding
MSTRG.17114.1	0.867365	coding
MSTRG.17114.2	-0.458669	noncoding
MSTRG.17115.2	1.29132	coding
MSTRG.17110.3	0.447087	coding
MSTRG.17110.1	0.775578	coding
MSTRG.17110.2	0.779353	coding
MSTRG.17110.4	0.329992	coding
MSTRG.17110.5	0.283596	coding
MSTRG.17110.6	0.245872	coding
MSTRG.17116.1	0.953947	coding
MSTRG.17117.1	1.35459	coding
MSTRG.17117.3	1.43548	coding
MSTRG.17119.2	1.16599	coding
MSTRG.17119.3	1.31534	coding
MSTRG.17119.5	0.951355	coding
MSTRG.17131.1	1.62083	coding
MSTRG.17138.2	-2.26231	noncoding
MSTRG.17138.3	-0.230073	noncoding
MSTRG.17138.4	-0.32461	noncoding
MSTRG.17138.6	-0.219119	noncoding
MSTRG.17140.2	-1.17868	noncoding
MSTRG.17134.1	1.81509	coding
MSTRG.17134.2	1.98856	coding
MSTRG.17145.1	-2.30274	noncoding
MSTRG.17150.1	-1.12126	noncoding
MSTRG.17150.2	-0.987686	noncoding
MSTRG.17151.4	1.52478	coding
MSTRG.17152.2	-0.374105	noncoding
MSTRG.17155.1	0.727136	coding
MSTRG.17155.4	0.482234	coding
MSTRG.17166.1	-0.134721	noncoding
MSTRG.17166.2	-2.18714	noncoding

MSTRG.17168.2	1.90345	coding
MSTRG.17168.3	1.4717	coding
MSTRG.17158.1	-1.23804	noncoding
MSTRG.17172.2	-0.892181	noncoding
MSTRG.17172.3	-0.643095	noncoding
MSTRG.17192.2	1.12512	coding
MSTRG.17192.1	1.14354	coding
MSTRG.17192.3	1.15575	coding
MSTRG.17192.4	1.76783	coding
MSTRG.17193.1	0.361907	coding
MSTRG.17202.1	-0.610266	noncoding
MSTRG.17207.1	-0.533365	noncoding
MSTRG.17215.2	-1.02609	noncoding
MSTRG.17225.1	-1.79519	noncoding
MSTRG.17175.1	-1.15809	noncoding
MSTRG.17175.2	-0.87437	noncoding
MSTRG.17176.1	-2.11587	noncoding
MSTRG.17177.4	0.646549	coding
MSTRG.17177.3	1.84895	coding
MSTRG.17177.2	2.07514	coding
MSTRG.17177.5	1.44699	coding
MSTRG.17177.6	1.21109	coding
MSTRG.17178.1	0.996591	coding
MSTRG.17178.2	-0.050037	noncoding
MSTRG.17177.7	1.02931	coding
MSTRG.17179.1	0.164512	coding
MSTRG.17180.1	-1.09842	noncoding
MSTRG.17398.2	0.211552	coding
MSTRG.17399.1	-0.607497	noncoding
MSTRG.17400.1	-0.707561	noncoding
MSTRG.17401.1	-0.860792	noncoding
MSTRG.17402.1	1.38595	coding
MSTRG.17402.2	-0.742324	noncoding
MSTRG.17402.3	-0.8356	noncoding
MSTRG.17402.5	-2.24965	noncoding
MSTRG.17402.6	-1.53188	noncoding
MSTRG.17402.11	0.349618	coding
MSTRG.17403.1	-1.59374	noncoding
MSTRG.17402.13	-0.129994	noncoding
MSTRG.17405.1	0.688761	coding
MSTRG.17402.14	0.090504	coding
MSTRG.17402.15	1.60571	coding
MSTRG.17402.16	-0.38997	noncoding
MSTRG.17402.19	1.91148	coding
MSTRG.17402.17	-1.01171	noncoding
MSTRG.17402.20	1.19874	coding
MSTRG.17402.21	1.75394	coding
MSTRG.17402.24	2.06751	coding
MSTRG.17402.23	0.656708	coding
MSTRG.17402.25	1.09807	coding
MSTRG.17402.29	0.617317	coding
MSTRG.17402.27	1.67624	coding
MSTRG.17402.31	0.67982	coding
MSTRG.17402.32	0.34571	coding

MSTRG.17406.1	-1.76505	noncoding
MSTRG.17407.1	0.154416	coding
MSTRG.17402.34	-1.63691	noncoding
MSTRG.17402.35	-1.14101	noncoding
MSTRG.17402.36	-1.08394	noncoding
MSTRG.17402.37	2.26377	coding
MSTRG.17408.1	-1.19601	noncoding
MSTRG.17409.1	-2.18817	noncoding
MSTRG.17409.2	-2.44181	noncoding
MSTRG.17409.3	-2.32524	noncoding
MSTRG.17410.1	-2.61867	noncoding
MSTRG.17411.1	-2.64934	noncoding
MSTRG.17412.1	-1.27813	noncoding
MSTRG.17413.1	-0.144572	noncoding
MSTRG.17414.1	-2.60991	noncoding
MSTRG.17402.38	-0.849365	noncoding
MSTRG.17415.1	-0.1	noncoding
MSTRG.17416.1	-1.84805	noncoding
MSTRG.17417.1	-1.22427	noncoding
MSTRG.17407.2	0.072652	coding
MSTRG.17407.3	0.943279	coding
MSTRG.17407.4	1.09509	coding
MSTRG.17418.1	-2.44633	noncoding
MSTRG.17419.1	-1.07282	noncoding
MSTRG.17420.1	-1.47987	noncoding
MSTRG.17421.2	0.899115	coding
MSTRG.17421.4	-0.909505	noncoding
MSTRG.17422.1	0.551528	coding
MSTRG.17400.2	-1.05081	noncoding
MSTRG.17402.68	-1.16712	noncoding
MSTRG.17402.75	1.47422	coding
MSTRG.17402.77	-0.952482	noncoding
MSTRG.17424.1	-1.34402	noncoding
MSTRG.17407.10	1.96968	coding
MSTRG.17407.12	2.11163	coding
MSTRG.17428.1	-1.5346	noncoding
MSTRG.17421.5	0.514695	coding
MSTRG.17432.1	-2.01793	noncoding
MSTRG.17421.13	2.11387	coding
MSTRG.17432.2	-0.427126	noncoding
MSTRG.17187.1	-0.531728	noncoding
MSTRG.17189.1	-1.32912	noncoding
MSTRG.17189.2	-2.23628	noncoding
MSTRG.17276.3	-0.269819	noncoding
MSTRG.17276.4	0.575711	coding
MSTRG.17276.5	0.668394	coding
MSTRG.17276.6	0.449177	coding
MSTRG.17276.7	-1.0442	noncoding
MSTRG.17278.1	0.254902	coding
MSTRG.17278.3	0.833013	coding
MSTRG.17278.2	0.633607	coding
MSTRG.17278.4	0.408346	coding
MSTRG.17279.4	0.284799	coding
MSTRG.17279.3	2.29724	coding

MSTRG.17280.5	1.74307	coding
MSTRG.17280.4	1.62576	coding
MSTRG.17190.1	2.72177	coding
MSTRG.17263.3	0.236401	coding
MSTRG.17264.1	0.039174	coding
MSTRG.17264.2	-0.703548	noncoding
MSTRG.17265.1	-1.63797	noncoding
MSTRG.17266.1	0.989043	coding
MSTRG.17266.3	2.06498	coding
MSTRG.17266.5	0.966875	coding
MSTRG.17266.6	1.19243	coding
MSTRG.17191.1	-1.21338	noncoding
MSTRG.17270.1	0.904078	coding
MSTRG.17269.4	1.37752	coding
MSTRG.17271.1	0.891137	coding
MSTRG.17292.1	-1.21623	noncoding
MSTRG.17292.2	-1.87363	noncoding
MSTRG.17294.1	1.8992	coding
MSTRG.17294.2	1.8727	coding
MSTRG.17294.3	1.41349	coding
MSTRG.17294.5	0.522923	coding
MSTRG.17294.4	0.992319	coding
MSTRG.17294.6	1.05526	coding
MSTRG.17294.7	0.14777	coding
MSTRG.17294.9	0.05556	coding
MSTRG.17294.8	-0.099289	noncoding
MSTRG.17294.10	1.26778	coding
MSTRG.17294.12	-0.250889	noncoding
MSTRG.17294.13	0.489195	coding
MSTRG.17294.14	0.530193	coding
MSTRG.17291.1	-2.66452	noncoding
MSTRG.17299.2	1.40443	coding
MSTRG.17299.3	1.09621	coding
MSTRG.17301.3	0.909561	coding
MSTRG.17297.2	0.662318	coding
MSTRG.17307.1	-1.67084	noncoding
MSTRG.17308.3	0.997033	coding
MSTRG.17308.6	0.886059	coding
MSTRG.17304.1	-2.22502	noncoding
MSTRG.17306.1	-1.02941	noncoding
MSTRG.17309.2	0.878948	coding
MSTRG.17310.1	-2.02967	noncoding
MSTRG.17310.2	-1.92326	noncoding
MSTRG.17311.2	1.96921	coding
MSTRG.17313.1	-1.30142	noncoding
MSTRG.17313.2	-2.01386	noncoding
MSTRG.17315.2	1.47015	coding
MSTRG.17326.3	1.35384	coding
MSTRG.17329.1	1.60806	coding
MSTRG.17333.3	0.448375	coding
MSTRG.17333.2	-0.097199	noncoding
MSTRG.17334.2	1.03282	coding
MSTRG.17337.1	0.907684	coding
MSTRG.17339.1	-2.1051	noncoding

MSTRG.17336.6	-2.32388	noncoding
MSTRG.17336.7	-2.43376	noncoding
MSTRG.17340.1	-1.95929	noncoding
MSTRG.17341.2	1.06103	coding
MSTRG.17341.6	1.41376	coding
MSTRG.17343.1	-1.51989	noncoding
MSTRG.17349.5	1.58862	coding
MSTRG.17349.4	1.40616	coding
MSTRG.17350.1	-1.66457	noncoding
MSTRG.17349.9	1.48877	coding
MSTRG.17349.12	1.39761	coding
MSTRG.17349.8	1.10318	coding
MSTRG.17349.7	0.807577	coding
MSTRG.17349.10	1.80619	coding
MSTRG.17349.11	1.63965	coding
MSTRG.17349.13	1.16282	coding
MSTRG.17349.14	0.679479	coding
MSTRG.17349.15	1.35795	coding
MSTRG.17351.1	0.168395	coding
MSTRG.17352.1	-0.527157	noncoding
MSTRG.17347.1	-1.57607	noncoding
MSTRG.17348.1	3.10978	coding
MSTRG.17348.2	3.15068	coding
MSTRG.17348.4	2.99014	coding
MSTRG.17368.10	1.06117	coding
MSTRG.17368.1	1.59748	coding
MSTRG.17368.4	1.5606	coding
MSTRG.17368.5	1.57623	coding
MSTRG.17368.7	1.48326	coding
MSTRG.17368.12	1.56943	coding
MSTRG.17369.1	-0.519615	noncoding
MSTRG.17368.13	-1.62085	noncoding
MSTRG.17368.14	0.728831	coding
MSTRG.17364.3	0.833237	coding
MSTRG.17366.1	1.21815	coding
MSTRG.17370.2	0.91195	coding
MSTRG.17371.2	0.113037	coding
MSTRG.17372.1	0.611488	coding
MSTRG.17370.3	-0.642873	noncoding
MSTRG.17373.1	1.14093	coding
MSTRG.17374.1	1.35068	coding
MSTRG.17370.4	0.030379	coding
MSTRG.17370.5	-0.056653	noncoding
MSTRG.17370.6	0.223975	coding
MSTRG.17370.7	-1.20489	noncoding
MSTRG.17370.8	0.751791	coding
MSTRG.17370.9	0.804129	coding
MSTRG.17375.1	-1.50163	noncoding
MSTRG.17370.10	0.371156	coding
MSTRG.17379.1	0.928262	coding
MSTRG.17379.13	-1.60601	noncoding
MSTRG.17379.14	1.39678	coding
MSTRG.17379.12	0.717504	coding
MSTRG.17379.8	0.880564	coding

MSTRG.17379.10	0.890343	coding
MSTRG.17379.11	1.02044	coding
MSTRG.17379.15	0.966583	coding
MSTRG.17380.1	-0.414829	noncoding
MSTRG.17381.1	-0.214862	noncoding
MSTRG.17387.2	-0.5553	noncoding
MSTRG.17397.1	-1.49879	noncoding
MSTRG.17438.3	1.72392	coding
MSTRG.17438.6	2.28567	coding
MSTRG.17438.7	2.30735	coding
MSTRG.17438.8	2.2212	coding
MSTRG.17438.9	1.14352	coding
MSTRG.17438.10	1.5729	coding
MSTRG.17438.11	1.02852	coding
MSTRG.17437.1	-0.782264	noncoding
MSTRG.17441.1	1.4343	coding
MSTRG.17441.2	1.47341	coding
MSTRG.17441.3	-1.54547	noncoding
MSTRG.17462.1	-0.022479	noncoding
MSTRG.17463.1	-1.14236	noncoding
MSTRG.17464.1	0.186693	coding
MSTRG.17464.4	0.092383	coding
MSTRG.17465.1	-0.331341	noncoding
MSTRG.17467.1	-0.393225	noncoding
MSTRG.17469.1	0.327677	coding
MSTRG.17470.1	-0.489696	noncoding
MSTRG.17471.1	-1.01377	noncoding
MSTRG.17474.1	1.00534	coding
MSTRG.17475.1	0.278457	coding
MSTRG.17475.2	0.293216	coding
MSTRG.17475.3	-0.082199	noncoding
MSTRG.17476.1	0.452811	coding
MSTRG.17477.1	-0.051491	noncoding
MSTRG.17478.1	-0.034152	noncoding
MSTRG.17464.12	1.35846	coding
MSTRG.17464.11	1.88848	coding
MSTRG.17464.10	1.83832	coding
MSTRG.17464.14	1.80938	coding
MSTRG.17464.16	-0.110181	noncoding
MSTRG.17480.1	-1.61486	noncoding
MSTRG.17481.1	1.93418	coding
MSTRG.17481.4	2.07676	coding
MSTRG.17481.5	1.87759	coding
MSTRG.17481.7	2.01845	coding
MSTRG.17481.6	1.93302	coding
MSTRG.17446.1	-0.534673	noncoding
MSTRG.17450.1	1.87129	coding
MSTRG.17454.1	0.971391	coding
MSTRG.17454.3	0.264784	coding
MSTRG.17456.2	0.6301	coding
MSTRG.17456.3	0.599895	coding
MSTRG.17456.4	0.785321	coding
MSTRG.17456.5	0.813998	coding
MSTRG.17453.2	-0.256137	noncoding

MSTRG.17458.3	0.889206	coding
MSTRG.17524.1	0.786997	coding
MSTRG.17528.1	1.99952	coding
MSTRG.17528.3	2.09113	coding
MSTRG.17528.2	2.16454	coding
MSTRG.17528.5	1.9532	coding
MSTRG.17529.1	-1.57184	noncoding
MSTRG.17530.1	0.201844	coding
MSTRG.17531.1	-1.11369	noncoding
MSTRG.17528.6	0.403581	coding
MSTRG.17528.7	-0.089246	noncoding
MSTRG.17528.8	0.360781	coding
MSTRG.17528.9	-0.136846	noncoding
MSTRG.17532.1	0.052511	coding
MSTRG.17532.2	-0.384474	noncoding
MSTRG.17532.3	0.290797	coding
MSTRG.17528.10	-1.41636	noncoding
MSTRG.17528.11	-0.113905	noncoding
MSTRG.17533.1	-1.5803	noncoding
MSTRG.17528.12	0.169656	coding
MSTRG.17528.13	0.405145	coding
MSTRG.17528.14	-0.007467	noncoding
MSTRG.17528.15	0.210818	coding
MSTRG.17528.16	0.205464	coding
MSTRG.17528.17	0.41053	coding
MSTRG.17528.18	0.225323	coding
MSTRG.17535.1	-2.03629	noncoding
MSTRG.17528.19	-1.30425	noncoding
MSTRG.17528.20	-2.38227	noncoding
MSTRG.17536.1	-0.324345	noncoding
MSTRG.17528.21	-1.03886	noncoding
MSTRG.17459.2	-0.472146	noncoding
MSTRG.17482.3	-0.635752	noncoding
MSTRG.17482.4	0.987749	coding
MSTRG.17490.2	2.49373	coding
MSTRG.17490.5	-0.073756	noncoding
MSTRG.17490.4	0.446957	coding
MSTRG.17460.3	-0.285096	noncoding
MSTRG.17496.2	0.731598	coding
MSTRG.17498.2	0.974188	coding
MSTRG.17498.3	1.28974	coding
MSTRG.17539.1	2.07054	coding
MSTRG.17539.2	1.44087	coding
MSTRG.17541.1	0.096387	coding
MSTRG.17541.2	0.655166	coding
MSTRG.17541.3	0.418702	coding
MSTRG.17541.4	0.24684	coding
MSTRG.17541.5	0.880478	coding
MSTRG.17541.6	0.182177	coding
MSTRG.17541.7	0.995107	coding
MSTRG.17546.1	0.543665	coding
MSTRG.17541.8	0.542177	coding
MSTRG.17548.1	-0.507351	noncoding
MSTRG.17550.1	-0.593885	noncoding

MSTRG.17551.1	0.891088	coding
MSTRG.17551.2	-0.147004	noncoding
MSTRG.17552.1	-1.55468	noncoding
MSTRG.17553.1	-0.219159	noncoding
MSTRG.17557.1	1.64567	coding
MSTRG.17512.2	1.25849	coding
MSTRG.17515.1	-1.1632	noncoding
MSTRG.17516.3	-0.375831	noncoding
MSTRG.17516.4	2.33804	coding
MSTRG.17516.5	2.28767	coding
MSTRG.17523.6	0.187947	coding
MSTRG.17523.4	0.313649	coding
MSTRG.17523.7	0.338613	coding
MSTRG.17523.5	2.86972	coding
MSTRG.17523.8	2.96431	coding
MSTRG.17559.1	2.76289	coding
MSTRG.17559.3	1.36803	coding
MSTRG.17559.4	0.949952	coding
MSTRG.17559.5	0.958976	coding
MSTRG.17559.7	1.61231	coding
MSTRG.17559.6	1.42941	coding
MSTRG.17559.8	0.758345	coding
MSTRG.17560.1	-0.702409	noncoding
MSTRG.17560.2	-1.96204	noncoding
MSTRG.17566.3	0.795863	coding
MSTRG.17566.5	0.563677	coding
MSTRG.17567.1	0.261907	coding
MSTRG.17567.2	1.03405	coding
MSTRG.17568.1	-0.515977	noncoding
MSTRG.17579.1	-0.480276	noncoding
MSTRG.17580.1	2.32363	coding
MSTRG.17580.3	2.04607	coding
MSTRG.17580.2	2.05472	coding
MSTRG.17580.4	1.97729	coding
MSTRG.17580.6	2.12188	coding
MSTRG.17581.2	-1.61495	noncoding
MSTRG.17581.1	-1.46278	noncoding
MSTRG.17581.3	-1.35545	noncoding
MSTRG.17582.1	0.650949	coding
MSTRG.17582.2	1.15247	coding
MSTRG.17580.8	0.930688	coding
MSTRG.17580.9	0.700008	coding
MSTRG.17580.10	-0.192062	noncoding
MSTRG.17580.11	0.103041	coding
MSTRG.17571.1	0.510072	coding
MSTRG.17572.1	-1.50748	noncoding
MSTRG.17571.3	0.552741	coding
MSTRG.17571.4	0.448628	coding
MSTRG.17571.5	0.567931	coding
MSTRG.17575.1	-2.12389	noncoding
MSTRG.17577.1	-0.780794	noncoding
MSTRG.17577.2	-1.05472	noncoding
MSTRG.17584.1	-0.929061	noncoding
MSTRG.17585.9	0.577192	coding

MSTRG.17585.8	1.34517	coding
MSTRG.17585.5	1.84945	coding
MSTRG.17585.12	1.27631	coding
MSTRG.17585.15	0.599097	coding
MSTRG.17585.16	0.625853	coding
MSTRG.17585.13	1.77447	coding
MSTRG.17585.14	1.75871	coding
MSTRG.17585.17	1.31658	coding
MSTRG.17585.18	0.210109	coding
MSTRG.17590.2	0.800792	coding
MSTRG.17590.3	1.62399	coding
MSTRG.17590.6	1.91748	coding
MSTRG.17592.1	-0.157998	noncoding
MSTRG.17597.2	1.97837	coding
MSTRG.17602.1	-1.38108	noncoding
MSTRG.17615.1	0.011745	coding
MSTRG.17615.2	0.316873	coding
MSTRG.17615.15	2.55555	coding
MSTRG.17615.5	2.36522	coding
MSTRG.17615.18	-0.135514	noncoding
MSTRG.17605.2	0.927217	coding
MSTRG.17605.3	0.469844	coding
MSTRG.17608.6	1.07852	coding
MSTRG.17608.8	1.22727	coding
MSTRG.17608.4	1.15814	coding
MSTRG.17608.5	1.09512	coding
MSTRG.17608.7	0.914716	coding
MSTRG.17608.9	0.649103	coding
MSTRG.17608.2	1.32089	coding
MSTRG.17607.1	-0.368007	noncoding
MSTRG.17619.1	-0.055482	noncoding
MSTRG.17622.1	0.767649	coding
MSTRG.17622.2	0.803552	coding
MSTRG.17619.3	-0.155294	noncoding
MSTRG.17622.8	1.24847	coding
MSTRG.17622.9	1.23121	coding
MSTRG.17622.11	0.526381	coding
MSTRG.17628.2	1.09876	coding
MSTRG.17629.1	1.81484	coding
MSTRG.17631.2	0.724914	coding
MSTRG.17631.3	1.20447	coding
MSTRG.17631.4	1.00536	coding
MSTRG.17624.1	0.438965	coding
MSTRG.17635.2	1.71123	coding
MSTRG.17635.1	1.67612	coding
MSTRG.17635.3	1.74757	coding
MSTRG.17636.1	-2.03341	noncoding
MSTRG.17635.4	1.41582	coding
MSTRG.17635.6	1.69271	coding
MSTRG.17635.7	1.69271	coding
MSTRG.17635.9	1.56526	coding
MSTRG.17635.10	-1.58806	noncoding
MSTRG.17638.1	-0.395731	noncoding
MSTRG.17639.1	-0.831316	noncoding

MSTRG.17640.1	-2.07428	noncoding
MSTRG.17641.1	-1.02591	noncoding
MSTRG.17635.12	-0.425161	noncoding
MSTRG.17632.2	2.24567	coding
MSTRG.17632.3	2.12178	coding
MSTRG.17643.2	1.03974	coding
MSTRG.17643.3	0.224356	coding
MSTRG.17645.3	2.22684	coding
MSTRG.17645.7	0.249228	coding
MSTRG.17647.1	1.08329	coding
MSTRG.17647.2	1.90644	coding
MSTRG.17647.3	1.37945	coding
MSTRG.17647.4	1.77311	coding
MSTRG.17648.2	1.44679	coding
MSTRG.17653.1	-0.19686	noncoding
MSTRG.17654.1	-0.104443	noncoding
MSTRG.17660.1	0.348017	coding
MSTRG.17661.3	-0.215367	noncoding
MSTRG.17661.4	0.090984	coding
MSTRG.17663.2	0.83998	coding
MSTRG.17665.2	-1.42653	noncoding
MSTRG.17667.2	-0.46276	noncoding
MSTRG.17669.1	-1.9814	noncoding
MSTRG.17670.1	-1.36187	noncoding
MSTRG.17672.1	-1.87434	noncoding
MSTRG.17674.2	-0.386839	noncoding
MSTRG.17674.1	-0.721894	noncoding
MSTRG.17674.3	-0.534197	noncoding
MSTRG.17677.1	0.383613	coding
MSTRG.17677.3	0.351643	coding
MSTRG.17679.2	0.815547	coding
MSTRG.17676.2	-1.25166	noncoding
MSTRG.17681.1	0.10345	coding
MSTRG.17681.3	0.304038	coding
MSTRG.17682.2	1.49004	coding
MSTRG.17685.2	0.726569	coding
MSTRG.17686.1	-0.737831	noncoding
MSTRG.17706.6	1.30924	coding
MSTRG.17706.4	1.3678	coding
MSTRG.17706.5	1.48323	coding
MSTRG.17706.8	1.50231	coding
MSTRG.17706.7	1.51447	coding
MSTRG.17706.9	1.39389	coding
MSTRG.17706.12	1.39192	coding
MSTRG.17706.14	1.47631	coding
MSTRG.17706.11	1.49896	coding
MSTRG.17706.10	1.49609	coding
MSTRG.17706.16	1.49058	coding
MSTRG.17706.18	1.1786	coding
MSTRG.17706.17	1.48583	coding
MSTRG.17706.19	1.48249	coding
MSTRG.17706.20	1.50234	coding
MSTRG.17706.22	-0.064741	noncoding
MSTRG.17706.23	-0.723601	noncoding

MSTRG.17695.1	1.46951	coding
MSTRG.17697.1	-1.10276	noncoding
MSTRG.17698.2	0.206708	coding
MSTRG.17698.3	-0.749252	noncoding
MSTRG.17701.2	0.538457	coding
MSTRG.17702.1	-1.77786	noncoding
MSTRG.17710.3	1.22167	coding
MSTRG.17710.4	1.18814	coding
MSTRG.17710.6	1.63178	coding
MSTRG.17711.4	-0.258533	noncoding
MSTRG.17711.2	2.03486	coding
MSTRG.17709.1	-2.1847	noncoding
MSTRG.17714.1	0.660845	coding
MSTRG.17714.4	0.757556	coding
MSTRG.17718.2	1.27141	coding
MSTRG.17718.3	0.640067	coding
MSTRG.17730.1	0.185688	coding
MSTRG.17731.1	0.138461	coding
MSTRG.17731.2	-1.65162	noncoding
MSTRG.17729.6	-0.181045	noncoding
MSTRG.17729.4	1.36221	coding
MSTRG.17729.5	1.45722	coding
MSTRG.17729.7	1.60507	coding
MSTRG.17729.8	1.52478	coding
MSTRG.17729.9	0.108104	coding
MSTRG.17732.1	0.459494	coding
MSTRG.17729.10	-0.476129	noncoding
MSTRG.17733.7	1.91334	coding
MSTRG.17733.10	2.4553	coding
MSTRG.17733.12	0.380429	coding
MSTRG.17733.11	1.3583	coding
MSTRG.17725.2	0.309523	coding
MSTRG.17728.1	-2.06735	noncoding
MSTRG.17735.2	2.10838	coding
MSTRG.17740.2	0.743008	coding
MSTRG.17741.4	0.247275	coding
MSTRG.17741.5	0.441814	coding
MSTRG.17741.6	-0.523759	noncoding
MSTRG.17744.1	-2.16504	noncoding
MSTRG.17751.2	2.437	coding
MSTRG.17755.2	1.48147	coding
MSTRG.17755.3	1.49732	coding
MSTRG.17747.1	-1.17272	noncoding
MSTRG.17762.1	-2.21252	noncoding
MSTRG.17775.2	-0.468418	noncoding
MSTRG.17776.2	0.70728	coding
MSTRG.17779.1	0.411297	coding
MSTRG.17779.2	0.333494	coding
MSTRG.17779.4	0.351357	coding
MSTRG.17779.5	0.46102	coding
MSTRG.17779.10	0.307212	coding
MSTRG.17779.11	0.319068	coding
MSTRG.17768.1	2.17685	coding
MSTRG.17769.3	-0.320851	noncoding

MSTRG.17770.2	0.899743	coding
MSTRG.17770.3	1.25527	coding
MSTRG.17773.1	0.402187	coding
MSTRG.17773.2	1.74922	coding
MSTRG.17773.3	0.615387	coding
MSTRG.17774.1	-1.63628	noncoding
MSTRG.17781.2	0.357186	coding
MSTRG.17781.3	1.00388	coding
MSTRG.17782.1	-0.873131	noncoding
MSTRG.17784.3	2.63533	coding
MSTRG.17785.2	-0.34741	noncoding
MSTRG.17785.3	-0.070681	noncoding
MSTRG.17789.1	0.20776	coding
MSTRG.17789.4	0.212556	coding
MSTRG.17789.6	0.22756	coding
MSTRG.17791.6	2.76677	coding
MSTRG.17791.5	2.69647	coding
MSTRG.17791.4	2.61777	coding
MSTRG.17792.1	-0.211717	noncoding
MSTRG.17796.2	2.60829	coding
MSTRG.17794.1	1.42362	coding
MSTRG.17802.1	-0.097339	noncoding
MSTRG.17807.1	1.01181	coding
MSTRG.17806.1	-1.69749	noncoding
MSTRG.17814.1	1.32432	coding
MSTRG.17814.2	0.605672	coding
MSTRG.17816.1	0.024692	coding
MSTRG.17814.6	1.8101	coding
MSTRG.17814.4	1.74125	coding
MSTRG.17814.5	1.79018	coding
MSTRG.17817.2	-0.870338	noncoding
MSTRG.17817.1	-0.157752	noncoding
MSTRG.17817.3	-0.418627	noncoding
MSTRG.17810.2	1.40939	coding
MSTRG.17811.3	-0.242278	noncoding
MSTRG.17825.4	1.53205	coding
MSTRG.17826.4	1.50649	coding
MSTRG.17826.3	1.39779	coding
MSTRG.17826.2	1.49653	coding
MSTRG.17826.7	0.019186	coding
MSTRG.17826.8	0.197385	coding
MSTRG.17826.6	0.071683	coding
MSTRG.17826.9	0.105536	coding
MSTRG.17828.6	0.578841	coding
MSTRG.17828.7	-0.051914	noncoding
MSTRG.17828.3	0.758933	coding
MSTRG.17829.1	-0.963102	noncoding
MSTRG.17830.4	1.00158	coding
MSTRG.17830.5	0.982061	coding
MSTRG.17830.6	1.33833	coding
MSTRG.17832.2	1.87921	coding
MSTRG.17833.1	-1.79133	noncoding
MSTRG.17835.2	1.00942	coding
MSTRG.17836.2	1.7089	coding

MSTRG.17836.3	1.67102	coding
MSTRG.17837.3	0.911062	coding
MSTRG.17837.4	0.901073	coding
MSTRG.17839.4	1.31304	coding
MSTRG.17819.1	-0.28933	noncoding
MSTRG.17821.2	0.825675	coding
MSTRG.17841.2	1.98268	coding
MSTRG.17841.3	1.86578	coding
MSTRG.17841.4	2.34061	coding
MSTRG.17841.5	1.94094	coding
MSTRG.17841.6	2.38179	coding
MSTRG.17842.1	2.4278	coding
MSTRG.17842.4	1.92097	coding
MSTRG.17840.1	2.01255	coding
MSTRG.17840.2	1.56635	coding
MSTRG.17845.4	0.473543	coding
MSTRG.17847.1	1.20186	coding
MSTRG.17847.3	0.369854	coding
MSTRG.17849.1	-1.61112	noncoding
MSTRG.17848.5	2.03417	coding
MSTRG.17856.1	0.383225	coding
MSTRG.17863.4	0.858534	coding
MSTRG.17863.2	0.517482	coding
MSTRG.17863.3	0.868319	coding
MSTRG.17863.5	0.996445	coding
MSTRG.17873.4	-0.436825	noncoding
MSTRG.17873.3	0.786499	coding
MSTRG.17878.1	0.767762	coding
MSTRG.17878.2	0.9664	coding
MSTRG.17878.4	0.806028	coding
MSTRG.17878.5	1.01668	coding
MSTRG.17878.6	-0.258295	noncoding
MSTRG.17879.2	-1.39283	noncoding
MSTRG.17878.7	-0.72528	noncoding
MSTRG.17865.1	-1.8098	noncoding
MSTRG.17866.1	-0.805582	noncoding
MSTRG.17867.1	0.538847	coding
MSTRG.17891.1	1.75713	coding
MSTRG.17892.2	2.84132	coding
MSTRG.17892.3	2.96046	coding
MSTRG.17892.4	2.17442	coding
MSTRG.17892.5	2.43949	coding
MSTRG.17892.7	0.595179	coding
MSTRG.17903.1	-1.32099	noncoding
MSTRG.17904.6	2.75641	coding
MSTRG.17904.5	2.65196	coding
MSTRG.17904.7	1.2105	coding
MSTRG.17908.1	0.215514	coding
MSTRG.17906.1	-1.92807	noncoding
MSTRG.17907.2	-0.234836	noncoding
MSTRG.17912.2	-1.62402	noncoding
MSTRG.17913.1	-0.489652	noncoding
MSTRG.17921.1	1.09197	coding
MSTRG.17921.5	1.47914	coding

MSTRG.17923.1	1.26011	coding
MSTRG.17924.1	1.56544	coding
MSTRG.17923.2	-0.982004	noncoding
MSTRG.17923.3	1.4101	coding
MSTRG.17923.4	0.817163	coding
MSTRG.17923.5	-1.07986	noncoding
MSTRG.17918.1	-0.806466	noncoding
MSTRG.17929.1	-0.730446	noncoding
MSTRG.17919.1	-2.09903	noncoding
MSTRG.17931.1	-0.224547	noncoding
MSTRG.17931.3	-1.09535	noncoding
MSTRG.17931.4	-1.17724	noncoding
MSTRG.17941.1	1.67473	coding
MSTRG.17942.3	1.72608	coding
MSTRG.17943.4	1.50273	coding
MSTRG.17943.2	1.21868	coding
MSTRG.17943.3	0.905682	coding
MSTRG.17954.3	0.880202	coding
MSTRG.17952.2	1.86654	coding
MSTRG.17958.1	1.00807	coding
MSTRG.17963.2	-1.12053	noncoding
MSTRG.17963.1	-1.15996	noncoding
MSTRG.17972.5	1.49924	coding
MSTRG.17972.7	1.48325	coding
MSTRG.17972.6	1.16406	coding
MSTRG.17972.10	1.48516	coding
MSTRG.17972.8	1.51624	coding
MSTRG.17972.9	1.5226	coding
MSTRG.17972.11	1.51335	coding
MSTRG.17973.1	-0.622729	noncoding
MSTRG.17975.1	2.17575	coding
MSTRG.17978.2	3.29518	coding
MSTRG.17980.2	1.84141	coding
MSTRG.17980.3	1.28355	coding
MSTRG.17981.1	-0.730127	noncoding
MSTRG.17982.2	0.697412	coding
MSTRG.17984.8	1.31628	coding
MSTRG.17984.7	1.28753	coding
MSTRG.17984.10	1.33194	coding
MSTRG.17985.1	-1.11547	noncoding
MSTRG.17986.2	1.08405	coding
MSTRG.17987.3	0.925358	coding
MSTRG.17987.4	1.15902	coding
MSTRG.17987.5	1.59653	coding
MSTRG.17988.1	-1.59589	noncoding
MSTRG.17989.1	-0.335561	noncoding
MSTRG.17999.2	2.14013	coding
MSTRG.17999.3	1.95631	coding
MSTRG.17999.5	1.5047	coding
MSTRG.17995.1	-1.26157	noncoding
MSTRG.17996.1	-0.311464	noncoding
MSTRG.17998.1	-1.20825	noncoding
MSTRG.18002.1	0.368906	coding
MSTRG.18015.1	0.192025	coding

MSTRG.18019.1	-1.05664	noncoding
MSTRG.18024.4	1.0105	coding
MSTRG.18024.9	1.15427	coding
MSTRG.18024.11	0.068702	coding
MSTRG.18024.12	0.160312	coding
MSTRG.18024.13	0.413449	coding
MSTRG.18025.1	0.489139	coding
MSTRG.18026.2	0.443418	coding
MSTRG.18023.2	2.23853	coding
MSTRG.18031.3	1.36895	coding
MSTRG.18031.4	1.52024	coding
MSTRG.18032.1	1.18084	coding
MSTRG.18033.1	0.746533	coding
MSTRG.18037.2	-0.0715	noncoding
MSTRG.18038.3	1.67778	coding
MSTRG.18038.2	2.41478	coding
MSTRG.18040.7	0.643147	coding
MSTRG.18040.10	1.67968	coding
MSTRG.18040.9	1.50567	coding
MSTRG.18040.12	1.32204	coding
MSTRG.18040.13	1.55243	coding
MSTRG.18040.4	1.2738	coding
MSTRG.18040.8	1.39736	coding
MSTRG.18046.1	-1.35068	noncoding
MSTRG.18050.1	0.299272	coding
MSTRG.18051.1	-2.05976	noncoding
MSTRG.18054.3	0.016881	coding
MSTRG.18052.1	-0.22247	noncoding
MSTRG.18063.1	1.45063	coding
MSTRG.18063.2	1.50908	coding
MSTRG.18063.3	1.3906	coding
MSTRG.18063.5	1.7646	coding
MSTRG.18064.2	1.05273	coding
MSTRG.18066.7	1.10796	coding
MSTRG.18066.5	1.05064	coding
MSTRG.18066.6	0.990036	coding
MSTRG.18060.1	1.08841	coding
MSTRG.18068.1	-0.091181	noncoding
MSTRG.18068.3	-0.191261	noncoding
MSTRG.18068.4	-0.001788	noncoding
MSTRG.18069.1	1.59619	coding
MSTRG.18069.2	-0.388481	noncoding
MSTRG.18070.1	-2.22227	noncoding
MSTRG.18069.4	1.5519	coding
MSTRG.18069.5	1.62369	coding
MSTRG.18073.1	0.318356	coding
MSTRG.18072.2	1.35388	coding
MSTRG.18072.4	0.621223	coding
MSTRG.18072.8	0.414586	coding
MSTRG.18074.2	0.902739	coding
MSTRG.18093.6	0.189298	coding
MSTRG.18093.2	-0.594558	noncoding
MSTRG.18093.4	-0.195544	noncoding
MSTRG.18093.8	-0.831719	noncoding

MSTRG.18095.4	1.25213	coding
MSTRG.18098.1	0.685431	coding
MSTRG.18099.2	1.04924	coding
MSTRG.18101.1	0.017693	coding
MSTRG.18101.2	-0.270524	noncoding
MSTRG.18102.3	-0.566381	noncoding
MSTRG.18103.1	0.017212	coding
MSTRG.18105.1	-0.205834	noncoding
MSTRG.18106.1	-1.1551	noncoding
MSTRG.18083.2	0.142068	coding
MSTRG.18083.3	0.175396	coding
MSTRG.18085.2	-1.4679	noncoding
MSTRG.18089.1	-0.253554	noncoding
MSTRG.18091.3	0.683669	coding
MSTRG.18089.2	-0.66288	noncoding
MSTRG.18122.1	-0.871782	noncoding
MSTRG.18129.1	2.72589	coding
MSTRG.18130.3	1.46471	coding
MSTRG.18130.4	1.37169	coding
MSTRG.18131.5	2.49476	coding
MSTRG.18131.4	2.33058	coding
MSTRG.18131.3	2.32867	coding
MSTRG.18131.6	1.58617	coding
MSTRG.18132.1	-1.14965	noncoding
MSTRG.18135.1	2.12899	coding
MSTRG.18137.1	-1.14304	noncoding
MSTRG.18137.2	-1.14123	noncoding
MSTRG.18138.2	-1.10551	noncoding
MSTRG.18138.1	-0.491636	noncoding
MSTRG.18148.9	-0.538856	noncoding
MSTRG.18149.1	-2.46523	noncoding
MSTRG.18151.1	1.33597	coding
MSTRG.18152.2	1.6731	coding
MSTRG.18152.3	0.483866	coding
MSTRG.18152.4	-0.167016	noncoding
MSTRG.18153.1	2.02604	coding
MSTRG.18153.2	2.38087	coding
MSTRG.18142.3	0.827615	coding
MSTRG.18147.2	2.04888	coding
MSTRG.18140.1	-2.17184	noncoding
MSTRG.18140.2	-1.7918	noncoding
MSTRG.18155.1	0.65303	coding
MSTRG.18155.2	-0.274583	noncoding
MSTRG.18156.1	-0.167616	noncoding
MSTRG.18157.1	-0.166895	noncoding
MSTRG.18158.3	-0.197077	noncoding
MSTRG.18159.1	0.197075	coding
MSTRG.18161.4	1.51135	coding
MSTRG.18162.2	1.59979	coding
MSTRG.18164.1	2.64097	coding
MSTRG.18169.1	0.707724	coding
MSTRG.18170.3	0.907347	coding
MSTRG.18174.1	-1.54144	noncoding
MSTRG.18174.5	-1.99222	noncoding

MSTRG.18173.1	-1.37356	noncoding
MSTRG.18173.2	-1.45501	noncoding
MSTRG.18176.2	-2.00079	noncoding
MSTRG.18177.2	-0.298278	noncoding
MSTRG.18183.4	-0.335079	noncoding
MSTRG.18191.1	-1.56554	noncoding
MSTRG.18191.2	-0.982101	noncoding
MSTRG.18191.3	-1.03678	noncoding
MSTRG.18191.4	-1.46455	noncoding
MSTRG.18185.1	-0.259633	noncoding
MSTRG.18196.2	-0.884404	noncoding
MSTRG.18196.3	-1.88338	noncoding
MSTRG.18196.4	-1.61978	noncoding
MSTRG.18198.1	-1.07015	noncoding
MSTRG.18200.2	-0.571078	noncoding
MSTRG.18200.3	-0.474236	noncoding
MSTRG.18200.4	-0.626455	noncoding
MSTRG.18200.5	-0.780175	noncoding
MSTRG.18201.2	1.49669	coding
MSTRG.18219.2	1.27517	coding
MSTRG.18219.1	1.23343	coding
MSTRG.18219.3	1.63763	coding
MSTRG.18219.4	1.71796	coding
MSTRG.18219.5	1.52592	coding
MSTRG.18219.6	1.82748	coding
MSTRG.18219.8	1.87197	coding
MSTRG.18210.1	-0.550624	noncoding
MSTRG.18211.4	1.33111	coding
MSTRG.18212.2	-0.298961	noncoding
MSTRG.18214.1	1.39229	coding
MSTRG.18214.2	0.708398	coding
MSTRG.18215.1	-0.443587	noncoding
MSTRG.18220.2	2.7223	coding
MSTRG.18226.2	1.72508	coding
MSTRG.18229.2	2.71507	coding
MSTRG.18229.1	2.64884	coding
MSTRG.18229.5	2.44507	coding
MSTRG.18234.1	-2.37428	noncoding
MSTRG.18240.1	1.85076	coding
MSTRG.18240.8	2.08206	coding
MSTRG.18240.7	1.89576	coding
MSTRG.18240.9	2.0473	coding
MSTRG.18240.10	1.97687	coding
MSTRG.18240.11	1.85938	coding
MSTRG.18241.1	0.344837	coding
MSTRG.18240.12	1.67427	coding
MSTRG.18242.2	2.52526	coding
MSTRG.18242.4	2.15231	coding
MSTRG.18245.1	-0.991045	noncoding
MSTRG.18245.2	-0.560452	noncoding
MSTRG.18238.2	2.74454	coding
MSTRG.18239.1	-0.253009	noncoding
MSTRG.18246.2	1.59516	coding
MSTRG.18246.3	1.6366	coding

MSTRG.18252.2	2.67256	coding
MSTRG.18253.2	2.92436	coding
MSTRG.18251.1	-0.62679	noncoding
MSTRG.18259.2	1.32886	coding
MSTRG.18261.1	-0.436217	noncoding
MSTRG.18269.10	-1.59095	noncoding
MSTRG.18269.8	-1.56486	noncoding
MSTRG.18269.6	-1.50489	noncoding
MSTRG.18269.13	-1.31512	noncoding
MSTRG.18269.3	-1.10183	noncoding
MSTRG.18269.7	-0.551009	noncoding
MSTRG.18269.11	-1.23663	noncoding
MSTRG.18269.12	-0.68396	noncoding
MSTRG.18269.1	0.434518	coding
MSTRG.18269.14	0.175818	coding
MSTRG.18269.2	0.657764	coding
MSTRG.18269.4	0.443497	coding
MSTRG.18269.5	0.167609	coding
MSTRG.18269.9	0.517447	coding
MSTRG.18275.1	0.280246	coding
MSTRG.18276.4	0.500061	coding
MSTRG.18276.3	0.467009	coding
MSTRG.18277.1	1.63844	coding
MSTRG.18277.4	1.49627	coding
MSTRG.18278.1	-0.511267	noncoding
MSTRG.18270.2	2.37101	coding
MSTRG.18274.1	-0.999522	noncoding
MSTRG.18274.2	0.075646	coding
MSTRG.18274.3	-0.169757	noncoding
MSTRG.18274.4	-0.018849	noncoding
MSTRG.18273.2	-0.726694	noncoding
MSTRG.18292.2	2.24592	coding
MSTRG.18292.3	2.05174	coding
MSTRG.18294.2	0.290549	coding
MSTRG.18294.5	1.14837	coding
MSTRG.18329.1	-1.11397	noncoding
MSTRG.18346.1	-1.27697	noncoding
MSTRG.18346.2	-0.573258	noncoding
MSTRG.18346.3	-0.560819	noncoding
MSTRG.18347.1	0.458689	coding
MSTRG.18348.1	2.44558	coding
MSTRG.18348.2	0.674661	coding
MSTRG.18349.1	0.313093	coding
MSTRG.18350.1	0.189478	coding
MSTRG.18350.2	0.222574	coding
MSTRG.18350.3	0.482598	coding
MSTRG.18351.1	0.315208	coding
MSTRG.18348.3	2.27299	coding
MSTRG.18352.1	0.029893	coding
MSTRG.18353.1	-0.186582	noncoding
MSTRG.18354.1	-0.559959	noncoding
MSTRG.18355.1	0.299031	coding
MSTRG.18297.2	2.9181	coding
MSTRG.18297.3	2.89999	coding

MSTRG.18297.6	1.85429	coding
MSTRG.18297.5	1.70334	coding
MSTRG.18299.2	-0.386619	noncoding
MSTRG.18299.3	0.518273	coding
MSTRG.18304.1	-2.37933	noncoding
MSTRG.18306.2	-0.122051	noncoding
MSTRG.18306.3	-0.568866	noncoding
MSTRG.18307.1	-1.95227	noncoding
MSTRG.18321.1	-0.276736	noncoding
MSTRG.18369.2	1.77421	coding
MSTRG.18369.5	0.888779	coding
MSTRG.18369.4	1.34737	coding
MSTRG.18369.3	1.29037	coding
MSTRG.18371.2	-0.536958	noncoding
MSTRG.18373.1	-1.68785	noncoding
MSTRG.18378.2	0.878665	coding
MSTRG.18379.6	0.87199	coding
MSTRG.18381.3	1.03842	coding
MSTRG.18381.4	0.994067	coding
MSTRG.18382.3	1.13418	coding
MSTRG.18382.4	1.59822	coding
MSTRG.18383.3	1.11839	coding
MSTRG.18383.1	0.987535	coding
MSTRG.18386.1	0.907992	coding
MSTRG.18386.2	0.750724	coding
MSTRG.18398.5	2.1576	coding
MSTRG.18400.4	0.853929	coding
MSTRG.18400.3	1.75379	coding
MSTRG.18400.5	1.58533	coding
MSTRG.18400.7	1.56714	coding
MSTRG.18400.6	1.65219	coding
MSTRG.18400.9	1.59634	coding
MSTRG.18401.1	0.437976	coding
MSTRG.18405.1	0.209146	coding
MSTRG.18406.1	2.21715	coding
MSTRG.18408.1	0.365467	coding
MSTRG.18408.3	-0.102698	noncoding
MSTRG.18408.4	-0.58769	noncoding
MSTRG.18413.2	0.564492	coding
MSTRG.18413.3	0.408875	coding
MSTRG.18414.1	1.81356	coding
MSTRG.18416.4	0.98522	coding
MSTRG.18416.5	0.590127	coding
MSTRG.18416.6	0.43565	coding
MSTRG.18417.1	1.13233	coding
MSTRG.18417.3	0.282806	coding
MSTRG.18433.6	1.37336	coding
MSTRG.18433.7	1.38926	coding
MSTRG.18434.3	1.67581	coding
MSTRG.18435.1	2.47686	coding
MSTRG.18435.2	2.29468	coding
MSTRG.18438.1	-0.139732	noncoding
MSTRG.18438.2	-0.146821	noncoding
MSTRG.18447.3	1.12615	coding

MSTRG.18448.1	0.047677	coding
MSTRG.18448.2	-1.10792	noncoding
MSTRG.18447.5	1.61581	coding
MSTRG.18424.4	1.2334	coding
MSTRG.18424.3	1.53728	coding
MSTRG.18424.2	1.59458	coding
MSTRG.18425.1	0.261878	coding
MSTRG.18425.2	-0.380335	noncoding
MSTRG.18425.4	0.700147	coding
MSTRG.18425.3	0.500044	coding
MSTRG.18428.3	0.953481	coding
MSTRG.18428.2	1.66802	coding
MSTRG.18428.4	-0.260963	noncoding
MSTRG.18428.5	-0.292641	noncoding
MSTRG.18452.2	-1.20569	noncoding
MSTRG.18449.1	-1.27199	noncoding
MSTRG.18449.2	-1.48055	noncoding
MSTRG.18453.2	-0.661813	noncoding
MSTRG.18454.2	-0.284867	noncoding
MSTRG.18454.1	-0.438559	noncoding
MSTRG.18454.3	-0.407414	noncoding
MSTRG.18459.1	0.037896	coding
MSTRG.18462.1	1.75329	coding
MSTRG.18462.5	1.84379	coding
MSTRG.18462.7	1.78905	coding
MSTRG.18455.1	1.2719	coding
MSTRG.18464.1	-0.369998	noncoding
MSTRG.18466.1	-0.19262	noncoding
MSTRG.18465.3	1.95877	coding
MSTRG.18465.2	1.9026	coding
MSTRG.18465.5	2.03898	coding
MSTRG.18465.4	2.03103	coding
MSTRG.18468.2	1.21749	coding
MSTRG.18470.4	1.47776	coding
MSTRG.18470.6	0.201733	coding
MSTRG.18470.8	-0.814282	noncoding
MSTRG.18470.9	0.983469	coding
MSTRG.18470.11	-0.108579	noncoding
MSTRG.18469.2	1.90077	coding
MSTRG.18477.2	2.7243	coding
MSTRG.18477.1	2.69254	coding
MSTRG.18477.3	2.79292	coding
MSTRG.18477.4	2.55861	coding
MSTRG.18477.5	2.77536	coding
MSTRG.18477.6	2.73934	coding
MSTRG.18477.7	2.67725	coding
MSTRG.18477.8	2.78205	coding
MSTRG.18477.9	2.69171	coding
MSTRG.18477.10	2.77702	coding
MSTRG.18477.11	-2.33088	noncoding
MSTRG.18477.12	1.88691	coding
MSTRG.18478.1	-0.714356	noncoding
MSTRG.18477.14	1.50363	coding
MSTRG.18479.1	-0.320076	noncoding

MSTRG.18479.2	-0.462644	noncoding
MSTRG.18472.8	1.84147	coding
MSTRG.18473.1	-0.677222	noncoding
MSTRG.18472.12	1.64205	coding
MSTRG.18474.2	-0.948302	noncoding
MSTRG.18481.1	2.91923	coding
MSTRG.18482.1	-0.75478	noncoding
MSTRG.18483.2	-2.04588	noncoding
MSTRG.18484.1	-1.07445	noncoding
MSTRG.18484.2	-0.853545	noncoding
MSTRG.18484.3	-0.906438	noncoding
MSTRG.18480.2	1.74144	coding
MSTRG.18485.1	-1.89736	noncoding
MSTRG.18487.1	-2.11706	noncoding
MSTRG.18487.2	0.421388	coding
MSTRG.18492.2	1.79455	coding
MSTRG.18490.1	-1.33181	noncoding
MSTRG.18499.2	0.96697	coding
MSTRG.18500.1	-1.77149	noncoding
MSTRG.18504.1	0.14534	coding
MSTRG.18507.4	1.32359	coding
MSTRG.18507.5	1.1597	coding
MSTRG.18507.6	0.371706	coding
MSTRG.18508.2	-0.104056	noncoding
MSTRG.18508.3	-0.511662	noncoding
MSTRG.18511.1	1.38678	coding
MSTRG.18511.2	1.93582	coding
MSTRG.18511.3	2.04624	coding
MSTRG.18511.4	2.04134	coding
MSTRG.18513.3	1.57237	coding
MSTRG.18513.2	1.28883	coding
MSTRG.18516.2	2.67639	coding
MSTRG.18516.5	2.66355	coding
MSTRG.18517.1	-0.378231	noncoding
MSTRG.18514.2	1.54149	coding
MSTRG.18514.3	1.52271	coding
MSTRG.18514.4	1.8669	coding
MSTRG.18519.1	-2.54978	noncoding
MSTRG.18524.1	1.73924	coding
MSTRG.18524.2	3.07535	coding
MSTRG.18524.4	1.89968	coding
MSTRG.18527.1	2.93639	coding
MSTRG.18527.2	-1.46851	noncoding
MSTRG.18529.3	1.8219	coding
MSTRG.18529.5	0.836769	coding
MSTRG.18529.4	1.18024	coding
MSTRG.18530.1	-0.186244	noncoding
MSTRG.18530.2	-0.172693	noncoding
MSTRG.18531.1	0.33048	coding
MSTRG.18536.1	-2.38387	noncoding
MSTRG.18538.1	-2.41092	noncoding
MSTRG.18540.1	-2.09983	noncoding
MSTRG.18600.2	0.072363	coding
MSTRG.18614.1	-0.326605	noncoding

MSTRG.18615.1	-1.1565	noncoding
MSTRG.18626.2	-1.0279	noncoding
MSTRG.18622.6	-0.066087	noncoding
MSTRG.18622.7	-0.828775	noncoding
MSTRG.18631.2	0.335617	coding
MSTRG.18631.3	-0.257657	noncoding
MSTRG.18632.1	-0.939276	noncoding
MSTRG.18631.4	-0.123193	noncoding
MSTRG.18633.1	-2.32505	noncoding
MSTRG.18622.8	-0.594201	noncoding
MSTRG.18622.14	-0.641327	noncoding
MSTRG.18631.6	-0.058054	noncoding
MSTRG.18631.7	-2.30098	noncoding
MSTRG.18622.16	-2.05584	noncoding
MSTRG.18622.17	1.05452	coding
MSTRG.18631.9	-0.03572	noncoding
MSTRG.18631.8	0.268799	coding
MSTRG.18622.20	0.681584	coding
MSTRG.18622.21	0.670784	coding
MSTRG.18622.22	-1.73458	noncoding
MSTRG.18631.10	-0.666837	noncoding
MSTRG.18622.34	0.281292	coding
MSTRG.18638.1	-1.28839	noncoding
MSTRG.18637.1	-2.24272	noncoding
MSTRG.18542.3	2.08466	coding
MSTRG.18546.4	0.571189	coding
MSTRG.18546.5	0.535419	coding
MSTRG.18546.6	0.372742	coding
MSTRG.18546.7	-0.127115	noncoding
MSTRG.18546.8	0.444261	coding
MSTRG.18546.9	0.482949	coding
MSTRG.18546.10	0.549243	coding
MSTRG.18546.11	0.564658	coding
MSTRG.18546.12	0.583051	coding
MSTRG.18546.13	0.967625	coding
MSTRG.18546.14	1.20877	coding
MSTRG.18548.2	-2.32885	noncoding
MSTRG.18551.1	0.134157	coding
MSTRG.18552.1	-0.302835	noncoding
MSTRG.18552.2	-0.040033	noncoding
MSTRG.18555.1	1.38415	coding
MSTRG.18555.2	0.722132	coding
MSTRG.18557.4	0.998087	coding
MSTRG.18558.1	-1.41332	noncoding
MSTRG.18559.1	1.51124	coding
MSTRG.18559.4	1.19818	coding
MSTRG.18559.6	1.54485	coding
MSTRG.18561.1	2.28655	coding
MSTRG.18561.3	0.394017	coding
MSTRG.18562.1	0.199312	coding
MSTRG.18562.2	0.282058	coding
MSTRG.18562.3	1.03239	coding
MSTRG.18563.1	-1.97792	noncoding
MSTRG.18564.1	2.20266	coding

MSTRG.18564.2	2.25663	coding
MSTRG.18565.1	-0.233607	noncoding
MSTRG.18565.2	0.177616	coding
MSTRG.18568.1	3.51193	coding
MSTRG.18568.3	3.06779	coding
MSTRG.18568.6	2.40156	coding
MSTRG.18572.1	1.57622	coding
MSTRG.18572.4	1.56523	coding
MSTRG.18574.4	2.14898	coding
MSTRG.18574.5	2.2211	coding
MSTRG.18574.6	2.22771	coding
MSTRG.18574.7	1.12458	coding
MSTRG.18567.2	1.15135	coding
MSTRG.18589.1	-2.12935	noncoding
MSTRG.18590.1	-2.3325	noncoding
MSTRG.18594.2	-0.427634	noncoding
MSTRG.18594.3	-1.12839	noncoding
MSTRG.18594.5	-0.542979	noncoding
MSTRG.18594.6	-0.886277	noncoding
MSTRG.18594.7	-0.689044	noncoding
MSTRG.18654.3	0.142277	coding
MSTRG.18654.4	2.67524	coding
MSTRG.18656.1	-1.96111	noncoding
MSTRG.18645.1	0.566712	coding
MSTRG.18647.2	0.426827	coding
MSTRG.18653.1	-2.19202	noncoding
MSTRG.18659.1	1.73206	coding
MSTRG.18659.5	0.989718	coding
MSTRG.18662.5	2.47959	coding
MSTRG.18662.3	2.64998	coding
MSTRG.18666.8	0.256719	coding
MSTRG.18666.9	0.237506	coding
MSTRG.18666.4	0.955251	coding
MSTRG.18666.5	0.901831	coding
MSTRG.18666.6	0.882843	coding
MSTRG.18666.7	0.758879	coding
MSTRG.18666.10	0.423922	coding
MSTRG.18667.2	0.693599	coding
MSTRG.18677.1	-1.75321	noncoding
MSTRG.18679.3	-1.72304	noncoding
MSTRG.18681.1	-2.20427	noncoding
MSTRG.18684.2	1.07256	coding
MSTRG.18685.1	-2.2629	noncoding
MSTRG.18698.1	-0.253684	noncoding
MSTRG.18697.4	0.372456	coding
MSTRG.18702.2	1.30437	coding
MSTRG.18703.1	0.107794	coding
MSTRG.18704.1	0.149625	coding
MSTRG.18705.1	-1.13331	noncoding
MSTRG.18706.1	0.605743	coding
MSTRG.18707.1	0.370323	coding
MSTRG.18709.2	1.52188	coding
MSTRG.18709.3	1.52522	coding
MSTRG.18709.4	0.689424	coding

MSTRG.18711.4	0.902442	coding
MSTRG.18713.1	-2.39702	noncoding
MSTRG.18715.2	0.143745	coding
MSTRG.18715.4	0.546516	coding
MSTRG.18722.1	-0.469171	noncoding
MSTRG.18725.6	1.90772	coding
MSTRG.18725.7	1.93299	coding
MSTRG.18726.1	-1.95567	noncoding
MSTRG.18718.1	-1.29577	noncoding
MSTRG.18732.2	0.496103	coding
MSTRG.18733.1	0.226777	coding
MSTRG.18734.1	0.400852	coding
MSTRG.18734.2	1.07516	coding
MSTRG.18741.3	0.982433	coding
MSTRG.18743.3	-0.748048	noncoding
MSTRG.18744.1	1.41214	coding
MSTRG.18749.1	3.28139	coding
MSTRG.18749.2	2.99832	coding
MSTRG.18749.3	2.89964	coding
MSTRG.18750.1	-0.450157	noncoding
MSTRG.18751.2	-0.157941	noncoding
MSTRG.18751.4	-0.583054	noncoding
MSTRG.18752.1	0.901219	coding
MSTRG.18752.2	0.606563	coding
MSTRG.18762.2	0.658476	coding
MSTRG.18762.1	0.92016	coding
MSTRG.18763.1	2.69178	coding
MSTRG.18762.3	-0.152088	noncoding
MSTRG.18764.1	-0.726761	noncoding
MSTRG.18762.4	-0.135322	noncoding
MSTRG.18763.2	2.16211	coding
MSTRG.18763.4	2.41909	coding
MSTRG.18763.5	2.41573	coding
MSTRG.18763.7	1.88735	coding
MSTRG.18768.2	2.75108	coding
MSTRG.18756.1	-0.074234	noncoding
MSTRG.18761.1	1.44671	coding
MSTRG.18769.5	1.25017	coding
MSTRG.18769.6	1.37829	coding
MSTRG.18770.1	0.411406	coding
MSTRG.18771.1	-0.029769	noncoding
MSTRG.18772.1	-3.11948	noncoding
MSTRG.18773.1	-1.52983	noncoding
MSTRG.18776.1	-1.00034	noncoding
MSTRG.18805.1	0.714703	coding
MSTRG.18805.2	0.032845	coding
MSTRG.18816.4	0.301814	coding
MSTRG.18816.5	0.116278	coding
MSTRG.18818.2	0.419389	coding
MSTRG.18825.7	1.10588	coding
MSTRG.18829.1	-0.023163	noncoding
MSTRG.18821.1	-0.853791	noncoding
MSTRG.18822.4	1.38427	coding
MSTRG.18830.2	0.822532	coding

MSTRG.18830.3	-1.93816	noncoding
MSTRG.18838.1	-1.36029	noncoding
MSTRG.18841.1	-0.833439	noncoding
MSTRG.18850.1	-2.63627	noncoding
MSTRG.18852.1	2.51269	coding
MSTRG.18861.1	-1.04341	noncoding
MSTRG.18862.1	-2.26277	noncoding
MSTRG.18872.1	-1.92301	noncoding
MSTRG.18882.1	-1.84774	noncoding
MSTRG.18894.1	-1.90038	noncoding
MSTRG.18894.4	-1.78703	noncoding
MSTRG.18894.6	-1.76214	noncoding
MSTRG.18894.7	-1.48219	noncoding
MSTRG.18894.3	-1.928	noncoding
MSTRG.18894.5	-1.86931	noncoding
MSTRG.18906.1	0.427257	coding
MSTRG.18907.1	-0.117639	noncoding
MSTRG.18908.1	-1.81244	noncoding
MSTRG.18911.1	-2.18813	noncoding
MSTRG.18917.2	2.79421	coding
MSTRG.18917.3	2.25933	coding
MSTRG.18958.1	-0.924947	noncoding
MSTRG.18967.1	-0.121799	noncoding
MSTRG.18967.3	1.87355	coding
MSTRG.18955.1	-0.468599	noncoding
MSTRG.18979.1	-0.973397	noncoding
MSTRG.18983.2	0.955879	coding
MSTRG.18986.1	0.378102	coding
MSTRG.18987.1	1.22471	coding
MSTRG.18984.2	-1.68904	noncoding
MSTRG.18997.1	-0.175298	noncoding
MSTRG.18997.2	-0.17052	noncoding
MSTRG.18999.1	-2.19666	noncoding
MSTRG.18999.2	-0.238582	noncoding
MSTRG.19005.1	-0.096649	noncoding
MSTRG.19006.1	0.531612	coding
MSTRG.19014.6	2.57379	coding
MSTRG.19014.5	2.67603	coding
MSTRG.19015.1	-1.15171	noncoding
MSTRG.19015.2	-2.00762	noncoding
MSTRG.19015.3	-1.84989	noncoding
MSTRG.19009.1	-2.58283	noncoding
MSTRG.19024.1	-2.03147	noncoding
MSTRG.19041.1	-2.40622	noncoding
MSTRG.19049.1	0.446273	coding
MSTRG.19049.2	0.585234	coding
MSTRG.19049.3	-2.67197	noncoding
MSTRG.19049.4	-0.460618	noncoding
MSTRG.19052.2	2.34678	coding
MSTRG.19034.1	-0.826322	noncoding
MSTRG.19038.1	-1.35526	noncoding
MSTRG.19039.3	1.31262	coding
MSTRG.19053.2	1.63339	coding
MSTRG.19053.3	1.37097	coding

MSTRG.19059.4	2.82247	coding
MSTRG.19059.3	2.99665	coding
MSTRG.19061.1	0.654242	coding
MSTRG.19063.1	-1.97219	noncoding
MSTRG.19066.1	-0.350257	noncoding
MSTRG.19068.2	-1.84376	noncoding
MSTRG.19069.1	1.3264	coding
MSTRG.19069.2	1.4562	coding
MSTRG.19069.4	1.79647	coding
MSTRG.19069.6	1.17596	coding
MSTRG.19072.1	2.66414	coding
MSTRG.19072.2	2.2875	coding
MSTRG.19072.3	2.00252	coding
MSTRG.19072.4	2.27012	coding
MSTRG.19073.3	-2.39244	noncoding
MSTRG.19075.1	0.151637	coding
MSTRG.19089.2	1.9321	coding
MSTRG.19089.3	2.56185	coding
MSTRG.19090.2	1.36675	coding
MSTRG.19094.1	2.66205	coding
MSTRG.19094.2	2.9817	coding
MSTRG.19094.3	2.85952	coding
MSTRG.19094.5	1.50883	coding
MSTRG.19095.1	-1.29988	noncoding
MSTRG.19096.1	3.11457	coding
MSTRG.19099.1	-2.21363	noncoding
MSTRG.19100.1	-1.79702	noncoding
MSTRG.19101.1	-1.31922	noncoding
MSTRG.19078.2	1.45899	coding
MSTRG.19078.3	1.55071	coding
MSTRG.19079.1	0.979661	coding
MSTRG.19081.1	0.72097	coding
MSTRG.19083.1	-1.86517	noncoding
MSTRG.19084.3	1.63254	coding
MSTRG.19085.1	-1.52804	noncoding
MSTRG.19107.3	2.66232	coding
MSTRG.19110.1	-1.084	noncoding
MSTRG.19113.4	0.459845	coding
MSTRG.19115.4	2.50953	coding
MSTRG.19115.5	2.34843	coding
MSTRG.19116.2	2.69678	coding
MSTRG.19116.4	-2.16815	noncoding
MSTRG.19117.2	2.53151	coding
MSTRG.19121.1	-1.82337	noncoding
MSTRG.19122.1	-1.78437	noncoding
MSTRG.19126.2	1.05461	coding
MSTRG.19127.1	-0.806297	noncoding
MSTRG.19129.1	0.727791	coding
MSTRG.19129.2	0.81389	coding
MSTRG.19129.4	0.585087	coding
MSTRG.19129.5	1.03448	coding
MSTRG.19131.8	-0.303245	noncoding
MSTRG.19131.7	1.09591	coding
MSTRG.19131.4	1.28709	coding

MSTRG.19131.5	1.10781	coding
MSTRG.19131.6	1.16061	coding
MSTRG.19133.3	-0.008005	noncoding
MSTRG.19133.4	0.158495	coding
MSTRG.19134.3	1.56413	coding
MSTRG.19134.4	1.43792	coding
MSTRG.19136.1	-1.29191	noncoding
MSTRG.19138.1	1.94301	coding
MSTRG.19142.5	1.42966	coding
MSTRG.19148.3	1.24317	coding
MSTRG.19148.4	1.203	coding
MSTRG.19150.1	0.77435	coding
MSTRG.19150.2	1.29149	coding
MSTRG.19152.2	2.39845	coding
MSTRG.19144.1	-2.2	noncoding
MSTRG.19146.1	-0.731535	noncoding
MSTRG.19155.1	-0.961414	noncoding
MSTRG.19156.1	-2.25123	noncoding
MSTRG.19158.4	2.97381	coding
MSTRG.19175.1	-1.1258	noncoding
MSTRG.19177.4	0.263466	coding
MSTRG.19177.1	1.0223	coding
MSTRG.19177.3	1.2091	coding
MSTRG.19177.5	0.324362	coding
MSTRG.19177.6	-1.09913	noncoding
MSTRG.19177.7	1.40545	coding
MSTRG.19177.8	0.775396	coding
MSTRG.19177.9	0.51898	coding
MSTRG.19177.10	1.73046	coding
MSTRG.19177.12	0.038998	coding
MSTRG.19177.13	-0.529314	noncoding
MSTRG.19183.1	-0.950394	noncoding
MSTRG.19183.2	-1.85566	noncoding
MSTRG.19189.2	0.539206	coding
MSTRG.19166.2	0.696656	coding
MSTRG.19166.3	2.86623	coding
MSTRG.19168.1	-0.305074	noncoding
MSTRG.19169.4	1.71313	coding
MSTRG.19171.7	-0.676668	noncoding
MSTRG.19171.2	0.734279	coding
MSTRG.19171.3	0.925738	coding
MSTRG.19171.4	0.811026	coding
MSTRG.19171.5	0.78768	coding
MSTRG.19171.6	0.55522	coding
MSTRG.19194.2	-0.865861	noncoding
MSTRG.19196.1	-0.295533	noncoding
MSTRG.19196.2	-0.243864	noncoding
MSTRG.19203.1	1.37886	coding
MSTRG.19203.2	-0.269292	noncoding
MSTRG.19204.3	1.54747	coding
MSTRG.19205.1	1.08389	coding
MSTRG.19205.2	1.0516	coding
MSTRG.19205.3	0.348799	coding
MSTRG.19206.1	-2.68826	noncoding

MSTRG.19207.2	-0.694989	noncoding
MSTRG.19210.5	1.68906	coding
MSTRG.19210.6	0.670169	coding
MSTRG.19210.4	1.84274	coding
MSTRG.19210.7	1.59293	coding
MSTRG.19210.3	1.88283	coding
MSTRG.19210.8	0.408434	coding
MSTRG.19197.1	-0.820569	noncoding
MSTRG.19202.1	-1.45617	noncoding
MSTRG.19213.1	0.25898	coding
MSTRG.19213.2	-0.218153	noncoding
MSTRG.19215.1	-0.40162	noncoding
MSTRG.19217.4	1.60059	coding
MSTRG.19217.2	1.82109	coding
MSTRG.19217.5	1.52545	coding
MSTRG.19218.1	-1.3553	noncoding
MSTRG.19318.1	0.196458	coding
MSTRG.19319.3	0.827275	coding
MSTRG.19323.1	-1.59112	noncoding
MSTRG.19319.4	0.556536	coding
MSTRG.19319.5	0.928722	coding
MSTRG.19319.6	-0.521327	noncoding
MSTRG.19325.1	-0.401532	noncoding
MSTRG.19325.2	0.326387	coding
MSTRG.19326.1	0.243419	coding
MSTRG.19328.1	0.71923	coding
MSTRG.19338.1	-1.21191	noncoding
MSTRG.19339.1	-0.152535	noncoding
MSTRG.19340.1	-0.131547	noncoding
MSTRG.19342.1	-0.993501	noncoding
MSTRG.19347.2	0.006624	coding
MSTRG.19339.2	1.01391	coding
MSTRG.19339.3	0.766463	coding
MSTRG.19339.4	0.578162	coding
MSTRG.19339.5	0.416964	coding
MSTRG.19339.6	0.21608	coding
MSTRG.19339.7	0.068076	coding
MSTRG.19339.8	1.1603	coding
MSTRG.19349.1	-0.714276	noncoding
MSTRG.19221.1	0.688128	coding
MSTRG.19221.2	-0.20334	noncoding
MSTRG.19230.1	-0.441836	noncoding
MSTRG.19235.1	-1.23257	noncoding
MSTRG.19249.1	-1.88176	noncoding
MSTRG.19249.2	-1.4897	noncoding
MSTRG.19249.3	-1.98308	noncoding
MSTRG.19249.4	0.608779	coding
MSTRG.19237.1	0.427827	coding
MSTRG.19252.4	-0.132141	noncoding
MSTRG.19253.1	1.35014	coding
MSTRG.19253.4	0.996825	coding
MSTRG.19253.5	-0.273414	noncoding
MSTRG.19254.1	0.412323	coding
MSTRG.19258.1	-1.20206	noncoding

MSTRG.19258.2	-1.77925	noncoding
MSTRG.19264.1	0.322455	coding
MSTRG.19265.1	0.014574	coding
MSTRG.19266.2	2.14467	coding
MSTRG.19270.2	1.79086	coding
MSTRG.19278.1	-0.592283	noncoding
MSTRG.19281.2	1.94148	coding
MSTRG.19285.2	0.781972	coding
MSTRG.19285.3	0.542294	coding
MSTRG.19285.4	0.886795	coding
MSTRG.19291.1	-0.432393	noncoding
MSTRG.19291.2	-0.735992	noncoding
MSTRG.19283.1	0.722441	coding
MSTRG.19283.3	1.07476	coding
MSTRG.19305.4	0.642646	coding
MSTRG.19311.1	1.57721	coding
MSTRG.19311.2	3.21699	coding
MSTRG.19311.4	-2.06484	noncoding
MSTRG.19311.5	2.4542	coding
MSTRG.19311.6	2.05027	coding
MSTRG.19312.1	-1.30606	noncoding
MSTRG.19314.1	0.98125	coding
MSTRG.19314.3	0.913639	coding
MSTRG.19314.4	0.855428	coding
MSTRG.19316.1	2.52909	coding
MSTRG.19314.6	0.728244	coding
MSTRG.19316.2	2.49766	coding
MSTRG.19316.3	2.54876	coding
MSTRG.19316.4	2.84342	coding
MSTRG.19317.1	1.17491	coding
MSTRG.19313.1	1.24448	coding
MSTRG.19352.1	1.03948	coding
MSTRG.19352.3	2.83197	coding
MSTRG.19352.4	1.62208	coding
MSTRG.19352.5	1.18022	coding
MSTRG.19352.12	0.350846	coding
MSTRG.19352.10	0.678577	coding
MSTRG.19352.13	0.504206	coding
MSTRG.19352.9	0.656457	coding
MSTRG.19352.8	0.718963	coding
MSTRG.19352.11	1.02499	coding
MSTRG.19352.6	0.754224	coding
MSTRG.19354.1	0.034254	coding
MSTRG.19359.2	0.783618	coding
MSTRG.19359.3	0.308088	coding
MSTRG.19359.4	0.675785	coding
MSTRG.19359.5	0.063123	coding
MSTRG.19361.1	1.54743	coding
MSTRG.19361.2	1.72552	coding
MSTRG.19361.3	1.69158	coding
MSTRG.19361.4	1.91495	coding
MSTRG.19361.5	1.5228	coding
MSTRG.19361.6	0.833739	coding
MSTRG.19361.10	-0.050611	noncoding

MSTRG.19361.7	2.3817	coding
MSTRG.19361.8	2.90513	coding
MSTRG.19361.9	2.32239	coding
MSTRG.19361.11	-0.146129	noncoding
MSTRG.19361.12	-0.54263	noncoding
MSTRG.19361.13	-0.641494	noncoding
MSTRG.19361.14	-1.18906	noncoding
MSTRG.19363.1	-1.11612	noncoding
MSTRG.19401.1	0.003078	coding
MSTRG.19402.1	-1.94957	noncoding
MSTRG.19368.1	-2.32333	noncoding
MSTRG.19369.1	-2.39847	noncoding
MSTRG.19370.1	-2.17718	noncoding
MSTRG.19407.1	-1.55605	noncoding
MSTRG.19408.1	1.46638	coding
MSTRG.19414.1	1.98215	coding
MSTRG.19414.2	1.81065	coding
MSTRG.19415.1	1.54748	coding
MSTRG.19415.3	1.6364	coding
MSTRG.19415.4	1.66238	coding
MSTRG.19416.1	2.02186	coding
MSTRG.19416.2	0.146789	coding
MSTRG.19413.1	-0.609138	noncoding
MSTRG.19420.2	1.9445	coding
MSTRG.19422.1	1.95579	coding
MSTRG.19422.3	2.21095	coding
MSTRG.19422.2	2.25902	coding
MSTRG.19422.4	1.49996	coding
MSTRG.19422.5	1.98158	coding
MSTRG.19423.1	0.425104	coding
MSTRG.19425.1	2.69307	coding
MSTRG.19425.4	1.4908	coding
MSTRG.19428.1	2.34837	coding
MSTRG.19432.1	-0.834631	noncoding
MSTRG.19434.1	1.83279	coding
MSTRG.19435.1	0.44184	coding
MSTRG.19436.1	-1.75803	noncoding
MSTRG.19440.2	0.852534	coding
MSTRG.19440.1	-1.23474	noncoding
MSTRG.19441.1	-0.704851	noncoding
MSTRG.19446.2	1.88502	coding
MSTRG.19464.1	2.44341	coding
MSTRG.19464.6	-0.798754	noncoding
MSTRG.19478.2	0.442389	coding
MSTRG.19479.1	-0.924077	noncoding
MSTRG.19448.1	-0.553057	noncoding
MSTRG.19458.2	0.372905	coding
MSTRG.19458.4	0.63493	coding
MSTRG.19458.3	-0.496996	noncoding
MSTRG.19458.5	2.6191	coding
MSTRG.19459.1	-0.698965	noncoding
MSTRG.19481.1	0.633502	coding
MSTRG.19480.2	1.75682	coding
MSTRG.19485.1	0.924263	coding

MSTRG.19485.2	1.88154	coding
MSTRG.19485.3	-0.336044	noncoding
MSTRG.19486.1	0.655718	coding
MSTRG.19490.5	2.20314	coding
MSTRG.19490.8	-0.125079	noncoding
MSTRG.19491.1	-2.10306	noncoding
MSTRG.19492.1	0.918637	coding
MSTRG.19493.1	-2.77885	noncoding
MSTRG.19494.1	-1.90607	noncoding
MSTRG.19495.1	0.071911	coding
MSTRG.19496.1	-1.16687	noncoding
MSTRG.19505.2	0.178642	coding
MSTRG.19505.3	-0.303486	noncoding
MSTRG.19505.4	0.30828	coding
MSTRG.19506.1	-0.728312	noncoding
MSTRG.19507.4	0.634343	coding
MSTRG.19508.3	-1.05433	noncoding
MSTRG.19509.2	1.66176	coding
MSTRG.19515.3	-0.836637	noncoding
MSTRG.19515.2	0.507426	coding
MSTRG.19515.4	-1.23358	noncoding
MSTRG.19521.1	1.37672	coding
MSTRG.19521.2	1.10221	coding
MSTRG.19522.4	0.966004	coding
MSTRG.19526.1	-2.30023	noncoding
MSTRG.19530.1	0.5827	coding
MSTRG.19531.1	-1.68541	noncoding
MSTRG.19526.2	0.021922	coding
MSTRG.19532.1	-1.3572	noncoding
MSTRG.19516.1	-0.965301	noncoding
MSTRG.19533.5	0.000768	coding
MSTRG.19533.6	-1.14406	noncoding
MSTRG.19534.1	0.465546	coding
MSTRG.19533.7	3.27079	coding
MSTRG.19535.1	-1.04285	noncoding
MSTRG.19538.1	-1.34329	noncoding
MSTRG.19540.1	-0.855826	noncoding
MSTRG.19543.4	2.32068	coding
MSTRG.19543.5	2.13896	coding
MSTRG.19543.6	2.61482	coding
MSTRG.19544.1	-0.731489	noncoding
MSTRG.19547.1	-0.146158	noncoding
MSTRG.19551.2	0.729323	coding
MSTRG.19555.1	0.198749	coding
MSTRG.19559.7	3.33449	coding
MSTRG.19559.8	3.25442	coding
MSTRG.19559.5	3.28502	coding
MSTRG.19559.6	3.25846	coding
MSTRG.19560.1	0.838839	coding
MSTRG.19561.1	0.28548	coding
MSTRG.19562.2	0.111805	coding
MSTRG.19562.1	-1.60507	noncoding
MSTRG.19562.4	0.107787	coding
MSTRG.19562.5	0.160658	coding

MSTRG.19562.3	0.176215	coding
MSTRG.19562.6	0.159934	coding
MSTRG.19559.9	1.31598	coding
MSTRG.19563.1	0.475238	coding
MSTRG.19568.1	1.77334	coding
MSTRG.19568.2	0.20534	coding
MSTRG.19568.3	-0.558309	noncoding
MSTRG.19568.4	-0.037321	noncoding
MSTRG.19572.9	1.69389	coding
MSTRG.19572.8	2.04359	coding
MSTRG.19572.5	3.0856	coding
MSTRG.19572.7	2.56746	coding
MSTRG.19572.6	1.54376	coding
MSTRG.19573.1	-0.183066	noncoding
MSTRG.19572.10	0.623011	coding
MSTRG.19572.11	0.736851	coding
MSTRG.19574.1	-1.75989	noncoding
MSTRG.19572.12	0.838393	coding
MSTRG.19570.2	2.60185	coding
MSTRG.19570.3	1.16306	coding
MSTRG.19570.4	1.94985	coding
MSTRG.19571.2	2.39813	coding
MSTRG.19577.2	-0.254942	noncoding
MSTRG.19579.1	2.7729	coding
MSTRG.19579.2	2.19495	coding
MSTRG.19582.2	1.62145	coding
MSTRG.19582.4	1.5609	coding
MSTRG.19582.5	1.49728	coding
MSTRG.19582.6	1.70227	coding
MSTRG.19582.3	1.5846	coding
MSTRG.19582.7	1.61566	coding
MSTRG.19581.1	-1.19939	noncoding
MSTRG.19583.1	1.80278	coding
MSTRG.19590.1	0.59054	coding
MSTRG.19590.2	-0.061384	noncoding
MSTRG.19591.1	-1.12342	noncoding
MSTRG.19591.2	-0.431157	noncoding
MSTRG.19593.1	-0.572947	noncoding
MSTRG.19596.4	2.35316	coding
MSTRG.19598.3	1.85327	coding
MSTRG.19598.5	2.21424	coding
MSTRG.19598.6	1.5719	coding
MSTRG.19600.1	-0.16173	noncoding
MSTRG.19602.1	1.84709	coding
MSTRG.19603.1	-2.58354	noncoding
MSTRG.19602.3	1.81321	coding
MSTRG.19602.4	1.91011	coding
MSTRG.19602.5	1.66686	coding
MSTRG.19602.6	1.71841	coding
MSTRG.19604.6	2.16865	coding
MSTRG.19604.4	2.22321	coding
MSTRG.19604.8	1.86976	coding
MSTRG.19622.1	0.505375	coding
MSTRG.19624.7	1.7727	coding

MSTRG.19624.9	1.74943	coding
MSTRG.19616.2	1.06546	coding
MSTRG.19614.1	1.04126	coding
MSTRG.19614.2	1.04953	coding
MSTRG.19629.1	1.83531	coding
MSTRG.19628.1	-2.45989	noncoding
MSTRG.19628.2	-2.29654	noncoding
MSTRG.19638.4	1.85162	coding
MSTRG.19638.2	1.79294	coding
MSTRG.19638.1	1.96489	coding
MSTRG.19639.1	-0.004843	noncoding
MSTRG.19640.1	-0.091258	noncoding
MSTRG.19642.5	0.829875	coding
MSTRG.19642.6	0.874918	coding
MSTRG.19642.7	0.703079	coding
MSTRG.19643.1	3.11068	coding
MSTRG.19648.1	1.79373	coding
MSTRG.19649.3	1.1656	coding
MSTRG.19650.1	-2.01257	noncoding
MSTRG.19671.9	2.20028	coding
MSTRG.19666.1	0.485028	coding
MSTRG.19672.2	-0.124346	noncoding
MSTRG.19672.3	-0.759911	noncoding
MSTRG.19672.4	-0.873867	noncoding
MSTRG.19674.1	1.53871	coding
MSTRG.19675.1	1.32181	coding
MSTRG.19676.2	0.471564	coding
MSTRG.19678.5	2.70458	coding
MSTRG.19678.8	2.30737	coding
MSTRG.19681.1	-1.16202	noncoding
MSTRG.19682.1	-0.357899	noncoding
MSTRG.19683.1	-0.515643	noncoding
MSTRG.19684.1	1.2027	coding
MSTRG.19684.2	1.06072	coding
MSTRG.19701.1	-1.24463	noncoding
MSTRG.19707.2	1.96687	coding
MSTRG.19708.1	-1.51882	noncoding
MSTRG.19707.3	0.132706	coding
MSTRG.19713.2	0.801347	coding
MSTRG.19718.1	-0.533876	noncoding
MSTRG.19720.3	1.15057	coding
MSTRG.19721.1	-0.311935	noncoding
MSTRG.19720.4	-0.195252	noncoding
MSTRG.19720.5	-0.177153	noncoding
MSTRG.19722.1	1.46328	coding
MSTRG.19723.1	1.61343	coding
MSTRG.19723.4	0.165824	coding
MSTRG.19723.3	1.99024	coding
MSTRG.19723.5	1.42651	coding
MSTRG.19723.6	1.12654	coding
MSTRG.19733.1	0.949369	coding
MSTRG.19743.1	-1.68978	noncoding
MSTRG.19744.2	0.856504	coding
MSTRG.19744.4	-1.02732	noncoding

MSTRG.19744.5	-0.224353	noncoding
MSTRG.19744.6	-0.386044	noncoding
MSTRG.19744.7	-1.90682	noncoding
MSTRG.19744.8	-1.24338	noncoding
MSTRG.19744.9	-1.91812	noncoding
MSTRG.19744.10	-1.81794	noncoding
MSTRG.19744.11	-1.85115	noncoding
MSTRG.19744.12	-1.89001	noncoding
MSTRG.19744.13	-1.58516	noncoding
MSTRG.19744.14	-0.732223	noncoding
MSTRG.19744.15	-0.224142	noncoding
MSTRG.19744.16	-2.04884	noncoding
MSTRG.19744.17	-1.97171	noncoding
MSTRG.19744.18	-2.03318	noncoding
MSTRG.19744.19	-2.01475	noncoding
MSTRG.19744.20	-1.71819	noncoding
MSTRG.19744.21	-2.0824	noncoding
MSTRG.19744.22	-2.06519	noncoding
MSTRG.19744.23	-1.97883	noncoding
MSTRG.19744.24	-2.05168	noncoding
MSTRG.19744.25	-2.13963	noncoding
MSTRG.19744.26	-2.15822	noncoding
MSTRG.19744.27	-2.0644	noncoding
MSTRG.19744.28	-1.95455	noncoding
MSTRG.19744.29	-2.15345	noncoding
MSTRG.19744.30	-1.77327	noncoding
MSTRG.19744.31	-2.07283	noncoding
MSTRG.19744.32	-2.10595	noncoding
MSTRG.19739.1	-1.64929	noncoding
MSTRG.19751.1	0.43277	coding
MSTRG.19752.1	-0.756049	noncoding
MSTRG.19752.2	-0.570211	noncoding
MSTRG.19751.2	-1.08316	noncoding
MSTRG.19751.3	-2.09082	noncoding
MSTRG.19755.1	1.24549	coding
MSTRG.19755.2	1.38503	coding
MSTRG.19755.3	1.52878	coding
MSTRG.19755.4	1.53971	coding
MSTRG.19755.5	1.53878	coding
MSTRG.19761.1	0.135957	coding
MSTRG.19765.1	-2.48846	noncoding
MSTRG.19766.1	-0.361055	noncoding
MSTRG.19767.3	0.139036	coding
MSTRG.19768.1	0.507675	coding
MSTRG.19757.2	1.85621	coding
MSTRG.19774.1	2.5229	coding
MSTRG.19779.2	-0.056953	noncoding
MSTRG.19784.2	0.744646	coding
MSTRG.19785.3	0.331671	coding
MSTRG.19788.1	-0.031521	noncoding
MSTRG.19791.1	-1.47193	noncoding
MSTRG.19803.2	0.211662	coding
MSTRG.19803.6	1.05068	coding
MSTRG.19803.7	0.87013	coding

MSTRG.19803.5	0.840495	coding
MSTRG.19804.1	-1.65176	noncoding
MSTRG.19805.1	0.039321	coding
MSTRG.19806.1	-2.10396	noncoding
MSTRG.19807.3	1.79402	coding
MSTRG.19809.1	-2.30286	noncoding
MSTRG.19811.1	-1.3135	noncoding
MSTRG.19821.1	-0.068298	noncoding
MSTRG.19822.1	-1.93608	noncoding
MSTRG.19821.2	-1.26267	noncoding
MSTRG.19821.3	-1.43026	noncoding
MSTRG.19821.4	-1.4591	noncoding
MSTRG.19821.5	-0.197127	noncoding
MSTRG.19821.7	-0.28366	noncoding
MSTRG.19821.6	-1.0524	noncoding
MSTRG.19821.8	-1.12721	noncoding
MSTRG.19821.9	-1.70379	noncoding
MSTRG.19825.1	1.14617	coding
MSTRG.19827.1	-0.714226	noncoding
MSTRG.19831.2	-0.184213	noncoding
MSTRG.19840.1	-2.13565	noncoding
MSTRG.19841.1	2.64207	coding
MSTRG.19848.2	1.43113	coding
MSTRG.19856.8	1.36479	coding
MSTRG.19856.9	1.43913	coding
MSTRG.19856.11	0.459296	coding
MSTRG.19857.2	1.99528	coding
MSTRG.19861.2	-0.070807	noncoding
MSTRG.19863.3	1.28081	coding
MSTRG.19863.2	1.14872	coding
MSTRG.19863.4	1.13739	coding
MSTRG.19868.1	-0.569569	noncoding
MSTRG.19868.2	-1.23395	noncoding
MSTRG.19879.1	-1.46277	noncoding
MSTRG.19884.1	0.534675	coding
MSTRG.19884.2	-1.53237	noncoding
MSTRG.19884.3	0.080126	coding
MSTRG.19884.4	-0.800788	noncoding
MSTRG.19885.1	0.151568	coding
MSTRG.19888.1	-2.4256	noncoding
MSTRG.19893.3	0.896588	coding
MSTRG.19906.4	1.46025	coding
MSTRG.19907.1	-2.23995	noncoding
MSTRG.19908.2	0.376576	coding
MSTRG.19909.5	1.04201	coding
MSTRG.19909.7	1.06588	coding
MSTRG.19909.6	0.951436	coding
MSTRG.19904.1	1.29614	coding
MSTRG.19904.2	1.41909	coding
MSTRG.19904.3	0.761092	coding
MSTRG.19905.1	1.63059	coding
MSTRG.19905.2	1.25417	coding
MSTRG.19917.1	0.775183	coding
MSTRG.19918.3	1.00375	coding

MSTRG.19912.3	0.964565	coding
MSTRG.19912.4	0.552325	coding
MSTRG.19913.2	0.687484	coding
MSTRG.19914.2	3.06373	coding
MSTRG.19923.1	-1.84133	noncoding
MSTRG.19924.1	-0.445434	noncoding
MSTRG.19924.2	-0.459683	noncoding
MSTRG.19925.3	2.47295	coding
MSTRG.19925.5	1.58689	coding
MSTRG.19926.5	1.13124	coding
MSTRG.19926.4	1.03872	coding
MSTRG.19926.3	1.61137	coding
MSTRG.19930.1	-0.081172	noncoding
MSTRG.19931.1	-2.31266	noncoding
MSTRG.19937.1	-0.954125	noncoding
MSTRG.19938.1	-1.91489	noncoding
MSTRG.19939.4	-1.6499	noncoding
MSTRG.19941.1	0.529886	coding
MSTRG.19951.1	0.785363	coding
MSTRG.19954.2	0.973601	coding
MSTRG.19958.1	0.104497	coding
MSTRG.19959.1	1.37467	coding
MSTRG.19959.3	1.43698	coding
MSTRG.19959.5	1.49691	coding
MSTRG.19959.2	1.43967	coding
MSTRG.19959.8	1.45635	coding
MSTRG.19959.9	1.42069	coding
MSTRG.19959.10	1.47038	coding
MSTRG.19959.11	1.25907	coding
MSTRG.19959.12	1.05051	coding
MSTRG.19959.13	1.45251	coding
MSTRG.19942.1	1.78453	coding
MSTRG.19945.1	-0.148309	noncoding
MSTRG.19947.1	-1.27754	noncoding
MSTRG.19946.2	0.984131	coding
MSTRG.19967.1	-0.047264	noncoding
MSTRG.19967.5	-0.021584	noncoding
MSTRG.19967.4	0.337007	coding
MSTRG.19967.8	-1.41121	noncoding
MSTRG.19965.2	2.086	coding
MSTRG.19978.1	1.77931	coding
MSTRG.19978.3	1.79473	coding
MSTRG.19978.2	1.67292	coding
MSTRG.19978.4	1.68371	coding
MSTRG.19978.5	1.88505	coding
MSTRG.19979.1	-0.610505	noncoding
MSTRG.19980.1	2.20917	coding
MSTRG.19983.1	0.023439	coding
MSTRG.19985.1	-0.44644	noncoding
MSTRG.19981.1	-1.45106	noncoding
MSTRG.19990.1	-0.802058	noncoding
MSTRG.19990.2	-0.887339	noncoding
MSTRG.20000.1	0.473355	coding
MSTRG.20003.1	-0.769839	noncoding

MSTRG.20006.1	-0.663805	noncoding
MSTRG.20008.1	-1.45479	noncoding
MSTRG.20013.4	2.95335	coding
MSTRG.20013.5	-0.803051	noncoding
MSTRG.20015.3	1.43494	coding
MSTRG.20015.5	1.4003	coding
MSTRG.20015.6	0.141063	coding
MSTRG.20018.1	0.283401	coding
MSTRG.20018.2	-0.076007	noncoding
MSTRG.20018.3	0.937063	coding
MSTRG.20027.1	1.22992	coding
MSTRG.20033.1	-0.119911	noncoding
MSTRG.20037.1	-0.963165	noncoding
MSTRG.20038.4	-1.4035	noncoding
MSTRG.20047.1	-0.190442	noncoding
MSTRG.20056.1	0.97083	coding
MSTRG.20056.4	1.06606	coding
MSTRG.20057.3	1.46676	coding
MSTRG.20057.4	0.868655	coding
MSTRG.20059.1	1.52683	coding
MSTRG.20059.5	-1.3898	noncoding
MSTRG.20061.3	0.97854	coding
MSTRG.20061.2	1.84112	coding
MSTRG.20063.1	-2.18251	noncoding
MSTRG.20066.1	-0.483169	noncoding
MSTRG.20069.4	-0.175968	noncoding
MSTRG.20074.2	1.92406	coding
MSTRG.20074.4	1.84975	coding
MSTRG.20075.1	-0.170118	noncoding
MSTRG.20079.1	0.911283	coding
MSTRG.20086.5	2.08807	coding
MSTRG.20086.6	1.7778	coding
MSTRG.20086.9	1.98076	coding
MSTRG.20087.1	1.31376	coding
MSTRG.20086.10	2.00686	coding
MSTRG.20088.2	0.959072	coding
MSTRG.20090.1	-1.54832	noncoding
MSTRG.20092.2	2.10701	coding
MSTRG.20094.2	0.631271	coding
MSTRG.20097.2	2.08573	coding
MSTRG.20098.1	-0.430754	noncoding
MSTRG.20107.1	-1.73907	noncoding
MSTRG.20109.1	-0.803956	noncoding
MSTRG.20110.1	1.15914	coding
MSTRG.20110.2	1.57237	coding
MSTRG.20110.3	1.14006	coding
MSTRG.20114.1	2.21358	coding
MSTRG.20114.2	0.40923	coding
MSTRG.20114.4	1.95584	coding
MSTRG.20115.1	0.591784	coding
MSTRG.20112.1	-2.25423	noncoding
MSTRG.20118.1	-1.57754	noncoding
MSTRG.20117.3	0.697277	coding
MSTRG.20121.2	-1.81097	noncoding

MSTRG.20121.1	-0.291582	noncoding
MSTRG.20119.1	-1.28355	noncoding
MSTRG.20120.1	1.77668	coding
MSTRG.20126.2	1.07326	coding
MSTRG.20126.4	-0.245729	noncoding
MSTRG.20126.3	0.578363	coding
MSTRG.20128.2	0.049299	coding
MSTRG.20132.3	0.053699	coding
MSTRG.20132.4	0.29881	coding
MSTRG.20135.1	1.97727	coding
MSTRG.20148.1	0.521572	coding
MSTRG.20158.1	0.286458	coding
MSTRG.20158.2	0.106327	coding
MSTRG.20158.5	2.56415	coding
MSTRG.20158.6	-0.226137	noncoding
MSTRG.20162.4	2.47204	coding
MSTRG.20163.1	-0.728448	noncoding
MSTRG.20164.1	-0.832433	noncoding
MSTRG.20168.1	0.432548	coding
MSTRG.20154.2	1.16494	coding
MSTRG.20155.1	1.71457	coding
MSTRG.20156.1	0.554389	coding
MSTRG.20157.1	0.064497	coding
MSTRG.20154.3	0.543299	coding
MSTRG.20172.3	0.365893	coding
MSTRG.20173.1	-0.952634	noncoding
MSTRG.20177.2	1.15124	coding
MSTRG.20179.1	-1.18369	noncoding
MSTRG.20180.1	-1.58004	noncoding
MSTRG.20186.1	1.32498	coding
MSTRG.20188.1	0.212572	coding
MSTRG.20189.2	-1.89625	noncoding
MSTRG.20189.1	-2.36014	noncoding
MSTRG.20190.1	-1.18539	noncoding
MSTRG.20193.2	-0.199139	noncoding
MSTRG.20193.3	0.144147	coding
MSTRG.20198.2	2.05278	coding
MSTRG.20198.1	2.20385	coding
MSTRG.20199.1	-0.747875	noncoding
MSTRG.20200.3	2.65048	coding
MSTRG.20202.1	-0.817792	noncoding
MSTRG.20203.2	0.949233	coding
MSTRG.20210.4	1.37202	coding
MSTRG.20210.3	1.37832	coding
MSTRG.20210.5	1.10464	coding
MSTRG.20210.6	0.677024	coding
MSTRG.20212.1	0.363481	coding
MSTRG.20212.2	-0.071361	noncoding
MSTRG.20212.3	-1.67188	noncoding
MSTRG.20212.4	-0.086229	noncoding
MSTRG.20210.7	-0.504587	noncoding
MSTRG.20207.2	1.58396	coding
MSTRG.20207.3	1.6389	coding
MSTRG.20207.4	1.64865	coding

MSTRG.20208.3	1.75792	coding
MSTRG.20213.5	0.076027	coding
MSTRG.20213.6	-0.52604	noncoding
MSTRG.20214.1	0.250049	coding
MSTRG.20215.1	-2.44895	noncoding
MSTRG.20216.1	0.621719	coding
MSTRG.20218.4	0.643794	coding
MSTRG.20218.5	0.225549	coding
MSTRG.20220.1	-0.580863	noncoding
MSTRG.20225.1	-1.88473	noncoding
MSTRG.20225.2	-1.85153	noncoding
MSTRG.20226.2	0.759806	coding
MSTRG.20226.3	1.30677	coding
MSTRG.20226.4	0.960477	coding
MSTRG.20227.6	2.18544	coding
MSTRG.20227.5	2.26723	coding
MSTRG.20227.3	2.58065	coding
MSTRG.20227.4	2.57439	coding
MSTRG.20227.8	2.15861	coding
MSTRG.20227.10	2.21051	coding
MSTRG.20227.9	2.15085	coding
MSTRG.20227.11	2.39025	coding
MSTRG.20227.12	2.18489	coding
MSTRG.20228.1	-1.28432	noncoding
MSTRG.20229.1	2.44845	coding
MSTRG.20222.3	1.23842	coding
MSTRG.20222.4	0.743271	coding
MSTRG.20223.1	0.869955	coding
MSTRG.20223.2	0.611543	coding
MSTRG.20241.1	2.00962	coding
MSTRG.20241.2	1.96143	coding
MSTRG.20241.3	1.96693	coding
MSTRG.20241.5	1.99652	coding
MSTRG.20241.4	2.00993	coding
MSTRG.20241.6	2.2551	coding
MSTRG.20231.1	-1.31887	noncoding
MSTRG.20231.2	-1.32201	noncoding
MSTRG.20232.1	-1.47494	noncoding
MSTRG.20233.1	0.708987	coding
MSTRG.20242.4	1.75769	coding
MSTRG.20242.1	2.59974	coding
MSTRG.20242.2	2.59775	coding
MSTRG.20242.3	2.39325	coding
MSTRG.20242.5	1.6515	coding
MSTRG.20242.8	1.79548	coding
MSTRG.20244.2	1.39798	coding
MSTRG.20245.1	0.551819	coding
MSTRG.20246.1	0.831727	coding
MSTRG.20246.3	0.387342	coding
MSTRG.20249.4	1.40839	coding
MSTRG.20250.2	2.33855	coding
MSTRG.20250.4	2.56047	coding
MSTRG.20250.7	2.25253	coding
MSTRG.20250.8	2.16305	coding

MSTRG.20251.1	-1.83055	noncoding
MSTRG.20247.1	2.04126	coding
MSTRG.20247.2	2.22308	coding
MSTRG.20248.1	-0.168176	noncoding
MSTRG.20257.1	2.04286	coding
MSTRG.20257.2	-0.120396	noncoding
MSTRG.20269.2	-0.235672	noncoding
MSTRG.20270.1	0.849802	coding
MSTRG.20270.2	0.899764	coding
MSTRG.20270.3	0.602948	coding
MSTRG.20271.2	2.75378	coding
MSTRG.20272.1	0.77792	coding
MSTRG.20274.1	-1.65822	noncoding
MSTRG.20274.5	1.97527	coding
MSTRG.20274.4	2.05229	coding
MSTRG.20274.6	-0.359788	noncoding
MSTRG.20277.1	-0.263101	noncoding
MSTRG.20278.2	1.29123	coding
MSTRG.20279.1	2.34178	coding
MSTRG.20281.2	1.56615	coding
MSTRG.20281.4	1.70588	coding
MSTRG.20287.3	1.98593	coding
MSTRG.20288.2	1.0393	coding
MSTRG.20288.3	0.618039	coding
MSTRG.20290.1	0.084814	coding
MSTRG.20291.3	0.262235	coding
MSTRG.20292.1	1.87632	coding
MSTRG.20292.2	1.88247	coding
MSTRG.20292.3	1.90759	coding
MSTRG.20292.4	2.51486	coding
MSTRG.20292.7	2.11222	coding
MSTRG.20293.1	-1.58618	noncoding
MSTRG.20298.3	1.6255	coding
MSTRG.20298.4	1.75887	coding
MSTRG.20298.5	1.54518	coding
MSTRG.20298.6	1.56004	coding
MSTRG.20298.7	1.38373	coding
MSTRG.20299.1	0.361244	coding
MSTRG.20315.2	1.71879	coding
MSTRG.20315.4	1.52069	coding
MSTRG.20315.6	1.85634	coding
MSTRG.20315.5	2.5049	coding
MSTRG.20315.7	-0.219076	noncoding
MSTRG.20315.11	0.203482	coding
MSTRG.20315.14	0.128792	coding
MSTRG.20315.16	0.689161	coding
MSTRG.20318.1	-1.59986	noncoding
MSTRG.20308.1	-1.78766	noncoding
MSTRG.20310.1	1.2837	coding
MSTRG.20313.1	0.881052	coding
MSTRG.20313.2	2.20333	coding
MSTRG.20313.3	2.23536	coding
MSTRG.20313.4	-0.940986	noncoding
MSTRG.20313.5	0.56453	coding

MSTRG.20334.1	1.62091	coding
MSTRG.20335.3	-0.985499	noncoding
MSTRG.20338.1	-1.07139	noncoding
MSTRG.20338.2	-1.54093	noncoding
MSTRG.20339.3	-0.68145	noncoding
MSTRG.20339.4	-0.746807	noncoding
MSTRG.20345.2	-0.468578	noncoding
MSTRG.20346.2	0.277722	coding
MSTRG.20347.4	-0.198427	noncoding
MSTRG.20321.1	-2.52508	noncoding
MSTRG.20328.4	-0.300915	noncoding
MSTRG.20328.5	-0.546158	noncoding
MSTRG.20328.6	-0.278185	noncoding
MSTRG.20330.1	-2.3035	noncoding
MSTRG.20332.6	0.008005	coding
MSTRG.20354.1	-1.92783	noncoding
MSTRG.20361.1	-2.32198	noncoding
MSTRG.20362.1	-2.3274	noncoding
MSTRG.20362.2	-2.36278	noncoding
MSTRG.20362.3	-2.3341	noncoding
MSTRG.20366.1	1.16765	coding
MSTRG.20367.3	1.09603	coding
MSTRG.20367.4	1.12576	coding
MSTRG.20367.6	0.713675	coding
MSTRG.20367.5	0.868933	coding
MSTRG.20376.4	1.71445	coding
MSTRG.20376.5	1.79329	coding
MSTRG.20376.7	1.83878	coding
MSTRG.20376.8	1.2152	coding
MSTRG.20376.6	-0.397267	noncoding
MSTRG.20376.9	0.068796	coding
MSTRG.20376.10	-0.423094	noncoding
MSTRG.20378.2	1.7544	coding
MSTRG.20382.3	2.6244	coding
MSTRG.20382.5	2.59423	coding
MSTRG.20382.8	2.54887	coding
MSTRG.20387.2	2.21835	coding
MSTRG.20387.8	-1.44353	noncoding
MSTRG.20387.7	2.45917	coding
MSTRG.20388.1	-0.045402	noncoding
MSTRG.20393.1	0.148956	coding
MSTRG.20394.1	1.67612	coding
MSTRG.20395.1	1.42449	coding
MSTRG.20395.2	1.44224	coding
MSTRG.20395.3	1.33496	coding
MSTRG.20395.4	1.19957	coding
MSTRG.20395.14	1.77043	coding
MSTRG.20395.10	2.13526	coding
MSTRG.20395.11	2.18073	coding
MSTRG.20395.7	2.07698	coding
MSTRG.20395.8	2.06655	coding
MSTRG.20395.12	1.77959	coding
MSTRG.20395.13	2.17532	coding
MSTRG.20395.9	2.43162	coding

MSTRG.20396.1	-1.74602	noncoding
MSTRG.20397.1	1.30105	coding
MSTRG.20396.2	-0.24414	noncoding
MSTRG.20396.3	-0.427992	noncoding
MSTRG.20396.4	-0.731569	noncoding
MSTRG.20390.2	2.11858	coding
MSTRG.20392.1	2.78646	coding
MSTRG.20400.7	0.330995	coding
MSTRG.20401.1	-0.417739	noncoding
MSTRG.20402.1	-0.300367	noncoding
MSTRG.20403.1	-0.916928	noncoding
MSTRG.20407.1	0.303092	coding
MSTRG.20414.2	2.22785	coding
MSTRG.20415.1	-0.053048	noncoding
MSTRG.20416.1	1.43147	coding
MSTRG.20421.1	0.444605	coding
MSTRG.20421.2	0.454082	coding
MSTRG.20421.3	1.27199	coding
MSTRG.20424.1	-1.33081	noncoding
MSTRG.20426.1	1.40035	coding
MSTRG.20426.2	1.42256	coding
MSTRG.20429.2	0.212585	coding
MSTRG.20430.1	-2.33707	noncoding
MSTRG.20433.4	2.61956	coding
MSTRG.20434.1	0.575661	coding
MSTRG.20433.7	0.578064	coding
MSTRG.20431.2	1.85966	coding
MSTRG.20431.3	0.668076	coding
MSTRG.20431.4	0.567322	coding
MSTRG.20438.4	1.15728	coding
MSTRG.20439.1	0.672369	coding
MSTRG.20439.2	-0.061024	noncoding
MSTRG.20439.3	0.85155	coding
MSTRG.20440.1	0.555371	coding
MSTRG.20440.2	0.336079	coding
MSTRG.20446.2	-0.864877	noncoding
MSTRG.20451.2	0.424715	coding
MSTRG.20452.1	-1.66842	noncoding
MSTRG.20453.1	0.299085	coding
MSTRG.20455.2	-0.130151	noncoding
MSTRG.20457.2	1.42286	coding
MSTRG.20457.4	1.31348	coding
MSTRG.20457.7	1.43454	coding
MSTRG.20457.6	1.42342	coding
MSTRG.20457.8	0.795219	coding
MSTRG.20469.1	0.964337	coding
MSTRG.20469.2	1.02811	coding
MSTRG.20471.1	-0.660098	noncoding
MSTRG.20471.2	-1.1331	noncoding
MSTRG.20473.2	-1.80534	noncoding
MSTRG.20474.1	-1.70182	noncoding
MSTRG.20476.1	-0.706854	noncoding
MSTRG.20482.6	-0.284034	noncoding
MSTRG.20483.1	0.285985	coding

MSTRG.20484.1	-0.547627	noncoding
MSTRG.20486.1	-0.944725	noncoding
MSTRG.20479.1	-1.98257	noncoding
MSTRG.20489.1	-0.181336	noncoding
MSTRG.20504.2	-1.03611	noncoding
MSTRG.20505.1	1.74006	coding
MSTRG.20506.1	0.091389	coding
MSTRG.20512.1	-1.05188	noncoding
MSTRG.20512.2	-1.0311	noncoding
MSTRG.20512.3	-0.195869	noncoding
MSTRG.20512.4	-0.752889	noncoding
MSTRG.20512.5	-0.457547	noncoding
MSTRG.20512.6	-1.64063	noncoding
MSTRG.20514.1	-2.44618	noncoding
MSTRG.20516.1	-2.3992	noncoding
MSTRG.20526.1	-0.458735	noncoding
MSTRG.20528.1	-0.0468	noncoding
MSTRG.20547.1	-1.22084	noncoding
MSTRG.20552.1	-0.11905	noncoding
MSTRG.20565.1	-1.39897	noncoding
MSTRG.20566.1	-0.455025	noncoding
MSTRG.20568.2	1.07645	coding
MSTRG.20569.1	-1.81763	noncoding
MSTRG.20570.1	-0.879627	noncoding
MSTRG.20571.1	0.843785	coding
MSTRG.20574.1	-0.720205	noncoding
MSTRG.20577.2	1.6731	coding
MSTRG.20577.3	0.702046	coding
MSTRG.20579.2	1.0831	coding
MSTRG.20579.4	1.56145	coding
MSTRG.20580.1	-1.21981	noncoding
MSTRG.20581.1	-0.423339	noncoding
MSTRG.20583.1	1.76908	coding
MSTRG.20584.1	-0.693598	noncoding
MSTRG.20593.2	1.95434	coding
MSTRG.20593.3	2.78179	coding
MSTRG.20608.7	1.35064	coding
MSTRG.20608.6	2.54256	coding
MSTRG.20609.1	-0.362854	noncoding
MSTRG.20610.1	-0.214197	noncoding
MSTRG.20608.8	2.12368	coding
MSTRG.20611.1	-0.435053	noncoding
MSTRG.20612.1	-0.032814	noncoding
MSTRG.20608.9	1.29556	coding
MSTRG.20603.1	-0.503358	noncoding
MSTRG.20603.2	-0.638084	noncoding
MSTRG.20603.3	0.543737	coding
MSTRG.20613.1	1.28923	coding
MSTRG.20613.2	1.96443	coding
MSTRG.20614.1	-2.57361	noncoding
MSTRG.20616.1	-2.09356	noncoding
MSTRG.20618.2	1.35458	coding
MSTRG.20620.2	-2.2846	noncoding
MSTRG.20619.4	1.80979	coding

MSTRG.20621.1	0.038989	coding
MSTRG.20628.2	2.19254	coding
MSTRG.20629.1	-1.75158	noncoding
MSTRG.20629.2	-2.15185	noncoding
MSTRG.20631.2	1.52509	coding
MSTRG.20631.3	1.04794	coding
MSTRG.20634.1	1.85143	coding
MSTRG.20634.2	1.89732	coding
MSTRG.20634.3	0.859848	coding
MSTRG.20637.3	1.26567	coding
MSTRG.20637.5	0.893671	coding
MSTRG.20637.4	0.853287	coding
MSTRG.20638.1	0.045956	coding
MSTRG.20642.1	0.288805	coding
MSTRG.20654.1	-1.70873	noncoding
MSTRG.20654.2	2.77421	coding
MSTRG.20654.4	0.423614	coding
MSTRG.20654.3	2.0709	coding
MSTRG.20654.5	1.26932	coding
MSTRG.20654.12	0.680198	coding
MSTRG.20654.8	1.99704	coding
MSTRG.20654.10	1.86253	coding
MSTRG.20654.11	2.8762	coding
MSTRG.20654.9	2.83007	coding
MSTRG.20654.6	2.70737	coding
MSTRG.20654.7	2.70117	coding
MSTRG.20654.13	0.685104	coding
MSTRG.20656.1	0.139227	coding
MSTRG.20657.1	-0.905593	noncoding
MSTRG.20658.1	-0.912476	noncoding
MSTRG.20654.14	-0.103041	noncoding
MSTRG.20660.2	2.89624	coding
MSTRG.20644.1	0.870646	coding
MSTRG.20644.3	1.16408	coding
MSTRG.20644.6	0.514918	coding
MSTRG.20646.1	1.70074	coding
MSTRG.20649.2	2.49076	coding
MSTRG.20649.3	2.87985	coding
MSTRG.20662.1	-2.67085	noncoding
MSTRG.20662.2	-1.33204	noncoding
MSTRG.20662.3	1.24178	coding
MSTRG.20662.4	1.25731	coding
MSTRG.20662.5	1.26002	coding
MSTRG.20662.6	1.09213	coding
MSTRG.20664.1	2.9983	coding
MSTRG.20664.2	1.67551	coding
MSTRG.20667.2	-0.531523	noncoding
MSTRG.20668.1	-0.713866	noncoding
MSTRG.20669.2	0.57202	coding
MSTRG.20670.1	0.092601	coding
MSTRG.20672.6	2.36202	coding
MSTRG.20672.8	1.73048	coding
MSTRG.20674.1	1.77348	coding
MSTRG.20674.3	1.78187	coding

MSTRG.20674.5	2.48793	coding
MSTRG.20674.6	-0.191704	noncoding
MSTRG.20678.2	1.87372	coding
MSTRG.20678.3	-0.601489	noncoding
MSTRG.20678.4	2.01934	coding
MSTRG.20679.1	-0.332905	noncoding
MSTRG.20677.1	-0.1477	noncoding
MSTRG.20681.1	-0.707592	noncoding
MSTRG.20682.1	-3.29254	noncoding
MSTRG.20682.2	-3.07498	noncoding
MSTRG.20688.2	0.749366	coding
MSTRG.20705.1	0.476065	coding
MSTRG.20705.2	0.489369	coding
MSTRG.20710.3	1.74808	coding
MSTRG.20710.5	0.740807	coding
MSTRG.20711.1	-1.79672	noncoding
MSTRG.20713.1	-0.902892	noncoding
MSTRG.20708.2	1.81306	coding
MSTRG.20709.1	-0.259048	noncoding
MSTRG.20714.1	2.08673	coding
MSTRG.20725.3	1.63286	coding
MSTRG.20725.5	-0.336645	noncoding
MSTRG.20725.4	1.67749	coding
MSTRG.20725.6	1.62923	coding
MSTRG.20725.7	-2.11018	noncoding
MSTRG.20725.8	1.6718	coding
MSTRG.20725.9	0.076605	coding
MSTRG.20725.10	1.02856	coding
MSTRG.20725.11	1.48857	coding
MSTRG.20725.12	0.838624	coding
MSTRG.20725.14	-0.126251	noncoding
MSTRG.20726.1	-0.097575	noncoding
MSTRG.20727.1	0.34521	coding
MSTRG.20728.1	0.832278	coding
MSTRG.20730.12	-1.79733	noncoding
MSTRG.20730.6	1.6838	coding
MSTRG.20730.8	1.68983	coding
MSTRG.20730.11	2.21269	coding
MSTRG.20730.10	1.99141	coding
MSTRG.20730.7	1.98204	coding
MSTRG.20730.4	2.03321	coding
MSTRG.20730.5	2.03368	coding
MSTRG.20730.9	2.18044	coding
MSTRG.20730.13	1.83534	coding
MSTRG.20730.14	1.81155	coding
MSTRG.20718.1	-1.26923	noncoding
MSTRG.20720.2	-1.7129	noncoding
MSTRG.20734.1	-0.102856	noncoding
MSTRG.20740.1	2.0079	coding
MSTRG.20742.2	1.53611	coding
MSTRG.20742.3	0.877428	coding
MSTRG.20742.4	-0.04523	noncoding
MSTRG.20743.1	0.592938	coding
MSTRG.20746.1	-1.20787	noncoding

MSTRG.20737.1	0.285002	coding
MSTRG.20737.2	0.653055	coding
MSTRG.20751.2	0.510809	coding
MSTRG.20753.3	1.30931	coding
MSTRG.20753.4	0.749815	coding
MSTRG.20750.3	1.75598	coding
MSTRG.20750.1	2.05508	coding
MSTRG.20758.1	-0.130004	noncoding
MSTRG.20763.10	-1.74177	noncoding
MSTRG.20763.8	1.53485	coding
MSTRG.20763.13	1.56746	coding
MSTRG.20774.1	0.600735	coding
MSTRG.20775.4	0.942834	coding
MSTRG.20775.6	0.881413	coding
MSTRG.20775.7	0.892438	coding
MSTRG.20775.8	1.2257	coding
MSTRG.20775.9	-1.29196	noncoding
MSTRG.20776.1	0.380034	coding
MSTRG.20776.2	0.401556	coding
MSTRG.20777.1	-0.498579	noncoding
MSTRG.20778.1	0.659483	coding
MSTRG.20780.1	-1.3913	noncoding
MSTRG.20781.1	-0.895753	noncoding
MSTRG.20782.1	0.29138	coding
MSTRG.20783.1	1.01294	coding
MSTRG.20781.2	-0.376764	noncoding
MSTRG.20784.1	0.56594	coding
MSTRG.20785.1	1.0222	coding
MSTRG.20786.1	-0.451579	noncoding
MSTRG.20775.10	0.600861	coding
MSTRG.20787.1	0.674961	coding
MSTRG.20787.2	0.403051	coding
MSTRG.20792.1	0.700618	coding
MSTRG.20793.1	-0.096643	noncoding
MSTRG.20794.1	0.463775	coding
MSTRG.20800.1	0.166813	coding
MSTRG.20800.2	-0.471268	noncoding
MSTRG.20801.1	-1.76084	noncoding
MSTRG.20797.1	-1.79865	noncoding
MSTRG.20798.4	1.58622	coding
MSTRG.20819.4	2.14755	coding
MSTRG.20819.8	2.39292	coding
MSTRG.20822.1	-2.29082	noncoding
MSTRG.20816.5	1.17534	coding
MSTRG.20816.3	1.42346	coding
MSTRG.20816.4	1.20973	coding
MSTRG.20816.2	1.31223	coding
MSTRG.20823.3	1.25037	coding
MSTRG.20823.4	2.13479	coding
MSTRG.20823.2	2.42751	coding
MSTRG.20823.1	3.01382	coding
MSTRG.20817.2	1.56347	coding
MSTRG.20817.1	1.92659	coding
MSTRG.20828.4	2.5175	coding

MSTRG.20828.5	-0.062872	noncoding
MSTRG.20829.1	2.12637	coding
MSTRG.20829.2	2.30827	coding
MSTRG.20829.5	2.30857	coding
MSTRG.20830.4	0.515854	coding
MSTRG.20825.3	0.806113	coding
MSTRG.20825.4	0.808754	coding
MSTRG.20826.2	-0.478119	noncoding
MSTRG.20831.1	0.98618	coding
MSTRG.20832.3	1.42052	coding
MSTRG.20836.2	2.95711	coding
MSTRG.20836.3	2.94566	coding
MSTRG.20836.4	2.92279	coding
MSTRG.20840.1	0.540638	coding
MSTRG.20845.4	1.76835	coding
MSTRG.20845.7	1.64896	coding
MSTRG.20845.6	1.82949	coding
MSTRG.20845.9	0.325201	coding
MSTRG.20844.2	0.461599	coding
MSTRG.20850.1	0.970349	coding
MSTRG.20850.3	0.298246	coding
MSTRG.20852.4	1.83113	coding
MSTRG.20854.1	-0.148172	noncoding
MSTRG.20854.2	-0.064081	noncoding
MSTRG.20866.1	-1.66887	noncoding
MSTRG.20867.2	1.55859	coding
MSTRG.20873.1	2.77917	coding
MSTRG.20873.2	2.75108	coding
MSTRG.20873.3	2.89085	coding
MSTRG.20873.4	2.98405	coding
MSTRG.20875.3	-0.108463	noncoding
MSTRG.20875.2	1.89962	coding
MSTRG.20876.1	0.104074	coding
MSTRG.20877.1	-0.726915	noncoding
MSTRG.20878.1	0.430526	coding
MSTRG.20872.1	-0.746709	noncoding
MSTRG.20882.1	1.02079	coding
MSTRG.20883.1	-1.50817	noncoding
MSTRG.20884.2	0.913014	coding
MSTRG.20884.3	1.29699	coding
MSTRG.20884.4	0.763538	coding
MSTRG.20891.1	0.517876	coding
MSTRG.20891.2	0.91039	coding
MSTRG.20891.3	0.660414	coding
MSTRG.20892.1	0.251922	coding
MSTRG.20895.1	1.00497	coding
MSTRG.20900.4	0.056241	coding
MSTRG.20900.3	0.428821	coding
MSTRG.20912.2	0.15446	coding
MSTRG.20913.3	2.75288	coding
MSTRG.20913.4	3.19547	coding
MSTRG.20913.5	2.66088	coding
MSTRG.20915.1	-2.23815	noncoding
MSTRG.20919.1	1.71711	coding

MSTRG.20914.1	-0.707784	noncoding
MSTRG.20914.2	-1.96758	noncoding
MSTRG.20914.3	-1.95138	noncoding
MSTRG.20923.1	-1.53536	noncoding
MSTRG.20921.1	-1.91229	noncoding
MSTRG.20921.2	-0.879902	noncoding
MSTRG.20932.2	-0.010009	noncoding
MSTRG.20934.1	-1.74497	noncoding
MSTRG.20930.1	1.55538	coding
MSTRG.20943.1	-1.52663	noncoding
MSTRG.20944.1	-0.550766	noncoding
MSTRG.20940.1	-1.11724	noncoding
MSTRG.20949.2	2.57717	coding
MSTRG.20951.1	1.03721	coding
MSTRG.20952.2	0.621723	coding
MSTRG.20953.4	0.627231	coding
MSTRG.20953.2	-0.247267	noncoding
MSTRG.20953.5	-0.079213	noncoding
MSTRG.20950.2	2.48511	coding
MSTRG.20955.3	1.4935	coding
MSTRG.20955.5	2.20634	coding
MSTRG.20955.4	1.98085	coding
MSTRG.20955.1	1.81945	coding
MSTRG.20957.1	-0.579914	noncoding
MSTRG.20959.1	-2.3074	noncoding
MSTRG.20967.3	1.29392	coding
MSTRG.20967.7	1.44881	coding
MSTRG.20967.9	1.52074	coding
MSTRG.20967.10	1.50571	coding
MSTRG.20967.8	1.66402	coding
MSTRG.20967.11	1.72268	coding
MSTRG.20967.13	1.22159	coding
MSTRG.20967.14	1.33186	coding
MSTRG.20969.2	-0.955412	noncoding
MSTRG.20963.4	2.02001	coding
MSTRG.20963.5	1.99516	coding
MSTRG.20963.8	2.38973	coding
MSTRG.20963.6	1.95589	coding
MSTRG.20975.2	0.218494	coding
MSTRG.20979.2	2.42712	coding
MSTRG.20981.2	1.76016	coding
MSTRG.20977.2	1.35256	coding
MSTRG.20978.1	1.48547	coding
MSTRG.20978.3	0.598176	coding
MSTRG.20986.2	1.57457	coding
MSTRG.20986.5	1.86708	coding
MSTRG.20986.1	1.61746	coding
MSTRG.20986.3	1.57279	coding
MSTRG.20986.7	1.48421	coding
MSTRG.20986.8	1.26771	coding
MSTRG.20989.1	0.733008	coding
MSTRG.20990.1	-0.215287	noncoding
MSTRG.20990.2	0.011018	coding
MSTRG.20994.3	1.40898	coding

MSTRG.20999.2	1.59851	coding
MSTRG.20999.3	1.60999	coding
MSTRG.21000.1	-1.27989	noncoding
MSTRG.21002.1	0.268717	coding
MSTRG.21011.4	2.36615	coding
MSTRG.21017.1	-2.03331	noncoding
MSTRG.21018.1	1.4404	coding
MSTRG.21018.2	1.42606	coding
MSTRG.21018.3	1.47457	coding
MSTRG.21024.5	1.64757	coding
MSTRG.21024.6	1.30746	coding
MSTRG.21024.7	1.36418	coding
MSTRG.21022.1	-1.06481	noncoding
MSTRG.21027.4	0.328179	coding
MSTRG.21027.6	0.280517	coding
MSTRG.21027.5	0.093277	coding
MSTRG.21027.8	0.211901	coding
MSTRG.21037.2	1.49155	coding
MSTRG.21038.2	1.05321	coding
MSTRG.21038.5	-1.39393	noncoding
MSTRG.21038.6	-0.49455	noncoding
MSTRG.21039.2	0.495244	coding
MSTRG.21048.3	1.40964	coding
MSTRG.21048.5	1.81545	coding
MSTRG.21049.2	0.523841	coding
MSTRG.21061.1	1.93374	coding
MSTRG.21061.2	1.54741	coding
MSTRG.21062.1	0.420115	coding
MSTRG.21061.3	0.859273	coding
MSTRG.21066.1	-0.034895	noncoding
MSTRG.21068.3	3.02856	coding
MSTRG.21074.1	0.559541	coding
MSTRG.21076.4	-0.714671	noncoding
MSTRG.21079.1	-0.869277	noncoding
MSTRG.21080.3	1.62909	coding
MSTRG.21080.4	-0.355546	noncoding
MSTRG.21082.1	-1.11013	noncoding
MSTRG.21083.1	0.398534	coding
MSTRG.21086.1	-0.943911	noncoding
MSTRG.21087.3	0.404575	coding
MSTRG.21090.1	0.721973	coding
MSTRG.21089.3	0.300838	coding
MSTRG.21096.4	0.652417	coding
MSTRG.21092.2	-0.009653	noncoding
MSTRG.21093.3	2.46238	coding
MSTRG.21104.3	0.474193	coding
MSTRG.21104.4	0.257231	coding
MSTRG.21105.1	-0.126079	noncoding
MSTRG.21106.5	1.38515	coding
MSTRG.21106.7	2.26848	coding
MSTRG.21106.6	2.40977	coding
MSTRG.21106.2	1.46463	coding
MSTRG.21106.4	1.46781	coding
MSTRG.21106.8	1.26173	coding

MSTRG.21106.9	1.49817	coding
MSTRG.21106.10	1.0701	coding
MSTRG.21106.11	0.95702	coding
MSTRG.21101.3	1.72753	coding
MSTRG.21098.1	-0.23716	noncoding
MSTRG.21102.2	0.399461	coding
MSTRG.21107.2	1.93625	coding
MSTRG.21107.3	2.19192	coding
MSTRG.21108.1	0.488184	coding
MSTRG.21109.1	-0.710593	noncoding
MSTRG.21107.4	1.22949	coding
MSTRG.21111.2	1.49867	coding
MSTRG.21112.1	0.839836	coding
MSTRG.21112.2	0.704187	coding
MSTRG.21115.1	-1.08364	noncoding
MSTRG.21117.1	-2.32607	noncoding
MSTRG.21120.1	-1.27403	noncoding
MSTRG.21126.2	1.00078	coding
MSTRG.21123.1	-0.906682	noncoding
MSTRG.21130.3	0.527631	coding
MSTRG.21127.1	1.99873	coding
MSTRG.21127.2	2.0011	coding
MSTRG.21128.1	-2.8145	noncoding
MSTRG.21146.3	0.744753	coding
MSTRG.21147.1	-0.466247	noncoding
MSTRG.21146.4	0.688481	coding
MSTRG.21146.6	0.73358	coding
MSTRG.21146.5	0.808356	coding
MSTRG.21146.7	1.44423	coding
MSTRG.21146.8	0.552985	coding
MSTRG.21146.9	-0.301489	noncoding
MSTRG.21146.10	0.472693	coding
MSTRG.21146.11	0.264211	coding
MSTRG.21146.12	-0.017959	noncoding
MSTRG.21148.10	1.1153	coding
MSTRG.21148.12	1.14166	coding
MSTRG.21148.6	1.2688	coding
MSTRG.21148.8	1.11058	coding
MSTRG.21148.9	1.37711	coding
MSTRG.21134.1	0.42704	coding
MSTRG.21151.1	-1.21248	noncoding
MSTRG.21152.1	2.16105	coding
MSTRG.21153.1	2.06637	coding
MSTRG.21154.1	1.99076	coding
MSTRG.21154.2	0.029083	coding
MSTRG.21155.1	-1.14081	noncoding
MSTRG.21156.2	1.70974	coding
MSTRG.21154.3	0.673575	coding
MSTRG.21154.4	2.04625	coding
MSTRG.21159.3	2.29329	coding
MSTRG.21159.4	1.92752	coding
MSTRG.21159.5	1.46707	coding
MSTRG.21159.6	1.56679	coding
MSTRG.21159.7	-0.343759	noncoding

MSTRG.21162.6	1.42733	coding
MSTRG.21162.7	2.20671	coding
MSTRG.21163.1	-0.719047	noncoding
MSTRG.21163.2	0.578014	coding
MSTRG.21173.1	2.71684	coding
MSTRG.21169.1	-0.012698	noncoding
MSTRG.21176.1	-1.19656	noncoding
MSTRG.21176.2	-1.25546	noncoding
MSTRG.21179.3	2.62294	coding
MSTRG.21179.2	2.60964	coding
MSTRG.21179.5	2.56701	coding
MSTRG.21179.6	2.59155	coding
MSTRG.21181.2	1.71251	coding
MSTRG.21198.1	-0.033008	noncoding
MSTRG.21198.2	0.706764	coding
MSTRG.21198.3	1.31534	coding
MSTRG.21212.1	1.87356	coding
MSTRG.21212.2	1.83401	coding
MSTRG.21213.2	1.83653	coding
MSTRG.21216.2	0.957467	coding
MSTRG.21218.2	-0.178289	noncoding
MSTRG.21218.3	-0.072231	noncoding
MSTRG.21220.3	0.378342	coding
MSTRG.21220.4	0.18379	coding
MSTRG.21224.1	-0.591486	noncoding
MSTRG.21235.3	2.81359	coding
MSTRG.21234.1	-2.04491	noncoding
MSTRG.21244.1	-1.64923	noncoding
MSTRG.21248.2	-1.2678	noncoding
MSTRG.21249.1	-1.14299	noncoding
MSTRG.21250.2	1.20577	coding
MSTRG.21251.1	-1.10309	noncoding
MSTRG.21251.2	-0.555002	noncoding
MSTRG.21252.1	-1.60384	noncoding
MSTRG.21252.2	-1.67686	noncoding
MSTRG.21254.1	-1.12578	noncoding
MSTRG.21256.5	0.696685	coding
MSTRG.21256.6	-0.470071	noncoding
MSTRG.21257.1	-1.10873	noncoding
MSTRG.21260.1	1.67169	coding
MSTRG.21262.1	-0.236898	noncoding
MSTRG.21268.1	-1.26115	noncoding
MSTRG.21269.1	0.641781	coding
MSTRG.21269.2	-1.14668	noncoding
MSTRG.21271.2	0.983642	coding
MSTRG.21272.1	-1.53318	noncoding
MSTRG.21278.2	1.88347	coding
MSTRG.21301.3	2.85169	coding
MSTRG.21302.1	0.126247	coding
MSTRG.21308.1	0.22983	coding
MSTRG.21309.2	1.86665	coding
MSTRG.21310.3	1.90051	coding
MSTRG.21315.2	1.78498	coding
MSTRG.21316.1	-0.991974	noncoding

MSTRG.21317.1	-2.50452	noncoding
MSTRG.21318.1	-0.93185	noncoding
MSTRG.21315.4	0.138812	coding
MSTRG.21314.1	1.02368	coding
MSTRG.21319.1	1.57887	coding
MSTRG.21323.1	2.52692	coding
MSTRG.21323.5	2.62039	coding
MSTRG.21323.3	2.62827	coding
MSTRG.21323.8	2.81252	coding
MSTRG.21323.7	2.77062	coding
MSTRG.21323.9	2.75766	coding
MSTRG.21321.1	2.53825	coding
MSTRG.21321.2	2.58945	coding
MSTRG.21321.3	2.553	coding
MSTRG.21324.2	0.79352	coding
MSTRG.21324.3	1.26089	coding
MSTRG.21326.1	-0.593091	noncoding
MSTRG.21327.3	1.75642	coding
MSTRG.21327.6	1.49368	coding
MSTRG.21327.8	1.97756	coding
MSTRG.21330.2	-0.797263	noncoding
MSTRG.21333.4	0.05133	coding
MSTRG.21334.5	1.50768	coding
MSTRG.21334.6	1.60184	coding
MSTRG.21334.7	1.71723	coding
MSTRG.21334.8	1.63564	coding
MSTRG.21334.9	1.64422	coding
MSTRG.21334.10	1.99457	coding
MSTRG.21335.1	0.199179	coding
MSTRG.21336.1	0.785658	coding
MSTRG.21336.2	0.852016	coding
MSTRG.21336.3	0.83986	coding
MSTRG.21338.4	0.07612	coding
MSTRG.21338.6	0.761571	coding
MSTRG.21338.7	0.901263	coding
MSTRG.21338.9	-0.257028	noncoding
MSTRG.21338.10	-0.122751	noncoding
MSTRG.21338.13	0.64312	coding
MSTRG.21338.12	1.18942	coding
MSTRG.21342.1	-0.275333	noncoding
MSTRG.21343.3	-0.147791	noncoding
MSTRG.21348.4	1.10014	coding
MSTRG.21348.1	0.814722	coding
MSTRG.21348.2	0.824036	coding
MSTRG.21349.1	1.49272	coding
MSTRG.21351.1	-1.18767	noncoding
MSTRG.21345.1	0.019928	coding
MSTRG.21371.3	2.01264	coding
MSTRG.21371.5	2.31515	coding
MSTRG.21371.4	3.0422	coding
MSTRG.21371.6	2.29619	coding
MSTRG.21371.7	1.55313	coding
MSTRG.21371.9	1.81676	coding
MSTRG.21371.10	2.19623	coding

MSTRG.21371.11	2.15129	coding
MSTRG.21371.13	2.28115	coding
MSTRG.21371.12	2.12482	coding
MSTRG.21371.15	2.10015	coding
MSTRG.21371.16	2.34343	coding
MSTRG.21371.17	1.91036	coding
MSTRG.21371.18	2.57419	coding
MSTRG.21372.1	-0.598356	noncoding
MSTRG.21373.1	-0.807029	noncoding
MSTRG.21372.2	-1.69569	noncoding
MSTRG.21356.1	1.59181	coding
MSTRG.21359.1	1.0447	coding
MSTRG.21361.1	2.1088	coding
MSTRG.21362.1	-0.586108	noncoding
MSTRG.21363.1	1.5552	coding
MSTRG.21368.2	2.27357	coding
MSTRG.21368.3	1.28591	coding
MSTRG.21369.1	-2.14695	noncoding
MSTRG.21377.1	-1.99746	noncoding
MSTRG.21375.1	-1.60699	noncoding
MSTRG.21384.1	-0.164272	noncoding
MSTRG.21385.5	1.28846	coding
MSTRG.21385.4	2.95158	coding
MSTRG.21385.7	0.206567	coding
MSTRG.21385.8	-1.61804	noncoding
MSTRG.21385.9	1.16721	coding
MSTRG.21385.10	-0.579365	noncoding
MSTRG.21395.3	1.49097	coding
MSTRG.21396.3	2.62854	coding
MSTRG.21396.4	0.699338	coding
MSTRG.21406.1	1.41369	coding
MSTRG.21406.2	0.431127	coding
MSTRG.21400.1	-1.31799	noncoding
MSTRG.21402.1	-1.54245	noncoding
MSTRG.21403.1	-2.23227	noncoding
MSTRG.21414.1	-2.517	noncoding
MSTRG.21415.1	-0.014081	noncoding
MSTRG.21416.1	-0.413953	noncoding
MSTRG.21433.1	2.34803	coding
MSTRG.21433.4	2.2503	coding
MSTRG.21433.3	2.29319	coding
MSTRG.21433.5	2.01741	coding
MSTRG.21433.6	1.33549	coding
MSTRG.21433.7	1.86047	coding
MSTRG.21433.8	1.85841	coding
MSTRG.21433.9	1.06095	coding
MSTRG.21434.1	0.071289	coding
MSTRG.21433.10	2.0583	coding
MSTRG.21433.11	0.773844	coding
MSTRG.21433.13	2.13495	coding
MSTRG.21433.14	2.18422	coding
MSTRG.21433.15	2.04344	coding
MSTRG.21433.16	2.16202	coding
MSTRG.21433.18	2.06799	coding

MSTRG.21433.19	0.910456	coding
MSTRG.21433.20	1.54715	coding
MSTRG.21433.22	-1.10771	noncoding
MSTRG.21441.2	0.527717	coding
MSTRG.21419.1	2.92197	coding
MSTRG.21425.1	-2.11329	noncoding
MSTRG.21427.2	0.3464	coding
MSTRG.21427.1	0.752848	coding
MSTRG.21428.1	-2.0538	noncoding
MSTRG.21429.1	-0.260717	noncoding
MSTRG.21432.1	-1.68002	noncoding
MSTRG.21449.1	0.970389	coding
MSTRG.21449.4	2.04683	coding
MSTRG.21450.1	1.12415	coding
MSTRG.21450.2	-0.923229	noncoding
MSTRG.21451.1	-1.53698	noncoding
MSTRG.21449.5	-0.001179	noncoding
MSTRG.21452.1	0.655493	coding
MSTRG.21453.1	0.73483	coding
MSTRG.21454.1	0.129906	coding
MSTRG.21454.2	-0.138586	noncoding
MSTRG.21446.2	1.87089	coding
MSTRG.21447.4	0.039426	coding
MSTRG.21447.2	0.141011	coding
MSTRG.21471.1	2.53329	coding
MSTRG.21471.3	2.50298	coding
MSTRG.21472.1	-2.01353	noncoding
MSTRG.21470.3	-1.09138	noncoding
MSTRG.21474.3	0.859599	coding
MSTRG.21484.4	1.95915	coding
MSTRG.21486.2	0.73087	coding
MSTRG.21502.2	1.10466	coding
MSTRG.21502.7	0.76331	coding
MSTRG.21502.4	0.739343	coding
MSTRG.21502.6	1.18095	coding
MSTRG.21502.5	1.14846	coding
MSTRG.21503.1	-1.74755	noncoding
MSTRG.21511.1	0.233963	coding
MSTRG.21520.1	-0.569049	noncoding
MSTRG.21526.1	-1.30225	noncoding
MSTRG.21533.1	-1.6053	noncoding
MSTRG.21534.1	-1.75823	noncoding
MSTRG.21532.2	1.64352	coding
MSTRG.21535.1	0.65658	coding
MSTRG.21532.3	0.691567	coding
MSTRG.21536.1	-3.16196	noncoding
MSTRG.21537.1	0.582167	coding
MSTRG.21532.4	0.634121	coding
MSTRG.21538.1	-0.746852	noncoding
MSTRG.21499.2	0.238099	coding
MSTRG.21499.3	-0.009452	noncoding
MSTRG.21552.1	0.09164	coding
MSTRG.21553.1	0.088193	coding
MSTRG.21555.1	0.834158	coding

MSTRG.21556.1	0.511629	coding
MSTRG.21569.1	-1.74433	noncoding
MSTRG.21572.1	-0.057092	noncoding
MSTRG.21572.2	-0.642141	noncoding
MSTRG.21573.5	1.28006	coding
MSTRG.21573.6	2.02555	coding
MSTRG.21573.7	1.85673	coding
MSTRG.21573.9	1.875	coding
MSTRG.21573.14	1.54201	coding
MSTRG.21575.1	1.48468	coding
MSTRG.21580.1	0.473357	coding
MSTRG.21580.2	1.14387	coding
MSTRG.21580.3	-0.884984	noncoding
MSTRG.21598.1	-1.95798	noncoding
MSTRG.21612.1	-1.82739	noncoding
MSTRG.21649.1	-0.365569	noncoding
MSTRG.21656.9	1.68484	coding
MSTRG.21657.1	-1.50314	noncoding
MSTRG.21670.1	-1.22049	noncoding
MSTRG.21670.2	-2.33307	noncoding
MSTRG.21695.4	1.00898	coding
MSTRG.21696.1	-0.233941	noncoding
MSTRG.21695.6	0.626187	coding
MSTRG.21697.1	-1.44972	noncoding
MSTRG.21695.7	0.353467	coding
MSTRG.21698.1	-0.442233	noncoding
MSTRG.21695.10	1.25426	coding
MSTRG.21708.1	-2.32121	noncoding
MSTRG.21674.1	-0.575368	noncoding
MSTRG.21676.1	-1.56934	noncoding
MSTRG.21680.1	-1.1018	noncoding
MSTRG.21729.1	-1.59037	noncoding
MSTRG.21725.1	-0.650724	noncoding
MSTRG.21739.1	0.327452	coding
MSTRG.21741.2	0.231239	coding
MSTRG.21741.1	0.586451	coding
MSTRG.21749.1	2.53505	coding
MSTRG.21749.4	1.74215	coding
MSTRG.21749.5	0.489401	coding
MSTRG.21749.6	0.388071	coding
MSTRG.21749.7	-2.74324	noncoding
MSTRG.21751.1	-0.868697	noncoding
MSTRG.21752.1	-1.19977	noncoding
MSTRG.21749.8	-0.87548	noncoding
MSTRG.21753.1	1.27424	coding
MSTRG.21754.1	-0.284961	noncoding
MSTRG.21754.2	0.931979	coding
MSTRG.21749.9	1.31793	coding
MSTRG.21755.1	-1.72316	noncoding
MSTRG.21755.2	-1.6688	noncoding
MSTRG.21760.1	1.1859	coding
MSTRG.21771.6	1.7342	coding
MSTRG.21772.1	0.071646	coding
MSTRG.21771.19	1.87898	coding

MSTRG.21771.20	1.59821	coding
MSTRG.21771.21	1.59837	coding
MSTRG.21771.22	1.15642	coding
MSTRG.21771.23	1.72278	coding
MSTRG.21773.1	0.7516	coding
MSTRG.21775.1	-0.362642	noncoding
MSTRG.21771.24	2.61443	coding
MSTRG.21771.25	2.79022	coding
MSTRG.21771.26	2.75025	coding
MSTRG.21771.27	2.6793	coding
MSTRG.21771.28	1.15949	coding
MSTRG.21771.29	2.56498	coding
MSTRG.21763.1	-2.95231	noncoding
MSTRG.21779.2	2.09998	coding
MSTRG.21779.1	2.10268	coding
MSTRG.21790.1	-1.11824	noncoding
MSTRG.21791.2	1.52178	coding
MSTRG.21795.1	-0.111663	noncoding
MSTRG.21795.3	0.434532	coding
MSTRG.21795.4	0.845142	coding
MSTRG.21795.5	0.394498	coding
MSTRG.21795.6	0.505366	coding
MSTRG.21796.1	-0.536732	noncoding
MSTRG.21797.1	1.78554	coding
MSTRG.21797.2	1.92903	coding
MSTRG.21794.1	-2.3119	noncoding
MSTRG.21806.2	1.34049	coding
MSTRG.21812.2	-0.43644	noncoding
MSTRG.21812.1	0.864856	coding
MSTRG.21812.4	1.20033	coding
MSTRG.21808.2	1.30978	coding
MSTRG.21815.1	-0.569001	noncoding
MSTRG.21817.2	-1.55054	noncoding
MSTRG.21818.3	-0.228123	noncoding
MSTRG.21819.1	0.704253	coding
MSTRG.21820.1	-0.155672	noncoding
MSTRG.21820.2	-0.551715	noncoding
MSTRG.21827.2	0.330674	coding
MSTRG.21827.3	1.45316	coding
MSTRG.21829.2	2.53675	coding
MSTRG.21829.3	2.62607	coding
MSTRG.21831.1	2.24427	coding
MSTRG.21831.10	2.10469	coding
MSTRG.21831.11	2.23057	coding
MSTRG.21831.3	2.36709	coding
MSTRG.21831.5	2.36411	coding
MSTRG.21831.7	2.22978	coding
MSTRG.21831.9	2.01589	coding
MSTRG.21831.12	-0.884045	noncoding
MSTRG.21831.13	0.326893	coding
MSTRG.21831.14	0.607962	coding
MSTRG.21830.1	-2.20608	noncoding
MSTRG.21834.3	1.31508	coding
MSTRG.21834.2	1.27141	coding

MSTRG.21837.5	2.0341	coding
MSTRG.21837.6	2.09134	coding
MSTRG.21837.8	2.03595	coding
MSTRG.21837.9	2.08368	coding
MSTRG.21842.2	2.67982	coding
MSTRG.21842.6	0.675973	coding
MSTRG.21841.1	-2.16177	noncoding
MSTRG.21846.1	1.94433	coding
MSTRG.21846.2	0.404987	coding
MSTRG.21846.3	1.68414	coding
MSTRG.21856.2	0.932095	coding
MSTRG.21863.1	-1.12073	noncoding
MSTRG.21864.2	0.530096	coding
MSTRG.21865.1	-0.11953	noncoding
MSTRG.21850.3	0.531002	coding
MSTRG.21850.2	0.715634	coding
MSTRG.21867.1	1.22056	coding
MSTRG.21870.1	-1.84527	noncoding
MSTRG.21874.1	-0.339489	noncoding
MSTRG.21876.1	-2.00364	noncoding
MSTRG.21880.1	1.2671	coding
MSTRG.21880.2	1.2616	coding
MSTRG.21880.3	0.538937	coding
MSTRG.21880.4	-0.104964	noncoding
MSTRG.21880.5	0.453797	coding
MSTRG.21880.6	0.312852	coding
MSTRG.21881.1	-2.24069	noncoding
MSTRG.21880.7	-0.09312	noncoding
MSTRG.21880.8	0.594685	coding
MSTRG.21880.9	0.370081	coding
MSTRG.21885.4	0.89903	coding
MSTRG.21885.3	1.14842	coding
MSTRG.21886.1	-0.737223	noncoding
MSTRG.21887.1	2.84015	coding
MSTRG.21889.1	-1.30989	noncoding
MSTRG.21890.2	0.131454	coding
MSTRG.21890.3	0.006888	coding
MSTRG.21890.4	-0.433311	noncoding
MSTRG.21890.5	-0.077171	noncoding
MSTRG.21890.6	0.336572	coding
MSTRG.21890.7	-0.211497	noncoding
MSTRG.21890.8	0.046997	coding
MSTRG.21890.12	-0.641311	noncoding
MSTRG.21890.20	-0.8174	noncoding
MSTRG.21890.15	-0.738384	noncoding
MSTRG.21890.19	-0.981019	noncoding
MSTRG.21890.10	-0.594344	noncoding
MSTRG.21890.21	-0.814777	noncoding
MSTRG.21890.22	-0.249238	noncoding
MSTRG.21882.1	-2.43153	noncoding
MSTRG.21883.1	-1.0457	noncoding
MSTRG.21897.10	2.03028	coding
MSTRG.21897.11	2.01755	coding
MSTRG.21897.12	2.01646	coding

MSTRG.21897.13	2.03862	coding
MSTRG.21897.15	2.01525	coding
MSTRG.21897.16	2.0208	coding
MSTRG.21897.17	2.02783	coding
MSTRG.21897.18	2.00683	coding
MSTRG.21897.3	2.0198	coding
MSTRG.21897.4	2.01703	coding
MSTRG.21897.5	1.98862	coding
MSTRG.21897.6	2.0084	coding
MSTRG.21897.7	2.01574	coding
MSTRG.21897.8	2.01158	coding
MSTRG.21897.9	2.00786	coding
MSTRG.21898.2	-0.739177	noncoding
MSTRG.21898.1	-0.942327	noncoding
MSTRG.21899.1	-2.24504	noncoding
MSTRG.21897.19	1.65472	coding
MSTRG.21897.20	1.83642	coding
MSTRG.21897.21	1.0178	coding
MSTRG.21897.22	1.62522	coding
MSTRG.21897.23	1.58587	coding
MSTRG.21897.24	1.54674	coding
MSTRG.21897.25	1.56401	coding
MSTRG.21897.26	1.24578	coding
MSTRG.21902.1	-2.00423	noncoding
MSTRG.21903.2	-0.10094	noncoding
MSTRG.21903.5	-1.36583	noncoding
MSTRG.21903.3	-1.98773	noncoding
MSTRG.21903.4	-0.828709	noncoding
MSTRG.21903.7	-1.61263	noncoding
MSTRG.21905.2	0.124933	coding
MSTRG.21905.3	0.27187	coding
MSTRG.21905.6	0.299606	coding
MSTRG.21907.2	2.08343	coding
MSTRG.21907.3	1.7232	coding
MSTRG.21912.1	1.13323	coding
MSTRG.21912.2	0.781701	coding
MSTRG.21900.5	0.34995	coding
MSTRG.21916.3	3.13414	coding
MSTRG.21917.1	-0.069406	noncoding
MSTRG.21918.2	0.266727	coding
MSTRG.21919.4	-1.07555	noncoding
MSTRG.21919.5	-1.55721	noncoding
MSTRG.21920.7	1.56847	coding
MSTRG.21925.1	2.2187	coding
MSTRG.21926.1	-0.924874	noncoding
MSTRG.21925.2	1.90117	coding
MSTRG.21925.3	2.15462	coding
MSTRG.21927.1	-2.72979	noncoding
MSTRG.21927.2	-1.34553	noncoding
MSTRG.21925.4	2.14421	coding
MSTRG.21925.5	2.19536	coding
MSTRG.21928.2	1.15047	coding
MSTRG.21921.2	0.365633	coding
MSTRG.21932.2	1.82731	coding

MSTRG.21932.3	1.6349	coding
MSTRG.21934.2	1.54139	coding
MSTRG.21935.2	-1.0861	noncoding
MSTRG.21941.1	2.35847	coding
MSTRG.21941.2	2.22578	coding
MSTRG.21942.1	1.82529	coding
MSTRG.21942.5	1.30808	coding
MSTRG.21939.2	3.00454	coding
MSTRG.21940.1	-1.51239	noncoding
MSTRG.21939.3	2.84356	coding
MSTRG.21939.4	2.9199	coding
MSTRG.21943.1	2.10146	coding
MSTRG.21943.2	2.11945	coding
MSTRG.21949.1	0.806875	coding
MSTRG.21953.1	-2.92615	noncoding
MSTRG.21955.1	-0.540813	noncoding
MSTRG.21958.4	-1.84186	noncoding
MSTRG.21958.5	0.101995	coding
MSTRG.21958.10	0.676859	coding
MSTRG.21958.3	-0.873522	noncoding
MSTRG.21958.6	-0.155427	noncoding
MSTRG.21958.7	0.744412	coding
MSTRG.21958.8	0.15583	coding
MSTRG.21958.9	0.481417	coding
MSTRG.21959.1	-1.30005	noncoding
MSTRG.21961.2	2.48571	coding
MSTRG.21961.3	2.52393	coding
MSTRG.21961.4	1.84182	coding
MSTRG.21961.5	1.66812	coding
MSTRG.21963.3	1.39034	coding
MSTRG.21977.2	1.26364	coding
MSTRG.21978.2	1.82823	coding
MSTRG.21980.1	-0.50344	noncoding
MSTRG.21985.2	1.62997	coding
MSTRG.21985.4	1.49034	coding
MSTRG.21985.5	1.48071	coding
MSTRG.21985.6	2.2236	coding
MSTRG.21985.7	2.25678	coding
MSTRG.21985.8	0.580241	coding
MSTRG.21987.2	0.115076	coding
MSTRG.21987.3	-0.954086	noncoding
MSTRG.21965.1	2.48535	coding
MSTRG.21965.2	-0.495471	noncoding
MSTRG.21966.1	-1.39987	noncoding
MSTRG.21967.1	0.417793	coding
MSTRG.21967.2	0.513767	coding
MSTRG.21967.4	0.341074	coding
MSTRG.21967.5	0.284846	coding
MSTRG.21967.6	0.462416	coding
MSTRG.21968.3	-0.575546	noncoding
MSTRG.21971.1	0.463859	coding
MSTRG.21971.2	0.679658	coding
MSTRG.21972.1	0.196541	coding
MSTRG.21994.4	-0.06026	noncoding

MSTRG.21994.3	-0.018263	noncoding
MSTRG.21994.5	-0.422283	noncoding
MSTRG.21996.12	1.28791	coding
MSTRG.21996.17	1.13007	coding
MSTRG.21996.14	2.08914	coding
MSTRG.21996.16	2.04201	coding
MSTRG.21996.9	1.66873	coding
MSTRG.21996.10	2.0941	coding
MSTRG.21996.13	1.62594	coding
MSTRG.21996.6	2.16732	coding
MSTRG.21996.8	2.09186	coding
MSTRG.21996.15	2.03774	coding
MSTRG.21997.1	0.486291	coding
MSTRG.22002.2	1.91832	coding
MSTRG.22005.4	1.6391	coding
MSTRG.22008.1	-0.034418	noncoding
MSTRG.22008.2	0.331243	coding
MSTRG.22008.3	0.17116	coding
MSTRG.22010.3	2.01453	coding
MSTRG.22010.4	2.13074	coding
MSTRG.22010.5	2.24442	coding
MSTRG.22021.1	-0.690254	noncoding
MSTRG.22014.1	-1.56972	noncoding
MSTRG.22026.1	1.56729	coding
MSTRG.22028.1	0.621857	coding
MSTRG.22027.4	-0.068703	noncoding
MSTRG.22029.1	-1.41105	noncoding
MSTRG.22027.6	2.31259	coding
MSTRG.22034.2	1.54515	coding
MSTRG.22034.5	0.628454	coding
MSTRG.22030.3	1.98103	coding
MSTRG.22030.2	1.91533	coding
MSTRG.22030.4	1.83808	coding
MSTRG.22038.3	2.76584	coding
MSTRG.22041.3	1.32412	coding
MSTRG.22041.4	0.19595	coding
MSTRG.22042.1	-2.43652	noncoding
MSTRG.22043.1	1.03755	coding
MSTRG.22043.3	1.29206	coding
MSTRG.22043.4	1.16123	coding
MSTRG.22045.2	0.97913	coding
MSTRG.22046.2	0.708688	coding
MSTRG.22050.3	1.70421	coding
MSTRG.22051.1	-2.44141	noncoding
MSTRG.22051.2	-2.45438	noncoding
MSTRG.22050.4	1.0853	coding
MSTRG.22056.1	-0.700747	noncoding
MSTRG.22058.1	-1.92086	noncoding
MSTRG.22063.3	2.06173	coding
MSTRG.22063.4	2.43435	coding
MSTRG.22063.5	1.32087	coding
MSTRG.22059.1	-0.329137	noncoding
MSTRG.22059.2	-1.0694	noncoding
MSTRG.22064.3	1.79656	coding

MSTRG.22064.4	2.03732	coding
MSTRG.22065.2	0.671172	coding
MSTRG.22068.1	2.52449	coding
MSTRG.22071.2	1.6017	coding
MSTRG.22072.1	2.4017	coding
MSTRG.22072.3	1.82448	coding
MSTRG.22074.1	-1.05759	noncoding
MSTRG.22070.1	0.658519	coding
MSTRG.22070.2	0.697509	coding
MSTRG.22075.2	1.16775	coding
MSTRG.22075.3	1.0601	coding
MSTRG.22075.4	1.12365	coding
MSTRG.22076.1	-1.87291	noncoding
MSTRG.22078.3	1.66595	coding
MSTRG.22080.1	0.513439	coding
MSTRG.22080.6	-0.093059	noncoding
MSTRG.22113.6	2.75893	coding
MSTRG.22113.5	2.8786	coding
MSTRG.22113.7	2.75182	coding
MSTRG.22114.1	0.692446	coding
MSTRG.22113.9	2.77418	coding
MSTRG.22115.3	0.929683	coding
MSTRG.22116.1	0.263449	coding
MSTRG.22117.1	-2.02104	noncoding
MSTRG.22118.4	1.76687	coding
MSTRG.22118.2	1.88877	coding
MSTRG.22118.3	0.597183	coding
MSTRG.22118.5	1.29244	coding
MSTRG.22126.1	0.766983	coding
MSTRG.22126.2	0.686035	coding
MSTRG.22126.3	0.727925	coding
MSTRG.22126.4	0.70995	coding
MSTRG.22126.5	1.34668	coding
MSTRG.22126.10	0.69706	coding
MSTRG.22082.3	2.71338	coding
MSTRG.22082.5	2.15643	coding
MSTRG.22082.4	2.64306	coding
MSTRG.22084.2	1.29335	coding
MSTRG.22086.1	0.031071	coding
MSTRG.22089.3	1.16849	coding
MSTRG.22089.4	1.12181	coding
MSTRG.22089.5	0.631566	coding
MSTRG.22091.1	-1.26972	noncoding
MSTRG.22091.3	-1.03367	noncoding
MSTRG.22098.2	-0.460506	noncoding
MSTRG.22099.2	1.55699	coding
MSTRG.22103.2	0.647581	coding
MSTRG.22139.5	1.16435	coding
MSTRG.22139.6	0.638947	coding
MSTRG.22141.1	0.133638	coding
MSTRG.22141.2	-0.708812	noncoding
MSTRG.22141.3	-0.813665	noncoding
MSTRG.22142.1	3.3643	coding
MSTRG.22145.2	-0.208738	noncoding

MSTRG.22146.1	1.43353	coding
MSTRG.22146.2	1.40821	coding
MSTRG.22146.3	1.37654	coding
MSTRG.22147.2	1.6993	coding
MSTRG.22147.3	1.87922	coding
MSTRG.22155.1	2.89614	coding
MSTRG.22155.2	2.98205	coding
MSTRG.22155.4	2.94975	coding
MSTRG.22155.3	3.04794	coding
MSTRG.22155.7	0.602907	coding
MSTRG.22150.1	-0.266581	noncoding
MSTRG.22152.2	1.78288	coding
MSTRG.22152.3	1.77112	coding
MSTRG.22152.5	1.56242	coding
MSTRG.22153.1	1.7953	coding
MSTRG.22168.1	-0.374382	noncoding
MSTRG.22179.1	0.483157	coding
MSTRG.22179.2	0.225602	coding
MSTRG.22179.3	-0.038345	noncoding
MSTRG.22179.4	0.125343	coding
MSTRG.22179.5	0.081273	coding
MSTRG.22179.7	0.139013	coding
MSTRG.22179.8	-0.012164	noncoding
MSTRG.22179.9	-0.198328	noncoding
MSTRG.22179.6	-0.003954	noncoding
MSTRG.22179.10	0.1231	coding
MSTRG.22179.11	0.013138	coding
MSTRG.22179.13	0.102273	coding
MSTRG.22179.12	-0.096224	noncoding
MSTRG.22179.14	-0.199032	noncoding
MSTRG.22179.15	0.088207	coding
MSTRG.22179.16	-0.12801	noncoding
MSTRG.22181.1	0.083505	coding
MSTRG.22181.2	0.263187	coding
MSTRG.22181.3	0.097873	coding
MSTRG.22181.4	0.241721	coding
MSTRG.22181.5	0.397572	coding
MSTRG.22181.6	0.559604	coding
MSTRG.22182.1	1.47344	coding
MSTRG.22182.3	0.899887	coding
MSTRG.22181.7	0.572329	coding
MSTRG.22184.1	1.20201	coding
MSTRG.22184.2	0.286584	coding
MSTRG.22184.3	1.16662	coding
MSTRG.22184.5	0.898734	coding
MSTRG.22184.6	1.22974	coding
MSTRG.22185.1	-1.08881	noncoding
MSTRG.22170.2	0.618438	coding
MSTRG.22172.3	0.835124	coding
MSTRG.22175.1	1.97744	coding
MSTRG.22175.2	2.00738	coding
MSTRG.22189.2	-1.28722	noncoding
MSTRG.22190.1	-0.958934	noncoding
MSTRG.22190.2	-0.272615	noncoding

MSTRG.22191.2	-0.485111	noncoding
MSTRG.22192.3	1.39679	coding
MSTRG.22195.3	2.27464	coding
MSTRG.22195.2	2.13859	coding
MSTRG.22178.2	-0.345294	noncoding
MSTRG.22178.3	-0.435333	noncoding
MSTRG.22188.1	-1.39235	noncoding
MSTRG.22197.1	-2.43196	noncoding
MSTRG.22219.1	2.01825	coding
MSTRG.22220.1	-0.794748	noncoding
MSTRG.22219.2	0.638132	coding
MSTRG.22219.5	1.38148	coding
MSTRG.22219.3	1.26839	coding
MSTRG.22223.5	-0.588589	noncoding
MSTRG.22224.1	-0.03206	noncoding
MSTRG.22226.1	0.054682	coding
MSTRG.22228.1	1.17795	coding
MSTRG.22228.2	-0.925968	noncoding
MSTRG.22223.6	1.06942	coding
MSTRG.22230.1	-0.111876	noncoding
MSTRG.22199.1	-1.20139	noncoding
MSTRG.22200.1	-0.054344	noncoding
MSTRG.22203.2	0.077483	coding
MSTRG.22204.1	-1.0858	noncoding
MSTRG.22205.1	1.46225	coding
MSTRG.22205.2	1.24123	coding
MSTRG.22205.4	0.607428	coding
MSTRG.22207.3	1.6082	coding
MSTRG.22207.7	1.4187	coding
MSTRG.22207.9	1.63846	coding
MSTRG.22208.4	0.803429	coding
MSTRG.22211.5	0.800559	coding
MSTRG.22212.2	2.2104	coding
MSTRG.22212.3	2.36348	coding
MSTRG.22213.4	1.43096	coding
MSTRG.22213.5	1.35976	coding
MSTRG.22213.2	1.11429	coding
MSTRG.22213.3	1.45914	coding
MSTRG.22214.4	0.075384	coding
MSTRG.22216.7	2.68411	coding
MSTRG.22217.1	-1.75034	noncoding
MSTRG.22238.4	0.062282	coding
MSTRG.22238.6	-0.192646	noncoding
MSTRG.22238.7	-0.69343	noncoding
MSTRG.22239.1	0.436004	coding
MSTRG.22240.1	-1.42765	noncoding
MSTRG.22241.1	0.512231	coding
MSTRG.22242.2	1.3806	coding
MSTRG.22242.3	0.920054	coding
MSTRG.22242.4	1.34238	coding
MSTRG.22245.2	1.722	coding
MSTRG.22246.3	0.024588	coding
MSTRG.22251.3	1.01717	coding
MSTRG.22251.2	2.41186	coding

MSTRG.22251.4	2.88215	coding
MSTRG.22261.1	0.766076	coding
MSTRG.22261.2	1.35714	coding
MSTRG.22262.2	-0.531422	noncoding
MSTRG.22262.3	0.327409	coding
MSTRG.22262.4	0.471706	coding
MSTRG.22262.5	-0.511294	noncoding
MSTRG.22265.1	-1.28503	noncoding
MSTRG.22264.3	2.69133	coding
MSTRG.22264.4	2.79466	coding
MSTRG.22276.2	0.17245	coding
MSTRG.22277.1	1.22629	coding
MSTRG.22277.3	1.32352	coding
MSTRG.22277.10	1.07335	coding
MSTRG.22277.5	1.08533	coding
MSTRG.22277.9	1.46885	coding
MSTRG.22277.7	1.74862	coding
MSTRG.22277.11	-0.141292	noncoding
MSTRG.22269.2	0.398419	coding
MSTRG.22269.3	0.202457	coding
MSTRG.22271.1	-1.72502	noncoding
MSTRG.22282.1	-0.537847	noncoding
MSTRG.22282.2	-0.069597	noncoding
MSTRG.22282.3	-0.104148	noncoding
MSTRG.22283.3	0.1941	coding
MSTRG.22284.2	2.03397	coding
MSTRG.22285.1	0.999155	coding
MSTRG.22286.3	1.52004	coding
MSTRG.22286.6	1.39772	coding
MSTRG.22301.1	-1.32088	noncoding
MSTRG.22309.1	-1.2368	noncoding
MSTRG.22310.1	-0.062182	noncoding
MSTRG.22312.3	0.109341	coding
MSTRG.22312.2	0.538131	coding
MSTRG.22306.4	-0.25602	noncoding
MSTRG.22315.1	-1.32666	noncoding
MSTRG.22306.5	-1.02788	noncoding
MSTRG.22306.7	-0.731726	noncoding
MSTRG.22306.8	-0.573512	noncoding
MSTRG.22306.6	-0.251342	noncoding
MSTRG.22306.9	-0.461466	noncoding
MSTRG.22306.11	0.021112	coding
MSTRG.22295.1	1.02864	coding
MSTRG.22295.3	-0.405301	noncoding
MSTRG.22299.3	1.71996	coding
MSTRG.22300.1	-0.581533	noncoding
MSTRG.22300.3	0.151217	coding
MSTRG.22300.4	-0.539941	noncoding
MSTRG.22316.1	0.712439	coding
MSTRG.22316.2	1.11042	coding
MSTRG.22319.2	0.650599	coding
MSTRG.22319.3	0.765413	coding
MSTRG.22332.7	0.30931	coding
MSTRG.22326.2	0.7342	coding

MSTRG.22327.1	1.32209	coding
MSTRG.22327.3	1.29706	coding
MSTRG.22325.1	0.055426	coding
MSTRG.22335.2	2.68131	coding
MSTRG.22335.3	1.72881	coding
MSTRG.22341.2	-0.516659	noncoding
MSTRG.22341.3	-0.831493	noncoding
MSTRG.22344.1	0.810693	coding
MSTRG.22344.2	1.07637	coding
MSTRG.22344.4	1.02098	coding
MSTRG.22344.5	-0.675899	noncoding
MSTRG.22344.7	0.472954	coding
MSTRG.22344.9	1.14624	coding
MSTRG.22346.1	-1.88062	noncoding
MSTRG.22344.11	0.356415	coding
MSTRG.22338.2	0.186095	coding
MSTRG.22338.4	-0.563876	noncoding
MSTRG.22340.2	-2.22954	noncoding
MSTRG.22348.3	-0.32926	noncoding
MSTRG.22355.2	0.926858	coding
MSTRG.22356.1	1.84693	coding
MSTRG.22356.4	1.32812	coding
MSTRG.22356.5	1.74959	coding
MSTRG.22356.3	1.63063	coding
MSTRG.22351.2	1.58247	coding
MSTRG.22351.3	1.48729	coding
MSTRG.22358.2	-2.44312	noncoding
MSTRG.22359.2	3.13659	coding
MSTRG.22361.2	-1.12515	noncoding
MSTRG.22370.2	2.05488	coding
MSTRG.22370.4	2.13544	coding
MSTRG.22378.3	2.29326	coding
MSTRG.22378.5	2.41243	coding
MSTRG.22378.6	2.40827	coding
MSTRG.22378.7	1.81002	coding
MSTRG.22367.3	-0.879908	noncoding
MSTRG.22367.4	0.693003	coding
MSTRG.22367.5	-0.352113	noncoding
MSTRG.22382.1	0.226715	coding
MSTRG.22382.4	0.511759	coding
MSTRG.22382.5	0.36648	coding
MSTRG.22382.8	1.21172	coding
MSTRG.22382.6	-0.053333	noncoding
MSTRG.22382.7	0.432587	coding
MSTRG.22382.9	0.651167	coding
MSTRG.22383.1	0.65746	coding
MSTRG.22383.2	0.65746	coding
MSTRG.22383.3	0.65746	coding
MSTRG.22383.4	0.738662	coding
MSTRG.22390.8	0.717701	coding
MSTRG.22390.7	0.874946	coding
MSTRG.22390.6	1.41134	coding
MSTRG.22390.2	1.68709	coding
MSTRG.22391.2	0.231379	coding

MSTRG.22391.4	0.302515	coding
MSTRG.22392.2	-0.291798	noncoding
MSTRG.22392.4	-1.70037	noncoding
MSTRG.22394.2	-1.92265	noncoding
MSTRG.22394.3	0.292019	coding
MSTRG.22385.2	1.02594	coding
MSTRG.22386.2	1.19498	coding
MSTRG.22397.2	0.404285	coding
MSTRG.22397.3	0.337312	coding
MSTRG.22397.4	0.14145	coding
MSTRG.22398.1	1.19219	coding
MSTRG.22399.1	1.90161	coding
MSTRG.22399.2	1.85946	coding
MSTRG.22400.1	-0.73446	noncoding
MSTRG.22402.2	0.220493	coding
MSTRG.22402.3	0.284661	coding
MSTRG.22403.1	0.354224	coding
MSTRG.22406.2	-0.031035	noncoding
MSTRG.22406.5	0.358374	coding
MSTRG.22411.2	1.45083	coding
MSTRG.22411.1	0.526287	coding
MSTRG.22407.1	0.812003	coding
MSTRG.22407.2	0.783671	coding
MSTRG.22408.1	0.030256	coding
MSTRG.22419.1	-0.625968	noncoding
MSTRG.22420.1	-1.43007	noncoding
MSTRG.22417.1	-1.97183	noncoding
MSTRG.22417.2	-0.988948	noncoding
MSTRG.22426.1	-2.93788	noncoding
MSTRG.22428.2	2.61295	coding
MSTRG.22430.2	1.7839	coding
MSTRG.22435.2	1.5749	coding
MSTRG.22431.1	2.01253	coding
MSTRG.22444.7	1.49942	coding
MSTRG.22444.5	1.37517	coding
MSTRG.22449.1	2.42279	coding
MSTRG.22444.10	-1.96716	noncoding
MSTRG.22449.3	2.16186	coding
MSTRG.22449.4	2.12414	coding
MSTRG.22449.5	2.12083	coding
MSTRG.22449.9	2.14374	coding
MSTRG.22449.10	2.27025	coding
MSTRG.22451.3	1.11919	coding
MSTRG.22451.8	1.28828	coding
MSTRG.22451.5	1.33528	coding
MSTRG.22451.9	1.35771	coding
MSTRG.22451.13	-0.290325	noncoding
MSTRG.22451.14	1.86058	coding
MSTRG.22451.11	1.84968	coding
MSTRG.22451.12	1.85237	coding
MSTRG.22442.1	-0.744836	noncoding
MSTRG.22459.3	0.490335	coding
MSTRG.22459.1	0.518178	coding
MSTRG.22459.4	0.409076	coding

MSTRG.22459.5	0.309062	coding
MSTRG.22459.6	1.60329	coding
MSTRG.22460.1	1.06673	coding
MSTRG.22460.2	-0.3825	noncoding
MSTRG.22461.1	0.031855	coding
MSTRG.22461.2	0.490139	coding
MSTRG.22461.4	-0.632322	noncoding
MSTRG.22461.5	-0.217609	noncoding
MSTRG.22461.6	0.433258	coding
MSTRG.22454.1	1.18569	coding
MSTRG.22454.2	1.23849	coding
MSTRG.22455.3	1.82814	coding
MSTRG.22464.1	0.374345	coding
MSTRG.22465.1	-0.681173	noncoding
MSTRG.22466.1	0.759031	coding
MSTRG.22478.3	2.76348	coding
MSTRG.22478.2	1.84832	coding
MSTRG.22478.4	2.58065	coding
MSTRG.22478.5	1.84438	coding
MSTRG.22478.7	1.79821	coding
MSTRG.22478.6	1.95828	coding
MSTRG.22478.10	2.53749	coding
MSTRG.22478.11	2.68551	coding
MSTRG.22480.1	-0.152161	noncoding
MSTRG.22482.1	-2.13115	noncoding
MSTRG.22490.1	1.3892	coding
MSTRG.22490.2	-0.356362	noncoding
MSTRG.22490.3	0.458381	coding
MSTRG.22490.4	0.010197	coding
MSTRG.22490.5	0.950601	coding
MSTRG.22490.6	0.811712	coding
MSTRG.22490.7	1.54192	coding
MSTRG.22490.9	0.555622	coding
MSTRG.22468.1	-1.11173	noncoding
MSTRG.22469.1	-0.748755	noncoding
MSTRG.22476.1	-1.54342	noncoding
MSTRG.22477.1	-0.597769	noncoding
MSTRG.22519.1	1.44936	coding
MSTRG.22521.1	-1.7184	noncoding
MSTRG.22517.1	0.069449	coding
MSTRG.22517.2	1.06613	coding
MSTRG.22517.3	0.500689	coding
MSTRG.22524.1	-2.25825	noncoding
MSTRG.22526.1	2.33292	coding
MSTRG.22539.1	-1.23382	noncoding
MSTRG.22542.2	2.41139	coding
MSTRG.22544.1	-1.59003	noncoding
MSTRG.22544.2	-2.16759	noncoding
MSTRG.22528.1	-2.39077	noncoding
MSTRG.22530.1	-1.51645	noncoding
MSTRG.22549.1	-0.636674	noncoding
MSTRG.22549.2	-0.876256	noncoding
MSTRG.22551.3	-1.17992	noncoding
MSTRG.22551.2	-0.686583	noncoding

MSTRG.22551.4	-1.28232	noncoding
MSTRG.22551.5	-1.63231	noncoding
MSTRG.22552.2	1.05676	coding
MSTRG.22550.2	1.46759	coding
MSTRG.22553.3	0.205025	coding
MSTRG.22563.1	2.26947	coding
MSTRG.22563.3	-0.207927	noncoding
MSTRG.22564.3	1.23849	coding
MSTRG.22572.1	2.38321	coding
MSTRG.22574.1	-0.523934	noncoding
MSTRG.22572.2	2.49596	coding
MSTRG.22572.3	2.58551	coding
MSTRG.22576.1	-0.135668	noncoding
MSTRG.22580.1	-1.39129	noncoding
MSTRG.22579.3	-1.70913	noncoding
MSTRG.22579.4	-1.65486	noncoding
MSTRG.22583.2	-0.121176	noncoding
MSTRG.22583.5	-0.166655	noncoding
MSTRG.22592.2	0.884222	coding
MSTRG.22592.3	0.889227	coding
MSTRG.22592.4	0.545101	coding
MSTRG.22585.2	0.287708	coding
MSTRG.22585.1	-0.103775	noncoding
MSTRG.22593.4	2.52135	coding
MSTRG.22598.2	1.39558	coding
MSTRG.22598.4	1.40624	coding
MSTRG.22599.1	0.781881	coding
MSTRG.22599.2	0.572312	coding
MSTRG.22599.3	0.586047	coding
MSTRG.22599.4	-1.17746	noncoding
MSTRG.22599.5	0.175669	coding
MSTRG.22605.1	-0.381521	noncoding
MSTRG.22607.3	0.606054	coding
MSTRG.22609.1	0.465611	coding
MSTRG.22608.2	2.14126	coding
MSTRG.22610.1	-0.576499	noncoding
MSTRG.22610.2	-0.066479	noncoding
MSTRG.22611.1	-0.456652	noncoding
MSTRG.22612.1	-0.783769	noncoding
MSTRG.22608.4	1.71529	coding
MSTRG.22617.3	1.62834	coding
MSTRG.22617.1	1.67779	coding
MSTRG.22617.4	1.58011	coding
MSTRG.22618.1	-1.49117	noncoding
MSTRG.22627.2	0.78949	coding
MSTRG.22627.3	2.48277	coding
MSTRG.22627.4	2.57351	coding
MSTRG.22627.5	2.50985	coding
MSTRG.22627.6	2.31398	coding
MSTRG.22627.7	2.47108	coding
MSTRG.22627.8	2.50703	coding
MSTRG.22621.2	-0.701706	noncoding
MSTRG.22624.2	2.77249	coding
MSTRG.22633.4	0.103997	coding

MSTRG.22634.3	-0.560985	noncoding
MSTRG.22644.1	0.474216	coding
MSTRG.22644.4	-0.711635	noncoding
MSTRG.22644.5	-0.559947	noncoding
MSTRG.22644.3	-0.123836	noncoding
MSTRG.22646.1	1.25883	coding
MSTRG.22646.2	1.74699	coding
MSTRG.22646.4	0.624571	coding
MSTRG.22650.1	-1.05186	noncoding
MSTRG.22650.5	-0.507894	noncoding
MSTRG.22650.6	-0.566922	noncoding
MSTRG.22650.3	-0.499635	noncoding
MSTRG.22650.4	-0.402341	noncoding
MSTRG.22650.7	-0.452026	noncoding
MSTRG.22639.2	1.43933	coding
MSTRG.22651.3	1.62386	coding
MSTRG.22651.4	1.40218	coding
MSTRG.22651.5	1.22911	coding
MSTRG.22651.6	1.30116	coding
MSTRG.22651.7	1.32895	coding
MSTRG.22654.1	-0.649758	noncoding
MSTRG.22656.1	-0.939677	noncoding
MSTRG.22660.2	-0.948476	noncoding
MSTRG.22668.2	1.55233	coding
MSTRG.22664.1	1.97435	coding
MSTRG.22683.4	1.19183	coding
MSTRG.22684.1	-0.563961	noncoding
MSTRG.22686.2	2.40165	coding
MSTRG.22686.3	2.28437	coding
MSTRG.22689.1	0.20835	coding
MSTRG.22679.1	-0.37078	noncoding
MSTRG.22703.3	0.204606	coding
MSTRG.22703.2	0.42859	coding
MSTRG.22709.2	1.51901	coding
MSTRG.22713.1	1.06822	coding
MSTRG.22713.5	2.45258	coding
MSTRG.22713.4	2.48311	coding
MSTRG.22714.1	-0.141326	noncoding
MSTRG.22694.2	0.664747	coding
MSTRG.22718.1	0.43894	coding
MSTRG.22718.2	1.03288	coding
MSTRG.22718.3	0.850265	coding
MSTRG.22718.4	1.61526	coding
MSTRG.22721.1	0.613951	coding
MSTRG.22722.1	0.1099	coding
MSTRG.22725.1	-0.599151	noncoding
MSTRG.22731.2	2.01121	coding
MSTRG.22733.1	0.061989	coding
MSTRG.22733.2	-0.040394	noncoding
MSTRG.22737.2	1.0176	coding
MSTRG.22739.1	-1.53153	noncoding
MSTRG.22740.1	0.23634	coding
MSTRG.22740.2	1.14933	coding
MSTRG.22740.4	0.276954	coding

MSTRG.22742.1	1.70174	coding
MSTRG.22742.2	0.518044	coding
MSTRG.22744.2	2.31687	coding
MSTRG.22744.3	2.19671	coding
MSTRG.22744.5	1.15738	coding
MSTRG.22744.4	2.34144	coding
MSTRG.22744.6	2.30762	coding
MSTRG.22744.8	0.012812	coding
MSTRG.22746.1	2.67221	coding
MSTRG.22747.1	0.473457	coding
MSTRG.22747.3	1.31303	coding
MSTRG.22747.4	1.67756	coding
MSTRG.22749.4	0.975775	coding
MSTRG.22750.5	0.530356	coding
MSTRG.22755.1	-0.737156	noncoding
MSTRG.22763.2	2.6336	coding
MSTRG.22763.4	2.73155	coding
MSTRG.22763.5	2.53211	coding
MSTRG.22763.6	2.54684	coding
MSTRG.22764.1	0.834793	coding
MSTRG.22764.3	-0.047062	noncoding
MSTRG.22770.2	1.20223	coding
MSTRG.22774.1	-1.93563	noncoding
MSTRG.22776.1	-1.5581	noncoding
MSTRG.22779.1	0.06684	coding
MSTRG.22780.1	-1.38136	noncoding
MSTRG.22790.3	2.84556	coding
MSTRG.22790.4	2.57681	coding
MSTRG.22790.5	2.45613	coding
MSTRG.22793.1	-1.50232	noncoding
MSTRG.22798.3	2.59588	coding
MSTRG.22800.5	1.06543	coding
MSTRG.22800.3	1.2782	coding
MSTRG.22784.1	1.36274	coding
MSTRG.22785.4	2.15518	coding
MSTRG.22785.5	0.698841	coding
MSTRG.22785.6	1.02387	coding
MSTRG.22786.1	-0.809155	noncoding
MSTRG.22787.1	0.705194	coding
MSTRG.22789.2	-0.292709	noncoding
MSTRG.22803.1	-2.3711	noncoding
MSTRG.22804.1	1.04917	coding
MSTRG.22804.3	0.788662	coding
MSTRG.22804.4	0.76827	coding
MSTRG.22806.2	-0.787182	noncoding
MSTRG.22811.1	2.21929	coding
MSTRG.22812.3	1.0997	coding
MSTRG.22813.1	-0.096209	noncoding
MSTRG.22814.2	1.52537	coding
MSTRG.22818.2	2.07667	coding
MSTRG.22819.2	1.20895	coding
MSTRG.22819.1	1.23451	coding
MSTRG.22822.2	0.71326	coding
MSTRG.22822.3	-0.714846	noncoding

MSTRG.22825.1	0.787523	coding
MSTRG.22825.7	0.347831	coding
MSTRG.22825.5	0.294542	coding
MSTRG.22825.6	0.714869	coding
MSTRG.22827.1	-1.07059	noncoding
MSTRG.22829.3	0.951709	coding
MSTRG.22829.4	0.977512	coding
MSTRG.22829.2	1.52816	coding
MSTRG.22824.1	0.610629	coding
MSTRG.22832.5	1.06259	coding
MSTRG.22832.6	1.09425	coding
MSTRG.22832.4	1.41223	coding
MSTRG.22833.2	-0.029935	noncoding
MSTRG.22833.3	0.296418	coding
MSTRG.22841.2	1.57567	coding
MSTRG.22841.3	1.38427	coding
MSTRG.22842.8	1.24395	coding
MSTRG.22842.9	1.11692	coding
MSTRG.22842.10	0.887537	coding
MSTRG.22842.12	1.09331	coding
MSTRG.22842.13	1.12603	coding
MSTRG.22835.1	1.28487	coding
MSTRG.22835.2	1.60853	coding
MSTRG.22836.1	-0.487792	noncoding
MSTRG.22838.4	0.344308	coding
MSTRG.22838.5	0.210531	coding
MSTRG.22838.3	0.739102	coding
MSTRG.22838.2	0.658788	coding
MSTRG.22845.2	0.602579	coding
MSTRG.22847.3	0.889094	coding
MSTRG.22857.1	0.285393	coding
MSTRG.22857.2	1.47959	coding
MSTRG.22858.3	1.84457	coding
MSTRG.22858.5	1.83302	coding
MSTRG.22858.6	1.82427	coding
MSTRG.22858.7	1.84069	coding
MSTRG.22858.8	1.84637	coding
MSTRG.22858.10	1.83111	coding
MSTRG.22858.4	1.84843	coding
MSTRG.22858.9	1.84471	coding
MSTRG.22867.1	2.67528	coding
MSTRG.22871.1	-0.990994	noncoding
MSTRG.22871.2	-1.52154	noncoding
MSTRG.22881.2	2.22442	coding
MSTRG.22881.3	2.13216	coding
MSTRG.22892.2	1.17024	coding
MSTRG.22892.4	1.18621	coding
MSTRG.22892.5	0.177055	coding
MSTRG.22893.1	-1.68682	noncoding
MSTRG.22895.2	-0.110631	noncoding
MSTRG.22896.1	0.652432	coding
MSTRG.22896.4	1.43764	coding
MSTRG.22898.2	1.85291	coding
MSTRG.22899.1	1.28517	coding

MSTRG.22909.2	0.312688	coding
MSTRG.22911.3	2.04159	coding
MSTRG.22912.1	-0.942286	noncoding
MSTRG.22913.2	1.50157	coding
MSTRG.22914.3	1.31138	coding
MSTRG.22919.1	2.57453	coding
MSTRG.22921.1	-3.02532	noncoding
MSTRG.22922.1	1.02707	coding
MSTRG.22922.3	0.326158	coding
MSTRG.22924.1	1.56972	coding
MSTRG.22922.6	-0.095975	noncoding
MSTRG.22924.3	0.306577	coding
MSTRG.22924.2	1.22169	coding
MSTRG.22928.2	1.52568	coding
MSTRG.22931.5	1.23136	coding
MSTRG.22931.6	1.68354	coding
MSTRG.22933.1	0.435557	coding
MSTRG.22935.1	0.939383	coding
MSTRG.22936.1	0.166725	coding
MSTRG.22937.2	0.448918	coding
MSTRG.22937.3	0.581243	coding
MSTRG.22941.4	1.83358	coding
MSTRG.22943.1	0.587042	coding
MSTRG.22944.2	0.549449	coding
MSTRG.22925.3	1.26594	coding
MSTRG.22946.1	1.59392	coding
MSTRG.22946.4	0.65477	coding
MSTRG.22947.2	1.6256	coding
MSTRG.22948.3	2.78551	coding
MSTRG.22948.4	2.93773	coding
MSTRG.22948.2	2.58611	coding
MSTRG.22948.5	2.47439	coding
MSTRG.22951.1	2.00112	coding
MSTRG.22951.2	2.08787	coding
MSTRG.22953.1	1.82102	coding
MSTRG.22953.2	2.26944	coding
MSTRG.22953.8	1.56757	coding
MSTRG.22953.4	2.20078	coding
MSTRG.22953.5	1.523	coding
MSTRG.22953.6	2.18754	coding
MSTRG.22953.7	1.91389	coding
MSTRG.22953.3	2.80237	coding
MSTRG.22954.1	-0.932151	noncoding
MSTRG.22955.1	-2.58717	noncoding
MSTRG.22965.3	0.053112	coding
MSTRG.22965.5	0.956032	coding
MSTRG.22965.4	1.76572	coding
MSTRG.22965.6	1.78416	coding
MSTRG.22965.8	0.238909	coding
MSTRG.22976.4	-0.217571	noncoding
MSTRG.22977.2	1.52591	coding
MSTRG.22979.2	1.50678	coding
MSTRG.22979.3	1.25375	coding
MSTRG.22983.2	2.23762	coding

MSTRG.22984.5	-0.108899	noncoding
MSTRG.22984.1	0.297095	coding
MSTRG.22984.3	1.12856	coding
MSTRG.22984.4	0.815446	coding
MSTRG.22985.2	-0.545948	noncoding
MSTRG.22986.3	1.78511	coding
MSTRG.22986.4	1.00304	coding
MSTRG.22986.5	0.758186	coding
MSTRG.22966.6	1.42649	coding
MSTRG.22966.2	1.20344	coding
MSTRG.22966.3	1.19504	coding
MSTRG.22966.4	1.23509	coding
MSTRG.22966.5	0.388144	coding
MSTRG.22967.1	0.062404	coding
MSTRG.22968.1	0.249061	coding
MSTRG.22967.2	0.138895	coding
MSTRG.22966.7	0.821653	coding
MSTRG.22966.8	1.05173	coding
MSTRG.22966.9	1.57269	coding
MSTRG.22966.10	1.22339	coding
MSTRG.22972.1	0.876941	coding
MSTRG.23003.1	-1.79945	noncoding
MSTRG.23003.2	-2.14208	noncoding
MSTRG.23003.3	-1.87131	noncoding
MSTRG.23015.1	3.93899	coding
MSTRG.23015.2	4.0351	coding
MSTRG.23016.1	-0.791713	noncoding
MSTRG.23015.3	4.07257	coding
MSTRG.23017.1	0.073344	coding
MSTRG.23018.1	-0.849098	noncoding
MSTRG.23006.1	-2.22777	noncoding
MSTRG.23007.1	1.68916	coding
MSTRG.23007.3	1.56573	coding
MSTRG.23009.1	1.38292	coding
MSTRG.23009.2	1.19993	coding
MSTRG.23009.3	1.12421	coding
MSTRG.23049.1	-0.533605	noncoding
MSTRG.23050.1	-1.34965	noncoding
MSTRG.23048.5	2.13822	coding
MSTRG.23048.4	2.13578	coding
MSTRG.23048.6	2.0906	coding
MSTRG.23052.2	1.04331	coding
MSTRG.23055.1	1.40921	coding
MSTRG.23055.5	1.46657	coding
MSTRG.23054.3	-0.001014	noncoding
MSTRG.23057.1	-1.18441	noncoding
MSTRG.23058.1	-0.481409	noncoding
MSTRG.23061.1	0.504713	coding
MSTRG.23061.2	-1.11279	noncoding
MSTRG.23071.1	0.703761	coding
MSTRG.23080.2	0.309105	coding
MSTRG.23080.1	-1.36985	noncoding
MSTRG.23082.1	2.89154	coding
MSTRG.23082.2	0.763987	coding

MSTRG.23082.3	2.94173	coding
MSTRG.23082.4	2.93395	coding
MSTRG.23084.1	-0.766939	noncoding
MSTRG.23072.2	0.439414	coding
MSTRG.23074.1	2.065	coding
MSTRG.23074.2	2.19248	coding
MSTRG.23075.1	-0.878101	noncoding
MSTRG.23076.1	-0.244248	noncoding
MSTRG.23087.3	1.3627	coding
MSTRG.23079.2	3.68884	coding
MSTRG.23089.2	-0.915195	noncoding
MSTRG.23089.1	-1.78588	noncoding
MSTRG.23094.2	1.15595	coding
MSTRG.23094.4	0.604591	coding
MSTRG.23094.5	0.703517	coding
MSTRG.23094.6	0.519581	coding
MSTRG.23101.1	1.73596	coding
MSTRG.23101.2	1.67982	coding
MSTRG.23106.3	1.11648	coding
MSTRG.23106.4	0.779695	coding
MSTRG.23106.5	1.25292	coding
MSTRG.23108.1	1.89766	coding
MSTRG.23105.2	1.60262	coding
MSTRG.23110.2	2.46971	coding
MSTRG.23110.3	2.19819	coding
MSTRG.23122.1	1.65615	coding
MSTRG.23122.2	1.60213	coding
MSTRG.23122.3	1.85785	coding
MSTRG.23127.1	-1.99483	noncoding
MSTRG.23127.2	-0.506667	noncoding
MSTRG.23128.1	-1.89249	noncoding
MSTRG.23130.1	-0.380785	noncoding
MSTRG.23134.1	-0.068086	noncoding
MSTRG.23139.2	1.16738	coding
MSTRG.23139.4	-0.477525	noncoding
MSTRG.23139.5	-0.497583	noncoding
MSTRG.23139.6	-0.526921	noncoding
MSTRG.23139.7	-0.756454	noncoding
MSTRG.23142.2	-0.190124	noncoding
MSTRG.23142.1	-0.249636	noncoding
MSTRG.23145.1	-0.097091	noncoding
MSTRG.23145.2	-0.406004	noncoding
MSTRG.23146.1	0.243495	coding
MSTRG.23153.2	2.24916	coding
MSTRG.23153.3	2.13392	coding
MSTRG.23150.2	2.29592	coding
MSTRG.23151.1	-0.338488	noncoding
MSTRG.23154.1	-0.794104	noncoding
MSTRG.23159.3	0.492749	coding
MSTRG.23160.1	2.1844	coding
MSTRG.23158.1	-0.772086	noncoding
MSTRG.23158.2	-0.522594	noncoding
MSTRG.23166.1	-1.09576	noncoding
MSTRG.23169.1	-0.280958	noncoding

MSTRG.23173.1	-2.01933	noncoding
MSTRG.23175.1	-0.322351	noncoding
MSTRG.23175.2	-0.767175	noncoding
MSTRG.23174.1	-1.54431	noncoding
MSTRG.23178.1	1.51157	coding
MSTRG.23182.1	1.86252	coding
MSTRG.23187.1	-0.791095	noncoding
MSTRG.23188.1	-0.069035	noncoding
MSTRG.23188.2	-0.287999	noncoding
MSTRG.23188.4	-0.339499	noncoding
MSTRG.23188.3	-0.252269	noncoding
MSTRG.23189.2	2.54974	coding
MSTRG.23191.3	0.107916	coding
MSTRG.23191.4	0.094441	coding
MSTRG.23191.2	0.111278	coding
MSTRG.23192.4	-0.573582	noncoding
MSTRG.23186.2	0.443179	coding
MSTRG.23194.1	2.04933	coding
MSTRG.23194.2	2.05027	coding
MSTRG.23195.3	0.713398	coding
MSTRG.23195.4	0.682143	coding
MSTRG.23195.2	0.71713	coding
MSTRG.23195.1	0.58459	coding
MSTRG.23196.1	-0.914637	noncoding
MSTRG.23193.1	1.78501	coding
MSTRG.23197.2	-1.09875	noncoding
MSTRG.23202.1	0.399428	coding
MSTRG.23204.3	2.22124	coding
MSTRG.23201.2	0.660078	coding
MSTRG.23207.1	0.561663	coding
MSTRG.23207.4	0.369441	coding
MSTRG.23212.1	-0.203319	noncoding
MSTRG.23213.1	0.58341	coding
MSTRG.23221.3	1.56791	coding
MSTRG.23221.2	0.777013	coding
MSTRG.23222.1	-1.41845	noncoding
MSTRG.23223.1	1.4807	coding
MSTRG.23223.2	0.970625	coding
MSTRG.23223.3	1.26431	coding
MSTRG.23223.4	0.788725	coding
MSTRG.23224.1	-0.331175	noncoding
MSTRG.23234.1	-0.408351	noncoding
MSTRG.23237.1	1.53431	coding
MSTRG.23237.3	1.50499	coding
MSTRG.23239.1	1.25338	coding
MSTRG.23241.1	0.604473	coding
MSTRG.23253.1	0.232058	coding
MSTRG.23255.2	2.15091	coding
MSTRG.23255.4	2.63529	coding
MSTRG.23255.5	2.29994	coding
MSTRG.23255.6	2.67642	coding
MSTRG.23257.1	-0.324976	noncoding
MSTRG.23255.7	1.949	coding
MSTRG.23255.8	1.18396	coding

MSTRG.23265.1	2.28607	coding
MSTRG.23265.2	2.20801	coding
MSTRG.23265.3	0.605527	coding
MSTRG.23265.12	2.04433	coding
MSTRG.23265.11	1.92685	coding
MSTRG.23265.13	2.23453	coding
MSTRG.23265.7	2.26085	coding
MSTRG.23265.15	0.477002	coding
MSTRG.23262.1	0.523386	coding
MSTRG.23264.1	0.837923	coding
MSTRG.23258.1	-1.68137	noncoding
MSTRG.23259.1	-1.2279	noncoding
MSTRG.23268.1	2.07167	coding
MSTRG.23272.1	-0.913502	noncoding
MSTRG.23272.2	-0.768317	noncoding
MSTRG.23272.3	-0.500534	noncoding
MSTRG.23272.4	-0.494596	noncoding
MSTRG.23272.5	-1.3324	noncoding
MSTRG.23271.3	0.619275	coding
MSTRG.23273.3	3.24698	coding
MSTRG.23275.3	1.19395	coding
MSTRG.23280.2	1.76579	coding
MSTRG.23284.2	1.09204	coding
MSTRG.23285.3	2.16459	coding
MSTRG.23285.4	1.99778	coding
MSTRG.23285.5	1.86635	coding
MSTRG.23285.6	2.01305	coding
MSTRG.23285.8	1.48079	coding
MSTRG.23279.1	-0.621424	noncoding
MSTRG.23291.2	-0.633665	noncoding
MSTRG.23291.3	-0.860798	noncoding
MSTRG.23292.3	1.78785	coding
MSTRG.23292.4	1.75291	coding
MSTRG.23292.5	1.15737	coding
MSTRG.23292.6	1.84266	coding
MSTRG.23292.8	1.08342	coding
MSTRG.23292.9	1.0639	coding
MSTRG.23292.10	1.18937	coding
MSTRG.23297.4	2.8903	coding
MSTRG.23297.3	3.19023	coding
MSTRG.23295.1	0.679255	coding
MSTRG.23296.2	-0.200581	noncoding
MSTRG.23296.3	-0.55468	noncoding
MSTRG.23301.1	0.169202	coding
MSTRG.23307.1	1.27725	coding
MSTRG.23307.2	2.38893	coding
MSTRG.23307.3	1.67578	coding
MSTRG.23307.7	2.44377	coding
MSTRG.23318.4	1.59594	coding
MSTRG.23318.6	1.65313	coding
MSTRG.23318.7	1.64935	coding
MSTRG.23318.8	1.87529	coding
MSTRG.23318.9	1.85455	coding
MSTRG.23321.3	0.262942	coding

MSTRG.23321.2	0.496249	coding
MSTRG.23322.1	0.873327	coding
MSTRG.23322.4	-0.1596	noncoding
MSTRG.23322.5	-0.33021	noncoding
MSTRG.23322.3	0.831218	coding
MSTRG.23323.3	1.44583	coding
MSTRG.23314.1	2.07895	coding
MSTRG.23324.2	1.53838	coding
MSTRG.23325.1	1.87395	coding
MSTRG.23332.2	1.70258	coding
MSTRG.23333.1	0.418408	coding
MSTRG.23333.3	0.583267	coding
MSTRG.23327.1	0.131676	coding
MSTRG.23327.2	0.521981	coding
MSTRG.23327.3	-0.566938	noncoding
MSTRG.23327.4	0.495184	coding
MSTRG.23339.3	1.78542	coding
MSTRG.23347.2	2.20298	coding
MSTRG.23347.3	2.09221	coding
MSTRG.23348.2	0.770133	coding
MSTRG.23348.1	0.358521	coding
MSTRG.23349.1	0.700091	coding
MSTRG.23349.2	0.307728	coding
MSTRG.23349.3	0.425559	coding
MSTRG.23349.4	0.436847	coding
MSTRG.23349.5	0.506247	coding
MSTRG.23350.1	1.80432	coding
MSTRG.23350.4	1.92989	coding
MSTRG.23355.2	1.9341	coding
MSTRG.23357.1	0.810343	coding
MSTRG.23360.1	-0.449255	noncoding
MSTRG.23360.2	-0.643491	noncoding
MSTRG.23360.3	-0.397719	noncoding
MSTRG.23361.1	-1.0804	noncoding
MSTRG.23352.3	2.1926	coding
MSTRG.23352.4	2.01197	coding
MSTRG.23352.5	1.79847	coding
MSTRG.23365.2	0.010464	coding
MSTRG.23369.1	-0.307925	noncoding
MSTRG.23369.9	0.027417	coding
MSTRG.23369.10	0.459016	coding
MSTRG.23367.1	-2.3421	noncoding
MSTRG.23367.3	-0.215215	noncoding
MSTRG.23370.3	-0.404899	noncoding
MSTRG.23373.2	-0.169642	noncoding
MSTRG.23374.3	2.21872	coding
MSTRG.23377.2	1.27002	coding
MSTRG.23377.3	1.20559	coding
MSTRG.23379.1	0.495462	coding
MSTRG.23380.1	-0.580558	noncoding
MSTRG.23379.3	0.197722	coding
MSTRG.23380.2	-0.485727	noncoding
MSTRG.23380.3	-0.587554	noncoding
MSTRG.23380.4	-0.540052	noncoding

MSTRG.23379.4	0.301263	coding
MSTRG.23380.5	-0.665887	noncoding
MSTRG.23380.6	-0.646654	noncoding
MSTRG.23380.7	-0.794991	noncoding
MSTRG.23380.8	-0.645238	noncoding
MSTRG.23382.4	1.8765	coding
MSTRG.23384.2	1.30779	coding
MSTRG.23387.2	0.414571	coding
MSTRG.23388.2	0.721913	coding
MSTRG.23391.1	1.45929	coding
MSTRG.23391.2	0.302871	coding
MSTRG.23391.4	1.31881	coding
MSTRG.23392.1	0.142688	coding
MSTRG.23396.1	0.015187	coding
MSTRG.23397.2	1.85801	coding
MSTRG.23401.5	0.199918	coding
MSTRG.23399.3	0.689455	coding
MSTRG.23407.2	0.784133	coding
MSTRG.23412.4	0.110635	coding
MSTRG.23412.2	1.57685	coding
MSTRG.23412.3	1.55411	coding
MSTRG.23413.1	-0.395113	noncoding
MSTRG.23406.1	1.05903	coding
MSTRG.23414.1	-0.662043	noncoding
MSTRG.23415.1	1.95181	coding
MSTRG.23415.2	0.554374	coding
MSTRG.23415.3	0.58477	coding
MSTRG.23415.5	-0.253675	noncoding
MSTRG.23415.4	1.3583	coding
MSTRG.23416.1	0.776311	coding
MSTRG.23416.2	1.46609	coding
MSTRG.23420.1	2.47657	coding
MSTRG.23430.2	0.396814	coding
MSTRG.23433.1	0.267143	coding
MSTRG.23433.2	1.14242	coding
MSTRG.23435.1	0.120449	coding
MSTRG.23443.5	1.94523	coding
MSTRG.23443.6	2.45435	coding
MSTRG.23443.7	1.59381	coding
MSTRG.23438.3	0.197522	coding
MSTRG.23451.2	-0.295498	noncoding
MSTRG.23451.3	-0.246043	noncoding
MSTRG.23454.2	-0.735436	noncoding
MSTRG.23455.1	-0.82335	noncoding
MSTRG.23456.1	1.47522	coding
MSTRG.23461.2	1.41854	coding
MSTRG.23462.1	1.42669	coding
MSTRG.23457.2	0.416025	coding
MSTRG.23463.2	-0.687875	noncoding
MSTRG.23467.2	2.78127	coding
MSTRG.23467.3	2.46949	coding
MSTRG.23467.5	2.18853	coding
MSTRG.23467.4	2.21558	coding
MSTRG.23466.3	0.649778	coding

MSTRG.23470.1	1.7738	coding
MSTRG.23470.2	2.03276	coding
MSTRG.23474.2	1.90291	coding
MSTRG.23479.8	1.51504	coding
MSTRG.23479.5	1.96798	coding
MSTRG.23479.7	1.85541	coding
MSTRG.23479.9	1.66718	coding
MSTRG.23479.3	1.55232	coding
MSTRG.23479.10	1.57631	coding
MSTRG.23479.11	1.60251	coding
MSTRG.23482.3	1.67638	coding
MSTRG.23482.4	1.79861	coding
MSTRG.23484.1	1.35171	coding
MSTRG.23485.2	1.07089	coding
MSTRG.23486.1	1.57066	coding
MSTRG.23488.2	0.986624	coding
MSTRG.23488.4	0.209761	coding
MSTRG.23491.2	-0.036135	noncoding
MSTRG.23492.2	1.76784	coding
MSTRG.23492.5	1.18829	coding
MSTRG.23495.1	1.86836	coding
MSTRG.23505.2	0.857663	coding
MSTRG.23510.2	0.946635	coding
MSTRG.23515.4	0.908681	coding
MSTRG.23520.1	0.136083	coding
MSTRG.23520.2	0.234146	coding
MSTRG.23520.3	0.32191	coding
MSTRG.23520.4	0.367147	coding
MSTRG.23513.1	0.684014	coding
MSTRG.23522.5	0.88436	coding
MSTRG.23522.3	1.07925	coding
MSTRG.23522.4	1.08747	coding
MSTRG.23525.2	1.71418	coding
MSTRG.23528.2	1.32276	coding
MSTRG.23529.9	2.29485	coding
MSTRG.23530.1	1.44897	coding
MSTRG.23531.2	1.69031	coding
MSTRG.23531.1	1.64595	coding
MSTRG.23530.3	0.969709	coding
MSTRG.23532.1	-1.56217	noncoding
MSTRG.23535.3	2.01088	coding
MSTRG.23535.2	2.012	coding
MSTRG.23535.4	1.52928	coding
MSTRG.23535.6	1.86767	coding
MSTRG.23534.2	-0.057506	noncoding
MSTRG.23545.2	2.67628	coding
MSTRG.23550.1	2.14654	coding
MSTRG.23550.3	1.94183	coding
MSTRG.23550.4	2.21634	coding
MSTRG.23551.4	0.656775	coding
MSTRG.23552.4	-0.242061	noncoding
MSTRG.23552.5	0.035093	coding
MSTRG.23552.2	0.067042	coding
MSTRG.23552.3	0.287976	coding

MSTRG.23553.4	1.08969	coding
MSTRG.23567.3	3.26652	coding
MSTRG.23568.2	1.48517	coding
MSTRG.23568.3	-0.934958	noncoding
MSTRG.23572.3	0.01343	coding
MSTRG.23572.4	-0.012145	noncoding
MSTRG.23574.4	0.648538	coding
MSTRG.23575.2	0.00234	coding
MSTRG.23559.4	2.10113	coding
MSTRG.23559.5	2.16081	coding
MSTRG.23560.2	-1.11856	noncoding
MSTRG.23561.1	1.57896	coding
MSTRG.23561.2	0.466445	coding
MSTRG.23556.1	-0.527246	noncoding
MSTRG.23558.2	2.3565	coding
MSTRG.23562.1	-0.865754	noncoding
MSTRG.23577.2	0.413657	coding
MSTRG.23579.1	2.04668	coding
MSTRG.23580.1	0.559069	coding
MSTRG.23582.1	0.444968	coding
MSTRG.23583.2	3.23084	coding
MSTRG.23585.4	-0.45943	noncoding
MSTRG.23586.1	1.21783	coding
MSTRG.23586.2	0.658141	coding
MSTRG.23586.3	1.2147	coding
MSTRG.23585.6	-0.091949	noncoding
MSTRG.23585.7	0.13777	coding
MSTRG.23585.8	0.333758	coding
MSTRG.23585.9	0.092503	coding
MSTRG.23585.10	0.226465	coding
MSTRG.23585.11	0.238462	coding
MSTRG.23587.1	-0.045459	noncoding
MSTRG.23587.2	-0.209914	noncoding
MSTRG.23592.1	0.859665	coding
MSTRG.23593.1	-0.040299	noncoding
MSTRG.23594.1	-0.136648	noncoding
MSTRG.23594.2	-0.420332	noncoding
MSTRG.23594.3	0.481314	coding
MSTRG.23594.4	0.120248	coding
MSTRG.23595.1	-0.370524	noncoding
MSTRG.23595.2	-0.61785	noncoding
MSTRG.23595.3	-0.221862	noncoding
MSTRG.23595.4	-0.341515	noncoding
MSTRG.23594.5	-1.00911	noncoding
MSTRG.23597.2	1.23127	coding
MSTRG.23597.3	0.443318	coding
MSTRG.23598.1	1.26678	coding
MSTRG.23598.2	1.57842	coding
MSTRG.23598.3	1.42857	coding
MSTRG.23598.4	0.858967	coding
MSTRG.23603.2	1.79392	coding
MSTRG.23607.2	-1.9715	noncoding
MSTRG.23608.2	-1.2635	noncoding
MSTRG.23619.1	-0.256158	noncoding

MSTRG.23621.5	1.18286	coding
MSTRG.23621.4	2.43415	coding
MSTRG.23621.1	2.73155	coding
MSTRG.23621.6	2.73344	coding
MSTRG.23621.3	2.24991	coding
MSTRG.23622.1	-1.23056	noncoding
MSTRG.23621.7	1.35577	coding
MSTRG.23621.9	2.45412	coding
MSTRG.23621.11	1.90855	coding
MSTRG.23621.17	-0.032393	noncoding
MSTRG.23621.18	0.93229	coding
MSTRG.23623.1	-1.50489	noncoding
MSTRG.23629.1	0.094615	coding
MSTRG.23630.1	0.170128	coding
MSTRG.23630.2	0.026354	coding
MSTRG.23630.3	-0.609377	noncoding
MSTRG.23630.4	0.879913	coding
MSTRG.23631.1	-0.252778	noncoding
MSTRG.23616.2	0.348283	coding
MSTRG.23753.1	-0.395453	noncoding
MSTRG.23754.2	1.08258	coding
MSTRG.23754.1	1.61105	coding
MSTRG.23754.4	0.636537	coding
MSTRG.23754.3	1.2723	coding
MSTRG.23754.5	-0.274437	noncoding
MSTRG.23754.7	1.43357	coding
MSTRG.23754.13	1.2097	coding
MSTRG.23754.14	0.802874	coding
MSTRG.23754.16	0.589662	coding
MSTRG.23758.1	-2.42822	noncoding
MSTRG.23763.1	0.396809	coding
MSTRG.23769.5	1.07207	coding
MSTRG.23769.6	0.940023	coding
MSTRG.23769.8	1.50987	coding
MSTRG.23770.2	1.48476	coding
MSTRG.23770.1	1.51002	coding
MSTRG.23770.3	1.61572	coding
MSTRG.23770.4	0.766495	coding
MSTRG.23770.5	1.17477	coding
MSTRG.23770.6	1.43695	coding
MSTRG.23770.7	1.2615	coding
MSTRG.23770.8	1.16373	coding
MSTRG.23770.10	1.43676	coding
MSTRG.23770.9	1.53667	coding
MSTRG.23773.2	0.356337	coding
MSTRG.23769.11	-0.310385	noncoding
MSTRG.23765.13	-1.19051	noncoding
MSTRG.23765.15	-1.17509	noncoding
MSTRG.23765.10	-1.1764	noncoding
MSTRG.23765.12	-1.17302	noncoding
MSTRG.23765.9	-1.15185	noncoding
MSTRG.23765.8	-0.346693	noncoding
MSTRG.23765.11	-0.666368	noncoding
MSTRG.23765.14	-0.60717	noncoding

MSTRG.23765.4	-0.403332	noncoding
MSTRG.23765.7	-0.533322	noncoding
MSTRG.23765.5	-0.795549	noncoding
MSTRG.23765.6	-0.626601	noncoding
MSTRG.23770.13	1.08602	coding
MSTRG.23774.1	1.47238	coding
MSTRG.23770.14	-1.3018	noncoding
MSTRG.23775.1	-1.86507	noncoding
MSTRG.23776.1	-0.70657	noncoding
MSTRG.23777.1	2.02604	coding
MSTRG.23777.2	1.39367	coding
MSTRG.23777.3	1.69376	coding
MSTRG.23778.1	-0.164501	noncoding
MSTRG.23778.2	1.37533	coding
MSTRG.23779.1	0.672524	coding
MSTRG.23779.2	-0.613729	noncoding
MSTRG.23779.3	-0.511807	noncoding
MSTRG.23783.1	-0.744523	noncoding
MSTRG.23785.1	-1.69421	noncoding
MSTRG.23786.1	-1.79512	noncoding
MSTRG.23788.2	0.060207	coding
MSTRG.23788.3	-0.271215	noncoding
MSTRG.23788.4	0.099179	coding
MSTRG.23788.5	0.33786	coding
MSTRG.23788.7	0.282931	coding
MSTRG.23788.6	-0.828654	noncoding
MSTRG.23788.8	-0.783903	noncoding
MSTRG.23788.9	-1.2033	noncoding
MSTRG.23789.1	1.14458	coding
MSTRG.23790.1	-1.68612	noncoding
MSTRG.23790.2	-1.27612	noncoding
MSTRG.23789.2	0.349315	coding
MSTRG.23789.3	-0.106723	noncoding
MSTRG.23791.1	2.09432	coding
MSTRG.23791.3	1.56608	coding
MSTRG.23791.5	1.99022	coding
MSTRG.23791.6	2.08204	coding
MSTRG.23791.4	2.00123	coding
MSTRG.23791.7	1.43264	coding
MSTRG.23791.8	2.00781	coding
MSTRG.23791.9	1.42427	coding
MSTRG.23791.11	1.4364	coding
MSTRG.23791.10	1.40372	coding
MSTRG.23632.1	0.257257	coding
MSTRG.23632.3	0.91914	coding
MSTRG.23632.4	1.27813	coding
MSTRG.23632.5	1.15441	coding
MSTRG.23632.6	1.18938	coding
MSTRG.23632.7	0.871488	coding
MSTRG.23632.8	1.14528	coding
MSTRG.23632.9	0.830069	coding
MSTRG.23632.10	-1.39655	noncoding
MSTRG.23632.11	0.47774	coding
MSTRG.23635.2	0.162107	coding

MSTRG.23635.3	0.121277	coding
MSTRG.23639.1	0.767096	coding
MSTRG.23651.1	0.512059	coding
MSTRG.23652.1	-2.32131	noncoding
MSTRG.23653.1	-2.00954	noncoding
MSTRG.23654.1	0.943224	coding
MSTRG.23659.1	-0.608015	noncoding
MSTRG.23651.2	-1.30194	noncoding
MSTRG.23660.1	-1.17275	noncoding
MSTRG.23651.3	-2.23396	noncoding
MSTRG.23651.4	0.303989	coding
MSTRG.23651.5	-1.96092	noncoding
MSTRG.23663.1	-0.825417	noncoding
MSTRG.23664.1	-1.359	noncoding
MSTRG.23651.6	1.79255	coding
MSTRG.23651.7	-0.330774	noncoding
MSTRG.23651.8	-1.90641	noncoding
MSTRG.23666.1	-1.87217	noncoding
MSTRG.23667.1	-0.874087	noncoding
MSTRG.23668.1	-2.22052	noncoding
MSTRG.23670.1	1.51916	coding
MSTRG.23670.2	1.23594	coding
MSTRG.23670.3	1.25621	coding
MSTRG.23671.1	-2.1784	noncoding
MSTRG.23644.2	-0.508269	noncoding
MSTRG.23644.1	-1.31775	noncoding
MSTRG.23644.3	-0.820348	noncoding
MSTRG.23644.4	-0.745088	noncoding
MSTRG.23677.1	1.67441	coding
MSTRG.23677.2	1.17668	coding
MSTRG.23678.6	1.85748	coding
MSTRG.23681.4	1.01075	coding
MSTRG.23681.5	0.995521	coding
MSTRG.23681.6	1.57772	coding
MSTRG.23683.1	0.674542	coding
MSTRG.23683.2	-0.027991	noncoding
MSTRG.23683.3	-0.080537	noncoding
MSTRG.23690.1	-0.823964	noncoding
MSTRG.23694.7	0.605042	coding
MSTRG.23694.5	1.12543	coding
MSTRG.23694.6	0.764263	coding
MSTRG.23694.4	1.28701	coding
MSTRG.23696.1	1.95603	coding
MSTRG.23696.3	1.86996	coding
MSTRG.23687.1	-0.145385	noncoding
MSTRG.23688.1	-1.21637	noncoding
MSTRG.23698.3	0.864152	coding
MSTRG.23701.3	1.2092	coding
MSTRG.23706.1	2.10232	coding
MSTRG.23706.3	2.61682	coding
MSTRG.23706.2	2.11421	coding
MSTRG.23699.1	0.518195	coding
MSTRG.23709.3	1.97841	coding
MSTRG.23709.4	2.27529	coding

MSTRG.23709.5	2.14614	coding
MSTRG.23709.6	1.90083	coding
MSTRG.23709.7	1.24605	coding
MSTRG.23712.1	-0.72605	noncoding
MSTRG.23711.2	-1.06872	noncoding
MSTRG.23716.1	1.42455	coding
MSTRG.23716.2	1.43574	coding
MSTRG.23716.6	0.538354	coding
MSTRG.23716.7	0.334467	coding
MSTRG.23717.2	-0.869271	noncoding
MSTRG.23717.3	0.029814	coding
MSTRG.23718.2	1.81491	coding
MSTRG.23720.4	1.74911	coding
MSTRG.23720.5	1.73911	coding
MSTRG.23727.3	1.03277	coding
MSTRG.23728.1	-1.27725	noncoding
MSTRG.23728.2	-0.434177	noncoding
MSTRG.23731.2	2.0906	coding
MSTRG.23732.2	2.21589	coding
MSTRG.23732.3	2.17682	coding
MSTRG.23736.1	0.570638	coding
MSTRG.23743.2	1.87278	coding
MSTRG.23744.2	0.259107	coding
MSTRG.23744.3	0.298421	coding
MSTRG.23797.2	1.08622	coding
MSTRG.23801.4	0.706926	coding
MSTRG.23801.3	1.14617	coding
MSTRG.23801.5	1.06312	coding
MSTRG.23809.2	1.47457	coding
MSTRG.23794.1	0.92655	coding
MSTRG.23794.3	0.645211	coding
MSTRG.23814.1	1.17855	coding
MSTRG.23817.2	-0.447814	noncoding
MSTRG.23841.3	0.040404	coding
MSTRG.23841.2	1.90335	coding
MSTRG.23846.1	-0.067063	noncoding
MSTRG.23846.2	-0.012945	noncoding
MSTRG.23846.3	-0.002499	noncoding
MSTRG.23847.1	1.01634	coding
MSTRG.23849.1	-0.864548	noncoding
MSTRG.23855.2	0.339381	coding
MSTRG.23857.1	-0.282287	noncoding
MSTRG.23857.2	0.425942	coding
MSTRG.23855.3	0.618337	coding
MSTRG.23855.4	0.993495	coding
MSTRG.23860.1	0.245623	coding
MSTRG.23860.2	0.474614	coding
MSTRG.23857.3	-1.68918	noncoding
MSTRG.23855.9	1.12174	coding
MSTRG.23824.1	-1.73367	noncoding
MSTRG.23825.1	0.942262	coding
MSTRG.23825.3	1.52809	coding
MSTRG.23825.4	1.0653	coding
MSTRG.23825.5	0.472	coding

MSTRG.23827.1	-0.604162	noncoding
MSTRG.23828.1	-1.56799	noncoding
MSTRG.23833.1	0.527135	coding
MSTRG.23834.1	1.82237	coding
MSTRG.23834.2	2.03039	coding
MSTRG.23834.3	1.42194	coding
MSTRG.23834.4	1.79472	coding
MSTRG.23834.5	1.61634	coding
MSTRG.23868.2	1.82164	coding
MSTRG.23868.4	1.83574	coding
MSTRG.23872.6	-0.206367	noncoding
MSTRG.23872.7	0.767777	coding
MSTRG.23872.3	0.673078	coding
MSTRG.23872.1	0.923469	coding
MSTRG.23873.1	-1.03414	noncoding
MSTRG.23872.8	-0.324287	noncoding
MSTRG.23872.9	-0.408615	noncoding
MSTRG.23875.1	0.222613	coding
MSTRG.23872.10	1.83151	coding
MSTRG.23877.6	2.24624	coding
MSTRG.23878.2	0.108779	coding
MSTRG.23879.2	1.68693	coding
MSTRG.23879.3	1.62461	coding
MSTRG.23879.4	0.614196	coding
MSTRG.23880.1	0.721804	coding
MSTRG.23880.3	1.23098	coding
MSTRG.23881.3	1.21845	coding
MSTRG.23881.5	1.13916	coding
MSTRG.23883.1	-2.07782	noncoding
MSTRG.23840.2	1.634	coding
MSTRG.23890.1	0.512928	coding
MSTRG.23894.3	1.28106	coding
MSTRG.23898.2	1.31959	coding
MSTRG.23927.1	-1.07408	noncoding
MSTRG.23929.1	-1.82019	noncoding
MSTRG.23931.3	0.849995	coding
MSTRG.23933.2	0.778158	coding
MSTRG.23944.2	-1.23453	noncoding
MSTRG.23946.1	-2.12616	noncoding
MSTRG.23947.1	1.04597	coding
MSTRG.23947.2	0.514774	coding
MSTRG.23949.1	1.8862	coding
MSTRG.23949.2	1.94357	coding
MSTRG.23950.1	-1.67683	noncoding
MSTRG.23950.2	-1.91414	noncoding
MSTRG.23952.2	1.2536	coding
MSTRG.23952.3	1.06613	coding
MSTRG.23952.5	0.185295	coding
MSTRG.23952.4	0.914103	coding
MSTRG.23954.3	1.48464	coding
MSTRG.23954.5	1.0496	coding
MSTRG.23962.5	-0.855102	noncoding
MSTRG.23962.8	0.097841	coding
MSTRG.23962.4	-0.351625	noncoding

MSTRG.23962.6	0.436608	coding
MSTRG.23962.1	0.326669	coding
MSTRG.23962.3	1.25191	coding
MSTRG.23963.1	-0.523926	noncoding
MSTRG.23964.1	-0.213053	noncoding
MSTRG.23962.11	0.404216	coding
MSTRG.23962.12	0.259631	coding
MSTRG.23965.1	-0.403328	noncoding
MSTRG.23966.1	-0.52322	noncoding
MSTRG.23962.13	-0.312833	noncoding
MSTRG.23967.1	1.04444	coding
MSTRG.23968.1	-0.564781	noncoding
MSTRG.23958.1	-1.17366	noncoding
MSTRG.23970.2	0.10216	coding
MSTRG.23976.1	1.93697	coding
MSTRG.23977.1	2.43661	coding
MSTRG.23981.3	2.33673	coding
MSTRG.23981.4	1.13558	coding
MSTRG.23981.5	1.1675	coding
MSTRG.23981.6	2.38887	coding
MSTRG.23981.7	2.39068	coding
MSTRG.23981.8	1.95359	coding
MSTRG.23983.4	0.907207	coding
MSTRG.23983.3	2.30025	coding
MSTRG.23998.5	1.70854	coding
MSTRG.23998.6	1.73034	coding
MSTRG.23998.7	1.74897	coding
MSTRG.23999.11	-0.399066	noncoding
MSTRG.24000.2	-0.117597	noncoding
MSTRG.24004.1	-1.8815	noncoding
MSTRG.23986.1	-0.824628	noncoding
MSTRG.23986.2	-1.67885	noncoding
MSTRG.23987.2	-0.102195	noncoding
MSTRG.23988.1	-1.13459	noncoding
MSTRG.23988.2	0.529905	coding
MSTRG.23989.1	0.779152	coding
MSTRG.23988.3	1.05601	coding
MSTRG.23988.4	0.768631	coding
MSTRG.23990.1	-0.631393	noncoding
MSTRG.23990.2	-0.539585	noncoding
MSTRG.23990.3	-0.131863	noncoding
MSTRG.23991.1	0.689428	coding
MSTRG.23992.1	-1.24947	noncoding
MSTRG.23984.1	1.05686	coding
MSTRG.23995.1	1.54631	coding
MSTRG.23995.4	0.232592	coding
MSTRG.23996.4	-0.845267	noncoding
MSTRG.23997.1	-1.54821	noncoding
MSTRG.24007.1	-1.99569	noncoding
MSTRG.24020.1	2.98737	coding
MSTRG.24020.3	-0.124675	noncoding
MSTRG.24020.4	-0.248398	noncoding
MSTRG.24023.1	0.094959	coding
MSTRG.24022.3	0.702791	coding

MSTRG.24026.3	2.32417	coding
MSTRG.24026.1	1.06624	coding
MSTRG.24026.4	0.265218	coding
MSTRG.24027.1	1.61111	coding
MSTRG.24011.2	2.8541	coding
MSTRG.24013.3	2.35486	coding
MSTRG.24013.5	2.10248	coding
MSTRG.24014.1	-1.99302	noncoding
MSTRG.24015.1	0.530854	coding
MSTRG.24016.2	1.29867	coding
MSTRG.24017.3	1.66835	coding
MSTRG.24019.1	2.59148	coding
MSTRG.24019.2	-0.559292	noncoding
MSTRG.24031.2	0.73186	coding
MSTRG.24035.1	2.12754	coding
MSTRG.24035.3	-0.991127	noncoding
MSTRG.24036.1	-1.538	noncoding
MSTRG.24037.1	-0.361652	noncoding
MSTRG.24049.1	2.29376	coding
MSTRG.24049.2	2.21305	coding
MSTRG.24049.8	1.81769	coding
MSTRG.24049.9	2.02566	coding
MSTRG.24049.10	2.21334	coding
MSTRG.24049.12	1.51531	coding
MSTRG.24050.2	1.84292	coding
MSTRG.24050.3	1.73663	coding
MSTRG.24051.1	-0.38394	noncoding
MSTRG.24041.2	1.07596	coding
MSTRG.24057.3	1.65376	coding
MSTRG.24057.4	1.93795	coding
MSTRG.24058.1	-0.659088	noncoding
MSTRG.24058.2	-1.65752	noncoding
MSTRG.24062.1	1.28329	coding
MSTRG.24062.5	1.53798	coding
MSTRG.24062.6	1.54059	coding
MSTRG.24062.8	1.24384	coding
MSTRG.24062.9	1.46447	coding
MSTRG.24059.1	0.020836	coding
MSTRG.24059.3	0.142768	coding
MSTRG.24059.4	0.345296	coding
MSTRG.24063.1	0.163451	coding
MSTRG.24063.2	0.157489	coding
MSTRG.24063.3	0.0433	coding
MSTRG.24063.6	0.047662	coding
MSTRG.24071.2	-0.074587	noncoding
MSTRG.24071.1	0.295727	coding
MSTRG.24081.1	0.603299	coding
MSTRG.24081.2	0.640472	coding
MSTRG.24081.9	-0.143701	noncoding
MSTRG.24081.5	-0.266314	noncoding
MSTRG.24081.7	0.129816	coding
MSTRG.24081.10	-0.228808	noncoding
MSTRG.24075.2	0.800365	coding
MSTRG.24082.1	1.21274	coding

MSTRG.24082.4	2.04346	coding
MSTRG.24082.3	2.046	coding
MSTRG.24086.1	2.82759	coding
MSTRG.24093.3	-0.503243	noncoding
MSTRG.24095.3	2.28621	coding
MSTRG.24095.2	2.4481	coding
MSTRG.24098.3	0.388843	coding
MSTRG.24098.4	0.143272	coding
MSTRG.24099.1	-1.62347	noncoding
MSTRG.24102.1	-0.534921	noncoding
MSTRG.24104.1	-0.543709	noncoding
MSTRG.24105.1	-1.08782	noncoding
MSTRG.24105.2	-0.97233	noncoding
MSTRG.24107.1	-1.57366	noncoding
MSTRG.24108.1	-2.06037	noncoding
MSTRG.24111.2	0.381891	coding
MSTRG.24112.1	-0.766452	noncoding
MSTRG.24113.3	0.510025	coding
MSTRG.24114.2	0.423371	coding
MSTRG.24115.1	2.62696	coding
MSTRG.24115.3	1.60324	coding
MSTRG.24117.2	2.55901	coding
MSTRG.24117.3	2.48696	coding
MSTRG.24125.1	-2.38599	noncoding
MSTRG.24125.2	-2.32636	noncoding
MSTRG.24123.1	-0.499397	noncoding
MSTRG.24127.1	1.14777	coding
MSTRG.24134.2	0.65805	coding
MSTRG.24135.4	1.89195	coding
MSTRG.24135.2	3.31805	coding
MSTRG.24135.6	1.77039	coding
MSTRG.24136.8	0.354607	coding
MSTRG.24136.9	-0.395499	noncoding
MSTRG.24139.1	0.181231	coding
MSTRG.24140.1	-1.30414	noncoding
MSTRG.24147.2	0.014242	coding
MSTRG.24173.1	3.01269	coding
MSTRG.24173.3	3.09716	coding
MSTRG.24173.4	3.07829	coding
MSTRG.24173.2	3.05762	coding
MSTRG.24173.6	3.18537	coding
MSTRG.24173.7	3.14991	coding
MSTRG.24173.5	3.03762	coding
MSTRG.24173.10	2.87782	coding
MSTRG.24173.8	3.05413	coding
MSTRG.24173.9	3.07252	coding
MSTRG.24173.13	3.2023	coding
MSTRG.24173.14	3.28696	coding
MSTRG.24173.11	3.15856	coding
MSTRG.24173.12	3.17734	coding
MSTRG.24173.15	3.17983	coding
MSTRG.24173.16	3.11401	coding
MSTRG.24173.17	3.19183	coding
MSTRG.24175.1	-0.566764	noncoding

MSTRG.24148.2	1.77797	coding
MSTRG.24152.1	-1.73358	noncoding
MSTRG.24162.1	-0.762504	noncoding
MSTRG.24163.1	0.516903	coding
MSTRG.24164.1	-1.76885	noncoding
MSTRG.24163.2	2.89928	coding
MSTRG.24163.4	1.28151	coding
MSTRG.24163.5	1.54694	coding
MSTRG.24163.6	-0.169138	noncoding
MSTRG.24158.1	1.64112	coding
MSTRG.24153.1	1.00921	coding
MSTRG.24168.1	-0.610685	noncoding
MSTRG.24171.1	-0.000363	noncoding
MSTRG.24172.1	2.6609	coding
MSTRG.24172.2	2.06765	coding
MSTRG.24184.1	0.834089	coding
MSTRG.24184.3	0.481355	coding
MSTRG.24184.4	0.191153	coding
MSTRG.24185.3	2.03451	coding
MSTRG.24187.1	-0.466141	noncoding
MSTRG.24187.2	0.848375	coding
MSTRG.24187.3	-0.485112	noncoding
MSTRG.24187.4	-0.412713	noncoding
MSTRG.24187.5	-0.832647	noncoding
MSTRG.24183.1	-1.96796	noncoding
MSTRG.24190.6	-0.591689	noncoding
MSTRG.24195.1	-2.41418	noncoding
MSTRG.24197.1	0.669787	coding
MSTRG.24198.1	1.58984	coding
MSTRG.24198.2	1.89217	coding
MSTRG.24201.3	1.9067	coding
MSTRG.24204.4	1.31225	coding
MSTRG.24209.1	1.32689	coding
MSTRG.24209.3	1.44284	coding
MSTRG.24209.4	1.34873	coding
MSTRG.24211.2	1.7042	coding
MSTRG.24211.3	1.09145	coding
MSTRG.24212.1	-1.92172	noncoding
MSTRG.24207.5	0.579457	coding
MSTRG.24207.4	-0.4247	noncoding
MSTRG.24215.1	-1.23154	noncoding
MSTRG.24214.3	2.48357	coding
MSTRG.24214.4	2.48663	coding
MSTRG.24216.1	1.5809	coding
MSTRG.24222.1	-0.703838	noncoding
MSTRG.24223.1	0.00567	coding
MSTRG.24224.1	0.611024	coding
MSTRG.24225.2	1.96091	coding
MSTRG.24228.5	0.538749	coding
MSTRG.24228.6	0.781223	coding
MSTRG.24228.7	-0.184983	noncoding
MSTRG.24229.7	1.11565	coding
MSTRG.24229.10	0.99656	coding
MSTRG.24218.3	0.389736	coding

MSTRG.24230.2	1.82859	coding
MSTRG.24232.3	0.905051	coding
MSTRG.24233.3	2.56822	coding
MSTRG.24236.3	0.907532	coding
MSTRG.24252.5	1.83103	coding
MSTRG.24252.6	2.10854	coding
MSTRG.24252.7	0.405141	coding
MSTRG.24252.8	1.25427	coding
MSTRG.24252.9	1.17709	coding
MSTRG.24250.1	1.04395	coding
MSTRG.24250.3	0.581734	coding
MSTRG.24250.5	0.793462	coding
MSTRG.24250.8	0.812818	coding
MSTRG.24250.4	0.722329	coding
MSTRG.24250.2	0.768467	coding
MSTRG.24250.7	0.778115	coding
MSTRG.24257.5	1.75615	coding
MSTRG.24257.4	1.95064	coding
MSTRG.24257.3	2.31743	coding
MSTRG.24258.5	2.07068	coding
MSTRG.24258.4	1.97147	coding
MSTRG.24260.1	0.151825	coding
MSTRG.24262.1	-1.71904	noncoding
MSTRG.24265.2	0.645634	coding
MSTRG.24265.3	0.552953	coding
MSTRG.24265.5	0.317239	coding
MSTRG.24265.6	0.193318	coding
MSTRG.24268.2	-0.550288	noncoding
MSTRG.24269.3	-1.71362	noncoding
MSTRG.24269.2	1.0586	coding
MSTRG.24273.1	0.486842	coding
MSTRG.24275.2	0.432321	coding
MSTRG.24276.2	1.93245	coding
MSTRG.24276.3	2.21396	coding
MSTRG.24285.2	1.70559	coding
MSTRG.24285.3	1.69105	coding
MSTRG.24286.2	0.695733	coding
MSTRG.24286.3	-0.151313	noncoding
MSTRG.24288.2	0.416888	coding
MSTRG.24288.3	0.611815	coding
MSTRG.24288.4	0.917218	coding
MSTRG.24283.3	0.523844	coding
MSTRG.24283.5	1.44365	coding
MSTRG.24283.6	0.583046	coding
MSTRG.24282.2	-1.50243	noncoding
MSTRG.24289.3	1.51467	coding
MSTRG.24289.2	1.3753	coding
MSTRG.24290.3	1.23256	coding
MSTRG.24291.1	0.166622	coding
MSTRG.24296.1	-0.192618	noncoding
MSTRG.24297.2	-0.195095	noncoding
MSTRG.24297.1	1.63929	coding
MSTRG.24297.3	1.58336	coding
MSTRG.24297.6	1.51814	coding

MSTRG.24297.9	1.07271	coding
MSTRG.24295.1	-2.02519	noncoding
MSTRG.24302.1	-1.89389	noncoding
MSTRG.24303.1	1.67466	coding
MSTRG.24313.2	2.03296	coding
MSTRG.24315.1	1.1857	coding
MSTRG.24314.2	1.40672	coding
MSTRG.24318.1	2.05932	coding
MSTRG.24318.2	2.08023	coding
MSTRG.24318.3	2.02179	coding
MSTRG.24318.4	1.89827	coding
MSTRG.24306.2	1.39446	coding
MSTRG.24306.3	1.62508	coding
MSTRG.24307.1	0.210001	coding
MSTRG.24307.2	1.38836	coding
MSTRG.24307.3	0.678163	coding
MSTRG.24307.4	0.332922	coding
MSTRG.24309.1	1.74758	coding
MSTRG.24305.1	-0.059864	noncoding
MSTRG.24304.4	0.205429	coding
MSTRG.24328.2	2.53147	coding
MSTRG.24328.5	0.150607	coding
MSTRG.24328.7	0.643381	coding
MSTRG.24328.8	1.84125	coding
MSTRG.24336.3	-0.799864	noncoding
MSTRG.24338.6	1.61124	coding
MSTRG.24339.1	1.91539	coding
MSTRG.24340.1	-1.59068	noncoding
MSTRG.24341.3	2.47518	coding
MSTRG.24344.1	-1.28348	noncoding
MSTRG.24346.1	0.876226	coding
MSTRG.24346.6	0.973759	coding
MSTRG.24346.2	0.864279	coding
MSTRG.24346.3	0.975131	coding
MSTRG.24346.5	0.890299	coding
MSTRG.24346.4	0.913352	coding
MSTRG.24346.7	0.93808	coding
MSTRG.24348.3	0.880427	coding
MSTRG.24350.3	1.8115	coding
MSTRG.24350.4	1.60169	coding
MSTRG.24350.5	1.78787	coding
MSTRG.24351.7	0.166447	coding
MSTRG.24351.5	0.65478	coding
MSTRG.24351.4	0.400768	coding
MSTRG.24351.1	0.493766	coding
MSTRG.24351.3	1.18899	coding
MSTRG.24351.2	1.2311	coding
MSTRG.24352.1	-0.902635	noncoding
MSTRG.24356.3	0.747922	coding
MSTRG.24357.1	0.978943	coding
MSTRG.24357.3	1.34896	coding
MSTRG.24358.5	1.04863	coding
MSTRG.24364.1	1.17938	coding
MSTRG.24365.4	3.23441	coding

MSTRG.24365.8	3.30632	coding
MSTRG.24365.9	3.37602	coding
MSTRG.24365.10	3.19329	coding
MSTRG.24365.11	3.31489	coding
MSTRG.24365.6	3.1602	coding
MSTRG.24365.3	3.32334	coding
MSTRG.24365.5	3.27681	coding
MSTRG.24365.1	3.19397	coding
MSTRG.24365.12	3.29329	coding
MSTRG.24365.13	3.24084	coding
MSTRG.24365.14	3.22511	coding
MSTRG.24366.1	-1.92868	noncoding
MSTRG.24367.1	-1.86383	noncoding
MSTRG.24374.2	0.784242	coding
MSTRG.24376.1	1.05047	coding
MSTRG.24376.2	0.687506	coding
MSTRG.24376.5	0.778384	coding
MSTRG.24376.6	0.671335	coding
MSTRG.24378.4	0.795644	coding
MSTRG.24378.5	0.649561	coding
MSTRG.24379.1	0.141898	coding
MSTRG.24379.2	0.220087	coding
MSTRG.24380.1	0.928947	coding
MSTRG.24359.2	1.09434	coding
MSTRG.24359.3	0.80525	coding
MSTRG.24361.2	1.73641	coding
MSTRG.24382.5	2.31191	coding
MSTRG.24387.6	-0.897374	noncoding
MSTRG.24387.1	-1.03166	noncoding
MSTRG.24387.3	-0.33485	noncoding
MSTRG.24387.4	-1.60251	noncoding
MSTRG.24389.1	-0.900712	noncoding
MSTRG.24387.7	-0.930422	noncoding
MSTRG.24390.8	3.06049	coding
MSTRG.24384.3	2.39231	coding
MSTRG.24384.2	2.20606	coding
MSTRG.24397.1	-0.948221	noncoding
MSTRG.24398.1	-1.75044	noncoding
MSTRG.24397.3	-0.704319	noncoding
MSTRG.24398.4	-1.14538	noncoding
MSTRG.24400.1	1.01788	coding
MSTRG.24405.1	0.077205	coding
MSTRG.24405.3	-0.204266	noncoding
MSTRG.24405.4	-0.151344	noncoding
MSTRG.24406.3	0.312641	coding
MSTRG.24408.1	-1.38163	noncoding
MSTRG.24408.2	-1.77396	noncoding
MSTRG.24410.3	1.03002	coding
MSTRG.24412.1	3.48506	coding
MSTRG.24412.2	3.42733	coding
MSTRG.24413.1	-0.355054	noncoding
MSTRG.24401.1	0.20324	coding
MSTRG.24404.2	0.556337	coding
MSTRG.24415.7	0.118074	coding

MSTRG.24415.3	0.163071	coding
MSTRG.24415.5	0.071566	coding
MSTRG.24415.4	0.163493	coding
MSTRG.24415.6	0.425424	coding
MSTRG.24415.2	0.774812	coding
MSTRG.24417.1	0.74379	coding
MSTRG.24417.6	0.451216	coding
MSTRG.24417.5	0.639413	coding
MSTRG.24419.1	0.683289	coding
MSTRG.24419.2	-0.408147	noncoding
MSTRG.24420.1	-0.592249	noncoding
MSTRG.24421.1	-1.41257	noncoding
MSTRG.24424.2	1.2046	coding
MSTRG.24436.1	1.26319	coding
MSTRG.24442.2	2.89766	coding
MSTRG.24443.1	1.545	coding
MSTRG.24429.7	-0.990845	noncoding
MSTRG.24429.5	-1.74801	noncoding
MSTRG.24429.6	-1.55852	noncoding
MSTRG.24429.1	-1.74467	noncoding
MSTRG.24429.2	-1.4401	noncoding
MSTRG.24429.3	-1.54769	noncoding
MSTRG.24429.4	-1.80355	noncoding
MSTRG.24429.8	-1.96594	noncoding
MSTRG.24454.1	-0.313692	noncoding
MSTRG.24456.2	1.61745	coding
MSTRG.24458.1	-2.53829	noncoding
MSTRG.24460.1	-0.346998	noncoding
MSTRG.24460.2	-0.367414	noncoding
MSTRG.24460.3	-0.144781	noncoding
MSTRG.24460.4	0.077588	coding
MSTRG.24460.5	0.392645	coding
MSTRG.24460.6	-0.044847	noncoding
MSTRG.24461.1	-1.57924	noncoding
MSTRG.24462.1	-0.878396	noncoding
MSTRG.24464.3	2.06561	coding
MSTRG.24464.2	2.01599	coding
MSTRG.24464.1	1.7937	coding
MSTRG.24465.1	0.498474	coding
MSTRG.24463.1	1.90394	coding
MSTRG.24468.1	2.13073	coding
MSTRG.24468.2	1.7563	coding
MSTRG.24484.2	-0.334351	noncoding
MSTRG.24484.3	-0.196905	noncoding
MSTRG.24484.4	-0.226641	noncoding
MSTRG.24484.5	-0.314005	noncoding
MSTRG.24484.6	0.191837	coding
MSTRG.24484.7	-0.250159	noncoding
MSTRG.24485.3	-0.101939	noncoding
MSTRG.24485.2	-0.533566	noncoding
MSTRG.24486.1	0.710506	coding
MSTRG.24487.10	2.11773	coding
MSTRG.24487.3	2.06156	coding
MSTRG.24487.4	2.08227	coding

MSTRG.24487.5	2.07435	coding
MSTRG.24487.6	2.04575	coding
MSTRG.24487.7	2.03707	coding
MSTRG.24487.9	2.1031	coding
MSTRG.24487.8	1.94173	coding
MSTRG.24487.11	1.92954	coding
MSTRG.24487.17	2.09414	coding
MSTRG.24487.18	2.09855	coding
MSTRG.24487.19	2.07907	coding
MSTRG.24488.7	0.846717	coding
MSTRG.24488.10	0.446756	coding
MSTRG.24488.8	1.16786	coding
MSTRG.24488.14	-2.31614	noncoding
MSTRG.24489.2	-1.02613	noncoding
MSTRG.24472.3	1.61939	coding
MSTRG.24472.5	1.80344	coding
MSTRG.24477.1	1.34536	coding
MSTRG.24477.10	1.47014	coding
MSTRG.24477.8	1.652	coding
MSTRG.24477.7	1.1971	coding
MSTRG.24477.9	1.23008	coding
MSTRG.24478.1	1.16168	coding
MSTRG.24495.3	2.47614	coding
MSTRG.24510.1	2.51475	coding
MSTRG.24503.1	-0.385254	noncoding
MSTRG.24504.1	-1.32368	noncoding
MSTRG.24514.2	0.108695	coding
MSTRG.24516.2	-1.60317	noncoding
MSTRG.24521.6	0.96536	coding
MSTRG.24523.3	1.73301	coding
MSTRG.24523.4	0.460764	coding
MSTRG.24520.2	1.8071	coding
MSTRG.24520.3	1.57508	coding
MSTRG.24520.4	1.31562	coding
MSTRG.24520.6	1.67517	coding
MSTRG.24528.4	-0.168079	noncoding
MSTRG.24528.3	0.02133	coding
MSTRG.24528.2	0.155595	coding
MSTRG.24528.5	0.555558	coding
MSTRG.24528.6	-0.153987	noncoding
MSTRG.24531.2	-0.017114	noncoding
MSTRG.24539.3	0.293551	coding
MSTRG.24539.4	1.5078	coding
MSTRG.24541.2	1.49905	coding
MSTRG.24543.1	0.266445	coding
MSTRG.24548.1	0.271944	coding
MSTRG.24548.3	0.204432	coding
MSTRG.24554.1	2.66562	coding
MSTRG.24554.4	0.972257	coding
MSTRG.24554.7	1.38626	coding
MSTRG.24554.9	1.73649	coding
MSTRG.24555.1	-2.0155	noncoding
MSTRG.24553.1	1.7592	coding
MSTRG.24557.1	1.73841	coding

MSTRG.24557.3	1.65484	coding
MSTRG.24557.4	1.40447	coding
MSTRG.24557.5	0.715898	coding
MSTRG.24566.2	1.83628	coding
MSTRG.24566.3	1.65381	coding
MSTRG.24568.2	0.366604	coding
MSTRG.24571.1	2.27512	coding
MSTRG.24574.4	-0.330933	noncoding
MSTRG.24574.5	-0.019448	noncoding
MSTRG.24574.3	-0.663292	noncoding
MSTRG.24574.2	-0.781095	noncoding
MSTRG.24574.6	-0.161145	noncoding
MSTRG.24575.1	-1.02169	noncoding
MSTRG.24580.2	0.325855	coding
MSTRG.24584.2	1.7369	coding
MSTRG.24584.3	1.38591	coding
MSTRG.24585.2	-0.067627	noncoding
MSTRG.24586.1	0.939291	coding
MSTRG.24588.3	-0.010841	noncoding
MSTRG.24589.5	0.367394	coding
MSTRG.24589.6	0.185335	coding
MSTRG.24589.4	0.887084	coding
MSTRG.24589.7	0.698007	coding
MSTRG.24591.10	1.95028	coding
MSTRG.24591.3	2.02563	coding
MSTRG.24591.4	1.83192	coding
MSTRG.24591.5	1.72591	coding
MSTRG.24591.6	1.64257	coding
MSTRG.24591.7	1.91389	coding
MSTRG.24591.8	2.06821	coding
MSTRG.24591.9	2.11166	coding
MSTRG.24594.1	0.295686	coding
MSTRG.24594.2	0.711746	coding
MSTRG.24595.3	1.34587	coding
MSTRG.24595.4	2.14573	coding
MSTRG.24597.2	1.50838	coding
MSTRG.24599.3	0.918459	coding
MSTRG.24599.4	0.798597	coding
MSTRG.24599.5	0.860416	coding
MSTRG.24604.2	-0.025076	noncoding
MSTRG.24604.1	0.054454	coding
MSTRG.24604.3	0.215498	coding
MSTRG.24602.1	-1.83303	noncoding
MSTRG.24609.7	1.49859	coding
MSTRG.24619.1	-2.0361	noncoding
MSTRG.24620.2	2.72164	coding
MSTRG.24620.3	2.49503	coding
MSTRG.24620.4	2.57849	coding
MSTRG.24620.5	1.59912	coding
MSTRG.24621.1	0.219701	coding
MSTRG.24623.3	2.2513	coding
MSTRG.24625.4	1.44544	coding
MSTRG.24625.5	1.41424	coding
MSTRG.24629.1	1.60104	coding

MSTRG.24629.3	1.4632	coding
MSTRG.24630.1	1.55162	coding
MSTRG.24633.1	-1.48095	noncoding
MSTRG.24637.1	1.3122	coding
MSTRG.24637.3	0.919462	coding
MSTRG.24637.2	1.01815	coding
MSTRG.24637.4	1.39141	coding
MSTRG.24640.2	0.025405	coding
MSTRG.24648.1	-0.755886	noncoding
MSTRG.24649.1	-1.42496	noncoding
MSTRG.24651.1	0.534205	coding
MSTRG.24651.2	0.67444	coding
MSTRG.24642.6	1.65249	coding
MSTRG.24642.7	1.78193	coding
MSTRG.24644.1	2.58504	coding
MSTRG.24656.3	1.04837	coding
MSTRG.24659.3	0.573282	coding
MSTRG.24659.4	0.321742	coding
MSTRG.24662.2	2.43363	coding
MSTRG.24663.2	1.20207	coding
MSTRG.24664.2	1.77398	coding
MSTRG.24666.3	0.658998	coding
MSTRG.24661.3	1.21186	coding
MSTRG.24661.4	1.20951	coding
MSTRG.24670.1	-0.818903	noncoding
MSTRG.24669.3	2.23066	coding
MSTRG.24669.6	-0.035406	noncoding
MSTRG.24673.2	2.53217	coding
MSTRG.24672.1	-1.37304	noncoding
MSTRG.24676.1	-1.149	noncoding
MSTRG.24676.2	-1.10998	noncoding
MSTRG.24677.1	-0.933052	noncoding
MSTRG.24693.3	1.89862	coding
MSTRG.24696.1	-0.143224	noncoding
MSTRG.24696.2	0.788787	coding
MSTRG.24697.2	2.63861	coding
MSTRG.24697.5	2.40801	coding
MSTRG.24724.1	0.368886	coding
MSTRG.24724.6	0.735161	coding
MSTRG.24724.5	0.725699	coding
MSTRG.24724.7	0.755389	coding
MSTRG.24724.2	0.800171	coding
MSTRG.24724.3	0.777393	coding
MSTRG.24724.4	1.41854	coding
MSTRG.24725.1	1.17683	coding
MSTRG.24725.2	1.08908	coding
MSTRG.24725.3	1.04354	coding
MSTRG.24725.4	2.29889	coding
MSTRG.24725.5	1.21342	coding
MSTRG.24729.1	1.35051	coding
MSTRG.24729.2	1.39848	coding
MSTRG.24729.3	1.03236	coding
MSTRG.24729.4	1.18282	coding
MSTRG.24729.5	1.03868	coding

MSTRG.24729.6	1.37244	coding
MSTRG.24732.1	1.90018	coding
MSTRG.24732.2	1.88173	coding
MSTRG.24732.4	1.11676	coding
MSTRG.24735.4	2.12106	coding
MSTRG.24710.4	1.91406	coding
MSTRG.24710.5	2.16483	coding
MSTRG.24706.1	0.927312	coding
MSTRG.24711.3	1.97189	coding
MSTRG.24711.4	1.97183	coding
MSTRG.24717.2	-0.99759	noncoding
MSTRG.24721.1	1.60528	coding
MSTRG.24721.2	0.58987	coding
MSTRG.24721.4	0.334933	coding
MSTRG.24722.2	2.62469	coding
MSTRG.24722.4	2.24644	coding
MSTRG.24714.2	1.28783	coding
MSTRG.24715.2	0.987335	coding
MSTRG.24741.2	1.17717	coding
MSTRG.24742.3	1.08775	coding
MSTRG.24743.2	0.656679	coding
MSTRG.24743.3	0.648847	coding
MSTRG.24745.1	-0.331638	noncoding
MSTRG.24745.2	-0.370295	noncoding
MSTRG.24745.3	-0.923487	noncoding
MSTRG.24748.1	0.084895	coding
MSTRG.24763.1	0.908336	coding
MSTRG.24763.3	1.18943	coding
MSTRG.24768.3	1.26415	coding
MSTRG.24768.7	1.2806	coding
MSTRG.24769.1	-1.21455	noncoding
MSTRG.24772.2	1.34992	coding
MSTRG.24774.5	2.78986	coding
MSTRG.24774.6	2.92718	coding
MSTRG.24784.2	1.53411	coding
MSTRG.24784.3	1.66782	coding
MSTRG.24785.3	1.82379	coding
MSTRG.24788.1	-1.51878	noncoding
MSTRG.24789.1	-0.959546	noncoding
MSTRG.24792.2	1.00229	coding
MSTRG.24793.1	-1.92395	noncoding
MSTRG.24793.2	-1.69899	noncoding
MSTRG.24778.1	0.609742	coding
MSTRG.24782.1	2.09037	coding
MSTRG.24795.2	1.13104	coding
MSTRG.24801.1	1.08808	coding
MSTRG.24801.2	0.927104	coding
MSTRG.24805.2	0.282571	coding
MSTRG.24807.1	-1.47541	noncoding
MSTRG.24809.1	1.7878	coding
MSTRG.24810.1	-1.0289	noncoding
MSTRG.24814.1	1.30161	coding
MSTRG.24815.2	0.30864	coding
MSTRG.24816.1	-0.812105	noncoding

MSTRG.24816.2	-1.91543	noncoding
MSTRG.24817.1	1.23261	coding
MSTRG.24817.2	0.357149	coding
MSTRG.24820.2	1.30483	coding
MSTRG.24821.1	-1.53144	noncoding
MSTRG.24825.1	-1.54676	noncoding
MSTRG.24826.4	1.8178	coding
MSTRG.24826.6	1.92791	coding
MSTRG.24826.2	1.91626	coding
MSTRG.24826.3	1.76304	coding
MSTRG.24826.5	1.8866	coding
MSTRG.24826.7	1.93728	coding
MSTRG.24826.8	1.16896	coding
MSTRG.24827.6	1.23569	coding
MSTRG.24827.7	0.456086	coding
MSTRG.24827.8	0.339834	coding
MSTRG.24827.9	0.298023	coding
MSTRG.24827.10	0.012287	coding
MSTRG.24827.11	0.437433	coding
MSTRG.24828.1	-0.308989	noncoding
MSTRG.24830.1	-2.02749	noncoding
MSTRG.24833.1	-0.5237	noncoding
MSTRG.24835.1	1.15948	coding
MSTRG.24836.1	-1.49117	noncoding
MSTRG.24837.1	1.8324	coding
MSTRG.24837.2	0.71955	coding
MSTRG.24837.3	1.18343	coding
MSTRG.24837.4	1.17832	coding
MSTRG.24837.5	1.18243	coding
MSTRG.24841.2	1.03632	coding
MSTRG.24841.1	1.09066	coding
MSTRG.24834.3	1.49784	coding
MSTRG.24844.1	1.43912	coding
MSTRG.24844.2	1.51539	coding
MSTRG.24844.3	1.76987	coding
MSTRG.24844.8	1.55011	coding
MSTRG.24844.9	1.58754	coding
MSTRG.24844.10	1.20522	coding
MSTRG.24846.1	-1.81343	noncoding
MSTRG.24847.1	-2.29809	noncoding
MSTRG.24842.4	-0.283556	noncoding
MSTRG.24850.1	0.476986	coding
MSTRG.24849.3	0.178111	coding
MSTRG.24849.2	1.49259	coding
MSTRG.24852.1	0.410492	coding
MSTRG.24853.1	-0.256798	noncoding
MSTRG.24854.1	0.188875	coding
MSTRG.24855.1	-0.663891	noncoding
MSTRG.24856.1	-1.28404	noncoding
MSTRG.24858.2	2.05971	coding
MSTRG.24858.3	2.11553	coding
MSTRG.24858.4	0.598976	coding
MSTRG.24860.3	1.91503	coding
MSTRG.24861.2	2.03414	coding

MSTRG.24861.3	1.93336	coding
MSTRG.24861.4	2.03399	coding
MSTRG.24861.5	2.04728	coding
MSTRG.24862.1	1.6051	coding
MSTRG.24866.1	3.02678	coding
MSTRG.24866.2	3.01533	coding
MSTRG.24866.3	3.06692	coding
MSTRG.24866.7	2.13797	coding
MSTRG.24867.2	0.005783	coding
MSTRG.24867.4	0.354799	coding
MSTRG.24867.3	1.39142	coding
MSTRG.24867.5	1.25616	coding
MSTRG.24867.11	0.849342	coding
MSTRG.24867.7	0.773377	coding
MSTRG.24867.8	0.882973	coding
MSTRG.24878.1	1.85238	coding
MSTRG.24879.2	1.01006	coding
MSTRG.24864.1	3.04297	coding
MSTRG.24865.1	-1.13612	noncoding
MSTRG.24865.2	-1.1934	noncoding
MSTRG.24869.2	-0.861894	noncoding
MSTRG.24869.4	0.376078	coding
MSTRG.24869.3	-0.740227	noncoding
MSTRG.24871.2	2.66412	coding
MSTRG.24890.1	1.07338	coding
MSTRG.24892.1	2.46272	coding
MSTRG.24892.3	-0.354561	noncoding
MSTRG.24892.4	0.334349	coding
MSTRG.24892.5	2.41756	coding
MSTRG.24892.6	1.58475	coding
MSTRG.24893.3	1.78823	coding
MSTRG.24885.7	2.39483	coding
MSTRG.24885.6	2.48063	coding
MSTRG.24885.5	2.84747	coding
MSTRG.24885.3	2.86581	coding
MSTRG.24885.10	1.06776	coding
MSTRG.24885.11	1.65998	coding
MSTRG.24885.8	1.95182	coding
MSTRG.24885.9	2.22185	coding
MSTRG.24898.1	0.99339	coding
MSTRG.24898.2	0.728368	coding
MSTRG.24898.3	1.4909	coding
MSTRG.24898.4	1.47573	coding
MSTRG.24898.5	0.776295	coding
MSTRG.24898.6	0.687455	coding
MSTRG.24903.1	-1.45747	noncoding
MSTRG.24913.2	1.28012	coding
MSTRG.24914.2	-0.593961	noncoding
MSTRG.24911.4	-0.1434	noncoding
MSTRG.24920.1	-0.68575	noncoding
MSTRG.24922.1	-2.3963	noncoding
MSTRG.24924.2	1.5233	coding
MSTRG.24925.1	0.427414	coding
MSTRG.24925.2	-0.072911	noncoding

MSTRG.24930.1	-1.01614	noncoding
MSTRG.24932.1	-1.83748	noncoding
MSTRG.24936.1	0.692174	coding
MSTRG.24936.5	0.998633	coding
MSTRG.24936.4	0.980394	coding
MSTRG.24936.7	0.589891	coding
MSTRG.24958.2	1.45271	coding
MSTRG.24958.1	1.36018	coding
MSTRG.24959.1	1.6444	coding
MSTRG.24959.2	1.64876	coding
MSTRG.24960.1	1.82447	coding
MSTRG.24960.2	0.927265	coding
MSTRG.24960.4	1.65724	coding
MSTRG.24960.5	1.29773	coding
MSTRG.24961.1	-2.48144	noncoding
MSTRG.24962.2	1.072	coding
MSTRG.24963.1	-0.456243	noncoding
MSTRG.24969.4	-0.670435	noncoding
MSTRG.24971.1	-0.355676	noncoding
MSTRG.24971.2	0.004024	coding
MSTRG.24971.3	-0.017016	noncoding
MSTRG.24971.4	0.677602	coding
MSTRG.24960.6	2.01867	coding
MSTRG.24960.7	1.90102	coding
MSTRG.24944.2	-0.790827	noncoding
MSTRG.24946.4	0.847119	coding
MSTRG.24946.3	0.784107	coding
MSTRG.24946.5	1.02627	coding
MSTRG.24948.2	0.505192	coding
MSTRG.24954.3	0.932399	coding
MSTRG.24954.6	0.818789	coding
MSTRG.24976.3	0.954697	coding
MSTRG.24976.2	2.41948	coding
MSTRG.24976.4	0.075227	coding
MSTRG.25009.1	0.082169	coding
MSTRG.25010.1	1.27317	coding
MSTRG.25010.8	1.82973	coding
MSTRG.25010.10	2.20292	coding
MSTRG.25010.11	2.23314	coding
MSTRG.25010.12	2.26677	coding
MSTRG.25010.13	2.11661	coding
MSTRG.25010.6	0.987379	coding
MSTRG.25010.4	1.31343	coding
MSTRG.25010.7	1.56686	coding
MSTRG.25010.3	2.0437	coding
MSTRG.25010.5	2.27552	coding
MSTRG.25012.1	-1.37611	noncoding
MSTRG.25010.15	2.33973	coding
MSTRG.25010.16	2.44866	coding
MSTRG.24992.2	1.80757	coding
MSTRG.24993.1	2.22368	coding
MSTRG.24993.3	2.19028	coding
MSTRG.24993.4	2.15774	coding
MSTRG.24993.5	2.15566	coding

MSTRG.24986.1	1.42384	coding
MSTRG.24996.1	-0.55369	noncoding
MSTRG.25001.2	1.39579	coding
MSTRG.25001.4	1.75615	coding
MSTRG.25003.2	0.725621	coding
MSTRG.25006.1	1.60407	coding
MSTRG.25006.3	0.325194	coding
MSTRG.25020.1	1.55983	coding
MSTRG.25020.4	1.53026	coding
MSTRG.25020.5	1.24905	coding
MSTRG.25021.1	3.13868	coding
MSTRG.25021.2	3.05236	coding
MSTRG.25021.3	3.21123	coding
MSTRG.25027.4	0.826809	coding
MSTRG.25027.7	1.88997	coding
MSTRG.25027.9	1.65557	coding
MSTRG.25027.8	1.65197	coding
MSTRG.25028.2	2.04231	coding
MSTRG.25029.1	-1.76154	noncoding
MSTRG.25031.2	-1.36659	noncoding
MSTRG.25031.3	-1.51024	noncoding
MSTRG.25031.1	-0.871506	noncoding
MSTRG.25031.4	-1.59491	noncoding
MSTRG.25033.2	0.898531	coding
MSTRG.25032.4	0.601866	coding
MSTRG.25039.3	1.46234	coding
MSTRG.25041.2	0.109219	coding
MSTRG.25038.1	-1.83299	noncoding
MSTRG.25042.1	0.045888	coding
MSTRG.25045.3	0.390017	coding
MSTRG.25049.1	-1.45122	noncoding
MSTRG.25050.2	0.879428	coding
MSTRG.25050.4	0.889204	coding
MSTRG.25050.5	0.740875	coding
MSTRG.25051.3	-0.016289	noncoding
MSTRG.25051.4	-0.144919	noncoding
MSTRG.25051.6	-0.622608	noncoding
MSTRG.25053.6	2.01222	coding
MSTRG.25058.1	1.31167	coding
MSTRG.25058.3	0.980733	coding
MSTRG.25059.1	1.30778	coding
MSTRG.25055.1	-0.678344	noncoding
MSTRG.25056.1	1.74605	coding
MSTRG.25060.3	0.841245	coding
MSTRG.25063.2	1.55976	coding
MSTRG.25063.1	1.82868	coding
MSTRG.25064.2	0.870505	coding
MSTRG.25067.2	-0.550933	noncoding
MSTRG.25067.3	-1.00574	noncoding
MSTRG.25070.1	-0.49732	noncoding
MSTRG.25070.2	-0.640636	noncoding
MSTRG.25070.4	-0.623354	noncoding
MSTRG.25072.4	2.18704	coding
MSTRG.25072.3	1.99598	coding

MSTRG.25074.2	2.30869	coding
MSTRG.25074.3	2.44248	coding
MSTRG.25071.3	-0.164561	noncoding
MSTRG.25078.3	2.39424	coding
MSTRG.25079.3	0.922836	coding
MSTRG.25079.2	0.791185	coding
MSTRG.25078.4	1.6781	coding
MSTRG.25080.2	1.80676	coding
MSTRG.25076.2	-0.411382	noncoding
MSTRG.25076.3	-0.037119	noncoding
MSTRG.25085.2	1.00752	coding
MSTRG.25085.3	1.26369	coding
MSTRG.25081.1	0.975977	coding
MSTRG.25090.3	0.600921	coding
MSTRG.25108.4	0.873069	coding
MSTRG.25108.7	0.622746	coding
MSTRG.25109.1	-0.992648	noncoding
MSTRG.25110.1	1.40626	coding
MSTRG.25103.2	1.13526	coding
MSTRG.25103.3	1.27314	coding
MSTRG.25103.5	1.15801	coding
MSTRG.25103.4	1.04023	coding
MSTRG.25103.6	0.148785	coding
MSTRG.25105.1	-0.243078	noncoding
MSTRG.25104.1	-2.79316	noncoding
MSTRG.25107.1	-1.4478	noncoding
MSTRG.25116.1	1.1612	coding
MSTRG.25116.3	1.02755	coding
MSTRG.25115.1	-1.62047	noncoding
MSTRG.25118.2	1.59563	coding
MSTRG.25120.1	0.245801	coding
MSTRG.25123.1	0.055636	coding
MSTRG.25124.1	2.62806	coding
MSTRG.25124.2	1.97926	coding
MSTRG.25125.1	-0.682989	noncoding
MSTRG.25143.1	0.744197	coding
MSTRG.25143.2	0.489135	coding
MSTRG.25143.3	0.642569	coding
MSTRG.25128.1	-0.184742	noncoding
MSTRG.25138.1	-1.18372	noncoding
MSTRG.25139.1	-1.91764	noncoding
MSTRG.25150.4	1.2339	coding
MSTRG.25166.1	-1.13033	noncoding
MSTRG.25167.1	2.06997	coding
MSTRG.25167.3	2.12235	coding
MSTRG.25168.1	1.69182	coding
MSTRG.25164.2	0.075463	coding
MSTRG.25164.1	-0.497118	noncoding
MSTRG.25164.4	0.514172	coding
MSTRG.25170.1	-0.577927	noncoding
MSTRG.25174.2	0.126742	coding
MSTRG.25174.3	-0.661692	noncoding
MSTRG.25176.4	0.896811	coding
MSTRG.25176.5	0.938956	coding

MSTRG.25181.1	0.299628	coding
MSTRG.25185.1	0.42138	coding
MSTRG.25195.1	0.375867	coding
MSTRG.25195.2	-0.03277	noncoding
MSTRG.25196.3	1.65348	coding
MSTRG.25196.4	2.09637	coding
MSTRG.25196.5	0.791682	coding
MSTRG.25196.6	0.415361	coding
MSTRG.25198.1	-2.24168	noncoding
MSTRG.25190.3	0.721611	coding
MSTRG.25205.4	2.1026	coding
MSTRG.25205.5	2.38114	coding
MSTRG.25206.1	-0.61708	noncoding
MSTRG.25205.6	1.98954	coding
MSTRG.25207.1	-0.295411	noncoding
MSTRG.25207.2	-0.237672	noncoding
MSTRG.25207.3	-0.184866	noncoding
MSTRG.25207.4	-0.827373	noncoding
MSTRG.25208.1	-1.94507	noncoding
MSTRG.25211.1	-0.534106	noncoding
MSTRG.25214.1	1.22952	coding
MSTRG.25214.2	0.843436	coding
MSTRG.25217.1	-0.152481	noncoding
MSTRG.25232.1	-1.77343	noncoding
MSTRG.25248.1	-0.241141	noncoding
MSTRG.25254.2	1.18578	coding
MSTRG.25221.1	-2.67718	noncoding
MSTRG.25224.3	-1.99637	noncoding
MSTRG.25226.1	-0.149404	noncoding
MSTRG.25226.3	-0.06282	noncoding
MSTRG.25226.5	-0.271212	noncoding
MSTRG.25226.4	-0.919892	noncoding
MSTRG.25226.8	-0.511776	noncoding
MSTRG.25255.4	2.06638	coding
MSTRG.25255.1	1.65146	coding
MSTRG.25256.1	0.235075	coding
MSTRG.25257.1	-0.563105	noncoding
MSTRG.25258.1	-0.367105	noncoding
MSTRG.25255.6	-0.244444	noncoding
MSTRG.25268.6	1.99285	coding
MSTRG.25269.1	-0.928046	noncoding
MSTRG.25280.2	-1.18495	noncoding
MSTRG.25261.1	1.60069	coding
MSTRG.25262.1	-1.28979	noncoding
MSTRG.25262.2	-0.684132	noncoding
MSTRG.25265.1	-2.53429	noncoding
MSTRG.25281.1	-0.91502	noncoding
MSTRG.25285.2	-1.10858	noncoding
MSTRG.25290.3	1.84384	coding
MSTRG.25297.2	-1.57814	noncoding
MSTRG.25298.1	0.409511	coding
MSTRG.25302.1	-2.06223	noncoding
MSTRG.25310.1	-1.36555	noncoding
MSTRG.25318.1	0.980567	coding

MSTRG.25315.1	0.103424	coding
MSTRG.25327.1	1.7923	coding
MSTRG.25327.2	1.93501	coding
MSTRG.25327.3	2.02941	coding
MSTRG.25328.1	-0.759918	noncoding
MSTRG.25329.1	-3.086	noncoding
MSTRG.25335.1	1.57429	coding
MSTRG.25335.2	-1.32904	noncoding
MSTRG.25342.1	2.01341	coding
MSTRG.25342.4	2.22486	coding
MSTRG.25342.5	1.89634	coding
MSTRG.25343.1	-0.203409	noncoding
MSTRG.25348.2	2.66916	coding
MSTRG.25350.2	-0.922647	noncoding
MSTRG.25351.2	2.71018	coding
MSTRG.25351.3	2.71342	coding
MSTRG.25351.4	2.66222	coding
MSTRG.25356.2	-0.944392	noncoding
MSTRG.25359.1	1.49623	coding
MSTRG.25359.4	1.69597	coding
MSTRG.25353.1	0.142968	coding
MSTRG.25353.3	0.193249	coding
MSTRG.25354.1	-2.42551	noncoding
MSTRG.25354.2	-1.84487	noncoding
MSTRG.25361.1	0.350034	coding
MSTRG.25361.3	-0.226119	noncoding
MSTRG.25364.1	-0.139092	noncoding
MSTRG.25362.2	0.921985	coding
MSTRG.25372.1	-1.10284	noncoding
MSTRG.25384.1	1.05622	coding
MSTRG.25384.2	0.995062	coding
MSTRG.25384.3	0.7255	coding
MSTRG.25384.6	-0.075095	noncoding
MSTRG.25388.1	2.33528	coding
MSTRG.25388.5	1.88315	coding
MSTRG.25388.6	2.10852	coding
MSTRG.25388.7	-0.605731	noncoding
MSTRG.25389.1	0.130211	coding
MSTRG.25390.1	0.613894	coding
MSTRG.25389.2	-1.45208	noncoding
MSTRG.25389.3	-0.738622	noncoding
MSTRG.25409.4	1.36473	coding
MSTRG.25409.5	1.78818	coding
MSTRG.25409.7	1.41513	coding
MSTRG.25410.2	1.98384	coding
MSTRG.25413.2	2.56043	coding
MSTRG.25413.3	2.96905	coding
MSTRG.25413.4	2.95453	coding
MSTRG.25413.5	2.91501	coding
MSTRG.25413.6	1.04212	coding
MSTRG.25413.7	2.1863	coding
MSTRG.25415.1	1.80673	coding
MSTRG.25415.2	1.21561	coding
MSTRG.25415.3	-1.69946	noncoding

MSTRG.25415.4	2.37515	coding
MSTRG.25417.1	-1.122	noncoding
MSTRG.25399.1	1.38231	coding
MSTRG.25391.1	-2.26301	noncoding
MSTRG.25405.2	0.16063	coding
MSTRG.25406.1	-0.046045	noncoding
MSTRG.25405.3	-1.96429	noncoding
MSTRG.25433.2	0.109942	coding
MSTRG.25434.1	-1.73379	noncoding
MSTRG.25435.3	1.71178	coding
MSTRG.25436.1	1.38451	coding
MSTRG.25436.2	1.6531	coding
MSTRG.25436.5	1.28284	coding
MSTRG.25437.1	-1.9121	noncoding
MSTRG.25441.2	0.95999	coding
MSTRG.25447.2	1.46588	coding
MSTRG.25447.6	1.51075	coding
MSTRG.25447.7	1.18903	coding
MSTRG.25448.1	0.674286	coding
MSTRG.25444.1	-0.594977	noncoding
MSTRG.25451.4	0.893423	coding
MSTRG.25462.1	-1.57534	noncoding
MSTRG.25463.1	0.489683	coding
MSTRG.25465.1	-0.491346	noncoding
MSTRG.25472.5	-0.038948	noncoding
MSTRG.25472.6	1.54065	coding
MSTRG.25472.7	1.43088	coding
MSTRG.25472.8	1.45393	coding
MSTRG.25477.1	2.11344	coding
MSTRG.25477.2	2.26325	coding
MSTRG.25478.1	0.433205	coding
MSTRG.25478.3	0.690393	coding
MSTRG.25478.5	0.406723	coding
MSTRG.25480.3	1.47514	coding
MSTRG.25480.4	2.53503	coding
MSTRG.25483.1	-1.86141	noncoding
MSTRG.25483.2	-0.98513	noncoding
MSTRG.25484.2	-0.560004	noncoding
MSTRG.25489.2	0.797363	coding
MSTRG.25489.3	1.12355	coding
MSTRG.25491.1	1.54222	coding
MSTRG.25492.4	1.16571	coding
MSTRG.25492.3	1.24296	coding
MSTRG.25492.5	1.09563	coding
MSTRG.25499.3	1.72547	coding
MSTRG.25499.2	0.882846	coding
MSTRG.25499.6	1.48183	coding
MSTRG.25499.7	0.903048	coding
MSTRG.25499.8	0.98095	coding
MSTRG.25499.10	-0.484933	noncoding
MSTRG.25508.1	0.345198	coding
MSTRG.25508.3	0.657327	coding
MSTRG.25508.4	0.46769	coding
MSTRG.25508.5	1.01534	coding

MSTRG.25508.6	0.675059	coding
MSTRG.25508.7	0.001705	coding
MSTRG.25509.1	0.054378	coding
MSTRG.25508.9	1.82387	coding
MSTRG.25508.8	0.863291	coding
MSTRG.25510.1	-2.28076	noncoding
MSTRG.25508.10	0.804685	coding
MSTRG.25508.11	0.495158	coding
MSTRG.25508.12	0.666747	coding
MSTRG.25508.13	0.320505	coding
MSTRG.25511.1	-0.701555	noncoding
MSTRG.25512.1	0.20757	coding
MSTRG.25512.2	-0.261082	noncoding
MSTRG.25513.1	-0.846979	noncoding
MSTRG.25514.1	-0.20276	noncoding
MSTRG.25508.14	0.144391	coding
MSTRG.25517.1	0.801042	coding
MSTRG.25517.3	0.583704	coding
MSTRG.25517.4	0.242174	coding
MSTRG.25518.2	2.30004	coding
MSTRG.25528.2	0.887344	coding
MSTRG.25528.3	1.29245	coding
MSTRG.25529.1	-0.092979	noncoding
MSTRG.25528.4	0.585754	coding
MSTRG.25530.1	0.535302	coding
MSTRG.25531.1	-1.42438	noncoding
MSTRG.25532.1	0.450165	coding
MSTRG.25537.2	0.663952	coding
MSTRG.25537.1	0.641788	coding
MSTRG.25537.3	0.617151	coding
MSTRG.25540.1	1.25299	coding
MSTRG.25546.1	1.2008	coding
MSTRG.25550.3	2.14977	coding
MSTRG.25552.1	1.88341	coding
MSTRG.25552.2	1.82679	coding
MSTRG.25552.3	1.83375	coding
MSTRG.25552.4	1.94575	coding
MSTRG.25552.5	1.61387	coding
MSTRG.25552.6	1.68527	coding
MSTRG.25552.7	1.83969	coding
MSTRG.25552.9	2.34056	coding
MSTRG.25552.10	2.27324	coding
MSTRG.25556.3	0.012183	coding
MSTRG.25557.2	2.20038	coding
MSTRG.25571.1	0.526381	coding
MSTRG.25577.7	2.4687	coding
MSTRG.25577.8	2.48547	coding
MSTRG.25582.3	1.90271	coding
MSTRG.25593.2	0.960624	coding
MSTRG.25594.1	-0.831924	noncoding
MSTRG.25595.1	2.42511	coding
MSTRG.25601.2	1.62866	coding
MSTRG.25597.2	2.22108	coding
MSTRG.25597.3	-0.999623	noncoding

MSTRG.25598.2	-1.87983	noncoding
MSTRG.25598.1	-1.78542	noncoding
MSTRG.25599.1	-1.45909	noncoding
MSTRG.25597.4	0.649289	coding
MSTRG.25602.1	1.74038	coding
MSTRG.25602.2	2.38261	coding
MSTRG.25602.4	1.5737	coding
MSTRG.25602.5	0.873703	coding
MSTRG.25604.3	2.11979	coding
MSTRG.25604.2	1.7965	coding
MSTRG.25615.2	2.47885	coding
MSTRG.25615.6	2.2822	coding
MSTRG.25615.7	2.20442	coding
MSTRG.25615.9	2.26101	coding
MSTRG.25615.10	1.32475	coding
MSTRG.25607.5	2.11422	coding
MSTRG.25608.1	-1.22614	noncoding
MSTRG.25609.1	-0.425799	noncoding
MSTRG.25610.1	-1.15561	noncoding
MSTRG.25610.2	0.597511	coding
MSTRG.25611.1	-0.853743	noncoding
MSTRG.25614.1	-1.67991	noncoding
MSTRG.25616.2	1.71421	coding
MSTRG.25616.3	1.32373	coding
MSTRG.25620.2	1.92103	coding
MSTRG.25626.1	-1.18728	noncoding
MSTRG.25622.1	-1.50594	noncoding
MSTRG.25622.2	-2.45746	noncoding
MSTRG.25623.1	-2.20251	noncoding
MSTRG.25630.1	-3.0594	noncoding
MSTRG.25631.2	0.146321	coding
MSTRG.25632.2	0.993578	coding
MSTRG.25632.4	1.51668	coding
MSTRG.25635.1	-0.03316	noncoding
MSTRG.25637.2	1.34616	coding
MSTRG.25638.1	1.20502	coding
MSTRG.25638.2	-1.64781	noncoding
MSTRG.25642.1	-1.75662	noncoding
MSTRG.25642.2	-2.43619	noncoding
MSTRG.25639.1	-2.06302	noncoding
MSTRG.25653.1	-0.401326	noncoding
MSTRG.25654.2	0.881772	coding
MSTRG.25654.3	2.20431	coding
MSTRG.25657.6	2.26281	coding
MSTRG.25657.7	1.93437	coding
MSTRG.25664.2	1.86349	coding
MSTRG.25667.1	-1.56711	noncoding
MSTRG.25711.1	-0.942719	noncoding
MSTRG.25711.2	-1.06198	noncoding
MSTRG.25711.3	-0.006563	noncoding
MSTRG.25711.4	-0.956663	noncoding
MSTRG.25712.1	0.63852	coding
MSTRG.25711.5	-0.271526	noncoding
MSTRG.25711.6	0.007514	coding

MSTRG.25711.7	-0.416405	noncoding
MSTRG.25707.1	-1.16957	noncoding
MSTRG.25718.1	-0.979849	noncoding
MSTRG.25723.2	0.395438	coding
MSTRG.25725.1	-1.24984	noncoding
MSTRG.25726.3	0.857893	coding
MSTRG.25730.2	0.544442	coding
MSTRG.25734.2	1.22571	coding
MSTRG.25734.3	1.35306	coding
MSTRG.25736.1	-1.74815	noncoding
MSTRG.25734.4	-0.12934	noncoding
MSTRG.25737.2	1.60452	coding
MSTRG.25747.4	2.75674	coding
MSTRG.25747.2	2.86052	coding
MSTRG.25748.1	0.029316	coding
MSTRG.25748.2	0.81497	coding
MSTRG.25739.1	-1.66228	noncoding
MSTRG.25749.1	-1.1551	noncoding
MSTRG.25750.1	-1.60703	noncoding
MSTRG.25750.2	-1.24142	noncoding
MSTRG.25752.1	0.833909	coding
MSTRG.25760.4	2.40903	coding
MSTRG.25757.2	0.423954	coding
MSTRG.25771.2	1.64169	coding
MSTRG.25772.1	-0.970796	noncoding
MSTRG.25771.3	1.03863	coding
MSTRG.25771.4	0.890249	coding
MSTRG.25771.5	0.790778	coding
MSTRG.25778.1	0.669411	coding
MSTRG.25779.2	-0.197913	noncoding
MSTRG.25779.3	0.614907	coding
MSTRG.25773.1	-0.894785	noncoding
MSTRG.25781.2	2.79	coding
MSTRG.25782.1	-1.79801	noncoding
MSTRG.25782.2	-1.67504	noncoding
MSTRG.25784.1	-0.116353	noncoding
MSTRG.25785.2	0.166982	coding
MSTRG.25790.1	-0.989238	noncoding
MSTRG.25796.1	0.070978	coding
MSTRG.25796.2	0.549846	coding
MSTRG.25798.1	0.331025	coding
MSTRG.25799.1	-1.10804	noncoding
MSTRG.25802.1	-1.48704	noncoding
MSTRG.25802.2	-0.441827	noncoding
MSTRG.25803.1	-1.75515	noncoding
MSTRG.25808.1	-0.800066	noncoding
MSTRG.25809.1	-1.26272	noncoding
MSTRG.25809.2	-1.02829	noncoding
MSTRG.25809.3	-1.01027	noncoding
MSTRG.25806.1	0.275613	coding
MSTRG.25817.2	1.1818	coding
MSTRG.25817.3	1.31746	coding
MSTRG.25828.1	0.617056	coding
MSTRG.25824.4	1.5201	coding

MSTRG.25847.1	-2.46971	noncoding
MSTRG.25848.1	-1.06364	noncoding
MSTRG.25848.2	-1.00701	noncoding
MSTRG.25849.1	-1.34077	noncoding
MSTRG.25857.1	-0.566764	noncoding
MSTRG.25858.1	-1.2844	noncoding
MSTRG.25857.2	-1.86946	noncoding
MSTRG.25861.1	-1.74104	noncoding
MSTRG.25862.1	-2.7113	noncoding
MSTRG.25869.1	-2.50341	noncoding
MSTRG.25872.1	-2.01841	noncoding
MSTRG.25897.4	-0.11819	noncoding
MSTRG.25897.6	-0.076849	noncoding
MSTRG.25897.3	0.694132	coding
MSTRG.25897.7	0.015363	coding
MSTRG.25898.1	-0.502477	noncoding
MSTRG.25897.11	-0.180538	noncoding
MSTRG.25897.12	-0.482701	noncoding
MSTRG.25897.13	-0.494223	noncoding
MSTRG.25897.14	-0.388958	noncoding
MSTRG.25874.2	2.37687	coding
MSTRG.25874.1	2.78891	coding
MSTRG.25907.2	1.28801	coding
MSTRG.25907.3	0.704455	coding
MSTRG.25911.2	0.936301	coding
MSTRG.25912.2	2.26078	coding
MSTRG.25916.1	0.419011	coding
MSTRG.25916.2	0.36906	coding
MSTRG.25917.1	-1.81555	noncoding
MSTRG.25923.1	-1.15422	noncoding
MSTRG.25926.4	0.93333	coding
MSTRG.25926.5	0.602925	coding
MSTRG.25927.1	-0.444101	noncoding
MSTRG.25928.1	-1.0193	noncoding
MSTRG.25926.6	-0.981754	noncoding
MSTRG.25929.1	-0.236547	noncoding
MSTRG.25940.1	0.759696	coding
MSTRG.25940.5	0.919023	coding
MSTRG.25940.3	1.08622	coding
MSTRG.25949.3	2.11579	coding
MSTRG.25950.1	0.304117	coding
MSTRG.25951.1	0.104691	coding
MSTRG.25949.5	2.55625	coding
MSTRG.25949.6	1.74576	coding
MSTRG.25949.7	1.24118	coding
MSTRG.25949.8	2.2972	coding
MSTRG.25956.1	-1.56615	noncoding
MSTRG.25958.1	0.335442	coding
MSTRG.25958.2	-0.026598	noncoding
MSTRG.25958.3	0.139281	coding
MSTRG.25959.1	-0.060945	noncoding
MSTRG.25959.2	0.646351	coding
MSTRG.25959.3	0.574406	coding
MSTRG.25959.5	0.387899	coding

MSTRG.25959.4	0.633704	coding
MSTRG.25959.6	-0.266411	noncoding
MSTRG.25959.7	0.397191	coding
MSTRG.25959.8	0.420409	coding
MSTRG.25960.1	1.71146	coding
MSTRG.25972.1	-2.08062	noncoding
MSTRG.25973.2	0.538437	coding
MSTRG.25973.3	0.762183	coding
MSTRG.25973.4	0.969145	coding
MSTRG.25973.6	-0.021938	noncoding
MSTRG.25977.1	0.581807	coding
MSTRG.25977.2	0.43706	coding
MSTRG.25977.3	-0.503178	noncoding
MSTRG.25978.2	-0.235119	noncoding
MSTRG.25975.1	-1.9118	noncoding
MSTRG.25980.2	0.710333	coding
MSTRG.25997.5	1.62595	coding
MSTRG.25997.4	2.08267	coding
MSTRG.25997.1	2.12997	coding
MSTRG.25997.2	2.12312	coding
MSTRG.25997.6	1.91879	coding
MSTRG.25998.1	-1.49097	noncoding
MSTRG.25997.8	2.36935	coding
MSTRG.25997.7	2.34472	coding
MSTRG.25997.9	2.44491	coding
MSTRG.25997.10	2.36985	coding
MSTRG.25997.11	2.36115	coding
MSTRG.25997.12	2.56057	coding
MSTRG.25997.13	0.737431	coding
MSTRG.25997.14	1.04896	coding
MSTRG.25997.18	0.584189	coding
MSTRG.25997.19	-0.04585	noncoding
MSTRG.25997.20	0.084954	coding
MSTRG.25997.21	-0.378314	noncoding
MSTRG.25989.1	0.124599	coding
MSTRG.25989.2	0.181285	coding
MSTRG.25989.3	0.18633	coding
MSTRG.25989.5	0.241217	coding
MSTRG.25989.6	0.115075	coding
MSTRG.25991.1	0.180005	coding
MSTRG.25991.2	0.106498	coding
MSTRG.25991.3	0.224894	coding
MSTRG.25991.4	0.230654	coding
MSTRG.25991.5	0.194899	coding
MSTRG.25985.2	1.01077	coding
MSTRG.25986.1	-0.48475	noncoding
MSTRG.25992.2	2.96983	coding
MSTRG.25993.1	-0.368011	noncoding
MSTRG.25994.1	0.047804	coding
MSTRG.25994.2	0.705362	coding
MSTRG.25995.1	0.245747	coding
MSTRG.25992.3	0.428201	coding
MSTRG.25992.4	0.445017	coding
MSTRG.26010.2	1.20091	coding

MSTRG.26010.3	0.360025	coding
MSTRG.26016.1	-0.290231	noncoding
MSTRG.26018.1	-0.791663	noncoding
MSTRG.26021.1	-0.609846	noncoding
MSTRG.26026.5	-0.179116	noncoding
MSTRG.26026.4	-0.324564	noncoding
MSTRG.26026.2	-0.431604	noncoding
MSTRG.26026.3	-0.22931	noncoding
MSTRG.26028.1	0.9523	coding
MSTRG.26033.1	2.13664	coding
MSTRG.26034.1	-2.3674	noncoding
MSTRG.26034.3	-0.13372	noncoding
MSTRG.26034.2	-0.077737	noncoding
MSTRG.26034.4	0.46418	coding
MSTRG.26034.5	0.073275	coding
MSTRG.26034.6	-0.136963	noncoding
MSTRG.26034.7	0.087347	coding
MSTRG.26031.2	1.55848	coding
MSTRG.26031.3	-0.446261	noncoding
MSTRG.26035.2	1.97417	coding
MSTRG.26035.3	1.93317	coding
MSTRG.26036.1	1.32535	coding
MSTRG.26038.2	2.28095	coding
MSTRG.26038.3	1.92925	coding
MSTRG.26037.1	1.72689	coding
MSTRG.26041.2	2.20931	coding
MSTRG.26042.1	2.12785	coding
MSTRG.26042.2	2.01776	coding
MSTRG.26051.1	2.59966	coding
MSTRG.26052.1	0.89597	coding
MSTRG.26053.3	-1.64862	noncoding
MSTRG.26053.4	1.0129	coding
MSTRG.26045.2	0.60825	coding
MSTRG.26048.1	-2.28731	noncoding
MSTRG.26047.2	-0.176509	noncoding
MSTRG.26059.1	2.39006	coding
MSTRG.26059.2	2.5909	coding
MSTRG.26083.4	1.47324	coding
MSTRG.26083.5	1.465	coding
MSTRG.26087.7	0.742732	coding
MSTRG.26087.6	0.483619	coding
MSTRG.26087.5	2.31781	coding
MSTRG.26088.1	-0.408374	noncoding
MSTRG.26089.1	-0.202528	noncoding
MSTRG.26091.1	1.69227	coding
MSTRG.26091.4	2.21925	coding
MSTRG.26091.2	2.28668	coding
MSTRG.26093.1	-0.598391	noncoding
MSTRG.26096.1	-0.140581	noncoding
MSTRG.26102.2	1.38613	coding
MSTRG.26106.1	-0.144814	noncoding
MSTRG.26106.2	-0.014101	noncoding
MSTRG.26107.2	-0.407946	noncoding
MSTRG.26108.1	-0.987057	noncoding

MSTRG.26109.1	-0.328648	noncoding
MSTRG.26111.4	1.87606	coding
MSTRG.26112.2	-1.23887	noncoding
MSTRG.26112.3	0.117765	coding
MSTRG.26112.4	0.135245	coding
MSTRG.26113.1	-0.99104	noncoding
MSTRG.26118.3	-0.402579	noncoding
MSTRG.26118.11	0.558076	coding
MSTRG.26118.12	0.856228	coding
MSTRG.26118.13	1.12233	coding
MSTRG.26120.1	-0.627283	noncoding
MSTRG.26121.1	0.24284	coding
MSTRG.26123.1	0.052318	coding
MSTRG.26123.2	0.650703	coding
MSTRG.26123.8	1.0459	coding
MSTRG.26123.10	0.796227	coding
MSTRG.26123.12	0.884541	coding
MSTRG.26123.11	0.716056	coding
MSTRG.26123.9	0.883852	coding
MSTRG.26123.14	0.381302	coding
MSTRG.26123.13	0.298734	coding
MSTRG.26124.2	0.46064	coding
MSTRG.26126.2	2.3273	coding
MSTRG.26126.4	0.222979	coding
MSTRG.26122.2	1.97752	coding
MSTRG.26138.1	-1.19266	noncoding
MSTRG.26139.1	-0.03445	noncoding
MSTRG.26142.2	1.16937	coding
MSTRG.26142.3	0.856005	coding
MSTRG.26147.3	2.24506	coding
MSTRG.26148.1	0.507032	coding
MSTRG.26147.6	1.93667	coding
MSTRG.26154.2	-0.526036	noncoding
MSTRG.26154.3	-0.425949	noncoding
MSTRG.26158.3	0.952215	coding
MSTRG.26163.1	-1.73208	noncoding
MSTRG.26171.1	1.1206	coding
MSTRG.26171.3	-0.062464	noncoding
MSTRG.26171.4	1.36559	coding
MSTRG.26171.5	1.98072	coding
MSTRG.26180.1	0.962025	coding
MSTRG.26180.2	2.2687	coding
MSTRG.26180.5	2.74065	coding
MSTRG.26180.6	2.63698	coding
MSTRG.26180.13	2.2716	coding
MSTRG.26181.1	-0.701105	noncoding
MSTRG.26180.14	0.568185	coding
MSTRG.26180.17	-1.69559	noncoding
MSTRG.26180.18	0.272725	coding
MSTRG.26180.19	0.576726	coding
MSTRG.26180.20	0.353155	coding
MSTRG.26180.21	0.152173	coding
MSTRG.26184.2	2.14466	coding
MSTRG.26184.3	0.240943	coding

MSTRG.26184.5	0.605904	coding
MSTRG.26185.1	1.65342	coding
MSTRG.26185.3	1.52747	coding
MSTRG.26186.1	-1.75801	noncoding
MSTRG.26187.1	-1.28436	noncoding
MSTRG.26182.2	0.424311	coding
MSTRG.26192.1	-2.05326	noncoding
MSTRG.26194.17	-0.405422	noncoding
MSTRG.26189.2	-0.626949	noncoding
MSTRG.26190.1	-1.33165	noncoding
MSTRG.26203.1	-2.33259	noncoding
MSTRG.26212.1	0.214875	coding
MSTRG.26213.1	0.445625	coding
MSTRG.26213.2	-0.637842	noncoding
MSTRG.26213.3	0.08725	coding
MSTRG.26213.5	-0.6148	noncoding
MSTRG.26213.6	-0.19452	noncoding
MSTRG.26214.3	1.43526	coding
MSTRG.26215.3	0.740883	coding
MSTRG.26216.1	-0.029769	noncoding
MSTRG.26220.2	1.32319	coding
MSTRG.26224.1	3.05221	coding
MSTRG.26224.2	3.17593	coding
MSTRG.26224.3	3.27226	coding
MSTRG.26224.4	3.04563	coding
MSTRG.26224.5	3.1334	coding
MSTRG.26226.1	3.08028	coding
MSTRG.26226.2	1.08599	coding
MSTRG.26227.1	-0.970323	noncoding
MSTRG.26230.2	1.35692	coding
MSTRG.26232.3	2.80637	coding
MSTRG.26232.4	2.74484	coding
MSTRG.26232.5	2.56342	coding
MSTRG.26240.5	0.909389	coding
MSTRG.26240.6	0.954544	coding
MSTRG.26240.7	0.956014	coding
MSTRG.26241.1	-0.213958	noncoding
MSTRG.26242.1	-0.049902	noncoding
MSTRG.26244.1	-0.598662	noncoding
MSTRG.26244.2	-0.378075	noncoding
MSTRG.26245.1	-0.570468	noncoding
MSTRG.26246.1	0.295559	coding
MSTRG.26240.8	0.724194	coding
MSTRG.26247.1	-2.23959	noncoding
MSTRG.26247.2	-1.64653	noncoding
MSTRG.26249.1	-2.55809	noncoding
MSTRG.26239.1	2.39146	coding
MSTRG.26239.2	2.45908	coding
MSTRG.26239.4	2.55558	coding
MSTRG.26259.5	0.44365	coding
MSTRG.26260.1	0.572888	coding
MSTRG.26257.1	-1.15487	noncoding
MSTRG.26257.2	-0.806383	noncoding
MSTRG.26265.1	-0.564835	noncoding

MSTRG.26266.1	1.72845	coding
MSTRG.26266.2	1.05249	coding
MSTRG.26266.3	0.482769	coding
MSTRG.26281.1	-1.64782	noncoding
MSTRG.26282.2	-0.439609	noncoding
MSTRG.26286.2	1.55679	coding
MSTRG.26287.5	-0.696937	noncoding
MSTRG.26287.3	-0.651106	noncoding
MSTRG.26287.4	1.33769	coding
MSTRG.26278.1	-0.001045	noncoding
MSTRG.26291.3	1.59666	coding
MSTRG.26291.6	1.80143	coding
MSTRG.26291.4	1.23884	coding
MSTRG.26292.1	-1.27568	noncoding
MSTRG.26297.3	0.556436	coding
MSTRG.26297.4	-2.40945	noncoding
MSTRG.26299.1	-0.888207	noncoding
MSTRG.26301.1	-1.79786	noncoding
MSTRG.26303.1	1.1009	coding
MSTRG.26308.1	-1.95185	noncoding
MSTRG.26308.2	-2.08688	noncoding
MSTRG.26315.1	-0.408198	noncoding
MSTRG.26316.1	-1.0421	noncoding
MSTRG.26321.1	1.16142	coding
MSTRG.26321.2	1.19191	coding
MSTRG.26321.3	0.612547	coding
MSTRG.26321.4	1.14187	coding
MSTRG.26321.5	-1.04719	noncoding
MSTRG.26322.1	0.804181	coding
MSTRG.26323.1	-2.06977	noncoding
MSTRG.26324.1	-2.07339	noncoding
MSTRG.26325.1	-0.58919	noncoding
MSTRG.26338.1	-1.25021	noncoding
MSTRG.26340.1	2.26629	coding
MSTRG.26340.2	2.14565	coding
MSTRG.26337.1	-1.06613	noncoding
MSTRG.26337.2	-2.02613	noncoding
MSTRG.26343.1	1.93538	coding
MSTRG.26344.1	0.575504	coding
MSTRG.26345.1	-1.82531	noncoding
MSTRG.26345.2	0.056802	coding
MSTRG.26346.1	0.446116	coding
MSTRG.26347.1	-0.554707	noncoding
MSTRG.26348.1	0.424229	coding
MSTRG.26349.1	-0.179399	noncoding
MSTRG.26369.3	2.33694	coding
MSTRG.26369.2	1.47211	coding
MSTRG.26369.4	2.16569	coding
MSTRG.26369.5	2.18301	coding
MSTRG.26370.2	1.84975	coding
MSTRG.26373.2	2.08712	coding
MSTRG.26373.3	1.84787	coding
MSTRG.26374.1	0.792451	coding
MSTRG.26375.1	1.49907	coding

MSTRG.26376.1	-2.08316	noncoding
MSTRG.26377.1	-1.65599	noncoding
MSTRG.26374.5	1.24934	coding
MSTRG.26374.6	1.04892	coding
MSTRG.26374.7	0.500423	coding
MSTRG.26374.8	0.90399	coding
MSTRG.26375.2	-0.180645	noncoding
MSTRG.26378.2	0.070837	coding
MSTRG.26379.7	2.72383	coding
MSTRG.26379.6	3.10401	coding
MSTRG.26379.9	3.1593	coding
MSTRG.26386.1	0.933668	coding
MSTRG.26391.2	0.16501	coding
MSTRG.26391.3	-0.664079	noncoding
MSTRG.26391.4	0.778173	coding
MSTRG.26391.5	0.86342	coding
MSTRG.26394.3	2.46998	coding
MSTRG.26394.4	2.57334	coding
MSTRG.26394.5	2.97448	coding
MSTRG.26394.8	2.99181	coding
MSTRG.26395.1	1.14997	coding
MSTRG.26394.10	0.08846	coding
MSTRG.26394.9	0.054507	coding
MSTRG.26389.1	-1.98317	noncoding
MSTRG.26428.1	0.738045	coding
MSTRG.26428.4	0.305154	coding
MSTRG.26433.1	0.176447	coding
MSTRG.26434.1	-0.012483	noncoding
MSTRG.26433.2	0.197342	coding
MSTRG.26428.7	1.70125	coding
MSTRG.26399.8	-0.595182	noncoding
MSTRG.26399.7	0.803276	coding
MSTRG.26405.1	2.43127	coding
MSTRG.26405.2	2.17011	coding
MSTRG.26405.5	1.24915	coding
MSTRG.26405.4	1.45705	coding
MSTRG.26405.6	1.08794	coding
MSTRG.26411.4	1.09649	coding
MSTRG.26411.5	2.23689	coding
MSTRG.26411.3	2.55158	coding
MSTRG.26412.1	-2.40329	noncoding
MSTRG.26413.1	0.378605	coding
MSTRG.26415.1	-0.940795	noncoding
MSTRG.26408.1	-1.85713	noncoding
MSTRG.26439.1	0.906356	coding
MSTRG.26448.3	3.1771	coding
MSTRG.26448.1	2.9293	coding
MSTRG.26448.2	3.24888	coding
MSTRG.26448.5	2.82863	coding
MSTRG.26448.6	2.20081	coding
MSTRG.26448.7	0.327091	coding
MSTRG.26452.3	1.17148	coding
MSTRG.26453.1	-1.48983	noncoding
MSTRG.26455.1	0.353339	coding

MSTRG.26446.4	-0.203809	noncoding
MSTRG.26446.5	-0.198868	noncoding
MSTRG.26477.1	-1.53163	noncoding
MSTRG.26491.1	0.624731	coding
MSTRG.26493.1	0.814692	coding
MSTRG.26495.1	-1.32872	noncoding
MSTRG.26498.1	-0.526188	noncoding
MSTRG.26498.2	0.999973	coding
MSTRG.26461.4	1.7051	coding
MSTRG.26461.2	2.15181	coding
MSTRG.26461.3	2.05787	coding
MSTRG.26461.5	1.07258	coding
MSTRG.26469.2	0.628202	coding
MSTRG.26470.2	0.147518	coding
MSTRG.26470.3	-0.855575	noncoding
MSTRG.26470.4	-0.066535	noncoding
MSTRG.26515.1	-2.33133	noncoding
MSTRG.26515.2	-2.52102	noncoding
MSTRG.26515.3	-2.4331	noncoding
MSTRG.26521.1	-2.22547	noncoding
MSTRG.26533.1	-1.49533	noncoding
MSTRG.26541.1	-1.81118	noncoding
MSTRG.26567.1	-1.74704	noncoding
MSTRG.26569.2	0.06042	coding
MSTRG.26569.5	0.198519	coding
MSTRG.26569.6	0.157969	coding
MSTRG.26569.7	0.067433	coding
MSTRG.26569.8	0.474954	coding
MSTRG.26569.3	0.577956	coding
MSTRG.26569.4	0.39369	coding
MSTRG.26569.10	0.088687	coding
MSTRG.26569.11	0.21036	coding
MSTRG.26569.12	0.471582	coding
MSTRG.26569.9	0.241555	coding
MSTRG.26570.1	-0.378722	noncoding
MSTRG.26571.1	0.109316	coding
MSTRG.26571.2	-0.059574	noncoding
MSTRG.26573.1	1.28001	coding
MSTRG.26574.1	-0.698385	noncoding
MSTRG.26574.2	-0.124976	noncoding
MSTRG.26571.3	0.255167	coding
MSTRG.26572.4	-0.477064	noncoding
MSTRG.26572.5	-0.443937	noncoding
MSTRG.26572.6	0.709739	coding
MSTRG.26572.7	-0.251227	noncoding
MSTRG.26572.8	0.475442	coding
MSTRG.26572.9	-1.14765	noncoding
MSTRG.26572.10	0.359485	coding
MSTRG.26572.11	0.068373	coding
MSTRG.26572.12	1.04202	coding
MSTRG.26572.13	0.932098	coding
MSTRG.26572.14	-0.206236	noncoding
MSTRG.26572.15	-1.2435	noncoding
MSTRG.26572.16	-1.50287	noncoding

MSTRG.26572.17	-1.53456	noncoding
MSTRG.26575.1	1.10316	coding
MSTRG.26542.4	2.5784	coding
MSTRG.26542.3	2.5876	coding
MSTRG.26542.6	-1.08567	noncoding
MSTRG.26552.3	1.11235	coding
MSTRG.26552.5	0.952793	coding
MSTRG.26553.2	1.67328	coding
MSTRG.26553.1	1.45844	coding
MSTRG.26554.2	1.18798	coding
MSTRG.26554.1	1.11782	coding
MSTRG.26554.4	1.17108	coding
MSTRG.26554.3	1.1821	coding
MSTRG.26554.5	1.07616	coding
MSTRG.26555.3	2.18795	coding
MSTRG.26557.2	1.87153	coding
MSTRG.26546.1	1.66489	coding
MSTRG.26547.1	0.338118	coding
MSTRG.26547.2	-0.09791	noncoding
MSTRG.26548.3	1.90792	coding
MSTRG.26548.5	2.21345	coding
MSTRG.26560.1	0.661822	coding
MSTRG.26563.1	2.37319	coding
MSTRG.26586.3	3.27317	coding
MSTRG.26593.1	1.26256	coding
MSTRG.26585.2	1.37852	coding
MSTRG.26594.3	1.87434	coding
MSTRG.26595.1	2.67648	coding
MSTRG.26596.1	-0.54749	noncoding
MSTRG.26598.1	1.39479	coding
MSTRG.26602.2	2.26218	coding
MSTRG.26602.3	1.85807	coding
MSTRG.26601.2	1.53011	coding
MSTRG.26611.2	0.072371	coding
MSTRG.26614.1	-0.189908	noncoding
MSTRG.26615.1	-1.72081	noncoding
MSTRG.26615.2	-2.37055	noncoding
MSTRG.26616.1	-0.65794	noncoding
MSTRG.26616.2	-1.34086	noncoding
MSTRG.26617.2	1.35194	coding
MSTRG.26617.3	0.382474	coding
MSTRG.26617.4	1.45027	coding
MSTRG.26620.4	-0.596276	noncoding
MSTRG.26624.4	2.03955	coding
MSTRG.26623.3	0.864131	coding
MSTRG.26630.3	1.71027	coding
MSTRG.26630.2	2.39758	coding
MSTRG.26630.4	2.76937	coding
MSTRG.26636.2	0.486695	coding
MSTRG.26637.4	1.08251	coding
MSTRG.26637.5	1.93102	coding
MSTRG.26644.1	2.04518	coding
MSTRG.26645.1	-0.915016	noncoding
MSTRG.26644.2	2.06675	coding

MSTRG.26644.5	1.36906	coding
MSTRG.26642.1	0.700488	coding
MSTRG.26643.1	-0.054996	noncoding
MSTRG.26646.1	-0.390582	noncoding
MSTRG.26647.1	-0.34798	noncoding
MSTRG.26647.2	0.236055	coding
MSTRG.26651.1	-2.49328	noncoding
MSTRG.26652.1	-0.935323	noncoding
MSTRG.26657.2	0.938194	coding
MSTRG.26658.2	-0.724388	noncoding
MSTRG.26660.2	2.22386	coding
MSTRG.26660.1	2.08105	coding
MSTRG.26660.8	1.5032	coding
MSTRG.26660.6	1.41797	coding
MSTRG.26660.10	1.88207	coding
MSTRG.26660.11	0.147173	coding
MSTRG.26660.12	1.13699	coding
MSTRG.26665.1	-1.16737	noncoding
MSTRG.26662.1	-1.67054	noncoding
MSTRG.26667.1	-2.46194	noncoding
MSTRG.26668.1	-1.05454	noncoding
MSTRG.26671.1	-0.649547	noncoding
MSTRG.26675.2	1.89836	coding
MSTRG.26675.6	1.98574	coding
MSTRG.26675.7	1.69339	coding
MSTRG.26675.5	1.70034	coding
MSTRG.26675.8	2.29643	coding
MSTRG.26675.11	1.43749	coding
MSTRG.26675.12	0.606666	coding
MSTRG.26677.2	0.674609	coding
MSTRG.26677.1	-2.00832	noncoding
MSTRG.26680.2	1.17883	coding
MSTRG.26684.1	1.37632	coding
MSTRG.26684.2	0.919652	coding
MSTRG.26684.4	0.670408	coding
MSTRG.26684.6	1.86285	coding
MSTRG.26685.3	2.07065	coding
MSTRG.26685.2	2.4501	coding
MSTRG.26686.1	1.57727	coding
MSTRG.26688.1	0.225183	coding
MSTRG.26707.1	-2.1577	noncoding
MSTRG.26709.1	-0.566501	noncoding
MSTRG.26710.1	1.82563	coding
MSTRG.26710.3	1.33122	coding
MSTRG.26710.11	2.61665	coding
MSTRG.26710.12	2.10033	coding
MSTRG.26711.1	-1.07737	noncoding
MSTRG.26708.1	-0.07872	noncoding
MSTRG.26715.1	0.305437	coding
MSTRG.26715.2	-0.456203	noncoding
MSTRG.26715.3	-0.040117	noncoding
MSTRG.26715.4	0.152252	coding
MSTRG.26715.7	0.192053	coding
MSTRG.26715.8	0.167829	coding

MSTRG.26715.9	-0.178733	noncoding
MSTRG.26720.2	0.142617	coding
MSTRG.26720.5	1.1717	coding
MSTRG.26720.6	0.846281	coding
MSTRG.26720.7	1.01978	coding
MSTRG.26712.4	1.3064	coding
MSTRG.26712.3	1.46313	coding
MSTRG.26712.2	1.53691	coding
MSTRG.26713.1	-0.547484	noncoding
MSTRG.26722.2	0.365766	coding
MSTRG.26722.3	0.227449	coding
MSTRG.26722.4	-0.623678	noncoding
MSTRG.26725.5	1.58776	coding
MSTRG.26725.6	1.44304	coding
MSTRG.26725.8	0.894259	coding
MSTRG.26726.2	1.1287	coding
MSTRG.26726.1	-0.324395	noncoding
MSTRG.26727.1	0.623888	coding
MSTRG.26727.2	0.51863	coding
MSTRG.26727.4	1.12275	coding
MSTRG.26727.3	1.88122	coding
MSTRG.26727.5	1.72527	coding
MSTRG.26728.1	-0.054304	noncoding
MSTRG.26728.2	0.71584	coding
MSTRG.26729.1	1.81481	coding
MSTRG.26739.3	1.22547	coding
MSTRG.26739.4	2.1832	coding
MSTRG.26739.5	2.36863	coding
MSTRG.26741.1	-0.696042	noncoding
MSTRG.26744.1	1.93242	coding
MSTRG.26744.4	2.53535	coding
MSTRG.26742.2	1.1882	coding
MSTRG.26742.3	1.12457	coding
MSTRG.26748.1	0.651197	coding
MSTRG.26749.1	1.3814	coding
MSTRG.26749.2	1.21375	coding
MSTRG.26751.1	1.22793	coding
MSTRG.26751.4	-1.02257	noncoding
MSTRG.26751.6	0.212975	coding
MSTRG.26751.5	0.911333	coding
MSTRG.26751.7	1.00179	coding
MSTRG.26751.3	0.871243	coding
MSTRG.26751.8	-0.616585	noncoding
MSTRG.26753.1	0.474713	coding
MSTRG.26751.9	0.492325	coding
MSTRG.26754.1	0.247043	coding
MSTRG.26757.2	1.55326	coding
MSTRG.26773.20	-0.874974	noncoding
MSTRG.26774.2	0.99915	coding
MSTRG.26776.1	-1.30341	noncoding
MSTRG.26777.2	2.18848	coding
MSTRG.26777.3	2.13845	coding
MSTRG.26765.1	-2.46979	noncoding
MSTRG.26770.1	-0.127204	noncoding

MSTRG.26770.2	-0.243973	noncoding
MSTRG.26790.1	1.78707	coding
MSTRG.26790.3	2.29055	coding
MSTRG.26788.2	-0.388497	noncoding
MSTRG.26786.1	0.901162	coding
MSTRG.26792.3	1.4467	coding
MSTRG.26792.4	1.48199	coding
MSTRG.26793.2	-1.26985	noncoding
MSTRG.26793.3	-1.28877	noncoding
MSTRG.26795.2	0.508826	coding
MSTRG.26795.3	0.832078	coding
MSTRG.26796.1	-0.024717	noncoding
MSTRG.26796.2	0.456062	coding
MSTRG.26796.3	0.227448	coding
MSTRG.26798.2	0.324101	coding
MSTRG.26803.3	1.87102	coding
MSTRG.26804.1	-0.78291	noncoding
MSTRG.26805.1	-1.44085	noncoding
MSTRG.26806.7	0.393566	coding
MSTRG.26806.8	0.392171	coding
MSTRG.26806.4	0.262508	coding
MSTRG.26806.5	0.196503	coding
MSTRG.26808.1	0.662148	coding
MSTRG.26808.2	0.68939	coding
MSTRG.26821.1	0.622081	coding
MSTRG.26821.3	-1.28127	noncoding
MSTRG.26821.4	-1.09099	noncoding
MSTRG.26821.5	-1.18991	noncoding
MSTRG.26821.6	-0.895768	noncoding
MSTRG.26830.1	0.15204	coding
MSTRG.26830.2	-0.198444	noncoding
MSTRG.26830.3	-0.008883	noncoding
MSTRG.26828.2	1.52687	coding
MSTRG.26835.2	-0.598863	noncoding
MSTRG.26836.2	2.28958	coding
MSTRG.26836.3	-1.15849	noncoding
MSTRG.26837.2	3.09677	coding
MSTRG.26837.4	2.66749	coding
MSTRG.26837.5	1.45666	coding
MSTRG.26841.1	-0.956185	noncoding
MSTRG.26843.2	1.30458	coding
MSTRG.26844.1	0.368626	coding
MSTRG.26845.1	0.128298	coding
MSTRG.26853.1	-0.250654	noncoding
MSTRG.26838.1	-0.387668	noncoding
MSTRG.26855.1	-0.942427	noncoding
MSTRG.26856.1	-1.95885	noncoding
MSTRG.26863.4	0.08671	coding
MSTRG.26863.5	1.14766	coding
MSTRG.26864.1	-0.603587	noncoding
MSTRG.26865.1	-0.335435	noncoding
MSTRG.26866.1	-0.664285	noncoding
MSTRG.26867.1	-0.309494	noncoding
MSTRG.26868.1	-1.17105	noncoding

MSTRG.26868.2	0.189952	coding
MSTRG.26868.3	-0.119636	noncoding
MSTRG.26870.1	-1.56369	noncoding
MSTRG.26858.1	-2.64927	noncoding
MSTRG.26861.1	-2.04093	noncoding
MSTRG.26861.2	-1.19528	noncoding
MSTRG.26889.8	1.18822	coding
MSTRG.26889.9	1.48464	coding
MSTRG.26889.4	1.48957	coding
MSTRG.26889.7	1.59047	coding
MSTRG.26889.6	1.74091	coding
MSTRG.26889.5	1.70744	coding
MSTRG.26889.3	1.37481	coding
MSTRG.26889.2	2.07332	coding
MSTRG.26889.10	-1.06684	noncoding
MSTRG.26889.11	0.707159	coding
MSTRG.26890.1	-1.57667	noncoding
MSTRG.26889.12	0.977802	coding
MSTRG.26891.1	0.594424	coding
MSTRG.26892.1	0.385853	coding
MSTRG.26894.1	-0.242245	noncoding
MSTRG.26879.2	1.30321	coding
MSTRG.26879.3	0.740554	coding
MSTRG.26880.2	0.250559	coding
MSTRG.26898.1	-0.187045	noncoding
MSTRG.26903.2	1.53706	coding
MSTRG.26904.1	-1.31739	noncoding
MSTRG.26902.1	1.85578	coding
MSTRG.26910.3	0.004818	coding
MSTRG.26910.4	0.330613	coding
MSTRG.26910.5	0.17102	coding
MSTRG.26919.8	2.10026	coding
MSTRG.26919.5	2.55748	coding
MSTRG.26919.1	2.85026	coding
MSTRG.26919.2	2.85154	coding
MSTRG.26919.3	2.89997	coding
MSTRG.26919.7	2.80048	coding
MSTRG.26919.9	2.7733	coding
MSTRG.26919.10	2.9293	coding
MSTRG.26920.1	-0.548695	noncoding
MSTRG.26932.1	0.995284	coding
MSTRG.26932.4	1.21558	coding
MSTRG.26932.5	1.37332	coding
MSTRG.26932.7	2.16494	coding
MSTRG.26932.6	2.3119	coding
MSTRG.26932.8	1.70631	coding
MSTRG.26932.9	1.80577	coding
MSTRG.26932.10	1.11486	coding
MSTRG.26933.3	2.15998	coding
MSTRG.26937.2	2.8455	coding
MSTRG.26937.5	2.53791	coding
MSTRG.26929.1	2.30784	coding
MSTRG.26929.2	1.93755	coding
MSTRG.26930.1	-1.11047	noncoding

MSTRG.26941.9	1.36945	coding
MSTRG.26941.8	2.71871	coding
MSTRG.26941.10	2.73292	coding
MSTRG.26941.11	2.73258	coding
MSTRG.26941.13	2.17295	coding
MSTRG.26941.14	0.199814	coding
MSTRG.26941.15	0.822676	coding
MSTRG.26942.1	1.39191	coding
MSTRG.26943.1	-0.463209	noncoding
MSTRG.26942.3	1.25793	coding
MSTRG.26945.1	2.41257	coding
MSTRG.26944.11	-0.262404	noncoding
MSTRG.26953.3	0.282729	coding
MSTRG.26953.2	1.35541	coding
MSTRG.26954.1	0.805281	coding
MSTRG.26955.2	1.92605	coding
MSTRG.26955.1	2.37422	coding
MSTRG.26955.3	1.5866	coding
MSTRG.26955.5	2.49751	coding
MSTRG.26957.1	-0.122993	noncoding
MSTRG.26962.1	-3.02126	noncoding
MSTRG.26974.1	3.11669	coding
MSTRG.26988.1	1.05762	coding
MSTRG.26988.2	-0.341724	noncoding
MSTRG.26990.1	0.827688	coding
MSTRG.26990.2	1.31831	coding
MSTRG.26990.3	1.16213	coding
MSTRG.26994.1	0.254153	coding
MSTRG.26990.4	1.6465	coding
MSTRG.26990.6	-0.725973	noncoding
MSTRG.26990.5	1.18465	coding
MSTRG.26997.1	0.540618	coding
MSTRG.26998.1	-0.838768	noncoding
MSTRG.27003.4	1.97314	coding
MSTRG.27006.6	0.450527	coding
MSTRG.27006.8	0.547403	coding
MSTRG.27006.9	0.459857	coding
MSTRG.27006.7	0.671178	coding
MSTRG.27006.5	1.22302	coding
MSTRG.27006.4	1.66962	coding
MSTRG.27006.2	1.46665	coding
MSTRG.27007.1	-0.799818	noncoding
MSTRG.27008.1	-0.74882	noncoding
MSTRG.27011.1	-1.14757	noncoding
MSTRG.27002.4	2.10387	coding
MSTRG.27014.1	-0.473198	noncoding
MSTRG.27017.6	1.11865	coding
MSTRG.27017.5	0.650187	coding
MSTRG.27017.7	1.66813	coding
MSTRG.27018.1	-0.266751	noncoding
MSTRG.27017.9	1.73066	coding
MSTRG.27017.10	-1.06384	noncoding
MSTRG.27017.11	-1.18753	noncoding
MSTRG.27012.3	1.82682	coding

MSTRG.27012.2	1.87488	coding
MSTRG.27019.2	1.0546	coding
MSTRG.27021.2	0.205965	coding
MSTRG.27023.1	0.414444	coding
MSTRG.27027.1	-1.77193	noncoding
MSTRG.27027.2	-1.87845	noncoding
MSTRG.27029.1	-1.87595	noncoding
MSTRG.27031.2	2.44185	coding
MSTRG.27035.4	-1.31307	noncoding
MSTRG.27035.6	0.58728	coding
MSTRG.27035.3	1.99333	coding
MSTRG.27035.5	1.92731	coding
MSTRG.27035.7	0.169293	coding
MSTRG.27038.2	0.713339	coding
MSTRG.27038.3	0.792891	coding
MSTRG.27038.4	0.777383	coding
MSTRG.27038.5	0.73984	coding
MSTRG.27039.1	-1.02635	noncoding
MSTRG.27039.2	-1.43059	noncoding
MSTRG.27069.1	0.16497	coding
MSTRG.27069.2	1.48476	coding
MSTRG.27069.3	0.816308	coding
MSTRG.27042.2	1.51967	coding
MSTRG.27043.1	1.19718	coding
MSTRG.27045.4	1.13521	coding
MSTRG.27045.5	1.2517	coding
MSTRG.27045.6	2.09204	coding
MSTRG.27045.7	1.8962	coding
MSTRG.27045.8	1.68916	coding
MSTRG.27073.2	1.2267	coding
MSTRG.27073.5	1.1876	coding
MSTRG.27073.6	1.50228	coding
MSTRG.27073.7	1.34492	coding
MSTRG.27073.8	1.33948	coding
MSTRG.27073.9	1.04383	coding
MSTRG.27051.1	-2.03431	noncoding
MSTRG.27078.1	0.598996	coding
MSTRG.27083.2	-0.412978	noncoding
MSTRG.27083.3	-0.511773	noncoding
MSTRG.27085.4	2.29955	coding
MSTRG.27086.2	0.162092	coding
MSTRG.27098.1	0.598884	coding
MSTRG.27098.2	0.408621	coding
MSTRG.27098.3	0.553303	coding
MSTRG.27099.1	-0.765421	noncoding
MSTRG.27099.2	-1.35634	noncoding
MSTRG.27101.3	2.33178	coding
MSTRG.27101.2	2.44873	coding
MSTRG.27101.1	2.50186	coding
MSTRG.27101.4	2.43761	coding
MSTRG.27101.5	2.68863	coding
MSTRG.27101.10	2.38583	coding
MSTRG.27101.11	2.48181	coding
MSTRG.27101.12	2.40176	coding

MSTRG.27101.8	2.40209	coding
MSTRG.27101.14	2.82585	coding
MSTRG.27101.16	2.50658	coding
MSTRG.27103.1	-1.43485	noncoding
MSTRG.27104.1	1.26117	coding
MSTRG.27104.2	1.49962	coding
MSTRG.27104.3	2.04091	coding
MSTRG.27104.4	2.04169	coding
MSTRG.27104.5	1.25026	coding
MSTRG.27104.6	1.22078	coding
MSTRG.27101.17	1.79701	coding
MSTRG.27091.2	0.677451	coding
MSTRG.27092.1	-0.005696	noncoding
MSTRG.27092.2	-0.132525	noncoding
MSTRG.27092.3	-0.190846	noncoding
MSTRG.27092.4	-0.042586	noncoding
MSTRG.27092.5	0.279672	coding
MSTRG.27092.6	-0.090723	noncoding
MSTRG.27094.1	-0.789421	noncoding
MSTRG.27092.7	-0.038998	noncoding
MSTRG.27114.1	1.41358	coding
MSTRG.27118.1	1.43024	coding
MSTRG.27118.2	-0.800017	noncoding
MSTRG.27119.1	0.268649	coding
MSTRG.27119.2	0.188375	coding
MSTRG.27118.3	0.745224	coding
MSTRG.27118.4	0.580453	coding
MSTRG.27123.1	-1.06502	noncoding
MSTRG.27124.2	2.24707	coding
MSTRG.27124.5	-0.13742	noncoding
MSTRG.27125.1	-0.659644	noncoding
MSTRG.27124.6	2.101	coding
MSTRG.27124.7	-0.972717	noncoding
MSTRG.27106.1	0.390059	coding
MSTRG.27109.1	0.349486	coding
MSTRG.27129.1	-2.30961	noncoding
MSTRG.27143.1	-2.16773	noncoding
MSTRG.27144.1	-1.04689	noncoding
MSTRG.27150.1	0.088224	coding
MSTRG.27156.1	-0.645678	noncoding
MSTRG.27157.2	-0.038945	noncoding
MSTRG.27179.1	-1.49267	noncoding
MSTRG.27191.1	1.60825	coding
MSTRG.27192.1	0.71157	coding
MSTRG.27192.3	0.849002	coding
MSTRG.27192.2	1.26634	coding
MSTRG.27194.1	-0.496204	noncoding
MSTRG.27198.1	1.0333	coding
MSTRG.27202.5	1.96277	coding
MSTRG.27217.1	-0.989981	noncoding
MSTRG.27217.2	0.283099	coding
MSTRG.27212.1	-0.668126	noncoding
MSTRG.27221.2	1.71587	coding
MSTRG.27222.1	-0.594863	noncoding

MSTRG.27223.1	-0.319177	noncoding
MSTRG.27226.1	1.78291	coding
MSTRG.27226.4	0.611948	coding
MSTRG.27226.5	-0.267312	noncoding
MSTRG.27230.2	0.333179	coding
MSTRG.27230.4	-1.67815	noncoding
MSTRG.27230.9	0.662898	coding
MSTRG.27230.16	-0.999729	noncoding
MSTRG.27230.17	-1.04186	noncoding
MSTRG.27239.1	0.777244	coding
MSTRG.27239.2	0.236802	coding
MSTRG.27282.1	0.182298	coding
MSTRG.27282.3	0.991073	coding
MSTRG.27282.11	-0.411084	noncoding
MSTRG.27282.10	-0.722809	noncoding
MSTRG.27282.12	0.346161	coding
MSTRG.27282.14	0.087683	coding
MSTRG.27282.15	-0.132978	noncoding
MSTRG.27285.1	0.397733	coding
MSTRG.27282.16	-0.474168	noncoding
MSTRG.27282.20	0.886162	coding
MSTRG.27282.21	0.157678	coding
MSTRG.27282.18	1.47011	coding
MSTRG.27288.1	-1.65854	noncoding
MSTRG.27291.1	2.04342	coding
MSTRG.27291.2	-0.427087	noncoding
MSTRG.27292.1	-0.306979	noncoding
MSTRG.27293.1	-1.27465	noncoding
MSTRG.27291.7	2.50587	coding
MSTRG.27291.8	2.49927	coding
MSTRG.27247.3	0.746545	coding
MSTRG.27247.2	0.723585	coding
MSTRG.27248.1	1.05688	coding
MSTRG.27250.1	-0.360189	noncoding
MSTRG.27251.1	1.45555	coding
MSTRG.27255.2	2.11565	coding
MSTRG.27256.1	-0.873148	noncoding
MSTRG.27263.1	0.088435	coding
MSTRG.27264.1	-0.897851	noncoding
MSTRG.27263.2	-0.171612	noncoding
MSTRG.27263.3	1.99399	coding
MSTRG.27268.1	-1.36381	noncoding
MSTRG.27272.1	-0.694035	noncoding
MSTRG.27276.1	0.12225	coding
MSTRG.27275.2	1.02509	coding
MSTRG.27304.3	0.354839	coding
MSTRG.27305.1	0.702014	coding
MSTRG.27305.2	0.659464	coding
MSTRG.27305.3	0.418046	coding
MSTRG.27305.4	0.586582	coding
MSTRG.27305.5	0.657539	coding
MSTRG.27305.6	-0.267967	noncoding
MSTRG.27304.6	0.136899	coding
MSTRG.27304.7	1.01465	coding

MSTRG.27304.8	0.249339	coding
MSTRG.27304.9	0.237358	coding
MSTRG.27304.10	2.67304	coding
MSTRG.27304.11	1.53796	coding
MSTRG.27304.12	0.143069	coding
MSTRG.27307.1	0.557811	coding
MSTRG.27304.13	0.229673	coding
MSTRG.27308.1	0.875386	coding
MSTRG.27308.2	-0.379169	noncoding
MSTRG.27304.14	1.23637	coding
MSTRG.27309.1	0.111584	coding
MSTRG.27309.2	0.292987	coding
MSTRG.27310.1	0.382129	coding
MSTRG.27311.1	-1.82925	noncoding
MSTRG.27277.1	-0.798734	noncoding
MSTRG.27278.1	-0.623322	noncoding
MSTRG.27300.3	2.06294	coding
MSTRG.27300.4	2.0316	coding
MSTRG.27300.5	2.16476	coding
MSTRG.27302.1	-0.201017	noncoding
MSTRG.27303.5	-0.415052	noncoding
MSTRG.27312.1	0.955991	coding
MSTRG.27314.2	0.515901	coding
MSTRG.27314.3	-1.76582	noncoding
MSTRG.27329.1	0.309145	coding
MSTRG.27329.2	-0.085718	noncoding
MSTRG.27328.4	-0.33733	noncoding
MSTRG.27330.1	0.888224	coding
MSTRG.27368.2	-1.65284	noncoding
MSTRG.27368.3	-0.477754	noncoding
MSTRG.27368.4	0.983102	coding
MSTRG.27368.5	-0.626786	noncoding
MSTRG.27368.6	0.798976	coding
MSTRG.27370.1	0.040687	coding
MSTRG.27368.7	0.749412	coding
MSTRG.27373.1	0.917391	coding
MSTRG.27337.1	-0.636493	noncoding
MSTRG.27338.3	2.01211	coding
MSTRG.27338.4	2.32336	coding
MSTRG.27339.1	-1.49557	noncoding
MSTRG.27340.1	-0.205646	noncoding
MSTRG.27343.3	0.020554	coding
MSTRG.27343.4	-0.01132	noncoding
MSTRG.27343.6	0.670835	coding
MSTRG.27343.8	0.386858	coding
MSTRG.27359.4	1.26733	coding
MSTRG.27362.3	0.668549	coding
MSTRG.27391.4	2.09075	coding
MSTRG.27391.2	1.90847	coding
MSTRG.27391.7	2.02502	coding
MSTRG.27391.8	1.78765	coding
MSTRG.27391.10	1.31355	coding
MSTRG.27391.9	0.831906	coding
MSTRG.27393.3	0.384249	coding

MSTRG.27393.4	1.00205	coding
MSTRG.27400.3	2.71632	coding
MSTRG.27400.4	1.43845	coding
MSTRG.27401.1	1.34854	coding
MSTRG.27401.2	1.36645	coding
MSTRG.27401.3	1.20637	coding
MSTRG.27401.4	1.26242	coding
MSTRG.27404.1	1.15912	coding
MSTRG.27411.2	0.742754	coding
MSTRG.27412.1	-0.778041	noncoding
MSTRG.27411.3	2.50056	coding
MSTRG.27413.1	0.69883	coding
MSTRG.27413.2	0.112665	coding
MSTRG.27413.3	0.106365	coding
MSTRG.27411.5	2.29748	coding
MSTRG.27378.6	0.195263	coding
MSTRG.27378.7	1.48453	coding
MSTRG.27378.8	1.04545	coding
MSTRG.27378.9	0.707826	coding
MSTRG.27387.1	-0.871813	noncoding
MSTRG.27387.2	-1.33319	noncoding
MSTRG.27387.3	-1.45042	noncoding
MSTRG.27387.4	-2.25715	noncoding
MSTRG.27387.5	-2.26304	noncoding
MSTRG.27387.6	-2.2108	noncoding
MSTRG.27387.7	0.509806	coding
MSTRG.27387.8	-0.094412	noncoding
MSTRG.27387.9	-2.20258	noncoding
MSTRG.27387.10	-2.19169	noncoding
MSTRG.27387.11	-1.91925	noncoding
MSTRG.27420.1	1.01098	coding
MSTRG.27420.2	2.48094	coding
MSTRG.27431.1	-2.57046	noncoding
MSTRG.27435.3	1.80951	coding
MSTRG.27436.1	0.663846	coding
MSTRG.27440.1	-2.36084	noncoding
MSTRG.27427.9	2.36685	coding
MSTRG.27427.8	3.09894	coding
MSTRG.27427.7	3.31208	coding
MSTRG.27427.10	2.49363	coding
MSTRG.27427.11	2.15375	coding
MSTRG.27427.12	2.76729	coding
MSTRG.27427.13	2.71133	coding
MSTRG.27428.2	-1.10798	noncoding
MSTRG.27428.1	-1.32402	noncoding
MSTRG.27428.3	-1.64618	noncoding
MSTRG.27428.4	-1.1818	noncoding
MSTRG.27445.2	-1.01871	noncoding
MSTRG.27446.1	-0.78782	noncoding
MSTRG.27449.1	1.1521	coding
MSTRG.27454.3	2.35719	coding
MSTRG.27454.2	2.28179	coding
MSTRG.27459.1	-0.017497	noncoding
MSTRG.27459.2	-0.380317	noncoding

MSTRG.27458.8	0.800628	coding
MSTRG.27461.1	3.47991	coding
MSTRG.27461.3	3.44004	coding
MSTRG.27461.5	2.81076	coding
MSTRG.27461.6	2.80615	coding
MSTRG.27461.7	2.91521	coding
MSTRG.27461.10	3.21221	coding
MSTRG.27461.11	3.27964	coding
MSTRG.27462.1	-0.273175	noncoding
MSTRG.27462.2	-0.285639	noncoding
MSTRG.27462.3	0.852858	coding
MSTRG.27457.3	2.00956	coding
MSTRG.27474.4	2.0383	coding
MSTRG.27474.2	1.98648	coding
MSTRG.27474.6	1.60067	coding
MSTRG.27475.1	0.939559	coding
MSTRG.27479.1	2.54131	coding
MSTRG.27480.2	3.30365	coding
MSTRG.27480.1	3.19849	coding
MSTRG.27480.4	3.23075	coding
MSTRG.27480.5	3.05728	coding
MSTRG.27480.6	3.25889	coding
MSTRG.27480.8	3.35231	coding
MSTRG.27480.9	3.57143	coding
MSTRG.27480.7	3.31308	coding
MSTRG.27480.10	1.1371	coding
MSTRG.27480.12	1.08039	coding
MSTRG.27463.1	-1.55626	noncoding
MSTRG.27466.2	0.930946	coding
MSTRG.27466.3	0.723772	coding
MSTRG.27466.4	0.768857	coding
MSTRG.27467.2	2.1078	coding
MSTRG.27469.3	1.34644	coding
MSTRG.27471.2	-0.059013	noncoding
MSTRG.27471.6	0.275116	coding
MSTRG.27473.1	-1.8548	noncoding
MSTRG.27471.7	-1.75761	noncoding
MSTRG.27471.8	-0.035358	noncoding
MSTRG.27486.2	1.13748	coding
MSTRG.27486.3	1.32537	coding
MSTRG.27487.2	0.782104	coding
MSTRG.27489.4	0.673805	coding
MSTRG.27489.5	0.418541	coding
MSTRG.27489.6	-1.88402	noncoding
MSTRG.27489.7	0.583698	coding
MSTRG.27483.1	1.78635	coding
MSTRG.27507.2	1.67133	coding
MSTRG.27507.3	0.533305	coding
MSTRG.27507.4	-0.20228	noncoding
MSTRG.27507.5	0.574916	coding
MSTRG.27507.7	1.56473	coding
MSTRG.27507.8	1.5324	coding
MSTRG.27508.1	-0.739213	noncoding
MSTRG.27510.1	0.922304	coding

MSTRG.27512.12	-0.283604	noncoding
MSTRG.27510.3	2.41582	coding
MSTRG.27510.4	1.81861	coding
MSTRG.27510.5	1.8931	coding
MSTRG.27510.6	-0.002907	noncoding
MSTRG.27514.1	2.99123	coding
MSTRG.27514.2	2.86342	coding
MSTRG.27515.1	-0.632182	noncoding
MSTRG.27521.1	2.38372	coding
MSTRG.27521.2	2.36008	coding
MSTRG.27521.3	2.45352	coding
MSTRG.27524.2	1.65318	coding
MSTRG.27526.1	1.60811	coding
MSTRG.27526.4	1.33054	coding
MSTRG.27497.1	1.09384	coding
MSTRG.27499.2	0.530123	coding
MSTRG.27499.4	0.423128	coding
MSTRG.27505.1	1.00581	coding
MSTRG.27505.5	1.18015	coding
MSTRG.27502.1	1.66739	coding
MSTRG.27533.4	2.10013	coding
MSTRG.27540.1	0.102911	coding
MSTRG.27572.2	-0.222487	noncoding
MSTRG.27573.4	-0.946527	noncoding
MSTRG.27573.3	-0.476475	noncoding
MSTRG.27573.2	-0.549444	noncoding
MSTRG.27580.6	-0.786887	noncoding
MSTRG.27580.7	-0.31548	noncoding
MSTRG.27580.8	-0.469605	noncoding
MSTRG.27580.5	-0.149687	noncoding
MSTRG.27580.9	0.751052	coding
MSTRG.27581.3	0.679669	coding
MSTRG.27581.6	0.592363	coding
MSTRG.27581.5	0.415904	coding
MSTRG.27584.1	1.27758	coding
MSTRG.27584.10	1.38997	coding
MSTRG.27584.11	1.41258	coding
MSTRG.27584.9	1.39897	coding
MSTRG.27584.12	1.90781	coding
MSTRG.27584.13	1.49123	coding
MSTRG.27584.14	1.42793	coding
MSTRG.27584.15	1.40194	coding
MSTRG.27587.3	-0.862759	noncoding
MSTRG.27587.2	0.686092	coding
MSTRG.27588.4	1.40023	coding
MSTRG.27589.2	1.71047	coding
MSTRG.27589.4	2.44859	coding
MSTRG.27590.5	1.77642	coding
MSTRG.27541.1	0.133224	coding
MSTRG.27541.2	-0.781254	noncoding
MSTRG.27544.2	0.961347	coding
MSTRG.27544.3	0.353009	coding
MSTRG.27547.1	2.46871	coding
MSTRG.27548.2	1.70543	coding

MSTRG.27550.1	-1.65053	noncoding
MSTRG.27554.1	-1.03203	noncoding
MSTRG.27555.1	-0.126578	noncoding
MSTRG.27555.2	-1.56512	noncoding
MSTRG.27555.3	-1.61885	noncoding
MSTRG.27556.1	-0.043328	noncoding
MSTRG.27557.1	2.41437	coding
MSTRG.27558.2	1.87813	coding
MSTRG.27559.4	-0.193779	noncoding
MSTRG.27559.5	-0.160642	noncoding
MSTRG.27560.1	0.807663	coding
MSTRG.27563.1	-0.042484	noncoding
MSTRG.27563.10	1.18282	coding
MSTRG.27563.9	0.191554	coding
MSTRG.27563.6	1.23069	coding
MSTRG.27563.7	0.908817	coding
MSTRG.27563.8	0.891099	coding
MSTRG.27563.11	0.731088	coding
MSTRG.27563.12	1.22579	coding
MSTRG.27563.13	0.755581	coding
MSTRG.27563.14	-1.89791	noncoding
MSTRG.27563.15	0.909132	coding
MSTRG.27565.1	-0.301183	noncoding
MSTRG.27563.16	0.917784	coding
MSTRG.27563.17	0.591027	coding
MSTRG.27567.5	3.04194	coding
MSTRG.27567.6	3.02028	coding
MSTRG.27569.2	2.62062	coding
MSTRG.27596.1	2.53527	coding
MSTRG.27596.3	2.28825	coding
MSTRG.27596.4	2.2823	coding
MSTRG.27597.2	0.749158	coding
MSTRG.27617.2	1.10768	coding
MSTRG.27617.3	0.593819	coding
MSTRG.27617.4	0.921631	coding
MSTRG.27620.8	1.76388	coding
MSTRG.27620.2	2.2133	coding
MSTRG.27620.3	2.14488	coding
MSTRG.27620.4	2.1307	coding
MSTRG.27620.5	2.3103	coding
MSTRG.27620.6	2.21807	coding
MSTRG.27620.7	2.21833	coding
MSTRG.27620.9	2.31386	coding
MSTRG.27620.10	2.02335	coding
MSTRG.27620.11	2.08365	coding
MSTRG.27620.12	-0.257397	noncoding
MSTRG.27622.2	2.39525	coding
MSTRG.27624.2	-1.47579	noncoding
MSTRG.27624.3	-0.153935	noncoding
MSTRG.27598.2	1.30121	coding
MSTRG.27598.3	1.08145	coding
MSTRG.27601.1	-2.015	noncoding
MSTRG.27602.1	-1.22304	noncoding
MSTRG.27605.1	-1.45654	noncoding

MSTRG.27605.5	-0.517262	noncoding
MSTRG.27605.2	-0.172522	noncoding
MSTRG.27605.4	-0.120894	noncoding
MSTRG.27606.1	-1.15427	noncoding
MSTRG.27599.2	0.894379	coding
MSTRG.27630.1	-0.919525	noncoding
MSTRG.27633.1	-0.011094	noncoding
MSTRG.27626.1	1.75467	coding
MSTRG.27626.2	-1.786	noncoding
MSTRG.27626.3	-1.48303	noncoding
MSTRG.27627.1	-0.129229	noncoding
MSTRG.27628.1	-0.315256	noncoding
MSTRG.27651.1	2.66834	coding
MSTRG.27651.2	2.49066	coding
MSTRG.27651.3	0.375841	coding
MSTRG.27655.1	0.383423	coding
MSTRG.27655.2	0.074694	coding
MSTRG.27666.2	-0.165924	noncoding
MSTRG.27667.1	-0.54288	noncoding
MSTRG.27668.6	0.153561	coding
MSTRG.27668.7	0.834434	coding
MSTRG.27668.8	1.17491	coding
MSTRG.27668.9	0.441726	coding
MSTRG.27668.10	-0.854435	noncoding
MSTRG.27669.5	0.218985	coding
MSTRG.27669.6	0.54045	coding
MSTRG.27669.7	-0.458799	noncoding
MSTRG.27669.8	0.105302	coding
MSTRG.27659.1	-0.754743	noncoding
MSTRG.27673.1	-0.542597	noncoding
MSTRG.27676.3	1.29309	coding
MSTRG.27676.4	1.1725	coding
MSTRG.27676.6	1.24049	coding
MSTRG.27676.7	1.22087	coding
MSTRG.27676.8	1.14795	coding
MSTRG.27676.10	0.79969	coding
MSTRG.27680.1	1.69094	coding
MSTRG.27680.3	1.63511	coding
MSTRG.27680.5	0.708845	coding
MSTRG.27680.4	1.75002	coding
MSTRG.27684.4	0.576136	coding
MSTRG.27684.3	0.651423	coding
MSTRG.27684.2	0.866713	coding
MSTRG.27689.1	-2.22786	noncoding
MSTRG.27690.2	-0.441844	noncoding
MSTRG.27695.1	0.702479	coding
MSTRG.27695.3	0.569195	coding
MSTRG.27704.1	-0.259074	noncoding
MSTRG.27706.1	-0.555443	noncoding
MSTRG.27707.2	-0.019643	noncoding
MSTRG.27707.1	0.288921	coding
MSTRG.27707.4	1.31041	coding
MSTRG.27708.2	-0.427726	noncoding
MSTRG.27708.3	-0.063952	noncoding

MSTRG.27717.5	1.38177	coding
MSTRG.27717.1	1.47253	coding
MSTRG.27717.2	1.43711	coding
MSTRG.27720.1	-0.898305	noncoding
MSTRG.27722.1	-0.281938	noncoding
MSTRG.27718.2	1.18661	coding
MSTRG.27719.1	-1.69695	noncoding
MSTRG.27724.4	1.833	coding
MSTRG.27724.5	1.88614	coding
MSTRG.27730.2	0.094911	coding
MSTRG.27731.3	1.19256	coding
MSTRG.27732.2	1.10635	coding
MSTRG.27725.1	-1.38992	noncoding
MSTRG.27737.2	1.87294	coding
MSTRG.27738.1	-0.976702	noncoding
MSTRG.27747.1	0.939877	coding
MSTRG.27747.2	2.11694	coding
MSTRG.27747.3	2.13619	coding
MSTRG.27747.4	2.06839	coding
MSTRG.27748.1	-0.120821	noncoding
MSTRG.27748.2	-0.224439	noncoding
MSTRG.27751.1	-1.72049	noncoding
MSTRG.27747.5	1.63602	coding
MSTRG.27752.1	-1.25738	noncoding
MSTRG.27753.1	-2.16886	noncoding
MSTRG.27747.6	1.86754	coding
MSTRG.27747.8	1.84743	coding
MSTRG.27754.1	0.015603	coding
MSTRG.27744.2	1.29497	coding
MSTRG.27745.1	0.115168	coding
MSTRG.27745.3	0.437324	coding
MSTRG.27769.1	-0.865078	noncoding
MSTRG.27772.3	1.01631	coding
MSTRG.27772.4	1.37706	coding
MSTRG.27772.6	1.47204	coding
MSTRG.27778.2	0.846587	coding
MSTRG.27781.2	2.65543	coding
MSTRG.27755.1	2.2869	coding
MSTRG.27757.1	1.14897	coding
MSTRG.27782.3	0.554105	coding
MSTRG.27783.1	-0.918253	noncoding
MSTRG.27784.1	1.48166	coding
MSTRG.27784.3	1.31848	coding
MSTRG.27784.2	1.20189	coding
MSTRG.27786.1	1.59044	coding
MSTRG.27793.1	-1.45795	noncoding
MSTRG.27794.1	-1.1378	noncoding
MSTRG.27795.1	0.087104	coding
MSTRG.27796.4	2.65663	coding
MSTRG.27798.3	0.127619	coding
MSTRG.27800.1	-0.179543	noncoding
MSTRG.27802.2	0.198275	coding
MSTRG.27802.3	0.259248	coding
MSTRG.27802.4	-0.100093	noncoding

MSTRG.27802.5	0.128964	coding
MSTRG.27802.6	0.56101	coding
MSTRG.27804.2	-1.52576	noncoding
MSTRG.27804.3	-1.10638	noncoding
MSTRG.27801.1	-0.646163	noncoding
MSTRG.27801.2	-1.13001	noncoding
MSTRG.27807.2	1.75849	coding
MSTRG.27808.4	0.382761	coding
MSTRG.27810.2	-2.32839	noncoding
MSTRG.27814.1	1.05067	coding
MSTRG.27814.4	0.442311	coding
MSTRG.27814.5	0.834409	coding
MSTRG.27814.12	0.431017	coding
MSTRG.27814.14	0.68201	coding
MSTRG.27818.1	-1.08635	noncoding
MSTRG.27814.15	0.468137	coding
MSTRG.27814.16	0.427493	coding
MSTRG.27814.17	0.381546	coding
MSTRG.27838.3	0.660646	coding
MSTRG.27838.2	0.722517	coding
MSTRG.27838.4	0.671786	coding
MSTRG.27839.1	-1.50909	noncoding
MSTRG.27840.1	0.968809	coding
MSTRG.27842.1	-1.85586	noncoding
MSTRG.27842.2	-1.99653	noncoding
MSTRG.27846.1	0.438858	coding
MSTRG.27846.2	0.248501	coding
MSTRG.27846.3	0.144019	coding
MSTRG.27846.4	2.17436	coding
MSTRG.27846.5	1.3326	coding
MSTRG.27846.7	2.59412	coding
MSTRG.27846.9	1.85161	coding
MSTRG.27846.8	2.12677	coding
MSTRG.27846.11	1.53812	coding
MSTRG.27846.12	1.71219	coding
MSTRG.27847.1	0.578057	coding
MSTRG.27848.1	-1.13575	noncoding
MSTRG.27846.13	1.40011	coding
MSTRG.27846.15	1.14082	coding
MSTRG.27844.2	-0.389136	noncoding
MSTRG.27884.3	2.81836	coding
MSTRG.27884.6	2.59608	coding
MSTRG.27885.1	0.265708	coding
MSTRG.27884.11	2.8514	coding
MSTRG.27884.9	2.79048	coding
MSTRG.27884.8	2.77733	coding
MSTRG.27884.12	2.80599	coding
MSTRG.27884.14	2.79264	coding
MSTRG.27884.15	2.65194	coding
MSTRG.27884.16	2.71778	coding
MSTRG.27884.17	2.70795	coding
MSTRG.27884.18	2.62313	coding
MSTRG.27884.19	2.26726	coding
MSTRG.27884.20	1.44214	coding

MSTRG.27854.1	-2.5436	noncoding
MSTRG.27852.1	0.656557	coding
MSTRG.27862.1	-1.55401	noncoding
MSTRG.27863.2	2.37225	coding
MSTRG.27864.1	-0.898153	noncoding
MSTRG.27865.1	-0.716354	noncoding
MSTRG.27870.1	2.01514	coding
MSTRG.27870.3	2.08793	coding
MSTRG.27870.4	1.95106	coding
MSTRG.27870.5	0.392181	coding
MSTRG.27875.2	-1.35326	noncoding
MSTRG.27876.1	1.98316	coding
MSTRG.27878.5	1.71478	coding
MSTRG.27880.3	1.25548	coding
MSTRG.27882.2	0.110184	coding
MSTRG.27882.4	0.06306	coding
MSTRG.27882.3	0.4513	coding
MSTRG.27893.3	1.29614	coding
MSTRG.27901.7	1.9399	coding
MSTRG.27901.8	1.8896	coding
MSTRG.27904.1	0.052866	coding
MSTRG.27905.5	0.73508	coding
MSTRG.27908.4	0.481182	coding
MSTRG.27908.5	0.593144	coding
MSTRG.27909.1	2.10555	coding
MSTRG.27909.4	1.51125	coding
MSTRG.27909.3	2.04448	coding
MSTRG.27909.5	1.52271	coding
MSTRG.27910.1	-0.487923	noncoding
MSTRG.27896.1	0.760173	coding
MSTRG.27896.2	0.764526	coding
MSTRG.27897.1	0.16606	coding
MSTRG.27897.2	0.535591	coding
MSTRG.27897.5	0.948142	coding
MSTRG.27900.2	0.991765	coding
MSTRG.27922.2	-0.439114	noncoding
MSTRG.27913.2	0.547636	coding
MSTRG.27925.1	0.222461	coding
MSTRG.27925.3	0.192832	coding
MSTRG.27928.1	0.279927	coding
MSTRG.27934.2	1.71598	coding
MSTRG.27935.1	0.523167	coding
MSTRG.27936.1	-0.995707	noncoding
MSTRG.27941.3	1.53865	coding
MSTRG.27942.1	-1.97566	noncoding
MSTRG.27942.4	-2.27157	noncoding
MSTRG.27942.3	-2.22625	noncoding
MSTRG.27942.2	-1.64184	noncoding
MSTRG.27943.2	-0.86146	noncoding
MSTRG.27943.1	-0.900812	noncoding
MSTRG.27943.3	-0.811113	noncoding
MSTRG.27945.3	1.08263	coding
MSTRG.27945.5	0.958321	coding
MSTRG.27940.2	2.01589	coding

MSTRG.27949.1	1.44235	coding
MSTRG.27949.2	1.12713	coding
MSTRG.27950.3	1.61036	coding
MSTRG.27950.4	1.50899	coding
MSTRG.27951.1	-1.09551	noncoding
MSTRG.27950.6	1.13968	coding
MSTRG.27952.1	0.814083	coding
MSTRG.27953.1	0.005609	coding
MSTRG.27955.1	-1.1147	noncoding
MSTRG.27954.5	0.222101	coding
MSTRG.27956.1	1.96218	coding
MSTRG.27956.5	1.96102	coding
MSTRG.27957.4	0.647376	coding
MSTRG.27957.3	0.943096	coding
MSTRG.27957.5	1.45412	coding
MSTRG.27957.6	1.69576	coding
MSTRG.27957.7	1.47519	coding
MSTRG.27957.8	1.85983	coding
MSTRG.27957.9	1.61017	coding
MSTRG.27957.10	1.50171	coding
MSTRG.27959.1	0.741593	coding
MSTRG.27960.1	0.273572	coding
MSTRG.27960.2	-1.37981	noncoding
MSTRG.27961.1	-0.315452	noncoding
MSTRG.27963.2	2.68637	coding
MSTRG.27967.4	1.20004	coding
MSTRG.27967.5	1.10983	coding
MSTRG.27967.6	1.36183	coding
MSTRG.27967.7	0.676825	coding
MSTRG.27968.3	1.33425	coding
MSTRG.27968.4	0.053523	coding
MSTRG.27964.2	1.55083	coding
MSTRG.27969.3	1.09833	coding
MSTRG.27969.4	1.06634	coding
MSTRG.27970.2	0.749625	coding
MSTRG.27975.5	-0.137342	noncoding
MSTRG.27975.4	-0.330038	noncoding
MSTRG.27975.7	-0.003383	noncoding
MSTRG.27983.2	0.543986	coding
MSTRG.27985.1	-1.11082	noncoding
MSTRG.27988.3	1.42034	coding
MSTRG.27995.1	1.20138	coding
MSTRG.27995.3	0.377899	coding
MSTRG.27996.1	1.10972	coding
MSTRG.27996.2	1.19696	coding
MSTRG.27997.1	0.43358	coding
MSTRG.28003.6	1.14089	coding
MSTRG.28004.1	-2.27867	noncoding
MSTRG.28005.1	0.366806	coding
MSTRG.28005.2	0.463917	coding
MSTRG.28003.7	0.809143	coding
MSTRG.28003.8	0.772162	coding
MSTRG.28003.9	-0.281819	noncoding
MSTRG.28007.2	1.0904	coding

MSTRG.28008.1	3.23304	coding
MSTRG.28008.2	3.22317	coding
MSTRG.28008.3	3.08843	coding
MSTRG.28009.1	-1.80862	noncoding
MSTRG.28011.1	-1.77818	noncoding
MSTRG.28012.5	2.18067	coding
MSTRG.28012.4	2.21871	coding
MSTRG.28013.3	0.718408	coding
MSTRG.28018.2	-1.15681	noncoding
MSTRG.28033.6	-0.349377	noncoding
MSTRG.28033.8	-0.117236	noncoding
MSTRG.28033.5	-0.180035	noncoding
MSTRG.28033.7	-0.138589	noncoding
MSTRG.28034.2	2.098	coding
MSTRG.28034.4	2.10996	coding
MSTRG.28034.9	1.99831	coding
MSTRG.28034.5	1.97566	coding
MSTRG.28034.6	1.98068	coding
MSTRG.28025.1	-0.619959	noncoding
MSTRG.28024.4	1.45886	coding
MSTRG.28027.1	-0.690195	noncoding
MSTRG.28027.2	-0.824345	noncoding
MSTRG.28040.1	1.44443	coding
MSTRG.28040.2	1.13827	coding
MSTRG.28040.4	1.29411	coding
MSTRG.28045.3	1.80804	coding
MSTRG.28035.2	0.244707	coding
MSTRG.28055.1	-0.050434	noncoding
MSTRG.28058.1	0.207935	coding
MSTRG.28059.1	-1.04425	noncoding
MSTRG.28051.1	0.498933	coding
MSTRG.28051.4	0.636088	coding
MSTRG.28051.7	0.626609	coding
MSTRG.28062.1	-2.39989	noncoding
MSTRG.28064.1	0.262548	coding
MSTRG.28070.1	1.88295	coding
MSTRG.28072.3	0.636104	coding
MSTRG.28076.1	-1.55399	noncoding
MSTRG.28075.1	0.207575	coding
MSTRG.28081.1	1.63632	coding
MSTRG.28085.6	1.05798	coding
MSTRG.28091.4	-0.01225	noncoding
MSTRG.28091.2	1.41431	coding
MSTRG.28093.2	2.40681	coding
MSTRG.28095.3	0.296751	coding
MSTRG.28095.2	0.923191	coding
MSTRG.28097.2	-1.20483	noncoding
MSTRG.28098.2	0.361834	coding
MSTRG.28098.1	0.529951	coding
MSTRG.28098.3	-0.413933	noncoding
MSTRG.28099.3	3.3807	coding
MSTRG.28100.1	1.61543	coding
MSTRG.28100.5	1.63458	coding
MSTRG.28100.6	1.49679	coding

MSTRG.28100.7	1.61021	coding
MSTRG.28100.8	1.59423	coding
MSTRG.28105.5	2.28868	coding
MSTRG.28105.6	2.39823	coding
MSTRG.28107.1	3.00136	coding
MSTRG.28109.3	-0.784719	noncoding
MSTRG.28109.4	-0.780949	noncoding
MSTRG.28109.2	-1.17116	noncoding
MSTRG.28109.5	-0.834655	noncoding
MSTRG.28121.1	0.436036	coding
MSTRG.28122.1	-2.25695	noncoding
MSTRG.28115.4	-0.584265	noncoding
MSTRG.28114.2	2.1695	coding
MSTRG.28127.1	-1.82148	noncoding
MSTRG.28132.2	0.578841	coding
MSTRG.28137.1	0.012784	coding
MSTRG.28131.3	-0.330433	noncoding
MSTRG.28141.1	-2.45974	noncoding
MSTRG.28142.2	1.74231	coding
MSTRG.28147.1	1.45035	coding
MSTRG.28154.1	-2.22751	noncoding
MSTRG.28160.1	1.87835	coding
MSTRG.28160.5	0.813998	coding
MSTRG.28161.1	-0.793237	noncoding
MSTRG.28162.1	0.165591	coding
MSTRG.28168.3	1.34496	coding
MSTRG.28174.1	0.754236	coding
MSTRG.28174.2	0.530667	coding
MSTRG.28175.1	-2.1466	noncoding
MSTRG.28177.1	-1.29345	noncoding
MSTRG.28178.1	0.471323	coding
MSTRG.28171.1	-2.56364	noncoding
MSTRG.28186.1	-0.256094	noncoding
MSTRG.28191.1	0.656792	coding
MSTRG.28191.8	-0.022088	noncoding
MSTRG.28192.3	-0.64849	noncoding
MSTRG.28204.2	-0.347663	noncoding
MSTRG.28207.1	-2.13507	noncoding
MSTRG.28207.2	-0.855072	noncoding
MSTRG.28207.3	-2.22562	noncoding
MSTRG.28211.2	0.739661	coding
MSTRG.28213.2	0.351337	coding
MSTRG.28194.2	0.322872	coding
MSTRG.28196.2	-0.711946	noncoding
MSTRG.28197.1	-1.21878	noncoding
MSTRG.28214.1	-1.58433	noncoding
MSTRG.28220.1	1.06658	coding
MSTRG.28220.2	0.888525	coding
MSTRG.28223.2	0.428785	coding
MSTRG.28224.2	1.68559	coding
MSTRG.28217.1	-1.41602	noncoding
MSTRG.28217.2	-1.40582	noncoding
MSTRG.28225.1	0.807987	coding
MSTRG.28227.3	1.85844	coding

MSTRG.28249.2	2.08183	coding
MSTRG.28249.3	1.92632	coding
MSTRG.28249.4	2.06553	coding
MSTRG.28249.5	1.86239	coding
MSTRG.28249.7	1.10843	coding
MSTRG.28249.8	1.06173	coding
MSTRG.28249.6	1.96842	coding
MSTRG.28249.9	1.87522	coding
MSTRG.28249.11	1.71578	coding
MSTRG.28250.1	0.203261	coding
MSTRG.28251.1	1.36242	coding
MSTRG.28251.2	1.30239	coding
MSTRG.28251.5	1.17843	coding
MSTRG.28251.6	0.264312	coding
MSTRG.28249.13	1.81821	coding
MSTRG.28232.2	2.00011	coding
MSTRG.28233.2	-0.378682	noncoding
MSTRG.28233.3	0.116005	coding
MSTRG.28233.4	-0.407419	noncoding
MSTRG.28236.2	2.58246	coding
MSTRG.28236.4	2.5863	coding
MSTRG.28229.1	1.43678	coding
MSTRG.28239.1	0.462098	coding
MSTRG.28244.2	2.13797	coding
MSTRG.28244.3	-0.639821	noncoding
MSTRG.28244.6	1.96193	coding
MSTRG.28246.2	-0.157722	noncoding
MSTRG.28247.1	0.043286	coding
MSTRG.28247.3	0.551367	coding
MSTRG.28247.5	0.403099	coding
MSTRG.28247.2	0.055716	coding
MSTRG.28241.1	-1.04881	noncoding
MSTRG.28242.1	1.02598	coding
MSTRG.28242.3	1.23523	coding
MSTRG.28242.5	1.56031	coding
MSTRG.28243.1	1.28675	coding
MSTRG.28255.2	0.100522	coding
MSTRG.28256.5	1.92929	coding
MSTRG.28257.2	1.99571	coding
MSTRG.28257.3	2.13834	coding
MSTRG.28257.4	2.04441	coding
MSTRG.28300.1	-0.72283	noncoding
MSTRG.28300.2	0.891756	coding
MSTRG.28301.1	-1.39252	noncoding
MSTRG.28300.3	1.39003	coding
MSTRG.28300.4	0.757402	coding
MSTRG.28300.5	0.797228	coding
MSTRG.28300.6	2.00916	coding
MSTRG.28300.8	0.648187	coding
MSTRG.28300.9	1.46394	coding
MSTRG.28300.10	1.22307	coding
MSTRG.28308.1	1.30752	coding
MSTRG.28308.6	1.22825	coding
MSTRG.28312.1	2.74438	coding

MSTRG.28312.3	2.6256	coding
MSTRG.28312.2	2.67867	coding
MSTRG.28312.4	2.72464	coding
MSTRG.28315.1	-2.45425	noncoding
MSTRG.28316.8	1.34815	coding
MSTRG.28319.1	0.484974	coding
MSTRG.28319.2	0.483331	coding
MSTRG.28319.4	0.780286	coding
MSTRG.28319.6	0.7531	coding
MSTRG.28321.1	1.23445	coding
MSTRG.28325.2	0.806584	coding
MSTRG.28325.5	0.008221	coding
MSTRG.28324.3	1.88735	coding
MSTRG.28326.1	2.92026	coding
MSTRG.28328.2	-0.559395	noncoding
MSTRG.28329.3	1.498	coding
MSTRG.28332.2	-1.1541	noncoding
MSTRG.28300.13	-0.291654	noncoding
MSTRG.28266.4	1.40164	coding
MSTRG.28266.5	1.02049	coding
MSTRG.28266.7	-0.754653	noncoding
MSTRG.28267.1	-0.935831	noncoding
MSTRG.28267.2	-1.20774	noncoding
MSTRG.28266.17	-1.24298	noncoding
MSTRG.28274.1	0.589311	coding
MSTRG.28274.2	0.224884	coding
MSTRG.28270.1	1.34306	coding
MSTRG.28271.1	-0.094467	noncoding
MSTRG.28281.2	2.16861	coding
MSTRG.28281.3	2.31194	coding
MSTRG.28281.4	2.50095	coding
MSTRG.28281.5	2.28822	coding
MSTRG.28281.6	2.30065	coding
MSTRG.28281.7	2.67709	coding
MSTRG.28281.8	2.37871	coding
MSTRG.28281.9	0.711373	coding
MSTRG.28281.10	1.71629	coding
MSTRG.28294.2	1.15417	coding
MSTRG.28297.3	-1.06021	noncoding
MSTRG.28335.1	2.64663	coding
MSTRG.28336.1	-2.5709	noncoding
MSTRG.28335.2	2.04538	coding
MSTRG.28335.3	1.83867	coding
MSTRG.28338.1	1.55175	coding
MSTRG.28339.3	0.983766	coding
MSTRG.28341.2	0.711037	coding
MSTRG.28341.4	1.54376	coding
MSTRG.28342.3	1.75773	coding
MSTRG.28342.4	1.22899	coding
MSTRG.28343.1	-1.58068	noncoding
MSTRG.28367.1	0.741761	coding
MSTRG.28367.2	0.578904	coding
MSTRG.28368.2	0.722653	coding
MSTRG.28370.1	-0.02295	noncoding

MSTRG.28371.1	-0.714809	noncoding
MSTRG.28372.1	-0.470557	noncoding
MSTRG.28375.1	-1.15091	noncoding
MSTRG.28377.1	0.685724	coding
MSTRG.28371.2	0.4593	coding
MSTRG.28371.3	0.369468	coding
MSTRG.28378.1	0.156667	coding
MSTRG.28379.1	1.77316	coding
MSTRG.28380.1	0.71019	coding
MSTRG.28386.3	1.22074	coding
MSTRG.28386.2	1.07869	coding
MSTRG.28388.1	0.329651	coding
MSTRG.28386.4	0.265108	coding
MSTRG.28388.2	0.619909	coding
MSTRG.28389.1	1.39824	coding
MSTRG.28379.2	0.439392	coding
MSTRG.28388.3	1.21503	coding
MSTRG.28371.4	0.964829	coding
MSTRG.28359.7	-0.667977	noncoding
MSTRG.28359.6	0.128978	coding
MSTRG.28359.3	0.886139	coding
MSTRG.28359.5	1.09511	coding
MSTRG.28359.8	1.09246	coding
MSTRG.28359.9	0.795715	coding
MSTRG.28359.10	-0.305355	noncoding
MSTRG.28361.1	0.113146	coding
MSTRG.28362.1	-0.160036	noncoding
MSTRG.28359.11	0.281504	coding
MSTRG.28363.1	0.384547	coding
MSTRG.28363.2	0.071491	coding
MSTRG.28363.3	0.414748	coding
MSTRG.28366.4	0.753227	coding
MSTRG.28366.5	1.55617	coding
MSTRG.28366.6	0.605839	coding
MSTRG.28393.1	1.89607	coding
MSTRG.28393.3	2.2641	coding
MSTRG.28393.4	2.17045	coding
MSTRG.28393.6	2.04848	coding
MSTRG.28393.2	2.10024	coding
MSTRG.28397.1	0.366986	coding
MSTRG.28399.2	0.606333	coding
MSTRG.28400.1	1.12991	coding
MSTRG.28400.2	1.19577	coding
MSTRG.28400.3	1.01318	coding
MSTRG.28401.1	-1.33871	noncoding
MSTRG.28391.2	0.576095	coding
MSTRG.28403.1	-2.44784	noncoding
MSTRG.28404.1	0.193203	coding
MSTRG.28409.2	-0.442989	noncoding
MSTRG.28409.1	0.01376	coding
MSTRG.28409.3	-1.73544	noncoding
MSTRG.28409.4	-1.27438	noncoding
MSTRG.28409.6	-1.26845	noncoding
MSTRG.28409.5	-0.172474	noncoding

MSTRG.28409.7	-0.551481	noncoding
MSTRG.28409.8	-0.666142	noncoding
MSTRG.28410.1	-1.0356	noncoding
MSTRG.28409.9	0.612355	coding
MSTRG.28409.10	-0.752928	noncoding
MSTRG.28411.1	-0.864651	noncoding
MSTRG.28409.11	-1.04958	noncoding
MSTRG.28449.3	0.22928	coding
MSTRG.28457.1	1.19219	coding
MSTRG.28457.2	0.839541	coding
MSTRG.28457.5	0.929246	coding
MSTRG.28457.6	1.68901	coding
MSTRG.28459.1	-0.126987	noncoding
MSTRG.28460.1	0.731134	coding
MSTRG.28463.1	-0.608682	noncoding
MSTRG.28464.1	0.894579	coding
MSTRG.28466.1	-0.489577	noncoding
MSTRG.28467.1	0.337599	coding
MSTRG.28470.1	-0.15191	noncoding
MSTRG.28470.2	-0.189735	noncoding
MSTRG.28471.2	0.776591	coding
MSTRG.28471.3	0.392057	coding
MSTRG.28474.1	0.810569	coding
MSTRG.28471.4	0.560369	coding
MSTRG.28476.2	1.46281	coding
MSTRG.28430.1	-0.694597	noncoding
MSTRG.28430.2	-0.180383	noncoding
MSTRG.28432.1	0.418116	coding
MSTRG.28433.1	-2.27164	noncoding
MSTRG.28439.1	2.73521	coding
MSTRG.28443.1	-2.00421	noncoding
MSTRG.28442.2	1.56474	coding
MSTRG.28445.1	1.88967	coding
MSTRG.28480.2	-1.7945	noncoding
MSTRG.28480.1	-1.14038	noncoding
MSTRG.28480.3	-0.362157	noncoding
MSTRG.28481.3	1.77262	coding
MSTRG.28485.1	0.017737	coding
MSTRG.28485.2	-0.036176	noncoding
MSTRG.28485.3	0.658048	coding
MSTRG.28485.4	0.249631	coding
MSTRG.28485.5	0.091574	coding
MSTRG.28485.6	-0.853682	noncoding
MSTRG.28487.2	0.180971	coding
MSTRG.28487.3	0.265774	coding
MSTRG.28488.1	2.25688	coding
MSTRG.28488.2	2.45057	coding
MSTRG.28488.3	2.44553	coding
MSTRG.28488.4	2.54054	coding
MSTRG.28488.5	1.79976	coding
MSTRG.28488.7	0.849517	coding
MSTRG.28489.1	-0.036324	noncoding
MSTRG.28488.8	0.068278	coding
MSTRG.28488.9	-0.146545	noncoding

MSTRG.28488.10	-0.065263	noncoding
MSTRG.28488.11	0.120291	coding
MSTRG.28491.1	-0.808323	noncoding
MSTRG.28492.1	-2.08403	noncoding
MSTRG.28495.1	-0.799334	noncoding
MSTRG.28495.2	-0.625665	noncoding
MSTRG.28499.3	1.36477	coding
MSTRG.28501.3	0.929667	coding
MSTRG.28501.4	0.740237	coding
MSTRG.28502.1	-2.00946	noncoding
MSTRG.28505.3	0.717302	coding
MSTRG.28509.4	-0.006291	noncoding
MSTRG.28509.5	0.092121	coding
MSTRG.28509.2	0.345114	coding
MSTRG.28509.3	0.314992	coding
MSTRG.28510.1	-1.21893	noncoding
MSTRG.28514.5	1.35019	coding
MSTRG.28515.1	0.411557	coding
MSTRG.28515.2	-0.308341	noncoding
MSTRG.28515.3	0.013208	coding
MSTRG.28517.1	-2.10316	noncoding
MSTRG.28517.2	-2.53141	noncoding
MSTRG.28517.3	-1.81651	noncoding
MSTRG.28517.4	-1.68738	noncoding
MSTRG.28517.5	-1.87986	noncoding
MSTRG.28520.2	0.017247	coding
MSTRG.28522.2	-0.156749	noncoding
MSTRG.28522.3	0.07741	coding
MSTRG.28522.4	0.028889	coding
MSTRG.28522.6	0.125991	coding
MSTRG.28522.8	-0.038026	noncoding
MSTRG.28525.2	0.78085	coding
MSTRG.28525.3	0.184268	coding
MSTRG.28532.3	1.49104	coding
MSTRG.28532.5	1.00665	coding
MSTRG.28533.1	-1.70446	noncoding
MSTRG.28538.2	3.3725	coding
MSTRG.28546.1	1.14997	coding
MSTRG.28546.2	0.033621	coding
MSTRG.28546.3	0.439492	coding
MSTRG.28546.4	0.255257	coding
MSTRG.28546.12	0.996395	coding
MSTRG.28546.10	0.300945	coding
MSTRG.28546.8	-0.021813	noncoding
MSTRG.28539.2	2.34178	coding
MSTRG.28539.3	2.37149	coding
MSTRG.28539.5	2.28649	coding
MSTRG.28539.6	2.2092	coding
MSTRG.28539.7	2.2186	coding
MSTRG.28539.8	2.35815	coding
MSTRG.28550.3	-1.26282	noncoding
MSTRG.28551.1	-0.505012	noncoding
MSTRG.28552.1	0.519425	coding
MSTRG.28553.1	-0.312759	noncoding

MSTRG.28554.1	-0.227541	noncoding
MSTRG.28555.2	-0.853846	noncoding
MSTRG.28555.3	-1.32135	noncoding
MSTRG.28556.1	-0.694516	noncoding
MSTRG.28557.1	-1.27063	noncoding
MSTRG.28555.4	-1.83827	noncoding
MSTRG.28559.1	0.000024	coding
MSTRG.28561.3	1.3921	coding
MSTRG.28563.1	2.09567	coding
MSTRG.28563.5	1.71021	coding
MSTRG.28564.1	-0.054877	noncoding
MSTRG.28565.1	-0.008579	noncoding
MSTRG.28566.1	-0.902486	noncoding
MSTRG.28567.1	-0.529643	noncoding
MSTRG.28568.1	-0.918451	noncoding
MSTRG.28571.2	-0.32631	noncoding
MSTRG.28571.3	0.066984	coding
MSTRG.28572.1	-0.394052	noncoding
MSTRG.28579.2	1.96394	coding
MSTRG.28586.1	2.16387	coding
MSTRG.28586.3	1.69143	coding
MSTRG.28586.4	1.83511	coding
MSTRG.28586.5	1.83159	coding
MSTRG.28587.2	0.124616	coding
MSTRG.28587.3	0.298818	coding
MSTRG.28588.1	0.845449	coding
MSTRG.28590.1	-2.4499	noncoding
MSTRG.28588.2	0.023859	coding
MSTRG.28594.2	-0.072399	noncoding
MSTRG.28581.2	1.19334	coding
MSTRG.28585.1	-0.563715	noncoding
MSTRG.28595.1	-1.92604	noncoding
MSTRG.28600.2	0.705646	coding
MSTRG.28600.3	0.580858	coding
MSTRG.28600.6	0.433519	coding
MSTRG.28600.5	1.42094	coding
MSTRG.28600.4	0.89515	coding
MSTRG.28600.7	1.5654	coding
MSTRG.28600.8	0.626154	coding
MSTRG.28600.9	1.25901	coding
MSTRG.28602.1	-2.19471	noncoding
MSTRG.28603.1	0.467971	coding
MSTRG.28604.1	-1.29882	noncoding
MSTRG.28605.1	-1.45081	noncoding
MSTRG.28606.1	-0.439076	noncoding
MSTRG.28597.1	-0.480008	noncoding
MSTRG.28613.7	-0.095055	noncoding
MSTRG.28613.6	0.469898	coding
MSTRG.28613.8	0.442077	coding
MSTRG.28613.9	0.472281	coding
MSTRG.28613.10	-0.799372	noncoding
MSTRG.28614.1	-0.715604	noncoding
MSTRG.28609.1	-2.17027	noncoding
MSTRG.28618.1	-2.16514	noncoding

MSTRG.28620.4	1.48482	coding
MSTRG.28620.3	1.57977	coding
MSTRG.28623.2	0.133768	coding
MSTRG.28625.1	0.931546	coding
MSTRG.28625.2	1.6584	coding
MSTRG.28625.3	0.707121	coding
MSTRG.28625.4	0.785175	coding
MSTRG.28627.4	1.43801	coding
MSTRG.28627.3	1.41632	coding
MSTRG.28627.5	1.40626	coding
MSTRG.28628.1	-0.351058	noncoding
MSTRG.28631.2	-0.989975	noncoding
MSTRG.28631.3	0.840381	coding
MSTRG.28633.1	-0.965509	noncoding
MSTRG.28642.2	1.1746	coding
MSTRG.28642.8	1.61289	coding
MSTRG.28642.7	1.54367	coding
MSTRG.28642.5	1.36588	coding
MSTRG.28642.6	1.56604	coding
MSTRG.28642.3	1.3551	coding
MSTRG.28638.1	-2.21857	noncoding
MSTRG.28638.2	-2.39798	noncoding
MSTRG.28644.1	0.27912	coding
MSTRG.28645.2	1.1891	coding
MSTRG.28646.3	-1.37354	noncoding
MSTRG.28646.4	-1.50407	noncoding
MSTRG.28646.5	-1.29514	noncoding
MSTRG.28647.1	0.355821	coding
MSTRG.28648.1	-1.33754	noncoding
MSTRG.28648.2	-1.70298	noncoding
MSTRG.28648.3	-2.4854	noncoding
MSTRG.28650.1	-0.380224	noncoding
MSTRG.28650.3	-1.6424	noncoding
MSTRG.28650.2	-1.15501	noncoding
MSTRG.28652.1	-2.0773	noncoding
MSTRG.28653.1	3.86829	coding
MSTRG.28654.1	0.451604	coding
MSTRG.28656.1	-2.22299	noncoding
MSTRG.28657.3	1.23623	coding
MSTRG.28658.1	-0.591125	noncoding
MSTRG.28662.4	0.845756	coding
MSTRG.28662.3	0.878672	coding
MSTRG.28661.1	-1.07365	noncoding
MSTRG.28661.2	-1.70861	noncoding
MSTRG.28663.1	1.37526	coding
MSTRG.28663.2	1.55473	coding
MSTRG.28669.2	-0.644473	noncoding
MSTRG.28672.1	-1.99714	noncoding
MSTRG.28672.2	-1.72686	noncoding
MSTRG.28672.3	-1.5019	noncoding
MSTRG.28677.1	-1.23802	noncoding
MSTRG.28677.2	-2.19646	noncoding
MSTRG.28682.2	2.51485	coding
MSTRG.28688.2	1.40431	coding

MSTRG.28693.1	-1.93818	noncoding
MSTRG.28702.1	-2.10071	noncoding
MSTRG.28705.1	-1.62617	noncoding
MSTRG.28707.1	-0.027493	noncoding
MSTRG.28712.2	-1.14213	noncoding
MSTRG.28712.3	-1.33081	noncoding
MSTRG.28714.1	-2.14879	noncoding
MSTRG.28716.1	-0.744157	noncoding
MSTRG.28718.2	1.29537	coding
MSTRG.28715.1	-2.28755	noncoding
MSTRG.28720.1	-1.31137	noncoding
MSTRG.28731.1	-0.716838	noncoding
MSTRG.28732.1	-1.75506	noncoding
MSTRG.28742.4	1.74987	coding
MSTRG.28743.1	-0.850943	noncoding
MSTRG.28744.1	-0.067001	noncoding
MSTRG.28745.1	0.54321	coding
MSTRG.28745.3	0.829852	coding
MSTRG.28745.5	0.520744	coding
MSTRG.28746.1	0.849231	coding
MSTRG.28746.3	1.21242	coding
MSTRG.28746.4	-0.037221	noncoding
MSTRG.28746.5	-0.445064	noncoding
MSTRG.28737.2	1.50872	coding
MSTRG.28737.3	1.17053	coding
MSTRG.28739.3	1.16338	coding
MSTRG.28739.4	0.603319	coding
MSTRG.28739.5	0.570612	coding
MSTRG.28739.6	-0.330332	noncoding
MSTRG.28740.1	-0.199068	noncoding
MSTRG.28755.1	-1.40468	noncoding
MSTRG.28765.1	1.77272	coding
MSTRG.28770.1	0.970944	coding
MSTRG.28770.3	1.09488	coding
MSTRG.28780.1	2.51722	coding
MSTRG.28780.3	2.94334	coding
MSTRG.28784.1	1.4177	coding
MSTRG.28784.2	1.29091	coding
MSTRG.28783.1	0.855654	coding
MSTRG.28790.1	-2.70992	noncoding
MSTRG.28790.2	-2.76826	noncoding
MSTRG.28794.1	-2.12489	noncoding
MSTRG.28799.2	2.11927	coding
MSTRG.28822.1	-0.741961	noncoding
MSTRG.28830.1	-1.92036	noncoding
MSTRG.28839.1	1.95559	coding
MSTRG.28839.2	1.94742	coding
MSTRG.28839.4	-0.076695	noncoding
MSTRG.28842.1	-0.24677	noncoding
MSTRG.28844.6	1.51339	coding
MSTRG.28844.5	1.83284	coding
MSTRG.28846.3	2.15901	coding
MSTRG.28853.2	2.44494	coding
MSTRG.28853.3	1.39256	coding

MSTRG.28853.5	2.43861	coding
MSTRG.28853.6	1.14902	coding
MSTRG.28854.1	-2.88387	noncoding
MSTRG.28853.10	-0.63052	noncoding
MSTRG.28853.9	2.6187	coding
MSTRG.28856.1	-0.472958	noncoding
MSTRG.28857.6	1.21017	coding
MSTRG.28857.5	1.07386	coding
MSTRG.28857.8	1.5132	coding
MSTRG.28857.7	1.146	coding
MSTRG.28858.2	2.07245	coding
MSTRG.28859.1	-0.415299	noncoding
MSTRG.28862.2	1.74799	coding
MSTRG.28862.3	1.42922	coding
MSTRG.28862.4	1.50014	coding
MSTRG.28862.5	1.75024	coding
MSTRG.28862.6	1.72415	coding
MSTRG.28862.7	0.336545	coding
MSTRG.28860.2	-0.075094	noncoding
MSTRG.28866.2	2.50456	coding
MSTRG.28866.3	-0.345477	noncoding
MSTRG.28869.1	-1.99512	noncoding
MSTRG.28879.1	0.480005	coding
MSTRG.28879.4	-0.544387	noncoding
MSTRG.28879.3	-0.415113	noncoding
MSTRG.28879.5	-0.253343	noncoding
MSTRG.28880.1	0.561556	coding
MSTRG.28880.3	1.32887	coding
MSTRG.28880.2	2.23474	coding
MSTRG.28880.4	0.62628	coding
MSTRG.28882.1	-0.087132	noncoding
MSTRG.28883.1	0.38996	coding
MSTRG.28884.1	-1.63621	noncoding
MSTRG.28885.2	-0.428098	noncoding
MSTRG.28885.3	-0.374156	noncoding
MSTRG.28886.2	1.22118	coding
MSTRG.28887.1	1.88783	coding
MSTRG.28887.2	1.56283	coding
MSTRG.28887.4	1.95741	coding
MSTRG.28888.1	2.0196	coding
MSTRG.28873.1	-0.431293	noncoding
MSTRG.28876.1	1.20126	coding
MSTRG.28890.1	2.14116	coding
MSTRG.28890.5	2.20451	coding
MSTRG.28890.6	2.34571	coding
MSTRG.28890.4	1.41599	coding
MSTRG.28890.3	1.49101	coding
MSTRG.28893.1	0.731585	coding
MSTRG.28892.2	1.038	coding
MSTRG.28894.1	-1.36363	noncoding
MSTRG.28894.2	-2.14695	noncoding
MSTRG.28900.3	0.904882	coding
MSTRG.28905.2	1.50996	coding
MSTRG.28907.1	0.924339	coding

MSTRG.28908.1	0.375473	coding
MSTRG.28914.1	1.33299	coding
MSTRG.28914.2	1.60292	coding
MSTRG.28914.3	1.64291	coding
MSTRG.28917.1	-0.45626	noncoding
MSTRG.28920.1	-0.47853	noncoding
MSTRG.28923.4	1.84863	coding
MSTRG.28923.3	1.8395	coding
MSTRG.28923.5	1.76178	coding
MSTRG.28923.7	1.80731	coding
MSTRG.28923.6	1.66189	coding
MSTRG.28923.8	1.75933	coding
MSTRG.28934.1	0.493296	coding
MSTRG.28935.1	0.407222	coding
MSTRG.28936.1	0.216826	coding
MSTRG.28937.1	-0.917229	noncoding
MSTRG.28933.3	-0.469826	noncoding
MSTRG.28941.1	-1.30764	noncoding
MSTRG.28942.1	-0.089369	noncoding
MSTRG.28940.4	0.494376	coding
MSTRG.28949.2	1.08405	coding
MSTRG.28949.4	1.00098	coding
MSTRG.28951.2	1.24381	coding
MSTRG.28951.1	2.60081	coding
MSTRG.28951.6	1.46255	coding
MSTRG.28951.5	2.06454	coding
MSTRG.28952.1	-0.744403	noncoding
MSTRG.28953.1	0.065167	coding
MSTRG.28947.1	2.56002	coding
MSTRG.28961.6	1.68393	coding
MSTRG.28961.8	1.6388	coding
MSTRG.28961.4	1.72418	coding
MSTRG.28961.5	1.77758	coding
MSTRG.28961.3	1.57592	coding
MSTRG.28961.7	1.68291	coding
MSTRG.28961.10	1.57414	coding
MSTRG.28961.12	1.6704	coding
MSTRG.28961.9	1.50031	coding
MSTRG.28961.13	-0.898462	noncoding
MSTRG.28961.14	-2.32251	noncoding
MSTRG.28961.15	-2.32232	noncoding
MSTRG.28961.16	-2.32184	noncoding
MSTRG.28963.1	1.19111	coding
MSTRG.28967.1	-0.63494	noncoding
MSTRG.28975.1	-2.58895	noncoding
MSTRG.28980.2	-0.337259	noncoding
MSTRG.28976.2	0.69449	coding
MSTRG.28976.3	0.575724	coding
MSTRG.28977.1	-2.15169	noncoding
MSTRG.28976.4	-0.344546	noncoding
MSTRG.28987.2	0.569076	coding
MSTRG.28988.2	1.30002	coding
MSTRG.28988.3	1.77468	coding
MSTRG.28990.1	0.574746	coding

MSTRG.28988.4	-0.519821	noncoding
MSTRG.28988.5	1.36506	coding
MSTRG.28988.6	1.39179	coding
MSTRG.28988.8	1.91482	coding
MSTRG.28988.9	1.90288	coding
MSTRG.28988.10	1.72779	coding
MSTRG.28988.11	2.26268	coding
MSTRG.28988.12	1.84091	coding
MSTRG.28993.2	1.30576	coding
MSTRG.28993.3	2.03322	coding
MSTRG.29016.1	-1.30216	noncoding
MSTRG.29017.8	1.29063	coding
MSTRG.29017.9	1.8111	coding
MSTRG.29017.10	1.11884	coding
MSTRG.29017.12	0.825413	coding
MSTRG.29017.13	0.80286	coding
MSTRG.29017.11	1.94348	coding
MSTRG.29000.1	-0.095205	noncoding
MSTRG.29008.1	0.264549	coding
MSTRG.29009.1	0.17486	coding
MSTRG.29013.3	0.787474	coding
MSTRG.28996.1	0.109217	coding
MSTRG.29020.2	1.55759	coding
MSTRG.29020.3	0.625957	coding
MSTRG.29021.1	0.17022	coding
MSTRG.29022.1	-1.9937	noncoding
MSTRG.29019.1	-1.55175	noncoding
MSTRG.29024.2	0.267204	coding
MSTRG.29024.4	0.226839	coding
MSTRG.29028.3	1.98741	coding
MSTRG.29026.3	0.561161	coding
MSTRG.29027.1	-0.693387	noncoding
MSTRG.29029.1	0.525861	coding
MSTRG.29034.1	-0.493349	noncoding
MSTRG.29035.1	1.86305	coding
MSTRG.29037.1	-1.27029	noncoding
MSTRG.29041.1	0.549107	coding
MSTRG.29041.5	1.81907	coding
MSTRG.29041.4	1.64649	coding
MSTRG.29041.7	1.96445	coding
MSTRG.29043.1	0.391504	coding
MSTRG.29043.2	1.23151	coding
MSTRG.29043.3	1.55101	coding
MSTRG.29045.1	1.50997	coding
MSTRG.29049.4	1.2817	coding
MSTRG.29051.2	-1.54322	noncoding
MSTRG.29054.4	2.08433	coding
MSTRG.29054.5	1.46687	coding
MSTRG.29055.1	0.339124	coding
MSTRG.29064.3	1.01312	coding
MSTRG.29064.4	0.933443	coding
MSTRG.29064.6	0.813459	coding
MSTRG.29065.1	0.074783	coding
MSTRG.29065.2	-0.933175	noncoding

MSTRG.29068.1	-0.557927	noncoding
MSTRG.29068.3	-0.262431	noncoding
MSTRG.29079.2	-1.37745	noncoding
MSTRG.29079.1	2.86185	coding
MSTRG.29079.4	-1.55776	noncoding
MSTRG.29079.3	2.89663	coding
MSTRG.29079.5	2.85921	coding
MSTRG.29080.1	0.015645	coding
MSTRG.29086.2	0.804816	coding
MSTRG.29086.3	0.784016	coding
MSTRG.29082.3	1.82698	coding
MSTRG.29082.4	2.02076	coding
MSTRG.29083.1	-0.753573	noncoding
MSTRG.29085.1	0.878366	coding
MSTRG.29092.2	1.3848	coding
MSTRG.29092.6	1.53353	coding
MSTRG.29092.7	1.1316	coding
MSTRG.29092.5	1.35284	coding
MSTRG.29093.4	0.87203	coding
MSTRG.29094.1	-1.84091	noncoding
MSTRG.29099.2	1.3208	coding
MSTRG.29101.1	-0.265349	noncoding
MSTRG.29101.2	-0.265033	noncoding
MSTRG.29107.1	-2.07626	noncoding
MSTRG.29108.1	-2.18454	noncoding
MSTRG.29111.3	1.59583	coding
MSTRG.29111.4	1.66115	coding
MSTRG.29111.2	1.50751	coding
MSTRG.29120.3	0.22215	coding
MSTRG.29120.4	0.346521	coding
MSTRG.29121.1	1.67897	coding
MSTRG.29121.3	1.53417	coding
MSTRG.29113.1	-1.00213	noncoding
MSTRG.29115.1	-3.04341	noncoding
MSTRG.29128.1	0.601827	coding
MSTRG.29129.1	-1.09274	noncoding
MSTRG.29129.2	-0.990739	noncoding
MSTRG.29130.1	0.256679	coding
MSTRG.29122.1	-1.64453	noncoding
MSTRG.29126.1	-2.22468	noncoding
MSTRG.29133.1	-0.594859	noncoding
MSTRG.29136.2	2.35053	coding
MSTRG.29136.5	1.90279	coding
MSTRG.29136.1	2.0203	coding
MSTRG.29137.1	-1.77212	noncoding
MSTRG.29155.4	1.45614	coding
MSTRG.29155.6	1.53523	coding
MSTRG.29155.1	1.57322	coding
MSTRG.29155.8	1.62044	coding
MSTRG.29155.11	1.25253	coding
MSTRG.29155.15	0.80926	coding
MSTRG.29155.16	1.37332	coding
MSTRG.29155.17	1.40702	coding
MSTRG.29155.19	0.346108	coding

MSTRG.29157.1	0.39692	coding
MSTRG.29155.22	0.310789	coding
MSTRG.29160.1	0.628651	coding
MSTRG.29161.1	-1.78998	noncoding
MSTRG.29142.1	-0.175012	noncoding
MSTRG.29143.1	0.240994	coding
MSTRG.29144.3	1.87363	coding
MSTRG.29149.2	1.74195	coding
MSTRG.29149.8	1.62206	coding
MSTRG.29149.9	1.4913	coding
MSTRG.29149.10	0.50811	coding
MSTRG.29149.11	0.578925	coding
MSTRG.29150.1	2.49743	coding
MSTRG.29150.2	2.41631	coding
MSTRG.29150.3	2.32427	coding
MSTRG.29150.4	2.24067	coding
MSTRG.29150.5	2.10988	coding
MSTRG.29150.6	2.33793	coding
MSTRG.29150.7	2.68591	coding
MSTRG.29150.8	2.49669	coding
MSTRG.29165.3	0.637319	coding
MSTRG.29162.1	-1.79236	noncoding
MSTRG.29162.2	-2.09614	noncoding
MSTRG.29163.1	-0.983398	noncoding
MSTRG.29172.1	-2.28146	noncoding
MSTRG.29191.1	-1.33449	noncoding
MSTRG.29192.1	-0.114528	noncoding
MSTRG.29195.1	-0.078267	noncoding
MSTRG.29195.2	-0.451143	noncoding
MSTRG.29195.3	-1.25203	noncoding
MSTRG.29195.4	-1.31593	noncoding
MSTRG.29201.1	-2.59612	noncoding
MSTRG.29202.4	2.54175	coding
MSTRG.29202.6	0.849665	coding
MSTRG.29202.7	1.78474	coding
MSTRG.29202.8	-0.740064	noncoding
MSTRG.29202.9	0.106428	coding
MSTRG.29196.1	-0.870944	noncoding
MSTRG.29198.1	1.3091	coding
MSTRG.29198.2	1.3677	coding
MSTRG.29198.4	-0.058003	noncoding
MSTRG.29207.2	2.09656	coding
MSTRG.29207.3	2.11721	coding
MSTRG.29208.1	-2.16315	noncoding
MSTRG.29213.1	-2.32636	noncoding
MSTRG.29214.1	0.176606	coding
MSTRG.29215.1	0.16349	coding
MSTRG.29219.1	-1.40039	noncoding
MSTRG.29231.1	-0.925157	noncoding
MSTRG.29231.2	-1.10468	noncoding
MSTRG.29229.16	-0.876513	noncoding
MSTRG.29232.1	-0.364378	noncoding
MSTRG.29232.2	-1.30178	noncoding
MSTRG.29221.2	-0.157782	noncoding

MSTRG.29224.2	0.584997	coding
MSTRG.29226.2	-0.299265	noncoding
MSTRG.29226.3	-0.299265	noncoding
MSTRG.29233.3	0.069794	coding
MSTRG.29238.2	0.359521	coding
MSTRG.29239.3	1.68662	coding
MSTRG.29240.1	0.173953	coding
MSTRG.29242.2	1.88412	coding
MSTRG.29242.3	2.00753	coding
MSTRG.29242.5	1.75612	coding
MSTRG.29242.6	1.28444	coding
MSTRG.29246.3	1.86252	coding
MSTRG.29257.4	-0.082338	noncoding
MSTRG.29257.2	-0.731139	noncoding
MSTRG.29258.1	-0.598683	noncoding
MSTRG.29258.2	-0.439104	noncoding
MSTRG.29258.4	-0.517849	noncoding
MSTRG.29258.5	-0.53677	noncoding
MSTRG.29258.6	-1.19451	noncoding
MSTRG.29258.9	-0.477384	noncoding
MSTRG.29260.1	-1.18626	noncoding
MSTRG.29262.2	-0.027088	noncoding
MSTRG.29254.1	0.825157	coding
MSTRG.29255.4	0.35658	coding
MSTRG.29268.1	-0.919646	noncoding
MSTRG.29271.3	0.412468	coding
MSTRG.29271.5	0.629047	coding
MSTRG.29276.1	2.16693	coding
MSTRG.29276.3	2.12985	coding
MSTRG.29278.1	1.09169	coding
MSTRG.29278.2	0.736054	coding
MSTRG.29278.3	0.755528	coding
MSTRG.29279.2	-0.166651	noncoding
MSTRG.29281.3	1.3196	coding
MSTRG.29282.2	0.669798	coding
MSTRG.29283.2	-1.10724	noncoding
MSTRG.29283.3	-0.898929	noncoding
MSTRG.29283.4	-1.13359	noncoding
MSTRG.29283.5	-1.22845	noncoding
MSTRG.29301.1	-1.52556	noncoding
MSTRG.29302.3	2.23397	coding
MSTRG.29302.1	2.82289	coding
MSTRG.29305.2	1.59082	coding
MSTRG.29305.3	1.1173	coding
MSTRG.29307.3	0.308167	coding
MSTRG.29308.4	1.60215	coding
MSTRG.29308.5	1.1895	coding
MSTRG.29308.6	1.21668	coding
MSTRG.29308.7	1.18247	coding
MSTRG.29293.2	0.936083	coding
MSTRG.29298.1	0.425734	coding
MSTRG.29325.2	1.21738	coding
MSTRG.29328.7	-0.369426	noncoding
MSTRG.29328.9	-0.437406	noncoding

MSTRG.29328.8	1.16683	coding
MSTRG.29333.2	2.43366	coding
MSTRG.29334.1	1.97778	coding
MSTRG.29334.5	-1.00775	noncoding
MSTRG.29341.3	1.24688	coding
MSTRG.29342.1	1.12965	coding
MSTRG.29342.2	0.89023	coding
MSTRG.29343.1	0.373787	coding
MSTRG.29342.4	0.487554	coding
MSTRG.29356.21	-1.04407	noncoding
MSTRG.29356.18	-0.475595	noncoding
MSTRG.29356.9	-0.641779	noncoding
MSTRG.29356.12	-0.292206	noncoding
MSTRG.29356.13	-0.605733	noncoding
MSTRG.29356.14	-0.595977	noncoding
MSTRG.29356.16	-0.556553	noncoding
MSTRG.29356.5	-0.563	noncoding
MSTRG.29356.6	-0.652813	noncoding
MSTRG.29356.8	-0.588192	noncoding
MSTRG.29356.15	-0.587051	noncoding
MSTRG.29356.19	-0.599056	noncoding
MSTRG.29356.22	-0.368929	noncoding
MSTRG.29356.23	-0.630341	noncoding
MSTRG.29356.24	-0.343622	noncoding
MSTRG.29347.1	1.37615	coding
MSTRG.29353.1	-1.04173	noncoding
MSTRG.29354.1	-0.516685	noncoding
MSTRG.29359.2	1.50392	coding
MSTRG.29361.1	-1.19737	noncoding
MSTRG.29363.1	-2.01045	noncoding
MSTRG.29364.1	-0.212536	noncoding
MSTRG.29367.2	-0.225318	noncoding
MSTRG.29370.2	1.43552	coding
MSTRG.29375.2	1.1088	coding
MSTRG.29376.3	0.212939	coding
MSTRG.29387.1	1.31377	coding
MSTRG.29387.6	1.50375	coding
MSTRG.29389.1	0.834431	coding
MSTRG.29388.6	1.10413	coding
MSTRG.29378.1	-1.5052	noncoding
MSTRG.29380.1	0.013479	coding
MSTRG.29379.3	2.33857	coding
MSTRG.29393.3	-0.079024	noncoding
MSTRG.29393.2	-0.002222	noncoding
MSTRG.29393.4	-0.234278	noncoding
MSTRG.29393.5	-0.177044	noncoding
MSTRG.29393.6	-0.299516	noncoding
MSTRG.29393.7	-0.091176	noncoding
MSTRG.29405.2	1.873	coding
MSTRG.29409.2	0.857567	coding
MSTRG.29409.3	0.93995	coding
MSTRG.29411.1	0.2716	coding
MSTRG.29412.1	1.84781	coding
MSTRG.29417.3	0.544024	coding

MSTRG.29417.4	0.458654	coding
MSTRG.29418.4	0.956733	coding
MSTRG.29420.2	0.637441	coding
MSTRG.29420.1	1.17938	coding
MSTRG.29425.2	1.16764	coding
MSTRG.29425.5	1.21449	coding
MSTRG.29425.6	1.17505	coding
MSTRG.29425.7	1.02153	coding
MSTRG.29426.3	1.40998	coding
MSTRG.29426.4	1.33287	coding
MSTRG.29426.5	1.40224	coding
MSTRG.29426.6	1.3412	coding
MSTRG.29395.2	1.09933	coding
MSTRG.29404.5	0.658012	coding
MSTRG.29404.3	0.545184	coding
MSTRG.29404.6	0.760793	coding
MSTRG.29404.7	0.671558	coding
MSTRG.29428.3	1.3697	coding
MSTRG.29436.3	0.31766	coding
MSTRG.29432.3	2.26093	coding
MSTRG.29445.1	0.918262	coding
MSTRG.29447.1	0.80304	coding
MSTRG.29447.2	-0.509721	noncoding
MSTRG.29449.1	-1.68368	noncoding
MSTRG.29450.1	-2.44106	noncoding
MSTRG.29447.6	1.10782	coding
MSTRG.29447.7	1.65015	coding
MSTRG.29440.2	1.37739	coding
MSTRG.29455.4	1.89083	coding
MSTRG.29455.6	2.32848	coding
MSTRG.29455.7	0.39135	coding
MSTRG.29454.1	-1.69018	noncoding
MSTRG.29458.1	-1.31151	noncoding
MSTRG.29457.2	-0.494925	noncoding
MSTRG.29463.1	0.618056	coding
MSTRG.29463.7	0.713832	coding
MSTRG.29463.6	0.045811	coding
MSTRG.29466.1	0.204011	coding
MSTRG.29466.3	0.364606	coding
MSTRG.29467.2	1.87905	coding
MSTRG.29467.3	1.43236	coding
MSTRG.29467.4	-1.31094	noncoding
MSTRG.29467.5	-1.46969	noncoding
MSTRG.29481.2	1.20053	coding
MSTRG.29482.2	-0.862345	noncoding
MSTRG.29481.3	1.3083	coding
MSTRG.29470.1	-1.61388	noncoding
MSTRG.29470.2	-1.70031	noncoding
MSTRG.29471.1	-0.146923	noncoding
MSTRG.29484.1	-2.33916	noncoding
MSTRG.29495.2	0.716394	coding
MSTRG.29495.3	1.8383	coding
MSTRG.29497.1	1.74276	coding
MSTRG.29499.4	0.763375	coding

MSTRG.29497.4	1.68053	coding
MSTRG.29497.3	1.8138	coding
MSTRG.29500.1	0.380975	coding
MSTRG.29504.3	2.28692	coding
MSTRG.29504.4	2.25353	coding
MSTRG.29504.2	2.22982	coding
MSTRG.29506.3	0.715674	coding
MSTRG.29502.2	0.687217	coding
MSTRG.29502.3	0.391687	coding
MSTRG.29502.4	1.22066	coding
MSTRG.29509.1	2.54553	coding
MSTRG.29509.2	2.51935	coding
MSTRG.29509.3	2.16325	coding
MSTRG.29511.2	1.37382	coding
MSTRG.29511.3	1.40456	coding
MSTRG.29511.4	1.43036	coding
MSTRG.29511.5	1.11811	coding
MSTRG.29513.1	-1.24622	noncoding
MSTRG.29510.1	-2.05123	noncoding
MSTRG.29512.1	-1.53121	noncoding
MSTRG.29521.2	0.322795	coding
MSTRG.29521.3	0.931501	coding
MSTRG.29521.4	2.70619	coding
MSTRG.29524.1	-1.80543	noncoding
MSTRG.29517.1	1.6676	coding
MSTRG.29525.1	0.196203	coding
MSTRG.29525.3	-0.010693	noncoding
MSTRG.29525.4	0.374477	coding
MSTRG.29526.1	-0.068789	noncoding
MSTRG.29532.5	1.32444	coding
MSTRG.29533.5	0.394385	coding
MSTRG.29533.6	0.610489	coding
MSTRG.29535.2	0.207022	coding
MSTRG.29535.3	-0.000372	noncoding
MSTRG.29535.5	0.757498	coding
MSTRG.29535.4	0.72393	coding
MSTRG.29536.1	1.30011	coding
MSTRG.29530.1	1.43243	coding
MSTRG.29530.3	0.684957	coding
MSTRG.29531.3	-0.743323	noncoding
MSTRG.29537.3	0.476464	coding
MSTRG.29537.4	0.416267	coding
MSTRG.29557.1	0.492753	coding
MSTRG.29557.2	-0.00054	noncoding
MSTRG.29557.3	0.107539	coding
MSTRG.29557.4	0.716183	coding
MSTRG.29557.5	0.90207	coding
MSTRG.29557.6	0.678514	coding
MSTRG.29557.7	0.375055	coding
MSTRG.29557.8	0.363306	coding
MSTRG.29557.10	1.22608	coding
MSTRG.29558.5	1.50475	coding
MSTRG.29558.6	-2.01321	noncoding
MSTRG.29558.7	-1.32853	noncoding

MSTRG.29559.3	0.879488	coding
MSTRG.29559.4	0.818465	coding
MSTRG.29560.1	0.437426	coding
MSTRG.29561.1	0.015102	coding
MSTRG.29565.1	2.89762	coding
MSTRG.29566.1	-1.22695	noncoding
MSTRG.29566.2	0.766676	coding
MSTRG.29567.1	1.12895	coding
MSTRG.29566.3	-0.289919	noncoding
MSTRG.29544.1	-1.15928	noncoding
MSTRG.29543.2	2.84436	coding
MSTRG.29545.1	1.12578	coding
MSTRG.29546.1	-0.424203	noncoding
MSTRG.29550.1	1.90605	coding
MSTRG.29550.2	1.66519	coding
MSTRG.29551.1	0.993576	coding
MSTRG.29552.2	1.45319	coding
MSTRG.29553.1	0.310894	coding
MSTRG.29553.2	0.206505	coding
MSTRG.29553.3	-0.249738	noncoding
MSTRG.29553.4	-0.015041	noncoding
MSTRG.29553.5	-0.182575	noncoding
MSTRG.29553.6	-0.181142	noncoding
MSTRG.29553.7	0.088627	coding
MSTRG.29554.2	0.547923	coding
MSTRG.29556.1	0.854713	coding
MSTRG.29548.2	1.60379	coding
MSTRG.29548.1	1.77975	coding
MSTRG.29547.1	-0.527289	noncoding
MSTRG.29571.1	-0.99904	noncoding
MSTRG.29576.3	1.13768	coding
MSTRG.29576.4	0.718731	coding
MSTRG.29578.3	0.23087	coding
MSTRG.29580.1	-2.09958	noncoding
MSTRG.29583.2	2.45618	coding
MSTRG.29583.4	1.82391	coding
MSTRG.29584.1	-1.53223	noncoding
MSTRG.29585.2	2.31502	coding
MSTRG.29593.2	0.920897	coding
MSTRG.29594.3	0.03964	coding
MSTRG.29596.1	-1.91653	noncoding
MSTRG.29588.2	2.28063	coding
MSTRG.29598.1	1.56933	coding
MSTRG.29600.2	1.16624	coding
MSTRG.29601.1	-0.389217	noncoding
MSTRG.29599.1	-2.01684	noncoding
MSTRG.29599.2	-1.75282	noncoding
MSTRG.29603.3	2.07629	coding
MSTRG.29607.6	-0.060755	noncoding
MSTRG.29607.11	-1.55001	noncoding
MSTRG.29607.10	0.768746	coding
MSTRG.29607.5	1.44802	coding
MSTRG.29608.1	0.495238	coding
MSTRG.29620.1	1.2634	coding

MSTRG.29624.1	-1.32904	noncoding
MSTRG.29625.1	-1.10777	noncoding
MSTRG.29625.2	-0.756717	noncoding
MSTRG.29627.1	-1.13183	noncoding
MSTRG.29633.2	-0.867355	noncoding
MSTRG.29634.2	1.95018	coding
MSTRG.29635.2	1.82283	coding
MSTRG.29652.1	-1.30712	noncoding
MSTRG.29654.1	1.32807	coding
MSTRG.29654.4	0.939893	coding
MSTRG.29654.2	0.990951	coding
MSTRG.29654.5	1.01228	coding
MSTRG.29657.1	3.34862	coding
MSTRG.29657.4	3.41302	coding
MSTRG.29657.9	2.15626	coding
MSTRG.29657.11	3.69951	coding
MSTRG.29644.1	0.376525	coding
MSTRG.29647.2	-0.062945	noncoding
MSTRG.29647.3	-0.133436	noncoding
MSTRG.29663.2	1.03953	coding
MSTRG.29664.2	0.130963	coding
MSTRG.29666.5	1.7778	coding
MSTRG.29667.1	0.453958	coding
MSTRG.29667.2	0.508967	coding
MSTRG.29667.3	0.465632	coding
MSTRG.29667.4	0.033729	coding
MSTRG.29668.3	1.09229	coding
MSTRG.29672.2	2.04023	coding
MSTRG.29673.1	-0.369952	noncoding
MSTRG.29673.3	-0.735943	noncoding
MSTRG.29658.7	-0.676952	noncoding
MSTRG.29658.5	-0.861158	noncoding
MSTRG.29675.2	2.04684	coding
MSTRG.29675.5	0.969175	coding
MSTRG.29676.2	0.106235	coding
MSTRG.29676.1	0.096707	coding
MSTRG.29676.3	-0.036732	noncoding
MSTRG.29676.4	-0.409572	noncoding
MSTRG.29676.5	-0.524867	noncoding
MSTRG.29676.6	0.05906	coding
MSTRG.29677.3	0.945911	coding
MSTRG.29677.6	0.959514	coding
MSTRG.29677.8	2.16556	coding
MSTRG.29677.5	1.90555	coding
MSTRG.29677.7	1.89018	coding
MSTRG.29677.2	1.62063	coding
MSTRG.29678.3	1.1308	coding
MSTRG.29678.2	0.86717	coding
MSTRG.29682.4	1.13543	coding
MSTRG.29687.2	1.23743	coding
MSTRG.29687.3	1.43908	coding
MSTRG.29681.1	0.674585	coding
MSTRG.29692.2	1.01678	coding
MSTRG.29692.3	0.874292	coding

MSTRG.29693.2	-1.41187	noncoding
MSTRG.29695.2	0.321633	coding
MSTRG.29696.1	1.31296	coding
MSTRG.29696.2	-0.004206	noncoding
MSTRG.29696.3	-2.01957	noncoding
MSTRG.29704.3	1.94239	coding
MSTRG.29699.3	-0.88413	noncoding
MSTRG.29700.1	-0.247856	noncoding
MSTRG.29700.2	0.201807	coding
MSTRG.29701.3	-1.92333	noncoding
MSTRG.29722.1	1.29417	coding
MSTRG.29722.2	0.010286	coding
MSTRG.29723.1	0.203358	coding
MSTRG.29723.3	-1.95713	noncoding
MSTRG.29724.1	-0.71455	noncoding
MSTRG.29725.1	-0.435053	noncoding
MSTRG.29726.1	-2.34346	noncoding
MSTRG.29724.4	0.226653	coding
MSTRG.29724.5	-0.238441	noncoding
MSTRG.29724.6	1.14893	coding
MSTRG.29727.1	-2.22893	noncoding
MSTRG.29724.7	1.10464	coding
MSTRG.29724.10	1.79747	coding
MSTRG.29724.11	1.81985	coding
MSTRG.29730.1	0.962022	coding
MSTRG.29735.6	2.25585	coding
MSTRG.29707.1	0.93753	coding
MSTRG.29710.1	-1.06991	noncoding
MSTRG.29713.2	-1.25275	noncoding
MSTRG.29715.3	0.846697	coding
MSTRG.29738.1	0.30021	coding
MSTRG.29738.2	0.265474	coding
MSTRG.29738.3	0.508253	coding
MSTRG.29739.1	0.642086	coding
MSTRG.29741.2	1.3368	coding
MSTRG.29747.1	1.22441	coding
MSTRG.29748.1	-0.447508	noncoding
MSTRG.29755.2	2.66035	coding
MSTRG.29756.5	-1.44735	noncoding
MSTRG.29757.1	0.680127	coding
MSTRG.29759.1	0.863737	coding
MSTRG.29758.1	0.911922	coding
MSTRG.29763.1	3.62003	coding
MSTRG.29763.4	1.07185	coding
MSTRG.29767.1	1.56288	coding
MSTRG.29767.2	1.16782	coding
MSTRG.29767.3	0.834073	coding
MSTRG.29767.4	0.933692	coding
MSTRG.29767.5	2.06938	coding
MSTRG.29773.2	-0.018405	noncoding
MSTRG.29774.1	0.014445	coding
MSTRG.29774.4	0.541075	coding
MSTRG.29774.10	0.07921	coding
MSTRG.29774.9	0.675208	coding

MSTRG.29774.7	0.802684	coding
MSTRG.29774.12	-0.059053	noncoding
MSTRG.29774.13	0.629206	coding
MSTRG.29774.15	0.661134	coding
MSTRG.29776.1	-0.22481	noncoding
MSTRG.29769.2	1.08298	coding
MSTRG.29849.2	1.81939	coding
MSTRG.29849.4	1.83677	coding
MSTRG.29849.3	1.96698	coding
MSTRG.29851.1	0.467501	coding
MSTRG.29852.1	-0.169121	noncoding
MSTRG.29853.1	-0.082433	noncoding
MSTRG.29854.1	-1.71571	noncoding
MSTRG.29856.1	0.182734	coding
MSTRG.29857.1	0.353149	coding
MSTRG.29858.1	0.039317	coding
MSTRG.29860.1	-0.23597	noncoding
MSTRG.29861.1	1.08992	coding
MSTRG.29861.2	1.02405	coding
MSTRG.29861.3	0.737286	coding
MSTRG.29849.5	0.231623	coding
MSTRG.29849.6	0.511288	coding
MSTRG.29849.7	0.304273	coding
MSTRG.29849.8	0.508533	coding
MSTRG.29849.9	0.684719	coding
MSTRG.29849.10	-0.274886	noncoding
MSTRG.29791.15	1.03398	coding
MSTRG.29791.16	1.24958	coding
MSTRG.29791.7	1.68179	coding
MSTRG.29791.8	2.21916	coding
MSTRG.29791.13	2.28355	coding
MSTRG.29791.10	2.04675	coding
MSTRG.29791.11	1.95155	coding
MSTRG.29791.9	2.32732	coding
MSTRG.29791.17	1.5253	coding
MSTRG.29791.18	1.1057	coding
MSTRG.29792.1	-0.75127	noncoding
MSTRG.29786.2	1.76158	coding
MSTRG.29787.1	1.20358	coding
MSTRG.29787.3	0.99742	coding
MSTRG.29795.2	1.07807	coding
MSTRG.29796.1	-0.220676	noncoding
MSTRG.29797.3	-0.123916	noncoding
MSTRG.29799.1	1.29387	coding
MSTRG.29799.2	0.174756	coding
MSTRG.29799.3	0.30919	coding
MSTRG.29810.3	1.35131	coding
MSTRG.29810.4	1.39946	coding
MSTRG.29810.7	1.36779	coding
MSTRG.29810.8	1.3597	coding
MSTRG.29810.6	1.06297	coding
MSTRG.29810.2	1.346	coding
MSTRG.29810.9	0.797249	coding
MSTRG.29800.5	-0.649547	noncoding

MSTRG.29800.6	-0.742199	noncoding
MSTRG.29801.3	0.753826	coding
MSTRG.29802.1	-0.961574	noncoding
MSTRG.29815.4	0.860315	coding
MSTRG.29815.2	0.645684	coding
MSTRG.29815.5	0.922884	coding
MSTRG.29815.6	1.4202	coding
MSTRG.29821.4	0.860965	coding
MSTRG.29821.5	0.630579	coding
MSTRG.29825.2	0.226927	coding
MSTRG.29825.3	0.369693	coding
MSTRG.29827.1	-0.166198	noncoding
MSTRG.29830.1	2.62705	coding
MSTRG.29830.2	2.65074	coding
MSTRG.29834.1	1.61503	coding
MSTRG.29835.2	-0.423707	noncoding
MSTRG.29836.2	1.35955	coding
MSTRG.29846.1	2.07298	coding
MSTRG.29846.8	1.62284	coding
MSTRG.29846.7	1.5444	coding
MSTRG.29846.9	1.55906	coding
MSTRG.29846.10	0.316786	coding
MSTRG.29846.11	2.34323	coding
MSTRG.29846.12	0.423591	coding
MSTRG.29846.13	0.333867	coding
MSTRG.29846.14	0.194811	coding
MSTRG.29847.1	-0.178452	noncoding
MSTRG.29845.1	0.862526	coding
MSTRG.29868.1	0.080549	coding
MSTRG.29869.1	0.569555	coding
MSTRG.29869.3	-0.93579	noncoding
MSTRG.29869.5	0.076089	coding
MSTRG.29869.4	-0.548448	noncoding
MSTRG.29869.6	0.157115	coding
MSTRG.29869.7	0.45201	coding
MSTRG.29866.2	-1.79787	noncoding
MSTRG.29867.1	-0.269067	noncoding
MSTRG.29867.2	-1.25858	noncoding
MSTRG.29879.4	2.30279	coding
MSTRG.29879.6	1.47396	coding
MSTRG.29879.7	1.58394	coding
MSTRG.29879.3	1.91756	coding
MSTRG.29880.2	2.75367	coding
MSTRG.29880.3	2.59418	coding
MSTRG.29881.2	1.04793	coding
MSTRG.29884.1	-0.544652	noncoding
MSTRG.29884.2	-0.539118	noncoding
MSTRG.29884.3	-0.434003	noncoding
MSTRG.29885.3	2.12477	coding
MSTRG.29890.4	-0.280968	noncoding
MSTRG.29891.1	0.687301	coding
MSTRG.29891.2	0.99129	coding
MSTRG.29891.3	2.69391	coding
MSTRG.29891.4	2.68262	coding

MSTRG.29891.6	2.19952	coding
MSTRG.29891.7	2.02402	coding
MSTRG.29894.2	0.833561	coding
MSTRG.29894.3	1.88332	coding
MSTRG.29894.4	1.48314	coding
MSTRG.29896.7	0.210604	coding
MSTRG.29896.6	-0.779984	noncoding
MSTRG.29896.4	0.351259	coding
MSTRG.29896.5	0.559964	coding
MSTRG.29897.1	-0.841419	noncoding
MSTRG.29897.2	-1.51428	noncoding
MSTRG.29897.3	-1.32277	noncoding
MSTRG.29889.2	-0.460944	noncoding
MSTRG.29900.1	1.77738	coding
MSTRG.29903.3	1.73861	coding
MSTRG.29903.1	1.73637	coding
MSTRG.29904.1	0.628049	coding
MSTRG.29902.1	-0.623391	noncoding
MSTRG.29902.2	0.343692	coding
MSTRG.29902.3	-0.032271	noncoding
MSTRG.29910.3	0.372365	coding
MSTRG.29910.2	0.477245	coding
MSTRG.29912.2	0.219785	coding
MSTRG.29912.3	-0.04378	noncoding
MSTRG.29912.4	-0.088279	noncoding
MSTRG.29912.5	-0.028185	noncoding
MSTRG.29912.6	0.179027	coding
MSTRG.29906.2	0.920132	coding
MSTRG.29906.4	0.731508	coding
MSTRG.29908.4	0.941843	coding
MSTRG.29916.1	-0.40547	noncoding
MSTRG.29917.1	0.799047	coding
MSTRG.29917.3	0.788346	coding
MSTRG.29918.1	-1.84036	noncoding
MSTRG.29920.1	0.608446	coding
MSTRG.29923.5	0.093552	coding
MSTRG.29923.6	0.529479	coding
MSTRG.29921.1	1.36471	coding
MSTRG.29921.4	2.37801	coding
MSTRG.29921.5	2.2747	coding
MSTRG.29926.3	1.82007	coding
MSTRG.29926.2	1.83079	coding
MSTRG.29927.3	0.721819	coding
MSTRG.29934.4	0.467193	coding
MSTRG.29934.5	0.63442	coding
MSTRG.29934.8	-0.437687	noncoding
MSTRG.29941.3	1.18806	coding
MSTRG.29941.4	1.43463	coding
MSTRG.29943.5	1.87646	coding
MSTRG.29943.3	3.49751	coding
MSTRG.29943.2	3.54594	coding
MSTRG.29943.4	3.5569	coding
MSTRG.29938.1	-0.593032	noncoding
MSTRG.29946.2	0.637942	coding

MSTRG.29947.1	-0.235368	noncoding
MSTRG.29949.1	0.492432	coding
MSTRG.29948.2	-0.142615	noncoding
MSTRG.29950.4	1.18234	coding
MSTRG.29950.5	0.998727	coding
MSTRG.29950.6	1.33134	coding
MSTRG.29965.1	1.52053	coding
MSTRG.29965.5	1.47183	coding
MSTRG.29967.2	1.2759	coding
MSTRG.29970.1	1.9612	coding
MSTRG.29970.2	1.70859	coding
MSTRG.29970.3	1.62599	coding
MSTRG.29970.4	1.88407	coding
MSTRG.29970.5	1.51838	coding
MSTRG.29970.7	1.64503	coding
MSTRG.29970.8	1.51666	coding
MSTRG.29970.9	1.39993	coding
MSTRG.29974.3	1.3287	coding
MSTRG.29974.2	1.56115	coding
MSTRG.29975.1	-1.02406	noncoding
MSTRG.29954.1	-2.70338	noncoding
MSTRG.29955.1	0.538811	coding
MSTRG.29957.2	1.73089	coding
MSTRG.29961.2	-0.58087	noncoding
MSTRG.29980.5	-0.840439	noncoding
MSTRG.29978.3	1.09052	coding
MSTRG.29978.1	0.549752	coding
MSTRG.29978.2	-0.323971	noncoding
MSTRG.29985.3	2.14126	coding
MSTRG.29985.4	1.74742	coding
MSTRG.29990.1	2.69863	coding
MSTRG.29990.2	2.70021	coding
MSTRG.29990.5	2.28328	coding
MSTRG.29990.6	2.72048	coding
MSTRG.29990.7	2.69791	coding
MSTRG.29990.8	2.56037	coding
MSTRG.29990.9	2.44549	coding
MSTRG.29990.10	2.52539	coding
MSTRG.29991.1	0.204023	coding
MSTRG.29991.2	-0.741764	noncoding
MSTRG.29997.1	1.20703	coding
MSTRG.29997.2	0.933396	coding
MSTRG.30003.7	1.73252	coding
MSTRG.30003.2	1.99972	coding
MSTRG.30003.5	2.09271	coding
MSTRG.30003.4	2.09858	coding
MSTRG.30003.3	2.01851	coding
MSTRG.30003.6	2.0539	coding
MSTRG.30003.8	2.13915	coding
MSTRG.30003.9	-0.444677	noncoding
MSTRG.29998.1	-2.62945	noncoding
MSTRG.29999.1	-1.4501	noncoding
MSTRG.30009.10	1.93791	coding
MSTRG.30009.11	1.90486	coding

MSTRG.30009.6	1.93703	coding
MSTRG.30009.7	2.0604	coding
MSTRG.30009.15	1.29802	coding
MSTRG.30009.8	1.26917	coding
MSTRG.30009.17	1.86642	coding
MSTRG.30009.18	2.00331	coding
MSTRG.30009.19	1.33453	coding
MSTRG.30009.20	1.9227	coding
MSTRG.30009.21	1.96796	coding
MSTRG.30009.22	1.98593	coding
MSTRG.30009.23	2.02284	coding
MSTRG.30009.24	0.931573	coding
MSTRG.30005.3	1.81499	coding
MSTRG.30010.3	1.31301	coding
MSTRG.30010.1	1.30516	coding
MSTRG.30010.2	1.28283	coding
MSTRG.30011.2	1.72392	coding
MSTRG.30016.2	1.32852	coding
MSTRG.30016.3	1.44681	coding
MSTRG.30016.5	1.92385	coding
MSTRG.30016.4	1.46893	coding
MSTRG.30018.1	-2.30532	noncoding
MSTRG.30020.2	0.650388	coding
MSTRG.30020.3	0.657175	coding
MSTRG.30013.1	-0.160792	noncoding
MSTRG.30014.1	-0.616457	noncoding
MSTRG.30014.2	-1.11547	noncoding
MSTRG.30014.3	-1.10189	noncoding
MSTRG.30014.4	-0.615328	noncoding
MSTRG.30024.3	-0.438911	noncoding
MSTRG.30033.1	-2.18243	noncoding
MSTRG.30035.2	0.848952	coding
MSTRG.30036.2	1.10563	coding
MSTRG.30036.3	0.86897	coding
MSTRG.30036.4	1.30057	coding
MSTRG.30054.11	1.27384	coding
MSTRG.30054.13	1.26126	coding
MSTRG.30054.2	1.04619	coding
MSTRG.30054.7	1.08942	coding
MSTRG.30054.8	1.93286	coding
MSTRG.30054.17	0.994804	coding
MSTRG.30054.19	0.637969	coding
MSTRG.30054.21	1.03749	coding
MSTRG.30054.20	0.148977	coding
MSTRG.30054.18	0.372328	coding
MSTRG.30040.2	1.45167	coding
MSTRG.30040.3	0.274915	coding
MSTRG.30039.1	0.949124	coding
MSTRG.30046.1	-1.39934	noncoding
MSTRG.30046.2	-0.885277	noncoding
MSTRG.30049.3	1.17576	coding
MSTRG.30049.2	1.00821	coding
MSTRG.30055.1	-0.439028	noncoding
MSTRG.30064.5	-1.37193	noncoding

MSTRG.30064.6	-1.09175	noncoding
MSTRG.30068.1	-2.22179	noncoding
MSTRG.30068.2	-1.81067	noncoding
MSTRG.30087.1	-1.91924	noncoding
MSTRG.30091.1	-1.75317	noncoding
MSTRG.30114.1	0.872843	coding
MSTRG.30114.3	1.06433	coding
MSTRG.30102.1	0.200173	coding
MSTRG.30102.2	1.49867	coding
MSTRG.30102.3	-1.89986	noncoding
MSTRG.30126.5	0.50175	coding
MSTRG.30127.1	-1.44596	noncoding
MSTRG.30128.1	0.27889	coding
MSTRG.30129.1	0.83081	coding
MSTRG.30130.1	0.786254	coding
MSTRG.30132.1	-1.00315	noncoding
MSTRG.30121.2	1.25223	coding
MSTRG.30122.3	-2.031	noncoding
MSTRG.30124.1	-1.98279	noncoding
MSTRG.30123.2	0.85532	coding
MSTRG.30138.1	0.837044	coding
MSTRG.30143.1	-1.98359	noncoding
MSTRG.30133.1	-1.72823	noncoding
MSTRG.30137.1	0.238013	coding
MSTRG.30165.3	-2.08926	noncoding
MSTRG.30164.1	-0.327288	noncoding
MSTRG.30164.2	-0.517261	noncoding
MSTRG.30175.1	-0.328867	noncoding
MSTRG.30176.1	0.185186	coding
MSTRG.30177.1	1.98986	coding
MSTRG.30177.4	1.10363	coding
MSTRG.30178.1	-0.168023	noncoding
MSTRG.30177.5	-0.364299	noncoding
MSTRG.30177.6	-0.369389	noncoding
MSTRG.30171.6	1.09352	coding
MSTRG.30174.1	-0.576641	noncoding
MSTRG.30182.1	0.602725	coding
MSTRG.30186.3	0.581612	coding
MSTRG.30190.2	3.04973	coding
MSTRG.30198.1	2.57657	coding
MSTRG.30198.3	0.412969	coding
MSTRG.30198.4	0.659983	coding
MSTRG.30199.2	1.10538	coding
MSTRG.30199.3	1.16594	coding
MSTRG.30204.1	0.311536	coding
MSTRG.30204.2	0.413955	coding
MSTRG.30189.2	0.527777	coding
MSTRG.30211.1	-1.37309	noncoding
MSTRG.30212.3	1.77675	coding
MSTRG.30212.4	2.00459	coding
MSTRG.30213.1	-0.522506	noncoding
MSTRG.30214.1	0.39272	coding
MSTRG.30215.2	3.1938	coding
MSTRG.30215.4	0.562327	coding

MSTRG.30216.1	-2.16556	noncoding
MSTRG.30217.2	1.55784	coding
MSTRG.30219.1	1.46271	coding
MSTRG.30227.1	-2.00391	noncoding
MSTRG.30227.2	-1.57803	noncoding
MSTRG.30240.2	2.08476	coding
MSTRG.30241.1	0.179831	coding
MSTRG.30245.1	-0.050975	noncoding
MSTRG.30235.1	1.09664	coding
MSTRG.30239.1	-0.708241	noncoding
MSTRG.30248.3	1.15394	coding
MSTRG.30248.2	1.27649	coding
MSTRG.30248.4	-1.92445	noncoding
MSTRG.30248.5	-2.07414	noncoding
MSTRG.30248.7	1.09887	coding
MSTRG.30248.9	1.19672	coding
MSTRG.30248.8	1.02753	coding
MSTRG.30249.2	1.09438	coding
MSTRG.30258.2	2.16111	coding
MSTRG.30258.3	1.38673	coding
MSTRG.30258.4	1.95548	coding
MSTRG.30260.1	0.802652	coding
MSTRG.30258.5	2.17963	coding
MSTRG.30252.5	1.80818	coding
MSTRG.30271.2	1.26277	coding
MSTRG.30271.3	3.25812	coding
MSTRG.30272.1	-2.56596	noncoding
MSTRG.30275.2	0.648332	coding
MSTRG.30275.6	1.34638	coding
MSTRG.30275.7	1.11014	coding
MSTRG.30275.8	0.185196	coding
MSTRG.30275.9	1.24914	coding
MSTRG.30275.12	-0.519768	noncoding
MSTRG.30278.1	-0.035955	noncoding
MSTRG.30264.3	1.18413	coding
MSTRG.30265.1	0.546347	coding
MSTRG.30281.1	2.07287	coding
MSTRG.30281.2	1.99085	coding
MSTRG.30282.2	2.10251	coding
MSTRG.30282.3	2.09962	coding
MSTRG.30287.2	-0.021592	noncoding
MSTRG.30287.4	0.212983	coding
MSTRG.30288.2	-1.11836	noncoding
MSTRG.30290.1	0.042764	coding
MSTRG.30291.5	-0.373594	noncoding
MSTRG.30291.4	-0.209463	noncoding
MSTRG.30291.6	-0.314043	noncoding
MSTRG.30283.1	-2.01607	noncoding
MSTRG.30302.5	1.22864	coding
MSTRG.30315.3	0.428163	coding
MSTRG.30315.2	0.761966	coding
MSTRG.30315.4	0.510594	coding
MSTRG.30315.5	0.439341	coding
MSTRG.30320.1	1.83556	coding

MSTRG.30321.3	0.6758	coding
MSTRG.30321.4	1.1177	coding
MSTRG.30321.5	1.14076	coding
MSTRG.30321.6	1.34662	coding
MSTRG.30321.7	0.707358	coding
MSTRG.30312.1	-1.86608	noncoding
MSTRG.30313.1	-0.402677	noncoding
MSTRG.30325.2	2.70125	coding
MSTRG.30326.1	-0.515074	noncoding
MSTRG.30326.2	-1.38346	noncoding
MSTRG.30322.3	0.519013	coding
MSTRG.30329.2	1.29572	coding
MSTRG.30331.2	1.40582	coding
MSTRG.30332.2	-0.054139	noncoding
MSTRG.30332.3	0.141473	coding
MSTRG.30343.1	1.6214	coding
MSTRG.30343.6	0.520937	coding
MSTRG.30343.4	2.06115	coding
MSTRG.30344.1	1.66171	coding
MSTRG.30345.1	-0.46191	noncoding
MSTRG.30348.1	-1.79772	noncoding
MSTRG.30350.5	0.069387	coding
MSTRG.30354.2	2.00235	coding
MSTRG.30357.2	1.75043	coding
MSTRG.30359.5	-0.16525	noncoding
MSTRG.30359.6	1.99204	coding
MSTRG.30361.7	1.82925	coding
MSTRG.30361.2	1.73441	coding
MSTRG.30361.9	1.6065	coding
MSTRG.30361.11	2.35736	coding
MSTRG.30361.5	0.876195	coding
MSTRG.30361.4	1.48715	coding
MSTRG.30361.14	2.38367	coding
MSTRG.30361.15	1.67324	coding
MSTRG.30361.12	1.73259	coding
MSTRG.30361.6	2.24572	coding
MSTRG.30361.16	0.334135	coding
MSTRG.30362.5	1.08656	coding
MSTRG.30363.8	2.48637	coding
MSTRG.30363.3	2.59484	coding
MSTRG.30363.5	2.55542	coding
MSTRG.30363.6	2.56271	coding
MSTRG.30363.2	2.59078	coding
MSTRG.30363.4	2.54535	coding
MSTRG.30363.7	2.55283	coding
MSTRG.30365.1	1.05004	coding
MSTRG.30367.1	0.151898	coding
MSTRG.30367.4	0.479981	coding
MSTRG.30368.7	1.57586	coding
MSTRG.30368.4	1.83323	coding
MSTRG.30368.9	1.76825	coding
MSTRG.30368.10	1.62863	coding
MSTRG.30375.1	0.777241	coding
MSTRG.30375.5	1.53686	coding

MSTRG.30375.6	1.57633	coding
MSTRG.30375.9	1.29937	coding
MSTRG.30375.7	1.54707	coding
MSTRG.30375.8	1.29188	coding
MSTRG.30375.12	1.54796	coding
MSTRG.30375.11	0.487884	coding
MSTRG.30375.14	1.6395	coding
MSTRG.30375.15	1.66848	coding
MSTRG.30378.1	1.09949	coding
MSTRG.30371.1	-0.38882	noncoding
MSTRG.30371.2	-1.1225	noncoding
MSTRG.30372.1	-2.0986	noncoding
MSTRG.30382.2	-0.763635	noncoding
MSTRG.30386.3	2.04123	coding
MSTRG.30388.1	-2.19737	noncoding
MSTRG.30386.5	1.89988	coding
MSTRG.30386.6	1.90811	coding
MSTRG.30386.8	1.57286	coding
MSTRG.30393.2	1.5911	coding
MSTRG.30393.8	2.14701	coding
MSTRG.30393.5	1.81741	coding
MSTRG.30393.9	1.71098	coding
MSTRG.30393.7	1.98371	coding
MSTRG.30393.3	2.25011	coding
MSTRG.30394.1	-0.005256	noncoding
MSTRG.30395.1	0.593568	coding
MSTRG.30398.2	-0.171524	noncoding
MSTRG.30398.3	-0.266415	noncoding
MSTRG.30399.3	1.9819	coding
MSTRG.30403.3	1.42202	coding
MSTRG.30403.4	1.49133	coding
MSTRG.30403.5	1.89218	coding
MSTRG.30403.6	1.505	coding
MSTRG.30404.1	-1.88257	noncoding
MSTRG.30416.1	-0.969776	noncoding
MSTRG.30419.5	1.36027	coding
MSTRG.30409.1	-0.306601	noncoding
MSTRG.30409.2	-0.762475	noncoding
MSTRG.30410.1	-2.04426	noncoding
MSTRG.30411.3	-0.421042	noncoding
MSTRG.30411.4	-0.3529	noncoding
MSTRG.30411.2	-0.247355	noncoding
MSTRG.30411.6	0.303594	coding
MSTRG.30411.5	-0.2606	noncoding
MSTRG.30408.1	-2.05082	noncoding
MSTRG.30436.1	-1.62342	noncoding
MSTRG.30437.1	-1.31984	noncoding
MSTRG.30438.1	2.51861	coding
MSTRG.30439.1	0.005143	coding
MSTRG.30442.1	0.322451	coding
MSTRG.30442.2	-0.310277	noncoding
MSTRG.30442.3	-0.719954	noncoding
MSTRG.30444.2	1.8497	coding
MSTRG.30445.2	0.82828	coding

MSTRG.30448.1	-0.446951	noncoding
MSTRG.30451.1	0.122607	coding
MSTRG.30453.3	2.51586	coding
MSTRG.30453.4	2.30994	coding
MSTRG.30453.5	1.98799	coding
MSTRG.30456.2	-0.145654	noncoding
MSTRG.30456.1	0.348287	coding
MSTRG.30456.7	-0.033583	noncoding
MSTRG.30456.4	-0.037841	noncoding
MSTRG.30438.2	-2.36714	noncoding
MSTRG.30426.1	-1.62765	noncoding
MSTRG.30467.1	-3.11871	noncoding
MSTRG.30469.1	-1.22948	noncoding
MSTRG.30483.1	-1.46825	noncoding
MSTRG.30496.1	0.691734	coding
MSTRG.30496.2	0.799929	coding
MSTRG.30497.1	-0.271526	noncoding
MSTRG.30497.2	1.16962	coding
MSTRG.30496.3	-0.07953	noncoding
MSTRG.30473.1	-2.98167	noncoding
MSTRG.30475.10	0.760603	coding
MSTRG.30475.11	0.63698	coding
MSTRG.30476.1	-2.09181	noncoding
MSTRG.30479.1	-0.808394	noncoding
MSTRG.30481.1	0.891201	coding
MSTRG.30481.2	0.701201	coding
MSTRG.30481.3	0.273733	coding
MSTRG.30477.2	0.425475	coding
MSTRG.30503.1	1.19498	coding
MSTRG.30505.4	0.403377	coding
MSTRG.30506.1	0.198023	coding
MSTRG.30506.4	0.073358	coding
MSTRG.30507.2	1.41917	coding
MSTRG.30518.1	1.00668	coding
MSTRG.30518.2	0.871017	coding
MSTRG.30518.6	0.918064	coding
MSTRG.30517.2	0.581283	coding
MSTRG.30522.5	-0.561263	noncoding
MSTRG.30522.6	0.38446	coding
MSTRG.30523.1	0.289575	coding
MSTRG.30524.1	0.779276	coding
MSTRG.30525.1	1.99549	coding
MSTRG.30529.1	1.919	coding
MSTRG.30530.1	0.42087	coding
MSTRG.30530.2	0.49359	coding
MSTRG.30530.3	0.085237	coding
MSTRG.30531.3	1.39283	coding
MSTRG.30536.1	-1.95573	noncoding
MSTRG.30539.1	-1.21702	noncoding
MSTRG.30534.2	1.61476	coding
MSTRG.30534.3	2.27839	coding
MSTRG.30534.4	0.513045	coding
MSTRG.30547.1	1.5761	coding
MSTRG.30547.2	2.56072	coding

MSTRG.30547.6	2.9772	coding
MSTRG.30547.5	2.95402	coding
MSTRG.30547.7	1.80743	coding
MSTRG.30548.1	-0.133023	noncoding
MSTRG.30548.2	0.101301	coding
MSTRG.30548.3	-2.02311	noncoding
MSTRG.30549.1	-0.442471	noncoding
MSTRG.30550.1	0.289519	coding
MSTRG.30551.1	-1.15245	noncoding
MSTRG.30553.1	-0.724887	noncoding
MSTRG.30554.1	-1.39443	noncoding
MSTRG.30555.1	0.192871	coding
MSTRG.30556.1	2.92499	coding
MSTRG.30556.2	2.32701	coding
MSTRG.30545.1	-2.60215	noncoding
MSTRG.30559.1	0.503331	coding
MSTRG.30559.3	0.423082	coding
MSTRG.30561.2	1.79386	coding
MSTRG.30561.3	1.41178	coding
MSTRG.30562.2	2.22045	coding
MSTRG.30565.1	1.35463	coding
MSTRG.30565.2	2.28779	coding
MSTRG.30565.4	1.96332	coding
MSTRG.30566.1	-1.07782	noncoding
MSTRG.30569.1	-0.688263	noncoding
MSTRG.30571.1	-2.7147	noncoding
MSTRG.30572.1	-0.88529	noncoding
MSTRG.30575.1	0.848479	coding
MSTRG.30575.2	1.19915	coding
MSTRG.30575.3	0.526295	coding
MSTRG.30575.5	1.04263	coding
MSTRG.30576.1	-1.92396	noncoding
MSTRG.30583.2	2.36092	coding
MSTRG.30583.3	1.99121	coding
MSTRG.30583.4	1.98209	coding
MSTRG.30584.1	0.145343	coding
MSTRG.30595.3	1.41842	coding
MSTRG.30595.5	0.829563	coding
MSTRG.30595.4	0.955058	coding
MSTRG.30596.1	2.04186	coding
MSTRG.30597.1	2.06697	coding
MSTRG.30597.3	2.67151	coding
MSTRG.30597.5	2.47312	coding
MSTRG.30599.4	1.25297	coding
MSTRG.30614.8	1.55178	coding
MSTRG.30616.7	2.22486	coding
MSTRG.30616.3	2.55134	coding
MSTRG.30616.6	2.70141	coding
MSTRG.30617.1	0.265593	coding
MSTRG.30616.8	1.41395	coding
MSTRG.30616.9	0.988143	coding
MSTRG.30616.10	0.687862	coding
MSTRG.30618.1	-1.77346	noncoding
MSTRG.30620.1	1.59601	coding

MSTRG.30620.3	0.38786	coding
MSTRG.30623.4	1.61743	coding
MSTRG.30623.5	1.30428	coding
MSTRG.30624.1	-2.21494	noncoding
MSTRG.30625.1	1.01058	coding
MSTRG.30625.2	0.998554	coding
MSTRG.30625.3	0.980835	coding
MSTRG.30625.4	0.897098	coding
MSTRG.30625.5	1.02677	coding
MSTRG.30625.6	0.987662	coding
MSTRG.30628.1	-1.63636	noncoding
MSTRG.30631.2	0.300907	coding
MSTRG.30643.1	0.684727	coding
MSTRG.30645.3	0.249113	coding
MSTRG.30646.1	1.33704	coding
MSTRG.30646.2	0.99097	coding
MSTRG.30637.1	-0.176176	noncoding
MSTRG.30654.4	0.446158	coding
MSTRG.30657.1	-1.13196	noncoding
MSTRG.30658.2	1.09726	coding
MSTRG.30658.3	1.04034	coding
MSTRG.30658.4	1.49604	coding
MSTRG.30669.1	-0.672571	noncoding
MSTRG.30668.5	0.032454	coding
MSTRG.30668.3	1.4423	coding
MSTRG.30668.4	1.41811	coding
MSTRG.30668.6	1.67565	coding
MSTRG.30670.1	-1.23799	noncoding
MSTRG.30671.10	0.165512	coding
MSTRG.30676.4	1.75785	coding
MSTRG.30678.1	1.79227	coding
MSTRG.30685.1	0.812106	coding
MSTRG.30685.4	0.790615	coding
MSTRG.30685.5	0.353712	coding
MSTRG.30685.6	0.33562	coding
MSTRG.30685.7	0.62582	coding
MSTRG.30685.8	0.662951	coding
MSTRG.30685.9	0.686393	coding
MSTRG.30685.10	1.04922	coding
MSTRG.30685.11	-0.859945	noncoding
MSTRG.30685.12	0.424619	coding
MSTRG.30685.13	-0.248634	noncoding
MSTRG.30685.14	-0.1022	noncoding
MSTRG.30685.15	0.342564	coding
MSTRG.30688.1	-0.744871	noncoding
MSTRG.30688.2	0.13518	coding
MSTRG.30693.1	1.33788	coding
MSTRG.30693.6	1.04356	coding
MSTRG.30693.2	1.24533	coding
MSTRG.30693.3	1.23894	coding
MSTRG.30693.4	1.24019	coding
MSTRG.30693.7	1.36118	coding
MSTRG.30693.9	1.1369	coding
MSTRG.30695.1	-1.20119	noncoding

MSTRG.30698.1	-1.06114	noncoding
MSTRG.30698.2	-1.89151	noncoding
MSTRG.30699.1	-0.190424	noncoding
MSTRG.30701.1	-0.865521	noncoding
MSTRG.30703.1	-0.878562	noncoding
MSTRG.30703.2	-1.89844	noncoding
MSTRG.30704.1	-0.768835	noncoding
MSTRG.30705.2	-0.715928	noncoding
MSTRG.30706.1	0.153998	coding
MSTRG.30712.1	-1.15033	noncoding
MSTRG.30712.2	-1.02833	noncoding
MSTRG.30716.1	2.04278	coding
MSTRG.30718.1	-0.009419	noncoding
MSTRG.30718.2	0.038007	coding
MSTRG.30719.1	-0.190819	noncoding
MSTRG.30720.5	-0.533276	noncoding
MSTRG.30720.4	0.954017	coding
MSTRG.30720.3	1.18121	coding
MSTRG.30720.2	1.04074	coding
MSTRG.30736.1	2.66352	coding
MSTRG.30736.3	2.95048	coding
MSTRG.30736.4	2.94377	coding
MSTRG.30737.1	-2.2666	noncoding
MSTRG.30725.2	1.09056	coding
MSTRG.30727.1	-1.76459	noncoding
MSTRG.30728.1	-0.32978	noncoding
MSTRG.30740.1	0.559362	coding
MSTRG.30741.1	-1.41141	noncoding
MSTRG.30743.6	-0.343594	noncoding
MSTRG.30743.4	0.040396	coding
MSTRG.30743.8	-0.162107	noncoding
MSTRG.30743.9	-1.28937	noncoding
MSTRG.30743.10	0.060195	coding
MSTRG.30743.7	0.101452	coding
MSTRG.30747.5	0.83111	coding
MSTRG.30747.6	0.400222	coding
MSTRG.30758.2	1.09785	coding
MSTRG.30759.1	-2.18083	noncoding
MSTRG.30760.1	-0.013104	noncoding
MSTRG.30760.3	-0.076462	noncoding
MSTRG.30760.4	0.005978	coding
MSTRG.30760.5	1.51047	coding
MSTRG.30760.2	1.69735	coding
MSTRG.30762.1	-0.553618	noncoding
MSTRG.30763.1	0.580193	coding
MSTRG.30760.7	-0.714969	noncoding
MSTRG.30760.8	-1.63604	noncoding
MSTRG.30765.1	-1.55031	noncoding
MSTRG.30768.1	-2.31856	noncoding
MSTRG.30771.1	0.270956	coding
MSTRG.30772.1	0.622831	coding
MSTRG.30772.2	0.800151	coding
MSTRG.30753.7	1.16284	coding
MSTRG.30757.1	-0.840348	noncoding

MSTRG.30774.1	1.68948	coding
MSTRG.30776.1	-1.7336	noncoding
MSTRG.30787.1	0.278813	coding
MSTRG.30791.1	-2.14407	noncoding
MSTRG.30792.2	-1.84208	noncoding
MSTRG.30793.1	-0.926195	noncoding
MSTRG.30792.3	-0.856648	noncoding
MSTRG.30792.4	-0.877109	noncoding
MSTRG.30792.5	-2.11184	noncoding
MSTRG.30792.6	-2.47034	noncoding
MSTRG.30794.1	-2.40455	noncoding
MSTRG.30795.4	0.270064	coding
MSTRG.30831.1	-0.52046	noncoding
MSTRG.30832.2	-2.21872	noncoding
MSTRG.30798.1	-0.061196	noncoding
MSTRG.30801.1	-1.13705	noncoding
MSTRG.30802.1	-2.3935	noncoding
MSTRG.30803.2	-0.227281	noncoding
MSTRG.30805.2	2.07083	coding
MSTRG.30805.4	2.93971	coding
MSTRG.30806.1	0.358029	coding
MSTRG.30807.1	0.554454	coding
MSTRG.30808.1	0.257068	coding
MSTRG.30808.2	-1.01091	noncoding
MSTRG.30805.6	1.03285	coding
MSTRG.30809.1	1.11595	coding
MSTRG.30812.1	2.24636	coding
MSTRG.30812.2	2.4185	coding
MSTRG.30812.4	2.54913	coding
MSTRG.30815.2	-2.10566	noncoding
MSTRG.30817.1	0.053363	coding
MSTRG.30826.2	1.35096	coding
MSTRG.30826.3	1.41323	coding
MSTRG.30826.5	0.03485	coding
MSTRG.30826.6	0.752813	coding
MSTRG.30833.1	-1.70286	noncoding
MSTRG.30866.1	0.043043	coding
MSTRG.30867.1	0.90157	coding
MSTRG.30866.2	-0.588201	noncoding
MSTRG.30868.1	-1.28708	noncoding
MSTRG.30866.3	-0.109508	noncoding
MSTRG.30866.4	-0.084638	noncoding
MSTRG.30866.5	-0.075772	noncoding
MSTRG.30866.6	-0.586906	noncoding
MSTRG.30866.7	-0.165847	noncoding
MSTRG.30866.8	0.383241	coding
MSTRG.30835.1	1.35824	coding
MSTRG.30836.1	-0.886828	noncoding
MSTRG.30836.4	-0.887995	noncoding
MSTRG.30836.3	-1.13382	noncoding
MSTRG.30836.2	-0.732889	noncoding
MSTRG.30836.5	-1.27196	noncoding
MSTRG.30836.6	-1.27159	noncoding
MSTRG.30837.1	-0.824375	noncoding

MSTRG.30889.5	1.47906	coding
MSTRG.30889.7	2.70828	coding
MSTRG.30889.10	-0.486241	noncoding
MSTRG.30889.14	-0.632885	noncoding
MSTRG.30892.1	-1.1906	noncoding
MSTRG.30892.2	-1.08427	noncoding
MSTRG.30892.3	-1.50496	noncoding
MSTRG.30892.4	0.122737	coding
MSTRG.30893.1	0.084768	coding
MSTRG.30894.1	0.200607	coding
MSTRG.30889.16	-0.36	noncoding
MSTRG.30889.17	0.255605	coding
MSTRG.30895.1	-1.12754	noncoding
MSTRG.30889.19	0.206346	coding
MSTRG.30889.18	0.410014	coding
MSTRG.30896.1	-0.328147	noncoding
MSTRG.30889.20	0.134892	coding
MSTRG.30897.1	0.187321	coding
MSTRG.30889.21	2.95551	coding
MSTRG.30871.1	-0.438427	noncoding
MSTRG.30874.1	0.95282	coding
MSTRG.30874.2	1.59087	coding
MSTRG.30876.3	-0.094409	noncoding
MSTRG.30877.4	2.26442	coding
MSTRG.30877.6	2.04557	coding
MSTRG.30878.2	1.28783	coding
MSTRG.30878.3	1.0341	coding
MSTRG.30880.2	0.957646	coding
MSTRG.30883.2	-0.542024	noncoding
MSTRG.30882.1	-1.25301	noncoding
MSTRG.30888.2	0.34191	coding
MSTRG.30899.2	2.01896	coding
MSTRG.30900.4	0.995436	coding
MSTRG.30900.5	1.00295	coding
MSTRG.30900.6	1.89695	coding
MSTRG.30900.9	0.940725	coding
MSTRG.30900.7	2.21511	coding
MSTRG.30900.8	2.19804	coding
MSTRG.30900.10	2.28833	coding
MSTRG.30905.4	1.62706	coding
MSTRG.30906.1	-2.56467	noncoding
MSTRG.30907.2	0.613376	coding
MSTRG.30908.1	1.17963	coding
MSTRG.30909.1	1.29325	coding
MSTRG.30909.2	0.68049	coding
MSTRG.30909.3	0.887613	coding
MSTRG.30909.4	1.49585	coding
MSTRG.30909.5	1.38761	coding
MSTRG.30909.6	1.37052	coding
MSTRG.30910.2	1.03474	coding
MSTRG.30910.3	1.19206	coding
MSTRG.30910.4	1.12067	coding
MSTRG.30910.5	0.473554	coding
MSTRG.30910.8	0.427524	coding

MSTRG.30910.9	0.240631	coding
MSTRG.30911.3	-1.88678	noncoding
MSTRG.30914.1	-2.03747	noncoding
MSTRG.30920.1	-0.174956	noncoding
MSTRG.30937.1	-1.14341	noncoding
MSTRG.30937.2	-0.542677	noncoding
MSTRG.30937.5	0.715468	coding
MSTRG.30937.6	0.249501	coding
MSTRG.30937.7	1.04851	coding
MSTRG.30937.8	0.485907	coding
MSTRG.30937.9	0.174497	coding
MSTRG.30937.10	0.625774	coding
MSTRG.30939.1	0.264788	coding
MSTRG.30943.1	-0.239106	noncoding
MSTRG.30922.1	0.615143	coding
MSTRG.30922.2	0.424847	coding
MSTRG.30922.3	0.677591	coding
MSTRG.30945.1	-1.03444	noncoding
MSTRG.30948.11	0.57769	coding
MSTRG.30948.8	1.24569	coding
MSTRG.30948.18	1.40384	coding
MSTRG.30948.12	1.56465	coding
MSTRG.30948.13	1.51916	coding
MSTRG.30948.14	1.502	coding
MSTRG.30948.15	1.87193	coding
MSTRG.30948.16	1.57454	coding
MSTRG.30948.9	1.52476	coding
MSTRG.30948.7	1.43386	coding
MSTRG.30948.10	1.58765	coding
MSTRG.30948.17	1.53833	coding
MSTRG.30948.6	1.56901	coding
MSTRG.30948.19	-0.235878	noncoding
MSTRG.30949.1	-0.346127	noncoding
MSTRG.30950.2	2.13819	coding
MSTRG.30951.1	-2.27939	noncoding
MSTRG.30952.1	-2.52083	noncoding
MSTRG.30950.5	0.986961	coding
MSTRG.30950.7	0.820778	coding
MSTRG.30929.3	2.04963	coding
MSTRG.30961.3	2.09089	coding
MSTRG.30961.4	2.09501	coding
MSTRG.30965.1	1.29742	coding
MSTRG.30965.2	2.49222	coding
MSTRG.30965.3	0.865997	coding
MSTRG.30965.4	0.396196	coding
MSTRG.30965.6	1.3622	coding
MSTRG.30965.5	2.2759	coding
MSTRG.30966.1	-0.826094	noncoding
MSTRG.30965.8	2.21553	coding
MSTRG.30965.9	-0.468115	noncoding
MSTRG.30965.10	0.721027	coding
MSTRG.30967.1	-1.1477	noncoding
MSTRG.30967.3	-0.823776	noncoding
MSTRG.30971.1	-1.59453	noncoding

MSTRG.30971.2	-1.63303	noncoding
MSTRG.30968.1	-1.94512	noncoding
MSTRG.31004.1	0.670898	coding
MSTRG.31004.2	-0.005444	noncoding
MSTRG.31004.4	-0.111334	noncoding
MSTRG.31004.5	0.76044	coding
MSTRG.31006.1	-0.744813	noncoding
MSTRG.31007.1	1.24376	coding
MSTRG.31011.1	-1.66601	noncoding
MSTRG.31011.2	-2.06398	noncoding
MSTRG.31011.3	-1.6082	noncoding
MSTRG.31011.4	-1.86341	noncoding
MSTRG.31011.5	-1.88924	noncoding
MSTRG.31015.3	1.33995	coding
MSTRG.31015.4	0.688478	coding
MSTRG.31015.5	1.65076	coding
MSTRG.31015.6	0.745836	coding
MSTRG.31021.2	0.696211	coding
MSTRG.31019.1	-1.38731	noncoding
MSTRG.31027.1	-2.11918	noncoding
MSTRG.31029.2	0.070378	coding
MSTRG.31030.1	1.07449	coding
MSTRG.31035.1	1.14804	coding
MSTRG.31035.4	0.694919	coding
MSTRG.31035.3	1.23323	coding
MSTRG.31035.5	0.616007	coding
MSTRG.31035.6	0.40262	coding
MSTRG.31035.7	1.02964	coding
MSTRG.31035.8	0.897187	coding
MSTRG.31031.1	-0.529712	noncoding
MSTRG.31036.2	0.715019	coding
MSTRG.31043.1	0.750924	coding
MSTRG.31044.1	-0.946882	noncoding
MSTRG.31045.13	-0.20993	noncoding
MSTRG.31045.4	1.73055	coding
MSTRG.31045.7	1.96679	coding
MSTRG.31045.11	2.12949	coding
MSTRG.31045.15	2.10956	coding
MSTRG.31045.16	0.238077	coding
MSTRG.31045.8	2.16065	coding
MSTRG.31045.14	0.488042	coding
MSTRG.31045.12	2.04113	coding
MSTRG.31045.9	2.02507	coding
MSTRG.31045.10	0.63446	coding
MSTRG.31045.6	2.0183	coding
MSTRG.31046.1	-0.766111	noncoding
MSTRG.31047.1	-1.18155	noncoding
MSTRG.31051.1	-1.05474	noncoding
MSTRG.31052.1	0.308865	coding
MSTRG.31053.5	2.06927	coding
MSTRG.31058.1	1.12494	coding
MSTRG.31071.1	-0.572055	noncoding
MSTRG.31071.2	-1.60441	noncoding
MSTRG.31083.2	2.20002	coding

MSTRG.31105.3	1.50124	coding
MSTRG.31105.4	1.37202	coding
MSTRG.31111.2	0.764833	coding
MSTRG.31112.1	0.855303	coding
MSTRG.31116.2	0.139923	coding
MSTRG.31116.3	1.66183	coding
MSTRG.31116.4	2.16829	coding
MSTRG.31116.5	1.97076	coding
MSTRG.31117.1	-2.0504	noncoding
MSTRG.31117.2	-1.85863	noncoding
MSTRG.31116.6	1.19932	coding
MSTRG.31119.2	1.64276	coding
MSTRG.31120.8	2.36884	coding
MSTRG.31121.1	0.156669	coding
MSTRG.31122.4	0.912188	coding
MSTRG.31122.3	2.01688	coding
MSTRG.31114.4	1.28679	coding
MSTRG.31125.1	1.47583	coding
MSTRG.31125.2	1.36838	coding
MSTRG.31128.3	1.69924	coding
MSTRG.31132.3	2.09648	coding
MSTRG.31134.3	0.08554	coding
MSTRG.31134.4	-1.72328	noncoding
MSTRG.31134.5	-1.00914	noncoding
MSTRG.31134.6	-0.690403	noncoding
MSTRG.31133.1	-0.985354	noncoding
MSTRG.31142.2	0.161569	coding
MSTRG.31143.2	1.33364	coding
MSTRG.31143.3	1.33143	coding
MSTRG.31146.1	2.61948	coding
MSTRG.31146.2	-0.438816	noncoding
MSTRG.31146.4	-0.272641	noncoding
MSTRG.31146.3	-0.337244	noncoding
MSTRG.31146.5	-0.388332	noncoding
MSTRG.31148.2	2.45526	coding
MSTRG.31149.2	0.121967	coding
MSTRG.31144.1	0.943055	coding
MSTRG.31144.2	0.991458	coding
MSTRG.31144.5	-0.545691	noncoding
MSTRG.31144.6	-0.571594	noncoding
MSTRG.31145.2	1.69953	coding
MSTRG.31157.1	-1.19546	noncoding
MSTRG.31157.2	-1.29563	noncoding
MSTRG.31158.2	1.05005	coding
MSTRG.31158.3	1.39874	coding
MSTRG.31158.4	0.699956	coding
MSTRG.31159.1	-0.510268	noncoding
MSTRG.31160.2	0.502634	coding
MSTRG.31160.3	1.40922	coding
MSTRG.31160.4	0.322073	coding
MSTRG.31161.1	-2.40871	noncoding
MSTRG.31165.1	1.24035	coding
MSTRG.31165.3	0.010871	coding
MSTRG.31155.1	-0.78653	noncoding

MSTRG.31155.2	-1.51141	noncoding
MSTRG.31156.1	1.02877	coding
MSTRG.31167.1	1.69991	coding
MSTRG.31167.2	-0.608208	noncoding
MSTRG.31169.5	2.62497	coding
MSTRG.31169.6	2.72139	coding
MSTRG.31169.7	1.52854	coding
MSTRG.31171.1	1.37418	coding
MSTRG.31172.3	2.32311	coding
MSTRG.31175.2	1.30938	coding
MSTRG.31175.3	0.326761	coding
MSTRG.31174.1	-1.39991	noncoding
MSTRG.31187.3	1.7714	coding
MSTRG.31188.4	3.28894	coding
MSTRG.31189.2	0.7501	coding
MSTRG.31189.3	1.25579	coding
MSTRG.31189.4	0.963569	coding
MSTRG.31193.1	-0.776707	noncoding
MSTRG.31194.1	0.196238	coding
MSTRG.31196.2	0.436677	coding
MSTRG.31196.1	0.846971	coding
MSTRG.31197.1	-0.56539	noncoding
MSTRG.31201.1	-0.1414	noncoding
MSTRG.31202.1	-1.41021	noncoding
MSTRG.31205.1	-0.456914	noncoding
MSTRG.31210.1	0.08247	coding
MSTRG.31211.1	1.07218	coding
MSTRG.31215.1	0.617855	coding
MSTRG.31215.2	1.92845	coding
MSTRG.31218.2	0.442455	coding
MSTRG.31219.2	-1.11905	noncoding
MSTRG.31222.3	1.03931	coding
MSTRG.31223.1	-0.900175	noncoding
MSTRG.31231.2	0.080133	coding
MSTRG.31231.3	-0.175473	noncoding
MSTRG.31231.4	0.447402	coding
MSTRG.31231.5	-1.36601	noncoding
MSTRG.31231.6	1.20651	coding
MSTRG.31233.1	-0.579997	noncoding
MSTRG.31235.1	-0.721979	noncoding
MSTRG.31236.1	-0.007298	noncoding
MSTRG.31237.1	0.691566	coding
MSTRG.31238.1	0.240054	coding
MSTRG.31231.7	1.50933	coding
MSTRG.31239.1	0.582205	coding
MSTRG.31240.1	1.16137	coding
MSTRG.31241.1	0.812045	coding
MSTRG.31242.1	-0.492099	noncoding
MSTRG.31240.2	0.847003	coding
MSTRG.31244.1	0.468741	coding
MSTRG.31244.2	-0.077149	noncoding
MSTRG.31245.2	-0.849711	noncoding
MSTRG.31230.1	-2.25015	noncoding
MSTRG.31246.1	-1.23611	noncoding

MSTRG.31246.2	-1.37863	noncoding
MSTRG.31247.2	0.207074	coding
MSTRG.31251.1	0.915353	coding
MSTRG.31251.3	-0.668557	noncoding
MSTRG.31251.2	-0.471155	noncoding
MSTRG.31251.4	1.41003	coding
MSTRG.31251.5	-1.08021	noncoding
MSTRG.31271.1	-0.510378	noncoding
MSTRG.31271.2	-0.437672	noncoding
MSTRG.31263.1	0.041669	coding
MSTRG.31273.1	-0.867977	noncoding
MSTRG.31278.3	0.380521	coding
MSTRG.31278.4	1.50285	coding
MSTRG.31278.5	0.939999	coding
MSTRG.31279.1	-1.09866	noncoding
MSTRG.31286.4	-2.57125	noncoding
MSTRG.31287.3	1.11968	coding
MSTRG.31285.1	0.114856	coding
MSTRG.31289.1	-0.178931	noncoding
MSTRG.31291.1	-0.774373	noncoding
MSTRG.31301.1	0.327212	coding
MSTRG.31301.2	-2.41592	noncoding
MSTRG.31301.3	-0.601601	noncoding
MSTRG.31301.4	-0.006274	noncoding
MSTRG.31301.5	-0.027703	noncoding
MSTRG.31301.6	-1.35572	noncoding
MSTRG.31301.7	-1.91476	noncoding
MSTRG.31307.1	-1.31376	noncoding
MSTRG.31316.2	0.565989	coding
MSTRG.31317.1	-1.04825	noncoding
MSTRG.31320.1	-1.18626	noncoding
MSTRG.31320.3	0.129334	coding
MSTRG.31320.2	0.144459	coding
MSTRG.31320.7	-0.918256	noncoding
MSTRG.31320.4	0.250621	coding
MSTRG.31320.5	0.376267	coding
MSTRG.31320.6	0.245546	coding
MSTRG.31320.8	-0.389686	noncoding
MSTRG.31320.9	0.52734	coding
MSTRG.31320.10	-0.022961	noncoding
MSTRG.31320.11	-1.13369	noncoding
MSTRG.31320.12	-0.347315	noncoding
MSTRG.31304.1	-2.07173	noncoding
MSTRG.31324.2	1.80008	coding
MSTRG.31333.2	1.73504	coding
MSTRG.31336.3	2.50711	coding
MSTRG.31337.1	1.6335	coding
MSTRG.31341.1	-0.528079	noncoding
MSTRG.31347.1	1.57338	coding
MSTRG.31348.1	-2.24609	noncoding
MSTRG.31350.2	1.22025	coding
MSTRG.31351.3	2.70671	coding
MSTRG.31363.1	-2.0782	noncoding
MSTRG.31364.5	0.559824	coding

MSTRG.31364.6	0.900651	coding
MSTRG.31365.1	0.775475	coding
MSTRG.31367.2	0.970823	coding
MSTRG.31371.1	-1.39909	noncoding
MSTRG.31372.2	2.86023	coding
MSTRG.31374.1	-1.26133	noncoding
MSTRG.31375.1	-0.138724	noncoding
MSTRG.31376.1	0.716173	coding
MSTRG.31378.4	1.4411	coding
MSTRG.31379.1	-1.91196	noncoding
MSTRG.31380.2	0.593474	coding
MSTRG.31380.5	0.995886	coding
MSTRG.31381.1	2.6672	coding
MSTRG.31383.1	-0.173579	noncoding
MSTRG.31384.2	1.83678	coding
MSTRG.31385.1	-0.312217	noncoding
MSTRG.31386.1	-0.841895	noncoding
MSTRG.31390.1	-2.48584	noncoding
MSTRG.31401.2	1.75031	coding
MSTRG.31401.1	1.78182	coding
MSTRG.31401.3	1.78377	coding
MSTRG.31407.1	0.674995	coding
MSTRG.31408.1	-1.30189	noncoding
MSTRG.31407.2	-0.95556	noncoding
MSTRG.31419.1	0.329381	coding
MSTRG.31421.1	-0.008045	noncoding
MSTRG.31426.1	2.49731	coding
MSTRG.31426.2	2.19169	coding
MSTRG.31431.1	-0.725271	noncoding
MSTRG.31435.6	0.802237	coding
MSTRG.31435.10	0.446221	coding
MSTRG.31435.2	0.935241	coding
MSTRG.31435.8	1.10194	coding
MSTRG.31436.1	-0.607259	noncoding
MSTRG.31440.1	-1.64842	noncoding
MSTRG.31459.1	-0.797637	noncoding
MSTRG.31474.1	2.11746	coding
MSTRG.31474.2	1.25097	coding
MSTRG.31476.1	-2.06018	noncoding
MSTRG.31477.1	0.747064	coding
MSTRG.31474.3	0.298737	coding
MSTRG.31478.1	0.538907	coding
MSTRG.31477.2	-0.053885	noncoding
MSTRG.31479.1	0.271356	coding
MSTRG.31474.4	-0.066636	noncoding
MSTRG.31474.6	-1.83707	noncoding
MSTRG.31474.7	-0.017064	noncoding
MSTRG.31480.1	-2.82982	noncoding
MSTRG.31468.2	2.00936	coding
MSTRG.31468.4	0.128867	coding
MSTRG.31468.5	-0.757561	noncoding
MSTRG.31484.1	2.05734	coding
MSTRG.31485.1	0.585255	coding
MSTRG.31485.2	0.528536	coding

MSTRG.31493.1	1.068	coding
MSTRG.31493.2	0.84433	coding
MSTRG.31505.1	-0.156879	noncoding
MSTRG.31506.1	-0.027888	noncoding
MSTRG.31505.2	-0.267157	noncoding
MSTRG.31505.4	0.837667	coding
MSTRG.31505.3	0.375724	coding
MSTRG.31507.1	0.495618	coding
MSTRG.31505.5	-0.575923	noncoding
MSTRG.31495.1	-0.608088	noncoding
MSTRG.31511.1	0.059806	coding
MSTRG.31511.2	0.127092	coding
MSTRG.31512.1	0.952915	coding
MSTRG.31510.4	0.04966	coding
MSTRG.31510.5	0.670359	coding
MSTRG.31510.7	0.609477	coding
MSTRG.31510.8	0.683551	coding
MSTRG.31510.9	-0.601404	noncoding
MSTRG.31513.1	0.632833	coding
MSTRG.31515.1	0.593107	coding
MSTRG.31517.1	-2.2859	noncoding
MSTRG.31522.1	-1.79544	noncoding
MSTRG.31525.1	-0.275276	noncoding
MSTRG.31525.2	0.613093	coding
MSTRG.31526.1	-2.02646	noncoding
MSTRG.31527.1	-0.792644	noncoding
MSTRG.31531.1	1.13827	coding
MSTRG.31531.2	1.18088	coding
MSTRG.31532.2	3.19763	coding
MSTRG.31531.4	2.85821	coding
MSTRG.31533.1	0.355476	coding
MSTRG.31531.8	1.22851	coding
MSTRG.31536.1	-1.46306	noncoding
MSTRG.31537.13	1.07574	coding
MSTRG.31543.1	1.79707	coding
MSTRG.31544.3	-0.889566	noncoding
MSTRG.31546.5	2.26441	coding
MSTRG.31546.7	2.53582	coding
MSTRG.31548.1	0.327623	coding
MSTRG.31548.2	0.325744	coding
MSTRG.31548.3	0.344558	coding
MSTRG.31548.4	0.347599	coding
MSTRG.31548.5	0.326906	coding
MSTRG.31549.3	-0.51599	noncoding
MSTRG.31551.3	0.646057	coding
MSTRG.31551.2	2.29582	coding
MSTRG.31552.1	-0.785302	noncoding
MSTRG.31551.4	1.80243	coding
MSTRG.31559.1	2.98812	coding
MSTRG.31559.3	2.44332	coding
MSTRG.31559.4	2.43627	coding
MSTRG.31559.5	2.48888	coding
MSTRG.31559.6	1.64746	coding
MSTRG.31563.1	2.40293	coding

MSTRG.31564.2	2.5617	coding
MSTRG.31565.1	-2.51882	noncoding
MSTRG.31566.2	-0.422226	noncoding
MSTRG.31570.4	0.588891	coding
MSTRG.31570.5	2.41893	coding
MSTRG.31570.3	2.40063	coding
MSTRG.31570.6	2.28156	coding
MSTRG.31570.9	1.3852	coding
MSTRG.31570.8	0.609004	coding
MSTRG.31570.7	2.15258	coding
MSTRG.31571.2	-1.03332	noncoding
MSTRG.31571.1	-0.281071	noncoding
MSTRG.31571.3	-0.324728	noncoding
MSTRG.31571.4	-0.834795	noncoding
MSTRG.31567.2	0.870533	coding
MSTRG.31576.1	-2.19344	noncoding
MSTRG.31581.4	1.10245	coding
MSTRG.31586.5	-1.57657	noncoding
MSTRG.31584.2	1.40988	coding
MSTRG.31591.1	-1.10992	noncoding
MSTRG.31598.1	1.79006	coding
MSTRG.31598.2	1.94902	coding
MSTRG.31598.3	1.94555	coding
MSTRG.31598.5	1.84593	coding
MSTRG.31598.4	1.83731	coding
MSTRG.31599.3	1.8174	coding
MSTRG.31599.4	1.79619	coding
MSTRG.31597.1	1.03386	coding
MSTRG.31597.2	1.31248	coding
MSTRG.31603.2	1.55917	coding
MSTRG.31603.3	1.16917	coding
MSTRG.31603.4	0.657023	coding
MSTRG.31603.8	0.583422	coding
MSTRG.31603.11	0.914032	coding
MSTRG.31602.2	0.800772	coding
MSTRG.31606.1	1.57865	coding
MSTRG.31606.2	1.10777	coding
MSTRG.31606.3	-0.803983	noncoding
MSTRG.31606.4	-0.326076	noncoding
MSTRG.31607.1	0.277551	coding
MSTRG.31606.6	0.7124	coding
MSTRG.31606.7	1.2938	coding
MSTRG.31606.8	1.35873	coding
MSTRG.31608.1	1.28839	coding
MSTRG.31606.10	1.37992	coding
MSTRG.31606.11	1.12982	coding
MSTRG.31617.1	1.50836	coding
MSTRG.31618.1	0.798245	coding
MSTRG.31618.4	0.553975	coding
MSTRG.31618.5	0.85834	coding
MSTRG.31619.1	1.65575	coding
MSTRG.31620.1	-1.04122	noncoding
MSTRG.31622.2	2.9348	coding
MSTRG.31623.4	1.36909	coding

MSTRG.31625.1	-1.57294	noncoding
MSTRG.31626.3	1.06854	coding
MSTRG.31626.4	1.22046	coding
MSTRG.31626.5	0.970246	coding
MSTRG.31627.1	-1.35005	noncoding
MSTRG.31613.2	2.13021	coding
MSTRG.31613.3	1.33562	coding
MSTRG.31614.1	-0.285078	noncoding
MSTRG.31630.5	1.59755	coding
MSTRG.31630.6	1.24054	coding
MSTRG.31633.1	-1.34936	noncoding
MSTRG.31633.2	-1.12967	noncoding
MSTRG.31629.1	-0.317517	noncoding
MSTRG.31636.5	-0.176443	noncoding
MSTRG.31637.2	-0.006709	noncoding
MSTRG.31637.3	1.54483	coding
MSTRG.31638.1	0.773826	coding
MSTRG.31640.1	0.736405	coding
MSTRG.31641.1	-0.029983	noncoding
MSTRG.31642.1	-0.373189	noncoding
MSTRG.31643.1	0.287312	coding
MSTRG.31637.6	-0.481845	noncoding
MSTRG.31644.2	0.985952	coding
MSTRG.31644.5	3.20908	coding
MSTRG.31648.1	-0.546002	noncoding
MSTRG.31648.2	-0.691006	noncoding
MSTRG.31648.3	1.14662	coding
MSTRG.31648.5	1.40473	coding
MSTRG.31650.1	-2.59115	noncoding
MSTRG.31651.2	1.62605	coding
MSTRG.31661.1	-0.254867	noncoding
MSTRG.31662.1	-0.789025	noncoding
MSTRG.31672.2	-0.235982	noncoding
MSTRG.31672.3	1.58134	coding
MSTRG.31673.1	-0.470447	noncoding
MSTRG.31678.2	-0.515555	noncoding
MSTRG.31678.1	-0.511361	noncoding
MSTRG.31679.2	1.74586	coding
MSTRG.31682.1	-0.630558	noncoding
MSTRG.31690.1	-0.696218	noncoding
MSTRG.31691.1	2.29125	coding
MSTRG.31691.2	2.30816	coding
MSTRG.31692.1	-1.21058	noncoding
MSTRG.31691.3	2.28758	coding
MSTRG.31700.1	-0.274494	noncoding
MSTRG.31703.2	-1.43926	noncoding
MSTRG.31694.1	-0.90427	noncoding
MSTRG.31721.4	-0.169423	noncoding
MSTRG.31722.2	-1.79765	noncoding
MSTRG.31723.5	0.136127	coding
MSTRG.31723.6	0.538593	coding
MSTRG.31725.2	2.18122	coding
MSTRG.31725.3	2.11077	coding
MSTRG.31725.4	1.42318	coding

MSTRG.31729.1	-1.75849	noncoding
MSTRG.31742.2	0.678552	coding
MSTRG.31740.1	1.15307	coding
MSTRG.31743.1	0.294263	coding
MSTRG.31743.2	0.017003	coding
MSTRG.31743.3	0.212149	coding
MSTRG.31745.3	1.09608	coding
MSTRG.31745.6	1.73541	coding
MSTRG.31745.7	1.07152	coding
MSTRG.31746.1	0.110034	coding
MSTRG.31747.1	-0.302702	noncoding
MSTRG.31745.10	0.503529	coding
MSTRG.31748.1	-1.89474	noncoding
MSTRG.31748.2	-1.82297	noncoding
MSTRG.31748.3	-1.76465	noncoding
MSTRG.31749.1	-1.62007	noncoding
MSTRG.31750.1	-1.85612	noncoding
MSTRG.31753.1	0.265799	coding
MSTRG.31757.4	2.31441	coding
MSTRG.31757.5	1.66099	coding
MSTRG.31758.1	1.39609	coding
MSTRG.31766.1	0.591776	coding
MSTRG.31777.1	-1.99719	noncoding
MSTRG.31763.1	-1.28955	noncoding
MSTRG.31786.3	0.249335	coding
MSTRG.31786.2	0.357008	coding
MSTRG.31787.1	-2.20479	noncoding
MSTRG.31786.4	-1.71363	noncoding
MSTRG.31786.7	-1.74875	noncoding
MSTRG.31786.10	-1.23164	noncoding
MSTRG.31786.11	-2.01534	noncoding
MSTRG.31786.12	-1.8477	noncoding
MSTRG.31796.5	1.5397	coding
MSTRG.31796.4	1.9134	coding
MSTRG.31796.6	1.91158	coding
MSTRG.31797.1	0.239744	coding
MSTRG.31799.1	0.940384	coding
MSTRG.31801.1	0.736106	coding
MSTRG.31801.2	0.036757	coding
MSTRG.31801.3	-0.223358	noncoding
MSTRG.31801.4	0.356521	coding
MSTRG.31801.5	0.580646	coding
MSTRG.31801.6	0.090836	coding
MSTRG.31801.7	-0.536647	noncoding
MSTRG.31801.8	0.198291	coding
MSTRG.31789.1	0.984629	coding
MSTRG.31789.2	1.4786	coding
MSTRG.31790.1	0.304278	coding
MSTRG.31792.2	1.65475	coding
MSTRG.31792.3	0.602789	coding
MSTRG.31793.1	-2.32835	noncoding
MSTRG.31793.2	-2.32788	noncoding
MSTRG.31792.4	0.03921	coding
MSTRG.31803.1	2.66131	coding

MSTRG.31803.3	1.33097	coding
MSTRG.31804.1	-2.137	noncoding
MSTRG.31805.5	-2.08812	noncoding
MSTRG.31805.3	-1.5942	noncoding
MSTRG.31805.1	-1.67135	noncoding
MSTRG.31805.4	-1.76143	noncoding
MSTRG.31806.1	1.02841	coding
MSTRG.31810.1	-2.25873	noncoding
MSTRG.31812.5	0.937847	coding
MSTRG.31812.9	-0.394391	noncoding
MSTRG.31812.8	-0.38106	noncoding
MSTRG.31813.1	-1.17826	noncoding
MSTRG.31812.12	0.043082	coding
MSTRG.31812.10	-0.037715	noncoding
MSTRG.31815.4	2.74983	coding
MSTRG.31815.2	2.52343	coding
MSTRG.31815.3	2.5256	coding
MSTRG.31815.5	0.820895	coding
MSTRG.31818.2	1.23324	coding
MSTRG.31818.3	1.89802	coding
MSTRG.31818.7	2.05282	coding
MSTRG.31820.2	0.111778	coding
MSTRG.31820.3	-0.258681	noncoding
MSTRG.31820.4	0.106646	coding
MSTRG.31820.5	-0.40131	noncoding
MSTRG.31820.6	0.020601	coding
MSTRG.31820.7	-0.331913	noncoding
MSTRG.31820.8	0.010176	coding
MSTRG.31820.9	-0.154979	noncoding
MSTRG.31822.2	2.48429	coding
MSTRG.31824.1	-0.859309	noncoding
MSTRG.31826.1	0.238658	coding
MSTRG.31829.1	-1.53406	noncoding
MSTRG.31833.4	0.118003	coding
MSTRG.31838.1	-0.17283	noncoding
MSTRG.31839.2	0.922509	coding
MSTRG.31865.1	0.129774	coding
MSTRG.31867.3	0.645686	coding
MSTRG.31867.5	0.329959	coding
MSTRG.31867.7	0.383856	coding
MSTRG.31867.6	0.439541	coding
MSTRG.31868.1	-0.128019	noncoding
MSTRG.31867.8	-0.470689	noncoding
MSTRG.31859.4	1.48639	coding
MSTRG.31859.3	1.39423	coding
MSTRG.31861.2	1.18451	coding
MSTRG.31875.1	-1.20542	noncoding
MSTRG.31883.1	-2.40569	noncoding
MSTRG.31885.3	1.00406	coding
MSTRG.31885.4	0.496223	coding
MSTRG.31886.1	-1.26483	noncoding
MSTRG.31886.2	-0.962001	noncoding
MSTRG.31897.1	0.655064	coding
MSTRG.31898.1	0.06357	coding

MSTRG.31897.2	0.03801	coding
MSTRG.31904.1	-2.09202	noncoding
MSTRG.31907.1	-1.30094	noncoding
MSTRG.31905.1	-2.30277	noncoding
MSTRG.31909.1	-1.32796	noncoding
MSTRG.31910.1	1.54786	coding
MSTRG.31911.2	0.633634	coding
MSTRG.31914.2	-0.698965	noncoding
MSTRG.31914.3	-0.456183	noncoding
MSTRG.31917.7	-1.05282	noncoding
MSTRG.31919.1	1.52873	coding
MSTRG.31920.6	1.45881	coding
MSTRG.31920.8	1.23901	coding
MSTRG.31920.9	1.19479	coding
MSTRG.31923.1	-2.23049	noncoding
MSTRG.31923.2	-2.4409	noncoding
MSTRG.31994.3	-0.364604	noncoding
MSTRG.31998.2	0.786675	coding
MSTRG.31999.1	0.505534	coding
MSTRG.32003.1	-0.013406	noncoding
MSTRG.32003.2	0.007501	coding
MSTRG.32016.1	-1.99742	noncoding
MSTRG.32029.1	-1.09556	noncoding
MSTRG.32030.1	-0.988868	noncoding
MSTRG.32051.1	-1.66229	noncoding
MSTRG.32052.1	0.2743	coding
MSTRG.32052.2	0.270014	coding
MSTRG.31935.1	1.80218	coding
MSTRG.31935.2	0.981747	coding
MSTRG.31935.6	1.39667	coding
MSTRG.31935.8	1.43779	coding
MSTRG.31935.9	2.00259	coding
MSTRG.31935.10	0.064801	coding
MSTRG.31936.3	-2.52974	noncoding
MSTRG.31939.1	-1.15513	noncoding
MSTRG.31939.2	-0.991851	noncoding
MSTRG.31939.3	-1.55789	noncoding
MSTRG.31939.4	-0.952806	noncoding
MSTRG.31940.1	-0.327575	noncoding
MSTRG.31941.1	0.583515	coding
MSTRG.31941.2	0.693202	coding
MSTRG.31941.3	0.865137	coding
MSTRG.31941.4	0.591034	coding
MSTRG.31942.4	-0.249177	noncoding
MSTRG.31928.2	0.660677	coding
MSTRG.31930.1	-1.37451	noncoding
MSTRG.31930.2	0.632829	coding
MSTRG.31948.1	-0.746301	noncoding
MSTRG.31963.1	-2.03899	noncoding
MSTRG.31968.1	-0.702901	noncoding
MSTRG.31981.1	0.494575	coding
MSTRG.31990.1	-0.248274	noncoding
MSTRG.31992.2	3.06425	coding
MSTRG.32053.1	0.608654	coding

MSTRG.32054.1	-0.924751	noncoding
MSTRG.32056.2	0.273783	coding
MSTRG.32056.3	0.927	coding
MSTRG.32058.1	-0.59543	noncoding
MSTRG.32059.1	1.78947	coding
MSTRG.32068.2	0.623736	coding
MSTRG.32055.2	-0.488002	noncoding
MSTRG.32069.2	-0.71541	noncoding
MSTRG.32070.3	-0.84548	noncoding
MSTRG.32072.3	-0.464583	noncoding
MSTRG.32076.5	0.561584	coding
MSTRG.32076.7	0.377559	coding
MSTRG.32076.3	1.01058	coding
MSTRG.32076.4	1.02753	coding
MSTRG.32076.6	0.997344	coding
MSTRG.32077.1	-2.38017	noncoding
MSTRG.32078.1	0.432423	coding
MSTRG.32084.1	-2.03226	noncoding
MSTRG.32085.1	-1.76485	noncoding
MSTRG.32086.1	-1.47494	noncoding
MSTRG.32091.1	-0.289966	noncoding
MSTRG.32091.2	-1.22003	noncoding
MSTRG.32104.3	0.262769	coding
MSTRG.32105.1	-0.372987	noncoding
MSTRG.32106.1	-2.03953	noncoding
MSTRG.32107.1	-0.972751	noncoding
MSTRG.32108.2	-1.15126	noncoding
MSTRG.32108.1	-0.283244	noncoding
MSTRG.32109.1	-1.73876	noncoding
MSTRG.32110.1	0.072495	coding
MSTRG.32104.4	0.806065	coding
MSTRG.32111.1	0.10839	coding
MSTRG.32112.1	0.300505	coding
MSTRG.32094.1	-1.85231	noncoding
MSTRG.32094.2	-1.96611	noncoding
MSTRG.32113.1	-1.49946	noncoding
MSTRG.32114.2	-0.559137	noncoding
MSTRG.32115.1	-2.68883	noncoding
MSTRG.32116.2	1.29255	coding
MSTRG.32119.1	-1.26439	noncoding
MSTRG.32123.1	-2.26384	noncoding
MSTRG.32128.1	-0.237896	noncoding
MSTRG.32129.1	-0.621358	noncoding
MSTRG.32130.2	-0.974539	noncoding
MSTRG.32130.1	-0.63783	noncoding
MSTRG.32130.3	-0.664568	noncoding
MSTRG.32133.1	0.17615	coding
MSTRG.32134.1	2.17953	coding
MSTRG.32134.2	1.97629	coding
MSTRG.32134.4	0.143573	coding
MSTRG.32137.1	2.01328	coding
MSTRG.32137.2	2.04367	coding
MSTRG.32137.3	2.05314	coding
MSTRG.32136.1	-1.17883	noncoding

MSTRG.32140.1	-1.7869	noncoding
MSTRG.32141.1	0.913734	coding
MSTRG.32142.1	-0.668533	noncoding
MSTRG.32143.4	1.33531	coding
MSTRG.32146.1	1.69059	coding
MSTRG.32154.2	2.23488	coding
MSTRG.32155.1	2.35917	coding
MSTRG.32155.2	1.91087	coding
MSTRG.32158.3	-0.731198	noncoding
MSTRG.32163.1	-1.22123	noncoding
MSTRG.32164.1	0.776159	coding
MSTRG.32150.2	0.74427	coding
MSTRG.32150.3	1.01934	coding
MSTRG.32167.2	-1.11513	noncoding
MSTRG.32167.3	-1.37838	noncoding
MSTRG.32168.1	-1.39653	noncoding
MSTRG.32169.2	0.170478	coding
MSTRG.32166.1	-1.79213	noncoding
MSTRG.32172.2	1.2841	coding
MSTRG.32176.4	0.971398	coding
MSTRG.32176.3	0.512295	coding
MSTRG.32177.1	-0.466223	noncoding
MSTRG.32180.1	1.5872	coding
MSTRG.32181.4	1.14933	coding
MSTRG.32183.6	-0.490421	noncoding
MSTRG.32184.1	-0.673764	noncoding
MSTRG.32184.3	0.565532	coding
MSTRG.32199.1	0.20377	coding
MSTRG.32199.2	-0.928511	noncoding
MSTRG.32199.3	-0.318293	noncoding
MSTRG.32201.1	-1.10142	noncoding
MSTRG.32203.2	2.69516	coding
MSTRG.32203.4	2.45752	coding
MSTRG.32206.2	1.20345	coding
MSTRG.32206.4	0.852147	coding
MSTRG.32206.5	1.10503	coding
MSTRG.32208.2	1.13992	coding
MSTRG.32204.2	-0.401879	noncoding
MSTRG.32212.1	-0.384505	noncoding
MSTRG.32225.1	-1.85036	noncoding
MSTRG.32227.1	-2.87927	noncoding
MSTRG.32229.2	0.453278	coding
MSTRG.32232.1	2.56587	coding
MSTRG.32232.2	2.03681	coding
MSTRG.32231.1	-2.27324	noncoding
MSTRG.32231.2	-2.28094	noncoding
MSTRG.32237.2	0.402714	coding
MSTRG.32238.1	-0.892041	noncoding
MSTRG.32239.1	-0.399832	noncoding
MSTRG.32241.1	-1.79863	noncoding
MSTRG.32242.1	0.162022	coding
MSTRG.32246.2	2.25848	coding
MSTRG.32246.3	2.23304	coding
MSTRG.32249.1	2.07619	coding

MSTRG.32249.5	1.15618	coding
MSTRG.32250.1	-1.03015	noncoding
MSTRG.32251.1	0.622975	coding
MSTRG.32251.2	0.847059	coding
MSTRG.32252.1	-1.3185	noncoding
MSTRG.32254.1	0.765555	coding
MSTRG.32257.1	-0.847298	noncoding
MSTRG.32260.2	0.451876	coding
MSTRG.32265.1	0.145176	coding
MSTRG.32266.1	-0.80356	noncoding
MSTRG.32267.1	-0.197447	noncoding
MSTRG.32270.2	-1.14054	noncoding
MSTRG.32275.3	-0.097145	noncoding
MSTRG.32275.1	-0.034347	noncoding
MSTRG.32275.2	0.546212	coding
MSTRG.32275.6	0.220313	coding
MSTRG.32275.7	-0.07128	noncoding
MSTRG.32276.4	0.159959	coding
MSTRG.32278.3	1.40802	coding
MSTRG.32278.4	1.59467	coding
MSTRG.32277.1	2.08651	coding
MSTRG.32283.1	-1.92076	noncoding
MSTRG.32283.2	-1.87113	noncoding
MSTRG.32285.1	-1.06581	noncoding
MSTRG.32286.1	-0.137561	noncoding
MSTRG.32289.1	2.78355	coding
MSTRG.32291.2	0.262408	coding
MSTRG.32292.1	2.7753	coding
MSTRG.32292.3	2.77405	coding
MSTRG.32293.1	0.877148	coding
MSTRG.32300.3	0.480004	coding
MSTRG.32300.5	0.644774	coding
MSTRG.32301.3	1.57012	coding
MSTRG.32305.2	0.03315	coding
MSTRG.32324.12	1.35285	coding
MSTRG.32324.17	1.3341	coding
MSTRG.32324.18	1.21625	coding
MSTRG.32324.20	1.36831	coding
MSTRG.32324.21	1.35181	coding
MSTRG.32324.3	1.72579	coding
MSTRG.32324.33	1.48259	coding
MSTRG.32324.35	1.50981	coding
MSTRG.32324.38	1.39354	coding
MSTRG.32324.40	1.38091	coding
MSTRG.32324.43	1.38781	coding
MSTRG.32324.47	1.15857	coding
MSTRG.32324.49	1.2273	coding
MSTRG.32324.50	1.30737	coding
MSTRG.32324.53	1.6408	coding
MSTRG.32324.55	1.32918	coding
MSTRG.32324.56	1.20599	coding
MSTRG.32324.7	1.43642	coding
MSTRG.32324.9	1.2141	coding
MSTRG.32310.3	2.18655	coding

MSTRG.32310.4	2.07755	coding
MSTRG.32322.7	2.29308	coding
MSTRG.32322.10	2.10526	coding
MSTRG.32322.12	2.04104	coding
MSTRG.32322.8	1.90768	coding
MSTRG.32322.11	1.88872	coding
MSTRG.32322.3	1.90189	coding
MSTRG.32322.4	2.38342	coding
MSTRG.32322.9	2.21447	coding
MSTRG.32322.2	2.26206	coding
MSTRG.32322.16	2.2805	coding
MSTRG.32323.1	0.07683	coding
MSTRG.32320.3	0.918563	coding
MSTRG.32331.1	1.28552	coding
MSTRG.32331.3	1.17999	coding
MSTRG.32331.4	1.16736	coding
MSTRG.32331.5	1.85133	coding
MSTRG.32331.6	2.09852	coding
MSTRG.32334.1	1.67352	coding
MSTRG.32334.2	1.6975	coding
MSTRG.32334.3	1.89124	coding
MSTRG.32338.1	-1.7216	noncoding
MSTRG.32339.2	-0.264191	noncoding
MSTRG.32339.1	0.095509	coding
MSTRG.32339.3	-0.576658	noncoding
MSTRG.32338.4	1.0757	coding
MSTRG.32338.6	1.01576	coding
MSTRG.32338.7	0.964856	coding
MSTRG.32329.1	1.10447	coding
MSTRG.32329.2	1.30455	coding
MSTRG.32329.3	1.27601	coding
MSTRG.32329.5	1.44862	coding
MSTRG.32329.6	1.05575	coding
MSTRG.32329.7	1.33843	coding
MSTRG.32340.2	1.74365	coding
MSTRG.32340.4	2.05078	coding
MSTRG.32342.1	0.691981	coding
MSTRG.32343.2	-0.575918	noncoding
MSTRG.32344.1	0.066882	coding
MSTRG.32350.3	1.81828	coding
MSTRG.32354.2	1.56678	coding
MSTRG.32354.3	1.74024	coding
MSTRG.32356.1	2.3718	coding
MSTRG.32358.2	1.3924	coding
MSTRG.32358.3	1.57027	coding
MSTRG.32358.4	1.55617	coding
MSTRG.32364.3	0.445984	coding
MSTRG.32365.1	-0.479881	noncoding
MSTRG.32365.2	-0.915382	noncoding
MSTRG.32366.5	-0.875272	noncoding
MSTRG.32369.4	-0.334062	noncoding
MSTRG.32369.3	0.258007	coding
MSTRG.32371.2	1.49946	coding
MSTRG.32371.3	1.77868	coding

MSTRG.32359.2	-0.67309	noncoding
MSTRG.32372.1	-0.166459	noncoding
MSTRG.32377.1	-0.806113	noncoding
MSTRG.32381.7	1.79613	coding
MSTRG.32381.11	1.72362	coding
MSTRG.32381.10	1.52976	coding
MSTRG.32381.2	1.69543	coding
MSTRG.32381.3	1.84522	coding
MSTRG.32381.1	1.83483	coding
MSTRG.32381.8	1.71849	coding
MSTRG.32381.12	1.72625	coding
MSTRG.32381.4	2.16513	coding
MSTRG.32381.6	1.86536	coding
MSTRG.32381.14	-2.14154	noncoding
MSTRG.32378.1	-1.38885	noncoding
MSTRG.32384.10	1.21726	coding
MSTRG.32384.11	1.29205	coding
MSTRG.32384.5	1.33693	coding
MSTRG.32388.1	1.1937	coding
MSTRG.32395.5	1.70477	coding
MSTRG.32395.2	1.56786	coding
MSTRG.32395.3	1.56334	coding
MSTRG.32395.4	1.60041	coding
MSTRG.32395.7	1.39666	coding
MSTRG.32394.2	1.26181	coding
MSTRG.32397.2	0.786958	coding
MSTRG.32397.3	0.753359	coding
MSTRG.32397.4	1.04345	coding
MSTRG.32397.5	0.938082	coding
MSTRG.32397.6	0.955543	coding
MSTRG.32402.1	1.89894	coding
MSTRG.32405.3	0.775585	coding
MSTRG.32407.1	-2.00797	noncoding
MSTRG.32411.2	2.02278	coding
MSTRG.32412.2	2.01477	coding
MSTRG.32411.3	1.28914	coding
MSTRG.32411.4	1.13362	coding
MSTRG.32411.5	1.2711	coding
MSTRG.32415.1	0.627376	coding
MSTRG.32416.3	1.86273	coding
MSTRG.32418.6	2.629	coding
MSTRG.32418.9	2.5621	coding
MSTRG.32419.2	2.50791	coding
MSTRG.32424.1	1.09689	coding
MSTRG.32427.1	-0.660848	noncoding
MSTRG.32434.2	0.396604	coding
MSTRG.32441.1	0.008839	coding
MSTRG.32441.2	0.22245	coding
MSTRG.32441.3	0.015012	coding
MSTRG.32444.1	-0.429884	noncoding
MSTRG.32444.2	-1.49768	noncoding
MSTRG.32456.3	2.35688	coding
MSTRG.32456.2	2.33345	coding
MSTRG.32447.2	0.075807	coding

MSTRG.32451.2	1.38487	coding
MSTRG.32457.1	-0.966058	noncoding
MSTRG.32484.5	1.79207	coding
MSTRG.32484.6	1.80952	coding
MSTRG.32484.1	2.36456	coding
MSTRG.32484.4	2.31009	coding
MSTRG.32484.3	1.69049	coding
MSTRG.32484.2	2.2691	coding
MSTRG.32484.7	2.31007	coding
MSTRG.32484.8	1.9163	coding
MSTRG.32484.9	2.13838	coding
MSTRG.32484.11	1.0079	coding
MSTRG.32489.1	-0.277717	noncoding
MSTRG.32489.2	-0.252063	noncoding
MSTRG.32492.2	1.91905	coding
MSTRG.32492.5	2.17364	coding
MSTRG.32494.1	-1.90625	noncoding
MSTRG.32468.1	1.82923	coding
MSTRG.32468.4	2.2766	coding
MSTRG.32468.5	2.20919	coding
MSTRG.32468.6	-0.17661	noncoding
MSTRG.32469.1	0.94875	coding
MSTRG.32474.2	-0.834974	noncoding
MSTRG.32474.3	-0.730834	noncoding
MSTRG.32480.1	0.720373	coding
MSTRG.32497.2	0.106655	coding
MSTRG.32501.2	0.921496	coding
MSTRG.32501.3	0.563497	coding
MSTRG.32507.4	1.77896	coding
MSTRG.32507.3	1.81999	coding
MSTRG.32507.6	0.909338	coding
MSTRG.32509.1	1.12062	coding
MSTRG.32509.3	0.962073	coding
MSTRG.32513.1	-1.00902	noncoding
MSTRG.32513.2	-0.717418	noncoding
MSTRG.32514.4	2.81979	coding
MSTRG.32514.5	1.05549	coding
MSTRG.32515.1	0.078301	coding
MSTRG.32519.1	1.34745	coding
MSTRG.32519.3	1.00168	coding
MSTRG.32519.4	1.24912	coding
MSTRG.32519.5	0.831806	coding
MSTRG.32519.6	0.520049	coding
MSTRG.32519.7	1.20253	coding
MSTRG.32519.8	1.15511	coding
MSTRG.32519.9	0.365894	coding
MSTRG.32520.1	0.012215	coding
MSTRG.32520.2	-0.241362	noncoding
MSTRG.32520.3	0.075832	coding
MSTRG.32520.4	-0.747179	noncoding
MSTRG.32520.5	0.0437	coding
MSTRG.32523.3	0.107318	coding
MSTRG.32528.2	-0.592864	noncoding
MSTRG.32528.1	-1.47003	noncoding

MSTRG.32534.11	0.557143	coding
MSTRG.32534.10	0.182966	coding
MSTRG.32534.3	-0.132952	noncoding
MSTRG.32534.4	-0.153601	noncoding
MSTRG.32534.6	-0.130648	noncoding
MSTRG.32534.7	-0.137169	noncoding
MSTRG.32534.13	1.71857	coding
MSTRG.32534.5	2.62426	coding
MSTRG.32534.12	2.7766	coding
MSTRG.32534.8	2.82456	coding
MSTRG.32542.1	-0.753844	noncoding
MSTRG.32542.3	-0.64789	noncoding
MSTRG.32545.1	-1.59312	noncoding
MSTRG.32546.2	2.19501	coding
MSTRG.32547.4	0.864234	coding
MSTRG.32547.5	0.952414	coding
MSTRG.32547.6	0.725849	coding
MSTRG.32549.1	-0.083958	noncoding
MSTRG.32549.2	0.056652	coding
MSTRG.32549.3	-0.073987	noncoding
MSTRG.32550.1	-0.555846	noncoding
MSTRG.32552.1	-1.5338	noncoding
MSTRG.32554.2	-1.31677	noncoding
MSTRG.32554.1	-1.08953	noncoding
MSTRG.32554.3	-0.687107	noncoding
MSTRG.32538.2	-0.305621	noncoding
MSTRG.32538.4	-0.439495	noncoding
MSTRG.32538.5	1.40633	coding
MSTRG.32541.1	1.98558	coding
MSTRG.32541.4	2.06616	coding
MSTRG.32557.1	-1.77943	noncoding
MSTRG.32558.1	1.16212	coding
MSTRG.32558.2	1.1916	coding
MSTRG.32561.2	1.4637	coding
MSTRG.32561.3	1.54155	coding
MSTRG.32561.4	1.29877	coding
MSTRG.32561.5	1.5166	coding
MSTRG.32564.1	1.71355	coding
MSTRG.32564.2	1.52901	coding
MSTRG.32562.1	-0.021217	noncoding
MSTRG.32566.3	0.100393	coding
MSTRG.32566.4	0.21957	coding
MSTRG.32566.5	-0.451184	noncoding
MSTRG.32568.3	0.131311	coding
MSTRG.32575.3	1.00861	coding
MSTRG.32575.4	1.11579	coding
MSTRG.32575.5	1.83996	coding
MSTRG.32575.6	1.11272	coding
MSTRG.32575.8	1.42366	coding
MSTRG.32575.10	1.52952	coding
MSTRG.32575.9	1.26043	coding
MSTRG.32575.11	1.50907	coding
MSTRG.32575.12	1.55703	coding
MSTRG.32574.3	0.540483	coding

MSTRG.32576.2	-0.30813	noncoding
MSTRG.32582.1	0.569002	coding
MSTRG.32588.3	2.6189	coding
MSTRG.32592.1	1.27021	coding
MSTRG.32592.3	1.03893	coding
MSTRG.32596.2	1.93462	coding
MSTRG.32598.2	-1.17266	noncoding
MSTRG.32606.2	1.5463	coding
MSTRG.32606.4	1.49777	coding
MSTRG.32606.3	1.63306	coding
MSTRG.32607.2	0.52244	coding
MSTRG.32607.3	-1.07252	noncoding
MSTRG.32604.3	1.78256	coding
MSTRG.32609.2	1.14068	coding
MSTRG.32611.1	0.353358	coding
MSTRG.32613.1	-0.081947	noncoding
MSTRG.32614.1	0.43109	coding
MSTRG.32614.2	0.738101	coding
MSTRG.32614.3	0.789963	coding
MSTRG.32614.4	0.856322	coding
MSTRG.32614.5	0.470481	coding
MSTRG.32614.6	0.674765	coding
MSTRG.32617.1	0.063939	coding
MSTRG.32619.2	0.32601	coding
MSTRG.32621.1	-1.78097	noncoding
MSTRG.32628.1	0.037132	coding
MSTRG.32628.2	-1.54785	noncoding
MSTRG.32640.1	1.34307	coding
MSTRG.32641.1	0.337665	coding
MSTRG.32641.2	0.465496	coding
MSTRG.32641.3	0.558492	coding
MSTRG.32641.4	0.074	coding
MSTRG.32634.1	1.43444	coding
MSTRG.32634.2	1.52684	coding
MSTRG.32646.2	-2.02143	noncoding
MSTRG.32646.3	-1.8995	noncoding
MSTRG.32658.1	-0.281203	noncoding
MSTRG.32658.2	-0.053274	noncoding
MSTRG.32658.4	-0.598439	noncoding
MSTRG.32658.3	-0.370909	noncoding
MSTRG.32660.1	-1.78066	noncoding
MSTRG.32661.2	1.69085	coding
MSTRG.32662.1	-0.631112	noncoding
MSTRG.32663.1	-0.16085	noncoding
MSTRG.32664.1	-0.35646	noncoding
MSTRG.32665.1	-1.24394	noncoding
MSTRG.32666.2	-0.850257	noncoding
MSTRG.32656.1	1.5945	coding
MSTRG.32656.3	0.572895	coding
MSTRG.32668.5	2.63191	coding
MSTRG.32668.6	2.54322	coding
MSTRG.32674.2	0.478789	coding
MSTRG.32672.1	0.299336	coding
MSTRG.32681.3	2.62351	coding

MSTRG.32683.1	1.11443	coding
MSTRG.32682.3	0.590552	coding
MSTRG.32682.4	0.222158	coding
MSTRG.32686.1	3.63206	coding
MSTRG.32686.3	3.88327	coding
MSTRG.32687.1	0.401017	coding
MSTRG.32688.1	-1.39537	noncoding
MSTRG.32689.1	-0.271968	noncoding
MSTRG.32690.1	1.03323	coding
MSTRG.32691.1	-0.463142	noncoding
MSTRG.32692.1	-1.53233	noncoding
MSTRG.32697.1	-2.23331	noncoding
MSTRG.32698.1	-1.49576	noncoding
MSTRG.32699.1	1.14264	coding
MSTRG.32699.2	0.838838	coding
MSTRG.32699.3	0.83079	coding
MSTRG.32699.4	0.460938	coding
MSTRG.32699.5	0.699686	coding
MSTRG.32709.11	-1.1369	noncoding
MSTRG.32712.1	1.38345	coding
MSTRG.32719.2	0.910248	coding
MSTRG.32720.1	0.141639	coding
MSTRG.32718.2	0.864283	coding
MSTRG.32723.2	1.74777	coding
MSTRG.32723.3	1.80594	coding
MSTRG.32723.10	1.55291	coding
MSTRG.32723.8	1.42985	coding
MSTRG.32723.7	1.42477	coding
MSTRG.32723.11	1.56025	coding
MSTRG.32723.9	1.69974	coding
MSTRG.32723.12	1.51486	coding
MSTRG.32723.14	1.5113	coding
MSTRG.32723.16	1.60177	coding
MSTRG.32723.13	-0.086437	noncoding
MSTRG.32723.15	1.34398	coding
MSTRG.32723.17	1.04514	coding
MSTRG.32723.18	0.544761	coding
MSTRG.32721.5	0.900489	coding
MSTRG.32721.6	1.1207	coding
MSTRG.32730.1	0.556334	coding
MSTRG.32731.1	-2.19828	noncoding
MSTRG.32732.1	-0.84198	noncoding
MSTRG.32730.3	-1.19828	noncoding
MSTRG.32738.1	0.387662	coding
MSTRG.32738.2	0.49441	coding
MSTRG.32738.4	1.77637	coding
MSTRG.32738.5	0.518702	coding
MSTRG.32738.6	0.496207	coding
MSTRG.32738.7	0.999074	coding
MSTRG.32739.1	2.17018	coding
MSTRG.32740.1	-2.30403	noncoding
MSTRG.32747.4	0.556407	coding
MSTRG.32747.9	0.209654	coding
MSTRG.32749.1	-0.717265	noncoding

MSTRG.32751.3	1.58677	coding
MSTRG.32751.2	1.21842	coding
MSTRG.32752.1	-1.67631	noncoding
MSTRG.32749.2	-0.815227	noncoding
MSTRG.32752.2	0.306159	coding
MSTRG.32752.4	0.154708	coding
MSTRG.32752.5	-0.480448	noncoding
MSTRG.32752.6	-0.480448	noncoding
MSTRG.32752.7	-0.395487	noncoding
MSTRG.32752.8	-0.402964	noncoding
MSTRG.32752.9	-0.962047	noncoding
MSTRG.32754.1	-1.75514	noncoding
MSTRG.32754.2	-2.32745	noncoding
MSTRG.32754.3	-2.27262	noncoding
MSTRG.32755.1	-1.39719	noncoding
MSTRG.32754.4	-1.91089	noncoding
MSTRG.32756.1	-3.08981	noncoding
MSTRG.32757.1	-1.13241	noncoding
MSTRG.32760.1	-1.18856	noncoding
MSTRG.32765.1	0.514031	coding
MSTRG.32773.1	-0.098616	noncoding
MSTRG.32767.1	1.33557	coding
MSTRG.32781.1	0.821548	coding
MSTRG.32782.1	0.917498	coding
MSTRG.32796.3	-1.71013	noncoding
MSTRG.32796.5	-1.71013	noncoding
MSTRG.32796.4	-1.2522	noncoding
MSTRG.32796.6	-0.492641	noncoding
MSTRG.32796.7	1.11119	coding
MSTRG.32796.9	0.421384	coding
MSTRG.32796.10	1.11087	coding
MSTRG.32796.8	1.11269	coding
MSTRG.32796.11	0.951311	coding
MSTRG.32796.12	1.05848	coding
MSTRG.32796.14	-1.97025	noncoding
MSTRG.32796.13	1.31255	coding
MSTRG.32796.15	0.868236	coding
MSTRG.32796.16	0.358379	coding
MSTRG.32796.17	0.804748	coding
MSTRG.32796.18	1.26683	coding
MSTRG.32796.19	1.24625	coding
MSTRG.32796.20	1.0564	coding
MSTRG.32798.3	0.430694	coding
MSTRG.32798.4	0.765478	coding
MSTRG.32798.5	0.502702	coding
MSTRG.32798.2	0.480139	coding
MSTRG.32798.6	0.412503	coding
MSTRG.32798.8	-0.180348	noncoding
MSTRG.32789.1	2.24837	coding
MSTRG.32792.1	-1.5574	noncoding
MSTRG.32801.1	1.25975	coding
MSTRG.32801.3	0.780562	coding
MSTRG.32801.4	0.238048	coding
MSTRG.32802.1	-1.79483	noncoding

MSTRG.32814.2	2.19267	coding
MSTRG.32815.1	-1.86364	noncoding
MSTRG.32821.1	3.24728	coding
MSTRG.32821.2	3.09334	coding
MSTRG.32821.3	2.28697	coding
MSTRG.32821.4	2.57704	coding
MSTRG.32821.5	2.67734	coding
MSTRG.32819.1	-2.16624	noncoding
MSTRG.32820.2	-2.3266	noncoding
MSTRG.32827.1	-0.378188	noncoding
MSTRG.32828.1	-1.10025	noncoding
MSTRG.32832.1	-2.37159	noncoding
MSTRG.32832.2	-2.6793	noncoding
MSTRG.32836.1	3.3863	coding
MSTRG.32837.1	-1.89296	noncoding
MSTRG.32841.1	0.044024	coding
MSTRG.32842.7	-1.25948	noncoding
MSTRG.32842.8	-1.25948	noncoding
MSTRG.32842.6	-1.27453	noncoding
MSTRG.32842.2	-1.23336	noncoding
MSTRG.32839.1	-2.56975	noncoding
MSTRG.32839.2	-3.13622	noncoding
MSTRG.32839.3	-2.98873	noncoding
MSTRG.32840.1	-2.41161	noncoding
MSTRG.32849.1	-1.87036	noncoding
MSTRG.32859.1	-1.56933	noncoding
MSTRG.32860.1	0.376564	coding
MSTRG.32862.1	-1.29069	noncoding
MSTRG.32859.2	-0.592216	noncoding
MSTRG.32855.1	-2.04331	noncoding
MSTRG.32866.1	-0.304094	noncoding
MSTRG.32867.1	0.208891	coding
MSTRG.32867.2	-0.331891	noncoding
MSTRG.32867.3	-0.038746	noncoding
MSTRG.32867.4	0.09274	coding
MSTRG.32867.5	0.426517	coding
MSTRG.32867.6	-0.829775	noncoding
MSTRG.32867.7	-0.107501	noncoding
MSTRG.32863.5	0.689812	coding
MSTRG.32863.6	1.91748	coding
MSTRG.32864.2	0.556921	coding
MSTRG.32874.3	1.30839	coding
MSTRG.32874.5	1.26963	coding
MSTRG.32874.6	0.666074	coding
MSTRG.32876.1	-0.668413	noncoding
MSTRG.32877.1	-0.030485	noncoding
MSTRG.32869.1	-0.619724	noncoding
MSTRG.32880.1	-1.85793	noncoding
MSTRG.32884.1	-0.032847	noncoding
MSTRG.32888.1	-1.25374	noncoding
MSTRG.32889.4	0.44676	coding
MSTRG.32889.3	0.656533	coding
MSTRG.32889.6	0.151331	coding
MSTRG.32889.5	0.581462	coding

MSTRG.32889.7	0.711618	coding
MSTRG.32889.8	0.542561	coding
MSTRG.32889.9	-0.10927	noncoding
MSTRG.32892.1	2.46359	coding
MSTRG.32892.5	0.235553	coding
MSTRG.32893.4	1.47628	coding
MSTRG.32895.3	0.614054	coding
MSTRG.32897.1	-2.05436	noncoding
MSTRG.32899.1	-2.03039	noncoding
MSTRG.32906.3	0.748621	coding
MSTRG.32906.4	1.00568	coding
MSTRG.32906.6	0.653296	coding
MSTRG.32906.5	1.40017	coding
MSTRG.32907.1	-1.82241	noncoding
MSTRG.32916.1	0.026034	coding
MSTRG.32921.1	-0.233287	noncoding
MSTRG.32922.2	1.15322	coding
MSTRG.32922.4	1.22072	coding
MSTRG.32925.1	-2.20167	noncoding
MSTRG.32925.2	0.859027	coding
MSTRG.32927.1	0.674718	coding
MSTRG.32929.1	1.06262	coding
MSTRG.32930.2	1.9472	coding
MSTRG.32933.1	-0.289567	noncoding
MSTRG.32936.6	-0.136644	noncoding
MSTRG.32936.7	0.180581	coding
MSTRG.32936.8	0.109082	coding
MSTRG.32936.9	0.082486	coding
MSTRG.32934.2	-0.525324	noncoding
MSTRG.32934.3	-0.041225	noncoding
MSTRG.32934.4	-0.400562	noncoding
MSTRG.32934.5	-0.338675	noncoding
MSTRG.32935.1	-0.338313	noncoding
MSTRG.32937.2	0.012403	coding
MSTRG.32939.2	2.80532	coding
MSTRG.32943.1	-2.08779	noncoding
MSTRG.32945.2	-0.300934	noncoding
MSTRG.32946.1	-0.276353	noncoding
MSTRG.32951.4	1.83908	coding
MSTRG.32953.1	2.35201	coding
MSTRG.32957.5	1.63094	coding
MSTRG.32957.3	1.66361	coding
MSTRG.32954.2	0.961097	coding
MSTRG.32961.2	2.14068	coding
MSTRG.32964.1	2.18635	coding
MSTRG.32968.2	1.96976	coding
MSTRG.32984.2	0.97425	coding
MSTRG.32984.4	0.436563	coding
MSTRG.32984.3	0.536343	coding
MSTRG.32984.5	0.99766	coding
MSTRG.32981.1	0.144118	coding
MSTRG.32988.1	-1.9542	noncoding
MSTRG.32992.1	-2.53255	noncoding
MSTRG.33013.3	1.48771	coding

MSTRG.33013.4	0.980591	coding
MSTRG.33014.1	1.05475	coding
MSTRG.33018.1	-0.850269	noncoding
MSTRG.33003.1	-2.19076	noncoding
MSTRG.33021.1	1.7241	coding
MSTRG.33021.2	2.32886	coding
MSTRG.33021.3	1.2908	coding
MSTRG.33023.1	-0.019789	noncoding
MSTRG.33024.1	-2.13725	noncoding
MSTRG.33025.1	-1.01097	noncoding
MSTRG.33031.5	0.717702	coding
MSTRG.33031.6	0.169789	coding
MSTRG.33033.2	1.45902	coding
MSTRG.33036.1	-1.00083	noncoding
MSTRG.33036.3	0.128257	coding
MSTRG.33050.3	-1.16621	noncoding
MSTRG.33050.2	-0.897911	noncoding
MSTRG.33050.5	-1.2126	noncoding
MSTRG.33054.1	0.262594	coding
MSTRG.33056.1	0.401845	coding
MSTRG.33063.2	1.32761	coding
MSTRG.33063.3	0.90654	coding
MSTRG.33068.1	-0.467957	noncoding
MSTRG.33069.1	1.00214	coding
MSTRG.33083.6	0.49493	coding
MSTRG.33088.1	0.482312	coding
MSTRG.33090.2	0.42843	coding
MSTRG.33090.3	-1.00497	noncoding
MSTRG.33097.2	0.187096	coding
MSTRG.33101.1	1.69612	coding
MSTRG.33102.5	1.12728	coding
MSTRG.33102.4	1.37784	coding
MSTRG.33102.6	1.5814	coding
MSTRG.33114.1	2.23429	coding
MSTRG.33114.2	2.23429	coding
MSTRG.33114.3	-1.14569	noncoding
MSTRG.33114.4	2.29136	coding
MSTRG.33114.5	2.38245	coding
MSTRG.33114.6	2.25965	coding
MSTRG.33114.7	2.12606	coding
MSTRG.33114.8	1.96346	coding
MSTRG.33114.9	2.03776	coding
MSTRG.33109.1	-2.55568	noncoding
MSTRG.33108.3	-0.195396	noncoding
MSTRG.33120.2	0.483837	coding
MSTRG.33124.3	-0.013311	noncoding
MSTRG.33124.1	-0.345297	noncoding
MSTRG.33127.4	0.903092	coding
MSTRG.33127.5	1.30126	coding
MSTRG.33128.1	1.83583	coding
MSTRG.33131.1	-0.609402	noncoding
MSTRG.33132.1	-0.430656	noncoding
MSTRG.33131.2	-0.832752	noncoding
MSTRG.33131.4	-0.476301	noncoding

MSTRG.33133.1	-1.11645	noncoding
MSTRG.33129.2	-0.0191	noncoding
MSTRG.33135.1	0.320396	coding
MSTRG.33138.2	0.207853	coding
MSTRG.33141.2	0.555083	coding
MSTRG.33144.1	0.629306	coding
MSTRG.33144.2	0.484977	coding
MSTRG.33150.2	0.111824	coding
MSTRG.33151.1	0.299433	coding
MSTRG.33148.1	0.962088	coding
MSTRG.33155.2	-1.151	noncoding
MSTRG.33158.1	1.18856	coding
MSTRG.33161.1	2.24787	coding
MSTRG.33161.2	1.54652	coding
MSTRG.33161.3	1.81637	coding
MSTRG.33164.1	-0.577086	noncoding
MSTRG.33165.1	-0.577086	noncoding
MSTRG.33166.1	-0.471143	noncoding
MSTRG.33166.2	-0.324146	noncoding
MSTRG.33168.1	-2.16856	noncoding
MSTRG.33172.1	-2.41895	noncoding
MSTRG.33179.1	0.092464	coding
MSTRG.33189.1	-2.4446	noncoding
MSTRG.33224.2	1.51722	coding
MSTRG.33224.3	1.58822	coding
MSTRG.33223.1	-2.06729	noncoding
MSTRG.33234.1	-2.0396	noncoding
MSTRG.33248.1	-0.907814	noncoding
MSTRG.33249.1	-1.19931	noncoding
MSTRG.33245.1	0.964969	coding
MSTRG.33278.2	0.785486	coding
MSTRG.33280.1	0.999306	coding
MSTRG.33282.4	1.75413	coding
MSTRG.33284.1	2.43545	coding
MSTRG.33287.3	0.548296	coding
MSTRG.33301.2	2.67425	coding
MSTRG.33306.3	0.543887	coding
MSTRG.33309.1	0.901506	coding
MSTRG.33309.3	2.84157	coding
MSTRG.33319.1	-1.92633	noncoding
MSTRG.33322.3	0.792534	coding
MSTRG.33322.4	0.820154	coding
MSTRG.33322.5	-0.148038	noncoding
MSTRG.33323.1	-0.260489	noncoding
MSTRG.33325.2	2.15535	coding
MSTRG.33325.4	2.36791	coding
MSTRG.33325.5	2.21919	coding
MSTRG.33333.1	1.08983	coding
MSTRG.33333.2	1.74326	coding
MSTRG.33333.5	1.02288	coding
MSTRG.33333.4	0.35902	coding
MSTRG.33328.1	0.723444	coding
MSTRG.33329.1	1.46173	coding
MSTRG.33329.2	1.60883	coding

MSTRG.33329.4	1.63246	coding
MSTRG.33329.5	0.237852	coding
MSTRG.33338.1	0.836941	coding
MSTRG.33338.3	-0.664238	noncoding
MSTRG.33339.1	-2.31744	noncoding
MSTRG.33348.1	-2.15355	noncoding
MSTRG.33367.7	2.06768	coding
MSTRG.33373.2	2.35498	coding
MSTRG.33373.3	2.24851	coding
MSTRG.33373.5	1.80629	coding
MSTRG.33373.4	2.14345	coding
MSTRG.33373.8	2.02372	coding
MSTRG.33374.1	-1.88636	noncoding
MSTRG.33369.2	0.406515	coding
MSTRG.33369.3	0.337405	coding
MSTRG.33376.1	-0.42798	noncoding
MSTRG.33386.1	-1.22746	noncoding
MSTRG.33388.1	-1.78789	noncoding
MSTRG.33389.2	1.88416	coding
MSTRG.33391.1	1.80253	coding
MSTRG.33398.2	2.12594	coding
MSTRG.33398.1	1.95421	coding
MSTRG.33400.2	0.256691	coding
MSTRG.33400.1	0.24255	coding
MSTRG.33400.5	0.014001	coding
MSTRG.33403.5	0.958372	coding
MSTRG.33404.1	-1.28526	noncoding
MSTRG.33405.2	1.32284	coding
MSTRG.33405.3	1.04653	coding
MSTRG.33417.1	-1.86158	noncoding
MSTRG.33419.13	-0.918602	noncoding
MSTRG.33419.20	-0.604432	noncoding
MSTRG.33419.23	-0.543147	noncoding
MSTRG.33419.24	-0.498601	noncoding
MSTRG.33408.3	1.74333	coding
MSTRG.33423.3	2.09148	coding
MSTRG.33423.4	2.30013	coding
MSTRG.33424.1	2.05536	coding
MSTRG.33424.2	2.0909	coding
MSTRG.33424.3	1.94344	coding
MSTRG.33443.13	2.46663	coding
MSTRG.33443.9	2.26424	coding
MSTRG.33443.3	2.2344	coding
MSTRG.33443.11	2.35378	coding
MSTRG.33443.14	2.29517	coding
MSTRG.33443.6	2.31424	coding
MSTRG.33443.5	2.28337	coding
MSTRG.33443.15	1.5244	coding
MSTRG.33448.8	1.78654	coding
MSTRG.33450.1	1.44962	coding
MSTRG.33451.1	0.142323	coding
MSTRG.33452.1	-0.535298	noncoding
MSTRG.33453.1	2.34535	coding
MSTRG.33453.2	2.36149	coding

MSTRG.33453.3	2.36859	coding
MSTRG.33453.4	2.39453	coding
MSTRG.33453.5	2.38328	coding
MSTRG.33453.6	2.23038	coding
MSTRG.33430.1	-2.09257	noncoding
MSTRG.33439.2	2.6432	coding
MSTRG.33439.3	1.62848	coding
MSTRG.33444.1	-1.24375	noncoding
MSTRG.33466.2	0.702503	coding
MSTRG.33467.1	-2.3414	noncoding
MSTRG.33482.1	1.87975	coding
MSTRG.33482.3	1.6304	coding
MSTRG.33483.1	-1.00596	noncoding
MSTRG.33482.5	2.21418	coding
MSTRG.33482.6	1.61748	coding
MSTRG.33482.7	1.70203	coding
MSTRG.33482.8	1.4555	coding
MSTRG.33519.1	0.897598	coding
MSTRG.33521.1	1.5827	coding
MSTRG.33525.1	-1.19745	noncoding
MSTRG.33534.1	-1.13397	noncoding
MSTRG.33534.2	-1.42784	noncoding
MSTRG.33535.4	0.916642	coding
MSTRG.33535.5	1.6643	coding
MSTRG.33535.6	1.5767	coding
MSTRG.33536.1	-0.818794	noncoding
MSTRG.33535.7	1.41655	coding
MSTRG.33537.1	-0.102964	noncoding
MSTRG.33535.8	-0.682825	noncoding
MSTRG.33535.9	1.13198	coding
MSTRG.33546.1	2.23787	coding
MSTRG.33546.3	2.28024	coding
MSTRG.33548.4	2.59615	coding
MSTRG.33553.1	1.8684	coding
MSTRG.33553.2	1.91337	coding
MSTRG.33553.3	1.9349	coding
MSTRG.33555.1	-0.638422	noncoding
MSTRG.33553.8	1.99641	coding
MSTRG.33550.2	0.607527	coding
MSTRG.33559.4	1.13954	coding
MSTRG.33568.1	-2.75143	noncoding
MSTRG.33573.1	0.977696	coding
MSTRG.33577.2	0.358701	coding
MSTRG.33577.1	0.873167	coding
MSTRG.33587.3	-1.98804	noncoding
MSTRG.33609.2	1.66276	coding
MSTRG.33614.1	1.56681	coding
MSTRG.33613.2	0.132661	coding
MSTRG.33619.1	0.720447	coding
MSTRG.33619.2	0.444362	coding
MSTRG.33627.2	0.698966	coding
MSTRG.33629.2	2.80447	coding
MSTRG.33629.1	2.21051	coding
MSTRG.33629.4	2.30006	coding

MSTRG.33629.6	2.25396	coding
MSTRG.33629.3	2.11738	coding
MSTRG.33629.5	2.27118	coding
MSTRG.33629.7	2.17214	coding
MSTRG.33629.10	2.18358	coding
MSTRG.33629.8	2.27284	coding
MSTRG.33629.9	2.15493	coding
MSTRG.33629.11	2.13143	coding
MSTRG.33634.2	3.07618	coding
MSTRG.33634.4	1.05878	coding
MSTRG.33639.6	0.922565	coding
MSTRG.33639.7	1.22184	coding
MSTRG.33648.2	0.25042	coding
MSTRG.33648.5	0.489507	coding
MSTRG.33649.1	0.916909	coding
MSTRG.33649.3	0.93077	coding
MSTRG.33654.2	1.43371	coding
MSTRG.33658.1	0.964166	coding
MSTRG.33662.3	1.39962	coding
MSTRG.33662.4	1.22741	coding
MSTRG.33661.1	0.615808	coding
MSTRG.33664.6	2.05357	coding
MSTRG.33664.2	2.42819	coding
MSTRG.33664.3	2.34127	coding
MSTRG.33664.4	2.33066	coding
MSTRG.33664.7	2.33765	coding
MSTRG.33664.8	2.00028	coding
MSTRG.33664.9	1.96115	coding
MSTRG.33664.10	-0.058853	noncoding
MSTRG.33666.1	-0.490963	noncoding
MSTRG.33669.2	-0.228838	noncoding
MSTRG.33669.1	0.798257	coding
MSTRG.33670.2	2.09506	coding
MSTRG.33676.1	1.74973	coding
MSTRG.33676.2	1.70259	coding
MSTRG.33676.3	1.19887	coding
MSTRG.33679.1	-1.29854	noncoding
MSTRG.33679.2	-1.42469	noncoding
MSTRG.33681.1	1.52282	coding
MSTRG.33682.1	-1.82617	noncoding
MSTRG.33683.1	0.674257	coding
MSTRG.33683.2	0.306728	coding
MSTRG.33683.3	-0.09505	noncoding
MSTRG.33686.2	1.06545	coding
MSTRG.33687.3	1.14534	coding
MSTRG.33688.2	1.47132	coding
MSTRG.33690.1	-0.028436	noncoding
MSTRG.33690.2	-0.36001	noncoding
MSTRG.33692.1	0.139029	coding
MSTRG.33692.2	0.143431	coding
MSTRG.33692.3	0.617493	coding
MSTRG.33709.1	0.845225	coding
MSTRG.33709.2	0.951524	coding
MSTRG.33709.3	0.833047	coding

MSTRG.33709.4	0.718532	coding
MSTRG.33709.5	0.915483	coding
MSTRG.33709.6	0.836146	coding
MSTRG.33712.1	-0.724586	noncoding
MSTRG.33712.2	0.899846	coding
MSTRG.33713.1	-0.277246	noncoding
MSTRG.33714.1	1.29767	coding
MSTRG.33729.1	1.72974	coding
MSTRG.33731.1	0.96632	coding
MSTRG.33744.3	0.743882	coding
MSTRG.33754.2	-1.20877	noncoding
MSTRG.33754.1	-1.51974	noncoding
MSTRG.33754.4	-1.56321	noncoding
MSTRG.33754.5	-1.56321	noncoding
MSTRG.33754.3	-1.30637	noncoding
MSTRG.33754.6	-1.71193	noncoding
MSTRG.33756.2	2.8684	coding
MSTRG.33760.1	-1.9033	noncoding
MSTRG.33760.2	-1.64518	noncoding
MSTRG.33764.1	-0.41285	noncoding
MSTRG.33758.1	-1.5484	noncoding
MSTRG.33768.1	1.70176	coding
MSTRG.33768.2	1.11801	coding
MSTRG.33768.3	0.550427	coding
MSTRG.33768.4	1.87683	coding
MSTRG.33769.1	-0.084925	noncoding
MSTRG.33770.1	0.39147	coding
MSTRG.33768.5	0.48978	coding
MSTRG.33768.6	0.964586	coding
MSTRG.33771.1	0.596849	coding
MSTRG.33772.1	-0.661485	noncoding
MSTRG.33773.1	0.253973	coding
MSTRG.33787.1	-1.22383	noncoding
MSTRG.33789.1	-0.928968	noncoding
MSTRG.33792.1	-2.07985	noncoding
MSTRG.33793.1	-0.439104	noncoding
MSTRG.33791.1	-0.746603	noncoding
MSTRG.33813.2	2.06021	coding
MSTRG.33813.4	2.02768	coding
MSTRG.33813.5	1.50113	coding
MSTRG.33813.6	1.55596	coding
MSTRG.33815.1	0.761627	coding
MSTRG.33817.1	-0.920956	noncoding
MSTRG.33819.2	0.792008	coding
MSTRG.33820.1	0.441593	coding
MSTRG.33820.2	0.488796	coding
MSTRG.33820.4	0.532094	coding
MSTRG.33822.1	-1.42079	noncoding
MSTRG.33824.1	-1.81813	noncoding
MSTRG.33817.2	2.73022	coding
MSTRG.33817.4	0.200066	coding
MSTRG.33804.1	-2.21702	noncoding
MSTRG.33803.1	-2.32637	noncoding
MSTRG.33805.1	-2.32819	noncoding

MSTRG.33806.1	-2.28652	noncoding
MSTRG.33806.2	-2.32327	noncoding
MSTRG.33809.1	-2.25945	noncoding
MSTRG.33810.1	-2.32135	noncoding
MSTRG.33811.1	-2.36129	noncoding
MSTRG.33812.1	-2.38646	noncoding
MSTRG.33812.2	-2.26564	noncoding
MSTRG.33846.1	0.281109	coding
MSTRG.33829.1	-1.30741	noncoding
MSTRG.33831.1	-1.39376	noncoding
MSTRG.33831.2	-1.41591	noncoding
MSTRG.33831.3	-1.80627	noncoding
MSTRG.33831.4	-1.38879	noncoding
MSTRG.33832.1	0.213131	coding
MSTRG.33832.2	0.130774	coding
MSTRG.33838.1	-2.13779	noncoding
MSTRG.33839.1	-2.14478	noncoding
MSTRG.33839.2	-2.27513	noncoding
MSTRG.33841.1	-0.25914	noncoding
MSTRG.33843.1	0.222624	coding
MSTRG.33851.1	-1.60927	noncoding
MSTRG.33852.1	-1.49562	noncoding
MSTRG.33858.1	-2.34608	noncoding
MSTRG.33860.1	-2.38913	noncoding
MSTRG.33861.1	-2.44074	noncoding
MSTRG.33863.1	-2.4749	noncoding
MSTRG.33865.1	-1.74872	noncoding
MSTRG.33868.1	-0.490526	noncoding
MSTRG.33868.2	-0.932697	noncoding
MSTRG.33869.1	-2.33283	noncoding
MSTRG.33869.2	-2.29851	noncoding
MSTRG.33875.1	0.580758	coding
MSTRG.33875.2	0.209541	coding
MSTRG.33875.4	0.728973	coding
MSTRG.33875.6	-0.231654	noncoding
MSTRG.33874.1	-0.946927	noncoding
MSTRG.33879.1	-0.925372	noncoding
MSTRG.33882.1	1.87589	coding
MSTRG.33883.1	-2.17051	noncoding
MSTRG.33886.1	-1.50155	noncoding
MSTRG.33880.1	-0.466939	noncoding
MSTRG.33881.1	-2.28092	noncoding
MSTRG.33896.2	-0.497861	noncoding
MSTRG.33896.3	-0.597672	noncoding
MSTRG.33896.1	-0.508556	noncoding
MSTRG.33896.4	-0.121622	noncoding
MSTRG.33896.5	0.088955	coding
MSTRG.33896.6	-0.425472	noncoding
MSTRG.33894.2	-0.467253	noncoding
MSTRG.33898.1	-1.94932	noncoding
MSTRG.33898.2	-1.94932	noncoding
MSTRG.33898.3	-0.334938	noncoding
MSTRG.33898.4	-2.2055	noncoding
MSTRG.33898.5	-0.915087	noncoding

MSTRG.33898.6	-0.908046	noncoding
MSTRG.33899.1	-1.33157	noncoding
MSTRG.33899.2	-0.874595	noncoding
MSTRG.33899.3	-0.872248	noncoding
MSTRG.33899.4	-0.872248	noncoding
MSTRG.33898.7	-1.73587	noncoding
MSTRG.33901.1	-1.86455	noncoding
MSTRG.33901.2	-1.94159	noncoding
MSTRG.33901.3	-1.85015	noncoding
MSTRG.33901.4	-1.63202	noncoding
MSTRG.33901.5	-1.72537	noncoding
MSTRG.33901.6	-1.84762	noncoding
MSTRG.33901.7	-2.50603	noncoding
MSTRG.33901.8	-2.27832	noncoding
MSTRG.33901.9	-1.76242	noncoding
MSTRG.33903.1	0.99684	coding
MSTRG.33910.1	-2.32614	noncoding
MSTRG.33910.2	-2.32516	noncoding
MSTRG.33914.2	0.794912	coding
MSTRG.33914.1	0.67868	coding
MSTRG.33914.3	0.810087	coding
MSTRG.33917.1	-1.61262	noncoding
MSTRG.33911.1	-2.30909	noncoding
MSTRG.33920.2	0.048143	coding
MSTRG.33920.3	-0.079005	noncoding
MSTRG.33921.2	-0.544953	noncoding
MSTRG.33921.3	-0.71947	noncoding
MSTRG.33922.1	-1.24672	noncoding
MSTRG.33922.2	-1.94916	noncoding
MSTRG.33949.1	-1.09995	noncoding
MSTRG.33949.2	-2.06036	noncoding
MSTRG.33949.3	-0.865878	noncoding
MSTRG.33950.2	0.302846	coding
MSTRG.33950.5	0.350743	coding
MSTRG.33950.6	0.258034	coding
MSTRG.33950.7	0.257273	coding
MSTRG.33950.12	-0.786077	noncoding
MSTRG.33950.10	0.365348	coding
MSTRG.33950.13	0.644543	coding
MSTRG.33950.14	0.711204	coding
MSTRG.33950.9	0.696798	coding
MSTRG.33950.11	0.241128	coding
MSTRG.33950.15	-0.354375	noncoding
MSTRG.33950.17	-0.612996	noncoding
MSTRG.33950.19	-0.815366	noncoding
MSTRG.33953.1	1.13257	coding
MSTRG.33931.1	-1.34135	noncoding
MSTRG.33932.1	-1.93045	noncoding
MSTRG.33931.2	-1.69729	noncoding
MSTRG.33933.1	-1.50689	noncoding
MSTRG.33934.1	-2.65287	noncoding
MSTRG.33928.1	-1.66901	noncoding
MSTRG.33929.1	-0.554273	noncoding
MSTRG.33935.1	-1.02894	noncoding

MSTRG.33941.2	-0.439462	noncoding
MSTRG.33944.1	-1.9862	noncoding
MSTRG.33946.1	-2.24658	noncoding
MSTRG.33946.2	-2.24658	noncoding
MSTRG.33946.3	-2.28808	noncoding
MSTRG.33947.1	-2.24406	noncoding
MSTRG.33960.1	0.147167	coding
MSTRG.33960.2	0.87234	coding
MSTRG.33960.7	-0.600753	noncoding
MSTRG.33960.8	-0.368256	noncoding
MSTRG.33960.9	0.707457	coding
MSTRG.33960.10	0.162493	coding
MSTRG.33960.11	0.116832	coding
MSTRG.33963.1	-1.84781	noncoding
MSTRG.33964.1	-1.27086	noncoding
MSTRG.33965.1	-1.25072	noncoding
MSTRG.33965.2	-2.37105	noncoding
MSTRG.33966.1	-2.46638	noncoding
MSTRG.33962.1	-2.28152	noncoding
MSTRG.33962.2	-2.2881	noncoding
MSTRG.33967.1	-2.14278	noncoding
MSTRG.33985.4	0.329866	coding
MSTRG.33985.7	0.151147	coding
MSTRG.33985.10	0.178766	coding
MSTRG.33985.9	0.928077	coding
MSTRG.33985.11	0.154028	coding
MSTRG.33979.1	-2.35932	noncoding
MSTRG.33986.1	-1.4659	noncoding
MSTRG.33986.2	-1.69453	noncoding
MSTRG.33986.3	-1.93767	noncoding
MSTRG.33986.4	-1.72448	noncoding
MSTRG.33987.1	-2.07734	noncoding
MSTRG.33986.5	-1.68535	noncoding
MSTRG.33992.1	-2.47262	noncoding
MSTRG.33993.1	-2.06333	noncoding
MSTRG.33993.2	-2.08501	noncoding
MSTRG.33998.1	-2.2806	noncoding
MSTRG.33999.1	-2.32182	noncoding
MSTRG.34000.2	-0.662141	noncoding
MSTRG.34000.3	-0.220483	noncoding
MSTRG.34000.1	-0.603102	noncoding
MSTRG.34000.4	-0.122196	noncoding
MSTRG.34000.5	-0.122196	noncoding
MSTRG.34000.6	-0.002016	noncoding
MSTRG.34016.1	-2.20137	noncoding
MSTRG.34016.2	-2.1217	noncoding
MSTRG.34016.4	-2.30638	noncoding
MSTRG.34016.5	-0.612433	noncoding
MSTRG.34016.6	-0.612433	noncoding
MSTRG.34016.3	-0.859661	noncoding
MSTRG.34016.7	-2.2979	noncoding
MSTRG.34016.8	-2.38707	noncoding
MSTRG.34016.9	-1.8473	noncoding
MSTRG.34016.10	-1.05629	noncoding

MSTRG.34016.11	-0.761317	noncoding
MSTRG.34016.12	-0.81799	noncoding
MSTRG.34016.13	-1.38273	noncoding
MSTRG.34016.15	-0.776819	noncoding
MSTRG.34016.14	-0.708367	noncoding
MSTRG.34016.16	-0.82042	noncoding
MSTRG.34016.17	-1.78388	noncoding
MSTRG.34016.18	-1.74873	noncoding
MSTRG.34012.1	0.054583	coding
MSTRG.34013.1	-1.94236	noncoding
MSTRG.34017.1	-1.90099	noncoding
MSTRG.34017.3	-2.39624	noncoding
MSTRG.34017.2	-1.16843	noncoding
MSTRG.34017.4	-1.91253	noncoding
MSTRG.34017.5	-0.911799	noncoding
MSTRG.34018.1	-1.49775	noncoding
MSTRG.34026.2	-1.74579	noncoding
MSTRG.34028.1	-1.39693	noncoding
MSTRG.34035.1	-0.510423	noncoding
MSTRG.34037.1	-2.21836	noncoding
MSTRG.34038.1	-1.84576	noncoding
MSTRG.34046.1	1.49103	coding
MSTRG.34046.2	1.48861	coding
MSTRG.34046.3	1.41184	coding
MSTRG.34046.4	2.35548	coding
MSTRG.34046.5	1.10639	coding
MSTRG.34046.6	1.84173	coding
MSTRG.34047.2	-1.05549	noncoding
MSTRG.34052.1	-1.54828	noncoding
MSTRG.34053.1	-1.45649	noncoding
MSTRG.34053.2	-1.61257	noncoding
MSTRG.34055.1	-0.509382	noncoding
MSTRG.34056.2	0.214491	coding
MSTRG.34066.1	0.253552	coding
MSTRG.34072.1	-2.32591	noncoding
MSTRG.34072.2	-2.32594	noncoding
MSTRG.34072.3	-2.32608	noncoding
MSTRG.34072.4	-2.32595	noncoding
MSTRG.34072.5	-2.326	noncoding
MSTRG.34088.1	-2.32636	noncoding
MSTRG.34090.1	-2.3263	noncoding
MSTRG.34088.2	-2.32639	noncoding
MSTRG.34084.1	-2.32628	noncoding
MSTRG.34085.1	-2.32638	noncoding
MSTRG.34093.1	-0.130367	noncoding
MSTRG.34094.1	0.089943	coding
MSTRG.34101.1	-1.01357	noncoding
MSTRG.34097.1	-2.44893	noncoding
MSTRG.34108.2	0.084424	coding
MSTRG.34110.1	-1.47832	noncoding
MSTRG.34110.2	-2.12196	noncoding
MSTRG.34129.1	-1.79842	noncoding
MSTRG.34130.3	-1.63014	noncoding
MSTRG.34130.1	-1.63245	noncoding

MSTRG.34130.2	-1.63395	noncoding
MSTRG.34131.1	-1.88013	noncoding
MSTRG.34131.2	-1.99955	noncoding
MSTRG.34130.4	-1.5778	noncoding
MSTRG.34130.5	-1.63014	noncoding
MSTRG.34135.3	-1.50226	noncoding
MSTRG.34135.1	-1.41661	noncoding
MSTRG.34135.2	-1.41661	noncoding
MSTRG.34135.4	-1.37479	noncoding
MSTRG.34136.1	-1.01294	noncoding
MSTRG.34135.5	-1.69977	noncoding
MSTRG.34137.3	-0.575558	noncoding
MSTRG.34137.1	-0.544652	noncoding
MSTRG.34137.2	-0.544652	noncoding
MSTRG.34135.6	-1.77676	noncoding
MSTRG.34133.1	-2.33476	noncoding
MSTRG.34146.1	1.58131	coding
MSTRG.34149.1	0.154455	coding
MSTRG.34152.1	-0.799107	noncoding
MSTRG.34153.1	-2.47525	noncoding
MSTRG.34185.7	0.588675	coding
MSTRG.34185.8	-0.888079	noncoding
MSTRG.34185.10	-1.06191	noncoding
MSTRG.34185.9	-0.962977	noncoding
MSTRG.34185.11	-1.00091	noncoding
MSTRG.34185.12	-1.04311	noncoding
MSTRG.34185.13	-1.13434	noncoding
MSTRG.34185.15	-0.451847	noncoding
MSTRG.34185.14	-0.455512	noncoding
MSTRG.34185.16	-1.00546	noncoding
MSTRG.34185.17	-0.36194	noncoding
MSTRG.34185.18	-0.35168	noncoding
MSTRG.34185.19	-1.31132	noncoding
MSTRG.34185.20	-0.584835	noncoding
MSTRG.34185.21	-0.594183	noncoding
MSTRG.34185.22	-0.430093	noncoding
MSTRG.34185.23	-1.56583	noncoding
MSTRG.34154.1	-2.07664	noncoding
MSTRG.34155.1	-2.37887	noncoding
MSTRG.34154.2	-2.1391	noncoding
MSTRG.34161.2	0.06161	coding
MSTRG.34161.3	-0.415768	noncoding
MSTRG.34159.1	-1.74106	noncoding
MSTRG.34164.1	-1.82975	noncoding
MSTRG.34164.2	-1.04103	noncoding
MSTRG.34171.2	1.66245	coding
MSTRG.34171.5	1.2023	coding
MSTRG.34171.6	0.700948	coding
MSTRG.34174.1	-1.98734	noncoding
MSTRG.34178.1	-0.926786	noncoding
MSTRG.34183.2	1.06729	coding
MSTRG.34183.3	0.777724	coding
MSTRG.34183.4	-1.43129	noncoding
MSTRG.34190.1	1.47182	coding

MSTRG.34190.2	1.52252	coding
MSTRG.34190.6	1.8484	coding
MSTRG.34190.9	1.57085	coding
MSTRG.34169.1	-2.18193	noncoding
MSTRG.34196.1	-0.972427	noncoding
MSTRG.34198.3	-0.522288	noncoding
MSTRG.34198.4	-0.677223	noncoding
MSTRG.34206.1	-1.05051	noncoding
MSTRG.34202.1	0.243957	coding
MSTRG.34209.1	-1.02256	noncoding
MSTRG.34209.2	-1.12885	noncoding
MSTRG.34210.1	-1.18279	noncoding
MSTRG.34210.2	-1.46105	noncoding
MSTRG.34219.1	-2.40512	noncoding
MSTRG.34220.1	-2.25018	noncoding
MSTRG.34219.2	-2.40512	noncoding
MSTRG.34220.2	-2.25313	noncoding
MSTRG.34220.3	-2.29994	noncoding
MSTRG.34219.3	-2.41617	noncoding
MSTRG.34225.1	-2.32806	noncoding
MSTRG.34226.1	-2.38276	noncoding
MSTRG.34235.1	-2.64641	noncoding
MSTRG.34238.1	-1.62273	noncoding
MSTRG.34252.1	-1.77434	noncoding
MSTRG.34252.2	-1.86059	noncoding
MSTRG.34252.3	-1.0341	noncoding
MSTRG.34252.4	-1.0341	noncoding
MSTRG.34272.1	-0.525333	noncoding
MSTRG.34272.2	-0.442777	noncoding
MSTRG.34272.4	-0.642857	noncoding
MSTRG.34272.3	-0.844148	noncoding
MSTRG.34272.6	-0.855802	noncoding
MSTRG.34272.5	-0.655513	noncoding
MSTRG.34272.7	-1.0446	noncoding
MSTRG.34272.8	-0.876078	noncoding
MSTRG.34272.9	-0.855098	noncoding
MSTRG.34293.1	-3.58103	noncoding
MSTRG.34294.1	-1.21843	noncoding
MSTRG.34294.2	-0.962124	noncoding
MSTRG.34295.1	-0.477313	noncoding
MSTRG.34295.2	-0.19073	noncoding
MSTRG.34295.4	-0.861253	noncoding
MSTRG.34295.3	-0.470391	noncoding
MSTRG.34295.5	-1.51254	noncoding
MSTRG.34294.3	-0.612962	noncoding
MSTRG.34294.4	-0.324697	noncoding
MSTRG.34294.6	-0.509399	noncoding
MSTRG.34294.5	-0.521106	noncoding
MSTRG.34295.6	-0.260313	noncoding
MSTRG.34294.8	-2.54865	noncoding
MSTRG.34294.7	-0.506083	noncoding
MSTRG.34294.9	-0.063731	noncoding
MSTRG.34295.7	-2.43674	noncoding
MSTRG.34294.10	-0.425231	noncoding

MSTRG.34294.11	-1.1189	noncoding
MSTRG.34298.1	-2.17639	noncoding
MSTRG.34298.2	-2.22636	noncoding
MSTRG.34299.1	-1.91669	noncoding
MSTRG.34282.1	-1.55315	noncoding
MSTRG.34283.1	-2.32616	noncoding
MSTRG.34284.1	-2.12404	noncoding
MSTRG.34287.1	-2.56757	noncoding
MSTRG.34300.1	-1.94803	noncoding
MSTRG.34302.1	-1.3469	noncoding
MSTRG.34303.1	-1.60795	noncoding
MSTRG.34314.1	-1.81355	noncoding
MSTRG.34314.3	-1.1038	noncoding
MSTRG.34314.4	-0.897054	noncoding
MSTRG.34314.2	-0.767603	noncoding
MSTRG.34315.1	0.412795	coding
MSTRG.34315.2	0.7576	coding
MSTRG.34315.3	0.162234	coding
MSTRG.34315.4	0.590224	coding
MSTRG.34317.2	0.396339	coding
MSTRG.34317.3	-0.566208	noncoding
MSTRG.34315.5	0.440132	coding
MSTRG.34315.6	0.021136	coding
MSTRG.34315.7	-1.21031	noncoding
MSTRG.34315.8	0.180104	coding
MSTRG.34315.10	0.004939	coding
MSTRG.34315.11	-0.065857	noncoding
MSTRG.34315.9	-0.084918	noncoding
MSTRG.34304.1	-2.32414	noncoding
MSTRG.34305.1	-2.45421	noncoding
MSTRG.34306.2	-2.33123	noncoding
MSTRG.34306.1	-2.32425	noncoding
MSTRG.34307.1	-2.33032	noncoding
MSTRG.34307.2	-2.32756	noncoding
MSTRG.34308.1	-2.33523	noncoding
MSTRG.34321.1	-0.108946	noncoding
MSTRG.34328.1	-2.3141	noncoding
MSTRG.34330.1	-1.99887	noncoding
MSTRG.34327.1	-0.390972	noncoding
MSTRG.34334.2	-0.941571	noncoding
MSTRG.34346.1	-1.60427	noncoding
MSTRG.34346.2	-0.372587	noncoding
MSTRG.34346.3	-1.38784	noncoding
MSTRG.34346.4	-1.38784	noncoding
MSTRG.34342.1	-1.93856	noncoding
MSTRG.34342.2	-1.93856	noncoding
MSTRG.34351.1	-1.39329	noncoding
MSTRG.34351.3	-1.95379	noncoding
MSTRG.34351.2	-1.77538	noncoding
MSTRG.34351.4	-1.68125	noncoding
MSTRG.34351.5	-1.61748	noncoding
MSTRG.34351.6	-1.66727	noncoding
MSTRG.34351.7	-1.81694	noncoding
MSTRG.34351.8	-1.76992	noncoding

MSTRG.34349.1	-1.67123	noncoding
MSTRG.34352.1	-1.38853	noncoding
MSTRG.34352.2	-1.953	noncoding
MSTRG.34369.1	1.74778	coding
MSTRG.34369.2	1.32369	coding
MSTRG.34369.3	0.923292	coding
MSTRG.34369.4	1.1267	coding
MSTRG.34369.5	1.1077	coding
MSTRG.34369.7	1.9258	coding
MSTRG.34369.8	2.3188	coding
MSTRG.34369.9	0.130987	coding
MSTRG.34369.10	1.53847	coding
MSTRG.34369.11	1.21899	coding
MSTRG.34356.1	-2.08025	noncoding
MSTRG.34356.2	-1.58398	noncoding
MSTRG.34357.1	-1.96376	noncoding
MSTRG.34355.1	1.01635	coding
MSTRG.34363.2	0.684326	coding
MSTRG.34363.3	1.56646	coding
MSTRG.34365.2	0.695326	coding
MSTRG.34365.3	-0.216696	noncoding
MSTRG.34365.4	-2.19774	noncoding
MSTRG.34361.1	-2.21254	noncoding
MSTRG.34373.1	-2.4541	noncoding
MSTRG.34373.2	-2.50585	noncoding
MSTRG.34373.3	-2.4541	noncoding
MSTRG.34374.1	-2.47987	noncoding
MSTRG.34374.2	-2.2356	noncoding
MSTRG.34374.3	-2.39162	noncoding
MSTRG.34375.1	-0.110391	noncoding
MSTRG.34375.2	-0.136661	noncoding
MSTRG.34373.4	-1.38333	noncoding
MSTRG.34373.5	-1.38333	noncoding
MSTRG.34376.5	-0.321512	noncoding
MSTRG.34376.2	-0.775235	noncoding
MSTRG.34376.4	-0.408971	noncoding
MSTRG.34376.3	-0.433398	noncoding
MSTRG.34376.6	-0.31165	noncoding
MSTRG.34376.7	-0.363153	noncoding
MSTRG.34376.1	-0.398249	noncoding
MSTRG.34383.1	-2.31035	noncoding
MSTRG.34383.2	-2.32699	noncoding
MSTRG.34383.3	-2.26441	noncoding
MSTRG.34384.1	-2.40719	noncoding
MSTRG.34389.1	-2.12474	noncoding
MSTRG.34390.1	-1.87493	noncoding
MSTRG.34391.1	-2.17198	noncoding
MSTRG.34393.1	0.962565	coding
MSTRG.34395.2	1.25928	coding
MSTRG.34394.1	0.238253	coding
MSTRG.34394.3	0.071386	coding
MSTRG.34400.1	-1.43069	noncoding
MSTRG.34401.2	-2.46442	noncoding
MSTRG.34401.1	-2.47426	noncoding

MSTRG.34402.1	-1.71676	noncoding
MSTRG.34402.2	-1.71676	noncoding
MSTRG.34401.3	-2.02175	noncoding
MSTRG.34402.3	-1.50499	noncoding
MSTRG.34400.2	-0.306451	noncoding
MSTRG.34411.1	-1.71726	noncoding
MSTRG.34414.1	0.091537	coding
MSTRG.34414.2	-0.352683	noncoding
MSTRG.34414.3	-0.35936	noncoding
MSTRG.34416.1	-2.35081	noncoding
MSTRG.34435.10	0.66927	coding
MSTRG.34435.7	0.604485	coding
MSTRG.34435.8	0.661153	coding
MSTRG.34435.9	0.66927	coding
MSTRG.34435.2	0.637702	coding
MSTRG.34418.1	-2.32632	noncoding
MSTRG.34420.1	-2.23703	noncoding
MSTRG.34421.1	-2.05197	noncoding
MSTRG.34422.1	-2.32623	noncoding
MSTRG.34427.1	-1.72039	noncoding
MSTRG.34427.2	-0.99934	noncoding
MSTRG.34427.3	-1.01446	noncoding
MSTRG.34429.2	-1.90037	noncoding
MSTRG.34429.1	-1.60915	noncoding
MSTRG.34438.1	-1.69767	noncoding
MSTRG.34439.1	-1.61429	noncoding
MSTRG.34449.1	-1.52997	noncoding
MSTRG.34449.2	-1.31728	noncoding
MSTRG.34449.3	-1.55079	noncoding
MSTRG.34449.4	-1.66642	noncoding
MSTRG.34449.5	-1.28748	noncoding
MSTRG.34449.6	-1.33872	noncoding
MSTRG.34449.7	-1.29944	noncoding
MSTRG.34449.9	-1.21788	noncoding
MSTRG.34449.8	-1.22544	noncoding
MSTRG.34449.10	-0.951299	noncoding
MSTRG.34446.1	-1.69572	noncoding
MSTRG.34446.2	-2.31255	noncoding
MSTRG.34452.1	-1.9283	noncoding
MSTRG.34452.2	-2.05139	noncoding
MSTRG.34458.1	-1.85683	noncoding
MSTRG.34460.1	-1.86144	noncoding
MSTRG.34455.1	-2.28794	noncoding
MSTRG.34455.2	-2.25415	noncoding
MSTRG.34467.1	-1.86047	noncoding
MSTRG.34471.1	-2.32451	noncoding
MSTRG.34478.1	0.688974	coding
MSTRG.34482.3	-1.03843	noncoding
MSTRG.34481.1	-1.52537	noncoding
MSTRG.34486.1	-2.38943	noncoding
MSTRG.34489.1	-1.22802	noncoding
MSTRG.34501.1	-1.32607	noncoding
MSTRG.34505.1	0.954322	coding
MSTRG.34511.1	-1.05566	noncoding

MSTRG.34511.2	-1.98556	noncoding
MSTRG.34511.3	-1.98556	noncoding
MSTRG.34511.4	-2.5337	noncoding
MSTRG.34512.1	-2.02604	noncoding
MSTRG.34513.1	-1.45379	noncoding
MSTRG.34514.1	-2.2611	noncoding
MSTRG.34515.1	-2.32017	noncoding
MSTRG.34519.2	-0.829928	noncoding
MSTRG.34519.1	-0.641875	noncoding
MSTRG.34519.3	-1.07351	noncoding
MSTRG.34519.4	-1.07351	noncoding
MSTRG.34519.5	-0.086529	noncoding
MSTRG.34519.6	-0.089338	noncoding
MSTRG.34520.1	-1.85861	noncoding
MSTRG.34521.1	-2.41257	noncoding
MSTRG.34519.7	-1.07351	noncoding
MSTRG.34524.1	-1.36208	noncoding
MSTRG.34524.2	-1.27985	noncoding
MSTRG.34524.3	-0.990145	noncoding
MSTRG.34524.4	-0.85284	noncoding
MSTRG.34524.5	-0.820084	noncoding
MSTRG.34528.2	-2.28199	noncoding
MSTRG.34528.1	-1.69195	noncoding
MSTRG.34529.1	-1.46988	noncoding
MSTRG.34537.1	-0.662307	noncoding
MSTRG.34537.3	-1.25464	noncoding
MSTRG.34537.2	-2.02057	noncoding
MSTRG.34537.4	-1.14928	noncoding
MSTRG.34532.1	-2.28708	noncoding
MSTRG.34554.1	0.762487	coding
MSTRG.34556.1	-0.512032	noncoding
MSTRG.34554.2	-0.231412	noncoding
MSTRG.34557.2	0.22927	coding
MSTRG.34557.1	1.04124	coding
MSTRG.34557.4	1.82819	coding
MSTRG.34550.1	-2.32005	noncoding
MSTRG.34550.2	-2.26728	noncoding
MSTRG.34561.1	-2.32546	noncoding
MSTRG.34561.2	-2.32604	noncoding
MSTRG.34563.1	-1.06676	noncoding
MSTRG.34621.2	-0.344117	noncoding
MSTRG.34621.1	-0.040482	noncoding
MSTRG.34621.3	0.524952	coding
MSTRG.34621.4	-1.42964	noncoding
MSTRG.34621.5	-0.762179	noncoding
MSTRG.34622.1	-0.018464	noncoding
MSTRG.34621.8	-0.351284	noncoding
MSTRG.34621.6	-0.29306	noncoding
MSTRG.34621.7	-0.223946	noncoding
MSTRG.34621.11	-0.203612	noncoding
MSTRG.34621.12	-0.106698	noncoding
MSTRG.34621.10	-0.035922	noncoding
MSTRG.34621.9	-0.34898	noncoding
MSTRG.34621.13	0.653253	coding

MSTRG.34621.14	-0.465393	noncoding
MSTRG.34621.15	-1.39938	noncoding
MSTRG.34621.17	-1.21631	noncoding
MSTRG.34621.16	-1.80296	noncoding
MSTRG.34621.18	-1.30813	noncoding
MSTRG.34622.3	-0.173799	noncoding
MSTRG.34622.4	-0.275883	noncoding
MSTRG.34622.5	-0.279371	noncoding
MSTRG.34622.6	-0.173421	noncoding
MSTRG.34622.7	0.031905	coding
MSTRG.34622.8	-0.434745	noncoding
MSTRG.34622.10	-0.189298	noncoding
MSTRG.34622.9	-0.113406	noncoding
MSTRG.34622.11	-0.39677	noncoding
MSTRG.34622.12	-0.217972	noncoding
MSTRG.34622.13	-0.21571	noncoding
MSTRG.34622.14	-0.262081	noncoding
MSTRG.34622.15	-0.395741	noncoding
MSTRG.34622.16	-0.216785	noncoding
MSTRG.34568.1	-1.3159	noncoding
MSTRG.34568.2	-1.45687	noncoding
MSTRG.34569.1	-1.9755	noncoding
MSTRG.34570.1	-2.12176	noncoding
MSTRG.34572.1	-1.24441	noncoding
MSTRG.34574.1	-2.26304	noncoding
MSTRG.34575.1	-2.29427	noncoding
MSTRG.34576.1	-2.34598	noncoding
MSTRG.34579.2	0.557506	coding
MSTRG.34580.1	-1.58932	noncoding
MSTRG.34583.1	-0.918623	noncoding
MSTRG.34583.2	0.031072	coding
MSTRG.34587.1	-2.32628	noncoding
MSTRG.34592.2	-1.68468	noncoding
MSTRG.34592.1	-1.76784	noncoding
MSTRG.34592.3	-1.78157	noncoding
MSTRG.34592.4	-1.68896	noncoding
MSTRG.34592.5	-1.81575	noncoding
MSTRG.34595.7	-0.783807	noncoding
MSTRG.34595.3	-0.643413	noncoding
MSTRG.34595.5	-0.643413	noncoding
MSTRG.34595.6	-0.643413	noncoding
MSTRG.34595.8	-1.49277	noncoding
MSTRG.34595.2	-0.769402	noncoding
MSTRG.34595.4	-1.02103	noncoding
MSTRG.34595.9	-0.657905	noncoding
MSTRG.34595.10	-1.54982	noncoding
MSTRG.34595.11	-1.54955	noncoding
MSTRG.34595.12	-1.02103	noncoding
MSTRG.34595.13	-0.786981	noncoding
MSTRG.34595.14	-1.59022	noncoding
MSTRG.34595.15	-1.50042	noncoding
MSTRG.34595.16	-1.58313	noncoding
MSTRG.34600.1	-1.57029	noncoding
MSTRG.34601.2	-1.68924	noncoding

MSTRG.34601.3	-1.7779	noncoding
MSTRG.34601.1	-1.78113	noncoding
MSTRG.34603.1	-2.90558	noncoding
MSTRG.34600.2	-0.977615	noncoding
MSTRG.34600.3	-1.38687	noncoding
MSTRG.34606.1	-1.66152	noncoding
MSTRG.34600.5	-1.36826	noncoding
MSTRG.34600.4	-1.21886	noncoding
MSTRG.34600.6	-1.66926	noncoding
MSTRG.34597.1	-2.06376	noncoding
MSTRG.34612.1	-0.768245	noncoding
MSTRG.34613.1	-1.88194	noncoding
MSTRG.34611.1	-2.1675	noncoding
MSTRG.34616.1	-1.89968	noncoding
MSTRG.34616.2	-1.89968	noncoding
MSTRG.34616.3	-2.0295	noncoding
MSTRG.34616.4	-1.84123	noncoding
MSTRG.34616.5	-1.89968	noncoding
MSTRG.34615.1	-2.24958	noncoding
MSTRG.34619.1	-0.484891	noncoding
MSTRG.34631.3	-0.126281	noncoding
MSTRG.34631.2	-0.565843	noncoding
MSTRG.34632.1	-2.11503	noncoding
MSTRG.34633.1	-1.66703	noncoding
MSTRG.34632.2	-2.34178	noncoding
MSTRG.34638.2	0.250602	coding
MSTRG.34638.1	-0.842745	noncoding
MSTRG.34637.2	0.722654	coding
MSTRG.34637.4	0.796825	coding
MSTRG.34653.1	0.550756	coding
MSTRG.34655.2	0.042547	coding
MSTRG.34655.1	-0.120482	noncoding
MSTRG.34656.2	-1.9581	noncoding
MSTRG.34656.1	-1.63651	noncoding
MSTRG.34657.1	-1.49155	noncoding
MSTRG.34660.1	-2.07153	noncoding
MSTRG.34661.1	-2.52984	noncoding
MSTRG.34653.2	-1.00421	noncoding
MSTRG.34664.1	-0.248465	noncoding
MSTRG.34666.1	-2.13068	noncoding
MSTRG.34671.1	0.373296	coding
MSTRG.34646.1	-2.32087	noncoding
MSTRG.34647.3	0.313393	coding
MSTRG.34647.1	0.644167	coding
MSTRG.34675.3	-1.2668	noncoding
MSTRG.34675.2	-1.16896	noncoding
MSTRG.34675.4	-1.32416	noncoding
MSTRG.34675.5	-1.46942	noncoding
MSTRG.34681.2	0.068014	coding
MSTRG.34681.4	0.594375	coding
MSTRG.34681.3	1.07036	coding
MSTRG.34682.7	0.26778	coding
MSTRG.34682.9	0.189941	coding
MSTRG.34682.11	0.780961	coding

MSTRG.34682.12	0.780961	coding
MSTRG.34683.1	-2.09145	noncoding
MSTRG.34707.1	-2.02195	noncoding
MSTRG.34708.1	-2.194	noncoding
MSTRG.34712.1	-1.50195	noncoding
MSTRG.34714.1	-1.23157	noncoding
MSTRG.34715.1	0.927313	coding
MSTRG.34716.1	-2.45909	noncoding
MSTRG.34717.1	-0.615288	noncoding
MSTRG.34715.2	0.96829	coding
MSTRG.34715.3	1.05206	coding
MSTRG.34718.1	-1.20744	noncoding
MSTRG.34719.1	-2.07845	noncoding
MSTRG.34720.1	-0.595296	noncoding
MSTRG.34721.1	-0.142477	noncoding
MSTRG.34722.1	-2.43185	noncoding
MSTRG.34715.4	-0.0074	noncoding
MSTRG.34723.1	-2.1018	noncoding
MSTRG.34723.2	-1.73374	noncoding
MSTRG.34724.1	1.06332	coding
MSTRG.34725.1	-1.26908	noncoding
MSTRG.34719.2	0.139781	coding
MSTRG.34717.2	-1.48874	noncoding
MSTRG.34717.3	-2.07514	noncoding
MSTRG.34726.1	-0.278763	noncoding
MSTRG.34715.5	0.080927	coding
MSTRG.34727.1	-1.26721	noncoding
MSTRG.34687.1	-2.45756	noncoding
MSTRG.34689.2	-1.41584	noncoding
MSTRG.34690.2	2.97007	coding
MSTRG.34692.1	-2.09672	noncoding
MSTRG.34701.1	-1.41689	noncoding
MSTRG.34702.1	-0.794424	noncoding
MSTRG.34729.1	-1.33868	noncoding
MSTRG.34743.1	0.904338	coding
MSTRG.34743.2	1.51602	coding
MSTRG.34744.2	1.75353	coding
MSTRG.34744.3	1.50866	coding
MSTRG.34744.4	0.807825	coding
MSTRG.34744.5	1.88462	coding
MSTRG.34751.1	0.501527	coding
MSTRG.34755.2	-0.267004	noncoding
MSTRG.34755.1	0.275922	coding
MSTRG.34761.2	0.347559	coding
MSTRG.34763.2	1.89032	coding
MSTRG.34764.1	-0.700346	noncoding
MSTRG.34771.9	-0.851235	noncoding
MSTRG.34771.13	-0.778321	noncoding
MSTRG.34778.1	-0.959934	noncoding
MSTRG.34778.3	-1.44088	noncoding
MSTRG.34778.4	-1.44088	noncoding
MSTRG.34778.5	-0.990423	noncoding
MSTRG.34778.6	-0.990423	noncoding
