# Supplementary material for: Identification of Novel lncRNA and Differentially Expressed Genes (DEGs) of Testicular Tissues among Cattle, Yak, and Cattle-Yak Associated with Male Infertility
Source: Animals (Basel). 2021 Aug 17;11(8):2420. doi: 10.3390/ani11082420 (PMC8388754; doi:10.3390/ani11082420)
Supplement: Supplementary file 1 [file animals-11-02420-s001.zip › Table S5 DE_lncRNA and DE mRNA .pdf]

Table S1 DE lncRNA and DE mRNA among cattle,yak and cattle-yak

DE lncRNA	MSTRG.18068.3,MSTRG.308.1,MSTRG.33132.1,MSTRG.34206.1,MSTRG.34210.1,MSTRG.4843.1,XR_003032659.1,XR_003033672.1,XR_233800.4
DE mRNA	NM_001015538.1,NM_001015609.1,NM_001037624.1,NM_001038065.1,NM_001038208.1,NM_001040492.2,NM_001075673.1,NM_001076805.1,NM_001081544.1,NM_001102057.1,NM_001191163.2,NM_001199006.1,NM_001205607.1,NM_001206310.2,NM_174180.3,NM_174518.1,XM_005206728.4,XM_005209090.4,XM_005216683.4,XM_005216684.4,XM_005217380.4,XM_005218713.4,XM_005221063.3,XM_005226096.4,XM_005227099.4,XM_010803980.3,XM_010811409.3,XM_010811994.3,XM_010813031.2,XM_010820324.3,XM_010820326.3,XM_024978047.1,XM_024978483.1,XM_024978484.1,XM_024979836.1,XM_024980446.1,XM_024982910.1,XM_024983649.1,XM_024985389.1,XM_024988619.1,XM_024988946.1,XM_024991809.1,XM_024993546.1,XM_024996459.1,XM_024996471.1,XM_024999382.1
