# Supplementary material for: Identification of Novel lncRNA and Differentially Expressed Genes (DEGs) of Testicular Tissues among Cattle, Yak, and Cattle-Yak Associated with Male Infertility
Source: Animals (Basel). 2021 Aug 17;11(8):2420. doi: 10.3390/ani11082420 (PMC8388754; doi:10.3390/ani11082420)
Supplement: Supplementary file 1 [file animals-11-02420-s001.zip › Table S7_GO terms of mRNA target genes of cattle.pdf]

Table S7 The significantly enriched GO description of BP, CC and MF for the upregulated mRNA target genes of cattle

Ontology	Term	GO.ID	Significant	Annotated	Qvalue
BP	cellular component organization or biogenesis	GO:0071840	658	5228	1.14E-11
BP	reproductive process	GO:0022414	187	1188	3.10E-08
BP	reproduction	GO:0000003	187	1190	3.40E-08
BP	metabolic process	GO:0008152	1067	9982	0.002728
BP	cellular process	GO:0009987	1505	14842	0.092403
BP	localization	GO:0051179	556	5065	0.094813
BP	multi-organism process	GO:0051704	242	2045	0.095071
BP	establishment of localization	GO:0051234	433	3965	0.357635
CC	organelle	GO:0043226	1476	12576	4.94E-28
CC	organelle part	GO:0044422	988	7853	1.39E-20
CC	membrane-enclosed lumen	GO:0031974	471	3755	6.06E-07
CC	supramolecular fiber	GO:0099512	135	841	8.82E-07
CC	cell part	GO:0044464	1728	16747	0.001307
CC	cell	GO:0005623	1728	16775	0.002938
CC	protein-containing complex	GO:0032991	493	4316	0.008014
CC	synapse	GO:0045202	82	716	0.762281
CC	synapse part	GO:0044456	67	582	0.820478
CC	nucleoid	GO:0009295	7	43	0.856805
MF	catalytic activity	GO:0003824	713	5764	7.85E-17
MF	binding	GO:0005488	1152	10991	2.38E-08
MF	translation regulator activity	GO:0045182	11	39	0.064563
