# Supplementary material for: Identification of Novel lncRNA and Differentially Expressed Genes (DEGs) of Testicular Tissues among Cattle, Yak, and Cattle-Yak Associated with Male Infertility
Source: Animals (Basel). 2021 Aug 17;11(8):2420. doi: 10.3390/ani11082420 (PMC8388754; doi:10.3390/ani11082420)
Supplement: Supplementary file 1 [file animals-11-02420-s001.zip › Table S8_GO terms of mRNA target genes of yak.pdf]

Table S8 The significantly enriched GO description of BP, CC and MF for the upregulated mRNA target genes of yak

Ontology	Term	GO.ID	Significant	Annotated	Qvalue
BP	positive regulation of biological process	GO:0048518	15	4757	1
BP	cellular component organization or biogenesis	GO:0071840	16	5228	1
BP	metabolic process	GO:0008152	27	9982	1
BP	localization	GO:0051179	14	5065	1
BP	cellular process	GO:0009987	37	14842	1
BP	regulation of biological process	GO:0050789	26	10466	1
BP	biological regulation	GO:0065007	27	11042	1
BP	multicellular organismal process	GO:0032501	14	6664	1
BP	response to stimulus	GO:0050896	18	8387	1
BP	signaling	GO:0023052	12	6065	1
CC	organelle	GO:0043226	36	12576	1
CC	organelle part	GO:0044422	25	7853	1
CC	supramolecular fiber	GO:0099512	5	841	1
CC	membrane-enclosed lumen	GO:0031974	13	3755	1
CC	cell part	GO:0044464	42	16747	1
CC	protein-containing complex	GO:0032991	14	4316	1
CC	cell	GO:0005623	42	16775	1
CC	extracellular region	GO:0005576	7	4337	1
CC	membrane	GO:0016020	18	9946	1
CC	membrane part	GO:0044425	12	7766	1
MF	transcription factor activity, protein binding	GO:0000988	4	520	1
MF	binding	GO:0005488	31	10991	1
MF	catalytic activity	GO:0003824	18	5764	1
MF	structural molecule activity	GO:0005198	3	706	1
MF	channel regulator activity	GO:0016247	1	117	1
MF	molecular function regulator	GO:0098772	5	1506	1
MF	enzyme regulator activity	GO:0030234	3	890	1
MF	transporter activity	GO:0005215	2	1273	1
MF	signal transducer activity	GO:0004871	1	2456	1
