# Supplementary material for: Identification of Novel lncRNA and Differentially Expressed Genes (DEGs) of Testicular Tissues among Cattle, Yak, and Cattle-Yak Associated with Male Infertility
Source: Animals (Basel). 2021 Aug 17;11(8):2420. doi: 10.3390/ani11082420 (PMC8388754; doi:10.3390/ani11082420)
Supplement: Supplementary file 1 [file animals-11-02420-s001.zip › Table S9-S12_ enriched pathways.pdf]

Table S9　The significantly enriched pathway description for the up-regulated lncRNA targert genes of cattle-yak

Pathway Id	Description	Annotated	Pvalue	Qvalue
ko04210	Apoptosis	225/8066	0.055015	0.049557

Table S10　The significantly enriched pathway description for the up-regulated lncRNA targert genes of cattle

Pathway Id	Description	Annotated	Pvalue	Qvalue
ko04512	ECM-receptor interaction	146/8066	0.001906	0.00602
ko04510	Focal adhesion	330/8066	0.009479	0.014967
ko04151	PI3K-Akt signaling pathway	493/8066	0.020597	0.021681
ko04066	HIF-1 signaling pathway	147/8066	0.070943	0.056007
ko04120	Ubiquitin mediated proteolysis	290/8066	0.136266	0.071719

Table S11　The significantly enriched pathway description for the up-regulated mRNA targert genes of cattle

Pathway Id	Description	Significant	Annotated	Pvalue	Qvalue
ko04120	Ubiquitin mediated proteolysis	51/836	290/8066	9.57E-05	0.014715
ko04340	Hedgehog signaling pathway	25/836	116/8066	0.000277	0.021253
ko04141	Protein processing in endoplasmic reticulum	44/836	267/8066	0.001177	0.060271
ko04114	Oocyte meiosis	30/836	171/8066	0.002635	0.087095
ko00521	Streptomycin biosynthesis	5/836	11/8066	0.003201	0.087095
ko00564	Glycerophospholipid metabolism	23/836	124/8066	0.003918	0.087095
ko04330	Notch signaling pathway	18/836	89/8066	0.003967	0.087095
ko04341	Hedgehog signaling pathway - Fly	11/836	49/8066	0.010149	0.176362
ko00010	Glycolysis / Gluconeogenesis	14/836	70/8066	0.011476	0.176362
ko03460	Fanconi anemia pathway	14/836	70/8066	0.011476	0.176362
ko00520	Amino sugar and nucleotide sugar metabolism	13/836	66/8066	0.016467	0.218762
ko04110	Cell cycle	30/836	196/8066	0.018421	0.218762
ko03022	Basal transcription factors	12/836	60/8066	0.018505	0.218762
ko00250	Alanine, aspartate and glutamate metabolism	10/836	48/8066	0.023128	0.253884

ko00051	Fructose and mannose metabolism	8/836	36/8066	0.028159	0.279824
ko04144	Endocytosis	57/836	430/8066	0.029132	0.279824
ko00061	Fatty acid biosynthesis	4/836	13/8066	0.038218	0.345498
ko00562	Inositol phosphate metabolism	14/836	84/8066	0.049012	0.409839

Table S12 The significantly enriched pathway description for the up-regulated mRNA targert genes of yak

Pathway Id	Description	Annotated	Pvalue	Qvalue
ko04150	mTOR signaling pathway	214/8066	0.011195	0.149375
ko04550	Signaling pathways regulating pluripotency of stem cells	243/8066	0.015767	0.149375
ko04064	NF-kappa B signaling pathway	138/8066	0.037175	0.192707
ko04668	TNF signaling pathway	145/8066	0.040683	0.192707
