# Supplementary material for: Identification of Novel lncRNA and Differentially Expressed Genes (DEGs) of Testicular Tissues among Cattle, Yak, and Cattle-Yak Associated with Male Infertility
Source: Animals (Basel). 2021 Aug 17;11(8):2420. doi: 10.3390/ani11082420 (PMC8388754; doi:10.3390/ani11082420)
Supplement: Supplementary file 1 [file animals-11-02420-s001.zip › Table_S6 GO terms of lncRNA target genes of cattle.pdf]

Table S6 The significantly enriched GO description of BP, CC and MF for up-regulated lncRNA target genes of cattle

Ontology	Term	GO.ID	Significant	Annotated	Qvalue
BP	reproductive process	GO:0022414	2	1188	1
BP	reproduction	GO:0000003	2	1190	1
BP	multicellular organismal process	GO:0032501	3	6664	1
BP	positive regulation of biological process	GO:0048518	2	4757	1
BP	localization	GO:0051179	2	5065	1
BP	developmental process	GO:0032502	2	5268	1
BP	signaling	GO:0023052	2	6065	1
BP	regulation of biological process	GO:0050789	3	10466	1
BP	biological regulation	GO:0065007	3	11042	1
BP	cellular process	GO:0009987	3	14842	1
CC	extracellular region	GO:0005576	3	4337	1
CC	extracellular region part	GO:0044421	2	3507	1
CC	organelle	GO:0043226	5	12576	1
CC	membrane	GO:0016020	3	9946	1
CC	membrane-enclosed lumen	GO:0031974	1	3755	1
CC	protein-containing complex	GO:0032991	1	4316	1
CC	cell part	GO:0044464	5	16747	1
CC	cell	GO:0005623	5	16775	1
CC	membrane part	GO:0044425	1	7766	1
CC	organelle part	GO:0044422	1	7853	1
MF	catalytic activity	GO:0003824	2	5764	1
MF	binding	GO:0005488	1	10991	1
